# Initiator tRNA genes template the 3′ CCA end at high frequencies in bacteria

**DOI:** 10.1186/s12864-016-3314-x

**Published:** 2016-12-08

**Authors:** David H. Ardell, Ya-Ming Hou

**Affiliations:** 1 Program in Quantitative and Systems Biology, University of California, 5200 North Lake Road, CA 95343 Merced, USA; 2 Molecular and Cell Biology Unit, School of Natural Sciences, University of California, 5200 North Lake Road, Merced, CA 95343 USA; 3 Department of Biochemistry and Molecular Biology, Thomas Jefferson University, 233 South 10th Street, BLSB 220, Philadelphia, PA 19107 USA

**Keywords:** Initiator tRNA, Bacteria, CCA

## Abstract

**Background:**

While the CCA sequence at the mature 3′ end of tRNAs is conserved and critical for translational function, a genetic template for this sequence is not always contained in tRNA genes. In eukaryotes and Archaea, the CCA ends of tRNAs are synthesized post-transcriptionally by CCA-adding enzymes. In Bacteria, tRNA genes template CCA sporadically.

**Results:**

In order to understand the variation in how prokaryotic tRNA genes template CCA, we re-annotated tRNA genes in tRNAdb-CE database version 0.8. Among 132,129 prokaryotic tRNA genes, initiator tRNA genes template CCA at the highest average frequency (74.1%) over all functional classes except selenocysteine and pyrrolysine tRNA genes (88.1% and 100% respectively). Across bacterial phyla and a wide range of genome sizes, many lineages exist in which predominantly initiator tRNA genes template CCA. Convergent and parallel retention of CCA templating in initiator tRNA genes evolved in independent histories of reductive genome evolution in Bacteria. Also, in a majority of cyanobacterial and actinobacterial genera, predominantly initiator tRNA genes template CCA. We also found that a surprising fraction of archaeal tRNA genes template CCA.

**Conclusions:**

We suggest that cotranscriptional synthesis of initiator tRNA CCA 3′ ends can complement inefficient processing of initiator tRNA precursors, “bootstrap” rapid initiation of protein synthesis from a non-growing state, or contribute to an increase in cellular growth rates by reducing overheads of mass and energy to maintain nonfunctional tRNA precursor pools. More generally, CCA templating in structurally non-conforming tRNA genes can afford cells robustness and greater plasticity to respond rapidly to environmental changes and stimuli.

**Electronic supplementary material:**

The online version of this article (doi:10.1186/s12864-016-3314-x) contains supplementary material, which is available to authorized users.

## Background

All active tRNA molecules must contain a CCA sequence at the 3′-end as the site for amino acid attachment and for interaction with the ribosome during protein synthesis [1–3]. While essential for tRNA activities, the CCA sequence is generally not contained in tRNA genes but is added post-transcriptionally. Exceptions are found in Bacteria, where some tRNA genes contain a template of the CCA sequence for direct synthesis at the time of transcription. However, CCA-templating is not necessarily conserved among tRNA genes with different functional identities or among bacterial species across different phyla. To explore whether there is potential selective pressure for tRNA genes to template CCA in Bacteria, we undertook a reannotation of publicly available tRNA gene data.

One source of error in the annotation of tRNA genes concerns the functional classification of genes for tRNAs with CAU anticodons. These include genes for initiator tRNA^fMet^, elongator tRNA^Met^ and elongator tRNA^Ile^_CAU_ isoacceptors that read AUA codons in Bacteria and Archaea. In this last case of elongator tRNA^Ile^_CAU_, the cytidines at the wobble anticodon position 34 (of the Sprinzl coordinate system for tRNA structure [4]) in transcribed CAU anticodons (C34) are post-transcriptionally modified to lysidine in most Bacteria [5, 6], changing the decoding specificity of these tRNAs from AUG to AUA codons. Additionally, this L34 lysidine modification changes the amino acid charging specificity of these tRNAs from methionine to isoleucine by serving as a recognition determinant for aminoacylation of tRNA^Ile^ by the cognate isoleucyl-tRNA synthetase [7]. Archaea use a similar system to decode AUA to isoleucine with a different kind of modification at the same anticodon position [8]. However, currently available tRNA gene-finders annotate all three classes as elongator tRNA^Met^ genes [9]. The TFAM tRNA functional classifier, which uses profile-based models of whole tRNA sequences [10, 11], can differentiate all three tRNA functional classes with generally high specificity and sensitivity (uniformly greater than 98% depending on data and models used) [12]. However, the tRNA^Ile^_CAU_ class evolves more rapidly than other classes, so that even though the TFAM 1.4 proteobacterial-specific model generalizes well to some other bacterial phyla, this model does not generalize well to all [13]. An alternative TFAM model [14], for just genes with tRNAs with CAU anticodons, is based on a custom annotation of such genes in a wide sampling of bacterial taxa [12]. Although this alternative model is imperfect in its sensitivity and specificity [12], as discussed further below, its performance is satisfactory and suitable for the present study.

Here we apply this alternative “Silva TFAM model” to improve the functional annotation of tRNA genes with CAU anticodons in the high quality public database tRNAdb-CE [15, 16]. In our analysis, we found that genes for the initiator class of tRNAs across the bacterial domain consistently template CCA with significantly higher frequencies than elongator tRNA genes. This CCA-templating can provide unique advantages to initiator tRNA for rapid maturation, aminoacylation, and initiation of protein synthesis.

## Results

### Functional reannotation of bacterial genes in tRNAdb-CE v.8

The tRNAdb-CE v0.8 database contains 132,129 total prokaryotic gene records, of which 129,989 gene records are bacterial. Among the bacterial sequences, 9,917 contain a template for anticodon with sequence CAU. For a substantial fraction of its records, the tRNAdb-CE v0.8 database uses TFAM 1.4 for functional classification of bacterial tRNA genes (as described in http://trna.ie.niigata-u.ac.jp/trnadb/method.html). However, the Proteobacterial model for the tRNA^Ile^_CAU_ elongator class that comes with TFAM 1.4 does not generalize well to all bacterial phyla [13]. Therefore, we reannotated the 9,917 bacterial genes for tRNAs with CAU anticodons in tRNAdb-CE v0.8 using the more general Silva TFAM model derived from the analysis of Silva et al. [12]. This model is also provided as supplementary data in the present work. By applying the Silva TFAM model to 9,917 bacterial CAU-anticodon containing tRNA genes, we revised the functional annotations of 62 of 3,918 previously annotated genes (*≈*1.58%) and provided new annotations for 5,999 additional genes that were previously unspecified (*≈*60.4%). Reclassification frequencies are presented in Table 1, showing that no previously classified genes were reannotated as initiator tRNA^fMet^ genes and that our reannotation efforts resulted in approximately equal frequencies of the two elongator tRNA gene classes. These reannotated data are also provided in Additional files 1 and 2.

**Table 1:** Reannotation of bacterial tRNAs with CAU anticodons in tRNAdb-CE v.8 using a custom model based on the analysis of Silva et al. [12]. “kIle” means Isoleucine tRNA elongators with CAU anticodon. “???” means that no specific annotation was provided in tRNAdb-CE v.8

		Met	Ini	kIle	Sum
Annotations	Met	1055	0	51	1106
In tRNAdb-CE	Ini	0	1824	0	1824
v.8	kIle	11	0	977	988
	???	1696	2614	1689	5999
	Sum	2762	4438	2717	9917

### Structural reannotation of bacterial genes in tRNAdb-CE v.8

A well-designed feature of the tRNAdb-CE v0.8 data model lies in that its gene records contain not only annotated gene sequences but also ten bases of genomic context both up- and downstream. Inspection of tRNAdb-CE v0.8 data revealed multiple genes with an annotated 3′-end sequence other than CCA, followed by 3′-trailer sequences that begin with the sequence CCA. To confidently assess whether these genes might template a 3′-CCA-end for their gene products, we assigned Sprinzl coordinates [4] to these bases for each gene sequence. These coordinates were not provided in tRNAdb-CE v0.8. We did this by implementing a dynamic programming algorithm to optimize base-pairing of the acceptor 3′-end region against the database-annotated 5′-end. Our dynamic programming algorithm with parameters given in methods (which were chosen somewhat arbitrarily and not optimized) almost always annotated acceptor stems identically to those given in the database. Also, we never used our algorithm to reannotate acceptor stems, only to confidently and consistently assign Sprinzl coordinates to the 3′-end region of each gene. Using this technique, we annotated an additional 2,866 bacterial tRNA genes out of 129,989 (or 2.2%) records as containing the CCA template at the 3′-end in the sequence framework of Sprinzl coordinates 74-76.

To clarify why we could identify an additional 2,866 tRNA genes in tRNAdb-CE v0.8 that template CCA, we ran tRNA gene-finding programs on the database records, suspecting that perhaps user error with tRNA gene-finders may have caused these misannotations. We used ARAGORN v1.0 [17] and tRNAscan-SE v.1.23 [18] in default eukaryotic tRNA gene-finding mode, and tRNAscan-SE v.1.23 in Bacterial mode (with the -B option). We found that tRNAscan-SE v.1.23, when run in its default eukaryotic gene-finding mode, never annotates nucleotides at positions 74-76 irrespective of their sequence. An exception to this rule was with selenocysteine tRNA genes, for which tRNAscan-SE in eukaryotic mode does annotate positions 74–76 if they contain the CCA sequence. From these observations we conclude that a likely cause of misannotations in tRNAdb-CE is user error in genome annotation pipelines. This is particularly notable when users of tRNAscan-SE apply its default eukaryotic gene-finding mode on prokaryotic genomes. Such errors may then be incessantly propagated in public and private databases.

### Frequencies of CCA-templating in bacterial tRNA genes

**Fig. 1:**
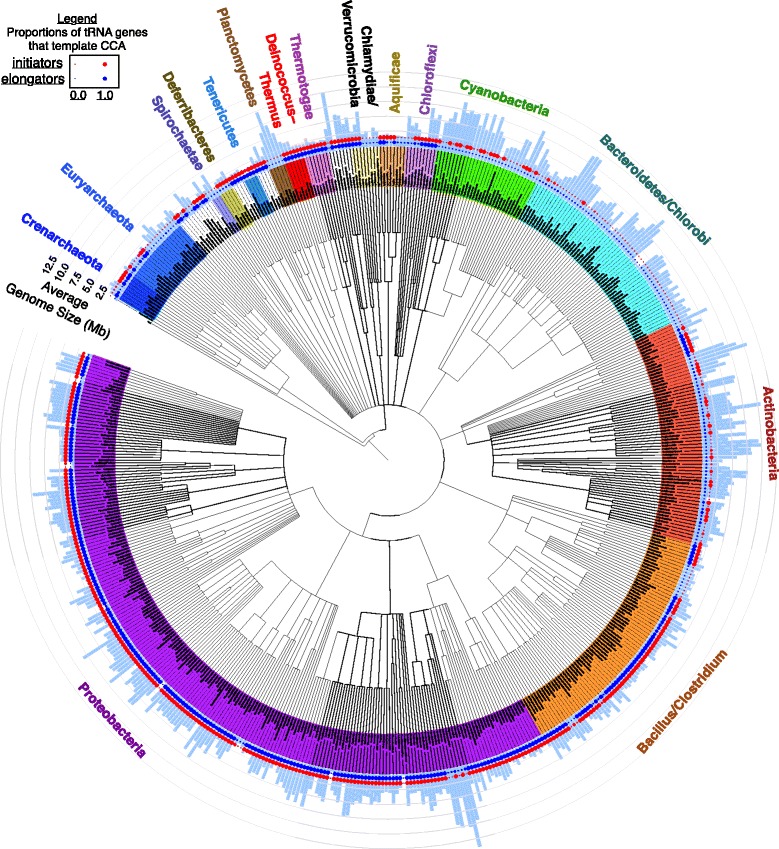
Summarized frequencies at which elongator tRNA genes and initiator tRNA genes template 3’-CCA against average genome size in different prokaryotic genera. NCBI-Taxonomy - based cladogram of prokaryotic genera in tRNAdb-CE v.8 showing average genome size (radial light blue bars) and average fractions at which elongator tRNA genes template 3’-CCA (blue circles) and initiator tRNA genes template 3’-CCA (red circles)

With our reannotated tRNAdb-CE data in hand, we calculated frequencies of CCA-templating in tRNA genes across different tRNA functional classes and taxonomic groupings as defined by NCBI Taxonomy [19]. Figure 1 shows our data summarized by prokaryotic genus. Prokaryotic clades exhibit all four possible patterns: 1) most or all tRNAs genes template CCA, 2) few or no tRNA genes template CCA, 3) primarily initiator genes template CCA, or — most rarely — 4) primarily elongator tRNA genes template CCA.

The five best-sampled phyla in our dataset, as defined by number of distinct genera with at least one genome sequenced, are Proteobacteria, Bacillus/Clostridium, Actinobacteria, Bacteroidetes/Clorobi, and Cyanobacteria. These five phyla exhibit three of four patterns described above in a strikingly consistent pattern by phylum. Practically all tRNA genes template CCA in Proteobacteria and Bacillus/Clostridium, except in certain reduced genomes, most of which template CCA only in initiator tRNA genes, or in no tRNA genes at all. In Cyanobacteria and Actinobacteria, on the other hand, primarily only the initiator tRNA genes template CCA, with certain exceptions. For example, a clade of Actinobacteria with relatively small genomes exists in which both initiator and elongator tRNA genes template CCA at high frequencies. In the Bacteroidetes/Chlorobi group, most tRNA genes do not template CCA, except for one lineage, the Solitalea, in which only initiator tRNA genes template CCA. In all five of the most-sampled phyla, there exist both small and moderately-sized genomes in which only initiator tRNA genes template CCA, or no tRNA genes at all template CCA. Certain Myxococcales, among the Deltaproteobacteria, are exceptional in having among the largest genomes that we observed and yet no tRNA genes or only initiator tRNA genes template CCA.

Less-sampled phyla are also quite heterogeneous in our dataset. In the Thermotogae, Deinococcus/Thermus and Tenericutes, all tRNA genes template CCA. Spirochaetae do not template CCA in any genes, while in Deferribacteres, only initiator tRNA genes template CCA. In the most rare pattern we observed, in only a few archaeal or bacterial genera, primarily elongator tRNA genes and not initiator tRNA genes template CCA.

**Fig. 2:**
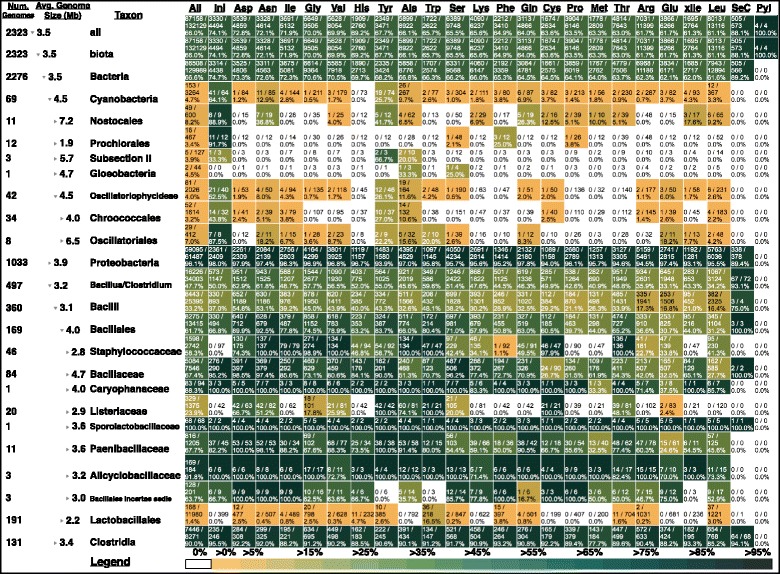
Summarized genome size and CCA frequency data in Bacterial clades broken out by tRNA functional class. Except for columns labelled “All,” “SeC,” and “Pyl,” all columns of frequency data are sorted in decreasing order from left to right by the frequency at which tRNA gene classes template CCA over all prokaryotic genomes that we sampled. Clades are defined as in NCBI Taxonomy. Column labels correspond to IUPAC three-letter amino acid charging identity except for “Ini” (initiators) and “xIle” (AUA-codon-reading isoleucine isoacceptors). “All” summarizes frequency data over all tRNA classes

In order to better visualize these data down to individual genomes and separating different elongator classes, we created an interactive javascript-based taxonomic navigator for our results visualized with heatmaps in any ordinary web browser. The full interactive data navigator is available as Additional files 1, 2, 3, 4, 5, 6, 7, 8, 9, 10, 11, 12, and 13 to this work. A static view on these data is also provided as a searchable PDF in Additional files 1, 2, 3, 4, 5, 6, 7, 8, 9, 10, 11, 12, 13 and 14. Figure 2 presents a snapshot from this browser with some notable detailed results for Bacterial tRNA genes. The figure shows columns of frequency data, corresponding to functional classes of tRNA genes, sorted left to right by decreasing average frequency at which tRNA genes template CCA over all prokaryotic genomic sequences in our sample. This analysis reveals that, in Bacteria and Archaea, initiator tRNA genes (labeled as Ini) template CCA at the highest frequency (74.1%), versus 66.2% for elongator tRNA genes generally. In Bacteria, initiator tRNA genes template CCA at a frequency of 74.7%, second only to selenocysteinyl tRNA genes (tRNA^Sec^, Sec = selenocysteine), with a frequency of 89.2%. We also found that genes for tRNA^Asp^ and tRNA^Asn^ template CCA at the highest frequencies among all canonical elongator tRNA genes. Below we describe some of the notable results shown in Fig. 2.

#### Cyanobacteria

Among bacterial phyla we observed, Cyanobacteria have the most striking and consistent pattern in which specifically initiator and not elongator tRNA genes template CCA. The overall frequencies are 64.1% for initiator tRNA genes versus 25.7% for the next highest gene class, which are elongator tRNA^Tyr^ genes. But different cyanobacterial lineages exhibit considerable variation in this trait. For example, among Prochlorales and Nostocales genomes — comprising both the smallest and largest average genome sizes, respectively — the frequencies at which initiator tRNA genes template CCA are 91.7% and 88.9%, while elongator tRNA genes template CCA at only 3.4% and 8.2% respectively. In 11 out of 12 of *Prochlorococcus* genomes and 9 out of 13 *Synechococcus* genomes, only initiator tRNA genes template CCA. Initiator tRNA genes template CCA at very different frequencies in sister orders Oscillatoriales and Chroococcales within subclass Oscillatoriophycideae: 87.5 and 43.8% respectively.

The Cyanobacteria are also unusual in that different strains and groups feature specific elongator tRNA gene classes that also template CCA at intermediate frequencies (above 10%) while other elongator classes template CCA at lower frequencies (below 10%). Usually, if in any one genome the initiator tRNA gene or genes template CCA, at least one elongator tRNA gene class will also template CCA at an intermediate frequency. The elongator gene class that templates CCA most consistently across the phylum is the tRNA^Tyr^ gene class. In *Nostocales*, tRNA^Tyr^ genes template CCA at a frequency of (41.7%), while tRNA^Asn^ and tRNA^Gln^ elongator genes also template CCA at a high relative frequency (36.8% and 23.7%).

#### Proteobacteria

All proteobacterial tRNA genes generally template CCA at consistently high frequencies: 96.1% overall (Fig. 2). Yet proteobacterial initiator tRNA genes template CCA at 98.0%, significantly higher than proteobacterial elongators (*G* = 29.039, d.f. = 1, *p <* 10^*−*6^ by a G-test of independence computed in R 3.3.1 using function “G.test” in package “RVAideMemoire” version 0.9–60). Closer examination of the proteobacterial variation (Additional file 1, 2, 3, 4, 5, 6, 7, 8, 9, 10, 11, 12, 13 and 14) reveals that while many free-living proteobacteria template CCA at high frequencies, endosymbiotic γ-proteobacteria and α-proteobacteria with reduced genomes show similar patterns to those described above for cyanobacteria with reduced genomes. In these cases, initiator tRNA genes appear to be the only class to consistently template CCA, while several elongator classes also template CCA. For example, in most *Buchnera aphidicola* genomes, about eight or nine additional elongator tRNA classes template CCA at intermediate to high frequencies while other classes do not template CCA, as previously reported [20]. Remarkably, in all *Buchnera* strain genomes except one, initiator tRNA genes always template CCA. Furthermore, like in the Cyanobacteria, in the smallest of the *Buchnera* genomes, only initiator tRNA genes template CCA. This same pattern holds in other endosymbiotic γ-proteobacteria genomes such as Ca. *Blochmannia, Wigglesworthia, Glossina*, Ca. *Baumannia,* Ca. *Carsonella,* Ca. *Portiera,* as well as α-proteobacteria endosymbionts such as *Wolbachia*. In contrast, among the smallest γ-proteobacterial genomes like Ca. *Hodgkinia,* none of the tRNA genes template CCA.

#### Other bacterial phyla

Many diverse genera and classes of Bacteria preferentially template CCA in their initiator tRNA genes (Additional file 1, 2, 3, 4, 5, 6, 7, 8, 9, 10, 11, 12, 13 and 14). Examples include *Geobacillus*, *Thermoaerobacter, Ruminococcus, Thermomicrobiales, Deferribacter,* Thermodesulfobacteria*, Mycobacterium, Propionibacterium, Frankia,* and *Bifidobacterium.* As shown in Fig. 2, within the Bacillus/Clostridium phylum, frequency variation in this genomic trait is highly extensive. An unusual pattern is found in the pathogenic Staphylococcaeceae and Listeraceae families, and also the Lactobacillales, which contain both pathogens and non-pathogens, in which initiator tRNA genes never template CCA, even while elongator tRNA genes do template CCA at intermediate frequencies. For example, in Staphylococcaceae about 60% of elongator tRNA genes template CCA and in Listeraceae about 24% of elongator tRNA genes template CCA, while in Lactobacillales, 1.4% of elongator genes template CCA. Yet among the 257 genome representatives of these three families in our dataset, not one initiator tRNA gene templates CCA.

#### Archaea

**Fig. 3:**
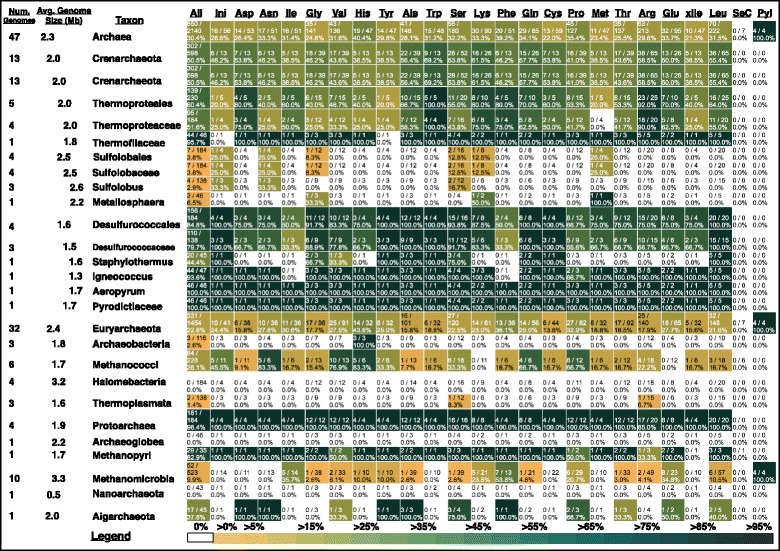
Summarized genome size and CCA frequency data in Archaeal clades broken out by tRNA functional class. Annotations are the same as in Fig. 2

We found no need for structural or functional reannotation of archaeal tRNA genes in tRNAdb-CE v.8. Figure 3 presents a snapshot from our data browser (Additional file 11) with some of our most notable results for archaeal tRNA genes. While there are fairly high frequencies of CCA-templating in archaeal tRNA genes overall, at 30.4%, we found that initiator tRNA genes in Archaea do not template CCA at any especially high frequency among tRNA genes, which presents a major difference from Bacteria. Other than this, we observed extensive phyletic variation in this trait across Archaea. Crenarchaeota tRNA genes template CCA at a frequency of 50.5%, while Euryarchaeota tRNA genes template CCA at about half of that frequency. Within Crenarchaeota, tRNA genes in the Sulfolobales template CCA at 3.8%, but in the Desulforococcales this frequency is 84.8%. All four tRNA^Pyl^ (Pyl = pyrrolysine) genes template CCA in the Methanomicrobia. Contrary to the generalization that Archaea tRNA genes do not template CCA, there exist lineages in both the Crenarchaeota and Euryarchaeota in which all or nearly all tRNA genes template CCA, for example in the Desulfurococcales, Protoarchaea, and Methanopyri. Although variation exists across tRNA functional classes in a phyletic pattern, no obvious overall pattern emerges.

## Discussion

With the rapid accumulation of whole-genome sequencing data in recent years, it has become increasingly critical to design strategies that aid in proper genome annotation at large scales. One of the most challenging issues is consistent annotation of tRNA genes. We describe here a strategy that addresses this challenge, particularly with the goal of consistently annotating initiator *vs*. elongator tRNA^Met^ genes. Using our new strategy, we observed widespread phyletic variation in the frequencies and patterns at which tRNA genes template CCA across functional classes in prokaryotic genomes. Across diverse bacterial and archaeal clades, frequencies range between 0 to 100%. The key finding is that initiator tRNA genes have the greatest class-specific frequency of CCA-templating in Bacteria after tRNA^Sec^ genes. Furthermore, in diverse bacterial lineages, especially among the reduced genomes of free-living Cyanobacteria and host-associated endosymbiotic Proteobacteria, initiator tRNA genes template CCA at uniquely high frequencies. In Proteobacteria, all tRNA genes template CCA at high frequencies, but initiator tRNA genes have the highest overall frequency, second only to tRNA^Sec^ genes.

The tRNA gene reannotations that led to our results are meaningful. The most likely source of reannotation errors would be from our reclassification of tRNA gene function (Table 1). Note that no previously annotated initiator tRNA genes were reclassified in our analysis, but rather a substantial fraction of previously unclassified genes were annotated. With respect to these new classifications, detection of initiator tRNA genes by TFAM has high sensitivity and specificity, of less than 1% false negatives or positives respectively [10, 12]. This is because initiator tRNAs contain features that distinguish them from elongator tRNAs in highly conserved and domain-specific ways [21]. As shown in Tables 2, 3 and 4, our initiator tRNA gene predictions confirm and extend previously described rules and exceptions for bacterial initiator tRNA genes [21], including distinct patterns of mismatching at coordinates 1 and 72 in the acceptor stem (Table 2), distinct nucleotide frequencies at the 11:24 base-pair in the D-stem (Table 3), and unique anticodon-stem statistics at positions 29–31 and 39–41 (Table 4).

**Table 2:** Frequencies of bases at coordinates 1 and 72 in the acceptor stem of reannotated tRNA genes of tRNAdb-CE v.8

		Met	Ini	kIle	Sum
**1:72**	**C A**	2	3392	1	3395
	**C U**	0	626	0	626
	**U U**	1	207	1	209
	**C C**	0	90	2	92
	U:A	2	63	1	66
	**A A**	0	39	2	41
	U.G	1	9	2	12
	**G A**	0	7	0	7
	**U C**	1	3	3	7
	**A C**	0	1	6	7
	**A G**	0	1	1	2
	G.U	1371	0	103	1474
	G:C	1287	0	2511	3798
	C:G	66	0	77	143
	A:U	30	0	6	36
	S C	1	0	0	1
	**G G**	0	0	1	1
**Sum**		2762	4438	2717	9917

Watson-Crick matching base-pairs are underlined and mismatches are in bold-face

**Table 3:** Frequencies of base-pairs at coordinates 11:24 in the D-stem of reannotated tRNA genes of tRNAdb-CE v.8

		Met	Ini	kIle	Sum
11:24	A:U	0	4430	0	4430
	C:G	2759	3	2706	5468
	G:C	0	3	0	3
	– –	1	1	0	2
	U:A	1	1	8	10
	U.G	1	0	2	3
	C A	0	0	1	1
Sum		2762	4438	2717	9917

Watson-Crick matching base-pairs are underlined

**Table 4:** Frequency of base-pairing sequences at coordinates 29–31 and 39–41 in the coding stem of reannotated tRNA genes of tRNAdb-CE v.8. All sequences that appear more than 100 times in total are shown; complete data are shown for initiator tRNA genes

		Met	Ini	kIle	Sum
29-31,39-41	GGG,CCC	2	3849	1	3852
	AGG,CCU	1	522	1	524
	AGG,UCU	6	34	0	40
	GGG,UCC	0	18	0	18
	CGG,CCG	869	0	974	1843
	UCA,UGA	405	0	0	405
	GCA,UGC	369	0	0	369
	GGA,UCC	335	0	583	918
	CGA,UCG	213	0	654	867
	GGA,CCC	114	0	75	189
Sum		2314	4438	2286	9038

Furthermore, our results are robust to missing data. For example, we were unable to annotate any initiator tRNA genes in one of the Cyanobacterial lineages shown in Fig. 2 — *Gloeobacter*. In our analysis of the tRNA gene complements of 2,323 prokaryotic genomes in tRNAdb-CE v.8, we were unable to annotate initiator tRNAs genes in 15 genomes (0.6%; Additional files 1, 2, 3, 4, 5, 6, 7, 8, 9, 10, 11, 12, 13 and 14). We spot-checked several of these aberrant tRNA gene complements by examining their score distributions with TFAM 0.4 to verify that there were no viable candidates for initiator tRNA genes according to our models. All of the tRNA genes in these gene complements that we checked scored inside of the normal background distribution for the initiator tFAM model [10, 12]. We believe that initiator tRNA genes may simply be missing from the genome annotations that were aggregated in tRNAdb-CE v.8. Importantly, in these genomes, the pattern by which tRNA genes template CCA is phylogenetically consistent with related genomes (Fig. 2 and Additional files 1, 2, 3, 4, 5, 6, 7, 8, 9, 10, 11, 12, 13 and 14).

The initiator tRNA of protein synthesis in Bacteria is known as tRNA^fMet^ because its charged methionine moiety contains a formyl group attached to the *α*-amino group. By templating CCA in tRNA^fMet^ genes, Bacteria can directly synthesize tRNA^fMet^ with the CCA sequence at the 3′-end. Below we hypothesize five non-mutually exclusive potential advantages for tRNA genes to template CCA.

Our first hypothesis is that certain tRNA classes, particularly initiator tRNAs, may have relatively non-conforming structures that lead to inefficient processing in shared tRNA maturation pathways. For example, tRNA^fMet^ in Bacteria is exceptional in that it almost always contains a mismatch pair at the 1–72 position of the acceptor end. In our data (Table 2), 98.6% of our predicted bacterial initiators contain a mismatch at coordinates 1 and 72, and among those, 77.5% contain the C1-A72 mismatch, which has been specifically shown to promote recognition by initiation factors to initiate protein synthesis in *E. coli* [22]. Only 66 of 4438 (1.4%) of predicted initiators contain a Watson-Crick (W-C) or wobble match at these positions, and these all contain the U1–A72 W-C match, which is almost completely absent in any elongator tRNAs (only 3 of 5179 predicted elongators or about 0.05%). In contrast, 99.5% of our predicted elongator tRNAs contain a Watson-Crick (W-C) or wobble base pair at the 1–72 position which may be important for them to be discriminated against by initiation factors. Critically, because the C1-A72 mismatch motif of tRNA^fMet^ is known to compromise the efficiency of processing at the 5′-end [23], we suggest that direct transcription of the CCA sequence can serve as a determinant to help with 5’-end processing [24, 25]. In contrast, initiator tRNAs in Archaea have Watson-Crick base-pairs in the 1–72 position [21] so we conjecture that they do not share this “Achilles heel” problem with Bacterial initiator tRNAs, but instead are efficiently processed at the 5′-end without any requirement for a 3′-end CCA sequence. This is consistent with our observation that initiator tRNA genes in Archaea do not have a particularly high frequency of CCA-templating. In contast, we find that both tRNA^Sec^ genes and tRNA^Pyl^ genes have high frequencies of CCA-templating in bacteria, consistent with the notion that both tRNA^Sec^ and tRNA^Pyl^ have unusual structures that should compromise 5’-end processing. Specifically, tRNA^Sec^ is unique in having 8 base-pairs in the acceptor stem, in contrast to 7 base-pairs in all other tRNAs. Similarly, tRNA^Pyl^ is characterized by the absence of the conserved G18-G19 bases in the D-loop and the absence of the TC sequence in the T loop. All of these unusual features can alter the structure of the acceptor-T stem loop, leading to discrimination by the 5′-end processing enzyme RNase P [26]. By having the CCA-template, both tRNA^Sec^ and tRNA^Pyl^ can use the transcribed CCA sequence to mitigate the reduced efficiency with 5′-end processing. Additionally, because the CCA-adding enzyme (conserved in evolution) recognizes the acceptor-T stem-loop structure to catalyze CCA addition [27], the alteration of the structure by abberant features in tRNA^fMet^, tRNA^Sec^, and tRNA^Pyl^ may compromise the activity of the CCA enzyme. Thus direct templating of CCA provides a solution to circumvent the problem.

Second, direct templating of the CCA sequence in tRNAs can potentially increase the maximal growth rate of cells. Under conditions of rapid growth, the co-transcriptional synthesis of 3′-terminal CCA in tRNAs can increase the allocation of cellular resources directly to the synthesis of new proteomic biomass and growth in two ways: first, by reducing steady-state cellular pools of species of nonfunctional tRNA precursors, which reduces the mass and energy overhead of the translational machinery itself, and second, by reducing the steady-state fraction of ribosomes devoted to synthesizing tRNA-affiliated proteins such as CCA-adding enzyme [28, 29].

Third, given that translational initiation is rate-limiting in protein synthesis [30], and therefore a key determinant of maximal growth rate [28, 31, 32], cells selected for a high maximum growth rate may need to efficiently maintain high concentrations of mature initiator tRNA^fMet^ for rapid growth. The costs of maturation of a tRNA to a growing cell should increase proportionally with the concentration of that tRNA, and initiator tRNA concentration increases more with growth rate in *E. coli* than elongator tRNA concentration [33]. Thus, the fitness impact of templating CCA on initiator tRNAs should be greater than on elongator tRNAs in rapidly growing cells. For example, the record-high growth rates reported among *Vibrio* species [34] is associated with very high initiator tRNA gene copy numbers in *Vibrio* genomes [10]. Consistent with the above, all initiator tRNA genes in *Vibrio* template CCA in the present analysis.

Fourth, rapid synthesis of initiator tRNAs through co-transcriptional synthesis of CCA could reduce the lag phase associated with the transition to growth by reducing the waiting time to increase initiator tRNA concentration. Importantly, this “bootstrapping” trait may be important for all cells, including free-living and endosymbiotic Bacteria under reductive genome evolution, and not just for cells capable of rapid growth. Many such cells could have an advantage in the rapid initiation of protein synthesis from a quiescent state in response to environmental change. Indeed, we have shown that each nucleotide addition for post-transcriptional synthesis of CCA requires the CCA enzyme to proofread tRNA integrity [35, 36], which likely delays maturation of newly transcribed tRNAs.

Fifth, for elongator tRNA genes, direct templating of CCA can facilitate more rapid synthesis of the corresponding elongator tRNAs to help cells avoid transient depletion of specific ternary complexes and the detrimental consequences that such shortages may have on the accuracy of protein synthesis and proteomic integrity. The supply-demand theory of tRNA charging dynamics [37] predicts wide variability in the sensitivity of charging levels of tRNA species to perturbations, such as amino acid starvation, affecting specific elongator tRNAs for both proteomically abundant and rare amino acids such as Leucine, Tyrosine and Phenylalanine. Stalled ribosomes caused by shortages of specific ternary complexes increase translational misreading at corresponding “hungry” codons [38, 39], including frame-shift errors [40], all of which can cause protein misfolding, aggregation, and damage [41].

While many cyanobacteria with reduced genomes are not fast-growing, they may generally be subject to multiple constraints of chronic nutrient limitation and a heavy burden of a large fraction of proteome dedicated to autotrophic functions [42]. When combined, these factors may lead to “proteomic constraints” from small cell sizes, an exacerbation of macromolecular crowding, and increased sensitivity to mistranslation of the most abundant parts of the proteome [42]. We suggest that the relatively high frequency at which tRNA^Tyr^ genes template CCA in Cyanobacteria (Fig. 2) is associated with a unique biological sensitivity to depletion of charged tRNA^Tyr^. Tyrosine residues are critically important for both catalysis and stability of RuBisCo [43], one of the most abundant proteins in Cyanobacteria [44]. It is remarkable that there exists a D-Tyr-tRNA^Tyr^ deacylase that is conserved and apparently unique to Cyanobacteria [45], which helps maintain the accuracy of tRNA^Tyr^ charging. Competition experiments that model biologically relevant conditions with Cyanobacterial strains with or without CCA-templating for tRNA^Tyr^, as well as biochemical assays, could test this hypothesis.

We further suggest that the advantages of avoiding supply shortages and streamlining tRNA biogenesis pathways may extend to other elongator tRNAs that we found to template CCA in an often lineage-specific manner. Selenocysteine and Pyrrolysine tRNAs both have complex biosynthetic/maturation pathways and both template CCA at high frequencies in our analysis. Similarly, biosynthesis of Asn-tRNA^Asn^ involves two steps, first by synthesizing a mispaired Asp-tRNA^Asn^, followed by conversion of Asp to Asn [46–48]. Indeed, genes for both tRNA^Asp^ and tRNA^Asn^ template CCA at high frequencies. Although the synthesis of Gln-tRNA^Gln^ also relies on a two-step pathway involving transamidation of Glu on Glu-tRNA^Gln^ [49], the frequencies for tRNA^Glu^ and tRNA^Gln^ are among the lowest we observed in Bacteria overall. Further analysis and experiments will be necessary to fully understand the patterns reported in this paper.

We have not examined trends of CCA-templating in eukaryotic tRNA genes as these data are not currently included in tRNAdb-CE. Although the hypotheses we have described for our results in Bacteria are all potentially applicable to eukaryotes, we anticipate that eukaryotic tRNA genes may exhibit entirely distinct trends in structural variation. In bacteria, our hypotheses could be further investigated computationally through phylogenetically informed comparative analysis of bacterial traits including metagenome data, and through the genomically informed simulation of whole-cell models of gene expression in bacteria.

## Conclusions

Re-annotation of tRNA gene sequences was essential to our discovery that CCA-templating is a major feature of initiator tRNA genes in Bacteria. This shows the importance for genome annotation projects of using tRNA gene-finders with taxonomically correct models. More generally, this work demonstrates the importance of using bioinformatic assets carefully to maximize scientific returns.

## Methods

### Data

Version 8 (October, 2014) of the tRNAdb-CE database [16] was downloaded on November 4, 2014. NCBI Taxonomy data [19] was downloaded on November 13, 2014.

### Functional reannotation of CAU-anticodon tRNAs

We classified bacterial CAU-anticodon-templating tRNA genes as templating methionine elongators, lysidinylated isoleucine elongators or initiators using TFAM version 1.4 [10, 11] with a general bacterial model for this purpose based on a previously published analysis [12].

### Structural annotation of 3′- ends

To annotate Sprinzl coordinates to the 3′-end of each tRNAdb-CE sequence record, we implemented a dynamic programming algorithm to optimize base-pairing of the annotated 3′-end of the mature tRNA in each record against its own annotated 5′-end and trailer sequence.

For each sequence record we obtained the 5′-most seven bases of the annotated acceptor stem sequence and reversed it to obtain sequence *x*. Given sequence *x*, we computed its optimal pairing against a second sequence *y* defined by the last 12 bases of the annotated 3′-end and the first five bases of the annotated 3′-trailer using the simple dynamic programming algorithm described here.

Let *x* and *y* be finite sequences over the alphabet Σ = {*A, C, G, U*}, with lengths *m* and *n* respectively. We compute a matrix *H* whose elements are specified as follows:

$$ \begin{array}{l}H\left(i,1\right)=0\kern.5em \forall i\kern.3em \in \kern.3em \left[1,m\right];\kern1em \\ {}H\left(1,j\right)=0\kern.5em \forall j\kern.3em \in \kern.3em \left[1,n\right];\kern1em \\ {}H\left(i,j\right)= \max \left(D,U,L\right)\kern.5em \forall i\kern.3em \in \kern.3em \left[2,m\right],\kern.3em \forall j\kern.3em \in \kern.3em \left[2,n\right];\kern1em \\ {}D=H\left(i-1,j-1\right)+s\left({x}_i,{y}_j\right)+a\left({x}_i,{y}_j\right);\kern1em \\ {}U=H\left(i-1,j\right)+g;\kern1em \\ {}L=H\left(i,j-1\right)+g.\kern1em \end{array} $$

where *i* and *j* are integers﻿, *x*_*i*_ is the *i*th base in sequence *x* of length *m = 7, y*_*j*_ is the *j*th base in sequence *y* of length *n = 17, s(x*_*i*_ *, y*_*j*_ *) =* 4, for *(x*_*i*_ *, y*_*j*_ *) ∈ {(A,U), (U,A), (C,G), (G,C), (G,U), (U,G)}* and *s(x*_*i*_ *, y*_*j*_ *) =* 1 otherwise, *a(x*_*i*_ *, y*_*j*_ *)* is an annotation bonus if *x*_*i*_ and *y*_*j*_ were annotated as paired in tRNAdb-CE, *g* = –5 is a linear gap penalty, and *H(i, j)* is the maximum base-pairing score obtained on sequence prefixes *x*[*1,i*] and *y*[*1,j*]. We compared results both with and without an annotation bonus, *i.e.* we recomputed *H(m, n)* for every record using the bonus *a(x*_*i*_ *, y*_*j*_ *) = 1* or no bonus *a(x*_*i*_ *, y*_*j*_ *) = 0.*

### Statistics and visualization of genome size and CCA-templating data

After reannotation, we considered a tRNA gene to template CCA if Sprinzl bases 74 through 76 contained the sequence CCA. We used genome size data downloaded as a “genome report” from NCBI Genome on October 26, 2015 [19] and visualized data using the Interactive Tree of Life [50, 51].

### Statistics of features in CAU-anticodon tRNAs

We structurally aligned tRNA data using COVEA [52] with the TRNA2-prok.cm model from tRNAscan-SE v.1.23 [18] and analyzed feature statistics using FAST [53]. Complete commands to reproduce data in Tables 1, 2, 3 and 4 are provided in the Supplement.

## Additional files

Additional file 1: Reannotated tRNAdb-CE v.8 data in FastA format including TFAM scores and CCA reannotations. (TXT 43 MB)
Additional file 2: Structurally aligned data for subset of tRNAs with CAU anticodons, also containing TFAM scores with Silva model in “aligned FastA” format. (FAS 3 MB)
Additional file 3: Expanded description of each additional file with usage. (TXT 3 kb)
Additional file 4: Prokaryotic genome size data from NCBI, downloaded on October 26, 2015. (TXT 10 MB)
Additional file 5: NCBI taxonomy data required by compute_heatmap.pl (GZ 14 MB)
Additional file 6: Installation code-base for the TFAM program. (ZIP 819 kb)
Additional file 7: TFAM model used to reannotate tRNA functions in COVE format. To use this model file with TFAM, give its name as an option argument to the “-m” option. (TXT 1 MB)
Additional file 8: Perl script for structural reannotation of 3’ends. (TXT 5 kb)
Additional file 9: Perl script to generate full data browser and iTol input. (TXT 18 kb)
Additional file 10: Color definitions for clades to generate Fig. 1. (TXT 625 bytes)
Additional file 11: Phylogenetic tree in New Hampshire format of NCBI taxon IDs from NCBI Taxonomy corresponding to addresses_v4.txt.gz (TXT 8 kb)
Additional file 12: Web-browser based navigator of full dataset of results on frequencies of CCA-templating by tRNA functional class, organized by clades in NCBI taxonomy. (ZIP 548 kb)
Additional file 13: Static and searchable PDF with full dataset of results on frequencies of CCA-templating by tRNA functional class, organized by clades in NCBI taxonomy. (PDF 1 MB)
Additional file 14: List of FAST commands to reproduce frequency data shown in Tables 1, 2, 3 and 4. (TXT 4 kb)

## Abbreviations

Asn: Asparagine
AUA/G: Codon sequences in messenger RNA
CAU: The anticodon sequence of initiator tRNAs and certain elongator tRNAs decoding codons as Met or Ile
CCA: The polyribonucleotide sequence ordinarily at the 3-prime end of mature tRNAs
fMet: N-Formylmethionine
Gln: Glutamine
Ile: Isoleucine
kIle: Bacterial isoleucine tRNA elongators with CAU anticodons in which wobble position C34 is modified to lysidine
Met: Methionine
NCBI: National Center for Biotechnology Information
PDF: Portable Document Format
Pyl: Pyrrolysine
Sec: Selenocysteine
TFAM: The "tRNA Family" tRNA function classifier
tRNA: transfer ribonucleic acid
tRNAdb-CE: tRNA database — Curated by Experts
Tyr: Tyrosine
