# Supplementary material for: Initiator tRNA genes template the 3′ CCA end at high frequencies in bacteria
Source: BMC Genomics. 2016 Dec 8;17:1003. doi: 10.1186/s12864-016-3314-x (PMC5143459; doi:10.1186/s12864-016-3314-x)
Supplement: Additional file 12: — Web-browser based navigator of full dataset of results on frequencies of CCA-templating by tRNA functional class, organized by clades in NCBI taxonomy. (ZIP 548 kb) [file 12864_2016_3314_MOESM12_ESM.zip › CCA-HEATMAP-TAX-BROWSER/index.html]

 
   
       Analysis of CCA templating in  tRNADB-CE ver. 8.0, Oct., 2014 release by David H. Ardell 
         
         
         
         

   
   
  Title: Analysis of CCA templating in  tRNADB-CE ver. 8.0, Oct., 2014 release by David H. Ardell    
  Date: 2015-11-30 17:25:19
    
  Input: tCE-Nov5-2014.3.fas or tCE-Nov5-2014.taxsort.model_cca_U0_tfam.fas    
  Generated By: perl compute_heatmap.pl tCE-Nov5-2014.3.fas > Ardell_Hou_2015.html    
  Execution dependencies: addresses_v4.txt.gz and     
  Open in: any javascript-capable web-browser    
  Function dependencies: jquery.js, jquery.treeTable.css, jquery.treeTable.js, toggle-collapse-dark.png, toggle-collapse-light.png, toggle-expand-dark.png, toggle-expand-light.png    
  Description:  
Supplementary data with frequency analytics for Ardell and Hou (2015) 
Analysis of CCA-templating in Bacterial and Archaeal tRNA genes from a reannotation of Version 8 of tRNAdb-CE. 

Uses NCBI Taxonomy data 

Two reannotations were carried out:    Reannotation of function
on CAU-anticodon tRNAs, assignment to classes M (elongator Met), X
(initiator), or J (LAU-anticodon eleongator Ile) using TFAM and the
Silva model as described in Ardell and Hou 2015 "High Frequencies of
CCA-Templating in Bacterial Initiator tRNA Genes"  
 Reannotation of CCA-templating, with verification of the modeling
of the acceptor stem using dynamic programming. We assumed that if CCA
occurs just after the annotated acceptor stem, that the gene templates
CCA.
 
 

The dynamic programming algorithm works as follows: seven bases of the
 annotated 5' end are taken from the database entries and the best
 pairing segment near the annotated 3' end is calculated by dynamic
 programming. The following parameters were used to generate these
 specific results:

  
 
 
Number of bases of annotated 3' end searched
 
 
12
 
 
Number of bases of annotated 3' trailer
 
 
5
 
 
Match bonus (including G:U and U:G)
 
 
4
 
 
Mismatch bonus
 
 
1
 
 
Annotation bonus (pairing within boundaries annotated in tRNAdb-CE)
 
 
1 or 0
 
 
Gap penalty
 
 
-5
 
  
  
The algorithm segments the 17 bases from the 3'end into segments: [3'stem][id-base][cca?][trailer]
Certain conditions were tallied and appear at the end of the rows.
Here are the meanings of these conditions.
 
 
A
 
 
W:CCApredicted: if [cca?] eq "CCA", correctly annotated in tCE
 
 
B
 
 
"W:cca" if [cca?] =~ /cca/i. All A are also B, so our tRNAdb-CE CCA-templating false negatives are "B and not A"
 
 
C
 
 
"W:cc" if [cca?] =~ /^cc/
 
 
D
 
 
"W:shortLength" if predicted FPE of Acceptor stem is  
 
E
 
 
"W:pos89nTA" if the tCE-annoted 8th and 9th bases are not "T" and "A"
 
  
  
Reclassifications of bacterial data based on TFAM with Silva model: 
 
 
 
  
 M 
 X 
 J 
 
 
 
 
 M 
 2751 
 2614 
 1737 
 
 
 X 
 0 
 1824 
 0 
 
 
 J 
 11 
 0 
 977 
 
 
  
 
	 
		 
			 No. Genomes 
			 Avg. Genome Size (MB) 
			 Taxon 
			 All 
			 Ini 
			 Asp 
			 Asn 
			 Ile 
			 Gly 
			 Val 
			 His 
			 Tyr 
			 Ala 
			 Trp 
			 Ser 
			 Lys 
			 Phe 
			 Gln 
			 Cys 
			 Pro 
			 Met 
			 Thr 
			 Arg 
			 Glu 
			 xIle 
			 Leu 
			 SeC 
			 Pyl 
			 Pseudo 
			 Stop 
		 
	 
	 
		 
			 2323 
			  3.5 
			 all 
			  87158 / 132129 66.0% 
			   3330 / 4494   74.1% 
			   3539 / 4859   72.8% 
			   3328 / 4614   72.1% 
			   3691 / 5132   71.9% 
			   6649 / 9505   70.0% 
			   5628 / 8054   69.9% 
			   1909 / 2760   69.2% 
			   2349 / 3471   67.7% 
			   5899 / 8922   66.1% 
			   1722 / 2622   65.7% 
			   6389 / 9748   65.5% 
			   4090 / 6237   65.6% 
			   2212 / 3410   64.9% 
			   3113 / 4866   64.0% 
			   1674 / 2634   63.6% 
			   3904 / 6146   63.5% 
			   1778 / 2809   63.3% 
			   4814 / 7643   63.0% 
			   7031 / 11399  61.7% 
			   3866 / 6266   61.7% 
			   1695 / 2764   61.3% 
			   8013 / 13116  61.1% 
			    505 / 573    88.1% 
			      4 / 4      100.0% 
			     19 / 68     27.9% 
			      6 / 10     60.0% 
			  54763 / 132129 41.4%AB 
			  27920 / 132129 21.1%ABE 
			  21294 / 132129 16.1%E 
			  21263 / 132129 16.1% 
			   2866 / 132129  2.2%B 
			   1609 / 132129  1.2%BE 
			   1260 / 132129  1.0%C 
			   1145 / 132129  0.9%CE 
			      4 / 132129  0.0%GG 
			      3 / 132129  0.0%D 
			      2 / 132129  0.0%EGG 
		 
		 
			 2323 
			  3.5 
			 biota 
			  87158 / 132129 66.0% 
			   3330 / 4494   74.1% 
			   3539 / 4859   72.8% 
			   3328 / 4614   72.1% 
			   3691 / 5132   71.9% 
			   6649 / 9505   70.0% 
			   5628 / 8054   69.9% 
			   1909 / 2760   69.2% 
			   2349 / 3471   67.7% 
			   5899 / 8922   66.1% 
			   1722 / 2622   65.7% 
			   6389 / 9748   65.5% 
			   4090 / 6237   65.6% 
			   2212 / 3410   64.9% 
			   3113 / 4866   64.0% 
			   1674 / 2634   63.6% 
			   3904 / 6146   63.5% 
			   1778 / 2809   63.3% 
			   4814 / 7643   63.0% 
			   7031 / 11399  61.7% 
			   3866 / 6266   61.7% 
			   1695 / 2764   61.3% 
			   8013 / 13116  61.1% 
			    505 / 573    88.1% 
			      4 / 4      100.0% 
			     19 / 68     27.9% 
			      6 / 10     60.0% 
			  54763 / 132129 41.4%AB 
			  27920 / 132129 21.1%ABE 
			  21294 / 132129 16.1%E 
			  21263 / 132129 16.1% 
			   2866 / 132129  2.2%B 
			   1609 / 132129  1.2%BE 
			   1260 / 132129  1.0%C 
			   1145 / 132129  0.9%CE 
			      4 / 132129  0.0%GG 
			      3 / 132129  0.0%D 
			      2 / 132129  0.0%EGG 
		 
		 
			 2276 
			  3.5 
			 Bacteria 
			  86508 / 129989 66.6% 
			   3314 / 4438   74.7% 
			   3525 / 4806   73.3% 
			   3311 / 4563   72.6% 
			   3675 / 5081   72.3% 
			   6614 / 9364   70.6% 
			   5585 / 7918   70.5% 
			   1890 / 2713   69.7% 
			   2335 / 3424   68.2% 
			   5858 / 8776   66.8% 
			   1707 / 2574   66.3% 
			   6331 / 9568   66.2% 
			   4060 / 6147   66.0% 
			   2192 / 3359   65.3% 
			   3084 / 4781   64.5% 
			   1661 / 2575   64.5% 
			   3859 / 6019   64.1% 
			   1767 / 2762   64.0% 
			   4779 / 7506   63.7% 
			   6968 / 11186  62.3% 
			   3834 / 6171   62.1% 
			   1685 / 2717   62.0% 
			   7943 / 12894  61.6% 
			    505 / 566    89.2% 
			      0 / 0       0.0% 
			     19 / 68     27.9% 
			      6 / 10     60.0% 
			  54413 / 129989 41.9%AB 
			  27620 / 129989 21.2%ABE 
			  20629 / 129989 15.9%E 
			  20502 / 129989 15.8% 
			   2866 / 129989  2.2%B 
			   1609 / 129989  1.2%BE 
			   1225 / 129989  0.9%C 
			   1116 / 129989  0.9%CE 
			      4 / 129989  0.0%GG 
			      3 / 129989  0.0%D 
			      2 / 129989  0.0%EGG 
		 
		 
			 69 
			  4.5 
			 Cyanobacteria 
			    153 / 3264    4.7% 
			     41 / 64     64.1% 
			      1 / 84      1.2% 
			     11 / 85     12.9% 
			      4 / 144     2.8% 
			      1 / 211     0.5% 
			      3 / 179     1.7% 
			      0 / 73      0.0% 
			     19 / 74     25.7% 
			     26 / 267     9.7% 
			      2 / 77      2.6% 
			      3 / 304     1.0% 
			      2 / 111     1.8% 
			      3 / 80      3.8% 
			      6 / 87      6.9% 
			      3 / 82      3.7% 
			      3 / 213     1.4% 
			      1 / 56      1.8% 
			      2 / 230     0.9% 
			      2 / 287     0.7% 
			      3 / 82      3.7% 
			      4 / 93      4.3% 
			     12 / 367     3.3% 
			      0 / 0       0.0% 
			      0 / 0       0.0% 
			      0 / 12      0.0% 
			      0 / 1       0.0% 
			   1636 / 3264   50.1% 
			   1327 / 3264   40.7%E 
			    103 / 3264    3.2%C 
			    101 / 3264    3.1%AB 
			     44 / 3264    1.3%CE 
			     42 / 3264    1.3%ABE 
			      7 / 3264    0.2%B 
			      3 / 3264    0.1%BE 
			      1 / 3264    0.0%EGG 
		 
		 
			 11 
			  7.2 
			 Nostocales 
			     49 / 600     8.2% 
			      8 / 9      88.9% 
			      0 / 15      0.0% 
			      7 / 19     36.8% 
			      0 / 28      0.0% 
			      0 / 35      0.0% 
			      1 / 25      4.0% 
			      0 / 12      0.0% 
			      5 / 12     41.7% 
			      4 / 62      6.5% 
			      0 / 13      0.0% 
			      0 / 50      0.0% 
			      2 / 29      6.9% 
			      0 / 17      0.0% 
			      5 / 19     26.3% 
			      2 / 16     12.5% 
			      2 / 39      5.1% 
			      1 / 10     10.0% 
			      2 / 39      5.1% 
			      0 / 48      0.0% 
			      0 / 15      0.0% 
			      3 / 17     17.6% 
			      6 / 65      9.2% 
			      0 / 0       0.0% 
			      0 / 0       0.0% 
			      0 / 5       0.0% 
			      0 / 0       0.0% 
			    268 / 600    44.7% 
			    236 / 600    39.3%E 
			     40 / 600     6.7%C 
			     31 / 600     5.2%AB 
			     12 / 600     2.0%ABE 
			      7 / 600     1.2%CE 
			      4 / 600     0.7%B 
			      2 / 600     0.3%BE 
		 
		 
			 8 
			  7.1 
			 Nostocaceae 
			     35 / 445     7.9% 
			      5 / 6      83.3% 
			      0 / 11      0.0% 
			      6 / 14     42.9% 
			      0 / 22      0.0% 
			      0 / 27      0.0% 
			      1 / 18      5.6% 
			      0 / 9       0.0% 
			      4 / 9      44.4% 
			      1 / 48      2.1% 
			      0 / 9       0.0% 
			      0 / 35      0.0% 
			      2 / 21      9.5% 
			      0 / 12      0.0% 
			      3 / 14     21.4% 
			      1 / 12      8.3% 
			      1 / 29      3.4% 
			      1 / 9      11.1% 
			      1 / 28      3.6% 
			      0 / 35      0.0% 
			      0 / 12      0.0% 
			      2 / 11     18.2% 
			      6 / 49     12.2% 
			      0 / 0       0.0% 
			      0 / 0       0.0% 
			      0 / 4       0.0% 
			      0 / 0       0.0% 
			    204 / 445    45.8% 
			    167 / 445    37.5%E 
			     32 / 445     7.2%C 
			     22 / 445     4.9%AB 
			      7 / 445     1.6%CE 
			      7 / 445     1.6%ABE 
			      4 / 445     0.9%B 
			      2 / 445     0.4%BE 
		 
		 
			 2 
			  7.1 
			 Anabaena 
			      9 / 105     8.6% 
			      2 / 2      100.0% 
			      0 / 3       0.0% 
			      2 / 4      50.0% 
			      0 / 6       0.0% 
			      0 / 7       0.0% 
			      0 / 4       0.0% 
			      0 / 2       0.0% 
			      1 / 2      50.0% 
			      0 / 10      0.0% 
			      0 / 3       0.0% 
			      0 / 8       0.0% 
			      2 / 6      33.3% 
			      0 / 3       0.0% 
			      0 / 3       0.0% 
			      1 / 3      33.3% 
			      1 / 7      14.3% 
			      0 / 2       0.0% 
			      0 / 6       0.0% 
			      0 / 8       0.0% 
			      0 / 2       0.0% 
			      0 / 2       0.0% 
			      0 / 11      0.0% 
			      0 / 0       0.0% 
			      0 / 0       0.0% 
			      0 / 1       0.0% 
			      0 / 0       0.0% 
			     51 / 105    48.6% 
			     38 / 105    36.2%E 
			      7 / 105     6.7%AB 
			      6 / 105     5.7%C 
			      2 / 105     1.9%ABE 
			      1 / 105     1.0%CE 
		 
		 
			 1 
			  7.1 
			 Anabaena cylindrica 
			      7 / 57     12.3% 
			      1 / 1      100.0% 
			      0 / 2       0.0% 
			      1 / 2      50.0% 
			      0 / 3       0.0% 
			      0 / 4       0.0% 
			      0 / 2       0.0% 
			      0 / 1       0.0% 
			      1 / 1      100.0% 
			      0 / 4       0.0% 
			      0 / 2       0.0% 
			      0 / 4       0.0% 
			      2 / 4      50.0% 
			      0 / 2       0.0% 
			      0 / 2       0.0% 
			      1 / 2      50.0% 
			      1 / 4      25.0% 
			      0 / 1       0.0% 
			      0 / 3       0.0% 
			      0 / 4       0.0% 
			      0 / 1       0.0% 
			      0 / 1       0.0% 
			      0 / 6       0.0% 
			      0 / 0       0.0% 
			      0 / 0       0.0% 
			      0 / 1       0.0% 
			      0 / 0       0.0% 
			     24 / 57     42.1% 
			     23 / 57     40.4%E 
			      5 / 57      8.8%AB 
			      3 / 57      5.3%C 
			      2 / 57      3.5%ABE 
		 
		 
			 1 
			  7.1 
			 Anabaena aequalis UTEX 'B 1609' 
			      7 / 57     12.3% 
			      1 / 1      100.0% 
			      0 / 2       0.0% 
			      1 / 2      50.0% 
			      0 / 3       0.0% 
			      0 / 4       0.0% 
			      0 / 2       0.0% 
			      0 / 1       0.0% 
			      1 / 1      100.0% 
			      0 / 4       0.0% 
			      0 / 2       0.0% 
			      0 / 4       0.0% 
			      2 / 4      50.0% 
			      0 / 2       0.0% 
			      0 / 2       0.0% 
			      1 / 2      50.0% 
			      1 / 4      25.0% 
			      0 / 1       0.0% 
			      0 / 3       0.0% 
			      0 / 4       0.0% 
			      0 / 1       0.0% 
			      0 / 1       0.0% 
			      0 / 6       0.0% 
			      0 / 0       0.0% 
			      0 / 0       0.0% 
			      0 / 1       0.0% 
			      0 / 0       0.0% 
			     24 / 57     42.1% 
			     23 / 57     40.4%E 
			      5 / 57      8.8%AB 
			      3 / 57      5.3%C 
			      2 / 57      3.5%ABE 
		 
		 
			 1 
			  7.1 
			 Anabaena variabilis 
			      2 / 48      4.2% 
			      1 / 1      100.0% 
			      0 / 1       0.0% 
			      1 / 2      50.0% 
			      0 / 3       0.0% 
			      0 / 3       0.0% 
			      0 / 2       0.0% 
			      0 / 1       0.0% 
			      0 / 1       0.0% 
			      0 / 6       0.0% 
			      0 / 1       0.0% 
			      0 / 4       0.0% 
			      0 / 2       0.0% 
			      0 / 1       0.0% 
			      0 / 1       0.0% 
			      0 / 1       0.0% 
			      0 / 3       0.0% 
			      0 / 1       0.0% 
			      0 / 3       0.0% 
			      0 / 4       0.0% 
			      0 / 1       0.0% 
			      0 / 1       0.0% 
			      0 / 5       0.0% 
			      0 / 0       0.0% 
			      0 / 0       0.0% 
			      0 / 0       0.0% 
			      0 / 0       0.0% 
			     27 / 48     56.2% 
			     15 / 48     31.2%E 
			      3 / 48      6.2%C 
			      2 / 48      4.2%AB 
			      1 / 48      2.1%CE 
		 
		 
			 1 
			  7.1 
			 Anabaena ATCC29413 
			      2 / 48      4.2% 
			      1 / 1      100.0% 
			      0 / 1       0.0% 
			      1 / 2      50.0% 
			      0 / 3       0.0% 
			      0 / 3       0.0% 
			      0 / 2       0.0% 
			      0 / 1       0.0% 
			      0 / 1       0.0% 
			      0 / 6       0.0% 
			      0 / 1       0.0% 
			      0 / 4       0.0% 
			      0 / 2       0.0% 
			      0 / 1       0.0% 
			      0 / 1       0.0% 
			      0 / 1       0.0% 
			      0 / 3       0.0% 
			      0 / 1       0.0% 
			      0 / 3       0.0% 
			      0 / 4       0.0% 
			      0 / 1       0.0% 
			      0 / 1       0.0% 
			      0 / 5       0.0% 
			      0 / 0       0.0% 
			      0 / 0       0.0% 
			      0 / 0       0.0% 
			      0 / 0       0.0% 
			     27 / 48     56.2% 
			     15 / 48     31.2%E 
			      3 / 48      6.2%C 
			      2 / 48      4.2%AB 
			      1 / 48      2.1%CE 
		 
		 
			 4 
			  7.3 
			 Amorphonostoc 
			     19 / 234     8.1% 
			      3 / 3      100.0% 
			      0 / 6       0.0% 
			      3 / 7      42.9% 
			      0 / 13      0.0% 
			      0 / 13      0.0% 
			      1 / 9      11.1% 
			      0 / 5       0.0% 
			      1 / 5      20.0% 
			      0 / 27      0.0% 
			      0 / 4       0.0% 
			      0 / 18      0.0% 
			      0 / 10      0.0% 
			      0 / 6       0.0% 
			      2 / 8      25.0% 
			      0 / 6       0.0% 
			      0 / 15      0.0% 
			      1 / 5      20.0% 
			      0 / 14      0.0% 
			      0 / 18      0.0% 
			      0 / 7       0.0% 
			      2 / 6      33.3% 
			      5 / 27     18.5% 
			      0 / 0       0.0% 
			      0 / 0       0.0% 
			      0 / 1       0.0% 
			      0 / 0       0.0% 
			     99 / 234    42.3% 
			     89 / 234    38.0%E 
			     22 / 234     9.4%C 
			     11 / 234     4.7%AB 
			      5 / 234     2.1%CE 
			      4 / 234     1.7%B 
			      2 / 234     0.9%ABE 
			      2 / 234     0.9%BE 
		 
		 
			 1 
			  6.7 
			 NOSTOC SP. (STRAIN PCC 7524) 
			      0 / 45      0.0% 
			      0 / 0       0.0% 
			      0 / 1       0.0% 
			      0 / 1       0.0% 
			      0 / 3       0.0% 
			      0 / 3       0.0% 
			      0 / 2       0.0% 
			      0 / 1       0.0% 
			      0 / 1       0.0% 
			      0 / 6       0.0% 
			      0 / 1       0.0% 
			      0 / 4       0.0% 
			      0 / 2       0.0% 
			      0 / 1       0.0% 
			      0 / 1       0.0% 
			      0 / 1       0.0% 
			      0 / 3       0.0% 
			      0 / 1       0.0% 
			      0 / 3       0.0% 
			      0 / 4       0.0% 
			      0 / 1       0.0% 
			      0 / 1       0.0% 
			      0 / 4       0.0% 
			      0 / 0       0.0% 
			      0 / 0       0.0% 
			      0 / 0       0.0% 
			      0 / 0       0.0% 
			     20 / 45     44.4%E 
			     18 / 45     40.0% 
			      6 / 45     13.3%C 
			      1 / 45      2.2%CE 
		 
		 
			 1 
			  7.2 
			 Anabaena 7120 
			      2 / 49      4.1% 
			      1 / 1      100.0% 
			      0 / 1       0.0% 
			      1 / 2      50.0% 
			      0 / 3       0.0% 
			      0 / 3       0.0% 
			      0 / 2       0.0% 
			      0 / 1       0.0% 
			      0 / 1       0.0% 
			      0 / 6       0.0% 
			      0 / 1       0.0% 
			      0 / 4       0.0% 
			      0 / 2       0.0% 
			      0 / 1       0.0% 
			      0 / 1       0.0% 
			      0 / 1       0.0% 
			      0 / 3       0.0% 
			      0 / 1       0.0% 
			      0 / 3       0.0% 
			      0 / 4       0.0% 
			      0 / 1       0.0% 
			      0 / 1       0.0% 
			      0 / 6       0.0% 
			      0 / 0       0.0% 
			      0 / 0       0.0% 
			      0 / 0       0.0% 
			      0 / 0       0.0% 
			     28 / 49     57.1% 
			     15 / 49     30.6%E 
			      3 / 49      6.1%C 
			      2 / 49      4.1%AB 
			      1 / 49      2.0%CE 
		 
		 
			 1 
			  9.1 
			 Nostoc punctiforme 
			      9 / 69     13.0% 
			      1 / 1      100.0% 
			      0 / 2       0.0% 
			      1 / 2      50.0% 
			      0 / 4       0.0% 
			      0 / 3       0.0% 
			      0 / 2       0.0% 
			      0 / 1       0.0% 
			      1 / 1      100.0% 
			      0 / 8       0.0% 
			      0 / 1       0.0% 
			      0 / 5       0.0% 
			      0 / 3       0.0% 
			      0 / 2       0.0% 
			      1 / 3      33.3% 
			      0 / 2       0.0% 
			      0 / 5       0.0% 
			      1 / 2      50.0% 
			      0 / 4       0.0% 
			      0 / 5       0.0% 
			      0 / 2       0.0% 
			      1 / 2      50.0% 
			      3 / 9      33.3% 
			      0 / 0       0.0% 
			      0 / 0       0.0% 
			      0 / 0       0.0% 
			      0 / 0       0.0% 
			     27 / 69     39.1%E 
			     26 / 69     37.7% 
			      7 / 69     10.1%C 
			      4 / 69      5.8%B 
			      2 / 69      2.9%BE 
			      2 / 69      2.9%AB 
			      1 / 69      1.4%ABE 
		 
		 
			 1 
			  9.1 
			 Nostoc ATCC 29133 
			      9 / 69     13.0% 
			      1 / 1      100.0% 
			      0 / 2       0.0% 
			      1 / 2      50.0% 
			      0 / 4       0.0% 
			      0 / 3       0.0% 
			      0 / 2       0.0% 
			      0 / 1       0.0% 
			      1 / 1      100.0% 
			      0 / 8       0.0% 
			      0 / 1       0.0% 
			      0 / 5       0.0% 
			      0 / 3       0.0% 
			      0 / 2       0.0% 
			      1 / 3      33.3% 
			      0 / 2       0.0% 
			      0 / 5       0.0% 
			      1 / 2      50.0% 
			      0 / 4       0.0% 
			      0 / 5       0.0% 
			      0 / 2       0.0% 
			      1 / 2      50.0% 
			      3 / 9      33.3% 
			      0 / 0       0.0% 
			      0 / 0       0.0% 
			      0 / 0       0.0% 
			      0 / 0       0.0% 
			     27 / 69     39.1%E 
			     26 / 69     37.7% 
			      7 / 69     10.1%C 
			      4 / 69      5.8%B 
			      2 / 69      2.9%BE 
			      2 / 69      2.9%AB 
			      1 / 69      1.4%ABE 
		 
		 
			 1 
			  6.3 
			 Nostoc sp. PCC 7107 
			      8 / 71     11.3% 
			      1 / 1      100.0% 
			      0 / 2       0.0% 
			      1 / 2      50.0% 
			      0 / 3       0.0% 
			      0 / 4       0.0% 
			      1 / 3      33.3% 
			      0 / 2       0.0% 
			      0 / 2       0.0% 
			      0 / 7       0.0% 
			      0 / 1       0.0% 
			      0 / 5       0.0% 
			      0 / 3       0.0% 
			      0 / 2       0.0% 
			      1 / 3      33.3% 
			      0 / 2       0.0% 
			      0 / 4       0.0% 
			      0 / 1       0.0% 
			      0 / 4       0.0% 
			      0 / 5       0.0% 
			      0 / 3       0.0% 
			      1 / 2      50.0% 
			      2 / 8      25.0% 
			      0 / 0       0.0% 
			      0 / 0       0.0% 
			      0 / 1       0.0% 
			      0 / 0       0.0% 
			     27 / 71     38.0% 
			     27 / 71     38.0%E 
			      7 / 71      9.9%AB 
			      6 / 71      8.5%C 
			      3 / 71      4.2%CE 
			      1 / 71      1.4%ABE 
		 
		 
			 1 
			  7.6 
			 Cronbergia 
			      6 / 64      9.4% 
			      0 / 0       0.0% 
			      0 / 1       0.0% 
			      1 / 2      50.0% 
			      0 / 2       0.0% 
			      0 / 4       0.0% 
			      0 / 3       0.0% 
			      0 / 1       0.0% 
			      1 / 1      100.0% 
			      1 / 7      14.3% 
			      0 / 1       0.0% 
			      0 / 5       0.0% 
			      0 / 3       0.0% 
			      0 / 2       0.0% 
			      1 / 2      50.0% 
			      0 / 2       0.0% 
			      0 / 4       0.0% 
			      0 / 1       0.0% 
			      1 / 5      20.0% 
			      0 / 5       0.0% 
			      0 / 2       0.0% 
			      0 / 2       0.0% 
			      1 / 7      14.3% 
			      0 / 0       0.0% 
			      0 / 0       0.0% 
			      0 / 2       0.0% 
			      0 / 0       0.0% 
			     30 / 64     46.9% 
			     23 / 64     35.9%E 
			      4 / 64      6.2%C 
			      4 / 64      6.2%AB 
			      2 / 64      3.1%ABE 
			      1 / 64      1.6%CE 
		 
		 
			 1 
			  7.6 
			 Cylindrospermum stagnale 
			      6 / 64      9.4% 
			      0 / 0       0.0% 
			      0 / 1       0.0% 
			      1 / 2      50.0% 
			      0 / 2       0.0% 
			      0 / 4       0.0% 
			      0 / 3       0.0% 
			      0 / 1       0.0% 
			      1 / 1      100.0% 
			      1 / 7      14.3% 
			      0 / 1       0.0% 
			      0 / 5       0.0% 
			      0 / 3       0.0% 
			      0 / 2       0.0% 
			      1 / 2      50.0% 
			      0 / 2       0.0% 
			      0 / 4       0.0% 
			      0 / 1       0.0% 
			      1 / 5      20.0% 
			      0 / 5       0.0% 
			      0 / 2       0.0% 
			      0 / 2       0.0% 
			      1 / 7      14.3% 
			      0 / 0       0.0% 
			      0 / 0       0.0% 
			      0 / 2       0.0% 
			      0 / 0       0.0% 
			     30 / 64     46.9% 
			     23 / 64     35.9%E 
			      4 / 64      6.2%AB 
			      4 / 64      6.2%C 
			      2 / 64      3.1%ABE 
			      1 / 64      1.6%CE 
		 
		 
			 1 
			  7.6 
			 Cylindrospermum ATCC 29204 
			      6 / 64      9.4% 
			      0 / 0       0.0% 
			      0 / 1       0.0% 
			      1 / 2      50.0% 
			      0 / 2       0.0% 
			      0 / 4       0.0% 
			      0 / 3       0.0% 
			      0 / 1       0.0% 
			      1 / 1      100.0% 
			      1 / 7      14.3% 
			      0 / 1       0.0% 
			      0 / 5       0.0% 
			      0 / 3       0.0% 
			      0 / 2       0.0% 
			      1 / 2      50.0% 
			      0 / 2       0.0% 
			      0 / 4       0.0% 
			      0 / 1       0.0% 
			      1 / 5      20.0% 
			      0 / 5       0.0% 
			      0 / 2       0.0% 
			      0 / 2       0.0% 
			      1 / 7      14.3% 
			      0 / 0       0.0% 
			      0 / 0       0.0% 
			      0 / 2       0.0% 
			      0 / 0       0.0% 
			     30 / 64     46.9% 
			     23 / 64     35.9%E 
			      4 / 64      6.2%AB 
			      4 / 64      6.2%C 
			      2 / 64      3.1%ABE 
			      1 / 64      1.6%CE 
		 
		 
			 1 
			  5.5 
			 Trichormus 
			      1 / 42      2.4% 
			      0 / 1       0.0% 
			      0 / 1       0.0% 
			      0 / 1       0.0% 
			      0 / 1       0.0% 
			      0 / 3       0.0% 
			      0 / 2       0.0% 
			      0 / 1       0.0% 
			      1 / 1      100.0% 
			      0 / 4       0.0% 
			      0 / 1       0.0% 
			      0 / 4       0.0% 
			      0 / 2       0.0% 
			      0 / 1       0.0% 
			      0 / 1       0.0% 
			      0 / 1       0.0% 
			      0 / 3       0.0% 
			      0 / 1       0.0% 
			      0 / 3       0.0% 
			      0 / 4       0.0% 
			      0 / 1       0.0% 
			      0 / 1       0.0% 
			      0 / 4       0.0% 
			      0 / 0       0.0% 
			      0 / 0       0.0% 
			      0 / 0       0.0% 
			      0 / 0       0.0% 
			     24 / 42     57.1% 
			     17 / 42     40.5%E 
			      1 / 42      2.4%ABE 
		 
		 
			 1 
			  5.5 
			 Anabaena azollae 
			      1 / 42      2.4% 
			      0 / 1       0.0% 
			      0 / 1       0.0% 
			      0 / 1       0.0% 
			      0 / 1       0.0% 
			      0 / 3       0.0% 
			      0 / 2       0.0% 
			      0 / 1       0.0% 
			      1 / 1      100.0% 
			      0 / 4       0.0% 
			      0 / 1       0.0% 
			      0 / 4       0.0% 
			      0 / 2       0.0% 
			      0 / 1       0.0% 
			      0 / 1       0.0% 
			      0 / 1       0.0% 
			      0 / 3       0.0% 
			      0 / 1       0.0% 
			      0 / 3       0.0% 
			      0 / 4       0.0% 
			      0 / 1       0.0% 
			      0 / 1       0.0% 
			      0 / 4       0.0% 
			      0 / 0       0.0% 
			      0 / 0       0.0% 
			      0 / 0       0.0% 
			      0 / 0       0.0% 
			     24 / 42     57.1% 
			     17 / 42     40.5%E 
			      1 / 42      2.4%ABE 
		 
		 
			 1 
			  5.5 
			 'Nostoc azollae' 0708 
			      1 / 42      2.4% 
			      0 / 1       0.0% 
			      0 / 1       0.0% 
			      0 / 1       0.0% 
			      0 / 1       0.0% 
			      0 / 3       0.0% 
			      0 / 2       0.0% 
			      0 / 1       0.0% 
			      1 / 1      100.0% 
			      0 / 4       0.0% 
			      0 / 1       0.0% 
			      0 / 4       0.0% 
			      0 / 2       0.0% 
			      0 / 1       0.0% 
			      0 / 1       0.0% 
			      0 / 1       0.0% 
			      0 / 3       0.0% 
			      0 / 1       0.0% 
			      0 / 3       0.0% 
			      0 / 4       0.0% 
			      0 / 1       0.0% 
			      0 / 1       0.0% 
			      0 / 4       0.0% 
			      0 / 0       0.0% 
			      0 / 0       0.0% 
			      0 / 0       0.0% 
			      0 / 0       0.0% 
			     24 / 42     57.1% 
			     17 / 42     40.5%E 
			      1 / 42      2.4%ABE 
		 
		 
			 3 
			  7.6 
			 Rivulariaceae 
			     14 / 155     9.0% 
			      3 / 3      100.0% 
			      0 / 4       0.0% 
			      1 / 5      20.0% 
			      0 / 6       0.0% 
			      0 / 8       0.0% 
			      0 / 7       0.0% 
			      0 / 3       0.0% 
			      1 / 3      33.3% 
			      3 / 14     21.4% 
			      0 / 4       0.0% 
			      0 / 15      0.0% 
			      0 / 8       0.0% 
			      0 / 5       0.0% 
			      2 / 5      40.0% 
			      1 / 4      25.0% 
			      1 / 10     10.0% 
			      0 / 1       0.0% 
			      1 / 11      9.1% 
			      0 / 13      0.0% 
			      0 / 3       0.0% 
			      1 / 6      16.7% 
			      0 / 16      0.0% 
			      0 / 0       0.0% 
			      0 / 0       0.0% 
			      0 / 1       0.0% 
			      0 / 0       0.0% 
			     69 / 155    44.5%E 
			     64 / 155    41.3% 
			      9 / 155     5.8%AB 
			      8 / 155     5.2%C 
			      5 / 155     3.2%ABE 
		 
		 
			 2 
			  7.0 
			 Calothrix 
			      9 / 106     8.5% 
			      2 / 2      100.0% 
			      0 / 3       0.0% 
			      1 / 3      33.3% 
			      0 / 3       0.0% 
			      0 / 6       0.0% 
			      0 / 5       0.0% 
			      0 / 2       0.0% 
			      1 / 2      50.0% 
			      0 / 10      0.0% 
			      0 / 3       0.0% 
			      0 / 10      0.0% 
			      0 / 6       0.0% 
			      0 / 4       0.0% 
			      2 / 4      50.0% 
			      1 / 3      33.3% 
			      1 / 7      14.3% 
			      0 / 1       0.0% 
			      1 / 8      12.5% 
			      0 / 8       0.0% 
			      0 / 2       0.0% 
			      0 / 3       0.0% 
			      0 / 11      0.0% 
			      0 / 0       0.0% 
			      0 / 0       0.0% 
			      0 / 0       0.0% 
			      0 / 0       0.0% 
			     48 / 106    45.3%E 
			     45 / 106    42.5% 
			      5 / 106     4.7%ABE 
			      4 / 106     3.8%C 
			      4 / 106     3.8%AB 
		 
		 
			 1 
			  7.0 
			 Calothrix parietina 
			      1 / 40      2.5% 
			      1 / 1      100.0% 
			      0 / 1       0.0% 
			      0 / 1       0.0% 
			      0 / 1       0.0% 
			      0 / 2       0.0% 
			      0 / 2       0.0% 
			      0 / 1       0.0% 
			      0 / 1       0.0% 
			      0 / 3       0.0% 
			      0 / 1       0.0% 
			      0 / 4       0.0% 
			      0 / 2       0.0% 
			      0 / 1       0.0% 
			      0 / 1       0.0% 
			      0 / 1       0.0% 
			      0 / 3       0.0% 
			      0 / 0       0.0% 
			      0 / 3       0.0% 
			      0 / 4       0.0% 
			      0 / 1       0.0% 
			      0 / 2       0.0% 
			      0 / 4       0.0% 
			      0 / 0       0.0% 
			      0 / 0       0.0% 
			      0 / 0       0.0% 
			      0 / 0       0.0% 
			     20 / 40     50.0%E 
			     18 / 40     45.0% 
			      1 / 40      2.5%C 
			      1 / 40      2.5%AB 
		 
		 
			 1 
			  7.0 
			 Calothrix parietina 1018 
			      1 / 40      2.5% 
			      1 / 1      100.0% 
			      0 / 1       0.0% 
			      0 / 1       0.0% 
			      0 / 1       0.0% 
			      0 / 2       0.0% 
			      0 / 2       0.0% 
			      0 / 1       0.0% 
			      0 / 1       0.0% 
			      0 / 3       0.0% 
			      0 / 1       0.0% 
			      0 / 4       0.0% 
			      0 / 2       0.0% 
			      0 / 1       0.0% 
			      0 / 1       0.0% 
			      0 / 1       0.0% 
			      0 / 3       0.0% 
			      0 / 0       0.0% 
			      0 / 3       0.0% 
			      0 / 4       0.0% 
			      0 / 1       0.0% 
			      0 / 2       0.0% 
			      0 / 4       0.0% 
			      0 / 0       0.0% 
			      0 / 0       0.0% 
			      0 / 0       0.0% 
			      0 / 0       0.0% 
			     20 / 40     50.0%E 
			     18 / 40     45.0% 
			      1 / 40      2.5%AB 
			      1 / 40      2.5%C 
		 
		 
			 1 
			  7.0 
			 Calothrix PCC7507 
			      8 / 66     12.1% 
			      1 / 1      100.0% 
			      0 / 2       0.0% 
			      1 / 2      50.0% 
			      0 / 2       0.0% 
			      0 / 4       0.0% 
			      0 / 3       0.0% 
			      0 / 1       0.0% 
			      1 / 1      100.0% 
			      0 / 7       0.0% 
			      0 / 2       0.0% 
			      0 / 6       0.0% 
			      0 / 4       0.0% 
			      0 / 3       0.0% 
			      2 / 3      66.7% 
			      1 / 2      50.0% 
			      1 / 4      25.0% 
			      0 / 1       0.0% 
			      1 / 5      20.0% 
			      0 / 4       0.0% 
			      0 / 1       0.0% 
			      0 / 1       0.0% 
			      0 / 7       0.0% 
			      0 / 0       0.0% 
			      0 / 0       0.0% 
			      0 / 0       0.0% 
			      0 / 0       0.0% 
			     28 / 66     42.4%E 
			     27 / 66     40.9% 
			      5 / 66      7.6%ABE 
			      3 / 66      4.5%C 
			      3 / 66      4.5%AB 
		 
		 
			 1 
			  8.7 
			 Rivularia 
			      5 / 49     10.2% 
			      1 / 1      100.0% 
			      0 / 1       0.0% 
			      0 / 2       0.0% 
			      0 / 3       0.0% 
			      0 / 2       0.0% 
			      0 / 2       0.0% 
			      0 / 1       0.0% 
			      0 / 1       0.0% 
			      3 / 4      75.0% 
			      0 / 1       0.0% 
			      0 / 5       0.0% 
			      0 / 2       0.0% 
			      0 / 1       0.0% 
			      0 / 1       0.0% 
			      0 / 1       0.0% 
			      0 / 3       0.0% 
			      0 / 0       0.0% 
			      0 / 3       0.0% 
			      0 / 5       0.0% 
			      0 / 1       0.0% 
			      1 / 3      33.3% 
			      0 / 5       0.0% 
			      0 / 0       0.0% 
			      0 / 0       0.0% 
			      0 / 1       0.0% 
			      0 / 0       0.0% 
			     21 / 49     42.9%E 
			     19 / 49     38.8% 
			      5 / 49     10.2%AB 
			      4 / 49      8.2%C 
		 
		 
			 1 
			  8.7 
			 Calothrix sp. ATCC 29111 
			      5 / 49     10.2% 
			      1 / 1      100.0% 
			      0 / 1       0.0% 
			      0 / 2       0.0% 
			      0 / 3       0.0% 
			      0 / 2       0.0% 
			      0 / 2       0.0% 
			      0 / 1       0.0% 
			      0 / 1       0.0% 
			      3 / 4      75.0% 
			      0 / 1       0.0% 
			      0 / 5       0.0% 
			      0 / 2       0.0% 
			      0 / 1       0.0% 
			      0 / 1       0.0% 
			      0 / 1       0.0% 
			      0 / 3       0.0% 
			      0 / 0       0.0% 
			      0 / 3       0.0% 
			      0 / 5       0.0% 
			      0 / 1       0.0% 
			      1 / 3      33.3% 
			      0 / 5       0.0% 
			      0 / 0       0.0% 
			      0 / 0       0.0% 
			      0 / 1       0.0% 
			      0 / 0       0.0% 
			     21 / 49     42.9%E 
			     19 / 49     38.8% 
			      5 / 49     10.2%AB 
			      4 / 49      8.2%C 
		 
		 
			 12 
			  1.9 
			 "Prochlorales" Lewin 1977 
			     16 / 467     3.4% 
			     11 / 12     91.7% 
			      0 / 12      0.0% 
			      0 / 12      0.0% 
			      0 / 14      0.0% 
			      0 / 30      0.0% 
			      0 / 26      0.0% 
			      0 / 12      0.0% 
			      0 / 12      0.0% 
			      0 / 28      0.0% 
			      0 / 12      0.0% 
			      1 / 48      2.1% 
			      0 / 12      0.0% 
			      3 / 12     25.0% 
			      0 / 12      0.0% 
			      0 / 12      0.0% 
			      1 / 26      3.8% 
			      0 / 12      0.0% 
			      0 / 39      0.0% 
			      0 / 48      0.0% 
			      0 / 12      0.0% 
			      0 / 12      0.0% 
			      0 / 52      0.0% 
			      0 / 0       0.0% 
			      0 / 0       0.0% 
			      0 / 0       0.0% 
			      0 / 0       0.0% 
			    241 / 467    51.6% 
			    199 / 467    42.6%E 
			     14 / 467     3.0%AB 
			      7 / 467     1.5%C 
			      4 / 467     0.9%CE 
			      2 / 467     0.4%ABE 
		 
		 
			 12 
			  1.9 
			 'Prochlorococcaceae' 
			     16 / 467     3.4% 
			     11 / 12     91.7% 
			      0 / 12      0.0% 
			      0 / 12      0.0% 
			      0 / 14      0.0% 
			      0 / 30      0.0% 
			      0 / 26      0.0% 
			      0 / 12      0.0% 
			      0 / 12      0.0% 
			      0 / 28      0.0% 
			      0 / 12      0.0% 
			      1 / 48      2.1% 
			      0 / 12      0.0% 
			      3 / 12     25.0% 
			      0 / 12      0.0% 
			      0 / 12      0.0% 
			      1 / 26      3.8% 
			      0 / 12      0.0% 
			      0 / 39      0.0% 
			      0 / 48      0.0% 
			      0 / 12      0.0% 
			      0 / 12      0.0% 
			      0 / 52      0.0% 
			      0 / 0       0.0% 
			      0 / 0       0.0% 
			      0 / 0       0.0% 
			      0 / 0       0.0% 
			    241 / 467    51.6% 
			    199 / 467    42.6%E 
			     14 / 467     3.0%AB 
			      7 / 467     1.5%C 
			      4 / 467     0.9%CE 
			      2 / 467     0.4%ABE 
		 
		 
			 12 
			  1.9 
			 Prochlorococcus 
			     16 / 467     3.4% 
			     11 / 12     91.7% 
			      0 / 12      0.0% 
			      0 / 12      0.0% 
			      0 / 14      0.0% 
			      0 / 30      0.0% 
			      0 / 26      0.0% 
			      0 / 12      0.0% 
			      0 / 12      0.0% 
			      0 / 28      0.0% 
			      0 / 12      0.0% 
			      1 / 48      2.1% 
			      0 / 12      0.0% 
			      3 / 12     25.0% 
			      0 / 12      0.0% 
			      0 / 12      0.0% 
			      1 / 26      3.8% 
			      0 / 12      0.0% 
			      0 / 39      0.0% 
			      0 / 48      0.0% 
			      0 / 12      0.0% 
			      0 / 12      0.0% 
			      0 / 52      0.0% 
			      0 / 0       0.0% 
			      0 / 0       0.0% 
			      0 / 0       0.0% 
			      0 / 0       0.0% 
			    241 / 467    51.6% 
			    199 / 467    42.6%E 
			     14 / 467     3.0%AB 
			      7 / 467     1.5%C 
			      4 / 467     0.9%CE 
			      2 / 467     0.4%ABE 
		 
		 
			 12 
			  1.9 
			 CCMP1375 
			     16 / 467     3.4% 
			     11 / 12     91.7% 
			      0 / 12      0.0% 
			      0 / 12      0.0% 
			      0 / 14      0.0% 
			      0 / 30      0.0% 
			      0 / 26      0.0% 
			      0 / 12      0.0% 
			      0 / 12      0.0% 
			      0 / 28      0.0% 
			      0 / 12      0.0% 
			      1 / 48      2.1% 
			      0 / 12      0.0% 
			      3 / 12     25.0% 
			      0 / 12      0.0% 
			      0 / 12      0.0% 
			      1 / 26      3.8% 
			      0 / 12      0.0% 
			      0 / 39      0.0% 
			      0 / 48      0.0% 
			      0 / 12      0.0% 
			      0 / 12      0.0% 
			      0 / 52      0.0% 
			      0 / 0       0.0% 
			      0 / 0       0.0% 
			      0 / 0       0.0% 
			      0 / 0       0.0% 
			    241 / 467    51.6% 
			    199 / 467    42.6%E 
			     14 / 467     3.0%AB 
			      7 / 467     1.5%C 
			      4 / 467     0.9%CE 
			      2 / 467     0.4%ABE 
		 
		 
			 1 
			  1.8 
			 Prochlorococcus marinus str. NATL2A 
			      2 / 38      5.3% 
			      1 / 1      100.0% 
			      0 / 1       0.0% 
			      0 / 1       0.0% 
			      0 / 1       0.0% 
			      0 / 3       0.0% 
			      0 / 2       0.0% 
			      0 / 1       0.0% 
			      0 / 1       0.0% 
			      0 / 2       0.0% 
			      0 / 1       0.0% 
			      0 / 4       0.0% 
			      0 / 1       0.0% 
			      1 / 1      100.0% 
			      0 / 1       0.0% 
			      0 / 1       0.0% 
			      0 / 2       0.0% 
			      0 / 1       0.0% 
			      0 / 3       0.0% 
			      0 / 4       0.0% 
			      0 / 1       0.0% 
			      0 / 1       0.0% 
			      0 / 4       0.0% 
			      0 / 0       0.0% 
			      0 / 0       0.0% 
			      0 / 0       0.0% 
			      0 / 0       0.0% 
			     22 / 38     57.9% 
			     14 / 38     36.8%E 
			      2 / 38      5.3%AB 
		 
		 
			 1 
			  2.7 
			 Prochlorococcus marinus str. MIT 9303 
			      1 / 44      2.3% 
			      1 / 1      100.0% 
			      0 / 1       0.0% 
			      0 / 1       0.0% 
			      0 / 2       0.0% 
			      0 / 3       0.0% 
			      0 / 3       0.0% 
			      0 / 1       0.0% 
			      0 / 1       0.0% 
			      0 / 4       0.0% 
			      0 / 1       0.0% 
			      0 / 4       0.0% 
			      0 / 1       0.0% 
			      0 / 1       0.0% 
			      0 / 1       0.0% 
			      0 / 1       0.0% 
			      0 / 3       0.0% 
			      0 / 1       0.0% 
			      0 / 3       0.0% 
			      0 / 4       0.0% 
			      0 / 1       0.0% 
			      0 / 1       0.0% 
			      0 / 5       0.0% 
			      0 / 0       0.0% 
			      0 / 0       0.0% 
			      0 / 0       0.0% 
			      0 / 0       0.0% 
			     26 / 44     59.1% 
			     16 / 44     36.4%E 
			      1 / 44      2.3%AB 
			      1 / 44      2.3%CE 
		 
		 
			 1 
			  1.7 
			 Prochlorococcus marinus str. MIT 9312 
			      2 / 37      5.4% 
			      1 / 1      100.0% 
			      0 / 1       0.0% 
			      0 / 1       0.0% 
			      0 / 1       0.0% 
			      0 / 2       0.0% 
			      0 / 2       0.0% 
			      0 / 1       0.0% 
			      0 / 1       0.0% 
			      0 / 2       0.0% 
			      0 / 1       0.0% 
			      0 / 4       0.0% 
			      0 / 1       0.0% 
			      0 / 1       0.0% 
			      0 / 1       0.0% 
			      0 / 1       0.0% 
			      1 / 2      50.0% 
			      0 / 1       0.0% 
			      0 / 3       0.0% 
			      0 / 4       0.0% 
			      0 / 1       0.0% 
			      0 / 1       0.0% 
			      0 / 4       0.0% 
			      0 / 0       0.0% 
			      0 / 0       0.0% 
			      0 / 0       0.0% 
			      0 / 0       0.0% 
			     20 / 37     54.1% 
			     14 / 37     37.8%E 
			      1 / 37      2.7%ABE 
			      1 / 37      2.7%AB 
			      1 / 37      2.7%C 
		 
		 
			 1 
			  2.4 
			 Prochlorococcus marinus MIT9313 
			      0 / 44      0.0% 
			      0 / 1       0.0% 
			      0 / 1       0.0% 
			      0 / 1       0.0% 
			      0 / 2       0.0% 
			      0 / 3       0.0% 
			      0 / 3       0.0% 
			      0 / 1       0.0% 
			      0 / 1       0.0% 
			      0 / 4       0.0% 
			      0 / 1       0.0% 
			      0 / 4       0.0% 
			      0 / 1       0.0% 
			      0 / 1       0.0% 
			      0 / 1       0.0% 
			      0 / 1       0.0% 
			      0 / 3       0.0% 
			      0 / 1       0.0% 
			      0 / 3       0.0% 
			      0 / 4       0.0% 
			      0 / 1       0.0% 
			      0 / 1       0.0% 
			      0 / 5       0.0% 
			      0 / 0       0.0% 
			      0 / 0       0.0% 
			      0 / 0       0.0% 
			      0 / 0       0.0% 
			     26 / 44     59.1% 
			     16 / 44     36.4%E 
			      1 / 44      2.3%CE 
			      1 / 44      2.3%C 
		 
		 
			 1 
			  1.7 
			 Prochlorococcus marinus str. MIT 9211 
			      1 / 40      2.5% 
			      1 / 1      100.0% 
			      0 / 1       0.0% 
			      0 / 1       0.0% 
			      0 / 1       0.0% 
			      0 / 3       0.0% 
			      0 / 2       0.0% 
			      0 / 1       0.0% 
			      0 / 1       0.0% 
			      0 / 2       0.0% 
			      0 / 1       0.0% 
			      0 / 4       0.0% 
			      0 / 1       0.0% 
			      0 / 1       0.0% 
			      0 / 1       0.0% 
			      0 / 1       0.0% 
			      0 / 2       0.0% 
			      0 / 1       0.0% 
			      0 / 4       0.0% 
			      0 / 4       0.0% 
			      0 / 1       0.0% 
			      0 / 1       0.0% 
			      0 / 5       0.0% 
			      0 / 0       0.0% 
			      0 / 0       0.0% 
			      0 / 0       0.0% 
			      0 / 0       0.0% 
			     26 / 40     65.0%E 
			     12 / 40     30.0% 
			      1 / 40      2.5%CE 
			      1 / 40      2.5%AB 
		 
		 
			 1 
			  1.7 
			 Prochlorococcus marinus MIT 9215 
			      1 / 38      2.6% 
			      1 / 1      100.0% 
			      0 / 1       0.0% 
			      0 / 1       0.0% 
			      0 / 1       0.0% 
			      0 / 2       0.0% 
			      0 / 2       0.0% 
			      0 / 1       0.0% 
			      0 / 1       0.0% 
			      0 / 2       0.0% 
			      0 / 1       0.0% 
			      0 / 4       0.0% 
			      0 / 1       0.0% 
			      0 / 1       0.0% 
			      0 / 1       0.0% 
			      0 / 1       0.0% 
			      0 / 2       0.0% 
			      0 / 1       0.0% 
			      0 / 4       0.0% 
			      0 / 4       0.0% 
			      0 / 1       0.0% 
			      0 / 1       0.0% 
			      0 / 4       0.0% 
			      0 / 0       0.0% 
			      0 / 0       0.0% 
			      0 / 0       0.0% 
			      0 / 0       0.0% 
			     26 / 38     68.4%E 
			     10 / 38     26.3% 
			      1 / 38      2.6%AB 
			      1 / 38      2.6%C 
		 
		 
			 1 
			  1.7 
			 ATCC 700925 
			      2 / 37      5.4% 
			      1 / 1      100.0% 
			      0 / 1       0.0% 
			      0 / 1       0.0% 
			      0 / 1       0.0% 
			      0 / 2       0.0% 
			      0 / 2       0.0% 
			      0 / 1       0.0% 
			      0 / 1       0.0% 
			      0 / 2       0.0% 
			      0 / 1       0.0% 
			      1 / 4      25.0% 
			      0 / 1       0.0% 
			      0 / 1       0.0% 
			      0 / 1       0.0% 
			      0 / 1       0.0% 
			      0 / 2       0.0% 
			      0 / 1       0.0% 
			      0 / 3       0.0% 
			      0 / 4       0.0% 
			      0 / 1       0.0% 
			      0 / 1       0.0% 
			      0 / 4       0.0% 
			      0 / 0       0.0% 
			      0 / 0       0.0% 
			      0 / 0       0.0% 
			      0 / 0       0.0% 
			     20 / 37     54.1% 
			     14 / 37     37.8%E 
			      1 / 37      2.7%C 
			      1 / 37      2.7%AB 
			      1 / 37      2.7%ABE 
		 
		 
			 1 
			  1.7 
			 Prochlorococcus marinus MED4 
			      2 / 37      5.4% 
			      1 / 1      100.0% 
			      0 / 1       0.0% 
			      0 / 1       0.0% 
			      0 / 1       0.0% 
			      0 / 2       0.0% 
			      0 / 2       0.0% 
			      0 / 1       0.0% 
			      0 / 1       0.0% 
			      0 / 2       0.0% 
			      0 / 1       0.0% 
			      1 / 4      25.0% 
			      0 / 1       0.0% 
			      0 / 1       0.0% 
			      0 / 1       0.0% 
			      0 / 1       0.0% 
			      0 / 2       0.0% 
			      0 / 1       0.0% 
			      0 / 3       0.0% 
			      0 / 4       0.0% 
			      0 / 1       0.0% 
			      0 / 1       0.0% 
			      0 / 4       0.0% 
			      0 / 0       0.0% 
			      0 / 0       0.0% 
			      0 / 0       0.0% 
			      0 / 0       0.0% 
			     20 / 37     54.1% 
			     14 / 37     37.8%E 
			      1 / 37      2.7%C 
			      1 / 37      2.7%AB 
			      1 / 37      2.7%ABE 
		 
		 
			 1 
			  1.8 
			 Prochlorococcus marinus subsp. marinus 
			      2 / 39      5.1% 
			      1 / 1      100.0% 
			      0 / 1       0.0% 
			      0 / 1       0.0% 
			      0 / 1       0.0% 
			      0 / 3       0.0% 
			      0 / 2       0.0% 
			      0 / 1       0.0% 
			      0 / 1       0.0% 
			      0 / 2       0.0% 
			      0 / 1       0.0% 
			      0 / 4       0.0% 
			      0 / 1       0.0% 
			      1 / 1      100.0% 
			      0 / 1       0.0% 
			      0 / 1       0.0% 
			      0 / 2       0.0% 
			      0 / 1       0.0% 
			      0 / 3       0.0% 
			      0 / 4       0.0% 
			      0 / 1       0.0% 
			      0 / 1       0.0% 
			      0 / 5       0.0% 
			      0 / 0       0.0% 
			      0 / 0       0.0% 
			      0 / 0       0.0% 
			      0 / 0       0.0% 
			     22 / 39     56.4% 
			     14 / 39     35.9%E 
			      2 / 39      5.1%AB 
			      1 / 39      2.6%CE 
		 
		 
			 1 
			  1.8 
			 Prochlorococcus marinus SS120 
			      2 / 39      5.1% 
			      1 / 1      100.0% 
			      0 / 1       0.0% 
			      0 / 1       0.0% 
			      0 / 1       0.0% 
			      0 / 3       0.0% 
			      0 / 2       0.0% 
			      0 / 1       0.0% 
			      0 / 1       0.0% 
			      0 / 2       0.0% 
			      0 / 1       0.0% 
			      0 / 4       0.0% 
			      0 / 1       0.0% 
			      1 / 1      100.0% 
			      0 / 1       0.0% 
			      0 / 1       0.0% 
			      0 / 2       0.0% 
			      0 / 1       0.0% 
			      0 / 3       0.0% 
			      0 / 4       0.0% 
			      0 / 1       0.0% 
			      0 / 1       0.0% 
			      0 / 5       0.0% 
			      0 / 0       0.0% 
			      0 / 0       0.0% 
			      0 / 0       0.0% 
			      0 / 0       0.0% 
			     22 / 39     56.4% 
			     14 / 39     35.9%E 
			      2 / 39      5.1%AB 
			      1 / 39      2.6%CE 
		 
		 
			 1 
			  1.7 
			 Prochlorococcus marinus str. AS9601 
			      1 / 38      2.6% 
			      1 / 1      100.0% 
			      0 / 1       0.0% 
			      0 / 1       0.0% 
			      0 / 1       0.0% 
			      0 / 2       0.0% 
			      0 / 2       0.0% 
			      0 / 1       0.0% 
			      0 / 1       0.0% 
			      0 / 2       0.0% 
			      0 / 1       0.0% 
			      0 / 4       0.0% 
			      0 / 1       0.0% 
			      0 / 1       0.0% 
			      0 / 1       0.0% 
			      0 / 1       0.0% 
			      0 / 2       0.0% 
			      0 / 1       0.0% 
			      0 / 4       0.0% 
			      0 / 4       0.0% 
			      0 / 1       0.0% 
			      0 / 1       0.0% 
			      0 / 4       0.0% 
			      0 / 0       0.0% 
			      0 / 0       0.0% 
			      0 / 0       0.0% 
			      0 / 0       0.0% 
			     21 / 38     55.3% 
			     15 / 38     39.5%E 
			      1 / 38      2.6%AB 
			      1 / 38      2.6%C 
		 
		 
			 1 
			  1.7 
			 Prochlorococcus marinus str. MIT 9515 
			      1 / 37      2.7% 
			      1 / 1      100.0% 
			      0 / 1       0.0% 
			      0 / 1       0.0% 
			      0 / 1       0.0% 
			      0 / 2       0.0% 
			      0 / 2       0.0% 
			      0 / 1       0.0% 
			      0 / 1       0.0% 
			      0 / 2       0.0% 
			      0 / 1       0.0% 
			      0 / 4       0.0% 
			      0 / 1       0.0% 
			      0 / 1       0.0% 
			      0 / 1       0.0% 
			      0 / 1       0.0% 
			      0 / 2       0.0% 
			      0 / 1       0.0% 
			      0 / 3       0.0% 
			      0 / 4       0.0% 
			      0 / 1       0.0% 
			      0 / 1       0.0% 
			      0 / 4       0.0% 
			      0 / 0       0.0% 
			      0 / 0       0.0% 
			      0 / 0       0.0% 
			      0 / 0       0.0% 
			     20 / 37     54.1% 
			     15 / 37     40.5%E 
			      1 / 37      2.7%AB 
			      1 / 37      2.7%C 
		 
		 
			 1 
			  1.6 
			 Prochlorococcus marinus str. MIT 9301 
			      1 / 37      2.7% 
			      1 / 1      100.0% 
			      0 / 1       0.0% 
			      0 / 1       0.0% 
			      0 / 1       0.0% 
			      0 / 2       0.0% 
			      0 / 2       0.0% 
			      0 / 1       0.0% 
			      0 / 1       0.0% 
			      0 / 2       0.0% 
			      0 / 1       0.0% 
			      0 / 4       0.0% 
			      0 / 1       0.0% 
			      0 / 1       0.0% 
			      0 / 1       0.0% 
			      0 / 1       0.0% 
			      0 / 2       0.0% 
			      0 / 1       0.0% 
			      0 / 3       0.0% 
			      0 / 4       0.0% 
			      0 / 1       0.0% 
			      0 / 1       0.0% 
			      0 / 4       0.0% 
			      0 / 0       0.0% 
			      0 / 0       0.0% 
			      0 / 0       0.0% 
			      0 / 0       0.0% 
			     20 / 37     54.1% 
			     15 / 37     40.5%E 
			      1 / 37      2.7%C 
			      1 / 37      2.7%AB 
		 
		 
			 1 
			  1.9 
			 Prochlorococcus marinus str. NATL1A 
			      2 / 38      5.3% 
			      1 / 1      100.0% 
			      0 / 1       0.0% 
			      0 / 1       0.0% 
			      0 / 1       0.0% 
			      0 / 3       0.0% 
			      0 / 2       0.0% 
			      0 / 1       0.0% 
			      0 / 1       0.0% 
			      0 / 2       0.0% 
			      0 / 1       0.0% 
			      0 / 4       0.0% 
			      0 / 1       0.0% 
			      1 / 1      100.0% 
			      0 / 1       0.0% 
			      0 / 1       0.0% 
			      0 / 2       0.0% 
			      0 / 1       0.0% 
			      0 / 3       0.0% 
			      0 / 4       0.0% 
			      0 / 1       0.0% 
			      0 / 1       0.0% 
			      0 / 4       0.0% 
			      0 / 0       0.0% 
			      0 / 0       0.0% 
			      0 / 0       0.0% 
			      0 / 0       0.0% 
			     22 / 38     57.9% 
			     14 / 38     36.8%E 
			      2 / 38      5.3%AB 
		 
		 
			 3 
			  5.7 
			 Cyanobacteria Subsection II 
			      5 / 127     3.9% 
			      1 / 3      33.3% 
			      0 / 3       0.0% 
			      0 / 3       0.0% 
			      0 / 7       0.0% 
			      0 / 8       0.0% 
			      0 / 7       0.0% 
			      0 / 3       0.0% 
			      2 / 3      66.7% 
			      2 / 10     20.0% 
			      0 / 3       0.0% 
			      0 / 12      0.0% 
			      0 / 5       0.0% 
			      0 / 3       0.0% 
			      0 / 3       0.0% 
			      0 / 3       0.0% 
			      0 / 9       0.0% 
			      0 / 2       0.0% 
			      0 / 9       0.0% 
			      0 / 10      0.0% 
			      0 / 3       0.0% 
			      0 / 4       0.0% 
			      0 / 14      0.0% 
			      0 / 0       0.0% 
			      0 / 0       0.0% 
			      0 / 0       0.0% 
			      0 / 0       0.0% 
			     60 / 127    47.2% 
			     53 / 127    41.7%E 
			      7 / 127     5.5%C 
			      3 / 127     2.4%AB 
			      2 / 127     1.6%CE 
			      2 / 127     1.6%ABE 
		 
		 
			 1 
			  5.0 
			 Pleurocapsa 
			      1 / 42      2.4% 
			      0 / 1       0.0% 
			      0 / 1       0.0% 
			      0 / 1       0.0% 
			      0 / 2       0.0% 
			      0 / 3       0.0% 
			      0 / 3       0.0% 
			      0 / 1       0.0% 
			      1 / 1      100.0% 
			      0 / 3       0.0% 
			      0 / 1       0.0% 
			      0 / 4       0.0% 
			      0 / 2       0.0% 
			      0 / 1       0.0% 
			      0 / 1       0.0% 
			      0 / 1       0.0% 
			      0 / 3       0.0% 
			      0 / 0       0.0% 
			      0 / 3       0.0% 
			      0 / 3       0.0% 
			      0 / 1       0.0% 
			      0 / 2       0.0% 
			      0 / 4       0.0% 
			      0 / 0       0.0% 
			      0 / 0       0.0% 
			      0 / 0       0.0% 
			      0 / 0       0.0% 
			     21 / 42     50.0% 
			     16 / 42     38.1%E 
			      2 / 42      4.8%CE 
			      2 / 42      4.8%C 
			      1 / 42      2.4%ABE 
		 
		 
			 1 
			  5.0 
			 Pleurocapsa minor 
			      1 / 42      2.4% 
			      0 / 1       0.0% 
			      0 / 1       0.0% 
			      0 / 1       0.0% 
			      0 / 2       0.0% 
			      0 / 3       0.0% 
			      0 / 3       0.0% 
			      0 / 1       0.0% 
			      1 / 1      100.0% 
			      0 / 3       0.0% 
			      0 / 1       0.0% 
			      0 / 4       0.0% 
			      0 / 2       0.0% 
			      0 / 1       0.0% 
			      0 / 1       0.0% 
			      0 / 1       0.0% 
			      0 / 3       0.0% 
			      0 / 0       0.0% 
			      0 / 3       0.0% 
			      0 / 3       0.0% 
			      0 / 1       0.0% 
			      0 / 2       0.0% 
			      0 / 4       0.0% 
			      0 / 0       0.0% 
			      0 / 0       0.0% 
			      0 / 0       0.0% 
			      0 / 0       0.0% 
			     21 / 42     50.0% 
			     16 / 42     38.1%E 
			      2 / 42      4.8%C 
			      2 / 42      4.8%CE 
			      1 / 42      2.4%ABE 
		 
		 
			 1 
			  5.0 
			 Pleurocapsa PCC7327 
			      1 / 42      2.4% 
			      0 / 1       0.0% 
			      0 / 1       0.0% 
			      0 / 1       0.0% 
			      0 / 2       0.0% 
			      0 / 3       0.0% 
			      0 / 3       0.0% 
			      0 / 1       0.0% 
			      1 / 1      100.0% 
			      0 / 3       0.0% 
			      0 / 1       0.0% 
			      0 / 4       0.0% 
			      0 / 2       0.0% 
			      0 / 1       0.0% 
			      0 / 1       0.0% 
			      0 / 1       0.0% 
			      0 / 3       0.0% 
			      0 / 0       0.0% 
			      0 / 3       0.0% 
			      0 / 3       0.0% 
			      0 / 1       0.0% 
			      0 / 2       0.0% 
			      0 / 4       0.0% 
			      0 / 0       0.0% 
			      0 / 0       0.0% 
			      0 / 0       0.0% 
			      0 / 0       0.0% 
			     21 / 42     50.0% 
			     16 / 42     38.1%E 
			      2 / 42      4.8%C 
			      2 / 42      4.8%CE 
			      1 / 42      2.4%ABE 
		 
		 
			 1 
			  6.7 
			 Chroococcidiopsis 
			      1 / 43      2.3% 
			      1 / 1      100.0% 
			      0 / 1       0.0% 
			      0 / 1       0.0% 
			      0 / 3       0.0% 
			      0 / 3       0.0% 
			      0 / 2       0.0% 
			      0 / 1       0.0% 
			      0 / 1       0.0% 
			      0 / 3       0.0% 
			      0 / 1       0.0% 
			      0 / 4       0.0% 
			      0 / 1       0.0% 
			      0 / 1       0.0% 
			      0 / 1       0.0% 
			      0 / 1       0.0% 
			      0 / 3       0.0% 
			      0 / 1       0.0% 
			      0 / 3       0.0% 
			      0 / 4       0.0% 
			      0 / 1       0.0% 
			      0 / 1       0.0% 
			      0 / 5       0.0% 
			      0 / 0       0.0% 
			      0 / 0       0.0% 
			      0 / 0       0.0% 
			      0 / 0       0.0% 
			     23 / 43     53.5% 
			     16 / 43     37.2%E 
			      3 / 43      7.0%C 
			      1 / 43      2.3%AB 
		 
		 
			 1 
			  6.7 
			 Chroococcidiopsis thermalis 
			      1 / 43      2.3% 
			      1 / 1      100.0% 
			      0 / 1       0.0% 
			      0 / 1       0.0% 
			      0 / 3       0.0% 
			      0 / 3       0.0% 
			      0 / 2       0.0% 
			      0 / 1       0.0% 
			      0 / 1       0.0% 
			      0 / 3       0.0% 
			      0 / 1       0.0% 
			      0 / 4       0.0% 
			      0 / 1       0.0% 
			      0 / 1       0.0% 
			      0 / 1       0.0% 
			      0 / 1       0.0% 
			      0 / 3       0.0% 
			      0 / 1       0.0% 
			      0 / 3       0.0% 
			      0 / 4       0.0% 
			      0 / 1       0.0% 
			      0 / 1       0.0% 
			      0 / 5       0.0% 
			      0 / 0       0.0% 
			      0 / 0       0.0% 
			      0 / 0       0.0% 
			      0 / 0       0.0% 
			     23 / 43     53.5% 
			     16 / 43     37.2%E 
			      3 / 43      7.0%C 
			      1 / 43      2.3%AB 
		 
		 
			 1 
			  6.7 
			 Chroococcidiopsis sp. ATCC 27900 
			      1 / 43      2.3% 
			      1 / 1      100.0% 
			      0 / 1       0.0% 
			      0 / 1       0.0% 
			      0 / 3       0.0% 
			      0 / 3       0.0% 
			      0 / 2       0.0% 
			      0 / 1       0.0% 
			      0 / 1       0.0% 
			      0 / 3       0.0% 
			      0 / 1       0.0% 
			      0 / 4       0.0% 
			      0 / 1       0.0% 
			      0 / 1       0.0% 
			      0 / 1       0.0% 
			      0 / 1       0.0% 
			      0 / 3       0.0% 
			      0 / 1       0.0% 
			      0 / 3       0.0% 
			      0 / 4       0.0% 
			      0 / 1       0.0% 
			      0 / 1       0.0% 
			      0 / 5       0.0% 
			      0 / 0       0.0% 
			      0 / 0       0.0% 
			      0 / 0       0.0% 
			      0 / 0       0.0% 
			     23 / 43     53.5% 
			     16 / 43     37.2%E 
			      3 / 43      7.0%C 
			      1 / 43      2.3%AB 
		 
		 
			 1 
			  5.5 
			 Stanieria 
			      3 / 42      7.1% 
			      0 / 1       0.0% 
			      0 / 1       0.0% 
			      0 / 1       0.0% 
			      0 / 2       0.0% 
			      0 / 2       0.0% 
			      0 / 2       0.0% 
			      0 / 1       0.0% 
			      1 / 1      100.0% 
			      2 / 4      50.0% 
			      0 / 1       0.0% 
			      0 / 4       0.0% 
			      0 / 2       0.0% 
			      0 / 1       0.0% 
			      0 / 1       0.0% 
			      0 / 1       0.0% 
			      0 / 3       0.0% 
			      0 / 1       0.0% 
			      0 / 3       0.0% 
			      0 / 3       0.0% 
			      0 / 1       0.0% 
			      0 / 1       0.0% 
			      0 / 5       0.0% 
			      0 / 0       0.0% 
			      0 / 0       0.0% 
			      0 / 0       0.0% 
			      0 / 0       0.0% 
			     21 / 42     50.0%E 
			     16 / 42     38.1% 
			      2 / 42      4.8%AB 
			      2 / 42      4.8%C 
			      1 / 42      2.4%ABE 
		 
		 
			 1 
			  5.5 
			 PCC 7437 
			      3 / 42      7.1% 
			      0 / 1       0.0% 
			      0 / 1       0.0% 
			      0 / 1       0.0% 
			      0 / 2       0.0% 
			      0 / 2       0.0% 
			      0 / 2       0.0% 
			      0 / 1       0.0% 
			      1 / 1      100.0% 
			      2 / 4      50.0% 
			      0 / 1       0.0% 
			      0 / 4       0.0% 
			      0 / 2       0.0% 
			      0 / 1       0.0% 
			      0 / 1       0.0% 
			      0 / 1       0.0% 
			      0 / 3       0.0% 
			      0 / 1       0.0% 
			      0 / 3       0.0% 
			      0 / 3       0.0% 
			      0 / 1       0.0% 
			      0 / 1       0.0% 
			      0 / 5       0.0% 
			      0 / 0       0.0% 
			      0 / 0       0.0% 
			      0 / 0       0.0% 
			      0 / 0       0.0% 
			     21 / 42     50.0%E 
			     16 / 42     38.1% 
			      2 / 42      4.8%AB 
			      2 / 42      4.8%C 
			      1 / 42      2.4%ABE 
		 
		 
			 1 
			  5.5 
			 Chroococcidiopsis cyanosphaera 'Hindak 1965/25' 
			      3 / 42      7.1% 
			      0 / 1       0.0% 
			      0 / 1       0.0% 
			      0 / 1       0.0% 
			      0 / 2       0.0% 
			      0 / 2       0.0% 
			      0 / 2       0.0% 
			      0 / 1       0.0% 
			      1 / 1      100.0% 
			      2 / 4      50.0% 
			      0 / 1       0.0% 
			      0 / 4       0.0% 
			      0 / 2       0.0% 
			      0 / 1       0.0% 
			      0 / 1       0.0% 
			      0 / 1       0.0% 
			      0 / 3       0.0% 
			      0 / 1       0.0% 
			      0 / 3       0.0% 
			      0 / 3       0.0% 
			      0 / 1       0.0% 
			      0 / 1       0.0% 
			      0 / 5       0.0% 
			      0 / 0       0.0% 
			      0 / 0       0.0% 
			      0 / 0       0.0% 
			      0 / 0       0.0% 
			     21 / 42     50.0%E 
			     16 / 42     38.1% 
			      2 / 42      4.8%C 
			      2 / 42      4.8%AB 
			      1 / 42      2.4%ABE 
		 
		 
			 1 
			  4.7 
			 Gloeobacteria 
			      2 / 44      4.5% 
			      0 / 0       0.0% 
			      0 / 1       0.0% 
			      0 / 1       0.0% 
			      0 / 1       0.0% 
			      0 / 3       0.0% 
			      0 / 3       0.0% 
			      0 / 1       0.0% 
			      0 / 1       0.0% 
			      1 / 3      33.3% 
			      0 / 1       0.0% 
			      1 / 4      25.0% 
			      0 / 2       0.0% 
			      0 / 1       0.0% 
			      0 / 2       0.0% 
			      0 / 1       0.0% 
			      0 / 3       0.0% 
			      0 / 0       0.0% 
			      0 / 3       0.0% 
			      0 / 4       0.0% 
			      0 / 2       0.0% 
			      0 / 2       0.0% 
			      0 / 5       0.0% 
			      0 / 0       0.0% 
			      0 / 0       0.0% 
			      0 / 0       0.0% 
			      0 / 0       0.0% 
			     23 / 44     52.3% 
			     18 / 44     40.9%E 
			      1 / 44      2.3%ABE 
			      1 / 44      2.3%AB 
			      1 / 44      2.3%C 
		 
		 
			 1 
			  4.7 
			 Gloeobacterales 
			      2 / 44      4.5% 
			      0 / 0       0.0% 
			      0 / 1       0.0% 
			      0 / 1       0.0% 
			      0 / 1       0.0% 
			      0 / 3       0.0% 
			      0 / 3       0.0% 
			      0 / 1       0.0% 
			      0 / 1       0.0% 
			      1 / 3      33.3% 
			      0 / 1       0.0% 
			      1 / 4      25.0% 
			      0 / 2       0.0% 
			      0 / 1       0.0% 
			      0 / 2       0.0% 
			      0 / 1       0.0% 
			      0 / 3       0.0% 
			      0 / 0       0.0% 
			      0 / 3       0.0% 
			      0 / 4       0.0% 
			      0 / 2       0.0% 
			      0 / 2       0.0% 
			      0 / 5       0.0% 
			      0 / 0       0.0% 
			      0 / 0       0.0% 
			      0 / 0       0.0% 
			      0 / 0       0.0% 
			     23 / 44     52.3% 
			     18 / 44     40.9%E 
			      1 / 44      2.3%C 
			      1 / 44      2.3%AB 
			      1 / 44      2.3%ABE 
		 
		 
			 1 
			  4.7 
			 Gloeobacter 
			      2 / 44      4.5% 
			      0 / 0       0.0% 
			      0 / 1       0.0% 
			      0 / 1       0.0% 
			      0 / 1       0.0% 
			      0 / 3       0.0% 
			      0 / 3       0.0% 
			      0 / 1       0.0% 
			      0 / 1       0.0% 
			      1 / 3      33.3% 
			      0 / 1       0.0% 
			      1 / 4      25.0% 
			      0 / 2       0.0% 
			      0 / 1       0.0% 
			      0 / 2       0.0% 
			      0 / 1       0.0% 
			      0 / 3       0.0% 
			      0 / 0       0.0% 
			      0 / 3       0.0% 
			      0 / 4       0.0% 
			      0 / 2       0.0% 
			      0 / 2       0.0% 
			      0 / 5       0.0% 
			      0 / 0       0.0% 
			      0 / 0       0.0% 
			      0 / 0       0.0% 
			      0 / 0       0.0% 
			     23 / 44     52.3% 
			     18 / 44     40.9%E 
			      1 / 44      2.3%AB 
			      1 / 44      2.3%C 
			      1 / 44      2.3%ABE 
		 
		 
			 1 
			  4.7 
			 Gloeobacter violaceus 
			      2 / 44      4.5% 
			      0 / 0       0.0% 
			      0 / 1       0.0% 
			      0 / 1       0.0% 
			      0 / 1       0.0% 
			      0 / 3       0.0% 
			      0 / 3       0.0% 
			      0 / 1       0.0% 
			      0 / 1       0.0% 
			      1 / 3      33.3% 
			      0 / 1       0.0% 
			      1 / 4      25.0% 
			      0 / 2       0.0% 
			      0 / 1       0.0% 
			      0 / 2       0.0% 
			      0 / 1       0.0% 
			      0 / 3       0.0% 
			      0 / 0       0.0% 
			      0 / 3       0.0% 
			      0 / 4       0.0% 
			      0 / 2       0.0% 
			      0 / 2       0.0% 
			      0 / 5       0.0% 
			      0 / 0       0.0% 
			      0 / 0       0.0% 
			      0 / 0       0.0% 
			      0 / 0       0.0% 
			     23 / 44     52.3% 
			     18 / 44     40.9%E 
			      1 / 44      2.3%AB 
			      1 / 44      2.3%C 
			      1 / 44      2.3%ABE 
		 
		 
			 1 
			  4.7 
			 Gloeobacter violaceus ATCC 29082 
			      2 / 44      4.5% 
			      0 / 0       0.0% 
			      0 / 1       0.0% 
			      0 / 1       0.0% 
			      0 / 1       0.0% 
			      0 / 3       0.0% 
			      0 / 3       0.0% 
			      0 / 1       0.0% 
			      0 / 1       0.0% 
			      1 / 3      33.3% 
			      0 / 1       0.0% 
			      1 / 4      25.0% 
			      0 / 2       0.0% 
			      0 / 1       0.0% 
			      0 / 2       0.0% 
			      0 / 1       0.0% 
			      0 / 3       0.0% 
			      0 / 0       0.0% 
			      0 / 3       0.0% 
			      0 / 4       0.0% 
			      0 / 2       0.0% 
			      0 / 2       0.0% 
			      0 / 5       0.0% 
			      0 / 0       0.0% 
			      0 / 0       0.0% 
			      0 / 0       0.0% 
			      0 / 0       0.0% 
			     23 / 44     52.3% 
			     18 / 44     40.9%E 
			      1 / 44      2.3%AB 
			      1 / 44      2.3%C 
			      1 / 44      2.3%ABE 
		 
		 
			 42 
			  4.5 
			 Oscillatoriophycideae 
			     81 / 2026    4.0% 
			     21 / 40     52.5% 
			      1 / 53      1.9% 
			      4 / 50      8.0% 
			      4 / 94      4.3% 
			      1 / 135     0.7% 
			      2 / 118     1.7% 
			      0 / 45      0.0% 
			     12 / 46     26.1% 
			     19 / 164    11.6% 
			      2 / 48      4.2% 
			      1 / 190     0.5% 
			      0 / 63      0.0% 
			      0 / 47      0.0% 
			      1 / 51      2.0% 
			      1 / 50      2.0% 
			      0 / 136     0.0% 
			      0 / 32      0.0% 
			      0 / 140     0.0% 
			      2 / 177     1.1% 
			      3 / 50      6.0% 
			      1 / 58      1.7% 
			      6 / 231     2.6% 
			      0 / 0       0.0% 
			      0 / 0       0.0% 
			      0 / 7       0.0% 
			      0 / 1       0.0% 
			   1044 / 2026   51.5% 
			    821 / 2026   40.5%E 
			     52 / 2026    2.6%AB 
			     48 / 2026    2.4%C 
			     31 / 2026    1.5%CE 
			     25 / 2026    1.2%ABE 
			      3 / 2026    0.1%B 
			      1 / 2026    0.0%EGG 
			      1 / 2026    0.0%BE 
		 
		 
			 34 
			  4.0 
			 Chroococcales 
			     52 / 1614    3.2% 
			     14 / 32     43.8% 
			      1 / 41      2.4% 
			      2 / 39      5.1% 
			      3 / 79      3.8% 
			      0 / 107     0.0% 
			      0 / 95      0.0% 
			      0 / 37      0.0% 
			     10 / 37     27.0% 
			     14 / 132    10.6% 
			      0 / 38      0.0% 
			      0 / 151     0.0% 
			      0 / 47      0.0% 
			      0 / 37      0.0% 
			      0 / 39      0.0% 
			      1 / 40      2.5% 
			      0 / 110     0.0% 
			      0 / 29      0.0% 
			      0 / 112     0.0% 
			      2 / 145     1.4% 
			      1 / 39      2.6% 
			      0 / 45      0.0% 
			      4 / 183     2.2% 
			      0 / 0       0.0% 
			      0 / 0       0.0% 
			      0 / 0       0.0% 
			      0 / 0       0.0% 
			    858 / 1614   53.2% 
			    642 / 1614   39.8%E 
			     39 / 1614    2.4%C 
			     29 / 1614    1.8%AB 
			     22 / 1614    1.4%CE 
			     19 / 1614    1.2%ABE 
			      3 / 1614    0.2%B 
			      1 / 1614    0.1%EGG 
			      1 / 1614    0.1%BE 
		 
		 
			 1 
			  5.8 
			 Microcystis 
			      1 / 42      2.4% 
			      0 / 1       0.0% 
			      0 / 1       0.0% 
			      0 / 1       0.0% 
			      0 / 2       0.0% 
			      0 / 3       0.0% 
			      0 / 2       0.0% 
			      0 / 1       0.0% 
			      1 / 1      100.0% 
			      0 / 3       0.0% 
			      0 / 1       0.0% 
			      0 / 4       0.0% 
			      0 / 1       0.0% 
			      0 / 1       0.0% 
			      0 / 1       0.0% 
			      0 / 1       0.0% 
			      0 / 3       0.0% 
			      0 / 1       0.0% 
			      0 / 3       0.0% 
			      0 / 4       0.0% 
			      0 / 1       0.0% 
			      0 / 1       0.0% 
			      0 / 5       0.0% 
			      0 / 0       0.0% 
			      0 / 0       0.0% 
			      0 / 0       0.0% 
			      0 / 0       0.0% 
			     19 / 42     45.2% 
			     18 / 42     42.9%E 
			      3 / 42      7.1%C 
			      1 / 42      2.4%CE 
			      1 / 42      2.4%ABE 
		 
		 
			 1 
			  5.8 
			 IAM M-247 
			      1 / 42      2.4% 
			      0 / 1       0.0% 
			      0 / 1       0.0% 
			      0 / 1       0.0% 
			      0 / 2       0.0% 
			      0 / 3       0.0% 
			      0 / 2       0.0% 
			      0 / 1       0.0% 
			      1 / 1      100.0% 
			      0 / 3       0.0% 
			      0 / 1       0.0% 
			      0 / 4       0.0% 
			      0 / 1       0.0% 
			      0 / 1       0.0% 
			      0 / 1       0.0% 
			      0 / 1       0.0% 
			      0 / 3       0.0% 
			      0 / 1       0.0% 
			      0 / 3       0.0% 
			      0 / 4       0.0% 
			      0 / 1       0.0% 
			      0 / 1       0.0% 
			      0 / 5       0.0% 
			      0 / 0       0.0% 
			      0 / 0       0.0% 
			      0 / 0       0.0% 
			      0 / 0       0.0% 
			     19 / 42     45.2% 
			     18 / 42     42.9%E 
			      3 / 42      7.1%C 
			      1 / 42      2.4%CE 
			      1 / 42      2.4%ABE 
		 
		 
			 1 
			  5.8 
			 Microcystis aeruginosa NIES-843 
			      1 / 42      2.4% 
			      0 / 1       0.0% 
			      0 / 1       0.0% 
			      0 / 1       0.0% 
			      0 / 2       0.0% 
			      0 / 3       0.0% 
			      0 / 2       0.0% 
			      0 / 1       0.0% 
			      1 / 1      100.0% 
			      0 / 3       0.0% 
			      0 / 1       0.0% 
			      0 / 4       0.0% 
			      0 / 1       0.0% 
			      0 / 1       0.0% 
			      0 / 1       0.0% 
			      0 / 1       0.0% 
			      0 / 3       0.0% 
			      0 / 1       0.0% 
			      0 / 3       0.0% 
			      0 / 4       0.0% 
			      0 / 1       0.0% 
			      0 / 1       0.0% 
			      0 / 5       0.0% 
			      0 / 0       0.0% 
			      0 / 0       0.0% 
			      0 / 0       0.0% 
			      0 / 0       0.0% 
			     19 / 42     45.2% 
			     18 / 42     42.9%E 
			      3 / 42      7.1%C 
			      1 / 42      2.4%CE 
			      1 / 42      2.4%ABE 
		 
		 
			 13 
			  2.8 
			 Anacystis 
			     24 / 568     4.2% 
			      9 / 13     69.2% 
			      0 / 13      0.0% 
			      0 / 13      0.0% 
			      0 / 24      0.0% 
			      0 / 38      0.0% 
			      0 / 37      0.0% 
			      0 / 13      0.0% 
			      3 / 13     23.1% 
			     10 / 50     20.0% 
			      0 / 13      0.0% 
			      0 / 53      0.0% 
			      0 / 15      0.0% 
			      0 / 13      0.0% 
			      0 / 13      0.0% 
			      0 / 15      0.0% 
			      0 / 38      0.0% 
			      0 / 10      0.0% 
			      0 / 39      0.0% 
			      0 / 51      0.0% 
			      0 / 13      0.0% 
			      0 / 16      0.0% 
			      2 / 65      3.1% 
			      0 / 0       0.0% 
			      0 / 0       0.0% 
			      0 / 0       0.0% 
			      0 / 0       0.0% 
			    328 / 568    57.7% 
			    203 / 568    35.7%E 
			     18 / 568     3.2%AB 
			      8 / 568     1.4%CE 
			      5 / 568     0.9%C 
			      5 / 568     0.9%ABE 
			      1 / 568     0.2%B 
		 
		 
			 2 
			  2.7 
			 PCC 6301 
			      6 / 90      6.7% 
			      2 / 2      100.0% 
			      0 / 2       0.0% 
			      0 / 2       0.0% 
			      0 / 4       0.0% 
			      0 / 6       0.0% 
			      0 / 6       0.0% 
			      0 / 2       0.0% 
			      0 / 2       0.0% 
			      4 / 8      50.0% 
			      0 / 2       0.0% 
			      0 / 8       0.0% 
			      0 / 2       0.0% 
			      0 / 2       0.0% 
			      0 / 2       0.0% 
			      0 / 4       0.0% 
			      0 / 6       0.0% 
			      0 / 0       0.0% 
			      0 / 6       0.0% 
			      0 / 8       0.0% 
			      0 / 2       0.0% 
			      0 / 4       0.0% 
			      0 / 10      0.0% 
			      0 / 0       0.0% 
			      0 / 0       0.0% 
			      0 / 0       0.0% 
			      0 / 0       0.0% 
			     54 / 90     60.0% 
			     24 / 90     26.7%E 
			      6 / 90      6.7%AB 
			      4 / 90      4.4%C 
			      2 / 90      2.2%CE 
		 
		 
			 1 
			  2.7 
			 Anacystis nidulans R2 
			      3 / 45      6.7% 
			      1 / 1      100.0% 
			      0 / 1       0.0% 
			      0 / 1       0.0% 
			      0 / 2       0.0% 
			      0 / 3       0.0% 
			      0 / 3       0.0% 
			      0 / 1       0.0% 
			      0 / 1       0.0% 
			      2 / 4      50.0% 
			      0 / 1       0.0% 
			      0 / 4       0.0% 
			      0 / 1       0.0% 
			      0 / 1       0.0% 
			      0 / 1       0.0% 
			      0 / 2       0.0% 
			      0 / 3       0.0% 
			      0 / 0       0.0% 
			      0 / 3       0.0% 
			      0 / 4       0.0% 
			      0 / 1       0.0% 
			      0 / 2       0.0% 
			      0 / 5       0.0% 
			      0 / 0       0.0% 
			      0 / 0       0.0% 
			      0 / 0       0.0% 
			      0 / 0       0.0% 
			     27 / 45     60.0% 
			     12 / 45     26.7%E 
			      3 / 45      6.7%AB 
			      2 / 45      4.4%C 
			      1 / 45      2.2%CE 
		 
		 
			 1 
			  2.7 
			 Anacystis nidulans SAUG 1402-1 
			      3 / 45      6.7% 
			      1 / 1      100.0% 
			      0 / 1       0.0% 
			      0 / 1       0.0% 
			      0 / 2       0.0% 
			      0 / 3       0.0% 
			      0 / 3       0.0% 
			      0 / 1       0.0% 
			      0 / 1       0.0% 
			      2 / 4      50.0% 
			      0 / 1       0.0% 
			      0 / 4       0.0% 
			      0 / 1       0.0% 
			      0 / 1       0.0% 
			      0 / 1       0.0% 
			      0 / 2       0.0% 
			      0 / 3       0.0% 
			      0 / 0       0.0% 
			      0 / 3       0.0% 
			      0 / 4       0.0% 
			      0 / 1       0.0% 
			      0 / 2       0.0% 
			      0 / 5       0.0% 
			      0 / 0       0.0% 
			      0 / 0       0.0% 
			      0 / 0       0.0% 
			      0 / 0       0.0% 
			     27 / 45     60.0% 
			     12 / 45     26.7%E 
			      3 / 45      6.7%AB 
			      2 / 45      4.4%C 
			      1 / 45      2.2%CE 
		 
		 
			 1 
			  3.4 
			 Agmenellum quadruplicatum 
			      4 / 42      9.5% 
			      1 / 1      100.0% 
			      0 / 1       0.0% 
			      0 / 1       0.0% 
			      0 / 2       0.0% 
			      0 / 3       0.0% 
			      0 / 2       0.0% 
			      0 / 1       0.0% 
			      1 / 1      100.0% 
			      2 / 4      50.0% 
			      0 / 1       0.0% 
			      0 / 4       0.0% 
			      0 / 1       0.0% 
			      0 / 1       0.0% 
			      0 / 1       0.0% 
			      0 / 1       0.0% 
			      0 / 3       0.0% 
			      0 / 1       0.0% 
			      0 / 3       0.0% 
			      0 / 3       0.0% 
			      0 / 1       0.0% 
			      0 / 1       0.0% 
			      0 / 5       0.0% 
			      0 / 0       0.0% 
			      0 / 0       0.0% 
			      0 / 0       0.0% 
			      0 / 0       0.0% 
			     20 / 42     47.6%E 
			     17 / 42     40.5% 
			      2 / 42      4.8%AB 
			      1 / 42      2.4%B 
			      1 / 42      2.4%ABE 
			      1 / 42      2.4%CE 
		 
		 
			 1 
			  2.4 
			 Synechococcus WH7803 
			      1 / 44      2.3% 
			      1 / 1      100.0% 
			      0 / 1       0.0% 
			      0 / 1       0.0% 
			      0 / 2       0.0% 
			      0 / 3       0.0% 
			      0 / 3       0.0% 
			      0 / 1       0.0% 
			      0 / 1       0.0% 
			      0 / 4       0.0% 
			      0 / 1       0.0% 
			      0 / 4       0.0% 
			      0 / 1       0.0% 
			      0 / 1       0.0% 
			      0 / 1       0.0% 
			      0 / 1       0.0% 
			      0 / 3       0.0% 
			      0 / 1       0.0% 
			      0 / 3       0.0% 
			      0 / 4       0.0% 
			      0 / 1       0.0% 
			      0 / 1       0.0% 
			      0 / 5       0.0% 
			      0 / 0       0.0% 
			      0 / 0       0.0% 
			      0 / 0       0.0% 
			      0 / 0       0.0% 
			     28 / 44     63.6% 
			     15 / 44     34.1%E 
			      1 / 44      2.3%AB 
		 
		 
			 1 
			  2.6 
			 Synechococcus sp. CC9311 
			      1 / 44      2.3% 
			      1 / 1      100.0% 
			      0 / 1       0.0% 
			      0 / 1       0.0% 
			      0 / 2       0.0% 
			      0 / 3       0.0% 
			      0 / 3       0.0% 
			      0 / 1       0.0% 
			      0 / 1       0.0% 
			      0 / 4       0.0% 
			      0 / 1       0.0% 
			      0 / 4       0.0% 
			      0 / 1       0.0% 
			      0 / 1       0.0% 
			      0 / 1       0.0% 
			      0 / 1       0.0% 
			      0 / 3       0.0% 
			      0 / 1       0.0% 
			      0 / 3       0.0% 
			      0 / 4       0.0% 
			      0 / 1       0.0% 
			      0 / 1       0.0% 
			      0 / 5       0.0% 
			      0 / 0       0.0% 
			      0 / 0       0.0% 
			      0 / 0       0.0% 
			      0 / 0       0.0% 
			     29 / 44     65.9% 
			     14 / 44     31.8%E 
			      1 / 44      2.3%AB 
		 
		 
			 1 
			  2.4 
			 Synechococcus WH8102 
			      1 / 44      2.3% 
			      1 / 1      100.0% 
			      0 / 1       0.0% 
			      0 / 1       0.0% 
			      0 / 2       0.0% 
			      0 / 3       0.0% 
			      0 / 3       0.0% 
			      0 / 1       0.0% 
			      0 / 1       0.0% 
			      0 / 4       0.0% 
			      0 / 1       0.0% 
			      0 / 4       0.0% 
			      0 / 1       0.0% 
			      0 / 1       0.0% 
			      0 / 1       0.0% 
			      0 / 1       0.0% 
			      0 / 3       0.0% 
			      0 / 1       0.0% 
			      0 / 3       0.0% 
			      0 / 4       0.0% 
			      0 / 1       0.0% 
			      0 / 1       0.0% 
			      0 / 5       0.0% 
			      0 / 0       0.0% 
			      0 / 0       0.0% 
			      0 / 0       0.0% 
			      0 / 0       0.0% 
			     26 / 44     59.1% 
			     17 / 44     38.6%E 
			      1 / 44      2.3%AB 
		 
		 
			 1 
			  2.5 
			 Synechococcus sp. CC9605 
			      2 / 44      4.5% 
			      1 / 1      100.0% 
			      0 / 1       0.0% 
			      0 / 1       0.0% 
			      0 / 2       0.0% 
			      0 / 3       0.0% 
			      0 / 3       0.0% 
			      0 / 1       0.0% 
			      0 / 1       0.0% 
			      0 / 4       0.0% 
			      0 / 1       0.0% 
			      0 / 4       0.0% 
			      0 / 1       0.0% 
			      0 / 1       0.0% 
			      0 / 1       0.0% 
			      0 / 1       0.0% 
			      0 / 3       0.0% 
			      0 / 1       0.0% 
			      0 / 3       0.0% 
			      0 / 4       0.0% 
			      0 / 1       0.0% 
			      0 / 1       0.0% 
			      1 / 5      20.0% 
			      0 / 0       0.0% 
			      0 / 0       0.0% 
			      0 / 0       0.0% 
			      0 / 0       0.0% 
			     25 / 44     56.8% 
			     17 / 44     38.6%E 
			      1 / 44      2.3%ABE 
			      1 / 44      2.3%AB 
		 
		 
			 1 
			  3.7 
			 Synechococcus sp. ATCC 27167 
			      0 / 41      0.0% 
			      0 / 1       0.0% 
			      0 / 1       0.0% 
			      0 / 1       0.0% 
			      0 / 1       0.0% 
			      0 / 3       0.0% 
			      0 / 3       0.0% 
			      0 / 1       0.0% 
			      0 / 1       0.0% 
			      0 / 3       0.0% 
			      0 / 1       0.0% 
			      0 / 4       0.0% 
			      0 / 1       0.0% 
			      0 / 1       0.0% 
			      0 / 1       0.0% 
			      0 / 1       0.0% 
			      0 / 3       0.0% 
			      0 / 1       0.0% 
			      0 / 3       0.0% 
			      0 / 4       0.0% 
			      0 / 1       0.0% 
			      0 / 1       0.0% 
			      0 / 4       0.0% 
			      0 / 0       0.0% 
			      0 / 0       0.0% 
			      0 / 0       0.0% 
			      0 / 0       0.0% 
			     19 / 41     46.3%E 
			     18 / 41     43.9% 
			      4 / 41      9.8%CE 
		 
		 
			 1 
			  2.2 
			 Synechococcus sp. MINSyn016-15m-01 substr. AC6A1 
			      1 / 42      2.4% 
			      1 / 1      100.0% 
			      0 / 1       0.0% 
			      0 / 1       0.0% 
			      0 / 1       0.0% 
			      0 / 3       0.0% 
			      0 / 3       0.0% 
			      0 / 1       0.0% 
			      0 / 1       0.0% 
			      0 / 3       0.0% 
			      0 / 1       0.0% 
			      0 / 4       0.0% 
			      0 / 1       0.0% 
			      0 / 1       0.0% 
			      0 / 1       0.0% 
			      0 / 1       0.0% 
			      0 / 3       0.0% 
			      0 / 1       0.0% 
			      0 / 3       0.0% 
			      0 / 4       0.0% 
			      0 / 1       0.0% 
			      0 / 1       0.0% 
			      0 / 5       0.0% 
			      0 / 0       0.0% 
			      0 / 0       0.0% 
			      0 / 0       0.0% 
			      0 / 0       0.0% 
			     27 / 42     64.3% 
			     13 / 42     31.0%E 
			      1 / 42      2.4%AB 
			      1 / 42      2.4%C 
		 
		 
			 1 
			  2.2 
			 Synechococcus sp. CC9902 
			      1 / 44      2.3% 
			      1 / 1      100.0% 
			      0 / 1       0.0% 
			      0 / 1       0.0% 
			      0 / 2       0.0% 
			      0 / 3       0.0% 
			      0 / 3       0.0% 
			      0 / 1       0.0% 
			      0 / 1       0.0% 
			      0 / 4       0.0% 
			      0 / 1       0.0% 
			      0 / 4       0.0% 
			      0 / 1       0.0% 
			      0 / 1       0.0% 
			      0 / 1       0.0% 
			      0 / 1       0.0% 
			      0 / 3       0.0% 
			      0 / 1       0.0% 
			      0 / 3       0.0% 
			      0 / 4       0.0% 
			      0 / 1       0.0% 
			      0 / 1       0.0% 
			      0 / 5       0.0% 
			      0 / 0       0.0% 
			      0 / 0       0.0% 
			      0 / 0       0.0% 
			      0 / 0       0.0% 
			     26 / 44     59.1% 
			     16 / 44     36.4%E 
			      1 / 44      2.3%CE 
			      1 / 44      2.3%AB 
		 
		 
			 1 
			  2.9 
			 Cyanobacteria bacterium Yellowstone A' 
			      3 / 47      6.4% 
			      0 / 1       0.0% 
			      0 / 1       0.0% 
			      0 / 1       0.0% 
			      0 / 2       0.0% 
			      0 / 3       0.0% 
			      0 / 3       0.0% 
			      0 / 1       0.0% 
			      1 / 1      100.0% 
			      2 / 4      50.0% 
			      0 / 1       0.0% 
			      0 / 5       0.0% 
			      0 / 2       0.0% 
			      0 / 1       0.0% 
			      0 / 1       0.0% 
			      0 / 1       0.0% 
			      0 / 3       0.0% 
			      0 / 1       0.0% 
			      0 / 3       0.0% 
			      0 / 4       0.0% 
			      0 / 1       0.0% 
			      0 / 1       0.0% 
			      0 / 6       0.0% 
			      0 / 0       0.0% 
			      0 / 0       0.0% 
			      0 / 0       0.0% 
			      0 / 0       0.0% 
			     30 / 47     63.8% 
			     14 / 47     29.8%E 
			      2 / 47      4.3%AB 
			      1 / 47      2.1%ABE 
		 
		 
			 1 
			  3.0 
			 Cyanobacteria bacterium Yellowstone B' 
			      3 / 44      6.8% 
			      0 / 1       0.0% 
			      0 / 1       0.0% 
			      0 / 1       0.0% 
			      0 / 2       0.0% 
			      0 / 3       0.0% 
			      0 / 3       0.0% 
			      0 / 1       0.0% 
			      1 / 1      100.0% 
			      2 / 4      50.0% 
			      0 / 1       0.0% 
			      0 / 4       0.0% 
			      0 / 1       0.0% 
			      0 / 1       0.0% 
			      0 / 1       0.0% 
			      0 / 1       0.0% 
			      0 / 3       0.0% 
			      0 / 0       0.0% 
			      0 / 3       0.0% 
			      0 / 4       0.0% 
			      0 / 1       0.0% 
			      0 / 2       0.0% 
			      0 / 5       0.0% 
			      0 / 0       0.0% 
			      0 / 0       0.0% 
			      0 / 0       0.0% 
			      0 / 0       0.0% 
			     28 / 44     63.6% 
			     13 / 44     29.5%E 
			      2 / 44      4.5%AB 
			      1 / 44      2.3%ABE 
		 
		 
			 1 
			  3.6 
			 Synechococcus sp. PCC 7502 
			      1 / 42      2.4% 
			      0 / 1       0.0% 
			      0 / 1       0.0% 
			      0 / 1       0.0% 
			      0 / 2       0.0% 
			      0 / 2       0.0% 
			      0 / 2       0.0% 
			      0 / 1       0.0% 
			      0 / 1       0.0% 
			      0 / 4       0.0% 
			      0 / 1       0.0% 
			      0 / 4       0.0% 
			      0 / 2       0.0% 
			      0 / 1       0.0% 
			      0 / 1       0.0% 
			      0 / 1       0.0% 
			      0 / 2       0.0% 
			      0 / 1       0.0% 
			      0 / 3       0.0% 
			      0 / 4       0.0% 
			      0 / 1       0.0% 
			      0 / 1       0.0% 
			      1 / 5      20.0% 
			      0 / 0       0.0% 
			      0 / 0       0.0% 
			      0 / 0       0.0% 
			      0 / 0       0.0% 
			     21 / 42     50.0%E 
			     20 / 42     47.6% 
			      1 / 42      2.4%ABE 
		 
		 
			 4 
			  3.7 
			 Synechococystis 
			      0 / 247     0.0% 
			      0 / 1       0.0% 
			      0 / 6       0.0% 
			      0 / 6       0.0% 
			      0 / 12      0.0% 
			      0 / 18      0.0% 
			      0 / 12      0.0% 
			      0 / 6       0.0% 
			      0 / 6       0.0% 
			      0 / 18      0.0% 
			      0 / 6       0.0% 
			      0 / 24      0.0% 
			      0 / 6       0.0% 
			      0 / 6       0.0% 
			      0 / 6       0.0% 
			      0 / 6       0.0% 
			      0 / 18      0.0% 
			      0 / 6       0.0% 
			      0 / 18      0.0% 
			      0 / 24      0.0% 
			      0 / 6       0.0% 
			      0 / 6       0.0% 
			      0 / 30      0.0% 
			      0 / 0       0.0% 
			      0 / 0       0.0% 
			      0 / 0       0.0% 
			      0 / 0       0.0% 
			    137 / 247    55.5% 
			     92 / 247    37.2%E 
			     12 / 247     4.9%C 
			      5 / 247     2.0%CE 
			      1 / 247     0.4%EGG 
		 
		 
			 4 
			  3.7 
			 Aphanocapsa sp. (strain N-1) 
			      0 / 247     0.0% 
			      0 / 1       0.0% 
			      0 / 6       0.0% 
			      0 / 6       0.0% 
			      0 / 12      0.0% 
			      0 / 18      0.0% 
			      0 / 12      0.0% 
			      0 / 6       0.0% 
			      0 / 6       0.0% 
			      0 / 18      0.0% 
			      0 / 6       0.0% 
			      0 / 24      0.0% 
			      0 / 6       0.0% 
			      0 / 6       0.0% 
			      0 / 6       0.0% 
			      0 / 6       0.0% 
			      0 / 18      0.0% 
			      0 / 6       0.0% 
			      0 / 18      0.0% 
			      0 / 24      0.0% 
			      0 / 6       0.0% 
			      0 / 6       0.0% 
			      0 / 30      0.0% 
			      0 / 0       0.0% 
			      0 / 0       0.0% 
			      0 / 0       0.0% 
			      0 / 0       0.0% 
			    137 / 247    55.5% 
			     92 / 247    37.2%E 
			     12 / 247     4.9%C 
			      5 / 247     2.0%CE 
			      1 / 247     0.4%EGG 
		 
		 
			 1 
			  3.6 
			 Synechocystis sp. PCC 6803 substr. GT-I 
			      0 / 41      0.0% 
			      0 / 0       0.0% 
			      0 / 1       0.0% 
			      0 / 1       0.0% 
			      0 / 2       0.0% 
			      0 / 3       0.0% 
			      0 / 2       0.0% 
			      0 / 1       0.0% 
			      0 / 1       0.0% 
			      0 / 3       0.0% 
			      0 / 1       0.0% 
			      0 / 4       0.0% 
			      0 / 1       0.0% 
			      0 / 1       0.0% 
			      0 / 1       0.0% 
			      0 / 1       0.0% 
			      0 / 3       0.0% 
			      0 / 1       0.0% 
			      0 / 3       0.0% 
			      0 / 4       0.0% 
			      0 / 1       0.0% 
			      0 / 1       0.0% 
			      0 / 5       0.0% 
			      0 / 0       0.0% 
			      0 / 0       0.0% 
			      0 / 0       0.0% 
			      0 / 0       0.0% 
			     22 / 41     53.7% 
			     16 / 41     39.0%E 
			      2 / 41      4.9%C 
			      1 / 41      2.4%CE 
		 
		 
			 1 
			  3.6 
			 Synechocystis sp. PCC 6803 substr. PCC-N 
			      0 / 41      0.0% 
			      0 / 0       0.0% 
			      0 / 1       0.0% 
			      0 / 1       0.0% 
			      0 / 2       0.0% 
			      0 / 3       0.0% 
			      0 / 2       0.0% 
			      0 / 1       0.0% 
			      0 / 1       0.0% 
			      0 / 3       0.0% 
			      0 / 1       0.0% 
			      0 / 4       0.0% 
			      0 / 1       0.0% 
			      0 / 1       0.0% 
			      0 / 1       0.0% 
			      0 / 1       0.0% 
			      0 / 3       0.0% 
			      0 / 1       0.0% 
			      0 / 3       0.0% 
			      0 / 4       0.0% 
			      0 / 1       0.0% 
			      0 / 1       0.0% 
			      0 / 5       0.0% 
			      0 / 0       0.0% 
			      0 / 0       0.0% 
			      0 / 0       0.0% 
			      0 / 0       0.0% 
			     22 / 41     53.7% 
			     16 / 41     39.0%E 
			      2 / 41      4.9%C 
			      1 / 41      2.4%CE 
		 
		 
			 1 
			  3.6 
			 Synechocystis sp. PCC 6803 substr. PCC-P 
			      0 / 41      0.0% 
			      0 / 0       0.0% 
			      0 / 1       0.0% 
			      0 / 1       0.0% 
			      0 / 2       0.0% 
			      0 / 3       0.0% 
			      0 / 2       0.0% 
			      0 / 1       0.0% 
			      0 / 1       0.0% 
			      0 / 3       0.0% 
			      0 / 1       0.0% 
			      0 / 4       0.0% 
			      0 / 1       0.0% 
			      0 / 1       0.0% 
			      0 / 1       0.0% 
			      0 / 1       0.0% 
			      0 / 3       0.0% 
			      0 / 1       0.0% 
			      0 / 3       0.0% 
			      0 / 4       0.0% 
			      0 / 1       0.0% 
			      0 / 1       0.0% 
			      0 / 5       0.0% 
			      0 / 0       0.0% 
			      0 / 0       0.0% 
			      0 / 0       0.0% 
			      0 / 0       0.0% 
			     22 / 41     53.7% 
			     16 / 41     39.0%E 
			      2 / 41      4.9%C 
			      1 / 41      2.4%CE 
		 
		 
			 1 
			  3.8 
			 Dactylococcopsis 
			      0 / 42      0.0% 
			      0 / 1       0.0% 
			      0 / 1       0.0% 
			      0 / 1       0.0% 
			      0 / 2       0.0% 
			      0 / 2       0.0% 
			      0 / 2       0.0% 
			      0 / 1       0.0% 
			      0 / 1       0.0% 
			      0 / 4       0.0% 
			      0 / 1       0.0% 
			      0 / 4       0.0% 
			      0 / 1       0.0% 
			      0 / 1       0.0% 
			      0 / 1       0.0% 
			      0 / 1       0.0% 
			      0 / 3       0.0% 
			      0 / 0       0.0% 
			      0 / 3       0.0% 
			      0 / 4       0.0% 
			      0 / 1       0.0% 
			      0 / 2       0.0% 
			      0 / 5       0.0% 
			      0 / 0       0.0% 
			      0 / 0       0.0% 
			      0 / 0       0.0% 
			      0 / 0       0.0% 
			     22 / 42     52.4% 
			     20 / 42     47.6%E 
		 
		 
			 1 
			  3.8 
			 Dactylococcopsis salina 
			      0 / 42      0.0% 
			      0 / 1       0.0% 
			      0 / 1       0.0% 
			      0 / 1       0.0% 
			      0 / 2       0.0% 
			      0 / 2       0.0% 
			      0 / 2       0.0% 
			      0 / 1       0.0% 
			      0 / 1       0.0% 
			      0 / 4       0.0% 
			      0 / 1       0.0% 
			      0 / 4       0.0% 
			      0 / 1       0.0% 
			      0 / 1       0.0% 
			      0 / 1       0.0% 
			      0 / 1       0.0% 
			      0 / 3       0.0% 
			      0 / 0       0.0% 
			      0 / 3       0.0% 
			      0 / 4       0.0% 
			      0 / 1       0.0% 
			      0 / 2       0.0% 
			      0 / 5       0.0% 
			      0 / 0       0.0% 
			      0 / 0       0.0% 
			      0 / 0       0.0% 
			      0 / 0       0.0% 
			     22 / 42     52.4% 
			     20 / 42     47.6%E 
		 
		 
			 1 
			  3.8 
			 Dactylococcopsis PCC 8305 
			      0 / 42      0.0% 
			      0 / 1       0.0% 
			      0 / 1       0.0% 
			      0 / 1       0.0% 
			      0 / 2       0.0% 
			      0 / 2       0.0% 
			      0 / 2       0.0% 
			      0 / 1       0.0% 
			      0 / 1       0.0% 
			      0 / 4       0.0% 
			      0 / 1       0.0% 
			      0 / 4       0.0% 
			      0 / 1       0.0% 
			      0 / 1       0.0% 
			      0 / 1       0.0% 
			      0 / 1       0.0% 
			      0 / 3       0.0% 
			      0 / 0       0.0% 
			      0 / 3       0.0% 
			      0 / 4       0.0% 
			      0 / 1       0.0% 
			      0 / 2       0.0% 
			      0 / 5       0.0% 
			      0 / 0       0.0% 
			      0 / 0       0.0% 
			      0 / 0       0.0% 
			      0 / 0       0.0% 
			     22 / 42     52.4% 
			     20 / 42     47.6%E 
		 
		 
			 6 
			  5.9 
			 Cyanothece 
			     10 / 305     3.3% 
			      1 / 7      14.3% 
			      0 / 7       0.0% 
			      0 / 7       0.0% 
			      3 / 17     17.6% 
			      0 / 21      0.0% 
			      0 / 19      0.0% 
			      0 / 7       0.0% 
			      6 / 7      85.7% 
			      0 / 22      0.0% 
			      0 / 7       0.0% 
			      0 / 28      0.0% 
			      0 / 13      0.0% 
			      0 / 7       0.0% 
			      0 / 7       0.0% 
			      0 / 7       0.0% 
			      0 / 22      0.0% 
			      0 / 7       0.0% 
			      0 / 21      0.0% 
			      0 / 24      0.0% 
			      0 / 7       0.0% 
			      0 / 8       0.0% 
			      0 / 33      0.0% 
			      0 / 0       0.0% 
			      0 / 0       0.0% 
			      0 / 0       0.0% 
			      0 / 0       0.0% 
			    158 / 305    51.8% 
			    119 / 305    39.0%E 
			     16 / 305     5.2%C 
			      5 / 305     1.6%ABE 
			      4 / 305     1.3%AB 
			      2 / 305     0.7%CE 
			      1 / 305     0.3%BE 
		 
		 
			 1 
			  4.8 
			 Cyanothece PCC 8801 
			      1 / 42      2.4% 
			      0 / 1       0.0% 
			      0 / 1       0.0% 
			      0 / 1       0.0% 
			      0 / 2       0.0% 
			      0 / 3       0.0% 
			      0 / 3       0.0% 
			      0 / 1       0.0% 
			      1 / 1      100.0% 
			      0 / 3       0.0% 
			      0 / 1       0.0% 
			      0 / 4       0.0% 
			      0 / 2       0.0% 
			      0 / 1       0.0% 
			      0 / 1       0.0% 
			      0 / 1       0.0% 
			      0 / 3       0.0% 
			      0 / 1       0.0% 
			      0 / 3       0.0% 
			      0 / 3       0.0% 
			      0 / 1       0.0% 
			      0 / 1       0.0% 
			      0 / 4       0.0% 
			      0 / 0       0.0% 
			      0 / 0       0.0% 
			      0 / 0       0.0% 
			      0 / 0       0.0% 
			     20 / 42     47.6% 
			     19 / 42     45.2%E 
			      2 / 42      4.8%C 
			      1 / 42      2.4%ABE 
		 
		 
			 1 
			  5.5 
			 Cyanothece (strain ATCC 51142) 
			      1 / 42      2.4% 
			      0 / 1       0.0% 
			      0 / 1       0.0% 
			      0 / 1       0.0% 
			      0 / 2       0.0% 
			      0 / 3       0.0% 
			      0 / 2       0.0% 
			      0 / 1       0.0% 
			      1 / 1      100.0% 
			      0 / 3       0.0% 
			      0 / 1       0.0% 
			      0 / 4       0.0% 
			      0 / 1       0.0% 
			      0 / 1       0.0% 
			      0 / 1       0.0% 
			      0 / 1       0.0% 
			      0 / 3       0.0% 
			      0 / 2       0.0% 
			      0 / 3       0.0% 
			      0 / 3       0.0% 
			      0 / 1       0.0% 
			      0 / 1       0.0% 
			      0 / 5       0.0% 
			      0 / 0       0.0% 
			      0 / 0       0.0% 
			      0 / 0       0.0% 
			      0 / 0       0.0% 
			     20 / 42     47.6%E 
			     19 / 42     45.2% 
			      2 / 42      4.8%C 
			      1 / 42      2.4%BE 
		 
		 
			 1 
			  6.6 
			 Cyanothece PCC 7424 
			      2 / 43      4.7% 
			      0 / 1       0.0% 
			      0 / 1       0.0% 
			      0 / 1       0.0% 
			      1 / 3      33.3% 
			      0 / 3       0.0% 
			      0 / 2       0.0% 
			      0 / 1       0.0% 
			      1 / 1      100.0% 
			      0 / 3       0.0% 
			      0 / 1       0.0% 
			      0 / 4       0.0% 
			      0 / 2       0.0% 
			      0 / 1       0.0% 
			      0 / 1       0.0% 
			      0 / 1       0.0% 
			      0 / 3       0.0% 
			      0 / 1       0.0% 
			      0 / 3       0.0% 
			      0 / 3       0.0% 
			      0 / 1       0.0% 
			      0 / 1       0.0% 
			      0 / 5       0.0% 
			      0 / 0       0.0% 
			      0 / 0       0.0% 
			      0 / 0       0.0% 
			      0 / 0       0.0% 
			     23 / 43     53.5% 
			     16 / 43     37.2%E 
			      2 / 43      4.7%C 
			      1 / 43      2.3%ABE 
			      1 / 43      2.3%AB 
		 
		 
			 1 
			  5.8 
			 Cyanothece sp. ATCC 29141 
			      3 / 46      6.5% 
			      1 / 1      100.0% 
			      0 / 1       0.0% 
			      0 / 1       0.0% 
			      2 / 2      100.0% 
			      0 / 3       0.0% 
			      0 / 3       0.0% 
			      0 / 1       0.0% 
			      0 / 1       0.0% 
			      0 / 4       0.0% 
			      0 / 1       0.0% 
			      0 / 4       0.0% 
			      0 / 2       0.0% 
			      0 / 1       0.0% 
			      0 / 1       0.0% 
			      0 / 1       0.0% 
			      0 / 4       0.0% 
			      0 / 0       0.0% 
			      0 / 3       0.0% 
			      0 / 4       0.0% 
			      0 / 1       0.0% 
			      0 / 2       0.0% 
			      0 / 5       0.0% 
			      0 / 0       0.0% 
			      0 / 0       0.0% 
			      0 / 0       0.0% 
			      0 / 0       0.0% 
			     26 / 46     56.5% 
			     15 / 46     32.6%E 
			      3 / 46      6.5%AB 
			      2 / 46      4.3%CE 
		 
		 
			 1 
			  4.8 
			 Cyanothece sp. PCC 8802 
			      1 / 42      2.4% 
			      0 / 1       0.0% 
			      0 / 1       0.0% 
			      0 / 1       0.0% 
			      0 / 2       0.0% 
			      0 / 3       0.0% 
			      0 / 3       0.0% 
			      0 / 1       0.0% 
			      1 / 1      100.0% 
			      0 / 3       0.0% 
			      0 / 1       0.0% 
			      0 / 4       0.0% 
			      0 / 2       0.0% 
			      0 / 1       0.0% 
			      0 / 1       0.0% 
			      0 / 1       0.0% 
			      0 / 3       0.0% 
			      0 / 1       0.0% 
			      0 / 3       0.0% 
			      0 / 3       0.0% 
			      0 / 1       0.0% 
			      0 / 1       0.0% 
			      0 / 4       0.0% 
			      0 / 0       0.0% 
			      0 / 0       0.0% 
			      0 / 0       0.0% 
			      0 / 0       0.0% 
			     20 / 42     47.6% 
			     19 / 42     45.2%E 
			      2 / 42      4.8%C 
			      1 / 42      2.4%ABE 
		 
		 
			 1 
			  7.8 
			 Cyanothece sp. PCC 7822 
			      2 / 90      2.2% 
			      0 / 2       0.0% 
			      0 / 2       0.0% 
			      0 / 2       0.0% 
			      0 / 6       0.0% 
			      0 / 6       0.0% 
			      0 / 6       0.0% 
			      0 / 2       0.0% 
			      2 / 2      100.0% 
			      0 / 6       0.0% 
			      0 / 2       0.0% 
			      0 / 8       0.0% 
			      0 / 4       0.0% 
			      0 / 2       0.0% 
			      0 / 2       0.0% 
			      0 / 2       0.0% 
			      0 / 6       0.0% 
			      0 / 2       0.0% 
			      0 / 6       0.0% 
			      0 / 8       0.0% 
			      0 / 2       0.0% 
			      0 / 2       0.0% 
			      0 / 10      0.0% 
			      0 / 0       0.0% 
			      0 / 0       0.0% 
			      0 / 0       0.0% 
			      0 / 0       0.0% 
			     50 / 90     55.6% 
			     30 / 90     33.3%E 
			      8 / 90      8.9%C 
			      2 / 90      2.2%ABE 
		 
		 
			 1 
			  4.2 
			 Halothece cluster 
			      0 / 45      0.0% 
			      0 / 1       0.0% 
			      0 / 2       0.0% 
			      0 / 1       0.0% 
			      0 / 3       0.0% 
			      0 / 3       0.0% 
			      0 / 2       0.0% 
			      0 / 1       0.0% 
			      0 / 1       0.0% 
			      0 / 4       0.0% 
			      0 / 1       0.0% 
			      0 / 4       0.0% 
			      0 / 1       0.0% 
			      0 / 1       0.0% 
			      0 / 1       0.0% 
			      0 / 1       0.0% 
			      0 / 3       0.0% 
			      0 / 0       0.0% 
			      0 / 3       0.0% 
			      0 / 4       0.0% 
			      0 / 1       0.0% 
			      0 / 2       0.0% 
			      0 / 5       0.0% 
			      0 / 0       0.0% 
			      0 / 0       0.0% 
			      0 / 0       0.0% 
			      0 / 0       0.0% 
			     24 / 45     53.3%E 
			     21 / 45     46.7% 
		 
		 
			 1 
			  4.2 
			 Halothece 
			      0 / 45      0.0% 
			      0 / 1       0.0% 
			      0 / 2       0.0% 
			      0 / 1       0.0% 
			      0 / 3       0.0% 
			      0 / 3       0.0% 
			      0 / 2       0.0% 
			      0 / 1       0.0% 
			      0 / 1       0.0% 
			      0 / 4       0.0% 
			      0 / 1       0.0% 
			      0 / 4       0.0% 
			      0 / 1       0.0% 
			      0 / 1       0.0% 
			      0 / 1       0.0% 
			      0 / 1       0.0% 
			      0 / 3       0.0% 
			      0 / 0       0.0% 
			      0 / 3       0.0% 
			      0 / 4       0.0% 
			      0 / 1       0.0% 
			      0 / 2       0.0% 
			      0 / 5       0.0% 
			      0 / 0       0.0% 
			      0 / 0       0.0% 
			      0 / 0       0.0% 
			      0 / 0       0.0% 
			     24 / 45     53.3%E 
			     21 / 45     46.7% 
		 
		 
			 1 
			  4.2 
			 Aphanothece halophytica 'Solar Lake' 
			      0 / 45      0.0% 
			      0 / 1       0.0% 
			      0 / 2       0.0% 
			      0 / 1       0.0% 
			      0 / 3       0.0% 
			      0 / 3       0.0% 
			      0 / 2       0.0% 
			      0 / 1       0.0% 
			      0 / 1       0.0% 
			      0 / 4       0.0% 
			      0 / 1       0.0% 
			      0 / 4       0.0% 
			      0 / 1       0.0% 
			      0 / 1       0.0% 
			      0 / 1       0.0% 
			      0 / 1       0.0% 
			      0 / 3       0.0% 
			      0 / 0       0.0% 
			      0 / 3       0.0% 
			      0 / 4       0.0% 
			      0 / 1       0.0% 
			      0 / 2       0.0% 
			      0 / 5       0.0% 
			      0 / 0       0.0% 
			      0 / 0       0.0% 
			      0 / 0       0.0% 
			      0 / 0       0.0% 
			     24 / 45     53.3%E 
			     21 / 45     46.7% 
		 
		 
			 1 
			  5.9 
			 Bichatia 
			      1 / 41      2.4% 
			      1 / 1      100.0% 
			      0 / 1       0.0% 
			      0 / 1       0.0% 
			      0 / 2       0.0% 
			      0 / 3       0.0% 
			      0 / 3       0.0% 
			      0 / 1       0.0% 
			      0 / 1       0.0% 
			      0 / 3       0.0% 
			      0 / 1       0.0% 
			      0 / 4       0.0% 
			      0 / 1       0.0% 
			      0 / 1       0.0% 
			      0 / 1       0.0% 
			      0 / 1       0.0% 
			      0 / 3       0.0% 
			      0 / 1       0.0% 
			      0 / 3       0.0% 
			      0 / 3       0.0% 
			      0 / 1       0.0% 
			      0 / 1       0.0% 
			      0 / 4       0.0% 
			      0 / 0       0.0% 
			      0 / 0       0.0% 
			      0 / 0       0.0% 
			      0 / 0       0.0% 
			     20 / 41     48.8% 
			     16 / 41     39.0%E 
			      2 / 41      4.9%C 
			      2 / 41      4.9%CE 
			      1 / 41      2.4%AB 
		 
		 
			 1 
			  5.9 
			 Chroococcus sp. Cey un 
			      1 / 41      2.4% 
			      1 / 1      100.0% 
			      0 / 1       0.0% 
			      0 / 1       0.0% 
			      0 / 2       0.0% 
			      0 / 3       0.0% 
			      0 / 3       0.0% 
			      0 / 1       0.0% 
			      0 / 1       0.0% 
			      0 / 3       0.0% 
			      0 / 1       0.0% 
			      0 / 4       0.0% 
			      0 / 1       0.0% 
			      0 / 1       0.0% 
			      0 / 1       0.0% 
			      0 / 1       0.0% 
			      0 / 3       0.0% 
			      0 / 1       0.0% 
			      0 / 3       0.0% 
			      0 / 3       0.0% 
			      0 / 1       0.0% 
			      0 / 1       0.0% 
			      0 / 4       0.0% 
			      0 / 0       0.0% 
			      0 / 0       0.0% 
			      0 / 0       0.0% 
			      0 / 0       0.0% 
			     20 / 41     48.8% 
			     16 / 41     39.0%E 
			      2 / 41      4.9%CE 
			      2 / 41      4.9%C 
			      1 / 41      2.4%AB 
		 
		 
			 2 
			  3.7 
			 Cyanobacterium 
			      1 / 86      1.2% 
			      0 / 2       0.0% 
			      0 / 2       0.0% 
			      0 / 2       0.0% 
			      0 / 6       0.0% 
			      0 / 4       0.0% 
			      0 / 4       0.0% 
			      0 / 2       0.0% 
			      0 / 2       0.0% 
			      0 / 10      0.0% 
			      0 / 2       0.0% 
			      0 / 8       0.0% 
			      0 / 2       0.0% 
			      0 / 2       0.0% 
			      0 / 2       0.0% 
			      0 / 2       0.0% 
			      0 / 5       0.0% 
			      0 / 2       0.0% 
			      0 / 6       0.0% 
			      1 / 8      12.5% 
			      0 / 2       0.0% 
			      0 / 2       0.0% 
			      0 / 9       0.0% 
			      0 / 0       0.0% 
			      0 / 0       0.0% 
			      0 / 0       0.0% 
			      0 / 0       0.0% 
			     45 / 86     52.3%E 
			     40 / 86     46.5% 
			      1 / 86      1.2%ABE 
		 
		 
			 1 
			  3.2 
			 Cyanobacterium stanieri 
			      0 / 43      0.0% 
			      0 / 1       0.0% 
			      0 / 1       0.0% 
			      0 / 1       0.0% 
			      0 / 3       0.0% 
			      0 / 2       0.0% 
			      0 / 2       0.0% 
			      0 / 1       0.0% 
			      0 / 1       0.0% 
			      0 / 5       0.0% 
			      0 / 1       0.0% 
			      0 / 4       0.0% 
			      0 / 1       0.0% 
			      0 / 1       0.0% 
			      0 / 1       0.0% 
			      0 / 1       0.0% 
			      0 / 2       0.0% 
			      0 / 1       0.0% 
			      0 / 3       0.0% 
			      0 / 4       0.0% 
			      0 / 1       0.0% 
			      0 / 1       0.0% 
			      0 / 5       0.0% 
			      0 / 0       0.0% 
			      0 / 0       0.0% 
			      0 / 0       0.0% 
			      0 / 0       0.0% 
			     25 / 43     58.1%E 
			     18 / 43     41.9% 
		 
		 
			 1 
			  3.2 
			 Cyanobacterium stanieri PCC 7202 
			      0 / 43      0.0% 
			      0 / 1       0.0% 
			      0 / 1       0.0% 
			      0 / 1       0.0% 
			      0 / 3       0.0% 
			      0 / 2       0.0% 
			      0 / 2       0.0% 
			      0 / 1       0.0% 
			      0 / 1       0.0% 
			      0 / 5       0.0% 
			      0 / 1       0.0% 
			      0 / 4       0.0% 
			      0 / 1       0.0% 
			      0 / 1       0.0% 
			      0 / 1       0.0% 
			      0 / 1       0.0% 
			      0 / 2       0.0% 
			      0 / 1       0.0% 
			      0 / 3       0.0% 
			      0 / 4       0.0% 
			      0 / 1       0.0% 
			      0 / 1       0.0% 
			      0 / 5       0.0% 
			      0 / 0       0.0% 
			      0 / 0       0.0% 
			      0 / 0       0.0% 
			      0 / 0       0.0% 
			     25 / 43     58.1%E 
			     18 / 43     41.9% 
		 
		 
			 1 
			  4.2 
			 Cyanobacterium aponinum 
			      1 / 43      2.3% 
			      0 / 1       0.0% 
			      0 / 1       0.0% 
			      0 / 1       0.0% 
			      0 / 3       0.0% 
			      0 / 2       0.0% 
			      0 / 2       0.0% 
			      0 / 1       0.0% 
			      0 / 1       0.0% 
			      0 / 5       0.0% 
			      0 / 1       0.0% 
			      0 / 4       0.0% 
			      0 / 1       0.0% 
			      0 / 1       0.0% 
			      0 / 1       0.0% 
			      0 / 1       0.0% 
			      0 / 3       0.0% 
			      0 / 1       0.0% 
			      0 / 3       0.0% 
			      1 / 4      25.0% 
			      0 / 1       0.0% 
			      0 / 1       0.0% 
			      0 / 4       0.0% 
			      0 / 0       0.0% 
			      0 / 0       0.0% 
			      0 / 0       0.0% 
			      0 / 0       0.0% 
			     22 / 43     51.2% 
			     20 / 43     46.5%E 
			      1 / 43      2.3%ABE 
		 
		 
			 1 
			  4.2 
			 Cyanobacterium aponinum PCC 10605 
			      1 / 43      2.3% 
			      0 / 1       0.0% 
			      0 / 1       0.0% 
			      0 / 1       0.0% 
			      0 / 3       0.0% 
			      0 / 2       0.0% 
			      0 / 2       0.0% 
			      0 / 1       0.0% 
			      0 / 1       0.0% 
			      0 / 5       0.0% 
			      0 / 1       0.0% 
			      0 / 4       0.0% 
			      0 / 1       0.0% 
			      0 / 1       0.0% 
			      0 / 1       0.0% 
			      0 / 1       0.0% 
			      0 / 3       0.0% 
			      0 / 1       0.0% 
			      0 / 3       0.0% 
			      1 / 4      25.0% 
			      0 / 1       0.0% 
			      0 / 1       0.0% 
			      0 / 4       0.0% 
			      0 / 0       0.0% 
			      0 / 0       0.0% 
			      0 / 0       0.0% 
			      0 / 0       0.0% 
			     22 / 43     51.2% 
			     20 / 43     46.5%E 
			      1 / 43      2.3%ABE 
		 
		 
			 1 
			  2.6 
			 Thermosynechococcus 
			      1 / 41      2.4% 
			      0 / 1       0.0% 
			      0 / 1       0.0% 
			      0 / 1       0.0% 
			      0 / 1       0.0% 
			      0 / 3       0.0% 
			      0 / 3       0.0% 
			      0 / 1       0.0% 
			      0 / 1       0.0% 
			      0 / 3       0.0% 
			      0 / 1       0.0% 
			      0 / 4       0.0% 
			      0 / 1       0.0% 
			      0 / 1       0.0% 
			      0 / 1       0.0% 
			      0 / 1       0.0% 
			      0 / 3       0.0% 
			      0 / 0       0.0% 
			      0 / 3       0.0% 
			      0 / 4       0.0% 
			      0 / 1       0.0% 
			      0 / 1       0.0% 
			      1 / 5      20.0% 
			      0 / 0       0.0% 
			      0 / 0       0.0% 
			      0 / 0       0.0% 
			      0 / 0       0.0% 
			     27 / 41     65.9% 
			     13 / 41     31.7%E 
			      1 / 41      2.4%ABE 
		 
		 
			 1 
			  2.6 
			 Thermosynechococcus elongatus 
			      1 / 41      2.4% 
			      0 / 1       0.0% 
			      0 / 1       0.0% 
			      0 / 1       0.0% 
			      0 / 1       0.0% 
			      0 / 3       0.0% 
			      0 / 3       0.0% 
			      0 / 1       0.0% 
			      0 / 1       0.0% 
			      0 / 3       0.0% 
			      0 / 1       0.0% 
			      0 / 4       0.0% 
			      0 / 1       0.0% 
			      0 / 1       0.0% 
			      0 / 1       0.0% 
			      0 / 1       0.0% 
			      0 / 3       0.0% 
			      0 / 0       0.0% 
			      0 / 3       0.0% 
			      0 / 4       0.0% 
			      0 / 1       0.0% 
			      0 / 1       0.0% 
			      1 / 5      20.0% 
			      0 / 0       0.0% 
			      0 / 0       0.0% 
			      0 / 0       0.0% 
			      0 / 0       0.0% 
			     27 / 41     65.9% 
			     13 / 41     31.7%E 
			      1 / 41      2.4%ABE 
		 
		 
			 1 
			  2.6 
			 Thermosynechococcus elongatus BP-1 
			      1 / 41      2.4% 
			      0 / 1       0.0% 
			      0 / 1       0.0% 
			      0 / 1       0.0% 
			      0 / 1       0.0% 
			      0 / 3       0.0% 
			      0 / 3       0.0% 
			      0 / 1       0.0% 
			      0 / 1       0.0% 
			      0 / 3       0.0% 
			      0 / 1       0.0% 
			      0 / 4       0.0% 
			      0 / 1       0.0% 
			      0 / 1       0.0% 
			      0 / 1       0.0% 
			      0 / 1       0.0% 
			      0 / 3       0.0% 
			      0 / 0       0.0% 
			      0 / 3       0.0% 
			      0 / 4       0.0% 
			      0 / 1       0.0% 
			      0 / 1       0.0% 
			      1 / 5      20.0% 
			      0 / 0       0.0% 
			      0 / 0       0.0% 
			      0 / 0       0.0% 
			      0 / 0       0.0% 
			     27 / 41     65.9% 
			     13 / 41     31.7%E 
			      1 / 41      2.4%ABE 
		 
		 
			 1 
			  8.4 
			 Acaryochloris 
			      8 / 63     12.7% 
			      1 / 1      100.0% 
			      0 / 2       0.0% 
			      1 / 2      50.0% 
			      0 / 2       0.0% 
			      0 / 4       0.0% 
			      0 / 4       0.0% 
			      0 / 1       0.0% 
			      0 / 1       0.0% 
			      2 / 4      50.0% 
			      0 / 2       0.0% 
			      0 / 5       0.0% 
			      0 / 2       0.0% 
			      0 / 1       0.0% 
			      0 / 3       0.0% 
			      1 / 2      50.0% 
			      0 / 4       0.0% 
			      0 / 0       0.0% 
			      0 / 4       0.0% 
			      1 / 7      14.3% 
			      1 / 2      50.0% 
			      0 / 2       0.0% 
			      1 / 8      12.5% 
			      0 / 0       0.0% 
			      0 / 0       0.0% 
			      0 / 0       0.0% 
			      0 / 0       0.0% 
			     28 / 63     44.4% 
			     26 / 63     41.3%E 
			      3 / 63      4.8%AB 
			      3 / 63      4.8%ABE 
			      2 / 63      3.2%B 
			      1 / 63      1.6%C 
		 
		 
			 1 
			  8.4 
			 Acaryochloris marina 
			      8 / 63     12.7% 
			      1 / 1      100.0% 
			      0 / 2       0.0% 
			      1 / 2      50.0% 
			      0 / 2       0.0% 
			      0 / 4       0.0% 
			      0 / 4       0.0% 
			      0 / 1       0.0% 
			      0 / 1       0.0% 
			      2 / 4      50.0% 
			      0 / 2       0.0% 
			      0 / 5       0.0% 
			      0 / 2       0.0% 
			      0 / 1       0.0% 
			      0 / 3       0.0% 
			      1 / 2      50.0% 
			      0 / 4       0.0% 
			      0 / 0       0.0% 
			      0 / 4       0.0% 
			      1 / 7      14.3% 
			      1 / 2      50.0% 
			      0 / 2       0.0% 
			      1 / 8      12.5% 
			      0 / 0       0.0% 
			      0 / 0       0.0% 
			      0 / 0       0.0% 
			      0 / 0       0.0% 
			     28 / 63     44.4% 
			     26 / 63     41.3%E 
			      3 / 63      4.8%ABE 
			      3 / 63      4.8%AB 
			      2 / 63      3.2%B 
			      1 / 63      1.6%C 
		 
		 
			 1 
			  8.4 
			 Acaryochloris marina MBIC11017 
			      8 / 63     12.7% 
			      1 / 1      100.0% 
			      0 / 2       0.0% 
			      1 / 2      50.0% 
			      0 / 2       0.0% 
			      0 / 4       0.0% 
			      0 / 4       0.0% 
			      0 / 1       0.0% 
			      0 / 1       0.0% 
			      2 / 4      50.0% 
			      0 / 2       0.0% 
			      0 / 5       0.0% 
			      0 / 2       0.0% 
			      0 / 1       0.0% 
			      0 / 3       0.0% 
			      1 / 2      50.0% 
			      0 / 4       0.0% 
			      0 / 0       0.0% 
			      0 / 4       0.0% 
			      1 / 7      14.3% 
			      1 / 2      50.0% 
			      0 / 2       0.0% 
			      1 / 8      12.5% 
			      0 / 0       0.0% 
			      0 / 0       0.0% 
			      0 / 0       0.0% 
			      0 / 0       0.0% 
			     28 / 63     44.4% 
			     26 / 63     41.3%E 
			      3 / 63      4.8%AB 
			      3 / 63      4.8%ABE 
			      2 / 63      3.2%B 
			      1 / 63      1.6%C 
		 
		 
			 1 
			  3.3 
			 Cyanobium 
			      3 / 44      6.8% 
			      1 / 1      100.0% 
			      0 / 1       0.0% 
			      0 / 1       0.0% 
			      0 / 2       0.0% 
			      0 / 3       0.0% 
			      0 / 3       0.0% 
			      0 / 1       0.0% 
			      0 / 1       0.0% 
			      2 / 4      50.0% 
			      0 / 1       0.0% 
			      0 / 4       0.0% 
			      0 / 1       0.0% 
			      0 / 1       0.0% 
			      0 / 1       0.0% 
			      0 / 1       0.0% 
			      0 / 3       0.0% 
			      0 / 1       0.0% 
			      0 / 3       0.0% 
			      0 / 4       0.0% 
			      0 / 1       0.0% 
			      0 / 1       0.0% 
			      0 / 5       0.0% 
			      0 / 0       0.0% 
			      0 / 0       0.0% 
			      0 / 0       0.0% 
			      0 / 0       0.0% 
			     23 / 44     52.3%E 
			     17 / 44     38.6% 
			      2 / 44      4.5%ABE 
			      1 / 44      2.3%AB 
			      1 / 44      2.3%CE 
		 
		 
			 1 
			  3.3 
			 Cyanobium gracile 
			      3 / 44      6.8% 
			      1 / 1      100.0% 
			      0 / 1       0.0% 
			      0 / 1       0.0% 
			      0 / 2       0.0% 
			      0 / 3       0.0% 
			      0 / 3       0.0% 
			      0 / 1       0.0% 
			      0 / 1       0.0% 
			      2 / 4      50.0% 
			      0 / 1       0.0% 
			      0 / 4       0.0% 
			      0 / 1       0.0% 
			      0 / 1       0.0% 
			      0 / 1       0.0% 
			      0 / 1       0.0% 
			      0 / 3       0.0% 
			      0 / 1       0.0% 
			      0 / 3       0.0% 
			      0 / 4       0.0% 
			      0 / 1       0.0% 
			      0 / 1       0.0% 
			      0 / 5       0.0% 
			      0 / 0       0.0% 
			      0 / 0       0.0% 
			      0 / 0       0.0% 
			      0 / 0       0.0% 
			     23 / 44     52.3%E 
			     17 / 44     38.6% 
			      2 / 44      4.5%ABE 
			      1 / 44      2.3%CE 
			      1 / 44      2.3%AB 
		 
		 
			 1 
			  3.3 
			 Coccochloris peniocystis 1020 
			      3 / 44      6.8% 
			      1 / 1      100.0% 
			      0 / 1       0.0% 
			      0 / 1       0.0% 
			      0 / 2       0.0% 
			      0 / 3       0.0% 
			      0 / 3       0.0% 
			      0 / 1       0.0% 
			      0 / 1       0.0% 
			      2 / 4      50.0% 
			      0 / 1       0.0% 
			      0 / 4       0.0% 
			      0 / 1       0.0% 
			      0 / 1       0.0% 
			      0 / 1       0.0% 
			      0 / 1       0.0% 
			      0 / 3       0.0% 
			      0 / 1       0.0% 
			      0 / 3       0.0% 
			      0 / 4       0.0% 
			      0 / 1       0.0% 
			      0 / 1       0.0% 
			      0 / 5       0.0% 
			      0 / 0       0.0% 
			      0 / 0       0.0% 
			      0 / 0       0.0% 
			      0 / 0       0.0% 
			     23 / 44     52.3%E 
			     17 / 44     38.6% 
			      2 / 44      4.5%ABE 
			      1 / 44      2.3%CE 
			      1 / 44      2.3%AB 
		 
		 
			 1 
			  6.8 
			 Chamaesiphon 
			      3 / 53      5.7% 
			      1 / 1      100.0% 
			      1 / 3      33.3% 
			      1 / 2      50.0% 
			      0 / 4       0.0% 
			      0 / 3       0.0% 
			      0 / 2       0.0% 
			      0 / 1       0.0% 
			      0 / 1       0.0% 
			      0 / 4       0.0% 
			      0 / 1       0.0% 
			      0 / 5       0.0% 
			      0 / 2       0.0% 
			      0 / 1       0.0% 
			      0 / 1       0.0% 
			      0 / 1       0.0% 
			      0 / 3       0.0% 
			      0 / 0       0.0% 
			      0 / 3       0.0% 
			      0 / 5       0.0% 
			      0 / 2       0.0% 
			      0 / 2       0.0% 
			      0 / 6       0.0% 
			      0 / 0       0.0% 
			      0 / 0       0.0% 
			      0 / 0       0.0% 
			      0 / 0       0.0% 
			     25 / 53     47.2% 
			     24 / 53     45.3%E 
			      2 / 53      3.8%AB 
			      1 / 53      1.9%CE 
			      1 / 53      1.9%ABE 
		 
		 
			 1 
			  6.8 
			 Chamaesiphon minutus 
			      3 / 53      5.7% 
			      1 / 1      100.0% 
			      1 / 3      33.3% 
			      1 / 2      50.0% 
			      0 / 4       0.0% 
			      0 / 3       0.0% 
			      0 / 2       0.0% 
			      0 / 1       0.0% 
			      0 / 1       0.0% 
			      0 / 4       0.0% 
			      0 / 1       0.0% 
			      0 / 5       0.0% 
			      0 / 2       0.0% 
			      0 / 1       0.0% 
			      0 / 1       0.0% 
			      0 / 1       0.0% 
			      0 / 3       0.0% 
			      0 / 0       0.0% 
			      0 / 3       0.0% 
			      0 / 5       0.0% 
			      0 / 2       0.0% 
			      0 / 2       0.0% 
			      0 / 6       0.0% 
			      0 / 0       0.0% 
			      0 / 0       0.0% 
			      0 / 0       0.0% 
			      0 / 0       0.0% 
			     25 / 53     47.2% 
			     24 / 53     45.3%E 
			      2 / 53      3.8%AB 
			      1 / 53      1.9%CE 
			      1 / 53      1.9%ABE 
		 
		 
			 1 
			  6.8 
			 Chamaesiphon minutus PCC 6605 
			      3 / 53      5.7% 
			      1 / 1      100.0% 
			      1 / 3      33.3% 
			      1 / 2      50.0% 
			      0 / 4       0.0% 
			      0 / 3       0.0% 
			      0 / 2       0.0% 
			      0 / 1       0.0% 
			      0 / 1       0.0% 
			      0 / 4       0.0% 
			      0 / 1       0.0% 
			      0 / 5       0.0% 
			      0 / 2       0.0% 
			      0 / 1       0.0% 
			      0 / 1       0.0% 
			      0 / 1       0.0% 
			      0 / 3       0.0% 
			      0 / 0       0.0% 
			      0 / 3       0.0% 
			      0 / 5       0.0% 
			      0 / 2       0.0% 
			      0 / 2       0.0% 
			      0 / 6       0.0% 
			      0 / 0       0.0% 
			      0 / 0       0.0% 
			      0 / 0       0.0% 
			      0 / 0       0.0% 
			     25 / 53     47.2% 
			     24 / 53     45.3%E 
			      2 / 53      3.8%AB 
			      1 / 53      1.9%ABE 
			      1 / 53      1.9%CE 
		 
		 
			 1 
			  1.4 
			 "Candidatus Atelocyanobacterium" Thompson et al. 2012 
			      0 / 37      0.0% 
			      0 / 1       0.0% 
			      0 / 1       0.0% 
			      0 / 1       0.0% 
			      0 / 2       0.0% 
			      0 / 2       0.0% 
			      0 / 2       0.0% 
			      0 / 1       0.0% 
			      0 / 1       0.0% 
			      0 / 3       0.0% 
			      0 / 1       0.0% 
			      0 / 4       0.0% 
			      0 / 1       0.0% 
			      0 / 1       0.0% 
			      0 / 1       0.0% 
			      0 / 1       0.0% 
			      0 / 2       0.0% 
			      0 / 1       0.0% 
			      0 / 3       0.0% 
			      0 / 3       0.0% 
			      0 / 1       0.0% 
			      0 / 1       0.0% 
			      0 / 3       0.0% 
			      0 / 0       0.0% 
			      0 / 0       0.0% 
			      0 / 0       0.0% 
			      0 / 0       0.0% 
			     19 / 37     51.4%E 
			     16 / 37     43.2% 
			      2 / 37      5.4%CE 
		 
		 
			 1 
			  1.4 
			 "Candidatus Atelocyanobacterium thalassa" Thompson et al. 2012 
			      0 / 37      0.0% 
			      0 / 1       0.0% 
			      0 / 1       0.0% 
			      0 / 1       0.0% 
			      0 / 2       0.0% 
			      0 / 2       0.0% 
			      0 / 2       0.0% 
			      0 / 1       0.0% 
			      0 / 1       0.0% 
			      0 / 3       0.0% 
			      0 / 1       0.0% 
			      0 / 4       0.0% 
			      0 / 1       0.0% 
			      0 / 1       0.0% 
			      0 / 1       0.0% 
			      0 / 1       0.0% 
			      0 / 2       0.0% 
			      0 / 1       0.0% 
			      0 / 3       0.0% 
			      0 / 3       0.0% 
			      0 / 1       0.0% 
			      0 / 1       0.0% 
			      0 / 3       0.0% 
			      0 / 0       0.0% 
			      0 / 0       0.0% 
			      0 / 0       0.0% 
			      0 / 0       0.0% 
			     19 / 37     51.4%E 
			     16 / 37     43.2% 
			      2 / 37      5.4%CE 
		 
		 
			 8 
			  6.5 
			 Oscillatoriales 
			     29 / 412     7.0% 
			      7 / 8      87.5% 
			      0 / 12      0.0% 
			      2 / 11     18.2% 
			      1 / 15      6.7% 
			      1 / 28      3.6% 
			      2 / 23      8.7% 
			      0 / 8       0.0% 
			      2 / 9      22.2% 
			      5 / 32     15.6% 
			      2 / 10     20.0% 
			      1 / 39      2.6% 
			      0 / 16      0.0% 
			      0 / 10      0.0% 
			      1 / 12      8.3% 
			      0 / 10      0.0% 
			      0 / 26      0.0% 
			      0 / 3       0.0% 
			      0 / 28      0.0% 
			      0 / 32      0.0% 
			      2 / 11     18.2% 
			      1 / 13      7.7% 
			      2 / 48      4.2% 
			      0 / 0       0.0% 
			      0 / 0       0.0% 
			      0 / 7       0.0% 
			      0 / 1       0.0% 
			    186 / 412    45.1% 
			    179 / 412    43.4%E 
			     23 / 412     5.6%AB 
			      9 / 412     2.2%C 
			      9 / 412     2.2%CE 
			      6 / 412     1.5%ABE 
		 
		 
			 1 
			  4.9 
			 Pseudanabaena 
			      2 / 44      4.5% 
			      0 / 1       0.0% 
			      0 / 1       0.0% 
			      0 / 1       0.0% 
			      0 / 2       0.0% 
			      0 / 3       0.0% 
			      0 / 3       0.0% 
			      0 / 1       0.0% 
			      0 / 1       0.0% 
			      2 / 4      50.0% 
			      0 / 1       0.0% 
			      0 / 4       0.0% 
			      0 / 2       0.0% 
			      0 / 1       0.0% 
			      0 / 1       0.0% 
			      0 / 1       0.0% 
			      0 / 3       0.0% 
			      0 / 1       0.0% 
			      0 / 3       0.0% 
			      0 / 4       0.0% 
			      0 / 1       0.0% 
			      0 / 1       0.0% 
			      0 / 4       0.0% 
			      0 / 0       0.0% 
			      0 / 0       0.0% 
			      0 / 0       0.0% 
			      0 / 0       0.0% 
			     23 / 44     52.3%E 
			     18 / 44     40.9% 
			      2 / 44      4.5%AB 
			      1 / 44      2.3%CE 
		 
		 
			 1 
			  4.9 
			 Pseudanabaena PCC7367 
			      2 / 44      4.5% 
			      0 / 1       0.0% 
			      0 / 1       0.0% 
			      0 / 1       0.0% 
			      0 / 2       0.0% 
			      0 / 3       0.0% 
			      0 / 3       0.0% 
			      0 / 1       0.0% 
			      0 / 1       0.0% 
			      2 / 4      50.0% 
			      0 / 1       0.0% 
			      0 / 4       0.0% 
			      0 / 2       0.0% 
			      0 / 1       0.0% 
			      0 / 1       0.0% 
			      0 / 1       0.0% 
			      0 / 3       0.0% 
			      0 / 1       0.0% 
			      0 / 3       0.0% 
			      0 / 4       0.0% 
			      0 / 1       0.0% 
			      0 / 1       0.0% 
			      0 / 4       0.0% 
			      0 / 0       0.0% 
			      0 / 0       0.0% 
			      0 / 0       0.0% 
			      0 / 0       0.0% 
			     23 / 44     52.3%E 
			     18 / 44     40.9% 
			      2 / 44      4.5%AB 
			      1 / 44      2.3%CE 
		 
		 
			 2 
			  8.0 
			 Oscillatoria 
			     15 / 132    11.4% 
			      3 / 3      100.0% 
			      0 / 4       0.0% 
			      1 / 4      25.0% 
			      0 / 3       0.0% 
			      0 / 8       0.0% 
			      1 / 7      14.3% 
			      0 / 2       0.0% 
			      1 / 3      33.3% 
			      1 / 10     10.0% 
			      1 / 3      33.3% 
			      1 / 13      7.7% 
			      0 / 6       0.0% 
			      0 / 3       0.0% 
			      1 / 5      20.0% 
			      0 / 3       0.0% 
			      0 / 8       0.0% 
			      0 / 0       0.0% 
			      0 / 8       0.0% 
			      0 / 9       0.0% 
			      2 / 3      66.7% 
			      1 / 3      33.3% 
			      2 / 18     11.1% 
			      0 / 0       0.0% 
			      0 / 0       0.0% 
			      0 / 5       0.0% 
			      0 / 1       0.0% 
			     58 / 132    43.9%E 
			     52 / 132    39.4% 
			     10 / 132     7.6%AB 
			      5 / 132     3.8%ABE 
			      4 / 132     3.0%CE 
			      3 / 132     2.3%C 
		 
		 
			 1 
			  7.8 
			 Oscillatoria acuminata 
			      8 / 65     12.3% 
			      1 / 1      100.0% 
			      0 / 2       0.0% 
			      0 / 2       0.0% 
			      0 / 1       0.0% 
			      0 / 4       0.0% 
			      1 / 4      25.0% 
			      0 / 1       0.0% 
			      1 / 2      50.0% 
			      0 / 4       0.0% 
			      1 / 2      50.0% 
			      1 / 8      12.5% 
			      0 / 3       0.0% 
			      0 / 1       0.0% 
			      0 / 2       0.0% 
			      0 / 1       0.0% 
			      0 / 4       0.0% 
			      0 / 0       0.0% 
			      0 / 4       0.0% 
			      0 / 3       0.0% 
			      1 / 2      50.0% 
			      1 / 2      50.0% 
			      1 / 8      12.5% 
			      0 / 0       0.0% 
			      0 / 0       0.0% 
			      0 / 4       0.0% 
			      0 / 0       0.0% 
			     30 / 65     46.2%E 
			     24 / 65     36.9% 
			      4 / 65      6.2%AB 
			      4 / 65      6.2%ABE 
			      2 / 65      3.1%CE 
			      1 / 65      1.5%C 
		 
		 
			 1 
			  7.8 
			 Microcoleus vaginatus KFRI M-7.1.1 
			      8 / 65     12.3% 
			      1 / 1      100.0% 
			      0 / 2       0.0% 
			      0 / 2       0.0% 
			      0 / 1       0.0% 
			      0 / 4       0.0% 
			      1 / 4      25.0% 
			      0 / 1       0.0% 
			      1 / 2      50.0% 
			      0 / 4       0.0% 
			      1 / 2      50.0% 
			      1 / 8      12.5% 
			      0 / 3       0.0% 
			      0 / 1       0.0% 
			      0 / 2       0.0% 
			      0 / 1       0.0% 
			      0 / 4       0.0% 
			      0 / 0       0.0% 
			      0 / 4       0.0% 
			      0 / 3       0.0% 
			      1 / 2      50.0% 
			      1 / 2      50.0% 
			      1 / 8      12.5% 
			      0 / 0       0.0% 
			      0 / 0       0.0% 
			      0 / 4       0.0% 
			      0 / 0       0.0% 
			     30 / 65     46.2%E 
			     24 / 65     36.9% 
			      4 / 65      6.2%AB 
			      4 / 65      6.2%ABE 
			      2 / 65      3.1%CE 
			      1 / 65      1.5%C 
		 
		 
			 1 
			  8.3 
			 Oscillatoria nigro-viridis 
			      7 / 67     10.4% 
			      2 / 2      100.0% 
			      0 / 2       0.0% 
			      1 / 2      50.0% 
			      0 / 2       0.0% 
			      0 / 4       0.0% 
			      0 / 3       0.0% 
			      0 / 1       0.0% 
			      0 / 1       0.0% 
			      1 / 6      16.7% 
			      0 / 1       0.0% 
			      0 / 5       0.0% 
			      0 / 3       0.0% 
			      0 / 2       0.0% 
			      1 / 3      33.3% 
			      0 / 2       0.0% 
			      0 / 4       0.0% 
			      0 / 0       0.0% 
			      0 / 4       0.0% 
			      0 / 6       0.0% 
			      1 / 1      100.0% 
			      0 / 1       0.0% 
			      1 / 10     10.0% 
			      0 / 0       0.0% 
			      0 / 0       0.0% 
			      0 / 1       0.0% 
			      0 / 1       0.0% 
			     28 / 67     41.8%E 
			     28 / 67     41.8% 
			      6 / 67      9.0%AB 
			      2 / 67      3.0%C 
			      2 / 67      3.0%CE 
			      1 / 67      1.5%ABE 
		 
		 
			 1 
			  8.3 
			 Oscillatoria PCC 7112 
			      7 / 67     10.4% 
			      2 / 2      100.0% 
			      0 / 2       0.0% 
			      1 / 2      50.0% 
			      0 / 2       0.0% 
			      0 / 4       0.0% 
			      0 / 3       0.0% 
			      0 / 1       0.0% 
			      0 / 1       0.0% 
			      1 / 6      16.7% 
			      0 / 1       0.0% 
			      0 / 5       0.0% 
			      0 / 3       0.0% 
			      0 / 2       0.0% 
			      1 / 3      33.3% 
			      0 / 2       0.0% 
			      0 / 4       0.0% 
			      0 / 0       0.0% 
			      0 / 4       0.0% 
			      0 / 6       0.0% 
			      1 / 1      100.0% 
			      0 / 1       0.0% 
			      1 / 10     10.0% 
			      0 / 0       0.0% 
			      0 / 0       0.0% 
			      0 / 1       0.0% 
			      0 / 1       0.0% 
			     28 / 67     41.8% 
			     28 / 67     41.8%E 
			      6 / 67      9.0%AB 
			      2 / 67      3.0%CE 
			      2 / 67      3.0%C 
			      1 / 67      1.5%ABE 
		 
		 
			 1 
			  7.8 
			 Trichodesmium 
			      0 / 38      0.0% 
			      0 / 0       0.0% 
			      0 / 1       0.0% 
			      0 / 1       0.0% 
			      0 / 2       0.0% 
			      0 / 2       0.0% 
			      0 / 2       0.0% 
			      0 / 1       0.0% 
			      0 / 1       0.0% 
			      0 / 3       0.0% 
			      0 / 1       0.0% 
			      0 / 4       0.0% 
			      0 / 1       0.0% 
			      0 / 1       0.0% 
			      0 / 1       0.0% 
			      0 / 1       0.0% 
			      0 / 2       0.0% 
			      0 / 0       0.0% 
			      0 / 2       0.0% 
			      0 / 3       0.0% 
			      0 / 1       0.0% 
			      0 / 3       0.0% 
			      0 / 5       0.0% 
			      0 / 0       0.0% 
			      0 / 0       0.0% 
			      0 / 0       0.0% 
			      0 / 0       0.0% 
			     20 / 38     52.6% 
			     16 / 38     42.1%E 
			      2 / 38      5.3%C 
		 
		 
			 1 
			  7.8 
			 Trichodesmium erythraeum 
			      0 / 38      0.0% 
			      0 / 0       0.0% 
			      0 / 1       0.0% 
			      0 / 1       0.0% 
			      0 / 2       0.0% 
			      0 / 2       0.0% 
			      0 / 2       0.0% 
			      0 / 1       0.0% 
			      0 / 1       0.0% 
			      0 / 3       0.0% 
			      0 / 1       0.0% 
			      0 / 4       0.0% 
			      0 / 1       0.0% 
			      0 / 1       0.0% 
			      0 / 1       0.0% 
			      0 / 1       0.0% 
			      0 / 2       0.0% 
			      0 / 0       0.0% 
			      0 / 2       0.0% 
			      0 / 3       0.0% 
			      0 / 1       0.0% 
			      0 / 3       0.0% 
			      0 / 5       0.0% 
			      0 / 0       0.0% 
			      0 / 0       0.0% 
			      0 / 0       0.0% 
			      0 / 0       0.0% 
			     20 / 38     52.6% 
			     16 / 38     42.1%E 
			      2 / 38      5.3%C 
		 
		 
			 1 
			  7.8 
			 Trichodesmium erythraeum IMS101 
			      0 / 38      0.0% 
			      0 / 0       0.0% 
			      0 / 1       0.0% 
			      0 / 1       0.0% 
			      0 / 2       0.0% 
			      0 / 2       0.0% 
			      0 / 2       0.0% 
			      0 / 1       0.0% 
			      0 / 1       0.0% 
			      0 / 3       0.0% 
			      0 / 1       0.0% 
			      0 / 4       0.0% 
			      0 / 1       0.0% 
			      0 / 1       0.0% 
			      0 / 1       0.0% 
			      0 / 1       0.0% 
			      0 / 2       0.0% 
			      0 / 0       0.0% 
			      0 / 2       0.0% 
			      0 / 3       0.0% 
			      0 / 1       0.0% 
			      0 / 3       0.0% 
			      0 / 5       0.0% 
			      0 / 0       0.0% 
			      0 / 0       0.0% 
			      0 / 0       0.0% 
			      0 / 0       0.0% 
			     20 / 38     52.6% 
			     16 / 38     42.1%E 
			      2 / 38      5.3%C 
		 
		 
			 1 
			  8.0 
			 Microcoleus 
			      5 / 68      7.4% 
			      1 / 1      100.0% 
			      0 / 3       0.0% 
			      1 / 2      50.0% 
			      0 / 2       0.0% 
			      1 / 5      20.0% 
			      1 / 4      25.0% 
			      0 / 1       0.0% 
			      0 / 1       0.0% 
			      0 / 4       0.0% 
			      1 / 2      50.0% 
			      0 / 6       0.0% 
			      0 / 3       0.0% 
			      0 / 2       0.0% 
			      0 / 2       0.0% 
			      0 / 2       0.0% 
			      0 / 4       0.0% 
			      0 / 0       0.0% 
			      0 / 5       0.0% 
			      0 / 5       0.0% 
			      0 / 3       0.0% 
			      0 / 2       0.0% 
			      0 / 7       0.0% 
			      0 / 0       0.0% 
			      0 / 0       0.0% 
			      0 / 2       0.0% 
			      0 / 0       0.0% 
			     33 / 68     48.5% 
			     27 / 68     39.7%E 
			      5 / 68      7.4%AB 
			      3 / 68      4.4%CE 
		 
		 
			 1 
			  8.0 
			 Microcoleus sp. PCC 7113 
			      5 / 68      7.4% 
			      1 / 1      100.0% 
			      0 / 3       0.0% 
			      1 / 2      50.0% 
			      0 / 2       0.0% 
			      1 / 5      20.0% 
			      1 / 4      25.0% 
			      0 / 1       0.0% 
			      0 / 1       0.0% 
			      0 / 4       0.0% 
			      1 / 2      50.0% 
			      0 / 6       0.0% 
			      0 / 3       0.0% 
			      0 / 2       0.0% 
			      0 / 2       0.0% 
			      0 / 2       0.0% 
			      0 / 4       0.0% 
			      0 / 0       0.0% 
			      0 / 5       0.0% 
			      0 / 5       0.0% 
			      0 / 3       0.0% 
			      0 / 2       0.0% 
			      0 / 7       0.0% 
			      0 / 0       0.0% 
			      0 / 0       0.0% 
			      0 / 2       0.0% 
			      0 / 0       0.0% 
			     33 / 68     48.5% 
			     27 / 68     39.7%E 
			      5 / 68      7.4%AB 
			      3 / 68      4.4%CE 
		 
		 
			 1 
			  5.1 
			 Leptolyngbya 
			      3 / 42      7.1% 
			      1 / 1      100.0% 
			      0 / 1       0.0% 
			      0 / 1       0.0% 
			      0 / 2       0.0% 
			      0 / 3       0.0% 
			      0 / 2       0.0% 
			      0 / 1       0.0% 
			      0 / 1       0.0% 
			      2 / 4      50.0% 
			      0 / 1       0.0% 
			      0 / 4       0.0% 
			      0 / 1       0.0% 
			      0 / 1       0.0% 
			      0 / 1       0.0% 
			      0 / 1       0.0% 
			      0 / 3       0.0% 
			      0 / 1       0.0% 
			      0 / 3       0.0% 
			      0 / 3       0.0% 
			      0 / 1       0.0% 
			      0 / 1       0.0% 
			      0 / 5       0.0% 
			      0 / 0       0.0% 
			      0 / 0       0.0% 
			      0 / 0       0.0% 
			      0 / 0       0.0% 
			     24 / 42     57.1% 
			     15 / 42     35.7%E 
			      3 / 42      7.1%AB 
		 
		 
			 1 
			  5.1 
			 Leptolyngbya PCC7376 
			      3 / 42      7.1% 
			      1 / 1      100.0% 
			      0 / 1       0.0% 
			      0 / 1       0.0% 
			      0 / 2       0.0% 
			      0 / 3       0.0% 
			      0 / 2       0.0% 
			      0 / 1       0.0% 
			      0 / 1       0.0% 
			      2 / 4      50.0% 
			      0 / 1       0.0% 
			      0 / 4       0.0% 
			      0 / 1       0.0% 
			      0 / 1       0.0% 
			      0 / 1       0.0% 
			      0 / 1       0.0% 
			      0 / 3       0.0% 
			      0 / 1       0.0% 
			      0 / 3       0.0% 
			      0 / 3       0.0% 
			      0 / 1       0.0% 
			      0 / 1       0.0% 
			      0 / 5       0.0% 
			      0 / 0       0.0% 
			      0 / 0       0.0% 
			      0 / 0       0.0% 
			      0 / 0       0.0% 
			     24 / 42     57.1% 
			     15 / 42     35.7%E 
			      3 / 42      7.1%AB 
		 
		 
			 1 
			  4.7 
			 Geitlerinema 
			      2 / 46      4.3% 
			      1 / 1      100.0% 
			      0 / 1       0.0% 
			      0 / 1       0.0% 
			      0 / 3       0.0% 
			      0 / 4       0.0% 
			      0 / 3       0.0% 
			      0 / 1       0.0% 
			      1 / 1      100.0% 
			      0 / 5       0.0% 
			      0 / 1       0.0% 
			      0 / 4       0.0% 
			      0 / 1       0.0% 
			      0 / 1       0.0% 
			      0 / 1       0.0% 
			      0 / 1       0.0% 
			      0 / 3       0.0% 
			      0 / 0       0.0% 
			      0 / 3       0.0% 
			      0 / 4       0.0% 
			      0 / 1       0.0% 
			      0 / 2       0.0% 
			      0 / 4       0.0% 
			      0 / 0       0.0% 
			      0 / 0       0.0% 
			      0 / 0       0.0% 
			      0 / 0       0.0% 
			     22 / 46     47.8% 
			     19 / 46     41.3%E 
			      3 / 46      6.5%C 
			      1 / 46      2.2%AB 
			      1 / 46      2.2%ABE 
		 
		 
			 1 
			  4.7 
			 Geitlerinema sp. ATCC 29126 
			      2 / 46      4.3% 
			      1 / 1      100.0% 
			      0 / 1       0.0% 
			      0 / 1       0.0% 
			      0 / 3       0.0% 
			      0 / 4       0.0% 
			      0 / 3       0.0% 
			      0 / 1       0.0% 
			      1 / 1      100.0% 
			      0 / 5       0.0% 
			      0 / 1       0.0% 
			      0 / 4       0.0% 
			      0 / 1       0.0% 
			      0 / 1       0.0% 
			      0 / 1       0.0% 
			      0 / 1       0.0% 
			      0 / 3       0.0% 
			      0 / 0       0.0% 
			      0 / 3       0.0% 
			      0 / 4       0.0% 
			      0 / 1       0.0% 
			      0 / 2       0.0% 
			      0 / 4       0.0% 
			      0 / 0       0.0% 
			      0 / 0       0.0% 
			      0 / 0       0.0% 
			      0 / 0       0.0% 
			     22 / 46     47.8% 
			     19 / 46     41.3%E 
			      3 / 46      6.5%C 
			      1 / 46      2.2%AB 
			      1 / 46      2.2%ABE 
		 
		 
			 1 
			  5.6 
			 Crinalium 
			      2 / 42      4.8% 
			      1 / 1      100.0% 
			      0 / 1       0.0% 
			      0 / 1       0.0% 
			      1 / 1      100.0% 
			      0 / 3       0.0% 
			      0 / 2       0.0% 
			      0 / 1       0.0% 
			      0 / 1       0.0% 
			      0 / 2       0.0% 
			      0 / 1       0.0% 
			      0 / 4       0.0% 
			      0 / 2       0.0% 
			      0 / 1       0.0% 
			      0 / 1       0.0% 
			      0 / 1       0.0% 
			      0 / 3       0.0% 
			      0 / 1       0.0% 
			      0 / 4       0.0% 
			      0 / 4       0.0% 
			      0 / 1       0.0% 
			      0 / 1       0.0% 
			      0 / 5       0.0% 
			      0 / 0       0.0% 
			      0 / 0       0.0% 
			      0 / 0       0.0% 
			      0 / 0       0.0% 
			     21 / 42     50.0%E 
			     17 / 42     40.5% 
			      2 / 42      4.8%AB 
			      1 / 42      2.4%CE 
			      1 / 42      2.4%C 
		 
		 
			 1 
			  5.6 
			 ATCC 49662 
			      2 / 42      4.8% 
			      1 / 1      100.0% 
			      0 / 1       0.0% 
			      0 / 1       0.0% 
			      1 / 1      100.0% 
			      0 / 3       0.0% 
			      0 / 2       0.0% 
			      0 / 1       0.0% 
			      0 / 1       0.0% 
			      0 / 2       0.0% 
			      0 / 1       0.0% 
			      0 / 4       0.0% 
			      0 / 2       0.0% 
			      0 / 1       0.0% 
			      0 / 1       0.0% 
			      0 / 1       0.0% 
			      0 / 3       0.0% 
			      0 / 1       0.0% 
			      0 / 4       0.0% 
			      0 / 4       0.0% 
			      0 / 1       0.0% 
			      0 / 1       0.0% 
			      0 / 5       0.0% 
			      0 / 0       0.0% 
			      0 / 0       0.0% 
			      0 / 0       0.0% 
			      0 / 0       0.0% 
			     21 / 42     50.0%E 
			     17 / 42     40.5% 
			      2 / 42      4.8%AB 
			      1 / 42      2.4%C 
			      1 / 42      2.4%CE 
		 
		 
			 1 
			  5.6 
			 Crinalium epipsammum PCC 9333 
			      2 / 42      4.8% 
			      1 / 1      100.0% 
			      0 / 1       0.0% 
			      0 / 1       0.0% 
			      1 / 1      100.0% 
			      0 / 3       0.0% 
			      0 / 2       0.0% 
			      0 / 1       0.0% 
			      0 / 1       0.0% 
			      0 / 2       0.0% 
			      0 / 1       0.0% 
			      0 / 4       0.0% 
			      0 / 2       0.0% 
			      0 / 1       0.0% 
			      0 / 1       0.0% 
			      0 / 1       0.0% 
			      0 / 3       0.0% 
			      0 / 1       0.0% 
			      0 / 4       0.0% 
			      0 / 4       0.0% 
			      0 / 1       0.0% 
			      0 / 1       0.0% 
			      0 / 5       0.0% 
			      0 / 0       0.0% 
			      0 / 0       0.0% 
			      0 / 0       0.0% 
			      0 / 0       0.0% 
			     21 / 42     50.0%E 
			     17 / 42     40.5% 
			      2 / 42      4.8%AB 
			      1 / 42      2.4%C 
			      1 / 42      2.4%CE 
		 
		 
			 1033 
			  3.9 
			 Proteobacteria 
			  59095 / 61487  96.1% 
			   2361 / 2409   98.0% 
			   2261 / 2309   97.9% 
			   2084 / 2139   97.4% 
			   2756 / 2803   98.3% 
			   4164 / 4299   96.9% 
			   3801 / 3925   96.8% 
			   1119 / 1157   96.7% 
			   1483 / 1580   93.9% 
			   4395 / 4529   97.0% 
			   1097 / 1145   95.8% 
			   4050 / 4234   95.7% 
			   2691 / 2814   95.6% 
			   1346 / 1414   95.2% 
			   2132 / 2180   97.8% 
			   1089 / 1158   94.0% 
			   2680 / 2789   96.1% 
			   1257 / 1313   95.7% 
			   3127 / 3305   94.6% 
			   5159 / 5461   94.5% 
			   2741 / 2815   97.4% 
			   1192 / 1281   93.1% 
			   5763 / 6036   95.5% 
			    338 / 378    89.4% 
			      0 / 0       0.0% 
			      4 / 8      50.0% 
			      5 / 6      83.3% 
			  36489 / 61487  59.3%AB 
			  19265 / 61487  31.3%ABE 
			   2124 / 61487   3.5%B 
			   1217 / 61487   2.0%BE 
			   1153 / 61487   1.9% 
			   1062 / 61487   1.7%E 
			    112 / 61487   0.2%C 
			     64 / 61487   0.1%CE 
			      1 / 61487   0.0%GG 
		 
		 
			 514 
			  4.1 
			 Gammaproteobacteria 
			  35257 / 36399  96.9% 
			   1485 / 1498   99.1% 
			   1439 / 1471   97.8% 
			   1448 / 1474   98.2% 
			   1484 / 1505   98.6% 
			   2561 / 2636   97.2% 
			   2414 / 2469   97.8% 
			    599 / 618    96.9% 
			    992 / 1041   95.3% 
			   2344 / 2400   97.7% 
			    587 / 615    95.4% 
			   2221 / 2317   95.9% 
			   1814 / 1872   96.9% 
			    843 / 871    96.8% 
			   1371 / 1397   98.1% 
			    581 / 616    94.3% 
			   1440 / 1482   97.2% 
			    792 / 819    96.7% 
			   1712 / 1809   94.6% 
			   3101 / 3267   94.9% 
			   1778 / 1813   98.1% 
			    679 / 715    95.0% 
			   3327 / 3443   96.6% 
			    241 / 245    98.4% 
			      0 / 0       0.0% 
			      0 / 1       0.0% 
			      4 / 5      80.0% 
			  21414 / 36399  58.8%AB 
			  11869 / 36399  32.6%ABE 
			   1196 / 36399   3.3%B 
			    778 / 36399   2.1%BE 
			    609 / 36399   1.7%E 
			    504 / 36399   1.4% 
			     19 / 36399   0.1%C 
			      9 / 36399   0.0%CE 
			      1 / 36399   0.0%GG 
		 
		 
			 23 
			  2.0 
			 Beggiatoales 
			    921 / 923    99.8% 
			     24 / 24     100.0% 
			     28 / 28     100.0% 
			     24 / 24     100.0% 
			     67 / 67     100.0% 
			     69 / 69     100.0% 
			     69 / 69     100.0% 
			     24 / 24     100.0% 
			     24 / 24     100.0% 
			     72 / 72     100.0% 
			     24 / 24     100.0% 
			     72 / 72     100.0% 
			     28 / 28     100.0% 
			     24 / 24     100.0% 
			     24 / 24     100.0% 
			     24 / 24     100.0% 
			     29 / 29     100.0% 
			     24 / 24     100.0% 
			     49 / 49     100.0% 
			     75 / 76     98.7% 
			     27 / 27     100.0% 
			     24 / 24     100.0% 
			     96 / 97     99.0% 
			      0 / 0       0.0% 
			      0 / 0       0.0% 
			      0 / 0       0.0% 
			      0 / 0       0.0% 
			    581 / 923    62.9%AB 
			    282 / 923    30.6%ABE 
			     45 / 923     4.9%B 
			     13 / 923     1.4%BE 
			      1 / 923     0.1% 
			      1 / 923     0.1%E 
		 
		 
			 18 
			  1.9 
			 Francisella group 
			    722 / 722    100.0% 
			     19 / 19     100.0% 
			     19 / 19     100.0% 
			     19 / 19     100.0% 
			     55 / 55     100.0% 
			     57 / 57     100.0% 
			     57 / 57     100.0% 
			     19 / 19     100.0% 
			     19 / 19     100.0% 
			     55 / 55     100.0% 
			     19 / 19     100.0% 
			     57 / 57     100.0% 
			     22 / 22     100.0% 
			     19 / 19     100.0% 
			     19 / 19     100.0% 
			     19 / 19     100.0% 
			     19 / 19     100.0% 
			     19 / 19     100.0% 
			     38 / 38     100.0% 
			     57 / 57     100.0% 
			     20 / 20     100.0% 
			     19 / 19     100.0% 
			     76 / 76     100.0% 
			      0 / 0       0.0% 
			      0 / 0       0.0% 
			      0 / 0       0.0% 
			      0 / 0       0.0% 
			    448 / 722    62.0%AB 
			    216 / 722    29.9%ABE 
			     45 / 722     6.2%B 
			     13 / 722     1.8%BE 
		 
		 
			 18 
			  1.9 
			 Francisella 
			    722 / 722    100.0% 
			     19 / 19     100.0% 
			     19 / 19     100.0% 
			     19 / 19     100.0% 
			     55 / 55     100.0% 
			     57 / 57     100.0% 
			     57 / 57     100.0% 
			     19 / 19     100.0% 
			     19 / 19     100.0% 
			     55 / 55     100.0% 
			     19 / 19     100.0% 
			     57 / 57     100.0% 
			     22 / 22     100.0% 
			     19 / 19     100.0% 
			     19 / 19     100.0% 
			     19 / 19     100.0% 
			     19 / 19     100.0% 
			     19 / 19     100.0% 
			     38 / 38     100.0% 
			     57 / 57     100.0% 
			     20 / 20     100.0% 
			     19 / 19     100.0% 
			     76 / 76     100.0% 
			      0 / 0       0.0% 
			      0 / 0       0.0% 
			      0 / 0       0.0% 
			      0 / 0       0.0% 
			    448 / 722    62.0%AB 
			    216 / 722    29.9%ABE 
			     45 / 722     6.2%B 
			     13 / 722     1.8%BE 
		 
		 
			 15 
			  1.9 
			 "Bacterium tularense" McCoy and Chapin 1912 
			    609 / 609    100.0% 
			     16 / 16     100.0% 
			     16 / 16     100.0% 
			     16 / 16     100.0% 
			     48 / 48     100.0% 
			     48 / 48     100.0% 
			     48 / 48     100.0% 
			     16 / 16     100.0% 
			     16 / 16     100.0% 
			     48 / 48     100.0% 
			     16 / 16     100.0% 
			     48 / 48     100.0% 
			     16 / 16     100.0% 
			     16 / 16     100.0% 
			     16 / 16     100.0% 
			     16 / 16     100.0% 
			     16 / 16     100.0% 
			     16 / 16     100.0% 
			     32 / 32     100.0% 
			     48 / 48     100.0% 
			     17 / 17     100.0% 
			     16 / 16     100.0% 
			     64 / 64     100.0% 
			      0 / 0       0.0% 
			      0 / 0       0.0% 
			      0 / 0       0.0% 
			      0 / 0       0.0% 
			    384 / 609    63.1%AB 
			    184 / 609    30.2%ABE 
			     32 / 609     5.3%B 
			      9 / 609     1.5%BE 
		 
		 
			 3 
			  1.9 
			 "Francisella tularensis subsp. novicida" (Larson et al. 1955) Sjostedt 2005 
			    114 / 114    100.0% 
			      3 / 3      100.0% 
			      3 / 3      100.0% 
			      3 / 3      100.0% 
			      9 / 9      100.0% 
			      9 / 9      100.0% 
			      9 / 9      100.0% 
			      3 / 3      100.0% 
			      3 / 3      100.0% 
			      9 / 9      100.0% 
			      3 / 3      100.0% 
			      9 / 9      100.0% 
			      3 / 3      100.0% 
			      3 / 3      100.0% 
			      3 / 3      100.0% 
			      3 / 3      100.0% 
			      3 / 3      100.0% 
			      3 / 3      100.0% 
			      6 / 6      100.0% 
			      9 / 9      100.0% 
			      3 / 3      100.0% 
			      3 / 3      100.0% 
			     12 / 12     100.0% 
			      0 / 0       0.0% 
			      0 / 0       0.0% 
			      0 / 0       0.0% 
			      0 / 0       0.0% 
			     78 / 114    68.4%AB 
			     36 / 114    31.6%ABE 
		 
		 
			 1 
			  1.9 
			 Francisella novicida U112 
			     38 / 38     100.0% 
			      1 / 1      100.0% 
			      1 / 1      100.0% 
			      1 / 1      100.0% 
			      3 / 3      100.0% 
			      3 / 3      100.0% 
			      3 / 3      100.0% 
			      1 / 1      100.0% 
			      1 / 1      100.0% 
			      3 / 3      100.0% 
			      1 / 1      100.0% 
			      3 / 3      100.0% 
			      1 / 1      100.0% 
			      1 / 1      100.0% 
			      1 / 1      100.0% 
			      1 / 1      100.0% 
			      1 / 1      100.0% 
			      1 / 1      100.0% 
			      2 / 2      100.0% 
			      3 / 3      100.0% 
			      1 / 1      100.0% 
			      1 / 1      100.0% 
			      4 / 4      100.0% 
			      0 / 0       0.0% 
			      0 / 0       0.0% 
			      0 / 0       0.0% 
			      0 / 0       0.0% 
			     26 / 38     68.4%AB 
			     12 / 38     31.6%ABE 
		 
		 
			 1 
			  1.9 
			 Francisella cf. novicida 3523 
			     38 / 38     100.0% 
			      1 / 1      100.0% 
			      1 / 1      100.0% 
			      1 / 1      100.0% 
			      3 / 3      100.0% 
			      3 / 3      100.0% 
			      3 / 3      100.0% 
			      1 / 1      100.0% 
			      1 / 1      100.0% 
			      3 / 3      100.0% 
			      1 / 1      100.0% 
			      3 / 3      100.0% 
			      1 / 1      100.0% 
			      1 / 1      100.0% 
			      1 / 1      100.0% 
			      1 / 1      100.0% 
			      1 / 1      100.0% 
			      1 / 1      100.0% 
			      2 / 2      100.0% 
			      3 / 3      100.0% 
			      1 / 1      100.0% 
			      1 / 1      100.0% 
			      4 / 4      100.0% 
			      0 / 0       0.0% 
			      0 / 0       0.0% 
			      0 / 0       0.0% 
			      0 / 0       0.0% 
			     26 / 38     68.4%AB 
			     12 / 38     31.6%ABE 
		 
		 
			 1 
			  1.9 
			 Francisella cf. novicida Fx1 
			     38 / 38     100.0% 
			      1 / 1      100.0% 
			      1 / 1      100.0% 
			      1 / 1      100.0% 
			      3 / 3      100.0% 
			      3 / 3      100.0% 
			      3 / 3      100.0% 
			      1 / 1      100.0% 
			      1 / 1      100.0% 
			      3 / 3      100.0% 
			      1 / 1      100.0% 
			      3 / 3      100.0% 
			      1 / 1      100.0% 
			      1 / 1      100.0% 
			      1 / 1      100.0% 
			      1 / 1      100.0% 
			      1 / 1      100.0% 
			      1 / 1      100.0% 
			      2 / 2      100.0% 
			      3 / 3      100.0% 
			      1 / 1      100.0% 
			      1 / 1      100.0% 
			      4 / 4      100.0% 
			      0 / 0       0.0% 
			      0 / 0       0.0% 
			      0 / 0       0.0% 
			      0 / 0       0.0% 
			     26 / 38     68.4%AB 
			     12 / 38     31.6%ABE 
		 
		 
			 6 
			  1.9 
			 "Francisella tularensis subsp. nearctica" Olsufjev 1970 
			    229 / 229    100.0% 
			      6 / 6      100.0% 
			      6 / 6      100.0% 
			      6 / 6      100.0% 
			     18 / 18     100.0% 
			     18 / 18     100.0% 
			     18 / 18     100.0% 
			      6 / 6      100.0% 
			      6 / 6      100.0% 
			     18 / 18     100.0% 
			      6 / 6      100.0% 
			     18 / 18     100.0% 
			      6 / 6      100.0% 
			      6 / 6      100.0% 
			      6 / 6      100.0% 
			      6 / 6      100.0% 
			      6 / 6      100.0% 
			      6 / 6      100.0% 
			     12 / 12     100.0% 
			     18 / 18     100.0% 
			      7 / 7      100.0% 
			      6 / 6      100.0% 
			     24 / 24     100.0% 
			      0 / 0       0.0% 
			      0 / 0       0.0% 
			      0 / 0       0.0% 
			      0 / 0       0.0% 
			    156 / 229    68.1%AB 
			     73 / 229    31.9%ABE 
		 
		 
			 1 
			  1.9 
			 Francisella tularensis Biovar A str. SCHU S4 
			     38 / 38     100.0% 
			      1 / 1      100.0% 
			      1 / 1      100.0% 
			      1 / 1      100.0% 
			      3 / 3      100.0% 
			      3 / 3      100.0% 
			      3 / 3      100.0% 
			      1 / 1      100.0% 
			      1 / 1      100.0% 
			      3 / 3      100.0% 
			      1 / 1      100.0% 
			      3 / 3      100.0% 
			      1 / 1      100.0% 
			      1 / 1      100.0% 
			      1 / 1      100.0% 
			      1 / 1      100.0% 
			      1 / 1      100.0% 
			      1 / 1      100.0% 
			      2 / 2      100.0% 
			      3 / 3      100.0% 
			      1 / 1      100.0% 
			      1 / 1      100.0% 
			      4 / 4      100.0% 
			      0 / 0       0.0% 
			      0 / 0       0.0% 
			      0 / 0       0.0% 
			      0 / 0       0.0% 
			     26 / 38     68.4%AB 
			     12 / 38     31.6%ABE 
		 
		 
			 1 
			  1.9 
			 Francisella tularensis subsp. tularensis FSC 198 
			     38 / 38     100.0% 
			      1 / 1      100.0% 
			      1 / 1      100.0% 
			      1 / 1      100.0% 
			      3 / 3      100.0% 
			      3 / 3      100.0% 
			      3 / 3      100.0% 
			      1 / 1      100.0% 
			      1 / 1      100.0% 
			      3 / 3      100.0% 
			      1 / 1      100.0% 
			      3 / 3      100.0% 
			      1 / 1      100.0% 
			      1 / 1      100.0% 
			      1 / 1      100.0% 
			      1 / 1      100.0% 
			      1 / 1      100.0% 
			      1 / 1      100.0% 
			      2 / 2      100.0% 
			      3 / 3      100.0% 
			      1 / 1      100.0% 
			      1 / 1      100.0% 
			      4 / 4      100.0% 
			      0 / 0       0.0% 
			      0 / 0       0.0% 
			      0 / 0       0.0% 
			      0 / 0       0.0% 
			     26 / 38     68.4%AB 
			     12 / 38     31.6%ABE 
		 
		 
			 1 
			  1.9 
			 Francisella tularensis subsp. tularensis WY96-3418 
			     38 / 38     100.0% 
			      1 / 1      100.0% 
			      1 / 1      100.0% 
			      1 / 1      100.0% 
			      3 / 3      100.0% 
			      3 / 3      100.0% 
			      3 / 3      100.0% 
			      1 / 1      100.0% 
			      1 / 1      100.0% 
			      3 / 3      100.0% 
			      1 / 1      100.0% 
			      3 / 3      100.0% 
			      1 / 1      100.0% 
			      1 / 1      100.0% 
			      1 / 1      100.0% 
			      1 / 1      100.0% 
			      1 / 1      100.0% 
			      1 / 1      100.0% 
			      2 / 2      100.0% 
			      3 / 3      100.0% 
			      1 / 1      100.0% 
			      1 / 1      100.0% 
			      4 / 4      100.0% 
			      0 / 0       0.0% 
			      0 / 0       0.0% 
			      0 / 0       0.0% 
			      0 / 0       0.0% 
			     26 / 38     68.4%AB 
			     12 / 38     31.6%ABE 
		 
		 
			 1 
			  1.9 
			 Francisella tularensis subsp. tularensis NE061598 
			     38 / 38     100.0% 
			      1 / 1      100.0% 
			      1 / 1      100.0% 
			      1 / 1      100.0% 
			      3 / 3      100.0% 
			      3 / 3      100.0% 
			      3 / 3      100.0% 
			      1 / 1      100.0% 
			      1 / 1      100.0% 
			      3 / 3      100.0% 
			      1 / 1      100.0% 
			      3 / 3      100.0% 
			      1 / 1      100.0% 
			      1 / 1      100.0% 
			      1 / 1      100.0% 
			      1 / 1      100.0% 
			      1 / 1      100.0% 
			      1 / 1      100.0% 
			      2 / 2      100.0% 
			      3 / 3      100.0% 
			      1 / 1      100.0% 
			      1 / 1      100.0% 
			      4 / 4      100.0% 
			      0 / 0       0.0% 
			      0 / 0       0.0% 
			      0 / 0       0.0% 
			      0 / 0       0.0% 
			     26 / 38     68.4%AB 
			     12 / 38     31.6%ABE 
		 
		 
			 2 
			  1.9 
			 Francisella tularensis TI0902 
			     77 / 77     100.0% 
			      2 / 2      100.0% 
			      2 / 2      100.0% 
			      2 / 2      100.0% 
			      6 / 6      100.0% 
			      6 / 6      100.0% 
			      6 / 6      100.0% 
			      2 / 2      100.0% 
			      2 / 2      100.0% 
			      6 / 6      100.0% 
			      2 / 2      100.0% 
			      6 / 6      100.0% 
			      2 / 2      100.0% 
			      2 / 2      100.0% 
			      2 / 2      100.0% 
			      2 / 2      100.0% 
			      2 / 2      100.0% 
			      2 / 2      100.0% 
			      4 / 4      100.0% 
			      6 / 6      100.0% 
			      3 / 3      100.0% 
			      2 / 2      100.0% 
			      8 / 8      100.0% 
			      0 / 0       0.0% 
			      0 / 0       0.0% 
			      0 / 0       0.0% 
			      0 / 0       0.0% 
			     52 / 77     67.5%AB 
			     25 / 77     32.5%ABE 
		 
		 
			 1 
			  2.0 
			 Francisella tularensis TIGB03 
			     39 / 39     100.0% 
			      1 / 1      100.0% 
			      1 / 1      100.0% 
			      1 / 1      100.0% 
			      3 / 3      100.0% 
			      3 / 3      100.0% 
			      3 / 3      100.0% 
			      1 / 1      100.0% 
			      1 / 1      100.0% 
			      3 / 3      100.0% 
			      1 / 1      100.0% 
			      3 / 3      100.0% 
			      1 / 1      100.0% 
			      1 / 1      100.0% 
			      1 / 1      100.0% 
			      1 / 1      100.0% 
			      1 / 1      100.0% 
			      1 / 1      100.0% 
			      2 / 2      100.0% 
			      3 / 3      100.0% 
			      2 / 2      100.0% 
			      1 / 1      100.0% 
			      4 / 4      100.0% 
			      0 / 0       0.0% 
			      0 / 0       0.0% 
			      0 / 0       0.0% 
			      0 / 0       0.0% 
			     26 / 39     66.7%AB 
			     13 / 39     33.3%ABE 
		 
		 
			 5 
			  1.9 
			 "Francisella tularensis subsp. holarctica" Olsufjev 1970 
			    228 / 228    100.0% 
			      6 / 6      100.0% 
			      6 / 6      100.0% 
			      6 / 6      100.0% 
			     18 / 18     100.0% 
			     18 / 18     100.0% 
			     18 / 18     100.0% 
			      6 / 6      100.0% 
			      6 / 6      100.0% 
			     18 / 18     100.0% 
			      6 / 6      100.0% 
			     18 / 18     100.0% 
			      6 / 6      100.0% 
			      6 / 6      100.0% 
			      6 / 6      100.0% 
			      6 / 6      100.0% 
			      6 / 6      100.0% 
			      6 / 6      100.0% 
			     12 / 12     100.0% 
			     18 / 18     100.0% 
			      6 / 6      100.0% 
			      6 / 6      100.0% 
			     24 / 24     100.0% 
			      0 / 0       0.0% 
			      0 / 0       0.0% 
			      0 / 0       0.0% 
			      0 / 0       0.0% 
			    142 / 228    62.3%AB 
			     67 / 228    29.4%ABE 
			     14 / 228     6.1%B 
			      5 / 228     2.2%BE 
		 
		 
			 1 
			  1.9 
			 Francisella tularensis subsp. holarctica FSC200 
			     38 / 38     100.0% 
			      1 / 1      100.0% 
			      1 / 1      100.0% 
			      1 / 1      100.0% 
			      3 / 3      100.0% 
			      3 / 3      100.0% 
			      3 / 3      100.0% 
			      1 / 1      100.0% 
			      1 / 1      100.0% 
			      3 / 3      100.0% 
			      1 / 1      100.0% 
			      3 / 3      100.0% 
			      1 / 1      100.0% 
			      1 / 1      100.0% 
			      1 / 1      100.0% 
			      1 / 1      100.0% 
			      1 / 1      100.0% 
			      1 / 1      100.0% 
			      2 / 2      100.0% 
			      3 / 3      100.0% 
			      1 / 1      100.0% 
			      1 / 1      100.0% 
			      4 / 4      100.0% 
			      0 / 0       0.0% 
			      0 / 0       0.0% 
			      0 / 0       0.0% 
			      0 / 0       0.0% 
			     26 / 38     68.4%AB 
			     12 / 38     31.6%ABE 
		 
		 
			 1 
			  1.9 
			 Francisella tularensis subsp. holarctica LVS 
			     38 / 38     100.0% 
			      1 / 1      100.0% 
			      1 / 1      100.0% 
			      1 / 1      100.0% 
			      3 / 3      100.0% 
			      3 / 3      100.0% 
			      3 / 3      100.0% 
			      1 / 1      100.0% 
			      1 / 1      100.0% 
			      3 / 3      100.0% 
			      1 / 1      100.0% 
			      3 / 3      100.0% 
			      1 / 1      100.0% 
			      1 / 1      100.0% 
			      1 / 1      100.0% 
			      1 / 1      100.0% 
			      1 / 1      100.0% 
			      1 / 1      100.0% 
			      2 / 2      100.0% 
			      3 / 3      100.0% 
			      1 / 1      100.0% 
			      1 / 1      100.0% 
			      4 / 4      100.0% 
			      0 / 0       0.0% 
			      0 / 0       0.0% 
			      0 / 0       0.0% 
			      0 / 0       0.0% 
			     26 / 38     68.4%AB 
			     12 / 38     31.6%ABE 
		 
		 
			 1 
			  1.9 
			 Francisella tularensis subsp. holarctica OSU18 
			     76 / 76     100.0% 
			      2 / 2      100.0% 
			      2 / 2      100.0% 
			      2 / 2      100.0% 
			      6 / 6      100.0% 
			      6 / 6      100.0% 
			      6 / 6      100.0% 
			      2 / 2      100.0% 
			      2 / 2      100.0% 
			      6 / 6      100.0% 
			      2 / 2      100.0% 
			      6 / 6      100.0% 
			      2 / 2      100.0% 
			      2 / 2      100.0% 
			      2 / 2      100.0% 
			      2 / 2      100.0% 
			      2 / 2      100.0% 
			      2 / 2      100.0% 
			      4 / 4      100.0% 
			      6 / 6      100.0% 
			      2 / 2      100.0% 
			      2 / 2      100.0% 
			      8 / 8      100.0% 
			      0 / 0       0.0% 
			      0 / 0       0.0% 
			      0 / 0       0.0% 
			      0 / 0       0.0% 
			     52 / 76     68.4%AB 
			     24 / 76     31.6%ABE 
		 
		 
			 1 
			  1.9 
			 Francisella tularensis subsp. holarctica FTA 
			     38 / 38     100.0% 
			      1 / 1      100.0% 
			      1 / 1      100.0% 
			      1 / 1      100.0% 
			      3 / 3      100.0% 
			      3 / 3      100.0% 
			      3 / 3      100.0% 
			      1 / 1      100.0% 
			      1 / 1      100.0% 
			      3 / 3      100.0% 
			      1 / 1      100.0% 
			      3 / 3      100.0% 
			      1 / 1      100.0% 
			      1 / 1      100.0% 
			      1 / 1      100.0% 
			      1 / 1      100.0% 
			      1 / 1      100.0% 
			      1 / 1      100.0% 
			      2 / 2      100.0% 
			      3 / 3      100.0% 
			      1 / 1      100.0% 
			      1 / 1      100.0% 
			      4 / 4      100.0% 
			      0 / 0       0.0% 
			      0 / 0       0.0% 
			      0 / 0       0.0% 
			      0 / 0       0.0% 
			     14 / 38     36.8%B 
			     12 / 38     31.6%AB 
			      7 / 38     18.4%ABE 
			      5 / 38     13.2%BE 
		 
		 
			 1 
			  1.9 
			 Francisella tularensis subsp. holarctica F92 
			     38 / 38     100.0% 
			      1 / 1      100.0% 
			      1 / 1      100.0% 
			      1 / 1      100.0% 
			      3 / 3      100.0% 
			      3 / 3      100.0% 
			      3 / 3      100.0% 
			      1 / 1      100.0% 
			      1 / 1      100.0% 
			      3 / 3      100.0% 
			      1 / 1      100.0% 
			      3 / 3      100.0% 
			      1 / 1      100.0% 
			      1 / 1      100.0% 
			      1 / 1      100.0% 
			      1 / 1      100.0% 
			      1 / 1      100.0% 
			      1 / 1      100.0% 
			      2 / 2      100.0% 
			      3 / 3      100.0% 
			      1 / 1      100.0% 
			      1 / 1      100.0% 
			      4 / 4      100.0% 
			      0 / 0       0.0% 
			      0 / 0       0.0% 
			      0 / 0       0.0% 
			      0 / 0       0.0% 
			     26 / 38     68.4%AB 
			     12 / 38     31.6%ABE 
		 
		 
			 1 
			  1.9 
			 "Francisella tularensis subsp. mediasiatica" (Aikimbaev 1966) Olsufjev and Meshcheryakova 1982 
			     38 / 38     100.0% 
			      1 / 1      100.0% 
			      1 / 1      100.0% 
			      1 / 1      100.0% 
			      3 / 3      100.0% 
			      3 / 3      100.0% 
			      3 / 3      100.0% 
			      1 / 1      100.0% 
			      1 / 1      100.0% 
			      3 / 3      100.0% 
			      1 / 1      100.0% 
			      3 / 3      100.0% 
			      1 / 1      100.0% 
			      1 / 1      100.0% 
			      1 / 1      100.0% 
			      1 / 1      100.0% 
			      1 / 1      100.0% 
			      1 / 1      100.0% 
			      2 / 2      100.0% 
			      3 / 3      100.0% 
			      1 / 1      100.0% 
			      1 / 1      100.0% 
			      4 / 4      100.0% 
			      0 / 0       0.0% 
			      0 / 0       0.0% 
			      0 / 0       0.0% 
			      0 / 0       0.0% 
			     18 / 38     47.4%B 
			      8 / 38     21.1%ABE 
			      8 / 38     21.1%AB 
			      4 / 38     10.5%BE 
		 
		 
			 1 
			  1.9 
			 Francisella tularensis subsp. mediasiatica FSC147 
			     38 / 38     100.0% 
			      1 / 1      100.0% 
			      1 / 1      100.0% 
			      1 / 1      100.0% 
			      3 / 3      100.0% 
			      3 / 3      100.0% 
			      3 / 3      100.0% 
			      1 / 1      100.0% 
			      1 / 1      100.0% 
			      3 / 3      100.0% 
			      1 / 1      100.0% 
			      3 / 3      100.0% 
			      1 / 1      100.0% 
			      1 / 1      100.0% 
			      1 / 1      100.0% 
			      1 / 1      100.0% 
			      1 / 1      100.0% 
			      1 / 1      100.0% 
			      2 / 2      100.0% 
			      3 / 3      100.0% 
			      1 / 1      100.0% 
			      1 / 1      100.0% 
			      4 / 4      100.0% 
			      0 / 0       0.0% 
			      0 / 0       0.0% 
			      0 / 0       0.0% 
			      0 / 0       0.0% 
			     18 / 38     47.4%B 
			      8 / 38     21.1%AB 
			      8 / 38     21.1%ABE 
			      4 / 38     10.5%BE 
		 
		 
			 1 
			  2.0 
			 'Philomiragia' bacterium 
			     39 / 39     100.0% 
			      1 / 1      100.0% 
			      1 / 1      100.0% 
			      1 / 1      100.0% 
			      3 / 3      100.0% 
			      3 / 3      100.0% 
			      3 / 3      100.0% 
			      1 / 1      100.0% 
			      1 / 1      100.0% 
			      3 / 3      100.0% 
			      1 / 1      100.0% 
			      3 / 3      100.0% 
			      2 / 2      100.0% 
			      1 / 1      100.0% 
			      1 / 1      100.0% 
			      1 / 1      100.0% 
			      1 / 1      100.0% 
			      1 / 1      100.0% 
			      2 / 2      100.0% 
			      3 / 3      100.0% 
			      1 / 1      100.0% 
			      1 / 1      100.0% 
			      4 / 4      100.0% 
			      0 / 0       0.0% 
			      0 / 0       0.0% 
			      0 / 0       0.0% 
			      0 / 0       0.0% 
			     14 / 39     35.9%AB 
			     13 / 39     33.3%B 
			      8 / 39     20.5%ABE 
			      4 / 39     10.3%BE 
		 
		 
			 1 
			  2.0 
			 Francisella philomiragia subsp. philomiragia ATCC 25017 
			     39 / 39     100.0% 
			      1 / 1      100.0% 
			      1 / 1      100.0% 
			      1 / 1      100.0% 
			      3 / 3      100.0% 
			      3 / 3      100.0% 
			      3 / 3      100.0% 
			      1 / 1      100.0% 
			      1 / 1      100.0% 
			      3 / 3      100.0% 
			      1 / 1      100.0% 
			      3 / 3      100.0% 
			      2 / 2      100.0% 
			      1 / 1      100.0% 
			      1 / 1      100.0% 
			      1 / 1      100.0% 
			      1 / 1      100.0% 
			      1 / 1      100.0% 
			      2 / 2      100.0% 
			      3 / 3      100.0% 
			      1 / 1      100.0% 
			      1 / 1      100.0% 
			      4 / 4      100.0% 
			      0 / 0       0.0% 
			      0 / 0       0.0% 
			      0 / 0       0.0% 
			      0 / 0       0.0% 
			     14 / 39     35.9%AB 
			     13 / 39     33.3%B 
			      8 / 39     20.5%ABE 
			      4 / 39     10.3%BE 
		 
		 
			 1 
			  2.0 
			 Francisella sp. TX077308 
			     39 / 39     100.0% 
			      1 / 1      100.0% 
			      1 / 1      100.0% 
			      1 / 1      100.0% 
			      3 / 3      100.0% 
			      3 / 3      100.0% 
			      3 / 3      100.0% 
			      1 / 1      100.0% 
			      1 / 1      100.0% 
			      3 / 3      100.0% 
			      1 / 1      100.0% 
			      3 / 3      100.0% 
			      2 / 2      100.0% 
			      1 / 1      100.0% 
			      1 / 1      100.0% 
			      1 / 1      100.0% 
			      1 / 1      100.0% 
			      1 / 1      100.0% 
			      2 / 2      100.0% 
			      3 / 3      100.0% 
			      1 / 1      100.0% 
			      1 / 1      100.0% 
			      4 / 4      100.0% 
			      0 / 0       0.0% 
			      0 / 0       0.0% 
			      0 / 0       0.0% 
			      0 / 0       0.0% 
			     27 / 39     69.2%AB 
			     12 / 39     30.8%ABE 
		 
		 
			 1 
			  1.8 
			 DSM 23596 
			     35 / 35     100.0% 
			      1 / 1      100.0% 
			      1 / 1      100.0% 
			      1 / 1      100.0% 
			      1 / 1      100.0% 
			      3 / 3      100.0% 
			      3 / 3      100.0% 
			      1 / 1      100.0% 
			      1 / 1      100.0% 
			      1 / 1      100.0% 
			      1 / 1      100.0% 
			      3 / 3      100.0% 
			      2 / 2      100.0% 
			      1 / 1      100.0% 
			      1 / 1      100.0% 
			      1 / 1      100.0% 
			      1 / 1      100.0% 
			      1 / 1      100.0% 
			      2 / 2      100.0% 
			      3 / 3      100.0% 
			      1 / 1      100.0% 
			      1 / 1      100.0% 
			      4 / 4      100.0% 
			      0 / 0       0.0% 
			      0 / 0       0.0% 
			      0 / 0       0.0% 
			      0 / 0       0.0% 
			     23 / 35     65.7%AB 
			     12 / 35     34.3%ABE 
		 
		 
			 1 
			  1.8 
			 'Francisella asiatica' 
			     35 / 35     100.0% 
			      1 / 1      100.0% 
			      1 / 1      100.0% 
			      1 / 1      100.0% 
			      1 / 1      100.0% 
			      3 / 3      100.0% 
			      3 / 3      100.0% 
			      1 / 1      100.0% 
			      1 / 1      100.0% 
			      1 / 1      100.0% 
			      1 / 1      100.0% 
			      3 / 3      100.0% 
			      2 / 2      100.0% 
			      1 / 1      100.0% 
			      1 / 1      100.0% 
			      1 / 1      100.0% 
			      1 / 1      100.0% 
			      1 / 1      100.0% 
			      2 / 2      100.0% 
			      3 / 3      100.0% 
			      1 / 1      100.0% 
			      1 / 1      100.0% 
			      4 / 4      100.0% 
			      0 / 0       0.0% 
			      0 / 0       0.0% 
			      0 / 0       0.0% 
			      0 / 0       0.0% 
			     23 / 35     65.7%AB 
			     12 / 35     34.3%ABE 
		 
		 
			 1 
			  1.8 
			 Francisella noatunensis subsp. orientalis Toba 04 
			     35 / 35     100.0% 
			      1 / 1      100.0% 
			      1 / 1      100.0% 
			      1 / 1      100.0% 
			      1 / 1      100.0% 
			      3 / 3      100.0% 
			      3 / 3      100.0% 
			      1 / 1      100.0% 
			      1 / 1      100.0% 
			      1 / 1      100.0% 
			      1 / 1      100.0% 
			      3 / 3      100.0% 
			      2 / 2      100.0% 
			      1 / 1      100.0% 
			      1 / 1      100.0% 
			      1 / 1      100.0% 
			      1 / 1      100.0% 
			      1 / 1      100.0% 
			      2 / 2      100.0% 
			      3 / 3      100.0% 
			      1 / 1      100.0% 
			      1 / 1      100.0% 
			      4 / 4      100.0% 
			      0 / 0       0.0% 
			      0 / 0       0.0% 
			      0 / 0       0.0% 
			      0 / 0       0.0% 
			     23 / 35     65.7%AB 
			     12 / 35     34.3%ABE 
		 
		 
			 5 
			  2.5 
			 Piscirickettsia group 
			    199 / 201    99.0% 
			      5 / 5      100.0% 
			      9 / 9      100.0% 
			      5 / 5      100.0% 
			     12 / 12     100.0% 
			     12 / 12     100.0% 
			     12 / 12     100.0% 
			      5 / 5      100.0% 
			      5 / 5      100.0% 
			     17 / 17     100.0% 
			      5 / 5      100.0% 
			     15 / 15     100.0% 
			      6 / 6      100.0% 
			      5 / 5      100.0% 
			      5 / 5      100.0% 
			      5 / 5      100.0% 
			     10 / 10     100.0% 
			      5 / 5      100.0% 
			     11 / 11     100.0% 
			     18 / 19     94.7% 
			      7 / 7      100.0% 
			      5 / 5      100.0% 
			     20 / 21     95.2% 
			      0 / 0       0.0% 
			      0 / 0       0.0% 
			      0 / 0       0.0% 
			      0 / 0       0.0% 
			    133 / 201    66.2%AB 
			     66 / 201    32.8%ABE 
			      1 / 201     0.5%E 
			      1 / 201     0.5% 
		 
		 
			 1 
			  2.4 
			 Thiomicrospira 
			     43 / 43     100.0% 
			      1 / 1      100.0% 
			      2 / 2      100.0% 
			      1 / 1      100.0% 
			      3 / 3      100.0% 
			      3 / 3      100.0% 
			      3 / 3      100.0% 
			      1 / 1      100.0% 
			      1 / 1      100.0% 
			      4 / 4      100.0% 
			      1 / 1      100.0% 
			      3 / 3      100.0% 
			      2 / 2      100.0% 
			      1 / 1      100.0% 
			      1 / 1      100.0% 
			      1 / 1      100.0% 
			      2 / 2      100.0% 
			      1 / 1      100.0% 
			      2 / 2      100.0% 
			      3 / 3      100.0% 
			      2 / 2      100.0% 
			      1 / 1      100.0% 
			      4 / 4      100.0% 
			      0 / 0       0.0% 
			      0 / 0       0.0% 
			      0 / 0       0.0% 
			      0 / 0       0.0% 
			     29 / 43     67.4%AB 
			     14 / 43     32.6%ABE 
		 
		 
			 1 
			  2.4 
			 ATCC 35932 
			     43 / 43     100.0% 
			      1 / 1      100.0% 
			      2 / 2      100.0% 
			      1 / 1      100.0% 
			      3 / 3      100.0% 
			      3 / 3      100.0% 
			      3 / 3      100.0% 
			      1 / 1      100.0% 
			      1 / 1      100.0% 
			      4 / 4      100.0% 
			      1 / 1      100.0% 
			      3 / 3      100.0% 
			      2 / 2      100.0% 
			      1 / 1      100.0% 
			      1 / 1      100.0% 
			      1 / 1      100.0% 
			      2 / 2      100.0% 
			      1 / 1      100.0% 
			      2 / 2      100.0% 
			      3 / 3      100.0% 
			      2 / 2      100.0% 
			      1 / 1      100.0% 
			      4 / 4      100.0% 
			      0 / 0       0.0% 
			      0 / 0       0.0% 
			      0 / 0       0.0% 
			      0 / 0       0.0% 
			     29 / 43     67.4%AB 
			     14 / 43     32.6%ABE 
		 
		 
			 1 
			  2.4 
			 Thiomicrospira crunogena XCL-2 
			     43 / 43     100.0% 
			      1 / 1      100.0% 
			      2 / 2      100.0% 
			      1 / 1      100.0% 
			      3 / 3      100.0% 
			      3 / 3      100.0% 
			      3 / 3      100.0% 
			      1 / 1      100.0% 
			      1 / 1      100.0% 
			      4 / 4      100.0% 
			      1 / 1      100.0% 
			      3 / 3      100.0% 
			      2 / 2      100.0% 
			      1 / 1      100.0% 
			      1 / 1      100.0% 
			      1 / 1      100.0% 
			      2 / 2      100.0% 
			      1 / 1      100.0% 
			      2 / 2      100.0% 
			      3 / 3      100.0% 
			      2 / 2      100.0% 
			      1 / 1      100.0% 
			      4 / 4      100.0% 
			      0 / 0       0.0% 
			      0 / 0       0.0% 
			      0 / 0       0.0% 
			      0 / 0       0.0% 
			     29 / 43     67.4%AB 
			     14 / 43     32.6%ABE 
		 
		 
			 1 
			  2.4 
			 Cycloclasticus 
			     35 / 35     100.0% 
			      1 / 1      100.0% 
			      1 / 1      100.0% 
			      1 / 1      100.0% 
			      1 / 1      100.0% 
			      2 / 2      100.0% 
			      2 / 2      100.0% 
			      1 / 1      100.0% 
			      1 / 1      100.0% 
			      2 / 2      100.0% 
			      1 / 1      100.0% 
			      3 / 3      100.0% 
			      1 / 1      100.0% 
			      1 / 1      100.0% 
			      1 / 1      100.0% 
			      1 / 1      100.0% 
			      2 / 2      100.0% 
			      1 / 1      100.0% 
			      3 / 3      100.0% 
			      4 / 4      100.0% 
			      1 / 1      100.0% 
			      1 / 1      100.0% 
			      3 / 3      100.0% 
			      0 / 0       0.0% 
			      0 / 0       0.0% 
			      0 / 0       0.0% 
			      0 / 0       0.0% 
			     24 / 35     68.6%AB 
			     11 / 35     31.4%ABE 
		 
		 
			 1 
			  2.4 
			 Cycloclasticus sp. P1 
			     35 / 35     100.0% 
			      1 / 1      100.0% 
			      1 / 1      100.0% 
			      1 / 1      100.0% 
			      1 / 1      100.0% 
			      2 / 2      100.0% 
			      2 / 2      100.0% 
			      1 / 1      100.0% 
			      1 / 1      100.0% 
			      2 / 2      100.0% 
			      1 / 1      100.0% 
			      3 / 3      100.0% 
			      1 / 1      100.0% 
			      1 / 1      100.0% 
			      1 / 1      100.0% 
			      1 / 1      100.0% 
			      2 / 2      100.0% 
			      1 / 1      100.0% 
			      3 / 3      100.0% 
			      4 / 4      100.0% 
			      1 / 1      100.0% 
			      1 / 1      100.0% 
			      3 / 3      100.0% 
			      0 / 0       0.0% 
			      0 / 0       0.0% 
			      0 / 0       0.0% 
			      0 / 0       0.0% 
			     24 / 35     68.6%AB 
			     11 / 35     31.4%ABE 
		 
		 
			 2 
			  2.9 
			 Methyliphaga 
			     82 / 82     100.0% 
			      2 / 2      100.0% 
			      4 / 4      100.0% 
			      2 / 2      100.0% 
			      6 / 6      100.0% 
			      4 / 4      100.0% 
			      4 / 4      100.0% 
			      2 / 2      100.0% 
			      2 / 2      100.0% 
			      8 / 8      100.0% 
			      2 / 2      100.0% 
			      6 / 6      100.0% 
			      2 / 2      100.0% 
			      2 / 2      100.0% 
			      2 / 2      100.0% 
			      2 / 2      100.0% 
			      4 / 4      100.0% 
			      2 / 2      100.0% 
			      4 / 4      100.0% 
			      8 / 8      100.0% 
			      2 / 2      100.0% 
			      2 / 2      100.0% 
			     10 / 10     100.0% 
			      0 / 0       0.0% 
			      0 / 0       0.0% 
			      0 / 0       0.0% 
			      0 / 0       0.0% 
			     55 / 82     67.1%AB 
			     27 / 82     32.9%ABE 
		 
		 
			 1 
			  3.1 
			 ATCC BAA-2433 
			     41 / 41     100.0% 
			      1 / 1      100.0% 
			      2 / 2      100.0% 
			      1 / 1      100.0% 
			      3 / 3      100.0% 
			      2 / 2      100.0% 
			      2 / 2      100.0% 
			      1 / 1      100.0% 
			      1 / 1      100.0% 
			      4 / 4      100.0% 
			      1 / 1      100.0% 
			      3 / 3      100.0% 
			      1 / 1      100.0% 
			      1 / 1      100.0% 
			      1 / 1      100.0% 
			      1 / 1      100.0% 
			      2 / 2      100.0% 
			      1 / 1      100.0% 
			      2 / 2      100.0% 
			      4 / 4      100.0% 
			      1 / 1      100.0% 
			      1 / 1      100.0% 
			      5 / 5      100.0% 
			      0 / 0       0.0% 
			      0 / 0       0.0% 
			      0 / 0       0.0% 
			      0 / 0       0.0% 
			     27 / 41     65.9%AB 
			     14 / 41     34.1%ABE 
		 
		 
			 1 
			  2.7 
			 ATCC BAA-2434 
			     41 / 41     100.0% 
			      1 / 1      100.0% 
			      2 / 2      100.0% 
			      1 / 1      100.0% 
			      3 / 3      100.0% 
			      2 / 2      100.0% 
			      2 / 2      100.0% 
			      1 / 1      100.0% 
			      1 / 1      100.0% 
			      4 / 4      100.0% 
			      1 / 1      100.0% 
			      3 / 3      100.0% 
			      1 / 1      100.0% 
			      1 / 1      100.0% 
			      1 / 1      100.0% 
			      1 / 1      100.0% 
			      2 / 2      100.0% 
			      1 / 1      100.0% 
			      2 / 2      100.0% 
			      4 / 4      100.0% 
			      1 / 1      100.0% 
			      1 / 1      100.0% 
			      5 / 5      100.0% 
			      0 / 0       0.0% 
			      0 / 0       0.0% 
			      0 / 0       0.0% 
			      0 / 0       0.0% 
			     28 / 41     68.3%AB 
			     13 / 41     31.7%ABE 
		 
		 
			 1 
			  1.9 
			 Thialkalimicrobium 
			     39 / 41     95.1% 
			      1 / 1      100.0% 
			      2 / 2      100.0% 
			      1 / 1      100.0% 
			      2 / 2      100.0% 
			      3 / 3      100.0% 
			      3 / 3      100.0% 
			      1 / 1      100.0% 
			      1 / 1      100.0% 
			      3 / 3      100.0% 
			      1 / 1      100.0% 
			      3 / 3      100.0% 
			      1 / 1      100.0% 
			      1 / 1      100.0% 
			      1 / 1      100.0% 
			      1 / 1      100.0% 
			      2 / 2      100.0% 
			      1 / 1      100.0% 
			      2 / 2      100.0% 
			      3 / 4      75.0% 
			      2 / 2      100.0% 
			      1 / 1      100.0% 
			      3 / 4      75.0% 
			      0 / 0       0.0% 
			      0 / 0       0.0% 
			      0 / 0       0.0% 
			      0 / 0       0.0% 
			     25 / 41     61.0%AB 
			     14 / 41     34.1%ABE 
			      1 / 41      2.4% 
			      1 / 41      2.4%E 
		 
		 
			 1 
			  1.9 
			 DSM 14477 
			     39 / 41     95.1% 
			      1 / 1      100.0% 
			      2 / 2      100.0% 
			      1 / 1      100.0% 
			      2 / 2      100.0% 
			      3 / 3      100.0% 
			      3 / 3      100.0% 
			      1 / 1      100.0% 
			      1 / 1      100.0% 
			      3 / 3      100.0% 
			      1 / 1      100.0% 
			      3 / 3      100.0% 
			      1 / 1      100.0% 
			      1 / 1      100.0% 
			      1 / 1      100.0% 
			      1 / 1      100.0% 
			      2 / 2      100.0% 
			      1 / 1      100.0% 
			      2 / 2      100.0% 
			      3 / 4      75.0% 
			      2 / 2      100.0% 
			      1 / 1      100.0% 
			      3 / 4      75.0% 
			      0 / 0       0.0% 
			      0 / 0       0.0% 
			      0 / 0       0.0% 
			      0 / 0       0.0% 
			     25 / 41     61.0%AB 
			     14 / 41     34.1%ABE 
			      1 / 41      2.4%E 
			      1 / 41      2.4% 
		 
		 
			 1 
			  1.9 
			 Thioalkalimicrobium cyclicum ALM1 
			     39 / 41     95.1% 
			      1 / 1      100.0% 
			      2 / 2      100.0% 
			      1 / 1      100.0% 
			      2 / 2      100.0% 
			      3 / 3      100.0% 
			      3 / 3      100.0% 
			      1 / 1      100.0% 
			      1 / 1      100.0% 
			      3 / 3      100.0% 
			      1 / 1      100.0% 
			      3 / 3      100.0% 
			      1 / 1      100.0% 
			      1 / 1      100.0% 
			      1 / 1      100.0% 
			      1 / 1      100.0% 
			      2 / 2      100.0% 
			      1 / 1      100.0% 
			      2 / 2      100.0% 
			      3 / 4      75.0% 
			      2 / 2      100.0% 
			      1 / 1      100.0% 
			      3 / 4      75.0% 
			      0 / 0       0.0% 
			      0 / 0       0.0% 
			      0 / 0       0.0% 
			      0 / 0       0.0% 
			     25 / 41     61.0%AB 
			     14 / 41     34.1%ABE 
			      1 / 41      2.4%E 
			      1 / 41      2.4% 
		 
		 
			 69 
			  5.2 
			 Pseudomonaceae/Moraxellaceae group 
			   4597 / 4626   99.4% 
			    190 / 191    99.5% 
			    222 / 222    100.0% 
			    175 / 175    100.0% 
			    327 / 327    100.0% 
			    334 / 334    100.0% 
			    256 / 256    100.0% 
			     97 / 97     100.0% 
			     71 / 71     100.0% 
			    449 / 451    99.6% 
			    105 / 105    100.0% 
			    277 / 278    99.6% 
			    148 / 148    100.0% 
			     91 / 91     100.0% 
			    148 / 148    100.0% 
			     71 / 71     100.0% 
			    200 / 200    100.0% 
			     89 / 89     100.0% 
			    201 / 201    100.0% 
			    373 / 398    93.7% 
			    260 / 260    100.0% 
			     72 / 72     100.0% 
			    410 / 410    100.0% 
			     31 / 31     100.0% 
			      0 / 0       0.0% 
			      0 / 0       0.0% 
			      0 / 0       0.0% 
			   2969 / 4626   64.2%AB 
			   1443 / 4626   31.2%ABE 
			    128 / 4626    2.8%B 
			     57 / 4626    1.2%BE 
			     26 / 4626    0.6% 
			      1 / 4626    0.0%CE 
			      1 / 4626    0.0%E 
			      1 / 4626    0.0%C 
		 
		 
			 22 
			  3.7 
			 Acinetobacteraceae 
			   1530 / 1545   99.0% 
			     56 / 56     100.0% 
			     56 / 56     100.0% 
			     80 / 80     100.0% 
			    124 / 124    100.0% 
			     89 / 89     100.0% 
			     84 / 84     100.0% 
			     24 / 24     100.0% 
			     24 / 24     100.0% 
			    148 / 148    100.0% 
			     58 / 58     100.0% 
			     91 / 91     100.0% 
			     46 / 46     100.0% 
			     41 / 41     100.0% 
			     73 / 73     100.0% 
			     24 / 24     100.0% 
			     49 / 49     100.0% 
			     42 / 42     100.0% 
			     64 / 64     100.0% 
			    119 / 134    88.8% 
			     82 / 82     100.0% 
			     25 / 25     100.0% 
			    131 / 131    100.0% 
			      0 / 0       0.0% 
			      0 / 0       0.0% 
			      0 / 0       0.0% 
			      0 / 0       0.0% 
			    924 / 1545   59.8%AB 
			    491 / 1545   31.8%ABE 
			     78 / 1545    5.0%B 
			     37 / 1545    2.4%BE 
			     15 / 1545    1.0% 
		 
		 
			 17 
			  4.0 
			 Acinetobacter 
			   1231 / 1246   98.8% 
			     50 / 50     100.0% 
			     42 / 42     100.0% 
			     68 / 68     100.0% 
			     99 / 99     100.0% 
			     71 / 71     100.0% 
			     64 / 64     100.0% 
			     18 / 18     100.0% 
			     18 / 18     100.0% 
			    117 / 117    100.0% 
			     52 / 52     100.0% 
			     71 / 71     100.0% 
			     34 / 34     100.0% 
			     34 / 34     100.0% 
			     61 / 61     100.0% 
			     18 / 18     100.0% 
			     37 / 37     100.0% 
			     36 / 36     100.0% 
			     52 / 52     100.0% 
			     96 / 111    86.5% 
			     69 / 69     100.0% 
			     19 / 19     100.0% 
			    105 / 105    100.0% 
			      0 / 0       0.0% 
			      0 / 0       0.0% 
			      0 / 0       0.0% 
			      0 / 0       0.0% 
			    735 / 1246   59.0%AB 
			    399 / 1246   32.0%ABE 
			     63 / 1246    5.1%B 
			     34 / 1246    2.7%BE 
			     15 / 1246    1.2% 
		 
		 
			 1 
			  3.6 
			 Acinetobacter baylyi ADP1 
			     75 / 76     98.7% 
			      3 / 3      100.0% 
			      3 / 3      100.0% 
			      4 / 4      100.0% 
			      7 / 7      100.0% 
			      4 / 4      100.0% 
			      4 / 4      100.0% 
			      1 / 1      100.0% 
			      1 / 1      100.0% 
			      8 / 8      100.0% 
			      2 / 2      100.0% 
			      4 / 4      100.0% 
			      2 / 2      100.0% 
			      2 / 2      100.0% 
			      5 / 5      100.0% 
			      1 / 1      100.0% 
			      2 / 2      100.0% 
			      2 / 2      100.0% 
			      3 / 3      100.0% 
			      5 / 6      83.3% 
			      5 / 5      100.0% 
			      1 / 1      100.0% 
			      6 / 6      100.0% 
			      0 / 0       0.0% 
			      0 / 0       0.0% 
			      0 / 0       0.0% 
			      0 / 0       0.0% 
			     49 / 76     64.5%AB 
			     26 / 76     34.2%ABE 
			      1 / 76      1.3% 
		 
		 
			 15 
			  4.0 
			 Acinetobacter calcoaceticus/baumannii complex 
			   1086 / 1099   98.8% 
			     44 / 44     100.0% 
			     36 / 36     100.0% 
			     60 / 60     100.0% 
			     86 / 86     100.0% 
			     63 / 63     100.0% 
			     56 / 56     100.0% 
			     16 / 16     100.0% 
			     16 / 16     100.0% 
			    102 / 102    100.0% 
			     47 / 47     100.0% 
			     63 / 63     100.0% 
			     30 / 30     100.0% 
			     30 / 30     100.0% 
			     54 / 54     100.0% 
			     16 / 16     100.0% 
			     33 / 33     100.0% 
			     32 / 32     100.0% 
			     46 / 46     100.0% 
			     86 / 99     86.9% 
			     60 / 60     100.0% 
			     17 / 17     100.0% 
			     93 / 93     100.0% 
			      0 / 0       0.0% 
			      0 / 0       0.0% 
			      0 / 0       0.0% 
			      0 / 0       0.0% 
			    639 / 1099   58.1%AB 
			    350 / 1099   31.8%ABE 
			     63 / 1099    5.7%B 
			     34 / 1099    3.1%BE 
			     13 / 1099    1.2% 
		 
		 
			 14 
			  4.0 
			 ATCC 19606 
			   1020 / 1032   98.8% 
			     41 / 41     100.0% 
			     34 / 34     100.0% 
			     56 / 56     100.0% 
			     84 / 84     100.0% 
			     60 / 60     100.0% 
			     52 / 52     100.0% 
			     15 / 15     100.0% 
			     15 / 15     100.0% 
			     99 / 99     100.0% 
			     43 / 43     100.0% 
			     59 / 59     100.0% 
			     27 / 27     100.0% 
			     28 / 28     100.0% 
			     49 / 49     100.0% 
			     15 / 15     100.0% 
			     31 / 31     100.0% 
			     30 / 30     100.0% 
			     43 / 43     100.0% 
			     81 / 93     87.1% 
			     56 / 56     100.0% 
			     16 / 16     100.0% 
			     86 / 86     100.0% 
			      0 / 0       0.0% 
			      0 / 0       0.0% 
			      0 / 0       0.0% 
			      0 / 0       0.0% 
			    601 / 1032   58.2%AB 
			    322 / 1032   31.2%ABE 
			     63 / 1032    6.1%B 
			     34 / 1032    3.3%BE 
			     12 / 1032    1.2% 
		 
		 
			 1 
			  4.0 
			 Acinetobacter baumannii ATCC 17978 
			     69 / 70     98.6% 
			      2 / 2      100.0% 
			      3 / 3      100.0% 
			      4 / 4      100.0% 
			      5 / 5      100.0% 
			      4 / 4      100.0% 
			      4 / 4      100.0% 
			      1 / 1      100.0% 
			      1 / 1      100.0% 
			      6 / 6      100.0% 
			      3 / 3      100.0% 
			      4 / 4      100.0% 
			      2 / 2      100.0% 
			      2 / 2      100.0% 
			      4 / 4      100.0% 
			      1 / 1      100.0% 
			      2 / 2      100.0% 
			      2 / 2      100.0% 
			      3 / 3      100.0% 
			      5 / 6      83.3% 
			      4 / 4      100.0% 
			      1 / 1      100.0% 
			      6 / 6      100.0% 
			      0 / 0       0.0% 
			      0 / 0       0.0% 
			      0 / 0       0.0% 
			      0 / 0       0.0% 
			     45 / 70     64.3%AB 
			     24 / 70     34.3%ABE 
			      1 / 70      1.4% 
		 
		 
			 1 
			  4.0 
			 Acinetobacter baumannii ACICU 
			     63 / 64     98.4% 
			      3 / 3      100.0% 
			      0 / 0       0.0% 
			      4 / 4      100.0% 
			      6 / 6      100.0% 
			      3 / 3      100.0% 
			      2 / 2      100.0% 
			      1 / 1      100.0% 
			      1 / 1      100.0% 
			      7 / 7      100.0% 
			      3 / 3      100.0% 
			      4 / 4      100.0% 
			      2 / 2      100.0% 
			      2 / 2      100.0% 
			      1 / 1      100.0% 
			      1 / 1      100.0% 
			      2 / 2      100.0% 
			      2 / 2      100.0% 
			      3 / 3      100.0% 
			      5 / 6      83.3% 
			      4 / 4      100.0% 
			      1 / 1      100.0% 
			      6 / 6      100.0% 
			      0 / 0       0.0% 
			      0 / 0       0.0% 
			      0 / 0       0.0% 
			      0 / 0       0.0% 
			     23 / 64     35.9%AB 
			     18 / 64     28.1%B 
			     13 / 64     20.3%ABE 
			      9 / 64     14.1%BE 
			      1 / 64      1.6% 
		 
		 
			 1 
			  4.1 
			 Acinetobacter baumannii AB0057 
			     73 / 73     100.0% 
			      3 / 3      100.0% 
			      3 / 3      100.0% 
			      4 / 4      100.0% 
			      6 / 6      100.0% 
			      4 / 4      100.0% 
			      4 / 4      100.0% 
			      1 / 1      100.0% 
			      1 / 1      100.0% 
			      7 / 7      100.0% 
			      3 / 3      100.0% 
			      4 / 4      100.0% 
			      1 / 1      100.0% 
			      2 / 2      100.0% 
			      4 / 4      100.0% 
			      1 / 1      100.0% 
			      2 / 2      100.0% 
			      2 / 2      100.0% 
			      3 / 3      100.0% 
			      7 / 7      100.0% 
			      4 / 4      100.0% 
			      1 / 1      100.0% 
			      6 / 6      100.0% 
			      0 / 0       0.0% 
			      0 / 0       0.0% 
			      0 / 0       0.0% 
			      0 / 0       0.0% 
			     47 / 73     64.4%AB 
			     26 / 73     35.6%ABE 
		 
		 
			 1 
			  4.0 
			 Acinetobacter baumannii MDR-ZJ06 
			     68 / 69     98.6% 
			      2 / 2      100.0% 
			      3 / 3      100.0% 
			      2 / 2      100.0% 
			      6 / 6      100.0% 
			      4 / 4      100.0% 
			      4 / 4      100.0% 
			      1 / 1      100.0% 
			      1 / 1      100.0% 
			      7 / 7      100.0% 
			      3 / 3      100.0% 
			      4 / 4      100.0% 
			      2 / 2      100.0% 
			      1 / 1      100.0% 
			      4 / 4      100.0% 
			      1 / 1      100.0% 
			      2 / 2      100.0% 
			      2 / 2      100.0% 
			      3 / 3      100.0% 
			      5 / 6      83.3% 
			      4 / 4      100.0% 
			      1 / 1      100.0% 
			      6 / 6      100.0% 
			      0 / 0       0.0% 
			      0 / 0       0.0% 
			      0 / 0       0.0% 
			      0 / 0       0.0% 
			     44 / 69     63.8%AB 
			     24 / 69     34.8%ABE 
			      1 / 69      1.4% 
		 
		 
			 1 
			  3.8 
			 Acinetobacter baumannii A307 
			     72 / 73     98.6% 
			      3 / 3      100.0% 
			      3 / 3      100.0% 
			      4 / 4      100.0% 
			      6 / 6      100.0% 
			      4 / 4      100.0% 
			      4 / 4      100.0% 
			      1 / 1      100.0% 
			      1 / 1      100.0% 
			      7 / 7      100.0% 
			      3 / 3      100.0% 
			      4 / 4      100.0% 
			      2 / 2      100.0% 
			      2 / 2      100.0% 
			      4 / 4      100.0% 
			      1 / 1      100.0% 
			      2 / 2      100.0% 
			      2 / 2      100.0% 
			      3 / 3      100.0% 
			      5 / 6      83.3% 
			      4 / 4      100.0% 
			      1 / 1      100.0% 
			      6 / 6      100.0% 
			      0 / 0       0.0% 
			      0 / 0       0.0% 
			      0 / 0       0.0% 
			      0 / 0       0.0% 
			     47 / 73     64.4%AB 
			     25 / 73     34.2%ABE 
			      1 / 73      1.4% 
		 
		 
			 1 
			  4.0 
			 Acinetobacter baumannii 1656-2 
			     70 / 71     98.6% 
			      3 / 3      100.0% 
			      2 / 2      100.0% 
			      4 / 4      100.0% 
			      6 / 6      100.0% 
			      4 / 4      100.0% 
			      3 / 3      100.0% 
			      1 / 1      100.0% 
			      1 / 1      100.0% 
			      7 / 7      100.0% 
			      3 / 3      100.0% 
			      4 / 4      100.0% 
			      2 / 2      100.0% 
			      2 / 2      100.0% 
			      4 / 4      100.0% 
			      1 / 1      100.0% 
			      2 / 2      100.0% 
			      2 / 2      100.0% 
			      3 / 3      100.0% 
			      5 / 6      83.3% 
			      4 / 4      100.0% 
			      1 / 1      100.0% 
			      6 / 6      100.0% 
			      0 / 0       0.0% 
			      0 / 0       0.0% 
			      0 / 0       0.0% 
			      0 / 0       0.0% 
			     45 / 71     63.4%AB 
			     25 / 71     35.2%ABE 
			      1 / 71      1.4% 
		 
		 
			 1 
			  4.2 
			 Acinetobacter baumannii MDR-TJ 
			     73 / 74     98.6% 
			      3 / 3      100.0% 
			      3 / 3      100.0% 
			      4 / 4      100.0% 
			      6 / 6      100.0% 
			      4 / 4      100.0% 
			      4 / 4      100.0% 
			      1 / 1      100.0% 
			      1 / 1      100.0% 
			      7 / 7      100.0% 
			      3 / 3      100.0% 
			      4 / 4      100.0% 
			      2 / 2      100.0% 
			      2 / 2      100.0% 
			      4 / 4      100.0% 
			      1 / 1      100.0% 
			      2 / 2      100.0% 
			      2 / 2      100.0% 
			      3 / 3      100.0% 
			      6 / 7      85.7% 
			      4 / 4      100.0% 
			      1 / 1      100.0% 
			      6 / 6      100.0% 
			      0 / 0       0.0% 
			      0 / 0       0.0% 
			      0 / 0       0.0% 
			      0 / 0       0.0% 
			     47 / 74     63.5%AB 
			     26 / 74     35.1%ABE 
			      1 / 74      1.4% 
		 
		 
			 1 
			  3.7 
			 Acinetobacter baumannii D1279779 
			     63 / 64     98.4% 
			      3 / 3      100.0% 
			      1 / 1      100.0% 
			      4 / 4      100.0% 
			      6 / 6      100.0% 
			      4 / 4      100.0% 
			      3 / 3      100.0% 
			      1 / 1      100.0% 
			      1 / 1      100.0% 
			      7 / 7      100.0% 
			      2 / 2      100.0% 
			      4 / 4      100.0% 
			      2 / 2      100.0% 
			      2 / 2      100.0% 
			      1 / 1      100.0% 
			      1 / 1      100.0% 
			      2 / 2      100.0% 
			      2 / 2      100.0% 
			      3 / 3      100.0% 
			      5 / 6      83.3% 
			      4 / 4      100.0% 
			      1 / 1      100.0% 
			      4 / 4      100.0% 
			      0 / 0       0.0% 
			      0 / 0       0.0% 
			      0 / 0       0.0% 
			      0 / 0       0.0% 
			     44 / 64     68.8%AB 
			     19 / 64     29.7%ABE 
			      1 / 64      1.6% 
		 
		 
			 1 
			  4.2 
			 Acinetobacter baumannii TCDC-AB0715 
			     41 / 42     97.6% 
			      1 / 1      100.0% 
			      0 / 0       0.0% 
			      4 / 4      100.0% 
			      4 / 4      100.0% 
			      1 / 1      100.0% 
			      1 / 1      100.0% 
			      1 / 1      100.0% 
			      1 / 1      100.0% 
			      5 / 5      100.0% 
			      2 / 2      100.0% 
			      4 / 4      100.0% 
			      0 / 0       0.0% 
			      1 / 1      100.0% 
			      0 / 0       0.0% 
			      1 / 1      100.0% 
			      2 / 2      100.0% 
			      2 / 2      100.0% 
			      2 / 2      100.0% 
			      3 / 4      75.0% 
			      0 / 0       0.0% 
			      2 / 2      100.0% 
			      4 / 4      100.0% 
			      0 / 0       0.0% 
			      0 / 0       0.0% 
			      0 / 0       0.0% 
			      0 / 0       0.0% 
			     28 / 42     66.7%AB 
			     13 / 42     31.0%ABE 
			      1 / 42      2.4% 
		 
		 
			 1 
			  4.0 
			 Acinetobacter baumannii BJAB07104 
			     73 / 74     98.6% 
			      3 / 3      100.0% 
			      3 / 3      100.0% 
			      4 / 4      100.0% 
			      6 / 6      100.0% 
			      4 / 4      100.0% 
			      4 / 4      100.0% 
			      1 / 1      100.0% 
			      1 / 1      100.0% 
			      7 / 7      100.0% 
			      3 / 3      100.0% 
			      4 / 4      100.0% 
			      2 / 2      100.0% 
			      2 / 2      100.0% 
			      4 / 4      100.0% 
			      1 / 1      100.0% 
			      2 / 2      100.0% 
			      2 / 2      100.0% 
			      3 / 3      100.0% 
			      6 / 7      85.7% 
			      4 / 4      100.0% 
			      1 / 1      100.0% 
			      6 / 6      100.0% 
			      0 / 0       0.0% 
			      0 / 0       0.0% 
			      0 / 0       0.0% 
			      0 / 0       0.0% 
			     47 / 74     63.5%AB 
			     26 / 74     35.1%ABE 
			      1 / 74      1.4% 
		 
		 
			 1 
			  4.1 
			 Acinetobacter baumannii BJAB0715 
			     72 / 73     98.6% 
			      3 / 3      100.0% 
			      3 / 3      100.0% 
			      4 / 4      100.0% 
			      6 / 6      100.0% 
			      4 / 4      100.0% 
			      4 / 4      100.0% 
			      1 / 1      100.0% 
			      1 / 1      100.0% 
			      7 / 7      100.0% 
			      3 / 3      100.0% 
			      4 / 4      100.0% 
			      2 / 2      100.0% 
			      2 / 2      100.0% 
			      5 / 5      100.0% 
			      1 / 1      100.0% 
			      2 / 2      100.0% 
			      2 / 2      100.0% 
			      2 / 2      100.0% 
			      5 / 6      83.3% 
			      4 / 4      100.0% 
			      1 / 1      100.0% 
			      6 / 6      100.0% 
			      0 / 0       0.0% 
			      0 / 0       0.0% 
			      0 / 0       0.0% 
			      0 / 0       0.0% 
			     46 / 73     63.0%AB 
			     26 / 73     35.6%ABE 
			      1 / 73      1.4% 
		 
		 
			 1 
			  4.0 
			 Acinetobacter baumannii BJAB0868 
			     75 / 75     100.0% 
			      3 / 3      100.0% 
			      3 / 3      100.0% 
			      4 / 4      100.0% 
			      6 / 6      100.0% 
			      4 / 4      100.0% 
			      4 / 4      100.0% 
			      1 / 1      100.0% 
			      1 / 1      100.0% 
			      7 / 7      100.0% 
			      3 / 3      100.0% 
			      4 / 4      100.0% 
			      2 / 2      100.0% 
			      2 / 2      100.0% 
			      4 / 4      100.0% 
			      1 / 1      100.0% 
			      2 / 2      100.0% 
			      2 / 2      100.0% 
			      3 / 3      100.0% 
			      8 / 8      100.0% 
			      4 / 4      100.0% 
			      1 / 1      100.0% 
			      6 / 6      100.0% 
			      0 / 0       0.0% 
			      0 / 0       0.0% 
			      0 / 0       0.0% 
			      0 / 0       0.0% 
			     48 / 75     64.0%AB 
			     27 / 75     36.0%ABE 
		 
		 
			 1 
			  4.0 
			 Acinetobacter baumannii TYTH-1 
			     73 / 74     98.6% 
			      3 / 3      100.0% 
			      3 / 3      100.0% 
			      4 / 4      100.0% 
			      4 / 4      100.0% 
			      8 / 8      100.0% 
			      4 / 4      100.0% 
			      1 / 1      100.0% 
			      1 / 1      100.0% 
			      5 / 5      100.0% 
			      3 / 3      100.0% 
			      4 / 4      100.0% 
			      2 / 2      100.0% 
			      2 / 2      100.0% 
			      4 / 4      100.0% 
			      1 / 1      100.0% 
			      2 / 2      100.0% 
			      2 / 2      100.0% 
			      3 / 3      100.0% 
			      6 / 7      85.7% 
			      4 / 4      100.0% 
			      1 / 1      100.0% 
			      6 / 6      100.0% 
			      0 / 0       0.0% 
			      0 / 0       0.0% 
			      0 / 0       0.0% 
			      0 / 0       0.0% 
			     47 / 74     63.5%AB 
			     26 / 74     35.1%ABE 
			      1 / 74      1.4% 
		 
		 
			 1 
			  3.9 
			 "Micrococcus calco-aceticus" Beijerinck 1911 
			     66 / 67     98.5% 
			      3 / 3      100.0% 
			      2 / 2      100.0% 
			      4 / 4      100.0% 
			      2 / 2      100.0% 
			      3 / 3      100.0% 
			      4 / 4      100.0% 
			      1 / 1      100.0% 
			      1 / 1      100.0% 
			      3 / 3      100.0% 
			      4 / 4      100.0% 
			      4 / 4      100.0% 
			      3 / 3      100.0% 
			      2 / 2      100.0% 
			      5 / 5      100.0% 
			      1 / 1      100.0% 
			      2 / 2      100.0% 
			      2 / 2      100.0% 
			      3 / 3      100.0% 
			      5 / 6      83.3% 
			      4 / 4      100.0% 
			      1 / 1      100.0% 
			      7 / 7      100.0% 
			      0 / 0       0.0% 
			      0 / 0       0.0% 
			      0 / 0       0.0% 
			      0 / 0       0.0% 
			     38 / 67     56.7%AB 
			     28 / 67     41.8%ABE 
			      1 / 67      1.5% 
		 
		 
			 1 
			  3.9 
			 Acinetobacter calcoaceticus PHEA-2 
			     66 / 67     98.5% 
			      3 / 3      100.0% 
			      2 / 2      100.0% 
			      4 / 4      100.0% 
			      2 / 2      100.0% 
			      3 / 3      100.0% 
			      4 / 4      100.0% 
			      1 / 1      100.0% 
			      1 / 1      100.0% 
			      3 / 3      100.0% 
			      4 / 4      100.0% 
			      4 / 4      100.0% 
			      3 / 3      100.0% 
			      2 / 2      100.0% 
			      5 / 5      100.0% 
			      1 / 1      100.0% 
			      2 / 2      100.0% 
			      2 / 2      100.0% 
			      3 / 3      100.0% 
			      5 / 6      83.3% 
			      4 / 4      100.0% 
			      1 / 1      100.0% 
			      7 / 7      100.0% 
			      0 / 0       0.0% 
			      0 / 0       0.0% 
			      0 / 0       0.0% 
			      0 / 0       0.0% 
			     38 / 67     56.7%AB 
			     28 / 67     41.8%ABE 
			      1 / 67      1.5% 
		 
		 
			 1 
			  4.2 
			 "Acinetobacter oleivorans" Kang et al. 2011 
			     70 / 71     98.6% 
			      3 / 3      100.0% 
			      3 / 3      100.0% 
			      4 / 4      100.0% 
			      6 / 6      100.0% 
			      4 / 4      100.0% 
			      4 / 4      100.0% 
			      1 / 1      100.0% 
			      1 / 1      100.0% 
			      7 / 7      100.0% 
			      3 / 3      100.0% 
			      4 / 4      100.0% 
			      2 / 2      100.0% 
			      2 / 2      100.0% 
			      2 / 2      100.0% 
			      1 / 1      100.0% 
			      2 / 2      100.0% 
			      2 / 2      100.0% 
			      3 / 3      100.0% 
			      5 / 6      83.3% 
			      4 / 4      100.0% 
			      1 / 1      100.0% 
			      6 / 6      100.0% 
			      0 / 0       0.0% 
			      0 / 0       0.0% 
			      0 / 0       0.0% 
			      0 / 0       0.0% 
			     47 / 71     66.2%AB 
			     23 / 71     32.4%ABE 
			      1 / 71      1.4% 
		 
		 
			 1 
			  4.2 
			 Acinetobacter oleivorans DR1 
			     70 / 71     98.6% 
			      3 / 3      100.0% 
			      3 / 3      100.0% 
			      4 / 4      100.0% 
			      6 / 6      100.0% 
			      4 / 4      100.0% 
			      4 / 4      100.0% 
			      1 / 1      100.0% 
			      1 / 1      100.0% 
			      7 / 7      100.0% 
			      3 / 3      100.0% 
			      4 / 4      100.0% 
			      2 / 2      100.0% 
			      2 / 2      100.0% 
			      2 / 2      100.0% 
			      1 / 1      100.0% 
			      2 / 2      100.0% 
			      2 / 2      100.0% 
			      3 / 3      100.0% 
			      5 / 6      83.3% 
			      4 / 4      100.0% 
			      1 / 1      100.0% 
			      6 / 6      100.0% 
			      0 / 0       0.0% 
			      0 / 0       0.0% 
			      0 / 0       0.0% 
			      0 / 0       0.0% 
			     47 / 71     66.2%AB 
			     23 / 71     32.4%ABE 
			      1 / 71      1.4% 
		 
		 
			 1 
			  1.8 
			 "Diplobacillus" McNab 1904 
			     50 / 50     100.0% 
			      1 / 1      100.0% 
			      2 / 2      100.0% 
			      2 / 2      100.0% 
			      4 / 4      100.0% 
			      3 / 3      100.0% 
			      3 / 3      100.0% 
			      1 / 1      100.0% 
			      1 / 1      100.0% 
			      5 / 5      100.0% 
			      1 / 1      100.0% 
			      4 / 4      100.0% 
			      2 / 2      100.0% 
			      2 / 2      100.0% 
			      2 / 2      100.0% 
			      1 / 1      100.0% 
			      2 / 2      100.0% 
			      1 / 1      100.0% 
			      2 / 2      100.0% 
			      3 / 3      100.0% 
			      2 / 2      100.0% 
			      1 / 1      100.0% 
			      5 / 5      100.0% 
			      0 / 0       0.0% 
			      0 / 0       0.0% 
			      0 / 0       0.0% 
			      0 / 0       0.0% 
			     33 / 50     66.0%AB 
			     17 / 50     34.0%ABE 
		 
		 
			 1 
			  1.8 
			 Branhamella 
			     50 / 50     100.0% 
			      1 / 1      100.0% 
			      2 / 2      100.0% 
			      2 / 2      100.0% 
			      4 / 4      100.0% 
			      3 / 3      100.0% 
			      3 / 3      100.0% 
			      1 / 1      100.0% 
			      1 / 1      100.0% 
			      5 / 5      100.0% 
			      1 / 1      100.0% 
			      4 / 4      100.0% 
			      2 / 2      100.0% 
			      2 / 2      100.0% 
			      2 / 2      100.0% 
			      1 / 1      100.0% 
			      2 / 2      100.0% 
			      1 / 1      100.0% 
			      2 / 2      100.0% 
			      3 / 3      100.0% 
			      2 / 2      100.0% 
			      1 / 1      100.0% 
			      5 / 5      100.0% 
			      0 / 0       0.0% 
			      0 / 0       0.0% 
			      0 / 0       0.0% 
			      0 / 0       0.0% 
			     33 / 50     66.0%AB 
			     17 / 50     34.0%ABE 
		 
		 
			 1 
			  1.8 
			 "Mikrokkokus catarrhalis" (sic) Frosch and Kolle in Flugge 1896 
			     50 / 50     100.0% 
			      1 / 1      100.0% 
			      2 / 2      100.0% 
			      2 / 2      100.0% 
			      4 / 4      100.0% 
			      3 / 3      100.0% 
			      3 / 3      100.0% 
			      1 / 1      100.0% 
			      1 / 1      100.0% 
			      5 / 5      100.0% 
			      1 / 1      100.0% 
			      4 / 4      100.0% 
			      2 / 2      100.0% 
			      2 / 2      100.0% 
			      2 / 2      100.0% 
			      1 / 1      100.0% 
			      2 / 2      100.0% 
			      1 / 1      100.0% 
			      2 / 2      100.0% 
			      3 / 3      100.0% 
			      2 / 2      100.0% 
			      1 / 1      100.0% 
			      5 / 5      100.0% 
			      0 / 0       0.0% 
			      0 / 0       0.0% 
			      0 / 0       0.0% 
			      0 / 0       0.0% 
			     33 / 50     66.0%AB 
			     17 / 50     34.0%ABE 
		 
		 
			 1 
			  1.8 
			 Moraxella catarrhalis RH4 
			     50 / 50     100.0% 
			      1 / 1      100.0% 
			      2 / 2      100.0% 
			      2 / 2      100.0% 
			      4 / 4      100.0% 
			      3 / 3      100.0% 
			      3 / 3      100.0% 
			      1 / 1      100.0% 
			      1 / 1      100.0% 
			      5 / 5      100.0% 
			      1 / 1      100.0% 
			      4 / 4      100.0% 
			      2 / 2      100.0% 
			      2 / 2      100.0% 
			      2 / 2      100.0% 
			      1 / 1      100.0% 
			      2 / 2      100.0% 
			      1 / 1      100.0% 
			      2 / 2      100.0% 
			      3 / 3      100.0% 
			      2 / 2      100.0% 
			      1 / 1      100.0% 
			      5 / 5      100.0% 
			      0 / 0       0.0% 
			      0 / 0       0.0% 
			      0 / 0       0.0% 
			      0 / 0       0.0% 
			     33 / 50     66.0%AB 
			     17 / 50     34.0%ABE 
		 
		 
			 4 
			  3.0 
			 Psychrobacter 
			    249 / 249    100.0% 
			      5 / 5      100.0% 
			     12 / 12     100.0% 
			     10 / 10     100.0% 
			     21 / 21     100.0% 
			     15 / 15     100.0% 
			     17 / 17     100.0% 
			      5 / 5      100.0% 
			      5 / 5      100.0% 
			     26 / 26     100.0% 
			      5 / 5      100.0% 
			     16 / 16     100.0% 
			     10 / 10     100.0% 
			      5 / 5      100.0% 
			     10 / 10     100.0% 
			      5 / 5      100.0% 
			     10 / 10     100.0% 
			      5 / 5      100.0% 
			     10 / 10     100.0% 
			     20 / 20     100.0% 
			     11 / 11     100.0% 
			      5 / 5      100.0% 
			     21 / 21     100.0% 
			      0 / 0       0.0% 
			      0 / 0       0.0% 
			      0 / 0       0.0% 
			      0 / 0       0.0% 
			    156 / 249    62.7%AB 
			     75 / 249    30.1%ABE 
			     15 / 249     6.0%B 
			      3 / 249     1.2%BE 
		 
		 
			 1 
			  3.1 
			 DSM 17306 
			     48 / 48     100.0% 
			      1 / 1      100.0% 
			      2 / 2      100.0% 
			      2 / 2      100.0% 
			      4 / 4      100.0% 
			      3 / 3      100.0% 
			      3 / 3      100.0% 
			      1 / 1      100.0% 
			      1 / 1      100.0% 
			      5 / 5      100.0% 
			      1 / 1      100.0% 
			      3 / 3      100.0% 
			      2 / 2      100.0% 
			      1 / 1      100.0% 
			      2 / 2      100.0% 
			      1 / 1      100.0% 
			      2 / 2      100.0% 
			      1 / 1      100.0% 
			      2 / 2      100.0% 
			      4 / 4      100.0% 
			      2 / 2      100.0% 
			      1 / 1      100.0% 
			      4 / 4      100.0% 
			      0 / 0       0.0% 
			      0 / 0       0.0% 
			      0 / 0       0.0% 
			      0 / 0       0.0% 
			     33 / 48     68.8%AB 
			     15 / 48     31.2%ABE 
		 
		 
			 1 
			  3.1 
			 Psychrobacter cryohalolentis K5 
			     48 / 48     100.0% 
			      1 / 1      100.0% 
			      2 / 2      100.0% 
			      2 / 2      100.0% 
			      4 / 4      100.0% 
			      3 / 3      100.0% 
			      3 / 3      100.0% 
			      1 / 1      100.0% 
			      1 / 1      100.0% 
			      5 / 5      100.0% 
			      1 / 1      100.0% 
			      3 / 3      100.0% 
			      2 / 2      100.0% 
			      1 / 1      100.0% 
			      2 / 2      100.0% 
			      1 / 1      100.0% 
			      2 / 2      100.0% 
			      1 / 1      100.0% 
			      2 / 2      100.0% 
			      4 / 4      100.0% 
			      2 / 2      100.0% 
			      1 / 1      100.0% 
			      4 / 4      100.0% 
			      0 / 0       0.0% 
			      0 / 0       0.0% 
			      0 / 0       0.0% 
			      0 / 0       0.0% 
			     33 / 48     68.8%AB 
			     15 / 48     31.2%ABE 
		 
		 
			 1 
			  2.7 
			 DSM 17307 
			     96 / 96     100.0% 
			      2 / 2      100.0% 
			      4 / 4      100.0% 
			      4 / 4      100.0% 
			      8 / 8      100.0% 
			      6 / 6      100.0% 
			      6 / 6      100.0% 
			      2 / 2      100.0% 
			      2 / 2      100.0% 
			     10 / 10     100.0% 
			      2 / 2      100.0% 
			      6 / 6      100.0% 
			      4 / 4      100.0% 
			      2 / 2      100.0% 
			      4 / 4      100.0% 
			      2 / 2      100.0% 
			      4 / 4      100.0% 
			      2 / 2      100.0% 
			      4 / 4      100.0% 
			      8 / 8      100.0% 
			      4 / 4      100.0% 
			      2 / 2      100.0% 
			      8 / 8      100.0% 
			      0 / 0       0.0% 
			      0 / 0       0.0% 
			      0 / 0       0.0% 
			      0 / 0       0.0% 
			     51 / 96     53.1%AB 
			     27 / 96     28.1%ABE 
			     15 / 96     15.6%B 
			      3 / 96      3.1%BE 
		 
		 
			 1 
			  2.7 
			 Psychrobacter arcticum 273-4 
			     96 / 96     100.0% 
			      2 / 2      100.0% 
			      4 / 4      100.0% 
			      4 / 4      100.0% 
			      8 / 8      100.0% 
			      6 / 6      100.0% 
			      6 / 6      100.0% 
			      2 / 2      100.0% 
			      2 / 2      100.0% 
			     10 / 10     100.0% 
			      2 / 2      100.0% 
			      6 / 6      100.0% 
			      4 / 4      100.0% 
			      2 / 2      100.0% 
			      4 / 4      100.0% 
			      2 / 2      100.0% 
			      4 / 4      100.0% 
			      2 / 2      100.0% 
			      4 / 4      100.0% 
			      8 / 8      100.0% 
			      4 / 4      100.0% 
			      2 / 2      100.0% 
			      8 / 8      100.0% 
			      0 / 0       0.0% 
			      0 / 0       0.0% 
			      0 / 0       0.0% 
			      0 / 0       0.0% 
			     51 / 96     53.1%AB 
			     27 / 96     28.1%ABE 
			     15 / 96     15.6%B 
			      3 / 96      3.1%BE 
		 
		 
			 1 
			  3.0 
			 Psychrobacter sp. PRwf-1 
			     57 / 57     100.0% 
			      1 / 1      100.0% 
			      4 / 4      100.0% 
			      2 / 2      100.0% 
			      5 / 5      100.0% 
			      3 / 3      100.0% 
			      5 / 5      100.0% 
			      1 / 1      100.0% 
			      1 / 1      100.0% 
			      6 / 6      100.0% 
			      1 / 1      100.0% 
			      4 / 4      100.0% 
			      2 / 2      100.0% 
			      1 / 1      100.0% 
			      2 / 2      100.0% 
			      1 / 1      100.0% 
			      2 / 2      100.0% 
			      1 / 1      100.0% 
			      2 / 2      100.0% 
			      4 / 4      100.0% 
			      3 / 3      100.0% 
			      1 / 1      100.0% 
			      5 / 5      100.0% 
			      0 / 0       0.0% 
			      0 / 0       0.0% 
			      0 / 0       0.0% 
			      0 / 0       0.0% 
			     39 / 57     68.4%AB 
			     18 / 57     31.6%ABE 
		 
		 
			 1 
			  3.1 
			 Psychrobacter sp. G 
			     48 / 48     100.0% 
			      1 / 1      100.0% 
			      2 / 2      100.0% 
			      2 / 2      100.0% 
			      4 / 4      100.0% 
			      3 / 3      100.0% 
			      3 / 3      100.0% 
			      1 / 1      100.0% 
			      1 / 1      100.0% 
			      5 / 5      100.0% 
			      1 / 1      100.0% 
			      3 / 3      100.0% 
			      2 / 2      100.0% 
			      1 / 1      100.0% 
			      2 / 2      100.0% 
			      1 / 1      100.0% 
			      2 / 2      100.0% 
			      1 / 1      100.0% 
			      2 / 2      100.0% 
			      4 / 4      100.0% 
			      2 / 2      100.0% 
			      1 / 1      100.0% 
			      4 / 4      100.0% 
			      0 / 0       0.0% 
			      0 / 0       0.0% 
			      0 / 0       0.0% 
			      0 / 0       0.0% 
			     33 / 48     68.8%AB 
			     15 / 48     31.2%ABE 
		 
		 
			 47 
			  5.9 
			 Pseudomonadaceae 
			   3067 / 3081   99.5% 
			    134 / 135    99.3% 
			    166 / 166    100.0% 
			     95 / 95     100.0% 
			    203 / 203    100.0% 
			    245 / 245    100.0% 
			    172 / 172    100.0% 
			     73 / 73     100.0% 
			     47 / 47     100.0% 
			    301 / 303    99.3% 
			     47 / 47     100.0% 
			    186 / 187    99.5% 
			    102 / 102    100.0% 
			     50 / 50     100.0% 
			     75 / 75     100.0% 
			     47 / 47     100.0% 
			    151 / 151    100.0% 
			     47 / 47     100.0% 
			    137 / 137    100.0% 
			    254 / 264    96.2% 
			    178 / 178    100.0% 
			     47 / 47     100.0% 
			    279 / 279    100.0% 
			     31 / 31     100.0% 
			      0 / 0       0.0% 
			      0 / 0       0.0% 
			      0 / 0       0.0% 
			   2045 / 3081   66.4%AB 
			    952 / 3081   30.9%ABE 
			     50 / 3081    1.6%B 
			     20 / 3081    0.6%BE 
			     11 / 3081    0.4% 
			      1 / 3081    0.0%CE 
			      1 / 3081    0.0%E 
			      1 / 3081    0.0%C 
		 
		 
			 1 
			  4.6 
			 "Cellvibrio" Winogradsky 1929 
			     46 / 46     100.0% 
			      1 / 1      100.0% 
			      2 / 2      100.0% 
			      1 / 1      100.0% 
			      3 / 3      100.0% 
			      3 / 3      100.0% 
			      3 / 3      100.0% 
			      1 / 1      100.0% 
			      1 / 1      100.0% 
			      4 / 4      100.0% 
			      1 / 1      100.0% 
			      3 / 3      100.0% 
			      2 / 2      100.0% 
			      1 / 1      100.0% 
			      1 / 1      100.0% 
			      1 / 1      100.0% 
			      3 / 3      100.0% 
			      1 / 1      100.0% 
			      2 / 2      100.0% 
			      4 / 4      100.0% 
			      2 / 2      100.0% 
			      1 / 1      100.0% 
			      5 / 5      100.0% 
			      0 / 0       0.0% 
			      0 / 0       0.0% 
			      0 / 0       0.0% 
			      0 / 0       0.0% 
			     24 / 46     52.2%AB 
			      9 / 46     19.6%ABE 
			      8 / 46     17.4%B 
			      5 / 46     10.9%BE 
		 
		 
			 1 
			  4.6 
			 "Cellvibrio cellulosa" Nagy et al. 2002 
			     46 / 46     100.0% 
			      1 / 1      100.0% 
			      2 / 2      100.0% 
			      1 / 1      100.0% 
			      3 / 3      100.0% 
			      3 / 3      100.0% 
			      3 / 3      100.0% 
			      1 / 1      100.0% 
			      1 / 1      100.0% 
			      4 / 4      100.0% 
			      1 / 1      100.0% 
			      3 / 3      100.0% 
			      2 / 2      100.0% 
			      1 / 1      100.0% 
			      1 / 1      100.0% 
			      1 / 1      100.0% 
			      3 / 3      100.0% 
			      1 / 1      100.0% 
			      2 / 2      100.0% 
			      4 / 4      100.0% 
			      2 / 2      100.0% 
			      1 / 1      100.0% 
			      5 / 5      100.0% 
			      0 / 0       0.0% 
			      0 / 0       0.0% 
			      0 / 0       0.0% 
			      0 / 0       0.0% 
			     24 / 46     52.2%AB 
			      9 / 46     19.6%ABE 
			      8 / 46     17.4%B 
			      5 / 46     10.9%BE 
		 
		 
			 1 
			  4.6 
			 Cellvibrio japonicus Ueda107 
			     46 / 46     100.0% 
			      1 / 1      100.0% 
			      2 / 2      100.0% 
			      1 / 1      100.0% 
			      3 / 3      100.0% 
			      3 / 3      100.0% 
			      3 / 3      100.0% 
			      1 / 1      100.0% 
			      1 / 1      100.0% 
			      4 / 4      100.0% 
			      1 / 1      100.0% 
			      3 / 3      100.0% 
			      2 / 2      100.0% 
			      1 / 1      100.0% 
			      1 / 1      100.0% 
			      1 / 1      100.0% 
			      3 / 3      100.0% 
			      1 / 1      100.0% 
			      2 / 2      100.0% 
			      4 / 4      100.0% 
			      2 / 2      100.0% 
			      1 / 1      100.0% 
			      5 / 5      100.0% 
			      0 / 0       0.0% 
			      0 / 0       0.0% 
			      0 / 0       0.0% 
			      0 / 0       0.0% 
			     24 / 46     52.2%AB 
			      9 / 46     19.6%ABE 
			      8 / 46     17.4%B 
			      5 / 46     10.9%BE 
		 
		 
			 43 
			  6.0 
			 "Chlorobacterium" Guillebeau 1890, nom. rejic. Opin. 6 (not "Chlorobacterium" Lauterborn 1916) 
			   2829 / 2843   99.5% 
			    127 / 128    99.2% 
			    158 / 158    100.0% 
			     88 / 88     100.0% 
			    182 / 182    100.0% 
			    227 / 227    100.0% 
			    160 / 160    100.0% 
			     69 / 69     100.0% 
			     43 / 43     100.0% 
			    273 / 275    99.3% 
			     43 / 43     100.0% 
			    171 / 172    99.4% 
			     94 / 94     100.0% 
			     43 / 43     100.0% 
			     68 / 68     100.0% 
			     43 / 43     100.0% 
			    139 / 139    100.0% 
			     43 / 43     100.0% 
			    126 / 126    100.0% 
			    235 / 245    95.9% 
			    167 / 167    100.0% 
			     43 / 43     100.0% 
			    256 / 256    100.0% 
			     31 / 31     100.0% 
			      0 / 0       0.0% 
			      0 / 0       0.0% 
			      0 / 0       0.0% 
			   1889 / 2843   66.4%AB 
			    883 / 2843   31.1%ABE 
			     42 / 2843    1.5%B 
			     15 / 2843    0.5%BE 
			     11 / 2843    0.4% 
			      1 / 2843    0.0%CE 
			      1 / 2843    0.0%E 
			      1 / 2843    0.0%C 
		 
		 
			 11 
			  6.2 
			 Pseudomonas aeruginosa group 
			    694 / 703    98.7% 
			     24 / 24     100.0% 
			     42 / 42     100.0% 
			     24 / 24     100.0% 
			     44 / 44     100.0% 
			     57 / 57     100.0% 
			     33 / 33     100.0% 
			     20 / 20     100.0% 
			     11 / 11     100.0% 
			     68 / 68     100.0% 
			     11 / 11     100.0% 
			     44 / 44     100.0% 
			     22 / 22     100.0% 
			     11 / 11     100.0% 
			     13 / 13     100.0% 
			     11 / 11     100.0% 
			     33 / 33     100.0% 
			     11 / 11     100.0% 
			     34 / 34     100.0% 
			     57 / 66     86.4% 
			     37 / 37     100.0% 
			     11 / 11     100.0% 
			     66 / 66     100.0% 
			     10 / 10     100.0% 
			      0 / 0       0.0% 
			      0 / 0       0.0% 
			      0 / 0       0.0% 
			    474 / 703    67.4%AB 
			    216 / 703    30.7%ABE 
			      9 / 703     1.3% 
			      4 / 703     0.6%BE 
		 
		 
			 9 
			  6.4 
			 "Bacillus aeruginosus" (Schroeter 1872) Trevisan 1885 
			    564 / 573    98.4% 
			     18 / 18     100.0% 
			     36 / 36     100.0% 
			     20 / 20     100.0% 
			     36 / 36     100.0% 
			     47 / 47     100.0% 
			     27 / 27     100.0% 
			     18 / 18     100.0% 
			      9 / 9      100.0% 
			     54 / 54     100.0% 
			      9 / 9      100.0% 
			     36 / 36     100.0% 
			     18 / 18     100.0% 
			      9 / 9      100.0% 
			      9 / 9      100.0% 
			      9 / 9      100.0% 
			     27 / 27     100.0% 
			      9 / 9      100.0% 
			     29 / 29     100.0% 
			     45 / 54     83.3% 
			     27 / 27     100.0% 
			      9 / 9      100.0% 
			     54 / 54     100.0% 
			      9 / 9      100.0% 
			      0 / 0       0.0% 
			      0 / 0       0.0% 
			      0 / 0       0.0% 
			    391 / 573    68.2%AB 
			    169 / 573    29.5%ABE 
			      9 / 573     1.6% 
			      4 / 573     0.7%BE 
		 
		 
			 1 
			  6.5 
			 Pseudomonas aeruginosa UCBPP-PA14 
			     62 / 63     98.4% 
			      2 / 2      100.0% 
			      4 / 4      100.0% 
			      2 / 2      100.0% 
			      4 / 4      100.0% 
			      5 / 5      100.0% 
			      3 / 3      100.0% 
			      2 / 2      100.0% 
			      1 / 1      100.0% 
			      6 / 6      100.0% 
			      1 / 1      100.0% 
			      4 / 4      100.0% 
			      2 / 2      100.0% 
			      1 / 1      100.0% 
			      1 / 1      100.0% 
			      1 / 1      100.0% 
			      3 / 3      100.0% 
			      1 / 1      100.0% 
			      3 / 3      100.0% 
			      5 / 6      83.3% 
			      3 / 3      100.0% 
			      1 / 1      100.0% 
			      6 / 6      100.0% 
			      1 / 1      100.0% 
			      0 / 0       0.0% 
			      0 / 0       0.0% 
			      0 / 0       0.0% 
			     43 / 63     68.3%AB 
			     17 / 63     27.0%ABE 
			      2 / 63      3.2%BE 
			      1 / 63      1.6% 
		 
		 
			 1 
			  6.1 
			 Pseudomonas aeruginosa PA01 
			     62 / 63     98.4% 
			      2 / 2      100.0% 
			      4 / 4      100.0% 
			      2 / 2      100.0% 
			      4 / 4      100.0% 
			      5 / 5      100.0% 
			      3 / 3      100.0% 
			      2 / 2      100.0% 
			      1 / 1      100.0% 
			      6 / 6      100.0% 
			      1 / 1      100.0% 
			      4 / 4      100.0% 
			      2 / 2      100.0% 
			      1 / 1      100.0% 
			      1 / 1      100.0% 
			      1 / 1      100.0% 
			      3 / 3      100.0% 
			      1 / 1      100.0% 
			      3 / 3      100.0% 
			      5 / 6      83.3% 
			      3 / 3      100.0% 
			      1 / 1      100.0% 
			      6 / 6      100.0% 
			      1 / 1      100.0% 
			      0 / 0       0.0% 
			      0 / 0       0.0% 
			      0 / 0       0.0% 
			     43 / 63     68.3%AB 
			     18 / 63     28.6%ABE 
			      1 / 63      1.6%BE 
			      1 / 63      1.6% 
		 
		 
			 1 
			  6.6 
			 Pseudomonas aeruginosa PA7 
			     62 / 63     98.4% 
			      2 / 2      100.0% 
			      4 / 4      100.0% 
			      2 / 2      100.0% 
			      4 / 4      100.0% 
			      5 / 5      100.0% 
			      3 / 3      100.0% 
			      2 / 2      100.0% 
			      1 / 1      100.0% 
			      6 / 6      100.0% 
			      1 / 1      100.0% 
			      4 / 4      100.0% 
			      2 / 2      100.0% 
			      1 / 1      100.0% 
			      1 / 1      100.0% 
			      1 / 1      100.0% 
			      3 / 3      100.0% 
			      1 / 1      100.0% 
			      3 / 3      100.0% 
			      5 / 6      83.3% 
			      3 / 3      100.0% 
			      1 / 1      100.0% 
			      6 / 6      100.0% 
			      1 / 1      100.0% 
			      0 / 0       0.0% 
			      0 / 0       0.0% 
			      0 / 0       0.0% 
			     43 / 63     68.3%AB 
			     18 / 63     28.6%ABE 
			      1 / 63      1.6% 
			      1 / 63      1.6%BE 
		 
		 
			 1 
			  6.6 
			 Pseudomonas aeruginosa LESB58 
			     65 / 66     98.5% 
			      2 / 2      100.0% 
			      4 / 4      100.0% 
			      3 / 3      100.0% 
			      4 / 4      100.0% 
			      6 / 6      100.0% 
			      3 / 3      100.0% 
			      2 / 2      100.0% 
			      1 / 1      100.0% 
			      6 / 6      100.0% 
			      1 / 1      100.0% 
			      4 / 4      100.0% 
			      2 / 2      100.0% 
			      1 / 1      100.0% 
			      1 / 1      100.0% 
			      1 / 1      100.0% 
			      3 / 3      100.0% 
			      1 / 1      100.0% 
			      4 / 4      100.0% 
			      5 / 6      83.3% 
			      3 / 3      100.0% 
			      1 / 1      100.0% 
			      6 / 6      100.0% 
			      1 / 1      100.0% 
			      0 / 0       0.0% 
			      0 / 0       0.0% 
			      0 / 0       0.0% 
			     45 / 66     68.2%AB 
			     20 / 66     30.3%ABE 
			      1 / 66      1.5% 
		 
		 
			 1 
			  6.3 
			 Pseudomonas aeruginosa M18 
			     62 / 63     98.4% 
			      2 / 2      100.0% 
			      4 / 4      100.0% 
			      2 / 2      100.0% 
			      4 / 4      100.0% 
			      5 / 5      100.0% 
			      3 / 3      100.0% 
			      2 / 2      100.0% 
			      1 / 1      100.0% 
			      6 / 6      100.0% 
			      1 / 1      100.0% 
			      4 / 4      100.0% 
			      2 / 2      100.0% 
			      1 / 1      100.0% 
			      1 / 1      100.0% 
			      1 / 1      100.0% 
			      3 / 3      100.0% 
			      1 / 1      100.0% 
			      3 / 3      100.0% 
			      5 / 6      83.3% 
			      3 / 3      100.0% 
			      1 / 1      100.0% 
			      6 / 6      100.0% 
			      1 / 1      100.0% 
			      0 / 0       0.0% 
			      0 / 0       0.0% 
			      0 / 0       0.0% 
			     43 / 63     68.3%AB 
			     19 / 63     30.2%ABE 
			      1 / 63      1.6% 
		 
		 
			 1 
			  6.8 
			 Pseudomonas aeruginosa NCGM2.S1 
			     65 / 66     98.5% 
			      2 / 2      100.0% 
			      4 / 4      100.0% 
			      3 / 3      100.0% 
			      4 / 4      100.0% 
			      6 / 6      100.0% 
			      3 / 3      100.0% 
			      2 / 2      100.0% 
			      1 / 1      100.0% 
			      6 / 6      100.0% 
			      1 / 1      100.0% 
			      4 / 4      100.0% 
			      2 / 2      100.0% 
			      1 / 1      100.0% 
			      1 / 1      100.0% 
			      1 / 1      100.0% 
			      3 / 3      100.0% 
			      1 / 1      100.0% 
			      4 / 4      100.0% 
			      5 / 6      83.3% 
			      3 / 3      100.0% 
			      1 / 1      100.0% 
			      6 / 6      100.0% 
			      1 / 1      100.0% 
			      0 / 0       0.0% 
			      0 / 0       0.0% 
			      0 / 0       0.0% 
			     45 / 66     68.2%AB 
			     20 / 66     30.3%ABE 
			      1 / 66      1.5% 
		 
		 
			 1 
			  6.4 
			 Pseudomonas aeruginosa CF333-2007-apr0 
			     62 / 63     98.4% 
			      2 / 2      100.0% 
			      4 / 4      100.0% 
			      2 / 2      100.0% 
			      4 / 4      100.0% 
			      5 / 5      100.0% 
			      3 / 3      100.0% 
			      2 / 2      100.0% 
			      1 / 1      100.0% 
			      6 / 6      100.0% 
			      1 / 1      100.0% 
			      4 / 4      100.0% 
			      2 / 2      100.0% 
			      1 / 1      100.0% 
			      1 / 1      100.0% 
			      1 / 1      100.0% 
			      3 / 3      100.0% 
			      1 / 1      100.0% 
			      3 / 3      100.0% 
			      5 / 6      83.3% 
			      3 / 3      100.0% 
			      1 / 1      100.0% 
			      6 / 6      100.0% 
			      1 / 1      100.0% 
			      0 / 0       0.0% 
			      0 / 0       0.0% 
			      0 / 0       0.0% 
			     43 / 63     68.3%AB 
			     19 / 63     30.2%ABE 
			      1 / 63      1.6% 
		 
		 
			 1 
			  6.4 
			 Pseudomonas aeruginosa B136-33 
			     62 / 63     98.4% 
			      2 / 2      100.0% 
			      4 / 4      100.0% 
			      2 / 2      100.0% 
			      4 / 4      100.0% 
			      5 / 5      100.0% 
			      3 / 3      100.0% 
			      2 / 2      100.0% 
			      1 / 1      100.0% 
			      6 / 6      100.0% 
			      1 / 1      100.0% 
			      4 / 4      100.0% 
			      2 / 2      100.0% 
			      1 / 1      100.0% 
			      1 / 1      100.0% 
			      1 / 1      100.0% 
			      3 / 3      100.0% 
			      1 / 1      100.0% 
			      3 / 3      100.0% 
			      5 / 6      83.3% 
			      3 / 3      100.0% 
			      1 / 1      100.0% 
			      6 / 6      100.0% 
			      1 / 1      100.0% 
			      0 / 0       0.0% 
			      0 / 0       0.0% 
			      0 / 0       0.0% 
			     43 / 63     68.3%AB 
			     19 / 63     30.2%ABE 
			      1 / 63      1.6% 
		 
		 
			 1 
			  6.3 
			 Pseudomonas aeruginosa RP73 
			     62 / 63     98.4% 
			      2 / 2      100.0% 
			      4 / 4      100.0% 
			      2 / 2      100.0% 
			      4 / 4      100.0% 
			      5 / 5      100.0% 
			      3 / 3      100.0% 
			      2 / 2      100.0% 
			      1 / 1      100.0% 
			      6 / 6      100.0% 
			      1 / 1      100.0% 
			      4 / 4      100.0% 
			      2 / 2      100.0% 
			      1 / 1      100.0% 
			      1 / 1      100.0% 
			      1 / 1      100.0% 
			      3 / 3      100.0% 
			      1 / 1      100.0% 
			      3 / 3      100.0% 
			      5 / 6      83.3% 
			      3 / 3      100.0% 
			      1 / 1      100.0% 
			      6 / 6      100.0% 
			      1 / 1      100.0% 
			      0 / 0       0.0% 
			      0 / 0       0.0% 
			      0 / 0       0.0% 
			     43 / 63     68.3%AB 
			     19 / 63     30.2%ABE 
			      1 / 63      1.6% 
		 
		 
			 2 
			  5.3 
			 ATCC 25411 
			    130 / 130    100.0% 
			      6 / 6      100.0% 
			      6 / 6      100.0% 
			      4 / 4      100.0% 
			      8 / 8      100.0% 
			     10 / 10     100.0% 
			      6 / 6      100.0% 
			      2 / 2      100.0% 
			      2 / 2      100.0% 
			     14 / 14     100.0% 
			      2 / 2      100.0% 
			      8 / 8      100.0% 
			      4 / 4      100.0% 
			      2 / 2      100.0% 
			      4 / 4      100.0% 
			      2 / 2      100.0% 
			      6 / 6      100.0% 
			      2 / 2      100.0% 
			      5 / 5      100.0% 
			     12 / 12     100.0% 
			     10 / 10     100.0% 
			      2 / 2      100.0% 
			     12 / 12     100.0% 
			      1 / 1      100.0% 
			      0 / 0       0.0% 
			      0 / 0       0.0% 
			      0 / 0       0.0% 
			     83 / 130    63.8%AB 
			     47 / 130    36.2%ABE 
		 
		 
			 1 
			  5.1 
			 Pseudomonas mendocina str. ymp 
			     65 / 65     100.0% 
			      3 / 3      100.0% 
			      3 / 3      100.0% 
			      2 / 2      100.0% 
			      4 / 4      100.0% 
			      5 / 5      100.0% 
			      3 / 3      100.0% 
			      1 / 1      100.0% 
			      1 / 1      100.0% 
			      7 / 7      100.0% 
			      1 / 1      100.0% 
			      4 / 4      100.0% 
			      2 / 2      100.0% 
			      1 / 1      100.0% 
			      2 / 2      100.0% 
			      1 / 1      100.0% 
			      3 / 3      100.0% 
			      1 / 1      100.0% 
			      3 / 3      100.0% 
			      6 / 6      100.0% 
			      5 / 5      100.0% 
			      1 / 1      100.0% 
			      6 / 6      100.0% 
			      0 / 0       0.0% 
			      0 / 0       0.0% 
			      0 / 0       0.0% 
			      0 / 0       0.0% 
			     42 / 65     64.6%AB 
			     23 / 65     35.4%ABE 
		 
		 
			 1 
			  5.4 
			 Pseudomonas mendocina NK-01 
			     65 / 65     100.0% 
			      3 / 3      100.0% 
			      3 / 3      100.0% 
			      2 / 2      100.0% 
			      4 / 4      100.0% 
			      5 / 5      100.0% 
			      3 / 3      100.0% 
			      1 / 1      100.0% 
			      1 / 1      100.0% 
			      7 / 7      100.0% 
			      1 / 1      100.0% 
			      4 / 4      100.0% 
			      2 / 2      100.0% 
			      1 / 1      100.0% 
			      2 / 2      100.0% 
			      1 / 1      100.0% 
			      3 / 3      100.0% 
			      1 / 1      100.0% 
			      2 / 2      100.0% 
			      6 / 6      100.0% 
			      5 / 5      100.0% 
			      1 / 1      100.0% 
			      6 / 6      100.0% 
			      1 / 1      100.0% 
			      0 / 0       0.0% 
			      0 / 0       0.0% 
			      0 / 0       0.0% 
			     41 / 65     63.1%AB 
			     24 / 65     36.9%ABE 
		 
		 
			 7 
			  6.5 
			 Pseudomonas fluorescens group 
			    473 / 473    100.0% 
			     21 / 21     100.0% 
			     28 / 28     100.0% 
			     14 / 14     100.0% 
			     37 / 37     100.0% 
			     38 / 38     100.0% 
			     28 / 28     100.0% 
			     11 / 11     100.0% 
			      7 / 7      100.0% 
			     52 / 52     100.0% 
			      7 / 7      100.0% 
			     28 / 28     100.0% 
			     14 / 14     100.0% 
			      7 / 7      100.0% 
			     10 / 10     100.0% 
			      7 / 7      100.0% 
			     21 / 21     100.0% 
			      7 / 7      100.0% 
			     18 / 18     100.0% 
			     36 / 36     100.0% 
			     32 / 32     100.0% 
			      7 / 7      100.0% 
			     42 / 42     100.0% 
			      1 / 1      100.0% 
			      0 / 0       0.0% 
			      0 / 0       0.0% 
			      0 / 0       0.0% 
			    328 / 473    69.3%AB 
			    145 / 473    30.7%ABE 
		 
		 
			 4 
			  6.5 
			 "Bacillus fluorescens liquefaciens" Flugge 1886 
			    270 / 270    100.0% 
			     12 / 12     100.0% 
			     16 / 16     100.0% 
			      8 / 8      100.0% 
			     22 / 22     100.0% 
			     21 / 21     100.0% 
			     16 / 16     100.0% 
			      7 / 7      100.0% 
			      4 / 4      100.0% 
			     30 / 30     100.0% 
			      4 / 4      100.0% 
			     16 / 16     100.0% 
			      8 / 8      100.0% 
			      4 / 4      100.0% 
			      5 / 5      100.0% 
			      4 / 4      100.0% 
			     12 / 12     100.0% 
			      4 / 4      100.0% 
			     10 / 10     100.0% 
			     21 / 21     100.0% 
			     17 / 17     100.0% 
			      4 / 4      100.0% 
			     24 / 24     100.0% 
			      1 / 1      100.0% 
			      0 / 0       0.0% 
			      0 / 0       0.0% 
			      0 / 0       0.0% 
			    189 / 270    70.0%AB 
			     81 / 270    30.0%ABE 
		 
		 
			 1 
			  6.4 
			 Pseudomonas fluorescens Pf0-1 
			     73 / 73     100.0% 
			      3 / 3      100.0% 
			      4 / 4      100.0% 
			      2 / 2      100.0% 
			      6 / 6      100.0% 
			      6 / 6      100.0% 
			      4 / 4      100.0% 
			      2 / 2      100.0% 
			      1 / 1      100.0% 
			      8 / 8      100.0% 
			      1 / 1      100.0% 
			      4 / 4      100.0% 
			      2 / 2      100.0% 
			      1 / 1      100.0% 
			      2 / 2      100.0% 
			      1 / 1      100.0% 
			      3 / 3      100.0% 
			      1 / 1      100.0% 
			      3 / 3      100.0% 
			      6 / 6      100.0% 
			      5 / 5      100.0% 
			      1 / 1      100.0% 
			      6 / 6      100.0% 
			      1 / 1      100.0% 
			      0 / 0       0.0% 
			      0 / 0       0.0% 
			      0 / 0       0.0% 
			     50 / 73     68.5%AB 
			     23 / 73     31.5%ABE 
		 
		 
			 1 
			  6.7 
			 Pseudomonas fluorescens SBW25 
			     65 / 65     100.0% 
			      3 / 3      100.0% 
			      4 / 4      100.0% 
			      2 / 2      100.0% 
			      5 / 5      100.0% 
			      5 / 5      100.0% 
			      4 / 4      100.0% 
			      2 / 2      100.0% 
			      1 / 1      100.0% 
			      7 / 7      100.0% 
			      1 / 1      100.0% 
			      4 / 4      100.0% 
			      2 / 2      100.0% 
			      1 / 1      100.0% 
			      1 / 1      100.0% 
			      1 / 1      100.0% 
			      3 / 3      100.0% 
			      1 / 1      100.0% 
			      2 / 2      100.0% 
			      5 / 5      100.0% 
			      4 / 4      100.0% 
			      1 / 1      100.0% 
			      6 / 6      100.0% 
			      0 / 0       0.0% 
			      0 / 0       0.0% 
			      0 / 0       0.0% 
			      0 / 0       0.0% 
			     46 / 65     70.8%AB 
			     19 / 65     29.2%ABE 
		 
		 
			 1 
			  6.0 
			 Pseudomonas fluorescens A506 
			     68 / 68     100.0% 
			      3 / 3      100.0% 
			      4 / 4      100.0% 
			      2 / 2      100.0% 
			      6 / 6      100.0% 
			      5 / 5      100.0% 
			      4 / 4      100.0% 
			      2 / 2      100.0% 
			      1 / 1      100.0% 
			      8 / 8      100.0% 
			      1 / 1      100.0% 
			      4 / 4      100.0% 
			      2 / 2      100.0% 
			      1 / 1      100.0% 
			      1 / 1      100.0% 
			      1 / 1      100.0% 
			      3 / 3      100.0% 
			      1 / 1      100.0% 
			      3 / 3      100.0% 
			      5 / 5      100.0% 
			      4 / 4      100.0% 
			      1 / 1      100.0% 
			      6 / 6      100.0% 
			      0 / 0       0.0% 
			      0 / 0       0.0% 
			      0 / 0       0.0% 
			      0 / 0       0.0% 
			     49 / 68     72.1%AB 
			     19 / 68     27.9%ABE 
		 
		 
			 1 
			  6.8 
			 Pseudomonas fluorescens F113 
			     64 / 64     100.0% 
			      3 / 3      100.0% 
			      4 / 4      100.0% 
			      2 / 2      100.0% 
			      5 / 5      100.0% 
			      5 / 5      100.0% 
			      4 / 4      100.0% 
			      1 / 1      100.0% 
			      1 / 1      100.0% 
			      7 / 7      100.0% 
			      1 / 1      100.0% 
			      4 / 4      100.0% 
			      2 / 2      100.0% 
			      1 / 1      100.0% 
			      1 / 1      100.0% 
			      1 / 1      100.0% 
			      3 / 3      100.0% 
			      1 / 1      100.0% 
			      2 / 2      100.0% 
			      5 / 5      100.0% 
			      4 / 4      100.0% 
			      1 / 1      100.0% 
			      6 / 6      100.0% 
			      0 / 0       0.0% 
			      0 / 0       0.0% 
			      0 / 0       0.0% 
			      0 / 0       0.0% 
			     44 / 64     68.8%AB 
			     20 / 64     31.2%ABE 
		 
		 
			 1 
			  5.5 
			 DSM 14936 
			     65 / 65     100.0% 
			      3 / 3      100.0% 
			      4 / 4      100.0% 
			      2 / 2      100.0% 
			      5 / 5      100.0% 
			      5 / 5      100.0% 
			      4 / 4      100.0% 
			      2 / 2      100.0% 
			      1 / 1      100.0% 
			      7 / 7      100.0% 
			      1 / 1      100.0% 
			      4 / 4      100.0% 
			      2 / 2      100.0% 
			      1 / 1      100.0% 
			      1 / 1      100.0% 
			      1 / 1      100.0% 
			      3 / 3      100.0% 
			      1 / 1      100.0% 
			      2 / 2      100.0% 
			      5 / 5      100.0% 
			      4 / 4      100.0% 
			      1 / 1      100.0% 
			      6 / 6      100.0% 
			      0 / 0       0.0% 
			      0 / 0       0.0% 
			      0 / 0       0.0% 
			      0 / 0       0.0% 
			     46 / 65     70.8%AB 
			     19 / 65     29.2%ABE 
		 
		 
			 1 
			  5.5 
			 Pseudomonas poae RE*1-1-14 
			     65 / 65     100.0% 
			      3 / 3      100.0% 
			      4 / 4      100.0% 
			      2 / 2      100.0% 
			      5 / 5      100.0% 
			      5 / 5      100.0% 
			      4 / 4      100.0% 
			      2 / 2      100.0% 
			      1 / 1      100.0% 
			      7 / 7      100.0% 
			      1 / 1      100.0% 
			      4 / 4      100.0% 
			      2 / 2      100.0% 
			      1 / 1      100.0% 
			      1 / 1      100.0% 
			      1 / 1      100.0% 
			      3 / 3      100.0% 
			      1 / 1      100.0% 
			      2 / 2      100.0% 
			      5 / 5      100.0% 
			      4 / 4      100.0% 
			      1 / 1      100.0% 
			      6 / 6      100.0% 
			      0 / 0       0.0% 
			      0 / 0       0.0% 
			      0 / 0       0.0% 
			      0 / 0       0.0% 
			     46 / 65     70.8%AB 
			     19 / 65     29.2%ABE 
		 
		 
			 2 
			  7.0 
			 CFBP 6595 
			    138 / 138    100.0% 
			      6 / 6      100.0% 
			      8 / 8      100.0% 
			      4 / 4      100.0% 
			     10 / 10     100.0% 
			     12 / 12     100.0% 
			      8 / 8      100.0% 
			      2 / 2      100.0% 
			      2 / 2      100.0% 
			     15 / 15     100.0% 
			      2 / 2      100.0% 
			      8 / 8      100.0% 
			      4 / 4      100.0% 
			      2 / 2      100.0% 
			      4 / 4      100.0% 
			      2 / 2      100.0% 
			      6 / 6      100.0% 
			      2 / 2      100.0% 
			      6 / 6      100.0% 
			     10 / 10     100.0% 
			     11 / 11     100.0% 
			      2 / 2      100.0% 
			     12 / 12     100.0% 
			      0 / 0       0.0% 
			      0 / 0       0.0% 
			      0 / 0       0.0% 
			      0 / 0       0.0% 
			     93 / 138    67.4%AB 
			     45 / 138    32.6%ABE 
		 
		 
			 1 
			  7.1 
			 Pseudomonas fluorescens Pf-5 
			     70 / 70     100.0% 
			      3 / 3      100.0% 
			      4 / 4      100.0% 
			      2 / 2      100.0% 
			      5 / 5      100.0% 
			      6 / 6      100.0% 
			      4 / 4      100.0% 
			      1 / 1      100.0% 
			      1 / 1      100.0% 
			      8 / 8      100.0% 
			      1 / 1      100.0% 
			      4 / 4      100.0% 
			      2 / 2      100.0% 
			      1 / 1      100.0% 
			      2 / 2      100.0% 
			      1 / 1      100.0% 
			      3 / 3      100.0% 
			      1 / 1      100.0% 
			      3 / 3      100.0% 
			      5 / 5      100.0% 
			      6 / 6      100.0% 
			      1 / 1      100.0% 
			      6 / 6      100.0% 
			      0 / 0       0.0% 
			      0 / 0       0.0% 
			      0 / 0       0.0% 
			      0 / 0       0.0% 
			     47 / 70     67.1%AB 
			     23 / 70     32.9%ABE 
		 
		 
			 1 
			  6.9 
			 Pseudomonas fluorescens CHA0 
			     68 / 68     100.0% 
			      3 / 3      100.0% 
			      4 / 4      100.0% 
			      2 / 2      100.0% 
			      5 / 5      100.0% 
			      6 / 6      100.0% 
			      4 / 4      100.0% 
			      1 / 1      100.0% 
			      1 / 1      100.0% 
			      7 / 7      100.0% 
			      1 / 1      100.0% 
			      4 / 4      100.0% 
			      2 / 2      100.0% 
			      1 / 1      100.0% 
			      2 / 2      100.0% 
			      1 / 1      100.0% 
			      3 / 3      100.0% 
			      1 / 1      100.0% 
			      3 / 3      100.0% 
			      5 / 5      100.0% 
			      5 / 5      100.0% 
			      1 / 1      100.0% 
			      6 / 6      100.0% 
			      0 / 0       0.0% 
			      0 / 0       0.0% 
			      0 / 0       0.0% 
			      0 / 0       0.0% 
			     46 / 68     67.6%AB 
			     22 / 68     32.4%ABE 
		 
		 
			 1 
			  5.7 
			 Pseudomonas pertucinogena group 
			     63 / 63     100.0% 
			      3 / 3      100.0% 
			      3 / 3      100.0% 
			      2 / 2      100.0% 
			      5 / 5      100.0% 
			      5 / 5      100.0% 
			      3 / 3      100.0% 
			      1 / 1      100.0% 
			      1 / 1      100.0% 
			      7 / 7      100.0% 
			      1 / 1      100.0% 
			      4 / 4      100.0% 
			      2 / 2      100.0% 
			      1 / 1      100.0% 
			      2 / 2      100.0% 
			      1 / 1      100.0% 
			      3 / 3      100.0% 
			      1 / 1      100.0% 
			      3 / 3      100.0% 
			      5 / 5      100.0% 
			      3 / 3      100.0% 
			      1 / 1      100.0% 
			      5 / 5      100.0% 
			      1 / 1      100.0% 
			      0 / 0       0.0% 
			      0 / 0       0.0% 
			      0 / 0       0.0% 
			     43 / 63     68.3%AB 
			     20 / 63     31.7%ABE 
		 
		 
			 1 
			  5.7 
			 "Pseudomonas denitrificans" (Christensen 1903) Bergey et al. 1923, nom. rejic. 
			     63 / 63     100.0% 
			      3 / 3      100.0% 
			      3 / 3      100.0% 
			      2 / 2      100.0% 
			      5 / 5      100.0% 
			      5 / 5      100.0% 
			      3 / 3      100.0% 
			      1 / 1      100.0% 
			      1 / 1      100.0% 
			      7 / 7      100.0% 
			      1 / 1      100.0% 
			      4 / 4      100.0% 
			      2 / 2      100.0% 
			      1 / 1      100.0% 
			      2 / 2      100.0% 
			      1 / 1      100.0% 
			      3 / 3      100.0% 
			      1 / 1      100.0% 
			      3 / 3      100.0% 
			      5 / 5      100.0% 
			      3 / 3      100.0% 
			      1 / 1      100.0% 
			      5 / 5      100.0% 
			      1 / 1      100.0% 
			      0 / 0       0.0% 
			      0 / 0       0.0% 
			      0 / 0       0.0% 
			     43 / 63     68.3%AB 
			     20 / 63     31.7%ABE 
		 
		 
			 1 
			  5.7 
			 Pseudomonas denitrificans ATCC 13867 
			     63 / 63     100.0% 
			      3 / 3      100.0% 
			      3 / 3      100.0% 
			      2 / 2      100.0% 
			      5 / 5      100.0% 
			      5 / 5      100.0% 
			      3 / 3      100.0% 
			      1 / 1      100.0% 
			      1 / 1      100.0% 
			      7 / 7      100.0% 
			      1 / 1      100.0% 
			      4 / 4      100.0% 
			      2 / 2      100.0% 
			      1 / 1      100.0% 
			      2 / 2      100.0% 
			      1 / 1      100.0% 
			      3 / 3      100.0% 
			      1 / 1      100.0% 
			      3 / 3      100.0% 
			      5 / 5      100.0% 
			      3 / 3      100.0% 
			      1 / 1      100.0% 
			      5 / 5      100.0% 
			      1 / 1      100.0% 
			      0 / 0       0.0% 
			      0 / 0       0.0% 
			      0 / 0       0.0% 
			     43 / 63     68.3%AB 
			     20 / 63     31.7%ABE 
		 
		 
			 12 
			  6.0 
			 Pseudomonas putida group 
			    838 / 841    99.6% 
			     43 / 43     100.0% 
			     52 / 52     100.0% 
			     24 / 24     100.0% 
			     38 / 38     100.0% 
			     72 / 72     100.0% 
			     55 / 55     100.0% 
			     23 / 23     100.0% 
			     12 / 12     100.0% 
			     65 / 66     98.5% 
			     12 / 12     100.0% 
			     47 / 48     97.9% 
			     32 / 32     100.0% 
			     12 / 12     100.0% 
			     24 / 24     100.0% 
			     12 / 12     100.0% 
			     44 / 44     100.0% 
			     12 / 12     100.0% 
			     36 / 36     100.0% 
			     76 / 77     98.7% 
			     52 / 52     100.0% 
			     12 / 12     100.0% 
			     72 / 72     100.0% 
			     11 / 11     100.0% 
			      0 / 0       0.0% 
			      0 / 0       0.0% 
			      0 / 0       0.0% 
			    534 / 841    63.5%AB 
			    251 / 841    29.8%ABE 
			     42 / 841     5.0%B 
			     11 / 841     1.3%BE 
			      1 / 841     0.1%CE 
			      1 / 841     0.1%C 
			      1 / 841     0.1% 
		 
		 
			 11 
			  6.1 
			 "Bacillus fluorescens putidus" Flugge 1886 
			    775 / 778    99.6% 
			     40 / 40     100.0% 
			     49 / 49     100.0% 
			     22 / 22     100.0% 
			     34 / 34     100.0% 
			     67 / 67     100.0% 
			     52 / 52     100.0% 
			     22 / 22     100.0% 
			     11 / 11     100.0% 
			     59 / 60     98.3% 
			     11 / 11     100.0% 
			     43 / 44     97.7% 
			     30 / 30     100.0% 
			     11 / 11     100.0% 
			     22 / 22     100.0% 
			     11 / 11     100.0% 
			     41 / 41     100.0% 
			     11 / 11     100.0% 
			     33 / 33     100.0% 
			     70 / 71     98.6% 
			     48 / 48     100.0% 
			     11 / 11     100.0% 
			     66 / 66     100.0% 
			     11 / 11     100.0% 
			      0 / 0       0.0% 
			      0 / 0       0.0% 
			      0 / 0       0.0% 
			    492 / 778    63.2%AB 
			    230 / 778    29.6%ABE 
			     42 / 778     5.4%B 
			     11 / 778     1.4%BE 
			      1 / 778     0.1%CE 
			      1 / 778     0.1% 
			      1 / 778     0.1%C 
		 
		 
			 1 
			  6.1 
			 Pseudomonas putida GB-1 
			     75 / 75     100.0% 
			      4 / 4      100.0% 
			      5 / 5      100.0% 
			      2 / 2      100.0% 
			      3 / 3      100.0% 
			      7 / 7      100.0% 
			      5 / 5      100.0% 
			      2 / 2      100.0% 
			      1 / 1      100.0% 
			      6 / 6      100.0% 
			      1 / 1      100.0% 
			      4 / 4      100.0% 
			      3 / 3      100.0% 
			      1 / 1      100.0% 
			      2 / 2      100.0% 
			      1 / 1      100.0% 
			      4 / 4      100.0% 
			      1 / 1      100.0% 
			      3 / 3      100.0% 
			      7 / 7      100.0% 
			      5 / 5      100.0% 
			      1 / 1      100.0% 
			      6 / 6      100.0% 
			      1 / 1      100.0% 
			      0 / 0       0.0% 
			      0 / 0       0.0% 
			      0 / 0       0.0% 
			     33 / 75     44.0%AB 
			     19 / 75     25.3%B 
			     16 / 75     21.3%ABE 
			      7 / 75      9.3%BE 
		 
		 
			 1 
			  6.2 
			 Pseudomonas putida (strain KT2440) 
			     73 / 74     98.6% 
			      4 / 4      100.0% 
			      5 / 5      100.0% 
			      2 / 2      100.0% 
			      3 / 3      100.0% 
			      6 / 6      100.0% 
			      5 / 5      100.0% 
			      2 / 2      100.0% 
			      1 / 1      100.0% 
			      6 / 6      100.0% 
			      1 / 1      100.0% 
			      4 / 4      100.0% 
			      3 / 3      100.0% 
			      1 / 1      100.0% 
			      2 / 2      100.0% 
			      1 / 1      100.0% 
			      4 / 4      100.0% 
			      1 / 1      100.0% 
			      3 / 3      100.0% 
			      6 / 7      85.7% 
			      5 / 5      100.0% 
			      1 / 1      100.0% 
			      6 / 6      100.0% 
			      1 / 1      100.0% 
			      0 / 0       0.0% 
			      0 / 0       0.0% 
			      0 / 0       0.0% 
			     50 / 74     67.6%AB 
			     23 / 74     31.1%ABE 
			      1 / 74      1.4% 
		 
		 
			 1 
			  6.2 
			 Pseudomonas putida ND6 
			     76 / 76     100.0% 
			      4 / 4      100.0% 
			      5 / 5      100.0% 
			      2 / 2      100.0% 
			      4 / 4      100.0% 
			      6 / 6      100.0% 
			      5 / 5      100.0% 
			      2 / 2      100.0% 
			      1 / 1      100.0% 
			      7 / 7      100.0% 
			      1 / 1      100.0% 
			      4 / 4      100.0% 
			      3 / 3      100.0% 
			      1 / 1      100.0% 
			      2 / 2      100.0% 
			      1 / 1      100.0% 
			      4 / 4      100.0% 
			      1 / 1      100.0% 
			      3 / 3      100.0% 
			      7 / 7      100.0% 
			      5 / 5      100.0% 
			      1 / 1      100.0% 
			      6 / 6      100.0% 
			      1 / 1      100.0% 
			      0 / 0       0.0% 
			      0 / 0       0.0% 
			      0 / 0       0.0% 
			     53 / 76     69.7%AB 
			     23 / 76     30.3%ABE 
		 
		 
			 1 
			  6.0 
			 Pseudomonas putida F1 
			     76 / 76     100.0% 
			      4 / 4      100.0% 
			      5 / 5      100.0% 
			      2 / 2      100.0% 
			      4 / 4      100.0% 
			      6 / 6      100.0% 
			      5 / 5      100.0% 
			      2 / 2      100.0% 
			      1 / 1      100.0% 
			      7 / 7      100.0% 
			      1 / 1      100.0% 
			      4 / 4      100.0% 
			      3 / 3      100.0% 
			      1 / 1      100.0% 
			      2 / 2      100.0% 
			      1 / 1      100.0% 
			      4 / 4      100.0% 
			      1 / 1      100.0% 
			      3 / 3      100.0% 
			      7 / 7      100.0% 
			      5 / 5      100.0% 
			      1 / 1      100.0% 
			      6 / 6      100.0% 
			      1 / 1      100.0% 
			      0 / 0       0.0% 
			      0 / 0       0.0% 
			      0 / 0       0.0% 
			     53 / 76     69.7%AB 
			     23 / 76     30.3%ABE 
		 
		 
			 1 
			  5.8 
			 Pseudomonas putida W619 
			     75 / 75     100.0% 
			      4 / 4      100.0% 
			      5 / 5      100.0% 
			      2 / 2      100.0% 
			      5 / 5      100.0% 
			      6 / 6      100.0% 
			      5 / 5      100.0% 
			      2 / 2      100.0% 
			      1 / 1      100.0% 
			      8 / 8      100.0% 
			      1 / 1      100.0% 
			      4 / 4      100.0% 
			      2 / 2      100.0% 
			      1 / 1      100.0% 
			      2 / 2      100.0% 
			      1 / 1      100.0% 
			      3 / 3      100.0% 
			      1 / 1      100.0% 
			      3 / 3      100.0% 
			      6 / 6      100.0% 
			      5 / 5      100.0% 
			      1 / 1      100.0% 
			      6 / 6      100.0% 
			      1 / 1      100.0% 
			      0 / 0       0.0% 
			      0 / 0       0.0% 
			      0 / 0       0.0% 
			     29 / 75     38.7%AB 
			     23 / 75     30.7%B 
			     19 / 75     25.3%ABE 
			      4 / 75      5.3%BE 
		 
		 
			 1 
			  5.7 
			 Pseudomonas putida BIRD-1 
			     64 / 64     100.0% 
			      3 / 3      100.0% 
			      3 / 3      100.0% 
			      2 / 2      100.0% 
			      3 / 3      100.0% 
			      6 / 6      100.0% 
			      4 / 4      100.0% 
			      2 / 2      100.0% 
			      1 / 1      100.0% 
			      4 / 4      100.0% 
			      1 / 1      100.0% 
			      4 / 4      100.0% 
			      2 / 2      100.0% 
			      1 / 1      100.0% 
			      2 / 2      100.0% 
			      1 / 1      100.0% 
			      3 / 3      100.0% 
			      1 / 1      100.0% 
			      3 / 3      100.0% 
			      7 / 7      100.0% 
			      3 / 3      100.0% 
			      1 / 1      100.0% 
			      6 / 6      100.0% 
			      1 / 1      100.0% 
			      0 / 0       0.0% 
			      0 / 0       0.0% 
			      0 / 0       0.0% 
			     44 / 64     68.8%AB 
			     20 / 64     31.2%ABE 
		 
		 
			 1 
			  6.0 
			 Pseudomonas putida S16 
			     64 / 66     97.0% 
			      3 / 3      100.0% 
			      5 / 5      100.0% 
			      2 / 2      100.0% 
			      0 / 0       0.0% 
			      6 / 6      100.0% 
			      5 / 5      100.0% 
			      2 / 2      100.0% 
			      1 / 1      100.0% 
			      2 / 3      66.7% 
			      1 / 1      100.0% 
			      3 / 4      75.0% 
			      3 / 3      100.0% 
			      1 / 1      100.0% 
			      2 / 2      100.0% 
			      1 / 1      100.0% 
			      4 / 4      100.0% 
			      1 / 1      100.0% 
			      3 / 3      100.0% 
			      6 / 6      100.0% 
			      5 / 5      100.0% 
			      1 / 1      100.0% 
			      6 / 6      100.0% 
			      1 / 1      100.0% 
			      0 / 0       0.0% 
			      0 / 0       0.0% 
			      0 / 0       0.0% 
			     43 / 66     65.2%AB 
			     21 / 66     31.8%ABE 
			      1 / 66      1.5%CE 
			      1 / 66      1.5%C 
		 
		 
			 1 
			  6.3 
			 Pseudomonas putida DOT-T1E 
			     59 / 59     100.0% 
			      3 / 3      100.0% 
			      2 / 2      100.0% 
			      2 / 2      100.0% 
			      2 / 2      100.0% 
			      6 / 6      100.0% 
			      4 / 4      100.0% 
			      2 / 2      100.0% 
			      1 / 1      100.0% 
			      2 / 2      100.0% 
			      1 / 1      100.0% 
			      4 / 4      100.0% 
			      2 / 2      100.0% 
			      1 / 1      100.0% 
			      2 / 2      100.0% 
			      1 / 1      100.0% 
			      3 / 3      100.0% 
			      1 / 1      100.0% 
			      3 / 3      100.0% 
			      6 / 6      100.0% 
			      3 / 3      100.0% 
			      1 / 1      100.0% 
			      6 / 6      100.0% 
			      1 / 1      100.0% 
			      0 / 0       0.0% 
			      0 / 0       0.0% 
			      0 / 0       0.0% 
			     39 / 59     66.1%AB 
			     20 / 59     33.9%ABE 
		 
		 
			 1 
			  6.2 
			 Pseudomonas putida NBRC 14164 
			     75 / 75     100.0% 
			      4 / 4      100.0% 
			      5 / 5      100.0% 
			      2 / 2      100.0% 
			      4 / 4      100.0% 
			      6 / 6      100.0% 
			      5 / 5      100.0% 
			      2 / 2      100.0% 
			      1 / 1      100.0% 
			      7 / 7      100.0% 
			      1 / 1      100.0% 
			      4 / 4      100.0% 
			      3 / 3      100.0% 
			      1 / 1      100.0% 
			      2 / 2      100.0% 
			      1 / 1      100.0% 
			      4 / 4      100.0% 
			      1 / 1      100.0% 
			      3 / 3      100.0% 
			      6 / 6      100.0% 
			      5 / 5      100.0% 
			      1 / 1      100.0% 
			      6 / 6      100.0% 
			      1 / 1      100.0% 
			      0 / 0       0.0% 
			      0 / 0       0.0% 
			      0 / 0       0.0% 
			     52 / 75     69.3%AB 
			     23 / 75     30.7%ABE 
		 
		 
			 1 
			  6.0 
			 Pseudomonas putida HB3267 
			     70 / 70     100.0% 
			      3 / 3      100.0% 
			      5 / 5      100.0% 
			      2 / 2      100.0% 
			      3 / 3      100.0% 
			      6 / 6      100.0% 
			      5 / 5      100.0% 
			      2 / 2      100.0% 
			      1 / 1      100.0% 
			      5 / 5      100.0% 
			      1 / 1      100.0% 
			      4 / 4      100.0% 
			      3 / 3      100.0% 
			      1 / 1      100.0% 
			      2 / 2      100.0% 
			      1 / 1      100.0% 
			      4 / 4      100.0% 
			      1 / 1      100.0% 
			      3 / 3      100.0% 
			      6 / 6      100.0% 
			      4 / 4      100.0% 
			      1 / 1      100.0% 
			      6 / 6      100.0% 
			      1 / 1      100.0% 
			      0 / 0       0.0% 
			      0 / 0       0.0% 
			      0 / 0       0.0% 
			     49 / 70     70.0%AB 
			     21 / 70     30.0%ABE 
		 
		 
			 1 
			  6.9 
			 Pseudomonas putida H8234 
			     68 / 68     100.0% 
			      4 / 4      100.0% 
			      4 / 4      100.0% 
			      2 / 2      100.0% 
			      3 / 3      100.0% 
			      6 / 6      100.0% 
			      4 / 4      100.0% 
			      2 / 2      100.0% 
			      1 / 1      100.0% 
			      5 / 5      100.0% 
			      1 / 1      100.0% 
			      4 / 4      100.0% 
			      3 / 3      100.0% 
			      1 / 1      100.0% 
			      2 / 2      100.0% 
			      1 / 1      100.0% 
			      4 / 4      100.0% 
			      1 / 1      100.0% 
			      3 / 3      100.0% 
			      6 / 6      100.0% 
			      3 / 3      100.0% 
			      1 / 1      100.0% 
			      6 / 6      100.0% 
			      1 / 1      100.0% 
			      0 / 0       0.0% 
			      0 / 0       0.0% 
			      0 / 0       0.0% 
			     47 / 68     69.1%AB 
			     21 / 68     30.9%ABE 
		 
		 
			 1 
			  4.9 
			 ATCC 31418 
			     63 / 63     100.0% 
			      3 / 3      100.0% 
			      3 / 3      100.0% 
			      2 / 2      100.0% 
			      4 / 4      100.0% 
			      5 / 5      100.0% 
			      3 / 3      100.0% 
			      1 / 1      100.0% 
			      1 / 1      100.0% 
			      6 / 6      100.0% 
			      1 / 1      100.0% 
			      4 / 4      100.0% 
			      2 / 2      100.0% 
			      1 / 1      100.0% 
			      2 / 2      100.0% 
			      1 / 1      100.0% 
			      3 / 3      100.0% 
			      1 / 1      100.0% 
			      3 / 3      100.0% 
			      6 / 6      100.0% 
			      4 / 4      100.0% 
			      1 / 1      100.0% 
			      6 / 6      100.0% 
			      0 / 0       0.0% 
			      0 / 0       0.0% 
			      0 / 0       0.0% 
			      0 / 0       0.0% 
			     42 / 63     66.7%AB 
			     21 / 63     33.3%ABE 
		 
		 
			 1 
			  4.9 
			 Pseudomonas fulva 12-X 
			     63 / 63     100.0% 
			      3 / 3      100.0% 
			      3 / 3      100.0% 
			      2 / 2      100.0% 
			      4 / 4      100.0% 
			      5 / 5      100.0% 
			      3 / 3      100.0% 
			      1 / 1      100.0% 
			      1 / 1      100.0% 
			      6 / 6      100.0% 
			      1 / 1      100.0% 
			      4 / 4      100.0% 
			      2 / 2      100.0% 
			      1 / 1      100.0% 
			      2 / 2      100.0% 
			      1 / 1      100.0% 
			      3 / 3      100.0% 
			      1 / 1      100.0% 
			      3 / 3      100.0% 
			      6 / 6      100.0% 
			      4 / 4      100.0% 
			      1 / 1      100.0% 
			      6 / 6      100.0% 
			      0 / 0       0.0% 
			      0 / 0       0.0% 
			      0 / 0       0.0% 
			      0 / 0       0.0% 
			     42 / 63     66.7%AB 
			     21 / 63     33.3%ABE 
		 
		 
			 6 
			  4.5 
			 Pseudomonas stutzeri group 
			    358 / 359    99.7% 
			     18 / 18     100.0% 
			     12 / 12     100.0% 
			     12 / 12     100.0% 
			     24 / 24     100.0% 
			     24 / 24     100.0% 
			     18 / 18     100.0% 
			      6 / 6      100.0% 
			      6 / 6      100.0% 
			     35 / 36     97.2% 
			      6 / 6      100.0% 
			     24 / 24     100.0% 
			     12 / 12     100.0% 
			      6 / 6      100.0% 
			     11 / 11     100.0% 
			      6 / 6      100.0% 
			     18 / 18     100.0% 
			      6 / 6      100.0% 
			     20 / 20     100.0% 
			     29 / 29     100.0% 
			     18 / 18     100.0% 
			      6 / 6      100.0% 
			     35 / 35     100.0% 
			      6 / 6      100.0% 
			      0 / 0       0.0% 
			      0 / 0       0.0% 
			      0 / 0       0.0% 
			    228 / 359    63.5%AB 
			    130 / 359    36.2%ABE 
			      1 / 359     0.3% 
		 
		 
			 6 
			  4.5 
			 Pseudomonas stutzeri subgroup 
			    358 / 359    99.7% 
			     18 / 18     100.0% 
			     12 / 12     100.0% 
			     12 / 12     100.0% 
			     24 / 24     100.0% 
			     24 / 24     100.0% 
			     18 / 18     100.0% 
			      6 / 6      100.0% 
			      6 / 6      100.0% 
			     35 / 36     97.2% 
			      6 / 6      100.0% 
			     24 / 24     100.0% 
			     12 / 12     100.0% 
			      6 / 6      100.0% 
			     11 / 11     100.0% 
			      6 / 6      100.0% 
			     18 / 18     100.0% 
			      6 / 6      100.0% 
			     20 / 20     100.0% 
			     29 / 29     100.0% 
			     18 / 18     100.0% 
			      6 / 6      100.0% 
			     35 / 35     100.0% 
			      6 / 6      100.0% 
			      0 / 0       0.0% 
			      0 / 0       0.0% 
			      0 / 0       0.0% 
			    228 / 359    63.5%AB 
			    130 / 359    36.2%ABE 
			      1 / 359     0.3% 
		 
		 
			 6 
			  4.5 
			 "Achromobacter sewerinii" Bergey et al. 1923 
			    358 / 359    99.7% 
			     18 / 18     100.0% 
			     12 / 12     100.0% 
			     12 / 12     100.0% 
			     24 / 24     100.0% 
			     24 / 24     100.0% 
			     18 / 18     100.0% 
			      6 / 6      100.0% 
			      6 / 6      100.0% 
			     35 / 36     97.2% 
			      6 / 6      100.0% 
			     24 / 24     100.0% 
			     12 / 12     100.0% 
			      6 / 6      100.0% 
			     11 / 11     100.0% 
			      6 / 6      100.0% 
			     18 / 18     100.0% 
			      6 / 6      100.0% 
			     20 / 20     100.0% 
			     29 / 29     100.0% 
			     18 / 18     100.0% 
			      6 / 6      100.0% 
			     35 / 35     100.0% 
			      6 / 6      100.0% 
			      0 / 0       0.0% 
			      0 / 0       0.0% 
			      0 / 0       0.0% 
			    228 / 359    63.5%AB 
			    130 / 359    36.2%ABE 
			      1 / 359     0.3% 
		 
		 
			 1 
			  4.5 
			 Pseudomonas stutzeri ATCC 17588 
			     59 / 59     100.0% 
			      3 / 3      100.0% 
			      2 / 2      100.0% 
			      2 / 2      100.0% 
			      4 / 4      100.0% 
			      4 / 4      100.0% 
			      3 / 3      100.0% 
			      1 / 1      100.0% 
			      1 / 1      100.0% 
			      6 / 6      100.0% 
			      1 / 1      100.0% 
			      4 / 4      100.0% 
			      2 / 2      100.0% 
			      1 / 1      100.0% 
			      2 / 2      100.0% 
			      1 / 1      100.0% 
			      3 / 3      100.0% 
			      1 / 1      100.0% 
			      3 / 3      100.0% 
			      4 / 4      100.0% 
			      3 / 3      100.0% 
			      1 / 1      100.0% 
			      6 / 6      100.0% 
			      1 / 1      100.0% 
			      0 / 0       0.0% 
			      0 / 0       0.0% 
			      0 / 0       0.0% 
			     37 / 59     62.7%AB 
			     22 / 59     37.3%ABE 
		 
		 
			 1 
			  4.6 
			 Pseudomonas stutzeri A1501 
			     60 / 60     100.0% 
			      3 / 3      100.0% 
			      2 / 2      100.0% 
			      2 / 2      100.0% 
			      4 / 4      100.0% 
			      4 / 4      100.0% 
			      3 / 3      100.0% 
			      1 / 1      100.0% 
			      1 / 1      100.0% 
			      6 / 6      100.0% 
			      1 / 1      100.0% 
			      4 / 4      100.0% 
			      2 / 2      100.0% 
			      1 / 1      100.0% 
			      2 / 2      100.0% 
			      1 / 1      100.0% 
			      3 / 3      100.0% 
			      1 / 1      100.0% 
			      3 / 3      100.0% 
			      5 / 5      100.0% 
			      3 / 3      100.0% 
			      1 / 1      100.0% 
			      6 / 6      100.0% 
			      1 / 1      100.0% 
			      0 / 0       0.0% 
			      0 / 0       0.0% 
			      0 / 0       0.0% 
			     38 / 60     63.3%AB 
			     22 / 60     36.7%ABE 
		 
		 
			 1 
			  4.6 
			 Pseudomonas stutzeri RCH2 
			     58 / 59     98.3% 
			      3 / 3      100.0% 
			      2 / 2      100.0% 
			      2 / 2      100.0% 
			      4 / 4      100.0% 
			      4 / 4      100.0% 
			      3 / 3      100.0% 
			      1 / 1      100.0% 
			      1 / 1      100.0% 
			      5 / 6      83.3% 
			      1 / 1      100.0% 
			      4 / 4      100.0% 
			      2 / 2      100.0% 
			      1 / 1      100.0% 
			      1 / 1      100.0% 
			      1 / 1      100.0% 
			      3 / 3      100.0% 
			      1 / 1      100.0% 
			      3 / 3      100.0% 
			      5 / 5      100.0% 
			      3 / 3      100.0% 
			      1 / 1      100.0% 
			      6 / 6      100.0% 
			      1 / 1      100.0% 
			      0 / 0       0.0% 
			      0 / 0       0.0% 
			      0 / 0       0.0% 
			     37 / 59     62.7%AB 
			     21 / 59     35.6%ABE 
			      1 / 59      1.7% 
		 
		 
			 1 
			  4.7 
			 Pseudomonas stutzeri CMT.9.A 
			     60 / 60     100.0% 
			      3 / 3      100.0% 
			      2 / 2      100.0% 
			      2 / 2      100.0% 
			      4 / 4      100.0% 
			      4 / 4      100.0% 
			      3 / 3      100.0% 
			      1 / 1      100.0% 
			      1 / 1      100.0% 
			      6 / 6      100.0% 
			      1 / 1      100.0% 
			      4 / 4      100.0% 
			      2 / 2      100.0% 
			      1 / 1      100.0% 
			      2 / 2      100.0% 
			      1 / 1      100.0% 
			      3 / 3      100.0% 
			      1 / 1      100.0% 
			      4 / 4      100.0% 
			      5 / 5      100.0% 
			      3 / 3      100.0% 
			      1 / 1      100.0% 
			      5 / 5      100.0% 
			      1 / 1      100.0% 
			      0 / 0       0.0% 
			      0 / 0       0.0% 
			      0 / 0       0.0% 
			     39 / 60     65.0%AB 
			     21 / 60     35.0%ABE 
		 
		 
			 1 
			  4.2 
			 Pseudomonas stutzeri DSM 10701 
			     60 / 60     100.0% 
			      3 / 3      100.0% 
			      2 / 2      100.0% 
			      2 / 2      100.0% 
			      4 / 4      100.0% 
			      4 / 4      100.0% 
			      3 / 3      100.0% 
			      1 / 1      100.0% 
			      1 / 1      100.0% 
			      6 / 6      100.0% 
			      1 / 1      100.0% 
			      4 / 4      100.0% 
			      2 / 2      100.0% 
			      1 / 1      100.0% 
			      2 / 2      100.0% 
			      1 / 1      100.0% 
			      3 / 3      100.0% 
			      1 / 1      100.0% 
			      3 / 3      100.0% 
			      5 / 5      100.0% 
			      3 / 3      100.0% 
			      1 / 1      100.0% 
			      6 / 6      100.0% 
			      1 / 1      100.0% 
			      0 / 0       0.0% 
			      0 / 0       0.0% 
			      0 / 0       0.0% 
			     38 / 60     63.3%AB 
			     22 / 60     36.7%ABE 
		 
		 
			 1 
			  4.7 
			 Pseudomonas stutzeri CCUG 29243 
			     61 / 61     100.0% 
			      3 / 3      100.0% 
			      2 / 2      100.0% 
			      2 / 2      100.0% 
			      4 / 4      100.0% 
			      4 / 4      100.0% 
			      3 / 3      100.0% 
			      1 / 1      100.0% 
			      1 / 1      100.0% 
			      6 / 6      100.0% 
			      1 / 1      100.0% 
			      4 / 4      100.0% 
			      2 / 2      100.0% 
			      1 / 1      100.0% 
			      2 / 2      100.0% 
			      1 / 1      100.0% 
			      3 / 3      100.0% 
			      1 / 1      100.0% 
			      4 / 4      100.0% 
			      5 / 5      100.0% 
			      3 / 3      100.0% 
			      1 / 1      100.0% 
			      6 / 6      100.0% 
			      1 / 1      100.0% 
			      0 / 0       0.0% 
			      0 / 0       0.0% 
			      0 / 0       0.0% 
			     39 / 61     63.9%AB 
			     22 / 61     36.1%ABE 
		 
		 
			 3 
			  6.2 
			 Pseudomonas syringae group 
			    189 / 190    99.5% 
			      8 / 9      88.9% 
			      9 / 9      100.0% 
			      6 / 6      100.0% 
			     15 / 15     100.0% 
			     15 / 15     100.0% 
			     11 / 11     100.0% 
			      3 / 3      100.0% 
			      3 / 3      100.0% 
			     21 / 21     100.0% 
			      3 / 3      100.0% 
			     12 / 12     100.0% 
			      6 / 6      100.0% 
			      3 / 3      100.0% 
			      3 / 3      100.0% 
			      3 / 3      100.0% 
			     11 / 11     100.0% 
			      3 / 3      100.0% 
			      6 / 6      100.0% 
			     15 / 15     100.0% 
			     12 / 12     100.0% 
			      3 / 3      100.0% 
			     18 / 18     100.0% 
			      0 / 0       0.0% 
			      0 / 0       0.0% 
			      0 / 0       0.0% 
			      0 / 0       0.0% 
			    133 / 190    70.0%AB 
			     56 / 190    29.5%ABE 
			      1 / 190     0.5%E 
		 
		 
			 1 
			  6.1 
			 Pseudomonas syringae group genomosp. 1 
			     64 / 64     100.0% 
			      3 / 3      100.0% 
			      3 / 3      100.0% 
			      2 / 2      100.0% 
			      5 / 5      100.0% 
			      5 / 5      100.0% 
			      4 / 4      100.0% 
			      1 / 1      100.0% 
			      1 / 1      100.0% 
			      7 / 7      100.0% 
			      1 / 1      100.0% 
			      4 / 4      100.0% 
			      2 / 2      100.0% 
			      1 / 1      100.0% 
			      1 / 1      100.0% 
			      1 / 1      100.0% 
			      4 / 4      100.0% 
			      1 / 1      100.0% 
			      2 / 2      100.0% 
			      5 / 5      100.0% 
			      4 / 4      100.0% 
			      1 / 1      100.0% 
			      6 / 6      100.0% 
			      0 / 0       0.0% 
			      0 / 0       0.0% 
			      0 / 0       0.0% 
			      0 / 0       0.0% 
			     45 / 64     70.3%AB 
			     19 / 64     29.7%ABE 
		 
		 
			 1 
			  6.1 
			 ATCC 19310 
			     64 / 64     100.0% 
			      3 / 3      100.0% 
			      3 / 3      100.0% 
			      2 / 2      100.0% 
			      5 / 5      100.0% 
			      5 / 5      100.0% 
			      4 / 4      100.0% 
			      1 / 1      100.0% 
			      1 / 1      100.0% 
			      7 / 7      100.0% 
			      1 / 1      100.0% 
			      4 / 4      100.0% 
			      2 / 2      100.0% 
			      1 / 1      100.0% 
			      1 / 1      100.0% 
			      1 / 1      100.0% 
			      4 / 4      100.0% 
			      1 / 1      100.0% 
			      2 / 2      100.0% 
			      5 / 5      100.0% 
			      4 / 4      100.0% 
			      1 / 1      100.0% 
			      6 / 6      100.0% 
			      0 / 0       0.0% 
			      0 / 0       0.0% 
			      0 / 0       0.0% 
			      0 / 0       0.0% 
			     45 / 64     70.3%AB 
			     19 / 64     29.7%ABE 
		 
		 
			 1 
			  6.1 
			 Pseudomonas syringae (PV. SYRINGAE) 
			     64 / 64     100.0% 
			      3 / 3      100.0% 
			      3 / 3      100.0% 
			      2 / 2      100.0% 
			      5 / 5      100.0% 
			      5 / 5      100.0% 
			      4 / 4      100.0% 
			      1 / 1      100.0% 
			      1 / 1      100.0% 
			      7 / 7      100.0% 
			      1 / 1      100.0% 
			      4 / 4      100.0% 
			      2 / 2      100.0% 
			      1 / 1      100.0% 
			      1 / 1      100.0% 
			      1 / 1      100.0% 
			      4 / 4      100.0% 
			      1 / 1      100.0% 
			      2 / 2      100.0% 
			      5 / 5      100.0% 
			      4 / 4      100.0% 
			      1 / 1      100.0% 
			      6 / 6      100.0% 
			      0 / 0       0.0% 
			      0 / 0       0.0% 
			      0 / 0       0.0% 
			      0 / 0       0.0% 
			     45 / 64     70.3%AB 
			     19 / 64     29.7%ABE 
		 
		 
			 1 
			  6.1 
			 Pseudomonas syringae pv. syringae B728a 
			     64 / 64     100.0% 
			      3 / 3      100.0% 
			      3 / 3      100.0% 
			      2 / 2      100.0% 
			      5 / 5      100.0% 
			      5 / 5      100.0% 
			      4 / 4      100.0% 
			      1 / 1      100.0% 
			      1 / 1      100.0% 
			      7 / 7      100.0% 
			      1 / 1      100.0% 
			      4 / 4      100.0% 
			      2 / 2      100.0% 
			      1 / 1      100.0% 
			      1 / 1      100.0% 
			      1 / 1      100.0% 
			      4 / 4      100.0% 
			      1 / 1      100.0% 
			      2 / 2      100.0% 
			      5 / 5      100.0% 
			      4 / 4      100.0% 
			      1 / 1      100.0% 
			      6 / 6      100.0% 
			      0 / 0       0.0% 
			      0 / 0       0.0% 
			      0 / 0       0.0% 
			      0 / 0       0.0% 
			     45 / 64     70.3%AB 
			     19 / 64     29.7%ABE 
		 
		 
			 1 
			  6.1 
			 Pseudomonas syringae group genomosp. 2 
			     62 / 63     98.4% 
			      2 / 3      66.7% 
			      3 / 3      100.0% 
			      2 / 2      100.0% 
			      5 / 5      100.0% 
			      5 / 5      100.0% 
			      3 / 3      100.0% 
			      1 / 1      100.0% 
			      1 / 1      100.0% 
			      7 / 7      100.0% 
			      1 / 1      100.0% 
			      4 / 4      100.0% 
			      2 / 2      100.0% 
			      1 / 1      100.0% 
			      1 / 1      100.0% 
			      1 / 1      100.0% 
			      4 / 4      100.0% 
			      1 / 1      100.0% 
			      2 / 2      100.0% 
			      5 / 5      100.0% 
			      4 / 4      100.0% 
			      1 / 1      100.0% 
			      6 / 6      100.0% 
			      0 / 0       0.0% 
			      0 / 0       0.0% 
			      0 / 0       0.0% 
			      0 / 0       0.0% 
			     44 / 63     69.8%AB 
			     18 / 63     28.6%ABE 
			      1 / 63      1.6%E 
		 
		 
			 1 
			  6.1 
			 ATCC 13522 
			     62 / 63     98.4% 
			      2 / 3      66.7% 
			      3 / 3      100.0% 
			      2 / 2      100.0% 
			      5 / 5      100.0% 
			      5 / 5      100.0% 
			      3 / 3      100.0% 
			      1 / 1      100.0% 
			      1 / 1      100.0% 
			      7 / 7      100.0% 
			      1 / 1      100.0% 
			      4 / 4      100.0% 
			      2 / 2      100.0% 
			      1 / 1      100.0% 
			      1 / 1      100.0% 
			      1 / 1      100.0% 
			      4 / 4      100.0% 
			      1 / 1      100.0% 
			      2 / 2      100.0% 
			      5 / 5      100.0% 
			      4 / 4      100.0% 
			      1 / 1      100.0% 
			      6 / 6      100.0% 
			      0 / 0       0.0% 
			      0 / 0       0.0% 
			      0 / 0       0.0% 
			      0 / 0       0.0% 
			     44 / 63     69.8%AB 
			     18 / 63     28.6%ABE 
			      1 / 63      1.6%E 
		 
		 
			 1 
			  6.1 
			 Pseudomonas savastanoi pv. phaseolicola 
			     62 / 63     98.4% 
			      2 / 3      66.7% 
			      3 / 3      100.0% 
			      2 / 2      100.0% 
			      5 / 5      100.0% 
			      5 / 5      100.0% 
			      3 / 3      100.0% 
			      1 / 1      100.0% 
			      1 / 1      100.0% 
			      7 / 7      100.0% 
			      1 / 1      100.0% 
			      4 / 4      100.0% 
			      2 / 2      100.0% 
			      1 / 1      100.0% 
			      1 / 1      100.0% 
			      1 / 1      100.0% 
			      4 / 4      100.0% 
			      1 / 1      100.0% 
			      2 / 2      100.0% 
			      5 / 5      100.0% 
			      4 / 4      100.0% 
			      1 / 1      100.0% 
			      6 / 6      100.0% 
			      0 / 0       0.0% 
			      0 / 0       0.0% 
			      0 / 0       0.0% 
			      0 / 0       0.0% 
			     44 / 63     69.8%AB 
			     18 / 63     28.6%ABE 
			      1 / 63      1.6%E 
		 
		 
			 1 
			  6.1 
			 Pseudomonas syringae pv. phaseolicola 1448A 
			     62 / 63     98.4% 
			      2 / 3      66.7% 
			      3 / 3      100.0% 
			      2 / 2      100.0% 
			      5 / 5      100.0% 
			      5 / 5      100.0% 
			      3 / 3      100.0% 
			      1 / 1      100.0% 
			      1 / 1      100.0% 
			      7 / 7      100.0% 
			      1 / 1      100.0% 
			      4 / 4      100.0% 
			      2 / 2      100.0% 
			      1 / 1      100.0% 
			      1 / 1      100.0% 
			      1 / 1      100.0% 
			      4 / 4      100.0% 
			      1 / 1      100.0% 
			      2 / 2      100.0% 
			      5 / 5      100.0% 
			      4 / 4      100.0% 
			      1 / 1      100.0% 
			      6 / 6      100.0% 
			      0 / 0       0.0% 
			      0 / 0       0.0% 
			      0 / 0       0.0% 
			      0 / 0       0.0% 
			     44 / 63     69.8%AB 
			     18 / 63     28.6%ABE 
			      1 / 63      1.6%E 
		 
		 
			 1 
			  6.5 
			 Pseudomonas syringae group genomosp. 3 
			     63 / 63     100.0% 
			      3 / 3      100.0% 
			      3 / 3      100.0% 
			      2 / 2      100.0% 
			      5 / 5      100.0% 
			      5 / 5      100.0% 
			      4 / 4      100.0% 
			      1 / 1      100.0% 
			      1 / 1      100.0% 
			      7 / 7      100.0% 
			      1 / 1      100.0% 
			      4 / 4      100.0% 
			      2 / 2      100.0% 
			      1 / 1      100.0% 
			      1 / 1      100.0% 
			      1 / 1      100.0% 
			      3 / 3      100.0% 
			      1 / 1      100.0% 
			      2 / 2      100.0% 
			      5 / 5      100.0% 
			      4 / 4      100.0% 
			      1 / 1      100.0% 
			      6 / 6      100.0% 
			      0 / 0       0.0% 
			      0 / 0       0.0% 
			      0 / 0       0.0% 
			      0 / 0       0.0% 
			     44 / 63     69.8%AB 
			     19 / 63     30.2%ABE 
		 
		 
			 1 
			  6.5 
			 Pseudomonas syringae (PV. TOMATO) 
			     63 / 63     100.0% 
			      3 / 3      100.0% 
			      3 / 3      100.0% 
			      2 / 2      100.0% 
			      5 / 5      100.0% 
			      5 / 5      100.0% 
			      4 / 4      100.0% 
			      1 / 1      100.0% 
			      1 / 1      100.0% 
			      7 / 7      100.0% 
			      1 / 1      100.0% 
			      4 / 4      100.0% 
			      2 / 2      100.0% 
			      1 / 1      100.0% 
			      1 / 1      100.0% 
			      1 / 1      100.0% 
			      3 / 3      100.0% 
			      1 / 1      100.0% 
			      2 / 2      100.0% 
			      5 / 5      100.0% 
			      4 / 4      100.0% 
			      1 / 1      100.0% 
			      6 / 6      100.0% 
			      0 / 0       0.0% 
			      0 / 0       0.0% 
			      0 / 0       0.0% 
			      0 / 0       0.0% 
			     44 / 63     69.8%AB 
			     19 / 63     30.2%ABE 
		 
		 
			 1 
			  6.5 
			 Pseudomonas syringae DC3000 
			     63 / 63     100.0% 
			      3 / 3      100.0% 
			      3 / 3      100.0% 
			      2 / 2      100.0% 
			      5 / 5      100.0% 
			      5 / 5      100.0% 
			      4 / 4      100.0% 
			      1 / 1      100.0% 
			      1 / 1      100.0% 
			      7 / 7      100.0% 
			      1 / 1      100.0% 
			      4 / 4      100.0% 
			      2 / 2      100.0% 
			      1 / 1      100.0% 
			      1 / 1      100.0% 
			      1 / 1      100.0% 
			      3 / 3      100.0% 
			      1 / 1      100.0% 
			      2 / 2      100.0% 
			      5 / 5      100.0% 
			      4 / 4      100.0% 
			      1 / 1      100.0% 
			      6 / 6      100.0% 
			      0 / 0       0.0% 
			      0 / 0       0.0% 
			      0 / 0       0.0% 
			      0 / 0       0.0% 
			     44 / 63     69.8%AB 
			     19 / 63     30.2%ABE 
		 
		 
			 1 
			  5.9 
			 "Pseudomonas entomophila" Vodovar et al. 2005 
			     78 / 78     100.0% 
			      4 / 4      100.0% 
			      4 / 4      100.0% 
			      2 / 2      100.0% 
			      7 / 7      100.0% 
			      6 / 6      100.0% 
			      4 / 4      100.0% 
			      2 / 2      100.0% 
			      1 / 1      100.0% 
			     10 / 10     100.0% 
			      1 / 1      100.0% 
			      4 / 4      100.0% 
			      2 / 2      100.0% 
			      1 / 1      100.0% 
			      2 / 2      100.0% 
			      1 / 1      100.0% 
			      3 / 3      100.0% 
			      1 / 1      100.0% 
			      3 / 3      100.0% 
			      7 / 7      100.0% 
			      5 / 5      100.0% 
			      1 / 1      100.0% 
			      6 / 6      100.0% 
			      1 / 1      100.0% 
			      0 / 0       0.0% 
			      0 / 0       0.0% 
			      0 / 0       0.0% 
			     55 / 78     70.5%AB 
			     23 / 78     29.5%ABE 
		 
		 
			 1 
			  5.9 
			 Pseudomonas entomophila L48 
			     78 / 78     100.0% 
			      4 / 4      100.0% 
			      4 / 4      100.0% 
			      2 / 2      100.0% 
			      7 / 7      100.0% 
			      6 / 6      100.0% 
			      4 / 4      100.0% 
			      2 / 2      100.0% 
			      1 / 1      100.0% 
			     10 / 10     100.0% 
			      1 / 1      100.0% 
			      4 / 4      100.0% 
			      2 / 2      100.0% 
			      1 / 1      100.0% 
			      2 / 2      100.0% 
			      1 / 1      100.0% 
			      3 / 3      100.0% 
			      1 / 1      100.0% 
			      3 / 3      100.0% 
			      7 / 7      100.0% 
			      5 / 5      100.0% 
			      1 / 1      100.0% 
			      6 / 6      100.0% 
			      1 / 1      100.0% 
			      0 / 0       0.0% 
			      0 / 0       0.0% 
			      0 / 0       0.0% 
			     55 / 78     70.5%AB 
			     23 / 78     29.5%ABE 
		 
		 
			 1 
			  6.8 
			 CFBP 11706 
			     65 / 65     100.0% 
			      3 / 3      100.0% 
			      4 / 4      100.0% 
			      2 / 2      100.0% 
			      5 / 5      100.0% 
			      5 / 5      100.0% 
			      4 / 4      100.0% 
			      1 / 1      100.0% 
			      1 / 1      100.0% 
			      7 / 7      100.0% 
			      1 / 1      100.0% 
			      4 / 4      100.0% 
			      2 / 2      100.0% 
			      1 / 1      100.0% 
			      1 / 1      100.0% 
			      1 / 1      100.0% 
			      3 / 3      100.0% 
			      1 / 1      100.0% 
			      3 / 3      100.0% 
			      5 / 5      100.0% 
			      4 / 4      100.0% 
			      1 / 1      100.0% 
			      6 / 6      100.0% 
			      0 / 0       0.0% 
			      0 / 0       0.0% 
			      0 / 0       0.0% 
			      0 / 0       0.0% 
			     45 / 65     69.2%AB 
			     20 / 65     30.8%ABE 
		 
		 
			 1 
			  6.8 
			 Pseudomonas brassicacearum subsp. brassicacearum 
			     65 / 65     100.0% 
			      3 / 3      100.0% 
			      4 / 4      100.0% 
			      2 / 2      100.0% 
			      5 / 5      100.0% 
			      5 / 5      100.0% 
			      4 / 4      100.0% 
			      1 / 1      100.0% 
			      1 / 1      100.0% 
			      7 / 7      100.0% 
			      1 / 1      100.0% 
			      4 / 4      100.0% 
			      2 / 2      100.0% 
			      1 / 1      100.0% 
			      1 / 1      100.0% 
			      1 / 1      100.0% 
			      3 / 3      100.0% 
			      1 / 1      100.0% 
			      3 / 3      100.0% 
			      5 / 5      100.0% 
			      4 / 4      100.0% 
			      1 / 1      100.0% 
			      6 / 6      100.0% 
			      0 / 0       0.0% 
			      0 / 0       0.0% 
			      0 / 0       0.0% 
			      0 / 0       0.0% 
			     45 / 65     69.2%AB 
			     20 / 65     30.8%ABE 
		 
		 
			 1 
			  6.8 
			 Pseudomonas brassicacearum subsp. brassicacearum CFBP 11874 
			     65 / 65     100.0% 
			      3 / 3      100.0% 
			      4 / 4      100.0% 
			      2 / 2      100.0% 
			      5 / 5      100.0% 
			      5 / 5      100.0% 
			      4 / 4      100.0% 
			      1 / 1      100.0% 
			      1 / 1      100.0% 
			      7 / 7      100.0% 
			      1 / 1      100.0% 
			      4 / 4      100.0% 
			      2 / 2      100.0% 
			      1 / 1      100.0% 
			      1 / 1      100.0% 
			      1 / 1      100.0% 
			      3 / 3      100.0% 
			      1 / 1      100.0% 
			      3 / 3      100.0% 
			      5 / 5      100.0% 
			      4 / 4      100.0% 
			      1 / 1      100.0% 
			      6 / 6      100.0% 
			      0 / 0       0.0% 
			      0 / 0       0.0% 
			      0 / 0       0.0% 
			      0 / 0       0.0% 
			     45 / 65     69.2%AB 
			     20 / 65     30.8%ABE 
		 
		 
			 1 
			  6.2 
			 Pseudomonas putida UW4 
			     71 / 71     100.0% 
			      3 / 3      100.0% 
			      4 / 4      100.0% 
			      2 / 2      100.0% 
			      7 / 7      100.0% 
			      5 / 5      100.0% 
			      4 / 4      100.0% 
			      2 / 2      100.0% 
			      1 / 1      100.0% 
			      8 / 8      100.0% 
			      1 / 1      100.0% 
			      4 / 4      100.0% 
			      2 / 2      100.0% 
			      1 / 1      100.0% 
			      2 / 2      100.0% 
			      1 / 1      100.0% 
			      3 / 3      100.0% 
			      1 / 1      100.0% 
			      3 / 3      100.0% 
			      5 / 5      100.0% 
			      4 / 4      100.0% 
			      1 / 1      100.0% 
			      6 / 6      100.0% 
			      1 / 1      100.0% 
			      0 / 0       0.0% 
			      0 / 0       0.0% 
			      0 / 0       0.0% 
			     49 / 71     69.0%AB 
			     22 / 71     31.0%ABE 
		 
		 
			 3 
			  5.4 
			 Azotobacter group 
			    192 / 192    100.0% 
			      6 / 6      100.0% 
			      6 / 6      100.0% 
			      6 / 6      100.0% 
			     18 / 18     100.0% 
			     15 / 15     100.0% 
			      9 / 9      100.0% 
			      3 / 3      100.0% 
			      3 / 3      100.0% 
			     24 / 24     100.0% 
			      3 / 3      100.0% 
			     12 / 12     100.0% 
			      6 / 6      100.0% 
			      6 / 6      100.0% 
			      6 / 6      100.0% 
			      3 / 3      100.0% 
			      9 / 9      100.0% 
			      3 / 3      100.0% 
			      9 / 9      100.0% 
			     15 / 15     100.0% 
			      9 / 9      100.0% 
			      3 / 3      100.0% 
			     18 / 18     100.0% 
			      0 / 0       0.0% 
			      0 / 0       0.0% 
			      0 / 0       0.0% 
			      0 / 0       0.0% 
			    132 / 192    68.8%AB 
			     60 / 192    31.2%ABE 
		 
		 
			 3 
			  5.4 
			 "Azotomonas" Orla-Jensen 1909 
			    192 / 192    100.0% 
			      6 / 6      100.0% 
			      6 / 6      100.0% 
			      6 / 6      100.0% 
			     18 / 18     100.0% 
			     15 / 15     100.0% 
			      9 / 9      100.0% 
			      3 / 3      100.0% 
			      3 / 3      100.0% 
			     24 / 24     100.0% 
			      3 / 3      100.0% 
			     12 / 12     100.0% 
			      6 / 6      100.0% 
			      6 / 6      100.0% 
			      6 / 6      100.0% 
			      3 / 3      100.0% 
			      9 / 9      100.0% 
			      3 / 3      100.0% 
			      9 / 9      100.0% 
			     15 / 15     100.0% 
			      9 / 9      100.0% 
			      3 / 3      100.0% 
			     18 / 18     100.0% 
			      0 / 0       0.0% 
			      0 / 0       0.0% 
			      0 / 0       0.0% 
			      0 / 0       0.0% 
			    132 / 192    68.8%AB 
			     60 / 192    31.2%ABE 
		 
		 
			 3 
			  5.4 
			 ATCC 478 
			    192 / 192    100.0% 
			      6 / 6      100.0% 
			      6 / 6      100.0% 
			      6 / 6      100.0% 
			     18 / 18     100.0% 
			     15 / 15     100.0% 
			      9 / 9      100.0% 
			      3 / 3      100.0% 
			      3 / 3      100.0% 
			     24 / 24     100.0% 
			      3 / 3      100.0% 
			     12 / 12     100.0% 
			      6 / 6      100.0% 
			      6 / 6      100.0% 
			      6 / 6      100.0% 
			      3 / 3      100.0% 
			      9 / 9      100.0% 
			      3 / 3      100.0% 
			      9 / 9      100.0% 
			     15 / 15     100.0% 
			      9 / 9      100.0% 
			      3 / 3      100.0% 
			     18 / 18     100.0% 
			      0 / 0       0.0% 
			      0 / 0       0.0% 
			      0 / 0       0.0% 
			      0 / 0       0.0% 
			    132 / 192    68.8%AB 
			     60 / 192    31.2%ABE 
		 
		 
			 1 
			  5.4 
			 Azotobacter vinelandii ATCC BAA-1303 
			     64 / 64     100.0% 
			      2 / 2      100.0% 
			      2 / 2      100.0% 
			      2 / 2      100.0% 
			      6 / 6      100.0% 
			      5 / 5      100.0% 
			      3 / 3      100.0% 
			      1 / 1      100.0% 
			      1 / 1      100.0% 
			      8 / 8      100.0% 
			      1 / 1      100.0% 
			      4 / 4      100.0% 
			      2 / 2      100.0% 
			      2 / 2      100.0% 
			      2 / 2      100.0% 
			      1 / 1      100.0% 
			      3 / 3      100.0% 
			      1 / 1      100.0% 
			      3 / 3      100.0% 
			      5 / 5      100.0% 
			      3 / 3      100.0% 
			      1 / 1      100.0% 
			      6 / 6      100.0% 
			      0 / 0       0.0% 
			      0 / 0       0.0% 
			      0 / 0       0.0% 
			      0 / 0       0.0% 
			     44 / 64     68.8%AB 
			     20 / 64     31.2%ABE 
		 
		 
			 2 
			  5.3 
			 Azotobacter vinelandii CA 
			    128 / 128    100.0% 
			      4 / 4      100.0% 
			      4 / 4      100.0% 
			      4 / 4      100.0% 
			     12 / 12     100.0% 
			     10 / 10     100.0% 
			      6 / 6      100.0% 
			      2 / 2      100.0% 
			      2 / 2      100.0% 
			     16 / 16     100.0% 
			      2 / 2      100.0% 
			      8 / 8      100.0% 
			      4 / 4      100.0% 
			      4 / 4      100.0% 
			      4 / 4      100.0% 
			      2 / 2      100.0% 
			      6 / 6      100.0% 
			      2 / 2      100.0% 
			      6 / 6      100.0% 
			     10 / 10     100.0% 
			      6 / 6      100.0% 
			      2 / 2      100.0% 
			     12 / 12     100.0% 
			      0 / 0       0.0% 
			      0 / 0       0.0% 
			      0 / 0       0.0% 
			      0 / 0       0.0% 
			     88 / 128    68.8%AB 
			     40 / 128    31.2%ABE 
		 
		 
			 1 
			  5.3 
			 Azotobacter vinelandii CA6 
			     64 / 64     100.0% 
			      2 / 2      100.0% 
			      2 / 2      100.0% 
			      2 / 2      100.0% 
			      6 / 6      100.0% 
			      5 / 5      100.0% 
			      3 / 3      100.0% 
			      1 / 1      100.0% 
			      1 / 1      100.0% 
			      8 / 8      100.0% 
			      1 / 1      100.0% 
			      4 / 4      100.0% 
			      2 / 2      100.0% 
			      2 / 2      100.0% 
			      2 / 2      100.0% 
			      1 / 1      100.0% 
			      3 / 3      100.0% 
			      1 / 1      100.0% 
			      3 / 3      100.0% 
			      5 / 5      100.0% 
			      3 / 3      100.0% 
			      1 / 1      100.0% 
			      6 / 6      100.0% 
			      0 / 0       0.0% 
			      0 / 0       0.0% 
			      0 / 0       0.0% 
			      0 / 0       0.0% 
			     44 / 64     68.8%AB 
			     20 / 64     31.2%ABE 
		 
		 
			 215 
			  4.5 
			 'Enterobacteriales' 
			  16318 / 16982  96.1% 
			    707 / 710    99.6% 
			    588 / 610    96.4% 
			    749 / 765    97.9% 
			    584 / 596    98.0% 
			   1175 / 1203   97.7% 
			   1199 / 1238   96.8% 
			    208 / 219    95.0% 
			    522 / 535    97.6% 
			    956 / 995    96.1% 
			    202 / 221    91.4% 
			    980 / 1040   94.2% 
			    987 / 1017   97.1% 
			    395 / 414    95.4% 
			    708 / 724    97.8% 
			    202 / 223    90.6% 
			    601 / 617    97.4% 
			    394 / 407    96.8% 
			    813 / 868    93.7% 
			   1530 / 1643   93.1% 
			    775 / 797    97.2% 
			    378 / 404    93.6% 
			   1496 / 1566   95.5% 
			    166 / 166    100.0% 
			      0 / 0       0.0% 
			      0 / 1       0.0% 
			      3 / 3      100.0% 
			   9807 / 16982  57.7%AB 
			   5648 / 16982  33.3%ABE 
			    484 / 16982   2.9%B 
			    379 / 16982   2.2%BE 
			    365 / 16982   2.1%E 
			    289 / 16982   1.7% 
			      5 / 16982   0.0%CE 
			      4 / 16982   0.0%C 
			      1 / 16982   0.0%GG 
		 
		 
			 215 
			  4.5 
			 Enterobacteraceae 
			  16318 / 16982  96.1% 
			    707 / 710    99.6% 
			    588 / 610    96.4% 
			    749 / 765    97.9% 
			    584 / 596    98.0% 
			   1175 / 1203   97.7% 
			   1199 / 1238   96.8% 
			    208 / 219    95.0% 
			    522 / 535    97.6% 
			    956 / 995    96.1% 
			    202 / 221    91.4% 
			    980 / 1040   94.2% 
			    987 / 1017   97.1% 
			    395 / 414    95.4% 
			    708 / 724    97.8% 
			    202 / 223    90.6% 
			    601 / 617    97.4% 
			    394 / 407    96.8% 
			    813 / 868    93.7% 
			   1530 / 1643   93.1% 
			    775 / 797    97.2% 
			    378 / 404    93.6% 
			   1496 / 1566   95.5% 
			    166 / 166    100.0% 
			      0 / 0       0.0% 
			      0 / 1       0.0% 
			      3 / 3      100.0% 
			   9807 / 16982  57.7%AB 
			   5648 / 16982  33.3%ABE 
			    484 / 16982   2.9%B 
			    379 / 16982   2.2%BE 
			    365 / 16982   2.1%E 
			    289 / 16982   1.7% 
			      5 / 16982   0.0%CE 
			      4 / 16982   0.0%C 
			      1 / 16982   0.0%GG 
		 
		 
			 2 
			  5.1 
			 Citrobacter 
			    165 / 165    100.0% 
			      8 / 8      100.0% 
			      6 / 6      100.0% 
			      8 / 8      100.0% 
			      5 / 5      100.0% 
			     10 / 10     100.0% 
			     10 / 10     100.0% 
			      2 / 2      100.0% 
			      6 / 6      100.0% 
			     12 / 12     100.0% 
			      2 / 2      100.0% 
			     10 / 10     100.0% 
			      9 / 9      100.0% 
			      4 / 4      100.0% 
			      8 / 8      100.0% 
			      2 / 2      100.0% 
			      6 / 6      100.0% 
			      4 / 4      100.0% 
			      9 / 9      100.0% 
			     15 / 15     100.0% 
			      9 / 9      100.0% 
			      2 / 2      100.0% 
			     16 / 16     100.0% 
			      2 / 2      100.0% 
			      0 / 0       0.0% 
			      0 / 0       0.0% 
			      0 / 0       0.0% 
			     68 / 165    41.2%AB 
			     52 / 165    31.5%ABE 
			     30 / 165    18.2%B 
			     15 / 165     9.1%BE 
		 
		 
			 1 
			  4.7 
			 "Aerobacter diversum" (sic) Burkey 1928 
			     82 / 82     100.0% 
			      4 / 4      100.0% 
			      3 / 3      100.0% 
			      4 / 4      100.0% 
			      2 / 2      100.0% 
			      5 / 5      100.0% 
			      6 / 6      100.0% 
			      1 / 1      100.0% 
			      3 / 3      100.0% 
			      4 / 4      100.0% 
			      1 / 1      100.0% 
			      5 / 5      100.0% 
			      5 / 5      100.0% 
			      2 / 2      100.0% 
			      4 / 4      100.0% 
			      1 / 1      100.0% 
			      3 / 3      100.0% 
			      2 / 2      100.0% 
			      5 / 5      100.0% 
			      7 / 7      100.0% 
			      5 / 5      100.0% 
			      1 / 1      100.0% 
			      8 / 8      100.0% 
			      1 / 1      100.0% 
			      0 / 0       0.0% 
			      0 / 0       0.0% 
			      0 / 0       0.0% 
			     30 / 82     36.6%B 
			     19 / 82     23.2%ABE 
			     18 / 82     22.0%AB 
			     15 / 82     18.3%BE 
		 
		 
			 1 
			  4.7 
			 Citrobacter (diversus) koseri ATCC BAA-895 
			     82 / 82     100.0% 
			      4 / 4      100.0% 
			      3 / 3      100.0% 
			      4 / 4      100.0% 
			      2 / 2      100.0% 
			      5 / 5      100.0% 
			      6 / 6      100.0% 
			      1 / 1      100.0% 
			      3 / 3      100.0% 
			      4 / 4      100.0% 
			      1 / 1      100.0% 
			      5 / 5      100.0% 
			      5 / 5      100.0% 
			      2 / 2      100.0% 
			      4 / 4      100.0% 
			      1 / 1      100.0% 
			      3 / 3      100.0% 
			      2 / 2      100.0% 
			      5 / 5      100.0% 
			      7 / 7      100.0% 
			      5 / 5      100.0% 
			      1 / 1      100.0% 
			      8 / 8      100.0% 
			      1 / 1      100.0% 
			      0 / 0       0.0% 
			      0 / 0       0.0% 
			      0 / 0       0.0% 
			     30 / 82     36.6%B 
			     19 / 82     23.2%ABE 
			     18 / 82     22.0%AB 
			     15 / 82     18.3%BE 
		 
		 
			 1 
			  5.4 
			 ATCC 51116 
			     83 / 83     100.0% 
			      4 / 4      100.0% 
			      3 / 3      100.0% 
			      4 / 4      100.0% 
			      3 / 3      100.0% 
			      5 / 5      100.0% 
			      4 / 4      100.0% 
			      1 / 1      100.0% 
			      3 / 3      100.0% 
			      8 / 8      100.0% 
			      1 / 1      100.0% 
			      5 / 5      100.0% 
			      4 / 4      100.0% 
			      2 / 2      100.0% 
			      4 / 4      100.0% 
			      1 / 1      100.0% 
			      3 / 3      100.0% 
			      2 / 2      100.0% 
			      4 / 4      100.0% 
			      8 / 8      100.0% 
			      4 / 4      100.0% 
			      1 / 1      100.0% 
			      8 / 8      100.0% 
			      1 / 1      100.0% 
			      0 / 0       0.0% 
			      0 / 0       0.0% 
			      0 / 0       0.0% 
			     50 / 83     60.2%AB 
			     33 / 83     39.8%ABE 
		 
		 
			 1 
			  5.4 
			 Citrobacter rodentium ICC168 
			     83 / 83     100.0% 
			      4 / 4      100.0% 
			      3 / 3      100.0% 
			      4 / 4      100.0% 
			      3 / 3      100.0% 
			      5 / 5      100.0% 
			      4 / 4      100.0% 
			      1 / 1      100.0% 
			      3 / 3      100.0% 
			      8 / 8      100.0% 
			      1 / 1      100.0% 
			      5 / 5      100.0% 
			      4 / 4      100.0% 
			      2 / 2      100.0% 
			      4 / 4      100.0% 
			      1 / 1      100.0% 
			      3 / 3      100.0% 
			      2 / 2      100.0% 
			      4 / 4      100.0% 
			      8 / 8      100.0% 
			      4 / 4      100.0% 
			      1 / 1      100.0% 
			      8 / 8      100.0% 
			      1 / 1      100.0% 
			      0 / 0       0.0% 
			      0 / 0       0.0% 
			      0 / 0       0.0% 
			     50 / 83     60.2%AB 
			     33 / 83     39.8%ABE 
		 
		 
			 10 
			  5.1 
			 "Aerobacter" Hormaeche and Edwards 1958 
			    822 / 828    99.3% 
			     34 / 34     100.0% 
			     31 / 31     100.0% 
			     43 / 43     100.0% 
			     31 / 31     100.0% 
			     57 / 57     100.0% 
			     62 / 62     100.0% 
			     10 / 10     100.0% 
			     30 / 30     100.0% 
			     51 / 51     100.0% 
			     11 / 11     100.0% 
			     49 / 49     100.0% 
			     51 / 51     100.0% 
			     20 / 20     100.0% 
			     40 / 40     100.0% 
			     11 / 11     100.0% 
			     30 / 30     100.0% 
			     20 / 20     100.0% 
			     42 / 43     97.7% 
			     63 / 68     92.6% 
			     39 / 39     100.0% 
			     10 / 10     100.0% 
			     77 / 77     100.0% 
			     10 / 10     100.0% 
			      0 / 0       0.0% 
			      0 / 0       0.0% 
			      0 / 0       0.0% 
			    506 / 828    61.1%AB 
			    315 / 828    38.0%ABE 
			      5 / 828     0.6% 
			      1 / 828     0.1%BE 
			      1 / 828     0.1%E 
		 
		 
			 2 
			  5.4 
			 "Aerobacter aerogenes" Hormaeche and Edwards 1958 
			    168 / 169    99.4% 
			      8 / 8      100.0% 
			      6 / 6      100.0% 
			      9 / 9      100.0% 
			      6 / 6      100.0% 
			     11 / 11     100.0% 
			     15 / 15     100.0% 
			      2 / 2      100.0% 
			      6 / 6      100.0% 
			     10 / 10     100.0% 
			      2 / 2      100.0% 
			      9 / 9      100.0% 
			     11 / 11     100.0% 
			      4 / 4      100.0% 
			      8 / 8      100.0% 
			      3 / 3      100.0% 
			      6 / 6      100.0% 
			      4 / 4      100.0% 
			      8 / 9      88.9% 
			     14 / 14     100.0% 
			      8 / 8      100.0% 
			      2 / 2      100.0% 
			     14 / 14     100.0% 
			      2 / 2      100.0% 
			      0 / 0       0.0% 
			      0 / 0       0.0% 
			      0 / 0       0.0% 
			    104 / 169    61.5%AB 
			     64 / 169    37.9%ABE 
			      1 / 169     0.6% 
		 
		 
			 1 
			  5.6 
			 Enterobacter aerogenes EA1509E 
			     86 / 86     100.0% 
			      4 / 4      100.0% 
			      3 / 3      100.0% 
			      5 / 5      100.0% 
			      3 / 3      100.0% 
			      5 / 5      100.0% 
			      8 / 8      100.0% 
			      1 / 1      100.0% 
			      3 / 3      100.0% 
			      5 / 5      100.0% 
			      1 / 1      100.0% 
			      4 / 4      100.0% 
			      6 / 6      100.0% 
			      2 / 2      100.0% 
			      4 / 4      100.0% 
			      2 / 2      100.0% 
			      3 / 3      100.0% 
			      2 / 2      100.0% 
			      5 / 5      100.0% 
			      7 / 7      100.0% 
			      4 / 4      100.0% 
			      1 / 1      100.0% 
			      7 / 7      100.0% 
			      1 / 1      100.0% 
			      0 / 0       0.0% 
			      0 / 0       0.0% 
			      0 / 0       0.0% 
			     54 / 86     62.8%AB 
			     32 / 86     37.2%ABE 
		 
		 
			 1 
			  5.3 
			 Enterobacter aerogenes KCTC 2190 
			     82 / 83     98.8% 
			      4 / 4      100.0% 
			      3 / 3      100.0% 
			      4 / 4      100.0% 
			      3 / 3      100.0% 
			      6 / 6      100.0% 
			      7 / 7      100.0% 
			      1 / 1      100.0% 
			      3 / 3      100.0% 
			      5 / 5      100.0% 
			      1 / 1      100.0% 
			      5 / 5      100.0% 
			      5 / 5      100.0% 
			      2 / 2      100.0% 
			      4 / 4      100.0% 
			      1 / 1      100.0% 
			      3 / 3      100.0% 
			      2 / 2      100.0% 
			      3 / 4      75.0% 
			      7 / 7      100.0% 
			      4 / 4      100.0% 
			      1 / 1      100.0% 
			      7 / 7      100.0% 
			      1 / 1      100.0% 
			      0 / 0       0.0% 
			      0 / 0       0.0% 
			      0 / 0       0.0% 
			     50 / 83     60.2%AB 
			     32 / 83     38.6%ABE 
			      1 / 83      1.2% 
		 
		 
			 6 
			  5.0 
			 Enterobacter cloacae complex 
			    485 / 489    99.2% 
			     19 / 19     100.0% 
			     18 / 18     100.0% 
			     26 / 26     100.0% 
			     18 / 18     100.0% 
			     34 / 34     100.0% 
			     35 / 35     100.0% 
			      6 / 6      100.0% 
			     17 / 17     100.0% 
			     30 / 30     100.0% 
			      6 / 6      100.0% 
			     30 / 30     100.0% 
			     30 / 30     100.0% 
			     12 / 12     100.0% 
			     24 / 24     100.0% 
			      6 / 6      100.0% 
			     18 / 18     100.0% 
			     12 / 12     100.0% 
			     26 / 26     100.0% 
			     36 / 40     90.0% 
			     23 / 23     100.0% 
			      6 / 6      100.0% 
			     47 / 47     100.0% 
			      6 / 6      100.0% 
			      0 / 0       0.0% 
			      0 / 0       0.0% 
			      0 / 0       0.0% 
			    298 / 489    60.9%AB 
			    187 / 489    38.2%ABE 
			      4 / 489     0.8% 
		 
		 
			 4 
			  5.0 
			 "Aerobacter cloacae" (Jordan 1890) Bergey et al. 1923 
			    322 / 324    99.4% 
			     12 / 12     100.0% 
			     12 / 12     100.0% 
			     18 / 18     100.0% 
			     12 / 12     100.0% 
			     23 / 23     100.0% 
			     22 / 22     100.0% 
			      4 / 4      100.0% 
			     11 / 11     100.0% 
			     20 / 20     100.0% 
			      4 / 4      100.0% 
			     20 / 20     100.0% 
			     20 / 20     100.0% 
			      8 / 8      100.0% 
			     16 / 16     100.0% 
			      4 / 4      100.0% 
			     12 / 12     100.0% 
			      8 / 8      100.0% 
			     18 / 18     100.0% 
			     24 / 26     92.3% 
			     15 / 15     100.0% 
			      4 / 4      100.0% 
			     31 / 31     100.0% 
			      4 / 4      100.0% 
			      0 / 0       0.0% 
			      0 / 0       0.0% 
			      0 / 0       0.0% 
			    199 / 324    61.4%AB 
			    123 / 324    38.0%ABE 
			      2 / 324     0.6% 
		 
		 
			 1 
			  5.0 
			 "Aerobacter dissolvens" (Rosen 1922) Waldee 1945 
			     78 / 79     98.7% 
			      2 / 2      100.0% 
			      3 / 3      100.0% 
			      4 / 4      100.0% 
			      2 / 2      100.0% 
			      6 / 6      100.0% 
			      6 / 6      100.0% 
			      1 / 1      100.0% 
			      3 / 3      100.0% 
			      4 / 4      100.0% 
			      1 / 1      100.0% 
			      5 / 5      100.0% 
			      5 / 5      100.0% 
			      2 / 2      100.0% 
			      4 / 4      100.0% 
			      1 / 1      100.0% 
			      3 / 3      100.0% 
			      2 / 2      100.0% 
			      4 / 4      100.0% 
			      6 / 7      85.7% 
			      4 / 4      100.0% 
			      1 / 1      100.0% 
			      8 / 8      100.0% 
			      1 / 1      100.0% 
			      0 / 0       0.0% 
			      0 / 0       0.0% 
			      0 / 0       0.0% 
			     48 / 79     60.8%AB 
			     30 / 79     38.0%ABE 
			      1 / 79      1.3% 
		 
		 
			 1 
			  5.0 
			 Enterobacter cloacae subsp. dissolvens SDM 
			     78 / 79     98.7% 
			      2 / 2      100.0% 
			      3 / 3      100.0% 
			      4 / 4      100.0% 
			      2 / 2      100.0% 
			      6 / 6      100.0% 
			      6 / 6      100.0% 
			      1 / 1      100.0% 
			      3 / 3      100.0% 
			      4 / 4      100.0% 
			      1 / 1      100.0% 
			      5 / 5      100.0% 
			      5 / 5      100.0% 
			      2 / 2      100.0% 
			      4 / 4      100.0% 
			      1 / 1      100.0% 
			      3 / 3      100.0% 
			      2 / 2      100.0% 
			      4 / 4      100.0% 
			      6 / 7      85.7% 
			      4 / 4      100.0% 
			      1 / 1      100.0% 
			      8 / 8      100.0% 
			      1 / 1      100.0% 
			      0 / 0       0.0% 
			      0 / 0       0.0% 
			      0 / 0       0.0% 
			     48 / 79     60.8%AB 
			     30 / 79     38.0%ABE 
			      1 / 79      1.3% 
		 
		 
			 2 
			  5.2 
			 Enterobacter cloacae subsp. cloacae 
			    162 / 163    99.4% 
			      7 / 7      100.0% 
			      6 / 6      100.0% 
			     10 / 10     100.0% 
			      7 / 7      100.0% 
			     11 / 11     100.0% 
			     10 / 10     100.0% 
			      2 / 2      100.0% 
			      5 / 5      100.0% 
			     11 / 11     100.0% 
			      2 / 2      100.0% 
			     10 / 10     100.0% 
			     10 / 10     100.0% 
			      4 / 4      100.0% 
			      8 / 8      100.0% 
			      2 / 2      100.0% 
			      6 / 6      100.0% 
			      4 / 4      100.0% 
			     10 / 10     100.0% 
			     11 / 12     91.7% 
			      7 / 7      100.0% 
			      2 / 2      100.0% 
			     15 / 15     100.0% 
			      2 / 2      100.0% 
			      0 / 0       0.0% 
			      0 / 0       0.0% 
			      0 / 0       0.0% 
			    100 / 163    61.3%AB 
			     62 / 163    38.0%ABE 
			      1 / 163     0.6% 
		 
		 
			 1 
			  5.6 
			 Enterobacter cloacae subsp. cloacae ATCC 13047 
			     82 / 82     100.0% 
			      4 / 4      100.0% 
			      3 / 3      100.0% 
			      6 / 6      100.0% 
			      4 / 4      100.0% 
			      5 / 5      100.0% 
			      4 / 4      100.0% 
			      1 / 1      100.0% 
			      2 / 2      100.0% 
			      6 / 6      100.0% 
			      1 / 1      100.0% 
			      5 / 5      100.0% 
			      5 / 5      100.0% 
			      2 / 2      100.0% 
			      4 / 4      100.0% 
			      1 / 1      100.0% 
			      3 / 3      100.0% 
			      2 / 2      100.0% 
			      6 / 6      100.0% 
			      5 / 5      100.0% 
			      3 / 3      100.0% 
			      1 / 1      100.0% 
			      8 / 8      100.0% 
			      1 / 1      100.0% 
			      0 / 0       0.0% 
			      0 / 0       0.0% 
			      0 / 0       0.0% 
			     50 / 82     61.0%AB 
			     32 / 82     39.0%ABE 
		 
		 
			 1 
			  4.7 
			 Enterobacter cloacae subsp. cloacae ENHKU01 
			     80 / 81     98.8% 
			      3 / 3      100.0% 
			      3 / 3      100.0% 
			      4 / 4      100.0% 
			      3 / 3      100.0% 
			      6 / 6      100.0% 
			      6 / 6      100.0% 
			      1 / 1      100.0% 
			      3 / 3      100.0% 
			      5 / 5      100.0% 
			      1 / 1      100.0% 
			      5 / 5      100.0% 
			      5 / 5      100.0% 
			      2 / 2      100.0% 
			      4 / 4      100.0% 
			      1 / 1      100.0% 
			      3 / 3      100.0% 
			      2 / 2      100.0% 
			      4 / 4      100.0% 
			      6 / 7      85.7% 
			      4 / 4      100.0% 
			      1 / 1      100.0% 
			      7 / 7      100.0% 
			      1 / 1      100.0% 
			      0 / 0       0.0% 
			      0 / 0       0.0% 
			      0 / 0       0.0% 
			     50 / 81     61.7%AB 
			     30 / 81     37.0%ABE 
			      1 / 81      1.2% 
		 
		 
			 1 
			  4.8 
			 Enterobacter cloacae EcWSU1 
			     82 / 82     100.0% 
			      3 / 3      100.0% 
			      3 / 3      100.0% 
			      4 / 4      100.0% 
			      3 / 3      100.0% 
			      6 / 6      100.0% 
			      6 / 6      100.0% 
			      1 / 1      100.0% 
			      3 / 3      100.0% 
			      5 / 5      100.0% 
			      1 / 1      100.0% 
			      5 / 5      100.0% 
			      5 / 5      100.0% 
			      2 / 2      100.0% 
			      4 / 4      100.0% 
			      1 / 1      100.0% 
			      3 / 3      100.0% 
			      2 / 2      100.0% 
			      4 / 4      100.0% 
			      7 / 7      100.0% 
			      4 / 4      100.0% 
			      1 / 1      100.0% 
			      8 / 8      100.0% 
			      1 / 1      100.0% 
			      0 / 0       0.0% 
			      0 / 0       0.0% 
			      0 / 0       0.0% 
			     51 / 82     62.2%AB 
			     31 / 82     37.8%ABE 
		 
		 
			 1 
			  5.0 
			 ATCC 35953 
			     82 / 83     98.8% 
			      4 / 4      100.0% 
			      3 / 3      100.0% 
			      4 / 4      100.0% 
			      3 / 3      100.0% 
			      6 / 6      100.0% 
			      6 / 6      100.0% 
			      1 / 1      100.0% 
			      3 / 3      100.0% 
			      5 / 5      100.0% 
			      1 / 1      100.0% 
			      5 / 5      100.0% 
			      5 / 5      100.0% 
			      2 / 2      100.0% 
			      4 / 4      100.0% 
			      1 / 1      100.0% 
			      3 / 3      100.0% 
			      2 / 2      100.0% 
			      4 / 4      100.0% 
			      6 / 7      85.7% 
			      4 / 4      100.0% 
			      1 / 1      100.0% 
			      8 / 8      100.0% 
			      1 / 1      100.0% 
			      0 / 0       0.0% 
			      0 / 0       0.0% 
			      0 / 0       0.0% 
			     50 / 83     60.2%AB 
			     32 / 83     38.6%ABE 
			      1 / 83      1.2% 
		 
		 
			 1 
			  5.0 
			 Enterobacter asburiae LF7a 
			     82 / 83     98.8% 
			      4 / 4      100.0% 
			      3 / 3      100.0% 
			      4 / 4      100.0% 
			      3 / 3      100.0% 
			      6 / 6      100.0% 
			      6 / 6      100.0% 
			      1 / 1      100.0% 
			      3 / 3      100.0% 
			      5 / 5      100.0% 
			      1 / 1      100.0% 
			      5 / 5      100.0% 
			      5 / 5      100.0% 
			      2 / 2      100.0% 
			      4 / 4      100.0% 
			      1 / 1      100.0% 
			      3 / 3      100.0% 
			      2 / 2      100.0% 
			      4 / 4      100.0% 
			      6 / 7      85.7% 
			      4 / 4      100.0% 
			      1 / 1      100.0% 
			      8 / 8      100.0% 
			      1 / 1      100.0% 
			      0 / 0       0.0% 
			      0 / 0       0.0% 
			      0 / 0       0.0% 
			     50 / 83     60.2%AB 
			     32 / 83     38.6%ABE 
			      1 / 83      1.2% 
		 
		 
			 1 
			  4.8 
			 "Enterobacter lignolyticus" Deangelis et al. 2011 
			     81 / 82     98.8% 
			      3 / 3      100.0% 
			      3 / 3      100.0% 
			      4 / 4      100.0% 
			      3 / 3      100.0% 
			      5 / 5      100.0% 
			      7 / 7      100.0% 
			      1 / 1      100.0% 
			      3 / 3      100.0% 
			      5 / 5      100.0% 
			      1 / 1      100.0% 
			      5 / 5      100.0% 
			      5 / 5      100.0% 
			      2 / 2      100.0% 
			      4 / 4      100.0% 
			      1 / 1      100.0% 
			      3 / 3      100.0% 
			      2 / 2      100.0% 
			      4 / 4      100.0% 
			      6 / 7      85.7% 
			      4 / 4      100.0% 
			      1 / 1      100.0% 
			      8 / 8      100.0% 
			      1 / 1      100.0% 
			      0 / 0       0.0% 
			      0 / 0       0.0% 
			      0 / 0       0.0% 
			     49 / 82     59.8%AB 
			     32 / 82     39.0%ABE 
			      1 / 82      1.2% 
		 
		 
			 1 
			  4.8 
			 Enterobacter cloacae SCF1 
			     81 / 82     98.8% 
			      3 / 3      100.0% 
			      3 / 3      100.0% 
			      4 / 4      100.0% 
			      3 / 3      100.0% 
			      5 / 5      100.0% 
			      7 / 7      100.0% 
			      1 / 1      100.0% 
			      3 / 3      100.0% 
			      5 / 5      100.0% 
			      1 / 1      100.0% 
			      5 / 5      100.0% 
			      5 / 5      100.0% 
			      2 / 2      100.0% 
			      4 / 4      100.0% 
			      1 / 1      100.0% 
			      3 / 3      100.0% 
			      2 / 2      100.0% 
			      4 / 4      100.0% 
			      6 / 7      85.7% 
			      4 / 4      100.0% 
			      1 / 1      100.0% 
			      8 / 8      100.0% 
			      1 / 1      100.0% 
			      0 / 0       0.0% 
			      0 / 0       0.0% 
			      0 / 0       0.0% 
			     49 / 82     59.8%AB 
			     32 / 82     39.0%ABE 
			      1 / 82      1.2% 
		 
		 
			 1 
			  4.7 
			 Enterobacter sp. 638 
			     83 / 83     100.0% 
			      4 / 4      100.0% 
			      3 / 3      100.0% 
			      4 / 4      100.0% 
			      3 / 3      100.0% 
			      6 / 6      100.0% 
			      6 / 6      100.0% 
			      1 / 1      100.0% 
			      3 / 3      100.0% 
			      5 / 5      100.0% 
			      1 / 1      100.0% 
			      5 / 5      100.0% 
			      5 / 5      100.0% 
			      2 / 2      100.0% 
			      4 / 4      100.0% 
			      1 / 1      100.0% 
			      3 / 3      100.0% 
			      2 / 2      100.0% 
			      4 / 4      100.0% 
			      7 / 7      100.0% 
			      4 / 4      100.0% 
			      1 / 1      100.0% 
			      8 / 8      100.0% 
			      1 / 1      100.0% 
			      0 / 0       0.0% 
			      0 / 0       0.0% 
			      0 / 0       0.0% 
			     51 / 83     61.4%AB 
			     31 / 83     37.3%ABE 
			      1 / 83      1.2%BE 
		 
		 
			 1 
			  5.2 
			 Enterobacter sp. R4-368 
			     86 / 87     98.9% 
			      3 / 3      100.0% 
			      4 / 4      100.0% 
			      4 / 4      100.0% 
			      4 / 4      100.0% 
			      6 / 6      100.0% 
			      6 / 6      100.0% 
			      1 / 1      100.0% 
			      4 / 4      100.0% 
			      6 / 6      100.0% 
			      2 / 2      100.0% 
			      5 / 5      100.0% 
			      5 / 5      100.0% 
			      2 / 2      100.0% 
			      4 / 4      100.0% 
			      1 / 1      100.0% 
			      3 / 3      100.0% 
			      2 / 2      100.0% 
			      4 / 4      100.0% 
			      6 / 7      85.7% 
			      4 / 4      100.0% 
			      1 / 1      100.0% 
			      8 / 8      100.0% 
			      1 / 1      100.0% 
			      0 / 0       0.0% 
			      0 / 0       0.0% 
			      0 / 0       0.0% 
			     53 / 87     60.9%AB 
			     33 / 87     37.9%ABE 
			      1 / 87      1.1%E 
		 
		 
			 7 
			  4.2 
			 Erwinia 
			    534 / 537    99.4% 
			     28 / 28     100.0% 
			     21 / 21     100.0% 
			     25 / 25     100.0% 
			     23 / 23     100.0% 
			     41 / 41     100.0% 
			     41 / 41     100.0% 
			      7 / 7      100.0% 
			     14 / 14     100.0% 
			     37 / 37     100.0% 
			      7 / 7      100.0% 
			     35 / 35     100.0% 
			     29 / 29     100.0% 
			     14 / 14     100.0% 
			     22 / 22     100.0% 
			      7 / 7      100.0% 
			     21 / 21     100.0% 
			     14 / 14     100.0% 
			     20 / 20     100.0% 
			     40 / 43     93.0% 
			     26 / 26     100.0% 
			      7 / 7      100.0% 
			     54 / 54     100.0% 
			      1 / 1      100.0% 
			      0 / 0       0.0% 
			      0 / 0       0.0% 
			      0 / 0       0.0% 
			    292 / 537    54.4%AB 
			    201 / 537    37.4%ABE 
			     29 / 537     5.4%B 
			     12 / 537     2.2%BE 
			      3 / 537     0.6% 
		 
		 
			 2 
			  3.9 
			 "Bacillus amylovorus" (Burrill 1882) Trevisan 1889 
			    151 / 153    98.7% 
			      8 / 8      100.0% 
			      6 / 6      100.0% 
			      6 / 6      100.0% 
			      7 / 7      100.0% 
			     12 / 12     100.0% 
			     12 / 12     100.0% 
			      2 / 2      100.0% 
			      4 / 4      100.0% 
			     11 / 11     100.0% 
			      2 / 2      100.0% 
			     10 / 10     100.0% 
			      8 / 8      100.0% 
			      4 / 4      100.0% 
			      6 / 6      100.0% 
			      2 / 2      100.0% 
			      6 / 6      100.0% 
			      4 / 4      100.0% 
			      6 / 6      100.0% 
			     10 / 12     83.3% 
			      7 / 7      100.0% 
			      2 / 2      100.0% 
			     16 / 16     100.0% 
			      0 / 0       0.0% 
			      0 / 0       0.0% 
			      0 / 0       0.0% 
			      0 / 0       0.0% 
			     90 / 153    58.8%AB 
			     61 / 153    39.9%ABE 
			      2 / 153     1.3% 
		 
		 
			 1 
			  3.8 
			 Erwinia amylovora CFBP1430 
			     75 / 76     98.7% 
			      4 / 4      100.0% 
			      3 / 3      100.0% 
			      3 / 3      100.0% 
			      3 / 3      100.0% 
			      6 / 6      100.0% 
			      6 / 6      100.0% 
			      1 / 1      100.0% 
			      2 / 2      100.0% 
			      5 / 5      100.0% 
			      1 / 1      100.0% 
			      5 / 5      100.0% 
			      4 / 4      100.0% 
			      2 / 2      100.0% 
			      3 / 3      100.0% 
			      1 / 1      100.0% 
			      3 / 3      100.0% 
			      2 / 2      100.0% 
			      3 / 3      100.0% 
			      5 / 6      83.3% 
			      4 / 4      100.0% 
			      1 / 1      100.0% 
			      8 / 8      100.0% 
			      0 / 0       0.0% 
			      0 / 0       0.0% 
			      0 / 0       0.0% 
			      0 / 0       0.0% 
			     44 / 76     57.9%AB 
			     31 / 76     40.8%ABE 
			      1 / 76      1.3% 
		 
		 
			 1 
			  3.9 
			 Erwinia amylovora 27-3 
			     76 / 77     98.7% 
			      4 / 4      100.0% 
			      3 / 3      100.0% 
			      3 / 3      100.0% 
			      4 / 4      100.0% 
			      6 / 6      100.0% 
			      6 / 6      100.0% 
			      1 / 1      100.0% 
			      2 / 2      100.0% 
			      6 / 6      100.0% 
			      1 / 1      100.0% 
			      5 / 5      100.0% 
			      4 / 4      100.0% 
			      2 / 2      100.0% 
			      3 / 3      100.0% 
			      1 / 1      100.0% 
			      3 / 3      100.0% 
			      2 / 2      100.0% 
			      3 / 3      100.0% 
			      5 / 6      83.3% 
			      3 / 3      100.0% 
			      1 / 1      100.0% 
			      8 / 8      100.0% 
			      0 / 0       0.0% 
			      0 / 0       0.0% 
			      0 / 0       0.0% 
			      0 / 0       0.0% 
			     46 / 77     59.7%AB 
			     30 / 77     39.0%ABE 
			      1 / 77      1.3% 
		 
		 
			 2 
			  4.1 
			 CFBP 4172 
			    150 / 150    100.0% 
			      8 / 8      100.0% 
			      6 / 6      100.0% 
			      8 / 8      100.0% 
			      6 / 6      100.0% 
			     12 / 12     100.0% 
			     10 / 10     100.0% 
			      2 / 2      100.0% 
			      4 / 4      100.0% 
			     10 / 10     100.0% 
			      2 / 2      100.0% 
			     10 / 10     100.0% 
			      8 / 8      100.0% 
			      4 / 4      100.0% 
			      6 / 6      100.0% 
			      2 / 2      100.0% 
			      6 / 6      100.0% 
			      4 / 4      100.0% 
			      6 / 6      100.0% 
			     12 / 12     100.0% 
			      8 / 8      100.0% 
			      2 / 2      100.0% 
			     14 / 14     100.0% 
			      0 / 0       0.0% 
			      0 / 0       0.0% 
			      0 / 0       0.0% 
			      0 / 0       0.0% 
			     90 / 150    60.0%AB 
			     60 / 150    40.0%ABE 
		 
		 
			 1 
			  4.1 
			 Erwinia pyrifoliae 16/96 
			     75 / 75     100.0% 
			      4 / 4      100.0% 
			      3 / 3      100.0% 
			      4 / 4      100.0% 
			      3 / 3      100.0% 
			      6 / 6      100.0% 
			      5 / 5      100.0% 
			      1 / 1      100.0% 
			      2 / 2      100.0% 
			      5 / 5      100.0% 
			      1 / 1      100.0% 
			      5 / 5      100.0% 
			      4 / 4      100.0% 
			      2 / 2      100.0% 
			      3 / 3      100.0% 
			      1 / 1      100.0% 
			      3 / 3      100.0% 
			      2 / 2      100.0% 
			      3 / 3      100.0% 
			      6 / 6      100.0% 
			      4 / 4      100.0% 
			      1 / 1      100.0% 
			      7 / 7      100.0% 
			      0 / 0       0.0% 
			      0 / 0       0.0% 
			      0 / 0       0.0% 
			      0 / 0       0.0% 
			     45 / 75     60.0%AB 
			     30 / 75     40.0%ABE 
		 
		 
			 1 
			  5.4 
			 CIP 106121 
			     77 / 77     100.0% 
			      4 / 4      100.0% 
			      3 / 3      100.0% 
			      3 / 3      100.0% 
			      3 / 3      100.0% 
			      5 / 5      100.0% 
			      6 / 6      100.0% 
			      1 / 1      100.0% 
			      2 / 2      100.0% 
			      5 / 5      100.0% 
			      1 / 1      100.0% 
			      5 / 5      100.0% 
			      4 / 4      100.0% 
			      2 / 2      100.0% 
			      4 / 4      100.0% 
			      1 / 1      100.0% 
			      3 / 3      100.0% 
			      2 / 2      100.0% 
			      2 / 2      100.0% 
			      7 / 7      100.0% 
			      4 / 4      100.0% 
			      1 / 1      100.0% 
			      8 / 8      100.0% 
			      1 / 1      100.0% 
			      0 / 0       0.0% 
			      0 / 0       0.0% 
			      0 / 0       0.0% 
			     46 / 77     59.7%AB 
			     31 / 77     40.3%ABE 
		 
		 
			 1 
			  5.4 
			 Erwinia billingiae 661 
			     77 / 77     100.0% 
			      4 / 4      100.0% 
			      3 / 3      100.0% 
			      3 / 3      100.0% 
			      3 / 3      100.0% 
			      5 / 5      100.0% 
			      6 / 6      100.0% 
			      1 / 1      100.0% 
			      2 / 2      100.0% 
			      5 / 5      100.0% 
			      1 / 1      100.0% 
			      5 / 5      100.0% 
			      4 / 4      100.0% 
			      2 / 2      100.0% 
			      4 / 4      100.0% 
			      1 / 1      100.0% 
			      3 / 3      100.0% 
			      2 / 2      100.0% 
			      2 / 2      100.0% 
			      7 / 7      100.0% 
			      4 / 4      100.0% 
			      1 / 1      100.0% 
			      8 / 8      100.0% 
			      1 / 1      100.0% 
			      0 / 0       0.0% 
			      0 / 0       0.0% 
			      0 / 0       0.0% 
			     46 / 77     59.7%AB 
			     31 / 77     40.3%ABE 
		 
		 
			 1 
			  4.0 
			 Erwinia sp. Ejp617 
			     75 / 76     98.7% 
			      4 / 4      100.0% 
			      3 / 3      100.0% 
			      4 / 4      100.0% 
			      3 / 3      100.0% 
			      6 / 6      100.0% 
			      5 / 5      100.0% 
			      1 / 1      100.0% 
			      2 / 2      100.0% 
			      5 / 5      100.0% 
			      1 / 1      100.0% 
			      5 / 5      100.0% 
			      4 / 4      100.0% 
			      2 / 2      100.0% 
			      3 / 3      100.0% 
			      1 / 1      100.0% 
			      3 / 3      100.0% 
			      2 / 2      100.0% 
			      3 / 3      100.0% 
			      5 / 6      83.3% 
			      4 / 4      100.0% 
			      1 / 1      100.0% 
			      8 / 8      100.0% 
			      0 / 0       0.0% 
			      0 / 0       0.0% 
			      0 / 0       0.0% 
			      0 / 0       0.0% 
			     44 / 76     57.9%AB 
			     31 / 76     40.8%ABE 
			      1 / 76      1.3% 
		 
		 
			 1 
			  4.1 
			 DSM 17950 
			     81 / 81     100.0% 
			      4 / 4      100.0% 
			      3 / 3      100.0% 
			      4 / 4      100.0% 
			      4 / 4      100.0% 
			      6 / 6      100.0% 
			      8 / 8      100.0% 
			      1 / 1      100.0% 
			      2 / 2      100.0% 
			      6 / 6      100.0% 
			      1 / 1      100.0% 
			      5 / 5      100.0% 
			      5 / 5      100.0% 
			      2 / 2      100.0% 
			      3 / 3      100.0% 
			      1 / 1      100.0% 
			      3 / 3      100.0% 
			      2 / 2      100.0% 
			      3 / 3      100.0% 
			      6 / 6      100.0% 
			      3 / 3      100.0% 
			      1 / 1      100.0% 
			      8 / 8      100.0% 
			      0 / 0       0.0% 
			      0 / 0       0.0% 
			      0 / 0       0.0% 
			      0 / 0       0.0% 
			     29 / 81     35.8%B 
			     22 / 81     27.2%AB 
			     18 / 81     22.2%ABE 
			     12 / 81     14.8%BE 
		 
		 
			 58 
			  5.1 
			 Escherchia 
			   5347 / 5375   99.5% 
			    228 / 228    100.0% 
			    179 / 180    99.4% 
			    245 / 245    100.0% 
			    183 / 183    100.0% 
			    375 / 375    100.0% 
			    411 / 411    100.0% 
			     60 / 60     100.0% 
			    178 / 178    100.0% 
			    296 / 297    99.7% 
			     60 / 60     100.0% 
			    298 / 298    100.0% 
			    351 / 351    100.0% 
			    119 / 119    100.0% 
			    231 / 231    100.0% 
			     60 / 60     100.0% 
			    180 / 180    100.0% 
			    118 / 118    100.0% 
			    254 / 258    98.4% 
			    575 / 597    96.3% 
			    227 / 227    100.0% 
			    194 / 194    100.0% 
			    463 / 463    100.0% 
			     60 / 60     100.0% 
			      0 / 0       0.0% 
			      0 / 0       0.0% 
			      2 / 2      100.0% 
			   3343 / 5375   62.2%AB 
			   1805 / 5375   33.6%ABE 
			    105 / 5375    2.0%B 
			     94 / 5375    1.7%BE 
			     16 / 5375    0.3%E 
			     11 / 5375    0.2% 
			      1 / 5375    0.0%GG 
		 
		 
			 57 
			  5.1 
			 "Bacillus coli" Migula 1895 
			   5263 / 5290   99.5% 
			    224 / 224    100.0% 
			    176 / 177    99.4% 
			    241 / 241    100.0% 
			    180 / 180    100.0% 
			    369 / 369    100.0% 
			    404 / 404    100.0% 
			     59 / 59     100.0% 
			    175 / 175    100.0% 
			    291 / 292    99.7% 
			     59 / 59     100.0% 
			    293 / 293    100.0% 
			    345 / 345    100.0% 
			    117 / 117    100.0% 
			    227 / 227    100.0% 
			     59 / 59     100.0% 
			    177 / 177    100.0% 
			    116 / 116    100.0% 
			    250 / 254    98.4% 
			    569 / 590    96.4% 
			    223 / 223    100.0% 
			    193 / 193    100.0% 
			    455 / 455    100.0% 
			     59 / 59     100.0% 
			      0 / 0       0.0% 
			      0 / 0       0.0% 
			      2 / 2      100.0% 
			   3291 / 5290   62.2%AB 
			   1773 / 5290   33.5%ABE 
			    105 / 5290    2.0%B 
			     94 / 5290    1.8%BE 
			     16 / 5290    0.3%E 
			     10 / 5290    0.2% 
			      1 / 5290    0.0%GG 
		 
		 
			 1 
			  4.6 
			 Escherichia coli (strain B) 
			     85 / 86     98.8% 
			      4 / 4      100.0% 
			      3 / 3      100.0% 
			      4 / 4      100.0% 
			      3 / 3      100.0% 
			      6 / 6      100.0% 
			      7 / 7      100.0% 
			      1 / 1      100.0% 
			      3 / 3      100.0% 
			      5 / 5      100.0% 
			      1 / 1      100.0% 
			      5 / 5      100.0% 
			      6 / 6      100.0% 
			      2 / 2      100.0% 
			      4 / 4      100.0% 
			      1 / 1      100.0% 
			      3 / 3      100.0% 
			      2 / 2      100.0% 
			      4 / 4      100.0% 
			      7 / 8      87.5% 
			      4 / 4      100.0% 
			      1 / 1      100.0% 
			      8 / 8      100.0% 
			      1 / 1      100.0% 
			      0 / 0       0.0% 
			      0 / 0       0.0% 
			      0 / 0       0.0% 
			     53 / 86     61.6%AB 
			     32 / 86     37.2%ABE 
			      1 / 86      1.2%E 
		 
		 
			 1 
			  4.6 
			 Escherichia coli B (REL606 strain) 
			     85 / 86     98.8% 
			      4 / 4      100.0% 
			      3 / 3      100.0% 
			      4 / 4      100.0% 
			      3 / 3      100.0% 
			      6 / 6      100.0% 
			      7 / 7      100.0% 
			      1 / 1      100.0% 
			      3 / 3      100.0% 
			      5 / 5      100.0% 
			      1 / 1      100.0% 
			      5 / 5      100.0% 
			      6 / 6      100.0% 
			      2 / 2      100.0% 
			      4 / 4      100.0% 
			      1 / 1      100.0% 
			      3 / 3      100.0% 
			      2 / 2      100.0% 
			      4 / 4      100.0% 
			      7 / 8      87.5% 
			      4 / 4      100.0% 
			      1 / 1      100.0% 
			      8 / 8      100.0% 
			      1 / 1      100.0% 
			      0 / 0       0.0% 
			      0 / 0       0.0% 
			      0 / 0       0.0% 
			     53 / 86     61.6%AB 
			     32 / 86     37.2%ABE 
			      1 / 86      1.2%E 
		 
		 
			 5 
			  4.5 
			 Escherichia coli K-12 
			    429 / 430    99.8% 
			     20 / 20     100.0% 
			     15 / 15     100.0% 
			     20 / 20     100.0% 
			     15 / 15     100.0% 
			     30 / 30     100.0% 
			     35 / 35     100.0% 
			      5 / 5      100.0% 
			     15 / 15     100.0% 
			     25 / 25     100.0% 
			      5 / 5      100.0% 
			     25 / 25     100.0% 
			     31 / 31     100.0% 
			     10 / 10     100.0% 
			     20 / 20     100.0% 
			      5 / 5      100.0% 
			     15 / 15     100.0% 
			     10 / 10     100.0% 
			     20 / 20     100.0% 
			     35 / 36     97.2% 
			     19 / 19     100.0% 
			      9 / 9      100.0% 
			     40 / 40     100.0% 
			      5 / 5      100.0% 
			      0 / 0       0.0% 
			      0 / 0       0.0% 
			      0 / 0       0.0% 
			    253 / 430    58.8%AB 
			    136 / 430    31.6%ABE 
			     23 / 430     5.3%BE 
			     17 / 430     4.0%B 
			      1 / 430     0.2%E 
		 
		 
			 1 
			  4.7 
			 Escherichia coli DH10B 
			     86 / 86     100.0% 
			      4 / 4      100.0% 
			      3 / 3      100.0% 
			      4 / 4      100.0% 
			      3 / 3      100.0% 
			      6 / 6      100.0% 
			      7 / 7      100.0% 
			      1 / 1      100.0% 
			      3 / 3      100.0% 
			      5 / 5      100.0% 
			      1 / 1      100.0% 
			      5 / 5      100.0% 
			      6 / 6      100.0% 
			      2 / 2      100.0% 
			      4 / 4      100.0% 
			      1 / 1      100.0% 
			      3 / 3      100.0% 
			      2 / 2      100.0% 
			      4 / 4      100.0% 
			      7 / 7      100.0% 
			      4 / 4      100.0% 
			      2 / 2      100.0% 
			      8 / 8      100.0% 
			      1 / 1      100.0% 
			      0 / 0       0.0% 
			      0 / 0       0.0% 
			      0 / 0       0.0% 
			     37 / 86     43.0%AB 
			     21 / 86     24.4%BE 
			     17 / 86     19.8%B 
			     11 / 86     12.8%ABE 
		 
		 
			 1 
			  4.6 
			 Escherichia coli W3110 
			     86 / 86     100.0% 
			      4 / 4      100.0% 
			      3 / 3      100.0% 
			      4 / 4      100.0% 
			      3 / 3      100.0% 
			      6 / 6      100.0% 
			      7 / 7      100.0% 
			      1 / 1      100.0% 
			      3 / 3      100.0% 
			      5 / 5      100.0% 
			      1 / 1      100.0% 
			      5 / 5      100.0% 
			      6 / 6      100.0% 
			      2 / 2      100.0% 
			      4 / 4      100.0% 
			      1 / 1      100.0% 
			      3 / 3      100.0% 
			      2 / 2      100.0% 
			      4 / 4      100.0% 
			      7 / 7      100.0% 
			      4 / 4      100.0% 
			      2 / 2      100.0% 
			      8 / 8      100.0% 
			      1 / 1      100.0% 
			      0 / 0       0.0% 
			      0 / 0       0.0% 
			      0 / 0       0.0% 
			     54 / 86     62.8%AB 
			     31 / 86     36.0%ABE 
			      1 / 86      1.2%BE 
		 
		 
			 1 
			  4.6 
			 Escherichia coli BW2952 
			     86 / 87     98.9% 
			      4 / 4      100.0% 
			      3 / 3      100.0% 
			      4 / 4      100.0% 
			      3 / 3      100.0% 
			      6 / 6      100.0% 
			      7 / 7      100.0% 
			      1 / 1      100.0% 
			      3 / 3      100.0% 
			      5 / 5      100.0% 
			      1 / 1      100.0% 
			      5 / 5      100.0% 
			      7 / 7      100.0% 
			      2 / 2      100.0% 
			      4 / 4      100.0% 
			      1 / 1      100.0% 
			      3 / 3      100.0% 
			      2 / 2      100.0% 
			      4 / 4      100.0% 
			      7 / 8      87.5% 
			      3 / 3      100.0% 
			      2 / 2      100.0% 
			      8 / 8      100.0% 
			      1 / 1      100.0% 
			      0 / 0       0.0% 
			      0 / 0       0.0% 
			      0 / 0       0.0% 
			     55 / 87     63.2%AB 
			     31 / 87     35.6%ABE 
			      1 / 87      1.1%E 
		 
		 
			 1 
			  4.0 
			 Escherichia coli MDS42 
			     85 / 85     100.0% 
			      4 / 4      100.0% 
			      3 / 3      100.0% 
			      4 / 4      100.0% 
			      3 / 3      100.0% 
			      6 / 6      100.0% 
			      7 / 7      100.0% 
			      1 / 1      100.0% 
			      3 / 3      100.0% 
			      5 / 5      100.0% 
			      1 / 1      100.0% 
			      5 / 5      100.0% 
			      6 / 6      100.0% 
			      2 / 2      100.0% 
			      4 / 4      100.0% 
			      1 / 1      100.0% 
			      3 / 3      100.0% 
			      2 / 2      100.0% 
			      4 / 4      100.0% 
			      7 / 7      100.0% 
			      4 / 4      100.0% 
			      1 / 1      100.0% 
			      8 / 8      100.0% 
			      1 / 1      100.0% 
			      0 / 0       0.0% 
			      0 / 0       0.0% 
			      0 / 0       0.0% 
			     53 / 85     62.4%AB 
			     32 / 85     37.6%ABE 
		 
		 
			 4 
			  5.6 
			 Escherichia coli 0157:H7 
			    411 / 414    99.3% 
			     16 / 16     100.0% 
			     12 / 12     100.0% 
			     16 / 16     100.0% 
			     12 / 12     100.0% 
			     24 / 24     100.0% 
			     28 / 28     100.0% 
			      4 / 4      100.0% 
			     12 / 12     100.0% 
			     19 / 19     100.0% 
			      4 / 4      100.0% 
			     20 / 20     100.0% 
			     20 / 20     100.0% 
			      8 / 8      100.0% 
			     16 / 16     100.0% 
			      4 / 4      100.0% 
			     12 / 12     100.0% 
			      8 / 8      100.0% 
			     16 / 16     100.0% 
			     75 / 78     96.2% 
			     16 / 16     100.0% 
			     38 / 38     100.0% 
			     27 / 27     100.0% 
			      4 / 4      100.0% 
			      0 / 0       0.0% 
			      0 / 0       0.0% 
			      0 / 0       0.0% 
			    288 / 414    69.6%AB 
			    121 / 414    29.2%ABE 
			      2 / 414     0.5%BE 
			      2 / 414     0.5%E 
			      1 / 414     0.2%GG 
		 
		 
			 1 
			  5.6 
			 Escherichia coli 0157:H7 EDL933 
			     98 / 100    98.0% 
			      4 / 4      100.0% 
			      3 / 3      100.0% 
			      4 / 4      100.0% 
			      3 / 3      100.0% 
			      6 / 6      100.0% 
			      7 / 7      100.0% 
			      1 / 1      100.0% 
			      3 / 3      100.0% 
			      4 / 4      100.0% 
			      1 / 1      100.0% 
			      5 / 5      100.0% 
			      5 / 5      100.0% 
			      2 / 2      100.0% 
			      4 / 4      100.0% 
			      1 / 1      100.0% 
			      3 / 3      100.0% 
			      2 / 2      100.0% 
			      4 / 4      100.0% 
			     16 / 18     88.9% 
			      4 / 4      100.0% 
			      9 / 9      100.0% 
			      6 / 6      100.0% 
			      1 / 1      100.0% 
			      0 / 0       0.0% 
			      0 / 0       0.0% 
			      0 / 0       0.0% 
			     68 / 100    68.0%AB 
			     29 / 100    29.0%ABE 
			      1 / 100     1.0%E 
			      1 / 100     1.0%BE 
			      1 / 100     1.0%GG 
		 
		 
			 1 
			  5.6 
			 Escherichia coli O157:H7 str. Sakai 
			    101 / 102    99.0% 
			      4 / 4      100.0% 
			      3 / 3      100.0% 
			      4 / 4      100.0% 
			      3 / 3      100.0% 
			      6 / 6      100.0% 
			      7 / 7      100.0% 
			      1 / 1      100.0% 
			      3 / 3      100.0% 
			      5 / 5      100.0% 
			      1 / 1      100.0% 
			      5 / 5      100.0% 
			      5 / 5      100.0% 
			      2 / 2      100.0% 
			      4 / 4      100.0% 
			      1 / 1      100.0% 
			      3 / 3      100.0% 
			      2 / 2      100.0% 
			      4 / 4      100.0% 
			     17 / 18     94.4% 
			      4 / 4      100.0% 
			      9 / 9      100.0% 
			      7 / 7      100.0% 
			      1 / 1      100.0% 
			      0 / 0       0.0% 
			      0 / 0       0.0% 
			      0 / 0       0.0% 
			     70 / 102    68.6%AB 
			     30 / 102    29.4%ABE 
			      1 / 102     1.0%E 
			      1 / 102     1.0%BE 
		 
		 
			 1 
			  5.7 
			 Escherichia coli O157:H7 str. EC4115 
			    106 / 106    100.0% 
			      4 / 4      100.0% 
			      3 / 3      100.0% 
			      4 / 4      100.0% 
			      3 / 3      100.0% 
			      6 / 6      100.0% 
			      7 / 7      100.0% 
			      1 / 1      100.0% 
			      3 / 3      100.0% 
			      5 / 5      100.0% 
			      1 / 1      100.0% 
			      5 / 5      100.0% 
			      5 / 5      100.0% 
			      2 / 2      100.0% 
			      4 / 4      100.0% 
			      1 / 1      100.0% 
			      3 / 3      100.0% 
			      2 / 2      100.0% 
			      4 / 4      100.0% 
			     21 / 21     100.0% 
			      4 / 4      100.0% 
			     10 / 10     100.0% 
			      7 / 7      100.0% 
			      1 / 1      100.0% 
			      0 / 0       0.0% 
			      0 / 0       0.0% 
			      0 / 0       0.0% 
			     75 / 106    70.8%AB 
			     31 / 106    29.2%ABE 
		 
		 
			 1 
			  5.6 
			 Escherichia coli O157:H7 str. TW14359 
			    106 / 106    100.0% 
			      4 / 4      100.0% 
			      3 / 3      100.0% 
			      4 / 4      100.0% 
			      3 / 3      100.0% 
			      6 / 6      100.0% 
			      7 / 7      100.0% 
			      1 / 1      100.0% 
			      3 / 3      100.0% 
			      5 / 5      100.0% 
			      1 / 1      100.0% 
			      5 / 5      100.0% 
			      5 / 5      100.0% 
			      2 / 2      100.0% 
			      4 / 4      100.0% 
			      1 / 1      100.0% 
			      3 / 3      100.0% 
			      2 / 2      100.0% 
			      4 / 4      100.0% 
			     21 / 21     100.0% 
			      4 / 4      100.0% 
			     10 / 10     100.0% 
			      7 / 7      100.0% 
			      1 / 1      100.0% 
			      0 / 0       0.0% 
			      0 / 0       0.0% 
			      0 / 0       0.0% 
			     75 / 106    70.8%AB 
			     31 / 106    29.2%ABE 
		 
		 
			 1 
			  5.1 
			 Escherichia coli 0127:H6 
			     88 / 89     98.9% 
			      4 / 4      100.0% 
			      3 / 3      100.0% 
			      5 / 5      100.0% 
			      3 / 3      100.0% 
			      6 / 6      100.0% 
			      7 / 7      100.0% 
			      1 / 1      100.0% 
			      2 / 2      100.0% 
			      5 / 5      100.0% 
			      1 / 1      100.0% 
			      5 / 5      100.0% 
			      7 / 7      100.0% 
			      2 / 2      100.0% 
			      3 / 3      100.0% 
			      1 / 1      100.0% 
			      3 / 3      100.0% 
			      2 / 2      100.0% 
			      5 / 5      100.0% 
			      8 / 9      88.9% 
			      4 / 4      100.0% 
			      3 / 3      100.0% 
			      7 / 7      100.0% 
			      1 / 1      100.0% 
			      0 / 0       0.0% 
			      0 / 0       0.0% 
			      0 / 0       0.0% 
			     58 / 89     65.2%AB 
			     30 / 89     33.7%ABE 
			      1 / 89      1.1% 
		 
		 
			 1 
			  5.1 
			 Escherichia coli 0127:H6 str. E2348/69 
			     88 / 89     98.9% 
			      4 / 4      100.0% 
			      3 / 3      100.0% 
			      5 / 5      100.0% 
			      3 / 3      100.0% 
			      6 / 6      100.0% 
			      7 / 7      100.0% 
			      1 / 1      100.0% 
			      2 / 2      100.0% 
			      5 / 5      100.0% 
			      1 / 1      100.0% 
			      5 / 5      100.0% 
			      7 / 7      100.0% 
			      2 / 2      100.0% 
			      3 / 3      100.0% 
			      1 / 1      100.0% 
			      3 / 3      100.0% 
			      2 / 2      100.0% 
			      5 / 5      100.0% 
			      8 / 9      88.9% 
			      4 / 4      100.0% 
			      3 / 3      100.0% 
			      7 / 7      100.0% 
			      1 / 1      100.0% 
			      0 / 0       0.0% 
			      0 / 0       0.0% 
			      0 / 0       0.0% 
			     58 / 89     65.2%AB 
			     30 / 89     33.7%ABE 
			      1 / 89      1.1% 
		 
		 
			 1 
			  5.8 
			 Escherichia coli 0111:H- 
			    105 / 106    99.1% 
			      4 / 4      100.0% 
			      3 / 3      100.0% 
			      5 / 5      100.0% 
			      3 / 3      100.0% 
			      6 / 6      100.0% 
			      7 / 7      100.0% 
			      1 / 1      100.0% 
			      3 / 3      100.0% 
			      5 / 5      100.0% 
			      1 / 1      100.0% 
			      5 / 5      100.0% 
			      6 / 6      100.0% 
			      2 / 2      100.0% 
			      4 / 4      100.0% 
			      1 / 1      100.0% 
			      3 / 3      100.0% 
			      2 / 2      100.0% 
			      5 / 5      100.0% 
			     18 / 19     94.7% 
			      4 / 4      100.0% 
			      8 / 8      100.0% 
			      8 / 8      100.0% 
			      1 / 1      100.0% 
			      0 / 0       0.0% 
			      0 / 0       0.0% 
			      0 / 0       0.0% 
			     72 / 106    67.9%AB 
			     33 / 106    31.1%ABE 
			      1 / 106     0.9% 
		 
		 
			 1 
			  5.8 
			 Escherichia coli O111:H- str. 11128 
			    105 / 106    99.1% 
			      4 / 4      100.0% 
			      3 / 3      100.0% 
			      5 / 5      100.0% 
			      3 / 3      100.0% 
			      6 / 6      100.0% 
			      7 / 7      100.0% 
			      1 / 1      100.0% 
			      3 / 3      100.0% 
			      5 / 5      100.0% 
			      1 / 1      100.0% 
			      5 / 5      100.0% 
			      6 / 6      100.0% 
			      2 / 2      100.0% 
			      4 / 4      100.0% 
			      1 / 1      100.0% 
			      3 / 3      100.0% 
			      2 / 2      100.0% 
			      5 / 5      100.0% 
			     18 / 19     94.7% 
			      4 / 4      100.0% 
			      8 / 8      100.0% 
			      8 / 8      100.0% 
			      1 / 1      100.0% 
			      0 / 0       0.0% 
			      0 / 0       0.0% 
			      0 / 0       0.0% 
			     72 / 106    67.9%AB 
			     33 / 106    31.1%ABE 
			      1 / 106     0.9% 
		 
		 
			 1 
			  5.2 
			 Escherichia coli CFT073 
			     86 / 86     100.0% 
			      4 / 4      100.0% 
			      3 / 3      100.0% 
			      4 / 4      100.0% 
			      1 / 1      100.0% 
			      6 / 6      100.0% 
			      7 / 7      100.0% 
			      1 / 1      100.0% 
			      3 / 3      100.0% 
			      3 / 3      100.0% 
			      1 / 1      100.0% 
			      5 / 5      100.0% 
			      6 / 6      100.0% 
			      2 / 2      100.0% 
			      4 / 4      100.0% 
			      1 / 1      100.0% 
			      3 / 3      100.0% 
			      2 / 2      100.0% 
			      3 / 3      100.0% 
			     11 / 11     100.0% 
			      5 / 5      100.0% 
			      2 / 2      100.0% 
			      8 / 8      100.0% 
			      1 / 1      100.0% 
			      0 / 0       0.0% 
			      0 / 0       0.0% 
			      0 / 0       0.0% 
			     53 / 86     61.6%AB 
			     32 / 86     37.2%ABE 
			      1 / 86      1.2%BE 
		 
		 
			 1 
			  5.9 
			 Escherichia coli O26:H11 
			    100 / 101    99.0% 
			      4 / 4      100.0% 
			      3 / 3      100.0% 
			      4 / 4      100.0% 
			      3 / 3      100.0% 
			      6 / 6      100.0% 
			      7 / 7      100.0% 
			      1 / 1      100.0% 
			      3 / 3      100.0% 
			      5 / 5      100.0% 
			      1 / 1      100.0% 
			      5 / 5      100.0% 
			      6 / 6      100.0% 
			      2 / 2      100.0% 
			      4 / 4      100.0% 
			      1 / 1      100.0% 
			      3 / 3      100.0% 
			      2 / 2      100.0% 
			      4 / 4      100.0% 
			     16 / 17     94.1% 
			      4 / 4      100.0% 
			      7 / 7      100.0% 
			      8 / 8      100.0% 
			      1 / 1      100.0% 
			      0 / 0       0.0% 
			      0 / 0       0.0% 
			      0 / 0       0.0% 
			     68 / 101    67.3%AB 
			     32 / 101    31.7%ABE 
			      1 / 101     1.0% 
		 
		 
			 1 
			  5.9 
			 Escherichia coli O26:H11 11368 
			    100 / 101    99.0% 
			      4 / 4      100.0% 
			      3 / 3      100.0% 
			      4 / 4      100.0% 
			      3 / 3      100.0% 
			      6 / 6      100.0% 
			      7 / 7      100.0% 
			      1 / 1      100.0% 
			      3 / 3      100.0% 
			      5 / 5      100.0% 
			      1 / 1      100.0% 
			      5 / 5      100.0% 
			      6 / 6      100.0% 
			      2 / 2      100.0% 
			      4 / 4      100.0% 
			      1 / 1      100.0% 
			      3 / 3      100.0% 
			      2 / 2      100.0% 
			      4 / 4      100.0% 
			     16 / 17     94.1% 
			      4 / 4      100.0% 
			      7 / 7      100.0% 
			      8 / 8      100.0% 
			      1 / 1      100.0% 
			      0 / 0       0.0% 
			      0 / 0       0.0% 
			      0 / 0       0.0% 
			     68 / 101    67.3%AB 
			     32 / 101    31.7%ABE 
			      1 / 101     1.0% 
		 
		 
			 2 
			  5.5 
			 Escherichia coli O55:H7 
			    195 / 197    99.0% 
			      8 / 8      100.0% 
			      6 / 6      100.0% 
			     10 / 10     100.0% 
			      6 / 6      100.0% 
			     14 / 14     100.0% 
			     14 / 14     100.0% 
			      2 / 2      100.0% 
			      6 / 6      100.0% 
			     10 / 10     100.0% 
			      2 / 2      100.0% 
			     10 / 10     100.0% 
			     12 / 12     100.0% 
			      4 / 4      100.0% 
			      8 / 8      100.0% 
			      2 / 2      100.0% 
			      6 / 6      100.0% 
			      4 / 4      100.0% 
			      8 / 8      100.0% 
			     25 / 27     92.6% 
			      8 / 8      100.0% 
			     12 / 12     100.0% 
			     16 / 16     100.0% 
			      2 / 2      100.0% 
			      0 / 0       0.0% 
			      0 / 0       0.0% 
			      0 / 0       0.0% 
			    127 / 197    64.5%AB 
			     68 / 197    34.5%ABE 
			      2 / 197     1.0% 
		 
		 
			 1 
			  5.5 
			 Escherichia coli O55:H7 CB9615 
			     98 / 99     99.0% 
			      4 / 4      100.0% 
			      3 / 3      100.0% 
			      5 / 5      100.0% 
			      3 / 3      100.0% 
			      7 / 7      100.0% 
			      7 / 7      100.0% 
			      1 / 1      100.0% 
			      3 / 3      100.0% 
			      5 / 5      100.0% 
			      1 / 1      100.0% 
			      5 / 5      100.0% 
			      6 / 6      100.0% 
			      2 / 2      100.0% 
			      4 / 4      100.0% 
			      1 / 1      100.0% 
			      3 / 3      100.0% 
			      2 / 2      100.0% 
			      4 / 4      100.0% 
			     13 / 14     92.9% 
			      4 / 4      100.0% 
			      6 / 6      100.0% 
			      8 / 8      100.0% 
			      1 / 1      100.0% 
			      0 / 0       0.0% 
			      0 / 0       0.0% 
			      0 / 0       0.0% 
			     64 / 99     64.6%AB 
			     34 / 99     34.3%ABE 
			      1 / 99      1.0% 
		 
		 
			 1 
			  5.4 
			 Escherichia coli O55:H7 str. RM12579 
			     97 / 98     99.0% 
			      4 / 4      100.0% 
			      3 / 3      100.0% 
			      5 / 5      100.0% 
			      3 / 3      100.0% 
			      7 / 7      100.0% 
			      7 / 7      100.0% 
			      1 / 1      100.0% 
			      3 / 3      100.0% 
			      5 / 5      100.0% 
			      1 / 1      100.0% 
			      5 / 5      100.0% 
			      6 / 6      100.0% 
			      2 / 2      100.0% 
			      4 / 4      100.0% 
			      1 / 1      100.0% 
			      3 / 3      100.0% 
			      2 / 2      100.0% 
			      4 / 4      100.0% 
			     12 / 13     92.3% 
			      4 / 4      100.0% 
			      6 / 6      100.0% 
			      8 / 8      100.0% 
			      1 / 1      100.0% 
			      0 / 0       0.0% 
			      0 / 0       0.0% 
			      0 / 0       0.0% 
			     63 / 98     64.3%AB 
			     34 / 98     34.7%ABE 
			      1 / 98      1.0% 
		 
		 
			 1 
			  5.3 
			 Escherichia coli ETEC H10407 
			     88 / 89     98.9% 
			      4 / 4      100.0% 
			      3 / 3      100.0% 
			      4 / 4      100.0% 
			      3 / 3      100.0% 
			      6 / 6      100.0% 
			      7 / 7      100.0% 
			      1 / 1      100.0% 
			      4 / 4      100.0% 
			      4 / 5      80.0% 
			      1 / 1      100.0% 
			      5 / 5      100.0% 
			      6 / 6      100.0% 
			      2 / 2      100.0% 
			      4 / 4      100.0% 
			      1 / 1      100.0% 
			      3 / 3      100.0% 
			      2 / 2      100.0% 
			      4 / 4      100.0% 
			      8 / 8      100.0% 
			      4 / 4      100.0% 
			      3 / 3      100.0% 
			      8 / 8      100.0% 
			      1 / 1      100.0% 
			      0 / 0       0.0% 
			      0 / 0       0.0% 
			      0 / 0       0.0% 
			     55 / 89     61.8%AB 
			     33 / 89     37.1%ABE 
			      1 / 89      1.1% 
		 
		 
			 1 
			  5.2 
			 Escherichia coli E24377A 
			     88 / 88     100.0% 
			      4 / 4      100.0% 
			      3 / 3      100.0% 
			      4 / 4      100.0% 
			      3 / 3      100.0% 
			      8 / 8      100.0% 
			      6 / 6      100.0% 
			      1 / 1      100.0% 
			      3 / 3      100.0% 
			      5 / 5      100.0% 
			      1 / 1      100.0% 
			      5 / 5      100.0% 
			      5 / 5      100.0% 
			      2 / 2      100.0% 
			      3 / 3      100.0% 
			      1 / 1      100.0% 
			      3 / 3      100.0% 
			      2 / 2      100.0% 
			      6 / 6      100.0% 
			      7 / 7      100.0% 
			      4 / 4      100.0% 
			      3 / 3      100.0% 
			      8 / 8      100.0% 
			      1 / 1      100.0% 
			      0 / 0       0.0% 
			      0 / 0       0.0% 
			      0 / 0       0.0% 
			     39 / 88     44.3%AB 
			     20 / 88     22.7%BE 
			     16 / 88     18.2%B 
			     13 / 88     14.8%ABE 
		 
		 
			 1 
			  4.6 
			 Escherichia coli HS 
			     87 / 87     100.0% 
			      5 / 5      100.0% 
			      3 / 3      100.0% 
			      4 / 4      100.0% 
			      3 / 3      100.0% 
			      6 / 6      100.0% 
			      7 / 7      100.0% 
			      1 / 1      100.0% 
			      3 / 3      100.0% 
			      5 / 5      100.0% 
			      1 / 1      100.0% 
			      5 / 5      100.0% 
			      6 / 6      100.0% 
			      2 / 2      100.0% 
			      4 / 4      100.0% 
			      1 / 1      100.0% 
			      3 / 3      100.0% 
			      2 / 2      100.0% 
			      4 / 4      100.0% 
			      7 / 7      100.0% 
			      4 / 4      100.0% 
			      2 / 2      100.0% 
			      8 / 8      100.0% 
			      1 / 1      100.0% 
			      0 / 0       0.0% 
			      0 / 0       0.0% 
			      0 / 0       0.0% 
			     38 / 87     43.7%AB 
			     21 / 87     24.1%BE 
			     16 / 87     18.4%B 
			     12 / 87     13.8%ABE 
		 
		 
			 1 
			  4.9 
			 Escherichia coli 536 
			     80 / 80     100.0% 
			      4 / 4      100.0% 
			      3 / 3      100.0% 
			      4 / 4      100.0% 
			      1 / 1      100.0% 
			      6 / 6      100.0% 
			      7 / 7      100.0% 
			      1 / 1      100.0% 
			      3 / 3      100.0% 
			      3 / 3      100.0% 
			      1 / 1      100.0% 
			      5 / 5      100.0% 
			      6 / 6      100.0% 
			      2 / 2      100.0% 
			      2 / 2      100.0% 
			      1 / 1      100.0% 
			      3 / 3      100.0% 
			      1 / 1      100.0% 
			      4 / 4      100.0% 
			      8 / 8      100.0% 
			      6 / 6      100.0% 
			      1 / 1      100.0% 
			      7 / 7      100.0% 
			      1 / 1      100.0% 
			      0 / 0       0.0% 
			      0 / 0       0.0% 
			      0 / 0       0.0% 
			     48 / 80     60.0%AB 
			     31 / 80     38.8%ABE 
			      1 / 80      1.2%BE 
		 
		 
			 1 
			  5.2 
			 Escherichia coli UTI89 
			     88 / 89     98.9% 
			      4 / 4      100.0% 
			      3 / 3      100.0% 
			      4 / 4      100.0% 
			      3 / 3      100.0% 
			      6 / 6      100.0% 
			      7 / 7      100.0% 
			      1 / 1      100.0% 
			      3 / 3      100.0% 
			      5 / 5      100.0% 
			      1 / 1      100.0% 
			      5 / 5      100.0% 
			      6 / 6      100.0% 
			      2 / 2      100.0% 
			      4 / 4      100.0% 
			      1 / 1      100.0% 
			      3 / 3      100.0% 
			      2 / 2      100.0% 
			      5 / 5      100.0% 
			      8 / 9      88.9% 
			      4 / 4      100.0% 
			      2 / 2      100.0% 
			      8 / 8      100.0% 
			      1 / 1      100.0% 
			      0 / 0       0.0% 
			      0 / 0       0.0% 
			      0 / 0       0.0% 
			     55 / 89     61.8%AB 
			     32 / 89     36.0%ABE 
			      1 / 89      1.1%E 
			      1 / 89      1.1%BE 
		 
		 
			 1 
			  5.5 
			 Escherichia coli O103:H2 
			     98 / 98     100.0% 
			      4 / 4      100.0% 
			      3 / 3      100.0% 
			      4 / 4      100.0% 
			      3 / 3      100.0% 
			      6 / 6      100.0% 
			      6 / 6      100.0% 
			      1 / 1      100.0% 
			      3 / 3      100.0% 
			      5 / 5      100.0% 
			      1 / 1      100.0% 
			      5 / 5      100.0% 
			      6 / 6      100.0% 
			      2 / 2      100.0% 
			      4 / 4      100.0% 
			      1 / 1      100.0% 
			      3 / 3      100.0% 
			      2 / 2      100.0% 
			      4 / 4      100.0% 
			     16 / 16     100.0% 
			      4 / 4      100.0% 
			      6 / 6      100.0% 
			      8 / 8      100.0% 
			      1 / 1      100.0% 
			      0 / 0       0.0% 
			      0 / 0       0.0% 
			      0 / 0       0.0% 
			     66 / 98     67.3%AB 
			     32 / 98     32.7%ABE 
		 
		 
			 1 
			  5.5 
			 Escherichia coli O103:H2 str. 12009 
			     98 / 98     100.0% 
			      4 / 4      100.0% 
			      3 / 3      100.0% 
			      4 / 4      100.0% 
			      3 / 3      100.0% 
			      6 / 6      100.0% 
			      6 / 6      100.0% 
			      1 / 1      100.0% 
			      3 / 3      100.0% 
			      5 / 5      100.0% 
			      1 / 1      100.0% 
			      5 / 5      100.0% 
			      6 / 6      100.0% 
			      2 / 2      100.0% 
			      4 / 4      100.0% 
			      1 / 1      100.0% 
			      3 / 3      100.0% 
			      2 / 2      100.0% 
			      4 / 4      100.0% 
			     16 / 16     100.0% 
			      4 / 4      100.0% 
			      6 / 6      100.0% 
			      8 / 8      100.0% 
			      1 / 1      100.0% 
			      0 / 0       0.0% 
			      0 / 0       0.0% 
			      0 / 0       0.0% 
			     66 / 98     67.3%AB 
			     32 / 98     32.7%ABE 
		 
		 
			 1 
			  5.5 
			 Escherichia coli APEC O1 
			     91 / 92     98.9% 
			      4 / 4      100.0% 
			      3 / 3      100.0% 
			      4 / 4      100.0% 
			      3 / 3      100.0% 
			      8 / 8      100.0% 
			      7 / 7      100.0% 
			      1 / 1      100.0% 
			      3 / 3      100.0% 
			      5 / 5      100.0% 
			      1 / 1      100.0% 
			      5 / 5      100.0% 
			      6 / 6      100.0% 
			      2 / 2      100.0% 
			      4 / 4      100.0% 
			      1 / 1      100.0% 
			      3 / 3      100.0% 
			      2 / 2      100.0% 
			      6 / 6      100.0% 
			      9 / 10     90.0% 
			      3 / 3      100.0% 
			      3 / 3      100.0% 
			      7 / 7      100.0% 
			      1 / 1      100.0% 
			      0 / 0       0.0% 
			      0 / 0       0.0% 
			      0 / 0       0.0% 
			     59 / 92     64.1%AB 
			     31 / 92     33.7%ABE 
			      1 / 92      1.1%BE 
			      1 / 92      1.1%E 
		 
		 
			 1 
			  5.2 
			 Escherichia coli SE11 
			     87 / 88     98.9% 
			      4 / 4      100.0% 
			      3 / 3      100.0% 
			      4 / 4      100.0% 
			      4 / 4      100.0% 
			      7 / 7      100.0% 
			      7 / 7      100.0% 
			      1 / 1      100.0% 
			      3 / 3      100.0% 
			      6 / 6      100.0% 
			      1 / 1      100.0% 
			      5 / 5      100.0% 
			      6 / 6      100.0% 
			      2 / 2      100.0% 
			      4 / 4      100.0% 
			      1 / 1      100.0% 
			      3 / 3      100.0% 
			      2 / 2      100.0% 
			      4 / 4      100.0% 
			      7 / 8      87.5% 
			      3 / 3      100.0% 
			      1 / 1      100.0% 
			      8 / 8      100.0% 
			      1 / 1      100.0% 
			      0 / 0       0.0% 
			      0 / 0       0.0% 
			      0 / 0       0.0% 
			     55 / 88     62.5%AB 
			     32 / 88     36.4%ABE 
			      1 / 88      1.1%E 
		 
		 
			 1 
			  4.8 
			 Escherichia coli SE15 
			     85 / 85     100.0% 
			      4 / 4      100.0% 
			      3 / 3      100.0% 
			      4 / 4      100.0% 
			      3 / 3      100.0% 
			      6 / 6      100.0% 
			      7 / 7      100.0% 
			      1 / 1      100.0% 
			      3 / 3      100.0% 
			      5 / 5      100.0% 
			      1 / 1      100.0% 
			      5 / 5      100.0% 
			      6 / 6      100.0% 
			      2 / 2      100.0% 
			      4 / 4      100.0% 
			      1 / 1      100.0% 
			      3 / 3      100.0% 
			      2 / 2      100.0% 
			      4 / 4      100.0% 
			      7 / 7      100.0% 
			      4 / 4      100.0% 
			      1 / 1      100.0% 
			      8 / 8      100.0% 
			      1 / 1      100.0% 
			      0 / 0       0.0% 
			      0 / 0       0.0% 
			      0 / 0       0.0% 
			     53 / 85     62.4%AB 
			     32 / 85     37.6%ABE 
		 
		 
			 1 
			  5.2 
			 Escherichia coli SECEC SMS-3-5 
			     89 / 89     100.0% 
			      4 / 4      100.0% 
			      3 / 3      100.0% 
			      4 / 4      100.0% 
			      2 / 2      100.0% 
			      7 / 7      100.0% 
			      7 / 7      100.0% 
			      1 / 1      100.0% 
			      3 / 3      100.0% 
			      4 / 4      100.0% 
			      1 / 1      100.0% 
			      5 / 5      100.0% 
			      7 / 7      100.0% 
			      2 / 2      100.0% 
			      4 / 4      100.0% 
			      1 / 1      100.0% 
			      3 / 3      100.0% 
			      2 / 2      100.0% 
			      5 / 5      100.0% 
			      7 / 7      100.0% 
			      5 / 5      100.0% 
			      3 / 3      100.0% 
			      8 / 8      100.0% 
			      1 / 1      100.0% 
			      0 / 0       0.0% 
			      0 / 0       0.0% 
			      0 / 0       0.0% 
			     37 / 89     41.6%AB 
			     20 / 89     22.5%ABE 
			     18 / 89     20.2%B 
			     14 / 89     15.7%BE 
		 
		 
			 1 
			  4.6 
			 Escherichia coli BL21(DE3) 
			     85 / 86     98.8% 
			      4 / 4      100.0% 
			      3 / 3      100.0% 
			      4 / 4      100.0% 
			      3 / 3      100.0% 
			      6 / 6      100.0% 
			      7 / 7      100.0% 
			      1 / 1      100.0% 
			      3 / 3      100.0% 
			      5 / 5      100.0% 
			      1 / 1      100.0% 
			      5 / 5      100.0% 
			      6 / 6      100.0% 
			      2 / 2      100.0% 
			      4 / 4      100.0% 
			      1 / 1      100.0% 
			      3 / 3      100.0% 
			      2 / 2      100.0% 
			      4 / 4      100.0% 
			      7 / 8      87.5% 
			      4 / 4      100.0% 
			      1 / 1      100.0% 
			      8 / 8      100.0% 
			      1 / 1      100.0% 
			      0 / 0       0.0% 
			      0 / 0       0.0% 
			      0 / 0       0.0% 
			     53 / 86     61.6%AB 
			     32 / 86     37.2%ABE 
			      1 / 86      1.2%E 
		 
		 
			 1 
			  4.7 
			 Escherichia coli C 
			     86 / 86     100.0% 
			      4 / 4      100.0% 
			      3 / 3      100.0% 
			      4 / 4      100.0% 
			      3 / 3      100.0% 
			      6 / 6      100.0% 
			      7 / 7      100.0% 
			      1 / 1      100.0% 
			      3 / 3      100.0% 
			      5 / 5      100.0% 
			      1 / 1      100.0% 
			      5 / 5      100.0% 
			      6 / 6      100.0% 
			      2 / 2      100.0% 
			      4 / 4      100.0% 
			      1 / 1      100.0% 
			      3 / 3      100.0% 
			      2 / 2      100.0% 
			      4 / 4      100.0% 
			      7 / 7      100.0% 
			      4 / 4      100.0% 
			      2 / 2      100.0% 
			      8 / 8      100.0% 
			      1 / 1      100.0% 
			      0 / 0       0.0% 
			      0 / 0       0.0% 
			      0 / 0       0.0% 
			     38 / 86     44.2%B 
			     22 / 86     25.6%ABE 
			     16 / 86     18.6%AB 
			     10 / 86     11.6%BE 
		 
		 
			 1 
			  4.7 
			 Escherichia coli ATCC 8739 
			     86 / 86     100.0% 
			      4 / 4      100.0% 
			      3 / 3      100.0% 
			      4 / 4      100.0% 
			      3 / 3      100.0% 
			      6 / 6      100.0% 
			      7 / 7      100.0% 
			      1 / 1      100.0% 
			      3 / 3      100.0% 
			      5 / 5      100.0% 
			      1 / 1      100.0% 
			      5 / 5      100.0% 
			      6 / 6      100.0% 
			      2 / 2      100.0% 
			      4 / 4      100.0% 
			      1 / 1      100.0% 
			      3 / 3      100.0% 
			      2 / 2      100.0% 
			      4 / 4      100.0% 
			      7 / 7      100.0% 
			      4 / 4      100.0% 
			      2 / 2      100.0% 
			      8 / 8      100.0% 
			      1 / 1      100.0% 
			      0 / 0       0.0% 
			      0 / 0       0.0% 
			      0 / 0       0.0% 
			     38 / 86     44.2%B 
			     22 / 86     25.6%ABE 
			     16 / 86     18.6%AB 
			     10 / 86     11.6%BE 
		 
		 
			 1 
			  4.6 
			 Escherichia coli BL21 
			     80 / 81     98.8% 
			      4 / 4      100.0% 
			      3 / 3      100.0% 
			      4 / 4      100.0% 
			      3 / 3      100.0% 
			      6 / 6      100.0% 
			      7 / 7      100.0% 
			      1 / 1      100.0% 
			      3 / 3      100.0% 
			      5 / 5      100.0% 
			      1 / 1      100.0% 
			      5 / 5      100.0% 
			      6 / 6      100.0% 
			      2 / 2      100.0% 
			      2 / 2      100.0% 
			      1 / 1      100.0% 
			      3 / 3      100.0% 
			      1 / 1      100.0% 
			      4 / 4      100.0% 
			      7 / 8      87.5% 
			      4 / 4      100.0% 
			      1 / 1      100.0% 
			      6 / 6      100.0% 
			      1 / 1      100.0% 
			      0 / 0       0.0% 
			      0 / 0       0.0% 
			      0 / 0       0.0% 
			     52 / 81     64.2%AB 
			     28 / 81     34.6%ABE 
			      1 / 81      1.2%E 
		 
		 
			 1 
			  4.6 
			 Escherichia coli ATCC 33849 
			    169 / 171    98.8% 
			      8 / 8      100.0% 
			      6 / 6      100.0% 
			      8 / 8      100.0% 
			      6 / 6      100.0% 
			     12 / 12     100.0% 
			     14 / 14     100.0% 
			      2 / 2      100.0% 
			      6 / 6      100.0% 
			     10 / 10     100.0% 
			      2 / 2      100.0% 
			      9 / 9      100.0% 
			     12 / 12     100.0% 
			      4 / 4      100.0% 
			      6 / 6      100.0% 
			      2 / 2      100.0% 
			      6 / 6      100.0% 
			      4 / 4      100.0% 
			      6 / 8      75.0% 
			     14 / 14     100.0% 
			      8 / 8      100.0% 
			      4 / 4      100.0% 
			     16 / 16     100.0% 
			      2 / 2      100.0% 
			      0 / 0       0.0% 
			      0 / 0       0.0% 
			      2 / 2      100.0% 
			    106 / 171    62.0%AB 
			     63 / 171    36.8%ABE 
			      2 / 171     1.2%E 
		 
		 
			 1 
			  4.9 
			 Escherichia coli ATCC 9637 
			    174 / 174    100.0% 
			      8 / 8      100.0% 
			      6 / 6      100.0% 
			      8 / 8      100.0% 
			      6 / 6      100.0% 
			     14 / 14     100.0% 
			     14 / 14     100.0% 
			      2 / 2      100.0% 
			      6 / 6      100.0% 
			     10 / 10     100.0% 
			      2 / 2      100.0% 
			     10 / 10     100.0% 
			     12 / 12     100.0% 
			      4 / 4      100.0% 
			      8 / 8      100.0% 
			      2 / 2      100.0% 
			      6 / 6      100.0% 
			      4 / 4      100.0% 
			     10 / 10     100.0% 
			     14 / 14     100.0% 
			      8 / 8      100.0% 
			      2 / 2      100.0% 
			     16 / 16     100.0% 
			      2 / 2      100.0% 
			      0 / 0       0.0% 
			      0 / 0       0.0% 
			      0 / 0       0.0% 
			    108 / 174    62.1%AB 
			     66 / 174    37.9%ABE 
		 
		 
			 1 
			  4.7 
			 Escherichia coli IAI1 
			     85 / 85     100.0% 
			      4 / 4      100.0% 
			      3 / 3      100.0% 
			      4 / 4      100.0% 
			      3 / 3      100.0% 
			      6 / 6      100.0% 
			      7 / 7      100.0% 
			      1 / 1      100.0% 
			      3 / 3      100.0% 
			      5 / 5      100.0% 
			      1 / 1      100.0% 
			      5 / 5      100.0% 
			      6 / 6      100.0% 
			      2 / 2      100.0% 
			      4 / 4      100.0% 
			      1 / 1      100.0% 
			      3 / 3      100.0% 
			      2 / 2      100.0% 
			      4 / 4      100.0% 
			      6 / 6      100.0% 
			      4 / 4      100.0% 
			      2 / 2      100.0% 
			      8 / 8      100.0% 
			      1 / 1      100.0% 
			      0 / 0       0.0% 
			      0 / 0       0.0% 
			      0 / 0       0.0% 
			     53 / 85     62.4%AB 
			     32 / 85     37.6%ABE 
		 
		 
			 1 
			  5.2 
			 Escherichia coli S88 
			     91 / 91     100.0% 
			      4 / 4      100.0% 
			      3 / 3      100.0% 
			      4 / 4      100.0% 
			      3 / 3      100.0% 
			      6 / 6      100.0% 
			      7 / 7      100.0% 
			      1 / 1      100.0% 
			      3 / 3      100.0% 
			      5 / 5      100.0% 
			      1 / 1      100.0% 
			      5 / 5      100.0% 
			      6 / 6      100.0% 
			      2 / 2      100.0% 
			      4 / 4      100.0% 
			      1 / 1      100.0% 
			      3 / 3      100.0% 
			      2 / 2      100.0% 
			      4 / 4      100.0% 
			     11 / 11     100.0% 
			      4 / 4      100.0% 
			      3 / 3      100.0% 
			      8 / 8      100.0% 
			      1 / 1      100.0% 
			      0 / 0       0.0% 
			      0 / 0       0.0% 
			      0 / 0       0.0% 
			     59 / 91     64.8%AB 
			     32 / 91     35.2%ABE 
		 
		 
			 1 
			  5.2 
			 Escherichia coli 55989 
			     94 / 95     98.9% 
			      4 / 4      100.0% 
			      3 / 3      100.0% 
			      4 / 4      100.0% 
			      3 / 3      100.0% 
			      6 / 6      100.0% 
			      7 / 7      100.0% 
			      1 / 1      100.0% 
			      3 / 3      100.0% 
			      5 / 5      100.0% 
			      1 / 1      100.0% 
			      5 / 5      100.0% 
			      6 / 6      100.0% 
			      2 / 2      100.0% 
			      4 / 4      100.0% 
			      1 / 1      100.0% 
			      3 / 3      100.0% 
			      2 / 2      100.0% 
			      4 / 4      100.0% 
			     12 / 13     92.3% 
			      4 / 4      100.0% 
			      5 / 5      100.0% 
			      8 / 8      100.0% 
			      1 / 1      100.0% 
			      0 / 0       0.0% 
			      0 / 0       0.0% 
			      0 / 0       0.0% 
			     62 / 95     65.3%AB 
			     32 / 95     33.7%ABE 
			      1 / 95      1.1%E 
		 
		 
			 1 
			  5.4 
			 Escherichia coli UMN026 
			     87 / 88     98.9% 
			      5 / 5      100.0% 
			      3 / 3      100.0% 
			      4 / 4      100.0% 
			      3 / 3      100.0% 
			      6 / 6      100.0% 
			      7 / 7      100.0% 
			      1 / 1      100.0% 
			      3 / 3      100.0% 
			      5 / 5      100.0% 
			      1 / 1      100.0% 
			      5 / 5      100.0% 
			      6 / 6      100.0% 
			      2 / 2      100.0% 
			      4 / 4      100.0% 
			      1 / 1      100.0% 
			      3 / 3      100.0% 
			      2 / 2      100.0% 
			      4 / 4      100.0% 
			      7 / 8      87.5% 
			      4 / 4      100.0% 
			      2 / 2      100.0% 
			      8 / 8      100.0% 
			      1 / 1      100.0% 
			      0 / 0       0.0% 
			      0 / 0       0.0% 
			      0 / 0       0.0% 
			     54 / 88     61.4%AB 
			     33 / 88     37.5%ABE 
			      1 / 88      1.1%E 
		 
		 
			 1 
			  5.1 
			 Escherichia coli IAI39 
			     87 / 87     100.0% 
			      3 / 3      100.0% 
			      3 / 3      100.0% 
			      4 / 4      100.0% 
			      3 / 3      100.0% 
			      6 / 6      100.0% 
			      8 / 8      100.0% 
			      1 / 1      100.0% 
			      3 / 3      100.0% 
			      5 / 5      100.0% 
			      1 / 1      100.0% 
			      5 / 5      100.0% 
			      6 / 6      100.0% 
			      2 / 2      100.0% 
			      4 / 4      100.0% 
			      1 / 1      100.0% 
			      3 / 3      100.0% 
			      2 / 2      100.0% 
			      4 / 4      100.0% 
			      8 / 8      100.0% 
			      4 / 4      100.0% 
			      2 / 2      100.0% 
			      8 / 8      100.0% 
			      1 / 1      100.0% 
			      0 / 0       0.0% 
			      0 / 0       0.0% 
			      0 / 0       0.0% 
			     56 / 87     64.4%AB 
			     31 / 87     35.6%ABE 
		 
		 
			 1 
			  5.2 
			 Escherichia coli ED1a 
			     89 / 89     100.0% 
			      4 / 4      100.0% 
			      3 / 3      100.0% 
			      4 / 4      100.0% 
			      3 / 3      100.0% 
			      6 / 6      100.0% 
			      7 / 7      100.0% 
			      1 / 1      100.0% 
			      3 / 3      100.0% 
			      4 / 4      100.0% 
			      1 / 1      100.0% 
			      5 / 5      100.0% 
			      6 / 6      100.0% 
			      2 / 2      100.0% 
			      4 / 4      100.0% 
			      1 / 1      100.0% 
			      3 / 3      100.0% 
			      2 / 2      100.0% 
			      4 / 4      100.0% 
			     10 / 10     100.0% 
			      4 / 4      100.0% 
			      3 / 3      100.0% 
			      8 / 8      100.0% 
			      1 / 1      100.0% 
			      0 / 0       0.0% 
			      0 / 0       0.0% 
			      0 / 0       0.0% 
			     57 / 89     64.0%AB 
			     32 / 89     36.0%ABE 
		 
		 
			 1 
			  4.8 
			 Escherichia coli LF82 
			     85 / 85     100.0% 
			      4 / 4      100.0% 
			      3 / 3      100.0% 
			      4 / 4      100.0% 
			      3 / 3      100.0% 
			      6 / 6      100.0% 
			      6 / 6      100.0% 
			      1 / 1      100.0% 
			      3 / 3      100.0% 
			      6 / 6      100.0% 
			      1 / 1      100.0% 
			      5 / 5      100.0% 
			      6 / 6      100.0% 
			      2 / 2      100.0% 
			      4 / 4      100.0% 
			      1 / 1      100.0% 
			      3 / 3      100.0% 
			      2 / 2      100.0% 
			      4 / 4      100.0% 
			      7 / 7      100.0% 
			      4 / 4      100.0% 
			      1 / 1      100.0% 
			      8 / 8      100.0% 
			      1 / 1      100.0% 
			      0 / 0       0.0% 
			      0 / 0       0.0% 
			      0 / 0       0.0% 
			     53 / 85     62.4%AB 
			     32 / 85     37.6%ABE 
		 
		 
			 2 
			  5.0 
			 Escherichia coli ATCC 55124 
			    169 / 170    99.4% 
			      6 / 6      100.0% 
			      5 / 6      83.3% 
			      8 / 8      100.0% 
			      6 / 6      100.0% 
			     13 / 13     100.0% 
			     14 / 14     100.0% 
			      2 / 2      100.0% 
			      6 / 6      100.0% 
			      9 / 9      100.0% 
			      2 / 2      100.0% 
			     10 / 10     100.0% 
			     12 / 12     100.0% 
			      4 / 4      100.0% 
			      8 / 8      100.0% 
			      2 / 2      100.0% 
			      6 / 6      100.0% 
			      4 / 4      100.0% 
			     10 / 10     100.0% 
			     14 / 14     100.0% 
			      8 / 8      100.0% 
			      2 / 2      100.0% 
			     16 / 16     100.0% 
			      2 / 2      100.0% 
			      0 / 0       0.0% 
			      0 / 0       0.0% 
			      0 / 0       0.0% 
			    105 / 170    61.8%AB 
			     64 / 170    37.6%ABE 
			      1 / 170     0.6% 
		 
		 
			 1 
			  5.1 
			 Escherichia coli ABU 83972 
			     88 / 88     100.0% 
			      4 / 4      100.0% 
			      3 / 3      100.0% 
			      4 / 4      100.0% 
			      3 / 3      100.0% 
			      6 / 6      100.0% 
			      7 / 7      100.0% 
			      1 / 1      100.0% 
			      3 / 3      100.0% 
			      5 / 5      100.0% 
			      1 / 1      100.0% 
			      5 / 5      100.0% 
			      5 / 5      100.0% 
			      2 / 2      100.0% 
			      4 / 4      100.0% 
			      1 / 1      100.0% 
			      3 / 3      100.0% 
			      2 / 2      100.0% 
			      4 / 4      100.0% 
			     10 / 10     100.0% 
			      4 / 4      100.0% 
			      2 / 2      100.0% 
			      8 / 8      100.0% 
			      1 / 1      100.0% 
			      0 / 0       0.0% 
			      0 / 0       0.0% 
			      0 / 0       0.0% 
			     55 / 88     62.5%AB 
			     33 / 88     37.5%ABE 
		 
		 
			 1 
			  4.9 
			 Escherichia coli O83:H1 
			     82 / 82     100.0% 
			      3 / 3      100.0% 
			      3 / 3      100.0% 
			      4 / 4      100.0% 
			      3 / 3      100.0% 
			      6 / 6      100.0% 
			      7 / 7      100.0% 
			      1 / 1      100.0% 
			      3 / 3      100.0% 
			      5 / 5      100.0% 
			      1 / 1      100.0% 
			      5 / 5      100.0% 
			      6 / 6      100.0% 
			      2 / 2      100.0% 
			      4 / 4      100.0% 
			      1 / 1      100.0% 
			      3 / 3      100.0% 
			      2 / 2      100.0% 
			      4 / 4      100.0% 
			      6 / 6      100.0% 
			      4 / 4      100.0% 
			      1 / 1      100.0% 
			      7 / 7      100.0% 
			      1 / 1      100.0% 
			      0 / 0       0.0% 
			      0 / 0       0.0% 
			      0 / 0       0.0% 
			     52 / 82     63.4%AB 
			     30 / 82     36.6%ABE 
		 
		 
			 1 
			  4.9 
			 Escherichia coli O83:H1 str. NRG 857C 
			     82 / 82     100.0% 
			      3 / 3      100.0% 
			      3 / 3      100.0% 
			      4 / 4      100.0% 
			      3 / 3      100.0% 
			      6 / 6      100.0% 
			      7 / 7      100.0% 
			      1 / 1      100.0% 
			      3 / 3      100.0% 
			      5 / 5      100.0% 
			      1 / 1      100.0% 
			      5 / 5      100.0% 
			      6 / 6      100.0% 
			      2 / 2      100.0% 
			      4 / 4      100.0% 
			      1 / 1      100.0% 
			      3 / 3      100.0% 
			      2 / 2      100.0% 
			      4 / 4      100.0% 
			      6 / 6      100.0% 
			      4 / 4      100.0% 
			      1 / 1      100.0% 
			      7 / 7      100.0% 
			      1 / 1      100.0% 
			      0 / 0       0.0% 
			      0 / 0       0.0% 
			      0 / 0       0.0% 
			     52 / 82     63.4%AB 
			     30 / 82     36.6%ABE 
		 
		 
			 1 
			  5.7 
			 Escherichia coli UMNK88 
			     92 / 94     97.9% 
			      3 / 3      100.0% 
			      3 / 3      100.0% 
			      4 / 4      100.0% 
			      6 / 6      100.0% 
			      8 / 8      100.0% 
			      7 / 7      100.0% 
			      1 / 1      100.0% 
			      3 / 3      100.0% 
			      8 / 8      100.0% 
			      1 / 1      100.0% 
			      5 / 5      100.0% 
			      6 / 6      100.0% 
			      2 / 2      100.0% 
			      4 / 4      100.0% 
			      1 / 1      100.0% 
			      3 / 3      100.0% 
			      2 / 2      100.0% 
			      4 / 6      66.7% 
			      8 / 8      100.0% 
			      1 / 1      100.0% 
			      4 / 4      100.0% 
			      7 / 7      100.0% 
			      1 / 1      100.0% 
			      0 / 0       0.0% 
			      0 / 0       0.0% 
			      0 / 0       0.0% 
			     63 / 94     67.0%AB 
			     29 / 94     30.9%ABE 
			      2 / 94      2.1% 
		 
		 
			 1 
			  5.1 
			 Escherichia coli IHE3034 
			     97 / 97     100.0% 
			      4 / 4      100.0% 
			      3 / 3      100.0% 
			      4 / 4      100.0% 
			      3 / 3      100.0% 
			      8 / 8      100.0% 
			      7 / 7      100.0% 
			      1 / 1      100.0% 
			      3 / 3      100.0% 
			      5 / 5      100.0% 
			      1 / 1      100.0% 
			      5 / 5      100.0% 
			      7 / 7      100.0% 
			      2 / 2      100.0% 
			      4 / 4      100.0% 
			      1 / 1      100.0% 
			      3 / 3      100.0% 
			      2 / 2      100.0% 
			      6 / 6      100.0% 
			     11 / 11     100.0% 
			      4 / 4      100.0% 
			      4 / 4      100.0% 
			      8 / 8      100.0% 
			      1 / 1      100.0% 
			      0 / 0       0.0% 
			      0 / 0       0.0% 
			      0 / 0       0.0% 
			     62 / 97     63.9%AB 
			     35 / 97     36.1%ABE 
		 
		 
			 1 
			  5.5 
			 Escherichia coli Xuzhou21 
			     93 / 93     100.0% 
			      2 / 2      100.0% 
			      3 / 3      100.0% 
			      4 / 4      100.0% 
			      3 / 3      100.0% 
			      5 / 5      100.0% 
			      5 / 5      100.0% 
			      1 / 1      100.0% 
			      3 / 3      100.0% 
			      4 / 4      100.0% 
			      1 / 1      100.0% 
			      5 / 5      100.0% 
			      5 / 5      100.0% 
			      2 / 2      100.0% 
			      4 / 4      100.0% 
			      1 / 1      100.0% 
			      3 / 3      100.0% 
			      2 / 2      100.0% 
			      4 / 4      100.0% 
			     17 / 17     100.0% 
			      4 / 4      100.0% 
			      8 / 8      100.0% 
			      6 / 6      100.0% 
			      1 / 1      100.0% 
			      0 / 0       0.0% 
			      0 / 0       0.0% 
			      0 / 0       0.0% 
			     65 / 93     69.9%AB 
			     28 / 93     30.1%ABE 
		 
		 
			 1 
			  5.4 
			 Escherichia coli O44:H18 
			     93 / 93     100.0% 
			      4 / 4      100.0% 
			      3 / 3      100.0% 
			      4 / 4      100.0% 
			      3 / 3      100.0% 
			      7 / 7      100.0% 
			      7 / 7      100.0% 
			      1 / 1      100.0% 
			      3 / 3      100.0% 
			      5 / 5      100.0% 
			      1 / 1      100.0% 
			      5 / 5      100.0% 
			      6 / 6      100.0% 
			      2 / 2      100.0% 
			      4 / 4      100.0% 
			      1 / 1      100.0% 
			      3 / 3      100.0% 
			      2 / 2      100.0% 
			      5 / 5      100.0% 
			     10 / 10     100.0% 
			      4 / 4      100.0% 
			      4 / 4      100.0% 
			      8 / 8      100.0% 
			      1 / 1      100.0% 
			      0 / 0       0.0% 
			      0 / 0       0.0% 
			      0 / 0       0.0% 
			     60 / 93     64.5%AB 
			     33 / 93     35.5%ABE 
		 
		 
			 1 
			  5.4 
			 EAEC 042 
			     93 / 93     100.0% 
			      4 / 4      100.0% 
			      3 / 3      100.0% 
			      4 / 4      100.0% 
			      3 / 3      100.0% 
			      7 / 7      100.0% 
			      7 / 7      100.0% 
			      1 / 1      100.0% 
			      3 / 3      100.0% 
			      5 / 5      100.0% 
			      1 / 1      100.0% 
			      5 / 5      100.0% 
			      6 / 6      100.0% 
			      2 / 2      100.0% 
			      4 / 4      100.0% 
			      1 / 1      100.0% 
			      3 / 3      100.0% 
			      2 / 2      100.0% 
			      5 / 5      100.0% 
			     10 / 10     100.0% 
			      4 / 4      100.0% 
			      4 / 4      100.0% 
			      8 / 8      100.0% 
			      1 / 1      100.0% 
			      0 / 0       0.0% 
			      0 / 0       0.0% 
			      0 / 0       0.0% 
			     60 / 93     64.5%AB 
			     33 / 93     35.5%ABE 
		 
		 
			 1 
			  5.1 
			 Escherichia coli UM146 
			     82 / 82     100.0% 
			      4 / 4      100.0% 
			      3 / 3      100.0% 
			      5 / 5      100.0% 
			      3 / 3      100.0% 
			      6 / 6      100.0% 
			      5 / 5      100.0% 
			      1 / 1      100.0% 
			      3 / 3      100.0% 
			      5 / 5      100.0% 
			      1 / 1      100.0% 
			      5 / 5      100.0% 
			      2 / 2      100.0% 
			      2 / 2      100.0% 
			      4 / 4      100.0% 
			      1 / 1      100.0% 
			      3 / 3      100.0% 
			      2 / 2      100.0% 
			      5 / 5      100.0% 
			      8 / 8      100.0% 
			      4 / 4      100.0% 
			      2 / 2      100.0% 
			      7 / 7      100.0% 
			      1 / 1      100.0% 
			      0 / 0       0.0% 
			      0 / 0       0.0% 
			      0 / 0       0.0% 
			     50 / 82     61.0%AB 
			     32 / 82     39.0%ABE 
		 
		 
			 1 
			  5.0 
			 Escherichia coli clone D i14 
			     88 / 88     100.0% 
			      4 / 4      100.0% 
			      3 / 3      100.0% 
			      4 / 4      100.0% 
			      3 / 3      100.0% 
			      6 / 6      100.0% 
			      6 / 6      100.0% 
			      1 / 1      100.0% 
			      3 / 3      100.0% 
			      4 / 4      100.0% 
			      1 / 1      100.0% 
			      5 / 5      100.0% 
			      6 / 6      100.0% 
			      2 / 2      100.0% 
			      4 / 4      100.0% 
			      1 / 1      100.0% 
			      3 / 3      100.0% 
			      2 / 2      100.0% 
			      4 / 4      100.0% 
			     11 / 11     100.0% 
			      4 / 4      100.0% 
			      2 / 2      100.0% 
			      8 / 8      100.0% 
			      1 / 1      100.0% 
			      0 / 0       0.0% 
			      0 / 0       0.0% 
			      0 / 0       0.0% 
			     55 / 88     62.5%AB 
			     33 / 88     37.5%ABE 
		 
		 
			 1 
			  5.0 
			 Escherichia coli clone D i2 
			     88 / 88     100.0% 
			      4 / 4      100.0% 
			      3 / 3      100.0% 
			      4 / 4      100.0% 
			      3 / 3      100.0% 
			      6 / 6      100.0% 
			      6 / 6      100.0% 
			      1 / 1      100.0% 
			      3 / 3      100.0% 
			      4 / 4      100.0% 
			      1 / 1      100.0% 
			      5 / 5      100.0% 
			      6 / 6      100.0% 
			      2 / 2      100.0% 
			      4 / 4      100.0% 
			      1 / 1      100.0% 
			      3 / 3      100.0% 
			      2 / 2      100.0% 
			      4 / 4      100.0% 
			     11 / 11     100.0% 
			      4 / 4      100.0% 
			      2 / 2      100.0% 
			      8 / 8      100.0% 
			      1 / 1      100.0% 
			      0 / 0       0.0% 
			      0 / 0       0.0% 
			      0 / 0       0.0% 
			     55 / 88     62.5%AB 
			     33 / 88     37.5%ABE 
		 
		 
			 1 
			  4.9 
			 Escherichia coli P12b 
			     82 / 82     100.0% 
			      4 / 4      100.0% 
			      3 / 3      100.0% 
			      5 / 5      100.0% 
			      3 / 3      100.0% 
			      5 / 5      100.0% 
			      7 / 7      100.0% 
			      1 / 1      100.0% 
			      3 / 3      100.0% 
			      5 / 5      100.0% 
			      1 / 1      100.0% 
			      5 / 5      100.0% 
			      6 / 6      100.0% 
			      2 / 2      100.0% 
			      4 / 4      100.0% 
			      1 / 1      100.0% 
			      3 / 3      100.0% 
			      2 / 2      100.0% 
			      3 / 3      100.0% 
			      5 / 5      100.0% 
			      4 / 4      100.0% 
			      1 / 1      100.0% 
			      8 / 8      100.0% 
			      1 / 1      100.0% 
			      0 / 0       0.0% 
			      0 / 0       0.0% 
			      0 / 0       0.0% 
			     49 / 82     59.8%AB 
			     33 / 82     40.2%ABE 
		 
		 
			 1 
			  5.0 
			 Escherichia coli NA114 
			     66 / 67     98.5% 
			      3 / 3      100.0% 
			      3 / 3      100.0% 
			      3 / 3      100.0% 
			      1 / 1      100.0% 
			      5 / 5      100.0% 
			      7 / 7      100.0% 
			      1 / 1      100.0% 
			      1 / 1      100.0% 
			      3 / 3      100.0% 
			      1 / 1      100.0% 
			      4 / 4      100.0% 
			      4 / 4      100.0% 
			      1 / 1      100.0% 
			      3 / 3      100.0% 
			      1 / 1      100.0% 
			      3 / 3      100.0% 
			      2 / 2      100.0% 
			      2 / 2      100.0% 
			      7 / 8      87.5% 
			      1 / 1      100.0% 
			      2 / 2      100.0% 
			      7 / 7      100.0% 
			      1 / 1      100.0% 
			      0 / 0       0.0% 
			      0 / 0       0.0% 
			      0 / 0       0.0% 
			     44 / 67     65.7%AB 
			     22 / 67     32.8%ABE 
			      1 / 67      1.5% 
		 
		 
			 3 
			  5.4 
			 Escherichia coli O104:H4 
			    279 / 282    98.9% 
			      7 / 7      100.0% 
			      9 / 9      100.0% 
			     12 / 12     100.0% 
			     12 / 12     100.0% 
			     16 / 16     100.0% 
			     21 / 21     100.0% 
			      3 / 3      100.0% 
			      9 / 9      100.0% 
			     18 / 18     100.0% 
			      3 / 3      100.0% 
			     15 / 15     100.0% 
			     18 / 18     100.0% 
			      6 / 6      100.0% 
			     12 / 12     100.0% 
			      3 / 3      100.0% 
			      9 / 9      100.0% 
			      6 / 6      100.0% 
			     12 / 12     100.0% 
			     36 / 39     92.3% 
			      9 / 9      100.0% 
			     16 / 16     100.0% 
			     24 / 24     100.0% 
			      3 / 3      100.0% 
			      0 / 0       0.0% 
			      0 / 0       0.0% 
			      0 / 0       0.0% 
			    191 / 282    67.7%AB 
			     88 / 282    31.2%ABE 
			      3 / 282     1.1%E 
		 
		 
			 1 
			  5.4 
			 Escherichia coli O104:H4 str. 2011C-3493 
			     92 / 92     100.0% 
			      2 / 2      100.0% 
			      3 / 3      100.0% 
			      4 / 4      100.0% 
			      6 / 6      100.0% 
			      4 / 4      100.0% 
			      7 / 7      100.0% 
			      1 / 1      100.0% 
			      3 / 3      100.0% 
			      8 / 8      100.0% 
			      1 / 1      100.0% 
			      5 / 5      100.0% 
			      6 / 6      100.0% 
			      2 / 2      100.0% 
			      4 / 4      100.0% 
			      1 / 1      100.0% 
			      3 / 3      100.0% 
			      2 / 2      100.0% 
			      4 / 4      100.0% 
			     11 / 11     100.0% 
			      1 / 1      100.0% 
			      5 / 5      100.0% 
			      8 / 8      100.0% 
			      1 / 1      100.0% 
			      0 / 0       0.0% 
			      0 / 0       0.0% 
			      0 / 0       0.0% 
			     65 / 92     70.7%AB 
			     27 / 92     29.3%ABE 
		 
		 
			 1 
			  5.4 
			 Escherichia coli O104:H4 str. 2009EL-2071 
			     95 / 97     97.9% 
			      3 / 3      100.0% 
			      3 / 3      100.0% 
			      4 / 4      100.0% 
			      3 / 3      100.0% 
			      6 / 6      100.0% 
			      7 / 7      100.0% 
			      1 / 1      100.0% 
			      3 / 3      100.0% 
			      5 / 5      100.0% 
			      1 / 1      100.0% 
			      5 / 5      100.0% 
			      6 / 6      100.0% 
			      2 / 2      100.0% 
			      4 / 4      100.0% 
			      1 / 1      100.0% 
			      3 / 3      100.0% 
			      2 / 2      100.0% 
			      4 / 4      100.0% 
			     13 / 15     86.7% 
			      4 / 4      100.0% 
			      6 / 6      100.0% 
			      8 / 8      100.0% 
			      1 / 1      100.0% 
			      0 / 0       0.0% 
			      0 / 0       0.0% 
			      0 / 0       0.0% 
			     64 / 97     66.0%AB 
			     31 / 97     32.0%ABE 
			      2 / 97      2.1%E 
		 
		 
			 1 
			  5.4 
			 Escherichia coli O104:H4 str. 2009EL-2050 
			     92 / 93     98.9% 
			      2 / 2      100.0% 
			      3 / 3      100.0% 
			      4 / 4      100.0% 
			      3 / 3      100.0% 
			      6 / 6      100.0% 
			      7 / 7      100.0% 
			      1 / 1      100.0% 
			      3 / 3      100.0% 
			      5 / 5      100.0% 
			      1 / 1      100.0% 
			      5 / 5      100.0% 
			      6 / 6      100.0% 
			      2 / 2      100.0% 
			      4 / 4      100.0% 
			      1 / 1      100.0% 
			      3 / 3      100.0% 
			      2 / 2      100.0% 
			      4 / 4      100.0% 
			     12 / 13     92.3% 
			      4 / 4      100.0% 
			      5 / 5      100.0% 
			      8 / 8      100.0% 
			      1 / 1      100.0% 
			      0 / 0       0.0% 
			      0 / 0       0.0% 
			      0 / 0       0.0% 
			     62 / 93     66.7%AB 
			     30 / 93     32.3%ABE 
			      1 / 93      1.1%E 
		 
		 
			 1 
			  5.6 
			 Escherichia coli UMNF18 
			     95 / 95     100.0% 
			      3 / 3      100.0% 
			      3 / 3      100.0% 
			      4 / 4      100.0% 
			      3 / 3      100.0% 
			     10 / 10     100.0% 
			      6 / 6      100.0% 
			      1 / 1      100.0% 
			      3 / 3      100.0% 
			      5 / 5      100.0% 
			      1 / 1      100.0% 
			      5 / 5      100.0% 
			      6 / 6      100.0% 
			      2 / 2      100.0% 
			      4 / 4      100.0% 
			      1 / 1      100.0% 
			      3 / 3      100.0% 
			      2 / 2      100.0% 
			      8 / 8      100.0% 
			      8 / 8      100.0% 
			      3 / 3      100.0% 
			      5 / 5      100.0% 
			      8 / 8      100.0% 
			      1 / 1      100.0% 
			      0 / 0       0.0% 
			      0 / 0       0.0% 
			      0 / 0       0.0% 
			     61 / 95     64.2%AB 
			     34 / 95     35.8%ABE 
		 
		 
			 1 
			  5.4 
			 Escherichia coli O7:K1 
			     91 / 91     100.0% 
			      4 / 4      100.0% 
			      3 / 3      100.0% 
			      4 / 4      100.0% 
			      3 / 3      100.0% 
			      6 / 6      100.0% 
			      7 / 7      100.0% 
			      1 / 1      100.0% 
			      3 / 3      100.0% 
			      5 / 5      100.0% 
			      1 / 1      100.0% 
			      5 / 5      100.0% 
			      6 / 6      100.0% 
			      2 / 2      100.0% 
			      4 / 4      100.0% 
			      1 / 1      100.0% 
			      3 / 3      100.0% 
			      2 / 2      100.0% 
			      4 / 4      100.0% 
			     11 / 11     100.0% 
			      4 / 4      100.0% 
			      4 / 4      100.0% 
			      7 / 7      100.0% 
			      1 / 1      100.0% 
			      0 / 0       0.0% 
			      0 / 0       0.0% 
			      0 / 0       0.0% 
			     60 / 91     65.9%AB 
			     31 / 91     34.1%ABE 
		 
		 
			 1 
			  5.4 
			 Escherichia coli O7:K1 str. CE10 
			     91 / 91     100.0% 
			      4 / 4      100.0% 
			      3 / 3      100.0% 
			      4 / 4      100.0% 
			      3 / 3      100.0% 
			      6 / 6      100.0% 
			      7 / 7      100.0% 
			      1 / 1      100.0% 
			      3 / 3      100.0% 
			      5 / 5      100.0% 
			      1 / 1      100.0% 
			      5 / 5      100.0% 
			      6 / 6      100.0% 
			      2 / 2      100.0% 
			      4 / 4      100.0% 
			      1 / 1      100.0% 
			      3 / 3      100.0% 
			      2 / 2      100.0% 
			      4 / 4      100.0% 
			     11 / 11     100.0% 
			      4 / 4      100.0% 
			      4 / 4      100.0% 
			      7 / 7      100.0% 
			      1 / 1      100.0% 
			      0 / 0       0.0% 
			      0 / 0       0.0% 
			      0 / 0       0.0% 
			     60 / 91     65.9%AB 
			     31 / 91     34.1%ABE 
		 
		 
			 1 
			  4.8 
			 Escherichia coli APEC O78 
			     86 / 86     100.0% 
			      4 / 4      100.0% 
			      3 / 3      100.0% 
			      4 / 4      100.0% 
			      6 / 6      100.0% 
			      6 / 6      100.0% 
			      7 / 7      100.0% 
			      1 / 1      100.0% 
			      3 / 3      100.0% 
			      7 / 7      100.0% 
			      1 / 1      100.0% 
			      5 / 5      100.0% 
			      6 / 6      100.0% 
			      2 / 2      100.0% 
			      4 / 4      100.0% 
			      1 / 1      100.0% 
			      3 / 3      100.0% 
			      2 / 2      100.0% 
			      4 / 4      100.0% 
			      7 / 7      100.0% 
			      0 / 0       0.0% 
			      1 / 1      100.0% 
			      8 / 8      100.0% 
			      1 / 1      100.0% 
			      0 / 0       0.0% 
			      0 / 0       0.0% 
			      0 / 0       0.0% 
			     58 / 86     67.4%AB 
			     28 / 86     32.6%ABE 
		 
		 
			 1 
			  4.6 
			 ATCC 35469 
			     84 / 85     98.8% 
			      4 / 4      100.0% 
			      3 / 3      100.0% 
			      4 / 4      100.0% 
			      3 / 3      100.0% 
			      6 / 6      100.0% 
			      7 / 7      100.0% 
			      1 / 1      100.0% 
			      3 / 3      100.0% 
			      5 / 5      100.0% 
			      1 / 1      100.0% 
			      5 / 5      100.0% 
			      6 / 6      100.0% 
			      2 / 2      100.0% 
			      4 / 4      100.0% 
			      1 / 1      100.0% 
			      3 / 3      100.0% 
			      2 / 2      100.0% 
			      4 / 4      100.0% 
			      6 / 7      85.7% 
			      4 / 4      100.0% 
			      1 / 1      100.0% 
			      8 / 8      100.0% 
			      1 / 1      100.0% 
			      0 / 0       0.0% 
			      0 / 0       0.0% 
			      0 / 0       0.0% 
			     52 / 85     61.2%AB 
			     32 / 85     37.6%ABE 
			      1 / 85      1.2% 
		 
		 
			 1 
			  4.6 
			 Escherichia fergusonii ATCC 35469 
			     84 / 85     98.8% 
			      4 / 4      100.0% 
			      3 / 3      100.0% 
			      4 / 4      100.0% 
			      3 / 3      100.0% 
			      6 / 6      100.0% 
			      7 / 7      100.0% 
			      1 / 1      100.0% 
			      3 / 3      100.0% 
			      5 / 5      100.0% 
			      1 / 1      100.0% 
			      5 / 5      100.0% 
			      6 / 6      100.0% 
			      2 / 2      100.0% 
			      4 / 4      100.0% 
			      1 / 1      100.0% 
			      3 / 3      100.0% 
			      2 / 2      100.0% 
			      4 / 4      100.0% 
			      6 / 7      85.7% 
			      4 / 4      100.0% 
			      1 / 1      100.0% 
			      8 / 8      100.0% 
			      1 / 1      100.0% 
			      0 / 0       0.0% 
			      0 / 0       0.0% 
			      0 / 0       0.0% 
			     52 / 85     61.2%AB 
			     32 / 85     37.6%ABE 
			      1 / 85      1.2% 
		 
		 
			 10 
			  5.8 
			 "Donovania" Anderson et al. 1944 
			    827 / 833    99.3% 
			     37 / 37     100.0% 
			     30 / 30     100.0% 
			     42 / 42     100.0% 
			     26 / 26     100.0% 
			     56 / 56     100.0% 
			     74 / 74     100.0% 
			     10 / 10     100.0% 
			     28 / 28     100.0% 
			     45 / 45     100.0% 
			     10 / 10     100.0% 
			     49 / 49     100.0% 
			     55 / 55     100.0% 
			     20 / 20     100.0% 
			     38 / 38     100.0% 
			     10 / 10     100.0% 
			     30 / 30     100.0% 
			     19 / 19     100.0% 
			     38 / 40     95.0% 
			     68 / 72     94.4% 
			     44 / 44     100.0% 
			     11 / 11     100.0% 
			     77 / 77     100.0% 
			     10 / 10     100.0% 
			      0 / 0       0.0% 
			      0 / 0       0.0% 
			      0 / 0       0.0% 
			    489 / 833    58.7%AB 
			    306 / 833    36.7%ABE 
			     18 / 833     2.2%BE 
			     14 / 833     1.7%B 
			      5 / 833     0.6% 
			      1 / 833     0.1%C 
		 
		 
			 2 
			  6.3 
			 "Bacillus oxytocus perniciosus" Flugge 1886 
			    165 / 167    98.8% 
			      8 / 8      100.0% 
			      6 / 6      100.0% 
			      8 / 8      100.0% 
			      6 / 6      100.0% 
			     11 / 11     100.0% 
			     14 / 14     100.0% 
			      2 / 2      100.0% 
			      6 / 6      100.0% 
			     10 / 10     100.0% 
			      2 / 2      100.0% 
			     10 / 10     100.0% 
			     10 / 10     100.0% 
			      4 / 4      100.0% 
			      8 / 8      100.0% 
			      2 / 2      100.0% 
			      6 / 6      100.0% 
			      4 / 4      100.0% 
			      9 / 9      100.0% 
			     12 / 14     85.7% 
			      8 / 8      100.0% 
			      2 / 2      100.0% 
			     15 / 15     100.0% 
			      2 / 2      100.0% 
			      0 / 0       0.0% 
			      0 / 0       0.0% 
			      0 / 0       0.0% 
			    100 / 167    59.9%AB 
			     65 / 167    38.9%ABE 
			      2 / 167     1.2% 
		 
		 
			 1 
			  6.0 
			 Klebsiella oxytoca ATCC 8724 
			     83 / 84     98.8% 
			      4 / 4      100.0% 
			      3 / 3      100.0% 
			      4 / 4      100.0% 
			      3 / 3      100.0% 
			      6 / 6      100.0% 
			      7 / 7      100.0% 
			      1 / 1      100.0% 
			      3 / 3      100.0% 
			      5 / 5      100.0% 
			      1 / 1      100.0% 
			      5 / 5      100.0% 
			      5 / 5      100.0% 
			      2 / 2      100.0% 
			      4 / 4      100.0% 
			      1 / 1      100.0% 
			      3 / 3      100.0% 
			      2 / 2      100.0% 
			      4 / 4      100.0% 
			      6 / 7      85.7% 
			      4 / 4      100.0% 
			      1 / 1      100.0% 
			      8 / 8      100.0% 
			      1 / 1      100.0% 
			      0 / 0       0.0% 
			      0 / 0       0.0% 
			      0 / 0       0.0% 
			     50 / 84     59.5%AB 
			     33 / 84     39.3%ABE 
			      1 / 84      1.2% 
		 
		 
			 1 
			  6.6 
			 Klebsiella oxytoca E718 
			     82 / 83     98.8% 
			      4 / 4      100.0% 
			      3 / 3      100.0% 
			      4 / 4      100.0% 
			      3 / 3      100.0% 
			      5 / 5      100.0% 
			      7 / 7      100.0% 
			      1 / 1      100.0% 
			      3 / 3      100.0% 
			      5 / 5      100.0% 
			      1 / 1      100.0% 
			      5 / 5      100.0% 
			      5 / 5      100.0% 
			      2 / 2      100.0% 
			      4 / 4      100.0% 
			      1 / 1      100.0% 
			      3 / 3      100.0% 
			      2 / 2      100.0% 
			      5 / 5      100.0% 
			      6 / 7      85.7% 
			      4 / 4      100.0% 
			      1 / 1      100.0% 
			      7 / 7      100.0% 
			      1 / 1      100.0% 
			      0 / 0       0.0% 
			      0 / 0       0.0% 
			      0 / 0       0.0% 
			     50 / 83     60.2%AB 
			     32 / 83     38.6%ABE 
			      1 / 83      1.2% 
		 
		 
			 7 
			  5.7 
			 "Bacillus pneumoniae" (Schroeter 1886) Flugge 1886 
			    578 / 581    99.5% 
			     26 / 26     100.0% 
			     21 / 21     100.0% 
			     30 / 30     100.0% 
			     17 / 17     100.0% 
			     39 / 39     100.0% 
			     52 / 52     100.0% 
			      7 / 7      100.0% 
			     20 / 20     100.0% 
			     30 / 30     100.0% 
			      7 / 7      100.0% 
			     34 / 34     100.0% 
			     39 / 39     100.0% 
			     14 / 14     100.0% 
			     26 / 26     100.0% 
			      7 / 7      100.0% 
			     21 / 21     100.0% 
			     13 / 13     100.0% 
			     25 / 26     96.2% 
			     49 / 51     96.1% 
			     32 / 32     100.0% 
			      8 / 8      100.0% 
			     54 / 54     100.0% 
			      7 / 7      100.0% 
			      0 / 0       0.0% 
			      0 / 0       0.0% 
			      0 / 0       0.0% 
			    336 / 581    57.8%AB 
			    210 / 581    36.1%ABE 
			     18 / 581     3.1%BE 
			     14 / 581     2.4%B 
			      2 / 581     0.3% 
			      1 / 581     0.2%C 
		 
		 
			 4 
			  5.6 
			 Klebsiella pneumoniae subsp. pneumoniae 
			    337 / 337    100.0% 
			     15 / 15     100.0% 
			     12 / 12     100.0% 
			     16 / 16     100.0% 
			     10 / 10     100.0% 
			     24 / 24     100.0% 
			     30 / 30     100.0% 
			      4 / 4      100.0% 
			     12 / 12     100.0% 
			     18 / 18     100.0% 
			      4 / 4      100.0% 
			     19 / 19     100.0% 
			     23 / 23     100.0% 
			      8 / 8      100.0% 
			     16 / 16     100.0% 
			      4 / 4      100.0% 
			     12 / 12     100.0% 
			      8 / 8      100.0% 
			     16 / 16     100.0% 
			     28 / 28     100.0% 
			     18 / 18     100.0% 
			      5 / 5      100.0% 
			     31 / 31     100.0% 
			      4 / 4      100.0% 
			      0 / 0       0.0% 
			      0 / 0       0.0% 
			      0 / 0       0.0% 
			    191 / 337    56.7%AB 
			    114 / 337    33.8%ABE 
			     18 / 337     5.3%BE 
			     14 / 337     4.2%B 
		 
		 
			 1 
			  5.7 
			 Klebsiella pneumoniae MCG 78578 
			     86 / 86     100.0% 
			      4 / 4      100.0% 
			      3 / 3      100.0% 
			      4 / 4      100.0% 
			      3 / 3      100.0% 
			      6 / 6      100.0% 
			      8 / 8      100.0% 
			      1 / 1      100.0% 
			      3 / 3      100.0% 
			      5 / 5      100.0% 
			      1 / 1      100.0% 
			      5 / 5      100.0% 
			      6 / 6      100.0% 
			      2 / 2      100.0% 
			      4 / 4      100.0% 
			      1 / 1      100.0% 
			      3 / 3      100.0% 
			      2 / 2      100.0% 
			      4 / 4      100.0% 
			      7 / 7      100.0% 
			      4 / 4      100.0% 
			      1 / 1      100.0% 
			      8 / 8      100.0% 
			      1 / 1      100.0% 
			      0 / 0       0.0% 
			      0 / 0       0.0% 
			      0 / 0       0.0% 
			     39 / 86     45.3%AB 
			     18 / 86     20.9%BE 
			     15 / 86     17.4%ABE 
			     14 / 86     16.3%B 
		 
		 
			 1 
			  5.5 
			 Klebsiella pneumoniae NTUH-K2044 
			     86 / 86     100.0% 
			      4 / 4      100.0% 
			      3 / 3      100.0% 
			      4 / 4      100.0% 
			      3 / 3      100.0% 
			      6 / 6      100.0% 
			      8 / 8      100.0% 
			      1 / 1      100.0% 
			      3 / 3      100.0% 
			      5 / 5      100.0% 
			      1 / 1      100.0% 
			      5 / 5      100.0% 
			      6 / 6      100.0% 
			      2 / 2      100.0% 
			      4 / 4      100.0% 
			      1 / 1      100.0% 
			      3 / 3      100.0% 
			      2 / 2      100.0% 
			      4 / 4      100.0% 
			      7 / 7      100.0% 
			      4 / 4      100.0% 
			      1 / 1      100.0% 
			      8 / 8      100.0% 
			      1 / 1      100.0% 
			      0 / 0       0.0% 
			      0 / 0       0.0% 
			      0 / 0       0.0% 
			     53 / 86     61.6%AB 
			     33 / 86     38.4%ABE 
		 
		 
			 1 
			  5.7 
			 Klebsiella pneumoniae subsp. pneumoniae HS11286 
			     87 / 87     100.0% 
			      4 / 4      100.0% 
			      3 / 3      100.0% 
			      4 / 4      100.0% 
			      3 / 3      100.0% 
			      6 / 6      100.0% 
			      8 / 8      100.0% 
			      1 / 1      100.0% 
			      3 / 3      100.0% 
			      5 / 5      100.0% 
			      1 / 1      100.0% 
			      5 / 5      100.0% 
			      6 / 6      100.0% 
			      2 / 2      100.0% 
			      4 / 4      100.0% 
			      1 / 1      100.0% 
			      3 / 3      100.0% 
			      2 / 2      100.0% 
			      4 / 4      100.0% 
			      7 / 7      100.0% 
			      4 / 4      100.0% 
			      2 / 2      100.0% 
			      8 / 8      100.0% 
			      1 / 1      100.0% 
			      0 / 0       0.0% 
			      0 / 0       0.0% 
			      0 / 0       0.0% 
			     53 / 87     60.9%AB 
			     34 / 87     39.1%ABE 
		 
		 
			 1 
			   ND 
			 Klebsiella pneumoniae subsp. pneumoniae 1084 
			     78 / 78     100.0% 
			      3 / 3      100.0% 
			      3 / 3      100.0% 
			      4 / 4      100.0% 
			      1 / 1      100.0% 
			      6 / 6      100.0% 
			      6 / 6      100.0% 
			      1 / 1      100.0% 
			      3 / 3      100.0% 
			      3 / 3      100.0% 
			      1 / 1      100.0% 
			      4 / 4      100.0% 
			      5 / 5      100.0% 
			      2 / 2      100.0% 
			      4 / 4      100.0% 
			      1 / 1      100.0% 
			      3 / 3      100.0% 
			      2 / 2      100.0% 
			      4 / 4      100.0% 
			      7 / 7      100.0% 
			      6 / 6      100.0% 
			      1 / 1      100.0% 
			      7 / 7      100.0% 
			      1 / 1      100.0% 
			      0 / 0       0.0% 
			      0 / 0       0.0% 
			      0 / 0       0.0% 
			     46 / 78     59.0%AB 
			     32 / 78     41.0%ABE 
		 
		 
			 1 
			  5.9 
			 Klebsiella pneumoniae 342 
			     88 / 88     100.0% 
			      4 / 4      100.0% 
			      3 / 3      100.0% 
			      5 / 5      100.0% 
			      3 / 3      100.0% 
			      6 / 6      100.0% 
			      8 / 8      100.0% 
			      1 / 1      100.0% 
			      3 / 3      100.0% 
			      5 / 5      100.0% 
			      1 / 1      100.0% 
			      5 / 5      100.0% 
			      6 / 6      100.0% 
			      2 / 2      100.0% 
			      4 / 4      100.0% 
			      1 / 1      100.0% 
			      3 / 3      100.0% 
			      2 / 2      100.0% 
			      4 / 4      100.0% 
			      8 / 8      100.0% 
			      4 / 4      100.0% 
			      1 / 1      100.0% 
			      8 / 8      100.0% 
			      1 / 1      100.0% 
			      0 / 0       0.0% 
			      0 / 0       0.0% 
			      0 / 0       0.0% 
			     54 / 88     61.4%AB 
			     34 / 88     38.6%ABE 
		 
		 
			 1 
			   ND 
			 Klebsiella pneumoniae KCTC 2242 
			     87 / 88     98.9% 
			      4 / 4      100.0% 
			      3 / 3      100.0% 
			      5 / 5      100.0% 
			      3 / 3      100.0% 
			      6 / 6      100.0% 
			      8 / 8      100.0% 
			      1 / 1      100.0% 
			      3 / 3      100.0% 
			      5 / 5      100.0% 
			      1 / 1      100.0% 
			      5 / 5      100.0% 
			      6 / 6      100.0% 
			      2 / 2      100.0% 
			      4 / 4      100.0% 
			      1 / 1      100.0% 
			      3 / 3      100.0% 
			      2 / 2      100.0% 
			      3 / 4      75.0% 
			      8 / 8      100.0% 
			      4 / 4      100.0% 
			      1 / 1      100.0% 
			      8 / 8      100.0% 
			      1 / 1      100.0% 
			      0 / 0       0.0% 
			      0 / 0       0.0% 
			      0 / 0       0.0% 
			     53 / 88     60.2%AB 
			     34 / 88     38.6%ABE 
			      1 / 88      1.1% 
		 
		 
			 1 
			  5.5 
			 ATCC BAA-830 
			     84 / 85     98.8% 
			      3 / 3      100.0% 
			      3 / 3      100.0% 
			      4 / 4      100.0% 
			      3 / 3      100.0% 
			      6 / 6      100.0% 
			      8 / 8      100.0% 
			      1 / 1      100.0% 
			      2 / 2      100.0% 
			      5 / 5      100.0% 
			      1 / 1      100.0% 
			      5 / 5      100.0% 
			      6 / 6      100.0% 
			      2 / 2      100.0% 
			      4 / 4      100.0% 
			      1 / 1      100.0% 
			      3 / 3      100.0% 
			      2 / 2      100.0% 
			      4 / 5      80.0% 
			      7 / 7      100.0% 
			      4 / 4      100.0% 
			      1 / 1      100.0% 
			      8 / 8      100.0% 
			      1 / 1      100.0% 
			      0 / 0       0.0% 
			      0 / 0       0.0% 
			      0 / 0       0.0% 
			     53 / 85     62.4%AB 
			     31 / 85     36.5%ABE 
			      1 / 85      1.2% 
		 
		 
			 1 
			  5.5 
			 Klebsiella variicola At-22 
			     84 / 85     98.8% 
			      3 / 3      100.0% 
			      3 / 3      100.0% 
			      4 / 4      100.0% 
			      3 / 3      100.0% 
			      6 / 6      100.0% 
			      8 / 8      100.0% 
			      1 / 1      100.0% 
			      2 / 2      100.0% 
			      5 / 5      100.0% 
			      1 / 1      100.0% 
			      5 / 5      100.0% 
			      6 / 6      100.0% 
			      2 / 2      100.0% 
			      4 / 4      100.0% 
			      1 / 1      100.0% 
			      3 / 3      100.0% 
			      2 / 2      100.0% 
			      4 / 5      80.0% 
			      7 / 7      100.0% 
			      4 / 4      100.0% 
			      1 / 1      100.0% 
			      8 / 8      100.0% 
			      1 / 1      100.0% 
			      0 / 0       0.0% 
			      0 / 0       0.0% 
			      0 / 0       0.0% 
			     53 / 85     62.4%AB 
			     31 / 85     36.5%ABE 
			      1 / 85      1.2% 
		 
		 
			 1 
			  3.8 
			 "Liquidobacterium" Orla-Jensen 1908 
			     83 / 83     100.0% 
			      4 / 4      100.0% 
			      3 / 3      100.0% 
			      4 / 4      100.0% 
			      3 / 3      100.0% 
			      6 / 6      100.0% 
			      6 / 6      100.0% 
			      1 / 1      100.0% 
			      2 / 2      100.0% 
			      5 / 5      100.0% 
			      1 / 1      100.0% 
			      5 / 5      100.0% 
			      6 / 6      100.0% 
			      2 / 2      100.0% 
			      3 / 3      100.0% 
			      1 / 1      100.0% 
			      3 / 3      100.0% 
			      2 / 2      100.0% 
			      5 / 5      100.0% 
			      7 / 7      100.0% 
			      4 / 4      100.0% 
			      1 / 1      100.0% 
			      8 / 8      100.0% 
			      1 / 1      100.0% 
			      0 / 0       0.0% 
			      0 / 0       0.0% 
			      0 / 0       0.0% 
			     35 / 83     42.2%AB 
			     20 / 83     24.1%B 
			     14 / 83     16.9%BE 
			     14 / 83     16.9%ABE 
		 
		 
			 1 
			  3.8 
			 ATCC 29906 
			     83 / 83     100.0% 
			      4 / 4      100.0% 
			      3 / 3      100.0% 
			      4 / 4      100.0% 
			      3 / 3      100.0% 
			      6 / 6      100.0% 
			      6 / 6      100.0% 
			      1 / 1      100.0% 
			      2 / 2      100.0% 
			      5 / 5      100.0% 
			      1 / 1      100.0% 
			      5 / 5      100.0% 
			      6 / 6      100.0% 
			      2 / 2      100.0% 
			      3 / 3      100.0% 
			      1 / 1      100.0% 
			      3 / 3      100.0% 
			      2 / 2      100.0% 
			      5 / 5      100.0% 
			      7 / 7      100.0% 
			      4 / 4      100.0% 
			      1 / 1      100.0% 
			      8 / 8      100.0% 
			      1 / 1      100.0% 
			      0 / 0       0.0% 
			      0 / 0       0.0% 
			      0 / 0       0.0% 
			     35 / 83     42.2%AB 
			     20 / 83     24.1%B 
			     14 / 83     16.9%BE 
			     14 / 83     16.9%ABE 
		 
		 
			 1 
			  4.4 
			 Providencia 
			     71 / 72     98.6% 
			      4 / 4      100.0% 
			      3 / 3      100.0% 
			      3 / 3      100.0% 
			      3 / 3      100.0% 
			      5 / 5      100.0% 
			      5 / 5      100.0% 
			      1 / 1      100.0% 
			      2 / 2      100.0% 
			      4 / 4      100.0% 
			      1 / 1      100.0% 
			      4 / 4      100.0% 
			      3 / 4      75.0% 
			      2 / 2      100.0% 
			      3 / 3      100.0% 
			      1 / 1      100.0% 
			      3 / 3      100.0% 
			      2 / 2      100.0% 
			      4 / 4      100.0% 
			      4 / 4      100.0% 
			      4 / 4      100.0% 
			      1 / 1      100.0% 
			      8 / 8      100.0% 
			      1 / 1      100.0% 
			      0 / 0       0.0% 
			      0 / 0       0.0% 
			      0 / 0       0.0% 
			     45 / 72     62.5%AB 
			     26 / 72     36.1%ABE 
			      1 / 72      1.4% 
		 
		 
			 1 
			  4.4 
			 "Proteus stuartii" Buttiaux et al. 1954 
			     71 / 72     98.6% 
			      4 / 4      100.0% 
			      3 / 3      100.0% 
			      3 / 3      100.0% 
			      3 / 3      100.0% 
			      5 / 5      100.0% 
			      5 / 5      100.0% 
			      1 / 1      100.0% 
			      2 / 2      100.0% 
			      4 / 4      100.0% 
			      1 / 1      100.0% 
			      4 / 4      100.0% 
			      3 / 4      75.0% 
			      2 / 2      100.0% 
			      3 / 3      100.0% 
			      1 / 1      100.0% 
			      3 / 3      100.0% 
			      2 / 2      100.0% 
			      4 / 4      100.0% 
			      4 / 4      100.0% 
			      4 / 4      100.0% 
			      1 / 1      100.0% 
			      8 / 8      100.0% 
			      1 / 1      100.0% 
			      0 / 0       0.0% 
			      0 / 0       0.0% 
			      0 / 0       0.0% 
			     45 / 72     62.5%AB 
			     26 / 72     36.1%ABE 
			      1 / 72      1.4% 
		 
		 
			 1 
			  4.4 
			 Providencia stuartii MRSN 2154 
			     71 / 72     98.6% 
			      4 / 4      100.0% 
			      3 / 3      100.0% 
			      3 / 3      100.0% 
			      3 / 3      100.0% 
			      5 / 5      100.0% 
			      5 / 5      100.0% 
			      1 / 1      100.0% 
			      2 / 2      100.0% 
			      4 / 4      100.0% 
			      1 / 1      100.0% 
			      4 / 4      100.0% 
			      3 / 4      75.0% 
			      2 / 2      100.0% 
			      3 / 3      100.0% 
			      1 / 1      100.0% 
			      3 / 3      100.0% 
			      2 / 2      100.0% 
			      4 / 4      100.0% 
			      4 / 4      100.0% 
			      4 / 4      100.0% 
			      1 / 1      100.0% 
			      8 / 8      100.0% 
			      1 / 1      100.0% 
			      0 / 0       0.0% 
			      0 / 0       0.0% 
			      0 / 0       0.0% 
			     45 / 72     62.5%AB 
			     26 / 72     36.1%ABE 
			      1 / 72      1.4% 
		 
		 
			 30 
			  4.8 
			 Salmonella 
			   2463 / 2472   99.6% 
			     90 / 90     100.0% 
			     89 / 90     98.9% 
			    120 / 120    100.0% 
			     91 / 91     100.0% 
			    172 / 172    100.0% 
			    172 / 172    100.0% 
			     30 / 30     100.0% 
			     80 / 80     100.0% 
			    148 / 148    100.0% 
			     30 / 30     100.0% 
			    149 / 150    99.3% 
			    136 / 140    97.1% 
			     60 / 60     100.0% 
			    113 / 113    100.0% 
			     30 / 30     100.0% 
			     90 / 90     100.0% 
			     59 / 60     98.3% 
			    122 / 122    100.0% 
			    273 / 274    99.6% 
			    120 / 120    100.0% 
			     30 / 30     100.0% 
			    228 / 229    99.6% 
			     30 / 30     100.0% 
			      0 / 0       0.0% 
			      0 / 0       0.0% 
			      1 / 1      100.0% 
			   1433 / 2472   58.0%AB 
			    783 / 2472   31.7%ABE 
			    125 / 2472    5.1%B 
			    122 / 2472    4.9%BE 
			      6 / 2472    0.2% 
			      3 / 2472    0.1%E 
		 
		 
			 29 
			  4.9 
			 "Bacillus cholerae-suis" Smith 1894 
			   2380 / 2389   99.6% 
			     87 / 87     100.0% 
			     86 / 87     98.9% 
			    116 / 116    100.0% 
			     88 / 88     100.0% 
			    167 / 167    100.0% 
			    166 / 166    100.0% 
			     29 / 29     100.0% 
			     77 / 77     100.0% 
			    143 / 143    100.0% 
			     29 / 29     100.0% 
			    144 / 145    99.3% 
			    131 / 135    97.0% 
			     58 / 58     100.0% 
			    109 / 109    100.0% 
			     29 / 29     100.0% 
			     87 / 87     100.0% 
			     57 / 58     98.3% 
			    118 / 118    100.0% 
			    264 / 265    99.6% 
			    116 / 116    100.0% 
			     29 / 29     100.0% 
			    220 / 221    99.5% 
			     29 / 29     100.0% 
			      0 / 0       0.0% 
			      0 / 0       0.0% 
			      1 / 1      100.0% 
			   1381 / 2389   57.8%AB 
			    752 / 2389   31.5%ABE 
			    125 / 2389    5.2%B 
			    122 / 2389    5.1%BE 
			      6 / 2389    0.3% 
			      3 / 2389    0.1%E 
		 
		 
			 29 
			  4.9 
			 Salmonella cholerae-suis subsp. cholerae-suis 
			   2380 / 2389   99.6% 
			     87 / 87     100.0% 
			     86 / 87     98.9% 
			    116 / 116    100.0% 
			     88 / 88     100.0% 
			    167 / 167    100.0% 
			    166 / 166    100.0% 
			     29 / 29     100.0% 
			     77 / 77     100.0% 
			    143 / 143    100.0% 
			     29 / 29     100.0% 
			    144 / 145    99.3% 
			    131 / 135    97.0% 
			     58 / 58     100.0% 
			    109 / 109    100.0% 
			     29 / 29     100.0% 
			     87 / 87     100.0% 
			     57 / 58     98.3% 
			    118 / 118    100.0% 
			    264 / 265    99.6% 
			    116 / 116    100.0% 
			     29 / 29     100.0% 
			    220 / 221    99.5% 
			     29 / 29     100.0% 
			      0 / 0       0.0% 
			      0 / 0       0.0% 
			      1 / 1      100.0% 
			   1381 / 2389   57.8%AB 
			    752 / 2389   31.5%ABE 
			    125 / 2389    5.2%B 
			    122 / 2389    5.1%BE 
			      6 / 2389    0.3% 
			      3 / 2389    0.1%E 
		 
		 
			 1 
			  4.7 
			 Salmonella enterica serovar Gallinarum 
			     74 / 75     98.7% 
			      3 / 3      100.0% 
			      3 / 3      100.0% 
			      4 / 4      100.0% 
			      3 / 3      100.0% 
			      6 / 6      100.0% 
			      5 / 5      100.0% 
			      1 / 1      100.0% 
			      2 / 2      100.0% 
			      4 / 4      100.0% 
			      1 / 1      100.0% 
			      5 / 5      100.0% 
			      3 / 3      100.0% 
			      2 / 2      100.0% 
			      3 / 3      100.0% 
			      1 / 1      100.0% 
			      3 / 3      100.0% 
			      1 / 2      50.0% 
			      4 / 4      100.0% 
			      8 / 8      100.0% 
			      4 / 4      100.0% 
			      1 / 1      100.0% 
			      6 / 6      100.0% 
			      1 / 1      100.0% 
			      0 / 0       0.0% 
			      0 / 0       0.0% 
			      0 / 0       0.0% 
			     47 / 75     62.7%AB 
			     27 / 75     36.0%ABE 
			      1 / 75      1.3% 
		 
		 
			 1 
			  4.7 
			 Salmonella enterica subsp. enterica serovar Gallinarum str. 287/91 
			     74 / 75     98.7% 
			      3 / 3      100.0% 
			      3 / 3      100.0% 
			      4 / 4      100.0% 
			      3 / 3      100.0% 
			      6 / 6      100.0% 
			      5 / 5      100.0% 
			      1 / 1      100.0% 
			      2 / 2      100.0% 
			      4 / 4      100.0% 
			      1 / 1      100.0% 
			      5 / 5      100.0% 
			      3 / 3      100.0% 
			      2 / 2      100.0% 
			      3 / 3      100.0% 
			      1 / 1      100.0% 
			      3 / 3      100.0% 
			      1 / 2      50.0% 
			      4 / 4      100.0% 
			      8 / 8      100.0% 
			      4 / 4      100.0% 
			      1 / 1      100.0% 
			      6 / 6      100.0% 
			      1 / 1      100.0% 
			      0 / 0       0.0% 
			      0 / 0       0.0% 
			      0 / 0       0.0% 
			     47 / 75     62.7%AB 
			     27 / 75     36.0%ABE 
			      1 / 75      1.3% 
		 
		 
			 1 
			  4.6 
			 Salmonella enterica subsp. enterica serovar Gallinarum/pullorum 
			     73 / 73     100.0% 
			      3 / 3      100.0% 
			      3 / 3      100.0% 
			      4 / 4      100.0% 
			      3 / 3      100.0% 
			      6 / 6      100.0% 
			      4 / 4      100.0% 
			      1 / 1      100.0% 
			      2 / 2      100.0% 
			      4 / 4      100.0% 
			      1 / 1      100.0% 
			      5 / 5      100.0% 
			      2 / 2      100.0% 
			      2 / 2      100.0% 
			      2 / 2      100.0% 
			      1 / 1      100.0% 
			      3 / 3      100.0% 
			      2 / 2      100.0% 
			      4 / 4      100.0% 
			      8 / 8      100.0% 
			      4 / 4      100.0% 
			      1 / 1      100.0% 
			      6 / 6      100.0% 
			      1 / 1      100.0% 
			      0 / 0       0.0% 
			      0 / 0       0.0% 
			      1 / 1      100.0% 
			     46 / 73     63.0%AB 
			     27 / 73     37.0%ABE 
		 
		 
			 3 
			  4.9 
			 Salmonella enterica subsp. enterica serovar Heidelberg 
			    250 / 250    100.0% 
			      9 / 9      100.0% 
			      9 / 9      100.0% 
			     12 / 12     100.0% 
			      9 / 9      100.0% 
			     18 / 18     100.0% 
			     18 / 18     100.0% 
			      3 / 3      100.0% 
			      9 / 9      100.0% 
			     15 / 15     100.0% 
			      3 / 3      100.0% 
			     15 / 15     100.0% 
			     15 / 15     100.0% 
			      6 / 6      100.0% 
			     12 / 12     100.0% 
			      3 / 3      100.0% 
			      9 / 9      100.0% 
			      6 / 6      100.0% 
			     12 / 12     100.0% 
			     27 / 27     100.0% 
			     12 / 12     100.0% 
			      3 / 3      100.0% 
			     22 / 22     100.0% 
			      3 / 3      100.0% 
			      0 / 0       0.0% 
			      0 / 0       0.0% 
			      0 / 0       0.0% 
			    144 / 250    57.6%AB 
			     72 / 250    28.8%ABE 
			     19 / 250     7.6%BE 
			     15 / 250     6.0%B 
		 
		 
			 1 
			  5.0 
			 Salmonella enterica subsp. enterica serovar Heidelberg str. SL476 
			     84 / 84     100.0% 
			      3 / 3      100.0% 
			      3 / 3      100.0% 
			      4 / 4      100.0% 
			      3 / 3      100.0% 
			      6 / 6      100.0% 
			      6 / 6      100.0% 
			      1 / 1      100.0% 
			      3 / 3      100.0% 
			      5 / 5      100.0% 
			      1 / 1      100.0% 
			      5 / 5      100.0% 
			      5 / 5      100.0% 
			      2 / 2      100.0% 
			      4 / 4      100.0% 
			      1 / 1      100.0% 
			      3 / 3      100.0% 
			      2 / 2      100.0% 
			      4 / 4      100.0% 
			      9 / 9      100.0% 
			      4 / 4      100.0% 
			      1 / 1      100.0% 
			      8 / 8      100.0% 
			      1 / 1      100.0% 
			      0 / 0       0.0% 
			      0 / 0       0.0% 
			      0 / 0       0.0% 
			     38 / 84     45.2%AB 
			     19 / 84     22.6%BE 
			     15 / 84     17.9%B 
			     12 / 84     14.3%ABE 
		 
		 
			 1 
			  5.0 
			 Salmonella enterica subsp. enterica serovar Heidelberg str. 41578 
			     84 / 84     100.0% 
			      3 / 3      100.0% 
			      3 / 3      100.0% 
			      4 / 4      100.0% 
			      3 / 3      100.0% 
			      6 / 6      100.0% 
			      6 / 6      100.0% 
			      1 / 1      100.0% 
			      3 / 3      100.0% 
			      5 / 5      100.0% 
			      1 / 1      100.0% 
			      5 / 5      100.0% 
			      5 / 5      100.0% 
			      2 / 2      100.0% 
			      4 / 4      100.0% 
			      1 / 1      100.0% 
			      3 / 3      100.0% 
			      2 / 2      100.0% 
			      4 / 4      100.0% 
			      9 / 9      100.0% 
			      4 / 4      100.0% 
			      1 / 1      100.0% 
			      8 / 8      100.0% 
			      1 / 1      100.0% 
			      0 / 0       0.0% 
			      0 / 0       0.0% 
			      0 / 0       0.0% 
			     53 / 84     63.1%AB 
			     31 / 84     36.9%ABE 
		 
		 
			 1 
			  4.8 
			 Salmonella enterica subsp. enterica serovar Heidelberg str. B182 
			     82 / 82     100.0% 
			      3 / 3      100.0% 
			      3 / 3      100.0% 
			      4 / 4      100.0% 
			      3 / 3      100.0% 
			      6 / 6      100.0% 
			      6 / 6      100.0% 
			      1 / 1      100.0% 
			      3 / 3      100.0% 
			      5 / 5      100.0% 
			      1 / 1      100.0% 
			      5 / 5      100.0% 
			      5 / 5      100.0% 
			      2 / 2      100.0% 
			      4 / 4      100.0% 
			      1 / 1      100.0% 
			      3 / 3      100.0% 
			      2 / 2      100.0% 
			      4 / 4      100.0% 
			      9 / 9      100.0% 
			      4 / 4      100.0% 
			      1 / 1      100.0% 
			      6 / 6      100.0% 
			      1 / 1      100.0% 
			      0 / 0       0.0% 
			      0 / 0       0.0% 
			      0 / 0       0.0% 
			     53 / 82     64.6%AB 
			     29 / 82     35.4%ABE 
		 
		 
			 1 
			  4.6 
			 "Bacterium paratyphi typhus A" Brion and Kaiser 1902 
			     81 / 81     100.0% 
			      3 / 3      100.0% 
			      3 / 3      100.0% 
			      4 / 4      100.0% 
			      3 / 3      100.0% 
			      5 / 5      100.0% 
			      6 / 6      100.0% 
			      1 / 1      100.0% 
			      3 / 3      100.0% 
			      4 / 4      100.0% 
			      1 / 1      100.0% 
			      5 / 5      100.0% 
			      5 / 5      100.0% 
			      2 / 2      100.0% 
			      4 / 4      100.0% 
			      1 / 1      100.0% 
			      3 / 3      100.0% 
			      2 / 2      100.0% 
			      4 / 4      100.0% 
			      8 / 8      100.0% 
			      4 / 4      100.0% 
			      1 / 1      100.0% 
			      8 / 8      100.0% 
			      1 / 1      100.0% 
			      0 / 0       0.0% 
			      0 / 0       0.0% 
			      0 / 0       0.0% 
			     25 / 81     30.9%AB 
			     25 / 81     30.9%B 
			     18 / 81     22.2%ABE 
			     13 / 81     16.0%BE 
		 
		 
			 1 
			  4.6 
			 Salmonella enterica subsp. enterica serovar Paratyphi A str. AKU_12601 
			     81 / 81     100.0% 
			      3 / 3      100.0% 
			      3 / 3      100.0% 
			      4 / 4      100.0% 
			      3 / 3      100.0% 
			      5 / 5      100.0% 
			      6 / 6      100.0% 
			      1 / 1      100.0% 
			      3 / 3      100.0% 
			      4 / 4      100.0% 
			      1 / 1      100.0% 
			      5 / 5      100.0% 
			      5 / 5      100.0% 
			      2 / 2      100.0% 
			      4 / 4      100.0% 
			      1 / 1      100.0% 
			      3 / 3      100.0% 
			      2 / 2      100.0% 
			      4 / 4      100.0% 
			      8 / 8      100.0% 
			      4 / 4      100.0% 
			      1 / 1      100.0% 
			      8 / 8      100.0% 
			      1 / 1      100.0% 
			      0 / 0       0.0% 
			      0 / 0       0.0% 
			      0 / 0       0.0% 
			     25 / 81     30.9%AB 
			     25 / 81     30.9%B 
			     18 / 81     22.2%ABE 
			     13 / 81     16.0%BE 
		 
		 
			 1 
			  4.9 
			 Salmonella enterica subsp. enterica serovar Paratyphi B 
			     85 / 85     100.0%
[truncated: 3,956,133 more chars]
