# Supplementary figures and images for: Initiator tRNA genes template the 3′ CCA end at high frequencies in bacteria
Source: BMC Genomics. 2016 Dec 8;17:1003. doi: 10.1186/s12864-016-3314-x (PMC5143459; doi:10.1186/s12864-016-3314-x)

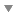

Supplement: Additional file 12: — Web-browser based navigator of full dataset of results on frequencies of CCA-templating by tRNA functional class, organized by clades in NCBI taxonomy. (ZIP 548 kb) [file 12864_2016_3314_MOESM12_ESM.zip › CCA-HEATMAP-TAX-BROWSER/toggle-collapse-dark.png]

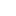

Supplement: Additional file 12: — Web-browser based navigator of full dataset of results on frequencies of CCA-templating by tRNA functional class, organized by clades in NCBI taxonomy. (ZIP 548 kb) [file 12864_2016_3314_MOESM12_ESM.zip › CCA-HEATMAP-TAX-BROWSER/toggle-collapse-light.png]

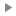

Supplement: Additional file 12: — Web-browser based navigator of full dataset of results on frequencies of CCA-templating by tRNA functional class, organized by clades in NCBI taxonomy. (ZIP 548 kb) [file 12864_2016_3314_MOESM12_ESM.zip › CCA-HEATMAP-TAX-BROWSER/toggle-expand-dark.png]

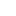

Supplement: Additional file 12: — Web-browser based navigator of full dataset of results on frequencies of CCA-templating by tRNA functional class, organized by clades in NCBI taxonomy. (ZIP 548 kb) [file 12864_2016_3314_MOESM12_ESM.zip › CCA-HEATMAP-TAX-BROWSER/toggle-expand-light.png]
