# Supplementary material for: Initiator tRNA genes template the 3′ CCA end at high frequencies in bacteria
Source: BMC Genomics. 2016 Dec 8;17:1003. doi: 10.1186/s12864-016-3314-x (PMC5143459; doi:10.1186/s12864-016-3314-x)
Supplement: Additional file 13: — Static and searchable PDF with full dataset of results on frequencies of CCA-templating by tRNA functional class, organized by clades in NCBI taxonomy. (PDF 1 MB) [file 12864_2016_3314_MOESM13_ESM.pdf]

Title: Analysis of CCA templating in tRNADB-CE ver. 8.0, Oct., 2014 release by David H. Ardell

Date: 2015-07-07 16:30:51

Input: tCE-Nov5-2014.3.fas or tCE-Nov5-2014.taxsort.model_cca_U0_tfam.fas

Generated By: perl compute_CCA_mss_figure.pl tCE-Nov5-2014.3.fas > cca_mss.html

Execution dependencies: addresses_v3.txt.gz

Open in: any javascript-capable web-browser

Require files in same directory as this file: jquery.js, jquery.treeTable.css, jquery.treeTable.js, toggle-collapse-dark.png, toggle-collapse-light.png, toggle-expand-dark.png, toggle-expand-light.png

Description: Supplementary data with frequency analytics for Ardell and Hou (2015) Analysis of CCA-templating in Bacterial and Archaeal tRNA genes from a reannotation of Version 8 of tRNAdb-CE. Uses NCBI Taxonomy data Two reannotations were carried out:

1. Reannotation of function on CAU-anticodon tRNAs, assignment to classes M (elongator Met), X (initiator), or J (LAU-anticodon eleongator Ile) using TFAM and the Silva model as described in Ardell and Hou 2015 "High Frequencies of CCA-Templating in Bacterial Initiator tRNA Genes"
2. Reannotation of CCA-templating, with verification of the modeling of the acceptor stem using dynamic programming. We assumed that if CCA occurs just after the annotated acceptor stem, that the gene templates CCA.

The dynamic programming algorithm works as follows: seven bases of the annotated 5' end are taken from the database entries and the best pairing segment near the annotated 3' end is calculated by dynamic programming. The following parameters were used to generate these specific results:

Number of bases of annotated 3' end searched
12
Number of bases of annotated 3' trailer
5
Match bonus (including G:U and U:G)
4
Mismatch bonus
1
Annotation bonus (pairing within boundaries annotated in tRNAdb-CE)
1 or 0
Gap penalty
-5

The algorithm segments the 17 bases from the 3'end into segments: [3'stem][id-base][cca?][trailer] Certain conditions were tallied and appear at the end of the rows. Here are the meanings of these conditions.

A
W:CCApredicted: if [cca?] eq "CCA", correctly annotated in tCE
B
"W:cca" if [cca?] =~ /cca/i. All A are also B, so our tRNAdb-CE CCA-templating false negatives are "B and not A"
C
"W:cc" if [cca?] =~ /^cc/
D
"W:shortLength" if predicted FPE of Acceptor stem is < 7
E
"W:pos89nTA" if the tCE-annoted 8th and 9th bases are not "T" and "A"

Reclassifications of bacterial data based on TFAM with Silva model:

	M	X	J
M	2751	2614	1737
X	0	1824	0
J	11	0	977

No. Genomes	Taxon	All	Ini	Asp	Asn	Ile	Gly	Val	His	Tyr	Ala	Trp	Ser	Lys	Phe	Gln	Cys	Pro	Met
2323	all	87158 / 132129 66.0%	3330 / 4494 74.1%	3539 / 4859 72.8%	3328 / 4614 72.1%	3691 / 5132 71.9%	6649 / 9505 70.0%	5628 / 8054 69.9%	1909 / 2760 69.2%	2349 / 3471 67.7%	5899 / 8922 66.1%	1722 / 2622 65.7%	6389 / 9748 65.5%	4090 / 6237 65.6%	2212 / 3410 64.9%	3113 / 4866 64.0%	1674 / 2634 63.6%	3904 / 6146 63.5%	1778 / 2809 63.3%
2323	biota	87158 / 132129 66.0%	3330 / 4494 74.1%	3539 / 4859 72.8%	3328 / 4614 72.1%	3691 / 5132 71.9%	6649 / 9505 70.0%	5628 / 8054 69.9%	1909 / 2760 69.2%	2349 / 3471 67.7%	5899 / 8922 66.1%	1722 / 2622 65.7%	6389 / 9748 65.5%	4090 / 6237 65.6%	2212 / 3410 64.9%	3113 / 4866 64.0%	1674 / 2634 63.6%	3904 / 6146 63.5%	1778 / 2809 63.3%
2276	Bacteria	86508 / 129989 66.6%	3314 / 4438 74.7%	3525 / 4806 73.3%	3311 / 4563 72.6%	3675 / 5081 72.3%	6614 / 9364 70.6%	5585 / 7918 70.5%	1890 / 2713 69.7%	2335 / 3424 68.2%	5858 / 8776 66.8%	1707 / 2574 66.3%	6331 / 9568 66.2%	4060 / 6147 66.0%	2192 / 3359 65.3%	3084 / 4781 64.5%	1661 / 2575 64.5%	3859 / 6019 64.1%	1767 / 2762 64.0%
69	Cyanobacteria	153 / 3264 4.7%	41 / 64 64.1%	1 / 84 1.2%	11 / 85 12.9%	4 / 144 2.8%	1 / 211 0.5%	3 / 179 1.7%	0 / 73 0.0%	19 / 74 25.7%	26 / 267 9.7%	2 / 77 2.6%	3 / 304 1.0%	2 / 111 1.8%	3 / 80 3.8%	6 / 87 6.9%	3 / 82 3.7%	3 / 213 1.4%	1 / 56 1.8%
11	Nostocales	49 / 600 8.2%	8 / 9 88.9%	0 / 15 0.0%	7 / 19 36.8%	0 / 28 0.0%	0 / 35 0.0%	1 / 25 4.0%	0 / 12 0.0%	5 / 12 41.7%	4 / 62 6.5%	0 / 13 0.0%	0 / 50 0.0%	2 / 29 6.9%	0 / 17 0.0%	5 / 19 26.3%	2 / 16 12.5%	2 / 39 5.1%	1 / 10 10.0%
8	Nostocaceae	35 / 445 7.9%	5 / 6 83.3%	0 / 11 0.0%	6 / 14 42.9%	0 / 22 0.0%	0 / 27 0.0%	1 / 18 5.6%	0 / 9 0.0%	4 / 9 44.4%	1 / 48 2.1%	0 / 9 0.0%	0 / 35 0.0%	2 / 21 9.5%	0 / 12 0.0%	3 / 14 21.4%	1 / 12 8.3%	1 / 29 3.4%	1 / 9 11.1%
2	Anabaena	9 / 105 8.6%	2 / 2 100.0%	0 / 3 0.0%	2 / 4 50.0%	0 / 6 0.0%	0 / 7 0.0%	0 / 4 0.0%	0 / 2 0.0%	1 / 2 50.0%	0 / 10 0.0%	0 / 3 0.0%	0 / 8 0.0%	2 / 6 33.3%	0 / 3 0.0%	0 / 3 0.0%	1 / 3 33.3%	1 / 7 14.3%	0 / 2 0.0%
1	Anabaena cylindrica	7 / 57 12.3%	1 / 1 100.0%	0 / 2 0.0%	1 / 2 50.0%	0 / 3 0.0%	0 / 4 0.0%	0 / 2 0.0%	0 / 1 0.0%	1 / 1 100.0%	0 / 4 0.0%	0 / 2 0.0%	0 / 4 0.0%	2 / 4 50.0%	0 / 2 0.0%	0 / 2 0.0%	1 / 2 50.0%	1 / 4 25.0%	0 / 1 0.0%
1	Anabaena aequalis UTEX 'B 1609'	7 / 57 12.3%	1 / 1 100.0%	0 / 2 0.0%	1 / 2 50.0%	0 / 3 0.0%	0 / 4 0.0%	0 / 2 0.0%	0 / 1 0.0%	1 / 1 100.0%	0 / 4 0.0%	0 / 2 0.0%	0 / 4 0.0%	2 / 4 50.0%	0 / 2 0.0%	0 / 2 0.0%	1 / 2 50.0%	1 / 4 25.0%	0 / 1 0.0%
1	Anabaena variabilis	2 / 48 4.2%	1 / 1 100.0%	0 / 1 0.0%	1 / 2 50.0%	0 / 3 0.0%	0 / 3 0.0%	0 / 2 0.0%	0 / 1 0.0%	0 / 1 0.0%	0 / 6 0.0%	0 / 1 0.0%	0 / 4 0.0%	0 / 2 0.0%	0 / 1 0.0%	0 / 1 0.0%	0 / 1 0.0%	0 / 3 0.0%	0 / 1 0.0%
1	Anabaena ATCC29413	2 / 48 4.2%	1 / 1 100.0%	0 / 1 0.0%	1 / 2 50.0%	0 / 3 0.0%	0 / 3 0.0%	0 / 2 0.0%	0 / 1 0.0%	0 / 1 0.0%	0 / 6 0.0%	0 / 1 0.0%	0 / 4 0.0%	0 / 2 0.0%	0 / 1 0.0%	0 / 1 0.0%	0 / 1 0.0%	0 / 3 0.0%	0 / 1 0.0%
4	Amorphonostoc	19 / 234 8.1%	3 / 3 100.0%	0 / 6 0.0%	3 / 7 42.9%	0 / 13 0.0%	0 / 13 0.0%	1 / 9 11.1%	0 / 5 0.0%	1 / 5 20.0%	0 / 27 0.0%	0 / 4 0.0%	0 / 18 0.0%	0 / 10 0.0%	0 / 6 0.0%	2 / 8 25.0%	0 / 6 0.0%	0 / 15 0.0%	1 / 5 20.0%
1	NOSTOC SP. (STRAIN PCC 7524)	0 / 45 0.0%	0 / 0 0.0%	0 / 1 0.0%	0 / 1 0.0%	0 / 3 0.0%	0 / 3 0.0%	0 / 2 0.0%	0 / 1 0.0%	0 / 1 0.0%	0 / 6 0.0%	0 / 1 0.0%	0 / 4 0.0%	0 / 2 0.0%	0 / 1 0.0%	0 / 1 0.0%	0 / 1 0.0%	0 / 3 0.0%	0 / 1 0.0%
1	Anabaena 7120	2 / 49 4.1%	1 / 1 100.0%	0 / 1 0.0%	1 / 2 50.0%	0 / 3 0.0%	0 / 3 0.0%	0 / 2 0.0%	0 / 1 0.0%	0 / 1 0.0%	0 / 6 0.0%	0 / 1 0.0%	0 / 4 0.0%	0 / 2 0.0%	0 / 1 0.0%	0 / 1 0.0%	0 / 1 0.0%	0 / 3 0.0%	0 / 1 0.0%
1	Nostoc punctiforme	9 / 69 13.0%	1 / 1 100.0%	0 / 2 0.0%	1 / 2 50.0%	0 / 4 0.0%	0 / 3 0.0%	0 / 2 0.0%	0 / 1 0.0%	1 / 1 100.0%	0 / 8 0.0%	0 / 1 0.0%	0 / 5 0.0%	0 / 3 0.0%	0 / 2 0.0%	1 / 3 33.3%	0 / 2 0.0%	0 / 5 0.0%	1 / 2 50.0%
1	Nostoc ATCC 29133	9 / 69 13.0%	1 / 1 100.0%	0 / 2 0.0%	1 / 2 50.0%	0 / 4 0.0%	0 / 3 0.0%	0 / 2 0.0%	0 / 1 0.0%	1 / 1 100.0%	0 / 8 0.0%	0 / 1 0.0%	0 / 5 0.0%	0 / 3 0.0%	0 / 2 0.0%	1 / 3 33.3%	0 / 2 0.0%	0 / 5 0.0%	1 / 2 50.0%
1	Nostoc sp. PCC 7107	8 / 71 11.3%	1 / 1 100.0%	0 / 2 0.0%	1 / 2 50.0%	0 / 3 0.0%	0 / 4 0.0%	1 / 3 33.3%	0 / 2 0.0%	0 / 2 0.0%	0 / 7 0.0%	0 / 1 0.0%	0 / 5 0.0%	0 / 3 0.0%	0 / 2 0.0%	1 / 3 33.3%	0 / 2 0.0%	0 / 4 0.0%	0 / 1 0.0%
1	Cronbergia	6 / 64 9.4%	0 / 0 0.0%	0 / 1 0.0%	1 / 2 50.0%	0 / 2 0.0%	0 / 4 0.0%	0 / 3 0.0%	0 / 1 0.0%	1 / 1 100.0%	1 / 7 14.3%	0 / 1 0.0%	0 / 5 0.0%	0 / 3 0.0%	0 / 2 0.0%	1 / 2 50.0%	0 / 2 0.0%	0 / 4 0.0%	0 / 1 0.0%
1	Cylindrospermum stagnale	6 / 64 9.4%	0 / 0 0.0%	0 / 1 0.0%	1 / 2 50.0%	0 / 2 0.0%	0 / 4 0.0%	0 / 3 0.0%	0 / 1 0.0%	1 / 1 100.0%	1 / 7 14.3%	0 / 1 0.0%	0 / 5 0.0%	0 / 3 0.0%	0 / 2 0.0%	1 / 2 50.0%	0 / 2 0.0%	0 / 4 0.0%	0 / 1 0.0%
1	Cylindrospermum ATCC 29204	6 / 64 9.4%	0 / 0 0.0%	0 / 1 0.0%	1 / 2 50.0%	0 / 2 0.0%	0 / 4 0.0%	0 / 3 0.0%	0 / 1 0.0%	1 / 1 100.0%	1 / 7 14.3%	0 / 1 0.0%	0 / 5 0.0%	0 / 3 0.0%	0 / 2 0.0%	1 / 2 50.0%	0 / 2 0.0%	0 / 4 0.0%	0 / 1 0.0%
1	Trichormus	1 / 42 2.4%	0 / 1 0.0%	0 / 1 0.0%	0 / 1 0.0%	0 / 1 0.0%	0 / 3 0.0%	0 / 2 0.0%	0 / 1 0.0%	1 / 1 100.0%	0 / 4 0.0%	0 / 1 0.0%	0 / 4 0.0%	0 / 2 0.0%	0 / 1 0.0%	0 / 1 0.0%	0 / 1 0.0%	0 / 3 0.0%	0 / 1 0.0%
1	Anabaena azollae	1 / 42 2.4%	0 / 1 0.0%	0 / 1 0.0%	0 / 1 0.0%	0 / 1 0.0%	0 / 3 0.0%	0 / 2 0.0%	0 / 1 0.0%	1 / 1 100.0%	0 / 4 0.0%	0 / 1 0.0%	0 / 4 0.0%	0 / 2 0.0%	0 / 1 0.0%	0 / 1 0.0%	0 / 1 0.0%	0 / 3 0.0%	0 / 1 0.0%
1	'Nostoc azollae' 0708	1 / 42 2.4%	0 / 1 0.0%	0 / 1 0.0%	0 / 1 0.0%	0 / 1 0.0%	0 / 3 0.0%	0 / 2 0.0%	0 / 1 0.0%	1 / 1 100.0%	0 / 4 0.0%	0 / 1 0.0%	0 / 4 0.0%	0 / 2 0.0%	0 / 1 0.0%	0 / 1 0.0%	0 / 1 0.0%	0 / 3 0.0%	0 / 1 0.0%
3	Rivulariaceae	14 / 155 9.0%	3 / 3 100.0%	0 / 4 0.0%	1 / 5 20.0%	0 / 6 0.0%	0 / 8 0.0%	0 / 7 0.0%	0 / 3 0.0%	1 / 3 33.3%	3 / 14 21.4%	0 / 4 0.0%	0 / 15 0.0%	0 / 8 0.0%	0 / 5 0.0%	2 / 5 40.0%	1 / 4 25.0%	1 / 10 10.0%	0 / 1 0.0%
2	Calothrix	9 / 106 8.5%	2 / 2 100.0%	0 / 3 0.0%	1 / 3 33.3%	0 / 3 0.0%	0 / 6 0.0%	0 / 5 0.0%	0 / 2 0.0%	1 / 2 50.0%	0 / 10 0.0%	0 / 3 0.0%	0 / 10 0.0%	0 / 6 0.0%	0 / 4 0.0%	2 / 4 50.0%	1 / 3 33.3%	1 / 7 14.3%	0 / 1 0.0%
1	Calothrix parietina	1 / 40 2.5%	1 / 1 100.0%	0 / 1 0.0%	0 / 1 0.0%	0 / 1 0.0%	0 / 2 0.0%	0 / 2 0.0%	0 / 1 0.0%	0 / 1 0.0%	0 / 3 0.0%	0 / 1 0.0%	0 / 4 0.0%	0 / 2 0.0%	0 / 1 0.0%	0 / 1 0.0%	0 / 1 0.0%	0 / 3 0.0%	0 / 0 0.0%
1	Calothrix parietina 1018	1 / 40 2.5%	1 / 1 100.0%	0 / 1 0.0%	0 / 1 0.0%	0 / 1 0.0%	0 / 2 0.0%	0 / 2 0.0%	0 / 1 0.0%	0 / 1 0.0%	0 / 3 0.0%	0 / 1 0.0%	0 / 4 0.0%	0 / 2 0.0%	0 / 1 0.0%	0 / 1 0.0%	0 / 1 0.0%	0 / 3 0.0%	0 / 0 0.0%
1	Calothrix PCC7507	8 / 66 12.1%	1 / 1 100.0%	0 / 2 0.0%	1 / 2 50.0%	0 / 2 0.0%	0 / 4 0.0%	0 / 3 0.0%	0 / 1 0.0%	1 / 1 100.0%	0 / 7 0.0%	0 / 2 0.0%	0 / 6 0.0%	0 / 4 0.0%	0 / 3 0.0%	2 / 3 66.7%	1 / 2 50.0%	1 / 4 25.0%	0 / 1 0.0%
1	Rivularia	5 / 49 10.2%	1 / 1 100.0%	0 / 1 0.0%	0 / 2 0.0%	0 / 3 0.0%	0 / 2 0.0%	0 / 2 0.0%	0 / 1 0.0%	0 / 1 0.0%	3 / 4 75.0%	0 / 1 0.0%	0 / 5 0.0%	0 / 2 0.0%	0 / 1 0.0%	0 / 1 0.0%	0 / 1 0.0%	0 / 3 0.0%	0 / 0 0.0%
1	Calothrix sp. ATCC 29111	5 / 49 10.2%	1 / 1 100.0%	0 / 1 0.0%	0 / 2 0.0%	0 / 3 0.0%	0 / 2 0.0%	0 / 2 0.0%	0 / 1 0.0%	0 / 1 0.0%	3 / 4 75.0%	0 / 1 0.0%	0 / 5 0.0%	0 / 2 0.0%	0 / 1 0.0%	0 / 1 0.0%	0 / 1 0.0%	0 / 3 0.0%	0 / 0 0.0%
12	"Prochlorales" Lewin 1977	16 / 467 3.4%	11 / 12 91.7%	0 / 12 0.0%	0 / 12 0.0%	0 / 14 0.0%	0 / 30 0.0%	0 / 26 0.0%	0 / 12 0.0%	0 / 12 0.0%	0 / 28 0.0%	0 / 12 0.0%	1 / 48 2.1%	0 / 12 0.0%	3 / 12 25.0%	0 / 12 0.0%	0 / 12 0.0%	1 / 26 3.8%	0 / 12 0.0%
12	'Prochlorococcaceae'	16 / 467 3.4%	11 / 12 91.7%	0 / 12 0.0%	0 / 12 0.0%	0 / 14 0.0%	0 / 30 0.0%	0 / 26 0.0%	0 / 12 0.0%	0 / 12 0.0%	0 / 28 0.0%	0 / 12 0.0%	1 / 48 2.1%	0 / 12 0.0%	3 / 12 25.0%	0 / 12 0.0%	0 / 12 0.0%	1 / 26 3.8%	0 / 12 0.0%
12	Prochlorococcus	16 / 467 3.4%	11 / 12 91.7%	0 / 12 0.0%	0 / 12 0.0%	0 / 14 0.0%	0 / 30 0.0%	0 / 26 0.0%	0 / 12 0.0%	0 / 12 0.0%	0 / 28 0.0%	0 / 12 0.0%	1 / 48 2.1%	0 / 12 0.0%	3 / 12 25.0%	0 / 12 0.0%	0 / 12 0.0%	1 / 26 3.8%	0 / 12 0.0%
12	CCMP1375	16 / 467 3.4%	11 / 12 91.7%	0 / 12 0.0%	0 / 12 0.0%	0 / 14 0.0%	0 / 30 0.0%	0 / 26 0.0%	0 / 12 0.0%	0 / 12 0.0%	0 / 28 0.0%	0 / 12 0.0%	1 / 48 2.1%	0 / 12 0.0%	3 / 12 25.0%	0 / 12 0.0%	0 / 12 0.0%	1 / 26 3.8%	0 / 12 0.0%
1	Prochlorococcus marinus str. NATL2A	2 / 38 5.3%	1 / 1 100.0%	0 / 1 0.0%	0 / 1 0.0%	0 / 1 0.0%	0 / 3 0.0%	0 / 2 0.0%	0 / 1 0.0%	0 / 1 0.0%	0 / 2 0.0%	0 / 1 0.0%	0 / 4 0.0%	0 / 1 0.0%	1 / 1 100.0%	0 / 1 0.0%	0 / 1 0.0%	0 / 2 0.0%	0 / 1 0.0%
1	Prochlorococcus marinus str. MIT 9303	1 / 44 2.3%	1 / 1 100.0%	0 / 1 0.0%	0 / 1 0.0%	0 / 2 0.0%	0 / 3 0.0%	0 / 3 0.0%	0 / 1 0.0%	0 / 1 0.0%	0 / 4 0.0%	0 / 1 0.0%	0 / 4 0.0%	0 / 1 0.0%	0 / 1 0.0%	0 / 1 0.0%	0 / 1 0.0%	0 / 3 0.0%	0 / 1 0.0%
1	Prochlorococcus marinus str. MIT 9312	2 / 37 5.4%	1 / 1 100.0%	0 / 1 0.0%	0 / 1 0.0%	0 / 1 0.0%	0 / 2 0.0%	0 / 2 0.0%	0 / 1 0.0%	0 / 1 0.0%	0 / 2 0.0%	0 / 1 0.0%	0 / 4 0.0%	0 / 1 0.0%	0 / 1 0.0%	0 / 1 0.0%	0 / 1 0.0%	1 / 2 50.0%	0 / 1 0.0%
1	Prochlorococcus marinus MIT9313	0 / 44 0.0%	0 / 1 0.0%	0 / 1 0.0%	0 / 1 0.0%	0 / 2 0.0%	0 / 3 0.0%	0 / 3 0.0%	0 / 1 0.0%	0 / 1 0.0%	0 / 4 0.0%	0 / 1 0.0%	0 / 4 0.0%	0 / 1 0.0%	0 / 1 0.0%	0 / 1 0.0%	0 / 1 0.0%	0 / 3 0.0%	0 / 1 0.0%
1	Prochlorococcus marinus str. MIT 9211	1 / 40 2.5%	1 / 1 100.0%	0 / 1 0.0%	0 / 1 0.0%	0 / 1 0.0%	0 / 3 0.0%	0 / 2 0.0%	0 / 1 0.0%	0 / 1 0.0%	0 / 2 0.0%	0 / 1 0.0%	0 / 4 0.0%	0 / 1 0.0%	0 / 1 0.0%	0 / 1 0.0%	0 / 1 0.0%	0 / 2 0.0%	0 / 1 0.0%
1	Prochlorococcus marinus MIT 9215	1 / 38 2.6%	1 / 1 100.0%	0 / 1 0.0%	0 / 1 0.0%	0 / 1 0.0%	0 / 2 0.0%	0 / 2 0.0%	0 / 1 0.0%	0 / 1 0.0%	0 / 2 0.0%	0 / 1 0.0%	0 / 4 0.0%	0 / 1 0.0%	0 / 1 0.0%	0 / 1 0.0%	0 / 1 0.0%	0 / 2 0.0%	0 / 1 0.0%
1	ATCC 700925	2 / 37 5.4%	1 / 1 100.0%	0 / 1 0.0%	0 / 1 0.0%	0 / 1 0.0%	0 / 2 0.0%	0 / 2 0.0%	0 / 1 0.0%	0 / 1 0.0%	0 / 2 0.0%	0 / 1 0.0%	1 / 4 25.0%	0 / 1 0.0%	0 / 1 0.0%	0 / 1 0.0%	0 / 1 0.0%	0 / 2 0.0%	0 / 1 0.0%
1	Prochlorococcus marinus MED4	2 / 37 5.4%	1 / 1 100.0%	0 / 1 0.0%	0 / 1 0.0%	0 / 1 0.0%	0 / 2 0.0%	0 / 2 0.0%	0 / 1 0.0%	0 / 1 0.0%	0 / 2 0.0%	0 / 1 0.0%	1 / 4 25.0%	0 / 1 0.0%	0 / 1 0.0%	0 / 1 0.0%	0 / 1 0.0%	0 / 2 0.0%	0 / 1 0.0%
1	Prochlorococcus marinus subsp. marinus	2 / 39 5.1%	1 / 1 100.0%	0 / 1 0.0%	0 / 1 0.0%	0 / 1 0.0%	0 / 3 0.0%	0 / 2 0.0%	0 / 1 0.0%	0 / 1 0.0%	0 / 2 0.0%	0 / 1 0.0%	0 / 4 0.0%	0 / 1 0.0%	1 / 1 100.0%	0 / 1 0.0%	0 / 1 0.0%	0 / 2 0.0%	0 / 1 0.0%
1	Prochlorococcus marinus SS120	2 / 39 5.1%	1 / 1 100.0%	0 / 1 0.0%	0 / 1 0.0%	0 / 1 0.0%	0 / 3 0.0%	0 / 2 0.0%	0 / 1 0.0%	0 / 1 0.0%	0 / 2 0.0%	0 / 1 0.0%	0 / 4 0.0%	0 / 1 0.0%	1 / 1 100.0%	0 / 1 0.0%	0 / 1 0.0%	0 / 2 0.0%	0 / 1 0.0%
1	Prochlorococcus marinus str. AS9601	1 / 38 2.6%	1 / 1 100.0%	0 / 1 0.0%	0 / 1 0.0%	0 / 1 0.0%	0 / 2 0.0%	0 / 2 0.0%	0 / 1 0.0%	0 / 1 0.0%	0 / 2 0.0%	0 / 1 0.0%	0 / 4 0.0%	0 / 1 0.0%	0 / 1 0.0%	0 / 1 0.0%	0 / 1 0.0%	0 / 2 0.0%	0 / 1 0.0%
1	Prochlorococcus marinus str. MIT 9515	1 / 37 2.7%	1 / 1 100.0%	0 / 1 0.0%	0 / 1 0.0%	0 / 1 0.0%	0 / 2 0.0%	0 / 2 0.0%	0 / 1 0.0%	0 / 1 0.0%	0 / 2 0.0%	0 / 1 0.0%	0 / 4 0.0%	0 / 1 0.0%	0 / 1 0.0%	0 / 1 0.0%	0 / 1 0.0%	0 / 2 0.0%	0 / 1 0.0%
1	Prochlorococcus marinus str. MIT 9301	1 / 37 2.7%	1 / 1 100.0%	0 / 1 0.0%	0 / 1 0.0%	0 / 1 0.0%	0 / 2 0.0%	0 / 2 0.0%	0 / 1 0.0%	0 / 1 0.0%	0 / 2 0.0%	0 / 1 0.0%	0 / 4 0.0%	0 / 1 0.0%	0 / 1 0.0%	0 / 1 0.0%	0 / 1 0.0%	0 / 2 0.0%	0 / 1 0.0%
1	Prochlorococcus marinus str. NATL1A	2 / 38 5.3%	1 / 1 100.0%	0 / 1 0.0%	0 / 1 0.0%	0 / 1 0.0%	0 / 3 0.0%	0 / 2 0.0%	0 / 1 0.0%	0 / 1 0.0%	0 / 2 0.0%	0 / 1 0.0%	0 / 4 0.0%	0 / 1 0.0%	1 / 1 100.0%	0 / 1 0.0%	0 / 1 0.0%	0 / 2 0.0%	0 / 1 0.0%
3	Cyanobacteria Subsection II	5 / 127 3.9%	1 / 3 33.3%	0 / 3 0.0%	0 / 3 0.0%	0 / 7 0.0%	0 / 8 0.0%	0 / 7 0.0%	0 / 3 0.0%	2 / 3 66.7%	2 / 10 20.0%	0 / 3 0.0%	0 / 12 0.0%	0 / 5 0.0%	0 / 3 0.0%	0 / 3 0.0%	0 / 3 0.0%	0 / 9 0.0%	0 / 2 0.0%
1	Pleurocapsa	1 / 42 2.4%	0 / 1 0.0%	0 / 1 0.0%	0 / 1 0.0%	0 / 2 0.0%	0 / 3 0.0%	0 / 3 0.0%	0 / 1 0.0%	1 / 1 100.0%	0 / 3 0.0%	0 / 1 0.0%	0 / 4 0.0%	0 / 2 0.0%	0 / 1 0.0%	0 / 1 0.0%	0 / 1 0.0%	0 / 3 0.0%	0 / 0 0.0%
1	Pleurocapsa minor	1 / 42 2.4%	0 / 1 0.0%	0 / 1 0.0%	0 / 1 0.0%	0 / 2 0.0%	0 / 3 0.0%	0 / 3 0.0%	0 / 1 0.0%	1 / 1 100.0%	0 / 3 0.0%	0 / 1 0.0%	0 / 4 0.0%	0 / 2 0.0%	0 / 1 0.0%	0 / 1 0.0%	0 / 1 0.0%	0 / 3 0.0%	0 / 0 0.0%
1	Pleurocapsa PCC7327	1 / 42 2.4%	0 / 1 0.0%	0 / 1 0.0%	0 / 1 0.0%	0 / 2 0.0%	0 / 3 0.0%	0 / 3 0.0%	0 / 1 0.0%	1 / 1 100.0%	0 / 3 0.0%	0 / 1 0.0%	0 / 4 0.0%	0 / 2 0.0%	0 / 1 0.0%	0 / 1 0.0%	0 / 1 0.0%	0 / 3 0.0%	0 / 0 0.0%
1	Chroococcidiopsis	1 / 43 2.3%	1 / 1 100.0%	0 / 1 0.0%	0 / 1 0.0%	0 / 3 0.0%	0 / 3 0.0%	0 / 2 0.0%	0 / 1 0.0%	0 / 1 0.0%	0 / 3 0.0%	0 / 1 0.0%	0 / 4 0.0%	0 / 1 0.0%	0 / 1 0.0%	0 / 1 0.0%	0 / 1 0.0%	0 / 3 0.0%	0 / 1 0.0%
1	Chroococcidiopsis thermalis	1 / 43 2.3%	1 / 1 100.0%	0 / 1 0.0%	0 / 1 0.0%	0 / 3 0.0%	0 / 3 0.0%	0 / 2 0.0%	0 / 1 0.0%	0 / 1 0.0%	0 / 3 0.0%	0 / 1 0.0%	0 / 4 0.0%	0 / 1 0.0%	0 / 1 0.0%	0 / 1 0.0%	0 / 1 0.0%	0 / 3 0.0%	0 / 1 0.0%
1	Chroococcidiopsis sp. ATCC 27900	1 / 43 [illegible]	1 / 1 [illegible]	0 / 1 [illegible]	0 / 1 [illegible]	0 / 3 [illegible]	0 / 3 [illegible]	0 / 2 [illegible]	0 / 1 [illegible]	0 / 1 [illegible]	0 / 3 [illegible]	0 / 1 [illegible]	0 / 4 [illegible]	0 / 1 [illegible]	0 / 1 [illegible]	0 / 1 [illegible]	0 / 1 [illegible]	0 / 3 [illegible]	0 / 1 [illegible]

No. Genomes	Taxon	Thr	Arg	Glu	xIle	Leu	SeC	Pyl	Pseudo	Stop											
2323	all	4814 / 7643 63.0%	7031 / 11399 61.7%	3866 / 6266 61.7%	1695 / 2764 61.3%	8013 / 13116 61.1%	505 / 573 88.1%	4 / 4 100.0%	19 / 68 27.9%	6 / 10 60.0%	54763 / 132129 41.4%AB	27920 / 132129 21.1%ABE	21294 / 132129 16.1%E	21263 / 132129 16.1%	2866 / 132129 2.2%B	1609 / 132129 1.2%BE	1260 / 132129 1.0%C	1145 / 132129 0.9%CE	4 / 132129 0.0%GG	3 / 132129 0.0%D	2 / 132129 0.0%EGG
2323	biota	4814 / 7643 63.0%	7031 / 11399 61.7%	3866 / 6266 61.7%	1695 / 2764 61.3%	8013 / 13116 61.1%	505 / 573 88.1%	4 / 4 100.0%	19 / 68 27.9%	6 / 10 60.0%	54763 / 132129 41.4%AB	27920 / 132129 21.1%ABE	21294 / 132129 16.1%E	21263 / 132129 16.1%	2866 / 132129 2.2%B	1609 / 132129 1.2%BE	1260 / 132129 1.0%C	1145 / 132129 0.9%CE	4 / 132129 0.0%GG	3 / 132129 0.0%D	2 / 132129 0.0%EGG
2276	Bacteria	4779 / 7506 63.7%	6968 / 11186 62.3%	3834 / 6171 62.1%	1685 / 2717 62.0%	7943 / 12894 61.6%	505 / 566 89.2%	0 / 0 0.0%	19 / 68 27.9%	6 / 10 60.0%	54413 / 129989 41.9%AB	27620 / 129989 21.2%ABE	20629 / 129989 15.9%E	20502 / 129989 15.8%	2866 / 129989 2.2%B	1609 / 129989 1.2%BE	1225 / 129989 0.9%C	1116 / 129989 0.9%CE	4 / 129989 0.0%GG	3 / 129989 0.0%D	2 / 129989 0.0%EGG
69	Cyanobacteria	2 / 230 0.9%	2 / 287 0.7%	3 / 82 3.7%	4 / 93 4.3%	12 / 367 3.3%	0 / 0 0.0%	0 / 0 0.0%	0 / 12 0.0%	0 / 1 0.0%	1636 / 3264 50.1%	1327 / 3264 40.7%E	103 / 3264 3.2%C	101 / 3264 3.1%AB	44 / 3264 1.3%CE	42 / 3264 1.3%ABE	7 / 3264 0.2%B	3 / 3264 0.1%BE	1 / 3264 0.0%EGG		
11	Nostocales	2 / 39 5.1%	0 / 48 0.0%	0 / 15 0.0%	3 / 17 17.6%	6 / 65 9.2%	0 / 0 0.0%	0 / 0 0.0%	0 / 5 0.0%	0 / 0 0.0%	268 / 600 44.7%	236 / 600 39.3%E	40 / 600 6.7%C	31 / 600 5.2%AB	12 / 600 2.0%ABE	7 / 600 1.2%CE	4 / 600 0.7%B	2 / 600 0.3%BE			
8	Nostocaceae	1 / 28 3.6%	0 / 35 0.0%	0 / 12 0.0%	2 / 11 18.2%	6 / 49 12.2%	0 / 0 0.0%	0 / 0 0.0%	0 / 4 0.0%	0 / 0 0.0%	204 / 445 45.8%	167 / 445 37.5%E	32 / 445 7.2%C	22 / 445 4.9%AB	7 / 445 1.6%CE	7 / 445 1.6%ABE	4 / 445 0.9%B	2 / 445 0.4%BE			
2	Anabaena	0 / 6 0.0%	0 / 8 0.0%	0 / 2 0.0%	0 / 2 0.0%	0 / 11 0.0%	0 / 0 0.0%	0 / 0 0.0%	0 / 1 0.0%	0 / 0 0.0%	51 / 105 48.6%	38 / 105 36.2%E	7 / 105 6.7%AB	6 / 105 5.7%C	2 / 105 1.9%ABE	1 / 105 1.0%CE					
1	Anabaena cylindrica	0 / 3 0.0%	0 / 4 0.0%	0 / 1 0.0%	0 / 1 0.0%	0 / 6 0.0%	0 / 0 0.0%	0 / 0 0.0%	0 / 1 0.0%	0 / 0 0.0%	24 / 57 42.1%	23 / 57 40.4%E	5 / 57 8.8%AB	3 / 57 5.3%C	2 / 57 3.5%ABE						
1	Anabaena aequalis UTEX 'B 1609'	0 / 3 0.0%	0 / 4 0.0%	0 / 1 0.0%	0 / 1 0.0%	0 / 6 0.0%	0 / 0 0.0%	0 / 0 0.0%	0 / 1 0.0%	0 / 0 0.0%	24 / 57 42.1%	23 / 57 40.4%E	5 / 57 8.8%AB	3 / 57 5.3%C	2 / 57 3.5%ABE						
1	Anabaena variabilis	0 / 3 0.0%	0 / 4 0.0%	0 / 1 0.0%	0 / 1 0.0%	0 / 5 0.0%	0 / 0 0.0%	0 / 0 0.0%	0 / 0 0.0%	0 / 0 0.0%	27 / 48 56.2%	15 / 48 31.2%E	3 / 48 6.2%C	2 / 48 4.2%AB	1 / 48 2.1%CE						
1	Anabaena ATCC29413	0 / 3 0.0%	0 / 4 0.0%	0 / 1 0.0%	0 / 1 0.0%	0 / 5 0.0%	0 / 0 0.0%	0 / 0 0.0%	0 / 0 0.0%	0 / 0 0.0%	27 / 48 56.2%	15 / 48 31.2%E	3 / 48 6.2%C	2 / 48 4.2%AB	1 / 48 2.1%CE						
4	Amorphonostoc	0 / 14 0.0%	0 / 18 0.0%	0 / 7 0.0%	2 / 6 33.3%	5 / 27 18.5%	0 / 0 0.0%	0 / 0 0.0%	0 / 1 0.0%	0 / 0 0.0%	99 / 234 42.3%	89 / 234 38.0%E	22 / 234 9.4%C	11 / 234 4.7%AB	5 / 234 2.1%CE	4 / 234 1.7%B	2 / 234 0.9%ABE	2 / 234 0.9%BE			
1	NOSTOC SP. (STRAIN PCC 7524)	0 / 3 0.0%	0 / 4 0.0%	0 / 1 0.0%	0 / 1 0.0%	0 / 4 0.0%	0 / 0 0.0%	0 / 0 0.0%	0 / 0 0.0%	0 / 0 0.0%	20 / 45 44.4%E	18 / 45 40.0%	6 / 45 13.3%C	1 / 45 2.2%CE							
1	Anabaena 7120	0 / 3 0.0%	0 / 4 0.0%	0 / 1 0.0%	0 / 1 0.0%	0 / 6 0.0%	0 / 0 0.0%	0 / 0 0.0%	0 / 0 0.0%	0 / 0 0.0%	28 / 49 57.1%	15 / 49 30.6%E	3 / 49 6.1%C	2 / 49 4.1%AB	1 / 49 2.0%CE						
1	Nostoc punctiforme	0 / 4 0.0%	0 / 5 0.0%	0 / 2 0.0%	1 / 2 50.0%	3 / 9 33.3%	0 / 0 0.0%	0 / 0 0.0%	0 / 0 0.0%	0 / 0 0.0%	27 / 69 39.1%E	26 / 69 37.7%	7 / 69 10.1%C	4 / 69 5.8%B	2 / 69 2.9%AB	2 / 69 2.9%BE	1 / 69 1.4%ABE				
1	Nostoc ATCC 29133	0 / 4 0.0%	0 / 5 0.0%	0 / 2 0.0%	1 / 2 50.0%	3 / 9 33.3%	0 / 0 0.0%	0 / 0 0.0%	0 / 0 0.0%	0 / 0 0.0%	27 / 69 39.1%E	26 / 69 37.7%	7 / 69 10.1%C	4 / 69 5.8%B	2 / 69 2.9%BE	2 / 69 2.9%AB	1 / 69 1.4%ABE				
1	Nostoc sp. PCC 7107	0 / 4 0.0%	0 / 5 0.0%	0 / 3 0.0%	1 / 2 50.0%	2 / 8 25.0%	0 / 0 0.0%	0 / 0 0.0%	0 / 1 0.0%	0 / 0 0.0%	27 / 71 38.0%	27 / 71 38.0%E	7 / 71 9.9%AB	6 / 71 8.5%C	3 / 71 4.2%CE	1 / 71 1.4%ABE					
1	Cronbergia	1 / 5 20.0%	0 / 5 0.0%	0 / 2 0.0%	0 / 2 0.0%	1 / 7 14.3%	0 / 0 0.0%	0 / 0 0.0%	0 / 2 0.0%	0 / 0 0.0%	30 / 64 46.9%	23 / 64 35.9%E	4 / 64 6.2%C	4 / 64 6.2%AB	2 / 64 3.1%ABE	1 / 64 1.6%CE					
1	Cylindrospermum stagnale	1 / 5 20.0%	0 / 5 0.0%	0 / 2 0.0%	0 / 2 0.0%	1 / 7 14.3%	0 / 0 0.0%	0 / 0 0.0%	0 / 2 0.0%	0 / 0 0.0%	30 / 64 46.9%	23 / 64 35.9%E	4 / 64 6.2%AB	4 / 64 6.2%C	2 / 64 3.1%ABE	1 / 64 1.6%CE					
1	Cylindrospermum ATCC 29204	1 / 5 20.0%	0 / 5 0.0%	0 / 2 0.0%	0 / 2 0.0%	1 / 7 14.3%	0 / 0 0.0%	0 / 0 0.0%	0 / 2 0.0%	0 / 0 0.0%	30 / 64 46.9%	23 / 64 35.9%E	4 / 64 6.2%C	4 / 64 6.2%AB	2 / 64 3.1%ABE	1 / 64 1.6%CE					
1	Trichormus	0 / 3 0.0%	0 / 4 0.0%	0 / 1 0.0%	0 / 1 0.0%	0 / 4 0.0%	0 / 0 0.0%	0 / 0 0.0%	0 / 0 0.0%	0 / 0 0.0%	24 / 42 57.1%	17 / 42 40.5%E	1 / 42 2.4%ABE								
1	Anabaena azollae	0 / 3 0.0%	0 / 4 0.0%	0 / 1 0.0%	0 / 1 0.0%	0 / 4 0.0%	0 / 0 0.0%	0 / 0 0.0%	0 / 0 0.0%	0 / 0 0.0%	24 / 42 57.1%	17 / 42 40.5%E	1 / 42 2.4%ABE								
1	'Nostoc azollae' 0708	0 / 3 0.0%	0 / 4 0.0%	0 / 1 0.0%	0 / 1 0.0%	0 / 4 0.0%	0 / 0 0.0%	0 / 0 0.0%	0 / 0 0.0%	0 / 0 0.0%	24 / 42 57.1%	17 / 42 40.5%E	1 / 42 2.4%ABE								
3	Rivulariaceae	1 / 11 9.1%	0 / 13 0.0%	0 / 3 0.0%	1 / 6 16.7%	0 / 16 0.0%	0 / 0 0.0%	0 / 0 0.0%	0 / 1 0.0%	0 / 0 0.0%	69 / 155 44.5%E	64 / 155 41.3%	9 / 155 5.8%AB	8 / 155 5.2%C	5 / 155 3.2%ABE						
2	Calothrix	1 / 8 12.5%	0 / 8 0.0%	0 / 2 0.0%	0 / 3 0.0%	0 / 11 0.0%	0 / 0 0.0%	0 / 0 0.0%	0 / 0 0.0%	0 / 0 0.0%	48 / 106 45.3%E	45 / 106 42.5%	5 / 106 4.7%ABE	4 / 106 3.8%C	4 / 106 3.8%AB						
1	Calothrix parietina	0 / 3 0.0%	0 / 4 0.0%	0 / 1 0.0%	0 / 2 0.0%	0 / 4 0.0%	0 / 0 0.0%	0 / 0 0.0%	0 / 0 0.0%	0 / 0 0.0%	20 / 40 50.0%E	18 / 40 45.0%	1 / 40 2.5%AB	1 / 40 2.5%C							
1	Calothrix parietina 1018	0 / 3 0.0%	0 / 4 0.0%	0 / 1 0.0%	0 / 2 0.0%	0 / 4 0.0%	0 / 0 0.0%	0 / 0 0.0%	0 / 0 0.0%	0 / 0 0.0%	20 / 40 50.0%E	18 / 40 45.0%	1 / 40 2.5%C	1 / 40 2.5%AB							
1	Calothrix PCC7507	1 / 5 20.0%	0 / 4 0.0%	0 / 1 0.0%	0 / 1 0.0%	0 / 7 0.0%	0 / 0 0.0%	0 / 0 0.0%	0 / 0 0.0%	0 / 0 0.0%	28 / 66 42.4%E	27 / 66 40.9%	5 / 66 7.6%ABE	3 / 66 4.5%C	3 / 66 4.5%AB						
1	Rivularia	0 / 3 0.0%	0 / 5 0.0%	0 / 1 0.0%	1 / 3 33.3%	0 / 5 0.0%	0 / 0 0.0%	0 / 0 0.0%	0 / 1 0.0%	0 / 0 0.0%	21 / 49 42.9%E	19 / 49 38.8%	5 / 49 10.2%AB	4 / 49 8.2%C							
1	Calothrix sp. ATCC 29111	0 / 3 0.0%	0 / 5 0.0%	0 / 1 0.0%	1 / 3 33.3%	0 / 5 0.0%	0 / 0 0.0%	0 / 0 0.0%	0 / 1 0.0%	0 / 0 0.0%	21 / 49 42.9%E	19 / 49 38.8%	5 / 49 10.2%AB	4 / 49 8.2%C							
12	"Prochlorales" Lewin 1977	0 / 39 0.0%	0 / 48 0.0%	0 / 12 0.0%	0 / 12 0.0%	0 / 52 0.0%	0 / 0 0.0%	0 / 0 0.0%	0 / 0 0.0%	0 / 0 0.0%	241 / 467 51.6%	199 / 467 42.6%E	14 / 467 3.0%AB	7 / 467 1.5%C	4 / 467 0.9%CE	2 / 467 0.4%ABE					
12	'Prochlorococcaceae'	0 / 39 0.0%	0 / 48 0.0%	0 / 12 0.0%	0 / 12 0.0%	0 / 52 0.0%	0 / 0 0.0%	0 / 0 0.0%	0 / 0 0.0%	0 / 0 0.0%	241 / 467 51.6%	199 / 467 42.6%E	14 / 467 3.0%AB	7 / 467 1.5%C	4 / 467 0.9%CE	2 / 467 0.4%ABE					
12	Prochlorococcus	0 / 39 0.0%	0 / 48 0.0%	0 / 12 0.0%	0 / 12 0.0%	0 / 52 0.0%	0 / 0 0.0%	0 / 0 0.0%	0 / 0 0.0%	0 / 0 0.0%	241 / 467 51.6%	199 / 467 42.6%E	14 / 467 3.0%AB	7 / 467 1.5%C	4 / 467 0.9%CE	2 / 467 0.4%ABE					
12	CCMP1375	0 / 39 0.0%	0 / 48 0.0%	0 / 12 0.0%	0 / 12 0.0%	0 / 52 0.0%	0 / 0 0.0%	0 / 0 0.0%	0 / 0 0.0%	0 / 0 0.0%	241 / 467 51.6%	199 / 467 42.6%E	14 / 467 3.0%AB	7 / 467 1.5%C	4 / 467 0.9%CE	2 / 467 0.4%ABE					
1	Prochlorococcus marinus str. NATL2A	0 / 3 0.0%	0 / 4 0.0%	0 / 1 0.0%	0 / 1 0.0%	0 / 4 0.0%	0 / 0 0.0%	0 / 0 0.0%	0 / 0 0.0%	0 / 0 0.0%	22 / 38 57.9%	14 / 38 36.8%E	2 / 38 5.3%AB								
1	Prochlorococcus marinus str. MIT 9303	0 / 3 0.0%	0 / 4 0.0%	0 / 1 0.0%	0 / 1 0.0%	0 / 5 0.0%	0 / 0 0.0%	0 / 0 0.0%	0 / 0 0.0%	0 / 0 0.0%	26 / 44 59.1%	16 / 44 36.4%E	1 / 44 2.3%CE	1 / 44 2.3%AB							
1	Prochlorococcus marinus str. MIT 9312	0 / 3 0.0%	0 / 4 0.0%	0 / 1 0.0%	0 / 1 0.0%	0 / 4 0.0%	0 / 0 0.0%	0 / 0 0.0%	0 / 0 0.0%	0 / 0 0.0%	20 / 37 54.1%	14 / 37 37.8%E	1 / 37 2.7%ABE	1 / 37 2.7%AB	1 / 37 2.7%C						
1	Prochlorococcus marinus MIT9313	0 / 3 0.0%	0 / 4 0.0%	0 / 1 0.0%	0 / 1 0.0%	0 / 5 0.0%	0 / 0 0.0%	0 / 0 0.0%	0 / 0 0.0%	0 / 0 0.0%	26 / 44 59.1%	16 / 44 36.4%E	1 / 44 2.3%CE	1 / 44 2.3%C							
1	Prochlorococcus marinus str. MIT 9211	0 / 4 0.0%	0 / 4 0.0%	0 / 1 0.0%	0 / 1 0.0%	0 / 5 0.0%	0 / 0 0.0%	0 / 0 0.0%	0 / 0 0.0%	0 / 0 0.0%	26 / 40 65.0%E	12 / 40 30.0%	1 / 40 2.5%AB	1 / 40 2.5%CE							
1	Prochlorococcus marinus MIT 9215	0 / 4 0.0%	0 / 4 0.0%	0 / 1 0.0%	0 / 1 0.0%	0 / 4 0.0%	0 / 0 0.0%	0 / 0 0.0%	0 / 0 0.0%	0 / 0 0.0%	26 / 38 68.4%E	10 / 38 26.3%	1 / 38 2.6%C	1 / 38 2.6%AB							
1	ATCC 700925	0 / 3 0.0%	0 / 4 0.0%	0 / 1 0.0%	0 / 1 0.0%	0 / 4 0.0%	0 / 0 0.0%	0 / 0 0.0%	0 / 0 0.0%	0 / 0 0.0%	20 / 37 54.1%	14 / 37 37.8%E	1 / 37 2.7%C	1 / 37 2.7%AB	1 / 37 2.7%ABE						
1	Prochlorococcus marinus MED4	0 / 3 0.0%	0 / 4 0.0%	0 / 1 0.0%	0 / 1 0.0%	0 / 4 0.0%	0 / 0 0.0%	0 / 0 0.0%	0 / 0 0.0%	0 / 0 0.0%	20 / 37 54.1%	14 / 37 37.8%E	1 / 37 2.7%AB	1 / 37 2.7%C	1 / 37 2.7%ABE						
1	Prochlorococcus marinus subsp. marinus	0 / 3 0.0%	0 / 4 0.0%	0 / 1 0.0%	0 / 1 0.0%	0 / 5 0.0%	0 / 0 0.0%	0 / 0 0.0%	0 / 0 0.0%	0 / 0 0.0%	22 / 39 56.4%	14 / 39 35.9%E	2 / 39 5.1%AB	1 / 39 2.6%CE							
1	Prochlorococcus marinus SS120	0 / 3 0.0%	0 / 4 0.0%	0 / 1 0.0%	0 / 1 0.0%	0 / 5 0.0%	0 / 0 0.0%	0 / 0 0.0%	0 / 0 0.0%	0 / 0 0.0%	22 / 39 56.4%	14 / 39 35.9%E	2 / 39 5.1%AB	1 / 39 2.6%CE							
1	Prochlorococcus marinus str. AS9601	0 / 4 0.0%	0 / 4 0.0%	0 / 1 0.0%	0 / 1 0.0%	0 / 4 0.0%	0 / 0 0.0%	0 / 0 0.0%	0 / 0 0.0%	0 / 0 0.0%	21 / 38 55.3%	15 / 38 39.5%E	1 / 38 2.6%C	1 / 38 2.6%AB							
1	Prochlorococcus marinus str. MIT 9515	0 / 3 0.0%	0 / 4 0.0%	0 / 1 0.0%	0 / 1 0.0%	0 / 4 0.0%	0 / 0 0.0%	0 / 0 0.0%	0 / 0 0.0%	0 / 0 0.0%	20 / 37 54.1%	15 / 37 40.5%E	1 / 37 2.7%AB	1 / 37 2.7%C							
1	Prochlorococcus marinus str. MIT 9301	0 / 3 0.0%	0 / 4 0.0%	0 / 1 0.0%	0 / 1 0.0%	0 / 4 0.0%	0 / 0 0.0%	0 / 0 0.0%	0 / 0 0.0%	0 / 0 0.0%	20 / 37 54.1%	15 / 37 40.5%E	1 / 37 2.7%AB	1 / 37 2.7%C							
1	Prochlorococcus marinus str. NATL1A	0 / 3 0.0%	0 / 4 0.0%	0 / 1 0.0%	0 / 1 0.0%	0 / 4 0.0%	0 / 0 0.0%	0 / 0 0.0%	0 / 0 0.0%	0 / 0 0.0%	22 / 38 57.9%	14 / 38 36.8%E	2 / 38 5.3%AB								
3	Cyanobacteria Subsection II	0 / 9 0.0%	0 / 10 0.0%	0 / 3 0.0%	0 / 4 0.0%	0 / 14 0.0%	0 / 0 0.0%	0 / 0 0.0%	0 / 0 0.0%	0 / 0 0.0%	60 / 127 47.2%	53 / 127 41.7%E	7 / 127 5.5%C	3 / 127 2.4%AB	2 / 127 1.6%CE	2 / 127 1.6%ABE					
1	Pleurocapsa	0 / 3 0.0%	0 / 3 0.0%	0 / 1 0.0%	0 / 2 0.0%	0 / 4 0.0%	0 / 0 0.0%	0 / 0 0.0%	0 / 0 0.0%	0 / 0 0.0%	21 / 42 50.0%	16 / 42 38.1%E	2 / 42 4.8%C	2 / 42 4.8%CE	1 / 42 2.4%ABE						
1	Pleurocapsa minor	0 / 3 0.0%	0 / 3 0.0%	0 / 1 0.0%	0 / 2 0.0%	0 / 4 0.0%	0 / 0 0.0%	0 / 0 0.0%	0 / 0 0.0%	0 / 0 0.0%	21 / 42 50.0%	16 / 42 38.1%E	2 / 42 4.8%CE	2 / 42 4.8%C	1 / 42 2.4%ABE						
1	Pleurocapsa PCC7327	0 / 3 0.0%	0 / 3 0.0%	0 / 1 0.0%	0 / 2 0.0%	0 / 4 0.0%	0 / 0 0.0%	0 / 0 0.0%	0 / 0 0.0%	0 / 0 0.0%	21 / 42 50.0%	16 / 42 38.1%E	2 / 42 4.8%CE	2 / 42 4.8%C	1 / 42 2.4%ABE						
1	Chroococcidiopsis	0 / 3 0.0%	0 / 4 0.0%	0 / 1 0.0%	0 / 1 0.0%	0 / 5 0.0%	0 / 0 0.0%	0 / 0 0.0%	0 / 0 0.0%	0 / 0 0.0%	23 / 43 53.5%	16 / 43 37.2%E	3 / 43 7.0%C	1 / 43 2.3%AB							
1	Chroococcidiopsis thermalis	0 / 3 0.0%	0 / 4 0.0%	0 / 1 0.0%	0 / 1 0.0%	0 / 5 0.0%	0 / 0 0.0%	0 / 0 0.0%	0 / 0 0.0%	0 / 0 0.0%	23 / 43 53.5%	16 / 43 37.2%E	3 / 43 7.0%C	1 / 43 2.3%AB							
1	Chroococcidiopsis sp. ATCC 27900	0 / 3 [illegible]	0 / 4 [illegible]	0 / 1 [illegible]	0 / 1 [illegible]	0 / 5 [illegible]	0 / 0 [illegible]	0 / 0 [illegible]	0 / 0 [illegible]	0 / 0 [illegible]	23 / 43 [illegible]	16 / 43 [illegible]	3 / 43 [illegible]	1 / 43 [illegible]							

1	Stanieria	3 / 42 7.1%	0 / 1 0.0%	0 / 1 0.0%	0 / 1 0.0%	0 / 2 0.0%	0 / 2 0.0%	0 / 2 0.0%	0 / 1 0.0%	1 / 1 100.0%	2 / 4 50.0%	0 / 1 0.0%	0 / 4 0.0%	0 / 2 0.0%	0 / 1 0.0%	0 / 1 0.0%	0 / 1 0.0%	0 / 3 0.0%	0 / 1 0.0%	0 / 3 0.0%	0 / 3 0.0%	0 / 1 0.0%	0 / 1 0.0%	0 / 5 0.0%	0 / 0 0.0%	0 / 0 0.0%	0 / 0 0.0%	0 / 0 0.0%	21 / 42 50.0%E	16 / 42 38.1%	2 / 42 4.8%C	2 / 42 4.8%AB	1 / 42 2.4%ABE				
1	PCC 7437	3 / 42 7.1%	0 / 1 0.0%	0 / 1 0.0%	0 / 1 0.0%	0 / 2 0.0%	0 / 2 0.0%	0 / 2 0.0%	0 / 1 0.0%	1 / 1 100.0%	2 / 4 50.0%	0 / 1 0.0%	0 / 4 0.0%	0 / 2 0.0%	0 / 1 0.0%	0 / 1 0.0%	0 / 1 0.0%	0 / 3 0.0%	0 / 1 0.0%	0 / 3 0.0%	0 / 3 0.0%	0 / 1 0.0%	0 / 1 0.0%	0 / 5 0.0%	0 / 0 0.0%	0 / 0 0.0%	0 / 0 0.0%	0 / 0 0.0%	21 / 42 50.0%E	16 / 42 38.1%	2 / 42 4.8%C	2 / 42 4.8%AB	1 / 42 2.4%ABE				
1	Chroococcidiopsis cyanosphaera 'Hindak 1965/25'	3 / 42 7.1%	0 / 1 0.0%	0 / 1 0.0%	0 / 1 0.0%	0 / 2 0.0%	0 / 2 0.0%	0 / 2 0.0%	0 / 1 0.0%	1 / 1 100.0%	2 / 4 50.0%	0 / 1 0.0%	0 / 4 0.0%	0 / 2 0.0%	0 / 1 0.0%	0 / 1 0.0%	0 / 1 0.0%	0 / 3 0.0%	0 / 1 0.0%	0 / 3 0.0%	0 / 3 0.0%	0 / 1 0.0%	0 / 1 0.0%	0 / 5 0.0%	0 / 0 0.0%	0 / 0 0.0%	0 / 0 0.0%	0 / 0 0.0%	21 / 42 50.0%E	16 / 42 38.1%	2 / 42 4.8%AB	2 / 42 4.8%C	1 / 42 2.4%ABE				
1	Gloeobacteria	2 / 44 4.5%	0 / 0 0.0%	0 / 1 0.0%	0 / 1 0.0%	0 / 1 0.0%	0 / 3 0.0%	0 / 3 0.0%	0 / 1 0.0%	0 / 1 0.0%	1 / 3 33.3%	0 / 1 0.0%	1 / 4 25.0%	0 / 2 0.0%	0 / 1 0.0%	0 / 2 0.0%	0 / 1 0.0%	0 / 3 0.0%	0 / 0 0.0%	0 / 3 0.0%	0 / 4 0.0%	0 / 2 0.0%	0 / 2 0.0%	0 / 5 0.0%	0 / 0 0.0%	0 / 0 0.0%	0 / 0 0.0%	0 / 0 0.0%	23 / 44 52.3%	18 / 44 40.9%E	1 / 44 2.3%C	1 / 44 2.3%AB	1 / 44 2.3%ABE				
1	Gloeobacterales	2 / 44 4.5%	0 / 0 0.0%	0 / 1 0.0%	0 / 1 0.0%	0 / 1 0.0%	0 / 3 0.0%	0 / 3 0.0%	0 / 1 0.0%	0 / 1 0.0%	1 / 3 33.3%	0 / 1 0.0%	1 / 4 25.0%	0 / 2 0.0%	0 / 1 0.0%	0 / 2 0.0%	0 / 1 0.0%	0 / 3 0.0%	0 / 0 0.0%	0 / 3 0.0%	0 / 4 0.0%	0 / 2 0.0%	0 / 2 0.0%	0 / 5 0.0%	0 / 0 0.0%	0 / 0 0.0%	0 / 0 0.0%	0 / 0 0.0%	23 / 44 52.3%	18 / 44 40.9%E	1 / 44 2.3%C	1 / 44 2.3%AB	1 / 44 2.3%ABE				
1	Gloeobacter	2 / 44 4.5%	0 / 0 0.0%	0 / 1 0.0%	0 / 1 0.0%	0 / 1 0.0%	0 / 3 0.0%	0 / 3 0.0%	0 / 1 0.0%	0 / 1 0.0%	1 / 3 33.3%	0 / 1 0.0%	1 / 4 25.0%	0 / 2 0.0%	0 / 1 0.0%	0 / 2 0.0%	0 / 1 0.0%	0 / 3 0.0%	0 / 0 0.0%	0 / 3 0.0%	0 / 4 0.0%	0 / 2 0.0%	0 / 2 0.0%	0 / 5 0.0%	0 / 0 0.0%	0 / 0 0.0%	0 / 0 0.0%	0 / 0 0.0%	23 / 44 52.3%	18 / 44 40.9%E	1 / 44 2.3%AB	1 / 44 2.3%C	1 / 44 2.3%ABE				
1	Gloeobacter violaceus	2 / 44 4.5%	0 / 0 0.0%	0 / 1 0.0%	0 / 1 0.0%	0 / 1 0.0%	0 / 3 0.0%	0 / 3 0.0%	0 / 1 0.0%	0 / 1 0.0%	1 / 3 33.3%	0 / 1 0.0%	1 / 4 25.0%	0 / 2 0.0%	0 / 1 0.0%	0 / 2 0.0%	0 / 1 0.0%	0 / 3 0.0%	0 / 0 0.0%	0 / 3 0.0%	0 / 4 0.0%	0 / 2 0.0%	0 / 2 0.0%	0 / 5 0.0%	0 / 0 0.0%	0 / 0 0.0%	0 / 0 0.0%	0 / 0 0.0%	23 / 44 52.3%	18 / 44 40.9%E	1 / 44 2.3%ABE	1 / 44 2.3%C	1 / 44 2.3%AB				
1	Gloeobacter violaceus ATCC 29082	2 / 44 4.5%	0 / 0 0.0%	0 / 1 0.0%	0 / 1 0.0%	0 / 1 0.0%	0 / 3 0.0%	0 / 3 0.0%	0 / 1 0.0%	0 / 1 0.0%	1 / 3 33.3%	0 / 1 0.0%	1 / 4 25.0%	0 / 2 0.0%	0 / 1 0.0%	0 / 2 0.0%	0 / 1 0.0%	0 / 3 0.0%	0 / 0 0.0%	0 / 3 0.0%	0 / 4 0.0%	0 / 2 0.0%	0 / 2 0.0%	0 / 5 0.0%	0 / 0 0.0%	0 / 0 0.0%	0 / 0 0.0%	0 / 0 0.0%	23 / 44 52.3%	18 / 44 40.9%E	1 / 44 2.3%ABE	1 / 44 2.3%C	1 / 44 2.3%AB				
42	Oscillatoriophycideae	81 / 2026 4.0%	21 / 40 52.5%	1 / 53 1.9%	4 / 50 8.0%	4 / 94 4.3%	1 / 135 0.7%	2 / 118 1.7%	0 / 45 0.0%	12 / 46 26.1%	19 / 164 11.6%	2 / 48 4.2%	1 / 190 0.5%	0 / 63 0.0%	0 / 47 0.0%	1 / 51 2.0%	1 / 50 2.0%	0 / 136 0.0%	0 / 32 0.0%	0 / 140 0.0%	2 / 177 1.1%	3 / 50 6.0%	1 / 58 1.7%	6 / 231 2.6%	0 / 0 0.0%	0 / 0 0.0%	0 / 7 0.0%	0 / 1 0.0%	1044 / 2026 51.5%	821 / 2026 40.5%E	52 / 2026 2.6%AB	48 / 2026 2.4%C	31 / 2026 1.5%CE	25 / 2026 1.2%ABE	3 / 2026 0.1%B	1 / 2026 0.0%BE	1 / 2026 0.0%EGG
34	Chroococcales	52 / 1614 3.2%	14 / 32 43.8%	1 / 41 2.4%	2 / 39 5.1%	3 / 79 3.8%	0 / 107 0.0%	0 / 95 0.0%	0 / 37 0.0%	10 / 37 27.0%	14 / 132 10.6%	0 / 38 0.0%	0 / 151 0.0%	0 / 47 0.0%	0 / 37 0.0%	0 / 39 0.0%	1 / 40 2.5%	0 / 110 0.0%	0 / 29 0.0%	0 / 112 0.0%	2 / 145 1.4%	1 / 39 2.6%	0 / 45 0.0%	4 / 183 2.2%	0 / 0 0.0%	0 / 0 0.0%	0 / 0 0.0%	0 / 0 0.0%	858 / 1614 53.2%	642 / 1614 39.8%E	39 / 1614 2.4%C	29 / 1614 1.8%AB	22 / 1614 1.4%CE	19 / 1614 1.2%ABE	3 / 1614 0.2%B	1 / 1614 0.1%EGG	1 / 1614 0.1%BE
1	Microcystis	1 / 42 2.4%	0 / 1 0.0%	0 / 1 0.0%	0 / 1 0.0%	0 / 2 0.0%	0 / 3 0.0%	0 / 2 0.0%	0 / 1 0.0%	1 / 1 100.0%	0 / 3 0.0%	0 / 1 0.0%	0 / 4 0.0%	0 / 1 0.0%	0 / 1 0.0%	0 / 1 0.0%	0 / 1 0.0%	0 / 3 0.0%	0 / 1 0.0%	0 / 3 0.0%	0 / 4 0.0%	0 / 1 0.0%	0 / 1 0.0%	0 / 5 0.0%	0 / 0 0.0%	0 / 0 0.0%	0 / 0 0.0%	0 / 0 0.0%	19 / 42 45.2%	18 / 42 42.9%E	3 / 42 7.1%C	1 / 42 2.4%CE	1 / 42 2.4%ABE				
1	IAM M-247	1 / 42 2.4%	0 / 1 0.0%	0 / 1 0.0%	0 / 1 0.0%	0 / 2 0.0%	0 / 3 0.0%	0 / 2 0.0%	0 / 1 0.0%	1 / 1 100.0%	0 / 3 0.0%	0 / 1 0.0%	0 / 4 0.0%	0 / 1 0.0%	0 / 1 0.0%	0 / 1 0.0%	0 / 1 0.0%	0 / 3 0.0%	0 / 1 0.0%	0 / 3 0.0%	0 / 4 0.0%	0 / 1 0.0%	0 / 1 0.0%	0 / 5 0.0%	0 / 0 0.0%	0 / 0 0.0%	0 / 0 0.0%	0 / 0 0.0%	19 / 42 45.2%	18 / 42 42.9%E	3 / 42 7.1%C	1 / 42 2.4%ABE	1 / 42 2.4%CE				
1	Microcystis aeruginosa NIES-843	1 / 42 2.4%	0 / 1 0.0%	0 / 1 0.0%	0 / 1 0.0%	0 / 2 0.0%	0 / 3 0.0%	0 / 2 0.0%	0 / 1 0.0%	1 / 1 100.0%	0 / 3 0.0%	0 / 1 0.0%	0 / 4 0.0%	0 / 1 0.0%	0 / 1 0.0%	0 / 1 0.0%	0 / 1 0.0%	0 / 3 0.0%	0 / 1 0.0%	0 / 3 0.0%	0 / 4 0.0%	0 / 1 0.0%	0 / 1 0.0%	0 / 5 0.0%	0 / 0 0.0%	0 / 0 0.0%	0 / 0 0.0%	0 / 0 0.0%	19 / 42 45.2%	18 / 42 42.9%E	3 / 42 7.1%C	1 / 42 2.4%CE	1 / 42 2.4%ABE				
13	Anacystis	24 / 568 4.2%	9 / 13 69.2%	0 / 13 0.0%	0 / 13 0.0%	0 / 24 0.0%	0 / 38 0.0%	0 / 37 0.0%	0 / 13 0.0%	3 / 13 23.1%	10 / 50 20.0%	0 / 13 0.0%	0 / 53 0.0%	0 / 15 0.0%	0 / 13 0.0%	0 / 13 0.0%	0 / 15 0.0%	0 / 38 0.0%	0 / 10 0.0%	0 / 39 0.0%	0 / 51 0.0%	0 / 13 0.0%	0 / 16 0.0%	2 / 65 3.1%	0 / 0 0.0%	0 / 0 0.0%	0 / 0 0.0%	0 / 0 0.0%	328 / 568 57.7%	203 / 568 35.7%E	18 / 568 3.2%AB	8 / 568 1.4%CE	5 / 568 0.9%ABE	5 / 568 0.9%C	1 / 568 0.2%B		
2	PCC 6301	6 / 90 6.7%	2 / 2 100.0%	0 / 2 0.0%	0 / 2 0.0%	0 / 4 0.0%	0 / 6 0.0%	0 / 6 0.0%	0 / 2 0.0%	0 / 2 0.0%	4 / 8 50.0%	0 / 2 0.0%	0 / 8 0.0%	0 / 2 0.0%	0 / 2 0.0%	0 / 2 0.0%	0 / 4 0.0%	0 / 6 0.0%	0 / 0 0.0%	0 / 6 0.0%	0 / 8 0.0%	0 / 2 0.0%	0 / 4 0.0%	0 / 10 0.0%	0 / 0 0.0%	0 / 0 0.0%	0 / 0 0.0%	0 / 0 0.0%	54 / 90 60.0%	24 / 90 26.7%E	6 / 90 6.7%AB	4 / 90 4.4%C	2 / 90 2.2%CE				
1	Anacystis nidulans R2	3 / 45 6.7%	1 / 1 100.0%	0 / 1 0.0%	0 / 1 0.0%	0 / 2 0.0%	0 / 3 0.0%	0 / 3 0.0%	0 / 1 0.0%	0 / 1 0.0%	2 / 4 50.0%	0 / 1 0.0%	0 / 4 0.0%	0 / 1 0.0%	0 / 1 0.0%	0 / 1 0.0%	0 / 2 0.0%	0 / 3 0.0%	0 / 0 0.0%	0 / 3 0.0%	0 / 4 0.0%	0 / 1 0.0%	0 / 2 0.0%	0 / 5 0.0%	0 / 0 0.0%	0 / 0 0.0%	0 / 0 0.0%	0 / 0 0.0%	27 / 45 60.0%	12 / 45 26.7%E	3 / 45 6.7%AB	2 / 45 4.4%C	1 / 45 2.2%CE				
1	Anacystis nidulans SAUG 1402-1	3 / 45 6.7%	1 / 1 100.0%	0 / 1 0.0%	0 / 1 0.0%	0 / 2 0.0%	0 / 3 0.0%	0 / 3 0.0%	0 / 1 0.0%	0 / 1 0.0%	2 / 4 50.0%	0 / 1 0.0%	0 / 4 0.0%	0 / 1 0.0%	0 / 1 0.0%	0 / 1 0.0%	0 / 2 0.0%	0 / 3 0.0%	0 / 0 0.0%	0 / 3 0.0%	0 / 4 0.0%	0 / 1 0.0%	0 / 2 0.0%	0 / 5 0.0%	0 / 0 0.0%	0 / 0 0.0%	0 / 0 0.0%	0 / 0 0.0%	27 / 45 60.0%	12 / 45 26.7%E	3 / 45 6.7%AB	2 / 45 4.4%C	1 / 45 2.2%CE				
1	Agmenellum quadruplicatum	4 / 42 9.5%	1 / 1 100.0%	0 / 1 0.0%	0 / 1 0.0%	0 / 2 0.0%	0 / 3 0.0%	0 / 2 0.0%	0 / 1 0.0%	1 / 1 100.0%	2 / 4 50.0%	0 / 1 0.0%	0 / 4 0.0%	0 / 1 0.0%	0 / 1 0.0%	0 / 1 0.0%	0 / 1 0.0%	0 / 3 0.0%	0 / 1 0.0%	0 / 3 0.0%	0 / 3 0.0%	0 / 1 0.0%	0 / 1 0.0%	0 / 5 0.0%	0 / 0 0.0%	0 / 0 0.0%	0 / 0 0.0%	0 / 0 0.0%	20 / 42 47.6%E	17 / 42 40.5%	2 / 42 4.8%AB	1 / 42 2.4%ABE	1 / 42 2.4%B	1 / 42 2.4%CE			
1	Synechococcus WH7803	1 / 44 2.3%	1 / 1 100.0%	0 / 1 0.0%	0 / 1 0.0%	0 / 2 0.0%	0 / 3 0.0%	0 / 3 0.0%	0 / 1 0.0%	0 / 1 0.0%	0 / 4 0.0%	0 / 1 0.0%	0 / 4 0.0%	0 / 1 0.0%	0 / 1 0.0%	0 / 1 0.0%	0 / 1 0.0%	0 / 3 0.0%	0 / 1 0.0%	0 / 3 0.0%	0 / 4 0.0%	0 / 1 0.0%	0 / 1 0.0%	0 / 5 0.0%	0 / 0 0.0%	0 / 0 0.0%	0 / 0 0.0%	0 / 0 0.0%	28 / 44 63.6%	15 / 44 34.1%E	1 / 44 2.3%AB						
1	Synechococcus sp. CC9311	1 / 44 2.3%	1 / 1 100.0%	0 / 1 0.0%	0 / 1 0.0%	0 / 2 0.0%	0 / 3 0.0%	0 / 3 0.0%	0 / 1 0.0%	0 / 1 0.0%	0 / 4 0.0%	0 / 1 0.0%	0 / 4 0.0%	0 / 1 0.0%	0 / 1 0.0%	0 / 1 0.0%	0 / 1 0.0%	0 / 3 0.0%	0 / 1 0.0%	0 / 3 0.0%	0 / 4 0.0%	0 / 1 0.0%	0 / 1 0.0%	0 / 5 0.0%	0 / 0 0.0%	0 / 0 0.0%	0 / 0 0.0%	0 / 0 0.0%	29 / 44 65.9%	14 / 44 31.8%E	1 / 44 2.3%AB						
1	Synechococcus WH8102	1 / 44 2.3%	1 / 1 100.0%	0 / 1 0.0%	0 / 1 0.0%	0 / 2 0.0%	0 / 3 0.0%	0 / 3 0.0%	0 / 1 0.0%	0 / 1 0.0%	0 / 4 0.0%	0 / 1 0.0%	0 / 4 0.0%	0 / 1 0.0%	0 / 1 0.0%	0 / 1 0.0%	0 / 1 0.0%	0 / 3 0.0%	0 / 1 0.0%	0 / 3 0.0%	0 / 4 0.0%	0 / 1 0.0%	0 / 1 0.0%	0 / 5 0.0%	0 / 0 0.0%	0 / 0 0.0%	0 / 0 0.0%	0 / 0 0.0%	26 / 44 59.1%	17 / 44 38.6%E	1 / 44 2.3%AB						
1	Synechococcus sp. CC9605	2 / 44 4.5%	1 / 1 100.0%	0 / 1 0.0%	0 / 1 0.0%	0 / 2 0.0%	0 / 3 0.0%	0 / 3 0.0%	0 / 1 0.0%	0 / 1 0.0%	0 / 4 0.0%	0 / 1 0.0%	0 / 4 0.0%	0 / 1 0.0%	0 / 1 0.0%	0 / 1 0.0%	0 / 1 0.0%	0 / 3 0.0%	0 / 1 0.0%	0 / 3 0.0%	0 / 4 0.0%	0 / 1 0.0%	0 / 1 0.0%	1 / 5 20.0%	0 / 0 0.0%	0 / 0 0.0%	0 / 0 0.0%	0 / 0 0.0%	25 / 44 56.8%	17 / 44 38.6%E	1 / 44 2.3%ABE	1 / 44 2.3%AB					
1	Synechococcus sp. ATCC 27167	0 / 41 0.0%	0 / 1 0.0%	0 / 1 0.0%	0 / 1 0.0%	0 / 1 0.0%	0 / 3 0.0%	0 / 3 0.0%	0 / 1 0.0%	0 / 1 0.0%	0 / 3 0.0%	0 / 1 0.0%	0 / 4 0.0%	0 / 1 0.0%	0 / 1 0.0%	0 / 1 0.0%	0 / 1 0.0%	0 / 3 0.0%	0 / 1 0.0%	0 / 3 0.0%	0 / 4 0.0%	0 / 1 0.0%	0 / 1 0.0%	0 / 4 0.0%	0 / 0 0.0%	0 / 0 0.0%	0 / 0 0.0%	0 / 0 0.0%	19 / 41 46.3%E	18 / 41 43.9%	4 / 41 9.8%CE						
1	Synechococcus sp. MINSyn016-15m-01 substr. AC6A1	1 / 42 2.4%	1 / 1 100.0%	0 / 1 0.0%	0 / 1 0.0%	0 / 1 0.0%	0 / 3 0.0%	0 / 3 0.0%	0 / 1 0.0%	0 / 1 0.0%	0 / 3 0.0%	0 / 1 0.0%	0 / 4 0.0%	0 / 1 0.0%	0 / 1 0.0%	0 / 1 0.0%	0 / 1 0.0%	0 / 3 0.0%	0 / 1 0.0%	0 / 3 0.0%	0 / 4 0.0%	0 / 1 0.0%	0 / 1 0.0%	0 / 5 0.0%	0 / 0 0.0%	0 / 0 0.0%	0 / 0 0.0%	0 / 0 0.0%	27 / 42 64.3%	13 / 42 31.0%E	1 / 42 2.4%AB	1 / 42 2.4%C					
1	Synechococcus sp. CC9902	1 / 44 2.3%	1 / 1 100.0%	0 / 1 0.0%	0 / 1 0.0%	0 / 2 0.0%	0 / 3 0.0%	0 / 3 0.0%	0 / 1 0.0%	0 / 1 0.0%	0 / 4 0.0%	0 / 1 0.0%	0 / 4 0.0%	0 / 1 0.0%	0 / 1 0.0%	0 / 1 0.0%	0 / 1 0.0%	0 / 3 0.0%	0 / 1 0.0%	0 / 3 0.0%	0 / 4 0.0%	0 / 1 0.0%	0 / 1 0.0%	0 / 5 0.0%	0 / 0 0.0%	0 / 0 0.0%	0 / 0 0.0%	0 / 0 0.0%	26 / 44 59.1%	16 / 44 36.4%E	1 / 44 2.3%CE	1 / 44 2.3%AB					
1	Cyanobacteria bacterium Yellowstone A'	3 / 47 6.4%	0 / 1 0.0%	0 / 1 0.0%	0 / 1 0.0%	0 / 2 0.0%	0 / 3 0.0%	0 / 3 0.0%	0 / 1 0.0%	1 / 1 100.0%	2 / 4 50.0%	0 / 1 0.0%	0 / 5 0.0%	0 / 2 0.0%	0 / 1 0.0%	0 / 1 0.0%	0 / 1 0.0%	0 / 3 0.0%	0 / 1 0.0%	0 / 3 0.0%	0 / 4 0.0%	0 / 1 0.0%	0 / 1 0.0%	0 / 6 0.0%	0 / 0 0.0%	0 / 0 0.0%	0 / 0 0.0%	0 / 0 0.0%	30 / 47 63.8%	14 / 47 29.8%E	2 / 47 4.3%AB	1 / 47 2.1%ABE					
1	Cyanobacteria bacterium Yellowstone B'	3 / 44 6.8%	0 / 1 0.0%	0 / 1 0.0%	0 / 1 0.0%	0 / 2 0.0%	0 / 3 0.0%	0 / 3 0.0%	0 / 1 0.0%	1 / 1 100.0%	2 / 4 50.0%	0 / 1 0.0%	0 / 4 0.0%	0 / 1 0.0%	0 / 1 0.0%	0 / 1 0.0%	0 / 1 0.0%	0 / 3 0.0%	0 / 0 0.0%	0 / 3 0.0%	0 / 4 0.0%	0 / 1 0.0%	0 / 2 0.0%	0 / 5 0.0%	0 / 0 0.0%	0 / 0 0.0%	0 / 0 0.0%	0 / 0 0.0%	28 / 44 63.6%	13 / 44 29.5%E	2 / 44 4.5%AB	1 / 44 2.3%ABE					
1	Synechococcus sp. PCC 7502	1 / 42 2.4%	0 / 1 0.0%	0 / 1 0.0%	0 / 1 0.0%	0 / 2 0.0%	0 / 2 0.0%	0 / 2 0.0%	0 / 1 0.0%	0 / 1 0.0%	0 / 4 0.0%	0 / 1 0.0%	0 / 4 0.0%	0 / 2 0.0%	0 / 1 0.0%	0 / 1 0.0%	0 / 1 0.0%	0 / 2 0.0%	0 / 1 0.0%	0 / 3 0.0%	0 / 4 0.0%	0 / 1 0.0%	0 / 1 0.0%	1 / 5 20.0%	0 / 0 0.0%	0 / 0 0.0%	0 / 0 0.0%	0 / 0 0.0%	21 / 42 50.0%E	20 / 42 47.6%	1 / 42 2.4%ABE						
4	Synechococystis	0 / 247 0.0%	0 / 1 0.0%	0 / 6 0.0%	0 / 6 0.0%	0 / 12 0.0%	0 / 18 0.0%	0 / 12 0.0%	0 / 6 0.0%	0 / 6 0.0%	0 / 18 0.0%	0 / 6 0.0%	0 / 24 0.0%	0 / 6 0.0%	0 / 6 0.0%	0 / 6 0.0%	0 / 6 0.0%	0 / 18 0.0%	0 / 6 0.0%	0 / 18 0.0%	0 / 24 0.0%	0 / 6 0.0%	0 / 6 0.0%	0 / 30 0.0%	0 / 0 0.0%	0 / 0 0.0%	0 / 0 0.0%	0 / 0 0.0%	137 / 247 55.5%	92 / 247 37.2%E	12 / 247 4.9%C	5 / 247 2.0%CE	1 / 247 0.4%EGG				
4	Aphanocapsa sp. (strain N-1)	0 / 247 0.0%	0 / 1 0.0%	0 / 6 0.0%	0 / 6 0.0%	0 / 12 0.0%	0 / 18 0.0%	0 / 12 0.0%	0 / 6 0.0%	0 / 6 0.0%	0 / 18 0.0%	0 / 6 0.0%	0 / 24 0.0%	0 / 6 0.0%	0 / 6 0.0%	0 / 6 0.0%	0 / 6 0.0%	0 / 18 0.0%	0 / 6 0.0%	0 / 18 0.0%	0 / 24 0.0%	0 / 6 0.0%	0 / 6 0.0%	0 / 30 0.0%	0 / 0 0.0%	0 / 0 0.0%	0 / 0 0.0%	0 / 0 0.0%	137 / 247 55.5%	92 / 247 37.2%E	12 / 247 4.9%C	5 / 247 2.0%CE	1 / 247 0.4%EGG				
1	Synechocystis sp. PCC 6803 substr. GT-I	0 / 41 0.0%	0 / 0 0.0%	0 / 1 0.0%	0 / 1 0.0%	0 / 2 0.0%	0 / 3 0.0%	0 / 2 0.0%	0 / 1 0.0%	0 / 1 0.0%	0 / 3 0.0%	0 / 1 0.0%	0 / 4 0.0%	0 / 1 0.0%	0 / 1 0.0%	0 / 1 0.0%	0 / 1 0.0%	0 / 3 0.0%	0 / 1 0.0%	0 / 3 0.0%	0 / 4 0.0%	0 / 1 0.0%	0 / 1 0.0%	0 / 5 0.0%	0 / 0 0.0%	0 / 0 0.0%	0 / 0 0.0%	0 / 0 0.0%	22 / 41 53.7%	16 / 41 39.0%E	2 / 41 4.9%C	1 / 41 2.4%CE					
1	Synechocystis sp. PCC 6803 substr. PCC-N	0 / 41 0.0%	0 / 0 0.0%	0 / 1 0.0%	0 / 1 0.0%	0 / 2 0.0%	0 / 3 0.0%	0 / 2 0.0%	0 / 1 0.0%	0 / 1 0.0%	0 / 3 0.0%	0 / 1 0.0%	0 / 4 0.0%	0 / 1 0.0%	0 / 1 0.0%	0 / 1 0.0%	0 / 1 0.0%	0 / 3 0.0%	0 / 1 0.0%	0 / 3 0.0%	0 / 4 0.0%	0 / 1 0.0%	0 / 1 0.0%	0 / 5 0.0%	0 / 0 0.0%	0 / 0 0.0%	0 / 0 0.0%	0 / 0 0.0%	22 / 41 53.7%	16 / 41 39.0%E	2 / 41 4.9%C	1 / 41 2.4%CE					
1	Synechocystis sp. PCC 6803 substr. PCC-P	0 / 41 0.0%	0 / 0 0.0%	0 / 1 0.0%	0 / 1 0.0%	0 / 2 0.0%	0 / 3 0.0%	0 / 2 0.0%	0 / 1 0.0%	0 / 1 0.0%	0 / 3 0.0%	0 / 1 0.0%	0 / 4 0.0%	0 / 1 0.0%	0 / 1 0.0%	0 / 1 0.0%	0 / 1 0.0%	0 / 3 0.0%	0 / 1 0.0%	0 / 3 0.0%	0 / 4 0.0%	0 / 1 0.0%	0 / 1 0.0%	0 / 5 0.0%	0 / 0 0.0%	0 / 0 0.0%	0 / 0 0.0%	0 / 0 0.0%	22 / 41 53.7%	16 / 41 39.0%E	2 / 41 4.9%C	1 / 41 2.4%CE					
1	Dactylococcopsis	0 / 42 0.0%	0 / 1 0.0%	0 / 1 0.0%	0 / 1 0.0%	0 / 2 0.0%	0 / 2 0.0%	0 / 2 0.0%	0 / 1 0.0%	0 / 1 0.0%	0 / 4 0.0%	0 / 1 0.0%	0 / 4 0.0%	0 / 1 0.0%	0 / 1 0.0%	0 / 1 0.0%	0 / 1 0.0%	0 / 3 0.0%	0 / 0 0.0%	0 / 3 0.0%	0 / 4 0.0%	0 / 1 0.0%	0 / 2 0.0%	0 / 5 0.0%	0 / 0 0.0%	0 / 0 0.0%	0 / 0 0.0%	0 / 0 0.0%	22 / 42 52.4%	20 / 42 47.6%E							
1	Dactylococcopsis salina	0 / 42 0.0%	0 / 1 0.0%	0 / 1 0.0%	0 / 1 0.0%	0 / 2 0.0%	0 / 2 0.0%	0 / 2 0.0%	0 / 1 0.0%	0 / 1 0.0%	0 / 4 0.0%	0 / 1 0.0%	0 / 4 0.0%	0 / 1 0.0%	0 / 1 0.0%	0 / 1 0.0%	0 / 1 0.0%	0 / 3 0.0%	0 / 0 0.0%	0 / 3 0.0%	0 / 4 0.0%	0 / 1 0.0%	0 / 2 0.0%	0 / 5 0.0%	0 / 0 0.0%	0 / 0 0.0%	0 / 0 0.0%	0 / 0 0.0%	22 / 42 52.4%	20 / 42 47.6%E							
1	Dactylococcopsis PCC 8305	0 / 42 0.0%	0 / 1 0.0%	0 / 1 0.0%	0 / 1 0.0%	0 / 2 0.0%	0 / 2 0.0%	0 / 2 0.0%	0 / 1 0.0%	0 / 1 0.0%	0 / 4 0.0%	0 / 1 0.0%	0 / 4 0.0%	0 / 1 0.0%	0 / 1 0.0%	0 / 1 0.0%	0 / 1 0.0%	0 / 3 0.0%	0 / 0 0.0%	0 / 3 0.0%	0 / 4 0.0%	0 / 1 0.0%	0 / 2 0.0%	0 / 5 0.0%	0 / 0 0.0%	0 / 0 0.0%	0 / 0 0.0%	0 / 0 0.0%	22 / 42 52.4%	20 / 42 47.6%E							
6	Cyanothece	10 / 305 3.3%	1 / 7 14.3%	0 / 7 0.0%	0 / 7 0.0%	3 / 17 17.6%	0 / 21 0.0%	0 / 19 0.0%	0 / 7 0.0%	6 / 7 85.7%	0 / 22 0.0%	0 / 7 0.0%	0 / 28 0.0%	0 / 13 0.0%	0 / 7 0.0%	0 / 7 0.0%	0 / 7 0.0%	0 / 22 0.0%	0 / 7 0.0%	0 / 21 0.0%	0 / 24 0.0%	0 / 7 0.0%	0 / 8 0.0%	0 / 33 0.0%	0 / 0 0.0%	0 / 0 0.0%	0 / 0 0.0%	0 / 0 0.0%	158 / 305 51.8%	119 / 305 39.0%E	16 / 305 5.2%C	5 / 305 1.6%ABE	4 / 305 1.3%AB	2 / 305 0.7%CE	1 / 305 0.3%BE		
1	Cyanothece PCC 8801	1 / 42 2.4%	0 / 1 0.0%	0 / 1 0.0%	0 / 1 0.0%	0 / 2 0.0%	0 / 3 0.0%	0 / 3 0.0%	0 / 1 0.0%	1 / 1 100.0%	0 / 3 0.0%	0 / 1 0.0%	0 / 4 0.0%	0 / 2 0.0%	0 / 1 0.0%	0 / 1 0.0%	0 / 1 0.0%	0 / 3 0.0%	0 / 1 0.0%	0 / 3 0.0%	0 / 3 0.0%	0 / 1 0.0%	0 / 1 0.0%	0 / 4 0.0%	0 / 0 0.0%	0 / 0 0.0%	0 / 0 0.0%	0 / 0 0.0%	20 / 42 47.6%	19 / 42 45.2%E	2 / 42 4.8%C	1 / 42 2.4%ABE					
1	Cyanothece (strain ATCC 51142)	1 / 42 2.4%	0 / 1 0.0%	0 / 1 0.0%	0 / 1 0.0%	0 / 2 0.0%	0 / 3 0.0%	0 / 2 0.0%	0 / 1 0.0%	1 / 1 100.0%	0 / 3 0.0%	0 / 1 0.0%	0 / 4 0.0%	0 / 1 0.0%	0 / 1 0.0%	0 / 1 0.0%	0 / 1 0.0%	0 / 3 0.0%	0 / 2 0.0%	0 / 3 0.0%	0 / 3 0.0%	0 / 1 0.0%	0 / 1 0.0%	0 / 5 0.0%	0 / 0 0.0%	0 / 0 0.0%	0 / 0 0.0%	0 / 0 0.0%	20 / 42 47.6%E	19 / 42 45.2%	2 / 42 4.8%C	1 / 42 2.4%BE					
1	Cyanothece PCC 7424	2 / 43 4.7%	0 / 1 0.0%	0 / 1 0.0%	0 / 1 0.0%	1 / 3 33.3%	0 / 3 0.0%	0 / 2 0.0%	0 / 1 0.0%	1 / 1 100.0%	0 / 3 0.0%	0 / 1 0.0%	0 / 4 0.0%	0 / 2 0.0%	0 / 1 0.0%	0 / 1 0.0%	0 / 1 0.0%	0 / 3 0.0%	0 / 1 0.0%	0 / 3 0.0%	0 / 3 0.0%	0 / 1 0.0%	0 / 1 0.0%	0 / 5 0.0%	0 / 0 0.0%	0 / 0 0.0%	0 / 0 0.0%	0 / 0 0.0%	23 / 43 53.5%	16 / 43 37.2%E	2 / 43 4.7%C	1 / 43 2.3%ABE	1 / 43 2.3%AB				
1	Cyanothece sp. ATCC 29141	3 / 46 6.5%	1 / 1 100.0%	0 / 1 0.0%	0 / 1 0.0%	2 / 2 100.0%	0 / 3 0.0%	0 / 3 0.0%	0 / 1 0.0%	0 / 1 0.0%	0 / 4 0.0%	0 / 1 0.0%	0 / 4 0.0%	0 / 1 0.0%	0 / 1 0.0%	0 / 1 0.0%	0 / 1 0.0%	0 / 4 0.0%	0 / 0 0.0%	0 / 3 0.0%	0 / 4 0.0%	0 / 1 0.0%	0 / 2 0.0%	0 / 5 0.0%	0 / 0 0.0%	0 / 0 0.0%	0 / 0 0.0%	0 / 0 0.0%	26 / 46 56.5%	15 / 46 32.6%E	3 / 46 6.5%AB	2 / 46 4.3%CE					
1	Cyanothece sp. PCC 8802	1 / 42 2.4%	0 / 1 0.0%	0 / 1 0.0%	0 / 1 0.0%	0 / 2 0.0%	0 / 3 0.0%	0 / 3 0.0%	0 / 1 0.0%	1 / 1 100.0%	0 / 3 0.0%	0 / 1 0.0%	0 / 4 0.0%	0 / 2 0.0%	0 / 1 0.0%	0 / 1 0.0%	0 / 1 0.0%	0 / 3 0.0%	0 / 1 0.0%	0 / 3 0.0%	0 / 3 0.0%	0 / 1 0.0%	0 / 1 0.0%	0 / 4 0.0%	0 / 0 0.0%	0 / 0 0.0%	0 / 0 0.0%	0 / 0 0.0%	20 / 42 47.6%	19 / 42 45.2%E	2 / 42 4.8%C	1 / 42 2.4%ABE					
1	Cyanothece sp. PCC 7822	2 / 90 2.2%	0 / 2 0.0%	0 / 2 0.0%	0 / 2 0.0%	0 / 6 0.0%	0 / 6 0.0%	0 / 6 0.0%	0 / 2 0.0%	2 / 2 100.0%	0 / 6 0.0%	0 / 2 0.0%	0 / 8 0.0%	0 / 4 0.0%	0 / 2 0.0%	0 / 2 0.0%	0 / 2 0.0%	0 / 6 0.0%	0 / 2 0.0%	0 / 6 0.0%	0 / 8 0.0%	0 / 2 0.0%	0 / 2 0.0%	0 / 10 0.0%	0 / 0 0.0%	0 / 0 0.0%	0 / 0 0.0%	0 / 0 0.0%	50 / 90 55.6%	30 / 90 33.3%E	8 / 90 8.9%C	2 / 90 2.2%ABE					
1	Halothece cluster	0 / 45 0.0%	0 / 1 0.0%	0 / 2 0.0%	0 / 1 0.0%	0 / 3 0.0%	0 / 3 0.0%	0 / 2 0.0%	0 / 1 0.0%	0 / 1 0.0%	0 / 4 0.0%	0 / 1 0.0%	0 / 4 0.0%	0 / 1 0.0%	0 / 1 0.0%	0 / 1 0.0%	0 / 1 0.0%	0 / 3 0.0%	0 / 0 0.0%	0 / 3 0.0%	0 / 4 0.0%	0 / 1 0.0%	0 / 2 0.0%	0 / 5 0.0%	0 / 0 0.0%	0 / 0 0.0%	0 / 0 0.0%	0 / 0 0.0%	24 / 45 53.3%E	21 / 45 46.7%							
1	Halothece	0 / 45 0.0%	0 / 1 0.0%	0 / 2 0.0%	0 / 1 0.0%	0 / 3 0.0%	0 / 3 0.0%	0 / 2 0.0%	0 / 1 0.0%	0 / 1 0.0%	0 / 4 0.0%	0 / 1 0.0%	0 / 4 0.0%	0 / 1 0.0%	0 / 1 0.0%	0 / 1 0.0%	0 / 1 0.0%	0 / 3 0.0%	0 / 0 0.0%	0 / 3 0.0%	0 / 4 0.0%	0 / 1 0.0%	0 / 2 0.0%	0 / 5 0.0%	0 / 0 0.0%	0 / 0 0.0%	0 / 0 0.0%	0 / 0 0.0%	24 / 45 53.3%E	21 / 45 46.7%							
1	Aphanothece halophytica 'Solar Lake'	0 / 45 0.0%	0 / 1 0.0%	0 / 2 0.0%	0 / 1 0.0%	0 / 3 0.0%	0 / 3 0.0%	0 / 2 0.0%	0 / 1 0.0%	0 / 1 0.0%	0 / 4 0.0%	0 / 1 0.0%	0 / 4 0.0%	0 / 1 0.0%	0 / 1 0.0%	0 / 1 0.0%	0 / 1 0.0%	0 / 3 0.0%	0 / 0 0.0%	0 / 3 0.0%	0 / 4 0.0%	0 / 1 0.0%	0 / 2 0.0%	0 / 5 0.0%	0 / 0 0.0%	0 / 0 0.0%	0 / 0 0.0%	0 / 0 0.0%	24 / 45 53.3%E	21 / 45 46.7%							
1	Bichatia	1 / 41 2.4%	1 / 1 100.0%	0 / 1 0.0%	0 / 1 0.0%	0 / 2 0.0%	0 / 3 0.0%	0 / 3 0.0%	0 / 1 0.0%	0 / 1 0.0%	0 / 3 0.0%	0 / 1 0.0%	0 / 4 0.0%	0 / 1 0.0%	0 / 1 0.0%	0 / 1 0.0%	0 / 1 0.0%	0 / 3 0.0%	0 / 1 0.0%	0 / 3 0.0%	0 / 3 0.0%	0 / 1 0.0%	0 / 1 0.0%	0 / 4 0.0%	0 / 0 0.0%	0 / 0 0.0%	0 / 0 0.0%	0 / 0 0.0%	20 / 41 48.8%	16 / 41 39.0%E	2 / 41 4.9%CE	2 / 41 4.9%C	1 / 41 2.4%AB				
1	Chroococcus sp. Cey un	1 / 41 2.4%	1 / 1 100.0%	0 / 1 0.0%	0 / 1 0.0%	0 / 2 0.0%	0 / 3 0.0%	0 / 3 0.0%	0 / 1 0.0%	0 / 1 0.0%	0 / 3 0.0%	0 / 1 0.0%	0 / 4 0.0%	0 / 1 0.0%	0 / 1 0.0%	0 / 1 0.0%	0 / 1 0.0%	0 / 3 0.0%	0 / 1 0.0%	0 / 3 0.0%	0 / 3 0.0%	0 / 1 0.0%	0 / 1 0.0%	0 / 4 0.0%	0 / 0 0.0%	0 / 0 0.0%	0 / 0 0.0%	0 / 0 0.0%	20 / 41 48.8%	16 / 41 39.0%E	2 / 41 4.9%CE	2 / 41 4.9%C	1 / 41 2.4%AB				
2	Cyanobacterium	1 / 86 1.2%	0 / 2 0.0%	0 / 2 0.0%	0 / 2 0.0%	0 / 6 0.0%	0 / 4 0.0%	0 / 4 0.0%	0 / 2 0.0%	0 / 2 0.0%	0 / 10 0.0%	0 / 2 0.0%	0 / 8 0.0%	0 / 2 0.0%	0 / 2 0.0%	0 / 2 0.0%	0 / 2 0.0%	0 / 5 0.0%	0 / 2 0.0%	0 / 6 0.0%	1 / 8 12.5%	0 / 2 0.0%	0 / 2 0.0%	0 / 9 0.0%	0 / 0 0.0%	0 / 0 0.0%	0 / 0 0.0%	0 / 0 0.0%	45 / 86 52.3%E	40 / 86 46.5%	1 / 86 1.2%ABE						
1	Cyanobacterium stanieri	0 / 43 0.0%	0 / 1 0.0%	0 / 1 0.0%	0 / 1 0.0%	0 / 3 0.0%	0 / 2 0.0%	0 / 2 0.0%	0 / 1 0.0%	0 / 1 0.0%	0 / 5 0.0%	0 / 1 0.0%	0 / 4 0.0%	0 / 1 0.0%	0 / 1 0.0%	0 / 1 0.0%	0 / 1 0.0%	0 / 2 0.0%	0 / 1 0.0%	0 / 3 0.0%	0 / 4 0.0%	0 / 1 0.0%	0 / 1 0.0%	0 / 5 0.0%	0 / 0 0.0%	0 / 0 0.0%	0 / 0 0.0%	0 / 0 0.0%	25 / 43 58.1%E	18 / 43 41.9%							
1	Cyanobacterium stanieri PCC 7202	0 / 43 0.0%	0 / 1 0.0%	0 / 1 0.0%	0 / 1 0.0%	0 / 3 0.0%	0 / 2 0.0%	0 / 2 0.0%	0 / 1 0.0%	0 / 1 0.0%	0 / 5 0.0%	0 / 1 0.0%	0 / 4 0.0%	0 / 1 0.0%	0 / 1 0.0%	0 / 1 0.0%	0 / 1 0.0%	0 / 2 0.0%	0 / 1 0.0%	0 / 3 0.0%	0 / 4 0.0%	0 / 1 0.0%	0 / 1 0.0%	0 / 5 0.0%	0 / 0 0.0%	0 / 0 0.0%	0 / 0 0.0%	0 / 0 0.0%	25 / 43 58.1%E	18 / 43 41.9%							
1	Cyanobacterium aponinum	1 / 43 2.3%	0 / 1 0.0%	0 / 1 0.0%	0 / 1 0.0%	0 / 3 0.0%	0 / 2 0.0%	0 / 2 0.0%	0 / 1 0.0%	0 / 1 0.0%	0 / 5 0.0%	0 / 1 0.0%	0 / 4 0.0%	0 / 1 0.0%	0 / 1 0.0%	0 / 1 0.0%	0 / 1 0.0%	0 / 3 0.0%	0 / 1 0.0%	0 / 3 0.0%	1 / 4 25.0%	0 / 1 0.0%	0 / 1 0.0%	0 / 4 0.0%	0 / 0 0.0%	0 / 0 0.0%	0 / 0 0.0%	0 / 0 0.0%	22 / 43 51.2%	20 / 43 46.5%E	1 / 43 2.3%ABE						
1	Cyanobacterium aponinum PCC 10605	1 / 43 2.3%	0 / 1 0.0%	0 / 1 0.0%	0 / 1 0.0%	0 / 3 0.0%	0 / 2 0.0%	0 / 2 0.0%	0 / 1 0.0%	0 / 1 0.0%	0 / 5 0.0%	0 / 1 0.0%	0 / 4 0.0%	0 / 1 0.0%	0 / 1 0.0%	0 / 1 0.0%	0 / 1 0.0%	0 / 3 0.0%	0 / 1 0.0%	0 / 3 0.0%	1 / 4 25.0%	0 / 1 0.0%	0 / 1 0.0%	0 / 4 0.0%	0 / 0 0.0%	0 / 0 0.0%	0 / 0 0.0%	0 / 0 0.0%	22 / 43 51.2%	20 / 43 46.5%E	1 / 43 2.3%ABE						
1	Thermosynechococcus	1 / 41 2.4%	0 / 1 0.0%	0 / 1 0.0%	0 / 1 0.0%	0 / 1 0.0%	0 / 3 0.0%	0 / 3 0.0%	0 / 1 0.0%	0 / 1 0.0%	0 / 3 0.0%	0 / 1 0.0%	0 / 4 0.0%	0 / 1 0.0%	0 / 1 0.0%	0 / 1 0.0%	0 / 1 0.0%	0 / 3 0.0%	0 / 0 0.0%	0 / 3 0.0%	0 / 4 0.0%	0 / 1 0.0%	0 / 1 0.0%	1 / 5 20.0%	0 / 0 0.0%	0 / 0 0.0%	0 / 0 0.0%	0 / 0 0.0%	27 / 41 65.9%	13 / 41 31.7%E	1 / 41 2.4%ABE						
1	Thermosynechococcus elongatus	1 / 41 2.4%	0 / 1 0.0%	0 / 1 0.0%	0 / 1 0.0%	0 / 1 0.0%	0 / 3 0.0%	0 / 3 0.0%	0 / 1 0.0%	0 / 1 0.0%	0 / 3 0.0%	0 / 1 0.0%	0 / 4 0.0%	0 / 1 0.0%	0 / 1 0.0%	0 / 1 0.0%	0 / 1 0.0%	0 / 3 0.0%	0 / 0 0.0%	0 / 3 0.0%	0 / 4 0.0%	0 / 1 0.0%	0 / 1 0.0%	1 / 5 20.0%	0 / 0 0.0%	0 / 0 0.0%	0 / 0 0.0%	0 / 0 0.0%	27 / 41 65.9%	13 / 41 31.7%E	1 / 41 2.4%ABE						
1	Thermosynechococcus elongatus BP-1	1 / 41 2.4%	0 / 1 0.0%	0 / 1 0.0%	0 / 1 0.0%	0 / 1 0.0%	0 / 3 0.0%	0 / 3 0.0%	0 / 1 0.0%	0 / 1 0.0%	0 / 3 0.0%	0 / 1 0.0%	0 / 4 0.0%	0 / 1 0.0%	0 / 1 0.0%	0 / 1 0.0%	0 / 1 0.0%	0 / 3 0.0%	0 / 0 0.0%	0 / 3 0.0%	0 / 4 0.0%	0 / 1 0.0%	0 / 1 0.0%	1 / 5 20.0%	0 / 0 0.0%	0 / 0 0.0%	0 / 0 0.0%	0 / 0 0.0%	27 / 41 65.9%	13 / 41 31.7%E	1 / 41 2.4%ABE						
1	Acaryochloris	8 / 63 12.7%	1 / 1 100.0%	0 / 2 0.0%	1 / 2 50.0%	0 / 2 0.0%	0 / 4 0.0%	0 / 4 0.0%	0 / 1 0.0%	0 / 1 0.0%	2 / 4 50.0%	0 / 2 0.0%	0 / 5 0.0%	0 / 2 0.0%	0 / 1 0.0%	0 / 3 0.0%	1 / 2 50.0%	0 / 4 0.0%	0 / 0 0.0%	0 / 4 0.0%	1 / 7 14.3%	1 / 2 50.0%	0 / 2 0.0%	1 / 8 12.5%	0 / 0 0.0%	0 / 0 0.0%	0 / 0 0.0%	0 / 0 0.0%	28 / 63 44.4%	26 / 63 41.3%E	3 / 63 4.8%ABE	3 / 63 4.8%AB	2 / 63 3.2%B	1 / 63 1.6%C			
1	Acaryochloris marina	8 / 63 12.7%	1 / 1 100.0%	0 / 2 0.0%	1 / 2 50.0%	0 / 2 0.0%	0 / 4 0.0%	0 / 4 0.0%	0 / 1 0.0%	0 / 1 0.0%	2 / 4 50.0%	0 / 2 0.0%	0 / 5 0.0%	0 / 2 0.0%	0 / 1 0.0%	0 / 3 0.0%	1 / 2 50.0%	0 / 4 0.0%	0 / 0 0.0%	0 / 4 0.0%	1 / 7 14.3%	1 / 2 50.0%	0 / 2 0.0%	1 / 8 12.5%	0 / 0 0.0%	0 / 0 0.0%	0 / 0 0.0%	0 / 0 0.0%	28 / 63 44.4%	26 / 63 41.3%E	3 / 63 4.8%ABE	3 / 63 4.8%AB	2 / 63 3.2%B	1 / 63 1.6%C			
1	Acaryochloris marina MBIC11017	8 / 63 12.7%	1 / 1 100.0%	0 / 2 0.0%	1 / 2 50.0%	0 / 2 0.0%	0 / 4 0.0%	0 / 4 0.0%	0 / 1 0.0%	0 / 1 0.0%	2 / 4 50.0%	0 / 2 0.0%	0 / 5 0.0%	0 / 2 0.0%	0 / 1 0.0%	0 / 3 0.0%	1 / 2 50.0%	0 / 4 0.0%	0 / 0 0.0%	0 / 4 0.0%	1 / 7 14.3%	1 / 2 50.0%	0 / 2 0.0%	1 / 8 12.5%	0 / 0 0.0%	0 / 0 0.0%	0 / 0 0.0%	0 / 0 0.0%	28 / 63 44.4%	26 / 63 41.3%E	3 / 63 4.8%AB	3 / 63 4.8%ABE	2 / 63 3.2%B	1 / 63 1.6%C			
1	Cyanobium	3 / 44 6.8%	1 / 1 100.0%	0 / 1 0.0%	0 / 1 0.0%	0 / 2 0.0%	0 / 3 0.0%	0 / 3 0.0%	0 / 1 0.0%	0 / 1 0.0%	2 / 4 50.0%	0 / 1 0.0%	0 / 4 0.0%	0 / 1 0.0%	0 / 1 0.0%	0 / 1 0.0%	0 / 1 0.0%	0 / 3 0.0%	0 / 1 0.0%	0 / 3 0.0%	0 / 4 0.0%	0 / 1 0.0%	0 / 1 0.0%	0 / 5 0.0%	0 / 0 0.0%	0 / 0 0.0%	0 / 0 0.0%	0 / 0 0.0%	23 / 44 52.3%E	17 / 44 38.6%	2 / 44 4.5%ABE	1 / 44 2.3%CE	1 / 44 2.3%AB				
1	Cyanobium gracile	3 / 44 6.8%	1 / 1 100.0%	0 / 1 0.0%	0 / 1 0.0%	0 / 2 0.0%	0 / 3 0.0%	0 / 3 0.0%	0 / 1 0.0%	0 / 1 0.0%	2 / 4 50.0%	0 / 1 0.0%	0 / 4 0.0%	0 / 1 0.0%	0 / 1 0.0%	0 / 1 0.0%	0 / 1 0.0%	0 / 3 0.0%	0 / 1 0.0%	0 / 3 0.0%	0 / 4 0.0%	0 / 1 0.0%	0 / 1 0.0%	0 / 5 0.0%	0 / 0 0.0%	0 / 0 0.0%	0 / 0 0.0%	0 / 0 0.0%	23 / 44 52.3%E	17 / 44 38.6%	2 / 44 4.5%ABE	1 / 44 2.3%CE	1 / 44 2.3%AB				
1	Coccochloris peniocystis 1020	3 / 44 6.8%	1 / 1 100.0%	0 / 1 0.0%	0 / 1 0.0%	0 / 2 0.0%	0 / 3 0.0%	0 / 3 0.0%	0 / 1 0.0%	0 / 1 0.0%	2 / 4 50.0%	0 / 1 0.0%	0 / 4 0.0%	0 / 1 0.0%	0 / 1 0.0%	0 / 1 0.0%	0 / 1 0.0%	0 / 3 0.0%	0 / 1 0.0%	0 / 3 0.0%	0 / 4 0.0%	0 / 1 0.0%	0 / 1 0.0%	0 / 5 0.0%	0 / 0 0.0%	0 / 0 0.0%	0 / 0 0.0%	0 / 0 0.0%	23 / 44 52.3%E	17 / 44 38.6%	2 / 44 4.5%ABE	1 / 44 2.3%CE	1 / 44 2.3%AB				
1	Chamaesiphon	3 / 53 5.7%	1 / 1 100.0%	1 / 3 33.3%	1 / 2 50.0%	0 / 4 0.0%	0 / 3 0.0%	0 / 2 0.0%	0 / 1 0.0%	0 / 1 0.0%	0 / 4 0.0%	0 / 1 0.0%	0 / 5 0.0%	0 / 2 0.0%	0 / 1 0.0%	0 / 1 0.0%	0 / 1 0.0%	0 / 3 0.0%	0 / 0 0.0%	0 / 3 0.0%	0 / 5 0.0%	0 / 2 0.0%	0 / 2 0.0%	0 / 6 0.0%	0 / 0 0.0%	0 / 0 0.0%	0 / 0 0.0%	0 / 0 0.0%	25 / 53 47.2%	24 / 53 45.3%E	2 / 53 3.8%AB	1 / 53 1.9%ABE	1 / 53 1.9%CE				
1	Chamaesiphon minutus	3 / 53 5.7%	1 / 1 100.0%	1 / 3 33.3%	1 / 2 50.0%	0 / 4 0.0%	0 / 3 0.0%	0 / 2 0.0%	0 / 1 0.0%	0 / 1 0.0%	0 / 4 0.0%	0 / 1 0.0%	0 / 5 0.0%	0 / 2 0.0%	0 / 1 0.0%	0 / 1 0.0%	0 / 1 0.0%	0 / 3 0.0%	0 / 0 0.0%	0 / 3 0.0%	0 / 5 0.0%	0 / 2 0.0%	0 / 2 0.0%	0 / 6 0.0%	0 / 0 0.0%	0 / 0 0.0%	0 / 0 0.0%	0 / 0 0.0%	25 / 53 47.2%	24 / 53 45.3%E	2 / 53 3.8%AB	1 / 53 1.9%ABE	1 / 53 1.9%CE				
1	Chamaesiphon minutus PCC 6605	3 / 53 5.7%	1 / 1 100.0%	1 / 3 33.3%	1 / 2 50.0%	0 / 4 0.0%	0 / 3 0.0%	0 / 2 0.0%	0 / 1 0.0%	0 / 1 0.0%	0 / 4 0.0%	0 / 1 0.0%	0 / 5 0.0%	0 / 2 0.0%	0 / 1 0.0%	0 / 1 0.0%	0 / 1 0.0%	0 / 3 0.0%	0 / 0 0.0%	0 / 3 0.0%	0 / 5 0.0%	0 / 2 0.0%	0 / 2 0.0%	0 / 6 0.0%	0 / 0 0.0%	0 / 0 0.0%	0 / 0 0.0%	0 / 0 0.0%	25 / 53 47.2%	24 / 53 45.3%E	2 / 53 3.8%AB	1 / 53 1.9%ABE	1 / 53 1.9%CE				
1	"Candidatus Atelocyanobacterium" Thompson et al. 2012	0 / 37 0.0%	0 / 1 0.0%	0 / 1 0.0%	0 / 1 0.0%	0 / 2 0.0%	0 / 2 0.0%	0 / 2 0.0%	0 / 1 0.0%	0 / 1 0.0%	0 / 3 0.0%	0 / 1 0.0%	0 / 4 0.0%	0 / 1 0.0%	0 / 1 0.0%	0 / 1 0.0%	0 / 1 0.0%	0 / 2 0.0%	0 / 1 0.0%	0 / 3 0.0%	0 / 3 0.0%	0 / 1 0.0%	0 / 1 0.0%	0 / 3 0.0%	0 / 0 0.0%	0 / 0 0.0%	0 / 0 0.0%	0 / 0 0.0%	19 / 37 51.4%E	16 / 37 43.2%	2 / 37 5.4%CE						
1	"Candidatus Atelocyanobacterium thalassa" Thompson et al. 2012	0 / 37 0.0%	0 / 1 0.0%	0 / 1 0.0%	0 / 1 0.0%	0 / 2 0.0%	0 / 2 0.0%	0 / 2 0.0%	0 / 1 0.0%	0 / 1 0.0%	0 / 3 0.0%	0 / 1 0.0%	0 / 4 0.0%	0 / 1 0.0%	0 / 1 0.0%	0 / 1 0.0%	0 / 1 0.0%	0 / 2 0.0%	0 / 1 0.0%	0 / 3 0.0%	0 / 3 0.0%	0 / 1 0.0%	0 / 1 0.0%	0 / 3 0.0%	0 / 0 0.0%	0 / 0 0.0%	0 / 0 0.0%	0 / 0 0.0%	19 / 37 51.4%E	16 / 37 43.2%	2 / 37 5.4%CE						
8	Oscillatoriales	29 / 412 7.0%	7 / 8 87.5%	0 / 12 0.0%	2 / 11 18.2%	1 / 15 6.7%	1 / 28 3.6%	2 / 23 8.7%	0 / 8 0.0%	2 / 9 22.2%	5 / 32 15.6%	2 / 10 20.0%	1 / 39 2.6%	0 / 16 0.0%	0 / 10 0.0%	1 / 12 8.3%	0 / 10 0.0%	0 / 26 0.0%	0 / 3 0.0%	0 / 28 0.0%	0 / 32 0.0%	2 / 11 18.2%	1 / 13 7.7%	2 / 48 4.2%	0 / 0 0.0%	0 / 0 0.0%	0 / 7 0.0%	0 / 1 0.0%	186 / 412 45.1%	179 / 412 43.4%E	23 / 412 5.6%AB	9 / 412 2.2%C	9 / 412 2.2%CE	6 / 412 1.5%ABE			
1	Pseudanabaena	2 / 44 4.5%	0 / 1 0.0%	0 / 1 0.0%	0 / 1 0.0%	0 / 2 0.0%	0 / 3 0.0%	0 / 3 0.0%	0 / 1 0.0%	0 / 1 0.0%	2 / 4 50.0%	0 / 1 0.0%	0 / 4 0.0%	0 / 2 0.0%	0 / 1 0.0%	0 / 1 0.0%	0 / 1 0.0%	0 / 3 0.0%	0 / 1 0.0%	0 / 3 0.0%	0 / 4 0.0%	0 / 1 0.0%	0 / 1 0.0%	0 / 4 0.0%	0 / 0 0.0%	0 / 0 0.0%	0 / 0 0.0%	0 / 0 0.0%	23 / 44 52.3%E	18 / 44 40.9%	2 / 44 4.5%AB	1 / 44 2.3%CE					
1	Pseudanabaena PCC7367	2 / 44 4.5%	0 / 1 0.0%	0 / 1 0.0%	0 / 1 0.0%	0 / 2 0.0%	0 / 3 0.0%	0 / 3 0.0%	0 / 1 0.0%	0 / 1 0.0%	2 / 4 50.0%	0 / 1 0.0%	0 / 4 0.0%	0 / 2 0.0%	0 / 1 0.0%	0 / 1 0.0%	0 / 1 0.0%	0 / 3 0.0%	0 / 1 0.0%	0 / 3 0.0%	0 / 4 0.0%	0 / 1 0.0%	0 / 1 0.0%	0 / 4 0.0%	0 / 0 0.0%	0 / 0 0.0%	0 / 0 0.0%	0 / 0 0.0%	23 / 44 52.3%E	18 / 44 40.9%	2 / 44 4.5%AB	1 / 44 2.3%CE					
2	Oscillatoria	15 / 132 11.4%	3 / 3 100.0%	0 / 4 0.0%	1 / 4 25.0%	0 / 3 0.0%	0 / 8 0.0%	1 / 7 14.3%	0 / 2 0.0%	1 / 3 33.3%	1 / 10 10.0%	1 / 3 33.3%	1 / 13 7.7%	0 / 6 0.0%	0 / 3 0.0%	1 / 5 20.0%	0 / 3 0.0%	0 / 8 0.0%	0 / 0 0.0%	0 / 8 0.0%	0 / 9 0.0%	2 / 3 66.7%	1 / 3 33.3%	2 / 18 11.1%	0 / 0 0.0%	0 / 0 0.0%	0 / 5 0.0%	0 / 1 0.0%	58 / 132 43.9%E	52 / 132 39.4%	10 / 132 7.6%AB	5 / 132 3.8%ABE	4 / 132 3.0%CE	3 / 132 2.3%C			
1	Oscillatoria acuminata	8 / 65 12.3%	1 / 1 100.0%	0 / 2 0.0%	0 / 2 0.0%	0 / 1 0.0%	0 / 4 0.0%	1 / 4 25.0%	0 / 1 0.0%	1 / 2 50.0%	0 / 4 0.0%	1 / 2 50.0%	1 / 8 12.5%	0 / 3 0.0%	0 / 1 0.0%	0 / 2 0.0%	0 / 1 0.0%	0 / 4 0.0%	0 / 0 0.0%	0 / 4 0.0%	0 / 3 0.0%	1 / 2 50.0%	1 / 2 50.0%	1 / 8 12.5%	0 / 0 0.0%	0 / 0 0.0%	0 / 4 0.0%	0 / 0 0.0%	30 / 65 46.2%E	24 / 65 36.9%	4 / 65 6.2%ABE	4 / 65 6.2%AB	2 / 65 3.1%CE	1 / 65 1.5%C			
1	Microcoleus vaginatus KFRI M-7.1.1	8 / 65 12.3%	1 / 1 100.0%	0 / 2 0.0%	0 / 2 0.0%	0 / 1 0.0%	0 / 4 0.0%	1 / 4 25.0%	0 / 1 0.0%	1 / 2 50.0%	0 / 4 0.0%	1 / 2 50.0%	1 / 8 12.5%	0 / 3 0.0%	0 / 1 0.0%	0 / 2 0.0%	0 / 1 0.0%	0 / 4 0.0%	0 / 0 0.0%	0 / 4 0.0%	0 / 3 0.0%	1 / 2 50.0%	1 / 2 50.0%	1 / 8 12.5%	0 / 0 0.0%	0 / 0 0.0%	0 / 4 0.0%	0 / 0 0.0%	30 / 65 46.2%E	24 / 65 36.9%	4 / 65 6.2%AB	4 / 65 6.2%ABE	2 / 65 3.1%CE	1 / 65 1.5%C			
1	Oscillatoria nigro-viridis	7 / 67 10.4%	2 / 2 100.0%	0 / 2 0.0%	1 / 2 50.0%	0 / 2 0.0%	0 / 4 0.0%	0 / 3 0.0%	0 / 1 0.0%	0 / 1 0.0%	1 / 6 16.7%	0 / 1 0.0%	0 / 5 0.0%	0 / 3 0.0%	0 / 2 0.0%	1 / 3 33.3%	0 / 2 0.0%	0 / 4 0.0%	0 / 0 0.0%	0 / 4 0.0%	0 / 6 0.0%	1 / 1 100.0%	0 / 1 0.0%	1 / 10 10.0%	0 / 0 0.0%	0 / 0 0.0%	0 / 1 0.0%	0 / 1 0.0%	28 / 67 41.8%E	28 / 67 41.8%	6 / 67 9.0%AB	2 / 67 3.0%C	2 / 67 3.0%CE	1 / 67 1.5%ABE			
1	Oscillatoria PCC 7112	7 / 67 10.4%	2 / 2 100.0%	0 / 2 0.0%	1 / 2 50.0%	0 / 2 0.0%	0 / 4 0.0%	0 / 3 0.0%	0 / 1 0.0%	0 / 1 0.0%	1 / 6 16.7%	0 / 1 0.0%	0 / 5 0.0%	0 / 3 0.0%	0 / 2 0.0%	1 / 3 33.3%	0 / 2 0.0%	0 / 4 0.0%	0 / 0 0.0%	0 / 4 0.0%	0 / 6 0.0%	1 / 1 100.0%	0 / 1 0.0%	1 / 10 10.0%	0 / 0 0.0%	0 / 0 0.0%	0 / 1 0.0%	0 / 1 0.0%	28 / 67 41.8%	28 / 67 41.8%E	6 / 67 9.0%AB	2 / 67 3.0%CE	2 / 67 3.0%C	1 / 67 1.5%ABE			
1	Trichodesmium	0 / 38 0.0%	0 / 0 0.0%	0 / 1 0.0%	0 / 1 0.0%	0 / 2 0.0%	0 / 2 0.0%	0 / 2 0.0%	0 / 1 0.0%	0 / 1 0.0%	0 / 3 0.0%	0 / 1 0.0%	0 / 4 0.0%	0 / 1 0.0%	0 / 1 0.0%	0 / 1 0.0%	0 / 1 0.0%	0 / 2 0.0%	0 / 0 0.0%	0 / 2 0.0%	0 / 3 0.0%	0 / 1 0.0%	0 / 3 0.0%	0 / 5 0.0%	0 / 0 0.0%	0 / 0 0.0%	0 / 0 0.0%	0 / 0 0.0%	20 / 38 52.6%	16 / 38 42.1%E	2 / 38 5.3%C						
1	Trichodesmium erythraeum	0 / 38 0.0%	0 / 0 0.0%	0 / 1 0.0%	0 / 1 0.0%	0 / 2 0.0%	0 / 2 0.0%	0 / 2 0.0%	0 / 1 0.0%	0 / 1 0.0%	0 / 3 0.0%	0 / 1 0.0%	0 / 4 0.0%	0 / 1 0.0%	0 / 1 0.0%	0 / 1 0.0%	0 / 1 0.0%	0 / 2 0.0%	0 / 0 0.0%	0 / 2 0.0%	0 / 3 0.0%	0 / 1 0.0%	0 / 3 0.0%	0 / 5 0.0%	0 / 0 0.0%	0 / 0 0.0%	0 / 0 0.0%	0 / 0 0.0%	20 / 38 52.6%	16 / 38 42.1%E	2 / 38 5.3%C						
1	Trichodesmium erythraeum IMS101	0 / 38 0.0%	0 / 0 0.0%	0 / 1 0.0%	0 / 1 0.0%	0 / 2 0.0%	0 / 2 0.0%	0 / 2 0.0%	0 / 1 0.0%	0 / 1 0.0%	0 / 3 0.0%	0 / 1 0.0%	0 / 4 0.0%	0 / 1 0.0%	0 / 1 0.0%	0 / 1 0.0%	0 / 1 0.0%	0 / 2 0.0%	0 / 0 0.0%	0 / 2 0.0%	0 / 3 0.0%	0 / 1 0.0%	0 / 3 0.0%	0 / 5 0.0%	0 / 0 0.0%	0 / 0 0.0%	0 / 0 0.0%	0 / 0 0.0%	20 / 38 52.6%	16 / 38 42.1%E	2 / 38 5.3%C						
1	Microcoleus	5 / 68 7.4%	1 / 1 100.0%	0 / 3 0.0%	1 / 2 50.0%	0 / 2 0.0%	1 / 5 20.0%	1 / 4 25.0%	0 / 1 0.0%	0 / 1 0.0%	0 / 4 0.0%	1 / 2 50.0%	0 / 6 0.0%	0 / 3 0.0%	0 / 2 0.0%	0 / 2 0.0%	0 / 2 0.0%	0 / 4 0.0%	0 / 0 0.0%	0 / 5 0.0%	0 / 5 0.0%	0 / 3 0.0%	0 / 2 0.0%	0 / 7 0.0%	0 / 0 0.0%	0 / 0 0.0%	0 / 2 0.0%	0 / 0 0.0%	33 / 68 48.5%	27 / 68 39.7%E	5 / 68 7.4%AB	3 / 68 4.4%CE					
1	Microcoleus sp. PCC 7113	5 / 68 7.4%	1 / 1 100.0%	0 / 3 0.0%	1 / 2 50.0%	0 / 2 0.0%	1 / 5 20.0%	1 / 4 25.0%	0 / 1 0.0%	0 / 1 0.0%	0 / 4 0.0%	1 / 2 50.0%	0 / 6 0.0%	0 / 3 0.0%	0 / 2 0.0%	0 / 2 0.0%	0 / 2 0.0%	0 / 4 0.0%	0 / 0 0.0%	0 / 5 0.0%	0 / 5 0.0%	0 / 3 0.0%	0 / 2 0.0%	0 / 7 0.0%	0 / 0 0.0%	0 / 0 0.0%	0 / 2 0.0%	0 / 0 0.0%	33 / 68 48.5%	27 / 68 39.7%E	5 / 68 7.4%AB	3 / 68 4.4%CE					
1	Leptolyngbya	3 / 42 7.1%	1 / 1 100.0%	0 / 1 0.0%	0 / 1 0.0%	0 / 2 0.0%	0 / 3 0.0%	0 / 2 0.0%	0 / 1 0.0%	0 / 1 0.0%	2 / 4 50.0%	0 / 1 0.0%	0 / 4 0.0%	0 / 1 0.0%	0 / 1 0.0%	0 / 1 0.0%	0 / 1 0.0%	0 / 3 0.0%	0 / 1 0.0%	0 / 3 0.0%	0 / 3 0.0%	0 / 1 0.0%	0 / 1 0.0%	0 / 5 0.0%	0 / 0 0.0%	0 / 0 0.0%	0 / 0 0.0%	0 / 0 0.0%	24 / 42 57.1%	15 / 42 35.7%E	3 / 42 7.1%AB						
1	Leptolyngbya PCC7376	3 / 42 7.1%	1 / 1 100.0%	0 / 1 0.0%	0 / 1 0.0%	0 / 2 0.0%	0 / 3 0.0%	0 / 2 0.0%	0 / 1 0.0%	0 / 1 0.0%	2 / 4 50.0%	0 / 1 0.0%	0 / 4 0.0%	0 / 1 0.0%	0 / 1 0.0%	0 / 1 0.0%	0 / 1 0.0%	0 / 3 0.0%	0 / 1 0.0%	0 / 3 0.0%	0 / 3 0.0%	0 / 1 0.0%	0 / 1 0.0%	0 / 5 0.0%	0 / 0 0.0%	0 / 0 0.0%	0 / 0 0.0%	0 / 0 0.0%	24 / 42 57.1%	15 / 42 35.7%E	3 / 42 7.1%AB						
1	Geitlerinema	2 / 46 4.3%	1 / 1 100.0%	0 / 1 0.0%	0 / 1 0.0%	0 / 3 0.0%	0 / 4 0.0%	0 / 3 0.0%	0 / 1 0.0%	1 / 1 100.0%	0 / 5 0.0%	0 / 1 0.0%	0 / 4 0.0%	0 / 1 0.0%	0 / 1 0.0%	0 / 1 0.0%	0 / 1 0.0%	0 / 3 0.0%	0 / 0 0.0%	0 / 3 0.0%	0 / 4 0.0%	0 / 1 0.0%	0 / 2 0.0%	0 / 4 0.0%	0 / 0 0.0%	0 / 0 0.0%	0 / 0 0.0%	0 / 0 0.0%	22 / 46 47.8%	19 / 46 41.3%E	3 / 46 6.5%C	1 / 46 2.2%ABE	1 / 46 2.2%AB				
1	Geitlerinema sp. ATCC 29126	2 / 46 4.3%	1 / 1 100.0%	0 / 1 0.0%	0 / 1 0.0%	0 / 3 0.0%	0 / 4 0.0%	0 / 3 0.0%	0 / 1 0.0%	1 / 1 100.0%	0 / 5 0.0%	0 / 1 0.0%	0 / 4 0.0%	0 / 1 0.0%	0 / 1 0.0%	0 / 1 0.0%	0 / 1 0.0%	0 / 3 0.0%	0 / 0 0.0%	0 / 3 0.0%	0 / 4 0.0%	0 / 1 0.0%	0 / 2 0.0%	0 / 4 0.0%	0 / 0 0.0%	0 / 0 0.0%	0 / 0 0.0%	0 / 0 0.0%	22 / 46 47.8%	19 / 46 41.3%E	3 / 46 6.5%C	1 / 46 2.2%ABE	1 / 46 2.2%AB				
1	Crinalium	2 / 42 4.8%	1 / 1 100.0%	0 / 1 0.0%	0 / 1 0.0%	1 / 1 100.0%	0 / 3 0.0%	0 / 2 0.0%	0 / 1 0.0%	0 / 1 0.0%	0 / 2 0.0%	0 / 1 0.0%	0 / 4 0.0%	0 / 2 0.0%	0 / 1 0.0%	0 / 1 0.0%	0 / 1 0.0%	0 / 3 0.0%	0 / 1 0.0%	0 / 4 0.0%	0 / 4 0.0%	0 / 1 0.0%	0 / 1 0.0%	0 / 5 0.0%	0 / 0 0.0%	0 / 0 0.0%	0 / 0 0.0%	0 / 0 0.0%	21 / 42 50.0%E	17 / 42 40.5%	2 / 42 4.8%AB	1 / 42 2.4%CE	1 / 42 2.4%C				
1	ATCC 49662	2 / 42 4.8%	1 / 1 100.0%	0 / 1 0.0%	0 / 1 0.0%	1 / 1 100.0%	0 / 3 0.0%	0 / 2 0.0%	0 / 1 0.0%	0 / 1 0.0%	0 / 2 0.0%	0 / 1 0.0%	0 / 4 0.0%	0 / 2 0.0%	0 / 1 0.0%	0 / 1 0.0%	0 / 1 0.0%	0 / 3 0.0%	0 / 1 0.0%	0 / 4 0.0%	0 / 4 0.0%	0 / 1 0.0%	0 / 1 0.0%	0 / 5 0.0%	0 / 0 0.0%	0 / 0 0.0%	0 / 0 0.0%	0 / 0 0.0%	21 / 42 50.0%E	17 / 42 40.5%	2 / 42 4.8%AB	1 / 42 2.4%C	1 / 42 2.4%CE				
1	Crinalium epipsammum PCC 9333	2 / 42 4.8%	1 / 1 100.0%	0 / 1 0.0%	0 / 1 0.0%	1 / 1 100.0%	0 / 3 0.0%	0 / 2 0.0%	0 / 1 0.0%	0 / 1 0.0%	0 / 2 0.0%	0 / 1 0.0%	0 / 4 0.0%	0 / 2 0.0%	0 / 1 0.0%	0 / 1 0.0%	0 / 1 0.0%	0 / 3 0.0%	0 / 1 0.0%	0 / 4 0.0%	0 / 4 0.0%	0 / 1 0.0%	0 / 1 0.0%	0 / 5 0.0%	0 / 0 0.0%	0 / 0 0.0%	0 / 0 0.0%	0 / 0 0.0%	21 / 42 50.0%E	17 / 42 40.5%	2 / 42 4.8%AB	1 / 42 2.4%CE	1 / 42 2.4%C				
1033	Proteobacteria	59095 / 61487 96.1%	2361 / 2409 98.0%	2261 / 2309 97.9%	2084 / 2139 97.4%	2756 / 2803 98.3%	4164 / 4299 96.9%	3801 / 3925 96.8%	1119 / 1157 96.7%	1483 / 1580 93.9%	4395 / 4529 97.0%	1097 / 1145 95.8%	4050 / 4234 95.7%	2691 / 2814 95.6%	1346 / 1414 95.2%	2132 / 2180 97.8%	1089 / 1158 94.0%	2680 / 2789 96.1%	1257 / 1313 95.7%	3127 / 3305 94.6%	5159 / 5461 94.5%	2741 / 2815 97.4%	1192 / 1281 93.1%	5763 / 6036 95.5%	338 / 378 89.4%	0 / 0 0.0%	4 / 8 50.0%	5 / 6 83.3%	36489 / 61487 59.3%AB	19265 / 61487 31.3%ABE	2124 / 61487 3.5%B	1217 / 61487 2.0%BE	1153 / 61487 1.9%	1062 / 61487 1.7%E	112 / 61487 0.2%C	64 / 61487 0.1%CE	1 / 61487 0.0%GG
		35257 / [illegible]	1485 / [illegible]	1439 / [illegible]	1448 / [illegible]	1484 / [illegible]	2561 / [illegible]	2414 / [illegible]	599 / [illegible]	992 / [illegible]	2344 / [illegible]	587 / [illegible]	2221 / [illegible]	1814 / [illegible]	843 / [illegible]	1371 / [illegible]	581 / [illegible]	1440 / [illegible]	792 / [illegible]	1712 / [illegible]	3101 / [illegible]	1778 / [illegible]	679 / [illegible]	3327 / [illegible]	241 / [illegible]			[illegible]	21414 / [illegible]	11869 / [illegible]	1196 / [illegible]	778 / [illegible]	609 / [illegible]	504 / [illegible]	19 / [illegible]	[illegible]	[illegible]

514	Gammaproteobacteria	36399 96.9%	1498 99.1%	1471 97.8%	1474 98.2%	1505 98.6%	2636 97.2%	2469 97.8%	618 96.9%	1041 95.3%	2400 97.7%	615 95.4%	2317 95.9%	1872 96.9%	871 96.8%	1397 98.1%	616 94.3%	1482 97.2%	819 96.7%	1809 94.6%	3267 94.9%	1813 98.1%	715 95.0%	3443 96.6%	245 98.4%	0 / 0 0.0%	0 / 1 0.0%	4 / 5 80.0%	36399 58.8%AB	36399 32.6%ABE	36399 3.3%B	36399 2.1%BE	36399 1.7%E	36399 1.4%	36399 0.1%C	9 / 36399 0.0%CE	1 / 36399 0.0%GG
23	Beggiatoales	921 / 923 99.8%	24 / 24 100.0%	28 / 28 100.0%	24 / 24 100.0%	67 / 67 100.0%	69 / 69 100.0%	69 / 69 100.0%	24 / 24 100.0%	24 / 24 100.0%	72 / 72 100.0%	24 / 24 100.0%	72 / 72 100.0%	28 / 28 100.0%	24 / 24 100.0%	24 / 24 100.0%	24 / 24 100.0%	29 / 29 100.0%	24 / 24 100.0%	49 / 49 100.0%	75 / 76 98.7%	27 / 27 100.0%	24 / 24 100.0%	96 / 97 99.0%	0 / 0 0.0%	0 / 0 0.0%	0 / 0 0.0%	0 / 0 0.0%	581 / 923 62.9%AB	282 / 923 30.6%ABE	45 / 923 4.9%B	13 / 923 1.4%BE	1 / 923 0.1%E	1 / 923 0.1%			
18	Francisella group	722 / 722 100.0%	19 / 19 100.0%	19 / 19 100.0%	19 / 19 100.0%	55 / 55 100.0%	57 / 57 100.0%	57 / 57 100.0%	19 / 19 100.0%	19 / 19 100.0%	55 / 55 100.0%	19 / 19 100.0%	57 / 57 100.0%	22 / 22 100.0%	19 / 19 100.0%	19 / 19 100.0%	19 / 19 100.0%	19 / 19 100.0%	19 / 19 100.0%	38 / 38 100.0%	57 / 57 100.0%	20 / 20 100.0%	19 / 19 100.0%	76 / 76 100.0%	0 / 0 0.0%	0 / 0 0.0%	0 / 0 0.0%	0 / 0 0.0%	448 / 722 62.0%AB	216 / 722 29.9%ABE	45 / 722 6.2%B	13 / 722 1.8%BE					
18	Francisella	722 / 722 100.0%	19 / 19 100.0%	19 / 19 100.0%	19 / 19 100.0%	55 / 55 100.0%	57 / 57 100.0%	57 / 57 100.0%	19 / 19 100.0%	19 / 19 100.0%	55 / 55 100.0%	19 / 19 100.0%	57 / 57 100.0%	22 / 22 100.0%	19 / 19 100.0%	19 / 19 100.0%	19 / 19 100.0%	19 / 19 100.0%	19 / 19 100.0%	38 / 38 100.0%	57 / 57 100.0%	20 / 20 100.0%	19 / 19 100.0%	76 / 76 100.0%	0 / 0 0.0%	0 / 0 0.0%	0 / 0 0.0%	0 / 0 0.0%	448 / 722 62.0%AB	216 / 722 29.9%ABE	45 / 722 6.2%B	13 / 722 1.8%BE					
15	"Bacterium tularense" McCoy and Chapin 1912	609 / 609 100.0%	16 / 16 100.0%	16 / 16 100.0%	16 / 16 100.0%	48 / 48 100.0%	48 / 48 100.0%	48 / 48 100.0%	16 / 16 100.0%	16 / 16 100.0%	48 / 48 100.0%	16 / 16 100.0%	48 / 48 100.0%	16 / 16 100.0%	16 / 16 100.0%	16 / 16 100.0%	16 / 16 100.0%	16 / 16 100.0%	16 / 16 100.0%	32 / 32 100.0%	48 / 48 100.0%	17 / 17 100.0%	16 / 16 100.0%	64 / 64 100.0%	0 / 0 0.0%	0 / 0 0.0%	0 / 0 0.0%	0 / 0 0.0%	384 / 609 63.1%AB	184 / 609 30.2%ABE	32 / 609 5.3%B	9 / 609 1.5%BE					
3	"Francisella tularensis subsp. novicida" (Larson et al. 1955) Sjostedt 2005	114 / 114 100.0%	3 / 3 100.0%	3 / 3 100.0%	3 / 3 100.0%	9 / 9 100.0%	9 / 9 100.0%	9 / 9 100.0%	3 / 3 100.0%	3 / 3 100.0%	9 / 9 100.0%	3 / 3 100.0%	9 / 9 100.0%	3 / 3 100.0%	3 / 3 100.0%	3 / 3 100.0%	3 / 3 100.0%	3 / 3 100.0%	3 / 3 100.0%	6 / 6 100.0%	9 / 9 100.0%	3 / 3 100.0%	3 / 3 100.0%	12 / 12 100.0%	0 / 0 0.0%	0 / 0 0.0%	0 / 0 0.0%	0 / 0 0.0%	78 / 114 68.4%AB	36 / 114 31.6%ABE							
1	Francisella novicida U112	38 / 38 100.0%	1 / 1 100.0%	1 / 1 100.0%	1 / 1 100.0%	3 / 3 100.0%	3 / 3 100.0%	3 / 3 100.0%	1 / 1 100.0%	1 / 1 100.0%	3 / 3 100.0%	1 / 1 100.0%	3 / 3 100.0%	1 / 1 100.0%	1 / 1 100.0%	1 / 1 100.0%	1 / 1 100.0%	1 / 1 100.0%	1 / 1 100.0%	2 / 2 100.0%	3 / 3 100.0%	1 / 1 100.0%	1 / 1 100.0%	4 / 4 100.0%	0 / 0 0.0%	0 / 0 0.0%	0 / 0 0.0%	0 / 0 0.0%	26 / 38 68.4%AB	12 / 38 31.6%ABE							
1	Francisella cf. novicida 3523	38 / 38 100.0%	1 / 1 100.0%	1 / 1 100.0%	1 / 1 100.0%	3 / 3 100.0%	3 / 3 100.0%	3 / 3 100.0%	1 / 1 100.0%	1 / 1 100.0%	3 / 3 100.0%	1 / 1 100.0%	3 / 3 100.0%	1 / 1 100.0%	1 / 1 100.0%	1 / 1 100.0%	1 / 1 100.0%	1 / 1 100.0%	1 / 1 100.0%	2 / 2 100.0%	3 / 3 100.0%	1 / 1 100.0%	1 / 1 100.0%	4 / 4 100.0%	0 / 0 0.0%	0 / 0 0.0%	0 / 0 0.0%	0 / 0 0.0%	26 / 38 68.4%AB	12 / 38 31.6%ABE							
1	Francisella cf. novicida Fx1	38 / 38 100.0%	1 / 1 100.0%	1 / 1 100.0%	1 / 1 100.0%	3 / 3 100.0%	3 / 3 100.0%	3 / 3 100.0%	1 / 1 100.0%	1 / 1 100.0%	3 / 3 100.0%	1 / 1 100.0%	3 / 3 100.0%	1 / 1 100.0%	1 / 1 100.0%	1 / 1 100.0%	1 / 1 100.0%	1 / 1 100.0%	1 / 1 100.0%	2 / 2 100.0%	3 / 3 100.0%	1 / 1 100.0%	1 / 1 100.0%	4 / 4 100.0%	0 / 0 0.0%	0 / 0 0.0%	0 / 0 0.0%	0 / 0 0.0%	26 / 38 68.4%AB	12 / 38 31.6%ABE							
6	"Francisella tularensis subsp. nearctica" Olsufjev 1970	229 / 229 100.0%	6 / 6 100.0%	6 / 6 100.0%	6 / 6 100.0%	18 / 18 100.0%	18 / 18 100.0%	18 / 18 100.0%	6 / 6 100.0%	6 / 6 100.0%	18 / 18 100.0%	6 / 6 100.0%	18 / 18 100.0%	6 / 6 100.0%	6 / 6 100.0%	6 / 6 100.0%	6 / 6 100.0%	6 / 6 100.0%	6 / 6 100.0%	12 / 12 100.0%	18 / 18 100.0%	7 / 7 100.0%	6 / 6 100.0%	24 / 24 100.0%	0 / 0 0.0%	0 / 0 0.0%	0 / 0 0.0%	0 / 0 0.0%	156 / 229 68.1%AB	73 / 229 31.9%ABE							
1	Francisella tularensis Biovar A str. SCHU S4	38 / 38 100.0%	1 / 1 100.0%	1 / 1 100.0%	1 / 1 100.0%	3 / 3 100.0%	3 / 3 100.0%	3 / 3 100.0%	1 / 1 100.0%	1 / 1 100.0%	3 / 3 100.0%	1 / 1 100.0%	3 / 3 100.0%	1 / 1 100.0%	1 / 1 100.0%	1 / 1 100.0%	1 / 1 100.0%	1 / 1 100.0%	1 / 1 100.0%	2 / 2 100.0%	3 / 3 100.0%	1 / 1 100.0%	1 / 1 100.0%	4 / 4 100.0%	0 / 0 0.0%	0 / 0 0.0%	0 / 0 0.0%	0 / 0 0.0%	26 / 38 68.4%AB	12 / 38 31.6%ABE							
1	Francisella tularensis subsp. tularensis FSC 198	38 / 38 100.0%	1 / 1 100.0%	1 / 1 100.0%	1 / 1 100.0%	3 / 3 100.0%	3 / 3 100.0%	3 / 3 100.0%	1 / 1 100.0%	1 / 1 100.0%	3 / 3 100.0%	1 / 1 100.0%	3 / 3 100.0%	1 / 1 100.0%	1 / 1 100.0%	1 / 1 100.0%	1 / 1 100.0%	1 / 1 100.0%	1 / 1 100.0%	2 / 2 100.0%	3 / 3 100.0%	1 / 1 100.0%	1 / 1 100.0%	4 / 4 100.0%	0 / 0 0.0%	0 / 0 0.0%	0 / 0 0.0%	0 / 0 0.0%	26 / 38 68.4%AB	12 / 38 31.6%ABE							
1	Francisella tularensis subsp. tularensis WY96-3418	38 / 38 100.0%	1 / 1 100.0%	1 / 1 100.0%	1 / 1 100.0%	3 / 3 100.0%	3 / 3 100.0%	3 / 3 100.0%	1 / 1 100.0%	1 / 1 100.0%	3 / 3 100.0%	1 / 1 100.0%	3 / 3 100.0%	1 / 1 100.0%	1 / 1 100.0%	1 / 1 100.0%	1 / 1 100.0%	1 / 1 100.0%	1 / 1 100.0%	2 / 2 100.0%	3 / 3 100.0%	1 / 1 100.0%	1 / 1 100.0%	4 / 4 100.0%	0 / 0 0.0%	0 / 0 0.0%	0 / 0 0.0%	0 / 0 0.0%	26 / 38 68.4%AB	12 / 38 31.6%ABE							
1	Francisella tularensis subsp. tularensis NE061598	38 / 38 100.0%	1 / 1 100.0%	1 / 1 100.0%	1 / 1 100.0%	3 / 3 100.0%	3 / 3 100.0%	3 / 3 100.0%	1 / 1 100.0%	1 / 1 100.0%	3 / 3 100.0%	1 / 1 100.0%	3 / 3 100.0%	1 / 1 100.0%	1 / 1 100.0%	1 / 1 100.0%	1 / 1 100.0%	1 / 1 100.0%	1 / 1 100.0%	2 / 2 100.0%	3 / 3 100.0%	1 / 1 100.0%	1 / 1 100.0%	4 / 4 100.0%	0 / 0 0.0%	0 / 0 0.0%	0 / 0 0.0%	0 / 0 0.0%	26 / 38 68.4%AB	12 / 38 31.6%ABE							
2	Francisella tularensis TI0902	77 / 77 100.0%	2 / 2 100.0%	2 / 2 100.0%	2 / 2 100.0%	6 / 6 100.0%	6 / 6 100.0%	6 / 6 100.0%	2 / 2 100.0%	2 / 2 100.0%	6 / 6 100.0%	2 / 2 100.0%	6 / 6 100.0%	2 / 2 100.0%	2 / 2 100.0%	2 / 2 100.0%	2 / 2 100.0%	2 / 2 100.0%	2 / 2 100.0%	4 / 4 100.0%	6 / 6 100.0%	3 / 3 100.0%	2 / 2 100.0%	8 / 8 100.0%	0 / 0 0.0%	0 / 0 0.0%	0 / 0 0.0%	0 / 0 0.0%	52 / 77 67.5%AB	25 / 77 32.5%ABE							
1	Francisella tularensis TIGB03	39 / 39 100.0%	1 / 1 100.0%	1 / 1 100.0%	1 / 1 100.0%	3 / 3 100.0%	3 / 3 100.0%	3 / 3 100.0%	1 / 1 100.0%	1 / 1 100.0%	3 / 3 100.0%	1 / 1 100.0%	3 / 3 100.0%	1 / 1 100.0%	1 / 1 100.0%	1 / 1 100.0%	1 / 1 100.0%	1 / 1 100.0%	1 / 1 100.0%	2 / 2 100.0%	3 / 3 100.0%	2 / 2 100.0%	1 / 1 100.0%	4 / 4 100.0%	0 / 0 0.0%	0 / 0 0.0%	0 / 0 0.0%	0 / 0 0.0%	26 / 39 66.7%AB	13 / 39 33.3%ABE							
5	"Francisella tularensis subsp. holarctica" Olsufjev 1970	228 / 228 100.0%	6 / 6 100.0%	6 / 6 100.0%	6 / 6 100.0%	18 / 18 100.0%	18 / 18 100.0%	18 / 18 100.0%	6 / 6 100.0%	6 / 6 100.0%	18 / 18 100.0%	6 / 6 100.0%	18 / 18 100.0%	6 / 6 100.0%	6 / 6 100.0%	6 / 6 100.0%	6 / 6 100.0%	6 / 6 100.0%	6 / 6 100.0%	12 / 12 100.0%	18 / 18 100.0%	6 / 6 100.0%	6 / 6 100.0%	24 / 24 100.0%	0 / 0 0.0%	0 / 0 0.0%	0 / 0 0.0%	0 / 0 0.0%	142 / 228 62.3%AB	67 / 228 29.4%ABE	14 / 228 6.1%B	5 / 228 2.2%BE					
1	Francisella tularensis subsp. holarctica FSC200	38 / 38 100.0%	1 / 1 100.0%	1 / 1 100.0%	1 / 1 100.0%	3 / 3 100.0%	3 / 3 100.0%	3 / 3 100.0%	1 / 1 100.0%	1 / 1 100.0%	3 / 3 100.0%	1 / 1 100.0%	3 / 3 100.0%	1 / 1 100.0%	1 / 1 100.0%	1 / 1 100.0%	1 / 1 100.0%	1 / 1 100.0%	1 / 1 100.0%	2 / 2 100.0%	3 / 3 100.0%	1 / 1 100.0%	1 / 1 100.0%	4 / 4 100.0%	0 / 0 0.0%	0 / 0 0.0%	0 / 0 0.0%	0 / 0 0.0%	26 / 38 68.4%AB	12 / 38 31.6%ABE							
1	Francisella tularensis subsp. holarctica LVS	38 / 38 100.0%	1 / 1 100.0%	1 / 1 100.0%	1 / 1 100.0%	3 / 3 100.0%	3 / 3 100.0%	3 / 3 100.0%	1 / 1 100.0%	1 / 1 100.0%	3 / 3 100.0%	1 / 1 100.0%	3 / 3 100.0%	1 / 1 100.0%	1 / 1 100.0%	1 / 1 100.0%	1 / 1 100.0%	1 / 1 100.0%	1 / 1 100.0%	2 / 2 100.0%	3 / 3 100.0%	1 / 1 100.0%	1 / 1 100.0%	4 / 4 100.0%	0 / 0 0.0%	0 / 0 0.0%	0 / 0 0.0%	0 / 0 0.0%	26 / 38 68.4%AB	12 / 38 31.6%ABE							
1	Francisella tularensis subsp. holarctica OSU18	76 / 76 100.0%	2 / 2 100.0%	2 / 2 100.0%	2 / 2 100.0%	6 / 6 100.0%	6 / 6 100.0%	6 / 6 100.0%	2 / 2 100.0%	2 / 2 100.0%	6 / 6 100.0%	2 / 2 100.0%	6 / 6 100.0%	2 / 2 100.0%	2 / 2 100.0%	2 / 2 100.0%	2 / 2 100.0%	2 / 2 100.0%	2 / 2 100.0%	4 / 4 100.0%	6 / 6 100.0%	2 / 2 100.0%	2 / 2 100.0%	8 / 8 100.0%	0 / 0 0.0%	0 / 0 0.0%	0 / 0 0.0%	0 / 0 0.0%	52 / 76 68.4%AB	24 / 76 31.6%ABE							
1	Francisella tularensis subsp. holarctica FTA	38 / 38 100.0%	1 / 1 100.0%	1 / 1 100.0%	1 / 1 100.0%	3 / 3 100.0%	3 / 3 100.0%	3 / 3 100.0%	1 / 1 100.0%	1 / 1 100.0%	3 / 3 100.0%	1 / 1 100.0%	3 / 3 100.0%	1 / 1 100.0%	1 / 1 100.0%	1 / 1 100.0%	1 / 1 100.0%	1 / 1 100.0%	1 / 1 100.0%	2 / 2 100.0%	3 / 3 100.0%	1 / 1 100.0%	1 / 1 100.0%	4 / 4 100.0%	0 / 0 0.0%	0 / 0 0.0%	0 / 0 0.0%	0 / 0 0.0%	14 / 38 36.8%B	12 / 38 31.6%AB	7 / 38 18.4%ABE	5 / 38 13.2%BE					
1	Francisella tularensis subsp. holarctica F92	38 / 38 100.0%	1 / 1 100.0%	1 / 1 100.0%	1 / 1 100.0%	3 / 3 100.0%	3 / 3 100.0%	3 / 3 100.0%	1 / 1 100.0%	1 / 1 100.0%	3 / 3 100.0%	1 / 1 100.0%	3 / 3 100.0%	1 / 1 100.0%	1 / 1 100.0%	1 / 1 100.0%	1 / 1 100.0%	1 / 1 100.0%	1 / 1 100.0%	2 / 2 100.0%	3 / 3 100.0%	1 / 1 100.0%	1 / 1 100.0%	4 / 4 100.0%	0 / 0 0.0%	0 / 0 0.0%	0 / 0 0.0%	0 / 0 0.0%	26 / 38 68.4%AB	12 / 38 31.6%ABE							
1	"Francisella tularensis subsp. mediasiatica" (Aikimbaev 1966) Olsufjev and Meshcheryakova 1982	38 / 38 100.0%	1 / 1 100.0%	1 / 1 100.0%	1 / 1 100.0%	3 / 3 100.0%	3 / 3 100.0%	3 / 3 100.0%	1 / 1 100.0%	1 / 1 100.0%	3 / 3 100.0%	1 / 1 100.0%	3 / 3 100.0%	1 / 1 100.0%	1 / 1 100.0%	1 / 1 100.0%	1 / 1 100.0%	1 / 1 100.0%	1 / 1 100.0%	2 / 2 100.0%	3 / 3 100.0%	1 / 1 100.0%	1 / 1 100.0%	4 / 4 100.0%	0 / 0 0.0%	0 / 0 0.0%	0 / 0 0.0%	0 / 0 0.0%	18 / 38 47.4%B	8 / 38 21.1%AB	8 / 38 21.1%ABE	4 / 38 10.5%BE					
1	Francisella tularensis subsp. mediasiatica FSC147	38 / 38 100.0%	1 / 1 100.0%	1 / 1 100.0%	1 / 1 100.0%	3 / 3 100.0%	3 / 3 100.0%	3 / 3 100.0%	1 / 1 100.0%	1 / 1 100.0%	3 / 3 100.0%	1 / 1 100.0%	3 / 3 100.0%	1 / 1 100.0%	1 / 1 100.0%	1 / 1 100.0%	1 / 1 100.0%	1 / 1 100.0%	1 / 1 100.0%	2 / 2 100.0%	3 / 3 100.0%	1 / 1 100.0%	1 / 1 100.0%	4 / 4 100.0%	0 / 0 0.0%	0 / 0 0.0%	0 / 0 0.0%	0 / 0 0.0%	18 / 38 47.4%B	8 / 38 21.1%AB	8 / 38 21.1%ABE	4 / 38 10.5%BE					
1	'Philomiragia' bacterium	39 / 39 100.0%	1 / 1 100.0%	1 / 1 100.0%	1 / 1 100.0%	3 / 3 100.0%	3 / 3 100.0%	3 / 3 100.0%	1 / 1 100.0%	1 / 1 100.0%	3 / 3 100.0%	1 / 1 100.0%	3 / 3 100.0%	2 / 2 100.0%	1 / 1 100.0%	1 / 1 100.0%	1 / 1 100.0%	1 / 1 100.0%	1 / 1 100.0%	2 / 2 100.0%	3 / 3 100.0%	1 / 1 100.0%	1 / 1 100.0%	4 / 4 100.0%	0 / 0 0.0%	0 / 0 0.0%	0 / 0 0.0%	0 / 0 0.0%	14 / 39 35.9%AB	13 / 39 33.3%B	8 / 39 20.5%ABE	4 / 39 10.3%BE					
1	Francisella philomiragia subsp. philomiragia ATCC 25017	39 / 39 100.0%	1 / 1 100.0%	1 / 1 100.0%	1 / 1 100.0%	3 / 3 100.0%	3 / 3 100.0%	3 / 3 100.0%	1 / 1 100.0%	1 / 1 100.0%	3 / 3 100.0%	1 / 1 100.0%	3 / 3 100.0%	2 / 2 100.0%	1 / 1 100.0%	1 / 1 100.0%	1 / 1 100.0%	1 / 1 100.0%	1 / 1 100.0%	2 / 2 100.0%	3 / 3 100.0%	1 / 1 100.0%	1 / 1 100.0%	4 / 4 100.0%	0 / 0 0.0%	0 / 0 0.0%	0 / 0 0.0%	0 / 0 0.0%	14 / 39 35.9%AB	13 / 39 33.3%B	8 / 39 20.5%ABE	4 / 39 10.3%BE					
1	Francisella sp. TX077308	39 / 39 100.0%	1 / 1 100.0%	1 / 1 100.0%	1 / 1 100.0%	3 / 3 100.0%	3 / 3 100.0%	3 / 3 100.0%	1 / 1 100.0%	1 / 1 100.0%	3 / 3 100.0%	1 / 1 100.0%	3 / 3 100.0%	2 / 2 100.0%	1 / 1 100.0%	1 / 1 100.0%	1 / 1 100.0%	1 / 1 100.0%	1 / 1 100.0%	2 / 2 100.0%	3 / 3 100.0%	1 / 1 100.0%	1 / 1 100.0%	4 / 4 100.0%	0 / 0 0.0%	0 / 0 0.0%	0 / 0 0.0%	0 / 0 0.0%	27 / 39 69.2%AB	12 / 39 30.8%ABE							
1	DSM 23596	35 / 35 100.0%	1 / 1 100.0%	1 / 1 100.0%	1 / 1 100.0%	1 / 1 100.0%	3 / 3 100.0%	3 / 3 100.0%	1 / 1 100.0%	1 / 1 100.0%	1 / 1 100.0%	1 / 1 100.0%	3 / 3 100.0%	2 / 2 100.0%	1 / 1 100.0%	1 / 1 100.0%	1 / 1 100.0%	1 / 1 100.0%	1 / 1 100.0%	2 / 2 100.0%	3 / 3 100.0%	1 / 1 100.0%	1 / 1 100.0%	4 / 4 100.0%	0 / 0 0.0%	0 / 0 0.0%	0 / 0 0.0%	0 / 0 0.0%	23 / 35 65.7%AB	12 / 35 34.3%ABE							
1	'Francisella asiatica'	35 / 35 100.0%	1 / 1 100.0%	1 / 1 100.0%	1 / 1 100.0%	1 / 1 100.0%	3 / 3 100.0%	3 / 3 100.0%	1 / 1 100.0%	1 / 1 100.0%	1 / 1 100.0%	1 / 1 100.0%	3 / 3 100.0%	2 / 2 100.0%	1 / 1 100.0%	1 / 1 100.0%	1 / 1 100.0%	1 / 1 100.0%	1 / 1 100.0%	2 / 2 100.0%	3 / 3 100.0%	1 / 1 100.0%	1 / 1 100.0%	4 / 4 100.0%	0 / 0 0.0%	0 / 0 0.0%	0 / 0 0.0%	0 / 0 0.0%	23 / 35 65.7%AB	12 / 35 34.3%ABE							
1	Francisella noatunensis subsp. orientalis Toba 04	35 / 35 100.0%	1 / 1 100.0%	1 / 1 100.0%	1 / 1 100.0%	1 / 1 100.0%	3 / 3 100.0%	3 / 3 100.0%	1 / 1 100.0%	1 / 1 100.0%	1 / 1 100.0%	1 / 1 100.0%	3 / 3 100.0%	2 / 2 100.0%	1 / 1 100.0%	1 / 1 100.0%	1 / 1 100.0%	1 / 1 100.0%	1 / 1 100.0%	2 / 2 100.0%	3 / 3 100.0%	1 / 1 100.0%	1 / 1 100.0%	4 / 4 100.0%	0 / 0 0.0%	0 / 0 0.0%	0 / 0 0.0%	0 / 0 0.0%	23 / 35 65.7%AB	12 / 35 34.3%ABE							
5	Piscirickettsia group	199 / 201 99.0%	5 / 5 100.0%	9 / 9 100.0%	5 / 5 100.0%	12 / 12 100.0%	12 / 12 100.0%	12 / 12 100.0%	5 / 5 100.0%	5 / 5 100.0%	17 / 17 100.0%	5 / 5 100.0%	15 / 15 100.0%	6 / 6 100.0%	5 / 5 100.0%	5 / 5 100.0%	5 / 5 100.0%	10 / 10 100.0%	5 / 5 100.0%	11 / 11 100.0%	18 / 19 94.7%	7 / 7 100.0%	5 / 5 100.0%	20 / 21 95.2%	0 / 0 0.0%	0 / 0 0.0%	0 / 0 0.0%	0 / 0 0.0%	133 / 201 66.2%AB	66 / 201 32.8%ABE	1 / 201 0.5%	1 / 201 0.5%E					
1	Thiomicrospira	43 / 43 100.0%	1 / 1 100.0%	2 / 2 100.0%	1 / 1 100.0%	3 / 3 100.0%	3 / 3 100.0%	3 / 3 100.0%	1 / 1 100.0%	1 / 1 100.0%	4 / 4 100.0%	1 / 1 100.0%	3 / 3 100.0%	2 / 2 100.0%	1 / 1 100.0%	1 / 1 100.0%	1 / 1 100.0%	2 / 2 100.0%	1 / 1 100.0%	2 / 2 100.0%	3 / 3 100.0%	2 / 2 100.0%	1 / 1 100.0%	4 / 4 100.0%	0 / 0 0.0%	0 / 0 0.0%	0 / 0 0.0%	0 / 0 0.0%	29 / 43 67.4%AB	14 / 43 32.6%ABE							
1	ATCC 35932	43 / 43 100.0%	1 / 1 100.0%	2 / 2 100.0%	1 / 1 100.0%	3 / 3 100.0%	3 / 3 100.0%	3 / 3 100.0%	1 / 1 100.0%	1 / 1 100.0%	4 / 4 100.0%	1 / 1 100.0%	3 / 3 100.0%	2 / 2 100.0%	1 / 1 100.0%	1 / 1 100.0%	1 / 1 100.0%	2 / 2 100.0%	1 / 1 100.0%	2 / 2 100.0%	3 / 3 100.0%	2 / 2 100.0%	1 / 1 100.0%	4 / 4 100.0%	0 / 0 0.0%	0 / 0 0.0%	0 / 0 0.0%	0 / 0 0.0%	29 / 43 67.4%AB	14 / 43 32.6%ABE							
1	Thiomicrospira crunogena XCL-2	43 / 43 100.0%	1 / 1 100.0%	2 / 2 100.0%	1 / 1 100.0%	3 / 3 100.0%	3 / 3 100.0%	3 / 3 100.0%	1 / 1 100.0%	1 / 1 100.0%	4 / 4 100.0%	1 / 1 100.0%	3 / 3 100.0%	2 / 2 100.0%	1 / 1 100.0%	1 / 1 100.0%	1 / 1 100.0%	2 / 2 100.0%	1 / 1 100.0%	2 / 2 100.0%	3 / 3 100.0%	2 / 2 100.0%	1 / 1 100.0%	4 / 4 100.0%	0 / 0 0.0%	0 / 0 0.0%	0 / 0 0.0%	0 / 0 0.0%	29 / 43 67.4%AB	14 / 43 32.6%ABE							
1	Cycloclasticus	35 / 35 100.0%	1 / 1 100.0%	1 / 1 100.0%	1 / 1 100.0%	1 / 1 100.0%	2 / 2 100.0%	2 / 2 100.0%	1 / 1 100.0%	1 / 1 100.0%	2 / 2 100.0%	1 / 1 100.0%	3 / 3 100.0%	1 / 1 100.0%	1 / 1 100.0%	1 / 1 100.0%	1 / 1 100.0%	2 / 2 100.0%	1 / 1 100.0%	3 / 3 100.0%	4 / 4 100.0%	1 / 1 100.0%	1 / 1 100.0%	3 / 3 100.0%	0 / 0 0.0%	0 / 0 0.0%	0 / 0 0.0%	0 / 0 0.0%	24 / 35 68.6%AB	11 / 35 31.4%ABE							
1	Cycloclasticus sp. P1	35 / 35 100.0%	1 / 1 100.0%	1 / 1 100.0%	1 / 1 100.0%	1 / 1 100.0%	2 / 2 100.0%	2 / 2 100.0%	1 / 1 100.0%	1 / 1 100.0%	2 / 2 100.0%	1 / 1 100.0%	3 / 3 100.0%	1 / 1 100.0%	1 / 1 100.0%	1 / 1 100.0%	1 / 1 100.0%	2 / 2 100.0%	1 / 1 100.0%	3 / 3 100.0%	4 / 4 100.0%	1 / 1 100.0%	1 / 1 100.0%	3 / 3 100.0%	0 / 0 0.0%	0 / 0 0.0%	0 / 0 0.0%	0 / 0 0.0%	24 / 35 68.6%AB	11 / 35 31.4%ABE							
2	Methyliphaga	82 / 82 100.0%	2 / 2 100.0%	4 / 4 100.0%	2 / 2 100.0%	6 / 6 100.0%	4 / 4 100.0%	4 / 4 100.0%	2 / 2 100.0%	2 / 2 100.0%	8 / 8 100.0%	2 / 2 100.0%	6 / 6 100.0%	2 / 2 100.0%	2 / 2 100.0%	2 / 2 100.0%	2 / 2 100.0%	4 / 4 100.0%	2 / 2 100.0%	4 / 4 100.0%	8 / 8 100.0%	2 / 2 100.0%	2 / 2 100.0%	10 / 10 100.0%	0 / 0 0.0%	0 / 0 0.0%	0 / 0 0.0%	0 / 0 0.0%	55 / 82 67.1%AB	27 / 82 32.9%ABE							
1	ATCC BAA-2433	41 / 41 100.0%	1 / 1 100.0%	2 / 2 100.0%	1 / 1 100.0%	3 / 3 100.0%	2 / 2 100.0%	2 / 2 100.0%	1 / 1 100.0%	1 / 1 100.0%	4 / 4 100.0%	1 / 1 100.0%	3 / 3 100.0%	1 / 1 100.0%	1 / 1 100.0%	1 / 1 100.0%	1 / 1 100.0%	2 / 2 100.0%	1 / 1 100.0%	2 / 2 100.0%	4 / 4 100.0%	1 / 1 100.0%	1 / 1 100.0%	5 / 5 100.0%	0 / 0 0.0%	0 / 0 0.0%	0 / 0 0.0%	0 / 0 0.0%	27 / 41 65.9%AB	14 / 41 34.1%ABE							
1	ATCC BAA-2434	41 / 41 100.0%	1 / 1 100.0%	2 / 2 100.0%	1 / 1 100.0%	3 / 3 100.0%	2 / 2 100.0%	2 / 2 100.0%	1 / 1 100.0%	1 / 1 100.0%	4 / 4 100.0%	1 / 1 100.0%	3 / 3 100.0%	1 / 1 100.0%	1 / 1 100.0%	1 / 1 100.0%	1 / 1 100.0%	2 / 2 100.0%	1 / 1 100.0%	2 / 2 100.0%	4 / 4 100.0%	1 / 1 100.0%	1 / 1 100.0%	5 / 5 100.0%	0 / 0 0.0%	0 / 0 0.0%	0 / 0 0.0%	0 / 0 0.0%	28 / 41 68.3%AB	13 / 41 31.7%ABE							
1	Thialkalimicrobium	39 / 41 95.1%	1 / 1 100.0%	2 / 2 100.0%	1 / 1 100.0%	2 / 2 100.0%	3 / 3 100.0%	3 / 3 100.0%	1 / 1 100.0%	1 / 1 100.0%	3 / 3 100.0%	1 / 1 100.0%	3 / 3 100.0%	1 / 1 100.0%	1 / 1 100.0%	1 / 1 100.0%	1 / 1 100.0%	2 / 2 100.0%	1 / 1 100.0%	2 / 2 100.0%	3 / 4 75.0%	2 / 2 100.0%	1 / 1 100.0%	3 / 4 75.0%	0 / 0 0.0%	0 / 0 0.0%	0 / 0 0.0%	0 / 0 0.0%	25 / 41 61.0%AB	14 / 41 34.1%ABE	1 / 41 2.4%	1 / 41 2.4%E					
1	DSM 14477	39 / 41 95.1%	1 / 1 100.0%	2 / 2 100.0%	1 / 1 100.0%	2 / 2 100.0%	3 / 3 100.0%	3 / 3 100.0%	1 / 1 100.0%	1 / 1 100.0%	3 / 3 100.0%	1 / 1 100.0%	3 / 3 100.0%	1 / 1 100.0%	1 / 1 100.0%	1 / 1 100.0%	1 / 1 100.0%	2 / 2 100.0%	1 / 1 100.0%	2 / 2 100.0%	3 / 4 75.0%	2 / 2 100.0%	1 / 1 100.0%	3 / 4 75.0%	0 / 0 0.0%	0 / 0 0.0%	0 / 0 0.0%	0 / 0 0.0%	25 / 41 61.0%AB	14 / 41 34.1%ABE	1 / 41 2.4%	1 / 41 2.4%E					
1	Thioalkalimicrobium cyclicum ALM1	39 / 41 95.1%	1 / 1 100.0%	2 / 2 100.0%	1 / 1 100.0%	2 / 2 100.0%	3 / 3 100.0%	3 / 3 100.0%	1 / 1 100.0%	1 / 1 100.0%	3 / 3 100.0%	1 / 1 100.0%	3 / 3 100.0%	1 / 1 100.0%	1 / 1 100.0%	1 / 1 100.0%	1 / 1 100.0%	2 / 2 100.0%	1 / 1 100.0%	2 / 2 100.0%	3 / 4 75.0%	2 / 2 100.0%	1 / 1 100.0%	3 / 4 75.0%	0 / 0 0.0%	0 / 0 0.0%	0 / 0 0.0%	0 / 0 0.0%	25 / 41 61.0%AB	14 / 41 34.1%ABE	1 / 41 2.4%	1 / 41 2.4%E					
69	Pseudomonaceae/Moraxellaceae group	4597 / 4626 99.4%	190 / 191 99.5%	222 / 222 100.0%	175 / 175 100.0%	327 / 327 100.0%	334 / 334 100.0%	256 / 256 100.0%	97 / 97 100.0%	71 / 71 100.0%	449 / 451 99.6%	105 / 105 100.0%	277 / 278 99.6%	148 / 148 100.0%	91 / 91 100.0%	148 / 148 100.0%	71 / 71 100.0%	200 / 200 100.0%	89 / 89 100.0%	201 / 201 100.0%	373 / 398 93.7%	260 / 260 100.0%	72 / 72 100.0%	410 / 410 100.0%	31 / 31 100.0%	0 / 0 0.0%	0 / 0 0.0%	0 / 0 0.0%	2969 / 4626 64.2%AB	1443 / 4626 31.2%ABE	128 / 4626 2.8%B	57 / 4626 1.2%BE	26 / 4626 0.6%	1 / 4626 0.0%C	1 / 4626 0.0%CE	1 / 4626 0.0%E	
22	Acinetobacteraceae	1530 / 1545 99.0%	56 / 56 100.0%	56 / 56 100.0%	80 / 80 100.0%	124 / 124 100.0%	89 / 89 100.0%	84 / 84 100.0%	24 / 24 100.0%	24 / 24 100.0%	148 / 148 100.0%	58 / 58 100.0%	91 / 91 100.0%	46 / 46 100.0%	41 / 41 100.0%	73 / 73 100.0%	24 / 24 100.0%	49 / 49 100.0%	42 / 42 100.0%	64 / 64 100.0%	119 / 134 88.8%	82 / 82 100.0%	25 / 25 100.0%	131 / 131 100.0%	0 / 0 0.0%	0 / 0 0.0%	0 / 0 0.0%	0 / 0 0.0%	924 / 1545 59.8%AB	491 / 1545 31.8%ABE	78 / 1545 5.0%B	37 / 1545 2.4%BE	15 / 1545 1.0%				
17	Acinetobacter	1231 / 1246 98.8%	50 / 50 100.0%	42 / 42 100.0%	68 / 68 100.0%	99 / 99 100.0%	71 / 71 100.0%	64 / 64 100.0%	18 / 18 100.0%	18 / 18 100.0%	117 / 117 100.0%	52 / 52 100.0%	71 / 71 100.0%	34 / 34 100.0%	34 / 34 100.0%	61 / 61 100.0%	18 / 18 100.0%	37 / 37 100.0%	36 / 36 100.0%	52 / 52 100.0%	96 / 111 86.5%	69 / 69 100.0%	19 / 19 100.0%	105 / 105 100.0%	0 / 0 0.0%	0 / 0 0.0%	0 / 0 0.0%	0 / 0 0.0%	735 / 1246 59.0%AB	399 / 1246 32.0%ABE	63 / 1246 5.1%B	34 / 1246 2.7%BE	15 / 1246 1.2%				
1	Acinetobacter baylyi ADP1	75 / 76 98.7%	3 / 3 100.0%	3 / 3 100.0%	4 / 4 100.0%	7 / 7 100.0%	4 / 4 100.0%	4 / 4 100.0%	1 / 1 100.0%	1 / 1 100.0%	8 / 8 100.0%	2 / 2 100.0%	4 / 4 100.0%	2 / 2 100.0%	2 / 2 100.0%	5 / 5 100.0%	1 / 1 100.0%	2 / 2 100.0%	2 / 2 100.0%	3 / 3 100.0%	5 / 6 83.3%	5 / 5 100.0%	1 / 1 100.0%	6 / 6 100.0%	0 / 0 0.0%	0 / 0 0.0%	0 / 0 0.0%	0 / 0 0.0%	49 / 76 64.5%AB	26 / 76 34.2%ABE	1 / 76 1.3%						
15	Acinetobacter calcoaceticus/baumannii complex	1086 / 1099 98.8%	44 / 44 100.0%	36 / 36 100.0%	60 / 60 100.0%	86 / 86 100.0%	63 / 63 100.0%	56 / 56 100.0%	16 / 16 100.0%	16 / 16 100.0%	102 / 102 100.0%	47 / 47 100.0%	63 / 63 100.0%	30 / 30 100.0%	30 / 30 100.0%	54 / 54 100.0%	16 / 16 100.0%	33 / 33 100.0%	32 / 32 100.0%	46 / 46 100.0%	86 / 99 86.9%	60 / 60 100.0%	17 / 17 100.0%	93 / 93 100.0%	0 / 0 0.0%	0 / 0 0.0%	0 / 0 0.0%	0 / 0 0.0%	639 / 1099 58.1%AB	350 / 1099 31.8%ABE	63 / 1099 5.7%B	34 / 1099 3.1%BE	13 / 1099 1.2%				
14	ATCC 19606	1020 / 1032 98.8%	41 / 41 100.0%	34 / 34 100.0%	56 / 56 100.0%	84 / 84 100.0%	60 / 60 100.0%	52 / 52 100.0%	15 / 15 100.0%	15 / 15 100.0%	99 / 99 100.0%	43 / 43 100.0%	59 / 59 100.0%	27 / 27 100.0%	28 / 28 100.0%	49 / 49 100.0%	15 / 15 100.0%	31 / 31 100.0%	30 / 30 100.0%	43 / 43 100.0%	81 / 93 87.1%	56 / 56 100.0%	16 / 16 100.0%	86 / 86 100.0%	0 / 0 0.0%	0 / 0 0.0%	0 / 0 0.0%	0 / 0 0.0%	601 / 1032 58.2%AB	322 / 1032 31.2%ABE	63 / 1032 6.1%B	34 / 1032 3.3%BE	12 / 1032 1.2%				
1	Acinetobacter baumannii ATCC 17978	69 / 70 98.6%	2 / 2 100.0%	3 / 3 100.0%	4 / 4 100.0%	5 / 5 100.0%	4 / 4 100.0%	4 / 4 100.0%	1 / 1 100.0%	1 / 1 100.0%	6 / 6 100.0%	3 / 3 100.0%	4 / 4 100.0%	2 / 2 100.0%	2 / 2 100.0%	4 / 4 100.0%	1 / 1 100.0%	2 / 2 100.0%	2 / 2 100.0%	3 / 3 100.0%	5 / 6 83.3%	4 / 4 100.0%	1 / 1 100.0%	6 / 6 100.0%	0 / 0 0.0%	0 / 0 0.0%	0 / 0 0.0%	0 / 0 0.0%	45 / 70 64.3%AB	24 / 70 34.3%ABE	1 / 70 1.4%						
1	Acinetobacter baumannii ACICU	63 / 64 98.4%	3 / 3 100.0%	0 / 0 0.0%	4 / 4 100.0%	6 / 6 100.0%	3 / 3 100.0%	2 / 2 100.0%	1 / 1 100.0%	1 / 1 100.0%	7 / 7 100.0%	3 / 3 100.0%	4 / 4 100.0%	2 / 2 100.0%	2 / 2 100.0%	1 / 1 100.0%	1 / 1 100.0%	2 / 2 100.0%	2 / 2 100.0%	3 / 3 100.0%	5 / 6 83.3%	4 / 4 100.0%	1 / 1 100.0%	6 / 6 100.0%	0 / 0 0.0%	0 / 0 0.0%	0 / 0 0.0%	0 / 0 0.0%	23 / 64 35.9%AB	18 / 64 28.1%B	13 / 64 20.3%ABE	9 / 64 14.1%BE	1 / 64 1.6%				
1	Acinetobacter baumannii AB0057	73 / 73 100.0%	3 / 3 100.0%	3 / 3 100.0%	4 / 4 100.0%	6 / 6 100.0%	4 / 4 100.0%	4 / 4 100.0%	1 / 1 100.0%	1 / 1 100.0%	7 / 7 100.0%	3 / 3 100.0%	4 / 4 100.0%	1 / 1 100.0%	2 / 2 100.0%	4 / 4 100.0%	1 / 1 100.0%	2 / 2 100.0%	2 / 2 100.0%	3 / 3 100.0%	7 / 7 100.0%	4 / 4 100.0%	1 / 1 100.0%	6 / 6 100.0%	0 / 0 0.0%	0 / 0 0.0%	0 / 0 0.0%	0 / 0 0.0%	47 / 73 64.4%AB	26 / 73 35.6%ABE							
1	Acinetobacter baumannii MDR-ZJ06	68 / 69 98.6%	2 / 2 100.0%	3 / 3 100.0%	2 / 2 100.0%	6 / 6 100.0%	4 / 4 100.0%	4 / 4 100.0%	1 / 1 100.0%	1 / 1 100.0%	7 / 7 100.0%	3 / 3 100.0%	4 / 4 100.0%	2 / 2 100.0%	1 / 1 100.0%	4 / 4 100.0%	1 / 1 100.0%	2 / 2 100.0%	2 / 2 100.0%	3 / 3 100.0%	5 / 6 83.3%	4 / 4 100.0%	1 / 1 100.0%	6 / 6 100.0%	0 / 0 0.0%	0 / 0 0.0%	0 / 0 0.0%	0 / 0 0.0%	44 / 69 63.8%AB	24 / 69 34.8%ABE	1 / 69 1.4%						
1	Acinetobacter baumannii A307	72 / 73 98.6%	3 / 3 100.0%	3 / 3 100.0%	4 / 4 100.0%	6 / 6 100.0%	4 / 4 100.0%	4 / 4 100.0%	1 / 1 100.0%	1 / 1 100.0%	7 / 7 100.0%	3 / 3 100.0%	4 / 4 100.0%	2 / 2 100.0%	2 / 2 100.0%	4 / 4 100.0%	1 / 1 100.0%	2 / 2 100.0%	2 / 2 100.0%	3 / 3 100.0%	5 / 6 83.3%	4 / 4 100.0%	1 / 1 100.0%	6 / 6 100.0%	0 / 0 0.0%	0 / 0 0.0%	0 / 0 0.0%	0 / 0 0.0%	47 / 73 64.4%AB	25 / 73 34.2%ABE	1 / 73 1.4%						
1	Acinetobacter baumannii 1656-2	70 / 71 98.6%	3 / 3 100.0%	2 / 2 100.0%	4 / 4 100.0%	6 / 6 100.0%	4 / 4 100.0%	3 / 3 100.0%	1 / 1 100.0%	1 / 1 100.0%	7 / 7 100.0%	3 / 3 100.0%	4 / 4 100.0%	2 / 2 100.0%	2 / 2 100.0%	4 / 4 100.0%	1 / 1 100.0%	2 / 2 100.0%	2 / 2 100.0%	3 / 3 100.0%	5 / 6 83.3%	4 / 4 100.0%	1 / 1 100.0%	6 / 6 100.0%	0 / 0 0.0%	0 / 0 0.0%	0 / 0 0.0%	0 / 0 0.0%	45 / 71 63.4%AB	25 / 71 35.2%ABE	1 / 71 1.4%						
1	Acinetobacter baumannii MDR-TJ	73 / 74 98.6%	3 / 3 100.0%	3 / 3 100.0%	4 / 4 100.0%	6 / 6 100.0%	4 / 4 100.0%	4 / 4 100.0%	1 / 1 100.0%	1 / 1 100.0%	7 / 7 100.0%	3 / 3 100.0%	4 / 4 100.0%	2 / 2 100.0%	2 / 2 100.0%	4 / 4 100.0%	1 / 1 100.0%	2 / 2 100.0%	2 / 2 100.0%	3 / 3 100.0%	6 / 7 85.7%	4 / 4 100.0%	1 / 1 100.0%	6 / 6 100.0%	0 / 0 0.0%	0 / 0 0.0%	0 / 0 0.0%	0 / 0 0.0%	47 / 74 63.5%AB	26 / 74 35.1%ABE	1 / 74 1.4%						
1	Acinetobacter baumannii D1279779	63 / 64 98.4%	3 / 3 100.0%	1 / 1 100.0%	4 / 4 100.0%	6 / 6 100.0%	4 / 4 100.0%	3 / 3 100.0%	1 / 1 100.0%	1 / 1 100.0%	7 / 7 100.0%	2 / 2 100.0%	4 / 4 100.0%	2 / 2 100.0%	2 / 2 100.0%	1 / 1 100.0%	1 / 1 100.0%	2 / 2 100.0%	2 / 2 100.0%	3 / 3 100.0%	5 / 6 83.3%	4 / 4 100.0%	1 / 1 100.0%	4 / 4 100.0%	0 / 0 0.0%	0 / 0 0.0%	0 / 0 0.0%	0 / 0 0.0%	44 / 64 68.8%AB	19 / 64 29.7%ABE	1 / 64 1.6%						
1	Acinetobacter baumannii TCDC-AB0715	41 / 42 97.6%	1 / 1 100.0%	0 / 0 0.0%	4 / 4 100.0%	4 / 4 100.0%	1 / 1 100.0%	1 / 1 100.0%	1 / 1 100.0%	1 / 1 100.0%	5 / 5 100.0%	2 / 2 100.0%	4 / 4 100.0%	0 / 0 0.0%	1 / 1 100.0%	0 / 0 0.0%	1 / 1 100.0%	2 / 2 100.0%	2 / 2 100.0%	2 / 2 100.0%	3 / 4 75.0%	0 / 0 0.0%	2 / 2 100.0%	4 / 4 100.0%	0 / 0 0.0%	0 / 0 0.0%	0 / 0 0.0%	0 / 0 0.0%	28 / 42 66.7%AB	13 / 42 31.0%ABE	1 / 42 2.4%						
1	Acinetobacter baumannii BJAB07104	73 / 74 98.6%	3 / 3 100.0%	3 / 3 100.0%	4 / 4 100.0%	6 / 6 100.0%	4 / 4 100.0%	4 / 4 100.0%	1 / 1 100.0%	1 / 1 100.0%	7 / 7 100.0%	3 / 3 100.0%	4 / 4 100.0%	2 / 2 100.0%	2 / 2 100.0%	4 / 4 100.0%	1 / 1 100.0%	2 / 2 100.0%	2 / 2 100.0%	3 / 3 100.0%	6 / 7 85.7%	4 / 4 100.0%	1 / 1 100.0%	6 / 6 100.0%	0 / 0 0.0%	0 / 0 0.0%	0 / 0 0.0%	0 / 0 0.0%	47 / 74 63.5%AB	26 / 74 35.1%ABE	1 / 74 1.4%						
1	Acinetobacter baumannii BJAB0715	72 / 73 98.6%	3 / 3 100.0%	3 / 3 100.0%	4 / 4 100.0%	6 / 6 100.0%	4 / 4 100.0%	4 / 4 100.0%	1 / 1 100.0%	1 / 1 100.0%	7 / 7 100.0%	3 / 3 100.0%	4 / 4 100.0%	2 / 2 100.0%	2 / 2 100.0%	5 / 5 100.0%	1 / 1 100.0%	2 / 2 100.0%	2 / 2 100.0%	2 / 2 100.0%	5 / 6 83.3%	4 / 4 100.0%	1 / 1 100.0%	6 / 6 100.0%	0 / 0 0.0%	0 / 0 0.0%	0 / 0 0.0%	0 / 0 0.0%	46 / 73 63.0%AB	26 / 73 35.6%ABE	1 / 73 1.4%						
1	Acinetobacter baumannii BJAB0868	75 / 75 100.0%	3 / 3 100.0%	3 / 3 100.0%	4 / 4 100.0%	6 / 6 100.0%	4 / 4 100.0%	4 / 4 100.0%	1 / 1 100.0%	1 / 1 100.0%	7 / 7 100.0%	3 / 3 100.0%	4 / 4 100.0%	2 / 2 100.0%	2 / 2 100.0%	4 / 4 100.0%	1 / 1 100.0%	2 / 2 100.0%	2 / 2 100.0%	3 / 3 100.0%	8 / 8 100.0%	4 / 4 100.0%	1 / 1 100.0%	6 / 6 100.0%	0 / 0 0.0%	0 / 0 0.0%	0 / 0 0.0%	0 / 0 0.0%	48 / 75 64.0%AB	27 / 75 36.0%ABE							
1	Acinetobacter baumannii TYTH-1	73 / 74 98.6%	3 / 3 100.0%	3 / 3 100.0%	4 / 4 100.0%	4 / 4 100.0%	8 / 8 100.0%	4 / 4 100.0%	1 / 1 100.0%	1 / 1 100.0%	5 / 5 100.0%	3 / 3 100.0%	4 / 4 100.0%	2 / 2 100.0%	2 / 2 100.0%	4 / 4 100.0%	1 / 1 100.0%	2 / 2 100.0%	2 / 2 100.0%	3 / 3 100.0%	6 / 7 85.7%	4 / 4 100.0%	1 / 1 100.0%	6 / 6 100.0%	0 / 0 0.0%	0 / 0 0.0%	0 / 0 0.0%	0 / 0 0.0%	47 / 74 63.5%AB	26 / 74 35.1%ABE	1 / 74 1.4%						
1	"Micrococcus calco-aceticus" Beijerinck 1911	66 / 67 98.5%	3 / 3 100.0%	2 / 2 100.0%	4 / 4 100.0%	2 / 2 100.0%	3 / 3 100.0%	4 / 4 100.0%	1 / 1 100.0%	1 / 1 100.0%	3 / 3 100.0%	4 / 4 100.0%	4 / 4 100.0%	3 / 3 100.0%	2 / 2 100.0%	5 / 5 100.0%	1 / 1 100.0%	2 / 2 100.0%	2 / 2 100.0%	3 / 3 100.0%	5 / 6 83.3%	4 / 4 100.0%	1 / 1 100.0%	7 / 7 100.0%	0 / 0 0.0%	0 / 0 0.0%	0 / 0 0.0%	0 / 0 0.0%	38 / 67 56.7%AB	28 / 67 41.8%ABE	1 / 67 1.5%						
1	Acinetobacter calcoaceticus PHEA-2	66 / 67 98.5%	3 / 3 100.0%	2 / 2 100.0%	4 / 4 100.0%	2 / 2 100.0%	3 / 3 100.0%	4 / 4 100.0%	1 / 1 100.0%	1 / 1 100.0%	3 / 3 100.0%	4 / 4 100.0%	4 / 4 100.0%	3 / 3 100.0%	2 / 2 100.0%	5 / 5 100.0%	1 / 1 100.0%	2 / 2 100.0%	2 / 2 100.0%	3 / 3 100.0%	5 / 6 83.3%	4 / 4 100.0%	1 / 1 100.0%	7 / 7 100.0%	0 / 0 0.0%	0 / 0 0.0%	0 / 0 0.0%	0 / 0 0.0%	38 / 67 56.7%AB	28 / 67 41.8%ABE	1 / 67 1.5%						
1	"Acinetobacter oleivorans" Kang et al. 2011	70 / 71 98.6%	3 / 3 100.0%	3 / 3 100.0%	4 / 4 100.0%	6 / 6 100.0%	4 / 4 100.0%	4 / 4 100.0%	1 / 1 100.0%	1 / 1 100.0%	7 / 7 100.0%	3 / 3 100.0%	4 / 4 100.0%	2 / 2 100.0%	2 / 2 100.0%	2 / 2 100.0%	1 / 1 100.0%	2 / 2 100.0%	2 / 2 100.0%	3 / 3 100.0%	5 / 6 83.3%	4 / 4 100.0%	1 / 1 100.0%	6 / 6 100.0%	0 / 0 0.0%	0 / 0 0.0%	0 / 0 0.0%	0 / 0 0.0%	47 / 71 66.2%AB	23 / 71 32.4%ABE	1 / 71 1.4%						
1	Acinetobacter oleivorans DR1	70 / 71 98.6%	3 / 3 100.0%	3 / 3 100.0%	4 / 4 100.0%	6 / 6 100.0%	4 / 4 100.0%	4 / 4 100.0%	1 / 1 100.0%	1 / 1 100.0%	7 / 7 100.0%	3 / 3 100.0%	4 / 4 100.0%	2 / 2 100.0%	2 / 2 100.0%	2 / 2 100.0%	1 / 1 100.0%	2 / 2 100.0%	2 / 2 100.0%	3 / 3 100.0%	5 / 6 83.3%	4 / 4 100.0%	1 / 1 100.0%	6 / 6 100.0%	0 / 0 0.0%	0 / 0 0.0%	0 / 0 0.0%	0 / 0 0.0%	47 / 71 66.2%AB	23 / 71 32.4%ABE	1 / 71 1.4%						
1	"Diplobacillus" McNab 1904	50 / 50 100.0%	1 / 1 100.0%	2 / 2 100.0%	2 / 2 100.0%	4 / 4 100.0%	3 / 3 100.0%	3 / 3 100.0%	1 / 1 100.0%	1 / 1 100.0%	5 / 5 100.0%	1 / 1 100.0%	4 / 4 100.0%	2 / 2 100.0%	2 / 2 100.0%	2 / 2 100.0%	1 / 1 100.0%	2 / 2 100.0%	1 / 1 100.0%	2 / 2 100.0%	3 / 3 100.0%	2 / 2 100.0%	1 / 1 100.0%	5 / 5 100.0%	0 / 0 0.0%	0 / 0 0.0%	0 / 0 0.0%	0 / 0 0.0%	33 / 50 66.0%AB	17 / 50 34.0%ABE							
1	Branhamella	50 / 50 100.0%	1 / 1 100.0%	2 / 2 100.0%	2 / 2 100.0%	4 / 4 100.0%	3 / 3 100.0%	3 / 3 100.0%	1 / 1 100.0%	1 / 1 100.0%	5 / 5 100.0%	1 / 1 100.0%	4 / 4 100.0%	2 / 2 100.0%	2 / 2 100.0%	2 / 2 100.0%	1 / 1 100.0%	2 / 2 100.0%	1 / 1 100.0%	2 / 2 100.0%	3 / 3 100.0%	2 / 2 100.0%	1 / 1 100.0%	5 / 5 100.0%	0 / 0 0.0%	0 / 0 0.0%	0 / 0 0.0%	0 / 0 0.0%	33 / 50 66.0%AB	17 / 50 34.0%ABE							
1	"Mikrokkokus catarrhalis" (sic) Frosch and Kolle in Flugge 1896	50 / 50 100.0%	1 / 1 100.0%	2 / 2 100.0%	2 / 2 100.0%	4 / 4 100.0%	3 / 3 100.0%	3 / 3 100.0%	1 / 1 100.0%	1 / 1 100.0%	5 / 5 100.0%	1 / 1 100.0%	4 / 4 100.0%	2 / 2 100.0%	2 / 2 100.0%	2 / 2 100.0%	1 / 1 100.0%	2 / 2 100.0%	1 / 1 100.0%	2 / 2 100.0%	3 / 3 100.0%	2 / 2 100.0%	1 / 1 100.0%	5 / 5 100.0%	0 / 0 0.0%	0 / 0 0.0%	0 / 0 0.0%	0 / 0 0.0%	33 / 50 66.0%AB	17 / 50 34.0%ABE							
1	Moraxella catarrhalis RH4	50 / 50 100.0%	1 / 1 100.0%	2 / 2 100.0%	2 / 2 100.0%	4 / 4 100.0%	3 / 3 100.0%	3 / 3 100.0%	1 / 1 100.0%	1 / 1 100.0%	5 / 5 100.0%	1 / 1 100.0%	4 / 4 100.0%	2 / 2 100.0%	2 / 2 100.0%	2 / 2 100.0%	1 / 1 100.0%	2 / 2 100.0%	1 / 1 100.0%	2 / 2 100.0%	3 / 3 100.0%	2 / 2 100.0%	1 / 1 100.0%	5 / 5 100.0%	0 / 0 0.0%	0 / 0 0.0%	0 / 0 0.0%	0 / 0 0.0%	33 / 50 66.0%AB	17 / 50 34.0%ABE							
4	Psychrobacter	249 / 249 100.0%	5 / 5 100.0%	12 / 12 100.0%	10 / 10 100.0%	21 / 21 100.0%	15 / 15 100.0%	17 / 17 100.0%	5 / 5 100.0%	5 / 5 100.0%	26 / 26 100.0%	5 / 5 100.0%	16 / 16 100.0%	10 / 10 100.0%	5 / 5 100.0%	10 / 10 100.0%	5 / 5 100.0%	10 / 10 100.0%	5 / 5 100.0%	10 / 10 100.0%	20 / 20 100.0%	11 / 11 100.0%	5 / 5 100.0%	21 / 21 100.0%	0 / 0 0.0%	0 / 0 0.0%	0 / 0 0.0%	0 / 0 0.0%	156 / 249 62.7%AB	75 / 249 30.1%ABE	15 / 249 6.0%B	3 / 249 1.2%BE					
1	DSM 17306	48 / 48 100.0%	1 / 1 100.0%	2 / 2 100.0%	2 / 2 100.0%	4 / 4 100.0%	3 / 3 100.0%	3 / 3 100.0%	1 / 1 100.0%	1 / 1 100.0%	5 / 5 100.0%	1 / 1 100.0%	3 / 3 100.0%	2 / 2 100.0%	1 / 1 100.0%	2 / 2 100.0%	1 / 1 100.0%	2 / 2 100.0%	1 / 1 100.0%	2 / 2 100.0%	4 / 4 100.0%	2 / 2 100.0%	1 / 1 100.0%	4 / 4 100.0%	0 / 0 0.0%	0 / 0 0.0%	0 / 0 0.0%	0 / 0 0.0%	33 / 48 68.8%AB	15 / 48 31.2%ABE							
1	Psychrobacter cryohalolentis K5	48 / 48 100.0%	1 / 1 100.0%	2 / 2 100.0%	2 / 2 100.0%	4 / 4 100.0%	3 / 3 100.0%	3 / 3 100.0%	1 / 1 100.0%	1 / 1 100.0%	5 / 5 100.0%	1 / 1 100.0%	3 / 3 100.0%	2 / 2 100.0%	1 / 1 100.0%	2 / 2 100.0%	1 / 1 100.0%	2 / 2 100.0%	1 / 1 100.0%	2 / 2 100.0%	4 / 4 100.0%	2 / 2 100.0%	1 / 1 100.0%	4 / 4 100.0%	0 / 0 0.0%	0 / 0 0.0%	0 / 0 0.0%	0 / 0 0.0%	33 / 48 68.8%AB	15 / 48 31.2%ABE							
1	DSM 17307	96 / 96 100.0%	2 / 2 100.0%	4 / 4 100.0%	4 / 4 100.0%	8 / 8 100.0%	6 / 6 100.0%	6 / 6 100.0%	2 / 2 100.0%	2 / 2 100.0%	10 / 10 100.0%	2 / 2 100.0%	6 / 6 100.0%	4 / 4 100.0%	2 / 2 100.0%	4 / 4 100.0%	2 / 2 100.0%	4 / 4 100.0%	2 / 2 100.0%	4 / 4 100.0%	8 / 8 100.0%	4 / 4 100.0%	2 / 2 100.0%	8 / 8 100.0%	0 / 0 0.0%	0 / 0 0.0%	0 / 0 0.0%	0 / 0 0.0%	51 / 96 53.1%AB	27 / 96 28.1%ABE	15 / 96 15.6%B	3 / 96 3.1%BE					
1	Psychrobacter arcticum 273-4	96 / 96 100.0%	2 / 2 100.0%	4 / 4 100.0%	4 / 4 100.0%	8 / 8 100.0%	6 / 6 100.0%	6 / 6 100.0%	2 / 2 100.0%	2 / 2 100.0%	10 / 10 100.0%	2 / 2 100.0%	6 / 6 100.0%	4 / 4 100.0%	2 / 2 100.0%	4 / 4 100.0%	2 / 2 100.0%	4 / 4 100.0%	2 / 2 100.0%	4 / 4 100.0%	8 / 8 100.0%	4 / 4 100.0%	2 / 2 100.0%	8 / 8 100.0%	0 / 0 0.0%	0 / 0 0.0%	0 / 0 0.0%	0 / 0 0.0%	51 / 96 53.1%AB	27 / 96 28.1%ABE	15 / 96 15.6%B	3 / 96 3.1%BE					
1	Psychrobacter sp. PRwf-1	57 / 57 100.0%	1 / 1 100.0%	4 / 4 100.0%	2 / 2 100.0%	5 / 5 100.0%	3 / 3 100.0%	5 / 5 100.0%	1 / 1 100.0%	1 / 1 100.0%	6 / 6 100.0%	1 / 1 100.0%	4 / 4 100.0%	2 / 2 100.0%	1 / 1 100.0%	2 / 2 100.0%	1 / 1 100.0%	2 / 2 100.0%	1 / 1 100.0%	2 / 2 100.0%	4 / 4 100.0%	3 / 3 100.0%	1 / 1 100.0%	5 / 5 100.0%	0 / 0 0.0%	0 / 0 0.0%	0 / 0 0.0%	0 / 0 0.0%	39 / 57 68.4%AB	18 / 57 31.6%ABE							
1	Psychrobacter sp. G	48 / 48 100.0%	1 / 1 100.0%	2 / 2 100.0%	2 / 2 100.0%	4 / 4 100.0%	3 / 3 100.0%	3 / 3 100.0%	1 / 1 100.0%	1 / 1 100.0%	5 / 5 100.0%	1 / 1 100.0%	3 / 3 100.0%	2 / 2 100.0%	1 / 1 100.0%	2 / 2 100.0%	1 / 1 100.0%	2 / 2 100.0%	1 / 1 100.0%	2 / 2 100.0%	4 / 4 100.0%	2 / 2 100.0%	1 / 1 100.0%	4 / 4 100.0%	0 / 0 0.0%	0 / 0 0.0%	0 / 0 0.0%	0 / 0 0.0%	33 / 48 68.8%AB	15 / 48 31.2%ABE							
47	Pseudomonadaceae	3067 / 3081 99.5%	134 / 135 99.3%	166 / 166 100.0%	95 / 95 100.0%	203 / 203 100.0%	245 / 245 100.0%	172 / 172 100.0%	73 / 73 100.0%	47 / 47 100.0%	301 / 303 99.3%	47 / 47 100.0%	186 / 187 99.5%	102 / 102 100.0%	50 / 50 100.0%	75 / 75 100.0%	47 / 47 100.0%	151 / 151 100.0%	47 / 47 100.0%	137 / 137 100.0%	254 / 264 96.2%	178 / 178 100.0%	47 / 47 100.0%	279 / 279 100.0%	31 / 31 100.0%	0 / 0 0.0%	0 / 0 0.0%	0 / 0 0.0%	2045 / 3081 66.4%AB	952 / 3081 30.9%ABE	50 / 3081 1.6%B	20 / 3081 0.6%BE	11 / 3081 0.4%	1 / 3081 0.0%C	1 / 3081 0.0%E	1 / 3081 0.0%CE	
1	"Cellvibrio" Winogradsky 1929	46 / 46 100.0%	1 / 1 100.0%	2 / 2 100.0%	1 / 1 100.0%	3 / 3 100.0%	3 / 3 100.0%	3 / 3 100.0%	1 / 1 100.0%	1 / 1 100.0%	4 / 4 100.0%	1 / 1 100.0%	3 / 3 100.0%	2 / 2 100.0%	1 / 1 100.0%	1 / 1 100.0%	1 / 1 100.0%	3 / 3 100.0%	1 / 1 100.0%	2 / 2 100.0%	4 / 4 100.0%	2 / 2 100.0%	1 / 1 100.0%	5 / 5 100.0%	0 / 0 0.0%	0 / 0 0.0%	0 / 0 0.0%	0 / 0 0.0%	24 / 46 52.2%AB	9 / 46 19.6%ABE	8 / 46 17.4%B	5 / 46 10.9%BE					
1	"Cellvibrio cellulosa" Nagy et al. 2002	46 / 46 100.0%	1 / 1 100.0%	2 / 2 100.0%	1 / 1 100.0%	3 / 3 100.0%	3 / 3 100.0%	3 / 3 100.0%	1 / 1 100.0%	1 / 1 100.0%	4 / 4 100.0%	1 / 1 100.0%	3 / 3 100.0%	2 / 2 100.0%	1 / 1 100.0%	1 / 1 100.0%	1 / 1 100.0%	3 / 3 100.0%	1 / 1 100.0%	2 / 2 100.0%	4 / 4 100.0%	2 / 2 100.0%	1 / 1 100.0%	5 / 5 100.0%	0 / 0 0.0%	0 / 0 0.0%	0 / 0 0.0%	0 / 0 0.0%	24 / 46 52.2%AB	9 / 46 19.6%ABE	8 / 46 17.4%B	5 / 46 10.9%BE					
1	Cellvibrio japonicus Ueda107	46 / 46 100.0%	1 / 1 100.0%	2 / 2 100.0%	1 / 1 100.0%	3 / 3 100.0%	3 / 3 100.0%	3 / 3 100.0%	1 / 1 100.0%	1 / 1 100.0%	4 / 4 100.0%	1 / 1 100.0%	3 / 3 100.0%	2 / 2 100.0%	1 / 1 100.0%	1 / 1 100.0%	1 / 1 100.0%	3 / 3 100.0%	1 / 1 100.0%	2 / 2 100.0%	4 / 4 100.0%	2 / 2 100.0%	1 / 1 100.0%	5 / 5 100.0%	0 / 0 0.0%	0 / 0 0.0%	0 / 0 0.0%	0 / 0 0.0%	24 / 46 52.2%AB	9 / 46 19.6%ABE	8 / 46 17.4%B	5 / 46 10.9%BE					
[illegible]	"Chlorobacterium" Guillebeau 1890,	[illegible]																																				

43	nom. rejic. Opin. 6 (not "Chlorobacterium" Lauterborn 1916)	2829 / 2843 99.5%	127 / 128 99.2%	158 / 158 100.0%	88 / 88 100.0%	182 / 182 100.0%	227 / 227 100.0%	160 / 160 100.0%	69 / 69 100.0%	43 / 43 100.0%	273 / 275 99.3%	43 / 43 100.0%	171 / 172 99.4%	94 / 94 100.0%	43 / 43 100.0%	68 / 68 100.0%	43 / 43 100.0%	139 / 139 100.0%	43 / 43 100.0%	126 / 126 100.0%	235 / 245 95.9%	167 / 167 100.0%	43 / 43 100.0%	256 / 256 100.0%	31 / 31 100.0%	0 / 0 0.0%	0 / 0 0.0%	0 / 0 0.0%	1889 / 2843 66.4%AB	883 / 2843 31.1%ABE	42 / 2843 1.5%B	15 / 2843 0.5%BE	11 / 2843 0.4%	1 / 2843 0.0%C	1 / 2843 0.0%CE	1 / 2843 0.0%E
11	Pseudomonas aeruginosa group	694 / 703 98.7%	24 / 24 100.0%	42 / 42 100.0%	24 / 24 100.0%	44 / 44 100.0%	57 / 57 100.0%	33 / 33 100.0%	20 / 20 100.0%	11 / 11 100.0%	68 / 68 100.0%	11 / 11 100.0%	44 / 44 100.0%	22 / 22 100.0%	11 / 11 100.0%	13 / 13 100.0%	11 / 11 100.0%	33 / 33 100.0%	11 / 11 100.0%	34 / 34 100.0%	57 / 66 86.4%	37 / 37 100.0%	11 / 11 100.0%	66 / 66 100.0%	10 / 10 100.0%	0 / 0 0.0%	0 / 0 0.0%	0 / 0 0.0%	474 / 703 67.4%AB	216 / 703 30.7%ABE	9 / 703 1.3%	4 / 703 0.6%BE				
9	"Bacillus aeruginosus" (Schroeter 1872) Trevisan 1885	564 / 573 98.4%	18 / 18 100.0%	36 / 36 100.0%	20 / 20 100.0%	36 / 36 100.0%	47 / 47 100.0%	27 / 27 100.0%	18 / 18 100.0%	9 / 9 100.0%	54 / 54 100.0%	9 / 9 100.0%	36 / 36 100.0%	18 / 18 100.0%	9 / 9 100.0%	9 / 9 100.0%	9 / 9 100.0%	27 / 27 100.0%	9 / 9 100.0%	29 / 29 100.0%	45 / 54 83.3%	27 / 27 100.0%	9 / 9 100.0%	54 / 54 100.0%	9 / 9 100.0%	0 / 0 0.0%	0 / 0 0.0%	0 / 0 0.0%	391 / 573 68.2%AB	169 / 573 29.5%ABE	9 / 573 1.6%	4 / 573 0.7%BE				
1	Pseudomonas aeruginosa UCBPP-PA14	62 / 63 98.4%	2 / 2 100.0%	4 / 4 100.0%	2 / 2 100.0%	4 / 4 100.0%	5 / 5 100.0%	3 / 3 100.0%	2 / 2 100.0%	1 / 1 100.0%	6 / 6 100.0%	1 / 1 100.0%	4 / 4 100.0%	2 / 2 100.0%	1 / 1 100.0%	1 / 1 100.0%	1 / 1 100.0%	3 / 3 100.0%	1 / 1 100.0%	3 / 3 100.0%	5 / 6 83.3%	3 / 3 100.0%	1 / 1 100.0%	6 / 6 100.0%	1 / 1 100.0%	0 / 0 0.0%	0 / 0 0.0%	0 / 0 0.0%	43 / 63 68.3%AB	17 / 63 27.0%ABE	2 / 63 3.2%BE	1 / 63 1.6%				
1	Pseudomonas aeruginosa PA01	62 / 63 98.4%	2 / 2 100.0%	4 / 4 100.0%	2 / 2 100.0%	4 / 4 100.0%	5 / 5 100.0%	3 / 3 100.0%	2 / 2 100.0%	1 / 1 100.0%	6 / 6 100.0%	1 / 1 100.0%	4 / 4 100.0%	2 / 2 100.0%	1 / 1 100.0%	1 / 1 100.0%	1 / 1 100.0%	3 / 3 100.0%	1 / 1 100.0%	3 / 3 100.0%	5 / 6 83.3%	3 / 3 100.0%	1 / 1 100.0%	6 / 6 100.0%	1 / 1 100.0%	0 / 0 0.0%	0 / 0 0.0%	0 / 0 0.0%	43 / 63 68.3%AB	18 / 63 28.6%ABE	1 / 63 1.6%BE	1 / 63 1.6%				
1	Pseudomonas aeruginosa PA7	62 / 63 98.4%	2 / 2 100.0%	4 / 4 100.0%	2 / 2 100.0%	4 / 4 100.0%	5 / 5 100.0%	3 / 3 100.0%	2 / 2 100.0%	1 / 1 100.0%	6 / 6 100.0%	1 / 1 100.0%	4 / 4 100.0%	2 / 2 100.0%	1 / 1 100.0%	1 / 1 100.0%	1 / 1 100.0%	3 / 3 100.0%	1 / 1 100.0%	3 / 3 100.0%	5 / 6 83.3%	3 / 3 100.0%	1 / 1 100.0%	6 / 6 100.0%	1 / 1 100.0%	0 / 0 0.0%	0 / 0 0.0%	0 / 0 0.0%	43 / 63 68.3%AB	18 / 63 28.6%ABE	1 / 63 1.6%	1 / 63 1.6%BE				
1	Pseudomonas aeruginosa LESB58	65 / 66 98.5%	2 / 2 100.0%	4 / 4 100.0%	3 / 3 100.0%	4 / 4 100.0%	6 / 6 100.0%	3 / 3 100.0%	2 / 2 100.0%	1 / 1 100.0%	6 / 6 100.0%	1 / 1 100.0%	4 / 4 100.0%	2 / 2 100.0%	1 / 1 100.0%	1 / 1 100.0%	1 / 1 100.0%	3 / 3 100.0%	1 / 1 100.0%	4 / 4 100.0%	5 / 6 83.3%	3 / 3 100.0%	1 / 1 100.0%	6 / 6 100.0%	1 / 1 100.0%	0 / 0 0.0%	0 / 0 0.0%	0 / 0 0.0%	45 / 66 68.2%AB	20 / 66 30.3%ABE	1 / 66 1.5%					
1	Pseudomonas aeruginosa M18	62 / 63 98.4%	2 / 2 100.0%	4 / 4 100.0%	2 / 2 100.0%	4 / 4 100.0%	5 / 5 100.0%	3 / 3 100.0%	2 / 2 100.0%	1 / 1 100.0%	6 / 6 100.0%	1 / 1 100.0%	4 / 4 100.0%	2 / 2 100.0%	1 / 1 100.0%	1 / 1 100.0%	1 / 1 100.0%	3 / 3 100.0%	1 / 1 100.0%	3 / 3 100.0%	5 / 6 83.3%	3 / 3 100.0%	1 / 1 100.0%	6 / 6 100.0%	1 / 1 100.0%	0 / 0 0.0%	0 / 0 0.0%	0 / 0 0.0%	43 / 63 68.3%AB	19 / 63 30.2%ABE	1 / 63 1.6%					
1	Pseudomonas aeruginosa NCGM2.S1	65 / 66 98.5%	2 / 2 100.0%	4 / 4 100.0%	3 / 3 100.0%	4 / 4 100.0%	6 / 6 100.0%	3 / 3 100.0%	2 / 2 100.0%	1 / 1 100.0%	6 / 6 100.0%	1 / 1 100.0%	4 / 4 100.0%	2 / 2 100.0%	1 / 1 100.0%	1 / 1 100.0%	1 / 1 100.0%	3 / 3 100.0%	1 / 1 100.0%	4 / 4 100.0%	5 / 6 83.3%	3 / 3 100.0%	1 / 1 100.0%	6 / 6 100.0%	1 / 1 100.0%	0 / 0 0.0%	0 / 0 0.0%	0 / 0 0.0%	45 / 66 68.2%AB	20 / 66 30.3%ABE	1 / 66 1.5%					
1	Pseudomonas aeruginosa CF333-2007-apr0	62 / 63 98.4%	2 / 2 100.0%	4 / 4 100.0%	2 / 2 100.0%	4 / 4 100.0%	5 / 5 100.0%	3 / 3 100.0%	2 / 2 100.0%	1 / 1 100.0%	6 / 6 100.0%	1 / 1 100.0%	4 / 4 100.0%	2 / 2 100.0%	1 / 1 100.0%	1 / 1 100.0%	1 / 1 100.0%	3 / 3 100.0%	1 / 1 100.0%	3 / 3 100.0%	5 / 6 83.3%	3 / 3 100.0%	1 / 1 100.0%	6 / 6 100.0%	1 / 1 100.0%	0 / 0 0.0%	0 / 0 0.0%	0 / 0 0.0%	43 / 63 68.3%AB	19 / 63 30.2%ABE	1 / 63 1.6%					
1	Pseudomonas aeruginosa B136-33	62 / 63 98.4%	2 / 2 100.0%	4 / 4 100.0%	2 / 2 100.0%	4 / 4 100.0%	5 / 5 100.0%	3 / 3 100.0%	2 / 2 100.0%	1 / 1 100.0%	6 / 6 100.0%	1 / 1 100.0%	4 / 4 100.0%	2 / 2 100.0%	1 / 1 100.0%	1 / 1 100.0%	1 / 1 100.0%	3 / 3 100.0%	1 / 1 100.0%	3 / 3 100.0%	5 / 6 83.3%	3 / 3 100.0%	1 / 1 100.0%	6 / 6 100.0%	1 / 1 100.0%	0 / 0 0.0%	0 / 0 0.0%	0 / 0 0.0%	43 / 63 68.3%AB	19 / 63 30.2%ABE	1 / 63 1.6%					
1	Pseudomonas aeruginosa RP73	62 / 63 98.4%	2 / 2 100.0%	4 / 4 100.0%	2 / 2 100.0%	4 / 4 100.0%	5 / 5 100.0%	3 / 3 100.0%	2 / 2 100.0%	1 / 1 100.0%	6 / 6 100.0%	1 / 1 100.0%	4 / 4 100.0%	2 / 2 100.0%	1 / 1 100.0%	1 / 1 100.0%	1 / 1 100.0%	3 / 3 100.0%	1 / 1 100.0%	3 / 3 100.0%	5 / 6 83.3%	3 / 3 100.0%	1 / 1 100.0%	6 / 6 100.0%	1 / 1 100.0%	0 / 0 0.0%	0 / 0 0.0%	0 / 0 0.0%	43 / 63 68.3%AB	19 / 63 30.2%ABE	1 / 63 1.6%					
2	ATCC 25411	130 / 130 100.0%	6 / 6 100.0%	6 / 6 100.0%	4 / 4 100.0%	8 / 8 100.0%	10 / 10 100.0%	6 / 6 100.0%	2 / 2 100.0%	2 / 2 100.0%	14 / 14 100.0%	2 / 2 100.0%	8 / 8 100.0%	4 / 4 100.0%	2 / 2 100.0%	4 / 4 100.0%	2 / 2 100.0%	6 / 6 100.0%	2 / 2 100.0%	5 / 5 100.0%	12 / 12 100.0%	10 / 10 100.0%	2 / 2 100.0%	12 / 12 100.0%	1 / 1 100.0%	0 / 0 0.0%	0 / 0 0.0%	0 / 0 0.0%	83 / 130 63.8%AB	47 / 130 36.2%ABE						
1	Pseudomonas mendocina str. ymp	65 / 65 100.0%	3 / 3 100.0%	3 / 3 100.0%	2 / 2 100.0%	4 / 4 100.0%	5 / 5 100.0%	3 / 3 100.0%	1 / 1 100.0%	1 / 1 100.0%	7 / 7 100.0%	1 / 1 100.0%	4 / 4 100.0%	2 / 2 100.0%	1 / 1 100.0%	2 / 2 100.0%	1 / 1 100.0%	3 / 3 100.0%	1 / 1 100.0%	3 / 3 100.0%	6 / 6 100.0%	5 / 5 100.0%	1 / 1 100.0%	6 / 6 100.0%	0 / 0 0.0%	0 / 0 0.0%	0 / 0 0.0%	0 / 0 0.0%	42 / 65 64.6%AB	23 / 65 35.4%ABE						
1	Pseudomonas mendocina NK-01	65 / 65 100.0%	3 / 3 100.0%	3 / 3 100.0%	2 / 2 100.0%	4 / 4 100.0%	5 / 5 100.0%	3 / 3 100.0%	1 / 1 100.0%	1 / 1 100.0%	7 / 7 100.0%	1 / 1 100.0%	4 / 4 100.0%	2 / 2 100.0%	1 / 1 100.0%	2 / 2 100.0%	1 / 1 100.0%	3 / 3 100.0%	1 / 1 100.0%	2 / 2 100.0%	6 / 6 100.0%	5 / 5 100.0%	1 / 1 100.0%	6 / 6 100.0%	1 / 1 100.0%	0 / 0 0.0%	0 / 0 0.0%	0 / 0 0.0%	41 / 65 63.1%AB	24 / 65 36.9%ABE						
7	Pseudomonas fluorescens group	473 / 473 100.0%	21 / 21 100.0%	28 / 28 100.0%	14 / 14 100.0%	37 / 37 100.0%	38 / 38 100.0%	28 / 28 100.0%	11 / 11 100.0%	7 / 7 100.0%	52 / 52 100.0%	7 / 7 100.0%	28 / 28 100.0%	14 / 14 100.0%	7 / 7 100.0%	10 / 10 100.0%	7 / 7 100.0%	21 / 21 100.0%	7 / 7 100.0%	18 / 18 100.0%	36 / 36 100.0%	32 / 32 100.0%	7 / 7 100.0%	42 / 42 100.0%	1 / 1 100.0%	0 / 0 0.0%	0 / 0 0.0%	0 / 0 0.0%	328 / 473 69.3%AB	145 / 473 30.7%ABE						
4	"Bacillus fluorescens liquefaciens" Flugge 1886	270 / 270 100.0%	12 / 12 100.0%	16 / 16 100.0%	8 / 8 100.0%	22 / 22 100.0%	21 / 21 100.0%	16 / 16 100.0%	7 / 7 100.0%	4 / 4 100.0%	30 / 30 100.0%	4 / 4 100.0%	16 / 16 100.0%	8 / 8 100.0%	4 / 4 100.0%	5 / 5 100.0%	4 / 4 100.0%	12 / 12 100.0%	4 / 4 100.0%	10 / 10 100.0%	21 / 21 100.0%	17 / 17 100.0%	4 / 4 100.0%	24 / 24 100.0%	1 / 1 100.0%	0 / 0 0.0%	0 / 0 0.0%	0 / 0 0.0%	189 / 270 70.0%AB	81 / 270 30.0%ABE						
1	Pseudomonas fluorescens Pf0-1	73 / 73 100.0%	3 / 3 100.0%	4 / 4 100.0%	2 / 2 100.0%	6 / 6 100.0%	6 / 6 100.0%	4 / 4 100.0%	2 / 2 100.0%	1 / 1 100.0%	8 / 8 100.0%	1 / 1 100.0%	4 / 4 100.0%	2 / 2 100.0%	1 / 1 100.0%	2 / 2 100.0%	1 / 1 100.0%	3 / 3 100.0%	1 / 1 100.0%	3 / 3 100.0%	6 / 6 100.0%	5 / 5 100.0%	1 / 1 100.0%	6 / 6 100.0%	1 / 1 100.0%	0 / 0 0.0%	0 / 0 0.0%	0 / 0 0.0%	50 / 73 68.5%AB	23 / 73 31.5%ABE						
1	Pseudomonas fluorescens SBW25	65 / 65 100.0%	3 / 3 100.0%	4 / 4 100.0%	2 / 2 100.0%	5 / 5 100.0%	5 / 5 100.0%	4 / 4 100.0%	2 / 2 100.0%	1 / 1 100.0%	7 / 7 100.0%	1 / 1 100.0%	4 / 4 100.0%	2 / 2 100.0%	1 / 1 100.0%	1 / 1 100.0%	1 / 1 100.0%	3 / 3 100.0%	1 / 1 100.0%	2 / 2 100.0%	5 / 5 100.0%	4 / 4 100.0%	1 / 1 100.0%	6 / 6 100.0%	0 / 0 0.0%	0 / 0 0.0%	0 / 0 0.0%	0 / 0 0.0%	46 / 65 70.8%AB	19 / 65 29.2%ABE						
1	Pseudomonas fluorescens A506	68 / 68 100.0%	3 / 3 100.0%	4 / 4 100.0%	2 / 2 100.0%	6 / 6 100.0%	5 / 5 100.0%	4 / 4 100.0%	2 / 2 100.0%	1 / 1 100.0%	8 / 8 100.0%	1 / 1 100.0%	4 / 4 100.0%	2 / 2 100.0%	1 / 1 100.0%	1 / 1 100.0%	1 / 1 100.0%	3 / 3 100.0%	1 / 1 100.0%	3 / 3 100.0%	5 / 5 100.0%	4 / 4 100.0%	1 / 1 100.0%	6 / 6 100.0%	0 / 0 0.0%	0 / 0 0.0%	0 / 0 0.0%	0 / 0 0.0%	49 / 68 72.1%AB	19 / 68 27.9%ABE						
1	Pseudomonas fluorescens F113	64 / 64 100.0%	3 / 3 100.0%	4 / 4 100.0%	2 / 2 100.0%	5 / 5 100.0%	5 / 5 100.0%	4 / 4 100.0%	1 / 1 100.0%	1 / 1 100.0%	7 / 7 100.0%	1 / 1 100.0%	4 / 4 100.0%	2 / 2 100.0%	1 / 1 100.0%	1 / 1 100.0%	1 / 1 100.0%	3 / 3 100.0%	1 / 1 100.0%	2 / 2 100.0%	5 / 5 100.0%	4 / 4 100.0%	1 / 1 100.0%	6 / 6 100.0%	0 / 0 0.0%	0 / 0 0.0%	0 / 0 0.0%	0 / 0 0.0%	44 / 64 68.8%AB	20 / 64 31.2%ABE						
1	DSM 14936	65 / 65 100.0%	3 / 3 100.0%	4 / 4 100.0%	2 / 2 100.0%	5 / 5 100.0%	5 / 5 100.0%	4 / 4 100.0%	2 / 2 100.0%	1 / 1 100.0%	7 / 7 100.0%	1 / 1 100.0%	4 / 4 100.0%	2 / 2 100.0%	1 / 1 100.0%	1 / 1 100.0%	1 / 1 100.0%	3 / 3 100.0%	1 / 1 100.0%	2 / 2 100.0%	5 / 5 100.0%	4 / 4 100.0%	1 / 1 100.0%	6 / 6 100.0%	0 / 0 0.0%	0 / 0 0.0%	0 / 0 0.0%	0 / 0 0.0%	46 / 65 70.8%AB	19 / 65 29.2%ABE						
1	Pseudomonas poae RE*1-1-14	65 / 65 100.0%	3 / 3 100.0%	4 / 4 100.0%	2 / 2 100.0%	5 / 5 100.0%	5 / 5 100.0%	4 / 4 100.0%	2 / 2 100.0%	1 / 1 100.0%	7 / 7 100.0%	1 / 1 100.0%	4 / 4 100.0%	2 / 2 100.0%	1 / 1 100.0%	1 / 1 100.0%	1 / 1 100.0%	3 / 3 100.0%	1 / 1 100.0%	2 / 2 100.0%	5 / 5 100.0%	4 / 4 100.0%	1 / 1 100.0%	6 / 6 100.0%	0 / 0 0.0%	0 / 0 0.0%	0 / 0 0.0%	0 / 0 0.0%	46 / 65 70.8%AB	19 / 65 29.2%ABE						
2	CFBP 6595	138 / 138 100.0%	6 / 6 100.0%	8 / 8 100.0%	4 / 4 100.0%	10 / 10 100.0%	12 / 12 100.0%	8 / 8 100.0%	2 / 2 100.0%	2 / 2 100.0%	15 / 15 100.0%	2 / 2 100.0%	8 / 8 100.0%	4 / 4 100.0%	2 / 2 100.0%	4 / 4 100.0%	2 / 2 100.0%	6 / 6 100.0%	2 / 2 100.0%	6 / 6 100.0%	10 / 10 100.0%	11 / 11 100.0%	2 / 2 100.0%	12 / 12 100.0%	0 / 0 0.0%	0 / 0 0.0%	0 / 0 0.0%	0 / 0 0.0%	93 / 138 67.4%AB	45 / 138 32.6%ABE						
1	Pseudomonas fluorescens Pf-5	70 / 70 100.0%	3 / 3 100.0%	4 / 4 100.0%	2 / 2 100.0%	5 / 5 100.0%	6 / 6 100.0%	4 / 4 100.0%	1 / 1 100.0%	1 / 1 100.0%	8 / 8 100.0%	1 / 1 100.0%	4 / 4 100.0%	2 / 2 100.0%	1 / 1 100.0%	2 / 2 100.0%	1 / 1 100.0%	3 / 3 100.0%	1 / 1 100.0%	3 / 3 100.0%	5 / 5 100.0%	6 / 6 100.0%	1 / 1 100.0%	6 / 6 100.0%	0 / 0 0.0%	0 / 0 0.0%	0 / 0 0.0%	0 / 0 0.0%	47 / 70 67.1%AB	23 / 70 32.9%ABE						
1	Pseudomonas fluorescens CHA0	68 / 68 100.0%	3 / 3 100.0%	4 / 4 100.0%	2 / 2 100.0%	5 / 5 100.0%	6 / 6 100.0%	4 / 4 100.0%	1 / 1 100.0%	1 / 1 100.0%	7 / 7 100.0%	1 / 1 100.0%	4 / 4 100.0%	2 / 2 100.0%	1 / 1 100.0%	2 / 2 100.0%	1 / 1 100.0%	3 / 3 100.0%	1 / 1 100.0%	3 / 3 100.0%	5 / 5 100.0%	5 / 5 100.0%	1 / 1 100.0%	6 / 6 100.0%	0 / 0 0.0%	0 / 0 0.0%	0 / 0 0.0%	0 / 0 0.0%	46 / 68 67.6%AB	22 / 68 32.4%ABE						
1	Pseudomonas pertucinogena group	63 / 63 100.0%	3 / 3 100.0%	3 / 3 100.0%	2 / 2 100.0%	5 / 5 100.0%	5 / 5 100.0%	3 / 3 100.0%	1 / 1 100.0%	1 / 1 100.0%	7 / 7 100.0%	1 / 1 100.0%	4 / 4 100.0%	2 / 2 100.0%	1 / 1 100.0%	2 / 2 100.0%	1 / 1 100.0%	3 / 3 100.0%	1 / 1 100.0%	3 / 3 100.0%	5 / 5 100.0%	3 / 3 100.0%	1 / 1 100.0%	5 / 5 100.0%	1 / 1 100.0%	0 / 0 0.0%	0 / 0 0.0%	0 / 0 0.0%	43 / 63 68.3%AB	20 / 63 31.7%ABE						
1	"Pseudomonas denitrificans" (Christensen 1903) Bergey et al. 1923, nom. rejic.	63 / 63 100.0%	3 / 3 100.0%	3 / 3 100.0%	2 / 2 100.0%	5 / 5 100.0%	5 / 5 100.0%	3 / 3 100.0%	1 / 1 100.0%	1 / 1 100.0%	7 / 7 100.0%	1 / 1 100.0%	4 / 4 100.0%	2 / 2 100.0%	1 / 1 100.0%	2 / 2 100.0%	1 / 1 100.0%	3 / 3 100.0%	1 / 1 100.0%	3 / 3 100.0%	5 / 5 100.0%	3 / 3 100.0%	1 / 1 100.0%	5 / 5 100.0%	1 / 1 100.0%	0 / 0 0.0%	0 / 0 0.0%	0 / 0 0.0%	43 / 63 68.3%AB	20 / 63 31.7%ABE						
1	Pseudomonas denitrificans ATCC 13867	63 / 63 100.0%	3 / 3 100.0%	3 / 3 100.0%	2 / 2 100.0%	5 / 5 100.0%	5 / 5 100.0%	3 / 3 100.0%	1 / 1 100.0%	1 / 1 100.0%	7 / 7 100.0%	1 / 1 100.0%	4 / 4 100.0%	2 / 2 100.0%	1 / 1 100.0%	2 / 2 100.0%	1 / 1 100.0%	3 / 3 100.0%	1 / 1 100.0%	3 / 3 100.0%	5 / 5 100.0%	3 / 3 100.0%	1 / 1 100.0%	5 / 5 100.0%	1 / 1 100.0%	0 / 0 0.0%	0 / 0 0.0%	0 / 0 0.0%	43 / 63 68.3%AB	20 / 63 31.7%ABE						
12	Pseudomonas putida group	838 / 841 99.6%	43 / 43 100.0%	52 / 52 100.0%	24 / 24 100.0%	38 / 38 100.0%	72 / 72 100.0%	55 / 55 100.0%	23 / 23 100.0%	12 / 12 100.0%	65 / 66 98.5%	12 / 12 100.0%	47 / 48 97.9%	32 / 32 100.0%	12 / 12 100.0%	24 / 24 100.0%	12 / 12 100.0%	44 / 44 100.0%	12 / 12 100.0%	36 / 36 100.0%	76 / 77 98.7%	52 / 52 100.0%	12 / 12 100.0%	72 / 72 100.0%	11 / 11 100.0%	0 / 0 0.0%	0 / 0 0.0%	0 / 0 0.0%	534 / 841 63.5%AB	251 / 841 29.8%ABE	42 / 841 5.0%B	11 / 841 1.3%BE	1 / 841 0.1%	1 / 841 0.1%CE	1 / 841 0.1%C	
11	"Bacillus fluorescens putidus" Flugge 1886	775 / 778 99.6%	40 / 40 100.0%	49 / 49 100.0%	22 / 22 100.0%	34 / 34 100.0%	67 / 67 100.0%	52 / 52 100.0%	22 / 22 100.0%	11 / 11 100.0%	59 / 60 98.3%	11 / 11 100.0%	43 / 44 97.7%	30 / 30 100.0%	11 / 11 100.0%	22 / 22 100.0%	11 / 11 100.0%	41 / 41 100.0%	11 / 11 100.0%	33 / 33 100.0%	70 / 71 98.6%	48 / 48 100.0%	11 / 11 100.0%	66 / 66 100.0%	11 / 11 100.0%	0 / 0 0.0%	0 / 0 0.0%	0 / 0 0.0%	492 / 778 63.2%AB	230 / 778 29.6%ABE	42 / 778 5.4%B	11 / 778 1.4%BE	1 / 778 0.1%C	1 / 778 0.1%	1 / 778 0.1%CE	
1	Pseudomonas putida GB-1	75 / 75 100.0%	4 / 4 100.0%	5 / 5 100.0%	2 / 2 100.0%	3 / 3 100.0%	7 / 7 100.0%	5 / 5 100.0%	2 / 2 100.0%	1 / 1 100.0%	6 / 6 100.0%	1 / 1 100.0%	4 / 4 100.0%	3 / 3 100.0%	1 / 1 100.0%	2 / 2 100.0%	1 / 1 100.0%	4 / 4 100.0%	1 / 1 100.0%	3 / 3 100.0%	7 / 7 100.0%	5 / 5 100.0%	1 / 1 100.0%	6 / 6 100.0%	1 / 1 100.0%	0 / 0 0.0%	0 / 0 0.0%	0 / 0 0.0%	33 / 75 44.0%AB	19 / 75 25.3%B	16 / 75 21.3%ABE	7 / 75 9.3%BE				
1	Pseudomonas putida (strain KT2440)	73 / 74 98.6%	4 / 4 100.0%	5 / 5 100.0%	2 / 2 100.0%	3 / 3 100.0%	6 / 6 100.0%	5 / 5 100.0%	2 / 2 100.0%	1 / 1 100.0%	6 / 6 100.0%	1 / 1 100.0%	4 / 4 100.0%	3 / 3 100.0%	1 / 1 100.0%	2 / 2 100.0%	1 / 1 100.0%	4 / 4 100.0%	1 / 1 100.0%	3 / 3 100.0%	6 / 7 85.7%	5 / 5 100.0%	1 / 1 100.0%	6 / 6 100.0%	1 / 1 100.0%	0 / 0 0.0%	0 / 0 0.0%	0 / 0 0.0%	50 / 74 67.6%AB	23 / 74 31.1%ABE	1 / 74 1.4%					
1	Pseudomonas putida ND6	76 / 76 100.0%	4 / 4 100.0%	5 / 5 100.0%	2 / 2 100.0%	4 / 4 100.0%	6 / 6 100.0%	5 / 5 100.0%	2 / 2 100.0%	1 / 1 100.0%	7 / 7 100.0%	1 / 1 100.0%	4 / 4 100.0%	3 / 3 100.0%	1 / 1 100.0%	2 / 2 100.0%	1 / 1 100.0%	4 / 4 100.0%	1 / 1 100.0%	3 / 3 100.0%	7 / 7 100.0%	5 / 5 100.0%	1 / 1 100.0%	6 / 6 100.0%	1 / 1 100.0%	0 / 0 0.0%	0 / 0 0.0%	0 / 0 0.0%	53 / 76 69.7%AB	23 / 76 30.3%ABE						
1	Pseudomonas putida F1	76 / 76 100.0%	4 / 4 100.0%	5 / 5 100.0%	2 / 2 100.0%	4 / 4 100.0%	6 / 6 100.0%	5 / 5 100.0%	2 / 2 100.0%	1 / 1 100.0%	7 / 7 100.0%	1 / 1 100.0%	4 / 4 100.0%	3 / 3 100.0%	1 / 1 100.0%	2 / 2 100.0%	1 / 1 100.0%	4 / 4 100.0%	1 / 1 100.0%	3 / 3 100.0%	7 / 7 100.0%	5 / 5 100.0%	1 / 1 100.0%	6 / 6 100.0%	1 / 1 100.0%	0 / 0 0.0%	0 / 0 0.0%	0 / 0 0.0%	53 / 76 69.7%AB	23 / 76 30.3%ABE						
1	Pseudomonas putida W619	75 / 75 100.0%	4 / 4 100.0%	5 / 5 100.0%	2 / 2 100.0%	5 / 5 100.0%	6 / 6 100.0%	5 / 5 100.0%	2 / 2 100.0%	1 / 1 100.0%	8 / 8 100.0%	1 / 1 100.0%	4 / 4 100.0%	2 / 2 100.0%	1 / 1 100.0%	2 / 2 100.0%	1 / 1 100.0%	3 / 3 100.0%	1 / 1 100.0%	3 / 3 100.0%	6 / 6 100.0%	5 / 5 100.0%	1 / 1 100.0%	6 / 6 100.0%	1 / 1 100.0%	0 / 0 0.0%	0 / 0 0.0%	0 / 0 0.0%	29 / 75 38.7%AB	23 / 75 30.7%B	19 / 75 25.3%ABE	4 / 75 5.3%BE				
1	Pseudomonas putida BIRD-1	64 / 64 100.0%	3 / 3 100.0%	3 / 3 100.0%	2 / 2 100.0%	3 / 3 100.0%	6 / 6 100.0%	4 / 4 100.0%	2 / 2 100.0%	1 / 1 100.0%	4 / 4 100.0%	1 / 1 100.0%	4 / 4 100.0%	2 / 2 100.0%	1 / 1 100.0%	2 / 2 100.0%	1 / 1 100.0%	3 / 3 100.0%	1 / 1 100.0%	3 / 3 100.0%	7 / 7 100.0%	3 / 3 100.0%	1 / 1 100.0%	6 / 6 100.0%	1 / 1 100.0%	0 / 0 0.0%	0 / 0 0.0%	0 / 0 0.0%	44 / 64 68.8%AB	20 / 64 31.2%ABE						
1	Pseudomonas putida S16	64 / 66 97.0%	3 / 3 100.0%	5 / 5 100.0%	2 / 2 100.0%	0 / 0 0.0%	6 / 6 100.0%	5 / 5 100.0%	2 / 2 100.0%	1 / 1 100.0%	2 / 3 66.7%	1 / 1 100.0%	3 / 4 75.0%	3 / 3 100.0%	1 / 1 100.0%	2 / 2 100.0%	1 / 1 100.0%	4 / 4 100.0%	1 / 1 100.0%	3 / 3 100.0%	6 / 6 100.0%	5 / 5 100.0%	1 / 1 100.0%	6 / 6 100.0%	1 / 1 100.0%	0 / 0 0.0%	0 / 0 0.0%	0 / 0 0.0%	43 / 66 65.2%AB	21 / 66 31.8%ABE	1 / 66 1.5%CE	1 / 66 1.5%C				
1	Pseudomonas putida DOT-T1E	59 / 59 100.0%	3 / 3 100.0%	2 / 2 100.0%	2 / 2 100.0%	2 / 2 100.0%	6 / 6 100.0%	4 / 4 100.0%	2 / 2 100.0%	1 / 1 100.0%	2 / 2 100.0%	1 / 1 100.0%	4 / 4 100.0%	2 / 2 100.0%	1 / 1 100.0%	2 / 2 100.0%	1 / 1 100.0%	3 / 3 100.0%	1 / 1 100.0%	3 / 3 100.0%	6 / 6 100.0%	3 / 3 100.0%	1 / 1 100.0%	6 / 6 100.0%	1 / 1 100.0%	0 / 0 0.0%	0 / 0 0.0%	0 / 0 0.0%	39 / 59 66.1%AB	20 / 59 33.9%ABE						
1	Pseudomonas putida NBRC 14164	75 / 75 100.0%	4 / 4 100.0%	5 / 5 100.0%	2 / 2 100.0%	4 / 4 100.0%	6 / 6 100.0%	5 / 5 100.0%	2 / 2 100.0%	1 / 1 100.0%	7 / 7 100.0%	1 / 1 100.0%	4 / 4 100.0%	3 / 3 100.0%	1 / 1 100.0%	2 / 2 100.0%	1 / 1 100.0%	4 / 4 100.0%	1 / 1 100.0%	3 / 3 100.0%	6 / 6 100.0%	5 / 5 100.0%	1 / 1 100.0%	6 / 6 100.0%	1 / 1 100.0%	0 / 0 0.0%	0 / 0 0.0%	0 / 0 0.0%	52 / 75 69.3%AB	23 / 75 30.7%ABE						
1	Pseudomonas putida HB3267	70 / 70 100.0%	3 / 3 100.0%	5 / 5 100.0%	2 / 2 100.0%	3 / 3 100.0%	6 / 6 100.0%	5 / 5 100.0%	2 / 2 100.0%	1 / 1 100.0%	5 / 5 100.0%	1 / 1 100.0%	4 / 4 100.0%	3 / 3 100.0%	1 / 1 100.0%	2 / 2 100.0%	1 / 1 100.0%	4 / 4 100.0%	1 / 1 100.0%	3 / 3 100.0%	6 / 6 100.0%	4 / 4 100.0%	1 / 1 100.0%	6 / 6 100.0%	1 / 1 100.0%	0 / 0 0.0%	0 / 0 0.0%	0 / 0 0.0%	49 / 70 70.0%AB	21 / 70 30.0%ABE						
1	Pseudomonas putida H8234	68 / 68 100.0%	4 / 4 100.0%	4 / 4 100.0%	2 / 2 100.0%	3 / 3 100.0%	6 / 6 100.0%	4 / 4 100.0%	2 / 2 100.0%	1 / 1 100.0%	5 / 5 100.0%	1 / 1 100.0%	4 / 4 100.0%	3 / 3 100.0%	1 / 1 100.0%	2 / 2 100.0%	1 / 1 100.0%	4 / 4 100.0%	1 / 1 100.0%	3 / 3 100.0%	6 / 6 100.0%	3 / 3 100.0%	1 / 1 100.0%	6 / 6 100.0%	1 / 1 100.0%	0 / 0 0.0%	0 / 0 0.0%	0 / 0 0.0%	47 / 68 69.1%AB	21 / 68 30.9%ABE						
1	ATCC 31418	63 / 63 100.0%	3 / 3 100.0%	3 / 3 100.0%	2 / 2 100.0%	4 / 4 100.0%	5 / 5 100.0%	3 / 3 100.0%	1 / 1 100.0%	1 / 1 100.0%	6 / 6 100.0%	1 / 1 100.0%	4 / 4 100.0%	2 / 2 100.0%	1 / 1 100.0%	2 / 2 100.0%	1 / 1 100.0%	3 / 3 100.0%	1 / 1 100.0%	3 / 3 100.0%	6 / 6 100.0%	4 / 4 100.0%	1 / 1 100.0%	6 / 6 100.0%	0 / 0 0.0%	0 / 0 0.0%	0 / 0 0.0%	0 / 0 0.0%	42 / 63 66.7%AB	21 / 63 33.3%ABE						
1	Pseudomonas fulva 12-X	63 / 63 100.0%	3 / 3 100.0%	3 / 3 100.0%	2 / 2 100.0%	4 / 4 100.0%	5 / 5 100.0%	3 / 3 100.0%	1 / 1 100.0%	1 / 1 100.0%	6 / 6 100.0%	1 / 1 100.0%	4 / 4 100.0%	2 / 2 100.0%	1 / 1 100.0%	2 / 2 100.0%	1 / 1 100.0%	3 / 3 100.0%	1 / 1 100.0%	3 / 3 100.0%	6 / 6 100.0%	4 / 4 100.0%	1 / 1 100.0%	6 / 6 100.0%	0 / 0 0.0%	0 / 0 0.0%	0 / 0 0.0%	0 / 0 0.0%	42 / 63 66.7%AB	21 / 63 33.3%ABE						
6	Pseudomonas stutzeri group	358 / 359 99.7%	18 / 18 100.0%	12 / 12 100.0%	12 / 12 100.0%	24 / 24 100.0%	24 / 24 100.0%	18 / 18 100.0%	6 / 6 100.0%	6 / 6 100.0%	35 / 36 97.2%	6 / 6 100.0%	24 / 24 100.0%	12 / 12 100.0%	6 / 6 100.0%	11 / 11 100.0%	6 / 6 100.0%	18 / 18 100.0%	6 / 6 100.0%	20 / 20 100.0%	29 / 29 100.0%	18 / 18 100.0%	6 / 6 100.0%	35 / 35 100.0%	6 / 6 100.0%	0 / 0 0.0%	0 / 0 0.0%	0 / 0 0.0%	228 / 359 63.5%AB	130 / 359 36.2%ABE	1 / 359 0.3%					
6	Pseudomonas stutzeri subgroup	358 / 359 99.7%	18 / 18 100.0%	12 / 12 100.0%	12 / 12 100.0%	24 / 24 100.0%	24 / 24 100.0%	18 / 18 100.0%	6 / 6 100.0%	6 / 6 100.0%	35 / 36 97.2%	6 / 6 100.0%	24 / 24 100.0%	12 / 12 100.0%	6 / 6 100.0%	11 / 11 100.0%	6 / 6 100.0%	18 / 18 100.0%	6 / 6 100.0%	20 / 20 100.0%	29 / 29 100.0%	18 / 18 100.0%	6 / 6 100.0%	35 / 35 100.0%	6 / 6 100.0%	0 / 0 0.0%	0 / 0 0.0%	0 / 0 0.0%	228 / 359 63.5%AB	130 / 359 36.2%ABE	1 / 359 0.3%					
6	"Achromobacter sewerinii" Bergey et al. 1923	358 / 359 99.7%	18 / 18 100.0%	12 / 12 100.0%	12 / 12 100.0%	24 / 24 100.0%	24 / 24 100.0%	18 / 18 100.0%	6 / 6 100.0%	6 / 6 100.0%	35 / 36 97.2%	6 / 6 100.0%	24 / 24 100.0%	12 / 12 100.0%	6 / 6 100.0%	11 / 11 100.0%	6 / 6 100.0%	18 / 18 100.0%	6 / 6 100.0%	20 / 20 100.0%	29 / 29 100.0%	18 / 18 100.0%	6 / 6 100.0%	35 / 35 100.0%	6 / 6 100.0%	0 / 0 0.0%	0 / 0 0.0%	0 / 0 0.0%	228 / 359 63.5%AB	130 / 359 36.2%ABE	1 / 359 0.3%					
1	Pseudomonas stutzeri ATCC 17588	59 / 59 100.0%	3 / 3 100.0%	2 / 2 100.0%	2 / 2 100.0%	4 / 4 100.0%	4 / 4 100.0%	3 / 3 100.0%	1 / 1 100.0%	1 / 1 100.0%	6 / 6 100.0%	1 / 1 100.0%	4 / 4 100.0%	2 / 2 100.0%	1 / 1 100.0%	2 / 2 100.0%	1 / 1 100.0%	3 / 3 100.0%	1 / 1 100.0%	3 / 3 100.0%	4 / 4 100.0%	3 / 3 100.0%	1 / 1 100.0%	6 / 6 100.0%	1 / 1 100.0%	0 / 0 0.0%	0 / 0 0.0%	0 / 0 0.0%	37 / 59 62.7%AB	22 / 59 37.3%ABE						
1	Pseudomonas stutzeri A1501	60 / 60 100.0%	3 / 3 100.0%	2 / 2 100.0%	2 / 2 100.0%	4 / 4 100.0%	4 / 4 100.0%	3 / 3 100.0%	1 / 1 100.0%	1 / 1 100.0%	6 / 6 100.0%	1 / 1 100.0%	4 / 4 100.0%	2 / 2 100.0%	1 / 1 100.0%	2 / 2 100.0%	1 / 1 100.0%	3 / 3 100.0%	1 / 1 100.0%	3 / 3 100.0%	5 / 5 100.0%	3 / 3 100.0%	1 / 1 100.0%	6 / 6 100.0%	1 / 1 100.0%	0 / 0 0.0%	0 / 0 0.0%	0 / 0 0.0%	38 / 60 63.3%AB	22 / 60 36.7%ABE						
1	Pseudomonas stutzeri RCH2	58 / 59 98.3%	3 / 3 100.0%	2 / 2 100.0%	2 / 2 100.0%	4 / 4 100.0%	4 / 4 100.0%	3 / 3 100.0%	1 / 1 100.0%	1 / 1 100.0%	5 / 6 83.3%	1 / 1 100.0%	4 / 4 100.0%	2 / 2 100.0%	1 / 1 100.0%	1 / 1 100.0%	1 / 1 100.0%	3 / 3 100.0%	1 / 1 100.0%	3 / 3 100.0%	5 / 5 100.0%	3 / 3 100.0%	1 / 1 100.0%	6 / 6 100.0%	1 / 1 100.0%	0 / 0 0.0%	0 / 0 0.0%	0 / 0 0.0%	37 / 59 62.7%AB	21 / 59 35.6%ABE	1 / 59 1.7%					
1	Pseudomonas stutzeri CMT.9.A	60 / 60 100.0%	3 / 3 100.0%	2 / 2 100.0%	2 / 2 100.0%	4 / 4 100.0%	4 / 4 100.0%	3 / 3 100.0%	1 / 1 100.0%	1 / 1 100.0%	6 / 6 100.0%	1 / 1 100.0%	4 / 4 100.0%	2 / 2 100.0%	1 / 1 100.0%	2 / 2 100.0%	1 / 1 100.0%	3 / 3 100.0%	1 / 1 100.0%	4 / 4 100.0%	5 / 5 100.0%	3 / 3 100.0%	1 / 1 100.0%	5 / 5 100.0%	1 / 1 100.0%	0 / 0 0.0%	0 / 0 0.0%	0 / 0 0.0%	39 / 60 65.0%AB	21 / 60 35.0%ABE						
1	Pseudomonas stutzeri DSM 10701	60 / 60 100.0%	3 / 3 100.0%	2 / 2 100.0%	2 / 2 100.0%	4 / 4 100.0%	4 / 4 100.0%	3 / 3 100.0%	1 / 1 100.0%	1 / 1 100.0%	6 / 6 100.0%	1 / 1 100.0%	4 / 4 100.0%	2 / 2 100.0%	1 / 1 100.0%	2 / 2 100.0%	1 / 1 100.0%	3 / 3 100.0%	1 / 1 100.0%	3 / 3 100.0%	5 / 5 100.0%	3 / 3 100.0%	1 / 1 100.0%	6 / 6 100.0%	1 / 1 100.0%	0 / 0 0.0%	0 / 0 0.0%	0 / 0 0.0%	38 / 60 63.3%AB	22 / 60 36.7%ABE						
1	Pseudomonas stutzeri CCUG 29243	61 / 61 100.0%	3 / 3 100.0%	2 / 2 100.0%	2 / 2 100.0%	4 / 4 100.0%	4 / 4 100.0%	3 / 3 100.0%	1 / 1 100.0%	1 / 1 100.0%	6 / 6 100.0%	1 / 1 100.0%	4 / 4 100.0%	2 / 2 100.0%	1 / 1 100.0%	2 / 2 100.0%	1 / 1 100.0%	3 / 3 100.0%	1 / 1 100.0%	4 / 4 100.0%	5 / 5 100.0%	3 / 3 100.0%	1 / 1 100.0%	6 / 6 100.0%	1 / 1 100.0%	0 / 0 0.0%	0 / 0 0.0%	0 / 0 0.0%	39 / 61 63.9%AB	22 / 61 36.1%ABE						
3	Pseudomonas syringae group	189 / 190 99.5%	8 / 9 88.9%	9 / 9 100.0%	6 / 6 100.0%	15 / 15 100.0%	15 / 15 100.0%	11 / 11 100.0%	3 / 3 100.0%	3 / 3 100.0%	21 / 21 100.0%	3 / 3 100.0%	12 / 12 100.0%	6 / 6 100.0%	3 / 3 100.0%	3 / 3 100.0%	3 / 3 100.0%	11 / 11 100.0%	3 / 3 100.0%	6 / 6 100.0%	15 / 15 100.0%	12 / 12 100.0%	3 / 3 100.0%	18 / 18 100.0%	0 / 0 0.0%	0 / 0 0.0%	0 / 0 0.0%	0 / 0 0.0%	133 / 190 70.0%AB	56 / 190 29.5%ABE	1 / 190 0.5%E					
1	Pseudomonas syringae group genomosp. 1	64 / 64 100.0%	3 / 3 100.0%	3 / 3 100.0%	2 / 2 100.0%	5 / 5 100.0%	5 / 5 100.0%	4 / 4 100.0%	1 / 1 100.0%	1 / 1 100.0%	7 / 7 100.0%	1 / 1 100.0%	4 / 4 100.0%	2 / 2 100.0%	1 / 1 100.0%	1 / 1 100.0%	1 / 1 100.0%	4 / 4 100.0%	1 / 1 100.0%	2 / 2 100.0%	5 / 5 100.0%	4 / 4 100.0%	1 / 1 100.0%	6 / 6 100.0%	0 / 0 0.0%	0 / 0 0.0%	0 / 0 0.0%	0 / 0 0.0%	45 / 64 70.3%AB	19 / 64 29.7%ABE						
1	ATCC 19310	64 / 64 100.0%	3 / 3 100.0%	3 / 3 100.0%	2 / 2 100.0%	5 / 5 100.0%	5 / 5 100.0%	4 / 4 100.0%	1 / 1 100.0%	1 / 1 100.0%	7 / 7 100.0%	1 / 1 100.0%	4 / 4 100.0%	2 / 2 100.0%	1 / 1 100.0%	1 / 1 100.0%	1 / 1 100.0%	4 / 4 100.0%	1 / 1 100.0%	2 / 2 100.0%	5 / 5 100.0%	4 / 4 100.0%	1 / 1 100.0%	6 / 6 100.0%	0 / 0 0.0%	0 / 0 0.0%	0 / 0 0.0%	0 / 0 0.0%	45 / 64 70.3%AB	19 / 64 29.7%ABE						
1	Pseudomonas syringae (PV. SYRINGAE)	64 / 64 100.0%	3 / 3 100.0%	3 / 3 100.0%	2 / 2 100.0%	5 / 5 100.0%	5 / 5 100.0%	4 / 4 100.0%	1 / 1 100.0%	1 / 1 100.0%	7 / 7 100.0%	1 / 1 100.0%	4 / 4 100.0%	2 / 2 100.0%	1 / 1 100.0%	1 / 1 100.0%	1 / 1 100.0%	4 / 4 100.0%	1 / 1 100.0%	2 / 2 100.0%	5 / 5 100.0%	4 / 4 100.0%	1 / 1 100.0%	6 / 6 100.0%	0 / 0 0.0%	0 / 0 0.0%	0 / 0 0.0%	0 / 0 0.0%	45 / 64 70.3%AB	19 / 64 29.7%ABE						
1	Pseudomonas syringae pv. syringae B728a	64 / 64 100.0%	3 / 3 100.0%	3 / 3 100.0%	2 / 2 100.0%	5 / 5 100.0%	5 / 5 100.0%	4 / 4 100.0%	1 / 1 100.0%	1 / 1 100.0%	7 / 7 100.0%	1 / 1 100.0%	4 / 4 100.0%	2 / 2 100.0%	1 / 1 100.0%	1 / 1 100.0%	1 / 1 100.0%	4 / 4 100.0%	1 / 1 100.0%	2 / 2 100.0%	5 / 5 100.0%	4 / 4 100.0%	1 / 1 100.0%	6 / 6 100.0%	0 / 0 0.0%	0 / 0 0.0%	0 / 0 0.0%	0 / 0 0.0%	45 / 64 70.3%AB	19 / 64 29.7%ABE						
1	Pseudomonas syringae group genomosp. 2	62 / 63 98.4%	2 / 3 66.7%	3 / 3 100.0%	2 / 2 100.0%	5 / 5 100.0%	5 / 5 100.0%	3 / 3 100.0%	1 / 1 100.0%	1 / 1 100.0%	7 / 7 100.0%	1 / 1 100.0%	4 / 4 100.0%	2 / 2 100.0%	1 / 1 100.0%	1 / 1 100.0%	1 / 1 100.0%	4 / 4 100.0%	1 / 1 100.0%	2 / 2 100.0%	5 / 5 100.0%	4 / 4 100.0%	1 / 1 100.0%	6 / 6 100.0%	0 / 0 0.0%	0 / 0 0.0%	0 / 0 0.0%	0 / 0 0.0%	44 / 63 69.8%AB	18 / 63 28.6%ABE	1 / 63 1.6%E					
1	ATCC 13522	62 / 63 98.4%	2 / 3 66.7%	3 / 3 100.0%	2 / 2 100.0%	5 / 5 100.0%	5 / 5 100.0%	3 / 3 100.0%	1 / 1 100.0%	1 / 1 100.0%	7 / 7 100.0%	1 / 1 100.0%	4 / 4 100.0%	2 / 2 100.0%	1 / 1 100.0%	1 / 1 100.0%	1 / 1 100.0%	4 / 4 100.0%	1 / 1 100.0%	2 / 2 100.0%	5 / 5 100.0%	4 / 4 100.0%	1 / 1 100.0%	6 / 6 100.0%	0 / 0 0.0%	0 / 0 0.0%	0 / 0 0.0%	0 / 0 0.0%	44 / 63 69.8%AB	18 / 63 28.6%ABE	1 / 63 1.6%E					
1	Pseudomonas savastanoi pv. phaseolicola	62 / 63 98.4%	2 / 3 66.7%	3 / 3 100.0%	2 / 2 100.0%	5 / 5 100.0%	5 / 5 100.0%	3 / 3 100.0%	1 / 1 100.0%	1 / 1 100.0%	7 / 7 100.0%	1 / 1 100.0%	4 / 4 100.0%	2 / 2 100.0%	1 / 1 100.0%	1 / 1 100.0%	1 / 1 100.0%	4 / 4 100.0%	1 / 1 100.0%	2 / 2 100.0%	5 / 5 100.0%	4 / 4 100.0%	1 / 1 100.0%	6 / 6 100.0%	0 / 0 0.0%	0 / 0 0.0%	0 / 0 0.0%	0 / 0 0.0%	44 / 63 69.8%AB	18 / 63 28.6%ABE	1 / 63 1.6%E					
1	Pseudomonas syringae pv. phaseolicola 1448A	62 / 63 98.4%	2 / 3 66.7%	3 / 3 100.0%	2 / 2 100.0%	5 / 5 100.0%	5 / 5 100.0%	3 / 3 100.0%	1 / 1 100.0%	1 / 1 100.0%	7 / 7 100.0%	1 / 1 100.0%	4 / 4 100.0%	2 / 2 100.0%	1 / 1 100.0%	1 / 1 100.0%	1 / 1 100.0%	4 / 4 100.0%	1 / 1 100.0%	2 / 2 100.0%	5 / 5 100.0%	4 / 4 100.0%	1 / 1 100.0%	6 / 6 100.0%	0 / 0 0.0%	0 / 0 0.0%	0 / 0 0.0%	0 / 0 0.0%	44 / 63 69.8%AB	18 / 63 28.6%ABE	1 / 63 1.6%E					
1	Pseudomonas syringae group genomosp. 3	63 / 63 100.0%	3 / 3 100.0%	3 / 3 100.0%	2 / 2 100.0%	5 / 5 100.0%	5 / 5 100.0%	4 / 4 100.0%	1 / 1 100.0%	1 / 1 100.0%	7 / 7 100.0%	1 / 1 100.0%	4 / 4 100.0%	2 / 2 100.0%	1 / 1 100.0%	1 / 1 100.0%	1 / 1 100.0%	3 / 3 100.0%	1 / 1 100.0%	2 / 2 100.0%	5 / 5 100.0%	4 / 4 100.0%	1 / 1 100.0%	6 / 6 100.0%	0 / 0 0.0%	0 / 0 0.0%	0 / 0 0.0%	0 / 0 0.0%	44 / 63 69.8%AB	19 / 63 30.2%ABE						
1	Pseudomonas syringae (PV. TOMATO)	63 / 63 100.0%	3 / 3 100.0%	3 / 3 100.0%	2 / 2 100.0%	5 / 5 100.0%	5 / 5 100.0%	4 / 4 100.0%	1 / 1 100.0%	1 / 1 100.0%	7 / 7 100.0%	1 / 1 100.0%	4 / 4 100.0%	2 / 2 100.0%	1 / 1 100.0%	1 / 1 100.0%	1 / 1 100.0%	3 / 3 100.0%	1 / 1 100.0%	2 / 2 100.0%	5 / 5 100.0%	4 / 4 100.0%	1 / 1 100.0%	6 / 6 100.0%	0 / 0 0.0%	0 / 0 0.0%	0 / 0 0.0%	0 / 0 0.0%	44 / 63 69.8%AB	19 / 63 30.2%ABE						
1	Pseudomonas syringae DC3000	63 / 63 100.0%	3 / 3 100.0%	3 / 3 100.0%	2 / 2 100.0%	5 / 5 100.0%	5 / 5 100.0%	4 / 4 100.0%	1 / 1 100.0%	1 / 1 100.0%	7 / 7 100.0%	1 / 1 100.0%	4 / 4 100.0%	2 / 2 100.0%	1 / 1 100.0%	1 / 1 100.0%	1 / 1 100.0%	3 / 3 100.0%	1 / 1 100.0%	2 / 2 100.0%	5 / 5 100.0%	4 / 4 100.0%	1 / 1 100.0%	6 / 6 100.0%	0 / 0 0.0%	0 / 0 0.0%	0 / 0 0.0%	0 / 0 0.0%	44 / 63 69.8%AB	19 / 63 30.2%ABE						
1	"Pseudomonas entomophila" Vodovar et al. 2005	78 / 78 100.0%	4 / 4 100.0%	4 / 4 100.0%	2 / 2 100.0%	7 / 7 100.0%	6 / 6 100.0%	4 / 4 100.0%	2 / 2 100.0%	1 / 1 100.0%	10 / 10 100.0%	1 / 1 100.0%	4 / 4 100.0%	2 / 2 100.0%	1 / 1 100.0%	2 / 2 100.0%	1 / 1 100.0%	3 / 3 100.0%	1 / 1 100.0%	3 / 3 100.0%	7 / 7 100.0%	5 / 5 100.0%	1 / 1 100.0%	6 / 6 100.0%	1 / 1 100.0%	0 / 0 0.0%	0 / 0 0.0%	0 / 0 0.0%	55 / 78 70.5%AB	23 / 78 29.5%ABE						
1	Pseudomonas entomophila L48	78 / 78 100.0%	4 / 4 100.0%	4 / 4 100.0%	2 / 2 100.0%	7 / 7 100.0%	6 / 6 100.0%	4 / 4 100.0%	2 / 2 100.0%	1 / 1 100.0%	10 / 10 100.0%	1 / 1 100.0%	4 / 4 100.0%	2 / 2 100.0%	1 / 1 100.0%	2 / 2 100.0%	1 / 1 100.0%	3 / 3 100.0%	1 / 1 100.0%	3 / 3 100.0%	7 / 7 100.0%	5 / 5 100.0%	1 / 1 100.0%	6 / 6 100.0%	1 / 1 100.0%	0 / 0 0.0%	0 / 0 0.0%	0 / 0 0.0%	55 / 78 70.5%AB	23 / 78 29.5%ABE						
1	CFBP 11706	65 / 65 100.0%	3 / 3 100.0%	4 / 4 100.0%	2 / 2 100.0%	5 / 5 100.0%	5 / 5 100.0%	4 / 4 100.0%	1 / 1 100.0%	1 / 1 100.0%	7 / 7 100.0%	1 / 1 100.0%	4 / 4 100.0%	2 / 2 100.0%	1 / 1 100.0%	1 / 1 100.0%	1 / 1 100.0%	3 / 3 100.0%	1 / 1 100.0%	3 / 3 100.0%	5 / 5 100.0%	4 / 4 100.0%	1 / 1 100.0%	6 / 6 100.0%	0 / 0 0.0%	0 / 0 0.0%	0 / 0 0.0%	0 / 0 0.0%	45 / 65 69.2%AB	20 / 65 30.8%ABE						
1	Pseudomonas brassicacearum subsp. brassicacearum	65 / 65 100.0%	3 / 3 100.0%	4 / 4 100.0%	2 / 2 100.0%	5 / 5 100.0%	5 / 5 100.0%	4 / 4 100.0%	1 / 1 100.0%	1 / 1 100.0%	7 / 7 100.0%	1 / 1 100.0%	4 / 4 100.0%	2 / 2 100.0%	1 / 1 100.0%	1 / 1 100.0%	1 / 1 100.0%	3 / 3 100.0%	1 / 1 100.0%	3 / 3 100.0%	5 / 5 100.0%	4 / 4 100.0%	1 / 1 100.0%	6 / 6 100.0%	0 / 0 0.0%	0 / 0 0.0%	0 / 0 0.0%	0 / 0 0.0%	45 / 65 69.2%AB	20 / 65 30.8%ABE						
1	Pseudomonas brassicacearum subsp. brassicacearum CFBP 11874	65 / 65 100.0%	3 / 3 100.0%	4 / 4 100.0%	2 / 2 100.0%	5 / 5 100.0%	5 / 5 100.0%	4 / 4 100.0%	1 / 1 100.0%	1 / 1 100.0%	7 / 7 100.0%	1 / 1 100.0%	4 / 4 100.0%	2 / 2 100.0%	1 / 1 100.0%	1 / 1 100.0%	1 / 1 100.0%	3 / 3 100.0%	1 / 1 100.0%	3 / 3 100.0%	5 / 5 100.0%	4 / 4 100.0%	1 / 1 100.0%	6 / 6 100.0%	0 / 0 0.0%	0 / 0 0.0%	0 / 0 0.0%	0 / 0 0.0%	45 / 65 69.2%AB	20 / 65 30.8%ABE						
1	Pseudomonas putida UW4	71 / 71 100.0%	3 / 3 100.0%	4 / 4 100.0%	2 / 2 100.0%	7 / 7 100.0%	5 / 5 100.0%	4 / 4 100.0%	2 / 2 100.0%	1 / 1 100.0%	8 / 8 100.0%	1 / 1 100.0%	4 / 4 100.0%	2 / 2 100.0%	1 / 1 100.0%	2 / 2 100.0%	1 / 1 100.0%	3 / 3 100.0%	1 / 1 100.0%	3 / 3 100.0%	5 / 5 100.0%	4 / 4 100.0%	1 / 1 100.0%	6 / 6 100.0%	1 / 1 100.0%	0 / 0 0.0%	0 / 0 0.0%	0 / 0 0.0%	49 / 71 69.0%AB	22 / 71 31.0%ABE						
3	Azotobacter group	192 / 192 100.0%	6 / 6 100.0%	6 / 6 100.0%	6 / 6 100.0%	18 / 18 100.0%	15 / 15 100.0%	9 / 9 100.0%	3 / 3 100.0%	3 / 3 100.0%	24 / 24 100.0%	3 / 3 100.0%	12 / 12 100.0%	6 / 6 100.0%	6 / 6 100.0%	6 / 6 100.0%	3 / 3 100.0%	9 / 9 100.0%	3 / 3 100.0%	9 / 9 100.0%	15 / 15 100.0%	9 / 9 100.0%	3 / 3 100.0%	18 / 18 100.0%	0 / 0 0.0%	0 / 0 0.0%	0 / 0 0.0%	0 / 0 0.0%	132 / 192 68.8%AB	60 / 192 31.2%ABE						
3	"Azotomonas" Orla-Jensen 1909	192 / 192 100.0%	6 / 6 100.0%	6 / 6 100.0%	6 / 6 100.0%	18 / 18 100.0%	15 / 15 100.0%	9 / 9 100.0%	3 / 3 100.0%	3 / 3 100.0%	24 / 24 100.0%	3 / 3 100.0%	12 / 12 100.0%	6 / 6 100.0%	6 / 6 100.0%	6 / 6 100.0%	3 / 3 100.0%	9 / 9 100.0%	3 / 3 100.0%	9 / 9 100.0%	15 / 15 100.0%	9 / 9 100.0%	3 / 3 100.0%	18 / 18 100.0%	0 / 0 0.0%	0 / 0 0.0%	0 / 0 0.0%	0 / 0 0.0%	132 / 192 68.8%AB	60 / 192 31.2%ABE						
3	ATCC 478	192 / 192 100.0%	6 / 6 100.0%	6 / 6 100.0%	6 / 6 100.0%	18 / 18 100.0%	15 / 15 100.0%	9 / 9 100.0%	3 / 3 100.0%	3 / 3 100.0%	24 / 24 100.0%	3 / 3 100.0%	12 / 12 100.0%	6 / 6 100.0%	6 / 6 100.0%	6 / 6 100.0%	3 / 3 100.0%	9 / 9 100.0%	3 / 3 100.0%	9 / 9 100.0%	15 / 15 100.0%	9 / 9 100.0%	3 / 3 100.0%	18 / 18 100.0%	0 / 0 0.0%	0 / 0 0.0%	0 / 0 0.0%	0 / 0 0.0%	132 / 192 68.8%AB	60 / 192 31.2%ABE						
1	Azotobacter vinelandii ATCC BAA-1303	64 / 64 100.0%	2 / 2 100.0%	2 / 2 100.0%	2 / 2 100.0%	6 / 6 100.0%	5 / 5 100.0%	3 / 3 100.0%	1 / 1 100.0%	1 / 1 100.0%	8 / 8 100.0%	1 / 1 100.0%	4 / 4 100.0%	2 / 2 100.0%	2 / 2 100.0%	2 / 2 100.0%	1 / 1 100.0%	3 / 3 100.0%	1 / 1 100.0%	3 / 3 100.0%	5 / 5 100.0%	3 / 3 100.0%	1 / 1 100.0%	6 / 6 100.0%	0 / 0 0.0%	0 / 0 0.0%	0 / 0 0.0%	0 / 0 0.0%	44 / 64 68.8%AB	20 / 64 31.2%ABE						
2	Azotobacter vinelandii CA	128 / 128 100.0%	4 / 4 100.0%	4 / 4 100.0%	4 / 4 100.0%	12 / 12 100.0%	10 / 10 100.0%	6 / 6 100.0%	2 / 2 100.0%	2 / 2 100.0%	16 / 16 100.0%	2 / 2 100.0%	8 / 8 100.0%	4 / 4 100.0%	4 / 4 100.0%	4 / 4 100.0%	2 / 2 100.0%	6 / 6 100.0%	2 / 2 100.0%	6 / 6 100.0%	10 / 10 100.0%	6 / 6 100.0%	2 / 2 100.0%	12 / 12 100.0%	0 / 0 0.0%	0 / 0 0.0%	0 / 0 0.0%	0 / 0 0.0%	88 / 128 68.8%AB	40 / 128 31.2%ABE						
1	Azotobacter vinelandii CA6	64 / 64 100.0%	2 / 2 100.0%	2 / 2 100.0%	2 / 2 100.0%	6 / 6 100.0%	5 / 5 100.0%	3 / 3 100.0%	1 / 1 100.0%	1 / 1 100.0%	8 / 8 100.0%	1 / 1 100.0%	4 / 4 100.0%	2 / 2 100.0%	2 / 2 100.0%	2 / 2 100.0%	1 / 1 100.0%	3 / 3 100.0%	1 / 1 100.0%	3 / 3 100.0%	5 / 5 100.0%	3 / 3 100.0%	1 / 1 100.0%	6 / 6 100.0%	0 / 0 0.0%	0 / 0 0.0%	0 / 0 0.0%	0 / 0 0.0%	44 / 64 68.8%AB	20 / 64 31.2%ABE						
215	'Enterobacteriales'	16318 / 16982 96.1%	707 / 710 99.6%	588 / 610 96.4%	749 / 765 97.9%	584 / 596 98.0%	1175 / 1203 97.7%	1199 / 1238 96.8%	208 / 219 95.0%	522 / 535 97.6%	956 / 995 96.1%	202 / 221 91.4%	980 / 1040 94.2%	987 / 1017 97.1%	395 / 414 95.4%	708 / 724 97.8%	202 / 223 90.6%	601 / 617 97.4%	394 / 407 96.8%	813 / 868 93.7%	1530 / 1643 93.1%	775 / 797 97.2%	378 / 404 93.6%	1496 / 1566 95.5%	166 / 166 100.0%	0 / 0 0.0%	0 / 1 0.0%	3 / 3 100.0%	9807 / 16982 57.7%AB	5648 / 16982 33.3%ABE	484 / 16982 2.9%B	379 / 16982 2.2%BE	365 / 16982 2.1%E	289 / 16982 1.7%	5 / 16982 0.0%CE	4 / 16982 0.0%C	1 / 16982 0.0%GG
215	Enterobacteraceae	16318 / 16982 96.1%	707 / 710 99.6%	588 / 610 96.4%	749 / 765 97.9%	584 / 596 98.0%	1175 / 1203 97.7%	1199 / 1238 96.8%	208 / 219 95.0%	522 / 535 97.6%	956 / 995 96.1%	202 / 221 91.4%	980 / 1040 94.2%	987 / 1017 97.1%	395 / 414 95.4%	708 / 724 97.8%	202 / 223 90.6%	601 / 617 97.4%	394 / 407 96.8%	813 / 868 93.7%	1530 / 1643 93.1%	775 / 797 97.2%	378 / 404 93.6%	1496 / 1566 95.5%	166 / 166 100.0%	0 / 0 0.0%	0 / 1 0.0%	3 / 3 100.0%	9807 / 16982 57.7%AB	5648 / 16982 33.3%ABE	484 / 16982 2.9%B	379 / 16982 2.2%BE	365 / 16982 2.1%E	289 / 16982 1.7%	5 / 16982 0.0%CE	4 / 16982 0.0%C	1 / 16982 0.0%GG
2	Citrobacter	165 / 165 [illegible]	8 / 8 [illegible]	6 / 6 [illegible]	8 / 8 [illegible]	5 / 5 [illegible]	10 / 10 [illegible]	10 / 10 [illegible]	2 / 2 [illegible]	6 / 6 [illegible]	12 / 12 [illegible]	2 / 2 [illegible]	10 / 10 [illegible]	9 / 9 [illegible]	4 / 4 [illegible]	8 / 8 [illegible]	2 / 2 [illegible]	6 / 6 [illegible]	4 / 4 [illegible]	9 / 9 [illegible]	15 / 15 [illegible]	9 / 9 [illegible]	2 / 2 [illegible]	16 / 16 [illegible]	2 / 2 [illegible]	0 / 0 [illegible]	0 / 0 [illegible]	0 / 0 [illegible]	68 / 165 [illegible]	52 / 165 [illegible]	30 / 165 [illegible]	15 / 165 [illegible]				

1	"Aerobacter diversum" (sic) Burkey 1928	82 / 82 100.0%	4 / 4 100.0%	3 / 3 100.0%	4 / 4 100.0%	2 / 2 100.0%	5 / 5 100.0%	6 / 6 100.0%	1 / 1 100.0%	3 / 3 100.0%	4 / 4 100.0%	1 / 1 100.0%	5 / 5 100.0%	5 / 5 100.0%	2 / 2 100.0%	4 / 4 100.0%	1 / 1 100.0%	3 / 3 100.0%	2 / 2 100.0%	5 / 5 100.0%	7 / 7 100.0%	5 / 5 100.0%	1 / 1 100.0%	8 / 8 100.0%	1 / 1 100.0%	0 / 0 0.0%	0 / 0 0.0%	0 / 0 0.0%	30 / 82 36.6%B	19 / 82 23.2%ABE	18 / 82 22.0%AB	15 / 82 18.3%BE			
1	Citrobacter (diversus) koseri ATCC BAA-895	82 / 82 100.0%	4 / 4 100.0%	3 / 3 100.0%	4 / 4 100.0%	2 / 2 100.0%	5 / 5 100.0%	6 / 6 100.0%	1 / 1 100.0%	3 / 3 100.0%	4 / 4 100.0%	1 / 1 100.0%	5 / 5 100.0%	5 / 5 100.0%	2 / 2 100.0%	4 / 4 100.0%	1 / 1 100.0%	3 / 3 100.0%	2 / 2 100.0%	5 / 5 100.0%	7 / 7 100.0%	5 / 5 100.0%	1 / 1 100.0%	8 / 8 100.0%	1 / 1 100.0%	0 / 0 0.0%	0 / 0 0.0%	0 / 0 0.0%	30 / 82 36.6%B	19 / 82 23.2%ABE	18 / 82 22.0%AB	15 / 82 18.3%BE			
1	ATCC 51116	83 / 83 100.0%	4 / 4 100.0%	3 / 3 100.0%	4 / 4 100.0%	3 / 3 100.0%	5 / 5 100.0%	4 / 4 100.0%	1 / 1 100.0%	3 / 3 100.0%	8 / 8 100.0%	1 / 1 100.0%	5 / 5 100.0%	4 / 4 100.0%	2 / 2 100.0%	4 / 4 100.0%	1 / 1 100.0%	3 / 3 100.0%	2 / 2 100.0%	4 / 4 100.0%	8 / 8 100.0%	4 / 4 100.0%	1 / 1 100.0%	8 / 8 100.0%	1 / 1 100.0%	0 / 0 0.0%	0 / 0 0.0%	0 / 0 0.0%	50 / 83 60.2%AB	33 / 83 39.8%ABE					
1	Citrobacter rodentium ICC168	83 / 83 100.0%	4 / 4 100.0%	3 / 3 100.0%	4 / 4 100.0%	3 / 3 100.0%	5 / 5 100.0%	4 / 4 100.0%	1 / 1 100.0%	3 / 3 100.0%	8 / 8 100.0%	1 / 1 100.0%	5 / 5 100.0%	4 / 4 100.0%	2 / 2 100.0%	4 / 4 100.0%	1 / 1 100.0%	3 / 3 100.0%	2 / 2 100.0%	4 / 4 100.0%	8 / 8 100.0%	4 / 4 100.0%	1 / 1 100.0%	8 / 8 100.0%	1 / 1 100.0%	0 / 0 0.0%	0 / 0 0.0%	0 / 0 0.0%	50 / 83 60.2%AB	33 / 83 39.8%ABE					
10	"Aerobacter" Hormaeche and Edwards 1958	822 / 828 99.3%	34 / 34 100.0%	31 / 31 100.0%	43 / 43 100.0%	31 / 31 100.0%	57 / 57 100.0%	62 / 62 100.0%	10 / 10 100.0%	30 / 30 100.0%	51 / 51 100.0%	11 / 11 100.0%	49 / 49 100.0%	51 / 51 100.0%	20 / 20 100.0%	40 / 40 100.0%	11 / 11 100.0%	30 / 30 100.0%	20 / 20 100.0%	42 / 43 97.7%	63 / 68 92.6%	39 / 39 100.0%	10 / 10 100.0%	77 / 77 100.0%	10 / 10 100.0%	0 / 0 0.0%	0 / 0 0.0%	0 / 0 0.0%	506 / 828 61.1%AB	315 / 828 38.0%ABE	5 / 828 0.6%	1 / 828 0.1%E	1 / 828 0.1%BE		
2	"Aerobacter aerogenes" Hormaeche and Edwards 1958	168 / 169 99.4%	8 / 8 100.0%	6 / 6 100.0%	9 / 9 100.0%	6 / 6 100.0%	11 / 11 100.0%	15 / 15 100.0%	2 / 2 100.0%	6 / 6 100.0%	10 / 10 100.0%	2 / 2 100.0%	9 / 9 100.0%	11 / 11 100.0%	4 / 4 100.0%	8 / 8 100.0%	3 / 3 100.0%	6 / 6 100.0%	4 / 4 100.0%	8 / 9 88.9%	14 / 14 100.0%	8 / 8 100.0%	2 / 2 100.0%	14 / 14 100.0%	2 / 2 100.0%	0 / 0 0.0%	0 / 0 0.0%	0 / 0 0.0%	104 / 169 61.5%AB	64 / 169 37.9%ABE	1 / 169 0.6%				
1	Enterobacter aerogenes EA1509E	86 / 86 100.0%	4 / 4 100.0%	3 / 3 100.0%	5 / 5 100.0%	3 / 3 100.0%	5 / 5 100.0%	8 / 8 100.0%	1 / 1 100.0%	3 / 3 100.0%	5 / 5 100.0%	1 / 1 100.0%	4 / 4 100.0%	6 / 6 100.0%	2 / 2 100.0%	4 / 4 100.0%	2 / 2 100.0%	3 / 3 100.0%	2 / 2 100.0%	5 / 5 100.0%	7 / 7 100.0%	4 / 4 100.0%	1 / 1 100.0%	7 / 7 100.0%	1 / 1 100.0%	0 / 0 0.0%	0 / 0 0.0%	0 / 0 0.0%	54 / 86 62.8%AB	32 / 86 37.2%ABE					
1	Enterobacter aerogenes KCTC 2190	82 / 83 98.8%	4 / 4 100.0%	3 / 3 100.0%	4 / 4 100.0%	3 / 3 100.0%	6 / 6 100.0%	7 / 7 100.0%	1 / 1 100.0%	3 / 3 100.0%	5 / 5 100.0%	1 / 1 100.0%	5 / 5 100.0%	5 / 5 100.0%	2 / 2 100.0%	4 / 4 100.0%	1 / 1 100.0%	3 / 3 100.0%	2 / 2 100.0%	3 / 4 75.0%	7 / 7 100.0%	4 / 4 100.0%	1 / 1 100.0%	7 / 7 100.0%	1 / 1 100.0%	0 / 0 0.0%	0 / 0 0.0%	0 / 0 0.0%	50 / 83 60.2%AB	32 / 83 38.6%ABE	1 / 83 1.2%				
6	Enterobacter cloacae complex	485 / 489 99.2%	19 / 19 100.0%	18 / 18 100.0%	26 / 26 100.0%	18 / 18 100.0%	34 / 34 100.0%	35 / 35 100.0%	6 / 6 100.0%	17 / 17 100.0%	30 / 30 100.0%	6 / 6 100.0%	30 / 30 100.0%	30 / 30 100.0%	12 / 12 100.0%	24 / 24 100.0%	6 / 6 100.0%	18 / 18 100.0%	12 / 12 100.0%	26 / 26 100.0%	36 / 40 90.0%	23 / 23 100.0%	6 / 6 100.0%	47 / 47 100.0%	6 / 6 100.0%	0 / 0 0.0%	0 / 0 0.0%	0 / 0 0.0%	298 / 489 60.9%AB	187 / 489 38.2%ABE	4 / 489 0.8%				
4	"Aerobacter cloacae" (Jordan 1890) Bergey et al. 1923	322 / 324 99.4%	12 / 12 100.0%	12 / 12 100.0%	18 / 18 100.0%	12 / 12 100.0%	23 / 23 100.0%	22 / 22 100.0%	4 / 4 100.0%	11 / 11 100.0%	20 / 20 100.0%	4 / 4 100.0%	20 / 20 100.0%	20 / 20 100.0%	8 / 8 100.0%	16 / 16 100.0%	4 / 4 100.0%	12 / 12 100.0%	8 / 8 100.0%	18 / 18 100.0%	24 / 26 92.3%	15 / 15 100.0%	4 / 4 100.0%	31 / 31 100.0%	4 / 4 100.0%	0 / 0 0.0%	0 / 0 0.0%	0 / 0 0.0%	199 / 324 61.4%AB	123 / 324 38.0%ABE	2 / 324 0.6%				
1	"Aerobacter dissolvens" (Rosen 1922) Waldee 1945	78 / 79 98.7%	2 / 2 100.0%	3 / 3 100.0%	4 / 4 100.0%	2 / 2 100.0%	6 / 6 100.0%	6 / 6 100.0%	1 / 1 100.0%	3 / 3 100.0%	4 / 4 100.0%	1 / 1 100.0%	5 / 5 100.0%	5 / 5 100.0%	2 / 2 100.0%	4 / 4 100.0%	1 / 1 100.0%	3 / 3 100.0%	2 / 2 100.0%	4 / 4 100.0%	6 / 7 85.7%	4 / 4 100.0%	1 / 1 100.0%	8 / 8 100.0%	1 / 1 100.0%	0 / 0 0.0%	0 / 0 0.0%	0 / 0 0.0%	48 / 79 60.8%AB	30 / 79 38.0%ABE	1 / 79 1.3%				
1	Enterobacter cloacae subsp. dissolvens SDM	78 / 79 98.7%	2 / 2 100.0%	3 / 3 100.0%	4 / 4 100.0%	2 / 2 100.0%	6 / 6 100.0%	6 / 6 100.0%	1 / 1 100.0%	3 / 3 100.0%	4 / 4 100.0%	1 / 1 100.0%	5 / 5 100.0%	5 / 5 100.0%	2 / 2 100.0%	4 / 4 100.0%	1 / 1 100.0%	3 / 3 100.0%	2 / 2 100.0%	4 / 4 100.0%	6 / 7 85.7%	4 / 4 100.0%	1 / 1 100.0%	8 / 8 100.0%	1 / 1 100.0%	0 / 0 0.0%	0 / 0 0.0%	0 / 0 0.0%	48 / 79 60.8%AB	30 / 79 38.0%ABE	1 / 79 1.3%				
2	Enterobacter cloacae subsp. cloacae	162 / 163 99.4%	7 / 7 100.0%	6 / 6 100.0%	10 / 10 100.0%	7 / 7 100.0%	11 / 11 100.0%	10 / 10 100.0%	2 / 2 100.0%	5 / 5 100.0%	11 / 11 100.0%	2 / 2 100.0%	10 / 10 100.0%	10 / 10 100.0%	4 / 4 100.0%	8 / 8 100.0%	2 / 2 100.0%	6 / 6 100.0%	4 / 4 100.0%	10 / 10 100.0%	11 / 12 91.7%	7 / 7 100.0%	2 / 2 100.0%	15 / 15 100.0%	2 / 2 100.0%	0 / 0 0.0%	0 / 0 0.0%	0 / 0 0.0%	100 / 163 61.3%AB	62 / 163 38.0%ABE	1 / 163 0.6%				
1	Enterobacter cloacae subsp. cloacae ATCC 13047	82 / 82 100.0%	4 / 4 100.0%	3 / 3 100.0%	6 / 6 100.0%	4 / 4 100.0%	5 / 5 100.0%	4 / 4 100.0%	1 / 1 100.0%	2 / 2 100.0%	6 / 6 100.0%	1 / 1 100.0%	5 / 5 100.0%	5 / 5 100.0%	2 / 2 100.0%	4 / 4 100.0%	1 / 1 100.0%	3 / 3 100.0%	2 / 2 100.0%	6 / 6 100.0%	5 / 5 100.0%	3 / 3 100.0%	1 / 1 100.0%	8 / 8 100.0%	1 / 1 100.0%	0 / 0 0.0%	0 / 0 0.0%	0 / 0 0.0%	50 / 82 61.0%AB	32 / 82 39.0%ABE					
1	Enterobacter cloacae subsp. cloacae ENHKU01	80 / 81 98.8%	3 / 3 100.0%	3 / 3 100.0%	4 / 4 100.0%	3 / 3 100.0%	6 / 6 100.0%	6 / 6 100.0%	1 / 1 100.0%	3 / 3 100.0%	5 / 5 100.0%	1 / 1 100.0%	5 / 5 100.0%	5 / 5 100.0%	2 / 2 100.0%	4 / 4 100.0%	1 / 1 100.0%	3 / 3 100.0%	2 / 2 100.0%	4 / 4 100.0%	6 / 7 85.7%	4 / 4 100.0%	1 / 1 100.0%	7 / 7 100.0%	1 / 1 100.0%	0 / 0 0.0%	0 / 0 0.0%	0 / 0 0.0%	50 / 81 61.7%AB	30 / 81 37.0%ABE	1 / 81 1.2%				
1	Enterobacter cloacae EcWSU1	82 / 82 100.0%	3 / 3 100.0%	3 / 3 100.0%	4 / 4 100.0%	3 / 3 100.0%	6 / 6 100.0%	6 / 6 100.0%	1 / 1 100.0%	3 / 3 100.0%	5 / 5 100.0%	1 / 1 100.0%	5 / 5 100.0%	5 / 5 100.0%	2 / 2 100.0%	4 / 4 100.0%	1 / 1 100.0%	3 / 3 100.0%	2 / 2 100.0%	4 / 4 100.0%	7 / 7 100.0%	4 / 4 100.0%	1 / 1 100.0%	8 / 8 100.0%	1 / 1 100.0%	0 / 0 0.0%	0 / 0 0.0%	0 / 0 0.0%	51 / 82 62.2%AB	31 / 82 37.8%ABE					
1	ATCC 35953	82 / 83 98.8%	4 / 4 100.0%	3 / 3 100.0%	4 / 4 100.0%	3 / 3 100.0%	6 / 6 100.0%	6 / 6 100.0%	1 / 1 100.0%	3 / 3 100.0%	5 / 5 100.0%	1 / 1 100.0%	5 / 5 100.0%	5 / 5 100.0%	2 / 2 100.0%	4 / 4 100.0%	1 / 1 100.0%	3 / 3 100.0%	2 / 2 100.0%	4 / 4 100.0%	6 / 7 85.7%	4 / 4 100.0%	1 / 1 100.0%	8 / 8 100.0%	1 / 1 100.0%	0 / 0 0.0%	0 / 0 0.0%	0 / 0 0.0%	50 / 83 60.2%AB	32 / 83 38.6%ABE	1 / 83 1.2%				
1	Enterobacter asburiae LF7a	82 / 83 98.8%	4 / 4 100.0%	3 / 3 100.0%	4 / 4 100.0%	3 / 3 100.0%	6 / 6 100.0%	6 / 6 100.0%	1 / 1 100.0%	3 / 3 100.0%	5 / 5 100.0%	1 / 1 100.0%	5 / 5 100.0%	5 / 5 100.0%	2 / 2 100.0%	4 / 4 100.0%	1 / 1 100.0%	3 / 3 100.0%	2 / 2 100.0%	4 / 4 100.0%	6 / 7 85.7%	4 / 4 100.0%	1 / 1 100.0%	8 / 8 100.0%	1 / 1 100.0%	0 / 0 0.0%	0 / 0 0.0%	0 / 0 0.0%	50 / 83 60.2%AB	32 / 83 38.6%ABE	1 / 83 1.2%				
1	"Enterobacter lignolyticus" Deangelis et al. 2011	81 / 82 98.8%	3 / 3 100.0%	3 / 3 100.0%	4 / 4 100.0%	3 / 3 100.0%	5 / 5 100.0%	7 / 7 100.0%	1 / 1 100.0%	3 / 3 100.0%	5 / 5 100.0%	1 / 1 100.0%	5 / 5 100.0%	5 / 5 100.0%	2 / 2 100.0%	4 / 4 100.0%	1 / 1 100.0%	3 / 3 100.0%	2 / 2 100.0%	4 / 4 100.0%	6 / 7 85.7%	4 / 4 100.0%	1 / 1 100.0%	8 / 8 100.0%	1 / 1 100.0%	0 / 0 0.0%	0 / 0 0.0%	0 / 0 0.0%	49 / 82 59.8%AB	32 / 82 39.0%ABE	1 / 82 1.2%				
1	Enterobacter cloacae SCF1	81 / 82 98.8%	3 / 3 100.0%	3 / 3 100.0%	4 / 4 100.0%	3 / 3 100.0%	5 / 5 100.0%	7 / 7 100.0%	1 / 1 100.0%	3 / 3 100.0%	5 / 5 100.0%	1 / 1 100.0%	5 / 5 100.0%	5 / 5 100.0%	2 / 2 100.0%	4 / 4 100.0%	1 / 1 100.0%	3 / 3 100.0%	2 / 2 100.0%	4 / 4 100.0%	6 / 7 85.7%	4 / 4 100.0%	1 / 1 100.0%	8 / 8 100.0%	1 / 1 100.0%	0 / 0 0.0%	0 / 0 0.0%	0 / 0 0.0%	49 / 82 59.8%AB	32 / 82 39.0%ABE	1 / 82 1.2%				
1	Enterobacter sp. 638	83 / 83 100.0%	4 / 4 100.0%	3 / 3 100.0%	4 / 4 100.0%	3 / 3 100.0%	6 / 6 100.0%	6 / 6 100.0%	1 / 1 100.0%	3 / 3 100.0%	5 / 5 100.0%	1 / 1 100.0%	5 / 5 100.0%	5 / 5 100.0%	2 / 2 100.0%	4 / 4 100.0%	1 / 1 100.0%	3 / 3 100.0%	2 / 2 100.0%	4 / 4 100.0%	7 / 7 100.0%	4 / 4 100.0%	1 / 1 100.0%	8 / 8 100.0%	1 / 1 100.0%	0 / 0 0.0%	0 / 0 0.0%	0 / 0 0.0%	51 / 83 61.4%AB	31 / 83 37.3%ABE	1 / 83 1.2%BE				
1	Enterobacter sp. R4-368	86 / 87 98.9%	3 / 3 100.0%	4 / 4 100.0%	4 / 4 100.0%	4 / 4 100.0%	6 / 6 100.0%	6 / 6 100.0%	1 / 1 100.0%	4 / 4 100.0%	6 / 6 100.0%	2 / 2 100.0%	5 / 5 100.0%	5 / 5 100.0%	2 / 2 100.0%	4 / 4 100.0%	1 / 1 100.0%	3 / 3 100.0%	2 / 2 100.0%	4 / 4 100.0%	6 / 7 85.7%	4 / 4 100.0%	1 / 1 100.0%	8 / 8 100.0%	1 / 1 100.0%	0 / 0 0.0%	0 / 0 0.0%	0 / 0 0.0%	53 / 87 60.9%AB	33 / 87 37.9%ABE	1 / 87 1.1%E				
7	Erwinia	534 / 537 99.4%	28 / 28 100.0%	21 / 21 100.0%	25 / 25 100.0%	23 / 23 100.0%	41 / 41 100.0%	41 / 41 100.0%	7 / 7 100.0%	14 / 14 100.0%	37 / 37 100.0%	7 / 7 100.0%	35 / 35 100.0%	29 / 29 100.0%	14 / 14 100.0%	22 / 22 100.0%	7 / 7 100.0%	21 / 21 100.0%	14 / 14 100.0%	20 / 20 100.0%	40 / 43 93.0%	26 / 26 100.0%	7 / 7 100.0%	54 / 54 100.0%	1 / 1 100.0%	0 / 0 0.0%	0 / 0 0.0%	0 / 0 0.0%	292 / 537 54.4%AB	201 / 537 37.4%ABE	29 / 537 5.4%B	12 / 537 2.2%BE	3 / 537 0.6%		
2	"Bacillus amylovorus" (Burrill 1882) Trevisan 1889	151 / 153 98.7%	8 / 8 100.0%	6 / 6 100.0%	6 / 6 100.0%	7 / 7 100.0%	12 / 12 100.0%	12 / 12 100.0%	2 / 2 100.0%	4 / 4 100.0%	11 / 11 100.0%	2 / 2 100.0%	10 / 10 100.0%	8 / 8 100.0%	4 / 4 100.0%	6 / 6 100.0%	2 / 2 100.0%	6 / 6 100.0%	4 / 4 100.0%	6 / 6 100.0%	10 / 12 83.3%	7 / 7 100.0%	2 / 2 100.0%	16 / 16 100.0%	0 / 0 0.0%	0 / 0 0.0%	0 / 0 0.0%	0 / 0 0.0%	90 / 153 58.8%AB	61 / 153 39.9%ABE	2 / 153 1.3%				
1	Erwinia amylovora CFBP1430	75 / 76 98.7%	4 / 4 100.0%	3 / 3 100.0%	3 / 3 100.0%	3 / 3 100.0%	6 / 6 100.0%	6 / 6 100.0%	1 / 1 100.0%	2 / 2 100.0%	5 / 5 100.0%	1 / 1 100.0%	5 / 5 100.0%	4 / 4 100.0%	2 / 2 100.0%	3 / 3 100.0%	1 / 1 100.0%	3 / 3 100.0%	2 / 2 100.0%	3 / 3 100.0%	5 / 6 83.3%	4 / 4 100.0%	1 / 1 100.0%	8 / 8 100.0%	0 / 0 0.0%	0 / 0 0.0%	0 / 0 0.0%	0 / 0 0.0%	44 / 76 57.9%AB	31 / 76 40.8%ABE	1 / 76 1.3%				
1	Erwinia amylovora 27-3	76 / 77 98.7%	4 / 4 100.0%	3 / 3 100.0%	3 / 3 100.0%	4 / 4 100.0%	6 / 6 100.0%	6 / 6 100.0%	1 / 1 100.0%	2 / 2 100.0%	6 / 6 100.0%	1 / 1 100.0%	5 / 5 100.0%	4 / 4 100.0%	2 / 2 100.0%	3 / 3 100.0%	1 / 1 100.0%	3 / 3 100.0%	2 / 2 100.0%	3 / 3 100.0%	5 / 6 83.3%	3 / 3 100.0%	1 / 1 100.0%	8 / 8 100.0%	0 / 0 0.0%	0 / 0 0.0%	0 / 0 0.0%	0 / 0 0.0%	46 / 77 59.7%AB	30 / 77 39.0%ABE	1 / 77 1.3%				
2	CFBP 4172	150 / 150 100.0%	8 / 8 100.0%	6 / 6 100.0%	8 / 8 100.0%	6 / 6 100.0%	12 / 12 100.0%	10 / 10 100.0%	2 / 2 100.0%	4 / 4 100.0%	10 / 10 100.0%	2 / 2 100.0%	10 / 10 100.0%	8 / 8 100.0%	4 / 4 100.0%	6 / 6 100.0%	2 / 2 100.0%	6 / 6 100.0%	4 / 4 100.0%	6 / 6 100.0%	12 / 12 100.0%	8 / 8 100.0%	2 / 2 100.0%	14 / 14 100.0%	0 / 0 0.0%	0 / 0 0.0%	0 / 0 0.0%	0 / 0 0.0%	90 / 150 60.0%AB	60 / 150 40.0%ABE					
1	Erwinia pyrifoliae 16/96	75 / 75 100.0%	4 / 4 100.0%	3 / 3 100.0%	4 / 4 100.0%	3 / 3 100.0%	6 / 6 100.0%	5 / 5 100.0%	1 / 1 100.0%	2 / 2 100.0%	5 / 5 100.0%	1 / 1 100.0%	5 / 5 100.0%	4 / 4 100.0%	2 / 2 100.0%	3 / 3 100.0%	1 / 1 100.0%	3 / 3 100.0%	2 / 2 100.0%	3 / 3 100.0%	6 / 6 100.0%	4 / 4 100.0%	1 / 1 100.0%	7 / 7 100.0%	0 / 0 0.0%	0 / 0 0.0%	0 / 0 0.0%	0 / 0 0.0%	45 / 75 60.0%AB	30 / 75 40.0%ABE					
1	CIP 106121	77 / 77 100.0%	4 / 4 100.0%	3 / 3 100.0%	3 / 3 100.0%	3 / 3 100.0%	5 / 5 100.0%	6 / 6 100.0%	1 / 1 100.0%	2 / 2 100.0%	5 / 5 100.0%	1 / 1 100.0%	5 / 5 100.0%	4 / 4 100.0%	2 / 2 100.0%	4 / 4 100.0%	1 / 1 100.0%	3 / 3 100.0%	2 / 2 100.0%	2 / 2 100.0%	7 / 7 100.0%	4 / 4 100.0%	1 / 1 100.0%	8 / 8 100.0%	1 / 1 100.0%	0 / 0 0.0%	0 / 0 0.0%	0 / 0 0.0%	46 / 77 59.7%AB	31 / 77 40.3%ABE					
1	Erwinia billingiae 661	77 / 77 100.0%	4 / 4 100.0%	3 / 3 100.0%	3 / 3 100.0%	3 / 3 100.0%	5 / 5 100.0%	6 / 6 100.0%	1 / 1 100.0%	2 / 2 100.0%	5 / 5 100.0%	1 / 1 100.0%	5 / 5 100.0%	4 / 4 100.0%	2 / 2 100.0%	4 / 4 100.0%	1 / 1 100.0%	3 / 3 100.0%	2 / 2 100.0%	2 / 2 100.0%	7 / 7 100.0%	4 / 4 100.0%	1 / 1 100.0%	8 / 8 100.0%	1 / 1 100.0%	0 / 0 0.0%	0 / 0 0.0%	0 / 0 0.0%	46 / 77 59.7%AB	31 / 77 40.3%ABE					
1	Erwinia sp. Ejp617	75 / 76 98.7%	4 / 4 100.0%	3 / 3 100.0%	4 / 4 100.0%	3 / 3 100.0%	6 / 6 100.0%	5 / 5 100.0%	1 / 1 100.0%	2 / 2 100.0%	5 / 5 100.0%	1 / 1 100.0%	5 / 5 100.0%	4 / 4 100.0%	2 / 2 100.0%	3 / 3 100.0%	1 / 1 100.0%	3 / 3 100.0%	2 / 2 100.0%	3 / 3 100.0%	5 / 6 83.3%	4 / 4 100.0%	1 / 1 100.0%	8 / 8 100.0%	0 / 0 0.0%	0 / 0 0.0%	0 / 0 0.0%	0 / 0 0.0%	44 / 76 57.9%AB	31 / 76 40.8%ABE	1 / 76 1.3%				
1	DSM 17950	81 / 81 100.0%	4 / 4 100.0%	3 / 3 100.0%	4 / 4 100.0%	4 / 4 100.0%	6 / 6 100.0%	8 / 8 100.0%	1 / 1 100.0%	2 / 2 100.0%	6 / 6 100.0%	1 / 1 100.0%	5 / 5 100.0%	5 / 5 100.0%	2 / 2 100.0%	3 / 3 100.0%	1 / 1 100.0%	3 / 3 100.0%	2 / 2 100.0%	3 / 3 100.0%	6 / 6 100.0%	3 / 3 100.0%	1 / 1 100.0%	8 / 8 100.0%	0 / 0 0.0%	0 / 0 0.0%	0 / 0 0.0%	0 / 0 0.0%	29 / 81 35.8%B	22 / 81 27.2%AB	18 / 81 22.2%ABE	12 / 81 14.8%BE			
58	Escherichia	5347 / 5375 99.5%	228 / 228 100.0%	179 / 180 99.4%	245 / 245 100.0%	183 / 183 100.0%	375 / 375 100.0%	411 / 411 100.0%	60 / 60 100.0%	178 / 178 100.0%	296 / 297 99.7%	60 / 60 100.0%	298 / 298 100.0%	351 / 351 100.0%	119 / 119 100.0%	231 / 231 100.0%	60 / 60 100.0%	180 / 180 100.0%	118 / 118 100.0%	254 / 258 98.4%	575 / 597 96.3%	227 / 227 100.0%	194 / 194 100.0%	463 / 463 100.0%	60 / 60 100.0%	0 / 0 0.0%	0 / 0 0.0%	2 / 2 100.0%	3343 / 5375 62.2%AB	1805 / 5375 33.6%ABE	105 / 5375 2.0%B	94 / 5375 1.7%BE	16 / 5375 0.3%E	11 / 5375 0.2%	1 / 5375 0.0%GG
57	"Bacillus coli" Migula 1895	5263 / 5290 99.5%	224 / 224 100.0%	176 / 177 99.4%	241 / 241 100.0%	180 / 180 100.0%	369 / 369 100.0%	404 / 404 100.0%	59 / 59 100.0%	175 / 175 100.0%	291 / 292 99.7%	59 / 59 100.0%	293 / 293 100.0%	345 / 345 100.0%	117 / 117 100.0%	227 / 227 100.0%	59 / 59 100.0%	177 / 177 100.0%	116 / 116 100.0%	250 / 254 98.4%	569 / 590 96.4%	223 / 223 100.0%	193 / 193 100.0%	455 / 455 100.0%	59 / 59 100.0%	0 / 0 0.0%	0 / 0 0.0%	2 / 2 100.0%	3291 / 5290 62.2%AB	1773 / 5290 33.5%ABE	105 / 5290 2.0%B	94 / 5290 1.8%BE	16 / 5290 0.3%E	10 / 5290 0.2%	1 / 5290 0.0%GG
1	Escherichia coli (strain B)	85 / 86 98.8%	4 / 4 100.0%	3 / 3 100.0%	4 / 4 100.0%	3 / 3 100.0%	6 / 6 100.0%	7 / 7 100.0%	1 / 1 100.0%	3 / 3 100.0%	5 / 5 100.0%	1 / 1 100.0%	5 / 5 100.0%	6 / 6 100.0%	2 / 2 100.0%	4 / 4 100.0%	1 / 1 100.0%	3 / 3 100.0%	2 / 2 100.0%	4 / 4 100.0%	7 / 8 87.5%	4 / 4 100.0%	1 / 1 100.0%	8 / 8 100.0%	1 / 1 100.0%	0 / 0 0.0%	0 / 0 0.0%	0 / 0 0.0%	53 / 86 61.6%AB	32 / 86 37.2%ABE	1 / 86 1.2%E				
1	Escherichia coli B (REL606 strain)	85 / 86 98.8%	4 / 4 100.0%	3 / 3 100.0%	4 / 4 100.0%	3 / 3 100.0%	6 / 6 100.0%	7 / 7 100.0%	1 / 1 100.0%	3 / 3 100.0%	5 / 5 100.0%	1 / 1 100.0%	5 / 5 100.0%	6 / 6 100.0%	2 / 2 100.0%	4 / 4 100.0%	1 / 1 100.0%	3 / 3 100.0%	2 / 2 100.0%	4 / 4 100.0%	7 / 8 87.5%	4 / 4 100.0%	1 / 1 100.0%	8 / 8 100.0%	1 / 1 100.0%	0 / 0 0.0%	0 / 0 0.0%	0 / 0 0.0%	53 / 86 61.6%AB	32 / 86 37.2%ABE	1 / 86 1.2%E				
5	Escherichia coli K-12	429 / 430 99.8%	20 / 20 100.0%	15 / 15 100.0%	20 / 20 100.0%	15 / 15 100.0%	30 / 30 100.0%	35 / 35 100.0%	5 / 5 100.0%	15 / 15 100.0%	25 / 25 100.0%	5 / 5 100.0%	25 / 25 100.0%	31 / 31 100.0%	10 / 10 100.0%	20 / 20 100.0%	5 / 5 100.0%	15 / 15 100.0%	10 / 10 100.0%	20 / 20 100.0%	35 / 36 97.2%	19 / 19 100.0%	9 / 9 100.0%	40 / 40 100.0%	5 / 5 100.0%	0 / 0 0.0%	0 / 0 0.0%	0 / 0 0.0%	253 / 430 58.8%AB	136 / 430 31.6%ABE	23 / 430 5.3%BE	17 / 430 4.0%B	1 / 430 0.2%E		
1	Escherichia coli DH10B	86 / 86 100.0%	4 / 4 100.0%	3 / 3 100.0%	4 / 4 100.0%	3 / 3 100.0%	6 / 6 100.0%	7 / 7 100.0%	1 / 1 100.0%	3 / 3 100.0%	5 / 5 100.0%	1 / 1 100.0%	5 / 5 100.0%	6 / 6 100.0%	2 / 2 100.0%	4 / 4 100.0%	1 / 1 100.0%	3 / 3 100.0%	2 / 2 100.0%	4 / 4 100.0%	7 / 7 100.0%	4 / 4 100.0%	2 / 2 100.0%	8 / 8 100.0%	1 / 1 100.0%	0 / 0 0.0%	0 / 0 0.0%	0 / 0 0.0%	37 / 86 43.0%AB	21 / 86 24.4%BE	17 / 86 19.8%B	11 / 86 12.8%ABE			
1	Escherichia coli W3110	86 / 86 100.0%	4 / 4 100.0%	3 / 3 100.0%	4 / 4 100.0%	3 / 3 100.0%	6 / 6 100.0%	7 / 7 100.0%	1 / 1 100.0%	3 / 3 100.0%	5 / 5 100.0%	1 / 1 100.0%	5 / 5 100.0%	6 / 6 100.0%	2 / 2 100.0%	4 / 4 100.0%	1 / 1 100.0%	3 / 3 100.0%	2 / 2 100.0%	4 / 4 100.0%	7 / 7 100.0%	4 / 4 100.0%	2 / 2 100.0%	8 / 8 100.0%	1 / 1 100.0%	0 / 0 0.0%	0 / 0 0.0%	0 / 0 0.0%	54 / 86 62.8%AB	31 / 86 36.0%ABE	1 / 86 1.2%BE				
1	Escherichia coli BW2952	86 / 87 98.9%	4 / 4 100.0%	3 / 3 100.0%	4 / 4 100.0%	3 / 3 100.0%	6 / 6 100.0%	7 / 7 100.0%	1 / 1 100.0%	3 / 3 100.0%	5 / 5 100.0%	1 / 1 100.0%	5 / 5 100.0%	7 / 7 100.0%	2 / 2 100.0%	4 / 4 100.0%	1 / 1 100.0%	3 / 3 100.0%	2 / 2 100.0%	4 / 4 100.0%	7 / 8 87.5%	3 / 3 100.0%	2 / 2 100.0%	8 / 8 100.0%	1 / 1 100.0%	0 / 0 0.0%	0 / 0 0.0%	0 / 0 0.0%	55 / 87 63.2%AB	31 / 87 35.6%ABE	1 / 87 1.1%E				
1	Escherichia coli MDS42	85 / 85 100.0%	4 / 4 100.0%	3 / 3 100.0%	4 / 4 100.0%	3 / 3 100.0%	6 / 6 100.0%	7 / 7 100.0%	1 / 1 100.0%	3 / 3 100.0%	5 / 5 100.0%	1 / 1 100.0%	5 / 5 100.0%	6 / 6 100.0%	2 / 2 100.0%	4 / 4 100.0%	1 / 1 100.0%	3 / 3 100.0%	2 / 2 100.0%	4 / 4 100.0%	7 / 7 100.0%	4 / 4 100.0%	1 / 1 100.0%	8 / 8 100.0%	1 / 1 100.0%	0 / 0 0.0%	0 / 0 0.0%	0 / 0 0.0%	53 / 85 62.4%AB	32 / 85 37.6%ABE					
4	Escherichia coli 0157:H7	411 / 414 99.3%	16 / 16 100.0%	12 / 12 100.0%	16 / 16 100.0%	12 / 12 100.0%	24 / 24 100.0%	28 / 28 100.0%	4 / 4 100.0%	12 / 12 100.0%	19 / 19 100.0%	4 / 4 100.0%	20 / 20 100.0%	20 / 20 100.0%	8 / 8 100.0%	16 / 16 100.0%	4 / 4 100.0%	12 / 12 100.0%	8 / 8 100.0%	16 / 16 100.0%	75 / 78 96.2%	16 / 16 100.0%	38 / 38 100.0%	27 / 27 100.0%	4 / 4 100.0%	0 / 0 0.0%	0 / 0 0.0%	0 / 0 0.0%	288 / 414 69.6%AB	121 / 414 29.2%ABE	2 / 414 0.5%E	2 / 414 0.5%BE	1 / 414 0.2%GG		
1	Escherichia coli 0157:H7 EDL933	98 / 100 98.0%	4 / 4 100.0%	3 / 3 100.0%	4 / 4 100.0%	3 / 3 100.0%	6 / 6 100.0%	7 / 7 100.0%	1 / 1 100.0%	3 / 3 100.0%	4 / 4 100.0%	1 / 1 100.0%	5 / 5 100.0%	5 / 5 100.0%	2 / 2 100.0%	4 / 4 100.0%	1 / 1 100.0%	3 / 3 100.0%	2 / 2 100.0%	4 / 4 100.0%	16 / 18 88.9%	4 / 4 100.0%	9 / 9 100.0%	6 / 6 100.0%	1 / 1 100.0%	0 / 0 0.0%	0 / 0 0.0%	0 / 0 0.0%	68 / 100 68.0%AB	29 / 100 29.0%ABE	1 / 100 1.0%E	1 / 100 1.0%BE	1 / 100 1.0%GG		
1	Escherichia coli O157:H7 str. Sakai	101 / 102 99.0%	4 / 4 100.0%	3 / 3 100.0%	4 / 4 100.0%	3 / 3 100.0%	6 / 6 100.0%	7 / 7 100.0%	1 / 1 100.0%	3 / 3 100.0%	5 / 5 100.0%	1 / 1 100.0%	5 / 5 100.0%	5 / 5 100.0%	2 / 2 100.0%	4 / 4 100.0%	1 / 1 100.0%	3 / 3 100.0%	2 / 2 100.0%	4 / 4 100.0%	17 / 18 94.4%	4 / 4 100.0%	9 / 9 100.0%	7 / 7 100.0%	1 / 1 100.0%	0 / 0 0.0%	0 / 0 0.0%	0 / 0 0.0%	70 / 102 68.6%AB	30 / 102 29.4%ABE	1 / 102 1.0%BE	1 / 102 1.0%E			
1	Escherichia coli O157:H7 str. EC4115	106 / 106 100.0%	4 / 4 100.0%	3 / 3 100.0%	4 / 4 100.0%	3 / 3 100.0%	6 / 6 100.0%	7 / 7 100.0%	1 / 1 100.0%	3 / 3 100.0%	5 / 5 100.0%	1 / 1 100.0%	5 / 5 100.0%	5 / 5 100.0%	2 / 2 100.0%	4 / 4 100.0%	1 / 1 100.0%	3 / 3 100.0%	2 / 2 100.0%	4 / 4 100.0%	21 / 21 100.0%	4 / 4 100.0%	10 / 10 100.0%	7 / 7 100.0%	1 / 1 100.0%	0 / 0 0.0%	0 / 0 0.0%	0 / 0 0.0%	75 / 106 70.8%AB	31 / 106 29.2%ABE					
1	Escherichia coli O157:H7 str. TW14359	106 / 106 100.0%	4 / 4 100.0%	3 / 3 100.0%	4 / 4 100.0%	3 / 3 100.0%	6 / 6 100.0%	7 / 7 100.0%	1 / 1 100.0%	3 / 3 100.0%	5 / 5 100.0%	1 / 1 100.0%	5 / 5 100.0%	5 / 5 100.0%	2 / 2 100.0%	4 / 4 100.0%	1 / 1 100.0%	3 / 3 100.0%	2 / 2 100.0%	4 / 4 100.0%	21 / 21 100.0%	4 / 4 100.0%	10 / 10 100.0%	7 / 7 100.0%	1 / 1 100.0%	0 / 0 0.0%	0 / 0 0.0%	0 / 0 0.0%	75 / 106 70.8%AB	31 / 106 29.2%ABE					
1	Escherichia coli 0127:H6	88 / 89 98.9%	4 / 4 100.0%	3 / 3 100.0%	5 / 5 100.0%	3 / 3 100.0%	6 / 6 100.0%	7 / 7 100.0%	1 / 1 100.0%	2 / 2 100.0%	5 / 5 100.0%	1 / 1 100.0%	5 / 5 100.0%	7 / 7 100.0%	2 / 2 100.0%	3 / 3 100.0%	1 / 1 100.0%	3 / 3 100.0%	2 / 2 100.0%	5 / 5 100.0%	8 / 9 88.9%	4 / 4 100.0%	3 / 3 100.0%	7 / 7 100.0%	1 / 1 100.0%	0 / 0 0.0%	0 / 0 0.0%	0 / 0 0.0%	58 / 89 65.2%AB	30 / 89 33.7%ABE	1 / 89 1.1%				
1	Escherichia coli 0127:H6 str. E2348/69	88 / 89 98.9%	4 / 4 100.0%	3 / 3 100.0%	5 / 5 100.0%	3 / 3 100.0%	6 / 6 100.0%	7 / 7 100.0%	1 / 1 100.0%	2 / 2 100.0%	5 / 5 100.0%	1 / 1 100.0%	5 / 5 100.0%	7 / 7 100.0%	2 / 2 100.0%	3 / 3 100.0%	1 / 1 100.0%	3 / 3 100.0%	2 / 2 100.0%	5 / 5 100.0%	8 / 9 88.9%	4 / 4 100.0%	3 / 3 100.0%	7 / 7 100.0%	1 / 1 100.0%	0 / 0 0.0%	0 / 0 0.0%	0 / 0 0.0%	58 / 89 65.2%AB	30 / 89 33.7%ABE	1 / 89 1.1%				
1	Escherichia coli 0111:H-	105 / 106 99.1%	4 / 4 100.0%	3 / 3 100.0%	5 / 5 100.0%	3 / 3 100.0%	6 / 6 100.0%	7 / 7 100.0%	1 / 1 100.0%	3 / 3 100.0%	5 / 5 100.0%	1 / 1 100.0%	5 / 5 100.0%	6 / 6 100.0%	2 / 2 100.0%	4 / 4 100.0%	1 / 1 100.0%	3 / 3 100.0%	2 / 2 100.0%	5 / 5 100.0%	18 / 19 94.7%	4 / 4 100.0%	8 / 8 100.0%	8 / 8 100.0%	1 / 1 100.0%	0 / 0 0.0%	0 / 0 0.0%	0 / 0 0.0%	72 / 106 67.9%AB	33 / 106 31.1%ABE	1 / 106 0.9%				
1	Escherichia coli O111:H- str. 11128	105 / 106 99.1%	4 / 4 100.0%	3 / 3 100.0%	5 / 5 100.0%	3 / 3 100.0%	6 / 6 100.0%	7 / 7 100.0%	1 / 1 100.0%	3 / 3 100.0%	5 / 5 100.0%	1 / 1 100.0%	5 / 5 100.0%	6 / 6 100.0%	2 / 2 100.0%	4 / 4 100.0%	1 / 1 100.0%	3 / 3 100.0%	2 / 2 100.0%	5 / 5 100.0%	18 / 19 94.7%	4 / 4 100.0%	8 / 8 100.0%	8 / 8 100.0%	1 / 1 100.0%	0 / 0 0.0%	0 / 0 0.0%	0 / 0 0.0%	72 / 106 67.9%AB	33 / 106 31.1%ABE	1 / 106 0.9%				
1	Escherichia coli CFT073	86 / 86 100.0%	4 / 4 100.0%	3 / 3 100.0%	4 / 4 100.0%	1 / 1 100.0%	6 / 6 100.0%	7 / 7 100.0%	1 / 1 100.0%	3 / 3 100.0%	3 / 3 100.0%	1 / 1 100.0%	5 / 5 100.0%	6 / 6 100.0%	2 / 2 100.0%	4 / 4 100.0%	1 / 1 100.0%	3 / 3 100.0%	2 / 2 100.0%	3 / 3 100.0%	11 / 11 100.0%	5 / 5 100.0%	2 / 2 100.0%	8 / 8 100.0%	1 / 1 100.0%	0 / 0 0.0%	0 / 0 0.0%	0 / 0 0.0%	53 / 86 61.6%AB	32 / 86 37.2%ABE	1 / 86 1.2%BE				
1	Escherichia coli O26:H11	100 / 101 99.0%	4 / 4 100.0%	3 / 3 100.0%	4 / 4 100.0%	3 / 3 100.0%	6 / 6 100.0%	7 / 7 100.0%	1 / 1 100.0%	3 / 3 100.0%	5 / 5 100.0%	1 / 1 100.0%	5 / 5 100.0%	6 / 6 100.0%	2 / 2 100.0%	4 / 4 100.0%	1 / 1 100.0%	3 / 3 100.0%	2 / 2 100.0%	4 / 4 100.0%	16 / 17 94.1%	4 / 4 100.0%	7 / 7 100.0%	8 / 8 100.0%	1 / 1 100.0%	0 / 0 0.0%	0 / 0 0.0%	0 / 0 0.0%	68 / 101 67.3%AB	32 / 101 31.7%ABE	1 / 101 1.0%				
1	Escherichia coli O26:H11 11368	100 / 101 99.0%	4 / 4 100.0%	3 / 3 100.0%	4 / 4 100.0%	3 / 3 100.0%	6 / 6 100.0%	7 / 7 100.0%	1 / 1 100.0%	3 / 3 100.0%	5 / 5 100.0%	1 / 1 100.0%	5 / 5 100.0%	6 / 6 100.0%	2 / 2 100.0%	4 / 4 100.0%	1 / 1 100.0%	3 / 3 100.0%	2 / 2 100.0%	4 / 4 100.0%	16 / 17 94.1%	4 / 4 100.0%	7 / 7 100.0%	8 / 8 100.0%	1 / 1 100.0%	0 / 0 0.0%	0 / 0 0.0%	0 / 0 0.0%	68 / 101 67.3%AB	32 / 101 31.7%ABE	1 / 101 1.0%				
2	Escherichia coli O55:H7	195 / 197 99.0%	8 / 8 100.0%	6 / 6 100.0%	10 / 10 100.0%	6 / 6 100.0%	14 / 14 100.0%	14 / 14 100.0%	2 / 2 100.0%	6 / 6 100.0%	10 / 10 100.0%	2 / 2 100.0%	10 / 10 100.0%	12 / 12 100.0%	4 / 4 100.0%	8 / 8 100.0%	2 / 2 100.0%	6 / 6 100.0%	4 / 4 100.0%	8 / 8 100.0%	25 / 27 92.6%	8 / 8 100.0%	12 / 12 100.0%	16 / 16 100.0%	2 / 2 100.0%	0 / 0 0.0%	0 / 0 0.0%	0 / 0 0.0%	127 / 197 64.5%AB	68 / 197 34.5%ABE	2 / 197 1.0%				
1	Escherichia coli O55:H7 CB9615	98 / 99 99.0%	4 / 4 100.0%	3 / 3 100.0%	5 / 5 100.0%	3 / 3 100.0%	7 / 7 100.0%	7 / 7 100.0%	1 / 1 100.0%	3 / 3 100.0%	5 / 5 100.0%	1 / 1 100.0%	5 / 5 100.0%	6 / 6 100.0%	2 / 2 100.0%	4 / 4 100.0%	1 / 1 100.0%	3 / 3 100.0%	2 / 2 100.0%	4 / 4 100.0%	13 / 14 92.9%	4 / 4 100.0%	6 / 6 100.0%	8 / 8 100.0%	1 / 1 100.0%	0 / 0 0.0%	0 / 0 0.0%	0 / 0 0.0%	64 / 99 64.6%AB	34 / 99 34.3%ABE	1 / 99 1.0%				
1	Escherichia coli O55:H7 str. RM12579	97 / 98 99.0%	4 / 4 100.0%	3 / 3 100.0%	5 / 5 100.0%	3 / 3 100.0%	7 / 7 100.0%	7 / 7 100.0%	1 / 1 100.0%	3 / 3 100.0%	5 / 5 100.0%	1 / 1 100.0%	5 / 5 100.0%	6 / 6 100.0%	2 / 2 100.0%	4 / 4 100.0%	1 / 1 100.0%	3 / 3 100.0%	2 / 2 100.0%	4 / 4 100.0%	12 / 13 92.3%	4 / 4 100.0%	6 / 6 100.0%	8 / 8 100.0%	1 / 1 100.0%	0 / 0 0.0%	0 / 0 0.0%	0 / 0 0.0%	63 / 98 64.3%AB	34 / 98 34.7%ABE	1 / 98 1.0%				
1	Escherichia coli ETEC H10407	88 / 89 98.9%	4 / 4 100.0%	3 / 3 100.0%	4 / 4 100.0%	3 / 3 100.0%	6 / 6 100.0%	7 / 7 100.0%	1 / 1 100.0%	4 / 4 100.0%	4 / 5 80.0%	1 / 1 100.0%	5 / 5 100.0%	6 / 6 100.0%	2 / 2 100.0%	4 / 4 100.0%	1 / 1 100.0%	3 / 3 100.0%	2 / 2 100.0%	4 / 4 100.0%	8 / 8 100.0%	4 / 4 100.0%	3 / 3 100.0%	8 / 8 100.0%	1 / 1 100.0%	0 / 0 0.0%	0 / 0 0.0%	0 / 0 0.0%	55 / 89 61.8%AB	33 / 89 37.1%ABE	1 / 89 1.1%				
1	Escherichia coli E24377A	88 / 88 100.0%	4 / 4 100.0%	3 / 3 100.0%	4 / 4 100.0%	3 / 3 100.0%	8 / 8 100.0%	6 / 6 100.0%	1 / 1 100.0%	3 / 3 100.0%	5 / 5 100.0%	1 / 1 100.0%	5 / 5 100.0%	5 / 5 100.0%	2 / 2 100.0%	3 / 3 100.0%	1 / 1 100.0%	3 / 3 100.0%	2 / 2 100.0%	6 / 6 100.0%	7 / 7 100.0%	4 / 4 100.0%	3 / 3 100.0%	8 / 8 100.0%	1 / 1 100.0%	0 / 0 0.0%	0 / 0 0.0%	0 / 0 0.0%	39 / 88 44.3%AB	20 / 88 22.7%BE	16 / 88 18.2%B	13 / 88 14.8%ABE			
1	Escherichia coli HS	87 / 87 100.0%	5 / 5 100.0%	3 / 3 100.0%	4 / 4 100.0%	3 / 3 100.0%	6 / 6 100.0%	7 / 7 100.0%	1 / 1 100.0%	3 / 3 100.0%	5 / 5 100.0%	1 / 1 100.0%	5 / 5 100.0%	6 / 6 100.0%	2 / 2 100.0%	4 / 4 100.0%	1 / 1 100.0%	3 / 3 100.0%	2 / 2 100.0%	4 / 4 100.0%	7 / 7 100.0%	4 / 4 100.0%	2 / 2 100.0%	8 / 8 100.0%	1 / 1 100.0%	0 / 0 0.0%	0 / 0 0.0%	0 / 0 0.0%	38 / 87 43.7%AB	21 / 87 24.1%BE	16 / 87 18.4%B	12 / 87 13.8%ABE			
1	Escherichia coli 536	80 / 80 100.0%	4 / 4 100.0%	3 / 3 100.0%	4 / 4 100.0%	1 / 1 100.0%	6 / 6 100.0%	7 / 7 100.0%	1 / 1 100.0%	3 / 3 100.0%	3 / 3 100.0%	1 / 1 100.0%	5 / 5 100.0%	6 / 6 100.0%	2 / 2 100.0%	2 / 2 100.0%	1 / 1 100.0%	3 / 3 100.0%	1 / 1 100.0%	4 / 4 100.0%	8 / 8 100.0%	6 / 6 100.0%	1 / 1 100.0%	7 / 7 100.0%	1 / 1 100.0%	0 / 0 0.0%	0 / 0 0.0%	0 / 0 0.0%	48 / 80 60.0%AB	31 / 80 38.8%ABE	1 / 80 1.2%BE				
1	Escherichia coli UTI89	88 / 89 98.9%	4 / 4 100.0%	3 / 3 100.0%	4 / 4 100.0%	3 / 3 100.0%	6 / 6 100.0%	7 / 7 100.0%	1 / 1 100.0%	3 / 3 100.0%	5 / 5 100.0%	1 / 1 100.0%	5 / 5 100.0%	6 / 6 100.0%	2 / 2 100.0%	4 / 4 100.0%	1 / 1 100.0%	3 / 3 100.0%	2 / 2 100.0%	5 / 5 100.0%	8 / 9 88.9%	4 / 4 100.0%	2 / 2 100.0%	8 / 8 100.0%	1 / 1 100.0%	0 / 0 0.0%	0 / 0 0.0%	0 / 0 0.0%	55 / 89 61.8%AB	32 / 89 36.0%ABE	1 / 89 1.1%BE	1 / 89 1.1%E			
1	Escherichia coli O103:H2	98 / 98 100.0%	4 / 4 100.0%	3 / 3 100.0%	4 / 4 100.0%	3 / 3 100.0%	6 / 6 100.0%	6 / 6 100.0%	1 / 1 100.0%	3 / 3 100.0%	5 / 5 100.0%	1 / 1 100.0%	5 / 5 100.0%	6 / 6 100.0%	2 / 2 100.0%	4 / 4 100.0%	1 / 1 100.0%	3 / 3 100.0%	2 / 2 100.0%	4 / 4 100.0%	16 / 16 100.0%	4 / 4 100.0%	6 / 6 100.0%	8 / 8 100.0%	1 / 1 100.0%	0 / 0 0.0%	0 / 0 0.0%	0 / 0 0.0%	66 / 98 67.3%AB	32 / 98 32.7%ABE					
1	Escherichia coli O103:H2 str. 12009	98 / 98 100.0%	4 / 4 100.0%	3 / 3 100.0%	4 / 4 100.0%	3 / 3 100.0%	6 / 6 100.0%	6 / 6 100.0%	1 / 1 100.0%	3 / 3 100.0%	5 / 5 100.0%	1 / 1 100.0%	5 / 5 100.0%	6 / 6 100.0%	2 / 2 100.0%	4 / 4 100.0%	1 / 1 100.0%	3 / 3 100.0%	2 / 2 100.0%	4 / 4 100.0%	16 / 16 100.0%	4 / 4 100.0%	6 / 6 100.0%	8 / 8 100.0%	1 / 1 100.0%	0 / 0 0.0%	0 / 0 0.0%	0 / 0 0.0%	66 / 98 67.3%AB	32 / 98 32.7%ABE					
1	Escherichia coli APEC O1	91 / 92 98.9%	4 / 4 100.0%	3 / 3 100.0%	4 / 4 100.0%	3 / 3 100.0%	8 / 8 100.0%	7 / 7 100.0%	1 / 1 100.0%	3 / 3 100.0%	5 / 5 100.0%	1 / 1 100.0%	5 / 5 100.0%	6 / 6 100.0%	2 / 2 100.0%	4 / 4 100.0%	1 / 1 100.0%	3 / 3 100.0%	2 / 2 100.0%	6 / 6 100.0%	9 / 10 90.0%	3 / 3 100.0%	3 / 3 100.0%	7 / 7 100.0%	1 / 1 100.0%	0 / 0 0.0%	0 / 0 0.0%	0 / 0 0.0%	59 / 92 64.1%AB	31 / 92 33.7%ABE	1 / 92 1.1%E	1 / 92 1.1%BE			
1	Escherichia coli SE11	87 / 88 98.9%	4 / 4 100.0%	3 / 3 100.0%	4 / 4 100.0%	4 / 4 100.0%	7 / 7 100.0%	7 / 7 100.0%	1 / 1 100.0%	3 / 3 100.0%	6 / 6 100.0%	1 / 1 100.0%	5 / 5 100.0%	6 / 6 100.0%	2 / 2 100.0%	4 / 4 100.0%	1 / 1 100.0%	3 / 3 100.0%	2 / 2 100.0%	4 / 4 100.0%	7 / 8 87.5%	3 / 3 100.0%	1 / 1 100.0%	8 / 8 100.0%	1 / 1 100.0%	0 / 0 0.0%	0 / 0 0.0%	0 / 0 0.0%	55 / 88 62.5%AB	32 / 88 36.4%ABE	1 / 88 1.1%E				
1	Escherichia coli SE15	85 / 85 100.0%	4 / 4 100.0%	3 / 3 100.0%	4 / 4 100.0%	3 / 3 100.0%	6 / 6 100.0%	7 / 7 100.0%	1 / 1 100.0%	3 / 3 100.0%	5 / 5 100.0%	1 / 1 100.0%	5 / 5 100.0%	6 / 6 100.0%	2 / 2 100.0%	4 / 4 100.0%	1 / 1 100.0%	3 / 3 100.0%	2 / 2 100.0%	4 / 4 100.0%	7 / 7 100.0%	4 / 4 100.0%	1 / 1 100.0%	8 / 8 100.0%	1 / 1 100.0%	0 / 0 0.0%	0 / 0 0.0%	0 / 0 0.0%	53 / 85 62.4%AB	32 / 85 37.6%ABE					
1	Escherichia coli SECEC SMS-3-5	89 / 89 100.0%	4 / 4 100.0%	3 / 3 100.0%	4 / 4 100.0%	2 / 2 100.0%	7 / 7 100.0%	7 / 7 100.0%	1 / 1 100.0%	3 / 3 100.0%	4 / 4 100.0%	1 / 1 100.0%	5 / 5 100.0%	7 / 7 100.0%	2 / 2 100.0%	4 / 4 100.0%	1 / 1 100.0%	3 / 3 100.0%	2 / 2 100.0%	5 / 5 100.0%	7 / 7 100.0%	5 / 5 100.0%	3 / 3 100.0%	8 / 8 100.0%	1 / 1 100.0%	0 / 0 0.0%	0 / 0 0.0%	0 / 0 0.0%	37 / 89 41.6%AB	20 / 89 22.5%ABE	18 / 89 20.2%B	14 / 89 15.7%BE			
1	Escherichia coli BL21(DE3)	85 / 86 98.8%	4 / 4 100.0%	3 / 3 100.0%	4 / 4 100.0%	3 / 3 100.0%	6 / 6 100.0%	7 / 7 100.0%	1 / 1 100.0%	3 / 3 100.0%	5 / 5 100.0%	1 / 1 100.0%	5 / 5 100.0%	6 / 6 100.0%	2 / 2 100.0%	4 / 4 100.0%	1 / 1 100.0%	3 / 3 100.0%	2 / 2 100.0%	4 / 4 100.0%	7 / 8 87.5%	4 / 4 100.0%	1 / 1 100.0%	8 / 8 100.0%	1 / 1 100.0%	0 / 0 0.0%	0 / 0 0.0%	0 / 0 0.0%	53 / 86 61.6%AB	32 / 86 37.2%ABE	1 / 86 1.2%E				
1	Escherichia coli C	86 / 86 100.0%	4 / 4 100.0%	3 / 3 100.0%	4 / 4 100.0%	3 / 3 100.0%	6 / 6 100.0%	7 / 7 100.0%	1 / 1 100.0%	3 / 3 100.0%	5 / 5 100.0%	1 / 1 100.0%	5 / 5 100.0%	6 / 6 100.0%	2 / 2 100.0%	4 / 4 100.0%	1 / 1 100.0%	3 / 3 100.0%	2 / 2 100.0%	4 / 4 100.0%	7 / 7 100.0%	4 / 4 100.0%	2 / 2 100.0%	8 / 8 100.0%	1 / 1 100.0%	0 / 0 0.0%	0 / 0 0.0%	0 / 0 0.0%	38 / 86 44.2%B	22 / 86 25.6%ABE	16 / 86 18.6%AB	10 / 86 11.6%BE			
1	Escherichia coli ATCC 8739	86 / 86 100.0%	4 / 4 100.0%	3 / 3 100.0%	4 / 4 100.0%	3 / 3 100.0%	6 / 6 100.0%	7 / 7 100.0%	1 / 1 100.0%	3 / 3 100.0%	5 / 5 100.0%	1 / 1 100.0%	5 / 5 100.0%	6 / 6 100.0%	2 / 2 100.0%	4 / 4 100.0%	1 / 1 100.0%	3 / 3 100.0%	2 / 2 100.0%	4 / 4 100.0%	7 / 7 100.0%	4 / 4 100.0%	2 / 2 100.0%	8 / 8 100.0%	1 / 1 100.0%	0 / 0 0.0%	0 / 0 0.0%	0 / 0 0.0%	38 / 86 44.2%B	22 / 86 25.6%ABE	16 / 86 18.6%AB	10 / 86 11.6%BE			
1	Escherichia coli BL21	80 / 81 98.8%	4 / 4 100.0%	3 / 3 100.0%	4 / 4 100.0%	3 / 3 100.0%	6 / 6 100.0%	7 / 7 100.0%	1 / 1 100.0%	3 / 3 100.0%	5 / 5 100.0%	1 / 1 100.0%	5 / 5 100.0%	6 / 6 100.0%	2 / 2 100.0%	2 / 2 100.0%	1 / 1 100.0%	3 / 3 100.0%	1 / 1 100.0%	4 / 4 100.0%	7 / 8 87.5%	4 / 4 100.0%	1 / 1 100.0%	6 / 6 100.0%	1 / 1 100.0%	0 / 0 0.0%	0 / 0 0.0%	0 / 0 0.0%	52 / 81 64.2%AB	28 / 81 34.6%ABE	1 / 81 1.2%E				
1	Escherichia coli ATCC 33849	169 / 171 98.8%	8 / 8 100.0%	6 / 6 100.0%	8 / 8 100.0%	6 / 6 100.0%	12 / 12 100.0%	14 / 14 100.0%	2 / 2 100.0%	6 / 6 100.0%	10 / 10 100.0%	2 / 2 100.0%	9 / 9 100.0%	12 / 12 100.0%	4 / 4 100.0%	6 / 6 100.0%	2 / 2 100.0%	6 / 6 100.0%	4 / 4 100.0%	6 / 8 75.0%	14 / 14 100.0%	8 / 8 100.0%	4 / 4 100.0%	16 / 16 100.0%	2 / 2 100.0%	0 / 0 0.0%	0 / 0 0.0%	2 / 2 100.0%	106 / 171 62.0%AB	63 / 171 36.8%ABE	2 / 171 1.2%E				
1	Escherichia coli ATCC 9637	174 / 174 100.0%	8 / 8 100.0%	6 / 6 100.0%	8 / 8 100.0%	6 / 6 100.0%	14 / 14 100.0%	14 / 14 100.0%	2 / 2 100.0%	6 / 6 100.0%	10 / 10 100.0%	2 / 2 100.0%	10 / 10 100.0%	12 / 12 100.0%	4 / 4 100.0%	8 / 8 100.0%	2 / 2 100.0%	6 / 6 100.0%	4 / 4 100.0%	10 / 10 100.0%	14 / 14 100.0%	8 / 8 100.0%	2 / 2 100.0%	16 / 16 100.0%	2 / 2 100.0%	0 / 0 0.0%	0 / 0 0.0%	0 / 0 0.0%	108 / 174 62.1%AB	66 / 174 37.9%ABE					
1	Escherichia coli IAI1	85 / 85 100.0%	4 / 4 100.0%	3 / 3 100.0%	4 / 4 100.0%	3 / 3 100.0%	6 / 6 100.0%	7 / 7 100.0%	1 / 1 100.0%	3 / 3 100.0%	5 / 5 100.0%	1 / 1 100.0%	5 / 5 100.0%	6 / 6 100.0%	2 / 2 100.0%	4 / 4 100.0%	1 / 1 100.0%	3 / 3 100.0%	2 / 2 100.0%	4 / 4 100.0%	6 / 6 100.0%	4 / 4 100.0%	2 / 2 100.0%	8 / 8 100.0%	1 / 1 100.0%	0 / 0 0.0%	0 / 0 0.0%	0 / 0 0.0%	53 / 85 62.4%AB	32 / 85 37.6%ABE					
1	Escherichia coli S88	91 / 91 100.0%	4 / 4 100.0%	3 / 3 100.0%	4 / 4 100.0%	3 / 3 100.0%	6 / 6 100.0%	7 / 7 100.0%	1 / 1 100.0%	3 / 3 100.0%	5 / 5 100.0%	1 / 1 100.0%	5 / 5 100.0%	6 / 6 100.0%	2 / 2 100.0%	4 / 4 100.0%	1 / 1 100.0%	3 / 3 100.0%	2 / 2 100.0%	4 / 4 100.0%	11 / 11 100.0%	4 / 4 100.0%	3 / 3 100.0%	8 / 8 100.0%	1 / 1 100.0%	0 / 0 0.0%	0 / 0 0.0%	0 / 0 0.0%	59 / 91 64.8%AB	32 / 91 35.2%ABE					
1	Escherichia coli 55989	94 / 95 98.9%	4 / 4 100.0%	3 / 3 100.0%	4 / 4 100.0%	3 / 3 100.0%	6 / 6 100.0%	7 / 7 100.0%	1 / 1 100.0%	3 / 3 100.0%	5 / 5 100.0%	1 / 1 100.0%	5 / 5 100.0%	6 / 6 100.0%	2 / 2 100.0%	4 / 4 100.0%	1 / 1 100.0%	3 / 3 100.0%	2 / 2 100.0%	4 / 4 100.0%	12 / 13 92.3%	4 / 4 100.0%	5 / 5 100.0%	8 / 8 100.0%	1 / 1 100.0%	0 / 0 0.0%	0 / 0 0.0%	0 / 0 0.0%	62 / 95 65.3%AB	32 / 95 33.7%ABE	1 / 95 1.1%E				
1	Escherichia coli UMN026	87 / 88 98.9%	5 / 5 100.0%	3 / 3 100.0%	4 / 4 100.0%	3 / 3 100.0%	6 / 6 100.0%	7 / 7 100.0%	1 / 1 100.0%	3 / 3 100.0%	5 / 5 100.0%	1 / 1 100.0%	5 / 5 100.0%	6 / 6 100.0%	2 / 2 100.0%	4 / 4 100.0%	1 / 1 100.0%	3 / 3 100.0%	2 / 2 100.0%	4 / 4 100.0%	7 / 8 87.5%	4 / 4 100.0%	2 / 2 100.0%	8 / 8 100.0%	1 / 1 100.0%	0 / 0 0.0%	0 / 0 0.0%	0 / 0 0.0%	54 / 88 61.4%AB	33 / 88 37.5%ABE	1 / 88 1.1%E				
1	Escherichia coli IAI39	87 / 87 100.0%	3 / 3 100.0%	3 / 3 100.0%	4 / 4 100.0%	3 / 3 100.0%	6 / 6 100.0%	8 / 8 100.0%	1 / 1 100.0%	3 / 3 100.0%	5 / 5 100.0%	1 / 1 100.0%	5 / 5 100.0%	6 / 6 100.0%	2 / 2 100.0%	4 / 4 100.0%	1 / 1 100.0%	3 / 3 100.0%	2 / 2 100.0%	4 / 4 100.0%	8 / 8 100.0%	4 / 4 100.0%	2 / 2 100.0%	8 / 8 100.0%	1 / 1 100.0%	0 / 0 0.0%	0 / 0 0.0%	0 / 0 0.0%	56 / 87 64.4%AB	31 / 87 35.6%ABE					
1	Escherichia coli ED1a	89 / 89 100.0%	4 / 4 100.0%	3 / 3 100.0%	4 / 4 100.0%	3 / 3 100.0%	6 / 6 100.0%	7 / 7 100.0%	1 / 1 100.0%	3 / 3 100.0%	4 / 4 100.0%	1 / 1 100.0%	5 / 5 100.0%	6 / 6 100.0%	2 / 2 100.0%	4 / 4 100.0%	1 / 1 100.0%	3 / 3 100.0%	2 / 2 100.0%	4 / 4 100.0%	10 / 10 100.0%	4 / 4 100.0%	3 / 3 100.0%	8 / 8 100.0%	1 / 1 100.0%	0 / 0 0.0%	0 / 0 0.0%	0 / 0 0.0%	57 / 89 64.0%AB	32 / 89 36.0%ABE					
1	Escherichia coli LF82	85 / 85 100.0%	4 / 4 100.0%	3 / 3 100.0%	4 / 4 100.0%	3 / 3 100.0%	6 / 6 100.0%	6 / 6 100.0%	1 / 1 100.0%	3 / 3 100.0%	6 / 6 100.0%	1 / 1 100.0%	5 / 5 100.0%	6 / 6 100.0%	2 / 2 100.0%	4 / 4 100.0%	1 / 1 100.0%	3 / 3 100.0%	2 / 2 100.0%	4 / 4 100.0%	7 / 7 100.0%	4 / 4 100.0%	1 / 1 100.0%	8 / 8 100.0%	1 / 1 100.0%	0 / 0 0.0%	0 / 0 0.0%	0 / 0 0.0%	53 / 85 62.4%AB	32 / 85 37.6%ABE					
2	Escherichia coli ATCC 55124	169 / 170 99.4%	6 / 6 100.0%	5 / 6 83.3%	8 / 8 100.0%	6 / 6 100.0%	13 / 13 100.0%	14 / 14 100.0%	2 / 2 100.0%	6 / 6 100.0%	9 / 9 100.0%	2 / 2 100.0%	10 / 10 100.0%	12 / 12 100.0%	4 / 4 100.0%	8 / 8 100.0%	2 / 2 100.0%	6 / 6 100.0%	4 / 4 100.0%	10 / 10 100.0%	14 / 14 100.0%	8 / 8 100.0%	2 / 2 100.0%	16 / 16 100.0%	2 / 2 100.0%	0 / 0 0.0%	0 / 0 0.0%	0 / 0 0.0%	105 / 170 61.8%AB	64 / 170 37.6%ABE	1 / 170 0.6%				
[illegible]	[illegible]	88 / 88 [illegible]	4 / 4 [illegible]	3 / 3 [illegible]	4 / 4 [illegible]	3 / 3 [illegible]	6 / 6 [illegible]	7 / 7 [illegible]	1 / 1 [illegible]	3 / 3 [illegible]	5 / 5 [illegible]	1 / 1 [illegible]	5 / 5 [illegible]	5 / 5 [illegible]	2 / 2 [illegible]	4 / 4 [illegible]	1 / 1 [illegible]	3 / 3 [illegible]	2 / 2 [illegible]	4 / 4 [illegible]	10 / 10 [illegible]	4 / 4 [illegible]	2 / 2 [illegible]	8 / 8 [illegible]	1 / 1 [illegible]	0 / 0 [illegible]	0 / 0 [illegible]	0 / 0 [illegible]	55 / 88 [illegible]	33 / 88 [illegible]					

1	Escherichia coli ABU 83972	100.0%	100.0%	100.0%	100.0%	100.0%	100.0%	100.0%	100.0%	100.0%	100.0%	100.0%	100.0%	100.0%	100.0%	100.0%	100.0%	100.0%	100.0%	100.0%	100.0%	100.0%	100.0%	100.0%	100.0%	0.0%	0.0%	0.0%	62.5%AB	37.5%ABE				
1	Escherichia coli O83:H1	82 / 82 100.0%	3 / 3 100.0%	3 / 3 100.0%	4 / 4 100.0%	3 / 3 100.0%	6 / 6 100.0%	7 / 7 100.0%	1 / 1 100.0%	3 / 3 100.0%	5 / 5 100.0%	1 / 1 100.0%	5 / 5 100.0%	6 / 6 100.0%	2 / 2 100.0%	4 / 4 100.0%	1 / 1 100.0%	3 / 3 100.0%	2 / 2 100.0%	4 / 4 100.0%	6 / 6 100.0%	4 / 4 100.0%	1 / 1 100.0%	7 / 7 100.0%	1 / 1 100.0%	0 / 0 0.0%	0 / 0 0.0%	0 / 0 0.0%	52 / 82 63.4%AB	30 / 82 36.6%ABE				
1	Escherichia coli O83:H1 str. NRG 857C	82 / 82 100.0%	3 / 3 100.0%	3 / 3 100.0%	4 / 4 100.0%	3 / 3 100.0%	6 / 6 100.0%	7 / 7 100.0%	1 / 1 100.0%	3 / 3 100.0%	5 / 5 100.0%	1 / 1 100.0%	5 / 5 100.0%	6 / 6 100.0%	2 / 2 100.0%	4 / 4 100.0%	1 / 1 100.0%	3 / 3 100.0%	2 / 2 100.0%	4 / 4 100.0%	6 / 6 100.0%	4 / 4 100.0%	1 / 1 100.0%	7 / 7 100.0%	1 / 1 100.0%	0 / 0 0.0%	0 / 0 0.0%	0 / 0 0.0%	52 / 82 63.4%AB	30 / 82 36.6%ABE				
1	Escherichia coli UMNK88	92 / 94 97.9%	3 / 3 100.0%	3 / 3 100.0%	4 / 4 100.0%	6 / 6 100.0%	8 / 8 100.0%	7 / 7 100.0%	1 / 1 100.0%	3 / 3 100.0%	8 / 8 100.0%	1 / 1 100.0%	5 / 5 100.0%	6 / 6 100.0%	2 / 2 100.0%	4 / 4 100.0%	1 / 1 100.0%	3 / 3 100.0%	2 / 2 100.0%	4 / 6 66.7%	8 / 8 100.0%	1 / 1 100.0%	4 / 4 100.0%	7 / 7 100.0%	1 / 1 100.0%	0 / 0 0.0%	0 / 0 0.0%	0 / 0 0.0%	63 / 94 67.0%AB	29 / 94 30.9%ABE	2 / 94 2.1%			
1	Escherichia coli IHE3034	97 / 97 100.0%	4 / 4 100.0%	3 / 3 100.0%	4 / 4 100.0%	3 / 3 100.0%	8 / 8 100.0%	7 / 7 100.0%	1 / 1 100.0%	3 / 3 100.0%	5 / 5 100.0%	1 / 1 100.0%	5 / 5 100.0%	7 / 7 100.0%	2 / 2 100.0%	4 / 4 100.0%	1 / 1 100.0%	3 / 3 100.0%	2 / 2 100.0%	6 / 6 100.0%	11 / 11 100.0%	4 / 4 100.0%	4 / 4 100.0%	8 / 8 100.0%	1 / 1 100.0%	0 / 0 0.0%	0 / 0 0.0%	0 / 0 0.0%	62 / 97 63.9%AB	35 / 97 36.1%ABE				
1	Escherichia coli Xuzhou21	93 / 93 100.0%	2 / 2 100.0%	3 / 3 100.0%	4 / 4 100.0%	3 / 3 100.0%	5 / 5 100.0%	5 / 5 100.0%	1 / 1 100.0%	3 / 3 100.0%	4 / 4 100.0%	1 / 1 100.0%	5 / 5 100.0%	5 / 5 100.0%	2 / 2 100.0%	4 / 4 100.0%	1 / 1 100.0%	3 / 3 100.0%	2 / 2 100.0%	4 / 4 100.0%	17 / 17 100.0%	4 / 4 100.0%	8 / 8 100.0%	6 / 6 100.0%	1 / 1 100.0%	0 / 0 0.0%	0 / 0 0.0%	0 / 0 0.0%	65 / 93 69.9%AB	28 / 93 30.1%ABE				
1	Escherichia coli O44:H18	93 / 93 100.0%	4 / 4 100.0%	3 / 3 100.0%	4 / 4 100.0%	3 / 3 100.0%	7 / 7 100.0%	7 / 7 100.0%	1 / 1 100.0%	3 / 3 100.0%	5 / 5 100.0%	1 / 1 100.0%	5 / 5 100.0%	6 / 6 100.0%	2 / 2 100.0%	4 / 4 100.0%	1 / 1 100.0%	3 / 3 100.0%	2 / 2 100.0%	5 / 5 100.0%	10 / 10 100.0%	4 / 4 100.0%	4 / 4 100.0%	8 / 8 100.0%	1 / 1 100.0%	0 / 0 0.0%	0 / 0 0.0%	0 / 0 0.0%	60 / 93 64.5%AB	33 / 93 35.5%ABE				
1	EAEC 042	93 / 93 100.0%	4 / 4 100.0%	3 / 3 100.0%	4 / 4 100.0%	3 / 3 100.0%	7 / 7 100.0%	7 / 7 100.0%	1 / 1 100.0%	3 / 3 100.0%	5 / 5 100.0%	1 / 1 100.0%	5 / 5 100.0%	6 / 6 100.0%	2 / 2 100.0%	4 / 4 100.0%	1 / 1 100.0%	3 / 3 100.0%	2 / 2 100.0%	5 / 5 100.0%	10 / 10 100.0%	4 / 4 100.0%	4 / 4 100.0%	8 / 8 100.0%	1 / 1 100.0%	0 / 0 0.0%	0 / 0 0.0%	0 / 0 0.0%	60 / 93 64.5%AB	33 / 93 35.5%ABE				
1	Escherichia coli UM146	82 / 82 100.0%	4 / 4 100.0%	3 / 3 100.0%	5 / 5 100.0%	3 / 3 100.0%	6 / 6 100.0%	5 / 5 100.0%	1 / 1 100.0%	3 / 3 100.0%	5 / 5 100.0%	1 / 1 100.0%	5 / 5 100.0%	2 / 2 100.0%	2 / 2 100.0%	4 / 4 100.0%	1 / 1 100.0%	3 / 3 100.0%	2 / 2 100.0%	5 / 5 100.0%	8 / 8 100.0%	4 / 4 100.0%	2 / 2 100.0%	7 / 7 100.0%	1 / 1 100.0%	0 / 0 0.0%	0 / 0 0.0%	0 / 0 0.0%	50 / 82 61.0%AB	32 / 82 39.0%ABE				
1	Escherichia coli clone D i14	88 / 88 100.0%	4 / 4 100.0%	3 / 3 100.0%	4 / 4 100.0%	3 / 3 100.0%	6 / 6 100.0%	6 / 6 100.0%	1 / 1 100.0%	3 / 3 100.0%	4 / 4 100.0%	1 / 1 100.0%	5 / 5 100.0%	6 / 6 100.0%	2 / 2 100.0%	4 / 4 100.0%	1 / 1 100.0%	3 / 3 100.0%	2 / 2 100.0%	4 / 4 100.0%	11 / 11 100.0%	4 / 4 100.0%	2 / 2 100.0%	8 / 8 100.0%	1 / 1 100.0%	0 / 0 0.0%	0 / 0 0.0%	0 / 0 0.0%	55 / 88 62.5%AB	33 / 88 37.5%ABE				
1	Escherichia coli clone D i2	88 / 88 100.0%	4 / 4 100.0%	3 / 3 100.0%	4 / 4 100.0%	3 / 3 100.0%	6 / 6 100.0%	6 / 6 100.0%	1 / 1 100.0%	3 / 3 100.0%	4 / 4 100.0%	1 / 1 100.0%	5 / 5 100.0%	6 / 6 100.0%	2 / 2 100.0%	4 / 4 100.0%	1 / 1 100.0%	3 / 3 100.0%	2 / 2 100.0%	4 / 4 100.0%	11 / 11 100.0%	4 / 4 100.0%	2 / 2 100.0%	8 / 8 100.0%	1 / 1 100.0%	0 / 0 0.0%	0 / 0 0.0%	0 / 0 0.0%	55 / 88 62.5%AB	33 / 88 37.5%ABE				
1	Escherichia coli P12b	82 / 82 100.0%	4 / 4 100.0%	3 / 3 100.0%	5 / 5 100.0%	3 / 3 100.0%	5 / 5 100.0%	7 / 7 100.0%	1 / 1 100.0%	3 / 3 100.0%	5 / 5 100.0%	1 / 1 100.0%	5 / 5 100.0%	6 / 6 100.0%	2 / 2 100.0%	4 / 4 100.0%	1 / 1 100.0%	3 / 3 100.0%	2 / 2 100.0%	3 / 3 100.0%	5 / 5 100.0%	4 / 4 100.0%	1 / 1 100.0%	8 / 8 100.0%	1 / 1 100.0%	0 / 0 0.0%	0 / 0 0.0%	0 / 0 0.0%	49 / 82 59.8%AB	33 / 82 40.2%ABE				
1	Escherichia coli NA114	66 / 67 98.5%	3 / 3 100.0%	3 / 3 100.0%	3 / 3 100.0%	1 / 1 100.0%	5 / 5 100.0%	7 / 7 100.0%	1 / 1 100.0%	1 / 1 100.0%	3 / 3 100.0%	1 / 1 100.0%	4 / 4 100.0%	4 / 4 100.0%	1 / 1 100.0%	3 / 3 100.0%	1 / 1 100.0%	3 / 3 100.0%	2 / 2 100.0%	2 / 2 100.0%	7 / 8 87.5%	1 / 1 100.0%	2 / 2 100.0%	7 / 7 100.0%	1 / 1 100.0%	0 / 0 0.0%	0 / 0 0.0%	0 / 0 0.0%	44 / 67 65.7%AB	22 / 67 32.8%ABE	1 / 67 1.5%			
3	Escherichia coli O104:H4	279 / 282 98.9%	7 / 7 100.0%	9 / 9 100.0%	12 / 12 100.0%	12 / 12 100.0%	16 / 16 100.0%	21 / 21 100.0%	3 / 3 100.0%	9 / 9 100.0%	18 / 18 100.0%	3 / 3 100.0%	15 / 15 100.0%	18 / 18 100.0%	6 / 6 100.0%	12 / 12 100.0%	3 / 3 100.0%	9 / 9 100.0%	6 / 6 100.0%	12 / 12 100.0%	36 / 39 92.3%	9 / 9 100.0%	16 / 16 100.0%	24 / 24 100.0%	3 / 3 100.0%	0 / 0 0.0%	0 / 0 0.0%	0 / 0 0.0%	191 / 282 67.7%AB	88 / 282 31.2%ABE	3 / 282 1.1%E			
1	Escherichia coli O104:H4 str. 2011C-3493	92 / 92 100.0%	2 / 2 100.0%	3 / 3 100.0%	4 / 4 100.0%	6 / 6 100.0%	4 / 4 100.0%	7 / 7 100.0%	1 / 1 100.0%	3 / 3 100.0%	8 / 8 100.0%	1 / 1 100.0%	5 / 5 100.0%	6 / 6 100.0%	2 / 2 100.0%	4 / 4 100.0%	1 / 1 100.0%	3 / 3 100.0%	2 / 2 100.0%	4 / 4 100.0%	11 / 11 100.0%	1 / 1 100.0%	5 / 5 100.0%	8 / 8 100.0%	1 / 1 100.0%	0 / 0 0.0%	0 / 0 0.0%	0 / 0 0.0%	65 / 92 70.7%AB	27 / 92 29.3%ABE				
1	Escherichia coli O104:H4 str. 2009EL-2071	95 / 97 97.9%	3 / 3 100.0%	3 / 3 100.0%	4 / 4 100.0%	3 / 3 100.0%	6 / 6 100.0%	7 / 7 100.0%	1 / 1 100.0%	3 / 3 100.0%	5 / 5 100.0%	1 / 1 100.0%	5 / 5 100.0%	6 / 6 100.0%	2 / 2 100.0%	4 / 4 100.0%	1 / 1 100.0%	3 / 3 100.0%	2 / 2 100.0%	4 / 4 100.0%	13 / 15 86.7%	4 / 4 100.0%	6 / 6 100.0%	8 / 8 100.0%	1 / 1 100.0%	0 / 0 0.0%	0 / 0 0.0%	0 / 0 0.0%	64 / 97 66.0%AB	31 / 97 32.0%ABE	2 / 97 2.1%E			
1	Escherichia coli O104:H4 str. 2009EL-2050	92 / 93 98.9%	2 / 2 100.0%	3 / 3 100.0%	4 / 4 100.0%	3 / 3 100.0%	6 / 6 100.0%	7 / 7 100.0%	1 / 1 100.0%	3 / 3 100.0%	5 / 5 100.0%	1 / 1 100.0%	5 / 5 100.0%	6 / 6 100.0%	2 / 2 100.0%	4 / 4 100.0%	1 / 1 100.0%	3 / 3 100.0%	2 / 2 100.0%	4 / 4 100.0%	12 / 13 92.3%	4 / 4 100.0%	5 / 5 100.0%	8 / 8 100.0%	1 / 1 100.0%	0 / 0 0.0%	0 / 0 0.0%	0 / 0 0.0%	62 / 93 66.7%AB	30 / 93 32.3%ABE	1 / 93 1.1%E			
1	Escherichia coli UMNF18	95 / 95 100.0%	3 / 3 100.0%	3 / 3 100.0%	4 / 4 100.0%	3 / 3 100.0%	10 / 10 100.0%	6 / 6 100.0%	1 / 1 100.0%	3 / 3 100.0%	5 / 5 100.0%	1 / 1 100.0%	5 / 5 100.0%	6 / 6 100.0%	2 / 2 100.0%	4 / 4 100.0%	1 / 1 100.0%	3 / 3 100.0%	2 / 2 100.0%	8 / 8 100.0%	8 / 8 100.0%	3 / 3 100.0%	5 / 5 100.0%	8 / 8 100.0%	1 / 1 100.0%	0 / 0 0.0%	0 / 0 0.0%	0 / 0 0.0%	61 / 95 64.2%AB	34 / 95 35.8%ABE				
1	Escherichia coli O7:K1	91 / 91 100.0%	4 / 4 100.0%	3 / 3 100.0%	4 / 4 100.0%	3 / 3 100.0%	6 / 6 100.0%	7 / 7 100.0%	1 / 1 100.0%	3 / 3 100.0%	5 / 5 100.0%	1 / 1 100.0%	5 / 5 100.0%	6 / 6 100.0%	2 / 2 100.0%	4 / 4 100.0%	1 / 1 100.0%	3 / 3 100.0%	2 / 2 100.0%	4 / 4 100.0%	11 / 11 100.0%	4 / 4 100.0%	4 / 4 100.0%	7 / 7 100.0%	1 / 1 100.0%	0 / 0 0.0%	0 / 0 0.0%	0 / 0 0.0%	60 / 91 65.9%AB	31 / 91 34.1%ABE				
1	Escherichia coli O7:K1 str. CE10	91 / 91 100.0%	4 / 4 100.0%	3 / 3 100.0%	4 / 4 100.0%	3 / 3 100.0%	6 / 6 100.0%	7 / 7 100.0%	1 / 1 100.0%	3 / 3 100.0%	5 / 5 100.0%	1 / 1 100.0%	5 / 5 100.0%	6 / 6 100.0%	2 / 2 100.0%	4 / 4 100.0%	1 / 1 100.0%	3 / 3 100.0%	2 / 2 100.0%	4 / 4 100.0%	11 / 11 100.0%	4 / 4 100.0%	4 / 4 100.0%	7 / 7 100.0%	1 / 1 100.0%	0 / 0 0.0%	0 / 0 0.0%	0 / 0 0.0%	60 / 91 65.9%AB	31 / 91 34.1%ABE				
1	Escherichia coli APEC O78	86 / 86 100.0%	4 / 4 100.0%	3 / 3 100.0%	4 / 4 100.0%	6 / 6 100.0%	6 / 6 100.0%	7 / 7 100.0%	1 / 1 100.0%	3 / 3 100.0%	7 / 7 100.0%	1 / 1 100.0%	5 / 5 100.0%	6 / 6 100.0%	2 / 2 100.0%	4 / 4 100.0%	1 / 1 100.0%	3 / 3 100.0%	2 / 2 100.0%	4 / 4 100.0%	7 / 7 100.0%	0 / 0 0.0%	1 / 1 100.0%	8 / 8 100.0%	1 / 1 100.0%	0 / 0 0.0%	0 / 0 0.0%	0 / 0 0.0%	58 / 86 67.4%AB	28 / 86 32.6%ABE				
1	ATCC 35469	84 / 85 98.8%	4 / 4 100.0%	3 / 3 100.0%	4 / 4 100.0%	3 / 3 100.0%	6 / 6 100.0%	7 / 7 100.0%	1 / 1 100.0%	3 / 3 100.0%	5 / 5 100.0%	1 / 1 100.0%	5 / 5 100.0%	6 / 6 100.0%	2 / 2 100.0%	4 / 4 100.0%	1 / 1 100.0%	3 / 3 100.0%	2 / 2 100.0%	4 / 4 100.0%	6 / 7 85.7%	4 / 4 100.0%	1 / 1 100.0%	8 / 8 100.0%	1 / 1 100.0%	0 / 0 0.0%	0 / 0 0.0%	0 / 0 0.0%	52 / 85 61.2%AB	32 / 85 37.6%ABE	1 / 85 1.2%			
1	Escherichia fergusonii ATCC 35469	84 / 85 98.8%	4 / 4 100.0%	3 / 3 100.0%	4 / 4 100.0%	3 / 3 100.0%	6 / 6 100.0%	7 / 7 100.0%	1 / 1 100.0%	3 / 3 100.0%	5 / 5 100.0%	1 / 1 100.0%	5 / 5 100.0%	6 / 6 100.0%	2 / 2 100.0%	4 / 4 100.0%	1 / 1 100.0%	3 / 3 100.0%	2 / 2 100.0%	4 / 4 100.0%	6 / 7 85.7%	4 / 4 100.0%	1 / 1 100.0%	8 / 8 100.0%	1 / 1 100.0%	0 / 0 0.0%	0 / 0 0.0%	0 / 0 0.0%	52 / 85 61.2%AB	32 / 85 37.6%ABE	1 / 85 1.2%			
10	"Donovania" Anderson et al. 1944	827 / 833 99.3%	37 / 37 100.0%	30 / 30 100.0%	42 / 42 100.0%	26 / 26 100.0%	56 / 56 100.0%	74 / 74 100.0%	10 / 10 100.0%	28 / 28 100.0%	45 / 45 100.0%	10 / 10 100.0%	49 / 49 100.0%	55 / 55 100.0%	20 / 20 100.0%	38 / 38 100.0%	10 / 10 100.0%	30 / 30 100.0%	19 / 19 100.0%	38 / 40 95.0%	68 / 72 94.4%	44 / 44 100.0%	11 / 11 100.0%	77 / 77 100.0%	10 / 10 100.0%	0 / 0 0.0%	0 / 0 0.0%	0 / 0 0.0%	489 / 833 58.7%AB	306 / 833 36.7%ABE	18 / 833 2.2%BE	14 / 833 1.7%B	5 / 833 0.6%	1 / 833 0.1%C
2	"Bacillus oxytocus perniciosus" Flugge 1886	165 / 167 98.8%	8 / 8 100.0%	6 / 6 100.0%	8 / 8 100.0%	6 / 6 100.0%	11 / 11 100.0%	14 / 14 100.0%	2 / 2 100.0%	6 / 6 100.0%	10 / 10 100.0%	2 / 2 100.0%	10 / 10 100.0%	10 / 10 100.0%	4 / 4 100.0%	8 / 8 100.0%	2 / 2 100.0%	6 / 6 100.0%	4 / 4 100.0%	9 / 9 100.0%	12 / 14 85.7%	8 / 8 100.0%	2 / 2 100.0%	15 / 15 100.0%	2 / 2 100.0%	0 / 0 0.0%	0 / 0 0.0%	0 / 0 0.0%	100 / 167 59.9%AB	65 / 167 38.9%ABE	2 / 167 1.2%			
1	Klebsiella oxytoca ATCC 8724	83 / 84 98.8%	4 / 4 100.0%	3 / 3 100.0%	4 / 4 100.0%	3 / 3 100.0%	6 / 6 100.0%	7 / 7 100.0%	1 / 1 100.0%	3 / 3 100.0%	5 / 5 100.0%	1 / 1 100.0%	5 / 5 100.0%	5 / 5 100.0%	2 / 2 100.0%	4 / 4 100.0%	1 / 1 100.0%	3 / 3 100.0%	2 / 2 100.0%	4 / 4 100.0%	6 / 7 85.7%	4 / 4 100.0%	1 / 1 100.0%	8 / 8 100.0%	1 / 1 100.0%	0 / 0 0.0%	0 / 0 0.0%	0 / 0 0.0%	50 / 84 59.5%AB	33 / 84 39.3%ABE	1 / 84 1.2%			
1	Klebsiella oxytoca E718	82 / 83 98.8%	4 / 4 100.0%	3 / 3 100.0%	4 / 4 100.0%	3 / 3 100.0%	5 / 5 100.0%	7 / 7 100.0%	1 / 1 100.0%	3 / 3 100.0%	5 / 5 100.0%	1 / 1 100.0%	5 / 5 100.0%	5 / 5 100.0%	2 / 2 100.0%	4 / 4 100.0%	1 / 1 100.0%	3 / 3 100.0%	2 / 2 100.0%	5 / 5 100.0%	6 / 7 85.7%	4 / 4 100.0%	1 / 1 100.0%	7 / 7 100.0%	1 / 1 100.0%	0 / 0 0.0%	0 / 0 0.0%	0 / 0 0.0%	50 / 83 60.2%AB	32 / 83 38.6%ABE	1 / 83 1.2%			
7	"Bacillus pneumoniae" (Schroeter 1886) Flugge 1886	578 / 581 99.5%	26 / 26 100.0%	21 / 21 100.0%	30 / 30 100.0%	17 / 17 100.0%	39 / 39 100.0%	52 / 52 100.0%	7 / 7 100.0%	20 / 20 100.0%	30 / 30 100.0%	7 / 7 100.0%	34 / 34 100.0%	39 / 39 100.0%	14 / 14 100.0%	26 / 26 100.0%	7 / 7 100.0%	21 / 21 100.0%	13 / 13 100.0%	25 / 26 96.2%	49 / 51 96.1%	32 / 32 100.0%	8 / 8 100.0%	54 / 54 100.0%	7 / 7 100.0%	0 / 0 0.0%	0 / 0 0.0%	0 / 0 0.0%	336 / 581 57.8%AB	210 / 581 36.1%ABE	18 / 581 3.1%BE	14 / 581 2.4%B	2 / 581 0.3%	1 / 581 0.2%C
4	Klebsiella pneumoniae subsp. pneumoniae	337 / 337 100.0%	15 / 15 100.0%	12 / 12 100.0%	16 / 16 100.0%	10 / 10 100.0%	24 / 24 100.0%	30 / 30 100.0%	4 / 4 100.0%	12 / 12 100.0%	18 / 18 100.0%	4 / 4 100.0%	19 / 19 100.0%	23 / 23 100.0%	8 / 8 100.0%	16 / 16 100.0%	4 / 4 100.0%	12 / 12 100.0%	8 / 8 100.0%	16 / 16 100.0%	28 / 28 100.0%	18 / 18 100.0%	5 / 5 100.0%	31 / 31 100.0%	4 / 4 100.0%	0 / 0 0.0%	0 / 0 0.0%	0 / 0 0.0%	191 / 337 56.7%AB	114 / 337 33.8%ABE	18 / 337 5.3%BE	14 / 337 4.2%B		
1	Klebsiella pneumoniae MCG 78578	86 / 86 100.0%	4 / 4 100.0%	3 / 3 100.0%	4 / 4 100.0%	3 / 3 100.0%	6 / 6 100.0%	8 / 8 100.0%	1 / 1 100.0%	3 / 3 100.0%	5 / 5 100.0%	1 / 1 100.0%	5 / 5 100.0%	6 / 6 100.0%	2 / 2 100.0%	4 / 4 100.0%	1 / 1 100.0%	3 / 3 100.0%	2 / 2 100.0%	4 / 4 100.0%	7 / 7 100.0%	4 / 4 100.0%	1 / 1 100.0%	8 / 8 100.0%	1 / 1 100.0%	0 / 0 0.0%	0 / 0 0.0%	0 / 0 0.0%	39 / 86 45.3%AB	18 / 86 20.9%BE	15 / 86 17.4%ABE	14 / 86 16.3%B		
1	Klebsiella pneumoniae NTUH-K2044	86 / 86 100.0%	4 / 4 100.0%	3 / 3 100.0%	4 / 4 100.0%	3 / 3 100.0%	6 / 6 100.0%	8 / 8 100.0%	1 / 1 100.0%	3 / 3 100.0%	5 / 5 100.0%	1 / 1 100.0%	5 / 5 100.0%	6 / 6 100.0%	2 / 2 100.0%	4 / 4 100.0%	1 / 1 100.0%	3 / 3 100.0%	2 / 2 100.0%	4 / 4 100.0%	7 / 7 100.0%	4 / 4 100.0%	1 / 1 100.0%	8 / 8 100.0%	1 / 1 100.0%	0 / 0 0.0%	0 / 0 0.0%	0 / 0 0.0%	53 / 86 61.6%AB	33 / 86 38.4%ABE				
1	Klebsiella pneumoniae subsp. pneumoniae HS11286	87 / 87 100.0%	4 / 4 100.0%	3 / 3 100.0%	4 / 4 100.0%	3 / 3 100.0%	6 / 6 100.0%	8 / 8 100.0%	1 / 1 100.0%	3 / 3 100.0%	5 / 5 100.0%	1 / 1 100.0%	5 / 5 100.0%	6 / 6 100.0%	2 / 2 100.0%	4 / 4 100.0%	1 / 1 100.0%	3 / 3 100.0%	2 / 2 100.0%	4 / 4 100.0%	7 / 7 100.0%	4 / 4 100.0%	2 / 2 100.0%	8 / 8 100.0%	1 / 1 100.0%	0 / 0 0.0%	0 / 0 0.0%	0 / 0 0.0%	53 / 87 60.9%AB	34 / 87 39.1%ABE				
1	Klebsiella pneumoniae subsp. pneumoniae 1084	78 / 78 100.0%	3 / 3 100.0%	3 / 3 100.0%	4 / 4 100.0%	1 / 1 100.0%	6 / 6 100.0%	6 / 6 100.0%	1 / 1 100.0%	3 / 3 100.0%	3 / 3 100.0%	1 / 1 100.0%	4 / 4 100.0%	5 / 5 100.0%	2 / 2 100.0%	4 / 4 100.0%	1 / 1 100.0%	3 / 3 100.0%	2 / 2 100.0%	4 / 4 100.0%	7 / 7 100.0%	6 / 6 100.0%	1 / 1 100.0%	7 / 7 100.0%	1 / 1 100.0%	0 / 0 0.0%	0 / 0 0.0%	0 / 0 0.0%	46 / 78 59.0%AB	32 / 78 41.0%ABE				
1	Klebsiella pneumoniae 342	88 / 88 100.0%	4 / 4 100.0%	3 / 3 100.0%	5 / 5 100.0%	3 / 3 100.0%	6 / 6 100.0%	8 / 8 100.0%	1 / 1 100.0%	3 / 3 100.0%	5 / 5 100.0%	1 / 1 100.0%	5 / 5 100.0%	6 / 6 100.0%	2 / 2 100.0%	4 / 4 100.0%	1 / 1 100.0%	3 / 3 100.0%	2 / 2 100.0%	4 / 4 100.0%	8 / 8 100.0%	4 / 4 100.0%	1 / 1 100.0%	8 / 8 100.0%	1 / 1 100.0%	0 / 0 0.0%	0 / 0 0.0%	0 / 0 0.0%	54 / 88 61.4%AB	34 / 88 38.6%ABE				
1	Klebsiella pneumoniae KCTC 2242	87 / 88 98.9%	4 / 4 100.0%	3 / 3 100.0%	5 / 5 100.0%	3 / 3 100.0%	6 / 6 100.0%	8 / 8 100.0%	1 / 1 100.0%	3 / 3 100.0%	5 / 5 100.0%	1 / 1 100.0%	5 / 5 100.0%	6 / 6 100.0%	2 / 2 100.0%	4 / 4 100.0%	1 / 1 100.0%	3 / 3 100.0%	2 / 2 100.0%	3 / 4 75.0%	8 / 8 100.0%	4 / 4 100.0%	1 / 1 100.0%	8 / 8 100.0%	1 / 1 100.0%	0 / 0 0.0%	0 / 0 0.0%	0 / 0 0.0%	53 / 88 60.2%AB	34 / 88 38.6%ABE	1 / 88 1.1%			
1	ATCC BAA-830	84 / 85 98.8%	3 / 3 100.0%	3 / 3 100.0%	4 / 4 100.0%	3 / 3 100.0%	6 / 6 100.0%	8 / 8 100.0%	1 / 1 100.0%	2 / 2 100.0%	5 / 5 100.0%	1 / 1 100.0%	5 / 5 100.0%	6 / 6 100.0%	2 / 2 100.0%	4 / 4 100.0%	1 / 1 100.0%	3 / 3 100.0%	2 / 2 100.0%	4 / 5 80.0%	7 / 7 100.0%	4 / 4 100.0%	1 / 1 100.0%	8 / 8 100.0%	1 / 1 100.0%	0 / 0 0.0%	0 / 0 0.0%	0 / 0 0.0%	53 / 85 62.4%AB	31 / 85 36.5%ABE	1 / 85 1.2%			
1	Klebsiella variicola At-22	84 / 85 98.8%	3 / 3 100.0%	3 / 3 100.0%	4 / 4 100.0%	3 / 3 100.0%	6 / 6 100.0%	8 / 8 100.0%	1 / 1 100.0%	2 / 2 100.0%	5 / 5 100.0%	1 / 1 100.0%	5 / 5 100.0%	6 / 6 100.0%	2 / 2 100.0%	4 / 4 100.0%	1 / 1 100.0%	3 / 3 100.0%	2 / 2 100.0%	4 / 5 80.0%	7 / 7 100.0%	4 / 4 100.0%	1 / 1 100.0%	8 / 8 100.0%	1 / 1 100.0%	0 / 0 0.0%	0 / 0 0.0%	0 / 0 0.0%	53 / 85 62.4%AB	31 / 85 36.5%ABE	1 / 85 1.2%			
1	"Liquidobacterium" Orla-Jensen 1908	83 / 83 100.0%	4 / 4 100.0%	3 / 3 100.0%	4 / 4 100.0%	3 / 3 100.0%	6 / 6 100.0%	6 / 6 100.0%	1 / 1 100.0%	2 / 2 100.0%	5 / 5 100.0%	1 / 1 100.0%	5 / 5 100.0%	6 / 6 100.0%	2 / 2 100.0%	3 / 3 100.0%	1 / 1 100.0%	3 / 3 100.0%	2 / 2 100.0%	5 / 5 100.0%	7 / 7 100.0%	4 / 4 100.0%	1 / 1 100.0%	8 / 8 100.0%	1 / 1 100.0%	0 / 0 0.0%	0 / 0 0.0%	0 / 0 0.0%	35 / 83 42.2%AB	20 / 83 24.1%B	14 / 83 16.9%ABE	14 / 83 16.9%BE		
1	ATCC 29906	83 / 83 100.0%	4 / 4 100.0%	3 / 3 100.0%	4 / 4 100.0%	3 / 3 100.0%	6 / 6 100.0%	6 / 6 100.0%	1 / 1 100.0%	2 / 2 100.0%	5 / 5 100.0%	1 / 1 100.0%	5 / 5 100.0%	6 / 6 100.0%	2 / 2 100.0%	3 / 3 100.0%	1 / 1 100.0%	3 / 3 100.0%	2 / 2 100.0%	5 / 5 100.0%	7 / 7 100.0%	4 / 4 100.0%	1 / 1 100.0%	8 / 8 100.0%	1 / 1 100.0%	0 / 0 0.0%	0 / 0 0.0%	0 / 0 0.0%	35 / 83 42.2%AB	20 / 83 24.1%B	14 / 83 16.9%ABE	14 / 83 16.9%BE		
1	Providencia	71 / 72 98.6%	4 / 4 100.0%	3 / 3 100.0%	3 / 3 100.0%	3 / 3 100.0%	5 / 5 100.0%	5 / 5 100.0%	1 / 1 100.0%	2 / 2 100.0%	4 / 4 100.0%	1 / 1 100.0%	4 / 4 100.0%	3 / 4 75.0%	2 / 2 100.0%	3 / 3 100.0%	1 / 1 100.0%	3 / 3 100.0%	2 / 2 100.0%	4 / 4 100.0%	4 / 4 100.0%	4 / 4 100.0%	1 / 1 100.0%	8 / 8 100.0%	1 / 1 100.0%	0 / 0 0.0%	0 / 0 0.0%	0 / 0 0.0%	45 / 72 62.5%AB	26 / 72 36.1%ABE	1 / 72 1.4%			
1	"Proteus stuartii" Buttiaux et al. 1954	71 / 72 98.6%	4 / 4 100.0%	3 / 3 100.0%	3 / 3 100.0%	3 / 3 100.0%	5 / 5 100.0%	5 / 5 100.0%	1 / 1 100.0%	2 / 2 100.0%	4 / 4 100.0%	1 / 1 100.0%	4 / 4 100.0%	3 / 4 75.0%	2 / 2 100.0%	3 / 3 100.0%	1 / 1 100.0%	3 / 3 100.0%	2 / 2 100.0%	4 / 4 100.0%	4 / 4 100.0%	4 / 4 100.0%	1 / 1 100.0%	8 / 8 100.0%	1 / 1 100.0%	0 / 0 0.0%	0 / 0 0.0%	0 / 0 0.0%	45 / 72 62.5%AB	26 / 72 36.1%ABE	1 / 72 1.4%			
1	Providencia stuartii MRSN 2154	71 / 72 98.6%	4 / 4 100.0%	3 / 3 100.0%	3 / 3 100.0%	3 / 3 100.0%	5 / 5 100.0%	5 / 5 100.0%	1 / 1 100.0%	2 / 2 100.0%	4 / 4 100.0%	1 / 1 100.0%	4 / 4 100.0%	3 / 4 75.0%	2 / 2 100.0%	3 / 3 100.0%	1 / 1 100.0%	3 / 3 100.0%	2 / 2 100.0%	4 / 4 100.0%	4 / 4 100.0%	4 / 4 100.0%	1 / 1 100.0%	8 / 8 100.0%	1 / 1 100.0%	0 / 0 0.0%	0 / 0 0.0%	0 / 0 0.0%	45 / 72 62.5%AB	26 / 72 36.1%ABE	1 / 72 1.4%			
30	Salmonella	2463 / 2472 99.6%	90 / 90 100.0%	89 / 90 98.9%	120 / 120 100.0%	91 / 91 100.0%	172 / 172 100.0%	172 / 172 100.0%	30 / 30 100.0%	80 / 80 100.0%	148 / 148 100.0%	30 / 30 100.0%	149 / 150 99.3%	136 / 140 97.1%	60 / 60 100.0%	113 / 113 100.0%	30 / 30 100.0%	90 / 90 100.0%	59 / 60 98.3%	122 / 122 100.0%	273 / 274 99.6%	120 / 120 100.0%	30 / 30 100.0%	228 / 229 99.6%	30 / 30 100.0%	0 / 0 0.0%	0 / 0 0.0%	1 / 1 100.0%	1433 / 2472 58.0%AB	783 / 2472 31.7%ABE	125 / 2472 5.1%B	122 / 2472 4.9%BE	6 / 2472 0.2%	3 / 2472 0.1%E
29	"Bacillus cholerae-suis" Smith 1894	2380 / 2389 99.6%	87 / 87 100.0%	86 / 87 98.9%	116 / 116 100.0%	88 / 88 100.0%	167 / 167 100.0%	166 / 166 100.0%	29 / 29 100.0%	77 / 77 100.0%	143 / 143 100.0%	29 / 29 100.0%	144 / 145 99.3%	131 / 135 97.0%	58 / 58 100.0%	109 / 109 100.0%	29 / 29 100.0%	87 / 87 100.0%	57 / 58 98.3%	118 / 118 100.0%	264 / 265 99.6%	116 / 116 100.0%	29 / 29 100.0%	220 / 221 99.5%	29 / 29 100.0%	0 / 0 0.0%	0 / 0 0.0%	1 / 1 100.0%	1381 / 2389 57.8%AB	752 / 2389 31.5%ABE	125 / 2389 5.2%B	122 / 2389 5.1%BE	6 / 2389 0.3%	3 / 2389 0.1%E
29	Salmonella cholerae-suis subsp. cholerae-suis	2380 / 2389 99.6%	87 / 87 100.0%	86 / 87 98.9%	116 / 116 100.0%	88 / 88 100.0%	167 / 167 100.0%	166 / 166 100.0%	29 / 29 100.0%	77 / 77 100.0%	143 / 143 100.0%	29 / 29 100.0%	144 / 145 99.3%	131 / 135 97.0%	58 / 58 100.0%	109 / 109 100.0%	29 / 29 100.0%	87 / 87 100.0%	57 / 58 98.3%	118 / 118 100.0%	264 / 265 99.6%	116 / 116 100.0%	29 / 29 100.0%	220 / 221 99.5%	29 / 29 100.0%	0 / 0 0.0%	0 / 0 0.0%	1 / 1 100.0%	1381 / 2389 57.8%AB	752 / 2389 31.5%ABE	125 / 2389 5.2%B	122 / 2389 5.1%BE	6 / 2389 0.3%	3 / 2389 0.1%E
1	Salmonella enterica serovar Gallinarum	74 / 75 98.7%	3 / 3 100.0%	3 / 3 100.0%	4 / 4 100.0%	3 / 3 100.0%	6 / 6 100.0%	5 / 5 100.0%	1 / 1 100.0%	2 / 2 100.0%	4 / 4 100.0%	1 / 1 100.0%	5 / 5 100.0%	3 / 3 100.0%	2 / 2 100.0%	3 / 3 100.0%	1 / 1 100.0%	3 / 3 100.0%	1 / 2 50.0%	4 / 4 100.0%	8 / 8 100.0%	4 / 4 100.0%	1 / 1 100.0%	6 / 6 100.0%	1 / 1 100.0%	0 / 0 0.0%	0 / 0 0.0%	0 / 0 0.0%	47 / 75 62.7%AB	27 / 75 36.0%ABE	1 / 75 1.3%			
1	Salmonella enterica subsp. enterica serovar Gallinarum str. 287/91	74 / 75 98.7%	3 / 3 100.0%	3 / 3 100.0%	4 / 4 100.0%	3 / 3 100.0%	6 / 6 100.0%	5 / 5 100.0%	1 / 1 100.0%	2 / 2 100.0%	4 / 4 100.0%	1 / 1 100.0%	5 / 5 100.0%	3 / 3 100.0%	2 / 2 100.0%	3 / 3 100.0%	1 / 1 100.0%	3 / 3 100.0%	1 / 2 50.0%	4 / 4 100.0%	8 / 8 100.0%	4 / 4 100.0%	1 / 1 100.0%	6 / 6 100.0%	1 / 1 100.0%	0 / 0 0.0%	0 / 0 0.0%	0 / 0 0.0%	47 / 75 62.7%AB	27 / 75 36.0%ABE	1 / 75 1.3%			
1	Salmonella enterica subsp. enterica serovar Gallinarum/pullorum	73 / 73 100.0%	3 / 3 100.0%	3 / 3 100.0%	4 / 4 100.0%	3 / 3 100.0%	6 / 6 100.0%	4 / 4 100.0%	1 / 1 100.0%	2 / 2 100.0%	4 / 4 100.0%	1 / 1 100.0%	5 / 5 100.0%	2 / 2 100.0%	2 / 2 100.0%	2 / 2 100.0%	1 / 1 100.0%	3 / 3 100.0%	2 / 2 100.0%	4 / 4 100.0%	8 / 8 100.0%	4 / 4 100.0%	1 / 1 100.0%	6 / 6 100.0%	1 / 1 100.0%	0 / 0 0.0%	0 / 0 0.0%	1 / 1 100.0%	46 / 73 63.0%AB	27 / 73 37.0%ABE				
3	Salmonella enterica subsp. enterica serovar Heidelberg	250 / 250 100.0%	9 / 9 100.0%	9 / 9 100.0%	12 / 12 100.0%	9 / 9 100.0%	18 / 18 100.0%	18 / 18 100.0%	3 / 3 100.0%	9 / 9 100.0%	15 / 15 100.0%	3 / 3 100.0%	15 / 15 100.0%	15 / 15 100.0%	6 / 6 100.0%	12 / 12 100.0%	3 / 3 100.0%	9 / 9 100.0%	6 / 6 100.0%	12 / 12 100.0%	27 / 27 100.0%	12 / 12 100.0%	3 / 3 100.0%	22 / 22 100.0%	3 / 3 100.0%	0 / 0 0.0%	0 / 0 0.0%	0 / 0 0.0%	144 / 250 57.6%AB	72 / 250 28.8%ABE	19 / 250 7.6%BE	15 / 250 6.0%B		
1	Salmonella enterica subsp. enterica serovar Heidelberg str. SL476	84 / 84 100.0%	3 / 3 100.0%	3 / 3 100.0%	4 / 4 100.0%	3 / 3 100.0%	6 / 6 100.0%	6 / 6 100.0%	1 / 1 100.0%	3 / 3 100.0%	5 / 5 100.0%	1 / 1 100.0%	5 / 5 100.0%	5 / 5 100.0%	2 / 2 100.0%	4 / 4 100.0%	1 / 1 100.0%	3 / 3 100.0%	2 / 2 100.0%	4 / 4 100.0%	9 / 9 100.0%	4 / 4 100.0%	1 / 1 100.0%	8 / 8 100.0%	1 / 1 100.0%	0 / 0 0.0%	0 / 0 0.0%	0 / 0 0.0%	38 / 84 45.2%AB	19 / 84 22.6%BE	15 / 84 17.9%B	12 / 84 14.3%ABE		
1	Salmonella enterica subsp. enterica serovar Heidelberg str. 41578	84 / 84 100.0%	3 / 3 100.0%	3 / 3 100.0%	4 / 4 100.0%	3 / 3 100.0%	6 / 6 100.0%	6 / 6 100.0%	1 / 1 100.0%	3 / 3 100.0%	5 / 5 100.0%	1 / 1 100.0%	5 / 5 100.0%	5 / 5 100.0%	2 / 2 100.0%	4 / 4 100.0%	1 / 1 100.0%	3 / 3 100.0%	2 / 2 100.0%	4 / 4 100.0%	9 / 9 100.0%	4 / 4 100.0%	1 / 1 100.0%	8 / 8 100.0%	1 / 1 100.0%	0 / 0 0.0%	0 / 0 0.0%	0 / 0 0.0%	53 / 84 63.1%AB	31 / 84 36.9%ABE				
1	Salmonella enterica subsp. enterica serovar Heidelberg str. B182	82 / 82 100.0%	3 / 3 100.0%	3 / 3 100.0%	4 / 4 100.0%	3 / 3 100.0%	6 / 6 100.0%	6 / 6 100.0%	1 / 1 100.0%	3 / 3 100.0%	5 / 5 100.0%	1 / 1 100.0%	5 / 5 100.0%	5 / 5 100.0%	2 / 2 100.0%	4 / 4 100.0%	1 / 1 100.0%	3 / 3 100.0%	2 / 2 100.0%	4 / 4 100.0%	9 / 9 100.0%	4 / 4 100.0%	1 / 1 100.0%	6 / 6 100.0%	1 / 1 100.0%	0 / 0 0.0%	0 / 0 0.0%	0 / 0 0.0%	53 / 82 64.6%AB	29 / 82 35.4%ABE				
1	"Bacterium paratyphi typhus A" Brion and Kaiser 1902	81 / 81 100.0%	3 / 3 100.0%	3 / 3 100.0%	4 / 4 100.0%	3 / 3 100.0%	5 / 5 100.0%	6 / 6 100.0%	1 / 1 100.0%	3 / 3 100.0%	4 / 4 100.0%	1 / 1 100.0%	5 / 5 100.0%	5 / 5 100.0%	2 / 2 100.0%	4 / 4 100.0%	1 / 1 100.0%	3 / 3 100.0%	2 / 2 100.0%	4 / 4 100.0%	8 / 8 100.0%	4 / 4 100.0%	1 / 1 100.0%	8 / 8 100.0%	1 / 1 100.0%	0 / 0 0.0%	0 / 0 0.0%	0 / 0 0.0%	25 / 81 30.9%B	25 / 81 30.9%AB	18 / 81 22.2%ABE	13 / 81 16.0%BE		
1	Salmonella enterica subsp. enterica serovar Paratyphi A str. AKU_12601	81 / 81 100.0%	3 / 3 100.0%	3 / 3 100.0%	4 / 4 100.0%	3 / 3 100.0%	5 / 5 100.0%	6 / 6 100.0%	1 / 1 100.0%	3 / 3 100.0%	4 / 4 100.0%	1 / 1 100.0%	5 / 5 100.0%	5 / 5 100.0%	2 / 2 100.0%	4 / 4 100.0%	1 / 1 100.0%	3 / 3 100.0%	2 / 2 100.0%	4 / 4 100.0%	8 / 8 100.0%	4 / 4 100.0%	1 / 1 100.0%	8 / 8 100.0%	1 / 1 100.0%	0 / 0 0.0%	0 / 0 0.0%	0 / 0 0.0%	25 / 81 30.9%B	25 / 81 30.9%AB	18 / 81 22.2%ABE	13 / 81 16.0%BE		
1	Salmonella enterica subsp. enterica serovar Paratyphi B	85 / 85 100.0%	3 / 3 100.0%	3 / 3 100.0%	4 / 4 100.0%	4 / 4 100.0%	6 / 6 100.0%	6 / 6 100.0%	1 / 1 100.0%	3 / 3 100.0%	6 / 6 100.0%	1 / 1 100.0%	5 / 5 100.0%	5 / 5 100.0%	2 / 2 100.0%	4 / 4 100.0%	1 / 1 100.0%	3 / 3 100.0%	2 / 2 100.0%	4 / 4 100.0%	9 / 9 100.0%	3 / 3 100.0%	1 / 1 100.0%	8 / 8 100.0%	1 / 1 100.0%	0 / 0 0.0%	0 / 0 0.0%	0 / 0 0.0%	28 / 85 32.9%B	27 / 85 31.8%AB	18 / 85 21.2%ABE	12 / 85 14.1%BE		
1	Salmonella enterica subsp. enterica serovar Paratyphi B str. SPB7	85 / 85 100.0%	3 / 3 100.0%	3 / 3 100.0%	4 / 4 100.0%	4 / 4 100.0%	6 / 6 100.0%	6 / 6 100.0%	1 / 1 100.0%	3 / 3 100.0%	6 / 6 100.0%	1 / 1 100.0%	5 / 5 100.0%	5 / 5 100.0%	2 / 2 100.0%	4 / 4 100.0%	1 / 1 100.0%	3 / 3 100.0%	2 / 2 100.0%	4 / 4 100.0%	9 / 9 100.0%	3 / 3 100.0%	1 / 1 100.0%	8 / 8 100.0%	1 / 1 100.0%	0 / 0 0.0%	0 / 0 0.0%	0 / 0 0.0%	28 / 85 32.9%B	27 / 85 31.8%AB	18 / 85 21.2%ABE	12 / 85 14.1%BE		
1	Salmonella enterica subsp. enterica serovar Paratyphi C	82 / 82 100.0%	3 / 3 100.0%	3 / 3 100.0%	4 / 4 100.0%	3 / 3 100.0%	6 / 6 100.0%	5 / 5 100.0%	1 / 1 100.0%	3 / 3 100.0%	5 / 5 100.0%	1 / 1 100.0%	5 / 5 100.0%	4 / 4 100.0%	2 / 2 100.0%	4 / 4 100.0%	1 / 1 100.0%	3 / 3 100.0%	2 / 2 100.0%	5 / 5 100.0%	8 / 8 100.0%	4 / 4 100.0%	1 / 1 100.0%	8 / 8 100.0%	1 / 1 100.0%	0 / 0 0.0%	0 / 0 0.0%	0 / 0 0.0%	50 / 82 61.0%AB	32 / 82 39.0%ABE				
1	Salmonella enterica subsp. enterica serovar Paratyphi C str. RKS4594	82 / 82 100.0%	3 / 3 100.0%	3 / 3 100.0%	4 / 4 100.0%	3 / 3 100.0%	6 / 6 100.0%	5 / 5 100.0%	1 / 1 100.0%	3 / 3 100.0%	5 / 5 100.0%	1 / 1 100.0%	5 / 5 100.0%	4 / 4 100.0%	2 / 2 100.0%	4 / 4 100.0%	1 / 1 100.0%	3 / 3 100.0%	2 / 2 100.0%	5 / 5 100.0%	8 / 8 100.0%	4 / 4 100.0%	1 / 1 100.0%	8 / 8 100.0%	1 / 1 100.0%	0 / 0 0.0%	0 / 0 0.0%	0 / 0 0.0%	50 / 82 61.0%AB	32 / 82 39.0%ABE				
1	Salmonella agona	84 / 84 100.0%	3 / 3 100.0%	3 / 3 100.0%	4 / 4 100.0%	3 / 3 100.0%	6 / 6 100.0%	6 / 6 100.0%	1 / 1 100.0%	3 / 3 100.0%	5 / 5 100.0%	1 / 1 100.0%	5 / 5 100.0%	5 / 5 100.0%	2 / 2 100.0%	4 / 4 100.0%	1 / 1 100.0%	3 / 3 100.0%	2 / 2 100.0%	4 / 4 100.0%	9 / 9 100.0%	4 / 4 100.0%	1 / 1 100.0%	8 / 8 100.0%	1 / 1 100.0%	0 / 0 0.0%	0 / 0 0.0%	0 / 0 0.0%	39 / 84 46.4%AB	19 / 84 22.6%BE	13 / 84 15.5%B	13 / 84 15.5%ABE		
1	Salmonella enterica subsp. enterica serovar Agona str. SL483	84 / 84 100.0%	3 / 3 100.0%	3 / 3 100.0%	4 / 4 100.0%	3 / 3 100.0%	6 / 6 100.0%	6 / 6 100.0%	1 / 1 100.0%	3 / 3 100.0%	5 / 5 100.0%	1 / 1 100.0%	5 / 5 100.0%	5 / 5 100.0%	2 / 2 100.0%	4 / 4 100.0%	1 / 1 100.0%	3 / 3 100.0%	2 / 2 100.0%	4 / 4 100.0%	9 / 9 100.0%	4 / 4 100.0%	1 / 1 100.0%	8 / 8 100.0%	1 / 1 100.0%	0 / 0 0.0%	0 / 0 0.0%	0 / 0 0.0%	39 / 84 46.4%AB	19 / 84 22.6%BE	13 / 84 15.5%B	13 / 84 15.5%ABE		
1	Salmonella bareilly	84 / 84 100.0%	3 / 3 100.0%	3 / 3 100.0%	4 / 4 100.0%	3 / 3 100.0%	6 / 6 100.0%	6 / 6 100.0%	1 / 1 100.0%	3 / 3 100.0%	5 / 5 100.0%	1 / 1 100.0%	5 / 5 100.0%	5 / 5 100.0%	2 / 2 100.0%	4 / 4 100.0%	1 / 1 100.0%	3 / 3 100.0%	2 / 2 100.0%	4 / 4 100.0%	9 / 9 100.0%	4 / 4 100.0%	1 / 1 100.0%	8 / 8 100.0%	1 / 1 100.0%	0 / 0 0.0%	0 / 0 0.0%	0 / 0 0.0%	53 / 84 63.1%AB	31 / 84 36.9%ABE				
1	Salmonella enterica subsp. enterica serovar Bareilly str. CFSAN000189	84 / 84 100.0%	3 / 3 100.0%	3 / 3 100.0%	4 / 4 100.0%	3 / 3 100.0%	6 / 6 100.0%	6 / 6 100.0%	1 / 1 100.0%	3 / 3 100.0%	5 / 5 100.0%	1 / 1 100.0%	5 / 5 100.0%	5 / 5 100.0%	2 / 2 100.0%	4 / 4 100.0%	1 / 1 100.0%	3 / 3 100.0%	2 / 2 100.0%	4 / 4 100.0%	9 / 9 100.0%	4 / 4 100.0%	1 / 1 100.0%	8 / 8 100.0%	1 / 1 100.0%	0 / 0 0.0%	0 / 0 0.0%	0 / 0 0.0%	53 / 84 63.1%AB	31 / 84 36.9%ABE				
4	"Bacillus typhi" Schroeter 1886	306 / 311 98.4%	12 / 12 100.0%	12 / 12 100.0%	16 / 16 100.0%	13 / 13 100.0%	20 / 20 100.0%	20 / 20 100.0%	4 / 4 100.0%	4 / 4 100.0%	21 / 21 100.0%	4 / 4 100.0%	20 / 20 100.0%	12 / 16 75.0%	8 / 8 100.0%	12 / 12 100.0%	4 / 4 100.0%	12 / 12 100.0%	8 / 8 100.0%	16 / 16 100.0%	36 / 36 100.0%	15 / 15 100.0%	4 / 4 100.0%	29 / 30 96.7%	4 / 4 100.0%	0 / 0 0.0%	0 / 0 0.0%	0 / 0 0.0%	197 / 311 63.3%AB	107 / 311 34.4%ABE	4 / 311 1.3%	2 / 311 0.6%BE	1 / 311 0.3%E	
1	Salmonella enterica subsp. enterica serovar Typhi Ty2	77 / 78 98.7%	3 / 3 100.0%	3 / 3 100.0%	4 / 4 100.0%	3 / 3 100.0%	5 / 5 100.0%	5 / 5 100.0%	1 / 1 100.0%	1 / 1 100.0%	5 / 5 100.0%	1 / 1 100.0%	5 / 5 100.0%	3 / 4 75.0%	2 / 2 100.0%	3 / 3 100.0%	1 / 1 100.0%	3 / 3 100.0%	2 / 2 100.0%	4 / 4 100.0%	9 / 9 100.0%	4 / 4 100.0%	1 / 1 100.0%	8 / 8 100.0%	1 / 1 100.0%	0 / 0 0.0%	0 / 0 0.0%	0 / 0 0.0%	49 / 78 62.8%AB	27 / 78 34.6%ABE	1 / 78 1.3%BE	1 / 78 1.3%		
1	Salmonella enterica subsp. enterica serovar Typhi str. Ty21a	77 / 78 98.7%	3 / 3 100.0%	3 / 3 100.0%	4 / 4 100.0%	3 / 3 100.0%	5 / 5 100.0%	5 / 5 100.0%	1 / 1 100.0%	1 / 1 100.0%	5 / 5 100.0%	1 / 1 100.0%	5 / 5 100.0%	3 / 4 75.0%	2 / 2 100.0%	3 / 3 100.0%	1 / 1 100.0%	3 / 3 100.0%	2 / 2 100.0%	4 / 4 100.0%	9 / 9 100.0%	4 / 4 100.0%	1 / 1 100.0%	8 / 8 100.0%	1 / 1 100.0%	0 / 0 0.0%	0 / 0 0.0%	0 / 0 0.0%	48 / 78 61.5%AB	29 / 78 37.2%ABE	1 / 78 1.3%			
1	Salmonella enterica subsp. enterica serovar Typhi str. P-stx-12	75 / 77 97.4%	3 / 3 100.0%	3 / 3 100.0%	4 / 4 100.0%	4 / 4 100.0%	5 / 5 100.0%	5 / 5 100.0%	1 / 1 100.0%	1 / 1 100.0%	6 / 6 100.0%	1 / 1 100.0%	5 / 5 100.0%	3 / 4 75.0%	2 / 2 100.0%	3 / 3 100.0%	1 / 1 100.0%	3 / 3 100.0%	2 / 2 100.0%	4 / 4 100.0%	9 / 9 100.0%	3 / 3 100.0%	1 / 1 100.0%	5 / 6 83.3%	1 / 1 100.0%	0 / 0 0.0%	0 / 0 0.0%	0 / 0 0.0%	51 / 77 66.2%AB	24 / 77 31.2%ABE	1 / 77 1.3%E	1 / 77 1.3%		
9	"Bacillus typhimurium" Loeffler 1892	760 / 761 99.9%	27 / 27 100.0%	26 / 27 96.3%	37 / 37 100.0%	27 / 27 100.0%	53 / 53 100.0%	54 / 54 100.0%	9 / 9 100.0%	27 / 27 100.0%	45 / 45 100.0%	9 / 9 100.0%	45 / 45 100.0%	45 / 45 100.0%	18 / 18 100.0%	36 / 36 100.0%	9 / 9 100.0%	27 / 27 100.0%	18 / 18 100.0%	37 / 37 100.0%	88 / 88 100.0%	36 / 36 100.0%	9 / 9 100.0%	69 / 69 100.0%	9 / 9 100.0%	0 / 0 0.0%	0 / 0 0.0%	0 / 0 0.0%	483 / 761 63.5%AB	276 / 761 36.3%ABE	1 / 761 0.1%BE	1 / 761 0.1%		
2	Salmonella enterica subsp. enterica serovar Typhimurium LT2	170 / 170 100.0%	6 / 6 100.0%	6 / 6 100.0%	8 / 8 100.0%	6 / 6 100.0%	12 / 12 100.0%	12 / 12 100.0%	2 / 2 100.0%	6 / 6 100.0%	10 / 10 100.0%	2 / 2 100.0%	10 / 10 100.0%	10 / 10 100.0%	4 / 4 100.0%	8 / 8 100.0%	2 / 2 100.0%	6 / 6 100.0%	4 / 4 100.0%	8 / 8 100.0%	20 / 20 100.0%	8 / 8 100.0%	2 / 2 100.0%	16 / 16 100.0%	2 / 2 100.0%	0 / 0 0.0%	0 / 0 0.0%	0 / 0 0.0%	108 / 170 63.5%AB	61 / 170 35.9%ABE	1 / 170 0.6%BE			
1	Salmonella enterica subsp. enterica serovar Typhimurium str. 14028S	85 / 85 100.0%	3 / 3 100.0%	3 / 3 100.0%	4 / 4 100.0%	3 / 3 100.0%	6 / 6 100.0%	6 / 6 100.0%	1 / 1 100.0%	3 / 3 100.0%	5 / 5 100.0%	1 / 1 100.0%	5 / 5 100.0%	5 / 5 100.0%	2 / 2 100.0%	4 / 4 100.0%	1 / 1 100.0%	3 / 3 100.0%	2 / 2 100.0%	4 / 4 100.0%	10 / 10 100.0%	4 / 4 100.0%	1 / 1 100.0%	8 / 8 100.0%	1 / 1 100.0%	0 / 0 0.0%	0 / 0 0.0%	0 / 0 0.0%	54 / 85 63.5%AB	31 / 85 36.5%ABE				
1	Salmonella enterica subsp. enterica serovar Typhimurium SL1344	85 / 85 100.0%	3 / 3 100.0%	3 / 3 100.0%	4 / 4 100.0%	3 / 3 100.0%	6 / 6 100.0%	6 / 6 100.0%	1 / 1 100.0%	3 / 3 100.0%	5 / 5 100.0%	1 / 1 100.0%	5 / 5 100.0%	5 / 5 100.0%	2 / 2 100.0%	4 / 4 100.0%	1 / 1 100.0%	3 / 3 100.0%	2 / 2 100.0%	4 / 4 100.0%	10 / 10 100.0%	4 / 4 100.0%	1 / 1 100.0%	8 / 8 100.0%	1 / 1 100.0%	0 / 0 0.0%	0 / 0 0.0%	0 / 0 0.0%	54 / 85 63.5%AB	31 / 85 36.5%ABE				
1	Salmonella enterica subsp. enterica serovar Typhimurium str. D23580	86 / 87 98.9%	3 / 3 100.0%	2 / 3 66.7%	5 / 5 100.0%	3 / 3 100.0%	6 / 6 100.0%	6 / 6 100.0%	1 / 1 100.0%	3 / 3 100.0%	5 / 5 100.0%	1 / 1 100.0%	5 / 5 100.0%	5 / 5 100.0%	2 / 2 100.0%	4 / 4 100.0%	1 / 1 100.0%	3 / 3 100.0%	2 / 2 100.0%	5 / 5 100.0%	10 / 10 100.0%	4 / 4 100.0%	1 / 1 100.0%	8 / 8 100.0%	1 / 1 100.0%	0 / 0 0.0%	0 / 0 0.0%	0 / 0 0.0%	54 / 87 62.1%AB	32 / 87 36.8%ABE	1 / 87 1.1%			
1	Salmonella enterica subsp. enterica serovar Typhimurium str. 4/74	85 / 85 100.0%	3 / 3 100.0%	3 / 3 100.0%	4 / 4 100.0%	3 / 3 100.0%	6 / 6 100.0%	6 / 6 100.0%	1 / 1 100.0%	3 / 3 100.0%	5 / 5 100.0%	1 / 1 100.0%	5 / 5 100.0%	5 / 5 100.0%	2 / 2 100.0%	4 / 4 100.0%	1 / 1 100.0%	3 / 3 100.0%	2 / 2 100.0%	4 / 4 100.0%	10 / 10 100.0%	4 / 4 100.0%	1 / 1 100.0%	8 / 8 100.0%	1 / 1 100.0%	0 / 0 0.0%	0 / 0 0.0%	0 / 0 0.0%	54 / 85 63.5%AB	31 / 85 36.5%ABE				
1	Salmonella enterica subsp. enterica serovar Typhimurium str. ATCC 68169	85 / 85 100.0%	3 / 3 100.0%	3 / 3 100.0%	4 / 4 100.0%	3 / 3 100.0%	6 / 6 100.0%	6 / 6 100.0%	1 / 1 100.0%	3 / 3 100.0%	5 / 5 100.0%	1 / 1 100.0%	5 / 5 100.0%	5 / 5 100.0%	2 / 2 100.0%	4 / 4 100.0%	1 / 1 100.0%	3 / 3 100.0%	2 / 2 100.0%	4 / 4 100.0%	10 / 10 100.0%	4 / 4 100.0%	1 / 1 100.0%	8 / 8 100.0%	1 / 1 100.0%	0 / 0 0.0%	0 / 0 0.0%	0 / 0 0.0%	54 / 85 63.5%AB	31 / 85 36.5%ABE				
1	Salmonella enterica subsp. enterica serovar Typhimurium str. 798	82 / 82 100.0%	3 / 3 100.0%	3 / 3 100.0%	4 / 4 100.0%	3 / 3 100.0%	5 / 5 100.0%	6 / 6 100.0%	1 / 1 100.0%	3 / 3 100.0%	5 / 5 100.0%	1 / 1 100.0%	5 / 5 100.0%	5 / 5 100.0%	2 / 2 100.0%	4 / 4 100.0%	1 / 1 100.0%	3 / 3 100.0%	2 / 2 100.0%	4 / 4 100.0%	10 / 10 100.0%	4 / 4 100.0%	1 / 1 100.0%	6 / 6 100.0%	1 / 1 100.0%	0 / 0 0.0%	0 / 0 0.0%	0 / 0 0.0%	53 / 82 64.6%AB	29 / 82 35.4%ABE				
1	Salmonella enterica subsp. enterica serovar Typhimurium str. U288	84 / 84 100.0%	3 / 3 100.0%	3 / 3 100.0%	4 / 4 100.0%	3 / 3 100.0%	6 / 6 100.0%	6 / 6 100.0%	1 / 1 100.0%	3 / 3 100.0%	5 / 5 100.0%	1 / 1 100.0%	5 / 5 100.0%	5 / 5 100.0%	2 / 2 100.0%	4 / 4 100.0%	1 / 1 100.0%	3 / 3 100.0%	2 / 2 100.0%	4 / 4 100.0%	9 / 9 100.0%	4 / 4 100.0%	1 / 1 100.0%	8 / 8 100.0%	1 / 1 100.0%	0 / 0 0.0%	0 / 0 0.0%	0 / 0 0.0%	53 / 84 63.1%AB	31 / 84 36.9%ABE				
1	Salmonella enterica subsp. enterica serovar Typhimurium var. 5- str. CFSAN001921	83 / 83 100.0%	3 / 3 100.0%	3 / 3 100.0%	4 / 4 100.0%	3 / 3 100.0%	6 / 6 100.0%	6 / 6 100.0%	1 / 1 100.0%	3 / 3 100.0%	5 / 5 100.0%	1 / 1 100.0%	5 / 5 100.0%	5 / 5 100.0%	2 / 2 100.0%	4 / 4 100.0%	1 / 1 100.0%	3 / 3 100.0%	2 / 2 100.0%	4 / 4 100.0%	9 / 9 100.0%	4 / 4 100.0%	1 / 1 100.0%	7 / 7 100.0%	1 / 1 100.0%	0 / 0 0.0%	0 / 0 0.0%	0 / 0 0.0%	53 / 83 63.9%AB	30 / 83 36.1%ABE				
1	Salmonella dublin	81 / 82 98.8%	3 / 3 100.0%	3 / 3 100.0%	3 / 3 100.0%	3 / 3 100.0%	6 / 6 100.0%	6 / 6 100.0%	1 / 1 100.0%	3 / 3 100.0%	5 / 5 100.0%	1 / 1 100.0%	4 / 5 80.0%	4 / 4 100.0%	2 / 2 100.0%	4 / 4 100.0%	1 / 1 100.0%	3 / 3 100.0%	2 / 2 100.0%	4 / 4 100.0%	9 / 9 100.0%	4 / 4 100.0%	1 / 1 100.0%	8 / 8 100.0%	1 / 1 100.0%	0 / 0 0.0%	0 / 0 0.0%	0 / 0 0.0%	38 / 82 46.3%AB	18 / 82 22.0%BE	13 / 82 15.9%B	12 / 82 14.6%ABE	1 / 82 1.2%E	

1	Salmonella enterica subsp. enterica serovar Dublin CT_02021853	81 / 82 98.8%	3 / 3 100.0%	3 / 3 100.0%	3 / 3 100.0%	3 / 3 100.0%	6 / 6 100.0%	6 / 6 100.0%	1 / 1 100.0%	3 / 3 100.0%	5 / 5 100.0%	1 / 1 100.0%	4 / 5 80.0%	4 / 4 100.0%	2 / 2 100.0%	4 / 4 100.0%	1 / 1 100.0%	3 / 3 100.0%	2 / 2 100.0%	4 / 4 100.0%	9 / 9 100.0%	4 / 4 100.0%	1 / 1 100.0%	8 / 8 100.0%	1 / 1 100.0%	0 / 0 0.0%	0 / 0 0.0%	0 / 0 0.0%	38 / 82 46.3%AB	18 / 82 22.0%BE	13 / 82 15.9%B	12 / 82 14.6%ABE	1 / 82 1.2%E		
1	Salmonella enterica serovar Newport	84 / 85 98.8%	3 / 3 100.0%	3 / 3 100.0%	4 / 4 100.0%	2 / 2 100.0%	6 / 6 100.0%	6 / 6 100.0%	1 / 1 100.0%	3 / 3 100.0%	4 / 4 100.0%	1 / 1 100.0%	5 / 5 100.0%	6 / 6 100.0%	2 / 2 100.0%	4 / 4 100.0%	1 / 1 100.0%	3 / 3 100.0%	2 / 2 100.0%	4 / 4 100.0%	9 / 10 90.0%	5 / 5 100.0%	1 / 1 100.0%	8 / 8 100.0%	1 / 1 100.0%	0 / 0 0.0%	0 / 0 0.0%	0 / 0 0.0%	36 / 85 42.4%AB	19 / 85 22.4%BE	16 / 85 18.8%B	13 / 85 15.3%ABE	1 / 85 1.2%E		
1	Salmonella enterica subsp. enterica serovar Newport SL254	84 / 85 98.8%	3 / 3 100.0%	3 / 3 100.0%	4 / 4 100.0%	2 / 2 100.0%	6 / 6 100.0%	6 / 6 100.0%	1 / 1 100.0%	3 / 3 100.0%	4 / 4 100.0%	1 / 1 100.0%	5 / 5 100.0%	6 / 6 100.0%	2 / 2 100.0%	4 / 4 100.0%	1 / 1 100.0%	3 / 3 100.0%	2 / 2 100.0%	4 / 4 100.0%	9 / 10 90.0%	5 / 5 100.0%	1 / 1 100.0%	8 / 8 100.0%	1 / 1 100.0%	0 / 0 0.0%	0 / 0 0.0%	0 / 0 0.0%	36 / 85 42.4%AB	19 / 85 22.4%BE	16 / 85 18.8%B	13 / 85 15.3%ABE	1 / 85 1.2%E		
1	"Bacillus enteritidis" Gaertner 1888	83 / 83 100.0%	3 / 3 100.0%	3 / 3 100.0%	4 / 4 100.0%	3 / 3 100.0%	6 / 6 100.0%	6 / 6 100.0%	1 / 1 100.0%	3 / 3 100.0%	5 / 5 100.0%	1 / 1 100.0%	5 / 5 100.0%	5 / 5 100.0%	2 / 2 100.0%	4 / 4 100.0%	1 / 1 100.0%	3 / 3 100.0%	2 / 2 100.0%	4 / 4 100.0%	8 / 8 100.0%	4 / 4 100.0%	1 / 1 100.0%	8 / 8 100.0%	1 / 1 100.0%	0 / 0 0.0%	0 / 0 0.0%	0 / 0 0.0%	52 / 83 62.7%AB	31 / 83 37.3%ABE					
1	Salmonella enterica subsp. enterica serovar Enteritidis str. P125109	83 / 83 100.0%	3 / 3 100.0%	3 / 3 100.0%	4 / 4 100.0%	3 / 3 100.0%	6 / 6 100.0%	6 / 6 100.0%	1 / 1 100.0%	3 / 3 100.0%	5 / 5 100.0%	1 / 1 100.0%	5 / 5 100.0%	5 / 5 100.0%	2 / 2 100.0%	4 / 4 100.0%	1 / 1 100.0%	3 / 3 100.0%	2 / 2 100.0%	4 / 4 100.0%	8 / 8 100.0%	4 / 4 100.0%	1 / 1 100.0%	8 / 8 100.0%	1 / 1 100.0%	0 / 0 0.0%	0 / 0 0.0%	0 / 0 0.0%	52 / 83 62.7%AB	31 / 83 37.3%ABE					
1	Salmonella enterica subsp. enterica serovar Schwarzengrund	83 / 83 100.0%	3 / 3 100.0%	3 / 3 100.0%	4 / 4 100.0%	3 / 3 100.0%	5 / 5 100.0%	6 / 6 100.0%	1 / 1 100.0%	3 / 3 100.0%	5 / 5 100.0%	1 / 1 100.0%	5 / 5 100.0%	5 / 5 100.0%	2 / 2 100.0%	4 / 4 100.0%	1 / 1 100.0%	3 / 3 100.0%	2 / 2 100.0%	4 / 4 100.0%	9 / 9 100.0%	4 / 4 100.0%	1 / 1 100.0%	8 / 8 100.0%	1 / 1 100.0%	0 / 0 0.0%	0 / 0 0.0%	0 / 0 0.0%	37 / 83 44.6%AB	19 / 83 22.9%BE	15 / 83 18.1%B	12 / 83 14.5%ABE			
1	Salmonella enterica subsp. enterica serovar Schwarzengrund CVM19633	83 / 83 100.0%	3 / 3 100.0%	3 / 3 100.0%	4 / 4 100.0%	3 / 3 100.0%	5 / 5 100.0%	6 / 6 100.0%	1 / 1 100.0%	3 / 3 100.0%	5 / 5 100.0%	1 / 1 100.0%	5 / 5 100.0%	5 / 5 100.0%	2 / 2 100.0%	4 / 4 100.0%	1 / 1 100.0%	3 / 3 100.0%	2 / 2 100.0%	4 / 4 100.0%	9 / 9 100.0%	4 / 4 100.0%	1 / 1 100.0%	8 / 8 100.0%	1 / 1 100.0%	0 / 0 0.0%	0 / 0 0.0%	0 / 0 0.0%	37 / 83 44.6%AB	19 / 83 22.9%BE	15 / 83 18.1%B	12 / 83 14.5%ABE			
1	Salmonella enterica subsp. enterica serovar Javiana	84 / 84 100.0%	3 / 3 100.0%	3 / 3 100.0%	4 / 4 100.0%	3 / 3 100.0%	6 / 6 100.0%	6 / 6 100.0%	1 / 1 100.0%	3 / 3 100.0%	5 / 5 100.0%	1 / 1 100.0%	5 / 5 100.0%	5 / 5 100.0%	2 / 2 100.0%	4 / 4 100.0%	1 / 1 100.0%	3 / 3 100.0%	2 / 2 100.0%	4 / 4 100.0%	9 / 9 100.0%	4 / 4 100.0%	1 / 1 100.0%	8 / 8 100.0%	1 / 1 100.0%	0 / 0 0.0%	0 / 0 0.0%	0 / 0 0.0%	53 / 84 63.1%AB	31 / 84 36.9%ABE					
1	Salmonella enterica subsp. enterica serovar Javiana str. AZ__PI1230515	84 / 84 100.0%	3 / 3 100.0%	3 / 3 100.0%	4 / 4 100.0%	3 / 3 100.0%	6 / 6 100.0%	6 / 6 100.0%	1 / 1 100.0%	3 / 3 100.0%	5 / 5 100.0%	1 / 1 100.0%	5 / 5 100.0%	5 / 5 100.0%	2 / 2 100.0%	4 / 4 100.0%	1 / 1 100.0%	3 / 3 100.0%	2 / 2 100.0%	4 / 4 100.0%	9 / 9 100.0%	4 / 4 100.0%	1 / 1 100.0%	8 / 8 100.0%	1 / 1 100.0%	0 / 0 0.0%	0 / 0 0.0%	0 / 0 0.0%	53 / 84 63.1%AB	31 / 84 36.9%ABE					
1	Salmonella enterica subsp. enterica serovar 1,4,(5),12:i:-	86 / 86 100.0%	3 / 3 100.0%	3 / 3 100.0%	4 / 4 100.0%	3 / 3 100.0%	6 / 6 100.0%	6 / 6 100.0%	1 / 1 100.0%	3 / 3 100.0%	5 / 5 100.0%	1 / 1 100.0%	5 / 5 100.0%	5 / 5 100.0%	2 / 2 100.0%	4 / 4 100.0%	1 / 1 100.0%	3 / 3 100.0%	2 / 2 100.0%	4 / 4 100.0%	10 / 10 100.0%	5 / 5 100.0%	1 / 1 100.0%	8 / 8 100.0%	1 / 1 100.0%	0 / 0 0.0%	0 / 0 0.0%	0 / 0 0.0%	54 / 86 62.8%AB	32 / 86 37.2%ABE					
1	Salmonella enterica subsp. enterica serovar 4,[5],12:i:- str. 08-1736	86 / 86 100.0%	3 / 3 100.0%	3 / 3 100.0%	4 / 4 100.0%	3 / 3 100.0%	6 / 6 100.0%	6 / 6 100.0%	1 / 1 100.0%	3 / 3 100.0%	5 / 5 100.0%	1 / 1 100.0%	5 / 5 100.0%	5 / 5 100.0%	2 / 2 100.0%	4 / 4 100.0%	1 / 1 100.0%	3 / 3 100.0%	2 / 2 100.0%	4 / 4 100.0%	10 / 10 100.0%	5 / 5 100.0%	1 / 1 100.0%	8 / 8 100.0%	1 / 1 100.0%	0 / 0 0.0%	0 / 0 0.0%	0 / 0 0.0%	54 / 86 62.8%AB	32 / 86 37.2%ABE					
1	ATCC 43975	83 / 83 100.0%	3 / 3 100.0%	3 / 3 100.0%	4 / 4 100.0%	3 / 3 100.0%	5 / 5 100.0%	6 / 6 100.0%	1 / 1 100.0%	3 / 3 100.0%	5 / 5 100.0%	1 / 1 100.0%	5 / 5 100.0%	5 / 5 100.0%	2 / 2 100.0%	4 / 4 100.0%	1 / 1 100.0%	3 / 3 100.0%	2 / 2 100.0%	4 / 4 100.0%	9 / 9 100.0%	4 / 4 100.0%	1 / 1 100.0%	8 / 8 100.0%	1 / 1 100.0%	0 / 0 0.0%	0 / 0 0.0%	0 / 0 0.0%	52 / 83 62.7%AB	31 / 83 37.3%ABE					
1	Salmonella bongori 12149	83 / 83 100.0%	3 / 3 100.0%	3 / 3 100.0%	4 / 4 100.0%	3 / 3 100.0%	5 / 5 100.0%	6 / 6 100.0%	1 / 1 100.0%	3 / 3 100.0%	5 / 5 100.0%	1 / 1 100.0%	5 / 5 100.0%	5 / 5 100.0%	2 / 2 100.0%	4 / 4 100.0%	1 / 1 100.0%	3 / 3 100.0%	2 / 2 100.0%	4 / 4 100.0%	9 / 9 100.0%	4 / 4 100.0%	1 / 1 100.0%	8 / 8 100.0%	1 / 1 100.0%	0 / 0 0.0%	0 / 0 0.0%	0 / 0 0.0%	52 / 83 62.7%AB	31 / 83 37.3%ABE					
10	Serratia	756 / 774 97.7%	36 / 36 100.0%	27 / 28 96.4%	37 / 37 100.0%	28 / 28 100.0%	55 / 55 100.0%	61 / 62 98.4%	10 / 10 100.0%	26 / 26 100.0%	47 / 47 100.0%	10 / 10 100.0%	53 / 54 98.1%	49 / 49 100.0%	19 / 19 100.0%	26 / 28 92.9%	9 / 10 90.0%	27 / 28 96.4%	18 / 19 94.7%	37 / 38 97.4%	55 / 58 94.8%	35 / 36 97.2%	9 / 10 90.0%	73 / 77 94.8%	9 / 9 100.0%	0 / 0 0.0%	0 / 0 0.0%	0 / 0 0.0%	465 / 774 60.1%AB	261 / 774 33.7%ABE	16 / 774 2.1%B	14 / 774 1.8%BE	11 / 774 1.4%E	7 / 774 0.9%	
1	"Aerobacter liquefaciens" Grimes and Hennerty 1931	77 / 78 98.7%	4 / 4 100.0%	3 / 3 100.0%	4 / 4 100.0%	2 / 2 100.0%	5 / 5 100.0%	7 / 7 100.0%	1 / 1 100.0%	2 / 2 100.0%	4 / 4 100.0%	1 / 1 100.0%	6 / 6 100.0%	5 / 5 100.0%	2 / 2 100.0%	3 / 3 100.0%	1 / 1 100.0%	3 / 3 100.0%	2 / 2 100.0%	2 / 2 100.0%	6 / 6 100.0%	4 / 5 80.0%	1 / 1 100.0%	8 / 8 100.0%	1 / 1 100.0%	0 / 0 0.0%	0 / 0 0.0%	0 / 0 0.0%	48 / 78 61.5%AB	29 / 78 37.2%ABE	1 / 78 1.3%E				
1	Serratia liquefaciens ATCC 27592	77 / 78 98.7%	4 / 4 100.0%	3 / 3 100.0%	4 / 4 100.0%	2 / 2 100.0%	5 / 5 100.0%	7 / 7 100.0%	1 / 1 100.0%	2 / 2 100.0%	4 / 4 100.0%	1 / 1 100.0%	6 / 6 100.0%	5 / 5 100.0%	2 / 2 100.0%	3 / 3 100.0%	1 / 1 100.0%	3 / 3 100.0%	2 / 2 100.0%	2 / 2 100.0%	6 / 6 100.0%	4 / 5 80.0%	1 / 1 100.0%	8 / 8 100.0%	1 / 1 100.0%	0 / 0 0.0%	0 / 0 0.0%	0 / 0 0.0%	48 / 78 61.5%AB	29 / 78 37.2%ABE	1 / 78 1.3%E				
2	"Bacillus marcescens" (Bizio 1823) Trevisan in de Toni and Trevisan 1889	162 / 162 100.0%	8 / 8 100.0%	6 / 6 100.0%	8 / 8 100.0%	6 / 6 100.0%	12 / 12 100.0%	12 / 12 100.0%	2 / 2 100.0%	6 / 6 100.0%	10 / 10 100.0%	2 / 2 100.0%	9 / 9 100.0%	9 / 9 100.0%	4 / 4 100.0%	6 / 6 100.0%	2 / 2 100.0%	6 / 6 100.0%	4 / 4 100.0%	10 / 10 100.0%	12 / 12 100.0%	8 / 8 100.0%	2 / 2 100.0%	16 / 16 100.0%	2 / 2 100.0%	0 / 0 0.0%	0 / 0 0.0%	0 / 0 0.0%	103 / 162 63.6%AB	59 / 162 36.4%ABE					
1	Serratia marcescens WW4	80 / 80 100.0%	4 / 4 100.0%	3 / 3 100.0%	4 / 4 100.0%	3 / 3 100.0%	6 / 6 100.0%	5 / 5 100.0%	1 / 1 100.0%	3 / 3 100.0%	5 / 5 100.0%	1 / 1 100.0%	5 / 5 100.0%	4 / 4 100.0%	2 / 2 100.0%	3 / 3 100.0%	1 / 1 100.0%	3 / 3 100.0%	2 / 2 100.0%	5 / 5 100.0%	6 / 6 100.0%	4 / 4 100.0%	1 / 1 100.0%	8 / 8 100.0%	1 / 1 100.0%	0 / 0 0.0%	0 / 0 0.0%	0 / 0 0.0%	50 / 80 62.5%AB	30 / 80 37.5%ABE					
1	Serratia marcescens FGI94	82 / 82 100.0%	4 / 4 100.0%	3 / 3 100.0%	4 / 4 100.0%	3 / 3 100.0%	6 / 6 100.0%	7 / 7 100.0%	1 / 1 100.0%	3 / 3 100.0%	5 / 5 100.0%	1 / 1 100.0%	4 / 4 100.0%	5 / 5 100.0%	2 / 2 100.0%	3 / 3 100.0%	1 / 1 100.0%	3 / 3 100.0%	2 / 2 100.0%	5 / 5 100.0%	6 / 6 100.0%	4 / 4 100.0%	1 / 1 100.0%	8 / 8 100.0%	1 / 1 100.0%	0 / 0 0.0%	0 / 0 0.0%	0 / 0 0.0%	53 / 82 64.6%AB	29 / 82 35.4%ABE					
1	"Erwinia proteamaculans" (Paine and Stansfield 1919) Dye 1966	83 / 83 100.0%	4 / 4 100.0%	3 / 3 100.0%	4 / 4 100.0%	3 / 3 100.0%	6 / 6 100.0%	7 / 7 100.0%	1 / 1 100.0%	3 / 3 100.0%	5 / 5 100.0%	1 / 1 100.0%	6 / 6 100.0%	5 / 5 100.0%	2 / 2 100.0%	3 / 3 100.0%	1 / 1 100.0%	3 / 3 100.0%	2 / 2 100.0%	4 / 4 100.0%	6 / 6 100.0%	4 / 4 100.0%	1 / 1 100.0%	8 / 8 100.0%	1 / 1 100.0%	0 / 0 0.0%	0 / 0 0.0%	0 / 0 0.0%	36 / 83 43.4%AB	17 / 83 20.5%ABE	16 / 83 19.3%B	14 / 83 16.9%BE			
1	Serratia proteamaculans 568	83 / 83 100.0%	4 / 4 100.0%	3 / 3 100.0%	4 / 4 100.0%	3 / 3 100.0%	6 / 6 100.0%	7 / 7 100.0%	1 / 1 100.0%	3 / 3 100.0%	5 / 5 100.0%	1 / 1 100.0%	6 / 6 100.0%	5 / 5 100.0%	2 / 2 100.0%	3 / 3 100.0%	1 / 1 100.0%	3 / 3 100.0%	2 / 2 100.0%	4 / 4 100.0%	6 / 6 100.0%	4 / 4 100.0%	1 / 1 100.0%	8 / 8 100.0%	1 / 1 100.0%	0 / 0 0.0%	0 / 0 0.0%	0 / 0 0.0%	36 / 83 43.4%AB	17 / 83 20.5%ABE	16 / 83 19.3%B	14 / 83 16.9%BE			
3	"Bacterium plymuthicum" (sic) Lehmann and Neumann 1896	248 / 248 100.0%	10 / 10 100.0%	9 / 9 100.0%	12 / 12 100.0%	9 / 9 100.0%	18 / 18 100.0%	21 / 21 100.0%	3 / 3 100.0%	8 / 8 100.0%	15 / 15 100.0%	3 / 3 100.0%	18 / 18 100.0%	18 / 18 100.0%	6 / 6 100.0%	8 / 8 100.0%	3 / 3 100.0%	9 / 9 100.0%	6 / 6 100.0%	12 / 12 100.0%	18 / 18 100.0%	12 / 12 100.0%	3 / 3 100.0%	24 / 24 100.0%	3 / 3 100.0%	0 / 0 0.0%	0 / 0 0.0%	0 / 0 0.0%	159 / 248 64.1%AB	89 / 248 35.9%ABE					
1	Serratia odorifera 4Rx13	80 / 80 100.0%	2 / 2 100.0%	3 / 3 100.0%	4 / 4 100.0%	3 / 3 100.0%	6 / 6 100.0%	7 / 7 100.0%	1 / 1 100.0%	2 / 2 100.0%	5 / 5 100.0%	1 / 1 100.0%	6 / 6 100.0%	6 / 6 100.0%	2 / 2 100.0%	2 / 2 100.0%	1 / 1 100.0%	3 / 3 100.0%	2 / 2 100.0%	4 / 4 100.0%	6 / 6 100.0%	4 / 4 100.0%	1 / 1 100.0%	8 / 8 100.0%	1 / 1 100.0%	0 / 0 0.0%	0 / 0 0.0%	0 / 0 0.0%	53 / 80 66.2%AB	27 / 80 33.8%ABE					
1	Serratia plymuthica AS9	84 / 84 100.0%	4 / 4 100.0%	3 / 3 100.0%	4 / 4 100.0%	3 / 3 100.0%	6 / 6 100.0%	7 / 7 100.0%	1 / 1 100.0%	3 / 3 100.0%	5 / 5 100.0%	1 / 1 100.0%	6 / 6 100.0%	6 / 6 100.0%	2 / 2 100.0%	3 / 3 100.0%	1 / 1 100.0%	3 / 3 100.0%	2 / 2 100.0%	4 / 4 100.0%	6 / 6 100.0%	4 / 4 100.0%	1 / 1 100.0%	8 / 8 100.0%	1 / 1 100.0%	0 / 0 0.0%	0 / 0 0.0%	0 / 0 0.0%	53 / 84 63.1%AB	31 / 84 36.9%ABE					
1	Serratia plymuthica S13	84 / 84 100.0%	4 / 4 100.0%	3 / 3 100.0%	4 / 4 100.0%	3 / 3 100.0%	6 / 6 100.0%	7 / 7 100.0%	1 / 1 100.0%	3 / 3 100.0%	5 / 5 100.0%	1 / 1 100.0%	6 / 6 100.0%	6 / 6 100.0%	2 / 2 100.0%	3 / 3 100.0%	1 / 1 100.0%	3 / 3 100.0%	2 / 2 100.0%	4 / 4 100.0%	6 / 6 100.0%	4 / 4 100.0%	1 / 1 100.0%	8 / 8 100.0%	1 / 1 100.0%	0 / 0 0.0%	0 / 0 0.0%	0 / 0 0.0%	53 / 84 63.1%AB	31 / 84 36.9%ABE					
1	"Candidatus Serratia symbiotica" Moran et al. 2005	18 / 35 51.4%	2 / 2 100.0%	0 / 1 0.0%	1 / 1 100.0%	1 / 1 100.0%	2 / 2 100.0%	1 / 2 50.0%	1 / 1 100.0%	1 / 1 100.0%	2 / 2 100.0%	1 / 1 100.0%	2 / 3 66.7%	0 / 0 0.0%	1 / 1 100.0%	0 / 2 0.0%	0 / 1 0.0%	0 / 1 0.0%	0 / 1 0.0%	1 / 2 50.0%	1 / 4 25.0%	0 / 0 0.0%	0 / 1 0.0%	1 / 5 20.0%	0 / 0 0.0%	0 / 0 0.0%	0 / 0 0.0%	0 / 0 0.0%	12 / 35 34.3%AB	10 / 35 28.6%E	7 / 35 20.0%	6 / 35 17.1%ABE			
1	Candidatus Serratia symbiotica Cinara cedri	18 / 35 51.4%	2 / 2 100.0%	0 / 1 0.0%	1 / 1 100.0%	1 / 1 100.0%	2 / 2 100.0%	1 / 2 50.0%	1 / 1 100.0%	1 / 1 100.0%	2 / 2 100.0%	1 / 1 100.0%	2 / 3 66.7%	0 / 0 0.0%	1 / 1 100.0%	0 / 2 0.0%	0 / 1 0.0%	0 / 1 0.0%	0 / 1 0.0%	1 / 2 50.0%	1 / 4 25.0%	0 / 0 0.0%	0 / 1 0.0%	1 / 5 20.0%	0 / 0 0.0%	0 / 0 0.0%	0 / 0 0.0%	0 / 0 0.0%	12 / 35 34.3%AB	10 / 35 28.6%E	7 / 35 20.0%	6 / 35 17.1%ABE			
1	Serratia sp. AS12	84 / 84 100.0%	4 / 4 100.0%	3 / 3 100.0%	4 / 4 100.0%	3 / 3 100.0%	6 / 6 100.0%	7 / 7 100.0%	1 / 1 100.0%	3 / 3 100.0%	5 / 5 100.0%	1 / 1 100.0%	6 / 6 100.0%	6 / 6 100.0%	2 / 2 100.0%	3 / 3 100.0%	1 / 1 100.0%	3 / 3 100.0%	2 / 2 100.0%	4 / 4 100.0%	6 / 6 100.0%	4 / 4 100.0%	1 / 1 100.0%	8 / 8 100.0%	1 / 1 100.0%	0 / 0 0.0%	0 / 0 0.0%	0 / 0 0.0%	53 / 84 63.1%AB	31 / 84 36.9%ABE					
1	Serratia sp. AS13	84 / 84 100.0%	4 / 4 100.0%	3 / 3 100.0%	4 / 4 100.0%	4 / 4 100.0%	6 / 6 100.0%	6 / 6 100.0%	1 / 1 100.0%	3 / 3 100.0%	6 / 6 100.0%	1 / 1 100.0%	6 / 6 100.0%	6 / 6 100.0%	2 / 2 100.0%	3 / 3 100.0%	1 / 1 100.0%	3 / 3 100.0%	2 / 2 100.0%	4 / 4 100.0%	6 / 6 100.0%	3 / 3 100.0%	1 / 1 100.0%	8 / 8 100.0%	1 / 1 100.0%	0 / 0 0.0%	0 / 0 0.0%	0 / 0 0.0%	54 / 84 64.3%AB	30 / 84 35.7%ABE					
9	Shigella	845 / 850 99.4%	31 / 31 100.0%	27 / 27 100.0%	35 / 35 100.0%	24 / 24 100.0%	80 / 80 100.0%	49 / 49 100.0%	9 / 9 100.0%	16 / 16 100.0%	40 / 40 100.0%	8 / 9 88.9%	45 / 45 100.0%	39 / 39 100.0%	18 / 18 100.0%	35 / 35 100.0%	9 / 9 100.0%	27 / 27 100.0%	18 / 18 100.0%	60 / 60 100.0%	97 / 99 98.0%	40 / 40 100.0%	57 / 59 96.6%	72 / 72 100.0%	9 / 9 100.0%	0 / 0 0.0%	0 / 0 0.0%	0 / 0 0.0%	518 / 850 60.9%AB	285 / 850 33.5%ABE	23 / 850 2.7%B	19 / 850 2.2%BE	2 / 850 0.2%E	2 / 850 0.2%	1 / 850 0.1%C
2	ATCC 8700	186 / 189 98.4%	8 / 8 100.0%	6 / 6 100.0%	8 / 8 100.0%	8 / 8 100.0%	14 / 14 100.0%	12 / 12 100.0%	2 / 2 100.0%	3 / 3 100.0%	12 / 12 100.0%	2 / 2 100.0%	10 / 10 100.0%	8 / 8 100.0%	4 / 4 100.0%	8 / 8 100.0%	2 / 2 100.0%	6 / 6 100.0%	4 / 4 100.0%	12 / 12 100.0%	22 / 24 91.7%	6 / 6 100.0%	11 / 12 91.7%	16 / 16 100.0%	2 / 2 100.0%	0 / 0 0.0%	0 / 0 0.0%	0 / 0 0.0%	102 / 189 54.0%AB	47 / 189 24.9%ABE	23 / 189 12.2%B	14 / 189 7.4%BE	2 / 189 1.1%E	1 / 189 0.5%	
1	Shigella boydii Sb227	90 / 90 100.0%	4 / 4 100.0%	3 / 3 100.0%	4 / 4 100.0%	5 / 5 100.0%	8 / 8 100.0%	5 / 5 100.0%	1 / 1 100.0%	1 / 1 100.0%	7 / 7 100.0%	1 / 1 100.0%	5 / 5 100.0%	3 / 3 100.0%	2 / 2 100.0%	4 / 4 100.0%	1 / 1 100.0%	3 / 3 100.0%	2 / 2 100.0%	6 / 6 100.0%	10 / 10 100.0%	2 / 2 100.0%	4 / 4 100.0%	8 / 8 100.0%	1 / 1 100.0%	0 / 0 0.0%	0 / 0 0.0%	0 / 0 0.0%	61 / 90 67.8%AB	28 / 90 31.1%ABE	1 / 90 1.1%BE				
1	Shigella boydii BS512	96 / 99 97.0%	4 / 4 100.0%	3 / 3 100.0%	4 / 4 100.0%	3 / 3 100.0%	6 / 6 100.0%	7 / 7 100.0%	1 / 1 100.0%	2 / 2 100.0%	5 / 5 100.0%	1 / 1 100.0%	5 / 5 100.0%	5 / 5 100.0%	2 / 2 100.0%	4 / 4 100.0%	1 / 1 100.0%	3 / 3 100.0%	2 / 2 100.0%	6 / 6 100.0%	12 / 14 85.7%	4 / 4 100.0%	7 / 8 87.5%	8 / 8 100.0%	1 / 1 100.0%	0 / 0 0.0%	0 / 0 0.0%	0 / 0 0.0%	41 / 99 41.4%AB	23 / 99 23.2%B	19 / 99 19.2%ABE	13 / 99 13.1%BE	2 / 99 2.0%E	1 / 99 1.0%	
1	"Bacillus dysenteriae" Shiga 1898	82 / 82 100.0%	3 / 3 100.0%	3 / 3 100.0%	4 / 4 100.0%	3 / 3 100.0%	6 / 6 100.0%	4 / 4 100.0%	1 / 1 100.0%	2 / 2 100.0%	4 / 4 100.0%	1 / 1 100.0%	5 / 5 100.0%	4 / 4 100.0%	2 / 2 100.0%	3 / 3 100.0%	1 / 1 100.0%	3 / 3 100.0%	2 / 2 100.0%	6 / 6 100.0%	9 / 9 100.0%	4 / 4 100.0%	4 / 4 100.0%	7 / 7 100.0%	1 / 1 100.0%	0 / 0 0.0%	0 / 0 0.0%	0 / 0 0.0%	53 / 82 64.6%AB	28 / 82 34.1%ABE	1 / 82 1.2%BE				
1	Shigella dysenteriae Sd197	82 / 82 100.0%	3 / 3 100.0%	3 / 3 100.0%	4 / 4 100.0%	3 / 3 100.0%	6 / 6 100.0%	4 / 4 100.0%	1 / 1 100.0%	2 / 2 100.0%	4 / 4 100.0%	1 / 1 100.0%	5 / 5 100.0%	4 / 4 100.0%	2 / 2 100.0%	3 / 3 100.0%	1 / 1 100.0%	3 / 3 100.0%	2 / 2 100.0%	6 / 6 100.0%	9 / 9 100.0%	4 / 4 100.0%	4 / 4 100.0%	7 / 7 100.0%	1 / 1 100.0%	0 / 0 0.0%	0 / 0 0.0%	0 / 0 0.0%	53 / 82 64.6%AB	28 / 82 34.1%ABE	1 / 82 1.2%BE				
4	"Shigella paradysenteriae" Weldin 1927	387 / 388 99.7%	12 / 12 100.0%	12 / 12 100.0%	15 / 15 100.0%	8 / 8 100.0%	46 / 46 100.0%	21 / 21 100.0%	4 / 4 100.0%	7 / 7 100.0%	15 / 15 100.0%	3 / 4 75.0%	20 / 20 100.0%	17 / 17 100.0%	8 / 8 100.0%	16 / 16 100.0%	4 / 4 100.0%	12 / 12 100.0%	8 / 8 100.0%	30 / 30 100.0%	43 / 43 100.0%	21 / 21 100.0%	30 / 30 100.0%	31 / 31 100.0%	4 / 4 100.0%	0 / 0 0.0%	0 / 0 0.0%	0 / 0 0.0%	242 / 388 62.4%AB	142 / 388 36.6%ABE	3 / 388 0.8%BE	1 / 388 0.3%C			
2	Shigella flexneri 2a	190 / 191 99.5%	6 / 6 100.0%	6 / 6 100.0%	7 / 7 100.0%	5 / 5 100.0%	22 / 22 100.0%	10 / 10 100.0%	2 / 2 100.0%	3 / 3 100.0%	9 / 9 100.0%	1 / 2 50.0%	10 / 10 100.0%	8 / 8 100.0%	4 / 4 100.0%	8 / 8 100.0%	2 / 2 100.0%	6 / 6 100.0%	4 / 4 100.0%	14 / 14 100.0%	22 / 22 100.0%	10 / 10 100.0%	14 / 14 100.0%	15 / 15 100.0%	2 / 2 100.0%	0 / 0 0.0%	0 / 0 0.0%	0 / 0 0.0%	120 / 191 62.8%AB	68 / 191 35.6%ABE	2 / 191 1.0%BE	1 / 191 0.5%C			
1	Shigella flexneri 2a str. 301	92 / 93 98.9%	3 / 3 100.0%	3 / 3 100.0%	3 / 3 100.0%	2 / 2 100.0%	11 / 11 100.0%	4 / 4 100.0%	1 / 1 100.0%	1 / 1 100.0%	4 / 4 100.0%	0 / 1 0.0%	5 / 5 100.0%	4 / 4 100.0%	2 / 2 100.0%	4 / 4 100.0%	1 / 1 100.0%	3 / 3 100.0%	2 / 2 100.0%	7 / 7 100.0%	11 / 11 100.0%	6 / 6 100.0%	7 / 7 100.0%	7 / 7 100.0%	1 / 1 100.0%	0 / 0 0.0%	0 / 0 0.0%	0 / 0 0.0%	57 / 93 61.3%AB	34 / 93 36.6%ABE	1 / 93 1.1%C	1 / 93 1.1%BE			
1	Shigella flexneri 2a str. 2457T	98 / 98 100.0%	3 / 3 100.0%	3 / 3 100.0%	4 / 4 100.0%	3 / 3 100.0%	11 / 11 100.0%	6 / 6 100.0%	1 / 1 100.0%	2 / 2 100.0%	5 / 5 100.0%	1 / 1 100.0%	5 / 5 100.0%	4 / 4 100.0%	2 / 2 100.0%	4 / 4 100.0%	1 / 1 100.0%	3 / 3 100.0%	2 / 2 100.0%	7 / 7 100.0%	11 / 11 100.0%	4 / 4 100.0%	7 / 7 100.0%	8 / 8 100.0%	1 / 1 100.0%	0 / 0 0.0%	0 / 0 0.0%	0 / 0 0.0%	63 / 98 64.3%AB	34 / 98 34.7%ABE	1 / 98 1.0%BE				
1	Shigella flexneri 5	96 / 96 100.0%	3 / 3 100.0%	3 / 3 100.0%	4 / 4 100.0%	0 / 0 0.0%	12 / 12 100.0%	5 / 5 100.0%	1 / 1 100.0%	2 / 2 100.0%	1 / 1 100.0%	1 / 1 100.0%	5 / 5 100.0%	4 / 4 100.0%	2 / 2 100.0%	4 / 4 100.0%	1 / 1 100.0%	3 / 3 100.0%	2 / 2 100.0%	8 / 8 100.0%	11 / 11 100.0%	7 / 7 100.0%	8 / 8 100.0%	8 / 8 100.0%	1 / 1 100.0%	0 / 0 0.0%	0 / 0 0.0%	0 / 0 0.0%	57 / 96 59.4%AB	38 / 96 39.6%ABE	1 / 96 1.0%BE				
1	Shigella flexneri 5 str. 8401	96 / 96 100.0%	3 / 3 100.0%	3 / 3 100.0%	4 / 4 100.0%	0 / 0 0.0%	12 / 12 100.0%	5 / 5 100.0%	1 / 1 100.0%	2 / 2 100.0%	1 / 1 100.0%	1 / 1 100.0%	5 / 5 100.0%	4 / 4 100.0%	2 / 2 100.0%	4 / 4 100.0%	1 / 1 100.0%	3 / 3 100.0%	2 / 2 100.0%	8 / 8 100.0%	11 / 11 100.0%	7 / 7 100.0%	8 / 8 100.0%	8 / 8 100.0%	1 / 1 100.0%	0 / 0 0.0%	0 / 0 0.0%	0 / 0 0.0%	57 / 96 59.4%AB	38 / 96 39.6%ABE	1 / 96 1.0%BE				
1	Shigella flexneri 2002017	101 / 101 100.0%	3 / 3 100.0%	3 / 3 100.0%	4 / 4 100.0%	3 / 3 100.0%	12 / 12 100.0%	6 / 6 100.0%	1 / 1 100.0%	2 / 2 100.0%	5 / 5 100.0%	1 / 1 100.0%	5 / 5 100.0%	5 / 5 100.0%	2 / 2 100.0%	4 / 4 100.0%	1 / 1 100.0%	3 / 3 100.0%	2 / 2 100.0%	8 / 8 100.0%	10 / 10 100.0%	4 / 4 100.0%	8 / 8 100.0%	8 / 8 100.0%	1 / 1 100.0%	0 / 0 0.0%	0 / 0 0.0%	0 / 0 0.0%	65 / 101 64.4%AB	36 / 101 35.6%ABE					
2	"Bacterium sonnei" Levine 1920	190 / 191 99.5%	8 / 8 100.0%	6 / 6 100.0%	8 / 8 100.0%	5 / 5 100.0%	14 / 14 100.0%	12 / 12 100.0%	2 / 2 100.0%	4 / 4 100.0%	9 / 9 100.0%	2 / 2 100.0%	10 / 10 100.0%	10 / 10 100.0%	4 / 4 100.0%	8 / 8 100.0%	2 / 2 100.0%	6 / 6 100.0%	4 / 4 100.0%	12 / 12 100.0%	23 / 23 100.0%	9 / 9 100.0%	12 / 13 92.3%	18 / 18 100.0%	2 / 2 100.0%	0 / 0 0.0%	0 / 0 0.0%	0 / 0 0.0%	121 / 191 63.4%AB	68 / 191 35.6%ABE	1 / 191 0.5%	1 / 191 0.5%BE			
1	Shigella sonnei 53G	94 / 95 98.9%	4 / 4 100.0%	3 / 3 100.0%	4 / 4 100.0%	3 / 3 100.0%	7 / 7 100.0%	6 / 6 100.0%	1 / 1 100.0%	2 / 2 100.0%	5 / 5 100.0%	1 / 1 100.0%	5 / 5 100.0%	5 / 5 100.0%	2 / 2 100.0%	4 / 4 100.0%	1 / 1 100.0%	3 / 3 100.0%	2 / 2 100.0%	6 / 6 100.0%	11 / 11 100.0%	4 / 4 100.0%	6 / 7 85.7%	8 / 8 100.0%	1 / 1 100.0%	0 / 0 0.0%	0 / 0 0.0%	0 / 0 0.0%	61 / 95 64.2%AB	33 / 95 34.7%ABE	1 / 95 1.1%				
1	Shigella sonnei Ss046	96 / 96 100.0%	4 / 4 100.0%	3 / 3 100.0%	4 / 4 100.0%	2 / 2 100.0%	7 / 7 100.0%	6 / 6 100.0%	1 / 1 100.0%	2 / 2 100.0%	4 / 4 100.0%	1 / 1 100.0%	5 / 5 100.0%	5 / 5 100.0%	2 / 2 100.0%	4 / 4 100.0%	1 / 1 100.0%	3 / 3 100.0%	2 / 2 100.0%	6 / 6 100.0%	12 / 12 100.0%	5 / 5 100.0%	6 / 6 100.0%	10 / 10 100.0%	1 / 1 100.0%	0 / 0 0.0%	0 / 0 0.0%	0 / 0 0.0%	60 / 96 62.5%AB	35 / 96 36.5%ABE	1 / 96 1.0%BE				
2	Xenorhabdus	161 / 161 100.0%	8 / 8 100.0%	6 / 6 100.0%	8 / 8 100.0%	7 / 7 100.0%	10 / 10 100.0%	12 / 12 100.0%	2 / 2 100.0%	4 / 4 100.0%	11 / 11 100.0%	2 / 2 100.0%	10 / 10 100.0%	11 / 11 100.0%	8 / 8 100.0%	6 / 6 100.0%	2 / 2 100.0%	6 / 6 100.0%	4 / 4 100.0%	8 / 8 100.0%	13 / 13 100.0%	7 / 7 100.0%	2 / 2 100.0%	14 / 14 100.0%	0 / 0 0.0%	0 / 0 0.0%	0 / 0 0.0%	0 / 0 0.0%	102 / 161 63.4%AB	59 / 161 36.6%ABE					
1	"Achromobacter nematophilus" Poinar and Thomas 1965	79 / 79 100.0%	4 / 4 100.0%	3 / 3 100.0%	4 / 4 100.0%	3 / 3 100.0%	5 / 5 100.0%	6 / 6 100.0%	1 / 1 100.0%	2 / 2 100.0%	5 / 5 100.0%	1 / 1 100.0%	5 / 5 100.0%	6 / 6 100.0%	2 / 2 100.0%	3 / 3 100.0%	1 / 1 100.0%	3 / 3 100.0%	2 / 2 100.0%	4 / 4 100.0%	7 / 7 100.0%	4 / 4 100.0%	1 / 1 100.0%	7 / 7 100.0%	0 / 0 0.0%	0 / 0 0.0%	0 / 0 0.0%	0 / 0 0.0%	51 / 79 64.6%AB	28 / 79 35.4%ABE					
1	Xenorhabdus nematophila ATCC 19061	79 / 79 100.0%	4 / 4 100.0%	3 / 3 100.0%	4 / 4 100.0%	3 / 3 100.0%	5 / 5 100.0%	6 / 6 100.0%	1 / 1 100.0%	2 / 2 100.0%	5 / 5 100.0%	1 / 1 100.0%	5 / 5 100.0%	6 / 6 100.0%	2 / 2 100.0%	3 / 3 100.0%	1 / 1 100.0%	3 / 3 100.0%	2 / 2 100.0%	4 / 4 100.0%	7 / 7 100.0%	4 / 4 100.0%	1 / 1 100.0%	7 / 7 100.0%	0 / 0 0.0%	0 / 0 0.0%	0 / 0 0.0%	0 / 0 0.0%	51 / 79 64.6%AB	28 / 79 35.4%ABE					
1	ATCC 35271	82 / 82 100.0%	4 / 4 100.0%	3 / 3 100.0%	4 / 4 100.0%	4 / 4 100.0%	5 / 5 100.0%	6 / 6 100.0%	1 / 1 100.0%	2 / 2 100.0%	6 / 6 100.0%	1 / 1 100.0%	5 / 5 100.0%	5 / 5 100.0%	6 / 6 100.0%	3 / 3 100.0%	1 / 1 100.0%	3 / 3 100.0%	2 / 2 100.0%	4 / 4 100.0%	6 / 6 100.0%	3 / 3 100.0%	1 / 1 100.0%	7 / 7 100.0%	0 / 0 0.0%	0 / 0 0.0%	0 / 0 0.0%	0 / 0 0.0%	51 / 82 62.2%AB	31 / 82 37.8%ABE					
1	Xenorhabdus bovienii SS-2004	82 / 82 100.0%	4 / 4 100.0%	3 / 3 100.0%	4 / 4 100.0%	4 / 4 100.0%	5 / 5 100.0%	6 / 6 100.0%	1 / 1 100.0%	2 / 2 100.0%	6 / 6 100.0%	1 / 1 100.0%	5 / 5 100.0%	5 / 5 100.0%	6 / 6 100.0%	3 / 3 100.0%	1 / 1 100.0%	3 / 3 100.0%	2 / 2 100.0%	4 / 4 100.0%	6 / 6 100.0%	3 / 3 100.0%	1 / 1 100.0%	7 / 7 100.0%	0 / 0 0.0%	0 / 0 0.0%	0 / 0 0.0%	0 / 0 0.0%	51 / 82 62.2%AB	31 / 82 37.8%ABE					
19	Yersinia	1377 / 1379 99.9%	63 / 63 100.0%	57 / 57 100.0%	53 / 53 100.0%	54 / 54 100.0%	95 / 95 100.0%	94 / 94 100.0%	19 / 19 100.0%	43 / 43 100.0%	87 / 87 100.0%	19 / 19 100.0%	91 / 93 97.8%	81 / 81 100.0%	38 / 38 100.0%	53 / 53 100.0%	19 / 19 100.0%	57 / 57 100.0%	38 / 38 100.0%	76 / 76 100.0%	104 / 104 100.0%	74 / 74 100.0%	19 / 19 100.0%	125 / 125 100.0%	18 / 18 100.0%	0 / 0 0.0%	0 / 0 0.0%	0 / 0 0.0%	759 / 1379 55.0%AB	473 / 1379 34.3%ABE	91 / 1379 6.6%B	54 / 1379 3.9%BE	2 / 1379 0.1%CE		
3	"Bacterium enterocoliticum" Schleifstein and Coleman 1939	220 / 220 100.0%	10 / 10 100.0%	9 / 9 100.0%	9 / 9 100.0%	10 / 10 100.0%	16 / 16 100.0%	13 / 13 100.0%	3 / 3 100.0%	7 / 7 100.0%	15 / 15 100.0%	3 / 3 100.0%	15 / 15 100.0%	11 / 11 100.0%	6 / 6 100.0%	9 / 9 100.0%	3 / 3 100.0%	9 / 9 100.0%	6 / 6 100.0%	13 / 13 100.0%	15 / 15 100.0%	11 / 11 100.0%	3 / 3 100.0%	21 / 21 100.0%	3 / 3 100.0%	0 / 0 0.0%	0 / 0 0.0%	0 / 0 0.0%	131 / 220 59.5%AB	88 / 220 40.0%ABE	1 / 220 0.5%BE				
1	Yersinia enterocolitica subsp. enterocolitica	79 / 79 100.0%	4 / 4 100.0%	3 / 3 100.0%	3 / 3 100.0%	3 / 3 100.0%	7 / 7 100.0%	6 / 6 100.0%	1 / 1 100.0%	2 / 2 100.0%	5 / 5 100.0%	1 / 1 100.0%	5 / 5 100.0%	5 / 5 100.0%	2 / 2 100.0%	3 / 3 100.0%	1 / 1 100.0%	3 / 3 100.0%	2 / 2 100.0%	5 / 5 100.0%	5 / 5 100.0%	4 / 4 100.0%	1 / 1 100.0%	7 / 7 100.0%	1 / 1 100.0%	0 / 0 0.0%	0 / 0 0.0%	0 / 0 0.0%	48 / 79 60.8%AB	30 / 79 38.0%ABE	1 / 79 1.3%BE				
1	Yersinia enterocolitica subsp. enterocolitica 8081	79 / 79 100.0%	4 / 4 100.0%	3 / 3 100.0%	3 / 3 100.0%	3 / 3 100.0%	7 / 7 100.0%	6 / 6 100.0%	1 / 1 100.0%	2 / 2 100.0%	5 / 5 100.0%	1 / 1 100.0%	5 / 5 100.0%	5 / 5 100.0%	2 / 2 100.0%	3 / 3 100.0%	1 / 1 100.0%	3 / 3 100.0%	2 / 2 100.0%	5 / 5 100.0%	5 / 5 100.0%	4 / 4 100.0%	1 / 1 100.0%	7 / 7 100.0%	1 / 1 100.0%	0 / 0 0.0%	0 / 0 0.0%	0 / 0 0.0%	48 / 79 60.8%AB	30 / 79 38.0%ABE	1 / 79 1.3%BE				
2	CCUG 52867	141 / 141 100.0%	6 / 6 100.0%	6 / 6 100.0%	6 / 6 100.0%	7 / 7 100.0%	9 / 9 100.0%	7 / 7 100.0%	2 / 2 100.0%	5 / 5 100.0%	10 / 10 100.0%	2 / 2 100.0%	10 / 10 100.0%	6 / 6 100.0%	4 / 4 100.0%	6 / 6 100.0%	2 / 2 100.0%	6 / 6 100.0%	4 / 4 100.0%	8 / 8 100.0%	10 / 10 100.0%	7 / 7 100.0%	2 / 2 100.0%	14 / 14 100.0%	2 / 2 100.0%	0 / 0 0.0%	0 / 0 0.0%	0 / 0 0.0%	83 / 141 58.9%AB	58 / 141 41.1%ABE					
1	Yersinia enterocolitica subsp. palearctica Y11	70 / 70 100.0%	2 / 2 100.0%	3 / 3 100.0%	3 / 3 100.0%	3 / 3 100.0%	5 / 5 100.0%	5 / 5 100.0%	1 / 1 100.0%	2 / 2 100.0%	4 / 4 100.0%	1 / 1 100.0%	5 / 5 100.0%	3 / 3 100.0%	2 / 2 100.0%	3 / 3 100.0%	1 / 1 100.0%	3 / 3 100.0%	2 / 2 100.0%	4 / 4 100.0%	5 / 5 100.0%	4 / 4 100.0%	1 / 1 100.0%	7 / 7 100.0%	1 / 1 100.0%	0 / 0 0.0%	0 / 0 0.0%	0 / 0 0.0%	42 / 70 60.0%AB	28 / 70 40.0%ABE					
1	Yersinia enterocolitica subsp. palearctica 105.5R(r)	71 / 71 100.0%	4 / 4 100.0%	3 / 3 100.0%	3 / 3 100.0%	4 / 4 100.0%	4 / 4 100.0%	2 / 2 100.0%	1 / 1 100.0%	3 / 3 100.0%	6 / 6 100.0%	1 / 1 100.0%	5 / 5 100.0%	3 / 3 100.0%	2 / 2 100.0%	3 / 3 100.0%	1 / 1 100.0%	3 / 3 100.0%	2 / 2 100.0%	4 / 4 100.0%	5 / 5 100.0%	3 / 3 100.0%	1 / 1 100.0%	7 / 7 100.0%	1 / 1 100.0%	0 / 0 0.0%	0 / 0 0.0%	0 / 0 0.0%	41 / 71 57.7%AB	30 / 71 42.3%ABE					
12	"Bacillus pestis" (Lehmann and Neumann 1896) Migula 1900	835 / 835 100.0%	37 / 37 100.0%	36 / 36 100.0%	32 / 32 100.0%	32 / 32 100.0%	55 / 55 100.0%	57 / 57 100.0%	12 / 12 100.0%	24 / 24 100.0%	52 / 52 100.0%	12 / 12 100.0%	58 / 58 100.0%	46 / 46 100.0%	24 / 24 100.0%	32 / 32 100.0%	12 / 12 100.0%	36 / 36 100.0%	24 / 24 100.0%	47 / 47 100.0%	65 / 65 100.0%	47 / 47 100.0%	12 / 12 100.0%	72 / 72 100.0%	11 / 11 100.0%	0 / 0 0.0%	0 / 0 0.0%	0 / 0 0.0%	504 / 835 60.4%AB	304 / 835 36.4%ABE	15 / 835 1.8%B	12 / 835 1.4%BE			
1	Yersinia pestis KIM	72 / 72 100.0%	3 / 3 100.0%	3 / 3 100.0%	3 / 3 100.0%	2 / 2 100.0%	5 / 5 100.0%	5 / 5 100.0%	1 / 1 100.0%	2 / 2 100.0%	4 / 4 100.0%	1 / 1 100.0%	5 / 5 100.0%	4 / 4 100.0%	2 / 2 100.0%	3 / 3 100.0%	1 / 1 100.0%	3 / 3 100.0%	2 / 2 100.0%	4 / 4 100.0%	6 / 6 100.0%	5 / 5 100.0%	1 / 1 100.0%	6 / 6 100.0%	1 / 1 100.0%	0 / 0 0.0%	0 / 0 0.0%	0 / 0 0.0%	44 / 72 61.1%AB	27 / 72 37.5%ABE	1 / 72 1.4%BE				
1	Yersinia pestis CO92	69 / 69 100.0%	3 / 3 100.0%	3 / 3 100.0%	3 / 3 100.0%	3 / 3 100.0%	4 / 4 100.0%	5 / 5 100.0%	1 / 1 100.0%	2 / 2 100.0%	5 / 5 100.0%	1 / 1 100.0%	5 / 5 100.0%	4 / 4 100.0%	2 / 2 100.0%	2 / 2 100.0%	1 / 1 100.0%	3 / 3 100.0%	2 / 2 100.0%	4 / 4 100.0%	5 / 5 100.0%	3 / 3 100.0%	1 / 1 100.0%	6 / 6 100.0%	1 / 1 100.0%	0 / 0 0.0%	0 / 0 0.0%	0 / 0 0.0%	44 / 69 63.8%AB	24 / 69 34.8%ABE	1 / 69 1.4%BE				
1	Yersinia pestis Angola	68 / 68 100.0%	3 / 3 100.0%	3 / 3 100.0%	3 / 3 100.0%	3 / 3 100.0%	5 / 5 100.0%	4 / 4 100.0%	1 / 1 100.0%	2 / 2 100.0%	4 / 4 100.0%	1 / 1 100.0%	4 / 4 100.0%	3 / 3 100.0%	2 / 2 100.0%	2 / 2 100.0%	1 / 1 100.0%	3 / 3 100.0%	2 / 2 100.0%	4 / 4 100.0%	6 / 6 100.0%	4 / 4 100.0%	1 / 1 100.0%	6 / 6 100.0%	1 / 1 100.0%	0 / 0 0.0%	0 / 0 0.0%	0 / 0 0.0%	28 / 68 41.2%AB	19 / 68 27.9%ABE	15 / 68 22.1%B	6 / 68 8.8%BE			
1	Yersinia pestis Antiqua	67 / 67 100.0%	3 / 3 100.0%	3 / 3 100.0%	3 / 3 100.0%	3 / 3 100.0%	5 / 5 100.0%	4 / 4 100.0%	1 / 1 100.0%	2 / 2 100.0%	4 / 4 100.0%	1 / 1 100.0%	4 / 4 100.0%	4 / 4 100.0%	2 / 2 100.0%	3 / 3 100.0%	1 / 1 100.0%	3 / 3 100.0%	2 / 2 100.0%	4 / 4 100.0%	3 / 3 100.0%	4 / 4 100.0%	1 / 1 100.0%	6 / 6 100.0%	1 / 1 100.0%	0 / 0 0.0%	0 / 0 0.0%	0 / 0 0.0%	41 / 67 61.2%AB	25 / 67 37.3%ABE	1 / 67 1.5%BE				
1	Yersinia pestis Nepal516	71 / 71 100.0%	3 / 3 100.0%	3 / 3 100.0%	2 / 2 100.0%	2 / 2 100.0%	5 / 5 100.0%	5 / 5 100.0%	1 / 1 100.0%	2 / 2 100.0%	4 / 4 100.0%	1 / 1 100.0%	5 / 5 100.0%	4 / 4 100.0%	2 / 2 100.0%	3 / 3 100.0%	1 / 1 100.0%	3 / 3 100.0%	2 / 2 100.0%	4 / 4 100.0%	6 / 6 100.0%	5 / 5 100.0%	1 / 1 100.0%	6 / 6 100.0%	1 / 1 100.0%	0 / 0 0.0%	0 / 0 0.0%	0 / 0 0.0%	43 / 71 60.6%AB	27 / 71 38.0%ABE	1 / 71 1.4%BE				
1	'Yersinia pestis subsp. pestis'	71 / 71 100.0%	3 / 3 100.0%	3 / 3 100.0%	2 / 2 100.0%	3 / 3 100.0%	5 / 5 100.0%	4 / 4 100.0%	1 / 1 100.0%	2 / 2 100.0%	5 / 5 100.0%	1 / 1 100.0%	5 / 5 100.0%	4 / 4 100.0%	2 / 2 100.0%	3 / 3 100.0%	1 / 1 100.0%	3 / 3 100.0%	2 / 2 100.0%	4 / 4 100.0%	6 / 6 100.0%	4 / 4 100.0%	1 / 1 100.0%	6 / 6 100.0%	1 / 1 100.0%	0 / 0 0.0%	0 / 0 0.0%	0 / 0 0.0%	44 / 71 62.0%AB	27 / 71 38.0%ABE					
1	Yersinia pestis subsp. pestis bv. Medievalis	71 / 71 100.0%	3 / 3 100.0%	3 / 3 100.0%	2 / 2 100.0%	3 / 3 100.0%	5 / 5 100.0%	4 / 4 100.0%	1 / 1 100.0%	2 / 2 100.0%	5 / 5 100.0%	1 / 1 100.0%	5 / 5 100.0%	4 / 4 100.0%	2 / 2 100.0%	3 / 3 100.0%	1 / 1 100.0%	3 / 3 100.0%	2 / 2 100.0%	4 / 4 100.0%	6 / 6 100.0%	4 / 4 100.0%	1 / 1 100.0%	6 / 6 100.0%	1 / 1 100.0%	0 / 0 0.0%	0 / 0 0.0%	0 / 0 0.0%	44 / 71 62.0%AB	27 / 71 38.0%ABE					
1	Yersinia pestis biovar Mediaevalis str. Harbin 35	71 / 71 100.0%	3 / 3 100.0%	3 / 3 100.0%	2 / 2 100.0%	3 / 3 100.0%	5 / 5 100.0%	4 / 4 100.0%	1 / 1 100.0%	2 / 2 100.0%	5 / 5 100.0%	1 / 1 100.0%	5 / 5 100.0%	4 / 4 100.0%	2 / 2 100.0%	3 / 3 100.0%	1 / 1 100.0%	3 / 3 100.0%	2 / 2 100.0%	4 / 4 100.0%	6 / 6 100.0%	4 / 4 100.0%	1 / 1 100.0%	6 / 6 100.0%	1 / 1 100.0%	0 / 0 0.0%	0 / 0 0.0%	0 / 0 0.0%	44 / 71 62.0%AB	27 / 71 38.0%ABE					
1	Yersinia pestis Pestoides F	70 / 70 100.0%	4 / 4 100.0%	3 / 3 100.0%	3 / 3 100.0%	2 / 2 100.0%	4 / 4 100.0%	5 / 5 100.0%	1 / 1 100.0%	2 / 2 100.0%	3 / 3 100.0%	1 / 1 100.0%	5 / 5 100.0%	4 / 4 100.0%	2 / 2 100.0%	3 / 3 100.0%	1 / 1 100.0%	3 / 3 100.0%	2 / 2 100.0%	4 / 4 100.0%	5 / 5 100.0%	5 / 5 100.0%	1 / 1 100.0%	6 / 6 100.0%	1 / 1 100.0%	0 / 0 0.0%	0 / 0 0.0%	0 / 0 0.0%	41 / 70 58.6%AB	28 / 70 40.0%ABE	1 / 70 1.4%BE				
1	Yersinia pestis D106004	69 / 69 100.0%	3 / 3 100.0%	3 / 3 100.0%	3 / 3 100.0%	1 / 1 100.0%	5 / 5 100.0%	5 / 5 100.0%	1 / 1 100.0%	2 / 2 100.0%	3 / 3 100.0%	1 / 1 100.0%	5 / 5 100.0%	4 / 4 100.0%	2 / 2 100.0%	3 / 3 100.0%	1 / 1 100.0%	3 / 3 100.0%	2 / 2 100.0%	4 / 4 100.0%	5 / 5 100.0%	5 / 5 100.0%	1 / 1 100.0%	6 / 6 100.0%	1 / 1 100.0%	0 / 0 0.0%	0 / 0 0.0%	0 / 0 0.0%	41 / 69 59.4%AB	28 / 69 40.6%ABE					
1	Yersinia pestis D182038	72 / 72 100.0%	3 / 3 100.0%	3 / 3 100.0%	3 / 3 100.0%	4 / 4 100.0%	5 / 5 100.0%	5 / 5 100.0%	1 / 1 100.0%	2 / 2 100.0%	6 / 6 100.0%	1 / 1 100.0%	5 / 5 100.0%	3 / 3 100.0%	2 / 2 100.0%	3 / 3 100.0%	1 / 1 100.0%	3 / 3 100.0%	2 / 2 100.0%	4 / 4 100.0%	6 / 6 100.0%	2 / 2 100.0%	1 / 1 100.0%	6 / 6 100.0%	1 / 1 100.0%	0 / 0 0.0%	0 / 0 0.0%	0 / 0 0.0%	47 / 72 65.3%AB	25 / 72 34.7%ABE					
1	Yersinia pestis Z176003	67 / 67 100.0%	3 / 3 100.0%	3 / 3 100.0%	2 / 2 100.0%	2 / 2 100.0%	4 / 4 100.0%	5 / 5 100.0%	1 / 1 100.0%	2 / 2 100.0%	4 / 4 100.0%	1 / 1 100.0%	5 / 5 100.0%	4 / 4 100.0%	2 / 2 100.0%	2 / 2 100.0%	1 / 1 100.0%	3 / 3 100.0%	2 / 2 100.0%	4 / 4 100.0%	6 / 6 100.0%	4 / 4 100.0%	1 / 1 100.0%	6 / 6 100.0%	0 / 0 0.0%	0 / 0 0.0%	0 / 0 0.0%	0 / 0 0.0%	42 / 67 62.7%AB	25 / 67 37.3%ABE					
1	Yersinia pestis A1122	68 / 68 100.0%	3 / 3 100.0%	3 / 3 100.0%	2 / 2 100.0%	3 / 3 100.0%	4 / 4 100.0%	5 / 5 100.0%	1 / 1 100.0%	2 / 2 100.0%	5 / 5 100.0%	1 / 1 100.0%	5 / 5 100.0%	4 / 4 100.0%	2 / 2 100.0%	2 / 2 100.0%	1 / 1 100.0%	3 / 3 100.0%	2 / 2 100.0%	3 / 3 100.0%	6 / 6 100.0%	3 / 3 100.0%	1 / 1 100.0%	6 / 6 100.0%	1 / 1 100.0%	0 / 0 0.0%	0 / 0 0.0%	0 / 0 0.0%	44 / 68 64.7%AB	24 / 68 35.3%ABE					
1	'Yersinia pestis subsp. microtus'	71 / 71 100.0%	3 / 3 100.0%	3 / 3 100.0%	3 / 3 100.0%	4 / 4 100.0%	4 / 4 100.0%	5 / 5 100.0%	1 / 1 100.0%	2 / 2 100.0%	5 / 5 100.0%	1 / 1 100.0%	5 / 5 100.0%	4 / 4 100.0%	2 / 2 100.0%	3 / 3 100.0%	1 / 1 100.0%	3 / 3 100.0%	2 / 2 100.0%	4 / 4 100.0%	5 / 5 100.0%	3 / 3 100.0%	1 / 1 100.0%	6 / 6 100.0%	1 / 1 100.0%	0 / 0 0.0%	0 / 0 0.0%	0 / 0 0.0%	45 / 71 63.4%AB	25 / 71 35.2%ABE	1 / 71 1.4%BE				
1	Yersinia pestis biovar Mediaevails str. 91001	71 / 71 100.0%	3 / 3 100.0%	3 / 3 100.0%	3 / 3 100.0%	4 / 4 100.0%	4 / 4 100.0%	5 / 5 100.0%	1 / 1 100.0%	2 / 2 100.0%	5 / 5 100.0%	1 / 1 100.0%	5 / 5 100.0%	4 / 4 100.0%	2 / 2 100.0%	3 / 3 100.0%	1 / 1 100.0%	3 / 3 100.0%	2 / 2 100.0%	4 / 4 100.0%	5 / 5 100.0%	3 / 3 100.0%	1 / 1 100.0%	6 / 6 100.0%	1 / 1 100.0%	0 / 0 0.0%	0 / 0 0.0%	0 / 0 0.0%	45 / 71 63.4%AB	25 / 71 35.2%ABE	1 / 71 1.4%BE				
4	"Bacillus pseudotuberkulosis" (sic) Pfeiffer 1889	322 / 324 99.4%	16 / 16 100.0%	12 / 12 100.0%	12 / 12 100.0%	12 / 12 100.0%	24 / 24 100.0%	24 / 24 100.0%	4 / 4 100.0%	12 / 12 100.0%	20 / 20 100.0%	4 / 4 100.0%	18 / 20 90.0%	24 / 24 100.0%	8 / 8 100.0%	12 / 12 100.0%	4 / 4 100.0%	12 / 12 100.0%	8 / 8 100.0%	16 / 16 100.0%	24 / 24 100.0%	16 / 16 100.0%	4 / 4 100.0%	32 / 32 100.0%	4 / 4 100.0%	0 / 0 0.0%	0 / 0 0.0%	0 / 0 0.0%	124 / 324 38.3%AB	81 / 324 25.0%ABE	76 / 324 23.5%B	41 / 324 12.7%BE	2 / 324 0.6%CE		
1	Yersinia pseudotuberculosis IP 32953	81 / 81 100.0%	4 / 4 100.0%	3 / 3 100.0%	3 / 3 100.0%	3 / 3 100.0%	6 / 6 100.0%	6 / 6 100.0%	1 / 1 100.0%	3 / 3 100.0%	5 / 5 100.0%	1 / 1 100.0%	5 / 5 100.0%	6 / 6 100.0%	2 / 2 100.0%	3 / 3 100.0%	1 / 1 100.0%	3 / 3 100.0%	2 / 2 100.0%	4 / 4 100.0%	6 / 6 100.0%	4 / 4 100.0%	1 / 1 100.0%	8 / 8 100.0%	1 / 1 100.0%	0 / 0 0.0%	0 / 0 0.0%	0 / 0 0.0%	50 / 81 61.7%AB	30 / 81 37.0%ABE	1 / 81 1.2%BE				
1	Yersinia pseudotuberculosis IP 31758	80 / 81 98.8%	4 / 4 100.0%	3 / 3 100.0%	3 / 3 100.0%	3 / 3 100.0%	6 / 6 100.0%	6 / 6 100.0%	1 / 1 100.0%	3 / 3 100.0%	5 / 5 100.0%	1 / 1 100.0%	4 / 5 80.0%	6 / 6 100.0%	2 / 2 100.0%	3 / 3 100.0%	1 / 1 100.0%	3 / 3 100.0%	2 / 2 100.0%	4 / 4 100.0%	6 / 6 100.0%	4 / 4 100.0%	1 / 1 100.0%	8 / 8 100.0%	1 / 1 100.0%	0 / 0 0.0%	0 / 0 0.0%	0 / 0 0.0%	28 / 81 34.6%B	22 / 81 27.2%AB	17 / 81 21.0%ABE	13 / 81 16.0%BE	1 / 81 1.2%CE		
1	Yersinia pseudotuberculosis YPIII	80 / 81 98.8%	4 / 4 100.0%	3 / 3 100.0%	3 / 3 100.0%	3 / 3 100.0%	6 / 6 100.0%	6 / 6 100.0%	1 / 1 100.0%	3 / 3 100.0%	5 / 5 100.0%	1 / 1 100.0%	4 / 5 80.0%	6 / 6 100.0%	2 / 2 100.0%	3 / 3 100.0%	1 / 1 100.0%	3 / 3 100.0%	2 / 2 100.0%	4 / 4 100.0%	6 / 6 100.0%	4 / 4 100.0%	1 / 1 100.0%	8 / 8 100.0%	1 / 1 100.0%	0 / 0 0.0%	0 / 0 0.0%	0 / 0 0.0%	30 / 81 37.0%B	20 / 81 24.7%AB	17 / 81 21.0%ABE	13 / 81 16.0%BE	1 / 81 1.2%CE		
1	Yersinia pseudotuberculosis PB1/+	81 / 81 100.0%	4 / 4 100.0%	3 / 3 100.0%	3 / 3 100.0%	3 / 3 100.0%	6 / 6 100.0%	6 / 6 100.0%	1 / 1 100.0%	3 / 3 100.0%	5 / 5 100.0%	1 / 1 100.0%	5 / 5 100.0%	6 / 6 100.0%	2 / 2 100.0%	3 / 3 100.0%	1 / 1 100.0%	3 / 3 100.0%	2 / 2 100.0%	4 / 4 100.0%	6 / 6 100.0%	4 / 4 100.0%	1 / 1 100.0%	8 / 8 100.0%	1 / 1 100.0%	0 / 0 0.0%	0 / 0 0.0%	0 / 0 0.0%	32 / 81 39.5%AB	18 / 81 22.2%B	17 / 81 21.0%ABE	14 / 81 17.3%BE			
[illegible]	"Asakusa group" Sakazaki and Murata [illegible]	377 / 377	16 / 16	15 / 15	23 / 23	7 / 7	29 / 29	31 / 31	4 / 4	12 / 12	23 / 23	4 / 4	20 / 20	31 / 31	8 / 8	16 / 16	8 / 8	16 / 16	8 / 8	16 / 16	24 / 24	25 / 25	5 / 5	32 / 32	4 / 4	0 / 0	0 / 0	0 / 0	238 / 377	139 / 377					

	1962	100.0%	100.0%	100.0%	100.0%	100.0%	100.0%	100.0%	100.0%	100.0%	100.0%	100.0%	100.0%	100.0%	100.0%	100.0%	100.0%	100.0%	100.0%	100.0%	100.0%	100.0%	100.0%	100.0%	100.0%	0.0%	0.0%	0.0%	63.1%AB	36.9%ABE			
2	"Paracolobactrum anguillimortiferum" Hoshina 1962	190 / 190 100.0%	8 / 8 100.0%	8 / 8 100.0%	12 / 12 100.0%	2 / 2 100.0%	14 / 14 100.0%	18 / 18 100.0%	2 / 2 100.0%	6 / 6 100.0%	10 / 10 100.0%	2 / 2 100.0%	10 / 10 100.0%	16 / 16 100.0%	4 / 4 100.0%	8 / 8 100.0%	4 / 4 100.0%	8 / 8 100.0%	4 / 4 100.0%	8 / 8 100.0%	12 / 12 100.0%	14 / 14 100.0%	2 / 2 100.0%	16 / 16 100.0%	2 / 2 100.0%	0 / 0 0.0%	0 / 0 0.0%	0 / 0 0.0%	119 / 190 62.6%AB	71 / 190 37.4%ABE			
1	Edwardsiella tarda EIB202	95 / 95 100.0%	4 / 4 100.0%	4 / 4 100.0%	6 / 6 100.0%	1 / 1 100.0%	7 / 7 100.0%	9 / 9 100.0%	1 / 1 100.0%	3 / 3 100.0%	5 / 5 100.0%	1 / 1 100.0%	5 / 5 100.0%	8 / 8 100.0%	2 / 2 100.0%	4 / 4 100.0%	2 / 2 100.0%	4 / 4 100.0%	2 / 2 100.0%	4 / 4 100.0%	6 / 6 100.0%	7 / 7 100.0%	1 / 1 100.0%	8 / 8 100.0%	1 / 1 100.0%	0 / 0 0.0%	0 / 0 0.0%	0 / 0 0.0%	60 / 95 63.2%AB	35 / 95 36.8%ABE			
1	Edwardsiella tarda FL6-60	95 / 95 100.0%	4 / 4 100.0%	4 / 4 100.0%	6 / 6 100.0%	1 / 1 100.0%	7 / 7 100.0%	9 / 9 100.0%	1 / 1 100.0%	3 / 3 100.0%	5 / 5 100.0%	1 / 1 100.0%	5 / 5 100.0%	8 / 8 100.0%	2 / 2 100.0%	4 / 4 100.0%	2 / 2 100.0%	4 / 4 100.0%	2 / 2 100.0%	4 / 4 100.0%	6 / 6 100.0%	7 / 7 100.0%	1 / 1 100.0%	8 / 8 100.0%	1 / 1 100.0%	0 / 0 0.0%	0 / 0 0.0%	0 / 0 0.0%	59 / 95 62.1%AB	36 / 95 37.9%ABE			
1	ATCC 33202	93 / 93 100.0%	4 / 4 100.0%	3 / 3 100.0%	5 / 5 100.0%	3 / 3 100.0%	7 / 7 100.0%	8 / 8 100.0%	1 / 1 100.0%	3 / 3 100.0%	7 / 7 100.0%	1 / 1 100.0%	5 / 5 100.0%	8 / 8 100.0%	2 / 2 100.0%	4 / 4 100.0%	2 / 2 100.0%	4 / 4 100.0%	2 / 2 100.0%	4 / 4 100.0%	5 / 5 100.0%	5 / 5 100.0%	1 / 1 100.0%	8 / 8 100.0%	1 / 1 100.0%	0 / 0 0.0%	0 / 0 0.0%	0 / 0 0.0%	60 / 93 64.5%AB	33 / 93 35.5%ABE			
1	Edwardsiella ictaluri 93-146	93 / 93 100.0%	4 / 4 100.0%	3 / 3 100.0%	5 / 5 100.0%	3 / 3 100.0%	7 / 7 100.0%	8 / 8 100.0%	1 / 1 100.0%	3 / 3 100.0%	7 / 7 100.0%	1 / 1 100.0%	5 / 5 100.0%	8 / 8 100.0%	2 / 2 100.0%	4 / 4 100.0%	2 / 2 100.0%	4 / 4 100.0%	2 / 2 100.0%	4 / 4 100.0%	5 / 5 100.0%	5 / 5 100.0%	1 / 1 100.0%	8 / 8 100.0%	1 / 1 100.0%	0 / 0 0.0%	0 / 0 0.0%	0 / 0 0.0%	60 / 93 64.5%AB	33 / 93 35.5%ABE			
1	CCUG 62929	94 / 94 100.0%	4 / 4 100.0%	4 / 4 100.0%	6 / 6 100.0%	2 / 2 100.0%	8 / 8 100.0%	5 / 5 100.0%	1 / 1 100.0%	3 / 3 100.0%	6 / 6 100.0%	1 / 1 100.0%	5 / 5 100.0%	7 / 7 100.0%	2 / 2 100.0%	4 / 4 100.0%	2 / 2 100.0%	4 / 4 100.0%	2 / 2 100.0%	4 / 4 100.0%	7 / 7 100.0%	6 / 6 100.0%	2 / 2 100.0%	8 / 8 100.0%	1 / 1 100.0%	0 / 0 0.0%	0 / 0 0.0%	0 / 0 0.0%	59 / 94 62.8%AB	35 / 94 37.2%ABE			
1	Edwardsiella piscicida C07-087	94 / 94 100.0%	4 / 4 100.0%	4 / 4 100.0%	6 / 6 100.0%	2 / 2 100.0%	8 / 8 100.0%	5 / 5 100.0%	1 / 1 100.0%	3 / 3 100.0%	6 / 6 100.0%	1 / 1 100.0%	5 / 5 100.0%	7 / 7 100.0%	2 / 2 100.0%	4 / 4 100.0%	2 / 2 100.0%	4 / 4 100.0%	2 / 2 100.0%	4 / 4 100.0%	7 / 7 100.0%	6 / 6 100.0%	2 / 2 100.0%	8 / 8 100.0%	1 / 1 100.0%	0 / 0 0.0%	0 / 0 0.0%	0 / 0 0.0%	59 / 94 62.8%AB	35 / 94 37.2%ABE			
2	Photorhabdus	232 / 233 99.6%	12 / 12 100.0%	9 / 9 100.0%	10 / 10 100.0%	9 / 9 100.0%	18 / 18 100.0%	15 / 15 100.0%	3 / 3 100.0%	6 / 6 100.0%	15 / 15 100.0%	3 / 3 100.0%	12 / 12 100.0%	15 / 15 100.0%	6 / 6 100.0%	9 / 9 100.0%	3 / 3 100.0%	9 / 9 100.0%	6 / 6 100.0%	12 / 12 100.0%	21 / 22 95.5%	12 / 12 100.0%	3 / 3 100.0%	21 / 21 100.0%	3 / 3 100.0%	0 / 0 0.0%	0 / 0 0.0%	0 / 0 0.0%	148 / 233 63.5%AB	83 / 233 35.6%ABE	1 / 233 0.4%BE	1 / 233 0.4%	
1	ATCC 29999	76 / 77 98.7%	4 / 4 100.0%	3 / 3 100.0%	4 / 4 100.0%	3 / 3 100.0%	6 / 6 100.0%	5 / 5 100.0%	1 / 1 100.0%	2 / 2 100.0%	5 / 5 100.0%	1 / 1 100.0%	4 / 4 100.0%	5 / 5 100.0%	2 / 2 100.0%	3 / 3 100.0%	1 / 1 100.0%	3 / 3 100.0%	2 / 2 100.0%	4 / 4 100.0%	5 / 6 83.3%	4 / 4 100.0%	1 / 1 100.0%	7 / 7 100.0%	1 / 1 100.0%	0 / 0 0.0%	0 / 0 0.0%	0 / 0 0.0%	48 / 77 62.3%AB	27 / 77 35.1%ABE	1 / 77 1.3%	1 / 77 1.3%BE	
1	CCM 7076	76 / 77 98.7%	4 / 4 100.0%	3 / 3 100.0%	4 / 4 100.0%	3 / 3 100.0%	6 / 6 100.0%	5 / 5 100.0%	1 / 1 100.0%	2 / 2 100.0%	5 / 5 100.0%	1 / 1 100.0%	4 / 4 100.0%	5 / 5 100.0%	2 / 2 100.0%	3 / 3 100.0%	1 / 1 100.0%	3 / 3 100.0%	2 / 2 100.0%	4 / 4 100.0%	5 / 6 83.3%	4 / 4 100.0%	1 / 1 100.0%	7 / 7 100.0%	1 / 1 100.0%	0 / 0 0.0%	0 / 0 0.0%	0 / 0 0.0%	48 / 77 62.3%AB	27 / 77 35.1%ABE	1 / 77 1.3%BE	1 / 77 1.3%	
1	Photorhabdus luminescens subsp. laumondii TTO1	76 / 77 98.7%	4 / 4 100.0%	3 / 3 100.0%	4 / 4 100.0%	3 / 3 100.0%	6 / 6 100.0%	5 / 5 100.0%	1 / 1 100.0%	2 / 2 100.0%	5 / 5 100.0%	1 / 1 100.0%	4 / 4 100.0%	5 / 5 100.0%	2 / 2 100.0%	3 / 3 100.0%	1 / 1 100.0%	3 / 3 100.0%	2 / 2 100.0%	4 / 4 100.0%	5 / 6 83.3%	4 / 4 100.0%	1 / 1 100.0%	7 / 7 100.0%	1 / 1 100.0%	0 / 0 0.0%	0 / 0 0.0%	0 / 0 0.0%	48 / 77 62.3%AB	27 / 77 35.1%ABE	1 / 77 1.3%	1 / 77 1.3%BE	
1	ATCC 43950	156 / 156 100.0%	8 / 8 100.0%	6 / 6 100.0%	6 / 6 100.0%	6 / 6 100.0%	12 / 12 100.0%	10 / 10 100.0%	2 / 2 100.0%	4 / 4 100.0%	10 / 10 100.0%	2 / 2 100.0%	8 / 8 100.0%	10 / 10 100.0%	4 / 4 100.0%	6 / 6 100.0%	2 / 2 100.0%	6 / 6 100.0%	4 / 4 100.0%	8 / 8 100.0%	16 / 16 100.0%	8 / 8 100.0%	2 / 2 100.0%	14 / 14 100.0%	2 / 2 100.0%	0 / 0 0.0%	0 / 0 0.0%	0 / 0 0.0%	100 / 156 64.1%AB	56 / 156 35.9%ABE			
13	Buchnera	99 / 411 24.1%	12 / 12 100.0%	0 / 13 0.0%	0 / 13 0.0%	10 / 13 76.9%	10 / 26 38.5%	4 / 26 15.4%	9 / 13 69.2%	7 / 13 53.8%	0 / 24 0.0%	2 / 13 15.4%	8 / 39 20.5%	0 / 13 0.0%	0 / 13 0.0%	9 / 13 69.2%	0 / 13 0.0%	10 / 13 76.9%	9 / 13 69.2%	0 / 26 0.0%	0 / 39 0.0%	0 / 12 0.0%	0 / 13 0.0%	9 / 38 23.7%	0 / 0 0.0%	0 / 0 0.0%	0 / 0 0.0%	0 / 0 0.0%	180 / 411 43.8%E	132 / 411 32.1%	61 / 411 14.8%AB	38 / 411 9.2%ABE	
13	Acyrthosiphon pisum symbiont P	99 / 411 24.1%	12 / 12 100.0%	0 / 13 0.0%	0 / 13 0.0%	10 / 13 76.9%	10 / 26 38.5%	4 / 26 15.4%	9 / 13 69.2%	7 / 13 53.8%	0 / 24 0.0%	2 / 13 15.4%	8 / 39 20.5%	0 / 13 0.0%	0 / 13 0.0%	9 / 13 69.2%	0 / 13 0.0%	10 / 13 76.9%	9 / 13 69.2%	0 / 26 0.0%	0 / 39 0.0%	0 / 12 0.0%	0 / 13 0.0%	9 / 38 23.7%	0 / 0 0.0%	0 / 0 0.0%	0 / 0 0.0%	0 / 0 0.0%	180 / 411 43.8%E	132 / 411 32.1%	61 / 411 14.8%AB	38 / 411 9.2%ABE	
1	Buchnera aphidicola (Schizaphis graminum)	9 / 32 28.1%	1 / 1 100.0%	0 / 1 0.0%	0 / 1 0.0%	1 / 1 100.0%	1 / 2 50.0%	1 / 2 50.0%	0 / 1 0.0%	0 / 1 0.0%	0 / 2 0.0%	1 / 1 100.0%	1 / 3 33.3%	0 / 1 0.0%	0 / 1 0.0%	1 / 1 100.0%	0 / 1 0.0%	1 / 1 100.0%	0 / 1 0.0%	0 / 2 0.0%	0 / 3 0.0%	0 / 1 0.0%	0 / 1 0.0%	1 / 3 33.3%	0 / 0 0.0%	0 / 0 0.0%	0 / 0 0.0%	0 / 0 0.0%	14 / 32 43.8%	9 / 32 28.1%E	6 / 32 18.8%AB	3 / 32 9.4%ABE	
1	Buchnera aphidicola str. Sg (Schizaphis graminum)	9 / 32 28.1%	1 / 1 100.0%	0 / 1 0.0%	0 / 1 0.0%	1 / 1 100.0%	1 / 2 50.0%	1 / 2 50.0%	0 / 1 0.0%	0 / 1 0.0%	0 / 2 0.0%	1 / 1 100.0%	1 / 3 33.3%	0 / 1 0.0%	0 / 1 0.0%	1 / 1 100.0%	0 / 1 0.0%	1 / 1 100.0%	0 / 1 0.0%	0 / 2 0.0%	0 / 3 0.0%	0 / 1 0.0%	0 / 1 0.0%	1 / 3 33.3%	0 / 0 0.0%	0 / 0 0.0%	0 / 0 0.0%	0 / 0 0.0%	14 / 32 43.8%	9 / 32 28.1%E	6 / 32 18.8%AB	3 / 32 9.4%ABE	
7	Acyrthosiphon pisum endosymbiont	68 / 221 30.8%	6 / 6 100.0%	0 / 7 0.0%	0 / 7 0.0%	7 / 7 100.0%	7 / 14 50.0%	0 / 14 0.0%	7 / 7 100.0%	7 / 7 100.0%	0 / 14 0.0%	0 / 7 0.0%	7 / 21 33.3%	0 / 7 0.0%	0 / 7 0.0%	7 / 7 100.0%	0 / 7 0.0%	7 / 7 100.0%	7 / 7 100.0%	0 / 14 0.0%	0 / 21 0.0%	0 / 6 0.0%	0 / 7 0.0%	6 / 20 30.0%	0 / 0 0.0%	0 / 0 0.0%	0 / 0 0.0%	0 / 0 0.0%	102 / 221 46.2%E	51 / 221 23.1%	41 / 221 18.6%AB	27 / 221 12.2%ABE	
2	Buchnera aphidicola str. APS (Acyrthosiphon pisum)	20 / 64 31.2%	2 / 2 100.0%	0 / 2 0.0%	0 / 2 0.0%	2 / 2 100.0%	2 / 4 50.0%	0 / 4 0.0%	2 / 2 100.0%	2 / 2 100.0%	0 / 4 0.0%	0 / 2 0.0%	2 / 6 33.3%	0 / 2 0.0%	0 / 2 0.0%	2 / 2 100.0%	0 / 2 0.0%	2 / 2 100.0%	2 / 2 100.0%	0 / 4 0.0%	0 / 6 0.0%	0 / 2 0.0%	0 / 2 0.0%	2 / 6 33.3%	0 / 0 0.0%	0 / 0 0.0%	0 / 0 0.0%	0 / 0 0.0%	24 / 64 37.5%E	20 / 64 31.2%	12 / 64 18.8%AB	8 / 64 12.5%ABE	
1	Buchnera aphidicola str. 5A (Acyrthosiphon pisum)	10 / 32 31.2%	1 / 1 100.0%	0 / 1 0.0%	0 / 1 0.0%	1 / 1 100.0%	1 / 2 50.0%	0 / 2 0.0%	1 / 1 100.0%	1 / 1 100.0%	0 / 2 0.0%	0 / 1 0.0%	1 / 3 33.3%	0 / 1 0.0%	0 / 1 0.0%	1 / 1 100.0%	0 / 1 0.0%	1 / 1 100.0%	1 / 1 100.0%	0 / 2 0.0%	0 / 3 0.0%	0 / 1 0.0%	0 / 1 0.0%	1 / 3 33.3%	0 / 0 0.0%	0 / 0 0.0%	0 / 0 0.0%	0 / 0 0.0%	16 / 32 50.0%E	6 / 32 18.8%AB	6 / 32 18.8%	4 / 32 12.5%ABE	
1	Buchnera aphidicola str. Tuc7 (Acyrthosiphon pisum)	10 / 32 31.2%	1 / 1 100.0%	0 / 1 0.0%	0 / 1 0.0%	1 / 1 100.0%	1 / 2 50.0%	0 / 2 0.0%	1 / 1 100.0%	1 / 1 100.0%	0 / 2 0.0%	0 / 1 0.0%	1 / 3 33.3%	0 / 1 0.0%	0 / 1 0.0%	1 / 1 100.0%	0 / 1 0.0%	1 / 1 100.0%	1 / 1 100.0%	0 / 2 0.0%	0 / 3 0.0%	0 / 1 0.0%	0 / 1 0.0%	1 / 3 33.3%	0 / 0 0.0%	0 / 0 0.0%	0 / 0 0.0%	0 / 0 0.0%	16 / 32 50.0%E	6 / 32 18.8%AB	6 / 32 18.8%	4 / 32 12.5%ABE	
1	Buchnera aphidicola str. JF98 (Acyrthosiphon pisum)	8 / 29 27.6%	0 / 0 0.0%	0 / 1 0.0%	0 / 1 0.0%	1 / 1 100.0%	1 / 2 50.0%	0 / 2 0.0%	1 / 1 100.0%	1 / 1 100.0%	0 / 2 0.0%	0 / 1 0.0%	1 / 3 33.3%	0 / 1 0.0%	0 / 1 0.0%	1 / 1 100.0%	0 / 1 0.0%	1 / 1 100.0%	1 / 1 100.0%	0 / 2 0.0%	0 / 3 0.0%	0 / 0 0.0%	0 / 1 0.0%	0 / 2 0.0%	0 / 0 0.0%	0 / 0 0.0%	0 / 0 0.0%	0 / 0 0.0%	14 / 29 48.3%E	7 / 29 24.1%	5 / 29 17.2%AB	3 / 29 10.3%ABE	
1	Buchnera aphidicola str. JF99 (Acyrthosiphon pisum)	10 / 32 31.2%	1 / 1 100.0%	0 / 1 0.0%	0 / 1 0.0%	1 / 1 100.0%	1 / 2 50.0%	0 / 2 0.0%	1 / 1 100.0%	1 / 1 100.0%	0 / 2 0.0%	0 / 1 0.0%	1 / 3 33.3%	0 / 1 0.0%	0 / 1 0.0%	1 / 1 100.0%	0 / 1 0.0%	1 / 1 100.0%	1 / 1 100.0%	0 / 2 0.0%	0 / 3 0.0%	0 / 1 0.0%	0 / 1 0.0%	1 / 3 33.3%	0 / 0 0.0%	0 / 0 0.0%	0 / 0 0.0%	0 / 0 0.0%	16 / 32 50.0%E	6 / 32 18.8%	6 / 32 18.8%AB	4 / 32 12.5%ABE	
1	Buchnera aphidicola str. TLW03 (Acyrthosiphon pisum)	10 / 32 31.2%	1 / 1 100.0%	0 / 1 0.0%	0 / 1 0.0%	1 / 1 100.0%	1 / 2 50.0%	0 / 2 0.0%	1 / 1 100.0%	1 / 1 100.0%	0 / 2 0.0%	0 / 1 0.0%	1 / 3 33.3%	0 / 1 0.0%	0 / 1 0.0%	1 / 1 100.0%	0 / 1 0.0%	1 / 1 100.0%	1 / 1 100.0%	0 / 2 0.0%	0 / 3 0.0%	0 / 1 0.0%	0 / 1 0.0%	1 / 3 33.3%	0 / 0 0.0%	0 / 0 0.0%	0 / 0 0.0%	0 / 0 0.0%	16 / 32 50.0%E	6 / 32 18.8%AB	6 / 32 18.8%	4 / 32 12.5%ABE	
1	Buchnera aphidicola str. LL01 (Acyrthosiphon pisum)	10 / 32 31.2%	1 / 1 100.0%	0 / 1 0.0%	0 / 1 0.0%	1 / 1 100.0%	1 / 2 50.0%	0 / 2 0.0%	1 / 1 100.0%	1 / 1 100.0%	0 / 2 0.0%	0 / 1 0.0%	1 / 3 33.3%	0 / 1 0.0%	0 / 1 0.0%	1 / 1 100.0%	0 / 1 0.0%	1 / 1 100.0%	1 / 1 100.0%	0 / 2 0.0%	0 / 3 0.0%	0 / 1 0.0%	0 / 1 0.0%	1 / 3 33.3%	0 / 0 0.0%	0 / 0 0.0%	0 / 0 0.0%	0 / 0 0.0%	16 / 32 50.0%E	6 / 32 18.8%AB	6 / 32 18.8%	4 / 32 12.5%ABE	
1	Buchnera aphidicola (Uroleucon ambrosiae)	7 / 32 21.9%	1 / 1 100.0%	0 / 1 0.0%	0 / 1 0.0%	1 / 1 100.0%	1 / 2 50.0%	1 / 2 50.0%	1 / 1 100.0%	0 / 1 0.0%	0 / 2 0.0%	0 / 1 0.0%	0 / 3 0.0%	0 / 1 0.0%	0 / 1 0.0%	0 / 1 0.0%	0 / 1 0.0%	1 / 1 100.0%	1 / 1 100.0%	0 / 2 0.0%	0 / 3 0.0%	0 / 1 0.0%	0 / 1 0.0%	0 / 3 0.0%	0 / 0 0.0%	0 / 0 0.0%	0 / 0 0.0%	0 / 0 0.0%	16 / 32 50.0%E	9 / 32 28.1%	6 / 32 18.8%AB	1 / 32 3.1%ABE	
1	Buchnera aphidicola str. Ua (Uroleucon ambrosiae)	7 / 32 21.9%	1 / 1 100.0%	0 / 1 0.0%	0 / 1 0.0%	1 / 1 100.0%	1 / 2 50.0%	1 / 2 50.0%	1 / 1 100.0%	0 / 1 0.0%	0 / 2 0.0%	0 / 1 0.0%	0 / 3 0.0%	0 / 1 0.0%	0 / 1 0.0%	0 / 1 0.0%	0 / 1 0.0%	1 / 1 100.0%	1 / 1 100.0%	0 / 2 0.0%	0 / 3 0.0%	0 / 1 0.0%	0 / 1 0.0%	0 / 3 0.0%	0 / 0 0.0%	0 / 0 0.0%	0 / 0 0.0%	0 / 0 0.0%	16 / 32 50.0%E	9 / 32 28.1%	6 / 32 18.8%AB	1 / 32 3.1%ABE	
1	Buchnera aphidicola (Baizongia pistaciae)	4 / 32 12.5%	1 / 1 100.0%	0 / 1 0.0%	0 / 1 0.0%	0 / 1 0.0%	1 / 2 50.0%	1 / 2 50.0%	0 / 1 0.0%	0 / 1 0.0%	0 / 2 0.0%	0 / 1 0.0%	0 / 3 0.0%	0 / 1 0.0%	0 / 1 0.0%	0 / 1 0.0%	0 / 1 0.0%	0 / 1 0.0%	0 / 1 0.0%	0 / 2 0.0%	0 / 3 0.0%	0 / 1 0.0%	0 / 1 0.0%	1 / 3 33.3%	0 / 0 0.0%	0 / 0 0.0%	0 / 0 0.0%	0 / 0 0.0%	20 / 32 62.5%	8 / 32 25.0%E	3 / 32 9.4%ABE	1 / 32 3.1%AB	
1	Buchnera aphidicola (subsp. Baizongia pistaciae)	4 / 32 12.5%	1 / 1 100.0%	0 / 1 0.0%	0 / 1 0.0%	0 / 1 0.0%	1 / 2 50.0%	1 / 2 50.0%	0 / 1 0.0%	0 / 1 0.0%	0 / 2 0.0%	0 / 1 0.0%	0 / 3 0.0%	0 / 1 0.0%	0 / 1 0.0%	0 / 1 0.0%	0 / 1 0.0%	0 / 1 0.0%	0 / 1 0.0%	0 / 2 0.0%	0 / 3 0.0%	0 / 1 0.0%	0 / 1 0.0%	1 / 3 33.3%	0 / 0 0.0%	0 / 0 0.0%	0 / 0 0.0%	0 / 0 0.0%	20 / 32 62.5%	8 / 32 25.0%E	3 / 32 9.4%ABE	1 / 32 3.1%AB	
1	Buchnera aphidicola (Cinara tujafilina)	1 / 31 3.2%	1 / 1 100.0%	0 / 1 0.0%	0 / 1 0.0%	0 / 1 0.0%	0 / 2 0.0%	0 / 2 0.0%	0 / 1 0.0%	0 / 1 0.0%	0 / 1 0.0%	0 / 1 0.0%	0 / 3 0.0%	0 / 1 0.0%	0 / 1 0.0%	0 / 1 0.0%	0 / 1 0.0%	0 / 1 0.0%	0 / 1 0.0%	0 / 2 0.0%	0 / 3 0.0%	0 / 1 0.0%	0 / 1 0.0%	0 / 3 0.0%	0 / 0 0.0%	0 / 0 0.0%	0 / 0 0.0%	0 / 0 0.0%	18 / 31 58.1%E	12 / 31 38.7%	1 / 31 3.2%ABE		
1	Buchnera aphidicola (Cinara cedri)	1 / 31 3.2%	1 / 1 100.0%	0 / 1 0.0%	0 / 1 0.0%	0 / 1 0.0%	0 / 2 0.0%	0 / 2 0.0%	0 / 1 0.0%	0 / 1 0.0%	0 / 1 0.0%	0 / 1 0.0%	0 / 3 0.0%	0 / 1 0.0%	0 / 1 0.0%	0 / 1 0.0%	0 / 1 0.0%	0 / 1 0.0%	0 / 1 0.0%	0 / 2 0.0%	0 / 3 0.0%	0 / 1 0.0%	0 / 1 0.0%	0 / 3 0.0%	0 / 0 0.0%	0 / 0 0.0%	0 / 0 0.0%	0 / 0 0.0%	19 / 31 61.3%	11 / 31 35.5%E	1 / 31 3.2%ABE		
1	Buchnera aphidicola BCc	1 / 31 3.2%	1 / 1 100.0%	0 / 1 0.0%	0 / 1 0.0%	0 / 1 0.0%	0 / 2 0.0%	0 / 2 0.0%	0 / 1 0.0%	0 / 1 0.0%	0 / 1 0.0%	0 / 1 0.0%	0 / 3 0.0%	0 / 1 0.0%	0 / 1 0.0%	0 / 1 0.0%	0 / 1 0.0%	0 / 1 0.0%	0 / 1 0.0%	0 / 2 0.0%	0 / 3 0.0%	0 / 1 0.0%	0 / 1 0.0%	0 / 3 0.0%	0 / 0 0.0%	0 / 0 0.0%	0 / 0 0.0%	0 / 0 0.0%	19 / 31 61.3%	11 / 31 35.5%E	1 / 31 3.2%ABE		
1	Buchnera aphidicola (Acyrthosiphon kondoi)	9 / 32 28.1%	1 / 1 100.0%	0 / 1 0.0%	0 / 1 0.0%	1 / 1 100.0%	0 / 2 0.0%	1 / 2 50.0%	1 / 1 100.0%	0 / 1 0.0%	0 / 2 0.0%	1 / 1 100.0%	0 / 3 0.0%	0 / 1 0.0%	0 / 1 0.0%	1 / 1 100.0%	0 / 1 0.0%	1 / 1 100.0%	1 / 1 100.0%	0 / 2 0.0%	0 / 3 0.0%	0 / 1 0.0%	0 / 1 0.0%	1 / 3 33.3%	0 / 0 0.0%	0 / 0 0.0%	0 / 0 0.0%	0 / 0 0.0%	16 / 32 50.0%E	7 / 32 21.9%	7 / 32 21.9%AB	2 / 32 6.2%ABE	
1	Buchnera aphidicola str. Ak (Acyrthosiphon kondoi)	9 / 32 28.1%	1 / 1 100.0%	0 / 1 0.0%	0 / 1 0.0%	1 / 1 100.0%	0 / 2 0.0%	1 / 2 50.0%	1 / 1 100.0%	0 / 1 0.0%	0 / 2 0.0%	1 / 1 100.0%	0 / 3 0.0%	0 / 1 0.0%	0 / 1 0.0%	1 / 1 100.0%	0 / 1 0.0%	1 / 1 100.0%	1 / 1 100.0%	0 / 2 0.0%	0 / 3 0.0%	0 / 1 0.0%	0 / 1 0.0%	1 / 3 33.3%	0 / 0 0.0%	0 / 0 0.0%	0 / 0 0.0%	0 / 0 0.0%	16 / 32 50.0%E	7 / 32 21.9%	7 / 32 21.9%AB	2 / 32 6.2%ABE	
3	Rahnella	228 / 228 100.0%	11 / 11 100.0%	9 / 9 100.0%	9 / 9 100.0%	8 / 8 100.0%	18 / 18 100.0%	15 / 15 100.0%	3 / 3 100.0%	6 / 6 100.0%	14 / 14 100.0%	3 / 3 100.0%	16 / 16 100.0%	15 / 15 100.0%	6 / 6 100.0%	8 / 8 100.0%	3 / 3 100.0%	6 / 6 100.0%	6 / 6 100.0%	14 / 14 100.0%	18 / 18 100.0%	13 / 13 100.0%	3 / 3 100.0%	24 / 24 100.0%	0 / 0 0.0%	0 / 0 0.0%	0 / 0 0.0%	0 / 0 0.0%	144 / 228 63.2%AB	84 / 228 36.8%ABE			
2	ATCC 33071	152 / 152 100.0%	8 / 8 100.0%	6 / 6 100.0%	6 / 6 100.0%	5 / 5 100.0%	12 / 12 100.0%	10 / 10 100.0%	2 / 2 100.0%	4 / 4 100.0%	9 / 9 100.0%	2 / 2 100.0%	11 / 11 100.0%	10 / 10 100.0%	4 / 4 100.0%	5 / 5 100.0%	2 / 2 100.0%	4 / 4 100.0%	4 / 4 100.0%	9 / 9 100.0%	12 / 12 100.0%	9 / 9 100.0%	2 / 2 100.0%	16 / 16 100.0%	0 / 0 0.0%	0 / 0 0.0%	0 / 0 0.0%	0 / 0 0.0%	95 / 152 62.5%AB	57 / 152 37.5%ABE			
1	Rahnella aquatilis ATCC 33071	76 / 76 100.0%	4 / 4 100.0%	3 / 3 100.0%	3 / 3 100.0%	3 / 3 100.0%	6 / 6 100.0%	5 / 5 100.0%	1 / 1 100.0%	2 / 2 100.0%	5 / 5 100.0%	1 / 1 100.0%	5 / 5 100.0%	5 / 5 100.0%	2 / 2 100.0%	3 / 3 100.0%	1 / 1 100.0%	2 / 2 100.0%	2 / 2 100.0%	4 / 4 100.0%	6 / 6 100.0%	4 / 4 100.0%	1 / 1 100.0%	8 / 8 100.0%	0 / 0 0.0%	0 / 0 0.0%	0 / 0 0.0%	0 / 0 0.0%	48 / 76 63.2%AB	28 / 76 36.8%ABE			
1	Rahnella aquatilis HX2	76 / 76 100.0%	4 / 4 100.0%	3 / 3 100.0%	3 / 3 100.0%	2 / 2 100.0%	6 / 6 100.0%	5 / 5 100.0%	1 / 1 100.0%	2 / 2 100.0%	4 / 4 100.0%	1 / 1 100.0%	6 / 6 100.0%	5 / 5 100.0%	2 / 2 100.0%	2 / 2 100.0%	1 / 1 100.0%	2 / 2 100.0%	2 / 2 100.0%	5 / 5 100.0%	6 / 6 100.0%	5 / 5 100.0%	1 / 1 100.0%	8 / 8 100.0%	0 / 0 0.0%	0 / 0 0.0%	0 / 0 0.0%	0 / 0 0.0%	47 / 76 61.8%AB	29 / 76 38.2%ABE			
1	Rahnella aquatilis Y9602	76 / 76 100.0%	3 / 3 100.0%	3 / 3 100.0%	3 / 3 100.0%	3 / 3 100.0%	6 / 6 100.0%	5 / 5 100.0%	1 / 1 100.0%	2 / 2 100.0%	5 / 5 100.0%	1 / 1 100.0%	5 / 5 100.0%	5 / 5 100.0%	2 / 2 100.0%	3 / 3 100.0%	1 / 1 100.0%	2 / 2 100.0%	2 / 2 100.0%	5 / 5 100.0%	6 / 6 100.0%	4 / 4 100.0%	1 / 1 100.0%	8 / 8 100.0%	0 / 0 0.0%	0 / 0 0.0%	0 / 0 0.0%	0 / 0 0.0%	49 / 76 64.5%AB	27 / 76 35.5%ABE			
2	Wigglesworthia	4 / 69 5.8%	2 / 2 100.0%	0 / 2 0.0%	0 / 2 0.0%	0 / 2 0.0%	0 / 4 0.0%	0 / 4 0.0%	0 / 2 0.0%	0 / 2 0.0%	0 / 4 0.0%	0 / 2 0.0%	0 / 6 0.0%	0 / 2 0.0%	0 / 2 0.0%	0 / 2 0.0%	0 / 2 0.0%	0 / 3 0.0%	0 / 2 0.0%	0 / 4 0.0%	0 / 6 0.0%	2 / 4 50.0%	0 / 2 0.0%	0 / 8 0.0%	0 / 0 0.0%	0 / 0 0.0%	0 / 0 0.0%	0 / 0 0.0%	34 / 69 49.3%E	31 / 69 44.9%	4 / 69 5.8%ABE		
2	Wigglesworthia glossinidia	4 / 69 5.8%	2 / 2 100.0%	0 / 2 0.0%	0 / 2 0.0%	0 / 2 0.0%	0 / 4 0.0%	0 / 4 0.0%	0 / 2 0.0%	0 / 2 0.0%	0 / 4 0.0%	0 / 2 0.0%	0 / 6 0.0%	0 / 2 0.0%	0 / 2 0.0%	0 / 2 0.0%	0 / 2 0.0%	0 / 3 0.0%	0 / 2 0.0%	0 / 4 0.0%	0 / 6 0.0%	2 / 4 50.0%	0 / 2 0.0%	0 / 8 0.0%	0 / 0 0.0%	0 / 0 0.0%	0 / 0 0.0%	0 / 0 0.0%	34 / 69 49.3%E	31 / 69 44.9%	4 / 69 5.8%ABE		
1	Glossina morsitans P-endosymbiont	3 / 35 8.6%	1 / 1 100.0%	0 / 1 0.0%	0 / 1 0.0%	0 / 1 0.0%	0 / 2 0.0%	0 / 2 0.0%	0 / 1 0.0%	0 / 1 0.0%	0 / 2 0.0%	0 / 1 0.0%	0 / 3 0.0%	0 / 1 0.0%	0 / 1 0.0%	0 / 1 0.0%	0 / 1 0.0%	0 / 2 0.0%	0 / 1 0.0%	0 / 2 0.0%	0 / 3 0.0%	2 / 2 100.0%	0 / 1 0.0%	0 / 4 0.0%	0 / 0 0.0%	0 / 0 0.0%	0 / 0 0.0%	0 / 0 0.0%	22 / 35 62.9%E	10 / 35 28.6%	3 / 35 8.6%ABE		
1	Glossina brevipalpis P-endosymbiont	1 / 34 2.9%	1 / 1 100.0%	0 / 1 0.0%	0 / 1 0.0%	0 / 1 0.0%	0 / 2 0.0%	0 / 2 0.0%	0 / 1 0.0%	0 / 1 0.0%	0 / 2 0.0%	0 / 1 0.0%	0 / 3 0.0%	0 / 1 0.0%	0 / 1 0.0%	0 / 1 0.0%	0 / 1 0.0%	0 / 1 0.0%	0 / 1 0.0%	0 / 2 0.0%	0 / 3 0.0%	0 / 2 0.0%	0 / 1 0.0%	0 / 4 0.0%	0 / 0 0.0%	0 / 0 0.0%	0 / 0 0.0%	0 / 0 0.0%	21 / 34 61.8%	12 / 34 35.3%E	1 / 34 2.9%ABE		
6	Pantoea	460 / 460 100.0%	21 / 21 100.0%	18 / 18 100.0%	19 / 19 100.0%	20 / 20 100.0%	27 / 27 100.0%	36 / 36 100.0%	7 / 7 100.0%	18 / 18 100.0%	33 / 33 100.0%	6 / 6 100.0%	24 / 24 100.0%	24 / 24 100.0%	12 / 12 100.0%	24 / 24 100.0%	6 / 6 100.0%	18 / 18 100.0%	12 / 12 100.0%	20 / 20 100.0%	40 / 40 100.0%	20 / 20 100.0%	6 / 6 100.0%	48 / 48 100.0%	1 / 1 100.0%	0 / 0 0.0%	0 / 0 0.0%	0 / 0 0.0%	279 / 460 60.7%AB	181 / 460 39.3%ABE			
4	"Bacillus ananas" Serrano 1928	303 / 303 100.0%	14 / 14 100.0%	12 / 12 100.0%	13 / 13 100.0%	13 / 13 100.0%	17 / 17 100.0%	24 / 24 100.0%	4 / 4 100.0%	12 / 12 100.0%	22 / 22 100.0%	4 / 4 100.0%	16 / 16 100.0%	16 / 16 100.0%	8 / 8 100.0%	16 / 16 100.0%	4 / 4 100.0%	12 / 12 100.0%	8 / 8 100.0%	12 / 12 100.0%	27 / 27 100.0%	13 / 13 100.0%	4 / 4 100.0%	32 / 32 100.0%	0 / 0 0.0%	0 / 0 0.0%	0 / 0 0.0%	0 / 0 0.0%	184 / 303 60.7%AB	119 / 303 39.3%ABE			
1	Pantoea ananatis LMG 20103	67 / 67 100.0%	3 / 3 100.0%	3 / 3 100.0%	1 / 1 100.0%	0 / 0 0.0%	2 / 2 100.0%	6 / 6 100.0%	1 / 1 100.0%	3 / 3 100.0%	3 / 3 100.0%	1 / 1 100.0%	4 / 4 100.0%	4 / 4 100.0%	2 / 2 100.0%	4 / 4 100.0%	1 / 1 100.0%	3 / 3 100.0%	2 / 2 100.0%	3 / 3 100.0%	7 / 7 100.0%	5 / 5 100.0%	1 / 1 100.0%	8 / 8 100.0%	0 / 0 0.0%	0 / 0 0.0%	0 / 0 0.0%	0 / 0 0.0%	36 / 67 53.7%AB	31 / 67 46.3%ABE			
1	Pantoea ananatis AJ13355	78 / 78 100.0%	4 / 4 100.0%	3 / 3 100.0%	4 / 4 100.0%	3 / 3 100.0%	5 / 5 100.0%	6 / 6 100.0%	1 / 1 100.0%	3 / 3 100.0%	5 / 5 100.0%	1 / 1 100.0%	4 / 4 100.0%	4 / 4 100.0%	2 / 2 100.0%	4 / 4 100.0%	1 / 1 100.0%	3 / 3 100.0%	2 / 2 100.0%	3 / 3 100.0%	7 / 7 100.0%	4 / 4 100.0%	1 / 1 100.0%	8 / 8 100.0%	0 / 0 0.0%	0 / 0 0.0%	0 / 0 0.0%	0 / 0 0.0%	47 / 78 60.3%AB	31 / 78 39.7%ABE			
1	Pantoea ananatis PA13	82 / 82 100.0%	4 / 4 100.0%	3 / 3 100.0%	4 / 4 100.0%	7 / 7 100.0%	5 / 5 100.0%	6 / 6 100.0%	1 / 1 100.0%	3 / 3 100.0%	9 / 9 100.0%	1 / 1 100.0%	4 / 4 100.0%	4 / 4 100.0%	2 / 2 100.0%	4 / 4 100.0%	1 / 1 100.0%	3 / 3 100.0%	2 / 2 100.0%	3 / 3 100.0%	7 / 7 100.0%	0 / 0 0.0%	1 / 1 100.0%	8 / 8 100.0%	0 / 0 0.0%	0 / 0 0.0%	0 / 0 0.0%	0 / 0 0.0%	55 / 82 67.1%AB	27 / 82 32.9%ABE			
1	Pantoea ananatis LMG 5342	76 / 76 100.0%	3 / 3 100.0%	3 / 3 100.0%	4 / 4 100.0%	3 / 3 100.0%	5 / 5 100.0%	6 / 6 100.0%	1 / 1 100.0%	3 / 3 100.0%	5 / 5 100.0%	1 / 1 100.0%	4 / 4 100.0%	4 / 4 100.0%	2 / 2 100.0%	4 / 4 100.0%	1 / 1 100.0%	3 / 3 100.0%	2 / 2 100.0%	3 / 3 100.0%	6 / 6 100.0%	4 / 4 100.0%	1 / 1 100.0%	8 / 8 100.0%	0 / 0 0.0%	0 / 0 0.0%	0 / 0 0.0%	0 / 0 0.0%	46 / 76 60.5%AB	30 / 76 39.5%ABE			
1	BCC 105	77 / 77 100.0%	4 / 4 100.0%	3 / 3 100.0%	3 / 3 100.0%	3 / 3 100.0%	5 / 5 100.0%	6 / 6 100.0%	1 / 1 100.0%	3 / 3 100.0%	5 / 5 100.0%	1 / 1 100.0%	4 / 4 100.0%	4 / 4 100.0%	2 / 2 100.0%	4 / 4 100.0%	1 / 1 100.0%	3 / 3 100.0%	2 / 2 100.0%	4 / 4 100.0%	6 / 6 100.0%	4 / 4 100.0%	1 / 1 100.0%	8 / 8 100.0%	0 / 0 0.0%	0 / 0 0.0%	0 / 0 0.0%	0 / 0 0.0%	46 / 77 59.7%AB	31 / 77 40.3%ABE			
1	Pantoea vagans C9-1	77 / 77 100.0%	4 / 4 100.0%	3 / 3 100.0%	3 / 3 100.0%	3 / 3 100.0%	5 / 5 100.0%	6 / 6 100.0%	1 / 1 100.0%	3 / 3 100.0%	5 / 5 100.0%	1 / 1 100.0%	4 / 4 100.0%	4 / 4 100.0%	2 / 2 100.0%	4 / 4 100.0%	1 / 1 100.0%	3 / 3 100.0%	2 / 2 100.0%	4 / 4 100.0%	6 / 6 100.0%	4 / 4 100.0%	1 / 1 100.0%	8 / 8 100.0%	0 / 0 0.0%	0 / 0 0.0%	0 / 0 0.0%	0 / 0 0.0%	46 / 77 59.7%AB	31 / 77 40.3%ABE			
1	Pantoea sp. At-9b	80 / 80 100.0%	3 / 3 100.0%	3 / 3 100.0%	3 / 3 100.0%	4 / 4 100.0%	5 / 5 100.0%	6 / 6 100.0%	2 / 2 100.0%	3 / 3 100.0%	6 / 6 100.0%	1 / 1 100.0%	4 / 4 100.0%	4 / 4 100.0%	2 / 2 100.0%	4 / 4 100.0%	1 / 1 100.0%	3 / 3 100.0%	2 / 2 100.0%	4 / 4 100.0%	7 / 7 100.0%	3 / 3 100.0%	1 / 1 100.0%	8 / 8 100.0%	1 / 1 100.0%	0 / 0 0.0%	0 / 0 0.0%	0 / 0 0.0%	49 / 80 61.2%AB	31 / 80 38.8%ABE			
1	Sodalis	66 / 66 100.0%	3 / 3 100.0%	3 / 3 100.0%	2 / 2 100.0%	4 / 4 100.0%	6 / 6 100.0%	4 / 4 100.0%	1 / 1 100.0%	1 / 1 100.0%	5 / 5 100.0%	1 / 1 100.0%	4 / 4 100.0%	4 / 4 100.0%	2 / 2 100.0%	3 / 3 100.0%	1 / 1 100.0%	3 / 3 100.0%	1 / 1 100.0%	4 / 4 100.0%	4 / 4 100.0%	3 / 3 100.0%	1 / 1 100.0%	6 / 6 100.0%	0 / 0 0.0%	0 / 0 0.0%	0 / 0 0.0%	0 / 0 0.0%	45 / 66 68.2%AB	21 / 66 31.8%ABE			
1	DSM 16929	66 / 66 100.0%	3 / 3 100.0%	3 / 3 100.0%	2 / 2 100.0%	4 / 4 100.0%	6 / 6 100.0%	4 / 4 100.0%	1 / 1 100.0%	1 / 1 100.0%	5 / 5 100.0%	1 / 1 100.0%	4 / 4 100.0%	4 / 4 100.0%	2 / 2 100.0%	3 / 3 100.0%	1 / 1 100.0%	3 / 3 100.0%	1 / 1 100.0%	4 / 4 100.0%	4 / 4 100.0%	3 / 3 100.0%	1 / 1 100.0%	6 / 6 100.0%	0 / 0 0.0%	0 / 0 0.0%	0 / 0 0.0%	0 / 0 0.0%	45 / 66 68.2%AB	21 / 66 31.8%ABE			
1	Sodalis glossinidius 'morsitans'	66 / 66 100.0%	3 / 3 100.0%	3 / 3 100.0%	2 / 2 100.0%	4 / 4 100.0%	6 / 6 100.0%	4 / 4 100.0%	1 / 1 100.0%	1 / 1 100.0%	5 / 5 100.0%	1 / 1 100.0%	4 / 4 100.0%	4 / 4 100.0%	2 / 2 100.0%	3 / 3 100.0%	1 / 1 100.0%	3 / 3 100.0%	1 / 1 100.0%	4 / 4 100.0%	4 / 4 100.0%	3 / 3 100.0%	1 / 1 100.0%	6 / 6 100.0%	0 / 0 0.0%	0 / 0 0.0%	0 / 0 0.0%	0 / 0 0.0%	45 / 66 68.2%AB	21 / 66 31.8%ABE			
5	Pectobacterium	381 / 381 100.0%	19 / 19 100.0%	15 / 15 100.0%	15 / 15 100.0%	15 / 15 100.0%	30 / 30 100.0%	25 / 25 100.0%	5 / 5 100.0%	10 / 10 100.0%	25 / 25 100.0%	5 / 5 100.0%	28 / 28 100.0%	20 / 20 100.0%	10 / 10 100.0%	15 / 15 100.0%	5 / 5 100.0%	15 / 15 100.0%	10 / 10 100.0%	20 / 20 100.0%	29 / 29 100.0%	20 / 20 100.0%	5 / 5 100.0%	40 / 40 100.0%	0 / 0 0.0%	0 / 0 0.0%	0 / 0 0.0%	0 / 0 0.0%	239 / 381 62.7%AB	142 / 381 37.3%ABE			
2	"Bacillus carotovorus" Jones 1901	153 / 153 100.0%	7 / 7 100.0%	6 / 6 100.0%	6 / 6 100.0%	6 / 6 100.0%	12 / 12 100.0%	10 / 10 100.0%	2 / 2 100.0%	4 / 4 100.0%	10 / 10 100.0%	2 / 2 100.0%	11 / 11 100.0%	8 / 8 100.0%	4 / 4 100.0%	6 / 6 100.0%	2 / 2 100.0%	6 / 6 100.0%	4 / 4 100.0%	8 / 8 100.0%	13 / 13 100.0%	8 / 8 100.0%	2 / 2 100.0%	16 / 16 100.0%	0 / 0 0.0%	0 / 0 0.0%	0 / 0 0.0%	0 / 0 0.0%	97 / 153 63.4%AB	56 / 153 36.6%ABE			
2	Erwinia carotovora carotovora	153 / 153 100.0%	7 / 7 100.0%	6 / 6 100.0%	6 / 6 100.0%	6 / 6 100.0%	12 / 12 100.0%	10 / 10 100.0%	2 / 2 100.0%	4 / 4 100.0%	10 / 10 100.0%	2 / 2 100.0%	11 / 11 100.0%	8 / 8 100.0%	4 / 4 100.0%	6 / 6 100.0%	2 / 2 100.0%	6 / 6 100.0%	4 / 4 100.0%	8 / 8 100.0%	13 / 13 100.0%	8 / 8 100.0%	2 / 2 100.0%	16 / 16 100.0%	0 / 0 0.0%	0 / 0 0.0%	0 / 0 0.0%	0 / 0 0.0%	97 / 153 63.4%AB	56 / 153 36.6%ABE			
1	Pectobacterium carotovorum subsp. carotovorum PC1	78 / 78 100.0%	4 / 4 100.0%	3 / 3 100.0%	3 / 3 100.0%	3 / 3 100.0%	6 / 6 100.0%	5 / 5 100.0%	1 / 1 100.0%	2 / 2 100.0%	5 / 5 100.0%	1 / 1 100.0%	6 / 6 100.0%	4 / 4 100.0%	2 / 2 100.0%	3 / 3 100.0%	1 / 1 100.0%	3 / 3 100.0%	2 / 2 100.0%	4 / 4 100.0%	7 / 7 100.0%	4 / 4 100.0%	1 / 1 100.0%	8 / 8 100.0%	0 / 0 0.0%	0 / 0 0.0%	0 / 0 0.0%	0 / 0 0.0%	49 / 78 62.8%AB	29 / 78 37.2%ABE			
1	Pectobacterium carotovorum subsp. carotovorum PCC21	75 / 75 100.0%	3 / 3 100.0%	3 / 3 100.0%	3 / 3 100.0%	3 / 3 100.0%	6 / 6 100.0%	5 / 5 100.0%	1 / 1 100.0%	2 / 2 100.0%	5 / 5 100.0%	1 / 1 100.0%	5 / 5 100.0%	4 / 4 100.0%	2 / 2 100.0%	3 / 3 100.0%	1 / 1 100.0%	3 / 3 100.0%	2 / 2 100.0%	4 / 4 100.0%	6 / 6 100.0%	4 / 4 100.0%	1 / 1 100.0%	8 / 8 100.0%	0 / 0 0.0%	0 / 0 0.0%	0 / 0 0.0%	0 / 0 0.0%	48 / 75 64.0%AB	27 / 75 36.0%ABE			
1	"Bacillus atrosepticus" van Hall 1902	76 / 76 100.0%	4 / 4 100.0%	3 / 3 100.0%	3 / 3 100.0%	3 / 3 100.0%	6 / 6 100.0%	5 / 5 100.0%	1 / 1 100.0%	2 / 2 100.0%	5 / 5 100.0%	1 / 1 100.0%	5 / 5 100.0%	4 / 4 100.0%	2 / 2 100.0%	3 / 3 100.0%	1 / 1 100.0%	3 / 3 100.0%	2 / 2 100.0%	4 / 4 100.0%	6 / 6 100.0%	4 / 4 100.0%	1 / 1 100.0%	8 / 8 100.0%	0 / 0 0.0%	0 / 0 0.0%	0 / 0 0.0%	0 / 0 0.0%	48 / 76 63.2%AB	28 / 76 36.8%ABE			
1	Erwinia carotovora subsp. atroseptica SCRI1043	76 / 76 100.0%	4 / 4 100.0%	3 / 3 100.0%	3 / 3 100.0%	3 / 3 100.0%	6 / 6 100.0%	5 / 5 100.0%	1 / 1 100.0%	2 / 2 100.0%	5 / 5 100.0%	1 / 1 100.0%	5 / 5 100.0%	4 / 4 100.0%	2 / 2 100.0%	3 / 3 100.0%	1 / 1 100.0%	3 / 3 100.0%	2 / 2 100.0%	4 / 4 100.0%	6 / 6 100.0%	4 / 4 100.0%	1 / 1 100.0%	8 / 8 100.0%	0 / 0 0.0%	0 / 0 0.0%	0 / 0 0.0%	0 / 0 0.0%	48 / 76 63.2%AB	28 / 76 36.8%ABE			
1	ATCC 43316	75 / 75 100.0%	4 / 4 100.0%	3 / 3 100.0%	3 / 3 100.0%	3 / 3 100.0%	6 / 6 100.0%	5 / 5 100.0%	1 / 1 100.0%	2 / 2 100.0%	5 / 5 100.0%	1 / 1 100.0%	6 / 6 100.0%	4 / 4 100.0%	2 / 2 100.0%	3 / 3 100.0%	1 / 1 100.0%	3 / 3 100.0%	2 / 2 100.0%	4 / 4 100.0%	4 / 4 100.0%	4 / 4 100.0%	1 / 1 100.0%	8 / 8 100.0%	0 / 0 0.0%	0 / 0 0.0%	0 / 0 0.0%	0 / 0 0.0%	46 / 75 61.3%AB	29 / 75 38.7%ABE			
1	Pectobacterium carotovorum subsp. wasabiae WPP163	75 / 75 100.0%	4 / 4 100.0%	3 / 3 100.0%	3 / 3 100.0%	3 / 3 100.0%	6 / 6 100.0%	5 / 5 100.0%	1 / 1 100.0%	2 / 2 100.0%	5 / 5 100.0%	1 / 1 100.0%	6 / 6 100.0%	4 / 4 100.0%	2 / 2 100.0%	3 / 3 100.0%	1 / 1 100.0%	3 / 3 100.0%	2 / 2 100.0%	4 / 4 100.0%	4 / 4 100.0%	4 / 4 100.0%	1 / 1 100.0%	8 / 8 100.0%	0 / 0 0.0%	0 / 0 0.0%	0 / 0 0.0%	0 / 0 0.0%	46 / 75 61.3%AB	29 / 75 38.7%ABE			
1	Pectobacterium sp. SCC3193	77 / 77 100.0%	4 / 4 100.0%	3 / 3 100.0%	3 / 3 100.0%	3 / 3 100.0%	6 / 6 100.0%	5 / 5 100.0%	1 / 1 100.0%	2 / 2 100.0%	5 / 5 100.0%	1 / 1 100.0%	6 / 6 100.0%	4 / 4 100.0%	2 / 2 100.0%	3 / 3 100.0%	1 / 1 100.0%	3 / 3 100.0%	2 / 2 100.0%	4 / 4 100.0%	6 / 6 100.0%	4 / 4 100.0%	1 / 1 100.0%	8 / 8 100.0%	0 / 0 0.0%	0 / 0 0.0%	0 / 0 0.0%	0 / 0 0.0%	48 / 77 62.3%AB	29 / 77 37.7%ABE			
1	Raoultella	79 / 79 100.0%	3 / 3 100.0%	3 / 3 100.0%	4 / 4 100.0%	0 / 0 0.0%	6 / 6 100.0%	7 / 7 100.0%	1 / 1 100.0%	3 / 3 100.0%	2 / 2 100.0%	1 / 1 100.0%	6 / 6 100.0%	5 / 5 100.0%	2 / 2 100.0%	4 / 4 100.0%	1 / 1 100.0%	3 / 3 100.0%	2 / 2 100.0%	4 / 4 100.0%	5 / 5 100.0%	8 / 8 100.0%	1 / 1 100.0%	7 / 7 100.0%	1 / 1 100.0%	0 / 0 0.0%	0 / 0 0.0%	0 / 0 0.0%	42 / 79 53.2%AB	37 / 79 46.8%ABE			
1	ATCC 31898	79 / 79 100.0%	3 / 3 100.0%	3 / 3 100.0%	4 / 4 100.0%	0 / 0 0.0%	6 / 6 100.0%	7 / 7 100.0%	1 / 1 100.0%	3 / 3 100.0%	2 / 2 100.0%	1 / 1 100.0%	6 / 6 100.0%	5 / 5 100.0%	2 / 2 100.0%	4 / 4 100.0%	1 / 1 100.0%	3 / 3 100.0%	2 / 2 100.0%	4 / 4 100.0%	5 / 5 100.0%	8 / 8 100.0%	1 / 1 100.0%	7 / 7 100.0%	1 / 1 100.0%	0 / 0 0.0%	0 / 0 0.0%	0 / 0 0.0%	42 / 79 53.2%AB	37 / 79 46.8%ABE			
1	Raoultella ornithinolytica B6	79 / 79 100.0%	3 / 3 100.0%	3 / 3 100.0%	4 / 4 100.0%	0 / 0 0.0%	6 / 6 100.0%	7 / 7 100.0%	1 / 1 100.0%	3 / 3 100.0%	2 / 2 100.0%	1 / 1 100.0%	6 / 6 100.0%	5 / 5 100.0%	2 / 2 100.0%	4 / 4 100.0%	1 / 1 100.0%	3 / 3 100.0%	2 / 2 100.0%	4 / 4 100.0%	5 / 5 100.0%	8 / 8 100.0%	1 / 1 100.0%	7 / 7 100.0%	1 / 1 100.0%	0 / 0 0.0%	0 / 0 0.0%	0 / 0 0.0%	42 / 79 53.2%AB	37 / 79 46.8%ABE			
7	unclassified Enterobacteriaceae	170 / 320 53.1%	7 / 9 77.8%	6 / 9 66.7%	10 / 10 100.0%	4 / 8 50.0%	11 / 17 64.7%	12 / 20 60.0%	3 / 7 42.9%	5 / 9 55.6%	7 / 14 50.0%	5 / 9 55.6%	13 / 28 46.4%	9 / 18 50.0%	5 / 8 62.5%	7 / 12 58.3%	3 / 7 42.9%	8 / 16 50.0%	4 / 8 50.0%	9 / 21 42.9%	14 / 31 45.2%	9 / 13 69.2%	2 / 7 28.6%	16 / 38 42.1%	1 / 1 100.0%	0 / 0 0.0%	0 / 0 0.0%	0 / 0 0.0%	107 / 320 33.4%AB	79 / 320 24.7%E	69 / 320 21.6%	63 / 320 19.7%ABE	2 / 320 0.6%CE
1	unclassified Enterobacteriaceae (miscellaneous)	84 / 84 100.0%	3 / 3 100.0%	3 / 3 100.0%	4 / 4 100.0%	2 / 2 100.0%	5 / 5 100.0%	8 / 8 100.0%	1 / 1 100.0%	3 / 3 100.0%	4 / 4 100.0%	1 / 1 100.0%	5 / 5 100.0%	6 / 6 100.0%	2 / 2 100.0%	4 / 4 100.0%	1 / 1 100.0%	3 / 3 100.0%	2 / 2 100.0%	4 / 4 100.0%	7 / 7 100.0%	6 / 6 100.0%	1 / 1 100.0%	8 / 8 100.0%	1 / 1 100.0%	0 / 0 0.0%	0 / 0 0.0%	0 / 0 0.0%	50 / 84 59.5%AB	34 / 84 40.5%ABE			
1	Cronobacter sp. FGI57	84 / 84 100.0%	3 / 3 100.0%	3 / 3 100.0%	4 / 4 100.0%	2 / 2 100.0%	5 / 5 100.0%	8 / 8 100.0%	1 / 1 100.0%	3 / 3 100.0%	4 / 4 100.0%	1 / 1 100.0%	5 / 5 100.0%	6 / 6 100.0%	2 / 2 100.0%	4 / 4 100.0%	1 / 1 100.0%	3 / 3 100.0%	2 / 2 100.0%	4 / 4 100.0%	7 / 7 100.0%	6 / 6 100.0%	1 / 1 100.0%	8 / 8 100.0%	1 / 1 100.0%	0 / 0 0.0%	0 / 0 0.0%	0 / 0 0.0%	50 / 84 59.5%AB	34 / 84 40.5%ABE			
6	ant, tsetse, mealybug, aphid, etc. endosymbionts	86 / 236 36.4%	4 / 6 66.7%	3 / 6 50.0%	6 / 6 100.0%	2 / 6 33.3%	6 / 12 50.0%	4 / 12 33.3%	2 / 6 33.3%	2 / 6 33.3%	3 / 10 30.0%	4 / 8 50.0%	8 / 23 34.8%	3 / 12 25.0%	3 / 6 50.0%	3 / 8 37.5%	2 / 6 33.3%	5 / 13 38.5%	2 / 6 33.3%	5 / 17 29.4%	7 / 24 29.2%	3 / 7 42.9%	1 / 6 16.7%	8 / 30 26.7%	0 / 0 0.0%	0 / 0 0.0%	0 / 0 0.0%	0 / 0 0.0%	79 / 236 33.5%E	69 / 236 29.2%	57 / 236 24.2%AB	29 / 236 12.3%ABE	2 / 236 0.8%CE
4	ant endosymbionts	14 / 154 9.1%	2 / 4 50.0%	1 / 4 25.0%	4 / 4 100.0%	0 / 4 0.0%	2 / 8 25.0%	0 / 8 0.0%	0 / 4 0.0%	0 / 4 0.0%	0 / 6 0.0%	0 / 4 0.0%	2 / 16 12.5%	0 / 8 0.0%	1 / 4 25.0%	0 / 4 0.0%	0 / 4 0.0%	0 / 8 0.0%	0 / 4 0.0%	2 / 12 16.7%	0 / 16 0.0%	0 / 4 0.0%	0 / 4 0.0%	0 / 20 0.0%	0 / 0 0.0%	0 / 0 0.0%	0 / 0 0.0%	0 / 0 0.0%	72 / 154 46.8%E	66 / 154 42.9%	10 / 154 6.5%AB	4 / 154 2.6%ABE	2 / 154 1.3%CE
4	"Candidatus Blochmannia" Sauer et al. 2000	14 / 154 9.1%	2 / 4 50.0%	1 / 4 25.0%	4 / 4 100.0%	0 / 4 0.0%	2 / 8 25.0%	0 / 8 0.0%	0 / 4 0.0%	0 / 4 0.0%	0 / 6 0.0%	0 / 4 0.0%	2 / 16 12.5%	0 / 8 0.0%	1 / 4 25.0%	0 / 4 0.0%	0 / 4 0.0%	0 / 8 0.0%	0 / 4 0.0%	2 / 12 16.7%	0 / 16 0.0%	0 / 4 0.0%	0 / 4 0.0%	0 / 20 0.0%	0 / 0 0.0%	0 / 0 0.0%	0 / 0 0.0%	0 / 0 0.0%	72 / 154 46.8%E	66 / 154 42.9%	10 / 154 6.5%AB	4 / 154 2.6%ABE	2 / 154 1.3%CE
1	'Candidatus Blochmannia pennsylvanicus'	4 / 40 10.0%	0 / 1 0.0%	0 / 1 0.0%	1 / 1 100.0%	0 / 1 0.0%	1 / 2 50.0%	0 / 2 0.0%	0 / 1 0.0%	0 / 1 0.0%	0 / 2 0.0%	0 / 1 0.0%	1 / 4 25.0%	0 / 2 0.0%	0 / 1 0.0%	0 / 1 0.0%	0 / 1 0.0%	0 / 3 0.0%	0 / 1 0.0%	1 / 3 33.3%	0 / 4 0.0%	0 / 1 0.0%	0 / 1 0.0%	0 / 5 0.0%	0 / 0 0.0%	0 / 0 0.0%	0 / 0 0.0%	0 / 0 0.0%	22 / 40 55.0%	13 / 40 32.5%E	3 / 40 7.5%AB	1 / 40 2.5%ABE	1 / 40 2.5%CE
1	Candidatus Blochmannia pennsylvanicus BPEN	4 / 40 10.0%	0 / 1 0.0%	0 / 1 0.0%	1 / 1 100.0%	0 / 1 0.0%	1 / 2 50.0%	0 / 2 0.0%	0 / 1 0.0%	0 / 1 0.0%	0 / 2 0.0%	0 / 1 0.0%	1 / 4 25.0%	0 / 2 0.0%	0 / 1 0.0%	0 / 1 0.0%	0 / 1 0.0%	0 / 3 0.0%	0 / 1 0.0%	1 / 3 33.3%	0 / 4 0.0%	0 / 1 0.0%	0 / 1 0.0%	0 / 5 0.0%	0 / 0 0.0%	0 / 0 0.0%	0 / 0 0.0%	0 / 0 0.0%	22 / 40 55.0%	13 / 40 32.5%E	3 / 40 7.5%AB	1 / 40 2.5%CE	1 / 40 2.5%ABE
1	"Candidatus Blochmannia floridanus" Sauer et al. 2000	4 / 37 10.8%	1 / 1 100.0%	1 / 1 100.0%	1 / 1 100.0%	0 / 1 0.0%	0 / 2 0.0%	0 / 2 0.0%	0 / 1 0.0%	0 / 1 0.0%	0 / 1 0.0%	0 / 1 0.0%	0 / 4 0.0%	0 / 2 0.0%	1 / 1 100.0%	0 / 1 0.0%	0 / 1 0.0%	0 / 1 0.0%	0 / 1 0.0%	0 / 3 0.0%	0 / 4 0.0%	0 / 1 0.0%	0 / 1 0.0%	0 / 5 0.0%	0 / 0 0.0%	0 / 0 0.0%	0 / 0 0.0%	0 / 0 0.0%	20 / 37 54.1%	13 / 37 35.1%E	3 / 37 8.1%AB	1 / 37 2.7%ABE	
1	'Candidatus Blochmannia vafer'	2 / 37 5.4%	1 / 1 100.0%	0 / 1 0.0%	1 / 1 100.0%	0 / 1 0.0%	0 / 2 0.0%	0 / 2 0.0%	0 / 1 0.0%	0 / 1 0.0%	0 / 1 0.0%	0 / 1 0.0%	0 / 4 0.0%	0 / 2 0.0%	0 / 1 0.0%	0 / 1 0.0%	0 / 1 0.0%	0 / 1 0.0%	0 / 1 0.0%	0 / 3 0.0%	0 / 4 0.0%	0 / 1 0.0%	0 / 1 0.0%	0 / 5 0.0%	0 / 0 0.0%	0 / 0 0.0%	0 / 0 0.0%	0 / 0 0.0%	25 / 37 67.6%E	10 / 37 27.0%	1 / 37 2.7%AB	1 / 37 2.7%ABE	

1	Candidatus Blochmannia vafer BVAF	5.4%	100.0%	0.0%	100.0%	0.0%	0.0%	0.0%	0.0%	0.0%	0.0%	0.0%	0.0%	0.0%	0.0%	0.0%	0.0%	0.0%	0.0%	0.0%	0.0%	0.0%	0.0%	0.0%	0.0%	0.0%	0.0%	0.0%	67.6%E	27.0%	2.7%AB	2.7%ABE				
1	'Candidatus Blochmannia chromaiodes'	4 / 40 10.0%	0 / 1 0.0%	0 / 1 0.0%	1 / 1 100.0%	0 / 1 0.0%	1 / 2 50.0%	0 / 2 0.0%	0 / 1 0.0%	0 / 1 0.0%	0 / 2 0.0%	0 / 1 0.0%	1 / 4 25.0%	0 / 2 0.0%	0 / 1 0.0%	0 / 1 0.0%	0 / 1 0.0%	0 / 3 0.0%	0 / 1 0.0%	1 / 3 33.3%	0 / 4 0.0%	0 / 1 0.0%	0 / 1 0.0%	0 / 5 0.0%	0 / 0 0.0%	0 / 0 0.0%	0 / 0 0.0%	0 / 0 0.0%	21 / 40 52.5%E	14 / 40 35.0%	3 / 40 7.5%AB	1 / 40 2.5%CE	1 / 40 2.5%ABE			
1	Candidatus Blochmannia chromaiodes str. 640	4 / 40 10.0%	0 / 1 0.0%	0 / 1 0.0%	1 / 1 100.0%	0 / 1 0.0%	1 / 2 50.0%	0 / 2 0.0%	0 / 1 0.0%	0 / 1 0.0%	0 / 2 0.0%	0 / 1 0.0%	1 / 4 25.0%	0 / 2 0.0%	0 / 1 0.0%	0 / 1 0.0%	0 / 1 0.0%	0 / 3 0.0%	0 / 1 0.0%	1 / 3 33.3%	0 / 4 0.0%	0 / 1 0.0%	0 / 1 0.0%	0 / 5 0.0%	0 / 0 0.0%	0 / 0 0.0%	0 / 0 0.0%	0 / 0 0.0%	21 / 40 52.5%E	14 / 40 35.0%	3 / 40 7.5%AB	1 / 40 2.5%ABE	1 / 40 2.5%CE			
2	aphid secondary symbionts	72 / 82 87.8%	2 / 2 100.0%	2 / 2 100.0%	2 / 2 100.0%	2 / 2 100.0%	4 / 4 100.0%	4 / 4 100.0%	2 / 2 100.0%	2 / 2 100.0%	3 / 4 75.0%	4 / 4 100.0%	6 / 7 85.7%	3 / 4 75.0%	2 / 2 100.0%	3 / 4 75.0%	2 / 2 100.0%	5 / 5 100.0%	2 / 2 100.0%	3 / 5 60.0%	7 / 8 87.5%	3 / 3 100.0%	1 / 2 50.0%	8 / 10 80.0%	0 / 0 0.0%	0 / 0 0.0%	0 / 0 0.0%	0 / 0 0.0%	47 / 82 57.3%AB	25 / 82 30.5%ABE	7 / 82 8.5%E	3 / 82 3.7%				
1	"Candidatus Hamiltonella" Moran et al. 2005	40 / 42 95.2%	1 / 1 100.0%	1 / 1 100.0%	1 / 1 100.0%	1 / 1 100.0%	2 / 2 100.0%	2 / 2 100.0%	1 / 1 100.0%	1 / 1 100.0%	2 / 2 100.0%	3 / 3 100.0%	3 / 3 100.0%	2 / 3 66.7%	1 / 1 100.0%	2 / 2 100.0%	1 / 1 100.0%	2 / 2 100.0%	1 / 1 100.0%	2 / 2 100.0%	4 / 4 100.0%	2 / 2 100.0%	1 / 1 100.0%	4 / 5 80.0%	0 / 0 0.0%	0 / 0 0.0%	0 / 0 0.0%	0 / 0 0.0%	28 / 42 66.7%AB	12 / 42 28.6%ABE	2 / 42 4.8%E					
1	"Candidatus Consessoris aphidicola" Darby et al. 2005	40 / 42 95.2%	1 / 1 100.0%	1 / 1 100.0%	1 / 1 100.0%	1 / 1 100.0%	2 / 2 100.0%	2 / 2 100.0%	1 / 1 100.0%	1 / 1 100.0%	2 / 2 100.0%	3 / 3 100.0%	3 / 3 100.0%	2 / 3 66.7%	1 / 1 100.0%	2 / 2 100.0%	1 / 1 100.0%	2 / 2 100.0%	1 / 1 100.0%	2 / 2 100.0%	4 / 4 100.0%	2 / 2 100.0%	1 / 1 100.0%	4 / 5 80.0%	0 / 0 0.0%	0 / 0 0.0%	0 / 0 0.0%	0 / 0 0.0%	28 / 42 66.7%AB	12 / 42 28.6%ABE	2 / 42 4.8%E					
1	Candidatus Hamiltonella defensa 5AT (Acyrthosiphon pisum)	40 / 42 95.2%	1 / 1 100.0%	1 / 1 100.0%	1 / 1 100.0%	1 / 1 100.0%	2 / 2 100.0%	2 / 2 100.0%	1 / 1 100.0%	1 / 1 100.0%	2 / 2 100.0%	3 / 3 100.0%	3 / 3 100.0%	2 / 3 66.7%	1 / 1 100.0%	2 / 2 100.0%	1 / 1 100.0%	2 / 2 100.0%	1 / 1 100.0%	2 / 2 100.0%	4 / 4 100.0%	2 / 2 100.0%	1 / 1 100.0%	4 / 5 80.0%	0 / 0 0.0%	0 / 0 0.0%	0 / 0 0.0%	0 / 0 0.0%	28 / 42 66.7%AB	12 / 42 28.6%ABE	2 / 42 4.8%E					
1	secondary endosymbiont of Ctenarytaina eucalypti	32 / 40 80.0%	1 / 1 100.0%	1 / 1 100.0%	1 / 1 100.0%	1 / 1 100.0%	2 / 2 100.0%	2 / 2 100.0%	1 / 1 100.0%	1 / 1 100.0%	1 / 2 50.0%	1 / 1 100.0%	3 / 4 75.0%	1 / 1 100.0%	1 / 1 100.0%	1 / 2 50.0%	1 / 1 100.0%	3 / 3 100.0%	1 / 1 100.0%	1 / 3 33.3%	3 / 4 75.0%	1 / 1 100.0%	0 / 1 0.0%	4 / 5 80.0%	0 / 0 0.0%	0 / 0 0.0%	0 / 0 0.0%	0 / 0 0.0%	19 / 40 47.5%AB	13 / 40 32.5%ABE	5 / 40 12.5%E	3 / 40 7.5%				
4	Dickeya	299 / 299 100.0%	12 / 12 100.0%	12 / 12 100.0%	12 / 12 100.0%	12 / 12 100.0%	24 / 24 100.0%	20 / 20 100.0%	4 / 4 100.0%	8 / 8 100.0%	20 / 20 100.0%	4 / 4 100.0%	20 / 20 100.0%	15 / 15 100.0%	8 / 8 100.0%	12 / 12 100.0%	4 / 4 100.0%	12 / 12 100.0%	8 / 8 100.0%	16 / 16 100.0%	24 / 24 100.0%	16 / 16 100.0%	4 / 4 100.0%	32 / 32 100.0%	0 / 0 0.0%	0 / 0 0.0%	0 / 0 0.0%	0 / 0 0.0%	183 / 299 61.2%AB	116 / 299 38.8%ABE						
3	CFBP 1269	225 / 225 100.0%	9 / 9 100.0%	9 / 9 100.0%	9 / 9 100.0%	10 / 10 100.0%	18 / 18 100.0%	15 / 15 100.0%	3 / 3 100.0%	6 / 6 100.0%	16 / 16 100.0%	3 / 3 100.0%	15 / 15 100.0%	11 / 11 100.0%	6 / 6 100.0%	9 / 9 100.0%	3 / 3 100.0%	9 / 9 100.0%	6 / 6 100.0%	12 / 12 100.0%	18 / 18 100.0%	11 / 11 100.0%	3 / 3 100.0%	24 / 24 100.0%	0 / 0 0.0%	0 / 0 0.0%	0 / 0 0.0%	0 / 0 0.0%	139 / 225 61.8%AB	86 / 225 38.2%ABE						
1	Dickeya dadantii 3937	75 / 75 100.0%	3 / 3 100.0%	3 / 3 100.0%	3 / 3 100.0%	3 / 3 100.0%	6 / 6 100.0%	5 / 5 100.0%	1 / 1 100.0%	2 / 2 100.0%	5 / 5 100.0%	1 / 1 100.0%	5 / 5 100.0%	4 / 4 100.0%	2 / 2 100.0%	3 / 3 100.0%	1 / 1 100.0%	3 / 3 100.0%	2 / 2 100.0%	4 / 4 100.0%	6 / 6 100.0%	4 / 4 100.0%	1 / 1 100.0%	8 / 8 100.0%	0 / 0 0.0%	0 / 0 0.0%	0 / 0 0.0%	0 / 0 0.0%	46 / 75 61.3%AB	29 / 75 38.7%ABE						
1	Dickeya dadantii Ech703	74 / 74 100.0%	3 / 3 100.0%	3 / 3 100.0%	3 / 3 100.0%	3 / 3 100.0%	6 / 6 100.0%	5 / 5 100.0%	1 / 1 100.0%	2 / 2 100.0%	5 / 5 100.0%	1 / 1 100.0%	5 / 5 100.0%	3 / 3 100.0%	2 / 2 100.0%	3 / 3 100.0%	1 / 1 100.0%	3 / 3 100.0%	2 / 2 100.0%	4 / 4 100.0%	6 / 6 100.0%	4 / 4 100.0%	1 / 1 100.0%	8 / 8 100.0%	0 / 0 0.0%	0 / 0 0.0%	0 / 0 0.0%	0 / 0 0.0%	45 / 74 60.8%AB	29 / 74 39.2%ABE						
1	Dickeya dadantii Ech586	76 / 76 100.0%	3 / 3 100.0%	3 / 3 100.0%	3 / 3 100.0%	4 / 4 100.0%	6 / 6 100.0%	5 / 5 100.0%	1 / 1 100.0%	2 / 2 100.0%	6 / 6 100.0%	1 / 1 100.0%	5 / 5 100.0%	4 / 4 100.0%	2 / 2 100.0%	3 / 3 100.0%	1 / 1 100.0%	3 / 3 100.0%	2 / 2 100.0%	4 / 4 100.0%	6 / 6 100.0%	3 / 3 100.0%	1 / 1 100.0%	8 / 8 100.0%	0 / 0 0.0%	0 / 0 0.0%	0 / 0 0.0%	0 / 0 0.0%	48 / 76 63.2%AB	28 / 76 36.8%ABE						
1	CFBP 2052	74 / 74 100.0%	3 / 3 100.0%	3 / 3 100.0%	3 / 3 100.0%	2 / 2 100.0%	6 / 6 100.0%	5 / 5 100.0%	1 / 1 100.0%	2 / 2 100.0%	4 / 4 100.0%	1 / 1 100.0%	5 / 5 100.0%	4 / 4 100.0%	2 / 2 100.0%	3 / 3 100.0%	1 / 1 100.0%	3 / 3 100.0%	2 / 2 100.0%	4 / 4 100.0%	6 / 6 100.0%	5 / 5 100.0%	1 / 1 100.0%	8 / 8 100.0%	0 / 0 0.0%	0 / 0 0.0%	0 / 0 0.0%	0 / 0 0.0%	44 / 74 59.5%AB	30 / 74 40.5%ABE						
1	Dickeya chrysanthemi Ech1591	74 / 74 100.0%	3 / 3 100.0%	3 / 3 100.0%	3 / 3 100.0%	2 / 2 100.0%	6 / 6 100.0%	5 / 5 100.0%	1 / 1 100.0%	2 / 2 100.0%	4 / 4 100.0%	1 / 1 100.0%	5 / 5 100.0%	4 / 4 100.0%	2 / 2 100.0%	3 / 3 100.0%	1 / 1 100.0%	3 / 3 100.0%	2 / 2 100.0%	4 / 4 100.0%	6 / 6 100.0%	5 / 5 100.0%	1 / 1 100.0%	8 / 8 100.0%	0 / 0 0.0%	0 / 0 0.0%	0 / 0 0.0%	0 / 0 0.0%	44 / 74 59.5%AB	30 / 74 40.5%ABE						
1	"Candidatus Riesia" Sasaki-Fukatsu et al. 2006	0 / 33 0.0%	0 / 1 0.0%	0 / 1 0.0%	0 / 1 0.0%	0 / 1 0.0%	0 / 2 0.0%	0 / 2 0.0%	0 / 1 0.0%	0 / 1 0.0%	0 / 1 0.0%	0 / 1 0.0%	0 / 2 0.0%	0 / 1 0.0%	0 / 1 0.0%	0 / 1 0.0%	0 / 1 0.0%	0 / 1 0.0%	0 / 1 0.0%	0 / 2 0.0%	0 / 4 0.0%	0 / 1 0.0%	0 / 1 0.0%	0 / 4 0.0%	0 / 0 0.0%	0 / 0 0.0%	0 / 1 0.0%	0 / 0 0.0%	21 / 33 63.6%E	11 / 33 33.3%	1 / 33 3.0%CE					
1	"Candidatus Riesia pediculicola" Sasaki-fukatsu et al. 2006	0 / 33 0.0%	0 / 1 0.0%	0 / 1 0.0%	0 / 1 0.0%	0 / 1 0.0%	0 / 2 0.0%	0 / 2 0.0%	0 / 1 0.0%	0 / 1 0.0%	0 / 1 0.0%	0 / 1 0.0%	0 / 2 0.0%	0 / 1 0.0%	0 / 1 0.0%	0 / 1 0.0%	0 / 1 0.0%	0 / 1 0.0%	0 / 1 0.0%	0 / 2 0.0%	0 / 4 0.0%	0 / 1 0.0%	0 / 1 0.0%	0 / 4 0.0%	0 / 0 0.0%	0 / 0 0.0%	0 / 1 0.0%	0 / 0 0.0%	21 / 33 63.6%E	11 / 33 33.3%	1 / 33 3.0%CE					
1	Candidatus Riesia pediculicola USDA	0 / 33 0.0%	0 / 1 0.0%	0 / 1 0.0%	0 / 1 0.0%	0 / 1 0.0%	0 / 2 0.0%	0 / 2 0.0%	0 / 1 0.0%	0 / 1 0.0%	0 / 1 0.0%	0 / 1 0.0%	0 / 2 0.0%	0 / 1 0.0%	0 / 1 0.0%	0 / 1 0.0%	0 / 1 0.0%	0 / 1 0.0%	0 / 1 0.0%	0 / 2 0.0%	0 / 4 0.0%	0 / 1 0.0%	0 / 1 0.0%	0 / 4 0.0%	0 / 0 0.0%	0 / 0 0.0%	0 / 1 0.0%	0 / 0 0.0%	21 / 33 63.6%E	11 / 33 33.3%	1 / 33 3.0%CE					
4	"Cronobacter" Iversen et al. 2007	326 / 327 99.7%	12 / 12 100.0%	12 / 12 100.0%	16 / 16 100.0%	14 / 14 100.0%	24 / 24 100.0%	24 / 24 100.0%	4 / 4 100.0%	12 / 12 100.0%	22 / 22 100.0%	4 / 4 100.0%	20 / 20 100.0%	20 / 20 100.0%	8 / 8 100.0%	16 / 16 100.0%	4 / 4 100.0%	12 / 12 100.0%	8 / 8 100.0%	15 / 16 93.8%	28 / 28 100.0%	13 / 13 100.0%	4 / 4 100.0%	30 / 30 100.0%	4 / 4 100.0%	0 / 0 0.0%	0 / 0 0.0%	0 / 0 0.0%	176 / 327 53.8%AB	104 / 327 31.8%ABE	31 / 327 9.5%B	15 / 327 4.6%BE	1 / 327 0.3%			
3	"Cronobacter sakazakii" (Farmer et al. 1980) Iversen et al. 2007	241 / 242 99.6%	9 / 9 100.0%	9 / 9 100.0%	12 / 12 100.0%	7 / 7 100.0%	18 / 18 100.0%	18 / 18 100.0%	3 / 3 100.0%	9 / 9 100.0%	13 / 13 100.0%	3 / 3 100.0%	15 / 15 100.0%	15 / 15 100.0%	6 / 6 100.0%	12 / 12 100.0%	3 / 3 100.0%	9 / 9 100.0%	6 / 6 100.0%	11 / 12 91.7%	21 / 21 100.0%	13 / 13 100.0%	3 / 3 100.0%	23 / 23 100.0%	3 / 3 100.0%	0 / 0 0.0%	0 / 0 0.0%	0 / 0 0.0%	117 / 242 48.3%AB	78 / 242 32.2%ABE	31 / 242 12.8%B	15 / 242 6.2%BE	1 / 242 0.4%			
1	Cronobacter sakazakii ATCC BAA-894	82 / 82 100.0%	3 / 3 100.0%	3 / 3 100.0%	4 / 4 100.0%	3 / 3 100.0%	6 / 6 100.0%	6 / 6 100.0%	1 / 1 100.0%	3 / 3 100.0%	5 / 5 100.0%	1 / 1 100.0%	5 / 5 100.0%	5 / 5 100.0%	2 / 2 100.0%	4 / 4 100.0%	1 / 1 100.0%	3 / 3 100.0%	2 / 2 100.0%	4 / 4 100.0%	7 / 7 100.0%	4 / 4 100.0%	1 / 1 100.0%	8 / 8 100.0%	1 / 1 100.0%	0 / 0 0.0%	0 / 0 0.0%	0 / 0 0.0%	31 / 82 37.8%B	20 / 82 24.4%AB	16 / 82 19.5%ABE	15 / 82 18.3%BE				
1	Cronobacter sakazakii SP291	81 / 81 100.0%	3 / 3 100.0%	3 / 3 100.0%	4 / 4 100.0%	3 / 3 100.0%	6 / 6 100.0%	6 / 6 100.0%	1 / 1 100.0%	3 / 3 100.0%	5 / 5 100.0%	1 / 1 100.0%	5 / 5 100.0%	5 / 5 100.0%	2 / 2 100.0%	4 / 4 100.0%	1 / 1 100.0%	3 / 3 100.0%	2 / 2 100.0%	4 / 4 100.0%	7 / 7 100.0%	3 / 3 100.0%	1 / 1 100.0%	8 / 8 100.0%	1 / 1 100.0%	0 / 0 0.0%	0 / 0 0.0%	0 / 0 0.0%	51 / 81 63.0%AB	30 / 81 37.0%ABE						
1	Cronobacter sakazakii ES15	78 / 79 98.7%	3 / 3 100.0%	3 / 3 100.0%	4 / 4 100.0%	1 / 1 100.0%	6 / 6 100.0%	6 / 6 100.0%	1 / 1 100.0%	3 / 3 100.0%	3 / 3 100.0%	1 / 1 100.0%	5 / 5 100.0%	5 / 5 100.0%	2 / 2 100.0%	4 / 4 100.0%	1 / 1 100.0%	3 / 3 100.0%	2 / 2 100.0%	3 / 4 75.0%	7 / 7 100.0%	6 / 6 100.0%	1 / 1 100.0%	7 / 7 100.0%	1 / 1 100.0%	0 / 0 0.0%	0 / 0 0.0%	0 / 0 0.0%	46 / 79 58.2%AB	32 / 79 40.5%ABE	1 / 79 1.3%					
1	"Cronobacter turicensis" Iversen et al. 2007	85 / 85 100.0%	3 / 3 100.0%	3 / 3 100.0%	4 / 4 100.0%	7 / 7 100.0%	6 / 6 100.0%	6 / 6 100.0%	1 / 1 100.0%	3 / 3 100.0%	9 / 9 100.0%	1 / 1 100.0%	5 / 5 100.0%	5 / 5 100.0%	2 / 2 100.0%	4 / 4 100.0%	1 / 1 100.0%	3 / 3 100.0%	2 / 2 100.0%	4 / 4 100.0%	7 / 7 100.0%	0 / 0 0.0%	1 / 1 100.0%	7 / 7 100.0%	1 / 1 100.0%	0 / 0 0.0%	0 / 0 0.0%	0 / 0 0.0%	59 / 85 69.4%AB	26 / 85 30.6%ABE						
1	Cronobacter turicensis DSM 18703	85 / 85 100.0%	3 / 3 100.0%	3 / 3 100.0%	4 / 4 100.0%	7 / 7 100.0%	6 / 6 100.0%	6 / 6 100.0%	1 / 1 100.0%	3 / 3 100.0%	9 / 9 100.0%	1 / 1 100.0%	5 / 5 100.0%	5 / 5 100.0%	2 / 2 100.0%	4 / 4 100.0%	1 / 1 100.0%	3 / 3 100.0%	2 / 2 100.0%	4 / 4 100.0%	7 / 7 100.0%	0 / 0 0.0%	1 / 1 100.0%	7 / 7 100.0%	1 / 1 100.0%	0 / 0 0.0%	0 / 0 0.0%	0 / 0 0.0%	59 / 85 69.4%AB	26 / 85 30.6%ABE						
2	"Candidatus Moranella" McCutcheon and von Dohlen 2011	58 / 82 70.7%	2 / 2 100.0%	4 / 4 100.0%	2 / 2 100.0%	0 / 2 0.0%	4 / 4 100.0%	2 / 4 50.0%	2 / 2 100.0%	2 / 2 100.0%	2 / 4 50.0%	2 / 2 100.0%	6 / 8 75.0%	2 / 2 100.0%	2 / 2 100.0%	2 / 4 50.0%	2 / 2 100.0%	6 / 6 100.0%	2 / 2 100.0%	4 / 6 66.7%	2 / 8 25.0%	0 / 2 0.0%	0 / 2 0.0%	8 / 10 80.0%	0 / 0 0.0%	0 / 0 0.0%	0 / 0 0.0%	0 / 0 0.0%	36 / 82 43.9%AB	22 / 82 26.8%ABE	18 / 82 22.0%E	4 / 82 4.9%	2 / 82 2.4%C			
2	"Candidatus Moranella endobia" McCutcheon and von Dohlen 2011	58 / 82 70.7%	2 / 2 100.0%	4 / 4 100.0%	2 / 2 100.0%	0 / 2 0.0%	4 / 4 100.0%	2 / 4 50.0%	2 / 2 100.0%	2 / 2 100.0%	2 / 4 50.0%	2 / 2 100.0%	6 / 8 75.0%	2 / 2 100.0%	2 / 2 100.0%	2 / 4 50.0%	2 / 2 100.0%	6 / 6 100.0%	2 / 2 100.0%	4 / 6 66.7%	2 / 8 25.0%	0 / 2 0.0%	0 / 2 0.0%	8 / 10 80.0%	0 / 0 0.0%	0 / 0 0.0%	0 / 0 0.0%	0 / 0 0.0%	36 / 82 43.9%AB	22 / 82 26.8%ABE	18 / 82 22.0%E	4 / 82 4.9%	2 / 82 2.4%C			
1	Candidatus Moranella endobia PCIT	29 / 41 70.7%	1 / 1 100.0%	2 / 2 100.0%	1 / 1 100.0%	0 / 1 0.0%	2 / 2 100.0%	1 / 2 50.0%	1 / 1 100.0%	1 / 1 100.0%	1 / 2 50.0%	1 / 1 100.0%	3 / 4 75.0%	1 / 1 100.0%	1 / 1 100.0%	1 / 2 50.0%	1 / 1 100.0%	3 / 3 100.0%	1 / 1 100.0%	2 / 3 66.7%	1 / 4 25.0%	0 / 1 0.0%	0 / 1 0.0%	4 / 5 80.0%	0 / 0 0.0%	0 / 0 0.0%	0 / 0 0.0%	0 / 0 0.0%	18 / 41 43.9%AB	11 / 41 26.8%ABE	9 / 41 22.0%E	2 / 41 4.9%	1 / 41 2.4%C			
1	Candidatus Moranella endobia PCVAL	29 / 41 70.7%	1 / 1 100.0%	2 / 2 100.0%	1 / 1 100.0%	0 / 1 0.0%	2 / 2 100.0%	1 / 2 50.0%	1 / 1 100.0%	1 / 1 100.0%	1 / 2 50.0%	1 / 1 100.0%	3 / 4 75.0%	1 / 1 100.0%	1 / 1 100.0%	1 / 2 50.0%	1 / 1 100.0%	3 / 3 100.0%	1 / 1 100.0%	2 / 3 66.7%	1 / 4 25.0%	0 / 1 0.0%	0 / 1 0.0%	4 / 5 80.0%	0 / 0 0.0%	0 / 0 0.0%	0 / 0 0.0%	0 / 0 0.0%	18 / 41 43.9%AB	11 / 41 26.8%ABE	9 / 41 22.0%E	2 / 41 4.9%	1 / 41 2.4%C			
1	Shimwellia	88 / 88 100.0%	4 / 4 100.0%	3 / 3 100.0%	4 / 4 100.0%	3 / 3 100.0%	6 / 6 100.0%	7 / 7 100.0%	1 / 1 100.0%	3 / 3 100.0%	5 / 5 100.0%	1 / 1 100.0%	5 / 5 100.0%	7 / 7 100.0%	2 / 2 100.0%	5 / 5 100.0%	1 / 1 100.0%	3 / 3 100.0%	2 / 2 100.0%	4 / 4 100.0%	7 / 7 100.0%	5 / 5 100.0%	1 / 1 100.0%	8 / 8 100.0%	1 / 1 100.0%	0 / 0 0.0%	0 / 0 0.0%	0 / 0 0.0%	54 / 88 61.4%AB	34 / 88 38.6%ABE						
1	ATCC 29907	88 / 88 100.0%	4 / 4 100.0%	3 / 3 100.0%	4 / 4 100.0%	3 / 3 100.0%	6 / 6 100.0%	7 / 7 100.0%	1 / 1 100.0%	3 / 3 100.0%	5 / 5 100.0%	1 / 1 100.0%	5 / 5 100.0%	7 / 7 100.0%	2 / 2 100.0%	5 / 5 100.0%	1 / 1 100.0%	3 / 3 100.0%	2 / 2 100.0%	4 / 4 100.0%	7 / 7 100.0%	5 / 5 100.0%	1 / 1 100.0%	8 / 8 100.0%	1 / 1 100.0%	0 / 0 0.0%	0 / 0 0.0%	0 / 0 0.0%	54 / 88 61.4%AB	34 / 88 38.6%ABE						
1	Escherichia blattae CIP 104942	88 / 88 100.0%	4 / 4 100.0%	3 / 3 100.0%	4 / 4 100.0%	3 / 3 100.0%	6 / 6 100.0%	7 / 7 100.0%	1 / 1 100.0%	3 / 3 100.0%	5 / 5 100.0%	1 / 1 100.0%	5 / 5 100.0%	7 / 7 100.0%	2 / 2 100.0%	5 / 5 100.0%	1 / 1 100.0%	3 / 3 100.0%	2 / 2 100.0%	4 / 4 100.0%	7 / 7 100.0%	5 / 5 100.0%	1 / 1 100.0%	8 / 8 100.0%	1 / 1 100.0%	0 / 0 0.0%	0 / 0 0.0%	0 / 0 0.0%	54 / 88 61.4%AB	34 / 88 38.6%ABE						
5	unclassified Gammaproteobacteria	166 / 199 83.4%	5 / 5 100.0%	5 / 6 83.3%	4 / 5 80.0%	8 / 9 88.9%	8 / 12 66.7%	9 / 10 90.0%	5 / 5 100.0%	4 / 5 80.0%	13 / 14 92.9%	4 / 5 80.0%	11 / 15 73.3%	5 / 6 83.3%	4 / 5 80.0%	4 / 6 66.7%	4 / 5 80.0%	10 / 12 83.3%	4 / 5 80.0%	10 / 12 83.3%	18 / 20 90.0%	5 / 9 55.6%	4 / 5 80.0%	22 / 23 95.7%	0 / 0 0.0%	0 / 0 0.0%	0 / 0 0.0%	0 / 0 0.0%	111 / 199 55.8%AB	55 / 199 27.6%ABE	16 / 199 8.0%	14 / 199 7.0%E	3 / 199 1.5%C			
2	sulfur-oxidizing symbionts	62 / 72 86.1%	2 / 2 100.0%	2 / 2 100.0%	2 / 2 100.0%	2 / 2 100.0%	1 / 4 25.0%	4 / 4 100.0%	2 / 2 100.0%	2 / 2 100.0%	4 / 4 100.0%	2 / 2 100.0%	5 / 6 83.3%	2 / 2 100.0%	2 / 2 100.0%	2 / 2 100.0%	2 / 2 100.0%	3 / 4 75.0%	1 / 2 50.0%	3 / 4 75.0%	7 / 8 87.5%	2 / 4 50.0%	2 / 2 100.0%	8 / 8 100.0%	0 / 0 0.0%	0 / 0 0.0%	0 / 0 0.0%	0 / 0 0.0%	39 / 72 54.2%AB	23 / 72 31.9%ABE	5 / 72 6.9%E	4 / 72 5.6%	1 / 72 1.4%C			
1	Calyptogena okutanii thioautotrophic gill symbiont	31 / 36 86.1%	1 / 1 100.0%	1 / 1 100.0%	1 / 1 100.0%	1 / 1 100.0%	1 / 2 50.0%	2 / 2 100.0%	1 / 1 100.0%	1 / 1 100.0%	2 / 2 100.0%	1 / 1 100.0%	2 / 3 66.7%	1 / 1 100.0%	1 / 1 100.0%	1 / 1 100.0%	1 / 1 100.0%	1 / 2 50.0%	1 / 1 100.0%	2 / 2 100.0%	3 / 4 75.0%	1 / 2 50.0%	1 / 1 100.0%	4 / 4 100.0%	0 / 0 0.0%	0 / 0 0.0%	0 / 0 0.0%	0 / 0 0.0%	20 / 36 55.6%AB	11 / 36 30.6%ABE	3 / 36 8.3%E	2 / 36 5.6%				
1	Calyptogena okutanii thioautotrophic gill symbiont HA	31 / 36 86.1%	1 / 1 100.0%	1 / 1 100.0%	1 / 1 100.0%	1 / 1 100.0%	1 / 2 50.0%	2 / 2 100.0%	1 / 1 100.0%	1 / 1 100.0%	2 / 2 100.0%	1 / 1 100.0%	2 / 3 66.7%	1 / 1 100.0%	1 / 1 100.0%	1 / 1 100.0%	1 / 1 100.0%	1 / 2 50.0%	1 / 1 100.0%	2 / 2 100.0%	3 / 4 75.0%	1 / 2 50.0%	1 / 1 100.0%	4 / 4 100.0%	0 / 0 0.0%	0 / 0 0.0%	0 / 0 0.0%	0 / 0 0.0%	20 / 36 55.6%AB	11 / 36 30.6%ABE	3 / 36 8.3%E	2 / 36 5.6%				
1	"Candidatus Ruthia" Newton et al. 2007	31 / 36 86.1%	1 / 1 100.0%	1 / 1 100.0%	1 / 1 100.0%	1 / 1 100.0%	0 / 2 0.0%	2 / 2 100.0%	1 / 1 100.0%	1 / 1 100.0%	2 / 2 100.0%	1 / 1 100.0%	3 / 3 100.0%	1 / 1 100.0%	1 / 1 100.0%	1 / 1 100.0%	1 / 1 100.0%	2 / 2 100.0%	0 / 1 0.0%	1 / 2 50.0%	4 / 4 100.0%	1 / 2 50.0%	1 / 1 100.0%	4 / 4 100.0%	0 / 0 0.0%	0 / 0 0.0%	0 / 0 0.0%	0 / 0 0.0%	19 / 36 52.8%AB	12 / 36 33.3%ABE	2 / 36 5.6%E	2 / 36 5.6%	1 / 36 2.8%C			
1	"Candidatus Ruthia magnifica" Newton et al. 2007	31 / 36 86.1%	1 / 1 100.0%	1 / 1 100.0%	1 / 1 100.0%	1 / 1 100.0%	0 / 2 0.0%	2 / 2 100.0%	1 / 1 100.0%	1 / 1 100.0%	2 / 2 100.0%	1 / 1 100.0%	3 / 3 100.0%	1 / 1 100.0%	1 / 1 100.0%	1 / 1 100.0%	1 / 1 100.0%	2 / 2 100.0%	0 / 1 0.0%	1 / 2 50.0%	4 / 4 100.0%	1 / 2 50.0%	1 / 1 100.0%	4 / 4 100.0%	0 / 0 0.0%	0 / 0 0.0%	0 / 0 0.0%	0 / 0 0.0%	19 / 36 52.8%AB	12 / 36 33.3%ABE	2 / 36 5.6%	2 / 36 5.6%E	1 / 36 2.8%C			
1	Candidatus Ruthia magnifica str. 'Calyptogena magnifica'	31 / 36 86.1%	1 / 1 100.0%	1 / 1 100.0%	1 / 1 100.0%	1 / 1 100.0%	0 / 2 0.0%	2 / 2 100.0%	1 / 1 100.0%	1 / 1 100.0%	2 / 2 100.0%	1 / 1 100.0%	3 / 3 100.0%	1 / 1 100.0%	1 / 1 100.0%	1 / 1 100.0%	1 / 1 100.0%	2 / 2 100.0%	0 / 1 0.0%	1 / 2 50.0%	4 / 4 100.0%	1 / 2 50.0%	1 / 1 100.0%	4 / 4 100.0%	0 / 0 0.0%	0 / 0 0.0%	0 / 0 0.0%	0 / 0 0.0%	19 / 36 52.8%AB	12 / 36 33.3%ABE	2 / 36 5.6%	2 / 36 5.6%E	1 / 36 2.8%C			
1	unclassified Gammaproteobacteria (miscellaneous)	47 / 47 100.0%	1 / 1 100.0%	2 / 2 100.0%	1 / 1 100.0%	3 / 3 100.0%	4 / 4 100.0%	2 / 2 100.0%	1 / 1 100.0%	1 / 1 100.0%	4 / 4 100.0%	1 / 1 100.0%	4 / 4 100.0%	1 / 1 100.0%	1 / 1 100.0%	1 / 1 100.0%	1 / 1 100.0%	3 / 3 100.0%	1 / 1 100.0%	3 / 3 100.0%	4 / 4 100.0%	2 / 2 100.0%	1 / 1 100.0%	5 / 5 100.0%	0 / 0 0.0%	0 / 0 0.0%	0 / 0 0.0%	0 / 0 0.0%	31 / 47 66.0%AB	16 / 47 34.0%ABE						
1	gamma proteobacterium HdN1	47 / 47 100.0%	1 / 1 100.0%	2 / 2 100.0%	1 / 1 100.0%	3 / 3 100.0%	4 / 4 100.0%	2 / 2 100.0%	1 / 1 100.0%	1 / 1 100.0%	4 / 4 100.0%	1 / 1 100.0%	4 / 4 100.0%	1 / 1 100.0%	1 / 1 100.0%	1 / 1 100.0%	1 / 1 100.0%	3 / 3 100.0%	1 / 1 100.0%	3 / 3 100.0%	4 / 4 100.0%	2 / 2 100.0%	1 / 1 100.0%	5 / 5 100.0%	0 / 0 0.0%	0 / 0 0.0%	0 / 0 0.0%	0 / 0 0.0%	31 / 47 66.0%AB	16 / 47 34.0%ABE						
1	"Candidatus Baumannia" Moran et al. 2003	17 / 39 43.6%	1 / 1 100.0%	1 / 1 100.0%	0 / 1 0.0%	0 / 1 0.0%	1 / 2 50.0%	1 / 2 50.0%	1 / 1 100.0%	0 / 1 0.0%	1 / 2 50.0%	0 / 1 0.0%	0 / 3 0.0%	0 / 1 0.0%	0 / 1 0.0%	0 / 2 0.0%	0 / 1 0.0%	1 / 2 50.0%	1 / 1 100.0%	2 / 3 66.7%	3 / 4 75.0%	0 / 2 0.0%	0 / 1 0.0%	4 / 5 80.0%	0 / 0 0.0%	0 / 0 0.0%	0 / 0 0.0%	0 / 0 0.0%	12 / 39 30.8%	11 / 39 28.2%AB	8 / 39 20.5%E	6 / 39 15.4%ABE	2 / 39 5.1%C			
1	"Candidatus Baumannia cicadellinicola" Moran et al. 2003	17 / 39 43.6%	1 / 1 100.0%	1 / 1 100.0%	0 / 1 0.0%	0 / 1 0.0%	1 / 2 50.0%	1 / 2 50.0%	1 / 1 100.0%	0 / 1 0.0%	1 / 2 50.0%	0 / 1 0.0%	0 / 3 0.0%	0 / 1 0.0%	0 / 1 0.0%	0 / 2 0.0%	0 / 1 0.0%	1 / 2 50.0%	1 / 1 100.0%	2 / 3 66.7%	3 / 4 75.0%	0 / 2 0.0%	0 / 1 0.0%	4 / 5 80.0%	0 / 0 0.0%	0 / 0 0.0%	0 / 0 0.0%	0 / 0 0.0%	12 / 39 30.8%	11 / 39 28.2%AB	8 / 39 20.5%E	6 / 39 15.4%ABE	2 / 39 5.1%C			
1	Baumannia cicadellinicola endosymbiont of Homalodisca coagulata	17 / 39 43.6%	1 / 1 100.0%	1 / 1 100.0%	0 / 1 0.0%	0 / 1 0.0%	1 / 2 50.0%	1 / 2 50.0%	1 / 1 100.0%	0 / 1 0.0%	1 / 2 50.0%	0 / 1 0.0%	0 / 3 0.0%	0 / 1 0.0%	0 / 1 0.0%	0 / 2 0.0%	0 / 1 0.0%	1 / 2 50.0%	1 / 1 100.0%	2 / 3 66.7%	3 / 4 75.0%	0 / 2 0.0%	0 / 1 0.0%	4 / 5 80.0%	0 / 0 0.0%	0 / 0 0.0%	0 / 0 0.0%	0 / 0 0.0%	12 / 39 30.8%	11 / 39 28.2%AB	8 / 39 20.5%E	6 / 39 15.4%ABE	2 / 39 5.1%C			
1	Simidua	40 / 41 97.6%	1 / 1 100.0%	0 / 1 0.0%	1 / 1 100.0%	3 / 3 100.0%	2 / 2 100.0%	2 / 2 100.0%	1 / 1 100.0%	1 / 1 100.0%	4 / 4 100.0%	1 / 1 100.0%	2 / 2 100.0%	2 / 2 100.0%	1 / 1 100.0%	1 / 1 100.0%	1 / 1 100.0%	3 / 3 100.0%	1 / 1 100.0%	2 / 2 100.0%	4 / 4 100.0%	1 / 1 100.0%	1 / 1 100.0%	5 / 5 100.0%	0 / 0 0.0%	0 / 0 0.0%	0 / 0 0.0%	0 / 0 0.0%	30 / 41 73.2%AB	10 / 41 24.4%ABE	1 / 41 2.4%E					
1	BCRC 17597	40 / 41 97.6%	1 / 1 100.0%	0 / 1 0.0%	1 / 1 100.0%	3 / 3 100.0%	2 / 2 100.0%	2 / 2 100.0%	1 / 1 100.0%	1 / 1 100.0%	4 / 4 100.0%	1 / 1 100.0%	2 / 2 100.0%	2 / 2 100.0%	1 / 1 100.0%	1 / 1 100.0%	1 / 1 100.0%	3 / 3 100.0%	1 / 1 100.0%	2 / 2 100.0%	4 / 4 100.0%	1 / 1 100.0%	1 / 1 100.0%	5 / 5 100.0%	0 / 0 0.0%	0 / 0 0.0%	0 / 0 0.0%	0 / 0 0.0%	30 / 41 73.2%AB	10 / 41 24.4%ABE	1 / 41 2.4%E					
1	Simiduia agarivorans DSM 21679	40 / 41 97.6%	1 / 1 100.0%	0 / 1 0.0%	1 / 1 100.0%	3 / 3 100.0%	2 / 2 100.0%	2 / 2 100.0%	1 / 1 100.0%	1 / 1 100.0%	4 / 4 100.0%	1 / 1 100.0%	2 / 2 100.0%	2 / 2 100.0%	1 / 1 100.0%	1 / 1 100.0%	1 / 1 100.0%	3 / 3 100.0%	1 / 1 100.0%	2 / 2 100.0%	4 / 4 100.0%	1 / 1 100.0%	1 / 1 100.0%	5 / 5 100.0%	0 / 0 0.0%	0 / 0 0.0%	0 / 0 0.0%	0 / 0 0.0%	30 / 41 73.2%AB	10 / 41 24.4%ABE	1 / 41 2.4%E					
18	Legionellaceae group	685 / 806 85.0%	19 / 19 100.0%	20 / 20 100.0%	19 / 19 100.0%	17 / 17 100.0%	32 / 56 57.1%	34 / 39 87.2%	19 / 19 100.0%	13 / 18 72.2%	47 / 52 90.4%	19 / 19 100.0%	62 / 62 100.0%	34 / 50 68.0%	19 / 19 100.0%	19 / 19 100.0%	14 / 19 73.7%	42 / 57 73.7%	14 / 19 73.7%	31 / 55 56.4%	70 / 76 92.1%	37 / 38 97.4%	19 / 19 100.0%	85 / 95 89.5%	0 / 0 0.0%	0 / 0 0.0%	0 / 0 0.0%	0 / 0 0.0%	405 / 806 50.2%AB	253 / 806 31.4%ABE	69 / 806 8.6%	46 / 806 5.7%E	16 / 806 2.0%B	11 / 806 1.4%BE	5 / 806 0.6%C	1 / 806 0.1%CE
13	Legionellaceae	556 / 596 93.3%	14 / 14 100.0%	15 / 15 100.0%	14 / 14 100.0%	12 / 12 100.0%	27 / 41 65.9%	29 / 29 100.0%	14 / 14 100.0%	13 / 13 100.0%	42 / 42 100.0%	14 / 14 100.0%	42 / 42 100.0%	29 / 40 72.5%	14 / 14 100.0%	14 / 14 100.0%	14 / 14 100.0%	42 / 42 100.0%	14 / 14 100.0%	26 / 40 65.0%	56 / 56 100.0%	27 / 28 96.4%	14 / 14 100.0%	70 / 70 100.0%	0 / 0 0.0%	0 / 0 0.0%	0 / 0 0.0%	0 / 0 0.0%	347 / 596 58.2%AB	209 / 596 35.1%ABE	25 / 596 4.2%	14 / 596 2.3%E	1 / 596 0.2%CE			
13	Legionella	556 / 596 93.3%	14 / 14 100.0%	15 / 15 100.0%	14 / 14 100.0%	12 / 12 100.0%	27 / 41 65.9%	29 / 29 100.0%	14 / 14 100.0%	13 / 13 100.0%	42 / 42 100.0%	14 / 14 100.0%	42 / 42 100.0%	29 / 40 72.5%	14 / 14 100.0%	14 / 14 100.0%	14 / 14 100.0%	42 / 42 100.0%	14 / 14 100.0%	26 / 40 65.0%	56 / 56 100.0%	27 / 28 96.4%	14 / 14 100.0%	70 / 70 100.0%	0 / 0 0.0%	0 / 0 0.0%	0 / 0 0.0%	0 / 0 0.0%	347 / 596 58.2%AB	209 / 596 35.1%ABE	25 / 596 4.2%	14 / 596 2.3%E	1 / 596 0.2%CE			
12	ATCC 33152	512 / 550 93.1%	13 / 13 100.0%	13 / 13 100.0%	13 / 13 100.0%	13 / 13 100.0%	11 / 11 100.0%	25 / 38 65.8%	26 / 26 100.0%	13 / 13 100.0%	12 / 12 100.0%	38 / 38 100.0%	13 / 13 100.0%	39 / 39 100.0%	26 / 37 70.3%	13 / 13 100.0%	13 / 13 100.0%	13 / 13 100.0%	39 / 39 100.0%	13 / 13 100.0%	24 / 37 64.9%	52 / 52 100.0%	25 / 26 96.2%	13 / 13 100.0%	65 / 65 100.0%	0 / 0 0.0%	0 / 0 0.0%	0 / 0 0.0%	0 / 0 0.0%	318 / 550 57.8%AB	194 / 550 35.3%ABE	23 / 550 4.2%	14 / 550 2.5%E	1 / 550 0.2%CE		
2	Legionella pneumophila serogroup 1	78 / 85 91.8%	2 / 2 100.0%	2 / 2 100.0%	2 / 2 100.0%	2 / 2 100.0%	4 / 6 66.7%	4 / 4 100.0%	2 / 2 100.0%	2 / 2 100.0%	5 / 5 100.0%	2 / 2 100.0%	6 / 6 100.0%	4 / 6 66.7%	2 / 2 100.0%	2 / 2 100.0%	2 / 2 100.0%	6 / 6 100.0%	2 / 2 100.0%	4 / 6 66.7%	8 / 8 100.0%	3 / 4 75.0%	2 / 2 100.0%	10 / 10 100.0%	0 / 0 0.0%	0 / 0 0.0%	0 / 0 0.0%	0 / 0 0.0%	48 / 85 56.5%AB	30 / 85 35.3%ABE	3 / 85 3.5%	3 / 85 3.5%E	1 / 85 1.2%CE			
1	Legionella pneumophila 2300/99	39 / 43 90.7%	1 / 1 100.0%	1 / 1 100.0%	1 / 1 100.0%	1 / 1 100.0%	2 / 3 66.7%	2 / 2 100.0%	1 / 1 100.0%	1 / 1 100.0%	3 / 3 100.0%	1 / 1 100.0%	3 / 3 100.0%	2 / 3 66.7%	1 / 1 100.0%	1 / 1 100.0%	1 / 1 100.0%	3 / 3 100.0%	1 / 1 100.0%	2 / 3 66.7%	4 / 4 100.0%	1 / 2 50.0%	1 / 1 100.0%	5 / 5 100.0%	0 / 0 0.0%	0 / 0 0.0%	0 / 0 0.0%	0 / 0 0.0%	24 / 43 55.8%AB	15 / 43 34.9%ABE	2 / 43 4.7%	1 / 43 2.3%CE	1 / 43 2.3%E			
1	Legionella pneumophila 130b	39 / 42 92.9%	1 / 1 100.0%	1 / 1 100.0%	1 / 1 100.0%	1 / 1 100.0%	2 / 3 66.7%	2 / 2 100.0%	1 / 1 100.0%	1 / 1 100.0%	2 / 2 100.0%	1 / 1 100.0%	3 / 3 100.0%	2 / 3 66.7%	1 / 1 100.0%	1 / 1 100.0%	1 / 1 100.0%	3 / 3 100.0%	1 / 1 100.0%	2 / 3 66.7%	4 / 4 100.0%	2 / 2 100.0%	1 / 1 100.0%	5 / 5 100.0%	0 / 0 0.0%	0 / 0 0.0%	0 / 0 0.0%	0 / 0 0.0%	24 / 42 57.1%AB	15 / 42 35.7%ABE	2 / 42 4.8%E	1 / 42 2.4%				
1	Legionella pneumophila serogroup 12	40 / 43 93.0%	1 / 1 100.0%	1 / 1 100.0%	1 / 1 100.0%	0 / 0 0.0%	2 / 3 66.7%	2 / 2 100.0%	1 / 1 100.0%	1 / 1 100.0%	4 / 4 100.0%	1 / 1 100.0%	3 / 3 100.0%	2 / 3 66.7%	1 / 1 100.0%	1 / 1 100.0%	1 / 1 100.0%	3 / 3 100.0%	1 / 1 100.0%	2 / 3 66.7%	4 / 4 100.0%	2 / 2 100.0%	1 / 1 100.0%	5 / 5 100.0%	0 / 0 0.0%	0 / 0 0.0%	0 / 0 0.0%	0 / 0 0.0%	25 / 43 58.1%AB	15 / 43 34.9%ABE	2 / 43 4.7%E	1 / 43 2.3%				
1	Legionella pneumophila subsp. pneumophila 570-CO-H	40 / 43 93.0%	1 / 1 100.0%	1 / 1 100.0%	1 / 1 100.0%	0 / 0 0.0%	2 / 3 66.7%	2 / 2 100.0%	1 / 1 100.0%	1 / 1 100.0%	4 / 4 100.0%	1 / 1 100.0%	3 / 3 100.0%	2 / 3 66.7%	1 / 1 100.0%	1 / 1 100.0%	1 / 1 100.0%	3 / 3 100.0%	1 / 1 100.0%	2 / 3 66.7%	4 / 4 100.0%	2 / 2 100.0%	1 / 1 100.0%	5 / 5 100.0%	0 / 0 0.0%	0 / 0 0.0%	0 / 0 0.0%	0 / 0 0.0%	25 / 43 58.1%AB	15 / 43 34.9%ABE	2 / 43 4.7%E	1 / 43 2.3%				
6	Legionella pneumophila subsp. pneumophila	274 / 293 93.5%	7 / 7 100.0%	7 / 7 100.0%	7 / 7 100.0%	6 / 6 100.0%	13 / 20 65.0%	14 / 14 100.0%	7 / 7 100.0%	6 / 6 100.0%	20 / 20 100.0%	7 / 7 100.0%	21 / 21 100.0%	14 / 19 73.7%	7 / 7 100.0%	7 / 7 100.0%	7 / 7 100.0%	21 / 21 100.0%	7 / 7 100.0%	12 / 19 63.2%	28 / 28 100.0%	14 / 14 100.0%	7 / 7 100.0%	35 / 35 100.0%	0 / 0 0.0%	0 / 0 0.0%	0 / 0 0.0%	0 / 0 0.0%	170 / 293 58.0%AB	104 / 293 35.5%ABE	10 / 293 3.4%	9 / 293 3.1%E				
3	Legionella pneumophila subsp. pneumophila 'Philadelphia 1'	120 / 127 94.5%	3 / 3 100.0%	3 / 3 100.0%	3 / 3 100.0%	3 / 3 100.0%	6 / 9 66.7%	6 / 6 100.0%	3 / 3 100.0%	3 / 3 100.0%	9 / 9 100.0%	3 / 3 100.0%	9 / 9 100.0%	6 / 7 85.7%	3 / 3 100.0%	3 / 3 100.0%	3 / 3 100.0%	9 / 9 100.0%	3 / 3 100.0%	6 / 9 66.7%	12 / 12 100.0%	6 / 6 100.0%	3 / 3 100.0%	15 / 15 100.0%	0 / 0 0.0%	0 / 0 0.0%	0 / 0 0.0%	0 / 0 0.0%	75 / 127 59.1%AB	45 / 127 35.4%ABE	5 / 127 3.9%	2 / 127 1.6%E				
2	Legionella pneumophila subsp. pneumophila Lp02	80 / 84 95.2%	2 / 2 100.0%	2 / 2 100.0%	2 / 2 100.0%	2 / 2 100.0%	4 / 6 66.7%	4 / 4 100.0%	2 / 2 100.0%	2 / 2 100.0%	6 / 6 100.0%	2 / 2 100.0%	6 / 6 100.0%	4 / 4 100.0%	2 / 2 100.0%	2 / 2 100.0%	2 / 2 100.0%	6 / 6 100.0%	2 / 2 100.0%	4 / 6 66.7%	8 / 8 100.0%	4 / 4 100.0%	2 / 2 100.0%	10 / 10 100.0%	0 / 0 0.0%	0 / 0 0.0%	0 / 0 0.0%	0 / 0 0.0%	50 / 84 59.5%AB	30 / 84 35.7%ABE	2 / 84 2.4%	2 / 84 2.4%E				
1	Legionella pneumophila subsp. pneumophila Hextuple_2q	40 / 42 95.2%	1 / 1 100.0%	1 / 1 100.0%	1 / 1 100.0%	1 / 1 100.0%	2 / 3 66.7%	2 / 2 100.0%	1 / 1 100.0%	1 / 1 100.0%	3 / 3 100.0%	1 / 1 100.0%	3 / 3 100.0%	2 / 2 100.0%	1 / 1 100.0%	1 / 1 100.0%	1 / 1 100.0%	3 / 3 100.0%	1 / 1 100.0%	2 / 3 66.7%	4 / 4 100.0%	2 / 2 100.0%	1 / 1 100.0%	5 / 5 100.0%	0 / 0 0.0%	0 / 0 0.0%	0 / 0 0.0%	0 / 0 0.0%	25 / 42 59.5%AB	15 / 42 35.7%ABE	1 / 42 2.4%E	1 / 42 2.4%				
1	Legionella pneumophila subsp. pneumophila Hextuple_3a	40 / 42 95.2%	1 / 1 100.0%	1 / 1 100.0%	1 / 1 100.0%	1 / 1 100.0%	2 / 3 66.7%	2 / 2 100.0%	1 / 1 100.0%	1 / 1 100.0%	3 / 3 100.0%	1 / 1 100.0%	3 / 3 100.0%	2 / 2 100.0%	1 / 1 100.0%	1 / 1 100.0%	1 / 1 100.0%	3 / 3 100.0%	1 / 1 100.0%	2 / 3 66.7%	4 / 4 100.0%	2 / 2 100.0%	1 / 1 100.0%	5 / 5 100.0%	0 / 0 0.0%	0 / 0 0.0%	0 / 0 0.0%	0 / 0 0.0%	25 / 42 59.5%AB	15 / 42 35.7%ABE	1 / 42 2.4%	1 / 42 2.4%E				
1	Legionella pneumophila Lp6	34 / 37 91.9%	1 / 1 100.0%	1 / 1 100.0%	1 / 1 100.0%	0 / 0 0.0%	1 / 2 50.0%	2 / 2 100.0%	1 / 1 100.0%	0 / 0 0.0%	2 / 2 100.0%	1 / 1 100.0%	3 / 3 100.0%	2 / 3 66.7%	1 / 1 100.0%	1 / 1 100.0%	1 / 1 100.0%	3 / 3 100.0%	1 / 1 100.0%	0 / 1 0.0%	4 / 4 100.0%	2 / 2 100.0%	1 / 1 100.0%	5 / 5 100.0%	0 / 0 0.0%	0 / 0 0.0%	0 / 0 0.0%	0 / 0 0.0%	20 / 37 54.1%AB	14 / 37 37.8%ABE	2 / 37 5.4%E	1 / 37 2.7%				
1	Legionella pneumophila subsp. pneumophila LPE509	40 / 43 93.0%	1 / 1 100.0%	1 / 1 100.0%	1 / 1 100.0%	1 / 1 100.0%	2 / 3 66.7%	2 / 2 100.0%	1 / 1 100.0%	1 / 1 100.0%	3 / 3 100.0%	1 / 1 100.0%	3 / 3 100.0%	2 / 3 66.7%	1 / 1 100.0%	1 / 1 100.0%	1 / 1 100.0%	3 / 3 100.0%	1 / 1 100.0%	2 / 3 66.7%	4 / 4 100.0%	2 / 2 100.0%	1 / 1 100.0%	5 / 5 100.0%	0 / 0 0.0%	0 / 0 0.0%	0 / 0 0.0%	0 / 0 0.0%	25 / 43 58.1%AB	15 / 43 34.9%ABE	2 / 43 4.7%	1 / 43 2.3%E				
1	Legionella pneumophila Lens	40 / 43 93.0%	1 / 1 100.0%	1 / 1 100.0%	1 / 1 100.0%	1 / 1 100.0%	2 / 3 66.7%	2 / 2 100.0%	1 / 1 100.0%	1 / 1 100.0%	3 / 3 100.0%	1 / 1 100.0%	3 / 3 100.0%	2 / 3 66.7%	1 / 1 100.0%	1 / 1 100.0%	1 / 1 100.0%	3 / 3 100.0%	1 / 1 100.0%	2 / 3 66.7%	4 / 4 100.0%	2 / 2 100.0%	1 / 1 100.0%	5 / 5 100.0%	0 / 0 0.0%	0 / 0 0.0%	0 / 0 0.0%	0 / 0 0.0%	25 / 43 58.1%AB	15 / 43 34.9%ABE	3 / 43 7.0%					
1	Legionella pneumophila Paris	40 / 43 93.0%	1 / 1 100.0%	1 / 1 100.0%	1 / 1 100.0%	1 / 1 100.0%	2 / 3 66.7%	2 / 2 100.0%	1 / 1 100.0%	1 / 1 100.0%	3 / 3 100.0%	1 / 1 100.0%	3 / 3 100.0%	2 / 3 66.7%	1 / 1 100.0%	1 / 1 100.0%	1 / 1 100.0%	3 / 3 100.0%	1 / 1 100.0%	2 / 3 66.7%	4 / 4 100.0%	2 / 2 100.0%	1 / 1 100.0%	5 / 5 100.0%	0 / 0 0.0%	0 / 0 0.0%	0 / 0 0.0%	0 / 0 0.0%	25 / 43 58.1%AB	15 / 43 34.9%ABE	3 / 43 7.0%					
1	Legionella pneumophila 'Corby'	40 / 43 93.0%	1 / 1 100.0%	1 / 1 100.0%	1 / 1 100.0%	1 / 1 100.0%	2 / 3 66.7%	2 / 2 100.0%	1 / 1 100.0%	1 / 1 100.0%	3 / 3 100.0%	1 / 1 100.0%	3 / 3 100.0%	2 / 3 66.7%	1 / 1 100.0%	1 / 1 100.0%	1 / 1 100.0%	3 / 3 100.0%	1 / 1 100.0%	2 / 3 66.7%	4 / 4 100.0%	2 / 2 100.0%	1 / 1 100.0%	5 / 5 100.0%	0 / 0 0.0%	0 / 0 0.0%	0 / 0 0.0%	0 / 0 0.0%	25 / 43 58.1%AB	15 / 43 34.9%ABE	3 / 43 7.0%					
1	ATCC 33462	44 / 46 95.7%	1 / 1 100.0%	2 / 2 100.0%	1 / 1 100.0%	1 / 1 100.0%	2 / 3 66.7%	3 / 3 100.0%	1 / 1 100.0%	1 / 1 100.0%	4 / 4 100.0%	1 / 1 100.0%	3 / 3 100.0%	3 / 3 100.0%	1 / 1 100.0%	1 / 1 100.0%	1 / 1 100.0%	3 / 3 100.0%	1 / 1 100.0%	2 / 3 66.7%	4 / 4 100.0%	2 / 2 100.0%	1 / 1 100.0%	5 / 5 100.0%	0 / 0 0.0%	0 / 0 0.0%	0 / 0 0.0%	0 / 0 0.0%	29 / 46 63.0%AB	15 / 46 32.6%ABE	2 / 46 4.3%					
1	Legionella longbeachae NSW150	44 / 46 95.7%	1 / 1 100.0%	2 / 2 100.0%	1 / 1 100.0%	1 / 1 100.0%	2 / 3 66.7%	3 / 3 100.0%	1 / 1 100.0%	1 / 1 100.0%	4 / 4 100.0%	1 / 1 100.0%	3 / 3 100.0%	3 / 3 100.0%	1 / 1 100.0%	1 / 1 100.0%	1 / 1 100.0%	3 / 3 100.0%	1 / 1 100.0%	2 / 3 66.7%	4 / 4 100.0%	2 / 2 100.0%	1 / 1 100.0%	5 / 5 100.0%	0 / 0 0.0%	0 / 0 0.0%	0 / 0 0.0%	0 / 0 0.0%	29 / 46 63.0%AB	15 / 46 32.6%ABE	2 / 46 4.3%					
5	Coxiella group	129 / 210 61.4%	5 / 5 100.0%	5 / 5 100.0%	5 / 5 100.0%	5 / 5 100.0%	5 / 15 33.3%	5 / 10 50.0%	5 / 5 100.0%	0 / 5 0.0%	5 / 10 50.0%	5 / 5 100.0%	20 / 20 100.0%	5 / 10 50.0%	5 / 5 100.0%	5 / 5 100.0%	0 / 5 0.0%	0 / 15 0.0%	0 / 5 0.0%	5 / 15 33.3%	14 / 20 70.0%	10 / 10 100.0%	5 / 5 100.0%	15 / 25 60.0%	0 / 0 0.0%	0 / 0 0.0%	0 / 0 0.0%	0 / 0 0.0%	58 / 210 27.6%AB	44 / 210 21.0%ABE	44 / 210 21.0%	32 / 210 15.2%E	16 / 210 7.6%B	11 / 210 5.2%BE	5 / 210 2.4%C	
5	Burnetia	129 / 210 61.4%	5 / 5 100.0%	5 / 5 100.0%	5 / 5 100.0%	5 / 5 100.0%	5 / 15 33.3%	5 / 10 50.0%	5 / 5 100.0%	0 / 5 0.0%	5 / 10 50.0%	5 / 5 100.0%	20 / 20 100.0%	5 / 10 50.0%	5 / 5 100.0%	5 / 5 100.0%	0 / 5 0.0%	0 / 15 0.0%	0 / 5 0.0%	5 / 15 33.3%	14 / 20 70.0%	10 / 10 100.0%	5 / 5 100.0%	15 / 25 60.0%	0 / 0 0.0%	0 / 0 0.0%	0 / 0 0.0%	0 / 0 0.0%	58 / 210 27.6%AB	44 / 210 21.0%ABE	44 / 210 21.0%	32 / 210 15.2%E	16 / 210 7.6%B	11 / 210 5.2%BE	5 / 210 2.4%C	
5	"Rickettsia burneti" (sic) Derrick 1939	129 / 210 61.4%	5 / 5 100.0%	5 / 5 100.0%	5 / 5 100.0%	5 / 5 100.0%	5 / 15 33.3%	5 / 10 50.0%	5 / 5 100.0%	0 / 5 0.0%	5 / 10 50.0%	5 / 5 100.0%	20 / 20 100.0%	5 / 10 50.0%	5 / 5 100.0%	5 / 5 100.0%	0 / 5 0.0%	0 / 15 0.0%	0 / 5 0.0%	5 / 15 33.3%	14 / 20 70.0%	10 / 10 100.0%	5 / 5 100.0%	15 / 25 60.0%	0 / 0 0.0%	0 / 0 0.0%	0 / 0 0.0%	0 / 0 0.0%	58 / 210 27.6%AB	44 / 210 21.0%	44 / 210 21.0%ABE	32 / 210 15.2%E	16 / 210 7.6%B	11 / 210 5.2%BE	5 / 210 2.4%C	
1	Coxiella burnetii RSA 493	26 / 42 61.9%	1 / 1 100.0%	1 / 1 100.0%	1 / 1 100.0%	1 / 1 100.0%	1 / 3 33.3%	1 / 2 50.0%	1 / 1 100.0%	0 / 1 0.0%	1 / 2 50.0%	1 / 1 100.0%	4 / 4 100.0%	1 / 2 50.0%	1 / 1 100.0%	1 / 1 100.0%	0 / 1 0.0%	0 / 3 0.0%	0 / 1 0.0%	1 / 3 33.3%	3 / 4 75.0%	2 / 2 100.0%	1 / 1 100.0%	3 / 5 60.0%	0 / 0 0.0%	0 / 0 0.0%	0 / 0 0.0%	0 / 0 0.0%	15 / 42 35.7%AB	11 / 42 26.2%	11 / 42 26.2%ABE	4 / 42 9.5%E	1 / 42 2.4%C			
1	Coxiella burnetii RSA 331	25 / 42 59.5%	1 / 1 100.0%	1 / 1 100.0%	1 / 1 100.0%	1 / 1 100.0%	1 / 3 33.3%	1 / 2 50.0%	1 / 1 100.0%	0 / 1 0.0%	1 / 2 50.0%	1 / 1 100.0%	4 / 4 100.0%	1 / 2 50.0%	1 / 1 100.0%	1 / 1 100.0%	0 / 1 0.0%	0 / 3 0.0%	0 / 1 0.0%	1 / 3 33.3%	2 / 4 50.0%	2 / 2 100.0%	1 / 1 100.0%	3 / 5 60.0%	0 / 0 0.0%	0 / 0 0.0%	0 / 0 0.0%	0 / 0 0.0%	9 / 42 21.4%	7 / 42 16.7%B	7 / 42 16.7%AB	7 / 42 16.7%E	7 / 42 16.7%BE	4 / 42 9.5%ABE	1 / 42 2.4%C	
1	Coxiella burnetii Dugway	26 / 42 61.9%	1 / 1 100.0%	1 / 1 100.0%	1 / 1 100.0%	1 / 1 100.0%	1 / 3 33.3%	1 / 2 50.0%	1 / 1 100.0%	0 / 1 0.0%	1 / 2 50.0%	1 / 1 100.0%	4 / 4 100.0%	1 / 2 50.0%	1 / 1 100.0%	1 / 1 100.0%	0 / 1 0.0%	0 / 3 0.0%	0 / 1 0.0%	1 / 3 33.3%	3 / 4 75.0%	2 / 2 100.0%	1 / 1 100.0%	3 / 5 60.0%	0 / 0 0.0%	0 / 0 0.0%	0 / 0 0.0%	0 / 0 0.0%	9 / 42 21.4%B	8 / 42 19.0%	7 / 42 16.7%E	7 / 42 16.7%ABE	6 / 42 14.3%AB	4 / 42 9.5%BE	1 / 42 2.4%C	
1	Coxiella burnetii CbuG_Q212	26 / 42 61.9%	1 / 1 100.0%	1 / 1 100.0%	1 / 1 100.0%	1 / 1 100.0%	1 / 3 33.3%	1 / 2 50.0%	1 / 1 100.0%	0 / 1 0.0%	1 / 2 50.0%	1 / 1 100.0%	4 / 4 100.0%	1 / 2 50.0%	1 / 1 100.0%	1 / 1 100.0%	0 / 1 0.0%	0 / 3 0.0%	0 / 1 0.0%	1 / 3 33.3%	3 / 4 75.0%	2 / 2 100.0%	1 / 1 100.0%	3 / 5 60.0%	0 / 0 0.0%	0 / 0 0.0%	0 / 0 0.0%	0 / 0 0.0%	15 / 42 35.7%AB	11 / 42 26.2%ABE	8 / 42 19.0%	7 / 42 16.7%E	1 / 42 2.4%C			
1	Coxiella burnetii CbuK_Q154	26 / 42 61.9%	1 / 1 100.0%	1 / 1 100.0%	1 / 1 100.0%	1 / 1 100.0%	1 / 3 33.3%	1 / 2 50.0%	1 / 1 100.0%	0 / 1 0.0%	1 / 2 50.0%	1 / 1 100.0%	4 / 4 100.0%	1 / 2 50.0%	1 / 1 100.0%	1 / 1 100.0%	0 / 1 0.0%	0 / 3 0.0%	0 / 1 0.0%	1 / 3 33.3%	3 / 4 75.0%	2 / 2 100.0%	1 / 1 100.0%	3 / 5 60.0%	0 / 0 0.0%	0 / 0 0.0%	0 / 0 0.0%	0 / 0 0.0%	15 / 42 35.7%AB	11 / 42 26.2%ABE	8 / 42 19.0%	7 / 42 16.7%E	1 / 42 2.4%C			
13	Chromatiaceae/Ectothiorhodospiraceae group	636 / 642 99.1%	15 / 15 100.0%	14 / 14 100.0%	14 / 14 100.0%	22 / 22 100.0%	43 / 43 100.0%	41 / 41 100.0%	14 / 14 100.0%	14 / 14 100.0%	51 / 51 100.0%	15 / 15 100.0%	54 / 55 98.2%	26 / 27 96.3%	14 / 14 100.0%	27 / 27 100.0%	14 / 14 100.0%	42 / 42 100.0%	14 / 14 100.0%	39 / 42 92.9%	55 / 56 98.2%	26 / 26 100.0%	14 / 14 100.0%	67 / 67 100.0%	1 / 1 100.0%	0 / 0 0.0%	0 / 0 0.0%	0 / 0 0.0%	413 / 642 64.3%AB	201 / 642 31.3%ABE	14 / 642 2.2%B	8 / 642 1.2%BE	3 / 642 0.5%C	2 / 642 0.3%	1 / 642 0.2%E	
6	Chromatiaceae	275 / 279 98.6%	6 / 6 100.0%	6 / 6 100.0%	6 / 6 100.0%	11 / 11 100.0%	18 / 18 100.0%	18 / 18 100.0%	6 / 6 100.0%	6 / 6 100.0%	23 / 23 100.0%	7 / 7 100.0%	24 / 24 100.0%	12 / 12 100.0%	6 / 6 100.0%	12 / 12 100.0%	6 / 6 100.0%	18 / 18 100.0%	6 / 6 100.0%	15 / 18 83.3%	23 / 24 95.8%	12 / 12 100.0%	6 / 6 100.0%	28 / 28 100.0%	0 / 0 0.0%	0 / 0 0.0%	0 / 0 0.0%	0 / 0 0.0%	185 / 279 66.3%AB	90 / 279 32.3%ABE	3 / 279 1.1%C	1 / 279 0.4%				

3	"Nitrosococcus" Winogradsky 1892 emend. Campbell et al. 2011	[illegible] 134 97.0%	3 / 3 100.0%	3 / 3 100.0%	3 / 3 100.0%	3 / 3 100.0%	9 / 9 100.0%	9 / 9 100.0%	3 / 3 100.0%	3 / 3 100.0%	9 / 9 100.0%	4 / 4 100.0%	12 / 12 100.0%	6 / 6 100.0%	3 / 3 100.0%	6 / 6 100.0%	3 / 3 100.0%	9 / 9 100.0%	3 / 3 100.0%	6 / 9 66.7%	11 / 12 91.7%	6 / 6 100.0%	3 / 3 100.0%	13 / 13 100.0%	0 / 0 0.0%	0 / 0 0.0%	0 / 0 0.0%	0 / 0 0.0%	87 / 134 64.9%AB	43 / 134 32.1%ABE	3 / 134 2.2%C	1 / 134 0.7%		
1	"Nitrosocystis oceanus" (sic) Watson 1965	44 / 45 97.8%	1 / 1 100.0%	1 / 1 100.0%	1 / 1 100.0%	1 / 1 100.0%	3 / 3 100.0%	3 / 3 100.0%	1 / 1 100.0%	1 / 1 100.0%	3 / 3 100.0%	1 / 1 100.0%	4 / 4 100.0%	2 / 2 100.0%	1 / 1 100.0%	2 / 2 100.0%	1 / 1 100.0%	3 / 3 100.0%	1 / 1 100.0%	2 / 3 66.7%	4 / 4 100.0%	2 / 2 100.0%	1 / 1 100.0%	5 / 5 100.0%	0 / 0 0.0%	0 / 0 0.0%	0 / 0 0.0%	0 / 0 0.0%	29 / 45 64.4%AB	15 / 45 33.3%ABE	1 / 45 2.2%C			
1	Nitrosococcus oceani ATCC 19707	44 / 45 97.8%	1 / 1 100.0%	1 / 1 100.0%	1 / 1 100.0%	1 / 1 100.0%	3 / 3 100.0%	3 / 3 100.0%	1 / 1 100.0%	1 / 1 100.0%	3 / 3 100.0%	1 / 1 100.0%	4 / 4 100.0%	2 / 2 100.0%	1 / 1 100.0%	2 / 2 100.0%	1 / 1 100.0%	3 / 3 100.0%	1 / 1 100.0%	2 / 3 66.7%	4 / 4 100.0%	2 / 2 100.0%	1 / 1 100.0%	5 / 5 100.0%	0 / 0 0.0%	0 / 0 0.0%	0 / 0 0.0%	0 / 0 0.0%	29 / 45 64.4%AB	15 / 45 33.3%ABE	1 / 45 2.2%C			
1	"Nitrosococcus halophilus" Koops et al. 1990	43 / 44 97.7%	1 / 1 100.0%	1 / 1 100.0%	1 / 1 100.0%	1 / 1 100.0%	3 / 3 100.0%	3 / 3 100.0%	1 / 1 100.0%	1 / 1 100.0%	3 / 3 100.0%	1 / 1 100.0%	4 / 4 100.0%	2 / 2 100.0%	1 / 1 100.0%	2 / 2 100.0%	1 / 1 100.0%	3 / 3 100.0%	1 / 1 100.0%	2 / 3 66.7%	4 / 4 100.0%	2 / 2 100.0%	1 / 1 100.0%	4 / 4 100.0%	0 / 0 0.0%	0 / 0 0.0%	0 / 0 0.0%	0 / 0 0.0%	29 / 44 65.9%AB	14 / 44 31.8%ABE	1 / 44 2.3%C			
1	Nitrosococcus halophilus Nc 4	43 / 44 97.7%	1 / 1 100.0%	1 / 1 100.0%	1 / 1 100.0%	1 / 1 100.0%	3 / 3 100.0%	3 / 3 100.0%	1 / 1 100.0%	1 / 1 100.0%	3 / 3 100.0%	1 / 1 100.0%	4 / 4 100.0%	2 / 2 100.0%	1 / 1 100.0%	2 / 2 100.0%	1 / 1 100.0%	3 / 3 100.0%	1 / 1 100.0%	2 / 3 66.7%	4 / 4 100.0%	2 / 2 100.0%	1 / 1 100.0%	4 / 4 100.0%	0 / 0 0.0%	0 / 0 0.0%	0 / 0 0.0%	0 / 0 0.0%	29 / 44 65.9%AB	14 / 44 31.8%ABE	1 / 44 2.3%C			
1	"Nitrosococcus watsonii" Campbell et al. 2011	43 / 45 95.6%	1 / 1 100.0%	1 / 1 100.0%	1 / 1 100.0%	1 / 1 100.0%	3 / 3 100.0%	3 / 3 100.0%	1 / 1 100.0%	1 / 1 100.0%	3 / 3 100.0%	2 / 2 100.0%	4 / 4 100.0%	2 / 2 100.0%	1 / 1 100.0%	2 / 2 100.0%	1 / 1 100.0%	3 / 3 100.0%	1 / 1 100.0%	2 / 3 66.7%	3 / 4 75.0%	2 / 2 100.0%	1 / 1 100.0%	4 / 4 100.0%	0 / 0 0.0%	0 / 0 0.0%	0 / 0 0.0%	0 / 0 0.0%	29 / 45 64.4%AB	14 / 45 31.1%ABE	1 / 45 2.2%C	1 / 45 2.2%		
1	Candidatus Nitrosococcus watsoni C-113	43 / 45 95.6%	1 / 1 100.0%	1 / 1 100.0%	1 / 1 100.0%	1 / 1 100.0%	3 / 3 100.0%	3 / 3 100.0%	1 / 1 100.0%	1 / 1 100.0%	3 / 3 100.0%	2 / 2 100.0%	4 / 4 100.0%	2 / 2 100.0%	1 / 1 100.0%	2 / 2 100.0%	1 / 1 100.0%	3 / 3 100.0%	1 / 1 100.0%	2 / 3 66.7%	3 / 4 75.0%	2 / 2 100.0%	1 / 1 100.0%	4 / 4 100.0%	0 / 0 0.0%	0 / 0 0.0%	0 / 0 0.0%	0 / 0 0.0%	29 / 45 64.4%AB	14 / 45 31.1%ABE	1 / 45 2.2%	1 / 45 2.2%C		
1	"Thiosphaera" Miyoshi 1897	49 / 49 100.0%	1 / 1 100.0%	1 / 1 100.0%	1 / 1 100.0%	3 / 3 100.0%	3 / 3 100.0%	3 / 3 100.0%	1 / 1 100.0%	1 / 1 100.0%	5 / 5 100.0%	1 / 1 100.0%	4 / 4 100.0%	2 / 2 100.0%	1 / 1 100.0%	2 / 2 100.0%	1 / 1 100.0%	3 / 3 100.0%	1 / 1 100.0%	3 / 3 100.0%	4 / 4 100.0%	2 / 2 100.0%	1 / 1 100.0%	5 / 5 100.0%	0 / 0 0.0%	0 / 0 0.0%	0 / 0 0.0%	0 / 0 0.0%	34 / 49 69.4%AB	15 / 49 30.6%ABE				
1	"Chromatium violaceum" Bergey et al. 1923	49 / 49 100.0%	1 / 1 100.0%	1 / 1 100.0%	1 / 1 100.0%	3 / 3 100.0%	3 / 3 100.0%	3 / 3 100.0%	1 / 1 100.0%	1 / 1 100.0%	5 / 5 100.0%	1 / 1 100.0%	4 / 4 100.0%	2 / 2 100.0%	1 / 1 100.0%	2 / 2 100.0%	1 / 1 100.0%	3 / 3 100.0%	1 / 1 100.0%	3 / 3 100.0%	4 / 4 100.0%	2 / 2 100.0%	1 / 1 100.0%	5 / 5 100.0%	0 / 0 0.0%	0 / 0 0.0%	0 / 0 0.0%	0 / 0 0.0%	34 / 49 69.4%AB	15 / 49 30.6%ABE				
1	Thiocystis violascens DSM 198	49 / 49 100.0%	1 / 1 100.0%	1 / 1 100.0%	1 / 1 100.0%	3 / 3 100.0%	3 / 3 100.0%	3 / 3 100.0%	1 / 1 100.0%	1 / 1 100.0%	5 / 5 100.0%	1 / 1 100.0%	4 / 4 100.0%	2 / 2 100.0%	1 / 1 100.0%	2 / 2 100.0%	1 / 1 100.0%	3 / 3 100.0%	1 / 1 100.0%	3 / 3 100.0%	4 / 4 100.0%	2 / 2 100.0%	1 / 1 100.0%	5 / 5 100.0%	0 / 0 0.0%	0 / 0 0.0%	0 / 0 0.0%	0 / 0 0.0%	34 / 49 69.4%AB	15 / 49 30.6%ABE				
1	Allochromatium	49 / 49 100.0%	1 / 1 100.0%	1 / 1 100.0%	1 / 1 100.0%	3 / 3 100.0%	3 / 3 100.0%	3 / 3 100.0%	1 / 1 100.0%	1 / 1 100.0%	5 / 5 100.0%	1 / 1 100.0%	4 / 4 100.0%	2 / 2 100.0%	1 / 1 100.0%	2 / 2 100.0%	1 / 1 100.0%	3 / 3 100.0%	1 / 1 100.0%	3 / 3 100.0%	4 / 4 100.0%	2 / 2 100.0%	1 / 1 100.0%	5 / 5 100.0%	0 / 0 0.0%	0 / 0 0.0%	0 / 0 0.0%	0 / 0 0.0%	33 / 49 67.3%AB	16 / 49 32.7%ABE				
1	"Bacillus vinosus" (Ehrenberg 1838) Trevisan 1889	49 / 49 100.0%	1 / 1 100.0%	1 / 1 100.0%	1 / 1 100.0%	3 / 3 100.0%	3 / 3 100.0%	3 / 3 100.0%	1 / 1 100.0%	1 / 1 100.0%	5 / 5 100.0%	1 / 1 100.0%	4 / 4 100.0%	2 / 2 100.0%	1 / 1 100.0%	2 / 2 100.0%	1 / 1 100.0%	3 / 3 100.0%	1 / 1 100.0%	3 / 3 100.0%	4 / 4 100.0%	2 / 2 100.0%	1 / 1 100.0%	5 / 5 100.0%	0 / 0 0.0%	0 / 0 0.0%	0 / 0 0.0%	0 / 0 0.0%	33 / 49 67.3%AB	16 / 49 32.7%ABE				
1	Allochromatium vinosum DSM 180	49 / 49 100.0%	1 / 1 100.0%	1 / 1 100.0%	1 / 1 100.0%	3 / 3 100.0%	3 / 3 100.0%	3 / 3 100.0%	1 / 1 100.0%	1 / 1 100.0%	5 / 5 100.0%	1 / 1 100.0%	4 / 4 100.0%	2 / 2 100.0%	1 / 1 100.0%	2 / 2 100.0%	1 / 1 100.0%	3 / 3 100.0%	1 / 1 100.0%	3 / 3 100.0%	4 / 4 100.0%	2 / 2 100.0%	1 / 1 100.0%	5 / 5 100.0%	0 / 0 0.0%	0 / 0 0.0%	0 / 0 0.0%	0 / 0 0.0%	33 / 49 67.3%AB	16 / 49 32.7%ABE				
1	Thioflavicoccus	47 / 47 100.0%	1 / 1 100.0%	1 / 1 100.0%	1 / 1 100.0%	2 / 2 100.0%	3 / 3 100.0%	3 / 3 100.0%	1 / 1 100.0%	1 / 1 100.0%	4 / 4 100.0%	1 / 1 100.0%	4 / 4 100.0%	2 / 2 100.0%	1 / 1 100.0%	2 / 2 100.0%	1 / 1 100.0%	3 / 3 100.0%	1 / 1 100.0%	3 / 3 100.0%	4 / 4 100.0%	2 / 2 100.0%	1 / 1 100.0%	5 / 5 100.0%	0 / 0 0.0%	0 / 0 0.0%	0 / 0 0.0%	0 / 0 0.0%	31 / 47 66.0%AB	16 / 47 34.0%ABE				
1	ATCC 700959	47 / 47 100.0%	1 / 1 100.0%	1 / 1 100.0%	1 / 1 100.0%	2 / 2 100.0%	3 / 3 100.0%	3 / 3 100.0%	1 / 1 100.0%	1 / 1 100.0%	4 / 4 100.0%	1 / 1 100.0%	4 / 4 100.0%	2 / 2 100.0%	1 / 1 100.0%	2 / 2 100.0%	1 / 1 100.0%	3 / 3 100.0%	1 / 1 100.0%	3 / 3 100.0%	4 / 4 100.0%	2 / 2 100.0%	1 / 1 100.0%	5 / 5 100.0%	0 / 0 0.0%	0 / 0 0.0%	0 / 0 0.0%	0 / 0 0.0%	31 / 47 66.0%AB	16 / 47 34.0%ABE				
1	Thioflavicoccus mobilis 8321	47 / 47 100.0%	1 / 1 100.0%	1 / 1 100.0%	1 / 1 100.0%	2 / 2 100.0%	3 / 3 100.0%	3 / 3 100.0%	1 / 1 100.0%	1 / 1 100.0%	4 / 4 100.0%	1 / 1 100.0%	4 / 4 100.0%	2 / 2 100.0%	1 / 1 100.0%	2 / 2 100.0%	1 / 1 100.0%	3 / 3 100.0%	1 / 1 100.0%	3 / 3 100.0%	4 / 4 100.0%	2 / 2 100.0%	1 / 1 100.0%	5 / 5 100.0%	0 / 0 0.0%	0 / 0 0.0%	0 / 0 0.0%	0 / 0 0.0%	31 / 47 66.0%AB	16 / 47 34.0%ABE				
6	Ectothiorhodospira group	318 / 320 99.4%	8 / 8 100.0%	7 / 7 100.0%	7 / 7 100.0%	9 / 9 100.0%	21 / 21 100.0%	21 / 21 100.0%	7 / 7 100.0%	7 / 7 100.0%	25 / 25 100.0%	7 / 7 100.0%	27 / 28 96.4%	13 / 14 92.9%	7 / 7 100.0%	14 / 14 100.0%	7 / 7 100.0%	21 / 21 100.0%	7 / 7 100.0%	21 / 21 100.0%	28 / 28 100.0%	12 / 12 100.0%	7 / 7 100.0%	34 / 34 100.0%	1 / 1 100.0%	0 / 0 0.0%	0 / 0 0.0%	0 / 0 0.0%	199 / 320 62.2%AB	97 / 320 30.3%ABE	14 / 320 4.4%B	8 / 320 2.5%BE	1 / 320 0.3%	1 / 320 0.3%E
1	Halorhodospira	45 / 46 97.8%	2 / 2 100.0%	1 / 1 100.0%	1 / 1 100.0%	1 / 1 100.0%	3 / 3 100.0%	3 / 3 100.0%	1 / 1 100.0%	1 / 1 100.0%	3 / 3 100.0%	1 / 1 100.0%	4 / 4 100.0%	1 / 2 50.0%	1 / 1 100.0%	2 / 2 100.0%	1 / 1 100.0%	3 / 3 100.0%	1 / 1 100.0%	3 / 3 100.0%	4 / 4 100.0%	2 / 2 100.0%	1 / 1 100.0%	5 / 5 100.0%	0 / 0 0.0%	0 / 0 0.0%	0 / 0 0.0%	0 / 0 0.0%	29 / 46 63.0%AB	16 / 46 34.8%ABE	1 / 46 2.2%			
1	DSM 244	45 / 46 97.8%	2 / 2 100.0%	1 / 1 100.0%	1 / 1 100.0%	1 / 1 100.0%	3 / 3 100.0%	3 / 3 100.0%	1 / 1 100.0%	1 / 1 100.0%	3 / 3 100.0%	1 / 1 100.0%	4 / 4 100.0%	1 / 2 50.0%	1 / 1 100.0%	2 / 2 100.0%	1 / 1 100.0%	3 / 3 100.0%	1 / 1 100.0%	3 / 3 100.0%	4 / 4 100.0%	2 / 2 100.0%	1 / 1 100.0%	5 / 5 100.0%	0 / 0 0.0%	0 / 0 0.0%	0 / 0 0.0%	0 / 0 0.0%	29 / 46 63.0%AB	16 / 46 34.8%ABE	1 / 46 2.2%			
1	Halorhodospira halophila DSM 244	45 / 46 97.8%	2 / 2 100.0%	1 / 1 100.0%	1 / 1 100.0%	1 / 1 100.0%	3 / 3 100.0%	3 / 3 100.0%	1 / 1 100.0%	1 / 1 100.0%	3 / 3 100.0%	1 / 1 100.0%	4 / 4 100.0%	1 / 2 50.0%	1 / 1 100.0%	2 / 2 100.0%	1 / 1 100.0%	3 / 3 100.0%	1 / 1 100.0%	3 / 3 100.0%	4 / 4 100.0%	2 / 2 100.0%	1 / 1 100.0%	5 / 5 100.0%	0 / 0 0.0%	0 / 0 0.0%	0 / 0 0.0%	0 / 0 0.0%	29 / 46 63.0%AB	16 / 46 34.8%ABE	1 / 46 2.2%			
3	Thialkalivibrio	133 / 133 100.0%	3 / 3 100.0%	3 / 3 100.0%	3 / 3 100.0%	3 / 3 100.0%	9 / 9 100.0%	9 / 9 100.0%	3 / 3 100.0%	3 / 3 100.0%	9 / 9 100.0%	3 / 3 100.0%	12 / 12 100.0%	6 / 6 100.0%	3 / 3 100.0%	6 / 6 100.0%	3 / 3 100.0%	9 / 9 100.0%	3 / 3 100.0%	9 / 9 100.0%	12 / 12 100.0%	4 / 4 100.0%	3 / 3 100.0%	14 / 14 100.0%	1 / 1 100.0%	0 / 0 0.0%	0 / 0 0.0%	0 / 0 0.0%	89 / 133 66.9%AB	44 / 133 33.1%ABE				
1	DSM 14787	45 / 45 100.0%	1 / 1 100.0%	1 / 1 100.0%	1 / 1 100.0%	1 / 1 100.0%	3 / 3 100.0%	3 / 3 100.0%	1 / 1 100.0%	1 / 1 100.0%	3 / 3 100.0%	1 / 1 100.0%	4 / 4 100.0%	2 / 2 100.0%	1 / 1 100.0%	2 / 2 100.0%	1 / 1 100.0%	3 / 3 100.0%	1 / 1 100.0%	3 / 3 100.0%	4 / 4 100.0%	1 / 1 100.0%	1 / 1 100.0%	5 / 5 100.0%	1 / 1 100.0%	0 / 0 0.0%	0 / 0 0.0%	0 / 0 0.0%	29 / 45 64.4%AB	16 / 45 35.6%ABE				
1	Thioalkalivibrio nitratireducens ALEN 2	45 / 45 100.0%	1 / 1 100.0%	1 / 1 100.0%	1 / 1 100.0%	1 / 1 100.0%	3 / 3 100.0%	3 / 3 100.0%	1 / 1 100.0%	1 / 1 100.0%	3 / 3 100.0%	1 / 1 100.0%	4 / 4 100.0%	2 / 2 100.0%	1 / 1 100.0%	2 / 2 100.0%	1 / 1 100.0%	3 / 3 100.0%	1 / 1 100.0%	3 / 3 100.0%	4 / 4 100.0%	1 / 1 100.0%	1 / 1 100.0%	5 / 5 100.0%	1 / 1 100.0%	0 / 0 0.0%	0 / 0 0.0%	0 / 0 0.0%	29 / 45 64.4%AB	16 / 45 35.6%ABE				
1	Thioalkalivibrio sp. K90mix	44 / 44 100.0%	1 / 1 100.0%	1 / 1 100.0%	1 / 1 100.0%	1 / 1 100.0%	3 / 3 100.0%	3 / 3 100.0%	1 / 1 100.0%	1 / 1 100.0%	3 / 3 100.0%	1 / 1 100.0%	4 / 4 100.0%	2 / 2 100.0%	1 / 1 100.0%	2 / 2 100.0%	1 / 1 100.0%	3 / 3 100.0%	1 / 1 100.0%	3 / 3 100.0%	4 / 4 100.0%	1 / 1 100.0%	1 / 1 100.0%	5 / 5 100.0%	0 / 0 0.0%	0 / 0 0.0%	0 / 0 0.0%	0 / 0 0.0%	31 / 44 70.5%AB	13 / 44 29.5%ABE				
1	"Thioalkalivibrio sulfidophilus" Muyzer et al. 2010	44 / 44 100.0%	1 / 1 100.0%	1 / 1 100.0%	1 / 1 100.0%	1 / 1 100.0%	3 / 3 100.0%	3 / 3 100.0%	1 / 1 100.0%	1 / 1 100.0%	3 / 3 100.0%	1 / 1 100.0%	4 / 4 100.0%	2 / 2 100.0%	1 / 1 100.0%	2 / 2 100.0%	1 / 1 100.0%	3 / 3 100.0%	1 / 1 100.0%	3 / 3 100.0%	4 / 4 100.0%	2 / 2 100.0%	1 / 1 100.0%	4 / 4 100.0%	0 / 0 0.0%	0 / 0 0.0%	0 / 0 0.0%	0 / 0 0.0%	29 / 44 65.9%AB	15 / 44 34.1%ABE				
1	Thioalkalivibrio sp. HL-EbGR7	44 / 44 100.0%	1 / 1 100.0%	1 / 1 100.0%	1 / 1 100.0%	1 / 1 100.0%	3 / 3 100.0%	3 / 3 100.0%	1 / 1 100.0%	1 / 1 100.0%	3 / 3 100.0%	1 / 1 100.0%	4 / 4 100.0%	2 / 2 100.0%	1 / 1 100.0%	2 / 2 100.0%	1 / 1 100.0%	3 / 3 100.0%	1 / 1 100.0%	3 / 3 100.0%	4 / 4 100.0%	2 / 2 100.0%	1 / 1 100.0%	4 / 4 100.0%	0 / 0 0.0%	0 / 0 0.0%	0 / 0 0.0%	0 / 0 0.0%	29 / 44 65.9%AB	15 / 44 34.1%ABE				
1	Alcalilimnicola	95 / 96 99.0%	2 / 2 100.0%	2 / 2 100.0%	2 / 2 100.0%	4 / 4 100.0%	6 / 6 100.0%	6 / 6 100.0%	2 / 2 100.0%	2 / 2 100.0%	10 / 10 100.0%	2 / 2 100.0%	7 / 8 87.5%	4 / 4 100.0%	2 / 2 100.0%	4 / 4 100.0%	2 / 2 100.0%	6 / 6 100.0%	2 / 2 100.0%	6 / 6 100.0%	8 / 8 100.0%	4 / 4 100.0%	2 / 2 100.0%	10 / 10 100.0%	0 / 0 0.0%	0 / 0 0.0%	0 / 0 0.0%	0 / 0 0.0%	50 / 96 52.1%AB	23 / 96 24.0%ABE	14 / 96 14.6%B	8 / 96 8.3%BE	1 / 96 1.0%E	
1	ATCC BAA-1101	95 / 96 99.0%	2 / 2 100.0%	2 / 2 100.0%	2 / 2 100.0%	4 / 4 100.0%	6 / 6 100.0%	6 / 6 100.0%	2 / 2 100.0%	2 / 2 100.0%	10 / 10 100.0%	2 / 2 100.0%	7 / 8 87.5%	4 / 4 100.0%	2 / 2 100.0%	4 / 4 100.0%	2 / 2 100.0%	6 / 6 100.0%	2 / 2 100.0%	6 / 6 100.0%	8 / 8 100.0%	4 / 4 100.0%	2 / 2 100.0%	10 / 10 100.0%	0 / 0 0.0%	0 / 0 0.0%	0 / 0 0.0%	0 / 0 0.0%	50 / 96 52.1%AB	23 / 96 24.0%ABE	14 / 96 14.6%B	8 / 96 8.3%BE	1 / 96 1.0%E	
1	Alkalilimnicola ehrlichei MLHE-1	95 / 96 99.0%	2 / 2 100.0%	2 / 2 100.0%	2 / 2 100.0%	4 / 4 100.0%	6 / 6 100.0%	6 / 6 100.0%	2 / 2 100.0%	2 / 2 100.0%	10 / 10 100.0%	2 / 2 100.0%	7 / 8 87.5%	4 / 4 100.0%	2 / 2 100.0%	4 / 4 100.0%	2 / 2 100.0%	6 / 6 100.0%	2 / 2 100.0%	6 / 6 100.0%	8 / 8 100.0%	4 / 4 100.0%	2 / 2 100.0%	10 / 10 100.0%	0 / 0 0.0%	0 / 0 0.0%	0 / 0 0.0%	0 / 0 0.0%	50 / 96 52.1%AB	23 / 96 24.0%ABE	14 / 96 14.6%B	8 / 96 8.3%BE	1 / 96 1.0%E	
1	Spiribacter	45 / 45 100.0%	1 / 1 100.0%	1 / 1 100.0%	1 / 1 100.0%	1 / 1 100.0%	3 / 3 100.0%	3 / 3 100.0%	1 / 1 100.0%	1 / 1 100.0%	3 / 3 100.0%	1 / 1 100.0%	4 / 4 100.0%	2 / 2 100.0%	1 / 1 100.0%	2 / 2 100.0%	1 / 1 100.0%	3 / 3 100.0%	1 / 1 100.0%	3 / 3 100.0%	4 / 4 100.0%	2 / 2 100.0%	1 / 1 100.0%	5 / 5 100.0%	0 / 0 0.0%	0 / 0 0.0%	0 / 0 0.0%	0 / 0 0.0%	31 / 45 68.9%AB	14 / 45 31.1%ABE				
1	CECT 8282	45 / 45 100.0%	1 / 1 100.0%	1 / 1 100.0%	1 / 1 100.0%	1 / 1 100.0%	3 / 3 100.0%	3 / 3 100.0%	1 / 1 100.0%	1 / 1 100.0%	3 / 3 100.0%	1 / 1 100.0%	4 / 4 100.0%	2 / 2 100.0%	1 / 1 100.0%	2 / 2 100.0%	1 / 1 100.0%	3 / 3 100.0%	1 / 1 100.0%	3 / 3 100.0%	4 / 4 100.0%	2 / 2 100.0%	1 / 1 100.0%	5 / 5 100.0%	0 / 0 0.0%	0 / 0 0.0%	0 / 0 0.0%	0 / 0 0.0%	31 / 45 68.9%AB	14 / 45 31.1%ABE				
1	Ectothiorhodospiraceae bacterium M19-40	45 / 45 100.0%	1 / 1 100.0%	1 / 1 100.0%	1 / 1 100.0%	1 / 1 100.0%	3 / 3 100.0%	3 / 3 100.0%	1 / 1 100.0%	1 / 1 100.0%	3 / 3 100.0%	1 / 1 100.0%	4 / 4 100.0%	2 / 2 100.0%	1 / 1 100.0%	2 / 2 100.0%	1 / 1 100.0%	3 / 3 100.0%	1 / 1 100.0%	3 / 3 100.0%	4 / 4 100.0%	2 / 2 100.0%	1 / 1 100.0%	5 / 5 100.0%	0 / 0 0.0%	0 / 0 0.0%	0 / 0 0.0%	0 / 0 0.0%	31 / 45 68.9%AB	14 / 45 31.1%ABE				
1	Halothiobacillaceae	43 / 43 100.0%	1 / 1 100.0%	1 / 1 100.0%	1 / 1 100.0%	2 / 2 100.0%	4 / 4 100.0%	2 / 2 100.0%	1 / 1 100.0%	1 / 1 100.0%	3 / 3 100.0%	1 / 1 100.0%	3 / 3 100.0%	1 / 1 100.0%	1 / 1 100.0%	1 / 1 100.0%	1 / 1 100.0%	3 / 3 100.0%	1 / 1 100.0%	3 / 3 100.0%	4 / 4 100.0%	2 / 2 100.0%	1 / 1 100.0%	5 / 5 100.0%	0 / 0 0.0%	0 / 0 0.0%	0 / 0 0.0%	0 / 0 0.0%	29 / 43 67.4%AB	14 / 43 32.6%ABE				
1	Halothiobacillus	43 / 43 100.0%	1 / 1 100.0%	1 / 1 100.0%	1 / 1 100.0%	2 / 2 100.0%	4 / 4 100.0%	2 / 2 100.0%	1 / 1 100.0%	1 / 1 100.0%	3 / 3 100.0%	1 / 1 100.0%	3 / 3 100.0%	1 / 1 100.0%	1 / 1 100.0%	1 / 1 100.0%	1 / 1 100.0%	3 / 3 100.0%	1 / 1 100.0%	3 / 3 100.0%	4 / 4 100.0%	2 / 2 100.0%	1 / 1 100.0%	5 / 5 100.0%	0 / 0 0.0%	0 / 0 0.0%	0 / 0 0.0%	0 / 0 0.0%	29 / 43 67.4%AB	14 / 43 32.6%ABE				
1	"Thiobacillus X" Parker and Prisk 1953	43 / 43 100.0%	1 / 1 100.0%	1 / 1 100.0%	1 / 1 100.0%	2 / 2 100.0%	4 / 4 100.0%	2 / 2 100.0%	1 / 1 100.0%	1 / 1 100.0%	3 / 3 100.0%	1 / 1 100.0%	3 / 3 100.0%	1 / 1 100.0%	1 / 1 100.0%	1 / 1 100.0%	1 / 1 100.0%	3 / 3 100.0%	1 / 1 100.0%	3 / 3 100.0%	4 / 4 100.0%	2 / 2 100.0%	1 / 1 100.0%	5 / 5 100.0%	0 / 0 0.0%	0 / 0 0.0%	0 / 0 0.0%	0 / 0 0.0%	29 / 43 67.4%AB	14 / 43 32.6%ABE				
1	Halothiobacillus neapolitanus ATCC 23641	43 / 43 100.0%	1 / 1 100.0%	1 / 1 100.0%	1 / 1 100.0%	2 / 2 100.0%	4 / 4 100.0%	2 / 2 100.0%	1 / 1 100.0%	1 / 1 100.0%	3 / 3 100.0%	1 / 1 100.0%	3 / 3 100.0%	1 / 1 100.0%	1 / 1 100.0%	1 / 1 100.0%	1 / 1 100.0%	3 / 3 100.0%	1 / 1 100.0%	3 / 3 100.0%	4 / 4 100.0%	2 / 2 100.0%	1 / 1 100.0%	5 / 5 100.0%	0 / 0 0.0%	0 / 0 0.0%	0 / 0 0.0%	0 / 0 0.0%	29 / 43 67.4%AB	14 / 43 32.6%ABE				
26	Xanthomonadales	1429 / 1444 99.0%	36 / 36 100.0%	56 / 56 100.0%	30 / 30 100.0%	61 / 61 100.0%	111 / 112 99.1%	83 / 83 100.0%	27 / 27 100.0%	26 / 26 100.0%	137 / 137 100.0%	26 / 26 100.0%	103 / 105 98.1%	61 / 63 96.8%	30 / 30 100.0%	53 / 53 100.0%	26 / 26 100.0%	83 / 84 98.8%	26 / 26 100.0%	79 / 79 100.0%	127 / 127 100.0%	75 / 75 100.0%	27 / 27 100.0%	142 / 151 94.0%	4 / 4 100.0%	0 / 0 0.0%	0 / 0 0.0%	0 / 0 0.0%	910 / 1444 63.0%AB	391 / 1444 27.1%ABE	79 / 1444 5.5%B	49 / 1444 3.4%BE	13 / 1444 0.9%E	2 / 1444 0.1%
26	Lysobacteraceae	1429 / 1444 99.0%	36 / 36 100.0%	56 / 56 100.0%	30 / 30 100.0%	61 / 61 100.0%	111 / 112 99.1%	83 / 83 100.0%	27 / 27 100.0%	26 / 26 100.0%	137 / 137 100.0%	26 / 26 100.0%	103 / 105 98.1%	61 / 63 96.8%	30 / 30 100.0%	53 / 53 100.0%	26 / 26 100.0%	83 / 84 98.8%	26 / 26 100.0%	79 / 79 100.0%	127 / 127 100.0%	75 / 75 100.0%	27 / 27 100.0%	142 / 151 94.0%	4 / 4 100.0%	0 / 0 0.0%	0 / 0 0.0%	0 / 0 0.0%	910 / 1444 63.0%AB	391 / 1444 27.1%ABE	79 / 1444 5.5%B	49 / 1444 3.4%BE	13 / 1444 0.9%E	2 / 1444 0.1%
13	"Phytomonas" Bergey et al. 1923	679 / 690 98.4%	13 / 13 100.0%	26 / 26 100.0%	13 / 13 100.0%	26 / 26 100.0%	51 / 52 98.1%	39 / 39 100.0%	13 / 13 100.0%	13 / 13 100.0%	65 / 65 100.0%	13 / 13 100.0%	53 / 53 100.0%	26 / 26 100.0%	13 / 13 100.0%	26 / 26 100.0%	13 / 13 100.0%	39 / 40 97.5%	13 / 13 100.0%	39 / 39 100.0%	65 / 65 100.0%	39 / 39 100.0%	13 / 13 100.0%	68 / 77 88.3%	0 / 0 0.0%	0 / 0 0.0%	0 / 0 0.0%	0 / 0 0.0%	439 / 690 63.6%AB	203 / 690 29.4%ABE	26 / 690 3.8%B	11 / 690 1.6%BE	9 / 690 1.3%E	2 / 690 0.3%
3	"Bacillus campestris" Pammel 1895	158 / 161 98.1%	3 / 3 100.0%	6 / 6 100.0%	3 / 3 100.0%	6 / 6 100.0%	12 / 12 100.0%	9 / 9 100.0%	3 / 3 100.0%	3 / 3 100.0%	15 / 15 100.0%	3 / 3 100.0%	12 / 12 100.0%	6 / 6 100.0%	3 / 3 100.0%	6 / 6 100.0%	3 / 3 100.0%	10 / 10 100.0%	3 / 3 100.0%	9 / 9 100.0%	16 / 16 100.0%	9 / 9 100.0%	3 / 3 100.0%	15 / 18 83.3%	0 / 0 0.0%	0 / 0 0.0%	0 / 0 0.0%	0 / 0 0.0%	109 / 161 67.7%AB	49 / 161 30.4%ABE	3 / 161 1.9%E			
2	Xanthomonas campestris (pv. campestris)	104 / 106 98.1%	2 / 2 100.0%	4 / 4 100.0%	2 / 2 100.0%	4 / 4 100.0%	8 / 8 100.0%	6 / 6 100.0%	2 / 2 100.0%	2 / 2 100.0%	10 / 10 100.0%	2 / 2 100.0%	8 / 8 100.0%	4 / 4 100.0%	2 / 2 100.0%	4 / 4 100.0%	2 / 2 100.0%	6 / 6 100.0%	2 / 2 100.0%	6 / 6 100.0%	10 / 10 100.0%	6 / 6 100.0%	2 / 2 100.0%	10 / 12 83.3%	0 / 0 0.0%	0 / 0 0.0%	0 / 0 0.0%	0 / 0 0.0%	71 / 106 67.0%AB	33 / 106 31.1%ABE	2 / 106 1.9%E			
1	Xanthomonas campestris pv. campestris str. ATCC 33913	52 / 53 98.1%	1 / 1 100.0%	2 / 2 100.0%	1 / 1 100.0%	2 / 2 100.0%	4 / 4 100.0%	3 / 3 100.0%	1 / 1 100.0%	1 / 1 100.0%	5 / 5 100.0%	1 / 1 100.0%	4 / 4 100.0%	2 / 2 100.0%	1 / 1 100.0%	2 / 2 100.0%	1 / 1 100.0%	3 / 3 100.0%	1 / 1 100.0%	3 / 3 100.0%	5 / 5 100.0%	3 / 3 100.0%	1 / 1 100.0%	5 / 6 83.3%	0 / 0 0.0%	0 / 0 0.0%	0 / 0 0.0%	0 / 0 0.0%	36 / 53 67.9%AB	16 / 53 30.2%ABE	1 / 53 1.9%E			
1	Xanthomonas campestris pv. campestris 8004	52 / 53 98.1%	1 / 1 100.0%	2 / 2 100.0%	1 / 1 100.0%	2 / 2 100.0%	4 / 4 100.0%	3 / 3 100.0%	1 / 1 100.0%	1 / 1 100.0%	5 / 5 100.0%	1 / 1 100.0%	4 / 4 100.0%	2 / 2 100.0%	1 / 1 100.0%	2 / 2 100.0%	1 / 1 100.0%	3 / 3 100.0%	1 / 1 100.0%	3 / 3 100.0%	5 / 5 100.0%	3 / 3 100.0%	1 / 1 100.0%	5 / 6 83.3%	0 / 0 0.0%	0 / 0 0.0%	0 / 0 0.0%	0 / 0 0.0%	35 / 53 66.0%AB	17 / 53 32.1%ABE	1 / 53 1.9%E			
1	Xanthomonas campestris pv. raphani	54 / 55 98.2%	1 / 1 100.0%	2 / 2 100.0%	1 / 1 100.0%	2 / 2 100.0%	4 / 4 100.0%	3 / 3 100.0%	1 / 1 100.0%	1 / 1 100.0%	5 / 5 100.0%	1 / 1 100.0%	4 / 4 100.0%	2 / 2 100.0%	1 / 1 100.0%	2 / 2 100.0%	1 / 1 100.0%	4 / 4 100.0%	1 / 1 100.0%	3 / 3 100.0%	6 / 6 100.0%	3 / 3 100.0%	1 / 1 100.0%	5 / 6 83.3%	0 / 0 0.0%	0 / 0 0.0%	0 / 0 0.0%	0 / 0 0.0%	38 / 55 69.1%AB	16 / 55 29.1%ABE	1 / 55 1.8%E			
1	Xanthomonas campestris pv. armoraciae 756C	54 / 55 98.2%	1 / 1 100.0%	2 / 2 100.0%	1 / 1 100.0%	2 / 2 100.0%	4 / 4 100.0%	3 / 3 100.0%	1 / 1 100.0%	1 / 1 100.0%	5 / 5 100.0%	1 / 1 100.0%	4 / 4 100.0%	2 / 2 100.0%	1 / 1 100.0%	2 / 2 100.0%	1 / 1 100.0%	4 / 4 100.0%	1 / 1 100.0%	3 / 3 100.0%	6 / 6 100.0%	3 / 3 100.0%	1 / 1 100.0%	5 / 6 83.3%	0 / 0 0.0%	0 / 0 0.0%	0 / 0 0.0%	0 / 0 0.0%	38 / 55 69.1%AB	16 / 55 29.1%ABE	1 / 55 1.8%E			
4	"Pseudomonas oryzae" Uyeda and Ishiyama in Ishiyama 1926	207 / 213 97.2%	4 / 4 100.0%	8 / 8 100.0%	4 / 4 100.0%	8 / 8 100.0%	15 / 16 93.8%	12 / 12 100.0%	4 / 4 100.0%	4 / 4 100.0%	20 / 20 100.0%	4 / 4 100.0%	17 / 17 100.0%	8 / 8 100.0%	4 / 4 100.0%	8 / 8 100.0%	4 / 4 100.0%	11 / 12 91.7%	4 / 4 100.0%	12 / 12 100.0%	20 / 20 100.0%	12 / 12 100.0%	4 / 4 100.0%	20 / 24 83.3%	0 / 0 0.0%	0 / 0 0.0%	0 / 0 0.0%	0 / 0 0.0%	116 / 213 54.5%AB	54 / 213 25.4%ABE	26 / 213 12.2%B	11 / 213 5.2%BE	4 / 213 1.9%E	2 / 213 0.9%
3	Xanthomonas campestris pv. oryza	155 / 160 96.9%	3 / 3 100.0%	6 / 6 100.0%	3 / 3 100.0%	6 / 6 100.0%	11 / 12 91.7%	9 / 9 100.0%	3 / 3 100.0%	3 / 3 100.0%	15 / 15 100.0%	3 / 3 100.0%	13 / 13 100.0%	6 / 6 100.0%	3 / 3 100.0%	6 / 6 100.0%	3 / 3 100.0%	8 / 9 88.9%	3 / 3 100.0%	9 / 9 100.0%	15 / 15 100.0%	9 / 9 100.0%	3 / 3 100.0%	15 / 18 83.3%	0 / 0 0.0%	0 / 0 0.0%	0 / 0 0.0%	0 / 0 0.0%	80 / 160 50.0%AB	38 / 160 23.8%ABE	26 / 160 16.2%B	11 / 160 6.9%BE	3 / 160 1.9%E	2 / 160 1.2%
1	Xanthomonas oryzae pv. oryzae KACC 10331	51 / 53 96.2%	1 / 1 100.0%	2 / 2 100.0%	1 / 1 100.0%	2 / 2 100.0%	3 / 4 75.0%	3 / 3 100.0%	1 / 1 100.0%	1 / 1 100.0%	5 / 5 100.0%	1 / 1 100.0%	4 / 4 100.0%	2 / 2 100.0%	1 / 1 100.0%	2 / 2 100.0%	1 / 1 100.0%	3 / 3 100.0%	1 / 1 100.0%	3 / 3 100.0%	5 / 5 100.0%	3 / 3 100.0%	1 / 1 100.0%	5 / 6 83.3%	0 / 0 0.0%	0 / 0 0.0%	0 / 0 0.0%	0 / 0 0.0%	35 / 53 66.0%AB	16 / 53 30.2%ABE	1 / 53 1.9%E	1 / 53 1.9%		
1	Xanthomonas oryzae pv. oryzae MAFF 311018	52 / 53 98.1%	1 / 1 100.0%	2 / 2 100.0%	1 / 1 100.0%	2 / 2 100.0%	4 / 4 100.0%	3 / 3 100.0%	1 / 1 100.0%	1 / 1 100.0%	5 / 5 100.0%	1 / 1 100.0%	4 / 4 100.0%	2 / 2 100.0%	1 / 1 100.0%	2 / 2 100.0%	1 / 1 100.0%	3 / 3 100.0%	1 / 1 100.0%	3 / 3 100.0%	5 / 5 100.0%	3 / 3 100.0%	1 / 1 100.0%	5 / 6 83.3%	0 / 0 0.0%	0 / 0 0.0%	0 / 0 0.0%	0 / 0 0.0%	36 / 53 67.9%AB	16 / 53 30.2%ABE	1 / 53 1.9%E			
1	Xanthomonas oryzae pv. oryzae PX099A	52 / 54 96.3%	1 / 1 100.0%	2 / 2 100.0%	1 / 1 100.0%	2 / 2 100.0%	4 / 4 100.0%	3 / 3 100.0%	1 / 1 100.0%	1 / 1 100.0%	5 / 5 100.0%	1 / 1 100.0%	5 / 5 100.0%	2 / 2 100.0%	1 / 1 100.0%	2 / 2 100.0%	1 / 1 100.0%	2 / 3 66.7%	1 / 1 100.0%	3 / 3 100.0%	5 / 5 100.0%	3 / 3 100.0%	1 / 1 100.0%	5 / 6 83.3%	0 / 0 0.0%	0 / 0 0.0%	0 / 0 0.0%	0 / 0 0.0%	26 / 54 48.1%B	11 / 54 20.4%BE	9 / 54 16.7%AB	6 / 54 11.1%ABE	1 / 54 1.9%E	1 / 54 1.9%
1	Xanthomonas campestris pv. oryzicola	52 / 53 98.1%	1 / 1 100.0%	2 / 2 100.0%	1 / 1 100.0%	2 / 2 100.0%	4 / 4 100.0%	3 / 3 100.0%	1 / 1 100.0%	1 / 1 100.0%	5 / 5 100.0%	1 / 1 100.0%	4 / 4 100.0%	2 / 2 100.0%	1 / 1 100.0%	2 / 2 100.0%	1 / 1 100.0%	3 / 3 100.0%	1 / 1 100.0%	3 / 3 100.0%	5 / 5 100.0%	3 / 3 100.0%	1 / 1 100.0%	5 / 6 83.3%	0 / 0 0.0%	0 / 0 0.0%	0 / 0 0.0%	0 / 0 0.0%	36 / 53 67.9%AB	16 / 53 30.2%ABE	1 / 53 1.9%E			
1	Xanthomonas oryzae pv. oryzicola BLS256	52 / 53 98.1%	1 / 1 100.0%	2 / 2 100.0%	1 / 1 100.0%	2 / 2 100.0%	4 / 4 100.0%	3 / 3 100.0%	1 / 1 100.0%	1 / 1 100.0%	5 / 5 100.0%	1 / 1 100.0%	4 / 4 100.0%	2 / 2 100.0%	1 / 1 100.0%	2 / 2 100.0%	1 / 1 100.0%	3 / 3 100.0%	1 / 1 100.0%	3 / 3 100.0%	5 / 5 100.0%	3 / 3 100.0%	1 / 1 100.0%	5 / 6 83.3%	0 / 0 0.0%	0 / 0 0.0%	0 / 0 0.0%	0 / 0 0.0%	36 / 53 67.9%AB	16 / 53 30.2%ABE	1 / 53 1.9%E			
1	"Agrobacterium albilineans" (Ashby 1929) Savulescu 1947	51 / 51 100.0%	1 / 1 100.0%	2 / 2 100.0%	1 / 1 100.0%	2 / 2 100.0%	4 / 4 100.0%	3 / 3 100.0%	1 / 1 100.0%	1 / 1 100.0%	5 / 5 100.0%	1 / 1 100.0%	4 / 4 100.0%	2 / 2 100.0%	1 / 1 100.0%	2 / 2 100.0%	1 / 1 100.0%	3 / 3 100.0%	1 / 1 100.0%	3 / 3 100.0%	4 / 4 100.0%	3 / 3 100.0%	1 / 1 100.0%	5 / 5 100.0%	0 / 0 0.0%	0 / 0 0.0%	0 / 0 0.0%	0 / 0 0.0%	35 / 51 68.6%AB	16 / 51 31.4%ABE				
1	"Xanthomonas axonoperis" (sic) Starr and Garces 1950	53 / 53 100.0%	1 / 1 100.0%	2 / 2 100.0%	1 / 1 100.0%	2 / 2 100.0%	4 / 4 100.0%	3 / 3 100.0%	1 / 1 100.0%	1 / 1 100.0%	5 / 5 100.0%	1 / 1 100.0%	4 / 4 100.0%	2 / 2 100.0%	1 / 1 100.0%	2 / 2 100.0%	1 / 1 100.0%	3 / 3 100.0%	1 / 1 100.0%	3 / 3 100.0%	5 / 5 100.0%	3 / 3 100.0%	1 / 1 100.0%	6 / 6 100.0%	0 / 0 0.0%	0 / 0 0.0%	0 / 0 0.0%	0 / 0 0.0%	36 / 53 67.9%AB	17 / 53 32.1%ABE				
1	Xanthomonas axonopodis Xac29-1	53 / 53 100.0%	1 / 1 100.0%	2 / 2 100.0%	1 / 1 100.0%	2 / 2 100.0%	4 / 4 100.0%	3 / 3 100.0%	1 / 1 100.0%	1 / 1 100.0%	5 / 5 100.0%	1 / 1 100.0%	4 / 4 100.0%	2 / 2 100.0%	1 / 1 100.0%	2 / 2 100.0%	1 / 1 100.0%	3 / 3 100.0%	1 / 1 100.0%	3 / 3 100.0%	5 / 5 100.0%	3 / 3 100.0%	1 / 1 100.0%	6 / 6 100.0%	0 / 0 0.0%	0 / 0 0.0%	0 / 0 0.0%	0 / 0 0.0%	36 / 53 67.9%AB	17 / 53 32.1%ABE				
1	"Xanthomonas alfalfae" (ex Riker et al. 1935) Dowson 1943	52 / 53 98.1%	1 / 1 100.0%	2 / 2 100.0%	1 / 1 100.0%	2 / 2 100.0%	4 / 4 100.0%	3 / 3 100.0%	1 / 1 100.0%	1 / 1 100.0%	5 / 5 100.0%	1 / 1 100.0%	4 / 4 100.0%	2 / 2 100.0%	1 / 1 100.0%	2 / 2 100.0%	1 / 1 100.0%	3 / 3 100.0%	1 / 1 100.0%	3 / 3 100.0%	5 / 5 100.0%	3 / 3 100.0%	1 / 1 100.0%	5 / 6 83.3%	0 / 0 0.0%	0 / 0 0.0%	0 / 0 0.0%	0 / 0 0.0%	36 / 53 67.9%AB	16 / 53 30.2%ABE	1 / 53 1.9%E			
1	"Xanthomonas alfalfae subsp. citrumelo" (ex Riker et al. 1935) Schaad et al. 2005	52 / 53 98.1%	1 / 1 100.0%	2 / 2 100.0%	1 / 1 100.0%	2 / 2 100.0%	4 / 4 100.0%	3 / 3 100.0%	1 / 1 100.0%	1 / 1 100.0%	5 / 5 100.0%	1 / 1 100.0%	4 / 4 100.0%	2 / 2 100.0%	1 / 1 100.0%	2 / 2 100.0%	1 / 1 100.0%	3 / 3 100.0%	1 / 1 100.0%	3 / 3 100.0%	5 / 5 100.0%	3 / 3 100.0%	1 / 1 100.0%	5 / 6 83.3%	0 / 0 0.0%	0 / 0 0.0%	0 / 0 0.0%	0 / 0 0.0%	36 / 53 67.9%AB	16 / 53 30.2%ABE	1 / 53 1.9%E			
1	Xanthomonas alfalfae subsp. citrumelonis F1	52 / 53 98.1%	1 / 1 100.0%	2 / 2 100.0%	1 / 1 100.0%	2 / 2 100.0%	4 / 4 100.0%	3 / 3 100.0%	1 / 1 100.0%	1 / 1 100.0%	5 / 5 100.0%	1 / 1 100.0%	4 / 4 100.0%	2 / 2 100.0%	1 / 1 100.0%	2 / 2 100.0%	1 / 1 100.0%	3 / 3 100.0%	1 / 1 100.0%	3 / 3 100.0%	5 / 5 100.0%	3 / 3 100.0%	1 / 1 100.0%	5 / 6 83.3%	0 / 0 0.0%	0 / 0 0.0%	0 / 0 0.0%	0 / 0 0.0%	36 / 53 67.9%AB	16 / 53 30.2%ABE	1 / 53 1.9%E			
1	"Bacterium vesicatorium" Doidge 1921	52 / 53 98.1%	1 / 1 100.0%	2 / 2 100.0%	1 / 1 100.0%	2 / 2 100.0%	4 / 4 100.0%	3 / 3 100.0%	1 / 1 100.0%	1 / 1 100.0%	5 / 5 100.0%	1 / 1 100.0%	4 / 4 100.0%	2 / 2 100.0%	1 / 1 100.0%	2 / 2 100.0%	1 / 1 100.0%	3 / 3 100.0%	1 / 1 100.0%	3 / 3 100.0%	5 / 5 100.0%	3 / 3 100.0%	1 / 1 100.0%	5 / 6 83.3%	0 / 0 0.0%	0 / 0 0.0%	0 / 0 0.0%	0 / 0 0.0%	35 / 53 66.0%AB	17 / 53 32.1%ABE	1 / 53 1.9%E			
2	Xanthomonas citri group	106 / 106 100.0%	2 / 2 100.0%	4 / 4 100.0%	2 / 2 100.0%	4 / 4 100.0%	8 / 8 100.0%	6 / 6 100.0%	2 / 2 100.0%	2 / 2 100.0%	10 / 10 100.0%	2 / 2 100.0%	8 / 8 100.0%	4 / 4 100.0%	2 / 2 100.0%	4 / 4 100.0%	2 / 2 100.0%	6 / 6 100.0%	2 / 2 100.0%	6 / 6 100.0%	10 / 10 100.0%	6 / 6 100.0%	2 / 2 100.0%	12 / 12 100.0%	0 / 0 0.0%	0 / 0 0.0%	0 / 0 0.0%	0 / 0 0.0%	72 / 106 67.9%AB	34 / 106 32.1%ABE				
2	"Xanthomonas citri" (Hasse 1915) Dowson 1939	106 / 106 100.0%	2 / 2 100.0%	4 / 4 100.0%	2 / 2 100.0%	4 / 4 100.0%	8 / 8 100.0%	6 / 6 100.0%	2 / 2 100.0%	2 / 2 100.0%	10 / 10 100.0%	2 / 2 100.0%	8 / 8 100.0%	4 / 4 100.0%	2 / 2 100.0%	4 / 4 100.0%	2 / 2 100.0%	6 / 6 100.0%	2 / 2 100.0%	6 / 6 100.0%	10 / 10 100.0%	6 / 6 100.0%	2 / 2 100.0%	12 / 12 100.0%	0 / 0 0.0%	0 / 0 0.0%	0 / 0 0.0%	0 / 0 0.0%	72 / 106 67.9%AB	34 / 106 32.1%ABE				
1	Xanthomonas citri pv. citri	53 / 53 100.0%	1 / 1 100.0%	2 / 2 100.0%	1 / 1 100.0%	2 / 2 100.0%	4 / 4 100.0%	3 / 3 100.0%	1 / 1 100.0%	1 / 1 100.0%	5 / 5 100.0%	1 / 1 100.0%	4 / 4 100.0%	2 / 2 100.0%	1 / 1 100.0%	2 / 2 100.0%	1 / 1 100.0%	3 / 3 100.0%	1 / 1 100.0%	3 / 3 100.0%	5 / 5 100.0%	3 / 3 100.0%	1 / 1 100.0%	6 / 6 100.0%	0 / 0 0.0%	0 / 0 0.0%	0 / 0 0.0%	0 / 0 0.0%	36 / 53 67.9%AB	17 / 53 32.1%ABE				
1	Xanthomonas axonopodis pv. citri str. 306	53 / 53 100.0%	1 / 1 100.0%	2 / 2 100.0%	1 / 1 100.0%	2 / 2 100.0%	4 / 4 100.0%	3 / 3 100.0%	1 / 1 100.0%	1 / 1 100.0%	5 / 5 100.0%	1 / 1 100.0%	4 / 4 100.0%	2 / 2 100.0%	1 / 1 100.0%	2 / 2 100.0%	1 / 1 100.0%	3 / 3 100.0%	1 / 1 100.0%	3 / 3 100.0%	5 / 5 100.0%	3 / 3 100.0%	1 / 1 100.0%	6 / 6 100.0%	0 / 0 0.0%	0 / 0 0.0%	0 / 0 0.0%	0 / 0 0.0%	36 / 53 67.9%AB	17 / 53 32.1%ABE				
1	Xanthomonas citri subsp. citri	53 / 53 100.0%	1 / 1 100.0%	2 / 2 100.0%	1 / 1 100.0%	2 / 2 100.0%	4 / 4 100.0%	3 / 3 100.0%	1 / 1 100.0%	1 / 1 100.0%	5 / 5 100.0%	1 / 1 100.0%	4 / 4 100.0%	2 / 2 100.0%	1 / 1 100.0%	2 / 2 100.0%	1 / 1 100.0%	3 / 3 100.0%	1 / 1 100.0%	3 / 3 100.0%	5 / 5 100.0%	3 / 3 100.0%	1 / 1 100.0%	6 / 6 100.0%	0 / 0 0.0%	0 / 0 0.0%	0 / 0 0.0%	0 / 0 0.0%	36 / 53 67.9%AB	17 / 53 32.1%ABE				
1	Xanthomonas citri subsp. citri Aw	53 / 53 100.0%	1 / 1 100.0%	2 / 2 100.0%	1 / 1 100.0%	2 / 2 100.0%	4 / 4 100.0%	3 / 3 100.0%	1 / 1 100.0%	1 / 1 100.0%	5 / 5 100.0%	1 / 1 100.0%	4 / 4 100.0%	2 / 2 100.0%	1 / 1 100.0%	2 / 2 100.0%	1 / 1 100.0%	3 / 3 100.0%	1 / 1 100.0%	3 / 3 100.0%	5 / 5 100.0%	3 / 3 100.0%	1 / 1 100.0%	6 / 6 100.0%	0 / 0 0.0%	0 / 0 0.0%	0 / 0 0.0%	0 / 0 0.0%	36 / 53 67.9%AB	17 / 53 32.1%ABE				
5	Xylella	242 / 244 99.2%	5 / 5 100.0%	5 / 5 100.0%	5 / 5 100.0%	10 / 10 100.0%	19 / 19 100.0%	15 / 15 100.0%	5 / 5 100.0%	5 / 5 100.0%	20 / 20 100.0%	5 / 5 100.0%	20 / 20 100.0%	13 / 15 86.7%	5 / 5 100.0%	10 / 10 100.0%	5 / 5 100.0%	15 / 15 100.0%	5 / 5 100.0%	15 / 15 100.0%	20 / 20 100.0%	10 / 10 100.0%	5 / 5 100.0%	25 / 25 100.0%	0 / 0 0.0%	0 / 0 0.0%	0 / 0 0.0%	0 / 0 0.0%	138 / 244 56.6%AB	55 / 244 22.5%ABE	29 / 244 11.9%B	20 / 244 8.2%BE	2 / 244 0.8%E	
5	ATCC 35879	242 / 244 99.2%	5 / 5 100.0%	5 / 5 100.0%	5 / 5 100.0%	10 / 10 100.0%	19 / 19 100.0%	15 / 15 100.0%	5 / 5 100.0%	5 / 5 100.0%	20 / 20 100.0%	5 / 5 100.0%	20 / 20 100.0%	13 / 15 86.7%	5 / 5 100.0%	10 / 10 100.0%	5 / 5 100.0%	15 / 15 100.0%	5 / 5 100.0%	15 / 15 100.0%	20 / 20 100.0%	10 / 10 100.0%	5 / 5 100.0%	25 / 25 100.0%	0 / 0 0.0%	0 / 0 0.0%	0 / 0 0.0%	0 / 0 0.0%	138 / 244 56.6%AB	55 / 244 22.5%ABE	29 / 244 11.9%B	20 / 244 8.2%BE	2 / 244 0.8%E	
1	Xylella fastidiosa 9a5c	48 / 48 100.0%	1 / 1 100.0%	1 / 1 100.0%	1 / 1 100.0%	2 / 2 100.0%	3 / 3 100.0%	3 / 3 100.0%	1 / 1 100.0%	1 / 1 100.0%	4 / 4 100.0%	1 / 1 100.0%	4 / 4 100.0%	3 / 3 100.0%	1 / 1 100.0%	2 / 2 100.0%	1 / 1 100.0%	3 / 3 100.0%	1 / 1 100.0%	3 / 3 100.0%	4 / 4 100.0%	2 / 2 100.0%	1 / 1 100.0%	5 / 5 100.0%	0 / 0 0.0%	0 / 0 0.0%	0 / 0 0.0%	0 / 0 0.0%	33 / 48 68.8%AB	15 / 48 31.2%ABE				
1	Xylella fastidiosa 'Pierce's disease strain'	49 / 49 100.0%	1 / 1 100.0%	1 / 1 100.0%	1 / 1 100.0%	2 / 2 100.0%	4 / 4 100.0%	3 / 3 100.0%	1 / 1 100.0%	1 / 1 100.0%	4 / 4 100.0%	1 / 1 100.0%	4 / 4 100.0%	3 / 3 100.0%	1 / 1 100.0%	2 / 2 100.0%	1 / 1 100.0%	3 / 3 100.0%	1 / 1 100.0%	3 / 3 100.0%	4 / 4 100.0%	2 / 2 100.0%	1 / 1 100.0%	5 / 5 100.0%	0 / 0 0.0%	0 / 0 0.0%	0 / 0 0.0%	0 / 0 0.0%	34 / 49 69.4%AB	15 / 49 30.6%ABE				
1	Xylella fastidiosa M12	49 / 49 100.0%	1 / 1 100.0%	1 / 1 100.0%	1 / 1 100.0%	2 / 2 100.0%	4 / 4 100.0%	3 / 3 100.0%	1 / 1 100.0%	1 / 1 100.0%	4 / 4 100.0%	1 / 1 100.0%	4 / 4 100.0%	3 / 3 100.0%	1 / 1 100.0%	2 / 2 100.0%	1 / 1 100.0%	3 / 3 100.0%	1 / 1 100.0%	3 / 3 100.0%	4 / 4 100.0%	2 / 2 100.0%	1 / 1 100.0%	5 / 5 100.0%	0 / 0 0.0%	0 / 0 0.0%	0 / 0 0.0%	0 / 0 0.0%	19 / 49 38.8%AB	15 / 49 30.6%B	10 / 49 20.4%BE	5 / 49 10.2%ABE		
1	Xylella fastidiosa M23	48 / 49 98.0%	1 / 1 100.0%	1 / 1 100.0%	1 / 1 100.0%	2 / 2 100.0%	4 / 4 100.0%	3 / 3 100.0%	1 / 1 100.0%	1 / 1 100.0%	4 / 4 100.0%	1 / 1 100.0%	4 / 4 100.0%	2 / 3 66.7%	1 / 1 100.0%	2 / 2 100.0%	1 / 1 100.0%	3 / 3 100.0%	1 / 1 100.0%	3 / 3 100.0%	4 / 4 100.0%	2 / 2 100.0%	1 / 1 100.0%	5 / 5 100.0%	0 / 0 0.0%	0 / 0 0.0%	0 / 0 0.0%	0 / 0 0.0%	19 / 49 38.8%AB	14 / 49 28.6%B	10 / 49 20.4%BE	5 / 49 10.2%ABE	1 / 49 2.0%E	
1	"Xylella fastidiosa subsp. piercei" Schaad et al. 2004	48 / 49 98.0%	1 / 1 100.0%	1 / 1 100.0%	1 / 1 100.0%	2 / 2 100.0%	4 / 4 100.0%	3 / 3 100.0%	1 / 1 100.0%	1 / 1 100.0%	4 / 4 100.0%	1 / 1 100.0%	4 / 4 100.0%	2 / 3 66.7%	1 / 1 100.0%	2 / 2 100.0%	1 / 1 100.0%	3 / 3 100.0%	1 / 1 100.0%	3 / 3 100.0%	4 / 4 100.0%	2 / 2 100.0%	1 / 1 100.0%	5 / 5 100.0%	0 / 0 0.0%	0 / 0 0.0%	0 / 0 0.0%	0 / 0 0.0%	33 / 49 67.3%AB	15 / 49 30.6%ABE	1 / 49 2.0%E			
1	Xylella fastidiosa GB514	48 / 49 98.0%	1 / 1 100.0%	1 / 1 100.0%	1 / 1 100.0%	2 / 2 100.0%	4 / 4 100.0%	3 / 3 100.0%	1 / 1 100.0%	1 / 1 100.0%	4 / 4 100.0%	1 / 1 100.0%	4 / 4 100.0%	2 / 3 66.7%	1 / 1 100.0%	2 / 2 100.0%	1 / 1 100.0%	3 / 3 100.0%	1 / 1 100.0%	3 / 3 100.0%	4 / 4 100.0%	2 / 2 100.0%	1 / 1 100.0%	5 / 5 100.0%	0 / 0 0.0%	0 / 0 0.0%	0 / 0 0.0%	0 / 0 0.0%	33 / 49 67.3%AB	15 / 49 30.6%ABE	1 / 49 2.0%E			
4	Stenotrophomonas	287 / 289 99.3%	9 / 9 100.0%	16 / 16 100.0%	8 / 8 100.0%	16 / 16 100.0%	24 / 24 100.0%	16 / 16 100.0%	4 / 4 100.0%	4 / 4 100.0%	32 / 32 100.0%	4 / 4 100.0%	14 / 16 87.5%	12 / 12 100.0%	8 / 8 100.0%	8 / 8 100.0%	4 / 4 100.0%	16 / 16 100.0%	4 / 4 100.0%	12 / 12 100.0%	24 / 24 100.0%	16 / 16 100.0%	4 / 4 100.0%	28 / 28 100.0%	4 / 4 100.0%	0 / 0 0.0%	0 / 0 0.0%	0 / 0 0.0%	182 / 289 63.0%AB	63 / 289 21.8%ABE	24 / 289 8.3%B	18 / 289 6.2%BE	2 / 289 0.7%E	
4	Stenotrophomonas maltophilia group	287 / 289 99.3%	9 / 9 100.0%	16 / 16 100.0%	8 / 8 100.0%	16 / 16 100.0%	24 / 24 100.0%	16 / 16 100.0%	4 / 4 100.0%	4 / 4 100.0%	32 / 32 100.0%	4 / 4 100.0%	14 / 16 87.5%	12 / 12 100.0%	8 / 8 100.0%	8 / 8 100.0%	4 / 4 100.0%	16 / 16 100.0%	4 / 4 100.0%	12 / 12 100.0%	24 / 24 100.0%	16 / 16 100.0%	4 / 4 100.0%	28 / 28 100.0%	4 / 4 100.0%	0 / 0 0.0%	0 / 0 0.0%	0 / 0 0.0%	182 / 289 63.0%AB	63 / 289 21.8%ABE	24 / 289 8.3%B	18 / 289 6.2%BE	2 / 289 0.7%E	
4	"Pseudomonas maltophilia" Hugh and Ryschenkow 1961	287 / 289 99.3%	9 / 9 100.0%	16 / 16 100.0%	8 / 8 100.0%	16 / 16 100.0%	24 / 24 100.0%	16 / 16 100.0%	4 / 4 100.0%	4 / 4 100.0%	32 / 32 100.0%	4 / 4 100.0%	14 / 16 87.5%	12 / 12 100.0%	8 / 8 100.0%	8 / 8 100.0%	4 / 4 100.0%	16 / 16 100.0%	4 / 4 100.0%	12 / 12 100.0%	24 / 24 100.0%	16 / 16 100.0%	4 / 4 100.0%	28 / 28 100.0%	4 / 4 100.0%	0 / 0 0.0%	0 / 0 0.0%	0 / 0 0.0%	182 / 289 63.0%AB	63 / 289 21.8%ABE	24 / 289 8.3%B	18 / 289 6.2%BE	2 / 289 0.7%E	
1	Stenotrophomonas maltophilia R551-3	71 / 72 98.6%	2 / 2 100.0%	4 / 4 100.0%	2 / 2 100.0%	4 / 4 100.0%	6 / 6 100.0%	4 / 4 100.0%	1 / 1 100.0%	1 / 1 100.0%	8 / 8 100.0%	1 / 1 100.0%	3 / 4 75.0%	3 / 3 100.0%	2 / 2 100.0%	2 / 2 100.0%	1 / 1 100.0%	4 / 4 100.0%	1 / 1 100.0%	3 / 3 100.0%	6 / 6 100.0%	4 / 4 100.0%	1 / 1 100.0%	7 / 7 100.0%	1 / 1 100.0%	0 / 0 0.0%	0 / 0 0.0%	0 / 0 0.0%	40 / 72 55.6%AB	12 / 72 16.7%B	10 / 72 13.9%ABE	9 / 72 12.5%BE	1 / 72 1.4%E	

1	Stenotrophomonas maltophilia K279a	72 / 73 98.6%	3 / 3 100.0%	4 / 4 100.0%	2 / 2 100.0%	4 / 4 100.0%	6 / 6 100.0%	4 / 4 100.0%	1 / 1 100.0%	1 / 1 100.0%	8 / 8 100.0%	1 / 1 100.0%	3 / 4 75.0%	3 / 3 100.0%	2 / 2 100.0%	2 / 2 100.0%	1 / 1 100.0%	4 / 4 100.0%	1 / 1 100.0%	3 / 3 100.0%	6 / 6 100.0%	4 / 4 100.0%	1 / 1 100.0%	7 / 7 100.0%	1 / 1 100.0%	0 / 0 0.0%	0 / 0 0.0%	0 / 0 0.0%	40 / 73 54.8%AB	12 / 73 16.4%B	11 / 73 15.1%ABE	9 / 73 12.3%BE	1 / 73 1.4%E	
1	Burkholderia sp. JV3	72 / 72 100.0%	2 / 2 100.0%	4 / 4 100.0%	2 / 2 100.0%	4 / 4 100.0%	6 / 6 100.0%	4 / 4 100.0%	1 / 1 100.0%	1 / 1 100.0%	8 / 8 100.0%	1 / 1 100.0%	4 / 4 100.0%	3 / 3 100.0%	2 / 2 100.0%	2 / 2 100.0%	1 / 1 100.0%	4 / 4 100.0%	1 / 1 100.0%	3 / 3 100.0%	6 / 6 100.0%	4 / 4 100.0%	1 / 1 100.0%	7 / 7 100.0%	1 / 1 100.0%	0 / 0 0.0%	0 / 0 0.0%	0 / 0 0.0%	51 / 72 70.8%AB	21 / 72 29.2%ABE				
1	Stenotrophomonas maltophilia D457	72 / 72 100.0%	2 / 2 100.0%	4 / 4 100.0%	2 / 2 100.0%	4 / 4 100.0%	6 / 6 100.0%	4 / 4 100.0%	1 / 1 100.0%	1 / 1 100.0%	8 / 8 100.0%	1 / 1 100.0%	4 / 4 100.0%	3 / 3 100.0%	2 / 2 100.0%	2 / 2 100.0%	1 / 1 100.0%	4 / 4 100.0%	1 / 1 100.0%	3 / 3 100.0%	6 / 6 100.0%	4 / 4 100.0%	1 / 1 100.0%	7 / 7 100.0%	1 / 1 100.0%	0 / 0 0.0%	0 / 0 0.0%	0 / 0 0.0%	51 / 72 70.8%AB	21 / 72 29.2%ABE				
1	Frateuria	71 / 71 100.0%	3 / 3 100.0%	4 / 4 100.0%	1 / 1 100.0%	4 / 4 100.0%	5 / 5 100.0%	4 / 4 100.0%	2 / 2 100.0%	1 / 1 100.0%	8 / 8 100.0%	1 / 1 100.0%	4 / 4 100.0%	4 / 4 100.0%	1 / 1 100.0%	3 / 3 100.0%	1 / 1 100.0%	4 / 4 100.0%	1 / 1 100.0%	4 / 4 100.0%	6 / 6 100.0%	4 / 4 100.0%	1 / 1 100.0%	5 / 5 100.0%	0 / 0 0.0%	0 / 0 0.0%	0 / 0 0.0%	0 / 0 0.0%	50 / 71 70.4%AB	21 / 71 29.6%ABE				
1	"Acetobacter aurantium" (sic) Kond and Ameyama 1958	71 / 71 100.0%	3 / 3 100.0%	4 / 4 100.0%	1 / 1 100.0%	4 / 4 100.0%	5 / 5 100.0%	4 / 4 100.0%	2 / 2 100.0%	1 / 1 100.0%	8 / 8 100.0%	1 / 1 100.0%	4 / 4 100.0%	4 / 4 100.0%	1 / 1 100.0%	3 / 3 100.0%	1 / 1 100.0%	4 / 4 100.0%	1 / 1 100.0%	4 / 4 100.0%	6 / 6 100.0%	4 / 4 100.0%	1 / 1 100.0%	5 / 5 100.0%	0 / 0 0.0%	0 / 0 0.0%	0 / 0 0.0%	0 / 0 0.0%	50 / 71 70.4%AB	21 / 71 29.6%ABE				
1	Frateuria aurantia DSM 6220	71 / 71 100.0%	3 / 3 100.0%	4 / 4 100.0%	1 / 1 100.0%	4 / 4 100.0%	5 / 5 100.0%	4 / 4 100.0%	2 / 2 100.0%	1 / 1 100.0%	8 / 8 100.0%	1 / 1 100.0%	4 / 4 100.0%	4 / 4 100.0%	1 / 1 100.0%	3 / 3 100.0%	1 / 1 100.0%	4 / 4 100.0%	1 / 1 100.0%	4 / 4 100.0%	6 / 6 100.0%	4 / 4 100.0%	1 / 1 100.0%	5 / 5 100.0%	0 / 0 0.0%	0 / 0 0.0%	0 / 0 0.0%	0 / 0 0.0%	50 / 71 70.4%AB	21 / 71 29.6%ABE				
1	Rhodanobacter	49 / 49 100.0%	1 / 1 100.0%	2 / 2 100.0%	1 / 1 100.0%	2 / 2 100.0%	4 / 4 100.0%	3 / 3 100.0%	1 / 1 100.0%	1 / 1 100.0%	4 / 4 100.0%	1 / 1 100.0%	4 / 4 100.0%	2 / 2 100.0%	1 / 1 100.0%	2 / 2 100.0%	1 / 1 100.0%	3 / 3 100.0%	1 / 1 100.0%	3 / 3 100.0%	4 / 4 100.0%	2 / 2 100.0%	1 / 1 100.0%	5 / 5 100.0%	0 / 0 0.0%	0 / 0 0.0%	0 / 0 0.0%	0 / 0 0.0%	34 / 49 69.4%AB	15 / 49 30.6%ABE				
1	DSM 23569	49 / 49 100.0%	1 / 1 100.0%	2 / 2 100.0%	1 / 1 100.0%	2 / 2 100.0%	4 / 4 100.0%	3 / 3 100.0%	1 / 1 100.0%	1 / 1 100.0%	4 / 4 100.0%	1 / 1 100.0%	4 / 4 100.0%	2 / 2 100.0%	1 / 1 100.0%	2 / 2 100.0%	1 / 1 100.0%	3 / 3 100.0%	1 / 1 100.0%	3 / 3 100.0%	4 / 4 100.0%	2 / 2 100.0%	1 / 1 100.0%	5 / 5 100.0%	0 / 0 0.0%	0 / 0 0.0%	0 / 0 0.0%	0 / 0 0.0%	34 / 49 69.4%AB	15 / 49 30.6%ABE				
2	Pseudoxanthomonas	101 / 101 100.0%	5 / 5 100.0%	3 / 3 100.0%	2 / 2 100.0%	3 / 3 100.0%	8 / 8 100.0%	6 / 6 100.0%	2 / 2 100.0%	2 / 2 100.0%	8 / 8 100.0%	2 / 2 100.0%	8 / 8 100.0%	4 / 4 100.0%	2 / 2 100.0%	4 / 4 100.0%	2 / 2 100.0%	6 / 6 100.0%	2 / 2 100.0%	6 / 6 100.0%	8 / 8 100.0%	4 / 4 100.0%	3 / 3 100.0%	11 / 11 100.0%	0 / 0 0.0%	0 / 0 0.0%	0 / 0 0.0%	0 / 0 0.0%	67 / 101 66.3%AB	34 / 101 33.7%ABE				
1	DSM 17175	52 / 52 100.0%	3 / 3 100.0%	1 / 1 100.0%	1 / 1 100.0%	2 / 2 100.0%	4 / 4 100.0%	3 / 3 100.0%	1 / 1 100.0%	1 / 1 100.0%	5 / 5 100.0%	1 / 1 100.0%	4 / 4 100.0%	2 / 2 100.0%	1 / 1 100.0%	2 / 2 100.0%	1 / 1 100.0%	3 / 3 100.0%	1 / 1 100.0%	3 / 3 100.0%	4 / 4 100.0%	2 / 2 100.0%	1 / 1 100.0%	6 / 6 100.0%	0 / 0 0.0%	0 / 0 0.0%	0 / 0 0.0%	0 / 0 0.0%	34 / 52 65.4%AB	18 / 52 34.6%ABE				
1	Pseudoxanthomonas suwonensis 11-1	52 / 52 100.0%	3 / 3 100.0%	1 / 1 100.0%	1 / 1 100.0%	2 / 2 100.0%	4 / 4 100.0%	3 / 3 100.0%	1 / 1 100.0%	1 / 1 100.0%	5 / 5 100.0%	1 / 1 100.0%	4 / 4 100.0%	2 / 2 100.0%	1 / 1 100.0%	2 / 2 100.0%	1 / 1 100.0%	3 / 3 100.0%	1 / 1 100.0%	3 / 3 100.0%	4 / 4 100.0%	2 / 2 100.0%	1 / 1 100.0%	6 / 6 100.0%	0 / 0 0.0%	0 / 0 0.0%	0 / 0 0.0%	0 / 0 0.0%	34 / 52 65.4%AB	18 / 52 34.6%ABE				
1	CCUG 53828	49 / 49 100.0%	2 / 2 100.0%	2 / 2 100.0%	1 / 1 100.0%	1 / 1 100.0%	4 / 4 100.0%	3 / 3 100.0%	1 / 1 100.0%	1 / 1 100.0%	3 / 3 100.0%	1 / 1 100.0%	4 / 4 100.0%	2 / 2 100.0%	1 / 1 100.0%	2 / 2 100.0%	1 / 1 100.0%	3 / 3 100.0%	1 / 1 100.0%	3 / 3 100.0%	4 / 4 100.0%	2 / 2 100.0%	2 / 2 100.0%	5 / 5 100.0%	0 / 0 0.0%	0 / 0 0.0%	0 / 0 0.0%	0 / 0 0.0%	33 / 49 67.3%AB	16 / 49 32.7%ABE				
1	Pseudoxanthomonas spadix BD-a59	49 / 49 100.0%	2 / 2 100.0%	2 / 2 100.0%	1 / 1 100.0%	1 / 1 100.0%	4 / 4 100.0%	3 / 3 100.0%	1 / 1 100.0%	1 / 1 100.0%	3 / 3 100.0%	1 / 1 100.0%	4 / 4 100.0%	2 / 2 100.0%	1 / 1 100.0%	2 / 2 100.0%	1 / 1 100.0%	3 / 3 100.0%	1 / 1 100.0%	3 / 3 100.0%	4 / 4 100.0%	2 / 2 100.0%	2 / 2 100.0%	5 / 5 100.0%	0 / 0 0.0%	0 / 0 0.0%	0 / 0 0.0%	0 / 0 0.0%	33 / 49 67.3%AB	16 / 49 32.7%ABE				
1	Cardiobacteriaceae group	44 / 44 100.0%	1 / 1 100.0%	2 / 2 100.0%	2 / 2 100.0%	3 / 3 100.0%	2 / 2 100.0%	2 / 2 100.0%	1 / 1 100.0%	1 / 1 100.0%	4 / 4 100.0%	1 / 1 100.0%	3 / 3 100.0%	2 / 2 100.0%	1 / 1 100.0%	1 / 1 100.0%	1 / 1 100.0%	3 / 3 100.0%	1 / 1 100.0%	2 / 2 100.0%	3 / 3 100.0%	2 / 2 100.0%	1 / 1 100.0%	5 / 5 100.0%	0 / 0 0.0%	0 / 0 0.0%	0 / 0 0.0%	0 / 0 0.0%	32 / 44 72.7%AB	12 / 44 27.3%ABE				
1	Cardiobacteriaceae	44 / 44 100.0%	1 / 1 100.0%	2 / 2 100.0%	2 / 2 100.0%	3 / 3 100.0%	2 / 2 100.0%	2 / 2 100.0%	1 / 1 100.0%	1 / 1 100.0%	4 / 4 100.0%	1 / 1 100.0%	3 / 3 100.0%	2 / 2 100.0%	1 / 1 100.0%	1 / 1 100.0%	1 / 1 100.0%	3 / 3 100.0%	1 / 1 100.0%	2 / 2 100.0%	3 / 3 100.0%	2 / 2 100.0%	1 / 1 100.0%	5 / 5 100.0%	0 / 0 0.0%	0 / 0 0.0%	0 / 0 0.0%	0 / 0 0.0%	32 / 44 72.7%AB	12 / 44 27.3%ABE				
1	Dichelobacter	44 / 44 100.0%	1 / 1 100.0%	2 / 2 100.0%	2 / 2 100.0%	3 / 3 100.0%	2 / 2 100.0%	2 / 2 100.0%	1 / 1 100.0%	1 / 1 100.0%	4 / 4 100.0%	1 / 1 100.0%	3 / 3 100.0%	2 / 2 100.0%	1 / 1 100.0%	1 / 1 100.0%	1 / 1 100.0%	3 / 3 100.0%	1 / 1 100.0%	2 / 2 100.0%	3 / 3 100.0%	2 / 2 100.0%	1 / 1 100.0%	5 / 5 100.0%	0 / 0 0.0%	0 / 0 0.0%	0 / 0 0.0%	0 / 0 0.0%	32 / 44 72.7%AB	12 / 44 27.3%ABE				
1	"Fusiformis nodosus" Beveridge 1941	44 / 44 100.0%	1 / 1 100.0%	2 / 2 100.0%	2 / 2 100.0%	3 / 3 100.0%	2 / 2 100.0%	2 / 2 100.0%	1 / 1 100.0%	1 / 1 100.0%	4 / 4 100.0%	1 / 1 100.0%	3 / 3 100.0%	2 / 2 100.0%	1 / 1 100.0%	1 / 1 100.0%	1 / 1 100.0%	3 / 3 100.0%	1 / 1 100.0%	2 / 2 100.0%	3 / 3 100.0%	2 / 2 100.0%	1 / 1 100.0%	5 / 5 100.0%	0 / 0 0.0%	0 / 0 0.0%	0 / 0 0.0%	0 / 0 0.0%	32 / 44 72.7%AB	12 / 44 27.3%ABE				
1	Dichelobacter nodosus VCS1703A	44 / 44 100.0%	1 / 1 100.0%	2 / 2 100.0%	2 / 2 100.0%	3 / 3 100.0%	2 / 2 100.0%	2 / 2 100.0%	1 / 1 100.0%	1 / 1 100.0%	4 / 4 100.0%	1 / 1 100.0%	3 / 3 100.0%	2 / 2 100.0%	1 / 1 100.0%	1 / 1 100.0%	1 / 1 100.0%	3 / 3 100.0%	1 / 1 100.0%	2 / 2 100.0%	3 / 3 100.0%	2 / 2 100.0%	1 / 1 100.0%	5 / 5 100.0%	0 / 0 0.0%	0 / 0 0.0%	0 / 0 0.0%	0 / 0 0.0%	32 / 44 72.7%AB	12 / 44 27.3%ABE				
2	Methylococcaceae group	91 / 91 100.0%	2 / 2 100.0%	3 / 3 100.0%	2 / 2 100.0%	5 / 5 100.0%	5 / 5 100.0%	6 / 6 100.0%	2 / 2 100.0%	2 / 2 100.0%	7 / 7 100.0%	2 / 2 100.0%	7 / 7 100.0%	3 / 3 100.0%	3 / 3 100.0%	3 / 3 100.0%	2 / 2 100.0%	5 / 5 100.0%	2 / 2 100.0%	6 / 6 100.0%	8 / 8 100.0%	4 / 4 100.0%	2 / 2 100.0%	10 / 10 100.0%	0 / 0 0.0%	0 / 0 0.0%	0 / 0 0.0%	0 / 0 0.0%	64 / 91 70.3%AB	27 / 91 29.7%ABE				
2	Methylococcaceae	91 / 91 100.0%	2 / 2 100.0%	3 / 3 100.0%	2 / 2 100.0%	5 / 5 100.0%	5 / 5 100.0%	6 / 6 100.0%	2 / 2 100.0%	2 / 2 100.0%	7 / 7 100.0%	2 / 2 100.0%	7 / 7 100.0%	3 / 3 100.0%	3 / 3 100.0%	3 / 3 100.0%	2 / 2 100.0%	5 / 5 100.0%	2 / 2 100.0%	6 / 6 100.0%	8 / 8 100.0%	4 / 4 100.0%	2 / 2 100.0%	10 / 10 100.0%	0 / 0 0.0%	0 / 0 0.0%	0 / 0 0.0%	0 / 0 0.0%	64 / 91 70.3%AB	27 / 91 29.7%ABE				
1	Methylococcus	46 / 46 100.0%	1 / 1 100.0%	1 / 1 100.0%	1 / 1 100.0%	2 / 2 100.0%	3 / 3 100.0%	3 / 3 100.0%	1 / 1 100.0%	1 / 1 100.0%	3 / 3 100.0%	1 / 1 100.0%	4 / 4 100.0%	2 / 2 100.0%	1 / 1 100.0%	2 / 2 100.0%	1 / 1 100.0%	3 / 3 100.0%	1 / 1 100.0%	3 / 3 100.0%	4 / 4 100.0%	2 / 2 100.0%	1 / 1 100.0%	5 / 5 100.0%	0 / 0 0.0%	0 / 0 0.0%	0 / 0 0.0%	0 / 0 0.0%	32 / 46 69.6%AB	14 / 46 30.4%ABE				
1	ACM 1292	46 / 46 100.0%	1 / 1 100.0%	1 / 1 100.0%	1 / 1 100.0%	2 / 2 100.0%	3 / 3 100.0%	3 / 3 100.0%	1 / 1 100.0%	1 / 1 100.0%	3 / 3 100.0%	1 / 1 100.0%	4 / 4 100.0%	2 / 2 100.0%	1 / 1 100.0%	2 / 2 100.0%	1 / 1 100.0%	3 / 3 100.0%	1 / 1 100.0%	3 / 3 100.0%	4 / 4 100.0%	2 / 2 100.0%	1 / 1 100.0%	5 / 5 100.0%	0 / 0 0.0%	0 / 0 0.0%	0 / 0 0.0%	0 / 0 0.0%	32 / 46 69.6%AB	14 / 46 30.4%ABE				
1	Methylococcus capsulatus ATCC 33009	46 / 46 100.0%	1 / 1 100.0%	1 / 1 100.0%	1 / 1 100.0%	2 / 2 100.0%	3 / 3 100.0%	3 / 3 100.0%	1 / 1 100.0%	1 / 1 100.0%	3 / 3 100.0%	1 / 1 100.0%	4 / 4 100.0%	2 / 2 100.0%	1 / 1 100.0%	2 / 2 100.0%	1 / 1 100.0%	3 / 3 100.0%	1 / 1 100.0%	3 / 3 100.0%	4 / 4 100.0%	2 / 2 100.0%	1 / 1 100.0%	5 / 5 100.0%	0 / 0 0.0%	0 / 0 0.0%	0 / 0 0.0%	0 / 0 0.0%	32 / 46 69.6%AB	14 / 46 30.4%ABE				
1	"Methamonas" Orla-Jensen 1909	45 / 45 100.0%	1 / 1 100.0%	2 / 2 100.0%	1 / 1 100.0%	3 / 3 100.0%	2 / 2 100.0%	3 / 3 100.0%	1 / 1 100.0%	1 / 1 100.0%	4 / 4 100.0%	1 / 1 100.0%	3 / 3 100.0%	1 / 1 100.0%	2 / 2 100.0%	1 / 1 100.0%	1 / 1 100.0%	2 / 2 100.0%	1 / 1 100.0%	3 / 3 100.0%	4 / 4 100.0%	2 / 2 100.0%	1 / 1 100.0%	5 / 5 100.0%	0 / 0 0.0%	0 / 0 0.0%	0 / 0 0.0%	0 / 0 0.0%	32 / 45 71.1%AB	13 / 45 28.9%ABE				
1	"Bacillus methanicus" Sohngen 1906	45 / 45 100.0%	1 / 1 100.0%	2 / 2 100.0%	1 / 1 100.0%	3 / 3 100.0%	2 / 2 100.0%	3 / 3 100.0%	1 / 1 100.0%	1 / 1 100.0%	4 / 4 100.0%	1 / 1 100.0%	3 / 3 100.0%	1 / 1 100.0%	2 / 2 100.0%	1 / 1 100.0%	1 / 1 100.0%	2 / 2 100.0%	1 / 1 100.0%	3 / 3 100.0%	4 / 4 100.0%	2 / 2 100.0%	1 / 1 100.0%	5 / 5 100.0%	0 / 0 0.0%	0 / 0 0.0%	0 / 0 0.0%	0 / 0 0.0%	32 / 45 71.1%AB	13 / 45 28.9%ABE				
1	Methylomonas methanica MC09	45 / 45 100.0%	1 / 1 100.0%	2 / 2 100.0%	1 / 1 100.0%	3 / 3 100.0%	2 / 2 100.0%	3 / 3 100.0%	1 / 1 100.0%	1 / 1 100.0%	4 / 4 100.0%	1 / 1 100.0%	3 / 3 100.0%	1 / 1 100.0%	2 / 2 100.0%	1 / 1 100.0%	1 / 1 100.0%	2 / 2 100.0%	1 / 1 100.0%	3 / 3 100.0%	4 / 4 100.0%	2 / 2 100.0%	1 / 1 100.0%	5 / 5 100.0%	0 / 0 0.0%	0 / 0 0.0%	0 / 0 0.0%	0 / 0 0.0%	32 / 45 71.1%AB	13 / 45 28.9%ABE				
18	Oceanospirillales	617 / 852 72.4%	29 / 36 80.6%	39 / 47 83.0%	19 / 27 70.4%	28 / 36 77.8%	47 / 63 74.6%	43 / 53 81.1%	12 / 20 60.0%	13 / 21 61.9%	42 / 51 82.4%	11 / 19 57.9%	33 / 59 55.9%	24 / 32 75.0%	13 / 21 61.9%	19 / 27 70.4%	12 / 20 60.0%	30 / 38 78.9%	15 / 23 65.2%	33 / 45 73.3%	50 / 68 73.5%	37 / 45 82.2%	10 / 19 52.6%	58 / 82 70.7%	0 / 0 0.0%	0 / 0 0.0%	0 / 0 0.0%	0 / 0 0.0%	422 / 852 49.5%AB	195 / 852 22.9%ABE	137 / 852 16.1%E	96 / 852 11.3%	2 / 852 0.2%CE	
10	Alcanivorax/Fundibacter group	135 / 369 36.6%	5 / 12 41.7%	6 / 14 42.9%	6 / 14 42.9%	9 / 17 52.9%	11 / 27 40.7%	8 / 18 44.4%	4 / 12 33.3%	2 / 10 20.0%	13 / 22 59.1%	2 / 10 20.0%	7 / 33 21.2%	5 / 13 38.5%	2 / 10 20.0%	4 / 12 33.3%	2 / 10 20.0%	6 / 14 42.9%	4 / 12 33.3%	6 / 18 33.3%	10 / 28 35.7%	7 / 15 46.7%	2 / 10 20.0%	14 / 38 36.8%	0 / 0 0.0%	0 / 0 0.0%	0 / 0 0.0%	0 / 0 0.0%	137 / 369 37.1%E	95 / 369 25.7%	94 / 369 25.5%AB	41 / 369 11.1%ABE	2 / 369 0.5%CE	
1	Deleya	65 / 66 98.5%	2 / 2 100.0%	3 / 3 100.0%	3 / 3 100.0%	4 / 4 100.0%	6 / 6 100.0%	4 / 4 100.0%	2 / 2 100.0%	1 / 1 100.0%	6 / 6 100.0%	1 / 1 100.0%	3 / 3 100.0%	2 / 2 100.0%	1 / 1 100.0%	2 / 2 100.0%	1 / 1 100.0%	3 / 3 100.0%	2 / 2 100.0%	3 / 3 100.0%	5 / 5 100.0%	3 / 4 75.0%	1 / 1 100.0%	7 / 7 100.0%	0 / 0 0.0%	0 / 0 0.0%	0 / 0 0.0%	0 / 0 0.0%	45 / 66 68.2%AB	20 / 66 30.3%ABE	1 / 66 1.5%CE			
1	ATCC 33173	65 / 66 98.5%	2 / 2 100.0%	3 / 3 100.0%	3 / 3 100.0%	4 / 4 100.0%	6 / 6 100.0%	4 / 4 100.0%	2 / 2 100.0%	1 / 1 100.0%	6 / 6 100.0%	1 / 1 100.0%	3 / 3 100.0%	2 / 2 100.0%	1 / 1 100.0%	2 / 2 100.0%	1 / 1 100.0%	3 / 3 100.0%	2 / 2 100.0%	3 / 3 100.0%	5 / 5 100.0%	3 / 4 75.0%	1 / 1 100.0%	7 / 7 100.0%	0 / 0 0.0%	0 / 0 0.0%	0 / 0 0.0%	0 / 0 0.0%	45 / 66 68.2%AB	20 / 66 30.3%ABE	1 / 66 1.5%CE			
1	Halomonas elongata ATCC 33173	65 / 66 98.5%	2 / 2 100.0%	3 / 3 100.0%	3 / 3 100.0%	4 / 4 100.0%	6 / 6 100.0%	4 / 4 100.0%	2 / 2 100.0%	1 / 1 100.0%	6 / 6 100.0%	1 / 1 100.0%	3 / 3 100.0%	2 / 2 100.0%	1 / 1 100.0%	2 / 2 100.0%	1 / 1 100.0%	3 / 3 100.0%	2 / 2 100.0%	3 / 3 100.0%	5 / 5 100.0%	3 / 4 75.0%	1 / 1 100.0%	7 / 7 100.0%	0 / 0 0.0%	0 / 0 0.0%	0 / 0 0.0%	0 / 0 0.0%	45 / 66 68.2%AB	20 / 66 30.3%ABE	1 / 66 1.5%CE			
1	Chromohalobacter	69 / 69 100.0%	2 / 2 100.0%	3 / 3 100.0%	3 / 3 100.0%	5 / 5 100.0%	5 / 5 100.0%	4 / 4 100.0%	2 / 2 100.0%	1 / 1 100.0%	7 / 7 100.0%	1 / 1 100.0%	4 / 4 100.0%	3 / 3 100.0%	1 / 1 100.0%	2 / 2 100.0%	1 / 1 100.0%	3 / 3 100.0%	2 / 2 100.0%	3 / 3 100.0%	5 / 5 100.0%	4 / 4 100.0%	1 / 1 100.0%	7 / 7 100.0%	0 / 0 0.0%	0 / 0 0.0%	0 / 0 0.0%	0 / 0 0.0%	49 / 69 71.0%AB	20 / 69 29.0%ABE				
1	ATCC BAA-138	69 / 69 100.0%	2 / 2 100.0%	3 / 3 100.0%	3 / 3 100.0%	5 / 5 100.0%	5 / 5 100.0%	4 / 4 100.0%	2 / 2 100.0%	1 / 1 100.0%	7 / 7 100.0%	1 / 1 100.0%	4 / 4 100.0%	3 / 3 100.0%	1 / 1 100.0%	2 / 2 100.0%	1 / 1 100.0%	3 / 3 100.0%	2 / 2 100.0%	3 / 3 100.0%	5 / 5 100.0%	4 / 4 100.0%	1 / 1 100.0%	7 / 7 100.0%	0 / 0 0.0%	0 / 0 0.0%	0 / 0 0.0%	0 / 0 0.0%	49 / 69 71.0%AB	20 / 69 29.0%ABE				
1	Chromohalobacter salexigens 1H11	69 / 69 100.0%	2 / 2 100.0%	3 / 3 100.0%	3 / 3 100.0%	5 / 5 100.0%	5 / 5 100.0%	4 / 4 100.0%	2 / 2 100.0%	1 / 1 100.0%	7 / 7 100.0%	1 / 1 100.0%	4 / 4 100.0%	3 / 3 100.0%	1 / 1 100.0%	2 / 2 100.0%	1 / 1 100.0%	3 / 3 100.0%	2 / 2 100.0%	3 / 3 100.0%	5 / 5 100.0%	4 / 4 100.0%	1 / 1 100.0%	7 / 7 100.0%	0 / 0 0.0%	0 / 0 0.0%	0 / 0 0.0%	0 / 0 0.0%	49 / 69 71.0%AB	20 / 69 29.0%ABE				
8	Zymobacter group	1 / 234 0.4%	1 / 8 12.5%	0 / 8 0.0%	0 / 8 0.0%	0 / 8 0.0%	0 / 16 0.0%	0 / 10 0.0%	0 / 8 0.0%	0 / 8 0.0%	0 / 9 0.0%	0 / 8 0.0%	0 / 26 0.0%	0 / 8 0.0%	0 / 8 0.0%	0 / 8 0.0%	0 / 8 0.0%	0 / 8 0.0%	0 / 8 0.0%	0 / 12 0.0%	0 / 18 0.0%	0 / 7 0.0%	0 / 8 0.0%	0 / 24 0.0%	0 / 0 0.0%	0 / 0 0.0%	0 / 0 0.0%	0 / 0 0.0%	137 / 234 58.5%E	95 / 234 40.6%	1 / 234 0.4%CE	1 / 234 0.4%ABE		
6	"Candidatus Carsonella" Thao et al. 2000	0 / 167 0.0%	0 / 6 0.0%	0 / 6 0.0%	0 / 6 0.0%	0 / 6 0.0%	0 / 12 0.0%	0 / 6 0.0%	0 / 6 0.0%	0 / 6 0.0%	0 / 6 0.0%	0 / 6 0.0%	0 / 18 0.0%	0 / 6 0.0%	0 / 6 0.0%	0 / 6 0.0%	0 / 6 0.0%	0 / 6 0.0%	0 / 6 0.0%	0 / 6 0.0%	0 / 12 0.0%	0 / 5 0.0%	0 / 6 0.0%	0 / 18 0.0%	0 / 0 0.0%	0 / 0 0.0%	0 / 0 0.0%	0 / 0 0.0%	101 / 167 60.5%E	65 / 167 38.9%	1 / 167 0.6%CE			
6	"Candidatus Carsonella ruddii" Thao et al. 2000	0 / 167 0.0%	0 / 6 0.0%	0 / 6 0.0%	0 / 6 0.0%	0 / 6 0.0%	0 / 12 0.0%	0 / 6 0.0%	0 / 6 0.0%	0 / 6 0.0%	0 / 6 0.0%	0 / 6 0.0%	0 / 18 0.0%	0 / 6 0.0%	0 / 6 0.0%	0 / 6 0.0%	0 / 6 0.0%	0 / 6 0.0%	0 / 6 0.0%	0 / 6 0.0%	0 / 12 0.0%	0 / 5 0.0%	0 / 6 0.0%	0 / 18 0.0%	0 / 0 0.0%	0 / 0 0.0%	0 / 0 0.0%	0 / 0 0.0%	101 / 167 60.5%E	65 / 167 38.9%	1 / 167 0.6%CE			
1	primary endosymbiont of Heteropsylla cubana	0 / 28 0.0%	0 / 1 0.0%	0 / 1 0.0%	0 / 1 0.0%	0 / 1 0.0%	0 / 2 0.0%	0 / 1 0.0%	0 / 1 0.0%	0 / 1 0.0%	0 / 1 0.0%	0 / 1 0.0%	0 / 3 0.0%	0 / 1 0.0%	0 / 1 0.0%	0 / 1 0.0%	0 / 1 0.0%	0 / 1 0.0%	0 / 1 0.0%	0 / 1 0.0%	0 / 2 0.0%	0 / 1 0.0%	0 / 1 0.0%	0 / 3 0.0%	0 / 0 0.0%	0 / 0 0.0%	0 / 0 0.0%	0 / 0 0.0%	19 / 28 67.9%E	9 / 28 32.1%				
1	Candidatus Carsonella ruddii HC isolate Thao2000	0 / 28 0.0%	0 / 1 0.0%	0 / 1 0.0%	0 / 1 0.0%	0 / 1 0.0%	0 / 2 0.0%	0 / 1 0.0%	0 / 1 0.0%	0 / 1 0.0%	0 / 1 0.0%	0 / 1 0.0%	0 / 3 0.0%	0 / 1 0.0%	0 / 1 0.0%	0 / 1 0.0%	0 / 1 0.0%	0 / 1 0.0%	0 / 1 0.0%	0 / 1 0.0%	0 / 2 0.0%	0 / 1 0.0%	0 / 1 0.0%	0 / 3 0.0%	0 / 0 0.0%	0 / 0 0.0%	0 / 0 0.0%	0 / 0 0.0%	19 / 28 67.9%E	9 / 28 32.1%				
1	Candidatus Carsonella ruddii PV	0 / 28 0.0%	0 / 1 0.0%	0 / 1 0.0%	0 / 1 0.0%	0 / 1 0.0%	0 / 2 0.0%	0 / 1 0.0%	0 / 1 0.0%	0 / 1 0.0%	0 / 1 0.0%	0 / 1 0.0%	0 / 3 0.0%	0 / 1 0.0%	0 / 1 0.0%	0 / 1 0.0%	0 / 1 0.0%	0 / 1 0.0%	0 / 1 0.0%	0 / 1 0.0%	0 / 2 0.0%	0 / 1 0.0%	0 / 1 0.0%	0 / 3 0.0%	0 / 0 0.0%	0 / 0 0.0%	0 / 0 0.0%	0 / 0 0.0%	21 / 28 75.0%	7 / 28 25.0%E				
1	Candidatus Carsonella ruddii CE isolate Thao2000	0 / 27 0.0%	0 / 1 0.0%	0 / 1 0.0%	0 / 1 0.0%	0 / 1 0.0%	0 / 2 0.0%	0 / 1 0.0%	0 / 1 0.0%	0 / 1 0.0%	0 / 1 0.0%	0 / 1 0.0%	0 / 3 0.0%	0 / 1 0.0%	0 / 1 0.0%	0 / 1 0.0%	0 / 1 0.0%	0 / 1 0.0%	0 / 1 0.0%	0 / 1 0.0%	0 / 2 0.0%	0 / 0 0.0%	0 / 1 0.0%	0 / 3 0.0%	0 / 0 0.0%	0 / 0 0.0%	0 / 0 0.0%	0 / 0 0.0%	20 / 27 74.1%E	7 / 27 25.9%				
1	Candidatus Carsonella ruddii CS isolate Thao2000	0 / 28 0.0%	0 / 1 0.0%	0 / 1 0.0%	0 / 1 0.0%	0 / 1 0.0%	0 / 2 0.0%	0 / 1 0.0%	0 / 1 0.0%	0 / 1 0.0%	0 / 1 0.0%	0 / 1 0.0%	0 / 3 0.0%	0 / 1 0.0%	0 / 1 0.0%	0 / 1 0.0%	0 / 1 0.0%	0 / 1 0.0%	0 / 1 0.0%	0 / 1 0.0%	0 / 2 0.0%	0 / 1 0.0%	0 / 1 0.0%	0 / 3 0.0%	0 / 0 0.0%	0 / 0 0.0%	0 / 0 0.0%	0 / 0 0.0%	20 / 28 71.4%E	8 / 28 28.6%				
1	Candidatus Carsonella ruddii HT isolate Thao2000	0 / 28 0.0%	0 / 1 0.0%	0 / 1 0.0%	0 / 1 0.0%	0 / 1 0.0%	0 / 2 0.0%	0 / 1 0.0%	0 / 1 0.0%	0 / 1 0.0%	0 / 1 0.0%	0 / 1 0.0%	0 / 3 0.0%	0 / 1 0.0%	0 / 1 0.0%	0 / 1 0.0%	0 / 1 0.0%	0 / 1 0.0%	0 / 1 0.0%	0 / 1 0.0%	0 / 2 0.0%	0 / 1 0.0%	0 / 1 0.0%	0 / 3 0.0%	0 / 0 0.0%	0 / 0 0.0%	0 / 0 0.0%	0 / 0 0.0%	18 / 28 64.3%E	10 / 28 35.7%				
1	Candidatus Carsonella ruddii PC isolate NHV	0 / 28 0.0%	0 / 1 0.0%	0 / 1 0.0%	0 / 1 0.0%	0 / 1 0.0%	0 / 2 0.0%	0 / 1 0.0%	0 / 1 0.0%	0 / 1 0.0%	0 / 1 0.0%	0 / 1 0.0%	0 / 3 0.0%	0 / 1 0.0%	0 / 1 0.0%	0 / 1 0.0%	0 / 1 0.0%	0 / 1 0.0%	0 / 1 0.0%	0 / 1 0.0%	0 / 2 0.0%	0 / 1 0.0%	0 / 1 0.0%	0 / 3 0.0%	0 / 0 0.0%	0 / 0 0.0%	0 / 0 0.0%	0 / 0 0.0%	17 / 28 60.7%E	10 / 28 35.7%	1 / 28 3.6%CE			
2	whitefly endosymbionts	1 / 67 1.5%	1 / 2 50.0%	0 / 2 0.0%	0 / 2 0.0%	0 / 2 0.0%	0 / 4 0.0%	0 / 4 0.0%	0 / 2 0.0%	0 / 2 0.0%	0 / 3 0.0%	0 / 2 0.0%	0 / 8 0.0%	0 / 2 0.0%	0 / 2 0.0%	0 / 2 0.0%	0 / 2 0.0%	0 / 2 0.0%	0 / 2 0.0%	0 / 6 0.0%	0 / 6 0.0%	0 / 2 0.0%	0 / 2 0.0%	0 / 6 0.0%	0 / 0 0.0%	0 / 0 0.0%	0 / 0 0.0%	0 / 0 0.0%	36 / 67 53.7%E	30 / 67 44.8%	1 / 67 1.5%ABE			
2	"Candidatus Portiera" Thao and Baumann 2004	1 / 67 1.5%	1 / 2 50.0%	0 / 2 0.0%	0 / 2 0.0%	0 / 2 0.0%	0 / 4 0.0%	0 / 4 0.0%	0 / 2 0.0%	0 / 2 0.0%	0 / 3 0.0%	0 / 2 0.0%	0 / 8 0.0%	0 / 2 0.0%	0 / 2 0.0%	0 / 2 0.0%	0 / 2 0.0%	0 / 2 0.0%	0 / 2 0.0%	0 / 6 0.0%	0 / 6 0.0%	0 / 2 0.0%	0 / 2 0.0%	0 / 6 0.0%	0 / 0 0.0%	0 / 0 0.0%	0 / 0 0.0%	0 / 0 0.0%	36 / 67 53.7%E	30 / 67 44.8%	1 / 67 1.5%ABE			
2	"Candidatus Portiera aleyrodidarum" Thao and Baumann 2004	1 / 67 1.5%	1 / 2 50.0%	0 / 2 0.0%	0 / 2 0.0%	0 / 2 0.0%	0 / 4 0.0%	0 / 4 0.0%	0 / 2 0.0%	0 / 2 0.0%	0 / 3 0.0%	0 / 2 0.0%	0 / 8 0.0%	0 / 2 0.0%	0 / 2 0.0%	0 / 2 0.0%	0 / 2 0.0%	0 / 2 0.0%	0 / 2 0.0%	0 / 6 0.0%	0 / 6 0.0%	0 / 2 0.0%	0 / 2 0.0%	0 / 6 0.0%	0 / 0 0.0%	0 / 0 0.0%	0 / 0 0.0%	0 / 0 0.0%	36 / 67 53.7%E	30 / 67 44.8%	1 / 67 1.5%ABE			
1	Candidatus Portiera aleyrodidarum BT-B	0 / 33 0.0%	0 / 1 0.0%	0 / 1 0.0%	0 / 1 0.0%	0 / 1 0.0%	0 / 2 0.0%	0 / 2 0.0%	0 / 1 0.0%	0 / 1 0.0%	0 / 1 0.0%	0 / 1 0.0%	0 / 4 0.0%	0 / 1 0.0%	0 / 1 0.0%	0 / 1 0.0%	0 / 1 0.0%	0 / 1 0.0%	0 / 1 0.0%	0 / 3 0.0%	0 / 3 0.0%	0 / 1 0.0%	0 / 1 0.0%	0 / 3 0.0%	0 / 0 0.0%	0 / 0 0.0%	0 / 0 0.0%	0 / 0 0.0%	19 / 33 57.6%E	14 / 33 42.4%				
1	Candidatus Portiera aleyrodidarum TV	1 / 34 2.9%	1 / 1 100.0%	0 / 1 0.0%	0 / 1 0.0%	0 / 1 0.0%	0 / 2 0.0%	0 / 2 0.0%	0 / 1 0.0%	0 / 1 0.0%	0 / 2 0.0%	0 / 1 0.0%	0 / 4 0.0%	0 / 1 0.0%	0 / 1 0.0%	0 / 1 0.0%	0 / 1 0.0%	0 / 1 0.0%	0 / 1 0.0%	0 / 3 0.0%	0 / 3 0.0%	0 / 1 0.0%	0 / 1 0.0%	0 / 3 0.0%	0 / 0 0.0%	0 / 0 0.0%	0 / 0 0.0%	0 / 0 0.0%	17 / 34 50.0%E	16 / 34 47.1%	1 / 34 2.9%ABE			
4	Alcanivorax/Fundibacter group	291 / 291 100.0%	18 / 18 100.0%	23 / 23 100.0%	8 / 8 100.0%	10 / 10 100.0%	23 / 23 100.0%	25 / 25 100.0%	4 / 4 100.0%	7 / 7 100.0%	16 / 16 100.0%	4 / 4 100.0%	12 / 12 100.0%	13 / 13 100.0%	6 / 6 100.0%	10 / 10 100.0%	5 / 5 100.0%	12 / 12 100.0%	7 / 7 100.0%	17 / 17 100.0%	23 / 23 100.0%	20 / 20 100.0%	4 / 4 100.0%	24 / 24 100.0%	0 / 0 0.0%	0 / 0 0.0%	0 / 0 0.0%	0 / 0 0.0%	201 / 291 69.1%AB	90 / 291 30.9%ABE				
3	Marinomonas	241 / 241 100.0%	16 / 16 100.0%	19 / 19 100.0%	6 / 6 100.0%	9 / 9 100.0%	19 / 19 100.0%	21 / 21 100.0%	3 / 3 100.0%	6 / 6 100.0%	14 / 14 100.0%	3 / 3 100.0%	9 / 9 100.0%	11 / 11 100.0%	5 / 5 100.0%	9 / 9 100.0%	4 / 4 100.0%	9 / 9 100.0%	6 / 6 100.0%	15 / 15 100.0%	18 / 18 100.0%	17 / 17 100.0%	3 / 3 100.0%	19 / 19 100.0%	0 / 0 0.0%	0 / 0 0.0%	0 / 0 0.0%	0 / 0 0.0%	167 / 241 69.3%AB	74 / 241 30.7%ABE				
1	ATCC 700492	78 / 78 100.0%	5 / 5 100.0%	7 / 7 100.0%	2 / 2 100.0%	3 / 3 100.0%	5 / 5 100.0%	7 / 7 100.0%	1 / 1 100.0%	2 / 2 100.0%	4 / 4 100.0%	1 / 1 100.0%	3 / 3 100.0%	4 / 4 100.0%	2 / 2 100.0%	3 / 3 100.0%	2 / 2 100.0%	3 / 3 100.0%	2 / 2 100.0%	5 / 5 100.0%	6 / 6 100.0%	3 / 3 100.0%	1 / 1 100.0%	7 / 7 100.0%	0 / 0 0.0%	0 / 0 0.0%	0 / 0 0.0%	0 / 0 0.0%	55 / 78 70.5%AB	23 / 78 29.5%ABE				
1	Marinomonas mediterranea MMB-1	78 / 78 100.0%	5 / 5 100.0%	7 / 7 100.0%	2 / 2 100.0%	3 / 3 100.0%	5 / 5 100.0%	7 / 7 100.0%	1 / 1 100.0%	2 / 2 100.0%	4 / 4 100.0%	1 / 1 100.0%	3 / 3 100.0%	4 / 4 100.0%	2 / 2 100.0%	3 / 3 100.0%	2 / 2 100.0%	3 / 3 100.0%	2 / 2 100.0%	5 / 5 100.0%	6 / 6 100.0%	3 / 3 100.0%	1 / 1 100.0%	7 / 7 100.0%	0 / 0 0.0%	0 / 0 0.0%	0 / 0 0.0%	0 / 0 0.0%	55 / 78 70.5%AB	23 / 78 29.5%ABE				
1	Marinomonas sp. MWYL1	83 / 83 100.0%	6 / 6 100.0%	6 / 6 100.0%	2 / 2 100.0%	3 / 3 100.0%	7 / 7 100.0%	7 / 7 100.0%	1 / 1 100.0%	2 / 2 100.0%	5 / 5 100.0%	1 / 1 100.0%	3 / 3 100.0%	4 / 4 100.0%	2 / 2 100.0%	3 / 3 100.0%	1 / 1 100.0%	3 / 3 100.0%	2 / 2 100.0%	5 / 5 100.0%	6 / 6 100.0%	7 / 7 100.0%	1 / 1 100.0%	6 / 6 100.0%	0 / 0 0.0%	0 / 0 0.0%	0 / 0 0.0%	0 / 0 0.0%	57 / 83 68.7%AB	26 / 83 31.3%ABE				
1	'Marinomonas posidonica'	80 / 80 100.0%	5 / 5 100.0%	6 / 6 100.0%	2 / 2 100.0%	3 / 3 100.0%	7 / 7 100.0%	7 / 7 100.0%	1 / 1 100.0%	2 / 2 100.0%	5 / 5 100.0%	1 / 1 100.0%	3 / 3 100.0%	3 / 3 100.0%	1 / 1 100.0%	3 / 3 100.0%	1 / 1 100.0%	3 / 3 100.0%	2 / 2 100.0%	5 / 5 100.0%	6 / 6 100.0%	7 / 7 100.0%	1 / 1 100.0%	6 / 6 100.0%	0 / 0 0.0%	0 / 0 0.0%	0 / 0 0.0%	0 / 0 0.0%	55 / 80 68.8%AB	25 / 80 31.2%ABE				
1	Marinomonas posidonica IVIA-Po-181	80 / 80 100.0%	5 / 5 100.0%	6 / 6 100.0%	2 / 2 100.0%	3 / 3 100.0%	7 / 7 100.0%	7 / 7 100.0%	1 / 1 100.0%	2 / 2 100.0%	5 / 5 100.0%	1 / 1 100.0%	3 / 3 100.0%	3 / 3 100.0%	1 / 1 100.0%	3 / 3 100.0%	1 / 1 100.0%	3 / 3 100.0%	2 / 2 100.0%	5 / 5 100.0%	6 / 6 100.0%	7 / 7 100.0%	1 / 1 100.0%	6 / 6 100.0%	0 / 0 0.0%	0 / 0 0.0%	0 / 0 0.0%	0 / 0 0.0%	55 / 80 68.8%AB	25 / 80 31.2%ABE				
1	Thalassolituus	50 / 50 100.0%	2 / 2 100.0%	4 / 4 100.0%	2 / 2 100.0%	1 / 1 100.0%	4 / 4 100.0%	4 / 4 100.0%	1 / 1 100.0%	1 / 1 100.0%	2 / 2 100.0%	1 / 1 100.0%	3 / 3 100.0%	2 / 2 100.0%	1 / 1 100.0%	1 / 1 100.0%	1 / 1 100.0%	3 / 3 100.0%	1 / 1 100.0%	2 / 2 100.0%	5 / 5 100.0%	3 / 3 100.0%	1 / 1 100.0%	5 / 5 100.0%	0 / 0 0.0%	0 / 0 0.0%	0 / 0 0.0%	0 / 0 0.0%	34 / 50 68.0%AB	16 / 50 32.0%ABE				
1	DSM 14913	50 / 50 100.0%	2 / 2 100.0%	4 / 4 100.0%	2 / 2 100.0%	1 / 1 100.0%	4 / 4 100.0%	4 / 4 100.0%	1 / 1 100.0%	1 / 1 100.0%	2 / 2 100.0%	1 / 1 100.0%	3 / 3 100.0%	2 / 2 100.0%	1 / 1 100.0%	1 / 1 100.0%	1 / 1 100.0%	3 / 3 100.0%	1 / 1 100.0%	2 / 2 100.0%	5 / 5 100.0%	3 / 3 100.0%	1 / 1 100.0%	5 / 5 100.0%	0 / 0 0.0%	0 / 0 0.0%	0 / 0 0.0%	0 / 0 0.0%	34 / 50 68.0%AB	16 / 50 32.0%ABE				
1	Thalassolituus oleivorans MIL-1	50 / 50 100.0%	2 / 2 100.0%	4 / 4 100.0%	2 / 2 100.0%	1 / 1 100.0%	4 / 4 100.0%	4 / 4 100.0%	1 / 1 100.0%	1 / 1 100.0%	2 / 2 100.0%	1 / 1 100.0%	3 / 3 100.0%	2 / 2 100.0%	1 / 1 100.0%	1 / 1 100.0%	1 / 1 100.0%	3 / 3 100.0%	1 / 1 100.0%	2 / 2 100.0%	5 / 5 100.0%	3 / 3 100.0%	1 / 1 100.0%	5 / 5 100.0%	0 / 0 0.0%	0 / 0 0.0%	0 / 0 0.0%	0 / 0 0.0%	34 / 50 68.0%AB	16 / 50 32.0%ABE				
3	Alcanivoracaceae	125 / 125 100.0%	3 / 3 100.0%	6 / 6 100.0%	3 / 3 100.0%	4 / 4 100.0%	10 / 10 100.0%	7 / 7 100.0%	3 / 3 100.0%	3 / 3 100.0%	7 / 7 100.0%	3 / 3 100.0%	9 / 9 100.0%	4 / 4 100.0%	3 / 3 100.0%	3 / 3 100.0%	3 / 3 100.0%	8 / 8 100.0%	3 / 3 100.0%	8 / 8 100.0%	12 / 12 100.0%	6 / 6 100.0%	3 / 3 100.0%	14 / 14 100.0%	0 / 0 0.0%	0 / 0 0.0%	0 / 0 0.0%	0 / 0 0.0%	84 / 125 67.2%AB	41 / 125 32.8%ABE				
2	Alcanivorax	84 / 84 100.0%	2 / 2 100.0%	4 / 4 100.0%	2 / 2 100.0%	2 / 2 100.0%	8 / 8 100.0%	4 / 4 100.0%	2 / 2 100.0%	2 / 2 100.0%	4 / 4 100.0%	2 / 2 100.0%	6 / 6 100.0%	2 / 2 100.0%	2 / 2 100.0%	2 / 2 100.0%	2 / 2 100.0%	6 / 6 100.0%	2 / 2 100.0%	6 / 6 100.0%	8 / 8 100.0%	4 / 4 100.0%	2 / 2 100.0%	10 / 10 100.0%	0 / 0 0.0%	0 / 0 0.0%	0 / 0 0.0%	0 / 0 0.0%	57 / 84 67.9%AB	27 / 84 32.1%ABE				
1	ATCC 700651	42 / 42 100.0%	1 / 1 100.0%	2 / 2 100.0%	1 / 1 100.0%	1 / 1 100.0%	4 / 4 100.0%	2 / 2 100.0%	1 / 1 100.0%	1 / 1 100.0%	2 / 2 100.0%	1 / 1 100.0%	3 / 3 100.0%	1 / 1 100.0%	1 / 1 100.0%	1 / 1 100.0%	1 / 1 100.0%	3 / 3 100.0%	1 / 1 100.0%	3 / 3 100.0%	4 / 4 100.0%	2 / 2 100.0%	1 / 1 100.0%	5 / 5 100.0%	0 / 0 0.0%	0 / 0 0.0%	0 / 0 0.0%	0 / 0 0.0%	29 / 42 69.0%AB	13 / 42 31.0%ABE				
1	Alcanivorax borkumensis SK2	42 / 42 100.0%	1 / 1 100.0%	2 / 2 100.0%	1 / 1 100.0%	1 / 1 100.0%	4 / 4 100.0%	2 / 2 100.0%	1 / 1 100.0%	1 / 1 100.0%	2 / 2 100.0%	1 / 1 100.0%	3 / 3 100.0%	1 / 1 100.0%	1 / 1 100.0%	1 / 1 100.0%	1 / 1 100.0%	3 / 3 100.0%	1 / 1 100.0%	3 / 3 100.0%	4 / 4 100.0%	2 / 2 100.0%	1 / 1 100.0%	5 / 5 100.0%	0 / 0 0.0%	0 / 0 0.0%	0 / 0 0.0%	0 / 0 0.0%	29 / 42 69.0%AB	13 / 42 31.0%ABE				
1	Alcanivorax dieselolei	42 / 42 100.0%	1 / 1 100.0%	2 / 2 100.0%	1 / 1 100.0%	1 / 1 100.0%	4 / 4 100.0%	2 / 2 100.0%	1 / 1 100.0%	1 / 1 100.0%	2 / 2 100.0%	1 / 1 100.0%	3 / 3 100.0%	1 / 1 100.0%	1 / 1 100.0%	1 / 1 100.0%	1 / 1 100.0%	3 / 3 100.0%	1 / 1 100.0%	3 / 3 100.0%	4 / 4 100.0%	2 / 2 100.0%	1 / 1 100.0%	5 / 5 100.0%	0 / 0 0.0%	0 / 0 0.0%	0 / 0 0.0%	0 / 0 0.0%	28 / 42 66.7%AB	14 / 42 33.3%ABE				
1	Alcanivorax dieselolei B5	42 / 42 100.0%	1 / 1 100.0%	2 / 2 100.0%	1 / 1 100.0%	1 / 1 100.0%	4 / 4 100.0%	2 / 2 100.0%	1 / 1 100.0%	1 / 1 100.0%	2 / 2 100.0%	1 / 1 100.0%	3 / 3 100.0%	1 / 1 100.0%	1 / 1 100.0%	1 / 1 100.0%	1 / 1 100.0%	3 / 3 100.0%	1 / 1 100.0%	3 / 3 100.0%	4 / 4 100.0%	2 / 2 100.0%	1 / 1 100.0%	5 / 5 100.0%	0 / 0 0.0%	0 / 0 0.0%	0 / 0 0.0%	0 / 0 0.0%	28 / 42 66.7%AB	14 / 42 33.3%ABE				
1	Kangiella	41 / 41 100.0%	1 / 1 100.0%	2 / 2 100.0%	1 / 1 100.0%	2 / 2 100.0%	2 / 2 100.0%	3 / 3 100.0%	1 / 1 100.0%	1 / 1 100.0%	3 / 3 100.0%	1 / 1 100.0%	3 / 3 100.0%	2 / 2 100.0%	1 / 1 100.0%	1 / 1 100.0%	1 / 1 100.0%	2 / 2 100.0%	1 / 1 100.0%	2 / 2 100.0%	4 / 4 100.0%	2 / 2 100.0%	1 / 1 100.0%	4 / 4 100.0%	0 / 0 0.0%	0 / 0 0.0%	0 / 0 0.0%	0 / 0 0.0%	27 / 41 65.9%AB	14 / 41 34.1%ABE				
1	DSM 16069	41 / 41 100.0%	1 / 1 100.0%	2 / 2 100.0%	1 / 1 100.0%	2 / 2 100.0%	2 / 2 100.0%	3 / 3 100.0%	1 / 1 100.0%	1 / 1 100.0%	3 / 3 100.0%	1 / 1 100.0%	3 / 3 100.0%	2 / 2 100.0%	1 / 1 100.0%	1 / 1 100.0%	1 / 1 100.0%	2 / 2 100.0%	1 / 1 100.0%	2 / 2 100.0%	4 / 4 100.0%	2 / 2 100.0%	1 / 1 100.0%	4 / 4 100.0%	0 / 0 0.0%	0 / 0 0.0%	0 / 0 0.0%	0 / 0 0.0%	27 / 41 65.9%AB	14 / 41 34.1%ABE				
1	Kangiella koreensis DSM 16069	41 / 41 100.0%	1 / 1 100.0%	2 / 2 100.0%	1 / 1 100.0%	2 / 2 100.0%	2 / 2 100.0%	3 / 3 100.0%	1 / 1 100.0%	1 / 1 100.0%	3 / 3 100.0%	1 / 1 100.0%	3 / 3 100.0%	2 / 2 100.0%	1 / 1 100.0%	1 / 1 100.0%	1 / 1 100.0%	2 / 2 100.0%	1 / 1 100.0%	2 / 2 100.0%	4 / 4 100.0%	2 / 2 100.0%	1 / 1 100.0%	4 / 4 100.0%	0 / 0 0.0%	0 / 0 0.0%	0 / 0 0.0%	0 / 0 0.0%	27 / 41 65.9%AB	14 / 41 34.1%ABE				
1	Hahellaceae	66 / 67 98.5%	3 / 3 100.0%	4 / 4 100.0%	2 / 2 100.0%	5 / 5 100.0%	3 / 3 100.0%	3 / 3 100.0%	1 / 1 100.0%	1 / 1 100.0%	6 / 6 100.0%	2 / 2 100.0%	5 / 5 100.0%	2 / 2 100.0%	2 / 2 100.0%	2 / 2 100.0%	2 / 2 100.0%	4 / 4 100.0%	1 / 1 100.0%	2 / 2 100.0%	5 / 5 100.0%	4 / 4 100.0%	1 / 2 50.0%	6 / 6 100.0%	0 / 0 0.0%	0 / 0 0.0%	0 / 0 0.0%	0 / 0 0.0%	43 / 67 64.2%AB	23 / 67 34.3%ABE	1 / 67 1.5%			
1	Hahella	66 / 67 98.5%	3 / 3 100.0%	4 / 4 100.0%	2 / 2 100.0%	5 / 5 100.0%	3 / 3 100.0%	3 / 3 100.0%	1 / 1 100.0%	1 / 1 100.0%	6 / 6 100.0%	2 / 2 100.0%	5 / 5 100.0%	2 / 2 100.0%	2 / 2 100.0%	2 / 2 100.0%	2 / 2 100.0%	4 / 4 100.0%	1 / 1 100.0%	2 / 2 100.0%	5 / 5 100.0%	4 / 4 100.0%	1 / 2 50.0%	6 / 6 100.0%	0 / 0 0.0%	0 / 0 0.0%	0 / 0 0.0%	0 / 0 0.0%	43 / 67 64.2%AB	23 / 67 34.3%ABE	1 / 67 1.5%			
1	Hahella chejuensis	66 / 67 98.5%	3 / 3 100.0%	4 / 4 100.0%	2 / 2 100.0%	5 / 5 100.0%	3 / 3 100.0%	3 / 3 100.0%	1 / 1 100.0%	1 / 1 100.0%	6 / 6 100.0%	2 / 2 100.0%	5 / 5 100.0%	2 / 2 100.0%	2 / 2 100.0%	2 / 2 100.0%	2 / 2 100.0%	4 / 4 100.0%	1 / 1 100.0%	2 / 2 100.0%	5 / 5 100.0%	4 / 4 100.0%	1 / 2 50.0%	6 / 6 100.0%	0 / 0 0.0%	0 / 0 0.0%	0 / 0 0.0%	0 / 0 0.0%	43 / 67 64.2%AB	23 / 67 34.3%ABE	1 / 67 1.5%			
1	Hahella chejuensis KCTC 2396	66 / 67 98.5%	3 / 3 100.0%	4 / 4 100.0%	2 / 2 100.0%	5 / 5 100.0%	3 / 3 100.0%	3 / 3 100.0%	1 / 1 100.0%	1 / 1 100.0%	6 / 6 100.0%	2 / 2 100.0%	5 / 5 100.0%	2 / 2 100.0%	2 / 2 100.0%	2 / 2 100.0%	2 / 2 100.0%	4 / 4 100.0%	1 / 1 100.0%	2 / 2 100.0%	5 / 5 100.0%	4 / 4 100.0%	1 / 2 50.0%	6 / 6 100.0%	0 / 0 0.0%	0 / 0 0.0%	0 / 0 0.0%	0 / 0 0.0%	43 / 67 64.2%AB	23 / 67 34.3%ABE	1 / 67 1.5%			
56	Alteromonadaceae group	4537 / 4558 99.5%	242 / 244 99.2%	206 / 206 100.0%	198 / 199 99.5%	168 / 168 100.0%	293 / 293 100.0%	332 / 332 100.0%	94 / 94 100.0%	131 / 149 87.9%	261 / 261 100.0%	80 / 80 100.0%	286 / 286 100.0%	269 / 269 100.0%	109 / 109 100.0%	157 / 157 100.0%	88 / 88 100.0%	203 / 203 100.0%	88 / 88 100.0%	193 / 193 100.0%	386 / 386 100.0%	281 / 281 100.0%	56 / 56 100.0%	406 / 406 100.0%	10 / 10 100.0%	0 / 0 0.0%	0 / 0 0.0%	0 / 0 0.0%	2603 / 4558 57.1%AB	1530 / 4558 33.6%ABE	253 / 4558 5.6%B	151 / 4558 3.3%BE	20 / 4558 0.4%E	1 / 4558 0.0%
22	Alteromonadaceae	1254 / 1255 99.9%	48 / 48 100.0%	66 / 66 100.0%	41 / 42 97.6%	69 / 69 100.0%	73 / 73 100.0%	69 / 69 100.0%	30 / 30 100.0%	21 / 21 100.0%	91 / 91 100.0%	38 / 38 100.0%	81 / 81 100.0%	55 / 55 100.0%	22 / 22 100.0%	57 / 57 100.0%	22 / 22 100.0%	69 / 69 100.0%	24 / 24 100.0%	56 / 56 100.0%	109 / 109 100.0%	62 / 62 100.0%	22 / 22 100.0%	129 / 129 100.0%	0 / 0 0.0%	0 / 0 0.0%	0 / 0 0.0%	0 / 0 0.0%	838 / 1255 66.8%AB	416 / 1255 33.1%ABE	1 / 1255 0.1%			
14	Alteromonas	843 / 844 99.9%	32 / 32 100.0%	49 / 49 100.0%	25 / 26 96.2%	41 / 41 100.0%	46 / 46 100.0%	47 / 47 100.0%	22 / 22 100.0%	13 / 13 100.0%	55 / 55 100.0%	27 / 27 100.0%	56 / 56 100.0%	37 / 37 100.0%	14 / 14 100.0%	43 / 43 100.0%	14 / 14 100.0%	47 / 47 100.0%	16 / 16 100.0%	38 / 38 100.0%	71 / 71 100.0%	47 / 47 100.0%	14 / 14 100.0%	89 / 89 100.0%	0 / 0 0.0%	0 / 0 0.0%	0 / 0 0.0%	0 / 0 0.0%	558 / 844 66.1%AB	285 / 844 33.8%ABE	1 / 844 0.1%			
13	ATCC 27126	779 / 780 99.9%	30 / 30 100.0%	45 / 45 100.0%	23 / 24 95.8%	38 / 38 100.0%	42 / 42 100.0%	42 / 42 100.0%	21 / 21 100.0%	12 / 12 100.0%	51 / 51 100.0%	25 / 25 100.0%	51 / 51 100.0%	34 / 34 100.0%	13 / 13 100.0%	40 / 40 100.0%	13 / 13 100.0%	44 / 44 100.0%	15 / 15 100.0%	35 / 35 100.0%	66 / 66 100.0%	43 / 43 100.0%	13 / 13 100.0%	83 / 83 100.0%	0 / 0 0.0%	0 / 0 0.0%	0 / 0 0.0%	0 / 0 0.0%	516 / 780 66.2%AB	263 / 780 33.7%ABE	1 / 780 0.1%			
1	Alteromonas macleodii 'Deep ecotype'	40 / 40 100.0%	3 / 3 100.0%	0 / 0 0.0%	1 / 1 100.0%	2 / 2 100.0%	2 / 2 100.0%	1 / 1 100.0%	0 / 0 0.0%	0 / 0 0.0%	3 / 3 100.0%	2 / 2 100.0%	4 / 4 100.0%	3 / 3 100.0%	1 / 1 100.0%	1 / 1 100.0%	1 / 1 100.0%	2 / 2 100.0%	0 / 0 0.0%	1 / 1 100.0%	5 / 5 100.0%	4 / 4 100.0%	1 / 1 100.0%	3 / 3 100.0%	0 / 0 0.0%	0 / 0 0.0%	0 / 0 0.0%	0 / 0 0.0%	24 / 40 60.0%AB	16 / 40 40.0%ABE				
1	Alteromonas macleodii ATCC 27126	49 / 50 98.0%	2 / 2 100.0%	3 / 3 100.0%	0 / 1 0.0%	3 / 3 100.0%	4 / 4 100.0%	1 / 1 100.0%	1 / 1 100.0%	1 / 1 100.0%	4 / 4 100.0%	2 / 2 100.0%	3 / 3 100.0%	2 / 2 100.0%	1 / 1 100.0%	3 / 3 100.0%	1 / 1 100.0%	2 / 2 100.0%	1 / 1 100.0%	3 / 3 100.0%	5 / 5 100.0%	2 / 2 100.0%	1 / 1 100.0%	4 / 4 100.0%	0 / 0 0.0%	0 / 0 0.0%	0 / 0 0.0%	0 / 0 0.0%	35 / 50 70.0%AB	14 / 50 28.0%ABE	1 / 50 2.0%			
1	Alteromonas macleodii str. 'Black Sea 11'	69 / 69 100.0%	3 / 3 100.0%	4 / 4 100.0%	2 / 2 100.0%	2 / 2 100.0%	4 / 4 100.0%	5 / 5 100.0%	2 / 2 100.0%	1 / 1 100.0%	3 / 3 100.0%	2 / 2 100.0%	5 / 5 100.0%	3 / 3 100.0%	1 / 1 100.0%	4 / 4 100.0%	1 / 1 100.0%	4 / 4 100.0%	2 / 2 100.0%	3 / 3 100.0%	5 / 5 100.0%	4 / 4 100.0%	1 / 1 100.0%	8 / 8 100.0%	0 / 0 0.0%	0 / 0 0.0%	0 / 0 0.0%	0 / 0 0.0%	44 / 69 63.8%AB	25 / 69 36.2%ABE				
1	Alteromonas macleodii AltDE1	67 / 67 100.0%	2 / 2 100.0%	4 / 4 100.0%	2 / 2 100.0%	3 / 3 100.0%	3 / 3 100.0%	4 / 4 100.0%	2 / 2 100.0%	1 / 1 100.0%	4 / 4 100.0%	2 / 2 100.0%	4 / 4 100.0%	3 / 3 100.0%	1 / 1 100.0%	4 / 4 100.0%	1 / 1 100.0%	4 / 4 100.0%	2 / 2 100.0%	3 / 3 100.0%	5 / 5 100.0%	4 / 4 100.0%	1 / 1 100.0%	8 / 8 100.0%	0 / 0 0.0%	0 / 0 0.0%	0 / 0 0.0%	0 / 0 0.0%	44 / 67 65.7%AB	23 / 67 34.3%ABE				
1	Alteromonas macleodii str. 'Balearic Sea AD45'	71 / 71 100.0%	3 / 3 100.0%	4 / 4 100.0%	2 / 2 100.0%	3 / 3 100.0%	4 / 4 100.0%	5 / 5 100.0%	2 / 2 100.0%	1 / 1 100.0%	4 / 4 100.0%	2 / 2 100.0%	5 / 5 100.0%	3 / 3 100.0%	1 / 1 100.0%	4 / 4 100.0%	1 / 1 100.0%	4 / 4 100.0%	2 / 2 100.0%	3 / 3 100.0%	5 / 5 100.0%	4 / 4 100.0%	1 / 1 100.0%	8 / 8 100.0%	0 / 0 0.0%	0 / 0 0.0%	0 / 0 0.0%	0 / 0 0.0%	46 / 71 64.8%AB	25 / 71 35.2%ABE				
1	Alteromonas macleodii str. 'English Channel 673'	75 / 75 100.0%	3 / 3 100.0%	4 / 4 100.0%	2 / 2 100.0%	5 / 5 100.0%	4 / 4 100.0%	5 / 5 100.0%	2 / 2 100.0%	1 / 1 100.0%	6 / 6 100.0%	2 / 2 100.0%	5 / 5 100.0%	3 / 3 100.0%	1 / 1 100.0%	4 / 4 100.0%	1 / 1 100.0%	4 / 4 100.0%	2 / 2 100.0%	3 / 3 100.0%	5 / 5 100.0%	4 / 4 100.0%	1 / 1 100.0%	8 / 8 100.0%	0 / 0 0.0%	0 / 0 0.0%	0 / 0 0.0%	0 / 0 0.0%	50 / 75 66.7%AB	25 / 75 33.3%ABE				
1	Alteromonas macleodii str. 'English Channel 615'	67 / 67 100.0%	3 / 3 100.0%	4 / 4 100.0%	2 / 2 100.0%	3 / 3 100.0%	4 / 4 100.0%	4 / 4 100.0%	2 / 2 100.0%	1 / 1 100.0%	4 / 4 100.0%	2 / 2 100.0%	3 / 3 100.0%	3 / 3 100.0%	1 / 1 100.0%	4 / 4 100.0%	1 / 1 100.0%	4 / 4 100.0%	2 / 2 100.0%	3 / 3 100.0%	5 / 5 100.0%	3 / 3 100.0%	1 / 1 100.0%	8 / 8 100.0%	0 / 0 0.0%	0 / 0 0.0%	0 / 0 0.0%	0 / 0 0.0%	45 / 67 67.2%AB	22 / 67 32.8%ABE				
[illegible]	Alteromonas macleodii str. 'Aegean [illegible]	[illegible]	[illegible]	[illegible]	[illegible]	[illegible]	[illegible]	[illegible]	[illegible]	[illegible]	[illegible]	[illegible]	[illegible]	[illegible]	[illegible]	[illegible]	[illegible]	[illegible]	[illegible]	[illegible]	[illegible]	[illegible]	[illegible]	[illegible]	[illegible]	[illegible]	[illegible]	[illegible]	[illegible]	[illegible]				

1	Sea MED64'	100.0%	100.0%	100.0%	100.0%	100.0%	100.0%	100.0%	100.0%	100.0%	100.0%	100.0%	100.0%	100.0%	100.0%	100.0%	100.0%	100.0%	100.0%	100.0%	100.0%	100.0%	100.0%	100.0%	0.0%	0.0%	0.0%	0.0%	71.0%AB	29.0%ABE			
1	Alteromonas macleodii str. 'Ionian Sea U4'	49 / 49 100.0%	3 / 3 100.0%	4 / 4 100.0%	2 / 2 100.0%	4 / 4 100.0%	1 / 1 100.0%	1 / 1 100.0%	2 / 2 100.0%	0 / 0 0.0%	5 / 5 100.0%	2 / 2 100.0%	4 / 4 100.0%	0 / 0 0.0%	1 / 1 100.0%	2 / 2 100.0%	1 / 1 100.0%	4 / 4 100.0%	0 / 0 0.0%	0 / 0 0.0%	6 / 6 100.0%	1 / 1 100.0%	1 / 1 100.0%	5 / 5 100.0%	0 / 0 0.0%	0 / 0 0.0%	0 / 0 0.0%	0 / 0 0.0%	32 / 49 65.3%AB	17 / 49 34.7%ABE			
1	Alteromonas macleodii str. 'Ionian Sea U7'	59 / 59 100.0%	2 / 2 100.0%	2 / 2 100.0%	2 / 2 100.0%	2 / 2 100.0%	4 / 4 100.0%	4 / 4 100.0%	1 / 1 100.0%	1 / 1 100.0%	3 / 3 100.0%	2 / 2 100.0%	4 / 4 100.0%	2 / 2 100.0%	1 / 1 100.0%	4 / 4 100.0%	1 / 1 100.0%	2 / 2 100.0%	1 / 1 100.0%	3 / 3 100.0%	5 / 5 100.0%	4 / 4 100.0%	1 / 1 100.0%	8 / 8 100.0%	0 / 0 0.0%	0 / 0 0.0%	0 / 0 0.0%	0 / 0 0.0%	38 / 59 64.4%AB	21 / 59 35.6%ABE			
1	Alteromonas macleodii str. 'Ionian Sea U8'	54 / 54 100.0%	0 / 0 0.0%	4 / 4 100.0%	2 / 2 100.0%	2 / 2 100.0%	3 / 3 100.0%	4 / 4 100.0%	2 / 2 100.0%	2 / 2 100.0%	3 / 3 100.0%	1 / 1 100.0%	2 / 2 100.0%	3 / 3 100.0%	1 / 1 100.0%	2 / 2 100.0%	1 / 1 100.0%	4 / 4 100.0%	0 / 0 0.0%	4 / 4 100.0%	4 / 4 100.0%	3 / 3 100.0%	1 / 1 100.0%	6 / 6 100.0%	0 / 0 0.0%	0 / 0 0.0%	0 / 0 0.0%	0 / 0 0.0%	37 / 54 68.5%AB	17 / 54 31.5%ABE			
1	Alteromonas macleodii str. 'Ionian Sea UM7'	60 / 60 100.0%	2 / 2 100.0%	4 / 4 100.0%	2 / 2 100.0%	3 / 3 100.0%	2 / 2 100.0%	1 / 1 100.0%	2 / 2 100.0%	1 / 1 100.0%	4 / 4 100.0%	2 / 2 100.0%	4 / 4 100.0%	3 / 3 100.0%	1 / 1 100.0%	3 / 3 100.0%	1 / 1 100.0%	4 / 4 100.0%	2 / 2 100.0%	3 / 3 100.0%	5 / 5 100.0%	3 / 3 100.0%	1 / 1 100.0%	7 / 7 100.0%	0 / 0 0.0%	0 / 0 0.0%	0 / 0 0.0%	0 / 0 0.0%	39 / 60 65.0%AB	21 / 60 35.0%ABE			
1	Alteromonas macleodii str. 'Ionian Sea UM4b'	57 / 57 100.0%	2 / 2 100.0%	4 / 4 100.0%	2 / 2 100.0%	3 / 3 100.0%	3 / 3 100.0%	2 / 2 100.0%	2 / 2 100.0%	1 / 1 100.0%	4 / 4 100.0%	2 / 2 100.0%	4 / 4 100.0%	2 / 2 100.0%	1 / 1 100.0%	2 / 2 100.0%	1 / 1 100.0%	3 / 3 100.0%	0 / 0 0.0%	3 / 3 100.0%	6 / 6 100.0%	4 / 4 100.0%	1 / 1 100.0%	5 / 5 100.0%	0 / 0 0.0%	0 / 0 0.0%	0 / 0 0.0%	0 / 0 0.0%	38 / 57 66.7%AB	19 / 57 33.3%ABE			
1	Alteromonas sp. SN2	64 / 64 100.0%	2 / 2 100.0%	4 / 4 100.0%	2 / 2 100.0%	3 / 3 100.0%	4 / 4 100.0%	5 / 5 100.0%	1 / 1 100.0%	1 / 1 100.0%	4 / 4 100.0%	2 / 2 100.0%	5 / 5 100.0%	3 / 3 100.0%	1 / 1 100.0%	3 / 3 100.0%	1 / 1 100.0%	3 / 3 100.0%	1 / 1 100.0%	3 / 3 100.0%	5 / 5 100.0%	4 / 4 100.0%	1 / 1 100.0%	6 / 6 100.0%	0 / 0 0.0%	0 / 0 0.0%	0 / 0 0.0%	0 / 0 0.0%	42 / 64 65.6%AB	22 / 64 34.4%ABE			
4	Marinobacter	199 / 199 100.0%	8 / 8 100.0%	8 / 8 100.0%	8 / 8 100.0%	12 / 12 100.0%	15 / 15 100.0%	12 / 12 100.0%	4 / 4 100.0%	4 / 4 100.0%	16 / 16 100.0%	4 / 4 100.0%	12 / 12 100.0%	9 / 9 100.0%	4 / 4 100.0%	4 / 4 100.0%	4 / 4 100.0%	12 / 12 100.0%	4 / 4 100.0%	7 / 7 100.0%	20 / 20 100.0%	8 / 8 100.0%	4 / 4 100.0%	20 / 20 100.0%	0 / 0 0.0%	0 / 0 0.0%	0 / 0 0.0%	0 / 0 0.0%	132 / 199 66.3%AB	67 / 199 33.7%ABE			
2	ATCC 49840	101 / 101 100.0%	4 / 4 100.0%	4 / 4 100.0%	4 / 4 100.0%	6 / 6 100.0%	8 / 8 100.0%	6 / 6 100.0%	2 / 2 100.0%	2 / 2 100.0%	8 / 8 100.0%	2 / 2 100.0%	6 / 6 100.0%	5 / 5 100.0%	2 / 2 100.0%	2 / 2 100.0%	2 / 2 100.0%	6 / 6 100.0%	2 / 2 100.0%	4 / 4 100.0%	10 / 10 100.0%	4 / 4 100.0%	2 / 2 100.0%	10 / 10 100.0%	0 / 0 0.0%	0 / 0 0.0%	0 / 0 0.0%	0 / 0 0.0%	67 / 101 66.3%AB	34 / 101 33.7%ABE			
1	Marinobacter aquaeolei VT8	51 / 51 100.0%	2 / 2 100.0%	2 / 2 100.0%	2 / 2 100.0%	3 / 3 100.0%	4 / 4 100.0%	3 / 3 100.0%	1 / 1 100.0%	1 / 1 100.0%	4 / 4 100.0%	1 / 1 100.0%	3 / 3 100.0%	3 / 3 100.0%	1 / 1 100.0%	1 / 1 100.0%	1 / 1 100.0%	3 / 3 100.0%	1 / 1 100.0%	2 / 2 100.0%	5 / 5 100.0%	2 / 2 100.0%	1 / 1 100.0%	5 / 5 100.0%	0 / 0 0.0%	0 / 0 0.0%	0 / 0 0.0%	0 / 0 0.0%	34 / 51 66.7%AB	17 / 51 33.3%ABE			
1	Marinobacter hydrocarbonoclasticus ATCC 49840	50 / 50 100.0%	2 / 2 100.0%	2 / 2 100.0%	2 / 2 100.0%	3 / 3 100.0%	4 / 4 100.0%	3 / 3 100.0%	1 / 1 100.0%	1 / 1 100.0%	4 / 4 100.0%	1 / 1 100.0%	3 / 3 100.0%	2 / 2 100.0%	1 / 1 100.0%	1 / 1 100.0%	1 / 1 100.0%	3 / 3 100.0%	1 / 1 100.0%	2 / 2 100.0%	5 / 5 100.0%	2 / 2 100.0%	1 / 1 100.0%	5 / 5 100.0%	0 / 0 0.0%	0 / 0 0.0%	0 / 0 0.0%	0 / 0 0.0%	33 / 50 66.0%AB	17 / 50 34.0%ABE			
1	Marinobacter sp. BSs20148	49 / 49 100.0%	2 / 2 100.0%	2 / 2 100.0%	2 / 2 100.0%	3 / 3 100.0%	3 / 3 100.0%	3 / 3 100.0%	1 / 1 100.0%	1 / 1 100.0%	4 / 4 100.0%	1 / 1 100.0%	3 / 3 100.0%	2 / 2 100.0%	1 / 1 100.0%	1 / 1 100.0%	1 / 1 100.0%	3 / 3 100.0%	1 / 1 100.0%	2 / 2 100.0%	5 / 5 100.0%	2 / 2 100.0%	1 / 1 100.0%	5 / 5 100.0%	0 / 0 0.0%	0 / 0 0.0%	0 / 0 0.0%	0 / 0 0.0%	32 / 49 65.3%AB	17 / 49 34.7%ABE			
1	"Marinobacter adhaerens" Gardes et al. 2010	49 / 49 100.0%	2 / 2 100.0%	2 / 2 100.0%	2 / 2 100.0%	3 / 3 100.0%	4 / 4 100.0%	3 / 3 100.0%	1 / 1 100.0%	1 / 1 100.0%	4 / 4 100.0%	1 / 1 100.0%	3 / 3 100.0%	2 / 2 100.0%	1 / 1 100.0%	1 / 1 100.0%	1 / 1 100.0%	3 / 3 100.0%	1 / 1 100.0%	1 / 1 100.0%	5 / 5 100.0%	2 / 2 100.0%	1 / 1 100.0%	5 / 5 100.0%	0 / 0 0.0%	0 / 0 0.0%	0 / 0 0.0%	0 / 0 0.0%	33 / 49 67.3%AB	16 / 49 32.7%ABE			
1	Marinobacter adhaerens HP15	49 / 49 100.0%	2 / 2 100.0%	2 / 2 100.0%	2 / 2 100.0%	3 / 3 100.0%	4 / 4 100.0%	3 / 3 100.0%	1 / 1 100.0%	1 / 1 100.0%	4 / 4 100.0%	1 / 1 100.0%	3 / 3 100.0%	2 / 2 100.0%	1 / 1 100.0%	1 / 1 100.0%	1 / 1 100.0%	3 / 3 100.0%	1 / 1 100.0%	1 / 1 100.0%	5 / 5 100.0%	2 / 2 100.0%	1 / 1 100.0%	5 / 5 100.0%	0 / 0 0.0%	0 / 0 0.0%	0 / 0 0.0%	0 / 0 0.0%	33 / 49 67.3%AB	16 / 49 32.7%ABE			
3	Glaciecola	172 / 172 100.0%	7 / 7 100.0%	7 / 7 100.0%	6 / 6 100.0%	15 / 15 100.0%	10 / 10 100.0%	7 / 7 100.0%	3 / 3 100.0%	3 / 3 100.0%	18 / 18 100.0%	6 / 6 100.0%	10 / 10 100.0%	7 / 7 100.0%	3 / 3 100.0%	9 / 9 100.0%	3 / 3 100.0%	8 / 8 100.0%	3 / 3 100.0%	9 / 9 100.0%	14 / 14 100.0%	6 / 6 100.0%	3 / 3 100.0%	15 / 15 100.0%	0 / 0 0.0%	0 / 0 0.0%	0 / 0 0.0%	0 / 0 0.0%	119 / 172 69.2%AB	53 / 172 30.8%ABE			
1	Glaciecola nitratireducens	54 / 54 100.0%	1 / 1 100.0%	3 / 3 100.0%	2 / 2 100.0%	4 / 4 100.0%	3 / 3 100.0%	3 / 3 100.0%	1 / 1 100.0%	1 / 1 100.0%	5 / 5 100.0%	2 / 2 100.0%	3 / 3 100.0%	2 / 2 100.0%	1 / 1 100.0%	3 / 3 100.0%	1 / 1 100.0%	3 / 3 100.0%	1 / 1 100.0%	3 / 3 100.0%	4 / 4 100.0%	2 / 2 100.0%	1 / 1 100.0%	5 / 5 100.0%	0 / 0 0.0%	0 / 0 0.0%	0 / 0 0.0%	0 / 0 0.0%	37 / 54 68.5%AB	17 / 54 31.5%ABE			
1	Glaciecola nitratireducens FR1064	54 / 54 100.0%	1 / 1 100.0%	3 / 3 100.0%	2 / 2 100.0%	4 / 4 100.0%	3 / 3 100.0%	3 / 3 100.0%	1 / 1 100.0%	1 / 1 100.0%	5 / 5 100.0%	2 / 2 100.0%	3 / 3 100.0%	2 / 2 100.0%	1 / 1 100.0%	3 / 3 100.0%	1 / 1 100.0%	3 / 3 100.0%	1 / 1 100.0%	3 / 3 100.0%	4 / 4 100.0%	2 / 2 100.0%	1 / 1 100.0%	5 / 5 100.0%	0 / 0 0.0%	0 / 0 0.0%	0 / 0 0.0%	0 / 0 0.0%	37 / 54 68.5%AB	17 / 54 31.5%ABE			
1	CGMCC 1.6130	59 / 59 100.0%	3 / 3 100.0%	1 / 1 100.0%	2 / 2 100.0%	6 / 6 100.0%	3 / 3 100.0%	2 / 2 100.0%	1 / 1 100.0%	1 / 1 100.0%	7 / 7 100.0%	2 / 2 100.0%	3 / 3 100.0%	3 / 3 100.0%	1 / 1 100.0%	3 / 3 100.0%	1 / 1 100.0%	3 / 3 100.0%	1 / 1 100.0%	3 / 3 100.0%	5 / 5 100.0%	2 / 2 100.0%	1 / 1 100.0%	5 / 5 100.0%	0 / 0 0.0%	0 / 0 0.0%	0 / 0 0.0%	0 / 0 0.0%	43 / 59 72.9%AB	16 / 59 27.1%ABE			
1	Glaciecola psychrophila 170	59 / 59 100.0%	3 / 3 100.0%	1 / 1 100.0%	2 / 2 100.0%	6 / 6 100.0%	3 / 3 100.0%	2 / 2 100.0%	1 / 1 100.0%	1 / 1 100.0%	7 / 7 100.0%	2 / 2 100.0%	3 / 3 100.0%	3 / 3 100.0%	1 / 1 100.0%	3 / 3 100.0%	1 / 1 100.0%	3 / 3 100.0%	1 / 1 100.0%	3 / 3 100.0%	5 / 5 100.0%	2 / 2 100.0%	1 / 1 100.0%	5 / 5 100.0%	0 / 0 0.0%	0 / 0 0.0%	0 / 0 0.0%	0 / 0 0.0%	43 / 59 72.9%AB	16 / 59 27.1%ABE			
1	Glaciecola agarilytica 4H-3-7+YE-5	59 / 59 100.0%	3 / 3 100.0%	3 / 3 100.0%	2 / 2 100.0%	5 / 5 100.0%	4 / 4 100.0%	2 / 2 100.0%	1 / 1 100.0%	1 / 1 100.0%	6 / 6 100.0%	2 / 2 100.0%	4 / 4 100.0%	2 / 2 100.0%	1 / 1 100.0%	3 / 3 100.0%	1 / 1 100.0%	2 / 2 100.0%	1 / 1 100.0%	3 / 3 100.0%	5 / 5 100.0%	2 / 2 100.0%	1 / 1 100.0%	5 / 5 100.0%	0 / 0 0.0%	0 / 0 0.0%	0 / 0 0.0%	0 / 0 0.0%	39 / 59 66.1%AB	20 / 59 33.9%ABE			
1	Saccharophagus	40 / 40 100.0%	1 / 1 100.0%	2 / 2 100.0%	2 / 2 100.0%	1 / 1 100.0%	2 / 2 100.0%	3 / 3 100.0%	1 / 1 100.0%	1 / 1 100.0%	2 / 2 100.0%	1 / 1 100.0%	3 / 3 100.0%	2 / 2 100.0%	1 / 1 100.0%	1 / 1 100.0%	1 / 1 100.0%	2 / 2 100.0%	1 / 1 100.0%	2 / 2 100.0%	4 / 4 100.0%	1 / 1 100.0%	1 / 1 100.0%	5 / 5 100.0%	0 / 0 0.0%	0 / 0 0.0%	0 / 0 0.0%	0 / 0 0.0%	29 / 40 72.5%AB	11 / 40 27.5%ABE			
1	ATCC 43961	40 / 40 100.0%	1 / 1 100.0%	2 / 2 100.0%	2 / 2 100.0%	1 / 1 100.0%	2 / 2 100.0%	3 / 3 100.0%	1 / 1 100.0%	1 / 1 100.0%	2 / 2 100.0%	1 / 1 100.0%	3 / 3 100.0%	2 / 2 100.0%	1 / 1 100.0%	1 / 1 100.0%	1 / 1 100.0%	2 / 2 100.0%	1 / 1 100.0%	2 / 2 100.0%	4 / 4 100.0%	1 / 1 100.0%	1 / 1 100.0%	5 / 5 100.0%	0 / 0 0.0%	0 / 0 0.0%	0 / 0 0.0%	0 / 0 0.0%	29 / 40 72.5%AB	11 / 40 27.5%ABE			
1	Microbulbifer degradans 2-40	40 / 40 100.0%	1 / 1 100.0%	2 / 2 100.0%	2 / 2 100.0%	1 / 1 100.0%	2 / 2 100.0%	3 / 3 100.0%	1 / 1 100.0%	1 / 1 100.0%	2 / 2 100.0%	1 / 1 100.0%	3 / 3 100.0%	2 / 2 100.0%	1 / 1 100.0%	1 / 1 100.0%	1 / 1 100.0%	2 / 2 100.0%	1 / 1 100.0%	2 / 2 100.0%	4 / 4 100.0%	1 / 1 100.0%	1 / 1 100.0%	5 / 5 100.0%	0 / 0 0.0%	0 / 0 0.0%	0 / 0 0.0%	0 / 0 0.0%	29 / 40 72.5%AB	11 / 40 27.5%ABE			
1	Alteromonadales genera incertae sedis	39 / 39 100.0%	1 / 1 100.0%	2 / 2 100.0%	1 / 1 100.0%	2 / 2 100.0%	2 / 2 100.0%	2 / 2 100.0%	1 / 1 100.0%	1 / 1 100.0%	3 / 3 100.0%	1 / 1 100.0%	3 / 3 100.0%	1 / 1 100.0%	1 / 1 100.0%	1 / 1 100.0%	1 / 1 100.0%	3 / 3 100.0%	1 / 1 100.0%	2 / 2 100.0%	3 / 3 100.0%	1 / 1 100.0%	1 / 1 100.0%	5 / 5 100.0%	0 / 0 0.0%	0 / 0 0.0%	0 / 0 0.0%	0 / 0 0.0%	26 / 39 66.7%AB	13 / 39 33.3%ABE			
1	"Teredinobacter" Waterbury et al. 1989	39 / 39 100.0%	1 / 1 100.0%	2 / 2 100.0%	1 / 1 100.0%	2 / 2 100.0%	2 / 2 100.0%	2 / 2 100.0%	1 / 1 100.0%	1 / 1 100.0%	3 / 3 100.0%	1 / 1 100.0%	3 / 3 100.0%	1 / 1 100.0%	1 / 1 100.0%	1 / 1 100.0%	1 / 1 100.0%	3 / 3 100.0%	1 / 1 100.0%	2 / 2 100.0%	3 / 3 100.0%	1 / 1 100.0%	1 / 1 100.0%	5 / 5 100.0%	0 / 0 0.0%	0 / 0 0.0%	0 / 0 0.0%	0 / 0 0.0%	26 / 39 66.7%AB	13 / 39 33.3%ABE			
1	"Teredinobacter turnerae" Waterbury et al. 1989	39 / 39 100.0%	1 / 1 100.0%	2 / 2 100.0%	1 / 1 100.0%	2 / 2 100.0%	2 / 2 100.0%	2 / 2 100.0%	1 / 1 100.0%	1 / 1 100.0%	3 / 3 100.0%	1 / 1 100.0%	3 / 3 100.0%	1 / 1 100.0%	1 / 1 100.0%	1 / 1 100.0%	1 / 1 100.0%	3 / 3 100.0%	1 / 1 100.0%	2 / 2 100.0%	3 / 3 100.0%	1 / 1 100.0%	1 / 1 100.0%	5 / 5 100.0%	0 / 0 0.0%	0 / 0 0.0%	0 / 0 0.0%	0 / 0 0.0%	26 / 39 66.7%AB	13 / 39 33.3%ABE			
1	Teredinibacter turnerae T7901	39 / 39 100.0%	1 / 1 100.0%	2 / 2 100.0%	1 / 1 100.0%	2 / 2 100.0%	2 / 2 100.0%	2 / 2 100.0%	1 / 1 100.0%	1 / 1 100.0%	3 / 3 100.0%	1 / 1 100.0%	3 / 3 100.0%	1 / 1 100.0%	1 / 1 100.0%	1 / 1 100.0%	1 / 1 100.0%	3 / 3 100.0%	1 / 1 100.0%	2 / 2 100.0%	3 / 3 100.0%	1 / 1 100.0%	1 / 1 100.0%	5 / 5 100.0%	0 / 0 0.0%	0 / 0 0.0%	0 / 0 0.0%	0 / 0 0.0%	26 / 39 66.7%AB	13 / 39 33.3%ABE			
3	Pseudoalteromonadaceae	229 / 229 100.0%	11 / 11 100.0%	15 / 15 100.0%	7 / 7 100.0%	8 / 8 100.0%	17 / 17 100.0%	19 / 19 100.0%	5 / 5 100.0%	6 / 6 100.0%	13 / 13 100.0%	6 / 6 100.0%	17 / 17 100.0%	12 / 12 100.0%	6 / 6 100.0%	8 / 8 100.0%	5 / 5 100.0%	9 / 9 100.0%	4 / 4 100.0%	10 / 10 100.0%	18 / 18 100.0%	11 / 11 100.0%	3 / 3 100.0%	19 / 19 100.0%	0 / 0 0.0%	0 / 0 0.0%	0 / 0 0.0%	0 / 0 0.0%	145 / 229 63.3%AB	84 / 229 36.7%ABE			
3	Pseudoalteromonas	229 / 229 100.0%	11 / 11 100.0%	15 / 15 100.0%	7 / 7 100.0%	8 / 8 100.0%	17 / 17 100.0%	19 / 19 100.0%	5 / 5 100.0%	6 / 6 100.0%	13 / 13 100.0%	6 / 6 100.0%	17 / 17 100.0%	12 / 12 100.0%	6 / 6 100.0%	8 / 8 100.0%	5 / 5 100.0%	9 / 9 100.0%	4 / 4 100.0%	10 / 10 100.0%	18 / 18 100.0%	11 / 11 100.0%	3 / 3 100.0%	19 / 19 100.0%	0 / 0 0.0%	0 / 0 0.0%	0 / 0 0.0%	0 / 0 0.0%	145 / 229 63.3%AB	84 / 229 36.7%ABE			
1	"Alteromonas marinopraesens" (ZoBell and Upham 1944) Baumann et al. 1972	105 / 105 100.0%	4 / 4 100.0%	11 / 11 100.0%	4 / 4 100.0%	2 / 2 100.0%	7 / 7 100.0%	9 / 9 100.0%	2 / 2 100.0%	3 / 3 100.0%	4 / 4 100.0%	2 / 2 100.0%	7 / 7 100.0%	8 / 8 100.0%	3 / 3 100.0%	4 / 4 100.0%	2 / 2 100.0%	4 / 4 100.0%	2 / 2 100.0%	4 / 4 100.0%	7 / 7 100.0%	6 / 6 100.0%	1 / 1 100.0%	9 / 9 100.0%	0 / 0 0.0%	0 / 0 0.0%	0 / 0 0.0%	0 / 0 0.0%	67 / 105 63.8%AB	38 / 105 36.2%ABE			
1	Pseudoalteromonas haloplanktis TAC125	105 / 105 100.0%	4 / 4 100.0%	11 / 11 100.0%	4 / 4 100.0%	2 / 2 100.0%	7 / 7 100.0%	9 / 9 100.0%	2 / 2 100.0%	3 / 3 100.0%	4 / 4 100.0%	2 / 2 100.0%	7 / 7 100.0%	8 / 8 100.0%	3 / 3 100.0%	4 / 4 100.0%	2 / 2 100.0%	4 / 4 100.0%	2 / 2 100.0%	4 / 4 100.0%	7 / 7 100.0%	6 / 6 100.0%	1 / 1 100.0%	9 / 9 100.0%	0 / 0 0.0%	0 / 0 0.0%	0 / 0 0.0%	0 / 0 0.0%	67 / 105 63.8%AB	38 / 105 36.2%ABE			
1	ATCC 19262	62 / 62 100.0%	3 / 3 100.0%	3 / 3 100.0%	2 / 2 100.0%	5 / 5 100.0%	4 / 4 100.0%	4 / 4 100.0%	1 / 1 100.0%	1 / 1 100.0%	6 / 6 100.0%	2 / 2 100.0%	4 / 4 100.0%	2 / 2 100.0%	1 / 1 100.0%	3 / 3 100.0%	1 / 1 100.0%	2 / 2 100.0%	1 / 1 100.0%	3 / 3 100.0%	5 / 5 100.0%	3 / 3 100.0%	1 / 1 100.0%	5 / 5 100.0%	0 / 0 0.0%	0 / 0 0.0%	0 / 0 0.0%	0 / 0 0.0%	41 / 62 66.1%AB	21 / 62 33.9%ABE			
1	Pseudoalteromonas atlantica T6c	62 / 62 100.0%	3 / 3 100.0%	3 / 3 100.0%	2 / 2 100.0%	5 / 5 100.0%	4 / 4 100.0%	4 / 4 100.0%	1 / 1 100.0%	1 / 1 100.0%	6 / 6 100.0%	2 / 2 100.0%	4 / 4 100.0%	2 / 2 100.0%	1 / 1 100.0%	3 / 3 100.0%	1 / 1 100.0%	2 / 2 100.0%	1 / 1 100.0%	3 / 3 100.0%	5 / 5 100.0%	3 / 3 100.0%	1 / 1 100.0%	5 / 5 100.0%	0 / 0 0.0%	0 / 0 0.0%	0 / 0 0.0%	0 / 0 0.0%	41 / 62 66.1%AB	21 / 62 33.9%ABE			
1	Pseudoalteromonas sp. SM9913	62 / 62 100.0%	4 / 4 100.0%	1 / 1 100.0%	1 / 1 100.0%	1 / 1 100.0%	6 / 6 100.0%	6 / 6 100.0%	2 / 2 100.0%	2 / 2 100.0%	3 / 3 100.0%	2 / 2 100.0%	6 / 6 100.0%	2 / 2 100.0%	2 / 2 100.0%	1 / 1 100.0%	2 / 2 100.0%	3 / 3 100.0%	1 / 1 100.0%	3 / 3 100.0%	6 / 6 100.0%	2 / 2 100.0%	1 / 1 100.0%	5 / 5 100.0%	0 / 0 0.0%	0 / 0 0.0%	0 / 0 0.0%	0 / 0 0.0%	37 / 62 59.7%AB	25 / 62 40.3%ABE			
1	Colwelliaceae	88 / 88 100.0%	6 / 6 100.0%	5 / 5 100.0%	4 / 4 100.0%	2 / 2 100.0%	5 / 5 100.0%	7 / 7 100.0%	2 / 2 100.0%	2 / 2 100.0%	8 / 8 100.0%	2 / 2 100.0%	5 / 5 100.0%	6 / 6 100.0%	2 / 2 100.0%	2 / 2 100.0%	1 / 1 100.0%	5 / 5 100.0%	2 / 2 100.0%	3 / 3 100.0%	5 / 5 100.0%	6 / 6 100.0%	1 / 1 100.0%	7 / 7 100.0%	0 / 0 0.0%	0 / 0 0.0%	0 / 0 0.0%	0 / 0 0.0%	59 / 88 67.0%AB	29 / 88 33.0%ABE			
1	Colwellia	88 / 88 100.0%	6 / 6 100.0%	5 / 5 100.0%	4 / 4 100.0%	2 / 2 100.0%	5 / 5 100.0%	7 / 7 100.0%	2 / 2 100.0%	2 / 2 100.0%	8 / 8 100.0%	2 / 2 100.0%	5 / 5 100.0%	6 / 6 100.0%	2 / 2 100.0%	2 / 2 100.0%	1 / 1 100.0%	5 / 5 100.0%	2 / 2 100.0%	3 / 3 100.0%	5 / 5 100.0%	6 / 6 100.0%	1 / 1 100.0%	7 / 7 100.0%	0 / 0 0.0%	0 / 0 0.0%	0 / 0 0.0%	0 / 0 0.0%	59 / 88 67.0%AB	29 / 88 33.0%ABE			
1	"Vibrio psychroerythrus" D'Aoust and Kushner 1972	88 / 88 100.0%	6 / 6 100.0%	5 / 5 100.0%	4 / 4 100.0%	2 / 2 100.0%	5 / 5 100.0%	7 / 7 100.0%	2 / 2 100.0%	2 / 2 100.0%	8 / 8 100.0%	2 / 2 100.0%	5 / 5 100.0%	6 / 6 100.0%	2 / 2 100.0%	2 / 2 100.0%	1 / 1 100.0%	5 / 5 100.0%	2 / 2 100.0%	3 / 3 100.0%	5 / 5 100.0%	6 / 6 100.0%	1 / 1 100.0%	7 / 7 100.0%	0 / 0 0.0%	0 / 0 0.0%	0 / 0 0.0%	0 / 0 0.0%	59 / 88 67.0%AB	29 / 88 33.0%ABE			
1	Colwellia psychrerythraea 34H	88 / 88 100.0%	6 / 6 100.0%	5 / 5 100.0%	4 / 4 100.0%	2 / 2 100.0%	5 / 5 100.0%	7 / 7 100.0%	2 / 2 100.0%	2 / 2 100.0%	8 / 8 100.0%	2 / 2 100.0%	5 / 5 100.0%	6 / 6 100.0%	2 / 2 100.0%	2 / 2 100.0%	1 / 1 100.0%	5 / 5 100.0%	2 / 2 100.0%	3 / 3 100.0%	5 / 5 100.0%	6 / 6 100.0%	1 / 1 100.0%	7 / 7 100.0%	0 / 0 0.0%	0 / 0 0.0%	0 / 0 0.0%	0 / 0 0.0%	59 / 88 67.0%AB	29 / 88 33.0%ABE			
24	Shewanellaceae	2522 / 2542 99.2%	149 / 151 98.7%	98 / 98 100.0%	127 / 127 100.0%	69 / 69 100.0%	170 / 170 100.0%	205 / 205 100.0%	48 / 48 100.0%	87 / 105 82.9%	119 / 119 100.0%	26 / 26 100.0%	159 / 159 100.0%	171 / 171 100.0%	69 / 69 100.0%	76 / 76 100.0%	53 / 53 100.0%	98 / 98 100.0%	49 / 49 100.0%	108 / 108 100.0%	221 / 221 100.0%	175 / 175 100.0%	24 / 24 100.0%	211 / 211 100.0%	10 / 10 100.0%	0 / 0 0.0%	0 / 0 0.0%	0 / 0 0.0%	1278 / 2542 50.3%AB	840 / 2542 33.0%ABE	253 / 2542 10.0%B	151 / 2542 5.9%BE	20 / 2542 0.8%E
24	Shewanella	2522 / 2542 99.2%	149 / 151 98.7%	98 / 98 100.0%	127 / 127 100.0%	69 / 69 100.0%	170 / 170 100.0%	205 / 205 100.0%	48 / 48 100.0%	87 / 105 82.9%	119 / 119 100.0%	26 / 26 100.0%	159 / 159 100.0%	171 / 171 100.0%	69 / 69 100.0%	76 / 76 100.0%	53 / 53 100.0%	98 / 98 100.0%	49 / 49 100.0%	108 / 108 100.0%	221 / 221 100.0%	175 / 175 100.0%	24 / 24 100.0%	211 / 211 100.0%	10 / 10 100.0%	0 / 0 0.0%	0 / 0 0.0%	0 / 0 0.0%	1278 / 2542 50.3%AB	840 / 2542 33.0%ABE	253 / 2542 10.0%B	151 / 2542 5.9%BE	20 / 2542 0.8%E
2	"Pseudomonas putrefaciens" (Derby and Hammer) Long and Hammer 1941	201 / 201 100.0%	11 / 11 100.0%	8 / 8 100.0%	10 / 10 100.0%	6 / 6 100.0%	14 / 14 100.0%	15 / 15 100.0%	4 / 4 100.0%	8 / 8 100.0%	10 / 10 100.0%	2 / 2 100.0%	12 / 12 100.0%	11 / 11 100.0%	6 / 6 100.0%	6 / 6 100.0%	4 / 4 100.0%	8 / 8 100.0%	4 / 4 100.0%	10 / 10 100.0%	18 / 18 100.0%	14 / 14 100.0%	2 / 2 100.0%	18 / 18 100.0%	0 / 0 0.0%	0 / 0 0.0%	0 / 0 0.0%	0 / 0 0.0%	122 / 201 60.7%AB	79 / 201 39.3%ABE			
1	Shewanella putrefaciens CN-32	101 / 101 100.0%	5 / 5 100.0%	4 / 4 100.0%	5 / 5 100.0%	3 / 3 100.0%	7 / 7 100.0%	8 / 8 100.0%	2 / 2 100.0%	4 / 4 100.0%	5 / 5 100.0%	1 / 1 100.0%	6 / 6 100.0%	6 / 6 100.0%	3 / 3 100.0%	3 / 3 100.0%	2 / 2 100.0%	4 / 4 100.0%	2 / 2 100.0%	5 / 5 100.0%	9 / 9 100.0%	7 / 7 100.0%	1 / 1 100.0%	9 / 9 100.0%	0 / 0 0.0%	0 / 0 0.0%	0 / 0 0.0%	0 / 0 0.0%	62 / 101 61.4%AB	39 / 101 38.6%ABE			
1	Shewanella putrefaciens 200	100 / 100 100.0%	6 / 6 100.0%	4 / 4 100.0%	5 / 5 100.0%	3 / 3 100.0%	7 / 7 100.0%	7 / 7 100.0%	2 / 2 100.0%	4 / 4 100.0%	5 / 5 100.0%	1 / 1 100.0%	6 / 6 100.0%	5 / 5 100.0%	3 / 3 100.0%	3 / 3 100.0%	2 / 2 100.0%	4 / 4 100.0%	2 / 2 100.0%	5 / 5 100.0%	9 / 9 100.0%	7 / 7 100.0%	1 / 1 100.0%	9 / 9 100.0%	0 / 0 0.0%	0 / 0 0.0%	0 / 0 0.0%	0 / 0 0.0%	60 / 100 60.0%AB	40 / 100 40.0%ABE			
1	ACAM 591	96 / 96 100.0%	6 / 6 100.0%	4 / 4 100.0%	5 / 5 100.0%	4 / 4 100.0%	6 / 6 100.0%	8 / 8 100.0%	2 / 2 100.0%	4 / 4 100.0%	5 / 5 100.0%	1 / 1 100.0%	6 / 6 100.0%	6 / 6 100.0%	2 / 2 100.0%	3 / 3 100.0%	2 / 2 100.0%	5 / 5 100.0%	2 / 2 100.0%	3 / 3 100.0%	7 / 7 100.0%	6 / 6 100.0%	1 / 1 100.0%	8 / 8 100.0%	0 / 0 0.0%	0 / 0 0.0%	0 / 0 0.0%	0 / 0 0.0%	58 / 96 60.4%AB	38 / 96 39.6%ABE			
1	Shewanella frigidimarina NCIMB 400	96 / 96 100.0%	6 / 6 100.0%	4 / 4 100.0%	5 / 5 100.0%	4 / 4 100.0%	6 / 6 100.0%	8 / 8 100.0%	2 / 2 100.0%	4 / 4 100.0%	5 / 5 100.0%	1 / 1 100.0%	6 / 6 100.0%	6 / 6 100.0%	2 / 2 100.0%	3 / 3 100.0%	2 / 2 100.0%	5 / 5 100.0%	2 / 2 100.0%	3 / 3 100.0%	7 / 7 100.0%	6 / 6 100.0%	1 / 1 100.0%	8 / 8 100.0%	0 / 0 0.0%	0 / 0 0.0%	0 / 0 0.0%	0 / 0 0.0%	58 / 96 60.4%AB	38 / 96 39.6%ABE			
1	Barotolerant bacterium DSS12	125 / 125 100.0%	10 / 10 100.0%	4 / 4 100.0%	7 / 7 100.0%	3 / 3 100.0%	7 / 7 100.0%	11 / 11 100.0%	2 / 2 100.0%	8 / 8 100.0%	5 / 5 100.0%	1 / 1 100.0%	8 / 8 100.0%	9 / 9 100.0%	3 / 3 100.0%	3 / 3 100.0%	2 / 2 100.0%	4 / 4 100.0%	2 / 2 100.0%	4 / 4 100.0%	14 / 14 100.0%	8 / 8 100.0%	1 / 1 100.0%	9 / 9 100.0%	0 / 0 0.0%	0 / 0 0.0%	0 / 0 0.0%	0 / 0 0.0%	74 / 125 59.2%AB	51 / 125 40.8%ABE			
1	Shewanella violacea DSS12	125 / 125 100.0%	10 / 10 100.0%	4 / 4 100.0%	7 / 7 100.0%	3 / 3 100.0%	7 / 7 100.0%	11 / 11 100.0%	2 / 2 100.0%	8 / 8 100.0%	5 / 5 100.0%	1 / 1 100.0%	8 / 8 100.0%	9 / 9 100.0%	3 / 3 100.0%	3 / 3 100.0%	2 / 2 100.0%	4 / 4 100.0%	2 / 2 100.0%	4 / 4 100.0%	14 / 14 100.0%	8 / 8 100.0%	1 / 1 100.0%	9 / 9 100.0%	0 / 0 0.0%	0 / 0 0.0%	0 / 0 0.0%	0 / 0 0.0%	74 / 125 59.2%AB	51 / 125 40.8%ABE			
1	ATCC 700329	101 / 101 100.0%	4 / 4 100.0%	4 / 4 100.0%	5 / 5 100.0%	3 / 3 100.0%	9 / 9 100.0%	6 / 6 100.0%	2 / 2 100.0%	4 / 4 100.0%	8 / 8 100.0%	1 / 1 100.0%	5 / 5 100.0%	8 / 8 100.0%	2 / 2 100.0%	3 / 3 100.0%	2 / 2 100.0%	5 / 5 100.0%	2 / 2 100.0%	4 / 4 100.0%	9 / 9 100.0%	7 / 7 100.0%	1 / 1 100.0%	7 / 7 100.0%	0 / 0 0.0%	0 / 0 0.0%	0 / 0 0.0%	0 / 0 0.0%	67 / 101 66.3%AB	34 / 101 33.7%ABE			
1	Shewanella amazonensis SB2B	101 / 101 100.0%	4 / 4 100.0%	4 / 4 100.0%	5 / 5 100.0%	3 / 3 100.0%	9 / 9 100.0%	6 / 6 100.0%	2 / 2 100.0%	4 / 4 100.0%	8 / 8 100.0%	1 / 1 100.0%	5 / 5 100.0%	8 / 8 100.0%	2 / 2 100.0%	3 / 3 100.0%	2 / 2 100.0%	5 / 5 100.0%	2 / 2 100.0%	4 / 4 100.0%	9 / 9 100.0%	7 / 7 100.0%	1 / 1 100.0%	7 / 7 100.0%	0 / 0 0.0%	0 / 0 0.0%	0 / 0 0.0%	0 / 0 0.0%	67 / 101 66.3%AB	34 / 101 33.7%ABE			
1	Shewanella sp. MR-4	101 / 101 100.0%	5 / 5 100.0%	4 / 4 100.0%	5 / 5 100.0%	2 / 2 100.0%	7 / 7 100.0%	8 / 8 100.0%	2 / 2 100.0%	4 / 4 100.0%	4 / 4 100.0%	1 / 1 100.0%	6 / 6 100.0%	7 / 7 100.0%	3 / 3 100.0%	3 / 3 100.0%	2 / 2 100.0%	4 / 4 100.0%	2 / 2 100.0%	5 / 5 100.0%	9 / 9 100.0%	7 / 7 100.0%	1 / 1 100.0%	9 / 9 100.0%	1 / 1 100.0%	0 / 0 0.0%	0 / 0 0.0%	0 / 0 0.0%	61 / 101 60.4%AB	39 / 101 38.6%ABE	1 / 101 1.0%BE		
1	Shewanella sp. MR-7	103 / 103 100.0%	5 / 5 100.0%	4 / 4 100.0%	5 / 5 100.0%	3 / 3 100.0%	7 / 7 100.0%	8 / 8 100.0%	2 / 2 100.0%	4 / 4 100.0%	5 / 5 100.0%	1 / 1 100.0%	6 / 6 100.0%	7 / 7 100.0%	3 / 3 100.0%	3 / 3 100.0%	2 / 2 100.0%	4 / 4 100.0%	2 / 2 100.0%	5 / 5 100.0%	9 / 9 100.0%	7 / 7 100.0%	1 / 1 100.0%	9 / 9 100.0%	1 / 1 100.0%	0 / 0 0.0%	0 / 0 0.0%	0 / 0 0.0%	63 / 103 61.2%AB	39 / 103 37.9%ABE	1 / 103 1.0%BE		
1	ATCC 51908	124 / 125 99.2%	7 / 7 100.0%	5 / 5 100.0%	9 / 9 100.0%	3 / 3 100.0%	7 / 7 100.0%	12 / 12 100.0%	2 / 2 100.0%	4 / 5 80.0%	5 / 5 100.0%	1 / 1 100.0%	8 / 8 100.0%	9 / 9 100.0%	3 / 3 100.0%	4 / 4 100.0%	3 / 3 100.0%	4 / 4 100.0%	2 / 2 100.0%	4 / 4 100.0%	11 / 11 100.0%	11 / 11 100.0%	1 / 1 100.0%	9 / 9 100.0%	0 / 0 0.0%	0 / 0 0.0%	0 / 0 0.0%	0 / 0 0.0%	56 / 125 44.8%B	25 / 125 20.0%BE	24 / 125 19.2%ABE	19 / 125 15.2%AB	1 / 125 0.8%E
1	Shewanella woodyi ATCC 51908	124 / 125 99.2%	7 / 7 100.0%	5 / 5 100.0%	9 / 9 100.0%	3 / 3 100.0%	7 / 7 100.0%	12 / 12 100.0%	2 / 2 100.0%	4 / 5 80.0%	5 / 5 100.0%	1 / 1 100.0%	8 / 8 100.0%	9 / 9 100.0%	3 / 3 100.0%	4 / 4 100.0%	3 / 3 100.0%	4 / 4 100.0%	2 / 2 100.0%	4 / 4 100.0%	11 / 11 100.0%	11 / 11 100.0%	1 / 1 100.0%	9 / 9 100.0%	0 / 0 0.0%	0 / 0 0.0%	0 / 0 0.0%	0 / 0 0.0%	56 / 125 44.8%B	25 / 125 20.0%BE	24 / 125 19.2%ABE	19 / 125 15.2%AB	1 / 125 0.8%E
7	CCUG 39356	715 / 723 98.9%	44 / 46 95.7%	28 / 28 100.0%	31 / 31 100.0%	21 / 21 100.0%	47 / 47 100.0%	53 / 53 100.0%	14 / 14 100.0%	21 / 27 77.8%	35 / 35 100.0%	7 / 7 100.0%	49 / 49 100.0%	49 / 49 100.0%	21 / 21 100.0%	21 / 21 100.0%	14 / 14 100.0%	28 / 28 100.0%	14 / 14 100.0%	35 / 35 100.0%	63 / 63 100.0%	46 / 46 100.0%	7 / 7 100.0%	63 / 63 100.0%	4 / 4 100.0%	0 / 0 0.0%	0 / 0 0.0%	0 / 0 0.0%	370 / 723 51.2%AB	233 / 723 32.2%ABE	62 / 723 8.6%B	50 / 723 6.9%BE	8 / 723 1.1%E
1	Shewanella baltica OS155	104 / 105 99.0%	6 / 7 85.7%	4 / 4 100.0%	4 / 4 100.0%	3 / 3 100.0%	7 / 7 100.0%	9 / 9 100.0%	2 / 2 100.0%	4 / 4 100.0%	5 / 5 100.0%	1 / 1 100.0%	7 / 7 100.0%	7 / 7 100.0%	3 / 3 100.0%	3 / 3 100.0%	2 / 2 100.0%	4 / 4 100.0%	2 / 2 100.0%	5 / 5 100.0%	9 / 9 100.0%	7 / 7 100.0%	1 / 1 100.0%	9 / 9 100.0%	0 / 0 0.0%	0 / 0 0.0%	0 / 0 0.0%	0 / 0 0.0%	63 / 105 60.0%AB	41 / 105 39.0%ABE	1 / 105 1.0%E		
1	Shewanella baltica OS195	101 / 104 97.1%	7 / 7 100.0%	4 / 4 100.0%	5 / 5 100.0%	3 / 3 100.0%	7 / 7 100.0%	6 / 6 100.0%	2 / 2 100.0%	1 / 4 25.0%	5 / 5 100.0%	1 / 1 100.0%	7 / 7 100.0%	7 / 7 100.0%	3 / 3 100.0%	3 / 3 100.0%	2 / 2 100.0%	4 / 4 100.0%	2 / 2 100.0%	5 / 5 100.0%	9 / 9 100.0%	7 / 7 100.0%	1 / 1 100.0%	9 / 9 100.0%	1 / 1 100.0%	0 / 0 0.0%	0 / 0 0.0%	0 / 0 0.0%	31 / 104 29.8%B	30 / 104 28.8%AB	25 / 104 24.0%BE	15 / 104 14.4%ABE	3 / 104 2.9%E
1	Shewanella baltica OS185	103 / 106 97.2%	6 / 6 100.0%	4 / 4 100.0%	5 / 5 100.0%	3 / 3 100.0%	7 / 7 100.0%	9 / 9 100.0%	2 / 2 100.0%	1 / 4 25.0%	5 / 5 100.0%	1 / 1 100.0%	7 / 7 100.0%	7 / 7 100.0%	3 / 3 100.0%	3 / 3 100.0%	2 / 2 100.0%	4 / 4 100.0%	2 / 2 100.0%	5 / 5 100.0%	9 / 9 100.0%	7 / 7 100.0%	1 / 1 100.0%	9 / 9 100.0%	1 / 1 100.0%	0 / 0 0.0%	0 / 0 0.0%	0 / 0 0.0%	33 / 106 31.1%AB	31 / 106 29.2%B	25 / 106 23.6%BE	14 / 106 13.2%ABE	3 / 106 2.8%E
1	Shewanella baltica OS223	107 / 107 100.0%	7 / 7 100.0%	4 / 4 100.0%	5 / 5 100.0%	3 / 3 100.0%	7 / 7 100.0%	9 / 9 100.0%	2 / 2 100.0%	4 / 4 100.0%	5 / 5 100.0%	1 / 1 100.0%	7 / 7 100.0%	7 / 7 100.0%	3 / 3 100.0%	3 / 3 100.0%	2 / 2 100.0%	4 / 4 100.0%	2 / 2 100.0%	5 / 5 100.0%	9 / 9 100.0%	7 / 7 100.0%	1 / 1 100.0%	9 / 9 100.0%	1 / 1 100.0%	0 / 0 0.0%	0 / 0 0.0%	0 / 0 0.0%	64 / 107 59.8%AB	43 / 107 40.2%ABE			
1	Shewanella baltica OS117	92 / 93 98.9%	4 / 5 80.0%	4 / 4 100.0%	4 / 4 100.0%	3 / 3 100.0%	5 / 5 100.0%	4 / 4 100.0%	2 / 2 100.0%	3 / 3 100.0%	5 / 5 100.0%	1 / 1 100.0%	7 / 7 100.0%	7 / 7 100.0%	3 / 3 100.0%	3 / 3 100.0%	2 / 2 100.0%	4 / 4 100.0%	2 / 2 100.0%	5 / 5 100.0%	9 / 9 100.0%	5 / 5 100.0%	1 / 1 100.0%	9 / 9 100.0%	0 / 0 0.0%	0 / 0 0.0%	0 / 0 0.0%	0 / 0 0.0%	56 / 93 60.2%AB	36 / 93 38.7%ABE	1 / 93 1.1%E		
1	Shewanella baltica OS678	102 / 102 100.0%	7 / 7 100.0%	4 / 4 100.0%	3 / 3 100.0%	3 / 3 100.0%	7 / 7 100.0%	7 / 7 100.0%	2 / 2 100.0%	4 / 4 100.0%	5 / 5 100.0%	1 / 1 100.0%	7 / 7 100.0%	7 / 7 100.0%	3 / 3 100.0%	3 / 3 100.0%	2 / 2 100.0%	4 / 4 100.0%	2 / 2 100.0%	5 / 5 100.0%	9 / 9 100.0%	6 / 6 100.0%	1 / 1 100.0%	9 / 9 100.0%	1 / 1 100.0%	0 / 0 0.0%	0 / 0 0.0%	0 / 0 0.0%	60 / 102 58.8%AB	42 / 102 41.2%ABE			
1	Shewanella baltica BA175	106 / 106 100.0%	7 / 7 100.0%	4 / 4 100.0%	5 / 5 100.0%	3 / 3 100.0%	7 / 7 100.0%	9 / 9 100.0%	2 / 2 100.0%	4 / 4 100.0%	5 / 5 100.0%	1 / 1 100.0%	7 / 7 100.0%	7 / 7 100.0%	3 / 3 100.0%	3 / 3 100.0%	2 / 2 100.0%	4 / 4 100.0%	2 / 2 100.0%	5 / 5 100.0%	9 / 9 100.0%	7 / 7 100.0%	1 / 1 100.0%	9 / 9 100.0%	0 / 0 0.0%	0 / 0 0.0%	0 / 0 0.0%	0 / 0 0.0%	64 / 106 60.4%AB	42 / 106 39.6%ABE			
1	ATCC 700550	101 / 101 100.0%	5 / 5 100.0%	4 / 4 100.0%	5 / 5 100.0%	3 / 3 100.0%	7 / 7 100.0%	7 / 7 100.0%	2 / 2 100.0%	4 / 4 100.0%	5 / 5 100.0%	1 / 1 100.0%	6 / 6 100.0%	7 / 7 100.0%	3 / 3 100.0%	3 / 3 100.0%	2 / 2 100.0%	4 / 4 100.0%	2 / 2 100.0%	5 / 5 100.0%	9 / 9 100.0%	6 / 6 100.0%	1 / 1 100.0%	9 / 9 100.0%	1 / 1 100.0%	0 / 0 0.0%	0 / 0 0.0%	0 / 0 0.0%	62 / 101 61.4%AB	38 / 101 37.6%ABE	1 / 101 1.0%BE		
1	Shewanella oneidensis ATCC 700550	101 / 101 100.0%	5 / 5 100.0%	4 / 4 100.0%	5 / 5 100.0%	3 / 3 100.0%	7 / 7 100.0%	7 / 7 100.0%	2 / 2 100.0%	4 / 4 100.0%	5 / 5 100.0%	1 / 1 100.0%	6 / 6 100.0%	7 / 7 100.0%	3 / 3 100.0%	3 / 3 100.0%	2 / 2 100.0%	4 / 4 100.0%	2 / 2 100.0%	5 / 5 100.0%	9 / 9 100.0%	6 / 6 100.0%	1 / 1 100.0%	9 / 9 100.0%	1 / 1 100.0%	0 / 0 0.0%	0 / 0 0.0%	0 / 0 0.0%	62 / 101 61.4%AB	38 / 101 37.6%ABE	1 / 101 1.0%BE		
1	ATCC 700345	143 / 143 100.0%	9 / 9 100.0%	6 / 6 100.0%	7 / 7 100.0%	3 / 3 100.0%	9 / 9 100.0%	13 / 13 100.0%	2 / 2 100.0%	6 / 6 100.0%	5 / 5 100.0%	2 / 2 100.0%	8 / 8 100.0%	12 / 12 100.0%	4 / 4 100.0%	5 / 5 100.0%	4 / 4 100.0%	4 / 4 100.0%	3 / 3 100.0%	5 / 5 100.0%	10 / 10 100.0%	13 / 13 100.0%	1 / 1 100.0%	11 / 11 100.0%	1 / 1 100.0%	0 / 0 0.0%	0 / 0 0.0%	0 / 0 0.0%	54 / 143 37.8%B	38 / 143 26.6%ABE	29 / 143 20.3%AB	22 / 143 15.4%BE	
1	Shewanella pealeana ATCC 700345	143 / 143 100.0%	9 / 9 100.0%	6 / 6 100.0%	7 / 7 100.0%	3 / 3 100.0%	9 / 9 100.0%	13 / 13 100.0%	2 / 2 100.0%	6 / 6 100.0%	5 / 5 100.0%	2 / 2 100.0%	8 / 8 100.0%	12 / 12 100.0%	4 / 4 100.0%	5 / 5 100.0%	4 / 4 100.0%	4 / 4 100.0%	3 / 3 100.0%	5 / 5 100.0%	10 / 10 100.0%	13 / 13 100.0%	1 / 1 100.0%	11 / 11 100.0%	1 / 1 100.0%	0 / 0 0.0%	0 / 0 0.0%	0 / 0 0.0%	54 / 143 37.8%B	38 / 143 26.6%ABE	29 / 143 20.3%AB	22 / 143 15.4%BE	
1	'Shewanella trabarsenatis'	103 / 103 100.0%	4 / 4 100.0%	4 / 4 100.0%	5 / 5 100.0%	3 / 3 100.0%	7 / 7 100.0%	9 / 9 100.0%	2 / 2 100.0%	4 / 4 100.0%	5 / 5 100.0%	1 / 1 100.0%	6 / 6 100.0%	7 / 7 100.0%	3 / 3 100.0%	3 / 3 100.0%	2 / 2 100.0%	4 / 4 100.0%	2 / 2 100.0%	5 / 5 100.0%	9 / 9 100.0%	7 / 7 100.0%	1 / 1 100.0%	9 / 9 100.0%	1 / 1 100.0%	0 / 0 0.0%	0 / 0 0.0%	0 / 0 0.0%	64 / 103 62.1%AB	38 / 103 36.9%ABE	1 / 103 1.0%BE		
1	DSM 15013	92 / 92 100.0%	5 / 5 100.0%	3 / 3 100.0%	5 / 5 100.0%	3 / 3 100.0%	6 / 6 100.0%	8 / 8 100.0%	2 / 2 100.0%	4 / 4 100.0%	5 / 5 100.0%	1 / 1 100.0%	6 / 6 100.0%	6 / 6 100.0%	2 / 2 100.0%	3 / 3 100.0%	2 / 2 100.0%	4 / 4 100.0%	2 / 2 100.0%	3 / 3 100.0%	7 / 7 100.0%	6 / 6 100.0%	1 / 1 100.0%	8 / 8 100.0%	0 / 0 0.0%	0 / 0 0.0%	0 / 0 0.0%	0 / 0 0.0%	54 / 92 58.7%AB	38 / 92 41.3%ABE			
1	Shewanella denitrificans OS-217	92 / 92 100.0%	5 / 5 100.0%	3 / 3 100.0%	5 / 5 100.0%	3 / 3 100.0%	6 / 6 100.0%	8 / 8 100.0%	2 / 2 100.0%	4 / 4 100.0%	5 / 5 100.0%	1 / 1 100.0%	6 / 6 100.0%	6 / 6 100.0%	2 / 2 100.0%	3 / 3 100.0%	2 / 2 100.0%	4 / 4 100.0%	2 / 2 100.0%	3 / 3 100.0%	7 / 7 100.0%	6 / 6 100.0%	1 / 1 100.0%	8 / 8 100.0%	0 / 0 0.0%	0 / 0 0.0%	0 / 0 0.0%	0 / 0 0.0%	54 / 92 58.7%AB	38 / 92 41.3%ABE			
1	DSM 17055	120 / 125 96.0%	9 / 9 100.0%	4 / 4 100.0%	7 / 7 100.0%	3 / 3 100.0%	8 / 8 100.0%	12 / 12 100.0%	2 / 2 100.0%	0 / 5 0.0%	5 / 5 100.0%	1 / 1 100.0%	8 / 8 100.0%	10 / 10 100.0%	3 / 3 100.0%	3 / 3 100.0%	3 / 3 100.0%	4 / 4 100.0%	2 / 2 100.0%	4 / 4 100.0%	12 / 12 100.0%	10 / 10 100.0%	1 / 1 100.0%	9 / 9 100.0%	0 / 0 0.0%	0 / 0 0.0%	0 / 0 0.0%	0 / 0 0.0%	43 / 125 34.4%AB	32 / 125 25.6%B	32 / 125 25.6%BE	13 / 125 10.4%ABE	5 / 125 4.0%E
1	Shewanella sediminis HAW-EB3	120 / 125 96.0%	9 / 9 100.0%	4 / 4 100.0%	7 / 7 100.0%	3 / 3 100.0%	8 / 8 100.0%	12 / 12 100.0%	2 / 2 100.0%	0 / 5 0.0%	5 / 5 100.0%	1 / 1 100.0%	8 / 8 100.0%	10 / 10 100.0%	3 / 3 100.0%	3 / 3 100.0%	3 / 3 100.0%	4 / 4 100.0%	2 / 2 100.0%	4 / 4 100.0%	12 / 12 100.0%	10 / 10 100.0%	1 / 1 100.0%	9 / 9 100.0%	0 / 0 0.0%	0 / 0 0.0%	0 / 0 0.0%	0 / 0 0.0%	43 / 125 34.4%AB	32 / 125 25.6%BE	32 / 125 25.6%B	13 / 125 10.4%ABE	5 / 125 4.0%E
1	DSM 17350	119 / 125 95.2%	9 / 9 100.0%	5 / 5 100.0%	7 / 7 100.0%	3 / 3 100.0%	8 / 8 100.0%	13 / 13 100.0%	2 / 2 100.0%	0 / 6 0.0%	5 / 5 100.0%	2 / 2 100.0%	8 / 8 100.0%	7 / 7 100.0%	4 / 4 100.0%	4 / 4 100.0%	3 / 3 100.0%	4 / 4 100.0%	2 / 2 100.0%	5 / 5 100.0%	10 / 10 100.0%	7 / 7 100.0%	1 / 1 100.0%	9 / 9 100.0%	1 / 1 100.0%	0 / 0 0.0%	0 / 0 0.0%	0 / 0 0.0%	49 / 125 39.2%B	26 / 125 20.8%ABE	26 / 125 20.8%AB	18 / 125 14.4%BE	6 / 125 4.8%E
1	Shewanella halifaxensis HAW-EB4	119 / 125 95.2%	9 / 9 100.0%	5 / 5 100.0%	7 / 7 100.0%	3 / 3 100.0%	8 / 8 100.0%	13 / 13 100.0%	2 / 2 100.0%	0 / 6 0.0%	5 / 5 100.0%	2 / 2 100.0%	8 / 8 100.0%	7 / 7 100.0%	4 / 4 100.0%	4 / 4 100.0%	3 / 3 100.0%	4 / 4 100.0%	2 / 2 100.0%	5 / 5 100.0%	10 / 10 100.0%	7 / 7 100.0%	1 / 1 100.0%	9 / 9 100.0%	1 / 1 100.0%	0 / 0 0.0%	0 / 0 0.0%	0 / 0 0.0%	49 / 125 39.2%B	26 / 125 20.8%ABE	26 / 125 20.8%AB	18 / 125 14.4%BE	6 / 125 4.8%E
1	Shewanella sp. W3-18-1	100 / 100 100.0%	5 / 5 100.0%	4 / 4 100.0%	5 / 5 100.0%	3 / 3 100.0%	7 / 7 100.0%	7 / 7 100.0%	2 / 2 100.0%	4 / 4 100.0%	5 / 5 100.0%	1 / 1 100.0%	6 / 6 100.0%	6 / 6 100.0%	3 / 3 100.0%	3 / 3 100.0%	2 / 2 100.0%	4 / 4 100.0%	2 / 2 100.0%	5 / 5 100.0%	9 / 9 100.0%	7 / 7 100.0%	1 / 1 100.0%	9 / 9 100.0%	0 / 0 0.0%	0 / 0 0.0%	0 / 0 0.0%	0 / 0 0.0%	61 / 100 61.0%AB	39 / 100 39.0%ABE			
1	ATCC BAA-1088	94 / 94 100.0%	5 / 5 100.0%	4 / 4 100.0%	4 / 4 100.0%	3 / 3 100.0%	7 / 7 100.0%	8 / 8 100.0%	2 / 2 100.0%	4 / 4 100.0%	5 / 5 100.0%	1 / 1 100.0%	5 / 5 100.0%	7 / 7 100.0%	2 / 2 100.0%	3 / 3 100.0%	2 / 2 100.0%	4 / 4 100.0%	2 / 2 100.0%	3 / 3 100.0%	7 / 7 100.0%	7 / 7 100.0%	1 / 1 100.0%	8 / 8 100.0%	0 / 0 0.0%	0 / 0 0.0%	0 / 0 0.0%	0 / 0 0.0%	58 / 94 61.7%AB	36 / 94 38.3%ABE			
1	Shewanella loihica PV-4	94 / 94 100.0%	5 / 5 100.0%	4 / 4 100.0%	4 / 4 100.0%	3 / 3 100.0%	7 / 7 100.0%	8 / 8 100.0%	2 / 2 100.0%	4 / 4 100.0%	5 / 5 100.0%	1 / 1 100.0%	5 / 5 100.0%	7 / 7 100.0%	2 / 2 100.0%	3 / 3 100.0%	2 / 2 100.0%	4 / 4 100.0%	2 / 2 100.0%	3 / 3 100.0%	7 / 7 100.0%	7 / 7 100.0%	1 / 1 100.0%	8 / 8 100.0%	0 / 0 0.0%	0 / 0 0.0%	0 / 0 0.0%	0 / 0 0.0%	58 / 94 61.7%AB	36 / 94 38.3%ABE			

1	CGMCC 1.6160	84 / 84 100.0%	6 / 6 100.0%	3 / 3 100.0%	5 / 5 100.0%	0 / 0 0.0%	7 / 7 100.0%	7 / 7 100.0%	2 / 2 100.0%	4 / 4 100.0%	2 / 2 100.0%	1 / 1 100.0%	6 / 6 100.0%	3 / 3 100.0%	2 / 2 100.0%	3 / 3 100.0%	2 / 2 100.0%	4 / 4 100.0%	2 / 2 100.0%	3 / 3 100.0%	8 / 8 100.0%	6 / 6 100.0%	1 / 1 100.0%	7 / 7 100.0%	0 / 0 0.0%	0 / 0 0.0%	0 / 0 0.0%	0 / 0 0.0%	47 / 84 56.0%AB	37 / 84 44.0%ABE				
1	Shewanella piezotolerans WP3	84 / 84 100.0%	6 / 6 100.0%	3 / 3 100.0%	5 / 5 100.0%	0 / 0 0.0%	7 / 7 100.0%	7 / 7 100.0%	2 / 2 100.0%	4 / 4 100.0%	2 / 2 100.0%	1 / 1 100.0%	6 / 6 100.0%	3 / 3 100.0%	2 / 2 100.0%	3 / 3 100.0%	2 / 2 100.0%	4 / 4 100.0%	2 / 2 100.0%	3 / 3 100.0%	8 / 8 100.0%	6 / 6 100.0%	1 / 1 100.0%	7 / 7 100.0%	0 / 0 0.0%	0 / 0 0.0%	0 / 0 0.0%	0 / 0 0.0%	47 / 84 56.0%AB	37 / 84 44.0%ABE				
1	Ferrimonadaceae	121 / 121 100.0%	10 / 10 100.0%	6 / 6 100.0%	4 / 4 100.0%	4 / 4 100.0%	9 / 9 100.0%	10 / 10 100.0%	2 / 2 100.0%	6 / 6 100.0%	9 / 9 100.0%	1 / 1 100.0%	5 / 5 100.0%	9 / 9 100.0%	3 / 3 100.0%	4 / 4 100.0%	2 / 2 100.0%	5 / 5 100.0%	2 / 2 100.0%	4 / 4 100.0%	9 / 9 100.0%	9 / 9 100.0%	1 / 1 100.0%	7 / 7 100.0%	0 / 0 0.0%	0 / 0 0.0%	0 / 0 0.0%	0 / 0 0.0%	76 / 121 62.8%AB	45 / 121 37.2%ABE				
1	Ferrimonas	121 / 121 100.0%	10 / 10 100.0%	6 / 6 100.0%	4 / 4 100.0%	4 / 4 100.0%	9 / 9 100.0%	10 / 10 100.0%	2 / 2 100.0%	6 / 6 100.0%	9 / 9 100.0%	1 / 1 100.0%	5 / 5 100.0%	9 / 9 100.0%	3 / 3 100.0%	4 / 4 100.0%	2 / 2 100.0%	5 / 5 100.0%	2 / 2 100.0%	4 / 4 100.0%	9 / 9 100.0%	9 / 9 100.0%	1 / 1 100.0%	7 / 7 100.0%	0 / 0 0.0%	0 / 0 0.0%	0 / 0 0.0%	0 / 0 0.0%	76 / 121 62.8%AB	45 / 121 37.2%ABE				
1	DSM 9799	121 / 121 100.0%	10 / 10 100.0%	6 / 6 100.0%	4 / 4 100.0%	4 / 4 100.0%	9 / 9 100.0%	10 / 10 100.0%	2 / 2 100.0%	6 / 6 100.0%	9 / 9 100.0%	1 / 1 100.0%	5 / 5 100.0%	9 / 9 100.0%	3 / 3 100.0%	4 / 4 100.0%	2 / 2 100.0%	5 / 5 100.0%	2 / 2 100.0%	4 / 4 100.0%	9 / 9 100.0%	9 / 9 100.0%	1 / 1 100.0%	7 / 7 100.0%	0 / 0 0.0%	0 / 0 0.0%	0 / 0 0.0%	0 / 0 0.0%	76 / 121 62.8%AB	45 / 121 37.2%ABE				
1	Ferrimonas balearica DSM 9799	121 / 121 100.0%	10 / 10 100.0%	6 / 6 100.0%	4 / 4 100.0%	4 / 4 100.0%	9 / 9 100.0%	10 / 10 100.0%	2 / 2 100.0%	6 / 6 100.0%	9 / 9 100.0%	1 / 1 100.0%	5 / 5 100.0%	9 / 9 100.0%	3 / 3 100.0%	4 / 4 100.0%	2 / 2 100.0%	5 / 5 100.0%	2 / 2 100.0%	4 / 4 100.0%	9 / 9 100.0%	9 / 9 100.0%	1 / 1 100.0%	7 / 7 100.0%	0 / 0 0.0%	0 / 0 0.0%	0 / 0 0.0%	0 / 0 0.0%	76 / 121 62.8%AB	45 / 121 37.2%ABE				
2	Idiomarinaceae	111 / 111 100.0%	4 / 4 100.0%	4 / 4 100.0%	4 / 4 100.0%	8 / 8 100.0%	8 / 8 100.0%	6 / 6 100.0%	2 / 2 100.0%	4 / 4 100.0%	10 / 10 100.0%	4 / 4 100.0%	6 / 6 100.0%	4 / 4 100.0%	2 / 2 100.0%	4 / 4 100.0%	2 / 2 100.0%	6 / 6 100.0%	2 / 2 100.0%	4 / 4 100.0%	9 / 9 100.0%	6 / 6 100.0%	2 / 2 100.0%	10 / 10 100.0%	0 / 0 0.0%	0 / 0 0.0%	0 / 0 0.0%	0 / 0 0.0%	75 / 111 67.6%AB	36 / 111 32.4%ABE				
2	Idiomarina	111 / 111 100.0%	4 / 4 100.0%	4 / 4 100.0%	4 / 4 100.0%	8 / 8 100.0%	8 / 8 100.0%	6 / 6 100.0%	2 / 2 100.0%	4 / 4 100.0%	10 / 10 100.0%	4 / 4 100.0%	6 / 6 100.0%	4 / 4 100.0%	2 / 2 100.0%	4 / 4 100.0%	2 / 2 100.0%	6 / 6 100.0%	2 / 2 100.0%	4 / 4 100.0%	9 / 9 100.0%	6 / 6 100.0%	2 / 2 100.0%	10 / 10 100.0%	0 / 0 0.0%	0 / 0 0.0%	0 / 0 0.0%	0 / 0 0.0%	75 / 111 67.6%AB	36 / 111 32.4%ABE				
2	ATCC BAA-735	111 / 111 100.0%	4 / 4 100.0%	4 / 4 100.0%	4 / 4 100.0%	8 / 8 100.0%	8 / 8 100.0%	6 / 6 100.0%	2 / 2 100.0%	4 / 4 100.0%	10 / 10 100.0%	4 / 4 100.0%	6 / 6 100.0%	4 / 4 100.0%	2 / 2 100.0%	4 / 4 100.0%	2 / 2 100.0%	6 / 6 100.0%	2 / 2 100.0%	4 / 4 100.0%	9 / 9 100.0%	6 / 6 100.0%	2 / 2 100.0%	10 / 10 100.0%	0 / 0 0.0%	0 / 0 0.0%	0 / 0 0.0%	0 / 0 0.0%	75 / 111 67.6%AB	36 / 111 32.4%ABE				
1	Idiomarina loihiensis L2TR	56 / 56 100.0%	2 / 2 100.0%	2 / 2 100.0%	2 / 2 100.0%	4 / 4 100.0%	4 / 4 100.0%	3 / 3 100.0%	1 / 1 100.0%	2 / 2 100.0%	5 / 5 100.0%	2 / 2 100.0%	3 / 3 100.0%	2 / 2 100.0%	1 / 1 100.0%	2 / 2 100.0%	1 / 1 100.0%	3 / 3 100.0%	1 / 1 100.0%	2 / 2 100.0%	5 / 5 100.0%	3 / 3 100.0%	1 / 1 100.0%	5 / 5 100.0%	0 / 0 0.0%	0 / 0 0.0%	0 / 0 0.0%	0 / 0 0.0%	38 / 56 67.9%AB	18 / 56 32.1%ABE				
1	Idiomarina loihiensis GSL 199	55 / 55 100.0%	2 / 2 100.0%	2 / 2 100.0%	2 / 2 100.0%	4 / 4 100.0%	4 / 4 100.0%	3 / 3 100.0%	1 / 1 100.0%	2 / 2 100.0%	5 / 5 100.0%	2 / 2 100.0%	3 / 3 100.0%	2 / 2 100.0%	1 / 1 100.0%	2 / 2 100.0%	1 / 1 100.0%	3 / 3 100.0%	1 / 1 100.0%	2 / 2 100.0%	4 / 4 100.0%	3 / 3 100.0%	1 / 1 100.0%	5 / 5 100.0%	0 / 0 0.0%	0 / 0 0.0%	0 / 0 0.0%	0 / 0 0.0%	37 / 55 67.3%AB	18 / 55 32.7%ABE				
2	Psychromonadaceae	173 / 173 100.0%	13 / 13 100.0%	10 / 10 100.0%	10 / 10 100.0%	6 / 6 100.0%	9 / 9 100.0%	14 / 14 100.0%	4 / 4 100.0%	4 / 4 100.0%	8 / 8 100.0%	2 / 2 100.0%	10 / 10 100.0%	11 / 11 100.0%	4 / 4 100.0%	5 / 5 100.0%	2 / 2 100.0%	8 / 8 100.0%	4 / 4 100.0%	6 / 6 100.0%	12 / 12 100.0%	11 / 11 100.0%	2 / 2 100.0%	18 / 18 100.0%	0 / 0 0.0%	0 / 0 0.0%	0 / 0 0.0%	0 / 0 0.0%	106 / 173 61.3%AB	67 / 173 38.7%ABE				
2	Psychromonas	173 / 173 100.0%	13 / 13 100.0%	10 / 10 100.0%	10 / 10 100.0%	6 / 6 100.0%	9 / 9 100.0%	14 / 14 100.0%	4 / 4 100.0%	4 / 4 100.0%	8 / 8 100.0%	2 / 2 100.0%	10 / 10 100.0%	11 / 11 100.0%	4 / 4 100.0%	5 / 5 100.0%	2 / 2 100.0%	8 / 8 100.0%	4 / 4 100.0%	6 / 6 100.0%	12 / 12 100.0%	11 / 11 100.0%	2 / 2 100.0%	18 / 18 100.0%	0 / 0 0.0%	0 / 0 0.0%	0 / 0 0.0%	0 / 0 0.0%	106 / 173 61.3%AB	67 / 173 38.7%ABE				
1	Psychromonas sp. CNPT3	88 / 88 100.0%	6 / 6 100.0%	5 / 5 100.0%	6 / 6 100.0%	3 / 3 100.0%	4 / 4 100.0%	6 / 6 100.0%	2 / 2 100.0%	2 / 2 100.0%	4 / 4 100.0%	1 / 1 100.0%	5 / 5 100.0%	6 / 6 100.0%	2 / 2 100.0%	3 / 3 100.0%	1 / 1 100.0%	4 / 4 100.0%	2 / 2 100.0%	3 / 3 100.0%	6 / 6 100.0%	7 / 7 100.0%	1 / 1 100.0%	9 / 9 100.0%	0 / 0 0.0%	0 / 0 0.0%	0 / 0 0.0%	0 / 0 0.0%	53 / 88 60.2%AB	35 / 88 39.8%ABE				
1	"Psychromonas ingrahamii" Breezee et al. 2004	85 / 85 100.0%	7 / 7 100.0%	5 / 5 100.0%	4 / 4 100.0%	3 / 3 100.0%	5 / 5 100.0%	8 / 8 100.0%	2 / 2 100.0%	2 / 2 100.0%	4 / 4 100.0%	1 / 1 100.0%	5 / 5 100.0%	5 / 5 100.0%	2 / 2 100.0%	2 / 2 100.0%	1 / 1 100.0%	4 / 4 100.0%	2 / 2 100.0%	3 / 3 100.0%	6 / 6 100.0%	4 / 4 100.0%	1 / 1 100.0%	9 / 9 100.0%	0 / 0 0.0%	0 / 0 0.0%	0 / 0 0.0%	0 / 0 0.0%	53 / 85 62.4%AB	32 / 85 37.6%ABE				
1	Psychromonas ingrahamii 37	85 / 85 100.0%	7 / 7 100.0%	5 / 5 100.0%	4 / 4 100.0%	3 / 3 100.0%	5 / 5 100.0%	8 / 8 100.0%	2 / 2 100.0%	2 / 2 100.0%	4 / 4 100.0%	1 / 1 100.0%	5 / 5 100.0%	5 / 5 100.0%	2 / 2 100.0%	2 / 2 100.0%	1 / 1 100.0%	4 / 4 100.0%	2 / 2 100.0%	3 / 3 100.0%	6 / 6 100.0%	4 / 4 100.0%	1 / 1 100.0%	9 / 9 100.0%	0 / 0 0.0%	0 / 0 0.0%	0 / 0 0.0%	0 / 0 0.0%	53 / 85 62.4%AB	32 / 85 37.6%ABE				
21	'Vibrionales'	2267 / 2273 99.7%	106 / 106 100.0%	125 / 125 100.0%	96 / 96 100.0%	59 / 59 100.0%	205 / 207 99.0%	108 / 108 100.0%	41 / 41 100.0%	101 / 105 96.2%	103 / 103 100.0%	39 / 39 100.0%	141 / 141 100.0%	67 / 67 100.0%	71 / 71 100.0%	97 / 97 100.0%	68 / 68 100.0%	76 / 76 100.0%	67 / 67 100.0%	133 / 133 100.0%	187 / 187 100.0%	95 / 95 100.0%	22 / 22 100.0%	260 / 260 100.0%	0 / 0 0.0%	0 / 0 0.0%	0 / 0 0.0%	0 / 0 0.0%	1230 / 2273 54.1%AB	876 / 2273 38.5%ABE	95 / 2273 4.2%B	66 / 2273 2.9%BE	4 / 2273 0.2%E	2 / 2273 0.1%
21	Vibrionaceae	2267 / 2273 99.7%	106 / 106 100.0%	125 / 125 100.0%	96 / 96 100.0%	59 / 59 100.0%	205 / 207 99.0%	108 / 108 100.0%	41 / 41 100.0%	101 / 105 96.2%	103 / 103 100.0%	39 / 39 100.0%	141 / 141 100.0%	67 / 67 100.0%	71 / 71 100.0%	97 / 97 100.0%	68 / 68 100.0%	76 / 76 100.0%	67 / 67 100.0%	133 / 133 100.0%	187 / 187 100.0%	95 / 95 100.0%	22 / 22 100.0%	260 / 260 100.0%	0 / 0 0.0%	0 / 0 0.0%	0 / 0 0.0%	0 / 0 0.0%	1230 / 2273 54.1%AB	876 / 2273 38.5%ABE	95 / 2273 4.2%B	66 / 2273 2.9%BE	4 / 2273 0.2%E	2 / 2273 0.1%
1	"Photobacter" Beijerinck 1900	162 / 162 100.0%	8 / 8 100.0%	9 / 9 100.0%	4 / 4 100.0%	3 / 3 100.0%	14 / 14 100.0%	11 / 11 100.0%	2 / 2 100.0%	8 / 8 100.0%	7 / 7 100.0%	3 / 3 100.0%	15 / 15 100.0%	6 / 6 100.0%	3 / 3 100.0%	7 / 7 100.0%	4 / 4 100.0%	6 / 6 100.0%	5 / 5 100.0%	7 / 7 100.0%	13 / 13 100.0%	7 / 7 100.0%	2 / 2 100.0%	18 / 18 100.0%	0 / 0 0.0%	0 / 0 0.0%	0 / 0 0.0%	0 / 0 0.0%	90 / 162 55.6%AB	72 / 162 44.4%ABE				
1	CIP 106289	162 / 162 100.0%	8 / 8 100.0%	9 / 9 100.0%	4 / 4 100.0%	3 / 3 100.0%	14 / 14 100.0%	11 / 11 100.0%	2 / 2 100.0%	8 / 8 100.0%	7 / 7 100.0%	3 / 3 100.0%	15 / 15 100.0%	6 / 6 100.0%	3 / 3 100.0%	7 / 7 100.0%	4 / 4 100.0%	6 / 6 100.0%	5 / 5 100.0%	7 / 7 100.0%	13 / 13 100.0%	7 / 7 100.0%	2 / 2 100.0%	18 / 18 100.0%	0 / 0 0.0%	0 / 0 0.0%	0 / 0 0.0%	0 / 0 0.0%	90 / 162 55.6%AB	72 / 162 44.4%ABE				
17	"Microspira" Schroeter 1886	1763 / 1767 99.8%	84 / 84 100.0%	94 / 94 100.0%	73 / 73 100.0%	49 / 49 100.0%	158 / 160 98.8%	81 / 81 100.0%	33 / 33 100.0%	78 / 80 97.5%	83 / 83 100.0%	27 / 27 100.0%	100 / 100 100.0%	50 / 50 100.0%	56 / 56 100.0%	75 / 75 100.0%	57 / 57 100.0%	61 / 61 100.0%	53 / 53 100.0%	104 / 104 100.0%	149 / 149 100.0%	73 / 73 100.0%	17 / 17 100.0%	208 / 208 100.0%	0 / 0 0.0%	0 / 0 0.0%	0 / 0 0.0%	0 / 0 0.0%	984 / 1767 55.7%AB	702 / 1767 39.7%ABE	44 / 1767 2.5%B	33 / 1767 1.9%BE	2 / 1767 0.1%	2 / 1767 0.1%E
7	"Bacillo virgola del Koch" Trevisan 1884	649 / 650 99.8%	34 / 34 100.0%	35 / 35 100.0%	26 / 26 100.0%	18 / 18 100.0%	55 / 56 98.2%	28 / 28 100.0%	13 / 13 100.0%	32 / 32 100.0%	29 / 29 100.0%	7 / 7 100.0%	35 / 35 100.0%	16 / 16 100.0%	18 / 18 100.0%	30 / 30 100.0%	21 / 21 100.0%	26 / 26 100.0%	19 / 19 100.0%	39 / 39 100.0%	56 / 56 100.0%	28 / 28 100.0%	7 / 7 100.0%	77 / 77 100.0%	0 / 0 0.0%	0 / 0 0.0%	0 / 0 0.0%	0 / 0 0.0%	372 / 650 57.2%AB	277 / 650 42.6%ABE	1 / 650 0.2%			
4	Vibrio cholerae 01	370 / 370 100.0%	20 / 20 100.0%	20 / 20 100.0%	16 / 16 100.0%	10 / 10 100.0%	32 / 32 100.0%	17 / 17 100.0%	7 / 7 100.0%	17 / 17 100.0%	15 / 15 100.0%	4 / 4 100.0%	20 / 20 100.0%	9 / 9 100.0%	11 / 11 100.0%	16 / 16 100.0%	12 / 12 100.0%	15 / 15 100.0%	10 / 10 100.0%	24 / 24 100.0%	32 / 32 100.0%	16 / 16 100.0%	4 / 4 100.0%	43 / 43 100.0%	0 / 0 0.0%	0 / 0 0.0%	0 / 0 0.0%	0 / 0 0.0%	214 / 370 57.8%AB	156 / 370 42.2%ABE				
1	'Vibrio eltor'	97 / 97 100.0%	5 / 5 100.0%	5 / 5 100.0%	4 / 4 100.0%	3 / 3 100.0%	8 / 8 100.0%	4 / 4 100.0%	2 / 2 100.0%	5 / 5 100.0%	4 / 4 100.0%	1 / 1 100.0%	5 / 5 100.0%	2 / 2 100.0%	3 / 3 100.0%	5 / 5 100.0%	3 / 3 100.0%	4 / 4 100.0%	3 / 3 100.0%	6 / 6 100.0%	8 / 8 100.0%	4 / 4 100.0%	1 / 1 100.0%	12 / 12 100.0%	0 / 0 0.0%	0 / 0 0.0%	0 / 0 0.0%	0 / 0 0.0%	55 / 97 56.7%AB	42 / 97 43.3%ABE				
1	Vibrio cholerae El Tor N16961	97 / 97 100.0%	5 / 5 100.0%	5 / 5 100.0%	4 / 4 100.0%	3 / 3 100.0%	8 / 8 100.0%	4 / 4 100.0%	2 / 2 100.0%	5 / 5 100.0%	4 / 4 100.0%	1 / 1 100.0%	5 / 5 100.0%	2 / 2 100.0%	3 / 3 100.0%	5 / 5 100.0%	3 / 3 100.0%	4 / 4 100.0%	3 / 3 100.0%	6 / 6 100.0%	8 / 8 100.0%	4 / 4 100.0%	1 / 1 100.0%	12 / 12 100.0%	0 / 0 0.0%	0 / 0 0.0%	0 / 0 0.0%	0 / 0 0.0%	55 / 97 56.7%AB	42 / 97 43.3%ABE				
1	Vibrio cholerae MJ-1236	98 / 98 100.0%	5 / 5 100.0%	5 / 5 100.0%	4 / 4 100.0%	2 / 2 100.0%	8 / 8 100.0%	5 / 5 100.0%	2 / 2 100.0%	5 / 5 100.0%	4 / 4 100.0%	1 / 1 100.0%	5 / 5 100.0%	3 / 3 100.0%	3 / 3 100.0%	5 / 5 100.0%	3 / 3 100.0%	4 / 4 100.0%	3 / 3 100.0%	6 / 6 100.0%	8 / 8 100.0%	4 / 4 100.0%	1 / 1 100.0%	12 / 12 100.0%	0 / 0 0.0%	0 / 0 0.0%	0 / 0 0.0%	0 / 0 0.0%	56 / 98 57.1%AB	42 / 98 42.9%ABE				
1	Vibrio cholerae 2010EL-1786	78 / 78 100.0%	5 / 5 100.0%	5 / 5 100.0%	4 / 4 100.0%	2 / 2 100.0%	8 / 8 100.0%	4 / 4 100.0%	1 / 1 100.0%	2 / 2 100.0%	3 / 3 100.0%	1 / 1 100.0%	5 / 5 100.0%	2 / 2 100.0%	2 / 2 100.0%	1 / 1 100.0%	3 / 3 100.0%	3 / 3 100.0%	1 / 1 100.0%	6 / 6 100.0%	8 / 8 100.0%	4 / 4 100.0%	1 / 1 100.0%	7 / 7 100.0%	0 / 0 0.0%	0 / 0 0.0%	0 / 0 0.0%	0 / 0 0.0%	48 / 78 61.5%AB	30 / 78 38.5%ABE				
1	Vibrio cholerae IEC224	97 / 97 100.0%	5 / 5 100.0%	5 / 5 100.0%	4 / 4 100.0%	3 / 3 100.0%	8 / 8 100.0%	4 / 4 100.0%	2 / 2 100.0%	5 / 5 100.0%	4 / 4 100.0%	1 / 1 100.0%	5 / 5 100.0%	2 / 2 100.0%	3 / 3 100.0%	5 / 5 100.0%	3 / 3 100.0%	4 / 4 100.0%	3 / 3 100.0%	6 / 6 100.0%	8 / 8 100.0%	4 / 4 100.0%	1 / 1 100.0%	12 / 12 100.0%	0 / 0 0.0%	0 / 0 0.0%	0 / 0 0.0%	0 / 0 0.0%	55 / 97 56.7%AB	42 / 97 43.3%ABE				
1	Vibrio cholerae O395	96 / 96 100.0%	5 / 5 100.0%	5 / 5 100.0%	4 / 4 100.0%	3 / 3 100.0%	8 / 8 100.0%	4 / 4 100.0%	2 / 2 100.0%	5 / 5 100.0%	5 / 5 100.0%	1 / 1 100.0%	5 / 5 100.0%	2 / 2 100.0%	3 / 3 100.0%	4 / 4 100.0%	3 / 3 100.0%	4 / 4 100.0%	3 / 3 100.0%	6 / 6 100.0%	8 / 8 100.0%	4 / 4 100.0%	1 / 1 100.0%	11 / 11 100.0%	0 / 0 0.0%	0 / 0 0.0%	0 / 0 0.0%	0 / 0 0.0%	56 / 96 58.3%AB	40 / 96 41.7%ABE				
1	Vibrio cholerae M66-2	97 / 97 100.0%	5 / 5 100.0%	5 / 5 100.0%	4 / 4 100.0%	3 / 3 100.0%	8 / 8 100.0%	4 / 4 100.0%	2 / 2 100.0%	5 / 5 100.0%	5 / 5 100.0%	1 / 1 100.0%	5 / 5 100.0%	2 / 2 100.0%	3 / 3 100.0%	5 / 5 100.0%	3 / 3 100.0%	4 / 4 100.0%	3 / 3 100.0%	6 / 6 100.0%	8 / 8 100.0%	3 / 3 100.0%	1 / 1 100.0%	12 / 12 100.0%	0 / 0 0.0%	0 / 0 0.0%	0 / 0 0.0%	0 / 0 0.0%	56 / 97 57.7%AB	41 / 97 42.3%ABE				
1	Vibrio cholerae LMA3894-4	86 / 87 98.9%	4 / 4 100.0%	5 / 5 100.0%	2 / 2 100.0%	2 / 2 100.0%	7 / 8 87.5%	3 / 3 100.0%	2 / 2 100.0%	5 / 5 100.0%	4 / 4 100.0%	1 / 1 100.0%	5 / 5 100.0%	3 / 3 100.0%	1 / 1 100.0%	5 / 5 100.0%	3 / 3 100.0%	3 / 3 100.0%	3 / 3 100.0%	3 / 3 100.0%	8 / 8 100.0%	5 / 5 100.0%	1 / 1 100.0%	11 / 11 100.0%	0 / 0 0.0%	0 / 0 0.0%	0 / 0 0.0%	0 / 0 0.0%	46 / 87 52.9%AB	40 / 87 46.0%ABE	1 / 87 1.1%			
3	ATCC 27562	331 / 333 99.4%	17 / 17 100.0%	19 / 19 100.0%	15 / 15 100.0%	13 / 13 100.0%	28 / 29 96.6%	16 / 16 100.0%	6 / 6 100.0%	9 / 10 90.0%	18 / 18 100.0%	6 / 6 100.0%	20 / 20 100.0%	11 / 11 100.0%	12 / 12 100.0%	14 / 14 100.0%	9 / 9 100.0%	12 / 12 100.0%	9 / 9 100.0%	18 / 18 100.0%	27 / 27 100.0%	13 / 13 100.0%	3 / 3 100.0%	36 / 36 100.0%	0 / 0 0.0%	0 / 0 0.0%	0 / 0 0.0%	0 / 0 0.0%	200 / 333 60.1%AB	131 / 333 39.3%ABE	1 / 333 0.3%	1 / 333 0.3%E		
1	Vibrio vulnificus YJ016	111 / 112 99.1%	6 / 6 100.0%	6 / 6 100.0%	5 / 5 100.0%	2 / 2 100.0%	10 / 10 100.0%	7 / 7 100.0%	2 / 2 100.0%	1 / 2 50.0%	4 / 4 100.0%	2 / 2 100.0%	7 / 7 100.0%	5 / 5 100.0%	4 / 4 100.0%	5 / 5 100.0%	3 / 3 100.0%	4 / 4 100.0%	3 / 3 100.0%	6 / 6 100.0%	9 / 9 100.0%	7 / 7 100.0%	1 / 1 100.0%	12 / 12 100.0%	0 / 0 0.0%	0 / 0 0.0%	0 / 0 0.0%	0 / 0 0.0%	66 / 112 58.9%AB	45 / 112 40.2%ABE	1 / 112 0.9%E			
1	Vibrio vulnificus CMCP6	110 / 111 99.1%	5 / 5 100.0%	6 / 6 100.0%	5 / 5 100.0%	3 / 3 100.0%	8 / 9 88.9%	6 / 6 100.0%	2 / 2 100.0%	4 / 4 100.0%	6 / 6 100.0%	2 / 2 100.0%	7 / 7 100.0%	5 / 5 100.0%	4 / 4 100.0%	4 / 4 100.0%	3 / 3 100.0%	4 / 4 100.0%	3 / 3 100.0%	6 / 6 100.0%	9 / 9 100.0%	5 / 5 100.0%	1 / 1 100.0%	12 / 12 100.0%	0 / 0 0.0%	0 / 0 0.0%	0 / 0 0.0%	0 / 0 0.0%	65 / 111 58.6%AB	45 / 111 40.5%ABE	1 / 111 0.9%			
1	Vibrio vulnificus M06-24/O	110 / 110 100.0%	6 / 6 100.0%	7 / 7 100.0%	5 / 5 100.0%	8 / 8 100.0%	10 / 10 100.0%	3 / 3 100.0%	2 / 2 100.0%	4 / 4 100.0%	8 / 8 100.0%	2 / 2 100.0%	6 / 6 100.0%	1 / 1 100.0%	4 / 4 100.0%	5 / 5 100.0%	3 / 3 100.0%	4 / 4 100.0%	3 / 3 100.0%	6 / 6 100.0%	9 / 9 100.0%	1 / 1 100.0%	1 / 1 100.0%	12 / 12 100.0%	0 / 0 0.0%	0 / 0 0.0%	0 / 0 0.0%	0 / 0 0.0%	69 / 110 62.7%AB	41 / 110 37.3%ABE				
1	ATCC 35016	98 / 98 100.0%	4 / 4 100.0%	5 / 5 100.0%	4 / 4 100.0%	3 / 3 100.0%	10 / 10 100.0%	4 / 4 100.0%	2 / 2 100.0%	5 / 5 100.0%	4 / 4 100.0%	2 / 2 100.0%	5 / 5 100.0%	2 / 2 100.0%	3 / 3 100.0%	5 / 5 100.0%	3 / 3 100.0%	4 / 4 100.0%	3 / 3 100.0%	6 / 6 100.0%	7 / 7 100.0%	3 / 3 100.0%	1 / 1 100.0%	13 / 13 100.0%	0 / 0 0.0%	0 / 0 0.0%	0 / 0 0.0%	0 / 0 0.0%	56 / 98 57.1%AB	42 / 98 42.9%ABE				
1	Vibrio furnissii NCTC 11218	98 / 98 100.0%	4 / 4 100.0%	5 / 5 100.0%	4 / 4 100.0%	3 / 3 100.0%	10 / 10 100.0%	4 / 4 100.0%	2 / 2 100.0%	5 / 5 100.0%	4 / 4 100.0%	2 / 2 100.0%	5 / 5 100.0%	2 / 2 100.0%	3 / 3 100.0%	5 / 5 100.0%	3 / 3 100.0%	4 / 4 100.0%	3 / 3 100.0%	6 / 6 100.0%	7 / 7 100.0%	3 / 3 100.0%	1 / 1 100.0%	13 / 13 100.0%	0 / 0 0.0%	0 / 0 0.0%	0 / 0 0.0%	0 / 0 0.0%	56 / 98 57.1%AB	42 / 98 42.9%ABE				
1	"Achromobacter ichthyodermis" Wells and ZoBell 1934	84 / 85 98.8%	1 / 1 100.0%	5 / 5 100.0%	4 / 4 100.0%	1 / 1 100.0%	5 / 5 100.0%	4 / 4 100.0%	2 / 2 100.0%	4 / 5 80.0%	4 / 4 100.0%	2 / 2 100.0%	6 / 6 100.0%	2 / 2 100.0%	3 / 3 100.0%	4 / 4 100.0%	2 / 2 100.0%	4 / 4 100.0%	3 / 3 100.0%	6 / 6 100.0%	7 / 7 100.0%	3 / 3 100.0%	1 / 1 100.0%	11 / 11 100.0%	0 / 0 0.0%	0 / 0 0.0%	0 / 0 0.0%	0 / 0 0.0%	50 / 85 58.8%AB	34 / 85 40.0%ABE	1 / 85 1.2%E			
1	Listonella anguillarum serovar O1	84 / 85 98.8%	1 / 1 100.0%	5 / 5 100.0%	4 / 4 100.0%	1 / 1 100.0%	5 / 5 100.0%	4 / 4 100.0%	2 / 2 100.0%	4 / 5 80.0%	4 / 4 100.0%	2 / 2 100.0%	6 / 6 100.0%	2 / 2 100.0%	3 / 3 100.0%	4 / 4 100.0%	2 / 2 100.0%	4 / 4 100.0%	3 / 3 100.0%	6 / 6 100.0%	7 / 7 100.0%	3 / 3 100.0%	1 / 1 100.0%	11 / 11 100.0%	0 / 0 0.0%	0 / 0 0.0%	0 / 0 0.0%	0 / 0 0.0%	50 / 85 58.8%AB	34 / 85 40.0%ABE	1 / 85 1.2%E			
1	Listonella anguillarum 775	84 / 85 98.8%	1 / 1 100.0%	5 / 5 100.0%	4 / 4 100.0%	1 / 1 100.0%	5 / 5 100.0%	4 / 4 100.0%	2 / 2 100.0%	4 / 5 80.0%	4 / 4 100.0%	2 / 2 100.0%	6 / 6 100.0%	2 / 2 100.0%	3 / 3 100.0%	4 / 4 100.0%	2 / 2 100.0%	4 / 4 100.0%	3 / 3 100.0%	6 / 6 100.0%	7 / 7 100.0%	3 / 3 100.0%	1 / 1 100.0%	11 / 11 100.0%	0 / 0 0.0%	0 / 0 0.0%	0 / 0 0.0%	0 / 0 0.0%	50 / 85 58.8%AB	34 / 85 40.0%ABE	1 / 85 1.2%E			
1	Vibrio sp. BV25Ex	124 / 124 100.0%	6 / 6 100.0%	6 / 6 100.0%	5 / 5 100.0%	3 / 3 100.0%	12 / 12 100.0%	6 / 6 100.0%	2 / 2 100.0%	4 / 4 100.0%	5 / 5 100.0%	2 / 2 100.0%	7 / 7 100.0%	4 / 4 100.0%	4 / 4 100.0%	6 / 6 100.0%	4 / 4 100.0%	3 / 3 100.0%	4 / 4 100.0%	7 / 7 100.0%	11 / 11 100.0%	6 / 6 100.0%	1 / 1 100.0%	16 / 16 100.0%	0 / 0 0.0%	0 / 0 0.0%	0 / 0 0.0%	0 / 0 0.0%	71 / 124 57.3%AB	53 / 124 42.7%ABE				
1	CAIM 634	113 / 113 100.0%	4 / 4 100.0%	6 / 6 100.0%	5 / 5 100.0%	1 / 1 100.0%	11 / 11 100.0%	5 / 5 100.0%	2 / 2 100.0%	8 / 8 100.0%	4 / 4 100.0%	2 / 2 100.0%	7 / 7 100.0%	3 / 3 100.0%	4 / 4 100.0%	4 / 4 100.0%	5 / 5 100.0%	3 / 3 100.0%	4 / 4 100.0%	7 / 7 100.0%	10 / 10 100.0%	3 / 3 100.0%	1 / 1 100.0%	14 / 14 100.0%	0 / 0 0.0%	0 / 0 0.0%	0 / 0 0.0%	0 / 0 0.0%	65 / 113 57.5%AB	48 / 113 42.5%ABE				
1	Vibrio lentus MEL32	113 / 113 100.0%	4 / 4 100.0%	6 / 6 100.0%	5 / 5 100.0%	1 / 1 100.0%	11 / 11 100.0%	5 / 5 100.0%	2 / 2 100.0%	8 / 8 100.0%	4 / 4 100.0%	2 / 2 100.0%	7 / 7 100.0%	3 / 3 100.0%	4 / 4 100.0%	4 / 4 100.0%	5 / 5 100.0%	3 / 3 100.0%	4 / 4 100.0%	7 / 7 100.0%	10 / 10 100.0%	3 / 3 100.0%	1 / 1 100.0%	14 / 14 100.0%	0 / 0 0.0%	0 / 0 0.0%	0 / 0 0.0%	0 / 0 0.0%	65 / 113 57.5%AB	48 / 113 42.5%ABE				
2	Vibrio harveyi clade	246 / 246 100.0%	12 / 12 100.0%	12 / 12 100.0%	9 / 9 100.0%	7 / 7 100.0%	27 / 27 100.0%	12 / 12 100.0%	4 / 4 100.0%	12 / 12 100.0%	13 / 13 100.0%	4 / 4 100.0%	13 / 13 100.0%	8 / 8 100.0%	8 / 8 100.0%	8 / 8 100.0%	9 / 9 100.0%	6 / 6 100.0%	7 / 7 100.0%	14 / 14 100.0%	21 / 21 100.0%	11 / 11 100.0%	2 / 2 100.0%	27 / 27 100.0%	0 / 0 0.0%	0 / 0 0.0%	0 / 0 0.0%	0 / 0 0.0%	100 / 246 40.7%AB	69 / 246 28.0%ABE	44 / 246 17.9%B	33 / 246 13.4%BE		
1	"Oceanomonas parahaemolytica" (Fujino et al. 1951) Miyamoto et al. 1961	126 / 126 100.0%	6 / 6 100.0%	6 / 6 100.0%	5 / 5 100.0%	2 / 2 100.0%	13 / 13 100.0%	6 / 6 100.0%	2 / 2 100.0%	7 / 7 100.0%	5 / 5 100.0%	2 / 2 100.0%	6 / 6 100.0%	4 / 4 100.0%	4 / 4 100.0%	6 / 6 100.0%	4 / 4 100.0%	3 / 3 100.0%	4 / 4 100.0%	7 / 7 100.0%	11 / 11 100.0%	6 / 6 100.0%	1 / 1 100.0%	16 / 16 100.0%	0 / 0 0.0%	0 / 0 0.0%	0 / 0 0.0%	0 / 0 0.0%	71 / 126 56.3%AB	55 / 126 43.7%ABE				
1	Vibrio parahaemolyticus RIMD 2210633	126 / 126 100.0%	6 / 6 100.0%	6 / 6 100.0%	5 / 5 100.0%	2 / 2 100.0%	13 / 13 100.0%	6 / 6 100.0%	2 / 2 100.0%	7 / 7 100.0%	5 / 5 100.0%	2 / 2 100.0%	6 / 6 100.0%	4 / 4 100.0%	4 / 4 100.0%	6 / 6 100.0%	4 / 4 100.0%	3 / 3 100.0%	4 / 4 100.0%	7 / 7 100.0%	11 / 11 100.0%	6 / 6 100.0%	1 / 1 100.0%	16 / 16 100.0%	0 / 0 0.0%	0 / 0 0.0%	0 / 0 0.0%	0 / 0 0.0%	71 / 126 56.3%AB	55 / 126 43.7%ABE				
1	ATCC 25920	120 / 120 100.0%	6 / 6 100.0%	6 / 6 100.0%	4 / 4 100.0%	5 / 5 100.0%	14 / 14 100.0%	6 / 6 100.0%	2 / 2 100.0%	5 / 5 100.0%	8 / 8 100.0%	2 / 2 100.0%	7 / 7 100.0%	4 / 4 100.0%	4 / 4 100.0%	2 / 2 100.0%	5 / 5 100.0%	3 / 3 100.0%	3 / 3 100.0%	7 / 7 100.0%	10 / 10 100.0%	5 / 5 100.0%	1 / 1 100.0%	11 / 11 100.0%	0 / 0 0.0%	0 / 0 0.0%	0 / 0 0.0%	0 / 0 0.0%	44 / 120 36.7%B	33 / 120 27.5%BE	29 / 120 24.2%AB	14 / 120 11.7%ABE		
1	Vibrio [harveyi] ATCC BAA-1116	120 / 120 100.0%	6 / 6 100.0%	6 / 6 100.0%	4 / 4 100.0%	5 / 5 100.0%	14 / 14 100.0%	6 / 6 100.0%	2 / 2 100.0%	5 / 5 100.0%	8 / 8 100.0%	2 / 2 100.0%	7 / 7 100.0%	4 / 4 100.0%	4 / 4 100.0%	2 / 2 100.0%	5 / 5 100.0%	3 / 3 100.0%	3 / 3 100.0%	7 / 7 100.0%	10 / 10 100.0%	5 / 5 100.0%	1 / 1 100.0%	11 / 11 100.0%	0 / 0 0.0%	0 / 0 0.0%	0 / 0 0.0%	0 / 0 0.0%	44 / 120 36.7%B	33 / 120 27.5%BE	29 / 120 24.2%AB	14 / 120 11.7%ABE		
1	Vibrio sp. EJY3	118 / 118 100.0%	6 / 6 100.0%	6 / 6 100.0%	5 / 5 100.0%	3 / 3 100.0%	10 / 10 100.0%	6 / 6 100.0%	2 / 2 100.0%	4 / 4 100.0%	6 / 6 100.0%	2 / 2 100.0%	7 / 7 100.0%	4 / 4 100.0%	4 / 4 100.0%	4 / 4 100.0%	4 / 4 100.0%	3 / 3 100.0%	4 / 4 100.0%	7 / 7 100.0%	10 / 10 100.0%	6 / 6 100.0%	1 / 1 100.0%	14 / 14 100.0%	0 / 0 0.0%	0 / 0 0.0%	0 / 0 0.0%	0 / 0 0.0%	70 / 118 59.3%AB	48 / 118 40.7%ABE				
3	Aliivibrio	342 / 344 99.4%	14 / 14 100.0%	22 / 22 100.0%	19 / 19 100.0%	7 / 7 100.0%	33 / 33 100.0%	16 / 16 100.0%	6 / 6 100.0%	15 / 17 88.2%	13 / 13 100.0%	9 / 9 100.0%	26 / 26 100.0%	11 / 11 100.0%	12 / 12 100.0%	15 / 15 100.0%	7 / 7 100.0%	9 / 9 100.0%	9 / 9 100.0%	22 / 22 100.0%	25 / 25 100.0%	15 / 15 100.0%	3 / 3 100.0%	34 / 34 100.0%	0 / 0 0.0%	0 / 0 0.0%	0 / 0 0.0%	0 / 0 0.0%	156 / 344 45.3%AB	102 / 344 29.7%ABE	51 / 344 14.8%B	33 / 344 9.6%BE	2 / 344 0.6%E	
2	"Achromobacter fischeri" (Beijerinck 1889) Bergey et al. 1930	240 / 240 100.0%	8 / 8 100.0%	15 / 15 100.0%	14 / 14 100.0%	5 / 5 100.0%	23 / 23 100.0%	11 / 11 100.0%	4 / 4 100.0%	13 / 13 100.0%	9 / 9 100.0%	6 / 6 100.0%	17 / 17 100.0%	7 / 7 100.0%	8 / 8 100.0%	11 / 11 100.0%	5 / 5 100.0%	6 / 6 100.0%	7 / 7 100.0%	16 / 16 100.0%	17 / 17 100.0%	11 / 11 100.0%	2 / 2 100.0%	25 / 25 100.0%	0 / 0 0.0%	0 / 0 0.0%	0 / 0 0.0%	0 / 0 0.0%	93 / 240 38.8%AB	63 / 240 26.2%ABE	51 / 240 21.2%B	33 / 240 13.8%BE		
1	Aliivibrio fischeri ES114	117 / 117 100.0%	4 / 4 100.0%	7 / 7 100.0%	7 / 7 100.0%	3 / 3 100.0%	10 / 10 100.0%	6 / 6 100.0%	2 / 2 100.0%	6 / 6 100.0%	5 / 5 100.0%	3 / 3 100.0%	8 / 8 100.0%	4 / 4 100.0%	4 / 4 100.0%	5 / 5 100.0%	2 / 2 100.0%	3 / 3 100.0%	3 / 3 100.0%	8 / 8 100.0%	9 / 9 100.0%	6 / 6 100.0%	1 / 1 100.0%	11 / 11 100.0%	0 / 0 0.0%	0 / 0 0.0%	0 / 0 0.0%	0 / 0 0.0%	72 / 117 61.5%AB	45 / 117 38.5%ABE				
1	Aiivibrio fischeri MJ11	123 / 123 100.0%	4 / 4 100.0%	8 / 8 100.0%	7 / 7 100.0%	2 / 2 100.0%	13 / 13 100.0%	5 / 5 100.0%	2 / 2 100.0%	7 / 7 100.0%	4 / 4 100.0%	3 / 3 100.0%	9 / 9 100.0%	3 / 3 100.0%	4 / 4 100.0%	6 / 6 100.0%	3 / 3 100.0%	3 / 3 100.0%	4 / 4 100.0%	8 / 8 100.0%	8 / 8 100.0%	5 / 5 100.0%	1 / 1 100.0%	14 / 14 100.0%	0 / 0 0.0%	0 / 0 0.0%	0 / 0 0.0%	0 / 0 0.0%	51 / 123 41.5%B	33 / 123 26.8%BE	21 / 123 17.1%AB	18 / 123 14.6%ABE		
1	ATCC 43839	102 / 104 98.1%	6 / 6 100.0%	7 / 7 100.0%	5 / 5 100.0%	2 / 2 100.0%	10 / 10 100.0%	5 / 5 100.0%	2 / 2 100.0%	2 / 4 50.0%	4 / 4 100.0%	3 / 3 100.0%	9 / 9 100.0%	4 / 4 100.0%	4 / 4 100.0%	4 / 4 100.0%	2 / 2 100.0%	3 / 3 100.0%	2 / 2 100.0%	6 / 6 100.0%	8 / 8 100.0%	4 / 4 100.0%	1 / 1 100.0%	9 / 9 100.0%	0 / 0 0.0%	0 / 0 0.0%	0 / 0 0.0%	0 / 0 0.0%	63 / 104 60.6%AB	39 / 104 37.5%ABE	2 / 104 1.9%E			
1	Aliivibrio salmonicida LFI1238	102 / 104 98.1%	6 / 6 100.0%	7 / 7 100.0%	5 / 5 100.0%	2 / 2 100.0%	10 / 10 100.0%	5 / 5 100.0%	2 / 2 100.0%	2 / 4 50.0%	4 / 4 100.0%	3 / 3 100.0%	9 / 9 100.0%	4 / 4 100.0%	4 / 4 100.0%	4 / 4 100.0%	2 / 2 100.0%	3 / 3 100.0%	2 / 2 100.0%	6 / 6 100.0%	8 / 8 100.0%	4 / 4 100.0%	1 / 1 100.0%	9 / 9 100.0%	0 / 0 0.0%	0 / 0 0.0%	0 / 0 0.0%	0 / 0 0.0%	63 / 104 60.6%AB	39 / 104 37.5%ABE	2 / 104 1.9%E			
6	Aeromonadaceae/Succinivibrionaceae group	623 / 624 99.8%	37 / 37 100.0%	22 / 22 100.0%	27 / 27 100.0%	17 / 17 100.0%	55 / 55 100.0%	48 / 48 100.0%	13 / 13 100.0%	28 / 28 100.0%	41 / 41 100.0%	11 / 11 100.0%	37 / 37 100.0%	25 / 25 100.0%	17 / 17 100.0%	25 / 25 100.0%	12 / 12 100.0%	28 / 28 100.0%	15 / 15 100.0%	23 / 24 95.8%	42 / 42 100.0%	36 / 36 100.0%	6 / 6 100.0%	57 / 57 100.0%	0 / 0 0.0%	0 / 0 0.0%	0 / 0 0.0%	1 / 1 100.0%	381 / 624 61.1%AB	242 / 624 38.8%ABE	1 / 624 0.2%C			
6	Aeromonadaceae	623 / 624 99.8%	37 / 37 100.0%	22 / 22 100.0%	27 / 27 100.0%	17 / 17 100.0%	55 / 55 100.0%	48 / 48 100.0%	13 / 13 100.0%	28 / 28 100.0%	41 / 41 100.0%	11 / 11 100.0%	37 / 37 100.0%	25 / 25 100.0%	17 / 17 100.0%	25 / 25 100.0%	12 / 12 100.0%	28 / 28 100.0%	15 / 15 100.0%	23 / 24 95.8%	42 / 42 100.0%	36 / 36 100.0%	6 / 6 100.0%	57 / 57 100.0%	0 / 0 0.0%	0 / 0 0.0%	0 / 0 0.0%	1 / 1 100.0%	381 / 624 61.1%AB	242 / 624 38.8%ABE	1 / 624 0.2%C			
4	Aeromonas	444 / 445 99.8%	28 / 28 100.0%	15 / 15 100.0%	20 / 20 100.0%	12 / 12 100.0%	40 / 40 100.0%	32 / 32 100.0%	8 / 8 100.0%	22 / 22 100.0%	30 / 30 100.0%	7 / 7 100.0%	28 / 28 100.0%	16 / 16 100.0%	12 / 12 100.0%	19 / 19 100.0%	8 / 8 100.0%	20 / 20 100.0%	11 / 11 100.0%	15 / 16 93.8%	29 / 29 100.0%	27 / 27 100.0%	4 / 4 100.0%	40 / 40 100.0%	0 / 0 0.0%	0 / 0 0.0%	0 / 0 0.0%	1 / 1 100.0%	263 / 445 59.1%AB	181 / 445 40.7%ABE	1 / 445 0.2%C			
2	"Aeromonas liquefaciens" Kluyver and van Niel 1936	236 / 236 100.0%	16 / 16 100.0%	8 / 8 100.0%	10 / 10 100.0%	6 / 6 100.0%	22 / 22 100.0%	19 / 19 100.0%	4 / 4 100.0%	11 / 11 100.0%	15 / 15 100.0%	4 / 4 100.0%	14 / 14 100.0%	11 / 11 100.0%	6 / 6 100.0%	10 / 10 100.0%	4 / 4 100.0%	10 / 10 100.0%	6 / 6 100.0%	8 / 8 100.0%	16 / 16 100.0%	14 / 14 100.0%	2 / 2 100.0%	20 / 20 100.0%	0 / 0 0.0%	0 / 0 0.0%	0 / 0 0.0%	0 / 0 0.0%	143 / 236 60.6%AB	93 / 236 39.4%ABE				
1	Aeromonas hydrophila subsp. hydrophila	125 / 125 100.0%	9 / 9 100.0%	4 / 4 100.0%	5 / 5 100.0%	3 / 3 100.0%	12 / 12 100.0%	12 / 12 100.0%	2 / 2 100.0%	6 / 6 100.0%	8 / 8 100.0%	2 / 2 100.0%	7 / 7 100.0%	7 / 7 100.0%	3 / 3 100.0%	5 / 5 100.0%	2 / 2 100.0%	5 / 5 100.0%	3 / 3 100.0%	4 / 4 100.0%	8 / 8 100.0%	7 / 7 100.0%	1 / 1 100.0%	10 / 10 100.0%	0 / 0 0.0%	0 / 0 0.0%	0 / 0 0.0%	0 / 0 0.0%	77 / 125 61.6%AB	48 / 125 38.4%ABE				
1	Aeromonas hydrophila subsp. hydrophila ATCC 7966	125 / 125 100.0%	9 / 9 100.0%	4 / 4 100.0%	5 / 5 100.0%	3 / 3 100.0%	12 / 12 100.0%	12 / 12 100.0%	2 / 2 100.0%	6 / 6 100.0%	8 / 8 100.0%	2 / 2 100.0%	7 / 7 100.0%	7 / 7 100.0%	3 / 3 100.0%	5 / 5 100.0%	2 / 2 100.0%	5 / 5 100.0%	3 / 3 100.0%	4 / 4 100.0%	8 / 8 100.0%	7 / 7 100.0%	1 / 1 100.0%	10 / 10 100.0%	0 / 0 0.0%	0 / 0 0.0%	0 / 0 0.0%	0 / 0 0.0%	77 / 125 61.6%AB	48 / 125 38.4%ABE				
1	Aeromonas hydrophila ML09-119	111 / 111 100.0%	7 / 7 100.0%	4 / 4 100.0%	5 / 5 100.0%	3 / 3 100.0%	10 / 10 100.0%	7 / 7 100.0%	2 / 2 100.0%	5 / 5 100.0%	7 / 7 100.0%	2 / 2 100.0%	7 / 7 100.0%	4 / 4 100.0%	3 / 3 100.0%	5 / 5 100.0%	2 / 2 100.0%	5 / 5 100.0%	3 / 3 100.0%	4 / 4 100.0%	8 / 8 100.0%	7 / 7 100.0%	1 / 1 100.0%	10 / 10 100.0%	0 / 0 0.0%	0 / 0 0.0%	0 / 0 0.0%	0 / 0 0.0%	66 / 111 59.5%AB	45 / 111 40.5%ABE				
1	"Bacillus salmonicida" (Lehmann and Neumann 1896) Kruse 1896	107 / 108 99.1%	8 / 8 100.0%	3 / 3 100.0%	5 / 5 100.0%	3 / 3 100.0%	10 / 10 100.0%	7 / 7 100.0%	2 / 2 100.0%	5 / 5 100.0%	7 / 7 100.0%	1 / 1 100.0%	7 / 7 100.0%	4 / 4 100.0%	3 / 3 100.0%	5 / 5 100.0%	2 / 2 100.0%	5 / 5 100.0%	3 / 3 100.0%	3 / 4 75.0%	6 / 6 100.0%	6 / 6 100.0%	1 / 1 100.0%	10 / 10 100.0%	0 / 0 0.0%	0 / 0 0.0%	0 / 0 0.0%	1 / 1 100.0%	61 / 108 56.5%AB	46 / 108 42.6%ABE	1 / 108 0.9%C			
1	Aeromonas salmonicida salmonicida	107 / 108 99.1%	8 / 8 100.0%	3 / 3 100.0%	5 / 5 100.0%	3 / 3 100.0%	10 / 10 100.0%	7 / 7 100.0%	2 / 2 100.0%	5 / 5 100.0%	7 / 7 100.0%	1 / 1 100.0%	7 / 7 100.0%	4 / 4 100.0%	3 / 3 100.0%	5 / 5 100.0%	2 / 2 100.0%	5 / 5 100.0%	3 / 3 100.0%	3 / 4 75.0%	6 / 6 100.0%	6 / 6 100.0%	1 / 1 100.0%	10 / 10 100.0%	0 / 0 0.0%	0 / 0 0.0%	0 / 0 0.0%	1 / 1 100.0%	61 / 108 56.5%AB	46 / 108 42.6%ABE	1 / 108 0.9%C			
1	Aeromonas salmonicida subsp. salmonicida A449	107 / 108 99.1%	8 / 8 100.0%	3 / 3 100.0%	5 / 5 100.0%	3 / 3 100.0%	10 / 10 100.0%	7 / 7 100.0%	2 / 2 100.0%	5 / 5 100.0%	7 / 7 100.0%	1 / 1 100.0%	7 / 7 100.0%	4 / 4 100.0%	3 / 3 100.0%	5 / 5 100.0%	2 / 2 100.0%	5 / 5 100.0%	3 / 3 100.0%	3 / 4 75.0%	6 / 6 100.0%	6 / 6 100.0%	1 / 1 100.0%	10 / 10 100.0%	0 / 0 0.0%	0 / 0 0.0%	0 / 0 0.0%	1 / 1 100.0%	61 / 108 56.5%AB	46 / 108 42.6%ABE	1 / 108 0.9%C			
1	ATCC 35624	101 / 101 100.0%	4 / 4 100.0%	4 / 4 100.0%	5 / 5 100.0%	3 / 3 100.0%	8 / 8 100.0%	6 / 6 100.0%	2 / 2 100.0%	6 / 6 100.0%	8 / 8 100.0%	2 / 2 100.0%	7 / 7 100.0%	1 / 1 100.0%	3 / 3 100.0%	4 / 4 100.0%	2 / 2 100.0%	5 / 5 100.0%	2 / 2 100.0%	4 / 4 100.0%	7 / 7 100.0%	7 / 7 100.0%	1 / 1 100.0%	10 / 10 100.0%	0 / 0 0.0%	0 / 0 0.0%	0 / 0 0.0%	0 / 0 0.0%	59 / 101 58.4%AB	42 / 101 41.6%ABE				
1	Aeromonas veronii B565	101 / 101 100.0%	4 / 4 100.0%	4 / 4 100.0%	5 / 5 100.0%	3 / 3 100.0%	8 / 8 100.0%	6 / 6 100.0%	2 / 2 100.0%	6 / 6 100.0%	8 / 8 100.0%	2 / 2 100.0%	7 / 7 100.0%	1 / 1 100.0%	3 / 3 100.0%	4 / 4 100.0%	2 / 2 100.0%	5 / 5 100.0%	2 / 2 100.0%	4 / 4 100.0%	7 / 7 100.0%	7 / 7 100.0%	1 / 1 100.0%	10 / 10 100.0%	0 / 0 0.0%	0 / 0 0.0%	0 / 0 0.0%	0 / 0 0.0%	59 / 101 58.4%AB	42 / 101 41.6%ABE				
1	Tolumonas	90 / 90 100.0%	4 / 4 100.0%	3 / 3 100.0%	4 / 4 100.0%	3 / 3 100.0%	9 / 9 100.0%	6 / 6 100.0%	3 / 3 100.0%	3 / 3 100.0%	5 / 5 100.0%	2 / 2 100.0%	4 / 4 100.0%	4 / 4 100.0%	3 / 3 100.0%	3 / 3 100.0%	2 / 2 100.0%	4 / 4 100.0%	2 / 2 100.0%	5 / 5 100.0%	7 / 7 100.0%	4 / 4 100.0%	1 / 1 100.0%	9 / 9 100.0%	0 / 0 0.0%	0 / 0 0.0%	0 / 0 0.0%	0 / 0 0.0%	59 / 90 65.6%AB	31 / 90 34.4%ABE				
1	DSM 9187	90 / 90 100.0%	4 / 4 100.0%	3 / 3 100.0%	4 / 4 100.0%	3 / 3 100.0%	9 / 9 100.0%	6 / 6 100.0%	3 / 3 100.0%	3 / 3 100.0%	5 / 5 100.0%	2 / 2 100.0%	4 / 4 100.0%	4 / 4 100.0%	3 / 3 100.0%	3 / 3 100.0%	2 / 2 100.0%	4 / 4 100.0%	2 / 2 100.0%	5 / 5 100.0%	7 / 7 100.0%	4 / 4 100.0%	1 / 1 100.0%	9 / 9 100.0%	0 / 0 0.0%	0 / 0 0.0%	0 / 0 0.0%	0 / 0 0.0%	59 / 90 65.6%AB	31 / 90 34.4%ABE				
1	Tolumonas auensis DSM 9187	90 / 90 100.0%	4 / 4 100.0%	3 / 3 100.0%	4 / 4 100.0%	3 / 3 100.0%	9 / 9 100.0%	6 / 6 100.0%	3 / 3 100.0%	3 / 3 100.0%	5 / 5 100.0%	2 / 2 100.0%	4 / 4 100.0%	4 / 4 100.0%	3 / 3 100.0%	3 / 3 100.0%	2 / 2 100.0%	4 / 4 100.0%	2 / 2 100.0%	5 / 5 100.0%	7 / 7 100.0%	4 / 4 100.0%	1 / 1 100.0%	9 / 9 100.0%	0 / 0 0.0%	0 / 0 0.0%	0 / 0 0.0%	0 / 0 0.0%	59 / 90 65.6%AB	31 / 90 34.4%ABE				
1	Oceanimonas	89 / 89 100.0%	5 / 5 100.0%	4 / 4 100.0%	3 / 3 100.0%	2 / 2 100.0%	6 / 6 100.0%	10 / 10 100.0%	2 / 2 100.0%	3 / 3 100.0%	6 / 6 100.0%	2 / 2 100.0%	5 / 5 100.0%	5 / 5 100.0%	2 / 2 100.0%	3 / 3 100.0%	2 / 2 100.0%	4 / 4 100.0%	2 / 2 100.0%	3 / 3 100.0%	6 / 6 100.0%	5 / 5 100.0%	1 / 1 100.0%	8 / 8 100.0%	0 / 0 0.0%	0 / 0 0.0%	0 / 0 0.0%	0 / 0 0.0%	59 / 89 66.3%AB	30 / 89 33.7%ABE				
1	Oceanimonas sp. GK1	89 / 89 100.0%	5 / 5 100.0%	4 / 4 100.0%	3 / 3 100.0%	2 / 2 100.0%	6 / 6 100.0%	10 / 10 100.0%	2 / 2 100.0%	3 / 3 100.0%	6 / 6 100.0%	2 / 2 100.0%	5 / 5 100.0%	5 / 5 100.0%	2 / 2 100.0%	3 / 3 100.0%	2 / 2 100.0%	4 / 4 100.0%	2 / 2 100.0%	3 / 3 100.0%	6 / 6 100.0%	5 / 5 100.0%	1 / 1 100.0%	8 / 8 100.0%	0 / 0 0.0%	0 / 0 0.0%	0 / 0 0.0%	0 / 0 0.0%	59 / 89 66.3%AB	30 / 89 33.7%ABE				
[illegible]	[illegible]	2110 / 2118 [illegible]	68 / 68 [illegible]	104 / 105 [illegible]	84 / 84 [illegible]	109 / 109 [illegible]	169 / 169 [illegible]	171 / 171 [illegible]	37 / 37 [illegible]	37 / 37 [illegible]	144 / 144 [illegible]	43 / 43 [illegible]	139 / 140 [illegible]	125 / 125 [illegible]	47 / 47 [illegible]	76 / 76 [illegible]	37 / 37 [illegible]	74 / 74 [illegible]	37 / 37 [illegible]	87 / 87 [illegible]	158 / 158 [illegible]	109 / 109 [illegible]	37 / 37 [illegible]	189 / 190 [illegible]	29 / 33 [illegible]	0 / 0 [illegible]	0 / 0 [illegible]	0 / 1 [illegible]	1356 / 2118 [illegible]	647 / 2118 [illegible]	69 / 2118 [illegible]	38 / 2118 [illegible]	6 / 2118 [illegible]	2 / 2118 [illegible]

37	Pasteurellaceae group	99.6%	100.0%	99.0%	100.0%	100.0%	100.0%	100.0%	100.0%	100.0%	100.0%	100.0%	99.3%	100.0%	100.0%	100.0%	100.0%	100.0%	100.0%	100.0%	100.0%	100.0%	100.0%	99.5%	87.9%	0.0%	0.0%	0.0%	64.0%AB	30.5%ABE	3.3%B	1.8%BE	0.3%E	0.1%C		
37	Pasteurellaceae	2110 / 2118 99.6%	68 / 68 100.0%	104 / 105 99.0%	84 / 84 100.0%	109 / 109 100.0%	169 / 169 100.0%	171 / 171 100.0%	37 / 37 100.0%	37 / 37 100.0%	144 / 144 100.0%	43 / 43 100.0%	139 / 140 99.3%	125 / 125 100.0%	47 / 47 100.0%	76 / 76 100.0%	37 / 37 100.0%	74 / 74 100.0%	37 / 37 100.0%	87 / 87 100.0%	158 / 158 100.0%	109 / 109 100.0%	37 / 37 100.0%	189 / 190 99.5%	29 / 33 87.9%	0 / 0 0.0%	0 / 0 0.0%	0 / 1 0.0%	1356 / 2118 64.0%AB	647 / 2118 30.5%ABE	69 / 2118 3.3%B	38 / 2118 1.8%BE	6 / 2118 0.3%E	2 / 2118 0.1%C		
5	Actinobacillus	302 / 303 99.7%	10 / 10 100.0%	14 / 15 93.3%	15 / 15 100.0%	17 / 17 100.0%	26 / 26 100.0%	21 / 21 100.0%	5 / 5 100.0%	5 / 5 100.0%	21 / 21 100.0%	5 / 5 100.0%	20 / 20 100.0%	15 / 15 100.0%	9 / 9 100.0%	12 / 12 100.0%	5 / 5 100.0%	10 / 10 100.0%	5 / 5 100.0%	15 / 15 100.0%	20 / 20 100.0%	13 / 13 100.0%	5 / 5 100.0%	29 / 29 100.0%	5 / 5 100.0%	0 / 0 0.0%	0 / 0 0.0%	0 / 0 0.0%	143 / 303 47.2%AB	77 / 303 25.4%ABE	59 / 303 19.5%B	23 / 303 7.6%BE	1 / 303 0.3%C			
3	ATCC 27088	185 / 185 100.0%	6 / 6 100.0%	9 / 9 100.0%	10 / 10 100.0%	11 / 11 100.0%	16 / 16 100.0%	15 / 15 100.0%	3 / 3 100.0%	3 / 3 100.0%	13 / 13 100.0%	3 / 3 100.0%	12 / 12 100.0%	8 / 8 100.0%	6 / 6 100.0%	7 / 7 100.0%	3 / 3 100.0%	6 / 6 100.0%	3 / 3 100.0%	9 / 9 100.0%	12 / 12 100.0%	7 / 7 100.0%	3 / 3 100.0%	17 / 17 100.0%	3 / 3 100.0%	0 / 0 0.0%	0 / 0 0.0%	0 / 0 0.0%	67 / 185 36.2%AB	59 / 185 31.9%B	37 / 185 20.0%ABE	22 / 185 11.9%BE				
1	Actinobacillus pleuropneumoniae 5	62 / 62 100.0%	2 / 2 100.0%	3 / 3 100.0%	3 / 3 100.0%	3 / 3 100.0%	5 / 5 100.0%	5 / 5 100.0%	1 / 1 100.0%	1 / 1 100.0%	4 / 4 100.0%	1 / 1 100.0%	4 / 4 100.0%	3 / 3 100.0%	2 / 2 100.0%	3 / 3 100.0%	1 / 1 100.0%	2 / 2 100.0%	1 / 1 100.0%	3 / 3 100.0%	4 / 4 100.0%	3 / 3 100.0%	1 / 1 100.0%	6 / 6 100.0%	1 / 1 100.0%	0 / 0 0.0%	0 / 0 0.0%	0 / 0 0.0%	41 / 62 66.1%AB	20 / 62 32.3%ABE	1 / 62 1.6%BE					
1	Actinobacillus pleuropneumoniae L20	62 / 62 100.0%	2 / 2 100.0%	3 / 3 100.0%	3 / 3 100.0%	3 / 3 100.0%	5 / 5 100.0%	5 / 5 100.0%	1 / 1 100.0%	1 / 1 100.0%	4 / 4 100.0%	1 / 1 100.0%	4 / 4 100.0%	3 / 3 100.0%	2 / 2 100.0%	3 / 3 100.0%	1 / 1 100.0%	2 / 2 100.0%	1 / 1 100.0%	3 / 3 100.0%	4 / 4 100.0%	3 / 3 100.0%	1 / 1 100.0%	6 / 6 100.0%	1 / 1 100.0%	0 / 0 0.0%	0 / 0 0.0%	0 / 0 0.0%	41 / 62 66.1%AB	20 / 62 32.3%ABE	1 / 62 1.6%BE					
1	Actinobacillus pleuropneumoniae 7	62 / 62 100.0%	2 / 2 100.0%	3 / 3 100.0%	4 / 4 100.0%	3 / 3 100.0%	6 / 6 100.0%	5 / 5 100.0%	1 / 1 100.0%	1 / 1 100.0%	4 / 4 100.0%	1 / 1 100.0%	4 / 4 100.0%	3 / 3 100.0%	2 / 2 100.0%	2 / 2 100.0%	1 / 1 100.0%	2 / 2 100.0%	1 / 1 100.0%	3 / 3 100.0%	4 / 4 100.0%	3 / 3 100.0%	1 / 1 100.0%	5 / 5 100.0%	1 / 1 100.0%	0 / 0 0.0%	0 / 0 0.0%	0 / 0 0.0%	30 / 62 48.4%B	12 / 62 19.4%AB	10 / 62 16.1%BE	10 / 62 16.1%ABE				
1	Actinobacillus pleuropneumoniae serovar 7 str. AP76	62 / 62 100.0%	2 / 2 100.0%	3 / 3 100.0%	4 / 4 100.0%	3 / 3 100.0%	6 / 6 100.0%	5 / 5 100.0%	1 / 1 100.0%	1 / 1 100.0%	4 / 4 100.0%	1 / 1 100.0%	4 / 4 100.0%	3 / 3 100.0%	2 / 2 100.0%	2 / 2 100.0%	1 / 1 100.0%	2 / 2 100.0%	1 / 1 100.0%	3 / 3 100.0%	4 / 4 100.0%	3 / 3 100.0%	1 / 1 100.0%	5 / 5 100.0%	1 / 1 100.0%	0 / 0 0.0%	0 / 0 0.0%	0 / 0 0.0%	30 / 62 48.4%B	12 / 62 19.4%AB	10 / 62 16.1%BE	10 / 62 16.1%ABE				
1	Actinobacillus pleuropneumoniae serovar 3	61 / 61 100.0%	2 / 2 100.0%	3 / 3 100.0%	3 / 3 100.0%	5 / 5 100.0%	5 / 5 100.0%	5 / 5 100.0%	1 / 1 100.0%	1 / 1 100.0%	5 / 5 100.0%	1 / 1 100.0%	4 / 4 100.0%	2 / 2 100.0%	2 / 2 100.0%	2 / 2 100.0%	1 / 1 100.0%	2 / 2 100.0%	1 / 1 100.0%	3 / 3 100.0%	4 / 4 100.0%	1 / 1 100.0%	1 / 1 100.0%	6 / 6 100.0%	1 / 1 100.0%	0 / 0 0.0%	0 / 0 0.0%	0 / 0 0.0%	29 / 61 47.5%B	14 / 61 23.0%AB	11 / 61 18.0%BE	7 / 61 11.5%ABE				
1	Actinobacillus pleuropneumoniae serovar 3 str. JL03	61 / 61 100.0%	2 / 2 100.0%	3 / 3 100.0%	3 / 3 100.0%	5 / 5 100.0%	5 / 5 100.0%	5 / 5 100.0%	1 / 1 100.0%	1 / 1 100.0%	5 / 5 100.0%	1 / 1 100.0%	4 / 4 100.0%	2 / 2 100.0%	2 / 2 100.0%	2 / 2 100.0%	1 / 1 100.0%	2 / 2 100.0%	1 / 1 100.0%	3 / 3 100.0%	4 / 4 100.0%	1 / 1 100.0%	1 / 1 100.0%	6 / 6 100.0%	1 / 1 100.0%	0 / 0 0.0%	0 / 0 0.0%	0 / 0 0.0%	29 / 61 47.5%B	14 / 61 23.0%AB	11 / 61 18.0%BE	7 / 61 11.5%ABE				
1	ATCC 33415	58 / 59 98.3%	2 / 2 100.0%	2 / 3 66.7%	3 / 3 100.0%	3 / 3 100.0%	6 / 6 100.0%	2 / 2 100.0%	1 / 1 100.0%	1 / 1 100.0%	4 / 4 100.0%	1 / 1 100.0%	4 / 4 100.0%	3 / 3 100.0%	2 / 2 100.0%	2 / 2 100.0%	1 / 1 100.0%	2 / 2 100.0%	1 / 1 100.0%	3 / 3 100.0%	4 / 4 100.0%	3 / 3 100.0%	1 / 1 100.0%	6 / 6 100.0%	1 / 1 100.0%	0 / 0 0.0%	0 / 0 0.0%	0 / 0 0.0%	38 / 59 64.4%AB	20 / 59 33.9%ABE	1 / 59 1.7%C					
1	Actinobacillus suis H91-0380	58 / 59 98.3%	2 / 2 100.0%	2 / 3 66.7%	3 / 3 100.0%	3 / 3 100.0%	6 / 6 100.0%	2 / 2 100.0%	1 / 1 100.0%	1 / 1 100.0%	4 / 4 100.0%	1 / 1 100.0%	4 / 4 100.0%	3 / 3 100.0%	2 / 2 100.0%	2 / 2 100.0%	1 / 1 100.0%	2 / 2 100.0%	1 / 1 100.0%	3 / 3 100.0%	4 / 4 100.0%	3 / 3 100.0%	1 / 1 100.0%	6 / 6 100.0%	1 / 1 100.0%	0 / 0 0.0%	0 / 0 0.0%	0 / 0 0.0%	38 / 59 64.4%AB	20 / 59 33.9%ABE	1 / 59 1.7%C					
1	ATCC 55618	59 / 59 100.0%	2 / 2 100.0%	3 / 3 100.0%	2 / 2 100.0%	3 / 3 100.0%	4 / 4 100.0%	4 / 4 100.0%	1 / 1 100.0%	1 / 1 100.0%	4 / 4 100.0%	1 / 1 100.0%	4 / 4 100.0%	4 / 4 100.0%	1 / 1 100.0%	3 / 3 100.0%	1 / 1 100.0%	2 / 2 100.0%	1 / 1 100.0%	3 / 3 100.0%	4 / 4 100.0%	3 / 3 100.0%	1 / 1 100.0%	6 / 6 100.0%	1 / 1 100.0%	0 / 0 0.0%	0 / 0 0.0%	0 / 0 0.0%	38 / 59 64.4%AB	20 / 59 33.9%ABE	1 / 59 1.7%BE					
1	Actinobacillus succinogenes 130Z	59 / 59 100.0%	2 / 2 100.0%	3 / 3 100.0%	2 / 2 100.0%	3 / 3 100.0%	4 / 4 100.0%	4 / 4 100.0%	1 / 1 100.0%	1 / 1 100.0%	4 / 4 100.0%	1 / 1 100.0%	4 / 4 100.0%	4 / 4 100.0%	1 / 1 100.0%	3 / 3 100.0%	1 / 1 100.0%	2 / 2 100.0%	1 / 1 100.0%	3 / 3 100.0%	4 / 4 100.0%	3 / 3 100.0%	1 / 1 100.0%	6 / 6 100.0%	1 / 1 100.0%	0 / 0 0.0%	0 / 0 0.0%	0 / 0 0.0%	38 / 59 64.4%AB	20 / 59 33.9%ABE	1 / 59 1.7%BE					
13	Haemophilus	733 / 737 99.5%	24 / 24 100.0%	36 / 36 100.0%	23 / 23 100.0%	37 / 37 100.0%	60 / 60 100.0%	65 / 65 100.0%	13 / 13 100.0%	13 / 13 100.0%	49 / 49 100.0%	14 / 14 100.0%	50 / 50 100.0%	50 / 50 100.0%	13 / 13 100.0%	25 / 25 100.0%	13 / 13 100.0%	26 / 26 100.0%	13 / 13 100.0%	27 / 27 100.0%	55 / 55 100.0%	40 / 40 100.0%	13 / 13 100.0%	66 / 67 98.5%	8 / 11 72.7%	0 / 0 0.0%	0 / 0 0.0%	0 / 0 0.0%	494 / 737 67.0%AB	234 / 737 31.8%ABE	5 / 737 0.7%BE	4 / 737 0.5%E				
9	"Bacterium influenzae" Lehmann and Neumann 1896	512 / 515 99.4%	18 / 18 100.0%	27 / 27 100.0%	18 / 18 100.0%	26 / 26 100.0%	39 / 39 100.0%	45 / 45 100.0%	9 / 9 100.0%	9 / 9 100.0%	35 / 35 100.0%	10 / 10 100.0%	36 / 36 100.0%	38 / 38 100.0%	9 / 9 100.0%	18 / 18 100.0%	9 / 9 100.0%	18 / 18 100.0%	9 / 9 100.0%	17 / 17 100.0%	36 / 36 100.0%	28 / 28 100.0%	9 / 9 100.0%	45 / 45 100.0%	4 / 7 57.1%	0 / 0 0.0%	0 / 0 0.0%	0 / 0 0.0%	345 / 515 67.0%AB	163 / 515 31.7%ABE	4 / 515 0.8%BE	3 / 515 0.6%E				
1	Haemophilus influenzae KW20	57 / 57 100.0%	2 / 2 100.0%	3 / 3 100.0%	2 / 2 100.0%	3 / 3 100.0%	4 / 4 100.0%	5 / 5 100.0%	1 / 1 100.0%	1 / 1 100.0%	4 / 4 100.0%	1 / 1 100.0%	4 / 4 100.0%	4 / 4 100.0%	1 / 1 100.0%	2 / 2 100.0%	1 / 1 100.0%	2 / 2 100.0%	1 / 1 100.0%	2 / 2 100.0%	4 / 4 100.0%	3 / 3 100.0%	1 / 1 100.0%	5 / 5 100.0%	1 / 1 100.0%	0 / 0 0.0%	0 / 0 0.0%	0 / 0 0.0%	38 / 57 66.7%AB	18 / 57 31.6%ABE	1 / 57 1.8%BE					
1	Haemophilus influenzae R2846	56 / 57 98.2%	2 / 2 100.0%	3 / 3 100.0%	2 / 2 100.0%	3 / 3 100.0%	4 / 4 100.0%	5 / 5 100.0%	1 / 1 100.0%	1 / 1 100.0%	4 / 4 100.0%	1 / 1 100.0%	4 / 4 100.0%	4 / 4 100.0%	1 / 1 100.0%	2 / 2 100.0%	1 / 1 100.0%	2 / 2 100.0%	1 / 1 100.0%	2 / 2 100.0%	4 / 4 100.0%	3 / 3 100.0%	1 / 1 100.0%	5 / 5 100.0%	0 / 1 0.0%	0 / 0 0.0%	0 / 0 0.0%	0 / 0 0.0%	38 / 57 66.7%AB	18 / 57 31.6%ABE	1 / 57 1.8%E					
1	Haemophilus influenzae R2866	58 / 59 98.3%	2 / 2 100.0%	3 / 3 100.0%	2 / 2 100.0%	3 / 3 100.0%	5 / 5 100.0%	5 / 5 100.0%	1 / 1 100.0%	1 / 1 100.0%	4 / 4 100.0%	1 / 1 100.0%	4 / 4 100.0%	5 / 5 100.0%	1 / 1 100.0%	2 / 2 100.0%	1 / 1 100.0%	2 / 2 100.0%	1 / 1 100.0%	2 / 2 100.0%	4 / 4 100.0%	3 / 3 100.0%	1 / 1 100.0%	5 / 5 100.0%	0 / 1 0.0%	0 / 0 0.0%	0 / 0 0.0%	0 / 0 0.0%	40 / 59 67.8%AB	18 / 59 30.5%ABE	1 / 59 1.7%E					
1	Haemophilus influenzae 86-028NP	58 / 58 100.0%	2 / 2 100.0%	3 / 3 100.0%	2 / 2 100.0%	3 / 3 100.0%	5 / 5 100.0%	5 / 5 100.0%	1 / 1 100.0%	1 / 1 100.0%	4 / 4 100.0%	1 / 1 100.0%	4 / 4 100.0%	4 / 4 100.0%	1 / 1 100.0%	2 / 2 100.0%	1 / 1 100.0%	2 / 2 100.0%	1 / 1 100.0%	2 / 2 100.0%	4 / 4 100.0%	3 / 3 100.0%	1 / 1 100.0%	5 / 5 100.0%	1 / 1 100.0%	0 / 0 0.0%	0 / 0 0.0%	0 / 0 0.0%	39 / 58 67.2%AB	18 / 58 31.0%ABE	1 / 58 1.7%BE					
1	Haemophilus influenzae PittEE	58 / 58 100.0%	2 / 2 100.0%	3 / 3 100.0%	2 / 2 100.0%	3 / 3 100.0%	5 / 5 100.0%	5 / 5 100.0%	1 / 1 100.0%	1 / 1 100.0%	4 / 4 100.0%	1 / 1 100.0%	4 / 4 100.0%	4 / 4 100.0%	1 / 1 100.0%	2 / 2 100.0%	1 / 1 100.0%	2 / 2 100.0%	1 / 1 100.0%	2 / 2 100.0%	4 / 4 100.0%	3 / 3 100.0%	1 / 1 100.0%	5 / 5 100.0%	1 / 1 100.0%	0 / 0 0.0%	0 / 0 0.0%	0 / 0 0.0%	39 / 58 67.2%AB	18 / 58 31.0%ABE	1 / 58 1.7%BE					
1	Haemophilus influenzae PittGG	55 / 55 100.0%	2 / 2 100.0%	3 / 3 100.0%	2 / 2 100.0%	2 / 2 100.0%	4 / 4 100.0%	5 / 5 100.0%	1 / 1 100.0%	1 / 1 100.0%	3 / 3 100.0%	1 / 1 100.0%	4 / 4 100.0%	4 / 4 100.0%	1 / 1 100.0%	2 / 2 100.0%	1 / 1 100.0%	2 / 2 100.0%	1 / 1 100.0%	1 / 1 100.0%	4 / 4 100.0%	4 / 4 100.0%	1 / 1 100.0%	5 / 5 100.0%	1 / 1 100.0%	0 / 0 0.0%	0 / 0 0.0%	0 / 0 0.0%	35 / 55 63.6%AB	19 / 55 34.5%ABE	1 / 55 1.8%BE					
1	Haemophilus influenzae 10810	56 / 57 98.2%	2 / 2 100.0%	3 / 3 100.0%	2 / 2 100.0%	3 / 3 100.0%	4 / 4 100.0%	5 / 5 100.0%	1 / 1 100.0%	1 / 1 100.0%	4 / 4 100.0%	1 / 1 100.0%	4 / 4 100.0%	4 / 4 100.0%	1 / 1 100.0%	2 / 2 100.0%	1 / 1 100.0%	2 / 2 100.0%	1 / 1 100.0%	2 / 2 100.0%	4 / 4 100.0%	3 / 3 100.0%	1 / 1 100.0%	5 / 5 100.0%	0 / 1 0.0%	0 / 0 0.0%	0 / 0 0.0%	0 / 0 0.0%	38 / 57 66.7%AB	18 / 57 31.6%ABE	1 / 57 1.8%E					
1	Haemophilus influenzae F3031	58 / 58 100.0%	2 / 2 100.0%	3 / 3 100.0%	2 / 2 100.0%	3 / 3 100.0%	4 / 4 100.0%	5 / 5 100.0%	1 / 1 100.0%	1 / 1 100.0%	4 / 4 100.0%	2 / 2 100.0%	4 / 4 100.0%	5 / 5 100.0%	1 / 1 100.0%	2 / 2 100.0%	1 / 1 100.0%	2 / 2 100.0%	1 / 1 100.0%	2 / 2 100.0%	4 / 4 100.0%	3 / 3 100.0%	1 / 1 100.0%	5 / 5 100.0%	0 / 0 0.0%	0 / 0 0.0%	0 / 0 0.0%	0 / 0 0.0%	40 / 58 69.0%AB	18 / 58 31.0%ABE						
1	Haemophilus influenzae F3047	56 / 56 100.0%	2 / 2 100.0%	3 / 3 100.0%	2 / 2 100.0%	3 / 3 100.0%	4 / 4 100.0%	5 / 5 100.0%	1 / 1 100.0%	1 / 1 100.0%	4 / 4 100.0%	1 / 1 100.0%	4 / 4 100.0%	4 / 4 100.0%	1 / 1 100.0%	2 / 2 100.0%	1 / 1 100.0%	2 / 2 100.0%	1 / 1 100.0%	2 / 2 100.0%	4 / 4 100.0%	3 / 3 100.0%	1 / 1 100.0%	5 / 5 100.0%	0 / 0 0.0%	0 / 0 0.0%	0 / 0 0.0%	0 / 0 0.0%	38 / 56 67.9%AB	18 / 56 32.1%ABE						
1	ATCC 33392	57 / 57 100.0%	2 / 2 100.0%	3 / 3 100.0%	2 / 2 100.0%	3 / 3 100.0%	5 / 5 100.0%	5 / 5 100.0%	1 / 1 100.0%	1 / 1 100.0%	4 / 4 100.0%	1 / 1 100.0%	3 / 3 100.0%	4 / 4 100.0%	1 / 1 100.0%	2 / 2 100.0%	1 / 1 100.0%	2 / 2 100.0%	1 / 1 100.0%	2 / 2 100.0%	4 / 4 100.0%	3 / 3 100.0%	1 / 1 100.0%	5 / 5 100.0%	1 / 1 100.0%	0 / 0 0.0%	0 / 0 0.0%	0 / 0 0.0%	39 / 57 68.4%AB	18 / 57 31.6%ABE						
1	Haemophilus parainfluenzae T3T1	57 / 57 100.0%	2 / 2 100.0%	3 / 3 100.0%	2 / 2 100.0%	3 / 3 100.0%	5 / 5 100.0%	5 / 5 100.0%	1 / 1 100.0%	1 / 1 100.0%	4 / 4 100.0%	1 / 1 100.0%	3 / 3 100.0%	4 / 4 100.0%	1 / 1 100.0%	2 / 2 100.0%	1 / 1 100.0%	2 / 2 100.0%	1 / 1 100.0%	2 / 2 100.0%	4 / 4 100.0%	3 / 3 100.0%	1 / 1 100.0%	5 / 5 100.0%	1 / 1 100.0%	0 / 0 0.0%	0 / 0 0.0%	0 / 0 0.0%	39 / 57 68.4%AB	18 / 57 31.6%ABE						
1	"Bacillus ulceris cancrosi" Kruse 1896	48 / 48 100.0%	2 / 2 100.0%	2 / 2 100.0%	1 / 1 100.0%	3 / 3 100.0%	4 / 4 100.0%	4 / 4 100.0%	1 / 1 100.0%	1 / 1 100.0%	4 / 4 100.0%	1 / 1 100.0%	3 / 3 100.0%	2 / 2 100.0%	1 / 1 100.0%	1 / 1 100.0%	1 / 1 100.0%	2 / 2 100.0%	1 / 1 100.0%	2 / 2 100.0%	3 / 3 100.0%	3 / 3 100.0%	1 / 1 100.0%	4 / 4 100.0%	1 / 1 100.0%	0 / 0 0.0%	0 / 0 0.0%	0 / 0 0.0%	32 / 48 66.7%AB	15 / 48 31.2%ABE	1 / 48 2.1%BE					
1	Haemophilus ducreyi 35000HP	48 / 48 100.0%	2 / 2 100.0%	2 / 2 100.0%	1 / 1 100.0%	3 / 3 100.0%	4 / 4 100.0%	4 / 4 100.0%	1 / 1 100.0%	1 / 1 100.0%	4 / 4 100.0%	1 / 1 100.0%	3 / 3 100.0%	2 / 2 100.0%	1 / 1 100.0%	1 / 1 100.0%	1 / 1 100.0%	2 / 2 100.0%	1 / 1 100.0%	2 / 2 100.0%	3 / 3 100.0%	3 / 3 100.0%	1 / 1 100.0%	4 / 4 100.0%	1 / 1 100.0%	0 / 0 0.0%	0 / 0 0.0%	0 / 0 0.0%	32 / 48 66.7%AB	15 / 48 31.2%ABE	1 / 48 2.1%BE					
2	ATCC 19417	116 / 117 99.1%	2 / 2 100.0%	4 / 4 100.0%	2 / 2 100.0%	5 / 5 100.0%	12 / 12 100.0%	11 / 11 100.0%	2 / 2 100.0%	2 / 2 100.0%	6 / 6 100.0%	2 / 2 100.0%	8 / 8 100.0%	6 / 6 100.0%	2 / 2 100.0%	4 / 4 100.0%	2 / 2 100.0%	4 / 4 100.0%	2 / 2 100.0%	6 / 6 100.0%	12 / 12 100.0%	6 / 6 100.0%	2 / 2 100.0%	12 / 13 92.3%	2 / 2 100.0%	0 / 0 0.0%	0 / 0 0.0%	0 / 0 0.0%	78 / 117 66.7%AB	38 / 117 32.5%ABE	1 / 117 0.9%E					
1	Haemophilus parasuis SH0165	55 / 55 100.0%	1 / 1 100.0%	2 / 2 100.0%	1 / 1 100.0%	2 / 2 100.0%	5 / 5 100.0%	5 / 5 100.0%	1 / 1 100.0%	1 / 1 100.0%	3 / 3 100.0%	1 / 1 100.0%	4 / 4 100.0%	3 / 3 100.0%	1 / 1 100.0%	2 / 2 100.0%	1 / 1 100.0%	2 / 2 100.0%	1 / 1 100.0%	3 / 3 100.0%	5 / 5 100.0%	4 / 4 100.0%	1 / 1 100.0%	5 / 5 100.0%	1 / 1 100.0%	0 / 0 0.0%	0 / 0 0.0%	0 / 0 0.0%	36 / 55 65.5%AB	19 / 55 34.5%ABE						
1	Haemophilus parasuis ZJ0906	61 / 62 98.4%	1 / 1 100.0%	2 / 2 100.0%	1 / 1 100.0%	3 / 3 100.0%	7 / 7 100.0%	6 / 6 100.0%	1 / 1 100.0%	1 / 1 100.0%	3 / 3 100.0%	1 / 1 100.0%	4 / 4 100.0%	3 / 3 100.0%	1 / 1 100.0%	2 / 2 100.0%	1 / 1 100.0%	2 / 2 100.0%	1 / 1 100.0%	3 / 3 100.0%	7 / 7 100.0%	2 / 2 100.0%	1 / 1 100.0%	7 / 8 87.5%	1 / 1 100.0%	0 / 0 0.0%	0 / 0 0.0%	0 / 0 0.0%	42 / 62 67.7%AB	19 / 62 30.6%ABE	1 / 62 1.6%E					
4	Pasteurella	220 / 221 99.5%	7 / 7 100.0%	12 / 12 100.0%	9 / 9 100.0%	10 / 10 100.0%	19 / 19 100.0%	18 / 18 100.0%	4 / 4 100.0%	4 / 4 100.0%	14 / 14 100.0%	4 / 4 100.0%	16 / 16 100.0%	9 / 9 100.0%	4 / 4 100.0%	8 / 8 100.0%	4 / 4 100.0%	8 / 8 100.0%	4 / 4 100.0%	8 / 8 100.0%	16 / 16 100.0%	14 / 14 100.0%	4 / 4 100.0%	20 / 20 100.0%	4 / 4 100.0%	0 / 0 0.0%	0 / 0 0.0%	0 / 1 0.0%	143 / 221 64.7%AB	76 / 221 34.4%ABE	1 / 221 0.5%C	1 / 221 0.5%BE				
4	"Bacterium multocidum" Lehmann and Neumann 1899	220 / 221 99.5%	7 / 7 100.0%	12 / 12 100.0%	9 / 9 100.0%	10 / 10 100.0%	19 / 19 100.0%	18 / 18 100.0%	4 / 4 100.0%	4 / 4 100.0%	14 / 14 100.0%	4 / 4 100.0%	16 / 16 100.0%	9 / 9 100.0%	4 / 4 100.0%	8 / 8 100.0%	4 / 4 100.0%	8 / 8 100.0%	4 / 4 100.0%	8 / 8 100.0%	16 / 16 100.0%	14 / 14 100.0%	4 / 4 100.0%	20 / 20 100.0%	4 / 4 100.0%	0 / 0 0.0%	0 / 0 0.0%	0 / 1 0.0%	143 / 221 64.7%AB	76 / 221 34.4%ABE	1 / 221 0.5%BE	1 / 221 0.5%C				
3	Pasteurella multocida multocida	167 / 168 99.4%	6 / 6 100.0%	9 / 9 100.0%	7 / 7 100.0%	7 / 7 100.0%	14 / 14 100.0%	15 / 15 100.0%	3 / 3 100.0%	3 / 3 100.0%	10 / 10 100.0%	3 / 3 100.0%	12 / 12 100.0%	7 / 7 100.0%	3 / 3 100.0%	6 / 6 100.0%	3 / 3 100.0%	6 / 6 100.0%	3 / 3 100.0%	6 / 6 100.0%	12 / 12 100.0%	11 / 11 100.0%	3 / 3 100.0%	15 / 15 100.0%	3 / 3 100.0%	0 / 0 0.0%	0 / 0 0.0%	0 / 1 0.0%	108 / 168 64.3%AB	58 / 168 34.5%ABE	1 / 168 0.6%C	1 / 168 0.6%BE				
1	Pasteurella multocida subsp. multocida Pm70	57 / 57 100.0%	2 / 2 100.0%	3 / 3 100.0%	2 / 2 100.0%	3 / 3 100.0%	5 / 5 100.0%	5 / 5 100.0%	1 / 1 100.0%	1 / 1 100.0%	4 / 4 100.0%	1 / 1 100.0%	4 / 4 100.0%	3 / 3 100.0%	1 / 1 100.0%	2 / 2 100.0%	1 / 1 100.0%	2 / 2 100.0%	1 / 1 100.0%	2 / 2 100.0%	4 / 4 100.0%	3 / 3 100.0%	1 / 1 100.0%	5 / 5 100.0%	1 / 1 100.0%	0 / 0 0.0%	0 / 0 0.0%	0 / 0 0.0%	38 / 57 66.7%AB	18 / 57 31.6%ABE	1 / 57 1.8%BE					
1	Pasteurella multocida subsp. multocida 3480	55 / 55 100.0%	2 / 2 100.0%	3 / 3 100.0%	2 / 2 100.0%	1 / 1 100.0%	5 / 5 100.0%	5 / 5 100.0%	1 / 1 100.0%	1 / 1 100.0%	2 / 2 100.0%	1 / 1 100.0%	4 / 4 100.0%	2 / 2 100.0%	1 / 1 100.0%	3 / 3 100.0%	1 / 1 100.0%	2 / 2 100.0%	1 / 1 100.0%	2 / 2 100.0%	4 / 4 100.0%	5 / 5 100.0%	1 / 1 100.0%	5 / 5 100.0%	1 / 1 100.0%	0 / 0 0.0%	0 / 0 0.0%	0 / 0 0.0%	33 / 55 60.0%AB	22 / 55 40.0%ABE						
1	Pasteurella multocida subsp. multocida HN06	55 / 56 98.2%	2 / 2 100.0%	3 / 3 100.0%	3 / 3 100.0%	3 / 3 100.0%	4 / 4 100.0%	5 / 5 100.0%	1 / 1 100.0%	1 / 1 100.0%	4 / 4 100.0%	1 / 1 100.0%	4 / 4 100.0%	2 / 2 100.0%	1 / 1 100.0%	1 / 1 100.0%	1 / 1 100.0%	2 / 2 100.0%	1 / 1 100.0%	2 / 2 100.0%	4 / 4 100.0%	3 / 3 100.0%	1 / 1 100.0%	5 / 5 100.0%	1 / 1 100.0%	0 / 0 0.0%	0 / 0 0.0%	0 / 1 0.0%	37 / 56 66.1%AB	18 / 56 32.1%ABE	1 / 56 1.8%C					
1	Pasteurella multocida 36950	53 / 53 100.0%	1 / 1 100.0%	3 / 3 100.0%	2 / 2 100.0%	3 / 3 100.0%	5 / 5 100.0%	3 / 3 100.0%	1 / 1 100.0%	1 / 1 100.0%	4 / 4 100.0%	1 / 1 100.0%	4 / 4 100.0%	2 / 2 100.0%	1 / 1 100.0%	2 / 2 100.0%	1 / 1 100.0%	2 / 2 100.0%	1 / 1 100.0%	2 / 2 100.0%	4 / 4 100.0%	3 / 3 100.0%	1 / 1 100.0%	5 / 5 100.0%	1 / 1 100.0%	0 / 0 0.0%	0 / 0 0.0%	0 / 0 0.0%	35 / 53 66.0%AB	18 / 53 34.0%ABE						
6	Mannheimia	369 / 369 100.0%	12 / 12 100.0%	17 / 17 100.0%	18 / 18 100.0%	21 / 21 100.0%	28 / 28 100.0%	30 / 30 100.0%	6 / 6 100.0%	6 / 6 100.0%	27 / 27 100.0%	11 / 11 100.0%	24 / 24 100.0%	17 / 17 100.0%	12 / 12 100.0%	12 / 12 100.0%	6 / 6 100.0%	12 / 12 100.0%	6 / 6 100.0%	18 / 18 100.0%	30 / 30 100.0%	15 / 15 100.0%	6 / 6 100.0%	29 / 29 100.0%	6 / 6 100.0%	0 / 0 0.0%	0 / 0 0.0%	0 / 0 0.0%	259 / 369 70.2%AB	110 / 369 29.8%ABE						
6	ATCC 33396	369 / 369 100.0%	12 / 12 100.0%	17 / 17 100.0%	18 / 18 100.0%	21 / 21 100.0%	28 / 28 100.0%	30 / 30 100.0%	6 / 6 100.0%	6 / 6 100.0%	27 / 27 100.0%	11 / 11 100.0%	24 / 24 100.0%	17 / 17 100.0%	12 / 12 100.0%	12 / 12 100.0%	6 / 6 100.0%	12 / 12 100.0%	6 / 6 100.0%	18 / 18 100.0%	30 / 30 100.0%	15 / 15 100.0%	6 / 6 100.0%	29 / 29 100.0%	6 / 6 100.0%	0 / 0 0.0%	0 / 0 0.0%	0 / 0 0.0%	259 / 369 70.2%AB	110 / 369 29.8%ABE						
1	Mannheimia haemolytica USDA-ARS-SAM-185	60 / 60 100.0%	2 / 2 100.0%	3 / 3 100.0%	3 / 3 100.0%	3 / 3 100.0%	5 / 5 100.0%	5 / 5 100.0%	1 / 1 100.0%	1 / 1 100.0%	4 / 4 100.0%	1 / 1 100.0%	4 / 4 100.0%	3 / 3 100.0%	2 / 2 100.0%	2 / 2 100.0%	1 / 1 100.0%	2 / 2 100.0%	1 / 1 100.0%	3 / 3 100.0%	4 / 4 100.0%	3 / 3 100.0%	1 / 1 100.0%	5 / 5 100.0%	1 / 1 100.0%	0 / 0 0.0%	0 / 0 0.0%	0 / 0 0.0%	41 / 60 68.3%AB	19 / 60 31.7%ABE						
1	Mannheimia haemolytica USDA-ARS-USMARC-183	64 / 64 100.0%	2 / 2 100.0%	3 / 3 100.0%	3 / 3 100.0%	3 / 3 100.0%	6 / 6 100.0%	5 / 5 100.0%	1 / 1 100.0%	1 / 1 100.0%	4 / 4 100.0%	2 / 2 100.0%	4 / 4 100.0%	3 / 3 100.0%	2 / 2 100.0%	2 / 2 100.0%	1 / 1 100.0%	2 / 2 100.0%	1 / 1 100.0%	3 / 3 100.0%	5 / 5 100.0%	3 / 3 100.0%	1 / 1 100.0%	6 / 6 100.0%	1 / 1 100.0%	0 / 0 0.0%	0 / 0 0.0%	0 / 0 0.0%	44 / 64 68.8%AB	20 / 64 31.2%ABE						
1	Mannheimia haemolytica D153	61 / 61 100.0%	2 / 2 100.0%	3 / 3 100.0%	3 / 3 100.0%	4 / 4 100.0%	4 / 4 100.0%	5 / 5 100.0%	1 / 1 100.0%	1 / 1 100.0%	5 / 5 100.0%	2 / 2 100.0%	4 / 4 100.0%	3 / 3 100.0%	2 / 2 100.0%	2 / 2 100.0%	1 / 1 100.0%	2 / 2 100.0%	1 / 1 100.0%	3 / 3 100.0%	5 / 5 100.0%	2 / 2 100.0%	1 / 1 100.0%	4 / 4 100.0%	1 / 1 100.0%	0 / 0 0.0%	0 / 0 0.0%	0 / 0 0.0%	44 / 61 72.1%AB	17 / 61 27.9%ABE						
1	Mannheimia haemolytica D171	59 / 59 100.0%	2 / 2 100.0%	3 / 3 100.0%	3 / 3 100.0%	3 / 3 100.0%	4 / 4 100.0%	5 / 5 100.0%	1 / 1 100.0%	1 / 1 100.0%	4 / 4 100.0%	1 / 1 100.0%	4 / 4 100.0%	2 / 2 100.0%	2 / 2 100.0%	2 / 2 100.0%	1 / 1 100.0%	2 / 2 100.0%	1 / 1 100.0%	3 / 3 100.0%	5 / 5 100.0%	3 / 3 100.0%	1 / 1 100.0%	5 / 5 100.0%	1 / 1 100.0%	0 / 0 0.0%	0 / 0 0.0%	0 / 0 0.0%	40 / 59 67.8%AB	19 / 59 32.2%ABE						
1	Mannheimia haemolytica D174	65 / 65 100.0%	2 / 2 100.0%	2 / 2 100.0%	3 / 3 100.0%	5 / 5 100.0%	5 / 5 100.0%	5 / 5 100.0%	1 / 1 100.0%	1 / 1 100.0%	6 / 6 100.0%	3 / 3 100.0%	4 / 4 100.0%	3 / 3 100.0%	2 / 2 100.0%	2 / 2 100.0%	1 / 1 100.0%	2 / 2 100.0%	1 / 1 100.0%	3 / 3 100.0%	6 / 6 100.0%	1 / 1 100.0%	1 / 1 100.0%	5 / 5 100.0%	1 / 1 100.0%	0 / 0 0.0%	0 / 0 0.0%	0 / 0 0.0%	48 / 65 73.8%AB	17 / 65 26.2%ABE						
1	Mannheimia haemolytica M42548	60 / 60 100.0%	2 / 2 100.0%	3 / 3 100.0%	3 / 3 100.0%	3 / 3 100.0%	4 / 4 100.0%	5 / 5 100.0%	1 / 1 100.0%	1 / 1 100.0%	4 / 4 100.0%	2 / 2 100.0%	4 / 4 100.0%	3 / 3 100.0%	2 / 2 100.0%	2 / 2 100.0%	1 / 1 100.0%	2 / 2 100.0%	1 / 1 100.0%	3 / 3 100.0%	5 / 5 100.0%	3 / 3 100.0%	1 / 1 100.0%	4 / 4 100.0%	1 / 1 100.0%	0 / 0 0.0%	0 / 0 0.0%	0 / 0 0.0%	42 / 60 70.0%AB	18 / 60 30.0%ABE						
1	Gallibacterium	57 / 57 100.0%	2 / 2 100.0%	3 / 3 100.0%	2 / 2 100.0%	0 / 0 0.0%	5 / 5 100.0%	5 / 5 100.0%	1 / 1 100.0%	1 / 1 100.0%	1 / 1 100.0%	1 / 1 100.0%	4 / 4 100.0%	4 / 4 100.0%	1 / 1 100.0%	3 / 3 100.0%	1 / 1 100.0%	2 / 2 100.0%	1 / 1 100.0%	2 / 2 100.0%	5 / 5 100.0%	6 / 6 100.0%	1 / 1 100.0%	5 / 5 100.0%	1 / 1 100.0%	0 / 0 0.0%	0 / 0 0.0%	0 / 0 0.0%	34 / 57 59.6%AB	23 / 57 40.4%ABE						
1	ATCC 43329	57 / 57 100.0%	2 / 2 100.0%	3 / 3 100.0%	2 / 2 100.0%	0 / 0 0.0%	5 / 5 100.0%	5 / 5 100.0%	1 / 1 100.0%	1 / 1 100.0%	1 / 1 100.0%	1 / 1 100.0%	4 / 4 100.0%	4 / 4 100.0%	1 / 1 100.0%	3 / 3 100.0%	1 / 1 100.0%	2 / 2 100.0%	1 / 1 100.0%	2 / 2 100.0%	5 / 5 100.0%	6 / 6 100.0%	1 / 1 100.0%	5 / 5 100.0%	1 / 1 100.0%	0 / 0 0.0%	0 / 0 0.0%	0 / 0 0.0%	34 / 57 59.6%AB	23 / 57 40.4%ABE						
1	Gallibacterium anatis UMN179	57 / 57 100.0%	2 / 2 100.0%	3 / 3 100.0%	2 / 2 100.0%	0 / 0 0.0%	5 / 5 100.0%	5 / 5 100.0%	1 / 1 100.0%	1 / 1 100.0%	1 / 1 100.0%	1 / 1 100.0%	4 / 4 100.0%	4 / 4 100.0%	1 / 1 100.0%	3 / 3 100.0%	1 / 1 100.0%	2 / 2 100.0%	1 / 1 100.0%	2 / 2 100.0%	5 / 5 100.0%	6 / 6 100.0%	1 / 1 100.0%	5 / 5 100.0%	1 / 1 100.0%	0 / 0 0.0%	0 / 0 0.0%	0 / 0 0.0%	34 / 57 59.6%AB	23 / 57 40.4%ABE						
2	Histophilus	97 / 97 100.0%	2 / 2 100.0%	6 / 6 100.0%	5 / 5 100.0%	4 / 4 100.0%	6 / 6 100.0%	8 / 8 100.0%	2 / 2 100.0%	2 / 2 100.0%	6 / 6 100.0%	2 / 2 100.0%	6 / 6 100.0%	6 / 6 100.0%	2 / 2 100.0%	4 / 4 100.0%	2 / 2 100.0%	4 / 4 100.0%	2 / 2 100.0%	4 / 4 100.0%	8 / 8 100.0%	6 / 6 100.0%	2 / 2 100.0%	8 / 8 100.0%	0 / 0 0.0%	0 / 0 0.0%	0 / 0 0.0%	0 / 0 0.0%	57 / 97 58.8%AB	22 / 97 22.7%ABE	10 / 97 10.3%B	8 / 97 8.2%BE				
2	"Haemophilus agni" Kennedy et al. 1958	97 / 97 100.0%	2 / 2 100.0%	6 / 6 100.0%	5 / 5 100.0%	4 / 4 100.0%	6 / 6 100.0%	8 / 8 100.0%	2 / 2 100.0%	2 / 2 100.0%	6 / 6 100.0%	2 / 2 100.0%	6 / 6 100.0%	6 / 6 100.0%	2 / 2 100.0%	4 / 4 100.0%	2 / 2 100.0%	4 / 4 100.0%	2 / 2 100.0%	4 / 4 100.0%	8 / 8 100.0%	6 / 6 100.0%	2 / 2 100.0%	8 / 8 100.0%	0 / 0 0.0%	0 / 0 0.0%	0 / 0 0.0%	0 / 0 0.0%	57 / 97 58.8%AB	22 / 97 22.7%ABE	10 / 97 10.3%B	8 / 97 8.2%BE				
1	Haemophilus somnus 129PT	49 / 49 100.0%	1 / 1 100.0%	3 / 3 100.0%	3 / 3 100.0%	2 / 2 100.0%	3 / 3 100.0%	4 / 4 100.0%	1 / 1 100.0%	1 / 1 100.0%	3 / 3 100.0%	1 / 1 100.0%	3 / 3 100.0%	3 / 3 100.0%	1 / 1 100.0%	2 / 2 100.0%	1 / 1 100.0%	2 / 2 100.0%	1 / 1 100.0%	2 / 2 100.0%	4 / 4 100.0%	3 / 3 100.0%	1 / 1 100.0%	4 / 4 100.0%	0 / 0 0.0%	0 / 0 0.0%	0 / 0 0.0%	0 / 0 0.0%	34 / 49 69.4%AB	15 / 49 30.6%ABE						
1	Haemophilus somnus 2336	48 / 48 100.0%	1 / 1 100.0%	3 / 3 100.0%	2 / 2 100.0%	2 / 2 100.0%	3 / 3 100.0%	4 / 4 100.0%	1 / 1 100.0%	1 / 1 100.0%	3 / 3 100.0%	1 / 1 100.0%	3 / 3 100.0%	3 / 3 100.0%	1 / 1 100.0%	2 / 2 100.0%	1 / 1 100.0%	2 / 2 100.0%	1 / 1 100.0%	2 / 2 100.0%	4 / 4 100.0%	3 / 3 100.0%	1 / 1 100.0%	4 / 4 100.0%	0 / 0 0.0%	0 / 0 0.0%	0 / 0 0.0%	0 / 0 0.0%	23 / 48 47.9%AB	10 / 48 20.8%B	8 / 48 16.7%BE	7 / 48 14.6%ABE				
4	Aggregatibacter	215 / 217 99.1%	8 / 8 100.0%	10 / 10 100.0%	8 / 8 100.0%	14 / 14 100.0%	15 / 15 100.0%	14 / 14 100.0%	4 / 4 100.0%	4 / 4 100.0%	18 / 18 100.0%	4 / 4 100.0%	11 / 12 91.7%	17 / 17 100.0%	4 / 4 100.0%	8 / 8 100.0%	4 / 4 100.0%	8 / 8 100.0%	4 / 4 100.0%	8 / 8 100.0%	16 / 16 100.0%	9 / 9 100.0%	4 / 4 100.0%	20 / 20 100.0%	3 / 4 75.0%	0 / 0 0.0%	0 / 0 0.0%	0 / 0 0.0%	148 / 217 68.2%AB	67 / 217 30.9%ABE	2 / 217 0.9%E					
3	"Bacterium acetinomycetum comitans" (sic) (Klinger 1912) Colebrook 1920	158 / 160 98.8%	6 / 6 100.0%	8 / 8 100.0%	6 / 6 100.0%	8 / 8 100.0%	12 / 12 100.0%	10 / 10 100.0%	3 / 3 100.0%	3 / 3 100.0%	11 / 11 100.0%	3 / 3 100.0%	8 / 9 88.9%	12 / 12 100.0%	3 / 3 100.0%	6 / 6 100.0%	3 / 3 100.0%	6 / 6 100.0%	3 / 3 100.0%	6 / 6 100.0%	12 / 12 100.0%	9 / 9 100.0%	3 / 3 100.0%	15 / 15 100.0%	2 / 3 66.7%	0 / 0 0.0%	0 / 0 0.0%	0 / 0 0.0%	106 / 160 66.2%AB	52 / 160 32.5%ABE	2 / 160 1.2%E					
1	Aggregatibacter actinomycetemcomitans D11S-1	54 / 55 98.2%	2 / 2 100.0%	3 / 3 100.0%	2 / 2 100.0%	3 / 3 100.0%	4 / 4 100.0%	4 / 4 100.0%	1 / 1 100.0%	1 / 1 100.0%	4 / 4 100.0%	1 / 1 100.0%	3 / 3 100.0%	4 / 4 100.0%	1 / 1 100.0%	2 / 2 100.0%	1 / 1 100.0%	2 / 2 100.0%	1 / 1 100.0%	2 / 2 100.0%	4 / 4 100.0%	3 / 3 100.0%	1 / 1 100.0%	5 / 5 100.0%	0 / 1 0.0%	0 / 0 0.0%	0 / 0 0.0%	0 / 0 0.0%	37 / 55 67.3%AB	17 / 55 30.9%ABE	1 / 55 1.8%E					
1	Aggregatibacter actinomycetemcomitans D7S-1	54 / 54 100.0%	2 / 2 100.0%	3 / 3 100.0%	2 / 2 100.0%	2 / 2 100.0%	4 / 4 100.0%	4 / 4 100.0%	1 / 1 100.0%	1 / 1 100.0%	3 / 3 100.0%	1 / 1 100.0%	3 / 3 100.0%	4 / 4 100.0%	1 / 1 100.0%	2 / 2 100.0%	1 / 1 100.0%	2 / 2 100.0%	1 / 1 100.0%	2 / 2 100.0%	4 / 4 100.0%	4 / 4 100.0%	1 / 1 100.0%	5 / 5 100.0%	1 / 1 100.0%	0 / 0 0.0%	0 / 0 0.0%	0 / 0 0.0%	35 / 54 64.8%AB	19 / 54 35.2%ABE						
1	Aggregatibacter actinomycetemcomitans ANH9381	50 / 51 98.0%	2 / 2 100.0%	2 / 2 100.0%	2 / 2 100.0%	3 / 3 100.0%	4 / 4 100.0%	2 / 2 100.0%	1 / 1 100.0%	1 / 1 100.0%	4 / 4 100.0%	1 / 1 100.0%	2 / 3 66.7%	4 / 4 100.0%	1 / 1 100.0%	2 / 2 100.0%	1 / 1 100.0%	2 / 2 100.0%	1 / 1 100.0%	2 / 2 100.0%	4 / 4 100.0%	2 / 2 100.0%	1 / 1 100.0%	5 / 5 100.0%	1 / 1 100.0%	0 / 0 0.0%	0 / 0 0.0%	0 / 0 0.0%	34 / 51 66.7%AB	16 / 51 31.4%ABE	1 / 51 2.0%E					
1	ATCC 33389	57 / 57 100.0%	2 / 2 100.0%	2 / 2 100.0%	2 / 2 100.0%	6 / 6 100.0%	3 / 3 100.0%	4 / 4 100.0%	1 / 1 100.0%	1 / 1 100.0%	7 / 7 100.0%	1 / 1 100.0%	3 / 3 100.0%	5 / 5 100.0%	1 / 1 100.0%	2 / 2 100.0%	1 / 1 100.0%	2 / 2 100.0%	1 / 1 100.0%	2 / 2 100.0%	4 / 4 100.0%	0 / 0 0.0%	1 / 1 100.0%	5 / 5 100.0%	1 / 1 100.0%	0 / 0 0.0%	0 / 0 0.0%	0 / 0 0.0%	42 / 57 73.7%AB	15 / 57 26.3%ABE						
1	Aggregatibacter aphrophilus NJ8700	57 / 57 100.0%	2 / 2 100.0%	2 / 2 100.0%	2 / 2 100.0%	6 / 6 100.0%	3 / 3 100.0%	4 / 4 100.0%	1 / 1 100.0%	1 / 1 100.0%	7 / 7 100.0%	1 / 1 100.0%	3 / 3 100.0%	5 / 5 100.0%	1 / 1 100.0%	2 / 2 100.0%	1 / 1 100.0%	2 / 2 100.0%	1 / 1 100.0%	2 / 2 100.0%	4 / 4 100.0%	0 / 0 0.0%	1 / 1 100.0%	5 / 5 100.0%	1 / 1 100.0%	0 / 0 0.0%	0 / 0 0.0%	0 / 0 0.0%	42 / 57 73.7%AB	15 / 57 26.3%ABE						
1	Bibersteinia	57 / 57 100.0%	1 / 1 100.0%	3 / 3 100.0%	2 / 2 100.0%	3 / 3 100.0%	5 / 5 100.0%	5 / 5 100.0%	1 / 1 100.0%	1 / 1 100.0%	4 / 4 100.0%	1 / 1 100.0%	4 / 4 100.0%	3 / 3 100.0%	1 / 1 100.0%	2 / 2 100.0%	1 / 1 100.0%	2 / 2 100.0%	1 / 1 100.0%	2 / 2 100.0%	4 / 4 100.0%	3 / 3 100.0%	1 / 1 100.0%	6 / 6 100.0%	1 / 1 100.0%	0 / 0 0.0%	0 / 0 0.0%	0 / 0 0.0%	38 / 57 66.7%AB	19 / 57 33.3%ABE						
1	ATCC 29703	57 / 57 100.0%	1 / 1 100.0%	3 / 3 100.0%	2 / 2 100.0%	3 / 3 100.0%	5 / 5 100.0%	5 / 5 100.0%	1 / 1 100.0%	1 / 1 100.0%	4 / 4 100.0%	1 / 1 100.0%	4 / 4 100.0%	3 / 3 100.0%	1 / 1 100.0%	2 / 2 100.0%	1 / 1 100.0%	2 / 2 100.0%	1 / 1 100.0%	2 / 2 100.0%	4 / 4 100.0%	3 / 3 100.0%	1 / 1 100.0%	6 / 6 100.0%	1 / 1 100.0%	0 / 0 0.0%	0 / 0 0.0%	0 / 0 0.0%	38 / 57 66.7%AB	19 / 57 33.3%ABE						
1	Bibersteinia trehalosi 192	57 / 57 100.0%	1 / 1 100.0%	3 / 3 100.0%	2 / 2 100.0%	3 / 3 100.0%	5 / 5 100.0%	5 / 5 100.0%	1 / 1 100.0%	1 / 1 100.0%	4 / 4 100.0%	1 / 1 100.0%	4 / 4 100.0%	3 / 3 100.0%	1 / 1 100.0%	2 / 2 100.0%	1 / 1 100.0%	2 / 2 100.0%	1 / 1 100.0%	2 / 2 100.0%	4 / 4 100.0%	3 / 3 100.0%	1 / 1 100.0%	6 / 6 100.0%	1 / 1 100.0%	0 / 0 0.0%	0 / 0 0.0%	0 / 0 0.0%	38 / 57 66.7%AB	19 / 57 33.3%ABE						
1	Basfia	60 / 60 100.0%	2 / 2 100.0%	3 / 3 100.0%	2 / 2 100.0%	3 / 3 100.0%	5 / 5 100.0%	5 / 5 100.0%	1 / 1 100.0%	1 / 1 100.0%	4 / 4 100.0%	1 / 1 100.0%	4 / 4 100.0%	4 / 4 100.0%	1 / 1 100.0%	2 / 2 100.0%	1 / 1 100.0%	2 / 2 100.0%	1 / 1 100.0%	3 / 3 100.0%	4 / 4 100.0%	3 / 3 100.0%	1 / 1 100.0%	6 / 6 100.0%	1 / 1 100.0%	0 / 0 0.0%	0 / 0 0.0%	0 / 0 0.0%	40 / 60 66.7%AB	19 / 60 31.7%ABE	1 / 60 1.7%BE					
1	"Mannheimia succiniciproducens" Lee et al. 2002	60 / 60 100.0%	2 / 2 100.0%	3 / 3 100.0%	2 / 2 100.0%	3 / 3 100.0%	5 / 5 100.0%	5 / 5 100.0%	1 / 1 100.0%	1 / 1 100.0%	4 / 4 100.0%	1 / 1 100.0%	4 / 4 100.0%	4 / 4 100.0%	1 / 1 100.0%	2 / 2 100.0%	1 / 1 100.0%	2 / 2 100.0%	1 / 1 100.0%	3 / 3 100.0%	4 / 4 100.0%	3 / 3 100.0%	1 / 1 100.0%	6 / 6 100.0%	1 / 1 100.0%	0 / 0 0.0%	0 / 0 0.0%	0 / 0 0.0%	40 / 60 66.7%AB	19 / 60 31.7%ABE	1 / 60 1.7%BE					
1	Mannheimia succiniciproducens MBEL55E	60 / 60 100.0%	2 / 2 100.0%	3 / 3 100.0%	2 / 2 100.0%	3 / 3 100.0%	5 / 5 100.0%	5 / 5 100.0%	1 / 1 100.0%	1 / 1 100.0%	4 / 4 100.0%	1 / 1 100.0%	4 / 4 100.0%	4 / 4 100.0%	1 / 1 100.0%	2 / 2 100.0%	1 / 1 100.0%	2 / 2 100.0%	1 / 1 100.0%	3 / 3 100.0%	4 / 4 100.0%	3 / 3 100.0%	1 / 1 100.0%	6 / 6 100.0%	1 / 1 100.0%	0 / 0 0.0%	0 / 0 0.0%	0 / 0 0.0%	40 / 60 66.7%AB	19 / 60 31.7%ABE	1 / 60 1.7%BE					
4	Acidithiobacillales	216 / 217 99.5%	4 / 4 100.0%	5 / 5 100.0%	5 / 5 100.0%	9 / 9 100.0%	13 / 13 100.0%	13 / 13 100.0%	5 / 5 100.0%	5 / 5 100.0%	17 / 17 100.0%	5 / 5 100.0%	16 / 17 94.1%	10 / 10 100.0%	5 / 5 100.0%	10 / 10 100.0%	6 / 6 100.0%	14 / 14 100.0%	2 / 2 100.0%	13 / 13 100.0%	19 / 19 100.0%	9 / 9 100.0%	7 / 7 100.0%	24 / 24 100.0%	0 / 0 0.0%	0 / 0 0.0%	0 / 0 0.0%	0 / 0 0.0%	130 / 217 59.9%AB	67 / 217 30.9%ABE	13 / 217 6.0%B	6 / 217 2.8%BE	1 / 217 0.5%E			
4	Acidithiobacillaceae	216 / 217 99.5%	4 / 4 100.0%	5 / 5 100.0%	5 / 5 100.0%	9 / 9 100.0%	13 / 13 100.0%	13 / 13 100.0%	5 / 5 100.0%	5 / 5 100.0%	17 / 17 100.0%	5 / 5 100.0%	16 / 17 94.1%	10 / 10 100.0%	5 / 5 100.0%	10 / 10 100.0%	6 / 6 100.0%	14 / 14 100.0%	2 / 2 100.0%	13 / 13 100.0%	19 / 19 100.0%	9 / 9 100.0%	7 / 7 100.0%	24 / 24 100.0%	0 / 0 0.0%	0 / 0 0.0%	0 / 0 0.0%	0 / 0 0.0%	130 / 217 59.9%AB	67 / 217 30.9%ABE	13 / 217 6.0%B	6 / 217 2.8%BE	1 / 217 0.5%E			
4	Acidithiobacillus	216 / 217 99.5%	4 / 4 100.0%	5 / 5 100.0%	5 / 5 100.0%	9 / 9 100.0%	13 / 13 100.0%	13 / 13 100.0%	5 / 5 100.0%	5 / 5 100.0%	17 / 17 100.0%	5 / 5 100.0%	16 / 17 94.1%	10 / 10 100.0%	5 / 5 100.0%	10 / 10 100.0%	6 / 6 100.0%	14 / 14 100.0%	2 / 2 100.0%	13 / 13 100.0%	19 / 19 100.0%	9 / 9 100.0%	7 / 7 100.0%	24 / 24 100.0%	0 / 0 0.0%	0 / 0 0.0%	0 / 0 0.0%	0 / 0 0.0%	130 / 217 59.9%AB	67 / 217 30.9%ABE	13 / 217 6.0%B	6 / 217 2.8%BE	1 / 217 0.5%E			
2	"Ferrobacillus ferrooxidans" Leathen and Braley 1954	124 / 125 99.2%	2 / 2 100.0%	3 / 3 100.0%	3 / 3 100.0%	5 / 5 100.0%	7 / 7 100.0%	7 / 7 100.0%	3 / 3 100.0%	3 / 3 100.0%	9 / 9 100.0%	3 / 3 100.0%	10 / 11 90.9%	6 / 6 100.0%	3 / 3 100.0%	6 / 6 100.0%	3 / 3 100.0%	8 / 8 100.0%	1 / 1 100.0%	8 / 8 100.0%	11 / 11 100.0%	5 / 5 100.0%	4 / 4 100.0%	14 / 14 100.0%	0 / 0 0.0%	0 / 0 0.0%	0 / 0 0.0%	0 / 0 0.0%	68 / 125 54.4%AB	37 / 125 29.6%ABE	13 / 125 10.4%B	6 / 125 4.8%BE	1 / 125 0.8%E			
1	Acidithiobacillus ferrooxidans ATCC 23270	78 / 78 100.0%	1 / 1 100.0%	2 / 2 100.0%	2 / 2 100.0%	3 / 3 100.0%	4 / 4 100.0%	4 / 4 100.0%	2 / 2 100.0%	2 / 2 100.0%	5 / 5 100.0%	2 / 2 100.0%	7 / 7 100.0%	4 / 4 100.0%	2 / 2 100.0%	4 / 4 100.0%	2 / 2 100.0%	5 / 5 100.0%	1 / 1 100.0%	5 / 5 100.0%	7 / 7 100.0%	3 / 3 100.0%	2 / 2 100.0%	9 / 9 100.0%	0 / 0 0.0%	0 / 0 0.0%	0 / 0 0.0%	0 / 0 0.0%	49 / 78 62.8%AB	29 / 78 37.2%ABE						
1	Acidithiobacillus ferrooxidans ATCC 53993	46 / 47 97.9%	1 / 1 100.0%	1 / 1 100.0%	1 / 1 100.0%	2 / 2 100.0%	3 / 3 100.0%	3 / 3 100.0%	1 / 1 100.0%	1 / 1 100.0%	4 / 4 100.0%	1 / 1 100.0%	3 / 4 75.0%	2 / 2 100.0%	1 / 1 100.0%	2 / 2 100.0%	1 / 1 100.0%	3 / 3 100.0%	0 / 0 0.0%	3 / 3 100.0%	4 / 4 100.0%	2 / 2 100.0%	2 / 2 100.0%	5 / 5 100.0%	0 / 0 0.0%	0 / 0 0.0%	0 / 0 0.0%	0 / 0 0.0%	19 / 47 40.4%AB	13 / 47 27.7%B	8 / 47 17.0%ABE	6 / 47 12.8%BE	1 / 47 2.1%E			
1	ATCC 51756	46 / 46 100.0%	1 / 1 100.0%	1 / 1 100.0%	1 / 1 100.0%	2 / 2 100.0%	3 / 3 100.0%	3 / 3 100.0%	1 / 1 100.0%	1 / 1 100.0%	4 / 4 100.0%	1 / 1 100.0%	3 / 3 100.0%	2 / 2 100.0%	1 / 1 100.0%	2 / 2 100.0%	2 / 2 100.0%	3 / 3 100.0%	0 / 0 0.0%	2 / 2 100.0%	4 / 4 100.0%	2 / 2 100.0%	2 / 2 100.0%	5 / 5 100.0%	0 / 0 0.0%	0 / 0 0.0%	0 / 0 0.0%	0 / 0 0.0%	30 / 46 65.2%AB	16 / 46 34.8%ABE						
1	Acidithiobacillus caldus SM-1	46 / 46 100.0%	1 / 1 100.0%	1 / 1 100.0%	1 / 1 100.0%	2 / 2 100.0%	3 / 3 100.0%	3 / 3 100.0%	1 / 1 100.0%	1 / 1 100.0%	4 / 4 100.0%	1 / 1 100.0%	3 / 3 100.0%	2 / 2 100.0%	1 / 1 100.0%	2 / 2 100.0%	2 / 2 100.0%	3 / 3 100.0%	0 / 0 0.0%	2 / 2 100.0%	4 / 4 100.0%	2 / 2 100.0%	2 / 2 100.0%	5 / 5 100.0%	0 / 0 0.0%	0 / 0 0.0%	0 / 0 0.0%	0 / 0 0.0%	30 / 46 65.2%AB	16 / 46 34.8%ABE						
1	Acidithiobacillus ferrivorans	46 / 46 100.0%	1 / 1 100.0%	1 / 1 100.0%	1 / 1 100.0%	2 / 2 100.0%	3 / 3 100.0%	3 / 3 100.0%	1 / 1 100.0%	1 / 1 100.0%	4 / 4 100.0%	1 / 1 100.0%	3 / 3 100.0%	2 / 2 100.0%	1 / 1 100.0%	2 / 2 100.0%	1 / 1 100.0%	3 / 3 100.0%	1 / 1 100.0%	3 / 3 100.0%	4 / 4 100.0%	2 / 2 100.0%	1 / 1 100.0%	5 / 5 100.0%	0 / 0 0.0%	0 / 0 0.0%	0 / 0 0.0%	0 / 0 0.0%	32 / 46 69.6%AB	14 / 46 30.4%ABE						
1	Acidithiobacillus ferrivorans SS3	46 / 46 100.0%	1 / 1 100.0%	1 / 1 100.0%	1 / 1 100.0%	2 / 2 100.0%	3 / 3 100.0%	3 / 3 100.0%	1 / 1 100.0%	1 / 1 100.0%	4 / 4 100.0%	1 / 1 100.0%	3 / 3 100.0%	2 / 2 100.0%	1 / 1 100.0%	2 / 2 100.0%	1 / 1 100.0%	3 / 3 100.0%	1 / 1 100.0%	3 / 3 100.0%	4 / 4 100.0%	2 / 2 100.0%	1 / 1 100.0%	5 / 5 100.0%	0 / 0 0.0%	0 / 0 0.0%	0 / 0 0.0%	0 / 0 0.0%	32 / 46 69.6%AB	14 / 46 30.4%ABE						
231	"Alphabacteria" Cavalier-Smith 1992	10065 / 10657 94.4%	480 / 495 97.0%	307 / 317 96.8%	229 / 247 92.7%	506 / 521 97.1%	634 / 671 94.5%	567 / 601 94.3%	225 / 235 95.7%	202 / 232 87.1%	857 / 874 98.1%	221 / 234 94.4%	818 / 862 94.9%	374 / 398 94.0%	210 / 232 90.5%	374 / 387 96.6%	214 / 232 92.2%	534 / 565 94.5%	215 / 235 91.5%	628 / 683 91.9%	830 / 879 94.4%	401 / 419 95.7%	214 / 243 88.1%	1007 / 1075 93.7%	15 / 17 88.2%	0 / 0 0.0%	2 / 2 100.0%	1 / 1 100.0%	6208 / 10657 58.3%AB	3233 / 10657 30.3%ABE	426 / 10657 4.0%B	294 / 10657 2.8%	232 / 10657 2.2%E	198 / 10657 1.9%BE	47 / 10657 0.4%C	19 / 10657 0.2%CE
94	Hyphomicrobiales	4829 / 4880 99.0%	243 / 246 98.8%	140 / 140 100.0%	98 / 100 98.0%	253 / 256 98.8%	302 / 304 99.3%	253 / 255 99.2%	96 / 97 99.0%	91 / 96 94.8%	430 / 432 99.5%	95 / 96 99.0%	383 / 386 99.2%	193 / 194 99.5%	94 / 96 97.9%	195 / 197 99.0%	95 / 97 97.9%	278 / 279 99.6%	96 / 98 98.0%	283 / 289 97.9%	383 / 385 99.5%	225 / 226 99.6%	101 / 106 95.3%	489 / 491 99.6%	13 / 14 92.9%	0 / 0 0.0%	0 / 0 0.0%	0 / 0 0.0%	2879 / 4880 59.0%AB	1515 / 4880 31.0%ABE	297 / 4880 6.1%B	138 / 4880 2.8%BE	19 / 4880 0.4%E	14 / 4880 0.3%	13 / 4880 0.3%C	5 / 4880 0.1%CE
[illegible]	[illegible]	365 / 380	19 / 19	9 / 9	10 / 10	18 / 18	19 / 19	17 / 18	9 / 9	9 / 9	27 / 27	8 / 9	35 / 36	18 / 18	7 / 9	16 / 17	9 / 9	18 / 18	8 / 9	22 / 27	27 / 27	9 / 9	8 / 9	43 / 45	0 / 0	0 / 0	0 / 0	0 / 0	227 / 380	118 / 380	14 / 380	10 / 380	6 / 380	3 / 380	1 / 380	1 / 380

[illegible]	Bartonella group	96.1%	100.0%	100.0%	100.0%	100.0%	100.0%	94.4%	100.0%	100.0%	100.0%	88.9%	97.2%	100.0%	77.8%	94.1%	100.0%	100.0%	88.9%	81.5%	100.0%	100.0%	88.9%	95.6%	0.0%	0.0%	0.0%	0.0%	59.7%AB	31.1%ABE	3.7%B	2.6%C	1.6%BE	0.8%E	0.3%CE	0.3%
9	"Bartonia" Strong et al. 1913	365 / 380 96.1%	19 / 19 100.0%	9 / 9 100.0%	10 / 10 100.0%	18 / 18 100.0%	19 / 19 100.0%	17 / 18 94.4%	9 / 9 100.0%	9 / 9 100.0%	27 / 27 100.0%	8 / 9 88.9%	35 / 36 97.2%	18 / 18 100.0%	7 / 9 77.8%	16 / 17 94.1%	9 / 9 100.0%	18 / 18 100.0%	8 / 9 88.9%	22 / 27 81.5%	27 / 27 100.0%	9 / 9 100.0%	8 / 9 88.9%	43 / 45 95.6%	0 / 0 0.0%	0 / 0 0.0%	0 / 0 0.0%	0 / 0 0.0%	227 / 380 59.7%AB	118 / 380 31.1%ABE	14 / 380 3.7%B	10 / 380 2.6%C	6 / 380 1.6%BE	3 / 380 0.8%E	1 / 380 0.3%	1 / 380 0.3%CE
1	"Bartonia bacilliformis" Strong et al. 1913	43 / 44 97.7%	2 / 2 100.0%	1 / 1 100.0%	2 / 2 100.0%	2 / 2 100.0%	2 / 2 100.0%	2 / 2 100.0%	1 / 1 100.0%	1 / 1 100.0%	3 / 3 100.0%	0 / 1 0.0%	4 / 4 100.0%	2 / 2 100.0%	1 / 1 100.0%	2 / 2 100.0%	2 / 2 100.0%	2 / 2 100.0%	1 / 1 100.0%	3 / 3 100.0%	3 / 3 100.0%	1 / 1 100.0%	1 / 1 100.0%	5 / 5 100.0%	0 / 0 0.0%	0 / 0 0.0%	0 / 0 0.0%	0 / 0 0.0%	28 / 44 63.6%AB	15 / 44 34.1%ABE	1 / 44 2.3%C					
1	Bartonella bacilliformis KC583	43 / 44 97.7%	2 / 2 100.0%	1 / 1 100.0%	2 / 2 100.0%	2 / 2 100.0%	2 / 2 100.0%	2 / 2 100.0%	1 / 1 100.0%	1 / 1 100.0%	3 / 3 100.0%	0 / 1 0.0%	4 / 4 100.0%	2 / 2 100.0%	1 / 1 100.0%	2 / 2 100.0%	2 / 2 100.0%	2 / 2 100.0%	1 / 1 100.0%	3 / 3 100.0%	3 / 3 100.0%	1 / 1 100.0%	1 / 1 100.0%	5 / 5 100.0%	0 / 0 0.0%	0 / 0 0.0%	0 / 0 0.0%	0 / 0 0.0%	28 / 44 63.6%AB	15 / 44 34.1%ABE	1 / 44 2.3%C					
2	"Burnetia (Rocha-limae) wolhynica" Macchiavello 1947	81 / 84 96.4%	4 / 4 100.0%	2 / 2 100.0%	2 / 2 100.0%	4 / 4 100.0%	4 / 4 100.0%	4 / 4 100.0%	2 / 2 100.0%	2 / 2 100.0%	6 / 6 100.0%	2 / 2 100.0%	8 / 8 100.0%	4 / 4 100.0%	1 / 2 50.0%	4 / 4 100.0%	2 / 2 100.0%	4 / 4 100.0%	2 / 2 100.0%	4 / 6 66.7%	6 / 6 100.0%	2 / 2 100.0%	2 / 2 100.0%	10 / 10 100.0%	0 / 0 0.0%	0 / 0 0.0%	0 / 0 0.0%	0 / 0 0.0%	53 / 84 63.1%AB	28 / 84 33.3%ABE	3 / 84 3.6%C					
1	Bartonella quintana str. Toulose	41 / 42 97.6%	2 / 2 100.0%	1 / 1 100.0%	1 / 1 100.0%	2 / 2 100.0%	2 / 2 100.0%	2 / 2 100.0%	1 / 1 100.0%	1 / 1 100.0%	3 / 3 100.0%	1 / 1 100.0%	4 / 4 100.0%	2 / 2 100.0%	1 / 1 100.0%	2 / 2 100.0%	1 / 1 100.0%	2 / 2 100.0%	1 / 1 100.0%	2 / 3 66.7%	3 / 3 100.0%	1 / 1 100.0%	1 / 1 100.0%	5 / 5 100.0%	0 / 0 0.0%	0 / 0 0.0%	0 / 0 0.0%	0 / 0 0.0%	27 / 42 64.3%AB	14 / 42 33.3%ABE	1 / 42 2.4%C					
1	Bartonella quintana RM-11	40 / 42 95.2%	2 / 2 100.0%	1 / 1 100.0%	1 / 1 100.0%	2 / 2 100.0%	2 / 2 100.0%	2 / 2 100.0%	1 / 1 100.0%	1 / 1 100.0%	3 / 3 100.0%	1 / 1 100.0%	4 / 4 100.0%	2 / 2 100.0%	0 / 1 0.0%	2 / 2 100.0%	1 / 1 100.0%	2 / 2 100.0%	1 / 1 100.0%	2 / 3 66.7%	3 / 3 100.0%	1 / 1 100.0%	1 / 1 100.0%	5 / 5 100.0%	0 / 0 0.0%	0 / 0 0.0%	0 / 0 0.0%	0 / 0 0.0%	26 / 42 61.9%AB	14 / 42 33.3%ABE	2 / 42 4.8%C					
1	ATCC 700132	40 / 42 95.2%	2 / 2 100.0%	1 / 1 100.0%	1 / 1 100.0%	2 / 2 100.0%	2 / 2 100.0%	2 / 2 100.0%	1 / 1 100.0%	1 / 1 100.0%	3 / 3 100.0%	1 / 1 100.0%	4 / 4 100.0%	2 / 2 100.0%	1 / 1 100.0%	1 / 2 50.0%	1 / 1 100.0%	2 / 2 100.0%	1 / 1 100.0%	2 / 3 66.7%	3 / 3 100.0%	1 / 1 100.0%	1 / 1 100.0%	5 / 5 100.0%	0 / 0 0.0%	0 / 0 0.0%	0 / 0 0.0%	0 / 0 0.0%	26 / 42 61.9%AB	14 / 42 33.3%ABE	1 / 42 2.4%E	1 / 42 2.4%C				
1	Bartonella grahamii as4aup	40 / 42 95.2%	2 / 2 100.0%	1 / 1 100.0%	1 / 1 100.0%	2 / 2 100.0%	2 / 2 100.0%	2 / 2 100.0%	1 / 1 100.0%	1 / 1 100.0%	3 / 3 100.0%	1 / 1 100.0%	4 / 4 100.0%	2 / 2 100.0%	1 / 1 100.0%	1 / 2 50.0%	1 / 1 100.0%	2 / 2 100.0%	1 / 1 100.0%	2 / 3 66.7%	3 / 3 100.0%	1 / 1 100.0%	1 / 1 100.0%	5 / 5 100.0%	0 / 0 0.0%	0 / 0 0.0%	0 / 0 0.0%	0 / 0 0.0%	26 / 42 61.9%AB	14 / 42 33.3%ABE	1 / 42 2.4%C	1 / 42 2.4%E				
1	ATCC VR-152	42 / 42 100.0%	2 / 2 100.0%	1 / 1 100.0%	1 / 1 100.0%	2 / 2 100.0%	2 / 2 100.0%	2 / 2 100.0%	1 / 1 100.0%	1 / 1 100.0%	3 / 3 100.0%	1 / 1 100.0%	4 / 4 100.0%	2 / 2 100.0%	1 / 1 100.0%	2 / 2 100.0%	1 / 1 100.0%	2 / 2 100.0%	1 / 1 100.0%	3 / 3 100.0%	3 / 3 100.0%	1 / 1 100.0%	1 / 1 100.0%	5 / 5 100.0%	0 / 0 0.0%	0 / 0 0.0%	0 / 0 0.0%	0 / 0 0.0%	28 / 42 66.7%AB	14 / 42 33.3%ABE						
1	ATCC 51672	42 / 42 100.0%	2 / 2 100.0%	1 / 1 100.0%	1 / 1 100.0%	2 / 2 100.0%	2 / 2 100.0%	2 / 2 100.0%	1 / 1 100.0%	1 / 1 100.0%	3 / 3 100.0%	1 / 1 100.0%	4 / 4 100.0%	2 / 2 100.0%	1 / 1 100.0%	2 / 2 100.0%	1 / 1 100.0%	2 / 2 100.0%	1 / 1 100.0%	3 / 3 100.0%	3 / 3 100.0%	1 / 1 100.0%	1 / 1 100.0%	5 / 5 100.0%	0 / 0 0.0%	0 / 0 0.0%	0 / 0 0.0%	0 / 0 0.0%	28 / 42 66.7%AB	14 / 42 33.3%ABE						
1	Bartonella vinsonii subsp. berkhoffii str. Winnie	42 / 42 100.0%	2 / 2 100.0%	1 / 1 100.0%	1 / 1 100.0%	2 / 2 100.0%	2 / 2 100.0%	2 / 2 100.0%	1 / 1 100.0%	1 / 1 100.0%	3 / 3 100.0%	1 / 1 100.0%	4 / 4 100.0%	2 / 2 100.0%	1 / 1 100.0%	2 / 2 100.0%	1 / 1 100.0%	2 / 2 100.0%	1 / 1 100.0%	3 / 3 100.0%	3 / 3 100.0%	1 / 1 100.0%	1 / 1 100.0%	5 / 5 100.0%	0 / 0 0.0%	0 / 0 0.0%	0 / 0 0.0%	0 / 0 0.0%	28 / 42 66.7%AB	14 / 42 33.3%ABE						
1	ATCC 49882	43 / 43 100.0%	3 / 3 100.0%	1 / 1 100.0%	1 / 1 100.0%	2 / 2 100.0%	3 / 3 100.0%	2 / 2 100.0%	1 / 1 100.0%	1 / 1 100.0%	3 / 3 100.0%	1 / 1 100.0%	4 / 4 100.0%	2 / 2 100.0%	1 / 1 100.0%	2 / 2 100.0%	0 / 0 0.0%	2 / 2 100.0%	1 / 1 100.0%	3 / 3 100.0%	3 / 3 100.0%	1 / 1 100.0%	1 / 1 100.0%	5 / 5 100.0%	0 / 0 0.0%	0 / 0 0.0%	0 / 0 0.0%	0 / 0 0.0%	29 / 43 67.4%AB	14 / 43 32.6%ABE						
1	ATCC 51734	35 / 41 85.4%	2 / 2 100.0%	1 / 1 100.0%	1 / 1 100.0%	2 / 2 100.0%	2 / 2 100.0%	1 / 2 50.0%	1 / 1 100.0%	1 / 1 100.0%	3 / 3 100.0%	1 / 1 100.0%	4 / 4 100.0%	2 / 2 100.0%	0 / 1 0.0%	1 / 1 100.0%	1 / 1 100.0%	2 / 2 100.0%	1 / 1 100.0%	2 / 3 66.7%	3 / 3 100.0%	1 / 1 100.0%	0 / 1 0.0%	3 / 5 60.0%	0 / 0 0.0%	0 / 0 0.0%	0 / 0 0.0%	0 / 0 0.0%	23 / 41 56.1%AB	12 / 41 29.3%ABE	3 / 41 7.3%C	1 / 41 2.4%E	1 / 41 2.4%CE	1 / 41 2.4%		
1	Bartonella tribochorum	41 / 42 97.6%	2 / 2 100.0%	1 / 1 100.0%	1 / 1 100.0%	2 / 2 100.0%	2 / 2 100.0%	2 / 2 100.0%	1 / 1 100.0%	1 / 1 100.0%	3 / 3 100.0%	1 / 1 100.0%	3 / 4 75.0%	2 / 2 100.0%	1 / 1 100.0%	2 / 2 100.0%	1 / 1 100.0%	2 / 2 100.0%	1 / 1 100.0%	3 / 3 100.0%	3 / 3 100.0%	1 / 1 100.0%	1 / 1 100.0%	5 / 5 100.0%	0 / 0 0.0%	0 / 0 0.0%	0 / 0 0.0%	0 / 0 0.0%	14 / 42 33.3%B	14 / 42 33.3%AB	7 / 42 16.7%ABE	6 / 42 14.3%BE	1 / 42 2.4%E			
1	Bartonella tribocorum CIP 105476	41 / 42 97.6%	2 / 2 100.0%	1 / 1 100.0%	1 / 1 100.0%	2 / 2 100.0%	2 / 2 100.0%	2 / 2 100.0%	1 / 1 100.0%	1 / 1 100.0%	3 / 3 100.0%	1 / 1 100.0%	3 / 4 75.0%	2 / 2 100.0%	1 / 1 100.0%	2 / 2 100.0%	1 / 1 100.0%	2 / 2 100.0%	1 / 1 100.0%	3 / 3 100.0%	3 / 3 100.0%	1 / 1 100.0%	1 / 1 100.0%	5 / 5 100.0%	0 / 0 0.0%	0 / 0 0.0%	0 / 0 0.0%	0 / 0 0.0%	14 / 42 33.3%AB	14 / 42 33.3%B	7 / 42 16.7%ABE	6 / 42 14.3%BE	1 / 42 2.4%E			
1	"Bartonella australis" Fournier et al. 2007	40 / 42 95.2%	2 / 2 100.0%	1 / 1 100.0%	1 / 1 100.0%	2 / 2 100.0%	2 / 2 100.0%	2 / 2 100.0%	1 / 1 100.0%	1 / 1 100.0%	3 / 3 100.0%	1 / 1 100.0%	4 / 4 100.0%	2 / 2 100.0%	1 / 1 100.0%	2 / 2 100.0%	1 / 1 100.0%	2 / 2 100.0%	0 / 1 0.0%	2 / 3 66.7%	3 / 3 100.0%	1 / 1 100.0%	1 / 1 100.0%	5 / 5 100.0%	0 / 0 0.0%	0 / 0 0.0%	0 / 0 0.0%	0 / 0 0.0%	26 / 42 61.9%AB	14 / 42 33.3%ABE	2 / 42 4.8%C					
1	Bartonella australis Aust/NH1	40 / 42 95.2%	2 / 2 100.0%	1 / 1 100.0%	1 / 1 100.0%	2 / 2 100.0%	2 / 2 100.0%	2 / 2 100.0%	1 / 1 100.0%	1 / 1 100.0%	3 / 3 100.0%	1 / 1 100.0%	4 / 4 100.0%	2 / 2 100.0%	1 / 1 100.0%	2 / 2 100.0%	1 / 1 100.0%	2 / 2 100.0%	0 / 1 0.0%	2 / 3 66.7%	3 / 3 100.0%	1 / 1 100.0%	1 / 1 100.0%	5 / 5 100.0%	0 / 0 0.0%	0 / 0 0.0%	0 / 0 0.0%	0 / 0 0.0%	26 / 42 61.9%AB	14 / 42 33.3%ABE	2 / 42 4.8%C					
1	'Methylocystaceae'	45 / 46 97.8%	1 / 1 100.0%	1 / 1 100.0%	1 / 1 100.0%	1 / 1 100.0%	3 / 3 100.0%	3 / 3 100.0%	1 / 1 100.0%	1 / 1 100.0%	3 / 3 100.0%	1 / 1 100.0%	4 / 4 100.0%	2 / 2 100.0%	1 / 1 100.0%	3 / 3 100.0%	0 / 1 0.0%	3 / 3 100.0%	1 / 1 100.0%	3 / 3 100.0%	5 / 5 100.0%	2 / 2 100.0%	1 / 1 100.0%	4 / 4 100.0%	0 / 0 0.0%	0 / 0 0.0%	0 / 0 0.0%	0 / 0 0.0%	32 / 46 69.6%AB	13 / 46 28.3%ABE	1 / 46 2.2%E					
1	Methylocystis	45 / 46 97.8%	1 / 1 100.0%	1 / 1 100.0%	1 / 1 100.0%	1 / 1 100.0%	3 / 3 100.0%	3 / 3 100.0%	1 / 1 100.0%	1 / 1 100.0%	3 / 3 100.0%	1 / 1 100.0%	4 / 4 100.0%	2 / 2 100.0%	1 / 1 100.0%	3 / 3 100.0%	0 / 1 0.0%	3 / 3 100.0%	1 / 1 100.0%	3 / 3 100.0%	5 / 5 100.0%	2 / 2 100.0%	1 / 1 100.0%	4 / 4 100.0%	0 / 0 0.0%	0 / 0 0.0%	0 / 0 0.0%	0 / 0 0.0%	32 / 46 69.6%AB	13 / 46 28.3%ABE	1 / 46 2.2%E					
1	Methylocystis sp. SC2	45 / 46 97.8%	1 / 1 100.0%	1 / 1 100.0%	1 / 1 100.0%	1 / 1 100.0%	3 / 3 100.0%	3 / 3 100.0%	1 / 1 100.0%	1 / 1 100.0%	3 / 3 100.0%	1 / 1 100.0%	4 / 4 100.0%	2 / 2 100.0%	1 / 1 100.0%	3 / 3 100.0%	0 / 1 0.0%	3 / 3 100.0%	1 / 1 100.0%	3 / 3 100.0%	5 / 5 100.0%	2 / 2 100.0%	1 / 1 100.0%	4 / 4 100.0%	0 / 0 0.0%	0 / 0 0.0%	0 / 0 0.0%	0 / 0 0.0%	32 / 46 69.6%AB	13 / 46 28.3%ABE	1 / 46 2.2%E					
17	'Bradyrhizobiaceae'	861 / 868 99.2%	21 / 21 100.0%	19 / 19 100.0%	18 / 19 94.7%	28 / 28 100.0%	55 / 55 100.0%	54 / 54 100.0%	18 / 18 100.0%	13 / 18 72.2%	64 / 64 100.0%	18 / 18 100.0%	73 / 73 100.0%	36 / 36 100.0%	18 / 18 100.0%	36 / 36 100.0%	18 / 18 100.0%	56 / 56 100.0%	18 / 18 100.0%	54 / 54 100.0%	93 / 93 100.0%	36 / 36 100.0%	22 / 23 95.7%	90 / 90 100.0%	3 / 3 100.0%	0 / 0 0.0%	0 / 0 0.0%	0 / 0 0.0%	552 / 868 63.6%AB	309 / 868 35.6%ABE	5 / 868 0.6%E	2 / 868 0.2%				
6	Agromonas	289 / 292 99.0%	7 / 7 100.0%	7 / 7 100.0%	6 / 7 85.7%	10 / 10 100.0%	18 / 18 100.0%	18 / 18 100.0%	6 / 6 100.0%	5 / 6 83.3%	22 / 22 100.0%	6 / 6 100.0%	24 / 24 100.0%	12 / 12 100.0%	6 / 6 100.0%	12 / 12 100.0%	6 / 6 100.0%	18 / 18 100.0%	6 / 6 100.0%	18 / 18 100.0%	30 / 30 100.0%	12 / 12 100.0%	10 / 11 90.9%	30 / 30 100.0%	0 / 0 0.0%	0 / 0 0.0%	0 / 0 0.0%	0 / 0 0.0%	186 / 292 63.7%AB	103 / 292 35.3%ABE	2 / 292 0.7%	1 / 292 0.3%E				
1	"Rhizobacterium japonicum" Kirchner 1896	50 / 51 98.0%	1 / 1 100.0%	1 / 1 100.0%	1 / 1 100.0%	2 / 2 100.0%	3 / 3 100.0%	3 / 3 100.0%	1 / 1 100.0%	1 / 1 100.0%	4 / 4 100.0%	1 / 1 100.0%	4 / 4 100.0%	2 / 2 100.0%	1 / 1 100.0%	2 / 2 100.0%	1 / 1 100.0%	3 / 3 100.0%	1 / 1 100.0%	3 / 3 100.0%	5 / 5 100.0%	2 / 2 100.0%	3 / 4 75.0%	5 / 5 100.0%	0 / 0 0.0%	0 / 0 0.0%	0 / 0 0.0%	0 / 0 0.0%	33 / 51 64.7%AB	17 / 51 33.3%ABE	1 / 51 2.0%					
1	Bradyrhizobium japonicum USDA 6	50 / 51 98.0%	1 / 1 100.0%	1 / 1 100.0%	1 / 1 100.0%	2 / 2 100.0%	3 / 3 100.0%	3 / 3 100.0%	1 / 1 100.0%	1 / 1 100.0%	4 / 4 100.0%	1 / 1 100.0%	4 / 4 100.0%	2 / 2 100.0%	1 / 1 100.0%	2 / 2 100.0%	1 / 1 100.0%	3 / 3 100.0%	1 / 1 100.0%	3 / 3 100.0%	5 / 5 100.0%	2 / 2 100.0%	3 / 4 75.0%	5 / 5 100.0%	0 / 0 0.0%	0 / 0 0.0%	0 / 0 0.0%	0 / 0 0.0%	33 / 51 64.7%AB	17 / 51 33.3%ABE	1 / 51 2.0%					
1	ATCC 43045	50 / 50 100.0%	2 / 2 100.0%	2 / 2 100.0%	1 / 1 100.0%	2 / 2 100.0%	3 / 3 100.0%	3 / 3 100.0%	1 / 1 100.0%	1 / 1 100.0%	4 / 4 100.0%	1 / 1 100.0%	4 / 4 100.0%	2 / 2 100.0%	1 / 1 100.0%	2 / 2 100.0%	1 / 1 100.0%	3 / 3 100.0%	1 / 1 100.0%	3 / 3 100.0%	5 / 5 100.0%	2 / 2 100.0%	1 / 1 100.0%	5 / 5 100.0%	0 / 0 0.0%	0 / 0 0.0%	0 / 0 0.0%	0 / 0 0.0%	31 / 50 62.0%AB	19 / 50 38.0%ABE						
1	Agromonas oligotrophica JCM 1494	50 / 50 100.0%	2 / 2 100.0%	2 / 2 100.0%	1 / 1 100.0%	2 / 2 100.0%	3 / 3 100.0%	3 / 3 100.0%	1 / 1 100.0%	1 / 1 100.0%	4 / 4 100.0%	1 / 1 100.0%	4 / 4 100.0%	2 / 2 100.0%	1 / 1 100.0%	2 / 2 100.0%	1 / 1 100.0%	3 / 3 100.0%	1 / 1 100.0%	3 / 3 100.0%	5 / 5 100.0%	2 / 2 100.0%	1 / 1 100.0%	5 / 5 100.0%	0 / 0 0.0%	0 / 0 0.0%	0 / 0 0.0%	0 / 0 0.0%	31 / 50 62.0%AB	19 / 50 38.0%ABE						
1	Bradyrhizobium sp. ORS 278	48 / 48 100.0%	1 / 1 100.0%	1 / 1 100.0%	1 / 1 100.0%	2 / 2 100.0%	3 / 3 100.0%	3 / 3 100.0%	1 / 1 100.0%	1 / 1 100.0%	4 / 4 100.0%	1 / 1 100.0%	4 / 4 100.0%	2 / 2 100.0%	1 / 1 100.0%	2 / 2 100.0%	1 / 1 100.0%	3 / 3 100.0%	1 / 1 100.0%	3 / 3 100.0%	5 / 5 100.0%	2 / 2 100.0%	1 / 1 100.0%	5 / 5 100.0%	0 / 0 0.0%	0 / 0 0.0%	0 / 0 0.0%	0 / 0 0.0%	31 / 48 64.6%AB	17 / 48 35.4%ABE						
1	Bradyrhizobium sp. BTAi1	48 / 48 100.0%	1 / 1 100.0%	1 / 1 100.0%	1 / 1 100.0%	2 / 2 100.0%	3 / 3 100.0%	3 / 3 100.0%	1 / 1 100.0%	1 / 1 100.0%	4 / 4 100.0%	1 / 1 100.0%	4 / 4 100.0%	2 / 2 100.0%	1 / 1 100.0%	2 / 2 100.0%	1 / 1 100.0%	3 / 3 100.0%	1 / 1 100.0%	3 / 3 100.0%	5 / 5 100.0%	2 / 2 100.0%	1 / 1 100.0%	5 / 5 100.0%	0 / 0 0.0%	0 / 0 0.0%	0 / 0 0.0%	0 / 0 0.0%	31 / 48 64.6%AB	17 / 48 35.4%ABE						
1	Bradyrhizobium sp. S23321	45 / 46 97.8%	1 / 1 100.0%	1 / 1 100.0%	1 / 1 100.0%	1 / 1 100.0%	3 / 3 100.0%	3 / 3 100.0%	1 / 1 100.0%	0 / 1 0.0%	3 / 3 100.0%	1 / 1 100.0%	4 / 4 100.0%	2 / 2 100.0%	1 / 1 100.0%	2 / 2 100.0%	1 / 1 100.0%	3 / 3 100.0%	1 / 1 100.0%	3 / 3 100.0%	5 / 5 100.0%	2 / 2 100.0%	1 / 1 100.0%	5 / 5 100.0%	0 / 0 0.0%	0 / 0 0.0%	0 / 0 0.0%	0 / 0 0.0%	29 / 46 63.0%AB	16 / 46 34.8%ABE	1 / 46 2.2%E					
1	'Bradyrhizobium diazoefficiens'	48 / 49 98.0%	1 / 1 100.0%	1 / 1 100.0%	1 / 2 50.0%	1 / 1 100.0%	3 / 3 100.0%	3 / 3 100.0%	1 / 1 100.0%	1 / 1 100.0%	3 / 3 100.0%	1 / 1 100.0%	4 / 4 100.0%	2 / 2 100.0%	1 / 1 100.0%	2 / 2 100.0%	1 / 1 100.0%	3 / 3 100.0%	1 / 1 100.0%	3 / 3 100.0%	5 / 5 100.0%	2 / 2 100.0%	3 / 3 100.0%	5 / 5 100.0%	0 / 0 0.0%	0 / 0 0.0%	0 / 0 0.0%	0 / 0 0.0%	31 / 49 63.3%AB	17 / 49 34.7%ABE	1 / 49 2.0%					
1	Bradyrhizobium diazoefficiens USDA 110	48 / 49 98.0%	1 / 1 100.0%	1 / 1 100.0%	1 / 2 50.0%	1 / 1 100.0%	3 / 3 100.0%	3 / 3 100.0%	1 / 1 100.0%	1 / 1 100.0%	3 / 3 100.0%	1 / 1 100.0%	4 / 4 100.0%	2 / 2 100.0%	1 / 1 100.0%	2 / 2 100.0%	1 / 1 100.0%	3 / 3 100.0%	1 / 1 100.0%	3 / 3 100.0%	5 / 5 100.0%	2 / 2 100.0%	3 / 3 100.0%	5 / 5 100.0%	0 / 0 0.0%	0 / 0 0.0%	0 / 0 0.0%	0 / 0 0.0%	31 / 49 63.3%AB	17 / 49 34.7%ABE	1 / 49 2.0%					
2	Nitrobacter	94 / 94 100.0%	2 / 2 100.0%	2 / 2 100.0%	2 / 2 100.0%	2 / 2 100.0%	6 / 6 100.0%	6 / 6 100.0%	2 / 2 100.0%	2 / 2 100.0%	6 / 6 100.0%	2 / 2 100.0%	8 / 8 100.0%	4 / 4 100.0%	2 / 2 100.0%	4 / 4 100.0%	2 / 2 100.0%	8 / 8 100.0%	2 / 2 100.0%	6 / 6 100.0%	10 / 10 100.0%	4 / 4 100.0%	2 / 2 100.0%	10 / 10 100.0%	0 / 0 0.0%	0 / 0 0.0%	0 / 0 0.0%	0 / 0 0.0%	62 / 94 66.0%AB	32 / 94 34.0%ABE						
1	DSM 10229	48 / 48 100.0%	1 / 1 100.0%	1 / 1 100.0%	1 / 1 100.0%	1 / 1 100.0%	3 / 3 100.0%	3 / 3 100.0%	1 / 1 100.0%	1 / 1 100.0%	3 / 3 100.0%	1 / 1 100.0%	4 / 4 100.0%	2 / 2 100.0%	1 / 1 100.0%	2 / 2 100.0%	1 / 1 100.0%	5 / 5 100.0%	1 / 1 100.0%	3 / 3 100.0%	5 / 5 100.0%	2 / 2 100.0%	1 / 1 100.0%	5 / 5 100.0%	0 / 0 0.0%	0 / 0 0.0%	0 / 0 0.0%	0 / 0 0.0%	32 / 48 66.7%AB	16 / 48 33.3%ABE						
1	Nitrobacter hamburgensis X14	48 / 48 100.0%	1 / 1 100.0%	1 / 1 100.0%	1 / 1 100.0%	1 / 1 100.0%	3 / 3 100.0%	3 / 3 100.0%	1 / 1 100.0%	1 / 1 100.0%	3 / 3 100.0%	1 / 1 100.0%	4 / 4 100.0%	2 / 2 100.0%	1 / 1 100.0%	2 / 2 100.0%	1 / 1 100.0%	5 / 5 100.0%	1 / 1 100.0%	3 / 3 100.0%	5 / 5 100.0%	2 / 2 100.0%	1 / 1 100.0%	5 / 5 100.0%	0 / 0 0.0%	0 / 0 0.0%	0 / 0 0.0%	0 / 0 0.0%	32 / 48 66.7%AB	16 / 48 33.3%ABE						
1	"Bacillus nirobakter" (sic) Castellani and Chamers 1919	46 / 46 100.0%	1 / 1 100.0%	1 / 1 100.0%	1 / 1 100.0%	1 / 1 100.0%	3 / 3 100.0%	3 / 3 100.0%	1 / 1 100.0%	1 / 1 100.0%	3 / 3 100.0%	1 / 1 100.0%	4 / 4 100.0%	2 / 2 100.0%	1 / 1 100.0%	2 / 2 100.0%	1 / 1 100.0%	3 / 3 100.0%	1 / 1 100.0%	3 / 3 100.0%	5 / 5 100.0%	2 / 2 100.0%	1 / 1 100.0%	5 / 5 100.0%	0 / 0 0.0%	0 / 0 0.0%	0 / 0 0.0%	0 / 0 0.0%	30 / 46 65.2%AB	16 / 46 34.8%ABE						
1	Nitrobacter winogradskyi Nb-255	46 / 46 100.0%	1 / 1 100.0%	1 / 1 100.0%	1 / 1 100.0%	1 / 1 100.0%	3 / 3 100.0%	3 / 3 100.0%	1 / 1 100.0%	1 / 1 100.0%	3 / 3 100.0%	1 / 1 100.0%	4 / 4 100.0%	2 / 2 100.0%	1 / 1 100.0%	2 / 2 100.0%	1 / 1 100.0%	3 / 3 100.0%	1 / 1 100.0%	3 / 3 100.0%	5 / 5 100.0%	2 / 2 100.0%	1 / 1 100.0%	5 / 5 100.0%	0 / 0 0.0%	0 / 0 0.0%	0 / 0 0.0%	0 / 0 0.0%	30 / 46 65.2%AB	16 / 46 34.8%ABE						
7	"Rhodobacillus" Molisch 1907	337 / 338 99.7%	9 / 9 100.0%	7 / 7 100.0%	7 / 7 100.0%	13 / 13 100.0%	22 / 22 100.0%	21 / 21 100.0%	7 / 7 100.0%	6 / 7 85.7%	27 / 27 100.0%	7 / 7 100.0%	29 / 29 100.0%	14 / 14 100.0%	7 / 7 100.0%	14 / 14 100.0%	7 / 7 100.0%	21 / 21 100.0%	7 / 7 100.0%	21 / 21 100.0%	35 / 35 100.0%	14 / 14 100.0%	7 / 7 100.0%	35 / 35 100.0%	0 / 0 0.0%	0 / 0 0.0%	0 / 0 0.0%	0 / 0 0.0%	217 / 338 64.2%AB	120 / 338 35.5%ABE	1 / 338 0.3%E					
7	"Rhodobacillus palustris" Molisch 1907	337 / 338 99.7%	9 / 9 100.0%	7 / 7 100.0%	7 / 7 100.0%	13 / 13 100.0%	22 / 22 100.0%	21 / 21 100.0%	7 / 7 100.0%	6 / 7 85.7%	27 / 27 100.0%	7 / 7 100.0%	29 / 29 100.0%	14 / 14 100.0%	7 / 7 100.0%	14 / 14 100.0%	7 / 7 100.0%	21 / 21 100.0%	7 / 7 100.0%	21 / 21 100.0%	35 / 35 100.0%	14 / 14 100.0%	7 / 7 100.0%	35 / 35 100.0%	0 / 0 0.0%	0 / 0 0.0%	0 / 0 0.0%	0 / 0 0.0%	217 / 338 64.2%AB	120 / 338 35.5%ABE	1 / 338 0.3%E					
1	Rhodopseudomonas palustris CGA009	48 / 48 100.0%	1 / 1 100.0%	1 / 1 100.0%	1 / 1 100.0%	2 / 2 100.0%	3 / 3 100.0%	3 / 3 100.0%	1 / 1 100.0%	1 / 1 100.0%	4 / 4 100.0%	1 / 1 100.0%	4 / 4 100.0%	2 / 2 100.0%	1 / 1 100.0%	2 / 2 100.0%	1 / 1 100.0%	3 / 3 100.0%	1 / 1 100.0%	3 / 3 100.0%	5 / 5 100.0%	2 / 2 100.0%	1 / 1 100.0%	5 / 5 100.0%	0 / 0 0.0%	0 / 0 0.0%	0 / 0 0.0%	0 / 0 0.0%	31 / 48 64.6%AB	17 / 48 35.4%ABE						
1	Rhodopseudomonas palustris BisA53	48 / 48 100.0%	1 / 1 100.0%	1 / 1 100.0%	1 / 1 100.0%	2 / 2 100.0%	3 / 3 100.0%	3 / 3 100.0%	1 / 1 100.0%	1 / 1 100.0%	4 / 4 100.0%	1 / 1 100.0%	4 / 4 100.0%	2 / 2 100.0%	1 / 1 100.0%	2 / 2 100.0%	1 / 1 100.0%	3 / 3 100.0%	1 / 1 100.0%	3 / 3 100.0%	5 / 5 100.0%	2 / 2 100.0%	1 / 1 100.0%	5 / 5 100.0%	0 / 0 0.0%	0 / 0 0.0%	0 / 0 0.0%	0 / 0 0.0%	32 / 48 66.7%AB	16 / 48 33.3%ABE						
1	Rhodopseudomonas palustris BisB18	47 / 48 97.9%	1 / 1 100.0%	1 / 1 100.0%	1 / 1 100.0%	2 / 2 100.0%	3 / 3 100.0%	3 / 3 100.0%	1 / 1 100.0%	0 / 1 0.0%	4 / 4 100.0%	1 / 1 100.0%	4 / 4 100.0%	2 / 2 100.0%	1 / 1 100.0%	2 / 2 100.0%	1 / 1 100.0%	3 / 3 100.0%	1 / 1 100.0%	3 / 3 100.0%	5 / 5 100.0%	2 / 2 100.0%	1 / 1 100.0%	5 / 5 100.0%	0 / 0 0.0%	0 / 0 0.0%	0 / 0 0.0%	0 / 0 0.0%	31 / 48 64.6%AB	16 / 48 33.3%ABE	1 / 48 2.1%E					
1	Rhodopseudomonas palustris BisB5	48 / 48 100.0%	1 / 1 100.0%	1 / 1 100.0%	1 / 1 100.0%	2 / 2 100.0%	3 / 3 100.0%	3 / 3 100.0%	1 / 1 100.0%	1 / 1 100.0%	4 / 4 100.0%	1 / 1 100.0%	4 / 4 100.0%	2 / 2 100.0%	1 / 1 100.0%	2 / 2 100.0%	1 / 1 100.0%	3 / 3 100.0%	1 / 1 100.0%	3 / 3 100.0%	5 / 5 100.0%	2 / 2 100.0%	1 / 1 100.0%	5 / 5 100.0%	0 / 0 0.0%	0 / 0 0.0%	0 / 0 0.0%	0 / 0 0.0%	31 / 48 64.6%AB	17 / 48 35.4%ABE						
1	Rhodopseudomonas palustris HaA2	48 / 48 100.0%	1 / 1 100.0%	1 / 1 100.0%	1 / 1 100.0%	1 / 1 100.0%	4 / 4 100.0%	3 / 3 100.0%	1 / 1 100.0%	1 / 1 100.0%	3 / 3 100.0%	1 / 1 100.0%	5 / 5 100.0%	2 / 2 100.0%	1 / 1 100.0%	2 / 2 100.0%	1 / 1 100.0%	3 / 3 100.0%	1 / 1 100.0%	3 / 3 100.0%	5 / 5 100.0%	2 / 2 100.0%	1 / 1 100.0%	5 / 5 100.0%	0 / 0 0.0%	0 / 0 0.0%	0 / 0 0.0%	0 / 0 0.0%	29 / 48 60.4%AB	19 / 48 39.6%ABE						
1	Rhodopseudomonas palustris TIE-1	50 / 50 100.0%	3 / 3 100.0%	1 / 1 100.0%	1 / 1 100.0%	2 / 2 100.0%	3 / 3 100.0%	3 / 3 100.0%	1 / 1 100.0%	1 / 1 100.0%	4 / 4 100.0%	1 / 1 100.0%	4 / 4 100.0%	2 / 2 100.0%	1 / 1 100.0%	2 / 2 100.0%	1 / 1 100.0%	3 / 3 100.0%	1 / 1 100.0%	3 / 3 100.0%	5 / 5 100.0%	2 / 2 100.0%	1 / 1 100.0%	5 / 5 100.0%	0 / 0 0.0%	0 / 0 0.0%	0 / 0 0.0%	0 / 0 0.0%	32 / 50 64.0%AB	18 / 50 36.0%ABE						
1	Rhodopseudomonas palustris DX-1	48 / 48 100.0%	1 / 1 100.0%	1 / 1 100.0%	1 / 1 100.0%	2 / 2 100.0%	3 / 3 100.0%	3 / 3 100.0%	1 / 1 100.0%	1 / 1 100.0%	4 / 4 100.0%	1 / 1 100.0%	4 / 4 100.0%	2 / 2 100.0%	1 / 1 100.0%	2 / 2 100.0%	1 / 1 100.0%	3 / 3 100.0%	1 / 1 100.0%	3 / 3 100.0%	5 / 5 100.0%	2 / 2 100.0%	1 / 1 100.0%	5 / 5 100.0%	0 / 0 0.0%	0 / 0 0.0%	0 / 0 0.0%	0 / 0 0.0%	31 / 48 64.6%AB	17 / 48 35.4%ABE						
2	Oligotropha	141 / 144 97.9%	3 / 3 100.0%	3 / 3 100.0%	3 / 3 100.0%	3 / 3 100.0%	9 / 9 100.0%	9 / 9 100.0%	3 / 3 100.0%	0 / 3 0.0%	9 / 9 100.0%	3 / 3 100.0%	12 / 12 100.0%	6 / 6 100.0%	3 / 3 100.0%	6 / 6 100.0%	3 / 3 100.0%	9 / 9 100.0%	3 / 3 100.0%	9 / 9 100.0%	18 / 18 100.0%	6 / 6 100.0%	3 / 3 100.0%	15 / 15 100.0%	3 / 3 100.0%	0 / 0 0.0%	0 / 0 0.0%	0 / 0 0.0%	87 / 144 60.4%AB	54 / 144 37.5%ABE	3 / 144 2.1%E					
2	"Pseudomonas carboxydovorans" Meyer and Schlegel 1978	141 / 144 97.9%	3 / 3 100.0%	3 / 3 100.0%	3 / 3 100.0%	3 / 3 100.0%	9 / 9 100.0%	9 / 9 100.0%	3 / 3 100.0%	0 / 3 0.0%	9 / 9 100.0%	3 / 3 100.0%	12 / 12 100.0%	6 / 6 100.0%	3 / 3 100.0%	6 / 6 100.0%	3 / 3 100.0%	9 / 9 100.0%	3 / 3 100.0%	9 / 9 100.0%	18 / 18 100.0%	6 / 6 100.0%	3 / 3 100.0%	15 / 15 100.0%	3 / 3 100.0%	0 / 0 0.0%	0 / 0 0.0%	0 / 0 0.0%	87 / 144 60.4%AB	54 / 144 37.5%ABE	3 / 144 2.1%E					
1	Oligotropha carboxidovorans DSM 1227	94 / 96 97.9%	2 / 2 100.0%	2 / 2 100.0%	2 / 2 100.0%	2 / 2 100.0%	6 / 6 100.0%	6 / 6 100.0%	2 / 2 100.0%	0 / 2 0.0%	6 / 6 100.0%	2 / 2 100.0%	8 / 8 100.0%	4 / 4 100.0%	2 / 2 100.0%	4 / 4 100.0%	2 / 2 100.0%	6 / 6 100.0%	2 / 2 100.0%	6 / 6 100.0%	12 / 12 100.0%	4 / 4 100.0%	2 / 2 100.0%	10 / 10 100.0%	2 / 2 100.0%	0 / 0 0.0%	0 / 0 0.0%	0 / 0 0.0%	58 / 96 60.4%AB	36 / 96 37.5%ABE	2 / 96 2.1%E					
1	Oligotropha carboxidovorans OM4	47 / 48 97.9%	1 / 1 100.0%	1 / 1 100.0%	1 / 1 100.0%	1 / 1 100.0%	3 / 3 100.0%	3 / 3 100.0%	1 / 1 100.0%	0 / 1 0.0%	3 / 3 100.0%	1 / 1 100.0%	4 / 4 100.0%	2 / 2 100.0%	1 / 1 100.0%	2 / 2 100.0%	1 / 1 100.0%	3 / 3 100.0%	1 / 1 100.0%	3 / 3 100.0%	6 / 6 100.0%	2 / 2 100.0%	1 / 1 100.0%	5 / 5 100.0%	1 / 1 100.0%	0 / 0 0.0%	0 / 0 0.0%	0 / 0 0.0%	29 / 48 60.4%AB	18 / 48 37.5%ABE	1 / 48 2.1%E					
5	Ancylobacter group	235 / 235 100.0%	5 / 5 100.0%	5 / 5 100.0%	5 / 5 100.0%	7 / 7 100.0%	16 / 16 100.0%	15 / 15 100.0%	5 / 5 100.0%	5 / 5 100.0%	17 / 17 100.0%	5 / 5 100.0%	20 / 20 100.0%	10 / 10 100.0%	5 / 5 100.0%	10 / 10 100.0%	5 / 5 100.0%	15 / 15 100.0%	5 / 5 100.0%	15 / 15 100.0%	23 / 23 100.0%	10 / 10 100.0%	5 / 5 100.0%	25 / 25 100.0%	2 / 2 100.0%	0 / 0 0.0%	0 / 0 0.0%	0 / 0 0.0%	157 / 235 66.8%AB	78 / 235 33.2%ABE						
3	Hyphomicrobium	140 / 140 100.0%	3 / 3 100.0%	3 / 3 100.0%	3 / 3 100.0%	3 / 3 100.0%	9 / 9 100.0%	9 / 9 100.0%	3 / 3 100.0%	3 / 3 100.0%	9 / 9 100.0%	3 / 3 100.0%	12 / 12 100.0%	6 / 6 100.0%	3 / 3 100.0%	6 / 6 100.0%	3 / 3 100.0%	9 / 9 100.0%	3 / 3 100.0%	9 / 9 100.0%	15 / 15 100.0%	6 / 6 100.0%	3 / 3 100.0%	15 / 15 100.0%	2 / 2 100.0%	0 / 0 0.0%	0 / 0 0.0%	0 / 0 0.0%	92 / 140 65.7%AB	48 / 140 34.3%ABE						
2	ATCC 51888	93 / 93 100.0%	2 / 2 100.0%	2 / 2 100.0%	2 / 2 100.0%	2 / 2 100.0%	6 / 6 100.0%	6 / 6 100.0%	2 / 2 100.0%	2 / 2 100.0%	6 / 6 100.0%	2 / 2 100.0%	8 / 8 100.0%	4 / 4 100.0%	2 / 2 100.0%	4 / 4 100.0%	2 / 2 100.0%	6 / 6 100.0%	2 / 2 100.0%	6 / 6 100.0%	10 / 10 100.0%	4 / 4 100.0%	2 / 2 100.0%	10 / 10 100.0%	1 / 1 100.0%	0 / 0 0.0%	0 / 0 0.0%	0 / 0 0.0%	62 / 93 66.7%AB	31 / 93 33.3%ABE						
1	Hyphomicrobium denitrificans ATCC 51888	46 / 46 100.0%	1 / 1 100.0%	1 / 1 100.0%	1 / 1 100.0%	1 / 1 100.0%	3 / 3 100.0%	3 / 3 100.0%	1 / 1 100.0%	1 / 1 100.0%	3 / 3 100.0%	1 / 1 100.0%	4 / 4 100.0%	2 / 2 100.0%	1 / 1 100.0%	2 / 2 100.0%	1 / 1 100.0%	3 / 3 100.0%	1 / 1 100.0%	3 / 3 100.0%	5 / 5 100.0%	2 / 2 100.0%	1 / 1 100.0%	5 / 5 100.0%	0 / 0 0.0%	0 / 0 0.0%	0 / 0 0.0%	0 / 0 0.0%	31 / 46 67.4%AB	15 / 46 32.6%ABE						
1	Hyphomicrobium denitrificans 1NES1	47 / 47 100.0%	1 / 1 100.0%	1 / 1 100.0%	1 / 1 100.0%	1 / 1 100.0%	3 / 3 100.0%	3 / 3 100.0%	1 / 1 100.0%	1 / 1 100.0%	3 / 3 100.0%	1 / 1 100.0%	4 / 4 100.0%	2 / 2 100.0%	1 / 1 100.0%	2 / 2 100.0%	1 / 1 100.0%	3 / 3 100.0%	1 / 1 100.0%	3 / 3 100.0%	5 / 5 100.0%	2 / 2 100.0%	1 / 1 100.0%	5 / 5 100.0%	1 / 1 100.0%	0 / 0 0.0%	0 / 0 0.0%	0 / 0 0.0%	31 / 47 66.0%AB	16 / 47 34.0%ABE						
1	Hyphomicrobium sp. MC1	47 / 47 100.0%	1 / 1 100.0%	1 / 1 100.0%	1 / 1 100.0%	1 / 1 100.0%	3 / 3 100.0%	3 / 3 100.0%	1 / 1 100.0%	1 / 1 100.0%	3 / 3 100.0%	1 / 1 100.0%	4 / 4 100.0%	2 / 2 100.0%	1 / 1 100.0%	2 / 2 100.0%	1 / 1 100.0%	3 / 3 100.0%	1 / 1 100.0%	3 / 3 100.0%	5 / 5 100.0%	2 / 2 100.0%	1 / 1 100.0%	5 / 5 100.0%	1 / 1 100.0%	0 / 0 0.0%	0 / 0 0.0%	0 / 0 0.0%	30 / 47 63.8%AB	17 / 47 36.2%ABE						
1	Rhodomicrobium	48 / 48 100.0%	1 / 1 100.0%	1 / 1 100.0%	1 / 1 100.0%	2 / 2 100.0%	4 / 4 100.0%	3 / 3 100.0%	1 / 1 100.0%	1 / 1 100.0%	4 / 4 100.0%	1 / 1 100.0%	4 / 4 100.0%	2 / 2 100.0%	1 / 1 100.0%	2 / 2 100.0%	1 / 1 100.0%	3 / 3 100.0%	1 / 1 100.0%	3 / 3 100.0%	4 / 4 100.0%	2 / 2 100.0%	1 / 1 100.0%	5 / 5 100.0%	0 / 0 0.0%	0 / 0 0.0%	0 / 0 0.0%	0 / 0 0.0%	33 / 48 68.8%AB	15 / 48 31.2%ABE						
1	ATCC 17100	48 / 48 100.0%	1 / 1 100.0%	1 / 1 100.0%	1 / 1 100.0%	2 / 2 100.0%	4 / 4 100.0%	3 / 3 100.0%	1 / 1 100.0%	1 / 1 100.0%	4 / 4 100.0%	1 / 1 100.0%	4 / 4 100.0%	2 / 2 100.0%	1 / 1 100.0%	2 / 2 100.0%	1 / 1 100.0%	3 / 3 100.0%	1 / 1 100.0%	3 / 3 100.0%	4 / 4 100.0%	2 / 2 100.0%	1 / 1 100.0%	5 / 5 100.0%	0 / 0 0.0%	0 / 0 0.0%	0 / 0 0.0%	0 / 0 0.0%	33 / 48 68.8%AB	15 / 48 31.2%ABE						
1	Rhodomicrobium vannielii ATCC 17100	48 / 48 100.0%	1 / 1 100.0%	1 / 1 100.0%	1 / 1 100.0%	2 / 2 100.0%	4 / 4 100.0%	3 / 3 100.0%	1 / 1 100.0%	1 / 1 100.0%	4 / 4 100.0%	1 / 1 100.0%	4 / 4 100.0%	2 / 2 100.0%	1 / 1 100.0%	2 / 2 100.0%	1 / 1 100.0%	3 / 3 100.0%	1 / 1 100.0%	3 / 3 100.0%	4 / 4 100.0%	2 / 2 100.0%	1 / 1 100.0%	5 / 5 100.0%	0 / 0 0.0%	0 / 0 0.0%	0 / 0 0.0%	0 / 0 0.0%	33 / 48 68.8%AB	15 / 48 31.2%ABE						
1	Pelagibacterium	47 / 47 100.0%	1 / 1 100.0%	1 / 1 100.0%	1 / 1 100.0%	2 / 2 100.0%	3 / 3 100.0%	3 / 3 100.0%	1 / 1 100.0%	1 / 1 100.0%	4 / 4 100.0%	1 / 1 100.0%	4 / 4 100.0%	2 / 2 100.0%	1 / 1 100.0%	2 / 2 100.0%	1 / 1 100.0%	3 / 3 100.0%	1 / 1 100.0%	3 / 3 100.0%	4 / 4 100.0%	2 / 2 100.0%	1 / 1 100.0%	5 / 5 100.0%	0 / 0 0.0%	0 / 0 0.0%	0 / 0 0.0%	0 / 0 0.0%	32 / 47 68.1%AB	15 / 47 31.9%ABE						
1	CGMCC 1.7692	47 / 47 100.0%	1 / 1 100.0%	1 / 1 100.0%	1 / 1 100.0%	2 / 2 100.0%	3 / 3 100.0%	3 / 3 100.0%	1 / 1 100.0%	1 / 1 100.0%	4 / 4 100.0%	1 / 1 100.0%	4 / 4 100.0%	2 / 2 100.0%	1 / 1 100.0%	2 / 2 100.0%	1 / 1 100.0%	3 / 3 100.0%	1 / 1 100.0%	3 / 3 100.0%	4 / 4 100.0%	2 / 2 100.0%	1 / 1 100.0%	5 / 5 100.0%	0 / 0 0.0%	0 / 0 0.0%	0 / 0 0.0%	0 / 0 0.0%	32 / 47 68.1%AB	15 / 47 31.9%ABE						
1	Devosia sp. B2	47 / 47 100.0%	1 / 1 100.0%	1 / 1 100.0%	1 / 1 100.0%	2 / 2 100.0%	3 / 3 100.0%	3 / 3 100.0%	1 / 1 100.0%	1 / 1 100.0%	4 / 4 100.0%	1 / 1 100.0%	4 / 4 100.0%	2 / 2 100.0%	1 / 1 100.0%	2 / 2 100.0%	1 / 1 100.0%	3 / 3 100.0%	1 / 1 100.0%	3 / 3 100.0%	4 / 4 100.0%	2 / 2 100.0%	1 / 1 100.0%	5 / 5 100.0%	0 / 0 0.0%	0 / 0 0.0%	0 / 0 0.0%	0 / 0 0.0%	32 / 47 68.1%AB	15 / 47 31.9%ABE						
2	'Beijerinckiaceae'	98 / 99 99.0%	4 / 4 100.0%	2 / 2 100.0%	2 / 2 100.0%	5 / 5 100.0%	6 / 6 100.0%	6 / 7 85.7%	2 / 2 100.0%	2 / 2 100.0%	9 / 9 100.0%	2 / 2 100.0%	8 / 8 100.0%	4 / 4 100.0%	2 / 2 100.0%	4 / 4 100.0%	2 / 2 100.0%	6 / 6 100.0%	2 / 2 100.0%	6 / 6 100.0%	8 / 8 100.0%	4 / 4 100.0%	2 / 2 100.0%	9 / 9 100.0%	1 / 1 100.0%	0 / 0 0.0%	0 / 0 0.0%	0 / 0 0.0%	47 / 99 47.5%AB	20 / 99 20.2%B	20 / 99 20.2%ABE	11 / 99 11.1%BE	1 / 99 1.0%			
1	Beijerinckia	50 / 51 98.0%	3 / 3 100.0%	1 / 1 100.0%	1 / 1 100.0%	3 / 3 100.0%	3 / 3 100.0%	3 / 4 75.0%	1 / 1 100.0%	1 / 1 100.0%	5 / 5 100.0%	1 / 1 100.0%	4 / 4 100.0%	2 / 2 100.0%	1 / 1 100.0%	2 / 2 100.0%	1 / 1 100.0%	3 / 3 100.0%	1 / 1 100.0%	3 / 3 100.0%	4 / 4 100.0%	2 / 2 100.0%	1 / 1 100.0%	4 / 4 100.0%	0 / 0 0.0%	0 / 0 0.0%	0 / 0 0.0%	0 / 0 0.0%	20 / 51 39.2%B	15 / 51 29.4%AB	10 / 51 19.6%BE	5 / 51 9.8%ABE	1 / 51 2.0%			
1	"Azotobacter acida" (sic) Roy 1958	50 / 51 98.0%	3 / 3 100.0%	1 / 1 100.0%	1 / 1 100.0%	3 / 3 100.0%	3 / 3 100.0%	3 / 4 75.0%	1 / 1 100.0%	1 / 1 100.0%	5 / 5 100.0%	1 / 1 100.0%	4 / 4 100.0%	2 / 2 100.0%	1 / 1 100.0%	2 / 2 100.0%	1 / 1 100.0%	3 / 3 100.0%	1 / 1 100.0%	3 / 3 100.0%	4 / 4 100.0%	2 / 2 100.0%	1 / 1 100.0%	4 / 4 100.0%	0 / 0 0.0%	0 / 0 0.0%	0 / 0 0.0%	0 / 0 0.0%	20 / 51 39.2%B	15 / 51 29.4%AB	10 / 51 19.6%BE	5 / 51 9.8%ABE	1 / 51 2.0%			
1	Azotobacter indicus	50 / 51 98.0%	3 / 3 100.0%	1 / 1 100.0%	1 / 1 100.0%	3 / 3 100.0%	3 / 3 100.0%	3 / 4 75.0%	1 / 1 100.0%	1 / 1 100.0%	5 / 5 100.0%	1 / 1 100.0%	4 / 4 100.0%	2 / 2 100.0%	1 / 1 100.0%	2 / 2 100.0%	1 / 1 100.0%	3 / 3 100.0%	1 / 1 100.0%	3 / 3 100.0%	4 / 4 100.0%	2 / 2 100.0%	1 / 1 100.0%	4 / 4 100.0%	0 / 0 0.0%	0 / 0 0.0%	0 / 0 0.0%	0 / 0 0.0%	20 / 51 39.2%B	15 / 51 29.4%AB	10 / 51 19.6%BE	5 / 51 9.8%ABE	1 / 51 2.0%			
1	Beijerinckia indica subsp. indica ATCC 9039	50 / 51 98.0%	3 / 3 100.0%	1 / 1 100.0%	1 / 1 100.0%	3 / 3 100.0%	3 / 3 100.0%	3 / 4 75.0%	1 / 1 100.0%	1 / 1 100.0%	5 / 5 100.0%	1 / 1 100.0%	4 / 4 100.0%	2 / 2 100.0%	1 / 1 100.0%	2 / 2 100.0%	1 / 1 100.0%	3 / 3 100.0%	1 / 1 100.0%	3 / 3 100.0%	4 / 4 100.0%	2 / 2 100.0%	1 / 1 100.0%	4 / 4 100.0%	0 / 0 0.0%	0 / 0 0.0%	0 / 0 0.0%	0 / 0 0.0%	20 / 51 39.2%B	15 / 51 29.4%AB	10 / 51 19.6%BE	5 / 51 9.8%ABE	1 / 51 2.0%			
1	Methylocella	48 / 48 100.0%	1 / 1 100.0%	1 / 1 100.0%	1 / 1 100.0%	2 / 2 100.0%	3 / 3 100.0%	3 / 3 100.0%	1 / 1 100.0%	1 / 1 100.0%	4 / 4 100.0%	1 / 1 100.0%	4 / 4 100.0%	2 / 2 100.0%	1 / 1 100.0%	2 / 2 100.0%	1 / 1 100.0%	3 / 3 100.0%	1 / 1 100.0%	3 / 3 100.0%	4 / 4 100.0%	2 / 2 100.0%	1 / 1 100.0%	5 / 5 100.0%	1 / 1 100.0%	0 / 0 0.0%	0 / 0 0.0%	0 / 0 0.0%	32 / 48 66.7%AB	15 / 48 31.2%ABE	1 / 48 2.1%BE					
1	DSM 15510	48 / 48 100.0%	1 / 1 100.0%	1 / 1 100.0%	1 / 1 100.0%	2 / 2 100.0%	3 / 3 100.0%	3 / 3 100.0%	1 / 1 100.0%	1 / 1 100.0%	4 / 4 100.0%	1 / 1 100.0%	4 / 4 100.0%	2 / 2 100.0%	1 / 1 100.0%	2 / 2 100.0%	1 / 1 100.0%	3 / 3 100.0%	1 / 1 100.0%	3 / 3 100.0%	4 / 4 100.0%	2 / 2 100.0%	1 / 1 100.0%	5 / 5 100.0%	1 / 1 100.0%	0 / 0 0.0%	0 / 0 0.0%	0 / 0 0.0%	32 / 48 66.7%AB	15 / 48 31.2%ABE	1 / 48 2.1%BE					
1	Methylocella silvestris BL2	48 / 48 100.0%	1 / 1 100.0%	1 / 1 100.0%	1 / 1 100.0%	2 / 2 100.0%	3 / 3 100.0%	3 / 3 100.0%	1 / 1 100.0%	1 / 1 100.0%	4 / 4 100.0%	1 / 1 100.0%	4 / 4 100.0%	2 / 2 100.0%	1 / 1 100.0%	2 / 2 100.0%	1 / 1 100.0%	3 / 3 100.0%	1 / 1 100.0%	3 / 3 100.0%	4 / 4 100.0%	2 / 2 100.0%	1 / 1 100.0%	5 / 5 100.0%	1 / 1 100.0%	0 / 0 0.0%	0 / 0 0.0%	0 / 0 0.0%	32 / 48 66.7%AB	15 / 48 31.2%ABE	1 / 48 2.1%BE					
5	'Phyllobacteriaceae'	245 / 248 98.8%	10 / 10 100.0%	5 / 5 100.0%	5 / 6 83.3%	10 / 10 100.0%	15 / 15 100.0%	15 / 15 100.0%	5 / 5 100.0%	5 / 5 100.0%	20 / 20 100.0%	5 / 5 100.0%	20 / 20 100.0%	10 / 10 100.0%	5 / 5 100.0%	14 / 14 100.0%	5 / 5 100.0%	15 / 15 100.0%	5 / 5 100.0%	15 / 15 100.0%	20 / 20 100.0%	10 / 10 100.0%	5 / 7 71.4%	26 / 26 100.0%	0 / 0 0.0%	0 / 0 0.0%	0 / 0 0.0%	0 / 0 0.0%	156 / 248 62.9%AB	89 / 248 35.9%ABE	2 / 248 0.8%	1 / 248 0.4%C				
4	Mesorhizobium	197 / 200 98.5%	8 / 8 100.0%	4 / 4 100.0%	4 / 5 80.0%	8 / 8 100.0%	12 / 12 100.0%	12 / 12 100.0%	4 / 4 100.0%	4 / 4 100.0%	16 / 16 100.0%	4 / 4 100.0%	16 / 16 100.0%	8 / 8 100.0%	4 / 4 100.0%	12 / 12 100.0%	4 / 4 100.0%	12 / 12 100.0%	4 / 4 100.0%	12 / 12 100.0%	16 / 16 100.0%	8 / 8 100.0%	4 / 6 66.7%	21 / 21 100.0%	0 / 0 0.0%	0 / 0 0.0%	0 / 0 0.0%	0 / 0 0.0%	124 / 200 62.0%AB	73 / 200 36.5%ABE	2 / 200 1.0%	1 / 200 0.5%C				
1	ATCC 33669	50 / 50 100.0%	2 / 2 100.0%	1 / 1 100.0%	1 / 1 100.0%	2 / 2 100.0%	3 / 3 100.0%	3 / 3 100.0%	1 / 1 100.0%	1 / 1 100.0%	4 / 4 100.0%	1 / 1 100.0%	4 / 4 100.0%	2 / 2 100.0%	1 / 1 100.0%	3 / 3 100.0%	1 / 1 100.0%	3 / 3 100.0%	1 / 1 100.0%	3 / 3 100.0%	4 / 4 100.0%	2 / 2 100.0%	1 / 1 100.0%	6 / 6 100.0%	0 / 0 0.0%	0 / 0 0.0%	0 / 0 0.0%	0 / 0 0.0%	31 / 50 62.0%AB	19 / 50 38.0%ABE						
1	Mesorhizobium loti MAFF303099	50 / 50 100.0%	2 / 2 100.0%	1 / 1 100.0%	1 / 1 100.0%	2 / 2 100.0%	3 / 3 100.0%	3 / 3 100.0%	1 / 1 100.0%	1 / 1 100.0%	4 / 4 100.0%	1 / 1 100.0%	4 / 4 100.0%	2 / 2 100.0%	1 / 1 100.0%	3 / 3 100.0%	1 / 1 100.0%	3 / 3 100.0%	1 / 1 100.0%	3 / 3 100.0%	4 / 4 100.0%	2 / 2 100.0%	1 / 1 100.0%	6 / 6 100.0%	0 / 0 0.0%	0 / 0 0.0%	0 / 0 0.0%	0 / 0 0.0%	31 / 50 62.0%AB	19 / 50 38.0%ABE						
1	ATCC 51585	49 / 50 98.0%	2 / 2 100.0%	1 / 1 100.0%	1 / 2 50.0%	2 / 2 100.0%	3 / 3 100.0%	3 / 3 100.0%	1 / 1 100.0%	1 / 1 100.0%	4 / 4 100.0%	1 / 1 100.0%	4 / 4 100.0%	2 / 2 100.0%	1 / 1 100.0%	3 / 3 100.0%	1 / 1 100.0%	3 / 3 100.0%	1 / 1 100.0%	3 / 3 100.0%	4 / 4 100.0%	2 / 2 100.0%	1 / 1 100.0%	5 / 5 100.0%	0 / 0 0.0%	0 / 0 0.0%	0 / 0 0.0%	0 / 0 0.0%	31 / 50 62.0%AB	18 / 50 36.0%ABE	1 / 50 2.0%C					
1	Mesorhizobium ciceri biovar biserrulae	49 / 50 98.0%	2 / 2 100.0%	1 / 1 100.0%	1 / 2 50.0%	2 / 2 100.0%	3 / 3 100.0%	3 / 3 100.0%	1 / 1 100.0%	1 / 1 100.0%	4 / 4 100.0%	1 / 1 100.0%	4 / 4 100.0%	2 / 2 100.0%	1 / 1 100.0%	3 / 3 100.0%	1 / 1 100.0%	3 / 3 100.0%	1 / 1 100.0%	3 / 3 100.0%	4 / 4 100.0%	2 / 2 100.0%	1 / 1 100.0%	5 / 5 100.0%	0 / 0 0.0%	0 / 0 0.0%	0 / 0 0.0%	0 / 0 0.0%	31 / 50 62.0%AB	18 / 50 36.0%ABE	1 / 50 2.0%C					
1	Mesorhizobium ciceri biovar biserrulae WSM1271	49 / 50 98.0%	2 / 2 100.0%	1 / 1 100.0%	1 / 2 50.0%	2 / 2 100.0%	3 / 3 100.0%	3 / 3 100.0%	1 / 1 100.0%	1 / 1 100.0%	4 / 4 100.0%	1 / 1 100.0%	4 / 4 100.0%	2 / 2 100.0%	1 / 1 100.0%	3 / 3 100.0%	1 / 1 100.0%	3 / 3 100.0%	1 / 1 100.0%	3 / 3 100.0%	4 / 4 100.0%	2 / 2 100.0%	1 / 1 100.0%	5 / 5 100.0%	0 / 0 0.0%	0 / 0 0.0%	0 / 0 0.0%	0 / 0 0.0%	31 / 50 62.0%AB	18 / 50 36.0%ABE	1 / 50 2.0%C					
1	HAMBI 3006	49 / 50 98.0%	2 / 2 100.0%	1 / 1 100.0%	1 / 1 100.0%	2 / 2 100.0%	3 / 3 100.0%	3 / 3 100.0%	1 / 1 100.0%	1 / 1 100.0%	4 / 4 100.0%	1 / 1 100.0%	4 / 4 100.0%	2 / 2 100.0%	1 / 1 100.0%	3 / 3 100.0%	1 / 1 100.0%	3 / 3 100.0%	1 / 1 100.0%	3 / 3 100.0%	4 / 4 100.0%	2 / 2 100.0%	1 / 2 50.0%	5 / 5 100.0%	0 / 0 0.0%	0 / 0 0.0%	0 / 0 0.0%	0 / 0 0.0%	31 / 50 62.0%AB	18 / 50 36.0%ABE	1 / 50 2.0%					
1	Mesorhizobium australicum LMG 24608	49 / 50 98.0%	2 / 2 100.0%	1 / 1 100.0%	1 / 1 100.0%	2 / 2 100.0%	3 / 3 100.0%	3 / 3 100.0%	1 / 1 100.0%	1 / 1 100.0%	4 / 4 100.0%	1 / 1 100.0%	4 / 4 100.0%	2 / 2 100.0%	1 / 1 100.0%	3 / 3 100.0%	1 / 1 100.0%	3 / 3 100.0%	1 / 1 100.0%	3 / 3 100.0%	4 / 4 100.0%	2 / 2 100.0%	1 / 2 50.0%	5 / 5 100.0%	0 / 0 0.0%	0 / 0 0.0%	0 / 0 0.0%	0 / 0 0.0%	31 / 50 62.0%AB	18 / 50 36.0%ABE	1 / 50 2.0%					
1	HAMBI 3007	49 / 50 98.0%	2 / 2 100.0%	1 / 1 100.0%	1 / 1 100.0%	2 / 2 100.0%	3 / 3 100.0%	3 / 3 100.0%	1 / 1 100.0%	1 / 1 100.0%	4 / 4 100.0%	1 / 1 100.0%	4 / 4 100.0%	2 / 2 100.0%	1 / 1 100.0%	3 / 3 100.0%	1 / 1 100.0%	3 / 3 100.0%	1 / 1 100.0%	3 / 3 100.0%	4 / 4 100.0%	2 / 2 100.0%	1 / 2 50.0%	5 / 5 100.0%	0 / 0 0.0%	0 / 0 0.0%	0 / 0 0.0%	0 / 0 0.0%	31 / 50 62.0%AB	18 / 50 36.0%ABE	1 / 50 2.0%					
1	Mesorhizobium opportunistum WSM2075	49 / 50 98.0%	2 / 2 100.0%	1 / 1 100.0%	1 / 1 100.0%	2 / 2 100.0%	3 / 3 100.0%	3 / 3 100.0%	1 / 1 100.0%	1 / 1 100.0%	4 / 4 100.0%	1 / 1 100.0%	4 / 4 100.0%	2 / 2 100.0%	1 / 1 100.0%	3 / 3 100.0%	1 / 1 100.0%	3 / 3 100.0%	1 / 1 100.0%	3 / 3 100.0%	4 / 4 100.0%	2 / 2 100.0%	1 / 2 50.0%	5 / 5 100.0%	0 / 0 0.0%	0 / 0 0.0%	0 / 0 0.0%	0 / 0 0.0%	31 / 50 62.0%AB	18 / 50 36.0%ABE	1 / 50 2.0%					
1	Chelativorans	48 / 48 100.0%	2 / 2 100.0%	1 / 1 100.0%	1 / 1 100.0%	2 / 2 100.0%	3 / 3 100.0%	3 / 3 100.0%	1 / 1 100.0%	1 / 1 100.0%	4 / 4 100.0%	1 / 1 100.0%	4 / 4 100.0%	2 / 2 100.0%	1 / 1 100.0%	2 / 2 100.0%	1 / 1 100.0%	3 / 3 100.0%	1 / 1 100.0%	3 / 3 100.0%	4 / 4 100.0%	2 / 2 100.0%	1 / 1 100.0%	5 / 5 100.0%	0 / 0 0.0%	0 / 0 0.0%	0 / 0 0.0%	0 / 0 0.0%	32 / 48 66.7%AB	16 / 48 33.3%ABE						
1	Chelativorans sp. BNC1	48 / 48 100.0%	2 / 2 100.0%	1 / 1 100.0%	1 / 1 100.0%	2 / 2 100.0%	3 / 3 100.0%	3 / 3 100.0%	1 / 1 100.0%	1 / 1 100.0%	4 / 4 100.0%	1 / 1 100.0%	4 / 4 100.0%	2 / 2 100.0%	1 / 1 100.0%	2 / 2 100.0%	1 / 1 100.0%	3 / 3 100.0%	1 / 1 100.0%	3 / 3 100.0%	4 / 4 100.0%	2 / 2 100.0%	1 / 1 100.0%	5 / 5 100.0%	0 / 0 0.0%	0 / 0 0.0%	0 / 0 0.0%	0 / 0 0.0%	32 / 48 66.7%AB	16 / 48 33.3%ABE						
24	Rhizobiaceae	1286 / 1291 99.6%	84 / 85 98.8%	47 / 47 100.0%	24 / 24 100.0%	79 / 80 98.8%	75 / 75 100.0%	50 / 50 100.0%	25 / 25 100.0%	25 / 25 100.0%	115 / 116 99.1%	25 / 25 100.0%	101 / 101 100.0%	50 / 50 100.0%	25 / 25 100.0%	50 / 50 100.0%	25 / 25 100.0%	71 / 71 100.0%	26 / 26 100.0%	75 / 75 100.0%	90 / 92 97.8%	77 / 77 100.0%	25 / 25 100.0%	121 / 121 100.0%	1 / 1 100.0%	0 / 0 0.0%	0 / 0 0.0%	0 / 0 0.0%	802 / 1291 62.1%AB	414 / 1291 32.1%ABE	41 / 1291 3.2%B	29 / 1291 2.2%BE	2 / 1291 0.2%E	1 / 1291 0.1%CE	1 / 1291 0.1%	1 / 1291 0.1%C
[illegible]	"Candidatus Liberobacter" Jagoueix et [illegible]	130 / 131	9 / 9	3 / 3	2 / 2	9 / 9	6 / 6	6 / 6	3 / 3	3 / 3	11 / 12	3 / 3	11 / 11	6 / 6	3 / 3	6 / 6	3 / 3	4 / 4	3 / 3	9 / 9	12 / 12	3 / 3	3 / 3	12 / 12	0 / 0	0 / 0	0 / 0	0 / 0	87 / 131	43 / 131	1 / 131					

[illegible]	[illegible]	[illegible] 99.2%	[illegible] 100.0%	[illegible] 100.0%	[illegible] 100.0%	[illegible] 100.0%	[illegible] 100.0%	[illegible] 100.0%	[illegible] 100.0%	[illegible] 100.0%	[illegible] 91.7%	[illegible] 100.0%	[illegible] 100.0%	[illegible] 100.0%	[illegible] 100.0%	[illegible] 100.0%	[illegible] 100.0%	[illegible] 100.0%	[illegible] 100.0%	[illegible] 100.0%	[illegible] 100.0%	[illegible] 100.0%	[illegible] 100.0%	[illegible] 100.0%	[illegible] 0.0%	[illegible] 0.0%	[illegible] 0.0%	[illegible] 0.0%	[illegible] 66.4%AB	[illegible] 32.8%ABE	[illegible] 0.8%C			
2	"Candidatus Liberobacter asiaticum" Jagoueix et al. 1994	86 / 87 98.9%	6 / 6 100.0%	2 / 2 100.0%	2 / 2 100.0%	6 / 6 100.0%	4 / 4 100.0%	4 / 4 100.0%	2 / 2 100.0%	2 / 2 100.0%	7 / 8 87.5%	2 / 2 100.0%	7 / 7 100.0%	4 / 4 100.0%	2 / 2 100.0%	4 / 4 100.0%	2 / 2 100.0%	2 / 2 100.0%	2 / 2 100.0%	6 / 6 100.0%	8 / 8 100.0%	2 / 2 100.0%	2 / 2 100.0%	8 / 8 100.0%	0 / 0 0.0%	0 / 0 0.0%	0 / 0 0.0%	0 / 0 0.0%	57 / 87 65.5%AB	29 / 87 33.3%ABE	1 / 87 1.1%C			
1	Candidatus Liberibacter asiaticus psy62	44 / 44 100.0%	3 / 3 100.0%	1 / 1 100.0%	1 / 1 100.0%	3 / 3 100.0%	2 / 2 100.0%	2 / 2 100.0%	1 / 1 100.0%	1 / 1 100.0%	4 / 4 100.0%	1 / 1 100.0%	4 / 4 100.0%	2 / 2 100.0%	1 / 1 100.0%	2 / 2 100.0%	1 / 1 100.0%	1 / 1 100.0%	1 / 1 100.0%	3 / 3 100.0%	4 / 4 100.0%	1 / 1 100.0%	1 / 1 100.0%	4 / 4 100.0%	0 / 0 0.0%	0 / 0 0.0%	0 / 0 0.0%	0 / 0 0.0%	29 / 44 65.9%AB	15 / 44 34.1%ABE				
1	Candidatus Liberibacter asiaticus str. gxpsy	42 / 43 97.7%	3 / 3 100.0%	1 / 1 100.0%	1 / 1 100.0%	3 / 3 100.0%	2 / 2 100.0%	2 / 2 100.0%	1 / 1 100.0%	1 / 1 100.0%	3 / 4 75.0%	1 / 1 100.0%	3 / 3 100.0%	2 / 2 100.0%	1 / 1 100.0%	2 / 2 100.0%	1 / 1 100.0%	1 / 1 100.0%	1 / 1 100.0%	3 / 3 100.0%	4 / 4 100.0%	1 / 1 100.0%	1 / 1 100.0%	4 / 4 100.0%	0 / 0 0.0%	0 / 0 0.0%	0 / 0 0.0%	0 / 0 0.0%	28 / 43 65.1%AB	14 / 43 32.6%ABE	1 / 43 2.3%C			
1	"Candidatus Liberibacter solanacearum" Liefting et al. 2009	44 / 44 100.0%	3 / 3 100.0%	1 / 1 100.0%	0 / 0 0.0%	3 / 3 100.0%	2 / 2 100.0%	2 / 2 100.0%	1 / 1 100.0%	1 / 1 100.0%	4 / 4 100.0%	1 / 1 100.0%	4 / 4 100.0%	2 / 2 100.0%	1 / 1 100.0%	2 / 2 100.0%	1 / 1 100.0%	2 / 2 100.0%	1 / 1 100.0%	3 / 3 100.0%	4 / 4 100.0%	1 / 1 100.0%	1 / 1 100.0%	4 / 4 100.0%	0 / 0 0.0%	0 / 0 0.0%	0 / 0 0.0%	0 / 0 0.0%	30 / 44 68.2%AB	14 / 44 31.8%ABE				
1	Candidatus Liberibacter solanacearum CLso-ZC1	44 / 44 100.0%	3 / 3 100.0%	1 / 1 100.0%	0 / 0 0.0%	3 / 3 100.0%	2 / 2 100.0%	2 / 2 100.0%	1 / 1 100.0%	1 / 1 100.0%	4 / 4 100.0%	1 / 1 100.0%	4 / 4 100.0%	2 / 2 100.0%	1 / 1 100.0%	2 / 2 100.0%	1 / 1 100.0%	2 / 2 100.0%	1 / 1 100.0%	3 / 3 100.0%	4 / 4 100.0%	1 / 1 100.0%	1 / 1 100.0%	4 / 4 100.0%	0 / 0 0.0%	0 / 0 0.0%	0 / 0 0.0%	0 / 0 0.0%	30 / 44 68.2%AB	14 / 44 31.8%ABE				
10	Rhizobium/Agrobacterium group	575 / 577 99.7%	40 / 41 97.6%	22 / 22 100.0%	11 / 11 100.0%	37 / 38 97.4%	36 / 36 100.0%	22 / 22 100.0%	11 / 11 100.0%	11 / 11 100.0%	49 / 49 100.0%	11 / 11 100.0%	45 / 45 100.0%	22 / 22 100.0%	11 / 11 100.0%	22 / 22 100.0%	11 / 11 100.0%	34 / 34 100.0%	11 / 11 100.0%	33 / 33 100.0%	41 / 41 100.0%	30 / 30 100.0%	11 / 11 100.0%	54 / 54 100.0%	0 / 0 0.0%	0 / 0 0.0%	0 / 0 0.0%	0 / 0 0.0%	336 / 577 58.2%AB	169 / 577 29.3%ABE	41 / 577 7.1%B	29 / 577 5.0%BE	1 / 577 0.2%CE	1 / 577 0.2%
4	"Polymonas" Lieske 1928	268 / 268 100.0%	21 / 21 100.0%	10 / 10 100.0%	5 / 5 100.0%	20 / 20 100.0%	18 / 18 100.0%	10 / 10 100.0%	5 / 5 100.0%	5 / 5 100.0%	25 / 25 100.0%	5 / 5 100.0%	20 / 20 100.0%	10 / 10 100.0%	5 / 5 100.0%	10 / 10 100.0%	5 / 5 100.0%	15 / 15 100.0%	5 / 5 100.0%	15 / 15 100.0%	17 / 17 100.0%	12 / 12 100.0%	5 / 5 100.0%	25 / 25 100.0%	0 / 0 0.0%	0 / 0 0.0%	0 / 0 0.0%	0 / 0 0.0%	148 / 268 55.2%AB	70 / 268 26.1%ABE	30 / 268 11.2%B	20 / 268 7.5%BE		
1	ATCC 49767	54 / 54 100.0%	4 / 4 100.0%	2 / 2 100.0%	1 / 1 100.0%	4 / 4 100.0%	3 / 3 100.0%	2 / 2 100.0%	1 / 1 100.0%	1 / 1 100.0%	5 / 5 100.0%	1 / 1 100.0%	4 / 4 100.0%	2 / 2 100.0%	1 / 1 100.0%	2 / 2 100.0%	1 / 1 100.0%	3 / 3 100.0%	1 / 1 100.0%	3 / 3 100.0%	4 / 4 100.0%	3 / 3 100.0%	1 / 1 100.0%	5 / 5 100.0%	0 / 0 0.0%	0 / 0 0.0%	0 / 0 0.0%	0 / 0 0.0%	36 / 54 66.7%AB	18 / 54 33.3%ABE				
1	Agrobacterium vitis S4	54 / 54 100.0%	4 / 4 100.0%	2 / 2 100.0%	1 / 1 100.0%	4 / 4 100.0%	3 / 3 100.0%	2 / 2 100.0%	1 / 1 100.0%	1 / 1 100.0%	5 / 5 100.0%	1 / 1 100.0%	4 / 4 100.0%	2 / 2 100.0%	1 / 1 100.0%	2 / 2 100.0%	1 / 1 100.0%	3 / 3 100.0%	1 / 1 100.0%	3 / 3 100.0%	4 / 4 100.0%	3 / 3 100.0%	1 / 1 100.0%	5 / 5 100.0%	0 / 0 0.0%	0 / 0 0.0%	0 / 0 0.0%	0 / 0 0.0%	36 / 54 66.7%AB	18 / 54 33.3%ABE				
1	Agrobacterium sp. H13-3	57 / 57 100.0%	6 / 6 100.0%	2 / 2 100.0%	1 / 1 100.0%	5 / 5 100.0%	4 / 4 100.0%	2 / 2 100.0%	1 / 1 100.0%	1 / 1 100.0%	6 / 6 100.0%	1 / 1 100.0%	4 / 4 100.0%	2 / 2 100.0%	1 / 1 100.0%	2 / 2 100.0%	1 / 1 100.0%	3 / 3 100.0%	1 / 1 100.0%	3 / 3 100.0%	3 / 3 100.0%	2 / 2 100.0%	1 / 1 100.0%	5 / 5 100.0%	0 / 0 0.0%	0 / 0 0.0%	0 / 0 0.0%	0 / 0 0.0%	38 / 57 66.7%AB	19 / 57 33.3%ABE				
2	Agrobacterium biovar 1	157 / 157 100.0%	11 / 11 100.0%	6 / 6 100.0%	3 / 3 100.0%	11 / 11 100.0%	11 / 11 100.0%	6 / 6 100.0%	3 / 3 100.0%	3 / 3 100.0%	14 / 14 100.0%	3 / 3 100.0%	12 / 12 100.0%	6 / 6 100.0%	3 / 3 100.0%	6 / 6 100.0%	3 / 3 100.0%	9 / 9 100.0%	3 / 3 100.0%	9 / 9 100.0%	10 / 10 100.0%	7 / 7 100.0%	3 / 3 100.0%	15 / 15 100.0%	0 / 0 0.0%	0 / 0 0.0%	0 / 0 0.0%	0 / 0 0.0%	74 / 157 47.1%AB	33 / 157 21.0%ABE	30 / 157 19.1%B	20 / 157 12.7%BE		
1	"Achromobacter radiobacter" (Beijerinck and van Delden 1902) Bergey et al. 1934	51 / 51 100.0%	3 / 3 100.0%	2 / 2 100.0%	1 / 1 100.0%	3 / 3 100.0%	3 / 3 100.0%	2 / 2 100.0%	1 / 1 100.0%	1 / 1 100.0%	4 / 4 100.0%	1 / 1 100.0%	4 / 4 100.0%	2 / 2 100.0%	1 / 1 100.0%	2 / 2 100.0%	1 / 1 100.0%	3 / 3 100.0%	1 / 1 100.0%	3 / 3 100.0%	4 / 4 100.0%	3 / 3 100.0%	1 / 1 100.0%	5 / 5 100.0%	0 / 0 0.0%	0 / 0 0.0%	0 / 0 0.0%	0 / 0 0.0%	34 / 51 66.7%AB	17 / 51 33.3%ABE				
1	Agrobacterium radiobacter K84	51 / 51 100.0%	3 / 3 100.0%	2 / 2 100.0%	1 / 1 100.0%	3 / 3 100.0%	3 / 3 100.0%	2 / 2 100.0%	1 / 1 100.0%	1 / 1 100.0%	4 / 4 100.0%	1 / 1 100.0%	4 / 4 100.0%	2 / 2 100.0%	1 / 1 100.0%	2 / 2 100.0%	1 / 1 100.0%	3 / 3 100.0%	1 / 1 100.0%	3 / 3 100.0%	4 / 4 100.0%	3 / 3 100.0%	1 / 1 100.0%	5 / 5 100.0%	0 / 0 0.0%	0 / 0 0.0%	0 / 0 0.0%	0 / 0 0.0%	34 / 51 66.7%AB	17 / 51 33.3%ABE				
1	"Agrobacterium fabrum" Lassalle et al. 2011	106 / 106 100.0%	8 / 8 100.0%	4 / 4 100.0%	2 / 2 100.0%	8 / 8 100.0%	8 / 8 100.0%	4 / 4 100.0%	2 / 2 100.0%	2 / 2 100.0%	10 / 10 100.0%	2 / 2 100.0%	8 / 8 100.0%	4 / 4 100.0%	2 / 2 100.0%	4 / 4 100.0%	2 / 2 100.0%	6 / 6 100.0%	2 / 2 100.0%	6 / 6 100.0%	6 / 6 100.0%	4 / 4 100.0%	2 / 2 100.0%	10 / 10 100.0%	0 / 0 0.0%	0 / 0 0.0%	0 / 0 0.0%	0 / 0 0.0%	40 / 106 37.7%AB	30 / 106 28.3%B	20 / 106 18.9%BE	16 / 106 15.1%ABE		
1	Agrobacterium fabrum str. C58	106 / 106 100.0%	8 / 8 100.0%	4 / 4 100.0%	2 / 2 100.0%	8 / 8 100.0%	8 / 8 100.0%	4 / 4 100.0%	2 / 2 100.0%	2 / 2 100.0%	10 / 10 100.0%	2 / 2 100.0%	8 / 8 100.0%	4 / 4 100.0%	2 / 2 100.0%	4 / 4 100.0%	2 / 2 100.0%	6 / 6 100.0%	2 / 2 100.0%	6 / 6 100.0%	6 / 6 100.0%	4 / 4 100.0%	2 / 2 100.0%	10 / 10 100.0%	0 / 0 0.0%	0 / 0 0.0%	0 / 0 0.0%	0 / 0 0.0%	40 / 106 37.7%AB	30 / 106 28.3%B	20 / 106 18.9%BE	16 / 106 15.1%ABE		
6	"Phytomyxa" Schroeter 1886	307 / 309 99.4%	19 / 20 95.0%	12 / 12 100.0%	6 / 6 100.0%	17 / 18 94.4%	18 / 18 100.0%	12 / 12 100.0%	6 / 6 100.0%	6 / 6 100.0%	24 / 24 100.0%	6 / 6 100.0%	25 / 25 100.0%	12 / 12 100.0%	6 / 6 100.0%	12 / 12 100.0%	6 / 6 100.0%	19 / 19 100.0%	6 / 6 100.0%	18 / 18 100.0%	24 / 24 100.0%	18 / 18 100.0%	6 / 6 100.0%	29 / 29 100.0%	0 / 0 0.0%	0 / 0 0.0%	0 / 0 0.0%	0 / 0 0.0%	188 / 309 60.8%AB	99 / 309 32.0%ABE	11 / 309 3.6%B	9 / 309 2.9%BE	1 / 309 0.3%CE	1 / 309 0.3%
3	"Bacillus francki" Matzuschita 1902	153 / 154 99.4%	9 / 10 90.0%	6 / 6 100.0%	3 / 3 100.0%	9 / 9 100.0%	9 / 9 100.0%	6 / 6 100.0%	3 / 3 100.0%	3 / 3 100.0%	12 / 12 100.0%	3 / 3 100.0%	13 / 13 100.0%	6 / 6 100.0%	3 / 3 100.0%	6 / 6 100.0%	3 / 3 100.0%	9 / 9 100.0%	3 / 3 100.0%	9 / 9 100.0%	12 / 12 100.0%	9 / 9 100.0%	3 / 3 100.0%	14 / 14 100.0%	0 / 0 0.0%	0 / 0 0.0%	0 / 0 0.0%	0 / 0 0.0%	99 / 154 64.3%AB	54 / 154 35.1%ABE	1 / 154 0.6%CE			
2	Rhizobium leguminosarum (BIOVAR TRIFOLII)	101 / 102 99.0%	6 / 7 85.7%	4 / 4 100.0%	2 / 2 100.0%	6 / 6 100.0%	6 / 6 100.0%	4 / 4 100.0%	2 / 2 100.0%	2 / 2 100.0%	8 / 8 100.0%	2 / 2 100.0%	8 / 8 100.0%	4 / 4 100.0%	2 / 2 100.0%	4 / 4 100.0%	2 / 2 100.0%	6 / 6 100.0%	2 / 2 100.0%	6 / 6 100.0%	8 / 8 100.0%	6 / 6 100.0%	2 / 2 100.0%	9 / 9 100.0%	0 / 0 0.0%	0 / 0 0.0%	0 / 0 0.0%	0 / 0 0.0%	66 / 102 64.7%AB	35 / 102 34.3%ABE	1 / 102 1.0%CE			
1	Rhizobium leguminosarum bv. trifolii WSM1325	51 / 51 100.0%	3 / 3 100.0%	2 / 2 100.0%	1 / 1 100.0%	3 / 3 100.0%	3 / 3 100.0%	2 / 2 100.0%	1 / 1 100.0%	1 / 1 100.0%	4 / 4 100.0%	1 / 1 100.0%	4 / 4 100.0%	2 / 2 100.0%	1 / 1 100.0%	2 / 2 100.0%	1 / 1 100.0%	3 / 3 100.0%	1 / 1 100.0%	3 / 3 100.0%	4 / 4 100.0%	3 / 3 100.0%	1 / 1 100.0%	5 / 5 100.0%	0 / 0 0.0%	0 / 0 0.0%	0 / 0 0.0%	0 / 0 0.0%	33 / 51 64.7%AB	18 / 51 35.3%ABE				
1	Rhizobium leguminosarum bv. trifolii WSM2304	50 / 51 98.0%	3 / 4 75.0%	2 / 2 100.0%	1 / 1 100.0%	3 / 3 100.0%	3 / 3 100.0%	2 / 2 100.0%	1 / 1 100.0%	1 / 1 100.0%	4 / 4 100.0%	1 / 1 100.0%	4 / 4 100.0%	2 / 2 100.0%	1 / 1 100.0%	2 / 2 100.0%	1 / 1 100.0%	3 / 3 100.0%	1 / 1 100.0%	3 / 3 100.0%	4 / 4 100.0%	3 / 3 100.0%	1 / 1 100.0%	4 / 4 100.0%	0 / 0 0.0%	0 / 0 0.0%	0 / 0 0.0%	0 / 0 0.0%	33 / 51 64.7%AB	17 / 51 33.3%ABE	1 / 51 2.0%CE			
1	Rhizobium leguminosarum (BIOVAR VICIAE)	52 / 52 100.0%	3 / 3 100.0%	2 / 2 100.0%	1 / 1 100.0%	3 / 3 100.0%	3 / 3 100.0%	2 / 2 100.0%	1 / 1 100.0%	1 / 1 100.0%	4 / 4 100.0%	1 / 1 100.0%	5 / 5 100.0%	2 / 2 100.0%	1 / 1 100.0%	2 / 2 100.0%	1 / 1 100.0%	3 / 3 100.0%	1 / 1 100.0%	3 / 3 100.0%	4 / 4 100.0%	3 / 3 100.0%	1 / 1 100.0%	5 / 5 100.0%	0 / 0 0.0%	0 / 0 0.0%	0 / 0 0.0%	0 / 0 0.0%	33 / 52 63.5%AB	19 / 52 36.5%ABE				
1	Rhizobium leguminosarum bv. viciae 3841	52 / 52 100.0%	3 / 3 100.0%	2 / 2 100.0%	1 / 1 100.0%	3 / 3 100.0%	3 / 3 100.0%	2 / 2 100.0%	1 / 1 100.0%	1 / 1 100.0%	4 / 4 100.0%	1 / 1 100.0%	5 / 5 100.0%	2 / 2 100.0%	1 / 1 100.0%	2 / 2 100.0%	1 / 1 100.0%	3 / 3 100.0%	1 / 1 100.0%	3 / 3 100.0%	4 / 4 100.0%	3 / 3 100.0%	1 / 1 100.0%	5 / 5 100.0%	0 / 0 0.0%	0 / 0 0.0%	0 / 0 0.0%	0 / 0 0.0%	33 / 52 63.5%AB	19 / 52 36.5%ABE				
1	ATCC 49672	53 / 53 100.0%	4 / 4 100.0%	2 / 2 100.0%	1 / 1 100.0%	3 / 3 100.0%	3 / 3 100.0%	2 / 2 100.0%	1 / 1 100.0%	1 / 1 100.0%	4 / 4 100.0%	1 / 1 100.0%	4 / 4 100.0%	2 / 2 100.0%	1 / 1 100.0%	2 / 2 100.0%	1 / 1 100.0%	4 / 4 100.0%	1 / 1 100.0%	3 / 3 100.0%	4 / 4 100.0%	3 / 3 100.0%	1 / 1 100.0%	5 / 5 100.0%	0 / 0 0.0%	0 / 0 0.0%	0 / 0 0.0%	0 / 0 0.0%	33 / 53 62.3%AB	20 / 53 37.7%ABE				
1	Rhizobium tropici CIAT 899	53 / 53 100.0%	4 / 4 100.0%	2 / 2 100.0%	1 / 1 100.0%	3 / 3 100.0%	3 / 3 100.0%	2 / 2 100.0%	1 / 1 100.0%	1 / 1 100.0%	4 / 4 100.0%	1 / 1 100.0%	4 / 4 100.0%	2 / 2 100.0%	1 / 1 100.0%	2 / 2 100.0%	1 / 1 100.0%	4 / 4 100.0%	1 / 1 100.0%	3 / 3 100.0%	4 / 4 100.0%	3 / 3 100.0%	1 / 1 100.0%	5 / 5 100.0%	0 / 0 0.0%	0 / 0 0.0%	0 / 0 0.0%	0 / 0 0.0%	33 / 53 62.3%AB	20 / 53 37.7%ABE				
2	ATCC 51251	101 / 102 99.0%	6 / 6 100.0%	4 / 4 100.0%	2 / 2 100.0%	5 / 6 83.3%	6 / 6 100.0%	4 / 4 100.0%	2 / 2 100.0%	2 / 2 100.0%	8 / 8 100.0%	2 / 2 100.0%	8 / 8 100.0%	4 / 4 100.0%	2 / 2 100.0%	4 / 4 100.0%	2 / 2 100.0%	6 / 6 100.0%	2 / 2 100.0%	6 / 6 100.0%	8 / 8 100.0%	6 / 6 100.0%	2 / 2 100.0%	10 / 10 100.0%	0 / 0 0.0%	0 / 0 0.0%	0 / 0 0.0%	0 / 0 0.0%	56 / 102 54.9%AB	25 / 102 24.5%ABE	11 / 102 10.8%B	9 / 102 8.8%BE	1 / 102 1.0%	
1	Rhizobium etli CFN 42	51 / 51 100.0%	3 / 3 100.0%	2 / 2 100.0%	1 / 1 100.0%	3 / 3 100.0%	3 / 3 100.0%	2 / 2 100.0%	1 / 1 100.0%	1 / 1 100.0%	4 / 4 100.0%	1 / 1 100.0%	4 / 4 100.0%	2 / 2 100.0%	1 / 1 100.0%	2 / 2 100.0%	1 / 1 100.0%	3 / 3 100.0%	1 / 1 100.0%	3 / 3 100.0%	4 / 4 100.0%	3 / 3 100.0%	1 / 1 100.0%	5 / 5 100.0%	0 / 0 0.0%	0 / 0 0.0%	0 / 0 0.0%	0 / 0 0.0%	34 / 51 66.7%AB	17 / 51 33.3%ABE				
1	Rhizobium etli CIAT 652	50 / 51 98.0%	3 / 3 100.0%	2 / 2 100.0%	1 / 1 100.0%	2 / 3 66.7%	3 / 3 100.0%	2 / 2 100.0%	1 / 1 100.0%	1 / 1 100.0%	4 / 4 100.0%	1 / 1 100.0%	4 / 4 100.0%	2 / 2 100.0%	1 / 1 100.0%	2 / 2 100.0%	1 / 1 100.0%	3 / 3 100.0%	1 / 1 100.0%	3 / 3 100.0%	4 / 4 100.0%	3 / 3 100.0%	1 / 1 100.0%	5 / 5 100.0%	0 / 0 0.0%	0 / 0 0.0%	0 / 0 0.0%	0 / 0 0.0%	22 / 51 43.1%AB	11 / 51 21.6%B	9 / 51 17.6%BE	8 / 51 15.7%ABE	1 / 51 2.0%	
11	Sinorhizobium/Ensifer group	581 / 583 99.7%	35 / 35 100.0%	22 / 22 100.0%	11 / 11 100.0%	33 / 33 100.0%	33 / 33 100.0%	22 / 22 100.0%	11 / 11 100.0%	11 / 11 100.0%	55 / 55 100.0%	11 / 11 100.0%	45 / 45 100.0%	22 / 22 100.0%	11 / 11 100.0%	22 / 22 100.0%	11 / 11 100.0%	33 / 33 100.0%	12 / 12 100.0%	33 / 33 100.0%	37 / 39 94.9%	44 / 44 100.0%	11 / 11 100.0%	55 / 55 100.0%	1 / 1 100.0%	0 / 0 0.0%	0 / 0 0.0%	0 / 0 0.0%	379 / 583 65.0%AB	202 / 583 34.6%ABE	2 / 583 0.3%E			
11	Sinorhizobium	581 / 583 99.7%	35 / 35 100.0%	22 / 22 100.0%	11 / 11 100.0%	33 / 33 100.0%	33 / 33 100.0%	22 / 22 100.0%	11 / 11 100.0%	11 / 11 100.0%	55 / 55 100.0%	11 / 11 100.0%	45 / 45 100.0%	22 / 22 100.0%	11 / 11 100.0%	22 / 22 100.0%	11 / 11 100.0%	33 / 33 100.0%	12 / 12 100.0%	33 / 33 100.0%	37 / 39 94.9%	44 / 44 100.0%	11 / 11 100.0%	55 / 55 100.0%	1 / 1 100.0%	0 / 0 0.0%	0 / 0 0.0%	0 / 0 0.0%	379 / 583 65.0%AB	202 / 583 34.6%ABE	2 / 583 0.3%E			
7	ATCC 9930	369 / 370 99.7%	23 / 23 100.0%	14 / 14 100.0%	7 / 7 100.0%	21 / 21 100.0%	21 / 21 100.0%	14 / 14 100.0%	7 / 7 100.0%	7 / 7 100.0%	35 / 35 100.0%	7 / 7 100.0%	29 / 29 100.0%	14 / 14 100.0%	7 / 7 100.0%	14 / 14 100.0%	7 / 7 100.0%	21 / 21 100.0%	7 / 7 100.0%	21 / 21 100.0%	22 / 23 95.7%	28 / 28 100.0%	7 / 7 100.0%	35 / 35 100.0%	1 / 1 100.0%	0 / 0 0.0%	0 / 0 0.0%	0 / 0 0.0%	240 / 370 64.9%AB	129 / 370 34.9%ABE	1 / 370 0.3%E			
1	Sinorhizobium meliloti 1021	52 / 53 98.1%	3 / 3 100.0%	2 / 2 100.0%	1 / 1 100.0%	3 / 3 100.0%	3 / 3 100.0%	2 / 2 100.0%	1 / 1 100.0%	1 / 1 100.0%	5 / 5 100.0%	1 / 1 100.0%	4 / 4 100.0%	2 / 2 100.0%	1 / 1 100.0%	2 / 2 100.0%	1 / 1 100.0%	3 / 3 100.0%	1 / 1 100.0%	3 / 3 100.0%	3 / 4 75.0%	4 / 4 100.0%	1 / 1 100.0%	5 / 5 100.0%	0 / 0 0.0%	0 / 0 0.0%	0 / 0 0.0%	0 / 0 0.0%	34 / 53 64.2%AB	18 / 53 34.0%ABE	1 / 53 1.9%E			
1	Ensifer meliloti AK83	55 / 55 100.0%	3 / 3 100.0%	2 / 2 100.0%	1 / 1 100.0%	3 / 3 100.0%	3 / 3 100.0%	2 / 2 100.0%	1 / 1 100.0%	1 / 1 100.0%	5 / 5 100.0%	1 / 1 100.0%	5 / 5 100.0%	2 / 2 100.0%	1 / 1 100.0%	2 / 2 100.0%	1 / 1 100.0%	3 / 3 100.0%	1 / 1 100.0%	3 / 3 100.0%	4 / 4 100.0%	4 / 4 100.0%	1 / 1 100.0%	5 / 5 100.0%	1 / 1 100.0%	0 / 0 0.0%	0 / 0 0.0%	0 / 0 0.0%	35 / 55 63.6%AB	20 / 55 36.4%ABE				
1	Ensifer meliloti BL225C	52 / 52 100.0%	3 / 3 100.0%	2 / 2 100.0%	1 / 1 100.0%	3 / 3 100.0%	3 / 3 100.0%	2 / 2 100.0%	1 / 1 100.0%	1 / 1 100.0%	5 / 5 100.0%	1 / 1 100.0%	4 / 4 100.0%	2 / 2 100.0%	1 / 1 100.0%	2 / 2 100.0%	1 / 1 100.0%	3 / 3 100.0%	1 / 1 100.0%	3 / 3 100.0%	3 / 3 100.0%	4 / 4 100.0%	1 / 1 100.0%	5 / 5 100.0%	0 / 0 0.0%	0 / 0 0.0%	0 / 0 0.0%	0 / 0 0.0%	34 / 52 65.4%AB	18 / 52 34.6%ABE				
1	Sinorhizobium meliloti SM11	52 / 52 100.0%	3 / 3 100.0%	2 / 2 100.0%	1 / 1 100.0%	3 / 3 100.0%	3 / 3 100.0%	2 / 2 100.0%	1 / 1 100.0%	1 / 1 100.0%	5 / 5 100.0%	1 / 1 100.0%	4 / 4 100.0%	2 / 2 100.0%	1 / 1 100.0%	2 / 2 100.0%	1 / 1 100.0%	3 / 3 100.0%	1 / 1 100.0%	3 / 3 100.0%	3 / 3 100.0%	4 / 4 100.0%	1 / 1 100.0%	5 / 5 100.0%	0 / 0 0.0%	0 / 0 0.0%	0 / 0 0.0%	0 / 0 0.0%	34 / 52 65.4%AB	18 / 52 34.6%ABE				
1	Sinorhizobium meliloti Rm41	54 / 54 100.0%	5 / 5 100.0%	2 / 2 100.0%	1 / 1 100.0%	3 / 3 100.0%	3 / 3 100.0%	2 / 2 100.0%	1 / 1 100.0%	1 / 1 100.0%	5 / 5 100.0%	1 / 1 100.0%	4 / 4 100.0%	2 / 2 100.0%	1 / 1 100.0%	2 / 2 100.0%	1 / 1 100.0%	3 / 3 100.0%	1 / 1 100.0%	3 / 3 100.0%	3 / 3 100.0%	4 / 4 100.0%	1 / 1 100.0%	5 / 5 100.0%	0 / 0 0.0%	0 / 0 0.0%	0 / 0 0.0%	0 / 0 0.0%	35 / 54 64.8%AB	19 / 54 35.2%ABE				
1	Sinorhizobium meliloti GR4	52 / 52 100.0%	3 / 3 100.0%	2 / 2 100.0%	1 / 1 100.0%	3 / 3 100.0%	3 / 3 100.0%	2 / 2 100.0%	1 / 1 100.0%	1 / 1 100.0%	5 / 5 100.0%	1 / 1 100.0%	4 / 4 100.0%	2 / 2 100.0%	1 / 1 100.0%	2 / 2 100.0%	1 / 1 100.0%	3 / 3 100.0%	1 / 1 100.0%	3 / 3 100.0%	3 / 3 100.0%	4 / 4 100.0%	1 / 1 100.0%	5 / 5 100.0%	0 / 0 0.0%	0 / 0 0.0%	0 / 0 0.0%	0 / 0 0.0%	34 / 52 65.4%AB	18 / 52 34.6%ABE				
1	Sinorhizobium meliloti 2011	52 / 52 100.0%	3 / 3 100.0%	2 / 2 100.0%	1 / 1 100.0%	3 / 3 100.0%	3 / 3 100.0%	2 / 2 100.0%	1 / 1 100.0%	1 / 1 100.0%	5 / 5 100.0%	1 / 1 100.0%	4 / 4 100.0%	2 / 2 100.0%	1 / 1 100.0%	2 / 2 100.0%	1 / 1 100.0%	3 / 3 100.0%	1 / 1 100.0%	3 / 3 100.0%	3 / 3 100.0%	4 / 4 100.0%	1 / 1 100.0%	5 / 5 100.0%	0 / 0 0.0%	0 / 0 0.0%	0 / 0 0.0%	0 / 0 0.0%	34 / 52 65.4%AB	18 / 52 34.6%ABE				
1	Ensifer medicae	52 / 53 98.1%	3 / 3 100.0%	2 / 2 100.0%	1 / 1 100.0%	3 / 3 100.0%	3 / 3 100.0%	2 / 2 100.0%	1 / 1 100.0%	1 / 1 100.0%	5 / 5 100.0%	1 / 1 100.0%	4 / 4 100.0%	2 / 2 100.0%	1 / 1 100.0%	2 / 2 100.0%	1 / 1 100.0%	3 / 3 100.0%	1 / 1 100.0%	3 / 3 100.0%	3 / 4 75.0%	4 / 4 100.0%	1 / 1 100.0%	5 / 5 100.0%	0 / 0 0.0%	0 / 0 0.0%	0 / 0 0.0%	0 / 0 0.0%	34 / 53 64.2%AB	18 / 53 34.0%ABE	1 / 53 1.9%E			
1	Ensifer medicae WSM419	52 / 53 98.1%	3 / 3 100.0%	2 / 2 100.0%	1 / 1 100.0%	3 / 3 100.0%	3 / 3 100.0%	2 / 2 100.0%	1 / 1 100.0%	1 / 1 100.0%	5 / 5 100.0%	1 / 1 100.0%	4 / 4 100.0%	2 / 2 100.0%	1 / 1 100.0%	2 / 2 100.0%	1 / 1 100.0%	3 / 3 100.0%	1 / 1 100.0%	3 / 3 100.0%	3 / 4 75.0%	4 / 4 100.0%	1 / 1 100.0%	5 / 5 100.0%	0 / 0 0.0%	0 / 0 0.0%	0 / 0 0.0%	0 / 0 0.0%	34 / 53 64.2%AB	18 / 53 34.0%ABE	1 / 53 1.9%E			
3	Sinorhizobium fredii group	160 / 160 100.0%	9 / 9 100.0%	6 / 6 100.0%	3 / 3 100.0%	9 / 9 100.0%	9 / 9 100.0%	6 / 6 100.0%	3 / 3 100.0%	3 / 3 100.0%	15 / 15 100.0%	3 / 3 100.0%	12 / 12 100.0%	6 / 6 100.0%	3 / 3 100.0%	6 / 6 100.0%	3 / 3 100.0%	9 / 9 100.0%	4 / 4 100.0%	9 / 9 100.0%	12 / 12 100.0%	12 / 12 100.0%	3 / 3 100.0%	15 / 15 100.0%	0 / 0 0.0%	0 / 0 0.0%	0 / 0 0.0%	0 / 0 0.0%	105 / 160 65.6%AB	55 / 160 34.4%ABE				
3	ATCC 35423	160 / 160 100.0%	9 / 9 100.0%	6 / 6 100.0%	3 / 3 100.0%	9 / 9 100.0%	9 / 9 100.0%	6 / 6 100.0%	3 / 3 100.0%	3 / 3 100.0%	15 / 15 100.0%	3 / 3 100.0%	12 / 12 100.0%	6 / 6 100.0%	3 / 3 100.0%	6 / 6 100.0%	3 / 3 100.0%	9 / 9 100.0%	4 / 4 100.0%	9 / 9 100.0%	12 / 12 100.0%	12 / 12 100.0%	3 / 3 100.0%	15 / 15 100.0%	0 / 0 0.0%	0 / 0 0.0%	0 / 0 0.0%	0 / 0 0.0%	105 / 160 65.6%AB	55 / 160 34.4%ABE				
1	Ensifer fredii NGR234	53 / 53 100.0%	3 / 3 100.0%	2 / 2 100.0%	1 / 1 100.0%	3 / 3 100.0%	3 / 3 100.0%	2 / 2 100.0%	1 / 1 100.0%	1 / 1 100.0%	5 / 5 100.0%	1 / 1 100.0%	4 / 4 100.0%	2 / 2 100.0%	1 / 1 100.0%	2 / 2 100.0%	1 / 1 100.0%	3 / 3 100.0%	1 / 1 100.0%	3 / 3 100.0%	4 / 4 100.0%	4 / 4 100.0%	1 / 1 100.0%	5 / 5 100.0%	0 / 0 0.0%	0 / 0 0.0%	0 / 0 0.0%	0 / 0 0.0%	35 / 53 66.0%AB	18 / 53 34.0%ABE				
1	Sinorhizobium fredii HH103	53 / 53 100.0%	3 / 3 100.0%	2 / 2 100.0%	1 / 1 100.0%	3 / 3 100.0%	3 / 3 100.0%	2 / 2 100.0%	1 / 1 100.0%	1 / 1 100.0%	5 / 5 100.0%	1 / 1 100.0%	4 / 4 100.0%	2 / 2 100.0%	1 / 1 100.0%	2 / 2 100.0%	1 / 1 100.0%	3 / 3 100.0%	1 / 1 100.0%	3 / 3 100.0%	4 / 4 100.0%	4 / 4 100.0%	1 / 1 100.0%	5 / 5 100.0%	0 / 0 0.0%	0 / 0 0.0%	0 / 0 0.0%	0 / 0 0.0%	35 / 53 66.0%AB	18 / 53 34.0%ABE				
1	Sinorhizobium fredii USDA 257	54 / 54 100.0%	3 / 3 100.0%	2 / 2 100.0%	1 / 1 100.0%	3 / 3 100.0%	3 / 3 100.0%	2 / 2 100.0%	1 / 1 100.0%	1 / 1 100.0%	5 / 5 100.0%	1 / 1 100.0%	4 / 4 100.0%	2 / 2 100.0%	1 / 1 100.0%	2 / 2 100.0%	1 / 1 100.0%	3 / 3 100.0%	2 / 2 100.0%	3 / 3 100.0%	4 / 4 100.0%	4 / 4 100.0%	1 / 1 100.0%	5 / 5 100.0%	0 / 0 0.0%	0 / 0 0.0%	0 / 0 0.0%	0 / 0 0.0%	35 / 54 64.8%AB	19 / 54 35.2%ABE				
18	Brucellaceae	1039 / 1041 99.8%	56 / 57 98.2%	38 / 38 100.0%	19 / 19 100.0%	58 / 58 100.0%	76 / 76 100.0%	57 / 57 100.0%	19 / 19 100.0%	19 / 19 100.0%	95 / 95 100.0%	19 / 19 100.0%	75 / 76 98.7%	38 / 38 100.0%	19 / 19 100.0%	38 / 38 100.0%	19 / 19 100.0%	57 / 57 100.0%	19 / 19 100.0%	57 / 57 100.0%	76 / 76 100.0%	53 / 53 100.0%	19 / 19 100.0%	112 / 112 100.0%	1 / 1 100.0%	0 / 0 0.0%	0 / 0 0.0%	0 / 0 0.0%	578 / 1041 55.5%AB	315 / 1041 30.3%ABE	108 / 1041 10.4%B	38 / 1041 3.7%BE	1 / 1041 0.1%E	1 / 1041 0.1%CE
17	Brucella	982 / 983 99.9%	51 / 52 98.1%	36 / 36 100.0%	18 / 18 100.0%	54 / 54 100.0%	72 / 72 100.0%	54 / 54 100.0%	18 / 18 100.0%	18 / 18 100.0%	90 / 90 100.0%	18 / 18 100.0%	72 / 72 100.0%	36 / 36 100.0%	18 / 18 100.0%	36 / 36 100.0%	18 / 18 100.0%	54 / 54 100.0%	18 / 18 100.0%	54 / 54 100.0%	72 / 72 100.0%	50 / 50 100.0%	18 / 18 100.0%	107 / 107 100.0%	0 / 0 0.0%	0 / 0 0.0%	0 / 0 0.0%	0 / 0 0.0%	561 / 983 57.1%AB	306 / 983 31.1%ABE	87 / 983 8.9%B	28 / 983 2.8%BE	1 / 983 0.1%CE	
4	"Bacillus of abortion" Bang 1897	220 / 220 100.0%	12 / 12 100.0%	8 / 8 100.0%	4 / 4 100.0%	12 / 12 100.0%	16 / 16 100.0%	12 / 12 100.0%	4 / 4 100.0%	4 / 4 100.0%	20 / 20 100.0%	4 / 4 100.0%	16 / 16 100.0%	8 / 8 100.0%	4 / 4 100.0%	8 / 8 100.0%	4 / 4 100.0%	12 / 12 100.0%	4 / 4 100.0%	12 / 12 100.0%	16 / 16 100.0%	12 / 12 100.0%	4 / 4 100.0%	24 / 24 100.0%	0 / 0 0.0%	0 / 0 0.0%	0 / 0 0.0%	0 / 0 0.0%	123 / 220 55.9%AB	69 / 220 31.4%ABE	21 / 220 9.5%B	7 / 220 3.2%BE		
1	Brucella abortus 1	55 / 55 100.0%	3 / 3 100.0%	2 / 2 100.0%	1 / 1 100.0%	3 / 3 100.0%	4 / 4 100.0%	3 / 3 100.0%	1 / 1 100.0%	1 / 1 100.0%	5 / 5 100.0%	1 / 1 100.0%	4 / 4 100.0%	2 / 2 100.0%	1 / 1 100.0%	2 / 2 100.0%	1 / 1 100.0%	3 / 3 100.0%	1 / 1 100.0%	3 / 3 100.0%	4 / 4 100.0%	3 / 3 100.0%	1 / 1 100.0%	6 / 6 100.0%	0 / 0 0.0%	0 / 0 0.0%	0 / 0 0.0%	0 / 0 0.0%	36 / 55 65.5%AB	19 / 55 34.5%ABE				
1	Brucella abortus biovar 1 str. 9-941	55 / 55 100.0%	3 / 3 100.0%	2 / 2 100.0%	1 / 1 100.0%	3 / 3 100.0%	4 / 4 100.0%	3 / 3 100.0%	1 / 1 100.0%	1 / 1 100.0%	5 / 5 100.0%	1 / 1 100.0%	4 / 4 100.0%	2 / 2 100.0%	1 / 1 100.0%	2 / 2 100.0%	1 / 1 100.0%	3 / 3 100.0%	1 / 1 100.0%	3 / 3 100.0%	4 / 4 100.0%	3 / 3 100.0%	1 / 1 100.0%	6 / 6 100.0%	0 / 0 0.0%	0 / 0 0.0%	0 / 0 0.0%	0 / 0 0.0%	36 / 55 65.5%AB	19 / 55 34.5%ABE				
1	Brucella abortus 2308	55 / 55 100.0%	3 / 3 100.0%	2 / 2 100.0%	1 / 1 100.0%	3 / 3 100.0%	4 / 4 100.0%	3 / 3 100.0%	1 / 1 100.0%	1 / 1 100.0%	5 / 5 100.0%	1 / 1 100.0%	4 / 4 100.0%	2 / 2 100.0%	1 / 1 100.0%	2 / 2 100.0%	1 / 1 100.0%	3 / 3 100.0%	1 / 1 100.0%	3 / 3 100.0%	4 / 4 100.0%	3 / 3 100.0%	1 / 1 100.0%	6 / 6 100.0%	0 / 0 0.0%	0 / 0 0.0%	0 / 0 0.0%	0 / 0 0.0%	36 / 55 65.5%AB	19 / 55 34.5%ABE				
1	Brucella abortus S19	55 / 55 100.0%	3 / 3 100.0%	2 / 2 100.0%	1 / 1 100.0%	3 / 3 100.0%	4 / 4 100.0%	3 / 3 100.0%	1 / 1 100.0%	1 / 1 100.0%	5 / 5 100.0%	1 / 1 100.0%	4 / 4 100.0%	2 / 2 100.0%	1 / 1 100.0%	2 / 2 100.0%	1 / 1 100.0%	3 / 3 100.0%	1 / 1 100.0%	3 / 3 100.0%	4 / 4 100.0%	3 / 3 100.0%	1 / 1 100.0%	6 / 6 100.0%	0 / 0 0.0%	0 / 0 0.0%	0 / 0 0.0%	0 / 0 0.0%	21 / 55 38.2%B	15 / 55 27.3%AB	12 / 55 21.8%ABE	7 / 55 12.7%BE		
1	Brucella abortus A13334	55 / 55 100.0%	3 / 3 100.0%	2 / 2 100.0%	1 / 1 100.0%	3 / 3 100.0%	4 / 4 100.0%	3 / 3 100.0%	1 / 1 100.0%	1 / 1 100.0%	5 / 5 100.0%	1 / 1 100.0%	4 / 4 100.0%	2 / 2 100.0%	1 / 1 100.0%	2 / 2 100.0%	1 / 1 100.0%	3 / 3 100.0%	1 / 1 100.0%	3 / 3 100.0%	4 / 4 100.0%	3 / 3 100.0%	1 / 1 100.0%	6 / 6 100.0%	0 / 0 0.0%	0 / 0 0.0%	0 / 0 0.0%	0 / 0 0.0%	36 / 55 65.5%AB	19 / 55 34.5%ABE				
1	ATCC 25840	53 / 53 100.0%	3 / 3 100.0%	2 / 2 100.0%	1 / 1 100.0%	3 / 3 100.0%	4 / 4 100.0%	3 / 3 100.0%	1 / 1 100.0%	1 / 1 100.0%	5 / 5 100.0%	1 / 1 100.0%	4 / 4 100.0%	2 / 2 100.0%	1 / 1 100.0%	2 / 2 100.0%	1 / 1 100.0%	3 / 3 100.0%	1 / 1 100.0%	3 / 3 100.0%	4 / 4 100.0%	2 / 2 100.0%	1 / 1 100.0%	5 / 5 100.0%	0 / 0 0.0%	0 / 0 0.0%	0 / 0 0.0%	0 / 0 0.0%	22 / 53 41.5%B	14 / 53 26.4%AB	10 / 53 18.9%ABE	7 / 53 13.2%BE		
1	Brucella ovis ATCC 25840	53 / 53 100.0%	3 / 3 100.0%	2 / 2 100.0%	1 / 1 100.0%	3 / 3 100.0%	4 / 4 100.0%	3 / 3 100.0%	1 / 1 100.0%	1 / 1 100.0%	5 / 5 100.0%	1 / 1 100.0%	4 / 4 100.0%	2 / 2 100.0%	1 / 1 100.0%	2 / 2 100.0%	1 / 1 100.0%	3 / 3 100.0%	1 / 1 100.0%	3 / 3 100.0%	4 / 4 100.0%	2 / 2 100.0%	1 / 1 100.0%	5 / 5 100.0%	0 / 0 0.0%	0 / 0 0.0%	0 / 0 0.0%	0 / 0 0.0%	22 / 53 41.5%B	14 / 53 26.4%AB	10 / 53 18.9%ABE	7 / 53 13.2%BE		
5	"Micrococcus melitensis" (Hughes 1893) Bruce 1893	270 / 271 99.6%	12 / 13 92.3%	10 / 10 100.0%	5 / 5 100.0%	15 / 15 100.0%	20 / 20 100.0%	15 / 15 100.0%	5 / 5 100.0%	5 / 5 100.0%	25 / 25 100.0%	5 / 5 100.0%	20 / 20 100.0%	10 / 10 100.0%	5 / 5 100.0%	10 / 10 100.0%	5 / 5 100.0%	15 / 15 100.0%	5 / 5 100.0%	15 / 15 100.0%	20 / 20 100.0%	13 / 13 100.0%	5 / 5 100.0%	30 / 30 100.0%	0 / 0 0.0%	0 / 0 0.0%	0 / 0 0.0%	0 / 0 0.0%	180 / 271 66.4%AB	90 / 271 33.2%ABE	1 / 271 0.4%CE			
1	Brucella melitensis biovar 1	54 / 54 100.0%	3 / 3 100.0%	2 / 2 100.0%	1 / 1 100.0%	3 / 3 100.0%	4 / 4 100.0%	3 / 3 100.0%	1 / 1 100.0%	1 / 1 100.0%	5 / 5 100.0%	1 / 1 100.0%	4 / 4 100.0%	2 / 2 100.0%	1 / 1 100.0%	2 / 2 100.0%	1 / 1 100.0%	3 / 3 100.0%	1 / 1 100.0%	3 / 3 100.0%	4 / 4 100.0%	2 / 2 100.0%	1 / 1 100.0%	6 / 6 100.0%	0 / 0 0.0%	0 / 0 0.0%	0 / 0 0.0%	0 / 0 0.0%	36 / 54 66.7%AB	18 / 54 33.3%ABE				
1	Brucella melitensis 16M	54 / 54 100.0%	3 / 3 100.0%	2 / 2 100.0%	1 / 1 100.0%	3 / 3 100.0%	4 / 4 100.0%	3 / 3 100.0%	1 / 1 100.0%	1 / 1 100.0%	5 / 5 100.0%	1 / 1 100.0%	4 / 4 100.0%	2 / 2 100.0%	1 / 1 100.0%	2 / 2 100.0%	1 / 1 100.0%	3 / 3 100.0%	1 / 1 100.0%	3 / 3 100.0%	4 / 4 100.0%	2 / 2 100.0%	1 / 1 100.0%	6 / 6 100.0%	0 / 0 0.0%	0 / 0 0.0%	0 / 0 0.0%	0 / 0 0.0%	36 / 54 66.7%AB	18 / 54 33.3%ABE				
1	Brucella melitensis biovar 2	55 / 55 100.0%	3 / 3 100.0%	2 / 2 100.0%	1 / 1 100.0%	3 / 3 100.0%	4 / 4 100.0%	3 / 3 100.0%	1 / 1 100.0%	1 / 1 100.0%	5 / 5 100.0%	1 / 1 100.0%	4 / 4 100.0%	2 / 2 100.0%	1 / 1 100.0%	2 / 2 100.0%	1 / 1 100.0%	3 / 3 100.0%	1 / 1 100.0%	3 / 3 100.0%	4 / 4 100.0%	3 / 3 100.0%	1 / 1 100.0%	6 / 6 100.0%	0 / 0 0.0%	0 / 0 0.0%	0 / 0 0.0%	0 / 0 0.0%	36 / 55 65.5%AB	19 / 55 34.5%ABE				
1	Brucella melitensis ATCC 23457	55 / 55 100.0%	3 / 3 100.0%	2 / 2 100.0%	1 / 1 100.0%	3 / 3 100.0%	4 / 4 100.0%	3 / 3 100.0%	1 / 1 100.0%	1 / 1 100.0%	5 / 5 100.0%	1 / 1 100.0%	4 / 4 100.0%	2 / 2 100.0%	1 / 1 100.0%	2 / 2 100.0%	1 / 1 100.0%	3 / 3 100.0%	1 / 1 100.0%	3 / 3 100.0%	4 / 4 100.0%	3 / 3 100.0%	1 / 1 100.0%	6 / 6 100.0%	0 / 0 0.0%	0 / 0 0.0%	0 / 0 0.0%	0 / 0 0.0%	36 / 55 65.5%AB	19 / 55 34.5%ABE				
2	Brucella melitensis M28	109 / 110 99.1%	5 / 6 83.3%	4 / 4 100.0%	2 / 2 100.0%	6 / 6 100.0%	8 / 8 100.0%	6 / 6 100.0%	2 / 2 100.0%	2 / 2 100.0%	10 / 10 100.0%	2 / 2 100.0%	8 / 8 100.0%	4 / 4 100.0%	2 / 2 100.0%	4 / 4 100.0%	2 / 2 100.0%	6 / 6 100.0%	2 / 2 100.0%	6 / 6 100.0%	8 / 8 100.0%	6 / 6 100.0%	2 / 2 100.0%	12 / 12 100.0%	0 / 0 0.0%	0 / 0 0.0%	0 / 0 0.0%	0 / 0 0.0%	72 / 110 65.5%AB	37 / 110 33.6%ABE	1 / 110 0.9%CE			
1	Brucella melitensis M5-90	54 / 55 98.2%	2 / 3 66.7%	2 / 2 100.0%	1 / 1 100.0%	3 / 3 100.0%	4 / 4 100.0%	3 / 3 100.0%	1 / 1 100.0%	1 / 1 100.0%	5 / 5 100.0%	1 / 1 100.0%	4 / 4 100.0%	2 / 2 100.0%	1 / 1 100.0%	2 / 2 100.0%	1 / 1 100.0%	3 / 3 100.0%	1 / 1 100.0%	3 / 3 100.0%	4 / 4 100.0%	3 / 3 100.0%	1 / 1 100.0%	6 / 6 100.0%	0 / 0 0.0%	0 / 0 0.0%	0 / 0 0.0%	0 / 0 0.0%	36 / 55 65.5%AB	18 / 55 32.7%ABE	1 / 55 1.8%CE			
1	Brucella melitensis NI	52 / 52 100.0%	1 / 1 100.0%	2 / 2 100.0%	1 / 1 100.0%	3 / 3 100.0%	4 / 4 100.0%	3 / 3 100.0%	1 / 1 100.0%	1 / 1 100.0%	5 / 5 100.0%	1 / 1 100.0%	4 / 4 100.0%	2 / 2 100.0%	1 / 1 100.0%	2 / 2 100.0%	1 / 1 100.0%	3 / 3 100.0%	1 / 1 100.0%	3 / 3 100.0%	4 / 4 100.0%	2 / 2 100.0%	1 / 1 100.0%	6 / 6 100.0%	0 / 0 0.0%	0 / 0 0.0%	0 / 0 0.0%	0 / 0 0.0%	36 / 52 69.2%AB	16 / 52 30.8%ABE				
3	"Organism resembling Bacillus abortus" Traum 1914	219 / 219 100.0%	12 / 12 100.0%	8 / 8 100.0%	4 / 4 100.0%	12 / 12 100.0%	16 / 16 100.0%	12 / 12 100.0%	4 / 4 100.0%	4 / 4 100.0%	20 / 20 100.0%	4 / 4 100.0%	16 / 16 100.0%	8 / 8 100.0%	4 / 4 100.0%	8 / 8 100.0%	4 / 4 100.0%	12 / 12 100.0%	4 / 4 100.0%	12 / 12 100.0%	16 / 16 100.0%	11 / 11 100.0%	4 / 4 100.0%	24 / 24 100.0%	0 / 0 0.0%	0 / 0 0.0%	0 / 0 0.0%	0 / 0 0.0%	122 / 219 55.7%AB	68 / 219 31.1%ABE	22 / 219 10.0%B	7 / 219 3.2%BE		
1	Brucella melitensis biovar Suis str. 1330	110 / 110 100.0%	6 / 6 100.0%	4 / 4 100.0%	2 / 2 100.0%	6 / 6 100.0%	8 / 8 100.0%	6 / 6 100.0%	2 / 2 100.0%	2 / 2 100.0%	10 / 10 100.0%	2 / 2 100.0%	8 / 8 100.0%	4 / 4 100.0%	2 / 2 100.0%	4 / 4 100.0%	2 / 2 100.0%	6 / 6 100.0%	2 / 2 100.0%	6 / 6 100.0%	8 / 8 100.0%	6 / 6 100.0%	2 / 2 100.0%	12 / 12 100.0%	0 / 0 0.0%	0 / 0 0.0%	0 / 0 0.0%	0 / 0 0.0%	72 / 110 65.5%AB	38 / 110 34.5%ABE				
1	Brucella suis biovar 2	54 / 54 100.0%	3 / 3 100.0%	2 / 2 100.0%	1 / 1 100.0%	3 / 3 100.0%	4 / 4 100.0%	3 / 3 100.0%	1 / 1 100.0%	1 / 1 100.0%	5 / 5 100.0%	1 / 1 100.0%	4 / 4 100.0%	2 / 2 100.0%	1 / 1 100.0%	2 / 2 100.0%	1 / 1 100.0%	3 / 3 100.0%	1 / 1 100.0%	3 / 3 100.0%	4 / 4 100.0%	2 / 2 100.0%	1 / 1 100.0%	6 / 6 100.0%	0 / 0 0.0%	0 / 0 0.0%	0 / 0 0.0%	0 / 0 0.0%	22 / 54 40.7%B	14 / 54 25.9%AB	11 / 54 20.4%ABE	7 / 54 13.0%BE		
1	Brucella suis ATCC 23445	54 / 54 100.0%	3 / 3 100.0%	2 / 2 100.0%	1 / 1 100.0%	3 / 3 100.0%	4 / 4 100.0%	3 / 3 100.0%	1 / 1 100.0%	1 / 1 100.0%	5 / 5 100.0%	1 / 1 100.0%	4 / 4 100.0%	2 / 2 100.0%	1 / 1 100.0%	2 / 2 100.0%	1 / 1 100.0%	3 / 3 100.0%	1 / 1 100.0%	3 / 3 100.0%	4 / 4 100.0%	2 / 2 100.0%	1 / 1 100.0%	6 / 6 100.0%	0 / 0 0.0%	0 / 0 0.0%	0 / 0 0.0%	0 / 0 0.0%	22 / 54 40.7%B	14 / 54 25.9%AB	11 / 54 20.4%ABE	7 / 54 13.0%BE		
1	Brucella suis VBI22	55 / 55 100.0%	3 / 3 100.0%	2 / 2 100.0%	1 / 1 100.0%	3 / 3 100.0%	4 / 4 100.0%	3 / 3 100.0%	1 / 1 100.0%	1 / 1 100.0%	5 / 5 100.0%	1 / 1 100.0%	4 / 4 100.0%	2 / 2 100.0%	1 / 1 100.0%	2 / 2 100.0%	1 / 1 100.0%	3 / 3 100.0%	1 / 1 100.0%	3 / 3 100.0%	4 / 4 100.0%	3 / 3 100.0%	1 / 1 100.0%	6 / 6 100.0%	0 / 0 0.0%	0 / 0 0.0%	0 / 0 0.0%	0 / 0 0.0%	36 / 55 65.5%AB	19 / 55 34.5%ABE				
2	ATCC 23365	110 / 110 100.0%	6 / 6 100.0%	4 / 4 100.0%	2 / 2 100.0%	6 / 6 100.0%	8 / 8 100.0%	6 / 6 100.0%	2 / 2 100.0%	2 / 2 100.0%	10 / 10 100.0%	2 / 2 100.0%	8 / 8 100.0%	4 / 4 100.0%	2 / 2 100.0%	4 / 4 100.0%	2 / 2 100.0%	6 / 6 100.0%	2 / 2 100.0%	6 / 6 100.0%	8 / 8 100.0%	6 / 6 100.0%	2 / 2 100.0%	12 / 12 100.0%	0 / 0 0.0%	0 / 0 0.0%	0 / 0 0.0%	0 / 0 0.0%	50 / 110 45.5%AB	31 / 110 28.2%ABE	22 / 110 20.0%B	7 / 110 6.4%BE		
1	Brucella canis ATCC 23365	55 / 55 100.0%	3 / 3 100.0%	2 / 2 100.0%	1 / 1 100.0%	3 / 3 100.0%	4 / 4 100.0%	3 / 3 100.0%	1 / 1 100.0%	1 / 1 100.0%	5 / 5 100.0%	1 / 1 100.0%	4 / 4 100.0%	2 / 2 100.0%	1 / 1 100.0%	2 / 2 100.0%	1 / 1 100.0%	3 / 3 100.0%	1 / 1 100.0%	3 / 3 100.0%	4 / 4 100.0%	3 / 3 100.0%	1 / 1 100.0%	6 / 6 100.0%	0 / 0 0.0%	0 / 0 0.0%	0 / 0 0.0%	0 / 0 0.0%	22 / 55 40.0%B	14 / 55 25.5%AB	12 / 55 21.8%ABE	7 / 55 12.7%BE		
1	Brucella canis HSK A52141	55 / 55 100.0%	3 / 3 100.0%	2 / 2 100.0%	1 / 1 100.0%	3 / 3 100.0%	4 / 4 100.0%	3 / 3 100.0%	1 / 1 100.0%	1 / 1 100.0%	5 / 5 100.0%	1 / 1 100.0%	4 / 4 100.0%	2 / 2 100.0%	1 / 1 100.0%	2 / 2 100.0%	1 / 1 100.0%	3 / 3 100.0%	1 / 1 100.0%	3 / 3 100.0%	4 / 4 100.0%	3 / 3 100.0%	1 / 1 100.0%	6 / 6 100.0%	0 / 0 0.0%	0 / 0 0.0%	0 / 0 0.0%	0 / 0 0.0%	36 / 55 65.5%AB	19 / 55 34.5%ABE				
1	"Brucella maris" Jahans et al. 1997	55 / 55 100.0%	3 / 3 100.0%	2 / 2 100.0%	1 / 1 100.0%	3 / 3 100.0%	4 / 4 100.0%	3 / 3 100.0%	1 / 1 100.0%	1 / 1 100.0%	5 / 5 100.0%	1 / 1 100.0%	4 / 4 100.0%	2 / 2 100.0%	1 / 1 100.0%	2 / 2 100.0%	1 / 1 100.0%	3 / 3 100.0%	1 / 1 100.0%	3 / 3 100.0%	4 / 4 100.0%	3 / 3 100.0%	1 / 1 100.0%	6 / 6 100.0%	0 / 0 0.0%	0 / 0 0.0%	0 / 0 0.0%	0 / 0 0.0%	36 / 55 65.5%AB	19 / 55 34.5%ABE				
1	Brucella pinnipedialis B2/94	55 / 55 100.0%	3 / 3 100.0%	2 / 2 100.0%	1 / 1 100.0%	3 / 3 100.0%	4 / 4 100.0%	3 / 3 100.0%	1 / 1 100.0%	1 / 1 100.0%	5 / 5 100.0%	1 / 1 100.0%	4 / 4 100.0%	2 / 2 100.0%	1 / 1 100.0%	2 / 2 100.0%	1 / 1 100.0%	3 / 3 100.0%	1 / 1 100.0%	3 / 3 100.0%	4 / 4 100.0%	3 / 3 100.0%	1 / 1 100.0%	6 / 6 100.0%	0 / 0 0.0%	0 / 0 0.0%	0 / 0 0.0%	0 / 0 0.0%	36 / 55 65.5%AB	19 / 55 34.5%ABE				
1	BCCN 07-01	55 / 55 100.0%	3 / 3 100.0%	2 / 2 100.0%	1 / 1 100.0%	3 / 3 100.0%	4 / 4 100.0%	3 / 3 100.0%	1 / 1 100.0%	1 / 1 100.0%	5 / 5 100.0%	1 / 1 100.0%	4 / 4 100.0%	2 / 2 100.0%	1 / 1 100.0%	2 / 2 100.0%	1 / 1 100.0%	3 / 3 100.0%	1 / 1 100.0%	3 / 3 100.0%	4 / 4 100.0%	3 / 3 100.0%	1 / 1 100.0%	6 / 6 100.0%	0 / 0 0.0%	0 / 0 0.0%	0 / 0 0.0%	0 / 0 0.0%	36 / 55 65.5%AB	19 / 55 34.5%ABE				
1	Brucella microti CCM 4915	55 / 55 100.0%	3 / 3 100.0%	2 / 2 100.0%	1 / 1 100.0%	3 / 3 100.0%	4 / 4 100.0%	3 / 3 100.0%	1 / 1 100.0%	1 / 1 100.0%	5 / 5 100.0%	1 / 1 100.0%	4 / 4 100.0%	2 / 2 100.0%	1 / 1 100.0%	2 / 2 100.0%	1 / 1 100.0%	3 / 3 100.0%	1 / 1 100.0%	3 / 3 100.0%	4 / 4 100.0%	3 / 3 100.0%	1 / 1 100.0%	6 / 6 100.0%	0 / 0 0.0%	0 / 0 0.0%	0 / 0 0.0%	0 / 0 0.0%	36 / 55 65.5%AB	19 / 55 34.5%ABE				
1	Ochrobactrum	57 / 58 98.3%	5 / 5 100.0%	2 / 2 100.0%	1 / 1 100.0%	4 / 4 100.0%	4 / 4 100.0%	3 / 3 100.0%	1 / 1 100.0%	1 / 1 100.0%	5 / 5 100.0%	1 / 1 100.0%	3 / 4 75.0%	2 / 2 100.0%	1 / 1 100.0%	2 / 2 100.0%	1 / 1 100.0%	3 / 3 100.0%	1 / 1 100.0%	3 / 3 100.0%	4 / 4 100.0%	3 / 3 100.0%	1 / 1 100.0%	5 / 5 100.0%	1 / 1 100.0%	0 / 0 0.0%	0 / 0 0.0%	0 / 0 0.0%	21 / 58 36.2%B	17 / 58 29.3%AB	10 / 58 17.2%BE	9 / 58 15.5%ABE	1 / 58 1.7%E	
1	ATCC 49188	57 / 58 98.3%	5 / 5 100.0%	2 / 2 100.0%	1 / 1 100.0%	4 / 4 100.0%	4 / 4 100.0%	3 / 3 100.0%	1 / 1 100.0%	1 / 1 100.0%	5 / 5 100.0%	1 / 1 100.0%	3 / 4 75.0%	2 / 2 100.0%	1 / 1 100.0%	2 / 2 100.0%	1 / 1 100.0%	3 / 3 100.0%	1 / 1 100.0%	3 / 3 100.0%	4 / 4 100.0%	3 / 3 100.0%	1 / 1 100.0%	5 / 5 100.0%	1 / 1 100.0%	0 / 0 0.0%	0 / 0 0.0%	0 / 0 0.0%	21 / 58 36.2%B	17 / 58 29.3%AB	10 / 58 17.2%BE	9 / 58 15.5%ABE	1 / 58 1.7%E	
1	Ochrobactrum anthropi ATCC 49188	57 / 58 98.3%	5 / 5 100.0%	2 / 2 100.0%	1 / 1 100.0%	4 / 4 100.0%	4 / 4 100.0%	3 / 3 100.0%	1 / 1 100.0%	1 / 1 100.0%	5 / 5 100.0%	1 / 1 100.0%	3 / 4 75.0%	2 / 2 100.0%	1 / 1 100.0%	2 / 2 100.0%	1 / 1 100.0%	3 / 3 100.0%	1 / 1 100.0%	3 / 3 100.0%	4 / 4 100.0%	3 / 3 100.0%	1 / 1 100.0%	5 / 5 100.0%	1 / 1 100.0%	0 / 0 0.0%	0 / 0 0.0%	0 / 0 0.0%	21 / 58 36.2%B	17 / 58 29.3%AB	10 / 58 17.2%BE	9 / 58 15.5%ABE	1 / 58 1.7%E	
1	unclassified Rhizobiaceae group	0 / 14 0.0%	0 / 1 0.0%	0 / 0 0.0%	0 / 0 0.0%	0 / 1 0.0%	0 / 2 0.0%	0 / 0 0.0%	0 / 1 0.0%	0 / 0 0.0%	0 / 1 0.0%	0 / 0 0.0%	0 / 0 0.0%	0 / 1 0.0%	0 / 0 0.0%	0 / 1 0.0%	0 / 1 0.0%	0 / 1 0.0%	0 / 1 0.0%	0 / 0 0.0%	0 / 0 0.0%	0 / 1 0.0%	0 / 1 0.0%	0 / 0 0.0%	0 / 1 0.0%	0 / 0 0.0%	0 / 0 0.0%	0 / 0 0.0%	7 / 14 50.0%E	6 / 14 42.9%	1 / 14 7.1%CE			
1	"Candidatus Hodgkinia" McCutcheon et al. 2009	0 / 14 0.0%	0 / 1 0.0%	0 / 0 0.0%	0 / 0 0.0%	0 / 1 0.0%	0 / 2 0.0%	0 / 0 0.0%	0 / 1 0.0%	0 / 0 0.0%	0 / 1 0.0%	0 / 0 0.0%	0 / 0 0.0%	0 / 1 0.0%	0 / 0 0.0%	0 / 1 0.0%	0 / 1 0.0%	0 / 1 0.0%	0 / 1 0.0%	0 / 0 0.0%	0 / 0 0.0%	0 / 1 0.0%	0 / 1 0.0%	0 / 0 0.0%	0 / 1 0.0%	0 / 0 0.0%	0 / 0 0.0%	0 / 0 0.0%	7 / 14 50.0%E	6 / 14 42.9%	1 / 14 7.1%CE			

1	"Candidatus Hodgkinia cicadicola" McCutcheon et al. 2009	0 / 14 0.0%	0 / 1 0.0%	0 / 0 0.0%	0 / 0 0.0%	0 / 1 0.0%	0 / 2 0.0%	0 / 0 0.0%	0 / 1 0.0%	0 / 0 0.0%	0 / 1 0.0%	0 / 0 0.0%	0 / 0 0.0%	0 / 1 0.0%	0 / 0 0.0%	0 / 1 0.0%	0 / 1 0.0%	0 / 1 0.0%	0 / 1 0.0%	0 / 0 0.0%	0 / 0 0.0%	0 / 1 0.0%	0 / 1 0.0%	0 / 0 0.0%	0 / 1 0.0%	0 / 0 0.0%	0 / 0 0.0%	0 / 0 0.0%	7 / 14 50.0%E	6 / 14 42.9%	1 / 14 7.1%CE					
1	Candidatus Hodgkinia cicadicola Dsem	0 / 14 0.0%	0 / 1 0.0%	0 / 0 0.0%	0 / 0 0.0%	0 / 1 0.0%	0 / 2 0.0%	0 / 0 0.0%	0 / 1 0.0%	0 / 0 0.0%	0 / 1 0.0%	0 / 0 0.0%	0 / 0 0.0%	0 / 1 0.0%	0 / 0 0.0%	0 / 1 0.0%	0 / 1 0.0%	0 / 1 0.0%	0 / 1 0.0%	0 / 0 0.0%	0 / 0 0.0%	0 / 1 0.0%	0 / 1 0.0%	0 / 0 0.0%	0 / 1 0.0%	0 / 0 0.0%	0 / 0 0.0%	0 / 0 0.0%	7 / 14 50.0%E	6 / 14 42.9%	1 / 14 7.1%CE					
1	'Rhodobiaceae'	44 / 45 97.8%	1 / 1 100.0%	1 / 1 100.0%	1 / 1 100.0%	1 / 1 100.0%	3 / 3 100.0%	3 / 3 100.0%	1 / 1 100.0%	1 / 1 100.0%	3 / 3 100.0%	1 / 1 100.0%	4 / 4 100.0%	2 / 2 100.0%	1 / 1 100.0%	2 / 2 100.0%	1 / 1 100.0%	3 / 3 100.0%	1 / 1 100.0%	2 / 3 66.7%	4 / 4 100.0%	2 / 2 100.0%	1 / 1 100.0%	5 / 5 100.0%	0 / 0 0.0%	0 / 0 0.0%	0 / 0 0.0%	0 / 0 0.0%	20 / 45 44.4%AB	11 / 45 24.4%ABE	8 / 45 17.8%B	5 / 45 11.1%BE	1 / 45 2.2%C			
1	Parvibaculum	44 / 45 97.8%	1 / 1 100.0%	1 / 1 100.0%	1 / 1 100.0%	1 / 1 100.0%	3 / 3 100.0%	3 / 3 100.0%	1 / 1 100.0%	1 / 1 100.0%	3 / 3 100.0%	1 / 1 100.0%	4 / 4 100.0%	2 / 2 100.0%	1 / 1 100.0%	2 / 2 100.0%	1 / 1 100.0%	3 / 3 100.0%	1 / 1 100.0%	2 / 3 66.7%	4 / 4 100.0%	2 / 2 100.0%	1 / 1 100.0%	5 / 5 100.0%	0 / 0 0.0%	0 / 0 0.0%	0 / 0 0.0%	0 / 0 0.0%	20 / 45 44.4%AB	11 / 45 24.4%ABE	8 / 45 17.8%B	5 / 45 11.1%BE	1 / 45 2.2%C			
1	DSM 13023	44 / 45 97.8%	1 / 1 100.0%	1 / 1 100.0%	1 / 1 100.0%	1 / 1 100.0%	3 / 3 100.0%	3 / 3 100.0%	1 / 1 100.0%	1 / 1 100.0%	3 / 3 100.0%	1 / 1 100.0%	4 / 4 100.0%	2 / 2 100.0%	1 / 1 100.0%	2 / 2 100.0%	1 / 1 100.0%	3 / 3 100.0%	1 / 1 100.0%	2 / 3 66.7%	4 / 4 100.0%	2 / 2 100.0%	1 / 1 100.0%	5 / 5 100.0%	0 / 0 0.0%	0 / 0 0.0%	0 / 0 0.0%	0 / 0 0.0%	20 / 45 44.4%AB	11 / 45 24.4%ABE	8 / 45 17.8%B	5 / 45 11.1%BE	1 / 45 2.2%C			
1	Parvibaculum lavamentivorans DS-1	44 / 45 97.8%	1 / 1 100.0%	1 / 1 100.0%	1 / 1 100.0%	1 / 1 100.0%	3 / 3 100.0%	3 / 3 100.0%	1 / 1 100.0%	1 / 1 100.0%	3 / 3 100.0%	1 / 1 100.0%	4 / 4 100.0%	2 / 2 100.0%	1 / 1 100.0%	2 / 2 100.0%	1 / 1 100.0%	3 / 3 100.0%	1 / 1 100.0%	2 / 3 66.7%	4 / 4 100.0%	2 / 2 100.0%	1 / 1 100.0%	5 / 5 100.0%	0 / 0 0.0%	0 / 0 0.0%	0 / 0 0.0%	0 / 0 0.0%	20 / 45 44.4%AB	11 / 45 24.4%ABE	8 / 45 17.8%B	5 / 45 11.1%BE	1 / 45 2.2%C			
8	'Methylobacteriaceae'	464 / 466 99.6%	39 / 39 100.0%	10 / 10 100.0%	10 / 10 100.0%	40 / 41 97.6%	25 / 25 100.0%	24 / 24 100.0%	8 / 8 100.0%	8 / 8 100.0%	63 / 63 100.0%	8 / 8 100.0%	31 / 32 96.9%	16 / 16 100.0%	8 / 8 100.0%	16 / 16 100.0%	8 / 8 100.0%	24 / 24 100.0%	8 / 8 100.0%	25 / 25 100.0%	25 / 25 100.0%	16 / 16 100.0%	10 / 10 100.0%	40 / 40 100.0%	2 / 2 100.0%	0 / 0 0.0%	0 / 0 0.0%	0 / 0 0.0%	236 / 466 50.6%AB	115 / 466 24.7%ABE	76 / 466 16.3%B	37 / 466 7.9%BE	1 / 466 0.2%CE	1 / 466 0.2%		
8	Methylobacterium	464 / 466 99.6%	39 / 39 100.0%	10 / 10 100.0%	10 / 10 100.0%	40 / 41 97.6%	25 / 25 100.0%	24 / 24 100.0%	8 / 8 100.0%	8 / 8 100.0%	63 / 63 100.0%	8 / 8 100.0%	31 / 32 96.9%	16 / 16 100.0%	8 / 8 100.0%	16 / 16 100.0%	8 / 8 100.0%	24 / 24 100.0%	8 / 8 100.0%	25 / 25 100.0%	25 / 25 100.0%	16 / 16 100.0%	10 / 10 100.0%	40 / 40 100.0%	2 / 2 100.0%	0 / 0 0.0%	0 / 0 0.0%	0 / 0 0.0%	236 / 466 50.6%AB	115 / 466 24.7%ABE	76 / 466 16.3%B	37 / 466 7.9%BE	1 / 466 0.2%	1 / 466 0.2%CE		
1	ATCC 27329	54 / 54 100.0%	3 / 3 100.0%	1 / 1 100.0%	1 / 1 100.0%	4 / 4 100.0%	3 / 3 100.0%	3 / 3 100.0%	1 / 1 100.0%	1 / 1 100.0%	7 / 7 100.0%	1 / 1 100.0%	4 / 4 100.0%	2 / 2 100.0%	1 / 1 100.0%	2 / 2 100.0%	1 / 1 100.0%	3 / 3 100.0%	1 / 1 100.0%	3 / 3 100.0%	3 / 3 100.0%	2 / 2 100.0%	1 / 1 100.0%	5 / 5 100.0%	1 / 1 100.0%	0 / 0 0.0%	0 / 0 0.0%	0 / 0 0.0%	18 / 54 33.3%B	17 / 54 31.5%AB	13 / 54 24.1%ABE	6 / 54 11.1%BE				
1	Methylobacterium radiotolerans ATCC 27329	54 / 54 100.0%	3 / 3 100.0%	1 / 1 100.0%	1 / 1 100.0%	4 / 4 100.0%	3 / 3 100.0%	3 / 3 100.0%	1 / 1 100.0%	1 / 1 100.0%	7 / 7 100.0%	1 / 1 100.0%	4 / 4 100.0%	2 / 2 100.0%	1 / 1 100.0%	2 / 2 100.0%	1 / 1 100.0%	3 / 3 100.0%	1 / 1 100.0%	3 / 3 100.0%	3 / 3 100.0%	2 / 2 100.0%	1 / 1 100.0%	5 / 5 100.0%	1 / 1 100.0%	0 / 0 0.0%	0 / 0 0.0%	0 / 0 0.0%	18 / 54 33.3%B	17 / 54 31.5%AB	13 / 54 24.1%ABE	6 / 54 11.1%BE				
1	CNCM I 2342	67 / 67 100.0%	7 / 7 100.0%	2 / 2 100.0%	2 / 2 100.0%	7 / 7 100.0%	4 / 4 100.0%	3 / 3 100.0%	1 / 1 100.0%	1 / 1 100.0%	10 / 10 100.0%	1 / 1 100.0%	4 / 4 100.0%	2 / 2 100.0%	1 / 1 100.0%	2 / 2 100.0%	1 / 1 100.0%	3 / 3 100.0%	1 / 1 100.0%	4 / 4 100.0%	3 / 3 100.0%	2 / 2 100.0%	1 / 1 100.0%	5 / 5 100.0%	0 / 0 0.0%	0 / 0 0.0%	0 / 0 0.0%	0 / 0 0.0%	47 / 67 70.1%AB	20 / 67 29.9%ABE						
1	Methylobacterium nodulans ORS 2060	67 / 67 100.0%	7 / 7 100.0%	2 / 2 100.0%	2 / 2 100.0%	7 / 7 100.0%	4 / 4 100.0%	3 / 3 100.0%	1 / 1 100.0%	1 / 1 100.0%	10 / 10 100.0%	1 / 1 100.0%	4 / 4 100.0%	2 / 2 100.0%	1 / 1 100.0%	2 / 2 100.0%	1 / 1 100.0%	3 / 3 100.0%	1 / 1 100.0%	4 / 4 100.0%	3 / 3 100.0%	2 / 2 100.0%	1 / 1 100.0%	5 / 5 100.0%	0 / 0 0.0%	0 / 0 0.0%	0 / 0 0.0%	0 / 0 0.0%	47 / 67 70.1%AB	20 / 67 29.9%ABE						
1	ATCC BAA-705	56 / 56 100.0%	5 / 5 100.0%	1 / 1 100.0%	1 / 1 100.0%	5 / 5 100.0%	3 / 3 100.0%	3 / 3 100.0%	1 / 1 100.0%	1 / 1 100.0%	7 / 7 100.0%	1 / 1 100.0%	4 / 4 100.0%	2 / 2 100.0%	1 / 1 100.0%	2 / 2 100.0%	1 / 1 100.0%	3 / 3 100.0%	1 / 1 100.0%	3 / 3 100.0%	3 / 3 100.0%	2 / 2 100.0%	1 / 1 100.0%	5 / 5 100.0%	0 / 0 0.0%	0 / 0 0.0%	0 / 0 0.0%	0 / 0 0.0%	19 / 56 33.9%AB	18 / 56 32.1%B	11 / 56 19.6%BE	8 / 56 14.3%ABE				
1	Methylobacterium populi BJ001	56 / 56 100.0%	5 / 5 100.0%	1 / 1 100.0%	1 / 1 100.0%	5 / 5 100.0%	3 / 3 100.0%	3 / 3 100.0%	1 / 1 100.0%	1 / 1 100.0%	7 / 7 100.0%	1 / 1 100.0%	4 / 4 100.0%	2 / 2 100.0%	1 / 1 100.0%	2 / 2 100.0%	1 / 1 100.0%	3 / 3 100.0%	1 / 1 100.0%	3 / 3 100.0%	3 / 3 100.0%	2 / 2 100.0%	1 / 1 100.0%	5 / 5 100.0%	0 / 0 0.0%	0 / 0 0.0%	0 / 0 0.0%	0 / 0 0.0%	19 / 56 33.9%AB	18 / 56 32.1%B	11 / 56 19.6%BE	8 / 56 14.3%ABE				
1	Methylobacterium sp. 4-46	61 / 62 98.4%	4 / 4 100.0%	2 / 2 100.0%	2 / 2 100.0%	4 / 4 100.0%	3 / 3 100.0%	3 / 3 100.0%	1 / 1 100.0%	1 / 1 100.0%	9 / 9 100.0%	1 / 1 100.0%	3 / 4 75.0%	2 / 2 100.0%	1 / 1 100.0%	2 / 2 100.0%	1 / 1 100.0%	3 / 3 100.0%	1 / 1 100.0%	3 / 3 100.0%	4 / 4 100.0%	2 / 2 100.0%	3 / 3 100.0%	5 / 5 100.0%	1 / 1 100.0%	0 / 0 0.0%	0 / 0 0.0%	0 / 0 0.0%	22 / 62 35.5%B	21 / 62 33.9%AB	10 / 62 16.1%BE	8 / 62 12.9%ABE	1 / 62 1.6%CE			
4	Methylobacterium extorquens group	226 / 227 99.6%	20 / 20 100.0%	4 / 4 100.0%	4 / 4 100.0%	20 / 21 95.2%	12 / 12 100.0%	12 / 12 100.0%	4 / 4 100.0%	4 / 4 100.0%	30 / 30 100.0%	4 / 4 100.0%	16 / 16 100.0%	8 / 8 100.0%	4 / 4 100.0%	8 / 8 100.0%	4 / 4 100.0%	12 / 12 100.0%	4 / 4 100.0%	12 / 12 100.0%	12 / 12 100.0%	8 / 8 100.0%	4 / 4 100.0%	20 / 20 100.0%	0 / 0 0.0%	0 / 0 0.0%	0 / 0 0.0%	0 / 0 0.0%	132 / 227 58.1%AB	66 / 227 29.1%ABE	18 / 227 7.9%B	10 / 227 4.4%BE	1 / 227 0.4%			
4	"Bacillus extorquens" Bassalik 1913	226 / 227 99.6%	20 / 20 100.0%	4 / 4 100.0%	4 / 4 100.0%	20 / 21 95.2%	12 / 12 100.0%	12 / 12 100.0%	4 / 4 100.0%	4 / 4 100.0%	30 / 30 100.0%	4 / 4 100.0%	16 / 16 100.0%	8 / 8 100.0%	4 / 4 100.0%	8 / 8 100.0%	4 / 4 100.0%	12 / 12 100.0%	4 / 4 100.0%	12 / 12 100.0%	12 / 12 100.0%	8 / 8 100.0%	4 / 4 100.0%	20 / 20 100.0%	0 / 0 0.0%	0 / 0 0.0%	0 / 0 0.0%	0 / 0 0.0%	132 / 227 58.1%AB	66 / 227 29.1%ABE	18 / 227 7.9%B	10 / 227 4.4%BE	1 / 227 0.4%			
1	Methylobacterium extorquens AM1	56 / 56 100.0%	5 / 5 100.0%	1 / 1 100.0%	1 / 1 100.0%	5 / 5 100.0%	3 / 3 100.0%	3 / 3 100.0%	1 / 1 100.0%	1 / 1 100.0%	7 / 7 100.0%	1 / 1 100.0%	4 / 4 100.0%	2 / 2 100.0%	1 / 1 100.0%	2 / 2 100.0%	1 / 1 100.0%	3 / 3 100.0%	1 / 1 100.0%	3 / 3 100.0%	3 / 3 100.0%	2 / 2 100.0%	1 / 1 100.0%	5 / 5 100.0%	0 / 0 0.0%	0 / 0 0.0%	0 / 0 0.0%	0 / 0 0.0%	37 / 56 66.1%AB	19 / 56 33.9%ABE						
1	Methylobacterium extorquens PA1	57 / 57 100.0%	5 / 5 100.0%	1 / 1 100.0%	1 / 1 100.0%	5 / 5 100.0%	3 / 3 100.0%	3 / 3 100.0%	1 / 1 100.0%	1 / 1 100.0%	8 / 8 100.0%	1 / 1 100.0%	4 / 4 100.0%	2 / 2 100.0%	1 / 1 100.0%	2 / 2 100.0%	1 / 1 100.0%	3 / 3 100.0%	1 / 1 100.0%	3 / 3 100.0%	3 / 3 100.0%	2 / 2 100.0%	1 / 1 100.0%	5 / 5 100.0%	0 / 0 0.0%	0 / 0 0.0%	0 / 0 0.0%	0 / 0 0.0%	20 / 57 35.1%AB	18 / 57 31.6%B	10 / 57 17.5%BE	9 / 57 15.8%ABE				
1	Methylobacterium chloromethanicum CM4	56 / 56 100.0%	5 / 5 100.0%	1 / 1 100.0%	1 / 1 100.0%	5 / 5 100.0%	3 / 3 100.0%	3 / 3 100.0%	1 / 1 100.0%	1 / 1 100.0%	7 / 7 100.0%	1 / 1 100.0%	4 / 4 100.0%	2 / 2 100.0%	1 / 1 100.0%	2 / 2 100.0%	1 / 1 100.0%	3 / 3 100.0%	1 / 1 100.0%	3 / 3 100.0%	3 / 3 100.0%	2 / 2 100.0%	1 / 1 100.0%	5 / 5 100.0%	0 / 0 0.0%	0 / 0 0.0%	0 / 0 0.0%	0 / 0 0.0%	37 / 56 66.1%AB	19 / 56 33.9%ABE						
1	Methylobacterium dichloromethanicum DM4	57 / 58 98.3%	5 / 5 100.0%	1 / 1 100.0%	1 / 1 100.0%	5 / 6 83.3%	3 / 3 100.0%	3 / 3 100.0%	1 / 1 100.0%	1 / 1 100.0%	8 / 8 100.0%	1 / 1 100.0%	4 / 4 100.0%	2 / 2 100.0%	1 / 1 100.0%	2 / 2 100.0%	1 / 1 100.0%	3 / 3 100.0%	1 / 1 100.0%	3 / 3 100.0%	3 / 3 100.0%	2 / 2 100.0%	1 / 1 100.0%	5 / 5 100.0%	0 / 0 0.0%	0 / 0 0.0%	0 / 0 0.0%	0 / 0 0.0%	38 / 58 65.5%AB	19 / 58 32.8%ABE	1 / 58 1.7%					
3	Xanthobacteraceae	147 / 147 100.0%	3 / 3 100.0%	3 / 3 100.0%	3 / 3 100.0%	6 / 6 100.0%	9 / 9 100.0%	9 / 9 100.0%	3 / 3 100.0%	3 / 3 100.0%	14 / 14 100.0%	3 / 3 100.0%	12 / 12 100.0%	7 / 7 100.0%	3 / 3 100.0%	6 / 6 100.0%	3 / 3 100.0%	10 / 10 100.0%	3 / 3 100.0%	9 / 9 100.0%	12 / 12 100.0%	6 / 6 100.0%	3 / 3 100.0%	14 / 14 100.0%	3 / 3 100.0%	0 / 0 0.0%	0 / 0 0.0%	0 / 0 0.0%	72 / 147 49.0%AB	33 / 147 22.4%ABE	30 / 147 20.4%B	12 / 147 8.2%BE				
1	Azorhizobium	53 / 53 100.0%	1 / 1 100.0%	1 / 1 100.0%	1 / 1 100.0%	3 / 3 100.0%	3 / 3 100.0%	3 / 3 100.0%	1 / 1 100.0%	1 / 1 100.0%	6 / 6 100.0%	1 / 1 100.0%	4 / 4 100.0%	3 / 3 100.0%	1 / 1 100.0%	2 / 2 100.0%	1 / 1 100.0%	4 / 4 100.0%	1 / 1 100.0%	3 / 3 100.0%	4 / 4 100.0%	2 / 2 100.0%	1 / 1 100.0%	5 / 5 100.0%	1 / 1 100.0%	0 / 0 0.0%	0 / 0 0.0%	0 / 0 0.0%	22 / 53 41.5%AB	16 / 53 30.2%B	8 / 53 15.1%ABE	7 / 53 13.2%BE				
1	ATCC 43989	53 / 53 100.0%	1 / 1 100.0%	1 / 1 100.0%	1 / 1 100.0%	3 / 3 100.0%	3 / 3 100.0%	3 / 3 100.0%	1 / 1 100.0%	1 / 1 100.0%	6 / 6 100.0%	1 / 1 100.0%	4 / 4 100.0%	3 / 3 100.0%	1 / 1 100.0%	2 / 2 100.0%	1 / 1 100.0%	4 / 4 100.0%	1 / 1 100.0%	3 / 3 100.0%	4 / 4 100.0%	2 / 2 100.0%	1 / 1 100.0%	5 / 5 100.0%	1 / 1 100.0%	0 / 0 0.0%	0 / 0 0.0%	0 / 0 0.0%	22 / 53 41.5%AB	16 / 53 30.2%B	8 / 53 15.1%ABE	7 / 53 13.2%BE				
1	Azorhizobium caulinodans ORS 571	53 / 53 100.0%	1 / 1 100.0%	1 / 1 100.0%	1 / 1 100.0%	3 / 3 100.0%	3 / 3 100.0%	3 / 3 100.0%	1 / 1 100.0%	1 / 1 100.0%	6 / 6 100.0%	1 / 1 100.0%	4 / 4 100.0%	3 / 3 100.0%	1 / 1 100.0%	2 / 2 100.0%	1 / 1 100.0%	4 / 4 100.0%	1 / 1 100.0%	3 / 3 100.0%	4 / 4 100.0%	2 / 2 100.0%	1 / 1 100.0%	5 / 5 100.0%	1 / 1 100.0%	0 / 0 0.0%	0 / 0 0.0%	0 / 0 0.0%	22 / 53 41.5%AB	16 / 53 30.2%B	8 / 53 15.1%ABE	7 / 53 13.2%BE				
1	Xanthobacter	48 / 48 100.0%	1 / 1 100.0%	1 / 1 100.0%	1 / 1 100.0%	2 / 2 100.0%	3 / 3 100.0%	3 / 3 100.0%	1 / 1 100.0%	1 / 1 100.0%	5 / 5 100.0%	1 / 1 100.0%	4 / 4 100.0%	2 / 2 100.0%	1 / 1 100.0%	2 / 2 100.0%	1 / 1 100.0%	3 / 3 100.0%	1 / 1 100.0%	3 / 3 100.0%	4 / 4 100.0%	2 / 2 100.0%	1 / 1 100.0%	4 / 4 100.0%	1 / 1 100.0%	0 / 0 0.0%	0 / 0 0.0%	0 / 0 0.0%	20 / 48 41.7%AB	14 / 48 29.2%B	9 / 48 18.8%ABE	5 / 48 10.4%BE				
1	"Corynebacterium autotrophicum" Baumgarten et al. 1974	48 / 48 100.0%	1 / 1 100.0%	1 / 1 100.0%	1 / 1 100.0%	2 / 2 100.0%	3 / 3 100.0%	3 / 3 100.0%	1 / 1 100.0%	1 / 1 100.0%	5 / 5 100.0%	1 / 1 100.0%	4 / 4 100.0%	2 / 2 100.0%	1 / 1 100.0%	2 / 2 100.0%	1 / 1 100.0%	3 / 3 100.0%	1 / 1 100.0%	3 / 3 100.0%	4 / 4 100.0%	2 / 2 100.0%	1 / 1 100.0%	4 / 4 100.0%	1 / 1 100.0%	0 / 0 0.0%	0 / 0 0.0%	0 / 0 0.0%	20 / 48 41.7%AB	14 / 48 29.2%B	9 / 48 18.8%ABE	5 / 48 10.4%BE				
1	Xanthobacter autotrophicus Py2	48 / 48 100.0%	1 / 1 100.0%	1 / 1 100.0%	1 / 1 100.0%	2 / 2 100.0%	3 / 3 100.0%	3 / 3 100.0%	1 / 1 100.0%	1 / 1 100.0%	5 / 5 100.0%	1 / 1 100.0%	4 / 4 100.0%	2 / 2 100.0%	1 / 1 100.0%	2 / 2 100.0%	1 / 1 100.0%	3 / 3 100.0%	1 / 1 100.0%	3 / 3 100.0%	4 / 4 100.0%	2 / 2 100.0%	1 / 1 100.0%	4 / 4 100.0%	1 / 1 100.0%	0 / 0 0.0%	0 / 0 0.0%	0 / 0 0.0%	20 / 48 41.7%AB	14 / 48 29.2%B	9 / 48 18.8%ABE	5 / 48 10.4%BE				
1	Starkeya	46 / 46 100.0%	1 / 1 100.0%	1 / 1 100.0%	1 / 1 100.0%	1 / 1 100.0%	3 / 3 100.0%	3 / 3 100.0%	1 / 1 100.0%	1 / 1 100.0%	3 / 3 100.0%	1 / 1 100.0%	4 / 4 100.0%	2 / 2 100.0%	1 / 1 100.0%	2 / 2 100.0%	1 / 1 100.0%	3 / 3 100.0%	1 / 1 100.0%	3 / 3 100.0%	4 / 4 100.0%	2 / 2 100.0%	1 / 1 100.0%	5 / 5 100.0%	1 / 1 100.0%	0 / 0 0.0%	0 / 0 0.0%	0 / 0 0.0%	30 / 46 65.2%AB	16 / 46 34.8%ABE						
1	ATCC 8093	46 / 46 100.0%	1 / 1 100.0%	1 / 1 100.0%	1 / 1 100.0%	1 / 1 100.0%	3 / 3 100.0%	3 / 3 100.0%	1 / 1 100.0%	1 / 1 100.0%	3 / 3 100.0%	1 / 1 100.0%	4 / 4 100.0%	2 / 2 100.0%	1 / 1 100.0%	2 / 2 100.0%	1 / 1 100.0%	3 / 3 100.0%	1 / 1 100.0%	3 / 3 100.0%	4 / 4 100.0%	2 / 2 100.0%	1 / 1 100.0%	5 / 5 100.0%	1 / 1 100.0%	0 / 0 0.0%	0 / 0 0.0%	0 / 0 0.0%	30 / 46 65.2%AB	16 / 46 34.8%ABE						
1	Starkeya novella DSM 506	46 / 46 100.0%	1 / 1 100.0%	1 / 1 100.0%	1 / 1 100.0%	1 / 1 100.0%	3 / 3 100.0%	3 / 3 100.0%	1 / 1 100.0%	1 / 1 100.0%	3 / 3 100.0%	1 / 1 100.0%	4 / 4 100.0%	2 / 2 100.0%	1 / 1 100.0%	2 / 2 100.0%	1 / 1 100.0%	3 / 3 100.0%	1 / 1 100.0%	3 / 3 100.0%	4 / 4 100.0%	2 / 2 100.0%	1 / 1 100.0%	5 / 5 100.0%	1 / 1 100.0%	0 / 0 0.0%	0 / 0 0.0%	0 / 0 0.0%	30 / 46 65.2%AB	16 / 46 34.8%ABE						
61	Rickettsiales	1605 / 2054 78.1%	50 / 61 82.0%	51 / 61 83.6%	45 / 61 73.8%	55 / 63 87.3%	90 / 125 72.0%	92 / 121 76.0%	52 / 61 85.2%	40 / 61 65.6%	94 / 104 90.4%	50 / 61 82.0%	142 / 183 77.6%	45 / 68 66.2%	45 / 61 73.8%	58 / 69 84.1%	44 / 60 73.3%	55 / 68 80.9%	46 / 63 73.0%	132 / 167 79.0%	155 / 200 77.5%	45 / 61 73.8%	50 / 59 84.7%	169 / 216 78.2%	0 / 0 0.0%	0 / 0 0.0%	0 / 0 0.0%	0 / 0 0.0%	1033 / 2054 50.3%AB	474 / 2054 23.1%ABE	239 / 2054 11.6%	168 / 2054 8.2%E	72 / 2054 3.5%B	29 / 2054 1.4%C	26 / 2054 1.3%BE	13 / 2054 0.6%CE
44	Rickettsiaceae	1443 / 1460 98.8%	44 / 44 100.0%	44 / 44 100.0%	44 / 44 100.0%	44 / 44 100.0%	86 / 88 97.7%	87 / 87 100.0%	44 / 44 100.0%	39 / 44 88.6%	88 / 88 100.0%	43 / 44 97.7%	129 / 132 97.7%	44 / 44 100.0%	44 / 44 100.0%	51 / 52 98.1%	43 / 43 100.0%	44 / 44 100.0%	44 / 44 100.0%	128 / 132 97.0%	131 / 132 99.2%	44 / 44 100.0%	44 / 44 100.0%	134 / 134 100.0%	0 / 0 0.0%	0 / 0 0.0%	0 / 0 0.0%	0 / 0 0.0%	936 / 1460 64.1%AB	414 / 1460 28.4%ABE	68 / 1460 4.7%B	25 / 1460 1.7%BE	8 / 1460 0.5%E	6 / 1460 0.4%C	2 / 1460 0.1%	1 / 1460 0.1%CE
44	Rickettsieae	1443 / 1460 98.8%	44 / 44 100.0%	44 / 44 100.0%	44 / 44 100.0%	44 / 44 100.0%	86 / 88 97.7%	87 / 87 100.0%	44 / 44 100.0%	39 / 44 88.6%	88 / 88 100.0%	43 / 44 97.7%	129 / 132 97.7%	44 / 44 100.0%	44 / 44 100.0%	51 / 52 98.1%	43 / 43 100.0%	44 / 44 100.0%	44 / 44 100.0%	128 / 132 97.0%	131 / 132 99.2%	44 / 44 100.0%	44 / 44 100.0%	134 / 134 100.0%	0 / 0 0.0%	0 / 0 0.0%	0 / 0 0.0%	0 / 0 0.0%	936 / 1460 64.1%AB	414 / 1460 28.4%ABE	68 / 1460 4.7%B	25 / 1460 1.7%BE	8 / 1460 0.5%E	6 / 1460 0.4%C	2 / 1460 0.1%	1 / 1460 0.1%CE
42	Rickettsia	1381 / 1392 99.2%	42 / 42 100.0%	42 / 42 100.0%	42 / 42 100.0%	42 / 42 100.0%	84 / 84 100.0%	83 / 83 100.0%	42 / 42 100.0%	37 / 42 88.1%	84 / 84 100.0%	42 / 42 100.0%	124 / 126 98.4%	42 / 42 100.0%	42 / 42 100.0%	49 / 50 98.0%	41 / 41 100.0%	42 / 42 100.0%	42 / 42 100.0%	124 / 126 98.4%	125 / 126 99.2%	42 / 42 100.0%	42 / 42 100.0%	126 / 126 100.0%	0 / 0 0.0%	0 / 0 0.0%	0 / 0 0.0%	0 / 0 0.0%	896 / 1392 64.4%AB	401 / 1392 28.8%ABE	65 / 1392 4.7%B	19 / 1392 1.4%BE	7 / 1392 0.5%E	2 / 1392 0.1%C	1 / 1392 0.1%CE	1 / 1392 0.1%
25	spotted fever group	825 / 832 99.2%	25 / 25 100.0%	25 / 25 100.0%	25 / 25 100.0%	25 / 25 100.0%	50 / 50 100.0%	50 / 50 100.0%	25 / 25 100.0%	21 / 25 84.0%	50 / 50 100.0%	25 / 25 100.0%	74 / 75 98.7%	25 / 25 100.0%	25 / 25 100.0%	32 / 33 97.0%	24 / 24 100.0%	25 / 25 100.0%	25 / 25 100.0%	75 / 75 100.0%	74 / 75 98.7%	25 / 25 100.0%	25 / 25 100.0%	75 / 75 100.0%	0 / 0 0.0%	0 / 0 0.0%	0 / 0 0.0%	0 / 0 0.0%	534 / 832 64.2%AB	238 / 832 28.6%ABE	39 / 832 4.7%B	14 / 832 1.7%BE	5 / 832 0.6%E	1 / 832 0.1%	1 / 832 0.1%CE	
1	ATCC VR-613	32 / 33 97.0%	1 / 1 100.0%	1 / 1 100.0%	1 / 1 100.0%	1 / 1 100.0%	2 / 2 100.0%	2 / 2 100.0%	1 / 1 100.0%	1 / 1 100.0%	2 / 2 100.0%	1 / 1 100.0%	3 / 3 100.0%	1 / 1 100.0%	1 / 1 100.0%	1 / 1 100.0%	1 / 1 100.0%	1 / 1 100.0%	1 / 1 100.0%	3 / 3 100.0%	2 / 3 66.7%	1 / 1 100.0%	1 / 1 100.0%	3 / 3 100.0%	0 / 0 0.0%	0 / 0 0.0%	0 / 0 0.0%	0 / 0 0.0%	22 / 33 66.7%AB	10 / 33 30.3%ABE	1 / 33 3.0%					
1	Rickettsia conorii 'Malish 7'	32 / 33 97.0%	1 / 1 100.0%	1 / 1 100.0%	1 / 1 100.0%	1 / 1 100.0%	2 / 2 100.0%	2 / 2 100.0%	1 / 1 100.0%	1 / 1 100.0%	2 / 2 100.0%	1 / 1 100.0%	3 / 3 100.0%	1 / 1 100.0%	1 / 1 100.0%	1 / 1 100.0%	1 / 1 100.0%	1 / 1 100.0%	1 / 1 100.0%	3 / 3 100.0%	2 / 3 66.7%	1 / 1 100.0%	1 / 1 100.0%	3 / 3 100.0%	0 / 0 0.0%	0 / 0 0.0%	0 / 0 0.0%	0 / 0 0.0%	22 / 33 66.7%AB	10 / 33 30.3%ABE	1 / 33 3.0%					
8	"Dermacentroxenus rickettsii" Wolbach 1919	269 / 271 99.3%	8 / 8 100.0%	8 / 8 100.0%	8 / 8 100.0%	8 / 8 100.0%	16 / 16 100.0%	16 / 16 100.0%	8 / 8 100.0%	6 / 8 75.0%	16 / 16 100.0%	8 / 8 100.0%	24 / 24 100.0%	8 / 8 100.0%	8 / 8 100.0%	16 / 16 100.0%	7 / 7 100.0%	8 / 8 100.0%	8 / 8 100.0%	24 / 24 100.0%	24 / 24 100.0%	8 / 8 100.0%	8 / 8 100.0%	24 / 24 100.0%	0 / 0 0.0%	0 / 0 0.0%	0 / 0 0.0%	0 / 0 0.0%	163 / 271 60.1%AB	78 / 271 28.8%ABE	20 / 271 7.4%B	8 / 271 3.0%BE	2 / 271 0.7%E			
1	Rickettsia rickettsii 'Sheila Smith'	33 / 34 97.1%	1 / 1 100.0%	1 / 1 100.0%	1 / 1 100.0%	1 / 1 100.0%	2 / 2 100.0%	2 / 2 100.0%	1 / 1 100.0%	0 / 1 0.0%	2 / 2 100.0%	1 / 1 100.0%	3 / 3 100.0%	1 / 1 100.0%	1 / 1 100.0%	2 / 2 100.0%	1 / 1 100.0%	1 / 1 100.0%	1 / 1 100.0%	3 / 3 100.0%	3 / 3 100.0%	1 / 1 100.0%	1 / 1 100.0%	3 / 3 100.0%	0 / 0 0.0%	0 / 0 0.0%	0 / 0 0.0%	0 / 0 0.0%	13 / 34 38.2%AB	10 / 34 29.4%B	6 / 34 17.6%ABE	4 / 34 11.8%BE	1 / 34 2.9%E			
1	Rickettsia rickettsii 'Iowa'	32 / 33 97.0%	1 / 1 100.0%	1 / 1 100.0%	1 / 1 100.0%	1 / 1 100.0%	2 / 2 100.0%	2 / 2 100.0%	1 / 1 100.0%	0 / 1 0.0%	2 / 2 100.0%	1 / 1 100.0%	3 / 3 100.0%	1 / 1 100.0%	1 / 1 100.0%	2 / 2 100.0%	0 / 0 0.0%	1 / 1 100.0%	1 / 1 100.0%	3 / 3 100.0%	3 / 3 100.0%	1 / 1 100.0%	1 / 1 100.0%	3 / 3 100.0%	0 / 0 0.0%	0 / 0 0.0%	0 / 0 0.0%	0 / 0 0.0%	12 / 33 36.4%AB	10 / 33 30.3%B	6 / 33 18.2%ABE	4 / 33 12.1%BE	1 / 33 3.0%E			
1	Rickettsia rickettsii str. Hino	34 / 34 100.0%	1 / 1 100.0%	1 / 1 100.0%	1 / 1 100.0%	1 / 1 100.0%	2 / 2 100.0%	2 / 2 100.0%	1 / 1 100.0%	1 / 1 100.0%	2 / 2 100.0%	1 / 1 100.0%	3 / 3 100.0%	1 / 1 100.0%	1 / 1 100.0%	2 / 2 100.0%	1 / 1 100.0%	1 / 1 100.0%	1 / 1 100.0%	3 / 3 100.0%	3 / 3 100.0%	1 / 1 100.0%	1 / 1 100.0%	3 / 3 100.0%	0 / 0 0.0%	0 / 0 0.0%	0 / 0 0.0%	0 / 0 0.0%	23 / 34 67.6%AB	11 / 34 32.4%ABE						
1	Rickettsia rickettsii str. Hlp#2	34 / 34 100.0%	1 / 1 100.0%	1 / 1 100.0%	1 / 1 100.0%	1 / 1 100.0%	2 / 2 100.0%	2 / 2 100.0%	1 / 1 100.0%	1 / 1 100.0%	2 / 2 100.0%	1 / 1 100.0%	3 / 3 100.0%	1 / 1 100.0%	1 / 1 100.0%	2 / 2 100.0%	1 / 1 100.0%	1 / 1 100.0%	1 / 1 100.0%	3 / 3 100.0%	3 / 3 100.0%	1 / 1 100.0%	1 / 1 100.0%	3 / 3 100.0%	0 / 0 0.0%	0 / 0 0.0%	0 / 0 0.0%	0 / 0 0.0%	23 / 34 67.6%AB	11 / 34 32.4%ABE						
1	Rickettsia rickettsii str. Colombia	34 / 34 100.0%	1 / 1 100.0%	1 / 1 100.0%	1 / 1 100.0%	1 / 1 100.0%	2 / 2 100.0%	2 / 2 100.0%	1 / 1 100.0%	1 / 1 100.0%	2 / 2 100.0%	1 / 1 100.0%	3 / 3 100.0%	1 / 1 100.0%	1 / 1 100.0%	2 / 2 100.0%	1 / 1 100.0%	1 / 1 100.0%	1 / 1 100.0%	3 / 3 100.0%	3 / 3 100.0%	1 / 1 100.0%	1 / 1 100.0%	3 / 3 100.0%	0 / 0 0.0%	0 / 0 0.0%	0 / 0 0.0%	0 / 0 0.0%	23 / 34 67.6%AB	11 / 34 32.4%ABE						
1	Rickettsia rickettsii str. Hauke	34 / 34 100.0%	1 / 1 100.0%	1 / 1 100.0%	1 / 1 100.0%	1 / 1 100.0%	2 / 2 100.0%	2 / 2 100.0%	1 / 1 100.0%	1 / 1 100.0%	2 / 2 100.0%	1 / 1 100.0%	3 / 3 100.0%	1 / 1 100.0%	1 / 1 100.0%	2 / 2 100.0%	1 / 1 100.0%	1 / 1 100.0%	1 / 1 100.0%	3 / 3 100.0%	3 / 3 100.0%	1 / 1 100.0%	1 / 1 100.0%	3 / 3 100.0%	0 / 0 0.0%	0 / 0 0.0%	0 / 0 0.0%	0 / 0 0.0%	23 / 34 67.6%AB	11 / 34 32.4%ABE						
1	Rickettsia rickettsii str. Brazil	34 / 34 100.0%	1 / 1 100.0%	1 / 1 100.0%	1 / 1 100.0%	1 / 1 100.0%	2 / 2 100.0%	2 / 2 100.0%	1 / 1 100.0%	1 / 1 100.0%	2 / 2 100.0%	1 / 1 100.0%	3 / 3 100.0%	1 / 1 100.0%	1 / 1 100.0%	2 / 2 100.0%	1 / 1 100.0%	1 / 1 100.0%	1 / 1 100.0%	3 / 3 100.0%	3 / 3 100.0%	1 / 1 100.0%	1 / 1 100.0%	3 / 3 100.0%	0 / 0 0.0%	0 / 0 0.0%	0 / 0 0.0%	0 / 0 0.0%	23 / 34 67.6%AB	11 / 34 32.4%ABE						
1	Rickettsia rickettsii str. Arizona	34 / 34 100.0%	1 / 1 100.0%	1 / 1 100.0%	1 / 1 100.0%	1 / 1 100.0%	2 / 2 100.0%	2 / 2 100.0%	1 / 1 100.0%	1 / 1 100.0%	2 / 2 100.0%	1 / 1 100.0%	3 / 3 100.0%	1 / 1 100.0%	1 / 1 100.0%	2 / 2 100.0%	1 / 1 100.0%	1 / 1 100.0%	1 / 1 100.0%	3 / 3 100.0%	3 / 3 100.0%	1 / 1 100.0%	1 / 1 100.0%	3 / 3 100.0%	0 / 0 0.0%	0 / 0 0.0%	0 / 0 0.0%	0 / 0 0.0%	23 / 34 67.6%AB	11 / 34 32.4%ABE						
1	"Dermacentroxenus murinus" Kulagin 1951	31 / 33 93.9%	1 / 1 100.0%	1 / 1 100.0%	1 / 1 100.0%	1 / 1 100.0%	2 / 2 100.0%	2 / 2 100.0%	1 / 1 100.0%	0 / 1 0.0%	2 / 2 100.0%	1 / 1 100.0%	2 / 3 66.7%	1 / 1 100.0%	1 / 1 100.0%	1 / 1 100.0%	1 / 1 100.0%	1 / 1 100.0%	1 / 1 100.0%	3 / 3 100.0%	3 / 3 100.0%	1 / 1 100.0%	1 / 1 100.0%	3 / 3 100.0%	0 / 0 0.0%	0 / 0 0.0%	0 / 0 0.0%	0 / 0 0.0%	13 / 33 39.4%AB	10 / 33 30.3%B	5 / 33 15.2%ABE	3 / 33 9.1%BE	2 / 33 6.1%E			
1	Rickettsia akari str. Hartford	31 / 33 93.9%	1 / 1 100.0%	1 / 1 100.0%	1 / 1 100.0%	1 / 1 100.0%	2 / 2 100.0%	2 / 2 100.0%	1 / 1 100.0%	0 / 1 0.0%	2 / 2 100.0%	1 / 1 100.0%	2 / 3 66.7%	1 / 1 100.0%	1 / 1 100.0%	1 / 1 100.0%	1 / 1 100.0%	1 / 1 100.0%	1 / 1 100.0%	3 / 3 100.0%	3 / 3 100.0%	1 / 1 100.0%	1 / 1 100.0%	3 / 3 100.0%	0 / 0 0.0%	0 / 0 0.0%	0 / 0 0.0%	0 / 0 0.0%	13 / 33 39.4%AB	10 / 33 30.3%B	5 / 33 15.2%ABE	3 / 33 9.1%BE	2 / 33 6.1%E			
1	NIAID Phillips 32	33 / 33 100.0%	1 / 1 100.0%	1 / 1 100.0%	1 / 1 100.0%	1 / 1 100.0%	2 / 2 100.0%	2 / 2 100.0%	1 / 1 100.0%	1 / 1 100.0%	2 / 2 100.0%	1 / 1 100.0%	3 / 3 100.0%	1 / 1 100.0%	1 / 1 100.0%	1 / 1 100.0%	1 / 1 100.0%	1 / 1 100.0%	1 / 1 100.0%	3 / 3 100.0%	3 / 3 100.0%	1 / 1 100.0%	1 / 1 100.0%	3 / 3 100.0%	0 / 0 0.0%	0 / 0 0.0%	0 / 0 0.0%	0 / 0 0.0%	23 / 33 69.7%AB	10 / 33 30.3%ABE						
1	Rickettsia australis str. Cutlack	33 / 33 100.0%	1 / 1 100.0%	1 / 1 100.0%	1 / 1 100.0%	1 / 1 100.0%	2 / 2 100.0%	2 / 2 100.0%	1 / 1 100.0%	1 / 1 100.0%	2 / 2 100.0%	1 / 1 100.0%	3 / 3 100.0%	1 / 1 100.0%	1 / 1 100.0%	1 / 1 100.0%	1 / 1 100.0%	1 / 1 100.0%	1 / 1 100.0%	3 / 3 100.0%	3 / 3 100.0%	1 / 1 100.0%	1 / 1 100.0%	3 / 3 100.0%	0 / 0 0.0%	0 / 0 0.0%	0 / 0 0.0%	0 / 0 0.0%	23 / 33 69.7%AB	10 / 33 30.3%ABE						
1	"Candidatus Rickettsia amblyommii" Labruna et al. 2004	33 / 33 100.0%	1 / 1 100.0%	1 / 1 100.0%	1 / 1 100.0%	1 / 1 100.0%	2 / 2 100.0%	2 / 2 100.0%	1 / 1 100.0%	1 / 1 100.0%	2 / 2 100.0%	1 / 1 100.0%	3 / 3 100.0%	1 / 1 100.0%	1 / 1 100.0%	1 / 1 100.0%	1 / 1 100.0%	1 / 1 100.0%	1 / 1 100.0%	3 / 3 100.0%	3 / 3 100.0%	1 / 1 100.0%	1 / 1 100.0%	3 / 3 100.0%	0 / 0 0.0%	0 / 0 0.0%	0 / 0 0.0%	0 / 0 0.0%	23 / 33 69.7%AB	10 / 33 30.3%ABE						
1	Candidatus Rickettsia amblyommii str. GAT-30V	33 / 33 100.0%	1 / 1 100.0%	1 / 1 100.0%	1 / 1 100.0%	1 / 1 100.0%	2 / 2 100.0%	2 / 2 100.0%	1 / 1 100.0%	1 / 1 100.0%	2 / 2 100.0%	1 / 1 100.0%	3 / 3 100.0%	1 / 1 100.0%	1 / 1 100.0%	1 / 1 100.0%	1 / 1 100.0%	1 / 1 100.0%	1 / 1 100.0%	3 / 3 100.0%	3 / 3 100.0%	1 / 1 100.0%	1 / 1 100.0%	3 / 3 100.0%	0 / 0 0.0%	0 / 0 0.0%	0 / 0 0.0%	0 / 0 0.0%	23 / 33 69.7%AB	10 / 33 30.3%ABE						
1	"Rickettsia montana" Lackman et al. 1965	33 / 33 100.0%	1 / 1 100.0%	1 / 1 100.0%	1 / 1 100.0%	1 / 1 100.0%	2 / 2 100.0%	2 / 2 100.0%	1 / 1 100.0%	1 / 1 100.0%	2 / 2 100.0%	1 / 1 100.0%	3 / 3 100.0%	1 / 1 100.0%	1 / 1 100.0%	1 / 1 100.0%	1 / 1 100.0%	1 / 1 100.0%	1 / 1 100.0%	3 / 3 100.0%	3 / 3 100.0%	1 / 1 100.0%	1 / 1 100.0%	3 / 3 100.0%	0 / 0 0.0%	0 / 0 0.0%	0 / 0 0.0%	0 / 0 0.0%	23 / 33 69.7%AB	10 / 33 30.3%ABE						
1	Rickettsia montanensis str. OSU 85-930	33 / 33 100.0%	1 / 1 100.0%	1 / 1 100.0%	1 / 1 100.0%	1 / 1 100.0%	2 / 2 100.0%	2 / 2 100.0%	1 / 1 100.0%	1 / 1 100.0%	2 / 2 100.0%	1 / 1 100.0%	3 / 3 100.0%	1 / 1 100.0%	1 / 1 100.0%	1 / 1 100.0%	1 / 1 100.0%	1 / 1 100.0%	1 / 1 100.0%	3 / 3 100.0%	3 / 3 100.0%	1 / 1 100.0%	1 / 1 100.0%	3 / 3 100.0%	0 / 0 0.0%	0 / 0 0.0%	0 / 0 0.0%	0 / 0 0.0%	23 / 33 69.7%AB	10 / 33 30.3%ABE						
1	"Rickettsia rhipicephali" Burgdorfer et al. 1978	33 / 33 100.0%	1 / 1 100.0%	1 / 1 100.0%	1 / 1 100.0%	1 / 1 100.0%	2 / 2 100.0%	2 / 2 100.0%	1 / 1 100.0%	1 / 1 100.0%	2 / 2 100.0%	1 / 1 100.0%	3 / 3 100.0%	1 / 1 100.0%	1 / 1 100.0%	1 / 1 100.0%	1 / 1 100.0%	1 / 1 100.0%	1 / 1 100.0%	3 / 3 100.0%	3 / 3 100.0%	1 / 1 100.0%	1 / 1 100.0%	3 / 3 100.0%	0 / 0 0.0%	0 / 0 0.0%	0 / 0 0.0%	0 / 0 0.0%	23 / 33 69.7%AB	10 / 33 30.3%ABE						
1	Rickettsia rhipicephali str. 3-7-female6-CWPP	33 / 33 100.0%	1 / 1 100.0%	1 / 1 100.0%	1 / 1 100.0%	1 / 1 100.0%	2 / 2 100.0%	2 / 2 100.0%	1 / 1 100.0%	1 / 1 100.0%	2 / 2 100.0%	1 / 1 100.0%	3 / 3 100.0%	1 / 1 100.0%	1 / 1 100.0%	1 / 1 100.0%	1 / 1 100.0%	1 / 1 100.0%	1 / 1 100.0%	3 / 3 100.0%	3 / 3 100.0%	1 / 1 100.0%	1 / 1 100.0%	3 / 3 100.0%	0 / 0 0.0%	0 / 0 0.0%	0 / 0 0.0%	0 / 0 0.0%	23 / 33 69.7%AB	10 / 33 30.3%ABE						
1	Rickettsia africae	32 / 33 97.0%	1 / 1 100.0%	1 / 1 100.0%	1 / 1 100.0%	1 / 1 100.0%	2 / 2 100.0%	2 / 2 100.0%	1 / 1 100.0%	1 / 1 100.0%	2 / 2 100.0%	1 / 1 100.0%	3 / 3 100.0%	1 / 1 100.0%	1 / 1 100.0%	0 / 1 0.0%	1 / 1 100.0%	1 / 1 100.0%	1 / 1 100.0%	3 / 3 100.0%	3 / 3 100.0%	1 / 1 100.0%	1 / 1 100.0%	3 / 3 100.0%	0 / 0 0.0%	0 / 0 0.0%	0 / 0 0.0%	0 / 0 0.0%	23 / 33 69.7%AB	9 / 33 27.3%ABE	1 / 33 3.0%CE					
1	Rickettsia africae ESF-5	32 / 33 97.0%	1 / 1 100.0%	1 / 1 100.0%	1 / 1 100.0%	1 / 1 100.0%	2 / 2 100.0%	2 / 2 100.0%	1 / 1 100.0%	1 / 1 100.0%	2 / 2 100.0%	1 / 1 100.0%	3 / 3 100.0%	1 / 1 100.0%	1 / 1 100.0%	0 / 1 0.0%	1 / 1 100.0%	1 / 1 100.0%	1 / 1 100.0%	3 / 3 100.0%	3 / 3 100.0%	1 / 1 100.0%	1 / 1 100.0%	3 / 3 100.0%	0 / 0 0.0%	0 / 0 0.0%	0 / 0 0.0%	0 / 0 0.0%	23 / 33 69.7%AB	9 / 33 27.3%ABE	1 / 33 3.0%CE					
1	ATCC VR-1363	33 / 33 100.0%	1 / 1 100.0%	1 / 1 100.0%	1 / 1 100.0%	1 / 1 100.0%	2 / 2 100.0%	2 / 2 100.0%	1 / 1 100.0%	1 / 1 100.0%	2 / 2 100.0%	1 / 1 100.0%	3 / 3 100.0%	1 / 1 100.0%	1 / 1 100.0%	1 / 1 100.0%	1 / 1 100.0%	1 / 1 100.0%	1 / 1 100.0%	3 / 3 100.0%	3 / 3 100.0%	1 / 1 100.0%	1 / 1 100.0%	3 / 3 100.0%	0 / 0 0.0%	0 / 0 0.0%	0 / 0 0.0%	0 / 0 0.0%	23 / 33 69.7%AB	10 / 33 30.3%ABE						
1	Rickettsia japonica ATCC VR-1363	33 / 33 100.0%	1 / 1 100.0%	1 / 1 100.0%	1 / 1 100.0%	1 / 1 100.0%	2 / 2 100.0%	2 / 2 100.0%	1 / 1 100.0%	1 / 1 100.0%	2 / 2 100.0%	1 / 1 100.0%	3 / 3 100.0%	1 / 1 100.0%	1 / 1 100.0%	1 / 1 100.0%	1 / 1 100.0%	1 / 1 100.0%	1 / 1 100.0%	3 / 3 100.0%	3 / 3 100.0%	1 / 1 100.0%	1 / 1 100.0%	3 / 3 100.0%	0 / 0 0.0%	0 / 0 0.0%	0 / 0 0.0%	0 / 0 0.0%	23 / 33 69.7%AB	10 / 33 30.3%ABE						
2	Rickettsia massiliae	65 / 66 98.5%	2 / 2 100.0%	2 / 2 100.0%	2 / 2 100.0%	2 / 2 100.0%	4 / 4 100.0%	4 / 4 100.0%	2 / 2 100.0%	1 / 2 50.0%	4 / 4 100.0%	2 / 2 100.0%	6 / 6 100.0%	2 / 2 100.0%	2 / 2 100.0%	2 / 2 100.0%	2 / 2 100.0%	2 / 2 100.0%	2 / 2 100.0%	6 / 6 100.0%	6 / 6 100.0%	2 / 2 100.0%	2 / 2 100.0%	6 / 6 100.0%	0 / 0 0.0%	0 / 0 0.0%	0 / 0 0.0%	0 / 0 0.0%	37 / 66 56.1%AB	16 / 66 24.2%ABE	9 / 66 13.6%B	3 / 66 4.5%BE	1 / 66 1.5%E			
1	Rickettsia massiliae MTU5	32 / 33 97.0%	1 / 1 100.0%	1 / 1 100.0%	1 / 1 100.0%	1 / 1 100.0%	2 / 2 100.0%	2 / 2 100.0%	1 / 1 100.0%	0 / 1 0.0%	2 / 2 100.0%	1 / 1 100.0%	3 / 3 100.0%	1 / 1 100.0%	1 / 1 100.0%	1 / 1 100.0%	1 / 1 100.0%	1 / 1 100.0%	1 / 1 100.0%	3 / 3 100.0%	3 / 3 100.0%	1 / 1 100.0%	1 / 1 100.0%	3 / 3 100.0%	0 / 0 0.0%	0 / 0 0.0%	0 / 0 0.0%	0 / 0 0.0%	14 / 33 42.4%AB	9 / 33 27.3%B	6 / 33 18.2%ABE	3 / 33 9.1%BE	1 / 33 3.0%E			
1	Rickettsia massiliae str. AZT80	33 / 33 100.0%	1 / 1 100.0%	1 / 1 100.0%	1 / 1 100.0%	1 / 1 100.0%	2 / 2 100.0%	2 / 2 100.0%	1 / 1 100.0%	1 / 1 100.0%	2 / 2 100.0%	1 / 1 100.0%	3 / 3 100.0%	1 / 1 100.0%	1 / 1 100.0%	1 / 1 100.0%	1 / 1 100.0%	1 / 1 100.0%	1 / 1 100.0%	3 / 3 100.0%	3 / 3 100.0%	1 / 1 100.0%	1 / 1 100.0%	3 / 3 100.0%	0 / 0 0.0%	0 / 0 0.0%	0 / 0 0.0%	0 / 0 0.0%	23 / 33 69.7%AB	10 / 33 30.3%ABE						
1	NIAID Maculatum 20	33 / 33 100.0%	1 / 1 100.0%	1 / 1 100.0%	1 / 1 100.0%	1 / 1 100.0%	2 / 2 100.0%	2 / 2 100.0%	1 / 1 100.0%	1 / 1 100.0%	2 / 2 100.0%	1 / 1 100.0%	3 / 3 100.0%	1 / 1 100.0%	1 / 1 100.0%	1 / 1 100.0%	1 / 1 100.0%	1 / 1 100.0%	1 / 1 100.0%	3 / 3 100.0%	3 / 3 100.0%	1 / 1 100.0%	1 / 1 100.0%	3 / 3 100.0%	0 / 0 0.0%	0 / 0 0.0%	0 / 0 0.0%	0 / 0 0.0%	23 / 33 69.7%AB	10 / 33 30.3%ABE						
1	Rickettsia parkeri str. Portsmouth	33 / 33 100.0%	1 / 1 100.0%	1 / 1 100.0%	1 / 1 100.0%	1 / 1 100.0%	2 / 2 100.0%	2 / 2 100.0%	1 / 1 100.0%	1 / 1 100.0%	2 / 2 100.0%	1 / 1 100.0%	3 / 3 100.0%	1 / 1 100.0%	1 / 1 100.0%	1 / 1 100.0%	1 / 1 100.0%	1 / 1 100.0%	1 / 1 100.0%	3 / 3 100.0%	3 / 3 100.0%	1 / 1 100.0%	1 / 1 100.0%	3 / 3 100.0%	0 / 0 0.0%	0 / 0 0.0%	0 / 0 0.0%	0 / 0 0.0%	23 / 33 69.7%AB	10 / 33 30.3%ABE						
2	Rickettsia slovaca	66 / 66 100.0%	2 / 2 100.0%	2 / 2 100.0%	2 / 2 100.0%	2 / 2 100.0%	4 / 4 100.0%	4 / 4 100.0%	2 / 2 100.0%	2 / 2 100.0%	4 / 4 100.0%	2 / 2 100.0%	6 / 6 100.0%	2 / 2 100.0%	2 / 2 100.0%	2 / 2 100.0%	2 / 2 100.0%	2 / 2 100.0%	2 / 2 100.0%	6 / 6 100.0%	6 / 6 100.0%	2 / 2 100.0%	2 / 2 100.0%	6 / 6 100.0%	0 / 0 0.0%	0 / 0 0.0%	0 / 0 0.0%	0 / 0 0.0%	46 / 66 69.7%AB	20 / 66 30.3%ABE						
1	Rickettsia slovaca 13-B	33 / 33 100.0%	1 / 1 100.0%	1 / 1 100.0%	1 / 1 100.0%	1 / 1 100.0%	2 / 2 100.0%	2 / 2 100.0%	1 / 1 100.0%	1 / 1 100.0%	2 / 2 100.0%	1 / 1 100.0%	3 / 3 100.0%	1 / 1 100.0%	1 / 1 100.0%	1 / 1 100.0%	1 / 1 100.0%	1 / 1 100.0%	1 / 1 100.0%	3 / 3 100.0%	3 / 3 100.0%	1 / 1 100.0%	1 / 1 100.0%	3 / 3 100.0%	0 / 0 0.0%	0 / 0 0.0%	0 / 0 0.0%	0 / 0 0.0%	23 / 33 69.7%AB	10 / 33 30.3%ABE						
1	Rickettsia slovaca str. D-CWPP	33 / 33 100.0%	1 / 1 100.0%	1 / 1 100.0%	1 / 1 100.0%	1 / 1 100.0%	2 / 2 100.0%	2 / 2 100.0%	1 / 1 100.0%	1 / 1 100.0%	2 / 2 100.0%	1 / 1 100.0%	3 / 3 100.0%	1 / 1 100.0%	1 / 1 100.0%	1 / 1 100.0%	1 / 1 100.0%	1 / 1 100.0%	1 / 1 100.0%	3 / 3 100.0%	3 / 3 100.0%	1 / 1 100.0%	1 / 1 100.0%	3 / 3 100.0%	0 / 0 0.0%	0 / 0 0.0%	0 / 0 0.0%	0 / 0 0.0%	23 / 33 69.7%AB	10 / 33 30.3%ABE						
1	"Rickettsia felis" Higgins et al. 1996	33 / 33 100.0%	1 / 1 100.0%	1 / 1 100.0%	1 / 1 100.0%	1 / 1 100.0%	2 / 2 100.0%	2 / 2 100.0%	1 / 1 100.0%	1 / 1 100.0%	2 / 2 100.0%	1 / 1 100.0%	3 / 3 100.0%	1 / 1 100.0%	1 / 1 100.0%	1 / 1 100.0%	1 / 1 100.0%	1 / 1 100.0%	1 / 1 100.0%	3 / 3 100.0%	3 / 3 100.0%	1 / 1 100.0%	1 / 1 100.0%	3 / 3 100.0%	0 / 0 0.0%	0 / 0 0.0%	0 / 0 0.0%	0 / 0 0.0%	23 / 33 69.7%AB	10 / 33 30.3%ABE						
1	Rickettsia felis URRWXCal2	33 / 33 100.0%	1 / 1 100.0%	1 / 1 100.0%	1 / 1 100.0%	1 / 1 100.0%	2 / 2 100.0%	2 / 2 100.0%	1 / 1 100.0%	1 / 1 100.0%	2 / 2 100.0%	1 / 1 100.0%	3 / 3 100.0%	1 / 1 100.0%	1 / 1 100.0%	1 / 1 100.0%	1 / 1 100.0%	1 / 1 100.0%	1 / 1 100.0%	3 / 3 100.0%	3 / 3 100.0%	1 / 1 100.0%	1 / 1 100.0%	3 / 3 100.0%	0 / 0 0.0%	0 / 0 0.0%	0 / 0 0.0%	0 / 0 0.0%	23 / 33 69.7%AB	10 / 33 30.3%ABE						
1	Rickettsia peacockia	33 / 33 100.0%	1 / 1 100.0%	1 / 1 100.0%	1 / 1 100.0%	1 / 1 100.0%	2 / 2 100.0%	2 / 2 100.0%	1 / 1 100.0%	1 / 1 100.0%	2 / 2 100.0%	1 / 1 100.0%	3 / 3 100.0%	1 / 1 100.0%	1 / 1 100.0%	1 / 1 100.0%	1 / 1 100.0%	1 / 1 100.0%	1 / 1 100.0%	3 / 3 100.0%	3 / 3 100.0%	1 / 1 100.0%	1 / 1 100.0%	3 / 3 100.0%	0 / 0 0.0%	0 / 0 0.0%	0 / 0 0.0%	0 / 0 0.0%	23 / 33 69.7%AB	10 / 33 30.3%ABE						
1	Rickettsia peacockii Rustic	33 / 33 100.0%	1 / 1 100.0%	1 / 1 100.0%	1 / 1 100.0%	1 / 1 100.0%	2 / 2 100.0%	2 / 2 100.0%	1 / 1 100.0%	1 / 1 100.0%	2 / 2 100.0%	1 / 1 100.0%	3 / 3 100.0%	1 / 1 100.0%	1 / 1 100.0%	1 / 1 100.0%	1 / 1 100.0%	1 / 1 100.0%	1 / 1 100.0%	3 / 3 100.0%	3 / 3 100.0%	1 / 1 100.0%	1 / 1 100.0%	3 / 3 100.0%	0 / 0 0.0%	0 / 0 0.0%	0 / 0 0.0%	0 / 0 0.0%	23 / 33 69.7%AB	10 / 33 30.3%ABE						
1	"Rickettsia heilongjiangii" Zhang et al. 2000	33 / 33 100.0%	1 / 1 100.0%	1 / 1 100.0%	1 / 1 100.0%	1 / 1 100.0%	2 / 2 100.0%	2 / 2 100.0%	1 / 1 100.0%	1 / 1 100.0%	2 / 2 100.0%	1 / 1 100.0%	3 / 3 100.0%	1 / 1 100.0%	1 / 1 100.0%	1 / 1 100.0%	1 / 1 100.0%	1 / 1 100.0%	1 / 1 100.0%	3 / 3 100.0%	3 / 3 100.0%	1 / 1 100.0%	1 / 1 100.0%	3 / 3 100.0%	0 / 0 0.0%	0 / 0 0.0%	0 / 0 0.0%	0 / 0 0.0%	23 / 33 69.7%AB	10 / 33 30.3%ABE						
1	Rickettsia heilongjiangensis 054	33 / 33 100.0%	1 / 1 100.0%	1 / 1 100.0%	1 / 1 100.0%	1 / 1 100.0%	2 / 2 100.0%	2 / 2 100.0%	1 / 1 100.0%	1 / 1 100.0%	2 / 2 100.0%	1 / 1 100.0%	3 / 3 100.0%	1 / 1 100.0%	1 / 1 100.0%	1 / 1 100.0%	1 / 1 100.0%	1 / 1 100.0%	1 / 1 100.0%	3 / 3 100.0%	3 / 3 100.0%	1 / 1 100.0%	1 / 1 100.0%	3 / 3 100.0%	0 / 0 0.0%	0 / 0 0.0%	0 / 0 0.0%	0 / 0 0.0%	23 / 33 69.7%AB	10 / 33 30.3%ABE						
1	"Rickettsia philipii" Kato et al. 2010	33 / 33 100.0%	1 / 1 100.0%	1 / 1 100.0%	1 / 1 100.0%	1 / 1 100.0%	2 / 2 100.0%	2 / 2 100.0%	1 / 1 100.0%	1 / 1 100.0%	2 / 2 100.0%	1 / 1 100.0%	3 / 3 100.0%	1 / 1 100.0%	1 / 1 100.0%	1 / 1 100.0%	1 / 1 100.0%	1 / 1 100.0%	1 / 1 100.0%	3 / 3 100.0%	3 / 3 100.0%	1 / 1 100.0%	1 / 1 100.0%	3 / 3 100.0%	0 / 0 0.0%	0 / 0 0.0%	0 / 0 0.0%	0 / 0 0.0%	23 / 33 69.7%AB	10 / 33 30.3%ABE						
1	Rickettsia philipii str. 364D	33 / 33 100.0%	1 / 1 100.0%	1 / 1 100.0%	1 / 1 100.0%	1 / 1 100.0%	2 / 2 100.0%	2 / 2 100.0%	1 / 1 100.0%	1 / 1 100.0%	2 / 2 100.0%	1 / 1 100.0%	3 / 3 100.0%	1 / 1 100.0%	1 / 1 100.0%	1 / 1 100.0%	1 / 1 100.0%	1 / 1 100.0%	1 / 1 100.0%	3 / 3 100.0%	3 / 3 100.0%	1 / 1 100.0%	1 / 1 100.0%	3 / 3 100.0%	0 / 0 0.0%	0 / 0 0.0%	0 / 0 0.0%	0 / 0 0.0%	23 / 33 69.7%AB	10 / 33 30.3%ABE						
13	typhus group	428 / 428 100.0%	13 / 13 100.0%	13 / 13 100.0%	13 / 13 100.0%	13 / 13 100.0%	26 / 26 100.0%	25 / 25 100.0%	13 / 13 100.0%	13 / 13 100.0%	26 / 26 100.0%	13 / 13 100.0%	39 / 39 100.0%	13 / 13 100.0%	13 / 13 100.0%	13 / 13 100.0%	13 / 13 100.0%	13 / 13 100.0%	13 / 13 100.0%	39 / 39 100.0%	39 / 39 100.0%	13 / 13 100.0%	13 / 13 100.0%	39 / 39 100.0%	0 / 0 0.0%	0 / 0 0.0%	0 / 0 0.0%	0 / 0 0.0%	298 / 428 69.6%AB	130 / 428 30.4%ABE						
10	ATCC VR-142	329 / 329 100.0%	10 / 10 100.0%	10 / 10 100.0%	10 / 10 100.0%	10 / 10 100.0%	20 / 20 100.0%	19 / 19 100.0%	10 / 10 100.0%	10 / 10 100.0%	20 / 20 100.0%	10 / 10 100.0%	30 / 30 100.0%	10 / 10 100.0%	10 / 10 100.0%	10 / 10 100.0%	10 / 10 100.0%	10 / 10 100.0%	10 / 10 100.0%	30 / 30 100.0%	30 / 30 100.0%	10 / 10 100.0%	10 / 10 100.0%	30 / 30 100.0%	0 / 0 0.0%	0 / 0 0.0%	0 / 0 0.0%	0 / 0 0.0%	229 / 329 69.6%AB	100 / 329 30.4%ABE						
1	Rickettsia prowazekii Rp22	33 / 33 100.0%	1 / 1 100.0%	1 / 1 100.0%	1 / 1 100.0%	1 / 1 100.0%	2 / 2 100.0%	2 / 2 100.0%	1 / 1 100.0%	1 / 1 100.0%	2 / 2 100.0%	1 / 1 100.0%	3 / 3 100.0%	1 / 1 100.0%	1 / 1 100.0%	1 / 1 100.0%	1 / 1 100.0%	1 / 1 100.0%	1 / 1 100.0%	3 / 3 100.0%	3 / 3 100.0%	1 / 1 100.0%	1 / 1 100.0%	3 / 3 100.0%	0 / 0 0.0%	0 / 0 0.0%	0 / 0 0.0%	0 / 0 0.0%	23 / 33 69.7%AB	10 / 33 30.3%ABE						
1	Rickettsia prowazekii str. Chernikova	33 / 33 100.0%	1 / 1 100.0%	1 / 1 100.0%	1 / 1 100.0%	1 / 1 100.0%	2 / 2 100.0%	2 / 2 100.0%	1 / 1 100.0%	1 / 1 100.0%	2 / 2 100.0%	1 / 1 100.0%	3 / 3 100.0%	1 / 1 100.0%	1 / 1 100.0%	1 / 1 100.0%	1 / 1 100.0%	1 / 1 100.0%	1 / 1 100.0%	3 / 3 100.0%	3 / 3 100.0%	1 / 1 100.0%	1 / 1 100.0%	3 / 3 100.0%	0 / 0 0.0%	0 / 0 0.0%	0 / 0 0.0%	0 / 0 0.0%	23 / 33 69.7%AB	10 / 33 30.3%ABE						
1	Rickettsia prowazekii str. Katsinyian	33 / 33 100.0%	1 / 1 100.0%	1 / 1 100.0%	1 / 1 100.0%	1 / 1 100.0%	2 / 2 100.0%	2 / 2 100.0%	1 / 1 100.0%	1 / 1 100.0%	2 / 2 100.0%	1 / 1 100.0%	3 / 3 100.0%	1 / 1 100.0%	1 / 1 100.0%	1 / 1 100.0%	1 / 1 100.0%	1 / 1 100.0%	1 / 1 100.0%	3 / 3 100.0%	3 / 3 100.0%	1 / 1 100.0%	1 / 1 100.0%	3 / 3 100.0%	0 / 0 0.0%	0 / 0 0.0%	0 / 0 0.0%	0 / 0 0.0%	23 / 33 69.7%AB	10 / 33 30.3%ABE						
1	Rickettsia prowazekii str. BuV67-CWPP	33 / 33 100.0%	1 / 1 100.0%	1 / 1 100.0%	1 / 1 100.0%	1 / 1 100.0%	2 / 2 100.0%	2 / 2 100.0%	1 / 1 100.0%	1 / 1 100.0%	2 / 2 100.0%	1 / 1 100.0%	3 / 3 100.0%	1 / 1 100.0%	1 / 1 100.0%	1 / 1 100.0%	1 / 1 100.0%	1 / 1 100.0%	1 / 1 100.0%	3 / 3 100.0%	3 / 3 100.0%	1 / 1 100.0%	1 / 1 100.0%	3 / 3 100.0%	0 / 0 0.0%	0 / 0 0.0%	0 / 0 0.0%	0 / 0 0.0%	23 / 33 69.7%AB	10 / 33 30.3%ABE						
1	Rickettsia prowazekii str. Dachau	33 / 33	1 / 1	1 / 1	1 / 1	1 / 1	2 / 2	2 / 2	1 / 1	1 / 1	2 / 2	1 / 1	3 / 3	1 / 1	1 / 1	1 / 1	1 / 1	1 / 1	1 / 1	3 / 3	3 / 3	1 / 1	1 / 1	3 / 3	0 / 0	0 / 0	0 / 0	0 / 0	23 / 33	10 / 33						

	[illegible]	100.0%	100.0%	100.0%	100.0%	100.0%	100.0%	100.0%	100.0%	100.0%	100.0%	100.0%	100.0%	100.0%	100.0%	100.0%	100.0%	100.0%	100.0%	100.0%	100.0%	100.0%	100.0%	100.0%	0.0%	0.0%	0.0%	0.0%	69.7%AB	30.3%ABE						
1	Rickettsia prowazekii str. GvV257	33 / 33 100.0%	1 / 1 100.0%	1 / 1 100.0%	1 / 1 100.0%	1 / 1 100.0%	2 / 2 100.0%	2 / 2 100.0%	1 / 1 100.0%	1 / 1 100.0%	2 / 2 100.0%	1 / 1 100.0%	3 / 3 100.0%	1 / 1 100.0%	1 / 1 100.0%	1 / 1 100.0%	1 / 1 100.0%	1 / 1 100.0%	1 / 1 100.0%	3 / 3 100.0%	3 / 3 100.0%	1 / 1 100.0%	1 / 1 100.0%	3 / 3 100.0%	0 / 0 0.0%	0 / 0 0.0%	0 / 0 0.0%	0 / 0 0.0%	23 / 33 69.7%AB	10 / 33 30.3%ABE						
1	Rickettsia prowazekii str. RpGvF24	33 / 33 100.0%	1 / 1 100.0%	1 / 1 100.0%	1 / 1 100.0%	1 / 1 100.0%	2 / 2 100.0%	2 / 2 100.0%	1 / 1 100.0%	1 / 1 100.0%	2 / 2 100.0%	1 / 1 100.0%	3 / 3 100.0%	1 / 1 100.0%	1 / 1 100.0%	1 / 1 100.0%	1 / 1 100.0%	1 / 1 100.0%	1 / 1 100.0%	3 / 3 100.0%	3 / 3 100.0%	1 / 1 100.0%	1 / 1 100.0%	3 / 3 100.0%	0 / 0 0.0%	0 / 0 0.0%	0 / 0 0.0%	0 / 0 0.0%	23 / 33 69.7%AB	10 / 33 30.3%ABE						
1	Rickettsia prowazekii NMRC Madrid E	33 / 33 100.0%	1 / 1 100.0%	1 / 1 100.0%	1 / 1 100.0%	1 / 1 100.0%	2 / 2 100.0%	2 / 2 100.0%	1 / 1 100.0%	1 / 1 100.0%	2 / 2 100.0%	1 / 1 100.0%	3 / 3 100.0%	1 / 1 100.0%	1 / 1 100.0%	1 / 1 100.0%	1 / 1 100.0%	1 / 1 100.0%	1 / 1 100.0%	3 / 3 100.0%	3 / 3 100.0%	1 / 1 100.0%	1 / 1 100.0%	3 / 3 100.0%	0 / 0 0.0%	0 / 0 0.0%	0 / 0 0.0%	0 / 0 0.0%	23 / 33 69.7%AB	10 / 33 30.3%ABE						
1	Rickettsia prowazekii Breinl	33 / 33 100.0%	1 / 1 100.0%	1 / 1 100.0%	1 / 1 100.0%	1 / 1 100.0%	2 / 2 100.0%	2 / 2 100.0%	1 / 1 100.0%	1 / 1 100.0%	2 / 2 100.0%	1 / 1 100.0%	3 / 3 100.0%	1 / 1 100.0%	1 / 1 100.0%	1 / 1 100.0%	1 / 1 100.0%	1 / 1 100.0%	1 / 1 100.0%	3 / 3 100.0%	3 / 3 100.0%	1 / 1 100.0%	1 / 1 100.0%	3 / 3 100.0%	0 / 0 0.0%	0 / 0 0.0%	0 / 0 0.0%	0 / 0 0.0%	23 / 33 69.7%AB	10 / 33 30.3%ABE						
3	"Dermacentroxenus typhi" Wolbach and Todd 1920	99 / 99 100.0%	3 / 3 100.0%	3 / 3 100.0%	3 / 3 100.0%	3 / 3 100.0%	6 / 6 100.0%	6 / 6 100.0%	3 / 3 100.0%	3 / 3 100.0%	6 / 6 100.0%	3 / 3 100.0%	9 / 9 100.0%	3 / 3 100.0%	3 / 3 100.0%	3 / 3 100.0%	3 / 3 100.0%	3 / 3 100.0%	3 / 3 100.0%	9 / 9 100.0%	9 / 9 100.0%	3 / 3 100.0%	3 / 3 100.0%	9 / 9 100.0%	0 / 0 0.0%	0 / 0 0.0%	0 / 0 0.0%	0 / 0 0.0%	69 / 99 69.7%AB	30 / 99 30.3%ABE						
1	Rickettsia typhi 'wilmington'	33 / 33 100.0%	1 / 1 100.0%	1 / 1 100.0%	1 / 1 100.0%	1 / 1 100.0%	2 / 2 100.0%	2 / 2 100.0%	1 / 1 100.0%	1 / 1 100.0%	2 / 2 100.0%	1 / 1 100.0%	3 / 3 100.0%	1 / 1 100.0%	1 / 1 100.0%	1 / 1 100.0%	1 / 1 100.0%	1 / 1 100.0%	1 / 1 100.0%	3 / 3 100.0%	3 / 3 100.0%	1 / 1 100.0%	1 / 1 100.0%	3 / 3 100.0%	0 / 0 0.0%	0 / 0 0.0%	0 / 0 0.0%	0 / 0 0.0%	23 / 33 69.7%AB	10 / 33 30.3%ABE						
1	Rickettsia typhi TH1527	33 / 33 100.0%	1 / 1 100.0%	1 / 1 100.0%	1 / 1 100.0%	1 / 1 100.0%	2 / 2 100.0%	2 / 2 100.0%	1 / 1 100.0%	1 / 1 100.0%	2 / 2 100.0%	1 / 1 100.0%	3 / 3 100.0%	1 / 1 100.0%	1 / 1 100.0%	1 / 1 100.0%	1 / 1 100.0%	1 / 1 100.0%	1 / 1 100.0%	3 / 3 100.0%	3 / 3 100.0%	1 / 1 100.0%	1 / 1 100.0%	3 / 3 100.0%	0 / 0 0.0%	0 / 0 0.0%	0 / 0 0.0%	0 / 0 0.0%	23 / 33 69.7%AB	10 / 33 30.3%ABE						
1	Rickettsia typhi B9991CWPP	33 / 33 100.0%	1 / 1 100.0%	1 / 1 100.0%	1 / 1 100.0%	1 / 1 100.0%	2 / 2 100.0%	2 / 2 100.0%	1 / 1 100.0%	1 / 1 100.0%	2 / 2 100.0%	1 / 1 100.0%	3 / 3 100.0%	1 / 1 100.0%	1 / 1 100.0%	1 / 1 100.0%	1 / 1 100.0%	1 / 1 100.0%	1 / 1 100.0%	3 / 3 100.0%	3 / 3 100.0%	1 / 1 100.0%	1 / 1 100.0%	3 / 3 100.0%	0 / 0 0.0%	0 / 0 0.0%	0 / 0 0.0%	0 / 0 0.0%	23 / 33 69.7%AB	10 / 33 30.3%ABE						
4	belli group	128 / 132 97.0%	4 / 4 100.0%	4 / 4 100.0%	4 / 4 100.0%	4 / 4 100.0%	8 / 8 100.0%	8 / 8 100.0%	4 / 4 100.0%	3 / 4 75.0%	8 / 8 100.0%	4 / 4 100.0%	11 / 12 91.7%	4 / 4 100.0%	4 / 4 100.0%	4 / 4 100.0%	4 / 4 100.0%	4 / 4 100.0%	4 / 4 100.0%	10 / 12 83.3%	12 / 12 100.0%	4 / 4 100.0%	4 / 4 100.0%	12 / 12 100.0%	0 / 0 0.0%	0 / 0 0.0%	0 / 0 0.0%	0 / 0 0.0%	64 / 132 48.5%AB	33 / 132 25.0%ABE	26 / 132 19.7%B	5 / 132 3.8%BE	2 / 132 1.5%C	2 / 132 1.5%E		
2	ATCC VR-610	64 / 66 97.0%	2 / 2 100.0%	2 / 2 100.0%	2 / 2 100.0%	2 / 2 100.0%	4 / 4 100.0%	4 / 4 100.0%	2 / 2 100.0%	1 / 2 50.0%	4 / 4 100.0%	2 / 2 100.0%	5 / 6 83.3%	2 / 2 100.0%	2 / 2 100.0%	2 / 2 100.0%	2 / 2 100.0%	2 / 2 100.0%	2 / 2 100.0%	6 / 6 100.0%	6 / 6 100.0%	2 / 2 100.0%	2 / 2 100.0%	6 / 6 100.0%	0 / 0 0.0%	0 / 0 0.0%	0 / 0 0.0%	0 / 0 0.0%	32 / 66 48.5%AB	17 / 66 25.8%ABE	14 / 66 21.2%B	2 / 66 3.0%E	1 / 66 1.5%BE			
1	Rickettsia canadensis str. McKiel	31 / 33 93.9%	1 / 1 100.0%	1 / 1 100.0%	1 / 1 100.0%	1 / 1 100.0%	2 / 2 100.0%	2 / 2 100.0%	1 / 1 100.0%	0 / 1 0.0%	2 / 2 100.0%	1 / 1 100.0%	2 / 3 66.7%	1 / 1 100.0%	1 / 1 100.0%	1 / 1 100.0%	1 / 1 100.0%	1 / 1 100.0%	1 / 1 100.0%	3 / 3 100.0%	3 / 3 100.0%	1 / 1 100.0%	1 / 1 100.0%	3 / 3 100.0%	0 / 0 0.0%	0 / 0 0.0%	0 / 0 0.0%	0 / 0 0.0%	14 / 33 42.4%B	9 / 33 27.3%AB	7 / 33 21.2%ABE	2 / 33 6.1%E	1 / 33 3.0%BE			
1	Rickettsia canadensis str. CA410	33 / 33 100.0%	1 / 1 100.0%	1 / 1 100.0%	1 / 1 100.0%	1 / 1 100.0%	2 / 2 100.0%	2 / 2 100.0%	1 / 1 100.0%	1 / 1 100.0%	2 / 2 100.0%	1 / 1 100.0%	3 / 3 100.0%	1 / 1 100.0%	1 / 1 100.0%	1 / 1 100.0%	1 / 1 100.0%	1 / 1 100.0%	1 / 1 100.0%	3 / 3 100.0%	3 / 3 100.0%	1 / 1 100.0%	1 / 1 100.0%	3 / 3 100.0%	0 / 0 0.0%	0 / 0 0.0%	0 / 0 0.0%	0 / 0 0.0%	23 / 33 69.7%AB	10 / 33 30.3%ABE						
2	RML 369-C	64 / 66 97.0%	2 / 2 100.0%	2 / 2 100.0%	2 / 2 100.0%	2 / 2 100.0%	4 / 4 100.0%	4 / 4 100.0%	2 / 2 100.0%	2 / 2 100.0%	4 / 4 100.0%	2 / 2 100.0%	6 / 6 100.0%	2 / 2 100.0%	2 / 2 100.0%	2 / 2 100.0%	2 / 2 100.0%	2 / 2 100.0%	2 / 2 100.0%	4 / 6 66.7%	6 / 6 100.0%	2 / 2 100.0%	2 / 2 100.0%	6 / 6 100.0%	0 / 0 0.0%	0 / 0 0.0%	0 / 0 0.0%	0 / 0 0.0%	32 / 66 48.5%AB	16 / 66 24.2%ABE	12 / 66 18.2%B	4 / 66 6.1%BE	2 / 66 3.0%C			
1	Rickettsia bellii RML369-C	32 / 33 97.0%	1 / 1 100.0%	1 / 1 100.0%	1 / 1 100.0%	1 / 1 100.0%	2 / 2 100.0%	2 / 2 100.0%	1 / 1 100.0%	1 / 1 100.0%	2 / 2 100.0%	1 / 1 100.0%	3 / 3 100.0%	1 / 1 100.0%	1 / 1 100.0%	1 / 1 100.0%	1 / 1 100.0%	1 / 1 100.0%	1 / 1 100.0%	2 / 3 66.7%	3 / 3 100.0%	1 / 1 100.0%	1 / 1 100.0%	3 / 3 100.0%	0 / 0 0.0%	0 / 0 0.0%	0 / 0 0.0%	0 / 0 0.0%	22 / 33 66.7%AB	10 / 33 30.3%ABE	1 / 33 3.0%C					
1	Rickettsia bellii OSU 85-389	32 / 33 97.0%	1 / 1 100.0%	1 / 1 100.0%	1 / 1 100.0%	1 / 1 100.0%	2 / 2 100.0%	2 / 2 100.0%	1 / 1 100.0%	1 / 1 100.0%	2 / 2 100.0%	1 / 1 100.0%	3 / 3 100.0%	1 / 1 100.0%	1 / 1 100.0%	1 / 1 100.0%	1 / 1 100.0%	1 / 1 100.0%	1 / 1 100.0%	2 / 3 66.7%	3 / 3 100.0%	1 / 1 100.0%	1 / 1 100.0%	3 / 3 100.0%	0 / 0 0.0%	0 / 0 0.0%	0 / 0 0.0%	0 / 0 0.0%	12 / 33 36.4%B	10 / 33 30.3%AB	6 / 33 18.2%ABE	4 / 33 12.1%BE	1 / 33 3.0%C			
2	Orientia	62 / 68 91.2%	2 / 2 100.0%	2 / 2 100.0%	2 / 2 100.0%	2 / 2 100.0%	2 / 4 50.0%	4 / 4 100.0%	2 / 2 100.0%	2 / 2 100.0%	4 / 4 100.0%	1 / 2 50.0%	5 / 6 83.3%	2 / 2 100.0%	2 / 2 100.0%	2 / 2 100.0%	2 / 2 100.0%	2 / 2 100.0%	2 / 2 100.0%	4 / 6 66.7%	6 / 6 100.0%	2 / 2 100.0%	2 / 2 100.0%	8 / 8 100.0%	0 / 0 0.0%	0 / 0 0.0%	0 / 0 0.0%	0 / 0 0.0%	40 / 68 58.8%AB	13 / 68 19.1%ABE	6 / 68 8.8%BE	4 / 68 5.9%C	3 / 68 4.4%B	1 / 68 1.5%	1 / 68 1.5%E	
2	"Rickettsia akamushi" Kawamura and Imagawa 1931	62 / 68 91.2%	2 / 2 100.0%	2 / 2 100.0%	2 / 2 100.0%	2 / 2 100.0%	2 / 4 50.0%	4 / 4 100.0%	2 / 2 100.0%	2 / 2 100.0%	4 / 4 100.0%	1 / 2 50.0%	5 / 6 83.3%	2 / 2 100.0%	2 / 2 100.0%	2 / 2 100.0%	2 / 2 100.0%	2 / 2 100.0%	2 / 2 100.0%	4 / 6 66.7%	6 / 6 100.0%	2 / 2 100.0%	2 / 2 100.0%	8 / 8 100.0%	0 / 0 0.0%	0 / 0 0.0%	0 / 0 0.0%	0 / 0 0.0%	40 / 68 58.8%AB	13 / 68 19.1%ABE	6 / 68 8.8%BE	4 / 68 5.9%C	3 / 68 4.4%B	1 / 68 1.5%E	1 / 68 1.5%	
1	Orientia tsutsugamushi Ikeda	30 / 34 88.2%	1 / 1 100.0%	1 / 1 100.0%	1 / 1 100.0%	1 / 1 100.0%	1 / 2 50.0%	2 / 2 100.0%	1 / 1 100.0%	1 / 1 100.0%	2 / 2 100.0%	0 / 1 0.0%	2 / 3 66.7%	1 / 1 100.0%	1 / 1 100.0%	1 / 1 100.0%	1 / 1 100.0%	1 / 1 100.0%	1 / 1 100.0%	2 / 3 66.7%	3 / 3 100.0%	1 / 1 100.0%	1 / 1 100.0%	4 / 4 100.0%	0 / 0 0.0%	0 / 0 0.0%	0 / 0 0.0%	0 / 0 0.0%	18 / 34 52.9%AB	6 / 34 17.6%BE	3 / 34 8.8%ABE	3 / 34 8.8%B	2 / 34 5.9%C	1 / 34 2.9%	1 / 34 2.9%E	
1	Orientia tsutsugamushi Boryong	32 / 34 94.1%	1 / 1 100.0%	1 / 1 100.0%	1 / 1 100.0%	1 / 1 100.0%	1 / 2 50.0%	2 / 2 100.0%	1 / 1 100.0%	1 / 1 100.0%	2 / 2 100.0%	1 / 1 100.0%	3 / 3 100.0%	1 / 1 100.0%	1 / 1 100.0%	1 / 1 100.0%	1 / 1 100.0%	1 / 1 100.0%	1 / 1 100.0%	2 / 3 66.7%	3 / 3 100.0%	1 / 1 100.0%	1 / 1 100.0%	4 / 4 100.0%	0 / 0 0.0%	0 / 0 0.0%	0 / 0 0.0%	0 / 0 0.0%	22 / 34 64.7%AB	10 / 34 29.4%ABE	2 / 34 5.9%C					
16	Anaplasmataceae	128 / 559 22.9%	5 / 16 31.2%	6 / 16 37.5%	0 / 16 0.0%	10 / 18 55.6%	2 / 35 5.7%	3 / 32 9.4%	7 / 16 43.8%	0 / 16 0.0%	4 / 14 28.6%	6 / 16 37.5%	10 / 48 20.8%	0 / 23 0.0%	0 / 16 0.0%	6 / 16 37.5%	1 / 16 6.2%	10 / 23 43.5%	1 / 18 5.6%	1 / 32 3.1%	20 / 64 31.2%	0 / 16 0.0%	5 / 14 35.7%	31 / 78 39.7%	0 / 0 0.0%	0 / 0 0.0%	0 / 0 0.0%	0 / 0 0.0%	236 / 559 42.2%	160 / 559 28.6%E	75 / 559 13.4%AB	48 / 559 8.6%ABE	23 / 559 4.1%C	12 / 559 2.1%CE	4 / 559 0.7%B	1 / 559 0.2%BE
4	Anaplasma	31 / 147 21.1%	0 / 4 0.0%	0 / 4 0.0%	0 / 4 0.0%	1 / 4 25.0%	0 / 11 0.0%	3 / 8 37.5%	1 / 4 25.0%	0 / 4 0.0%	0 / 3 0.0%	4 / 4 100.0%	4 / 12 33.3%	0 / 8 0.0%	0 / 4 0.0%	0 / 4 0.0%	0 / 4 0.0%	4 / 8 50.0%	0 / 5 0.0%	1 / 8 12.5%	4 / 16 25.0%	0 / 4 0.0%	0 / 4 0.0%	9 / 20 45.0%	0 / 0 0.0%	0 / 0 0.0%	0 / 0 0.0%	0 / 0 0.0%	65 / 147 44.2%	40 / 147 27.2%E	18 / 147 12.2%AB	13 / 147 8.8%ABE	6 / 147 4.1%C	5 / 147 3.4%CE		
1	"Anaplasma marginale subsp. centrale" Theiler 1911	8 / 37 21.6%	0 / 1 0.0%	0 / 1 0.0%	0 / 1 0.0%	0 / 1 0.0%	0 / 3 0.0%	1 / 2 50.0%	0 / 1 0.0%	0 / 1 0.0%	0 / 1 0.0%	1 / 1 100.0%	1 / 3 33.3%	0 / 2 0.0%	0 / 1 0.0%	0 / 1 0.0%	0 / 1 0.0%	1 / 2 50.0%	0 / 1 0.0%	0 / 2 0.0%	1 / 4 25.0%	0 / 1 0.0%	0 / 1 0.0%	3 / 5 60.0%	0 / 0 0.0%	0 / 0 0.0%	0 / 0 0.0%	0 / 0 0.0%	16 / 37 43.2%	9 / 37 24.3%E	4 / 37 10.8%AB	4 / 37 10.8%ABE	2 / 37 5.4%C	2 / 37 5.4%CE		
1	Anaplasma centrale str. Israel	8 / 37 21.6%	0 / 1 0.0%	0 / 1 0.0%	0 / 1 0.0%	0 / 1 0.0%	0 / 3 0.0%	1 / 2 50.0%	0 / 1 0.0%	0 / 1 0.0%	0 / 1 0.0%	1 / 1 100.0%	1 / 3 33.3%	0 / 2 0.0%	0 / 1 0.0%	0 / 1 0.0%	0 / 1 0.0%	1 / 2 50.0%	0 / 1 0.0%	0 / 2 0.0%	1 / 4 25.0%	0 / 1 0.0%	0 / 1 0.0%	3 / 5 60.0%	0 / 0 0.0%	0 / 0 0.0%	0 / 0 0.0%	0 / 0 0.0%	16 / 37 43.2%	9 / 37 24.3%E	4 / 37 10.8%AB	4 / 37 10.8%ABE	2 / 37 5.4%C	2 / 37 5.4%CE		
2	"Anaplasma argentium" Lignieres 1914	14 / 73 19.2%	0 / 2 0.0%	0 / 2 0.0%	0 / 2 0.0%	0 / 2 0.0%	0 / 6 0.0%	2 / 4 50.0%	0 / 2 0.0%	0 / 2 0.0%	0 / 1 0.0%	2 / 2 100.0%	2 / 6 33.3%	0 / 4 0.0%	0 / 2 0.0%	0 / 2 0.0%	0 / 2 0.0%	2 / 4 50.0%	0 / 2 0.0%	0 / 4 0.0%	2 / 8 25.0%	0 / 2 0.0%	0 / 2 0.0%	4 / 10 40.0%	0 / 0 0.0%	0 / 0 0.0%	0 / 0 0.0%	0 / 0 0.0%	32 / 73 43.8%	20 / 73 27.4%E	8 / 73 11.0%AB	6 / 73 8.2%ABE	4 / 73 5.5%C	3 / 73 4.1%CE		
1	Anaplasma marginale 'St. Maries'	7 / 37 18.9%	0 / 1 0.0%	0 / 1 0.0%	0 / 1 0.0%	0 / 1 0.0%	0 / 3 0.0%	1 / 2 50.0%	0 / 1 0.0%	0 / 1 0.0%	0 / 1 0.0%	1 / 1 100.0%	1 / 3 33.3%	0 / 2 0.0%	0 / 1 0.0%	0 / 1 0.0%	0 / 1 0.0%	1 / 2 50.0%	0 / 1 0.0%	0 / 2 0.0%	1 / 4 25.0%	0 / 1 0.0%	0 / 1 0.0%	2 / 5 40.0%	0 / 0 0.0%	0 / 0 0.0%	0 / 0 0.0%	0 / 0 0.0%	18 / 37 48.6%	9 / 37 24.3%E	4 / 37 10.8%AB	3 / 37 8.1%ABE	2 / 37 5.4%C	1 / 37 2.7%CE		
1	Anaplasma marginale 'Florida'	7 / 36 19.4%	0 / 1 0.0%	0 / 1 0.0%	0 / 1 0.0%	0 / 1 0.0%	0 / 3 0.0%	1 / 2 50.0%	0 / 1 0.0%	0 / 1 0.0%	0 / 0 0.0%	1 / 1 100.0%	1 / 3 33.3%	0 / 2 0.0%	0 / 1 0.0%	0 / 1 0.0%	0 / 1 0.0%	1 / 2 50.0%	0 / 1 0.0%	0 / 2 0.0%	1 / 4 25.0%	0 / 1 0.0%	0 / 1 0.0%	2 / 5 40.0%	0 / 0 0.0%	0 / 0 0.0%	0 / 0 0.0%	0 / 0 0.0%	14 / 36 38.9%	11 / 36 30.6%E	4 / 36 11.1%AB	3 / 36 8.3%ABE	2 / 36 5.6%CE	2 / 36 5.6%C		
1	phagocytophila group	9 / 37 24.3%	0 / 1 0.0%	0 / 1 0.0%	0 / 1 0.0%	1 / 1 100.0%	0 / 2 0.0%	0 / 2 0.0%	1 / 1 100.0%	0 / 1 0.0%	0 / 1 0.0%	1 / 1 100.0%	1 / 3 33.3%	0 / 2 0.0%	0 / 1 0.0%	0 / 1 0.0%	0 / 1 0.0%	1 / 2 50.0%	0 / 2 0.0%	1 / 2 50.0%	1 / 4 25.0%	0 / 1 0.0%	0 / 1 0.0%	2 / 5 40.0%	0 / 0 0.0%	0 / 0 0.0%	0 / 0 0.0%	0 / 0 0.0%	17 / 37 45.9%	11 / 37 29.7%E	6 / 37 16.2%AB	3 / 37 8.1%ABE				
1	"Cytoecetes bovis" Tuomi 1966	9 / 37 24.3%	0 / 1 0.0%	0 / 1 0.0%	0 / 1 0.0%	1 / 1 100.0%	0 / 2 0.0%	0 / 2 0.0%	1 / 1 100.0%	0 / 1 0.0%	0 / 1 0.0%	1 / 1 100.0%	1 / 3 33.3%	0 / 2 0.0%	0 / 1 0.0%	0 / 1 0.0%	0 / 1 0.0%	1 / 2 50.0%	0 / 2 0.0%	1 / 2 50.0%	1 / 4 25.0%	0 / 1 0.0%	0 / 1 0.0%	2 / 5 40.0%	0 / 0 0.0%	0 / 0 0.0%	0 / 0 0.0%	0 / 0 0.0%	17 / 37 45.9%	11 / 37 29.7%E	6 / 37 16.2%AB	3 / 37 8.1%ABE				
1	Anaplasma phagocytophila HZ	9 / 37 24.3%	0 / 1 0.0%	0 / 1 0.0%	0 / 1 0.0%	1 / 1 100.0%	0 / 2 0.0%	0 / 2 0.0%	1 / 1 100.0%	0 / 1 0.0%	0 / 1 0.0%	1 / 1 100.0%	1 / 3 33.3%	0 / 2 0.0%	0 / 1 0.0%	0 / 1 0.0%	0 / 1 0.0%	1 / 2 50.0%	0 / 2 0.0%	1 / 2 50.0%	1 / 4 25.0%	0 / 1 0.0%	0 / 1 0.0%	2 / 5 40.0%	0 / 0 0.0%	0 / 0 0.0%	0 / 0 0.0%	0 / 0 0.0%	17 / 37 45.9%	11 / 37 29.7%E	6 / 37 16.2%AB	3 / 37 8.1%ABE				
3	"Ehrlichia (subgen. Cowdria)" Moshkovski 1945	33 / 109 30.3%	0 / 3 0.0%	0 / 3 0.0%	0 / 3 0.0%	2 / 3 66.7%	2 / 6 33.3%	0 / 6 0.0%	1 / 3 33.3%	0 / 3 0.0%	0 / 3 0.0%	2 / 3 66.7%	6 / 9 66.7%	0 / 6 0.0%	0 / 3 0.0%	2 / 3 66.7%	1 / 3 33.3%	6 / 6 100.0%	1 / 4 25.0%	0 / 6 0.0%	4 / 12 33.3%	0 / 3 0.0%	0 / 3 0.0%	6 / 15 40.0%	0 / 0 0.0%	0 / 0 0.0%	0 / 0 0.0%	0 / 0 0.0%	51 / 109 46.8%	20 / 109 18.3%E	18 / 109 16.5%AB	15 / 109 13.8%ABE	4 / 109 3.7%C	1 / 109 0.9%CE		
3	canis group	33 / 109 30.3%	0 / 3 0.0%	0 / 3 0.0%	0 / 3 0.0%	2 / 3 66.7%	2 / 6 33.3%	0 / 6 0.0%	1 / 3 33.3%	0 / 3 0.0%	0 / 3 0.0%	2 / 3 66.7%	6 / 9 66.7%	0 / 6 0.0%	0 / 3 0.0%	2 / 3 66.7%	1 / 3 33.3%	6 / 6 100.0%	1 / 4 25.0%	0 / 6 0.0%	4 / 12 33.3%	0 / 3 0.0%	0 / 3 0.0%	6 / 15 40.0%	0 / 0 0.0%	0 / 0 0.0%	0 / 0 0.0%	0 / 0 0.0%	51 / 109 46.8%	20 / 109 18.3%E	18 / 109 16.5%AB	15 / 109 13.8%ABE	4 / 109 3.7%C	1 / 109 0.9%CE		
1	"Kurlovia ruminantium" (Cowdry 1925) Zhdanov 1953	11 / 36 30.6%	0 / 1 0.0%	0 / 1 0.0%	0 / 1 0.0%	1 / 1 100.0%	0 / 2 0.0%	0 / 2 0.0%	1 / 1 100.0%	0 / 1 0.0%	0 / 1 0.0%	1 / 1 100.0%	2 / 3 66.7%	0 / 2 0.0%	0 / 1 0.0%	1 / 1 100.0%	0 / 1 0.0%	2 / 2 100.0%	0 / 1 0.0%	0 / 2 0.0%	1 / 4 25.0%	0 / 1 0.0%	0 / 1 0.0%	2 / 5 40.0%	0 / 0 0.0%	0 / 0 0.0%	0 / 0 0.0%	0 / 0 0.0%	16 / 36 44.4%	8 / 36 22.2%E	7 / 36 19.4%AB	4 / 36 11.1%ABE	1 / 36 2.8%C			
1	Ehrlichia ruminantium str. Gardel	11 / 36 30.6%	0 / 1 0.0%	0 / 1 0.0%	0 / 1 0.0%	1 / 1 100.0%	0 / 2 0.0%	0 / 2 0.0%	1 / 1 100.0%	0 / 1 0.0%	0 / 1 0.0%	1 / 1 100.0%	2 / 3 66.7%	0 / 2 0.0%	0 / 1 0.0%	1 / 1 100.0%	0 / 1 0.0%	2 / 2 100.0%	0 / 1 0.0%	0 / 2 0.0%	1 / 4 25.0%	0 / 1 0.0%	0 / 1 0.0%	2 / 5 40.0%	0 / 0 0.0%	0 / 0 0.0%	0 / 0 0.0%	0 / 0 0.0%	16 / 36 44.4%	8 / 36 22.2%E	7 / 36 19.4%AB	4 / 36 11.1%ABE	1 / 36 2.8%C			
1	"Ehrlichia (Rickettsia) canis" (sic) Moshkovski 1937	11 / 36 30.6%	0 / 1 0.0%	0 / 1 0.0%	0 / 1 0.0%	1 / 1 100.0%	1 / 2 50.0%	0 / 2 0.0%	0 / 1 0.0%	0 / 1 0.0%	0 / 1 0.0%	1 / 1 100.0%	2 / 3 66.7%	0 / 2 0.0%	0 / 1 0.0%	0 / 1 0.0%	0 / 1 0.0%	2 / 2 100.0%	1 / 1 100.0%	0 / 2 0.0%	1 / 4 25.0%	0 / 1 0.0%	0 / 1 0.0%	2 / 5 40.0%	0 / 0 0.0%	0 / 0 0.0%	0 / 0 0.0%	0 / 0 0.0%	17 / 36 47.2%	7 / 36 19.4%AB	6 / 36 16.7%E	4 / 36 11.1%ABE	1 / 36 2.8%C	1 / 36 2.8%CE		
1	Ehrlichia canis 'Jake'	11 / 36 30.6%	0 / 1 0.0%	0 / 1 0.0%	0 / 1 0.0%	1 / 1 100.0%	1 / 2 50.0%	0 / 2 0.0%	0 / 1 0.0%	0 / 1 0.0%	0 / 1 0.0%	1 / 1 100.0%	2 / 3 66.7%	0 / 2 0.0%	0 / 1 0.0%	0 / 1 0.0%	0 / 1 0.0%	2 / 2 100.0%	1 / 1 100.0%	0 / 2 0.0%	1 / 4 25.0%	0 / 1 0.0%	0 / 1 0.0%	2 / 5 40.0%	0 / 0 0.0%	0 / 0 0.0%	0 / 0 0.0%	0 / 0 0.0%	17 / 36 47.2%	7 / 36 19.4%AB	6 / 36 16.7%E	4 / 36 11.1%ABE	1 / 36 2.8%CE	1 / 36 2.8%C		
1	ATCC CRL-10679	11 / 37 29.7%	0 / 1 0.0%	0 / 1 0.0%	0 / 1 0.0%	0 / 1 0.0%	1 / 2 50.0%	0 / 2 0.0%	0 / 1 0.0%	0 / 1 0.0%	0 / 1 0.0%	0 / 1 0.0%	2 / 3 66.7%	0 / 2 0.0%	0 / 1 0.0%	1 / 1 100.0%	1 / 1 100.0%	2 / 2 100.0%	0 / 2 0.0%	0 / 2 0.0%	2 / 4 50.0%	0 / 1 0.0%	0 / 1 0.0%	2 / 5 40.0%	0 / 0 0.0%	0 / 0 0.0%	0 / 0 0.0%	0 / 0 0.0%	18 / 37 48.6%	7 / 37 18.9%ABE	6 / 37 16.2%E	4 / 37 10.8%AB	2 / 37 5.4%C			
1	Ehrlichia chaffeensis Arkansas	11 / 37 29.7%	0 / 1 0.0%	0 / 1 0.0%	0 / 1 0.0%	0 / 1 0.0%	1 / 2 50.0%	0 / 2 0.0%	0 / 1 0.0%	0 / 1 0.0%	0 / 1 0.0%	0 / 1 0.0%	2 / 3 66.7%	0 / 2 0.0%	0 / 1 0.0%	1 / 1 100.0%	1 / 1 100.0%	2 / 2 100.0%	0 / 2 0.0%	0 / 2 0.0%	2 / 4 50.0%	0 / 1 0.0%	0 / 1 0.0%	2 / 5 40.0%	0 / 0 0.0%	0 / 0 0.0%	0 / 0 0.0%	0 / 0 0.0%	18 / 37 48.6%	7 / 37 18.9%ABE	6 / 37 16.2%E	4 / 37 10.8%AB	2 / 37 5.4%C			
7	Wolbachieae	57 / 237 24.1%	5 / 7 71.4%	4 / 7 57.1%	0 / 7 0.0%	7 / 7 100.0%	0 / 14 0.0%	0 / 14 0.0%	5 / 7 71.4%	0 / 7 0.0%	4 / 6 66.7%	0 / 7 0.0%	0 / 21 0.0%	0 / 7 0.0%	0 / 7 0.0%	4 / 7 57.1%	0 / 7 0.0%	0 / 7 0.0%	0 / 7 0.0%	0 / 14 0.0%	9 / 28 32.1%	0 / 7 0.0%	5 / 7 71.4%	14 / 35 40.0%	0 / 0 0.0%	0 / 0 0.0%	0 / 0 0.0%	0 / 0 0.0%	96 / 237 40.5%	77 / 237 32.5%E	34 / 237 14.3%AB	18 / 237 7.6%ABE	5 / 237 2.1%C	4 / 237 1.7%B	2 / 237 0.8%CE	1 / 237 0.4%BE
7	Wolbachia	57 / 237 24.1%	5 / 7 71.4%	4 / 7 57.1%	0 / 7 0.0%	7 / 7 100.0%	0 / 14 0.0%	0 / 14 0.0%	5 / 7 71.4%	0 / 7 0.0%	4 / 6 66.7%	0 / 7 0.0%	0 / 21 0.0%	0 / 7 0.0%	0 / 7 0.0%	4 / 7 57.1%	0 / 7 0.0%	0 / 7 0.0%	0 / 7 0.0%	0 / 14 0.0%	9 / 28 32.1%	0 / 7 0.0%	5 / 7 71.4%	14 / 35 40.0%	0 / 0 0.0%	0 / 0 0.0%	0 / 0 0.0%	0 / 0 0.0%	96 / 237 40.5%	77 / 237 32.5%E	34 / 237 14.3%AB	18 / 237 7.6%ABE	5 / 237 2.1%C	4 / 237 1.7%B	2 / 237 0.8%CE	1 / 237 0.4%BE
1	Wolbachia pipientis	9 / 34 26.5%	1 / 1 100.0%	1 / 1 100.0%	0 / 1 0.0%	1 / 1 100.0%	0 / 2 0.0%	0 / 2 0.0%	1 / 1 100.0%	0 / 1 0.0%	1 / 1 100.0%	0 / 1 0.0%	0 / 3 0.0%	0 / 1 0.0%	0 / 1 0.0%	0 / 1 0.0%	0 / 1 0.0%	0 / 1 0.0%	0 / 1 0.0%	0 / 2 0.0%	1 / 4 25.0%	0 / 1 0.0%	1 / 1 100.0%	2 / 5 40.0%	0 / 0 0.0%	0 / 0 0.0%	0 / 0 0.0%	0 / 0 0.0%	13 / 34 38.2%E	12 / 34 35.3%	4 / 34 11.8%B	2 / 34 5.9%AB	2 / 34 5.9%ABE	1 / 34 2.9%BE		
1	Wolbachia endosymbiont Drosophila simulans strain Riverside	9 / 33 27.3%	1 / 1 100.0%	0 / 1 0.0%	0 / 1 0.0%	1 / 1 100.0%	0 / 2 0.0%	0 / 2 0.0%	1 / 1 100.0%	0 / 1 0.0%	0 / 0 0.0%	0 / 1 0.0%	0 / 3 0.0%	0 / 1 0.0%	0 / 1 0.0%	1 / 1 100.0%	0 / 1 0.0%	0 / 1 0.0%	0 / 1 0.0%	0 / 2 0.0%	2 / 4 50.0%	0 / 1 0.0%	1 / 1 100.0%	2 / 5 40.0%	0 / 0 0.0%	0 / 0 0.0%	0 / 0 0.0%	0 / 0 0.0%	13 / 33 39.4%	10 / 33 30.3%E	6 / 33 18.2%AB	3 / 33 9.1%ABE	1 / 33 3.0%C			
2	Wolbachia endosymbiont of Drosophila simulans	20 / 68 29.4%	2 / 2 100.0%	2 / 2 100.0%	0 / 2 0.0%	2 / 2 100.0%	0 / 4 0.0%	0 / 4 0.0%	2 / 2 100.0%	0 / 2 0.0%	2 / 2 100.0%	0 / 2 0.0%	0 / 6 0.0%	0 / 2 0.0%	0 / 2 0.0%	1 / 2 50.0%	0 / 2 0.0%	0 / 2 0.0%	0 / 2 0.0%	0 / 4 0.0%	3 / 8 37.5%	0 / 2 0.0%	2 / 2 100.0%	4 / 10 40.0%	0 / 0 0.0%	0 / 0 0.0%	0 / 0 0.0%	0 / 0 0.0%	24 / 68 35.3%	23 / 68 33.8%E	14 / 68 20.6%AB	6 / 68 8.8%ABE	1 / 68 1.5%C			
1	Wolbachia endosymbiont of Drosophila simulans wNo	9 / 34 26.5%	1 / 1 100.0%	1 / 1 100.0%	0 / 1 0.0%	1 / 1 100.0%	0 / 2 0.0%	0 / 2 0.0%	1 / 1 100.0%	0 / 1 0.0%	1 / 1 100.0%	0 / 1 0.0%	0 / 3 0.0%	0 / 1 0.0%	0 / 1 0.0%	0 / 1 0.0%	0 / 1 0.0%	0 / 1 0.0%	0 / 1 0.0%	0 / 2 0.0%	1 / 4 25.0%	0 / 1 0.0%	1 / 1 100.0%	2 / 5 40.0%	0 / 0 0.0%	0 / 0 0.0%	0 / 0 0.0%	0 / 0 0.0%	13 / 34 38.2%E	12 / 34 35.3%	6 / 34 17.6%AB	3 / 34 8.8%ABE				
1	Wolbachia endosymbiont of Drosophila simulans wHa	11 / 34 32.4%	1 / 1 100.0%	1 / 1 100.0%	0 / 1 0.0%	1 / 1 100.0%	0 / 2 0.0%	0 / 2 0.0%	1 / 1 100.0%	0 / 1 0.0%	1 / 1 100.0%	0 / 1 0.0%	0 / 3 0.0%	0 / 1 0.0%	0 / 1 0.0%	1 / 1 100.0%	0 / 1 0.0%	0 / 1 0.0%	0 / 1 0.0%	0 / 2 0.0%	2 / 4 50.0%	0 / 1 0.0%	1 / 1 100.0%	2 / 5 40.0%	0 / 0 0.0%	0 / 0 0.0%	0 / 0 0.0%	0 / 0 0.0%	12 / 34 35.3%	10 / 34 29.4%E	8 / 34 23.5%AB	3 / 34 8.8%ABE	1 / 34 2.9%C			
1	"Wolbachia malayi" Pfarr et al. 2007	3 / 34 8.8%	0 / 1 0.0%	0 / 1 0.0%	0 / 1 0.0%	1 / 1 100.0%	0 / 2 0.0%	0 / 2 0.0%	0 / 1 0.0%	0 / 1 0.0%	0 / 1 0.0%	0 / 1 0.0%	0 / 3 0.0%	0 / 1 0.0%	0 / 1 0.0%	0 / 1 0.0%	0 / 1 0.0%	0 / 1 0.0%	0 / 1 0.0%	0 / 2 0.0%	0 / 4 0.0%	0 / 1 0.0%	0 / 1 0.0%	2 / 5 40.0%	0 / 0 0.0%	0 / 0 0.0%	0 / 0 0.0%	0 / 0 0.0%	20 / 34 58.8%	9 / 34 26.5%E	2 / 34 5.9%ABE	1 / 34 2.9%AB	1 / 34 2.9%C	1 / 34 2.9%CE		
1	Wolbachia endosymbiont strain TRS of Brugia malayi	3 / 34 8.8%	0 / 1 0.0%	0 / 1 0.0%	0 / 1 0.0%	1 / 1 100.0%	0 / 2 0.0%	0 / 2 0.0%	0 / 1 0.0%	0 / 1 0.0%	0 / 1 0.0%	0 / 1 0.0%	0 / 3 0.0%	0 / 1 0.0%	0 / 1 0.0%	0 / 1 0.0%	0 / 1 0.0%	0 / 1 0.0%	0 / 1 0.0%	0 / 2 0.0%	0 / 4 0.0%	0 / 1 0.0%	0 / 1 0.0%	2 / 5 40.0%	0 / 0 0.0%	0 / 0 0.0%	0 / 0 0.0%	0 / 0 0.0%	20 / 34 58.8%	9 / 34 26.5%E	2 / 34 5.9%ABE	1 / 34 2.9%AB	1 / 34 2.9%C	1 / 34 2.9%CE		
1	Wolbachia endosymbiont of Onchocerca ochengi	5 / 34 14.7%	0 / 1 0.0%	0 / 1 0.0%	0 / 1 0.0%	1 / 1 100.0%	0 / 2 0.0%	0 / 2 0.0%	0 / 1 0.0%	0 / 1 0.0%	0 / 1 0.0%	0 / 1 0.0%	0 / 3 0.0%	0 / 1 0.0%	0 / 1 0.0%	1 / 1 100.0%	0 / 1 0.0%	0 / 1 0.0%	0 / 1 0.0%	0 / 2 0.0%	1 / 4 25.0%	0 / 1 0.0%	0 / 1 0.0%	2 / 5 40.0%	0 / 0 0.0%	0 / 0 0.0%	0 / 0 0.0%	0 / 0 0.0%	14 / 34 41.2%	13 / 34 38.2%E	3 / 34 8.8%AB	2 / 34 5.9%ABE	1 / 34 2.9%C	1 / 34 2.9%CE		
1	Wolbach endosymbiont of Drosophila melanogaster	11 / 34 32.4%	1 / 1 100.0%	1 / 1 100.0%	0 / 1 0.0%	1 / 1 100.0%	0 / 2 0.0%	0 / 2 0.0%	1 / 1 100.0%	0 / 1 0.0%	1 / 1 100.0%	0 / 1 0.0%	0 / 3 0.0%	0 / 1 0.0%	0 / 1 0.0%	1 / 1 100.0%	0 / 1 0.0%	0 / 1 0.0%	0 / 1 0.0%	0 / 2 0.0%	2 / 4 50.0%	0 / 1 0.0%	1 / 1 100.0%	2 / 5 40.0%	0 / 0 0.0%	0 / 0 0.0%	0 / 0 0.0%	0 / 0 0.0%	13 / 34 38.2%	9 / 34 26.5%E	8 / 34 23.5%AB	3 / 34 8.8%ABE	1 / 34 2.9%C			
2	Neorickettsia	7 / 66 10.6%	0 / 2 0.0%	2 / 2 100.0%	0 / 2 0.0%	0 / 4 0.0%	0 / 4 0.0%	0 / 4 0.0%	0 / 2 0.0%	0 / 2 0.0%	0 / 2 0.0%	0 / 2 0.0%	0 / 6 0.0%	0 / 2 0.0%	0 / 2 0.0%	0 / 2 0.0%	0 / 2 0.0%	0 / 2 0.0%	0 / 2 0.0%	0 / 4 0.0%	3 / 8 37.5%	0 / 2 0.0%	0 / 0 0.0%	2 / 8 25.0%	0 / 0 0.0%	0 / 0 0.0%	0 / 0 0.0%	0 / 0 0.0%	24 / 66 36.4%	23 / 66 34.8%E	8 / 66 12.1%C	5 / 66 7.6%AB	4 / 66 6.1%CE	2 / 66 3.0%ABE		
1	ATCC VR-986	4 / 33 12.1%	0 / 1 0.0%	1 / 1 100.0%	0 / 1 0.0%	0 / 2 0.0%	0 / 2 0.0%	0 / 2 0.0%	0 / 1 0.0%	0 / 1 0.0%	0 / 1 0.0%	0 / 1 0.0%	0 / 3 0.0%	0 / 1 0.0%	0 / 1 0.0%	0 / 1 0.0%	0 / 1 0.0%	0 / 1 0.0%	0 / 1 0.0%	0 / 2 0.0%	2 / 4 50.0%	0 / 1 0.0%	0 / 0 0.0%	1 / 4 25.0%	0 / 0 0.0%	0 / 0 0.0%	0 / 0 0.0%	0 / 0 0.0%	13 / 33 39.4%E	10 / 33 30.3%	3 / 33 9.1%CE	3 / 33 9.1%C	3 / 33 9.1%AB	1 / 33 3.0%ABE		
1	Neorickettsia risticii 'Illinois'	4 / 33 12.1%	0 / 1 0.0%	1 / 1 100.0%	0 / 1 0.0%	0 / 2 0.0%	0 / 2 0.0%	0 / 2 0.0%	0 / 1 0.0%	0 / 1 0.0%	0 / 1 0.0%	0 / 1 0.0%	0 / 3 0.0%	0 / 1 0.0%	0 / 1 0.0%	0 / 1 0.0%	0 / 1 0.0%	0 / 1 0.0%	0 / 1 0.0%	0 / 2 0.0%	2 / 4 50.0%	0 / 1 0.0%	0 / 0 0.0%	1 / 4 25.0%	0 / 0 0.0%	0 / 0 0.0%	0 / 0 0.0%	0 / 0 0.0%	13 / 33 39.4%E	10 / 33 30.3%	3 / 33 9.1%AB	3 / 33 9.1%C	3 / 33 9.1%CE	1 / 33 3.0%ABE		
1	ATCC VR-367	3 / 33 9.1%	0 / 1 0.0%	1 / 1 100.0%	0 / 1 0.0%	0 / 2 0.0%	0 / 2 0.0%	0 / 2 0.0%	0 / 1 0.0%	0 / 1 0.0%	0 / 1 0.0%	0 / 1 0.0%	0 / 3 0.0%	0 / 1 0.0%	0 / 1 0.0%	0 / 1 0.0%	0 / 1 0.0%	0 / 1 0.0%	0 / 1 0.0%	0 / 2 0.0%	1 / 4 25.0%	0 / 1 0.0%	0 / 0 0.0%	1 / 4 25.0%	0 / 0 0.0%	0 / 0 0.0%	0 / 0 0.0%	0 / 0 0.0%	14 / 33 42.4%	10 / 33 30.3%E	5 / 33 15.2%C	2 / 33 6.1%AB	1 / 33 3.0%CE	1 / 33 3.0%ABE		
1	Neorickettsia sennetsu Miyayama	3 / 33 9.1%	0 / 1 0.0%	1 / 1 100.0%	0 / 1 0.0%	0 / 2 0.0%	0 / 2 0.0%	0 / 2 0.0%	0 / 1 0.0%	0 / 1 0.0%	0 / 1 0.0%	0 / 1 0.0%	0 / 3 0.0%	0 / 1 0.0%	0 / 1 0.0%	0 / 1 0.0%	0 / 1 0.0%	0 / 1 0.0%	0 / 1 0.0%	0 / 2 0.0%	1 / 4 25.0%	0 / 1 0.0%	0 / 0 0.0%	1 / 4 25.0%	0 / 0 0.0%	0 / 0 0.0%	0 / 0 0.0%	0 / 0 0.0%	14 / 33 42.4%	10 / 33 30.3%E	5 / 33 15.2%C	2 / 33 6.1%AB	1 / 33 3.0%CE	1 / 33 3.0%ABE		
1	"Candidatus Midichloriaceae" Montagna et al. 2013	34 / 35 97.1%	1 / 1 100.0%	1 / 1 100.0%	1 / 1 100.0%	1 / 1 100.0%	2 / 2 100.0%	2 / 2 100.0%	1 / 1 100.0%	1 / 1 100.0%	2 / 2 100.0%	1 / 1 100.0%	3 / 3 100.0%	1 / 1 100.0%	1 / 1 100.0%	1 / 1 100.0%	0 / 1 0.0%	1 / 1 100.0%	1 / 1 100.0%	3 / 3 100.0%	4 / 4 100.0%	1 / 1 100.0%	1 / 1 100.0%	4 / 4 100.0%	0 / 0 0.0%	0 / 0 0.0%	0 / 0 0.0%	0 / 0 0.0%	22 / 35 62.9%AB	12 / 35 34.3%ABE	1 / 35 2.9%					
1	"Candidatus Midichloria" Sassera et al. 2006	34 / 35 97.1%	1 / 1 100.0%	1 / 1 100.0%	1 / 1 100.0%	1 / 1 100.0%	2 / 2 100.0%	2 / 2 100.0%	1 / 1 100.0%	1 / 1 100.0%	2 / 2 100.0%	1 / 1 100.0%	3 / 3 100.0%	1 / 1 100.0%	1 / 1 100.0%	1 / 1 100.0%	0 / 1 0.0%	1 / 1 100.0%	1 / 1 100.0%	3 / 3 100.0%	4 / 4 100.0%	1 / 1 100.0%	1 / 1 100.0%	4 / 4 100.0%	0 / 0 0.0%	0 / 0 0.0%	0 / 0 0.0%	0 / 0 0.0%	22 / 35 62.9%AB	12 / 35 34.3%ABE	1 / 35 2.9%					
1	"Candidatus Midichloria mitochondrii" Sassera et al. 2006	34 / 35 97.1%	1 / 1 100.0%	1 / 1 100.0%	1 / 1 100.0%	1 / 1 100.0%	2 / 2 100.0%	2 / 2 100.0%	1 / 1 100.0%	1 / 1 100.0%	2 / 2 100.0%	1 / 1 100.0%	3 / 3 100.0%	1 / 1 100.0%	1 / 1 100.0%	1 / 1 100.0%	0 / 1 0.0%	1 / 1 100.0%	1 / 1 100.0%	3 / 3 100.0%	4 / 4 100.0%	1 / 1 100.0%	1 / 1 100.0%	4 / 4 100.0%	0 / 0 0.0%	0 / 0 0.0%	0 / 0 0.0%	0 / 0 0.0%	22 / 35 62.9%AB	12 / 35 34.3%ABE	1 / 35 2.9%					
1	Candidatus Midichloria mitochondrii IricVA	34 / 35 97.1%	1 / 1 100.0%	1 / 1 100.0%	1 / 1 100.0%	1 / 1 100.0%	2 / 2 100.0%	2 / 2 100.0%	1 / 1 100.0%	1 / 1 100.0%	2 / 2 100.0%	1 / 1 100.0%	3 / 3 100.0%	1 / 1 100.0%	1 / 1 100.0%	1 / 1 100.0%	0 / 1 0.0%	1 / 1 100.0%	1 / 1 100.0%	3 / 3 100.0%	4 / 4 100.0%	1 / 1 100.0%	1 / 1 100.0%	4 / 4 100.0%	0 / 0 0.0%	0 / 0 0.0%	0 / 0 0.0%	0 / 0 0.0%	22 / 35 62.9%AB	12 / 35 34.3%ABE	1 / 35 2.9%					
6	unclassified Alphaproteobacteria	227 / 227 100.0%	7 / 7 100.0%	7 / 7 100.0%	6 / 6 100.0%	7 / 7 100.0%	15 / 15 100.0%	12 / 12 100.0%	6 / 6 100.0%	6 / 6 100.0%	12 / 12 100.0%	6 / 6 100.0%	21 / 21 100.0%	7 / 7 100.0%	6 / 6 100.0%	7 / 7 100.0%	6 / 6 100.0%	13 / 13 100.0%	6 / 6 100.0%	16 / 16 100.0%	21 / 21 100.0%	7 / 7 100.0%	6 / 6 100.0%	27 / 27 100.0%	0 / 0 0.0%	0 / 0 0.0%	0 / 0 0.0%	0 / 0 0.0%	145 / 227 63.9%AB	82 / 227 36.1%ABE						
2	SAR 11 cluster	62 / 62 100.0%	2 / 2 100.0%	2 / 2 100.0%	2 / 2 100.0%	2 / 2 100.0%	4 / 4 100.0%	3 / 3 100.0%	2 / 2 100.0%	2 / 2 100.0%	2 / 2 100.0%	2 / 2 100.0%	6 / 6 100.0%	2 / 2 100.0%	2 / 2 100.0%	2 / 2 100.0%	2 / 2 100.0%	2 / 2 100.0%	2 / 2 100.0%	4 / 4 100.0%	6 / 6 100.0%	2 / 2 100.0%	2 / 2 100.0%	7 / 7 100.0%	0 / 0 0.0%	0 / 0 0.0%	0 / 0 0.0%	0 / 0 0.0%	40 / 62 64.5%AB	22 / 62 35.5%ABE						
2	"Candidatus Pelagibacter" Rappe et al. 2002	62 / 62 100.0%	2 / 2 100.0%	2 / 2 100.0%	2 / 2 100.0%	2 / 2 100.0%	4 / 4 100.0%	3 / 3 100.0%	2 / 2 100.0%	2 / 2 100.0%	2 / 2 100.0%	2 / 2 100.0%	6 / 6 100.0%	2 / 2 100.0%	2 / 2 100.0%	2 / 2 100.0%	2 / 2 100.0%	2 / 2 100.0%	2 / 2 100.0%	4 / 4 100.0%	6 / 6 100.0%	2 / 2 100.0%	2 / 2 100.0%	7 / 7 100.0%	0 / 0 0.0%	0 / 0 0.0%	0 / 0 0.0%	0 / 0 0.0%	40 / 62 64.5%AB	22 / 62 35.5%ABE						
1	"Candidatus Pelagibacter ubique" Rappe et al. 2002	31 / 31 100.0%	1 / 1 100.0%	1 / 1 100.0%	1 / 1 100.0%	1 / 1 100.0%	2 / 2 100.0%	2 / 2 100.0%	1 / 1 100.0%	1 / 1 100.0%	1 / 1 100.0%	1 / 1 100.0%	3 / 3 100.0%	1 / 1 100.0%	1 / 1 100.0%	1 / 1 100.0%	1 / 1 100.0%	1 / 1 100.0%	1 / 1 100.0%	2 / 2 100.0%	3 / 3 100.0%	1 / 1 100.0%	1 / 1 100.0%	3 / 3 100.0%	0 / 0 0.0%	0 / 0 0.0%	0 / 0 0.0%	0 / 0 0.0%	19 / 31 61.3%AB	12 / 31 38.7%ABE						
1	Candidatus Pelagibacter ubique HTCC1062	31 / 31 100.0%	1 / 1 100.0%	1 / 1 100.0%	1 / 1 100.0%	1 / 1 100.0%	2 / 2 100.0%	2 / 2 100.0%	1 / 1 100.0%	1 / 1 100.0%	1 / 1 100.0%	1 / 1 100.0%	3 / 3 100.0%	1 / 1 100.0%	1 / 1 100.0%	1 / 1 100.0%	1 / 1 100.0%	1 / 1 100.0%	1 / 1 100.0%	2 / 2 100.0%	3 / 3 100.0%	1 / 1 100.0%	1 / 1 100.0%	3 / 3 100.0%	0 / 0 0.0%	0 / 0 0.0%	0 / 0 0.0%	0 / 0 0.0%	19 / 31 61.3%AB	12 / 31 38.7%ABE						
1	Candidatus Pelagibacter sp. IMCC9063	31 / 31 100.0%	1 / 1 100.0%	1 / 1 100.0%	1 / 1 100.0%	1 / 1 100.0%	2 / 2 100.0%	1 / 1 100.0%	1 / 1 100.0%	1 / 1 100.0%	1 / 1 100.0%	1 / 1 100.0%	3 / 3 100.0%	1 / 1 100.0%	1 / 1 100.0%	1 / 1 100.0%	1 / 1 100.0%	1 / 1 100.0%	1 / 1 100.0%	2 / 2 100.0%	3 / 3 100.0%	1 / 1 100.0%	1 / 1 100.0%	4 / 4 100.0%	0 / 0 0.0%	0 / 0 0.0%	0 / 0 0.0%	0 / 0 0.0%	21 / 31 67.7%AB	10 / 31 32.3%ABE						
1	SAR116 cluster	36 / 36 100.0%	1 / 1 100.0%	1 / 1 100.0%	1 / 1 100.0%	1 / 1 100.0%	2 / 2 100.0%	2 / 2 100.0%	1 / 1 100.0%	1 / 1 100.0%	2 / 2 100.0%	1 / 1 100.0%	3 / 3 100.0%	1 / 1 100.0%	1 / 1 100.0%	1 / 1 100.0%	1 / 1 100.0%	2 / 2 100.0%	1 / 1 100.0%	3 / 3 100.0%	3 / 3 100.0%	1 / 1 100.0%	1 / 1 100.0%	5 / 5 100.0%	0 / 0 0.0%	0 / 0 0.0%	0 / 0 0.0%	0 / 0 0.0%	21 / 36 58.3%AB	15 / 36 41.7%ABE						
1	"Candidatus Puniceispirillum" Oh et al. 2010	36 / 36 100.0%	1 / 1 100.0%	1 / 1 100.0%	1 / 1 100.0%	1 / 1 100.0%	2 / 2 100.0%	2 / 2 100.0%	1 / 1 100.0%	1 / 1 100.0%	2 / 2 100.0%	1 / 1 100.0%	3 / 3 100.0%	1 / 1 100.0%	1 / 1 100.0%	1 / 1 100.0%	1 / 1 100.0%	2 / 2 100.0%	1 / 1 100.0%	3 / 3 100.0%	3 / 3 100.0%	1 / 1 100.0%	1 / 1 100.0%	5 / 5 100.0%	0 / 0 0.0%	0 / 0 0.0%	0 / 0 0.0%	0 / 0 0.0%	21 / 36 58.3%AB	15 / 36 41.7%ABE						
1	"Candidatus Puniceispirillum marinum" Oh et al. 2010	36 / 36 100.0%	1 / 1 100.0%	1 / 1 100.0%	1 / 1 100.0%	1 / 1 100.0%	2 / 2 100.0%	2 / 2 100.0%	1 / 1 100.0%	1 / 1 100.0%	2 / 2 100.0%	1 / 1 100.0%	3 / 3 100.0%	1 / 1 100.0%	1 / 1 100.0%	1 / 1 100.0%	1 / 1 100.0%	2 / 2 100.0%	1 / 1 100.0%	3 / 3 100.0%	3 / 3 100.0%	1 / 1 100.0%	1 / 1 100.0%	5 / 5 100.0%	0 / 0 0.0%	0 / 0 0.0%	0 / 0 0.0%	0 / 0 0.0%	21 / 36 58.3%AB	15 / 36 41.7%ABE						
1	Candidatus Puniceispirillum marinum IMCC1322	36 / 36 100.0%	1 / 1 100.0%	1 / 1 100.0%	1 / 1 100.0%	1 / 1 100.0%	2 / 2 100.0%	2 / 2 100.0%	1 / 1 100.0%	1 / 1 100.0%	2 / 2 100.0%	1 / 1 100.0%	3 / 3 100.0%	1 / 1 100.0%	1 / 1 100.0%	1 / 1 100.0%	1 / 1 100.0%	2 / 2 100.0%	1 / 1 100.0%	3 / 3 100.0%	3 / 3 100.0%	1 / 1 100.0%	1 / 1 100.0%	5 / 5 100.0%	0 / 0 0.0%	0 / 0 0.0%	0 / 0 0.0%	0 / 0 0.0%	21 / 36 58.3%AB	15 / 36 41.7%ABE						
2	Micavibrio	80 / 80 100.0%	2 / 2 100.0%	2 / 2 100.0%	2 / 2 100.0%	2 / 2 100.0%	6 / 6 100.0%	4 / 4 100.0%	2 / 2 100.0%	2 / 2 100.0%	4 / 4 100.0%	2 / 2 100.0%	8 / 8 100.0%	2 / 2 100.0%	2 / 2 100.0%	2 / 2 100.0%	2 / 2 100.0%	6 / 6 100.0%	2 / 2 100.0%	6 / 6 100.0%	8 / 8 100.0%	2 / 2 100.0%	2 / 2 100.0%	10 / 10 100.0%	0 / 0 0.0%	0 / 0 0.0%	0 / 0 0.0%	0 / 0 0.0%	50 / 80 62.5%AB	30 / 80 37.5%ABE						
2	"Micavibrio aeruginosavorus" Lambina et al. 1983	80 / 80 100.0%	2 / 2 100.0%	2 / 2 100.0%	2 / 2 100.0%	2 / 2 100.0%	6 / 6 100.0%	4 / 4 100.0%	2 / 2 100.0%	2 / 2 100.0%	4 / 4 100.0%	2 / 2 100.0%	8 / 8 100.0%	2 / 2 100.0%	2 / 2 100.0%	2 / 2 100.0%	2 / 2 100.0%	6 / 6 100.0%	2 / 2 100.0%	6 / 6 100.0%	8 / 8 100.0%	2 / 2 100.0%	2 / 2 100.0%	10 / 10 100.0%	0 / 0 0.0%	0 / 0 0.0%	0 / 0 0.0%	0 / 0 0.0%	50 / 80 62.5%AB	30 / 80 37.5%ABE						
1	Micavibrio aeruginosavorus EPB	40 / 40 100.0%	1 / 1 100.0%	1 / 1 100.0%	1 / 1 100.0%	1 / 1 100.0%	3 / 3 100.0%	2 / 2 100.0%	1 / 1 100.0%	1 / 1 100.0%	2 / 2 100.0%	1 / 1 100.0%	4 / 4 100.0%	1 / 1 100.0%	1 / 1 100.0%	1 / 1 100.0%	1 / 1 100.0%	3 / 3 100.0%	1 / 1 100.0%	3 / 3 100.0%	4 / 4 100.0%	1 / 1 100.0%	1 / 1 100.0%	5 / 5 100.0%	0 / 0 0.0%	0 / 0 0.0%	0 / 0 0.0%	0 / 0 0.0%	25 / 40 62.5%AB	15 / 40 37.5%ABE						
1	Micavibrio aeruginosavorus ARL-13	40 / 40 100.0%	1 / 1 100.0%	1 / 1 100.0%	1 / 1 100.0%	1 / 1 100.0%	3 / 3 100.0%	2 / 2 100.0%	1 / 1 100.0%	1 / 1 100.0%	2 / 2 100.0%	1 / 1 100.0%	4 / 4 100.0%	1 / 1 100.0%	1 / 1 100.0%	1 / 1 100.0%	1 / 1 100.0%	3 / 3 100.0%	1 / 1 100.0%	3 / 3 100.0%	4 / 4 100.0%	1 / 1 100.0%	1 / 1 100.0%	5 / 5 100.0%	0 / 0 0.0%	0 / 0 0.0%	0 / 0 0.0%	0 / 0 0.0%	25 / 40 62.5%AB	15 / 40 37.5%ABE						
1	'Polymorphum'	49 / 49 100.0%	2 / 2 100.0%	2 / 2 100.0%	1 / 1 100.0%	2 / 2 100.0%	3 / 3 100.0%	3 / 3 100.0%	1 / 1 100.0%	1 / 1 100.0%	4 / 4 100.0%	1 / 1 100.0%	4 / 4 100.0%	2 / 2 100.0%	1 / 1 100.0%	2 / 2 100.0%	1 / 1 100.0%	3 / 3 100.0%	1 / 1 100.0%	3 / 3 100.0%	4 / 4 100.0%	2 / 2 100.0%	1 / 1 100.0%	5 / 5 100.0%	0 / 0 0.0%	0 / 0 0.0%	0 / 0 0.0%	0 / 0 0.0%	34 / 49 69.4%AB	15 / 49 30.6%ABE						
1	'Polymorphum gilvum'	49 / 49 100.0%	2 / 2 100.0%	2 / 2 100.0%	1 / 1 100.0%	2 / 2 100.0%	3 / 3 100.0%	3 / 3 100.0%	1 / 1 100.0%	1 / 1 100.0%	4 / 4 100.0%	1 / 1 100.0%	4 / 4 100.0%	2 / 2 100.0%	1 / 1 100.0%	2 / 2 100.0%	1 / 1 100.0%	3 / 3 100.0%	1 / 1 100.0%	3 / 3 100.0%	4 / 4 100.0%	2 / 2 100.0%	1 / 1 100.0%	5 / 5 100.0%	0 / 0 0.0%	0 / 0 0.0%	0 / 0 0.0%	0 / 0 0.0%	34 / 49 69.4%AB	15 / 49 30.6%ABE						
1	Polymorphum gilvum SL003B-26A1	49 / 49 100.0%	2 / 2 100.0%	2 / 2 100.0%	1 / 1 100.0%	2 / 2 100.0%	3 / 3 100.0%	3 / 3 100.0%	1 / 1 100.0%	1 / 1 100.0%	4 / 4 100.0%	1 / 1 100.0%	4 / 4 100.0%	2 / 2 100.0%	1 / 1 100.0%	2 / 2 100.0%	1 / 1 100.0%	3 / 3 100.0%	1 / 1 100.0%	3 / 3 100.0%	4 / 4 100.0%	2 / 2 100.0%	1 / 1 100.0%	5 / 5 100.0%	0 / 0 0.0%	0 / 0 0.0%	0 / 0 0.0%	0 / 0 0.0%	34 / 49 69.4%AB	15 / 49 30.6%ABE						
24	Rhodospirillales	1181 / 1252 94.3%	79 / 80 98.8%	30 / 30 100.0%	24 / 24 100.0%	81 / 85 95.3%	75 / 75 100.0%	77 / 79 97.5%	25 / 25 100.0%	25 / 25 100.0%	131 / 136 96.3%	23 / 24 95.8%	90 / 90 100.0%	50 / 50 100.0%	20 / 21 95.2%	43 / 43 100.0%	24 / 24 100.0%	53 / 70 75.7%	22 / 23 95.7%	66 / 77 85.7%	83 / 85 97.6%	44 / 45 97.8%	10 / 23 43.5%	106 / 118 89.8%	0 / 0 0.0%	0 / 0 0.0%	0 / 0 0.0%	0 / 0 0.0%	774 / 1252 61.8%AB	383 / 1252 30.6%ABE	35 / 1252 2.8%	33 / 1252 2.6%E	14 / 1252 1.1%B	10 / 1252 0.8%BE	3 / 1252 0.2%C	
15	"Acetobacteraceae" Henrici 1939	765 / 824 92.8%	62 / 63 98.4%	18 / 18 100.0%	15 / 15 100.0%	60 / 64 93.8%	47 / 47 100.0%	51 / 53 96.2%	15 / 15 100.0%	15 / 15 100.0%	90 / 94 95.7%	15 / 15 100.0%	60 / 60 100.0%	30 / 30 100.0%	15 / 15 100.0%	30 / 30 100.0%	15 / 15 100.0%	31 / 45 68.9%	15 / 15 100.0%	37 / 45 82.2%	49 / 50 98.0%	30 / 30 100.0%	2 / 15 13.3%	63 / 75 84.0%	0 / 0 0.0%	0 / 0 0.0%	0 / 0 0.0%	0 / 0 0.0%	492 / 824 59.7%AB	249 / 824 30.2%ABE	32 / 824 3.9%	27 / 824 3.3%E	14 / 824 1.7%B	10 / 824 1.2%BE		
8	"Acetimonas" Orla-Jensen 1909	421 / 453 92.9%	39 / 39 100.0%	8 / 8 100.0%	8 / 8 100.0%	39 / 39 100.0%	24 / 24 100.0%	32 / 32 100.0%	8 / 8 100.0%	8 / 8 100.0%	55 / 55 100.0%	8 / 8 100.0%	32 / 32 100.0%	16 / 16 100.0%	8 / 8 100.0%	16 / 16 100.0%	8 / 8 100.0%	16 / 24 66.7%	8 / 8 100.0%	16 / 24 66.7%	24 / 24 100.0%	16 / 16 100.0%	0 / 8 0.0%	32 / 40 80.0%	0 / 0 0.0%	0 / 0 0.0%	0 / 0 0.0%	0 / 0 0.0%	278 / 453 61.4%AB	143 / 453 31.6%ABE	16 / 453 3.5%	16 / 453 3.5%E				
8	"Acetobacter acidum-mucosum" (sic) Tosic and Walker 1950	421 / 453 92.9%	39 / 39 100.0%	8 / 8 100.0%	8 / 8 100.0%	39 / 39 100.0%	24 / 24 100.0%	32 / 32 100.0%	8 / 8 100.0%	8 / 8 100.0%	55 / 55 100.0%	8 / 8 100.0%	32 / 32 100.0%	16 / 16 100.0%	8 / 8 100.0%	16 / 16 100.0%	8 / 8 100.0%	16 / 24 66.7%	8 / 8 100.0%	16 / 24 66.7%	24 / 24 100.0%	16 / 16 100.0%	0 / 8 0.0%	32 / 40 80.0%	0 / 0 0.0%	0 / 0 0.0%	0 / 0 0.0%	0 / 0 0.0%	278 / 453 61.4%AB	143 / 453 31.6%ABE	16 / 453 3.5%E	16 / 453 3.5%				
8	Acetobacter pasteurianus subsp. pasteurianus	421 / 453 92.9%	39 / 39 100.0%	8 / 8 100.0%	8 / 8 100.0%	39 / 39 100.0%	24 / 24 100.0%	32 / 32 100.0%	8 / 8 100.0%	8 / 8 100.0%	55 / 55 100.0%	8 / 8 100.0%	32 / 32 100.0%	16 / 16 100.0%	8 / 8 100.0%	16 / 16 100.0%	8 / 8 100.0%	16 / 24 66.7%	8 / 8 100.0%	16 / 24 66.7%	24 / 24 100.0%	16 / 16 100.0%	0 / 8 0.0%	32 / 40 80.0%	0 / 0 0.0%	0 / 0 0.0%	0 / 0 0.0%	0 / 0 0.0%	278 / 453 61.4%AB	143 / 453 31.6%ABE	16 / 453 3.5%	16 / 453 3.5%E				

1	Acetobacter pasteurianus IFO 3283-01	53 / 57 93.0%	5 / 5 100.0%	1 / 1 100.0%	1 / 1 100.0%	5 / 5 100.0%	3 / 3 100.0%	4 / 4 100.0%	1 / 1 100.0%	1 / 1 100.0%	7 / 7 100.0%	1 / 1 100.0%	4 / 4 100.0%	2 / 2 100.0%	1 / 1 100.0%	2 / 2 100.0%	1 / 1 100.0%	2 / 3 66.7%	1 / 1 100.0%	2 / 3 66.7%	3 / 3 100.0%	2 / 2 100.0%	0 / 1 0.0%	4 / 5 80.0%	0 / 0 0.0%	0 / 0 0.0%	0 / 0 0.0%	0 / 0 0.0%	35 / 57 61.4%AB	18 / 57 31.6%ABE	2 / 57 3.5%	2 / 57 3.5%E			
1	Acetobacter pasteurianus IFO 3283-03	53 / 57 93.0%	5 / 5 100.0%	1 / 1 100.0%	1 / 1 100.0%	5 / 5 100.0%	3 / 3 100.0%	4 / 4 100.0%	1 / 1 100.0%	1 / 1 100.0%	7 / 7 100.0%	1 / 1 100.0%	4 / 4 100.0%	2 / 2 100.0%	1 / 1 100.0%	2 / 2 100.0%	1 / 1 100.0%	2 / 3 66.7%	1 / 1 100.0%	2 / 3 66.7%	3 / 3 100.0%	2 / 2 100.0%	0 / 1 0.0%	4 / 5 80.0%	0 / 0 0.0%	0 / 0 0.0%	0 / 0 0.0%	0 / 0 0.0%	35 / 57 61.4%AB	18 / 57 31.6%ABE	2 / 57 3.5%	2 / 57 3.5%E			
1	Acetobacter pasteurianus IFO 3283-07	53 / 57 93.0%	5 / 5 100.0%	1 / 1 100.0%	1 / 1 100.0%	5 / 5 100.0%	3 / 3 100.0%	4 / 4 100.0%	1 / 1 100.0%	1 / 1 100.0%	7 / 7 100.0%	1 / 1 100.0%	4 / 4 100.0%	2 / 2 100.0%	1 / 1 100.0%	2 / 2 100.0%	1 / 1 100.0%	2 / 3 66.7%	1 / 1 100.0%	2 / 3 66.7%	3 / 3 100.0%	2 / 2 100.0%	0 / 1 0.0%	4 / 5 80.0%	0 / 0 0.0%	0 / 0 0.0%	0 / 0 0.0%	0 / 0 0.0%	35 / 57 61.4%AB	18 / 57 31.6%ABE	2 / 57 3.5%	2 / 57 3.5%E			
1	Acetobacter pasteurianus IFO 3283-22	53 / 57 93.0%	5 / 5 100.0%	1 / 1 100.0%	1 / 1 100.0%	5 / 5 100.0%	3 / 3 100.0%	4 / 4 100.0%	1 / 1 100.0%	1 / 1 100.0%	7 / 7 100.0%	1 / 1 100.0%	4 / 4 100.0%	2 / 2 100.0%	1 / 1 100.0%	2 / 2 100.0%	1 / 1 100.0%	2 / 3 66.7%	1 / 1 100.0%	2 / 3 66.7%	3 / 3 100.0%	2 / 2 100.0%	0 / 1 0.0%	4 / 5 80.0%	0 / 0 0.0%	0 / 0 0.0%	0 / 0 0.0%	0 / 0 0.0%	35 / 57 61.4%AB	18 / 57 31.6%ABE	2 / 57 3.5%	2 / 57 3.5%E			
1	Acetobacter pasteurianus IFO 3283-26	53 / 57 93.0%	5 / 5 100.0%	1 / 1 100.0%	1 / 1 100.0%	5 / 5 100.0%	3 / 3 100.0%	4 / 4 100.0%	1 / 1 100.0%	1 / 1 100.0%	7 / 7 100.0%	1 / 1 100.0%	4 / 4 100.0%	2 / 2 100.0%	1 / 1 100.0%	2 / 2 100.0%	1 / 1 100.0%	2 / 3 66.7%	1 / 1 100.0%	2 / 3 66.7%	3 / 3 100.0%	2 / 2 100.0%	0 / 1 0.0%	4 / 5 80.0%	0 / 0 0.0%	0 / 0 0.0%	0 / 0 0.0%	0 / 0 0.0%	35 / 57 61.4%AB	18 / 57 31.6%ABE	2 / 57 3.5%E	2 / 57 3.5%			
1	Acetobacter pasteurianus IFO 3283-32	53 / 57 93.0%	5 / 5 100.0%	1 / 1 100.0%	1 / 1 100.0%	5 / 5 100.0%	3 / 3 100.0%	4 / 4 100.0%	1 / 1 100.0%	1 / 1 100.0%	7 / 7 100.0%	1 / 1 100.0%	4 / 4 100.0%	2 / 2 100.0%	1 / 1 100.0%	2 / 2 100.0%	1 / 1 100.0%	2 / 3 66.7%	1 / 1 100.0%	2 / 3 66.7%	3 / 3 100.0%	2 / 2 100.0%	0 / 1 0.0%	4 / 5 80.0%	0 / 0 0.0%	0 / 0 0.0%	0 / 0 0.0%	0 / 0 0.0%	35 / 57 61.4%AB	18 / 57 31.6%ABE	2 / 57 3.5%E	2 / 57 3.5%			
1	Acetobacter pasteurianus IFO 3283-01-42C	50 / 54 92.6%	4 / 4 100.0%	1 / 1 100.0%	1 / 1 100.0%	4 / 4 100.0%	3 / 3 100.0%	4 / 4 100.0%	1 / 1 100.0%	1 / 1 100.0%	6 / 6 100.0%	1 / 1 100.0%	4 / 4 100.0%	2 / 2 100.0%	1 / 1 100.0%	2 / 2 100.0%	1 / 1 100.0%	2 / 3 66.7%	1 / 1 100.0%	2 / 3 66.7%	3 / 3 100.0%	2 / 2 100.0%	0 / 1 0.0%	4 / 5 80.0%	0 / 0 0.0%	0 / 0 0.0%	0 / 0 0.0%	0 / 0 0.0%	33 / 54 61.1%AB	17 / 54 31.5%ABE	2 / 54 3.7%	2 / 54 3.7%E			
1	Acetobacter pasteurianus IFO 3283-12	53 / 57 93.0%	5 / 5 100.0%	1 / 1 100.0%	1 / 1 100.0%	5 / 5 100.0%	3 / 3 100.0%	4 / 4 100.0%	1 / 1 100.0%	1 / 1 100.0%	7 / 7 100.0%	1 / 1 100.0%	4 / 4 100.0%	2 / 2 100.0%	1 / 1 100.0%	2 / 2 100.0%	1 / 1 100.0%	2 / 3 66.7%	1 / 1 100.0%	2 / 3 66.7%	3 / 3 100.0%	2 / 2 100.0%	0 / 1 0.0%	4 / 5 80.0%	0 / 0 0.0%	0 / 0 0.0%	0 / 0 0.0%	0 / 0 0.0%	35 / 57 61.4%AB	18 / 57 31.6%ABE	2 / 57 3.5%E	2 / 57 3.5%			
2	"Acetomonas" Leifson 1954	111 / 113 98.2%	8 / 8 100.0%	2 / 2 100.0%	2 / 2 100.0%	9 / 9 100.0%	8 / 8 100.0%	6 / 6 100.0%	2 / 2 100.0%	2 / 2 100.0%	13 / 13 100.0%	2 / 2 100.0%	8 / 8 100.0%	4 / 4 100.0%	2 / 2 100.0%	4 / 4 100.0%	2 / 2 100.0%	6 / 6 100.0%	2 / 2 100.0%	6 / 6 100.0%	8 / 8 100.0%	4 / 4 100.0%	0 / 2 0.0%	11 / 11 100.0%	0 / 0 0.0%	0 / 0 0.0%	0 / 0 0.0%	0 / 0 0.0%	74 / 113 65.5%AB	37 / 113 32.7%ABE	2 / 113 1.8%				
2	"Acetobacter capsulatum" (sic) Shimwell 1936	111 / 113 98.2%	8 / 8 100.0%	2 / 2 100.0%	2 / 2 100.0%	9 / 9 100.0%	8 / 8 100.0%	6 / 6 100.0%	2 / 2 100.0%	2 / 2 100.0%	13 / 13 100.0%	2 / 2 100.0%	8 / 8 100.0%	4 / 4 100.0%	2 / 2 100.0%	4 / 4 100.0%	2 / 2 100.0%	6 / 6 100.0%	2 / 2 100.0%	6 / 6 100.0%	8 / 8 100.0%	4 / 4 100.0%	0 / 2 0.0%	11 / 11 100.0%	0 / 0 0.0%	0 / 0 0.0%	0 / 0 0.0%	0 / 0 0.0%	74 / 113 65.5%AB	37 / 113 32.7%ABE	2 / 113 1.8%				
1	Gluconobacter oxydans 621H	54 / 55 98.2%	4 / 4 100.0%	1 / 1 100.0%	1 / 1 100.0%	4 / 4 100.0%	4 / 4 100.0%	3 / 3 100.0%	1 / 1 100.0%	1 / 1 100.0%	6 / 6 100.0%	1 / 1 100.0%	4 / 4 100.0%	2 / 2 100.0%	1 / 1 100.0%	2 / 2 100.0%	1 / 1 100.0%	3 / 3 100.0%	1 / 1 100.0%	3 / 3 100.0%	4 / 4 100.0%	2 / 2 100.0%	0 / 1 0.0%	5 / 5 100.0%	0 / 0 0.0%	0 / 0 0.0%	0 / 0 0.0%	0 / 0 0.0%	36 / 55 65.5%AB	18 / 55 32.7%ABE	1 / 55 1.8%				
1	Gluconobacter oxydans H24	57 / 58 98.3%	4 / 4 100.0%	1 / 1 100.0%	1 / 1 100.0%	5 / 5 100.0%	4 / 4 100.0%	3 / 3 100.0%	1 / 1 100.0%	1 / 1 100.0%	7 / 7 100.0%	1 / 1 100.0%	4 / 4 100.0%	2 / 2 100.0%	1 / 1 100.0%	2 / 2 100.0%	1 / 1 100.0%	3 / 3 100.0%	1 / 1 100.0%	3 / 3 100.0%	4 / 4 100.0%	2 / 2 100.0%	0 / 1 0.0%	6 / 6 100.0%	0 / 0 0.0%	0 / 0 0.0%	0 / 0 0.0%	0 / 0 0.0%	38 / 58 65.5%AB	19 / 58 32.8%ABE	1 / 58 1.7%				
2	Acidiphilium	77 / 94 81.9%	4 / 4 100.0%	2 / 2 100.0%	2 / 2 100.0%	0 / 4 0.0%	6 / 6 100.0%	4 / 6 66.7%	2 / 2 100.0%	2 / 2 100.0%	4 / 8 50.0%	2 / 2 100.0%	8 / 8 100.0%	4 / 4 100.0%	2 / 2 100.0%	4 / 4 100.0%	2 / 2 100.0%	4 / 6 66.7%	2 / 2 100.0%	6 / 6 100.0%	7 / 8 87.5%	4 / 4 100.0%	2 / 2 100.0%	4 / 8 50.0%	0 / 0 0.0%	0 / 0 0.0%	0 / 0 0.0%	0 / 0 0.0%	51 / 94 54.3%AB	26 / 94 27.7%ABE	9 / 94 9.6%E	8 / 94 8.5%			
1	ATCC 33463	39 / 47 83.0%	2 / 2 100.0%	1 / 1 100.0%	1 / 1 100.0%	0 / 2 0.0%	3 / 3 100.0%	2 / 3 66.7%	1 / 1 100.0%	1 / 1 100.0%	2 / 4 50.0%	1 / 1 100.0%	4 / 4 100.0%	2 / 2 100.0%	1 / 1 100.0%	2 / 2 100.0%	1 / 1 100.0%	2 / 3 66.7%	1 / 1 100.0%	3 / 3 100.0%	4 / 4 100.0%	2 / 2 100.0%	1 / 1 100.0%	2 / 4 50.0%	0 / 0 0.0%	0 / 0 0.0%	0 / 0 0.0%	0 / 0 0.0%	26 / 47 55.3%AB	13 / 47 27.7%ABE	5 / 47 10.6%	3 / 47 6.4%E			
1	Acidiphilium cryptum JF-5	39 / 47 83.0%	2 / 2 100.0%	1 / 1 100.0%	1 / 1 100.0%	0 / 2 0.0%	3 / 3 100.0%	2 / 3 66.7%	1 / 1 100.0%	1 / 1 100.0%	2 / 4 50.0%	1 / 1 100.0%	4 / 4 100.0%	2 / 2 100.0%	1 / 1 100.0%	2 / 2 100.0%	1 / 1 100.0%	2 / 3 66.7%	1 / 1 100.0%	3 / 3 100.0%	4 / 4 100.0%	2 / 2 100.0%	1 / 1 100.0%	2 / 4 50.0%	0 / 0 0.0%	0 / 0 0.0%	0 / 0 0.0%	0 / 0 0.0%	26 / 47 55.3%AB	13 / 47 27.7%ABE	5 / 47 10.6%	3 / 47 6.4%E			
1	Acidiphilium multivorum	38 / 47 80.9%	2 / 2 100.0%	1 / 1 100.0%	1 / 1 100.0%	0 / 2 0.0%	3 / 3 100.0%	2 / 3 66.7%	1 / 1 100.0%	1 / 1 100.0%	2 / 4 50.0%	1 / 1 100.0%	4 / 4 100.0%	2 / 2 100.0%	1 / 1 100.0%	2 / 2 100.0%	1 / 1 100.0%	2 / 3 66.7%	1 / 1 100.0%	3 / 3 100.0%	3 / 4 75.0%	2 / 2 100.0%	1 / 1 100.0%	2 / 4 50.0%	0 / 0 0.0%	0 / 0 0.0%	0 / 0 0.0%	0 / 0 0.0%	25 / 47 53.2%AB	13 / 47 27.7%ABE	6 / 47 12.8%E	3 / 47 6.4%			
1	Acidiphilium multivorum AIU301	38 / 47 80.9%	2 / 2 100.0%	1 / 1 100.0%	1 / 1 100.0%	0 / 2 0.0%	3 / 3 100.0%	2 / 3 66.7%	1 / 1 100.0%	1 / 1 100.0%	2 / 4 50.0%	1 / 1 100.0%	4 / 4 100.0%	2 / 2 100.0%	1 / 1 100.0%	2 / 2 100.0%	1 / 1 100.0%	2 / 3 66.7%	1 / 1 100.0%	3 / 3 100.0%	3 / 4 75.0%	2 / 2 100.0%	1 / 1 100.0%	2 / 4 50.0%	0 / 0 0.0%	0 / 0 0.0%	0 / 0 0.0%	0 / 0 0.0%	25 / 47 53.2%AB	13 / 47 27.7%ABE	6 / 47 12.8%E	3 / 47 6.4%			
1	Acetobacter (subgen. Gluconoacetobacter Yamada and Kondo 1985)	53 / 55 96.4%	4 / 4 100.0%	2 / 2 100.0%	1 / 1 100.0%	4 / 4 100.0%	3 / 3 100.0%	3 / 3 100.0%	1 / 1 100.0%	1 / 1 100.0%	6 / 6 100.0%	1 / 1 100.0%	4 / 4 100.0%	2 / 2 100.0%	1 / 1 100.0%	2 / 2 100.0%	1 / 1 100.0%	2 / 3 66.7%	1 / 1 100.0%	3 / 3 100.0%	3 / 3 100.0%	2 / 2 100.0%	0 / 1 0.0%	6 / 6 100.0%	0 / 0 0.0%	0 / 0 0.0%	0 / 0 0.0%	0 / 0 0.0%	20 / 55 36.4%AB	14 / 55 25.5%B	10 / 55 18.2%BE	9 / 55 16.4%ABE	2 / 55 3.6%		
1	ATCC 49037	53 / 55 96.4%	4 / 4 100.0%	2 / 2 100.0%	1 / 1 100.0%	4 / 4 100.0%	3 / 3 100.0%	3 / 3 100.0%	1 / 1 100.0%	1 / 1 100.0%	6 / 6 100.0%	1 / 1 100.0%	4 / 4 100.0%	2 / 2 100.0%	1 / 1 100.0%	2 / 2 100.0%	1 / 1 100.0%	2 / 3 66.7%	1 / 1 100.0%	3 / 3 100.0%	3 / 3 100.0%	2 / 2 100.0%	0 / 1 0.0%	6 / 6 100.0%	0 / 0 0.0%	0 / 0 0.0%	0 / 0 0.0%	0 / 0 0.0%	20 / 55 36.4%AB	14 / 55 25.5%B	10 / 55 18.2%BE	9 / 55 16.4%ABE	2 / 55 3.6%		
1	Gluconacetobacter diazotrophicus 'Dobereiner PA1 5'	53 / 55 96.4%	4 / 4 100.0%	2 / 2 100.0%	1 / 1 100.0%	4 / 4 100.0%	3 / 3 100.0%	3 / 3 100.0%	1 / 1 100.0%	1 / 1 100.0%	6 / 6 100.0%	1 / 1 100.0%	4 / 4 100.0%	2 / 2 100.0%	1 / 1 100.0%	2 / 2 100.0%	1 / 1 100.0%	2 / 3 66.7%	1 / 1 100.0%	3 / 3 100.0%	3 / 3 100.0%	2 / 2 100.0%	0 / 1 0.0%	6 / 6 100.0%	0 / 0 0.0%	0 / 0 0.0%	0 / 0 0.0%	0 / 0 0.0%	20 / 55 36.4%AB	14 / 55 25.5%B	10 / 55 18.2%BE	9 / 55 16.4%ABE	2 / 55 3.6%		
1	"Granulobacter (sic)" Greenberg et al. 2006	49 / 52 94.2%	3 / 3 100.0%	2 / 2 100.0%	1 / 1 100.0%	3 / 3 100.0%	3 / 3 100.0%	3 / 3 100.0%	1 / 1 100.0%	1 / 1 100.0%	5 / 5 100.0%	1 / 1 100.0%	4 / 4 100.0%	2 / 2 100.0%	1 / 1 100.0%	2 / 2 100.0%	1 / 1 100.0%	1 / 3 33.3%	1 / 1 100.0%	3 / 3 100.0%	4 / 4 100.0%	2 / 2 100.0%	0 / 1 0.0%	5 / 5 100.0%	0 / 0 0.0%	0 / 0 0.0%	0 / 0 0.0%	0 / 0 0.0%	33 / 52 63.5%AB	16 / 52 30.8%ABE	2 / 52 3.8%	1 / 52 1.9%E			
1	"Granulobacter (sic) bethesdensis" Greenberg et al. 2006	49 / 52 94.2%	3 / 3 100.0%	2 / 2 100.0%	1 / 1 100.0%	3 / 3 100.0%	3 / 3 100.0%	3 / 3 100.0%	1 / 1 100.0%	1 / 1 100.0%	5 / 5 100.0%	1 / 1 100.0%	4 / 4 100.0%	2 / 2 100.0%	1 / 1 100.0%	2 / 2 100.0%	1 / 1 100.0%	1 / 3 33.3%	1 / 1 100.0%	3 / 3 100.0%	4 / 4 100.0%	2 / 2 100.0%	0 / 1 0.0%	5 / 5 100.0%	0 / 0 0.0%	0 / 0 0.0%	0 / 0 0.0%	0 / 0 0.0%	33 / 52 63.5%AB	16 / 52 30.8%ABE	2 / 52 3.8%	1 / 52 1.9%E			
1	Granulibacter bethesdensis CGDNIH1	49 / 52 94.2%	3 / 3 100.0%	2 / 2 100.0%	1 / 1 100.0%	3 / 3 100.0%	3 / 3 100.0%	3 / 3 100.0%	1 / 1 100.0%	1 / 1 100.0%	5 / 5 100.0%	1 / 1 100.0%	4 / 4 100.0%	2 / 2 100.0%	1 / 1 100.0%	2 / 2 100.0%	1 / 1 100.0%	1 / 3 33.3%	1 / 1 100.0%	3 / 3 100.0%	4 / 4 100.0%	2 / 2 100.0%	0 / 1 0.0%	5 / 5 100.0%	0 / 0 0.0%	0 / 0 0.0%	0 / 0 0.0%	0 / 0 0.0%	33 / 52 63.5%AB	16 / 52 30.8%ABE	2 / 52 3.8%	1 / 52 1.9%E			
1	"Komagatabacter" Yamada et al. 2012	54 / 57 94.7%	4 / 5 80.0%	2 / 2 100.0%	1 / 1 100.0%	5 / 5 100.0%	3 / 3 100.0%	3 / 3 100.0%	1 / 1 100.0%	1 / 1 100.0%	7 / 7 100.0%	1 / 1 100.0%	4 / 4 100.0%	2 / 2 100.0%	1 / 1 100.0%	2 / 2 100.0%	1 / 1 100.0%	2 / 3 66.7%	1 / 1 100.0%	3 / 3 100.0%	3 / 3 100.0%	2 / 2 100.0%	0 / 1 0.0%	5 / 5 100.0%	0 / 0 0.0%	0 / 0 0.0%	0 / 0 0.0%	0 / 0 0.0%	36 / 57 63.2%AB	18 / 57 31.6%ABE	2 / 57 3.5%	1 / 57 1.8%E			
1	"Gluconacetobacter medellensis" Castro et al. 2012	54 / 57 94.7%	4 / 5 80.0%	2 / 2 100.0%	1 / 1 100.0%	5 / 5 100.0%	3 / 3 100.0%	3 / 3 100.0%	1 / 1 100.0%	1 / 1 100.0%	7 / 7 100.0%	1 / 1 100.0%	4 / 4 100.0%	2 / 2 100.0%	1 / 1 100.0%	2 / 2 100.0%	1 / 1 100.0%	2 / 3 66.7%	1 / 1 100.0%	3 / 3 100.0%	3 / 3 100.0%	2 / 2 100.0%	0 / 1 0.0%	5 / 5 100.0%	0 / 0 0.0%	0 / 0 0.0%	0 / 0 0.0%	0 / 0 0.0%	36 / 57 63.2%AB	18 / 57 31.6%ABE	2 / 57 3.5%	1 / 57 1.8%E			
1	Gluconacetobacter medellinensis NBRC 3288	54 / 57 94.7%	4 / 5 80.0%	2 / 2 100.0%	1 / 1 100.0%	5 / 5 100.0%	3 / 3 100.0%	3 / 3 100.0%	1 / 1 100.0%	1 / 1 100.0%	7 / 7 100.0%	1 / 1 100.0%	4 / 4 100.0%	2 / 2 100.0%	1 / 1 100.0%	2 / 2 100.0%	1 / 1 100.0%	2 / 3 66.7%	1 / 1 100.0%	3 / 3 100.0%	3 / 3 100.0%	2 / 2 100.0%	0 / 1 0.0%	5 / 5 100.0%	0 / 0 0.0%	0 / 0 0.0%	0 / 0 0.0%	0 / 0 0.0%	36 / 57 63.2%AB	18 / 57 31.6%ABE	2 / 57 3.5%	1 / 57 1.8%E			
9	Rhodospirillaceae	416 / 428 97.2%	17 / 17 100.0%	12 / 12 100.0%	9 / 9 100.0%	21 / 21 100.0%	28 / 28 100.0%	26 / 26 100.0%	10 / 10 100.0%	10 / 10 100.0%	41 / 42 97.6%	8 / 9 88.9%	30 / 30 100.0%	20 / 20 100.0%	5 / 6 83.3%	13 / 13 100.0%	9 / 9 100.0%	22 / 25 88.0%	7 / 8 87.5%	29 / 32 90.6%	34 / 35 97.1%	14 / 15 93.3%	8 / 8 100.0%	43 / 43 100.0%	0 / 0 0.0%	0 / 0 0.0%	0 / 0 0.0%	0 / 0 0.0%	282 / 428 65.9%AB	134 / 428 31.3%ABE	6 / 428 1.4%E	3 / 428 0.7%	3 / 428 0.7%C		
3	Azospirillum	135 / 135 100.0%	3 / 3 100.0%	3 / 3 100.0%	3 / 3 100.0%	5 / 5 100.0%	11 / 11 100.0%	8 / 8 100.0%	4 / 4 100.0%	4 / 4 100.0%	14 / 14 100.0%	3 / 3 100.0%	6 / 6 100.0%	8 / 8 100.0%	0 / 0 0.0%	2 / 2 100.0%	3 / 3 100.0%	8 / 8 100.0%	3 / 3 100.0%	11 / 11 100.0%	12 / 12 100.0%	5 / 5 100.0%	3 / 3 100.0%	16 / 16 100.0%	0 / 0 0.0%	0 / 0 0.0%	0 / 0 0.0%	0 / 0 0.0%	96 / 135 71.1%AB	39 / 135 28.9%ABE					
1	ATCC 29145	44 / 44 100.0%	0 / 0 0.0%	1 / 1 100.0%	1 / 1 100.0%	1 / 1 100.0%	3 / 3 100.0%	4 / 4 100.0%	2 / 2 100.0%	2 / 2 100.0%	4 / 4 100.0%	1 / 1 100.0%	2 / 2 100.0%	2 / 2 100.0%	0 / 0 0.0%	0 / 0 0.0%	1 / 1 100.0%	4 / 4 100.0%	1 / 1 100.0%	3 / 3 100.0%	4 / 4 100.0%	3 / 3 100.0%	1 / 1 100.0%	4 / 4 100.0%	0 / 0 0.0%	0 / 0 0.0%	0 / 0 0.0%	0 / 0 0.0%	32 / 44 72.7%AB	12 / 44 27.3%ABE					
1	Azospirillum brasilense Sp245	44 / 44 100.0%	0 / 0 0.0%	1 / 1 100.0%	1 / 1 100.0%	1 / 1 100.0%	3 / 3 100.0%	4 / 4 100.0%	2 / 2 100.0%	2 / 2 100.0%	4 / 4 100.0%	1 / 1 100.0%	2 / 2 100.0%	2 / 2 100.0%	0 / 0 0.0%	0 / 0 0.0%	1 / 1 100.0%	4 / 4 100.0%	1 / 1 100.0%	3 / 3 100.0%	4 / 4 100.0%	3 / 3 100.0%	1 / 1 100.0%	4 / 4 100.0%	0 / 0 0.0%	0 / 0 0.0%	0 / 0 0.0%	0 / 0 0.0%	32 / 44 72.7%AB	12 / 44 27.3%ABE					
2	"Spirillum lipoferum" Beijerinck 1925	91 / 91 100.0%	3 / 3 100.0%	2 / 2 100.0%	2 / 2 100.0%	4 / 4 100.0%	8 / 8 100.0%	4 / 4 100.0%	2 / 2 100.0%	2 / 2 100.0%	10 / 10 100.0%	2 / 2 100.0%	4 / 4 100.0%	6 / 6 100.0%	0 / 0 0.0%	2 / 2 100.0%	2 / 2 100.0%	4 / 4 100.0%	2 / 2 100.0%	8 / 8 100.0%	8 / 8 100.0%	2 / 2 100.0%	2 / 2 100.0%	12 / 12 100.0%	0 / 0 0.0%	0 / 0 0.0%	0 / 0 0.0%	0 / 0 0.0%	64 / 91 70.3%AB	27 / 91 29.7%ABE					
1	Azospirillum lipoferum B510	45 / 45 100.0%	1 / 1 100.0%	1 / 1 100.0%	1 / 1 100.0%	2 / 2 100.0%	4 / 4 100.0%	2 / 2 100.0%	1 / 1 100.0%	1 / 1 100.0%	5 / 5 100.0%	1 / 1 100.0%	2 / 2 100.0%	3 / 3 100.0%	0 / 0 0.0%	1 / 1 100.0%	1 / 1 100.0%	2 / 2 100.0%	1 / 1 100.0%	4 / 4 100.0%	4 / 4 100.0%	1 / 1 100.0%	1 / 1 100.0%	6 / 6 100.0%	0 / 0 0.0%	0 / 0 0.0%	0 / 0 0.0%	0 / 0 0.0%	32 / 45 71.1%AB	13 / 45 28.9%ABE					
1	Azospirillum lipoferum 4B	46 / 46 100.0%	2 / 2 100.0%	1 / 1 100.0%	1 / 1 100.0%	2 / 2 100.0%	4 / 4 100.0%	2 / 2 100.0%	1 / 1 100.0%	1 / 1 100.0%	5 / 5 100.0%	1 / 1 100.0%	2 / 2 100.0%	3 / 3 100.0%	0 / 0 0.0%	1 / 1 100.0%	1 / 1 100.0%	2 / 2 100.0%	1 / 1 100.0%	4 / 4 100.0%	4 / 4 100.0%	1 / 1 100.0%	1 / 1 100.0%	6 / 6 100.0%	0 / 0 0.0%	0 / 0 0.0%	0 / 0 0.0%	0 / 0 0.0%	32 / 46 69.6%AB	14 / 46 30.4%ABE					
3	Rhodospirillum	161 / 161 100.0%	9 / 9 100.0%	4 / 4 100.0%	3 / 3 100.0%	11 / 11 100.0%	9 / 9 100.0%	9 / 9 100.0%	3 / 3 100.0%	3 / 3 100.0%	19 / 19 100.0%	3 / 3 100.0%	12 / 12 100.0%	7 / 7 100.0%	3 / 3 100.0%	6 / 6 100.0%	3 / 3 100.0%	9 / 9 100.0%	3 / 3 100.0%	11 / 11 100.0%	12 / 12 100.0%	5 / 5 100.0%	3 / 3 100.0%	14 / 14 100.0%	0 / 0 0.0%	0 / 0 0.0%	0 / 0 0.0%	0 / 0 0.0%	109 / 161 67.7%AB	52 / 161 32.3%ABE					
2	"Dicrospirillum rubrum" Enderlein 1925	110 / 110 100.0%	6 / 6 100.0%	2 / 2 100.0%	2 / 2 100.0%	8 / 8 100.0%	6 / 6 100.0%	6 / 6 100.0%	2 / 2 100.0%	2 / 2 100.0%	14 / 14 100.0%	2 / 2 100.0%	8 / 8 100.0%	4 / 4 100.0%	2 / 2 100.0%	4 / 4 100.0%	2 / 2 100.0%	6 / 6 100.0%	2 / 2 100.0%	8 / 8 100.0%	8 / 8 100.0%	4 / 4 100.0%	2 / 2 100.0%	10 / 10 100.0%	0 / 0 0.0%	0 / 0 0.0%	0 / 0 0.0%	0 / 0 0.0%	74 / 110 67.3%AB	36 / 110 32.7%ABE					
1	Rhodospirillum rubrum ATCC 11170	55 / 55 100.0%	3 / 3 100.0%	1 / 1 100.0%	1 / 1 100.0%	4 / 4 100.0%	3 / 3 100.0%	3 / 3 100.0%	1 / 1 100.0%	1 / 1 100.0%	7 / 7 100.0%	1 / 1 100.0%	4 / 4 100.0%	2 / 2 100.0%	1 / 1 100.0%	2 / 2 100.0%	1 / 1 100.0%	3 / 3 100.0%	1 / 1 100.0%	4 / 4 100.0%	4 / 4 100.0%	2 / 2 100.0%	1 / 1 100.0%	5 / 5 100.0%	0 / 0 0.0%	0 / 0 0.0%	0 / 0 0.0%	0 / 0 0.0%	37 / 55 67.3%AB	18 / 55 32.7%ABE					
1	Rhodospirillum rubrum F11	55 / 55 100.0%	3 / 3 100.0%	1 / 1 100.0%	1 / 1 100.0%	4 / 4 100.0%	3 / 3 100.0%	3 / 3 100.0%	1 / 1 100.0%	1 / 1 100.0%	7 / 7 100.0%	1 / 1 100.0%	4 / 4 100.0%	2 / 2 100.0%	1 / 1 100.0%	2 / 2 100.0%	1 / 1 100.0%	3 / 3 100.0%	1 / 1 100.0%	4 / 4 100.0%	4 / 4 100.0%	2 / 2 100.0%	1 / 1 100.0%	5 / 5 100.0%	0 / 0 0.0%	0 / 0 0.0%	0 / 0 0.0%	0 / 0 0.0%	37 / 55 67.3%AB	18 / 55 32.7%ABE					
1	ATCC 43720	51 / 51 100.0%	3 / 3 100.0%	2 / 2 100.0%	1 / 1 100.0%	3 / 3 100.0%	3 / 3 100.0%	3 / 3 100.0%	1 / 1 100.0%	1 / 1 100.0%	5 / 5 100.0%	1 / 1 100.0%	4 / 4 100.0%	3 / 3 100.0%	1 / 1 100.0%	2 / 2 100.0%	1 / 1 100.0%	3 / 3 100.0%	1 / 1 100.0%	3 / 3 100.0%	4 / 4 100.0%	1 / 1 100.0%	1 / 1 100.0%	4 / 4 100.0%	0 / 0 0.0%	0 / 0 0.0%	0 / 0 0.0%	0 / 0 0.0%	35 / 51 68.6%AB	16 / 51 31.4%ABE					
1	Rhodocista centenaria SW	51 / 51 100.0%	3 / 3 100.0%	2 / 2 100.0%	1 / 1 100.0%	3 / 3 100.0%	3 / 3 100.0%	3 / 3 100.0%	1 / 1 100.0%	1 / 1 100.0%	5 / 5 100.0%	1 / 1 100.0%	4 / 4 100.0%	3 / 3 100.0%	1 / 1 100.0%	2 / 2 100.0%	1 / 1 100.0%	3 / 3 100.0%	1 / 1 100.0%	3 / 3 100.0%	4 / 4 100.0%	1 / 1 100.0%	1 / 1 100.0%	4 / 4 100.0%	0 / 0 0.0%	0 / 0 0.0%	0 / 0 0.0%	0 / 0 0.0%	35 / 51 68.6%AB	16 / 51 31.4%ABE					
1	Magnetospirillum	49 / 49 100.0%	2 / 2 100.0%	2 / 2 100.0%	1 / 1 100.0%	2 / 2 100.0%	3 / 3 100.0%	3 / 3 100.0%	1 / 1 100.0%	1 / 1 100.0%	4 / 4 100.0%	1 / 1 100.0%	4 / 4 100.0%	2 / 2 100.0%	1 / 1 100.0%	2 / 2 100.0%	1 / 1 100.0%	3 / 3 100.0%	1 / 1 100.0%	3 / 3 100.0%	4 / 4 100.0%	2 / 2 100.0%	1 / 1 100.0%	5 / 5 100.0%	0 / 0 0.0%	0 / 0 0.0%	0 / 0 0.0%	0 / 0 0.0%	32 / 49 65.3%AB	17 / 49 34.7%ABE					
1	'Magnetospirillum magneticum'	49 / 49 100.0%	2 / 2 100.0%	2 / 2 100.0%	1 / 1 100.0%	2 / 2 100.0%	3 / 3 100.0%	3 / 3 100.0%	1 / 1 100.0%	1 / 1 100.0%	4 / 4 100.0%	1 / 1 100.0%	4 / 4 100.0%	2 / 2 100.0%	1 / 1 100.0%	2 / 2 100.0%	1 / 1 100.0%	3 / 3 100.0%	1 / 1 100.0%	3 / 3 100.0%	4 / 4 100.0%	2 / 2 100.0%	1 / 1 100.0%	5 / 5 100.0%	0 / 0 0.0%	0 / 0 0.0%	0 / 0 0.0%	0 / 0 0.0%	32 / 49 65.3%AB	17 / 49 34.7%ABE					
1	Magnetospirillum magneticum AMB-1	49 / 49 100.0%	2 / 2 100.0%	2 / 2 100.0%	1 / 1 100.0%	2 / 2 100.0%	3 / 3 100.0%	3 / 3 100.0%	1 / 1 100.0%	1 / 1 100.0%	4 / 4 100.0%	1 / 1 100.0%	4 / 4 100.0%	2 / 2 100.0%	1 / 1 100.0%	2 / 2 100.0%	1 / 1 100.0%	3 / 3 100.0%	1 / 1 100.0%	3 / 3 100.0%	4 / 4 100.0%	2 / 2 100.0%	1 / 1 100.0%	5 / 5 100.0%	0 / 0 0.0%	0 / 0 0.0%	0 / 0 0.0%	0 / 0 0.0%	32 / 49 65.3%AB	17 / 49 34.7%ABE					
1	Tistrella	41 / 41 100.0%	2 / 2 100.0%	2 / 2 100.0%	1 / 1 100.0%	2 / 2 100.0%	3 / 3 100.0%	3 / 3 100.0%	1 / 1 100.0%	1 / 1 100.0%	2 / 2 100.0%	1 / 1 100.0%	4 / 4 100.0%	1 / 1 100.0%	1 / 1 100.0%	1 / 1 100.0%	1 / 1 100.0%	2 / 2 100.0%	0 / 0 0.0%	4 / 4 100.0%	4 / 4 100.0%	2 / 2 100.0%	0 / 0 0.0%	3 / 3 100.0%	0 / 0 0.0%	0 / 0 0.0%	0 / 0 0.0%	0 / 0 0.0%	27 / 41 65.9%AB	14 / 41 34.1%ABE					
1	IAM 14872	41 / 41 100.0%	2 / 2 100.0%	2 / 2 100.0%	1 / 1 100.0%	2 / 2 100.0%	3 / 3 100.0%	3 / 3 100.0%	1 / 1 100.0%	1 / 1 100.0%	2 / 2 100.0%	1 / 1 100.0%	4 / 4 100.0%	1 / 1 100.0%	1 / 1 100.0%	1 / 1 100.0%	1 / 1 100.0%	2 / 2 100.0%	0 / 0 0.0%	4 / 4 100.0%	4 / 4 100.0%	2 / 2 100.0%	0 / 0 0.0%	3 / 3 100.0%	0 / 0 0.0%	0 / 0 0.0%	0 / 0 0.0%	0 / 0 0.0%	27 / 41 65.9%AB	14 / 41 34.1%ABE					
1	Tistrella mobilis KA081020-065	41 / 41 100.0%	2 / 2 100.0%	2 / 2 100.0%	1 / 1 100.0%	2 / 2 100.0%	3 / 3 100.0%	3 / 3 100.0%	1 / 1 100.0%	1 / 1 100.0%	2 / 2 100.0%	1 / 1 100.0%	4 / 4 100.0%	1 / 1 100.0%	1 / 1 100.0%	1 / 1 100.0%	1 / 1 100.0%	2 / 2 100.0%	0 / 0 0.0%	4 / 4 100.0%	4 / 4 100.0%	2 / 2 100.0%	0 / 0 0.0%	3 / 3 100.0%	0 / 0 0.0%	0 / 0 0.0%	0 / 0 0.0%	0 / 0 0.0%	27 / 41 65.9%AB	14 / 41 34.1%ABE					
1	'Candidatus Endolissoclinum'	30 / 42 71.4%	1 / 1 100.0%	1 / 1 100.0%	1 / 1 100.0%	1 / 1 100.0%	2 / 2 100.0%	3 / 3 100.0%	1 / 1 100.0%	1 / 1 100.0%	2 / 3 66.7%	0 / 1 0.0%	4 / 4 100.0%	2 / 2 100.0%	0 / 1 0.0%	2 / 2 100.0%	1 / 1 100.0%	0 / 3 0.0%	0 / 1 0.0%	0 / 3 0.0%	2 / 3 66.7%	0 / 1 0.0%	1 / 1 100.0%	5 / 5 100.0%	0 / 0 0.0%	0 / 0 0.0%	0 / 0 0.0%	0 / 0 0.0%	18 / 42 42.9%AB	12 / 42 28.6%ABE	6 / 42 14.3%E	3 / 42 7.1%C	3 / 42 7.1%		
1	"Candidatus Endolissoclinum faulkneri" Kwan et al. 2012	30 / 42 71.4%	1 / 1 100.0%	1 / 1 100.0%	1 / 1 100.0%	1 / 1 100.0%	2 / 2 100.0%	3 / 3 100.0%	1 / 1 100.0%	1 / 1 100.0%	2 / 3 66.7%	0 / 1 0.0%	4 / 4 100.0%	2 / 2 100.0%	0 / 1 0.0%	2 / 2 100.0%	1 / 1 100.0%	0 / 3 0.0%	0 / 1 0.0%	0 / 3 0.0%	2 / 3 66.7%	0 / 1 0.0%	1 / 1 100.0%	5 / 5 100.0%	0 / 0 0.0%	0 / 0 0.0%	0 / 0 0.0%	0 / 0 0.0%	18 / 42 42.9%AB	12 / 42 28.6%ABE	6 / 42 14.3%E	3 / 42 7.1%C	3 / 42 7.1%		
1	Candidatus Endolissoclinum faulkneri L2	30 / 42 71.4%	1 / 1 100.0%	1 / 1 100.0%	1 / 1 100.0%	1 / 1 100.0%	2 / 2 100.0%	3 / 3 100.0%	1 / 1 100.0%	1 / 1 100.0%	2 / 3 66.7%	0 / 1 0.0%	4 / 4 100.0%	2 / 2 100.0%	0 / 1 0.0%	2 / 2 100.0%	1 / 1 100.0%	0 / 3 0.0%	0 / 1 0.0%	0 / 3 0.0%	2 / 3 66.7%	0 / 1 0.0%	1 / 1 100.0%	5 / 5 100.0%	0 / 0 0.0%	0 / 0 0.0%	0 / 0 0.0%	0 / 0 0.0%	18 / 42 42.9%AB	12 / 42 28.6%ABE	6 / 42 14.3%E	3 / 42 7.1%C	3 / 42 7.1%		
23	'Rhodobacterales'	1091 / 1106 98.6%	55 / 55 100.0%	45 / 45 100.0%	28 / 28 100.0%	60 / 60 100.0%	78 / 78 100.0%	64 / 65 98.5%	23 / 23 100.0%	18 / 21 85.7%	93 / 93 100.0%	24 / 24 100.0%	90 / 90 100.0%	29 / 29 100.0%	22 / 25 88.0%	29 / 29 100.0%	22 / 22 100.0%	64 / 64 100.0%	19 / 19 100.0%	61 / 64 95.3%	91 / 91 100.0%	36 / 36 100.0%	25 / 26 96.2%	111 / 114 97.4%	2 / 3 66.7%	0 / 0 0.0%	2 / 2 100.0%	0 / 0 0.0%	664 / 1106 60.0%AB	406 / 1106 36.7%ABE	13 / 1106 1.2%B	8 / 1106 0.7%BE	8 / 1106 0.7%E	6 / 1106 0.5%	1 / 1106 0.1%C
20	'Rhodobacteraceae'	966 / 980 98.6%	51 / 51 100.0%	42 / 42 100.0%	25 / 25 100.0%	55 / 55 100.0%	70 / 70 100.0%	56 / 57 98.2%	20 / 20 100.0%	15 / 18 83.3%	84 / 84 100.0%	21 / 21 100.0%	79 / 79 100.0%	25 / 25 100.0%	19 / 21 90.5%	26 / 26 100.0%	19 / 19 100.0%	56 / 56 100.0%	16 / 16 100.0%	53 / 56 94.6%	79 / 79 100.0%	33 / 33 100.0%	22 / 23 95.7%	97 / 100 97.0%	1 / 2 50.0%	0 / 0 0.0%	2 / 2 100.0%	0 / 0 0.0%	585 / 980 59.7%AB	360 / 980 36.7%ABE	13 / 980 1.3%B	8 / 980 0.8%E	8 / 980 0.8%BE	5 / 980 0.5%	1 / 980 0.1%C
2	Paracoccus	85 / 86 98.8%	4 / 4 100.0%	3 / 3 100.0%	2 / 2 100.0%	4 / 4 100.0%	4 / 4 100.0%	6 / 6 100.0%	2 / 2 100.0%	2 / 2 100.0%	8 / 8 100.0%	2 / 2 100.0%	9 / 9 100.0%	2 / 2 100.0%	2 / 2 100.0%	2 / 2 100.0%	1 / 1 100.0%	5 / 5 100.0%	2 / 2 100.0%	5 / 5 100.0%	8 / 8 100.0%	2 / 2 100.0%	2 / 2 100.0%	7 / 7 100.0%	1 / 2 50.0%	0 / 0 0.0%	0 / 0 0.0%	0 / 0 0.0%	57 / 86 66.3%AB	27 / 86 31.4%ABE	1 / 86 1.2%BE	1 / 86 1.2%E			
2	ATCC 17741	85 / 86 98.8%	4 / 4 100.0%	3 / 3 100.0%	2 / 2 100.0%	4 / 4 100.0%	4 / 4 100.0%	6 / 6 100.0%	2 / 2 100.0%	2 / 2 100.0%	8 / 8 100.0%	2 / 2 100.0%	9 / 9 100.0%	2 / 2 100.0%	2 / 2 100.0%	2 / 2 100.0%	1 / 1 100.0%	5 / 5 100.0%	2 / 2 100.0%	5 / 5 100.0%	8 / 8 100.0%	2 / 2 100.0%	2 / 2 100.0%	7 / 7 100.0%	1 / 2 50.0%	0 / 0 0.0%	0 / 0 0.0%	0 / 0 0.0%	57 / 86 66.3%AB	27 / 86 31.4%ABE	1 / 86 1.2%BE	1 / 86 1.2%E			
1	Paracoccus denitrificans PD1222	53 / 53 100.0%	3 / 3 100.0%	3 / 3 100.0%	1 / 1 100.0%	3 / 3 100.0%	3 / 3 100.0%	3 / 3 100.0%	1 / 1 100.0%	1 / 1 100.0%	5 / 5 100.0%	1 / 1 100.0%	5 / 5 100.0%	1 / 1 100.0%	1 / 1 100.0%	1 / 1 100.0%	1 / 1 100.0%	3 / 3 100.0%	1 / 1 100.0%	3 / 3 100.0%	4 / 4 100.0%	2 / 2 100.0%	1 / 1 100.0%	5 / 5 100.0%	1 / 1 100.0%	0 / 0 0.0%	0 / 0 0.0%	0 / 0 0.0%	35 / 53 66.0%AB	17 / 53 32.1%ABE	1 / 53 1.9%BE				
1	Paracoccus denitrificans SD1	32 / 33 97.0%	1 / 1 100.0%	0 / 0 0.0%	1 / 1 100.0%	1 / 1 100.0%	1 / 1 100.0%	3 / 3 100.0%	1 / 1 100.0%	1 / 1 100.0%	3 / 3 100.0%	1 / 1 100.0%	4 / 4 100.0%	1 / 1 100.0%	1 / 1 100.0%	1 / 1 100.0%	0 / 0 0.0%	2 / 2 100.0%	1 / 1 100.0%	2 / 2 100.0%	4 / 4 100.0%	0 / 0 0.0%	1 / 1 100.0%	2 / 2 100.0%	0 / 1 0.0%	0 / 0 0.0%	0 / 0 0.0%	0 / 0 0.0%	22 / 33 66.7%AB	10 / 33 30.3%ABE	1 / 33 3.0%E				
5	Rhodobacter	249 / 258 96.5%	18 / 18 100.0%	10 / 10 100.0%	4 / 4 100.0%	16 / 16 100.0%	18 / 18 100.0%	14 / 15 93.3%	5 / 5 100.0%	1 / 4 25.0%	26 / 26 100.0%	6 / 6 100.0%	19 / 19 100.0%	5 / 5 100.0%	5 / 5 100.0%	8 / 8 100.0%	5 / 5 100.0%	15 / 15 100.0%	5 / 5 100.0%	12 / 15 80.0%	20 / 20 100.0%	9 / 9 100.0%	3 / 4 75.0%	25 / 26 96.2%	0 / 0 0.0%	0 / 0 0.0%	0 / 0 0.0%	0 / 0 0.0%	154 / 258 59.7%AB	95 / 258 36.8%ABE	5 / 258 1.9%	3 / 258 1.2%E	1 / 258 0.4%C		
1	"Rhodonostoc capsulatum" Molisch 1907	51 / 51 100.0%	4 / 4 100.0%	2 / 2 100.0%	1 / 1 100.0%	4 / 4 100.0%	4 / 4 100.0%	3 / 3 100.0%	1 / 1 100.0%	0 / 0 0.0%	6 / 6 100.0%	1 / 1 100.0%	4 / 4 100.0%	1 / 1 100.0%	1 / 1 100.0%	1 / 1 100.0%	1 / 1 100.0%	3 / 3 100.0%	1 / 1 100.0%	3 / 3 100.0%	4 / 4 100.0%	1 / 1 100.0%	0 / 0 0.0%	5 / 5 100.0%	0 / 0 0.0%	0 / 0 0.0%	0 / 0 0.0%	0 / 0 0.0%	32 / 51 62.7%AB	19 / 51 37.3%ABE					
1	Rhodobacter capsulatus SB 1003	51 / 51 100.0%	4 / 4 100.0%	2 / 2 100.0%	1 / 1 100.0%	4 / 4 100.0%	4 / 4 100.0%	3 / 3 100.0%	1 / 1 100.0%	0 / 0 0.0%	6 / 6 100.0%	1 / 1 100.0%	4 / 4 100.0%	1 / 1 100.0%	1 / 1 100.0%	1 / 1 100.0%	1 / 1 100.0%	3 / 3 100.0%	1 / 1 100.0%	3 / 3 100.0%	4 / 4 100.0%	1 / 1 100.0%	0 / 0 0.0%	5 / 5 100.0%	0 / 0 0.0%	0 / 0 0.0%	0 / 0 0.0%	0 / 0 0.0%	32 / 51 62.7%AB	19 / 51 37.3%ABE					
4	"Rhodococcus capsulatus" Molisch 1907	198 / 207 95.7%	14 / 14 100.0%	8 / 8 100.0%	3 / 3 100.0%	12 / 12 100.0%	14 / 14 100.0%	11 / 12 91.7%	4 / 4 100.0%	1 / 4 25.0%	20 / 20 100.0%	5 / 5 100.0%	15 / 15 100.0%	4 / 4 100.0%	4 / 4 100.0%	7 / 7 100.0%	4 / 4 100.0%	12 / 12 100.0%	4 / 4 100.0%	9 / 12 75.0%	16 / 16 100.0%	8 / 8 100.0%	3 / 4 75.0%	20 / 21 95.2%	0 / 0 0.0%	0 / 0 0.0%	0 / 0 0.0%	0 / 0 0.0%	122 / 207 58.9%AB	76 / 207 36.7%ABE	5 / 207 2.4%	3 / 207 1.4%E	1 / 207 0.5%C		
1	Rhodobacter sphaeroides 2.4.1	50 / 52 96.2%	3 / 3 100.0%	2 / 2 100.0%	1 / 1 100.0%	3 / 3 100.0%	4 / 4 100.0%	3 / 3 100.0%	1 / 1 100.0%	0 / 1 0.0%	5 / 5 100.0%	1 / 1 100.0%	4 / 4 100.0%	1 / 1 100.0%	1 / 1 100.0%	2 / 2 100.0%	1 / 1 100.0%	3 / 3 100.0%	1 / 1 100.0%	2 / 3 66.7%	4 / 4 100.0%	2 / 2 100.0%	1 / 1 100.0%	5 / 5 100.0%	0 / 0 0.0%	0 / 0 0.0%	0 / 0 0.0%	0 / 0 0.0%	31 / 52 59.6%AB	19 / 52 36.5%ABE	1 / 52 1.9%E	1 / 52 1.9%			
1	Rhodobacter sphaeroides ATCC 17029	53 / 56 94.6%	4 / 4 100.0%	2 / 2 100.0%	1 / 1 100.0%	4 / 4 100.0%	4 / 4 100.0%	3 / 3 100.0%	1 / 1 100.0%	0 / 1 0.0%	6 / 6 100.0%	2 / 2 100.0%	4 / 4 100.0%	1 / 1 100.0%	1 / 1 100.0%	1 / 1 100.0%	1 / 1 100.0%	3 / 3 100.0%	1 / 1 100.0%	2 / 3 66.7%	4 / 4 100.0%	2 / 2 100.0%	1 / 1 100.0%	5 / 6 83.3%	0 / 0 0.0%	0 / 0 0.0%	0 / 0 0.0%	0 / 0 0.0%	34 / 56 60.7%AB	19 / 56 33.9%ABE	2 / 56 3.6%	1 / 56 1.8%E			
1	Rhodobacter sphaeroides ATCC 17025	43 / 44 97.7%	3 / 3 100.0%	2 / 2 100.0%	0 / 0 0.0%	1 / 1 100.0%	2 / 2 100.0%	3 / 3 100.0%	1 / 1 100.0%	0 / 1 0.0%	3 / 3 100.0%	1 / 1 100.0%	3 / 3 100.0%	1 / 1 100.0%	1 / 1 100.0%	2 / 2 100.0%	1 / 1 100.0%	3 / 3 100.0%	1 / 1 100.0%	3 / 3 100.0%	4 / 4 100.0%	2 / 2 100.0%	1 / 1 100.0%	5 / 5 100.0%	0 / 0 0.0%	0 / 0 0.0%	0 / 0 0.0%	0 / 0 0.0%	26 / 44 59.1%AB	17 / 44 38.6%ABE	1 / 44 2.3%E				
1	Rhodobacter sphaeroides KD131	52 / 55 94.5%	4 / 4 100.0%	2 / 2 100.0%	1 / 1 100.0%	4 / 4 100.0%	4 / 4 100.0%	2 / 3 66.7%	1 / 1 100.0%	1 / 1 100.0%	6 / 6 100.0%	1 / 1 100.0%	4 / 4 100.0%	1 / 1 100.0%	1 / 1 100.0%	2 / 2 100.0%	1 / 1 100.0%	3 / 3 100.0%	1 / 1 100.0%	2 / 3 66.7%	4 / 4 100.0%	2 / 2 100.0%	0 / 1 0.0%	5 / 5 100.0%	0 / 0 0.0%	0 / 0 0.0%	0 / 0 0.0%	0 / 0 0.0%	31 / 55 56.4%AB	21 / 55 38.2%ABE	2 / 55 3.6%	1 / 55 1.8%C			
2	Roseobacter	76 / 76 100.0%	2 / 2 100.0%	2 / 2 100.0%	2 / 2 100.0%	2 / 2 100.0%	6 / 6 100.0%	4 / 4 100.0%	2 / 2 100.0%	2 / 2 100.0%	4 / 4 100.0%	2 / 2 100.0%	8 / 8 100.0%	2 / 2 100.0%	2 / 2 100.0%	2 / 2 100.0%	2 / 2 100.0%	4 / 4 100.0%	0 / 0 0.0%	4 / 4 100.0%	8 / 8 100.0%	2 / 2 100.0%	4 / 4 100.0%	10 / 10 100.0%	0 / 0 0.0%	0 / 0 0.0%	0 / 0 0.0%	0 / 0 0.0%	42 / 76 55.3%AB	34 / 76 44.7%ABE					
1	ATCC 33942	38 / 38 100.0%	1 / 1 100.0%	1 / 1 100.0%	1 / 1 100.0%	1 / 1 100.0%	3 / 3 100.0%	2 / 2 100.0%	1 / 1 100.0%	1 / 1 100.0%	2 / 2 100.0%	1 / 1 100.0%	4 / 4 100.0%	1 / 1 100.0%	1 / 1 100.0%	1 / 1 100.0%	1 / 1 100.0%	2 / 2 100.0%	0 / 0 0.0%	2 / 2 100.0%	4 / 4 100.0%	1 / 1 100.0%	2 / 2 100.0%	5 / 5 100.0%	0 / 0 0.0%	0 / 0 0.0%	0 / 0 0.0%	0 / 0 0.0%	21 / 38 55.3%AB	17 / 38 44.7%ABE					
1	Erythrobacter sp. OCh114	38 / 38 100.0%	1 / 1 100.0%	1 / 1 100.0%	1 / 1 100.0%	1 / 1 100.0%	3 / 3 100.0%	2 / 2 100.0%	1 / 1 100.0%	1 / 1 100.0%	2 / 2 100.0%	1 / 1 100.0%	4 / 4 100.0%	1 / 1 100.0%	1 / 1 100.0%	1 / 1 100.0%	1 / 1 100.0%	2 / 2 100.0%	0 / 0 0.0%	2 / 2 100.0%	4 / 4 100.0%	1 / 1 100.0%	2 / 2 100.0%	5 / 5 100.0%	0 / 0 0.0%	0 / 0 0.0%	0 / 0 0.0%	0 / 0 0.0%	21 / 38 55.3%AB	17 / 38 44.7%ABE					
1	ATCC 49566	38 / 38 100.0%	1 / 1 100.0%	1 / 1 100.0%	1 / 1 100.0%	1 / 1 100.0%	3 / 3 100.0%	2 / 2 100.0%	1 / 1 100.0%	1 / 1 100.0%	2 / 2 100.0%	1 / 1 100.0%	4 / 4 100.0%	1 / 1 100.0%	1 / 1 100.0%	1 / 1 100.0%	1 / 1 100.0%	2 / 2 100.0%	0 / 0 0.0%	2 / 2 100.0%	4 / 4 100.0%	1 / 1 100.0%	2 / 2 100.0%	5 / 5 100.0%	0 / 0 0.0%	0 / 0 0.0%	0 / 0 0.0%	0 / 0 0.0%	21 / 38 55.3%AB	17 / 38 44.7%ABE					
1	Roseobacter litoralis Och 149	38 / 38 100.0%	1 / 1 100.0%	1 / 1 100.0%	1 / 1 100.0%	1 / 1 100.0%	3 / 3 100.0%	2 / 2 100.0%	1 / 1 100.0%	1 / 1 100.0%	2 / 2 100.0%	1 / 1 100.0%	4 / 4 100.0%	1 / 1 100.0%	1 / 1 100.0%	1 / 1 100.0%	1 / 1 100.0%	2 / 2 100.0%	0 / 0 0.0%	2 / 2 100.0%	4 / 4 100.0%	1 / 1 100.0%	2 / 2 100.0%	5 / 5 100.0%	0 / 0 0.0%	0 / 0 0.0%	0 / 0 0.0%	0 / 0 0.0%	21 / 38 55.3%AB	17 / 38 44.7%ABE					
2	Octadecabacter	85 / 85 100.0%	3 / 3 100.0%	4 / 4 100.0%	2 / 2 100.0%	4 / 4 100.0%	6 / 6 100.0%	4 / 4 100.0%	2 / 2 100.0%	0 / 0 0.0%	6 / 6 100.0%	2 / 2 100.0%	8 / 8 100.0%	2 / 2 100.0%	2 / 2 100.0%	2 / 2 100.0%	2 / 2 100.0%	4 / 4 100.0%	0 / 0 0.0%	6 / 6 100.0%	8 / 8 100.0%	2 / 2 100.0%	4 / 4 100.0%	10 / 10 100.0%	0 / 0 0.0%	0 / 0 0.0%	2 / 2 100.0%	0 / 0 0.0%	48 / 85 56.5%AB	37 / 85 43.5%ABE					
1	CIP 106732	42 / 42 100.0%	1 / 1 100.0%	2 / 2 100.0%	1 / 1 100.0%	2 / 2 100.0%	3 / 3 100.0%	2 / 2 100.0%	1 / 1 100.0%	0 / 0 0.0%	3 / 3 100.0%	1 / 1 100.0%	4 / 4 100.0%	1 / 1 100.0%	1 / 1 100.0%	1 / 1 100.0%	1 / 1 100.0%	2 / 2 100.0%	0 / 0 0.0%	3 / 3 100.0%	4 / 4 100.0%	1 / 1 100.0%	2 / 2 100.0%	5 / 5 100.0%	0 / 0 0.0%	0 / 0 0.0%	1 / 1 100.0%	0 / 0 0.0%	24 / 42 57.1%AB	18 / 42 42.9%ABE					
1	Octadecabacter antarcticus 238	42 / 42 100.0%	1 / 1 100.0%	2 / 2 100.0%	1 / 1 100.0%	2 / 2 100.0%	3 / 3 100.0%	2 / 2 100.0%	1 / 1 100.0%	0 / 0 0.0%	3 / 3 100.0%	1 / 1 100.0%	4 / 4 100.0%	1 / 1 100.0%	1 / 1 100.0%	1 / 1 100.0%	1 / 1 100.0%	2 / 2 100.0%	0 / 0 0.0%	3 / 3 100.0%	4 / 4 100.0%	1 / 1 100.0%	2 / 2 100.0%	5 / 5 100.0%	0 / 0 0.0%	0 / 0 0.0%	1 / 1 100.0%	0 / 0 0.0%	24 / 42 57.1%AB	18 / 42 42.9%ABE					
1	CIP 106731	43 / 43 100.0%	2 / 2 100.0%	2 / 2 100.0%	1 / 1 100.0%	2 / 2 100.0%	3 / 3 100.0%	2 / 2 100.0%	1 / 1 100.0%	0 / 0 0.0%	3 / 3 100.0%	1 / 1 100.0%	4 / 4 100.0%	1 / 1 100.0%	1 / 1 100.0%	1 / 1 100.0%	1 / 1 100.0%	2 / 2 100.0%	0 / 0 0.0%	3 / 3 100.0%	4 / 4 100.0%	1 / 1 100.0%	2 / 2 100.0%	5 / 5 100.0%	0 / 0 0.0%	0 / 0 0.0%	1 / 1 100.0%	0 / 0 0.0%	24 / 43 55.8%AB	19 / 43 44.2%ABE					
1	Octadecabacter antarcticus 307	43 / 43 100.0%	2 / 2 100.0%	2 / 2 100.0%	1 / 1 100.0%	2 / 2 100.0%	3 / 3 100.0%	2 / 2 100.0%	1 / 1 100.0%	0 / 0 0.0%	3 / 3 100.0%	1 / 1 100.0%	4 / 4 100.0%	1 / 1 100.0%	1 / 1 100.0%	1 / 1 100.0%	1 / 1 100.0%	2 / 2 100.0%	0 / 0 0.0%	3 / 3 100.0%	4 / 4 100.0%	1 / 1 100.0%	2 / 2 100.0%	5 / 5 100.0%	0 / 0 0.0%	0 / 0 0.0%	1 / 1 100.0%	0 / 0 0.0%	24 / 43 55.8%AB	19 / 43 44.2%ABE					
2	Ketogulonicigenium	110 / 112 98.2%	9 / 9 100.0%	4 / 4 100.0%	4 / 4 100.0%	8 / 8 100.0%	7 / 7 100.0%	6 / 6 100.0%	2 / 2 100.0%	2 / 2 100.0%	12 / 12 100.0%	2 / 2 100.0%	8 / 8 100.0%	4 / 4 100.0%	2 / 2 100.0%	2 / 2 100.0%	2 / 2 100.0%	6 / 6 100.0%	2 / 2 100.0%	6 / 6 100.0%	8 / 8 100.0%	4 / 4 100.0%	2 / 2 100.0%	8 / 10 80.0%	0 / 0 0.0%	0 / 0 0.0%	0 / 0 0.0%	0 / 0 0.0%	71 / 112 63.4%AB	39 / 112 34.8%ABE	2 / 112 1.8%E				
2	DSM 4025	110 / 112 98.2%	9 / 9 100.0%	4 / 4 100.0%	4 / 4 100.0%	8 / 8 100.0%	7 / 7 100.0%	6 / 6 100.0%	2 / 2 100.0%	2 / 2 100.0%	12 / 12 100.0%	2 / 2 100.0%	8 / 8 100.0%	4 / 4 100.0%	2 / 2 100.0%	2 / 2 100.0%	2 / 2 100.0%	6 / 6 100.0%	2 / 2 100.0%	6 / 6 100.0%	8 / 8 100.0%	4 / 4 100.0%	2 / 2 100.0%	8 / 10 80.0%	0 / 0 0.0%	0 / 0 0.0%	0 / 0 0.0%	0 / 0 0.0%	71 / 112 63.4%AB	39 / 112 34.8%ABE	2 / 112 1.8%E				
1	Ketogulonicigenium vulgare WSH-001	54 / 55 98.2%	4 / 4 100.0%	2 / 2 100.0%	2 / 2 100.0%	4 / 4 100.0%	3 / 3 100.0%	3 / 3 100.0%	1 / 1 100.0%	1 / 1 100.0%	6 / 6 100.0%	1 / 1 100.0%	4 / 4 100.0%	2 / 2 100.0%	1 / 1 100.0%	1 / 1 100.0%	1 / 1 100.0%	3 / 3 100.0%	1 / 1 100.0%	3 / 3 100.0%	4 / 4 100.0%	2 / 2 100.0%	1 / 1 100.0%	4 / 5 80.0%	0 / 0 0.0%	0 / 0 0.0%	0 / 0 0.0%	0 / 0 0.0%	35 / 55 63.6%AB	19 / 55 34.5%ABE	1 / 55 1.8%E				
1	Ketogulonicigenium vulgare Y25	56 / 57 98.2%	5 / 5 100.0%	2 / 2 100.0%	2 / 2 100.0%	4 / 4 100.0%	4 / 4 100.0%	3 / 3 100.0%	1 / 1 100.0%	1 / 1 100.0%	6 / 6 100.0%	1 / 1 100.0%	4 / 4 100.0%	2 / 2 100.0%	1 / 1 100.0%	1 / 1 100.0%	1 / 1 100.0%	3 / 3 100.0%	1 / 1 100.0%	3 / 3 100.0%	4 / 4 100.0%	2 / 2 100.0%	1 / 1 100.0%	4 / 5 80.0%	0 / 0 0.0%	0 / 0 0.0%	0 / 0 0.0%	0 / 0 0.0%	36 / 57 63.2%AB	20 / 57 35.1%ABE	1 / 57 1.8%E				
2	Ruegeria	92 / 94 97.9%	3 / 3 100.0%	5 / 5 100.0%	4 / 4 100.0%	4 / 4 100.0%	10 / 10 100.0%	6 / 6 100.0%	2 / 2 100.0%	2 / 2 100.0%	5 / 5 100.0%	2 / 2 100.0%	7 / 7 100.0%	2 / 2 100.0%	0 / 2 0.0%	3 / 3 100.0%	2 / 2 100.0%	6 / 6 100.0%	2 / 2 100.0%	4 / 4 100.0%	7 / 7 100.0%	4 / 4 100.0%	2 / 2 100.0%	10 / 10 100.0%	0 / 0 0.0%	0 / 0 0.0%	0 / 0 0.0%	0 / 0 0.0%	56 / 94 59.6%AB	36 / 94 38.3%ABE	2 / 94 2.1%E				
1	ATCC 700808	51 / 52 98.1%	2 / 2 100.0%	2 / 2 100.0%	2 / 2 100.0%	3 / 3 100.0%	5 / 5 100.0%	3 / 3 100.0%	1 / 1 100.0%	1 / 1 100.0%	4 / 4 100.0%	1 / 1 100.0%	4 / 4 100.0%	1 / 1 100.0%	0 / 1 0.0%	2 / 2 100.0%	1 / 1 100.0%	4 / 4 100.0%	1 / 1 100.0%	2 / 2 100.0%	4 / 4 100.0%	2 / 2 100.0%	1 / 1 100.0%	5 / 5 100.0%	0 / 0 0.0%	0 / 0 0.0%	0 / 0 0.0%	0 / 0 0.0%	31 / 52 59.6%AB	20 / 52 38.5%ABE	1 / 52 1.9%E				
1	Ruegeria pomeroyi DSS-3	51 / 52 98.1%	2 / 2 100.0%	2 / 2 100.0%	2 / 2 100.0%	3 / 3 100.0%	5 / 5 100.0%	3 / 3 100.0%	1 / 1 100.0%	1 / 1 100.0%	4 / 4 100.0%	1 / 1 100.0%	4 / 4 100.0%	1 / 1 100.0%	0 / 1 0.0%	2 / 2 100.0%	1 / 1 100.0%	4 / 4 100.0%	1 / 1 100.0%	2 / 2 100.0%	4 / 4 100.0%	2 / 2 100.0%	1 / 1 100.0%	5 / 5 100.0%	0 / 0 0.0%	0 / 0 0.0%	0 / 0 0.0%	0 / 0 0.0%	31 / 52 59.6%AB	20 / 52 38.5%ABE	1 / 52 1.9%E				
1	Ruegeria sp. TM1040	41 / 42 97.6%	1 / 1 100.0%	3 / 3 100.0%	2 / 2 100.0%	1 / 1 100.0%	5 / 5 100.0%	3 / 3 100.0%	1 / 1 100.0%	1 / 1 100.0%	1 / 1 100.0%	1 / 1 100.0%	3 / 3 100.0%	1 / 1 100.0%	0 / 1 0.0%	1 / 1 100.0%	1 / 1 100.0%	2 / 2 100.0%	1 / 1 100.0%	2 / 2 100.0%	3 / 3 100.0%	2 / 2 100.0%	1 / 1 100.0%	5 / 5 100.0%	0 / 0 0.0%	0 / 0 0.0%	0 / 0 0.0%	0 / 0 0.0%	25 / 42 59.5%AB	16 / 42 38.1%ABE	1 / 42 2.4%E				
1	Jannaschia	42 / 42 100.0%	1 / 1 100.0%	2 / 2 100.0%	1 / 1 100.0%	1 / 1 100.0%	3 / 3 100.0%	3 / 3 100.0%	1 / 1 100.0%	1 / 1 100.0%	2 / 2 100.0%	1 / 1 100.0%	4 / 4 100.0%	1 / 1 100.0%	1 / 1 100.0%	1 / 1 100.0%	1 / 1 100.0%	3 / 3 100.0%	1 / 1 100.0%	3 / 3 100.0%	4 / 4 100.0%	1 / 1 100.0%	1 / 1 100.0%	5 / 5 100.0%	0 / 0 0.0%	0 / 0 0.0%	0 / 0 0.0%	0 / 0 0.0%	23 / 42 54.8%AB	19 / 42 45.2%ABE					
1	Jannaschia sp. CCS1	42 / 42 100.0%	1 / 1 100.0%	2 / 2 100.0%	1 / 1 100.0%	1 / 1 100.0%	3 / 3 100.0%	3 / 3 100.0%	1 / 1 100.0%	1 / 1 100.0%	2 / 2 100.0%	1 / 1 100.0%	4 / 4 100.0%	1 / 1 100.0%	1 / 1 100.0%	1 / 1 100.0%	1 / 1 100.0%	3 / 3 100.0%	1 / 1 100.0%	3 / 3 100.0%	4 / 4 100.0%	1 / 1 100.0%	1 / 1 100.0%	5 / 5 100.0%	0 / 0 0.0%	0 / 0 0.0%	0 / 0 0.0%	0 / 0 0.0%	23 / 42 54.8%AB	19 / 42 45.2%ABE					
1	Pseudovibrio	69 / 69 100.0%	2 / 2 100.0%	4 / 4 100.0%	1 / 1 100.0%	6 / 6 100.0%	3 / 3 100.0%	4 / 4 100.0%	1 / 1 100.0%	2 / 2 100.0%	8 / 8 100.0%	1 / 1 100.0%	4 / 4 100.0%	4 / 4 100.0%	2 / 2 100.0%	3 / 3 100.0%	1 / 1 100.0%	4 / 4 100.0%	1 / 1 100.0%	4 / 4 100.0%	4 / 4 100.0%	4 / 4 100.0%	1 / 1 100.0%	5 / 5 100.0%	0 / 0 0.0%	0 / 0 0.0%	0 / 0 0.0%	0 / 0 0.0%	51 / 69 73.9%AB	18 / 69 26.1%ABE					
1	Pseudovibrio sp. FO-BEG1	69 / 69 100.0%	2 / 2 100.0%	4 / 4 100.0%	1 / 1 100.0%	6 / 6 100.0%	3 / 3 100.0%	4 / 4 100.0%	1 / 1 100.0%	2 / 2 100.0%	8 / 8 100.0%	1 / 1 100.0%	4 / 4 100.0%	4 / 4 100.0%	2 / 2 100.0%	3 / 3 100.0%	1 / 1 100.0%	4 / 4 100.0%	1 / 1 100.0%	4 / 4 100.0%	4 / 4 100.0%	4 / 4 100.0%	1 / 1 100.0%	5 / 5 100.0%	0 / 0 0.0%	0 / 0 0.0%	0 / 0 0.0%	0 / 0 0.0%	51 / 69 73.9%AB	18 / 69 26.1%ABE					
2	Phaeobacter	114 / 114 100.0%	8 / 8 100.0%	6 / 6 100.0%	4 / 4 100.0%	8 / 8 100.0%	10 / 10 100.0%	6 / 6 100.0%	2 / 2 100.0%	2 / 2 100.0%	10 / 10 100.0%	2 / 2 100.0%	8 / 8 100.0%	2 / 2 100.0%	2 / 2 100.0%	2 / 2 100.0%	2 / 2 100.0%	6 / 6 100.0%	2 / 2 100.0%	6 / 6 100.0%	8 / 8 100.0%	4 / 4 100.0%	2 / 2 100.0%	12 / 12 100.0%	0 / 0 0.0%	0 / 0 0.0%	0 / 0 0.0%	0 / 0 0.0%	68 / 114 59.6%AB	46 / 114 40.4%ABE					

1	ATCC 700781	57 / 57 100.0%	4 / 4 100.0%	3 / 3 100.0%	2 / 2 100.0%	4 / 4 100.0%	5 / 5 100.0%	3 / 3 100.0%	1 / 1 100.0%	1 / 1 100.0%	5 / 5 100.0%	1 / 1 100.0%	4 / 4 100.0%	1 / 1 100.0%	1 / 1 100.0%	1 / 1 100.0%	1 / 1 100.0%	3 / 3 100.0%	1 / 1 100.0%	3 / 3 100.0%	4 / 4 100.0%	2 / 2 100.0%	1 / 1 100.0%	6 / 6 100.0%	0 / 0 0.0%	0 / 0 0.0%	0 / 0 0.0%	0 / 0 0.0%	34 / 57 59.6%AB	23 / 57 40.4%ABE						
1	Phaeobacter gallaeciensis 2.10	57 / 57 100.0%	4 / 4 100.0%	3 / 3 100.0%	2 / 2 100.0%	4 / 4 100.0%	5 / 5 100.0%	3 / 3 100.0%	1 / 1 100.0%	1 / 1 100.0%	5 / 5 100.0%	1 / 1 100.0%	4 / 4 100.0%	1 / 1 100.0%	1 / 1 100.0%	1 / 1 100.0%	1 / 1 100.0%	3 / 3 100.0%	1 / 1 100.0%	3 / 3 100.0%	4 / 4 100.0%	2 / 2 100.0%	1 / 1 100.0%	6 / 6 100.0%	0 / 0 0.0%	0 / 0 0.0%	0 / 0 0.0%	0 / 0 0.0%	34 / 57 59.6%AB	23 / 57 40.4%ABE						
1	CECT 7251	57 / 57 100.0%	4 / 4 100.0%	3 / 3 100.0%	2 / 2 100.0%	4 / 4 100.0%	5 / 5 100.0%	3 / 3 100.0%	1 / 1 100.0%	1 / 1 100.0%	5 / 5 100.0%	1 / 1 100.0%	4 / 4 100.0%	1 / 1 100.0%	1 / 1 100.0%	1 / 1 100.0%	1 / 1 100.0%	3 / 3 100.0%	1 / 1 100.0%	3 / 3 100.0%	4 / 4 100.0%	2 / 2 100.0%	1 / 1 100.0%	6 / 6 100.0%	0 / 0 0.0%	0 / 0 0.0%	0 / 0 0.0%	0 / 0 0.0%	34 / 57 59.6%AB	23 / 57 40.4%ABE						
1	Phaeobacter gallaeciensis DSM 17395	57 / 57 100.0%	4 / 4 100.0%	3 / 3 100.0%	2 / 2 100.0%	4 / 4 100.0%	5 / 5 100.0%	3 / 3 100.0%	1 / 1 100.0%	1 / 1 100.0%	5 / 5 100.0%	1 / 1 100.0%	4 / 4 100.0%	1 / 1 100.0%	1 / 1 100.0%	1 / 1 100.0%	1 / 1 100.0%	3 / 3 100.0%	1 / 1 100.0%	3 / 3 100.0%	4 / 4 100.0%	2 / 2 100.0%	1 / 1 100.0%	6 / 6 100.0%	0 / 0 0.0%	0 / 0 0.0%	0 / 0 0.0%	0 / 0 0.0%	34 / 57 59.6%AB	23 / 57 40.4%ABE						
1	Dinoroseobacter	44 / 44 100.0%	1 / 1 100.0%	2 / 2 100.0%	1 / 1 100.0%	2 / 2 100.0%	3 / 3 100.0%	3 / 3 100.0%	1 / 1 100.0%	1 / 1 100.0%	3 / 3 100.0%	1 / 1 100.0%	4 / 4 100.0%	1 / 1 100.0%	1 / 1 100.0%	1 / 1 100.0%	1 / 1 100.0%	3 / 3 100.0%	1 / 1 100.0%	3 / 3 100.0%	4 / 4 100.0%	1 / 1 100.0%	1 / 1 100.0%	5 / 5 100.0%	0 / 0 0.0%	0 / 0 0.0%	0 / 0 0.0%	0 / 0 0.0%	15 / 44 34.1%AB	13 / 44 29.5%B	9 / 44 20.5%ABE	7 / 44 15.9%BE				
1	DSM 16493	44 / 44 100.0%	1 / 1 100.0%	2 / 2 100.0%	1 / 1 100.0%	2 / 2 100.0%	3 / 3 100.0%	3 / 3 100.0%	1 / 1 100.0%	1 / 1 100.0%	3 / 3 100.0%	1 / 1 100.0%	4 / 4 100.0%	1 / 1 100.0%	1 / 1 100.0%	1 / 1 100.0%	1 / 1 100.0%	3 / 3 100.0%	1 / 1 100.0%	3 / 3 100.0%	4 / 4 100.0%	1 / 1 100.0%	1 / 1 100.0%	5 / 5 100.0%	0 / 0 0.0%	0 / 0 0.0%	0 / 0 0.0%	0 / 0 0.0%	15 / 44 34.1%AB	13 / 44 29.5%B	9 / 44 20.5%ABE	7 / 44 15.9%BE				
1	Dinoroseobacter shibae DFL 12	44 / 44 100.0%	1 / 1 100.0%	2 / 2 100.0%	1 / 1 100.0%	2 / 2 100.0%	3 / 3 100.0%	3 / 3 100.0%	1 / 1 100.0%	1 / 1 100.0%	3 / 3 100.0%	1 / 1 100.0%	4 / 4 100.0%	1 / 1 100.0%	1 / 1 100.0%	1 / 1 100.0%	1 / 1 100.0%	3 / 3 100.0%	1 / 1 100.0%	3 / 3 100.0%	4 / 4 100.0%	1 / 1 100.0%	1 / 1 100.0%	5 / 5 100.0%	0 / 0 0.0%	0 / 0 0.0%	0 / 0 0.0%	0 / 0 0.0%	15 / 44 34.1%AB	13 / 44 29.5%B	9 / 44 20.5%ABE	7 / 44 15.9%BE				
3	Hyphomonadaceae	125 / 126 99.2%	4 / 4 100.0%	3 / 3 100.0%	3 / 3 100.0%	5 / 5 100.0%	8 / 8 100.0%	8 / 8 100.0%	3 / 3 100.0%	3 / 3 100.0%	9 / 9 100.0%	3 / 3 100.0%	11 / 11 100.0%	4 / 4 100.0%	3 / 4 75.0%	3 / 3 100.0%	3 / 3 100.0%	8 / 8 100.0%	3 / 3 100.0%	8 / 8 100.0%	12 / 12 100.0%	3 / 3 100.0%	3 / 3 100.0%	14 / 14 100.0%	1 / 1 100.0%	0 / 0 0.0%	0 / 0 0.0%	0 / 0 0.0%	79 / 126 62.7%AB	46 / 126 36.5%ABE	1 / 126 0.8%					
1	Hyphomonas	42 / 43 97.7%	1 / 1 100.0%	1 / 1 100.0%	1 / 1 100.0%	1 / 1 100.0%	3 / 3 100.0%	3 / 3 100.0%	1 / 1 100.0%	1 / 1 100.0%	2 / 2 100.0%	1 / 1 100.0%	4 / 4 100.0%	1 / 1 100.0%	1 / 2 50.0%	1 / 1 100.0%	1 / 1 100.0%	3 / 3 100.0%	1 / 1 100.0%	3 / 3 100.0%	4 / 4 100.0%	1 / 1 100.0%	1 / 1 100.0%	5 / 5 100.0%	1 / 1 100.0%	0 / 0 0.0%	0 / 0 0.0%	0 / 0 0.0%	26 / 43 60.5%AB	16 / 43 37.2%ABE	1 / 43 2.3%					
1	ATCC 15444	42 / 43 97.7%	1 / 1 100.0%	1 / 1 100.0%	1 / 1 100.0%	1 / 1 100.0%	3 / 3 100.0%	3 / 3 100.0%	1 / 1 100.0%	1 / 1 100.0%	2 / 2 100.0%	1 / 1 100.0%	4 / 4 100.0%	1 / 1 100.0%	1 / 2 50.0%	1 / 1 100.0%	1 / 1 100.0%	3 / 3 100.0%	1 / 1 100.0%	3 / 3 100.0%	4 / 4 100.0%	1 / 1 100.0%	1 / 1 100.0%	5 / 5 100.0%	1 / 1 100.0%	0 / 0 0.0%	0 / 0 0.0%	0 / 0 0.0%	26 / 43 60.5%AB	16 / 43 37.2%ABE	1 / 43 2.3%					
1	Hyphomonas neptunium ATCC 15444	42 / 43 97.7%	1 / 1 100.0%	1 / 1 100.0%	1 / 1 100.0%	1 / 1 100.0%	3 / 3 100.0%	3 / 3 100.0%	1 / 1 100.0%	1 / 1 100.0%	2 / 2 100.0%	1 / 1 100.0%	4 / 4 100.0%	1 / 1 100.0%	1 / 2 50.0%	1 / 1 100.0%	1 / 1 100.0%	3 / 3 100.0%	1 / 1 100.0%	3 / 3 100.0%	4 / 4 100.0%	1 / 1 100.0%	1 / 1 100.0%	5 / 5 100.0%	1 / 1 100.0%	0 / 0 0.0%	0 / 0 0.0%	0 / 0 0.0%	26 / 43 60.5%AB	16 / 43 37.2%ABE	1 / 43 2.3%					
1	Hirschia	38 / 38 100.0%	2 / 2 100.0%	1 / 1 100.0%	1 / 1 100.0%	2 / 2 100.0%	2 / 2 100.0%	2 / 2 100.0%	1 / 1 100.0%	1 / 1 100.0%	3 / 3 100.0%	1 / 1 100.0%	3 / 3 100.0%	1 / 1 100.0%	1 / 1 100.0%	1 / 1 100.0%	1 / 1 100.0%	2 / 2 100.0%	1 / 1 100.0%	2 / 2 100.0%	4 / 4 100.0%	1 / 1 100.0%	1 / 1 100.0%	4 / 4 100.0%	0 / 0 0.0%	0 / 0 0.0%	0 / 0 0.0%	0 / 0 0.0%	24 / 38 63.2%AB	14 / 38 36.8%ABE						
1	ATCC 49814	38 / 38 100.0%	2 / 2 100.0%	1 / 1 100.0%	1 / 1 100.0%	2 / 2 100.0%	2 / 2 100.0%	2 / 2 100.0%	1 / 1 100.0%	1 / 1 100.0%	3 / 3 100.0%	1 / 1 100.0%	3 / 3 100.0%	1 / 1 100.0%	1 / 1 100.0%	1 / 1 100.0%	1 / 1 100.0%	2 / 2 100.0%	1 / 1 100.0%	2 / 2 100.0%	4 / 4 100.0%	1 / 1 100.0%	1 / 1 100.0%	4 / 4 100.0%	0 / 0 0.0%	0 / 0 0.0%	0 / 0 0.0%	0 / 0 0.0%	24 / 38 63.2%AB	14 / 38 36.8%ABE						
1	Hirschia baltica ATCC 49814	38 / 38 100.0%	2 / 2 100.0%	1 / 1 100.0%	1 / 1 100.0%	2 / 2 100.0%	2 / 2 100.0%	2 / 2 100.0%	1 / 1 100.0%	1 / 1 100.0%	3 / 3 100.0%	1 / 1 100.0%	3 / 3 100.0%	1 / 1 100.0%	1 / 1 100.0%	1 / 1 100.0%	1 / 1 100.0%	2 / 2 100.0%	1 / 1 100.0%	2 / 2 100.0%	4 / 4 100.0%	1 / 1 100.0%	1 / 1 100.0%	4 / 4 100.0%	0 / 0 0.0%	0 / 0 0.0%	0 / 0 0.0%	0 / 0 0.0%	24 / 38 63.2%AB	14 / 38 36.8%ABE						
1	Maricaulis	45 / 45 100.0%	1 / 1 100.0%	1 / 1 100.0%	1 / 1 100.0%	2 / 2 100.0%	3 / 3 100.0%	3 / 3 100.0%	1 / 1 100.0%	1 / 1 100.0%	4 / 4 100.0%	1 / 1 100.0%	4 / 4 100.0%	2 / 2 100.0%	1 / 1 100.0%	1 / 1 100.0%	1 / 1 100.0%	3 / 3 100.0%	1 / 1 100.0%	3 / 3 100.0%	4 / 4 100.0%	1 / 1 100.0%	1 / 1 100.0%	5 / 5 100.0%	0 / 0 0.0%	0 / 0 0.0%	0 / 0 0.0%	0 / 0 0.0%	29 / 45 64.4%AB	16 / 45 35.6%ABE						
1	ATCC 15268	45 / 45 100.0%	1 / 1 100.0%	1 / 1 100.0%	1 / 1 100.0%	2 / 2 100.0%	3 / 3 100.0%	3 / 3 100.0%	1 / 1 100.0%	1 / 1 100.0%	4 / 4 100.0%	1 / 1 100.0%	4 / 4 100.0%	2 / 2 100.0%	1 / 1 100.0%	1 / 1 100.0%	1 / 1 100.0%	3 / 3 100.0%	1 / 1 100.0%	3 / 3 100.0%	4 / 4 100.0%	1 / 1 100.0%	1 / 1 100.0%	5 / 5 100.0%	0 / 0 0.0%	0 / 0 0.0%	0 / 0 0.0%	0 / 0 0.0%	29 / 45 64.4%AB	16 / 45 35.6%ABE						
1	Maricaulis maris MCS10	45 / 45 100.0%	1 / 1 100.0%	1 / 1 100.0%	1 / 1 100.0%	2 / 2 100.0%	3 / 3 100.0%	3 / 3 100.0%	1 / 1 100.0%	1 / 1 100.0%	4 / 4 100.0%	1 / 1 100.0%	4 / 4 100.0%	2 / 2 100.0%	1 / 1 100.0%	1 / 1 100.0%	1 / 1 100.0%	3 / 3 100.0%	1 / 1 100.0%	3 / 3 100.0%	4 / 4 100.0%	1 / 1 100.0%	1 / 1 100.0%	5 / 5 100.0%	0 / 0 0.0%	0 / 0 0.0%	0 / 0 0.0%	0 / 0 0.0%	29 / 45 64.4%AB	16 / 45 35.6%ABE						
14	'Sphingomonadales'	706 / 708 99.7%	30 / 30 100.0%	19 / 19 100.0%	16 / 16 100.0%	34 / 34 100.0%	42 / 42 100.0%	42 / 42 100.0%	14 / 14 100.0%	14 / 14 100.0%	63 / 63 100.0%	14 / 14 100.0%	56 / 56 100.0%	34 / 34 100.0%	14 / 14 100.0%	33 / 33 100.0%	14 / 14 100.0%	44 / 44 100.0%	14 / 14 100.0%	43 / 43 100.0%	60 / 60 100.0%	29 / 29 100.0%	14 / 14 100.0%	63 / 65 96.9%	0 / 0 0.0%	0 / 0 0.0%	0 / 0 0.0%	0 / 0 0.0%	471 / 708 66.5%AB	235 / 708 33.2%ABE	2 / 708 0.3%E					
13	Sphingomonadaceae	663 / 665 99.7%	29 / 29 100.0%	18 / 18 100.0%	15 / 15 100.0%	33 / 33 100.0%	39 / 39 100.0%	39 / 39 100.0%	13 / 13 100.0%	13 / 13 100.0%	61 / 61 100.0%	13 / 13 100.0%	52 / 52 100.0%	32 / 32 100.0%	13 / 13 100.0%	31 / 31 100.0%	13 / 13 100.0%	41 / 41 100.0%	13 / 13 100.0%	40 / 40 100.0%	56 / 56 100.0%	28 / 28 100.0%	13 / 13 100.0%	58 / 60 96.7%	0 / 0 0.0%	0 / 0 0.0%	0 / 0 0.0%	0 / 0 0.0%	443 / 665 66.6%AB	220 / 665 33.1%ABE	2 / 665 0.3%E					
5	Zymomonas	252 / 253 99.6%	14 / 14 100.0%	5 / 5 100.0%	5 / 5 100.0%	14 / 14 100.0%	15 / 15 100.0%	15 / 15 100.0%	5 / 5 100.0%	5 / 5 100.0%	24 / 24 100.0%	5 / 5 100.0%	20 / 20 100.0%	10 / 10 100.0%	5 / 5 100.0%	15 / 15 100.0%	5 / 5 100.0%	15 / 15 100.0%	5 / 5 100.0%	15 / 15 100.0%	20 / 20 100.0%	10 / 10 100.0%	5 / 5 100.0%	20 / 21 95.2%	0 / 0 0.0%	0 / 0 0.0%	0 / 0 0.0%	0 / 0 0.0%	168 / 253 66.4%AB	84 / 253 33.2%ABE	1 / 253 0.4%E					
5	"Achromobacter anaerobium" (sic) Shimwell 1937	252 / 253 99.6%	14 / 14 100.0%	5 / 5 100.0%	5 / 5 100.0%	14 / 14 100.0%	15 / 15 100.0%	15 / 15 100.0%	5 / 5 100.0%	5 / 5 100.0%	24 / 24 100.0%	5 / 5 100.0%	20 / 20 100.0%	10 / 10 100.0%	5 / 5 100.0%	15 / 15 100.0%	5 / 5 100.0%	15 / 15 100.0%	5 / 5 100.0%	15 / 15 100.0%	20 / 20 100.0%	10 / 10 100.0%	5 / 5 100.0%	20 / 21 95.2%	0 / 0 0.0%	0 / 0 0.0%	0 / 0 0.0%	0 / 0 0.0%	168 / 253 66.4%AB	84 / 253 33.2%ABE	1 / 253 0.4%E					
1	"Zymomonas anaerobia subsp. pomaceae" (sic) Millis 1956	51 / 51 100.0%	3 / 3 100.0%	1 / 1 100.0%	1 / 1 100.0%	3 / 3 100.0%	3 / 3 100.0%	3 / 3 100.0%	1 / 1 100.0%	1 / 1 100.0%	5 / 5 100.0%	1 / 1 100.0%	4 / 4 100.0%	2 / 2 100.0%	1 / 1 100.0%	3 / 3 100.0%	1 / 1 100.0%	3 / 3 100.0%	1 / 1 100.0%	3 / 3 100.0%	4 / 4 100.0%	2 / 2 100.0%	1 / 1 100.0%	4 / 4 100.0%	0 / 0 0.0%	0 / 0 0.0%	0 / 0 0.0%	0 / 0 0.0%	34 / 51 66.7%AB	17 / 51 33.3%ABE						
1	Zymomonas mobilis subsp. pomaceae ATCC 29192	51 / 51 100.0%	3 / 3 100.0%	1 / 1 100.0%	1 / 1 100.0%	3 / 3 100.0%	3 / 3 100.0%	3 / 3 100.0%	1 / 1 100.0%	1 / 1 100.0%	5 / 5 100.0%	1 / 1 100.0%	4 / 4 100.0%	2 / 2 100.0%	1 / 1 100.0%	3 / 3 100.0%	1 / 1 100.0%	3 / 3 100.0%	1 / 1 100.0%	3 / 3 100.0%	4 / 4 100.0%	2 / 2 100.0%	1 / 1 100.0%	4 / 4 100.0%	0 / 0 0.0%	0 / 0 0.0%	0 / 0 0.0%	0 / 0 0.0%	34 / 51 66.7%AB	17 / 51 33.3%ABE						
4	Zymomonas mobilis subsp. mobilis	201 / 202 99.5%	11 / 11 100.0%	4 / 4 100.0%	4 / 4 100.0%	11 / 11 100.0%	12 / 12 100.0%	12 / 12 100.0%	4 / 4 100.0%	4 / 4 100.0%	19 / 19 100.0%	4 / 4 100.0%	16 / 16 100.0%	8 / 8 100.0%	4 / 4 100.0%	12 / 12 100.0%	4 / 4 100.0%	12 / 12 100.0%	4 / 4 100.0%	12 / 12 100.0%	16 / 16 100.0%	8 / 8 100.0%	4 / 4 100.0%	16 / 17 94.1%	0 / 0 0.0%	0 / 0 0.0%	0 / 0 0.0%	0 / 0 0.0%	134 / 202 66.3%AB	67 / 202 33.2%ABE	1 / 202 0.5%E					
1	Zymomonas mobilis subsp. mobilis ATCC 31821	51 / 52 98.1%	3 / 3 100.0%	1 / 1 100.0%	1 / 1 100.0%	3 / 3 100.0%	3 / 3 100.0%	3 / 3 100.0%	1 / 1 100.0%	1 / 1 100.0%	5 / 5 100.0%	1 / 1 100.0%	4 / 4 100.0%	2 / 2 100.0%	1 / 1 100.0%	3 / 3 100.0%	1 / 1 100.0%	3 / 3 100.0%	1 / 1 100.0%	3 / 3 100.0%	4 / 4 100.0%	2 / 2 100.0%	1 / 1 100.0%	4 / 5 80.0%	0 / 0 0.0%	0 / 0 0.0%	0 / 0 0.0%	0 / 0 0.0%	34 / 52 65.4%AB	17 / 52 32.7%ABE	1 / 52 1.9%E					
1	Zymomonas mobilis subsp. mobilis ATCC 10988	48 / 48 100.0%	2 / 2 100.0%	1 / 1 100.0%	1 / 1 100.0%	2 / 2 100.0%	3 / 3 100.0%	3 / 3 100.0%	1 / 1 100.0%	1 / 1 100.0%	4 / 4 100.0%	1 / 1 100.0%	4 / 4 100.0%	2 / 2 100.0%	1 / 1 100.0%	3 / 3 100.0%	1 / 1 100.0%	3 / 3 100.0%	1 / 1 100.0%	3 / 3 100.0%	4 / 4 100.0%	2 / 2 100.0%	1 / 1 100.0%	4 / 4 100.0%	0 / 0 0.0%	0 / 0 0.0%	0 / 0 0.0%	0 / 0 0.0%	32 / 48 66.7%AB	16 / 48 33.3%ABE						
1	Zymomonas mobilis NCIMB 11163	51 / 51 100.0%	3 / 3 100.0%	1 / 1 100.0%	1 / 1 100.0%	3 / 3 100.0%	3 / 3 100.0%	3 / 3 100.0%	1 / 1 100.0%	1 / 1 100.0%	5 / 5 100.0%	1 / 1 100.0%	4 / 4 100.0%	2 / 2 100.0%	1 / 1 100.0%	3 / 3 100.0%	1 / 1 100.0%	3 / 3 100.0%	1 / 1 100.0%	3 / 3 100.0%	4 / 4 100.0%	2 / 2 100.0%	1 / 1 100.0%	4 / 4 100.0%	0 / 0 0.0%	0 / 0 0.0%	0 / 0 0.0%	0 / 0 0.0%	34 / 51 66.7%AB	17 / 51 33.3%ABE						
1	Zymomonas mobilis subsp. mobilis ATCC 29191	51 / 51 100.0%	3 / 3 100.0%	1 / 1 100.0%	1 / 1 100.0%	3 / 3 100.0%	3 / 3 100.0%	3 / 3 100.0%	1 / 1 100.0%	1 / 1 100.0%	5 / 5 100.0%	1 / 1 100.0%	4 / 4 100.0%	2 / 2 100.0%	1 / 1 100.0%	3 / 3 100.0%	1 / 1 100.0%	3 / 3 100.0%	1 / 1 100.0%	3 / 3 100.0%	4 / 4 100.0%	2 / 2 100.0%	1 / 1 100.0%	4 / 4 100.0%	0 / 0 0.0%	0 / 0 0.0%	0 / 0 0.0%	0 / 0 0.0%	34 / 51 66.7%AB	17 / 51 33.3%ABE						
2	Rhizomonas	96 / 97 99.0%	4 / 4 100.0%	3 / 3 100.0%	2 / 2 100.0%	4 / 4 100.0%	6 / 6 100.0%	6 / 6 100.0%	2 / 2 100.0%	2 / 2 100.0%	8 / 8 100.0%	2 / 2 100.0%	8 / 8 100.0%	4 / 4 100.0%	2 / 2 100.0%	4 / 4 100.0%	2 / 2 100.0%	7 / 7 100.0%	2 / 2 100.0%	6 / 6 100.0%	8 / 8 100.0%	4 / 4 100.0%	2 / 2 100.0%	8 / 9 88.9%	0 / 0 0.0%	0 / 0 0.0%	0 / 0 0.0%	0 / 0 0.0%	61 / 97 62.9%AB	35 / 97 36.1%ABE	1 / 97 1.0%E					
1	CCUG 31198	48 / 49 98.0%	2 / 2 100.0%	1 / 1 100.0%	1 / 1 100.0%	2 / 2 100.0%	3 / 3 100.0%	3 / 3 100.0%	1 / 1 100.0%	1 / 1 100.0%	4 / 4 100.0%	1 / 1 100.0%	4 / 4 100.0%	2 / 2 100.0%	1 / 1 100.0%	2 / 2 100.0%	1 / 1 100.0%	4 / 4 100.0%	1 / 1 100.0%	3 / 3 100.0%	4 / 4 100.0%	2 / 2 100.0%	1 / 1 100.0%	4 / 5 80.0%	0 / 0 0.0%	0 / 0 0.0%	0 / 0 0.0%	0 / 0 0.0%	30 / 49 61.2%AB	18 / 49 36.7%ABE	1 / 49 2.0%E					
1	Sphingomonas sp. RW1	48 / 49 98.0%	2 / 2 100.0%	1 / 1 100.0%	1 / 1 100.0%	2 / 2 100.0%	3 / 3 100.0%	3 / 3 100.0%	1 / 1 100.0%	1 / 1 100.0%	4 / 4 100.0%	1 / 1 100.0%	4 / 4 100.0%	2 / 2 100.0%	1 / 1 100.0%	2 / 2 100.0%	1 / 1 100.0%	4 / 4 100.0%	1 / 1 100.0%	3 / 3 100.0%	4 / 4 100.0%	2 / 2 100.0%	1 / 1 100.0%	4 / 5 80.0%	0 / 0 0.0%	0 / 0 0.0%	0 / 0 0.0%	0 / 0 0.0%	30 / 49 61.2%AB	18 / 49 36.7%ABE	1 / 49 2.0%E					
1	Sphingomonas sp. MM-1	48 / 48 100.0%	2 / 2 100.0%	2 / 2 100.0%	1 / 1 100.0%	2 / 2 100.0%	3 / 3 100.0%	3 / 3 100.0%	1 / 1 100.0%	1 / 1 100.0%	4 / 4 100.0%	1 / 1 100.0%	4 / 4 100.0%	2 / 2 100.0%	1 / 1 100.0%	2 / 2 100.0%	1 / 1 100.0%	3 / 3 100.0%	1 / 1 100.0%	3 / 3 100.0%	4 / 4 100.0%	2 / 2 100.0%	1 / 1 100.0%	4 / 4 100.0%	0 / 0 0.0%	0 / 0 0.0%	0 / 0 0.0%	0 / 0 0.0%	31 / 48 64.6%AB	17 / 48 35.4%ABE						
3	Sphingobium	159 / 159 100.0%	4 / 4 100.0%	5 / 5 100.0%	5 / 5 100.0%	8 / 8 100.0%	9 / 9 100.0%	9 / 9 100.0%	3 / 3 100.0%	3 / 3 100.0%	16 / 16 100.0%	3 / 3 100.0%	12 / 12 100.0%	9 / 9 100.0%	3 / 3 100.0%	6 / 6 100.0%	3 / 3 100.0%	10 / 10 100.0%	3 / 3 100.0%	10 / 10 100.0%	14 / 14 100.0%	6 / 6 100.0%	3 / 3 100.0%	15 / 15 100.0%	0 / 0 0.0%	0 / 0 0.0%	0 / 0 0.0%	0 / 0 0.0%	113 / 159 71.1%AB	46 / 159 28.9%ABE						
1	ATCC 33790	55 / 55 100.0%	1 / 1 100.0%	2 / 2 100.0%	2 / 2 100.0%	3 / 3 100.0%	3 / 3 100.0%	3 / 3 100.0%	1 / 1 100.0%	1 / 1 100.0%	6 / 6 100.0%	1 / 1 100.0%	4 / 4 100.0%	3 / 3 100.0%	1 / 1 100.0%	2 / 2 100.0%	1 / 1 100.0%	3 / 3 100.0%	1 / 1 100.0%	4 / 4 100.0%	5 / 5 100.0%	2 / 2 100.0%	1 / 1 100.0%	5 / 5 100.0%	0 / 0 0.0%	0 / 0 0.0%	0 / 0 0.0%	0 / 0 0.0%	40 / 55 72.7%AB	15 / 55 27.3%ABE						
1	FLAVOBACTERIUM SP. (STRAIN ATCC 39723)	55 / 55 100.0%	1 / 1 100.0%	2 / 2 100.0%	2 / 2 100.0%	3 / 3 100.0%	3 / 3 100.0%	3 / 3 100.0%	1 / 1 100.0%	1 / 1 100.0%	6 / 6 100.0%	1 / 1 100.0%	4 / 4 100.0%	3 / 3 100.0%	1 / 1 100.0%	2 / 2 100.0%	1 / 1 100.0%	3 / 3 100.0%	1 / 1 100.0%	4 / 4 100.0%	5 / 5 100.0%	2 / 2 100.0%	1 / 1 100.0%	5 / 5 100.0%	0 / 0 0.0%	0 / 0 0.0%	0 / 0 0.0%	0 / 0 0.0%	40 / 55 72.7%AB	15 / 55 27.3%ABE						
1	CCM 7287	55 / 55 100.0%	1 / 1 100.0%	2 / 2 100.0%	2 / 2 100.0%	3 / 3 100.0%	3 / 3 100.0%	3 / 3 100.0%	1 / 1 100.0%	1 / 1 100.0%	6 / 6 100.0%	1 / 1 100.0%	4 / 4 100.0%	4 / 4 100.0%	1 / 1 100.0%	2 / 2 100.0%	1 / 1 100.0%	3 / 3 100.0%	1 / 1 100.0%	3 / 3 100.0%	5 / 5 100.0%	2 / 2 100.0%	1 / 1 100.0%	5 / 5 100.0%	0 / 0 0.0%	0 / 0 0.0%	0 / 0 0.0%	0 / 0 0.0%	40 / 55 72.7%AB	15 / 55 27.3%ABE						
1	Sphingobium japonicum UT26	55 / 55 100.0%	1 / 1 100.0%	2 / 2 100.0%	2 / 2 100.0%	3 / 3 100.0%	3 / 3 100.0%	3 / 3 100.0%	1 / 1 100.0%	1 / 1 100.0%	6 / 6 100.0%	1 / 1 100.0%	4 / 4 100.0%	4 / 4 100.0%	1 / 1 100.0%	2 / 2 100.0%	1 / 1 100.0%	3 / 3 100.0%	1 / 1 100.0%	3 / 3 100.0%	5 / 5 100.0%	2 / 2 100.0%	1 / 1 100.0%	5 / 5 100.0%	0 / 0 0.0%	0 / 0 0.0%	0 / 0 0.0%	0 / 0 0.0%	40 / 55 72.7%AB	15 / 55 27.3%ABE						
1	Sphingobium sp. SYK-6	49 / 49 100.0%	2 / 2 100.0%	1 / 1 100.0%	1 / 1 100.0%	2 / 2 100.0%	3 / 3 100.0%	3 / 3 100.0%	1 / 1 100.0%	1 / 1 100.0%	4 / 4 100.0%	1 / 1 100.0%	4 / 4 100.0%	2 / 2 100.0%	1 / 1 100.0%	2 / 2 100.0%	1 / 1 100.0%	4 / 4 100.0%	1 / 1 100.0%	3 / 3 100.0%	4 / 4 100.0%	2 / 2 100.0%	1 / 1 100.0%	5 / 5 100.0%	0 / 0 0.0%	0 / 0 0.0%	0 / 0 0.0%	0 / 0 0.0%	33 / 49 67.3%AB	16 / 49 32.7%ABE						
2	Novosphingobium	111 / 111 100.0%	6 / 6 100.0%	4 / 4 100.0%	2 / 2 100.0%	6 / 6 100.0%	6 / 6 100.0%	6 / 6 100.0%	2 / 2 100.0%	2 / 2 100.0%	10 / 10 100.0%	2 / 2 100.0%	8 / 8 100.0%	7 / 7 100.0%	2 / 2 100.0%	4 / 4 100.0%	2 / 2 100.0%	6 / 6 100.0%	2 / 2 100.0%	6 / 6 100.0%	10 / 10 100.0%	6 / 6 100.0%	2 / 2 100.0%	10 / 10 100.0%	0 / 0 0.0%	0 / 0 0.0%	0 / 0 0.0%	0 / 0 0.0%	72 / 111 64.9%AB	39 / 111 35.1%ABE						
1	ATCC 700278	54 / 54 100.0%	3 / 3 100.0%	2 / 2 100.0%	1 / 1 100.0%	3 / 3 100.0%	3 / 3 100.0%	3 / 3 100.0%	1 / 1 100.0%	1 / 1 100.0%	5 / 5 100.0%	1 / 1 100.0%	4 / 4 100.0%	3 / 3 100.0%	1 / 1 100.0%	2 / 2 100.0%	1 / 1 100.0%	3 / 3 100.0%	1 / 1 100.0%	3 / 3 100.0%	5 / 5 100.0%	2 / 2 100.0%	1 / 1 100.0%	5 / 5 100.0%	0 / 0 0.0%	0 / 0 0.0%	0 / 0 0.0%	0 / 0 0.0%	36 / 54 66.7%AB	18 / 54 33.3%ABE						
1	Novosphingobium aromaticivorans DSM 12444	54 / 54 100.0%	3 / 3 100.0%	2 / 2 100.0%	1 / 1 100.0%	3 / 3 100.0%	3 / 3 100.0%	3 / 3 100.0%	1 / 1 100.0%	1 / 1 100.0%	5 / 5 100.0%	1 / 1 100.0%	4 / 4 100.0%	3 / 3 100.0%	1 / 1 100.0%	2 / 2 100.0%	1 / 1 100.0%	3 / 3 100.0%	1 / 1 100.0%	3 / 3 100.0%	5 / 5 100.0%	2 / 2 100.0%	1 / 1 100.0%	5 / 5 100.0%	0 / 0 0.0%	0 / 0 0.0%	0 / 0 0.0%	0 / 0 0.0%	36 / 54 66.7%AB	18 / 54 33.3%ABE						
1	Novosphingobium sp. PP1Y	57 / 57 100.0%	3 / 3 100.0%	2 / 2 100.0%	1 / 1 100.0%	3 / 3 100.0%	3 / 3 100.0%	3 / 3 100.0%	1 / 1 100.0%	1 / 1 100.0%	5 / 5 100.0%	1 / 1 100.0%	4 / 4 100.0%	4 / 4 100.0%	1 / 1 100.0%	2 / 2 100.0%	1 / 1 100.0%	3 / 3 100.0%	1 / 1 100.0%	3 / 3 100.0%	5 / 5 100.0%	4 / 4 100.0%	1 / 1 100.0%	5 / 5 100.0%	0 / 0 0.0%	0 / 0 0.0%	0 / 0 0.0%	0 / 0 0.0%	36 / 57 63.2%AB	21 / 57 36.8%ABE						
1	Sphingopyxis	45 / 45 100.0%	1 / 1 100.0%	1 / 1 100.0%	1 / 1 100.0%	1 / 1 100.0%	3 / 3 100.0%	3 / 3 100.0%	1 / 1 100.0%	1 / 1 100.0%	3 / 3 100.0%	1 / 1 100.0%	4 / 4 100.0%	2 / 2 100.0%	1 / 1 100.0%	2 / 2 100.0%	1 / 1 100.0%	3 / 3 100.0%	1 / 1 100.0%	3 / 3 100.0%	4 / 4 100.0%	2 / 2 100.0%	1 / 1 100.0%	5 / 5 100.0%	0 / 0 0.0%	0 / 0 0.0%	0 / 0 0.0%	0 / 0 0.0%	29 / 45 64.4%AB	16 / 45 35.6%ABE						
1	CCUG 45028	45 / 45 100.0%	1 / 1 100.0%	1 / 1 100.0%	1 / 1 100.0%	1 / 1 100.0%	3 / 3 100.0%	3 / 3 100.0%	1 / 1 100.0%	1 / 1 100.0%	3 / 3 100.0%	1 / 1 100.0%	4 / 4 100.0%	2 / 2 100.0%	1 / 1 100.0%	2 / 2 100.0%	1 / 1 100.0%	3 / 3 100.0%	1 / 1 100.0%	3 / 3 100.0%	4 / 4 100.0%	2 / 2 100.0%	1 / 1 100.0%	5 / 5 100.0%	0 / 0 0.0%	0 / 0 0.0%	0 / 0 0.0%	0 / 0 0.0%	29 / 45 64.4%AB	16 / 45 35.6%ABE						
1	Sphingopyxis alaskensis RB2256	45 / 45 100.0%	1 / 1 100.0%	1 / 1 100.0%	1 / 1 100.0%	1 / 1 100.0%	3 / 3 100.0%	3 / 3 100.0%	1 / 1 100.0%	1 / 1 100.0%	3 / 3 100.0%	1 / 1 100.0%	4 / 4 100.0%	2 / 2 100.0%	1 / 1 100.0%	2 / 2 100.0%	1 / 1 100.0%	3 / 3 100.0%	1 / 1 100.0%	3 / 3 100.0%	4 / 4 100.0%	2 / 2 100.0%	1 / 1 100.0%	5 / 5 100.0%	0 / 0 0.0%	0 / 0 0.0%	0 / 0 0.0%	0 / 0 0.0%	29 / 45 64.4%AB	16 / 45 35.6%ABE						
1	Erythrobacteraceae	43 / 43 100.0%	1 / 1 100.0%	1 / 1 100.0%	1 / 1 100.0%	1 / 1 100.0%	3 / 3 100.0%	3 / 3 100.0%	1 / 1 100.0%	1 / 1 100.0%	2 / 2 100.0%	1 / 1 100.0%	4 / 4 100.0%	2 / 2 100.0%	1 / 1 100.0%	2 / 2 100.0%	1 / 1 100.0%	3 / 3 100.0%	1 / 1 100.0%	3 / 3 100.0%	4 / 4 100.0%	1 / 1 100.0%	1 / 1 100.0%	5 / 5 100.0%	0 / 0 0.0%	0 / 0 0.0%	0 / 0 0.0%	0 / 0 0.0%	28 / 43 65.1%AB	15 / 43 34.9%ABE						
1	Erythrobacter	43 / 43 100.0%	1 / 1 100.0%	1 / 1 100.0%	1 / 1 100.0%	1 / 1 100.0%	3 / 3 100.0%	3 / 3 100.0%	1 / 1 100.0%	1 / 1 100.0%	2 / 2 100.0%	1 / 1 100.0%	4 / 4 100.0%	2 / 2 100.0%	1 / 1 100.0%	2 / 2 100.0%	1 / 1 100.0%	3 / 3 100.0%	1 / 1 100.0%	3 / 3 100.0%	4 / 4 100.0%	1 / 1 100.0%	1 / 1 100.0%	5 / 5 100.0%	0 / 0 0.0%	0 / 0 0.0%	0 / 0 0.0%	0 / 0 0.0%	28 / 43 65.1%AB	15 / 43 34.9%ABE						
1	ATCC 700002	43 / 43 100.0%	1 / 1 100.0%	1 / 1 100.0%	1 / 1 100.0%	1 / 1 100.0%	3 / 3 100.0%	3 / 3 100.0%	1 / 1 100.0%	1 / 1 100.0%	2 / 2 100.0%	1 / 1 100.0%	4 / 4 100.0%	2 / 2 100.0%	1 / 1 100.0%	2 / 2 100.0%	1 / 1 100.0%	3 / 3 100.0%	1 / 1 100.0%	3 / 3 100.0%	4 / 4 100.0%	1 / 1 100.0%	1 / 1 100.0%	5 / 5 100.0%	0 / 0 0.0%	0 / 0 0.0%	0 / 0 0.0%	0 / 0 0.0%	28 / 43 65.1%AB	15 / 43 34.9%ABE						
1	Erythrobacter litoralis HTCC2594	43 / 43 100.0%	1 / 1 100.0%	1 / 1 100.0%	1 / 1 100.0%	1 / 1 100.0%	3 / 3 100.0%	3 / 3 100.0%	1 / 1 100.0%	1 / 1 100.0%	2 / 2 100.0%	1 / 1 100.0%	4 / 4 100.0%	2 / 2 100.0%	1 / 1 100.0%	2 / 2 100.0%	1 / 1 100.0%	3 / 3 100.0%	1 / 1 100.0%	3 / 3 100.0%	4 / 4 100.0%	1 / 1 100.0%	1 / 1 100.0%	5 / 5 100.0%	0 / 0 0.0%	0 / 0 0.0%	0 / 0 0.0%	0 / 0 0.0%	28 / 43 65.1%AB	15 / 43 34.9%ABE						
7	Caulobacter group	338 / 342 98.8%	13 / 13 100.0%	13 / 13 100.0%	10 / 10 100.0%	14 / 14 100.0%	25 / 25 100.0%	21 / 21 100.0%	7 / 7 100.0%	6 / 7 85.7%	28 / 28 100.0%	7 / 7 100.0%	28 / 28 100.0%	14 / 14 100.0%	7 / 7 100.0%	7 / 7 100.0%	7 / 7 100.0%	21 / 21 100.0%	10 / 10 100.0%	21 / 21 100.0%	28 / 28 100.0%	12 / 12 100.0%	6 / 7 85.7%	32 / 34 94.1%	0 / 0 0.0%	0 / 0 0.0%	0 / 0 0.0%	1 / 1 100.0%	188 / 342 55.0%AB	104 / 342 30.4%ABE	30 / 342 8.8%B	16 / 342 4.7%BE	2 / 342 0.6%E	1 / 342 0.3%CE	1 / 342 0.3%C	
7	Caulobacteraceae	338 / 342 98.8%	13 / 13 100.0%	13 / 13 100.0%	10 / 10 100.0%	14 / 14 100.0%	25 / 25 100.0%	21 / 21 100.0%	7 / 7 100.0%	6 / 7 85.7%	28 / 28 100.0%	7 / 7 100.0%	28 / 28 100.0%	14 / 14 100.0%	7 / 7 100.0%	7 / 7 100.0%	7 / 7 100.0%	21 / 21 100.0%	10 / 10 100.0%	21 / 21 100.0%	28 / 28 100.0%	12 / 12 100.0%	6 / 7 85.7%	32 / 34 94.1%	0 / 0 0.0%	0 / 0 0.0%	0 / 0 0.0%	1 / 1 100.0%	188 / 342 55.0%AB	104 / 342 30.4%ABE	30 / 342 8.8%B	16 / 342 4.7%BE	2 / 342 0.6%E	1 / 342 0.3%CE	1 / 342 0.3%C	
1	Phenylobacterium	42 / 43 97.7%	1 / 1 100.0%	1 / 1 100.0%	1 / 1 100.0%	1 / 1 100.0%	3 / 3 100.0%	3 / 3 100.0%	1 / 1 100.0%	1 / 1 100.0%	3 / 3 100.0%	1 / 1 100.0%	4 / 4 100.0%	2 / 2 100.0%	1 / 1 100.0%	1 / 1 100.0%	1 / 1 100.0%	3 / 3 100.0%	1 / 1 100.0%	3 / 3 100.0%	4 / 4 100.0%	1 / 1 100.0%	1 / 1 100.0%	4 / 5 80.0%	0 / 0 0.0%	0 / 0 0.0%	0 / 0 0.0%	0 / 0 0.0%	15 / 43 34.9%B	13 / 43 30.2%AB	9 / 43 20.9%ABE	5 / 43 11.6%BE	1 / 43 2.3%E			
1	"Phenylobacterium zucineum" Zhang et al. 2007	42 / 43 97.7%	1 / 1 100.0%	1 / 1 100.0%	1 / 1 100.0%	1 / 1 100.0%	3 / 3 100.0%	3 / 3 100.0%	1 / 1 100.0%	1 / 1 100.0%	3 / 3 100.0%	1 / 1 100.0%	4 / 4 100.0%	2 / 2 100.0%	1 / 1 100.0%	1 / 1 100.0%	1 / 1 100.0%	3 / 3 100.0%	1 / 1 100.0%	3 / 3 100.0%	4 / 4 100.0%	1 / 1 100.0%	1 / 1 100.0%	4 / 5 80.0%	0 / 0 0.0%	0 / 0 0.0%	0 / 0 0.0%	0 / 0 0.0%	15 / 43 34.9%B	13 / 43 30.2%AB	9 / 43 20.9%ABE	5 / 43 11.6%BE	1 / 43 2.3%E			
1	Phenylobacterium zucineum HLK1	42 / 43 97.7%	1 / 1 100.0%	1 / 1 100.0%	1 / 1 100.0%	1 / 1 100.0%	3 / 3 100.0%	3 / 3 100.0%	1 / 1 100.0%	1 / 1 100.0%	3 / 3 100.0%	1 / 1 100.0%	4 / 4 100.0%	2 / 2 100.0%	1 / 1 100.0%	1 / 1 100.0%	1 / 1 100.0%	3 / 3 100.0%	1 / 1 100.0%	3 / 3 100.0%	4 / 4 100.0%	1 / 1 100.0%	1 / 1 100.0%	4 / 5 80.0%	0 / 0 0.0%	0 / 0 0.0%	0 / 0 0.0%	0 / 0 0.0%	15 / 43 34.9%B	13 / 43 30.2%AB	9 / 43 20.9%ABE	5 / 43 11.6%BE	1 / 43 2.3%E			
4	Caulobacter	202 / 204 99.0%	8 / 8 100.0%	9 / 9 100.0%	7 / 7 100.0%	8 / 8 100.0%	16 / 16 100.0%	12 / 12 100.0%	4 / 4 100.0%	3 / 4 75.0%	16 / 16 100.0%	4 / 4 100.0%	16 / 16 100.0%	8 / 8 100.0%	4 / 4 100.0%	4 / 4 100.0%	4 / 4 100.0%	12 / 12 100.0%	7 / 7 100.0%	12 / 12 100.0%	16 / 16 100.0%	8 / 8 100.0%	4 / 4 100.0%	19 / 20 95.0%	0 / 0 0.0%	0 / 0 0.0%	0 / 0 0.0%	1 / 1 100.0%	116 / 204 56.9%AB	60 / 204 29.4%ABE	15 / 204 7.4%B	11 / 204 5.4%BE	1 / 204 0.5%CE	1 / 204 0.5%E		
1	ATCC 21756	52 / 52 100.0%	2 / 2 100.0%	2 / 2 100.0%	2 / 2 100.0%	2 / 2 100.0%	4 / 4 100.0%	3 / 3 100.0%	1 / 1 100.0%	1 / 1 100.0%	4 / 4 100.0%	1 / 1 100.0%	4 / 4 100.0%	2 / 2 100.0%	1 / 1 100.0%	1 / 1 100.0%	1 / 1 100.0%	3 / 3 100.0%	2 / 2 100.0%	3 / 3 100.0%	4 / 4 100.0%	2 / 2 100.0%	1 / 1 100.0%	5 / 5 100.0%	0 / 0 0.0%	0 / 0 0.0%	0 / 0 0.0%	1 / 1 100.0%	33 / 52 63.5%AB	19 / 52 36.5%ABE						
1	Caulobacter segnis ATCC 21756	52 / 52 100.0%	2 / 2 100.0%	2 / 2 100.0%	2 / 2 100.0%	2 / 2 100.0%	4 / 4 100.0%	3 / 3 100.0%	1 / 1 100.0%	1 / 1 100.0%	4 / 4 100.0%	1 / 1 100.0%	4 / 4 100.0%	2 / 2 100.0%	1 / 1 100.0%	1 / 1 100.0%	1 / 1 100.0%	3 / 3 100.0%	2 / 2 100.0%	3 / 3 100.0%	4 / 4 100.0%	2 / 2 100.0%	1 / 1 100.0%	5 / 5 100.0%	0 / 0 0.0%	0 / 0 0.0%	0 / 0 0.0%	1 / 1 100.0%	33 / 52 63.5%AB	19 / 52 36.5%ABE						
2	CIP 106452	101 / 102 99.0%	4 / 4 100.0%	4 / 4 100.0%	4 / 4 100.0%	4 / 4 100.0%	8 / 8 100.0%	6 / 6 100.0%	2 / 2 100.0%	1 / 2 50.0%	8 / 8 100.0%	2 / 2 100.0%	8 / 8 100.0%	4 / 4 100.0%	2 / 2 100.0%	2 / 2 100.0%	2 / 2 100.0%	6 / 6 100.0%	4 / 4 100.0%	6 / 6 100.0%	8 / 8 100.0%	4 / 4 100.0%	2 / 2 100.0%	10 / 10 100.0%	0 / 0 0.0%	0 / 0 0.0%	0 / 0 0.0%	0 / 0 0.0%	66 / 102 64.7%AB	35 / 102 34.3%ABE	1 / 102 1.0%E					
2	Caulobacter crescentus CB 15	101 / 102 99.0%	4 / 4 100.0%	4 / 4 100.0%	4 / 4 100.0%	4 / 4 100.0%	8 / 8 100.0%	6 / 6 100.0%	2 / 2 100.0%	1 / 2 50.0%	8 / 8 100.0%	2 / 2 100.0%	8 / 8 100.0%	4 / 4 100.0%	2 / 2 100.0%	2 / 2 100.0%	2 / 2 100.0%	6 / 6 100.0%	4 / 4 100.0%	6 / 6 100.0%	8 / 8 100.0%	4 / 4 100.0%	2 / 2 100.0%	10 / 10 100.0%	0 / 0 0.0%	0 / 0 0.0%	0 / 0 0.0%	0 / 0 0.0%	66 / 102 64.7%AB	35 / 102 34.3%ABE	1 / 102 1.0%E					
1	Caulobacter crescentus NA1000	51 / 51 100.0%	2 / 2 100.0%	2 / 2 100.0%	2 / 2 100.0%	2 / 2 100.0%	4 / 4 100.0%	3 / 3 100.0%	1 / 1 100.0%	1 / 1 100.0%	4 / 4 100.0%	1 / 1 100.0%	4 / 4 100.0%	2 / 2 100.0%	1 / 1 100.0%	1 / 1 100.0%	1 / 1 100.0%	3 / 3 100.0%	2 / 2 100.0%	3 / 3 100.0%	4 / 4 100.0%	2 / 2 100.0%	1 / 1 100.0%	5 / 5 100.0%	0 / 0 0.0%	0 / 0 0.0%	0 / 0 0.0%	0 / 0 0.0%	33 / 51 64.7%AB	18 / 51 35.3%ABE						
1	Caulobacter sp. K31	49 / 50 98.0%	2 / 2 100.0%	3 / 3 100.0%	1 / 1 100.0%	2 / 2 100.0%	4 / 4 100.0%	3 / 3 100.0%	1 / 1 100.0%	1 / 1 100.0%	4 / 4 100.0%	1 / 1 100.0%	4 / 4 100.0%	2 / 2 100.0%	1 / 1 100.0%	1 / 1 100.0%	1 / 1 100.0%	3 / 3 100.0%	1 / 1 100.0%	3 / 3 100.0%	4 / 4 100.0%	2 / 2 100.0%	1 / 1 100.0%	4 / 5 80.0%	0 / 0 0.0%	0 / 0 0.0%	0 / 0 0.0%	0 / 0 0.0%	17 / 50 34.0%AB	15 / 50 30.0%B	11 / 50 22.0%BE	6 / 50 12.0%ABE	1 / 50 2.0%CE			
1	Brevundimonas	45 / 46 97.8%	2 / 2 100.0%	1 / 1 100.0%	1 / 1 100.0%	2 / 2 100.0%	3 / 3 100.0%	3 / 3 100.0%	1 / 1 100.0%	1 / 1 100.0%	4 / 4 100.0%	1 / 1 100.0%	4 / 4 100.0%	2 / 2 100.0%	1 / 1 100.0%	1 / 1 100.0%	1 / 1 100.0%	3 / 3 100.0%	1 / 1 100.0%	3 / 3 100.0%	4 / 4 100.0%	1 / 1 100.0%	0 / 1 0.0%	5 / 5 100.0%	0 / 0 0.0%	0 / 0 0.0%	0 / 0 0.0%	0 / 0 0.0%	27 / 46 58.7%AB	18 / 46 39.1%ABE	1 / 46 2.2%C					
1	ATCC 15264	45 / 46 97.8%	2 / 2 100.0%	1 / 1 100.0%	1 / 1 100.0%	2 / 2 100.0%	3 / 3 100.0%	3 / 3 100.0%	1 / 1 100.0%	1 / 1 100.0%	4 / 4 100.0%	1 / 1 100.0%	4 / 4 100.0%	2 / 2 100.0%	1 / 1 100.0%	1 / 1 100.0%	1 / 1 100.0%	3 / 3 100.0%	1 / 1 100.0%	3 / 3 100.0%	4 / 4 100.0%	1 / 1 100.0%	0 / 1 0.0%	5 / 5 100.0%	0 / 0 0.0%	0 / 0 0.0%	0 / 0 0.0%	0 / 0 0.0%	27 / 46 58.7%AB	18 / 46 39.1%ABE	1 / 46 2.2%C					
1	Brevundimonas subvibrioides ATCC 15264	45 / 46 97.8%	2 / 2 100.0%	1 / 1 100.0%	1 / 1 100.0%	2 / 2 100.0%	3 / 3 100.0%	3 / 3 100.0%	1 / 1 100.0%	1 / 1 100.0%	4 / 4 100.0%	1 / 1 100.0%	4 / 4 100.0%	2 / 2 100.0%	1 / 1 100.0%	1 / 1 100.0%	1 / 1 100.0%	3 / 3 100.0%	1 / 1 100.0%	3 / 3 100.0%	4 / 4 100.0%	1 / 1 100.0%	0 / 1 0.0%	5 / 5 100.0%	0 / 0 0.0%	0 / 0 0.0%	0 / 0 0.0%	0 / 0 0.0%	27 / 46 58.7%AB	18 / 46 39.1%ABE	1 / 46 2.2%C					
1	Asticcacaulis	49 / 49 100.0%	2 / 2 100.0%	2 / 2 100.0%	1 / 1 100.0%	3 / 3 100.0%	3 / 3 100.0%	3 / 3 100.0%	1 / 1 100.0%	1 / 1 100.0%	5 / 5 100.0%	1 / 1 100.0%	4 / 4 100.0%	2 / 2 100.0%	1 / 1 100.0%	1 / 1 100.0%	1 / 1 100.0%	3 / 3 100.0%	1 / 1 100.0%	3 / 3 100.0%	4 / 4 100.0%	2 / 2 100.0%	1 / 1 100.0%	4 / 4 100.0%	0 / 0 0.0%	0 / 0 0.0%	0 / 0 0.0%	0 / 0 0.0%	32 / 49 65.3%AB	17 / 49 34.7%ABE						
1	ATCC 15261	49 / 49 100.0%	2 / 2 100.0%	2 / 2 100.0%	1 / 1 100.0%	3 / 3 100.0%	3 / 3 100.0%	3 / 3 100.0%	1 / 1 100.0%	1 / 1 100.0%	5 / 5 100.0%	1 / 1 100.0%	4 / 4 100.0%	2 / 2 100.0%	1 / 1 100.0%	1 / 1 100.0%	1 / 1 100.0%	3 / 3 100.0%	1 / 1 100.0%	3 / 3 100.0%	4 / 4 100.0%	2 / 2 100.0%	1 / 1 100.0%	4 / 4 100.0%	0 / 0 0.0%	0 / 0 0.0%	0 / 0 0.0%	0 / 0 0.0%	32 / 49 65.3%AB	17 / 49 34.7%ABE						
1	Asticcacaulis excentricus ACM 1263	49 / 49 100.0%	2 / 2 100.0%	2 / 2 100.0%	1 / 1 100.0%	3 / 3 100.0%	3 / 3 100.0%	3 / 3 100.0%	1 / 1 100.0%	1 / 1 100.0%	5 / 5 100.0%	1 / 1 100.0%	4 / 4 100.0%	2 / 2 100.0%	1 / 1 100.0%	1 / 1 100.0%	1 / 1 100.0%	3 / 3 100.0%	1 / 1 100.0%	3 / 3 100.0%	4 / 4 100.0%	2 / 2 100.0%	1 / 1 100.0%	4 / 4 100.0%	0 / 0 0.0%	0 / 0 0.0%	0 / 0 0.0%	0 / 0 0.0%	32 / 49 65.3%AB	17 / 49 34.7%ABE						
1	'Parvularculales'	43 / 43 100.0%	1 / 1 100.0%	1 / 1 100.0%	1 / 1 100.0%	1 / 1 100.0%	3 / 3 100.0%	3 / 3 100.0%	1 / 1 100.0%	1 / 1 100.0%	3 / 3 100.0%	1 / 1 100.0%	4 / 4 100.0%	1 / 1 100.0%	1 / 1 100.0%	1 / 1 100.0%	1 / 1 100.0%	3 / 3 100.0%	1 / 1 100.0%	3 / 3 100.0%	5 / 5 100.0%	1 / 1 100.0%	1 / 1 100.0%	5 / 5 100.0%	0 / 0 0.0%	0 / 0 0.0%	0 / 0 0.0%	0 / 0 0.0%	29 / 43 67.4%AB	14 / 43 32.6%ABE						
1	'Parvularculaceae'	43 / 43 100.0%	1 / 1 100.0%	1 / 1 100.0%	1 / 1 100.0%	1 / 1 100.0%	3 / 3 100.0%	3 / 3 100.0%	1 / 1 100.0%	1 / 1 100.0%	3 / 3 100.0%	1 / 1 100.0%	4 / 4 100.0%	1 / 1 100.0%	1 / 1 100.0%	1 / 1 100.0%	1 / 1 100.0%	3 / 3 100.0%	1 / 1 100.0%	3 / 3 100.0%	5 / 5 100.0%	1 / 1 100.0%	1 / 1 100.0%	5 / 5 100.0%	0 / 0 0.0%	0 / 0 0.0%	0 / 0 0.0%	0 / 0 0.0%	29 / 43 67.4%AB	14 / 43 32.6%ABE						
1	Parvularcula	43 / 43 100.0%	1 / 1 100.0%	1 / 1 100.0%	1 / 1 100.0%	1 / 1 100.0%	3 / 3 100.0%	3 / 3 100.0%	1 / 1 100.0%	1 / 1 100.0%	3 / 3 100.0%	1 / 1 100.0%	4 / 4 100.0%	1 / 1 100.0%	1 / 1 100.0%	1 / 1 100.0%	1 / 1 100.0%	3 / 3 100.0%	1 / 1 100.0%	3 / 3 100.0%	5 / 5 100.0%	1 / 1 100.0%	1 / 1 100.0%	5 / 5 100.0%	0 / 0 0.0%	0 / 0 0.0%	0 / 0 0.0%	0 / 0 0.0%	29 / 43 67.4%AB	14 / 43 32.6%ABE						
1	ATCC BAA-594	43 / 43 100.0%	1 / 1 100.0%	1 / 1 100.0%	1 / 1 100.0%	1 / 1 100.0%	3 / 3 100.0%	3 / 3 100.0%	1 / 1 100.0%	1 / 1 100.0%	3 / 3 100.0%	1 / 1 100.0%	4 / 4 100.0%	1 / 1 100.0%	1 / 1 100.0%	1 / 1 100.0%	1 / 1 100.0%	3 / 3 100.0%	1 / 1 100.0%	3 / 3 100.0%	5 / 5 100.0%	1 / 1 100.0%	1 / 1 100.0%	5 / 5 100.0%	0 / 0 0.0%	0 / 0 0.0%	0 / 0 0.0%	0 / 0 0.0%	29 / 43 67.4%AB	14 / 43 32.6%ABE						
1	Parvularcula bermudensis HTCC2503	43 / 43 100.0%	1 / 1 100.0%	1 / 1 100.0%	1 / 1 100.0%	1 / 1 100.0%	3 / 3 100.0%	3 / 3 100.0%	1 / 1 100.0%	1 / 1 100.0%	3 / 3 100.0%	1 / 1 100.0%	4 / 4 100.0%	1 / 1 100.0%	1 / 1 100.0%	1 / 1 100.0%	1 / 1 100.0%	3 / 3 100.0%	1 / 1 100.0%	3 / 3 100.0%	5 / 5 100.0%	1 / 1 100.0%	1 / 1 100.0%	5 / 5 100.0%	0 / 0 0.0%	0 / 0 0.0%	0 / 0 0.0%	0 / 0 0.0%	29 / 43 67.4%AB	14 / 43 32.6%ABE						
1	Magnetococcales	45 / 45 100.0%	2 / 2 100.0%	1 / 1 100.0%	1 / 1 100.0%	1 / 1 100.0%	4 / 4 100.0%	3 / 3 100.0%	1 / 1 100.0%	1 / 1 100.0%	3 / 3 100.0%	1 / 1 100.0%	4 / 4 100.0%	1 / 1 100.0%	1 / 1 100.0%	1 / 1 100.0%	1 / 1 100.0%	3 / 3 100.0%	1 / 1 100.0%	3 / 3 100.0%	4 / 4 100.0%	2 / 2 100.0%	1 / 1 100.0%	5 / 5 100.0%	0 / 0 0.0%	0 / 0 0.0%	0 / 0 0.0%	0 / 0 0.0%	25 / 45 55.6%AB	20 / 45 44.4%ABE						
1	Magnetococcaceae	45 / 45 100.0%	2 / 2 100.0%	1 / 1 100.0%	1 / 1 100.0%	1 / 1 100.0%	4 / 4 100.0%	3 / 3 100.0%	1 / 1 100.0%	1 / 1 100.0%	3 / 3 100.0%	1 / 1 100.0%	4 / 4 100.0%	1 / 1 100.0%	1 / 1 100.0%	1 / 1 100.0%	1 / 1 100.0%	3 / 3 100.0%	1 / 1 100.0%	3 / 3 100.0%	4 / 4 100.0%	2 / 2 100.0%	1 / 1 100.0%	5 / 5 100.0%	0 / 0 0.0%	0 / 0 0.0%	0 / 0 0.0%	0 / 0 0.0%	25 / 45 55.6%AB	20 / 45 44.4%ABE						
1	Magnetococcus	45 / 45 100.0%	2 / 2 100.0%	1 / 1 100.0%	1 / 1 100.0%	1 / 1 100.0%	4 / 4 100.0%	3 / 3 100.0%	1 / 1 100.0%	1 / 1 100.0%	3 / 3 100.0%	1 / 1 100.0%	4 / 4 100.0%	1 / 1 100.0%	1 / 1 100.0%	1 / 1 100.0%	1 / 1 100.0%	3 / 3 100.0%	1 / 1 100.0%	3 / 3 100.0%	4 / 4 100.0%	2 / 2 100.0%	1 / 1 100.0%	5 / 5 100.0%	0 / 0 0.0%	0 / 0 0.0%	0 / 0 0.0%	0 / 0 0.0%	25 / 45 55.6%AB	20 / 45 44.4%ABE						
1	ATCC BAA-1437	45 / 45 100.0%	2 / 2 100.0%	1 / 1 100.0%	1 / 1 100.0%	1 / 1 100.0%	4 / 4 100.0%	3 / 3 100.0%	1 / 1 100.0%	1 / 1 100.0%	3 / 3 100.0%	1 / 1 100.0%	4 / 4 100.0%	1 / 1 100.0%	1 / 1 100.0%	1 / 1 100.0%	1 / 1 100.0%	3 / 3 100.0%	1 / 1 100.0%	3 / 3 100.0%	4 / 4 100.0%	2 / 2 100.0%	1 / 1 100.0%	5 / 5 100.0%	0 / 0 0.0%	0 / 0 0.0%	0 / 0 0.0%	0 / 0 0.0%	25 / 45 55.6%AB	20 / 45 44.4%ABE						
1	Magnetococcus marinus MC-1	45 / 45 100.0%	2 / 2 100.0%	1 / 1 100.0%	1 / 1 100.0%	1 / 1 100.0%	4 / 4 100.0%	3 / 3 100.0%	1 / 1 100.0%	1 / 1 100.0%	3 / 3 100.0%	1 / 1 100.0%	4 / 4 100.0%	1 / 1 100.0%	1 / 1 100.0%	1 / 1 100.0%	1 / 1 100.0%	3 / 3 100.0%	1 / 1 100.0%	3 / 3 100.0%	4 / 4 100.0%	2 / 2 100.0%	1 / 1 100.0%	5 / 5 100.0%	0 / 0 0.0%	0 / 0 0.0%	0 / 0 0.0%	0 / 0 0.0%	25 / 45 55.6%AB	20 / 45 44.4%ABE						
143	'Betaproteobacteria'	7654 / 7809 98.0%	224 / 230 97.4%	311 / 313 99.4%	245 / 247 99.2%	475 / 478 99.4%	558 / 565 98.8%	479 / 481 99.6%	150 / 151 99.3%	143 / 145 98.6%	739 / 758 97.5%	142 / 146 97.3%	548 / 563 97.3%	289 / 300 96.3%	145 / 148 98.0%	179 / 182 98.4%	155 / 159 97.5%	408 / 410 99.5%	107 / 109 98.2%	435 / 441 98.6%	638 / 669 95.4%	272 / 274 99.3%	167 / 171 97.7%	808 / 830 97.3%	37 / 39 94.9%	0 / 0 0.0%	0 / 0 0.0%	0 / 0 0.0%	4898 / 7809 62.7%AB	2298 / 7809 29.4%ABE	301 / 7809 3.9%B	157 / 7809 2.0%BE	98 / 7809 1.3%	47 / 7809 0.6%E	9 / 7809 0.1%CE	1 / 7809 0.0%C
5	'Nitrosomonadales'	200 / 201 99.5%	5 / 5 100.0%	5 / 5 100.0%	5 / 5 100.0%	5 / 5 100.0%	12 / 13 92.3%	13 / 13 100.0%	5 / 5 100.0%	5 / 5 100.0%	11 / 11 100.0%	5 / 5 100.0%	20 / 20 100.0%	6 / 6 100.0%	5 / 5 100.0%	5 / 5 100.0%	5 / 5 100.0%	13 / 13 100.0%	5 / 5 100.0%	15 / 15 100.0%	20 / 20 100.0%	5 / 5 100.0%	5 / 5 100.0%	25 / 25 100.0%	0 / 0 0.0%	0 / 0 0.0%	0 / 0 0.0%	0 / 0 0.0%	135 / 201 67.2%AB	65 / 201 32.3%ABE	1 / 201 0.5%					
5	'Nitrosomonadaceae'	200 / 201 99.5%	5 / 5 100.0%	5 / 5 100.0%	5 / 5 100.0%	5 / 5 100.0%	12 / 13 92.3%	13 / 13 100.0%	5 / 5 100.0%	5 / 5 100.0%	11 / 11 100.0%	5 / 5 100.0%	20 / 20 100.0%	6 / 6 100.0%	5 / 5 100.0%	5 / 5 100.0%	5 / 5 100.0%	13 / 13 100.0%	5 / 5 100.0%	15 / 15 100.0%	20 / 20 100.0%	5 / 5 100.0%	5 / 5 100.0%	25 / 25 100.0%	0 / 0 0.0%	0 / 0 0.0%	0 / 0 0.0%	0 / 0 0.0%	135 / 201 67.2%AB	65 / 201 32.3%ABE	1 / 201 0.5%					
4	"Nitrosomonas" Winogradsky 1892 emend. Campbell et al. 2011	157 / 158 99.4%	4 / 4 100.0%	4 / 4 100.0%	4 / 4 100.0%	4 / 4 100.0%	9 / 10 90.0%	10 / 10 100.0%	4 / 4 100.0%	4 / 4 100.0%	8 / 8 100.0%	4 / 4 100.0%	16 / 16 100.0%	4 / 4 100.0%	4 / 4 100.0%	4 / 4 100.0%	4 / 4 100.0%	10 / 10 100.0%	4 / 4 100.0%	12 / 12 100.0%	16 / 16 100.0%	4 / 4 100.0%	4 / 4 100.0%	20 / 20 100.0%	0 / 0 0.0%	0 / 0 0.0%	0 / 0 0.0%	0 / 0 0.0%	105 / 158 66.5%AB	52 / 158 32.9%ABE	1 / 158 0.6%					
1	"Bacterium nitrosomonas" Lehmann and Neumann 1899	41 / 41 100.0%	1 / 1 100.0%	1 / 1 100.0%	1 / 1 100.0%	1 / 1 100.0%	3 / 3 100.0%	3 / 3 100.0%	1 / 1 100.0%	1 / 1 100.0%	2 / 2 100.0%	1 / 1 100.0%	4 / 4 100.0%	1 / 1 100.0%	1 / 1 100.0%	1 / 1 100.0%	1 / 1 100.0%	3 / 3 100.0%	1 / 1 100.0%	3 / 3 100.0%	4 / 4 100.0%	1 / 1 100.0%	1 / 1 100.0%	5 / 5 100.0%	0 / 0 0.0%	0 / 0 0.0%	0 / 0 0.0%	0 / 0 0.0%	28 / 41 68.3%AB	13 / 41 31.7%ABE						
1	Nitrosomonas europaea ATCC 19718	41 / 41 100.0%	1 / 1 100.0%	1 / 1 100.0%	1 / 1 100.0%	1 / 1 100.0%	3 / 3 100.0%	3 / 3 100.0%	1 / 1 100.0%	1 / 1 100.0%	2 / 2 100.0%	1 / 1 100.0%	4 / 4 100.0%	1 / 1 100.0%	1 / 1 100.0%	1 / 1 100.0%	1 / 1 100.0%	3 / 3 100.0%	1 / 1 100.0%	3 / 3 100.0%	4 / 4 100.0%	1 / 1 100.0%	1 / 1 100.0%	5 / 5 100.0%	0 / 0 0.0%	0 / 0 0.0%	0 / 0 0.0%	0 / 0 0.0%	28 / 41 68.3%AB	13 / 41 31.7%ABE						
1	Nitrosomonas eutropha	40 / 41 97.6%	1 / 1 100.0%	1 / 1 100.0%	1 / 1 100.0%	1 / 1 100.0%	2 / 3 66.7%	3 / 3 100.0%	1 / 1 100.0%	1 / 1 100.0%	2 / 2 100.0%	1 / 1 100.0%	4 / 4 100.0%	1 / 1 100.0%	1 / 1 100.0%	1 / 1 100.0%	1 / 1 100.0%	3 / 3 100.0%	1 / 1 100.0%	3 / 3 100.0%	4 / 4 100.0%	1 / 1 100.0%	1 / 1 100.0%	5 / 5 100.0%	0 / 0 0.0%	0 / 0 0.0%	0 / 0 0.0%	0 / 0 0.0%	27 / 41 65.9%AB	13 / 41 31.7%ABE	1 / 41 2.4%					

1	Nitrosomonas eutropha C71	40 / 41 97.6%	1 / 1 100.0%	1 / 1 100.0%	1 / 1 100.0%	1 / 1 100.0%	2 / 3 66.7%	3 / 3 100.0%	1 / 1 100.0%	1 / 1 100.0%	2 / 2 100.0%	1 / 1 100.0%	4 / 4 100.0%	1 / 1 100.0%	1 / 1 100.0%	1 / 1 100.0%	1 / 1 100.0%	3 / 3 100.0%	1 / 1 100.0%	3 / 3 100.0%	4 / 4 100.0%	1 / 1 100.0%	1 / 1 100.0%	5 / 5 100.0%	0 / 0 0.0%	0 / 0 0.0%	0 / 0 0.0%	0 / 0 0.0%	27 / 41 65.9%AB	13 / 41 31.7%ABE	1 / 41 2.4%					
1	Nitrosomonas sp. AL212	38 / 38 100.0%	1 / 1 100.0%	1 / 1 100.0%	1 / 1 100.0%	1 / 1 100.0%	2 / 2 100.0%	2 / 2 100.0%	1 / 1 100.0%	1 / 1 100.0%	2 / 2 100.0%	1 / 1 100.0%	4 / 4 100.0%	1 / 1 100.0%	1 / 1 100.0%	1 / 1 100.0%	1 / 1 100.0%	2 / 2 100.0%	1 / 1 100.0%	3 / 3 100.0%	4 / 4 100.0%	1 / 1 100.0%	1 / 1 100.0%	5 / 5 100.0%	0 / 0 0.0%	0 / 0 0.0%	0 / 0 0.0%	0 / 0 0.0%	25 / 38 65.8%AB	13 / 38 34.2%ABE						
1	Nitrosomonas sp. Is79A3	38 / 38 100.0%	1 / 1 100.0%	1 / 1 100.0%	1 / 1 100.0%	1 / 1 100.0%	2 / 2 100.0%	2 / 2 100.0%	1 / 1 100.0%	1 / 1 100.0%	2 / 2 100.0%	1 / 1 100.0%	4 / 4 100.0%	1 / 1 100.0%	1 / 1 100.0%	1 / 1 100.0%	1 / 1 100.0%	2 / 2 100.0%	1 / 1 100.0%	3 / 3 100.0%	4 / 4 100.0%	1 / 1 100.0%	1 / 1 100.0%	5 / 5 100.0%	0 / 0 0.0%	0 / 0 0.0%	0 / 0 0.0%	0 / 0 0.0%	25 / 38 65.8%AB	13 / 38 34.2%ABE						
1	Nitrosolobus	43 / 43 100.0%	1 / 1 100.0%	1 / 1 100.0%	1 / 1 100.0%	1 / 1 100.0%	3 / 3 100.0%	3 / 3 100.0%	1 / 1 100.0%	1 / 1 100.0%	3 / 3 100.0%	1 / 1 100.0%	4 / 4 100.0%	2 / 2 100.0%	1 / 1 100.0%	1 / 1 100.0%	1 / 1 100.0%	3 / 3 100.0%	1 / 1 100.0%	3 / 3 100.0%	4 / 4 100.0%	1 / 1 100.0%	1 / 1 100.0%	5 / 5 100.0%	0 / 0 0.0%	0 / 0 0.0%	0 / 0 0.0%	0 / 0 0.0%	30 / 43 69.8%AB	13 / 43 30.2%ABE						
1	ATCC 25196	43 / 43 100.0%	1 / 1 100.0%	1 / 1 100.0%	1 / 1 100.0%	1 / 1 100.0%	3 / 3 100.0%	3 / 3 100.0%	1 / 1 100.0%	1 / 1 100.0%	3 / 3 100.0%	1 / 1 100.0%	4 / 4 100.0%	2 / 2 100.0%	1 / 1 100.0%	1 / 1 100.0%	1 / 1 100.0%	3 / 3 100.0%	1 / 1 100.0%	3 / 3 100.0%	4 / 4 100.0%	1 / 1 100.0%	1 / 1 100.0%	5 / 5 100.0%	0 / 0 0.0%	0 / 0 0.0%	0 / 0 0.0%	0 / 0 0.0%	30 / 43 69.8%AB	13 / 43 30.2%ABE						
1	Nitrosospira multiformis ATCC 25196	43 / 43 100.0%	1 / 1 100.0%	1 / 1 100.0%	1 / 1 100.0%	1 / 1 100.0%	3 / 3 100.0%	3 / 3 100.0%	1 / 1 100.0%	1 / 1 100.0%	3 / 3 100.0%	1 / 1 100.0%	4 / 4 100.0%	2 / 2 100.0%	1 / 1 100.0%	1 / 1 100.0%	1 / 1 100.0%	3 / 3 100.0%	1 / 1 100.0%	3 / 3 100.0%	4 / 4 100.0%	1 / 1 100.0%	1 / 1 100.0%	5 / 5 100.0%	0 / 0 0.0%	0 / 0 0.0%	0 / 0 0.0%	0 / 0 0.0%	30 / 43 69.8%AB	13 / 43 30.2%ABE						
92	'Burkholderiales'	5115 / 5196 98.4%	172 / 174 98.9%	217 / 218 99.5%	162 / 163 99.4%	313 / 315 99.4%	373 / 377 98.9%	329 / 330 99.7%	98 / 98 100.0%	93 / 94 98.9%	495 / 511 96.9%	94 / 95 98.9%	360 / 366 98.4%	193 / 197 98.0%	95 / 97 97.9%	99 / 101 98.0%	107 / 110 97.3%	276 / 277 99.6%	65 / 66 98.5%	275 / 277 99.3%	439 / 446 98.4%	169 / 170 99.4%	114 / 117 97.4%	541 / 560 96.6%	36 / 37 97.3%	0 / 0 0.0%	0 / 0 0.0%	0 / 0 0.0%	3188 / 5196 61.4%AB	1519 / 5196 29.2%ABE	265 / 5196 5.1%B	143 / 5196 2.8%BE	43 / 5196 0.8%	32 / 5196 0.6%E	5 / 5196 0.1%CE	1 / 5196 0.0%C
18	Alcaligenaceae	890 / 900 98.9%	30 / 30 100.0%	27 / 27 100.0%	33 / 33 100.0%	50 / 50 100.0%	65 / 66 98.5%	50 / 50 100.0%	18 / 18 100.0%	18 / 18 100.0%	88 / 88 100.0%	18 / 18 100.0%	58 / 61 95.1%	32 / 32 100.0%	19 / 19 100.0%	17 / 18 94.4%	20 / 20 100.0%	47 / 47 100.0%	1 / 1 100.0%	51 / 51 100.0%	86 / 88 97.7%	27 / 27 100.0%	34 / 35 97.1%	97 / 99 98.0%	4 / 4 100.0%	0 / 0 0.0%	0 / 0 0.0%	0 / 0 0.0%	587 / 900 65.2%AB	275 / 900 30.6%ABE	18 / 900 2.0%B	10 / 900 1.1%BE	6 / 900 0.7%E	4 / 900 0.4%		
2	Achromobacter	113 / 113 100.0%	4 / 4 100.0%	4 / 4 100.0%	4 / 4 100.0%	4 / 4 100.0%	10 / 10 100.0%	6 / 6 100.0%	2 / 2 100.0%	2 / 2 100.0%	10 / 10 100.0%	2 / 2 100.0%	8 / 8 100.0%	4 / 4 100.0%	3 / 3 100.0%	2 / 2 100.0%	4 / 4 100.0%	6 / 6 100.0%	0 / 0 0.0%	6 / 6 100.0%	12 / 12 100.0%	4 / 4 100.0%	4 / 4 100.0%	10 / 10 100.0%	2 / 2 100.0%	0 / 0 0.0%	0 / 0 0.0%	0 / 0 0.0%	75 / 113 66.4%AB	38 / 113 33.6%ABE						
2	"Achromobacter xylosoxidans" Yabuuchi and Ohyama 1971	113 / 113 100.0%	4 / 4 100.0%	4 / 4 100.0%	4 / 4 100.0%	4 / 4 100.0%	10 / 10 100.0%	6 / 6 100.0%	2 / 2 100.0%	2 / 2 100.0%	10 / 10 100.0%	2 / 2 100.0%	8 / 8 100.0%	4 / 4 100.0%	3 / 3 100.0%	2 / 2 100.0%	4 / 4 100.0%	6 / 6 100.0%	0 / 0 0.0%	6 / 6 100.0%	12 / 12 100.0%	4 / 4 100.0%	4 / 4 100.0%	10 / 10 100.0%	2 / 2 100.0%	0 / 0 0.0%	0 / 0 0.0%	0 / 0 0.0%	75 / 113 66.4%AB	38 / 113 33.6%ABE						
1	Achromobacter xylosoxidans A8	59 / 59 100.0%	2 / 2 100.0%	2 / 2 100.0%	2 / 2 100.0%	3 / 3 100.0%	5 / 5 100.0%	3 / 3 100.0%	1 / 1 100.0%	1 / 1 100.0%	6 / 6 100.0%	1 / 1 100.0%	4 / 4 100.0%	2 / 2 100.0%	2 / 2 100.0%	1 / 1 100.0%	2 / 2 100.0%	3 / 3 100.0%	0 / 0 0.0%	3 / 3 100.0%	6 / 6 100.0%	2 / 2 100.0%	2 / 2 100.0%	5 / 5 100.0%	1 / 1 100.0%	0 / 0 0.0%	0 / 0 0.0%	0 / 0 0.0%	40 / 59 67.8%AB	19 / 59 32.2%ABE						
1	Achromobacter xylosoxidans NH44784-1996	54 / 54 100.0%	2 / 2 100.0%	2 / 2 100.0%	2 / 2 100.0%	1 / 1 100.0%	5 / 5 100.0%	3 / 3 100.0%	1 / 1 100.0%	1 / 1 100.0%	4 / 4 100.0%	1 / 1 100.0%	4 / 4 100.0%	2 / 2 100.0%	1 / 1 100.0%	1 / 1 100.0%	2 / 2 100.0%	3 / 3 100.0%	0 / 0 0.0%	3 / 3 100.0%	6 / 6 100.0%	2 / 2 100.0%	2 / 2 100.0%	5 / 5 100.0%	1 / 1 100.0%	0 / 0 0.0%	0 / 0 0.0%	0 / 0 0.0%	35 / 54 64.8%AB	19 / 54 35.2%ABE						
11	Bordetella	586 / 591 99.2%	21 / 21 100.0%	18 / 18 100.0%	21 / 21 100.0%	33 / 33 100.0%	45 / 45 100.0%	33 / 33 100.0%	11 / 11 100.0%	11 / 11 100.0%	62 / 62 100.0%	11 / 11 100.0%	35 / 38 92.1%	22 / 22 100.0%	11 / 11 100.0%	11 / 11 100.0%	11 / 11 100.0%	33 / 33 100.0%	1 / 1 100.0%	33 / 33 100.0%	57 / 57 100.0%	18 / 18 100.0%	21 / 22 95.5%	66 / 67 98.5%	1 / 1 100.0%	0 / 0 0.0%	0 / 0 0.0%	0 / 0 0.0%	383 / 591 64.8%AB	175 / 591 29.6%ABE	18 / 591 3.0%B	10 / 591 1.7%BE	4 / 591 0.7%E	1 / 591 0.2%		
3	"Alcaligenes bronchicanis" (Ferry 1911) Haupt 1935	164 / 165 99.4%	6 / 6 100.0%	6 / 6 100.0%	6 / 6 100.0%	9 / 9 100.0%	13 / 13 100.0%	9 / 9 100.0%	3 / 3 100.0%	3 / 3 100.0%	18 / 18 100.0%	3 / 3 100.0%	9 / 10 90.0%	6 / 6 100.0%	3 / 3 100.0%	3 / 3 100.0%	3 / 3 100.0%	9 / 9 100.0%	1 / 1 100.0%	9 / 9 100.0%	15 / 15 100.0%	6 / 6 100.0%	6 / 6 100.0%	18 / 18 100.0%	0 / 0 0.0%	0 / 0 0.0%	0 / 0 0.0%	0 / 0 0.0%	113 / 165 68.5%AB	51 / 165 30.9%ABE	1 / 165 0.6%E					
1	Bordetella bronchiseptica 253	54 / 54 100.0%	2 / 2 100.0%	2 / 2 100.0%	2 / 2 100.0%	3 / 3 100.0%	4 / 4 100.0%	3 / 3 100.0%	1 / 1 100.0%	1 / 1 100.0%	6 / 6 100.0%	1 / 1 100.0%	3 / 3 100.0%	2 / 2 100.0%	1 / 1 100.0%	1 / 1 100.0%	1 / 1 100.0%	3 / 3 100.0%	0 / 0 0.0%	3 / 3 100.0%	5 / 5 100.0%	2 / 2 100.0%	2 / 2 100.0%	6 / 6 100.0%	0 / 0 0.0%	0 / 0 0.0%	0 / 0 0.0%	0 / 0 0.0%	37 / 54 68.5%AB	17 / 54 31.5%ABE						
1	Bordetella bronchiseptica MO149	54 / 54 100.0%	2 / 2 100.0%	2 / 2 100.0%	2 / 2 100.0%	3 / 3 100.0%	4 / 4 100.0%	3 / 3 100.0%	1 / 1 100.0%	1 / 1 100.0%	6 / 6 100.0%	1 / 1 100.0%	3 / 3 100.0%	2 / 2 100.0%	1 / 1 100.0%	1 / 1 100.0%	1 / 1 100.0%	3 / 3 100.0%	0 / 0 0.0%	3 / 3 100.0%	5 / 5 100.0%	2 / 2 100.0%	2 / 2 100.0%	6 / 6 100.0%	0 / 0 0.0%	0 / 0 0.0%	0 / 0 0.0%	0 / 0 0.0%	37 / 54 68.5%AB	17 / 54 31.5%ABE						
2	"Acinetobacter parapertussis" (Eldering and Kendrick 1938) Steel and Cowan 1964	105 / 108 97.2%	3 / 3 100.0%	4 / 4 100.0%	4 / 4 100.0%	6 / 6 100.0%	8 / 8 100.0%	6 / 6 100.0%	2 / 2 100.0%	2 / 2 100.0%	12 / 12 100.0%	2 / 2 100.0%	6 / 7 85.7%	4 / 4 100.0%	2 / 2 100.0%	2 / 2 100.0%	2 / 2 100.0%	6 / 6 100.0%	0 / 0 0.0%	6 / 6 100.0%	10 / 10 100.0%	4 / 4 100.0%	3 / 4 75.0%	11 / 12 91.7%	0 / 0 0.0%	0 / 0 0.0%	0 / 0 0.0%	0 / 0 0.0%	73 / 108 67.6%AB	32 / 108 29.6%ABE	2 / 108 1.9%E	1 / 108 0.9%				
1	Bordetella parapertussis Bpp5	53 / 54 98.1%	2 / 2 100.0%	2 / 2 100.0%	2 / 2 100.0%	3 / 3 100.0%	4 / 4 100.0%	3 / 3 100.0%	1 / 1 100.0%	1 / 1 100.0%	6 / 6 100.0%	1 / 1 100.0%	3 / 3 100.0%	2 / 2 100.0%	1 / 1 100.0%	1 / 1 100.0%	1 / 1 100.0%	3 / 3 100.0%	0 / 0 0.0%	3 / 3 100.0%	5 / 5 100.0%	2 / 2 100.0%	2 / 2 100.0%	5 / 6 83.3%	0 / 0 0.0%	0 / 0 0.0%	0 / 0 0.0%	0 / 0 0.0%	37 / 54 68.5%AB	16 / 54 29.6%ABE	1 / 54 1.9%E					
4	"Bacterium tussis-convulsivae" Lehmann and Neumann 1927	204 / 205 99.5%	8 / 8 100.0%	4 / 4 100.0%	8 / 8 100.0%	12 / 12 100.0%	16 / 16 100.0%	12 / 12 100.0%	4 / 4 100.0%	4 / 4 100.0%	20 / 20 100.0%	4 / 4 100.0%	12 / 13 92.3%	8 / 8 100.0%	4 / 4 100.0%	4 / 4 100.0%	4 / 4 100.0%	12 / 12 100.0%	0 / 0 0.0%	12 / 12 100.0%	20 / 20 100.0%	4 / 4 100.0%	8 / 8 100.0%	24 / 24 100.0%	0 / 0 0.0%	0 / 0 0.0%	0 / 0 0.0%	0 / 0 0.0%	140 / 205 68.3%AB	64 / 205 31.2%ABE	1 / 205 0.5%E					
1	Bordetella pertussis Tohama I	51 / 51 100.0%	2 / 2 100.0%	1 / 1 100.0%	2 / 2 100.0%	3 / 3 100.0%	4 / 4 100.0%	3 / 3 100.0%	1 / 1 100.0%	1 / 1 100.0%	5 / 5 100.0%	1 / 1 100.0%	3 / 3 100.0%	2 / 2 100.0%	1 / 1 100.0%	1 / 1 100.0%	1 / 1 100.0%	3 / 3 100.0%	0 / 0 0.0%	3 / 3 100.0%	5 / 5 100.0%	1 / 1 100.0%	2 / 2 100.0%	6 / 6 100.0%	0 / 0 0.0%	0 / 0 0.0%	0 / 0 0.0%	0 / 0 0.0%	35 / 51 68.6%AB	16 / 51 31.4%ABE						
1	Bordetella pertussis 18323	51 / 51 100.0%	2 / 2 100.0%	1 / 1 100.0%	2 / 2 100.0%	3 / 3 100.0%	4 / 4 100.0%	3 / 3 100.0%	1 / 1 100.0%	1 / 1 100.0%	5 / 5 100.0%	1 / 1 100.0%	3 / 3 100.0%	2 / 2 100.0%	1 / 1 100.0%	1 / 1 100.0%	1 / 1 100.0%	3 / 3 100.0%	0 / 0 0.0%	3 / 3 100.0%	5 / 5 100.0%	1 / 1 100.0%	2 / 2 100.0%	6 / 6 100.0%	0 / 0 0.0%	0 / 0 0.0%	0 / 0 0.0%	0 / 0 0.0%	35 / 51 68.6%AB	16 / 51 31.4%ABE						
1	Bordetella pertussis CS	51 / 51 100.0%	2 / 2 100.0%	1 / 1 100.0%	2 / 2 100.0%	3 / 3 100.0%	4 / 4 100.0%	3 / 3 100.0%	1 / 1 100.0%	1 / 1 100.0%	5 / 5 100.0%	1 / 1 100.0%	3 / 3 100.0%	2 / 2 100.0%	1 / 1 100.0%	1 / 1 100.0%	1 / 1 100.0%	3 / 3 100.0%	0 / 0 0.0%	3 / 3 100.0%	5 / 5 100.0%	1 / 1 100.0%	2 / 2 100.0%	6 / 6 100.0%	0 / 0 0.0%	0 / 0 0.0%	0 / 0 0.0%	0 / 0 0.0%	35 / 51 68.6%AB	16 / 51 31.4%ABE						
1	ATCC 35086	62 / 62 100.0%	2 / 2 100.0%	3 / 3 100.0%	2 / 2 100.0%	3 / 3 100.0%	4 / 4 100.0%	3 / 3 100.0%	1 / 1 100.0%	1 / 1 100.0%	7 / 7 100.0%	1 / 1 100.0%	4 / 4 100.0%	2 / 2 100.0%	1 / 1 100.0%	1 / 1 100.0%	1 / 1 100.0%	3 / 3 100.0%	0 / 0 0.0%	3 / 3 100.0%	7 / 7 100.0%	3 / 3 100.0%	2 / 2 100.0%	7 / 7 100.0%	1 / 1 100.0%	0 / 0 0.0%	0 / 0 0.0%	0 / 0 0.0%	41 / 62 66.1%AB	20 / 62 32.3%ABE	1 / 62 1.6%BE					
1	Bordetella avium 197N	62 / 62 100.0%	2 / 2 100.0%	3 / 3 100.0%	2 / 2 100.0%	3 / 3 100.0%	4 / 4 100.0%	3 / 3 100.0%	1 / 1 100.0%	1 / 1 100.0%	7 / 7 100.0%	1 / 1 100.0%	4 / 4 100.0%	2 / 2 100.0%	1 / 1 100.0%	1 / 1 100.0%	1 / 1 100.0%	3 / 3 100.0%	0 / 0 0.0%	3 / 3 100.0%	7 / 7 100.0%	3 / 3 100.0%	2 / 2 100.0%	7 / 7 100.0%	1 / 1 100.0%	0 / 0 0.0%	0 / 0 0.0%	0 / 0 0.0%	41 / 62 66.1%AB	20 / 62 32.3%ABE	1 / 62 1.6%BE					
1	Bordetella petri	51 / 51 100.0%	2 / 2 100.0%	1 / 1 100.0%	1 / 1 100.0%	3 / 3 100.0%	4 / 4 100.0%	3 / 3 100.0%	1 / 1 100.0%	1 / 1 100.0%	5 / 5 100.0%	1 / 1 100.0%	4 / 4 100.0%	2 / 2 100.0%	1 / 1 100.0%	1 / 1 100.0%	1 / 1 100.0%	3 / 3 100.0%	0 / 0 0.0%	3 / 3 100.0%	5 / 5 100.0%	1 / 1 100.0%	2 / 2 100.0%	6 / 6 100.0%	0 / 0 0.0%	0 / 0 0.0%	0 / 0 0.0%	0 / 0 0.0%	18 / 51 35.3%B	16 / 51 31.4%AB	9 / 51 17.6%BE	8 / 51 15.7%ABE				
3	Taylorella	111 / 114 97.4%	3 / 3 100.0%	3 / 3 100.0%	6 / 6 100.0%	9 / 9 100.0%	6 / 6 100.0%	6 / 6 100.0%	3 / 3 100.0%	3 / 3 100.0%	9 / 9 100.0%	3 / 3 100.0%	9 / 9 100.0%	3 / 3 100.0%	3 / 3 100.0%	3 / 3 100.0%	3 / 3 100.0%	3 / 3 100.0%	0 / 0 0.0%	6 / 6 100.0%	10 / 12 83.3%	3 / 3 100.0%	6 / 6 100.0%	11 / 12 91.7%	0 / 0 0.0%	0 / 0 0.0%	0 / 0 0.0%	0 / 0 0.0%	76 / 114 66.7%AB	35 / 114 30.7%ABE	2 / 114 1.8%	1 / 114 0.9%E				
2	ATCC 35865	74 / 76 97.4%	2 / 2 100.0%	2 / 2 100.0%	4 / 4 100.0%	6 / 6 100.0%	4 / 4 100.0%	4 / 4 100.0%	2 / 2 100.0%	2 / 2 100.0%	6 / 6 100.0%	2 / 2 100.0%	6 / 6 100.0%	2 / 2 100.0%	2 / 2 100.0%	2 / 2 100.0%	2 / 2 100.0%	2 / 2 100.0%	0 / 0 0.0%	4 / 4 100.0%	6 / 8 75.0%	2 / 2 100.0%	4 / 4 100.0%	8 / 8 100.0%	0 / 0 0.0%	0 / 0 0.0%	0 / 0 0.0%	0 / 0 0.0%	50 / 76 65.8%AB	24 / 76 31.6%ABE	2 / 76 2.6%					
1	Taylorella equigenitalis ATCC 35865	37 / 38 97.4%	1 / 1 100.0%	1 / 1 100.0%	2 / 2 100.0%	3 / 3 100.0%	2 / 2 100.0%	2 / 2 100.0%	1 / 1 100.0%	1 / 1 100.0%	3 / 3 100.0%	1 / 1 100.0%	3 / 3 100.0%	1 / 1 100.0%	1 / 1 100.0%	1 / 1 100.0%	1 / 1 100.0%	1 / 1 100.0%	0 / 0 0.0%	2 / 2 100.0%	3 / 4 75.0%	1 / 1 100.0%	2 / 2 100.0%	4 / 4 100.0%	0 / 0 0.0%	0 / 0 0.0%	0 / 0 0.0%	0 / 0 0.0%	25 / 38 65.8%AB	12 / 38 31.6%ABE	1 / 38 2.6%					
1	Taylorella equigenitalis MCE9	37 / 38 97.4%	1 / 1 100.0%	1 / 1 100.0%	2 / 2 100.0%	3 / 3 100.0%	2 / 2 100.0%	2 / 2 100.0%	1 / 1 100.0%	1 / 1 100.0%	3 / 3 100.0%	1 / 1 100.0%	3 / 3 100.0%	1 / 1 100.0%	1 / 1 100.0%	1 / 1 100.0%	1 / 1 100.0%	1 / 1 100.0%	0 / 0 0.0%	2 / 2 100.0%	3 / 4 75.0%	1 / 1 100.0%	2 / 2 100.0%	4 / 4 100.0%	0 / 0 0.0%	0 / 0 0.0%	0 / 0 0.0%	0 / 0 0.0%	25 / 38 65.8%AB	12 / 38 31.6%ABE	1 / 38 2.6%					
1	ATCC 700933	37 / 38 97.4%	1 / 1 100.0%	1 / 1 100.0%	2 / 2 100.0%	3 / 3 100.0%	2 / 2 100.0%	2 / 2 100.0%	1 / 1 100.0%	1 / 1 100.0%	3 / 3 100.0%	1 / 1 100.0%	3 / 3 100.0%	1 / 1 100.0%	1 / 1 100.0%	1 / 1 100.0%	1 / 1 100.0%	1 / 1 100.0%	0 / 0 0.0%	2 / 2 100.0%	4 / 4 100.0%	1 / 1 100.0%	2 / 2 100.0%	3 / 4 75.0%	0 / 0 0.0%	0 / 0 0.0%	0 / 0 0.0%	0 / 0 0.0%	26 / 38 68.4%AB	11 / 38 28.9%ABE	1 / 38 2.6%E					
1	Taylorella asinigenitalis MCE3	37 / 38 97.4%	1 / 1 100.0%	1 / 1 100.0%	2 / 2 100.0%	3 / 3 100.0%	2 / 2 100.0%	2 / 2 100.0%	1 / 1 100.0%	1 / 1 100.0%	3 / 3 100.0%	1 / 1 100.0%	3 / 3 100.0%	1 / 1 100.0%	1 / 1 100.0%	1 / 1 100.0%	1 / 1 100.0%	1 / 1 100.0%	0 / 0 0.0%	2 / 2 100.0%	4 / 4 100.0%	1 / 1 100.0%	2 / 2 100.0%	3 / 4 75.0%	0 / 0 0.0%	0 / 0 0.0%	0 / 0 0.0%	0 / 0 0.0%	26 / 38 68.4%AB	11 / 38 28.9%ABE	1 / 38 2.6%E					
1	Advenella	35 / 36 97.2%	1 / 1 100.0%	1 / 1 100.0%	1 / 1 100.0%	2 / 2 100.0%	1 / 1 100.0%	2 / 2 100.0%	1 / 1 100.0%	1 / 1 100.0%	3 / 3 100.0%	1 / 1 100.0%	3 / 3 100.0%	1 / 1 100.0%	1 / 1 100.0%	0 / 1 0.0%	1 / 1 100.0%	2 / 2 100.0%	0 / 0 0.0%	3 / 3 100.0%	3 / 3 100.0%	1 / 1 100.0%	1 / 1 100.0%	5 / 5 100.0%	0 / 0 0.0%	0 / 0 0.0%	0 / 0 0.0%	0 / 0 0.0%	23 / 36 63.9%AB	12 / 36 33.3%ABE	1 / 36 2.8%E					
1	Advenella kashmirensis	35 / 36 97.2%	1 / 1 100.0%	1 / 1 100.0%	1 / 1 100.0%	2 / 2 100.0%	1 / 1 100.0%	2 / 2 100.0%	1 / 1 100.0%	1 / 1 100.0%	3 / 3 100.0%	1 / 1 100.0%	3 / 3 100.0%	1 / 1 100.0%	1 / 1 100.0%	0 / 1 0.0%	1 / 1 100.0%	2 / 2 100.0%	0 / 0 0.0%	3 / 3 100.0%	3 / 3 100.0%	1 / 1 100.0%	1 / 1 100.0%	5 / 5 100.0%	0 / 0 0.0%	0 / 0 0.0%	0 / 0 0.0%	0 / 0 0.0%	23 / 36 63.9%AB	12 / 36 33.3%ABE	1 / 36 2.8%E					
1	Advenella kashmirensis WT001	35 / 36 97.2%	1 / 1 100.0%	1 / 1 100.0%	1 / 1 100.0%	2 / 2 100.0%	1 / 1 100.0%	2 / 2 100.0%	1 / 1 100.0%	1 / 1 100.0%	3 / 3 100.0%	1 / 1 100.0%	3 / 3 100.0%	1 / 1 100.0%	1 / 1 100.0%	0 / 1 0.0%	1 / 1 100.0%	2 / 2 100.0%	0 / 0 0.0%	3 / 3 100.0%	3 / 3 100.0%	1 / 1 100.0%	1 / 1 100.0%	5 / 5 100.0%	0 / 0 0.0%	0 / 0 0.0%	0 / 0 0.0%	0 / 0 0.0%	23 / 36 63.9%AB	12 / 36 33.3%ABE	1 / 36 2.8%E					
1	Pusillimonas	45 / 46 97.8%	1 / 1 100.0%	1 / 1 100.0%	1 / 1 100.0%	2 / 2 100.0%	3 / 4 75.0%	3 / 3 100.0%	1 / 1 100.0%	1 / 1 100.0%	4 / 4 100.0%	1 / 1 100.0%	3 / 3 100.0%	2 / 2 100.0%	1 / 1 100.0%	1 / 1 100.0%	1 / 1 100.0%	3 / 3 100.0%	0 / 0 0.0%	3 / 3 100.0%	4 / 4 100.0%	1 / 1 100.0%	2 / 2 100.0%	5 / 5 100.0%	1 / 1 100.0%	0 / 0 0.0%	0 / 0 0.0%	0 / 0 0.0%	30 / 46 65.2%AB	15 / 46 32.6%ABE	1 / 46 2.2%					
1	Pusillimonas sp. T7-7	45 / 46 97.8%	1 / 1 100.0%	1 / 1 100.0%	1 / 1 100.0%	2 / 2 100.0%	3 / 4 75.0%	3 / 3 100.0%	1 / 1 100.0%	1 / 1 100.0%	4 / 4 100.0%	1 / 1 100.0%	3 / 3 100.0%	2 / 2 100.0%	1 / 1 100.0%	1 / 1 100.0%	1 / 1 100.0%	3 / 3 100.0%	0 / 0 0.0%	3 / 3 100.0%	4 / 4 100.0%	1 / 1 100.0%	2 / 2 100.0%	5 / 5 100.0%	1 / 1 100.0%	0 / 0 0.0%	0 / 0 0.0%	0 / 0 0.0%	30 / 46 65.2%AB	15 / 46 32.6%ABE	1 / 46 2.2%					
5	'Oxalobacteraceae'	198 / 223 88.8%	5 / 6 83.3%	8 / 9 88.9%	5 / 6 83.3%	10 / 11 90.9%	15 / 17 88.2%	15 / 16 93.8%	4 / 4 100.0%	4 / 5 80.0%	15 / 16 93.8%	4 / 5 80.0%	16 / 18 88.9%	7 / 8 87.5%	4 / 6 66.7%	4 / 5 80.0%	4 / 5 80.0%	12 / 13 92.3%	4 / 5 80.0%	12 / 13 92.3%	16 / 18 88.9%	6 / 7 85.7%	5 / 6 83.3%	22 / 23 95.7%	1 / 1 100.0%	0 / 0 0.0%	0 / 0 0.0%	0 / 0 0.0%	140 / 223 62.8%AB	58 / 223 26.0%ABE	16 / 223 7.2%	9 / 223 4.0%E				
1	Herbaspirillum	55 / 55 100.0%	1 / 1 100.0%	2 / 2 100.0%	2 / 2 100.0%	3 / 3 100.0%	5 / 5 100.0%	4 / 4 100.0%	1 / 1 100.0%	1 / 1 100.0%	5 / 5 100.0%	1 / 1 100.0%	4 / 4 100.0%	3 / 3 100.0%	1 / 1 100.0%	1 / 1 100.0%	1 / 1 100.0%	3 / 3 100.0%	1 / 1 100.0%	3 / 3 100.0%	4 / 4 100.0%	2 / 2 100.0%	1 / 1 100.0%	6 / 6 100.0%	0 / 0 0.0%	0 / 0 0.0%	0 / 0 0.0%	0 / 0 0.0%	40 / 55 72.7%AB	15 / 55 27.3%ABE						
1	ATCC 35892	55 / 55 100.0%	1 / 1 100.0%	2 / 2 100.0%	2 / 2 100.0%	3 / 3 100.0%	5 / 5 100.0%	4 / 4 100.0%	1 / 1 100.0%	1 / 1 100.0%	5 / 5 100.0%	1 / 1 100.0%	4 / 4 100.0%	3 / 3 100.0%	1 / 1 100.0%	1 / 1 100.0%	1 / 1 100.0%	3 / 3 100.0%	1 / 1 100.0%	3 / 3 100.0%	4 / 4 100.0%	2 / 2 100.0%	1 / 1 100.0%	6 / 6 100.0%	0 / 0 0.0%	0 / 0 0.0%	0 / 0 0.0%	0 / 0 0.0%	40 / 55 72.7%AB	15 / 55 27.3%ABE						
1	Herbaspirillum seropedicae SmR1	55 / 55 100.0%	1 / 1 100.0%	2 / 2 100.0%	2 / 2 100.0%	3 / 3 100.0%	5 / 5 100.0%	4 / 4 100.0%	1 / 1 100.0%	1 / 1 100.0%	5 / 5 100.0%	1 / 1 100.0%	4 / 4 100.0%	3 / 3 100.0%	1 / 1 100.0%	1 / 1 100.0%	1 / 1 100.0%	3 / 3 100.0%	1 / 1 100.0%	3 / 3 100.0%	4 / 4 100.0%	2 / 2 100.0%	1 / 1 100.0%	6 / 6 100.0%	0 / 0 0.0%	0 / 0 0.0%	0 / 0 0.0%	0 / 0 0.0%	40 / 55 72.7%AB	15 / 55 27.3%ABE						
1	Janthinobacterium	46 / 46 100.0%	1 / 1 100.0%	2 / 2 100.0%	1 / 1 100.0%	2 / 2 100.0%	3 / 3 100.0%	4 / 4 100.0%	1 / 1 100.0%	1 / 1 100.0%	3 / 3 100.0%	1 / 1 100.0%	4 / 4 100.0%	1 / 1 100.0%	1 / 1 100.0%	1 / 1 100.0%	1 / 1 100.0%	3 / 3 100.0%	1 / 1 100.0%	3 / 3 100.0%	4 / 4 100.0%	1 / 1 100.0%	2 / 2 100.0%	5 / 5 100.0%	0 / 0 0.0%	0 / 0 0.0%	0 / 0 0.0%	0 / 0 0.0%	33 / 46 71.7%AB	13 / 46 28.3%ABE						
1	Janthinobacterium sp. Marseille	46 / 46 100.0%	1 / 1 100.0%	2 / 2 100.0%	1 / 1 100.0%	2 / 2 100.0%	3 / 3 100.0%	4 / 4 100.0%	1 / 1 100.0%	1 / 1 100.0%	3 / 3 100.0%	1 / 1 100.0%	4 / 4 100.0%	1 / 1 100.0%	1 / 1 100.0%	1 / 1 100.0%	1 / 1 100.0%	3 / 3 100.0%	1 / 1 100.0%	3 / 3 100.0%	4 / 4 100.0%	1 / 1 100.0%	2 / 2 100.0%	5 / 5 100.0%	0 / 0 0.0%	0 / 0 0.0%	0 / 0 0.0%	0 / 0 0.0%	33 / 46 71.7%AB	13 / 46 28.3%ABE						
1	Collimonas	52 / 52 100.0%	2 / 2 100.0%	2 / 2 100.0%	1 / 1 100.0%	3 / 3 100.0%	4 / 4 100.0%	3 / 3 100.0%	1 / 1 100.0%	1 / 1 100.0%	4 / 4 100.0%	1 / 1 100.0%	4 / 4 100.0%	2 / 2 100.0%	1 / 1 100.0%	1 / 1 100.0%	1 / 1 100.0%	3 / 3 100.0%	1 / 1 100.0%	3 / 3 100.0%	4 / 4 100.0%	2 / 2 100.0%	1 / 1 100.0%	6 / 6 100.0%	1 / 1 100.0%	0 / 0 0.0%	0 / 0 0.0%	0 / 0 0.0%	35 / 52 67.3%AB	17 / 52 32.7%ABE						
1	CCUG 48868	52 / 52 100.0%	2 / 2 100.0%	2 / 2 100.0%	1 / 1 100.0%	3 / 3 100.0%	4 / 4 100.0%	3 / 3 100.0%	1 / 1 100.0%	1 / 1 100.0%	4 / 4 100.0%	1 / 1 100.0%	4 / 4 100.0%	2 / 2 100.0%	1 / 1 100.0%	1 / 1 100.0%	1 / 1 100.0%	3 / 3 100.0%	1 / 1 100.0%	3 / 3 100.0%	4 / 4 100.0%	2 / 2 100.0%	1 / 1 100.0%	6 / 6 100.0%	1 / 1 100.0%	0 / 0 0.0%	0 / 0 0.0%	0 / 0 0.0%	35 / 52 67.3%AB	17 / 52 32.7%ABE						
1	Collimonas fungivorans Ter331	52 / 52 100.0%	2 / 2 100.0%	2 / 2 100.0%	1 / 1 100.0%	3 / 3 100.0%	4 / 4 100.0%	3 / 3 100.0%	1 / 1 100.0%	1 / 1 100.0%	4 / 4 100.0%	1 / 1 100.0%	4 / 4 100.0%	2 / 2 100.0%	1 / 1 100.0%	1 / 1 100.0%	1 / 1 100.0%	3 / 3 100.0%	1 / 1 100.0%	3 / 3 100.0%	4 / 4 100.0%	2 / 2 100.0%	1 / 1 100.0%	6 / 6 100.0%	1 / 1 100.0%	0 / 0 0.0%	0 / 0 0.0%	0 / 0 0.0%	35 / 52 67.3%AB	17 / 52 32.7%ABE						
1	Herminiimonas	45 / 45 100.0%	1 / 1 100.0%	2 / 2 100.0%	1 / 1 100.0%	2 / 2 100.0%	3 / 3 100.0%	4 / 4 100.0%	1 / 1 100.0%	1 / 1 100.0%	3 / 3 100.0%	1 / 1 100.0%	4 / 4 100.0%	1 / 1 100.0%	1 / 1 100.0%	1 / 1 100.0%	1 / 1 100.0%	3 / 3 100.0%	1 / 1 100.0%	3 / 3 100.0%	4 / 4 100.0%	1 / 1 100.0%	1 / 1 100.0%	5 / 5 100.0%	0 / 0 0.0%	0 / 0 0.0%	0 / 0 0.0%	0 / 0 0.0%	32 / 45 71.1%AB	13 / 45 28.9%ABE						
1	CCM 7303	45 / 45 100.0%	1 / 1 100.0%	2 / 2 100.0%	1 / 1 100.0%	2 / 2 100.0%	3 / 3 100.0%	4 / 4 100.0%	1 / 1 100.0%	1 / 1 100.0%	3 / 3 100.0%	1 / 1 100.0%	4 / 4 100.0%	1 / 1 100.0%	1 / 1 100.0%	1 / 1 100.0%	1 / 1 100.0%	3 / 3 100.0%	1 / 1 100.0%	3 / 3 100.0%	4 / 4 100.0%	1 / 1 100.0%	1 / 1 100.0%	5 / 5 100.0%	0 / 0 0.0%	0 / 0 0.0%	0 / 0 0.0%	0 / 0 0.0%	32 / 45 71.1%AB	13 / 45 28.9%ABE						
1	"Candidatus Zinderia" McCutcheon and Moran 2010	0 / 25 0.0%	0 / 1 0.0%	0 / 1 0.0%	0 / 1 0.0%	0 / 1 0.0%	0 / 2 0.0%	0 / 1 0.0%	0 / 0 0.0%	0 / 1 0.0%	0 / 1 0.0%	0 / 1 0.0%	0 / 2 0.0%	0 / 1 0.0%	0 / 2 0.0%	0 / 1 0.0%	0 / 1 0.0%	0 / 1 0.0%	0 / 1 0.0%	0 / 1 0.0%	0 / 2 0.0%	0 / 1 0.0%	0 / 1 0.0%	0 / 1 0.0%	0 / 0 0.0%	0 / 0 0.0%	0 / 0 0.0%	0 / 0 0.0%	16 / 25 64.0%	9 / 25 36.0%E						
1	"Candidatus Zinderia insecticola" McCutcheon and Moran 2010	0 / 25 0.0%	0 / 1 0.0%	0 / 1 0.0%	0 / 1 0.0%	0 / 1 0.0%	0 / 2 0.0%	0 / 1 0.0%	0 / 0 0.0%	0 / 1 0.0%	0 / 1 0.0%	0 / 1 0.0%	0 / 2 0.0%	0 / 1 0.0%	0 / 2 0.0%	0 / 1 0.0%	0 / 1 0.0%	0 / 1 0.0%	0 / 1 0.0%	0 / 1 0.0%	0 / 2 0.0%	0 / 1 0.0%	0 / 1 0.0%	0 / 1 0.0%	0 / 0 0.0%	0 / 0 0.0%	0 / 0 0.0%	0 / 0 0.0%	16 / 25 64.0%	9 / 25 36.0%E						
1	Candidatus Zinderia insecticola CARI	0 / 25 0.0%	0 / 1 0.0%	0 / 1 0.0%	0 / 1 0.0%	0 / 1 0.0%	0 / 2 0.0%	0 / 1 0.0%	0 / 0 0.0%	0 / 1 0.0%	0 / 1 0.0%	0 / 1 0.0%	0 / 2 0.0%	0 / 1 0.0%	0 / 2 0.0%	0 / 1 0.0%	0 / 1 0.0%	0 / 1 0.0%	0 / 1 0.0%	0 / 1 0.0%	0 / 2 0.0%	0 / 1 0.0%	0 / 1 0.0%	0 / 1 0.0%	0 / 0 0.0%	0 / 0 0.0%	0 / 0 0.0%	0 / 0 0.0%	16 / 25 64.0%	9 / 25 36.0%E						
17	Comamonadaceae	974 / 983 99.1%	22 / 23 95.7%	34 / 34 100.0%	34 / 34 100.0%	48 / 48 100.0%	75 / 76 98.7%	72 / 72 100.0%	23 / 23 100.0%	19 / 19 100.0%	82 / 85 96.5%	19 / 19 100.0%	75 / 76 98.7%	41 / 41 100.0%	19 / 19 100.0%	23 / 23 100.0%	19 / 20 95.0%	60 / 60 100.0%	12 / 12 100.0%	54 / 55 98.2%	82 / 83 98.8%	33 / 33 100.0%	22 / 22 100.0%	103 / 103 100.0%	3 / 3 100.0%	0 / 0 0.0%	0 / 0 0.0%	0 / 0 0.0%	623 / 983 63.4%AB	298 / 983 30.3%ABE	36 / 983 3.7%B	17 / 983 1.7%BE	4 / 983 0.4%	2 / 983 0.2%CE	2 / 983 0.2%E	1 / 983 0.1%C
1	"Comamonas" Davis and Park 1962	78 / 79 98.7%	3 / 3 100.0%	2 / 2 100.0%	4 / 4 100.0%	3 / 3 100.0%	6 / 7 85.7%	6 / 6 100.0%	2 / 2 100.0%	1 / 1 100.0%	5 / 5 100.0%	1 / 1 100.0%	5 / 5 100.0%	5 / 5 100.0%	2 / 2 100.0%	3 / 3 100.0%	1 / 1 100.0%	6 / 6 100.0%	2 / 2 100.0%	3 / 3 100.0%	7 / 7 100.0%	2 / 2 100.0%	2 / 2 100.0%	6 / 6 100.0%	1 / 1 100.0%	0 / 0 0.0%	0 / 0 0.0%	0 / 0 0.0%	55 / 79 69.6%AB	23 / 79 29.1%ABE	1 / 79 1.3%E					
1	ATCC 11996	78 / 79 98.7%	3 / 3 100.0%	2 / 2 100.0%	4 / 4 100.0%	3 / 3 100.0%	6 / 7 85.7%	6 / 6 100.0%	2 / 2 100.0%	1 / 1 100.0%	5 / 5 100.0%	1 / 1 100.0%	5 / 5 100.0%	5 / 5 100.0%	2 / 2 100.0%	3 / 3 100.0%	1 / 1 100.0%	6 / 6 100.0%	2 / 2 100.0%	3 / 3 100.0%	7 / 7 100.0%	2 / 2 100.0%	2 / 2 100.0%	6 / 6 100.0%	1 / 1 100.0%	0 / 0 0.0%	0 / 0 0.0%	0 / 0 0.0%	55 / 79 69.6%AB	23 / 79 29.1%ABE	1 / 79 1.3%E					
1	Comamonas sp. CNB-1	78 / 79 98.7%	3 / 3 100.0%	2 / 2 100.0%	4 / 4 100.0%	3 / 3 100.0%	6 / 7 85.7%	6 / 6 100.0%	2 / 2 100.0%	1 / 1 100.0%	5 / 5 100.0%	1 / 1 100.0%	5 / 5 100.0%	5 / 5 100.0%	2 / 2 100.0%	3 / 3 100.0%	1 / 1 100.0%	6 / 6 100.0%	2 / 2 100.0%	3 / 3 100.0%	7 / 7 100.0%	2 / 2 100.0%	2 / 2 100.0%	6 / 6 100.0%	1 / 1 100.0%	0 / 0 0.0%	0 / 0 0.0%	0 / 0 0.0%	55 / 79 69.6%AB	23 / 79 29.1%ABE	1 / 79 1.3%E					
1	Comamonas testosteroni CNB-2	78 / 79 98.7%	3 / 3 100.0%	2 / 2 100.0%	4 / 4 100.0%	3 / 3 100.0%	6 / 7 85.7%	6 / 6 100.0%	2 / 2 100.0%	1 / 1 100.0%	5 / 5 100.0%	1 / 1 100.0%	5 / 5 100.0%	5 / 5 100.0%	2 / 2 100.0%	3 / 3 100.0%	1 / 1 100.0%	6 / 6 100.0%	2 / 2 100.0%	3 / 3 100.0%	7 / 7 100.0%	2 / 2 100.0%	2 / 2 100.0%	6 / 6 100.0%	1 / 1 100.0%	0 / 0 0.0%	0 / 0 0.0%	0 / 0 0.0%	55 / 79 69.6%AB	23 / 79 29.1%ABE	1 / 79 1.3%E					
5	Acidivorax	258 / 258 100.0%	5 / 5 100.0%	9 / 9 100.0%	10 / 10 100.0%	15 / 15 100.0%	20 / 20 100.0%	20 / 20 100.0%	5 / 5 100.0%	5 / 5 100.0%	24 / 24 100.0%	5 / 5 100.0%	20 / 20 100.0%	10 / 10 100.0%	5 / 5 100.0%	5 / 5 100.0%	5 / 5 100.0%	15 / 15 100.0%	4 / 4 100.0%	15 / 15 100.0%	21 / 21 100.0%	8 / 8 100.0%	4 / 4 100.0%	28 / 28 100.0%	0 / 0 0.0%	0 / 0 0.0%	0 / 0 0.0%	0 / 0 0.0%	180 / 258 69.8%AB	77 / 258 29.8%ABE	1 / 258 0.4%BE					
1	"Phytomonas rubrilineans" Lee et al. 1925	52 / 52 100.0%	1 / 1 100.0%	2 / 2 100.0%	2 / 2 100.0%	3 / 3 100.0%	4 / 4 100.0%	4 / 4 100.0%	1 / 1 100.0%	1 / 1 100.0%	5 / 5 100.0%	1 / 1 100.0%	4 / 4 100.0%	2 / 2 100.0%	1 / 1 100.0%	1 / 1 100.0%	1 / 1 100.0%	3 / 3 100.0%	0 / 0 0.0%	3 / 3 100.0%	4 / 4 100.0%	2 / 2 100.0%	1 / 1 100.0%	6 / 6 100.0%	0 / 0 0.0%	0 / 0 0.0%	0 / 0 0.0%	0 / 0 0.0%	36 / 52 69.2%AB	16 / 52 30.8%ABE						
1	Acidivorax avenae subsp. avenae	52 / 52 100.0%	1 / 1 100.0%	2 / 2 100.0%	2 / 2 100.0%	3 / 3 100.0%	4 / 4 100.0%	4 / 4 100.0%	1 / 1 100.0%	1 / 1 100.0%	5 / 5 100.0%	1 / 1 100.0%	4 / 4 100.0%	2 / 2 100.0%	1 / 1 100.0%	1 / 1 100.0%	1 / 1 100.0%	3 / 3 100.0%	0 / 0 0.0%	3 / 3 100.0%	4 / 4 100.0%	2 / 2 100.0%	1 / 1 100.0%	6 / 6 100.0%	0 / 0 0.0%	0 / 0 0.0%	0 / 0 0.0%	0 / 0 0.0%	36 / 52 69.2%AB	16 / 52 30.8%ABE						
1	Acidovorax avenae subsp. avenae ATCC 19860	52 / 52 100.0%	1 / 1 100.0%	2 / 2 100.0%	2 / 2 100.0%	3 / 3 100.0%	4 / 4 100.0%	4 / 4 100.0%	1 / 1 100.0%	1 / 1 100.0%	5 / 5 100.0%	1 / 1 100.0%	4 / 4 100.0%	2 / 2 100.0%	1 / 1 100.0%	1 / 1 100.0%	1 / 1 100.0%	3 / 3 100.0%	0 / 0 0.0%	3 / 3 100.0%	4 / 4 100.0%	2 / 2 100.0%	1 / 1 100.0%	6 / 6 100.0%	0 / 0 0.0%	0 / 0 0.0%	0 / 0 0.0%	0 / 0 0.0%	36 / 52 69.2%AB	16 / 52 30.8%ABE						
1	ATCC 29625	53 / 53 100.0%	1 / 1 100.0%	2 / 2 100.0%	2 / 2 100.0%	3 / 3 100.0%	4 / 4 100.0%	4 / 4 100.0%	1 / 1 100.0%	1 / 1 100.0%	5 / 5 100.0%	1 / 1 100.0%	4 / 4 100.0%	2 / 2 100.0%	1 / 1 100.0%	1 / 1 100.0%	1 / 1 100.0%	3 / 3 100.0%	1 / 1 100.0%	3 / 3 100.0%	4 / 4 100.0%	2 / 2 100.0%	1 / 1 100.0%	6 / 6 100.0%	0 / 0 0.0%	0 / 0 0.0%	0 / 0 0.0%	0 / 0 0.0%	37 / 53 69.8%AB	16 / 53 30.2%ABE						
1	Acidovorax avenae subsp. citrulli AAC00-1	53 / 53 100.0%	1 / 1 100.0%	2 / 2 100.0%	2 / 2 100.0%	3 / 3 100.0%	4 / 4 100.0%	4 / 4 100.0%	1 / 1 100.0%	1 / 1 100.0%	5 / 5 100.0%	1 / 1 100.0%	4 / 4 100.0%	2 / 2 100.0%	1 / 1 100.0%	1 / 1 100.0%	1 / 1 100.0%	3 / 3 100.0%	1 / 1 100.0%	3 / 3 100.0%	4 / 4 100.0%	2 / 2 100.0%	1 / 1 100.0%	6 / 6 100.0%	0 / 0 0.0%	0 / 0 0.0%	0 / 0 0.0%	0 / 0 0.0%	37 / 53 69.8%AB	16 / 53 30.2%ABE						
1	Acidovorax sp. JS42	52 / 52 100.0%	1 / 1 100.0%	2 / 2 100.0%	2 / 2 100.0%	3 / 3 100.0%	4 / 4 100.0%	4 / 4 100.0%	1 / 1 100.0%	1 / 1 100.0%	5 / 5 100.0%	1 / 1 100.0%	4 / 4 100.0%	2 / 2 100.0%	1 / 1 100.0%	1 / 1 100.0%	1 / 1 100.0%	3 / 3 100.0%	1 / 1 100.0%	3 / 3 100.0%	5 / 5 100.0%	2 / 2 100.0%	0 / 0 0.0%	5 / 5 100.0%	0 / 0 0.0%	0 / 0 0.0%	0 / 0 0.0%	0 / 0 0.0%	36 / 52 69.2%AB	15 / 52 28.8%ABE	1 / 52 1.9%BE					
1	Acidovorax sp. KKS102	48 / 48 100.0%	1 / 1 100.0%	1 / 1 100.0%	2 / 2 100.0%	3 / 3 100.0%	4 / 4 100.0%	4 / 4 100.0%	1 / 1 100.0%	1 / 1 100.0%	4 / 4 100.0%	1 / 1 100.0%	4 / 4 100.0%	2 / 2 100.0%	1 / 1 100.0%	1 / 1 100.0%	1 / 1 100.0%	3 / 3 100.0%	1 / 1 100.0%	3 / 3 100.0%	4 / 4 100.0%	0 / 0 0.0%	1 / 1 100.0%	5 / 5 100.0%	0 / 0 0.0%	0 / 0 0.0%	0 / 0 0.0%	0 / 0 0.0%	35 / 48 72.9%AB	13 / 48 27.1%ABE						
1	"Acidovorax ebreus" Byrne-Bailey et al. 2010	53 / 53 100.0%	1 / 1 100.0%	2 / 2 100.0%	2 / 2 100.0%	3 / 3 100.0%	4 / 4 100.0%	4 / 4 100.0%	1 / 1 100.0%	1 / 1 100.0%	5 / 5 100.0%	1 / 1 100.0%	4 / 4 100.0%	2 / 2 100.0%	1 / 1 100.0%	1 / 1 100.0%	1 / 1 100.0%	3 / 3 100.0%	1 / 1 100.0%	3 / 3 100.0%	4 / 4 100.0%	2 / 2 100.0%	1 / 1 100.0%	6 / 6 100.0%	0 / 0 0.0%	0 / 0 0.0%	0 / 0 0.0%	0 / 0 0.0%	36 / 53 67.9%AB	17 / 53 32.1%ABE						
1	Acidovorax ebreus TPSY	53 / 53 100.0%	1 / 1 100.0%	2 / 2 100.0%	2 / 2 100.0%	3 / 3 100.0%	4 / 4 100.0%	4 / 4 100.0%	1 / 1 100.0%	1 / 1 100.0%	5 / 5 100.0%	1 / 1 100.0%	4 / 4 100.0%	2 / 2 100.0%	1 / 1 100.0%	1 / 1 100.0%	1 / 1 100.0%	3 / 3 100.0%	1 / 1 100.0%	3 / 3 100.0%	4 / 4 100.0%	2 / 2 100.0%	1 / 1 100.0%	6 / 6 100.0%	0 / 0 0.0%	0 / 0 0.0%	0 / 0 0.0%	0 / 0 0.0%	36 / 53 67.9%AB	17 / 53 32.1%ABE						
1	"Albidoferax" Ramana and Saskiala 2009	44 / 44 100.0%	1 / 1 100.0%	1 / 1 100.0%	1 / 1 100.0%	2 / 2 100.0%	3 / 3 100.0%	3 / 3 100.0%	1 / 1 100.0%	1 / 1 100.0%	4 / 4 100.0%	1 / 1 100.0%	4 / 4 100.0%	2 / 2 100.0%	1 / 1 100.0%	1 / 1 100.0%	1 / 1 100.0%	3 / 3 100.0%	0 / 0 0.0%	3 / 3 100.0%	4 / 4 100.0%	1 / 1 100.0%	1 / 1 100.0%	5 / 5 100.0%	0 / 0 0.0%	0 / 0 0.0%	0 / 0 0.0%	0 / 0 0.0%	27 / 44 61.4%AB	17 / 44 38.6%ABE						
1	"Albidoferax ferrireducens" (Finneran et al. 2003) Ramana and Sasikala 2009	44 / 44 100.0%	1 / 1 100.0%	1 / 1 100.0%	1 / 1 100.0%	2 / 2 100.0%	3 / 3 100.0%	3 / 3 100.0%	1 / 1 100.0%	1 / 1 100.0%	4 / 4 100.0%	1 / 1 100.0%	4 / 4 100.0%	2 / 2 100.0%	1 / 1 100.0%	1 / 1 100.0%	1 / 1 100.0%	3 / 3 100.0%	0 / 0 0.0%	3 / 3 100.0%	4 / 4 100.0%	1 / 1 100.0%	1 / 1 100.0%	5 / 5 100.0%	0 / 0 0.0%	0 / 0 0.0%	0 / 0 0.0%	0 / 0 0.0%	27 / 44 61.4%AB	17 / 44 38.6%ABE						
1	Albidiferax ferrireducens T118	44 / 44 100.0%	1 / 1 100.0%	1 / 1 100.0%	1 / 1 100.0%	2 / 2 100.0%	3 / 3 100.0%	3 / 3 100.0%	1 / 1 100.0%	1 / 1 100.0%	4 / 4 100.0%	1 / 1 100.0%	4 / 4 100.0%	2 / 2 100.0%	1 / 1 100.0%	1 / 1 100.0%	1 / 1 100.0%	3 / 3 100.0%	0 / 0 0.0%	3 / 3 100.0%	4 / 4 100.0%	1 / 1 100.0%	1 / 1 100.0%	5 / 5 100.0%	0 / 0 0.0%	0 / 0 0.0%	0 / 0 0.0%	0 / 0 0.0%	27 / 44 61.4%AB	17 / 44 38.6%ABE						
2	Variovorax	112 / 114 98.2%	3 / 3 100.0%	4 / 4 100.0%	3 / 3 100.0%	4 / 4 100.0%	8 / 8 100.0%	6 / 6 100.0%	4 / 4 100.0%	3 / 3 100.0%	6 / 8 75.0%	3 / 3 100.0%	9 / 9 100.0%	4 / 4 100.0%	2 / 2 100.0%	3 / 3 100.0%	3 / 3 100.0%	7 / 7 100.0%	0 / 0 0.0%	7 / 7 100.0%	11 / 11 100.0%	4 / 4 100.0%	4 / 4 100.0%	14 / 14 100.0%	0 / 0 0.0%	0 / 0 0.0%	0 / 0 0.0%	0 / 0 0.0%	72 / 114 63.2%AB	40 / 114 35.1%ABE	2 / 114 1.8%					
2	ATCC 17713	112 / 114 98.2%	3 / 3 100.0%	4 / 4 100.0%	3 / 3 100.0%	4 / 4 100.0%	8 / 8 100.0%	6 / 6 100.0%	4 / 4 100.0%	3 / 3 100.0%	6 / 8 75.0%	3 / 3 100.0%	9 / 9 100.0%	4 / 4 100.0%	2 / 2 100.0%	3 / 3 100.0%	3 / 3 100.0%	7 / 7 100.0%	0 / 0 0.0%	7 / 7 100.0%	11 / 11 100.0%	4 / 4 100.0%	4 / 4 100.0%	14 / 14 100.0%	0 / 0 0.0%	0 / 0 0.0%	0 / 0 0.0%	0 / 0 0.0%	72 / 114 63.2%AB	40 / 114 35.1%ABE	2 / 114 1.8%					
1	Variovorax paradoxus S110	57 / 58 98.3%	2 / 2 100.0%	2 / 2 100.0%	2 / 2 100.0%	2 / 2 100.0%	4 / 4 100.0%	3 / 3 100.0%	2 / 2 100.0%	1 / 1 100.0%	3 / 4 75.0%	2 / 2 100.0%	5 / 5 100.0%	2 / 2 100.0%	1 / 1 100.0%	2 / 2 100.0%	1 / 1 100.0%	4 / 4 100.0%	0 / 0 0.0%	4 / 4 100.0%	5 / 5 100.0%	2 / 2 100.0%	2 / 2 100.0%	6 / 6 100.0%	0 / 0 0.0%	0 / 0 0.0%	0 / 0 0.0%	0 / 0 0.0%	37 / 58 63.8%AB	20 / 58 34.5%ABE	1 / 58 1.7%					
1	Variovorax paradoxus EPS	55 / 56 98.2%	1 / 1 100.0%	2 / 2 100.0%	1 / 1 100.0%	2 / 2 100.0%	4 / 4 100.0%	3 / 3 100.0%	2 / 2 100.0%	2 / 2 100.0%	3 / 4 75.0%	1 / 1 100.0%	4 / 4 100.0%	2 / 2 100.0%	1 / 1 100.0%	1 / 1 100.0%	2 / 2 100.0%	3 / 3 100.0%	0 / 0 0.0%	3 / 3 100.0%	6 / 6 100.0%	2 / 2 100.0%	2 / 2 100.0%	8 / 8 100.0%	0 / 0 0.0%	0 / 0 0.0%	0 / 0 0.0%	0 / 0 0.0%	35 / 56 62.5%AB	20 / 56 35.7%ABE	1 / 56 1.8%					
2	Polaromonas	133 / 134 99.3%	3 / 3 100.0%	4 / 4 100.0%	3 / 3 100.0%	4 / 4 100.0%	12 / 12 100.0%	9 / 9 100.0%	3 / 3 100.0%	3 / 3 100.0%	9 / 9 100.0%	3 / 3 100.0%	11 / 12 91.7%	4 / 4 100.0%	3 / 3 100.0%	3 / 3 100.0%	4 / 4 100.0%	9 / 9 100.0%	3 / 3 100.0%	9 / 9 100.0%	12 / 12 100.0%	4 / 4 100.0%	3 / 3 100.0%	15 / 15 100.0%	0 / 0 0.0%	0 / 0 0.0%	0 / 0 0.0%	0 / 0 0.0%	71 / 134 53.0%AB	41 / 134 30.6%ABE	14 / 134 10.4%B	7 / 134 5.2%BE	1 / 134 0.7%CE			
1	ATCC BAA-779	47 / 48 97.9%	1 / 1 100.0%	2 / 2 100.0%	1 / 1 100.0%	2 / 2 100.0%	4 / 4 100.0%	3 / 3 100.0%	1 / 1 100.0%	1 / 1 100.0%	3 / 3 100.0%	1 / 1 100.0%	3 / 4 75.0%	2 / 2 100.0%	1 / 1 100.0%	1 / 1 100.0%	2 / 2 100.0%	3 / 3 100.0%	1 / 1 100.0%	3 / 3 100.0%	4 / 4 100.0%	2 / 2 100.0%	1 / 1 100.0%	5 / 5 100.0%	0 / 0 0.0%	0 / 0 0.0%	0 / 0 0.0%	0 / 0 0.0%	31 / 48 64.6%AB	16 / 48 33.3%ABE	1 / 48 2.1%CE					
1	Polaromonas naphthalenivorans CJ2	47 / 48 97.9%	1 / 1 100.0%	2 / 2 100.0%	1 / 1 100.0%	2 / 2 100.0%	4 / 4 100.0%	3 / 3 100.0%	1 / 1 100.0%	1 / 1 100.0%	3 / 3 100.0%	1 / 1 100.0%	3 / 4 75.0%	2 / 2 100.0%	1 / 1 100.0%	1 / 1 100.0%	2 / 2 100.0%	3 / 3 100.0%	1 / 1 100.0%	3 / 3 100.0%	4 / 4 100.0%	2 / 2 100.0%	1 / 1 100.0%	5 / 5 100.0%	0 / 0 0.0%	0 / 0 0.0%	0 / 0 0.0%	0 / 0 0.0%	31 / 48 64.6%AB	16 / 48 33.3%ABE	1 / 48 2.1%CE					
1	Polaromonas sp. JS666	86 / 86 100.0%	2 / 2 100.0%	2 / 2 100.0%	2 / 2 100.0%	2 / 2 100.0%	8 / 8 100.0%	6 / 6 100.0%	2 / 2 100.0%	2 / 2 100.0%	6 / 6 100.0%	2 / 2 100.0%	8 / 8 100.0%	2 / 2 100.0%	2 / 2 100.0%	2 / 2 100.0%	2 / 2 100.0%	6 / 6 100.0%	2 / 2 100.0%	6 / 6 100.0%	8 / 8 100.0%	2 / 2 100.0%	2 / 2 100.0%	10 / 10 100.0%	0 / 0 0.0%	0 / 0 0.0%	0 / 0 0.0%	0 / 0 0.0%	40 / 86 46.5%AB	25 / 86 29.1%ABE	14 / 86 16.3%B	7 / 86 8.1%BE				
2	Delftia	159 / 160 99.4%	3 / 4 75.0%	8 / 8 100.0%	6 / 6 100.0%	10 / 10 100.0%	12 / 12 100.0%	14 / 14 100.0%	4 / 4 100.0%	2 / 2 100.0%	16 / 16 100.0%	2 / 2 100.0%	10 / 10 100.0%	8 / 8 100.0%	2 / 2 100.0%	4 / 4 100.0%	2 / 2 100.0%	8 / 8 100.0%	0 / 0 0.0%	6 / 6 100.0%	12 / 12 100.0%	8 / 8 100.0%	4 / 4 100.0%	16 / 16 100.0%	2 / 2 100.0%	0 / 0 0.0%	0 / 0 0.0%	0 / 0 0.0%	88 / 160 55.0%AB	40 / 160 25.0%ABE	22 / 160 13.8%B	9 / 160 5.6%BE	1 / 160 0.6%E			
1	"Pseudomonas desmolytica" Gray and Thornton 1928	79 / 80 98.8%	1 / 2 50.0%	4 / 4 100.0%	3 / 3 100.0%	5 / 5 100.0%	6 / 6 100.0%	7 / 7 100.0%	2 / 2 100.0%	1 / 1 100.0%	8 / 8 100.0%	1 / 1 100.0%	5 / 5 100.0%	4 / 4 100.0%	1 / 1 100.0%	2 / 2 100.0%	1 / 1 100.0%	4 / 4 100.0%	0 / 0 0.0%	3 / 3 100.0%	6 / 6 100.0%	4 / 4 100.0%	2 / 2 100.0%	8 / 8 100.0%	1 / 1 100.0%	0 / 0 0.0%	0 / 0 0.0%	0 / 0 0.0%	33 / 80 41.2%AB	22 / 80 27.5%B	15 / 80 18.8%ABE	9 / 80 11.2%BE	1 / 80 1.2%E			
1	Delftia acidovorans SPH-1	79 / 80 98.8%	1 / 2 50.0%	4 / 4 100.0%	3 / 3 100.0%	5 / 5 100.0%	6 / 6 100.0%	7 / 7 100.0%	2 / 2 100.0%	1 / 1 100.0%	8 / 8 100.0%	1 / 1 100.0%	5 / 5 100.0%	4 / 4 100.0%	1 / 1 100.0%	2 / 2 100.0%	1 / 1 100.0%	4 / 4 100.0%	0 / 0 0.0%	3 / 3 100.0%	6 / 6 100.0%	4 / 4 100.0%	2 / 2 100.0%	8 / 8 100.0%	1 / 1 100.0%	0 / 0 0.0%	0 / 0 0.0%	0 / 0 0.0%	33 / 80 41.2%AB	22 / 80 27.5%B	15 / 80 18.8%ABE	9 / 80 11.2%BE	1 / 80 1.2%E			
1	Delftia sp. Cs1-4	80 / 80 100.0%	2 / 2 100.0%	4 / 4 100.0%	3 / 3 100.0%	5 / 5 100.0%	6 / 6 100.0%	7 / 7 100.0%	2 / 2 100.0%	1 / 1 100.0%	8 / 8 100.0%	1 / 1 100.0%	5 / 5 100.0%	4 / 4 100.0%	1 / 1 100.0%	2 / 2 100.0%	1 / 1 100.0%	4 / 4 100.0%	0 / 0 0.0%	3 / 3 100.0%	6 / 6 100.0%	4 / 4 100.0%	2 / 2 100.0%	8 / 8 100.0%	1 / 1 100.0%	0 / 0 0.0%	0 / 0 0.0%	0 / 0 0.0%	55 / 80 68.8%AB	25 / 80 31.2%ABE						
1	Ramlibacter	40 / 43 93.0%	1 / 1 100.0%	1 / 1 100.0%	1 / 1 100.0%	1 / 1 100.0%	3 / 3 100.0%	3 / 3 100.0%	1 / 1 100.0%	1 / 1 100.0%	2 / 3 66.7%	1 / 1 100.0%	4 / 4 100.0%	2 / 2 100.0%	1 / 1 100.0%	1 / 1 100.0%	1 / 1 100.0%	3 / 3 100.0%	0 / 0 0.0%	2 / 3 66.7%	3 / 4 75.0%	1 / 1 100.0%	2 / 2 100.0%	5 / 5 100.0%	0 / 0 0.0%	0 / 0 0.0%	0 / 0 0.0%	0 / 0 0.0%	25 / 43 58.1%AB	15 / 43 34.9%ABE	1 / 43 2.3%	1 / 43 2.3%CE	1 / 43 2.3%C			
1	ATCC BAA-407	40 / 43 93.0%	1 / 1 100.0%	1 / 1 100.0%	1 / 1 100.0%	1 / 1 100.0%	3 / 3 100.0%	3 / 3 100.0%	1 / 1 100.0%	1 / 1 100.0%	2 / 3 66.7%	1 / 1 100.0%	4 / 4 100.0%	2 / 2 100.0%	1 / 1 100.0%	1 / 1 100.0%	1 / 1 100.0%	3 / 3 100.0%	0 / 0 0.0%	2 / 3 66.7%	3 / 4 75.0%	1 / 1 100.0%	2 / 2 100.0%	5 / 5 100.0%	0 / 0 0.0%	0 / 0 0.0%	0 / 0 0.0%	0 / 0 0.0%	25 / 43 58.1%AB	15 / 43 34.9%ABE	1 / 43 2.3%C	1 / 43 2.3%	1 / 43 2.3%CE			
1	Acidivorax sp. TTB310	40 / 43 93.0%	1 / 1 100.0%	1 / 1 100.0%	1 / 1 100.0%	1 / 1 100.0%	3 / 3 100.0%	3 / 3 100.0%	1 / 1 100.0%	1 / 1 100.0%	2 / 3 66.7%	1 / 1 100.0%	4 / 4 100.0%	2 / 2 100.0%	1 / 1 100.0%	1 / 1 100.0%	1 / 1 100.0%	3 / 3 100.0%	0 / 0 0.0%	2 / 3 66.7%	3 / 4 75.0%	1 / 1 100.0%	2 / 2 100.0%	5 / 5 100.0%	0 / 0 0.0%	0 / 0 0.0%	0 / 0 0.0%	0 / 0 0.0%	25 / 43 58.1%AB	15 / 43 34.9%ABE	1 / 43 2.3%C	1 / 43 2.3%	1 / 43 2.3%CE			

2	Alicycliphilus	104 / 104 100.0%	2 / 2 100.0%	4 / 4 100.0%	4 / 4 100.0%	6 / 6 100.0%	8 / 8 100.0%	8 / 8 100.0%	2 / 2 100.0%	2 / 2 100.0%	12 / 12 100.0%	2 / 2 100.0%	8 / 8 100.0%	4 / 4 100.0%	2 / 2 100.0%	2 / 2 100.0%	2 / 2 100.0%	6 / 6 100.0%	2 / 2 100.0%	6 / 6 100.0%	8 / 8 100.0%	4 / 4 100.0%	1 / 1 100.0%	9 / 9 100.0%	0 / 0 0.0%	0 / 0 0.0%	0 / 0 0.0%	0 / 0 0.0%	73 / 104 70.2%AB	31 / 104 29.8%ABE					
2	Alicycliphilus denitrificans	104 / 104 100.0%	2 / 2 100.0%	4 / 4 100.0%	4 / 4 100.0%	6 / 6 100.0%	8 / 8 100.0%	8 / 8 100.0%	2 / 2 100.0%	2 / 2 100.0%	12 / 12 100.0%	2 / 2 100.0%	8 / 8 100.0%	4 / 4 100.0%	2 / 2 100.0%	2 / 2 100.0%	2 / 2 100.0%	6 / 6 100.0%	2 / 2 100.0%	6 / 6 100.0%	8 / 8 100.0%	4 / 4 100.0%	1 / 1 100.0%	9 / 9 100.0%	0 / 0 0.0%	0 / 0 0.0%	0 / 0 0.0%	0 / 0 0.0%	73 / 104 70.2%AB	31 / 104 29.8%ABE					
1	Alicycliphilus denitrificans BC	51 / 51 100.0%	1 / 1 100.0%	2 / 2 100.0%	2 / 2 100.0%	3 / 3 100.0%	4 / 4 100.0%	4 / 4 100.0%	1 / 1 100.0%	1 / 1 100.0%	6 / 6 100.0%	1 / 1 100.0%	4 / 4 100.0%	2 / 2 100.0%	1 / 1 100.0%	1 / 1 100.0%	1 / 1 100.0%	3 / 3 100.0%	1 / 1 100.0%	3 / 3 100.0%	4 / 4 100.0%	2 / 2 100.0%	0 / 0 0.0%	4 / 4 100.0%	0 / 0 0.0%	0 / 0 0.0%	0 / 0 0.0%	0 / 0 0.0%	36 / 51 70.6%AB	15 / 51 29.4%ABE					
1	Alicycliphilus denitrificans DSM 14773	53 / 53 100.0%	1 / 1 100.0%	2 / 2 100.0%	2 / 2 100.0%	3 / 3 100.0%	4 / 4 100.0%	4 / 4 100.0%	1 / 1 100.0%	1 / 1 100.0%	6 / 6 100.0%	1 / 1 100.0%	4 / 4 100.0%	2 / 2 100.0%	1 / 1 100.0%	1 / 1 100.0%	1 / 1 100.0%	3 / 3 100.0%	1 / 1 100.0%	3 / 3 100.0%	4 / 4 100.0%	2 / 2 100.0%	1 / 1 100.0%	5 / 5 100.0%	0 / 0 0.0%	0 / 0 0.0%	0 / 0 0.0%	0 / 0 0.0%	37 / 53 69.8%AB	16 / 53 30.2%ABE					
1	Verminephrobacter	46 / 47 97.9%	1 / 1 100.0%	1 / 1 100.0%	2 / 2 100.0%	3 / 3 100.0%	3 / 3 100.0%	3 / 3 100.0%	1 / 1 100.0%	1 / 1 100.0%	4 / 4 100.0%	1 / 1 100.0%	4 / 4 100.0%	2 / 2 100.0%	1 / 1 100.0%	1 / 1 100.0%	0 / 1 0.0%	3 / 3 100.0%	1 / 1 100.0%	3 / 3 100.0%	4 / 4 100.0%	1 / 1 100.0%	1 / 1 100.0%	5 / 5 100.0%	0 / 0 0.0%	0 / 0 0.0%	0 / 0 0.0%	0 / 0 0.0%	32 / 47 68.1%AB	14 / 47 29.8%ABE	1 / 47 2.1%				
1	ATCC BAA-1489	46 / 47 97.9%	1 / 1 100.0%	1 / 1 100.0%	2 / 2 100.0%	3 / 3 100.0%	3 / 3 100.0%	3 / 3 100.0%	1 / 1 100.0%	1 / 1 100.0%	4 / 4 100.0%	1 / 1 100.0%	4 / 4 100.0%	2 / 2 100.0%	1 / 1 100.0%	1 / 1 100.0%	0 / 1 0.0%	3 / 3 100.0%	1 / 1 100.0%	3 / 3 100.0%	4 / 4 100.0%	1 / 1 100.0%	1 / 1 100.0%	5 / 5 100.0%	0 / 0 0.0%	0 / 0 0.0%	0 / 0 0.0%	0 / 0 0.0%	32 / 47 68.1%AB	14 / 47 29.8%ABE	1 / 47 2.1%				
1	Verminephrobacter eiseniae EF01-2	46 / 47 97.9%	1 / 1 100.0%	1 / 1 100.0%	2 / 2 100.0%	3 / 3 100.0%	3 / 3 100.0%	3 / 3 100.0%	1 / 1 100.0%	1 / 1 100.0%	4 / 4 100.0%	1 / 1 100.0%	4 / 4 100.0%	2 / 2 100.0%	1 / 1 100.0%	1 / 1 100.0%	0 / 1 0.0%	3 / 3 100.0%	1 / 1 100.0%	3 / 3 100.0%	4 / 4 100.0%	1 / 1 100.0%	1 / 1 100.0%	5 / 5 100.0%	0 / 0 0.0%	0 / 0 0.0%	0 / 0 0.0%	0 / 0 0.0%	32 / 47 68.1%AB	14 / 47 29.8%ABE	1 / 47 2.1%				
47	'Burkholderiaceae'	2827 / 2861 98.8%	110 / 110 100.0%	142 / 142 100.0%	85 / 85 100.0%	197 / 198 99.5%	200 / 200 100.0%	177 / 177 100.0%	48 / 48 100.0%	47 / 47 100.0%	294 / 304 96.7%	48 / 48 100.0%	191 / 191 100.0%	102 / 105 97.1%	48 / 48 100.0%	50 / 50 100.0%	59 / 60 98.3%	142 / 142 100.0%	48 / 48 100.0%	143 / 143 100.0%	235 / 237 99.2%	96 / 96 100.0%	43 / 44 97.7%	295 / 310 95.2%	27 / 28 96.4%	0 / 0 0.0%	0 / 0 0.0%	0 / 0 0.0%	1704 / 2861 59.6%AB	821 / 2861 28.7%ABE	194 / 2861 6.8%B	108 / 2861 3.8%BE	17 / 2861 0.6%	15 / 2861 0.5%E	2 / 2861 0.1%CE
32	Burkholderia	1979 / 2012 98.4%	75 / 75 100.0%	103 / 103 100.0%	66 / 66 100.0%	149 / 150 99.3%	139 / 139 100.0%	126 / 126 100.0%	32 / 32 100.0%	32 / 32 100.0%	207 / 217 95.4%	32 / 32 100.0%	127 / 127 100.0%	66 / 69 95.7%	32 / 32 100.0%	34 / 34 100.0%	43 / 44 97.7%	96 / 96 100.0%	32 / 32 100.0%	95 / 95 100.0%	167 / 168 99.4%	71 / 71 100.0%	27 / 28 96.4%	201 / 216 93.1%	27 / 28 96.4%	0 / 0 0.0%	0 / 0 0.0%	0 / 0 0.0%	1229 / 2012 61.1%AB	569 / 2012 28.3%ABE	115 / 2012 5.7%B	66 / 2012 3.3%BE	16 / 2012 0.8%	15 / 2012 0.7%E	2 / 2012 0.1%CE
1	ATCC 33617	64 / 65 98.5%	2 / 2 100.0%	4 / 4 100.0%	2 / 2 100.0%	5 / 5 100.0%	4 / 4 100.0%	5 / 5 100.0%	1 / 1 100.0%	1 / 1 100.0%	7 / 8 87.5%	1 / 1 100.0%	4 / 4 100.0%	2 / 2 100.0%	1 / 1 100.0%	1 / 1 100.0%	1 / 1 100.0%	3 / 3 100.0%	1 / 1 100.0%	3 / 3 100.0%	4 / 4 100.0%	3 / 3 100.0%	1 / 1 100.0%	7 / 7 100.0%	1 / 1 100.0%	0 / 0 0.0%	0 / 0 0.0%	0 / 0 0.0%	43 / 65 66.2%AB	21 / 65 32.3%ABE	1 / 65 1.5%				
1	Burkholderia glumae BGR1	64 / 65 98.5%	2 / 2 100.0%	4 / 4 100.0%	2 / 2 100.0%	5 / 5 100.0%	4 / 4 100.0%	5 / 5 100.0%	1 / 1 100.0%	1 / 1 100.0%	7 / 8 87.5%	1 / 1 100.0%	4 / 4 100.0%	2 / 2 100.0%	1 / 1 100.0%	1 / 1 100.0%	1 / 1 100.0%	3 / 3 100.0%	1 / 1 100.0%	3 / 3 100.0%	4 / 4 100.0%	3 / 3 100.0%	1 / 1 100.0%	7 / 7 100.0%	1 / 1 100.0%	0 / 0 0.0%	0 / 0 0.0%	0 / 0 0.0%	43 / 65 66.2%AB	21 / 65 32.3%ABE	1 / 65 1.5%				
1	ATCC 10248	66 / 67 98.5%	2 / 2 100.0%	4 / 4 100.0%	2 / 2 100.0%	5 / 5 100.0%	4 / 4 100.0%	6 / 6 100.0%	1 / 1 100.0%	1 / 1 100.0%	7 / 8 87.5%	1 / 1 100.0%	4 / 4 100.0%	2 / 2 100.0%	1 / 1 100.0%	1 / 1 100.0%	2 / 2 100.0%	3 / 3 100.0%	1 / 1 100.0%	3 / 3 100.0%	5 / 5 100.0%	3 / 3 100.0%	1 / 1 100.0%	6 / 6 100.0%	1 / 1 100.0%	0 / 0 0.0%	0 / 0 0.0%	0 / 0 0.0%	46 / 67 68.7%AB	20 / 67 29.9%ABE	1 / 67 1.5%				
1	Burkholderia gladioli BSR3	66 / 67 98.5%	2 / 2 100.0%	4 / 4 100.0%	2 / 2 100.0%	5 / 5 100.0%	4 / 4 100.0%	6 / 6 100.0%	1 / 1 100.0%	1 / 1 100.0%	7 / 8 87.5%	1 / 1 100.0%	4 / 4 100.0%	2 / 2 100.0%	1 / 1 100.0%	1 / 1 100.0%	2 / 2 100.0%	3 / 3 100.0%	1 / 1 100.0%	3 / 3 100.0%	5 / 5 100.0%	3 / 3 100.0%	1 / 1 100.0%	6 / 6 100.0%	1 / 1 100.0%	0 / 0 0.0%	0 / 0 0.0%	0 / 0 0.0%	46 / 67 68.7%AB	20 / 67 29.9%ABE	1 / 67 1.5%				
1	Burkholderia xenovorans	60 / 64 93.8%	2 / 2 100.0%	3 / 3 100.0%	2 / 2 100.0%	6 / 6 100.0%	4 / 4 100.0%	4 / 4 100.0%	1 / 1 100.0%	1 / 1 100.0%	7 / 8 87.5%	1 / 1 100.0%	4 / 4 100.0%	2 / 3 66.7%	1 / 1 100.0%	1 / 1 100.0%	1 / 1 100.0%	3 / 3 100.0%	1 / 1 100.0%	3 / 3 100.0%	4 / 5 80.0%	2 / 2 100.0%	0 / 1 0.0%	6 / 6 100.0%	1 / 1 100.0%	0 / 0 0.0%	0 / 0 0.0%	0 / 0 0.0%	41 / 64 64.1%AB	19 / 64 29.7%ABE	3 / 64 4.7%	1 / 64 1.6%E			
1	Burkholderia cepacia LB400	60 / 64 93.8%	2 / 2 100.0%	3 / 3 100.0%	2 / 2 100.0%	6 / 6 100.0%	4 / 4 100.0%	4 / 4 100.0%	1 / 1 100.0%	1 / 1 100.0%	7 / 8 87.5%	1 / 1 100.0%	4 / 4 100.0%	2 / 3 66.7%	1 / 1 100.0%	1 / 1 100.0%	1 / 1 100.0%	3 / 3 100.0%	1 / 1 100.0%	3 / 3 100.0%	4 / 5 80.0%	2 / 2 100.0%	0 / 1 0.0%	6 / 6 100.0%	1 / 1 100.0%	0 / 0 0.0%	0 / 0 0.0%	0 / 0 0.0%	41 / 64 64.1%AB	19 / 64 29.7%ABE	3 / 64 4.7%	1 / 64 1.6%E			
9	Burkholderia cepacia complex	604 / 611 98.9%	29 / 29 100.0%	36 / 36 100.0%	20 / 20 100.0%	41 / 41 100.0%	47 / 47 100.0%	36 / 36 100.0%	9 / 9 100.0%	9 / 9 100.0%	59 / 59 100.0%	9 / 9 100.0%	36 / 36 100.0%	18 / 18 100.0%	9 / 9 100.0%	9 / 9 100.0%	10 / 11 90.9%	27 / 27 100.0%	9 / 9 100.0%	26 / 26 100.0%	54 / 54 100.0%	27 / 27 100.0%	9 / 9 100.0%	65 / 71 91.5%	10 / 10 100.0%	0 / 0 0.0%	0 / 0 0.0%	0 / 0 0.0%	341 / 611 55.8%AB	158 / 611 25.9%ABE	61 / 611 10.0%B	44 / 611 7.2%BE	5 / 611 0.8%E	1 / 611 0.2%	1 / 611 0.2%CE
1	"Pseudomonas cepacia" Burkholder 1950	57 / 57 100.0%	3 / 3 100.0%	4 / 4 100.0%	2 / 2 100.0%	1 / 1 100.0%	4 / 4 100.0%	4 / 4 100.0%	1 / 1 100.0%	1 / 1 100.0%	3 / 3 100.0%	1 / 1 100.0%	4 / 4 100.0%	2 / 2 100.0%	1 / 1 100.0%	1 / 1 100.0%	1 / 1 100.0%	3 / 3 100.0%	1 / 1 100.0%	2 / 2 100.0%	6 / 6 100.0%	3 / 3 100.0%	1 / 1 100.0%	7 / 7 100.0%	1 / 1 100.0%	0 / 0 0.0%	0 / 0 0.0%	0 / 0 0.0%	36 / 57 63.2%AB	21 / 57 36.8%ABE					
1	Burkholderia cepacia GG4	57 / 57 100.0%	3 / 3 100.0%	4 / 4 100.0%	2 / 2 100.0%	1 / 1 100.0%	4 / 4 100.0%	4 / 4 100.0%	1 / 1 100.0%	1 / 1 100.0%	3 / 3 100.0%	1 / 1 100.0%	4 / 4 100.0%	2 / 2 100.0%	1 / 1 100.0%	1 / 1 100.0%	1 / 1 100.0%	3 / 3 100.0%	1 / 1 100.0%	2 / 2 100.0%	6 / 6 100.0%	3 / 3 100.0%	1 / 1 100.0%	7 / 7 100.0%	1 / 1 100.0%	0 / 0 0.0%	0 / 0 0.0%	0 / 0 0.0%	36 / 57 63.2%AB	21 / 57 36.8%ABE					
1	ATCC BAA-248	68 / 69 98.6%	4 / 4 100.0%	4 / 4 100.0%	2 / 2 100.0%	5 / 5 100.0%	5 / 5 100.0%	4 / 4 100.0%	1 / 1 100.0%	1 / 1 100.0%	7 / 7 100.0%	1 / 1 100.0%	4 / 4 100.0%	2 / 2 100.0%	1 / 1 100.0%	1 / 1 100.0%	1 / 1 100.0%	3 / 3 100.0%	1 / 1 100.0%	3 / 3 100.0%	6 / 6 100.0%	3 / 3 100.0%	1 / 1 100.0%	7 / 8 87.5%	1 / 1 100.0%	0 / 0 0.0%	0 / 0 0.0%	0 / 0 0.0%	45 / 69 65.2%AB	23 / 69 33.3%ABE	1 / 69 1.4%E				
1	Burkholderia cepacia R1808	68 / 69 98.6%	4 / 4 100.0%	4 / 4 100.0%	2 / 2 100.0%	5 / 5 100.0%	5 / 5 100.0%	4 / 4 100.0%	1 / 1 100.0%	1 / 1 100.0%	7 / 7 100.0%	1 / 1 100.0%	4 / 4 100.0%	2 / 2 100.0%	1 / 1 100.0%	1 / 1 100.0%	1 / 1 100.0%	3 / 3 100.0%	1 / 1 100.0%	3 / 3 100.0%	6 / 6 100.0%	3 / 3 100.0%	1 / 1 100.0%	7 / 8 87.5%	1 / 1 100.0%	0 / 0 0.0%	0 / 0 0.0%	0 / 0 0.0%	45 / 69 65.2%AB	23 / 69 33.3%ABE	1 / 69 1.4%E				
4	ATCC BAA-245	275 / 278 98.9%	12 / 12 100.0%	16 / 16 100.0%	10 / 10 100.0%	20 / 20 100.0%	23 / 23 100.0%	16 / 16 100.0%	4 / 4 100.0%	4 / 4 100.0%	28 / 28 100.0%	4 / 4 100.0%	16 / 16 100.0%	8 / 8 100.0%	4 / 4 100.0%	4 / 4 100.0%	5 / 5 100.0%	12 / 12 100.0%	4 / 4 100.0%	12 / 12 100.0%	24 / 24 100.0%	12 / 12 100.0%	4 / 4 100.0%	29 / 32 90.6%	4 / 4 100.0%	0 / 0 0.0%	0 / 0 0.0%	0 / 0 0.0%	147 / 278 52.9%AB	60 / 278 21.6%ABE	39 / 278 14.0%B	29 / 278 10.4%BE	2 / 278 0.7%E	1 / 278 0.4%CE	
1	Burkholderia cenocepacia J2315	74 / 74 100.0%	3 / 3 100.0%	4 / 4 100.0%	4 / 4 100.0%	5 / 5 100.0%	8 / 8 100.0%	4 / 4 100.0%	1 / 1 100.0%	1 / 1 100.0%	7 / 7 100.0%	1 / 1 100.0%	4 / 4 100.0%	2 / 2 100.0%	1 / 1 100.0%	1 / 1 100.0%	2 / 2 100.0%	3 / 3 100.0%	1 / 1 100.0%	3 / 3 100.0%	6 / 6 100.0%	3 / 3 100.0%	1 / 1 100.0%	8 / 8 100.0%	1 / 1 100.0%	0 / 0 0.0%	0 / 0 0.0%	0 / 0 0.0%	30 / 74 40.5%AB	21 / 74 28.4%B	14 / 74 18.9%BE	9 / 74 12.2%ABE			
1	Burkholderia cenocepacia AU 1054	67 / 68 98.5%	3 / 3 100.0%	4 / 4 100.0%	2 / 2 100.0%	5 / 5 100.0%	5 / 5 100.0%	4 / 4 100.0%	1 / 1 100.0%	1 / 1 100.0%	7 / 7 100.0%	1 / 1 100.0%	4 / 4 100.0%	2 / 2 100.0%	1 / 1 100.0%	1 / 1 100.0%	1 / 1 100.0%	3 / 3 100.0%	1 / 1 100.0%	3 / 3 100.0%	6 / 6 100.0%	3 / 3 100.0%	1 / 1 100.0%	7 / 8 87.5%	1 / 1 100.0%	0 / 0 0.0%	0 / 0 0.0%	0 / 0 0.0%	45 / 68 66.2%AB	22 / 68 32.4%ABE	1 / 68 1.5%E				
1	Burkholderia cenocepacia HI2424	67 / 68 98.5%	3 / 3 100.0%	4 / 4 100.0%	2 / 2 100.0%	5 / 5 100.0%	5 / 5 100.0%	4 / 4 100.0%	1 / 1 100.0%	1 / 1 100.0%	7 / 7 100.0%	1 / 1 100.0%	4 / 4 100.0%	2 / 2 100.0%	1 / 1 100.0%	1 / 1 100.0%	1 / 1 100.0%	3 / 3 100.0%	1 / 1 100.0%	3 / 3 100.0%	6 / 6 100.0%	3 / 3 100.0%	1 / 1 100.0%	7 / 8 87.5%	1 / 1 100.0%	0 / 0 0.0%	0 / 0 0.0%	0 / 0 0.0%	45 / 68 66.2%AB	22 / 68 32.4%ABE	1 / 68 1.5%E				
1	Burkholderia cenocepacia MC0-3	67 / 68 98.5%	3 / 3 100.0%	4 / 4 100.0%	2 / 2 100.0%	5 / 5 100.0%	5 / 5 100.0%	4 / 4 100.0%	1 / 1 100.0%	1 / 1 100.0%	7 / 7 100.0%	1 / 1 100.0%	4 / 4 100.0%	2 / 2 100.0%	1 / 1 100.0%	1 / 1 100.0%	1 / 1 100.0%	3 / 3 100.0%	1 / 1 100.0%	3 / 3 100.0%	6 / 6 100.0%	3 / 3 100.0%	1 / 1 100.0%	7 / 8 87.5%	1 / 1 100.0%	0 / 0 0.0%	0 / 0 0.0%	0 / 0 0.0%	27 / 68 39.7%AB	18 / 68 26.5%B	15 / 68 22.1%BE	7 / 68 10.3%ABE	1 / 68 1.5%CE		
2	ATCC BAA-244	136 / 138 98.6%	7 / 7 100.0%	8 / 8 100.0%	4 / 4 100.0%	10 / 10 100.0%	10 / 10 100.0%	8 / 8 100.0%	2 / 2 100.0%	2 / 2 100.0%	14 / 14 100.0%	2 / 2 100.0%	8 / 8 100.0%	4 / 4 100.0%	2 / 2 100.0%	2 / 2 100.0%	2 / 3 66.7%	6 / 6 100.0%	2 / 2 100.0%	6 / 6 100.0%	12 / 12 100.0%	6 / 6 100.0%	2 / 2 100.0%	15 / 16 93.8%	2 / 2 100.0%	0 / 0 0.0%	0 / 0 0.0%	0 / 0 0.0%	68 / 138 49.3%AB	31 / 138 22.5%ABE	22 / 138 15.9%B	15 / 138 10.9%BE	1 / 138 0.7%E	1 / 138 0.7%	
1	Burkholderia ambifaria AMMD	68 / 69 98.6%	4 / 4 100.0%	4 / 4 100.0%	2 / 2 100.0%	5 / 5 100.0%	5 / 5 100.0%	4 / 4 100.0%	1 / 1 100.0%	1 / 1 100.0%	7 / 7 100.0%	1 / 1 100.0%	4 / 4 100.0%	2 / 2 100.0%	1 / 1 100.0%	1 / 1 100.0%	1 / 1 100.0%	3 / 3 100.0%	1 / 1 100.0%	3 / 3 100.0%	6 / 6 100.0%	3 / 3 100.0%	1 / 1 100.0%	7 / 8 87.5%	1 / 1 100.0%	0 / 0 0.0%	0 / 0 0.0%	0 / 0 0.0%	45 / 69 65.2%AB	22 / 69 31.9%ABE	1 / 69 1.4%BE	1 / 69 1.4%E			
1	Burkholderia ambifaria MC40-6	68 / 69 98.6%	3 / 3 100.0%	4 / 4 100.0%	2 / 2 100.0%	5 / 5 100.0%	5 / 5 100.0%	4 / 4 100.0%	1 / 1 100.0%	1 / 1 100.0%	7 / 7 100.0%	1 / 1 100.0%	4 / 4 100.0%	2 / 2 100.0%	1 / 1 100.0%	1 / 1 100.0%	1 / 2 50.0%	3 / 3 100.0%	1 / 1 100.0%	3 / 3 100.0%	6 / 6 100.0%	3 / 3 100.0%	1 / 1 100.0%	8 / 8 100.0%	1 / 1 100.0%	0 / 0 0.0%	0 / 0 0.0%	0 / 0 0.0%	23 / 69 33.3%AB	22 / 69 31.9%B	14 / 69 20.3%BE	9 / 69 13.0%ABE	1 / 69 1.4%		
1	ATCC 17760	68 / 69 98.6%	3 / 3 100.0%	4 / 4 100.0%	2 / 2 100.0%	5 / 5 100.0%	5 / 5 100.0%	4 / 4 100.0%	1 / 1 100.0%	1 / 1 100.0%	7 / 7 100.0%	1 / 1 100.0%	4 / 4 100.0%	2 / 2 100.0%	1 / 1 100.0%	1 / 1 100.0%	1 / 1 100.0%	3 / 3 100.0%	1 / 1 100.0%	3 / 3 100.0%	6 / 6 100.0%	3 / 3 100.0%	1 / 1 100.0%	7 / 8 87.5%	2 / 2 100.0%	0 / 0 0.0%	0 / 0 0.0%	0 / 0 0.0%	45 / 69 65.2%AB	23 / 69 33.3%ABE	1 / 69 1.4%E				
11	pseudomallei group	635 / 644 98.6%	22 / 22 100.0%	29 / 29 100.0%	22 / 22 100.0%	43 / 43 100.0%	42 / 42 100.0%	39 / 39 100.0%	11 / 11 100.0%	11 / 11 100.0%	65 / 65 100.0%	11 / 11 100.0%	43 / 43 100.0%	22 / 22 100.0%	11 / 11 100.0%	11 / 11 100.0%	20 / 20 100.0%	33 / 33 100.0%	11 / 11 100.0%	33 / 33 100.0%	55 / 55 100.0%	18 / 18 100.0%	10 / 10 100.0%	63 / 72 87.5%	10 / 10 100.0%	0 / 0 0.0%	0 / 0 0.0%	0 / 0 0.0%	435 / 644 67.5%AB	200 / 644 31.1%ABE	9 / 644 1.4%E				
4	"Acinetobacter mallei" (Zopf 1885) Steel and Cowan 1964	225 / 229 98.3%	8 / 8 100.0%	8 / 8 100.0%	8 / 8 100.0%	16 / 16 100.0%	14 / 14 100.0%	12 / 12 100.0%	4 / 4 100.0%	4 / 4 100.0%	24 / 24 100.0%	4 / 4 100.0%	16 / 16 100.0%	8 / 8 100.0%	4 / 4 100.0%	4 / 4 100.0%	8 / 8 100.0%	12 / 12 100.0%	4 / 4 100.0%	12 / 12 100.0%	20 / 20 100.0%	4 / 4 100.0%	4 / 4 100.0%	23 / 27 85.2%	4 / 4 100.0%	0 / 0 0.0%	0 / 0 0.0%	0 / 0 0.0%	154 / 229 67.2%AB	71 / 229 31.0%ABE	4 / 229 1.7%E				
1	Burkholderia mallei ATCC 23344	56 / 57 98.2%	2 / 2 100.0%	2 / 2 100.0%	2 / 2 100.0%	3 / 3 100.0%	4 / 4 100.0%	3 / 3 100.0%	1 / 1 100.0%	1 / 1 100.0%	6 / 6 100.0%	1 / 1 100.0%	4 / 4 100.0%	2 / 2 100.0%	1 / 1 100.0%	1 / 1 100.0%	2 / 2 100.0%	3 / 3 100.0%	1 / 1 100.0%	3 / 3 100.0%	5 / 5 100.0%	1 / 1 100.0%	1 / 1 100.0%	6 / 7 85.7%	1 / 1 100.0%	0 / 0 0.0%	0 / 0 0.0%	0 / 0 0.0%	38 / 57 66.7%AB	18 / 57 31.6%ABE	1 / 57 1.8%E				
1	Burkholderia mallei SAVP1	56 / 57 98.2%	2 / 2 100.0%	2 / 2 100.0%	2 / 2 100.0%	5 / 5 100.0%	3 / 3 100.0%	3 / 3 100.0%	1 / 1 100.0%	1 / 1 100.0%	6 / 6 100.0%	1 / 1 100.0%	4 / 4 100.0%	2 / 2 100.0%	1 / 1 100.0%	1 / 1 100.0%	2 / 2 100.0%	3 / 3 100.0%	1 / 1 100.0%	3 / 3 100.0%	5 / 5 100.0%	1 / 1 100.0%	1 / 1 100.0%	5 / 6 83.3%	1 / 1 100.0%	0 / 0 0.0%	0 / 0 0.0%	0 / 0 0.0%	39 / 57 68.4%AB	17 / 57 29.8%ABE	1 / 57 1.8%E				
1	Burkholderia mallei NCTC 10247	56 / 57 98.2%	2 / 2 100.0%	2 / 2 100.0%	2 / 2 100.0%	4 / 4 100.0%	3 / 3 100.0%	3 / 3 100.0%	1 / 1 100.0%	1 / 1 100.0%	6 / 6 100.0%	1 / 1 100.0%	4 / 4 100.0%	2 / 2 100.0%	1 / 1 100.0%	1 / 1 100.0%	2 / 2 100.0%	3 / 3 100.0%	1 / 1 100.0%	3 / 3 100.0%	5 / 5 100.0%	1 / 1 100.0%	1 / 1 100.0%	6 / 7 85.7%	1 / 1 100.0%	0 / 0 0.0%	0 / 0 0.0%	0 / 0 0.0%	38 / 57 66.7%AB	18 / 57 31.6%ABE	1 / 57 1.8%E				
1	Burkholderia mallei 10229	57 / 58 98.3%	2 / 2 100.0%	2 / 2 100.0%	2 / 2 100.0%	4 / 4 100.0%	4 / 4 100.0%	3 / 3 100.0%	1 / 1 100.0%	1 / 1 100.0%	6 / 6 100.0%	1 / 1 100.0%	4 / 4 100.0%	2 / 2 100.0%	1 / 1 100.0%	1 / 1 100.0%	2 / 2 100.0%	3 / 3 100.0%	1 / 1 100.0%	3 / 3 100.0%	5 / 5 100.0%	1 / 1 100.0%	1 / 1 100.0%	6 / 7 85.7%	1 / 1 100.0%	0 / 0 0.0%	0 / 0 0.0%	0 / 0 0.0%	39 / 58 67.2%AB	18 / 58 31.0%ABE	1 / 58 1.7%E				
6	"Bacillus pseudomallei" Whitmore 1913	352 / 356 98.9%	12 / 12 100.0%	18 / 18 100.0%	12 / 12 100.0%	23 / 23 100.0%	24 / 24 100.0%	23 / 23 100.0%	6 / 6 100.0%	6 / 6 100.0%	35 / 35 100.0%	6 / 6 100.0%	23 / 23 100.0%	12 / 12 100.0%	6 / 6 100.0%	6 / 6 100.0%	11 / 11 100.0%	18 / 18 100.0%	6 / 6 100.0%	18 / 18 100.0%	30 / 30 100.0%	12 / 12 100.0%	5 / 5 100.0%	35 / 39 89.7%	5 / 5 100.0%	0 / 0 0.0%	0 / 0 0.0%	0 / 0 0.0%	241 / 356 67.7%AB	111 / 356 31.2%ABE	4 / 356 1.1%E				
1	Burkholderia pseudomallei K96243	60 / 61 98.4%	2 / 2 100.0%	3 / 3 100.0%	2 / 2 100.0%	4 / 4 100.0%	4 / 4 100.0%	4 / 4 100.0%	1 / 1 100.0%	1 / 1 100.0%	6 / 6 100.0%	1 / 1 100.0%	4 / 4 100.0%	2 / 2 100.0%	1 / 1 100.0%	1 / 1 100.0%	2 / 2 100.0%	3 / 3 100.0%	1 / 1 100.0%	3 / 3 100.0%	5 / 5 100.0%	2 / 2 100.0%	1 / 1 100.0%	6 / 7 85.7%	1 / 1 100.0%	0 / 0 0.0%	0 / 0 0.0%	0 / 0 0.0%	41 / 61 67.2%AB	19 / 61 31.1%ABE	1 / 61 1.6%E				
1	Burkholderia pseudomallei 1710b	60 / 61 98.4%	2 / 2 100.0%	3 / 3 100.0%	2 / 2 100.0%	4 / 4 100.0%	4 / 4 100.0%	4 / 4 100.0%	1 / 1 100.0%	1 / 1 100.0%	6 / 6 100.0%	1 / 1 100.0%	4 / 4 100.0%	2 / 2 100.0%	1 / 1 100.0%	1 / 1 100.0%	2 / 2 100.0%	3 / 3 100.0%	1 / 1 100.0%	3 / 3 100.0%	5 / 5 100.0%	2 / 2 100.0%	1 / 1 100.0%	6 / 7 85.7%	1 / 1 100.0%	0 / 0 0.0%	0 / 0 0.0%	0 / 0 0.0%	41 / 61 67.2%AB	19 / 61 31.1%ABE	1 / 61 1.6%E				
1	Burkholderia pseudomallei 668	60 / 61 98.4%	2 / 2 100.0%	3 / 3 100.0%	2 / 2 100.0%	4 / 4 100.0%	4 / 4 100.0%	4 / 4 100.0%	1 / 1 100.0%	1 / 1 100.0%	6 / 6 100.0%	1 / 1 100.0%	4 / 4 100.0%	2 / 2 100.0%	1 / 1 100.0%	1 / 1 100.0%	2 / 2 100.0%	3 / 3 100.0%	1 / 1 100.0%	3 / 3 100.0%	5 / 5 100.0%	2 / 2 100.0%	1 / 1 100.0%	6 / 7 85.7%	1 / 1 100.0%	0 / 0 0.0%	0 / 0 0.0%	0 / 0 0.0%	41 / 61 67.2%AB	19 / 61 31.1%ABE	1 / 61 1.6%E				
1	Burkholderia pseudomallei 1106a	60 / 61 98.4%	2 / 2 100.0%	3 / 3 100.0%	2 / 2 100.0%	4 / 4 100.0%	4 / 4 100.0%	4 / 4 100.0%	1 / 1 100.0%	1 / 1 100.0%	6 / 6 100.0%	1 / 1 100.0%	4 / 4 100.0%	2 / 2 100.0%	1 / 1 100.0%	1 / 1 100.0%	2 / 2 100.0%	3 / 3 100.0%	1 / 1 100.0%	3 / 3 100.0%	5 / 5 100.0%	2 / 2 100.0%	1 / 1 100.0%	6 / 7 85.7%	1 / 1 100.0%	0 / 0 0.0%	0 / 0 0.0%	0 / 0 0.0%	41 / 61 67.2%AB	19 / 61 31.1%ABE	1 / 61 1.6%E				
1	Burkholderia pseudomallei MSHR346	52 / 52 100.0%	2 / 2 100.0%	3 / 3 100.0%	2 / 2 100.0%	3 / 3 100.0%	4 / 4 100.0%	3 / 3 100.0%	1 / 1 100.0%	1 / 1 100.0%	5 / 5 100.0%	1 / 1 100.0%	3 / 3 100.0%	2 / 2 100.0%	1 / 1 100.0%	1 / 1 100.0%	1 / 1 100.0%	3 / 3 100.0%	1 / 1 100.0%	3 / 3 100.0%	5 / 5 100.0%	2 / 2 100.0%	0 / 0 0.0%	5 / 5 100.0%	0 / 0 0.0%	0 / 0 0.0%	0 / 0 0.0%	0 / 0 0.0%	36 / 52 69.2%AB	16 / 52 30.8%ABE					
1	Burkholderia pseudomallei 1026b	60 / 60 100.0%	2 / 2 100.0%	3 / 3 100.0%	2 / 2 100.0%	4 / 4 100.0%	4 / 4 100.0%	4 / 4 100.0%	1 / 1 100.0%	1 / 1 100.0%	6 / 6 100.0%	1 / 1 100.0%	4 / 4 100.0%	2 / 2 100.0%	1 / 1 100.0%	1 / 1 100.0%	2 / 2 100.0%	3 / 3 100.0%	1 / 1 100.0%	3 / 3 100.0%	5 / 5 100.0%	2 / 2 100.0%	1 / 1 100.0%	6 / 6 100.0%	1 / 1 100.0%	0 / 0 0.0%	0 / 0 0.0%	0 / 0 0.0%	41 / 60 68.3%AB	19 / 60 31.7%ABE					
1	ATCC 700388	58 / 59 98.3%	2 / 2 100.0%	3 / 3 100.0%	2 / 2 100.0%	4 / 4 100.0%	4 / 4 100.0%	4 / 4 100.0%	1 / 1 100.0%	1 / 1 100.0%	6 / 6 100.0%	1 / 1 100.0%	4 / 4 100.0%	2 / 2 100.0%	1 / 1 100.0%	1 / 1 100.0%	1 / 1 100.0%	3 / 3 100.0%	1 / 1 100.0%	3 / 3 100.0%	5 / 5 100.0%	2 / 2 100.0%	1 / 1 100.0%	5 / 6 83.3%	1 / 1 100.0%	0 / 0 0.0%	0 / 0 0.0%	0 / 0 0.0%	40 / 59 67.8%AB	18 / 59 30.5%ABE	1 / 59 1.7%E				
1	Burkholderia thailandensis ATCC 70038	58 / 59 98.3%	2 / 2 100.0%	3 / 3 100.0%	2 / 2 100.0%	4 / 4 100.0%	4 / 4 100.0%	4 / 4 100.0%	1 / 1 100.0%	1 / 1 100.0%	6 / 6 100.0%	1 / 1 100.0%	4 / 4 100.0%	2 / 2 100.0%	1 / 1 100.0%	1 / 1 100.0%	1 / 1 100.0%	3 / 3 100.0%	1 / 1 100.0%	3 / 3 100.0%	5 / 5 100.0%	2 / 2 100.0%	1 / 1 100.0%	5 / 6 83.3%	1 / 1 100.0%	0 / 0 0.0%	0 / 0 0.0%	0 / 0 0.0%	40 / 59 67.8%AB	18 / 59 30.5%ABE	1 / 59 1.7%E				
1	Burkholderia phymatum	61 / 63 96.8%	2 / 2 100.0%	3 / 3 100.0%	2 / 2 100.0%	6 / 6 100.0%	4 / 4 100.0%	4 / 4 100.0%	1 / 1 100.0%	1 / 1 100.0%	8 / 9 88.9%	1 / 1 100.0%	4 / 4 100.0%	2 / 2 100.0%	1 / 1 100.0%	1 / 1 100.0%	1 / 1 100.0%	3 / 3 100.0%	1 / 1 100.0%	3 / 3 100.0%	5 / 5 100.0%	2 / 2 100.0%	0 / 0 0.0%	6 / 6 100.0%	0 / 1 0.0%	0 / 0 0.0%	0 / 0 0.0%	0 / 0 0.0%	30 / 63 47.6%AB	13 / 63 20.6%AB	10 / 63 15.9%BE	8 / 63 12.7%ABE	1 / 63 1.6%CE	1 / 63 1.6%	
1	Burkholderia phymatum STM815	61 / 63 96.8%	2 / 2 100.0%	3 / 3 100.0%	2 / 2 100.0%	6 / 6 100.0%	4 / 4 100.0%	4 / 4 100.0%	1 / 1 100.0%	1 / 1 100.0%	8 / 9 88.9%	1 / 1 100.0%	4 / 4 100.0%	2 / 2 100.0%	1 / 1 100.0%	1 / 1 100.0%	1 / 1 100.0%	3 / 3 100.0%	1 / 1 100.0%	3 / 3 100.0%	5 / 5 100.0%	2 / 2 100.0%	0 / 0 0.0%	6 / 6 100.0%	0 / 1 0.0%	0 / 0 0.0%	0 / 0 0.0%	0 / 0 0.0%	30 / 63 47.6%AB	13 / 63 20.6%AB	10 / 63 15.9%BE	8 / 63 12.7%ABE	1 / 63 1.6%	1 / 63 1.6%CE	
1	Burkholderia phytofirmans	62 / 63 98.4%	2 / 2 100.0%	3 / 3 100.0%	2 / 2 100.0%	6 / 6 100.0%	4 / 4 100.0%	4 / 4 100.0%	1 / 1 100.0%	1 / 1 100.0%	7 / 8 87.5%	1 / 1 100.0%	4 / 4 100.0%	2 / 2 100.0%	1 / 1 100.0%	1 / 1 100.0%	1 / 1 100.0%	3 / 3 100.0%	1 / 1 100.0%	3 / 3 100.0%	5 / 5 100.0%	2 / 2 100.0%	1 / 1 100.0%	6 / 6 100.0%	1 / 1 100.0%	0 / 0 0.0%	0 / 0 0.0%	0 / 0 0.0%	24 / 63 38.1%B	18 / 63 28.6%AB	12 / 63 19.0%BE	8 / 63 12.7%ABE	1 / 63 1.6%		
1	Burkholderia phytofirmans PsJN	62 / 63 98.4%	2 / 2 100.0%	3 / 3 100.0%	2 / 2 100.0%	6 / 6 100.0%	4 / 4 100.0%	4 / 4 100.0%	1 / 1 100.0%	1 / 1 100.0%	7 / 8 87.5%	1 / 1 100.0%	4 / 4 100.0%	2 / 2 100.0%	1 / 1 100.0%	1 / 1 100.0%	1 / 1 100.0%	3 / 3 100.0%	1 / 1 100.0%	3 / 3 100.0%	5 / 5 100.0%	2 / 2 100.0%	1 / 1 100.0%	6 / 6 100.0%	1 / 1 100.0%	0 / 0 0.0%	0 / 0 0.0%	0 / 0 0.0%	24 / 63 38.1%B	18 / 63 28.6%AB	12 / 63 19.0%BE	8 / 63 12.7%ABE	1 / 63 1.6%		
1	"Burkholderia rhizoxina" Scherlach et al 2006	45 / 46 97.8%	2 / 2 100.0%	1 / 1 100.0%	1 / 1 100.0%	2 / 3 66.7%	4 / 4 100.0%	3 / 3 100.0%	1 / 1 100.0%	1 / 1 100.0%	5 / 5 100.0%	1 / 1 100.0%	4 / 4 100.0%	2 / 2 100.0%	1 / 1 100.0%	1 / 1 100.0%	1 / 1 100.0%	3 / 3 100.0%	1 / 1 100.0%	2 / 2 100.0%	4 / 4 100.0%	1 / 1 100.0%	1 / 1 100.0%	3 / 3 100.0%	0 / 0 0.0%	0 / 0 0.0%	0 / 0 0.0%	0 / 0 0.0%	31 / 46 67.4%AB	14 / 46 30.4%ABE	1 / 46 2.2%				
1	Burkholderia rhizoxinica HKI 454	45 / 46 97.8%	2 / 2 100.0%	1 / 1 100.0%	1 / 1 100.0%	2 / 3 66.7%	4 / 4 100.0%	3 / 3 100.0%	1 / 1 100.0%	1 / 1 100.0%	5 / 5 100.0%	1 / 1 100.0%	4 / 4 100.0%	2 / 2 100.0%	1 / 1 100.0%	1 / 1 100.0%	1 / 1 100.0%	3 / 3 100.0%	1 / 1 100.0%	2 / 2 100.0%	4 / 4 100.0%	1 / 1 100.0%	1 / 1 100.0%	3 / 3 100.0%	0 / 0 0.0%	0 / 0 0.0%	0 / 0 0.0%	0 / 0 0.0%	31 / 46 67.4%AB	14 / 46 30.4%ABE	1 / 46 2.2%				
1	Burkholderia sp. KJ006	66 / 66 100.0%	2 / 2 100.0%	4 / 4 100.0%	2 / 2 100.0%	5 / 5 100.0%	5 / 5 100.0%	4 / 4 100.0%	1 / 1 100.0%	1 / 1 100.0%	7 / 7 100.0%	1 / 1 100.0%	4 / 4 100.0%	2 / 2 100.0%	1 / 1 100.0%	1 / 1 100.0%	1 / 1 100.0%	3 / 3 100.0%	1 / 1 100.0%	3 / 3 100.0%	6 / 6 100.0%	3 / 3 100.0%	1 / 1 100.0%	7 / 7 100.0%	1 / 1 100.0%	0 / 0 0.0%	0 / 0 0.0%	0 / 0 0.0%	45 / 66 68.2%AB	21 / 66 31.8%ABE					
1	Burkholderia sp. CCGE1001	61 / 62 98.4%	2 / 2 100.0%	3 / 3 100.0%	2 / 2 100.0%	6 / 6 100.0%	4 / 4 100.0%	4 / 4 100.0%	1 / 1 100.0%	1 / 1 100.0%	7 / 8 87.5%	1 / 1 100.0%	4 / 4 100.0%	3 / 3 100.0%	1 / 1 100.0%	1 / 1 100.0%	1 / 1 100.0%	3 / 3 100.0%	1 / 1 100.0%	3 / 3 100.0%	5 / 5 100.0%	2 / 2 100.0%	0 / 0 0.0%	6 / 6 100.0%	0 / 0 0.0%	0 / 0 0.0%	0 / 0 0.0%	0 / 0 0.0%	43 / 62 69.4%AB	18 / 62 29.0%ABE	1 / 62 1.6%				
1	Burkholderia sp. CCGE1002	69 / 72 95.8%	2 / 2 100.0%	4 / 4 100.0%	3 / 3 100.0%	7 / 7 100.0%	5 / 5 100.0%	4 / 4 100.0%	1 / 1 100.0%	1 / 1 100.0%	8 / 9 88.9%	1 / 1 100.0%	4 / 4 100.0%	2 / 4 50.0%	1 / 1 100.0%	2 / 2 100.0%	1 / 1 100.0%	3 / 3 100.0%	1 / 1 100.0%	4 / 4 100.0%	5 / 5 100.0%	2 / 2 100.0%	1 / 1 100.0%	7 / 7 100.0%	0 / 0 0.0%	0 / 0 0.0%	0 / 0 0.0%	0 / 0 0.0%	47 / 72 65.3%AB	22 / 72 30.6%ABE	3 / 72 4.2%				
1	Burkholderia sp. CCGE1003	62 / 63 98.4%	2 / 2 100.0%	3 / 3 100.0%	2 / 2 100.0%	6 / 6 100.0%	4 / 4 100.0%	5 / 5 100.0%	1 / 1 100.0%	1 / 1 100.0%	7 / 8 87.5%	1 / 1 100.0%	4 / 4 100.0%	3 / 3 100.0%	1 / 1 100.0%	1 / 1 100.0%	1 / 1 100.0%	3 / 3 100.0%	1 / 1 100.0%	3 / 3 100.0%	5 / 5 100.0%	2 / 2 100.0%	0 / 0 0.0%	6 / 6 100.0%	0 / 0 0.0%	0 / 0 0.0%	0 / 0 0.0%	0 / 0 0.0%	44 / 63 69.8%AB	18 / 63 28.6%ABE	1 / 63 1.6%				
1	Burkholderia sp. RPE64	61 / 62 98.4%	2 / 2 100.0%	3 / 3 100.0%	2 / 2 100.0%	5 / 5 100.0%	4 / 4 100.0%	4 / 4 100.0%	1 / 1 100.0%	1 / 1 100.0%	6 / 7 85.7%	1 / 1 100.0%	4 / 4 100.0%	2 / 2 100.0%	1 / 1 100.0%	2 / 2 100.0%	1 / 1 100.0%	3 / 3 100.0%	1 / 1 100.0%	3 / 3 100.0%	5 / 5 100.0%	2 / 2 100.0%	1 / 1 100.0%	6 / 6 100.0%	1 / 1 100.0%	0 / 0 0.0%	0 / 0 0.0%	0 / 0 0.0%	40 / 62 64.5%AB	21 / 62 33.9%ABE	1 / 62 1.6%				
1	Burkholderia sp. YI23	63 / 64 98.4%	2 / 2 100.0%	3 / 3 100.0%	2 / 2 100.0%	6 / 6 100.0%	4 / 4 100.0%	4 / 4 100.0%	1 / 1 100.0%	1 / 1 100.0%	7 / 8 87.5%	1 / 1 100.0%	4 / 4 100.0%	2 / 2 100.0%	1 / 1 100.0%	1 / 1 100.0%	1 / 1 100.0%	3 / 3 100.0%	1 / 1 100.0%	3 / 3 100.0%	5 / 5 100.0%	2 / 2 100.0%	1 / 1 100.0%	7 / 7 100.0%	1 / 1 100.0%	0 / 0 0.0%	0 / 0 0.0%	0 / 0 0.0%	42 / 64 65.6%AB	21 / 64 32.8%ABE	1 / 64 1.6%				
2	Polynucleibacter	75 / 75 100.0%	2 / 2 100.0%	2 / 2 100.0%	2 / 2 100.0%	2 / 2 100.0%	4 / 4 100.0%	4 / 4 100.0%	2 / 2 100.0%	2 / 2 100.0%	4 / 4 100.0%	2 / 2 100.0%	8 / 8 100.0%	2 / 2 100.0%	2 / 2 100.0%	2 / 2 100.0%	2 / 2 100.0%	4 / 4 100.0%	2 / 2 100.0%	5 / 5 100.0%	8 / 8 100.0%	2 / 2 100.0%	2 / 2 100.0%	10 / 10 100.0%	0 / 0 0.0%	0 / 0 0.0%	0 / 0 0.0%	0 / 0 0.0%	36 / 75 48.0%AB	24 / 75 32.0%ABE	9 / 75 12.0%B	6 / 75 8.0%BE			
2	ATCC 30859	75 / 75 100.0%	2 / 2 100.0%	2 / 2 100.0%	2 / 2 100.0%	2 / 2 100.0%	4 / 4 100.0%	4 / 4 100.0%	2 / 2 100.0%	2 / 2 100.0%	4 / 4 100.0%	2 / 2 100.0%	8 / 8 100.0%	2 / 2 100.0%	2 / 2 100.0%	2 / 2 100.0%	2 / 2 100.0%	4 / 4 100.0%	2 / 2 100.0%	5 / 5 100.0%	8 / 8 100.0%	2 / 2 100.0%	2 / 2 100.0%	10 / 10 100.0%	0 / 0 0.0%	0 / 0 0.0%	0 / 0 0.0%	0 / 0 0.0%	36 / 75 48.0%AB	24 / 75 32.0%ABE	9 / 75 12.0%B	6 / 75 8.0%BE			
1	Polynucleobacter necessarius subsp. necessarius	37 / 37 100.0%	1 / 1 100.0%	1 / 1 100.0%	1 / 1 100.0%	1 / 1 100.0%	2 / 2 100.0%	2 / 2 100.0%	1 / 1 100.0%	1 / 1 100.0%	2 / 2 100.0%	1 / 1 100.0%	4 / 4 100.0%	1 / 1 100.0%	1 / 1 100.0%	1 / 1 100.0%	1 / 1 100.0%	2 / 2 100.0%	1 / 1 100.0%	2 / 2 100.0%	4 / 4 100.0%	1 / 1 100.0%	1 / 1 100.0%	5 / 5 100.0%	0 / 0 0.0%	0 / 0 0.0%	0 / 0 0.0%	0 / 0 0.0%	13 / 37 35.1%AB	9 / 37 24.3%B	9 / 37 24.3%ABE	6 / 37 16.2%BE			
1	Polynucleobacter necessarius STIR1	37 / 37 100.0%	1 / 1 100.0%	1 / 1 100.0%	1 / 1 100.0%	1 / 1 100.0%	2 / 2 100.0%	2 / 2 100.0%	1 / 1 100.0%	1 / 1 100.0%	2 / 2 100.0%	1 / 1 100.0%	4 / 4 100.0%	1 / 1 100.0%	1 / 1 100.0%	1 / 1 100.0%	1 / 1 100.0%	2 / 2 100.0%	1 / 1 100.0%	2 / 2 100.0%	4 / 4 100.0%	1 / 1 100.0%	1 / 1 100.0%	5 / 5 100.0%	0 / 0 0.0%	0 / 0 0.0%	0 / 0 0.0%	0 / 0 0.0%	13 / 37 35.1%AB	9 / 37 24.3%B	9 / 37 24.3%ABE	6 / 37 16.2%BE			
1	Burkholderiaceae bacterium KF022	38 / 38 100.0%	1 / 1 100.0%	1 / 1 100.0%	1 / 1 100.0%	1 / 1 100.0%	2 / 2 100.0%	2 / 2 100.0%	1 / 1 100.0%	1 / 1 100.0%	2 / 2 100.0%	1 / 1 100.0%	4 / 4 100.0%	1 / 1 100.0%	1 / 1 100.0%	1 / 1 100.0%	1 / 1 100.0%	2 / 2 100.0%	1 / 1 100.0%	3 / 3 100.0%	4 / 4 100.0%	1 / 1 100.0%	1 / 1 100.0%	5 / 5 100.0%	0 / 0 0.0%	0 / 0 0.0%	0 / 0 0.0%	0 / 0 0.0%	23 / 38 60.5%AB	15 / 38 39.5%ABE					
1	Polynucleobacter necessarius subsp. asymbioticus QLW-P1DMWA-1	38 / 38 100.0%	1 / 1 100.0%	1 / 1 100.0%	1 / 1 100.0%	1 / 1 100.0%	2 / 2 100.0%	2 / 2 100.0%	1 / 1 100.0%	1 / 1 100.0%	2 / 2 100.0%	1 / 1 100.0%	4 / 4 100.0%	1 / 1 100.0%	1 / 1 100.0%	1 / 1 100.0%	1 / 1 100.0%	2 / 2 100.0%	1 / 1 100.0%	3 / 3 100.0%	4 / 4 100.0%	1 / 1 100.0%	1 / 1 100.0%	5 / 5 100.0%	0 / 0 0.0%	0 / 0 0.0%	0 / 0 0.0%	0 / 0 0.0%	23 / 38 60.5%AB	15 / 38 39.5%ABE					
8	Ralstonia	413 / 413 100.0%	15 / 15 100.0%	21 / 21 100.0%	9 / 9 100.0%	24 / 24 100.0%	31 / 31 100.0%	24 / 24 100.0%	8 / 8 100.0%	7 / 7 100.0%	45 / 45 100.0%	8 / 8 100.0%	32 / 32 100.0%	16 / 16 100.0%	8 / 8 100.0%	8 / 8 100.0%	8 / 8 100.0%	24 / 24 100.0%	8 / 8 100.0%	23 / 23 100.0%	32 / 32 100.0%	13 / 13 100.0%	8 / 8 100.0%	41 / 41 100.0%	0 / 0 0.0%	0 / 0 0.0%	0 / 0 0.0%	0 / 0 0.0%	254 / 413 61.5%AB	125 / 413 30.3%ABE	22 / 413 5.3%B	12 / 413 2.9%BE			
6	"Bacillus solanacearum" Smith 1896	309 / 309 100.0%	10 / 10 100.0%	17 / 17 100.0%	6 / 6 100.0%	18 / 18 100.0%	23 / 23 100.0%	18 / 18 100.0%	6 / 6 100.0%	5 / 5 100.0%	35 / 35 100.0%	6 / 6 100.0%	24 / 24 100.0%	12 / 12 100.0%	6 / 6 100.0%	6 / 6 100.0%	6 / 6 100.0%	18 / 18 100.0%	6 / 6 100.0%	17 / 17 100.0%	24 / 24 100.0%	11 / 11 100.0%	6 / 6 100.0%	29 / 29 100.0%	0 / 0 0.0%	0 / 0 0.0%	0 / 0 0.0%	0 / 0 0.0%	208 / 309 67.3%AB	101 / 309 32.7%ABE					
1	Ralstonia solanacearum CMR15	54 / 54 100.0%	3 / 3 100.0%	3 / 3 100.0%	1 / 1 100.0%	3 / 3 100.0%	4 / 4 100.0%	3 / 3 100.0%	1 / 1 100.0%	1 / 1 100.0%	6 / 6 100.0%	1 / 1 100.0%	4 / 4 100.0%	2 / 2 100.0%	1 / 1 100.0%	1 / 1 100.0%	1 / 1 100.0%	3 / 3 100.0%	1 / 1 100.0%	3 / 3 100.0%	4 / 4 100.0%	2 / 2 100.0%	1 / 1 100.0%	5 / 5 100.0%	0 / 0 0.0%	0 / 0 0.0%	0 / 0 0.0%	0 / 0 0.0%	36 / 54 66.7%AB	18 / 54 33.3%ABE					
1	Ralstonia solanacearum CFBP2957	52 / 52 100.0%	1 / 1 100.0%	3 / 3 100.0%	1 / 1 100.0%	3 / 3 100.0%	4 / 4 100.0%	3 / 3 100.0%	1 / 1 100.0%	1 / 1 100.0%	6 / 6 100.0%	1 / 1 100.0%	4 / 4 100.0%	2 / 2 100.0%	1 / 1 100.0%	1 / 1 100.0%	1 / 1 100.0%	3 / 3 100.0%	1 / 1 100.0%	3 / 3 100.0%	4 / 4 100.0%	2 / 2 100.0%	1 / 1 100.0%	5 / 5 100.0%	0 / 0 0.0%	0 / 0 0.0%	0 / 0 0.0%	0 / 0 0.0%	35 / 52 67.3%AB	17 / 52 32.7%ABE					
1	Ralstonia solanacearum PSI07	52 / 52 100.0%	1 / 1 100.0%	3 / 3 100.0%	1 / 1 100.0%	3 / 3 100.0%	4 / 4 100.0%	3 / 3 100.0%	1 / 1 100.0%	1 / 1 100.0%	6 / 6 100.0%	1 / 1 100.0%	4 / 4 100.0%	2 / 2 100.0%	1 / 1 100.0%	1 / 1 100.0%	1 / 1 100.0%	3 / 3 100.0%	1 / 1 100.0%	3 / 3 100.0%	4 / 4 100.0%	2 / 2 100.0%	1 / 1 100.0%	5 / 5 100.0%	0 / 0 0.0%	0 / 0 0.0%	0 / 0 0.0%	0 / 0 0.0%	35 / 52 67.3%AB	17 / 52 32.7%ABE					
1	Ralstonia solanacearum Po82	52 / 52 100.0%	1 / 1 100.0%	3 / 3 100.0%	1 / 1 100.0%	3 / 3 100.0%	4 / 4 100.0%	3 / 3 100.0%	1 / 1 100.0%	1 / 1 100.0%	6 / 6 100.0%	1 / 1 100.0%	4 / 4 100.0%	2 / 2 100.0%	1 / 1 100.0%	1 / 1 100.0%	1 / 1 100.0%	3 / 3 100.0%	1 / 1 100.0%	3 / 3 100.0%	4 / 4 100.0%	2 / 2 100.0%	1 / 1 100.0%	5 / 5 100.0%	0 / 0 0.0%	0 / 0 0.0%	0 / 0 0.0%	0 / 0 0.0%	35 / 52 67.3%AB	17 / 52 32.7%ABE					
1	Ralstonia solanacearum FQY_4	46 / 46 100.0%	2 / 2 100.0%	2 / 2 100.0%	1 / 1 100.0%	3 / 3 100.0%	3 / 3 100.0%	3 / 3 100.0%	1 / 1 100.0%	0 / 0 0.0%	5 / 5 100.0%	1 / 1 100.0%	4 / 4 100.0%	2 / 2 100.0%	1 / 1 100.0%	1 / 1 100.0%	1 / 1 100.0%	3 / 3 100.0%	1 / 1 100.0%	2 / 2 100.0%	4 / 4 100.0%	1 / 1 100.0%	1 / 1 100.0%	4 / 4 100.0%	0 / 0 0.0%	0 / 0 0.0%	0 / 0 0.0%	0 / 0 0.0%	32 / 46 69.6%AB	14 / 46 30.4%ABE					
2	ATCC 27511	104 / 104 100.0%	5 / 5 100.0%	4 / 4 100.0%	3 / 3 100.0%	6 / 6 100.0%	8 / 8 100.0%	6 / 6 100.0%	2 / 2 100.0%	2 / 2 100.0%	10 / 10 100.0%	2 / 2 100.0%	8 / 8 100.0%	4 / 4 100.0%	2 / 2 100.0%	2 / 2 100.0%	2 / 2 100.0%	6 / 6 100.0%	2 / 2 100.0%	6 / 6 100.0%	8 / 8 100.0%	2 / 2 100.0%	2 / 2 100.0%	12 / 12 100.0%	0 / 0 0.0%	0 / 0 0.0%	0 / 0 0.0%	0 / 0 0.0%	46 / 104 44.2%AB	24 / 104 23.1%ABE	22 / 104 21.2%B	12 / 104 11.5%BE			
1	Ralstonia pickettii 12J	53 / 53 100.0%	3 / 3 100.0%	2 / 2 100.0%	2 / 2 100.0%	3 / 3 100.0%	4 / 4 100.0%	3 / 3 100.0%	1 / 1 100.0%	1 / 1 100.0%	5 / 5 100.0%	1 / 1 100.0%	4 / 4 100.0%	2 / 2 100.0%	1 / 1 100.0%	1 / 1 100.0%	1 / 1 100.0%	3 / 3 100.0%	1 / 1 100.0%	3 / 3 100.0%	4 / 4 100.0%	1 / 1 100.0%	1 / 1 100.0%	6 / 6 100.0%	0 / 0 0.0%	0 / 0 0.0%	0 / 0 0.0%	0 / 0 0.0%	22 / 53 41.5%B	13 / 53 24.5%AB	12 / 53 22.6%BE	6 / 53 11.3%ABE			
1	Ralstonia pickettii 12D	51 / 51 100.0%	2 / 2 100.0%	2 / 2 100.0%	1 / 1 100.0%	3 / 3 100.0%	4 / 4 100.0%	3 / 3 100.0%	1 / 1 100.0%	1 / 1 100.0%	5 / 5 100.0%	1 / 1 100.0%	4 / 4 100.0%	2 / 2 100.0%	1 / 1 100.0%	1 / 1 100.0%	1 / 1 100.0%	3 / 3 100.0%	1 / 1 100.0%	3 / 3 100.0%	4 / 4 100.0%	1 / 1 100.0%	1 / 1 100.0%	6 / 6 100.0%	0 / 0 0.0%	0 / 0 0.0%	0 / 0 0.0%	0 / 0 0.0%	33 / 51 64.7%AB	18 / 51 35.3%ABE					
5	Cupravidus	360 / 361 99.7%	18 / 18 100.0%	16 / 16 100.0%	8 / 8 100.0%	22 / 22 100.0%	26 / 26 100.0%	23 / 23 100.0%	6 / 6 100.0%	6 / 6 100.0%	38 / 38 100.0%	6 / 6 100.0%	24 / 24 100.0%	18 / 18 100.0%	6 / 6 100.0%	6 / 6 100.0%	6 / 6 100.0%	18 / 18 100.0%	6 / 6 100.0%	20 / 20 100.0%	28 / 29 96.6%	10 / 10 100.0%	6 / 6 100.0%	43 / 43 100.0%	0 / 0 0.0%	0 / 0 0.0%	0 / 0 0.0%	0 / 0 0.0%	185 / 361 51.2%AB	103 / 361 28.5%ABE	48 / 361 13.3%B	24 / 361 6.6%BE	1 / 361 0.3%		
2	ATCC 43291	181 / 181 100.0%	10 / 10 100.0%	7 / 7 100.0%	3 / 3 100.0%	12 / 12 100.0%	12 / 12 100.0%	12 / 12 100.0%	3 / 3 100.0%	3 / 3 100.0%	19 / 19 100.0%	3 / 3 100.0%	12 / 12 100.0%	9 / 9 100.0%	3 / 3 100.0%	3 / 3 100.0%	3 / 3 100.0%	9 / 9 100.0%	3 / 3 100.0%	9 / 9 100.0%	15 / 15 100.0%	4 / 4 100.0%	3 / 3 100.0%	24 / 24 100.0%	0 / 0 0.0%	0 / 0 0.0%	0 / 0 0.0%	0 / 0 0.0%	94 / 181 51.9%AB	54 / 181 29.8%ABE	22 / 181 12.2%B	11 / 181 6.1%BE			
1	Alcaligenes eutropha H16	118 / 118 100.0%	6 / 6 100.0%	4 / 4 100.0%	2 / 2 100.0%	8 / 8 100.0%	8 / 8 100.0%	8 / 8 100.0%	2 / 2 100.0%	2 / 2 100.0%	12 / 12 100.0%	2 / 2 100.0%	8 / 8 100.0%	6 / 6 100.0%	2 / 2 100.0%	2 / 2 100.0%	2 / 2 100.0%	6 / 6 100.0%	2 / 2 100.0%	6 / 6 100.0%	10 / 10 100.0%	2 / 2 100.0%	2 / 2 100.0%	16 / 16 100.0%	0 / 0 0.0%	0 / 0 0.0%	0 / 0 0.0%	0 / 0 0.0%	54 / 118 45.8%AB	31 / 118 26.3%ABE	22 / 118 18.6%B	11 / 118 9.3%BE			
1	Cupriavidus necator N-1	63 / 63 100.0%	4 / 4 100.0%	3 / 3 100.0%	1 / 1 100.0%	4 / 4 100.0%	4 / 4 100.0%	4 / 4 100.0%	1 / 1 100.0%	1 / 1 100.0%	7 / 7 100.0%	1 / 1 100.0%	4 / 4 100.0%	3 / 3 100.0%	1 / 1 100.0%	1 / 1 100.0%	1 / 1 100.0%	3 / 3 100.0%	1 / 1 100.0%	3 / 3 100.0%	5 / 5 100.0%	2 / 2 100.0%	1 / 1 100.0%	8 / 8 100.0%	0 / 0 0.0%	0 / 0 0.0%	0 / 0 0.0%	0 / 0 0.0%	40 / 63 63.5%AB	23 / 63 36.5%ABE					
1	ATCC 43123	52 / 53 98.1%	3 / 3 100.0%	3 / 3 100.0%	1 / 1 100.0%	2 / 2 100.0%	4 / 4 100.0%	3 / 3 100.0%	1 / 1 100.0%	1 / 1 100.0%	5 / 5 100.0%	1 / 1 100.0%	4 / 4 100.0%	3 / 3 100.0%	1 / 1 100.0%	1 / 1 100.0%	1 / 1 100.0%	3 / 3 100.0%	1 / 1 100.0%	3 / 3 100.0%	3 / 4 75.0%	2 / 2 100.0%	1 / 1 100.0%	5 / 5 100.0%	0 / 0 0.0%	0 / 0 0.0%	0 / 0 0.0%	0 / 0 0.0%	33 / 53 62.3%AB	19 / 53 35.8%ABE	1 / 53 1.9%				
1	Cupriavidus metallidurans CH34	52 / 53 98.1%	3 / 3 100.0%	3 / 3 100.0%	1 / 1 100.0%	2 / 2 100.0%	4 / 4 100.0%	3 / 3 100.0%	1 / 1 100.0%	1 / 1 100.0%	5 / 5 100.0%	1 / 1 100.0%	4 / 4 100.0%	3 / 3 100.0%	1 / 1 100.0%	1 / 1 100.0%	1 / 1 100.0%	3 / 3 100.0%	1 / 1 100.0%	3 / 3 100.0%	3 / 4 75.0%	2 / 2 100.0%	1 / 1 100.0%	5 / 5 100.0%	0 / 0 0.0%	0 / 0 0.0%	0 / 0 0.0%	0 / 0 0.0%	33 / 53 62.3%AB	19 / 53 35.8%ABE	1 / 53 1.9%				
1	CCUG 44338	63 / 63 100.0%	2 / 2 100.0%	3 / 3 100.0%	2 / 2 100.0%	4 / 4 100.0%	5 / 5 100.0%	4 / 4 100.0%	1 / 1 100.0%	1 / 1 100.0%	7 / 7 100.0%	1 / 1 100.0%	4 / 4 100.0%	3 / 3 100.0%	1 / 1 100.0%	1 / 1 100.0%	1 / 1 100.0%	3 / 3 100.0%	1 / 1 100.0%	4 / 4 100.0%	5 / 5 100.0%	2 / 2 100.0%	1 / 1 100.0%	7 / 7 100.0%	0 / 0 0.0%	0 / 0 0.0%	0 / 0 0.0%	0 / 0 0.0%	26 / 63 41.3%B	16 / 63 25.4%AB	13 / 63 20.6%BE	8 / 63 12.7%ABE			
1	CIP 108725	64 / 64 100.0%	3 / 3 100.0%	3 / 3 100.0%	2 / 2 100.0%	4 / 4 100.0%	5 / 5 100.0%	4 / 4 100.0%	1 / 1 100.0%	1 / 1 100.0%	7 / 7 100.0%	1 / 1 100.0%	4 / 4 100.0%	3 / 3 100.0%	1 / 1 100.0%	1 / 1 100.0%	1 / 1 100.0%	3 / 3 100.0%	1 / 1 100.0%	4 / 4 100.0%	5 / 5 100.0%	2 / 2 100.0%	1 / 1 100.0%	7 / 7 100.0%	0 / 0 0.0%	0 / 0 0.0%	0 / 0 0.0%	0 / 0 0.0%	42 / 64 65.6%AB	22 / 64 34.4%ABE					
1	Cupriavidus necator JMP134	64 / 64 100.0%	3 / 3 100.0%	3 / 3 100.0%	2 / 2 100.0%	4 / 4 100.0%	5 / 5 100.0%	4 / 4 100.0%	1 / 1 100.0%	1 / 1 100.0%	7 / 7 100.0%	1 / 1 100.0%	4 / 4 100.0%	3 / 3 100.0%	1 / 1 100.0%	1 / 1 100.0%	1 / 1 100.0%	3 / 3 100.0%	1 / 1 100.0%	4 / 4 100.0%	5 / 5 100.0%	2 / 2 100.0%	1 / 1 100.0%	7 / 7 100.0%	0 / 0 0.0%	0 / 0 0.0%	0 / 0 0.0%	0 / 0 0.0%	42 / 64 65.6%AB	22 / 64 34.4%ABE					
5	unclassified Burkholderiales	226 / 229 98.7%	5 / 5 100.0%	6 / 6 100.0%	5 / 5 100.0%	8 / 8 100.0%	18 / 18 100.0%	15 / 15 100.0%	5 / 5 100.0%	5 / 5 100.0%	16 / 18 88.9%	5 / 5 100.0%	20 / 20 100.0%	11 / 11 100.0%	5 / 5 100.0%	5 / 5 100.0%	5 / 5 100.0%	15 / 15 100.0%	0 / 0 0.0%	15 / 15 100.0%	20 / 20 100.0%	7 / 7 100.0%	10 / 10 100.0%	24 / 25 96.0%	1 / 1 100.0%	0 / 0 0.0%	0 / 0 0.0%	0 / 0 0.0%	134 / 229 58.5%AB	67 / 229 29.3%ABE	17 / 229 7.4%B	8 / 229 3.5%BE	2 / 229 0.9%	1 / 229 0.4%CE	
5	Burkholderiales Genera incertae sedis	226 / 229 98.7%	5 / 5 100.0%	6 / 6 100.0%	5 / 5 100.0%	8 / 8 100.0%	18 / 18 100.0%	15 / 15 100.0%	5 / 5 100.0%	5 / 5 100.0%	16 / 18 88.9%	5 / 5 100.0%	20 / 20 100.0%	11 / 11 100.0%	5 / 5 100.0%	5 / 5 100.0%	5 / 5 100.0%	15 / 15 100.0%	0 / 0 0.0%	15 / 15 100.0%	20 / 20 100.0%	7 / 7 100.0%	10 / 10 100.0%	24 / 25 96.0%	1 / 1 100.0%	0 / 0 0.0%	0 / 0 0.0%	0 / 0 0.0%	134 / 229 58.5%AB	67 / 229 29.3%ABE	17 / 229 7.4%B	8 / 229 3.5%BE	2 / 229 0.9%	1 / 229 0.4%CE	
1	"Chlamydothrix" Migula 1900	49 / 50 98.0%	1 / 1 100.0%	2 / 2 100.0%	1 / 1 100.0%	2 / 2 100.0%	5 / 5 100.0%	3 / 3 100.0%	1 / 1 100.0%	1 / 1 100.0%	3 / 4 75.0%	1 / 1 100.0%	4 / 4 100.0%	3 / 3 100.0%	1 / 1 100.0%	1 / 1 100.0%	1 / 1 100.0%	3 / 3 100.0%	0 / 0 0.0%	3 / 3 100.0%	4 / 4 100.0%	2 / 2 100.0%	2 / 2 100.0%	5 / 5 100.0%	0 / 0 0.0%	0 / 0 0.0%	0 / 0 0.0%	0 / 0 0.0%	17 / 50 34.0%AB	17 / 50 34.0%B	8 / 50 16.0%BE	7 / 50 14.0%ABE	1 / 50 2.0%		
1	LMG 7171	49 / 50	1 / 1	2 / 2	1 / 1	2 / 2	5 / 5	3 / 3	1 / 1	1 / 1	3 / 4	1 / 1	4 / 4	3 / 3	1 / 1	1 / 1	1 / 1	3 / 3	0 / 0	3 / 3	4 / 4	2 / 2	2 / 2	5 / 5	0 / 0	0 / 0	0 / 0	0 / 0	17 / 50	17 / 50	8 / 50	7 / 50	1 / 50		

1	[illegible]	[illegible]	[illegible]	[illegible]	[illegible]	[illegible]	[illegible]	[illegible]	[illegible]	[illegible]	[illegible]	[illegible]	[illegible]	[illegible]	[illegible]	[illegible]	[illegible]	[illegible]	[illegible]	[illegible]	[illegible]	[illegible]	[illegible]	[illegible]	[illegible]	[illegible]	[illegible]	[illegible]	[illegible] 34.0%B	[illegible] 34.0%AB	[illegible] 16.0%BE	[illegible] 14.0%ABE	[illegible] 2.0%		
1	Leptothrix cholodnii SP-6	49 / 50 98.0%	1 / 1 100.0%	2 / 2 100.0%	1 / 1 100.0%	2 / 2 100.0%	5 / 5 100.0%	3 / 3 100.0%	1 / 1 100.0%	1 / 1 100.0%	3 / 4 75.0%	1 / 1 100.0%	4 / 4 100.0%	3 / 3 100.0%	1 / 1 100.0%	1 / 1 100.0%	1 / 1 100.0%	3 / 3 100.0%	0 / 0 0.0%	3 / 3 100.0%	4 / 4 100.0%	2 / 2 100.0%	2 / 2 100.0%	5 / 5 100.0%	0 / 0 0.0%	0 / 0 0.0%	0 / 0 0.0%	0 / 0 0.0%	17 / 50 34.0%B	17 / 50 34.0%AB	8 / 50 16.0%BE	7 / 50 14.0%ABE	1 / 50 2.0%		
1	Rubrivivax	50 / 50 100.0%	1 / 1 100.0%	1 / 1 100.0%	1 / 1 100.0%	3 / 3 100.0%	4 / 4 100.0%	3 / 3 100.0%	1 / 1 100.0%	1 / 1 100.0%	5 / 5 100.0%	1 / 1 100.0%	4 / 4 100.0%	2 / 2 100.0%	1 / 1 100.0%	1 / 1 100.0%	1 / 1 100.0%	3 / 3 100.0%	0 / 0 0.0%	3 / 3 100.0%	4 / 4 100.0%	2 / 2 100.0%	2 / 2 100.0%	5 / 5 100.0%	1 / 1 100.0%	0 / 0 0.0%	0 / 0 0.0%	0 / 0 0.0%	33 / 50 66.0%AB	17 / 50 34.0%ABE					
1	"Rhodocystis gelatinosa" Molisch 1907	50 / 50 100.0%	1 / 1 100.0%	1 / 1 100.0%	1 / 1 100.0%	3 / 3 100.0%	4 / 4 100.0%	3 / 3 100.0%	1 / 1 100.0%	1 / 1 100.0%	5 / 5 100.0%	1 / 1 100.0%	4 / 4 100.0%	2 / 2 100.0%	1 / 1 100.0%	1 / 1 100.0%	1 / 1 100.0%	3 / 3 100.0%	0 / 0 0.0%	3 / 3 100.0%	4 / 4 100.0%	2 / 2 100.0%	2 / 2 100.0%	5 / 5 100.0%	1 / 1 100.0%	0 / 0 0.0%	0 / 0 0.0%	0 / 0 0.0%	33 / 50 66.0%AB	17 / 50 34.0%ABE					
1	Rubrivivax gelatinosus IL144	50 / 50 100.0%	1 / 1 100.0%	1 / 1 100.0%	1 / 1 100.0%	3 / 3 100.0%	4 / 4 100.0%	3 / 3 100.0%	1 / 1 100.0%	1 / 1 100.0%	5 / 5 100.0%	1 / 1 100.0%	4 / 4 100.0%	2 / 2 100.0%	1 / 1 100.0%	1 / 1 100.0%	1 / 1 100.0%	3 / 3 100.0%	0 / 0 0.0%	3 / 3 100.0%	4 / 4 100.0%	2 / 2 100.0%	2 / 2 100.0%	5 / 5 100.0%	1 / 1 100.0%	0 / 0 0.0%	0 / 0 0.0%	0 / 0 0.0%	33 / 50 66.0%AB	17 / 50 34.0%ABE					
2	Thiomonas	86 / 86 100.0%	2 / 2 100.0%	2 / 2 100.0%	2 / 2 100.0%	2 / 2 100.0%	6 / 6 100.0%	6 / 6 100.0%	2 / 2 100.0%	2 / 2 100.0%	6 / 6 100.0%	2 / 2 100.0%	8 / 8 100.0%	4 / 4 100.0%	2 / 2 100.0%	2 / 2 100.0%	2 / 2 100.0%	6 / 6 100.0%	0 / 0 0.0%	6 / 6 100.0%	8 / 8 100.0%	2 / 2 100.0%	4 / 4 100.0%	10 / 10 100.0%	0 / 0 0.0%	0 / 0 0.0%	0 / 0 0.0%	0 / 0 0.0%	56 / 86 65.1%AB	30 / 86 34.9%ABE					
1	ATCC 15466	43 / 43 100.0%	1 / 1 100.0%	1 / 1 100.0%	1 / 1 100.0%	1 / 1 100.0%	3 / 3 100.0%	3 / 3 100.0%	1 / 1 100.0%	1 / 1 100.0%	3 / 3 100.0%	1 / 1 100.0%	4 / 4 100.0%	2 / 2 100.0%	1 / 1 100.0%	1 / 1 100.0%	1 / 1 100.0%	3 / 3 100.0%	0 / 0 0.0%	3 / 3 100.0%	4 / 4 100.0%	1 / 1 100.0%	2 / 2 100.0%	5 / 5 100.0%	0 / 0 0.0%	0 / 0 0.0%	0 / 0 0.0%	0 / 0 0.0%	28 / 43 65.1%AB	15 / 43 34.9%ABE					
1	Thiobacillus intermedius K12	43 / 43 100.0%	1 / 1 100.0%	1 / 1 100.0%	1 / 1 100.0%	1 / 1 100.0%	3 / 3 100.0%	3 / 3 100.0%	1 / 1 100.0%	1 / 1 100.0%	3 / 3 100.0%	1 / 1 100.0%	4 / 4 100.0%	2 / 2 100.0%	1 / 1 100.0%	1 / 1 100.0%	1 / 1 100.0%	3 / 3 100.0%	0 / 0 0.0%	3 / 3 100.0%	4 / 4 100.0%	1 / 1 100.0%	2 / 2 100.0%	5 / 5 100.0%	0 / 0 0.0%	0 / 0 0.0%	0 / 0 0.0%	0 / 0 0.0%	28 / 43 65.1%AB	15 / 43 34.9%ABE					
1	CIP 110005	43 / 43 100.0%	1 / 1 100.0%	1 / 1 100.0%	1 / 1 100.0%	1 / 1 100.0%	3 / 3 100.0%	3 / 3 100.0%	1 / 1 100.0%	1 / 1 100.0%	3 / 3 100.0%	1 / 1 100.0%	4 / 4 100.0%	2 / 2 100.0%	1 / 1 100.0%	1 / 1 100.0%	1 / 1 100.0%	3 / 3 100.0%	0 / 0 0.0%	3 / 3 100.0%	4 / 4 100.0%	1 / 1 100.0%	2 / 2 100.0%	5 / 5 100.0%	0 / 0 0.0%	0 / 0 0.0%	0 / 0 0.0%	0 / 0 0.0%	28 / 43 65.1%AB	15 / 43 34.9%ABE					
1	Methylibium	41 / 43 95.3%	1 / 1 100.0%	1 / 1 100.0%	1 / 1 100.0%	1 / 1 100.0%	3 / 3 100.0%	3 / 3 100.0%	1 / 1 100.0%	1 / 1 100.0%	2 / 3 66.7%	1 / 1 100.0%	4 / 4 100.0%	2 / 2 100.0%	1 / 1 100.0%	1 / 1 100.0%	1 / 1 100.0%	3 / 3 100.0%	0 / 0 0.0%	3 / 3 100.0%	4 / 4 100.0%	1 / 1 100.0%	2 / 2 100.0%	4 / 5 80.0%	0 / 0 0.0%	0 / 0 0.0%	0 / 0 0.0%	0 / 0 0.0%	28 / 43 65.1%AB	13 / 43 30.2%ABE	1 / 43 2.3%	1 / 43 2.3%CE			
1	ATCC BAA-1232	41 / 43 95.3%	1 / 1 100.0%	1 / 1 100.0%	1 / 1 100.0%	1 / 1 100.0%	3 / 3 100.0%	3 / 3 100.0%	1 / 1 100.0%	1 / 1 100.0%	2 / 3 66.7%	1 / 1 100.0%	4 / 4 100.0%	2 / 2 100.0%	1 / 1 100.0%	1 / 1 100.0%	1 / 1 100.0%	3 / 3 100.0%	0 / 0 0.0%	3 / 3 100.0%	4 / 4 100.0%	1 / 1 100.0%	2 / 2 100.0%	4 / 5 80.0%	0 / 0 0.0%	0 / 0 0.0%	0 / 0 0.0%	0 / 0 0.0%	28 / 43 65.1%AB	13 / 43 30.2%ABE	1 / 43 2.3%CE	1 / 43 2.3%			
1	MTBE-degrading bacterium PM1	41 / 43 95.3%	1 / 1 100.0%	1 / 1 100.0%	1 / 1 100.0%	1 / 1 100.0%	3 / 3 100.0%	3 / 3 100.0%	1 / 1 100.0%	1 / 1 100.0%	2 / 3 66.7%	1 / 1 100.0%	4 / 4 100.0%	2 / 2 100.0%	1 / 1 100.0%	1 / 1 100.0%	1 / 1 100.0%	3 / 3 100.0%	0 / 0 0.0%	3 / 3 100.0%	4 / 4 100.0%	1 / 1 100.0%	2 / 2 100.0%	4 / 5 80.0%	0 / 0 0.0%	0 / 0 0.0%	0 / 0 0.0%	0 / 0 0.0%	28 / 43 65.1%AB	13 / 43 30.2%ABE	1 / 43 2.3%CE	1 / 43 2.3%			
11	unclassified Betaproteobacteria	350 / 395 88.6%	7 / 11 63.6%	9 / 10 90.0%	8 / 9 88.9%	22 / 23 95.7%	18 / 20 90.0%	17 / 18 94.4%	8 / 9 88.9%	8 / 9 88.9%	42 / 43 97.7%	8 / 11 72.7%	33 / 38 86.8%	16 / 22 72.7%	8 / 9 88.9%	8 / 9 88.9%	8 / 9 88.9%	12 / 13 92.3%	1 / 2 50.0%	24 / 27 88.9%	31 / 36 86.1%	9 / 10 90.0%	14 / 15 93.3%	39 / 42 92.9%	0 / 0 0.0%	0 / 0 0.0%	0 / 0 0.0%	0 / 0 0.0%	228 / 395 57.7%AB	122 / 395 30.9%ABE	32 / 395 8.1%	12 / 395 3.0%E	1 / 395 0.3%CE		
7	"Kinetoplastibacterium" Du et al. 1994	301 / 302 99.7%	6 / 7 85.7%	7 / 7 100.0%	7 / 7 100.0%	20 / 20 100.0%	14 / 14 100.0%	14 / 14 100.0%	7 / 7 100.0%	7 / 7 100.0%	35 / 35 100.0%	7 / 7 100.0%	28 / 28 100.0%	14 / 14 100.0%	7 / 7 100.0%	7 / 7 100.0%	7 / 7 100.0%	9 / 9 100.0%	0 / 0 0.0%	21 / 21 100.0%	28 / 28 100.0%	7 / 7 100.0%	14 / 14 100.0%	35 / 35 100.0%	0 / 0 0.0%	0 / 0 0.0%	0 / 0 0.0%	0 / 0 0.0%	194 / 302 64.2%AB	107 / 302 35.4%ABE	1 / 302 0.3%CE				
2	"Candidatus Kinetoplastibacterium crithidii" Du et al. 1994 in Teixeira et al. 2011	87 / 87 100.0%	2 / 2 100.0%	2 / 2 100.0%	2 / 2 100.0%	5 / 5 100.0%	4 / 4 100.0%	4 / 4 100.0%	2 / 2 100.0%	2 / 2 100.0%	10 / 10 100.0%	2 / 2 100.0%	8 / 8 100.0%	4 / 4 100.0%	2 / 2 100.0%	2 / 2 100.0%	2 / 2 100.0%	4 / 4 100.0%	0 / 0 0.0%	6 / 6 100.0%	8 / 8 100.0%	2 / 2 100.0%	4 / 4 100.0%	10 / 10 100.0%	0 / 0 0.0%	0 / 0 0.0%	0 / 0 0.0%	0 / 0 0.0%	57 / 87 65.5%AB	30 / 87 34.5%ABE					
1	Candidatus Kinetoplastibacterium crithidii TCC036E	44 / 44 100.0%	1 / 1 100.0%	1 / 1 100.0%	1 / 1 100.0%	3 / 3 100.0%	2 / 2 100.0%	2 / 2 100.0%	1 / 1 100.0%	1 / 1 100.0%	5 / 5 100.0%	1 / 1 100.0%	4 / 4 100.0%	2 / 2 100.0%	1 / 1 100.0%	1 / 1 100.0%	1 / 1 100.0%	2 / 2 100.0%	0 / 0 0.0%	3 / 3 100.0%	4 / 4 100.0%	1 / 1 100.0%	2 / 2 100.0%	5 / 5 100.0%	0 / 0 0.0%	0 / 0 0.0%	0 / 0 0.0%	0 / 0 0.0%	29 / 44 65.9%AB	15 / 44 34.1%ABE					
1	Candidatus Kinetoplastibacterium crithidii (ex Angomonas deanei ATCC 30255)	43 / 43 100.0%	1 / 1 100.0%	1 / 1 100.0%	1 / 1 100.0%	2 / 2 100.0%	2 / 2 100.0%	2 / 2 100.0%	1 / 1 100.0%	1 / 1 100.0%	5 / 5 100.0%	1 / 1 100.0%	4 / 4 100.0%	2 / 2 100.0%	1 / 1 100.0%	1 / 1 100.0%	1 / 1 100.0%	2 / 2 100.0%	0 / 0 0.0%	3 / 3 100.0%	4 / 4 100.0%	1 / 1 100.0%	2 / 2 100.0%	5 / 5 100.0%	0 / 0 0.0%	0 / 0 0.0%	0 / 0 0.0%	0 / 0 0.0%	28 / 43 65.1%AB	15 / 43 34.9%ABE					
2	"Kinetoplastibacterium blastocrithidii" Du et al. 2004	86 / 86 100.0%	2 / 2 100.0%	2 / 2 100.0%	2 / 2 100.0%	6 / 6 100.0%	4 / 4 100.0%	4 / 4 100.0%	2 / 2 100.0%	2 / 2 100.0%	10 / 10 100.0%	2 / 2 100.0%	8 / 8 100.0%	4 / 4 100.0%	2 / 2 100.0%	2 / 2 100.0%	2 / 2 100.0%	2 / 2 100.0%	0 / 0 0.0%	6 / 6 100.0%	8 / 8 100.0%	2 / 2 100.0%	4 / 4 100.0%	10 / 10 100.0%	0 / 0 0.0%	0 / 0 0.0%	0 / 0 0.0%	0 / 0 0.0%	54 / 86 62.8%AB	32 / 86 37.2%ABE					
1	Candidatus Kinetoplastibacterium blastocrithidii TCC012E	43 / 43 100.0%	1 / 1 100.0%	1 / 1 100.0%	1 / 1 100.0%	3 / 3 100.0%	2 / 2 100.0%	2 / 2 100.0%	1 / 1 100.0%	1 / 1 100.0%	5 / 5 100.0%	1 / 1 100.0%	4 / 4 100.0%	2 / 2 100.0%	1 / 1 100.0%	1 / 1 100.0%	1 / 1 100.0%	1 / 1 100.0%	0 / 0 0.0%	3 / 3 100.0%	4 / 4 100.0%	1 / 1 100.0%	2 / 2 100.0%	5 / 5 100.0%	0 / 0 0.0%	0 / 0 0.0%	0 / 0 0.0%	0 / 0 0.0%	27 / 43 62.8%AB	16 / 43 37.2%ABE					
1	Candidatus Kinetoplastibacterium blastocrithidii (ex Strigomonas culicis)	43 / 43 100.0%	1 / 1 100.0%	1 / 1 100.0%	1 / 1 100.0%	3 / 3 100.0%	2 / 2 100.0%	2 / 2 100.0%	1 / 1 100.0%	1 / 1 100.0%	5 / 5 100.0%	1 / 1 100.0%	4 / 4 100.0%	2 / 2 100.0%	1 / 1 100.0%	1 / 1 100.0%	1 / 1 100.0%	1 / 1 100.0%	0 / 0 0.0%	3 / 3 100.0%	4 / 4 100.0%	1 / 1 100.0%	2 / 2 100.0%	5 / 5 100.0%	0 / 0 0.0%	0 / 0 0.0%	0 / 0 0.0%	0 / 0 0.0%	27 / 43 62.8%AB	16 / 43 37.2%ABE					
1	'Candidatus Kinetoplastibacterium desouzaii'	42 / 43 97.7%	0 / 1 0.0%	1 / 1 100.0%	1 / 1 100.0%	3 / 3 100.0%	2 / 2 100.0%	2 / 2 100.0%	1 / 1 100.0%	1 / 1 100.0%	5 / 5 100.0%	1 / 1 100.0%	4 / 4 100.0%	2 / 2 100.0%	1 / 1 100.0%	1 / 1 100.0%	1 / 1 100.0%	1 / 1 100.0%	0 / 0 0.0%	3 / 3 100.0%	4 / 4 100.0%	1 / 1 100.0%	2 / 2 100.0%	5 / 5 100.0%	0 / 0 0.0%	0 / 0 0.0%	0 / 0 0.0%	0 / 0 0.0%	28 / 43 65.1%AB	14 / 43 32.6%ABE	1 / 43 2.3%CE				
1	Candidatus Kinetoplastibacterium desouzaii TCC079E	42 / 43 97.7%	0 / 1 0.0%	1 / 1 100.0%	1 / 1 100.0%	3 / 3 100.0%	2 / 2 100.0%	2 / 2 100.0%	1 / 1 100.0%	1 / 1 100.0%	5 / 5 100.0%	1 / 1 100.0%	4 / 4 100.0%	2 / 2 100.0%	1 / 1 100.0%	1 / 1 100.0%	1 / 1 100.0%	1 / 1 100.0%	0 / 0 0.0%	3 / 3 100.0%	4 / 4 100.0%	1 / 1 100.0%	2 / 2 100.0%	5 / 5 100.0%	0 / 0 0.0%	0 / 0 0.0%	0 / 0 0.0%	0 / 0 0.0%	28 / 43 65.1%AB	14 / 43 32.6%ABE	1 / 43 2.3%CE				
1	'Candidatus Kinetoplastibacterium galatii'	43 / 43 100.0%	1 / 1 100.0%	1 / 1 100.0%	1 / 1 100.0%	3 / 3 100.0%	2 / 2 100.0%	2 / 2 100.0%	1 / 1 100.0%	1 / 1 100.0%	5 / 5 100.0%	1 / 1 100.0%	4 / 4 100.0%	2 / 2 100.0%	1 / 1 100.0%	1 / 1 100.0%	1 / 1 100.0%	1 / 1 100.0%	0 / 0 0.0%	3 / 3 100.0%	4 / 4 100.0%	1 / 1 100.0%	2 / 2 100.0%	5 / 5 100.0%	0 / 0 0.0%	0 / 0 0.0%	0 / 0 0.0%	0 / 0 0.0%	27 / 43 62.8%AB	16 / 43 37.2%ABE					
1	Candidatus Kinetoplastibacterium galatii TCC219	43 / 43 100.0%	1 / 1 100.0%	1 / 1 100.0%	1 / 1 100.0%	3 / 3 100.0%	2 / 2 100.0%	2 / 2 100.0%	1 / 1 100.0%	1 / 1 100.0%	5 / 5 100.0%	1 / 1 100.0%	4 / 4 100.0%	2 / 2 100.0%	1 / 1 100.0%	1 / 1 100.0%	1 / 1 100.0%	1 / 1 100.0%	0 / 0 0.0%	3 / 3 100.0%	4 / 4 100.0%	1 / 1 100.0%	2 / 2 100.0%	5 / 5 100.0%	0 / 0 0.0%	0 / 0 0.0%	0 / 0 0.0%	0 / 0 0.0%	27 / 43 62.8%AB	16 / 43 37.2%ABE					
1	'Candidatus Kinetoplastibacterium oncopeltii'	43 / 43 100.0%	1 / 1 100.0%	1 / 1 100.0%	1 / 1 100.0%	3 / 3 100.0%	2 / 2 100.0%	2 / 2 100.0%	1 / 1 100.0%	1 / 1 100.0%	5 / 5 100.0%	1 / 1 100.0%	4 / 4 100.0%	2 / 2 100.0%	1 / 1 100.0%	1 / 1 100.0%	1 / 1 100.0%	1 / 1 100.0%	0 / 0 0.0%	3 / 3 100.0%	4 / 4 100.0%	1 / 1 100.0%	2 / 2 100.0%	5 / 5 100.0%	0 / 0 0.0%	0 / 0 0.0%	0 / 0 0.0%	0 / 0 0.0%	28 / 43 65.1%AB	15 / 43 34.9%ABE					
1	Candidatus Kinetoplastibacterium oncopeltii TCC290E	43 / 43 100.0%	1 / 1 100.0%	1 / 1 100.0%	1 / 1 100.0%	3 / 3 100.0%	2 / 2 100.0%	2 / 2 100.0%	1 / 1 100.0%	1 / 1 100.0%	5 / 5 100.0%	1 / 1 100.0%	4 / 4 100.0%	2 / 2 100.0%	1 / 1 100.0%	1 / 1 100.0%	1 / 1 100.0%	1 / 1 100.0%	0 / 0 0.0%	3 / 3 100.0%	4 / 4 100.0%	1 / 1 100.0%	2 / 2 100.0%	5 / 5 100.0%	0 / 0 0.0%	0 / 0 0.0%	0 / 0 0.0%	0 / 0 0.0%	28 / 43 65.1%AB	15 / 43 34.9%ABE					
3	'Candidatus Tremblaya'	3 / 45 6.7%	0 / 3 0.0%	0 / 1 0.0%	0 / 1 0.0%	0 / 1 0.0%	0 / 2 0.0%	0 / 1 0.0%	0 / 1 0.0%	0 / 1 0.0%	2 / 3 66.7%	0 / 3 0.0%	1 / 6 16.7%	0 / 6 0.0%	0 / 1 0.0%	0 / 1 0.0%	0 / 1 0.0%	0 / 1 0.0%	0 / 1 0.0%	0 / 3 0.0%	0 / 4 0.0%	0 / 1 0.0%	0 / 1 0.0%	0 / 2 0.0%	0 / 0 0.0%	0 / 0 0.0%	0 / 0 0.0%	0 / 0 0.0%	31 / 45 68.9%	11 / 45 24.4%E	2 / 45 4.4%AB	1 / 45 2.2%ABE			
2	"Candidatus Tremblaya princeps" Thao et al. 2002	2 / 14 14.3%	0 / 2 0.0%	0 / 0 0.0%	0 / 0 0.0%	0 / 0 0.0%	0 / 0 0.0%	0 / 0 0.0%	0 / 0 0.0%	0 / 0 0.0%	2 / 2 100.0%	0 / 2 0.0%	0 / 2 0.0%	0 / 4 0.0%	0 / 0 0.0%	0 / 0 0.0%	0 / 0 0.0%	0 / 0 0.0%	0 / 0 0.0%	0 / 0 0.0%	0 / 2 0.0%	0 / 0 0.0%	0 / 0 0.0%	0 / 0 0.0%	0 / 0 0.0%	0 / 0 0.0%	0 / 0 0.0%	0 / 0 0.0%	12 / 14 85.7%	2 / 14 14.3%AB					
1	Candidatus Tremblaya princeps PCIT	1 / 7 14.3%	0 / 1 0.0%	0 / 0 0.0%	0 / 0 0.0%	0 / 0 0.0%	0 / 0 0.0%	0 / 0 0.0%	0 / 0 0.0%	0 / 0 0.0%	1 / 1 100.0%	0 / 1 0.0%	0 / 1 0.0%	0 / 2 0.0%	0 / 0 0.0%	0 / 0 0.0%	0 / 0 0.0%	0 / 0 0.0%	0 / 0 0.0%	0 / 0 0.0%	0 / 1 0.0%	0 / 0 0.0%	0 / 0 0.0%	0 / 0 0.0%	0 / 0 0.0%	0 / 0 0.0%	0 / 0 0.0%	0 / 0 0.0%	6 / 7 85.7%	1 / 7 14.3%AB					
1	Candidatus Tremblaya princeps PCVAL	1 / 7 14.3%	0 / 1 0.0%	0 / 0 0.0%	0 / 0 0.0%	0 / 0 0.0%	0 / 0 0.0%	0 / 0 0.0%	0 / 0 0.0%	0 / 0 0.0%	1 / 1 100.0%	0 / 1 0.0%	0 / 1 0.0%	0 / 2 0.0%	0 / 0 0.0%	0 / 0 0.0%	0 / 0 0.0%	0 / 0 0.0%	0 / 0 0.0%	0 / 0 0.0%	0 / 1 0.0%	0 / 0 0.0%	0 / 0 0.0%	0 / 0 0.0%	0 / 0 0.0%	0 / 0 0.0%	0 / 0 0.0%	0 / 0 0.0%	6 / 7 85.7%	1 / 7 14.3%AB					
1	"Candidatus Tremblaya phenacola" Gruwell et al. 2010	1 / 31 3.2%	0 / 1 0.0%	0 / 1 0.0%	0 / 1 0.0%	0 / 1 0.0%	0 / 2 0.0%	0 / 1 0.0%	0 / 1 0.0%	0 / 1 0.0%	0 / 1 0.0%	0 / 1 0.0%	1 / 4 25.0%	0 / 2 0.0%	0 / 1 0.0%	0 / 1 0.0%	0 / 1 0.0%	0 / 1 0.0%	0 / 1 0.0%	0 / 3 0.0%	0 / 2 0.0%	0 / 1 0.0%	0 / 1 0.0%	0 / 2 0.0%	0 / 0 0.0%	0 / 0 0.0%	0 / 0 0.0%	0 / 0 0.0%	19 / 31 61.3%	11 / 31 35.5%E	1 / 31 3.2%ABE				
1	Candidatus Tremblaya phenacola PAVE	1 / 31 3.2%	0 / 1 0.0%	0 / 1 0.0%	0 / 1 0.0%	0 / 1 0.0%	0 / 2 0.0%	0 / 1 0.0%	0 / 1 0.0%	0 / 1 0.0%	0 / 1 0.0%	0 / 1 0.0%	1 / 4 25.0%	0 / 2 0.0%	0 / 1 0.0%	0 / 1 0.0%	0 / 1 0.0%	0 / 1 0.0%	0 / 1 0.0%	0 / 3 0.0%	0 / 2 0.0%	0 / 1 0.0%	0 / 1 0.0%	0 / 2 0.0%	0 / 0 0.0%	0 / 0 0.0%	0 / 0 0.0%	0 / 0 0.0%	19 / 31 61.3%	11 / 31 35.5%E	1 / 31 3.2%ABE				
1	"Candidatus Accumulibacter" Hesselmann et al. 1999	46 / 48 95.8%	1 / 1 100.0%	2 / 2 100.0%	1 / 1 100.0%	2 / 2 100.0%	4 / 4 100.0%	3 / 3 100.0%	1 / 1 100.0%	1 / 1 100.0%	5 / 5 100.0%	1 / 1 100.0%	4 / 4 100.0%	2 / 2 100.0%	1 / 1 100.0%	1 / 1 100.0%	1 / 1 100.0%	3 / 3 100.0%	1 / 1 100.0%	3 / 3 100.0%	3 / 4 75.0%	2 / 2 100.0%	0 / 0 0.0%	4 / 5 80.0%	0 / 0 0.0%	0 / 0 0.0%	0 / 0 0.0%	0 / 0 0.0%	32 / 48 66.7%AB	14 / 48 29.2%ABE	1 / 48 2.1%E	1 / 48 2.1%			
1	"Candidatus Accumulibacter phosphatis" Hesselmann et al. 1999	46 / 48 95.8%	1 / 1 100.0%	2 / 2 100.0%	1 / 1 100.0%	2 / 2 100.0%	4 / 4 100.0%	3 / 3 100.0%	1 / 1 100.0%	1 / 1 100.0%	5 / 5 100.0%	1 / 1 100.0%	4 / 4 100.0%	2 / 2 100.0%	1 / 1 100.0%	1 / 1 100.0%	1 / 1 100.0%	3 / 3 100.0%	1 / 1 100.0%	3 / 3 100.0%	3 / 4 75.0%	2 / 2 100.0%	0 / 0 0.0%	4 / 5 80.0%	0 / 0 0.0%	0 / 0 0.0%	0 / 0 0.0%	0 / 0 0.0%	32 / 48 66.7%AB	14 / 48 29.2%ABE	1 / 48 2.1%	1 / 48 2.1%E			
1	Accumulibacter phosphatis UW-1	46 / 48 95.8%	1 / 1 100.0%	2 / 2 100.0%	1 / 1 100.0%	2 / 2 100.0%	4 / 4 100.0%	3 / 3 100.0%	1 / 1 100.0%	1 / 1 100.0%	5 / 5 100.0%	1 / 1 100.0%	4 / 4 100.0%	2 / 2 100.0%	1 / 1 100.0%	1 / 1 100.0%	1 / 1 100.0%	3 / 3 100.0%	1 / 1 100.0%	3 / 3 100.0%	3 / 4 75.0%	2 / 2 100.0%	0 / 0 0.0%	4 / 5 80.0%	0 / 0 0.0%	0 / 0 0.0%	0 / 0 0.0%	0 / 0 0.0%	32 / 48 66.7%AB	14 / 48 29.2%ABE	1 / 48 2.1%E	1 / 48 2.1%			
1	'Hydrogenophilales'	43 / 44 97.7%	1 / 1 100.0%	1 / 1 100.0%	1 / 1 100.0%	2 / 2 100.0%	3 / 3 100.0%	3 / 3 100.0%	1 / 1 100.0%	1 / 1 100.0%	4 / 4 100.0%	1 / 1 100.0%	3 / 4 75.0%	1 / 1 100.0%	1 / 1 100.0%	1 / 1 100.0%	1 / 1 100.0%	3 / 3 100.0%	1 / 1 100.0%	3 / 3 100.0%	4 / 4 100.0%	1 / 1 100.0%	1 / 1 100.0%	5 / 5 100.0%	0 / 0 0.0%	0 / 0 0.0%	0 / 0 0.0%	0 / 0 0.0%	30 / 44 68.2%AB	13 / 44 29.5%ABE	1 / 44 2.3%E				
1	'Hydrogenophilaceae'	43 / 44 97.7%	1 / 1 100.0%	1 / 1 100.0%	1 / 1 100.0%	2 / 2 100.0%	3 / 3 100.0%	3 / 3 100.0%	1 / 1 100.0%	1 / 1 100.0%	4 / 4 100.0%	1 / 1 100.0%	3 / 4 75.0%	1 / 1 100.0%	1 / 1 100.0%	1 / 1 100.0%	1 / 1 100.0%	3 / 3 100.0%	1 / 1 100.0%	3 / 3 100.0%	4 / 4 100.0%	1 / 1 100.0%	1 / 1 100.0%	5 / 5 100.0%	0 / 0 0.0%	0 / 0 0.0%	0 / 0 0.0%	0 / 0 0.0%	30 / 44 68.2%AB	13 / 44 29.5%ABE	1 / 44 2.3%E				
1	Thiobacillus	43 / 44 97.7%	1 / 1 100.0%	1 / 1 100.0%	1 / 1 100.0%	2 / 2 100.0%	3 / 3 100.0%	3 / 3 100.0%	1 / 1 100.0%	1 / 1 100.0%	4 / 4 100.0%	1 / 1 100.0%	3 / 4 75.0%	1 / 1 100.0%	1 / 1 100.0%	1 / 1 100.0%	1 / 1 100.0%	3 / 3 100.0%	1 / 1 100.0%	3 / 3 100.0%	4 / 4 100.0%	1 / 1 100.0%	1 / 1 100.0%	5 / 5 100.0%	0 / 0 0.0%	0 / 0 0.0%	0 / 0 0.0%	0 / 0 0.0%	30 / 44 68.2%AB	13 / 44 29.5%ABE	1 / 44 2.3%E				
1	"Thiobacillus denitrificans" Beijerinck 1904	43 / 44 97.7%	1 / 1 100.0%	1 / 1 100.0%	1 / 1 100.0%	2 / 2 100.0%	3 / 3 100.0%	3 / 3 100.0%	1 / 1 100.0%	1 / 1 100.0%	4 / 4 100.0%	1 / 1 100.0%	3 / 4 75.0%	1 / 1 100.0%	1 / 1 100.0%	1 / 1 100.0%	1 / 1 100.0%	3 / 3 100.0%	1 / 1 100.0%	3 / 3 100.0%	4 / 4 100.0%	1 / 1 100.0%	1 / 1 100.0%	5 / 5 100.0%	0 / 0 0.0%	0 / 0 0.0%	0 / 0 0.0%	0 / 0 0.0%	30 / 44 68.2%AB	13 / 44 29.5%ABE	1 / 44 2.3%E				
1	Thiobacillus denitrificans ATCC 25259	43 / 44 97.7%	1 / 1 100.0%	1 / 1 100.0%	1 / 1 100.0%	2 / 2 100.0%	3 / 3 100.0%	3 / 3 100.0%	1 / 1 100.0%	1 / 1 100.0%	4 / 4 100.0%	1 / 1 100.0%	3 / 4 75.0%	1 / 1 100.0%	1 / 1 100.0%	1 / 1 100.0%	1 / 1 100.0%	3 / 3 100.0%	1 / 1 100.0%	3 / 3 100.0%	4 / 4 100.0%	1 / 1 100.0%	1 / 1 100.0%	5 / 5 100.0%	0 / 0 0.0%	0 / 0 0.0%	0 / 0 0.0%	0 / 0 0.0%	30 / 44 68.2%AB	13 / 44 29.5%ABE	1 / 44 2.3%E				
5	'Methylophilales'	231 / 231 100.0%	5 / 5 100.0%	10 / 10 100.0%	9 / 9 100.0%	11 / 11 100.0%	18 / 18 100.0%	15 / 15 100.0%	5 / 5 100.0%	5 / 5 100.0%	16 / 16 100.0%	5 / 5 100.0%	18 / 18 100.0%	10 / 10 100.0%	5 / 5 100.0%	5 / 5 100.0%	5 / 5 100.0%	14 / 14 100.0%	5 / 5 100.0%	14 / 14 100.0%	19 / 19 100.0%	7 / 7 100.0%	5 / 5 100.0%	25 / 25 100.0%	0 / 0 0.0%	0 / 0 0.0%	0 / 0 0.0%	0 / 0 0.0%	160 / 231 69.3%AB	71 / 231 30.7%ABE					
5	'Methylophilaceae'	231 / 231 100.0%	5 / 5 100.0%	10 / 10 100.0%	9 / 9 100.0%	11 / 11 100.0%	18 / 18 100.0%	15 / 15 100.0%	5 / 5 100.0%	5 / 5 100.0%	16 / 16 100.0%	5 / 5 100.0%	18 / 18 100.0%	10 / 10 100.0%	5 / 5 100.0%	5 / 5 100.0%	5 / 5 100.0%	14 / 14 100.0%	5 / 5 100.0%	14 / 14 100.0%	19 / 19 100.0%	7 / 7 100.0%	5 / 5 100.0%	25 / 25 100.0%	0 / 0 0.0%	0 / 0 0.0%	0 / 0 0.0%	0 / 0 0.0%	160 / 231 69.3%AB	71 / 231 30.7%ABE					
1	Methylobacillus	46 / 46 100.0%	1 / 1 100.0%	2 / 2 100.0%	1 / 1 100.0%	2 / 2 100.0%	4 / 4 100.0%	3 / 3 100.0%	1 / 1 100.0%	1 / 1 100.0%	3 / 3 100.0%	1 / 1 100.0%	4 / 4 100.0%	2 / 2 100.0%	1 / 1 100.0%	1 / 1 100.0%	1 / 1 100.0%	2 / 2 100.0%	1 / 1 100.0%	3 / 3 100.0%	4 / 4 100.0%	2 / 2 100.0%	1 / 1 100.0%	5 / 5 100.0%	0 / 0 0.0%	0 / 0 0.0%	0 / 0 0.0%	0 / 0 0.0%	30 / 46 65.2%AB	16 / 46 34.8%ABE					
1	ATCC 51484	46 / 46 100.0%	1 / 1 100.0%	2 / 2 100.0%	1 / 1 100.0%	2 / 2 100.0%	4 / 4 100.0%	3 / 3 100.0%	1 / 1 100.0%	1 / 1 100.0%	3 / 3 100.0%	1 / 1 100.0%	4 / 4 100.0%	2 / 2 100.0%	1 / 1 100.0%	1 / 1 100.0%	1 / 1 100.0%	2 / 2 100.0%	1 / 1 100.0%	3 / 3 100.0%	4 / 4 100.0%	2 / 2 100.0%	1 / 1 100.0%	5 / 5 100.0%	0 / 0 0.0%	0 / 0 0.0%	0 / 0 0.0%	0 / 0 0.0%	30 / 46 65.2%AB	16 / 46 34.8%ABE					
1	Methylobacillus flagellatus KT	46 / 46 100.0%	1 / 1 100.0%	2 / 2 100.0%	1 / 1 100.0%	2 / 2 100.0%	4 / 4 100.0%	3 / 3 100.0%	1 / 1 100.0%	1 / 1 100.0%	3 / 3 100.0%	1 / 1 100.0%	4 / 4 100.0%	2 / 2 100.0%	1 / 1 100.0%	1 / 1 100.0%	1 / 1 100.0%	2 / 2 100.0%	1 / 1 100.0%	3 / 3 100.0%	4 / 4 100.0%	2 / 2 100.0%	1 / 1 100.0%	5 / 5 100.0%	0 / 0 0.0%	0 / 0 0.0%	0 / 0 0.0%	0 / 0 0.0%	30 / 46 65.2%AB	16 / 46 34.8%ABE					
2	Methylivorus	92 / 92 100.0%	2 / 2 100.0%	4 / 4 100.0%	4 / 4 100.0%	4 / 4 100.0%	7 / 7 100.0%	6 / 6 100.0%	2 / 2 100.0%	2 / 2 100.0%	6 / 6 100.0%	2 / 2 100.0%	8 / 8 100.0%	4 / 4 100.0%	2 / 2 100.0%	2 / 2 100.0%	2 / 2 100.0%	6 / 6 100.0%	2 / 2 100.0%	6 / 6 100.0%	7 / 7 100.0%	2 / 2 100.0%	2 / 2 100.0%	10 / 10 100.0%	0 / 0 0.0%	0 / 0 0.0%	0 / 0 0.0%	0 / 0 0.0%	64 / 92 69.6%AB	28 / 92 30.4%ABE					
1	ATCC 49758	47 / 47 100.0%	1 / 1 100.0%	2 / 2 100.0%	2 / 2 100.0%	2 / 2 100.0%	4 / 4 100.0%	3 / 3 100.0%	1 / 1 100.0%	1 / 1 100.0%	3 / 3 100.0%	1 / 1 100.0%	4 / 4 100.0%	2 / 2 100.0%	1 / 1 100.0%	1 / 1 100.0%	1 / 1 100.0%	3 / 3 100.0%	1 / 1 100.0%	3 / 3 100.0%	4 / 4 100.0%	1 / 1 100.0%	1 / 1 100.0%	5 / 5 100.0%	0 / 0 0.0%	0 / 0 0.0%	0 / 0 0.0%	0 / 0 0.0%	33 / 47 70.2%AB	14 / 47 29.8%ABE					
1	Methylotenera sp. SIP3-4	47 / 47 100.0%	1 / 1 100.0%	2 / 2 100.0%	2 / 2 100.0%	2 / 2 100.0%	4 / 4 100.0%	3 / 3 100.0%	1 / 1 100.0%	1 / 1 100.0%	3 / 3 100.0%	1 / 1 100.0%	4 / 4 100.0%	2 / 2 100.0%	1 / 1 100.0%	1 / 1 100.0%	1 / 1 100.0%	3 / 3 100.0%	1 / 1 100.0%	3 / 3 100.0%	4 / 4 100.0%	1 / 1 100.0%	1 / 1 100.0%	5 / 5 100.0%	0 / 0 0.0%	0 / 0 0.0%	0 / 0 0.0%	0 / 0 0.0%	33 / 47 70.2%AB	14 / 47 29.8%ABE					
1	Methylovorus sp. MP688	45 / 45 100.0%	1 / 1 100.0%	2 / 2 100.0%	2 / 2 100.0%	2 / 2 100.0%	3 / 3 100.0%	3 / 3 100.0%	1 / 1 100.0%	1 / 1 100.0%	3 / 3 100.0%	1 / 1 100.0%	4 / 4 100.0%	2 / 2 100.0%	1 / 1 100.0%	1 / 1 100.0%	1 / 1 100.0%	3 / 3 100.0%	1 / 1 100.0%	3 / 3 100.0%	3 / 3 100.0%	1 / 1 100.0%	1 / 1 100.0%	5 / 5 100.0%	0 / 0 0.0%	0 / 0 0.0%	0 / 0 0.0%	0 / 0 0.0%	31 / 45 68.9%AB	14 / 45 31.1%ABE					
2	Methylotenera	93 / 93 100.0%	2 / 2 100.0%	4 / 4 100.0%	4 / 4 100.0%	5 / 5 100.0%	7 / 7 100.0%	6 / 6 100.0%	2 / 2 100.0%	2 / 2 100.0%	7 / 7 100.0%	2 / 2 100.0%	6 / 6 100.0%	4 / 4 100.0%	2 / 2 100.0%	2 / 2 100.0%	2 / 2 100.0%	6 / 6 100.0%	2 / 2 100.0%	5 / 5 100.0%	8 / 8 100.0%	3 / 3 100.0%	2 / 2 100.0%	10 / 10 100.0%	0 / 0 0.0%	0 / 0 0.0%	0 / 0 0.0%	0 / 0 0.0%	66 / 93 71.0%AB	27 / 93 29.0%ABE					
1	ATCC BAA-1282	46 / 46 100.0%	1 / 1 100.0%	2 / 2 100.0%	2 / 2 100.0%	2 / 2 100.0%	4 / 4 100.0%	3 / 3 100.0%	1 / 1 100.0%	1 / 1 100.0%	3 / 3 100.0%	1 / 1 100.0%	3 / 3 100.0%	2 / 2 100.0%	1 / 1 100.0%	1 / 1 100.0%	1 / 1 100.0%	3 / 3 100.0%	1 / 1 100.0%	2 / 2 100.0%	4 / 4 100.0%	2 / 2 100.0%	1 / 1 100.0%	5 / 5 100.0%	0 / 0 0.0%	0 / 0 0.0%	0 / 0 0.0%	0 / 0 0.0%	32 / 46 69.6%AB	14 / 46 30.4%ABE					
1	Methylotenera mobilis JLW8	46 / 46 100.0%	1 / 1 100.0%	2 / 2 100.0%	2 / 2 100.0%	2 / 2 100.0%	4 / 4 100.0%	3 / 3 100.0%	1 / 1 100.0%	1 / 1 100.0%	3 / 3 100.0%	1 / 1 100.0%	3 / 3 100.0%	2 / 2 100.0%	1 / 1 100.0%	1 / 1 100.0%	1 / 1 100.0%	3 / 3 100.0%	1 / 1 100.0%	2 / 2 100.0%	4 / 4 100.0%	2 / 2 100.0%	1 / 1 100.0%	5 / 5 100.0%	0 / 0 0.0%	0 / 0 0.0%	0 / 0 0.0%	0 / 0 0.0%	32 / 46 69.6%AB	14 / 46 30.4%ABE					
1	"Methylotenera versatilis" Lapidus et al. 2011	47 / 47 100.0%	1 / 1 100.0%	2 / 2 100.0%	2 / 2 100.0%	3 / 3 100.0%	3 / 3 100.0%	3 / 3 100.0%	1 / 1 100.0%	1 / 1 100.0%	4 / 4 100.0%	1 / 1 100.0%	3 / 3 100.0%	2 / 2 100.0%	1 / 1 100.0%	1 / 1 100.0%	1 / 1 100.0%	3 / 3 100.0%	1 / 1 100.0%	3 / 3 100.0%	4 / 4 100.0%	1 / 1 100.0%	1 / 1 100.0%	5 / 5 100.0%	0 / 0 0.0%	0 / 0 0.0%	0 / 0 0.0%	0 / 0 0.0%	34 / 47 72.3%AB	13 / 47 27.7%ABE					
1	Methylotenera sp. 301	47 / 47 100.0%	1 / 1 100.0%	2 / 2 100.0%	2 / 2 100.0%	3 / 3 100.0%	3 / 3 100.0%	3 / 3 100.0%	1 / 1 100.0%	1 / 1 100.0%	4 / 4 100.0%	1 / 1 100.0%	3 / 3 100.0%	2 / 2 100.0%	1 / 1 100.0%	1 / 1 100.0%	1 / 1 100.0%	3 / 3 100.0%	1 / 1 100.0%	3 / 3 100.0%	4 / 4 100.0%	1 / 1 100.0%	1 / 1 100.0%	5 / 5 100.0%	0 / 0 0.0%	0 / 0 0.0%	0 / 0 0.0%	0 / 0 0.0%	34 / 47 72.3%AB	13 / 47 27.7%ABE					
21	Neisseriales	1274 / 1297 98.2%	24 / 24 100.0%	52 / 52 100.0%	46 / 46 100.0%	94 / 94 100.0%	99 / 99 100.0%	72 / 72 100.0%	24 / 24 100.0%	23 / 23 100.0%	124 / 124 100.0%	21 / 21 100.0%	82 / 85 96.5%	48 / 48 100.0%	23 / 23 100.0%	53 / 53 100.0%	21 / 21 100.0%	66 / 66 100.0%	22 / 22 100.0%	78 / 79 98.7%	89 / 107 83.2%	66 / 66 100.0%	20 / 20 100.0%	126 / 126 100.0%	1 / 2 50.0%	0 / 0 0.0%	0 / 0 0.0%	0 / 0 0.0%	847 / 1297 65.3%AB	378 / 1297 29.1%ABE	35 / 1297 2.7%B	18 / 1297 1.4%	14 / 1297 1.1%BE	3 / 1297 0.2%CE	2 / 1297 0.2%E
18	Neisseriaceae	1010 / 1032 97.9%	18 / 18 100.0%	33 / 33 100.0%	36 / 36 100.0%	71 / 71 100.0%	82 / 82 100.0%	51 / 51 100.0%	18 / 18 100.0%	18 / 18 100.0%	90 / 90 100.0%	18 / 18 100.0%	69 / 72 95.8%	36 / 36 100.0%	18 / 18 100.0%	49 / 49 100.0%	18 / 18 100.0%	54 / 54 100.0%	18 / 18 100.0%	66 / 67 98.5%	71 / 89 79.8%	53 / 53 100.0%	18 / 18 100.0%	105 / 105 100.0%	0 / 0 0.0%	0 / 0 0.0%	0 / 0 0.0%	0 / 0 0.0%	644 / 1032 62.4%AB	317 / 1032 30.7%ABE	35 / 1032 3.4%B	18 / 1032 1.7%	14 / 1032 1.4%BE	2 / 1032 0.2%CE	2 / 1032 0.2%E
18	"Gonococcus" Lindau 1898	1010 / 1032 97.9%	18 / 18 100.0%	33 / 33 100.0%	36 / 36 100.0%	71 / 71 100.0%	82 / 82 100.0%	51 / 51 100.0%	18 / 18 100.0%	18 / 18 100.0%	90 / 90 100.0%	18 / 18 100.0%	69 / 72 95.8%	36 / 36 100.0%	18 / 18 100.0%	49 / 49 100.0%	18 / 18 100.0%	54 / 54 100.0%	18 / 18 100.0%	66 / 67 98.5%	71 / 89 79.8%	53 / 53 100.0%	18 / 18 100.0%	105 / 105 100.0%	0 / 0 0.0%	0 / 0 0.0%	0 / 0 0.0%	0 / 0 0.0%	644 / 1032 62.4%AB	317 / 1032 30.7%ABE	35 / 1032 3.4%B	18 / 1032 1.7%	14 / 1032 1.4%BE	2 / 1032 0.2%CE	2 / 1032 0.2%E
3	"Diplococcus gonorrhoeae" (Zopf 1885) Lehmann and Neumann 1896	150 / 155 96.8%	3 / 3 100.0%	5 / 5 100.0%	6 / 6 100.0%	11 / 11 100.0%	9 / 9 100.0%	8 / 8 100.0%	3 / 3 100.0%	3 / 3 100.0%	15 / 15 100.0%	3 / 3 100.0%	10 / 12 83.3%	6 / 6 100.0%	3 / 3 100.0%	4 / 4 100.0%	3 / 3 100.0%	9 / 9 100.0%	3 / 3 100.0%	9 / 9 100.0%	11 / 14 78.6%	8 / 8 100.0%	3 / 3 100.0%	15 / 15 100.0%	0 / 0 0.0%	0 / 0 0.0%	0 / 0 0.0%	0 / 0 0.0%	89 / 155 57.4%AB	42 / 155 27.1%ABE	14 / 155 9.0%B	5 / 155 3.2%BE	3 / 155 1.9%	2 / 155 1.3%CE	
1	Neisseria gonorrhoeae FA 1090	52 / 54 96.3%	1 / 1 100.0%	2 / 2 100.0%	2 / 2 100.0%	3 / 3 100.0%	4 / 4 100.0%	3 / 3 100.0%	1 / 1 100.0%	1 / 1 100.0%	5 / 5 100.0%	1 / 1 100.0%	3 / 4 75.0%	2 / 2 100.0%	1 / 1 100.0%	2 / 2 100.0%	1 / 1 100.0%	3 / 3 100.0%	1 / 1 100.0%	3 / 3 100.0%	4 / 5 80.0%	3 / 3 100.0%	1 / 1 100.0%	5 / 5 100.0%	0 / 0 0.0%	0 / 0 0.0%	0 / 0 0.0%	0 / 0 0.0%	36 / 54 66.7%AB	16 / 54 29.6%ABE	1 / 54 1.9%	1 / 54 1.9%CE			
1	Neisseria gonorrhoeae NCCP11945	54 / 55 98.2%	1 / 1 100.0%	2 / 2 100.0%	2 / 2 100.0%	4 / 4 100.0%	4 / 4 100.0%	3 / 3 100.0%	1 / 1 100.0%	1 / 1 100.0%	5 / 5 100.0%	1 / 1 100.0%	4 / 4 100.0%	2 / 2 100.0%	1 / 1 100.0%	2 / 2 100.0%	1 / 1 100.0%	3 / 3 100.0%	1 / 1 100.0%	3 / 3 100.0%	4 / 5 80.0%	3 / 3 100.0%	1 / 1 100.0%	5 / 5 100.0%	0 / 0 0.0%	0 / 0 0.0%	0 / 0 0.0%	0 / 0 0.0%	23 / 55 41.8%AB	14 / 55 25.5%B	12 / 55 21.8%ABE	5 / 55 9.1%BE	1 / 55 1.8%		
1	Neisseria gonorrhoeae TCDC-NG08107	44 / 46 95.7%	1 / 1 100.0%	1 / 1 100.0%	2 / 2 100.0%	4 / 4 100.0%	1 / 1 100.0%	2 / 2 100.0%	1 / 1 100.0%	1 / 1 100.0%	5 / 5 100.0%	1 / 1 100.0%	3 / 4 75.0%	2 / 2 100.0%	1 / 1 100.0%	0 / 0 0.0%	1 / 1 100.0%	3 / 3 100.0%	1 / 1 100.0%	3 / 3 100.0%	3 / 4 75.0%	2 / 2 100.0%	1 / 1 100.0%	5 / 5 100.0%	0 / 0 0.0%	0 / 0 0.0%	0 / 0 0.0%	0 / 0 0.0%	30 / 46 65.2%AB	14 / 46 30.4%ABE	1 / 46 2.2%	1 / 46 2.2%CE			
1	"Neisseria lactamicus" (sic) Hollis et al. 1969	58 / 59 98.3%	1 / 1 100.0%	2 / 2 100.0%	2 / 2 100.0%	4 / 4 100.0%	5 / 5 100.0%	3 / 3 100.0%	1 / 1 100.0%	1 / 1 100.0%	5 / 5 100.0%	1 / 1 100.0%	4 / 4 100.0%	2 / 2 100.0%	1 / 1 100.0%	3 / 3 100.0%	1 / 1 100.0%	3 / 3 100.0%	1 / 1 100.0%	4 / 4 100.0%	4 / 5 80.0%	3 / 3 100.0%	1 / 1 100.0%	6 / 6 100.0%	0 / 0 0.0%	0 / 0 0.0%	0 / 0 0.0%	0 / 0 0.0%	39 / 59 66.1%AB	19 / 59 32.2%ABE	1 / 59 1.7%				
1	Neisseria lactamica 020-06	58 / 59 98.3%	1 / 1 100.0%	2 / 2 100.0%	2 / 2 100.0%	4 / 4 100.0%	5 / 5 100.0%	3 / 3 100.0%	1 / 1 100.0%	1 / 1 100.0%	5 / 5 100.0%	1 / 1 100.0%	4 / 4 100.0%	2 / 2 100.0%	1 / 1 100.0%	3 / 3 100.0%	1 / 1 100.0%	3 / 3 100.0%	1 / 1 100.0%	4 / 4 100.0%	4 / 5 80.0%	3 / 3 100.0%	1 / 1 100.0%	6 / 6 100.0%	0 / 0 0.0%	0 / 0 0.0%	0 / 0 0.0%	0 / 0 0.0%	39 / 59 66.1%AB	19 / 59 32.2%ABE	1 / 59 1.7%				
14	"Diplokokkus intracellularis meningitidis" (sic) Weichselbaum 1887	802 / 818 98.0%	14 / 14 100.0%	26 / 26 100.0%	28 / 28 100.0%	56 / 56 100.0%	68 / 68 100.0%	40 / 40 100.0%	14 / 14 100.0%	14 / 14 100.0%	70 / 70 100.0%	14 / 14 100.0%	55 / 56 98.2%	28 / 28 100.0%	14 / 14 100.0%	42 / 42 100.0%	14 / 14 100.0%	42 / 42 100.0%	14 / 14 100.0%	53 / 54 98.1%	56 / 70 80.0%	42 / 42 100.0%	14 / 14 100.0%	84 / 84 100.0%	0 / 0 0.0%	0 / 0 0.0%	0 / 0 0.0%	0 / 0 0.0%	516 / 818 63.1%AB	256 / 818 31.3%ABE	21 / 818 2.6%B	14 / 818 1.7%	9 / 818 1.1%BE	2 / 818 0.2%E	
3	Neisseria meningitidis (group B)	172 / 175 98.3%	3 / 3 100.0%	5 / 5 100.0%	6 / 6 100.0%	12 / 12 100.0%	15 / 15 100.0%	8 / 8 100.0%	3 / 3 100.0%	3 / 3 100.0%	15 / 15 100.0%	3 / 3 100.0%	12 / 12 100.0%	6 / 6 100.0%	3 / 3 100.0%	9 / 9 100.0%	3 / 3 100.0%	9 / 9 100.0%	3 / 3 100.0%	12 / 12 100.0%	12 / 15 80.0%	9 / 9 100.0%	3 / 3 100.0%	18 / 18 100.0%	0 / 0 0.0%	0 / 0 0.0%	0 / 0 0.0%	0 / 0 0.0%	115 / 175 65.7%AB	57 / 175 32.6%ABE	3 / 175 1.7%				
1	Neisseria meningitidis MC58	58 / 59 98.3%	1 / 1 100.0%	2 / 2 100.0%	2 / 2 100.0%	4 / 4 100.0%	5 / 5 100.0%	3 / 3 100.0%	1 / 1 100.0%	1 / 1 100.0%	5 / 5 100.0%	1 / 1 100.0%	4 / 4 100.0%	2 / 2 100.0%	1 / 1 100.0%	3 / 3 100.0%	1 / 1 100.0%	3 / 3 100.0%	1 / 1 100.0%	4 / 4 100.0%	4 / 5 80.0%	3 / 3 100.0%	1 / 1 100.0%	6 / 6 100.0%	0 / 0 0.0%	0 / 0 0.0%	0 / 0 0.0%	0 / 0 0.0%	39 / 59 66.1%AB	19 / 59 32.2%ABE	1 / 59 1.7%				
1	Neisseria meningitidis alpha710	56 / 57 98.2%	1 / 1 100.0%	1 / 1 100.0%	2 / 2 100.0%	4 / 4 100.0%	5 / 5 100.0%	2 / 2 100.0%	1 / 1 100.0%	1 / 1 100.0%	5 / 5 100.0%	1 / 1 100.0%	4 / 4 100.0%	2 / 2 100.0%	1 / 1 100.0%	3 / 3 100.0%	1 / 1 100.0%	3 / 3 100.0%	1 / 1 100.0%	4 / 4 100.0%	4 / 5 80.0%	3 / 3 100.0%	1 / 1 100.0%	6 / 6 100.0%	0 / 0 0.0%	0 / 0 0.0%	0 / 0 0.0%	0 / 0 0.0%	37 / 57 64.9%AB	19 / 57 33.3%ABE	1 / 57 1.8%				
1	Neisseria meningitidis H44-76	58 / 59 98.3%	1 / 1 100.0%	2 / 2 100.0%	2 / 2 100.0%	4 / 4 100.0%	5 / 5 100.0%	3 / 3 100.0%	1 / 1 100.0%	1 / 1 100.0%	5 / 5 100.0%	1 / 1 100.0%	4 / 4 100.0%	2 / 2 100.0%	1 / 1 100.0%	3 / 3 100.0%	1 / 1 100.0%	3 / 3 100.0%	1 / 1 100.0%	4 / 4 100.0%	4 / 5 80.0%	3 / 3 100.0%	1 / 1 100.0%	6 / 6 100.0%	0 / 0 0.0%	0 / 0 0.0%	0 / 0 0.0%	0 / 0 0.0%	39 / 59 66.1%AB	19 / 59 32.2%ABE	1 / 59 1.7%				
1	Neisseria meningitidis (serogroup A)	57 / 58 98.3%	1 / 1 100.0%	2 / 2 100.0%	2 / 2 100.0%	4 / 4 100.0%	5 / 5 100.0%	3 / 3 100.0%	1 / 1 100.0%	1 / 1 100.0%	5 / 5 100.0%	1 / 1 100.0%	4 / 4 100.0%	2 / 2 100.0%	1 / 1 100.0%	3 / 3 100.0%	1 / 1 100.0%	3 / 3 100.0%	1 / 1 100.0%	3 / 3 100.0%	4 / 5 80.0%	3 / 3 100.0%	1 / 1 100.0%	6 / 6 100.0%	0 / 0 0.0%	0 / 0 0.0%	0 / 0 0.0%	0 / 0 0.0%	38 / 58 65.5%AB	19 / 58 32.8%ABE	1 / 58 1.7%				
1	Neisseria meningitidis Z2491	57 / 58 98.3%	1 / 1 100.0%	2 / 2 100.0%	2 / 2 100.0%	4 / 4 100.0%	5 / 5 100.0%	3 / 3 100.0%	1 / 1 100.0%	1 / 1 100.0%	5 / 5 100.0%	1 / 1 100.0%	4 / 4 100.0%	2 / 2 100.0%	1 / 1 100.0%	3 / 3 100.0%	1 / 1 100.0%	3 / 3 100.0%	1 / 1 100.0%	3 / 3 100.0%	4 / 5 80.0%	3 / 3 100.0%	1 / 1 100.0%	6 / 6 100.0%	0 / 0 0.0%	0 / 0 0.0%	0 / 0 0.0%	0 / 0 0.0%	38 / 58 65.5%AB	19 / 58 32.8%ABE	1 / 58 1.7%				
3	Neisseria meningitidis (serogroup C)	174 / 177 98.3%	3 / 3 100.0%	6 / 6 100.0%	6 / 6 100.0%	12 / 12 100.0%	15 / 15 100.0%	9 / 9 100.0%	3 / 3 100.0%	3 / 3 100.0%	15 / 15 100.0%	3 / 3 100.0%	12 / 12 100.0%	6 / 6 100.0%	3 / 3 100.0%	9 / 9 100.0%	3 / 3 100.0%	9 / 9 100.0%	3 / 3 100.0%	12 / 12 100.0%	12 / 15 80.0%	9 / 9 100.0%	3 / 3 100.0%	18 / 18 100.0%	0 / 0 0.0%	0 / 0 0.0%	0 / 0 0.0%	0 / 0 0.0%	96 / 177 54.2%AB	48 / 177 27.1%ABE	21 / 177 11.9%B	9 / 177 5.1%BE	3 / 177 1.7%		
1	Neisseria meningitidis FAM18	58 / 59 98.3%	1 / 1 100.0%	2 / 2 100.0%	2 / 2 100.0%	4 / 4 100.0%	5 / 5 100.0%	3 / 3 100.0%	1 / 1 100.0%	1 / 1 100.0%	5 / 5 100.0%	1 / 1 100.0%	4 / 4 100.0%	2 / 2 100.0%	1 / 1 100.0%	3 / 3 100.0%	1 / 1 100.0%	3 / 3 100.0%	1 / 1 100.0%	4 / 4 100.0%	4 / 5 80.0%	3 / 3 100.0%	1 / 1 100.0%	6 / 6 100.0%	0 / 0 0.0%	0 / 0 0.0%	0 / 0 0.0%	0 / 0 0.0%	39 / 59 66.1%AB	19 / 59 32.2%ABE	1 / 59 1.7%				
1	Neisseria meningitidis 053442	58 / 59 98.3%	1 / 1 100.0%	2 / 2 100.0%	2 / 2 100.0%	4 / 4 100.0%	5 / 5 100.0%	3 / 3 100.0%	1 / 1 100.0%	1 / 1 100.0%	5 / 5 100.0%	1 / 1 100.0%	4 / 4 100.0%	2 / 2 100.0%	1 / 1 100.0%	3 / 3 100.0%	1 / 1 100.0%	3 / 3 100.0%	1 / 1 100.0%	4 / 4 100.0%	4 / 5 80.0%	3 / 3 100.0%	1 / 1 100.0%	6 / 6 100.0%	0 / 0 0.0%	0 / 0 0.0%	0 / 0 0.0%	0 / 0 0.0%	21 / 59 35.6%B	18 / 59 30.5%AB	10 / 59 16.9%ABE	9 / 59 15.3%BE	1 / 59 1.7%		
1	Neisseria meningitidis 8013	58 / 59 98.3%	1 / 1 100.0%	2 / 2 100.0%	2 / 2 100.0%	4 / 4 100.0%	5 / 5 100.0%	3 / 3 100.0%	1 / 1 100.0%	1 / 1 100.0%	5 / 5 100.0%	1 / 1 100.0%	4 / 4 100.0%	2 / 2 100.0%	1 / 1 100.0%	3 / 3 100.0%	1 / 1 100.0%	3 / 3 100.0%	1 / 1 100.0%	4 / 4 100.0%	4 / 5 80.0%	3 / 3 100.0%	1 / 1 100.0%	6 / 6 100.0%	0 / 0 0.0%	0 / 0 0.0%	0 / 0 0.0%	0 / 0 0.0%	39 / 59 66.1%AB	19 / 59 32.2%ABE	1 / 59 1.7%				
1	Neisseria meningitidis M01-240355	57 / 59 96.6%	1 / 1 100.0%	2 / 2 100.0%	2 / 2 100.0%	4 / 4 100.0%	5 / 5 100.0%	3 / 3 100.0%	1 / 1 100.0%	1 / 1 100.0%	5 / 5 100.0%	1 / 1 100.0%	3 / 4 75.0%	2 / 2 100.0%	1 / 1 100.0%	3 / 3 100.0%	1 / 1 100.0%	3 / 3 100.0%	1 / 1 100.0%	4 / 4 100.0%	4 / 5 80.0%	3 / 3 100.0%	1 / 1 100.0%	6 / 6 100.0%	0 / 0 0.0%	0 / 0 0.0%	0 / 0 0.0%	0 / 0 0.0%	39 / 59 66.1%AB	18 / 59 30.5%ABE	1 / 59 1.7%	1 / 59 1.7%E			
1	Neisseria meningitidis NZ-05/33	58 / 59 98.3%	1 / 1 100.0%	2 / 2 100.0%	2 / 2 100.0%	4 / 4 100.0%	5 / 5 100.0%	3 / 3 100.0%	1 / 1 100.0%	1 / 1 100.0%	5 / 5 100.0%	1 / 1 100.0%	4 / 4 100.0%	2 / 2 100.0%	1 / 1 100.0%	3 / 3 100.0%	1 / 1 100.0%	3 / 3 100.0%	1 / 1 100.0%	4 / 4 100.0%	4 / 5 80.0%	3 / 3 100.0%	1 / 1 100.0%	6 / 6 100.0%	0 / 0 0.0%	0 / 0 0.0%	0 / 0 0.0%	0 / 0 0.0%	39 / 59 66.1%AB	19 / 59 32.2%ABE	1 / 59 1.7%				
1	Neisseria meningitidis M01-240149	58 / 59 98.3%	1 / 1 100.0%	2 / 2 100.0%	2 / 2 100.0%	4 / 4 100.0%	5 / 5 100.0%	3 / 3 100.0%	1 / 1 100.0%	1 / 1 100.0%	5 / 5 100.0%	1 / 1 100.0%	4 / 4 100.0%	2 / 2 100.0%	1 / 1 100.0%	3 / 3 100.0%	1 / 1 100.0%	3 / 3 100.0%	1 / 1 100.0%	4 / 4 100.0%	4 / 5 80.0%	3 / 3 100.0%	1 / 1 100.0%	6 / 6 100.0%	0 / 0 0.0%	0 / 0 0.0%	0 / 0 0.0%	0 / 0 0.0%	39 / 59 66.1%AB	19 / 59 32.2%ABE	1 / 59 1.7%				
1	Neisseria meningitidis M04-240196	58 / 59 98.3%	1 / 1 100.0%	2 / 2 100.0%	2 / 2 100.0%	4 / 4 100.0%	5 / 5 100.0%	3 / 3 100.0%	1 / 1 100.0%	1 / 1 100.0%	5 / 5 100.0%	1 / 1 100.0%	4 / 4 100.0%	2 / 2 100.0%	1 / 1 100.0%	3 / 3 100.0%	1 / 1 100.0%	3 / 3 100.0%	1 / 1 100.0%	4 / 4 100.0%	4 / 5 80.0%	3 / 3 100.0%	1 / 1 100.0%	6 / 6 100.0%	0 / 0 0.0%	0 / 0 0.0%	0 / 0 0.0%	0 / 0 0.0%	39 / 59 66.1%AB	19 / 59 32.2%ABE	1 / 59 1.7%				
1	Neisseria meningitidis G2136	58 / 59 98.3%	1 / 1 100.0%	2 / 2 100.0%	2 / 2 100.0%	4 / 4 100.0%	5 / 5 100.0%	3 / 3 100.0%	1 / 1 100.0%	1 / 1 100.0%	5 / 5 100.0%	1 / 1 100.0%	4 / 4 100.0%	2 / 2 100.0%	1 / 1 100.0%	3 / 3 100.0%	1 / 1 100.0%	3 / 3 100.0%	1 / 1 100.0%	4 / 4 100.0%	4 / 5 80.0%	3 / 3 100.0%	1 / 1 100.0%	6 / 6 100.0%	0 / 0 0.0%	0 / 0 0.0%	0 / 0 0.0%	0 / 0 0.0%	39 / 59 66.1%AB	19 / 59 32.2%ABE	1 / 59 1.7%				
1	Neisseria meningitidis WUE 2594	53 / 55 [illegible]	1 / 1 [illegible]	1 / 1 [illegible]	2 / 2 [illegible]	4 / 4 [illegible]	3 / 3 [illegible]	2 / 2 [illegible]	1 / 1 [illegible]	1 / 1 [illegible]	5 / 5 [illegible]	1 / 1 [illegible]	4 / 4 [illegible]	2 / 2 [illegible]	1 / 1 [illegible]	3 / 3 [illegible]	1 / 1 [illegible]	3 / 3 [illegible]	1 / 1 [illegible]	3 / 4 [illegible]	4 / 5 [illegible]	3 / 3 [illegible]	1 / 1 [illegible]	6 / 6 [illegible]	0 / 0 [illegible]	0 / 0 [illegible]	0 / 0 [illegible]	0 / 0 [illegible]	34 / 55 [illegible]	19 / 55 [illegible]	1 / 55 [illegible]	1 / 55 [illegible]			

3	"Chromobacteriaceae" Adeolu and Gupta 2013	264 / 265 99.6%	6 / 6 100.0%	19 / 19 100.0%	10 / 10 100.0%	23 / 23 100.0%	17 / 17 100.0%	21 / 21 100.0%	6 / 6 100.0%	5 / 5 100.0%	34 / 34 100.0%	3 / 3 100.0%	13 / 13 100.0%	12 / 12 100.0%	5 / 5 100.0%	4 / 4 100.0%	3 / 3 100.0%	12 / 12 100.0%	4 / 4 100.0%	12 / 12 100.0%	18 / 18 100.0%	13 / 13 100.0%	2 / 2 100.0%	21 / 21 100.0%	1 / 2 50.0%	0 / 0 0.0%	0 / 0 0.0%	0 / 0 0.0%	203 / 265 76.6%AB	61 / 265 23.0%ABE	1 / 265 0.4%CE					
1	Chromobacterium group	98 / 98 100.0%	2 / 2 100.0%	7 / 7 100.0%	4 / 4 100.0%	8 / 8 100.0%	7 / 7 100.0%	8 / 8 100.0%	3 / 3 100.0%	2 / 2 100.0%	12 / 12 100.0%	1 / 1 100.0%	5 / 5 100.0%	5 / 5 100.0%	2 / 2 100.0%	2 / 2 100.0%	1 / 1 100.0%	4 / 4 100.0%	2 / 2 100.0%	4 / 4 100.0%	6 / 6 100.0%	4 / 4 100.0%	1 / 1 100.0%	8 / 8 100.0%	0 / 0 0.0%	0 / 0 0.0%	0 / 0 0.0%	0 / 0 0.0%	79 / 98 80.6%AB	19 / 98 19.4%ABE						
1	Chromobacterium	98 / 98 100.0%	2 / 2 100.0%	7 / 7 100.0%	4 / 4 100.0%	8 / 8 100.0%	7 / 7 100.0%	8 / 8 100.0%	3 / 3 100.0%	2 / 2 100.0%	12 / 12 100.0%	1 / 1 100.0%	5 / 5 100.0%	5 / 5 100.0%	2 / 2 100.0%	2 / 2 100.0%	1 / 1 100.0%	4 / 4 100.0%	2 / 2 100.0%	4 / 4 100.0%	6 / 6 100.0%	4 / 4 100.0%	1 / 1 100.0%	8 / 8 100.0%	0 / 0 0.0%	0 / 0 0.0%	0 / 0 0.0%	0 / 0 0.0%	79 / 98 80.6%AB	19 / 98 19.4%ABE						
1	"Chromobacterium janthinum" (Zopf) Ford 1927	98 / 98 100.0%	2 / 2 100.0%	7 / 7 100.0%	4 / 4 100.0%	8 / 8 100.0%	7 / 7 100.0%	8 / 8 100.0%	3 / 3 100.0%	2 / 2 100.0%	12 / 12 100.0%	1 / 1 100.0%	5 / 5 100.0%	5 / 5 100.0%	2 / 2 100.0%	2 / 2 100.0%	1 / 1 100.0%	4 / 4 100.0%	2 / 2 100.0%	4 / 4 100.0%	6 / 6 100.0%	4 / 4 100.0%	1 / 1 100.0%	8 / 8 100.0%	0 / 0 0.0%	0 / 0 0.0%	0 / 0 0.0%	0 / 0 0.0%	79 / 98 80.6%AB	19 / 98 19.4%ABE						
1	Chromobacterium violaceum ATCC 12472	98 / 98 100.0%	2 / 2 100.0%	7 / 7 100.0%	4 / 4 100.0%	8 / 8 100.0%	7 / 7 100.0%	8 / 8 100.0%	3 / 3 100.0%	2 / 2 100.0%	12 / 12 100.0%	1 / 1 100.0%	5 / 5 100.0%	5 / 5 100.0%	2 / 2 100.0%	2 / 2 100.0%	1 / 1 100.0%	4 / 4 100.0%	2 / 2 100.0%	4 / 4 100.0%	6 / 6 100.0%	4 / 4 100.0%	1 / 1 100.0%	8 / 8 100.0%	0 / 0 0.0%	0 / 0 0.0%	0 / 0 0.0%	0 / 0 0.0%	79 / 98 80.6%AB	19 / 98 19.4%ABE						
1	Laribacter	78 / 78 100.0%	2 / 2 100.0%	5 / 5 100.0%	3 / 3 100.0%	7 / 7 100.0%	6 / 6 100.0%	5 / 5 100.0%	1 / 1 100.0%	1 / 1 100.0%	10 / 10 100.0%	1 / 1 100.0%	4 / 4 100.0%	4 / 4 100.0%	1 / 1 100.0%	1 / 1 100.0%	1 / 1 100.0%	4 / 4 100.0%	1 / 1 100.0%	4 / 4 100.0%	6 / 6 100.0%	4 / 4 100.0%	0 / 0 0.0%	6 / 6 100.0%	1 / 1 100.0%	0 / 0 0.0%	0 / 0 0.0%	0 / 0 0.0%	59 / 78 75.6%AB	19 / 78 24.4%ABE						
1	CCUG 45813	78 / 78 100.0%	2 / 2 100.0%	5 / 5 100.0%	3 / 3 100.0%	7 / 7 100.0%	6 / 6 100.0%	5 / 5 100.0%	1 / 1 100.0%	1 / 1 100.0%	10 / 10 100.0%	1 / 1 100.0%	4 / 4 100.0%	4 / 4 100.0%	1 / 1 100.0%	1 / 1 100.0%	1 / 1 100.0%	4 / 4 100.0%	1 / 1 100.0%	4 / 4 100.0%	6 / 6 100.0%	4 / 4 100.0%	0 / 0 0.0%	6 / 6 100.0%	1 / 1 100.0%	0 / 0 0.0%	0 / 0 0.0%	0 / 0 0.0%	59 / 78 75.6%AB	19 / 78 24.4%ABE						
1	Laribacter hongkongensis HLHK9	78 / 78 100.0%	2 / 2 100.0%	5 / 5 100.0%	3 / 3 100.0%	7 / 7 100.0%	6 / 6 100.0%	5 / 5 100.0%	1 / 1 100.0%	1 / 1 100.0%	10 / 10 100.0%	1 / 1 100.0%	4 / 4 100.0%	4 / 4 100.0%	1 / 1 100.0%	1 / 1 100.0%	1 / 1 100.0%	4 / 4 100.0%	1 / 1 100.0%	4 / 4 100.0%	6 / 6 100.0%	4 / 4 100.0%	0 / 0 0.0%	6 / 6 100.0%	1 / 1 100.0%	0 / 0 0.0%	0 / 0 0.0%	0 / 0 0.0%	59 / 78 75.6%AB	19 / 78 24.4%ABE						
1	"Lutiella" Weber et al. 2007	88 / 89 98.9%	2 / 2 100.0%	7 / 7 100.0%	3 / 3 100.0%	8 / 8 100.0%	4 / 4 100.0%	8 / 8 100.0%	2 / 2 100.0%	2 / 2 100.0%	12 / 12 100.0%	1 / 1 100.0%	4 / 4 100.0%	3 / 3 100.0%	2 / 2 100.0%	1 / 1 100.0%	1 / 1 100.0%	4 / 4 100.0%	1 / 1 100.0%	4 / 4 100.0%	6 / 6 100.0%	5 / 5 100.0%	1 / 1 100.0%	7 / 7 100.0%	0 / 1 0.0%	0 / 0 0.0%	0 / 0 0.0%	0 / 0 0.0%	65 / 89 73.0%AB	23 / 89 25.8%ABE	1 / 89 1.1%CE					
1	Pseudogulbenkiania sp. NH8B	88 / 89 98.9%	2 / 2 100.0%	7 / 7 100.0%	3 / 3 100.0%	8 / 8 100.0%	4 / 4 100.0%	8 / 8 100.0%	2 / 2 100.0%	2 / 2 100.0%	12 / 12 100.0%	1 / 1 100.0%	4 / 4 100.0%	3 / 3 100.0%	2 / 2 100.0%	1 / 1 100.0%	1 / 1 100.0%	4 / 4 100.0%	1 / 1 100.0%	4 / 4 100.0%	6 / 6 100.0%	5 / 5 100.0%	1 / 1 100.0%	7 / 7 100.0%	0 / 1 0.0%	0 / 0 0.0%	0 / 0 0.0%	0 / 0 0.0%	65 / 89 73.0%AB	23 / 89 25.8%ABE	1 / 89 1.1%CE					
6	'Rhodocyclales'	347 / 351 98.9%	8 / 8 100.0%	14 / 14 100.0%	12 / 12 100.0%	23 / 23 100.0%	28 / 28 100.0%	24 / 24 100.0%	7 / 7 100.0%	6 / 6 100.0%	39 / 41 95.1%	6 / 6 100.0%	24 / 24 100.0%	11 / 12 91.7%	6 / 6 100.0%	6 / 6 100.0%	6 / 6 100.0%	18 / 18 100.0%	6 / 6 100.0%	20 / 20 100.0%	27 / 28 96.4%	12 / 12 100.0%	6 / 6 100.0%	38 / 38 100.0%	0 / 0 0.0%	0 / 0 0.0%	0 / 0 0.0%	0 / 0 0.0%	245 / 351 69.8%AB	101 / 351 28.8%ABE	4 / 351 1.1%	1 / 351 0.3%B				
6	'Rhodocyclaceae'	347 / 351 98.9%	8 / 8 100.0%	14 / 14 100.0%	12 / 12 100.0%	23 / 23 100.0%	28 / 28 100.0%	24 / 24 100.0%	7 / 7 100.0%	6 / 6 100.0%	39 / 41 95.1%	6 / 6 100.0%	24 / 24 100.0%	11 / 12 91.7%	6 / 6 100.0%	6 / 6 100.0%	6 / 6 100.0%	18 / 18 100.0%	6 / 6 100.0%	20 / 20 100.0%	27 / 28 96.4%	12 / 12 100.0%	6 / 6 100.0%	38 / 38 100.0%	0 / 0 0.0%	0 / 0 0.0%	0 / 0 0.0%	0 / 0 0.0%	245 / 351 69.8%AB	101 / 351 28.8%ABE	4 / 351 1.1%	1 / 351 0.3%B				
2	Azoarcus	118 / 120 98.3%	3 / 3 100.0%	4 / 4 100.0%	4 / 4 100.0%	9 / 9 100.0%	9 / 9 100.0%	9 / 9 100.0%	3 / 3 100.0%	2 / 2 100.0%	15 / 15 100.0%	2 / 2 100.0%	8 / 8 100.0%	3 / 4 75.0%	2 / 2 100.0%	2 / 2 100.0%	2 / 2 100.0%	6 / 6 100.0%	2 / 2 100.0%	6 / 6 100.0%	8 / 9 88.9%	4 / 4 100.0%	2 / 2 100.0%	13 / 13 100.0%	0 / 0 0.0%	0 / 0 0.0%	0 / 0 0.0%	0 / 0 0.0%	83 / 120 69.2%AB	34 / 120 28.3%ABE	2 / 120 1.7%	1 / 120 0.8%B				
1	Azoarcus sp. BH72	55 / 56 98.2%	1 / 1 100.0%	2 / 2 100.0%	2 / 2 100.0%	4 / 4 100.0%	4 / 4 100.0%	4 / 4 100.0%	1 / 1 100.0%	1 / 1 100.0%	7 / 7 100.0%	1 / 1 100.0%	4 / 4 100.0%	1 / 2 50.0%	1 / 1 100.0%	1 / 1 100.0%	1 / 1 100.0%	3 / 3 100.0%	1 / 1 100.0%	3 / 3 100.0%	4 / 4 100.0%	2 / 2 100.0%	1 / 1 100.0%	6 / 6 100.0%	0 / 0 0.0%	0 / 0 0.0%	0 / 0 0.0%	0 / 0 0.0%	38 / 56 67.9%AB	16 / 56 28.6%ABE	1 / 56 1.8%B	1 / 56 1.8%				
1	Azoarcus sp. KH32C	63 / 64 98.4%	2 / 2 100.0%	2 / 2 100.0%	2 / 2 100.0%	5 / 5 100.0%	5 / 5 100.0%	5 / 5 100.0%	2 / 2 100.0%	1 / 1 100.0%	8 / 8 100.0%	1 / 1 100.0%	4 / 4 100.0%	2 / 2 100.0%	1 / 1 100.0%	1 / 1 100.0%	1 / 1 100.0%	3 / 3 100.0%	1 / 1 100.0%	3 / 3 100.0%	4 / 5 80.0%	2 / 2 100.0%	1 / 1 100.0%	7 / 7 100.0%	0 / 0 0.0%	0 / 0 0.0%	0 / 0 0.0%	0 / 0 0.0%	45 / 64 70.3%AB	18 / 64 28.1%ABE	1 / 64 1.6%					
1	Thauera	58 / 58 100.0%	1 / 1 100.0%	2 / 2 100.0%	2 / 2 100.0%	4 / 4 100.0%	4 / 4 100.0%	4 / 4 100.0%	1 / 1 100.0%	1 / 1 100.0%	7 / 7 100.0%	1 / 1 100.0%	4 / 4 100.0%	2 / 2 100.0%	1 / 1 100.0%	1 / 1 100.0%	1 / 1 100.0%	3 / 3 100.0%	1 / 1 100.0%	3 / 3 100.0%	5 / 5 100.0%	2 / 2 100.0%	1 / 1 100.0%	7 / 7 100.0%	0 / 0 0.0%	0 / 0 0.0%	0 / 0 0.0%	0 / 0 0.0%	41 / 58 70.7%AB	17 / 58 29.3%ABE						
1	Thauera sp. MZ1T	58 / 58 100.0%	1 / 1 100.0%	2 / 2 100.0%	2 / 2 100.0%	4 / 4 100.0%	4 / 4 100.0%	4 / 4 100.0%	1 / 1 100.0%	1 / 1 100.0%	7 / 7 100.0%	1 / 1 100.0%	4 / 4 100.0%	2 / 2 100.0%	1 / 1 100.0%	1 / 1 100.0%	1 / 1 100.0%	3 / 3 100.0%	1 / 1 100.0%	3 / 3 100.0%	5 / 5 100.0%	2 / 2 100.0%	1 / 1 100.0%	7 / 7 100.0%	0 / 0 0.0%	0 / 0 0.0%	0 / 0 0.0%	0 / 0 0.0%	41 / 58 70.7%AB	17 / 58 29.3%ABE						
1	Dechloromonas	61 / 62 98.4%	2 / 2 100.0%	3 / 3 100.0%	2 / 2 100.0%	4 / 4 100.0%	6 / 6 100.0%	4 / 4 100.0%	1 / 1 100.0%	1 / 1 100.0%	6 / 7 85.7%	1 / 1 100.0%	4 / 4 100.0%	2 / 2 100.0%	1 / 1 100.0%	1 / 1 100.0%	1 / 1 100.0%	3 / 3 100.0%	1 / 1 100.0%	4 / 4 100.0%	5 / 5 100.0%	2 / 2 100.0%	1 / 1 100.0%	6 / 6 100.0%	0 / 0 0.0%	0 / 0 0.0%	0 / 0 0.0%	0 / 0 0.0%	43 / 62 69.4%AB	18 / 62 29.0%ABE	1 / 62 1.6%					
1	'Dechloromonas aromatica'	61 / 62 98.4%	2 / 2 100.0%	3 / 3 100.0%	2 / 2 100.0%	4 / 4 100.0%	6 / 6 100.0%	4 / 4 100.0%	1 / 1 100.0%	1 / 1 100.0%	6 / 7 85.7%	1 / 1 100.0%	4 / 4 100.0%	2 / 2 100.0%	1 / 1 100.0%	1 / 1 100.0%	1 / 1 100.0%	3 / 3 100.0%	1 / 1 100.0%	4 / 4 100.0%	5 / 5 100.0%	2 / 2 100.0%	1 / 1 100.0%	6 / 6 100.0%	0 / 0 0.0%	0 / 0 0.0%	0 / 0 0.0%	0 / 0 0.0%	43 / 62 69.4%AB	18 / 62 29.0%ABE	1 / 62 1.6%					
1	Dechloromonas aromatica RCB	61 / 62 98.4%	2 / 2 100.0%	3 / 3 100.0%	2 / 2 100.0%	4 / 4 100.0%	6 / 6 100.0%	4 / 4 100.0%	1 / 1 100.0%	1 / 1 100.0%	6 / 7 85.7%	1 / 1 100.0%	4 / 4 100.0%	2 / 2 100.0%	1 / 1 100.0%	1 / 1 100.0%	1 / 1 100.0%	3 / 3 100.0%	1 / 1 100.0%	4 / 4 100.0%	5 / 5 100.0%	2 / 2 100.0%	1 / 1 100.0%	6 / 6 100.0%	0 / 0 0.0%	0 / 0 0.0%	0 / 0 0.0%	0 / 0 0.0%	43 / 62 69.4%AB	18 / 62 29.0%ABE	1 / 62 1.6%					
1	Azospira	54 / 55 98.2%	1 / 1 100.0%	3 / 3 100.0%	2 / 2 100.0%	2 / 2 100.0%	5 / 5 100.0%	3 / 3 100.0%	1 / 1 100.0%	1 / 1 100.0%	4 / 5 80.0%	1 / 1 100.0%	4 / 4 100.0%	2 / 2 100.0%	1 / 1 100.0%	1 / 1 100.0%	1 / 1 100.0%	3 / 3 100.0%	1 / 1 100.0%	4 / 4 100.0%	5 / 5 100.0%	2 / 2 100.0%	1 / 1 100.0%	6 / 6 100.0%	0 / 0 0.0%	0 / 0 0.0%	0 / 0 0.0%	0 / 0 0.0%	39 / 55 70.9%AB	15 / 55 27.3%ABE	1 / 55 1.8%					
1	ATCC BAA-33 [[Dechlorosoma suillum]]	54 / 55 98.2%	1 / 1 100.0%	3 / 3 100.0%	2 / 2 100.0%	2 / 2 100.0%	5 / 5 100.0%	3 / 3 100.0%	1 / 1 100.0%	1 / 1 100.0%	4 / 5 80.0%	1 / 1 100.0%	4 / 4 100.0%	2 / 2 100.0%	1 / 1 100.0%	1 / 1 100.0%	1 / 1 100.0%	3 / 3 100.0%	1 / 1 100.0%	4 / 4 100.0%	5 / 5 100.0%	2 / 2 100.0%	1 / 1 100.0%	6 / 6 100.0%	0 / 0 0.0%	0 / 0 0.0%	0 / 0 0.0%	0 / 0 0.0%	39 / 55 70.9%AB	15 / 55 27.3%ABE	1 / 55 1.8%					
1	Azospira oryzae PS	54 / 55 98.2%	1 / 1 100.0%	3 / 3 100.0%	2 / 2 100.0%	2 / 2 100.0%	5 / 5 100.0%	3 / 3 100.0%	1 / 1 100.0%	1 / 1 100.0%	4 / 5 80.0%	1 / 1 100.0%	4 / 4 100.0%	2 / 2 100.0%	1 / 1 100.0%	1 / 1 100.0%	1 / 1 100.0%	3 / 3 100.0%	1 / 1 100.0%	4 / 4 100.0%	5 / 5 100.0%	2 / 2 100.0%	1 / 1 100.0%	6 / 6 100.0%	0 / 0 0.0%	0 / 0 0.0%	0 / 0 0.0%	0 / 0 0.0%	39 / 55 70.9%AB	15 / 55 27.3%ABE	1 / 55 1.8%					
1	"Aromatoleum" Trautwein et al. 2008	56 / 56 100.0%	1 / 1 100.0%	2 / 2 100.0%	2 / 2 100.0%	4 / 4 100.0%	4 / 4 100.0%	4 / 4 100.0%	1 / 1 100.0%	1 / 1 100.0%	7 / 7 100.0%	1 / 1 100.0%	4 / 4 100.0%	2 / 2 100.0%	1 / 1 100.0%	1 / 1 100.0%	1 / 1 100.0%	3 / 3 100.0%	1 / 1 100.0%	3 / 3 100.0%	4 / 4 100.0%	2 / 2 100.0%	1 / 1 100.0%	6 / 6 100.0%	0 / 0 0.0%	0 / 0 0.0%	0 / 0 0.0%	0 / 0 0.0%	39 / 56 69.6%AB	17 / 56 30.4%ABE						
1	"Aromatoleum aromaticum" Trautwein et al. 2008	56 / 56 100.0%	1 / 1 100.0%	2 / 2 100.0%	2 / 2 100.0%	4 / 4 100.0%	4 / 4 100.0%	4 / 4 100.0%	1 / 1 100.0%	1 / 1 100.0%	7 / 7 100.0%	1 / 1 100.0%	4 / 4 100.0%	2 / 2 100.0%	1 / 1 100.0%	1 / 1 100.0%	1 / 1 100.0%	3 / 3 100.0%	1 / 1 100.0%	3 / 3 100.0%	4 / 4 100.0%	2 / 2 100.0%	1 / 1 100.0%	6 / 6 100.0%	0 / 0 0.0%	0 / 0 0.0%	0 / 0 0.0%	0 / 0 0.0%	39 / 56 69.6%AB	17 / 56 30.4%ABE						
1	Aromatoleum aromaticum EbN1	56 / 56 100.0%	1 / 1 100.0%	2 / 2 100.0%	2 / 2 100.0%	4 / 4 100.0%	4 / 4 100.0%	4 / 4 100.0%	1 / 1 100.0%	1 / 1 100.0%	7 / 7 100.0%	1 / 1 100.0%	4 / 4 100.0%	2 / 2 100.0%	1 / 1 100.0%	1 / 1 100.0%	1 / 1 100.0%	3 / 3 100.0%	1 / 1 100.0%	3 / 3 100.0%	4 / 4 100.0%	2 / 2 100.0%	1 / 1 100.0%	6 / 6 100.0%	0 / 0 0.0%	0 / 0 0.0%	0 / 0 0.0%	0 / 0 0.0%	39 / 56 69.6%AB	17 / 56 30.4%ABE						
2	"Gallionellales" Weiss et al. 2007	94 / 94 100.0%	2 / 2 100.0%	3 / 3 100.0%	2 / 2 100.0%	5 / 5 100.0%	7 / 7 100.0%	6 / 6 100.0%	2 / 2 100.0%	2 / 2 100.0%	8 / 8 100.0%	2 / 2 100.0%	8 / 8 100.0%	4 / 4 100.0%	2 / 2 100.0%	2 / 2 100.0%	2 / 2 100.0%	6 / 6 100.0%	2 / 2 100.0%	6 / 6 100.0%	9 / 9 100.0%	3 / 3 100.0%	2 / 2 100.0%	9 / 9 100.0%	0 / 0 0.0%	0 / 0 0.0%	0 / 0 0.0%	0 / 0 0.0%	65 / 94 69.1%AB	29 / 94 30.9%ABE						
2	Gallionella group	94 / 94 100.0%	2 / 2 100.0%	3 / 3 100.0%	2 / 2 100.0%	5 / 5 100.0%	7 / 7 100.0%	6 / 6 100.0%	2 / 2 100.0%	2 / 2 100.0%	8 / 8 100.0%	2 / 2 100.0%	8 / 8 100.0%	4 / 4 100.0%	2 / 2 100.0%	2 / 2 100.0%	2 / 2 100.0%	6 / 6 100.0%	2 / 2 100.0%	6 / 6 100.0%	9 / 9 100.0%	3 / 3 100.0%	2 / 2 100.0%	9 / 9 100.0%	0 / 0 0.0%	0 / 0 0.0%	0 / 0 0.0%	0 / 0 0.0%	65 / 94 69.1%AB	29 / 94 30.9%ABE						
1	Gallionella	50 / 50 100.0%	1 / 1 100.0%	2 / 2 100.0%	1 / 1 100.0%	3 / 3 100.0%	4 / 4 100.0%	3 / 3 100.0%	1 / 1 100.0%	1 / 1 100.0%	4 / 4 100.0%	1 / 1 100.0%	4 / 4 100.0%	2 / 2 100.0%	1 / 1 100.0%	1 / 1 100.0%	1 / 1 100.0%	3 / 3 100.0%	1 / 1 100.0%	3 / 3 100.0%	5 / 5 100.0%	2 / 2 100.0%	1 / 1 100.0%	5 / 5 100.0%	0 / 0 0.0%	0 / 0 0.0%	0 / 0 0.0%	0 / 0 0.0%	34 / 50 68.0%AB	16 / 50 32.0%ABE						
1	"Gallionella capsiferriformans" Emerson et al. 2013	50 / 50 100.0%	1 / 1 100.0%	2 / 2 100.0%	1 / 1 100.0%	3 / 3 100.0%	4 / 4 100.0%	3 / 3 100.0%	1 / 1 100.0%	1 / 1 100.0%	4 / 4 100.0%	1 / 1 100.0%	4 / 4 100.0%	2 / 2 100.0%	1 / 1 100.0%	1 / 1 100.0%	1 / 1 100.0%	3 / 3 100.0%	1 / 1 100.0%	3 / 3 100.0%	5 / 5 100.0%	2 / 2 100.0%	1 / 1 100.0%	5 / 5 100.0%	0 / 0 0.0%	0 / 0 0.0%	0 / 0 0.0%	0 / 0 0.0%	34 / 50 68.0%AB	16 / 50 32.0%ABE						
1	Gallionella capsiferriformans ES-2	50 / 50 100.0%	1 / 1 100.0%	2 / 2 100.0%	1 / 1 100.0%	3 / 3 100.0%	4 / 4 100.0%	3 / 3 100.0%	1 / 1 100.0%	1 / 1 100.0%	4 / 4 100.0%	1 / 1 100.0%	4 / 4 100.0%	2 / 2 100.0%	1 / 1 100.0%	1 / 1 100.0%	1 / 1 100.0%	3 / 3 100.0%	1 / 1 100.0%	3 / 3 100.0%	5 / 5 100.0%	2 / 2 100.0%	1 / 1 100.0%	5 / 5 100.0%	0 / 0 0.0%	0 / 0 0.0%	0 / 0 0.0%	0 / 0 0.0%	34 / 50 68.0%AB	16 / 50 32.0%ABE						
1	"Siderooxidans" Emerson 2003	44 / 44 100.0%	1 / 1 100.0%	1 / 1 100.0%	1 / 1 100.0%	2 / 2 100.0%	3 / 3 100.0%	3 / 3 100.0%	1 / 1 100.0%	1 / 1 100.0%	4 / 4 100.0%	1 / 1 100.0%	4 / 4 100.0%	2 / 2 100.0%	1 / 1 100.0%	1 / 1 100.0%	1 / 1 100.0%	3 / 3 100.0%	1 / 1 100.0%	3 / 3 100.0%	4 / 4 100.0%	1 / 1 100.0%	1 / 1 100.0%	4 / 4 100.0%	0 / 0 0.0%	0 / 0 0.0%	0 / 0 0.0%	0 / 0 0.0%	31 / 44 70.5%AB	13 / 44 29.5%ABE						
1	"Sideroxydans lithotrophicus" Emerson et al. 2013	44 / 44 100.0%	1 / 1 100.0%	1 / 1 100.0%	1 / 1 100.0%	2 / 2 100.0%	3 / 3 100.0%	3 / 3 100.0%	1 / 1 100.0%	1 / 1 100.0%	4 / 4 100.0%	1 / 1 100.0%	4 / 4 100.0%	2 / 2 100.0%	1 / 1 100.0%	1 / 1 100.0%	1 / 1 100.0%	3 / 3 100.0%	1 / 1 100.0%	3 / 3 100.0%	4 / 4 100.0%	1 / 1 100.0%	1 / 1 100.0%	4 / 4 100.0%	0 / 0 0.0%	0 / 0 0.0%	0 / 0 0.0%	0 / 0 0.0%	31 / 44 70.5%AB	13 / 44 29.5%ABE						
1	Siderooxidans lithoautotrophicus ES-1	44 / 44 100.0%	1 / 1 100.0%	1 / 1 100.0%	1 / 1 100.0%	2 / 2 100.0%	3 / 3 100.0%	3 / 3 100.0%	1 / 1 100.0%	1 / 1 100.0%	4 / 4 100.0%	1 / 1 100.0%	4 / 4 100.0%	2 / 2 100.0%	1 / 1 100.0%	1 / 1 100.0%	1 / 1 100.0%	3 / 3 100.0%	1 / 1 100.0%	3 / 3 100.0%	4 / 4 100.0%	1 / 1 100.0%	1 / 1 100.0%	4 / 4 100.0%	0 / 0 0.0%	0 / 0 0.0%	0 / 0 0.0%	0 / 0 0.0%	31 / 44 70.5%AB	13 / 44 29.5%ABE						
145	delta/epsilon subdivisions	6119 / 6622 92.4%	172 / 186 92.5%	204 / 208 98.1%	162 / 171 94.7%	291 / 299 97.3%	411 / 427 96.3%	341 / 374 91.2%	145 / 153 94.8%	146 / 162 90.1%	455 / 497 91.5%	147 / 150 98.0%	463 / 492 94.1%	214 / 244 87.7%	148 / 163 90.8%	208 / 214 97.2%	139 / 151 92.1%	298 / 332 89.8%	143 / 150 95.3%	352 / 372 94.6%	590 / 646 91.3%	290 / 309 93.9%	132 / 152 86.8%	621 / 688 90.3%	45 / 77 58.4%	0 / 0 0.0%	2 / 5 40.0%	0 / 0 0.0%	3969 / 6622 59.9%AB	1865 / 6622 28.2%ABE	257 / 6622 3.9%	201 / 6622 3.0%B	174 / 6622 2.6%E	84 / 6622 1.3%BE	45 / 6622 0.7%C	27 / 6622 0.4%CE
58	"Deltabacteria" Cavalier-Smith 1992	2637 / 3103 85.0%	74 / 87 85.1%	91 / 95 95.8%	62 / 71 87.3%	137 / 145 94.5%	199 / 215 92.6%	142 / 175 81.1%	56 / 64 87.5%	53 / 69 76.8%	215 / 256 84.0%	58 / 61 95.1%	199 / 227 87.7%	91 / 119 76.5%	52 / 67 77.6%	109 / 115 94.8%	50 / 62 80.6%	142 / 176 80.7%	53 / 60 88.3%	164 / 184 89.1%	229 / 283 80.9%	114 / 131 87.0%	43 / 63 68.3%	259 / 326 79.4%	45 / 49 91.8%	0 / 0 0.0%	0 / 3 0.0%	0 / 0 0.0%	1634 / 3103 52.7%AB	898 / 3103 28.9%ABE	238 / 3103 7.7%	156 / 3103 5.0%E	66 / 3103 2.1%B	45 / 3103 1.5%C	39 / 3103 1.3%BE	27 / 3103 0.9%CE
12	Myxobacteria	226 / 680 33.2%	7 / 20 35.0%	13 / 17 76.5%	6 / 15 40.0%	23 / 31 74.2%	32 / 48 66.7%	4 / 37 10.8%	6 / 14 42.9%	1 / 17 5.9%	20 / 60 33.3%	9 / 12 75.0%	24 / 48 50.0%	1 / 28 3.6%	0 / 15 0.0%	18 / 24 75.0%	0 / 12 0.0%	12 / 45 26.7%	3 / 10 30.0%	18 / 37 48.6%	13 / 67 19.4%	7 / 24 29.2%	0 / 17 0.0%	4 / 70 5.7%	5 / 9 55.6%	0 / 0 0.0%	0 / 3 0.0%	0 / 0 0.0%	232 / 680 34.1%	150 / 680 22.1%E	124 / 680 18.2%AB	66 / 680 9.7%ABE	45 / 680 6.6%C	28 / 680 4.1%B	27 / 680 4.0%CE	8 / 680 1.2%BE
9	"Cystobacterineae" Reichenbach and Dworkin 1981	172 / 512 33.6%	4 / 16 25.0%	10 / 11 90.9%	4 / 10 40.0%	23 / 26 88.5%	26 / 35 74.3%	4 / 28 14.3%	1 / 9 11.1%	0 / 14 0.0%	17 / 49 34.7%	9 / 9 100.0%	20 / 36 55.6%	0 / 20 0.0%	0 / 12 0.0%	12 / 18 66.7%	0 / 9 0.0%	10 / 32 31.2%	0 / 7 0.0%	13 / 28 46.4%	10 / 50 20.0%	4 / 18 22.2%	0 / 14 0.0%	0 / 51 0.0%	5 / 9 55.6%	0 / 0 0.0%	0 / 1 0.0%	0 / 0 0.0%	200 / 512 39.1%	139 / 512 27.1%E	101 / 512 19.7%AB	45 / 512 8.8%ABE	23 / 512 4.5%B	3 / 512 0.6%BE	1 / 512 0.2%C	
4	Myxococcaceae	68 / 266 25.6%	0 / 10 0.0%	5 / 6 83.3%	0 / 5 0.0%	13 / 16 81.2%	12 / 20 60.0%	0 / 13 0.0%	0 / 4 0.0%	0 / 9 0.0%	4 / 29 13.8%	4 / 4 100.0%	4 / 16 25.0%	0 / 10 0.0%	0 / 7 0.0%	3 / 8 37.5%	0 / 4 0.0%	8 / 17 47.1%	0 / 3 0.0%	8 / 13 61.5%	4 / 25 16.0%	0 / 8 0.0%	0 / 8 0.0%	0 / 26 0.0%	3 / 4 75.0%	0 / 0 0.0%	0 / 1 0.0%	0 / 0 0.0%	124 / 266 46.6%	74 / 266 27.8%E	54 / 266 20.3%AB	14 / 266 5.3%ABE				
3	Myxococcus	50 / 207 24.2%	0 / 8 0.0%	3 / 4 75.0%	0 / 4 0.0%	10 / 13 76.9%	9 / 16 56.2%	0 / 10 0.0%	0 / 3 0.0%	0 / 8 0.0%	3 / 23 13.0%	3 / 3 100.0%	3 / 12 25.0%	0 / 8 0.0%	0 / 5 0.0%	2 / 6 33.3%	0 / 3 0.0%	6 / 13 46.2%	0 / 2 0.0%	6 / 10 60.0%	3 / 20 15.0%	0 / 6 0.0%	0 / 6 0.0%	0 / 20 0.0%	2 / 3 66.7%	0 / 0 0.0%	0 / 1 0.0%	0 / 0 0.0%	98 / 207 47.3%	59 / 207 28.5%E	40 / 207 19.3%AB	10 / 207 4.8%ABE				
1	"Micrococcus fulvus" Cohn 1875	18 / 67 26.9%	0 / 3 0.0%	1 / 1 100.0%	0 / 2 0.0%	3 / 3 100.0%	3 / 5 60.0%	0 / 3 0.0%	0 / 1 0.0%	0 / 2 0.0%	1 / 9 11.1%	1 / 1 100.0%	1 / 4 25.0%	0 / 3 0.0%	0 / 2 0.0%	1 / 2 50.0%	0 / 1 0.0%	2 / 5 40.0%	0 / 1 0.0%	2 / 3 66.7%	2 / 6 33.3%	0 / 2 0.0%	0 / 1 0.0%	0 / 6 0.0%	1 / 1 100.0%	0 / 0 0.0%	0 / 0 0.0%	0 / 0 0.0%	33 / 67 49.3%	16 / 67 23.9%E	14 / 67 20.9%AB	4 / 67 6.0%ABE				
1	Myxococcus fulvus HW-1	18 / 67 26.9%	0 / 3 0.0%	1 / 1 100.0%	0 / 2 0.0%	3 / 3 100.0%	3 / 5 60.0%	0 / 3 0.0%	0 / 1 0.0%	0 / 2 0.0%	1 / 9 11.1%	1 / 1 100.0%	1 / 4 25.0%	0 / 3 0.0%	0 / 2 0.0%	1 / 2 50.0%	0 / 1 0.0%	2 / 5 40.0%	0 / 1 0.0%	2 / 3 66.7%	2 / 6 33.3%	0 / 2 0.0%	0 / 1 0.0%	0 / 6 0.0%	1 / 1 100.0%	0 / 0 0.0%	0 / 0 0.0%	0 / 0 0.0%	33 / 67 49.3%	16 / 67 23.9%E	14 / 67 20.9%AB	4 / 67 6.0%ABE				
1	ATCC 25232	18 / 64 28.1%	0 / 3 0.0%	1 / 2 50.0%	0 / 1 0.0%	4 / 5 80.0%	3 / 5 60.0%	0 / 3 0.0%	0 / 1 0.0%	0 / 2 0.0%	1 / 6 16.7%	1 / 1 100.0%	1 / 4 25.0%	0 / 2 0.0%	0 / 1 0.0%	1 / 2 50.0%	0 / 1 0.0%	2 / 3 66.7%	0 / 1 0.0%	2 / 3 66.7%	1 / 6 16.7%	0 / 2 0.0%	0 / 2 0.0%	0 / 7 0.0%	1 / 1 100.0%	0 / 0 0.0%	0 / 0 0.0%	0 / 0 0.0%	30 / 64 46.9%	16 / 64 25.0%E	14 / 64 21.9%AB	4 / 64 6.2%ABE				
1	Myxococcus xanthus DK 1622	18 / 64 28.1%	0 / 3 0.0%	1 / 2 50.0%	0 / 1 0.0%	4 / 5 80.0%	3 / 5 60.0%	0 / 3 0.0%	0 / 1 0.0%	0 / 2 0.0%	1 / 6 16.7%	1 / 1 100.0%	1 / 4 25.0%	0 / 2 0.0%	0 / 1 0.0%	1 / 2 50.0%	0 / 1 0.0%	2 / 3 66.7%	0 / 1 0.0%	2 / 3 66.7%	1 / 6 16.7%	0 / 2 0.0%	0 / 2 0.0%	0 / 7 0.0%	1 / 1 100.0%	0 / 0 0.0%	0 / 0 0.0%	0 / 0 0.0%	30 / 64 46.9%	16 / 64 25.0%E	14 / 64 21.9%AB	4 / 64 6.2%ABE				
1	"Myxococcus ovalisporus" Krzemienievska and Krzemieniewski 1926	14 / 76 18.4%	0 / 2 0.0%	1 / 1 100.0%	0 / 1 0.0%	3 / 5 60.0%	3 / 6 50.0%	0 / 4 0.0%	0 / 1 0.0%	0 / 4 0.0%	1 / 8 12.5%	1 / 1 100.0%	1 / 4 25.0%	0 / 3 0.0%	0 / 2 0.0%	0 / 2 0.0%	0 / 1 0.0%	2 / 5 40.0%	0 / 0 0.0%	2 / 4 50.0%	0 / 8 0.0%	0 / 2 0.0%	0 / 3 0.0%	0 / 7 0.0%	0 / 1 0.0%	0 / 0 0.0%	0 / 1 0.0%	0 / 0 0.0%	35 / 76 46.1%	27 / 76 35.5%E	12 / 76 15.8%AB	2 / 76 2.6%ABE				
1	Myxococcus stipitatus DSM 14675	14 / 76 18.4%	0 / 2 0.0%	1 / 1 100.0%	0 / 1 0.0%	3 / 5 60.0%	3 / 6 50.0%	0 / 4 0.0%	0 / 1 0.0%	0 / 4 0.0%	1 / 8 12.5%	1 / 1 100.0%	1 / 4 25.0%	0 / 3 0.0%	0 / 2 0.0%	0 / 2 0.0%	0 / 1 0.0%	2 / 5 40.0%	0 / 0 0.0%	2 / 4 50.0%	0 / 8 0.0%	0 / 2 0.0%	0 / 3 0.0%	0 / 7 0.0%	0 / 1 0.0%	0 / 0 0.0%	0 / 1 0.0%	0 / 0 0.0%	35 / 76 46.1%	27 / 76 35.5%E	12 / 76 15.8%AB	2 / 76 2.6%ABE				
1	Chondrococcus	18 / 59 30.5%	0 / 2 0.0%	2 / 2 100.0%	0 / 1 0.0%	3 / 3 100.0%	3 / 4 75.0%	0 / 3 0.0%	0 / 1 0.0%	0 / 1 0.0%	1 / 6 16.7%	1 / 1 100.0%	1 / 4 25.0%	0 / 2 0.0%	0 / 2 0.0%	1 / 2 50.0%	0 / 1 0.0%	2 / 4 50.0%	0 / 1 0.0%	2 / 3 66.7%	1 / 5 20.0%	0 / 2 0.0%	0 / 2 0.0%	0 / 6 0.0%	1 / 1 100.0%	0 / 0 0.0%	0 / 0 0.0%	0 / 0 0.0%	26 / 59 44.1%	15 / 59 25.4%E	14 / 59 23.7%AB	4 / 59 6.8%ABE				
1	"Chondrococcus coralloides" (Thaxter 1892) Jahn 1924	18 / 59 30.5%	0 / 2 0.0%	2 / 2 100.0%	0 / 1 0.0%	3 / 3 100.0%	3 / 4 75.0%	0 / 3 0.0%	0 / 1 0.0%	0 / 1 0.0%	1 / 6 16.7%	1 / 1 100.0%	1 / 4 25.0%	0 / 2 0.0%	0 / 2 0.0%	1 / 2 50.0%	0 / 1 0.0%	2 / 4 50.0%	0 / 1 0.0%	2 / 3 66.7%	1 / 5 20.0%	0 / 2 0.0%	0 / 2 0.0%	0 / 6 0.0%	1 / 1 100.0%	0 / 0 0.0%	0 / 0 0.0%	0 / 0 0.0%	26 / 59 44.1%	15 / 59 25.4%E	14 / 59 23.7%AB	4 / 59 6.8%ABE				
1	Corallococcus coralloides ATCC 25202	18 / 59 30.5%	0 / 2 0.0%	2 / 2 100.0%	0 / 1 0.0%	3 / 3 100.0%	3 / 4 75.0%	0 / 3 0.0%	0 / 1 0.0%	0 / 1 0.0%	1 / 6 16.7%	1 / 1 100.0%	1 / 4 25.0%	0 / 2 0.0%	0 / 2 0.0%	1 / 2 50.0%	0 / 1 0.0%	2 / 4 50.0%	0 / 1 0.0%	2 / 3 66.7%	1 / 5 20.0%	0 / 2 0.0%	0 / 2 0.0%	0 / 6 0.0%	1 / 1 100.0%	0 / 0 0.0%	0 / 0 0.0%	0 / 0 0.0%	26 / 59 44.1%	15 / 59 25.4%E	14 / 59 23.7%AB	4 / 59 6.8%ABE				
1	Archangiaceae	12 / 50 24.0%	0 / 2 0.0%	1 / 1 100.0%	0 / 1 0.0%	2 / 2 100.0%	2 / 3 66.7%	0 / 3 0.0%	0 / 1 0.0%	0 / 1 0.0%	0 / 4 0.0%	1 / 1 100.0%	1 / 4 25.0%	0 / 2 0.0%	0 / 1 0.0%	1 / 2 50.0%	0 / 1 0.0%	2 / 3 66.7%	0 / 0 0.0%	2 / 3 66.7%	0 / 5 0.0%	0 / 2 0.0%	0 / 2 0.0%	0 / 5 0.0%	0 / 1 0.0%	0 / 0 0.0%	0 / 0 0.0%	0 / 0 0.0%	21 / 50 42.0%	16 / 50 32.0%E	9 / 50 18.0%AB	3 / 50 6.0%ABE	1 / 50 2.0%C			
1	Stigmatella	12 / 50 24.0%	0 / 2 0.0%	1 / 1 100.0%	0 / 1 0.0%	2 / 2 100.0%	2 / 3 66.7%	0 / 3 0.0%	0 / 1 0.0%	0 / 1 0.0%	0 / 4 0.0%	1 / 1 100.0%	1 / 4 25.0%	0 / 2 0.0%	0 / 1 0.0%	1 / 2 50.0%	0 / 1 0.0%	2 / 3 66.7%	0 / 0 0.0%	2 / 3 66.7%	0 / 5 0.0%	0 / 2 0.0%	0 / 2 0.0%	0 / 5 0.0%	0 / 1 0.0%	0 / 0 0.0%	0 / 0 0.0%	0 / 0 0.0%	21 / 50 42.0%	16 / 50 32.0%E	9 / 50 18.0%AB	3 / 50 6.0%ABE	1 / 50 2.0%C			
1	"Chondromyces aurantiacus" (Berkeley and Curtis) Thaxter 1892	12 / 50 24.0%	0 / 2 0.0%	1 / 1 100.0%	0 / 1 0.0%	2 / 2 100.0%	2 / 3 66.7%	0 / 3 0.0%	0 / 1 0.0%	0 / 1 0.0%	0 / 4 0.0%	1 / 1 100.0%	1 / 4 25.0%	0 / 2 0.0%	0 / 1 0.0%	1 / 2 50.0%	0 / 1 0.0%	2 / 3 66.7%	0 / 0 0.0%	2 / 3 66.7%	0 / 5 0.0%	0 / 2 0.0%	0 / 2 0.0%	0 / 5 0.0%	0 / 1 0.0%	0 / 0 0.0%	0 / 0 0.0%	0 / 0 0.0%	21 / 50 42.0%	16 / 50 32.0%E	9 / 50 18.0%AB	3 / 50 6.0%ABE	1 / 50 2.0%C			
1	Stigmatella aurantiaca DW4/3-1	12 / 50 24.0%	0 / 2 0.0%	1 / 1 100.0%	0 / 1 0.0%	2 / 2 100.0%	2 / 3 66.7%	0 / 3 0.0%	0 / 1 0.0%	0 / 1 0.0%	0 / 4 0.0%	1 / 1 100.0%	1 / 4 25.0%	0 / 2 0.0%	0 / 1 0.0%	1 / 2 50.0%	0 / 1 0.0%	2 / 3 66.7%	0 / 0 0.0%	2 / 3 66.7%	0 / 5 0.0%	0 / 2 0.0%	0 / 2 0.0%	0 / 5 0.0%	0 / 1 0.0%	0 / 0 0.0%	0 / 0 0.0%	0 / 0 0.0%	21 / 50 42.0%	16 / 50 32.0%E	9 / 50 18.0%AB	3 / 50 6.0%ABE	1 / 50 2.0%C			
4	Anaeromyxobacteraceae	92 / 196 46.9%	4 / 4 100.0%	4 / 4 100.0%	4 / 4 100.0%	8 / 8 100.0%	12 / 12 100.0%	4 / 12 33.3%	1 / 4 25.0%	0 / 4 0.0%	13 / 16 81.2%	4 / 4 100.0%	15 / 16 93.8%	0 / 8 0.0%	0 / 4 0.0%	8 / 8 100.0%	0 / 4 0.0%	0 / 12 0.0%	0 / 4 0.0%	3 / 12 25.0%	6 / 20 30.0%	4 / 8 50.0%	0 / 4 0.0%	0 / 20 0.0%	2 / 4 50.0%	0 / 0 0.0%	0 / 0 0.0%	0 / 0 0.0%	55 / 196 28.1%	49 / 196 25.0%E	38 / 196 19.4%AB	28 / 196 14.3%ABE	23 / 196 11.7%B	3 / 196 1.5%BE		
4	Anaeromyxobacter	92 / 196 46.9%	4 / 4 100.0%	4 / 4 100.0%	4 / 4 100.0%	8 / 8 100.0%	12 / 12 100.0%	4 / 12 33.3%	1 / 4 25.0%	0 / 4 0.0%	13 / 16 81.2%	4 / 4 100.0%	15 / 16 93.8%	0 / 8 0.0%	0 / 4 0.0%	8 / 8 100.0%	0 / 4 0.0%	0 / 12 0.0%	0 / 4 0.0%	3 / 12 25.0%	6 / 20 30.0%	4 / 8 50.0%	0 / 4 0.0%	0 / 20 0.0%	2 / 4 50.0%	0 / 0 0.0%	0 / 0 0.0%	0 / 0 0.0%	55 / 196 28.1%	49 / 196 25.0%E	38 / 196 19.4%AB	28 / 196 14.3%ABE	23 / 196 11.7%B	3 / 196 1.5%BE		
2	ATCC BAA-258	44 / 98 44.9%	2 / 2 100.0%	2 / 2 100.0%	2 / 2 100.0%	4 / 4 100.0%	6 / 6 100.0%	2 / 6 33.3%	0 / 2 0.0%	0 / 2 0.0%	6 / 8 75.0%	2 / 2 100.0%	8 / 8 100.0%	0 / 4 0.0%	0 / 2 0.0%	4 / 4 100.0%	0 / 2 0.0%	0 / 6 0.0%	0 / 2 0.0%	1 / 6 16.7%	2 / 10 20.0%	2 / 4 50.0%	0 / 2 0.0%	0 / 10 0.0%	1 / 2 50.0%	0 / 0 0.0%	0 / 0 0.0%	0 / 0 0.0%	29 / 98 29.6%	29 / 98 29.6%AB	25 / 98 25.5%E	15 / 98 15.3%ABE				
1	Anaeromyxobacter dehalogenans 2CP-C	22 / 49 44.9%	1 / 1 100.0%	1 / 1 100.0%	1 / 1 100.0%	2 / 2 100.0%	3 / 3 100.0%	1 / 3 33.3%	0 / 1 0.0%	0 / 1 0.0%	3 / 4 75.0%	1 / 1 100.0%	4 / 4 100.0%	0 / 2 0.0%	0 / 1 0.0%	2 / 2 100.0%	0 / 1 0.0%	0 / 3 0.0%	0 / 1 0.0%	1 / 3 33.3%	1 / 5 20.0%	1 / 2 50.0%	0 / 1 0.0%	0 / 5 0.0%	0 / 1 0.0%	0 / 0 0.0%	0 / 0 0.0%	0 / 0 0.0%	15 / 49 30.6%AB	14 / 49 28.6%	13 / 49 26.5%E	7 / 49 14.3%ABE				
1	Anaeromyxobacter dehalogenans 2CP-1	22 / 49 44.9%	1 / 1 100.0%	1 / 1 100.0%	1 / 1 100.0%	2 / 2 100.0%	3 / 3 100.0%	1 / 3 33.3%	0 / 1 0.0%	0 / 1 0.0%	3 / 4 75.0%	1 / 1 100.0%	4 / 4 100.0%	0 / 2 0.0%	0 / 1 0.0%	2 / 2 100.0%	0 / 1 0.0%	0 / 3 0.0%	0 / 1 0.0%	0 / 3 0.0%	1 / 5 20.0%	1 / 2 50.0%	0 / 1 0.0%	0 / 5 0.0%	1 / 1 100.0%	0 / 0 0.0%	0 / 0 0.0%	0 / 0 0.0%	15 / 49 30.6%	14 / 49 28.6%AB	12 / 49 24.5%E	8 / 49 16.3%ABE				
1	Anaeromyxobacter sp. Fw109-5	26 / 49 53.1%	1 / 1 100.0%	1 / 1 100.0%	1 / 1 100.0%	2 / 2 100.0%	3 / 3 100.0%	1 / 3 33.3%	1 / 1 100.0%	0 / 1 0.0%	4 / 4 100.0%	1 / 1 100.0%	3 / 4 75.0%	0 / 2 0.0%	0 / 1 0.0%	2 / 2 100.0%	0 / 1 0.0%	0 / 3 0.0%	0 / 1 0.0%	2 / 3 66.7%	3 / 5 60.0%	1 / 2 50.0%	0 / 1 0.0%	0 / 5 0.0%	0 / 1 0.0%	0 / 0 0.0%	0 / 0 0.0%	0 / 0 0.0%	14 / 49 28.6%B	12 / 49 24.5%E	11 / 49 22.4%	7 / 49 14.3%ABE	4 / 49 8.2%AB	1 / 49 2.0%BE		
1	Anaeromyxobacter sp. K	22 / 49 44.9%	1 / 1 100.0%	1 / 1 100.0%	1 / 1 100.0%	2 / 2 100.0%	3 / 3 100.0%	1 / 3 33.3%	0 / 1 0.0%	0 / 1 0.0%	3 / 4 75.0%	1 / 1 100.0%	4 / 4 100.0%	0 / 2 0.0%	0 / 1 0.0%	2 / 2 100.0%	0 / 1 0.0%	0 / 3 0.0%	0 / 1 0.0%	0 / 3 0.0%	1 / 5 20.0%	1 / 2 50.0%	0 / 1 0.0%	0 / 5 0.0%	1 / 1 100.0%	0 / 0 0.0%	0 / 0 0.0%	0 / 0 0.0%	15 / 49 30.6%	12 / 49 24.5%E	9 / 49 18.4%B	6 / 49 12.2%ABE	5 / 49 10.2%AB	2 / 49 4.1%BE		
2	"Sorangineae" Reichenbach and Dworkin 1981	34 / 122 27.9%	3 / 3 100.0%	3 / 5 60.0%	2 / 4 50.0%	0 / 4 0.0%	5 / 10 50.0%	0 / 6 0.0%	4 / 4 100.0%	0 / 2 0.0%	0 / 8 0.0%	0 / 2 0.0%	1 / 8 12.5%	1 / 6 16.7%	0 / 2 0.0%	4 / 4 100.0%	0 / 2 0.0%	1 / 10 10.0%	2 / 2 100.0%	3 / 6 50.0%	0 / 12 0.0%	2 / 4 50.0%	0 / 2 0.0%	3 / 14 21.4%	0 / 0 0.0%	0 / 0 0.0%	0 / 2 0.0%	0 / 0 0.0%	38 / 122 31.1%C	23 / 122 18.9%CE	20 / 122 16.4%	14 / 122 11.5%AB	10 / 122 8.2%ABE	7 / 122 5.7%E	5 / 122 4.1%B	5 / 122 4.1%BE
2	"Sorangiaceae" Jahn 1924	34 / 122 27.9%	3 / 3 100.0%	3 / 5 60.0%	2 / 4 50.0%	0 / 4 0.0%	5 / 10 50.0%	0 / 6 0.0%	4 / 4 100.0%	0 / 2 0.0%	0 / 8 0.0%	0 / 2 0.0%	1 / 8 12.5%	1 / 6 16.7%	0 / 2 0.0%	4 / 4 100.0%	0 / 2 0.0%	1 / 10 10.0%	2 / 2 100.0%	3 / 6 50.0%	0 / 12 0.0%	2 / 4 50.0%	0 / 2 0.0%	3 / 14 21.4%	0 / 0 0.0%	0 / 0 0.0%	0 / 2 0.0%	0 / 0 0.0%	38 / 122 31.1%C	23 / 122 18.9%CE	20 / 122 16.4%	14 / 122 11.5%AB	10 / 122 8.2%ABE	7 / 122 5.7%E	5 / 122 4.1%B	5 / 122 4.1%BE
2	Sorangium	34 / 122 27.9%	3 / 3 100.0%	3 / 5 60.0%	2 / 4 50.0%	0 / 4 0.0%	5 / 10 50.0%	0 / 6 0.0%	4 / 4 100.0%	0 / 2 0.0%	0 / 8 0.0%	0 / 2 0.0%	1 / 8 12.5%	1 / 6 16.7%	0 / 2 0.0%	4 / 4 100.0%	0 / 2 0.0%	1 / 10 10.0%	2 / 2 100.0%	3 / 6 50.0%	0 / 12 0.0%	2 / 4 50.0%	0 / 2 0.0%	3 / 14 21.4%	0 / 0 0.0%	0 / 0 0.0%	0 / 2 0.0%	0 / 0 0.0%	38 / 122 31.1%C	23 / 122 18.9%CE	20 / 122 16.4%	14 / 122 11.5%AB	10 / 122 8.2%ABE	7 / 122 5.7%E	5 / 122 4.1%BE	5 / 122 4.1%B
2	"Polyangium compositum" Thaxter 1904	34 / 122 27.9%	3 / 3 100.0%	3 / 5 60.0%	2 / 4 50.0%	0 / 4 0.0%	5 / 10 50.0%	0 / 6 0.0%	4 / 4 100.0%	0 / 2 0.0%	0 / 8 0.0%	0 / 2 0.0%	1 / 8 12.5%	1 / 6 16.7%	0 / 2 0.0%	4 / 4 100.0%	0 / 2 0.0%	1 / 10 10.0%	2 / 2 100.0%	3 / 6 50.0%	0 / 12 0.0%	2 / 4 50.0%	0 / 2 0.0%	3 / 14 21.4%	0 / 0 0.0%	0 / 0 0.0%	0 / 2 0.0%	0 / 0 0.0%	38 / 122 31.1%C	23 / 122 18.9%CE	20 / 122 16.4%	14 / 122 11.5%AB	10 / 122 8.2%ABE	7 / 122 5.7%E	5 / 122 4.1%BE	5 / 122 4.1%B
1	Sorangium cellulosum 'So ce 56'	16 / 61 26.2%	1 / 1 100.0%	2 / 3 66.7%	1 / 2 50.0%	0 / 2 0.0%	2 / 5 40.0%	0 / 3 0.0%	2 / 2 100.0%	0 / 1 0.0%	0 / 4 0.0%	0 / 1 0.0%	0 / 4 0.0%	1 / 3 33.3%	0 / 1 0.0%	2 / 2 100.0%	0 / 1 0.0%	0 / 6 0.0%	1 / 1 100.0%	1 / 3 33.3%	0 / 6 0.0%	1 / 2 50.0%	0 / 1 0.0%	2 / 7 28.6%	0 / 0 0.0%	0 / 0 0.0%	0 / 0 0.0%	0 / 0 0.0%	19 / 61 31.1%C	11 / 61 18.0%CE	11 / 61 18.0%	5 / 61 8.2%BE	5 / 61 8.2%B	4 / 61 6.6%E	4 / 61 6.6%AB	2 / 61 3.3%ABE
1	Sorangium cellulosum So0157-2	18 / 61 29.5%	2 / 2 100.0%	1 / 2 50.0%	1 / 2 50.0%	0 / 2 0.0%	3 / 5 60.0%	0 / 3 0.0%	2 / 2 100.0%	0 / 1 0.0%	0 / 4 0.0%	0 / 1 0.0%	1 / 4 25.0%	0 / 3 0.0%	0 / 1 0.0%	2 / 2 100.0%	0 / 1 0.0%	1 / 4 25.0%	1 / 1 100.0%	2 / 3 66.7%	0 / 6 0.0%	1 / 2 50.0%	0 / 1 0.0%	1 / 7 14.3%	0 / 0 0.0%	0 / 0 0.0%	0 / 2 0.0%	0 / 0 0.0%	19 / 61 31.1%C	12 / 61 19.7%CE	10 / 61 16.4%AB	9 / 61 14.8%	8 / 61 13.1%ABE	3 / 61 4.9%E		
1	Nannocystinea	20 / 46 43.5%	0 / 1 0.0%	0 / 1 0.0%	0 / 1 0.0%	0 / 1 0.0%	1 / 3 33.3%	0 / 3 0.0%	1 / 1 100.0%	1 / 1 100.0%	3 / 3 100.0%	0 / 1 0.0%	3 / 4 75.0%	0 / 2 0.0%	0 / 1 0.0%	2 / 2 100.0%	0 / 1 0.0%	1 / 3 33.3%	1 / 1 100.0%	2 / 3 66.7%	3 / 5 60.0%	1 / 2 50.0%	0 / 1 0.0%	1 / 5 20.0%	0 / 0 0.0%	0 / 0 0.0%	0 / 0 0.0%	0 / 0 0.0%	12 / 46 26.1%	11 / 46 23.9%ABE	9 / 46 19.6%AB	6 / 46 13.0%C	4 / 46 8.7%E	4 / 46 8.7%CE		
1	"Haliangiaceae" Garrity et al. 2003	20 / 46 43.5%	0 / 1 0.0%	0 / 1 0.0%	0 / 1 0.0%	0 / 1 0.0%	1 / 3 33.3%	0 / 3 0.0%	1 / 1 100.0%	1 / 1 100.0%	3 / 3 100.0%	0 / 1 0.0%	3 / 4 75.0%	0 / 2 0.0%	0 / 1 0.0%	2 / 2 100.0%	0 / 1 0.0%	1 / 3 33.3%	1 / 1 100.0%	2 / 3 66.7%	3 / 5 60.0%	1 / 2 50.0%	0 / 1 0.0%	1 / 5 20.0%	0 / 0 0.0%	0 / 0 0.0%	0 / 0 0.0%	0 / 0 0.0%	12 / 46 26.1%	11 / 46 23.9%ABE	9 / 46 19.6%AB	6 / 46 13.0%C	4 / 46 8.7%CE	4 / 46 8.7%E		
1	Haliangium	20 / 46 43.5%	0 / 1 0.0%	0 / 1 0.0%	0 / 1 0.0%	0 / 1 0.0%	1 / 3 33.3%	0 / 3 0.0%	1 / 1 100.0%	1 / 1 100.0%	3 / 3 100.0%	0 / 1 0.0%	3 / 4 75.0%	0 / 2 0.0%	0 / 1 0.0%	2 / 2 100.0%	0 / 1 0.0%	1 / 3 33.3%	1 / 1 100.0%	2 / 3 66.7%	3 / 5 60.0%	1 / 2 50.0%	0 / 1 0.0%	1 / 5 20.0%	0 / 0 0.0%	0 / 0 0.0%	0 / 0 0.0%	0 / 0 0.0%	12 / 46 26.1%	11 / 46 23.9%ABE	9 / 46 19.6%AB	6 / 46 13.0%C	4 / 46 8.7%E	4 / 46 8.7%CE		
1	AJ 13395	20 / 46 43.5%	0 / 1 0.0%	0 / 1 0.0%	0 / 1 0.0%	0 / 1 0.0%	1 / 3 33.3%	0 / 3 0.0%	1 / 1 100.0%	1 / 1 100.0%	3 / 3 100.0%	0 / 1 0.0%	3 / 4 75.0%	0 / 2 0.0%	0 / 1 0.0%	2 / 2 100.0%	0 / 1 0.0%	1 / 3 33.3%	1 / 1 100.0%	2 / 3 66.7%	3 / 5 60.0%	1 / 2 50.0%	0 / 1 0.0%	1 / 5 20.0%	0 / 0 0.0%	0 / 0 0.0%	0 / 0 0.0%	0 / 0 0.0%	12 / 46 26.1%	11 / 46 23.9%ABE	9 / 46 19.6%AB	6 / 46 13.0%C	4 / 46 8.7%E	4 / 46 8.7%CE		
1	Haliangium ochraceum DSM 14365	20 / 46 43.5%	0 / 1 0.0%	0 / 1 0.0%	0 / 1 0.0%	0 / 1 0.0%	1 / 3 33.3%	0 / 3 0.0%	1 / 1 100.0%	1 / 1 100.0%	3 / 3 100.0%	0 / 1 0.0%	3 / 4 75.0%	0 / 2 0.0%	0 / 1 0.0%	2 / 2 100.0%	0 / 1 0.0%	1 / 3 33.3%	1 / 1 100.0%	2 / 3 66.7%	3 / 5 60.0%	1 / 2 50.0%	0 / 1 0.0%	1 / 5 20.0%	0 / 0 0.0%	0 / 0 0.0%	0 / 0 0.0%	0 / 0 0.0%	12 / 46 26.1%	11 / 46 23.9%ABE	9 / 46 19.6%AB	6 / 46 13.0%C	4 / 46 8.7%CE	4 / 46 8.7%E		
11	"Desulfuromonadales" Kuever et al. 2005	567 / 568 99.8%	15 / 15 100.0%	23 / 23 100.0%	11 / 11 100.0%	30 / 30 100.0%	37 / 37 100.0%	34 / 34 100.0%	11 / 11 100.0%	11 / 11 100.0%	48 / 48 100.0%	11 / 11 100.0%	43 / 44 97.7%	16 / 16 100.0%	11 / 11 100.0%	21 / 21 100.0%	11 / 11 100.0%	33 / 33 100.0%	11 / 11 100.0%	34 / 34 100.0%	53 / 53 100.0%	23 / 23 100.0%	11 / 11 100.0%	59 / 59 100.0%	10 / 10 100.0%	0 / 0 0.0%	0 / 0 0.0%	0 / 0 0.0%	342 / 568 60.2%AB	185 / 568 32.6%ABE	23 / 568 4.0%B	17 / 568 3.0%BE	1 / 568 0.2%E			
9	Geobacteraceae	461 / 462 99.8%	12 / 12 100.0%	18 / 18 100.0%	9 / 9 100.0%	24 / 24 100.0%	30 / 30 100.0%	27 / 27 100.0%	9 / 9 100.0%	9 / 9 100.0%	39 / 39 100.0%	9 / 9 100.0%	35 / 36 97.2%	12 / 12 100.0%	9 / 9 100.0%	17 / 17 100.0%	9 / 9 100.0%	27 / 27 100.0%	9 / 9 100.0%	27 / 27 100.0%	44 / 44 100.0%	20 / 20 100.0%	9 / 9 100.0%	48 / 48 100.0%	9 / 9 100.0%	0 / 0 0.0%	0 / 0 0.0%	0 / 0 0.0%	272 / 462 58.9%AB	150 / 462 32.5%ABE	23 / 462 5.0%B	16 / 462 3.5%BE	1 / 462 0.2%E			
9	Geobacter	461 / 462 99.8%	12 / 12 100.0%	18 / 18 100.0%	9 / 9 100.0%	24 / 24 100.0%	30 / 30 100.0%	27 / 27 100.0%	9 / 9 100.0%	9 / 9 100.0%	39 / 39 100.0%	9 / 9 100.0%	35 / 36 97.2%	12 / 12 100.0%	9 / 9 100.0%	17 / 17 100.0%	9 / 9 100.0%	27 / 27 100.0%	9 / 9 100.0%	27 / 27 100.0%	44 / 44 100.0%	20 / 20 100.0%	9 / 9 100.0%	48 / 48 100.0%	9 / 9 100.0%	0 / 0 0.0%	0 / 0 0.0%	0 / 0 0.0%	272 / 462 58.9%AB	150 / 462 32.5%ABE	23 / 462 5.0%B	16 / 462 3.5%BE	1 / 462 0.2%E			
1	ATCC 53774	49 / 49 100.0%	1 / 1 100.0%	2 / 2 100.0%	1 / 1 100.0%	2 / 2 100.0%	3 / 3 100.0%	3 / 3 100.0%	1 / 1 100.0%	1 / 1 100.0%	4 / 4 100.0%	1 / 1 100.0%	4 / 4 100.0%	1 / 1 100.0%	1 / 1 100.0%	2 / 2 100.0%	1 / 1 100.0%	3 / 3 100.0%	1 / 1 100.0%	3 / 3 100.0%	5 / 5 100.0%	2 / 2 100.0%	1 / 1 100.0%	5 / 5 100.0%	1 / 1 100.0%	0 / 0 0.0%	0 / 0 0.0%	0 / 0 0.0%	32 / 49 65.3%AB	17 / 49 34.7%ABE						
1	Geobacter metallireducens GS-15	49 / 49 100.0%	1 / 1 100.0%	2 / 2 100.0%	1 / 1 100.0%	2 / 2 100.0%	3 / 3 100.0%	3 / 3 100.0%	1 / 1 100.0%	1 / 1 100.0%	4 / 4 100.0%	1 / 1 100.0%	4 / 4 100.0%	1 / 1 100.0%	1 / 1 100.0%	2 / 2 100.0%	1 / 1 100.0%	3 / 3 100.0%	1 / 1 100.0%	3 / 3 100.0%	5 / 5 100.0%	2 / 2 100.0%	1 / 1 100.0%	5 / 5 100.0%	1 / 1 100.0%	0 / 0 0.0%	0 / 0 0.0%	0 / 0 0.0%	32 / 49 65.3%AB	17 / 49 34.7%ABE						
2	ATCC 51573	96 / 96 100.0%	2 / 2 100.0%	4 / 4 100.0%	2 / 2 100.0%	4 / 4 100.0%	6 / 6 100.0%	6 / 6 100.0%	2 / 2 100.0%	2 / 2 100.0%	8 / 8 100.0%	2 / 2 100.0%	8 / 8 100.0%	2 / 2 100.0%	2 / 2 100.0%	4 / 4 100.0%	2 / 2 100.0%	6 / 6 100.0%	2 / 2 100.0%	6 / 6 100.0%	8 / 8 100.0%	4 / 4 100.0%	2 / 2 100.0%	10 / 10 100.0%	2 / 2 100.0%	0 / 0 0.0%	0 / 0 0.0%	0 / 0 0.0%	60 / 96 62.5%AB	35 / 96 36.5%ABE	1 / 96 1.0%BE					
1	Geobacter sulfurreducens PCA	48 / 48 100.0%	1 / 1 100.0%	2 / 2 100.0%	1 / 1 100.0%	2 / 2 100.0%	3 / 3 100.0%	3 / 3 100.0%	1 / 1 100.0%	1 / 1 100.0%	4 / 4 100.0%	1 / 1 100.0%	4 / 4 100.0%	1 / 1 100.0%	1 / 1 100.0%	2 / 2 100.0%	1 / 1 100.0%	3 / 3 100.0%	1 / 1 100.0%	3 / 3 100.0%	4 / 4 100.0%	2 / 2 100.0%	1 / 1 100.0%	5 / 5 100.0%	1 / 1 100.0%	0 / 0 0.0%	0 / 0 0.0%	0 / 0 0.0%	30 / 48 62.5%AB	17 / 48 35.4%ABE	1 / 48 2.1%BE					
1	Geobacter sulfurreducens KN400	48 / 48 100.0%	1 / 1 100.0%	2 / 2 100.0%	1 / 1 100.0%	2 / 2 100.0%	3 / 3 100.0%	3 / 3 100.0%	1 / 1 100.0%	1 / 1 100.0%	4 / 4 100.0%	1 / 1 100.0%	4 / 4 100.0%	1 / 1 100.0%	1 / 1 100.0%	2 / 2 100.0%	1 / 1 100.0%	3 / 3 100.0%	1 / 1 100.0%	3 / 3 100.0%	4 / 4 100.0%	2 / 2 100.0%	1 / 1 100.0%	5 / 5 100.0%	1 / 1 100.0%	0 / 0 0.0%	0 / 0 0.0%	0 / 0 0.0%	30 / 48 62.5%AB	18 / 48 37.5%ABE						
1	ATCC BAA-1014	57 / 58 98.3%	2 / 2 100.0%	2 / 2 100.0%	1 / 1 100.0%	4 / 4 100.0%	4 / 4 100.0%	3 / 3 100.0%	1 / 1 100.0%	1 / 1 100.0%	5 / 5 100.0%	1 / 1 100.0%	3 / 4 75.0%	2 / 2 100.0%	1 / 1 100.0%	2 / 2 100.0%	1 / 1 100.0%	3 / 3 100.0%	1 / 1 100.0%	3 / 3 100.0%	6 / 6 100.0%	3 / 3 100.0%	1 / 1 100.0%	6 / 6 100.0%	1 / 1 100.0%	0 / 0 0.0%	0 / 0 0.0%	0 / 0 0.0%	27 / 58 46.6%AB	11 / 58 19.0%ABE	10 / 58 17.2%B	9 / 58 15.5%BE	1 / 58 1.7%E			
1	Geobacter bemidjiensis Bem	57 / 58 98.3%	2 / 2 100.0%	2 / 2 100.0%	1 / 1 100.0%	4 / 4 100.0%	4 / 4 100.0%	3 / 3 100.0%	1 / 1 100.0%	1 / 1 100.0%	5 / 5 100.0%	1 / 1 100.0%	3 / 4 75.0%	2 / 2 100.0%	1 / 1 100.0%	2 / 2 100.0%	1 / 1 100.0%	3 / 3 100.0%	1 / 1 100.0%	3 / 3 100.0%	6 / 6 100.0%	3 / 3 100.0%	1 / 1 100.0%	6 / 6 100.0%	1 / 1 100.0%	0 / 0 0.0%	0 / 0 0.0%	0 / 0 0.0%	27 / 58 46.6%AB	11 / 58 19.0%ABE	10 / 58 17.2%B	9 / 58 15.5%BE	1 / 58 1.7%E			

1	ATCC BAA-1151	45 / 45 100.0%	1 / 1 100.0%	2 / 2 100.0%	1 / 1 100.0%	2 / 2 100.0%	3 / 3 100.0%	3 / 3 100.0%	1 / 1 100.0%	1 / 1 100.0%	3 / 3 100.0%	1 / 1 100.0%	4 / 4 100.0%	1 / 1 100.0%	1 / 1 100.0%	1 / 1 100.0%	1 / 1 100.0%	3 / 3 100.0%	1 / 1 100.0%	3 / 3 100.0%	4 / 4 100.0%	1 / 1 100.0%	1 / 1 100.0%	5 / 5 100.0%	1 / 1 100.0%	0 / 0 0.0%	0 / 0 0.0%	0 / 0 0.0%	17 / 45 37.8%AB	13 / 45 28.9%B	10 / 45 22.2%ABE	5 / 45 11.1%BE		
1	Geobacter lovleyi SZ	45 / 45 100.0%	1 / 1 100.0%	2 / 2 100.0%	1 / 1 100.0%	2 / 2 100.0%	3 / 3 100.0%	3 / 3 100.0%	1 / 1 100.0%	1 / 1 100.0%	3 / 3 100.0%	1 / 1 100.0%	4 / 4 100.0%	1 / 1 100.0%	1 / 1 100.0%	1 / 1 100.0%	1 / 1 100.0%	3 / 3 100.0%	1 / 1 100.0%	3 / 3 100.0%	4 / 4 100.0%	1 / 1 100.0%	1 / 1 100.0%	5 / 5 100.0%	1 / 1 100.0%	0 / 0 0.0%	0 / 0 0.0%	0 / 0 0.0%	17 / 45 37.8%AB	13 / 45 28.9%B	10 / 45 22.2%ABE	5 / 45 11.1%BE		
1	ATCC BAA-1134	49 / 49 100.0%	1 / 1 100.0%	2 / 2 100.0%	1 / 1 100.0%	2 / 2 100.0%	3 / 3 100.0%	3 / 3 100.0%	1 / 1 100.0%	1 / 1 100.0%	4 / 4 100.0%	1 / 1 100.0%	4 / 4 100.0%	1 / 1 100.0%	1 / 1 100.0%	2 / 2 100.0%	1 / 1 100.0%	3 / 3 100.0%	1 / 1 100.0%	3 / 3 100.0%	5 / 5 100.0%	2 / 2 100.0%	1 / 1 100.0%	5 / 5 100.0%	1 / 1 100.0%	0 / 0 0.0%	0 / 0 0.0%	0 / 0 0.0%	31 / 49 63.3%AB	17 / 49 34.7%ABE	1 / 49 2.0%BE			
1	Geobacter uraniireducens Rf4	49 / 49 100.0%	1 / 1 100.0%	2 / 2 100.0%	1 / 1 100.0%	2 / 2 100.0%	3 / 3 100.0%	3 / 3 100.0%	1 / 1 100.0%	1 / 1 100.0%	4 / 4 100.0%	1 / 1 100.0%	4 / 4 100.0%	1 / 1 100.0%	1 / 1 100.0%	2 / 2 100.0%	1 / 1 100.0%	3 / 3 100.0%	1 / 1 100.0%	3 / 3 100.0%	5 / 5 100.0%	2 / 2 100.0%	1 / 1 100.0%	5 / 5 100.0%	1 / 1 100.0%	0 / 0 0.0%	0 / 0 0.0%	0 / 0 0.0%	31 / 49 63.3%AB	17 / 49 34.7%ABE	1 / 49 2.0%BE			
1	Geobacter sp. M18	58 / 58 100.0%	2 / 2 100.0%	2 / 2 100.0%	1 / 1 100.0%	4 / 4 100.0%	4 / 4 100.0%	3 / 3 100.0%	1 / 1 100.0%	1 / 1 100.0%	6 / 6 100.0%	1 / 1 100.0%	4 / 4 100.0%	2 / 2 100.0%	1 / 1 100.0%	2 / 2 100.0%	1 / 1 100.0%	3 / 3 100.0%	1 / 1 100.0%	3 / 3 100.0%	5 / 5 100.0%	3 / 3 100.0%	1 / 1 100.0%	6 / 6 100.0%	1 / 1 100.0%	0 / 0 0.0%	0 / 0 0.0%	0 / 0 0.0%	37 / 58 63.8%AB	21 / 58 36.2%ABE				
1	Geobacter sp. M21	58 / 58 100.0%	2 / 2 100.0%	2 / 2 100.0%	1 / 1 100.0%	4 / 4 100.0%	4 / 4 100.0%	3 / 3 100.0%	1 / 1 100.0%	1 / 1 100.0%	5 / 5 100.0%	1 / 1 100.0%	4 / 4 100.0%	2 / 2 100.0%	1 / 1 100.0%	2 / 2 100.0%	1 / 1 100.0%	3 / 3 100.0%	1 / 1 100.0%	3 / 3 100.0%	6 / 6 100.0%	3 / 3 100.0%	1 / 1 100.0%	6 / 6 100.0%	1 / 1 100.0%	0 / 0 0.0%	0 / 0 0.0%	0 / 0 0.0%	37 / 58 63.8%AB	21 / 58 36.2%ABE				
1	DSM 22248	49 / 49 100.0%	1 / 1 100.0%	2 / 2 100.0%	1 / 1 100.0%	2 / 2 100.0%	3 / 3 100.0%	3 / 3 100.0%	1 / 1 100.0%	1 / 1 100.0%	4 / 4 100.0%	1 / 1 100.0%	4 / 4 100.0%	1 / 1 100.0%	1 / 1 100.0%	2 / 2 100.0%	1 / 1 100.0%	3 / 3 100.0%	1 / 1 100.0%	3 / 3 100.0%	5 / 5 100.0%	2 / 2 100.0%	1 / 1 100.0%	5 / 5 100.0%	1 / 1 100.0%	0 / 0 0.0%	0 / 0 0.0%	0 / 0 0.0%	31 / 49 63.3%AB	18 / 49 36.7%ABE				
1	Geobacter daltonii FRC-32	49 / 49 100.0%	1 / 1 100.0%	2 / 2 100.0%	1 / 1 100.0%	2 / 2 100.0%	3 / 3 100.0%	3 / 3 100.0%	1 / 1 100.0%	1 / 1 100.0%	4 / 4 100.0%	1 / 1 100.0%	4 / 4 100.0%	1 / 1 100.0%	1 / 1 100.0%	2 / 2 100.0%	1 / 1 100.0%	3 / 3 100.0%	1 / 1 100.0%	3 / 3 100.0%	5 / 5 100.0%	2 / 2 100.0%	1 / 1 100.0%	5 / 5 100.0%	1 / 1 100.0%	0 / 0 0.0%	0 / 0 0.0%	0 / 0 0.0%	31 / 49 63.3%AB	18 / 49 36.7%ABE				
2	'Pelobacteraceae'	106 / 106 100.0%	3 / 3 100.0%	5 / 5 100.0%	2 / 2 100.0%	6 / 6 100.0%	7 / 7 100.0%	7 / 7 100.0%	2 / 2 100.0%	2 / 2 100.0%	9 / 9 100.0%	2 / 2 100.0%	8 / 8 100.0%	4 / 4 100.0%	2 / 2 100.0%	4 / 4 100.0%	2 / 2 100.0%	6 / 6 100.0%	2 / 2 100.0%	7 / 7 100.0%	9 / 9 100.0%	3 / 3 100.0%	2 / 2 100.0%	11 / 11 100.0%	1 / 1 100.0%	0 / 0 0.0%	0 / 0 0.0%	0 / 0 0.0%	70 / 106 66.0%AB	35 / 106 33.0%ABE	1 / 106 0.9%BE			
2	Pelobacter	106 / 106 100.0%	3 / 3 100.0%	5 / 5 100.0%	2 / 2 100.0%	6 / 6 100.0%	7 / 7 100.0%	7 / 7 100.0%	2 / 2 100.0%	2 / 2 100.0%	9 / 9 100.0%	2 / 2 100.0%	8 / 8 100.0%	4 / 4 100.0%	2 / 2 100.0%	4 / 4 100.0%	2 / 2 100.0%	6 / 6 100.0%	2 / 2 100.0%	7 / 7 100.0%	9 / 9 100.0%	3 / 3 100.0%	2 / 2 100.0%	11 / 11 100.0%	1 / 1 100.0%	0 / 0 0.0%	0 / 0 0.0%	0 / 0 0.0%	70 / 106 66.0%AB	35 / 106 33.0%ABE	1 / 106 0.9%BE			
1	DSM 2380	53 / 53 100.0%	2 / 2 100.0%	3 / 3 100.0%	1 / 1 100.0%	2 / 2 100.0%	4 / 4 100.0%	4 / 4 100.0%	1 / 1 100.0%	1 / 1 100.0%	3 / 3 100.0%	1 / 1 100.0%	4 / 4 100.0%	2 / 2 100.0%	1 / 1 100.0%	2 / 2 100.0%	1 / 1 100.0%	3 / 3 100.0%	1 / 1 100.0%	3 / 3 100.0%	4 / 4 100.0%	2 / 2 100.0%	1 / 1 100.0%	6 / 6 100.0%	1 / 1 100.0%	0 / 0 0.0%	0 / 0 0.0%	0 / 0 0.0%	33 / 53 62.3%AB	19 / 53 35.8%ABE	1 / 53 1.9%BE			
1	Pelobacter carbinolicus DSM 2380	53 / 53 100.0%	2 / 2 100.0%	3 / 3 100.0%	1 / 1 100.0%	2 / 2 100.0%	4 / 4 100.0%	4 / 4 100.0%	1 / 1 100.0%	1 / 1 100.0%	3 / 3 100.0%	1 / 1 100.0%	4 / 4 100.0%	2 / 2 100.0%	1 / 1 100.0%	2 / 2 100.0%	1 / 1 100.0%	3 / 3 100.0%	1 / 1 100.0%	3 / 3 100.0%	4 / 4 100.0%	2 / 2 100.0%	1 / 1 100.0%	6 / 6 100.0%	1 / 1 100.0%	0 / 0 0.0%	0 / 0 0.0%	0 / 0 0.0%	33 / 53 62.3%AB	19 / 53 35.8%ABE	1 / 53 1.9%BE			
1	DSM 2379	53 / 53 100.0%	1 / 1 100.0%	2 / 2 100.0%	1 / 1 100.0%	4 / 4 100.0%	3 / 3 100.0%	3 / 3 100.0%	1 / 1 100.0%	1 / 1 100.0%	6 / 6 100.0%	1 / 1 100.0%	4 / 4 100.0%	2 / 2 100.0%	1 / 1 100.0%	2 / 2 100.0%	1 / 1 100.0%	3 / 3 100.0%	1 / 1 100.0%	4 / 4 100.0%	5 / 5 100.0%	1 / 1 100.0%	1 / 1 100.0%	5 / 5 100.0%	0 / 0 0.0%	0 / 0 0.0%	0 / 0 0.0%	0 / 0 0.0%	37 / 53 69.8%AB	16 / 53 30.2%ABE				
1	Pelobacter propionicus DSM 2379	53 / 53 100.0%	1 / 1 100.0%	2 / 2 100.0%	1 / 1 100.0%	4 / 4 100.0%	3 / 3 100.0%	3 / 3 100.0%	1 / 1 100.0%	1 / 1 100.0%	6 / 6 100.0%	1 / 1 100.0%	4 / 4 100.0%	2 / 2 100.0%	1 / 1 100.0%	2 / 2 100.0%	1 / 1 100.0%	3 / 3 100.0%	1 / 1 100.0%	4 / 4 100.0%	5 / 5 100.0%	1 / 1 100.0%	1 / 1 100.0%	5 / 5 100.0%	0 / 0 0.0%	0 / 0 0.0%	0 / 0 0.0%	0 / 0 0.0%	37 / 53 69.8%AB	16 / 53 30.2%ABE				
1	'Desulfurellales'	48 / 48 100.0%	1 / 1 100.0%	1 / 1 100.0%	1 / 1 100.0%	2 / 2 100.0%	3 / 3 100.0%	3 / 3 100.0%	1 / 1 100.0%	1 / 1 100.0%	4 / 4 100.0%	1 / 1 100.0%	4 / 4 100.0%	2 / 2 100.0%	1 / 1 100.0%	2 / 2 100.0%	1 / 1 100.0%	3 / 3 100.0%	1 / 1 100.0%	3 / 3 100.0%	5 / 5 100.0%	2 / 2 100.0%	1 / 1 100.0%	5 / 5 100.0%	0 / 0 0.0%	0 / 0 0.0%	0 / 0 0.0%	0 / 0 0.0%	34 / 48 70.8%AB	14 / 48 29.2%ABE				
1	'Desulfurellaceae'	48 / 48 100.0%	1 / 1 100.0%	1 / 1 100.0%	1 / 1 100.0%	2 / 2 100.0%	3 / 3 100.0%	3 / 3 100.0%	1 / 1 100.0%	1 / 1 100.0%	4 / 4 100.0%	1 / 1 100.0%	4 / 4 100.0%	2 / 2 100.0%	1 / 1 100.0%	2 / 2 100.0%	1 / 1 100.0%	3 / 3 100.0%	1 / 1 100.0%	3 / 3 100.0%	5 / 5 100.0%	2 / 2 100.0%	1 / 1 100.0%	5 / 5 100.0%	0 / 0 0.0%	0 / 0 0.0%	0 / 0 0.0%	0 / 0 0.0%	34 / 48 70.8%AB	14 / 48 29.2%ABE				
1	Hippea	48 / 48 100.0%	1 / 1 100.0%	1 / 1 100.0%	1 / 1 100.0%	2 / 2 100.0%	3 / 3 100.0%	3 / 3 100.0%	1 / 1 100.0%	1 / 1 100.0%	4 / 4 100.0%	1 / 1 100.0%	4 / 4 100.0%	2 / 2 100.0%	1 / 1 100.0%	2 / 2 100.0%	1 / 1 100.0%	3 / 3 100.0%	1 / 1 100.0%	3 / 3 100.0%	5 / 5 100.0%	2 / 2 100.0%	1 / 1 100.0%	5 / 5 100.0%	0 / 0 0.0%	0 / 0 0.0%	0 / 0 0.0%	0 / 0 0.0%	34 / 48 70.8%AB	14 / 48 29.2%ABE				
1	ATCC 700847	48 / 48 100.0%	1 / 1 100.0%	1 / 1 100.0%	1 / 1 100.0%	2 / 2 100.0%	3 / 3 100.0%	3 / 3 100.0%	1 / 1 100.0%	1 / 1 100.0%	4 / 4 100.0%	1 / 1 100.0%	4 / 4 100.0%	2 / 2 100.0%	1 / 1 100.0%	2 / 2 100.0%	1 / 1 100.0%	3 / 3 100.0%	1 / 1 100.0%	3 / 3 100.0%	5 / 5 100.0%	2 / 2 100.0%	1 / 1 100.0%	5 / 5 100.0%	0 / 0 0.0%	0 / 0 0.0%	0 / 0 0.0%	0 / 0 0.0%	34 / 48 70.8%AB	14 / 48 29.2%ABE				
1	Hippea maritima DSM 10411	48 / 48 100.0%	1 / 1 100.0%	1 / 1 100.0%	1 / 1 100.0%	2 / 2 100.0%	3 / 3 100.0%	3 / 3 100.0%	1 / 1 100.0%	1 / 1 100.0%	4 / 4 100.0%	1 / 1 100.0%	4 / 4 100.0%	2 / 2 100.0%	1 / 1 100.0%	2 / 2 100.0%	1 / 1 100.0%	3 / 3 100.0%	1 / 1 100.0%	3 / 3 100.0%	5 / 5 100.0%	2 / 2 100.0%	1 / 1 100.0%	5 / 5 100.0%	0 / 0 0.0%	0 / 0 0.0%	0 / 0 0.0%	0 / 0 0.0%	34 / 48 70.8%AB	14 / 48 29.2%ABE				
17	'Desulfovibrionales'	1024 / 1024 100.0%	31 / 31 100.0%	30 / 30 100.0%	26 / 26 100.0%	56 / 56 100.0%	77 / 77 100.0%	53 / 53 100.0%	20 / 20 100.0%	22 / 22 100.0%	96 / 96 100.0%	19 / 19 100.0%	70 / 70 100.0%	43 / 43 100.0%	20 / 20 100.0%	37 / 37 100.0%	20 / 20 100.0%	52 / 52 100.0%	20 / 20 100.0%	61 / 61 100.0%	84 / 84 100.0%	47 / 47 100.0%	17 / 17 100.0%	106 / 106 100.0%	17 / 17 100.0%	0 / 0 0.0%	0 / 0 0.0%	0 / 0 0.0%	667 / 1024 65.1%AB	353 / 1024 34.5%ABE	2 / 1024 0.2%BE	2 / 1024 0.2%B		
15	'Desulfovibrionaceae'	911 / 911 100.0%	28 / 28 100.0%	27 / 27 100.0%	23 / 23 100.0%	53 / 53 100.0%	66 / 66 100.0%	46 / 46 100.0%	17 / 17 100.0%	19 / 19 100.0%	88 / 88 100.0%	17 / 17 100.0%	63 / 63 100.0%	38 / 38 100.0%	17 / 17 100.0%	32 / 32 100.0%	17 / 17 100.0%	46 / 46 100.0%	18 / 18 100.0%	55 / 55 100.0%	74 / 74 100.0%	41 / 41 100.0%	15 / 15 100.0%	96 / 96 100.0%	15 / 15 100.0%	0 / 0 0.0%	0 / 0 0.0%	0 / 0 0.0%	591 / 911 64.9%AB	316 / 911 34.7%ABE	2 / 911 0.2%B	2 / 911 0.2%BE		
13	"Sporovibrio" Starkey 1938	823 / 823 100.0%	26 / 26 100.0%	25 / 25 100.0%	21 / 21 100.0%	49 / 49 100.0%	62 / 62 100.0%	40 / 40 100.0%	15 / 15 100.0%	17 / 17 100.0%	82 / 82 100.0%	15 / 15 100.0%	55 / 55 100.0%	34 / 34 100.0%	15 / 15 100.0%	28 / 28 100.0%	15 / 15 100.0%	42 / 42 100.0%	16 / 16 100.0%	49 / 49 100.0%	66 / 66 100.0%	39 / 39 100.0%	13 / 13 100.0%	86 / 86 100.0%	13 / 13 100.0%	0 / 0 0.0%	0 / 0 0.0%	0 / 0 0.0%	532 / 823 64.6%AB	288 / 823 35.0%ABE	2 / 823 0.2%BE	1 / 823 0.1%B		
1	ATCC 19996	57 / 57 100.0%	1 / 1 100.0%	2 / 2 100.0%	1 / 1 100.0%	3 / 3 100.0%	4 / 4 100.0%	3 / 3 100.0%	1 / 1 100.0%	1 / 1 100.0%	6 / 6 100.0%	1 / 1 100.0%	4 / 4 100.0%	3 / 3 100.0%	1 / 1 100.0%	2 / 2 100.0%	2 / 2 100.0%	3 / 3 100.0%	1 / 1 100.0%	3 / 3 100.0%	5 / 5 100.0%	2 / 2 100.0%	1 / 1 100.0%	6 / 6 100.0%	1 / 1 100.0%	0 / 0 0.0%	0 / 0 0.0%	0 / 0 0.0%	40 / 57 70.2%AB	17 / 57 29.8%ABE				
1	Desulfovibrio africanus ATCC 19997	57 / 57 100.0%	1 / 1 100.0%	2 / 2 100.0%	1 / 1 100.0%	3 / 3 100.0%	4 / 4 100.0%	3 / 3 100.0%	1 / 1 100.0%	1 / 1 100.0%	6 / 6 100.0%	1 / 1 100.0%	4 / 4 100.0%	3 / 3 100.0%	1 / 1 100.0%	2 / 2 100.0%	2 / 2 100.0%	3 / 3 100.0%	1 / 1 100.0%	3 / 3 100.0%	5 / 5 100.0%	2 / 2 100.0%	1 / 1 100.0%	6 / 6 100.0%	1 / 1 100.0%	0 / 0 0.0%	0 / 0 0.0%	0 / 0 0.0%	40 / 57 70.2%AB	17 / 57 29.8%ABE				
2	"Bacillus desulfuricans" (Beijerinck 1895) Saltet 1900	105 / 105 100.0%	3 / 3 100.0%	2 / 2 100.0%	2 / 2 100.0%	5 / 5 100.0%	9 / 9 100.0%	6 / 6 100.0%	2 / 2 100.0%	2 / 2 100.0%	10 / 10 100.0%	2 / 2 100.0%	7 / 7 100.0%	4 / 4 100.0%	2 / 2 100.0%	4 / 4 100.0%	2 / 2 100.0%	6 / 6 100.0%	2 / 2 100.0%	6 / 6 100.0%	8 / 8 100.0%	6 / 6 100.0%	2 / 2 100.0%	11 / 11 100.0%	2 / 2 100.0%	0 / 0 0.0%	0 / 0 0.0%	0 / 0 0.0%	67 / 105 63.8%AB	38 / 105 36.2%ABE				
1	Desulfovibrio desulfuricans subsp. desulfuricans	52 / 52 100.0%	1 / 1 100.0%	1 / 1 100.0%	1 / 1 100.0%	3 / 3 100.0%	4 / 4 100.0%	3 / 3 100.0%	1 / 1 100.0%	1 / 1 100.0%	5 / 5 100.0%	1 / 1 100.0%	4 / 4 100.0%	2 / 2 100.0%	1 / 1 100.0%	2 / 2 100.0%	1 / 1 100.0%	3 / 3 100.0%	1 / 1 100.0%	3 / 3 100.0%	4 / 4 100.0%	3 / 3 100.0%	1 / 1 100.0%	5 / 5 100.0%	1 / 1 100.0%	0 / 0 0.0%	0 / 0 0.0%	0 / 0 0.0%	35 / 52 67.3%AB	17 / 52 32.7%ABE				
1	Desulfovibrio desulfuricans subsp. desulfuricans ATCC 27774	52 / 52 100.0%	1 / 1 100.0%	1 / 1 100.0%	1 / 1 100.0%	3 / 3 100.0%	4 / 4 100.0%	3 / 3 100.0%	1 / 1 100.0%	1 / 1 100.0%	5 / 5 100.0%	1 / 1 100.0%	4 / 4 100.0%	2 / 2 100.0%	1 / 1 100.0%	2 / 2 100.0%	1 / 1 100.0%	3 / 3 100.0%	1 / 1 100.0%	3 / 3 100.0%	4 / 4 100.0%	3 / 3 100.0%	1 / 1 100.0%	5 / 5 100.0%	1 / 1 100.0%	0 / 0 0.0%	0 / 0 0.0%	0 / 0 0.0%	35 / 52 67.3%AB	17 / 52 32.7%ABE				
1	Desulfovibrio [desulfuricans] ND132	53 / 53 100.0%	2 / 2 100.0%	1 / 1 100.0%	1 / 1 100.0%	2 / 2 100.0%	5 / 5 100.0%	3 / 3 100.0%	1 / 1 100.0%	1 / 1 100.0%	5 / 5 100.0%	1 / 1 100.0%	3 / 3 100.0%	2 / 2 100.0%	1 / 1 100.0%	2 / 2 100.0%	1 / 1 100.0%	3 / 3 100.0%	1 / 1 100.0%	3 / 3 100.0%	4 / 4 100.0%	3 / 3 100.0%	1 / 1 100.0%	6 / 6 100.0%	1 / 1 100.0%	0 / 0 0.0%	0 / 0 0.0%	0 / 0 0.0%	32 / 53 60.4%AB	21 / 53 39.6%ABE				
1	"Microspira aestuarii" van Delden 1903	89 / 89 100.0%	3 / 3 100.0%	4 / 4 100.0%	3 / 3 100.0%	5 / 5 100.0%	7 / 7 100.0%	4 / 4 100.0%	2 / 2 100.0%	2 / 2 100.0%	7 / 7 100.0%	2 / 2 100.0%	6 / 6 100.0%	3 / 3 100.0%	2 / 2 100.0%	3 / 3 100.0%	2 / 2 100.0%	5 / 5 100.0%	2 / 2 100.0%	5 / 5 100.0%	5 / 5 100.0%	5 / 5 100.0%	1 / 1 100.0%	10 / 10 100.0%	1 / 1 100.0%	0 / 0 0.0%	0 / 0 0.0%	0 / 0 0.0%	53 / 89 59.6%AB	36 / 89 40.4%ABE				
1	Desulfovibrio salexigens ATCC 14822	89 / 89 100.0%	3 / 3 100.0%	4 / 4 100.0%	3 / 3 100.0%	5 / 5 100.0%	7 / 7 100.0%	4 / 4 100.0%	2 / 2 100.0%	2 / 2 100.0%	7 / 7 100.0%	2 / 2 100.0%	6 / 6 100.0%	3 / 3 100.0%	2 / 2 100.0%	3 / 3 100.0%	2 / 2 100.0%	5 / 5 100.0%	2 / 2 100.0%	5 / 5 100.0%	5 / 5 100.0%	5 / 5 100.0%	1 / 1 100.0%	10 / 10 100.0%	1 / 1 100.0%	0 / 0 0.0%	0 / 0 0.0%	0 / 0 0.0%	53 / 89 59.6%AB	36 / 89 40.4%ABE				
4	ATCC 29579	265 / 265 100.0%	8 / 8 100.0%	8 / 8 100.0%	8 / 8 100.0%	19 / 19 100.0%	20 / 20 100.0%	12 / 12 100.0%	4 / 4 100.0%	4 / 4 100.0%	31 / 31 100.0%	4 / 4 100.0%	16 / 16 100.0%	11 / 11 100.0%	4 / 4 100.0%	8 / 8 100.0%	4 / 4 100.0%	12 / 12 100.0%	4 / 4 100.0%	16 / 16 100.0%	24 / 24 100.0%	12 / 12 100.0%	4 / 4 100.0%	28 / 28 100.0%	4 / 4 100.0%	0 / 0 0.0%	0 / 0 0.0%	0 / 0 0.0%	174 / 265 65.7%AB	89 / 265 33.6%ABE	2 / 265 0.8%BE			
1	Desulfovibrio vulgaris (STRAIN HILDENBOROUGH)	67 / 67 100.0%	2 / 2 100.0%	2 / 2 100.0%	2 / 2 100.0%	5 / 5 100.0%	5 / 5 100.0%	3 / 3 100.0%	1 / 1 100.0%	1 / 1 100.0%	8 / 8 100.0%	1 / 1 100.0%	4 / 4 100.0%	3 / 3 100.0%	1 / 1 100.0%	2 / 2 100.0%	1 / 1 100.0%	3 / 3 100.0%	1 / 1 100.0%	4 / 4 100.0%	6 / 6 100.0%	3 / 3 100.0%	1 / 1 100.0%	7 / 7 100.0%	1 / 1 100.0%	0 / 0 0.0%	0 / 0 0.0%	0 / 0 0.0%	44 / 67 65.7%AB	22 / 67 32.8%ABE	1 / 67 1.5%BE			
1	Desulfovibrio vulgaris 'Miyazaki F'	64 / 64 100.0%	2 / 2 100.0%	2 / 2 100.0%	2 / 2 100.0%	4 / 4 100.0%	5 / 5 100.0%	3 / 3 100.0%	1 / 1 100.0%	1 / 1 100.0%	7 / 7 100.0%	1 / 1 100.0%	4 / 4 100.0%	2 / 2 100.0%	1 / 1 100.0%	2 / 2 100.0%	1 / 1 100.0%	3 / 3 100.0%	1 / 1 100.0%	4 / 4 100.0%	6 / 6 100.0%	3 / 3 100.0%	1 / 1 100.0%	7 / 7 100.0%	1 / 1 100.0%	0 / 0 0.0%	0 / 0 0.0%	0 / 0 0.0%	42 / 64 65.6%AB	22 / 64 34.4%ABE				
1	Desulfovibrio vulgaris DP4	67 / 67 100.0%	2 / 2 100.0%	2 / 2 100.0%	2 / 2 100.0%	5 / 5 100.0%	5 / 5 100.0%	3 / 3 100.0%	1 / 1 100.0%	1 / 1 100.0%	8 / 8 100.0%	1 / 1 100.0%	4 / 4 100.0%	3 / 3 100.0%	1 / 1 100.0%	2 / 2 100.0%	1 / 1 100.0%	3 / 3 100.0%	1 / 1 100.0%	4 / 4 100.0%	6 / 6 100.0%	3 / 3 100.0%	1 / 1 100.0%	7 / 7 100.0%	1 / 1 100.0%	0 / 0 0.0%	0 / 0 0.0%	0 / 0 0.0%	44 / 67 65.7%AB	22 / 67 32.8%ABE	1 / 67 1.5%BE			
1	Desulfovibrio vulgaris RCH1	67 / 67 100.0%	2 / 2 100.0%	2 / 2 100.0%	2 / 2 100.0%	5 / 5 100.0%	5 / 5 100.0%	3 / 3 100.0%	1 / 1 100.0%	1 / 1 100.0%	8 / 8 100.0%	1 / 1 100.0%	4 / 4 100.0%	3 / 3 100.0%	1 / 1 100.0%	2 / 2 100.0%	1 / 1 100.0%	3 / 3 100.0%	1 / 1 100.0%	4 / 4 100.0%	6 / 6 100.0%	3 / 3 100.0%	1 / 1 100.0%	7 / 7 100.0%	1 / 1 100.0%	0 / 0 0.0%	0 / 0 0.0%	0 / 0 0.0%	44 / 67 65.7%AB	23 / 67 34.3%ABE				
1	DSM 16109	66 / 66 100.0%	2 / 2 100.0%	2 / 2 100.0%	2 / 2 100.0%	4 / 4 100.0%	5 / 5 100.0%	3 / 3 100.0%	1 / 1 100.0%	1 / 1 100.0%	6 / 6 100.0%	1 / 1 100.0%	5 / 5 100.0%	3 / 3 100.0%	1 / 1 100.0%	2 / 2 100.0%	1 / 1 100.0%	3 / 3 100.0%	2 / 2 100.0%	4 / 4 100.0%	6 / 6 100.0%	3 / 3 100.0%	1 / 1 100.0%	7 / 7 100.0%	1 / 1 100.0%	0 / 0 0.0%	0 / 0 0.0%	0 / 0 0.0%	45 / 66 68.2%AB	20 / 66 30.3%ABE	1 / 66 1.5%B			
1	Desulfovibrio alaskensis G20	66 / 66 100.0%	2 / 2 100.0%	2 / 2 100.0%	2 / 2 100.0%	4 / 4 100.0%	5 / 5 100.0%	3 / 3 100.0%	1 / 1 100.0%	1 / 1 100.0%	6 / 6 100.0%	1 / 1 100.0%	5 / 5 100.0%	3 / 3 100.0%	1 / 1 100.0%	2 / 2 100.0%	1 / 1 100.0%	3 / 3 100.0%	2 / 2 100.0%	4 / 4 100.0%	6 / 6 100.0%	3 / 3 100.0%	1 / 1 100.0%	7 / 7 100.0%	1 / 1 100.0%	0 / 0 0.0%	0 / 0 0.0%	0 / 0 0.0%	45 / 66 68.2%AB	20 / 66 30.3%ABE	1 / 66 1.5%B			
1	ATCC 700646	54 / 54 100.0%	2 / 2 100.0%	1 / 1 100.0%	1 / 1 100.0%	2 / 2 100.0%	5 / 5 100.0%	3 / 3 100.0%	1 / 1 100.0%	1 / 1 100.0%	5 / 5 100.0%	1 / 1 100.0%	4 / 4 100.0%	2 / 2 100.0%	1 / 1 100.0%	2 / 2 100.0%	1 / 1 100.0%	3 / 3 100.0%	1 / 1 100.0%	4 / 4 100.0%	4 / 4 100.0%	2 / 2 100.0%	1 / 1 100.0%	6 / 6 100.0%	1 / 1 100.0%	0 / 0 0.0%	0 / 0 0.0%	0 / 0 0.0%	35 / 54 64.8%AB	19 / 54 35.2%ABE				
1	Desulfovibrio aespoeensis Aspo-2	54 / 54 100.0%	2 / 2 100.0%	1 / 1 100.0%	1 / 1 100.0%	2 / 2 100.0%	5 / 5 100.0%	3 / 3 100.0%	1 / 1 100.0%	1 / 1 100.0%	5 / 5 100.0%	1 / 1 100.0%	4 / 4 100.0%	2 / 2 100.0%	1 / 1 100.0%	2 / 2 100.0%	1 / 1 100.0%	3 / 3 100.0%	1 / 1 100.0%	4 / 4 100.0%	4 / 4 100.0%	2 / 2 100.0%	1 / 1 100.0%	6 / 6 100.0%	1 / 1 100.0%	0 / 0 0.0%	0 / 0 0.0%	0 / 0 0.0%	35 / 54 64.8%AB	19 / 54 35.2%ABE				
1	ATCC 700980	52 / 52 100.0%	1 / 1 100.0%	2 / 2 100.0%	1 / 1 100.0%	3 / 3 100.0%	3 / 3 100.0%	3 / 3 100.0%	1 / 1 100.0%	1 / 1 100.0%	5 / 5 100.0%	1 / 1 100.0%	4 / 4 100.0%	2 / 2 100.0%	1 / 1 100.0%	2 / 2 100.0%	1 / 1 100.0%	3 / 3 100.0%	1 / 1 100.0%	3 / 3 100.0%	5 / 5 100.0%	2 / 2 100.0%	1 / 1 100.0%	5 / 5 100.0%	1 / 1 100.0%	0 / 0 0.0%	0 / 0 0.0%	0 / 0 0.0%	35 / 52 67.3%AB	17 / 52 32.7%ABE				
1	Desulfovibrio magneticus 700980	52 / 52 100.0%	1 / 1 100.0%	2 / 2 100.0%	1 / 1 100.0%	3 / 3 100.0%	3 / 3 100.0%	3 / 3 100.0%	1 / 1 100.0%	1 / 1 100.0%	5 / 5 100.0%	1 / 1 100.0%	4 / 4 100.0%	2 / 2 100.0%	1 / 1 100.0%	2 / 2 100.0%	1 / 1 100.0%	3 / 3 100.0%	1 / 1 100.0%	3 / 3 100.0%	5 / 5 100.0%	2 / 2 100.0%	1 / 1 100.0%	5 / 5 100.0%	1 / 1 100.0%	0 / 0 0.0%	0 / 0 0.0%	0 / 0 0.0%	35 / 52 67.3%AB	17 / 52 32.7%ABE				
1	CIP 107303	77 / 77 100.0%	3 / 3 100.0%	3 / 3 100.0%	2 / 2 100.0%	5 / 5 100.0%	4 / 4 100.0%	3 / 3 100.0%	2 / 2 100.0%	4 / 4 100.0%	6 / 6 100.0%	2 / 2 100.0%	5 / 5 100.0%	4 / 4 100.0%	2 / 2 100.0%	3 / 3 100.0%	1 / 1 100.0%	4 / 4 100.0%	2 / 2 100.0%	4 / 4 100.0%	5 / 5 100.0%	4 / 4 100.0%	1 / 1 100.0%	7 / 7 100.0%	1 / 1 100.0%	0 / 0 0.0%	0 / 0 0.0%	0 / 0 0.0%	47 / 77 61.0%AB	30 / 77 39.0%ABE				
1	Desulfovibrio hydrothermalis AM13	77 / 77 100.0%	3 / 3 100.0%	3 / 3 100.0%	2 / 2 100.0%	5 / 5 100.0%	4 / 4 100.0%	3 / 3 100.0%	2 / 2 100.0%	4 / 4 100.0%	6 / 6 100.0%	2 / 2 100.0%	5 / 5 100.0%	4 / 4 100.0%	2 / 2 100.0%	3 / 3 100.0%	1 / 1 100.0%	4 / 4 100.0%	2 / 2 100.0%	4 / 4 100.0%	5 / 5 100.0%	4 / 4 100.0%	1 / 1 100.0%	7 / 7 100.0%	1 / 1 100.0%	0 / 0 0.0%	0 / 0 0.0%	0 / 0 0.0%	47 / 77 61.0%AB	30 / 77 39.0%ABE				
1	DSM 21447	58 / 58 100.0%	3 / 3 100.0%	1 / 1 100.0%	1 / 1 100.0%	3 / 3 100.0%	5 / 5 100.0%	3 / 3 100.0%	1 / 1 100.0%	1 / 1 100.0%	6 / 6 100.0%	1 / 1 100.0%	4 / 4 100.0%	2 / 2 100.0%	1 / 1 100.0%	2 / 2 100.0%	1 / 1 100.0%	3 / 3 100.0%	1 / 1 100.0%	4 / 4 100.0%	4 / 4 100.0%	3 / 3 100.0%	1 / 1 100.0%	6 / 6 100.0%	1 / 1 100.0%	0 / 0 0.0%	0 / 0 0.0%	0 / 0 0.0%	36 / 58 62.1%AB	22 / 58 37.9%ABE				
1	Desulfovibrio piezophilus C1TLV30	58 / 58 100.0%	3 / 3 100.0%	1 / 1 100.0%	1 / 1 100.0%	3 / 3 100.0%	5 / 5 100.0%	3 / 3 100.0%	1 / 1 100.0%	1 / 1 100.0%	6 / 6 100.0%	1 / 1 100.0%	4 / 4 100.0%	2 / 2 100.0%	1 / 1 100.0%	2 / 2 100.0%	1 / 1 100.0%	3 / 3 100.0%	1 / 1 100.0%	4 / 4 100.0%	4 / 4 100.0%	3 / 3 100.0%	1 / 1 100.0%	6 / 6 100.0%	1 / 1 100.0%	0 / 0 0.0%	0 / 0 0.0%	0 / 0 0.0%	36 / 58 62.1%AB	22 / 58 37.9%ABE				
2	Lawsonia	88 / 88 100.0%	2 / 2 100.0%	2 / 2 100.0%	2 / 2 100.0%	4 / 4 100.0%	4 / 4 100.0%	6 / 6 100.0%	2 / 2 100.0%	2 / 2 100.0%	6 / 6 100.0%	2 / 2 100.0%	8 / 8 100.0%	4 / 4 100.0%	2 / 2 100.0%	4 / 4 100.0%	2 / 2 100.0%	4 / 4 100.0%	2 / 2 100.0%	6 / 6 100.0%	8 / 8 100.0%	2 / 2 100.0%	2 / 2 100.0%	10 / 10 100.0%	2 / 2 100.0%	0 / 0 0.0%	0 / 0 0.0%	0 / 0 0.0%	59 / 88 67.0%AB	28 / 88 31.8%ABE	1 / 88 1.1%B			
2	"Candidatus intracellularis" Murray and Stackebrandt 1995	88 / 88 100.0%	2 / 2 100.0%	2 / 2 100.0%	2 / 2 100.0%	4 / 4 100.0%	4 / 4 100.0%	6 / 6 100.0%	2 / 2 100.0%	2 / 2 100.0%	6 / 6 100.0%	2 / 2 100.0%	8 / 8 100.0%	4 / 4 100.0%	2 / 2 100.0%	4 / 4 100.0%	2 / 2 100.0%	4 / 4 100.0%	2 / 2 100.0%	6 / 6 100.0%	8 / 8 100.0%	2 / 2 100.0%	2 / 2 100.0%	10 / 10 100.0%	2 / 2 100.0%	0 / 0 0.0%	0 / 0 0.0%	0 / 0 0.0%	59 / 88 67.0%AB	28 / 88 31.8%ABE	1 / 88 1.1%B			
1	Lawsonia intracellularis PHE/MN1-00	44 / 44 100.0%	1 / 1 100.0%	1 / 1 100.0%	1 / 1 100.0%	2 / 2 100.0%	2 / 2 100.0%	3 / 3 100.0%	1 / 1 100.0%	1 / 1 100.0%	3 / 3 100.0%	1 / 1 100.0%	4 / 4 100.0%	2 / 2 100.0%	1 / 1 100.0%	2 / 2 100.0%	1 / 1 100.0%	2 / 2 100.0%	1 / 1 100.0%	3 / 3 100.0%	4 / 4 100.0%	1 / 1 100.0%	1 / 1 100.0%	5 / 5 100.0%	1 / 1 100.0%	0 / 0 0.0%	0 / 0 0.0%	0 / 0 0.0%	29 / 44 65.9%AB	14 / 44 31.8%ABE	1 / 44 2.3%B			
1	Lawsonia intracellularis N343	44 / 44 100.0%	1 / 1 100.0%	1 / 1 100.0%	1 / 1 100.0%	2 / 2 100.0%	2 / 2 100.0%	3 / 3 100.0%	1 / 1 100.0%	1 / 1 100.0%	3 / 3 100.0%	1 / 1 100.0%	4 / 4 100.0%	2 / 2 100.0%	1 / 1 100.0%	2 / 2 100.0%	1 / 1 100.0%	2 / 2 100.0%	1 / 1 100.0%	3 / 3 100.0%	4 / 4 100.0%	1 / 1 100.0%	1 / 1 100.0%	5 / 5 100.0%	1 / 1 100.0%	0 / 0 0.0%	0 / 0 0.0%	0 / 0 0.0%	30 / 44 68.2%AB	14 / 44 31.8%ABE				
1	Desulfomicrobiaceae	64 / 64 100.0%	1 / 1 100.0%	2 / 2 100.0%	2 / 2 100.0%	1 / 1 100.0%	7 / 7 100.0%	4 / 4 100.0%	2 / 2 100.0%	2 / 2 100.0%	4 / 4 100.0%	1 / 1 100.0%	4 / 4 100.0%	3 / 3 100.0%	2 / 2 100.0%	3 / 3 100.0%	2 / 2 100.0%	3 / 3 100.0%	1 / 1 100.0%	3 / 3 100.0%	6 / 6 100.0%	4 / 4 100.0%	1 / 1 100.0%	5 / 5 100.0%	1 / 1 100.0%	0 / 0 0.0%	0 / 0 0.0%	0 / 0 0.0%	45 / 64 70.3%AB	19 / 64 29.7%ABE				
1	Desulfomicrobium	64 / 64 100.0%	1 / 1 100.0%	2 / 2 100.0%	2 / 2 100.0%	1 / 1 100.0%	7 / 7 100.0%	4 / 4 100.0%	2 / 2 100.0%	2 / 2 100.0%	4 / 4 100.0%	1 / 1 100.0%	4 / 4 100.0%	3 / 3 100.0%	2 / 2 100.0%	3 / 3 100.0%	2 / 2 100.0%	3 / 3 100.0%	1 / 1 100.0%	3 / 3 100.0%	6 / 6 100.0%	4 / 4 100.0%	1 / 1 100.0%	5 / 5 100.0%	1 / 1 100.0%	0 / 0 0.0%	0 / 0 0.0%	0 / 0 0.0%	45 / 64 70.3%AB	19 / 64 29.7%ABE				
1	DSM 4028	64 / 64 100.0%	1 / 1 100.0%	2 / 2 100.0%	2 / 2 100.0%	1 / 1 100.0%	7 / 7 100.0%	4 / 4 100.0%	2 / 2 100.0%	2 / 2 100.0%	4 / 4 100.0%	1 / 1 100.0%	4 / 4 100.0%	3 / 3 100.0%	2 / 2 100.0%	3 / 3 100.0%	2 / 2 100.0%	3 / 3 100.0%	1 / 1 100.0%	3 / 3 100.0%	6 / 6 100.0%	4 / 4 100.0%	1 / 1 100.0%	5 / 5 100.0%	1 / 1 100.0%	0 / 0 0.0%	0 / 0 0.0%	0 / 0 0.0%	45 / 64 70.3%AB	19 / 64 29.7%ABE				
1	Desulfomicrobium baculatum DSM 4028	64 / 64 100.0%	1 / 1 100.0%	2 / 2 100.0%	2 / 2 100.0%	1 / 1 100.0%	7 / 7 100.0%	4 / 4 100.0%	2 / 2 100.0%	2 / 2 100.0%	4 / 4 100.0%	1 / 1 100.0%	4 / 4 100.0%	3 / 3 100.0%	2 / 2 100.0%	3 / 3 100.0%	2 / 2 100.0%	3 / 3 100.0%	1 / 1 100.0%	3 / 3 100.0%	6 / 6 100.0%	4 / 4 100.0%	1 / 1 100.0%	5 / 5 100.0%	1 / 1 100.0%	0 / 0 0.0%	0 / 0 0.0%	0 / 0 0.0%	45 / 64 70.3%AB	19 / 64 29.7%ABE				
1	Desulfohalobiaceae	49 / 49 100.0%	2 / 2 100.0%	1 / 1 100.0%	1 / 1 100.0%	2 / 2 100.0%	4 / 4 100.0%	3 / 3 100.0%	1 / 1 100.0%	1 / 1 100.0%	4 / 4 100.0%	1 / 1 100.0%	3 / 3 100.0%	2 / 2 100.0%	1 / 1 100.0%	2 / 2 100.0%	1 / 1 100.0%	3 / 3 100.0%	1 / 1 100.0%	3 / 3 100.0%	4 / 4 100.0%	2 / 2 100.0%	1 / 1 100.0%	5 / 5 100.0%	1 / 1 100.0%	0 / 0 0.0%	0 / 0 0.0%	0 / 0 0.0%	31 / 49 63.3%AB	18 / 49 36.7%ABE				
1	Desulfatihalobium	49 / 49 100.0%	2 / 2 100.0%	1 / 1 100.0%	1 / 1 100.0%	2 / 2 100.0%	4 / 4 100.0%	3 / 3 100.0%	1 / 1 100.0%	1 / 1 100.0%	4 / 4 100.0%	1 / 1 100.0%	3 / 3 100.0%	2 / 2 100.0%	1 / 1 100.0%	2 / 2 100.0%	1 / 1 100.0%	3 / 3 100.0%	1 / 1 100.0%	3 / 3 100.0%	4 / 4 100.0%	2 / 2 100.0%	1 / 1 100.0%	5 / 5 100.0%	1 / 1 100.0%	0 / 0 0.0%	0 / 0 0.0%	0 / 0 0.0%	31 / 49 63.3%AB	18 / 49 36.7%ABE				
1	ATCC 49708	49 / 49 100.0%	2 / 2 100.0%	1 / 1 100.0%	1 / 1 100.0%	2 / 2 100.0%	4 / 4 100.0%	3 / 3 100.0%	1 / 1 100.0%	1 / 1 100.0%	4 / 4 100.0%	1 / 1 100.0%	3 / 3 100.0%	2 / 2 100.0%	1 / 1 100.0%	2 / 2 100.0%	1 / 1 100.0%	3 / 3 100.0%	1 / 1 100.0%	3 / 3 100.0%	4 / 4 100.0%	2 / 2 100.0%	1 / 1 100.0%	5 / 5 100.0%	1 / 1 100.0%	0 / 0 0.0%	0 / 0 0.0%	0 / 0 0.0%	31 / 49 63.3%AB	18 / 49 36.7%ABE				
1	Desulfohalobium retbaense DSM 5692	49 / 49 100.0%	2 / 2 100.0%	1 / 1 100.0%	1 / 1 100.0%	2 / 2 100.0%	4 / 4 100.0%	3 / 3 100.0%	1 / 1 100.0%	1 / 1 100.0%	4 / 4 100.0%	1 / 1 100.0%	3 / 3 100.0%	2 / 2 100.0%	1 / 1 100.0%	2 / 2 100.0%	1 / 1 100.0%	3 / 3 100.0%	1 / 1 100.0%	3 / 3 100.0%	4 / 4 100.0%	2 / 2 100.0%	1 / 1 100.0%	5 / 5 100.0%	1 / 1 100.0%	0 / 0 0.0%	0 / 0 0.0%	0 / 0 0.0%	31 / 49 63.3%AB	18 / 49 36.7%ABE				
8	'Desulfobacterales'	399 / 406 98.3%	11 / 11 100.0%	15 / 15 100.0%	8 / 8 100.0%	14 / 14 100.0%	27 / 27 100.0%	25 / 25 100.0%	9 / 9 100.0%	9 / 9 100.0%	24 / 24 100.0%	9 / 9 100.0%	27 / 30 90.0%	14 / 15 93.3%	11 / 11 100.0%	17 / 17 100.0%	8 / 8 100.0%	22 / 22 100.0%	9 / 9 100.0%	26 / 26 100.0%	39 / 39 100.0%	20 / 20 100.0%	6 / 8 75.0%	41 / 42 97.6%	8 / 8 100.0%	0 / 0 0.0%	0 / 0 0.0%	0 / 0 0.0%	238 / 406 58.6%AB	138 / 406 34.0%ABE	13 / 406 3.2%B	10 / 406 2.5%BE	5 / 406 1.2%E	2 / 406 0.5%
4	'Desulfobacteraceae'	195 / 201 97.0%	5 / 5 100.0%	6 / 6 100.0%	4 / 4 100.0%	5 / 5 100.0%	15 / 15 100.0%	11 / 11 100.0%	4 / 4 100.0%	4 / 4 100.0%	11 / 11 100.0%	5 / 5 100.0%	14 / 16 87.5%	7 / 8 87.5%	6 / 6 100.0%	9 / 9 100.0%	4 / 4 100.0%	12 / 12 100.0%	5 / 5 100.0%	13 / 13 100.0%	19 / 19 100.0%	10 / 10 100.0%	2 / 4 50.0%	20 / 21 95.2%	4 / 4 100.0%	0 / 0 0.0%	0 / 0 0.0%	0 / 0 0.0%	107 / 201 53.2%AB	66 / 201 32.8%ABE	13 / 201 6.5%B	9 / 201 4.5%BE	4 / 201 2.0%E	2 / 201 1.0%
1	Desulfococcus	44 / 47 93.6%	1 / 1 100.0%	1 / 1 100.0%	1 / 1 100.0%	1 / 1 100.0%	3 / 3 100.0%	3 / 3 100.0%	1 / 1 100.0%	1 / 1 100.0%	3 / 3 100.0%	1 / 1 100.0%	2 / 4 50.0%	2 / 2 100.0%	1 / 1 100.0%	2 / 2 100.0%	1 / 1 100.0%	3 / 3 100.0%	1 / 1 100.0%	3 / 3 100.0%	5 / 5 100.0%	2 / 2 100.0%	0 / 1 0.0%	5 / 5 100.0%	1 / 1 100.0%	0 / 0 0.0%	0 / 0 0.0%	0 / 0 0.0%	14 / 47 29.8%AB	13 / 47 27.7%B	9 / 47 19.1%BE	8 / 47 17.0%ABE	2 / 47 4.3%E	1 / 47 2.1%
1	"Desulfobacterium oleovorans" Aeckersberg et al. 1991	44 / 47 93.6%	1 / 1 100.0%	1 / 1 100.0%	1 / 1 100.0%	1 / 1 100.0%	3 / 3 100.0%	3 / 3 100.0%	1 / 1 100.0%	1 / 1 100.0%	3 / 3 100.0%	1 / 1 100.0%	2 / 4 50.0%	2 / 2 100.0%	1 / 1 100.0%	2 / 2 100.0%	1 / 1 100.0%	3 / 3 100.0%	1 / 1 100.0%	3 / 3 100.0%	5 / 5 100.0%	2 / 2 100.0%	0 / 1 0.0%	5 / 5 100.0%	1 / 1 100.0%	0 / 0 0.0%	0 / 0 0.0%	0 / 0 0.0%	14 / 47 29.8%AB	13 / 47 27.7%B	9 / 47 19.1%BE	8 / 47 17.0%ABE	2 / 47 4.3%E	1 / 47 2.1%
1	Candidatus Desulfococcus oleovorans Hxd3	44 / 47 93.6%	1 / 1 100.0%	1 / 1 100.0%	1 / 1 100.0%	1 / 1 100.0%	3 / 3 100.0%	3 / 3 100.0%	1 / 1 100.0%	1 / 1 100.0%	3 / 3 100.0%	1 / 1 100.0%	2 / 4 50.0%	2 / 2 100.0%	1 / 1 100.0%	2 / 2 100.0%	1 / 1 100.0%	3 / 3 100.0%	1 / 1 100.0%	3 / 3 100.0%	5 / 5 100.0%	2 / 2 100.0%	0 / 1 0.0%	5 / 5 100.0%	1 / 1 100.0%	0 / 0 0.0%	0 / 0 0.0%	0 / 0 0.0%	14 / 47 29.8%AB	13 / 47 27.7%B	9 / 47 19.1%BE	8 / 47 17.0%ABE	2 / 47 4.3%E	1 / 47 2.1%
1	Desulfobacterium	50 / 50 100.0%	2 / 2 100.0%	1 / 1 100.0%	1 / 1 100.0%	1 / 1 100.0%	4 / 4 100.0%	3 / 3 100.0%	1 / 1 100.0%	1 / 1 100.0%	2 / 2 100.0%	1 / 1 100.0%	4 / 4 100.0%	2 / 2 100.0%	2 / 2 100.0%	2 / 2 100.0%	1 / 1 100.0%	3 / 3 100.0%	1 / 1 100.0%	3 / 3 100.0%	5 / 5 100.0%	2 / 2 100.0%	1 / 1 100.0%	6 / 6 100.0%	1 / 1 100.0%	0 / 0 0.0%	0 / 0 0.0%	0 / 0 0.0%	30 / 50 60.0%AB	20 / 50 40.0%ABE				
1	ATCC 43914	50 / 50 100.0%	2 / 2 100.0%	1 / 1 100.0%	1 / 1 100.0%	1 / 1 100.0%	4 / 4 100.0%	3 / 3 100.0%	1 / 1 100.0%	1 / 1 100.0%	2 / 2 100.0%	1 / 1 100.0%	4 / 4 100.0%	2 / 2 100.0%	2 / 2 100.0%	2 / 2 100.0%	1 / 1 100.0%	3 / 3 100.0%	1 / 1 100.0%	3 / 3 100.0%	5 / 5 100.0%	2 / 2 100.0%	1 / 1 100.0%	6 / 6 100.0%	1 / 1 100.0%	0 / 0 0.0%	0 / 0 0.0%	0 / 0 0.0%	30 / 50 60.0%AB	20 / 50 40.0%ABE				
1	Desulfobacterium autotrophicum HRM2	50 / 50 100.0%	2 / 2 100.0%	1 / 1 100.0%	1 / 1 100.0%	1 / 1 100.0%	4 / 4 100.0%	3 / 3 100.0%	1 / 1 100.0%	1 / 1 100.0%	2 / 2 100.0%	1 / 1 100.0%	4 / 4 100.0%	2 / 2 100.0%	2 / 2 100.0%	2 / 2 100.0%	1 / 1 100.0%	3 / 3 100.0%	1 / 1 100.0%	3 / 3 100.0%	5 / 5 100.0%	2 / 2 100.0%	1 / 1 100.0%	6 / 6 100.0%	1 / 1 100.0%	0 / 0 0.0%	0 / 0 0.0%	0 / 0 0.0%	30 / 50 60.0%AB	20 / 50 40.0%ABE				
1	Desulfobacula	48 / 48 100.0%	1 / 1 100.0%	2 / 2 100.0%	1 / 1 100.0%	1 / 1 100.0%	4 / 4 100.0%	2 / 2 100.0%	1 / 1 100.0%	1 / 1 100.0%	2 / 2 100.0%	1 / 1 100.0%	3 / 3 100.0%	2 / 2 100.0%	2 / 2 100.0%	2 / 2 100.0%	1 / 1 100.0%	3 / 3 100.0%	1 / 1 100.0%	3 / 3 100.0%	5 / 5 100.0%	3 / 3 100.0%	1 / 1 100.0%	5 / 5 100.0%	1 / 1 100.0%	0 / 0 0.0%	0 / 0 0.0%	0 / 0 0.0%	30 / 48 62.5%AB	18 / 48 37.5%ABE				
1	DSM 7467	48 / 48 100.0%	1 / 1 100.0%	2 / 2 100.0%	1 / 1 100.0%	1 / 1 100.0%	4 / 4 100.0%	2 / 2 100.0%	1 / 1 100.0%	1 / 1 100.0%	2 / 2 100.0%	1 / 1 100.0%	3 / 3 100.0%	2 / 2 100.0%	2 / 2 100.0%	2 / 2 100.0%	1 / 1 100.0%	3 / 3 100.0%	1 / 1 100.0%	3 / 3 100.0%	5 / 5 100.0%	3 / 3 100.0%	1 / 1 100.0%	5 / 5 100.0%	1 / 1 100.0%	0 / 0 0.0%	0 / 0 0.0%	0 / 0 0.0%	30 / 48 62.5%AB	18 / 48 37.5%ABE				
1	Desulfobacula toluolica DSM 7467	48 / 48 100.0%	1 / 1 100.0%	2 / 2 100.0%	1 / 1 100.0%	1 / 1 100.0%	4 / 4 100.0%	2 / 2 100.0%	1 / 1 100.0%	1 / 1 100.0%	2 / 2 100.0%	1 / 1 100.0%	3 / 3 100.0%	2 / 2 100.0%	2 / 2 100.0%	2 / 2 100.0%	1 / 1 100.0%	3 / 3 100.0%	1 / 1 100.0%	3 / 3 100.0%	5 / 5 100.0%	3 / 3 100.0%	1 / 1 100.0%	5 / 5 100.0%	1 / 1 100.0%	0 / 0 0.0%	0 / 0 0.0%	0 / 0 0.0%	30 / 48 62.5%AB	18 / 48 37.5%ABE				
1	Desulfatibacillum	53 / 56 94.6%	1 / 1 100.0%	2 / 2 100.0%	1 / 1 100.0%	2 / 2 100.0%	4 / 4 100.0%	3 / 3 100.0%	1 / 1 100.0%	1 / 1 100.0%	4 / 4 100.0%	2 / 2 100.0%	5 / 5 100.0%	1 / 2 50.0%	1 / 1 100.0%	3 / 3 100.0%	1 / 1 100.0%	3 / 3 100.0%	2 / 2 100.0%	4 / 4 100.0%	4 / 4 100.0%	3 / 3 100.0%	0 / 1 0.0%	4 / 5 80.0%	1 / 1 100.0%	0 / 0 0.0%	0 / 0 0.0%	0 / 0 0.0%	33 / 56 58.9%AB	20 / 56 35.7%ABE	2 / 56 3.6%E	1 / 56 1.8%		
1	ATCC BAA-924	53 / 56 94.6%	1 / 1 100.0%	2 / 2 100.0%	1 / 1 100.0%	2 / 2 100.0%	4 / 4 100.0%	3 / 3 100.0%	1 / 1 100.0%	1 / 1 100.0%	4 / 4 100.0%	2 / 2 100.0%	5 / 5 100.0%	1 / 2 50.0%	1 / 1 100.0%	3 / 3 100.0%	1 / 1 100.0%	3 / 3 100.0%	2 / 2 100.0%	4 / 4 100.0%	4 / 4 100.0%	3 / 3 100.0%	0 / 1 0.0%	4 / 5 80.0%	1 / 1 100.0%	0 / 0 0.0%	0 / 0 0.0%	0 / 0 0.0%	33 / 56 58.9%AB	20 / 56 35.7%ABE	2 / 56 3.6%E	1 / 56 1.8%		
1	Desulfatibacillum alkenivorans AK-01	53 / 56 94.6%	1 / 1 100.0%	2 / 2 100.0%	1 / 1 100.0%	2 / 2 100.0%	4 / 4 100.0%	3 / 3 100.0%	1 / 1 100.0%	1 / 1 100.0%	4 / 4 100.0%	2 / 2 100.0%	5 / 5 100.0%	1 / 2 50.0%	1 / 1 100.0%	3 / 3 100.0%	1 / 1 100.0%	3 / 3 100.0%	2 / 2 100.0%	4 / 4 100.0%	4 / 4 100.0%	3 / 3 100.0%	0 / 1 0.0%	4 / 5 80.0%	1 / 1 100.0%	0 / 0 0.0%	0 / 0 0.0%	0 / 0 0.0%	33 / 56 58.9%AB	20 / 56 35.7%ABE	2 / 56 3.6%E	1 / 56 1.8%		
4	'Desulfobulbaceae'	204 / 205 99.5%	6 / 6 100.0%	9 / 9 100.0%	4 / 4 100.0%	9 / 9 100.0%	12 / 12 100.0%	14 / 14 100.0%	5 / 5 100.0%	5 / 5 100.0%	13 / 13 100.0%	4 / 4 100.0%	13 / 14 92.9%	7 / 7 100.0%	5 / 5 100.0%	8 / 8 100.0%	4 / 4 100.0%	10 / 10 100.0%	4 / 4 100.0%	13 / 13 100.0%	20 / 20 100.0%	10 / 10 100.0%	4 / 4 100.0%	21 / 21 100.0%	4 / 4 100.0%	0 / 0 0.0%	0 / 0 0.0%	0 / 0 0.0%	131 / 205 63.9%AB	72 / 205 35.1%ABE	1 / 205 0.5%BE	1 / 205 0.5%E		
1	Desulfobulbus	48 / 48 100.0%	1 / 1 100.0%	1 / 1 100.0%	1 / 1 100.0%	2 / 2 100.0%	3 / 3 100.0%	3 / 3 100.0%	1 / 1 100.0%	1 / 1 100.0%	3 / 3 100.0%	1 / 1 100.0%	4 / 4 100.0%	2 / 2 100.0%	1 / 1 100.0%	2 / 2 100.0%	1 / 1 100.0%	3 / 3 100.0%	1 / 1 100.0%	3 / 3 100.0%	5 / 5 100.0%	2 / 2 100.0%	1 / 1 100.0%	5 / 5 100.0%	1 / 1 100.0%	0 / 0 0.0%	0 / 0 0.0%	0 / 0 0.0%	31 / 48 64.6%AB	17 / 48 35.4%ABE				
1	ATCC 33891	48 / 48 100.0%	1 / 1 100.0%	1 / 1 100.0%	1 / 1 100.0%	2 / 2 100.0%	3 / 3 100.0%	3 / 3 100.0%	1 / 1 100.0%	1 / 1 100.0%	3 / 3 100.0%	1 / 1 100.0%	4 / 4 100.0%	2 / 2 100.0%	1 / 1 100.0%	2 / 2 100.0%	1 / 1 100.0%	3 / 3 100.0%	1 / 1 100.0%	3 / 3 100.0%	5 / 5 100.0%	2 / 2 100.0%	1 / 1 100.0%	5 / 5 100.0%	1 / 1 100.0%	0 / 0 0.0%	0 / 0 0.0%	0 / 0 0.0%	31 / 48 64.6%AB	17 / 48 35.4%ABE				
1	Desulfobulbus propionicus DSM 2032	48 / 48 100.0%	1 / 1 100.0%	1 / 1 100.0%	1 / 1 100.0%	2 / 2 100.0%	3 / 3 100.0%	3 / 3 100.0%	1 / 1 100.0%	1 / 1 100.0%	3 / 3 100.0%	1 / 1 100.0%	4 / 4 100.0%	2 / 2 100.0%	1 / 1 100.0%	2 / 2 100.0%	1 / 1 100.0%	3 / 3 100.0%	1 / 1 100.0%	3 / 3 100.0%	5 / 5 100.0%	2 / 2 100.0%	1 / 1 100.0%	5 / 5 100.0%	1 / 1 100.0%	0 / 0 0.0%	0 / 0 0.0%	0 / 0 0.0%	31 / 48 64.6%AB	17 / 48 35.4%ABE				
1	Desulfocapa	46 / 46 100.0%	1 / 1 100.0%	1 / 1 100.0%	1 / 1 100.0%	3 / 3 100.0%	3 / 3 100.0%	2 / 2 100.0%	1 / 1 100.0%	1 / 1 100.0%	4 / 4 100.0%	1 / 1 100.0%	3 / 3 100.0%	1 / 1 100.0%	1 / 1 100.0%	2 / 2 100.0%	1 / 1 100.0%	2 / 2 100.0%	1 / 1 100.0%	3 / 3 100.0%	5 / 5 100.0%	2 / 2 100.0%	1 / 1 100.0%	5 / 5 100.0%	1 / 1 100.0%	0 / 0 0.0%	0 / 0 0.0%	0 / 0 0.0%	32 / 46 69.6%AB	14 / 46 30.4%ABE				
1	"Desulfocapsa sulfoexigens" Finster et al. 1998	46 / 46 100.0%	1 / 1 100.0%	1 / 1 100.0%	1 / 1 100.0%	3 / 3 100.0%	3 / 3 100.0%	2 / 2 100.0%	1 / 1 100.0%	1 / 1 100.0%	4 / 4 100.0%	1 / 1 100.0%	3 / 3 100.0%	1 / 1 100.0%	1 / 1 100.0%	2 / 2 100.0%	1 / 1 100.0%	2 / 2 100.0%	1 / 1 100.0%	3 / 3 100.0%	5 / 5 100.0%	2 / 2 100.0%	1 / 1 100.0%	5 / 5 100.0%	1 / 1 100.0%	0 / 0 0.0%	0 / 0 0.0%	0 / 0 0.0%	32 / 46 69.6%AB	14 / 46 30.4%ABE				
1	Desulfocapsa sulfexigens DSM 10523	46 / 46 100.0%	1 / 1 100.0%	1 / 1 100.0%	1 / 1 100.0%	3 / 3 100.0%	3 / 3 100.0%	2 / 2 100.0%	1 / 1 100.0%	1 / 1 100.0%	4 / 4 100.0%	1 / 1 100.0%	3 / 3 100.0%	1 / 1 100.0%	1 / 1 100.0%	2 / 2 100.0%	1 / 1 100.0%	2 / 2 100.0%	1 / 1 100.0%	3 / 3 100.0%	5 / 5 100.0%	2 / 2 100.0%	1 / 1 100.0%	5 / 5 100.0%	1 / 1 100.0%	0 / 0 0.0%	0 / 0 0.0%	0 / 0 0.0%	32 / 46 69.6%AB	14 / 46 30.4%ABE				
1	Desulfotalea	63 / 64 98.4%	3 / 3 100.0%	6 / 6 100.0%	1 / 1 100.0%	2 / 2 100.0%	3 / 3 100.0%	7 / 7 100.0%	2 / 2 100.0%	2 / 2 100.0%	3 / 3 100.0%	1 / 1 100.0%	2 / 3 66.7%	2 / 2 100.0%	2 / 2 100.0%	2 / 2 100.0%	1 / 1 100.0%	2 / 2 100.0%	1 / 1 100.0%	4 / 4 100.0%	5 / 5 100.0%	4 / 4 100.0%	1 / 1 100.0%	6 / 6 100.0%	1 / 1 100.0%	0 / 0 0.0%	0 / 0 0.0%	0 / 0 0.0%	40 / 64 62.5%AB	22 / 64 34.4%ABE	1 / 64 1.6%E	1 / 64 1.6%BE		
1	DSM 12343	63 / 64 98.4%	3 / 3 100.0%	6 / 6 100.0%	1 / 1 100.0%	2 / 2 100.0%	3 / 3 100.0%	7 / 7 100.0%	2 / 2 100.0%	2 / 2 100.0%	3 / 3 100.0%	1 / 1 100.0%	2 / 3 66.7%	2 / 2 100.0%	2 / 2 100.0%	2 / 2 100.0%	1 / 1 100.0%	2 / 2 100.0%	1 / 1 100.0%	4 / 4 100.0%	5 / 5 100.0%	4 / 4 100.0%	1 / 1 100.0%	6 / 6 100.0%	1 / 1 100.0%	0 / 0 0.0%	0 / 0 0.0%	0 / 0 0.0%	40 / 64 62.5%AB	22 / 64 34.4%ABE	1 / 64 1.6%E	1 / 64 1.6%BE		
1	Desulfotalea psychrophila LSv54	63 / 64 98.4%	3 / 3 100.0%	6 / 6 100.0%	1 / 1 100.0%	2 / 2 100.0%	3 / 3 100.0%	7 / 7 100.0%	2 / 2 100.0%	2 / 2 100.0%	3 / 3 100.0%	1 / 1 100.0%	2 / 3 66.7%	2 / 2 100.0%	2 / 2 100.0%	2 / 2 100.0%	1 / 1 100.0%	2 / 2 100.0%	1 / 1 100.0%	4 / 4 100.0%	5 / 5 100.0%	4 / 4 100.0%	1 / 1 100.0%	6 / 6 100.0%	1 / 1 100.0%	0 / 0 0.0%	0 / 0 0.0%	0 / 0 0.0%	40 / 64 62.5%AB	22 / 64 34.4%ABE	1 / 64 1.6%E	1 / 64 1.6%BE		
1	Desulfurivibrio	47 / 47 100.0%	1 / 1 100.0%	1 / 1 100.0%	1 / 1 100.0%	2 / 2 100.0%	3 / 3 100.0%	2 / 2 100.0%	1 / 1 100.0%	1 / 1 100.0%	3 / 3 100.0%	1 / 1 100.0%	4 / 4 100.0%	2 / 2 100.0%	1 / 1 100.0%	2 / 2 100.0%	1 / 1 100.0%	3 / 3 100.0%	1 / 1 100.0%	3 / 3 100.0%	5 / 5 100.0%	2 / 2 100.0%	1 / 1 100.0%	5 / 5 100.0%	1 / 1 100.0%	0 / 0 0.0%	0 / 0 0.0%	0 / 0 0.0%	28 / 47 59.6%AB	19 / 47 40.4%ABE				
1	DSM 19089	47 / 47 100.0%	1 / 1 100.0%	1 / 1 100.0%	1 / 1 100.0%	2 / 2 100.0%	3 / 3 100.0%	2 / 2 100.0%	1 / 1 100.0%	1 / 1 100.0%	3 / 3 100.0%	1 / 1 100.0%	4 / 4 100.0%	2 / 2 100.0%	1 / 1 100.0%	2 / 2 100.0%	1 / 1 100.0%	3 / 3 100.0%	1 / 1 100.0%	3 / 3 100.0%	5 / 5 100.0%	2 / 2 100.0%	1 / 1 100.0%	5 / 5 100.0%	1 / 1 100.0%	0 / 0 0.0%	0 / 0 0.0%	0 / 0 0.0%	28 / 47 59.6%AB	19 / 47 40.4%ABE				
1	Desulfurivibrio alkaliphilus AHT2	47 / 47 100.0%	1 / 1 100.0%	1 / 1 100.0%	1 / 1 100.0%	2 / 2 100.0%	3 / 3 100.0%	2 / 2 100.0%	1 / 1 100.0%	1 / 1 100.0%	3 / 3 100.0%	1 / 1 100.0%	4 / 4 100.0%	2 / 2 100.0%	1 / 1 100.0%	2 / 2 100.0%	1 / 1 100.0%	3 / 3 100.0%	1 / 1 100.0%	3 / 3 100.0%	5 / 5 100.0%	2 / 2 100.0%	1 / 1 100.0%	5 / 5 100.0%	1 / 1 100.0%	0 / 0 0.0%	0 / 0 0.0%	0 / 0 0.0%	28 / 47 59.6%AB	19 / 47 40.4%ABE				
4	'Syntrophobacterales'	188 / 192 97.9%	4 / 4 100.0%	4 / 4 100.0%	5 / 5 100.0%	5 / 5 100.0%	12 / 12 100.0%	12 / 12 100.0%	4 / 4 100.0%	4 / 4 100.0%	13 / 14 92.9%	4 / 4 100.0%	16 / 16 100.0%	8 / 8 100.0%	4 / 4 100.0%	8 / 8 100.0%	5 / 5 100.0%	11 / 12 91.7%	4 / 4 100.0%	11 / 12 91.7%	19 / 19 100.0%	8 / 8 100.0%	3 / 4 75.0%	20 / 20 100.0%	4 / 4 100.0%	0 / 0 0.0%	0 / 0 0.0%	0 / 0 0.0%	120 / 192 62.5%AB	66 / 192 34.4%ABE	4 / 192 2.1%	2 / 192 1.0%BE		
1	'Syntrophobacteraceae'	46 / 50 92.0%	1 / 1 100.0%	1 / 1 100.0%	1 / 1 100.0%	2 / 2 100.0%	3 / 3 100.0%	3 / 3 100.0%	1 / 1 100.0%	1 / 1 100.0%	4 / 5 80.0%	1 / 1 100.0%	4 / 4 100.0%	2 / 2 100.0%	1 / 1 100.0%	2 / 2 100.0%	1 / 1 100.0%	2 / 3 66.7%	1 / 1 100.0%	2 / 3 66.7%	5 / 5 100.0%	2 / 2 100.0%	0 / 1 0.0%	5 / 5 100.0%	1 / 1 100.0%	0 / 0 0.0%	0 / 0 0.0%	0 / 0 0.0%	28 / 50 56.0%AB	17 / 50 34.0%ABE	4 / 50 8.0%	1 / 50 2.0%BE		
1	Syntrophobacter	46 / 50 [illegible]	1 / 1 [illegible]	1 / 1 [illegible]	1 / 1 [illegible]	2 / 2 [illegible]	3 / 3 [illegible]	3 / 3 [illegible]	1 / 1 [illegible]	1 / 1 [illegible]	4 / 5 [illegible]	1 / 1 [illegible]	4 / 4 [illegible]	2 / 2 [illegible]	1 / 1 [illegible]	2 / 2 [illegible]	1 / 1 [illegible]	2 / 3 [illegible]	1 / 1 [illegible]	2 / 3 [illegible]	5 / 5 [illegible]	2 / 2 [illegible]	0 / 1 [illegible]	5 / 5 [illegible]	1 / 1 [illegible]	0 / 0 [illegible]	0 / 0 [illegible]	0 / 0 [illegible]	28 / 50 [illegible]	17 / 50 [illegible]	4 / 50 [illegible]	1 / 50 [illegible]		

1	DSM 10017	46 / 50 92.0%	1 / 1 100.0%	1 / 1 100.0%	1 / 1 100.0%	2 / 2 100.0%	3 / 3 100.0%	3 / 3 100.0%	1 / 1 100.0%	1 / 1 100.0%	4 / 5 80.0%	1 / 1 100.0%	4 / 4 100.0%	2 / 2 100.0%	1 / 1 100.0%	2 / 2 100.0%	1 / 1 100.0%	2 / 3 66.7%	1 / 1 100.0%	2 / 3 66.7%	5 / 5 100.0%	2 / 2 100.0%	0 / 1 0.0%	5 / 5 100.0%	1 / 1 100.0%	0 / 0 0.0%	0 / 0 0.0%	0 / 0 0.0%	28 / 50 56.0%AB	17 / 50 34.0%ABE	4 / 50 8.0%	1 / 50 2.0%BE		
1	Syntrophobacter fumaroxidans DSM 10017	46 / 50 92.0%	1 / 1 100.0%	1 / 1 100.0%	1 / 1 100.0%	2 / 2 100.0%	3 / 3 100.0%	3 / 3 100.0%	1 / 1 100.0%	1 / 1 100.0%	4 / 5 80.0%	1 / 1 100.0%	4 / 4 100.0%	2 / 2 100.0%	1 / 1 100.0%	2 / 2 100.0%	1 / 1 100.0%	2 / 3 66.7%	1 / 1 100.0%	2 / 3 66.7%	5 / 5 100.0%	2 / 2 100.0%	0 / 1 0.0%	5 / 5 100.0%	1 / 1 100.0%	0 / 0 0.0%	0 / 0 0.0%	0 / 0 0.0%	28 / 50 56.0%AB	17 / 50 34.0%ABE	4 / 50 8.0%	1 / 50 2.0%BE		
3	'Syntrophaceae'	142 / 142 100.0%	3 / 3 100.0%	3 / 3 100.0%	4 / 4 100.0%	3 / 3 100.0%	9 / 9 100.0%	9 / 9 100.0%	3 / 3 100.0%	3 / 3 100.0%	9 / 9 100.0%	3 / 3 100.0%	12 / 12 100.0%	6 / 6 100.0%	3 / 3 100.0%	6 / 6 100.0%	4 / 4 100.0%	9 / 9 100.0%	3 / 3 100.0%	9 / 9 100.0%	14 / 14 100.0%	6 / 6 100.0%	3 / 3 100.0%	15 / 15 100.0%	3 / 3 100.0%	0 / 0 0.0%	0 / 0 0.0%	0 / 0 0.0%	92 / 142 64.8%AB	49 / 142 34.5%ABE	1 / 142 0.7%BE			
1	Desulfomonile	47 / 47 100.0%	1 / 1 100.0%	1 / 1 100.0%	1 / 1 100.0%	1 / 1 100.0%	3 / 3 100.0%	3 / 3 100.0%	1 / 1 100.0%	1 / 1 100.0%	3 / 3 100.0%	1 / 1 100.0%	4 / 4 100.0%	2 / 2 100.0%	1 / 1 100.0%	2 / 2 100.0%	1 / 1 100.0%	3 / 3 100.0%	1 / 1 100.0%	3 / 3 100.0%	5 / 5 100.0%	2 / 2 100.0%	1 / 1 100.0%	5 / 5 100.0%	1 / 1 100.0%	0 / 0 0.0%	0 / 0 0.0%	0 / 0 0.0%	30 / 47 63.8%AB	17 / 47 36.2%ABE				
1	ATCC 49306	47 / 47 100.0%	1 / 1 100.0%	1 / 1 100.0%	1 / 1 100.0%	1 / 1 100.0%	3 / 3 100.0%	3 / 3 100.0%	1 / 1 100.0%	1 / 1 100.0%	3 / 3 100.0%	1 / 1 100.0%	4 / 4 100.0%	2 / 2 100.0%	1 / 1 100.0%	2 / 2 100.0%	1 / 1 100.0%	3 / 3 100.0%	1 / 1 100.0%	3 / 3 100.0%	5 / 5 100.0%	2 / 2 100.0%	1 / 1 100.0%	5 / 5 100.0%	1 / 1 100.0%	0 / 0 0.0%	0 / 0 0.0%	0 / 0 0.0%	30 / 47 63.8%AB	17 / 47 36.2%ABE				
1	Desulfomonile tiedjei DCB-1	47 / 47 100.0%	1 / 1 100.0%	1 / 1 100.0%	1 / 1 100.0%	1 / 1 100.0%	3 / 3 100.0%	3 / 3 100.0%	1 / 1 100.0%	1 / 1 100.0%	3 / 3 100.0%	1 / 1 100.0%	4 / 4 100.0%	2 / 2 100.0%	1 / 1 100.0%	2 / 2 100.0%	1 / 1 100.0%	3 / 3 100.0%	1 / 1 100.0%	3 / 3 100.0%	5 / 5 100.0%	2 / 2 100.0%	1 / 1 100.0%	5 / 5 100.0%	1 / 1 100.0%	0 / 0 0.0%	0 / 0 0.0%	0 / 0 0.0%	30 / 47 63.8%AB	17 / 47 36.2%ABE				
1	Syntrophus	49 / 49 100.0%	1 / 1 100.0%	1 / 1 100.0%	2 / 2 100.0%	1 / 1 100.0%	3 / 3 100.0%	3 / 3 100.0%	1 / 1 100.0%	1 / 1 100.0%	3 / 3 100.0%	1 / 1 100.0%	4 / 4 100.0%	2 / 2 100.0%	1 / 1 100.0%	2 / 2 100.0%	2 / 2 100.0%	3 / 3 100.0%	1 / 1 100.0%	3 / 3 100.0%	5 / 5 100.0%	2 / 2 100.0%	1 / 1 100.0%	5 / 5 100.0%	1 / 1 100.0%	0 / 0 0.0%	0 / 0 0.0%	0 / 0 0.0%	32 / 49 65.3%AB	16 / 49 32.7%ABE	1 / 49 2.0%BE			
1	ATCC 700169	49 / 49 100.0%	1 / 1 100.0%	1 / 1 100.0%	2 / 2 100.0%	1 / 1 100.0%	3 / 3 100.0%	3 / 3 100.0%	1 / 1 100.0%	1 / 1 100.0%	3 / 3 100.0%	1 / 1 100.0%	4 / 4 100.0%	2 / 2 100.0%	1 / 1 100.0%	2 / 2 100.0%	2 / 2 100.0%	3 / 3 100.0%	1 / 1 100.0%	3 / 3 100.0%	5 / 5 100.0%	2 / 2 100.0%	1 / 1 100.0%	5 / 5 100.0%	1 / 1 100.0%	0 / 0 0.0%	0 / 0 0.0%	0 / 0 0.0%	32 / 49 65.3%AB	16 / 49 32.7%ABE	1 / 49 2.0%BE			
1	Syntrophus aciditrophicus SB	49 / 49 100.0%	1 / 1 100.0%	1 / 1 100.0%	2 / 2 100.0%	1 / 1 100.0%	3 / 3 100.0%	3 / 3 100.0%	1 / 1 100.0%	1 / 1 100.0%	3 / 3 100.0%	1 / 1 100.0%	4 / 4 100.0%	2 / 2 100.0%	1 / 1 100.0%	2 / 2 100.0%	2 / 2 100.0%	3 / 3 100.0%	1 / 1 100.0%	3 / 3 100.0%	5 / 5 100.0%	2 / 2 100.0%	1 / 1 100.0%	5 / 5 100.0%	1 / 1 100.0%	0 / 0 0.0%	0 / 0 0.0%	0 / 0 0.0%	32 / 49 65.3%AB	16 / 49 32.7%ABE	1 / 49 2.0%BE			
1	Desulfobacca	46 / 46 100.0%	1 / 1 100.0%	1 / 1 100.0%	1 / 1 100.0%	1 / 1 100.0%	3 / 3 100.0%	3 / 3 100.0%	1 / 1 100.0%	1 / 1 100.0%	3 / 3 100.0%	1 / 1 100.0%	4 / 4 100.0%	2 / 2 100.0%	1 / 1 100.0%	2 / 2 100.0%	1 / 1 100.0%	3 / 3 100.0%	1 / 1 100.0%	3 / 3 100.0%	4 / 4 100.0%	2 / 2 100.0%	1 / 1 100.0%	5 / 5 100.0%	1 / 1 100.0%	0 / 0 0.0%	0 / 0 0.0%	0 / 0 0.0%	30 / 46 65.2%AB	16 / 46 34.8%ABE				
1	ATCC 700848	46 / 46 100.0%	1 / 1 100.0%	1 / 1 100.0%	1 / 1 100.0%	1 / 1 100.0%	3 / 3 100.0%	3 / 3 100.0%	1 / 1 100.0%	1 / 1 100.0%	3 / 3 100.0%	1 / 1 100.0%	4 / 4 100.0%	2 / 2 100.0%	1 / 1 100.0%	2 / 2 100.0%	1 / 1 100.0%	3 / 3 100.0%	1 / 1 100.0%	3 / 3 100.0%	4 / 4 100.0%	2 / 2 100.0%	1 / 1 100.0%	5 / 5 100.0%	1 / 1 100.0%	0 / 0 0.0%	0 / 0 0.0%	0 / 0 0.0%	30 / 46 65.2%AB	16 / 46 34.8%ABE				
1	Desulfobacca acetoxidans DSM 11109	46 / 46 100.0%	1 / 1 100.0%	1 / 1 100.0%	1 / 1 100.0%	1 / 1 100.0%	3 / 3 100.0%	3 / 3 100.0%	1 / 1 100.0%	1 / 1 100.0%	3 / 3 100.0%	1 / 1 100.0%	4 / 4 100.0%	2 / 2 100.0%	1 / 1 100.0%	2 / 2 100.0%	1 / 1 100.0%	3 / 3 100.0%	1 / 1 100.0%	3 / 3 100.0%	4 / 4 100.0%	2 / 2 100.0%	1 / 1 100.0%	5 / 5 100.0%	1 / 1 100.0%	0 / 0 0.0%	0 / 0 0.0%	0 / 0 0.0%	30 / 46 65.2%AB	16 / 46 34.8%ABE				
4	Bdellovibrionales	140 / 140 100.0%	4 / 4 100.0%	4 / 4 100.0%	4 / 4 100.0%	6 / 6 100.0%	8 / 8 100.0%	8 / 8 100.0%	4 / 4 100.0%	4 / 4 100.0%	7 / 7 100.0%	4 / 4 100.0%	12 / 12 100.0%	5 / 5 100.0%	4 / 4 100.0%	4 / 4 100.0%	4 / 4 100.0%	6 / 6 100.0%	4 / 4 100.0%	8 / 8 100.0%	12 / 12 100.0%	5 / 5 100.0%	4 / 4 100.0%	19 / 19 100.0%	0 / 0 0.0%	0 / 0 0.0%	0 / 0 0.0%	0 / 0 0.0%	83 / 140 59.3%AB	57 / 140 40.7%ABE				
3	Bdellovibrionaceae	104 / 104 100.0%	3 / 3 100.0%	3 / 3 100.0%	3 / 3 100.0%	5 / 5 100.0%	6 / 6 100.0%	6 / 6 100.0%	3 / 3 100.0%	3 / 3 100.0%	5 / 5 100.0%	3 / 3 100.0%	9 / 9 100.0%	3 / 3 100.0%	3 / 3 100.0%	3 / 3 100.0%	3 / 3 100.0%	4 / 4 100.0%	3 / 3 100.0%	6 / 6 100.0%	9 / 9 100.0%	3 / 3 100.0%	3 / 3 100.0%	15 / 15 100.0%	0 / 0 0.0%	0 / 0 0.0%	0 / 0 0.0%	0 / 0 0.0%	63 / 104 60.6%AB	41 / 104 39.4%ABE				
3	Bdellovibrio	104 / 104 100.0%	3 / 3 100.0%	3 / 3 100.0%	3 / 3 100.0%	5 / 5 100.0%	6 / 6 100.0%	6 / 6 100.0%	3 / 3 100.0%	3 / 3 100.0%	5 / 5 100.0%	3 / 3 100.0%	9 / 9 100.0%	3 / 3 100.0%	3 / 3 100.0%	3 / 3 100.0%	3 / 3 100.0%	4 / 4 100.0%	3 / 3 100.0%	6 / 6 100.0%	9 / 9 100.0%	3 / 3 100.0%	3 / 3 100.0%	15 / 15 100.0%	0 / 0 0.0%	0 / 0 0.0%	0 / 0 0.0%	0 / 0 0.0%	63 / 104 60.6%AB	41 / 104 39.4%ABE				
2	ATCC 15356	71 / 71 100.0%	2 / 2 100.0%	2 / 2 100.0%	2 / 2 100.0%	4 / 4 100.0%	4 / 4 100.0%	4 / 4 100.0%	2 / 2 100.0%	2 / 2 100.0%	4 / 4 100.0%	2 / 2 100.0%	6 / 6 100.0%	2 / 2 100.0%	2 / 2 100.0%	2 / 2 100.0%	2 / 2 100.0%	3 / 3 100.0%	2 / 2 100.0%	4 / 4 100.0%	6 / 6 100.0%	2 / 2 100.0%	2 / 2 100.0%	10 / 10 100.0%	0 / 0 0.0%	0 / 0 0.0%	0 / 0 0.0%	0 / 0 0.0%	44 / 71 62.0%AB	27 / 71 38.0%ABE				
1	Bdellovibrio bacteriovorus str. Tiberius	35 / 35 100.0%	1 / 1 100.0%	1 / 1 100.0%	1 / 1 100.0%	2 / 2 100.0%	2 / 2 100.0%	2 / 2 100.0%	1 / 1 100.0%	1 / 1 100.0%	2 / 2 100.0%	1 / 1 100.0%	3 / 3 100.0%	1 / 1 100.0%	1 / 1 100.0%	1 / 1 100.0%	1 / 1 100.0%	1 / 1 100.0%	1 / 1 100.0%	2 / 2 100.0%	3 / 3 100.0%	1 / 1 100.0%	1 / 1 100.0%	5 / 5 100.0%	0 / 0 0.0%	0 / 0 0.0%	0 / 0 0.0%	0 / 0 0.0%	22 / 35 62.9%AB	13 / 35 37.1%ABE				
1	'Bdellovibrio exovorus'	33 / 33 100.0%	1 / 1 100.0%	1 / 1 100.0%	1 / 1 100.0%	1 / 1 100.0%	2 / 2 100.0%	2 / 2 100.0%	1 / 1 100.0%	1 / 1 100.0%	1 / 1 100.0%	1 / 1 100.0%	3 / 3 100.0%	1 / 1 100.0%	1 / 1 100.0%	1 / 1 100.0%	1 / 1 100.0%	1 / 1 100.0%	1 / 1 100.0%	2 / 2 100.0%	3 / 3 100.0%	1 / 1 100.0%	1 / 1 100.0%	5 / 5 100.0%	0 / 0 0.0%	0 / 0 0.0%	0 / 0 0.0%	0 / 0 0.0%	19 / 33 57.6%AB	14 / 33 42.4%ABE				
1	Bdellovibrio exovorus JSS	33 / 33 100.0%	1 / 1 100.0%	1 / 1 100.0%	1 / 1 100.0%	1 / 1 100.0%	2 / 2 100.0%	2 / 2 100.0%	1 / 1 100.0%	1 / 1 100.0%	1 / 1 100.0%	1 / 1 100.0%	3 / 3 100.0%	1 / 1 100.0%	1 / 1 100.0%	1 / 1 100.0%	1 / 1 100.0%	1 / 1 100.0%	1 / 1 100.0%	2 / 2 100.0%	3 / 3 100.0%	1 / 1 100.0%	1 / 1 100.0%	5 / 5 100.0%	0 / 0 0.0%	0 / 0 0.0%	0 / 0 0.0%	0 / 0 0.0%	19 / 33 57.6%AB	14 / 33 42.4%ABE				
1	Bacteriovoracaceae	36 / 36 100.0%	1 / 1 100.0%	1 / 1 100.0%	1 / 1 100.0%	1 / 1 100.0%	2 / 2 100.0%	2 / 2 100.0%	1 / 1 100.0%	1 / 1 100.0%	2 / 2 100.0%	1 / 1 100.0%	3 / 3 100.0%	2 / 2 100.0%	1 / 1 100.0%	1 / 1 100.0%	1 / 1 100.0%	2 / 2 100.0%	1 / 1 100.0%	2 / 2 100.0%	3 / 3 100.0%	2 / 2 100.0%	1 / 1 100.0%	4 / 4 100.0%	0 / 0 0.0%	0 / 0 0.0%	0 / 0 0.0%	0 / 0 0.0%	20 / 36 55.6%AB	16 / 36 44.4%ABE				
1	Bacteriolyticum	36 / 36 100.0%	1 / 1 100.0%	1 / 1 100.0%	1 / 1 100.0%	1 / 1 100.0%	2 / 2 100.0%	2 / 2 100.0%	1 / 1 100.0%	1 / 1 100.0%	2 / 2 100.0%	1 / 1 100.0%	3 / 3 100.0%	2 / 2 100.0%	1 / 1 100.0%	1 / 1 100.0%	1 / 1 100.0%	2 / 2 100.0%	1 / 1 100.0%	2 / 2 100.0%	3 / 3 100.0%	2 / 2 100.0%	1 / 1 100.0%	4 / 4 100.0%	0 / 0 0.0%	0 / 0 0.0%	0 / 0 0.0%	0 / 0 0.0%	20 / 36 55.6%AB	16 / 36 44.4%ABE				
1	ATCC BAA-682	36 / 36 100.0%	1 / 1 100.0%	1 / 1 100.0%	1 / 1 100.0%	1 / 1 100.0%	2 / 2 100.0%	2 / 2 100.0%	1 / 1 100.0%	1 / 1 100.0%	2 / 2 100.0%	1 / 1 100.0%	3 / 3 100.0%	2 / 2 100.0%	1 / 1 100.0%	1 / 1 100.0%	1 / 1 100.0%	2 / 2 100.0%	1 / 1 100.0%	2 / 2 100.0%	3 / 3 100.0%	2 / 2 100.0%	1 / 1 100.0%	4 / 4 100.0%	0 / 0 0.0%	0 / 0 0.0%	0 / 0 0.0%	0 / 0 0.0%	20 / 36 55.6%AB	16 / 36 44.4%ABE				
1	Bacteriovorax marinus SJ	36 / 36 100.0%	1 / 1 100.0%	1 / 1 100.0%	1 / 1 100.0%	1 / 1 100.0%	2 / 2 100.0%	2 / 2 100.0%	1 / 1 100.0%	1 / 1 100.0%	2 / 2 100.0%	1 / 1 100.0%	3 / 3 100.0%	2 / 2 100.0%	1 / 1 100.0%	1 / 1 100.0%	1 / 1 100.0%	2 / 2 100.0%	1 / 1 100.0%	2 / 2 100.0%	3 / 3 100.0%	2 / 2 100.0%	1 / 1 100.0%	4 / 4 100.0%	0 / 0 0.0%	0 / 0 0.0%	0 / 0 0.0%	0 / 0 0.0%	20 / 36 55.6%AB	16 / 36 44.4%ABE				
1	Desulfarculales	45 / 45 100.0%	1 / 1 100.0%	1 / 1 100.0%	1 / 1 100.0%	1 / 1 100.0%	3 / 3 100.0%	3 / 3 100.0%	1 / 1 100.0%	1 / 1 100.0%	3 / 3 100.0%	1 / 1 100.0%	3 / 3 100.0%	2 / 2 100.0%	1 / 1 100.0%	2 / 2 100.0%	1 / 1 100.0%	3 / 3 100.0%	1 / 1 100.0%	3 / 3 100.0%	4 / 4 100.0%	2 / 2 100.0%	1 / 1 100.0%	5 / 5 100.0%	1 / 1 100.0%	0 / 0 0.0%	0 / 0 0.0%	0 / 0 0.0%	26 / 45 57.8%AB	19 / 45 42.2%ABE				
1	Desulfarculaceae	45 / 45 100.0%	1 / 1 100.0%	1 / 1 100.0%	1 / 1 100.0%	1 / 1 100.0%	3 / 3 100.0%	3 / 3 100.0%	1 / 1 100.0%	1 / 1 100.0%	3 / 3 100.0%	1 / 1 100.0%	3 / 3 100.0%	2 / 2 100.0%	1 / 1 100.0%	2 / 2 100.0%	1 / 1 100.0%	3 / 3 100.0%	1 / 1 100.0%	3 / 3 100.0%	4 / 4 100.0%	2 / 2 100.0%	1 / 1 100.0%	5 / 5 100.0%	1 / 1 100.0%	0 / 0 0.0%	0 / 0 0.0%	0 / 0 0.0%	26 / 45 57.8%AB	19 / 45 42.2%ABE				
1	Desulfarculus	45 / 45 100.0%	1 / 1 100.0%	1 / 1 100.0%	1 / 1 100.0%	1 / 1 100.0%	3 / 3 100.0%	3 / 3 100.0%	1 / 1 100.0%	1 / 1 100.0%	3 / 3 100.0%	1 / 1 100.0%	3 / 3 100.0%	2 / 2 100.0%	1 / 1 100.0%	2 / 2 100.0%	1 / 1 100.0%	3 / 3 100.0%	1 / 1 100.0%	3 / 3 100.0%	4 / 4 100.0%	2 / 2 100.0%	1 / 1 100.0%	5 / 5 100.0%	1 / 1 100.0%	0 / 0 0.0%	0 / 0 0.0%	0 / 0 0.0%	26 / 45 57.8%AB	19 / 45 42.2%ABE				
1	ATCC 33931	45 / 45 100.0%	1 / 1 100.0%	1 / 1 100.0%	1 / 1 100.0%	1 / 1 100.0%	3 / 3 100.0%	3 / 3 100.0%	1 / 1 100.0%	1 / 1 100.0%	3 / 3 100.0%	1 / 1 100.0%	3 / 3 100.0%	2 / 2 100.0%	1 / 1 100.0%	2 / 2 100.0%	1 / 1 100.0%	3 / 3 100.0%	1 / 1 100.0%	3 / 3 100.0%	4 / 4 100.0%	2 / 2 100.0%	1 / 1 100.0%	5 / 5 100.0%	1 / 1 100.0%	0 / 0 0.0%	0 / 0 0.0%	0 / 0 0.0%	26 / 45 57.8%AB	19 / 45 42.2%ABE				
1	Desulfarculus baarsii 2st 14	45 / 45 100.0%	1 / 1 100.0%	1 / 1 100.0%	1 / 1 100.0%	1 / 1 100.0%	3 / 3 100.0%	3 / 3 100.0%	1 / 1 100.0%	1 / 1 100.0%	3 / 3 100.0%	1 / 1 100.0%	3 / 3 100.0%	2 / 2 100.0%	1 / 1 100.0%	2 / 2 100.0%	1 / 1 100.0%	3 / 3 100.0%	1 / 1 100.0%	3 / 3 100.0%	4 / 4 100.0%	2 / 2 100.0%	1 / 1 100.0%	5 / 5 100.0%	1 / 1 100.0%	0 / 0 0.0%	0 / 0 0.0%	0 / 0 0.0%	26 / 45 57.8%AB	19 / 45 42.2%ABE				
87	'Epsilonproteobacteria'	3482 / 3519 98.9%	98 / 99 99.0%	113 / 113 100.0%	100 / 100 100.0%	154 / 154 100.0%	212 / 212 100.0%	199 / 199 100.0%	89 / 89 100.0%	93 / 93 100.0%	240 / 241 99.6%	89 / 89 100.0%	264 / 265 99.6%	123 / 125 98.4%	96 / 96 100.0%	99 / 99 100.0%	89 / 89 100.0%	156 / 156 100.0%	90 / 90 100.0%	188 / 188 100.0%	361 / 363 99.4%	176 / 178 98.9%	89 / 89 100.0%	362 / 362 100.0%	0 / 28 0.0%	0 / 0 0.0%	2 / 2 100.0%	0 / 0 0.0%	2335 / 3519 66.4%AB	967 / 3519 27.5%ABE	135 / 3519 3.8%B	45 / 3519 1.3%BE	19 / 3519 0.5%	18 / 3519 0.5%E
2	unclassified Epsilonproteobacteria	88 / 89 98.9%	3 / 3 100.0%	3 / 3 100.0%	2 / 2 100.0%	4 / 4 100.0%	7 / 7 100.0%	5 / 5 100.0%	2 / 2 100.0%	2 / 2 100.0%	7 / 7 100.0%	2 / 2 100.0%	6 / 6 100.0%	4 / 4 100.0%	2 / 2 100.0%	3 / 3 100.0%	2 / 2 100.0%	4 / 4 100.0%	2 / 2 100.0%	5 / 5 100.0%	7 / 8 87.5%	5 / 5 100.0%	2 / 2 100.0%	9 / 9 100.0%	0 / 0 0.0%	0 / 0 0.0%	0 / 0 0.0%	0 / 0 0.0%	48 / 89 53.9%AB	22 / 89 24.7%ABE	12 / 89 13.5%B	6 / 89 6.7%BE	1 / 89 1.1%	
1	Sulfurovum	44 / 44 100.0%	1 / 1 100.0%	2 / 2 100.0%	1 / 1 100.0%	1 / 1 100.0%	4 / 4 100.0%	3 / 3 100.0%	1 / 1 100.0%	1 / 1 100.0%	3 / 3 100.0%	1 / 1 100.0%	3 / 3 100.0%	2 / 2 100.0%	1 / 1 100.0%	1 / 1 100.0%	1 / 1 100.0%	2 / 2 100.0%	1 / 1 100.0%	2 / 2 100.0%	4 / 4 100.0%	3 / 3 100.0%	1 / 1 100.0%	5 / 5 100.0%	0 / 0 0.0%	0 / 0 0.0%	0 / 0 0.0%	0 / 0 0.0%	24 / 44 54.5%AB	15 / 44 34.1%ABE	4 / 44 9.1%B	1 / 44 2.3%BE		
1	Sulfurovum sp. NBC37-1	44 / 44 100.0%	1 / 1 100.0%	2 / 2 100.0%	1 / 1 100.0%	1 / 1 100.0%	4 / 4 100.0%	3 / 3 100.0%	1 / 1 100.0%	1 / 1 100.0%	3 / 3 100.0%	1 / 1 100.0%	3 / 3 100.0%	2 / 2 100.0%	1 / 1 100.0%	1 / 1 100.0%	1 / 1 100.0%	2 / 2 100.0%	1 / 1 100.0%	2 / 2 100.0%	4 / 4 100.0%	3 / 3 100.0%	1 / 1 100.0%	5 / 5 100.0%	0 / 0 0.0%	0 / 0 0.0%	0 / 0 0.0%	0 / 0 0.0%	24 / 44 54.5%AB	15 / 44 34.1%ABE	4 / 44 9.1%B	1 / 44 2.3%BE		
1	Nitratiruptor	44 / 45 97.8%	2 / 2 100.0%	1 / 1 100.0%	1 / 1 100.0%	3 / 3 100.0%	3 / 3 100.0%	2 / 2 100.0%	1 / 1 100.0%	1 / 1 100.0%	4 / 4 100.0%	1 / 1 100.0%	3 / 3 100.0%	2 / 2 100.0%	1 / 1 100.0%	2 / 2 100.0%	1 / 1 100.0%	2 / 2 100.0%	1 / 1 100.0%	3 / 3 100.0%	3 / 4 75.0%	2 / 2 100.0%	1 / 1 100.0%	4 / 4 100.0%	0 / 0 0.0%	0 / 0 0.0%	0 / 0 0.0%	0 / 0 0.0%	24 / 45 53.3%AB	8 / 45 17.8%B	7 / 45 15.6%ABE	5 / 45 11.1%BE	1 / 45 2.2%	
1	Nitratiruptor sp. SB155-2	44 / 45 97.8%	2 / 2 100.0%	1 / 1 100.0%	1 / 1 100.0%	3 / 3 100.0%	3 / 3 100.0%	2 / 2 100.0%	1 / 1 100.0%	1 / 1 100.0%	4 / 4 100.0%	1 / 1 100.0%	3 / 3 100.0%	2 / 2 100.0%	1 / 1 100.0%	2 / 2 100.0%	1 / 1 100.0%	2 / 2 100.0%	1 / 1 100.0%	3 / 3 100.0%	3 / 4 75.0%	2 / 2 100.0%	1 / 1 100.0%	4 / 4 100.0%	0 / 0 0.0%	0 / 0 0.0%	0 / 0 0.0%	0 / 0 0.0%	24 / 45 53.3%AB	8 / 45 17.8%B	7 / 45 15.6%ABE	5 / 45 11.1%BE	1 / 45 2.2%	
84	'Campylobacterales'	3346 / 3381 99.0%	94 / 95 98.9%	108 / 108 100.0%	96 / 96 100.0%	146 / 146 100.0%	202 / 202 100.0%	191 / 191 100.0%	86 / 86 100.0%	90 / 90 100.0%	228 / 229 99.6%	86 / 86 100.0%	255 / 256 99.6%	117 / 119 98.3%	92 / 92 100.0%	95 / 95 100.0%	86 / 86 100.0%	151 / 151 100.0%	87 / 87 100.0%	180 / 180 100.0%	350 / 351 99.7%	169 / 171 98.8%	86 / 86 100.0%	349 / 349 100.0%	0 / 27 0.0%	0 / 0 0.0%	2 / 2 100.0%	0 / 0 0.0%	2250 / 3381 66.5%AB	934 / 3381 27.6%ABE	123 / 3381 3.6%B	39 / 3381 1.2%BE	18 / 3381 0.5%	17 / 3381 0.5%E
61	'Helicobacteraceae'	2272 / 2282 99.6%	64 / 65 98.5%	65 / 65 100.0%	65 / 65 100.0%	71 / 71 100.0%	125 / 125 100.0%	127 / 127 100.0%	62 / 62 100.0%	62 / 62 100.0%	133 / 133 100.0%	62 / 62 100.0%	185 / 186 99.5%	68 / 68 100.0%	64 / 64 100.0%	64 / 64 100.0%	62 / 62 100.0%	122 / 122 100.0%	62 / 62 100.0%	125 / 125 100.0%	247 / 247 100.0%	126 / 126 100.0%	62 / 62 100.0%	248 / 248 100.0%	0 / 8 0.0%	0 / 0 0.0%	1 / 1 100.0%	0 / 0 0.0%	1570 / 2282 68.8%AB	679 / 2282 29.8%ABE	17 / 2282 0.7%B	9 / 2282 0.4%E	6 / 2282 0.3%BE	1 / 2282 0.0%
57	Gastrospirillum	2096 / 2105 99.6%	57 / 58 98.3%	58 / 58 100.0%	58 / 58 100.0%	62 / 62 100.0%	116 / 116 100.0%	116 / 116 100.0%	58 / 58 100.0%	58 / 58 100.0%	120 / 120 100.0%	58 / 58 100.0%	173 / 174 99.4%	61 / 61 100.0%	58 / 58 100.0%	58 / 58 100.0%	58 / 58 100.0%	116 / 116 100.0%	58 / 58 100.0%	115 / 115 100.0%	232 / 232 100.0%	115 / 115 100.0%	58 / 58 100.0%	232 / 232 100.0%	0 / 7 0.0%	0 / 0 0.0%	1 / 1 100.0%	0 / 0 0.0%	1446 / 2105 68.7%AB	627 / 2105 29.8%ABE	17 / 2105 0.8%B	8 / 2105 0.4%E	6 / 2105 0.3%BE	1 / 2105 0.0%
48	ATCC 43504	1761 / 1763 99.9%	48 / 49 98.0%	49 / 49 100.0%	49 / 49 100.0%	49 / 49 100.0%	98 / 98 100.0%	98 / 98 100.0%	49 / 49 100.0%	49 / 49 100.0%	98 / 98 100.0%	49 / 49 100.0%	146 / 147 99.3%	49 / 49 100.0%	49 / 49 100.0%	49 / 49 100.0%	49 / 49 100.0%	98 / 98 100.0%	49 / 49 100.0%	97 / 97 100.0%	196 / 196 100.0%	98 / 98 100.0%	49 / 49 100.0%	196 / 196 100.0%	0 / 0 0.0%	0 / 0 0.0%	0 / 0 0.0%	0 / 0 0.0%	1206 / 1763 68.4%AB	532 / 1763 30.2%ABE	17 / 1763 1.0%B	6 / 1763 0.3%BE	1 / 1763 0.1%E	1 / 1763 0.1%
1	Helicobacter pylori (strain 26695)	72 / 72 100.0%	2 / 2 100.0%	2 / 2 100.0%	2 / 2 100.0%	2 / 2 100.0%	4 / 4 100.0%	4 / 4 100.0%	2 / 2 100.0%	2 / 2 100.0%	4 / 4 100.0%	2 / 2 100.0%	6 / 6 100.0%	2 / 2 100.0%	2 / 2 100.0%	2 / 2 100.0%	2 / 2 100.0%	4 / 4 100.0%	2 / 2 100.0%	4 / 4 100.0%	8 / 8 100.0%	4 / 4 100.0%	2 / 2 100.0%	8 / 8 100.0%	0 / 0 0.0%	0 / 0 0.0%	0 / 0 0.0%	0 / 0 0.0%	50 / 72 69.4%AB	22 / 72 30.6%ABE				
1	Helicobacter pylori J99	36 / 36 100.0%	1 / 1 100.0%	1 / 1 100.0%	1 / 1 100.0%	1 / 1 100.0%	2 / 2 100.0%	2 / 2 100.0%	1 / 1 100.0%	1 / 1 100.0%	2 / 2 100.0%	1 / 1 100.0%	3 / 3 100.0%	1 / 1 100.0%	1 / 1 100.0%	1 / 1 100.0%	1 / 1 100.0%	2 / 2 100.0%	1 / 1 100.0%	2 / 2 100.0%	4 / 4 100.0%	2 / 2 100.0%	1 / 1 100.0%	4 / 4 100.0%	0 / 0 0.0%	0 / 0 0.0%	0 / 0 0.0%	0 / 0 0.0%	25 / 36 69.4%AB	11 / 36 30.6%ABE				
1	Helicobacter pylori (strain F32)	36 / 36 100.0%	1 / 1 100.0%	1 / 1 100.0%	1 / 1 100.0%	1 / 1 100.0%	2 / 2 100.0%	2 / 2 100.0%	1 / 1 100.0%	1 / 1 100.0%	2 / 2 100.0%	1 / 1 100.0%	3 / 3 100.0%	1 / 1 100.0%	1 / 1 100.0%	1 / 1 100.0%	1 / 1 100.0%	2 / 2 100.0%	1 / 1 100.0%	2 / 2 100.0%	4 / 4 100.0%	2 / 2 100.0%	1 / 1 100.0%	4 / 4 100.0%	0 / 0 0.0%	0 / 0 0.0%	0 / 0 0.0%	0 / 0 0.0%	25 / 36 69.4%AB	11 / 36 30.6%ABE				
1	Helicobacter pylori 51	36 / 36 100.0%	1 / 1 100.0%	1 / 1 100.0%	1 / 1 100.0%	1 / 1 100.0%	2 / 2 100.0%	2 / 2 100.0%	1 / 1 100.0%	1 / 1 100.0%	2 / 2 100.0%	1 / 1 100.0%	3 / 3 100.0%	1 / 1 100.0%	1 / 1 100.0%	1 / 1 100.0%	1 / 1 100.0%	2 / 2 100.0%	1 / 1 100.0%	2 / 2 100.0%	4 / 4 100.0%	2 / 2 100.0%	1 / 1 100.0%	4 / 4 100.0%	0 / 0 0.0%	0 / 0 0.0%	0 / 0 0.0%	0 / 0 0.0%	25 / 36 69.4%AB	11 / 36 30.6%ABE				
1	Helicobacter pylori HPAG1	36 / 36 100.0%	1 / 1 100.0%	1 / 1 100.0%	1 / 1 100.0%	1 / 1 100.0%	2 / 2 100.0%	2 / 2 100.0%	1 / 1 100.0%	1 / 1 100.0%	2 / 2 100.0%	1 / 1 100.0%	3 / 3 100.0%	1 / 1 100.0%	1 / 1 100.0%	1 / 1 100.0%	1 / 1 100.0%	2 / 2 100.0%	1 / 1 100.0%	2 / 2 100.0%	4 / 4 100.0%	2 / 2 100.0%	1 / 1 100.0%	4 / 4 100.0%	0 / 0 0.0%	0 / 0 0.0%	0 / 0 0.0%	0 / 0 0.0%	25 / 36 69.4%AB	11 / 36 30.6%ABE				
1	Helicobacter pylori Shi470	34 / 36 94.4%	0 / 1 0.0%	1 / 1 100.0%	1 / 1 100.0%	1 / 1 100.0%	2 / 2 100.0%	2 / 2 100.0%	1 / 1 100.0%	1 / 1 100.0%	2 / 2 100.0%	1 / 1 100.0%	2 / 3 66.7%	1 / 1 100.0%	1 / 1 100.0%	1 / 1 100.0%	1 / 1 100.0%	2 / 2 100.0%	1 / 1 100.0%	2 / 2 100.0%	4 / 4 100.0%	2 / 2 100.0%	1 / 1 100.0%	4 / 4 100.0%	0 / 0 0.0%	0 / 0 0.0%	0 / 0 0.0%	0 / 0 0.0%	17 / 36 47.2%B	7 / 36 19.4%AB	6 / 36 16.7%BE	4 / 36 11.1%ABE	1 / 36 2.8%E	1 / 36 2.8%
1	Helicobacter pylori G27	36 / 36 100.0%	1 / 1 100.0%	1 / 1 100.0%	1 / 1 100.0%	1 / 1 100.0%	2 / 2 100.0%	2 / 2 100.0%	1 / 1 100.0%	1 / 1 100.0%	2 / 2 100.0%	1 / 1 100.0%	3 / 3 100.0%	1 / 1 100.0%	1 / 1 100.0%	1 / 1 100.0%	1 / 1 100.0%	2 / 2 100.0%	1 / 1 100.0%	2 / 2 100.0%	4 / 4 100.0%	2 / 2 100.0%	1 / 1 100.0%	4 / 4 100.0%	0 / 0 0.0%	0 / 0 0.0%	0 / 0 0.0%	0 / 0 0.0%	25 / 36 69.4%AB	11 / 36 30.6%ABE				
1	Helicobacter pylori P12	36 / 36 100.0%	1 / 1 100.0%	1 / 1 100.0%	1 / 1 100.0%	1 / 1 100.0%	2 / 2 100.0%	2 / 2 100.0%	1 / 1 100.0%	1 / 1 100.0%	2 / 2 100.0%	1 / 1 100.0%	3 / 3 100.0%	1 / 1 100.0%	1 / 1 100.0%	1 / 1 100.0%	1 / 1 100.0%	2 / 2 100.0%	1 / 1 100.0%	2 / 2 100.0%	4 / 4 100.0%	2 / 2 100.0%	1 / 1 100.0%	4 / 4 100.0%	0 / 0 0.0%	0 / 0 0.0%	0 / 0 0.0%	0 / 0 0.0%	25 / 36 69.4%AB	11 / 36 30.6%ABE				
1	Helicobacter pylori 35A	36 / 36 100.0%	1 / 1 100.0%	1 / 1 100.0%	1 / 1 100.0%	1 / 1 100.0%	2 / 2 100.0%	2 / 2 100.0%	1 / 1 100.0%	1 / 1 100.0%	2 / 2 100.0%	1 / 1 100.0%	3 / 3 100.0%	1 / 1 100.0%	1 / 1 100.0%	1 / 1 100.0%	1 / 1 100.0%	2 / 2 100.0%	1 / 1 100.0%	2 / 2 100.0%	4 / 4 100.0%	2 / 2 100.0%	1 / 1 100.0%	4 / 4 100.0%	0 / 0 0.0%	0 / 0 0.0%	0 / 0 0.0%	0 / 0 0.0%	25 / 36 69.4%AB	11 / 36 30.6%ABE				
1	Helicobacter pylori 83	36 / 36 100.0%	1 / 1 100.0%	1 / 1 100.0%	1 / 1 100.0%	1 / 1 100.0%	2 / 2 100.0%	2 / 2 100.0%	1 / 1 100.0%	1 / 1 100.0%	2 / 2 100.0%	1 / 1 100.0%	3 / 3 100.0%	1 / 1 100.0%	1 / 1 100.0%	1 / 1 100.0%	1 / 1 100.0%	2 / 2 100.0%	1 / 1 100.0%	2 / 2 100.0%	4 / 4 100.0%	2 / 2 100.0%	1 / 1 100.0%	4 / 4 100.0%	0 / 0 0.0%	0 / 0 0.0%	0 / 0 0.0%	0 / 0 0.0%	25 / 36 69.4%AB	11 / 36 30.6%ABE				
1	Helicobacter pylori B38	36 / 36 100.0%	1 / 1 100.0%	1 / 1 100.0%	1 / 1 100.0%	1 / 1 100.0%	2 / 2 100.0%	2 / 2 100.0%	1 / 1 100.0%	1 / 1 100.0%	2 / 2 100.0%	1 / 1 100.0%	3 / 3 100.0%	1 / 1 100.0%	1 / 1 100.0%	1 / 1 100.0%	1 / 1 100.0%	2 / 2 100.0%	1 / 1 100.0%	2 / 2 100.0%	4 / 4 100.0%	2 / 2 100.0%	1 / 1 100.0%	4 / 4 100.0%	0 / 0 0.0%	0 / 0 0.0%	0 / 0 0.0%	0 / 0 0.0%	25 / 36 69.4%AB	11 / 36 30.6%ABE				
1	Helicobacter pylori str. v225d	36 / 36 100.0%	1 / 1 100.0%	1 / 1 100.0%	1 / 1 100.0%	1 / 1 100.0%	2 / 2 100.0%	2 / 2 100.0%	1 / 1 100.0%	1 / 1 100.0%	2 / 2 100.0%	1 / 1 100.0%	3 / 3 100.0%	1 / 1 100.0%	1 / 1 100.0%	1 / 1 100.0%	1 / 1 100.0%	2 / 2 100.0%	1 / 1 100.0%	2 / 2 100.0%	4 / 4 100.0%	2 / 2 100.0%	1 / 1 100.0%	4 / 4 100.0%	0 / 0 0.0%	0 / 0 0.0%	0 / 0 0.0%	0 / 0 0.0%	25 / 36 69.4%AB	11 / 36 30.6%ABE				
1	Helicobacter pylori 52	36 / 36 100.0%	1 / 1 100.0%	1 / 1 100.0%	1 / 1 100.0%	1 / 1 100.0%	2 / 2 100.0%	2 / 2 100.0%	1 / 1 100.0%	1 / 1 100.0%	2 / 2 100.0%	1 / 1 100.0%	3 / 3 100.0%	1 / 1 100.0%	1 / 1 100.0%	1 / 1 100.0%	1 / 1 100.0%	2 / 2 100.0%	1 / 1 100.0%	2 / 2 100.0%	4 / 4 100.0%	2 / 2 100.0%	1 / 1 100.0%	4 / 4 100.0%	0 / 0 0.0%	0 / 0 0.0%	0 / 0 0.0%	0 / 0 0.0%	25 / 36 69.4%AB	11 / 36 30.6%ABE				
1	Helicobacter pylori B8	36 / 36 100.0%	1 / 1 100.0%	1 / 1 100.0%	1 / 1 100.0%	1 / 1 100.0%	2 / 2 100.0%	2 / 2 100.0%	1 / 1 100.0%	1 / 1 100.0%	2 / 2 100.0%	1 / 1 100.0%	3 / 3 100.0%	1 / 1 100.0%	1 / 1 100.0%	1 / 1 100.0%	1 / 1 100.0%	2 / 2 100.0%	1 / 1 100.0%	2 / 2 100.0%	4 / 4 100.0%	2 / 2 100.0%	1 / 1 100.0%	4 / 4 100.0%	0 / 0 0.0%	0 / 0 0.0%	0 / 0 0.0%	0 / 0 0.0%	25 / 36 69.4%AB	11 / 36 30.6%ABE				
1	Helicobacter pylori SJM180	36 / 36 100.0%	1 / 1 100.0%	1 / 1 100.0%	1 / 1 100.0%	1 / 1 100.0%	2 / 2 100.0%	2 / 2 100.0%	1 / 1 100.0%	1 / 1 100.0%	2 / 2 100.0%	1 / 1 100.0%	3 / 3 100.0%	1 / 1 100.0%	1 / 1 100.0%	1 / 1 100.0%	1 / 1 100.0%	2 / 2 100.0%	1 / 1 100.0%	2 / 2 100.0%	4 / 4 100.0%	2 / 2 100.0%	1 / 1 100.0%	4 / 4 100.0%	0 / 0 0.0%	0 / 0 0.0%	0 / 0 0.0%	0 / 0 0.0%	25 / 36 69.4%AB	11 / 36 30.6%ABE				
1	Helicobacter pylori PeCan4	36 / 36 100.0%	1 / 1 100.0%	1 / 1 100.0%	1 / 1 100.0%	1 / 1 100.0%	2 / 2 100.0%	2 / 2 100.0%	1 / 1 100.0%	1 / 1 100.0%	2 / 2 100.0%	1 / 1 100.0%	3 / 3 100.0%	1 / 1 100.0%	1 / 1 100.0%	1 / 1 100.0%	1 / 1 100.0%	2 / 2 100.0%	1 / 1 100.0%	2 / 2 100.0%	4 / 4 100.0%	2 / 2 100.0%	1 / 1 100.0%	4 / 4 100.0%	0 / 0 0.0%	0 / 0 0.0%	0 / 0 0.0%	0 / 0 0.0%	25 / 36 69.4%AB	11 / 36 30.6%ABE				
1	Helicobacter pylori Cuz20	36 / 36 100.0%	1 / 1 100.0%	1 / 1 100.0%	1 / 1 100.0%	1 / 1 100.0%	2 / 2 100.0%	2 / 2 100.0%	1 / 1 100.0%	1 / 1 100.0%	2 / 2 100.0%	1 / 1 100.0%	3 / 3 100.0%	1 / 1 100.0%	1 / 1 100.0%	1 / 1 100.0%	1 / 1 100.0%	2 / 2 100.0%	1 / 1 100.0%	2 / 2 100.0%	4 / 4 100.0%	2 / 2 100.0%	1 / 1 100.0%	4 / 4 100.0%	0 / 0 0.0%	0 / 0 0.0%	0 / 0 0.0%	0 / 0 0.0%	25 / 36 69.4%AB	11 / 36 30.6%ABE				
1	Helicobacter pylori Sat464	36 / 36 100.0%	1 / 1 100.0%	1 / 1 100.0%	1 / 1 100.0%	1 / 1 100.0%	2 / 2 100.0%	2 / 2 100.0%	1 / 1 100.0%	1 / 1 100.0%	2 / 2 100.0%	1 / 1 100.0%	3 / 3 100.0%	1 / 1 100.0%	1 / 1 100.0%	1 / 1 100.0%	1 / 1 100.0%	2 / 2 100.0%	1 / 1 100.0%	2 / 2 100.0%	4 / 4 100.0%	2 / 2 100.0%	1 / 1 100.0%	4 / 4 100.0%	0 / 0 0.0%	0 / 0 0.0%	0 / 0 0.0%	0 / 0 0.0%	25 / 36 69.4%AB	11 / 36 30.6%ABE				
1	Helicobacter pylori F16	36 / 36 100.0%	1 / 1 100.0%	1 / 1 100.0%	1 / 1 100.0%	1 / 1 100.0%	2 / 2 100.0%	2 / 2 100.0%	1 / 1 100.0%	1 / 1 100.0%	2 / 2 100.0%	1 / 1 100.0%	3 / 3 100.0%	1 / 1 100.0%	1 / 1 100.0%	1 / 1 100.0%	1 / 1 100.0%	2 / 2 100.0%	1 / 1 100.0%	2 / 2 100.0%	4 / 4 100.0%	2 / 2 100.0%	1 / 1 100.0%	4 / 4 100.0%	0 / 0 0.0%	0 / 0 0.0%	0 / 0 0.0%	0 / 0 0.0%	25 / 36 69.4%AB	11 / 36 30.6%ABE				
1	Helicobacter pylori F30	36 / 36 100.0%	1 / 1 100.0%	1 / 1 100.0%	1 / 1 100.0%	1 / 1 100.0%	2 / 2 100.0%	2 / 2 100.0%	1 / 1 100.0%	1 / 1 100.0%	2 / 2 100.0%	1 / 1 100.0%	3 / 3 100.0%	1 / 1 100.0%	1 / 1 100.0%	1 / 1 100.0%	1 / 1 100.0%	2 / 2 100.0%	1 / 1 100.0%	2 / 2 100.0%	4 / 4 100.0%	2 / 2 100.0%	1 / 1 100.0%	4 / 4 100.0%	0 / 0 0.0%	0 / 0 0.0%	0 / 0 0.0%	0 / 0 0.0%	25 / 36 69.4%AB	11 / 36 30.6%ABE				
1	Helicobacter pylori F57	36 / 36 100.0%	1 / 1 100.0%	1 / 1 100.0%	1 / 1 100.0%	1 / 1 100.0%	2 / 2 100.0%	2 / 2 100.0%	1 / 1 100.0%	1 / 1 100.0%	2 / 2 100.0%	1 / 1 100.0%	3 / 3 100.0%	1 / 1 100.0%	1 / 1 100.0%	1 / 1 100.0%	1 / 1 100.0%	2 / 2 100.0%	1 / 1 100.0%	2 / 2 100.0%	4 / 4 100.0%	2 / 2 100.0%	1 / 1 100.0%	4 / 4 100.0%	0 / 0 0.0%	0 / 0 0.0%	0 / 0 0.0%	0 / 0 0.0%	25 / 36 69.4%AB	11 / 36 30.6%ABE				
3	Helicobacter pylori 908	107 / 107 100.0%	3 / 3 100.0%	3 / 3 100.0%	3 / 3 100.0%	3 / 3 100.0%	6 / 6 100.0%	6 / 6 100.0%	3 / 3 100.0%	3 / 3 100.0%	6 / 6 100.0%	3 / 3 100.0%	9 / 9 100.0%	3 / 3 100.0%	3 / 3 100.0%	3 / 3 100.0%	3 / 3 100.0%	6 / 6 100.0%	3 / 3 100.0%	5 / 5 100.0%	12 / 12 100.0%	6 / 6 100.0%	3 / 3 100.0%	12 / 12 100.0%	0 / 0 0.0%	0 / 0 0.0%	0 / 0 0.0%	0 / 0 0.0%	74 / 107 69.2%AB	33 / 107 30.8%ABE				
1	Helicobacter pylori 2018	35 / 35 100.0%	1 / 1 100.0%	1 / 1 100.0%	1 / 1 100.0%	1 / 1 100.0%	2 / 2 100.0%	2 / 2 100.0%	1 / 1 100.0%	1 / 1 100.0%	2 / 2 100.0%	1 / 1 100.0%	3 / 3 100.0%	1 / 1 100.0%	1 / 1 100.0%	1 / 1 100.0%	1 / 1 100.0%	2 / 2 100.0%	1 / 1 100.0%	1 / 1 100.0%	4 / 4 100.0%	2 / 2 100.0%	1 / 1 100.0%	4 / 4 100.0%	0 / 0 0.0%	0 / 0 0.0%	0 / 0 0.0%	0 / 0 0.0%	24 / 35 68.6%AB	11 / 35 31.4%ABE				
1	Helicobacter pylori 2017	36 / 36 100.0%	1 / 1 100.0%	1 / 1 100.0%	1 / 1 100.0%	1 / 1 100.0%	2 / 2 100.0%	2 / 2 100.0%	1 / 1 100.0%	1 / 1 100.0%	2 / 2 100.0%	1 / 1 100.0%	3 / 3 100.0%	1 / 1 100.0%	1 / 1 100.0%	1 / 1 100.0%	1 / 1 100.0%	2 / 2 100.0%	1 / 1 100.0%	2 / 2 100.0%	4 / 4 100.0%	2 / 2 100.0%	1 / 1 100.0%	4 / 4 100.0%	0 / 0 0.0%	0 / 0 0.0%	0 / 0 0.0%	0 / 0 0.0%	25 / 36 69.4%AB	11 / 36 30.6%ABE				
1	Helicobacter pylori Lit75	36 / 36 100.0%	1 / 1 100.0%	1 / 1 100.0%	1 / 1 100.0%	1 / 1 100.0%	2 / 2 100.0%	2 / 2 100.0%	1 / 1 100.0%	1 / 1 100.0%	2 / 2 100.0%	1 / 1 100.0%	3 / 3 100.0%	1 / 1 100.0%	1 / 1 100.0%	1 / 1 100.0%	1 / 1 100.0%	2 / 2 100.0%	1 / 1 100.0%	2 / 2 100.0%	4 / 4 100.0%	2 / 2 100.0%	1 / 1 100.0%	4 / 4 100.0%	0 / 0 0.0%	0 / 0 0.0%	0 / 0 0.0%	0 / 0 0.0%	25 / 36 69.4%AB	11 / 36 30.6%ABE				
1	Helicobacter pylori Ind7	36 / 36 100.0%	1 / 1 100.0%	1 / 1 100.0%	1 / 1 100.0%	1 / 1 100.0%	2 / 2 100.0%	2 / 2 100.0%	1 / 1 100.0%	1 / 1 100.0%	2 / 2 100.0%	1 / 1 100.0%	3 / 3 100.0%	1 / 1 100.0%	1 / 1 100.0%	1 / 1 100.0%	1 / 1 100.0%	2 / 2 100.0%	1 / 1 100.0%	2 / 2 100.0%	4 / 4 100.0%	2 / 2 100.0%	1 / 1 100.0%	4 / 4 100.0%	0 / 0 0.0%	0 / 0 0.0%	0 / 0 0.0%	0 / 0 0.0%	25 / 36 69.4%AB	11 / 36 30.6%ABE				
1	Helicobacter pylori SAfr7	36 / 36 100.0%	1 / 1 100.0%	1 / 1 100.0%	1 / 1 100.0%	1 / 1 100.0%	2 / 2 100.0%	2 / 2 100.0%	1 / 1 100.0%	1 / 1 100.0%	2 / 2 100.0%	1 / 1 100.0%	3 / 3 100.0%	1 / 1 100.0%	1 / 1 100.0%	1 / 1 100.0%	1 / 1 100.0%	2 / 2 100.0%	1 / 1 100.0%	2 / 2 100.0%	4 / 4 100.0%	2 / 2 100.0%	1 / 1 100.0%	4 / 4 100.0%	0 / 0 0.0%	0 / 0 0.0%	0 / 0 0.0%	0 / 0 0.0%	25 / 36 69.4%AB	11 / 36 30.6%ABE				
1	Helicobacter pylori Gam94/24	36 / 36 100.0%	1 / 1 100.0%	1 / 1 100.0%	1 / 1 100.0%	1 / 1 100.0%	2 / 2 100.0%	2 / 2 100.0%	1 / 1 100.0%	1 / 1 100.0%	2 / 2 100.0%	1 / 1 100.0%	3 / 3 100.0%	1 / 1 100.0%	1 / 1 100.0%	1 / 1 100.0%	1 / 1 100.0%	2 / 2 100.0%	1 / 1 100.0%	2 / 2 100.0%	4 / 4 100.0%	2 / 2 100.0%	1 / 1 100.0%	4 / 4 100.0%	0 / 0 0.0%	0 / 0 0.0%	0 / 0 0.0%	0 / 0 0.0%	25 / 36 69.4%AB	11 / 36 30.6%ABE				
1	Helicobacter pylori ELS37	36 / 36 100.0%	1 / 1 100.0%	1 / 1 100.0%	1 / 1 100.0%	1 / 1 100.0%	2 / 2 100.0%	2 / 2 100.0%	1 / 1 100.0%	1 / 1 100.0%	2 / 2 100.0%	1 / 1 100.0%	3 / 3 100.0%	1 / 1 100.0%	1 / 1 100.0%	1 / 1 100.0%	1 / 1 100.0%	2 / 2 100.0%	1 / 1 100.0%	2 / 2 100.0%	4 / 4 100.0%	2 / 2 100.0%	1 / 1 100.0%	4 / 4 100.0%	0 / 0 0.0%	0 / 0 0.0%	0 / 0 0.0%	0 / 0 0.0%	25 / 36 69.4%AB	11 / 36 30.6%ABE				
1	Helicobacter pylori Puno120	36 / 36 100.0%	1 / 1 100.0%	1 / 1 100.0%	1 / 1 100.0%	1 / 1 100.0%	2 / 2 100.0%	2 / 2 100.0%	1 / 1 100.0%	1 / 1 100.0%	2 / 2 100.0%	1 / 1 100.0%	3 / 3 100.0%	1 / 1 100.0%	1 / 1 100.0%	1 / 1 100.0%	1 / 1 100.0%	2 / 2 100.0%	1 / 1 100.0%	2 / 2 100.0%	4 / 4 100.0%	2 / 2 100.0%	1 / 1 100.0%	4 / 4 100.0%	0 / 0 0.0%	0 / 0 0.0%	0 / 0 0.0%	0 / 0 0.0%	25 / 36 69.4%AB	11 / 36 30.6%ABE				
1	Helicobacter pylori Puno135	36 / 36 100.0%	1 / 1 100.0%	1 / 1 100.0%	1 / 1 100.0%	1 / 1 100.0%	2 / 2 100.0%	2 / 2 100.0%	1 / 1 100.0%	1 / 1 100.0%	2 / 2 100.0%	1 / 1 100.0%	3 / 3 100.0%	1 / 1 100.0%	1 / 1 100.0%	1 / 1 100.0%	1 / 1 100.0%	2 / 2 100.0%	1 / 1 100.0%	2 / 2 100.0%	4 / 4 100.0%	2 / 2 100.0%	1 / 1 100.0%	4 / 4 100.0%	0 / 0 0.0%	0 / 0 0.0%	0 / 0 0.0%	0 / 0 0.0%	25 / 36 69.4%AB	11 / 36 30.6%ABE				
1	Helicobacter pylori SNT49	36 / 36 100.0%	1 / 1 100.0%	1 / 1 100.0%	1 / 1 100.0%	1 / 1 100.0%	2 / 2 100.0%	2 / 2 100.0%	1 / 1 100.0%	1 / 1 100.0%	2 / 2 100.0%	1 / 1 100.0%	3 / 3 100.0%	1 / 1 100.0%	1 / 1 100.0%	1 / 1 100.0%	1 / 1 100.0%	2 / 2 100.0%	1 / 1 100.0%	2 / 2 100.0%	4 / 4 100.0%	2 / 2 100.0%	1 / 1 100.0%	4 / 4 100.0%	0 / 0 0.0%	0 / 0 0.0%	0 / 0 0.0%	0 / 0 0.0%	25 / 36 69.4%AB	11 / 36 30.6%ABE				
1	Helicobacter pylori Aklavik117	36 / 36 100.0%	1 / 1 100.0%	1 / 1 100.0%	1 / 1 100.0%	1 / 1 100.0%	2 / 2 100.0%	2 / 2 100.0%	1 / 1 100.0%	1 / 1 100.0%	2 / 2 100.0%	1 / 1 100.0%	3 / 3 100.0%	1 / 1 100.0%	1 / 1 100.0%	1 / 1 100.0%	1 / 1 100.0%	2 / 2 100.0%	1 / 1 100.0%	2 / 2 100.0%	4 / 4 100.0%	2 / 2 100.0%	1 / 1 100.0%	4 / 4 100.0%	0 / 0 0.0%	0 / 0 0.0%	0 / 0 0.0%	0 / 0 0.0%	25 / 36 69.4%AB	11 / 36 30.6%ABE				
1	Helicobacter pylori Aklavik86	36 / 36 100.0%	1 / 1 100.0%	1 / 1 100.0%	1 / 1 100.0%	1 / 1 100.0%	2 / 2 100.0%	2 / 2 100.0%	1 / 1 100.0%	1 / 1 100.0%	2 / 2 100.0%	1 / 1 100.0%	3 / 3 100.0%	1 / 1 100.0%	1 / 1 100.0%	1 / 1 100.0%	1 / 1 100.0%	2 / 2 100.0%	1 / 1 100.0%	2 / 2 100.0%	4 / 4 100.0%	2 / 2 100.0%	1 / 1 100.0%	4 / 4 100.0%	0 / 0 0.0%	0 / 0 0.0%	0 / 0 0.0%	0 / 0 0.0%	25 / 36 69.4%AB	11 / 36 30.6%ABE				
1	Helicobacter pylori XZ274	36 / 36 100.0%	1 / 1 100.0%	1 / 1 100.0%	1 / 1 100.0%	1 / 1 100.0%	2 / 2 100.0%	2 / 2 100.0%	1 / 1 100.0%	1 / 1 100.0%	2 / 2 100.0%	1 / 1 100.0%	3 / 3 100.0%	1 / 1 100.0%	1 / 1 100.0%	1 / 1 100.0%	1 / 1 100.0%	2 / 2 100.0%	1 / 1 100.0%	2 / 2 100.0%	4 / 4 100.0%	2 / 2 100.0%	1 / 1 100.0%	4 / 4 100.0%	0 / 0 0.0%	0 / 0 0.0%	0 / 0 0.0%	0 / 0 0.0%	25 / 36 69.4%AB	11 / 36 30.6%ABE				
1	Helicobacter pylori Shi417	36 / 36 100.0%	1 / 1 100.0%	1 / 1 100.0%	1 / 1 100.0%	1 / 1 100.0%	2 / 2 100.0%	2 / 2 100.0%	1 / 1 100.0%	1 / 1 100.0%	2 / 2 100.0%	1 / 1 100.0%	3 / 3 100.0%	1 / 1 100.0%	1 / 1 100.0%	1 / 1 100.0%	1 / 1 100.0%	2 / 2 100.0%	1 / 1 100.0%	2 / 2 100.0%	4 / 4 100.0%	2 / 2 100.0%	1 / 1 100.0%	4 / 4 100.0%	0 / 0 0.0%	0 / 0 0.0%	0 / 0 0.0%	0 / 0 0.0%	25 / 36 69.4%AB	11 / 36 30.6%ABE				
1	Helicobacter pylori Shi112	36 / 36 100.0%	1 / 1 100.0%	1 / 1 100.0%	1 / 1 100.0%	1 / 1 100.0%	2 / 2 100.0%	2 / 2 100.0%	1 / 1 100.0%	1 / 1 100.0%	2 / 2 100.0%	1 / 1 100.0%	3 / 3 100.0%	1 / 1 100.0%	1 / 1 100.0%	1 / 1 100.0%	1 / 1 100.0%	2 / 2 100.0%	1 / 1 100.0%	2 / 2 100.0%	4 / 4 100.0%	2 / 2 100.0%	1 / 1 100.0%	4 / 4 100.0%	0 / 0 0.0%	0 / 0 0.0%	0 / 0 0.0%	0 / 0 0.0%	25 / 36 69.4%AB	11 / 36 30.6%ABE				
1	Helicobacter pylori Shi169	36 / 36 100.0%	1 / 1 100.0%	1 / 1 100.0%	1 / 1 100.0%	1 / 1 100.0%	2 / 2 100.0%	2 / 2 100.0%	1 / 1 100.0%	1 / 1 100.0%	2 / 2 100.0%	1 / 1 100.0%	3 / 3 100.0%	1 / 1 100.0%	1 / 1 100.0%	1 / 1 100.0%	1 / 1 100.0%	2 / 2 100.0%	1 / 1 100.0%	2 / 2 100.0%	4 / 4 100.0%	2 / 2 100.0%	1 / 1 100.0%	4 / 4 100.0%	0 / 0 0.0%	0 / 0 0.0%	0 / 0 0.0%	0 / 0 0.0%	25 / 36 69.4%AB	11 / 36 30.6%ABE				
1	Helicobacter pylori PeCan18	36 / 36 100.0%	1 / 1 100.0%	1 / 1 100.0%	1 / 1 100.0%	1 / 1 100.0%	2 / 2 100.0%	2 / 2 100.0%	1 / 1 100.0%	1 / 1 100.0%	2 / 2 100.0%	1 / 1 100.0%	3 / 3 100.0%	1 / 1 100.0%	1 / 1 100.0%	1 / 1 100.0%	1 / 1 100.0%	2 / 2 100.0%	1 / 1 100.0%	2 / 2 100.0%	4 / 4 100.0%	2 / 2 100.0%	1 / 1 100.0%	4 / 4 100.0%	0 / 0 0.0%	0 / 0 0.0%	0 / 0 0.0%	0 / 0 0.0%	25 / 36 69.4%AB	11 / 36 30.6%ABE				
1	Helicobacter pylori HUP-B14	36 / 36 100.0%	1 / 1 100.0%	1 / 1 100.0%	1 / 1 100.0%	1 / 1 100.0%	2 / 2 100.0%	2 / 2 100.0%	1 / 1 100.0%	1 / 1 100.0%	2 / 2 100.0%	1 / 1 100.0%	3 / 3 100.0%	1 / 1 100.0%	1 / 1 100.0%	1 / 1 100.0%	1 / 1 100.0%	2 / 2 100.0%	1 / 1 100.0%	2 / 2 100.0%	4 / 4 100.0%	2 / 2 100.0%	1 / 1 100.0%	4 / 4 100.0%	0 / 0 0.0%	0 / 0 0.0%	0 / 0 0.0%	0 / 0 0.0%	25 / 36 69.4%AB	11 / 36 30.6%ABE				
1	Helicobacter pylori Rif1	36 / 36 100.0%	1 / 1 100.0%	1 / 1 100.0%	1 / 1 100.0%	1 / 1 100.0%	2 / 2 100.0%	2 / 2 100.0%	1 / 1 100.0%	1 / 1 100.0%	2 / 2 100.0%	1 / 1 100.0%	3 / 3 100.0%	1 / 1 100.0%	1 / 1 100.0%	1 / 1 100.0%	1 / 1 100.0%	2 / 2 100.0%	1 / 1 100.0%	2 / 2 100.0%	4 / 4 100.0%	2 / 2 100.0%	1 / 1 100.0%	4 / 4 100.0%	0 / 0 0.0%	0 / 0 0.0%	0 / 0 0.0%	0 / 0 0.0%	25 / 36 69.4%AB	11 / 36 30.6%ABE				
1	Helicobacter pylori Rif2	36 / 36 100.0%	1 / 1 100.0%	1 / 1 100.0%	1 / 1 100.0%	1 / 1 100.0%	2 / 2 100.0%	2 / 2 100.0%	1 / 1 100.0%	1 / 1 100.0%	2 / 2 100.0%	1 / 1 100.0%	3 / 3 100.0%	1 / 1 100.0%	1 / 1 100.0%	1 / 1 100.0%	1 / 1 100.0%	2 / 2 100.0%	1 / 1 100.0%	2 / 2 100.0%	4 / 4 100.0%	2 / 2 100.0%	1 / 1 100.0%	4 / 4 100.0%	0 / 0 0.0%	0 / 0 0.0%	0 / 0 0.0%	0 / 0 0.0%	25 / 36 69.4%AB	11 / 36 30.6%ABE				
1	Helicobacter pylori OK113	36 / 36 100.0%	1 / 1 100.0%	1 / 1 100.0%	1 / 1 100.0%	1 / 1 100.0%	2 / 2 100.0%	2 / 2 100.0%	1 / 1 100.0%	1 / 1 100.0%	2 / 2 100.0%	1 / 1 100.0%	3 / 3 100.0%	1 / 1 100.0%	1 / 1 100.0%	1 / 1 100.0%	1 / 1 100.0%	2 / 2 100.0%	1 / 1 100.0%	2 / 2 100.0%	4 / 4 100.0%	2 / 2 100.0%	1 / 1 100.0%	4 / 4 100.0%	0 / 0 0.0%	0 / 0 0.0%	0 / 0 0.0%	0 / 0 0.0%	25 / 36 69.4%AB	11 / 36 30.6%ABE				
1	Helicobacter pylori OK310	36 / 36 100.0%	1 / 1 100.0%	1 / 1 100.0%	1 / 1 100.0%	1 / 1 100.0%	2 / 2 100.0%	2 / 2 100.0%	1 / 1 100.0%	1 / 1 100.0%	2 / 2 100.0%	1 / 1 100.0%	3 / 3 100.0%	1 / 1 100.0%	1 / 1 100.0%	1 / 1 100.0%	1 / 1 100.0%	2 / 2 100.0%	1 / 1 100.0%	2 / 2 100.0%	4 / 4 100.0%	2 / 2 100.0%	1 / 1 100.0%	4 / 4 100.0%	0 / 0 0.0%	0 / 0 0.0%	0 / 0 0.0%	0 / 0 0.0%	25 / 36 69.4%AB	11 / 36 30.6%ABE				
1	Helicobacter pylori UM032	36 / 36 100.0%	1 / 1 100.0%	1 / 1 100.0%	1 / 1 100.0%	1 / 1 100.0%	2 / 2 100.0%	2 / 2 100.0%	1 / 1 100.0%	1 / 1 100.0%	2 / 2 100.0%	1 / 1 100.0%	3 / 3 100.0%	1 / 1 100.0%	1 / 1 100.0%	1 / 1 100.0%	1 / 1 100.0%	2 / 2 100.0%	1 / 1 100.0%	2 / 2 100.0%	4 / 4 100.0%	2 / 2 100.0%	1 / 1 100.0%	4 / 4 100.0%	0 / 0 0.0%	0 / 0 0.0%	0 / 0 0.0%	0 / 0 0.0%	25 / 36 69.4%AB	11 / 36 30.6%ABE				
1	Helicobacter pylori UM299	36 / 36 100.0%	1 / 1 100.0%	1 / 1 100.0%	1 / 1 100.0%	1 / 1 100.0%	2 / 2 100.0%	2 / 2 100.0%	1 / 1 100.0%	1 / 1 100.0%	2 / 2 100.0%	1 / 1 100.0%	3 / 3 100.0%	1 / 1 100.0%	1 / 1 100.0%	1 / 1 100.0%	1 / 1 100.0%	2 / 2 100.0%	1 / 1 100.0%	2 / 2 100.0%	4 / 4 100.0%	2 / 2 100.0%	1 / 1 100.0%	4 / 4 100.0%	0 / 0 0.0%	0 / 0 0.0%	0 / 0 0.0%	0 / 0 0.0%	25 / 36 69.4%AB	11 / 36 30.6%ABE				
1	Helicobacter pylori UM037	36 / 36 100.0%	1 / 1 100.0%	1 / 1 100.0%	1 / 1 100.0%	1 / 1 100.0%	2 / 2 100.0%	2 / 2 100.0%	1 / 1 100.0%	1 / 1 100.0%	2 / 2 100.0%	1 / 1 100.0%	3 / 3 100.0%	1 / 1 100.0%	1 / 1 100.0%	1 / 1 100.0%	1 / 1 100.0%	2 / 2 100.0%	1 / 1 100.0%	2 / 2 100.0%	4 / 4 100.0%	2 / 2 100.0%	1 / 1 100.0%	4 / 4 100.0%	0 / 0 0.0%	0 / 0 0.0%	0 / 0 0.0%	0 / 0 0.0%	25 / 36 69.4%AB	11 / 36 30.6%ABE				
1	Helicobacter pylori UM066	36 / 36 100.0%	1 / 1 100.0%	1 / 1 100.0%	1 / 1 100.0%	1 / 1 100.0%	2 / 2 100.0%	2 / 2 100.0%	1 / 1 100.0%	1 / 1 100.0%	2 / 2 100.0%	1 / 1 100.0%	3 / 3 100.0%	1 / 1 100.0%	1 / 1 100.0%	1 / 1 100.0%	1 / 1 100.0%	2 / 2 100.0%	1 / 1 100.0%	2 / 2 100.0%	4 / 4 100.0%	2 / 2 100.0%	1 / 1 100.0%	4 / 4 100.0%	0 / 0 0.0%	0 / 0 0.0%	0 / 0 0.0%	0 / 0 0.0%	25 / 36 69.4%AB	11 / 36 30.6%ABE				
1	ATCC 51101	36 / 36 100.0%	1 / 1 100.0%	1 / 1 100.0%	1 / 1 100.0%	1 / 1 100.0%	2 / 2 100.0%	2 / 2 100.0%	1 / 1 100.0%	1 / 1 100.0%	2 / 2 100.0%	1 / 1 100.0%	3 / 3 100.0%	1 / 1 100.0%	1 / 1 100.0%	1 / 1 100.0%	1 / 1 100.0%	2 / 2 100.0%	1 / 1 100.0%	2 / 2 100.0%	4 / 4 100.0%	2 / 2 100.0%	1 / 1 100.0%	4 / 4 100.0%	0 / 0 0.0%	0 / 0 0.0%	0 / 0 0.0%	0 / 0 0.0%	25 / 36 69.4%AB	11 / 36 30.6%ABE				
1	Helicobacter acinonychis Sheeba	36 / 36 100.0%	1 / 1 100.0%	1 / 1 100.0%	1 / 1 100.0%	1 / 1 100.0%	2 / 2 100.0%	2 / 2 100.0%	1 / 1 100.0%	1 / 1 100.0%	2 / 2 100.0%	1 / 1 100.0%	3 / 3 100.0%	1 / 1 100.0%	1 / 1 100.0%	1 / 1 100.0%	1 / 1 100.0%	2 / 2 100.0%	1 / 1 100.0%	2 / 2 100.0%	4 / 4 100.0%	2 / 2 100.0%	1 / 1 100.0%	4 / 4 100.0%	0 / 0 0.0%	0 / 0 0.0%	0 / 0 0.0%	0 / 0 0.0%	25 / 36 69.4%AB	11 / 36 30.6%ABE				
2	"Helicobacter westmeadii" Trivett-Moore et al. 1997	78 / 80 97.5%	2 / 2 100.0%	2 / 2 100.0%	2 / 2 100.0%	4 / 4 100.0%	4 / 4 100.0%	4 / 4 100.0%	2 / 2 100.0%	2 / 2 100.0%	6 / 6 100.0%	2 / 2 100.0%	6 / 6 100.0%	4 / 4 100.0%	2 / 2 100.0%	2 / 2 100.0%	2 / 2 100.0%	4 / 4 100.0%	2 / 2 100.0%	4 / 4 100.0%	8 / 8 100.0%	4 / 4 100.0%	2 / 2 100.0%	8 / 8 100.0%	0 / 2 0.0%	0 / 0 0.0%	0 / 0 0.0%	0 / 0 0.0%	58 / 80 72.5%AB	20 / 80 25.0%ABE	2 / 80 2.5%E			
[illegible]	Helicobacter cinaedi PAGU611	39 / 40	1 / 1	1 / 1	1 / 1	2 / 2	2 / 2	2 / 2	1 / 1	1 / 1	3 / 3	1 / 1	3 / 3	2 / 2	1 / 1	1 / 1	1 / 1	2 / 2	1 / 1	2 / 2	4 / 4	2 / 2	1 / 1	4 / 4	0 / 1	0 / 0	0 / 0	0 / 0	29 / 40	10 / 40	1 / 40			

1	Helicobacter cinaedi ATCC BAA-487	39 / 40 97.5%	1 / 1 100.0%	1 / 1 100.0%	1 / 1 100.0%	2 / 2 100.0%	2 / 2 100.0%	2 / 2 100.0%	1 / 1 100.0%	1 / 1 100.0%	3 / 3 100.0%	1 / 1 100.0%	3 / 3 100.0%	2 / 2 100.0%	1 / 1 100.0%	1 / 1 100.0%	1 / 1 100.0%	2 / 2 100.0%	1 / 1 100.0%	2 / 2 100.0%	4 / 4 100.0%	2 / 2 100.0%	1 / 1 100.0%	4 / 4 100.0%	0 / 1 0.0%	0 / 0 0.0%	0 / 0 0.0%	0 / 0 0.0%	29 / 40 72.5%AB	10 / 40 25.0%ABE	1 / 40 2.5%E						
1	ATCC 49179	36 / 37 97.3%	1 / 1 100.0%	1 / 1 100.0%	1 / 1 100.0%	1 / 1 100.0%	2 / 2 100.0%	2 / 2 100.0%	1 / 1 100.0%	1 / 1 100.0%	2 / 2 100.0%	1 / 1 100.0%	3 / 3 100.0%	1 / 1 100.0%	1 / 1 100.0%	1 / 1 100.0%	1 / 1 100.0%	2 / 2 100.0%	1 / 1 100.0%	2 / 2 100.0%	4 / 4 100.0%	2 / 2 100.0%	1 / 1 100.0%	4 / 4 100.0%	0 / 1 0.0%	0 / 0 0.0%	0 / 0 0.0%	0 / 0 0.0%	24 / 37 64.9%AB	12 / 37 32.4%ABE	1 / 37 2.7%E						
1	Helicobacter felis ATCC 49179	36 / 37 97.3%	1 / 1 100.0%	1 / 1 100.0%	1 / 1 100.0%	1 / 1 100.0%	2 / 2 100.0%	2 / 2 100.0%	1 / 1 100.0%	1 / 1 100.0%	2 / 2 100.0%	1 / 1 100.0%	3 / 3 100.0%	1 / 1 100.0%	1 / 1 100.0%	1 / 1 100.0%	1 / 1 100.0%	2 / 2 100.0%	1 / 1 100.0%	2 / 2 100.0%	4 / 4 100.0%	2 / 2 100.0%	1 / 1 100.0%	4 / 4 100.0%	0 / 1 0.0%	0 / 0 0.0%	0 / 0 0.0%	0 / 0 0.0%	24 / 37 64.9%AB	12 / 37 32.4%ABE	1 / 37 2.7%E						
1	ATCC 43772	38 / 38 100.0%	1 / 1 100.0%	1 / 1 100.0%	1 / 1 100.0%	2 / 2 100.0%	2 / 2 100.0%	2 / 2 100.0%	1 / 1 100.0%	1 / 1 100.0%	3 / 3 100.0%	1 / 1 100.0%	3 / 3 100.0%	1 / 1 100.0%	1 / 1 100.0%	1 / 1 100.0%	1 / 1 100.0%	2 / 2 100.0%	1 / 1 100.0%	2 / 2 100.0%	4 / 4 100.0%	1 / 1 100.0%	1 / 1 100.0%	4 / 4 100.0%	0 / 0 0.0%	0 / 0 0.0%	1 / 1 100.0%	0 / 0 0.0%	28 / 38 73.7%AB	10 / 38 26.3%ABE							
1	Helicobacter mustelae 12198	38 / 38 100.0%	1 / 1 100.0%	1 / 1 100.0%	1 / 1 100.0%	2 / 2 100.0%	2 / 2 100.0%	2 / 2 100.0%	1 / 1 100.0%	1 / 1 100.0%	3 / 3 100.0%	1 / 1 100.0%	3 / 3 100.0%	1 / 1 100.0%	1 / 1 100.0%	1 / 1 100.0%	1 / 1 100.0%	2 / 2 100.0%	1 / 1 100.0%	2 / 2 100.0%	4 / 4 100.0%	1 / 1 100.0%	1 / 1 100.0%	4 / 4 100.0%	0 / 0 0.0%	0 / 0 0.0%	1 / 1 100.0%	0 / 0 0.0%	28 / 38 73.7%AB	10 / 38 26.3%ABE							
1	ATCC 51448	37 / 38 97.4%	1 / 1 100.0%	1 / 1 100.0%	1 / 1 100.0%	1 / 1 100.0%	2 / 2 100.0%	2 / 2 100.0%	1 / 1 100.0%	1 / 1 100.0%	2 / 2 100.0%	1 / 1 100.0%	3 / 3 100.0%	2 / 2 100.0%	1 / 1 100.0%	1 / 1 100.0%	1 / 1 100.0%	2 / 2 100.0%	1 / 1 100.0%	2 / 2 100.0%	4 / 4 100.0%	2 / 2 100.0%	1 / 1 100.0%	4 / 4 100.0%	0 / 1 0.0%	0 / 0 0.0%	0 / 0 0.0%	0 / 0 0.0%	27 / 38 71.1%AB	10 / 38 26.3%ABE	1 / 38 2.6%E						
1	Helicobacter hepaticus ATCC 51449	37 / 38 97.4%	1 / 1 100.0%	1 / 1 100.0%	1 / 1 100.0%	1 / 1 100.0%	2 / 2 100.0%	2 / 2 100.0%	1 / 1 100.0%	1 / 1 100.0%	2 / 2 100.0%	1 / 1 100.0%	3 / 3 100.0%	2 / 2 100.0%	1 / 1 100.0%	1 / 1 100.0%	1 / 1 100.0%	2 / 2 100.0%	1 / 1 100.0%	2 / 2 100.0%	4 / 4 100.0%	2 / 2 100.0%	1 / 1 100.0%	4 / 4 100.0%	0 / 1 0.0%	0 / 0 0.0%	0 / 0 0.0%	0 / 0 0.0%	27 / 38 71.1%AB	10 / 38 26.3%ABE	1 / 38 2.6%E						
1	CCUG 35045	36 / 37 97.3%	1 / 1 100.0%	1 / 1 100.0%	1 / 1 100.0%	1 / 1 100.0%	2 / 2 100.0%	2 / 2 100.0%	1 / 1 100.0%	1 / 1 100.0%	2 / 2 100.0%	1 / 1 100.0%	3 / 3 100.0%	1 / 1 100.0%	1 / 1 100.0%	1 / 1 100.0%	1 / 1 100.0%	2 / 2 100.0%	1 / 1 100.0%	2 / 2 100.0%	4 / 4 100.0%	2 / 2 100.0%	1 / 1 100.0%	4 / 4 100.0%	0 / 1 0.0%	0 / 0 0.0%	0 / 0 0.0%	0 / 0 0.0%	26 / 37 70.3%AB	10 / 37 27.0%ABE	1 / 37 2.7%E						
1	Helicobacter bizzozeronii CIII-1	36 / 37 97.3%	1 / 1 100.0%	1 / 1 100.0%	1 / 1 100.0%	1 / 1 100.0%	2 / 2 100.0%	2 / 2 100.0%	1 / 1 100.0%	1 / 1 100.0%	2 / 2 100.0%	1 / 1 100.0%	3 / 3 100.0%	1 / 1 100.0%	1 / 1 100.0%	1 / 1 100.0%	1 / 1 100.0%	2 / 2 100.0%	1 / 1 100.0%	2 / 2 100.0%	4 / 4 100.0%	2 / 2 100.0%	1 / 1 100.0%	4 / 4 100.0%	0 / 1 0.0%	0 / 0 0.0%	0 / 0 0.0%	0 / 0 0.0%	26 / 37 70.3%AB	10 / 37 27.0%ABE	1 / 37 2.7%E						
2	ATCC BAA-540	74 / 76 97.4%	2 / 2 100.0%	2 / 2 100.0%	2 / 2 100.0%	3 / 3 100.0%	4 / 4 100.0%	4 / 4 100.0%	2 / 2 100.0%	2 / 2 100.0%	5 / 5 100.0%	2 / 2 100.0%	6 / 6 100.0%	2 / 2 100.0%	2 / 2 100.0%	2 / 2 100.0%	2 / 2 100.0%	4 / 4 100.0%	2 / 2 100.0%	4 / 4 100.0%	8 / 8 100.0%	4 / 4 100.0%	2 / 2 100.0%	8 / 8 100.0%	0 / 2 0.0%	0 / 0 0.0%	0 / 0 0.0%	0 / 0 0.0%	52 / 76 68.4%AB	22 / 76 28.9%ABE	2 / 76 2.6%E						
1	Helicobacter cetorum MIT 00-7128	38 / 39 97.4%	1 / 1 100.0%	1 / 1 100.0%	1 / 1 100.0%	2 / 2 100.0%	2 / 2 100.0%	2 / 2 100.0%	1 / 1 100.0%	1 / 1 100.0%	3 / 3 100.0%	1 / 1 100.0%	3 / 3 100.0%	1 / 1 100.0%	1 / 1 100.0%	1 / 1 100.0%	1 / 1 100.0%	2 / 2 100.0%	1 / 1 100.0%	2 / 2 100.0%	4 / 4 100.0%	2 / 2 100.0%	1 / 1 100.0%	4 / 4 100.0%	0 / 1 0.0%	0 / 0 0.0%	0 / 0 0.0%	0 / 0 0.0%	27 / 39 69.2%AB	11 / 39 28.2%ABE	1 / 39 2.6%E						
1	Helicobacter cetorum MIT 99-5656	36 / 37 97.3%	1 / 1 100.0%	1 / 1 100.0%	1 / 1 100.0%	1 / 1 100.0%	2 / 2 100.0%	2 / 2 100.0%	1 / 1 100.0%	1 / 1 100.0%	2 / 2 100.0%	1 / 1 100.0%	3 / 3 100.0%	1 / 1 100.0%	1 / 1 100.0%	1 / 1 100.0%	1 / 1 100.0%	2 / 2 100.0%	1 / 1 100.0%	2 / 2 100.0%	4 / 4 100.0%	2 / 2 100.0%	1 / 1 100.0%	4 / 4 100.0%	0 / 1 0.0%	0 / 0 0.0%	0 / 0 0.0%	0 / 0 0.0%	25 / 37 67.6%AB	11 / 37 29.7%ABE	1 / 37 2.7%E						
1	Wolinella	40 / 41 97.6%	1 / 1 100.0%	1 / 1 100.0%	1 / 1 100.0%	3 / 3 100.0%	2 / 2 100.0%	2 / 2 100.0%	1 / 1 100.0%	1 / 1 100.0%	4 / 4 100.0%	1 / 1 100.0%	3 / 3 100.0%	1 / 1 100.0%	1 / 1 100.0%	1 / 1 100.0%	1 / 1 100.0%	2 / 2 100.0%	1 / 1 100.0%	2 / 2 100.0%	4 / 4 100.0%	2 / 2 100.0%	1 / 1 100.0%	4 / 4 100.0%	0 / 1 0.0%	0 / 0 0.0%	0 / 0 0.0%	0 / 0 0.0%	30 / 41 73.2%AB	10 / 41 24.4%ABE	1 / 41 2.4%E						
1	ATCC 29543	40 / 41 97.6%	1 / 1 100.0%	1 / 1 100.0%	1 / 1 100.0%	3 / 3 100.0%	2 / 2 100.0%	2 / 2 100.0%	1 / 1 100.0%	1 / 1 100.0%	4 / 4 100.0%	1 / 1 100.0%	3 / 3 100.0%	1 / 1 100.0%	1 / 1 100.0%	1 / 1 100.0%	1 / 1 100.0%	2 / 2 100.0%	1 / 1 100.0%	2 / 2 100.0%	4 / 4 100.0%	2 / 2 100.0%	1 / 1 100.0%	4 / 4 100.0%	0 / 1 0.0%	0 / 0 0.0%	0 / 0 0.0%	0 / 0 0.0%	30 / 41 73.2%AB	10 / 41 24.4%ABE	1 / 41 2.4%E						
1	Wolinella succinogenes DSM 1740	40 / 41 97.6%	1 / 1 100.0%	1 / 1 100.0%	1 / 1 100.0%	3 / 3 100.0%	2 / 2 100.0%	2 / 2 100.0%	1 / 1 100.0%	1 / 1 100.0%	4 / 4 100.0%	1 / 1 100.0%	3 / 3 100.0%	1 / 1 100.0%	1 / 1 100.0%	1 / 1 100.0%	1 / 1 100.0%	2 / 2 100.0%	1 / 1 100.0%	2 / 2 100.0%	4 / 4 100.0%	2 / 2 100.0%	1 / 1 100.0%	4 / 4 100.0%	0 / 1 0.0%	0 / 0 0.0%	0 / 0 0.0%	0 / 0 0.0%	30 / 41 73.2%AB	10 / 41 24.4%ABE	1 / 41 2.4%E						
2	Sulfromonas	87 / 87 100.0%	4 / 4 100.0%	4 / 4 100.0%	4 / 4 100.0%	3 / 3 100.0%	4 / 4 100.0%	6 / 6 100.0%	2 / 2 100.0%	2 / 2 100.0%	5 / 5 100.0%	2 / 2 100.0%	6 / 6 100.0%	4 / 4 100.0%	4 / 4 100.0%	3 / 3 100.0%	2 / 2 100.0%	2 / 2 100.0%	2 / 2 100.0%	5 / 5 100.0%	7 / 7 100.0%	6 / 6 100.0%	2 / 2 100.0%	8 / 8 100.0%	0 / 0 0.0%	0 / 0 0.0%	0 / 0 0.0%	0 / 0 0.0%	58 / 87 66.7%AB	29 / 87 33.3%ABE							
1	ATCC 33889	44 / 44 100.0%	2 / 2 100.0%	2 / 2 100.0%	2 / 2 100.0%	1 / 1 100.0%	2 / 2 100.0%	3 / 3 100.0%	1 / 1 100.0%	1 / 1 100.0%	3 / 3 100.0%	1 / 1 100.0%	3 / 3 100.0%	2 / 2 100.0%	2 / 2 100.0%	2 / 2 100.0%	1 / 1 100.0%	1 / 1 100.0%	1 / 1 100.0%	3 / 3 100.0%	3 / 3 100.0%	3 / 3 100.0%	1 / 1 100.0%	4 / 4 100.0%	0 / 0 0.0%	0 / 0 0.0%	0 / 0 0.0%	0 / 0 0.0%	30 / 44 68.2%AB	14 / 44 31.8%ABE							
1	Sulfurimonas denitrificans DSM 1251	44 / 44 100.0%	2 / 2 100.0%	2 / 2 100.0%	2 / 2 100.0%	1 / 1 100.0%	2 / 2 100.0%	3 / 3 100.0%	1 / 1 100.0%	1 / 1 100.0%	3 / 3 100.0%	1 / 1 100.0%	3 / 3 100.0%	2 / 2 100.0%	2 / 2 100.0%	2 / 2 100.0%	1 / 1 100.0%	1 / 1 100.0%	1 / 1 100.0%	3 / 3 100.0%	3 / 3 100.0%	3 / 3 100.0%	1 / 1 100.0%	4 / 4 100.0%	0 / 0 0.0%	0 / 0 0.0%	0 / 0 0.0%	0 / 0 0.0%	30 / 44 68.2%AB	14 / 44 31.8%ABE							
1	ATCC BAA-671	43 / 43 100.0%	2 / 2 100.0%	2 / 2 100.0%	2 / 2 100.0%	2 / 2 100.0%	2 / 2 100.0%	3 / 3 100.0%	1 / 1 100.0%	1 / 1 100.0%	2 / 2 100.0%	1 / 1 100.0%	3 / 3 100.0%	2 / 2 100.0%	2 / 2 100.0%	1 / 1 100.0%	1 / 1 100.0%	1 / 1 100.0%	1 / 1 100.0%	2 / 2 100.0%	4 / 4 100.0%	3 / 3 100.0%	1 / 1 100.0%	4 / 4 100.0%	0 / 0 0.0%	0 / 0 0.0%	0 / 0 0.0%	0 / 0 0.0%	28 / 43 65.1%AB	15 / 43 34.9%ABE							
1	Sulfurimonas autotrophica DSM 16294	43 / 43 100.0%	2 / 2 100.0%	2 / 2 100.0%	2 / 2 100.0%	2 / 2 100.0%	2 / 2 100.0%	3 / 3 100.0%	1 / 1 100.0%	1 / 1 100.0%	2 / 2 100.0%	1 / 1 100.0%	3 / 3 100.0%	2 / 2 100.0%	2 / 2 100.0%	1 / 1 100.0%	1 / 1 100.0%	1 / 1 100.0%	1 / 1 100.0%	2 / 2 100.0%	4 / 4 100.0%	3 / 3 100.0%	1 / 1 100.0%	4 / 4 100.0%	0 / 0 0.0%	0 / 0 0.0%	0 / 0 0.0%	0 / 0 0.0%	28 / 43 65.1%AB	15 / 43 34.9%ABE							
1	Sulfuricurvum	49 / 49 100.0%	2 / 2 100.0%	2 / 2 100.0%	2 / 2 100.0%	3 / 3 100.0%	3 / 3 100.0%	3 / 3 100.0%	1 / 1 100.0%	1 / 1 100.0%	4 / 4 100.0%	1 / 1 100.0%	3 / 3 100.0%	2 / 2 100.0%	1 / 1 100.0%	2 / 2 100.0%	1 / 1 100.0%	2 / 2 100.0%	1 / 1 100.0%	3 / 3 100.0%	4 / 4 100.0%	3 / 3 100.0%	1 / 1 100.0%	4 / 4 100.0%	0 / 0 0.0%	0 / 0 0.0%	0 / 0 0.0%	0 / 0 0.0%	36 / 49 73.5%AB	13 / 49 26.5%ABE							
1	ATCC BAA-921	49 / 49 100.0%	2 / 2 100.0%	2 / 2 100.0%	2 / 2 100.0%	3 / 3 100.0%	3 / 3 100.0%	3 / 3 100.0%	1 / 1 100.0%	1 / 1 100.0%	4 / 4 100.0%	1 / 1 100.0%	3 / 3 100.0%	2 / 2 100.0%	1 / 1 100.0%	2 / 2 100.0%	1 / 1 100.0%	2 / 2 100.0%	1 / 1 100.0%	3 / 3 100.0%	4 / 4 100.0%	3 / 3 100.0%	1 / 1 100.0%	4 / 4 100.0%	0 / 0 0.0%	0 / 0 0.0%	0 / 0 0.0%	0 / 0 0.0%	36 / 49 73.5%AB	13 / 49 26.5%ABE							
1	Sulfuricurvum kujiense DSM 16994	49 / 49 100.0%	2 / 2 100.0%	2 / 2 100.0%	2 / 2 100.0%	3 / 3 100.0%	3 / 3 100.0%	3 / 3 100.0%	1 / 1 100.0%	1 / 1 100.0%	4 / 4 100.0%	1 / 1 100.0%	3 / 3 100.0%	2 / 2 100.0%	1 / 1 100.0%	2 / 2 100.0%	1 / 1 100.0%	2 / 2 100.0%	1 / 1 100.0%	3 / 3 100.0%	4 / 4 100.0%	3 / 3 100.0%	1 / 1 100.0%	4 / 4 100.0%	0 / 0 0.0%	0 / 0 0.0%	0 / 0 0.0%	0 / 0 0.0%	36 / 49 73.5%AB	13 / 49 26.5%ABE							
22	Campylobacter group	1030 / 1055 97.6%	29 / 29 100.0%	42 / 42 100.0%	30 / 30 100.0%	73 / 73 100.0%	74 / 74 100.0%	62 / 62 100.0%	23 / 23 100.0%	27 / 27 100.0%	92 / 93 98.9%	23 / 23 100.0%	66 / 66 100.0%	48 / 50 96.0%	27 / 27 100.0%	30 / 30 100.0%	23 / 23 100.0%	26 / 26 100.0%	24 / 24 100.0%	52 / 52 100.0%	98 / 99 99.0%	41 / 43 95.3%	23 / 23 100.0%	96 / 96 100.0%	0 / 19 0.0%	0 / 0 0.0%	1 / 1 100.0%	0 / 0 0.0%	651 / 1055 61.7%AB	240 / 1055 22.7%ABE	106 / 1055 10.0%B	33 / 1055 3.1%BE	17 / 1055 1.6%	8 / 1055 0.8%E			
16	Ampylobacter	729 / 752 96.9%	17 / 17 100.0%	32 / 32 100.0%	19 / 19 100.0%	49 / 49 100.0%	51 / 51 100.0%	49 / 49 100.0%	17 / 17 100.0%	17 / 17 100.0%	66 / 67 98.5%	17 / 17 100.0%	50 / 50 100.0%	35 / 37 94.6%	17 / 17 100.0%	17 / 17 100.0%	17 / 17 100.0%	18 / 18 100.0%	17 / 17 100.0%	34 / 34 100.0%	79 / 80 98.8%	26 / 28 92.9%	17 / 17 100.0%	68 / 68 100.0%	0 / 17 0.0%	0 / 0 0.0%	0 / 0 0.0%	0 / 0 0.0%	454 / 752 60.4%AB	161 / 752 21.4%ABE	85 / 752 11.3%B	29 / 752 3.9%BE	15 / 752 2.0%	8 / 752 1.1%E			
1	"Spirillum fetus" Lehmann and Neumann 1927	43 / 44 97.7%	1 / 1 100.0%	2 / 2 100.0%	2 / 2 100.0%	1 / 1 100.0%	3 / 3 100.0%	3 / 3 100.0%	1 / 1 100.0%	1 / 1 100.0%	3 / 3 100.0%	1 / 1 100.0%	3 / 3 100.0%	3 / 3 100.0%	1 / 1 100.0%	1 / 1 100.0%	1 / 1 100.0%	1 / 1 100.0%	1 / 1 100.0%	2 / 2 100.0%	4 / 4 100.0%	3 / 3 100.0%	1 / 1 100.0%	4 / 4 100.0%	0 / 1 0.0%	0 / 0 0.0%	0 / 0 0.0%	0 / 0 0.0%	31 / 44 70.5%AB	12 / 44 27.3%ABE	1 / 44 2.3%E						
1	"Vibrio fetus subsp. intestinalis" Florent 1959	43 / 44 97.7%	1 / 1 100.0%	2 / 2 100.0%	2 / 2 100.0%	1 / 1 100.0%	3 / 3 100.0%	3 / 3 100.0%	1 / 1 100.0%	1 / 1 100.0%	3 / 3 100.0%	1 / 1 100.0%	3 / 3 100.0%	3 / 3 100.0%	1 / 1 100.0%	1 / 1 100.0%	1 / 1 100.0%	1 / 1 100.0%	1 / 1 100.0%	2 / 2 100.0%	4 / 4 100.0%	3 / 3 100.0%	1 / 1 100.0%	4 / 4 100.0%	0 / 1 0.0%	0 / 0 0.0%	0 / 0 0.0%	0 / 0 0.0%	31 / 44 70.5%AB	12 / 44 27.3%ABE	1 / 44 2.3%E						
1	Campylobacter fetus subsp. fetus 82-40	43 / 44 97.7%	1 / 1 100.0%	2 / 2 100.0%	2 / 2 100.0%	1 / 1 100.0%	3 / 3 100.0%	3 / 3 100.0%	1 / 1 100.0%	1 / 1 100.0%	3 / 3 100.0%	1 / 1 100.0%	3 / 3 100.0%	3 / 3 100.0%	1 / 1 100.0%	1 / 1 100.0%	1 / 1 100.0%	1 / 1 100.0%	1 / 1 100.0%	2 / 2 100.0%	4 / 4 100.0%	3 / 3 100.0%	1 / 1 100.0%	4 / 4 100.0%	0 / 1 0.0%	0 / 0 0.0%	0 / 0 0.0%	0 / 0 0.0%	31 / 44 70.5%AB	12 / 44 27.3%ABE	1 / 44 2.3%E						
11	"Campylobacter fetus subsp. jejuni" Smibert 1974	513 / 525 97.7%	12 / 12 100.0%	23 / 23 100.0%	12 / 12 100.0%	36 / 36 100.0%	36 / 36 100.0%	35 / 35 100.0%	12 / 12 100.0%	12 / 12 100.0%	48 / 48 100.0%	12 / 12 100.0%	36 / 36 100.0%	23 / 23 100.0%	12 / 12 100.0%	12 / 12 100.0%	12 / 12 100.0%	12 / 12 100.0%	12 / 12 100.0%	24 / 24 100.0%	60 / 60 100.0%	12 / 12 100.0%	12 / 12 100.0%	48 / 48 100.0%	0 / 12 0.0%	0 / 0 0.0%	0 / 0 0.0%	0 / 0 0.0%	345 / 525 65.7%AB	125 / 525 23.8%ABE	36 / 525 6.9%B	12 / 525 2.3%	7 / 525 1.3%BE				
1	ATCC 49349	43 / 44 97.7%	1 / 1 100.0%	2 / 2 100.0%	1 / 1 100.0%	3 / 3 100.0%	3 / 3 100.0%	3 / 3 100.0%	1 / 1 100.0%	1 / 1 100.0%	4 / 4 100.0%	1 / 1 100.0%	3 / 3 100.0%	2 / 2 100.0%	1 / 1 100.0%	1 / 1 100.0%	1 / 1 100.0%	1 / 1 100.0%	1 / 1 100.0%	2 / 2 100.0%	5 / 5 100.0%	1 / 1 100.0%	1 / 1 100.0%	4 / 4 100.0%	0 / 1 0.0%	0 / 0 0.0%	0 / 0 0.0%	0 / 0 0.0%	22 / 44 50.0%B	10 / 44 22.7%AB	8 / 44 18.2%ABE	3 / 44 6.8%BE	1 / 44 2.3%				
1	Campylobacter jejuni subsp. doylei 269.97	43 / 44 97.7%	1 / 1 100.0%	2 / 2 100.0%	1 / 1 100.0%	3 / 3 100.0%	3 / 3 100.0%	3 / 3 100.0%	1 / 1 100.0%	1 / 1 100.0%	4 / 4 100.0%	1 / 1 100.0%	3 / 3 100.0%	2 / 2 100.0%	1 / 1 100.0%	1 / 1 100.0%	1 / 1 100.0%	1 / 1 100.0%	1 / 1 100.0%	2 / 2 100.0%	5 / 5 100.0%	1 / 1 100.0%	1 / 1 100.0%	4 / 4 100.0%	0 / 1 0.0%	0 / 0 0.0%	0 / 0 0.0%	0 / 0 0.0%	22 / 44 50.0%B	10 / 44 22.7%AB	8 / 44 18.2%ABE	3 / 44 6.8%BE	1 / 44 2.3%				
9	Campylobacter jejuni jejuni	427 / 437 97.7%	10 / 10 100.0%	19 / 19 100.0%	10 / 10 100.0%	30 / 30 100.0%	30 / 30 100.0%	29 / 29 100.0%	10 / 10 100.0%	10 / 10 100.0%	40 / 40 100.0%	10 / 10 100.0%	30 / 30 100.0%	19 / 19 100.0%	10 / 10 100.0%	10 / 10 100.0%	10 / 10 100.0%	10 / 10 100.0%	10 / 10 100.0%	20 / 20 100.0%	50 / 50 100.0%	10 / 10 100.0%	10 / 10 100.0%	40 / 40 100.0%	0 / 10 0.0%	0 / 0 0.0%	0 / 0 0.0%	0 / 0 0.0%	303 / 437 69.3%AB	106 / 437 24.3%ABE	14 / 437 3.2%B	10 / 437 2.3%	4 / 437 0.9%BE				
2	Campylobacter jejuni subsp. jejuni ATCC 700819	86 / 88 97.7%	2 / 2 100.0%	4 / 4 100.0%	2 / 2 100.0%	6 / 6 100.0%	6 / 6 100.0%	6 / 6 100.0%	2 / 2 100.0%	2 / 2 100.0%	8 / 8 100.0%	2 / 2 100.0%	6 / 6 100.0%	4 / 4 100.0%	2 / 2 100.0%	2 / 2 100.0%	2 / 2 100.0%	2 / 2 100.0%	2 / 2 100.0%	4 / 4 100.0%	10 / 10 100.0%	2 / 2 100.0%	2 / 2 100.0%	8 / 8 100.0%	0 / 2 0.0%	0 / 0 0.0%	0 / 0 0.0%	0 / 0 0.0%	64 / 88 72.7%AB	22 / 88 25.0%ABE	2 / 88 2.3%						
1	Campylobacter jejuni subsp. jejuni NCTC 11168-BN148	43 / 44 97.7%	1 / 1 100.0%	2 / 2 100.0%	1 / 1 100.0%	3 / 3 100.0%	3 / 3 100.0%	3 / 3 100.0%	1 / 1 100.0%	1 / 1 100.0%	4 / 4 100.0%	1 / 1 100.0%	3 / 3 100.0%	2 / 2 100.0%	1 / 1 100.0%	1 / 1 100.0%	1 / 1 100.0%	1 / 1 100.0%	1 / 1 100.0%	2 / 2 100.0%	5 / 5 100.0%	1 / 1 100.0%	1 / 1 100.0%	4 / 4 100.0%	0 / 1 0.0%	0 / 0 0.0%	0 / 0 0.0%	0 / 0 0.0%	32 / 44 72.7%AB	11 / 44 25.0%ABE	1 / 44 2.3%						
1	Campylobacter jejuni subsp. jejuni 81-176	43 / 44 97.7%	1 / 1 100.0%	2 / 2 100.0%	1 / 1 100.0%	3 / 3 100.0%	3 / 3 100.0%	3 / 3 100.0%	1 / 1 100.0%	1 / 1 100.0%	4 / 4 100.0%	1 / 1 100.0%	3 / 3 100.0%	2 / 2 100.0%	1 / 1 100.0%	1 / 1 100.0%	1 / 1 100.0%	1 / 1 100.0%	1 / 1 100.0%	2 / 2 100.0%	5 / 5 100.0%	1 / 1 100.0%	1 / 1 100.0%	4 / 4 100.0%	0 / 1 0.0%	0 / 0 0.0%	0 / 0 0.0%	0 / 0 0.0%	32 / 44 72.7%AB	11 / 44 25.0%ABE	1 / 44 2.3%						
1	Campylobacter jejuni subsp. jejuni 81116	43 / 44 97.7%	1 / 1 100.0%	2 / 2 100.0%	1 / 1 100.0%	3 / 3 100.0%	3 / 3 100.0%	3 / 3 100.0%	1 / 1 100.0%	1 / 1 100.0%	4 / 4 100.0%	1 / 1 100.0%	3 / 3 100.0%	2 / 2 100.0%	1 / 1 100.0%	1 / 1 100.0%	1 / 1 100.0%	1 / 1 100.0%	1 / 1 100.0%	2 / 2 100.0%	5 / 5 100.0%	1 / 1 100.0%	1 / 1 100.0%	4 / 4 100.0%	0 / 1 0.0%	0 / 0 0.0%	0 / 0 0.0%	0 / 0 0.0%	18 / 44 40.9%AB	14 / 44 31.8%B	7 / 44 15.9%ABE	4 / 44 9.1%BE	1 / 44 2.3%				
1	Campylobacter jejuni subsp. jejuni IA3902	43 / 44 97.7%	1 / 1 100.0%	2 / 2 100.0%	1 / 1 100.0%	3 / 3 100.0%	3 / 3 100.0%	3 / 3 100.0%	1 / 1 100.0%	1 / 1 100.0%	4 / 4 100.0%	1 / 1 100.0%	3 / 3 100.0%	2 / 2 100.0%	1 / 1 100.0%	1 / 1 100.0%	1 / 1 100.0%	1 / 1 100.0%	1 / 1 100.0%	2 / 2 100.0%	5 / 5 100.0%	1 / 1 100.0%	1 / 1 100.0%	4 / 4 100.0%	0 / 1 0.0%	0 / 0 0.0%	0 / 0 0.0%	0 / 0 0.0%	32 / 44 72.7%AB	11 / 44 25.0%ABE	1 / 44 2.3%						
1	Campylobacter jejuni subsp. jejuni M1	43 / 44 97.7%	1 / 1 100.0%	2 / 2 100.0%	1 / 1 100.0%	3 / 3 100.0%	3 / 3 100.0%	3 / 3 100.0%	1 / 1 100.0%	1 / 1 100.0%	4 / 4 100.0%	1 / 1 100.0%	3 / 3 100.0%	2 / 2 100.0%	1 / 1 100.0%	1 / 1 100.0%	1 / 1 100.0%	1 / 1 100.0%	1 / 1 100.0%	2 / 2 100.0%	5 / 5 100.0%	1 / 1 100.0%	1 / 1 100.0%	4 / 4 100.0%	0 / 1 0.0%	0 / 0 0.0%	0 / 0 0.0%	0 / 0 0.0%	32 / 44 72.7%AB	11 / 44 25.0%ABE	1 / 44 2.3%						
1	Campylobacter jejuni subsp. jejuni S3	43 / 44 97.7%	1 / 1 100.0%	2 / 2 100.0%	1 / 1 100.0%	3 / 3 100.0%	3 / 3 100.0%	3 / 3 100.0%	1 / 1 100.0%	1 / 1 100.0%	4 / 4 100.0%	1 / 1 100.0%	3 / 3 100.0%	2 / 2 100.0%	1 / 1 100.0%	1 / 1 100.0%	1 / 1 100.0%	1 / 1 100.0%	1 / 1 100.0%	2 / 2 100.0%	5 / 5 100.0%	1 / 1 100.0%	1 / 1 100.0%	4 / 4 100.0%	0 / 1 0.0%	0 / 0 0.0%	0 / 0 0.0%	0 / 0 0.0%	32 / 44 72.7%AB	11 / 44 25.0%ABE	1 / 44 2.3%						
1	Campylobacter jejuni subsp. jejuni ICDCCJ07001	86 / 88 97.7%	2 / 2 100.0%	4 / 4 100.0%	2 / 2 100.0%	6 / 6 100.0%	6 / 6 100.0%	6 / 6 100.0%	2 / 2 100.0%	2 / 2 100.0%	8 / 8 100.0%	2 / 2 100.0%	6 / 6 100.0%	4 / 4 100.0%	2 / 2 100.0%	2 / 2 100.0%	2 / 2 100.0%	2 / 2 100.0%	2 / 2 100.0%	4 / 4 100.0%	10 / 10 100.0%	2 / 2 100.0%	2 / 2 100.0%	8 / 8 100.0%	0 / 2 0.0%	0 / 0 0.0%	0 / 0 0.0%	0 / 0 0.0%	64 / 88 72.7%AB	22 / 88 25.0%ABE	2 / 88 2.3%						
1	Campylobacter jejuni subsp. jejuni PT14	40 / 41 97.6%	1 / 1 100.0%	1 / 1 100.0%	1 / 1 100.0%	3 / 3 100.0%	3 / 3 100.0%	2 / 2 100.0%	1 / 1 100.0%	1 / 1 100.0%	4 / 4 100.0%	1 / 1 100.0%	3 / 3 100.0%	1 / 1 100.0%	1 / 1 100.0%	1 / 1 100.0%	1 / 1 100.0%	1 / 1 100.0%	1 / 1 100.0%	2 / 2 100.0%	5 / 5 100.0%	1 / 1 100.0%	1 / 1 100.0%	4 / 4 100.0%	0 / 1 0.0%	0 / 0 0.0%	0 / 0 0.0%	0 / 0 0.0%	29 / 41 70.7%AB	11 / 41 26.8%ABE	1 / 41 2.4%						
1	Campylobacter jejuni RM1221	43 / 44 97.7%	1 / 1 100.0%	2 / 2 100.0%	1 / 1 100.0%	3 / 3 100.0%	3 / 3 100.0%	3 / 3 100.0%	1 / 1 100.0%	1 / 1 100.0%	4 / 4 100.0%	1 / 1 100.0%	3 / 3 100.0%	2 / 2 100.0%	1 / 1 100.0%	1 / 1 100.0%	1 / 1 100.0%	1 / 1 100.0%	1 / 1 100.0%	2 / 2 100.0%	5 / 5 100.0%	1 / 1 100.0%	1 / 1 100.0%	4 / 4 100.0%	0 / 1 0.0%	0 / 0 0.0%	0 / 0 0.0%	0 / 0 0.0%	32 / 44 72.7%AB	11 / 44 25.0%ABE	1 / 44 2.3%						
1	ATCC 33237	46 / 47 97.9%	1 / 1 100.0%	2 / 2 100.0%	1 / 1 100.0%	3 / 3 100.0%	3 / 3 100.0%	3 / 3 100.0%	1 / 1 100.0%	1 / 1 100.0%	4 / 4 100.0%	1 / 1 100.0%	3 / 3 100.0%	3 / 3 100.0%	1 / 1 100.0%	1 / 1 100.0%	1 / 1 100.0%	1 / 1 100.0%	1 / 1 100.0%	2 / 2 100.0%	4 / 4 100.0%	4 / 4 100.0%	1 / 1 100.0%	4 / 4 100.0%	0 / 1 0.0%	0 / 0 0.0%	0 / 0 0.0%	0 / 0 0.0%	21 / 47 44.7%AB	11 / 47 23.4%B	7 / 47 14.9%ABE	7 / 47 14.9%BE	1 / 47 2.1%E				
1	Campylobacter concisus 13826	46 / 47 97.9%	1 / 1 100.0%	2 / 2 100.0%	1 / 1 100.0%	3 / 3 100.0%	3 / 3 100.0%	3 / 3 100.0%	1 / 1 100.0%	1 / 1 100.0%	4 / 4 100.0%	1 / 1 100.0%	3 / 3 100.0%	3 / 3 100.0%	1 / 1 100.0%	1 / 1 100.0%	1 / 1 100.0%	1 / 1 100.0%	1 / 1 100.0%	2 / 2 100.0%	4 / 4 100.0%	4 / 4 100.0%	1 / 1 100.0%	4 / 4 100.0%	0 / 1 0.0%	0 / 0 0.0%	0 / 0 0.0%	0 / 0 0.0%	21 / 47 44.7%AB	11 / 47 23.4%B	7 / 47 14.9%BE	7 / 47 14.9%ABE	1 / 47 2.1%E				
1	ATCC 35224	46 / 47 97.9%	1 / 1 100.0%	2 / 2 100.0%	1 / 1 100.0%	3 / 3 100.0%	3 / 3 100.0%	3 / 3 100.0%	1 / 1 100.0%	1 / 1 100.0%	4 / 4 100.0%	1 / 1 100.0%	3 / 3 100.0%	3 / 3 100.0%	1 / 1 100.0%	1 / 1 100.0%	1 / 1 100.0%	1 / 1 100.0%	1 / 1 100.0%	2 / 2 100.0%	4 / 4 100.0%	4 / 4 100.0%	1 / 1 100.0%	4 / 4 100.0%	0 / 1 0.0%	0 / 0 0.0%	0 / 0 0.0%	0 / 0 0.0%	22 / 47 46.8%B	11 / 47 23.4%AB	10 / 47 21.3%BE	3 / 47 6.4%ABE	1 / 47 2.1%E				
1	Campylobacter curvus 525.92	46 / 47 97.9%	1 / 1 100.0%	2 / 2 100.0%	1 / 1 100.0%	3 / 3 100.0%	3 / 3 100.0%	3 / 3 100.0%	1 / 1 100.0%	1 / 1 100.0%	4 / 4 100.0%	1 / 1 100.0%	3 / 3 100.0%	3 / 3 100.0%	1 / 1 100.0%	1 / 1 100.0%	1 / 1 100.0%	1 / 1 100.0%	1 / 1 100.0%	2 / 2 100.0%	4 / 4 100.0%	4 / 4 100.0%	1 / 1 100.0%	4 / 4 100.0%	0 / 1 0.0%	0 / 0 0.0%	0 / 0 0.0%	0 / 0 0.0%	22 / 47 46.8%B	11 / 47 23.4%AB	10 / 47 21.3%BE	3 / 47 6.4%ABE	1 / 47 2.1%E				
1	ATCC 35221	45 / 46 97.8%	1 / 1 100.0%	2 / 2 100.0%	1 / 1 100.0%	3 / 3 100.0%	3 / 3 100.0%	3 / 3 100.0%	1 / 1 100.0%	1 / 1 100.0%	4 / 4 100.0%	1 / 1 100.0%	3 / 3 100.0%	3 / 3 100.0%	1 / 1 100.0%	1 / 1 100.0%	1 / 1 100.0%	1 / 1 100.0%	1 / 1 100.0%	2 / 2 100.0%	4 / 4 100.0%	3 / 3 100.0%	1 / 1 100.0%	4 / 4 100.0%	0 / 1 0.0%	0 / 0 0.0%	0 / 0 0.0%	0 / 0 0.0%	33 / 46 71.7%AB	12 / 46 26.1%ABE	1 / 46 2.2%						
1	Campylobacter lari RM2100	45 / 46 97.8%	1 / 1 100.0%	2 / 2 100.0%	1 / 1 100.0%	3 / 3 100.0%	3 / 3 100.0%	3 / 3 100.0%	1 / 1 100.0%	1 / 1 100.0%	4 / 4 100.0%	1 / 1 100.0%	3 / 3 100.0%	3 / 3 100.0%	1 / 1 100.0%	1 / 1 100.0%	1 / 1 100.0%	1 / 1 100.0%	1 / 1 100.0%	2 / 2 100.0%	4 / 4 100.0%	3 / 3 100.0%	1 / 1 100.0%	4 / 4 100.0%	0 / 1 0.0%	0 / 0 0.0%	0 / 0 0.0%	0 / 0 0.0%	33 / 46 71.7%AB	12 / 46 26.1%ABE	1 / 46 2.2%						
1	"Candidatus Campylobacter hominis" Lawson et al. 1998	36 / 43 83.7%	1 / 1 100.0%	1 / 1 100.0%	2 / 2 100.0%	3 / 3 100.0%	3 / 3 100.0%	2 / 2 100.0%	1 / 1 100.0%	1 / 1 100.0%	3 / 4 75.0%	1 / 1 100.0%	2 / 2 100.0%	0 / 2 0.0%	1 / 1 100.0%	1 / 1 100.0%	1 / 1 100.0%	2 / 2 100.0%	1 / 1 100.0%	2 / 2 100.0%	3 / 4 75.0%	0 / 2 0.0%	1 / 1 100.0%	4 / 4 100.0%	0 / 1 0.0%	0 / 0 0.0%	0 / 0 0.0%	0 / 0 0.0%	16 / 43 37.2%B	13 / 43 30.2%AB	5 / 43 11.6%E	5 / 43 11.6%BE	2 / 43 4.7%	2 / 43 4.7%ABE			
1	Campylobacter hominis ATCC BAA-381	36 / 43 83.7%	1 / 1 100.0%	1 / 1 100.0%	2 / 2 100.0%	3 / 3 100.0%	3 / 3 100.0%	2 / 2 100.0%	1 / 1 100.0%	1 / 1 100.0%	3 / 4 75.0%	1 / 1 100.0%	2 / 2 100.0%	0 / 2 0.0%	1 / 1 100.0%	1 / 1 100.0%	1 / 1 100.0%	2 / 2 100.0%	1 / 1 100.0%	2 / 2 100.0%	3 / 4 75.0%	0 / 2 0.0%	1 / 1 100.0%	4 / 4 100.0%	0 / 1 0.0%	0 / 0 0.0%	0 / 0 0.0%	0 / 0 0.0%	16 / 43 37.2%B	13 / 43 30.2%AB	5 / 43 11.6%BE	5 / 43 11.6%E	2 / 43 4.7%ABE	2 / 43 4.7%			
4	Arcibacter	217 / 219 99.1%	10 / 10 100.0%	8 / 8 100.0%	9 / 9 100.0%	19 / 19 100.0%	15 / 15 100.0%	9 / 9 100.0%	4 / 4 100.0%	8 / 8 100.0%	19 / 19 100.0%	4 / 4 100.0%	10 / 10 100.0%	9 / 9 100.0%	8 / 8 100.0%	11 / 11 100.0%	4 / 4 100.0%	4 / 4 100.0%	5 / 5 100.0%	14 / 14 100.0%	11 / 11 100.0%	11 / 11 100.0%	4 / 4 100.0%	20 / 20 100.0%	0 / 2 0.0%	0 / 0 0.0%	1 / 1 100.0%	0 / 0 0.0%	134 / 219 61.2%AB	58 / 219 26.5%ABE	21 / 219 9.6%B	4 / 219 1.8%BE	2 / 219 0.9%				
2	ATCC 49616	106 / 108 98.1%	4 / 4 100.0%	4 / 4 100.0%	4 / 4 100.0%	10 / 10 100.0%	8 / 8 100.0%	4 / 4 100.0%	2 / 2 100.0%	4 / 4 100.0%	10 / 10 100.0%	2 / 2 100.0%	4 / 4 100.0%	4 / 4 100.0%	4 / 4 100.0%	6 / 6 100.0%	2 / 2 100.0%	2 / 2 100.0%	2 / 2 100.0%	6 / 6 100.0%	6 / 6 100.0%	6 / 6 100.0%	2 / 2 100.0%	10 / 10 100.0%	0 / 2 0.0%	0 / 0 0.0%	0 / 0 0.0%	0 / 0 0.0%	55 / 108 50.9%AB	26 / 108 24.1%ABE	21 / 108 19.4%B	4 / 108 3.7%BE	2 / 108 1.9%				
1	Arcobacter butzleri RM4018	53 / 54 98.1%	2 / 2 100.0%	2 / 2 100.0%	2 / 2 100.0%	5 / 5 100.0%	4 / 4 100.0%	2 / 2 100.0%	1 / 1 100.0%	2 / 2 100.0%	5 / 5 100.0%	1 / 1 100.0%	2 / 2 100.0%	2 / 2 100.0%	2 / 2 100.0%	3 / 3 100.0%	1 / 1 100.0%	1 / 1 100.0%	1 / 1 100.0%	3 / 3 100.0%	3 / 3 100.0%	3 / 3 100.0%	1 / 1 100.0%	5 / 5 100.0%	0 / 1 0.0%	0 / 0 0.0%	0 / 0 0.0%	0 / 0 0.0%	21 / 54 38.9%B	17 / 54 31.5%AB	11 / 54 20.4%ABE	4 / 54 7.4%BE	1 / 54 1.9%				
1	Arcobacter butzleri ED-1	53 / 54 98.1%	2 / 2 100.0%	2 / 2 100.0%	2 / 2 100.0%	5 / 5 100.0%	4 / 4 100.0%	2 / 2 100.0%	1 / 1 100.0%	2 / 2 100.0%	5 / 5 100.0%	1 / 1 100.0%	2 / 2 100.0%	2 / 2 100.0%	2 / 2 100.0%	3 / 3 100.0%	1 / 1 100.0%	1 / 1 100.0%	1 / 1 100.0%	3 / 3 100.0%	3 / 3 100.0%	3 / 3 100.0%	1 / 1 100.0%	5 / 5 100.0%	0 / 1 0.0%	0 / 0 0.0%	0 / 0 0.0%	0 / 0 0.0%	38 / 54 70.4%AB	15 / 54 27.8%ABE	1 / 54 1.9%						
1	ATCC 33309	55 / 55 100.0%	4 / 4 100.0%	2 / 2 100.0%	2 / 2 100.0%	4 / 4 100.0%	3 / 3 100.0%	3 / 3 100.0%	1 / 1 100.0%	2 / 2 100.0%	4 / 4 100.0%	1 / 1 100.0%	4 / 4 100.0%	3 / 3 100.0%	2 / 2 100.0%	2 / 2 100.0%	1 / 1 100.0%	1 / 1 100.0%	1 / 1 100.0%	4 / 4 100.0%	2 / 2 100.0%	2 / 2 100.0%	1 / 1 100.0%	5 / 5 100.0%	0 / 0 0.0%	0 / 0 0.0%	1 / 1 100.0%	0 / 0 0.0%	38 / 55 69.1%AB	17 / 55 30.9%ABE							
1	Arcobacter nitrofigilis DSM 7299	55 / 55 100.0%	4 / 4 100.0%	2 / 2 100.0%	2 / 2 100.0%	4 / 4 100.0%	3 / 3 100.0%	3 / 3 100.0%	1 / 1 100.0%	2 / 2 100.0%	4 / 4 100.0%	1 / 1 100.0%	4 / 4 100.0%	3 / 3 100.0%	2 / 2 100.0%	2 / 2 100.0%	1 / 1 100.0%	1 / 1 100.0%	1 / 1 100.0%	4 / 4 100.0%	2 / 2 100.0%	2 / 2 100.0%	1 / 1 100.0%	5 / 5 100.0%	0 / 0 0.0%	0 / 0 0.0%	1 / 1 100.0%	0 / 0 0.0%	38 / 55 69.1%AB	17 / 55 30.9%ABE							
1	Arcobacter sp. L	56 / 56 100.0%	2 / 2 100.0%	2 / 2 100.0%	3 / 3 100.0%	5 / 5 100.0%	4 / 4 100.0%	2 / 2 100.0%	1 / 1 100.0%	2 / 2 100.0%	5 / 5 100.0%	1 / 1 100.0%	2 / 2 100.0%	2 / 2 100.0%	2 / 2 100.0%	3 / 3 100.0%	1 / 1 100.0%	1 / 1 100.0%	2 / 2 100.0%	4 / 4 100.0%	3 / 3 100.0%	3 / 3 100.0%	1 / 1 100.0%	5 / 5 100.0%	0 / 0 0.0%	0 / 0 0.0%	0 / 0 0.0%	0 / 0 0.0%	41 / 56 73.2%AB	15 / 56 26.8%ABE							
2	Dehalospirillum	84 / 84 100.0%	2 / 2 100.0%	2 / 2 100.0%	2 / 2 100.0%	5 / 5 100.0%	8 / 8 100.0%	4 / 4 100.0%	2 / 2 100.0%	2 / 2 100.0%	7 / 7 100.0%	2 / 2 100.0%	6 / 6 100.0%	4 / 4 100.0%	2 / 2 100.0%	2 / 2 100.0%	2 / 2 100.0%	4 / 4 100.0%	2 / 2 100.0%	4 / 4 100.0%	8 / 8 100.0%	4 / 4 100.0%	2 / 2 100.0%	8 / 8 100.0%	0 / 0 0.0%	0 / 0 0.0%	0 / 0 0.0%	0 / 0 0.0%	63 / 84 75.0%AB	21 / 84 25.0%ABE							
1	ATCC 700032	41 / 41 100.0%	1 / 1 100.0%	1 / 1 100.0%	1 / 1 100.0%	2 / 2 100.0%	4 / 4 100.0%	2 / 2 100.0%	1 / 1 100.0%	1 / 1 100.0%	3 / 3 100.0%	1 / 1 100.0%	3 / 3 100.0%	2 / 2 100.0%	1 / 1 100.0%	1 / 1 100.0%	1 / 1 100.0%	2 / 2 100.0%	1 / 1 100.0%	2 / 2 100.0%	4 / 4 100.0%	2 / 2 100.0%	1 / 1 100.0%	4 / 4 100.0%	0 / 0 0.0%	0 / 0 0.0%	0 / 0 0.0%	0 / 0 0.0%	30 / 41 73.2%AB	11 / 41 26.8%ABE							
1	Sulfurospirillum barnesii SES-3	41 / 41 100.0%	1 / 1 100.0%	1 / 1 100.0%	1 / 1 100.0%	2 / 2 100.0%	4 / 4 100.0%	2 / 2 100.0%	1 / 1 100.0%	1 / 1 100.0%	3 / 3 100.0%	1 / 1 100.0%	3 / 3 100.0%	2 / 2 100.0%	1 / 1 100.0%	1 / 1 100.0%	1 / 1 100.0%	2 / 2 100.0%	1 / 1 100.0%	2 / 2 100.0%	4 / 4 100.0%	2 / 2 100.0%	1 / 1 100.0%	4 / 4 100.0%	0 / 0 0.0%	0 / 0 0.0%	0 / 0 0.0%	0 / 0 0.0%	30 / 41 73.2%AB	11 / 41 26.8%ABE							
1	ATCC 51133	43 / 43 100.0%	1 / 1 100.0%	1 / 1 100.0%	1 / 1 100.0%	3 / 3 100.0%	4 / 4 100.0%	2 / 2 100.0%	1 / 1 100.0%	1 / 1 100.0%	4 / 4 100.0%	1 / 1 100.0%	3 / 3 100.0%	2 / 2 100.0%	1 / 1 100.0%	1 / 1 100.0%	1 / 1 100.0%	2 / 2 100.0%	1 / 1 100.0%	2 / 2 100.0%	4 / 4 100.0%	2 / 2 100.0%	1 / 1 100.0%	4 / 4 100.0%	0 / 0 0.0%	0 / 0 0.0%	0 / 0 0.0%	0 / 0 0.0%	33 / 43 76.7%AB	10 / 43 23.3%ABE							
1	Sulfurospirillum deleyianum DSM 6946	43 / 43 100.0%	1 / 1 100.0%	1 / 1 100.0%	1 / 1 100.0%	3 / 3 100.0%	4 / 4 100.0%	2 / 2 100.0%	1 / 1 100.0%	1 / 1 100.0%	4 / 4 100.0%	1 / 1 100.0%	3 / 3 100.0%	2 / 2 100.0%	1 / 1 100.0%	1 / 1 100.0%	1 / 1 100.0%	2 / 2 100.0%	1 / 1 100.0%	2 / 2 100.0%	4 / 4 100.0%	2 / 2 100.0%	1 / 1 100.0%	4 / 4 100.0%	0 / 0 0.0%	0 / 0 0.0%	0 / 0 0.0%	0 / 0 0.0%	33 / 43 76.7%AB	10 / 43 23.3%ABE							
1	unclassified Campylobacterales	44 / 44 100.0%	1 / 1 100.0%	1 / 1 100.0%	1 / 1 100.0%	2 / 2 100.0%	3 / 3 100.0%	2 / 2 100.0%	1 / 1 100.0%	1 / 1 100.0%	3 / 3 100.0%	1 / 1 100.0%	4 / 4 100.0%	1 / 1 100.0%	1 / 1 100.0%	1 / 1 100.0%	1 / 1 100.0%	3 / 3 100.0%	1 / 1 100.0%	3 / 3 100.0%	5 / 5 100.0%	2 / 2 100.0%	1 / 1 100.0%	5 / 5 100.0%	0 / 0 0.0%	0 / 0 0.0%	0 / 0 0.0%	0 / 0 0.0%	29 / 44 65.9%AB	15 / 44 34.1%ABE							
1	Nitratifractor	44 / 44 100.0%	1 / 1 100.0%	1 / 1 100.0%	1 / 1 100.0%	2 / 2 100.0%	3 / 3 100.0%	2 / 2 100.0%	1 / 1 100.0%	1 / 1 100.0%	3 / 3 100.0%	1 / 1 100.0%	4 / 4 100.0%	1 / 1 100.0%	1 / 1 100.0%	1 / 1 100.0%	1 / 1 100.0%	3 / 3 100.0%	1 / 1 100.0%	3 / 3 100.0%	5 / 5 100.0%	2 / 2 100.0%	1 / 1 100.0%	5 / 5 100.0%	0 / 0 0.0%	0 / 0 0.0%	0 / 0 0.0%	0 / 0 0.0%	29 / 44 65.9%AB	15 / 44 34.1%ABE							
1	DSM 16511	44 / 44 100.0%	1 / 1 100.0%	1 / 1 100.0%	1 / 1 100.0%	2 / 2 100.0%	3 / 3 100.0%	2 / 2 100.0%	1 / 1 100.0%	1 / 1 100.0%	3 / 3 100.0%	1 / 1 100.0%	4 / 4 100.0%	1 / 1 100.0%	1 / 1 100.0%	1 / 1 100.0%	1 / 1 100.0%	3 / 3 100.0%	1 / 1 100.0%	3 / 3 100.0%	5 / 5 100.0%	2 / 2 100.0%	1 / 1 100.0%	5 / 5 100.0%	0 / 0 0.0%	0 / 0 0.0%	0 / 0 0.0%	0 / 0 0.0%	29 / 44 65.9%AB	15 / 44 34.1%ABE							
1	Nitratifractor salsuginis DSM 16511	44 / 44 100.0%	1 / 1 100.0%	1 / 1 100.0%	1 / 1 100.0%	2 / 2 100.0%	3 / 3 100.0%	2 / 2 100.0%	1 / 1 100.0%	1 / 1 100.0%	3 / 3 100.0%	1 / 1 100.0%	4 / 4 100.0%	1 / 1 100.0%	1 / 1 100.0%	1 / 1 100.0%	1 / 1 100.0%	3 / 3 100.0%	1 / 1 100.0%	3 / 3 100.0%	5 / 5 100.0%	2 / 2 100.0%	1 / 1 100.0%	5 / 5 100.0%	0 / 0 0.0%	0 / 0 0.0%	0 / 0 0.0%	0 / 0 0.0%	29 / 44 65.9%AB	15 / 44 34.1%ABE							
1	Nautiliales	48 / 49 98.0%	1 / 1 100.0%	2 / 2 100.0%	2 / 2 100.0%	4 / 4 100.0%	3 / 3 100.0%	3 / 3 100.0%	1 / 1 100.0%	1 / 1 100.0%	5 / 5 100.0%	1 / 1 100.0%	3 / 3 100.0%	2 / 2 100.0%	2 / 2 100.0%	1 / 1 100.0%	1 / 1 100.0%	1 / 1 100.0%	1 / 1 100.0%	3 / 3 100.0%	4 / 4 100.0%	2 / 2 100.0%	1 / 1 100.0%	4 / 4 100.0%	0 / 1 0.0%	0 / 0 0.0%	0 / 0 0.0%	0 / 0 0.0%	37 / 49 75.5%AB	11 / 49 22.4%ABE	1 / 49 2.0%E						
1	Nautiliaceae	48 / 49 98.0%	1 / 1 100.0%	2 / 2 100.0%	2 / 2 100.0%	4 / 4 100.0%	3 / 3 100.0%	3 / 3 100.0%	1 / 1 100.0%	1 / 1 100.0%	5 / 5 100.0%	1 / 1 100.0%	3 / 3 100.0%	2 / 2 100.0%	2 / 2 100.0%	1 / 1 100.0%	1 / 1 100.0%	1 / 1 100.0%	1 / 1 100.0%	3 / 3 100.0%	4 / 4 100.0%	2 / 2 100.0%	1 / 1 100.0%	4 / 4 100.0%	0 / 1 0.0%	0 / 0 0.0%	0 / 0 0.0%	0 / 0 0.0%	37 / 49 75.5%AB	11 / 49 22.4%ABE	1 / 49 2.0%E						
1	Nautilia	48 / 49 98.0%	1 / 1 100.0%	2 / 2 100.0%	2 / 2 100.0%	4 / 4 100.0%	3 / 3 100.0%	3 / 3 100.0%	1 / 1 100.0%	1 / 1 100.0%	5 / 5 100.0%	1 / 1 100.0%	3 / 3 100.0%	2 / 2 100.0%	2 / 2 100.0%	1 / 1 100.0%	1 / 1 100.0%	1 / 1 100.0%	1 / 1 100.0%	3 / 3 100.0%	4 / 4 100.0%	2 / 2 100.0%	1 / 1 100.0%	4 / 4 100.0%	0 / 1 0.0%	0 / 0 0.0%	0 / 0 0.0%	0 / 0 0.0%	37 / 49 75.5%AB	11 / 49 22.4%ABE	1 / 49 2.0%E						
1	ATCC BAA-1463	48 / 49 98.0%	1 / 1 100.0%	2 / 2 100.0%	2 / 2 100.0%	4 / 4 100.0%	3 / 3 100.0%	3 / 3 100.0%	1 / 1 100.0%	1 / 1 100.0%	5 / 5 100.0%	1 / 1 100.0%	3 / 3 100.0%	2 / 2 100.0%	2 / 2 100.0%	1 / 1 100.0%	1 / 1 100.0%	1 / 1 100.0%	1 / 1 100.0%	3 / 3 100.0%	4 / 4 100.0%	2 / 2 100.0%	1 / 1 100.0%	4 / 4 100.0%	0 / 1 0.0%	0 / 0 0.0%	0 / 0 0.0%	0 / 0 0.0%	37 / 49 75.5%AB	11 / 49 22.4%ABE	1 / 49 2.0%E						
1	Nautilia profundicola AmH	48 / 49 98.0%	1 / 1 100.0%	2 / 2 100.0%	2 / 2 100.0%	4 / 4 100.0%	3 / 3 100.0%	3 / 3 100.0%	1 / 1 100.0%	1 / 1 100.0%	5 / 5 100.0%	1 / 1 100.0%	3 / 3 100.0%	2 / 2 100.0%	2 / 2 100.0%	1 / 1 100.0%	1 / 1 100.0%	1 / 1 100.0%	1 / 1 100.0%	3 / 3 100.0%	4 / 4 100.0%	2 / 2 100.0%	1 / 1 100.0%	4 / 4 100.0%	0 / 1 0.0%	0 / 0 0.0%	0 / 0 0.0%	0 / 0 0.0%	37 / 49 75.5%AB	11 / 49 22.4%ABE	1 / 49 2.0%E						
497	Bacillus/Clostridium group	16226 / 34003 47.7%	573 / 1147 50.0%	951 / 1512 62.9%	943 / 1525 61.8%	588 / 1207 48.7%	1544 / 2677 57.7%	1090 / 1930 56.5%	403 / 775 52.0%	564 / 1025 55.0%	921 / 2019 45.6%	349 / 586 59.6%	1246 / 2422 51.4%	868 / 1822 47.6%	501 / 1125 44.5%	619 / 1336 46.3%	285 / 571 49.9%	538 / 1264 42.6%	282 / 690 40.9%	951 / 1949 48.8%	934 / 2601 35.9%	645 / 1948 33.1%	283 / 653 43.3%	1067 / 3124 34.2%	67 / 72 93.1%	0 / 0 0.0%	14 / 21 66.7%	0 / 0 0.0%	11104 / 34003 32.7%AB	9831 / 34003 28.9%E	7813 / 34003 23.0%	4398 / 34003 12.9%ABE	472 / 34003 1.4%B	252 / 34003 0.7%BE	95 / 34003 0.3%CE	37 / 34003 0.1%C	1 / 34003 0.0%EGG
360	Bacilli	8443 / 25395 33.2%	330 / 893 37.0%	652 / 1189 54.8%	630 / 1186 53.1%	383 / 976 39.2%	878 / 1950 45.0%	620 / 1411 43.9%	234 / 585 40.0%	334 / 772 43.3%	511 / 1566 32.6%	208 / 432 48.1%	699 / 1828 38.2%	393 / 1301 30.2%	246 / 852 28.9%	331 / 1020 32.5%	112 / 384 29.2%	184 / 870 21.1%	131 / 498 26.3%	485 / 1431 33.9%	335 / 1941 17.3%	253 / 1506 16.8%	95 / 452 21.0%	382 / 2325 16.4%	3 / 4 75.0%	0 / 0 0.0%	14 / 21 66.7%	0 / 0 0.0%	9309 / 25395 36.7%E	7531 / 25395 29.7%	6477 / 25395 25.5%AB	1799 / 25395 7.1%ABE	139 / 25395 0.5%B	84 / 25395 0.3%CE	28 / 25395 0.1%C	28 / 25395 0.1%BE	
169	Bacillales	8275 / 13415 61.7%	330 / 494 66.8%	640 / 712 89.9%	628 / 679 92.5%	379 / 487 77.8%	858 / 1152 74.5%	618 / 783 78.9%	223 / 353 63.2%	324 / 387 83.7%	511 / 774 66.0%	172 / 214 80.4%	697 / 981 71.0%	393 / 679 57.9%	231 / 455 50.8%	327 / 519 63.0%	112 / 185 60.5%	184 / 463 39.7%	131 / 298 44.0%	474 / 727 65.2%	333 / 910 36.6%	253 / 825 30.7%	95 / 216 44.0%	345 / 1104 31.2%	3 / 3 100.0%	0 / 0 0.0%	14 / 15 93.3%	0 / 0 0.0%	6372 / 13415 47.5%AB	2774 / 13415 20.7%E	2283 / 13415 17.0%	1736 / 13415 12.9%ABE	139 / 13415 1.0%B	58 / 13415 0.4%CE	28 / 13415 0.2%BE	25 / 13415 0.2%C	
46	Staphylococcaceae	1598 / 2742 58.3%	0 / 97 0.0%	130 / 175 74.3%	137 / 137 100.0%	79 / 79 100.0%	271 / 274 98.9%	134 / 134 100.0%	44 / 94 46.8%	54 / 92 58.7%	134 / 134 100.0%	47 / 47 100.0%	97 / 229 42.4%	46 / 135 34.1%	1 / 92 1.1%	45 / 91 49.5%	46 / 47 97.9%	0 / 90 0.0%	0 / 47 0.0%	136 / 136 100.0%	41 / 181 22.7%	47 / 139 33.8%	0 / 47 0.0%	95 / 230 41.3%	0 / 0 0.0%	0 / 0 0.0%	14 / 15 93.3%	0 / 0 0.0%	1366 / 2742 49.8%AB	642 / 2742 23.4%E	501 / 2742 18.3%	184 / 2742 6.7%ABE	44 / 2742 1.6%B	4 / 2742 0.1%BE	1 / 2742 0.0%CE		
45	"Aurococcus" Winslow and Rogers 1906	1569 / 2694 58.2%	0 / 95 0.0%	128 / 172 74.4%	134 / 134 100.0%	78 / 78 100.0%	268 / 270 99.3%	132 / 132 100.0%	43 / 92 46.7%	52 / 90 57.8%	132 / 132 100.0%	46 / 46 100.0%	95 / 225 42.2%	45 / 133 33.8%	1 / 90 1.1%	44 / 89 49.4%	45 / 46 97.8%	0 / 89 0.0%	0 / 46 0.0%	134 / 134 100.0%	40 / 179 22.3%	45 / 135 33.3%	0 / 46 0.0%	93 / 226 41.2%	0 / 0 0.0%	0 / 0 0.0%	14 / 15 93.3%	0 / 0 0.0%	1344 / 2694 49.9%AB	630 / 2694 23.4%E	494 / 2694 18.3%	177 / 2694 6.6%ABE	44 / 2694 1.6%B	4 / 2694 0.1%BE	1 / 2694 0.0%CE		
35	"Micrococcus aureus" (Rosenbach 1884) Zopf 1885	1225 / 2104 58.2%	0 / 75 0.0%	104 / 138 75.4%	104 / 104 100.0%	63 / 63 100.0%	211 / 211 100.0%	102 / 102 100.0%	35 / 72 48.6%	34 / 70 48.6%	104 / 104 100.0%	36 / 36 100.0%	75 / 176 42.6%	36 / 103 35.0%	0 / 70 0.0%	34 / 69 49.3%	36 / 36 100.0%	0 / 69 0.0%	0 / 36 0.0%	104 / 104 100.0%	33 / 139 23.7%	34 / 103 33.0%	0 / 36 0.0%	68 / 176 38.6%	0 / 0 0.0%	0 / 0 0.0%	12 / 12 100.0%	0 / 0 0.0%	1044 / 2104 49.6%AB	515 / 2104 24.5%E	363 / 2104 17.3%	133 / 2104 6.3%ABE	44 / 2104 2.1%B	4 / 2104 0.2%BE	1 / 2104 0.0%CE		
29	Staphylococcus aureus aureus	1013 / 1742 58.2%	0 / 62 0.0%	86 / 114 75.4%	86 / 86 100.0%	52 / 52 100.0%	177 / 177 100.0%	84 / 84 100.0%	29 / 60 48.3%	28 / 58 48.3%	87 / 87 100.0%	30 / 30 100.0%	62 / 146 42.5%	30 / 85 35.3%	0 / 59 0.0%	28 / 57 49.1%	30 / 30 100.0%	0 / 57 0.0%	0 / 30 0.0%	86 / 86 100.0%	27 / 115 23.5%	28 / 85 32.9%	0 / 30 0.0%	56 / 145 38.6%	0 / 0 0.0%	0 / 0 0.0%	7 / 7 100.0%	0 / 0 0.0%	856 / 1742 49.1%AB	420 / 1742 24.1%E	308 / 1742 17.7%	109 / 1742 6.3%ABE	44 / 1742 2.5%B	4 / 1742 0.2%BE	1 / 1742 0.1%CE		
1	Staphylococcus aureus NCTC 8325	35 / 60 58.3%	0 / 2 0.0%	3 / 4 75.0%	3 / 3 100.0%	2 / 2 100.0%	6 / 6 100.0%	3 / 3 100.0%	1 / 2 50.0%	1 / 2 50.0%	3 / 3 100.0%	1 / 1 100.0%	2 / 5 40.0%	1 / 3 33.3%	0 / 2 0.0%	1 / 2 50.0%	1 / 1 100.0%	0 / 2 0.0%	0 / 1 0.0%	3 / 3 100.0%	1 / 4 25.0%	1 / 3 33.3%	0 / 1 0.0%	2 / 5 40.0%	0 / 0 0.0%	0 / 0 0.0%	0 / 0 0.0%	0 / 0 0.0%	31 / 60 51.7%AB	15 / 60 25.0%	10 / 60 16.7%E	4 / 60 6.7%ABE					
1	Staphylococcus aureus COL	30 / 53 56.6%	0 / 2 0.0%	3 / 4 75.0%	3 / 3 100.0%	2 / 2 100.0%	5 / 5 100.0%	2 / 2 100.0%	1 / 2 50.0%	1 / 2 50.0%	3 / 3 100.0%	1 / 1 100.0%	2 / 5 40.0%	1 / 2 50.0%	0 / 2 0.0%	1 / 2 50.0%	1 / 1 100.0%	0 / 1 0.0%	0 / 1 0.0%	2 / 2 100.0%	0 / 3 0.0%	1 / 3 33.3%	0 / 1 0.0%	1 / 4 25.0%	0 / 0 0.0%	0 / 0 0.0%	0 / 0 0.0%	0 / 0 0.0%	27 / 53 50.9%AB	13 / 53 24.5%	10 / 53 18.9%E	3 / 53 5.7%ABE					
1	Staphylococcus aureus (strain Mu50 / ATCC 700699)	34 / 59 57.6%	0 / 2 0.0%	2 / 3 66.7%	3 / 3 100.0%	2 / 2 100.0%	6 / 6 100.0%	3 / 3 100.0%	1 / 2 50.0%	1 / 2 50.0%	3 / 3 100.0%	1 / 1 100.0%	2 / 5 40.0%	1 / 3 33.3%	0 / 2 0.0%	1 / 2 50.0%	1 / 1 100.0%	0 / 2 0.0%	0 / 1 0.0%	3 / 3 100.0%	1 / 4 25.0%	1 / 3 33.3%	0 / 1 0.0%	2 / 5 40.0%	0 / 0 0.0%	0 / 0 0.0%	0 / 0 0.0%	0 / 0 0.0%	30 / 59 50.8%AB	15 / 59 25.4%	10 / 59 16.9%E	4 / 59 6.8%ABE					
1	Staphylococcus aureus (strain N315)	37 / 62 59.7%	0 / 2 0.0%	3 / 4 75.0%	3 / 3 100.0%	3 / 3 100.0%	6 / 6 100.0%	3 / 3 100.0%	1 / 2 50.0%	1 / 2 50.0%	4 / 4 100.0%	1 / 1 100.0%	2 / 5 40.0%	1 / 3 33.3%	0 / 2 0.0%	1 / 2 50.0%	1 / 1 100.0%	0 / 2 0.0%	0 / 1 0.0%	3 / 3 100.0%	1 / 4 25.0%	1 / 3 33.3%	0 / 1 0.0%	2 / 5 40.0%	0 / 0 0.0%	0 / 0 0.0%	0 / 0 0.0%	0 / 0 0.0%	33 / 62 53.2%AB	15 / 62 24.2%	10 / 62 16.1%E	4 / 62 6.5%ABE					
1	Staphylococcus aureus (strain MW2)	35 / 60 58.3%	0 / 2 0.0%	3 / 4 75.0%	3 / 3 100.0%	2 / 2 100.0%	6 / 6 100.0%	3 / 3 100.0%	1 / 2 50.0%	1 / 2 50.0%	3 / 3 100.0%	1 / 1 100.0%	2 / 5 40.0%	1 / 3 33.3%	0 / 2 0.0%	1 / 2 50.0%	1 / 1 100.0%	0 / 2 0.0%	0 / 1 0.0%	3 / 3 100.0%	1 / 4 25.0%	1 / 3 33.3%	0 / 1 0.0%	2 / 5 40.0%	0 / 0 0.0%	0 / 0 0.0%	0 / 0 0.0%	0 / 0 0.0%	31 / 60 51.7%AB	15 / 60 25.0%	10 / 60 16.7%E	4 / 60 6.7%ABE					

1	Staphylococcus aureus subsp. aureus MRSA252	35 / 60 58.3%	0 / 2 0.0%	3 / 4 75.0%	3 / 3 100.0%	2 / 2 100.0%	6 / 6 100.0%	3 / 3 100.0%	1 / 2 50.0%	1 / 2 50.0%	3 / 3 100.0%	1 / 1 100.0%	2 / 5 40.0%	1 / 3 33.3%	0 / 2 0.0%	1 / 2 50.0%	1 / 1 100.0%	0 / 2 0.0%	0 / 1 0.0%	3 / 3 100.0%	1 / 4 25.0%	1 / 3 33.3%	0 / 1 0.0%	2 / 5 40.0%	0 / 0 0.0%	0 / 0 0.0%	0 / 0 0.0%	0 / 0 0.0%	31 / 60 51.7%AB	15 / 60 25.0%	10 / 60 16.7%E	4 / 60 6.7%ABE				
1	Staphylococcus aureus subsp. aureus MSSA476	33 / 58 56.9%	0 / 2 0.0%	3 / 4 75.0%	3 / 3 100.0%	2 / 2 100.0%	4 / 4 100.0%	3 / 3 100.0%	1 / 2 50.0%	1 / 2 50.0%	3 / 3 100.0%	1 / 1 100.0%	2 / 5 40.0%	1 / 3 33.3%	0 / 2 0.0%	1 / 2 50.0%	1 / 1 100.0%	0 / 2 0.0%	0 / 1 0.0%	3 / 3 100.0%	1 / 4 25.0%	1 / 3 33.3%	0 / 1 0.0%	2 / 5 40.0%	0 / 0 0.0%	0 / 0 0.0%	0 / 0 0.0%	0 / 0 0.0%	29 / 58 50.0%AB	15 / 58 25.9%	10 / 58 17.2%E	4 / 58 6.9%ABE				
1	Staphylococcus aureus subsp. aureus JH9	34 / 59 57.6%	0 / 2 0.0%	3 / 4 75.0%	3 / 3 100.0%	1 / 1 100.0%	6 / 6 100.0%	3 / 3 100.0%	1 / 2 50.0%	1 / 2 50.0%	3 / 3 100.0%	1 / 1 100.0%	2 / 5 40.0%	1 / 3 33.3%	0 / 2 0.0%	1 / 2 50.0%	1 / 1 100.0%	0 / 2 0.0%	0 / 1 0.0%	3 / 3 100.0%	1 / 4 25.0%	1 / 3 33.3%	0 / 1 0.0%	2 / 5 40.0%	0 / 0 0.0%	0 / 0 0.0%	0 / 0 0.0%	0 / 0 0.0%	30 / 59 50.8%AB	15 / 59 25.4%	10 / 59 16.9%E	4 / 59 6.8%ABE				
1	Staphylococcus aureus subsp. aureus JH1	35 / 60 58.3%	0 / 2 0.0%	3 / 4 75.0%	3 / 3 100.0%	2 / 2 100.0%	6 / 6 100.0%	3 / 3 100.0%	1 / 2 50.0%	1 / 2 50.0%	3 / 3 100.0%	1 / 1 100.0%	2 / 5 40.0%	1 / 3 33.3%	0 / 2 0.0%	1 / 2 50.0%	1 / 1 100.0%	0 / 2 0.0%	0 / 1 0.0%	3 / 3 100.0%	1 / 4 25.0%	1 / 3 33.3%	0 / 1 0.0%	2 / 5 40.0%	0 / 0 0.0%	0 / 0 0.0%	0 / 0 0.0%	0 / 0 0.0%	31 / 60 51.7%AB	15 / 60 25.0%	10 / 60 16.7%E	4 / 60 6.7%ABE				
2	Staphylococcus aureus subsp. aureus USA300	66 / 114 57.9%	0 / 4 0.0%	6 / 8 75.0%	6 / 6 100.0%	4 / 4 100.0%	12 / 12 100.0%	5 / 5 100.0%	2 / 4 50.0%	2 / 4 50.0%	6 / 6 100.0%	2 / 2 100.0%	4 / 10 40.0%	2 / 5 40.0%	0 / 4 0.0%	2 / 4 50.0%	2 / 2 100.0%	0 / 3 0.0%	0 / 2 0.0%	5 / 5 100.0%	1 / 7 14.3%	2 / 6 33.3%	0 / 2 0.0%	3 / 9 33.3%	0 / 0 0.0%	0 / 0 0.0%	0 / 0 0.0%	0 / 0 0.0%	37 / 114 32.5%AB	26 / 114 22.8%E	22 / 114 19.3%	22 / 114 19.3%B	5 / 114 4.4%ABE	2 / 114 1.8%BE		
1	Staphylococcus aureus subsp. aureus USA300_FPR3757	30 / 53 56.6%	0 / 2 0.0%	3 / 4 75.0%	3 / 3 100.0%	2 / 2 100.0%	5 / 5 100.0%	2 / 2 100.0%	1 / 2 50.0%	1 / 2 50.0%	3 / 3 100.0%	1 / 1 100.0%	2 / 5 40.0%	1 / 2 50.0%	0 / 2 0.0%	1 / 2 50.0%	1 / 1 100.0%	0 / 1 0.0%	0 / 1 0.0%	2 / 2 100.0%	0 / 3 0.0%	1 / 3 33.3%	0 / 1 0.0%	1 / 4 25.0%	0 / 0 0.0%	0 / 0 0.0%	0 / 0 0.0%	0 / 0 0.0%	27 / 53 50.9%AB	13 / 53 24.5%	10 / 53 18.9%E	3 / 53 5.7%ABE				
1	Staphylococcus aureus subsp. aureus USA300-HOU-MR	36 / 61 59.0%	0 / 2 0.0%	3 / 4 75.0%	3 / 3 100.0%	2 / 2 100.0%	7 / 7 100.0%	3 / 3 100.0%	1 / 2 50.0%	1 / 2 50.0%	3 / 3 100.0%	1 / 1 100.0%	2 / 5 40.0%	1 / 3 33.3%	0 / 2 0.0%	1 / 2 50.0%	1 / 1 100.0%	0 / 2 0.0%	0 / 1 0.0%	3 / 3 100.0%	1 / 4 25.0%	1 / 3 33.3%	0 / 1 0.0%	2 / 5 40.0%	0 / 0 0.0%	0 / 0 0.0%	0 / 0 0.0%	0 / 0 0.0%	22 / 61 36.1%B	16 / 61 26.2%E	10 / 61 16.4%AB	9 / 61 14.8%	2 / 61 3.3%BE	2 / 61 3.3%ABE		
1	Staphylococcus aureus subsp. aureus Mu3	36 / 61 59.0%	0 / 2 0.0%	3 / 4 75.0%	3 / 3 100.0%	2 / 2 100.0%	7 / 7 100.0%	3 / 3 100.0%	1 / 2 50.0%	1 / 2 50.0%	3 / 3 100.0%	1 / 1 100.0%	2 / 5 40.0%	1 / 3 33.3%	0 / 2 0.0%	1 / 2 50.0%	1 / 1 100.0%	0 / 2 0.0%	0 / 1 0.0%	3 / 3 100.0%	1 / 4 25.0%	1 / 3 33.3%	0 / 1 0.0%	2 / 5 40.0%	0 / 0 0.0%	0 / 0 0.0%	0 / 0 0.0%	0 / 0 0.0%	22 / 61 36.1%B	17 / 61 27.9%E	10 / 61 16.4%AB	8 / 61 13.1%	2 / 61 3.3%BE	2 / 61 3.3%ABE		
1	Staphylococcus aureus subsp. aureus Newman	35 / 60 58.3%	0 / 2 0.0%	3 / 4 75.0%	3 / 3 100.0%	2 / 2 100.0%	6 / 6 100.0%	3 / 3 100.0%	1 / 2 50.0%	1 / 2 50.0%	3 / 3 100.0%	1 / 1 100.0%	2 / 5 40.0%	1 / 3 33.3%	0 / 2 0.0%	1 / 2 50.0%	1 / 1 100.0%	0 / 2 0.0%	0 / 1 0.0%	3 / 3 100.0%	1 / 4 25.0%	1 / 3 33.3%	0 / 1 0.0%	2 / 5 40.0%	0 / 0 0.0%	0 / 0 0.0%	0 / 0 0.0%	0 / 0 0.0%	31 / 60 51.7%AB	15 / 60 25.0%	10 / 60 16.7%E	4 / 60 6.7%ABE				
2	Staphylococcus aureus subsp. aureus 'sequence type 398'	68 / 118 57.6%	0 / 4 0.0%	6 / 8 75.0%	6 / 6 100.0%	2 / 2 100.0%	12 / 12 100.0%	6 / 6 100.0%	2 / 4 50.0%	2 / 4 50.0%	5 / 5 100.0%	2 / 2 100.0%	4 / 10 40.0%	2 / 6 33.3%	0 / 4 0.0%	2 / 4 50.0%	2 / 2 100.0%	0 / 4 0.0%	0 / 2 0.0%	6 / 6 100.0%	2 / 8 25.0%	2 / 6 33.3%	0 / 2 0.0%	4 / 10 40.0%	0 / 0 0.0%	0 / 0 0.0%	1 / 1 100.0%	0 / 0 0.0%	60 / 118 50.8%AB	34 / 118 28.8%E	16 / 118 13.6%	8 / 118 6.8%ABE				
1	Staphylococcus aureus subsp. aureus 71193	32 / 57 56.1%	0 / 2 0.0%	3 / 4 75.0%	3 / 3 100.0%	0 / 0 0.0%	6 / 6 100.0%	3 / 3 100.0%	1 / 2 50.0%	1 / 2 50.0%	2 / 2 100.0%	1 / 1 100.0%	2 / 5 40.0%	1 / 3 33.3%	0 / 2 0.0%	1 / 2 50.0%	1 / 1 100.0%	0 / 2 0.0%	0 / 1 0.0%	3 / 3 100.0%	1 / 4 25.0%	1 / 3 33.3%	0 / 1 0.0%	2 / 5 40.0%	0 / 0 0.0%	0 / 0 0.0%	0 / 0 0.0%	0 / 0 0.0%	28 / 57 49.1%AB	17 / 57 29.8%E	8 / 57 14.0%	4 / 57 7.0%ABE				
1	Staphylococcus aureus subsp. aureus JKD6008	49 / 84 58.3%	0 / 5 0.0%	5 / 6 83.3%	2 / 2 100.0%	3 / 3 100.0%	9 / 9 100.0%	3 / 3 100.0%	1 / 3 33.3%	0 / 2 0.0%	5 / 5 100.0%	2 / 2 100.0%	5 / 7 71.4%	2 / 3 66.7%	0 / 3 0.0%	0 / 2 0.0%	2 / 2 100.0%	0 / 3 0.0%	0 / 2 0.0%	5 / 5 100.0%	1 / 5 20.0%	1 / 2 50.0%	0 / 2 0.0%	3 / 8 37.5%	0 / 0 0.0%	0 / 0 0.0%	0 / 0 0.0%	0 / 0 0.0%	44 / 84 52.4%AB	24 / 84 28.6%E	11 / 84 13.1%	5 / 84 6.0%ABE				
1	Staphylococcus aureus subsp. aureus 60	36 / 61 59.0%	0 / 2 0.0%	3 / 4 75.0%	3 / 3 100.0%	2 / 2 100.0%	7 / 7 100.0%	3 / 3 100.0%	1 / 2 50.0%	1 / 2 50.0%	3 / 3 100.0%	1 / 1 100.0%	2 / 5 40.0%	1 / 3 33.3%	0 / 2 0.0%	1 / 2 50.0%	1 / 1 100.0%	0 / 2 0.0%	0 / 1 0.0%	3 / 3 100.0%	1 / 4 25.0%	1 / 3 33.3%	0 / 1 0.0%	2 / 5 40.0%	0 / 0 0.0%	0 / 0 0.0%	0 / 0 0.0%	0 / 0 0.0%	32 / 61 52.5%AB	17 / 61 27.9%E	8 / 61 13.1%	4 / 61 6.6%ABE				
1	Staphylococcus aureus subsp. aureus 0528	37 / 62 59.7%	0 / 3 0.0%	3 / 4 75.0%	3 / 3 100.0%	2 / 2 100.0%	6 / 6 100.0%	3 / 3 100.0%	1 / 2 50.0%	1 / 2 50.0%	3 / 3 100.0%	1 / 1 100.0%	3 / 5 60.0%	1 / 3 33.3%	0 / 2 0.0%	1 / 2 50.0%	1 / 1 100.0%	0 / 2 0.0%	0 / 1 0.0%	3 / 3 100.0%	1 / 4 25.0%	1 / 3 33.3%	0 / 1 0.0%	2 / 5 40.0%	0 / 0 0.0%	0 / 0 0.0%	1 / 1 100.0%	0 / 0 0.0%	33 / 62 53.2%AB	17 / 62 27.4%E	8 / 62 12.9%	4 / 62 6.5%ABE				
1	Staphylococcus aureus ED98	37 / 62 59.7%	0 / 2 0.0%	3 / 4 75.0%	3 / 3 100.0%	2 / 2 100.0%	6 / 6 100.0%	3 / 3 100.0%	1 / 2 50.0%	1 / 2 50.0%	3 / 3 100.0%	1 / 1 100.0%	2 / 5 40.0%	1 / 3 33.3%	0 / 2 0.0%	1 / 2 50.0%	1 / 1 100.0%	0 / 2 0.0%	0 / 1 0.0%	3 / 3 100.0%	1 / 4 25.0%	1 / 3 33.3%	0 / 1 0.0%	2 / 5 40.0%	0 / 0 0.0%	0 / 0 0.0%	2 / 2 100.0%	0 / 0 0.0%	33 / 62 53.2%AB	17 / 62 27.4%E	8 / 62 12.9%	4 / 62 6.5%ABE				
1	Staphylococcus aureus subsp. aureus ED133	35 / 60 58.3%	0 / 2 0.0%	3 / 4 75.0%	3 / 3 100.0%	1 / 1 100.0%	6 / 6 100.0%	3 / 3 100.0%	1 / 2 50.0%	1 / 2 50.0%	3 / 3 100.0%	1 / 1 100.0%	2 / 5 40.0%	1 / 3 33.3%	0 / 2 0.0%	1 / 2 50.0%	1 / 1 100.0%	0 / 2 0.0%	0 / 1 0.0%	3 / 3 100.0%	1 / 4 25.0%	1 / 3 33.3%	0 / 1 0.0%	2 / 5 40.0%	0 / 0 0.0%	0 / 0 0.0%	1 / 1 100.0%	0 / 0 0.0%	31 / 60 51.7%AB	17 / 60 28.3%E	8 / 60 13.3%	4 / 60 6.7%ABE				
1	Staphylococcus aureus JKD6159	33 / 58 56.9%	0 / 2 0.0%	3 / 4 75.0%	3 / 3 100.0%	0 / 0 0.0%	5 / 5 100.0%	3 / 3 100.0%	1 / 2 50.0%	1 / 2 50.0%	2 / 2 100.0%	1 / 1 100.0%	2 / 5 40.0%	1 / 3 33.3%	0 / 2 0.0%	1 / 2 50.0%	1 / 1 100.0%	0 / 2 0.0%	0 / 1 0.0%	3 / 3 100.0%	1 / 4 25.0%	1 / 3 33.3%	0 / 1 0.0%	2 / 5 40.0%	0 / 0 0.0%	0 / 0 0.0%	2 / 2 100.0%	0 / 0 0.0%	29 / 58 50.0%AB	17 / 58 29.3%E	8 / 58 13.8%	4 / 58 6.9%ABE				
1	Staphylococcus aureus subsp. aureus ECT-R 2	36 / 61 59.0%	0 / 2 0.0%	3 / 4 75.0%	3 / 3 100.0%	2 / 2 100.0%	7 / 7 100.0%	3 / 3 100.0%	1 / 2 50.0%	1 / 2 50.0%	3 / 3 100.0%	1 / 1 100.0%	2 / 5 40.0%	1 / 3 33.3%	0 / 2 0.0%	1 / 2 50.0%	1 / 1 100.0%	0 / 2 0.0%	0 / 1 0.0%	3 / 3 100.0%	1 / 4 25.0%	1 / 3 33.3%	0 / 1 0.0%	2 / 5 40.0%	0 / 0 0.0%	0 / 0 0.0%	0 / 0 0.0%	0 / 0 0.0%	32 / 61 52.5%AB	17 / 61 27.9%E	8 / 61 13.1%	4 / 61 6.6%ABE				
1	Staphylococcus aureus subsp. aureus MSHR1132	34 / 59 57.6%	0 / 2 0.0%	3 / 4 75.0%	3 / 3 100.0%	2 / 2 100.0%	7 / 7 100.0%	2 / 2 100.0%	1 / 2 50.0%	1 / 2 50.0%	3 / 3 100.0%	1 / 1 100.0%	2 / 5 40.0%	1 / 3 33.3%	0 / 2 0.0%	1 / 2 50.0%	1 / 1 100.0%	0 / 2 0.0%	0 / 1 0.0%	2 / 2 100.0%	1 / 4 25.0%	1 / 3 33.3%	0 / 1 0.0%	2 / 5 40.0%	0 / 0 0.0%	0 / 0 0.0%	0 / 0 0.0%	0 / 0 0.0%	30 / 59 50.8%AB	17 / 59 28.8%E	8 / 59 13.6%	4 / 59 6.8%ABE				
1	Staphylococcus aureus subsp. aureus LGA251	35 / 60 58.3%	0 / 2 0.0%	3 / 4 75.0%	3 / 3 100.0%	2 / 2 100.0%	6 / 6 100.0%	3 / 3 100.0%	1 / 2 50.0%	1 / 2 50.0%	3 / 3 100.0%	1 / 1 100.0%	2 / 5 40.0%	1 / 3 33.3%	0 / 2 0.0%	1 / 2 50.0%	1 / 1 100.0%	0 / 2 0.0%	0 / 1 0.0%	3 / 3 100.0%	1 / 4 25.0%	1 / 3 33.3%	0 / 1 0.0%	2 / 5 40.0%	0 / 0 0.0%	0 / 0 0.0%	0 / 0 0.0%	0 / 0 0.0%	31 / 60 51.7%AB	17 / 60 28.3%E	8 / 60 13.3%	4 / 60 6.7%ABE				
1	Staphylococcus aureus T0131	31 / 54 57.4%	0 / 2 0.0%	2 / 2 100.0%	3 / 3 100.0%	1 / 1 100.0%	6 / 6 100.0%	3 / 3 100.0%	1 / 2 50.0%	1 / 2 50.0%	2 / 2 100.0%	1 / 1 100.0%	2 / 4 50.0%	1 / 3 33.3%	0 / 2 0.0%	1 / 2 50.0%	1 / 1 100.0%	0 / 2 0.0%	0 / 1 0.0%	3 / 3 100.0%	1 / 4 25.0%	0 / 2 0.0%	0 / 1 0.0%	2 / 5 40.0%	0 / 0 0.0%	0 / 0 0.0%	0 / 0 0.0%	0 / 0 0.0%	28 / 54 51.9%AB	16 / 54 29.6%E	7 / 54 13.0%	3 / 54 5.6%ABE				
1	Staphylococcus aureus subsp. aureus VC40	35 / 60 58.3%	0 / 2 0.0%	3 / 4 75.0%	3 / 3 100.0%	2 / 2 100.0%	6 / 6 100.0%	3 / 3 100.0%	1 / 3 33.3%	1 / 2 50.0%	3 / 3 100.0%	1 / 1 100.0%	2 / 5 40.0%	1 / 3 33.3%	0 / 2 0.0%	1 / 1 100.0%	1 / 1 100.0%	0 / 2 0.0%	0 / 1 0.0%	3 / 3 100.0%	1 / 4 25.0%	1 / 3 33.3%	0 / 1 0.0%	2 / 5 40.0%	0 / 0 0.0%	0 / 0 0.0%	0 / 0 0.0%	0 / 0 0.0%	31 / 60 51.7%AB	17 / 60 28.3%E	8 / 60 13.3%	4 / 60 6.7%ABE				
1	Staphylococcus aureus subsp. aureus HO 5096 0412	35 / 60 58.3%	0 / 2 0.0%	3 / 4 75.0%	3 / 3 100.0%	2 / 2 100.0%	6 / 6 100.0%	3 / 3 100.0%	1 / 2 50.0%	1 / 2 50.0%	3 / 3 100.0%	1 / 1 100.0%	2 / 5 40.0%	1 / 3 33.3%	0 / 2 0.0%	1 / 2 50.0%	1 / 1 100.0%	0 / 2 0.0%	0 / 1 0.0%	3 / 3 100.0%	1 / 4 25.0%	1 / 3 33.3%	0 / 1 0.0%	2 / 5 40.0%	0 / 0 0.0%	0 / 0 0.0%	0 / 0 0.0%	0 / 0 0.0%	31 / 60 51.7%AB	17 / 60 28.3%E	8 / 60 13.3%	4 / 60 6.7%ABE				
1	Staphylococcus aureus subsp. aureus M013	34 / 59 57.6%	0 / 2 0.0%	2 / 3 66.7%	3 / 3 100.0%	2 / 2 100.0%	6 / 6 100.0%	3 / 3 100.0%	1 / 2 50.0%	1 / 2 50.0%	3 / 3 100.0%	1 / 1 100.0%	2 / 5 40.0%	1 / 3 33.3%	0 / 2 0.0%	1 / 2 50.0%	1 / 1 100.0%	0 / 2 0.0%	0 / 1 0.0%	3 / 3 100.0%	1 / 4 25.0%	1 / 3 33.3%	0 / 1 0.0%	2 / 5 40.0%	0 / 0 0.0%	0 / 0 0.0%	0 / 0 0.0%	0 / 0 0.0%	30 / 59 50.8%AB	16 / 59 27.1%E	8 / 59 13.6%	4 / 59 6.8%ABE	1 / 59 1.7%CE			
1	Staphylococcus aureus 11819-97	33 / 58 56.9%	0 / 2 0.0%	3 / 4 75.0%	3 / 3 100.0%	1 / 1 100.0%	6 / 6 100.0%	3 / 3 100.0%	1 / 2 50.0%	1 / 2 50.0%	3 / 3 100.0%	1 / 1 100.0%	2 / 5 40.0%	1 / 3 33.3%	0 / 2 0.0%	1 / 2 50.0%	1 / 1 100.0%	0 / 2 0.0%	0 / 1 0.0%	3 / 3 100.0%	1 / 4 25.0%	1 / 3 33.3%	0 / 1 0.0%	1 / 4 25.0%	0 / 0 0.0%	0 / 0 0.0%	0 / 0 0.0%	0 / 0 0.0%	30 / 58 51.7%AB	17 / 58 29.3%E	8 / 58 13.8%	3 / 58 5.2%ABE				
1	Staphylococcus aureus RF122	35 / 60 58.3%	0 / 2 0.0%	3 / 4 75.0%	3 / 3 100.0%	3 / 3 100.0%	5 / 5 100.0%	3 / 3 100.0%	1 / 2 50.0%	1 / 2 50.0%	3 / 3 100.0%	1 / 1 100.0%	2 / 5 40.0%	1 / 3 33.3%	0 / 2 0.0%	1 / 2 50.0%	1 / 1 100.0%	0 / 2 0.0%	0 / 1 0.0%	3 / 3 100.0%	1 / 4 25.0%	1 / 3 33.3%	0 / 1 0.0%	2 / 5 40.0%	0 / 0 0.0%	0 / 0 0.0%	0 / 0 0.0%	0 / 0 0.0%	31 / 60 51.7%AB	15 / 60 25.0%	10 / 60 16.7%E	4 / 60 6.7%ABE				
1	Staphylococcus aureus 04-02981	38 / 63 60.3%	0 / 2 0.0%	3 / 4 75.0%	3 / 3 100.0%	3 / 3 100.0%	6 / 6 100.0%	3 / 3 100.0%	1 / 2 50.0%	1 / 2 50.0%	4 / 4 100.0%	1 / 1 100.0%	2 / 5 40.0%	1 / 3 33.3%	0 / 2 0.0%	1 / 2 50.0%	1 / 1 100.0%	0 / 2 0.0%	0 / 1 0.0%	3 / 3 100.0%	1 / 4 25.0%	1 / 3 33.3%	0 / 1 0.0%	2 / 5 40.0%	0 / 0 0.0%	0 / 0 0.0%	1 / 1 100.0%	0 / 0 0.0%	34 / 63 54.0%AB	17 / 63 27.0%E	8 / 63 12.7%	4 / 63 6.3%ABE				
1	Staphylococcus aureus 08BA02176	33 / 58 56.9%	0 / 2 0.0%	3 / 4 75.0%	3 / 3 100.0%	1 / 1 100.0%	5 / 5 100.0%	3 / 3 100.0%	1 / 2 50.0%	1 / 2 50.0%	2 / 2 100.0%	1 / 1 100.0%	2 / 5 40.0%	1 / 3 33.3%	0 / 1 0.0%	1 / 2 50.0%	1 / 1 100.0%	0 / 2 0.0%	0 / 1 0.0%	3 / 3 100.0%	1 / 4 25.0%	1 / 3 33.3%	0 / 1 0.0%	2 / 6 33.3%	0 / 0 0.0%	0 / 0 0.0%	1 / 1 100.0%	0 / 0 0.0%	29 / 58 50.0%AB	17 / 58 29.3%E	8 / 58 13.8%	4 / 58 6.9%ABE				
1	Staphylococcus aureus M1	33 / 58 56.9%	0 / 2 0.0%	3 / 4 75.0%	3 / 3 100.0%	0 / 0 0.0%	6 / 6 100.0%	3 / 3 100.0%	1 / 2 50.0%	1 / 2 50.0%	2 / 2 100.0%	1 / 1 100.0%	2 / 5 40.0%	1 / 3 33.3%	0 / 2 0.0%	1 / 2 50.0%	1 / 1 100.0%	0 / 2 0.0%	0 / 1 0.0%	3 / 3 100.0%	1 / 4 25.0%	1 / 3 33.3%	0 / 1 0.0%	2 / 5 40.0%	0 / 0 0.0%	0 / 0 0.0%	1 / 1 100.0%	0 / 0 0.0%	29 / 58 50.0%AB	17 / 58 29.3%E	8 / 58 13.8%	4 / 58 6.9%ABE				
1	Staphylococcus aureus Bmb9393	37 / 62 59.7%	0 / 3 0.0%	3 / 4 75.0%	3 / 3 100.0%	2 / 2 100.0%	6 / 6 100.0%	3 / 3 100.0%	1 / 2 50.0%	1 / 2 50.0%	3 / 3 100.0%	1 / 1 100.0%	3 / 5 60.0%	1 / 3 33.3%	0 / 2 0.0%	1 / 2 50.0%	1 / 1 100.0%	0 / 2 0.0%	0 / 1 0.0%	3 / 3 100.0%	1 / 4 25.0%	1 / 3 33.3%	0 / 1 0.0%	2 / 5 40.0%	0 / 0 0.0%	0 / 0 0.0%	1 / 1 100.0%	0 / 0 0.0%	33 / 62 53.2%AB	17 / 62 27.4%E	8 / 62 12.9%	4 / 62 6.5%ABE				
1	Staphylococcus aureus CA-347	36 / 61 59.0%	0 / 2 0.0%	3 / 4 75.0%	3 / 3 100.0%	2 / 2 100.0%	6 / 6 100.0%	3 / 3 100.0%	1 / 2 50.0%	1 / 2 50.0%	3 / 3 100.0%	1 / 1 100.0%	2 / 5 40.0%	1 / 3 33.3%	0 / 2 0.0%	1 / 2 50.0%	1 / 1 100.0%	0 / 2 0.0%	0 / 1 0.0%	3 / 3 100.0%	1 / 4 25.0%	1 / 3 33.3%	0 / 1 0.0%	2 / 5 40.0%	0 / 0 0.0%	0 / 0 0.0%	1 / 1 100.0%	0 / 0 0.0%	32 / 61 52.5%AB	17 / 61 27.9%E	8 / 61 13.1%	4 / 61 6.6%ABE				
1	ATCC 51365	36 / 60 60.0%	0 / 2 0.0%	2 / 3 66.7%	3 / 3 100.0%	2 / 2 100.0%	6 / 6 100.0%	3 / 3 100.0%	1 / 2 50.0%	2 / 2 100.0%	3 / 3 100.0%	1 / 1 100.0%	2 / 6 33.3%	1 / 3 33.3%	1 / 2 50.0%	1 / 2 50.0%	1 / 1 100.0%	0 / 2 0.0%	0 / 1 0.0%	3 / 3 100.0%	1 / 4 25.0%	1 / 3 33.3%	0 / 1 0.0%	2 / 5 40.0%	0 / 0 0.0%	0 / 0 0.0%	0 / 0 0.0%	0 / 0 0.0%	32 / 60 53.3%AB	14 / 60 23.3%E	10 / 60 16.7%	4 / 60 6.7%ABE				
1	Staphylococcus carnosus subsp. carnosus	36 / 60 60.0%	0 / 2 0.0%	2 / 3 66.7%	3 / 3 100.0%	2 / 2 100.0%	6 / 6 100.0%	3 / 3 100.0%	1 / 2 50.0%	2 / 2 100.0%	3 / 3 100.0%	1 / 1 100.0%	2 / 6 33.3%	1 / 3 33.3%	1 / 2 50.0%	1 / 2 50.0%	1 / 1 100.0%	0 / 2 0.0%	0 / 1 0.0%	3 / 3 100.0%	1 / 4 25.0%	1 / 3 33.3%	0 / 1 0.0%	2 / 5 40.0%	0 / 0 0.0%	0 / 0 0.0%	0 / 0 0.0%	0 / 0 0.0%	32 / 60 53.3%AB	14 / 60 23.3%E	10 / 60 16.7%	4 / 60 6.7%ABE				
1	Staphylococcus carnosus subsp. carnosus TM300	36 / 60 60.0%	0 / 2 0.0%	2 / 3 66.7%	3 / 3 100.0%	2 / 2 100.0%	6 / 6 100.0%	3 / 3 100.0%	1 / 2 50.0%	2 / 2 100.0%	3 / 3 100.0%	1 / 1 100.0%	2 / 6 33.3%	1 / 3 33.3%	1 / 2 50.0%	1 / 2 50.0%	1 / 1 100.0%	0 / 2 0.0%	0 / 1 0.0%	3 / 3 100.0%	1 / 4 25.0%	1 / 3 33.3%	0 / 1 0.0%	2 / 5 40.0%	0 / 0 0.0%	0 / 0 0.0%	0 / 0 0.0%	0 / 0 0.0%	32 / 60 53.3%AB	14 / 60 23.3%E	10 / 60 16.7%	4 / 60 6.7%ABE				
2	"Albococcus epidermidis" Winslow and Winslow 1908	72 / 118 61.0%	0 / 4 0.0%	4 / 6 66.7%	6 / 6 100.0%	4 / 4 100.0%	12 / 12 100.0%	6 / 6 100.0%	2 / 4 50.0%	4 / 4 100.0%	6 / 6 100.0%	2 / 2 100.0%	4 / 10 40.0%	2 / 6 33.3%	0 / 4 0.0%	2 / 4 50.0%	2 / 2 100.0%	0 / 4 0.0%	0 / 2 0.0%	6 / 6 100.0%	2 / 8 25.0%	2 / 6 33.3%	0 / 2 0.0%	6 / 10 60.0%	0 / 0 0.0%	0 / 0 0.0%	0 / 0 0.0%	0 / 0 0.0%	62 / 118 52.5%AB	30 / 118 25.4%	16 / 118 13.6%E	10 / 118 8.5%ABE				
1	Staphylococcus epidermidis ATCC 35984	36 / 59 61.0%	0 / 2 0.0%	2 / 3 66.7%	3 / 3 100.0%	2 / 2 100.0%	6 / 6 100.0%	3 / 3 100.0%	1 / 2 50.0%	2 / 2 100.0%	3 / 3 100.0%	1 / 1 100.0%	2 / 5 40.0%	1 / 3 33.3%	0 / 2 0.0%	1 / 2 50.0%	1 / 1 100.0%	0 / 2 0.0%	0 / 1 0.0%	3 / 3 100.0%	1 / 4 25.0%	1 / 3 33.3%	0 / 1 0.0%	3 / 5 60.0%	0 / 0 0.0%	0 / 0 0.0%	0 / 0 0.0%	0 / 0 0.0%	31 / 59 52.5%AB	15 / 59 25.4%	8 / 59 13.6%E	5 / 59 8.5%ABE				
1	Staphylococcus epidermidis ATCC 12228	36 / 59 61.0%	0 / 2 0.0%	2 / 3 66.7%	3 / 3 100.0%	2 / 2 100.0%	6 / 6 100.0%	3 / 3 100.0%	1 / 2 50.0%	2 / 2 100.0%	3 / 3 100.0%	1 / 1 100.0%	2 / 5 40.0%	1 / 3 33.3%	0 / 2 0.0%	1 / 2 50.0%	1 / 1 100.0%	0 / 2 0.0%	0 / 1 0.0%	3 / 3 100.0%	1 / 4 25.0%	1 / 3 33.3%	0 / 1 0.0%	3 / 5 60.0%	0 / 0 0.0%	0 / 0 0.0%	0 / 0 0.0%	0 / 0 0.0%	31 / 59 52.5%AB	15 / 59 25.4%	8 / 59 13.6%E	5 / 59 8.5%ABE				
1	ATCC 29970	35 / 59 59.3%	0 / 2 0.0%	3 / 4 75.0%	3 / 3 100.0%	2 / 2 100.0%	5 / 5 100.0%	3 / 3 100.0%	1 / 2 50.0%	2 / 2 100.0%	3 / 3 100.0%	1 / 1 100.0%	2 / 5 40.0%	0 / 3 0.0%	0 / 2 0.0%	1 / 2 50.0%	1 / 1 100.0%	0 / 2 0.0%	0 / 1 0.0%	3 / 3 100.0%	0 / 4 0.0%	2 / 3 66.7%	0 / 1 0.0%	3 / 5 60.0%	0 / 0 0.0%	0 / 0 0.0%	0 / 0 0.0%	0 / 0 0.0%	29 / 59 49.2%AB	17 / 59 28.8%	7 / 59 11.9%E	6 / 59 10.2%ABE				
1	Staphylococcus haemolyticus JCSC1435	35 / 59 59.3%	0 / 2 0.0%	3 / 4 75.0%	3 / 3 100.0%	2 / 2 100.0%	5 / 5 100.0%	3 / 3 100.0%	1 / 2 50.0%	2 / 2 100.0%	3 / 3 100.0%	1 / 1 100.0%	2 / 5 40.0%	0 / 3 0.0%	0 / 2 0.0%	1 / 2 50.0%	1 / 1 100.0%	0 / 2 0.0%	0 / 1 0.0%	3 / 3 100.0%	0 / 4 0.0%	2 / 3 66.7%	0 / 1 0.0%	3 / 5 60.0%	0 / 0 0.0%	0 / 0 0.0%	0 / 0 0.0%	0 / 0 0.0%	29 / 59 49.2%AB	17 / 59 28.8%	7 / 59 11.9%E	6 / 59 10.2%ABE				
1	ATCC 27836	34 / 59 57.6%	0 / 2 0.0%	3 / 4 75.0%	3 / 3 100.0%	0 / 0 0.0%	6 / 6 100.0%	3 / 3 100.0%	1 / 2 50.0%	2 / 2 100.0%	2 / 2 100.0%	1 / 1 100.0%	1 / 5 20.0%	1 / 3 33.3%	0 / 2 0.0%	1 / 2 50.0%	1 / 1 100.0%	0 / 2 0.0%	0 / 1 0.0%	3 / 3 100.0%	1 / 4 25.0%	1 / 3 33.3%	0 / 1 0.0%	3 / 5 60.0%	0 / 0 0.0%	0 / 0 0.0%	1 / 2 50.0%	0 / 0 0.0%	29 / 59 49.2%AB	15 / 59 25.4%E	10 / 59 16.9%	5 / 59 8.5%ABE				
1	Staphylococcus warneri SG1	34 / 59 57.6%	0 / 2 0.0%	3 / 4 75.0%	3 / 3 100.0%	0 / 0 0.0%	6 / 6 100.0%	3 / 3 100.0%	1 / 2 50.0%	2 / 2 100.0%	2 / 2 100.0%	1 / 1 100.0%	1 / 5 20.0%	1 / 3 33.3%	0 / 2 0.0%	1 / 2 50.0%	1 / 1 100.0%	0 / 2 0.0%	0 / 1 0.0%	3 / 3 100.0%	1 / 4 25.0%	1 / 3 33.3%	0 / 1 0.0%	3 / 5 60.0%	0 / 0 0.0%	0 / 0 0.0%	1 / 2 50.0%	0 / 0 0.0%	29 / 59 49.2%AB	15 / 59 25.4%E	10 / 59 16.9%	5 / 59 8.5%ABE				
2	ATCC 43809	65 / 117 55.6%	0 / 4 0.0%	6 / 8 75.0%	6 / 6 100.0%	2 / 2 100.0%	9 / 11 81.8%	6 / 6 100.0%	2 / 4 50.0%	2 / 4 50.0%	5 / 5 100.0%	2 / 2 100.0%	4 / 10 40.0%	2 / 6 33.3%	0 / 4 0.0%	2 / 4 50.0%	2 / 2 100.0%	0 / 4 0.0%	0 / 2 0.0%	6 / 6 100.0%	0 / 8 0.0%	2 / 6 33.3%	0 / 2 0.0%	6 / 10 60.0%	0 / 0 0.0%	0 / 0 0.0%	1 / 1 100.0%	0 / 0 0.0%	55 / 117 47.0%AB	30 / 117 25.6%E	22 / 117 18.8%	10 / 117 8.5%ABE				
1	Staphylococcus lugdunensis HKU09-01	35 / 61 57.4%	0 / 2 0.0%	3 / 4 75.0%	3 / 3 100.0%	2 / 2 100.0%	5 / 6 83.3%	3 / 3 100.0%	1 / 2 50.0%	1 / 2 50.0%	3 / 3 100.0%	1 / 1 100.0%	2 / 5 40.0%	1 / 3 33.3%	0 / 2 0.0%	1 / 2 50.0%	1 / 1 100.0%	0 / 2 0.0%	0 / 1 0.0%	3 / 3 100.0%	0 / 4 0.0%	1 / 3 33.3%	0 / 1 0.0%	3 / 5 60.0%	0 / 0 0.0%	0 / 0 0.0%	1 / 1 100.0%	0 / 0 0.0%	30 / 61 49.2%AB	15 / 61 24.6%E	11 / 61 18.0%	5 / 61 8.2%ABE				
1	Staphylococcus lugdunensis N920143	30 / 56 53.6%	0 / 2 0.0%	3 / 4 75.0%	3 / 3 100.0%	0 / 0 0.0%	4 / 5 80.0%	3 / 3 100.0%	1 / 2 50.0%	1 / 2 50.0%	2 / 2 100.0%	1 / 1 100.0%	2 / 5 40.0%	1 / 3 33.3%	0 / 2 0.0%	1 / 2 50.0%	1 / 1 100.0%	0 / 2 0.0%	0 / 1 0.0%	3 / 3 100.0%	0 / 4 0.0%	1 / 3 33.3%	0 / 1 0.0%	3 / 5 60.0%	0 / 0 0.0%	0 / 0 0.0%	0 / 0 0.0%	0 / 0 0.0%	25 / 56 44.6%AB	15 / 56 26.8%E	11 / 56 19.6%	5 / 56 8.9%ABE				
1	"Staphylococcus saprophyticus" Fairbrother 1940	35 / 60 58.3%	0 / 2 0.0%	2 / 3 66.7%	3 / 3 100.0%	2 / 2 100.0%	7 / 7 100.0%	3 / 3 100.0%	1 / 2 50.0%	2 / 2 100.0%	3 / 3 100.0%	1 / 1 100.0%	3 / 5 60.0%	1 / 3 33.3%	0 / 2 0.0%	1 / 2 50.0%	0 / 1 0.0%	0 / 2 0.0%	0 / 1 0.0%	3 / 3 100.0%	1 / 4 25.0%	1 / 3 33.3%	0 / 1 0.0%	1 / 5 20.0%	0 / 0 0.0%	0 / 0 0.0%	0 / 0 0.0%	0 / 0 0.0%	32 / 60 53.3%AB	15 / 60 25.0%	10 / 60 16.7%E	3 / 60 5.0%ABE				
1	Staphylococcus saprophyticus ATCC 15305	35 / 60 58.3%	0 / 2 0.0%	2 / 3 66.7%	3 / 3 100.0%	2 / 2 100.0%	7 / 7 100.0%	3 / 3 100.0%	1 / 2 50.0%	2 / 2 100.0%	3 / 3 100.0%	1 / 1 100.0%	3 / 5 60.0%	1 / 3 33.3%	0 / 2 0.0%	1 / 2 50.0%	0 / 1 0.0%	0 / 2 0.0%	0 / 1 0.0%	3 / 3 100.0%	1 / 4 25.0%	1 / 3 33.3%	0 / 1 0.0%	1 / 5 20.0%	0 / 0 0.0%	0 / 0 0.0%	0 / 0 0.0%	0 / 0 0.0%	32 / 60 53.3%AB	15 / 60 25.0%	10 / 60 16.7%E	3 / 60 5.0%ABE				
1	Staphylococcus saprophyticus subsp. saprophyticus ATCC 15305	35 / 60 58.3%	0 / 2 0.0%	2 / 3 66.7%	3 / 3 100.0%	2 / 2 100.0%	7 / 7 100.0%	3 / 3 100.0%	1 / 2 50.0%	2 / 2 100.0%	3 / 3 100.0%	1 / 1 100.0%	3 / 5 60.0%	1 / 3 33.3%	0 / 2 0.0%	1 / 2 50.0%	0 / 1 0.0%	0 / 2 0.0%	0 / 1 0.0%	3 / 3 100.0%	1 / 4 25.0%	1 / 3 33.3%	0 / 1 0.0%	1 / 5 20.0%	0 / 0 0.0%	0 / 0 0.0%	0 / 0 0.0%	0 / 0 0.0%	32 / 60 53.3%AB	15 / 60 25.0%	10 / 60 16.7%E	3 / 60 5.0%ABE				
2	CCUG 49543	67 / 117 57.3%	0 / 4 0.0%	4 / 6 66.7%	6 / 6 100.0%	3 / 3 100.0%	12 / 12 100.0%	6 / 6 100.0%	0 / 4 0.0%	4 / 4 100.0%	6 / 6 100.0%	2 / 2 100.0%	4 / 8 50.0%	2 / 6 33.3%	0 / 4 0.0%	2 / 4 50.0%	2 / 2 100.0%	0 / 4 0.0%	0 / 2 0.0%	6 / 6 100.0%	2 / 8 25.0%	2 / 8 25.0%	0 / 2 0.0%	4 / 10 40.0%	0 / 0 0.0%	0 / 0 0.0%	0 / 0 0.0%	0 / 0 0.0%	61 / 117 52.1%AB	27 / 117 23.1%	23 / 117 19.7%E	6 / 117 5.1%ABE				
1	Staphylococcus pseudintermedius HKU10-03	34 / 59 57.6%	0 / 2 0.0%	2 / 3 66.7%	3 / 3 100.0%	2 / 2 100.0%	6 / 6 100.0%	3 / 3 100.0%	0 / 2 0.0%	2 / 2 100.0%	3 / 3 100.0%	1 / 1 100.0%	2 / 4 50.0%	1 / 3 33.3%	0 / 2 0.0%	1 / 2 50.0%	1 / 1 100.0%	0 / 2 0.0%	0 / 1 0.0%	3 / 3 100.0%	1 / 4 25.0%	1 / 4 25.0%	0 / 1 0.0%	2 / 5 40.0%	0 / 0 0.0%	0 / 0 0.0%	0 / 0 0.0%	0 / 0 0.0%	31 / 59 52.5%AB	13 / 59 22.0%	12 / 59 20.3%E	3 / 59 5.1%ABE				
1	Staphylococcus pseudintermedius ED99	33 / 58 56.9%	0 / 2 0.0%	2 / 3 66.7%	3 / 3 100.0%	1 / 1 100.0%	6 / 6 100.0%	3 / 3 100.0%	0 / 2 0.0%	2 / 2 100.0%	3 / 3 100.0%	1 / 1 100.0%	2 / 4 50.0%	1 / 3 33.3%	0 / 2 0.0%	1 / 2 50.0%	1 / 1 100.0%	0 / 2 0.0%	0 / 1 0.0%	3 / 3 100.0%	1 / 4 25.0%	1 / 4 25.0%	0 / 1 0.0%	2 / 5 40.0%	0 / 0 0.0%	0 / 0 0.0%	0 / 0 0.0%	0 / 0 0.0%	30 / 58 51.7%AB	14 / 58 24.1%	11 / 58 19.0%E	3 / 58 5.2%ABE				
1	Macrococcus	29 / 48 60.4%	0 / 2 0.0%	2 / 3 66.7%	3 / 3 100.0%	1 / 1 100.0%	3 / 4 75.0%	2 / 2 100.0%	1 / 2 50.0%	2 / 2 100.0%	2 / 2 100.0%	1 / 1 100.0%	2 / 4 50.0%	1 / 2 50.0%	0 / 2 0.0%	1 / 2 50.0%	1 / 1 100.0%	0 / 1 0.0%	0 / 1 0.0%	2 / 2 100.0%	1 / 2 50.0%	2 / 4 50.0%	0 / 1 0.0%	2 / 4 50.0%	0 / 0 0.0%	0 / 0 0.0%	0 / 0 0.0%	0 / 0 0.0%	22 / 48 45.8%AB	12 / 48 25.0%E	7 / 48 14.6%	7 / 48 14.6%ABE				
1	"Micrococcus caseolyticus" Evans 1916	29 / 48 60.4%	0 / 2 0.0%	2 / 3 66.7%	3 / 3 100.0%	1 / 1 100.0%	3 / 4 75.0%	2 / 2 100.0%	1 / 2 50.0%	2 / 2 100.0%	2 / 2 100.0%	1 / 1 100.0%	2 / 4 50.0%	1 / 2 50.0%	0 / 2 0.0%	1 / 2 50.0%	1 / 1 100.0%	0 / 1 0.0%	0 / 1 0.0%	2 / 2 100.0%	1 / 2 50.0%	2 / 4 50.0%	0 / 1 0.0%	2 / 4 50.0%	0 / 0 0.0%	0 / 0 0.0%	0 / 0 0.0%	0 / 0 0.0%	22 / 48 45.8%AB	12 / 48 25.0%E	7 / 48 14.6%ABE	7 / 48 14.6%				
1	Macrococcus caseolyticus JCSC5402	29 / 48 60.4%	0 / 2 0.0%	2 / 3 66.7%	3 / 3 100.0%	1 / 1 100.0%	3 / 4 75.0%	2 / 2 100.0%	1 / 2 50.0%	2 / 2 100.0%	2 / 2 100.0%	1 / 1 100.0%	2 / 4 50.0%	1 / 2 50.0%	0 / 2 0.0%	1 / 2 50.0%	1 / 1 100.0%	0 / 1 0.0%	0 / 1 0.0%	2 / 2 100.0%	1 / 2 50.0%	2 / 4 50.0%	0 / 1 0.0%	2 / 4 50.0%	0 / 0 0.0%	0 / 0 0.0%	0 / 0 0.0%	0 / 0 0.0%	22 / 48 45.8%AB	12 / 48 25.0%E	7 / 48 14.6%	7 / 48 14.6%ABE				
84	Bacillaceae	5084 / 7546 67.4%	276 / 290 95.2%	391 / 397 98.5%	369 / 379 97.4%	250 / 292 85.6%	460 / 629 73.1%	370 / 459 80.6%	143 / 170 84.1%	182 / 201 90.5%	240 / 468 51.3%	87 / 123 70.7%	487 / 506 96.2%	288 / 372 77.4%	194 / 267 72.7%	231 / 326 70.9%	24 / 90 26.7%	134 / 260 51.5%	109 / 176 61.9%	223 / 411 54.3%	213 / 507 42.0%	165 / 507 32.5%	84 / 129 65.1%	162 / 585 27.7%	2 / 2 100.0%	0 / 0 0.0%	0 / 0 0.0%	0 / 0 0.0%	3871 / 7546 51.3%AB	1305 / 7546 17.3%E	1109 / 7546 14.7%	1106 / 7546 14.7%ABE	88 / 7546 1.2%B	33 / 7546 0.4%CE	19 / 7546 0.3%BE	15 / 7546 0.2%C
69	Bacillus	4393 / 6312 69.6%	242 / 256 94.5%	342 / 345 99.1%	310 / 318 97.5%	232 / 237 97.9%	402 / 518 77.6%	314 / 388 80.9%	132 / 139 95.0%	163 / 170 95.9%	201 / 371 54.2%	73 / 106 68.9%	412 / 423 97.4%	259 / 317 81.7%	169 / 235 71.9%	184 / 272 67.6%	9 / 72 12.5%	119 / 202 58.9%	91 / 146 62.3%	184 / 342 53.8%	178 / 415 42.9%	156 / 435 35.9%	69 / 113 61.1%	150 / 490 30.6%	2 / 2 100.0%	0 / 0 0.0%	0 / 0 0.0%	0 / 0 0.0%	3294 / 6312 52.2%AB	1024 / 6312 16.2%ABE	999 / 6312 15.8%E	881 / 6312 14.0%	62 / 6312 1.0%B	32 / 6312 0.5%CE	13 / 6312 0.2%BE	7 / 6312 0.1%C
2	ATCC 7050	66 / 153 43.1%	0 / 6 0.0%	6 / 8 75.0%	6 / 8 75.0%	3 / 5 60.0%	9 / 12 75.0%	3 / 9 33.3%	2 / 4 50.0%	1 / 3 33.3%	3 / 10 30.0%	2 / 2 100.0%	7 / 11 63.6%	4 / 9 44.4%	4 / 6 66.7%	2 / 6 33.3%	2 / 2 100.0%	3 / 6 50.0%	0 / 4 0.0%	4 / 8 50.0%	0 / 10 0.0%	2 / 8 25.0%	2 / 2 100.0%	1 / 14 7.1%	0 / 0 0.0%	0 / 0 0.0%	0 / 0 0.0%	0 / 0 0.0%	59 / 153 38.6%AB	49 / 153 32.0%E	31 / 153 20.3%	7 / 153 4.6%ABE	6 / 153 3.9%C	1 / 153 0.7%CE		
1	Bacillus coagulans 36D1	38 / 84 45.2%	0 / 3 0.0%	3 / 4 75.0%	3 / 4 75.0%	2 / 3 66.7%	6 / 7 85.7%	2 / 5 40.0%	1 / 2 50.0%	1 / 2 50.0%	2 / 6 33.3%	1 / 1 100.0%	4 / 6 66.7%	2 / 5 40.0%	2 / 3 66.7%	1 / 3 33.3%	1 / 1 100.0%	2 / 4 50.0%	0 / 2 0.0%	2 / 4 50.0%	0 / 6 0.0%	1 / 4 25.0%	1 / 1 100.0%	1 / 8 12.5%	0 / 0 0.0%	0 / 0 0.0%	0 / 0 0.0%	0 / 0 0.0%	34 / 84 40.5%AB	26 / 84 31.0%E	16 / 84 19.0%	4 / 84 4.8%ABE	3 / 84 3.6%C	1 / 84 1.2%CE		
1	Bacillus coagulans 2-6	28 / 69 40.6%	0 / 3 0.0%	3 / 4 75.0%	3 / 4 75.0%	1 / 2 50.0%	3 / 5 60.0%	1 / 4 25.0%	1 / 2 50.0%	0 / 1 0.0%	1 / 4 25.0%	1 / 1 100.0%	3 / 5 60.0%	2 / 4 50.0%	2 / 3 66.7%	1 / 3 33.3%	1 / 1 100.0%	1 / 2 50.0%	0 / 2 0.0%	2 / 4 50.0%	0 / 4 0.0%	1 / 4 25.0%	1 / 1 100.0%	0 / 6 0.0%	0 / 0 0.0%	0 / 0 0.0%	0 / 0 0.0%	0 / 0 0.0%	25 / 69 36.2%AB	23 / 69 33.3%E	15 / 69 21.7%	3 / 69 4.3%C	3 / 69 4.3%ABE			
3	ATCC 14581	268 / 330 81.2%	17 / 17 100.0%	18 / 18 100.0%	19 / 19 100.0%	10 / 10 100.0%	26 / 29 89.7%	17 / 20 85.0%	9 / 9 100.0%	9 / 9 100.0%	15 / 18 83.3%	6 / 6 100.0%	24 / 24 100.0%	5 / 17 29.4%	9 / 12 75.0%	12 / 12 100.0%	3 / 3 100.0%	8 / 8 100.0%	6 / 11 54.5%	14 / 17 82.4%	15 / 19 78.9%	8 / 20 40.0%	5 / 5 100.0%	13 / 27 48.1%	0 / 0 0.0%	0 / 0 0.0%	0 / 0 0.0%	0 / 0 0.0%	186 / 330 56.4%AB	82 / 330 24.8%ABE	34 / 330 10.3%E	27 / 330 8.2%	1 / 330 0.3%CE			
1	Bacillus megaterium QM B1551	95 / 117 81.2%	6 / 6 100.0%	6 / 6 100.0%	7 / 7 100.0%	4 / 4 100.0%	9 / 10 90.0%	6 / 7 85.7%	3 / 3 100.0%	3 / 3 100.0%	6 / 7 85.7%	2 / 2 100.0%	8 / 8 100.0%	2 / 6 33.3%	3 / 4 75.0%	4 / 4 100.0%	1 / 1 100.0%	3 / 3 100.0%	2 / 4 50.0%	5 / 6 83.3%	5 / 7 71.4%	3 / 7 42.9%	2 / 2 100.0%	5 / 10 50.0%	0 / 0 0.0%	0 / 0 0.0%	0 / 0 0.0%	0 / 0 0.0%	67 / 117 57.3%AB	28 / 117 23.9%ABE	12 / 117 10.3%E	9 / 117 7.7%	1 / 117 0.9%CE			
1	Bacillus megaterium DSM 319	93 / 114 81.6%	6 / 6 100.0%	6 / 6 100.0%	6 / 6 100.0%	3 / 3 100.0%	9 / 10 90.0%	6 / 7 85.7%	3 / 3 100.0%	3 / 3 100.0%	5 / 6 83.3%	2 / 2 100.0%	9 / 9 100.0%	2 / 6 33.3%	3 / 4 75.0%	4 / 4 100.0%	1 / 1 100.0%	3 / 3 100.0%	2 / 4 50.0%	5 / 6 83.3%	6 / 7 85.7%	2 / 6 33.3%	2 / 2 100.0%	5 / 10 50.0%	0 / 0 0.0%	0 / 0 0.0%	0 / 0 0.0%	0 / 0 0.0%	65 / 114 57.0%AB	28 / 114 24.6%ABE	12 / 114 10.5%E	9 / 114 7.9%				
1	Bacillus megaterium WSH-002	80 / 99 80.8%	5 / 5 100.0%	6 / 6 100.0%	6 / 6 100.0%	3 / 3 100.0%	8 / 9 88.9%	5 / 6 83.3%	3 / 3 100.0%	3 / 3 100.0%	4 / 5 80.0%	2 / 2 100.0%	7 / 7 100.0%	1 / 5 20.0%	3 / 4 75.0%	4 / 4 100.0%	1 / 1 100.0%	2 / 2 100.0%	2 / 3 66.7%	4 / 5 80.0%	4 / 5 80.0%	3 / 7 42.9%	1 / 1 100.0%	3 / 7 42.9%	0 / 0 0.0%	0 / 0 0.0%	0 / 0 0.0%	0 / 0 0.0%	54 / 99 54.5%AB	26 / 99 26.3%ABE	10 / 99 10.1%E	9 / 99 9.1%				
1	ATCC 7061	43 / 68 63.2%	2 / 2 100.0%	4 / 4 100.0%	4 / 4 100.0%	2 / 2 100.0%	4 / 5 80.0%	3 / 4 75.0%	2 / 2 100.0%	2 / 2 100.0%	1 / 3 33.3%	1 / 1 100.0%	5 / 5 100.0%	3 / 3 100.0%	1 / 3 33.3%	1 / 4 25.0%	0 / 1 0.0%	1 / 1 100.0%	0 / 2 0.0%	1 / 3 33.3%	2 / 4 50.0%	2 / 6 33.3%	1 / 1 100.0%	1 / 6 16.7%	0 / 0 0.0%	0 / 0 0.0%	0 / 0 0.0%	0 / 0 0.0%	20 / 68 29.4%AB	15 / 68 22.1%B	15 / 68 22.1%E	9 / 68 13.2%	7 / 68 10.3%ABE	1 / 68 1.5%CE	1 / 68 1.5%BE	
1	Bacillus pumilus SAFR-032	43 / 68 63.2%	2 / 2 100.0%	4 / 4 100.0%	4 / 4 100.0%	2 / 2 100.0%	4 / 5 80.0%	3 / 4 75.0%	2 / 2 100.0%	2 / 2 100.0%	1 / 3 33.3%	1 / 1 100.0%	5 / 5 100.0%	3 / 3 100.0%	1 / 3 33.3%	1 / 4 25.0%	0 / 1 0.0%	1 / 1 100.0%	0 / 2 0.0%	1 / 3 33.3%	2 / 4 50.0%	2 / 6 33.3%	1 / 1 100.0%	1 / 6 16.7%	0 / 0 0.0%	0 / 0 0.0%	0 / 0 0.0%	0 / 0 0.0%	20 / 68 29.4%AB	15 / 68 22.1%B	15 / 68 22.1%E	9 / 68 13.2%	7 / 68 10.3%ABE	1 / 68 1.5%CE	1 / 68 1.5%BE	
1	Bacillus cellulosilyticus	78 / 78 100.0%	2 / 2 100.0%	4 / 4 100.0%	7 / 7 100.0%	3 / 3 100.0%	7 / 7 100.0%	4 / 4 100.0%	2 / 2 100.0%	2 / 2 100.0%	5 / 5 100.0%	2 / 2 100.0%	5 / 5 100.0%	3 / 3 100.0%	2 / 2 100.0%	4 / 4 100.0%	1 / 1 100.0%	2 / 2 100.0%	2 / 2 100.0%	4 / 4 100.0%	5 / 5 100.0%	5 / 5 100.0%	1 / 1 100.0%	6 / 6 100.0%	0 / 0 0.0%	0 / 0 0.0%	0 / 0 0.0%	0 / 0 0.0%	55 / 78 70.5%AB	23 / 78 29.5%ABE						
1	Bacillus cellulosilyticus DSM 2522	78 / 78 100.0%	2 / 2 100.0%	4 / 4 100.0%	7 / 7 100.0%	3 / 3 100.0%	7 / 7 100.0%	4 / 4 100.0%	2 / 2 100.0%	2 / 2 100.0%	5 / 5 100.0%	2 / 2 100.0%	5 / 5 100.0%	3 / 3 100.0%	2 / 2 100.0%	4 / 4 100.0%	1 / 1 100.0%	2 / 2 100.0%	2 / 2 100.0%	4 / 4 100.0%	5 / 5 100.0%	5 / 5 100.0%	1 / 1 100.0%	6 / 6 100.0%	0 / 0 0.0%	0 / 0 0.0%	0 / 0 0.0%	0 / 0 0.0%	55 / 78 70.5%AB	23 / 78 29.5%ABE						
1	ATCC 700160	45 / 75 60.0%	2 / 3 66.7%	3 / 3 100.0%	4 / 4 100.0%	2 / 3 66.7%	2 / 6 33.3%	4 / 5 80.0%	2 / 2 100.0%	2 / 2 100.0%	2 / 5 40.0%	1 / 1 100.0%	4 / 6 66.7%	2 / 3 66.7%	2 / 2 100.0%	3 / 3 100.0%	1 / 1 100.0%	2 / 2 100.0%	1 / 2 50.0%	1 / 4 25.0%	3 / 6 50.0%	1 / 4 25.0%	0 / 1 0.0%	1 / 7 14.3%	0 / 0 0.0%	0 / 0 0.0%	0 / 0 0.0%	0 / 0 0.0%	38 / 75 50.7%AB	19 / 75 25.3%	11 / 75 14.7%E	7 / 75 9.3%ABE				
1	Bacillus clausii KSM-K16	45 / 75 60.0%	2 / 3 66.7%	3 / 3 100.0%	4 / 4 100.0%	2 / 3 66.7%	2 / 6 33.3%	4 / 5 80.0%	2 / 2 100.0%	2 / 2 100.0%	2 / 5 40.0%	1 / 1 100.0%	4 / 6 66.7%	2 / 3 66.7%	2 / 2 100.0%	3 / 3 100.0%	1 / 1 100.0%	2 / 2 100.0%	1 / 2 50.0%	1 / 4 25.0%	3 / 6 50.0%	1 / 4 25.0%	0 / 1 0.0%	1 / 7 14.3%	0 / 0 0.0%	0 / 0 0.0%	0 / 0 0.0%	0 / 0 0.0%	38 / 75 50.7%AB	19 / 75 25.3%	11 / 75 14.7%E	7 / 75 9.3%ABE				
1	ATCC 700159	61 / 76 80.3%	2 / 3 66.7%	4 / 4 100.0%	5 / 5 100.0%	3 / 3 100.0%	3 / 6 50.0%	3 / 4 75.0%	2 / 3 66.7%	3 / 3 100.0%	3 / 4 75.0%	1 / 1 100.0%	6 / 6 100.0%	2 / 3 66.7%	2 / 2 100.0%	3 / 4 75.0%	1 / 2 50.0%	1 / 1 100.0%	1 / 2 50.0%	2 / 3 66.7%	5 / 5 100.0%	5 / 5 100.0%	1 / 1 100.0%	2 / 5 40.0%	1 / 1 100.0%	0 / 0 0.0%	0 / 0 0.0%	0 / 0 0.0%	43 / 76 56.6%AB	18 / 76 23.7%ABE	7 / 76 9.2%	6 / 76 7.9%E	2 / 76 2.6%CE			
1	Bacillus firmus OF4	61 / 76 80.3%	2 / 3 66.7%	4 / 4 100.0%	5 / 5 100.0%	3 / 3 100.0%	3 / 6 50.0%	3 / 4 75.0%	2 / 3 66.7%	3 / 3 100.0%	3 / 4 75.0%	1 / 1 100.0%	6 / 6 100.0%	2 / 3 66.7%	2 / 2 100.0%	3 / 4 75.0%	1 / 2 50.0%	1 / 1 100.0%	1 / 2 50.0%	2 / 3 66.7%	5 / 5 100.0%	5 / 5 100.0%	1 / 1 100.0%	2 / 5 40.0%	1 / 1 100.0%	0 / 0 0.0%	0 / 0 0.0%	0 / 0 0.0%	43 / 76 56.6%AB	18 / 76 23.7%ABE	7 / 76 9.2%	6 / 76 7.9%E	2 / 76 2.6%CE			
32	Bacillus cereus group	2195 / 3172 69.2%	139 / 140 99.3%	192 / 192 100.0%	160 / 161 99.4%	116 / 118 98.3%	173 / 268 64.6%	175 / 207 84.5%	61 / 61 100.0%	91 / 92 98.9%	71 / 164 43.3%	32 / 64 50.0%	220 / 221 99.5%	137 / 173 79.2%	95 / 127 74.8%	127 / 127 100.0%	0 / 33 0.0%	32 / 109 29.4%	29 / 61 47.5%	108 / 171 63.2%	33 / 169 19.5%	97 / 226 42.9%	29 / 71 40.8%	78 / 217 35.9%	0 / 0 0.0%	0 / 0 0.0%	0 / 0 0.0%	0 / 0 0.0%	1533 / 3172 48.3%AB	624 / 3172 19.7%ABE	491 / 3172 15.5%	473 / 3172 14.9%E	28 / 3172 0.9%B	13 / 3172 0.4%CE	10 / 3172 0.3%BE	
6	"Bacillus cereus var. anthracis" (Cohn 1872) Smith et al. 1946	399 / 573 69.6%	24 / 24 100.0%	36 / 36 100.0%	30 / 30 100.0%	24 / 24 100.0%	30 / 48 62.5%	30 / 36 83.3%	12 / 12 100.0%	14 / 14 100.0%	12 / 30 40.0%	6 / 12 50.0%	43 / 43 100.0%	24 / 30 80.0%	18 / 24 75.0%	24 / 24 100.0%	0 / 6 0.0%	6 / 18 33.3%	6 / 12 50.0%	18 / 30 60.0%	6 / 30 20.0%	18 / 42 42.9%	6 / 12 50.0%	12 / 36 33.3%	0 / 0 0.0%	0 / 0 0.0%	0 / 0 0.0%	0 / 0 0.0%	285 / 573 49.7%AB	114 / 573 19.9%ABE	99 / 573 17.3%	72 / 573 12.6%E	3 / 573 0.5%CE			
1	Bacillus anthracis (strain Ames)	66 / 95 69.5%	4 / 4 100.0%	6 / 6 100.0%	5 / 5 100.0%	4 / 4 100.0%	5 / 8 62.5%	5 / 6 83.3%	2 / 2 100.0%	2 / 2 100.0%	2 / 5 40.0%	1 / 2 50.0%	7 / 7 100.0%	4 / 5 80.0%	3 / 4 75.0%	4 / 4 100.0%	0 / 1 0.0%	1 / 3 33.3%	1 / 2 50.0%	3 / 5 60.0%	1 / 5 20.0%	3 / 7 42.9%	1 / 2 50.0%	2 / 6 33.3%	0 / 0 0.0%	0 / 0 0.0%	0 / 0 0.0%	0 / 0 0.0%	47 / 95 49.5%AB	19 / 95 20.0%	19 / 95 20.0%ABE	10 / 95 10.5%E				
1	Bacillus anthracis (strain Sterne)	66 / 95 69.5%	4 / 4 100.0%	6 / 6 100.0%	5 / 5 100.0%	4 / 4 100.0%	5 / 8 62.5%	5 / 6 83.3%	2 / 2 100.0%	2 / 2 100.0%	2 / 5 40.0%	1 / 2 50.0%	7 / 7 100.0%	4 / 5 80.0%	3 / 4 75.0%	4 / 4 100.0%	0 / 1 0.0%	1 / 3 33.3%	1 / 2 50.0%	3 / 5 60.0%	1 / 5 20.0%	3 / 7 42.9%	1 / 2 50.0%	2 / 6 33.3%	0 / 0 0.0%	0 / 0 0.0%	0 / 0 0.0%	0 / 0 0.0%	47 / 95 49.5%AB	19 / 95 20.0%	19 / 95 20.0%ABE	10 / 95 10.5%E				
1	Bacillus anthracis Ames "Ancestor"	66 / 95 69.5%	4 / 4 100.0%	6 / 6 100.0%	5 / 5 100.0%	4 / 4 100.0%	5 / 8 62.5%	5 / 6 83.3%	2 / 2 100.0%	2 / 2 100.0%	2 / 5 40.0%	1 / 2 50.0%	7 / 7 100.0%	4 / 5 80.0%	3 / 4 75.0%	4 / 4 100.0%	0 / 1 0.0%	1 / 3 33.3%	1 / 2 50.0%	3 / 5 60.0%	1 / 5 20.0%	3 / 7 42.9%	1 / 2 50.0%	2 / 6 33.3%	0 / 0 0.0%	0 / 0 0.0%	0 / 0 0.0%	0 / 0 0.0%	47 / 95 49.5%AB	19 / 95 20.0%ABE	19 / 95 20.0%	10 / 95 10.5%E				
1	Bacillus anthracis CDC 684	68 / 97 70.1%	4 / 4 100.0%	6 / 6 100.0%	5 / 5 100.0%	4 / 4 100.0%	5 / 8 62.5%	5 / 6 83.3%	2 / 2 100.0%	3 / 3 100.0%	2 / 5 40.0%	1 / 2 50.0%	8 / 8 100.0%	4 / 5 80.0%	3 / 4 75.0%	4 / 4 100.0%	0 / 1 0.0%	1 / 3 33.3%	1 / 2 50.0%	3 / 5 60.0%	1 / 5 20.0%	3 / 7 42.9%	1 / 2 50.0%	2 / 6 33.3%	0 / 0 0.0%	0 / 0 0.0%	0 / 0 0.0%	0 / 0 0.0%	49 / 97 50.5%AB	19 / 97 19.6%ABE	14 / 97 14.4%E	14 / 97 14.4%	1 / 97 1.0%CE			
1	Bacillus anthracis A0248	66 / 95 69.5%	4 / 4 100.0%	6 / 6 100.0%	5 / 5 100.0%	4 / 4 100.0%	5 / 8 62.5%	5 / 6 83.3%	2 / 2 100.0%	2 / 2 100.0%	2 / 5 40.0%	1 / 2 50.0%	7 / 7 100.0%	4 / 5 80.0%	3 / 4 75.0%	4 / 4 100.0%	0 / 1 0.0%	1 / 3 33.3%	1 / 2 50.0%	3 / 5 60.0%	1 / 5 20.0%	3 / 7 42.9%	1 / 2 50.0%	2 / 6 33.3%	0 / 0 0.0%	0 / 0 0.0%	0 / 0 0.0%	0 / 0 0.0%	47 / 95 49.5%AB	19 / 95 20.0%ABE	14 / 95 14.7%	14 / 95 14.7%E	1 / 95 1.1%CE			

1	Bacillus anthracis H9401	67 / 96 69.8%	4 / 4 100.0%	6 / 6 100.0%	5 / 5 100.0%	4 / 4 100.0%	5 / 8 62.5%	5 / 6 83.3%	2 / 2 100.0%	3 / 3 100.0%	2 / 5 40.0%	1 / 2 50.0%	7 / 7 100.0%	4 / 5 80.0%	3 / 4 75.0%	4 / 4 100.0%	0 / 1 0.0%	1 / 3 33.3%	1 / 2 50.0%	3 / 5 60.0%	1 / 5 20.0%	3 / 7 42.9%	1 / 2 50.0%	2 / 6 33.3%	0 / 0 0.0%	0 / 0 0.0%	0 / 0 0.0%	0 / 0 0.0%	48 / 96 50.0%AB	19 / 96 19.8%ABE	14 / 96 14.6%	14 / 96 14.6%E	1 / 96 1.0%CE			
13	ATCC 14579	915 / 1318 69.4%	55 / 55 100.0%	79 / 79 100.0%	65 / 65 100.0%	50 / 50 100.0%	73 / 112 65.2%	73 / 85 85.9%	26 / 26 100.0%	38 / 39 97.4%	32 / 72 44.4%	13 / 26 50.0%	90 / 90 100.0%	58 / 73 79.5%	39 / 52 75.0%	52 / 52 100.0%	0 / 13 0.0%	13 / 47 27.7%	13 / 26 50.0%	47 / 73 64.4%	13 / 70 18.6%	39 / 91 42.9%	13 / 28 46.4%	34 / 94 36.2%	0 / 0 0.0%	0 / 0 0.0%	0 / 0 0.0%	0 / 0 0.0%	656 / 1318 49.8%AB	259 / 1318 19.7%ABE	199 / 1318 15.1%	196 / 1318 14.9%E	8 / 1318 0.6%CE			
1	Bacillus cereus ATCC 10987	68 / 97 70.1%	4 / 4 100.0%	7 / 7 100.0%	5 / 5 100.0%	4 / 4 100.0%	5 / 8 62.5%	5 / 6 83.3%	2 / 2 100.0%	3 / 3 100.0%	2 / 5 40.0%	1 / 2 50.0%	7 / 7 100.0%	4 / 5 80.0%	3 / 4 75.0%	4 / 4 100.0%	0 / 1 0.0%	1 / 3 33.3%	1 / 2 50.0%	3 / 5 60.0%	1 / 5 20.0%	3 / 7 42.9%	1 / 2 50.0%	2 / 6 33.3%	0 / 0 0.0%	0 / 0 0.0%	0 / 0 0.0%	0 / 0 0.0%	48 / 97 49.5%AB	20 / 97 20.6%ABE	18 / 97 18.6%	11 / 97 11.3%E				
1	Bacillus cereus (strain ATCC 14579 / DSM 31)	73 / 106 68.9%	5 / 5 100.0%	6 / 6 100.0%	5 / 5 100.0%	4 / 4 100.0%	6 / 9 66.7%	6 / 7 85.7%	2 / 2 100.0%	3 / 3 100.0%	3 / 6 50.0%	1 / 2 50.0%	7 / 7 100.0%	4 / 6 66.7%	3 / 4 75.0%	4 / 4 100.0%	0 / 1 0.0%	1 / 4 25.0%	1 / 2 50.0%	4 / 6 66.7%	1 / 6 16.7%	3 / 7 42.9%	1 / 2 50.0%	3 / 8 37.5%	0 / 0 0.0%	0 / 0 0.0%	0 / 0 0.0%	0 / 0 0.0%	52 / 106 49.1%AB	21 / 106 19.8%ABE	21 / 106 19.8%	12 / 106 11.3%E				
1	Bacillus cereus E33L	67 / 96 69.8%	4 / 4 100.0%	6 / 6 100.0%	5 / 5 100.0%	4 / 4 100.0%	5 / 8 62.5%	5 / 6 83.3%	2 / 2 100.0%	3 / 3 100.0%	2 / 5 40.0%	1 / 2 50.0%	7 / 7 100.0%	4 / 5 80.0%	3 / 4 75.0%	4 / 4 100.0%	0 / 1 0.0%	1 / 3 33.3%	1 / 2 50.0%	3 / 5 60.0%	1 / 5 20.0%	3 / 7 42.9%	1 / 2 50.0%	2 / 6 33.3%	0 / 0 0.0%	0 / 0 0.0%	0 / 0 0.0%	0 / 0 0.0%	48 / 96 50.0%AB	19 / 96 19.8%	19 / 96 19.8%ABE	10 / 96 10.4%E				
1	Bacillus cereus NC7401	73 / 104 70.2%	4 / 4 100.0%	6 / 6 100.0%	5 / 5 100.0%	4 / 4 100.0%	6 / 9 66.7%	6 / 7 85.7%	2 / 2 100.0%	3 / 3 100.0%	3 / 6 50.0%	1 / 2 50.0%	7 / 7 100.0%	5 / 6 83.3%	3 / 4 75.0%	4 / 4 100.0%	0 / 1 0.0%	1 / 4 25.0%	1 / 2 50.0%	4 / 6 66.7%	1 / 5 20.0%	3 / 7 42.9%	1 / 2 50.0%	3 / 8 37.5%	0 / 0 0.0%	0 / 0 0.0%	0 / 0 0.0%	0 / 0 0.0%	53 / 104 51.0%AB	20 / 104 19.2%ABE	17 / 104 16.3%E	13 / 104 12.5%	1 / 104 1.0%CE			
1	Bacillus cereus F0837/76	73 / 105 69.5%	4 / 4 100.0%	6 / 6 100.0%	5 / 5 100.0%	4 / 4 100.0%	6 / 9 66.7%	6 / 7 85.7%	2 / 2 100.0%	3 / 3 100.0%	3 / 6 50.0%	1 / 2 50.0%	7 / 7 100.0%	5 / 6 83.3%	3 / 4 75.0%	4 / 4 100.0%	0 / 1 0.0%	1 / 4 25.0%	1 / 2 50.0%	4 / 6 66.7%	1 / 5 20.0%	3 / 7 42.9%	1 / 3 33.3%	3 / 8 37.5%	0 / 0 0.0%	0 / 0 0.0%	0 / 0 0.0%	0 / 0 0.0%	53 / 105 50.5%AB	20 / 105 19.0%ABE	17 / 105 16.2%E	14 / 105 13.3%	1 / 105 1.0%CE			
1	Bacillus cereus Q1	65 / 94 69.1%	4 / 4 100.0%	6 / 6 100.0%	5 / 5 100.0%	3 / 3 100.0%	5 / 8 62.5%	5 / 5 100.0%	2 / 2 100.0%	3 / 3 100.0%	1 / 5 20.0%	1 / 2 50.0%	7 / 7 100.0%	4 / 5 80.0%	3 / 4 75.0%	4 / 4 100.0%	0 / 1 0.0%	1 / 3 33.3%	1 / 2 50.0%	3 / 5 60.0%	1 / 5 20.0%	3 / 7 42.9%	1 / 2 50.0%	2 / 6 33.3%	0 / 0 0.0%	0 / 0 0.0%	0 / 0 0.0%	0 / 0 0.0%	46 / 94 48.9%AB	19 / 94 20.2%ABE	14 / 94 14.9%	14 / 94 14.9%E	1 / 94 1.1%CE			
1	Bacillus cereus G9842	68 / 97 70.1%	5 / 5 100.0%	6 / 6 100.0%	5 / 5 100.0%	4 / 4 100.0%	5 / 8 62.5%	5 / 6 83.3%	2 / 2 100.0%	3 / 3 100.0%	2 / 5 40.0%	1 / 2 50.0%	7 / 7 100.0%	4 / 5 80.0%	3 / 4 75.0%	4 / 4 100.0%	0 / 1 0.0%	1 / 3 33.3%	1 / 2 50.0%	3 / 5 60.0%	1 / 5 20.0%	3 / 7 42.9%	1 / 2 50.0%	2 / 6 33.3%	0 / 0 0.0%	0 / 0 0.0%	0 / 0 0.0%	0 / 0 0.0%	48 / 97 49.5%AB	20 / 97 20.6%ABE	15 / 97 15.5%E	14 / 97 14.4%				
1	Bacillus cereus B4264	73 / 106 68.9%	5 / 5 100.0%	6 / 6 100.0%	5 / 5 100.0%	4 / 4 100.0%	6 / 9 66.7%	6 / 7 85.7%	2 / 2 100.0%	3 / 3 100.0%	3 / 6 50.0%	1 / 2 50.0%	7 / 7 100.0%	4 / 6 66.7%	3 / 4 75.0%	4 / 4 100.0%	0 / 1 0.0%	1 / 4 25.0%	1 / 2 50.0%	4 / 6 66.7%	1 / 6 16.7%	3 / 7 42.9%	1 / 2 50.0%	3 / 8 37.5%	0 / 0 0.0%	0 / 0 0.0%	0 / 0 0.0%	0 / 0 0.0%	52 / 106 49.1%AB	21 / 106 19.8%ABE	18 / 106 17.0%E	15 / 106 14.2%				
1	Bacillus cereus AH187	73 / 105 69.5%	4 / 4 100.0%	6 / 6 100.0%	5 / 5 100.0%	4 / 4 100.0%	6 / 9 66.7%	6 / 7 85.7%	2 / 2 100.0%	3 / 3 100.0%	3 / 6 50.0%	1 / 2 50.0%	7 / 7 100.0%	5 / 6 83.3%	3 / 4 75.0%	4 / 4 100.0%	0 / 1 0.0%	1 / 4 25.0%	1 / 2 50.0%	4 / 6 66.7%	1 / 6 16.7%	3 / 7 42.9%	1 / 2 50.0%	3 / 8 37.5%	0 / 0 0.0%	0 / 0 0.0%	0 / 0 0.0%	0 / 0 0.0%	53 / 105 50.5%AB	20 / 105 19.0%ABE	17 / 105 16.2%E	14 / 105 13.3%	1 / 105 1.0%CE			
1	Bacillus cereus AH820	67 / 96 69.8%	4 / 4 100.0%	6 / 6 100.0%	5 / 5 100.0%	4 / 4 100.0%	5 / 8 62.5%	5 / 6 83.3%	2 / 2 100.0%	3 / 3 100.0%	2 / 5 40.0%	1 / 2 50.0%	7 / 7 100.0%	4 / 5 80.0%	3 / 4 75.0%	4 / 4 100.0%	0 / 1 0.0%	1 / 3 33.3%	1 / 2 50.0%	3 / 5 60.0%	1 / 5 20.0%	3 / 7 42.9%	1 / 2 50.0%	2 / 6 33.3%	0 / 0 0.0%	0 / 0 0.0%	0 / 0 0.0%	0 / 0 0.0%	48 / 96 50.0%AB	19 / 96 19.8%ABE	14 / 96 14.6%	14 / 96 14.6%E	1 / 96 1.0%CE			
1	Bacillus cereus 03BB102	73 / 106 68.9%	4 / 4 100.0%	6 / 6 100.0%	5 / 5 100.0%	4 / 4 100.0%	6 / 9 66.7%	6 / 7 85.7%	2 / 2 100.0%	3 / 3 100.0%	3 / 6 50.0%	1 / 2 50.0%	7 / 7 100.0%	5 / 6 83.3%	3 / 4 75.0%	4 / 4 100.0%	0 / 1 0.0%	1 / 4 25.0%	1 / 2 50.0%	4 / 6 66.7%	1 / 6 16.7%	3 / 7 42.9%	1 / 3 33.3%	3 / 8 37.5%	0 / 0 0.0%	0 / 0 0.0%	0 / 0 0.0%	0 / 0 0.0%	53 / 106 50.0%AB	20 / 106 18.9%ABE	17 / 106 16.0%E	15 / 106 14.2%	1 / 106 0.9%CE			
1	Bacillus cereus biovar anthracis	69 / 101 68.3%	4 / 4 100.0%	6 / 6 100.0%	5 / 5 100.0%	3 / 3 100.0%	6 / 9 66.7%	6 / 7 85.7%	2 / 2 100.0%	2 / 3 66.7%	2 / 5 40.0%	1 / 2 50.0%	6 / 6 100.0%	5 / 6 83.3%	3 / 4 75.0%	4 / 4 100.0%	0 / 1 0.0%	1 / 4 25.0%	1 / 2 50.0%	4 / 6 66.7%	1 / 5 20.0%	3 / 7 42.9%	1 / 2 50.0%	3 / 8 37.5%	0 / 0 0.0%	0 / 0 0.0%	0 / 0 0.0%	0 / 0 0.0%	49 / 101 48.5%AB	20 / 101 19.8%ABE	17 / 101 16.8%E	14 / 101 13.9%	1 / 101 1.0%CE			
1	Bacillus anthracis CI	69 / 101 68.3%	4 / 4 100.0%	6 / 6 100.0%	5 / 5 100.0%	3 / 3 100.0%	6 / 9 66.7%	6 / 7 85.7%	2 / 2 100.0%	2 / 3 66.7%	2 / 5 40.0%	1 / 2 50.0%	6 / 6 100.0%	5 / 6 83.3%	3 / 4 75.0%	4 / 4 100.0%	0 / 1 0.0%	1 / 4 25.0%	1 / 2 50.0%	4 / 6 66.7%	1 / 5 20.0%	3 / 7 42.9%	1 / 2 50.0%	3 / 8 37.5%	0 / 0 0.0%	0 / 0 0.0%	0 / 0 0.0%	0 / 0 0.0%	49 / 101 48.5%AB	20 / 101 19.8%ABE	17 / 101 16.8%E	14 / 101 13.9%	1 / 101 1.0%CE			
1	Bacillus cereus FRI-35	73 / 105 69.5%	4 / 4 100.0%	6 / 6 100.0%	5 / 5 100.0%	4 / 4 100.0%	6 / 9 66.7%	6 / 7 85.7%	2 / 2 100.0%	3 / 3 100.0%	3 / 6 50.0%	1 / 2 50.0%	7 / 7 100.0%	5 / 6 83.3%	3 / 4 75.0%	4 / 4 100.0%	0 / 1 0.0%	1 / 4 25.0%	1 / 2 50.0%	4 / 6 66.7%	1 / 6 16.7%	3 / 7 42.9%	1 / 2 50.0%	3 / 8 37.5%	0 / 0 0.0%	0 / 0 0.0%	0 / 0 0.0%	0 / 0 0.0%	53 / 105 50.5%AB	20 / 105 19.0%ABE	17 / 105 16.2%E	14 / 105 13.3%	1 / 105 1.0%CE			
11	"Bacillus cereus var. thuringiensis" Smith et al. 1952	733 / 1067 68.7%	50 / 51 98.0%	65 / 65 100.0%	56 / 56 100.0%	35 / 36 97.2%	58 / 90 64.4%	60 / 71 84.5%	19 / 19 100.0%	33 / 33 100.0%	21 / 50 42.0%	11 / 22 50.0%	74 / 74 100.0%	45 / 58 77.6%	32 / 43 74.4%	43 / 43 100.0%	0 / 12 0.0%	11 / 36 30.6%	8 / 19 42.1%	34 / 56 60.7%	11 / 57 19.3%	34 / 79 43.0%	8 / 27 29.6%	25 / 70 35.7%	0 / 0 0.0%	0 / 0 0.0%	0 / 0 0.0%	0 / 0 0.0%	515 / 1067 48.3%AB	218 / 1067 20.4%ABE	167 / 1067 15.7%	165 / 1067 15.5%E	2 / 1067 0.2%CE			
1	Bacillus thuringiensis serovar thuringiensis	59 / 84 70.2%	5 / 5 100.0%	6 / 6 100.0%	6 / 6 100.0%	2 / 2 100.0%	4 / 7 57.1%	5 / 6 83.3%	1 / 1 100.0%	3 / 3 100.0%	0 / 2 0.0%	1 / 2 50.0%	7 / 7 100.0%	3 / 4 75.0%	3 / 4 75.0%	4 / 4 100.0%	0 / 1 0.0%	1 / 2 50.0%	0 / 1 0.0%	2 / 4 50.0%	1 / 4 25.0%	4 / 8 50.0%	0 / 2 0.0%	1 / 3 33.3%	0 / 0 0.0%	0 / 0 0.0%	0 / 0 0.0%	0 / 0 0.0%	39 / 84 46.4%AB	20 / 84 23.8%ABE	13 / 84 15.5%	12 / 84 14.3%E				
1	Bacillus thuringiensis serovar thuringiensis str. IS5056	59 / 84 70.2%	5 / 5 100.0%	6 / 6 100.0%	6 / 6 100.0%	2 / 2 100.0%	4 / 7 57.1%	5 / 6 83.3%	1 / 1 100.0%	3 / 3 100.0%	0 / 2 0.0%	1 / 2 50.0%	7 / 7 100.0%	3 / 4 75.0%	3 / 4 75.0%	4 / 4 100.0%	0 / 1 0.0%	1 / 2 50.0%	0 / 1 0.0%	2 / 4 50.0%	1 / 4 25.0%	4 / 8 50.0%	0 / 2 0.0%	1 / 3 33.3%	0 / 0 0.0%	0 / 0 0.0%	0 / 0 0.0%	0 / 0 0.0%	39 / 84 46.4%AB	20 / 84 23.8%ABE	13 / 84 15.5%	12 / 84 14.3%E				
1	Bacillus thuringiensis (subsp. finitimus)	70 / 103 68.0%	4 / 4 100.0%	6 / 6 100.0%	5 / 5 100.0%	4 / 4 100.0%	6 / 9 66.7%	6 / 7 85.7%	2 / 2 100.0%	3 / 3 100.0%	3 / 6 50.0%	1 / 2 50.0%	7 / 7 100.0%	5 / 6 83.3%	3 / 4 75.0%	3 / 3 100.0%	0 / 1 0.0%	1 / 4 25.0%	1 / 2 50.0%	3 / 5 60.0%	1 / 5 20.0%	2 / 7 28.6%	1 / 3 33.3%	3 / 8 37.5%	0 / 0 0.0%	0 / 0 0.0%	0 / 0 0.0%	0 / 0 0.0%	50 / 103 48.5%AB	20 / 103 19.4%ABE	17 / 103 16.5%E	14 / 103 13.6%	2 / 103 1.9%CE			
1	Bacillus thuringiensis YBT-020	70 / 103 68.0%	4 / 4 100.0%	6 / 6 100.0%	5 / 5 100.0%	4 / 4 100.0%	6 / 9 66.7%	6 / 7 85.7%	2 / 2 100.0%	3 / 3 100.0%	3 / 6 50.0%	1 / 2 50.0%	7 / 7 100.0%	5 / 6 83.3%	3 / 4 75.0%	3 / 3 100.0%	0 / 1 0.0%	1 / 4 25.0%	1 / 2 50.0%	3 / 5 60.0%	1 / 5 20.0%	2 / 7 28.6%	1 / 3 33.3%	3 / 8 37.5%	0 / 0 0.0%	0 / 0 0.0%	0 / 0 0.0%	0 / 0 0.0%	50 / 103 48.5%AB	20 / 103 19.4%ABE	17 / 103 16.5%E	14 / 103 13.6%	2 / 103 1.9%CE			
2	Bacillus thuringiensis (SUBSP. KURSTAKI)	142 / 206 68.9%	10 / 10 100.0%	12 / 12 100.0%	10 / 10 100.0%	9 / 9 100.0%	11 / 17 64.7%	11 / 13 84.6%	4 / 4 100.0%	6 / 6 100.0%	6 / 12 50.0%	2 / 4 50.0%	14 / 14 100.0%	7 / 11 63.6%	6 / 8 75.0%	8 / 8 100.0%	0 / 2 0.0%	2 / 7 28.6%	2 / 4 50.0%	7 / 11 63.6%	2 / 11 18.2%	6 / 14 42.9%	2 / 5 40.0%	5 / 14 35.7%	0 / 0 0.0%	0 / 0 0.0%	0 / 0 0.0%	0 / 0 0.0%	101 / 206 49.0%AB	41 / 206 19.9%ABE	32 / 206 15.5%E	32 / 206 15.5%				
1	Bacillus thuringiensis BMB171	69 / 102 67.6%	5 / 5 100.0%	6 / 6 100.0%	5 / 5 100.0%	2 / 2 100.0%	6 / 9 66.7%	6 / 7 85.7%	2 / 2 100.0%	3 / 3 100.0%	1 / 4 25.0%	1 / 2 50.0%	7 / 7 100.0%	4 / 6 66.7%	3 / 4 75.0%	4 / 4 100.0%	0 / 1 0.0%	1 / 4 25.0%	1 / 2 50.0%	4 / 6 66.7%	1 / 6 16.7%	3 / 7 42.9%	1 / 2 50.0%	3 / 8 37.5%	0 / 0 0.0%	0 / 0 0.0%	0 / 0 0.0%	0 / 0 0.0%	48 / 102 47.1%AB	21 / 102 20.6%ABE	18 / 102 17.6%E	15 / 102 14.7%				
1	Bacillus thuringiensis serovar kurstaki str. HD73	73 / 104 70.2%	5 / 5 100.0%	6 / 6 100.0%	5 / 5 100.0%	7 / 7 100.0%	5 / 8 62.5%	5 / 6 83.3%	2 / 2 100.0%	3 / 3 100.0%	5 / 8 62.5%	1 / 2 50.0%	7 / 7 100.0%	3 / 5 60.0%	3 / 4 75.0%	4 / 4 100.0%	0 / 1 0.0%	1 / 3 33.3%	1 / 2 50.0%	3 / 5 60.0%	1 / 5 20.0%	3 / 7 42.9%	1 / 3 33.3%	2 / 6 33.3%	0 / 0 0.0%	0 / 0 0.0%	0 / 0 0.0%	0 / 0 0.0%	53 / 104 51.0%AB	20 / 104 19.2%ABE	17 / 104 16.3%	14 / 104 13.5%E				
1	Bacillus thuringiensis serovar konkukian	73 / 106 68.9%	4 / 4 100.0%	6 / 6 100.0%	5 / 5 100.0%	4 / 4 100.0%	6 / 9 66.7%	6 / 7 85.7%	2 / 2 100.0%	3 / 3 100.0%	3 / 6 50.0%	1 / 2 50.0%	7 / 7 100.0%	5 / 6 83.3%	3 / 4 75.0%	4 / 4 100.0%	0 / 2 0.0%	1 / 4 25.0%	1 / 2 50.0%	4 / 6 66.7%	1 / 6 16.7%	3 / 7 42.9%	1 / 2 50.0%	3 / 8 37.5%	0 / 0 0.0%	0 / 0 0.0%	0 / 0 0.0%	0 / 0 0.0%	53 / 106 50.0%AB	20 / 106 18.9%	20 / 106 18.9%ABE	13 / 106 12.3%E				
1	Bacillus thuringiensis serovar konkukian 97-27	73 / 106 68.9%	4 / 4 100.0%	6 / 6 100.0%	5 / 5 100.0%	4 / 4 100.0%	6 / 9 66.7%	6 / 7 85.7%	2 / 2 100.0%	3 / 3 100.0%	3 / 6 50.0%	1 / 2 50.0%	7 / 7 100.0%	5 / 6 83.3%	3 / 4 75.0%	4 / 4 100.0%	0 / 2 0.0%	1 / 4 25.0%	1 / 2 50.0%	4 / 6 66.7%	1 / 6 16.7%	3 / 7 42.9%	1 / 2 50.0%	3 / 8 37.5%	0 / 0 0.0%	0 / 0 0.0%	0 / 0 0.0%	0 / 0 0.0%	53 / 106 50.0%AB	20 / 106 18.9%	20 / 106 18.9%ABE	13 / 106 12.3%E				
1	Bacillus thuringiensis 'Al Hakam'	73 / 104 70.2%	4 / 4 100.0%	6 / 6 100.0%	5 / 5 100.0%	4 / 4 100.0%	6 / 9 66.7%	6 / 7 85.7%	2 / 2 100.0%	3 / 3 100.0%	3 / 5 60.0%	1 / 2 50.0%	7 / 7 100.0%	5 / 6 83.3%	3 / 4 75.0%	4 / 4 100.0%	0 / 1 0.0%	1 / 4 25.0%	1 / 2 50.0%	4 / 6 66.7%	1 / 6 16.7%	3 / 7 42.9%	1 / 2 50.0%	3 / 8 37.5%	0 / 0 0.0%	0 / 0 0.0%	0 / 0 0.0%	0 / 0 0.0%	53 / 104 51.0%AB	20 / 104 19.2%ABE	18 / 104 17.3%	13 / 104 12.5%E				
1	Bacillus thuringiensis Bt407	74 / 107 69.2%	5 / 5 100.0%	6 / 6 100.0%	5 / 5 100.0%	4 / 4 100.0%	6 / 9 66.7%	6 / 7 85.7%	2 / 2 100.0%	3 / 3 100.0%	3 / 6 50.0%	1 / 2 50.0%	7 / 7 100.0%	5 / 6 83.3%	3 / 4 75.0%	4 / 4 100.0%	0 / 1 0.0%	1 / 4 25.0%	1 / 2 50.0%	4 / 6 66.7%	1 / 6 16.7%	3 / 7 42.9%	1 / 3 33.3%	3 / 8 37.5%	0 / 0 0.0%	0 / 0 0.0%	0 / 0 0.0%	0 / 0 0.0%	53 / 107 49.5%AB	21 / 107 19.6%ABE	18 / 107 16.8%E	15 / 107 14.0%				
1	Bacillus thuringiensis serovar chinensis	58 / 83 69.9%	5 / 5 100.0%	6 / 6 100.0%	6 / 6 100.0%	2 / 2 100.0%	4 / 7 57.1%	5 / 6 83.3%	1 / 1 100.0%	3 / 3 100.0%	0 / 2 0.0%	1 / 2 50.0%	6 / 6 100.0%	3 / 4 75.0%	3 / 4 75.0%	4 / 4 100.0%	0 / 1 0.0%	1 / 2 50.0%	0 / 1 0.0%	2 / 4 50.0%	1 / 4 25.0%	4 / 8 50.0%	0 / 2 0.0%	1 / 3 33.3%	0 / 0 0.0%	0 / 0 0.0%	0 / 0 0.0%	0 / 0 0.0%	38 / 83 45.8%AB	20 / 83 24.1%ABE	13 / 83 15.7%	12 / 83 14.5%E				
1	Bacillus thuringiensis CT43	58 / 83 69.9%	5 / 5 100.0%	6 / 6 100.0%	6 / 6 100.0%	2 / 2 100.0%	4 / 7 57.1%	5 / 6 83.3%	1 / 1 100.0%	3 / 3 100.0%	0 / 2 0.0%	1 / 2 50.0%	6 / 6 100.0%	3 / 4 75.0%	3 / 4 75.0%	4 / 4 100.0%	0 / 1 0.0%	1 / 2 50.0%	0 / 1 0.0%	2 / 4 50.0%	1 / 4 25.0%	4 / 8 50.0%	0 / 2 0.0%	1 / 3 33.3%	0 / 0 0.0%	0 / 0 0.0%	0 / 0 0.0%	0 / 0 0.0%	38 / 83 45.8%AB	20 / 83 24.1%ABE	13 / 83 15.7%	12 / 83 14.5%E				
1	Bacillus thuringiensis MC28	48 / 73 65.8%	4 / 4 100.0%	5 / 5 100.0%	4 / 4 100.0%	1 / 1 100.0%	4 / 6 66.7%	4 / 5 80.0%	1 / 1 100.0%	3 / 3 100.0%	0 / 2 0.0%	1 / 2 50.0%	5 / 5 100.0%	3 / 4 75.0%	2 / 3 66.7%	4 / 4 100.0%	0 / 1 0.0%	1 / 2 50.0%	0 / 1 0.0%	1 / 3 33.3%	1 / 4 25.0%	3 / 7 42.9%	0 / 2 0.0%	1 / 4 25.0%	0 / 0 0.0%	0 / 0 0.0%	0 / 0 0.0%	0 / 0 0.0%	32 / 73 43.8%AB	16 / 73 21.9%ABE	14 / 73 19.2%E	11 / 73 15.1%				
1	Bacillus thuringiensis HD-789	69 / 104 66.3%	4 / 5 80.0%	6 / 6 100.0%	5 / 5 100.0%	2 / 2 100.0%	6 / 9 66.7%	6 / 7 85.7%	2 / 2 100.0%	3 / 3 100.0%	1 / 4 25.0%	1 / 2 50.0%	7 / 7 100.0%	5 / 6 83.3%	3 / 4 75.0%	4 / 4 100.0%	0 / 1 0.0%	1 / 4 25.0%	1 / 2 50.0%	4 / 6 66.7%	1 / 6 16.7%	3 / 7 42.9%	1 / 4 25.0%	3 / 8 37.5%	0 / 0 0.0%	0 / 0 0.0%	0 / 0 0.0%	0 / 0 0.0%	49 / 104 47.1%AB	20 / 104 19.2%ABE	19 / 104 18.3%E	16 / 104 15.4%				
1	Bacillus thuringiensis HD-771	67 / 97 69.1%	5 / 5 100.0%	6 / 6 100.0%	5 / 5 100.0%	3 / 4 75.0%	5 / 8 62.5%	5 / 6 83.3%	2 / 2 100.0%	3 / 3 100.0%	2 / 5 40.0%	1 / 2 50.0%	7 / 7 100.0%	4 / 5 80.0%	3 / 4 75.0%	4 / 4 100.0%	0 / 1 0.0%	1 / 3 33.3%	1 / 2 50.0%	3 / 5 60.0%	1 / 5 20.0%	3 / 7 42.9%	1 / 2 50.0%	2 / 6 33.3%	0 / 0 0.0%	0 / 0 0.0%	0 / 0 0.0%	0 / 0 0.0%	47 / 97 48.5%AB	20 / 97 20.6%ABE	15 / 97 15.5%E	15 / 97 15.5%				
1	Bacillus sp. TAN 119	76 / 108 70.4%	6 / 6 100.0%	6 / 6 100.0%	5 / 5 100.0%	4 / 4 100.0%	6 / 9 66.7%	6 / 8 75.0%	2 / 2 100.0%	3 / 3 100.0%	3 / 6 50.0%	1 / 2 50.0%	7 / 7 100.0%	5 / 6 83.3%	3 / 4 75.0%	4 / 4 100.0%	0 / 1 0.0%	1 / 4 25.0%	1 / 2 50.0%	5 / 6 83.3%	1 / 6 16.7%	3 / 7 42.9%	1 / 2 50.0%	3 / 8 37.5%	0 / 0 0.0%	0 / 0 0.0%	0 / 0 0.0%	0 / 0 0.0%	40 / 108 37.0%AB	19 / 108 17.6%E	17 / 108 15.7%ABE	14 / 108 13.0%B	13 / 108 12.0%	5 / 108 4.6%BE		
1	Bacillus cereus KBAB4	76 / 108 70.4%	6 / 6 100.0%	6 / 6 100.0%	5 / 5 100.0%	4 / 4 100.0%	6 / 9 66.7%	6 / 8 75.0%	2 / 2 100.0%	3 / 3 100.0%	3 / 6 50.0%	1 / 2 50.0%	7 / 7 100.0%	5 / 6 83.3%	3 / 4 75.0%	4 / 4 100.0%	0 / 1 0.0%	1 / 4 25.0%	1 / 2 50.0%	5 / 6 83.3%	1 / 6 16.7%	3 / 7 42.9%	1 / 2 50.0%	3 / 8 37.5%	0 / 0 0.0%	0 / 0 0.0%	0 / 0 0.0%	0 / 0 0.0%	40 / 108 37.0%AB	19 / 108 17.6%E	17 / 108 15.7%ABE	14 / 108 13.0%B	13 / 108 12.0%	5 / 108 4.6%BE		
1	"Bacillus cytotoxicus" Lapidus et al. 2008	72 / 106 67.9%	4 / 4 100.0%	6 / 6 100.0%	4 / 5 80.0%	3 / 4 75.0%	6 / 9 66.7%	6 / 7 85.7%	2 / 2 100.0%	3 / 3 100.0%	3 / 6 50.0%	1 / 2 50.0%	6 / 7 85.7%	5 / 6 83.3%	3 / 4 75.0%	4 / 4 100.0%	0 / 1 0.0%	1 / 4 25.0%	1 / 2 50.0%	4 / 6 66.7%	2 / 6 33.3%	3 / 7 42.9%	1 / 2 50.0%	4 / 9 44.4%	0 / 0 0.0%	0 / 0 0.0%	0 / 0 0.0%	0 / 0 0.0%	37 / 106 34.9%AB	21 / 106 19.8%E	16 / 106 15.1%ABE	14 / 106 13.2%B	13 / 106 12.3%	5 / 106 4.7%BE		
1	Bacillus cereus NVH 391-98	72 / 106 67.9%	4 / 4 100.0%	6 / 6 100.0%	4 / 5 80.0%	3 / 4 75.0%	6 / 9 66.7%	6 / 7 85.7%	2 / 2 100.0%	3 / 3 100.0%	3 / 6 50.0%	1 / 2 50.0%	6 / 7 85.7%	5 / 6 83.3%	3 / 4 75.0%	4 / 4 100.0%	0 / 1 0.0%	1 / 4 25.0%	1 / 2 50.0%	4 / 6 66.7%	2 / 6 33.3%	3 / 7 42.9%	1 / 2 50.0%	4 / 9 44.4%	0 / 0 0.0%	0 / 0 0.0%	0 / 0 0.0%	0 / 0 0.0%	37 / 106 34.9%AB	21 / 106 19.8%E	16 / 106 15.1%ABE	14 / 106 13.2%B	13 / 106 12.3%	5 / 106 4.7%BE		
1	"Bacillus alcalophilus subsp. halodurans" Boyer et al. 1973	63 / 78 80.8%	2 / 3 66.7%	3 / 3 100.0%	4 / 4 100.0%	3 / 3 100.0%	6 / 7 85.7%	4 / 5 80.0%	2 / 2 100.0%	3 / 3 100.0%	6 / 6 100.0%	1 / 1 100.0%	5 / 5 100.0%	3 / 3 100.0%	2 / 2 100.0%	3 / 4 75.0%	0 / 1 0.0%	2 / 2 100.0%	0 / 2 0.0%	1 / 5 20.0%	6 / 6 100.0%	2 / 4 50.0%	1 / 1 100.0%	4 / 6 66.7%	0 / 0 0.0%	0 / 0 0.0%	0 / 0 0.0%	0 / 0 0.0%	51 / 78 65.4%AB	12 / 78 15.4%ABE	10 / 78 12.8%	5 / 78 6.4%E				
1	Bacillus halodurans C-125	63 / 78 80.8%	2 / 3 66.7%	3 / 3 100.0%	4 / 4 100.0%	3 / 3 100.0%	6 / 7 85.7%	4 / 5 80.0%	2 / 2 100.0%	3 / 3 100.0%	6 / 6 100.0%	1 / 1 100.0%	5 / 5 100.0%	3 / 3 100.0%	2 / 2 100.0%	3 / 4 75.0%	0 / 1 0.0%	2 / 2 100.0%	0 / 2 0.0%	1 / 5 20.0%	6 / 6 100.0%	2 / 4 50.0%	1 / 1 100.0%	4 / 6 66.7%	0 / 0 0.0%	0 / 0 0.0%	0 / 0 0.0%	0 / 0 0.0%	51 / 78 65.4%AB	12 / 78 15.4%ABE	10 / 78 12.8%	5 / 78 6.4%E				
25	Bacillus subtilis group	1443 / 2108 68.5%	73 / 73 100.0%	100 / 101 99.0%	93 / 98 94.9%	84 / 84 100.0%	158 / 164 96.3%	92 / 119 77.3%	46 / 50 92.0%	46 / 50 92.0%	87 / 145 60.0%	25 / 26 96.2%	125 / 129 96.9%	93 / 94 98.9%	48 / 73 65.8%	25 / 100 25.0%	0 / 26 0.0%	62 / 65 95.4%	49 / 56 87.5%	43 / 117 36.8%	101 / 178 56.7%	30 / 146 20.5%	27 / 28 96.4%	36 / 186 19.4%	0 / 0 0.0%	0 / 0 0.0%	0 / 0 0.0%	0 / 0 0.0%	1201 / 2108 57.0%AB	380 / 2108 18.0%E	272 / 2108 12.9%	221 / 2108 10.5%ABE	19 / 2108 0.9%B	12 / 2108 0.6%CE	2 / 2108 0.1%BE	1 / 2108 0.0%C
10	"Bacillus amyloliquifaciens" (sic) Fukumoto 1943	586 / 850 68.9%	29 / 29 100.0%	40 / 40 100.0%	38 / 38 100.0%	31 / 31 100.0%	67 / 67 100.0%	40 / 50 80.0%	20 / 20 100.0%	20 / 20 100.0%	30 / 54 55.6%	10 / 10 100.0%	50 / 50 100.0%	39 / 39 100.0%	20 / 30 66.7%	10 / 40 25.0%	0 / 10 0.0%	27 / 27 100.0%	24 / 24 100.0%	19 / 47 40.4%	38 / 77 49.4%	14 / 59 23.7%	10 / 10 100.0%	10 / 78 12.8%	0 / 0 0.0%	0 / 0 0.0%	0 / 0 0.0%	0 / 0 0.0%	482 / 850 56.7%AB	172 / 850 20.2%E	92 / 850 10.8%	83 / 850 9.8%ABE	19 / 850 2.2%B	2 / 850 0.2%BE		
1	Bacillus amyloliquefaciens TA208	47 / 70 67.1%	3 / 3 100.0%	4 / 4 100.0%	3 / 3 100.0%	2 / 2 100.0%	5 / 5 100.0%	3 / 4 75.0%	2 / 2 100.0%	2 / 2 100.0%	1 / 3 33.3%	1 / 1 100.0%	5 / 5 100.0%	3 / 3 100.0%	2 / 3 66.7%	1 / 4 25.0%	0 / 1 0.0%	1 / 1 100.0%	2 / 2 100.0%	1 / 3 33.3%	2 / 6 33.3%	2 / 6 33.3%	1 / 1 100.0%	1 / 6 16.7%	0 / 0 0.0%	0 / 0 0.0%	0 / 0 0.0%	0 / 0 0.0%	38 / 70 54.3%AB	15 / 70 21.4%E	9 / 70 12.9%ABE	8 / 70 11.4%				
1	Bacillus amyloliquefaciens LL3	49 / 72 68.1%	3 / 3 100.0%	4 / 4 100.0%	3 / 3 100.0%	3 / 3 100.0%	5 / 5 100.0%	3 / 4 75.0%	2 / 2 100.0%	2 / 2 100.0%	2 / 4 50.0%	1 / 1 100.0%	5 / 5 100.0%	3 / 3 100.0%	2 / 3 66.7%	1 / 4 25.0%	0 / 1 0.0%	1 / 1 100.0%	2 / 2 100.0%	1 / 3 33.3%	2 / 6 33.3%	2 / 6 33.3%	1 / 1 100.0%	1 / 6 16.7%	0 / 0 0.0%	0 / 0 0.0%	0 / 0 0.0%	0 / 0 0.0%	40 / 72 55.6%AB	15 / 72 20.8%E	9 / 72 12.5%ABE	8 / 72 11.1%				
1	Bacillus amyloliquefaciens XH7	51 / 74 68.9%	3 / 3 100.0%	4 / 4 100.0%	4 / 4 100.0%	2 / 2 100.0%	6 / 6 100.0%	3 / 4 75.0%	2 / 2 100.0%	2 / 2 100.0%	1 / 3 33.3%	1 / 1 100.0%	5 / 5 100.0%	3 / 3 100.0%	2 / 3 66.7%	1 / 4 25.0%	0 / 1 0.0%	2 / 2 100.0%	2 / 2 100.0%	1 / 4 25.0%	3 / 7 42.9%	2 / 5 40.0%	1 / 1 100.0%	1 / 6 16.7%	0 / 0 0.0%	0 / 0 0.0%	0 / 0 0.0%	0 / 0 0.0%	42 / 74 56.8%AB	14 / 74 18.9%E	9 / 74 12.2%	9 / 74 12.2%ABE				
4	BGSC 10A6	247 / 359 68.8%	12 / 12 100.0%	16 / 16 100.0%	16 / 16 100.0%	12 / 12 100.0%	29 / 29 100.0%	17 / 21 81.0%	8 / 8 100.0%	8 / 8 100.0%	13 / 24 54.2%	4 / 4 100.0%	20 / 20 100.0%	17 / 17 100.0%	8 / 12 66.7%	4 / 16 25.0%	0 / 4 0.0%	13 / 13 100.0%	11 / 11 100.0%	9 / 21 42.9%	18 / 33 54.5%	4 / 24 16.7%	4 / 4 100.0%	4 / 34 11.8%	0 / 0 0.0%	0 / 0 0.0%	0 / 0 0.0%	0 / 0 0.0%	195 / 359 54.3%AB	73 / 359 20.3%E	39 / 359 10.9%	31 / 359 8.6%ABE	19 / 359 5.3%B	2 / 359 0.6%BE		
1	Bacillus amyloliquefaciens FZB42	60 / 88 68.2%	3 / 3 100.0%	4 / 4 100.0%	4 / 4 100.0%	3 / 3 100.0%	7 / 7 100.0%	4 / 5 80.0%	2 / 2 100.0%	2 / 2 100.0%	3 / 6 50.0%	1 / 1 100.0%	5 / 5 100.0%	4 / 4 100.0%	2 / 3 66.7%	1 / 4 25.0%	0 / 1 0.0%	3 / 3 100.0%	3 / 3 100.0%	2 / 5 40.0%	4 / 8 50.0%	1 / 6 16.7%	1 / 1 100.0%	1 / 8 12.5%	0 / 0 0.0%	0 / 0 0.0%	0 / 0 0.0%	0 / 0 0.0%	33 / 88 37.5%AB	19 / 88 21.6%B	18 / 88 20.5%E	10 / 88 11.4%	6 / 88 6.8%ABE	2 / 88 2.3%BE		
1	Bacillus amyloliquefaciens CAU-B946	66 / 95 69.5%	3 / 3 100.0%	4 / 4 100.0%	4 / 4 100.0%	3 / 3 100.0%	8 / 8 100.0%	5 / 6 83.3%	2 / 2 100.0%	2 / 2 100.0%	4 / 6 66.7%	1 / 1 100.0%	5 / 5 100.0%	5 / 5 100.0%	2 / 3 66.7%	1 / 4 25.0%	0 / 1 0.0%	4 / 4 100.0%	2 / 2 100.0%	3 / 6 50.0%	5 / 9 55.6%	1 / 6 16.7%	1 / 1 100.0%	1 / 10 10.0%	0 / 0 0.0%	0 / 0 0.0%	0 / 0 0.0%	0 / 0 0.0%	58 / 95 61.1%AB	20 / 95 21.1%E	9 / 95 9.5%	8 / 95 8.4%ABE				
1	Bacillus amyloliquefaciens subsp. plantarum UCMB5036	60 / 88 68.2%	3 / 3 100.0%	4 / 4 100.0%	4 / 4 100.0%	3 / 3 100.0%	7 / 7 100.0%	4 / 5 80.0%	2 / 2 100.0%	2 / 2 100.0%	3 / 6 50.0%	1 / 1 100.0%	5 / 5 100.0%	4 / 4 100.0%	2 / 3 66.7%	1 / 4 25.0%	0 / 1 0.0%	3 / 3 100.0%	3 / 3 100.0%	2 / 5 40.0%	4 / 8 50.0%	1 / 6 16.7%	1 / 1 100.0%	1 / 8 12.5%	0 / 0 0.0%	0 / 0 0.0%	0 / 0 0.0%	0 / 0 0.0%	52 / 88 59.1%AB	18 / 88 20.5%E	10 / 88 11.4%	8 / 88 9.1%ABE				
1	Bacillus amyloliquefaciens subsp. plantarum AS43.3	61 / 88 69.3%	3 / 3 100.0%	4 / 4 100.0%	4 / 4 100.0%	3 / 3 100.0%	7 / 7 100.0%	4 / 5 80.0%	2 / 2 100.0%	2 / 2 100.0%	3 / 6 50.0%	1 / 1 100.0%	5 / 5 100.0%	4 / 4 100.0%	2 / 3 66.7%	1 / 4 25.0%	0 / 1 0.0%	3 / 3 100.0%	3 / 3 100.0%	2 / 5 40.0%	5 / 8 62.5%	1 / 6 16.7%	1 / 1 100.0%	1 / 8 12.5%	0 / 0 0.0%	0 / 0 0.0%	0 / 0 0.0%	0 / 0 0.0%	52 / 88 59.1%AB	17 / 88 19.3%E	10 / 88 11.4%	9 / 88 10.2%ABE				
1	'Bacillus amyloliquefaciens subsp. amyloliquefaciens'	68 / 94 72.3%	2 / 2 100.0%	4 / 4 100.0%	4 / 4 100.0%	7 / 7 100.0%	7 / 7 100.0%	5 / 6 83.3%	2 / 2 100.0%	2 / 2 100.0%	7 / 9 77.8%	1 / 1 100.0%	5 / 5 100.0%	4 / 4 100.0%	2 / 3 66.7%	1 / 4 25.0%	0 / 1 0.0%	3 / 3 100.0%	2 / 2 100.0%	2 / 5 40.0%	4 / 8 50.0%	2 / 6 33.3%	1 / 1 100.0%	1 / 8 12.5%	0 / 0 0.0%	0 / 0 0.0%	0 / 0 0.0%	0 / 0 0.0%	59 / 94 62.8%AB	17 / 94 18.1%E	9 / 94 9.6%	9 / 94 9.6%ABE				
1	Bacillus amyloliquefaciens ATCC 23350	68 / 94 72.3%	2 / 2 100.0%	4 / 4 100.0%	4 / 4 100.0%	7 / 7 100.0%	7 / 7 100.0%	5 / 6 83.3%	2 / 2 100.0%	2 / 2 100.0%	7 / 9 77.8%	1 / 1 100.0%	5 / 5 100.0%	4 / 4 100.0%	2 / 3 66.7%	1 / 4 25.0%	0 / 1 0.0%	3 / 3 100.0%	2 / 2 100.0%	2 / 5 40.0%	4 / 8 50.0%	2 / 6 33.3%	1 / 1 100.0%	1 / 8 12.5%	0 / 0 0.0%	0 / 0 0.0%	0 / 0 0.0%	0 / 0 0.0%	59 / 94 62.8%AB	17 / 94 18.1%E	9 / 94 9.6%ABE	9 / 94 9.6%				
1	Bacillus amyloliquefaciens CNCM I-3800	66 / 95 69.5%	3 / 3 100.0%	4 / 4 100.0%	4 / 4 100.0%	3 / 3 100.0%	8 / 8 100.0%	5 / 6 83.3%	2 / 2 100.0%	2 / 2 100.0%	4 / 6 66.7%	1 / 1 100.0%	5 / 5 100.0%	5 / 5 100.0%	2 / 3 66.7%	1 / 4 25.0%	0 / 1 0.0%	4 / 4 100.0%	2 / 2 100.0%	3 / 6 50.0%	5 / 9 55.6%	1 / 6 16.7%	1 / 1 100.0%	1 / 10 10.0%	0 / 0 0.0%	0 / 0 0.0%	0 / 0 0.0%	0 / 0 0.0%	58 / 95 61.1%AB	20 / 95 21.1%E	9 / 95 9.5%	8 / 95 8.4%ABE				
1	Bacillus amyloliquefaciens Y2	58 / 86 67.4%	3 / 3 100.0%	4 / 4 100.0%	4 / 4 100.0%	2 / 2 100.0%	7 / 7 100.0%	4 / 5 80.0%	2 / 2 100.0%	2 / 2 100.0%	2 / 5 40.0%	1 / 1 100.0%	5 / 5 100.0%	4 / 4 100.0%	2 / 3 66.7%	1 / 4 25.0%	0 / 1 0.0%	3 / 3 100.0%	3 / 3 100.0%	2 / 5 40.0%	4 / 8 50.0%	1 / 6 16.7%	1 / 1 100.0%	1 / 8 12.5%	0 / 0 0.0%	0 / 0 0.0%	0 / 0 0.0%	0 / 0 0.0%	50 / 86 58.1%AB	18 / 86 20.9%E	10 / 86 11.6%	8 / 86 9.3%ABE				
3	"Clostridium licheniforme" Weigmann 1898	137 / 215 63.7%	9 / 9 100.0%	12 / 12 100.0%	12 / 12 100.0%	9 / 9 100.0%	12 / 15 80.0%	9 / 12 75.0%	3 / 6 50.0%	6 / 6 100.0%	6 / 12 50.0%	3 / 3 100.0%	15 / 15 100.0%	9 / 9 100.0%	6 / 9 66.7%	3 / 12 25.0%	0 / 3 0.0%	3 / 3 100.0%	0 / 6 0.0%	3 / 12 25.0%	6 / 15 40.0%	5 / 17 29.4%	3 / 3 100.0%	3 / 15 20.0%	0 / 0 0.0%	0 / 0 0.0%	0 / 0 0.0%	0 / 0 0.0%	114 / 215 53.0%AB	43 / 215 20.0%	35 / 215 16.3%E	23 / 215 10.7%ABE				
2	Bacillus licheniformis ATCC 14580	91 / 143 63.6%	6 / 6 100.0%	8 / 8 100.0%	8 / 8 100.0%	6 / 6 100.0%	8 / 10 80.0%	6 / 8 75.0%	2 / 4 50.0%	4 / 4 100.0%	4 / 8 50.0%	2 / 2 100.0%	10 / 10 100.0%	6 / 6 100.0%	4 / 6 66.7%	2 / 8 25.0%	0 / 2 0.0%	2 / 2 100.0%	0 / 4 0.0%	2 / 8 25.0%	4 / 10 40.0%	3 / 11 27.3%	2 / 2 100.0%	2 / 10 20.0%	0 / 0 0.0%	0 / 0 0.0%	0 / 0 0.0%	0 / 0 0.0%	76 / 143 53.1%AB	34 / 143 23.8%	18 / 143 12.6%E	15 / 143 10.5%ABE				
1	Bacillus licheniformis 9945A	46 / 72 63.9%	3 / 3 100.0%	4 / 4 100.0%	4 / 4 100.0%	3 / 3 100.0%	4 / 5 80.0%	3 / 4 75.0%	1 / 2 50.0%	2 / 2 100.0%	2 / 4 50.0%	1 / 1 100.0%	5 / 5 100.0%	3 / 3 100.0%	2 / 3 66.7%	1 / 4 25.0%	0 / 1 0.0%	1 / 1 100.0%	0 / 2 0.0%	1 / 4 25.0%	2 / 5 40.0%	2 / 6 33.3%	1 / 1 100.0%	1 / 5 20.0%	0 / 0 0.0%	0 / 0 0.0%	0 / 0 0.0%	0 / 0 0.0%	38 / 72 52.8%AB	17 / 72 23.6%E	9 / 72 12.5%	8 / 72 11.1%ABE				
11	"Vibrio subtilis" Ehrenberg 1835	669 / 968 69.1%	32 / 32 100.0%	44 / 45 97.8%	39 / 44 88.6%	41 / 41 100.0%	74 / 77 96.1%	40 / 53 75.5%	21 / 22 95.5%	18 / 22 81.8%	49 / 75 65.3%	10 / 11 90.9%	55 / 59 93.2%	42 / 43 97.7%	21 / 31 67.7%	11 / 44 25.0%	0 / 12 0.0%	31 / 34 91.2%	23 / 24 95.8%	20 / 54 37.0%	53 / 80 66.2%	10 / 64 15.6%	13 / 14 92.9%	22 / 87 25.3%	0 / 0 0.0%	0 / 0 0.0%	0 / 0 0.0%	0 / 0 0.0%	563 / 968 58.2%AB	159 / 968 16.4%E	128 / 968 13.2%	106 / 968 11.0%ABE	12 / 968 1.2%CE			
2	ATCC 6633	122 / 169 72.2%	6 / 6 100.0%	8 / 8 100.0%	9 / 9 100.0%	6 / 6 100.0%	14 / 14 100.0%	7 / 9 77.8%	4 / 4 100.0%	4 / 4 100.0%	8 / 12 66.7%	2 / 2 100.0%	10 / 10 100.0%	7 / 7 100.0%	4 / 6 66.7%	2 / 8 25.0%	0 / 2 0.0%	6 / 6 100.0%	4 / 4 100.0%	3 / 10 30.0%	10 / 14 71.4%	2 / 12 16.7%	2 / 2 100.0%	4 / 14 28.6%	0 / 0 0.0%	0 / 0 0.0%	0 / 0 0.0%	0 / 0 0.0%	102 / 169 60.4%AB	26 / 169 15.4%E	20 / 169 11.8%ABE	19 / 169 11.2%	2 / 169 1.2%CE			
1	Bacillus subtilis subsp. spizizenii str. W23	55 / 77 71.4%	3 / 3 100.0%	4 / 4 100.0%	4 / 4 100.0%	3 / 3 100.0%	6 / 6 100.0%	3 / 4 75.0%	2 / 2 100.0%	2 / 2 100.0%	3 / 5 60.0%	1 / 1 100.0%	5 / 5 100.0%	3 / 3 100.0%	2 / 3 66.7%	1 / 4 25.0%	0 / 1 0.0%	2 / 2 100.0%	2 / 2 100.0%	1 / 4 25.0%	4 / 6 66.7%	1 / 6 16.7%	1 / 1 100.0%	2 / 6 33.3%	0 / 0 0.0%	0 / 0 0.0%	0 / 0 0.0%	0 / 0 0.0%	45 / 77 58.4%AB	12 / 77 15.6%E	10 / 77 13.0%ABE	9 / 77 11.7%	1 / 77 1.3%CE			
1	Bacillus subtilis subsp. spizizenii TU-B-10	67 / 92 72.8%	3 / 3 100.0%	4 / 4 100.0%	5 / 5 100.0%	3 / 3 100.0%	8 / 8 100.0%	4 / 5 80.0%	2 / 2 100.0%	2 / 2 100.0%	5 / 7 71.4%	1 / 1 100.0%	5 / 5 100.0%	4 / 4 100.0%	2 / 3 66.7%	1 / 4 25.0%	0 / 1 0.0%	4 / 4 100.0%	2 / 2 100.0%	2 / 6 33.3%	6 / 8 75.0%	1 / 6 16.7%	1 / 1 100.0%	2 / 8 25.0%	0 / 0 0.0%	0 / 0 0.0%	0 / 0 0.0%	0 / 0 0.0%	57 / 92 62.0%AB	14 / 92 15.2%E	10 / 92 10.9%	10 / 92 10.9%ABE	1 / 92 1.1%CE			
5	Bacillus subtilis subsp. subtilis	308 / 428 72.0%	14 / 14 100.0%	20 / 20 100.0%	18 / 20 90.0%	22 / 22 100.0%	32 / 32 100.0%	19 / 24 79.2%	9 / 9 100.0%	9 / 10 90.0%	25 / 36 69.4%	4 / 4 100.0%	26 / 26 100.0%	19 / 19 100.0%	9 / 13 69.2%	5 / 19 26.3%	0 / 5 0.0%	14 / 14 100.0%	11 / 11 100.0%	9 / 23 39.1%	23 / 34 67.6%	4 / 29 13.8%	6 / 6 100.0%	10 / 38 26.3%	0 / 0 0.0%	0 / 0 0.0%	0 / 0 0.0%	0 / 0 0.0%	261 / 428 61.0%AB	64 / 428 15.0%E	52 / 428 12.1%	47 / 428 11.0%ABE	4 / 428 0.9%CE			
1	Bacillus subtilis 168	59 / 85 69.4%	3 / 3 100.0%	4 / 4 100.0%	3 / 4 75.0%	3 / 3 100.0%	7 / 7 100.0%	4 / 5 80.0%	2 / 2 100.0%	2 / 2 100.0%	4 / 6 66.7%	1 / 1 100.0%	5 / 5 100.0%	4 / 4 100.0%	2 / 3 66.7%	1 / 4 25.0%	0 / 1 0.0%	3 / 3 100.0%	2 / 2 100.0%	2 / 5 40.0%	4 / 7 57.1%	0 / 5 0.0%	1 / 1 100.0%	2 / 8 25.0%	0 / 0 0.0%	0 / 0 0.0%	0 / 0 0.0%	0 / 0 0.0%	51 / 85 60.0%AB	15 / 85 17.6%	11 / 85 12.9%E	8 / 85 9.4%ABE				
1	Bacillus subtilis subsp. subtilis str. ATCC 6051	62 / 86 72.1%	3 / 3 100.0%	4 / 4 100.0%	4 / 4 100.0%	3 / 3 100.0%	7 / 7 100.0%	4 / 5 80.0%	2 / 2 100.0%	2 / 2 100.0%	4 / 6 66.7%	1 / 1 100.0%	5 / 5 100.0%	4 / 4 100.0%	2 / 3 66.7%	1 / 4 25.0%	0 / 1 0.0%	3 / 3 100.0%	2 / 2 100.0%	2 / 5 40.0%	5 / 7 71.4%	1 / 6 16.7%	1 / 1 100.0%	2 / 8 25.0%	0 / 0 0.0%	0 / 0 0.0%	0 / 0 0.0%	0 / 0 0.0%	52 / 86 60.5%AB	14 / 86 16.3%E	10 / 86 11.6%ABE	9 / 86 10.5%	1 / 86 1.2%CE			
1	Bacillus subtilis subsp. subtilis 6051-HGW	62 / 86 72.1%	3 / 3 100.0%	4 / 4 100.0%	4 / 4 100.0%	3 / 3 100.0%	7 / 7 100.0%	4 / 5 80.0%	2 / 2 100.0%	2 / 2 100.0%	4 / 6 66.7%	1 / 1 100.0%	5 / 5 100.0%	4 / 4 100.0%	2 / 3 66.7%	1 / 4 25.0%	0 / 1 0.0%	3 / 3 100.0%	2 / 2 100.0%	2 / 5 40.0%	5 / 7 71.4%	1 / 6 16.7%	1 / 1 100.0%	2 / 8 25.0%	0 / 0 0.0%	0 / 0 0.0%	0 / 0 0.0%	0 / 0 0.0%	52 / 86 60.5%AB	14 / 86 16.3%E	10 / 86 11.6%ABE	9 / 86 10.5%	1 / 86 1.2%CE			
1	Bacillus subtilis subsp. subtilis RO-NN-1	62 / 86 72.1%	3 / 3 100.0%	4 / 4 100.0%	4 / 4 100.0%	3 / 3 100.0%	7 / 7 100.0%	4 / 5 80.0%	2 / 2 100.0%	2 / 2 100.0%	4 / 6 66.7%	1 / 1 100.0%	5 / 5 100.0%	4 / 4 100.0%	2 / 3 66.7%	1 / 4 25.0%	0 / 1 0.0%	3 / 3 100.0%	2 / 2 100.0%	2 / 5 40.0%	5 / 7 71.4%	1 / 6 16.7%	1 / 1 100.0%	2 / 8 25.0%	0 / 0 0.0%	0 / 0 0.0%	0 / 0 0.0%	0 / 0 0.0%	52 / 86 60.5%AB	14 / 86 16.3%E	10 / 86 11.6%ABE	9 / 86 10.5%	1 / 86 1.2%CE			
1	Bacillus subtilis subsp. subtilis BSP1	60 / 85 70.6%	3 / 3 100.0%	4 / 4 100.0%	4 / 5 80.0%	3 / 3 100.0%	7 / 7 100.0%	4 / 5 80.0%	1 / 1 100.0%	2 / 2 100.0%	4 / 6 66.7%	0 / 0 0.0%	5 / 5 100.0%	4 / 4 100.0%	2 / 3 66.7%	1 / 3 33.3%	0 / 1 0.0%	3 / 3 100.0%	2 / 2 100.0%	2 / 6 33.3%	5 / 7 71.4%	1 / 6 16.7%	1 / 1 100.0%	2 / 8 25.0%	0 / 0 0.0%	0 / 0 0.0%	0 / 0 0.0%	0 / 0 0.0%	51 / 85 60.0%AB	13 / 85 15.3%E	11 / 85 12.9%	9 / 85 10.6%ABE	1 / 85 1.2%CE			
1	Bacillus subtilis subsp. subtilis str. BAB-1	65 / 86 75.6%	2 / 2 100.0%	4 / 4 100.0%	3 / 3 100.0%	10 / 10 100.0%	4 / 4 100.0%	3 / 4 75.0%	2 / 2 100.0%	1 / 2 50.0%	9 / 12 75.0%	1 / 1 100.0%	6 / 6 100.0%	3 / 3 100.0%	1 / 1 100.0%	1 / 4 25.0%	0 / 1 0.0%	2 / 2 100.0%	3 / 3 100.0%	1 / 2 50.0%	4 / 6 66.7%	1 / 6 16.7%	2 / 2 100.0%	2 / 6 33.3%	0 / 0 0.0%	0 / 0 0.0%	0 / 0 0.0%	0 / 0 0.0%	55 / 86 64.0%AB	12 / 86 14.0%E	10 / 86 11.6%ABE	8 / 86 9.3%	1 / 86 1.2%CE			
1	Bacillus subtilis BSn5	60 / 81 74.1%	4 / 4 100.0%	5 / 5 100.0%	3 / 3 100.0%	4 / 4 100.0%	6 / 6 100.0%	3 / 4 75.0%	2 / 2 100.0%	1 / 2 50.0%	4 / 6 66.7%	1 / 1 100.0%	6 / 6 100.0%	3 / 3 100.0%	3 / 4 75.0%	1 / 4 25.0%	0 / 1 0.0%	2 / 2 100.0%	2 / 2 100.0%	1 / 3 33.3%	4 / 6 66.7%	1 / 6 16.7%	2 / 2 100.0%	2 / 5 40.0%	0 / 0 0.0%	0 / 0 0.0%	0 / 0 0.0%	0 / 0 0.0%	49 / 81 60.5%AB	11 / 81 13.6%ABE	11 / 81 13.6%E	8 / 81 9.9%	2 / 81 2.5%CE			
1	Bacillus subtilis BEST7613	66 / 129 51.2%	3 / 3 100.0%	4 / 5 80.0%	4 / 5 80.0%	3 / 3 100.0%	8 / 11 72.7%	4 / 7 57.1%	2 / 3 66.7%	2 / 3 66.7%	4 / 9 44.4%	1 / 2 50.0%	5 / 9 55.6%	5 / 6 83.3%	2 / 3 66.7%	1 / 5 20.0%	0 / 2 0.0%	3 / 6 50.0%	2 / 3 66.7%	3 / 9 33.3%	6 / 12 50.0%	1 / 7 14.3%	1 / 2 50.0%	2 / 14 14.3%	0 / 0 0.0%	0 / 0 0.0%	0 / 0 0.0%	0 / 0 0.0%	56 / 129 43.4%AB	31 / 129 24.0%E	30 / 129 23.3%	10 / 129 7.8%ABE	2 / 129 1.6%CE			
1	Bacillus subtilis QB928	61 / 86 70.9%	3 / 3 100.0%	4 / 4 100.0%	3 / 4 75.0%	3 / 3 100.0%	7 / 7 100.0%	4 / 5 80.0%	2 / 2 100.0%	2 / 2 100.0%	4 / 6 66.7%	1 / 1 100.0%	5 / 5 100.0%	4 / 4 100.0%	2 / 3 66.7%	1 / 4 25.0%	0 / 1 0.0%	3 / 3 100.0%	2 / 2 100.0%	2 / 5 40.0%	5 / 7 71.4%	1 / 6 16.7%	1 / 1 100.0%	2 / 8 25.0%	0 / 0 0.0%	0 / 0 0.0%	0 / 0 0.0%	0 / 0 0.0%	51 / 86 59.3%AB	14 / 86 16.3%E	10 / 86 11.6%ABE	10 / 86 11.6%	1 / 86 1.2%CE			
1	Bacillus subtilis XF-1	52 / 75 69.3%	2 / 2 100.0%	3 / 3 100.0%	2 / 3 66.7%	3 / 3 100.0%	7 / 7 100.0%	3 / 4 75.0%	2 / 2 100.0%	0 / 1 0.0%	4 / 6 66.7%	1 / 1 100.0%	3 / 3 100.0%	4 / 4 100.0%	1 / 2 50.0%	1 / 4 25.0%	0 / 1 0.0%	3 / 3 100.0%	2 / 2 100.0%	2 / 4 50.0%	5 / 7 71.4%	1 / 4 25.0%	1 / 1 100.0%	2 / 8 25.0%	0 / 0 0.0%	0 / 0 0.0%	0 / 0 0.0%	0 / 0 0.0%	44 / 75 58.7%AB	13 / 75 17.3%E	9 / 75 12.0%	8 / 75 10.7%ABE	1 / 75 1.3%CE			
1	ATCC 49337	51 / 75 68.0%	3 / 3 100.0%	4 / 4 100.0%	4 / 4 100.0%	3 / 3 100.0%	5 / 5 100.0%	3 / 4 75.0%	2 / 2 100.0%	2 / 2 100.0%	2 / 4 50.0%	2 / 2 100.0%	5 / 5 100.0%	3 / 3 100.0%	1 / 3 33.3%	1 / 4 25.0%	0 / 1 0.0%	1 / 1 100.0%	2 / 2 100.0%	1 / 4 25.0%	4 / 6 66.7%	1 / 6 16.7%	1 / 1 100.0%	1 / 6 16.7%	0 / 0 0.0%	0 / 0 0.0%	0 / 0 0.0%	0 / 0 0.0%	42 / 75 56.0%AB	14 / 75 18.7%E	9 / 75 12.0%	9 / 75 12.0%ABE	1 / 75 1.3%C			
1	Bacillus atrophaeus 1942	51 / 75 68.0%	3 / 3 100.0%	4 / 4 100.0%	4 / 4 100.0%	3 / 3 100.0%	5 / 5 100.0%	3 / 4 75.0%	2 / 2 100.0%	2 / 2 100.0%	2 / 4 50.0%	2 / 2 100.0%	5 / 5 100.0%	3 / 3 100.0%	1 / 3 33.3%	1 / 4 25.0%	0 / 1 0.0%	1 / 1 100.0%	2 / 2 100.0%	1 / 4 25.0%	4 / 6 66.7%	1 / 6 16.7%	1 / 1 100.0%	1 / 6 16.7%	0 / 0 0.0%	0 / 0 0.0%	0 / 0 0.0%	0 / 0 0.0%	42 / 75 56.0%AB	14 / 75 18.7%E	9 / 75 12.0%	9 / 75 12.0%ABE	1 / 75 1.3%C			
1	Bacillus sp. 1NLA3E	69 / 88 78.4%	0 / 4 0.0%	4 / 4 100.0%	4 / 4 100.0%	3 / 3 100.0%	7 / 7 100.0%	5 / 6 83.3%	2 / 2 100.0%	2 / 2 100.0%	4 / 5 80.0%	1 / 1 100.0%	6 / 6 100.0%	3 / 5 60.0%	2 / 3 66.7%	3 / 4 75.0%	1 / 1 100.0%	3 / 3 100.0%	1 / 2 50.0%	4 / 5 80.0%	3 / 6 50.0%	3 / 5 60.0%	1 / 1 100.0%	6 / 8 75.0%	1 / 1 100.0%	0 / 0 0.0%	0 / 0 0.0%	0 / 0 0.0%	56 / 88 63.6%AB	13 / 88 14.8%ABE	12 / 88 13.6%E	6 / 88 6.8%	1 / 88 1.1%CE			
		62 / 86 [illegible]	3 / 3 [illegible]	4 / 4 [illegible]	4 / 4 [illegible]	3 / 3 [illegible]	7 / 7 [illegible]	4 / 5 [illegible]	2 / 2 [illegible]	2 / 2 [illegible]	4 / 6 [illegible]	1 / 1 [illegible]	5 / 5 [illegible]	4 / 4 [illegible]	2 / 3 [illegible]	1 / 4 [illegible]	0 / 1 [illegible]	3 / 3 [illegible]	2 / 2 [illegible]	2 / 5 [illegible]	5 / 7 [illegible]	1 / 6 [illegible]	1 / 1 [illegible]	2 / 8 [illegible]	0 / 0 [illegible]	0 / 0 [illegible]	0 / 0 [illegible]	0 / 0 [illegible]	52 / 86 [illegible]	14 / 86 [illegible]	10 / 86 [illegible]	9 / 86 [illegible]	1 / 86 [illegible]			

1	Bacillus sp. JS	72.1%	100.0%	100.0%	100.0%	100.0%	100.0%	80.0%	100.0%	100.0%	66.7%	100.0%	100.0%	100.0%	66.7%	25.0%	0.0%	100.0%	100.0%	40.0%	71.4%	16.7%	100.0%	25.0%	0.0%	0.0%	0.0%	0.0%	60.5%AB	16.3%E	11.6%ABE	10.5%	1.2%CE	
1	Amphibacillus	42 / 55 76.4%	2 / 2 100.0%	3 / 3 100.0%	4 / 4 100.0%	2 / 2 100.0%	4 / 4 100.0%	2 / 3 66.7%	2 / 2 100.0%	2 / 2 100.0%	2 / 3 66.7%	0 / 1 0.0%	4 / 4 100.0%	1 / 2 50.0%	1 / 2 50.0%	2 / 3 66.7%	1 / 1 100.0%	1 / 1 100.0%	1 / 2 50.0%	1 / 3 33.3%	1 / 3 33.3%	3 / 3 100.0%	1 / 1 100.0%	2 / 4 50.0%	0 / 0 0.0%	0 / 0 0.0%	0 / 0 0.0%	0 / 0 0.0%	31 / 55 56.4%AB	11 / 55 20.0%ABE	8 / 55 14.5%	4 / 55 7.3%E	1 / 55 1.8%CE	
1	ATCC 51415	42 / 55 76.4%	2 / 2 100.0%	3 / 3 100.0%	4 / 4 100.0%	2 / 2 100.0%	4 / 4 100.0%	2 / 3 66.7%	2 / 2 100.0%	2 / 2 100.0%	2 / 3 66.7%	0 / 1 0.0%	4 / 4 100.0%	1 / 2 50.0%	1 / 2 50.0%	2 / 3 66.7%	1 / 1 100.0%	1 / 1 100.0%	1 / 2 50.0%	1 / 3 33.3%	1 / 3 33.3%	3 / 3 100.0%	1 / 1 100.0%	2 / 4 50.0%	0 / 0 0.0%	0 / 0 0.0%	0 / 0 0.0%	0 / 0 0.0%	31 / 55 56.4%AB	11 / 55 20.0%ABE	8 / 55 14.5%	4 / 55 7.3%E	1 / 55 1.8%CE	
1	Amphibacillus xylanus DSM 6626	42 / 55 76.4%	2 / 2 100.0%	3 / 3 100.0%	4 / 4 100.0%	2 / 2 100.0%	4 / 4 100.0%	2 / 3 66.7%	2 / 2 100.0%	2 / 2 100.0%	2 / 3 66.7%	0 / 1 0.0%	4 / 4 100.0%	1 / 2 50.0%	1 / 2 50.0%	2 / 3 66.7%	1 / 1 100.0%	1 / 1 100.0%	1 / 2 50.0%	1 / 3 33.3%	1 / 3 33.3%	3 / 3 100.0%	1 / 1 100.0%	2 / 4 50.0%	0 / 0 0.0%	0 / 0 0.0%	0 / 0 0.0%	0 / 0 0.0%	31 / 55 56.4%AB	11 / 55 20.0%ABE	8 / 55 14.5%	4 / 55 7.3%E	1 / 55 1.8%CE	
1	Halobacillus	49 / 67 73.1%	2 / 2 100.0%	4 / 4 100.0%	3 / 3 100.0%	2 / 2 100.0%	6 / 6 100.0%	3 / 4 75.0%	2 / 2 100.0%	2 / 2 100.0%	2 / 4 50.0%	0 / 1 0.0%	4 / 4 100.0%	2 / 3 66.7%	1 / 3 33.3%	3 / 3 100.0%	1 / 1 100.0%	2 / 2 100.0%	1 / 2 50.0%	3 / 4 75.0%	2 / 5 40.0%	2 / 4 50.0%	1 / 1 100.0%	1 / 5 20.0%	0 / 0 0.0%	0 / 0 0.0%	0 / 0 0.0%	0 / 0 0.0%	43 / 67 64.2%AB	11 / 67 16.4%	7 / 67 10.4%E	6 / 67 9.0%ABE		
1	ATCC 35676	49 / 67 73.1%	2 / 2 100.0%	4 / 4 100.0%	3 / 3 100.0%	2 / 2 100.0%	6 / 6 100.0%	3 / 4 75.0%	2 / 2 100.0%	2 / 2 100.0%	2 / 4 50.0%	0 / 1 0.0%	4 / 4 100.0%	2 / 3 66.7%	1 / 3 33.3%	3 / 3 100.0%	1 / 1 100.0%	2 / 2 100.0%	1 / 2 50.0%	3 / 4 75.0%	2 / 5 40.0%	2 / 4 50.0%	1 / 1 100.0%	1 / 5 20.0%	0 / 0 0.0%	0 / 0 0.0%	0 / 0 0.0%	0 / 0 0.0%	43 / 67 64.2%AB	11 / 67 16.4%	7 / 67 10.4%E	6 / 67 9.0%ABE		
1	Halobacillus halophilus DSM 2266	49 / 67 73.1%	2 / 2 100.0%	4 / 4 100.0%	3 / 3 100.0%	2 / 2 100.0%	6 / 6 100.0%	3 / 4 75.0%	2 / 2 100.0%	2 / 2 100.0%	2 / 4 50.0%	0 / 1 0.0%	4 / 4 100.0%	2 / 3 66.7%	1 / 3 33.3%	3 / 3 100.0%	1 / 1 100.0%	2 / 2 100.0%	1 / 2 50.0%	3 / 4 75.0%	2 / 5 40.0%	2 / 4 50.0%	1 / 1 100.0%	1 / 5 20.0%	0 / 0 0.0%	0 / 0 0.0%	0 / 0 0.0%	0 / 0 0.0%	43 / 67 64.2%AB	11 / 67 16.4%	7 / 67 10.4%E	6 / 67 9.0%ABE		
10	Geobacillus	424 / 884 48.0%	23 / 23 100.0%	30 / 33 90.9%	38 / 40 95.0%	4 / 38 10.5%	33 / 83 39.8%	40 / 50 80.0%	1 / 20 5.0%	8 / 20 40.0%	24 / 76 31.6%	10 / 11 90.9%	52 / 60 86.7%	20 / 40 50.0%	19 / 19 100.0%	33 / 39 84.6%	10 / 13 76.9%	5 / 48 10.4%	13 / 20 65.0%	26 / 50 52.0%	24 / 70 34.3%	0 / 50 0.0%	10 / 10 100.0%	1 / 71 1.4%	0 / 0 0.0%	0 / 0 0.0%	0 / 0 0.0%	0 / 0 0.0%	389 / 884 44.0%AB	267 / 884 30.2%E	185 / 884 20.9%	35 / 884 4.0%ABE	8 / 884 0.9%C	
1	'Geobacillus thermoglucosidans'	41 / 90 45.6%	3 / 3 100.0%	3 / 4 75.0%	3 / 4 75.0%	1 / 4 25.0%	4 / 9 44.4%	4 / 5 80.0%	0 / 2 0.0%	0 / 2 0.0%	3 / 7 42.9%	1 / 1 100.0%	6 / 6 100.0%	0 / 4 0.0%	2 / 2 100.0%	4 / 4 100.0%	1 / 2 50.0%	0 / 4 0.0%	0 / 2 0.0%	2 / 5 40.0%	3 / 7 42.9%	0 / 5 0.0%	1 / 1 100.0%	0 / 7 0.0%	0 / 0 0.0%	0 / 0 0.0%	0 / 0 0.0%	0 / 0 0.0%	37 / 90 41.1%AB	31 / 90 34.4%E	17 / 90 18.9%	4 / 90 4.4%ABE	1 / 90 1.1%C	
1	Geobacillus thermoglucosidasius C56-YS93	41 / 90 45.6%	3 / 3 100.0%	3 / 4 75.0%	3 / 4 75.0%	1 / 4 25.0%	4 / 9 44.4%	4 / 5 80.0%	0 / 2 0.0%	0 / 2 0.0%	3 / 7 42.9%	1 / 1 100.0%	6 / 6 100.0%	0 / 4 0.0%	2 / 2 100.0%	4 / 4 100.0%	1 / 2 50.0%	0 / 4 0.0%	0 / 2 0.0%	2 / 5 40.0%	3 / 7 42.9%	0 / 5 0.0%	1 / 1 100.0%	0 / 7 0.0%	0 / 0 0.0%	0 / 0 0.0%	0 / 0 0.0%	0 / 0 0.0%	37 / 90 41.1%AB	31 / 90 34.4%E	17 / 90 18.9%	4 / 90 4.4%ABE	1 / 90 1.1%C	
1	"Bacillus kaustophilus" Prickett 1928	41 / 86 47.7%	2 / 2 100.0%	3 / 3 100.0%	4 / 4 100.0%	0 / 3 0.0%	3 / 8 37.5%	4 / 5 80.0%	0 / 2 0.0%	1 / 2 50.0%	2 / 8 25.0%	1 / 1 100.0%	4 / 6 66.7%	3 / 4 75.0%	1 / 1 100.0%	3 / 4 75.0%	1 / 1 100.0%	1 / 5 20.0%	2 / 2 100.0%	3 / 5 60.0%	2 / 7 28.6%	0 / 5 0.0%	1 / 1 100.0%	0 / 7 0.0%	0 / 0 0.0%	0 / 0 0.0%	0 / 0 0.0%	0 / 0 0.0%	38 / 86 44.2%AB	29 / 86 33.7%	15 / 86 17.4%E	3 / 86 3.5%ABE	1 / 86 1.2%C	
1	Geobacillus kaustophilus HTA426	41 / 86 47.7%	2 / 2 100.0%	3 / 3 100.0%	4 / 4 100.0%	0 / 3 0.0%	3 / 8 37.5%	4 / 5 80.0%	0 / 2 0.0%	1 / 2 50.0%	2 / 8 25.0%	1 / 1 100.0%	4 / 6 66.7%	3 / 4 75.0%	1 / 1 100.0%	3 / 4 75.0%	1 / 1 100.0%	1 / 5 20.0%	2 / 2 100.0%	3 / 5 60.0%	2 / 7 28.6%	0 / 5 0.0%	1 / 1 100.0%	0 / 7 0.0%	0 / 0 0.0%	0 / 0 0.0%	0 / 0 0.0%	0 / 0 0.0%	38 / 86 44.2%AB	29 / 86 33.7%	15 / 86 17.4%E	3 / 86 3.5%ABE	1 / 86 1.2%C	
1	"Bacillus thermodenitrificans" Klaushofer and Hollaus 1970	42 / 88 47.7%	2 / 2 100.0%	3 / 3 100.0%	4 / 4 100.0%	1 / 4 25.0%	3 / 8 37.5%	4 / 5 80.0%	0 / 2 0.0%	1 / 2 50.0%	3 / 8 37.5%	1 / 1 100.0%	5 / 6 83.3%	2 / 4 50.0%	2 / 2 100.0%	3 / 4 75.0%	1 / 1 100.0%	0 / 5 0.0%	0 / 2 0.0%	3 / 5 60.0%	3 / 7 42.9%	0 / 5 0.0%	1 / 1 100.0%	0 / 7 0.0%	0 / 0 0.0%	0 / 0 0.0%	0 / 0 0.0%	0 / 0 0.0%	39 / 88 44.3%AB	31 / 88 35.2%	15 / 88 17.0%E	3 / 88 3.4%ABE		
1	Geobacillus thermodenitrificans NG80-2	42 / 88 47.7%	2 / 2 100.0%	3 / 3 100.0%	4 / 4 100.0%	1 / 4 25.0%	3 / 8 37.5%	4 / 5 80.0%	0 / 2 0.0%	1 / 2 50.0%	3 / 8 37.5%	1 / 1 100.0%	5 / 6 83.3%	2 / 4 50.0%	2 / 2 100.0%	3 / 4 75.0%	1 / 1 100.0%	0 / 5 0.0%	0 / 2 0.0%	3 / 5 60.0%	3 / 7 42.9%	0 / 5 0.0%	1 / 1 100.0%	0 / 7 0.0%	0 / 0 0.0%	0 / 0 0.0%	0 / 0 0.0%	0 / 0 0.0%	39 / 88 44.3%AB	31 / 88 35.2%	15 / 88 17.0%E	3 / 88 3.4%ABE		
1	Geobacillus sp. WCH70	47 / 92 51.1%	3 / 3 100.0%	3 / 4 75.0%	4 / 4 100.0%	1 / 3 33.3%	4 / 9 44.4%	4 / 5 80.0%	1 / 2 50.0%	1 / 2 50.0%	3 / 6 50.0%	1 / 2 50.0%	6 / 6 100.0%	0 / 4 0.0%	2 / 2 100.0%	4 / 5 80.0%	2 / 2 100.0%	1 / 5 20.0%	1 / 2 50.0%	1 / 5 20.0%	3 / 7 42.9%	0 / 5 0.0%	1 / 1 100.0%	1 / 8 12.5%	0 / 0 0.0%	0 / 0 0.0%	0 / 0 0.0%	0 / 0 0.0%	41 / 92 44.6%AB	24 / 92 26.1%E	21 / 92 22.8%	6 / 92 6.5%ABE		
1	Geobacillus sp. Y412MC61	42 / 87 48.3%	2 / 2 100.0%	3 / 3 100.0%	4 / 4 100.0%	0 / 4 0.0%	3 / 8 37.5%	4 / 5 80.0%	0 / 2 0.0%	1 / 2 50.0%	2 / 8 25.0%	1 / 1 100.0%	5 / 6 83.3%	3 / 4 75.0%	2 / 2 100.0%	3 / 3 100.0%	1 / 1 100.0%	0 / 5 0.0%	2 / 2 100.0%	3 / 5 60.0%	2 / 7 28.6%	0 / 5 0.0%	1 / 1 100.0%	0 / 7 0.0%	0 / 0 0.0%	0 / 0 0.0%	0 / 0 0.0%	0 / 0 0.0%	39 / 87 44.8%AB	30 / 87 34.5%E	14 / 87 16.1%	3 / 87 3.4%ABE	1 / 87 1.1%C	
1	Geobacillus sp. Y412MC52	42 / 87 48.3%	2 / 2 100.0%	3 / 3 100.0%	4 / 4 100.0%	0 / 4 0.0%	3 / 8 37.5%	4 / 5 80.0%	0 / 2 0.0%	1 / 2 50.0%	2 / 8 25.0%	1 / 1 100.0%	5 / 6 83.3%	3 / 4 75.0%	2 / 2 100.0%	3 / 3 100.0%	1 / 1 100.0%	0 / 5 0.0%	2 / 2 100.0%	3 / 5 60.0%	2 / 7 28.6%	0 / 5 0.0%	1 / 1 100.0%	0 / 7 0.0%	0 / 0 0.0%	0 / 0 0.0%	0 / 0 0.0%	0 / 0 0.0%	39 / 87 44.8%AB	30 / 87 34.5%E	14 / 87 16.1%	3 / 87 3.4%ABE	1 / 87 1.1%C	
1	Geobacillus sp. Y4.1MC1	41 / 90 45.6%	3 / 3 100.0%	3 / 4 75.0%	3 / 4 75.0%	1 / 4 25.0%	4 / 9 44.4%	4 / 5 80.0%	0 / 2 0.0%	0 / 2 0.0%	3 / 7 42.9%	1 / 1 100.0%	6 / 6 100.0%	0 / 4 0.0%	2 / 2 100.0%	4 / 4 100.0%	1 / 2 50.0%	0 / 4 0.0%	0 / 2 0.0%	2 / 5 40.0%	3 / 7 42.9%	0 / 5 0.0%	1 / 1 100.0%	0 / 7 0.0%	0 / 0 0.0%	0 / 0 0.0%	0 / 0 0.0%	0 / 0 0.0%	37 / 90 41.1%AB	29 / 90 32.2%E	19 / 90 21.1%	4 / 90 4.4%ABE	1 / 90 1.1%C	
1	Geobacillus sp. C56-T3	42 / 88 47.7%	2 / 2 100.0%	3 / 3 100.0%	4 / 4 100.0%	0 / 4 0.0%	3 / 8 37.5%	4 / 5 80.0%	0 / 2 0.0%	1 / 2 50.0%	2 / 8 25.0%	1 / 1 100.0%	5 / 6 83.3%	3 / 4 75.0%	2 / 2 100.0%	3 / 4 75.0%	0 / 1 0.0%	1 / 5 20.0%	2 / 2 100.0%	3 / 5 60.0%	2 / 7 28.6%	0 / 5 0.0%	1 / 1 100.0%	0 / 7 0.0%	0 / 0 0.0%	0 / 0 0.0%	0 / 0 0.0%	0 / 0 0.0%	39 / 88 44.3%AB	30 / 88 34.1%E	15 / 88 17.0%	3 / 88 3.4%ABE	1 / 88 1.1%C	
1	Geobacillus sp. GHH01	43 / 88 48.9%	2 / 2 100.0%	3 / 3 100.0%	4 / 4 100.0%	0 / 4 0.0%	3 / 8 37.5%	4 / 5 80.0%	0 / 2 0.0%	1 / 2 50.0%	2 / 8 25.0%	1 / 1 100.0%	5 / 6 83.3%	3 / 4 75.0%	2 / 2 100.0%	3 / 4 75.0%	1 / 1 100.0%	1 / 5 20.0%	2 / 2 100.0%	3 / 5 60.0%	2 / 7 28.6%	0 / 5 0.0%	1 / 1 100.0%	0 / 7 0.0%	0 / 0 0.0%	0 / 0 0.0%	0 / 0 0.0%	0 / 0 0.0%	40 / 88 45.5%AB	31 / 88 35.2%E	13 / 88 14.8%	3 / 88 3.4%ABE	1 / 88 1.1%C	
1	Geobacillus thermoleovorans group	43 / 88 48.9%	2 / 2 100.0%	3 / 3 100.0%	4 / 4 100.0%	0 / 4 0.0%	3 / 8 37.5%	4 / 5 80.0%	0 / 2 0.0%	1 / 2 50.0%	2 / 8 25.0%	1 / 1 100.0%	5 / 6 83.3%	3 / 4 75.0%	2 / 2 100.0%	3 / 4 75.0%	1 / 1 100.0%	1 / 5 20.0%	2 / 2 100.0%	3 / 5 60.0%	2 / 7 28.6%	0 / 5 0.0%	1 / 1 100.0%	0 / 7 0.0%	0 / 0 0.0%	0 / 0 0.0%	0 / 0 0.0%	0 / 0 0.0%	40 / 88 45.5%AB	32 / 88 36.4%E	12 / 88 13.6%	3 / 88 3.4%ABE	1 / 88 1.1%C	
1	ATCC 43513	43 / 88 48.9%	2 / 2 100.0%	3 / 3 100.0%	4 / 4 100.0%	0 / 4 0.0%	3 / 8 37.5%	4 / 5 80.0%	0 / 2 0.0%	1 / 2 50.0%	2 / 8 25.0%	1 / 1 100.0%	5 / 6 83.3%	3 / 4 75.0%	2 / 2 100.0%	3 / 4 75.0%	1 / 1 100.0%	1 / 5 20.0%	2 / 2 100.0%	3 / 5 60.0%	2 / 7 28.6%	0 / 5 0.0%	1 / 1 100.0%	0 / 7 0.0%	0 / 0 0.0%	0 / 0 0.0%	0 / 0 0.0%	0 / 0 0.0%	40 / 88 45.5%AB	32 / 88 36.4%E	12 / 88 13.6%	3 / 88 3.4%ABE	1 / 88 1.1%C	
1	Geobacillus thermoleovorans CCB_US3_UF5	43 / 88 48.9%	2 / 2 100.0%	3 / 3 100.0%	4 / 4 100.0%	0 / 4 0.0%	3 / 8 37.5%	4 / 5 80.0%	0 / 2 0.0%	1 / 2 50.0%	2 / 8 25.0%	1 / 1 100.0%	5 / 6 83.3%	3 / 4 75.0%	2 / 2 100.0%	3 / 4 75.0%	1 / 1 100.0%	1 / 5 20.0%	2 / 2 100.0%	3 / 5 60.0%	2 / 7 28.6%	0 / 5 0.0%	1 / 1 100.0%	0 / 7 0.0%	0 / 0 0.0%	0 / 0 0.0%	0 / 0 0.0%	0 / 0 0.0%	40 / 88 45.5%AB	32 / 88 36.4%E	12 / 88 13.6%	3 / 88 3.4%ABE	1 / 88 1.1%C	
1	Anoxybacillus	51 / 75 68.0%	3 / 3 100.0%	4 / 4 100.0%	4 / 4 100.0%	2 / 3 66.7%	3 / 6 50.0%	3 / 5 60.0%	2 / 2 100.0%	2 / 2 100.0%	3 / 4 75.0%	1 / 1 100.0%	6 / 6 100.0%	2 / 3 66.7%	1 / 2 50.0%	3 / 3 100.0%	1 / 1 100.0%	2 / 2 100.0%	1 / 2 50.0%	3 / 5 60.0%	2 / 4 50.0%	0 / 5 0.0%	1 / 2 50.0%	2 / 6 33.3%	0 / 0 0.0%	0 / 0 0.0%	0 / 0 0.0%	0 / 0 0.0%	41 / 75 54.7%AB	14 / 75 18.7%E	10 / 75 13.3%	10 / 75 13.3%ABE		
1	"Bacillus flavothermus (sic)" Heinen et al. 1982	51 / 75 68.0%	3 / 3 100.0%	4 / 4 100.0%	4 / 4 100.0%	2 / 3 66.7%	3 / 6 50.0%	3 / 5 60.0%	2 / 2 100.0%	2 / 2 100.0%	3 / 4 75.0%	1 / 1 100.0%	6 / 6 100.0%	2 / 3 66.7%	1 / 2 50.0%	3 / 3 100.0%	1 / 1 100.0%	2 / 2 100.0%	1 / 2 50.0%	3 / 5 60.0%	2 / 4 50.0%	0 / 5 0.0%	1 / 2 50.0%	2 / 6 33.3%	0 / 0 0.0%	0 / 0 0.0%	0 / 0 0.0%	0 / 0 0.0%	41 / 75 54.7%AB	14 / 75 18.7%E	10 / 75 13.3%ABE	10 / 75 13.3%		
1	Anoxybacillus flavithermus DSM 2641	51 / 75 68.0%	3 / 3 100.0%	4 / 4 100.0%	4 / 4 100.0%	2 / 3 66.7%	3 / 6 50.0%	3 / 5 60.0%	2 / 2 100.0%	2 / 2 100.0%	3 / 4 75.0%	1 / 1 100.0%	6 / 6 100.0%	2 / 3 66.7%	1 / 2 50.0%	3 / 3 100.0%	1 / 1 100.0%	2 / 2 100.0%	1 / 2 50.0%	3 / 5 60.0%	2 / 4 50.0%	0 / 5 0.0%	1 / 2 50.0%	2 / 6 33.3%	0 / 0 0.0%	0 / 0 0.0%	0 / 0 0.0%	0 / 0 0.0%	41 / 75 54.7%AB	14 / 75 18.7%E	10 / 75 13.3%	10 / 75 13.3%ABE		
1	Oceanobacillus	54 / 69 78.3%	2 / 2 100.0%	3 / 3 100.0%	4 / 4 100.0%	2 / 2 100.0%	6 / 6 100.0%	4 / 5 80.0%	1 / 2 50.0%	2 / 2 100.0%	2 / 4 50.0%	1 / 1 100.0%	3 / 3 100.0%	3 / 4 75.0%	0 / 2 0.0%	3 / 3 100.0%	1 / 1 100.0%	3 / 3 100.0%	1 / 2 50.0%	3 / 4 75.0%	3 / 6 50.0%	2 / 3 66.7%	1 / 1 100.0%	4 / 6 66.7%	0 / 0 0.0%	0 / 0 0.0%	0 / 0 0.0%	0 / 0 0.0%	44 / 69 63.8%AB	11 / 69 15.9%	10 / 69 14.5%ABE	4 / 69 5.8%E		
1	DSM 14371	54 / 69 78.3%	2 / 2 100.0%	3 / 3 100.0%	4 / 4 100.0%	2 / 2 100.0%	6 / 6 100.0%	4 / 5 80.0%	1 / 2 50.0%	2 / 2 100.0%	2 / 4 50.0%	1 / 1 100.0%	3 / 3 100.0%	3 / 4 75.0%	0 / 2 0.0%	3 / 3 100.0%	1 / 1 100.0%	3 / 3 100.0%	1 / 2 50.0%	3 / 4 75.0%	3 / 6 50.0%	2 / 3 66.7%	1 / 1 100.0%	4 / 6 66.7%	0 / 0 0.0%	0 / 0 0.0%	0 / 0 0.0%	0 / 0 0.0%	44 / 69 63.8%AB	11 / 69 15.9%	10 / 69 14.5%ABE	4 / 69 5.8%E		
1	Oceanobacillus iheyensis HTE831	54 / 69 78.3%	2 / 2 100.0%	3 / 3 100.0%	4 / 4 100.0%	2 / 2 100.0%	6 / 6 100.0%	4 / 5 80.0%	1 / 2 50.0%	2 / 2 100.0%	2 / 4 50.0%	1 / 1 100.0%	3 / 3 100.0%	3 / 4 75.0%	0 / 2 0.0%	3 / 3 100.0%	1 / 1 100.0%	3 / 3 100.0%	1 / 2 50.0%	3 / 4 75.0%	3 / 6 50.0%	2 / 3 66.7%	1 / 1 100.0%	4 / 6 66.7%	0 / 0 0.0%	0 / 0 0.0%	0 / 0 0.0%	0 / 0 0.0%	44 / 69 63.8%AB	11 / 69 15.9%	10 / 69 14.5%ABE	4 / 69 5.8%E		
1	Lysinibacillus	71 / 84 84.5%	2 / 2 100.0%	5 / 5 100.0%	6 / 6 100.0%	6 / 8 75.0%	6 / 6 100.0%	4 / 4 100.0%	3 / 3 100.0%	3 / 3 100.0%	6 / 6 100.0%	2 / 2 100.0%	6 / 6 100.0%	1 / 3 33.3%	3 / 4 75.0%	3 / 3 100.0%	1 / 1 100.0%	2 / 2 100.0%	1 / 2 50.0%	3 / 3 100.0%	3 / 4 75.0%	2 / 7 28.6%	1 / 1 100.0%	2 / 3 66.7%	0 / 0 0.0%	0 / 0 0.0%	0 / 0 0.0%	0 / 0 0.0%	29 / 84 34.5%AB	26 / 84 31.0%B	10 / 84 11.9%ABE	10 / 84 11.9%E	6 / 84 7.1%BE	3 / 84 3.6%
1	ATCC 14577	71 / 84 84.5%	2 / 2 100.0%	5 / 5 100.0%	6 / 6 100.0%	6 / 8 75.0%	6 / 6 100.0%	4 / 4 100.0%	3 / 3 100.0%	3 / 3 100.0%	6 / 6 100.0%	2 / 2 100.0%	6 / 6 100.0%	1 / 3 33.3%	3 / 4 75.0%	3 / 3 100.0%	1 / 1 100.0%	2 / 2 100.0%	1 / 2 50.0%	3 / 3 100.0%	3 / 4 75.0%	2 / 7 28.6%	1 / 1 100.0%	2 / 3 66.7%	0 / 0 0.0%	0 / 0 0.0%	0 / 0 0.0%	0 / 0 0.0%	29 / 84 34.5%AB	26 / 84 31.0%B	10 / 84 11.9%E	10 / 84 11.9%ABE	6 / 84 7.1%BE	3 / 84 3.6%
1	Bacillus sphaericus C3-41	71 / 84 84.5%	2 / 2 100.0%	5 / 5 100.0%	6 / 6 100.0%	6 / 8 75.0%	6 / 6 100.0%	4 / 4 100.0%	3 / 3 100.0%	3 / 3 100.0%	6 / 6 100.0%	2 / 2 100.0%	6 / 6 100.0%	1 / 3 33.3%	3 / 4 75.0%	3 / 3 100.0%	1 / 1 100.0%	2 / 2 100.0%	1 / 2 50.0%	3 / 3 100.0%	3 / 4 75.0%	2 / 7 28.6%	1 / 1 100.0%	2 / 3 66.7%	0 / 0 0.0%	0 / 0 0.0%	0 / 0 0.0%	0 / 0 0.0%	29 / 84 34.5%AB	26 / 84 31.0%B	10 / 84 11.9%E	10 / 84 11.9%ABE	6 / 84 7.1%BE	3 / 84 3.6%
1	Caryophanaceae	83 / 94 88.3%	3 / 3 100.0%	5 / 5 100.0%	7 / 7 100.0%	3 / 3 100.0%	8 / 8 100.0%	6 / 6 100.0%	2 / 2 100.0%	2 / 2 100.0%	3 / 3 100.0%	1 / 1 100.0%	7 / 7 100.0%	5 / 6 83.3%	4 / 4 100.0%	3 / 3 100.0%	1 / 1 100.0%	3 / 3 100.0%	1 / 3 33.3%	4 / 4 100.0%	5 / 7 71.4%	3 / 8 37.5%	1 / 1 100.0%	6 / 7 85.7%	0 / 0 0.0%	0 / 0 0.0%	0 / 0 0.0%	0 / 0 0.0%	63 / 94 67.0%AB	20 / 94 21.3%ABE	10 / 94 10.6%E	1 / 94 1.1%		
1	Solibacillus	83 / 94 88.3%	3 / 3 100.0%	5 / 5 100.0%	7 / 7 100.0%	3 / 3 100.0%	8 / 8 100.0%	6 / 6 100.0%	2 / 2 100.0%	2 / 2 100.0%	3 / 3 100.0%	1 / 1 100.0%	7 / 7 100.0%	5 / 6 83.3%	4 / 4 100.0%	3 / 3 100.0%	1 / 1 100.0%	3 / 3 100.0%	1 / 3 33.3%	4 / 4 100.0%	5 / 7 71.4%	3 / 8 37.5%	1 / 1 100.0%	6 / 7 85.7%	0 / 0 0.0%	0 / 0 0.0%	0 / 0 0.0%	0 / 0 0.0%	63 / 94 67.0%AB	20 / 94 21.3%ABE	10 / 94 10.6%E	1 / 94 1.1%		
1	ATCC BAA-269	83 / 94 88.3%	3 / 3 100.0%	5 / 5 100.0%	7 / 7 100.0%	3 / 3 100.0%	8 / 8 100.0%	6 / 6 100.0%	2 / 2 100.0%	2 / 2 100.0%	3 / 3 100.0%	1 / 1 100.0%	7 / 7 100.0%	5 / 6 83.3%	4 / 4 100.0%	3 / 3 100.0%	1 / 1 100.0%	3 / 3 100.0%	1 / 3 33.3%	4 / 4 100.0%	5 / 7 71.4%	3 / 8 37.5%	1 / 1 100.0%	6 / 7 85.7%	0 / 0 0.0%	0 / 0 0.0%	0 / 0 0.0%	0 / 0 0.0%	63 / 94 67.0%AB	20 / 94 21.3%ABE	10 / 94 10.6%E	1 / 94 1.1%		
1	Solibacillus silvestris StLB046	83 / 94 88.3%	3 / 3 100.0%	5 / 5 100.0%	7 / 7 100.0%	3 / 3 100.0%	8 / 8 100.0%	6 / 6 100.0%	2 / 2 100.0%	2 / 2 100.0%	3 / 3 100.0%	1 / 1 100.0%	7 / 7 100.0%	5 / 6 83.3%	4 / 4 100.0%	3 / 3 100.0%	1 / 1 100.0%	3 / 3 100.0%	1 / 3 33.3%	4 / 4 100.0%	5 / 7 71.4%	3 / 8 37.5%	1 / 1 100.0%	6 / 7 85.7%	0 / 0 0.0%	0 / 0 0.0%	0 / 0 0.0%	0 / 0 0.0%	63 / 94 67.0%AB	20 / 94 21.3%ABE	10 / 94 10.6%E	1 / 94 1.1%		
20	Listeriaceae	329 / 1375 23.9%	0 / 42 0.0%	42 / 63 66.7%	42 / 82 51.2%	0 / 62 0.0%	18 / 101 17.8%	21 / 81 25.9%	0 / 42 0.0%	42 / 42 100.0%	60 / 81 74.1%	21 / 21 100.0%	21 / 105 20.0%	0 / 81 0.0%	0 / 42 0.0%	0 / 42 0.0%	21 / 21 100.0%	0 / 39 0.0%	0 / 21 0.0%	39 / 81 48.1%	0 / 102 0.0%	2 / 83 2.4%	0 / 21 0.0%	0 / 120 0.0%	0 / 0 0.0%	0 / 0 0.0%	0 / 0 0.0%	0 / 0 0.0%	540 / 1375 39.3%E	491 / 1375 35.7%	285 / 1375 20.7%AB	44 / 1375 3.2%ABE	15 / 1375 1.1%CE	
20	"Listerella" Pirie 1927 (nom. rej. Opin 14)	329 / 1375 23.9%	0 / 42 0.0%	42 / 63 66.7%	42 / 82 51.2%	0 / 62 0.0%	18 / 101 17.8%	21 / 81 25.9%	0 / 42 0.0%	42 / 42 100.0%	60 / 81 74.1%	21 / 21 100.0%	21 / 105 20.0%	0 / 81 0.0%	0 / 42 0.0%	0 / 42 0.0%	21 / 21 100.0%	0 / 39 0.0%	0 / 21 0.0%	39 / 81 48.1%	0 / 102 0.0%	2 / 83 2.4%	0 / 21 0.0%	0 / 120 0.0%	0 / 0 0.0%	0 / 0 0.0%	0 / 0 0.0%	0 / 0 0.0%	540 / 1375 39.3%E	491 / 1375 35.7%	285 / 1375 20.7%AB	44 / 1375 3.2%ABE	15 / 1375 1.1%CE	
1	"Listeria bulgarica" Ivanov 1975	17 / 67 25.4%	0 / 2 0.0%	2 / 3 66.7%	2 / 4 50.0%	0 / 3 0.0%	1 / 5 20.0%	1 / 4 25.0%	0 / 2 0.0%	2 / 2 100.0%	3 / 4 75.0%	1 / 1 100.0%	1 / 5 20.0%	0 / 4 0.0%	0 / 2 0.0%	0 / 2 0.0%	1 / 1 100.0%	0 / 2 0.0%	0 / 1 0.0%	2 / 4 50.0%	0 / 5 0.0%	1 / 4 25.0%	0 / 1 0.0%	0 / 6 0.0%	0 / 0 0.0%	0 / 0 0.0%	0 / 0 0.0%	0 / 0 0.0%	27 / 67 40.3%E	23 / 67 34.3%	14 / 67 20.9%AB	3 / 67 4.5%ABE		
1	Listeria ivanovii subsp. ivanovii	17 / 67 25.4%	0 / 2 0.0%	2 / 3 66.7%	2 / 4 50.0%	0 / 3 0.0%	1 / 5 20.0%	1 / 4 25.0%	0 / 2 0.0%	2 / 2 100.0%	3 / 4 75.0%	1 / 1 100.0%	1 / 5 20.0%	0 / 4 0.0%	0 / 2 0.0%	0 / 2 0.0%	1 / 1 100.0%	0 / 2 0.0%	0 / 1 0.0%	2 / 4 50.0%	0 / 5 0.0%	1 / 4 25.0%	0 / 1 0.0%	0 / 6 0.0%	0 / 0 0.0%	0 / 0 0.0%	0 / 0 0.0%	0 / 0 0.0%	27 / 67 40.3%E	23 / 67 34.3%	14 / 67 20.9%AB	3 / 67 4.5%ABE		
1	Listeria ivanovii PAM55	17 / 67 25.4%	0 / 2 0.0%	2 / 3 66.7%	2 / 4 50.0%	0 / 3 0.0%	1 / 5 20.0%	1 / 4 25.0%	0 / 2 0.0%	2 / 2 100.0%	3 / 4 75.0%	1 / 1 100.0%	1 / 5 20.0%	0 / 4 0.0%	0 / 2 0.0%	0 / 2 0.0%	1 / 1 100.0%	0 / 2 0.0%	0 / 1 0.0%	2 / 4 50.0%	0 / 5 0.0%	1 / 4 25.0%	0 / 1 0.0%	0 / 6 0.0%	0 / 0 0.0%	0 / 0 0.0%	0 / 0 0.0%	0 / 0 0.0%	27 / 67 40.3%E	23 / 67 34.3%	14 / 67 20.9%AB	3 / 67 4.5%ABE		
16	"Bacterium monocytogenes hominis" Nyfeldt 1932	263 / 1109 23.7%	0 / 34 0.0%	34 / 51 66.7%	34 / 68 50.0%	0 / 50 0.0%	14 / 81 17.3%	17 / 65 26.2%	0 / 34 0.0%	34 / 34 100.0%	48 / 65 73.8%	17 / 17 100.0%	17 / 85 20.0%	0 / 65 0.0%	0 / 34 0.0%	0 / 34 0.0%	17 / 17 100.0%	0 / 31 0.0%	0 / 17 0.0%	31 / 65 47.7%	0 / 82 0.0%	0 / 67 0.0%	0 / 17 0.0%	0 / 96 0.0%	0 / 0 0.0%	0 / 0 0.0%	0 / 0 0.0%	0 / 0 0.0%	451 / 1109 40.7%E	380 / 1109 34.3%	229 / 1109 20.6%AB	34 / 1109 3.1%ABE	15 / 1109 1.4%CE	
1	Listeria monocytogenes serotype 4b str. F2365	15 / 67 22.4%	0 / 2 0.0%	2 / 3 66.7%	2 / 4 50.0%	0 / 3 0.0%	0 / 5 0.0%	1 / 4 25.0%	0 / 2 0.0%	2 / 2 100.0%	3 / 4 75.0%	1 / 1 100.0%	1 / 5 20.0%	0 / 4 0.0%	0 / 2 0.0%	0 / 2 0.0%	1 / 1 100.0%	0 / 2 0.0%	0 / 1 0.0%	2 / 4 50.0%	0 / 5 0.0%	0 / 4 0.0%	0 / 1 0.0%	0 / 6 0.0%	0 / 0 0.0%	0 / 0 0.0%	0 / 0 0.0%	0 / 0 0.0%	35 / 67 52.2%	17 / 67 25.4%E	13 / 67 19.4%AB	2 / 67 3.0%ABE		
1	Listeria monocytogenes FSL R2-561	16 / 67 23.9%	0 / 2 0.0%	2 / 3 66.7%	2 / 4 50.0%	0 / 3 0.0%	1 / 5 20.0%	1 / 4 25.0%	0 / 2 0.0%	2 / 2 100.0%	3 / 4 75.0%	1 / 1 100.0%	1 / 5 20.0%	0 / 4 0.0%	0 / 2 0.0%	0 / 2 0.0%	1 / 1 100.0%	0 / 2 0.0%	0 / 1 0.0%	2 / 4 50.0%	0 / 5 0.0%	0 / 4 0.0%	0 / 1 0.0%	0 / 6 0.0%	0 / 0 0.0%	0 / 0 0.0%	0 / 0 0.0%	0 / 0 0.0%	28 / 67 41.8%E	22 / 67 32.8%	14 / 67 20.9%AB	2 / 67 3.0%ABE	1 / 67 1.5%CE	
1	Listeria monocytogenes Finland 1988	16 / 67 23.9%	0 / 2 0.0%	2 / 3 66.7%	2 / 4 50.0%	0 / 3 0.0%	1 / 5 20.0%	1 / 4 25.0%	0 / 2 0.0%	2 / 2 100.0%	3 / 4 75.0%	1 / 1 100.0%	1 / 5 20.0%	0 / 4 0.0%	0 / 2 0.0%	0 / 2 0.0%	1 / 1 100.0%	0 / 2 0.0%	0 / 1 0.0%	2 / 4 50.0%	0 / 5 0.0%	0 / 4 0.0%	0 / 1 0.0%	0 / 6 0.0%	0 / 0 0.0%	0 / 0 0.0%	0 / 0 0.0%	0 / 0 0.0%	28 / 67 41.8%E	22 / 67 32.8%	14 / 67 20.9%AB	2 / 67 3.0%ABE	1 / 67 1.5%CE	
1	Listeria monocytogenes J0161	14 / 58 24.1%	0 / 2 0.0%	2 / 3 66.7%	2 / 4 50.0%	0 / 3 0.0%	1 / 4 25.0%	1 / 3 33.3%	0 / 2 0.0%	2 / 2 100.0%	2 / 3 66.7%	1 / 1 100.0%	1 / 5 20.0%	0 / 3 0.0%	0 / 2 0.0%	0 / 2 0.0%	1 / 1 100.0%	0 / 1 0.0%	0 / 1 0.0%	1 / 3 33.3%	0 / 4 0.0%	0 / 4 0.0%	0 / 1 0.0%	0 / 4 0.0%	0 / 0 0.0%	0 / 0 0.0%	0 / 0 0.0%	0 / 0 0.0%	25 / 58 43.1%E	18 / 58 31.0%	12 / 58 20.7%AB	2 / 58 3.4%ABE	1 / 58 1.7%CE	
1	Listeria monocytogenes 10403S	16 / 67 23.9%	0 / 2 0.0%	2 / 3 66.7%	2 / 4 50.0%	0 / 3 0.0%	1 / 5 20.0%	1 / 4 25.0%	0 / 2 0.0%	2 / 2 100.0%	3 / 4 75.0%	1 / 1 100.0%	1 / 5 20.0%	0 / 4 0.0%	0 / 2 0.0%	0 / 2 0.0%	1 / 1 100.0%	0 / 2 0.0%	0 / 1 0.0%	2 / 4 50.0%	0 / 5 0.0%	0 / 4 0.0%	0 / 1 0.0%	0 / 6 0.0%	0 / 0 0.0%	0 / 0 0.0%	0 / 0 0.0%	0 / 0 0.0%	28 / 67 41.8%E	22 / 67 32.8%	14 / 67 20.9%AB	2 / 67 3.0%ABE	1 / 67 1.5%CE	
1	Listeria monocytogenes HCC23	16 / 65 24.6%	0 / 2 0.0%	2 / 3 66.7%	2 / 4 50.0%	0 / 2 0.0%	1 / 5 20.0%	1 / 4 25.0%	0 / 2 0.0%	2 / 2 100.0%	3 / 4 75.0%	1 / 1 100.0%	1 / 5 20.0%	0 / 4 0.0%	0 / 2 0.0%	0 / 2 0.0%	1 / 1 100.0%	0 / 2 0.0%	0 / 1 0.0%	2 / 4 50.0%	0 / 5 0.0%	0 / 3 0.0%	0 / 1 0.0%	0 / 6 0.0%	0 / 0 0.0%	0 / 0 0.0%	0 / 0 0.0%	0 / 0 0.0%	28 / 65 43.1%E	20 / 65 30.8%	14 / 65 21.5%AB	2 / 65 3.1%ABE	1 / 65 1.5%CE	
1	Listeria monocytogenes L99	16 / 66 24.2%	0 / 2 0.0%	2 / 3 66.7%	2 / 4 50.0%	0 / 3 0.0%	1 / 4 25.0%	1 / 4 25.0%	0 / 2 0.0%	2 / 2 100.0%	3 / 4 75.0%	1 / 1 100.0%	1 / 5 20.0%	0 / 4 0.0%	0 / 2 0.0%	0 / 2 0.0%	1 / 1 100.0%	0 / 2 0.0%	0 / 1 0.0%	2 / 4 50.0%	0 / 5 0.0%	0 / 4 0.0%	0 / 1 0.0%	0 / 6 0.0%	0 / 0 0.0%	0 / 0 0.0%	0 / 0 0.0%	0 / 0 0.0%	29 / 66 43.9%E	20 / 66 30.3%	14 / 66 21.2%AB	2 / 66 3.0%ABE	1 / 66 1.5%CE	
1	Listeria monocytogenes Clip80459	16 / 67 23.9%	0 / 2 0.0%	2 / 3 66.7%	2 / 4 50.0%	0 / 3 0.0%	1 / 5 20.0%	1 / 4 25.0%	0 / 2 0.0%	2 / 2 100.0%	3 / 4 75.0%	1 / 1 100.0%	1 / 5 20.0%	0 / 4 0.0%	0 / 2 0.0%	0 / 2 0.0%	1 / 1 100.0%	0 / 2 0.0%	0 / 1 0.0%	2 / 4 50.0%	0 / 5 0.0%	0 / 4 0.0%	0 / 1 0.0%	0 / 6 0.0%	0 / 0 0.0%	0 / 0 0.0%	0 / 0 0.0%	0 / 0 0.0%	29 / 67 43.3%E	21 / 67 31.3%	14 / 67 20.9%AB	2 / 67 3.0%ABE	1 / 67 1.5%CE	
1	Listeria monocytogenes 08-5923	14 / 58 24.1%	0 / 2 0.0%	2 / 3 66.7%	2 / 4 50.0%	0 / 3 0.0%	1 / 4 25.0%	1 / 3 33.3%	0 / 2 0.0%	2 / 2 100.0%	2 / 3 66.7%	1 / 1 100.0%	1 / 5 20.0%	0 / 3 0.0%	0 / 2 0.0%	0 / 2 0.0%	1 / 1 100.0%	0 / 1 0.0%	0 / 1 0.0%	1 / 3 33.3%	0 / 4 0.0%	0 / 4 0.0%	0 / 1 0.0%	0 / 4 0.0%	0 / 0 0.0%	0 / 0 0.0%	0 / 0 0.0%	0 / 0 0.0%	25 / 58 43.1%E	18 / 58 31.0%	12 / 58 20.7%AB	2 / 58 3.4%ABE	1 / 58 1.7%CE	
1	Listeria monocytogenes 08-5578	14 / 58 24.1%	0 / 2 0.0%	2 / 3 66.7%	2 / 4 50.0%	0 / 3 0.0%	1 / 4 25.0%	1 / 3 33.3%	0 / 2 0.0%	2 / 2 100.0%	2 / 3 66.7%	1 / 1 100.0%	1 / 5 20.0%	0 / 3 0.0%	0 / 2 0.0%	0 / 2 0.0%	1 / 1 100.0%	0 / 1 0.0%	0 / 1 0.0%	1 / 3 33.3%	0 / 4 0.0%	0 / 4 0.0%	0 / 1 0.0%	0 / 4 0.0%	0 / 0 0.0%	0 / 0 0.0%	0 / 0 0.0%	0 / 0 0.0%	25 / 58 43.1%E	18 / 58 31.0%	12 / 58 20.7%AB	2 / 58 3.4%ABE	1 / 58 1.7%CE	
1	Listeria monocytogenes serotype 7 str. SLCC2482	16 / 67 23.9%	0 / 2 0.0%	2 / 3 66.7%	2 / 4 50.0%	0 / 3 0.0%	1 / 5 20.0%	1 / 4 25.0%	0 / 2 0.0%	2 / 2 100.0%	3 / 4 75.0%	1 / 1 100.0%	1 / 5 20.0%	0 / 4 0.0%	0 / 2 0.0%	0 / 2 0.0%	1 / 1 100.0%	0 / 2 0.0%	0 / 1 0.0%	2 / 4 50.0%	0 / 5 0.0%	0 / 4 0.0%	0 / 1 0.0%	0 / 6 0.0%	0 / 0 0.0%	0 / 0 0.0%	0 / 0 0.0%	0 / 0 0.0%	29 / 67 43.3%E	21 / 67 31.3%	14 / 67 20.9%AB	2 / 67 3.0%ABE	1 / 67 1.5%CE	
1	Listeria monocytogenes M7	16 / 67 23.9%	0 / 2 0.0%	2 / 3 66.7%	2 / 4 50.0%	0 / 3 0.0%	1 / 5 20.0%	1 / 4 25.0%	0 / 2 0.0%	2 / 2 100.0%	3 / 4 75.0%	1 / 1 100.0%	1 / 5 20.0%	0 / 4 0.0%	0 / 2 0.0%	0 / 2 0.0%	1 / 1 100.0%	0 / 2 0.0%	0 / 1 0.0%	2 / 4 50.0%	0 / 5 0.0%	0 / 4 0.0%	0 / 1 0.0%	0 / 6 0.0%	0 / 0 0.0%	0 / 0 0.0%	0 / 0 0.0%	0 / 0 0.0%	29 / 67 43.3%E	21 / 67 31.3%	14 / 67 20.9%AB	2 / 67 3.0%ABE	1 / 67 1.5%CE	
1	Listeria monocytogenes 07PF0776	15 / 67 22.4%	0 / 2 0.0%	2 / 3 66.7%	2 / 4 50.0%	0 / 3 0.0%	0 / 5 0.0%	1 / 4 25.0%	0 / 2 0.0%	2 / 2 100.0%	3 / 4 75.0%	1 / 1 100.0%	1 / 5 20.0%	0 / 4 0.0%	0 / 2 0.0%	0 / 2 0.0%	1 / 1 100.0%	0 / 2 0.0%	0 / 1 0.0%	2 / 4 50.0%	0 / 5 0.0%	0 / 4 0.0%	0 / 1 0.0%	0 / 6 0.0%	0 / 0 0.0%	0 / 0 0.0%	0 / 0 0.0%	0 / 0 0.0%	29 / 67 43.3%E	22 / 67 32.8%	13 / 67 19.4%AB	2 / 67 3.0%ABE	1 / 67 1.5%CE	
1	Listeria monocytogenes serotype 4b str. LL195	15 / 67 22.4%	0 / 2 0.0%	2 / 3 66.7%	2 / 4 50.0%	0 / 3 0.0%	0 / 5 0.0%	1 / 4 25.0%	0 / 2 0.0%	2 / 2 100.0%	3 / 4 75.0%	1 / 1 100.0%	1 / 5 20.0%	0 / 4 0.0%	0 / 2 0.0%	0 / 2 0.0%	1 / 1 100.0%	0 / 2 0.0%	0 / 1 0.0%	2 / 4 50.0%	0 / 5 0.0%	0 / 4 0.0%	0 / 1 0.0%	0 / 6 0.0%	0 / 0 0.0%	0 / 0 0.0%	0 / 0 0.0%	0 / 0 0.0%	29 / 67 43.3%E	22 / 67 32.8%	13 / 67 19.4%AB	2 / 67 3.0%ABE	1 / 67 1.5%CE	
1	Listeria monocytogenes N53-1	16 / 67 23.9%	0 / 2 0.0%	2 / 3 66.7%	2 / 4 50.0%	0 / 3 0.0%	1 / 5 20.0%	1 / 4 25.0%	0 / 2 0.0%	2 / 2 100.0%	3 / 4 75.0%	1 / 1 100.0%	1 / 5 20.0%	0 / 4 0.0%	0 / 2 0.0%	0 / 2 0.0%	1 / 1 100.0%	0 / 2 0.0%	0 / 1 0.0%	2 / 4 50.0%	0 / 5 0.0%	0 / 4 0.0%	0 / 1 0.0%	0 / 6 0.0%	0 / 0 0.0%	0 / 0 0.0%	0 / 0 0.0%	0 / 0 0.0%	28 / 67 41.8%E	22 / 67 32.8%	14 / 67 20.9%AB	2 / 67 3.0%ABE	1 / 67 1.5%CE	
1	ATCC 35967	17 / 67 25.4%	0 / 2 0.0%	2 / 3 66.7%	2 / 4 50.0%	0 / 3 0.0%	1 / 5 20.0%	1 / 4 25.0%	0 / 2 0.0%	2 / 2 100.0%	3 / 4 75.0%	1 / 1 100.0%	1 / 5 20.0%	0 / 4 0.0%	0 / 2 0.0%	0 / 2 0.0%	1 / 1 100.0%	0 / 2 0.0%	0 / 1 0.0%	2 / 4 50.0%	0 / 5 0.0%	1 / 4 25.0%	0 / 1 0.0%	0 / 6 0.0%	0 / 0 0.0%	0 / 0 0.0%	0 / 0 0.0%	0 / 0 0.0%	28 / 67 41.8%E	22 / 67 32.8%	14 / 67 20.9%AB	3 / 67 4.5%ABE		
1	Listeria seeligeri 1/2b strain SLCC3954	17 / 67 25.4%	0 / 2 0.0%	2 / 3 66.7%	2 / 4 50.0%	0 / 3 0.0%	1 / 5 20.0%	1 / 4 25.0%	0 / 2 0.0%	2 / 2 100.0%	3 / 4 75.0%	1 / 1 100.0%	1 / 5 20.0%	0 / 4 0.0%	0 / 2 0.0%	0 / 2 0.0%	1 / 1 100.0%	0 / 2 0.0%	0 / 1 0.0%	2 / 4 50.0%	0 / 5 0.0%	1 / 4 25.0%	0 / 1 0.0%	0 / 6 0.0%	0 / 0 0.0%	0 / 0 0.0%	0 / 0 0.0%	0 / 0 0.0%	28 / 67 41.8%E	22 / 67 32.8%	14 / 67 20.9%AB	3 / 67 4.5%ABE		
1	"Listeria innocua" Seeliger and Schoofs 1979	16 / 66 24.2%	0 / 2 0.0%	2 / 3 66.7%	2 / 3 66.7%	0 / 3 0.0%	1 / 5 20.0%	1 / 4 25.0%	0 / 2 0.0%	2 / 2 100.0%	3 / 4 75.0%	1 / 1 100.0%	1 / 5 20.0%	0 / 4 0.0%	0 / 2 0.0%	0 / 2 0.0%	1 / 1 100.0%	0 / 2 0.0%	0 / 1 0.0%	2 / 4 50.0%	0 / 5 0.0%	0 / 4 0.0%	0 / 1 0.0%	0 / 6 0.0%	0 / 0 0.0%	0 / 0 0.0%	0 / 0 0.0%	0 / 0 0.0%	33 / 66 50.0%	17 / 66 25.8%E	14 / 66 21.2%AB	2 / 66 3.0%ABE		
1	ATCC 35897	16 / 66 24.2%	0 / 2 0.0%	2 / 3 66.7%	2 / 3 66.7%	0 / 3 0.0%	1 / 5 20.0%	1 / 4 25.0%	0 / 2 0.0%	2 / 2 100.0%	3 / 4 75.0%	1 / 1 100.0%	1 / 5 20.0%	0 / 4 0.0%	0 / 2 0.0%	0 / 2 0.0%	1 / 1 100.0%	0 / 2 0.0%	0 / 1 0.0%	2 / 4 50.0%	0 / 5 0.0%	0 / 4 0.0%	0 / 1 0.0%	0 / 6 0.0%	0 / 0 0.0%	0 / 0 0.0%	0 / 0 0.0%	0 / 0 0.0%	33 / 66 50.0%	17 / 66 25.8%E	14 / 66 21.2%AB	2 / 66 3.0%ABE		
1	Listeria welshimeri serovar 6b SLCC5334	16 / 66 24.2%	0 / 2 0.0%	2 / 3 66.7%	2 / 3 66.7%	0 / 3 0.0%	1 / 5 20.0%	1 / 4 25.0%	0 / 2 0.0%	2 / 2 100.0%	3 / 4 75.0%	1 / 1 100.0%	1 / 5 20.0%	0 / 4 0.0%	0 / 2 0.0%	0 / 2 0.0%	1 / 1 100.0%	0 / 2 0.0%	0 / 1 0.0%	2 / 4 50.0%	0 / 5 0.0%	0 / 4 0.0%	0 / 1 0.0%	0 / 6 0.0%	0 / 0 0.0%	0 / 0 0.0%	0 / 0 0.0%	0 / 0 0.0%	33 / 66 50.0%	17 / 66 25.8%E	14 / 66 21.2%AB	2 / 66 3.0%ABE		
1	Sporolactobacillaceae	68 / 68 100.0%	2 / 2 100.0%	4 / 4 100.0%	4 / 4 100.0%	2 / 2 100.0%	5 / 5 100.0%	4 / 4 100.0%	2 / 2 100.0%	2 / 2 100.0%	4 / 4 100.0%	1 / 1 100.0%	4 / 4 100.0%	3 / 3 100.0%	2 / 2 100.0%	3 / 3 100.0%	1 / 1 100.0%	2 / 2 100.0%	2 / 2 100.0%	4 / 4 100.0%	5 / 5 100.0%	5 / 5 100.0%	1 / 1 100.0%	5 / 5 100.0%	1 / 1 100.0%	0 / 0 0.0%	0 / 0 0.0%	0 / 0 0.0%	47 / 68 69.1%AB	21 / 68 30.9%ABE				
1	unclassified Sporolactobacillaceae	68 / 68 100.0%	2 / 2 100.0%	4 / 4 100.0%	4 / 4 100.0%	2 / 2 100.0%	5 / 5 100.0%	4 / 4 100.0%	2 / 2 100.0%	2 / 2 100.0%	4 / 4 100.0%	1 / 1 100.0%	4 / 4 100.0%	3 / 3 100.0%	2 / 2 100.0%	3 / 3 100.0%	1 / 1 100.0%	2 / 2 100.0%	2 / 2 100.0%	4 / 4 100.0%	5 / 5 100.0%	5 / 5 100.0%	1 / 1 100.0%	5 / 5 100.0%	1 / 1 100.0%	0 / 0 0.0%	0 / 0 0.0%	0 / 0 0.0%	47 / 68 69.1%AB	21 / 68 30.9%ABE				
1	ATCC 700615	68 / 68 100.0%	2 / 2 100.0%	4 / 4 100.0%	4 / 4 100.0%	2 / 2 100.0%	5 / 5 100.0%	4 / 4 100.0%	2 / 2 100.0%	2 / 2 100.0%	4 / 4 100.0%	1 / 1 100.0%	4 / 4 100.0%	3 / 3 100.0%	2 / 2 100.0%	3 / 3 100.0%	1 / 1 100.0%	2 / 2 100.0%	2 / 2 100.0%	4 / 4 100.0%	5 / 5 100.0%	5 / 5 100.0%	1 / 1 100.0%	5 / 5 100.0%	1 / 1 100.0%	0 / 0 0.0%	0 / 0 0.0%	0 / 0 0.0%	47 / 68 69.1%AB	21 / 68 30.9%ABE				
1	Bacillus selenitireducens MLS10	68 / 68 100.0%	2 / 2 100.0%	4 / 4 100.0%	4 / 4 100.0%	2 / 2 100.0%	5 / 5 100.0%	4 / 4 100.0%	2 / 2 100.0%	2 / 2 100.0%	4 / 4 100.0%	1 / 1 100.0%	4 / 4 100.0%	3 / 3 100.0%	2 / 2 100.0%	3 / 3 100.0%	1 / 1 100.0%	2 / 2 100.0%	2 / 2 100.0%	4 / 4 100.0%	5 / 5 100.0%	5 / 5 100.0%	1 / 1 100.0%	5 / 5 100.0%	1 / 1 100.0%	0 / 0 0.0%	0 / 0 0.0%	0 / 0 0.0%	47 / 68 69.1%AB	21 / 68 30.9%ABE				
11	Paenibacillaceae	816 / 1205 67.7%	37 / 45 82.2%	53 / 53 100.0%	52 / 53 98.1%	30 / 34 88.2%	69 / 102 67.6%	68 / 77 88.3%	25 / 34 73.5%	38 / 38 100.0%	53 / 58 91.4%	12 / 15 80.0%	56 / 103 54.4%	39 / 66 59.1%	18 / 36 50.0%	38 / 42 90.5%	12 / 18 66.7%	30 / 54 55.6%	13 / 40 32.5%	48 / 62 77.4%	47 / 78 60.3%	15 / 61 24.6%	6 / 11 54.5%	57 / 125 45.6%	0 / 0 0.0%	0 / 0 0.0%	0 / 0 0.0%	0 / 0 0.0%	544 / 1205 45.1%AB	272 / 1205 22.6%ABE	240 / 1205 19.9%E	131 / 1205 10.9%	10 / 1205 0.8%C	8 / 1205 0.7%CE
9	Bacillus polymyxa group	643 / 1020 63.0%	30 / 38 78.9%	44 / 44 100.0%	44 / 45 97.8%	23 / 27 85.2%	54 / 87 62.1%	57 / 66 86.4%	20 / 29 69.0%	33 / 33 100.0%	43 / 48 89.6%	9 / 12 75.0%	45 / 91 49.5%	30 / 57 52.6%	14 / 31 45.2%	32 / 36 88.9%	9 / 15 60.0%	21 / 45 46.7%	9 / 36 25.0%	38 / 52 73.1%	36 / 64 56.2%	12 / 51 23.5%	4 / 9 44.4%	36 / 104 34.6%	0 / 0 0.0%	0 / 0 0.0%	0 / 0 0.0%	0 / 0 0.0%	426 / 1020 41.8%AB	235 / 1020 23.0%E	217 / 1020 21.3%ABE	128 / 1020 12.5%	9 / 1020 0.9%C	5 / 1020 0.5%CE
3	"Aerobacillus polymyxa" (Prazmowski 1880) Donker 1926	185 / 309 59.9%	6 / 11 54.5%	14 / 14 100.0%	17 / 17 100.0%	9 / 9 100.0%	11 / 25 44.0%	17 / 20 85.0%	0 / 6 0.0%	11 / 11 100.0%	17 / 17 100.0%	3 / 3 100.0%	20 / 23 87.0%	12 / 20 60.0%	0 / 9 0.0%	11 / 11 100.0%	3 / 6 50.0%	2 / 14 14.3%	3 / 6 50.0%	11 / 17 64.7%	12 / 20 60.0%	0 / 17 0.0%	0 / 3 0.0%	6 / 30 20.0%	0 / 0 0.0%	0 / 0 0.0%	0 / 0 0.0%	0 / 0 0.0%	139 / 309 45.0%AB	83 / 309 26.9%E	46 / 309 14.9%ABE	32 / 309 10.4%	6 / 309 1.9%C	3 / 309 1.0%CE
1	Paenibacillus polymyxa E681	55 / 91 60.4%	2 / 3 66.7%	4 / 4 100.0%	5 / 5 100.0%	3 / 3 100.0%	3 / 7 42.9%	5 / 6 83.3%	0 / 2 0.0%	3 / 3 100.0%	5 / 5 100.0%	1 / 1 100.0%	6 / 7 85.7%	4 / 6 66.7%	0 / 3 0.0%	3 / 3 100.0%	1 / 2 50.0%	0 / 4 0.0%	1 / 2 50.0%	3 / 5 60.0%	4 / 6 66.7%	0 / 5 0.0%	0 / 1 0.0%	2 / 8 25.0%	0 / 0 0.0%	0 / 0 0.0%	0 / 0 0.0%	0 / 0 0.0%	41 / 91 45.1%AB	23 / 91 25.3%E	14 / 91 15.4%ABE	10 / 91 11.0%	2 / 91 2.2%C	1 / 91 1.1%CE
1	Paenibacillus polymyxa SC2	65 / 109 59.6%	2 / 4 50.0%	5 / 5 100.0%	6 / 6 100.0%	3 / 3 100.0%	4 / 9 44.4%	6 / 7 85.7%	0 / 2 0.0%	4 / 4 100.0%	6 / 6 100.0%	1 / 1 100.0%	7 / 8 87.5%	4 / 7 57.1%	0 / 3 0.0%	4 / 4 100.0%	1 / 2 50.0%	1 / 5 20.0%	1 / 2 50.0%	4 / 6 66.7%	4 / 7 57.1%	0 / 6 0.0%	0 / 1 0.0%	2 / 11 18.2%	0 / 0 0.0%	0 / 0 0.0%	0 / 0 0.0%	0 / 0 0.0%	49 / 109 45.0%AB	30 / 109 27.5%E	16 / 109 14.7%ABE	11 / 109 10.1%	2 / 109 1.8%C	1 / 109 0.9%CE
1	Paenibacillus polymyxa M1	65 / 109 59.6%	2 / 4 50.0%	5 / 5 100.0%	6 / 6 100.0%	3 / 3 100.0%	4 / 9 44.4%	6 / 7 85.7%	0 / 2 0.0%	4 / 4 100.0%	6 / 6 100.0%	1 / 1 100.0%	7 / 8 87.5%	4 / 7 57.1%	0 / 3 0.0%	4 / 4 100.0%	1 / 2 50.0%	1 / 5 20.0%	1 / 2 50.0%	4 / 6 66.7%	4 / 7 57.1%	0 / 6 0.0%	0 / 1 0.0%	2 / 11 18.2%	0 / 0 0.0%	0 / 0 0.0%	0 / 0 0.0%	0 / 0 0.0%	49 / 109 45.0%AB	30 / 109 27.5%E	16 / 109 14.7%ABE	11 / 109 10.1%	2 / 109 1.8%C	1 / 109 0.9%CE
3	Bacillus mucilaginosus	274 / 464 59.1%	17 / 17 100.0%	19 / 19 100.0%	14 / 15 93.3%	6 / 10 60.0%	30 / 44 68.2%	26 / 29 89.7%	17 / 17 100.0%	14 / 14 100.0%	14 / 17 82.4%	3 / 6 50.0%	10 / 51 19.6%	9 / 23 39.1%	10 / 14 71.4%	13 / 17 76.5%	3 / 6 50.0%	15 / 21 71.4%	3 / 27 11.1%	15 / 19 78.9%	11 / 27 40.7%	3 / 20 15.0%	3 / 3 100.0%	19 / 48 39.6%	0 / 0 0.0%	0 / 0 0.0%	0 / 0 0.0%	0 / 0 0.0%	164 / 464 35.3%AB	111 / 464 23.9%E	110 / 464 23.7%ABE	78 / 464 16.8%	1 / 464 0.2%CE	
1	Bacillus mucilaginosus K02	100 / 185 54.1%	6 / 6 100.0%	7 / 7 100.0%	6 / 6 100.0%	3 / 4 75.0%	12 / 16 75.0%	9 / 10 90.0%	5 / 5 100.0%	5 / 5 100.0%	5 / 6 83.3%	1 / 2 50.0%	3 / 29 10.3%	3 / 9 33.3%	5 / 6 83.3%	5 / 6 83.3%	1 / 2 50.0%	6 / 7 85.7%	1 / 21 4.8%	5 / 6 83.3%	5 / 9 55.6%	1 / 7 14.3%	1 / 1 100.0%	5 / 15 33.3%	0 / 0 0.0%	0 / 0 0.0%	0 / 0 0.0%	0 / 0 0.0%	63 / 185 34.1%AB	49 / 185 26.5%E	37 / 185 20.0%ABE	36 / 185 19.5%		
1	Paenibacillus mucilaginosus KNP414	64 / 107 59.8%	3 / 3 100.0%	4 / 4 100.0%	2 / 3 66.7%	1 / 1 100.0%	9 / 14 64.3%	6 / 7 85.7%	5 / 5 100.0%	3 / 3 100.0%	3 / 4 75.0%	0 / 1 0.0%	3 / 8 37.5%	3 / 5 60.0%	2 / 4 50.0%	2 / 4 50.0%	1 / 2 50.0%	4 / 6 66.7%	0 / 2 0.0%	4 / 5 80.0%	3 / 8 37.5%	0 / 4 0.0%	1 / 1 100.0%	5 / 13 38.5%	0 / 0 0.0%	0 / 0 0.0%	0 / 0 0.0%	0 / 0 0.0%	37 / 107 34.6%AB	27 / 107 25.2%ABE	26 / 107 24.3%E	16 / 107 15.0%	1 / 107 0.9%CE	
1	Paenibacillus mucilaginosus 3016	110 / 172 64.0%	8 / 8 100.0%	8 / 8 100.0%	6 / 6 100.0%	2 / 5 40.0%	9 / 14 64.3%	11 / 12 91.7%	7 / 7 100.0%	6 / 6 100.0%	6 / 7 85.7%	2 / 3 66.7%	4 / 14 28.6%	3 / 9 33.3%	3 / 4 75.0%	6 / 7 85.7%	1 / 2 50.0%	5 / 8 62.5%	2 / 4 50.0%	6 / 8 75.0%	3 / 10 30.0%	2 / 9 22.2%	1 / 1 100.0%	9 / 20 45.0%	0 / 0 0.0%	0 / 0 0.0%	0 / 0 0.0%	0 / 0 0.0%	64 / 172 37.2%AB	46 / 172 26.7%ABE	36 / 172 20.9%E	26 / 172 15.1%		
1	JCM 11466	50 / 88 56.8%	0 / 3 0.0%	4 / 4 100.0%	5 / 5 100.0%	3 / 3 100.0%	3 / 7 42.9%	5 / 6 83.3%	0 / 2 0.0%	3 / 3 100.0%	4 / 5 80.0%	1 / 1 100.0%	5 / 6 83.3%	4 / 6 66.7%	0 / 3 0.0%	3 / 3 100.0%	1 / 1 100.0%	0 / 3 0.0%	1 / 1 100.0%	3 / 5 60.0%	4 / 6 66.7%	0 / 5 0.0%	0 / 1 0.0%	1 / 9 11.1%	0 / 0 0.0%	0 / 0 0.0%	0 / 0 0.0%	0 / 0 0.0%	39 / 88 44.3%AB	26 / 88 29.5%E	11 / 88 12.5%ABE	9 / 88 10.2%	2 / 88 2.3%C	1 / 88 1.1%CE
1	Paenibacillus sp. HPL-003	50 / 88 56.8%	0 / 3 0.0%	4 / 4 100.0%	5 / 5 100.0%	3 / 3 100.0%	3 / 7 42.9%	5 / 6 83.3%	0 / 2 0.0%	3 / 3 100.0%	4 / 5 80.0%	1 / 1 100.0%	5 / 6 83.3%	4 / 6 66.7%	0 / 3 0.0%	3 / 3 100.0%	1 / 1 100.0%	0 / 3 0.0%	1 / 1 100.0%	3 / 5 60.0%	4 / 6 66.7%	0 / 5 0.0%	0 / 1 0.0%	1 / 9 11.1%	0 / 0 0.0%	0 / 0 0.0%	0 / 0 0.0%	0 / 0 0.0%	39 / 88 44.3%AB	26 / 88 29.5%E	11 / 88 12.5%ABE	9 / 88 10.2%	2 / 88 2.3%C	1 / 88 1.1%CE
1	Paenibacillus sp. JDR-2	82 / 86 95.3%	4 / 4 100.0%	4 / 4 100.0%	4 / 4 100.0%	3 / 3 100.0%	6 / 6 100.0%	5 / 6 83.3%	2 / 2 100.0%	3 / 3 100.0%	5 / 5 100.0%	1 / 1 100.0%	5 / 5 100.0%	2 / 4 50.0%	3 / 3 100.0%	3 / 3 100.0%	1 / 1 100.0%	4 / 4 100.0%	1 / 1 100.0%	6 / 7 85.7%	5 / 5 100.0%	5 / 5 100.0%	1 / 1 100.0%	9 / 9 100.0%	0 / 0 0.0%	0 / 0 0.0%	0 / 0 0.0%	0 / 0 0.0%	50 / 86 58.1%AB	32 / 86 37.2%ABE	4 / 86 4.7%			
1	Geobacillus sp. Y412MC10	52 / 73 71.2%	3 / 3 100.0%	3 / 3 100.0%	4 / 4 100.0%	2 / 2 100.0%	4 / 5 80.0%	4 / 5 80.0%	1 / 2 50.0%	2 / 2 100.0%	3 / 4 75.0%	1 / 1 100.0%	5 / 6 83.3%	3 / 4 75.0%	1 / 2 50.0%	2 / 2 100.0%	1 / 1 100.0%	0 / 3 0.0%	1 / 1 100.0%	3 / 4 75.0%	4 / 6 66.7%	4 / 4 100.0%	0 / 1 0.0%	1 / 8 12.5%	0 / 0 0.0%	0 / 0 0.0%	0 / 0 0.0%	0 / 0 0.0%	34 / 73 46.6%AB	18 / 73 24.7%ABE	15 / 73 20.5%E	5 / 73 6.8%	1 / 73 1.4%C	
1	Bacillus brevis group	115 / 127 90.6%	4 / 4 100.0%	7 / 7 100.0%	5 / 5 100.0%	5 / 5 100.0%	10 / 10 100.0%	8 / 8 100.0%	4 / 4 100.0%	4 / 4 100.0%	7 / 7 100.0%	2 / 2 100.0%	7 / 8 87.5%	6 / 6 100.0%	3 / 4 75.0%	4 / 4 100.0%	2 / 2 100.0%	6 / 6 100.0%	3 / 3 100.0%	7 / 7 100.0%	6 / 9 66.7%	0 / 7 0.0%	1 / 1 100.0%	14 / 14 100.0%	0 / 0 0.0%	0 / 0 0.0%	0 / 0 0.0%	0 / 0 0.0%	82 / 127 64.6%AB	33 / 127 26.0%ABE	5 / 127 3.9%E	3 / 127 2.4%	3 / 127 2.4%CE	1 / 127 0.8%C
1	ATCC 8246	115 / 127 90.6%	4 / 4 100.0%	7 / 7 100.0%	5 / 5 100.0%	5 / 5 100.0%	10 / 10 100.0%	8 / 8 100.0%	4 / 4 100.0%	4 / 4 100.0%	7 / 7 100.0%	2 / 2 100.0%	7 / 8 87.5%	6 / 6 100.0%	3 / 4 75.0%	4 / 4 100.0%	2 / 2 100.0%	6 / 6 100.0%	3 / 3 100.0%	7 / 7 100.0%	6 / 9 66.7%	0 / 7 0.0%	1 / 1 100.0%	14 / 14 100.0%	0 / 0 0.0%	0 / 0 0.0%	0 / 0 0.0%	0 / 0 0.0%	82 / 127 64.6%AB	33 / 127 26.0%ABE	5 / 127 3.9%E	3 / 127 2.4%	3 / 127 2.4%CE	1 / 127 0.8%C
1	Brevibacillus brevis NBRC 100599	115 / 127 90.6%	4 / 4 100.0%	7 / 7 100.0%	5 / 5 100.0%	5 / 5 100.0%	10 / 10 100.0%	8 / 8 100.0%	4 / 4 100.0%	4 / 4 100.0%	7 / 7 100.0%	2 / 2 100.0%	7 / 8 87.5%	6 / 6 100.0%	3 / 4 75.0%	4 / 4 100.0%	2 / 2 100.0%	6 / 6 100.0%	3 / 3 100.0%	7 / 7 100.0%	6 / 9 66.7%	0 / 7 0.0%	1 / 1 100.0%	14 / 14 100.0%	0 / 0 0.0%	0 / 0 0.0%	0 / 0 0.0%	0 / 0 0.0%	82 / 127 64.6%AB	33 / 127 26.0%ABE	5 / 127 3.9%E	3 / 127 2.4%CE	3 / 127 2.4%	1 / 127 0.8%C
1	Thermobacillus	58 / 58 100.0%	3 / 3 100.0%	2 / 2 100.0%	3 / 3 100.0%	2 / 2 100.0%	5 / 5 100.0%	3 / 3 100.0%	1 / 1 100.0%	1 / 1 100.0%	3 / 3 100.0%	1 / 1 100.0%	4 / 4 100.0%	3 / 3 100.0%	1 / 1 100.0%	2 / 2 100.0%	1 / 1 100.0%	3 / 3 100.0%	1 / 1 100.0%	3 / 3 100.0%	5 / 5 100.0%	3 / 3 100.0%	1 / 1 100.0%	7 / 7 100.0%	0 / 0 0.0%	0 / 0 0.0%	0 / 0 0.0%	0 / 0 0.0%	36 / 58 62.1%AB	22 / 58 37.9%ABE				

1	DSM 18247	58 / 58 100.0%	3 / 3 100.0%	2 / 2 100.0%	3 / 3 100.0%	2 / 2 100.0%	5 / 5 100.0%	3 / 3 100.0%	1 / 1 100.0%	1 / 1 100.0%	3 / 3 100.0%	1 / 1 100.0%	4 / 4 100.0%	3 / 3 100.0%	1 / 1 100.0%	2 / 2 100.0%	1 / 1 100.0%	3 / 3 100.0%	1 / 1 100.0%	3 / 3 100.0%	5 / 5 100.0%	3 / 3 100.0%	1 / 1 100.0%	7 / 7 100.0%	0 / 0 0.0%	0 / 0 0.0%	0 / 0 0.0%	0 / 0 0.0%	36 / 58 62.1%AB	22 / 58 37.9%ABE					
1	Thermobacillus composti DSM 18247	58 / 58 100.0%	3 / 3 100.0%	2 / 2 100.0%	3 / 3 100.0%	2 / 2 100.0%	5 / 5 100.0%	3 / 3 100.0%	1 / 1 100.0%	1 / 1 100.0%	3 / 3 100.0%	1 / 1 100.0%	4 / 4 100.0%	3 / 3 100.0%	1 / 1 100.0%	2 / 2 100.0%	1 / 1 100.0%	3 / 3 100.0%	1 / 1 100.0%	3 / 3 100.0%	5 / 5 100.0%	3 / 3 100.0%	1 / 1 100.0%	7 / 7 100.0%	0 / 0 0.0%	0 / 0 0.0%	0 / 0 0.0%	0 / 0 0.0%	36 / 58 62.1%AB	22 / 58 37.9%ABE					
3	"Alicyclobacillaceae" da Costa and Rainey 2010 emend. Klenk et al. 2011	169 / 184 91.8%	6 / 6 100.0%	6 / 6 100.0%	8 / 8 100.0%	6 / 6 100.0%	17 / 17 100.0%	8 / 11 72.7%	3 / 3 100.0%	4 / 4 100.0%	12 / 12 100.0%	3 / 3 100.0%	13 / 13 100.0%	5 / 7 71.4%	6 / 6 100.0%	6 / 6 100.0%	4 / 4 100.0%	9 / 9 100.0%	3 / 3 100.0%	14 / 17 82.4%	15 / 15 100.0%	7 / 10 70.0%	3 / 3 100.0%	11 / 15 73.3%	0 / 0 0.0%	0 / 0 0.0%	0 / 0 0.0%	0 / 0 0.0%	115 / 184 62.5%AB	54 / 184 29.3%ABE	8 / 184 4.3%E	7 / 184 3.8%			
2	Alicyclobacillus	110 / 125 88.0%	4 / 4 100.0%	4 / 4 100.0%	6 / 6 100.0%	4 / 4 100.0%	12 / 12 100.0%	5 / 8 62.5%	2 / 2 100.0%	2 / 2 100.0%	8 / 8 100.0%	2 / 2 100.0%	8 / 8 100.0%	2 / 4 50.0%	4 / 4 100.0%	4 / 4 100.0%	2 / 2 100.0%	6 / 6 100.0%	2 / 2 100.0%	10 / 13 76.9%	10 / 10 100.0%	5 / 8 62.5%	2 / 2 100.0%	6 / 10 60.0%	0 / 0 0.0%	0 / 0 0.0%	0 / 0 0.0%	0 / 0 0.0%	73 / 125 58.4%AB	37 / 125 29.6%ABE	8 / 125 6.4%E	7 / 125 5.6%			
2	ATCC 27009	110 / 125 88.0%	4 / 4 100.0%	4 / 4 100.0%	6 / 6 100.0%	4 / 4 100.0%	12 / 12 100.0%	5 / 8 62.5%	2 / 2 100.0%	2 / 2 100.0%	8 / 8 100.0%	2 / 2 100.0%	8 / 8 100.0%	2 / 4 50.0%	4 / 4 100.0%	4 / 4 100.0%	2 / 2 100.0%	6 / 6 100.0%	2 / 2 100.0%	10 / 13 76.9%	10 / 10 100.0%	5 / 8 62.5%	2 / 2 100.0%	6 / 10 60.0%	0 / 0 0.0%	0 / 0 0.0%	0 / 0 0.0%	0 / 0 0.0%	73 / 125 58.4%AB	37 / 125 29.6%ABE	8 / 125 6.4%E	7 / 125 5.6%			
2	Alicyclobacillus acidocaldarius subsp. acidocaldarius	110 / 125 88.0%	4 / 4 100.0%	4 / 4 100.0%	6 / 6 100.0%	4 / 4 100.0%	12 / 12 100.0%	5 / 8 62.5%	2 / 2 100.0%	2 / 2 100.0%	8 / 8 100.0%	2 / 2 100.0%	8 / 8 100.0%	2 / 4 50.0%	4 / 4 100.0%	4 / 4 100.0%	2 / 2 100.0%	6 / 6 100.0%	2 / 2 100.0%	10 / 13 76.9%	10 / 10 100.0%	5 / 8 62.5%	2 / 2 100.0%	6 / 10 60.0%	0 / 0 0.0%	0 / 0 0.0%	0 / 0 0.0%	0 / 0 0.0%	73 / 125 58.4%AB	37 / 125 29.6%ABE	8 / 125 6.4%E	7 / 125 5.6%			
1	Alicyclobacillus acidocaldarius subsp. acidocaldarius ATCC 27009	54 / 62 87.1%	2 / 2 100.0%	2 / 2 100.0%	3 / 3 100.0%	2 / 2 100.0%	6 / 6 100.0%	2 / 4 50.0%	1 / 1 100.0%	1 / 1 100.0%	4 / 4 100.0%	1 / 1 100.0%	4 / 4 100.0%	1 / 2 50.0%	2 / 2 100.0%	2 / 2 100.0%	1 / 1 100.0%	3 / 3 100.0%	1 / 1 100.0%	5 / 6 83.3%	5 / 5 100.0%	2 / 4 50.0%	1 / 1 100.0%	3 / 5 60.0%	0 / 0 0.0%	0 / 0 0.0%	0 / 0 0.0%	0 / 0 0.0%	36 / 62 58.1%AB	18 / 62 29.0%ABE	4 / 62 6.5%	4 / 62 6.5%E			
1	Alicyclobacillus acidocaldarius subsp. acidocaldarius Tc-4-1	56 / 63 88.9%	2 / 2 100.0%	2 / 2 100.0%	3 / 3 100.0%	2 / 2 100.0%	6 / 6 100.0%	3 / 4 75.0%	1 / 1 100.0%	1 / 1 100.0%	4 / 4 100.0%	1 / 1 100.0%	4 / 4 100.0%	1 / 2 50.0%	2 / 2 100.0%	2 / 2 100.0%	1 / 1 100.0%	3 / 3 100.0%	1 / 1 100.0%	5 / 7 71.4%	5 / 5 100.0%	3 / 4 75.0%	1 / 1 100.0%	3 / 5 60.0%	0 / 0 0.0%	0 / 0 0.0%	0 / 0 0.0%	0 / 0 0.0%	37 / 63 58.7%AB	19 / 63 30.2%ABE	4 / 63 6.3%E	3 / 63 4.8%			
1	"Kyrpidia" Klenk et al. 2011	59 / 59 100.0%	2 / 2 100.0%	2 / 2 100.0%	2 / 2 100.0%	2 / 2 100.0%	5 / 5 100.0%	3 / 3 100.0%	1 / 1 100.0%	2 / 2 100.0%	4 / 4 100.0%	1 / 1 100.0%	5 / 5 100.0%	3 / 3 100.0%	2 / 2 100.0%	2 / 2 100.0%	2 / 2 100.0%	3 / 3 100.0%	1 / 1 100.0%	4 / 4 100.0%	5 / 5 100.0%	2 / 2 100.0%	1 / 1 100.0%	5 / 5 100.0%	0 / 0 0.0%	0 / 0 0.0%	0 / 0 0.0%	0 / 0 0.0%	42 / 59 71.2%AB	17 / 59 28.8%ABE					
1	"Kyrpidia tusciae" Klenk et al. 2011	59 / 59 100.0%	2 / 2 100.0%	2 / 2 100.0%	2 / 2 100.0%	2 / 2 100.0%	5 / 5 100.0%	3 / 3 100.0%	1 / 1 100.0%	2 / 2 100.0%	4 / 4 100.0%	1 / 1 100.0%	5 / 5 100.0%	3 / 3 100.0%	2 / 2 100.0%	2 / 2 100.0%	2 / 2 100.0%	3 / 3 100.0%	1 / 1 100.0%	4 / 4 100.0%	5 / 5 100.0%	2 / 2 100.0%	1 / 1 100.0%	5 / 5 100.0%	0 / 0 0.0%	0 / 0 0.0%	0 / 0 0.0%	0 / 0 0.0%	42 / 59 71.2%AB	17 / 59 28.8%ABE					
1	Bacillus tusciae DSM 2912	59 / 59 100.0%	2 / 2 100.0%	2 / 2 100.0%	2 / 2 100.0%	2 / 2 100.0%	5 / 5 100.0%	3 / 3 100.0%	1 / 1 100.0%	2 / 2 100.0%	4 / 4 100.0%	1 / 1 100.0%	5 / 5 100.0%	3 / 3 100.0%	2 / 2 100.0%	2 / 2 100.0%	2 / 2 100.0%	3 / 3 100.0%	1 / 1 100.0%	4 / 4 100.0%	5 / 5 100.0%	2 / 2 100.0%	1 / 1 100.0%	5 / 5 100.0%	0 / 0 0.0%	0 / 0 0.0%	0 / 0 0.0%	0 / 0 0.0%	42 / 59 71.2%AB	17 / 59 28.8%ABE					
3	Bacillales incertae sedis	128 / 201 63.7%	6 / 9 66.7%	9 / 9 100.0%	9 / 9 100.0%	9 / 9 100.0%	10 / 16 62.5%	7 / 11 63.6%	4 / 6 66.7%	0 / 6 0.0%	5 / 14 35.7%	0 / 3 0.0%	12 / 14 85.7%	7 / 9 77.8%	6 / 6 100.0%	1 / 6 16.7%	3 / 3 100.0%	6 / 6 100.0%	3 / 6 50.0%	6 / 12 50.0%	7 / 15 46.7%	9 / 12 75.0%	0 / 3 0.0%	9 / 17 52.9%	0 / 0 0.0%	0 / 0 0.0%	0 / 0 0.0%	0 / 0 0.0%	81 / 201 40.3%AB	43 / 201 21.4%	35 / 201 17.4%ABE	29 / 201 14.4%E	7 / 201 3.5%B	5 / 201 2.5%BE	1 / 201 0.5%CE
3	Bacillales Family XII. Incertae Sedis	128 / 201 63.7%	6 / 9 66.7%	9 / 9 100.0%	9 / 9 100.0%	9 / 9 100.0%	10 / 16 62.5%	7 / 11 63.6%	4 / 6 66.7%	0 / 6 0.0%	5 / 14 35.7%	0 / 3 0.0%	12 / 14 85.7%	7 / 9 77.8%	6 / 6 100.0%	1 / 6 16.7%	3 / 3 100.0%	6 / 6 100.0%	3 / 6 50.0%	6 / 12 50.0%	7 / 15 46.7%	9 / 12 75.0%	0 / 3 0.0%	9 / 17 52.9%	0 / 0 0.0%	0 / 0 0.0%	0 / 0 0.0%	0 / 0 0.0%	81 / 201 40.3%AB	43 / 201 21.4%	35 / 201 17.4%ABE	29 / 201 14.4%E	7 / 201 3.5%B	5 / 201 2.5%BE	1 / 201 0.5%CE
3	Exiguobacterium	128 / 201 63.7%	6 / 9 66.7%	9 / 9 100.0%	9 / 9 100.0%	9 / 9 100.0%	10 / 16 62.5%	7 / 11 63.6%	4 / 6 66.7%	0 / 6 0.0%	5 / 14 35.7%	0 / 3 0.0%	12 / 14 85.7%	7 / 9 77.8%	6 / 6 100.0%	1 / 6 16.7%	3 / 3 100.0%	6 / 6 100.0%	3 / 6 50.0%	6 / 12 50.0%	7 / 15 46.7%	9 / 12 75.0%	0 / 3 0.0%	9 / 17 52.9%	0 / 0 0.0%	0 / 0 0.0%	0 / 0 0.0%	0 / 0 0.0%	81 / 201 40.3%AB	43 / 201 21.4%	35 / 201 17.4%ABE	29 / 201 14.4%E	7 / 201 3.5%B	5 / 201 2.5%BE	1 / 201 0.5%CE
1	CCUG 47137	39 / 64 60.9%	2 / 3 66.7%	3 / 3 100.0%	3 / 3 100.0%	3 / 3 100.0%	2 / 4 50.0%	2 / 3 66.7%	1 / 2 50.0%	0 / 2 0.0%	1 / 4 25.0%	0 / 1 0.0%	4 / 4 100.0%	2 / 3 66.7%	2 / 2 100.0%	0 / 2 0.0%	1 / 1 100.0%	2 / 2 100.0%	1 / 2 50.0%	2 / 4 50.0%	2 / 5 40.0%	3 / 4 75.0%	0 / 1 0.0%	3 / 6 50.0%	0 / 0 0.0%	0 / 0 0.0%	0 / 0 0.0%	0 / 0 0.0%	26 / 64 40.6%AB	15 / 64 23.4%	13 / 64 20.3%ABE	10 / 64 15.6%E			
1	Exiguobacterium antarcticum B7	39 / 64 60.9%	2 / 3 66.7%	3 / 3 100.0%	3 / 3 100.0%	3 / 3 100.0%	2 / 4 50.0%	2 / 3 66.7%	1 / 2 50.0%	0 / 2 0.0%	1 / 4 25.0%	0 / 1 0.0%	4 / 4 100.0%	2 / 3 66.7%	2 / 2 100.0%	0 / 2 0.0%	1 / 1 100.0%	2 / 2 100.0%	1 / 2 50.0%	2 / 4 50.0%	2 / 5 40.0%	3 / 4 75.0%	0 / 1 0.0%	3 / 6 50.0%	0 / 0 0.0%	0 / 0 0.0%	0 / 0 0.0%	0 / 0 0.0%	26 / 64 40.6%AB	15 / 64 23.4%	13 / 64 20.3%ABE	10 / 64 15.6%E			
1	DSM 17290	41 / 69 59.4%	2 / 3 66.7%	3 / 3 100.0%	3 / 3 100.0%	3 / 3 100.0%	3 / 6 50.0%	2 / 4 50.0%	1 / 2 50.0%	0 / 2 0.0%	2 / 5 40.0%	0 / 1 0.0%	4 / 5 80.0%	2 / 3 66.7%	2 / 2 100.0%	0 / 2 0.0%	1 / 1 100.0%	2 / 2 100.0%	1 / 2 50.0%	2 / 4 50.0%	2 / 5 40.0%	3 / 4 75.0%	0 / 1 0.0%	3 / 6 50.0%	0 / 0 0.0%	0 / 0 0.0%	0 / 0 0.0%	0 / 0 0.0%	21 / 69 30.4%AB	15 / 69 21.7%	13 / 69 18.8%E	8 / 69 11.6%ABE	7 / 69 10.1%B	5 / 69 7.2%BE	
1	Exiguobacterium sibiricum 255-15	41 / 69 59.4%	2 / 3 66.7%	3 / 3 100.0%	3 / 3 100.0%	3 / 3 100.0%	3 / 6 50.0%	2 / 4 50.0%	1 / 2 50.0%	0 / 2 0.0%	2 / 5 40.0%	0 / 1 0.0%	4 / 5 80.0%	2 / 3 66.7%	2 / 2 100.0%	0 / 2 0.0%	1 / 1 100.0%	2 / 2 100.0%	1 / 2 50.0%	2 / 4 50.0%	2 / 5 40.0%	3 / 4 75.0%	0 / 1 0.0%	3 / 6 50.0%	0 / 0 0.0%	0 / 0 0.0%	0 / 0 0.0%	0 / 0 0.0%	21 / 69 30.4%AB	15 / 69 21.7%	13 / 69 18.8%E	8 / 69 11.6%ABE	7 / 69 10.1%B	5 / 69 7.2%BE	
1	Exiguobacterium sp. AT1b	48 / 68 70.6%	2 / 3 66.7%	3 / 3 100.0%	3 / 3 100.0%	3 / 3 100.0%	5 / 6 83.3%	3 / 4 75.0%	2 / 2 100.0%	0 / 2 0.0%	2 / 5 40.0%	0 / 1 0.0%	4 / 5 80.0%	3 / 3 100.0%	2 / 2 100.0%	1 / 2 50.0%	1 / 1 100.0%	2 / 2 100.0%	1 / 2 50.0%	2 / 4 50.0%	3 / 5 60.0%	3 / 4 75.0%	0 / 1 0.0%	3 / 5 60.0%	0 / 0 0.0%	0 / 0 0.0%	0 / 0 0.0%	0 / 0 0.0%	34 / 68 50.0%AB	14 / 68 20.6%ABE	13 / 68 19.1%	6 / 68 8.8%E	1 / 68 1.5%CE		
191	Lactobacillales	168 / 11980 1.4%	0 / 399 0.0%	12 / 477 2.5%	2 / 507 0.4%	4 / 489 0.8%	20 / 798 2.5%	2 / 628 0.3%	11 / 232 4.7%	10 / 385 2.6%	0 / 792 0.0%	36 / 218 16.5%	2 / 847 0.2%	0 / 622 0.0%	15 / 397 3.8%	4 / 501 0.8%	0 / 199 0.0%	0 / 407 0.0%	0 / 200 0.0%	11 / 704 1.6%	2 / 1031 0.2%	0 / 681 0.0%	0 / 236 0.0%	37 / 1221 3.0%	0 / 1 0.0%	0 / 0 0.0%	0 / 6 0.0%	0 / 0 0.0%	6535 / 11980 54.5%E	5248 / 11980 43.8%	105 / 11980 0.9%AB	63 / 11980 0.5%ABE	26 / 11980 0.2%CE	3 / 11980 0.0%C	
112	Streptococcaceae	9 / 6899 0.1%	0 / 239 0.0%	0 / 256 0.0%	0 / 277 0.0%	0 / 276 0.0%	0 / 421 0.0%	0 / 381 0.0%	0 / 120 0.0%	0 / 222 0.0%	0 / 565 0.0%	0 / 122 0.0%	0 / 475 0.0%	0 / 376 0.0%	0 / 230 0.0%	0 / 257 0.0%	0 / 112 0.0%	0 / 203 0.0%	0 / 108 0.0%	0 / 397 0.0%	0 / 604 0.0%	0 / 393 0.0%	0 / 120 0.0%	9 / 745 1.2%	0 / 0 0.0%	0 / 0 0.0%	0 / 0 0.0%	0 / 0 0.0%	3462 / 6899 50.2%E	3420 / 6899 49.6%	9 / 6899 0.1%ABE	8 / 6899 0.1%CE			
101	Streptococcus	0 / 6230 0.0%	0 / 209 0.0%	0 / 234 0.0%	0 / 255 0.0%	0 / 254 0.0%	0 / 377 0.0%	0 / 348 0.0%	0 / 109 0.0%	0 / 211 0.0%	0 / 501 0.0%	0 / 111 0.0%	0 / 427 0.0%	0 / 334 0.0%	0 / 208 0.0%	0 / 235 0.0%	0 / 101 0.0%	0 / 181 0.0%	0 / 97 0.0%	0 / 355 0.0%	0 / 549 0.0%	0 / 371 0.0%	0 / 99 0.0%	0 / 664 0.0%	0 / 0 0.0%	0 / 0 0.0%	0 / 0 0.0%	0 / 0 0.0%	3124 / 6230 50.1%	3100 / 6230 49.8%E	6 / 6230 0.1%CE				
1	ATCC 10558	0 / 59 0.0%	0 / 2 0.0%	0 / 2 0.0%	0 / 3 0.0%	0 / 2 0.0%	0 / 4 0.0%	0 / 3 0.0%	0 / 1 0.0%	0 / 2 0.0%	0 / 4 0.0%	0 / 1 0.0%	0 / 4 0.0%	0 / 3 0.0%	0 / 2 0.0%	0 / 2 0.0%	0 / 1 0.0%	0 / 2 0.0%	0 / 1 0.0%	0 / 4 0.0%	0 / 5 0.0%	0 / 3 0.0%	0 / 1 0.0%	0 / 7 0.0%	0 / 0 0.0%	0 / 0 0.0%	0 / 0 0.0%	0 / 0 0.0%	35 / 59 59.3%E	24 / 59 40.7%					
1	Streptococcus gordonii Challis	0 / 59 0.0%	0 / 2 0.0%	0 / 2 0.0%	0 / 3 0.0%	0 / 2 0.0%	0 / 4 0.0%	0 / 3 0.0%	0 / 1 0.0%	0 / 2 0.0%	0 / 4 0.0%	0 / 1 0.0%	0 / 4 0.0%	0 / 3 0.0%	0 / 2 0.0%	0 / 2 0.0%	0 / 1 0.0%	0 / 2 0.0%	0 / 1 0.0%	0 / 4 0.0%	0 / 5 0.0%	0 / 3 0.0%	0 / 1 0.0%	0 / 7 0.0%	0 / 0 0.0%	0 / 0 0.0%	0 / 0 0.0%	0 / 0 0.0%	35 / 59 59.3%E	24 / 59 40.7%					
1	Streptococcus gordonii ATCC 35105	0 / 59 0.0%	0 / 2 0.0%	0 / 2 0.0%	0 / 3 0.0%	0 / 2 0.0%	0 / 4 0.0%	0 / 3 0.0%	0 / 1 0.0%	0 / 2 0.0%	0 / 4 0.0%	0 / 1 0.0%	0 / 4 0.0%	0 / 3 0.0%	0 / 2 0.0%	0 / 2 0.0%	0 / 1 0.0%	0 / 2 0.0%	0 / 1 0.0%	0 / 4 0.0%	0 / 5 0.0%	0 / 3 0.0%	0 / 1 0.0%	0 / 7 0.0%	0 / 0 0.0%	0 / 0 0.0%	0 / 0 0.0%	0 / 0 0.0%	35 / 59 59.3%E	24 / 59 40.7%					
1	ATCC 35037	0 / 61 0.0%	0 / 2 0.0%	0 / 2 0.0%	0 / 2 0.0%	0 / 2 0.0%	0 / 4 0.0%	0 / 3 0.0%	0 / 1 0.0%	0 / 2 0.0%	0 / 4 0.0%	0 / 1 0.0%	0 / 5 0.0%	0 / 3 0.0%	0 / 2 0.0%	0 / 2 0.0%	0 / 1 0.0%	0 / 2 0.0%	0 / 1 0.0%	0 / 4 0.0%	0 / 5 0.0%	0 / 6 0.0%	0 / 1 0.0%	0 / 6 0.0%	0 / 0 0.0%	0 / 0 0.0%	0 / 0 0.0%	0 / 0 0.0%	36 / 61 59.0%E	25 / 61 41.0%					
1	Streptococcus oralis Uo5	0 / 61 0.0%	0 / 2 0.0%	0 / 2 0.0%	0 / 2 0.0%	0 / 2 0.0%	0 / 4 0.0%	0 / 3 0.0%	0 / 1 0.0%	0 / 2 0.0%	0 / 4 0.0%	0 / 1 0.0%	0 / 5 0.0%	0 / 3 0.0%	0 / 2 0.0%	0 / 2 0.0%	0 / 1 0.0%	0 / 2 0.0%	0 / 1 0.0%	0 / 4 0.0%	0 / 5 0.0%	0 / 6 0.0%	0 / 1 0.0%	0 / 6 0.0%	0 / 0 0.0%	0 / 0 0.0%	0 / 0 0.0%	0 / 0 0.0%	36 / 61 59.0%E	25 / 61 41.0%					
3	ATCC 7073	0 / 204 0.0%	0 / 6 0.0%	0 / 9 0.0%	0 / 9 0.0%	0 / 6 0.0%	0 / 12 0.0%	0 / 12 0.0%	0 / 3 0.0%	0 / 6 0.0%	0 / 18 0.0%	0 / 3 0.0%	0 / 12 0.0%	0 / 12 0.0%	0 / 6 0.0%	0 / 9 0.0%	0 / 3 0.0%	0 / 6 0.0%	0 / 3 0.0%	0 / 15 0.0%	0 / 15 0.0%	0 / 12 0.0%	0 / 3 0.0%	0 / 24 0.0%	0 / 0 0.0%	0 / 0 0.0%	0 / 0 0.0%	0 / 0 0.0%	130 / 204 63.7%E	74 / 204 36.3%					
1	Streptococcus salivarius JIM8777	0 / 68 0.0%	0 / 2 0.0%	0 / 3 0.0%	0 / 3 0.0%	0 / 2 0.0%	0 / 4 0.0%	0 / 4 0.0%	0 / 1 0.0%	0 / 2 0.0%	0 / 6 0.0%	0 / 1 0.0%	0 / 4 0.0%	0 / 4 0.0%	0 / 2 0.0%	0 / 3 0.0%	0 / 1 0.0%	0 / 2 0.0%	0 / 1 0.0%	0 / 5 0.0%	0 / 5 0.0%	0 / 4 0.0%	0 / 1 0.0%	0 / 8 0.0%	0 / 0 0.0%	0 / 0 0.0%	0 / 0 0.0%	0 / 0 0.0%	43 / 68 63.2%E	25 / 68 36.8%					
1	Streptococcus salivarius 57.I	0 / 68 0.0%	0 / 2 0.0%	0 / 3 0.0%	0 / 3 0.0%	0 / 2 0.0%	0 / 4 0.0%	0 / 4 0.0%	0 / 1 0.0%	0 / 2 0.0%	0 / 6 0.0%	0 / 1 0.0%	0 / 4 0.0%	0 / 4 0.0%	0 / 2 0.0%	0 / 3 0.0%	0 / 1 0.0%	0 / 2 0.0%	0 / 1 0.0%	0 / 5 0.0%	0 / 5 0.0%	0 / 4 0.0%	0 / 1 0.0%	0 / 8 0.0%	0 / 0 0.0%	0 / 0 0.0%	0 / 0 0.0%	0 / 0 0.0%	44 / 68 64.7%E	24 / 68 35.3%					
1	Streptococcus salivarius CCHSS3	0 / 68 0.0%	0 / 2 0.0%	0 / 3 0.0%	0 / 3 0.0%	0 / 2 0.0%	0 / 4 0.0%	0 / 4 0.0%	0 / 1 0.0%	0 / 2 0.0%	0 / 6 0.0%	0 / 1 0.0%	0 / 4 0.0%	0 / 4 0.0%	0 / 2 0.0%	0 / 3 0.0%	0 / 1 0.0%	0 / 2 0.0%	0 / 1 0.0%	0 / 5 0.0%	0 / 5 0.0%	0 / 4 0.0%	0 / 1 0.0%	0 / 8 0.0%	0 / 0 0.0%	0 / 0 0.0%	0 / 0 0.0%	0 / 0 0.0%	43 / 68 63.2%E	25 / 68 36.8%					
1	ATCC 10556	0 / 61 0.0%	0 / 2 0.0%	0 / 2 0.0%	0 / 3 0.0%	0 / 2 0.0%	0 / 4 0.0%	0 / 3 0.0%	0 / 1 0.0%	0 / 2 0.0%	0 / 4 0.0%	0 / 1 0.0%	0 / 4 0.0%	0 / 4 0.0%	0 / 2 0.0%	0 / 2 0.0%	0 / 1 0.0%	0 / 2 0.0%	0 / 1 0.0%	0 / 4 0.0%	0 / 6 0.0%	0 / 3 0.0%	0 / 1 0.0%	0 / 7 0.0%	0 / 0 0.0%	0 / 0 0.0%	0 / 0 0.0%	0 / 0 0.0%	47 / 61 77.0%	14 / 61 23.0%E					
1	Streptococcus sanguinis SK36	0 / 61 0.0%	0 / 2 0.0%	0 / 2 0.0%	0 / 3 0.0%	0 / 2 0.0%	0 / 4 0.0%	0 / 3 0.0%	0 / 1 0.0%	0 / 2 0.0%	0 / 4 0.0%	0 / 1 0.0%	0 / 4 0.0%	0 / 4 0.0%	0 / 2 0.0%	0 / 2 0.0%	0 / 1 0.0%	0 / 2 0.0%	0 / 1 0.0%	0 / 4 0.0%	0 / 6 0.0%	0 / 3 0.0%	0 / 1 0.0%	0 / 7 0.0%	0 / 0 0.0%	0 / 0 0.0%	0 / 0 0.0%	0 / 0 0.0%	47 / 61 77.0%	14 / 61 23.0%E					
16	ATCC 43765	0 / 887 0.0%	0 / 31 0.0%	0 / 31 0.0%	0 / 31 0.0%	0 / 33 0.0%	0 / 49 0.0%	0 / 48 0.0%	0 / 16 0.0%	0 / 33 0.0%	0 / 64 0.0%	0 / 16 0.0%	0 / 65 0.0%	0 / 46 0.0%	0 / 32 0.0%	0 / 33 0.0%	0 / 15 0.0%	0 / 30 0.0%	0 / 15 0.0%	0 / 47 0.0%	0 / 79 0.0%	0 / 47 0.0%	0 / 15 0.0%	0 / 111 0.0%	0 / 0 0.0%	0 / 0 0.0%	0 / 0 0.0%	0 / 0 0.0%	544 / 887 61.3%E	343 / 887 38.7%					
1	Streptococcus suis P1/7	0 / 56 0.0%	0 / 2 0.0%	0 / 2 0.0%	0 / 2 0.0%	0 / 2 0.0%	0 / 3 0.0%	0 / 3 0.0%	0 / 1 0.0%	0 / 2 0.0%	0 / 4 0.0%	0 / 1 0.0%	0 / 4 0.0%	0 / 3 0.0%	0 / 2 0.0%	0 / 2 0.0%	0 / 1 0.0%	0 / 2 0.0%	0 / 1 0.0%	0 / 3 0.0%	0 / 5 0.0%	0 / 3 0.0%	0 / 1 0.0%	0 / 7 0.0%	0 / 0 0.0%	0 / 0 0.0%	0 / 0 0.0%	0 / 0 0.0%	37 / 56 66.1%E	19 / 56 33.9%					
1	Streptococcus suis 05ZYH33	0 / 56 0.0%	0 / 2 0.0%	0 / 2 0.0%	0 / 2 0.0%	0 / 2 0.0%	0 / 3 0.0%	0 / 3 0.0%	0 / 1 0.0%	0 / 2 0.0%	0 / 4 0.0%	0 / 1 0.0%	0 / 4 0.0%	0 / 3 0.0%	0 / 2 0.0%	0 / 2 0.0%	0 / 1 0.0%	0 / 2 0.0%	0 / 1 0.0%	0 / 3 0.0%	0 / 5 0.0%	0 / 3 0.0%	0 / 1 0.0%	0 / 7 0.0%	0 / 0 0.0%	0 / 0 0.0%	0 / 0 0.0%	0 / 0 0.0%	41 / 56 73.2%	15 / 56 26.8%E					
1	Streptococcus suis 98HAH33	0 / 56 0.0%	0 / 2 0.0%	0 / 2 0.0%	0 / 2 0.0%	0 / 2 0.0%	0 / 3 0.0%	0 / 3 0.0%	0 / 1 0.0%	0 / 2 0.0%	0 / 4 0.0%	0 / 1 0.0%	0 / 4 0.0%	0 / 3 0.0%	0 / 2 0.0%	0 / 2 0.0%	0 / 1 0.0%	0 / 2 0.0%	0 / 1 0.0%	0 / 3 0.0%	0 / 5 0.0%	0 / 3 0.0%	0 / 1 0.0%	0 / 7 0.0%	0 / 0 0.0%	0 / 0 0.0%	0 / 0 0.0%	0 / 0 0.0%	41 / 56 73.2%	15 / 56 26.8%E					
1	Streptococcus suis GZ1	0 / 49 0.0%	0 / 2 0.0%	0 / 1 0.0%	0 / 2 0.0%	0 / 3 0.0%	0 / 3 0.0%	0 / 3 0.0%	0 / 1 0.0%	0 / 2 0.0%	0 / 4 0.0%	0 / 1 0.0%	0 / 4 0.0%	0 / 2 0.0%	0 / 2 0.0%	0 / 2 0.0%	0 / 1 0.0%	0 / 1 0.0%	0 / 0 0.0%	0 / 2 0.0%	0 / 4 0.0%	0 / 4 0.0%	0 / 0 0.0%	0 / 5 0.0%	0 / 0 0.0%	0 / 0 0.0%	0 / 0 0.0%	0 / 0 0.0%	35 / 49 71.4%E	14 / 49 28.6%					
1	Streptococcus suis SC84	0 / 56 0.0%	0 / 2 0.0%	0 / 2 0.0%	0 / 2 0.0%	0 / 2 0.0%	0 / 3 0.0%	0 / 3 0.0%	0 / 1 0.0%	0 / 2 0.0%	0 / 4 0.0%	0 / 1 0.0%	0 / 4 0.0%	0 / 3 0.0%	0 / 2 0.0%	0 / 2 0.0%	0 / 1 0.0%	0 / 2 0.0%	0 / 1 0.0%	0 / 3 0.0%	0 / 5 0.0%	0 / 3 0.0%	0 / 1 0.0%	0 / 7 0.0%	0 / 0 0.0%	0 / 0 0.0%	0 / 0 0.0%	0 / 0 0.0%	37 / 56 66.1%E	19 / 56 33.9%					
1	Streptococcus suis BM407	0 / 56 0.0%	0 / 2 0.0%	0 / 2 0.0%	0 / 2 0.0%	0 / 2 0.0%	0 / 3 0.0%	0 / 3 0.0%	0 / 1 0.0%	0 / 2 0.0%	0 / 4 0.0%	0 / 1 0.0%	0 / 4 0.0%	0 / 3 0.0%	0 / 2 0.0%	0 / 2 0.0%	0 / 1 0.0%	0 / 2 0.0%	0 / 1 0.0%	0 / 3 0.0%	0 / 5 0.0%	0 / 3 0.0%	0 / 1 0.0%	0 / 7 0.0%	0 / 0 0.0%	0 / 0 0.0%	0 / 0 0.0%	0 / 0 0.0%	37 / 56 66.1%E	19 / 56 33.9%					
1	Streptococcus suis JS14	0 / 59 0.0%	0 / 2 0.0%	0 / 2 0.0%	0 / 3 0.0%	0 / 3 0.0%	0 / 4 0.0%	0 / 2 0.0%	0 / 1 0.0%	0 / 2 0.0%	0 / 4 0.0%	0 / 1 0.0%	0 / 4 0.0%	0 / 2 0.0%	0 / 2 0.0%	0 / 2 0.0%	0 / 1 0.0%	0 / 2 0.0%	0 / 1 0.0%	0 / 3 0.0%	0 / 5 0.0%	0 / 5 0.0%	0 / 1 0.0%	0 / 7 0.0%	0 / 0 0.0%	0 / 0 0.0%	0 / 0 0.0%	0 / 0 0.0%	41 / 59 69.5%E	18 / 59 30.5%					
1	Streptococcus suis A7	0 / 53 0.0%	0 / 2 0.0%	0 / 2 0.0%	0 / 1 0.0%	0 / 2 0.0%	0 / 3 0.0%	0 / 3 0.0%	0 / 1 0.0%	0 / 3 0.0%	0 / 4 0.0%	0 / 1 0.0%	0 / 3 0.0%	0 / 3 0.0%	0 / 3 0.0%	0 / 2 0.0%	0 / 1 0.0%	0 / 2 0.0%	0 / 1 0.0%	0 / 3 0.0%	0 / 5 0.0%	0 / 0 0.0%	0 / 1 0.0%	0 / 7 0.0%	0 / 0 0.0%	0 / 0 0.0%	0 / 0 0.0%	0 / 0 0.0%	34 / 53 64.2%E	19 / 53 35.8%					
1	Streptococcus suis ST1	0 / 58 0.0%	0 / 2 0.0%	0 / 3 0.0%	0 / 2 0.0%	0 / 2 0.0%	0 / 3 0.0%	0 / 4 0.0%	0 / 1 0.0%	0 / 2 0.0%	0 / 4 0.0%	0 / 1 0.0%	0 / 4 0.0%	0 / 3 0.0%	0 / 2 0.0%	0 / 2 0.0%	0 / 1 0.0%	0 / 2 0.0%	0 / 1 0.0%	0 / 3 0.0%	0 / 5 0.0%	0 / 3 0.0%	0 / 1 0.0%	0 / 7 0.0%	0 / 0 0.0%	0 / 0 0.0%	0 / 0 0.0%	0 / 0 0.0%	38 / 58 65.5%E	20 / 58 34.5%					
1	Streptococcus suis D12	0 / 56 0.0%	0 / 2 0.0%	0 / 2 0.0%	0 / 2 0.0%	0 / 2 0.0%	0 / 3 0.0%	0 / 3 0.0%	0 / 1 0.0%	0 / 2 0.0%	0 / 4 0.0%	0 / 1 0.0%	0 / 4 0.0%	0 / 3 0.0%	0 / 2 0.0%	0 / 2 0.0%	0 / 1 0.0%	0 / 2 0.0%	0 / 1 0.0%	0 / 3 0.0%	0 / 5 0.0%	0 / 3 0.0%	0 / 1 0.0%	0 / 7 0.0%	0 / 0 0.0%	0 / 0 0.0%	0 / 0 0.0%	0 / 0 0.0%	36 / 56 64.3%E	20 / 56 35.7%					
1	Streptococcus suis SS12	0 / 59 0.0%	0 / 2 0.0%	0 / 2 0.0%	0 / 2 0.0%	0 / 2 0.0%	0 / 3 0.0%	0 / 3 0.0%	0 / 1 0.0%	0 / 2 0.0%	0 / 4 0.0%	0 / 1 0.0%	0 / 5 0.0%	0 / 3 0.0%	0 / 2 0.0%	0 / 3 0.0%	0 / 1 0.0%	0 / 2 0.0%	0 / 1 0.0%	0 / 3 0.0%	0 / 5 0.0%	0 / 3 0.0%	0 / 1 0.0%	0 / 8 0.0%	0 / 0 0.0%	0 / 0 0.0%	0 / 0 0.0%	0 / 0 0.0%	39 / 59 66.1%E	20 / 59 33.9%					
1	Streptococcus suis D9	0 / 53 0.0%	0 / 2 0.0%	0 / 2 0.0%	0 / 1 0.0%	0 / 2 0.0%	0 / 3 0.0%	0 / 3 0.0%	0 / 1 0.0%	0 / 2 0.0%	0 / 4 0.0%	0 / 1 0.0%	0 / 4 0.0%	0 / 3 0.0%	0 / 2 0.0%	0 / 2 0.0%	0 / 0 0.0%	0 / 2 0.0%	0 / 1 0.0%	0 / 3 0.0%	0 / 5 0.0%	0 / 2 0.0%	0 / 1 0.0%	0 / 7 0.0%	0 / 0 0.0%	0 / 0 0.0%	0 / 0 0.0%	0 / 0 0.0%	35 / 53 66.0%E	18 / 53 34.0%					
1	Streptococcus suis ST3	0 / 52 0.0%	0 / 1 0.0%	0 / 1 0.0%	0 / 2 0.0%	0 / 1 0.0%	0 / 3 0.0%	0 / 3 0.0%	0 / 1 0.0%	0 / 2 0.0%	0 / 4 0.0%	0 / 1 0.0%	0 / 5 0.0%	0 / 3 0.0%	0 / 1 0.0%	0 / 2 0.0%	0 / 1 0.0%	0 / 1 0.0%	0 / 1 0.0%	0 / 3 0.0%	0 / 5 0.0%	0 / 3 0.0%	0 / 1 0.0%	0 / 7 0.0%	0 / 0 0.0%	0 / 0 0.0%	0 / 0 0.0%	0 / 0 0.0%	35 / 52 67.3%E	17 / 52 32.7%					
1	Streptococcus suis S735	0 / 56 0.0%	0 / 2 0.0%	0 / 2 0.0%	0 / 2 0.0%	0 / 2 0.0%	0 / 3 0.0%	0 / 3 0.0%	0 / 1 0.0%	0 / 2 0.0%	0 / 4 0.0%	0 / 1 0.0%	0 / 4 0.0%	0 / 3 0.0%	0 / 2 0.0%	0 / 2 0.0%	0 / 1 0.0%	0 / 2 0.0%	0 / 1 0.0%	0 / 3 0.0%	0 / 5 0.0%	0 / 3 0.0%	0 / 1 0.0%	0 / 7 0.0%	0 / 0 0.0%	0 / 0 0.0%	0 / 0 0.0%	0 / 0 0.0%	37 / 56 66.1%E	19 / 56 33.9%					
1	Streptococcus suis SC070731	0 / 56 0.0%	0 / 2 0.0%	0 / 2 0.0%	0 / 2 0.0%	0 / 2 0.0%	0 / 3 0.0%	0 / 3 0.0%	0 / 1 0.0%	0 / 2 0.0%	0 / 4 0.0%	0 / 1 0.0%	0 / 4 0.0%	0 / 3 0.0%	0 / 2 0.0%	0 / 2 0.0%	0 / 1 0.0%	0 / 2 0.0%	0 / 1 0.0%	0 / 3 0.0%	0 / 5 0.0%	0 / 3 0.0%	0 / 1 0.0%	0 / 7 0.0%	0 / 0 0.0%	0 / 0 0.0%	0 / 0 0.0%	0 / 0 0.0%	37 / 56 66.1%E	19 / 56 33.9%					
1	Streptococcus suis TL13	0 / 56 0.0%	0 / 2 0.0%	0 / 2 0.0%	0 / 2 0.0%	0 / 2 0.0%	0 / 3 0.0%	0 / 3 0.0%	0 / 1 0.0%	0 / 2 0.0%	0 / 4 0.0%	0 / 1 0.0%	0 / 4 0.0%	0 / 3 0.0%	0 / 2 0.0%	0 / 2 0.0%	0 / 1 0.0%	0 / 2 0.0%	0 / 1 0.0%	0 / 3 0.0%	0 / 5 0.0%	0 / 3 0.0%	0 / 1 0.0%	0 / 7 0.0%	0 / 0 0.0%	0 / 0 0.0%	0 / 0 0.0%	0 / 0 0.0%	36 / 56 64.3%E	20 / 56 35.7%					
6	ATCC 19258	0 / 382 0.0%	0 / 12 0.0%	0 / 16 0.0%	0 / 18 0.0%	0 / 12 0.0%	0 / 22 0.0%	0 / 22 0.0%	0 / 6 0.0%	0 / 12 0.0%	0 / 34 0.0%	0 / 6 0.0%	0 / 24 0.0%	0 / 22 0.0%	0 / 12 0.0%	0 / 18 0.0%	0 / 6 0.0%	0 / 10 0.0%	0 / 6 0.0%	0 / 22 0.0%	0 / 28 0.0%	0 / 24 0.0%	0 / 6 0.0%	0 / 44 0.0%	0 / 0 0.0%	0 / 0 0.0%	0 / 0 0.0%	0 / 0 0.0%	203 / 382 53.1%	179 / 382 46.9%E					
1	Streptococcus thermophilus LMG 18311	0 / 67 0.0%	0 / 2 0.0%	0 / 3 0.0%	0 / 3 0.0%	0 / 2 0.0%	0 / 4 0.0%	0 / 4 0.0%	0 / 1 0.0%	0 / 2 0.0%	0 / 6 0.0%	0 / 1 0.0%	0 / 4 0.0%	0 / 4 0.0%	0 / 2 0.0%	0 / 3 0.0%	0 / 1 0.0%	0 / 2 0.0%	0 / 1 0.0%	0 / 4 0.0%	0 / 5 0.0%	0 / 4 0.0%	0 / 1 0.0%	0 / 8 0.0%	0 / 0 0.0%	0 / 0 0.0%	0 / 0 0.0%	0 / 0 0.0%	48 / 67 71.6%	19 / 67 28.4%E					
1	Streptococcus thermophilus CNRZ1066	0 / 67 0.0%	0 / 2 0.0%	0 / 3 0.0%	0 / 3 0.0%	0 / 2 0.0%	0 / 4 0.0%	0 / 4 0.0%	0 / 1 0.0%	0 / 2 0.0%	0 / 6 0.0%	0 / 1 0.0%	0 / 4 0.0%	0 / 4 0.0%	0 / 2 0.0%	0 / 3 0.0%	0 / 1 0.0%	0 / 2 0.0%	0 / 1 0.0%	0 / 4 0.0%	0 / 5 0.0%	0 / 4 0.0%	0 / 1 0.0%	0 / 8 0.0%	0 / 0 0.0%	0 / 0 0.0%	0 / 0 0.0%	0 / 0 0.0%	48 / 67 71.6%	19 / 67 28.4%E					
1	Streptococcus thermophilus LMD-9	0 / 67 0.0%	0 / 2 0.0%	0 / 3 0.0%	0 / 3 0.0%	0 / 2 0.0%	0 / 4 0.0%	0 / 4 0.0%	0 / 1 0.0%	0 / 2 0.0%	0 / 6 0.0%	0 / 1 0.0%	0 / 4 0.0%	0 / 4 0.0%	0 / 2 0.0%	0 / 3 0.0%	0 / 1 0.0%	0 / 2 0.0%	0 / 1 0.0%	0 / 4 0.0%	0 / 5 0.0%	0 / 4 0.0%	0 / 1 0.0%	0 / 8 0.0%	0 / 0 0.0%	0 / 0 0.0%	0 / 0 0.0%	0 / 0 0.0%	48 / 67 71.6%	19 / 67 28.4%E					
1	Streptococcus thermophilus ND03	0 / 57 0.0%	0 / 2 0.0%	0 / 2 0.0%	0 / 3 0.0%	0 / 2 0.0%	0 / 3 0.0%	0 / 3 0.0%	0 / 1 0.0%	0 / 2 0.0%	0 / 5 0.0%	0 / 1 0.0%	0 / 4 0.0%	0 / 3 0.0%	0 / 2 0.0%	0 / 3 0.0%	0 / 1 0.0%	0 / 1 0.0%	0 / 1 0.0%	0 / 3 0.0%	0 / 4 0.0%	0 / 4 0.0%	0 / 1 0.0%	0 / 6 0.0%	0 / 0 0.0%	0 / 0 0.0%	0 / 0 0.0%	0 / 0 0.0%	39 / 57 68.4%E	18 / 57 31.6%					
1	Streptococcus thermophilus JIM 8232	0 / 67 0.0%	0 / 2 0.0%	0 / 3 0.0%	0 / 3 0.0%	0 / 2 0.0%	0 / 4 0.0%	0 / 4 0.0%	0 / 1 0.0%	0 / 2 0.0%	0 / 6 0.0%	0 / 1 0.0%	0 / 4 0.0%	0 / 4 0.0%	0 / 2 0.0%	0 / 3 0.0%	0 / 1 0.0%	0 / 2 0.0%	0 / 1 0.0%	0 / 4 0.0%	0 / 5 0.0%	0 / 4 0.0%	0 / 1 0.0%	0 / 8 0.0%	0 / 0 0.0%	0 / 0 0.0%	0 / 0 0.0%	0 / 0 0.0%	44 / 67 65.7%E	23 / 67 34.3%					
1	Streptococcus thermophilus MN-ZLW-002	0 / 57 0.0%	0 / 2 0.0%	0 / 2 0.0%	0 / 3 0.0%	0 / 2 0.0%	0 / 3 0.0%	0 / 3 0.0%	0 / 1 0.0%	0 / 2 0.0%	0 / 5 0.0%	0 / 1 0.0%	0 / 4 0.0%	0 / 3 0.0%	0 / 2 0.0%	0 / 3 0.0%	0 / 1 0.0%	0 / 1 0.0%	0 / 1 0.0%	0 / 3 0.0%	0 / 4 0.0%	0 / 4 0.0%	0 / 1 0.0%	0 / 6 0.0%	0 / 0 0.0%	0 / 0 0.0%	0 / 0 0.0%	0 / 0 0.0%	39 / 57 68.4%E	18 / 57 31.6%					
3	ATCC 25175	0 / 192 0.0%	0 / 6 0.0%	0 / 9 0.0%	0 / 6 0.0%	0 / 6 0.0%	0 / 12 0.0%	0 / 12 0.0%	0 / 3 0.0%	0 / 6 0.0%	0 / 15 0.0%	0 / 3 0.0%	0 / 15 0.0%	0 / 12 0.0%	0 / 6 0.0%	0 / 6 0.0%	0 / 3 0.0%	0 / 6 0.0%	0 / 3 0.0%	0 / 15 0.0%	0 / 15 0.0%	0 / 6 0.0%	0 / 3 0.0%	0 / 24 0.0%	0 / 0 0.0%	0 / 0 0.0%	0 / 0 0.0%	0 / 0 0.0%	102 / 192 53.1%E	90 / 192 46.9%					
1	Streptococcus mutans UA159	0 / 64 0.0%	0 / 2 0.0%	0 / 3 0.0%	0 / 2 0.0%	0 / 2 0.0%	0 / 4 0.0%	0 / 4 0.0%	0 / 1 0.0%	0 / 2 0.0%	0 / 5 0.0%	0 / 1 0.0%	0 / 5 0.0%	0 / 4 0.0%	0 / 2 0.0%	0 / 2 0.0%	0 / 1 0.0%	0 / 2 0.0%	0 / 1 0.0%	0 / 5 0.0%	0 / 5 0.0%	0 / 2 0.0%	0 / 1 0.0%	0 / 8 0.0%	0 / 0 0.0%	0 / 0 0.0%	0 / 0 0.0%	0 / 0 0.0%	44 / 64 68.8%	20 / 64 31.2%E					
1	Streptococcus mutans NN2025	0 / 64 0.0%	0 / 2 0.0%	0 / 3 0.0%	0 / 2 0.0%	0 / 2 0.0%	0 / 4 0.0%	0 / 4 0.0%	0 / 1 0.0%	0 / 2 0.0%	0 / 5 0.0%	0 / 1 0.0%	0 / 5 0.0%	0 / 4 0.0%	0 / 2 0.0%	0 / 2 0.0%	0 / 1 0.0%	0 / 2 0.0%	0 / 1 0.0%	0 / 5 0.0%	0 / 5 0.0%	0 / 2 0.0%	0 / 1 0.0%	0 / 8 0.0%	0 / 0 0.0%	0 / 0 0.0%	0 / 0 0.0%	0 / 0 0.0%	41 / 64 64.1%E	23 / 64 35.9%					
1	Streptococcus mutans LJ23	0 / 64 0.0%	0 / 2 0.0%	0 / 3 0.0%	0 / 2 0.0%	0 / 2 0.0%	0 / 4 0.0%	0 / 4 0.0%	0 / 1 0.0%	0 / 2 0.0%	0 / 5 0.0%	0 / 1 0.0%	0 / 5 0.0%	0 / 4 0.0%	0 / 2 0.0%	0 / 2 0.0%	0 / 1 0.0%	0 / 2 0.0%	0 / 1 0.0%	0 / 5 0.0%	0 / 5 0.0%	0 / 2 0.0%	0 / 1 0.0%	0 / 8 0.0%	0 / 0 0.0%	0 / 0 0.0%	0 / 0 0.0%	0 / 0 0.0%	41 / 64 64.1%E	23 / 64 35.9%					
9	"Streptococcus de la mammite" Nocard and Mollereau 1887	0 / 701 0.0%	0 / 28 0.0%	0 / 24 0.0%	0 / 26 0.0%	0 / 35 0.0%	0 / 45 0.0%	0 / 45 0.0%	0 / 18 0.0%	0 / 27 0.0%	0 / 62 0.0%	0 / 18 0.0%	0 / 44 0.0%	0 / 33 0.0%	0 / 26 0.0%	0 / 36 0.0%	0 / 9 0.0%	0 / 19 0.0%	0 / 9 0.0%	0 / 33 0.0%	0 / 45 0.0%	0 / 44 0.0%	0 / 9 0.0%	0 / 66 0.0%	0 / 0 0.0%	0 / 0 0.0%	0 / 0 0.0%	0 / 0 0.0%	388 / 701 55.3%	313 / 701 44.7%E					
1	Streptococcus agalactiae (serotype V)	0 / 80 0.0%	0 / 3 0.0%	0 / 3 0.0%	0 / 3 0.0%	0 / 4 0.0%	0 / 5 0.0%	0 / 5 0.0%	0 / 2 0.0%	0 / 3 0.0%	0 / 7 0.0%	0 / 2 0.0%	0 / 5 0.0%	0 / 4 0.0%	0 / 3 0.0%	0 / 4 0.0%	0 / 1 0.0%	0 / 2 0.0%	0 / 1 0.0%	0 / 4 0.0%	0 / 5 0.0%	0 / 5 0.0%	0 / 1 0.0%	0 / 8 0.0%	0 / 0 0.0%	0 / 0 0.0%	0 / 0 0.0%	0 / 0 0.0%	61 / 80 76.2%	19 / 80 23.8%E					
1	Streptococcus agalactiae 2603V/R	0 / 80 0.0%	0 / 3 0.0%	0 / 3 0.0%	0 / 3 0.0%	0 / 4 0.0%	0 / 5 0.0%	0 / 5 0.0%	0 / 2 0.0%	0 / 3 0.0%	0 / 7 0.0%	0 / 2 0.0%	0 / 5 0.0%	0 / 4 0.0%	0 / 3 0.0%	0 / 4 0.0%	0 / 1 0.0%	0 / 2 0.0%	0 / 1 0.0%	0 / 4 0.0%	0 / 5 0.0%	0 / 5 0.0%	0 / 1 0.0%	0 / 8 0.0%	0 / 0 0.0%	0 / 0 0.0%	0 / 0 0.0%	0 / 0 0.0%	61 / 80 76.2%	19 / 80 23.8%E					
1	Streptococcus agalactiae (serotype III)	0 / 80 0.0%	0 / 3 0.0%	0 / 3 0.0%	0 / 3 0.0%	0 / 4 0.0%	0 / 5 0.0%	0 / 5 0.0%	0 / 2 0.0%	0 / 3 0.0%	0 / 7 0.0%	0 / 2 0.0%	0 / 5 0.0%	0 / 4 0.0%	0 / 3 0.0%	0 / 4 0.0%	0 / 1 0.0%	0 / 2 0.0%	0 / 1 0.0%	0 / 4 0.0%	0 / 5 0.0%	0 / 5 0.0%	0 / 1 0.0%	0 / 8 0.0%	0 / 0 0.0%	0 / 0 0.0%	0 / 0 0.0%	0 / 0 0.0%	61 / 80 76.2%	19 / 80 23.8%E					
1	Streptococcus agalactiae NEM316	0 / 80 0.0%	0 / 3 0.0%	0 / 3 0.0%	0 / 3 0.0%	0 / 4 0.0%	0 / 5 0.0%	0 / 5 0.0%	0 / 2 0.0%	0 / 3 0.0%	0 / 7 0.0%	0 / 2 0.0%	0 / 5 0.0%	0 / 4 0.0%	0 / 3 0.0%	0 / 4 0.0%	0 / 1 0.0%	0 / 2 0.0%	0 / 1 0.0%	0 / 4 0.0%	0 / 5 0.0%	0 / 5 0.0%	0 / 1 0.0%	0 / 8 0.0%	0 / 0 0.0%	0 / 0 0.0%	0 / 0 0.0%	0 / 0 0.0%	61 / 80 76.2%	19 / 80 23.8%E					
1	Streptococcus agalactiae serogroup Ia	0 / 80 0.0%	0 / 3 0.0%	0 / 3 0.0%	0 / 3 0.0%	0 / 4 0.0%	0 / 5 0.0%	0 / 5 0.0%	0 / 2 0.0%	0 / 3 0.0%	0 / 7 0.0%	0 / 2 0.0%	0 / 5 0.0%	0 / 4 0.0%	0 / 3 0.0%	0 / 4 0.0%	0 / 1 0.0%	0 / 2 0.0%	0 / 1 0.0%	0 / 4 0.0%	0 / 5 0.0%	0 / 5 0.0%	0 / 1 0.0%	0 / 8 0.0%	0 / 0 0.0%	0 / 0 0.0%	0 / 0 0.0%	0 / 0 0.0%	61 / 80 76.2%	19 / 80 23.8%E					
1	Streptococcus agalactiae A909	0 / 80 0.0%	0 / 3 0.0%	0 / 3 0.0%	0 / 3 0.0%	0 / 4 0.0%	0 / 5 0.0%	0 / 5 0.0%	0 / 2 0.0%	0 / 3 0.0%	0 / 7 0.0%	0 / 2 0.0%	0 / 5 0.0%	0 / 4 0.0%	0 / 3 0.0%	0 / 4 0.0%	0 / 1 0.0%	0 / 2 0.0%	0 / 1 0.0%	0 / 4 0.0%	0 / 5 0.0%	0 / 5 0.0%	0 / 1 0.0%	0 / 8 0.0%	0 / 0 0.0%	0 / 0 0.0%	0 / 0 0.0%	0 / 0 0.0%	61 / 80 76.2%	19 / 80 23.8%E					
1	Streptococcus agalactiae GD201008-001	0 / 76 0.0%	0 / 3 0.0%	0 / 2 0.0%	0 / 3 0.0%	0 / 4 0.0%	0 / 6 0.0%	0 / 5 0.0%	0 / 2 0.0%	0 / 3 0.0%	0 / 7 0.0%	0 / 2 0.0%	0 / 5 0.0%	0 / 3 0.0%	0 / 3 0.0%	0 / 4 0.0%	0 / 1 0.0%	0 / 2 0.0%	0 / 1 0.0%	0 / 3 0.0%	0 / 5 0.0%	0 / 5 0.0%	0 / 1 0.0%	0 / 6 0.0%	0 / 0 0.0%	0 / 0 0.0%	0 / 0 0.0%	0 / 0 0.0%	42 / 76 55.3%E	34 / 76 44.7%					
1	Streptococcus agalactiae SA20-06	0 / 78 0.0%	0 / 3 0.0%	0 / 3 0.0%	0 / 3 0.0%	0 / 4 0.0%	0 / 5 0.0%	0 / 5 0.0%	0 / 2 0.0%	0 / 3 0.0%	0 / 7 0.0%	0 / 2 0.0%	0 / 4 0.0%	0 / 4 0.0%	0 / 2 0.0%	0 / 4 0.0%	0 / 1 0.0%	0 / 2 0.0%	0 / 1 0.0%	0 / 4 0.0%	0 / 5 0.0%	0 / 5 0.0%	0 / 1 0.0%	0 / 8 0.0%	0 / 0 0.0%	0 / 0 0.0%	0 / 0 0.0%	0 / 0 0.0%	44 / 78 56.4%E	34 / 78 43.6%					
1	Streptococcus agalactiae 09mas018883	0 / 80 0.0%	0 / 3 0.0%	0 / 3 0.0%	0 / 3 0.0%	0 / 4 0.0%	0 / 5 0.0%	0 / 5 0.0%	0 / 2 0.0%	0 / 3 0.0%	0 / 7 0.0%	0 / 2 0.0%	0 / 5 0.0%	0 / 4 0.0%	0 / 3 0.0%	0 / 4 0.0%	0 / 1 0.0%	0 / 2 0.0%	0 / 1 0.0%	0 / 4 0.0%	0 / 5 0.0%	0 / 5 0.0%	0 / 1 0.0%	0 / 8 0.0%	0 / 0 0.0%	0 / 0 0.0%	0 / 0 0.0%	0 / 0 0.0%	44 / 80 55.0%E	36 / 80 45.0%					
1	Streptococcus agalactiae ILRI005	0 / 80 0.0%	0 / 3 0.0%	0 / 3 0.0%	0 / 3 0.0%	0 / 4 0.0%	0 / 5 0.0%	0 / 5 0.0%	0 / 2 0.0%	0 / 3 0.0%	0 / 7 0.0%	0 / 2 0.0%	0 / 5 0.0%	0 / 4 0.0%	0 / 3 0.0%	0 / 4 0.0%	0 / 1 0.0%	0 / 2 0.0%	0 / 1 0.0%	0 / 4 0.0%	0 / 5 0.0%	0 / 5 0.0%	0 / 1 0.0%	0 / 8 0.0%	0 / 0 0.0%	0 / 0 0.0%	0 / 0 0.0%	0 / 0 0.0%	44 / 80 55.0%E	36 / 80 45.0%					
1	Streptococcus agalactiae ILRI112	0 / 79 0.0%	0 / 3 0.0%	0 / 3 0.0%	0 / 3 0.0%	0 / 4 0.0%	0 / 5 0.0%	0 / 5 0.0%	0 / 2 0.0%	0 / 3 0.0%	0 / 7 0.0%	0 / 2 0.0%	0 / 5 0.0%	0 / 4 0.0%	0 / 3 0.0%	0 / 4 0.0%	0 / 1 0.0%	0 / 2 0.0%	0 / 1 0.0%	0 / 4 0.0%	0 / 5 0.0%	0 / 5 0.0%	0 / 1 0.0%	0 / 7 0.0%	0 / 0 0.0%	0 / 0 0.0%	0 / 0 0.0%	0 / 0 0.0%	43 / 79 54.4%E	36 / 79 45.6%					
20	"Diplococcus pneumoniae" (Klein 1884) Weichselbaum 1886	0 / 1151 0.0%	0 / 40 0.0%	0 / 40 0.0%	0 / 40 0.0%	0 / 40 0.0%	0 / 78 0.0%	0 / 60 0.0%	0 / 20 0.0%	0 / 40 0.0%	0 / 80 0.0%	0 / 20 0.0%	0 / 79 0.0%	0 / 60 0.0%	0 / 40 0.0%	0 / 40 0.0%	0 / 20 0.0%	0 / 39 0.0%	0 / 19 0.0%	0 / 59 0.0%	0 / 99 0.0%	0 / 99 0.0%	0 / 20 0.0%	0 / 119 0.0%	0 / 0 0.0%	0 / 0 0.0%	0 / 0 0.0%	0 / 0 0.0%	583 / 1151 50.7%E	568 / 1151 49.3%					
1	Streptococcus pneumoniae TIGR4	0 / 58 0.0%	0 / 2 0.0%	0 / 2 0.0%	0 / 2 0.0%	0 / 2 0.0%	0 / 4 0.0%	0 / 3 0.0%	0 / 1 0.0%	0 / 2 0.0%	0 / 4 0.0%	0 / 1 0.0%	0 / 4 0.0%	0 / 3 0.0%	0 / 2 0.0%	0 / 2 0.0%	0 / 1 0.0%	0 / 2 0.0%	0 / 1 0.0%	0 / 3 0.0%	0 / 5 0.0%	0 / 5 0.0%	0 / 1 0.0%	0 / 6 0.0%	0 / 0 0.0%	0 / 0 0.0%	0 / 0 0.0%	0 / 0 0.0%	44 / 58 75.9%	14 / 58 24.1%E					
1	Streptococcus pneumoniae (strain ATCC BAA-255 / R6)	0 / 58 0.0%	0 / 2 0.0%	0 / 2 0.0%	0 / 2 0.0%	0 / 2 0.0%	0 / 4 0.0%	0 / 3 0.0%	0 / 1 0.0%	0 / 2 0.0%	0 / 4 0.0%	0 / 1 0.0%	0 / 4 0.0%	0 / 3 0.0%	0 / 2 0.0%	0 / 2 0.0%	0 / 1 0.0%	0 / 2 0.0%	0 / 1 0.0%	0 / 3 0.0%	0 / 5 0.0%	0 / 5 0.0%	0 / 1 0.0%	0 / 6 0.0%	0 / 0 0.0%	0 / 0 0.0%	0 / 0 0.0%	0 / 0 0.0%	44 / 58 75.9%	14 / 58 24.1%E					
1	Streptococcus pneumoniae 670	0 / 58 0.0%	0 / 2 0.0%	0 / 2 0.0%	0 / 2 0.0%	0 / 2 0.0%	0 / 4 0.0%	0 / 3 0.0%	0 / 1 0.0%	0 / 2 0.0%	0 / 4 0.0%	0 / 1 0.0%	0 / 4 0.0%	0 / 3 0.0%	0 / 2 0.0%	0 / 2 0.0%	0 / 1 0.0%	0 / 2 0.0%	0 / 1 0.0%	0 / 3 0.0%	0 / 5 0.0%	0 / 5 0.0%	0 / 1 0.0%	0 / 6 0.0%	0 / 0 0.0%	0 / 0 0.0%	0 / 0 0.0%	0 / 0 0.0%	32 / 58 55.2%E	26 / 58 44.8%					
1	Streptococcus pneumoniae D39	0 / 58 0.0%	0 / 2 0.0%	0 / 2 0.0%	0 / 2 0.0%	0 / 2 0.0%	0 / 4 0.0%	0 / 3 0.0%	0 / 1 0.0%	0 / 2 0.0%	0 / 4 0.0%	0 / 1 0.0%	0 / 4 0.0%	0 / 3 0.0%	0 / 2 0.0%	0 / 2 0.0%	0 / 1 0.0%	0 / 2 0.0%	0 / 1 0.0%	0 / 3 0.0%	0 / 5 0.0%	0 / 5 0.0%	0 / 1 0.0%	0 / 6 0.0%	0 / 0 0.0%	0 / 0 0.0%	0 / 0 0.0%	0 / 0 0.0%	44 / 58 75.9%	14 / 58 24.1%E					
1	Streptococcus pneumoniae Taiwan19F-14	0 / 58 0.0%	0 / 2 0.0%	0 / 2 0.0%	0 / 2 0.0%	0 / 2 0.0%	0 / 4 0.0%	0 / 3 0.0%	0 / 1 0.0%	0 / 2 0.0%	0 / 4 0.0%	0 / 1 0.0%	0 / 4 0.0%	0 / 3 0.0%	0 / 2 0.0%	0 / 2 0.0%	0 / 1 0.0%	0 / 2 0.0%	0 / 1 0.0%	0 / 3 0.0%	0 / 5 0.0%	0 / 5 0.0%	0 / 1 0.0%	0 / 6 0.0%	0 / 0 0.0%	0 / 0 0.0%	0 / 0 0.0%	0 / 0 0.0%	32 / 58 55.2%E	26 / 58 44.8%					
1	Streptococcus pneumoniae Hungary19A-6	0 / 58 0.0%	0 / 2 0.0%	0 / 2 0.0%	0 / 2 0.0%	0 / 2 0.0%	0 / 4 0.0%	0 / 3 0.0%	0 / 1 0.0%	0 / 2 0.0%	0 / 4 0.0%	0 / 1 0.0%	0 / 4 0.0%	0 / 3 0.0%	0 / 2 0.0%	0 / 2 0.0%	0 / 1 0.0%	0 / 2 0.0%	0 / 1 0.0%	0 / 3 0.0%	0 / 5 0.0%	0 / 5 0.0%	0 / 1 0.0%	0 / 6 0.0%	0 / 0 0.0%	0 / 0 0.0%	0 / 0 0.0%	0 / 0 0.0%	33 / 58 56.9%E	25 / 58 43.1%					
1	Streptococcus pneumoniae 70585	0 / 58 0.0%	0 / 2 0.0%	0 / 2 0.0%	0 / 2 0.0%	0 / 2 0.0%	0 / 4 0.0%	0 / 3 0.0%	0 / 1 0.0%	0 / 2 0.0%	0 / 4 0.0%	0 / 1 0.0%	0 / 4 0.0%	0 / 3 0.0%	0 / 2 0.0%	0 / 2 0.0%	0 / 1 0.0%	0 / 2 0.0%	0 / 1 0.0%	0 / 3 0.0%	0 / 5 0.0%	0 / 5 0.0%	0 / 1 0.0%	0 / 6 0.0%	0 / 0 0.0%	0 / 0 0.0%	0 / 0 0.0%	0 / 0 0.0%	32 / 58 55.2%E	26 / 58 44.8%					
1	Streptococcus pneumoniae JJA	0 / 58 0.0%	0 / 2 0.0%	0 / 2 0.0%	0 / 2 0.0%	0 / 2 0.0%	0 / 4 0.0%	0 / 3 0.0%	0 / 1 0.0%	0 / 2 0.0%	0 / 4 0.0%	0 / 1 0.0%	0 / 4 0.0%	0 / 3 0.0%	0 / 2 0.0%	0 / 2 0.0%	0 / 1 0.0%	0 / 2 0.0%	0 / 1 0.0%	0 / 3 0.0%	0 / 5 0.0%	0 / 5 0.0%	0 / 1 0.0%	0 / 6 0.0%	0 / 0 0.0%	0 / 0 0.0%	0 / 0 0.0%	0 / 0 0.0%	32 / 58 55.2%E	26 / 58 44.8%					
1	Streptococcus pneumoniae P1031	0 / 58 0.0%	0 / 2 0.0%	0 / 2 0.0%	0 / 2 0.0%	0 / 2 0.0%	0 / 4 0.0%	0 / 3 0.0%	0 / 1 0.0%	0 / 2 0.0%	0 / 4 0.0%	0 / 1 0.0%	0 / 4 0.0%	0 / 3 0.0%	0 / 2 0.0%	0 / 2 0.0%	0 / 1 0.0%	0 / 2 0.0%	0 / 1 0.0%	0 / 3 0.0%	0 / 5 0.0%	0 / 5 0.0%	0 / 1 0.0%	0 / 6 0.0%	0 / 0 0.0%	0 / 0 0.0%	0 / 0 0.0%	0 / 0 0.0%	32 / 58 55.2%E	26 / 58 44.8%					
1	Streptococcus pneumoniae G54	0 / 56 0.0%	0 / 2 0.0%	0 / 2 0.0%	0 / 2 0.0%	0 / 2 0.0%	0 / 3 0.0%	0 / 3 0.0%	0 / 1 0.0%	0 / 2 0.0%	0 / 4 0.0%	0 / 1 0.0%	0 / 4 0.0%	0 / 3 0.0%	0 / 2 0.0%	0 / 2 0.0%	0 / 1 0.0%	0 / 2 0.0%	0 / 1 0.0%	0 / 3 0.0%	0 / 5 0.0%	0 / 5 0.0%	0 / 1 0.0%	0 / 5 0.0%	0 / 0 0.0%	0 / 0 0.0%	0 / 0 0.0%	0 / 0 0.0%	30 / 56 53.6%E	26 / 56 46.4%					
1	Streptococcus pneumoniae CGSP14	0 / 58 0.0%	0 / 2 0.0%	0 / 2 0.0%	0 / 2 0.0%	0 / 2 0.0%	0 / 4 0.0%	0 / 3 0.0%	0 / 1 0.0%	0 / 2 0.0%	0 / 4 0.0%	0 / 1 0.0%	0 / 4 0.0%	0 / 3 0.0%	0 / 2 0.0%	0 / 2 0.0%	0 / 1 0.0%	0 / 2 0.0%	0 / 1 0.0%	0 / 3 0.0%	0 / 5 0.0%	0 / 5 0.0%	0 / 1 0.0%	0 / 6 0.0%	0 / 0 0.0%	0 / 0 0.0%	0 / 0 0.0%	0 / 0 0.0%	32 / 58 55.2%E	26 / 58 44.8%					

1	Streptococcus pneumoniae TCH8431/19A	0 / 58 0.0%	0 / 2 0.0%	0 / 2 0.0%	0 / 2 0.0%	0 / 2 0.0%	0 / 4 0.0%	0 / 3 0.0%	0 / 1 0.0%	0 / 2 0.0%	0 / 4 0.0%	0 / 1 0.0%	0 / 4 0.0%	0 / 3 0.0%	0 / 2 0.0%	0 / 2 0.0%	0 / 1 0.0%	0 / 2 0.0%	0 / 1 0.0%	0 / 3 0.0%	0 / 5 0.0%	0 / 5 0.0%	0 / 1 0.0%	0 / 6 0.0%	0 / 0 0.0%	0 / 0 0.0%	0 / 0 0.0%	0 / 0 0.0%	32 / 58 55.2%E	26 / 58 44.8%	
1	Streptococcus pneumoniae ATCC 700669	0 / 58 0.0%	0 / 2 0.0%	0 / 2 0.0%	0 / 2 0.0%	0 / 2 0.0%	0 / 4 0.0%	0 / 3 0.0%	0 / 1 0.0%	0 / 2 0.0%	0 / 4 0.0%	0 / 1 0.0%	0 / 4 0.0%	0 / 3 0.0%	0 / 2 0.0%	0 / 2 0.0%	0 / 1 0.0%	0 / 2 0.0%	0 / 1 0.0%	0 / 3 0.0%	0 / 5 0.0%	0 / 5 0.0%	0 / 1 0.0%	0 / 6 0.0%	0 / 0 0.0%	0 / 0 0.0%	0 / 0 0.0%	0 / 0 0.0%	32 / 58 55.2%E	26 / 58 44.8%	
1	Streptococcus pneumoniae AP200	0 / 52 0.0%	0 / 2 0.0%	0 / 2 0.0%	0 / 2 0.0%	0 / 2 0.0%	0 / 3 0.0%	0 / 3 0.0%	0 / 1 0.0%	0 / 2 0.0%	0 / 4 0.0%	0 / 1 0.0%	0 / 3 0.0%	0 / 3 0.0%	0 / 2 0.0%	0 / 2 0.0%	0 / 1 0.0%	0 / 1 0.0%	0 / 0 0.0%	0 / 2 0.0%	0 / 4 0.0%	0 / 5 0.0%	0 / 1 0.0%	0 / 6 0.0%	0 / 0 0.0%	0 / 0 0.0%	0 / 0 0.0%	0 / 0 0.0%	30 / 52 57.7%E	22 / 52 42.3%	
1	Streptococcus pneumoniae gamPNI0373	0 / 57 0.0%	0 / 2 0.0%	0 / 2 0.0%	0 / 2 0.0%	0 / 2 0.0%	0 / 4 0.0%	0 / 3 0.0%	0 / 1 0.0%	0 / 2 0.0%	0 / 4 0.0%	0 / 1 0.0%	0 / 4 0.0%	0 / 3 0.0%	0 / 2 0.0%	0 / 2 0.0%	0 / 1 0.0%	0 / 2 0.0%	0 / 1 0.0%	0 / 3 0.0%	0 / 5 0.0%	0 / 4 0.0%	0 / 1 0.0%	0 / 6 0.0%	0 / 0 0.0%	0 / 0 0.0%	0 / 0 0.0%	0 / 0 0.0%	31 / 57 54.4%E	26 / 57 45.6%	
1	Streptococcus pneumoniae OXC141	0 / 58 0.0%	0 / 2 0.0%	0 / 2 0.0%	0 / 2 0.0%	0 / 2 0.0%	0 / 4 0.0%	0 / 3 0.0%	0 / 1 0.0%	0 / 2 0.0%	0 / 4 0.0%	0 / 1 0.0%	0 / 4 0.0%	0 / 3 0.0%	0 / 2 0.0%	0 / 2 0.0%	0 / 1 0.0%	0 / 2 0.0%	0 / 1 0.0%	0 / 3 0.0%	0 / 5 0.0%	0 / 5 0.0%	0 / 1 0.0%	0 / 6 0.0%	0 / 0 0.0%	0 / 0 0.0%	0 / 0 0.0%	0 / 0 0.0%	32 / 58 55.2%E	26 / 58 44.8%	
1	Streptococcus pneumoniae INV200	0 / 58 0.0%	0 / 2 0.0%	0 / 2 0.0%	0 / 2 0.0%	0 / 2 0.0%	0 / 4 0.0%	0 / 3 0.0%	0 / 1 0.0%	0 / 2 0.0%	0 / 4 0.0%	0 / 1 0.0%	0 / 4 0.0%	0 / 3 0.0%	0 / 2 0.0%	0 / 2 0.0%	0 / 1 0.0%	0 / 2 0.0%	0 / 1 0.0%	0 / 3 0.0%	0 / 5 0.0%	0 / 5 0.0%	0 / 1 0.0%	0 / 6 0.0%	0 / 0 0.0%	0 / 0 0.0%	0 / 0 0.0%	0 / 0 0.0%	32 / 58 55.2%E	26 / 58 44.8%	
1	Streptococcus pneumoniae INV104	0 / 58 0.0%	0 / 2 0.0%	0 / 2 0.0%	0 / 2 0.0%	0 / 2 0.0%	0 / 4 0.0%	0 / 3 0.0%	0 / 1 0.0%	0 / 2 0.0%	0 / 4 0.0%	0 / 1 0.0%	0 / 4 0.0%	0 / 3 0.0%	0 / 2 0.0%	0 / 2 0.0%	0 / 1 0.0%	0 / 2 0.0%	0 / 1 0.0%	0 / 3 0.0%	0 / 5 0.0%	0 / 5 0.0%	0 / 1 0.0%	0 / 6 0.0%	0 / 0 0.0%	0 / 0 0.0%	0 / 0 0.0%	0 / 0 0.0%	32 / 58 55.2%E	26 / 58 44.8%	
1	Streptococcus pneumoniae SPNA45	0 / 58 0.0%	0 / 2 0.0%	0 / 2 0.0%	0 / 2 0.0%	0 / 2 0.0%	0 / 4 0.0%	0 / 3 0.0%	0 / 1 0.0%	0 / 2 0.0%	0 / 4 0.0%	0 / 1 0.0%	0 / 4 0.0%	0 / 3 0.0%	0 / 2 0.0%	0 / 2 0.0%	0 / 1 0.0%	0 / 2 0.0%	0 / 1 0.0%	0 / 3 0.0%	0 / 5 0.0%	0 / 5 0.0%	0 / 1 0.0%	0 / 6 0.0%	0 / 0 0.0%	0 / 0 0.0%	0 / 0 0.0%	0 / 0 0.0%	33 / 58 56.9%E	25 / 58 43.1%	
1	Streptococcus pneumoniae ST556	0 / 58 0.0%	0 / 2 0.0%	0 / 2 0.0%	0 / 2 0.0%	0 / 2 0.0%	0 / 4 0.0%	0 / 3 0.0%	0 / 1 0.0%	0 / 2 0.0%	0 / 4 0.0%	0 / 1 0.0%	0 / 4 0.0%	0 / 3 0.0%	0 / 2 0.0%	0 / 2 0.0%	0 / 1 0.0%	0 / 2 0.0%	0 / 1 0.0%	0 / 3 0.0%	0 / 5 0.0%	0 / 5 0.0%	0 / 1 0.0%	0 / 6 0.0%	0 / 0 0.0%	0 / 0 0.0%	0 / 0 0.0%	0 / 0 0.0%	32 / 58 55.2%E	26 / 58 44.8%	
18	"Micrococcus scarlatinae" Klein 1884	0 / 1166 0.0%	0 / 35 0.0%	0 / 50 0.0%	0 / 54 0.0%	0 / 55 0.0%	0 / 69 0.0%	0 / 69 0.0%	0 / 18 0.0%	0 / 36 0.0%	0 / 105 0.0%	0 / 18 0.0%	0 / 72 0.0%	0 / 67 0.0%	0 / 35 0.0%	0 / 36 0.0%	0 / 18 0.0%	0 / 32 0.0%	0 / 17 0.0%	0 / 68 0.0%	0 / 122 0.0%	0 / 55 0.0%	0 / 17 0.0%	0 / 118 0.0%	0 / 0 0.0%	0 / 0 0.0%	0 / 0 0.0%	0 / 0 0.0%	784 / 1166 67.2%	382 / 1166 32.8%E	
3	Streptococcus pyogenes serotype M1	0 / 184 0.0%	0 / 6 0.0%	0 / 7 0.0%	0 / 9 0.0%	0 / 10 0.0%	0 / 11 0.0%	0 / 11 0.0%	0 / 3 0.0%	0 / 6 0.0%	0 / 17 0.0%	0 / 3 0.0%	0 / 12 0.0%	0 / 10 0.0%	0 / 6 0.0%	0 / 6 0.0%	0 / 3 0.0%	0 / 4 0.0%	0 / 2 0.0%	0 / 10 0.0%	0 / 19 0.0%	0 / 10 0.0%	0 / 2 0.0%	0 / 17 0.0%	0 / 0 0.0%	0 / 0 0.0%	0 / 0 0.0%	0 / 0 0.0%	124 / 184 67.4%	60 / 184 32.6%E	
1	Streptococcus pyogenes (serotype 1 / M1 GAS)	0 / 60 0.0%	0 / 2 0.0%	0 / 2 0.0%	0 / 3 0.0%	0 / 4 0.0%	0 / 4 0.0%	0 / 4 0.0%	0 / 1 0.0%	0 / 2 0.0%	0 / 6 0.0%	0 / 1 0.0%	0 / 4 0.0%	0 / 3 0.0%	0 / 2 0.0%	0 / 2 0.0%	0 / 1 0.0%	0 / 1 0.0%	0 / 0 0.0%	0 / 3 0.0%	0 / 6 0.0%	0 / 4 0.0%	0 / 0 0.0%	0 / 5 0.0%	0 / 0 0.0%	0 / 0 0.0%	0 / 0 0.0%	0 / 0 0.0%	47 / 60 78.3%	13 / 60 21.7%E	
1	Streptococcus pyogenes MGAS5005	0 / 67 0.0%	0 / 2 0.0%	0 / 3 0.0%	0 / 3 0.0%	0 / 3 0.0%	0 / 4 0.0%	0 / 4 0.0%	0 / 1 0.0%	0 / 2 0.0%	0 / 6 0.0%	0 / 1 0.0%	0 / 4 0.0%	0 / 4 0.0%	0 / 2 0.0%	0 / 2 0.0%	0 / 1 0.0%	0 / 2 0.0%	0 / 1 0.0%	0 / 4 0.0%	0 / 7 0.0%	0 / 3 0.0%	0 / 1 0.0%	0 / 7 0.0%	0 / 0 0.0%	0 / 0 0.0%	0 / 0 0.0%	0 / 0 0.0%	53 / 67 79.1%	14 / 67 20.9%E	
1	Streptococcus pyogenes M1 476	0 / 57 0.0%	0 / 2 0.0%	0 / 2 0.0%	0 / 3 0.0%	0 / 3 0.0%	0 / 3 0.0%	0 / 3 0.0%	0 / 1 0.0%	0 / 2 0.0%	0 / 5 0.0%	0 / 1 0.0%	0 / 4 0.0%	0 / 3 0.0%	0 / 2 0.0%	0 / 2 0.0%	0 / 1 0.0%	0 / 1 0.0%	0 / 1 0.0%	0 / 3 0.0%	0 / 6 0.0%	0 / 3 0.0%	0 / 1 0.0%	0 / 5 0.0%	0 / 0 0.0%	0 / 0 0.0%	0 / 0 0.0%	0 / 0 0.0%	33 / 57 57.9%E	24 / 57 42.1%	
2	Streptococcus pyogenes (serotype M3)	0 / 124 0.0%	0 / 4 0.0%	0 / 5 0.0%	0 / 6 0.0%	0 / 6 0.0%	0 / 7 0.0%	0 / 7 0.0%	0 / 2 0.0%	0 / 4 0.0%	0 / 11 0.0%	0 / 2 0.0%	0 / 8 0.0%	0 / 7 0.0%	0 / 4 0.0%	0 / 4 0.0%	0 / 2 0.0%	0 / 3 0.0%	0 / 2 0.0%	0 / 7 0.0%	0 / 13 0.0%	0 / 6 0.0%	0 / 2 0.0%	0 / 12 0.0%	0 / 0 0.0%	0 / 0 0.0%	0 / 0 0.0%	0 / 0 0.0%	98 / 124 79.0%	26 / 124 21.0%E	
1	Streptococcus pyogenes SSI-1	0 / 57 0.0%	0 / 2 0.0%	0 / 2 0.0%	0 / 3 0.0%	0 / 3 0.0%	0 / 3 0.0%	0 / 3 0.0%	0 / 1 0.0%	0 / 2 0.0%	0 / 5 0.0%	0 / 1 0.0%	0 / 4 0.0%	0 / 3 0.0%	0 / 2 0.0%	0 / 2 0.0%	0 / 1 0.0%	0 / 1 0.0%	0 / 1 0.0%	0 / 3 0.0%	0 / 6 0.0%	0 / 3 0.0%	0 / 1 0.0%	0 / 5 0.0%	0 / 0 0.0%	0 / 0 0.0%	0 / 0 0.0%	0 / 0 0.0%	45 / 57 78.9%	12 / 57 21.1%E	
1	Streptococcus pyogenes MGAS315	0 / 67 0.0%	0 / 2 0.0%	0 / 3 0.0%	0 / 3 0.0%	0 / 3 0.0%	0 / 4 0.0%	0 / 4 0.0%	0 / 1 0.0%	0 / 2 0.0%	0 / 6 0.0%	0 / 1 0.0%	0 / 4 0.0%	0 / 4 0.0%	0 / 2 0.0%	0 / 2 0.0%	0 / 1 0.0%	0 / 2 0.0%	0 / 1 0.0%	0 / 4 0.0%	0 / 7 0.0%	0 / 3 0.0%	0 / 1 0.0%	0 / 7 0.0%	0 / 0 0.0%	0 / 0 0.0%	0 / 0 0.0%	0 / 0 0.0%	53 / 67 79.1%	14 / 67 20.9%E	
1	Streptococcus pyogenes serotype M5	0 / 65 0.0%	0 / 1 0.0%	0 / 3 0.0%	0 / 3 0.0%	0 / 3 0.0%	0 / 4 0.0%	0 / 4 0.0%	0 / 1 0.0%	0 / 2 0.0%	0 / 6 0.0%	0 / 1 0.0%	0 / 4 0.0%	0 / 4 0.0%	0 / 1 0.0%	0 / 2 0.0%	0 / 1 0.0%	0 / 2 0.0%	0 / 1 0.0%	0 / 4 0.0%	0 / 7 0.0%	0 / 3 0.0%	0 / 1 0.0%	0 / 7 0.0%	0 / 0 0.0%	0 / 0 0.0%	0 / 0 0.0%	0 / 0 0.0%	52 / 65 80.0%	13 / 65 20.0%E	
1	Streptococcus pyogenes (serotype 5 / Manfredo)	0 / 65 0.0%	0 / 1 0.0%	0 / 3 0.0%	0 / 3 0.0%	0 / 3 0.0%	0 / 4 0.0%	0 / 4 0.0%	0 / 1 0.0%	0 / 2 0.0%	0 / 6 0.0%	0 / 1 0.0%	0 / 4 0.0%	0 / 4 0.0%	0 / 1 0.0%	0 / 2 0.0%	0 / 1 0.0%	0 / 2 0.0%	0 / 1 0.0%	0 / 4 0.0%	0 / 7 0.0%	0 / 3 0.0%	0 / 1 0.0%	0 / 7 0.0%	0 / 0 0.0%	0 / 0 0.0%	0 / 0 0.0%	0 / 0 0.0%	52 / 65 80.0%	13 / 65 20.0%E	
1	Streptococcus pyogenes serotype M6	0 / 67 0.0%	0 / 2 0.0%	0 / 3 0.0%	0 / 3 0.0%	0 / 3 0.0%	0 / 4 0.0%	0 / 4 0.0%	0 / 1 0.0%	0 / 2 0.0%	0 / 6 0.0%	0 / 1 0.0%	0 / 4 0.0%	0 / 4 0.0%	0 / 2 0.0%	0 / 2 0.0%	0 / 1 0.0%	0 / 2 0.0%	0 / 1 0.0%	0 / 4 0.0%	0 / 7 0.0%	0 / 3 0.0%	0 / 1 0.0%	0 / 7 0.0%	0 / 0 0.0%	0 / 0 0.0%	0 / 0 0.0%	0 / 0 0.0%	53 / 67 79.1%	14 / 67 20.9%E	
1	Streptococcus pyogenes MGAS10394	0 / 67 0.0%	0 / 2 0.0%	0 / 3 0.0%	0 / 3 0.0%	0 / 3 0.0%	0 / 4 0.0%	0 / 4 0.0%	0 / 1 0.0%	0 / 2 0.0%	0 / 6 0.0%	0 / 1 0.0%	0 / 4 0.0%	0 / 4 0.0%	0 / 2 0.0%	0 / 2 0.0%	0 / 1 0.0%	0 / 2 0.0%	0 / 1 0.0%	0 / 4 0.0%	0 / 7 0.0%	0 / 3 0.0%	0 / 1 0.0%	0 / 7 0.0%	0 / 0 0.0%	0 / 0 0.0%	0 / 0 0.0%	0 / 0 0.0%	53 / 67 79.1%	14 / 67 20.9%E	
1	Streptococcus pyogenes (serotype M18)	0 / 67 0.0%	0 / 2 0.0%	0 / 3 0.0%	0 / 3 0.0%	0 / 3 0.0%	0 / 4 0.0%	0 / 4 0.0%	0 / 1 0.0%	0 / 2 0.0%	0 / 6 0.0%	0 / 1 0.0%	0 / 4 0.0%	0 / 4 0.0%	0 / 2 0.0%	0 / 2 0.0%	0 / 1 0.0%	0 / 2 0.0%	0 / 1 0.0%	0 / 4 0.0%	0 / 7 0.0%	0 / 3 0.0%	0 / 1 0.0%	0 / 7 0.0%	0 / 0 0.0%	0 / 0 0.0%	0 / 0 0.0%	0 / 0 0.0%	53 / 67 79.1%	14 / 67 20.9%E	
1	Streptococcus pyogenes (serotype 18 / MGAS8232)	0 / 67 0.0%	0 / 2 0.0%	0 / 3 0.0%	0 / 3 0.0%	0 / 3 0.0%	0 / 4 0.0%	0 / 4 0.0%	0 / 1 0.0%	0 / 2 0.0%	0 / 6 0.0%	0 / 1 0.0%	0 / 4 0.0%	0 / 4 0.0%	0 / 2 0.0%	0 / 2 0.0%	0 / 1 0.0%	0 / 2 0.0%	0 / 1 0.0%	0 / 4 0.0%	0 / 7 0.0%	0 / 3 0.0%	0 / 1 0.0%	0 / 7 0.0%	0 / 0 0.0%	0 / 0 0.0%	0 / 0 0.0%	0 / 0 0.0%	53 / 67 79.1%	14 / 67 20.9%E	
1	Streptococcus pyogenes serotype M49	0 / 66 0.0%	0 / 2 0.0%	0 / 3 0.0%	0 / 3 0.0%	0 / 3 0.0%	0 / 4 0.0%	0 / 4 0.0%	0 / 1 0.0%	0 / 2 0.0%	0 / 6 0.0%	0 / 1 0.0%	0 / 4 0.0%	0 / 3 0.0%	0 / 2 0.0%	0 / 2 0.0%	0 / 1 0.0%	0 / 2 0.0%	0 / 1 0.0%	0 / 4 0.0%	0 / 7 0.0%	0 / 3 0.0%	0 / 1 0.0%	0 / 7 0.0%	0 / 0 0.0%	0 / 0 0.0%	0 / 0 0.0%	0 / 0 0.0%	38 / 66 57.6%E	28 / 66 42.4%	
1	Streptococcus pyogenes NZ131	0 / 66 0.0%	0 / 2 0.0%	0 / 3 0.0%	0 / 3 0.0%	0 / 3 0.0%	0 / 4 0.0%	0 / 4 0.0%	0 / 1 0.0%	0 / 2 0.0%	0 / 6 0.0%	0 / 1 0.0%	0 / 4 0.0%	0 / 3 0.0%	0 / 2 0.0%	0 / 2 0.0%	0 / 1 0.0%	0 / 2 0.0%	0 / 1 0.0%	0 / 4 0.0%	0 / 7 0.0%	0 / 3 0.0%	0 / 1 0.0%	0 / 7 0.0%	0 / 0 0.0%	0 / 0 0.0%	0 / 0 0.0%	0 / 0 0.0%	38 / 66 57.6%E	28 / 66 42.4%	
1	Streptococcus pyogenes serotype M28	0 / 67 0.0%	0 / 2 0.0%	0 / 3 0.0%	0 / 3 0.0%	0 / 3 0.0%	0 / 4 0.0%	0 / 4 0.0%	0 / 1 0.0%	0 / 2 0.0%	0 / 6 0.0%	0 / 1 0.0%	0 / 4 0.0%	0 / 4 0.0%	0 / 2 0.0%	0 / 2 0.0%	0 / 1 0.0%	0 / 2 0.0%	0 / 1 0.0%	0 / 4 0.0%	0 / 7 0.0%	0 / 3 0.0%	0 / 1 0.0%	0 / 7 0.0%	0 / 0 0.0%	0 / 0 0.0%	0 / 0 0.0%	0 / 0 0.0%	53 / 67 79.1%	14 / 67 20.9%E	
1	Streptococcus pyogenes MGAS6180	0 / 67 0.0%	0 / 2 0.0%	0 / 3 0.0%	0 / 3 0.0%	0 / 3 0.0%	0 / 4 0.0%	0 / 4 0.0%	0 / 1 0.0%	0 / 2 0.0%	0 / 6 0.0%	0 / 1 0.0%	0 / 4 0.0%	0 / 4 0.0%	0 / 2 0.0%	0 / 2 0.0%	0 / 1 0.0%	0 / 2 0.0%	0 / 1 0.0%	0 / 4 0.0%	0 / 7 0.0%	0 / 3 0.0%	0 / 1 0.0%	0 / 7 0.0%	0 / 0 0.0%	0 / 0 0.0%	0 / 0 0.0%	0 / 0 0.0%	53 / 67 79.1%	14 / 67 20.9%E	
2	Streptococcus pyogenes serotype M12	0 / 134 0.0%	0 / 4 0.0%	0 / 6 0.0%	0 / 6 0.0%	0 / 6 0.0%	0 / 8 0.0%	0 / 8 0.0%	0 / 2 0.0%	0 / 4 0.0%	0 / 12 0.0%	0 / 2 0.0%	0 / 8 0.0%	0 / 8 0.0%	0 / 4 0.0%	0 / 4 0.0%	0 / 2 0.0%	0 / 4 0.0%	0 / 2 0.0%	0 / 8 0.0%	0 / 14 0.0%	0 / 6 0.0%	0 / 2 0.0%	0 / 14 0.0%	0 / 0 0.0%	0 / 0 0.0%	0 / 0 0.0%	0 / 0 0.0%	106 / 134 79.1%	28 / 134 20.9%E	
1	Streptococcus pyogenes MGAS9429	0 / 67 0.0%	0 / 2 0.0%	0 / 3 0.0%	0 / 3 0.0%	0 / 3 0.0%	0 / 4 0.0%	0 / 4 0.0%	0 / 1 0.0%	0 / 2 0.0%	0 / 6 0.0%	0 / 1 0.0%	0 / 4 0.0%	0 / 4 0.0%	0 / 2 0.0%	0 / 2 0.0%	0 / 1 0.0%	0 / 2 0.0%	0 / 1 0.0%	0 / 4 0.0%	0 / 7 0.0%	0 / 3 0.0%	0 / 1 0.0%	0 / 7 0.0%	0 / 0 0.0%	0 / 0 0.0%	0 / 0 0.0%	0 / 0 0.0%	53 / 67 79.1%	14 / 67 20.9%E	
1	Streptococcus pyogenes MGAS2096	0 / 67 0.0%	0 / 2 0.0%	0 / 3 0.0%	0 / 3 0.0%	0 / 3 0.0%	0 / 4 0.0%	0 / 4 0.0%	0 / 1 0.0%	0 / 2 0.0%	0 / 6 0.0%	0 / 1 0.0%	0 / 4 0.0%	0 / 4 0.0%	0 / 2 0.0%	0 / 2 0.0%	0 / 1 0.0%	0 / 2 0.0%	0 / 1 0.0%	0 / 4 0.0%	0 / 7 0.0%	0 / 3 0.0%	0 / 1 0.0%	0 / 7 0.0%	0 / 0 0.0%	0 / 0 0.0%	0 / 0 0.0%	0 / 0 0.0%	53 / 67 79.1%	14 / 67 20.9%E	
1	Streptococcus pyogenes serotype M2	0 / 67 0.0%	0 / 2 0.0%	0 / 3 0.0%	0 / 3 0.0%	0 / 3 0.0%	0 / 4 0.0%	0 / 4 0.0%	0 / 1 0.0%	0 / 2 0.0%	0 / 6 0.0%	0 / 1 0.0%	0 / 4 0.0%	0 / 4 0.0%	0 / 2 0.0%	0 / 2 0.0%	0 / 1 0.0%	0 / 2 0.0%	0 / 1 0.0%	0 / 4 0.0%	0 / 7 0.0%	0 / 3 0.0%	0 / 1 0.0%	0 / 7 0.0%	0 / 0 0.0%	0 / 0 0.0%	0 / 0 0.0%	0 / 0 0.0%	53 / 67 79.1%	14 / 67 20.9%E	
1	Streptococcus pyogenes MGAS10270	0 / 67 0.0%	0 / 2 0.0%	0 / 3 0.0%	0 / 3 0.0%	0 / 3 0.0%	0 / 4 0.0%	0 / 4 0.0%	0 / 1 0.0%	0 / 2 0.0%	0 / 6 0.0%	0 / 1 0.0%	0 / 4 0.0%	0 / 4 0.0%	0 / 2 0.0%	0 / 2 0.0%	0 / 1 0.0%	0 / 2 0.0%	0 / 1 0.0%	0 / 4 0.0%	0 / 7 0.0%	0 / 3 0.0%	0 / 1 0.0%	0 / 7 0.0%	0 / 0 0.0%	0 / 0 0.0%	0 / 0 0.0%	0 / 0 0.0%	53 / 67 79.1%	14 / 67 20.9%E	
1	Streptococcus pyogenes serotype M4	0 / 67 0.0%	0 / 2 0.0%	0 / 3 0.0%	0 / 3 0.0%	0 / 3 0.0%	0 / 4 0.0%	0 / 4 0.0%	0 / 1 0.0%	0 / 2 0.0%	0 / 6 0.0%	0 / 1 0.0%	0 / 4 0.0%	0 / 4 0.0%	0 / 2 0.0%	0 / 2 0.0%	0 / 1 0.0%	0 / 2 0.0%	0 / 1 0.0%	0 / 4 0.0%	0 / 7 0.0%	0 / 3 0.0%	0 / 1 0.0%	0 / 7 0.0%	0 / 0 0.0%	0 / 0 0.0%	0 / 0 0.0%	0 / 0 0.0%	53 / 67 79.1%	14 / 67 20.9%E	
1	Streptococcus pyogenes MGAS10750	0 / 67 0.0%	0 / 2 0.0%	0 / 3 0.0%	0 / 3 0.0%	0 / 3 0.0%	0 / 4 0.0%	0 / 4 0.0%	0 / 1 0.0%	0 / 2 0.0%	0 / 6 0.0%	0 / 1 0.0%	0 / 4 0.0%	0 / 4 0.0%	0 / 2 0.0%	0 / 2 0.0%	0 / 1 0.0%	0 / 2 0.0%	0 / 1 0.0%	0 / 4 0.0%	0 / 7 0.0%	0 / 3 0.0%	0 / 1 0.0%	0 / 7 0.0%	0 / 0 0.0%	0 / 0 0.0%	0 / 0 0.0%	0 / 0 0.0%	53 / 67 79.1%	14 / 67 20.9%E	
1	Streptococcus pyogenes serotype M53	0 / 67 0.0%	0 / 2 0.0%	0 / 3 0.0%	0 / 3 0.0%	0 / 3 0.0%	0 / 4 0.0%	0 / 4 0.0%	0 / 1 0.0%	0 / 2 0.0%	0 / 6 0.0%	0 / 1 0.0%	0 / 4 0.0%	0 / 4 0.0%	0 / 2 0.0%	0 / 2 0.0%	0 / 1 0.0%	0 / 2 0.0%	0 / 1 0.0%	0 / 4 0.0%	0 / 7 0.0%	0 / 3 0.0%	0 / 1 0.0%	0 / 7 0.0%	0 / 0 0.0%	0 / 0 0.0%	0 / 0 0.0%	0 / 0 0.0%	38 / 67 56.7%E	29 / 67 43.3%	
1	Streptococcus pyogenes Alab49	0 / 67 0.0%	0 / 2 0.0%	0 / 3 0.0%	0 / 3 0.0%	0 / 3 0.0%	0 / 4 0.0%	0 / 4 0.0%	0 / 1 0.0%	0 / 2 0.0%	0 / 6 0.0%	0 / 1 0.0%	0 / 4 0.0%	0 / 4 0.0%	0 / 2 0.0%	0 / 2 0.0%	0 / 1 0.0%	0 / 2 0.0%	0 / 1 0.0%	0 / 4 0.0%	0 / 7 0.0%	0 / 3 0.0%	0 / 1 0.0%	0 / 7 0.0%	0 / 0 0.0%	0 / 0 0.0%	0 / 0 0.0%	0 / 0 0.0%	38 / 67 56.7%E	29 / 67 43.3%	
1	Streptococcus pyogenes serotype M59	0 / 57 0.0%	0 / 2 0.0%	0 / 2 0.0%	0 / 3 0.0%	0 / 3 0.0%	0 / 3 0.0%	0 / 3 0.0%	0 / 1 0.0%	0 / 2 0.0%	0 / 5 0.0%	0 / 1 0.0%	0 / 4 0.0%	0 / 3 0.0%	0 / 2 0.0%	0 / 2 0.0%	0 / 1 0.0%	0 / 1 0.0%	0 / 1 0.0%	0 / 3 0.0%	0 / 6 0.0%	0 / 3 0.0%	0 / 1 0.0%	0 / 5 0.0%	0 / 0 0.0%	0 / 0 0.0%	0 / 0 0.0%	0 / 0 0.0%	33 / 57 57.9%E	24 / 57 42.1%	
1	Streptococcus pyogenes BEI NR-33709	0 / 57 0.0%	0 / 2 0.0%	0 / 2 0.0%	0 / 3 0.0%	0 / 3 0.0%	0 / 3 0.0%	0 / 3 0.0%	0 / 1 0.0%	0 / 2 0.0%	0 / 5 0.0%	0 / 1 0.0%	0 / 4 0.0%	0 / 3 0.0%	0 / 2 0.0%	0 / 2 0.0%	0 / 1 0.0%	0 / 1 0.0%	0 / 1 0.0%	0 / 3 0.0%	0 / 6 0.0%	0 / 3 0.0%	0 / 1 0.0%	0 / 5 0.0%	0 / 0 0.0%	0 / 0 0.0%	0 / 0 0.0%	0 / 0 0.0%	33 / 57 57.9%E	24 / 57 42.1%	
1	Streptococcus pyogenes A20	0 / 67 0.0%	0 / 2 0.0%	0 / 3 0.0%	0 / 3 0.0%	0 / 3 0.0%	0 / 4 0.0%	0 / 4 0.0%	0 / 1 0.0%	0 / 2 0.0%	0 / 6 0.0%	0 / 1 0.0%	0 / 4 0.0%	0 / 4 0.0%	0 / 2 0.0%	0 / 2 0.0%	0 / 1 0.0%	0 / 2 0.0%	0 / 1 0.0%	0 / 4 0.0%	0 / 7 0.0%	0 / 3 0.0%	0 / 1 0.0%	0 / 7 0.0%	0 / 0 0.0%	0 / 0 0.0%	0 / 0 0.0%	0 / 0 0.0%	38 / 67 56.7%E	29 / 67 43.3%	
1	Streptococcus pyogenes HSC5	0 / 67 0.0%	0 / 2 0.0%	0 / 3 0.0%	0 / 3 0.0%	0 / 3 0.0%	0 / 4 0.0%	0 / 4 0.0%	0 / 1 0.0%	0 / 2 0.0%	0 / 6 0.0%	0 / 1 0.0%	0 / 4 0.0%	0 / 4 0.0%	0 / 2 0.0%	0 / 2 0.0%	0 / 1 0.0%	0 / 2 0.0%	0 / 1 0.0%	0 / 4 0.0%	0 / 7 0.0%	0 / 3 0.0%	0 / 1 0.0%	0 / 7 0.0%	0 / 0 0.0%	0 / 0 0.0%	0 / 0 0.0%	0 / 0 0.0%	38 / 67 56.7%E	29 / 67 43.3%	
2	ATCC 15912	0 / 122 0.0%	0 / 4 0.0%	0 / 4 0.0%	0 / 4 0.0%	0 / 4 0.0%	0 / 8 0.0%	0 / 6 0.0%	0 / 2 0.0%	0 / 4 0.0%	0 / 8 0.0%	0 / 2 0.0%	0 / 10 0.0%	0 / 6 0.0%	0 / 4 0.0%	0 / 4 0.0%	0 / 2 0.0%	0 / 4 0.0%	0 / 2 0.0%	0 / 8 0.0%	0 / 14 0.0%	0 / 6 0.0%	0 / 2 0.0%	0 / 14 0.0%	0 / 0 0.0%	0 / 0 0.0%	0 / 0 0.0%	0 / 0 0.0%	80 / 122 65.6%E	42 / 122 34.4%	
1	Streptococcus parasanguinis ATCC 15912	0 / 61 0.0%	0 / 2 0.0%	0 / 2 0.0%	0 / 2 0.0%	0 / 2 0.0%	0 / 4 0.0%	0 / 3 0.0%	0 / 1 0.0%	0 / 2 0.0%	0 / 4 0.0%	0 / 1 0.0%	0 / 5 0.0%	0 / 3 0.0%	0 / 2 0.0%	0 / 2 0.0%	0 / 1 0.0%	0 / 2 0.0%	0 / 1 0.0%	0 / 4 0.0%	0 / 7 0.0%	0 / 3 0.0%	0 / 1 0.0%	0 / 7 0.0%	0 / 0 0.0%	0 / 0 0.0%	0 / 0 0.0%	0 / 0 0.0%	40 / 61 65.6%E	21 / 61 34.4%	
1	Streptococcus parasanguinis FW213	0 / 61 0.0%	0 / 2 0.0%	0 / 2 0.0%	0 / 2 0.0%	0 / 2 0.0%	0 / 4 0.0%	0 / 3 0.0%	0 / 1 0.0%	0 / 2 0.0%	0 / 4 0.0%	0 / 1 0.0%	0 / 5 0.0%	0 / 3 0.0%	0 / 2 0.0%	0 / 2 0.0%	0 / 1 0.0%	0 / 2 0.0%	0 / 1 0.0%	0 / 4 0.0%	0 / 7 0.0%	0 / 3 0.0%	0 / 1 0.0%	0 / 7 0.0%	0 / 0 0.0%	0 / 0 0.0%	0 / 0 0.0%	0 / 0 0.0%	40 / 61 65.6%E	21 / 61 34.4%	
1	ATCC 29178	0 / 45 0.0%	0 / 1 0.0%	0 / 2 0.0%	0 / 3 0.0%	0 / 2 0.0%	0 / 2 0.0%	0 / 2 0.0%	0 / 0 0.0%	0 / 1 0.0%	0 / 4 0.0%	0 / 0 0.0%	0 / 4 0.0%	0 / 3 0.0%	0 / 1 0.0%	0 / 1 0.0%	0 / 1 0.0%	0 / 1 0.0%	0 / 1 0.0%	0 / 3 0.0%	0 / 6 0.0%	0 / 2 0.0%	0 / 1 0.0%	0 / 4 0.0%	0 / 0 0.0%	0 / 0 0.0%	0 / 0 0.0%	0 / 0 0.0%	25 / 45 55.6%E	20 / 45 44.4%	
1	Streptococcus iniae SF1	0 / 45 0.0%	0 / 1 0.0%	0 / 2 0.0%	0 / 3 0.0%	0 / 2 0.0%	0 / 2 0.0%	0 / 2 0.0%	0 / 0 0.0%	0 / 1 0.0%	0 / 4 0.0%	0 / 0 0.0%	0 / 4 0.0%	0 / 3 0.0%	0 / 1 0.0%	0 / 1 0.0%	0 / 1 0.0%	0 / 1 0.0%	0 / 1 0.0%	0 / 3 0.0%	0 / 6 0.0%	0 / 2 0.0%	0 / 1 0.0%	0 / 4 0.0%	0 / 0 0.0%	0 / 0 0.0%	0 / 0 0.0%	0 / 0 0.0%	25 / 45 55.6%E	20 / 45 44.4%	
1	CCUG 39954	0 / 60 0.0%	0 / 2 0.0%	0 / 2 0.0%	0 / 3 0.0%	0 / 3 0.0%	0 / 3 0.0%	0 / 3 0.0%	0 / 1 0.0%	0 / 2 0.0%	0 / 5 0.0%	0 / 2 0.0%	0 / 5 0.0%	0 / 3 0.0%	0 / 2 0.0%	0 / 2 0.0%	0 / 2 0.0%	0 / 1 0.0%	0 / 1 0.0%	0 / 3 0.0%	0 / 6 0.0%	0 / 3 0.0%	0 / 1 0.0%	0 / 5 0.0%	0 / 0 0.0%	0 / 0 0.0%	0 / 0 0.0%	0 / 0 0.0%	31 / 60 51.7%E	28 / 60 46.7%	1 / 60 1.7%CE
1	Streptococcus parauberis KCTC 11537	0 / 60 0.0%	0 / 2 0.0%	0 / 2 0.0%	0 / 3 0.0%	0 / 3 0.0%	0 / 3 0.0%	0 / 3 0.0%	0 / 1 0.0%	0 / 2 0.0%	0 / 5 0.0%	0 / 2 0.0%	0 / 5 0.0%	0 / 3 0.0%	0 / 2 0.0%	0 / 2 0.0%	0 / 2 0.0%	0 / 1 0.0%	0 / 1 0.0%	0 / 3 0.0%	0 / 6 0.0%	0 / 3 0.0%	0 / 1 0.0%	0 / 5 0.0%	0 / 0 0.0%	0 / 0 0.0%	0 / 0 0.0%	0 / 0 0.0%	31 / 60 51.7%E	28 / 60 46.7%	1 / 60 1.7%CE
1	ATCC 19436	0 / 58 0.0%	0 / 2 0.0%	0 / 2 0.0%	0 / 3 0.0%	0 / 3 0.0%	0 / 3 0.0%	0 / 3 0.0%	0 / 1 0.0%	0 / 2 0.0%	0 / 5 0.0%	0 / 1 0.0%	0 / 4 0.0%	0 / 3 0.0%	0 / 2 0.0%	0 / 2 0.0%	0 / 1 0.0%	0 / 1 0.0%	0 / 1 0.0%	0 / 4 0.0%	0 / 6 0.0%	0 / 3 0.0%	0 / 1 0.0%	0 / 5 0.0%	0 / 0 0.0%	0 / 0 0.0%	0 / 0 0.0%	0 / 0 0.0%	36 / 58 62.1%E	21 / 58 36.2%	1 / 58 1.7%CE
1	Streptococcus uberis 0140J	0 / 58 0.0%	0 / 2 0.0%	0 / 2 0.0%	0 / 3 0.0%	0 / 3 0.0%	0 / 3 0.0%	0 / 3 0.0%	0 / 1 0.0%	0 / 2 0.0%	0 / 5 0.0%	0 / 1 0.0%	0 / 4 0.0%	0 / 3 0.0%	0 / 2 0.0%	0 / 2 0.0%	0 / 1 0.0%	0 / 1 0.0%	0 / 1 0.0%	0 / 4 0.0%	0 / 6 0.0%	0 / 3 0.0%	0 / 1 0.0%	0 / 5 0.0%	0 / 0 0.0%	0 / 0 0.0%	0 / 0 0.0%	0 / 0 0.0%	36 / 58 62.1%E	21 / 58 36.2%	1 / 58 1.7%CE
1	ATCC 49456	0 / 60 0.0%	0 / 2 0.0%	0 / 2 0.0%	0 / 2 0.0%	0 / 2 0.0%	0 / 4 0.0%	0 / 3 0.0%	0 / 1 0.0%	0 / 2 0.0%	0 / 4 0.0%	0 / 1 0.0%	0 / 5 0.0%	0 / 3 0.0%	0 / 2 0.0%	0 / 2 0.0%	0 / 1 0.0%	0 / 2 0.0%	0 / 1 0.0%	0 / 4 0.0%	0 / 5 0.0%	0 / 5 0.0%	0 / 1 0.0%	0 / 6 0.0%	0 / 0 0.0%	0 / 0 0.0%	0 / 0 0.0%	0 / 0 0.0%	35 / 60 58.3%E	25 / 60 41.7%	
1	Streptococcus mitis B6	0 / 60 0.0%	0 / 2 0.0%	0 / 2 0.0%	0 / 2 0.0%	0 / 2 0.0%	0 / 4 0.0%	0 / 3 0.0%	0 / 1 0.0%	0 / 2 0.0%	0 / 4 0.0%	0 / 1 0.0%	0 / 5 0.0%	0 / 3 0.0%	0 / 2 0.0%	0 / 2 0.0%	0 / 1 0.0%	0 / 2 0.0%	0 / 1 0.0%	0 / 4 0.0%	0 / 5 0.0%	0 / 5 0.0%	0 / 1 0.0%	0 / 6 0.0%	0 / 0 0.0%	0 / 0 0.0%	0 / 0 0.0%	0 / 0 0.0%	35 / 60 58.3%E	25 / 60 41.7%	
1	ACA-DC 206	0 / 70 0.0%	0 / 2 0.0%	0 / 3 0.0%	0 / 3 0.0%	0 / 3 0.0%	0 / 4 0.0%	0 / 4 0.0%	0 / 1 0.0%	0 / 2 0.0%	0 / 6 0.0%	0 / 1 0.0%	0 / 5 0.0%	0 / 4 0.0%	0 / 2 0.0%	0 / 3 0.0%	0 / 1 0.0%	0 / 2 0.0%	0 / 1 0.0%	0 / 5 0.0%	0 / 5 0.0%	0 / 4 0.0%	0 / 1 0.0%	0 / 8 0.0%	0 / 0 0.0%	0 / 0 0.0%	0 / 0 0.0%	0 / 0 0.0%	41 / 70 58.6%E	29 / 70 41.4%	
1	Streptococcus macedonicus ACA-DC 198	0 / 70 0.0%	0 / 2 0.0%	0 / 3 0.0%	0 / 3 0.0%	0 / 3 0.0%	0 / 4 0.0%	0 / 4 0.0%	0 / 1 0.0%	0 / 2 0.0%	0 / 6 0.0%	0 / 1 0.0%	0 / 5 0.0%	0 / 4 0.0%	0 / 2 0.0%	0 / 3 0.0%	0 / 1 0.0%	0 / 2 0.0%	0 / 1 0.0%	0 / 5 0.0%	0 / 5 0.0%	0 / 4 0.0%	0 / 1 0.0%	0 / 8 0.0%	0 / 0 0.0%	0 / 0 0.0%	0 / 0 0.0%	0 / 0 0.0%	41 / 70 58.6%E	29 / 70 41.4%	
1	ATCC BAA-102	0 / 67 0.0%	0 / 2 0.0%	0 / 3 0.0%	0 / 4 0.0%	0 / 3 0.0%	0 / 4 0.0%	0 / 4 0.0%	0 / 1 0.0%	0 / 2 0.0%	0 / 6 0.0%	0 / 1 0.0%	0 / 4 0.0%	0 / 4 0.0%	0 / 2 0.0%	0 / 3 0.0%	0 / 1 0.0%	0 / 2 0.0%	0 / 1 0.0%	0 / 3 0.0%	0 / 5 0.0%	0 / 4 0.0%	0 / 1 0.0%	0 / 7 0.0%	0 / 0 0.0%	0 / 0 0.0%	0 / 0 0.0%	0 / 0 0.0%	43 / 67 64.2%E	24 / 67 35.8%	
1	Streptococcus infantarius subsp. infantarius	0 / 67 0.0%	0 / 2 0.0%	0 / 3 0.0%	0 / 4 0.0%	0 / 3 0.0%	0 / 4 0.0%	0 / 4 0.0%	0 / 1 0.0%	0 / 2 0.0%	0 / 6 0.0%	0 / 1 0.0%	0 / 4 0.0%	0 / 4 0.0%	0 / 2 0.0%	0 / 3 0.0%	0 / 1 0.0%	0 / 2 0.0%	0 / 1 0.0%	0 / 3 0.0%	0 / 5 0.0%	0 / 4 0.0%	0 / 1 0.0%	0 / 7 0.0%	0 / 0 0.0%	0 / 0 0.0%	0 / 0 0.0%	0 / 0 0.0%	43 / 67 64.2%E	24 / 67 35.8%	
1	Streptococcus infantarius subsp. infantarius CJ18	0 / 67 0.0%	0 / 2 0.0%	0 / 3 0.0%	0 / 4 0.0%	0 / 3 0.0%	0 / 4 0.0%	0 / 4 0.0%	0 / 1 0.0%	0 / 2 0.0%	0 / 6 0.0%	0 / 1 0.0%	0 / 4 0.0%	0 / 4 0.0%	0 / 2 0.0%	0 / 3 0.0%	0 / 1 0.0%	0 / 2 0.0%	0 / 1 0.0%	0 / 3 0.0%	0 / 5 0.0%	0 / 4 0.0%	0 / 1 0.0%	0 / 7 0.0%	0 / 0 0.0%	0 / 0 0.0%	0 / 0 0.0%	0 / 0 0.0%	43 / 67 64.2%E	24 / 67 35.8%	
8	Streptococcus dysgalactiae group	0 / 455 0.0%	0 / 16 0.0%	0 / 15 0.0%	0 / 24 0.0%	0 / 23 0.0%	0 / 24 0.0%	0 / 25 0.0%	0 / 8 0.0%	0 / 16 0.0%	0 / 41 0.0%	0 / 8 0.0%	0 / 32 0.0%	0 / 23 0.0%	0 / 16 0.0%	0 / 16 0.0%	0 / 8 0.0%	0 / 8 0.0%	0 / 7 0.0%	0 / 25 0.0%	0 / 49 0.0%	0 / 23 0.0%	0 / 8 0.0%	0 / 40 0.0%	0 / 0 0.0%	0 / 0 0.0%	0 / 0 0.0%	0 / 0 0.0%	242 / 455 53.2%E	209 / 455 45.9%	4 / 455 0.9%CE
4	"Group II" Minett 1934	0 / 225 0.0%	0 / 8 0.0%	0 / 8 0.0%	0 / 12 0.0%	0 / 12 0.0%	0 / 11 0.0%	0 / 12 0.0%	0 / 4 0.0%	0 / 8 0.0%	0 / 20 0.0%	0 / 4 0.0%	0 / 16 0.0%	0 / 12 0.0%	0 / 8 0.0%	0 / 8 0.0%	0 / 4 0.0%	0 / 3 0.0%	0 / 3 0.0%	0 / 12 0.0%	0 / 24 0.0%	0 / 12 0.0%	0 / 4 0.0%	0 / 20 0.0%	0 / 0 0.0%	0 / 0 0.0%	0 / 0 0.0%	0 / 0 0.0%	130 / 225 57.8%E	95 / 225 42.2%	
4	"Streptococcus equisimilis" Frost and Engelbrecht 1936 (human strains)	0 / 225 0.0%	0 / 8 0.0%	0 / 8 0.0%	0 / 12 0.0%	0 / 12 0.0%	0 / 11 0.0%	0 / 12 0.0%	0 / 4 0.0%	0 / 8 0.0%	0 / 20 0.0%	0 / 4 0.0%	0 / 16 0.0%	0 / 12 0.0%	0 / 8 0.0%	0 / 8 0.0%	0 / 4 0.0%	0 / 3 0.0%	0 / 3 0.0%	0 / 12 0.0%	0 / 24 0.0%	0 / 12 0.0%	0 / 4 0.0%	0 / 20 0.0%	0 / 0 0.0%	0 / 0 0.0%	0 / 0 0.0%	0 / 0 0.0%	130 / 225 57.8%E	95 / 225 42.2%	
1	Streptococcus dysgalactiae subsp. equisimilis GGS_124	0 / 56 0.0%	0 / 2 0.0%	0 / 2 0.0%	0 / 3 0.0%	0 / 3 0.0%	0 / 2 0.0%	0 / 3 0.0%	0 / 1 0.0%	0 / 2 0.0%	0 / 5 0.0%	0 / 1 0.0%	0 / 4 0.0%	0 / 3 0.0%	0 / 2 0.0%	0 / 2 0.0%	0 / 1 0.0%	0 / 1 0.0%	0 / 1 0.0%	0 / 3 0.0%	0 / 6 0.0%	0 / 3 0.0%	0 / 1 0.0%	0 / 5 0.0%	0 / 0 0.0%	0 / 0 0.0%	0 / 0 0.0%	0 / 0 0.0%	32 / 56 57.1%E	24 / 56 42.9%	
1	Streptococcus dysgalactiae subsp. equisimilis RE378	0 / 55 0.0%	0 / 2 0.0%	0 / 2 0.0%	0 / 3 0.0%	0 / 3 0.0%	0 / 3 0.0%	0 / 3 0.0%	0 / 1 0.0%	0 / 2 0.0%	0 / 5 0.0%	0 / 1 0.0%	0 / 4 0.0%	0 / 3 0.0%	0 / 2 0.0%	0 / 2 0.0%	0 / 1 0.0%	0 / 0 0.0%	0 / 0 0.0%	0 / 3 0.0%	0 / 6 0.0%	0 / 3 0.0%	0 / 1 0.0%	0 / 5 0.0%	0 / 0 0.0%	0 / 0 0.0%	0 / 0 0.0%	0 / 0 0.0%	32 / 55 58.2%E	23 / 55 41.8%	
1	Streptococcus dysgalactiae subsp. equisimilis ATCC 12394	0 / 57 0.0%	0 / 2 0.0%	0 / 2 0.0%	0 / 3 0.0%	0 / 3 0.0%	0 / 3 0.0%	0 / 3 0.0%	0 / 1 0.0%	0 / 2 0.0%	0 / 5 0.0%	0 / 1 0.0%	0 / 4 0.0%	0 / 3 0.0%	0 / 2 0.0%	0 / 2 0.0%	0 / 1 0.0%	0 / 1 0.0%	0 / 1 0.0%	0 / 3 0.0%	0 / 6 0.0%	0 / 3 0.0%	0 / 1 0.0%	0 / 5 0.0%	0 / 0 0.0%	0 / 0 0.0%	0 / 0 0.0%	0 / 0 0.0%	33 / 57 57.9%E	24 / 57 42.1%	
1	Streptococcus dysgalactiae subsp. equisimilis AC-2713	0 / 57 0.0%	0 / 2 0.0%	0 / 2 0.0%	0 / 3 0.0%	0 / 3 0.0%	0 / 3 0.0%	0 / 3 0.0%	0 / 1 0.0%	0 / 2 0.0%	0 / 5 0.0%	0 / 1 0.0%	0 / 4 0.0%	0 / 3 0.0%	0 / 2 0.0%	0 / 2 0.0%	0 / 1 0.0%	0 / 1 0.0%	0 / 1 0.0%	0 / 3 0.0%	0 / 6 0.0%	0 / 3 0.0%	0 / 1 0.0%	0 / 5 0.0%	0 / 0 0.0%	0 / 0 0.0%	0 / 0 0.0%	0 / 0 0.0%	33 / 57 57.9%E	24 / 57 42.1%	
4	ATCC 33398	0 / 230 0.0%	0 / 8 0.0%	0 / 7 0.0%	0 / 12 0.0%	0 / 11 0.0%	0 / 13 0.0%	0 / 13 0.0%	0 / 4 0.0%	0 / 8 0.0%	0 / 21 0.0%	0 / 4 0.0%	0 / 16 0.0%	0 / 11 0.0%	0 / 8 0.0%	0 / 8 0.0%	0 / 4 0.0%	0 / 5 0.0%	0 / 4 0.0%	0 / 13 0.0%	0 / 25 0.0%	0 / 11 0.0%	0 / 4 0.0%	0 / 20 0.0%	0 / 0 0.0%	0 / 0 0.0%	0 / 0 0.0%	0 / 0 0.0%	114 / 230 49.6%	112 / 230 48.7%E	4 / 230 1.7%CE
3	"Animal pyogenes, type A" Edwards 1934	0 / 165 0.0%	0 / 6 0.0%	0 / 4 0.0%	0 / 9 0.0%	0 / 9 0.0%	0 / 9 0.0%	0 / 9 0.0%	0 / 3 0.0%	0 / 6 0.0%	0 / 15 0.0%	0 / 3 0.0%	0 / 12 0.0%	0 / 7 0.0%	0 / 6 0.0%	0 / 6 0.0%	0 / 3 0.0%	0 / 3 0.0%	0 / 3 0.0%	0 / 9 0.0%	0 / 18 0.0%	0 / 9 0.0%	0 / 3 0.0%	0 / 13 0.0%	0 / 0 0.0%	0 / 0 0.0%	0 / 0 0.0%	0 / 0 0.0%	86 / 165 52.1%E	76 / 165 46.1%	3 / 165 1.8%CE
1	Streptococcus equi subsp. zooepidemicus MGCS10565	0 / 57 0.0%	0 / 2 0.0%	0 / 2 0.0%	0 / 3 0.0%	0 / 3 0.0%	0 / 3 0.0%	0 / 3 0.0%	0 / 1 0.0%	0 / 2 0.0%	0 / 5 0.0%	0 / 1 0.0%	0 / 4 0.0%	0 / 3 0.0%	0 / 2 0.0%	0 / 2 0.0%	0 / 1 0.0%	0 / 1 0.0%	0 / 1 0.0%	0 / 3 0.0%	0 / 6 0.0%	0 / 3 0.0%	0 / 1 0.0%	0 / 5 0.0%	0 / 0 0.0%	0 / 0 0.0%	0 / 0 0.0%	0 / 0 0.0%	31 / 57 54.4%E	25 / 57 43.9%	1 / 57 1.8%CE
1	Streptococcus equi subsp. zooepidemicus ATCC 35246	0 / 51 0.0%	0 / 2 0.0%	0 / 0 0.0%	0 / 3 0.0%	0 / 3 0.0%	0 / 3 0.0%	0 / 3 0.0%	0 / 1 0.0%	0 / 2 0.0%	0 / 5 0.0%	0 / 1 0.0%	0 / 4 0.0%	0 / 1 0.0%	0 / 2 0.0%	0 / 2 0.0%	0 / 1 0.0%	0 / 1 0.0%	0 / 1 0.0%	0 / 3 0.0%	0 / 6 0.0%	0 / 3 0.0%	0 / 1 0.0%	0 / 3 0.0%	0 / 0 0.0%	0 / 0 0.0%	0 / 0 0.0%	0 / 0 0.0%	26 / 51 51.0%	24 / 51 47.1%E	1 / 51 2.0%CE
1	Streptococcus equi subsp. equi	0 / 65 0.0%	0 / 2 0.0%	0 / 3 0.0%	0 / 3 0.0%	0 / 2 0.0%	0 / 4 0.0%	0 / 4 0.0%	0 / 1 0.0%	0 / 2 0.0%	0 / 6 0.0%	0 / 1 0.0%	0 / 4 0.0%	0 / 4 0.0%	0 / 2 0.0%	0 / 2 0.0%	0 / 1 0.0%	0 / 2 0.0%	0 / 1 0.0%	0 / 4 0.0%	0 / 7 0.0%	0 / 2 0.0%	0 / 1 0.0%	0 / 7 0.0%	0 / 0 0.0%	0 / 0 0.0%	0 / 0 0.0%	0 / 0 0.0%	38 / 65 58.5%	26 / 65 40.0%E	1 / 65 1.5%CE
1	Streptococcus equi subsp. equi 4047	0 / 65 0.0%	0 / 2 0.0%	0 / 3 0.0%	0 / 3 0.0%	0 / 2 0.0%	0 / 4 0.0%	0 / 4 0.0%	0 / 1 0.0%	0 / 2 0.0%	0 / 6 0.0%	0 / 1 0.0%	0 / 4 0.0%	0 / 4 0.0%	0 / 2 0.0%	0 / 2 0.0%	0 / 1 0.0%	0 / 2 0.0%	0 / 1 0.0%	0 / 4 0.0%	0 / 7 0.0%	0 / 2 0.0%	0 / 1 0.0%	0 / 7 0.0%	0 / 0 0.0%	0 / 0 0.0%	0 / 0 0.0%	0 / 0 0.0%	38 / 65 58.5%	26 / 65 40.0%E	1 / 65 1.5%CE
1	AS 1.3089	0 / 50 0.0%	0 / 2 0.0%	0 / 1 0.0%	0 / 2 0.0%	0 / 3 0.0%	0 / 4 0.0%	0 / 3 0.0%	0 / 1 0.0%	0 / 2 0.0%	0 / 4 0.0%	0 / 1 0.0%	0 / 4 0.0%	0 / 2 0.0%	0 / 2 0.0%	0 / 2 0.0%	0 / 1 0.0%	0 / 1 0.0%	0 / 0 0.0%	0 / 3 0.0%	0 / 4 0.0%	0 / 3 0.0%	0 / 0 0.0%	0 / 5 0.0%	0 / 0 0.0%	0 / 0 0.0%	0 / 0 0.0%	0 / 0 0.0%	32 / 50 64.0%E	18 / 50 36.0%	
1	Streptococcus oligofermentans AS 1.3089	0 / 50 0.0%	0 / 2 0.0%	0 / 1 0.0%	0 / 2 0.0%	0 / 3 0.0%	0 / 4 0.0%	0 / 3 0.0%	0 / 1 0.0%	0 / 2 0.0%	0 / 4 0.0%	0 / 1 0.0%	0 / 4 0.0%	0 / 2 0.0%	0 / 2 0.0%	0 / 2 0.0%	0 / 1 0.0%	0 / 1 0.0%	0 / 0 0.0%	0 / 3 0.0%	0 / 4 0.0%	0 / 3 0.0%	0 / 0 0.0%	0 / 5 0.0%	0 / 0 0.0%	0 / 0 0.0%	0 / 0 0.0%	0 / 0 0.0%	32 / 50 64.0%E	18 / 50 36.0%	
1	CCUG 46150	0 / 60 0.0%	0 / 2 0.0%	0 / 2 0.0%	0 / 3 0.0%	0 / 3 0.0%	0 / 3 0.0%	0 / 3 0.0%	0 / 1 0.0%	0 / 2 0.0%	0 / 5 0.0%	0 / 1 0.0%	0 / 5 0.0%	0 / 3 0.0%	0 / 2 0.0%	0 / 3 0.0%	0 / 1 0.0%	0 / 1 0.0%	0 / 1 0.0%	0 / 4 0.0%	0 / 4 0.0%	0 / 4 0.0%	0 / 1 0.0%	0 / 6 0.0%	0 / 0 0.0%	0 / 0 0.0%	0 / 0 0.0%	0 / 0 0.0%	34 / 60 56.7%E	26 / 60 43.3%	
1	Streptococcus gallolyticus subsp. pasteurianus ATCC 43144	0 / 60 0.0%	0 / 2 0.0%	0 / 2 0.0%	0 / 3 0.0%	0 / 3 0.0%	0 / 3 0.0%	0 / 3 0.0%	0 / 1 0.0%	0 / 2 0.0%	0 / 5 0.0%	0 / 1 0.0%	0 / 5 0.0%	0 / 3 0.0%	0 / 2 0.0%	0 / 3 0.0%	0 / 1 0.0%	0 / 1 0.0%	0 / 1 0.0%	0 / 4 0.0%	0 / 4 0.0%	0 / 4 0.0%	0 / 1 0.0%	0 / 6 0.0%	0 / 0 0.0%	0 / 0 0.0%	0 / 0 0.0%	0 / 0 0.0%	34 / 60 56.7%E	26 / 60 43.3%	
1	ATCC BAA-960	0 / 41 0.0%	0 / 2 0.0%	0 / 1 0.0%	0 / 1 0.0%	0 / 2 0.0%	0 / 3 0.0%	0 / 0 0.0%	0 / 1 0.0%	0 / 2 0.0%	0 / 1 0.0%	0 / 2 0.0%	0 / 4 0.0%	0 / 2 0.0%	0 / 2 0.0%	0 / 2 0.0%	0 / 1 0.0%	0 / 1 0.0%	0 / 1 0.0%	0 / 2 0.0%	0 / 4 0.0%	0 / 2 0.0%	0 / 1 0.0%	0 / 4 0.0%	0 / 0 0.0%	0 / 0 0.0%	0 / 0 0.0%	0 / 0 0.0%	23 / 41 56.1%E	18 / 41 43.9%	
1	Streptococcus pseudopneumoniae IS7493	0 / 41 0.0%	0 / 2 0.0%	0 / 1 0.0%	0 / 1 0.0%	0 / 2 0.0%	0 / 3 0.0%	0 / 0 0.0%	0 / 1 0.0%	0 / 2 0.0%	0 / 1 0.0%	0 / 2 0.0%	0 / 4 0.0%	0 / 2 0.0%	0 / 2 0.0%	0 / 2 0.0%	0 / 1 0.0%	0 / 1 0.0%	0 / 1 0.0%	0 / 2 0.0%	0 / 4 0.0%	0 / 2 0.0%	0 / 1 0.0%	0 / 4 0.0%	0 / 0 0.0%	0 / 0 0.0%	0 / 0 0.0%	0 / 0 0.0%	23 / 41 56.1%E	18 / 41 43.9%	
3	ACM 3611	0 / 211 0.0%	0 / 6 0.0%	0 / 9 0.0%	0 / 9 0.0%	0 / 9 0.0%	0 / 12 0.0%	0 / 12 0.0%	0 / 3 0.0%	0 / 6 0.0%	0 / 18 0.0%	0 / 3 0.0%	0 / 16 0.0%	0 / 12 0.0%	0 / 6 0.0%	0 / 9 0.0%	0 / 3 0.0%	0 / 6 0.0%	0 / 3 0.0%	0 / 15 0.0%	0 / 15 0.0%	0 / 12 0.0%	0 / 3 0.0%	0 / 24 0.0%	0 / 0 0.0%	0 / 0 0.0%	0 / 0 0.0%	0 / 0 0.0%	122 / 211 57.8%E	89 / 211 42.2%	
3	Streptococcus gallolyticus subsp. gallolyticus	0 / 211 0.0%	0 / 6 0.0%	0 / 9 0.0%	0 / 9 0.0%	0 / 9 0.0%	0 / 12 0.0%	0 / 12 0.0%	0 / 3 0.0%	0 / 6 0.0%	0 / 18 0.0%	0 / 3 0.0%	0 / 16 0.0%	0 / 12 0.0%	0 / 6 0.0%	0 / 9 0.0%	0 / 3 0.0%	0 / 6 0.0%	0 / 3 0.0%	0 / 15 0.0%	0 / 15 0.0%	0 / 12 0.0%	0 / 3 0.0%	0 / 24 0.0%	0 / 0 0.0%	0 / 0 0.0%	0 / 0 0.0%	0 / 0 0.0%	122 / 211 57.8%E	89 / 211 42.2%	
1	Streptococcus gallolyticus UCN34	0 / 71 0.0%	0 / 2 0.0%	0 / 3 0.0%	0 / 3 0.0%	0 / 3 0.0%	0 / 4 0.0%	0 / 4 0.0%	0 / 1 0.0%	0 / 2 0.0%	0 / 6 0.0%	0 / 1 0.0%	0 / 6 0.0%	0 / 4 0.0%	0 / 2 0.0%	0 / 3 0.0%	0 / 1 0.0%	0 / 2 0.0%	0 / 1 0.0%	0 / 5 0.0%	0 / 5 0.0%	0 / 4 0.0%	0 / 1 0.0%	0 / 8 0.0%	0 / 0 0.0%	0 / 0 0.0%	0 / 0 0.0%	0 / 0 0.0%	41 / 71 57.7%E	30 / 71 42.3%	
1	Streptococcus gallolyticus subsp. gallolyticus ATCC 43143	0 / 60 0.0%	0 / 2 0.0%	0 / 2 0.0%	0 / 3 0.0%	0 / 3 0.0%	0 / 3 0.0%	0 / 3 0.0%	0 / 1 0.0%	0 / 2 0.0%	0 / 5 0.0%	0 / 1 0.0%	0 / 5 0.0%	0 / 3 0.0%	0 / 2 0.0%	0 / 3 0.0%	0 / 1 0.0%	0 / 1 0.0%	0 / 1 0.0%	0 / 4 0.0%	0 / 4 0.0%	0 / 4 0.0%	0 / 1 0.0%	0 / 6 0.0%	0 / 0 0.0%	0 / 0 0.0%	0 / 0 0.0%	0 / 0 0.0%	35 / 60 58.3%E	25 / 60 41.7%	
1	Streptococcus gallolyticus subsp. gallolyticus ATCC BAA-2069	0 / 80 0.0%	0 / 2 0.0%	0 / 4 0.0%	0 / 3 0.0%	0 / 3 0.0%	0 / 5 0.0%	0 / 5 0.0%	0 / 1 0.0%	0 / 2 0.0%	0 / 7 0.0%	0 / 1 0.0%	0 / 5 0.0%	0 / 5 0.0%	0 / 2 0.0%	0 / 3 0.0%	0 / 1 0.0%	0 / 3 0.0%	0 / 1 0.0%	0 / 6 0.0%	0 / 6 0.0%	0 / 4 0.0%	0 / 1 0.0%	0 / 10 0.0%	0 / 0 0.0%	0 / 0 0.0%	0 / 0 0.0%	0 / 0 0.0%	46 / 80 57.5%E	34 / 80 42.5%	
1	Streptococcus anginosus group	0 / 67 0.0%	0 / 2 0.0%	0 / 3 0.0%	0 / 2 0.0%	0 / 1 0.0%	0 / 4 0.0%	0 / 3 0.0%	0 / 1 0.0%	0 / 2 0.0%	0 / 4 0.0%	0 / 1 0.0%	0 / 5 0.0%	0 / 4 0.0%	0 / 2 0.0%	0 / 2 0.0%	0 / 1 0.0%	0 / 3 0.0%	0 / 2 0.0%	0 / 5 0.0%	0 / 7 0.0%	0 / 1 0.0%	0 / 2 0.0%	0 / 10 0.0%	0 / 0 0.0%	0 / 0 0.0%	0 / 0 0.0%	0 / 0 0.0%	38 / 67 56.7%E	29 / 67 43.3%	
1	ATCC 27335	0 / 67 0.0%	0 / 2 0.0%	0 / 3 0.0%	0 / 2 0.0%	0 / 1 0.0%	0 / 4 0.0%	0 / 3 0.0%	0 / 1 0.0%	0 / 2 0.0%	0 / 4 0.0%	0 / 1 0.0%	0 / 5 0.0%	0 / 4 0.0%	0 / 2 0.0%	0 / 2 0.0%	0 / 1 0.0%	0 / 3 0.0%	0 / 2 0.0%	0 / 5 0.0%	0 / 7 0.0%	0 / 1 0.0%	0 / 2 0.0%	0 / 10 0.0%	0 / 0 0.0%	0 / 0 0.0%	0 / 0 0.0%	0 / 0 0.0%	38 / 67 56.7%E	29 / 67 43.3%	
1	Streptococcus intermedius JTH08	0 / 67 0.0%	0 / 2 0.0%	0 / 3 0.0%	0 / 2 0.0%	0 / 1 0.0%	0 / 4 0.0%	0 / 3 0.0%	0 / 1 0.0%	0 / 2 0.0%	0 / 4 0.0%	0 / 1 0.0%	0 / 5 0.0%	0 / 4 0.0%	0 / 2 0.0%	0 / 2 0.0%	0 / 1 0.0%	0 / 3 0.0%	0 / 2 0.0%	0 / 5 0.0%	0 / 7 0.0%	0 / 1 0.0%	0 / 2 0.0%	0 / 10 0.0%	0 / 0 0.0%	0 / 0 0.0%	0 / 0 0.0%	0 / 0 0.0%	38 / 67 56.7%E	29 / 67 43.3%	
		[illegible]	[illegible]	[illegible]	[illegible]	[illegible]	[illegible]	[illegible]	[illegible]	[illegible]	[illegible]	[illegible]	[illegible]	[illegible]	[illegible]	[illegible]	[illegible]	[illegible]	[illegible]	[illegible]	[illegible]	[illegible]	[illegible]	[illegible]	[illegible]	[illegible]	[illegible]	[illegible]	[illegible]	[illegible]	[illegible]

11	Lactococcus	1.3%	0.0%	0.0%	0.0%	0.0%	0.0%	0.0%	0.0%	0.0%	0.0%	0.0%	0.0%	0.0%	0.0%	0.0%	0.0%	0.0%	0.0%	0.0%	0.0%	0.0%	0.0%	11.1%	0.0%	0.0%	0.0%	0.0%	54.1%E	44.2%	1.3%ABE	0.3%CE		
9	"Bacterium lactis" Lister 1873	9 / 549 1.6%	0 / 22 0.0%	0 / 18 0.0%	0 / 18 0.0%	0 / 18 0.0%	0 / 36 0.0%	0 / 27 0.0%	0 / 9 0.0%	0 / 9 0.0%	0 / 54 0.0%	0 / 9 0.0%	0 / 40 0.0%	0 / 36 0.0%	0 / 18 0.0%	0 / 18 0.0%	0 / 9 0.0%	0 / 18 0.0%	0 / 9 0.0%	0 / 36 0.0%	0 / 45 0.0%	0 / 18 0.0%	0 / 15 0.0%	9 / 67 13.4%	0 / 0 0.0%	0 / 0 0.0%	0 / 0 0.0%	0 / 0 0.0%	308 / 549 56.1%E	232 / 549 42.3%	9 / 549 1.6%ABE			
5	"Streptococcus hollandicus" Scholl 1891	5 / 300 1.7%	0 / 12 0.0%	0 / 10 0.0%	0 / 10 0.0%	0 / 10 0.0%	0 / 20 0.0%	0 / 15 0.0%	0 / 5 0.0%	0 / 5 0.0%	0 / 30 0.0%	0 / 5 0.0%	0 / 21 0.0%	0 / 20 0.0%	0 / 10 0.0%	0 / 10 0.0%	0 / 5 0.0%	0 / 10 0.0%	0 / 5 0.0%	0 / 20 0.0%	0 / 25 0.0%	0 / 10 0.0%	0 / 7 0.0%	5 / 35 14.3%	0 / 0 0.0%	0 / 0 0.0%	0 / 0 0.0%	0 / 0 0.0%	159 / 300 53.0%E	136 / 300 45.3%	5 / 300 1.7%ABE			
1	Lactobacillus cremoris SK11	1 / 59 1.7%	0 / 2 0.0%	0 / 2 0.0%	0 / 2 0.0%	0 / 2 0.0%	0 / 4 0.0%	0 / 3 0.0%	0 / 1 0.0%	0 / 1 0.0%	0 / 6 0.0%	0 / 1 0.0%	0 / 4 0.0%	0 / 4 0.0%	0 / 2 0.0%	0 / 2 0.0%	0 / 1 0.0%	0 / 2 0.0%	0 / 1 0.0%	0 / 4 0.0%	0 / 5 0.0%	0 / 2 0.0%	0 / 1 0.0%	1 / 7 14.3%	0 / 0 0.0%	0 / 0 0.0%	0 / 0 0.0%	0 / 0 0.0%	43 / 59 72.9%	15 / 59 25.4%E	1 / 59 1.7%ABE			
1	Lactococcus lactis subsp. cremoris MG1363	1 / 61 1.6%	0 / 3 0.0%	0 / 2 0.0%	0 / 2 0.0%	0 / 2 0.0%	0 / 4 0.0%	0 / 3 0.0%	0 / 1 0.0%	0 / 1 0.0%	0 / 6 0.0%	0 / 1 0.0%	0 / 5 0.0%	0 / 4 0.0%	0 / 2 0.0%	0 / 2 0.0%	0 / 1 0.0%	0 / 2 0.0%	0 / 1 0.0%	0 / 4 0.0%	0 / 5 0.0%	0 / 2 0.0%	0 / 1 0.0%	1 / 7 14.3%	0 / 0 0.0%	0 / 0 0.0%	0 / 0 0.0%	0 / 0 0.0%	44 / 61 72.1%	16 / 61 26.2%E	1 / 61 1.6%ABE			
1	Lactococcus lactis subsp. cremoris NZ9000	1 / 61 1.6%	0 / 3 0.0%	0 / 2 0.0%	0 / 2 0.0%	0 / 2 0.0%	0 / 4 0.0%	0 / 3 0.0%	0 / 1 0.0%	0 / 1 0.0%	0 / 6 0.0%	0 / 1 0.0%	0 / 5 0.0%	0 / 4 0.0%	0 / 2 0.0%	0 / 2 0.0%	0 / 1 0.0%	0 / 2 0.0%	0 / 1 0.0%	0 / 4 0.0%	0 / 5 0.0%	0 / 2 0.0%	0 / 1 0.0%	1 / 7 14.3%	0 / 0 0.0%	0 / 0 0.0%	0 / 0 0.0%	0 / 0 0.0%	43 / 61 70.5%E	17 / 61 27.9%	1 / 61 1.6%ABE			
1	Lactococcus lactis subsp. cremoris A76	1 / 59 1.7%	0 / 2 0.0%	0 / 2 0.0%	0 / 2 0.0%	0 / 2 0.0%	0 / 4 0.0%	0 / 3 0.0%	0 / 1 0.0%	0 / 1 0.0%	0 / 6 0.0%	0 / 1 0.0%	0 / 3 0.0%	0 / 4 0.0%	0 / 2 0.0%	0 / 2 0.0%	0 / 1 0.0%	0 / 2 0.0%	0 / 1 0.0%	0 / 4 0.0%	0 / 5 0.0%	0 / 2 0.0%	0 / 2 0.0%	1 / 7 14.3%	0 / 0 0.0%	0 / 0 0.0%	0 / 0 0.0%	0 / 0 0.0%	42 / 59 71.2%E	16 / 59 27.1%	1 / 59 1.7%ABE			
1	Lactococcus lactis subsp. cremoris UC509.9	1 / 60 1.7%	0 / 2 0.0%	0 / 2 0.0%	0 / 2 0.0%	0 / 2 0.0%	0 / 4 0.0%	0 / 3 0.0%	0 / 1 0.0%	0 / 1 0.0%	0 / 6 0.0%	0 / 1 0.0%	0 / 4 0.0%	0 / 4 0.0%	0 / 2 0.0%	0 / 2 0.0%	0 / 1 0.0%	0 / 2 0.0%	0 / 1 0.0%	0 / 4 0.0%	0 / 5 0.0%	0 / 2 0.0%	0 / 2 0.0%	1 / 7 14.3%	0 / 0 0.0%	0 / 0 0.0%	0 / 0 0.0%	0 / 0 0.0%	43 / 60 71.7%E	16 / 60 26.7%	1 / 60 1.7%ABE			
4	"Lactococcus lactis subsp. diacitilactis (sic)" (Garvie and Farrow 1982) Schleifer et al. 1986	4 / 249 1.6%	0 / 10 0.0%	0 / 8 0.0%	0 / 8 0.0%	0 / 8 0.0%	0 / 16 0.0%	0 / 12 0.0%	0 / 4 0.0%	0 / 4 0.0%	0 / 24 0.0%	0 / 4 0.0%	0 / 19 0.0%	0 / 16 0.0%	0 / 8 0.0%	0 / 8 0.0%	0 / 4 0.0%	0 / 8 0.0%	0 / 4 0.0%	0 / 16 0.0%	0 / 20 0.0%	0 / 8 0.0%	0 / 8 0.0%	4 / 32 12.5%	0 / 0 0.0%	0 / 0 0.0%	0 / 0 0.0%	0 / 0 0.0%	149 / 249 59.8%E	96 / 249 38.6%	4 / 249 1.6%ABE			
1	Lactococcus lactis subsp. lactis Il1403	1 / 61 1.6%	0 / 1 0.0%	0 / 2 0.0%	0 / 2 0.0%	0 / 2 0.0%	0 / 4 0.0%	0 / 3 0.0%	0 / 1 0.0%	0 / 1 0.0%	0 / 6 0.0%	0 / 1 0.0%	0 / 5 0.0%	0 / 4 0.0%	0 / 2 0.0%	0 / 2 0.0%	0 / 1 0.0%	0 / 2 0.0%	0 / 1 0.0%	0 / 4 0.0%	0 / 5 0.0%	0 / 2 0.0%	0 / 2 0.0%	1 / 8 12.5%	0 / 0 0.0%	0 / 0 0.0%	0 / 0 0.0%	0 / 0 0.0%	44 / 61 72.1%	16 / 61 26.2%E	1 / 61 1.6%ABE			
1	Lactococcus lactis subsp. lactis KF147	1 / 64 1.6%	0 / 4 0.0%	0 / 2 0.0%	0 / 2 0.0%	0 / 2 0.0%	0 / 4 0.0%	0 / 3 0.0%	0 / 1 0.0%	0 / 1 0.0%	0 / 6 0.0%	0 / 1 0.0%	0 / 4 0.0%	0 / 4 0.0%	0 / 2 0.0%	0 / 2 0.0%	0 / 1 0.0%	0 / 2 0.0%	0 / 1 0.0%	0 / 4 0.0%	0 / 5 0.0%	0 / 2 0.0%	0 / 2 0.0%	1 / 9 11.1%	0 / 0 0.0%	0 / 0 0.0%	0 / 0 0.0%	0 / 0 0.0%	46 / 64 71.9%E	17 / 64 26.6%	1 / 64 1.6%ABE			
1	Lactococcus lactis subsp. lactis CV56	1 / 62 1.6%	0 / 3 0.0%	0 / 2 0.0%	0 / 2 0.0%	0 / 2 0.0%	0 / 4 0.0%	0 / 3 0.0%	0 / 1 0.0%	0 / 1 0.0%	0 / 6 0.0%	0 / 1 0.0%	0 / 5 0.0%	0 / 4 0.0%	0 / 2 0.0%	0 / 2 0.0%	0 / 1 0.0%	0 / 2 0.0%	0 / 1 0.0%	0 / 4 0.0%	0 / 5 0.0%	0 / 2 0.0%	0 / 2 0.0%	1 / 7 14.3%	0 / 0 0.0%	0 / 0 0.0%	0 / 0 0.0%	0 / 0 0.0%	44 / 62 71.0%E	17 / 62 27.4%	1 / 62 1.6%ABE			
1	Lactococcus lactis IO-1	1 / 62 1.6%	0 / 2 0.0%	0 / 2 0.0%	0 / 2 0.0%	0 / 2 0.0%	0 / 4 0.0%	0 / 3 0.0%	0 / 1 0.0%	0 / 1 0.0%	0 / 6 0.0%	0 / 1 0.0%	0 / 5 0.0%	0 / 4 0.0%	0 / 2 0.0%	0 / 2 0.0%	0 / 1 0.0%	0 / 2 0.0%	0 / 1 0.0%	0 / 4 0.0%	0 / 5 0.0%	0 / 2 0.0%	0 / 2 0.0%	1 / 8 12.5%	0 / 0 0.0%	0 / 0 0.0%	0 / 0 0.0%	0 / 0 0.0%	43 / 62 69.4%E	18 / 62 29.0%	1 / 62 1.6%ABE			
2	ATCC 43921	0 / 120 0.0%	0 / 8 0.0%	0 / 4 0.0%	0 / 4 0.0%	0 / 4 0.0%	0 / 8 0.0%	0 / 6 0.0%	0 / 2 0.0%	0 / 2 0.0%	0 / 10 0.0%	0 / 2 0.0%	0 / 8 0.0%	0 / 6 0.0%	0 / 4 0.0%	0 / 4 0.0%	0 / 2 0.0%	0 / 4 0.0%	0 / 2 0.0%	0 / 6 0.0%	0 / 10 0.0%	0 / 4 0.0%	0 / 6 0.0%	0 / 14 0.0%	0 / 0 0.0%	0 / 0 0.0%	0 / 0 0.0%	0 / 0 0.0%	64 / 120 53.3%	54 / 120 45.0%E	2 / 120 1.7%CE			
1	Lactococcus garvieae ATCC 49156	0 / 60 0.0%	0 / 4 0.0%	0 / 2 0.0%	0 / 2 0.0%	0 / 2 0.0%	0 / 4 0.0%	0 / 3 0.0%	0 / 1 0.0%	0 / 1 0.0%	0 / 5 0.0%	0 / 1 0.0%	0 / 4 0.0%	0 / 3 0.0%	0 / 2 0.0%	0 / 2 0.0%	0 / 1 0.0%	0 / 2 0.0%	0 / 1 0.0%	0 / 3 0.0%	0 / 5 0.0%	0 / 2 0.0%	0 / 3 0.0%	0 / 7 0.0%	0 / 0 0.0%	0 / 0 0.0%	0 / 0 0.0%	0 / 0 0.0%	32 / 60 53.3%	27 / 60 45.0%E	1 / 60 1.7%CE			
1	Lactococcus garvieae Lg2	0 / 60 0.0%	0 / 4 0.0%	0 / 2 0.0%	0 / 2 0.0%	0 / 2 0.0%	0 / 4 0.0%	0 / 3 0.0%	0 / 1 0.0%	0 / 1 0.0%	0 / 5 0.0%	0 / 1 0.0%	0 / 4 0.0%	0 / 3 0.0%	0 / 2 0.0%	0 / 2 0.0%	0 / 1 0.0%	0 / 2 0.0%	0 / 1 0.0%	0 / 3 0.0%	0 / 5 0.0%	0 / 2 0.0%	0 / 3 0.0%	0 / 7 0.0%	0 / 0 0.0%	0 / 0 0.0%	0 / 0 0.0%	0 / 0 0.0%	32 / 60 53.3%	27 / 60 45.0%E	1 / 60 1.7%CE			
55	Lactobacillaceae	92 / 3608 2.5%	0 / 119 0.0%	0 / 159 0.0%	2 / 164 1.2%	4 / 170 2.4%	20 / 287 7.0%	0 / 181 0.0%	11 / 84 13.1%	5 / 116 4.3%	0 / 147 0.0%	13 / 65 20.0%	0 / 247 0.0%	0 / 175 0.0%	5 / 121 4.1%	4 / 178 2.2%	0 / 62 0.0%	0 / 154 0.0%	0 / 62 0.0%	2 / 217 0.9%	2 / 309 0.6%	0 / 203 0.0%	0 / 62 0.0%	24 / 321 7.5%	0 / 0 0.0%	0 / 0 0.0%	0 / 3 0.0%	0 / 0 0.0%	2170 / 3608 60.1%E	1329 / 3608 36.8%	52 / 3608 1.4%AB	40 / 3608 1.1%ABE	14 / 3608 0.4%CE	3 / 3608 0.1%C
2	Pediococcus	4 / 112 3.6%	0 / 4 0.0%	0 / 6 0.0%	0 / 5 0.0%	0 / 6 0.0%	0 / 8 0.0%	0 / 6 0.0%	0 / 2 0.0%	2 / 2 100.0%	0 / 6 0.0%	2 / 2 100.0%	0 / 8 0.0%	0 / 4 0.0%	0 / 4 0.0%	0 / 4 0.0%	0 / 2 0.0%	0 / 4 0.0%	0 / 2 0.0%	0 / 8 0.0%	0 / 11 0.0%	0 / 6 0.0%	0 / 2 0.0%	0 / 10 0.0%	0 / 0 0.0%	0 / 0 0.0%	0 / 0 0.0%	0 / 0 0.0%	58 / 112 51.8%E	50 / 112 44.6%	4 / 112 3.6%AB			
1	"Pediococcus hennebergii" Sollied 1903	2 / 55 3.6%	0 / 2 0.0%	0 / 3 0.0%	0 / 2 0.0%	0 / 3 0.0%	0 / 4 0.0%	0 / 3 0.0%	0 / 1 0.0%	1 / 1 100.0%	0 / 3 0.0%	1 / 1 100.0%	0 / 4 0.0%	0 / 2 0.0%	0 / 2 0.0%	0 / 2 0.0%	0 / 1 0.0%	0 / 2 0.0%	0 / 1 0.0%	0 / 4 0.0%	0 / 5 0.0%	0 / 3 0.0%	0 / 1 0.0%	0 / 5 0.0%	0 / 0 0.0%	0 / 0 0.0%	0 / 0 0.0%	0 / 0 0.0%	35 / 55 63.6%	18 / 55 32.7%E	2 / 55 3.6%AB			
1	Pediococcus pentosaceus ATCC 25745	2 / 55 3.6%	0 / 2 0.0%	0 / 3 0.0%	0 / 2 0.0%	0 / 3 0.0%	0 / 4 0.0%	0 / 3 0.0%	0 / 1 0.0%	1 / 1 100.0%	0 / 3 0.0%	1 / 1 100.0%	0 / 4 0.0%	0 / 2 0.0%	0 / 2 0.0%	0 / 2 0.0%	0 / 1 0.0%	0 / 2 0.0%	0 / 1 0.0%	0 / 4 0.0%	0 / 5 0.0%	0 / 3 0.0%	0 / 1 0.0%	0 / 5 0.0%	0 / 0 0.0%	0 / 0 0.0%	0 / 0 0.0%	0 / 0 0.0%	35 / 55 63.6%	18 / 55 32.7%E	2 / 55 3.6%AB			
1	ATCC BAA-344	2 / 57 3.5%	0 / 2 0.0%	0 / 3 0.0%	0 / 3 0.0%	0 / 3 0.0%	0 / 4 0.0%	0 / 3 0.0%	0 / 1 0.0%	1 / 1 100.0%	0 / 3 0.0%	1 / 1 100.0%	0 / 4 0.0%	0 / 2 0.0%	0 / 2 0.0%	0 / 2 0.0%	0 / 1 0.0%	0 / 2 0.0%	0 / 1 0.0%	0 / 4 0.0%	0 / 6 0.0%	0 / 3 0.0%	0 / 1 0.0%	0 / 5 0.0%	0 / 0 0.0%	0 / 0 0.0%	0 / 0 0.0%	0 / 0 0.0%	40 / 57 70.2%E	15 / 57 26.3%	2 / 57 3.5%AB			
1	Pediococcus claussenii ATCC BAA-344	2 / 57 3.5%	0 / 2 0.0%	0 / 3 0.0%	0 / 3 0.0%	0 / 3 0.0%	0 / 4 0.0%	0 / 3 0.0%	0 / 1 0.0%	1 / 1 100.0%	0 / 3 0.0%	1 / 1 100.0%	0 / 4 0.0%	0 / 2 0.0%	0 / 2 0.0%	0 / 2 0.0%	0 / 1 0.0%	0 / 2 0.0%	0 / 1 0.0%	0 / 4 0.0%	0 / 6 0.0%	0 / 3 0.0%	0 / 1 0.0%	0 / 5 0.0%	0 / 0 0.0%	0 / 0 0.0%	0 / 0 0.0%	0 / 0 0.0%	40 / 57 70.2%E	15 / 57 26.3%	2 / 57 3.5%AB			
53	Lactobacillus	88 / 3496 2.5%	0 / 115 0.0%	0 / 153 0.0%	2 / 159 1.3%	4 / 164 2.4%	20 / 279 7.2%	0 / 175 0.0%	11 / 82 13.4%	3 / 114 2.6%	0 / 141 0.0%	11 / 63 17.5%	0 / 239 0.0%	0 / 171 0.0%	5 / 117 4.3%	4 / 174 2.3%	0 / 60 0.0%	0 / 150 0.0%	0 / 60 0.0%	2 / 209 1.0%	2 / 298 0.7%	0 / 197 0.0%	0 / 60 0.0%	24 / 311 7.7%	0 / 0 0.0%	0 / 0 0.0%	0 / 3 0.0%	0 / 0 0.0%	2112 / 3496 60.4%E	1279 / 3496 36.6%	48 / 3496 1.4%AB	40 / 3496 1.1%ABE	14 / 3496 0.4%CE	3 / 3496 0.1%C
3	"Bacillus acidophilus" Moro 1900	12 / 184 6.5%	0 / 6 0.0%	0 / 9 0.0%	0 / 9 0.0%	0 / 7 0.0%	6 / 16 37.5%	0 / 9 0.0%	3 / 6 50.0%	0 / 6 0.0%	0 / 4 0.0%	0 / 3 0.0%	0 / 15 0.0%	0 / 9 0.0%	0 / 6 0.0%	0 / 11 0.0%	0 / 3 0.0%	0 / 9 0.0%	0 / 3 0.0%	0 / 11 0.0%	1 / 15 6.7%	0 / 9 0.0%	0 / 3 0.0%	2 / 15 13.3%	0 / 0 0.0%	0 / 0 0.0%	0 / 0 0.0%	0 / 0 0.0%	100 / 184 54.3%E	72 / 184 39.1%	7 / 184 3.8%AB	5 / 184 2.7%ABE		
1	Lactobacillus acidophilus NCFM	4 / 61 6.6%	0 / 2 0.0%	0 / 3 0.0%	0 / 3 0.0%	0 / 2 0.0%	2 / 5 40.0%	0 / 3 0.0%	1 / 2 50.0%	0 / 2 0.0%	0 / 1 0.0%	0 / 1 0.0%	0 / 5 0.0%	0 / 3 0.0%	0 / 2 0.0%	0 / 4 0.0%	0 / 1 0.0%	0 / 3 0.0%	0 / 1 0.0%	0 / 4 0.0%	0 / 5 0.0%	0 / 3 0.0%	0 / 1 0.0%	1 / 5 20.0%	0 / 0 0.0%	0 / 0 0.0%	0 / 0 0.0%	0 / 0 0.0%	39 / 61 63.9%	18 / 61 29.5%E	2 / 61 3.3%ABE	2 / 61 3.3%AB		
1	Lactobacillus acidophilus 30SC	4 / 62 6.5%	0 / 2 0.0%	0 / 3 0.0%	0 / 3 0.0%	0 / 3 0.0%	2 / 6 33.3%	0 / 3 0.0%	1 / 2 50.0%	0 / 2 0.0%	0 / 2 0.0%	0 / 1 0.0%	0 / 5 0.0%	0 / 3 0.0%	0 / 2 0.0%	0 / 3 0.0%	0 / 1 0.0%	0 / 3 0.0%	0 / 1 0.0%	0 / 3 0.0%	1 / 5 20.0%	0 / 3 0.0%	0 / 1 0.0%	0 / 5 0.0%	0 / 0 0.0%	0 / 0 0.0%	0 / 0 0.0%	0 / 0 0.0%	41 / 62 66.1%E	17 / 62 27.4%	3 / 62 4.8%AB	1 / 62 1.6%ABE		
1	Lactobacillus acidophilus La-14	4 / 61 6.6%	0 / 2 0.0%	0 / 3 0.0%	0 / 3 0.0%	0 / 2 0.0%	2 / 5 40.0%	0 / 3 0.0%	1 / 2 50.0%	0 / 2 0.0%	0 / 1 0.0%	0 / 1 0.0%	0 / 5 0.0%	0 / 3 0.0%	0 / 2 0.0%	0 / 4 0.0%	0 / 1 0.0%	0 / 3 0.0%	0 / 1 0.0%	0 / 4 0.0%	0 / 5 0.0%	0 / 3 0.0%	0 / 1 0.0%	1 / 5 20.0%	0 / 0 0.0%	0 / 0 0.0%	0 / 0 0.0%	0 / 0 0.0%	41 / 61 67.2%E	16 / 61 26.2%	2 / 61 3.3%ABE	2 / 61 3.3%AB		
2	"Bacillus casei g" von Freudenreich and Thoni 1904	0 / 126 0.0%	0 / 5 0.0%	0 / 6 0.0%	0 / 4 0.0%	0 / 6 0.0%	0 / 8 0.0%	0 / 6 0.0%	0 / 2 0.0%	0 / 4 0.0%	0 / 6 0.0%	0 / 2 0.0%	0 / 8 0.0%	0 / 4 0.0%	0 / 4 0.0%	0 / 8 0.0%	0 / 2 0.0%	0 / 6 0.0%	0 / 2 0.0%	0 / 8 0.0%	0 / 14 0.0%	0 / 8 0.0%	0 / 3 0.0%	0 / 10 0.0%	0 / 0 0.0%	0 / 0 0.0%	0 / 0 0.0%	0 / 0 0.0%	69 / 126 54.8%E	57 / 126 45.2%				
1	Lactobacillus brevis ATCC 367	0 / 63 0.0%	0 / 3 0.0%	0 / 3 0.0%	0 / 2 0.0%	0 / 3 0.0%	0 / 4 0.0%	0 / 3 0.0%	0 / 1 0.0%	0 / 2 0.0%	0 / 3 0.0%	0 / 1 0.0%	0 / 4 0.0%	0 / 2 0.0%	0 / 2 0.0%	0 / 4 0.0%	0 / 1 0.0%	0 / 3 0.0%	0 / 1 0.0%	0 / 4 0.0%	0 / 7 0.0%	0 / 4 0.0%	0 / 1 0.0%	0 / 5 0.0%	0 / 0 0.0%	0 / 0 0.0%	0 / 0 0.0%	0 / 0 0.0%	44 / 63 69.8%	19 / 63 30.2%E				
1	Lactobacillus brevis KB290	0 / 63 0.0%	0 / 2 0.0%	0 / 3 0.0%	0 / 2 0.0%	0 / 3 0.0%	0 / 4 0.0%	0 / 3 0.0%	0 / 1 0.0%	0 / 2 0.0%	0 / 3 0.0%	0 / 1 0.0%	0 / 4 0.0%	0 / 2 0.0%	0 / 2 0.0%	0 / 4 0.0%	0 / 1 0.0%	0 / 3 0.0%	0 / 1 0.0%	0 / 4 0.0%	0 / 7 0.0%	0 / 4 0.0%	0 / 2 0.0%	0 / 5 0.0%	0 / 0 0.0%	0 / 0 0.0%	0 / 0 0.0%	0 / 0 0.0%	50 / 63 79.4%E	13 / 63 20.6%				
2	"Bacillus Buchneri" (sic) Henneberg 1903	4 / 124 3.2%	0 / 4 0.0%	0 / 6 0.0%	0 / 8 0.0%	4 / 6 66.7%	0 / 8 0.0%	0 / 6 0.0%	0 / 2 0.0%	0 / 4 0.0%	0 / 4 0.0%	0 / 2 0.0%	0 / 8 0.0%	0 / 6 0.0%	0 / 4 0.0%	0 / 7 0.0%	0 / 2 0.0%	0 / 6 0.0%	0 / 2 0.0%	0 / 8 0.0%	0 / 12 0.0%	0 / 7 0.0%	0 / 2 0.0%	0 / 10 0.0%	0 / 0 0.0%	0 / 0 0.0%	0 / 0 0.0%	0 / 0 0.0%	95 / 124 76.6%E	25 / 124 20.2%	4 / 124 3.2%ABE			
1	Lactobacillus buchneri NRRL B-30929	2 / 63 3.2%	0 / 2 0.0%	0 / 3 0.0%	0 / 4 0.0%	2 / 3 66.7%	0 / 4 0.0%	0 / 3 0.0%	0 / 1 0.0%	0 / 2 0.0%	0 / 2 0.0%	0 / 1 0.0%	0 / 4 0.0%	0 / 3 0.0%	0 / 2 0.0%	0 / 4 0.0%	0 / 1 0.0%	0 / 3 0.0%	0 / 1 0.0%	0 / 4 0.0%	0 / 6 0.0%	0 / 4 0.0%	0 / 1 0.0%	0 / 5 0.0%	0 / 0 0.0%	0 / 0 0.0%	0 / 0 0.0%	0 / 0 0.0%	48 / 63 76.2%E	13 / 63 20.6%	2 / 63 3.2%ABE			
1	Lactobacillus buchneri CD034	2 / 61 3.3%	0 / 2 0.0%	0 / 3 0.0%	0 / 4 0.0%	2 / 3 66.7%	0 / 4 0.0%	0 / 3 0.0%	0 / 1 0.0%	0 / 2 0.0%	0 / 2 0.0%	0 / 1 0.0%	0 / 4 0.0%	0 / 3 0.0%	0 / 2 0.0%	0 / 3 0.0%	0 / 1 0.0%	0 / 3 0.0%	0 / 1 0.0%	0 / 4 0.0%	0 / 6 0.0%	0 / 3 0.0%	0 / 1 0.0%	0 / 5 0.0%	0 / 0 0.0%	0 / 0 0.0%	0 / 0 0.0%	0 / 0 0.0%	47 / 61 77.0%E	12 / 61 19.7%	2 / 61 3.3%ABE			
4	"Bacillus Delbrucki" (sic) Leichmann 1896	4 / 380 1.1%	0 / 12 0.0%	0 / 16 0.0%	0 / 12 0.0%	0 / 20 0.0%	0 / 32 0.0%	0 / 24 0.0%	0 / 8 0.0%	0 / 12 0.0%	0 / 16 0.0%	0 / 8 0.0%	0 / 24 0.0%	0 / 24 0.0%	0 / 12 0.0%	0 / 16 0.0%	0 / 8 0.0%	0 / 16 0.0%	0 / 8 0.0%	0 / 20 0.0%	0 / 28 0.0%	0 / 24 0.0%	0 / 4 0.0%	4 / 36 11.1%	0 / 0 0.0%	0 / 0 0.0%	0 / 0 0.0%	0 / 0 0.0%	187 / 380 49.2%	186 / 380 48.9%E	4 / 380 1.1%ABE	3 / 380 0.8%C		
4	"Thermobacterium bulgaricum" Orla-Jensen 1919	4 / 380 1.1%	0 / 12 0.0%	0 / 16 0.0%	0 / 12 0.0%	0 / 20 0.0%	0 / 32 0.0%	0 / 24 0.0%	0 / 8 0.0%	0 / 12 0.0%	0 / 16 0.0%	0 / 8 0.0%	0 / 24 0.0%	0 / 24 0.0%	0 / 12 0.0%	0 / 16 0.0%	0 / 8 0.0%	0 / 16 0.0%	0 / 8 0.0%	0 / 20 0.0%	0 / 28 0.0%	0 / 24 0.0%	0 / 4 0.0%	4 / 36 11.1%	0 / 0 0.0%	0 / 0 0.0%	0 / 0 0.0%	0 / 0 0.0%	187 / 380 49.2%	186 / 380 48.9%E	4 / 380 1.1%ABE	3 / 380 0.8%C		
1	Lactobacillus delbrueckii subsp. bulgaricus ATCC BAA-365	1 / 98 1.0%	0 / 3 0.0%	0 / 4 0.0%	0 / 3 0.0%	0 / 6 0.0%	0 / 8 0.0%	0 / 6 0.0%	0 / 2 0.0%	0 / 3 0.0%	0 / 5 0.0%	0 / 2 0.0%	0 / 6 0.0%	0 / 7 0.0%	0 / 3 0.0%	0 / 4 0.0%	0 / 2 0.0%	0 / 4 0.0%	0 / 2 0.0%	0 / 5 0.0%	0 / 7 0.0%	0 / 6 0.0%	0 / 1 0.0%	1 / 9 11.1%	0 / 0 0.0%	0 / 0 0.0%	0 / 0 0.0%	0 / 0 0.0%	68 / 98 69.4%	28 / 98 28.6%E	1 / 98 1.0%C	1 / 98 1.0%ABE		
1	Lactobacillus delbrueckii subsp. bulgaricus 2038	1 / 89 1.1%	0 / 3 0.0%	0 / 4 0.0%	0 / 3 0.0%	0 / 2 0.0%	0 / 8 0.0%	0 / 6 0.0%	0 / 2 0.0%	0 / 3 0.0%	0 / 1 0.0%	0 / 2 0.0%	0 / 6 0.0%	0 / 6 0.0%	0 / 3 0.0%	0 / 4 0.0%	0 / 2 0.0%	0 / 4 0.0%	0 / 2 0.0%	0 / 5 0.0%	0 / 7 0.0%	0 / 6 0.0%	0 / 1 0.0%	1 / 9 11.1%	0 / 0 0.0%	0 / 0 0.0%	0 / 0 0.0%	0 / 0 0.0%	63 / 89 70.8%E	24 / 89 27.0%	1 / 89 1.1%ABE	1 / 89 1.1%C		
1	Lactobacillus delbrueckii subsp. bulgaricus ATCC 11842	1 / 95 1.1%	0 / 3 0.0%	0 / 4 0.0%	0 / 3 0.0%	0 / 5 0.0%	0 / 8 0.0%	0 / 6 0.0%	0 / 2 0.0%	0 / 3 0.0%	0 / 4 0.0%	0 / 2 0.0%	0 / 6 0.0%	0 / 6 0.0%	0 / 3 0.0%	0 / 4 0.0%	0 / 2 0.0%	0 / 4 0.0%	0 / 2 0.0%	0 / 5 0.0%	0 / 7 0.0%	0 / 6 0.0%	0 / 1 0.0%	1 / 9 11.1%	0 / 0 0.0%	0 / 0 0.0%	0 / 0 0.0%	0 / 0 0.0%	65 / 95 68.4%	28 / 95 29.5%E	1 / 95 1.1%C	1 / 95 1.1%ABE		
1	Lactobacillus delbrueckii subsp. bulgaricus ND02	1 / 98 1.0%	0 / 3 0.0%	0 / 4 0.0%	0 / 3 0.0%	0 / 7 0.0%	0 / 8 0.0%	0 / 6 0.0%	0 / 2 0.0%	0 / 3 0.0%	0 / 6 0.0%	0 / 2 0.0%	0 / 6 0.0%	0 / 5 0.0%	0 / 3 0.0%	0 / 4 0.0%	0 / 2 0.0%	0 / 4 0.0%	0 / 2 0.0%	0 / 5 0.0%	0 / 7 0.0%	0 / 6 0.0%	0 / 1 0.0%	1 / 9 11.1%	0 / 0 0.0%	0 / 0 0.0%	0 / 0 0.0%	0 / 0 0.0%	67 / 98 68.4%E	30 / 98 30.6%	1 / 98 1.0%ABE			
4	"Bacillus casei e" von Freudenreich and Thoni 1904	10 / 248 4.0%	0 / 8 0.0%	0 / 12 0.0%	0 / 12 0.0%	0 / 10 0.0%	4 / 20 20.0%	0 / 12 0.0%	4 / 8 50.0%	0 / 8 0.0%	0 / 6 0.0%	0 / 4 0.0%	0 / 20 0.0%	0 / 12 0.0%	0 / 8 0.0%	0 / 16 0.0%	0 / 4 0.0%	0 / 12 0.0%	0 / 4 0.0%	0 / 16 0.0%	0 / 20 0.0%	0 / 12 0.0%	0 / 4 0.0%	2 / 20 10.0%	0 / 0 0.0%	0 / 0 0.0%	0 / 0 0.0%	0 / 0 0.0%	172 / 248 69.4%E	66 / 248 26.6%	6 / 248 2.4%ABE	4 / 248 1.6%AB		
1	Lactobacillus helveticus CNRZ32	2 / 63 3.2%	0 / 2 0.0%	0 / 3 0.0%	0 / 3 0.0%	0 / 3 0.0%	1 / 5 20.0%	0 / 3 0.0%	1 / 2 50.0%	0 / 2 0.0%	0 / 2 0.0%	0 / 1 0.0%	0 / 5 0.0%	0 / 3 0.0%	0 / 2 0.0%	0 / 4 0.0%	0 / 1 0.0%	0 / 3 0.0%	0 / 1 0.0%	0 / 4 0.0%	0 / 5 0.0%	0 / 3 0.0%	0 / 1 0.0%	0 / 5 0.0%	0 / 0 0.0%	0 / 0 0.0%	0 / 0 0.0%	0 / 0 0.0%	44 / 63 69.8%E	17 / 63 27.0%	1 / 63 1.6%ABE	1 / 63 1.6%AB		
1	Lactobacillus helveticus DPC 4571	2 / 61 3.3%	0 / 2 0.0%	0 / 3 0.0%	0 / 3 0.0%	0 / 2 0.0%	1 / 5 20.0%	0 / 3 0.0%	1 / 2 50.0%	0 / 2 0.0%	0 / 1 0.0%	0 / 1 0.0%	0 / 5 0.0%	0 / 3 0.0%	0 / 2 0.0%	0 / 4 0.0%	0 / 1 0.0%	0 / 3 0.0%	0 / 1 0.0%	0 / 4 0.0%	0 / 5 0.0%	0 / 3 0.0%	0 / 1 0.0%	0 / 5 0.0%	0 / 0 0.0%	0 / 0 0.0%	0 / 0 0.0%	0 / 0 0.0%	43 / 61 70.5%E	16 / 61 26.2%	1 / 61 1.6%AB	1 / 61 1.6%ABE		
1	Lactobacillus helveticus H10	3 / 63 4.8%	0 / 2 0.0%	0 / 3 0.0%	0 / 3 0.0%	0 / 3 0.0%	1 / 5 20.0%	0 / 3 0.0%	1 / 2 50.0%	0 / 2 0.0%	0 / 2 0.0%	0 / 1 0.0%	0 / 5 0.0%	0 / 3 0.0%	0 / 2 0.0%	0 / 4 0.0%	0 / 1 0.0%	0 / 3 0.0%	0 / 1 0.0%	0 / 4 0.0%	0 / 5 0.0%	0 / 3 0.0%	0 / 1 0.0%	1 / 5 20.0%	0 / 0 0.0%	0 / 0 0.0%	0 / 0 0.0%	0 / 0 0.0%	43 / 63 68.3%E	17 / 63 27.0%	2 / 63 3.2%ABE	1 / 63 1.6%AB		
1	Lactobacillus helveticus R0052	3 / 61 4.9%	0 / 2 0.0%	0 / 3 0.0%	0 / 3 0.0%	0 / 2 0.0%	1 / 5 20.0%	0 / 3 0.0%	1 / 2 50.0%	0 / 2 0.0%	0 / 1 0.0%	0 / 1 0.0%	0 / 5 0.0%	0 / 3 0.0%	0 / 2 0.0%	0 / 4 0.0%	0 / 1 0.0%	0 / 3 0.0%	0 / 1 0.0%	0 / 4 0.0%	0 / 5 0.0%	0 / 3 0.0%	0 / 1 0.0%	1 / 5 20.0%	0 / 0 0.0%	0 / 0 0.0%	0 / 0 0.0%	0 / 0 0.0%	42 / 61 68.9%E	16 / 61 26.2%	2 / 61 3.3%ABE	1 / 61 1.6%AB		
6	"Lactobacillus arabinosus" Fred et al.	6 / 375 1.6%	0 / 12 0.0%	0 / 18 0.0%	0 / 25 0.0%	0 / 14 0.0%	0 / 30 0.0%	0 / 14 0.0%	0 / 12 0.0%	0 / 12 0.0%	0 / 14 0.0%	6 / 9 66.7%	0 / 24 0.0%	0 / 15 0.0%	0 / 12 0.0%	0 / 18 0.0%	0 / 6 0.0%	0 / 18 0.0%	0 / 6 0.0%	0 / 24 0.0%	0 / 30 0.0%	0 / 18 0.0%	0 / 8 0.0%	0 / 36 0.0%	0 / 0 0.0%	0 / 0 0.0%	0 / 0 0.0%	0 / 0 0.0%	257 / 375 68.5%E	112 / 375 29.9%	6 / 375 1.6%AB			
1	Lactobacillus plantarum WCFS1	1 / 68 1.5%	0 / 2 0.0%	0 / 3 0.0%	0 / 5 0.0%	0 / 3 0.0%	0 / 6 0.0%	0 / 3 0.0%	0 / 2 0.0%	0 / 2 0.0%	0 / 3 0.0%	1 / 2 50.0%	0 / 4 0.0%	0 / 3 0.0%	0 / 2 0.0%	0 / 3 0.0%	0 / 1 0.0%	0 / 3 0.0%	0 / 1 0.0%	0 / 4 0.0%	0 / 5 0.0%	0 / 3 0.0%	0 / 2 0.0%	0 / 6 0.0%	0 / 0 0.0%	0 / 0 0.0%	0 / 0 0.0%	0 / 0 0.0%	46 / 68 67.6%	21 / 68 30.9%E	1 / 68 1.5%AB			
2	Lactobacillus plantarum subsp. plantarum	2 / 127 1.6%	0 / 4 0.0%	0 / 6 0.0%	0 / 8 0.0%	0 / 6 0.0%	0 / 11 0.0%	0 / 4 0.0%	0 / 4 0.0%	0 / 4 0.0%	0 / 6 0.0%	2 / 3 66.7%	0 / 8 0.0%	0 / 4 0.0%	0 / 4 0.0%	0 / 6 0.0%	0 / 2 0.0%	0 / 6 0.0%	0 / 2 0.0%	0 / 8 0.0%	0 / 10 0.0%	0 / 6 0.0%	0 / 3 0.0%	0 / 12 0.0%	0 / 0 0.0%	0 / 0 0.0%	0 / 0 0.0%	0 / 0 0.0%	96 / 127 75.6%E	29 / 127 22.8%	2 / 127 1.6%AB			
1	Lactobacillus plantarum subsp. plantarum P-8	1 / 64 1.6%	0 / 2 0.0%	0 / 3 0.0%	0 / 4 0.0%	0 / 3 0.0%	0 / 5 0.0%	0 / 2 0.0%	0 / 2 0.0%	0 / 2 0.0%	0 / 3 0.0%	1 / 2 50.0%	0 / 4 0.0%	0 / 2 0.0%	0 / 2 0.0%	0 / 3 0.0%	0 / 1 0.0%	0 / 3 0.0%	0 / 1 0.0%	0 / 4 0.0%	0 / 5 0.0%	0 / 3 0.0%	0 / 2 0.0%	0 / 6 0.0%	0 / 0 0.0%	0 / 0 0.0%	0 / 0 0.0%	0 / 0 0.0%	49 / 64 76.6%E	14 / 64 21.9%	1 / 64 1.6%AB			
1	Lactobacillus plantarum subsp. plantarum ST-III	1 / 63 1.6%	0 / 2 0.0%	0 / 3 0.0%	0 / 4 0.0%	0 / 3 0.0%	0 / 6 0.0%	0 / 2 0.0%	0 / 2 0.0%	0 / 2 0.0%	0 / 3 0.0%	1 / 1 100.0%	0 / 4 0.0%	0 / 2 0.0%	0 / 2 0.0%	0 / 3 0.0%	0 / 1 0.0%	0 / 3 0.0%	0 / 1 0.0%	0 / 4 0.0%	0 / 5 0.0%	0 / 3 0.0%	0 / 1 0.0%	0 / 6 0.0%	0 / 0 0.0%	0 / 0 0.0%	0 / 0 0.0%	0 / 0 0.0%	47 / 63 74.6%E	15 / 63 23.8%	1 / 63 1.6%AB			
1	Lactobacillus plantarum JDM1	1 / 59 1.7%	0 / 2 0.0%	0 / 3 0.0%	0 / 4 0.0%	0 / 1 0.0%	0 / 4 0.0%	0 / 3 0.0%	0 / 2 0.0%	0 / 2 0.0%	0 / 1 0.0%	1 / 1 100.0%	0 / 4 0.0%	0 / 3 0.0%	0 / 2 0.0%	0 / 3 0.0%	0 / 1 0.0%	0 / 3 0.0%	0 / 1 0.0%	0 / 4 0.0%	0 / 5 0.0%	0 / 3 0.0%	0 / 1 0.0%	0 / 6 0.0%	0 / 0 0.0%	0 / 0 0.0%	0 / 0 0.0%	0 / 0 0.0%	46 / 59 78.0%E	12 / 59 20.3%	1 / 59 1.7%AB			
1	Lactobacillus plantarum ZJ316	1 / 58 1.7%	0 / 2 0.0%	0 / 3 0.0%	0 / 4 0.0%	0 / 1 0.0%	0 / 5 0.0%	0 / 2 0.0%	0 / 2 0.0%	0 / 2 0.0%	0 / 1 0.0%	1 / 1 100.0%	0 / 4 0.0%	0 / 2 0.0%	0 / 2 0.0%	0 / 3 0.0%	0 / 1 0.0%	0 / 3 0.0%	0 / 1 0.0%	0 / 4 0.0%	0 / 5 0.0%	0 / 3 0.0%	0 / 1 0.0%	0 / 6 0.0%	0 / 0 0.0%	0 / 0 0.0%	0 / 0 0.0%	0 / 0 0.0%	44 / 58 75.9%E	13 / 58 22.4%	1 / 58 1.7%AB			
1	Lactobacillus plantarum 16	1 / 63 1.6%	0 / 2 0.0%	0 / 3 0.0%	0 / 4 0.0%	0 / 3 0.0%	0 / 4 0.0%	0 / 2 0.0%	0 / 2 0.0%	0 / 2 0.0%	0 / 3 0.0%	1 / 2 50.0%	0 / 4 0.0%	0 / 3 0.0%	0 / 2 0.0%	0 / 3 0.0%	0 / 1 0.0%	0 / 3 0.0%	0 / 1 0.0%	0 / 4 0.0%	0 / 5 0.0%	0 / 3 0.0%	0 / 1 0.0%	0 / 6 0.0%	0 / 0 0.0%	0 / 0 0.0%	0 / 0 0.0%	0 / 0 0.0%	50 / 63 79.4%E	12 / 63 19.0%	1 / 63 1.6%AB			
1	ATCC 33323	5 / 76 6.6%	0 / 3 0.0%	0 / 3 0.0%	1 / 4 25.0%	0 / 4 0.0%	1 / 6 16.7%	0 / 3 0.0%	0 / 1 0.0%	0 / 2 0.0%	0 / 2 0.0%	0 / 1 0.0%	0 / 5 0.0%	0 / 4 0.0%	0 / 3 0.0%	0 / 6 0.0%	0 / 1 0.0%	0 / 3 0.0%	0 / 2 0.0%	0 / 6 0.0%	0 / 5 0.0%	0 / 3 0.0%	0 / 2 0.0%	3 / 7 42.9%	0 / 0 0.0%	0 / 0 0.0%	0 / 0 0.0%	0 / 0 0.0%	52 / 76 68.4%	19 / 76 25.0%E	3 / 76 3.9%ABE	2 / 76 2.6%AB		
1	Lactobacillus gasseri ATCC 33323	5 / 76 6.6%	0 / 3 0.0%	0 / 3 0.0%	1 / 4 25.0%	0 / 4 0.0%	1 / 6 16.7%	0 / 3 0.0%	0 / 1 0.0%	0 / 2 0.0%	0 / 2 0.0%	0 / 1 0.0%	0 / 5 0.0%	0 / 4 0.0%	0 / 3 0.0%	0 / 6 0.0%	0 / 1 0.0%	0 / 3 0.0%	0 / 2 0.0%	0 / 6 0.0%	0 / 5 0.0%	0 / 3 0.0%	0 / 2 0.0%	3 / 7 42.9%	0 / 0 0.0%	0 / 0 0.0%	0 / 0 0.0%	0 / 0 0.0%	52 / 76 68.4%	19 / 76 25.0%E	3 / 76 3.9%ABE	2 / 76 2.6%AB		
4	ATCC 23272	0 / 271 0.0%	0 / 8 0.0%	0 / 12 0.0%	0 / 8 0.0%	0 / 14 0.0%	0 / 24 0.0%	0 / 16 0.0%	0 / 8 0.0%	0 / 8 0.0%	0 / 10 0.0%	0 / 4 0.0%	0 / 16 0.0%	0 / 12 0.0%	0 / 8 0.0%	0 / 12 0.0%	0 / 4 0.0%	0 / 16 0.0%	0 / 4 0.0%	0 / 16 0.0%	0 / 23 0.0%	0 / 20 0.0%	0 / 4 0.0%	0 / 24 0.0%	0 / 0 0.0%	0 / 0 0.0%	0 / 0 0.0%	0 / 0 0.0%	157 / 271 57.9%E	114 / 271 42.1%				
1	Lactobacillus reuteri F 275	0 / 68 0.0%	0 / 2 0.0%	0 / 3 0.0%	0 / 2 0.0%	0 / 4 0.0%	0 / 6 0.0%	0 / 4 0.0%	0 / 2 0.0%	0 / 2 0.0%	0 / 3 0.0%	0 / 1 0.0%	0 / 4 0.0%	0 / 3 0.0%	0 / 2 0.0%	0 / 3 0.0%	0 / 1 0.0%	0 / 4 0.0%	0 / 1 0.0%	0 / 4 0.0%	0 / 5 0.0%	0 / 5 0.0%	0 / 1 0.0%	0 / 6 0.0%	0 / 0 0.0%	0 / 0 0.0%	0 / 0 0.0%	0 / 0 0.0%	54 / 68 79.4%	14 / 68 20.6%E				
1	Lactobacillus reuteri SD2112	0 / 69 0.0%	0 / 2 0.0%	0 / 3 0.0%	0 / 2 0.0%	0 / 4 0.0%	0 / 6 0.0%	0 / 4 0.0%	0 / 2 0.0%	0 / 2 0.0%	0 / 3 0.0%	0 / 1 0.0%	0 / 4 0.0%	0 / 3 0.0%	0 / 2 0.0%	0 / 3 0.0%	0 / 1 0.0%	0 / 4 0.0%	0 / 1 0.0%	0 / 4 0.0%	0 / 6 0.0%	0 / 5 0.0%	0 / 1 0.0%	0 / 6 0.0%	0 / 0 0.0%	0 / 0 0.0%	0 / 0 0.0%	0 / 0 0.0%	50 / 69 72.5%E	19 / 69 27.5%				
1	Lactobacillus reuteri JCM 1112	0 / 65 0.0%	0 / 2 0.0%	0 / 3 0.0%	0 / 2 0.0%	0 / 2 0.0%	0 / 6 0.0%	0 / 4 0.0%	0 / 2 0.0%	0 / 2 0.0%	0 / 1 0.0%	0 / 1 0.0%	0 / 4 0.0%	0 / 3 0.0%	0 / 2 0.0%	0 / 3 0.0%	0 / 1 0.0%	0 / 4 0.0%	0 / 1 0.0%	0 / 4 0.0%	0 / 6 0.0%	0 / 5 0.0%	0 / 1 0.0%	0 / 6 0.0%	0 / 0 0.0%	0 / 0 0.0%	0 / 0 0.0%	0 / 0 0.0%	45 / 65 69.2%E	20 / 65 30.8%				
1	Lactobacillus reuteri I5007	0 / 69 0.0%	0 / 2 0.0%	0 / 3 0.0%	0 / 2 0.0%	0 / 4 0.0%	0 / 6 0.0%	0 / 4 0.0%	0 / 2 0.0%	0 / 2 0.0%	0 / 3 0.0%	0 / 1 0.0%	0 / 4 0.0%	0 / 3 0.0%	0 / 2 0.0%	0 / 3 0.0%	0 / 1 0.0%	0 / 4 0.0%	0 / 1 0.0%	0 / 4 0.0%	0 / 6 0.0%	0 / 5 0.0%	0 / 1 0.0%	0 / 6 0.0%	0 / 0 0.0%	0 / 0 0.0%	0 / 0 0.0%	0 / 0 0.0%	48 / 69 69.6%E	21 / 69 30.4%				
1	ATCC 15521	0 / 63 0.0%	0 / 2 0.0%	0 / 3 0.0%	0 / 2 0.0%	0 / 2 0.0%	0 / 5 0.0%	0 / 3 0.0%	0 / 1 0.0%	0 / 2 0.0%	0 / 2 0.0%	0 / 1 0.0%	0 / 5 0.0%	0 / 3 0.0%	0 / 2 0.0%	0 / 4 0.0%	0 / 1 0.0%	0 / 3 0.0%	0 / 1 0.0%	0 / 4 0.0%	0 / 5 0.0%	0 / 4 0.0%	0 / 1 0.0%	0 / 7 0.0%	0 / 0 0.0%	0 / 0 0.0%	0 / 0 0.0%	0 / 0 0.0%	39 / 63 61.9%	24 / 63 38.1%E				
1	Lactobacillus sakei subsp. sakei	0 / 63 0.0%	0 / 2 0.0%	0 / 3 0.0%	0 / 2 0.0%	0 / 2 0.0%	0 / 5 0.0%	0 / 3 0.0%	0 / 1 0.0%	0 / 2 0.0%	0 / 2 0.0%	0 / 1 0.0%	0 / 5 0.0%	0 / 3 0.0%	0 / 2 0.0%	0 / 4 0.0%	0 / 1 0.0%	0 / 3 0.0%	0 / 1 0.0%	0 / 4 0.0%	0 / 5 0.0%	0 / 4 0.0%	0 / 1 0.0%	0 / 7 0.0%	0 / 0 0.0%	0 / 0 0.0%	0 / 0 0.0%	0 / 0 0.0%	39 / 63 61.9%	24 / 63 38.1%E				
1	Lactobacillus sakei str. 23K	0 / 63 0.0%	0 / 2 0.0%	0 / 3 0.0%	0 / 2 0.0%	0 / 2 0.0%	0 / 5 0.0%	0 / 3 0.0%	0 / 1 0.0%	0 / 2 0.0%	0 / 2 0.0%	0 / 1 0.0%	0 / 5 0.0%	0 / 3 0.0%	0 / 2 0.0%	0 / 4 0.0%	0 / 1 0.0%	0 / 3 0.0%	0 / 1 0.0%	0 / 4 0.0%	0 / 5 0.0%	0 / 4 0.0%	0 / 1 0.0%	0 / 7 0.0%	0 / 0 0.0%	0 / 0 0.0%	0 / 0 0.0%	0 / 0 0.0%	39 / 63 61.9%	24 / 63 38.1%E				
2	"Lactobacillus sobrius" Konstantinov et al. 2005	6 / 122 4.9%	0 / 4 0.0%	0 / 6 0.0%	0 / 6 0.0%	0 / 4 0.0%	3 / 10 30.0%	0 / 6 0.0%	2 / 4 50.0%	0 / 4 0.0%	0 / 2 0.0%	0 / 2 0.0%	0 / 10 0.0%	0 / 6 0.0%	0 / 4 0.0%	0 / 8 0.0%	0 / 2 0.0%	0 / 6 0.0%	0 / 2 0.0%	0 / 8 0.0%	1 / 10 10.0%	0 / 6 0.0%	0 / 2 0.0%	0 / 10 0.0%	0 / 0 0.0%	0 / 0 0.0%	0 / 0 0.0%	0 / 0 0.0%	81 / 122 66.4%E	35 / 122 28.7%	4 / 122 3.3%AB	2 / 122 1.6%ABE		
1	Lactobacillus amylovorus GRL 1112	2 / 60 3.3%	0 / 2 0.0%	0 / 3 0.0%	0 / 3 0.0%	0 / 2 0.0%	1 / 4 25.0%	0 / 3 0.0%	1 / 2 50.0%	0 / 2 0.0%	0 / 1 0.0%	0 / 1 0.0%	0 / 5 0.0%	0 / 3 0.0%	0 / 2 0.0%	0 / 4 0.0%	0 / 1 0.0%	0 / 3 0.0%	0 / 1 0.0%	0 / 4 0.0%	0 / 5 0.0%	0 / 3 0.0%	0 / 1 0.0%	0 / 5 0.0%	0 / 0 0.0%	0 / 0 0.0%	0 / 0 0.0%	0 / 0 0.0%	41 / 60 68.3%E	17 / 60 28.3%	1 / 60 1.7%ABE	1 / 60 1.7%AB		
1	Lactobacillus amylovorus GRL 1118	4 / 62 6.5%	0 / 2 0.0%	0 / 3 0.0%	0 / 3 0.0%	0 / 2 0.0%	2 / 6 33.3%	0 / 3 0.0%	1 / 2 50.0%	0 / 2 0.0%	0 / 1 0.0%	0 / 1 0.0%	0 / 5 0.0%	0 / 3 0.0%	0 / 2 0.0%	0 / 4 0.0%	0 / 1 0.0%	0 / 3 0.0%	0 / 1 0.0%	0 / 4 0.0%	1 / 5 20.0%	0 / 3 0.0%	0 / 1 0.0%	0 / 5 0.0%	0 / 0 0.0%	0 / 0 0.0%	0 / 0 0.0%	0 / 0 0.0%	40 / 62 64.5%E	18 / 62 29.0%	3 / 62 4.8%AB	1 / 62 1.6%ABE		
3	"Bacillus casei d" von Freudenreich and Thoni 1904	3 / 168 1.8%	0 / 6 0.0%	0 / 9 0.0%	0 / 3 0.0%	0 / 9 0.0%	0 / 12 0.0%	0 / 9 0.0%	0 / 3 0.0%	0 / 6 0.0%	0 / 6 0.0%	0 / 3 0.0%	0 / 11 0.0%	0 / 7 0.0%	0 / 6 0.0%	0 / 9 0.0%	0 / 3 0.0%	0 / 6 0.0%	0 / 3 0.0%	0 / 9 0.0%	0 / 15 0.0%	0 / 12 0.0%	0 / 3 0.0%	3 / 18 16.7%	0 / 0 0.0%	0 / 0 0.0%	0 / 0 0.0%	0 / 0 0.0%	114 / 168 67.9%E	51 / 168 30.4%	3 / 168 1.8%ABE			
1	Lactobacillus fermentum IFO 3956	1 / 56 1.8%	0 / 2 0.0%	0 / 3 0.0%	0 / 1 0.0%	0 / 3 0.0%	0 / 4 0.0%	0 / 3 0.0%	0 / 1 0.0%	0 / 2 0.0%	0 / 2 0.0%	0 / 1 0.0%	0 / 4 0.0%	0 / 2 0.0%	0 / 2 0.0%	0 / 3 0.0%	0 / 1 0.0%	0 / 2 0.0%	0 / 1 0.0%	0 / 3 0.0%	0 / 5 0.0%	0 / 4 0.0%	0 / 1 0.0%	1 / 6 16.7%	0 / 0 0.0%	0 / 0 0.0%	0 / 0 0.0%	0 / 0 0.0%	38 / 56 67.9%E	17 / 56 30.4%	1 / 56 1.8%ABE			
1	Lactobacillus fermentum CECT 5716	1 / 55 1.8%	0 / 2 0.0%	0 / 3 0.0%	0 / 1 0.0%	0 / 3 0.0%	0 / 4 0.0%	0 / 3 0.0%	0 / 1 0.0%	0 / 2 0.0%	0 / 2 0.0%	0 / 1 0.0%	0 / 3 0.0%	0 / 2 0.0%	0 / 2 0.0%	0 / 3 0.0%	0 / 1 0.0%	0 / 2 0.0%	0 / 1 0.0%	0 / 3 0.0%	0 / 5 0.0%	0 / 4 0.0%	0 / 1 0.0%	1 / 6 16.7%	0 / 0 0.0%	0 / 0 0.0%	0 / 0 0.0%	0 / 0 0.0%	37 / 55 67.3%E	17 / 55 30.9%	1 / 55 1.8%ABE			
1	Lactobacillus fermentum F-6	1 / 57 1.8%	0 / 2 0.0%	0 / 3 0.0%	0 / 1 0.0%	0 / 3 0.0%	0 / 4 0.0%	0 / 3 0.0%	0 / 1 0.0%	0 / 2 0.0%	0 / 2 0.0%	0 / 1 0.0%	0 / 4 0.0%	0 / 3 0.0%	0 / 2 0.0%	0 / 3 0.0%	0 / 1 0.0%	0 / 2 0.0%	0 / 1 0.0%	0 / 3 0.0%	0 / 5 0.0%	0 / 4 0.0%	0 / 1 0.0%	1 / 6 16.7%	0 / 0 0.0%	0 / 0 0.0%	0 / 0 0.0%	0 / 0 0.0%	39 / 57 68.4%E	17 / 57 29.8%	1 / 57 1.8%ABE			
1	ATCC 27780	6 / 66 9.1%	0 / 2 0.0%	0 / 3 0.0%	0 / 2 0.0%	0 / 3 0.0%	0 / 5 0.0%	0 / 4 0.0%	0 / 2 0.0%	1 / 2 50.0%	0 / 2 0.0%	1 / 1 100.0%	0 / 4 0.0%	0 / 4 0.0%	1 / 2 50.0%	0 / 3 0.0%	0 / 1 0.0%	0 / 3 0.0%	0 / 1 0.0%	2 / 4 50.0%	0 / 5 0.0%	0 / 4 0.0%	0 / 2 0.0%	1 / 7 14.3%	0 / 0 0.0%	0 / 0 0.0%	0 / 0 0.0%	0 / 0 0.0%	45 / 66 68.2%E	14 / 66 21.2%	4 / 66 6.1%AB	2 / 66 3.0%ABE	1 / 66 1.5%CE	
1	Lactobacillus ruminis ATCC 27782	6 / 66 9.1%	0 / 2 0.0%	0 / 3 0.0%	0 / 2 0.0%	0 / 3 0.0%	0 / 5 0.0%	0 / 4 0.0%	0 / 2 0.0%	1 / 2 50.0%	0 / 2 0.0%	1 / 1 100.0%	0 / 4 0.0%	0 / 4 0.0%	1 / 2 50.0%	0 / 3 0.0%	0 / 1 0.0%	0 / 3 0.0%	0 / 1 0.0%	2 / 4 50.0%	0 / 5 0.0%	0 / 4 0.0%	0 / 2 0.0%	1 / 7 14.3%	0 / 0 0.0%	0 / 0 0.0%	0 / 0 0.0%	0 / 0 0.0%	45 / 66 68.2%E	14 / 66 21.2%	4 / 66 6.1%AB	2 / 66 3.0%ABE	1 / 66 1.5%CE	
2	ATCC 11741	12 / 153 7.8%	0 / 4 0.0%	0 / 8 0.0%	0 / 6 0.0%	0 / 8 0.0%	0 / 10 0.0%	0 / 8 0.0%	0 / 4 0.0%	2 / 6 33.3%	0 / 6 0.0%	4 / 4 100.0%	0 / 10 0.0%	0 / 7 0.0%	4 / 6 66.7%	0 / 8 0.0%	0 / 4 0.0%	0 / 6 0.0%	0 / 2 0.0%	0 / 8 0.0%	0 / 11 0.0%	0 / 10 0.0%	0 / 2 0.0%	2 / 14 14.3%	0 / 0 0.0%	0 / 0 0.0%	0 / 1 0.0%	0 / 0 0.0%	80 / 153 52.3%E	60 / 153 39.2%	10 / 153 6.5%AB	2 / 153 1.3%ABE	1 / 153 0.7%CE	
1	Lactobacillus salivarius UCC118	6 / 77 7.8%	0 / 2 0.0%	0 / 4 0.0%	0 / 3 0.0%	0 / 4 0.0%	0 / 5 0.0%	0 / 4 0.0%	0 / 2 0.0%	1 / 3 33.3%	0 / 3 0.0%	2 / 2 100.0%	0 / 5 0.0%	0 / 4 0.0%	2 / 3 66.7%	0 / 4 0.0%	0 / 2 0.0%	0 / 3 0.0%	0 / 1 0.0%	0 / 4 0.0%	0 / 6 0.0%	0 / 5 0.0%	0 / 1 0.0%	1 / 7 14.3%	0 / 0 0.0%	0 / 0 0.0%	0 / 0 0.0%	0 / 0 0.0%	48 / 77 62.3%	23 / 77 29.9%E	5 / 77 6.5%AB	1 / 77 1.3%ABE		
1	Lactobacillus salivarius CECT 5713	6 / 76 7.9%	0 / 2 0.0%	0 / 4 0.0%	0 / 3 0.0%	0 / 4 0.0%	0 / 5 0.0%	0 / 4 0.0%	0 / 2 0.0%	1 / 3 33.3%	0 / 3 0.0%	2 / 2 100.0%	0 / 5 0.0%	0 / 3 0.0%	2 / 3 66.7%	0 / 4 0.0%	0 / 2 0.0%	0 / 3 0.0%	0 / 1 0.0%	0 / 4 0.0%	0 / 5 0.0%	0 / 5 0.0%	0 / 1 0.0%	1 / 7 14.3%	0 / 0 0.0%	0 / 0 0.0%	0 / 1 0.0%	0 / 0 0.0%	57 / 76 75.0%E	12 / 76 15.8%	5 / 76 6.6%AB	1 / 76 1.3%ABE	1 / 76 1.3%CE	
1	"Lactobacillus sanfrancisco" Kline and Sugihara 1971	0 / 60 0.0%	0 / 2 0.0%	0 / 3 0.0%	0 / 3 0.0%	0 / 3 0.0%	0 / 4 0.0%	0 / 3 0.0%	0 / 1 0.0%	0 / 2 0.0%	0 / 4 0.0%	0 / 1 0.0%	0 / 4 0.0%	0 / 4 0.0%	0 / 2 0.0%	0 / 2 0.0%	0 / 1 0.0%	0 / 2 0.0%	0 / 1 0.0%	0 / 4 0.0%	0 / 5 0.0%	0 / 3 0.0%	0 / 1 0.0%	0 / 5 0.0%	0 / 0 0.0%	0 / 0 0.0%	0 / 0 0.0%	0 / 0 0.0%	50 / 60 83.3%E	10 / 60 16.7%				
1	Lactobacillus sanfranciscensis TMW 1.1304	0 / 60 0.0%	0 / 2 0.0%	0 / 3 0.0%	0 / 3 0.0%	0 / 3 0.0%	0 / 4 0.0%	0 / 3 0.0%	0 / 1 0.0%	0 / 2 0.0%	0 / 4 0.0%	0 / 1 0.0%	0 / 4 0.0%	0 / 4 0.0%	0 / 2 0.0%	0 / 2 0.0%	0 / 1 0.0%	0 / 2 0.0%	0 / 1 0.0%	0 / 4 0.0%	0 / 5 0.0%	0 / 3 0.0%	0 / 1 0.0%	0 / 5 0.0%	0 / 0 0.0%	0 / 0 0.0%	0 / 0 0.0%	0 / 0 0.0%	50 / 60 83.3%E	10 / 60 16.7%				
3	ATCC 33200	11 / 187 5.9%	0 / 7 0.0%	0 / 7 0.0%	1 / 10 10.0%	0 / 8 0.0%	3 / 14 21.4%	0 / 7 0.0%	0 / 3 0.0%	0 / 6 0.0%	0 / 4 0.0%	0 / 3 0.0%	0 / 13 0.0%	0 / 9 0.0%	0 / 8 0.0%	0 / 12 0.0%	0 / 3 0.0%	0 / 6 0.0%	0 / 4 0.0%	0 / 16 0.0%	0 / 12 0.0%	0 / 12 0.0%	0 / 4 0.0%	7 / 17 41.2%	0 / 0 0.0%	0 / 0 0.0%	0 / 2 0.0%	0 / 0 0.0%	89 / 187 47.6%	87 / 187 46.5%E	7 / 187 3.7%ABE	4 / 187 2.1%AB		
1	Lactobacillus johnsonii NCC 533	5 / 76 6.6%	0 / 3 0.0%	0 / 3 0.0%	1 / 4 25.0%	0 / 4 0.0%	1 / 6 16.7%	0 / 3 0.0%	0 / 1 0.0%	0 / 2 0.0%	0 / 2 0.0%	0 / 1 0.0%	0 / 5 0.0%	0 / 4 0.0%	0 / 4 0.0%	0 / 4 0.0%	0 / 1 0.0%	0 / 2 0.0%	0 / 2 0.0%	0 / 6 0.0%	0 / 5 0.0%	0 / 5 0.0%	0 / 2 0.0%	3 / 7 42.9%	0 / 0 0.0%	0 / 0 0.0%	0 / 0 0.0%	0 / 0 0.0%	51 / 76 67.1%	20 / 76 26.3%E	3 / 76 3.9%ABE	2 / 76 2.6%AB		
1	Lactobacillus johnsonii FI9785	3 / 55 5.5%	0 / 2 0.0%	0 / 2 0.0%	0 / 3 0.0%	0 / 2 0.0%	1 / 4 25.0%	0 / 2 0.0%	0 / 1 0.0%	0 / 2 0.0%	0 / 1 0.0%	0 / 1 0.0%	0 / 4 0.0%	0 / 2 0.0%	0 / 2 0.0%	0 / 4 0.0%	0 / 1 0.0%	0 / 2 0.0%	0 / 1 0.0%	0 / 5 0.0%	0 / 3 0.0%	0 / 3 0.0%	0 / 1 0.0%	2 / 5 40.0%	0 / 0 0.0%	0 / 0 0.0%	0 / 2 0.0%	0 / 0 0.0%	32 / 55 58.2%E	20 / 55 36.4%	2 / 55 3.6%ABE	1 / 55 1.8%AB		
1	Lactobacillus johnsonii DPC 6026	3 / 56 5.4%	0 / 2 0.0%	0 / 2 0.0%	0 / 3 0.0%	0 / 2 0.0%	1 / 4 25.0%	0 / 2 0.0%	0 / 1 0.0%	0 / 2 0.0%	0 / 1 0.0%	0 / 1 0.0%	0 / 4 0.0%	0 / 3 0.0%	0 / 2 0.0%	0 / 4 0.0%	0 / 1 0.0%	0 / 2 0.0%	0 / 1 0.0%	0 / 5 0.0%	0 / 4 0.0%	0 / 4 0.0%	0 / 1 0.0%	2 / 5 40.0%	0 / 0 0.0%	0 / 0 0.0%	0 / 0 0.0%	0 / 0 0.0%	35 / 56 62.5%E	18 / 56 32.1%	2 / 56 3.6%ABE	1 / 56 1.8%AB		
5	ATCC 7469	3 / 353 0.8%	0 / 12 0.0%	0 / 12 0.0%	0 / 18 0.0%	0 / 17 0.0%	0 / 30 0.0%	0 / 18 0.0%	0 / 6 0.0%	0 / 12 0.0%	0 / 22 0.0%	0 / 6 0.0%	0 / 24 0.0%	0 / 18 0.0%	0 / 12 0.0%	3 / 12 25.0%	0 / 6 0.0%	0 / 12 0.0%	0 / 6 0.0%	0 / 18 0.0%	0 / 36 0.0%	0 / 18 0.0%	0 / 6 0.0%	0 / 30 0.0%	0 / 0 0.0%	0 / 0 0.0%	0 / 0 0.0%	0 / 0 0.0%	228 / 353 64.6%E	110 / 353 31.2%	12 / 353 3.4%CE	3 / 353 0.8%AB		
1	Lactobacillus rhamnosus ATCC 53103	0 / 116 0.0%	0 / 4 0.0%	0 / 4 0.0%	0 / 6 0.0%	0 / 5 0.0%	0 / 10 0.0%	0 / 6 0.0%	0 / 2 0.0%	0 / 4 0.0%	0 / 7 0.0%	0 / 2 0.0%	0 / 8 0.0%	0 / 6 0.0%	0 / 4 0.0%	0 / 4 0.0%	0 / 2 0.0%	0 / 4 0.0%	0 / 2 0.0%	0 / 6 0.0%	0 / 12 0.0%	0 / 6 0.0%	0 / 2 0.0%	0 / 10 0.0%	0 / 0 0.0%	0 / 0 0.0%	0 / 0 0.0%	0 / 0 0.0%	75 / 116 64.7%E	37 / 116 31.9%	4 / 116 3.4%CE			
1	Lactobacillus rhamnosus LC705	1 / 59 1.7%	0 / 2 0.0%	0 / 2 0.0%	0 / 3 0.0%	0 / 3 0.0%	0 / 5 0.0%	0 / 3 0.0%	0 / 1 0.0%	0 / 2 0.0%	0 / 3 0.0%	0 / 1 0.0%	0 / 4 0.0%	0 / 3 0.0%	0 / 2 0.0%	1 / 2 50.0%	0 / 1 0.0%	0 / 2 0.0%	0 / 1 0.0%	0 / 3 0.0%	0 / 6 0.0%	0 / 3 0.0%	0 / 1 0.0%	0 / 5 0.0%	0 / 0 0.0%	0 / 0 0.0%	0 / 0 0.0%	0 / 0 0.0%	38 / 59 64.4%E	18 / 59 30.5%	2 / 59 3.4%CE	1 / 59 1.7%AB		

1	Lactobacillus rhamnosus ATCC 8530	1 / 59 1.7%	0 / 2 0.0%	0 / 2 0.0%	0 / 3 0.0%	0 / 3 0.0%	0 / 5 0.0%	0 / 3 0.0%	0 / 1 0.0%	0 / 2 0.0%	0 / 4 0.0%	0 / 1 0.0%	0 / 4 0.0%	0 / 3 0.0%	0 / 2 0.0%	1 / 2 50.0%	0 / 1 0.0%	0 / 2 0.0%	0 / 1 0.0%	0 / 3 0.0%	0 / 6 0.0%	0 / 3 0.0%	0 / 1 0.0%	0 / 5 0.0%	0 / 0 0.0%	0 / 0 0.0%	0 / 0 0.0%	0 / 0 0.0%	38 / 59 64.4%E	18 / 59 30.5%	2 / 59 3.4%CE	1 / 59 1.7%AB					
1	Lactobacillus rhamnosus LOCK900	0 / 59 0.0%	0 / 2 0.0%	0 / 2 0.0%	0 / 3 0.0%	0 / 3 0.0%	0 / 5 0.0%	0 / 3 0.0%	0 / 1 0.0%	0 / 2 0.0%	0 / 4 0.0%	0 / 1 0.0%	0 / 4 0.0%	0 / 3 0.0%	0 / 2 0.0%	0 / 2 0.0%	0 / 1 0.0%	0 / 2 0.0%	0 / 1 0.0%	0 / 3 0.0%	0 / 6 0.0%	0 / 3 0.0%	0 / 1 0.0%	0 / 5 0.0%	0 / 0 0.0%	0 / 0 0.0%	0 / 0 0.0%	0 / 0 0.0%	39 / 59 66.1%E	18 / 59 30.5%	2 / 59 3.4%CE						
1	Lactobacillus rhamnosus LOCK908	1 / 60 1.7%	0 / 2 0.0%	0 / 2 0.0%	0 / 3 0.0%	0 / 3 0.0%	0 / 5 0.0%	0 / 3 0.0%	0 / 1 0.0%	0 / 2 0.0%	0 / 4 0.0%	0 / 1 0.0%	0 / 4 0.0%	0 / 3 0.0%	0 / 2 0.0%	1 / 2 50.0%	0 / 1 0.0%	0 / 2 0.0%	0 / 1 0.0%	0 / 3 0.0%	0 / 6 0.0%	0 / 3 0.0%	0 / 1 0.0%	0 / 5 0.0%	0 / 0 0.0%	0 / 0 0.0%	0 / 0 0.0%	0 / 0 0.0%	38 / 60 63.3%E	19 / 60 31.7%	2 / 60 3.3%CE	1 / 60 1.7%AB					
1	"Eubacterium crispatum" Brygoo and Aladame 1953	3 / 64 4.7%	0 / 2 0.0%	0 / 3 0.0%	0 / 3 0.0%	0 / 3 0.0%	2 / 6 33.3%	0 / 3 0.0%	1 / 2 50.0%	0 / 2 0.0%	0 / 2 0.0%	0 / 1 0.0%	0 / 5 0.0%	0 / 3 0.0%	0 / 2 0.0%	0 / 4 0.0%	0 / 1 0.0%	0 / 3 0.0%	0 / 1 0.0%	0 / 4 0.0%	0 / 5 0.0%	0 / 3 0.0%	0 / 1 0.0%	0 / 5 0.0%	0 / 0 0.0%	0 / 0 0.0%	0 / 0 0.0%	0 / 0 0.0%	44 / 64 68.8%E	17 / 64 26.6%	2 / 64 3.1%AB	1 / 64 1.6%ABE					
1	Lactobacillus crispatus ST1	3 / 64 4.7%	0 / 2 0.0%	0 / 3 0.0%	0 / 3 0.0%	0 / 3 0.0%	2 / 6 33.3%	0 / 3 0.0%	1 / 2 50.0%	0 / 2 0.0%	0 / 2 0.0%	0 / 1 0.0%	0 / 5 0.0%	0 / 3 0.0%	0 / 2 0.0%	0 / 4 0.0%	0 / 1 0.0%	0 / 3 0.0%	0 / 1 0.0%	0 / 4 0.0%	0 / 5 0.0%	0 / 3 0.0%	0 / 1 0.0%	0 / 5 0.0%	0 / 0 0.0%	0 / 0 0.0%	0 / 0 0.0%	0 / 0 0.0%	44 / 64 68.8%E	17 / 64 26.6%	2 / 64 3.1%AB	1 / 64 1.6%ABE					
1	ATCC 43761	2 / 60 3.3%	0 / 2 0.0%	0 / 3 0.0%	0 / 3 0.0%	0 / 2 0.0%	1 / 4 25.0%	0 / 3 0.0%	1 / 2 50.0%	0 / 2 0.0%	0 / 1 0.0%	0 / 1 0.0%	0 / 5 0.0%	0 / 3 0.0%	0 / 2 0.0%	0 / 4 0.0%	0 / 1 0.0%	0 / 3 0.0%	0 / 1 0.0%	0 / 4 0.0%	0 / 5 0.0%	0 / 3 0.0%	0 / 1 0.0%	0 / 5 0.0%	0 / 0 0.0%	0 / 0 0.0%	0 / 0 0.0%	0 / 0 0.0%	42 / 60 70.0%E	16 / 60 26.7%	1 / 60 1.7%AB	1 / 60 1.7%ABE					
1	Lactobacillus kefiranofaciens ZW3	2 / 60 3.3%	0 / 2 0.0%	0 / 3 0.0%	0 / 3 0.0%	0 / 2 0.0%	1 / 4 25.0%	0 / 3 0.0%	1 / 2 50.0%	0 / 2 0.0%	0 / 1 0.0%	0 / 1 0.0%	0 / 5 0.0%	0 / 3 0.0%	0 / 2 0.0%	0 / 4 0.0%	0 / 1 0.0%	0 / 3 0.0%	0 / 1 0.0%	0 / 4 0.0%	0 / 5 0.0%	0 / 3 0.0%	0 / 1 0.0%	0 / 5 0.0%	0 / 0 0.0%	0 / 0 0.0%	0 / 0 0.0%	0 / 0 0.0%	42 / 60 70.0%E	16 / 60 26.7%	1 / 60 1.7%ABE	1 / 60 1.7%AB					
7	Lactobacillus casei group	1 / 416 0.2%	0 / 14 0.0%	0 / 14 0.0%	0 / 21 0.0%	0 / 24 0.0%	0 / 35 0.0%	0 / 21 0.0%	0 / 7 0.0%	0 / 14 0.0%	0 / 28 0.0%	0 / 7 0.0%	0 / 28 0.0%	0 / 21 0.0%	0 / 14 0.0%	1 / 14 7.1%	0 / 7 0.0%	0 / 14 0.0%	0 / 7 0.0%	0 / 21 0.0%	0 / 42 0.0%	0 / 21 0.0%	0 / 7 0.0%	0 / 35 0.0%	0 / 0 0.0%	0 / 0 0.0%	0 / 0 0.0%	0 / 0 0.0%	262 / 416 63.0%E	153 / 416 36.8%	1 / 416 0.2%AB						
7	"Bacillus a" von Freudenreich 1890	1 / 416 0.2%	0 / 14 0.0%	0 / 14 0.0%	0 / 21 0.0%	0 / 24 0.0%	0 / 35 0.0%	0 / 21 0.0%	0 / 7 0.0%	0 / 14 0.0%	0 / 28 0.0%	0 / 7 0.0%	0 / 28 0.0%	0 / 21 0.0%	0 / 14 0.0%	1 / 14 7.1%	0 / 7 0.0%	0 / 14 0.0%	0 / 7 0.0%	0 / 21 0.0%	0 / 42 0.0%	0 / 21 0.0%	0 / 7 0.0%	0 / 35 0.0%	0 / 0 0.0%	0 / 0 0.0%	0 / 0 0.0%	0 / 0 0.0%	262 / 416 63.0%E	153 / 416 36.8%	1 / 416 0.2%AB						
1	Lactobacillus casei ATCC 334	1 / 59 1.7%	0 / 2 0.0%	0 / 2 0.0%	0 / 3 0.0%	0 / 3 0.0%	0 / 5 0.0%	0 / 3 0.0%	0 / 1 0.0%	0 / 2 0.0%	0 / 4 0.0%	0 / 1 0.0%	0 / 4 0.0%	0 / 3 0.0%	0 / 2 0.0%	1 / 2 50.0%	0 / 1 0.0%	0 / 2 0.0%	0 / 1 0.0%	0 / 3 0.0%	0 / 6 0.0%	0 / 3 0.0%	0 / 1 0.0%	0 / 5 0.0%	0 / 0 0.0%	0 / 0 0.0%	0 / 0 0.0%	0 / 0 0.0%	42 / 59 71.2%	16 / 59 27.1%E	1 / 59 1.7%AB						
1	Lactobacillus casei str. Zhang	0 / 59 0.0%	0 / 2 0.0%	0 / 2 0.0%	0 / 3 0.0%	0 / 3 0.0%	0 / 5 0.0%	0 / 3 0.0%	0 / 1 0.0%	0 / 2 0.0%	0 / 4 0.0%	0 / 1 0.0%	0 / 4 0.0%	0 / 3 0.0%	0 / 2 0.0%	0 / 2 0.0%	0 / 1 0.0%	0 / 2 0.0%	0 / 1 0.0%	0 / 3 0.0%	0 / 6 0.0%	0 / 3 0.0%	0 / 1 0.0%	0 / 5 0.0%	0 / 0 0.0%	0 / 0 0.0%	0 / 0 0.0%	0 / 0 0.0%	41 / 59 69.5%E	18 / 59 30.5%							
1	Lactobacillus casei BD-II	0 / 60 0.0%	0 / 2 0.0%	0 / 2 0.0%	0 / 3 0.0%	0 / 4 0.0%	0 / 5 0.0%	0 / 3 0.0%	0 / 1 0.0%	0 / 2 0.0%	0 / 4 0.0%	0 / 1 0.0%	0 / 4 0.0%	0 / 3 0.0%	0 / 2 0.0%	0 / 2 0.0%	0 / 1 0.0%	0 / 2 0.0%	0 / 1 0.0%	0 / 3 0.0%	0 / 6 0.0%	0 / 3 0.0%	0 / 1 0.0%	0 / 5 0.0%	0 / 0 0.0%	0 / 0 0.0%	0 / 0 0.0%	0 / 0 0.0%	41 / 60 68.3%E	19 / 60 31.7%							
1	Lactobacillus casei LC2W	0 / 59 0.0%	0 / 2 0.0%	0 / 2 0.0%	0 / 3 0.0%	0 / 3 0.0%	0 / 5 0.0%	0 / 3 0.0%	0 / 1 0.0%	0 / 2 0.0%	0 / 4 0.0%	0 / 1 0.0%	0 / 4 0.0%	0 / 3 0.0%	0 / 2 0.0%	0 / 2 0.0%	0 / 1 0.0%	0 / 2 0.0%	0 / 1 0.0%	0 / 3 0.0%	0 / 6 0.0%	0 / 3 0.0%	0 / 1 0.0%	0 / 5 0.0%	0 / 0 0.0%	0 / 0 0.0%	0 / 0 0.0%	0 / 0 0.0%	41 / 59 69.5%E	18 / 59 30.5%							
1	Lactobacillus casei W56	0 / 60 0.0%	0 / 2 0.0%	0 / 2 0.0%	0 / 3 0.0%	0 / 4 0.0%	0 / 5 0.0%	0 / 3 0.0%	0 / 1 0.0%	0 / 2 0.0%	0 / 4 0.0%	0 / 1 0.0%	0 / 4 0.0%	0 / 3 0.0%	0 / 2 0.0%	0 / 2 0.0%	0 / 1 0.0%	0 / 2 0.0%	0 / 1 0.0%	0 / 3 0.0%	0 / 6 0.0%	0 / 3 0.0%	0 / 1 0.0%	0 / 5 0.0%	0 / 0 0.0%	0 / 0 0.0%	0 / 0 0.0%	0 / 0 0.0%	41 / 60 68.3%E	19 / 60 31.7%							
1	Lactobacillus casei LOCK919	0 / 59 0.0%	0 / 2 0.0%	0 / 2 0.0%	0 / 3 0.0%	0 / 3 0.0%	0 / 5 0.0%	0 / 3 0.0%	0 / 1 0.0%	0 / 2 0.0%	0 / 4 0.0%	0 / 1 0.0%	0 / 4 0.0%	0 / 3 0.0%	0 / 2 0.0%	0 / 2 0.0%	0 / 1 0.0%	0 / 2 0.0%	0 / 1 0.0%	0 / 3 0.0%	0 / 6 0.0%	0 / 3 0.0%	0 / 1 0.0%	0 / 5 0.0%	0 / 0 0.0%	0 / 0 0.0%	0 / 0 0.0%	0 / 0 0.0%	41 / 59 69.5%E	18 / 59 30.5%							
10	Leuconostoc group	12 / 643 1.9%	0 / 19 0.0%	0 / 27 0.0%	0 / 30 0.0%	0 / 19 0.0%	0 / 38 0.0%	0 / 27 0.0%	0 / 10 0.0%	1 / 18 5.6%	0 / 38 0.0%	9 / 10 90.0%	2 / 64 3.1%	0 / 29 0.0%	0 / 19 0.0%	0 / 28 0.0%	0 / 10 0.0%	0 / 21 0.0%	0 / 18 0.0%	0 / 38 0.0%	0 / 46 0.0%	0 / 42 0.0%	0 / 22 0.0%	0 / 67 0.0%	0 / 0 0.0%	0 / 0 0.0%	0 / 3 0.0%	0 / 0 0.0%	420 / 643 65.3%E	210 / 643 32.7%	12 / 643 1.9%AB	1 / 643 0.2%CE					
8	"Betacoccus" Orla-Jensen 1919	10 / 545 1.8%	0 / 16 0.0%	0 / 24 0.0%	0 / 26 0.0%	0 / 16 0.0%	0 / 32 0.0%	0 / 23 0.0%	0 / 8 0.0%	0 / 16 0.0%	0 / 32 0.0%	8 / 8 100.0%	2 / 56 3.6%	0 / 24 0.0%	0 / 16 0.0%	0 / 24 0.0%	0 / 8 0.0%	0 / 17 0.0%	0 / 16 0.0%	0 / 32 0.0%	0 / 37 0.0%	0 / 37 0.0%	0 / 18 0.0%	0 / 56 0.0%	0 / 0 0.0%	0 / 0 0.0%	0 / 3 0.0%	0 / 0 0.0%	369 / 545 67.7%E	165 / 545 30.3%	10 / 545 1.8%AB	1 / 545 0.2%CE					
1	ATCC 49366	1 / 67 1.5%	0 / 2 0.0%	0 / 3 0.0%	0 / 3 0.0%	0 / 2 0.0%	0 / 4 0.0%	0 / 3 0.0%	0 / 1 0.0%	0 / 2 0.0%	0 / 4 0.0%	1 / 1 100.0%	0 / 7 0.0%	0 / 3 0.0%	0 / 2 0.0%	0 / 3 0.0%	0 / 1 0.0%	0 / 2 0.0%	0 / 2 0.0%	0 / 4 0.0%	0 / 4 0.0%	0 / 4 0.0%	0 / 2 0.0%	0 / 7 0.0%	0 / 0 0.0%	0 / 0 0.0%	0 / 1 0.0%	0 / 0 0.0%	50 / 67 74.6%E	16 / 67 23.9%	1 / 67 1.5%AB						
1	Leuconostoc gelidum JB7	1 / 67 1.5%	0 / 2 0.0%	0 / 3 0.0%	0 / 3 0.0%	0 / 2 0.0%	0 / 4 0.0%	0 / 3 0.0%	0 / 1 0.0%	0 / 2 0.0%	0 / 4 0.0%	1 / 1 100.0%	0 / 7 0.0%	0 / 3 0.0%	0 / 2 0.0%	0 / 3 0.0%	0 / 1 0.0%	0 / 2 0.0%	0 / 2 0.0%	0 / 4 0.0%	0 / 4 0.0%	0 / 4 0.0%	0 / 2 0.0%	0 / 7 0.0%	0 / 0 0.0%	0 / 0 0.0%	0 / 1 0.0%	0 / 0 0.0%	50 / 67 74.6%E	16 / 67 23.9%	1 / 67 1.5%AB						
2	"Ascococcus mesenteroides" Tsenkovskii 1878	3 / 140 2.1%	0 / 4 0.0%	0 / 6 0.0%	0 / 8 0.0%	0 / 4 0.0%	0 / 8 0.0%	0 / 6 0.0%	0 / 2 0.0%	0 / 4 0.0%	0 / 8 0.0%	2 / 2 100.0%	1 / 14 7.1%	0 / 6 0.0%	0 / 4 0.0%	0 / 6 0.0%	0 / 2 0.0%	0 / 4 0.0%	0 / 4 0.0%	0 / 8 0.0%	0 / 10 0.0%	0 / 10 0.0%	0 / 6 0.0%	0 / 14 0.0%	0 / 0 0.0%	0 / 0 0.0%	0 / 0 0.0%	0 / 0 0.0%	76 / 140 54.3%E	61 / 140 43.6%	3 / 140 2.1%AB						
2	Leuconostoc mesenteroides mesenteroides	3 / 140 2.1%	0 / 4 0.0%	0 / 6 0.0%	0 / 8 0.0%	0 / 4 0.0%	0 / 8 0.0%	0 / 6 0.0%	0 / 2 0.0%	0 / 4 0.0%	0 / 8 0.0%	2 / 2 100.0%	1 / 14 7.1%	0 / 6 0.0%	0 / 4 0.0%	0 / 6 0.0%	0 / 2 0.0%	0 / 4 0.0%	0 / 4 0.0%	0 / 8 0.0%	0 / 10 0.0%	0 / 10 0.0%	0 / 6 0.0%	0 / 14 0.0%	0 / 0 0.0%	0 / 0 0.0%	0 / 0 0.0%	0 / 0 0.0%	76 / 140 54.3%E	61 / 140 43.6%	3 / 140 2.1%AB						
1	Leuconostoc mesenteroides subsp. mesenteroides ATCC 8293	2 / 70 2.9%	0 / 2 0.0%	0 / 3 0.0%	0 / 4 0.0%	0 / 2 0.0%	0 / 4 0.0%	0 / 3 0.0%	0 / 1 0.0%	0 / 2 0.0%	0 / 4 0.0%	1 / 1 100.0%	1 / 7 14.3%	0 / 3 0.0%	0 / 2 0.0%	0 / 3 0.0%	0 / 1 0.0%	0 / 2 0.0%	0 / 2 0.0%	0 / 4 0.0%	0 / 5 0.0%	0 / 5 0.0%	0 / 3 0.0%	0 / 7 0.0%	0 / 0 0.0%	0 / 0 0.0%	0 / 0 0.0%	0 / 0 0.0%	43 / 70 61.4%	25 / 70 35.7%E	2 / 70 2.9%AB						
1	Leuconostoc mesenteroides subsp. mesenteroides J18	1 / 70 1.4%	0 / 2 0.0%	0 / 3 0.0%	0 / 4 0.0%	0 / 2 0.0%	0 / 4 0.0%	0 / 3 0.0%	0 / 1 0.0%	0 / 2 0.0%	0 / 4 0.0%	1 / 1 100.0%	0 / 7 0.0%	0 / 3 0.0%	0 / 2 0.0%	0 / 3 0.0%	0 / 1 0.0%	0 / 2 0.0%	0 / 2 0.0%	0 / 4 0.0%	0 / 5 0.0%	0 / 5 0.0%	0 / 3 0.0%	0 / 7 0.0%	0 / 0 0.0%	0 / 0 0.0%	0 / 0 0.0%	0 / 0 0.0%	51 / 70 72.9%E	18 / 70 25.7%	1 / 70 1.4%AB						
1	ATCC 49367	1 / 66 1.5%	0 / 2 0.0%	0 / 3 0.0%	0 / 3 0.0%	0 / 2 0.0%	0 / 4 0.0%	0 / 2 0.0%	0 / 1 0.0%	0 / 2 0.0%	0 / 4 0.0%	1 / 1 100.0%	0 / 7 0.0%	0 / 3 0.0%	0 / 2 0.0%	0 / 3 0.0%	0 / 1 0.0%	0 / 2 0.0%	0 / 2 0.0%	0 / 4 0.0%	0 / 4 0.0%	0 / 4 0.0%	0 / 2 0.0%	0 / 7 0.0%	0 / 0 0.0%	0 / 0 0.0%	0 / 1 0.0%	0 / 0 0.0%	50 / 66 75.8%E	15 / 66 22.7%	1 / 66 1.5%AB						
1	Leuconostoc carnosum JB16	1 / 66 1.5%	0 / 2 0.0%	0 / 3 0.0%	0 / 3 0.0%	0 / 2 0.0%	0 / 4 0.0%	0 / 2 0.0%	0 / 1 0.0%	0 / 2 0.0%	0 / 4 0.0%	1 / 1 100.0%	0 / 7 0.0%	0 / 3 0.0%	0 / 2 0.0%	0 / 3 0.0%	0 / 1 0.0%	0 / 2 0.0%	0 / 2 0.0%	0 / 4 0.0%	0 / 4 0.0%	0 / 4 0.0%	0 / 2 0.0%	0 / 7 0.0%	0 / 0 0.0%	0 / 0 0.0%	0 / 1 0.0%	0 / 0 0.0%	50 / 66 75.8%E	15 / 66 22.7%	1 / 66 1.5%AB						
1	ATCC 49370	1 / 69 1.4%	0 / 2 0.0%	0 / 3 0.0%	0 / 3 0.0%	0 / 2 0.0%	0 / 4 0.0%	0 / 3 0.0%	0 / 1 0.0%	0 / 2 0.0%	0 / 4 0.0%	1 / 1 100.0%	0 / 7 0.0%	0 / 3 0.0%	0 / 2 0.0%	0 / 3 0.0%	0 / 1 0.0%	0 / 3 0.0%	0 / 2 0.0%	0 / 4 0.0%	0 / 5 0.0%	0 / 5 0.0%	0 / 2 0.0%	0 / 7 0.0%	0 / 0 0.0%	0 / 0 0.0%	0 / 0 0.0%	0 / 0 0.0%	53 / 69 76.8%E	14 / 69 20.3%	1 / 69 1.4%CE	1 / 69 1.4%AB					
1	Leuconostoc citreum KM20	1 / 69 1.4%	0 / 2 0.0%	0 / 3 0.0%	0 / 3 0.0%	0 / 2 0.0%	0 / 4 0.0%	0 / 3 0.0%	0 / 1 0.0%	0 / 2 0.0%	0 / 4 0.0%	1 / 1 100.0%	0 / 7 0.0%	0 / 3 0.0%	0 / 2 0.0%	0 / 3 0.0%	0 / 1 0.0%	0 / 3 0.0%	0 / 2 0.0%	0 / 4 0.0%	0 / 5 0.0%	0 / 5 0.0%	0 / 2 0.0%	0 / 7 0.0%	0 / 0 0.0%	0 / 0 0.0%	0 / 0 0.0%	0 / 0 0.0%	53 / 69 76.8%E	14 / 69 20.3%	1 / 69 1.4%CE	1 / 69 1.4%AB					
1	CCUG 46042	1 / 67 1.5%	0 / 2 0.0%	0 / 3 0.0%	0 / 3 0.0%	0 / 2 0.0%	0 / 4 0.0%	0 / 3 0.0%	0 / 1 0.0%	0 / 2 0.0%	0 / 4 0.0%	1 / 1 100.0%	0 / 7 0.0%	0 / 3 0.0%	0 / 2 0.0%	0 / 3 0.0%	0 / 1 0.0%	0 / 2 0.0%	0 / 2 0.0%	0 / 4 0.0%	0 / 5 0.0%	0 / 4 0.0%	0 / 2 0.0%	0 / 7 0.0%	0 / 0 0.0%	0 / 0 0.0%	0 / 0 0.0%	0 / 0 0.0%	46 / 67 68.7%E	20 / 67 29.9%	1 / 67 1.5%AB						
1	Leuconostoc gasicomitatum CECT 5767	1 / 67 1.5%	0 / 2 0.0%	0 / 3 0.0%	0 / 3 0.0%	0 / 2 0.0%	0 / 4 0.0%	0 / 3 0.0%	0 / 1 0.0%	0 / 2 0.0%	0 / 4 0.0%	1 / 1 100.0%	0 / 7 0.0%	0 / 3 0.0%	0 / 2 0.0%	0 / 3 0.0%	0 / 1 0.0%	0 / 2 0.0%	0 / 2 0.0%	0 / 4 0.0%	0 / 5 0.0%	0 / 4 0.0%	0 / 2 0.0%	0 / 7 0.0%	0 / 0 0.0%	0 / 0 0.0%	0 / 0 0.0%	0 / 0 0.0%	46 / 67 68.7%E	20 / 67 29.9%	1 / 67 1.5%AB						
1	IMSNU 11154	1 / 68 1.5%	0 / 2 0.0%	0 / 3 0.0%	0 / 3 0.0%	0 / 2 0.0%	0 / 4 0.0%	0 / 3 0.0%	0 / 1 0.0%	0 / 2 0.0%	0 / 4 0.0%	1 / 1 100.0%	0 / 7 0.0%	0 / 3 0.0%	0 / 2 0.0%	0 / 3 0.0%	0 / 1 0.0%	0 / 2 0.0%	0 / 2 0.0%	0 / 4 0.0%	0 / 4 0.0%	0 / 5 0.0%	0 / 2 0.0%	0 / 7 0.0%	0 / 0 0.0%	0 / 0 0.0%	0 / 1 0.0%	0 / 0 0.0%	47 / 68 69.1%E	20 / 68 29.4%	1 / 68 1.5%AB						
1	Leuconostoc kimchii IMSNU 11154	1 / 68 1.5%	0 / 2 0.0%	0 / 3 0.0%	0 / 3 0.0%	0 / 2 0.0%	0 / 4 0.0%	0 / 3 0.0%	0 / 1 0.0%	0 / 2 0.0%	0 / 4 0.0%	1 / 1 100.0%	0 / 7 0.0%	0 / 3 0.0%	0 / 2 0.0%	0 / 3 0.0%	0 / 1 0.0%	0 / 2 0.0%	0 / 2 0.0%	0 / 4 0.0%	0 / 4 0.0%	0 / 5 0.0%	0 / 2 0.0%	0 / 7 0.0%	0 / 0 0.0%	0 / 0 0.0%	0 / 1 0.0%	0 / 0 0.0%	47 / 68 69.1%E	20 / 68 29.4%	1 / 68 1.5%AB						
1	Leuconostoc sp. C2	2 / 68 2.9%	0 / 2 0.0%	0 / 3 0.0%	0 / 3 0.0%	0 / 2 0.0%	0 / 4 0.0%	0 / 3 0.0%	0 / 1 0.0%	0 / 2 0.0%	0 / 4 0.0%	1 / 1 100.0%	1 / 7 14.3%	0 / 3 0.0%	0 / 2 0.0%	0 / 3 0.0%	0 / 1 0.0%	0 / 2 0.0%	0 / 2 0.0%	0 / 4 0.0%	0 / 5 0.0%	0 / 5 0.0%	0 / 2 0.0%	0 / 7 0.0%	0 / 0 0.0%	0 / 0 0.0%	0 / 0 0.0%	0 / 0 0.0%	47 / 68 69.1%E	19 / 68 27.9%	2 / 68 2.9%AB						
1	Oenococcus	1 / 43 2.3%	0 / 1 0.0%	0 / 1 0.0%	0 / 2 0.0%	0 / 1 0.0%	0 / 3 0.0%	0 / 1 0.0%	0 / 1 0.0%	0 / 1 0.0%	0 / 3 0.0%	1 / 1 100.0%	0 / 4 0.0%	0 / 2 0.0%	0 / 1 0.0%	0 / 2 0.0%	0 / 1 0.0%	0 / 2 0.0%	0 / 1 0.0%	0 / 3 0.0%	0 / 4 0.0%	0 / 2 0.0%	0 / 1 0.0%	0 / 5 0.0%	0 / 0 0.0%	0 / 0 0.0%	0 / 0 0.0%	0 / 0 0.0%	30 / 43 69.8%	12 / 43 27.9%E	1 / 43 2.3%AB						
1	ATCC 23279	1 / 43 2.3%	0 / 1 0.0%	0 / 1 0.0%	0 / 2 0.0%	0 / 1 0.0%	0 / 3 0.0%	0 / 1 0.0%	0 / 1 0.0%	0 / 1 0.0%	0 / 3 0.0%	1 / 1 100.0%	0 / 4 0.0%	0 / 2 0.0%	0 / 1 0.0%	0 / 2 0.0%	0 / 1 0.0%	0 / 2 0.0%	0 / 1 0.0%	0 / 3 0.0%	0 / 4 0.0%	0 / 2 0.0%	0 / 1 0.0%	0 / 5 0.0%	0 / 0 0.0%	0 / 0 0.0%	0 / 0 0.0%	0 / 0 0.0%	30 / 43 69.8%	12 / 43 27.9%E	1 / 43 2.3%AB						
1	Oenococcus oeni PSU-1	1 / 43 2.3%	0 / 1 0.0%	0 / 1 0.0%	0 / 2 0.0%	0 / 1 0.0%	0 / 3 0.0%	0 / 1 0.0%	0 / 1 0.0%	0 / 1 0.0%	0 / 3 0.0%	1 / 1 100.0%	0 / 4 0.0%	0 / 2 0.0%	0 / 1 0.0%	0 / 2 0.0%	0 / 1 0.0%	0 / 2 0.0%	0 / 1 0.0%	0 / 3 0.0%	0 / 4 0.0%	0 / 2 0.0%	0 / 1 0.0%	0 / 5 0.0%	0 / 0 0.0%	0 / 0 0.0%	0 / 0 0.0%	0 / 0 0.0%	30 / 43 69.8%	12 / 43 27.9%E	1 / 43 2.3%AB						
1	Leuconostoc paramesenteroides group	1 / 55 1.8%	0 / 2 0.0%	0 / 2 0.0%	0 / 2 0.0%	0 / 2 0.0%	0 / 3 0.0%	0 / 3 0.0%	0 / 1 0.0%	1 / 1 100.0%	0 / 3 0.0%	0 / 1 0.0%	0 / 4 0.0%	0 / 3 0.0%	0 / 2 0.0%	0 / 2 0.0%	0 / 1 0.0%	0 / 2 0.0%	0 / 1 0.0%	0 / 3 0.0%	0 / 5 0.0%	0 / 3 0.0%	0 / 3 0.0%	0 / 6 0.0%	0 / 0 0.0%	0 / 0 0.0%	0 / 0 0.0%	0 / 0 0.0%	39 / 55 70.9%E	15 / 55 27.3%	1 / 55 1.8%AB						
1	CCUG 47134	1 / 55 1.8%	0 / 2 0.0%	0 / 2 0.0%	0 / 2 0.0%	0 / 2 0.0%	0 / 3 0.0%	0 / 3 0.0%	0 / 1 0.0%	1 / 1 100.0%	0 / 3 0.0%	0 / 1 0.0%	0 / 4 0.0%	0 / 3 0.0%	0 / 2 0.0%	0 / 2 0.0%	0 / 1 0.0%	0 / 2 0.0%	0 / 1 0.0%	0 / 3 0.0%	0 / 5 0.0%	0 / 3 0.0%	0 / 3 0.0%	0 / 6 0.0%	0 / 0 0.0%	0 / 0 0.0%	0 / 0 0.0%	0 / 0 0.0%	39 / 55 70.9%E	15 / 55 27.3%	1 / 55 1.8%AB						
1	Weissella koreensis KACC 15510	1 / 55 1.8%	0 / 2 0.0%	0 / 2 0.0%	0 / 2 0.0%	0 / 2 0.0%	0 / 3 0.0%	0 / 3 0.0%	0 / 1 0.0%	1 / 1 100.0%	0 / 3 0.0%	0 / 1 0.0%	0 / 4 0.0%	0 / 3 0.0%	0 / 2 0.0%	0 / 2 0.0%	0 / 1 0.0%	0 / 2 0.0%	0 / 1 0.0%	0 / 3 0.0%	0 / 5 0.0%	0 / 3 0.0%	0 / 3 0.0%	0 / 6 0.0%	0 / 0 0.0%	0 / 0 0.0%	0 / 0 0.0%	0 / 0 0.0%	39 / 55 70.9%E	15 / 55 27.3%	1 / 55 1.8%AB						
12	Enterococcaceae	42 / 703 6.0%	0 / 19 0.0%	12 / 29 41.4%	0 / 31 0.0%	0 / 19 0.0%	0 / 45 0.0%	0 / 31 0.0%	0 / 15 0.0%	1 / 24 4.2%	0 / 35 0.0%	12 / 18 66.7%	0 / 54 0.0%	0 / 35 0.0%	8 / 22 36.4%	0 / 33 0.0%	0 / 12 0.0%	0 / 23 0.0%	0 / 10 0.0%	9 / 45 20.0%	0 / 62 0.0%	0 / 35 0.0%	0 / 30 0.0%	0 / 75 0.0%	0 / 1 0.0%	0 / 0 0.0%	0 / 0 0.0%	0 / 0 0.0%	415 / 703 59.0%E	243 / 703 34.6%	32 / 703 4.6%AB	10 / 703 1.4%ABE	3 / 703 0.4%CE				
9	"Enterococcus" Thiercelin and Jouhaud 1903	38 / 525 7.2%	0 / 14 0.0%	12 / 23 52.2%	0 / 24 0.0%	0 / 14 0.0%	0 / 33 0.0%	0 / 24 0.0%	0 / 12 0.0%	0 / 18 0.0%	0 / 22 0.0%	9 / 15 60.0%	0 / 40 0.0%	0 / 26 0.0%	8 / 16 50.0%	0 / 25 0.0%	0 / 9 0.0%	0 / 16 0.0%	0 / 7 0.0%	9 / 33 27.3%	0 / 47 0.0%	0 / 25 0.0%	0 / 27 0.0%	0 / 54 0.0%	0 / 1 0.0%	0 / 0 0.0%	0 / 0 0.0%	0 / 0 0.0%	299 / 525 57.0%E	188 / 525 35.8%	28 / 525 5.3%AB	10 / 525 1.9%ABE					
5	"Enterococcus proteiformis" Thiercelin and Jouhaud 1903	28 / 301 9.3%	0 / 10 0.0%	10 / 15 66.7%	0 / 11 0.0%	0 / 10 0.0%	0 / 19 0.0%	0 / 14 0.0%	0 / 5 0.0%	0 / 10 0.0%	0 / 15 0.0%	5 / 11 45.5%	0 / 25 0.0%	0 / 16 0.0%	4 / 10 40.0%	0 / 10 0.0%	0 / 6 0.0%	0 / 10 0.0%	0 / 5 0.0%	9 / 19 47.4%	0 / 25 0.0%	0 / 15 0.0%	0 / 12 0.0%	0 / 28 0.0%	0 / 0 0.0%	0 / 0 0.0%	0 / 0 0.0%	0 / 0 0.0%	146 / 301 48.5%E	127 / 301 42.2%	19 / 301 6.3%AB	9 / 301 3.0%ABE					
1	Enterococcus faecalis V583	5 / 66 7.6%	0 / 2 0.0%	2 / 3 66.7%	0 / 2 0.0%	0 / 2 0.0%	0 / 4 0.0%	0 / 3 0.0%	0 / 1 0.0%	0 / 2 0.0%	0 / 3 0.0%	1 / 3 33.3%	0 / 5 0.0%	0 / 4 0.0%	0 / 2 0.0%	0 / 2 0.0%	0 / 2 0.0%	0 / 2 0.0%	0 / 1 0.0%	2 / 4 50.0%	0 / 5 0.0%	0 / 3 0.0%	0 / 5 0.0%	0 / 6 0.0%	0 / 0 0.0%	0 / 0 0.0%	0 / 0 0.0%	0 / 0 0.0%	47 / 66 71.2%	14 / 66 21.2%E	4 / 66 6.1%AB	1 / 66 1.5%ABE					
1	Enterococcus faecalis OG1RF	6 / 58 10.3%	0 / 2 0.0%	2 / 3 66.7%	0 / 2 0.0%	0 / 2 0.0%	0 / 4 0.0%	0 / 3 0.0%	0 / 1 0.0%	0 / 2 0.0%	0 / 3 0.0%	1 / 1 100.0%	0 / 5 0.0%	0 / 3 0.0%	1 / 2 50.0%	0 / 2 0.0%	0 / 1 0.0%	0 / 2 0.0%	0 / 1 0.0%	2 / 4 50.0%	0 / 5 0.0%	0 / 3 0.0%	0 / 1 0.0%	0 / 6 0.0%	0 / 0 0.0%	0 / 0 0.0%	0 / 0 0.0%	0 / 0 0.0%	33 / 58 56.9%E	19 / 58 32.8%	4 / 58 6.9%AB	2 / 58 3.4%ABE					
1	Enterococcus faecalis 62	5 / 53 9.4%	0 / 2 0.0%	2 / 3 66.7%	0 / 2 0.0%	0 / 2 0.0%	0 / 3 0.0%	0 / 2 0.0%	0 / 1 0.0%	0 / 2 0.0%	0 / 3 0.0%	1 / 3 33.3%	0 / 5 0.0%	0 / 2 0.0%	1 / 2 50.0%	0 / 2 0.0%	0 / 1 0.0%	0 / 2 0.0%	0 / 1 0.0%	1 / 3 33.3%	0 / 4 0.0%	0 / 3 0.0%	0 / 1 0.0%	0 / 4 0.0%	0 / 0 0.0%	0 / 0 0.0%	0 / 0 0.0%	0 / 0 0.0%	30 / 53 56.6%E	18 / 53 34.0%	3 / 53 5.7%AB	2 / 53 3.8%ABE					
1	Enterococcus faecalis D32	6 / 61 9.8%	0 / 2 0.0%	2 / 3 66.7%	0 / 2 0.0%	0 / 2 0.0%	0 / 4 0.0%	0 / 3 0.0%	0 / 1 0.0%	0 / 2 0.0%	0 / 3 0.0%	1 / 2 50.0%	0 / 5 0.0%	0 / 3 0.0%	1 / 2 50.0%	0 / 2 0.0%	0 / 1 0.0%	0 / 2 0.0%	0 / 1 0.0%	2 / 4 50.0%	0 / 6 0.0%	0 / 3 0.0%	0 / 2 0.0%	0 / 6 0.0%	0 / 0 0.0%	0 / 0 0.0%	0 / 0 0.0%	0 / 0 0.0%	34 / 61 55.7%E	21 / 61 34.4%	4 / 61 6.6%AB	2 / 61 3.3%ABE					
1	Enterococcus faecalis str. Symbioflor 1	6 / 63 9.5%	0 / 2 0.0%	2 / 3 66.7%	0 / 3 0.0%	0 / 2 0.0%	0 / 4 0.0%	0 / 3 0.0%	0 / 1 0.0%	0 / 2 0.0%	0 / 3 0.0%	1 / 2 50.0%	0 / 5 0.0%	0 / 4 0.0%	1 / 2 50.0%	0 / 2 0.0%	0 / 1 0.0%	0 / 2 0.0%	0 / 1 0.0%	2 / 4 50.0%	0 / 5 0.0%	0 / 3 0.0%	0 / 3 0.0%	0 / 6 0.0%	0 / 0 0.0%	0 / 0 0.0%	0 / 0 0.0%	0 / 0 0.0%	35 / 63 55.6%E	22 / 63 34.9%	4 / 63 6.3%AB	2 / 63 3.2%ABE					
3	ATCC 19434	7 / 155 4.5%	0 / 2 0.0%	1 / 5 20.0%	0 / 10 0.0%	0 / 2 0.0%	0 / 9 0.0%	0 / 7 0.0%	0 / 5 0.0%	0 / 6 0.0%	0 / 4 0.0%	3 / 3 100.0%	0 / 10 0.0%	0 / 7 0.0%	3 / 4 75.0%	0 / 11 0.0%	0 / 2 0.0%	0 / 4 0.0%	0 / 1 0.0%	0 / 10 0.0%	0 / 16 0.0%	0 / 7 0.0%	0 / 11 0.0%	0 / 19 0.0%	0 / 0 0.0%	0 / 0 0.0%	0 / 0 0.0%	0 / 0 0.0%	102 / 155 65.8%E	46 / 155 29.7%	6 / 155 3.9%AB	1 / 155 0.6%ABE					
1	Enterococcus faecium ATCC BAA-472	2 / 62 3.2%	0 / 1 0.0%	0 / 2 0.0%	0 / 3 0.0%	0 / 1 0.0%	0 / 4 0.0%	0 / 3 0.0%	0 / 2 0.0%	0 / 2 0.0%	0 / 3 0.0%	1 / 1 100.0%	0 / 3 0.0%	0 / 3 0.0%	1 / 2 50.0%	0 / 4 0.0%	0 / 1 0.0%	0 / 2 0.0%	0 / 1 0.0%	0 / 4 0.0%	0 / 6 0.0%	0 / 2 0.0%	0 / 4 0.0%	0 / 8 0.0%	0 / 0 0.0%	0 / 0 0.0%	0 / 0 0.0%	0 / 0 0.0%	41 / 62 66.1%E	19 / 62 30.6%	2 / 62 3.2%AB						
1	Enterococcus faecium NRRL B-2354	3 / 47 6.4%	0 / 1 0.0%	1 / 2 50.0%	0 / 4 0.0%	0 / 1 0.0%	0 / 3 0.0%	0 / 2 0.0%	0 / 1 0.0%	0 / 2 0.0%	0 / 1 0.0%	1 / 1 100.0%	0 / 4 0.0%	0 / 2 0.0%	1 / 1 100.0%	0 / 3 0.0%	0 / 0 0.0%	0 / 1 0.0%	0 / 0 0.0%	0 / 3 0.0%	0 / 5 0.0%	0 / 3 0.0%	0 / 2 0.0%	0 / 5 0.0%	0 / 0 0.0%	0 / 0 0.0%	0 / 0 0.0%	0 / 0 0.0%	31 / 47 66.0%E	13 / 47 27.7%	2 / 47 4.3%AB	1 / 47 2.1%ABE					
1	Enterococcus faecium Aus0004	2 / 46 4.3%	0 / 0 0.0%	0 / 1 0.0%	0 / 3 0.0%	0 / 0 0.0%	0 / 2 0.0%	0 / 2 0.0%	0 / 2 0.0%	0 / 2 0.0%	0 / 0 0.0%	1 / 1 100.0%	0 / 3 0.0%	0 / 2 0.0%	1 / 1 100.0%	0 / 4 0.0%	0 / 1 0.0%	0 / 1 0.0%	0 / 0 0.0%	0 / 3 0.0%	0 / 5 0.0%	0 / 2 0.0%	0 / 5 0.0%	0 / 6 0.0%	0 / 0 0.0%	0 / 0 0.0%	0 / 0 0.0%	0 / 0 0.0%	30 / 46 65.2%E	14 / 46 30.4%	2 / 46 4.3%AB						
1	ATCC 8043	3 / 69 4.3%	0 / 2 0.0%	1 / 3 33.3%	0 / 3 0.0%	0 / 2 0.0%	0 / 5 0.0%	0 / 3 0.0%	0 / 2 0.0%	0 / 2 0.0%	0 / 3 0.0%	1 / 1 100.0%	0 / 5 0.0%	0 / 3 0.0%	1 / 2 50.0%	0 / 4 0.0%	0 / 1 0.0%	0 / 2 0.0%	0 / 1 0.0%	0 / 4 0.0%	0 / 6 0.0%	0 / 3 0.0%	0 / 4 0.0%	0 / 7 0.0%	0 / 1 0.0%	0 / 0 0.0%	0 / 0 0.0%	0 / 0 0.0%	51 / 69 73.9%E	15 / 69 21.7%	3 / 69 4.3%AB						
1	Enterococcus hirae ATCC 8043	3 / 69 4.3%	0 / 2 0.0%	1 / 3 33.3%	0 / 3 0.0%	0 / 2 0.0%	0 / 5 0.0%	0 / 3 0.0%	0 / 2 0.0%	0 / 2 0.0%	0 / 3 0.0%	1 / 1 100.0%	0 / 5 0.0%	0 / 3 0.0%	1 / 2 50.0%	0 / 4 0.0%	0 / 1 0.0%	0 / 2 0.0%	0 / 1 0.0%	0 / 4 0.0%	0 / 6 0.0%	0 / 3 0.0%	0 / 4 0.0%	0 / 7 0.0%	0 / 1 0.0%	0 / 0 0.0%	0 / 0 0.0%	0 / 0 0.0%	51 / 69 73.9%E	15 / 69 21.7%	3 / 69 4.3%AB						
2	Melisococcus	2 / 116 1.7%	0 / 3 0.0%	0 / 3 0.0%	0 / 4 0.0%	0 / 3 0.0%	0 / 7 0.0%	0 / 4 0.0%	0 / 2 0.0%	0 / 4 0.0%	0 / 10 0.0%	2 / 2 100.0%	0 / 9 0.0%	0 / 6 0.0%	0 / 4 0.0%	0 / 6 0.0%	0 / 2 0.0%	0 / 4 0.0%	0 / 2 0.0%	0 / 8 0.0%	0 / 10 0.0%	0 / 7 0.0%	0 / 2 0.0%	0 / 14 0.0%	0 / 0 0.0%	0 / 0 0.0%	0 / 0 0.0%	0 / 0 0.0%	76 / 116 65.5%E	35 / 116 30.2%	3 / 116 2.6%CE	2 / 116 1.7%AB					
2	"Streptococcus pluton" (White 1912) Gubler 1954	2 / 116 1.7%	0 / 3 0.0%	0 / 3 0.0%	0 / 4 0.0%	0 / 3 0.0%	0 / 7 0.0%	0 / 4 0.0%	0 / 2 0.0%	0 / 4 0.0%	0 / 10 0.0%	2 / 2 100.0%	0 / 9 0.0%	0 / 6 0.0%	0 / 4 0.0%	0 / 6 0.0%	0 / 2 0.0%	0 / 4 0.0%	0 / 2 0.0%	0 / 8 0.0%	0 / 10 0.0%	0 / 7 0.0%	0 / 2 0.0%	0 / 14 0.0%	0 / 0 0.0%	0 / 0 0.0%	0 / 0 0.0%	0 / 0 0.0%	76 / 116 65.5%E	35 / 116 30.2%	3 / 116 2.6%CE	2 / 116 1.7%AB					
1	Melissococcus plutonius ATCC 35311	1 / 61 1.6%	0 / 2 0.0%	0 / 2 0.0%	0 / 2 0.0%	0 / 2 0.0%	0 / 4 0.0%	0 / 2 0.0%	0 / 1 0.0%	0 / 2 0.0%	0 / 5 0.0%	1 / 1 100.0%	0 / 5 0.0%	0 / 3 0.0%	0 / 2 0.0%	0 / 3 0.0%	0 / 1 0.0%	0 / 2 0.0%	0 / 1 0.0%	0 / 4 0.0%	0 / 5 0.0%	0 / 4 0.0%	0 / 1 0.0%	0 / 7 0.0%	0 / 0 0.0%	0 / 0 0.0%	0 / 0 0.0%	0 / 0 0.0%	40 / 61 65.6%E	18 / 61 29.5%	2 / 61 3.3%CE	1 / 61 1.6%AB					
1	Melissococcus plutonius DAT561	1 / 55 1.8%	0 / 1 0.0%	0 / 1 0.0%	0 / 2 0.0%	0 / 1 0.0%	0 / 3 0.0%	0 / 2 0.0%	0 / 1 0.0%	0 / 2 0.0%	0 / 5 0.0%	1 / 1 100.0%	0 / 4 0.0%	0 / 3 0.0%	0 / 2 0.0%	0 / 3 0.0%	0 / 1 0.0%	0 / 2 0.0%	0 / 1 0.0%	0 / 4 0.0%	0 / 5 0.0%	0 / 3 0.0%	0 / 1 0.0%	0 / 7 0.0%	0 / 0 0.0%	0 / 0 0.0%	0 / 0 0.0%	0 / 0 0.0%	36 / 55 65.5%E	17 / 55 30.9%	1 / 55 1.8%CE	1 / 55 1.8%AB					
1	Tetragenococcus	2 / 62 3.2%	0 / 2 0.0%	0 / 3 0.0%	0 / 3 0.0%	0 / 2 0.0%	0 / 5 0.0%	0 / 3 0.0%	0 / 1 0.0%	1 / 2 50.0%	0 / 3 0.0%	1 / 1 100.0%	0 / 5 0.0%	0 / 3 0.0%	0 / 2 0.0%	0 / 2 0.0%	0 / 1 0.0%	0 / 3 0.0%	0 / 1 0.0%	0 / 4 0.0%	0 / 5 0.0%	0 / 3 0.0%	0 / 1 0.0%	0 / 7 0.0%	0 / 0 0.0%	0 / 0 0.0%	0 / 0 0.0%	0 / 0 0.0%	40 / 62 64.5%E	20 / 62 32.3%	2 / 62 3.2%AB						
1	"Pediococcus soyae" Sakaguchi 1958	2 / 62 3.2%	0 / 2 0.0%	0 / 3 0.0%	0 / 3 0.0%	0 / 2 0.0%	0 / 5 0.0%	0 / 3 0.0%	0 / 1 0.0%	1 / 2 50.0%	0 / 3 0.0%	1 / 1 100.0%	0 / 5 0.0%	0 / 3 0.0%	0 / 2 0.0%	0 / 2 0.0%	0 / 1 0.0%	0 / 3 0.0%	0 / 1 0.0%	0 / 4 0.0%	0 / 5 0.0%	0 / 3 0.0%	0 / 1 0.0%	0 / 7 0.0%	0 / 0 0.0%	0 / 0 0.0%	0 / 0 0.0%	0 / 0 0.0%	40 / 62 64.5%E	20 / 62 32.3%	2 / 62 3.2%AB						
1	Tetragenococcus halophilus NBRC 12172	2 / 62 3.2%	0 / 2 0.0%	0 / 3 0.0%	0 / 3 0.0%	0 / 2 0.0%	0 / 5 0.0%	0 / 3 0.0%	0 / 1 0.0%	1 / 2 50.0%	0 / 3 0.0%	1 / 1 100.0%	0 / 5 0.0%	0 / 3 0.0%	0 / 2 0.0%	0 / 2 0.0%	0 / 1 0.0%	0 / 3 0.0%	0 / 1 0.0%	0 / 4 0.0%	0 / 5 0.0%	0 / 3 0.0%	0 / 1 0.0%	0 / 7 0.0%	0 / 0 0.0%	0 / 0 0.0%	0 / 0 0.0%	0 / 0 0.0%	40 / 62 64.5%E	20 / 62 32.3%	2 / 62 3.2%AB						
1	Aerococcaceae	0 / 60 0.0%	0 / 2 0.0%	0 / 3 0.0%	0 / 2 0.0%	0 / 3 0.0%	0 / 4 0.0%	0 / 3 0.0%	0 / 1 0.0%	0 / 2 0.0%	0 / 3 0.0%	0 / 1 0.0%	0 / 4 0.0%	0 / 3 0.0%	0 / 2 0.0%	0 / 2 0.0%	0 / 1 0.0%	0 / 4 0.0%	0 / 1 0.0%	0 / 3 0.0%	0 / 5 0.0%	0 / 4 0.0%	0 / 1 0.0%	0 / 6 0.0%	0 / 0 0.0%	0 / 0 0.0%	0 / 0 0.0%	0 / 0 0.0%	36 / 60 60.0%E	24 / 60 40.0%							
1	Aerococcus	0 / 60 0.0%	0 / 2 0.0%	0 / 3 0.0%	0 / 2 0.0%	0 / 3 0.0%	0 / 4 0.0%	0 / 3 0.0%	0 / 1 0.0%	0 / 2 0.0%	0 / 3 0.0%	0 / 1 0.0%	0 / 4 0.0%	0 / 3 0.0%	0 / 2 0.0%	0 / 2 0.0%	0 / 1 0.0%	0 / 4 0.0%	0 / 1 0.0%	0 / 3 0.0%	0 / 5 0.0%	0 / 4 0.0%	0 / 1 0.0%	0 / 6 0.0%	0 / 0 0.0%	0 / 0 0.0%	0 / 0 0.0%	0 / 0 0.0%	36 / 60 60.0%E	24 / 60 40.0%							
1	ATCC 51268	0 / 60 0.0%	0 / 2 0.0%	0 / 3 0.0%	0 / 2 0.0%	0 / 3 0.0%	0 / 4 0.0%	0 / 3 0.0%	0 / 1 0.0%	0 / 2 0.0%	0 / 3 0.0%	0 / 1 0.0%	0 / 4 0.0%	0 / 3 0.0%	0 / 2 0.0%	0 / 2 0.0%	0 / 1 0.0%	0 / 4 0.0%	0 / 1 0.0%	0 / 3 0.0%	0 / 5 0.0%	0 / 4 0.0%	0 / 1 0.0%	0 / 6 0.0%	0 / 0 0.0%	0 / 0 0.0%	0 / 0 0.0%	0 / 0 0.0%	36 / 60 60.0%E	24 / 60 40.0%							
1	Aerococcus urinae ACS-120-V-Col10a	0 / 60 0.0%	0 / 2 0.0%	0 / 3 0.0%	0 / 2 0.0%	0 / 3 0.0%	0 / 4 0.0%	0 / 3 0.0%	0 / 1 0.0%	0 / 2 0.0%	0 / 3 0.0%	0 / 1 0.0%	0 / 4 0.0%	0 / 3 0.0%	0 / 2 0.0%	0 / 2 0.0%	0 / 1 0.0%	0 / 4 0.0%	0 / 1 0.0%	0 / 3 0.0%	0 / 5 0.0%	0 / 4 0.0%	0 / 1 0.0%	0 / 6 0.0%	0 / 0 0.0%	0 / 0 0.0%	0 / 0 0.0%	0 / 0 0.0%	36 / 60 60.0%E	24 / 60 40.0%							
1	Carnobacteriaceae	13 / 67 19.4%	0 / 1 0.0%	0 / 3 0.0%	0 / 3 0.0%	0 / 2 0.0%	0 / 3 0.0%	2 / 5 40.0%	0 / 2 0.0%	3 / 3 100.0%	0 / 4 0.0%	2 / 2 100.0%	0 / 3 0.0%	0 / 4 0.0%	2 / 3 66.7%	0 / 3 0.0%	0 / 2 0.0%	0 / 2 0.0%	0 / 1 0.0%	0 / 4 0.0%	0 / 5 0.0%	0 / 4 0.0%	0 / 1 0.0%	4 / 7 57.1%	0 / 0 0.0%	0 / 0 0.0%	0 / 0 0.0%	0 / 0 0.0%	32 / 67 47.8%E	22 / 67 32.8%	9 / 67 13.4%AB	4 / 67 6.0%ABE					
1	Carnibacterium	13 / 67 19.4%	0 / 1 0.0%	0 / 3 0.0%	0 / 3 0.0%	0 / 2 0.0%	0 / 3 0.0%	2 / 5 40.0%	0 / 2 0.0%	3 / 3 100.0%	0 / 4 0.0%	2 / 2 100.0%	0 / 3 0.0%	0 / 4 0.0%	2 / 3 66.7%	0 / 3 0.0%	0 / 2 0.0%	0 / 2 0.0%	0 / 1 0.0%	0 / 4 0.0%	0 / 5 0.0%	0 / 4 0.0%	0 / 1 0.0%	4 / 7 57.1%	0 / 0 0.0%	0 / 0 0.0%	0 / 0 0.0%	0 / 0 0.0%	32 / 67 47.8%E	22 / 67 32.8%	9 / 67 13.4%AB	4 / 67 6.0%ABE					
1	Carnobacterium sp. 17-4	13 / 67 19.4%	0 / 1 0.0%	0 / 3 0.0%	0 / 3 0.0%	0 / 2 0.0%	0 / 3 0.0%	2 / 5 40.0%	0 / 2 0.0%	3 / 3 100.0%	0 / 4 0.0%	2 / 2 100.0%	0 / 3 0.0%	0 / 4 0.0%	2 / 3 66.7%	0 / 3 0.0%	0 / 2 0.0%	0 / 2 0.0%	0 / 1 0.0%	0 / 4 0.0%	0 / 5 0.0%	0 / 4 0.0%	0 / 1 0.0%	4 / 7 57.1%	0 / 0 0.0%	0 / 0 0.0%	0 / 0 0.0%	0 / 0 0.0%	32 / 67 47.8%E	22 / 67 32.8%	9 / 67 13.4%AB	4 / 67 6.0%ABE					
131	Clostridia	7446 / 8271 90.0%	235 / 246 95.5%	284 / 308 92.2%	299 / 325 92.0%	195 / 221 88.2%	634 / 695 91.2%	449 / 498 90.2%	162 / 183 88.5%	222 / 245 90.6%	391 / 434 90.1%	134 / 147 91.2%	521 / 568 91.7%	458 / 504 90.9%	246 / 264 93.2%	276 / 304 90.8%	165 / 179 92.2%	339 / 379 89.4%	143 / 184 77.7%	447 / 499 89.6%	572 / 633 90.4%	374 / 424 88.2%	182 / 195 93.3%	654 / 768 85.2%	64 / 68 94.1%	0 / 0 0.0%	0 / 0 0.0%	0 / 0 0.0%	4395 / 8271 53.1%AB	2494 / 8271 30.2%ABE	522 / 8271 6.3%E	333 / 8271 4.0%B	282 / 8271 3.4%	224 / 8271 2.7%BE	11 / 8271 0.1%CE	9 / 8271 0.1%C	1 / 8271 0.0%EGG
5	Halanaerobiales	307 / 311 98.7%	12 / 12 100.0%	13 / 13 100.0%	10 / 10 100.0%	10 / 10 100.0%	21 / 22 95.5%	18 / 18 100.0%	8 / 8 100.0%	10 / 10 100.0%	15 / 15 100.0%	10 / 10 100.0%	20 / 21 95.2%	16 / 16 100.0%	10 / 11 90.9%	11 / 11 100.0%	6 / 6 100.0%	15 / 15 100.0%	5 / 5 100.0%	18 / 18 100.0%	21 / 21 100.0%	18 / 18 100.0%	6 / 6 100.0%	31 / 31 100.0%	3 / 4 75.0%	0 / 0 0.0%	0 / 0 0.0%	0 / 0 0.0%	202 / 311 65.0%AB	105 / 311 33.8%ABE	3 / 311 1.0%CE	1 / 311 0.3%C					
3	Halanaerobiaceae	167 / 168 99.4%	6 / 6 100.0%	6 / 6 100.0%	6 / 6 100.0%	7 / 7 100.0%	11 / 12 91.7%	9 / 9 100.0%	4 / 4 100.0%	6 / 6 100.0%	10 / 10 100.0%	6 / 6 100.0%	12 / 12 100.0%	7 / 7 100.0%	6 / 6 100.0%	4 / 4 100.0%	3 / 3 100.0%	8 / 8 100.0%	3 / 3 100.0%	10 / 10 100.0%	12 / 12 100.0%	9 / 9 100.0%	3 / 3 100.0%	17 / 17 100.0%	2 / 2 100.0%	0 / 0 0.0%	0 / 0 0.0%	0 / 0 0.0%	113 / 168 67.3%AB	54 / 168 32.1%ABE	1 / 168 0.6%C						
2	Halanaerobium	111 / 112 99.1%	4 / 4 100.0%	4 / 4 100.0%	4 / 4 100.0%	5 / 5 100.0%	7 / 8 87.5%	6 / 6 100.0%	3 / 3 100.0%	4 / 4 100.0%	7 / 7 100.0%	4 / 4 100.0%	8 / 8 100.0%	4 / 4 100.0%	4 / 4 100.0%	2 / 2 100.0%	2 / 2 100.0%	5 / 5 100.0%	2 / 2 100.0%	7 / 7 100.0%	8 / 8 100.0%	6 / 6 100.0%	2 / 2 100.0%	11 / 11 100.0%	2 / 2 100.0%	0 / 0 0.0%	0 / 0 0.0%	0 / 0 0.0%	77 / 112 68.8%AB	34 / 112 30.4%ABE	1 / 112 0.9%C						
1	ATCC 33744	55 / 55 100.0%	2 / 2 100.0%	2 / 2 100.0%	2 / 2 100.0%	2 / 2 100.0%	4 / 4 100.0%	3 / 3 100.0%	1 / 1 100.0%	2 / 2 100.0%	3 / 3 100.0%	2 / 2 100.0%	4 / 4 100.0%	2 / 2 100.0%	2 / 2 100.0%	1 / 1 100.0%	1 / 1 100.0%	2 / 2 100.0%	1 / 1 100.0%	4 / 4 100.0%	4 / 4 100.0%	3 / 3 100.0%	1 / 1 100.0%	5 / 5 100.0%	2 / 2 100.0%	0 / 0 0.0%	0 / 0 0.0%	0 / 0 0.0%	38 / 55 69.1%AB	17 / 55 30.9%ABE							
1	Halanaerobium praevalens ATCC 33744	55 / 55 100.0%	2 / 2 100.0%	2 / 2 100.0%	2 / 2 100.0%	2 / 2 100.0%	4 / 4 100.0%	3 / 3 100.0%	1 / 1 100.0%	2 / 2 100.0%	3 / 3 100.0%	2 / 2 100.0%	4 / 4 100.0%	2 / 2 100.0%	2 / 2 100.0%	1 / 1 100.0%	1 / 1 100.0%	2 / 2 100.0%	1 / 1 100.0%	4 / 4 100.0%	4 / 4 100.0%	3 / 3 100.0%	1 / 1 100.0%	5 / 5 100.0%	2 / 2 100.0%	0 / 0 0.0%	0 / 0 0.0%	0 / 0 0.0%	38 / 55 69.1%AB	17 / 55 30.9%ABE							
1	"Halanaerobium hydrogeniformans" Brown et al. 2011	56 / 57 98.2%	2 / 2 100.0%	2 / 2 100.0%	2 / 2 100.0%	3 / 3 100.0%	3 / 4 75.0%	3 / 3 100.0%	2 / 2 100.0%	2 / 2 100.0%	4 / 4 100.0%	2 / 2 100.0%	4 / 4 100.0%	2 / 2 100.0%	2 / 2 100.0%	1 / 1 100.0%	1 / 1 100.0%	3 / 3 100.0%	1 / 1 100.0%	3 / 3 100.0%	4 / 4 100.0%	3 / 3 100.0%	1 / 1 100.0%	6 / 6 100.0%	0 / 0 0.0%	0 / 0 0.0%	0 / 0 0.0%	0 / 0 0.0%	39 / 57 68.4%AB	17 / 57 29.8%ABE	1 / 57 1.8%C						
1	Halothermothrix	56 / 56 100.0%	2 / 2 100.0%	2 / 2 100.0%	2 / 2 100.0%	2 / 2 100.0%	4 / 4 100.0%	3 / 3 100.0%	1 / 1 100.0%	2 / 2 100.0%	3 / 3 100.0%	2 / 2 100.0%	4 / 4 100.0%	3 / 3 100.0%	2 / 2 100.0%	2 / 2 100.0%	1 / 1 100.0%	3 / 3 100.0%	1 / 1 100.0%	3 / 3 100.0%	4 / 4 100.0%	3 / 3 100.0%	1 / 1 100.0%	6 / 6 100.0%	0 / 0 0.0%	0 / 0 0.0%	0 / 0 0.0%	0 / 0 0.0%	36 / 56 64.3%AB	20 / 56 35.7%ABE							
1	DSM 9562	56 / 56 100.0%	2 / 2 100.0%	2 / 2 100.0%	2 / 2 100.0%	2 / 2 100.0%	4 / 4 100.0%	3 / 3 100.0%	1 / 1 100.0%	2 / 2 100.0%	3 / 3 100.0%	2 / 2 100.0%	4 / 4 100.0%	3 / 3 100.0%	2 / 2 100.0%	2 / 2 100.0%	1 / 1 100.0%	3 / 3 100.0%	1 / 1 100.0%	3 / 3 100.0%	4 / 4 100.0%	3 / 3 100.0%	1 / 1 100.0%	6 / 6 100.0%	0 / 0 0.0%	0 / 0 0.0%	0 / 0 0.0%	0 / 0 0.0%	36 / 56 64.3%AB	20 / 56 35.7%ABE							
1	Halothermothrix orenii H 168	56 / 56 100.0%	2 / 2 100.0%	2 / 2 100.0%	2 / 2 100.0%	2 / 2 100.0%	4 / 4 100.0%	3 / 3 100.0%	1 / 1 100.0%	2 / 2 100.0%	3 / 3 100.0%	2 / 2 100.0%	4 / 4 100.0%	3 / 3 100.0%	2 / 2 100.0%	2 / 2 100.0%	1 / 1 100.0%	3 / 3 100.0%	1 / 1 100.0%	3 / 3 100.0%	4 / 4 100.0%	3 / 3 100.0%	1 / 1 100.0%	6 / 6 100.0%	0 / 0 0.0%	0 / 0 0.0%	0 / 0 0.0%	0 / 0 0.0%	36 / 56 64.3%AB	20 / 56 35.7%ABE							
2	Halobacteroidaceae	140 / 143 97.9%	6 / 6 100.0%	7 / 7 100.0%	4 / 4 100.0%	3 / 3 100.0%	10 / 10 100.0%	9 / 9 100.0%	4 / 4 100.0%	4 / 4 100.0%	5 / 5 100.0%	4 / 4 100.0%	8 / 9 88.9%	9 / 9 100.0%	4 / 5 80.0%	7 / 7 100.0%	3 / 3 100.0%	7 / 7 100.0%	2 / 2 100.0%	8 / 8 100.0%	9 / 9 100.0%	9 / 9 100.0%	3 / 3 100.0%	14 / 14 100.0%	1 / 2 50.0%	0 / 0 0.0%	0 / 0 0.0%	0 / 0 0.0%	89 / 143 62.2%AB	51 / 143 35.7%ABE	3 / 143 2.1%CE						
1	Acetohalobium	67 / 67 100.0%	3 / 3 100.0%	3 / 3 100.0%	2 / 2 100.0%	2 / 2 100.0%	5 / 5 100.0%	4 / 4 100.0%	2 / 2 100.0%	2 / 2 100.0%	3 / 3 100.0%	1 / 1 100.0%	4 / 4 100.0%	4 / 4 100.0%	2 / 2 100.0%	4 / 4 100.0%	1 / 1 100.0%	4 / 4 100.0%	1 / 1 100.0%	4 / 4 100.0%	4 / 4 100.0%	4 / 4 100.0%	2 / 2 100.0%	5 / 5 100.0%	1 / 1 100.0%	0 / 0 0.0%	0 / 0 0.0%	0 / 0 0.0%	45 / 67 67.2%AB	22 / 67 32.8%ABE							
1	ATCC 49924	67 / 67 100.0%	3 / 3 100.0%	3 / 3 100.0%	2 / 2 100.0%	2 / 2 100.0%	5 / 5 100.0%	4 / 4 100.0%	2 / 2 100.0%	2 / 2 100.0%	3 / 3 100.0%	1 / 1 100.0%	4 / 4 100.0%	4 / 4 100.0%	2 / 2 100.0%	4 / 4 100.0%	1 / 1 100.0%	4 / 4 100.0%	1 / 1 100.0%	4 / 4 100.0%	4 / 4 100.0%	4 / 4 100.0%	2 / 2 100.0%	5 / 5 100.0%	1 / 1 100.0%	0 / 0 0.0%	0 / 0 0.0%	0 / 0 0.0%	45 / 67 67.2%AB	22 / 67 32.8%ABE							
1	Acetohalobium arabaticum DSM 5501	67 / 67 100.0%	3 / 3 100.0%	3 / 3 100.0%	2 / 2 100.0%	2 / 2 100.0%	5 / 5 100.0%	4 / 4 100.0%	2 / 2 100.0%	2 / 2 100.0%	3 / 3 100.0%	1 / 1 100.0%	4 / 4 100.0%	4 / 4 100.0%	2 / 2 100.0%	4 / 4 100.0%	1 / 1 100.0%	4 / 4 100.0%	1 / 1 100.0%	4 / 4 100.0%	4 / 4 100.0%	4 / 4 100.0%	2 / 2 100.0%	5 / 5 100.0%	1 / 1 100.0%	0 / 0 0.0%	0 / 0 0.0%	0 / 0 0.0%	45 / 67 67.2%AB	22 / 67 32.8%ABE							
1	Halobacteroides	73 / 76 96.1%	3 / 3 100.0%	4 / 4 100.0%	2 / 2 100.0%	1 / 1 100.0%	5 / 5 100.0%	5 / 5 100.0%	2 / 2 100.0%	2 / 2 100.0%	2 / 2 100.0%	3 / 3 100.0%	4 / 5 80.0%	5 / 5 100.0%	2 / 3 66.7%	3 / 3 100.0%	2 / 2 100.0%	3 / 3 100.0%	1 / 1 100.0%	4 / 4 100.0%	5 / 5 100.0%	5 / 5 100.0%	1 / 1 100.0%	9 / 9 100.0%	0 / 1 0.0%	0 / 0 0.0%	0 / 0 0.0%	0 / 0 0.0%	44 / 76 57.9%AB	29 / 76 38.2%ABE	3 / 76 3.9%CE						
1	ATCC 35273	73 / 76 96.1%	3 / 3 100.0%	4 / 4 100.0%	2 / 2 100.0%	1 / 1 100.0%	5 / 5 100.0%	5 / 5 100.0%	2 / 2 100.0%	2 / 2 100.0%	2 / 2 100.0%	3 / 3 100.0%	4 / 5 80.0%	5 / 5 100.0%	2 / 3 66.7%	3 / 3 100.0%	2 / 2 100.0%	3 / 3 100.0%	1 / 1 100.0%	4 / 4 100.0%	5 / 5 100.0%	5 / 5 100.0%	1 / 1 100.0%	9 / 9 100.0%	0 / 1 0.0%	0 / 0 0.0%	0 / 0 0.0%	0 / 0 0.0%	44 / 76 57.9%AB	29 / 76 38.2%ABE	3 / 76 3.9%CE						
1	Halobacteroides halobius ATCC 35273	73 / 76 96.1%	3 / 3 100.0%	4 / 4 100.0%	2 / 2 100.0%	1 / 1 100.0%	5 / 5 100.0%	5 / 5 100.0%	2 / 2 100.0%	2 / 2 100.0%	2 / 2 100.0%	3 / 3 100.0%	4 / 5 80.0%	5 / 5 100.0%	2 / 3 66.7%	3 / 3 100.0%	2 / 2 100.0%	3 / 3 100.0%	1 / 1 100.0%	4 / 4 100.0%	5 / 5 100.0%	5 / 5 100.0%	1 / 1 100.0%	9 / 9 100.0%	0 / 1 0.0%	0 / 0 0.0%	0 / 0 0.0%	0 / 0 0.0%	44 / 76 57.9%AB	29 / 76 38.2%ABE	3 / 76 3.9%CE						
30	Thermoanaerobacter group	1502 / 1539 97.6%	34 / 34 100.0%	43 / 43 100.0%	59 / 60 98.3%	37 / 41 90.2%	109 / 109 100.0%	92 / 94 97.9%	34 / 34 100.0%	44 / 45 97.8%	78 / 86 90.7%	30 / 30 100.0%	120 / 121 99.2%	80 / 85 94.1%	41 / 42 97.6%	60 / 60 100.0%	30 / 30 100.0%	90 / 90 100.0%	27 / 30 90.0%	88 / 91 96.7%	127 / 129 98.4%	84 / 86 97.7%	42 / 42 100.0%	145 / 149 97.3%	8 / 8 100.0%	0 / 0 0.0%	0 / 0 0.0%	0 / 0 0.0%	973 / 1539 63.2%AB	491 / 1539 31.9%ABE	26 / 1539 1.7%B	22 / 1539 1.4%	14 / 1539 0.9%E	12 / 1539 0.8%BE	1 / 1539 0.1%CE		
14	Thermoanaerobacteraceae	744 / 748 99.5%	17 / 17 100.0%	23 / 23 100.0%	35 / 36 97.2%	13 / 13 100.0%	55 / 55 100.0%	50 / 50 100.0%	14 / 14 100.0%	24 / 24 100.0%	43 / 43 100.0%	14 / 14 100.0%	55 / 56 98.2%	39 / 39 100.0%	22 / 22 100.0%	28 / 28 100.0%	14 / 14 100.0%	42 / 42 100.0%	14 / 14 100.0%	43 / 43 100.0%	64 / 64 100.0%	41 / 41 100.0%	22 / 22 100.0%	67 / 69 97.1%	5 / 5 100.0%	0 / 0 0.0%	0 / 0 0.0%	0 / 0 0.0%	472 / 748 63.1%AB	234 / 748 31.3%ABE	26 / 748 3.5%B	12 / 748 1.6%BE	3 / 748 0.4%E	1 / 748 0.1%CE			

7	Acetigenium	[illegible] / 392 99.2%	7 / 7 100.0%	14 / 14 100.0%	21 / 21 100.0%	6 / 6 100.0%	28 / 28 100.0%	28 / 28 100.0%	7 / 7 100.0%	14 / 14 100.0%	22 / 22 100.0%	7 / 7 100.0%	27 / 28 96.4%	21 / 21 100.0%	14 / 14 100.0%	14 / 14 100.0%	7 / 7 100.0%	21 / 21 100.0%	7 / 7 100.0%	21 / 21 100.0%	35 / 35 100.0%	21 / 21 100.0%	14 / 14 100.0%	33 / 35 94.3%	0 / 0 0.0%	0 / 0 0.0%	0 / 0 0.0%	0 / 0 0.0%	248 / 392 63.3%AB	104 / 392 26.5%ABE	26 / 392 6.6%B	11 / 392 2.8%BE	3 / 392 0.8%E				
1	ATCC 33075	56 / 56 100.0%	1 / 1 100.0%	2 / 2 100.0%	3 / 3 100.0%	1 / 1 100.0%	4 / 4 100.0%	4 / 4 100.0%	1 / 1 100.0%	2 / 2 100.0%	3 / 3 100.0%	1 / 1 100.0%	4 / 4 100.0%	3 / 3 100.0%	2 / 2 100.0%	2 / 2 100.0%	1 / 1 100.0%	3 / 3 100.0%	1 / 1 100.0%	3 / 3 100.0%	5 / 5 100.0%	3 / 3 100.0%	2 / 2 100.0%	5 / 5 100.0%	0 / 0 0.0%	0 / 0 0.0%	0 / 0 0.0%	0 / 0 0.0%	39 / 56 69.6%AB	17 / 56 30.4%ABE							
1	ATCC 43586	56 / 56 100.0%	1 / 1 100.0%	2 / 2 100.0%	3 / 3 100.0%	1 / 1 100.0%	4 / 4 100.0%	4 / 4 100.0%	1 / 1 100.0%	2 / 2 100.0%	3 / 3 100.0%	1 / 1 100.0%	4 / 4 100.0%	3 / 3 100.0%	2 / 2 100.0%	2 / 2 100.0%	1 / 1 100.0%	3 / 3 100.0%	1 / 1 100.0%	3 / 3 100.0%	5 / 5 100.0%	3 / 3 100.0%	2 / 2 100.0%	5 / 5 100.0%	0 / 0 0.0%	0 / 0 0.0%	0 / 0 0.0%	0 / 0 0.0%	39 / 56 69.6%AB	17 / 56 30.4%ABE							
1	Thermoanaerobacter brockii subsp. finnii Ako-1	56 / 56 100.0%	1 / 1 100.0%	2 / 2 100.0%	3 / 3 100.0%	1 / 1 100.0%	4 / 4 100.0%	4 / 4 100.0%	1 / 1 100.0%	2 / 2 100.0%	3 / 3 100.0%	1 / 1 100.0%	4 / 4 100.0%	3 / 3 100.0%	2 / 2 100.0%	2 / 2 100.0%	1 / 1 100.0%	3 / 3 100.0%	1 / 1 100.0%	3 / 3 100.0%	5 / 5 100.0%	3 / 3 100.0%	2 / 2 100.0%	5 / 5 100.0%	0 / 0 0.0%	0 / 0 0.0%	0 / 0 0.0%	0 / 0 0.0%	39 / 56 69.6%AB	17 / 56 30.4%ABE							
1	DSM 10319	57 / 57 100.0%	1 / 1 100.0%	2 / 2 100.0%	3 / 3 100.0%	1 / 1 100.0%	4 / 4 100.0%	4 / 4 100.0%	1 / 1 100.0%	2 / 2 100.0%	4 / 4 100.0%	1 / 1 100.0%	4 / 4 100.0%	3 / 3 100.0%	2 / 2 100.0%	2 / 2 100.0%	1 / 1 100.0%	3 / 3 100.0%	1 / 1 100.0%	3 / 3 100.0%	5 / 5 100.0%	3 / 3 100.0%	2 / 2 100.0%	5 / 5 100.0%	0 / 0 0.0%	0 / 0 0.0%	0 / 0 0.0%	0 / 0 0.0%	40 / 57 70.2%AB	17 / 57 29.8%ABE							
1	Thermoanaerobacter wiegelii Rt8.B1	57 / 57 100.0%	1 / 1 100.0%	2 / 2 100.0%	3 / 3 100.0%	1 / 1 100.0%	4 / 4 100.0%	4 / 4 100.0%	1 / 1 100.0%	2 / 2 100.0%	4 / 4 100.0%	1 / 1 100.0%	4 / 4 100.0%	3 / 3 100.0%	2 / 2 100.0%	2 / 2 100.0%	1 / 1 100.0%	3 / 3 100.0%	1 / 1 100.0%	3 / 3 100.0%	5 / 5 100.0%	3 / 3 100.0%	2 / 2 100.0%	5 / 5 100.0%	0 / 0 0.0%	0 / 0 0.0%	0 / 0 0.0%	0 / 0 0.0%	40 / 57 70.2%AB	17 / 57 29.8%ABE							
1	DSM 9252	54 / 54 100.0%	1 / 1 100.0%	2 / 2 100.0%	3 / 3 100.0%	0 / 0 0.0%	4 / 4 100.0%	4 / 4 100.0%	1 / 1 100.0%	2 / 2 100.0%	2 / 2 100.0%	1 / 1 100.0%	4 / 4 100.0%	3 / 3 100.0%	2 / 2 100.0%	2 / 2 100.0%	1 / 1 100.0%	3 / 3 100.0%	1 / 1 100.0%	3 / 3 100.0%	5 / 5 100.0%	3 / 3 100.0%	2 / 2 100.0%	5 / 5 100.0%	0 / 0 0.0%	0 / 0 0.0%	0 / 0 0.0%	0 / 0 0.0%	37 / 54 68.5%AB	17 / 54 31.5%ABE							
1	Thermoanaerobacter italicus Ab9	54 / 54 100.0%	1 / 1 100.0%	2 / 2 100.0%	3 / 3 100.0%	0 / 0 0.0%	4 / 4 100.0%	4 / 4 100.0%	1 / 1 100.0%	2 / 2 100.0%	2 / 2 100.0%	1 / 1 100.0%	4 / 4 100.0%	3 / 3 100.0%	2 / 2 100.0%	2 / 2 100.0%	1 / 1 100.0%	3 / 3 100.0%	1 / 1 100.0%	3 / 3 100.0%	5 / 5 100.0%	3 / 3 100.0%	2 / 2 100.0%	5 / 5 100.0%	0 / 0 0.0%	0 / 0 0.0%	0 / 0 0.0%	0 / 0 0.0%	37 / 54 68.5%AB	17 / 54 31.5%ABE							
1	Thermoanaerobacter ethanolicus X514	53 / 54 98.1%	1 / 1 100.0%	2 / 2 100.0%	3 / 3 100.0%	0 / 0 0.0%	4 / 4 100.0%	4 / 4 100.0%	1 / 1 100.0%	2 / 2 100.0%	2 / 2 100.0%	1 / 1 100.0%	4 / 4 100.0%	3 / 3 100.0%	2 / 2 100.0%	2 / 2 100.0%	1 / 1 100.0%	3 / 3 100.0%	1 / 1 100.0%	3 / 3 100.0%	5 / 5 100.0%	3 / 3 100.0%	2 / 2 100.0%	4 / 5 80.0%	0 / 0 0.0%	0 / 0 0.0%	0 / 0 0.0%	0 / 0 0.0%	27 / 54 50.0%AB	12 / 54 22.2%ABE	10 / 54 18.5%B	4 / 54 7.4%BE	1 / 54 1.9%E				
1	ATCC 33223	54 / 56 96.4%	1 / 1 100.0%	2 / 2 100.0%	3 / 3 100.0%	1 / 1 100.0%	4 / 4 100.0%	4 / 4 100.0%	1 / 1 100.0%	2 / 2 100.0%	3 / 3 100.0%	1 / 1 100.0%	3 / 4 75.0%	3 / 3 100.0%	2 / 2 100.0%	2 / 2 100.0%	1 / 1 100.0%	3 / 3 100.0%	1 / 1 100.0%	3 / 3 100.0%	5 / 5 100.0%	3 / 3 100.0%	2 / 2 100.0%	4 / 5 80.0%	0 / 0 0.0%	0 / 0 0.0%	0 / 0 0.0%	0 / 0 0.0%	23 / 56 41.1%AB	16 / 56 28.6%B	8 / 56 14.3%ABE	7 / 56 12.5%BE	2 / 56 3.6%E				
1	Clostridium thermohydrosulfuricum 39E	54 / 56 96.4%	1 / 1 100.0%	2 / 2 100.0%	3 / 3 100.0%	1 / 1 100.0%	4 / 4 100.0%	4 / 4 100.0%	1 / 1 100.0%	2 / 2 100.0%	3 / 3 100.0%	1 / 1 100.0%	3 / 4 75.0%	3 / 3 100.0%	2 / 2 100.0%	2 / 2 100.0%	1 / 1 100.0%	3 / 3 100.0%	1 / 1 100.0%	3 / 3 100.0%	5 / 5 100.0%	3 / 3 100.0%	2 / 2 100.0%	4 / 5 80.0%	0 / 0 0.0%	0 / 0 0.0%	0 / 0 0.0%	0 / 0 0.0%	23 / 56 41.1%AB	16 / 56 28.6%B	8 / 56 14.3%ABE	7 / 56 12.5%BE	2 / 56 3.6%E				
1	Thermoanaerobacter ethanolicus X513	56 / 56 100.0%	1 / 1 100.0%	2 / 2 100.0%	3 / 3 100.0%	1 / 1 100.0%	4 / 4 100.0%	4 / 4 100.0%	1 / 1 100.0%	2 / 2 100.0%	3 / 3 100.0%	1 / 1 100.0%	4 / 4 100.0%	3 / 3 100.0%	2 / 2 100.0%	2 / 2 100.0%	1 / 1 100.0%	3 / 3 100.0%	1 / 1 100.0%	3 / 3 100.0%	5 / 5 100.0%	3 / 3 100.0%	2 / 2 100.0%	5 / 5 100.0%	0 / 0 0.0%	0 / 0 0.0%	0 / 0 0.0%	0 / 0 0.0%	39 / 56 69.6%AB	17 / 56 30.4%ABE							
1	AIP 130-05	59 / 59 100.0%	1 / 1 100.0%	2 / 2 100.0%	3 / 3 100.0%	2 / 2 100.0%	4 / 4 100.0%	4 / 4 100.0%	1 / 1 100.0%	2 / 2 100.0%	5 / 5 100.0%	1 / 1 100.0%	4 / 4 100.0%	3 / 3 100.0%	2 / 2 100.0%	2 / 2 100.0%	1 / 1 100.0%	3 / 3 100.0%	1 / 1 100.0%	3 / 3 100.0%	5 / 5 100.0%	3 / 3 100.0%	2 / 2 100.0%	5 / 5 100.0%	0 / 0 0.0%	0 / 0 0.0%	0 / 0 0.0%	0 / 0 0.0%	43 / 59 72.9%AB	16 / 59 27.1%ABE							
1	Thermoanaerobacter mathranii subsp. mathranii	59 / 59 100.0%	1 / 1 100.0%	2 / 2 100.0%	3 / 3 100.0%	2 / 2 100.0%	4 / 4 100.0%	4 / 4 100.0%	1 / 1 100.0%	2 / 2 100.0%	5 / 5 100.0%	1 / 1 100.0%	4 / 4 100.0%	3 / 3 100.0%	2 / 2 100.0%	2 / 2 100.0%	1 / 1 100.0%	3 / 3 100.0%	1 / 1 100.0%	3 / 3 100.0%	5 / 5 100.0%	3 / 3 100.0%	2 / 2 100.0%	5 / 5 100.0%	0 / 0 0.0%	0 / 0 0.0%	0 / 0 0.0%	0 / 0 0.0%	43 / 59 72.9%AB	16 / 59 27.1%ABE							
1	Thermoanaerobacter mathranii subsp. mathranii A3	59 / 59 100.0%	1 / 1 100.0%	2 / 2 100.0%	3 / 3 100.0%	2 / 2 100.0%	4 / 4 100.0%	4 / 4 100.0%	1 / 1 100.0%	2 / 2 100.0%	5 / 5 100.0%	1 / 1 100.0%	4 / 4 100.0%	3 / 3 100.0%	2 / 2 100.0%	2 / 2 100.0%	1 / 1 100.0%	3 / 3 100.0%	1 / 1 100.0%	3 / 3 100.0%	5 / 5 100.0%	3 / 3 100.0%	2 / 2 100.0%	5 / 5 100.0%	0 / 0 0.0%	0 / 0 0.0%	0 / 0 0.0%	0 / 0 0.0%	43 / 59 72.9%AB	16 / 59 27.1%ABE							
2	Clostridium thermoautotrophicum group	98 / 98 100.0%	2 / 2 100.0%	3 / 3 100.0%	4 / 4 100.0%	2 / 2 100.0%	7 / 7 100.0%	6 / 6 100.0%	2 / 2 100.0%	2 / 2 100.0%	6 / 6 100.0%	2 / 2 100.0%	8 / 8 100.0%	5 / 5 100.0%	2 / 2 100.0%	4 / 4 100.0%	2 / 2 100.0%	6 / 6 100.0%	2 / 2 100.0%	6 / 6 100.0%	8 / 8 100.0%	5 / 5 100.0%	2 / 2 100.0%	10 / 10 100.0%	2 / 2 100.0%	0 / 0 0.0%	0 / 0 0.0%	0 / 0 0.0%	63 / 98 64.3%AB	34 / 98 34.7%ABE	1 / 98 1.0%BE						
1	Ammonifex	47 / 47 100.0%	1 / 1 100.0%	1 / 1 100.0%	2 / 2 100.0%	1 / 1 100.0%	3 / 3 100.0%	3 / 3 100.0%	1 / 1 100.0%	1 / 1 100.0%	3 / 3 100.0%	1 / 1 100.0%	4 / 4 100.0%	2 / 2 100.0%	1 / 1 100.0%	2 / 2 100.0%	1 / 1 100.0%	3 / 3 100.0%	1 / 1 100.0%	3 / 3 100.0%	4 / 4 100.0%	2 / 2 100.0%	1 / 1 100.0%	5 / 5 100.0%	1 / 1 100.0%	0 / 0 0.0%	0 / 0 0.0%	0 / 0 0.0%	30 / 47 63.8%AB	17 / 47 36.2%ABE							
1	Ammonifex degensii	47 / 47 100.0%	1 / 1 100.0%	1 / 1 100.0%	2 / 2 100.0%	1 / 1 100.0%	3 / 3 100.0%	3 / 3 100.0%	1 / 1 100.0%	1 / 1 100.0%	3 / 3 100.0%	1 / 1 100.0%	4 / 4 100.0%	2 / 2 100.0%	1 / 1 100.0%	2 / 2 100.0%	1 / 1 100.0%	3 / 3 100.0%	1 / 1 100.0%	3 / 3 100.0%	4 / 4 100.0%	2 / 2 100.0%	1 / 1 100.0%	5 / 5 100.0%	1 / 1 100.0%	0 / 0 0.0%	0 / 0 0.0%	0 / 0 0.0%	30 / 47 63.8%AB	17 / 47 36.2%ABE							
1	Ammonifex degensii KC4	47 / 47 100.0%	1 / 1 100.0%	1 / 1 100.0%	2 / 2 100.0%	1 / 1 100.0%	3 / 3 100.0%	3 / 3 100.0%	1 / 1 100.0%	1 / 1 100.0%	3 / 3 100.0%	1 / 1 100.0%	4 / 4 100.0%	2 / 2 100.0%	1 / 1 100.0%	2 / 2 100.0%	1 / 1 100.0%	3 / 3 100.0%	1 / 1 100.0%	3 / 3 100.0%	4 / 4 100.0%	2 / 2 100.0%	1 / 1 100.0%	5 / 5 100.0%	1 / 1 100.0%	0 / 0 0.0%	0 / 0 0.0%	0 / 0 0.0%	30 / 47 63.8%AB	17 / 47 36.2%ABE							
1	Moorella	51 / 51 100.0%	1 / 1 100.0%	2 / 2 100.0%	2 / 2 100.0%	1 / 1 100.0%	4 / 4 100.0%	3 / 3 100.0%	1 / 1 100.0%	1 / 1 100.0%	3 / 3 100.0%	1 / 1 100.0%	4 / 4 100.0%	3 / 3 100.0%	1 / 1 100.0%	2 / 2 100.0%	1 / 1 100.0%	3 / 3 100.0%	1 / 1 100.0%	3 / 3 100.0%	4 / 4 100.0%	3 / 3 100.0%	1 / 1 100.0%	5 / 5 100.0%	1 / 1 100.0%	0 / 0 0.0%	0 / 0 0.0%	0 / 0 0.0%	33 / 51 64.7%AB	17 / 51 33.3%ABE	1 / 51 2.0%BE						
1	ATCC 35608	51 / 51 100.0%	1 / 1 100.0%	2 / 2 100.0%	2 / 2 100.0%	1 / 1 100.0%	4 / 4 100.0%	3 / 3 100.0%	1 / 1 100.0%	1 / 1 100.0%	3 / 3 100.0%	1 / 1 100.0%	4 / 4 100.0%	3 / 3 100.0%	1 / 1 100.0%	2 / 2 100.0%	1 / 1 100.0%	3 / 3 100.0%	1 / 1 100.0%	3 / 3 100.0%	4 / 4 100.0%	3 / 3 100.0%	1 / 1 100.0%	5 / 5 100.0%	1 / 1 100.0%	0 / 0 0.0%	0 / 0 0.0%	0 / 0 0.0%	33 / 51 64.7%AB	17 / 51 33.3%ABE	1 / 51 2.0%BE						
1	Moorella thermoacetica ATCC 39073	51 / 51 100.0%	1 / 1 100.0%	2 / 2 100.0%	2 / 2 100.0%	1 / 1 100.0%	4 / 4 100.0%	3 / 3 100.0%	1 / 1 100.0%	1 / 1 100.0%	3 / 3 100.0%	1 / 1 100.0%	4 / 4 100.0%	3 / 3 100.0%	1 / 1 100.0%	2 / 2 100.0%	1 / 1 100.0%	3 / 3 100.0%	1 / 1 100.0%	3 / 3 100.0%	4 / 4 100.0%	3 / 3 100.0%	1 / 1 100.0%	5 / 5 100.0%	1 / 1 100.0%	0 / 0 0.0%	0 / 0 0.0%	0 / 0 0.0%	33 / 51 64.7%AB	17 / 51 33.3%ABE	1 / 51 2.0%BE						
1	Carboxydothermus	49 / 49 100.0%	1 / 1 100.0%	1 / 1 100.0%	2 / 2 100.0%	1 / 1 100.0%	5 / 5 100.0%	3 / 3 100.0%	1 / 1 100.0%	1 / 1 100.0%	3 / 3 100.0%	1 / 1 100.0%	4 / 4 100.0%	2 / 2 100.0%	1 / 1 100.0%	2 / 2 100.0%	1 / 1 100.0%	3 / 3 100.0%	1 / 1 100.0%	3 / 3 100.0%	4 / 4 100.0%	2 / 2 100.0%	1 / 1 100.0%	5 / 5 100.0%	1 / 1 100.0%	0 / 0 0.0%	0 / 0 0.0%	0 / 0 0.0%	29 / 49 59.2%AB	20 / 49 40.8%ABE							
1	ATCC BAA-161	49 / 49 100.0%	1 / 1 100.0%	1 / 1 100.0%	2 / 2 100.0%	1 / 1 100.0%	5 / 5 100.0%	3 / 3 100.0%	1 / 1 100.0%	1 / 1 100.0%	3 / 3 100.0%	1 / 1 100.0%	4 / 4 100.0%	2 / 2 100.0%	1 / 1 100.0%	2 / 2 100.0%	1 / 1 100.0%	3 / 3 100.0%	1 / 1 100.0%	3 / 3 100.0%	4 / 4 100.0%	2 / 2 100.0%	1 / 1 100.0%	5 / 5 100.0%	1 / 1 100.0%	0 / 0 0.0%	0 / 0 0.0%	0 / 0 0.0%	29 / 49 59.2%AB	20 / 49 40.8%ABE							
1	Carboxydothermus hydrogenoformans Z-2901	49 / 49 100.0%	1 / 1 100.0%	1 / 1 100.0%	2 / 2 100.0%	1 / 1 100.0%	5 / 5 100.0%	3 / 3 100.0%	1 / 1 100.0%	1 / 1 100.0%	3 / 3 100.0%	1 / 1 100.0%	4 / 4 100.0%	2 / 2 100.0%	1 / 1 100.0%	2 / 2 100.0%	1 / 1 100.0%	3 / 3 100.0%	1 / 1 100.0%	3 / 3 100.0%	4 / 4 100.0%	2 / 2 100.0%	1 / 1 100.0%	5 / 5 100.0%	1 / 1 100.0%	0 / 0 0.0%	0 / 0 0.0%	0 / 0 0.0%	29 / 49 59.2%AB	20 / 49 40.8%ABE							
1	Thermacetogenium	49 / 50 98.0%	2 / 2 100.0%	1 / 1 100.0%	1 / 2 50.0%	1 / 1 100.0%	3 / 3 100.0%	3 / 3 100.0%	1 / 1 100.0%	1 / 1 100.0%	3 / 3 100.0%	1 / 1 100.0%	4 / 4 100.0%	2 / 2 100.0%	1 / 1 100.0%	2 / 2 100.0%	1 / 1 100.0%	3 / 3 100.0%	1 / 1 100.0%	4 / 4 100.0%	5 / 5 100.0%	2 / 2 100.0%	1 / 1 100.0%	5 / 5 100.0%	1 / 1 100.0%	0 / 0 0.0%	0 / 0 0.0%	0 / 0 0.0%	31 / 50 62.0%AB	18 / 50 36.0%ABE	1 / 50 2.0%CE						
1	ATCC BAA-254	49 / 50 98.0%	2 / 2 100.0%	1 / 1 100.0%	1 / 2 50.0%	1 / 1 100.0%	3 / 3 100.0%	3 / 3 100.0%	1 / 1 100.0%	1 / 1 100.0%	3 / 3 100.0%	1 / 1 100.0%	4 / 4 100.0%	2 / 2 100.0%	1 / 1 100.0%	2 / 2 100.0%	1 / 1 100.0%	3 / 3 100.0%	1 / 1 100.0%	4 / 4 100.0%	5 / 5 100.0%	2 / 2 100.0%	1 / 1 100.0%	5 / 5 100.0%	1 / 1 100.0%	0 / 0 0.0%	0 / 0 0.0%	0 / 0 0.0%	31 / 50 62.0%AB	18 / 50 36.0%ABE	1 / 50 2.0%CE						
1	Clostridiaceae str. PB	49 / 50 98.0%	2 / 2 100.0%	1 / 1 100.0%	1 / 2 50.0%	1 / 1 100.0%	3 / 3 100.0%	3 / 3 100.0%	1 / 1 100.0%	1 / 1 100.0%	3 / 3 100.0%	1 / 1 100.0%	4 / 4 100.0%	2 / 2 100.0%	1 / 1 100.0%	2 / 2 100.0%	1 / 1 100.0%	3 / 3 100.0%	1 / 1 100.0%	4 / 4 100.0%	5 / 5 100.0%	2 / 2 100.0%	1 / 1 100.0%	5 / 5 100.0%	1 / 1 100.0%	0 / 0 0.0%	0 / 0 0.0%	0 / 0 0.0%	31 / 50 62.0%AB	18 / 50 36.0%ABE	1 / 50 2.0%CE						
1	Caldanaerobacter	56 / 56 100.0%	1 / 1 100.0%	2 / 2 100.0%	3 / 3 100.0%	1 / 1 100.0%	4 / 4 100.0%	4 / 4 100.0%	1 / 1 100.0%	2 / 2 100.0%	3 / 3 100.0%	1 / 1 100.0%	4 / 4 100.0%	3 / 3 100.0%	2 / 2 100.0%	2 / 2 100.0%	1 / 1 100.0%	3 / 3 100.0%	1 / 1 100.0%	3 / 3 100.0%	4 / 4 100.0%	3 / 3 100.0%	2 / 2 100.0%	5 / 5 100.0%	1 / 1 100.0%	0 / 0 0.0%	0 / 0 0.0%	0 / 0 0.0%	39 / 56 69.6%AB	17 / 56 30.4%ABE							
1	ATCC BAA-225	56 / 56 100.0%	1 / 1 100.0%	2 / 2 100.0%	3 / 3 100.0%	1 / 1 100.0%	4 / 4 100.0%	4 / 4 100.0%	1 / 1 100.0%	2 / 2 100.0%	3 / 3 100.0%	1 / 1 100.0%	4 / 4 100.0%	3 / 3 100.0%	2 / 2 100.0%	2 / 2 100.0%	1 / 1 100.0%	3 / 3 100.0%	1 / 1 100.0%	3 / 3 100.0%	4 / 4 100.0%	3 / 3 100.0%	2 / 2 100.0%	5 / 5 100.0%	1 / 1 100.0%	0 / 0 0.0%	0 / 0 0.0%	0 / 0 0.0%	39 / 56 69.6%AB	17 / 56 30.4%ABE							
1	AS 1.2430	56 / 56 100.0%	1 / 1 100.0%	2 / 2 100.0%	3 / 3 100.0%	1 / 1 100.0%	4 / 4 100.0%	4 / 4 100.0%	1 / 1 100.0%	2 / 2 100.0%	3 / 3 100.0%	1 / 1 100.0%	4 / 4 100.0%	3 / 3 100.0%	2 / 2 100.0%	2 / 2 100.0%	1 / 1 100.0%	3 / 3 100.0%	1 / 1 100.0%	3 / 3 100.0%	4 / 4 100.0%	3 / 3 100.0%	2 / 2 100.0%	5 / 5 100.0%	1 / 1 100.0%	0 / 0 0.0%	0 / 0 0.0%	0 / 0 0.0%	39 / 56 69.6%AB	17 / 56 30.4%ABE							
1	Caldanaerobacter subterraneus subsp. tengcongensis MB4	56 / 56 100.0%	1 / 1 100.0%	2 / 2 100.0%	3 / 3 100.0%	1 / 1 100.0%	4 / 4 100.0%	4 / 4 100.0%	1 / 1 100.0%	2 / 2 100.0%	3 / 3 100.0%	1 / 1 100.0%	4 / 4 100.0%	3 / 3 100.0%	2 / 2 100.0%	2 / 2 100.0%	1 / 1 100.0%	3 / 3 100.0%	1 / 1 100.0%	3 / 3 100.0%	4 / 4 100.0%	3 / 3 100.0%	2 / 2 100.0%	5 / 5 100.0%	1 / 1 100.0%	0 / 0 0.0%	0 / 0 0.0%	0 / 0 0.0%	39 / 56 69.6%AB	17 / 56 30.4%ABE							
2	Tepidanaerobacter	103 / 103 100.0%	4 / 4 100.0%	2 / 2 100.0%	4 / 4 100.0%	2 / 2 100.0%	8 / 8 100.0%	6 / 6 100.0%	2 / 2 100.0%	4 / 4 100.0%	6 / 6 100.0%	2 / 2 100.0%	8 / 8 100.0%	6 / 6 100.0%	2 / 2 100.0%	4 / 4 100.0%	2 / 2 100.0%	6 / 6 100.0%	2 / 2 100.0%	6 / 6 100.0%	8 / 8 100.0%	8 / 8 100.0%	2 / 2 100.0%	9 / 9 100.0%	0 / 0 0.0%	0 / 0 0.0%	0 / 0 0.0%	0 / 0 0.0%	62 / 103 60.2%AB	41 / 103 39.8%ABE							
2	"Tepidanaerobacter acetatoxydans" Westerholm et al. 2011	103 / 103 100.0%	4 / 4 100.0%	2 / 2 100.0%	4 / 4 100.0%	2 / 2 100.0%	8 / 8 100.0%	6 / 6 100.0%	2 / 2 100.0%	4 / 4 100.0%	6 / 6 100.0%	2 / 2 100.0%	8 / 8 100.0%	6 / 6 100.0%	2 / 2 100.0%	4 / 4 100.0%	2 / 2 100.0%	6 / 6 100.0%	2 / 2 100.0%	6 / 6 100.0%	8 / 8 100.0%	8 / 8 100.0%	2 / 2 100.0%	9 / 9 100.0%	0 / 0 0.0%	0 / 0 0.0%	0 / 0 0.0%	0 / 0 0.0%	62 / 103 60.2%AB	41 / 103 39.8%ABE							
2	Tepidanaerobacter acetatoxydans Re1	103 / 103 100.0%	4 / 4 100.0%	2 / 2 100.0%	4 / 4 100.0%	2 / 2 100.0%	8 / 8 100.0%	6 / 6 100.0%	2 / 2 100.0%	4 / 4 100.0%	6 / 6 100.0%	2 / 2 100.0%	8 / 8 100.0%	6 / 6 100.0%	2 / 2 100.0%	4 / 4 100.0%	2 / 2 100.0%	6 / 6 100.0%	2 / 2 100.0%	6 / 6 100.0%	8 / 8 100.0%	8 / 8 100.0%	2 / 2 100.0%	9 / 9 100.0%	0 / 0 0.0%	0 / 0 0.0%	0 / 0 0.0%	0 / 0 0.0%	62 / 103 60.2%AB	41 / 103 39.8%ABE							
2	Thermodesulfobiaceae	94 / 94 100.0%	2 / 2 100.0%	2 / 2 100.0%	2 / 2 100.0%	3 / 3 100.0%	6 / 6 100.0%	6 / 6 100.0%	2 / 2 100.0%	2 / 2 100.0%	6 / 6 100.0%	2 / 2 100.0%	8 / 8 100.0%	4 / 4 100.0%	2 / 2 100.0%	4 / 4 100.0%	2 / 2 100.0%	6 / 6 100.0%	2 / 2 100.0%	6 / 6 100.0%	9 / 9 100.0%	4 / 4 100.0%	2 / 2 100.0%	10 / 10 100.0%	2 / 2 100.0%	0 / 0 0.0%	0 / 0 0.0%	0 / 0 0.0%	59 / 94 62.8%AB	35 / 94 37.2%ABE							
1	Coprothermobacter	47 / 47 100.0%	1 / 1 100.0%	1 / 1 100.0%	1 / 1 100.0%	1 / 1 100.0%	3 / 3 100.0%	3 / 3 100.0%	1 / 1 100.0%	1 / 1 100.0%	3 / 3 100.0%	1 / 1 100.0%	4 / 4 100.0%	2 / 2 100.0%	1 / 1 100.0%	2 / 2 100.0%	1 / 1 100.0%	3 / 3 100.0%	1 / 1 100.0%	3 / 3 100.0%	5 / 5 100.0%	2 / 2 100.0%	1 / 1 100.0%	5 / 5 100.0%	1 / 1 100.0%	0 / 0 0.0%	0 / 0 0.0%	0 / 0 0.0%	27 / 47 57.4%AB	20 / 47 42.6%ABE							
1	ATCC 35245	47 / 47 100.0%	1 / 1 100.0%	1 / 1 100.0%	1 / 1 100.0%	1 / 1 100.0%	3 / 3 100.0%	3 / 3 100.0%	1 / 1 100.0%	1 / 1 100.0%	3 / 3 100.0%	1 / 1 100.0%	4 / 4 100.0%	2 / 2 100.0%	1 / 1 100.0%	2 / 2 100.0%	1 / 1 100.0%	3 / 3 100.0%	1 / 1 100.0%	3 / 3 100.0%	5 / 5 100.0%	2 / 2 100.0%	1 / 1 100.0%	5 / 5 100.0%	1 / 1 100.0%	0 / 0 0.0%	0 / 0 0.0%	0 / 0 0.0%	27 / 47 57.4%AB	20 / 47 42.6%ABE							
1	Coprothermobacter proteolyticus DSM 5265	47 / 47 100.0%	1 / 1 100.0%	1 / 1 100.0%	1 / 1 100.0%	1 / 1 100.0%	3 / 3 100.0%	3 / 3 100.0%	1 / 1 100.0%	1 / 1 100.0%	3 / 3 100.0%	1 / 1 100.0%	4 / 4 100.0%	2 / 2 100.0%	1 / 1 100.0%	2 / 2 100.0%	1 / 1 100.0%	3 / 3 100.0%	1 / 1 100.0%	3 / 3 100.0%	5 / 5 100.0%	2 / 2 100.0%	1 / 1 100.0%	5 / 5 100.0%	1 / 1 100.0%	0 / 0 0.0%	0 / 0 0.0%	0 / 0 0.0%	27 / 47 57.4%AB	20 / 47 42.6%ABE							
1	Thermodesulfobium	47 / 47 100.0%	1 / 1 100.0%	1 / 1 100.0%	1 / 1 100.0%	2 / 2 100.0%	3 / 3 100.0%	3 / 3 100.0%	1 / 1 100.0%	1 / 1 100.0%	3 / 3 100.0%	1 / 1 100.0%	4 / 4 100.0%	2 / 2 100.0%	1 / 1 100.0%	2 / 2 100.0%	1 / 1 100.0%	3 / 3 100.0%	1 / 1 100.0%	3 / 3 100.0%	4 / 4 100.0%	2 / 2 100.0%	1 / 1 100.0%	5 / 5 100.0%	1 / 1 100.0%	0 / 0 0.0%	0 / 0 0.0%	0 / 0 0.0%	32 / 47 68.1%AB	15 / 47 31.9%ABE							
1	DSM 14796	47 / 47 100.0%	1 / 1 100.0%	1 / 1 100.0%	1 / 1 100.0%	2 / 2 100.0%	3 / 3 100.0%	3 / 3 100.0%	1 / 1 100.0%	1 / 1 100.0%	3 / 3 100.0%	1 / 1 100.0%	4 / 4 100.0%	2 / 2 100.0%	1 / 1 100.0%	2 / 2 100.0%	1 / 1 100.0%	3 / 3 100.0%	1 / 1 100.0%	3 / 3 100.0%	4 / 4 100.0%	2 / 2 100.0%	1 / 1 100.0%	5 / 5 100.0%	1 / 1 100.0%	0 / 0 0.0%	0 / 0 0.0%	0 / 0 0.0%	32 / 47 68.1%AB	15 / 47 31.9%ABE							
1	Thermodesulfobium narugense DSM 14796	47 / 47 100.0%	1 / 1 100.0%	1 / 1 100.0%	1 / 1 100.0%	2 / 2 100.0%	3 / 3 100.0%	3 / 3 100.0%	1 / 1 100.0%	1 / 1 100.0%	3 / 3 100.0%	1 / 1 100.0%	4 / 4 100.0%	2 / 2 100.0%	1 / 1 100.0%	2 / 2 100.0%	1 / 1 100.0%	3 / 3 100.0%	1 / 1 100.0%	3 / 3 100.0%	4 / 4 100.0%	2 / 2 100.0%	1 / 1 100.0%	5 / 5 100.0%	1 / 1 100.0%	0 / 0 0.0%	0 / 0 0.0%	0 / 0 0.0%	32 / 47 68.1%AB	15 / 47 31.9%ABE							
13	Thermoanaerobacterales Family III. Incertae Sedis	617 / 650 94.9%	14 / 14 100.0%	17 / 17 100.0%	21 / 21 100.0%	20 / 24 83.3%	44 / 44 100.0%	33 / 35 94.3%	17 / 17 100.0%	17 / 18 94.4%	26 / 34 76.5%	13 / 13 100.0%	53 / 53 100.0%	34 / 39 87.2%	16 / 17 94.1%	26 / 26 100.0%	13 / 13 100.0%	39 / 39 100.0%	10 / 13 76.9%	36 / 39 92.3%	50 / 52 96.2%	37 / 39 94.9%	17 / 17 100.0%	63 / 65 96.9%	1 / 1 100.0%	0 / 0 0.0%	0 / 0 0.0%	0 / 0 0.0%	413 / 650 63.5%AB	204 / 650 31.4%ABE	22 / 650 3.4%	11 / 650 1.7%E					
4	Thermoanaerobacterium	195 / 228 85.5%	4 / 4 100.0%	8 / 8 100.0%	12 / 12 100.0%	4 / 8 50.0%	16 / 16 100.0%	14 / 16 87.5%	4 / 4 100.0%	7 / 8 87.5%	8 / 16 50.0%	4 / 4 100.0%	16 / 16 100.0%	7 / 12 58.3%	7 / 8 87.5%	8 / 8 100.0%	4 / 4 100.0%	12 / 12 100.0%	1 / 4 25.0%	9 / 12 75.0%	14 / 16 87.5%	10 / 12 83.3%	8 / 8 100.0%	18 / 20 90.0%	0 / 0 0.0%	0 / 0 0.0%	0 / 0 0.0%	0 / 0 0.0%	136 / 228 59.6%AB	59 / 228 25.9%ABE	22 / 228 9.6%	11 / 228 4.8%E					
2	"Clostridium tartarivorum" Mercer and Vaughn	101 / 114 88.6%	2 / 2 100.0%	4 / 4 100.0%	6 / 6 100.0%	2 / 4 50.0%	8 / 8 100.0%	7 / 8 87.5%	2 / 2 100.0%	4 / 4 100.0%	4 / 8 50.0%	2 / 2 100.0%	8 / 8 100.0%	4 / 6 66.7%	4 / 4 100.0%	4 / 4 100.0%	2 / 2 100.0%	6 / 6 100.0%	1 / 2 50.0%	6 / 6 100.0%	6 / 8 75.0%	6 / 6 100.0%	4 / 4 100.0%	9 / 10 90.0%	0 / 0 0.0%	0 / 0 0.0%	0 / 0 0.0%	0 / 0 0.0%	71 / 114 62.3%AB	30 / 114 26.3%ABE	7 / 114 6.1%	6 / 114 5.3%E					
1	Thermoanaerobacterium thermosaccharolyticum DSM 571	50 / 57 87.7%	1 / 1 100.0%	2 / 2 100.0%	3 / 3 100.0%	1 / 2 50.0%	4 / 4 100.0%	3 / 4 75.0%	1 / 1 100.0%	2 / 2 100.0%	2 / 4 50.0%	1 / 1 100.0%	4 / 4 100.0%	2 / 3 66.7%	2 / 2 100.0%	2 / 2 100.0%	1 / 1 100.0%	3 / 3 100.0%	0 / 1 0.0%	3 / 3 100.0%	3 / 4 75.0%	3 / 3 100.0%	2 / 2 100.0%	5 / 5 100.0%	0 / 0 0.0%	0 / 0 0.0%	0 / 0 0.0%	0 / 0 0.0%	34 / 57 59.6%AB	16 / 57 28.1%ABE	4 / 57 7.0%	3 / 57 5.3%E					
1	Thermoanaerobacterium thermosaccharolyticum 59	51 / 57 89.5%	1 / 1 100.0%	2 / 2 100.0%	3 / 3 100.0%	1 / 2 50.0%	4 / 4 100.0%	4 / 4 100.0%	1 / 1 100.0%	2 / 2 100.0%	2 / 4 50.0%	1 / 1 100.0%	4 / 4 100.0%	2 / 3 66.7%	2 / 2 100.0%	2 / 2 100.0%	1 / 1 100.0%	3 / 3 100.0%	1 / 1 100.0%	3 / 3 100.0%	3 / 4 75.0%	3 / 3 100.0%	2 / 2 100.0%	4 / 5 80.0%	0 / 0 0.0%	0 / 0 0.0%	0 / 0 0.0%	0 / 0 0.0%	37 / 57 64.9%AB	14 / 57 24.6%ABE	3 / 57 5.3%E	3 / 57 5.3%					
1	ATCC 49915	46 / 57 80.7%	1 / 1 100.0%	2 / 2 100.0%	3 / 3 100.0%	1 / 2 50.0%	4 / 4 100.0%	4 / 4 100.0%	1 / 1 100.0%	1 / 2 50.0%	2 / 4 50.0%	1 / 1 100.0%	4 / 4 100.0%	1 / 3 33.3%	2 / 2 100.0%	2 / 2 100.0%	1 / 1 100.0%	3 / 3 100.0%	0 / 1 0.0%	1 / 3 33.3%	4 / 4 100.0%	2 / 3 66.7%	2 / 2 100.0%	4 / 5 80.0%	0 / 0 0.0%	0 / 0 0.0%	0 / 0 0.0%	0 / 0 0.0%	32 / 57 56.1%AB	14 / 57 24.6%ABE	8 / 57 14.0%	3 / 57 5.3%E					
1	Thermoanaerobacterium saccharolyticum JW/SL-YS485	46 / 57 80.7%	1 / 1 100.0%	2 / 2 100.0%	3 / 3 100.0%	1 / 2 50.0%	4 / 4 100.0%	4 / 4 100.0%	1 / 1 100.0%	1 / 2 50.0%	2 / 4 50.0%	1 / 1 100.0%	4 / 4 100.0%	1 / 3 33.3%	2 / 2 100.0%	2 / 2 100.0%	1 / 1 100.0%	3 / 3 100.0%	0 / 1 0.0%	1 / 3 33.3%	4 / 4 100.0%	2 / 3 66.7%	2 / 2 100.0%	4 / 5 80.0%	0 / 0 0.0%	0 / 0 0.0%	0 / 0 0.0%	0 / 0 0.0%	32 / 57 56.1%AB	14 / 57 24.6%ABE	8 / 57 14.0%	3 / 57 5.3%E					
1	ATCC 49914	48 / 57 84.2%	1 / 1 100.0%	2 / 2 100.0%	3 / 3 100.0%	1 / 2 50.0%	4 / 4 100.0%	3 / 4 75.0%	1 / 1 100.0%	2 / 2 100.0%	2 / 4 50.0%	1 / 1 100.0%	4 / 4 100.0%	2 / 3 66.7%	1 / 2 50.0%	2 / 2 100.0%	1 / 1 100.0%	3 / 3 100.0%	0 / 1 0.0%	2 / 3 66.7%	4 / 4 100.0%	2 / 3 66.7%	2 / 2 100.0%	5 / 5 100.0%	0 / 0 0.0%	0 / 0 0.0%	0 / 0 0.0%	0 / 0 0.0%	33 / 57 57.9%AB	15 / 57 26.3%ABE	7 / 57 12.3%	2 / 57 3.5%E					
1	Thermoanaerobacterium xylanolyticum DSM 7097	48 / 57 84.2%	1 / 1 100.0%	2 / 2 100.0%	3 / 3 100.0%	1 / 2 50.0%	4 / 4 100.0%	3 / 4 75.0%	1 / 1 100.0%	2 / 2 100.0%	2 / 4 50.0%	1 / 1 100.0%	4 / 4 100.0%	2 / 3 66.7%	1 / 2 50.0%	2 / 2 100.0%	1 / 1 100.0%	3 / 3 100.0%	0 / 1 0.0%	2 / 3 66.7%	4 / 4 100.0%	2 / 3 66.7%	2 / 2 100.0%	5 / 5 100.0%	0 / 0 0.0%	0 / 0 0.0%	0 / 0 0.0%	0 / 0 0.0%	33 / 57 57.9%AB	15 / 57 26.3%ABE	7 / 57 12.3%	2 / 57 3.5%E					
8	Caldicellulosiruptor	371 / 371 100.0%	8 / 8 100.0%	8 / 8 100.0%	8 / 8 100.0%	15 / 15 100.0%	24 / 24 100.0%	16 / 16 100.0%	12 / 12 100.0%	8 / 8 100.0%	15 / 15 100.0%	8 / 8 100.0%	33 / 33 100.0%	24 / 24 100.0%	8 / 8 100.0%	16 / 16 100.0%	8 / 8 100.0%	24 / 24 100.0%	8 / 8 100.0%	24 / 24 100.0%	32 / 32 100.0%	24 / 24 100.0%	8 / 8 100.0%	40 / 40 100.0%	0 / 0 0.0%	0 / 0 0.0%	0 / 0 0.0%	0 / 0 0.0%	247 / 371 66.6%AB	124 / 371 33.4%ABE							
1	"Anaerocellum thermophilum" Svetlichnyi VA et al. 1990	47 / 47 100.0%	1 / 1 100.0%	1 / 1 100.0%	1 / 1 100.0%	2 / 2 100.0%	3 / 3 100.0%	2 / 2 100.0%	2 / 2 100.0%	1 / 1 100.0%	2 / 2 100.0%	1 / 1 100.0%	4 / 4 100.0%	3 / 3 100.0%	1 / 1 100.0%	2 / 2 100.0%	1 / 1 100.0%	3 / 3 100.0%	1 / 1 100.0%	3 / 3 100.0%	4 / 4 100.0%	3 / 3 100.0%	1 / 1 100.0%	5 / 5 100.0%	0 / 0 0.0%	0 / 0 0.0%	0 / 0 0.0%	0 / 0 0.0%	31 / 47 66.0%AB	16 / 47 34.0%ABE							
1	Anaerocellum thermophilum DSM 6725	47 / 47 100.0%	1 / 1 100.0%	1 / 1 100.0%	1 / 1 100.0%	2 / 2 100.0%	3 / 3 100.0%	2 / 2 100.0%	2 / 2 100.0%	1 / 1 100.0%	2 / 2 100.0%	1 / 1 100.0%	4 / 4 100.0%	3 / 3 100.0%	1 / 1 100.0%	2 / 2 100.0%	1 / 1 100.0%	3 / 3 100.0%	1 / 1 100.0%	3 / 3 100.0%	4 / 4 100.0%	3 / 3 100.0%	1 / 1 100.0%	5 / 5 100.0%	0 / 0 0.0%	0 / 0 0.0%	0 / 0 0.0%	0 / 0 0.0%	31 / 47 66.0%AB	16 / 47 34.0%ABE							
1	ATCC 43494	46 / 46 100.0%	1 / 1 100.0%	1 / 1 100.0%	1 / 1 100.0%	2 / 2 100.0%	3 / 3 100.0%	2 / 2 100.0%	1 / 1 100.0%	1 / 1 100.0%	2 / 2 100.0%	1 / 1 100.0%	4 / 4 100.0%	3 / 3 100.0%	1 / 1 100.0%	2 / 2 100.0%	1 / 1 100.0%	3 / 3 100.0%	1 / 1 100.0%	3 / 3 100.0%	4 / 4 100.0%	3 / 3 100.0%	1 / 1 100.0%	5 / 5 100.0%	0 / 0 0.0%	0 / 0 0.0%	0 / 0 0.0%	0 / 0 0.0%	31 / 46 67.4%AB	15 / 46 32.6%ABE							
1	Caldicellulosiruptor saccharolyticus DSM 8903	46 / 46 100.0%	1 / 1 100.0%	1 / 1 100.0%	1 / 1 100.0%	2 / 2 100.0%	3 / 3 100.0%	2 / 2 100.0%	1 / 1 100.0%	1 / 1 100.0%	2 / 2 100.0%	1 / 1 100.0%	4 / 4 100.0%	3 / 3 100.0%	1 / 1 100.0%	2 / 2 100.0%	1 / 1 100.0%	3 / 3 100.0%	1 / 1 100.0%	3 / 3 100.0%	4 / 4 100.0%	3 / 3 100.0%	1 / 1 100.0%	5 / 5 100.0%	0 / 0 0.0%	0 / 0 0.0%	0 / 0 0.0%	0 / 0 0.0%	31 / 46 67.4%AB	15 / 46 32.6%ABE							
1	ATCC 700853	47 / 47 100.0%	1 / 1 100.0%	1 / 1 100.0%	1 / 1 100.0%	2 / 2 100.0%	3 / 3 100.0%	2 / 2 100.0%	2 / 2 100.0%	1 / 1 100.0%	2 / 2 100.0%	1 / 1 100.0%	4 / 4 100.0%	3 / 3 100.0%	1 / 1 100.0%	2 / 2 100.0%	1 / 1 100.0%	3 / 3 100.0%	1 / 1 100.0%	3 / 3 100.0%	4 / 4 100.0%	3 / 3 100.0%	1 / 1 100.0%	5 / 5 100.0%	0 / 0 0.0%	0 / 0 0.0%	0 / 0 0.0%	0 / 0 0.0%	32 / 47 68.1%AB	15 / 47 31.9%ABE							
1	Caldicellulosiruptor kristjanssonii 177R1B	47 / 47 100.0%	1 / 1 100.0%	1 / 1 100.0%	1 / 1 100.0%	2 / 2 100.0%	3 / 3 100.0%	2 / 2 100.0%	2 / 2 100.0%	1 / 1 100.0%	2 / 2 100.0%	1 / 1 100.0%	4 / 4 100.0%	3 / 3 100.0%	1 / 1 100.0%	2 / 2 100.0%	1 / 1 100.0%	3 / 3 100.0%	1 / 1 100.0%	3 / 3 100.0%	4 / 4 100.0%	3 / 3 100.0%	1 / 1 100.0%	5 / 5 100.0%	0 / 0 0.0%	0 / 0 0.0%	0 / 0 0.0%	0 / 0 0.0%	32 / 47 68.1%AB	15 / 47 31.9%ABE							
1	Caldicellulosiruptor lactoaceticus	45 / 45 100.0%	1 / 1 100.0%	1 / 1 100.0%	1 / 1 100.0%	1 / 1 100.0%	3 / 3 100.0%	2 / 2 100.0%	1 / 1 100.0%	1 / 1 100.0%	1 / 1 100.0%	1 / 1 100.0%	5 / 5 100.0%	3 / 3 100.0%	1 / 1 100.0%	2 / 2 100.0%	1 / 1 100.0%	3 / 3 100.0%	1 / 1 100.0%	3 / 3 100.0%	4 / 4 100.0%	3 / 3 100.0%	1 / 1 100.0%	5 / 5 100.0%	0 / 0 0.0%	0 / 0 0.0%	0 / 0 0.0%	0 / 0 0.0%	29 / 45 64.4%AB	16 / 45 35.6%ABE							
1	Caldicellulosiruptor lactoaceticus 6A	45 / 45 100.0%	1 / 1 100.0%	1 / 1 100.0%	1 / 1 100.0%	1 / 1 100.0%	3 / 3 100.0%	2 / 2 100.0%	1 / 1 100.0%	1 / 1 100.0%	1 / 1 100.0%	1 / 1 100.0%	5 / 5 100.0%	3 / 3 100.0%	1 / 1 100.0%	2 / 2 100.0%	1 / 1 100.0%	3 / 3 100.0%	1 / 1 100.0%	3 / 3 100.0%	4 / 4 100.0%	3 / 3 100.0%	1 / 1 100.0%	5 / 5 100.0%	0 / 0 0.0%	0 / 0 0.0%	0 / 0 0.0%	0 / 0 0.0%	29 / 45 64.4%AB	16 / 45 35.6%ABE							
1	ATCC 700167	47 / 47 100.0%	1 / 1 100.0%	1 / 1 100.0%	1 / 1 100.0%	2 / 2 100.0%	3 / 3 100.0%	2 / 2 100.0%	2 / 2 100.0%	1 / 1 100.0%	2 / 2 100.0%	1 / 1 100.0%	4 / 4 100.0%	3 / 3 100.0%	1 / 1 100.0%	2 / 2 100.0%	1 / 1 100.0%	3 / 3 100.0%	1 / 1 100.0%	3 / 3 100.0%	4 / 4 100.0%	3 / 3 100.0%	1 / 1 100.0%	5 / 5 100.0%	0 / 0 0.0%	0 / 0 0.0%	0 / 0 0.0%	0 / 0 0.0%	31 / 47 66.0%AB	16 / 47 34.0%ABE							
1	Caldicellulosiruptor owensensis DSM 13100	47 / 47 100.0%	1 / 1 100.0%	1 / 1 100.0%	1 / 1 100.0%	2 / 2 100.0%	3 / 3 100.0%	2 / 2 100.0%	2 / 2 100.0%	1 / 1 100.0%	2 / 2 100.0%	1 / 1 100.0%	4 / 4 100.0%	3 / 3 100.0%	1 / 1 100.0%	2 / 2 100.0%	1 / 1 100.0%	3 / 3 100.0%	1 / 1 100.0%	3 / 3 100.0%	4 / 4 100.0%	3 / 3 100.0%	1 / 1 100.0%	5 / 5 100.0%	0 / 0 0.0%	0 / 0 0.0%	0 / 0 0.0%	0 / 0 0.0%	31 / 47 66.0%AB	16 / 47 34.0%ABE							
1	Caldicellulosiruptor hydrothermalis	47 / 47 100.0%	1 / 1 100.0%	1 / 1 100.0%	1 / 1 100.0%	2 / 2 100.0%	3 / 3 100.0%	2 / 2 100.0%	2 / 2 100.0%	1 / 1 100.0%	2 / 2 100.0%	1 / 1 100.0%	4 / 4 100.0%	3 / 3 100.0%	1 / 1 100.0%	2 / 2 100.0%	1 / 1 100.0%	3 / 3 100.0%	1 / 1 100.0%	3 / 3 100.0%	4 / 4 100.0%	3 / 3 100.0%	1 / 1 100.0%	5 / 5 100.0%	0 / 0 0.0%	0 / 0 0.0%	0 / 0 0.0%	0 / 0 0.0%	31 / 47 66.0%AB	16 / 47 34.0%ABE							
1	Caldicellulosiruptor hydrothermalis 108	47 / 47 100.0%	1 / 1 100.0%	1 / 1 100.0%	1 / 1 100.0%	2 / 2 100.0%	3 / 3 100.0%	2 / 2 100.0%	2 / 2 100.0%	1 / 1 100.0%	2 / 2 100.0%	1 / 1 100.0%	4 / 4 100.0%	3 / 3 100.0%	1 / 1 100.0%	2 / 2 100.0%	1 / 1 100.0%	3 / 3 100.0%	1 / 1 100.0%	3 / 3 100.0%	4 / 4 100.0%	3 / 3 100.0%	1 / 1 100.0%	5 / 5 100.0%	0 / 0 0.0%	0 / 0 0.0%	0 / 0 0.0%	0 / 0 0.0%	31 / 47 66.0%AB	16 / 47 34.0%ABE							
1	Caldicellulosiruptor kronotskiensis	46 / 46 100.0%	1 / 1 100.0%	1 / 1 100.0%	1 / 1 100.0%	2 / 2 100.0%	3 / 3 100.0%	2 / 2 100.0%	1 / 1 100.0%	1 / 1 100.0%	2 / 2 100.0%	1 / 1 100.0%	4 / 4 100.0%	3 / 3 100.0%	1 / 1 100.0%	2 / 2 100.0%	1 / 1 100.0%	3 / 3 100.0%	1 / 1 100.0%	3 / 3 100.0%	4 / 4 100.0%	3 / 3 100.0%	1 / 1 100.0%	5 / 5 100.0%	0 / 0 0.0%	0 / 0 0.0%	0 / 0 0.0%	0 / 0 0.0%	31 / 46 67.4%AB	15 / 46 32.6%ABE							
1	Caldicellulosiruptor kronotskyensis 2002	46 / 46 100.0%	1 / 1 100.0%	1 / 1 100.0%	1 / 1 100.0%	2 / 2 100.0%	3 / 3 100.0%	2 / 2 100.0%	1 / 1 100.0%	1 / 1 100.0%	2 / 2 100.0%	1 / 1 100.0%	4 / 4 100.0%	3 / 3 100.0%	1 / 1 100.0%	2 / 2 100.0%	1 / 1 100.0%	3 / 3 100.0%	1 / 1 100.0%	3 / 3 100.0%	4 / 4 100.0%	3 / 3 100.0%	1 / 1 100.0%	5 / 5 100.0%	0 / 0 0.0%	0 / 0 0.0%	0 / 0 0.0%	0 / 0 0.0%	31 / 46 67.4%AB	15 / 46 32.6%ABE							
1	"Caldicellulosiruptor obsidiansis" Hamilton-Brehm et al. 2010	46 / 46 100.0%	1 / 1 100.0%	1 / 1 100.0%	1 / 1 100.0%	2 / 2 100.0%	3 / 3 100.0%	2 / 2 100.0%	1 / 1 100.0%	1 / 1 100.0%	2 / 2 100.0%	1 / 1 100.0%	4 / 4 100.0%	3 / 3 100.0%	1 / 1 100.0%	2 / 2 100.0%	1 / 1 100.0%	3 / 3 100.0%	1 / 1 100.0%	3 / 3 100.0%	4 / 4 100.0%	3 / 3 100.0%	1 / 1 100.0%	5 / 5 100.0%	0 / 0 0.0%	0 / 0 0.0%	0 / 0 0.0%	0 / 0 0.0%	31 / 46 67.4%AB	15 / 46 32.6%ABE							
1	Caldicellulosiruptor obsidiansis OB47	46 / 46 100.0%	1 / 1 100.0%	1 / 1 100.0%	1 / 1 100.0%	2 / 2 100.0%	3 / 3 100.0%	2 / 2 100.0%	1 / 1 100.0%	1 / 1 100.0%	2 / 2 100.0%	1 / 1 100.0%	4 / 4 100.0%	3 / 3 100.0%	1 / 1 100.0%	2 / 2 100.0%	1 / 1 100.0%	3 / 3 100.0%	1 / 1 100.0%	3 / 3 100.0%	4 / 4 100.0%	3 / 3 100.0%	1 / 1 100.0%	5 / 5 100.0%	0 / 0 0.0%	0 / 0 0.0%	0 / 0 0.0%	0 / 0 0.0%	31 / 46 67.4%AB	15 / 46 32.6%ABE							
1	Thermosedimentibacter	51 / 51 100.0%	2 / 2 100.0%	1 / 1 100.0%	1 / 1 100.0%	1 / 1 100.0%	4 / 4 100.0%	3 / 3 100.0%	1 / 1 100.0%	2 / 2 100.0%	3 / 3 100.0%	1 / 1 100.0%	4 / 4 100.0%	3 / 3 100.0%	1 / 1 100.0%	2 / 2 100.0%	1 / 1 100.0%	3 / 3 100.0%	1 / 1 100.0%	3 / 3 100.0%	4 / 4 100.0%	3 / 3 100.0%	1 / 1 100.0%	5 / 5 100.0%	1 / 1 100.0%	0 / 0 0.0%	0 / 0 0.0%	0 / 0 0.0%	30 / 51 58.8%AB	21 / 51 41.2%ABE							
1	ATCC BAA-1034	51 / 51 100.0%	2 / 2 100.0%	1 / 1 100.0%	1 / 1 100.0%	1 / 1 100.0%	4 / 4 100.0%	3 / 3 100.0%	1 / 1 100.0%	2 / 2 100.0%	3 / 3 100.0%	1 / 1 100.0%	4 / 4 100.0%	3 / 3 100.0%	1 / 1 100.0%	2 / 2 100.0%	1 / 1 100.0%	3 / 3 100.0%	1 / 1 100.0%	3 / 3 100.0%	4 / 4 100.0%	3 / 3 100.0%	1 / 1 100.0%	5 / 5 100.0%	1 / 1 100.0%	0 / 0 0.0%	0 / 0 0.0%	0 / 0 0.0%	30 / 51 58.8%AB	21 / 51 41.2%ABE							
1	Thermosediminibacter oceani ATCC BAA-1034	51 / 51 100.0%	2 / 2 100.0%	1 / 1 100.0%	1 / 1 100.0%	1 / 1 100.0%	4 / 4 100.0%	3 / 3 100.0%	1 / 1 100.0%	2 / 2 100.0%	3 / 3 100.0%	1 / 1 100.0%	4 / 4 100.0%	3 / 3 100.0%	1 / 1 100.0%	2 / 2 100.0%	1 / 1 100.0%	3 / 3 100.0%	1 / 1 100.0%	3 / 3 100.0%	4 / 4 100.0%	3 / 3 100.0%	1 / 1 100.0%	5 / 5 100.0%	1 / 1 100.0%	0 / 0 0.0%	0 / 0 0.0%	0 / 0 0.0%	30 / 51 58.8%AB	21 / 51 41.2%ABE							
1	Thermoanaerobacterales Family IV. Incertae Sedis	47 / 47 100.0%	1 / 1 100.0%	1 / 1 100.0%	1 / 1 100.0%	1 / 1 100.0%	4 / 4 100.0%	3 / 3 100.0%	1 / 1 100.0%	1 / 1 100.0%	3 / 3 100.0%	1 / 1 100.0%	4 / 4 100.0%	3 / 3 100.0%	1 / 1 100.0%	2 / 2 100.0%	1 / 1 100.0%	3 / 3 100.0%	1 / 1 100.0%	3 / 3 100.0%	4 / 4 100.0%	2 / 2 100.0%	1 / 1 100.0%	5 / 5 100.0%	0 / 0 0.0%	0 / 0 0.0%	0 / 0 0.0%	0 / 0 0.0%	29 / 47 61.7%AB	18 / 47 38.3%ABE							
1	Mahella	47 / 47 100.0%	1 / 1 100.0%	1 / 1 100.0%	1 / 1 100.0%	1 / 1 100.0%	4 / 4 100.0%	3 / 3 100.0%	1 / 1 100.0%	1 / 1 100.0%	3 / 3 100.0%	1 / 1 100.0%	4 / 4 100.0%	3 / 3 100.0%	1 / 1 100.0%	2 / 2 100.0%	1 / 1 100.0%	3 / 3 100.0%	1 / 1 100.0%	3 / 3 100.0%	4 / 4 100.0%	2 / 2 100.0%	1 / 1 100.0%	5 / 5 100.0%	0 / 0 0.0%	0 / 0 0.0%	0 / 0 0.0%	0 / 0 0.0%	29 / 47 61.7%AB	18 / 47 38.3%ABE							
1	CIP 107919	47 / 47 100.0%	1 / 1 100.0%	1 / 1 100.0%	1 / 1 100.0%	1 / 1 100.0%	4 / 4 100.0%	3 / 3 100.0%	1 / 1 100.0%	1 / 1 100.0%	3 / 3 100.0%	1 / 1 100.0%	4 / 4 100.0%	3 / 3 100.0%	1 / 1 100.0%	2 / 2 100.0%	1 / 1 100.0%	3 / 3 100.0%	1 / 1 100.0%	3 / 3 100.0%	4 / 4 100.0%	2 / 2 100.0%	1 / 1 100.0%	5 / 5 100.0%	0 / 0 0.0%	0 / 0 0.0%	0 / 0 0.0%	0 / 0 0.0%	29 / 47 61.7%AB	18 / 47 38.3%ABE							
1	Mahella australiensis 50-1 BON	47 / 47 100.0%	1 / 1 100.0%	1 / 1 100.0%	1 / 1 100.0%	1 / 1 100.0%	4 / 4 100.0%	3 / 3 100.0%	1 / 1 100.0%	1 / 1 100.0%	3 / 3 100.0%	1 / 1 100.0%	4 / 4 100.0%	3 / 3 100.0%	1 / 1 100.0%	2 / 2 100.0%	1 / 1 100.0%	3 / 3 100.0%	1 / 1 100.0%	3 / 3 100.0%	4 / 4 100.0%	2 / 2 100.0%	1 / 1 100.0%	5 / 5 100.0%	0 / 0 0.0%	0 / 0 0.0%	0 / 0 0.0%	0 / 0 0.0%	29 / 47 61.7%AB	18 / 47 38.3%ABE							
95	Clostridiales	5586 / 6370 87.7%	187 / 198 94.4%	226 / 250 90.4%	228 / 253 90.1%	146 / 168 86.9%	500 / 560 89.3%	335 / 382 87.7%	119 / 140 85.0%	166 / 188 88.3%	295 / 330 89.4%	93 / 106 87.7%	377 / 422 89.3%	360 / 401 89.8%	194 / 210 92.4%	203 / 231 87.9%	128 / 142 90.1%	231 / 271 85.2%	110 / 148 74.3%	339 / 388 87.4%	420 / 479 87.7%	270 / 318 84.9%	133 / 146 91.1%	474 / 584 81.2%	52 / 55 94.5%	0 / 0 0.0%	0 / 0 0.0%	0 / 0 0.0%	3200 / 6370 50.2%AB	1887 / 6370 29.6%ABE	508 / 6370 8.0%E	292 / 6370 4.6%B	260 / 6370 4.1%	207 / 6370 3.2%BE	8 / 6370 0.1%C	7 / 6370 0.1%CE	1 / 6370 0.0%EGG
36	Clostridiaceae	2569 / 2653 96.8%	90 / 91 98.9%	102 / 104 98.1%	114 / 116 98.3%	69 / 72 95.8%	258 / 263 98.1%	139 / 146 95.2%	57 / 65 87.7%	71 / 75 94.7%	109 / 111 98.2%	44 / 45 97.8%	156 / 162 96.3%	187 / 193 96.9%	99 / 100 99.0%	94 / 96 97.9%	61 / 62 98.4%	92 / 98 93.9%	56 / 59 94.9%	156 / 159 98.1%	199 / 203 98.0%	113 / 122 92.6%	64 / 65 98.5%	222 / 229 96.9%	17 / 17 100.0%	0 / 0 0.0%	0 / 0 0.0%	0 / 0 0.0%	1313 / 2653 49.5%AB	836 / 2653 31.5%ABE	243 / 2653 9.2%B	177 / 2653 6.7%BE	54 / 2653 2.0%E	30 / 2653 1.1%			
31	Clostridium	2270 / 2349 96.6%	78 / 79 98.7%	87 / 89 97.8%	102 / 104 98.1%	59 / 62 95.2%	234 / 239 97.9%	120 / 127 94.5%	49 / 57 86.0%	65 / 66 98.5%	98 / 100 98.0%	38 / 39 97.4%	137 / 143 95.8%	173 / 179 96.6%	84 / 85 98.8%	85 / 87 97.7%	54 / 55 98.2%	81 / 87 93.1%	50 / 51 98.0%	139 / 142 97.9%	174 / 178 97.8%	97 / 106 91.5%	57 / 58 98.3%	194 / 201 96.5%	15 / 15 100.0%	0 / 0 0.0%	0 / 0 0.0%	0 / 0 0.0%	1157 / 2349 49.3%AB	760 / 2349 32.4%ABE	194 / 2349 8.3%B	159 / 2349 6.8%BE	51 / 2349 2.2%E	28 / 2349 1.2%			
3	"Clostridium acetobutyricum" Prevot 1940	199 / 220 90.5%	4 / 4 100.0%	9 / 9 100.0%	12 / 12 100.0%	0 / 0 0.0%	26 / 26 100.0%	9 / 12 75.0%	0 / 6 0.0%	6 / 6 100.0%	6 / 6 100.0%	3 / 3 100.0%	12 / 12 100.0%	15 / 18 83.3%	9 / 9 100.0%	9 / 9 100.0%	6 / 6 100.0%	9 / 12 75.0%	3 / 3 100.0%	15 / 15 100.0%	18 / 18 100.0%	3 / 9 33.3%	6 / 6 100.0%	19 / 19 100.0%	0 / 0 0.0%	0 / 0 0.0%	0 / 0 0.0%	0 / 0 0.0%	137 / 220 62.3%AB	62 / 220 28.2%ABE	15 / 220 6.8%	6 / 220 2.7%E					
1	Clostridium acetobutylicum ATCC 824	65 / 72 90.3%	1 / 1 100.0%	3 / 3 100.0%	4 / 4 100.0%	0 / 0 0.0%	8 / 8 100.0%	3 / 4 75.0%	0 / 2 0.0%	2 / 2 100.0%	2 / 2 100.0%	1 / 1 100.0%	4 / 4 100.0%	5 / 6 83.3%	3 / 3 100.0%	3 / 3 100.0%	2 / 2 100.0%	3 / 4 75.0%	1 / 1 100.0%	5 / 5 100.0%	6 / 6 100.0%	1 / 3 33.3%	2 / 2 100.0%	6 / 6 100.0%	0 / 0 0.0%	0 / 0 0.0%	0 / 0 0.0%	0 / 0 0.0%	45 / 72 62.5%AB	20 / 72 27.8%ABE	5 / 72 6.9%	2 / 72 2.8%E					
1	Clostridium acetobutylicum EA 2018	68 / 75 90.7%	2 / 2 100.0%	3 / 3 100.0%	4 / 4 100.0%	0 / 0 0.0%	9 / 9 100.0%	3 / 4 75.0%	0 / 2 0.0%	2 / 2 100.0%	2 / 2 100.0%	1 / 1 100.0%	4 / 4 100.0%	5 / 6 83.3%	3 / 3 100.0%	3 / 3 100.0%	2 / 2 100.0%	3 / 4 75.0%	1 / 1 100.0%	5 / 5 100.0%	6 / 6 100.0%	1 / 3 33.3%	2 / 2 100.0%	7 / 7 100.0%	0 / 0 0.0%	0 / 0 0.0%	0 / 0 0.0%	0 / 0 0.0%	46 / 75 61.3%AB	22 / 75 29.3%ABE	5 / 75 6.7%	2 / 75 2.7%E					
1	Clostridium acetobutylicum ATCC 4259	66 / 73 [illegible]	1 / 1 [illegible]	3 / 3 [illegible]	4 / 4 [illegible]	0 / 0 [illegible]	9 / 9 [illegible]	3 / 4 [illegible]	0 / 2 [illegible]	2 / 2 [illegible]	2 / 2 [illegible]	1 / 1 [illegible]	4 / 4 [illegible]	5 / 6 [illegible]	3 / 3 [illegible]	3 / 3 [illegible]	2 / 2 [illegible]	3 / 4 [illegible]	1 / 1 [illegible]	5 / 5 [illegible]	6 / 6 [illegible]	1 / 3 [illegible]	2 / 2 [illegible]	6 / 6 [illegible]	0 / 0 [illegible]	0 / 0 [illegible]	0 / 0 [illegible]	0 / 0 [illegible]	46 / 73 [illegible]	20 / 73 [illegible]	5 / 73 [illegible]	2 / 73 [illegible]					

13	"Bacillus botulinus" van Ermengem 1896	1028 / 1028 100.0%	39 / 39 100.0%	44 / 44 100.0%	41 / 41 100.0%	28 / 28 100.0%	100 / 100 100.0%	59 / 59 100.0%	26 / 26 100.0%	27 / 27 100.0%	40 / 40 100.0%	15 / 15 100.0%	65 / 65 100.0%	82 / 82 100.0%	35 / 35 100.0%	38 / 38 100.0%	26 / 26 100.0%	36 / 36 100.0%	24 / 24 100.0%	62 / 62 100.0%	81 / 81 100.0%	48 / 48 100.0%	25 / 25 100.0%	77 / 77 100.0%	10 / 10 100.0%	0 / 0 0.0%	0 / 0 0.0%	0 / 0 0.0%	400 / 1028 38.9%AB	300 / 1028 29.2%ABE	180 / 1028 17.5%B	148 / 1028 14.4%BE		
5	Clostridium botulinum A	403 / 403 100.0%	15 / 15 100.0%	20 / 20 100.0%	15 / 15 100.0%	11 / 11 100.0%	40 / 40 100.0%	25 / 25 100.0%	10 / 10 100.0%	10 / 10 100.0%	16 / 16 100.0%	5 / 5 100.0%	25 / 25 100.0%	30 / 30 100.0%	13 / 13 100.0%	15 / 15 100.0%	10 / 10 100.0%	15 / 15 100.0%	9 / 9 100.0%	25 / 25 100.0%	30 / 30 100.0%	20 / 20 100.0%	10 / 10 100.0%	29 / 29 100.0%	5 / 5 100.0%	0 / 0 0.0%	0 / 0 0.0%	0 / 0 0.0%	134 / 403 33.3%AB	98 / 403 24.3%ABE	90 / 403 22.3%B	81 / 403 20.1%BE		
1	Clostridium botulinum A str. ATCC 3502	79 / 79 100.0%	1 / 1 100.0%	4 / 4 100.0%	3 / 3 100.0%	3 / 3 100.0%	8 / 8 100.0%	5 / 5 100.0%	2 / 2 100.0%	2 / 2 100.0%	4 / 4 100.0%	1 / 1 100.0%	5 / 5 100.0%	6 / 6 100.0%	3 / 3 100.0%	3 / 3 100.0%	2 / 2 100.0%	3 / 3 100.0%	1 / 1 100.0%	5 / 5 100.0%	6 / 6 100.0%	4 / 4 100.0%	2 / 2 100.0%	5 / 5 100.0%	1 / 1 100.0%	0 / 0 0.0%	0 / 0 0.0%	0 / 0 0.0%	46 / 79 58.2%AB	32 / 79 40.5%ABE	1 / 79 1.3%BE			
1	Clostridium botulinum A ATCC 19397	81 / 81 100.0%	4 / 4 100.0%	4 / 4 100.0%	3 / 3 100.0%	2 / 2 100.0%	8 / 8 100.0%	5 / 5 100.0%	2 / 2 100.0%	2 / 2 100.0%	3 / 3 100.0%	1 / 1 100.0%	5 / 5 100.0%	6 / 6 100.0%	2 / 2 100.0%	3 / 3 100.0%	2 / 2 100.0%	3 / 3 100.0%	2 / 2 100.0%	5 / 5 100.0%	6 / 6 100.0%	4 / 4 100.0%	2 / 2 100.0%	6 / 6 100.0%	1 / 1 100.0%	0 / 0 0.0%	0 / 0 0.0%	0 / 0 0.0%	30 / 81 37.0%B	27 / 81 33.3%BE	14 / 81 17.3%AB	10 / 81 12.3%ABE		
1	Clostridium botulinum A str. Hall	81 / 81 100.0%	4 / 4 100.0%	4 / 4 100.0%	3 / 3 100.0%	2 / 2 100.0%	8 / 8 100.0%	5 / 5 100.0%	2 / 2 100.0%	2 / 2 100.0%	3 / 3 100.0%	1 / 1 100.0%	5 / 5 100.0%	6 / 6 100.0%	2 / 2 100.0%	3 / 3 100.0%	2 / 2 100.0%	3 / 3 100.0%	2 / 2 100.0%	5 / 5 100.0%	6 / 6 100.0%	4 / 4 100.0%	2 / 2 100.0%	6 / 6 100.0%	1 / 1 100.0%	0 / 0 0.0%	0 / 0 0.0%	0 / 0 0.0%	30 / 81 37.0%B	27 / 81 33.3%BE	14 / 81 17.3%AB	10 / 81 12.3%ABE		
1	Clostridium botulinum A3 str. Loch Maree	81 / 81 100.0%	3 / 3 100.0%	4 / 4 100.0%	3 / 3 100.0%	2 / 2 100.0%	8 / 8 100.0%	5 / 5 100.0%	2 / 2 100.0%	2 / 2 100.0%	3 / 3 100.0%	1 / 1 100.0%	5 / 5 100.0%	6 / 6 100.0%	3 / 3 100.0%	3 / 3 100.0%	2 / 2 100.0%	3 / 3 100.0%	2 / 2 100.0%	5 / 5 100.0%	6 / 6 100.0%	4 / 4 100.0%	2 / 2 100.0%	6 / 6 100.0%	1 / 1 100.0%	0 / 0 0.0%	0 / 0 0.0%	0 / 0 0.0%	30 / 81 37.0%B	26 / 81 32.1%BE	15 / 81 18.5%AB	10 / 81 12.3%ABE		
1	Clostridium botulinum A2 str. Kyoto	81 / 81 100.0%	3 / 3 100.0%	4 / 4 100.0%	3 / 3 100.0%	2 / 2 100.0%	8 / 8 100.0%	5 / 5 100.0%	2 / 2 100.0%	2 / 2 100.0%	3 / 3 100.0%	1 / 1 100.0%	5 / 5 100.0%	6 / 6 100.0%	3 / 3 100.0%	3 / 3 100.0%	2 / 2 100.0%	3 / 3 100.0%	2 / 2 100.0%	5 / 5 100.0%	6 / 6 100.0%	4 / 4 100.0%	2 / 2 100.0%	6 / 6 100.0%	1 / 1 100.0%	0 / 0 0.0%	0 / 0 0.0%	0 / 0 0.0%	45 / 81 55.6%AB	36 / 81 44.4%ABE				
3	Clostridium botulinum B	240 / 240 100.0%	10 / 10 100.0%	11 / 11 100.0%	10 / 10 100.0%	6 / 6 100.0%	21 / 21 100.0%	15 / 15 100.0%	6 / 6 100.0%	7 / 7 100.0%	9 / 9 100.0%	4 / 4 100.0%	15 / 15 100.0%	19 / 19 100.0%	8 / 8 100.0%	8 / 8 100.0%	6 / 6 100.0%	7 / 7 100.0%	6 / 6 100.0%	15 / 15 100.0%	19 / 19 100.0%	12 / 12 100.0%	6 / 6 100.0%	18 / 18 100.0%	2 / 2 100.0%	0 / 0 0.0%	0 / 0 0.0%	0 / 0 0.0%	88 / 240 36.7%AB	70 / 240 29.2%ABE	48 / 240 20.0%B	34 / 240 14.2%BE		
1	Clostridium botulinum Ba4 str. 657	81 / 81 100.0%	3 / 3 100.0%	4 / 4 100.0%	3 / 3 100.0%	2 / 2 100.0%	8 / 8 100.0%	5 / 5 100.0%	2 / 2 100.0%	2 / 2 100.0%	3 / 3 100.0%	1 / 1 100.0%	5 / 5 100.0%	6 / 6 100.0%	3 / 3 100.0%	3 / 3 100.0%	2 / 2 100.0%	3 / 3 100.0%	2 / 2 100.0%	5 / 5 100.0%	6 / 6 100.0%	4 / 4 100.0%	2 / 2 100.0%	6 / 6 100.0%	1 / 1 100.0%	0 / 0 0.0%	0 / 0 0.0%	0 / 0 0.0%	45 / 81 55.6%AB	36 / 81 44.4%ABE				
1	Clostridium botulinum B1	82 / 82 100.0%	4 / 4 100.0%	4 / 4 100.0%	3 / 3 100.0%	2 / 2 100.0%	8 / 8 100.0%	5 / 5 100.0%	2 / 2 100.0%	2 / 2 100.0%	3 / 3 100.0%	1 / 1 100.0%	5 / 5 100.0%	6 / 6 100.0%	3 / 3 100.0%	3 / 3 100.0%	2 / 2 100.0%	3 / 3 100.0%	2 / 2 100.0%	5 / 5 100.0%	6 / 6 100.0%	4 / 4 100.0%	2 / 2 100.0%	6 / 6 100.0%	1 / 1 100.0%	0 / 0 0.0%	0 / 0 0.0%	0 / 0 0.0%	30 / 82 36.6%B	27 / 82 32.9%BE	15 / 82 18.3%AB	10 / 82 12.2%ABE		
1	Clostridium botulinum B1 str. Okra	82 / 82 100.0%	4 / 4 100.0%	4 / 4 100.0%	3 / 3 100.0%	2 / 2 100.0%	8 / 8 100.0%	5 / 5 100.0%	2 / 2 100.0%	2 / 2 100.0%	3 / 3 100.0%	1 / 1 100.0%	5 / 5 100.0%	6 / 6 100.0%	3 / 3 100.0%	3 / 3 100.0%	2 / 2 100.0%	3 / 3 100.0%	2 / 2 100.0%	5 / 5 100.0%	6 / 6 100.0%	4 / 4 100.0%	2 / 2 100.0%	6 / 6 100.0%	1 / 1 100.0%	0 / 0 0.0%	0 / 0 0.0%	0 / 0 0.0%	30 / 82 36.6%B	27 / 82 32.9%BE	15 / 82 18.3%AB	10 / 82 12.2%ABE		
1	Clostridium botulinum B str. Eklund 17B	77 / 77 100.0%	3 / 3 100.0%	3 / 3 100.0%	4 / 4 100.0%	2 / 2 100.0%	5 / 5 100.0%	5 / 5 100.0%	2 / 2 100.0%	3 / 3 100.0%	3 / 3 100.0%	2 / 2 100.0%	5 / 5 100.0%	7 / 7 100.0%	2 / 2 100.0%	2 / 2 100.0%	2 / 2 100.0%	1 / 1 100.0%	2 / 2 100.0%	5 / 5 100.0%	7 / 7 100.0%	4 / 4 100.0%	2 / 2 100.0%	6 / 6 100.0%	0 / 0 0.0%	0 / 0 0.0%	0 / 0 0.0%	0 / 0 0.0%	28 / 77 36.4%AB	24 / 77 31.2%ABE	18 / 77 23.4%B	7 / 77 9.1%BE		
1	Clostridium botulinum E	79 / 79 100.0%	3 / 3 100.0%	3 / 3 100.0%	4 / 4 100.0%	2 / 2 100.0%	6 / 6 100.0%	5 / 5 100.0%	2 / 2 100.0%	3 / 3 100.0%	3 / 3 100.0%	2 / 2 100.0%	5 / 5 100.0%	7 / 7 100.0%	2 / 2 100.0%	2 / 2 100.0%	2 / 2 100.0%	2 / 2 100.0%	2 / 2 100.0%	5 / 5 100.0%	7 / 7 100.0%	4 / 4 100.0%	2 / 2 100.0%	6 / 6 100.0%	0 / 0 0.0%	0 / 0 0.0%	0 / 0 0.0%	0 / 0 0.0%	35 / 79 44.3%AB	25 / 79 31.6%ABE	12 / 79 15.2%B	7 / 79 8.9%BE		
1	Clostridium botulinum E3 str. Alaska E43	79 / 79 100.0%	3 / 3 100.0%	3 / 3 100.0%	4 / 4 100.0%	2 / 2 100.0%	6 / 6 100.0%	5 / 5 100.0%	2 / 2 100.0%	3 / 3 100.0%	3 / 3 100.0%	2 / 2 100.0%	5 / 5 100.0%	7 / 7 100.0%	2 / 2 100.0%	2 / 2 100.0%	2 / 2 100.0%	2 / 2 100.0%	2 / 2 100.0%	5 / 5 100.0%	7 / 7 100.0%	4 / 4 100.0%	2 / 2 100.0%	6 / 6 100.0%	0 / 0 0.0%	0 / 0 0.0%	0 / 0 0.0%	0 / 0 0.0%	35 / 79 44.3%AB	25 / 79 31.6%ABE	12 / 79 15.2%B	7 / 79 8.9%BE		
2	Clostridium botulinum F	151 / 151 100.0%	5 / 5 100.0%	5 / 5 100.0%	6 / 6 100.0%	4 / 4 100.0%	16 / 16 100.0%	8 / 8 100.0%	4 / 4 100.0%	4 / 4 100.0%	6 / 6 100.0%	2 / 2 100.0%	10 / 10 100.0%	12 / 12 100.0%	6 / 6 100.0%	6 / 6 100.0%	4 / 4 100.0%	6 / 6 100.0%	3 / 3 100.0%	9 / 9 100.0%	12 / 12 100.0%	6 / 6 100.0%	4 / 4 100.0%	11 / 11 100.0%	2 / 2 100.0%	0 / 0 0.0%	0 / 0 0.0%	0 / 0 0.0%	57 / 151 37.7%AB	38 / 151 25.2%ABE	30 / 151 19.9%B	26 / 151 17.2%BE		
1	Clostridium botulinum F str. Langeland	81 / 81 100.0%	3 / 3 100.0%	4 / 4 100.0%	3 / 3 100.0%	2 / 2 100.0%	8 / 8 100.0%	5 / 5 100.0%	2 / 2 100.0%	2 / 2 100.0%	3 / 3 100.0%	1 / 1 100.0%	5 / 5 100.0%	6 / 6 100.0%	3 / 3 100.0%	3 / 3 100.0%	2 / 2 100.0%	3 / 3 100.0%	2 / 2 100.0%	5 / 5 100.0%	6 / 6 100.0%	4 / 4 100.0%	2 / 2 100.0%	6 / 6 100.0%	1 / 1 100.0%	0 / 0 0.0%	0 / 0 0.0%	0 / 0 0.0%	30 / 81 37.0%B	26 / 81 32.1%BE	15 / 81 18.5%AB	10 / 81 12.3%ABE		
1	Clostridium botulinum F str. 230613	70 / 70 100.0%	2 / 2 100.0%	1 / 1 100.0%	3 / 3 100.0%	2 / 2 100.0%	8 / 8 100.0%	3 / 3 100.0%	2 / 2 100.0%	2 / 2 100.0%	3 / 3 100.0%	1 / 1 100.0%	5 / 5 100.0%	6 / 6 100.0%	3 / 3 100.0%	3 / 3 100.0%	2 / 2 100.0%	3 / 3 100.0%	1 / 1 100.0%	4 / 4 100.0%	6 / 6 100.0%	2 / 2 100.0%	2 / 2 100.0%	5 / 5 100.0%	1 / 1 100.0%	0 / 0 0.0%	0 / 0 0.0%	0 / 0 0.0%	42 / 70 60.0%AB	28 / 70 40.0%ABE				
1	Clostridium botulinum BKT015925	85 / 85 100.0%	3 / 3 100.0%	3 / 3 100.0%	4 / 4 100.0%	3 / 3 100.0%	9 / 9 100.0%	4 / 4 100.0%	2 / 2 100.0%	2 / 2 100.0%	4 / 4 100.0%	1 / 1 100.0%	5 / 5 100.0%	7 / 7 100.0%	3 / 3 100.0%	4 / 4 100.0%	2 / 2 100.0%	3 / 3 100.0%	2 / 2 100.0%	5 / 5 100.0%	7 / 7 100.0%	4 / 4 100.0%	1 / 1 100.0%	7 / 7 100.0%	0 / 0 0.0%	0 / 0 0.0%	0 / 0 0.0%	0 / 0 0.0%	45 / 85 52.9%AB	40 / 85 47.1%ABE				
1	Clostridium botulinum H04402 065	70 / 70 100.0%	3 / 3 100.0%	2 / 2 100.0%	2 / 2 100.0%	2 / 2 100.0%	8 / 8 100.0%	2 / 2 100.0%	2 / 2 100.0%	1 / 1 100.0%	2 / 2 100.0%	1 / 1 100.0%	5 / 5 100.0%	7 / 7 100.0%	3 / 3 100.0%	3 / 3 100.0%	2 / 2 100.0%	3 / 3 100.0%	2 / 2 100.0%	3 / 3 100.0%	6 / 6 100.0%	2 / 2 100.0%	2 / 2 100.0%	6 / 6 100.0%	1 / 1 100.0%	0 / 0 0.0%	0 / 0 0.0%	0 / 0 0.0%	41 / 70 58.6%AB	29 / 70 41.4%ABE				
1	ATCC 35296	79 / 80 98.8%	2 / 2 100.0%	2 / 2 100.0%	4 / 4 100.0%	2 / 2 100.0%	10 / 10 100.0%	4 / 4 100.0%	2 / 2 100.0%	1 / 1 100.0%	4 / 4 100.0%	1 / 1 100.0%	3 / 4 75.0%	7 / 7 100.0%	3 / 3 100.0%	4 / 4 100.0%	2 / 2 100.0%	3 / 3 100.0%	2 / 2 100.0%	3 / 3 100.0%	6 / 6 100.0%	4 / 4 100.0%	2 / 2 100.0%	8 / 8 100.0%	0 / 0 0.0%	0 / 0 0.0%	0 / 0 0.0%	0 / 0 0.0%	51 / 80 63.8%AB	28 / 80 35.0%ABE	1 / 80 1.2%E			
1	Clostridium cellulovorans 743B	79 / 80 98.8%	2 / 2 100.0%	2 / 2 100.0%	4 / 4 100.0%	2 / 2 100.0%	10 / 10 100.0%	4 / 4 100.0%	2 / 2 100.0%	1 / 1 100.0%	4 / 4 100.0%	1 / 1 100.0%	3 / 4 75.0%	7 / 7 100.0%	3 / 3 100.0%	4 / 4 100.0%	2 / 2 100.0%	3 / 3 100.0%	2 / 2 100.0%	3 / 3 100.0%	6 / 6 100.0%	4 / 4 100.0%	2 / 2 100.0%	8 / 8 100.0%	0 / 0 0.0%	0 / 0 0.0%	0 / 0 0.0%	0 / 0 0.0%	51 / 80 63.8%AB	28 / 80 35.0%ABE	1 / 80 1.2%E			
1	"Bacillus pasteurianus" (Winogradsky 1895) Lehmann and Neumann 1907	73 / 73 100.0%	2 / 2 100.0%	2 / 2 100.0%	3 / 3 100.0%	1 / 1 100.0%	9 / 9 100.0%	3 / 3 100.0%	2 / 2 100.0%	2 / 2 100.0%	2 / 2 100.0%	1 / 1 100.0%	4 / 4 100.0%	6 / 6 100.0%	3 / 3 100.0%	3 / 3 100.0%	2 / 2 100.0%	3 / 3 100.0%	2 / 2 100.0%	4 / 4 100.0%	6 / 6 100.0%	3 / 3 100.0%	2 / 2 100.0%	8 / 8 100.0%	0 / 0 0.0%	0 / 0 0.0%	0 / 0 0.0%	0 / 0 0.0%	45 / 73 61.6%AB	28 / 73 38.4%ABE				
1	Clostridia isolate BC1	73 / 73 100.0%	2 / 2 100.0%	2 / 2 100.0%	3 / 3 100.0%	1 / 1 100.0%	9 / 9 100.0%	3 / 3 100.0%	2 / 2 100.0%	2 / 2 100.0%	2 / 2 100.0%	1 / 1 100.0%	4 / 4 100.0%	6 / 6 100.0%	3 / 3 100.0%	3 / 3 100.0%	2 / 2 100.0%	3 / 3 100.0%	2 / 2 100.0%	4 / 4 100.0%	6 / 6 100.0%	3 / 3 100.0%	2 / 2 100.0%	8 / 8 100.0%	0 / 0 0.0%	0 / 0 0.0%	0 / 0 0.0%	0 / 0 0.0%	45 / 73 61.6%AB	28 / 73 38.4%ABE				
3	"Bacillus perfringens" Veillon and Zuber 1898	280 / 281 99.6%	12 / 12 100.0%	9 / 9 100.0%	12 / 12 100.0%	8 / 8 100.0%	35 / 36 97.2%	12 / 12 100.0%	6 / 6 100.0%	9 / 9 100.0%	13 / 13 100.0%	7 / 7 100.0%	16 / 16 100.0%	20 / 20 100.0%	12 / 12 100.0%	8 / 8 100.0%	6 / 6 100.0%	8 / 8 100.0%	6 / 6 100.0%	15 / 15 100.0%	20 / 20 100.0%	11 / 11 100.0%	5 / 5 100.0%	27 / 27 100.0%	3 / 3 100.0%	0 / 0 0.0%	0 / 0 0.0%	0 / 0 0.0%	163 / 281 58.0%AB	114 / 281 40.6%ABE	3 / 281 1.1%BE	1 / 281 0.4%E		
1	Clostridium perfringens 13	94 / 94 100.0%	4 / 4 100.0%	3 / 3 100.0%	4 / 4 100.0%	4 / 4 100.0%	11 / 11 100.0%	4 / 4 100.0%	2 / 2 100.0%	3 / 3 100.0%	6 / 6 100.0%	2 / 2 100.0%	6 / 6 100.0%	7 / 7 100.0%	4 / 4 100.0%	2 / 2 100.0%	2 / 2 100.0%	2 / 2 100.0%	2 / 2 100.0%	5 / 5 100.0%	6 / 6 100.0%	3 / 3 100.0%	2 / 2 100.0%	9 / 9 100.0%	1 / 1 100.0%	0 / 0 0.0%	0 / 0 0.0%	0 / 0 0.0%	57 / 94 60.6%AB	36 / 94 38.3%ABE	1 / 94 1.1%BE			
1	Clostridium perfringens ATCC 13124	92 / 93 98.9%	4 / 4 100.0%	3 / 3 100.0%	4 / 4 100.0%	2 / 2 100.0%	12 / 13 92.3%	4 / 4 100.0%	2 / 2 100.0%	3 / 3 100.0%	3 / 3 100.0%	3 / 3 100.0%	5 / 5 100.0%	6 / 6 100.0%	4 / 4 100.0%	3 / 3 100.0%	2 / 2 100.0%	3 / 3 100.0%	2 / 2 100.0%	5 / 5 100.0%	7 / 7 100.0%	4 / 4 100.0%	1 / 1 100.0%	9 / 9 100.0%	1 / 1 100.0%	0 / 0 0.0%	0 / 0 0.0%	0 / 0 0.0%	52 / 93 55.9%AB	39 / 93 41.9%ABE	1 / 93 1.1%BE	1 / 93 1.1%E		
1	Clostridium perfringens SM101	94 / 94 100.0%	4 / 4 100.0%	3 / 3 100.0%	4 / 4 100.0%	2 / 2 100.0%	12 / 12 100.0%	4 / 4 100.0%	2 / 2 100.0%	3 / 3 100.0%	4 / 4 100.0%	2 / 2 100.0%	5 / 5 100.0%	7 / 7 100.0%	4 / 4 100.0%	3 / 3 100.0%	2 / 2 100.0%	3 / 3 100.0%	2 / 2 100.0%	5 / 5 100.0%	7 / 7 100.0%	4 / 4 100.0%	2 / 2 100.0%	9 / 9 100.0%	1 / 1 100.0%	0 / 0 0.0%	0 / 0 0.0%	0 / 0 0.0%	54 / 94 57.4%AB	39 / 94 41.5%ABE	1 / 94 1.1%BE			
1	"Bacillus tetani" Flugge 1886	54 / 54 100.0%	2 / 2 100.0%	1 / 1 100.0%	3 / 3 100.0%	3 / 3 100.0%	4 / 4 100.0%	1 / 1 100.0%	1 / 1 100.0%	2 / 2 100.0%	3 / 3 100.0%	1 / 1 100.0%	4 / 4 100.0%	3 / 3 100.0%	2 / 2 100.0%	2 / 2 100.0%	2 / 2 100.0%	2 / 2 100.0%	1 / 1 100.0%	4 / 4 100.0%	4 / 4 100.0%	2 / 2 100.0%	2 / 2 100.0%	5 / 5 100.0%	0 / 0 0.0%	0 / 0 0.0%	0 / 0 0.0%	0 / 0 0.0%	32 / 54 59.3%AB	22 / 54 40.7%ABE				
1	Clostridium tetani E88	54 / 54 100.0%	2 / 2 100.0%	1 / 1 100.0%	3 / 3 100.0%	3 / 3 100.0%	4 / 4 100.0%	1 / 1 100.0%	1 / 1 100.0%	2 / 2 100.0%	3 / 3 100.0%	1 / 1 100.0%	4 / 4 100.0%	3 / 3 100.0%	2 / 2 100.0%	2 / 2 100.0%	2 / 2 100.0%	2 / 2 100.0%	1 / 1 100.0%	4 / 4 100.0%	4 / 4 100.0%	2 / 2 100.0%	2 / 2 100.0%	5 / 5 100.0%	0 / 0 0.0%	0 / 0 0.0%	0 / 0 0.0%	0 / 0 0.0%	32 / 54 59.3%AB	22 / 54 40.7%ABE				
1	"Clostridium rubrum" Ng and Vaughn 1963	92 / 94 97.9%	4 / 4 100.0%	4 / 4 100.0%	6 / 6 100.0%	2 / 3 66.7%	8 / 8 100.0%	5 / 5 100.0%	3 / 3 100.0%	3 / 3 100.0%	4 / 4 100.0%	2 / 2 100.0%	5 / 5 100.0%	7 / 7 100.0%	3 / 3 100.0%	3 / 3 100.0%	1 / 1 100.0%	3 / 3 100.0%	2 / 2 100.0%	6 / 6 100.0%	6 / 6 100.0%	5 / 5 100.0%	2 / 2 100.0%	8 / 9 88.9%	0 / 0 0.0%	0 / 0 0.0%	0 / 0 0.0%	0 / 0 0.0%	48 / 94 51.1%AB	44 / 94 46.8%ABE	2 / 94 2.1%E			
1	Clostridium [acetobutylicum] NCIMB 8052	92 / 94 97.9%	4 / 4 100.0%	4 / 4 100.0%	6 / 6 100.0%	2 / 3 66.7%	8 / 8 100.0%	5 / 5 100.0%	3 / 3 100.0%	3 / 3 100.0%	4 / 4 100.0%	2 / 2 100.0%	5 / 5 100.0%	7 / 7 100.0%	3 / 3 100.0%	3 / 3 100.0%	1 / 1 100.0%	3 / 3 100.0%	2 / 2 100.0%	6 / 6 100.0%	6 / 6 100.0%	5 / 5 100.0%	2 / 2 100.0%	8 / 9 88.9%	0 / 0 0.0%	0 / 0 0.0%	0 / 0 0.0%	0 / 0 0.0%	48 / 94 51.1%AB	44 / 94 46.8%ABE	2 / 94 2.1%E			
1	ATCC 35319	62 / 63 98.4%	1 / 1 100.0%	2 / 2 100.0%	3 / 3 100.0%	2 / 2 100.0%	5 / 5 100.0%	4 / 4 100.0%	1 / 1 100.0%	2 / 2 100.0%	5 / 5 100.0%	1 / 1 100.0%	4 / 5 80.0%	5 / 5 100.0%	2 / 2 100.0%	2 / 2 100.0%	1 / 1 100.0%	3 / 3 100.0%	1 / 1 100.0%	4 / 4 100.0%	4 / 4 100.0%	3 / 3 100.0%	2 / 2 100.0%	5 / 5 100.0%	0 / 0 0.0%	0 / 0 0.0%	0 / 0 0.0%	0 / 0 0.0%	47 / 63 74.6%AB	15 / 63 23.8%ABE	1 / 63 1.6%E			
1	Clostridium cellulolyticum H10	62 / 63 98.4%	1 / 1 100.0%	2 / 2 100.0%	3 / 3 100.0%	2 / 2 100.0%	5 / 5 100.0%	4 / 4 100.0%	1 / 1 100.0%	2 / 2 100.0%	5 / 5 100.0%	1 / 1 100.0%	4 / 5 80.0%	5 / 5 100.0%	2 / 2 100.0%	2 / 2 100.0%	1 / 1 100.0%	3 / 3 100.0%	1 / 1 100.0%	4 / 4 100.0%	4 / 4 100.0%	3 / 3 100.0%	2 / 2 100.0%	5 / 5 100.0%	0 / 0 0.0%	0 / 0 0.0%	0 / 0 0.0%	0 / 0 0.0%	47 / 63 74.6%AB	15 / 63 23.8%ABE	1 / 63 1.6%E			
2	ATCC 8527	120 / 120 100.0%	4 / 4 100.0%	6 / 6 100.0%	4 / 4 100.0%	4 / 4 100.0%	10 / 10 100.0%	8 / 8 100.0%	2 / 2 100.0%	4 / 4 100.0%	6 / 6 100.0%	2 / 2 100.0%	8 / 8 100.0%	6 / 6 100.0%	4 / 4 100.0%	4 / 4 100.0%	2 / 2 100.0%	2 / 2 100.0%	4 / 4 100.0%	10 / 10 100.0%	8 / 8 100.0%	6 / 6 100.0%	4 / 4 100.0%	12 / 12 100.0%	0 / 0 0.0%	0 / 0 0.0%	0 / 0 0.0%	0 / 0 0.0%	66 / 120 55.0%AB	32 / 120 26.7%ABE	14 / 120 11.7%B	8 / 120 6.7%BE		
1	Clostridium kluyveri DSM 555	60 / 60 100.0%	2 / 2 100.0%	3 / 3 100.0%	2 / 2 100.0%	2 / 2 100.0%	5 / 5 100.0%	4 / 4 100.0%	1 / 1 100.0%	2 / 2 100.0%	3 / 3 100.0%	1 / 1 100.0%	4 / 4 100.0%	3 / 3 100.0%	2 / 2 100.0%	2 / 2 100.0%	1 / 1 100.0%	1 / 1 100.0%	2 / 2 100.0%	5 / 5 100.0%	4 / 4 100.0%	3 / 3 100.0%	2 / 2 100.0%	6 / 6 100.0%	0 / 0 0.0%	0 / 0 0.0%	0 / 0 0.0%	0 / 0 0.0%	26 / 60 43.3%AB	14 / 60 23.3%B	12 / 60 20.0%ABE	8 / 60 13.3%BE		
1	Clostridium kluyveri NBRC 12016	60 / 60 100.0%	2 / 2 100.0%	3 / 3 100.0%	2 / 2 100.0%	2 / 2 100.0%	5 / 5 100.0%	4 / 4 100.0%	1 / 1 100.0%	2 / 2 100.0%	3 / 3 100.0%	1 / 1 100.0%	4 / 4 100.0%	3 / 3 100.0%	2 / 2 100.0%	2 / 2 100.0%	1 / 1 100.0%	1 / 1 100.0%	2 / 2 100.0%	5 / 5 100.0%	4 / 4 100.0%	3 / 3 100.0%	2 / 2 100.0%	6 / 6 100.0%	0 / 0 0.0%	0 / 0 0.0%	0 / 0 0.0%	0 / 0 0.0%	40 / 60 66.7%AB	20 / 60 33.3%ABE				
1	ATCC 55383	72 / 72 100.0%	3 / 3 100.0%	2 / 2 100.0%	3 / 3 100.0%	2 / 2 100.0%	6 / 6 100.0%	4 / 4 100.0%	1 / 1 100.0%	2 / 2 100.0%	3 / 3 100.0%	1 / 1 100.0%	4 / 4 100.0%	5 / 5 100.0%	3 / 3 100.0%	3 / 3 100.0%	2 / 2 100.0%	3 / 3 100.0%	1 / 1 100.0%	5 / 5 100.0%	5 / 5 100.0%	3 / 3 100.0%	2 / 2 100.0%	7 / 7 100.0%	2 / 2 100.0%	0 / 0 0.0%	0 / 0 0.0%	0 / 0 0.0%	43 / 72 59.7%AB	29 / 72 40.3%ABE				
1	Clostridium ljungdahlii ATCC 49587	72 / 72 100.0%	3 / 3 100.0%	2 / 2 100.0%	3 / 3 100.0%	2 / 2 100.0%	6 / 6 100.0%	4 / 4 100.0%	1 / 1 100.0%	2 / 2 100.0%	3 / 3 100.0%	1 / 1 100.0%	4 / 4 100.0%	5 / 5 100.0%	3 / 3 100.0%	3 / 3 100.0%	2 / 2 100.0%	3 / 3 100.0%	1 / 1 100.0%	5 / 5 100.0%	5 / 5 100.0%	3 / 3 100.0%	2 / 2 100.0%	7 / 7 100.0%	2 / 2 100.0%	0 / 0 0.0%	0 / 0 0.0%	0 / 0 0.0%	43 / 72 59.7%AB	29 / 72 40.3%ABE				
1	"Bacillus novyi" Migula 1894	81 / 81 100.0%	2 / 2 100.0%	3 / 3 100.0%	4 / 4 100.0%	3 / 3 100.0%	9 / 9 100.0%	4 / 4 100.0%	2 / 2 100.0%	2 / 2 100.0%	4 / 4 100.0%	1 / 1 100.0%	4 / 4 100.0%	6 / 6 100.0%	3 / 3 100.0%	4 / 4 100.0%	2 / 2 100.0%	3 / 3 100.0%	2 / 2 100.0%	5 / 5 100.0%	7 / 7 100.0%	4 / 4 100.0%	1 / 1 100.0%	6 / 6 100.0%	0 / 0 0.0%	0 / 0 0.0%	0 / 0 0.0%	0 / 0 0.0%	44 / 81 54.3%AB	37 / 81 45.7%ABE				
1	Clostridium novyi NT	81 / 81 100.0%	2 / 2 100.0%	3 / 3 100.0%	4 / 4 100.0%	3 / 3 100.0%	9 / 9 100.0%	4 / 4 100.0%	2 / 2 100.0%	2 / 2 100.0%	4 / 4 100.0%	1 / 1 100.0%	4 / 4 100.0%	6 / 6 100.0%	3 / 3 100.0%	4 / 4 100.0%	2 / 2 100.0%	3 / 3 100.0%	2 / 2 100.0%	5 / 5 100.0%	7 / 7 100.0%	4 / 4 100.0%	1 / 1 100.0%	6 / 6 100.0%	0 / 0 0.0%	0 / 0 0.0%	0 / 0 0.0%	0 / 0 0.0%	44 / 81 54.3%AB	37 / 81 45.7%ABE				
1	ATCC 27021	70 / 70 100.0%	2 / 2 100.0%	1 / 1 100.0%	4 / 4 100.0%	2 / 2 100.0%	7 / 7 100.0%	3 / 3 100.0%	2 / 2 100.0%	3 / 3 100.0%	4 / 4 100.0%	2 / 2 100.0%	4 / 4 100.0%	6 / 6 100.0%	3 / 3 100.0%	3 / 3 100.0%	1 / 1 100.0%	3 / 3 100.0%	1 / 1 100.0%	3 / 3 100.0%	5 / 5 100.0%	2 / 2 100.0%	2 / 2 100.0%	7 / 7 100.0%	0 / 0 0.0%	0 / 0 0.0%	0 / 0 0.0%	0 / 0 0.0%	36 / 70 51.4%AB	34 / 70 48.6%ABE				
1	Clostridium saccharoperbutylacetonicum ATCC 13564	70 / 70 100.0%	2 / 2 100.0%	1 / 1 100.0%	4 / 4 100.0%	2 / 2 100.0%	7 / 7 100.0%	3 / 3 100.0%	2 / 2 100.0%	3 / 3 100.0%	4 / 4 100.0%	2 / 2 100.0%	4 / 4 100.0%	6 / 6 100.0%	3 / 3 100.0%	3 / 3 100.0%	1 / 1 100.0%	3 / 3 100.0%	1 / 1 100.0%	3 / 3 100.0%	5 / 5 100.0%	2 / 2 100.0%	2 / 2 100.0%	7 / 7 100.0%	0 / 0 0.0%	0 / 0 0.0%	0 / 0 0.0%	0 / 0 0.0%	36 / 70 51.4%AB	34 / 70 48.6%ABE				
1	Clostridium saccharoperbutylacetonicum ATCC 27021	70 / 70 100.0%	2 / 2 100.0%	1 / 1 100.0%	4 / 4 100.0%	2 / 2 100.0%	7 / 7 100.0%	3 / 3 100.0%	2 / 2 100.0%	3 / 3 100.0%	4 / 4 100.0%	2 / 2 100.0%	4 / 4 100.0%	6 / 6 100.0%	3 / 3 100.0%	3 / 3 100.0%	1 / 1 100.0%	3 / 3 100.0%	1 / 1 100.0%	3 / 3 100.0%	5 / 5 100.0%	2 / 2 100.0%	2 / 2 100.0%	7 / 7 100.0%	0 / 0 0.0%	0 / 0 0.0%	0 / 0 0.0%	0 / 0 0.0%	36 / 70 51.4%AB	34 / 70 48.6%ABE				
1	Clostridium sp. BNL1100	60 / 60 100.0%	1 / 1 100.0%	2 / 2 100.0%	3 / 3 100.0%	2 / 2 100.0%	5 / 5 100.0%	4 / 4 100.0%	1 / 1 100.0%	2 / 2 100.0%	4 / 4 100.0%	1 / 1 100.0%	4 / 4 100.0%	5 / 5 100.0%	2 / 2 100.0%	2 / 2 100.0%	1 / 1 100.0%	3 / 3 100.0%	1 / 1 100.0%	3 / 3 100.0%	4 / 4 100.0%	3 / 3 100.0%	2 / 2 100.0%	5 / 5 100.0%	0 / 0 0.0%	0 / 0 0.0%	0 / 0 0.0%	0 / 0 0.0%	45 / 60 75.0%AB	15 / 60 25.0%ABE				
1	Clostridium sp. SY8519	0 / 53 0.0%	0 / 1 0.0%	0 / 2 0.0%	0 / 2 0.0%	0 / 2 0.0%	0 / 4 0.0%	0 / 4 0.0%	0 / 2 0.0%	0 / 1 0.0%	0 / 2 0.0%	0 / 1 0.0%	0 / 4 0.0%	0 / 3 0.0%	0 / 1 0.0%	0 / 2 0.0%	0 / 1 0.0%	0 / 3 0.0%	0 / 1 0.0%	0 / 3 0.0%	0 / 4 0.0%	0 / 3 0.0%	0 / 1 0.0%	0 / 6 0.0%	0 / 0 0.0%	0 / 0 0.0%	0 / 0 0.0%	0 / 0 0.0%	40 / 53 75.5%E	13 / 53 24.5%				
3	"Candidatus Arthromitus" Snel et al. 1995 emend Thompson et al. 2012	114 / 114 100.0%	3 / 3 100.0%	6 / 6 100.0%	5 / 5 100.0%	2 / 2 100.0%	9 / 9 100.0%	6 / 6 100.0%	3 / 3 100.0%	3 / 3 100.0%	3 / 3 100.0%	3 / 3 100.0%	9 / 9 100.0%	5 / 5 100.0%	6 / 6 100.0%	3 / 3 100.0%	3 / 3 100.0%	3 / 3 100.0%	3 / 3 100.0%	6 / 6 100.0%	12 / 12 100.0%	6 / 6 100.0%	3 / 3 100.0%	12 / 12 100.0%	0 / 0 0.0%	0 / 0 0.0%	0 / 0 0.0%	0 / 0 0.0%	75 / 114 65.8%AB	39 / 114 34.2%ABE				
2	Arthromitus sp. SFB-mouse	76 / 76 100.0%	2 / 2 100.0%	4 / 4 100.0%	3 / 3 100.0%	2 / 2 100.0%	6 / 6 100.0%	4 / 4 100.0%	2 / 2 100.0%	2 / 2 100.0%	2 / 2 100.0%	2 / 2 100.0%	6 / 6 100.0%	3 / 3 100.0%	4 / 4 100.0%	2 / 2 100.0%	2 / 2 100.0%	2 / 2 100.0%	2 / 2 100.0%	4 / 4 100.0%	8 / 8 100.0%	4 / 4 100.0%	2 / 2 100.0%	8 / 8 100.0%	0 / 0 0.0%	0 / 0 0.0%	0 / 0 0.0%	0 / 0 0.0%	50 / 76 65.8%AB	26 / 76 34.2%ABE				
1	Candidatus Arthromitus sp. SFB-mouse-Japan	39 / 39 100.0%	1 / 1 100.0%	2 / 2 100.0%	2 / 2 100.0%	1 / 1 100.0%	3 / 3 100.0%	2 / 2 100.0%	1 / 1 100.0%	1 / 1 100.0%	1 / 1 100.0%	1 / 1 100.0%	3 / 3 100.0%	2 / 2 100.0%	2 / 2 100.0%	1 / 1 100.0%	1 / 1 100.0%	1 / 1 100.0%	1 / 1 100.0%	2 / 2 100.0%	4 / 4 100.0%	2 / 2 100.0%	1 / 1 100.0%	4 / 4 100.0%	0 / 0 0.0%	0 / 0 0.0%	0 / 0 0.0%	0 / 0 0.0%	26 / 39 66.7%AB	13 / 39 33.3%ABE				
1	Candidatus Arthromitus sp. SFB-mouse-Yit	37 / 37 100.0%	1 / 1 100.0%	2 / 2 100.0%	1 / 1 100.0%	1 / 1 100.0%	3 / 3 100.0%	2 / 2 100.0%	1 / 1 100.0%	1 / 1 100.0%	1 / 1 100.0%	1 / 1 100.0%	3 / 3 100.0%	1 / 1 100.0%	2 / 2 100.0%	1 / 1 100.0%	1 / 1 100.0%	1 / 1 100.0%	1 / 1 100.0%	2 / 2 100.0%	4 / 4 100.0%	2 / 2 100.0%	1 / 1 100.0%	4 / 4 100.0%	0 / 0 0.0%	0 / 0 0.0%	0 / 0 0.0%	0 / 0 0.0%	24 / 37 64.9%AB	13 / 37 35.1%ABE				
1	Candidatus Arthromitus sp. SFB-rat-Yit	38 / 38 100.0%	1 / 1 100.0%	2 / 2 100.0%	2 / 2 100.0%	0 / 0 0.0%	3 / 3 100.0%	2 / 2 100.0%	1 / 1 100.0%	1 / 1 100.0%	1 / 1 100.0%	1 / 1 100.0%	3 / 3 100.0%	2 / 2 100.0%	2 / 2 100.0%	1 / 1 100.0%	1 / 1 100.0%	1 / 1 100.0%	1 / 1 100.0%	2 / 2 100.0%	4 / 4 100.0%	2 / 2 100.0%	1 / 1 100.0%	4 / 4 100.0%	0 / 0 0.0%	0 / 0 0.0%	0 / 0 0.0%	0 / 0 0.0%	25 / 38 65.8%AB	13 / 38 34.2%ABE				
2	Alkaliphilus	185 / 190 97.4%	9 / 9 100.0%	9 / 9 100.0%	7 / 7 100.0%	8 / 8 100.0%	15 / 15 100.0%	13 / 13 100.0%	5 / 5 100.0%	3 / 6 50.0%	8 / 8 100.0%	3 / 3 100.0%	10 / 10 100.0%	9 / 9 100.0%	9 / 9 100.0%	6 / 6 100.0%	4 / 4 100.0%	8 / 8 100.0%	3 / 5 60.0%	11 / 11 100.0%	13 / 13 100.0%	10 / 10 100.0%	4 / 4 100.0%	16 / 16 100.0%	2 / 2 100.0%	0 / 0 0.0%	0 / 0 0.0%	0 / 0 0.0%	81 / 190 42.6%AB	49 / 190 25.8%B	37 / 190 19.5%ABE	18 / 190 9.5%BE	3 / 190 1.6%E	2 / 190 1.1%
1	"Alkaliphilus metalliredigens" Ye et al. (2004)	104 / 104 100.0%	6 / 6 100.0%	5 / 5 100.0%	3 / 3 100.0%	5 / 5 100.0%	8 / 8 100.0%	7 / 7 100.0%	3 / 3 100.0%	3 / 3 100.0%	4 / 4 100.0%	2 / 2 100.0%	5 / 5 100.0%	5 / 5 100.0%	5 / 5 100.0%	3 / 3 100.0%	2 / 2 100.0%	5 / 5 100.0%	3 / 3 100.0%	6 / 6 100.0%	7 / 7 100.0%	6 / 6 100.0%	2 / 2 100.0%	8 / 8 100.0%	1 / 1 100.0%	0 / 0 0.0%	0 / 0 0.0%	0 / 0 0.0%	72 / 104 69.2%AB	31 / 104 29.8%ABE	1 / 104 1.0%BE			
1	Alkaliphilus metalliredigenes QYMF	104 / 104 100.0%	6 / 6 100.0%	5 / 5 100.0%	3 / 3 100.0%	5 / 5 100.0%	8 / 8 100.0%	7 / 7 100.0%	3 / 3 100.0%	3 / 3 100.0%	4 / 4 100.0%	2 / 2 100.0%	5 / 5 100.0%	5 / 5 100.0%	5 / 5 100.0%	3 / 3 100.0%	2 / 2 100.0%	5 / 5 100.0%	3 / 3 100.0%	6 / 6 100.0%	7 / 7 100.0%	6 / 6 100.0%	2 / 2 100.0%	8 / 8 100.0%	1 / 1 100.0%	0 / 0 0.0%	0 / 0 0.0%	0 / 0 0.0%	72 / 104 69.2%AB	31 / 104 29.8%ABE	1 / 104 1.0%BE			
1	ATCC BAA-1360	81 / 86 94.2%	3 / 3 100.0%	4 / 4 100.0%	4 / 4 100.0%	3 / 3 100.0%	7 / 7 100.0%	6 / 6 100.0%	2 / 2 100.0%	0 / 3 0.0%	4 / 4 100.0%	1 / 1 100.0%	5 / 5 100.0%	4 / 4 100.0%	4 / 4 100.0%	3 / 3 100.0%	2 / 2 100.0%	3 / 3 100.0%	0 / 2 0.0%	5 / 5 100.0%	6 / 6 100.0%	4 / 4 100.0%	2 / 2 100.0%	8 / 8 100.0%	1 / 1 100.0%	0 / 0 0.0%	0 / 0 0.0%	0 / 0 0.0%	49 / 86 57.0%B	17 / 86 19.8%BE	9 / 86 10.5%AB	6 / 86 7.0%ABE	3 / 86 3.5%E	2 / 86 2.3%
1	Alkaliphilus oremlandii OhILAs	81 / 86 94.2%	3 / 3 100.0%	4 / 4 100.0%	4 / 4 100.0%	3 / 3 100.0%	7 / 7 100.0%	6 / 6 100.0%	2 / 2 100.0%	0 / 3 0.0%	4 / 4 100.0%	1 / 1 100.0%	5 / 5 100.0%	4 / 4 100.0%	4 / 4 100.0%	3 / 3 100.0%	2 / 2 100.0%	3 / 3 100.0%	0 / 2 0.0%	5 / 5 100.0%	6 / 6 100.0%	4 / 4 100.0%	2 / 2 100.0%	8 / 8 100.0%	1 / 1 100.0%	0 / 0 0.0%	0 / 0 0.0%	0 / 0 0.0%	49 / 86 57.0%B	17 / 86 19.8%BE	9 / 86 10.5%AB	6 / 86 7.0%ABE	3 / 86 3.5%E	2 / 86 2.3%
1	'Heliobacteriaceae'	107 / 107 100.0%	4 / 4 100.0%	4 / 4 100.0%	4 / 4 100.0%	4 / 4 100.0%	9 / 9 100.0%	7 / 7 100.0%	2 / 2 100.0%	4 / 4 100.0%	8 / 8 100.0%	2 / 2 100.0%	6 / 6 100.0%	5 / 5 100.0%	3 / 3 100.0%	4 / 4 100.0%	2 / 2 100.0%	6 / 6 100.0%	3 / 3 100.0%	6 / 6 100.0%	7 / 7 100.0%	6 / 6 100.0%	1 / 1 100.0%	9 / 9 100.0%	1 / 1 100.0%	0 / 0 0.0%	0 / 0 0.0%	0 / 0 0.0%	36 / 107 33.6%AB	34 / 107 31.8%B	22 / 107 20.6%ABE	15 / 107 14.0%BE		
1	Heliobacterium	107 / 107 100.0%	4 / 4 100.0%	4 / 4 100.0%	4 / 4 100.0%	4 / 4 100.0%	9 / 9 100.0%	7 / 7 100.0%	2 / 2 100.0%	4 / 4 100.0%	8 / 8 100.0%	2 / 2 100.0%	6 / 6 100.0%	5 / 5 100.0%	3 / 3 100.0%	4 / 4 100.0%	2 / 2 100.0%	6 / 6 100.0%	3 / 3 100.0%	6 / 6 100.0%	7 / 7 100.0%	6 / 6 100.0%	1 / 1 100.0%	9 / 9 100.0%	1 / 1 100.0%	0 / 0 0.0%	0 / 0 0.0%	0 / 0 0.0%	36 / 107 33.6%AB	34 / 107 31.8%B	22 / 107 20.6%ABE	15 / 107 14.0%BE		
1	ATCC 51547	107 / 107 100.0%	4 / 4 100.0%	4 / 4 100.0%	4 / 4 100.0%	4 / 4 100.0%	9 / 9 100.0%	7 / 7 100.0%	2 / 2 100.0%	4 / 4 100.0%	8 / 8 100.0%	2 / 2 100.0%	6 / 6 100.0%	5 / 5 100.0%	3 / 3 100.0%	4 / 4 100.0%	2 / 2 100.0%	6 / 6 100.0%	3 / 3 100.0%	6 / 6 100.0%	7 / 7 100.0%	6 / 6 100.0%	1 / 1 100.0%	9 / 9 100.0%	1 / 1 100.0%	0 / 0 0.0%	0 / 0 0.0%	0 / 0 0.0%	36 / 107 33.6%AB	34 / 107 31.8%B	22 / 107 20.6%ABE	15 / 107 14.0%BE		
1	Heliobacterium modesticaldum Ice1	107 / 107 100.0%	4 / 4 100.0%	4 / 4 100.0%	4 / 4 100.0%	4 / 4 100.0%	9 / 9 100.0%	7 / 7 100.0%	2 / 2 100.0%	4 / 4 100.0%	8 / 8 100.0%	2 / 2 100.0%	6 / 6 100.0%	5 / 5 100.0%	3 / 3 100.0%	4 / 4 100.0%	2 / 2 100.0%	6 / 6 100.0%	3 / 3 100.0%	6 / 6 100.0%	7 / 7 100.0%	6 / 6 100.0%	1 / 1 100.0%	9 / 9 100.0%	1 / 1 100.0%	0 / 0 0.0%	0 / 0 0.0%	0 / 0 0.0%	36 / 107 33.6%AB	34 / 107 31.8%B	22 / 107 20.6%ABE	15 / 107 14.0%BE		
2	Syntrophomonadaceae	91 / 92 98.9%	2 / 2 100.0%	2 / 2 100.0%	2 / 2 100.0%	2 / 2 100.0%	7 / 7 100.0%	6 / 6 100.0%	2 / 2 100.0%	2 / 2 100.0%	6 / 6 100.0%	2 / 2 100.0%	8 / 8 100.0%	4 / 4 100.0%	2 / 2 100.0%	4 / 4 100.0%	2 / 2 100.0%	6 / 6 100.0%	2 / 2 100.0%	6 / 6 100.0%	8 / 8 100.0%	3 / 3 100.0%	2 / 2 100.0%	10 / 10 100.0%	1 / 2 50.0%	0 / 0 0.0%	0 / 0 0.0%	0 / 0 0.0%	50 / 92 54.3%AB	41 / 92 44.6%ABE	1 / 92 1.1%E			
1	Syntrophomonas	45 / 46 97.8%	1 / 1 100.0%	1 / 1 100.0%	1 / 1 100.0%	1 / 1 100.0%	3 / 3 100.0%	3 / 3 100.0%	1 / 1 100.0%	1 / 1 100.0%	3 / 3 100.0%	1 / 1 100.0%	4 / 4 100.0%	2 / 2 100.0%	1 / 1 100.0%	2 / 2 100.0%	1 / 1 100.0%	3 / 3 100.0%	1 / 1 100.0%	3 / 3 100.0%	4 / 4 100.0%	2 / 2 100.0%	1 / 1 100.0%	5 / 5 100.0%	0 / 1 0.0%	0 / 0 0.0%	0 / 0 0.0%	0 / 0 0.0%	24 / 46 52.2%AB	21 / 46 45.7%ABE	1 / 46 2.2%E			
1	DSM 2245	45 / 46 97.8%	1 / 1 100.0%	1 / 1 100.0%	1 / 1 100.0%	1 / 1 100.0%	3 / 3 100.0%	3 / 3 100.0%	1 / 1 100.0%	1 / 1 100.0%	3 / 3 100.0%	1 / 1 100.0%	4 / 4 100.0%	2 / 2 100.0%	1 / 1 100.0%	2 / 2 100.0%	1 / 1 100.0%	3 / 3 100.0%	1 / 1 100.0%	3 / 3 100.0%	4 / 4 100.0%	2 / 2 100.0%	1 / 1 100.0%	5 / 5 100.0%	0 / 1 0.0%	0 / 0 0.0%	0 / 0 0.0%	0 / 0 0.0%	24 / 46 52.2%AB	21 / 46 45.7%ABE	1 / 46 2.2%E			
1	Syntrophomonas wolfei subsp. wolfei	45 / 46 97.8%	1 / 1 100.0%	1 / 1 100.0%	1 / 1 100.0%	1 / 1 100.0%	3 / 3 100.0%	3 / 3 100.0%	1 / 1 100.0%	1 / 1 100.0%	3 / 3 100.0%	1 / 1 100.0%	4 / 4 100.0%	2 / 2 100.0%	1 / 1 100.0%	2 / 2 100.0%	1 / 1 100.0%	3 / 3 100.0%	1 / 1 100.0%	3 / 3 100.0%	4 / 4 100.0%	2 / 2 100.0%	1 / 1 100.0%	5 / 5 100.0%	0 / 1 0.0%	0 / 0 0.0%	0 / 0 0.0%	0 / 0 0.0%	24 / 46 52.2%AB	21 / 46 45.7%ABE	1 / 46 2.2%E			
1	Syntrophomonas wolfei Goettingen	45 / 46 97.8%	1 / 1 100.0%	1 / 1 100.0%	1 / 1 100.0%	1 / 1 100.0%	3 / 3 100.0%	3 / 3 100.0%	1 / 1 100.0%	1 / 1 100.0%	3 / 3 100.0%	1 / 1 100.0%	4 / 4 100.0%	2 / 2 100.0%	1 / 1 100.0%	2 / 2 100.0%	1 / 1 100.0%	3 / 3 100.0%	1 / 1 100.0%	3 / 3 100.0%	4 / 4 100.0%	2 / 2 100.0%	1 / 1 100.0%	5 / 5 100.0%	0 / 1 0.0%	0 / 0 0.0%	0 / 0 0.0%	0 / 0 0.0%	24 / 46 52.2%AB	21 / 46 45.7%ABE	1 / 46 2.2%E			
1	Syntrophothermus	46 / 46 100.0%	1 / 1 100.0%	1 / 1 100.0%	1 / 1 100.0%	1 / 1 100.0%	4 / 4 100.0%	3 / 3 100.0%	1 / 1 100.0%	1 / 1 100.0%	3 / 3 100.0%	1 / 1 100.0%	4 / 4 100.0%	2 / 2 100.0%	1 / 1 100.0%	2 / 2 100.0%	1 / 1 100.0%	3 / 3 100.0%	1 / 1 100.0%	3 / 3 100.0%	4 / 4 100.0%	1 / 1 100.0%	1 / 1 100.0%	5 / 5 100.0%	1 / 1 100.0%	0 / 0 0.0%	0 / 0 0.0%	0 / 0 0.0%	26 / 46 56.5%AB	20 / 46 43.5%ABE				
1	DSM 12680	46 / 46 100.0%	1 / 1 100.0%	1 / 1 100.0%	1 / 1 100.0%	1 / 1 100.0%	4 / 4 100.0%	3 / 3 100.0%	1 / 1 100.0%	1 / 1 100.0%	3 / 3 100.0%	1 / 1 100.0%	4 / 4 100.0%	2 / 2 100.0%	1 / 1 100.0%	2 / 2 100.0%	1 / 1 100.0%	3 / 3 100.0%	1 / 1 100.0%	3 / 3 100.0%	4 / 4 100.0%	1 / 1 100.0%	1 / 1 100.0%	5 / 5 100.0%	1 / 1 100.0%	0 / 0 0.0%	0 / 0 0.0%	0 / 0 0.0%	26 / 46 56.5%AB	20 / 46 43.5%ABE				
1	Syntrophothermus lipocalidus DSM 12680	46 / 46 100.0%	1 / 1 100.0%	1 / 1 100.0%	1 / 1 100.0%	1 / 1 100.0%	4 / 4 100.0%	3 / 3 100.0%	1 / 1 100.0%	1 / 1 100.0%	3 / 3 100.0%	1 / 1 100.0%	4 / 4 100.0%	2 / 2 100.0%	1 / 1 100.0%	2 / 2 100.0%	1 / 1 100.0%	3 / 3 100.0%	1 / 1 100.0%	3 / 3 100.0%	4 / 4 100.0%	1 / 1 100.0%	1 / 1 100.0%	5 / 5 100.0%	1 / 1 100.0%	0 / 0 0.0%	0 / 0 0.0%	0 / 0 0.0%	26 / 46 56.5%AB	20 / 46 43.5%ABE				
7	Lachnospiraceae	5 / 442 1.1%	2 / 10 20.0%	0 / 16 0.0%	0 / 17 0.0%	0 / 9 0.0%	0 / 38 0.0%	0 / 27 0.0%	0 / 9 0.0%	0 / 14 0.0%	1 / 24 4.2%	0 / 8 0.0%	0 / 28 0.0%	0 / 25 0.0%	1 / 13 7.7%	0 / 18 0.0%	0 / 9 0.0%	0 / 24 0.0%	0 / 12 0.0%	0 / 30 0.0%	1 / 33 3.0%	0 / 26 0.0%	0 / 7 0.0%	0 / 45 0.0%	0 / 0 0.0%	0 / 0 0.0%	0 / 0 0.0%	0 / 0 0.0%	311 / 442 70.4%E	124 / 442 28.1%	3 / 442 0.7%AB	2 / 442 0.5%ABE	1 / 442 0.2%EGG	1 / 442 0.2%CE
2	Butyrivibrio	0 / 106 0.0%	0 / 2 0.0%	0 / 5 0.0%	0 / 4 0.0%	0 / 1 0.0%	0 / 7 0.0%	0 / 6 0.0%	0 / 3 0.0%	0 / 3 0.0%	0 / 2 0.0%	0 / 2 0.0%	0 / 7 0.0%	0 / 5 0.0%	0 / 3 0.0%	0 / 4 0.0%	0 / 2 0.0%	0 / 6 0.0%	0 / 3 0.0%	0 / 9 0.0%	0 / 9 0.0%	0 / 7 0.0%	0 / 3 0.0%	0 / 13 0.0%	0 / 0 0.0%	0 / 0 0.0%	0 / 0 0.0%	0 / 0 0.0%	73 / 106 68.9%E	33 / 106 31.1%				
1	ATCC 19171	0 / 59 0.0%	0 / 1 0.0%	0 / 3 0.0%	0 / 2 0.0%	0 / 0 0.0%	0 / 2 0.0%	0 / 4 0.0%	0 / 2 0.0%	0 / 2 0.0%	0 / 1 0.0%	0 / 1 0.0%	0 / 3 0.0%	0 / 3 0.0%	0 / 2 0.0%	0 / 2 0.0%	0 / 1 0.0%	0 / 3 0.0%	0 / 2 0.0%	0 / 5 0.0%	0 / 6 0.0%	0 / 4 0.0%	0 / 2 0.0%	0 / 8 0.0%	0 / 0 0.0%	0 / 0 0.0%	0 / 0 0.0%	0 / 0 0.0%	44 / 59 74.6%E	15 / 59 25.4%				
1	Butyrivibrio fibrisolvens 16/4	0 / 59 0.0%	0 / 1 0.0%	0 / 3 0.0%	0 / 2 0.0%	0 / 0 0.0%	0 / 2 0.0%	0 / 4 0.0%	0 / 2 0.0%	0 / 2 0.0%	0 / 1 0.0%	0 / 1 0.0%	0 / 3 0.0%	0 / 3 0.0%	0 / 2 0.0%	0 / 2 0.0%	0 / 1 0.0%	0 / 3 0.0%	0 / 2 0.0%	0 / 5 0.0%	0 / 6 0.0%	0 / 4 0.0%	0 / 2 0.0%	0 / 8 0.0%	0 / 0 0.0%	0 / 0 0.0%	0 / 0 0.0%	0 / 0 0.0%	44 / 59 74.6%E	15 / 59 25.4%				
1	ATCC 51982	0 / 47 0.0%	0 / 1 0.0%	0 / 2 0.0%	0 / 2 0.0%	0 / 1 0.0%	0 / 5 0.0%	0 / 2 0.0%	0 / 1 0.0%	0 / 1 0.0%	0 / 1 0.0%	0 / 1 0.0%	0 / 4 0.0%	0 / 2 0.0%	0 / 1 0.0%	0 / 2 0.0%	0 / 1 0.0%	0 / 3 0.0%	0 / 1 0.0%	0 / 4 0.0%	0 / 3 0.0%	0 / 3 0.0%	0 / 1 0.0%	0 / 5 0.0%	0 / 0 0.0%	0 / 0 0.0%	0 / 0 0.0%	0 / 0 0.0%	29 / 47 61.7%E	18 / 47 38.3%				
1	Butyrivibrio proteoclasticus B316	0 / 47 0.0%	0 / 1 0.0%	0 / 2 0.0%	0 / 2 0.0%	0 / 1 0.0%	0 / 5 0.0%	0 / 2 0.0%	0 / 1 0.0%	0 / 1 0.0%	0 / 1 0.0%	0 / 1 0.0%	0 / 4 0.0%	0 / 2 0.0%	0 / 1 0.0%	0 / 2 0.0%	0 / 1 0.0%	0 / 3 0.0%	0 / 1 0.0%	0 / 4 0.0%	0 / 3 0.0%	0 / 3 0.0%	0 / 1 0.0%	0 / 5 0.0%	0 / 0 0.0%	0 / 0 0.0%	0 / 0 0.0%	0 / 0 0.0%	29 / 47 61.7%E	18 / 47 38.3%				
1	Roseburia	0 / 58 0.0%	0 / 2 0.0%	0 / 2 0.0%	0 / 2 0.0%	0 / 0 0.0%	0 / 5 0.0%	0 / 4 0.0%	0 / 1 0.0%	0 / 2 0.0%	0 / 5 0.0%	0 / 1 0.0%	0 / 4 0.0%	0 / 3 0.0%	0 / 2 0.0%	0 / 2 0.0%	0 / 2 0.0%	0 / 3 0.0%	0 / 1 0.0%	0 / 4 0.0%	0 / 4 0.0%	0 / 3 0.0%	0 / 1 0.0%	0 / 5 0.0%	0 / 0 0.0%	0 / 0 0.0%	0 / 0 0.0%	0 / 0 0.0%	40 / 58 69.0%E	18 / 58 31.0%				
1	DSM 16839	0 / 58 0.0%	0 / 2 0.0%	0 / 2 0.0%	0 / 2 0.0%	0 / 0 0.0%	0 / 5 0.0%	0 / 4 0.0%	0 / 1 0.0%	0 / 2 0.0%	0 / 5 0.0%	0 / 1 0.0%	0 / 4 0.0%	0 / 3 0.0%	0 / 2 0.0%	0 / 2 0.0%	0 / 2 0.0%	0 / 3 0.0%	0 / 1 0.0%	0 / 4 0.0%	0 / 4 0.0%	0 / 3 0.0%	0 / 1 0.0%	0 / 5 0.0%	0 / 0 0.0%	0 / 0 0.0%	0 / 0 0.0%	0 / 0 0.0%	40 / 58 69.0%E	18 / 58 31.0%				
1	Roseburia hominis A2-183	0 / 58 0.0%	0 / 2 0.0%	0 / 2 0.0%	0 / 2 0.0%	0 / 0 0.0%	0 / 5 0.0%	0 / 4 0.0%	0 / 1 0.0%	0 / 2 0.0%	0 / 5 0.0%	0 / 1 0.0%	0 / 4 0.0%	0 / 3 0.0%	0 / 2 0.0%	0 / 2 0.0%	0 / 2 0.0%	0 / 3 0.0%	0 / 1 0.0%	0 / 4 0.0%	0 / 4 0.0%	0 / 3 0.0%	0 / 1 0.0%	0 / 5 0.0%	0 / 0 0.0%	0 / 0 0.0%	0 / 0 0.0%	0 / 0 0.0%	40 / 58 69.0%E	18 / 58 31.0%				
1	Cellulosilyticum	3 / 104 2.9%	2 / 3 66.7%	0 / 4 0.0%	0 / 5 0.0%	0 / 4 0.0%	0 / 9 0.0%	0 / 6 0.0%	0 / 2 0.0%	0 / 3 0.0%	0 / 7 0.0%	0 / 2 0.0%	0 / 5 0.0%	0 / 6 0.0%	1 / 3 33.3%	0 / 4 0.0%	0 / 2 0.0%	0 / 5 0.0%	0 / 3 0.0%	0 / 6 0.0%	0 / 7 0.0%	0 / 6 0.0%	0 / 1 0.0%	0 / 11 0.0%	0 / 0 0.0%	0 / 0 0.0%	0 / 0 0.0%	0 / 0 0.0%	70 / 104 67.3%E	30 / 104 28.8%	2 / 104 1.9%ABE	1 / 104 1.0%EGG	1 / 104 1.0%AB	
1	ATCC 49066	3 / 104 2.9%	2 / 3 66.7%	0 / 4 0.0%	0 / 5 0.0%	0 / 4 0.0%	0 / 9 0.0%	0 / 6 0.0%	0 / 2 0.0%	0 / 3 0.0%	0 / 7 0.0%	0 / 2 0.0%	0 / 5 0.0%	0 / 6 0.0%	1 / 3 33.3%	0 / 4 0.0%	0 / 2 0.0%	0 / 5 0.0%	0 / 3 0.0%	0 / 6 0.0%	0 / 7 0.0%	0 / 6 0.0%	0 / 1 0.0%	0 / 11 0.0%	0 / 0 0.0%	0 / 0 0.0%	0 / 0 0.0%	0 / 0 0.0%	70 / 104 67.3%E	30 / 104 28.8%	2 / 104 1.9%ABE	1 / 104 1.0%AB	1 / 104 1.0%EGG	
1	Cellulosilyticum lentocellum DSM 5427	3 / 104 2.9%	2 / 3 66.7%	0 / 4 0.0%	0 / 5 0.0%	0 / 4 0.0%	0 / 9 0.0%	0 / 6 0.0%	0 / 2 0.0%	0 / 3 0.0%	0 / 7 0.0%	0 / 2 0.0%	0 / 5 0.0%	0 / 6 0.0%	1 / 3 33.3%	0 / 4 0.0%	0 / 2 0.0%	0 / 5 0.0%	0 / 3 0.0%	0 / 6 0.0%	0 / 7 0.0%	0 / 6 0.0%	0 / 1 0.0%	0 / 11 0.0%	0 / 0 0.0%	0 / 0 0.0%	0 / 0 0.0%	0 / 0 0.0%	70 / 104 67.3%E	30 / 104 28.8%	2 / 104 1.9%ABE	1 / 104 1.0%AB	1 / 104 1.0%EGG	
3	"Lachnoclostridium" Yutin and Galperin 2013	2 / 174 1.1%	0 / 3 0.0%	0 / 5 0.0%	0 / 6 0.0%	0 / 4 0.0%	0 / 17 0.0%	0 / 11 0.0%	0 / 3 0.0%	0 / 6 0.0%	1 / 10 10.0%	0 / 3 0.0%	0 / 12 0.0%	0 / 11 0.0%	0 / 5 0.0%	0 / 8 0.0%	0 / 3 0.0%	0 / 10 0.0%	0 / 5 0.0%	0 / 11 0.0%	1 / 13 7.7%	0 / 10 0.0%	0 / 2 0.0%	0 / 16 0.0%	0 / 0 0.0%	0 / 0 0.0%	0 / 0 0.0%	0 / 0 0.0%	128 / 174 73.6%E	43 / 174 24.7%	2 / 174 1.1%AB	1 / 174 0.6%CE		
1	"Lachnoclostridium phytofermentans" Yutin and Galperin 2013	0 / 59 0.0%	0 / 1 0.0%	0 / 2 0.0%	0 / 2 0.0%	0 / 2 0.0%	0 / 6 0.0%	0 / 3 0.0%	0 / 1 0.0%	0 / 2 0.0%	0 / 3 0.0%	0 / 1 0.0%	0 / 4 0.0%	0 / 4 0.0%	0 / 1 0.0%	0 / 2 0.0%	0 / 1 0.0%	0 / 3 0.0%	0 / 2 0.0%	0 / 4 0.0%	0 / 4 0.0%	0 / 4 0.0%	0 / 1 0.0%	0 / 6 0.0%	0 / 0 0.0%	0 / 0 0.0%	0 / 0 0.0%	0 / 0 0.0%	45 / 59 76.3%E	14 / 59 23.7%				
1	Clostridium phytofermentans ISDg	0 / 59 0.0%	0 / 1 0.0%	0 / 2 0.0%	0 / 2 0.0%	0 / 2 0.0%	0 / 6 0.0%	0 / 3 0.0%	0 / 1 0.0%	0 / 2 0.0%	0 / 3 0.0%	0 / 1 0.0%	0 / 4 0.0%	0 / 4 0.0%	0 / 1 0.0%	0 / 2 0.0%	0 / 1 0.0%	0 / 3 0.0%	0 / 2 0.0%	0 / 4 0.0%	0 / 4 0.0%	0 / 4 0.0%	0 / 1 0.0%	0 / 6 0.0%	0 / 0 0.0%	0 / 0 0.0%	0 / 0 0.0%	0 / 0 0.0%	45 / 59 76.3%E	14 / 59 23.7%				

2	ATCC 35040	2 / 115 1.7%	0 / 2 0.0%	0 / 3 0.0%	0 / 4 0.0%	0 / 2 0.0%	0 / 11 0.0%	0 / 8 0.0%	0 / 2 0.0%	0 / 4 0.0%	1 / 7 14.3%	0 / 2 0.0%	0 / 8 0.0%	0 / 7 0.0%	0 / 4 0.0%	0 / 6 0.0%	0 / 2 0.0%	0 / 7 0.0%	0 / 3 0.0%	0 / 7 0.0%	1 / 9 11.1%	0 / 6 0.0%	0 / 1 0.0%	0 / 10 0.0%	0 / 0 0.0%	0 / 0 0.0%	0 / 0 0.0%	0 / 0 0.0%	83 / 115 72.2%E	29 / 115 25.2%	2 / 115 1.7%AB	1 / 115 0.9%CE	
1	Clostridium saccharolyticum DSM 2544	0 / 68 0.0%	0 / 1 0.0%	0 / 2 0.0%	0 / 3 0.0%	0 / 2 0.0%	0 / 6 0.0%	0 / 5 0.0%	0 / 1 0.0%	0 / 2 0.0%	0 / 6 0.0%	0 / 1 0.0%	0 / 4 0.0%	0 / 3 0.0%	0 / 2 0.0%	0 / 3 0.0%	0 / 1 0.0%	0 / 3 0.0%	0 / 1 0.0%	0 / 4 0.0%	0 / 5 0.0%	0 / 4 0.0%	0 / 1 0.0%	0 / 8 0.0%	0 / 0 0.0%	0 / 0 0.0%	0 / 0 0.0%	0 / 0 0.0%	54 / 68 79.4%E	14 / 68 20.6%			
1	Clostridium cf. saccharolyticum K10	2 / 47 4.3%	0 / 1 0.0%	0 / 1 0.0%	0 / 1 0.0%	0 / 0 0.0%	0 / 5 0.0%	0 / 3 0.0%	0 / 1 0.0%	0 / 2 0.0%	1 / 1 100.0%	0 / 1 0.0%	0 / 4 0.0%	0 / 4 0.0%	0 / 2 0.0%	0 / 3 0.0%	0 / 1 0.0%	0 / 4 0.0%	0 / 2 0.0%	0 / 3 0.0%	1 / 4 25.0%	0 / 2 0.0%	0 / 0 0.0%	0 / 2 0.0%	0 / 0 0.0%	0 / 0 0.0%	0 / 0 0.0%	0 / 0 0.0%	29 / 47 61.7%E	15 / 47 31.9%	2 / 47 4.3%AB	1 / 47 2.1%CE	
11	Peptostreptococcaceae	706 / 767 92.0%	28 / 28 100.0%	34 / 34 100.0%	33 / 33 100.0%	8 / 8 100.0%	61 / 61 100.0%	55 / 55 100.0%	20 / 20 100.0%	25 / 25 100.0%	32 / 32 100.0%	6 / 6 100.0%	52 / 52 100.0%	45 / 45 100.0%	26 / 26 100.0%	24 / 24 100.0%	19 / 19 100.0%	21 / 21 100.0%	7 / 25 28.0%	47 / 47 100.0%	42 / 51 82.4%	44 / 44 100.0%	26 / 26 100.0%	41 / 75 54.7%	10 / 10 100.0%	0 / 0 0.0%	0 / 0 0.0%	0 / 0 0.0%	474 / 767 61.8%AB	232 / 767 30.2%ABE	42 / 767 5.5%E	19 / 767 2.5%	
1	Filifactor	47 / 47 100.0%	1 / 1 100.0%	2 / 2 100.0%	1 / 1 100.0%	2 / 2 100.0%	3 / 3 100.0%	3 / 3 100.0%	1 / 1 100.0%	2 / 2 100.0%	2 / 2 100.0%	1 / 1 100.0%	5 / 5 100.0%	3 / 3 100.0%	2 / 2 100.0%	1 / 1 100.0%	1 / 1 100.0%	1 / 1 100.0%	1 / 1 100.0%	4 / 4 100.0%	4 / 4 100.0%	2 / 2 100.0%	1 / 1 100.0%	4 / 4 100.0%	0 / 0 0.0%	0 / 0 0.0%	0 / 0 0.0%	0 / 0 0.0%	32 / 47 68.1%AB	15 / 47 31.9%ABE			
1	ATCC 35896	47 / 47 100.0%	1 / 1 100.0%	2 / 2 100.0%	1 / 1 100.0%	2 / 2 100.0%	3 / 3 100.0%	3 / 3 100.0%	1 / 1 100.0%	2 / 2 100.0%	2 / 2 100.0%	1 / 1 100.0%	5 / 5 100.0%	3 / 3 100.0%	2 / 2 100.0%	1 / 1 100.0%	1 / 1 100.0%	1 / 1 100.0%	1 / 1 100.0%	4 / 4 100.0%	4 / 4 100.0%	2 / 2 100.0%	1 / 1 100.0%	4 / 4 100.0%	0 / 0 0.0%	0 / 0 0.0%	0 / 0 0.0%	0 / 0 0.0%	32 / 47 68.1%AB	15 / 47 31.9%ABE			
1	Filifactor alocis ATCC 35896	47 / 47 100.0%	1 / 1 100.0%	2 / 2 100.0%	1 / 1 100.0%	2 / 2 100.0%	3 / 3 100.0%	3 / 3 100.0%	1 / 1 100.0%	2 / 2 100.0%	2 / 2 100.0%	1 / 1 100.0%	5 / 5 100.0%	3 / 3 100.0%	2 / 2 100.0%	1 / 1 100.0%	1 / 1 100.0%	1 / 1 100.0%	1 / 1 100.0%	4 / 4 100.0%	4 / 4 100.0%	2 / 2 100.0%	1 / 1 100.0%	4 / 4 100.0%	0 / 0 0.0%	0 / 0 0.0%	0 / 0 0.0%	0 / 0 0.0%	32 / 47 68.1%AB	15 / 47 31.9%ABE			
10	"Peptoclostridium" Yutin and Galperin 2013	659 / 720 91.5%	27 / 27 100.0%	32 / 32 100.0%	32 / 32 100.0%	6 / 6 100.0%	58 / 58 100.0%	52 / 52 100.0%	19 / 19 100.0%	23 / 23 100.0%	30 / 30 100.0%	5 / 5 100.0%	47 / 47 100.0%	42 / 42 100.0%	24 / 24 100.0%	23 / 23 100.0%	18 / 18 100.0%	20 / 20 100.0%	6 / 24 25.0%	43 / 43 100.0%	38 / 47 80.9%	42 / 42 100.0%	25 / 25 100.0%	37 / 71 52.1%	10 / 10 100.0%	0 / 0 0.0%	0 / 0 0.0%	0 / 0 0.0%	442 / 720 61.4%AB	217 / 720 30.1%ABE	42 / 720 5.8%E	19 / 720 2.6%	
9	"Bacillus difficilis" Hall and O'Toole 1935	600 / 661 90.8%	25 / 25 100.0%	29 / 29 100.0%	30 / 30 100.0%	4 / 4 100.0%	54 / 54 100.0%	48 / 48 100.0%	18 / 18 100.0%	21 / 21 100.0%	27 / 27 100.0%	4 / 4 100.0%	43 / 43 100.0%	39 / 39 100.0%	22 / 22 100.0%	21 / 21 100.0%	17 / 17 100.0%	17 / 17 100.0%	4 / 22 18.2%	40 / 40 100.0%	33 / 42 78.6%	39 / 39 100.0%	24 / 24 100.0%	32 / 66 48.5%	9 / 9 100.0%	0 / 0 0.0%	0 / 0 0.0%	0 / 0 0.0%	404 / 661 61.1%AB	196 / 661 29.7%ABE	42 / 661 6.4%E	19 / 661 2.9%	
1	Clostridium difficile 630	81 / 88 92.0%	3 / 3 100.0%	4 / 4 100.0%	4 / 4 100.0%	1 / 1 100.0%	7 / 7 100.0%	6 / 6 100.0%	2 / 2 100.0%	3 / 3 100.0%	5 / 5 100.0%	1 / 1 100.0%	6 / 6 100.0%	5 / 5 100.0%	3 / 3 100.0%	3 / 3 100.0%	2 / 2 100.0%	3 / 3 100.0%	1 / 3 33.3%	5 / 5 100.0%	4 / 5 80.0%	5 / 5 100.0%	3 / 3 100.0%	4 / 8 50.0%	1 / 1 100.0%	0 / 0 0.0%	0 / 0 0.0%	0 / 0 0.0%	56 / 88 63.6%AB	25 / 88 28.4%ABE	4 / 88 4.5%E	3 / 88 3.4%	
1	Clostridium difficile CD196	77 / 84 91.7%	3 / 3 100.0%	4 / 4 100.0%	4 / 4 100.0%	1 / 1 100.0%	7 / 7 100.0%	6 / 6 100.0%	2 / 2 100.0%	3 / 3 100.0%	3 / 3 100.0%	1 / 1 100.0%	6 / 6 100.0%	5 / 5 100.0%	3 / 3 100.0%	3 / 3 100.0%	2 / 2 100.0%	3 / 3 100.0%	1 / 3 33.3%	5 / 5 100.0%	4 / 5 80.0%	5 / 5 100.0%	2 / 2 100.0%	3 / 7 42.9%	1 / 1 100.0%	0 / 0 0.0%	0 / 0 0.0%	0 / 0 0.0%	53 / 84 63.1%AB	24 / 84 28.6%ABE	5 / 84 6.0%E	2 / 84 2.4%	
1	Clostridium difficile R20291	57 / 64 89.1%	3 / 3 100.0%	2 / 2 100.0%	3 / 3 100.0%	0 / 0 0.0%	6 / 6 100.0%	5 / 5 100.0%	2 / 2 100.0%	2 / 2 100.0%	2 / 2 100.0%	0 / 0 0.0%	4 / 4 100.0%	4 / 4 100.0%	2 / 2 100.0%	2 / 2 100.0%	2 / 2 100.0%	1 / 1 100.0%	0 / 2 0.0%	4 / 4 100.0%	3 / 4 75.0%	4 / 4 100.0%	2 / 2 100.0%	3 / 7 42.9%	1 / 1 100.0%	0 / 0 0.0%	0 / 0 0.0%	0 / 0 0.0%	37 / 64 57.8%AB	20 / 64 31.2%ABE	5 / 64 7.8%E	2 / 64 3.1%	
1	Clostridium difficile BI9	18 / 21 85.7%	0 / 0 0.0%	0 / 0 0.0%	0 / 0 0.0%	0 / 0 0.0%	1 / 1 100.0%	2 / 2 100.0%	1 / 1 100.0%	0 / 0 0.0%	0 / 0 0.0%	0 / 0 0.0%	2 / 2 100.0%	2 / 2 100.0%	1 / 1 100.0%	0 / 0 0.0%	1 / 1 100.0%	0 / 0 0.0%	0 / 1 0.0%	2 / 2 100.0%	2 / 3 66.7%	1 / 1 100.0%	1 / 1 100.0%	1 / 2 50.0%	1 / 1 100.0%	0 / 0 0.0%	0 / 0 0.0%	0 / 0 0.0%	12 / 21 57.1%AB	6 / 21 28.6%ABE	2 / 21 9.5%E	1 / 21 4.8%	
1	Clostridium difficile 2007855	59 / 66 89.4%	3 / 3 100.0%	3 / 3 100.0%	3 / 3 100.0%	0 / 0 0.0%	6 / 6 100.0%	5 / 5 100.0%	2 / 2 100.0%	2 / 2 100.0%	2 / 2 100.0%	0 / 0 0.0%	4 / 4 100.0%	4 / 4 100.0%	2 / 2 100.0%	2 / 2 100.0%	2 / 2 100.0%	1 / 1 100.0%	0 / 2 0.0%	4 / 4 100.0%	4 / 5 80.0%	4 / 4 100.0%	2 / 2 100.0%	3 / 7 42.9%	1 / 1 100.0%	0 / 0 0.0%	0 / 0 0.0%	0 / 0 0.0%	39 / 66 59.1%AB	20 / 66 30.3%ABE	5 / 66 7.6%E	2 / 66 3.0%	
1	Clostridium difficile BI1	70 / 77 90.9%	3 / 3 100.0%	4 / 4 100.0%	4 / 4 100.0%	0 / 0 0.0%	6 / 6 100.0%	6 / 6 100.0%	2 / 2 100.0%	2 / 2 100.0%	5 / 5 100.0%	0 / 0 0.0%	4 / 4 100.0%	4 / 4 100.0%	2 / 2 100.0%	2 / 2 100.0%	2 / 2 100.0%	1 / 1 100.0%	0 / 2 0.0%	5 / 5 100.0%	3 / 4 75.0%	5 / 5 100.0%	4 / 4 100.0%	5 / 9 55.6%	1 / 1 100.0%	0 / 0 0.0%	0 / 0 0.0%	0 / 0 0.0%	46 / 77 59.7%AB	24 / 77 31.2%ABE	5 / 77 6.5%E	2 / 77 2.6%	
1	Clostridium difficile M120	80 / 87 92.0%	3 / 3 100.0%	4 / 4 100.0%	4 / 4 100.0%	1 / 1 100.0%	7 / 7 100.0%	6 / 6 100.0%	2 / 2 100.0%	3 / 3 100.0%	4 / 4 100.0%	1 / 1 100.0%	6 / 6 100.0%	5 / 5 100.0%	3 / 3 100.0%	3 / 3 100.0%	2 / 2 100.0%	3 / 3 100.0%	1 / 3 33.3%	5 / 5 100.0%	4 / 5 80.0%	5 / 5 100.0%	3 / 3 100.0%	4 / 8 50.0%	1 / 1 100.0%	0 / 0 0.0%	0 / 0 0.0%	0 / 0 0.0%	55 / 87 63.2%AB	25 / 87 28.7%ABE	5 / 87 5.7%E	2 / 87 2.3%	
1	Clostridium difficile CF5	58 / 65 89.2%	3 / 3 100.0%	3 / 3 100.0%	3 / 3 100.0%	0 / 0 0.0%	6 / 6 100.0%	5 / 5 100.0%	2 / 2 100.0%	2 / 2 100.0%	2 / 2 100.0%	0 / 0 0.0%	4 / 4 100.0%	4 / 4 100.0%	2 / 2 100.0%	2 / 2 100.0%	2 / 2 100.0%	1 / 1 100.0%	0 / 2 0.0%	4 / 4 100.0%	3 / 4 75.0%	4 / 4 100.0%	2 / 2 100.0%	3 / 7 42.9%	1 / 1 100.0%	0 / 0 0.0%	0 / 0 0.0%	0 / 0 0.0%	38 / 65 58.5%AB	20 / 65 30.8%ABE	5 / 65 7.7%E	2 / 65 3.1%	
1	Clostridium difficile M68	100 / 109 91.7%	4 / 4 100.0%	5 / 5 100.0%	5 / 5 100.0%	1 / 1 100.0%	8 / 8 100.0%	7 / 7 100.0%	3 / 3 100.0%	4 / 4 100.0%	4 / 4 100.0%	1 / 1 100.0%	7 / 7 100.0%	6 / 6 100.0%	4 / 4 100.0%	4 / 4 100.0%	2 / 2 100.0%	4 / 4 100.0%	1 / 4 25.0%	6 / 6 100.0%	6 / 7 85.7%	6 / 6 100.0%	5 / 5 100.0%	6 / 11 54.5%	1 / 1 100.0%	0 / 0 0.0%	0 / 0 0.0%	0 / 0 0.0%	68 / 109 62.4%AB	32 / 109 29.4%ABE	6 / 109 5.5%E	3 / 109 2.8%	
1	ATCC 12662	59 / 59 100.0%	2 / 2 100.0%	3 / 3 100.0%	2 / 2 100.0%	2 / 2 100.0%	4 / 4 100.0%	4 / 4 100.0%	1 / 1 100.0%	2 / 2 100.0%	3 / 3 100.0%	1 / 1 100.0%	4 / 4 100.0%	3 / 3 100.0%	2 / 2 100.0%	2 / 2 100.0%	1 / 1 100.0%	3 / 3 100.0%	2 / 2 100.0%	3 / 3 100.0%	5 / 5 100.0%	3 / 3 100.0%	1 / 1 100.0%	5 / 5 100.0%	1 / 1 100.0%	0 / 0 0.0%	0 / 0 0.0%	0 / 0 0.0%	38 / 59 64.4%AB	21 / 59 35.6%ABE			
4	Eubacteriaceae	116 / 223 52.0%	3 / 5 60.0%	6 / 10 60.0%	4 / 8 50.0%	4 / 8 50.0%	7 / 16 43.8%	7 / 13 53.8%	2 / 4 50.0%	6 / 9 66.7%	8 / 13 61.5%	2 / 4 50.0%	6 / 11 54.5%	8 / 14 57.1%	4 / 7 57.1%	4 / 9 44.4%	2 / 4 50.0%	6 / 11 54.5%	2 / 5 40.0%	8 / 16 50.0%	8 / 16 50.0%	6 / 13 46.2%	2 / 4 50.0%	10 / 22 45.5%	1 / 1 100.0%	0 / 0 0.0%	0 / 0 0.0%	0 / 0 0.0%	69 / 223 30.9%E	69 / 223 30.9%AB	47 / 223 21.1%ABE	37 / 223 16.6%	1 / 223 0.4%CE
3	Eubacterium	57 / 164 34.8%	1 / 3 33.3%	3 / 7 42.9%	2 / 6 33.3%	2 / 6 33.3%	3 / 12 25.0%	3 / 9 33.3%	1 / 3 33.3%	3 / 6 50.0%	5 / 10 50.0%	1 / 3 33.3%	3 / 8 37.5%	4 / 10 40.0%	2 / 5 40.0%	2 / 7 28.6%	1 / 3 33.3%	3 / 8 37.5%	1 / 4 25.0%	4 / 12 33.3%	4 / 12 33.3%	3 / 10 30.0%	1 / 3 33.3%	5 / 17 29.4%	0 / 0 0.0%	0 / 0 0.0%	0 / 0 0.0%	0 / 0 0.0%	69 / 164 42.1%E	37 / 164 22.6%	34 / 164 20.7%AB	23 / 164 14.0%ABE	1 / 164 0.6%CE
1	"Bacteroides limosus" Eggerth 1935	57 / 57 100.0%	1 / 1 100.0%	3 / 3 100.0%	2 / 2 100.0%	2 / 2 100.0%	3 / 3 100.0%	3 / 3 100.0%	1 / 1 100.0%	3 / 3 100.0%	5 / 5 100.0%	1 / 1 100.0%	3 / 3 100.0%	4 / 4 100.0%	2 / 2 100.0%	2 / 2 100.0%	1 / 1 100.0%	3 / 3 100.0%	1 / 1 100.0%	4 / 4 100.0%	4 / 4 100.0%	3 / 3 100.0%	1 / 1 100.0%	5 / 5 100.0%	0 / 0 0.0%	0 / 0 0.0%	0 / 0 0.0%	0 / 0 0.0%	34 / 57 59.6%AB	23 / 57 40.4%ABE			
1	Eubacterium limosum KIST612	57 / 57 100.0%	1 / 1 100.0%	3 / 3 100.0%	2 / 2 100.0%	2 / 2 100.0%	3 / 3 100.0%	3 / 3 100.0%	1 / 1 100.0%	3 / 3 100.0%	5 / 5 100.0%	1 / 1 100.0%	3 / 3 100.0%	4 / 4 100.0%	2 / 2 100.0%	2 / 2 100.0%	1 / 1 100.0%	3 / 3 100.0%	1 / 1 100.0%	4 / 4 100.0%	4 / 4 100.0%	3 / 3 100.0%	1 / 1 100.0%	5 / 5 100.0%	0 / 0 0.0%	0 / 0 0.0%	0 / 0 0.0%	0 / 0 0.0%	34 / 57 59.6%AB	23 / 57 40.4%ABE			
1	ATCC 27750	0 / 48 0.0%	0 / 1 0.0%	0 / 2 0.0%	0 / 2 0.0%	0 / 2 0.0%	0 / 4 0.0%	0 / 2 0.0%	0 / 1 0.0%	0 / 1 0.0%	0 / 3 0.0%	0 / 1 0.0%	0 / 2 0.0%	0 / 2 0.0%	0 / 1 0.0%	0 / 2 0.0%	0 / 1 0.0%	0 / 2 0.0%	0 / 1 0.0%	0 / 4 0.0%	0 / 4 0.0%	0 / 3 0.0%	0 / 1 0.0%	0 / 6 0.0%	0 / 0 0.0%	0 / 0 0.0%	0 / 0 0.0%	0 / 0 0.0%	27 / 48 56.2%E	21 / 48 43.8%			
1	Eubacterium eligens ATCC 27750	0 / 48 0.0%	0 / 1 0.0%	0 / 2 0.0%	0 / 2 0.0%	0 / 2 0.0%	0 / 4 0.0%	0 / 2 0.0%	0 / 1 0.0%	0 / 1 0.0%	0 / 3 0.0%	0 / 1 0.0%	0 / 2 0.0%	0 / 2 0.0%	0 / 1 0.0%	0 / 2 0.0%	0 / 1 0.0%	0 / 2 0.0%	0 / 1 0.0%	0 / 4 0.0%	0 / 4 0.0%	0 / 3 0.0%	0 / 1 0.0%	0 / 6 0.0%	0 / 0 0.0%	0 / 0 0.0%	0 / 0 0.0%	0 / 0 0.0%	27 / 48 56.2%E	21 / 48 43.8%			
1	"Bacteroides rectalis" Hauduroy et al. 1937	0 / 59 0.0%	0 / 1 0.0%	0 / 2 0.0%	0 / 2 0.0%	0 / 2 0.0%	0 / 5 0.0%	0 / 4 0.0%	0 / 1 0.0%	0 / 2 0.0%	0 / 2 0.0%	0 / 1 0.0%	0 / 3 0.0%	0 / 4 0.0%	0 / 2 0.0%	0 / 3 0.0%	0 / 1 0.0%	0 / 3 0.0%	0 / 2 0.0%	0 / 4 0.0%	0 / 4 0.0%	0 / 4 0.0%	0 / 1 0.0%	0 / 6 0.0%	0 / 0 0.0%	0 / 0 0.0%	0 / 0 0.0%	0 / 0 0.0%	42 / 59 71.2%E	16 / 59 27.1%	1 / 59 1.7%CE		
1	Eubacterium rectale ATCC 33656	0 / 59 0.0%	0 / 1 0.0%	0 / 2 0.0%	0 / 2 0.0%	0 / 2 0.0%	0 / 5 0.0%	0 / 4 0.0%	0 / 1 0.0%	0 / 2 0.0%	0 / 2 0.0%	0 / 1 0.0%	0 / 3 0.0%	0 / 4 0.0%	0 / 2 0.0%	0 / 3 0.0%	0 / 1 0.0%	0 / 3 0.0%	0 / 2 0.0%	0 / 4 0.0%	0 / 4 0.0%	0 / 4 0.0%	0 / 1 0.0%	0 / 6 0.0%	0 / 0 0.0%	0 / 0 0.0%	0 / 0 0.0%	0 / 0 0.0%	42 / 59 71.2%E	16 / 59 27.1%	1 / 59 1.7%CE		
1	Acetobacterium	59 / 59 100.0%	2 / 2 100.0%	3 / 3 100.0%	2 / 2 100.0%	2 / 2 100.0%	4 / 4 100.0%	4 / 4 100.0%	1 / 1 100.0%	3 / 3 100.0%	3 / 3 100.0%	1 / 1 100.0%	3 / 3 100.0%	4 / 4 100.0%	2 / 2 100.0%	2 / 2 100.0%	1 / 1 100.0%	3 / 3 100.0%	1 / 1 100.0%	4 / 4 100.0%	4 / 4 100.0%	3 / 3 100.0%	1 / 1 100.0%	5 / 5 100.0%	1 / 1 100.0%	0 / 0 0.0%	0 / 0 0.0%	0 / 0 0.0%	35 / 59 59.3%AB	24 / 59 40.7%ABE			
1	ATCC 29683	59 / 59 100.0%	2 / 2 100.0%	3 / 3 100.0%	2 / 2 100.0%	2 / 2 100.0%	4 / 4 100.0%	4 / 4 100.0%	1 / 1 100.0%	3 / 3 100.0%	3 / 3 100.0%	1 / 1 100.0%	3 / 3 100.0%	4 / 4 100.0%	2 / 2 100.0%	2 / 2 100.0%	1 / 1 100.0%	3 / 3 100.0%	1 / 1 100.0%	4 / 4 100.0%	4 / 4 100.0%	3 / 3 100.0%	1 / 1 100.0%	5 / 5 100.0%	1 / 1 100.0%	0 / 0 0.0%	0 / 0 0.0%	0 / 0 0.0%	35 / 59 59.3%AB	24 / 59 40.7%ABE			
1	Acetobacterium woodii ATCC 29683	59 / 59 100.0%	2 / 2 100.0%	3 / 3 100.0%	2 / 2 100.0%	2 / 2 100.0%	4 / 4 100.0%	4 / 4 100.0%	1 / 1 100.0%	3 / 3 100.0%	3 / 3 100.0%	1 / 1 100.0%	3 / 3 100.0%	4 / 4 100.0%	2 / 2 100.0%	2 / 2 100.0%	1 / 1 100.0%	3 / 3 100.0%	1 / 1 100.0%	4 / 4 100.0%	4 / 4 100.0%	3 / 3 100.0%	1 / 1 100.0%	5 / 5 100.0%	1 / 1 100.0%	0 / 0 0.0%	0 / 0 0.0%	0 / 0 0.0%	35 / 59 59.3%AB	24 / 59 40.7%ABE			
19	Peptococcaceae	1170 / 1171 99.9%	34 / 34 100.0%	46 / 46 100.0%	43 / 43 100.0%	34 / 34 100.0%	98 / 98 100.0%	72 / 72 100.0%	20 / 20 100.0%	35 / 35 100.0%	77 / 78 98.7%	21 / 21 100.0%	85 / 85 100.0%	67 / 67 100.0%	33 / 33 100.0%	42 / 42 100.0%	25 / 25 100.0%	58 / 58 100.0%	23 / 23 100.0%	67 / 67 100.0%	92 / 92 100.0%	55 / 55 100.0%	18 / 18 100.0%	107 / 107 100.0%	18 / 18 100.0%	0 / 0 0.0%	0 / 0 0.0%	0 / 0 0.0%	699 / 1171 59.7%AB	446 / 1171 38.1%ABE	14 / 1171 1.2%B	11 / 1171 0.9%BE	1 / 1171 0.1%
6	Desulfotomaculum	369 / 369 100.0%	12 / 12 100.0%	15 / 15 100.0%	13 / 13 100.0%	12 / 12 100.0%	30 / 30 100.0%	24 / 24 100.0%	6 / 6 100.0%	11 / 11 100.0%	22 / 22 100.0%	6 / 6 100.0%	25 / 25 100.0%	20 / 20 100.0%	10 / 10 100.0%	16 / 16 100.0%	9 / 9 100.0%	19 / 19 100.0%	6 / 6 100.0%	21 / 21 100.0%	28 / 28 100.0%	18 / 18 100.0%	6 / 6 100.0%	34 / 34 100.0%	6 / 6 100.0%	0 / 0 0.0%	0 / 0 0.0%	0 / 0 0.0%	210 / 369 56.9%AB	159 / 369 43.1%ABE			
1	ATCC 23193	65 / 65 100.0%	2 / 2 100.0%	3 / 3 100.0%	2 / 2 100.0%	2 / 2 100.0%	6 / 6 100.0%	5 / 5 100.0%	1 / 1 100.0%	2 / 2 100.0%	3 / 3 100.0%	1 / 1 100.0%	4 / 4 100.0%	3 / 3 100.0%	2 / 2 100.0%	3 / 3 100.0%	2 / 2 100.0%	3 / 3 100.0%	1 / 1 100.0%	4 / 4 100.0%	5 / 5 100.0%	3 / 3 100.0%	1 / 1 100.0%	6 / 6 100.0%	1 / 1 100.0%	0 / 0 0.0%	0 / 0 0.0%	0 / 0 0.0%	34 / 65 52.3%AB	31 / 65 47.7%ABE			
1	Desulfotomaculum ruminis ATCC 23193	65 / 65 100.0%	2 / 2 100.0%	3 / 3 100.0%	2 / 2 100.0%	2 / 2 100.0%	6 / 6 100.0%	5 / 5 100.0%	1 / 1 100.0%	2 / 2 100.0%	3 / 3 100.0%	1 / 1 100.0%	4 / 4 100.0%	3 / 3 100.0%	2 / 2 100.0%	3 / 3 100.0%	2 / 2 100.0%	3 / 3 100.0%	1 / 1 100.0%	4 / 4 100.0%	5 / 5 100.0%	3 / 3 100.0%	1 / 1 100.0%	6 / 6 100.0%	1 / 1 100.0%	0 / 0 0.0%	0 / 0 0.0%	0 / 0 0.0%	34 / 65 52.3%AB	31 / 65 47.7%ABE			
1	DSM 6115	46 / 46 100.0%	1 / 1 100.0%	1 / 1 100.0%	1 / 1 100.0%	1 / 1 100.0%	3 / 3 100.0%	3 / 3 100.0%	1 / 1 100.0%	1 / 1 100.0%	3 / 3 100.0%	1 / 1 100.0%	4 / 4 100.0%	3 / 3 100.0%	1 / 1 100.0%	1 / 1 100.0%	1 / 1 100.0%	3 / 3 100.0%	1 / 1 100.0%	3 / 3 100.0%	4 / 4 100.0%	2 / 2 100.0%	1 / 1 100.0%	5 / 5 100.0%	1 / 1 100.0%	0 / 0 0.0%	0 / 0 0.0%	0 / 0 0.0%	27 / 46 58.7%AB	19 / 46 41.3%ABE			
1	Desulfotomaculum kuznetsovii 17	46 / 46 100.0%	1 / 1 100.0%	1 / 1 100.0%	1 / 1 100.0%	1 / 1 100.0%	3 / 3 100.0%	3 / 3 100.0%	1 / 1 100.0%	1 / 1 100.0%	3 / 3 100.0%	1 / 1 100.0%	4 / 4 100.0%	3 / 3 100.0%	1 / 1 100.0%	1 / 1 100.0%	1 / 1 100.0%	3 / 3 100.0%	1 / 1 100.0%	3 / 3 100.0%	4 / 4 100.0%	2 / 2 100.0%	1 / 1 100.0%	5 / 5 100.0%	1 / 1 100.0%	0 / 0 0.0%	0 / 0 0.0%	0 / 0 0.0%	27 / 46 58.7%AB	19 / 46 41.3%ABE			
1	ATCC 49208	66 / 66 100.0%	3 / 3 100.0%	3 / 3 100.0%	2 / 2 100.0%	3 / 3 100.0%	5 / 5 100.0%	4 / 4 100.0%	1 / 1 100.0%	2 / 2 100.0%	3 / 3 100.0%	1 / 1 100.0%	4 / 4 100.0%	4 / 4 100.0%	2 / 2 100.0%	5 / 5 100.0%	2 / 2 100.0%	3 / 3 100.0%	1 / 1 100.0%	3 / 3 100.0%	5 / 5 100.0%	3 / 3 100.0%	1 / 1 100.0%	5 / 5 100.0%	1 / 1 100.0%	0 / 0 0.0%	0 / 0 0.0%	0 / 0 0.0%	41 / 66 62.1%AB	25 / 66 37.9%ABE			
1	Desulfotomaculum acetoxidans DSM 771	66 / 66 100.0%	3 / 3 100.0%	3 / 3 100.0%	2 / 2 100.0%	3 / 3 100.0%	5 / 5 100.0%	4 / 4 100.0%	1 / 1 100.0%	2 / 2 100.0%	3 / 3 100.0%	1 / 1 100.0%	4 / 4 100.0%	4 / 4 100.0%	2 / 2 100.0%	5 / 5 100.0%	2 / 2 100.0%	3 / 3 100.0%	1 / 1 100.0%	3 / 3 100.0%	5 / 5 100.0%	3 / 3 100.0%	1 / 1 100.0%	5 / 5 100.0%	1 / 1 100.0%	0 / 0 0.0%	0 / 0 0.0%	0 / 0 0.0%	41 / 66 62.1%AB	25 / 66 37.9%ABE			
1	'Desulfotomaculum reducens'	69 / 69 100.0%	3 / 3 100.0%	3 / 3 100.0%	3 / 3 100.0%	2 / 2 100.0%	6 / 6 100.0%	4 / 4 100.0%	1 / 1 100.0%	2 / 2 100.0%	3 / 3 100.0%	1 / 1 100.0%	4 / 4 100.0%	4 / 4 100.0%	2 / 2 100.0%	3 / 3 100.0%	2 / 2 100.0%	4 / 4 100.0%	1 / 1 100.0%	4 / 4 100.0%	5 / 5 100.0%	4 / 4 100.0%	1 / 1 100.0%	6 / 6 100.0%	1 / 1 100.0%	0 / 0 0.0%	0 / 0 0.0%	0 / 0 0.0%	36 / 69 52.2%AB	33 / 69 47.8%ABE			
1	Desulfotomaculum reducens MI-1	69 / 69 100.0%	3 / 3 100.0%	3 / 3 100.0%	3 / 3 100.0%	2 / 2 100.0%	6 / 6 100.0%	4 / 4 100.0%	1 / 1 100.0%	2 / 2 100.0%	3 / 3 100.0%	1 / 1 100.0%	4 / 4 100.0%	4 / 4 100.0%	2 / 2 100.0%	3 / 3 100.0%	2 / 2 100.0%	4 / 4 100.0%	1 / 1 100.0%	4 / 4 100.0%	5 / 5 100.0%	4 / 4 100.0%	1 / 1 100.0%	6 / 6 100.0%	1 / 1 100.0%	0 / 0 0.0%	0 / 0 0.0%	0 / 0 0.0%	36 / 69 52.2%AB	33 / 69 47.8%ABE			
1	DSM 7213	58 / 58 100.0%	2 / 2 100.0%	2 / 2 100.0%	2 / 2 100.0%	2 / 2 100.0%	4 / 4 100.0%	3 / 3 100.0%	1 / 1 100.0%	2 / 2 100.0%	6 / 6 100.0%	1 / 1 100.0%	4 / 4 100.0%	3 / 3 100.0%	1 / 1 100.0%	2 / 2 100.0%	1 / 1 100.0%	3 / 3 100.0%	1 / 1 100.0%	3 / 3 100.0%	4 / 4 100.0%	3 / 3 100.0%	1 / 1 100.0%	6 / 6 100.0%	1 / 1 100.0%	0 / 0 0.0%	0 / 0 0.0%	0 / 0 0.0%	35 / 58 60.3%AB	23 / 58 39.7%ABE			
1	Desulfotomaculum gibsoniae DSM 7213	58 / 58 100.0%	2 / 2 100.0%	2 / 2 100.0%	2 / 2 100.0%	2 / 2 100.0%	4 / 4 100.0%	3 / 3 100.0%	1 / 1 100.0%	2 / 2 100.0%	6 / 6 100.0%	1 / 1 100.0%	4 / 4 100.0%	3 / 3 100.0%	1 / 1 100.0%	2 / 2 100.0%	1 / 1 100.0%	3 / 3 100.0%	1 / 1 100.0%	3 / 3 100.0%	4 / 4 100.0%	3 / 3 100.0%	1 / 1 100.0%	6 / 6 100.0%	1 / 1 100.0%	0 / 0 0.0%	0 / 0 0.0%	0 / 0 0.0%	35 / 58 60.3%AB	23 / 58 39.7%ABE			
1	DSM 14880	65 / 65 100.0%	1 / 1 100.0%	3 / 3 100.0%	3 / 3 100.0%	2 / 2 100.0%	6 / 6 100.0%	5 / 5 100.0%	1 / 1 100.0%	2 / 2 100.0%	4 / 4 100.0%	1 / 1 100.0%	5 / 5 100.0%	3 / 3 100.0%	2 / 2 100.0%	2 / 2 100.0%	1 / 1 100.0%	3 / 3 100.0%	1 / 1 100.0%	4 / 4 100.0%	5 / 5 100.0%	3 / 3 100.0%	1 / 1 100.0%	6 / 6 100.0%	1 / 1 100.0%	0 / 0 0.0%	0 / 0 0.0%	0 / 0 0.0%	37 / 65 56.9%AB	28 / 65 43.1%ABE			
1	Desulfotomaculum carboxydivorans CO-1-SRB	65 / 65 100.0%	1 / 1 100.0%	3 / 3 100.0%	3 / 3 100.0%	2 / 2 100.0%	6 / 6 100.0%	5 / 5 100.0%	1 / 1 100.0%	2 / 2 100.0%	4 / 4 100.0%	1 / 1 100.0%	5 / 5 100.0%	3 / 3 100.0%	2 / 2 100.0%	2 / 2 100.0%	1 / 1 100.0%	3 / 3 100.0%	1 / 1 100.0%	4 / 4 100.0%	5 / 5 100.0%	3 / 3 100.0%	1 / 1 100.0%	6 / 6 100.0%	1 / 1 100.0%	0 / 0 0.0%	0 / 0 0.0%	0 / 0 0.0%	37 / 65 56.9%AB	28 / 65 43.1%ABE			
4	Desulfitobacterium	279 / 280 99.6%	11 / 11 100.0%	11 / 11 100.0%	11 / 11 100.0%	9 / 9 100.0%	26 / 26 100.0%	15 / 15 100.0%	4 / 4 100.0%	11 / 11 100.0%	19 / 20 95.0%	4 / 4 100.0%	19 / 19 100.0%	15 / 15 100.0%	8 / 8 100.0%	7 / 7 100.0%	4 / 4 100.0%	12 / 12 100.0%	7 / 7 100.0%	15 / 15 100.0%	23 / 23 100.0%	15 / 15 100.0%	4 / 4 100.0%	23 / 23 100.0%	6 / 6 100.0%	0 / 0 0.0%	0 / 0 0.0%	0 / 0 0.0%	169 / 280 60.4%AB	108 / 280 38.6%ABE	2 / 280 0.7%BE	1 / 280 0.4%	
1	ATCC 51507	75 / 75 100.0%	3 / 3 100.0%	3 / 3 100.0%	3 / 3 100.0%	4 / 4 100.0%	7 / 7 100.0%	4 / 4 100.0%	1 / 1 100.0%	3 / 3 100.0%	4 / 4 100.0%	1 / 1 100.0%	5 / 5 100.0%	4 / 4 100.0%	2 / 2 100.0%	2 / 2 100.0%	1 / 1 100.0%	3 / 3 100.0%	2 / 2 100.0%	4 / 4 100.0%	6 / 6 100.0%	4 / 4 100.0%	1 / 1 100.0%	6 / 6 100.0%	2 / 2 100.0%	0 / 0 0.0%	0 / 0 0.0%	0 / 0 0.0%	46 / 75 61.3%AB	29 / 75 38.7%ABE			
1	Desulfitobacterium dehalogenans ATCC 51507	75 / 75 100.0%	3 / 3 100.0%	3 / 3 100.0%	3 / 3 100.0%	4 / 4 100.0%	7 / 7 100.0%	4 / 4 100.0%	1 / 1 100.0%	3 / 3 100.0%	4 / 4 100.0%	1 / 1 100.0%	5 / 5 100.0%	4 / 4 100.0%	2 / 2 100.0%	2 / 2 100.0%	1 / 1 100.0%	3 / 3 100.0%	2 / 2 100.0%	4 / 4 100.0%	6 / 6 100.0%	4 / 4 100.0%	1 / 1 100.0%	6 / 6 100.0%	2 / 2 100.0%	0 / 0 0.0%	0 / 0 0.0%	0 / 0 0.0%	46 / 75 61.3%AB	29 / 75 38.7%ABE			
2	DSM 10664	131 / 132 99.2%	5 / 5 100.0%	5 / 5 100.0%	5 / 5 100.0%	3 / 3 100.0%	12 / 12 100.0%	7 / 7 100.0%	2 / 2 100.0%	5 / 5 100.0%	10 / 11 90.9%	2 / 2 100.0%	9 / 9 100.0%	7 / 7 100.0%	4 / 4 100.0%	3 / 3 100.0%	2 / 2 100.0%	6 / 6 100.0%	3 / 3 100.0%	7 / 7 100.0%	11 / 11 100.0%	7 / 7 100.0%	2 / 2 100.0%	11 / 11 100.0%	3 / 3 100.0%	0 / 0 0.0%	0 / 0 0.0%	0 / 0 0.0%	78 / 132 59.1%AB	51 / 132 38.6%ABE	2 / 132 1.5%BE	1 / 132 0.8%	
1	Desulfitobacterium hafniense Y51	58 / 59 98.3%	2 / 2 100.0%	2 / 2 100.0%	2 / 2 100.0%	1 / 1 100.0%	5 / 5 100.0%	3 / 3 100.0%	1 / 1 100.0%	2 / 2 100.0%	5 / 6 83.3%	1 / 1 100.0%	4 / 4 100.0%	3 / 3 100.0%	2 / 2 100.0%	1 / 1 100.0%	1 / 1 100.0%	3 / 3 100.0%	1 / 1 100.0%	3 / 3 100.0%	5 / 5 100.0%	3 / 3 100.0%	1 / 1 100.0%	5 / 5 100.0%	2 / 2 100.0%	0 / 0 0.0%	0 / 0 0.0%	0 / 0 0.0%	34 / 59 57.6%AB	22 / 59 37.3%ABE	2 / 59 3.4%BE	1 / 59 1.7%	
1	Desulfitobacterium hafniense DCB-2	73 / 73 100.0%	3 / 3 100.0%	3 / 3 100.0%	3 / 3 100.0%	2 / 2 100.0%	7 / 7 100.0%	4 / 4 100.0%	1 / 1 100.0%	3 / 3 100.0%	5 / 5 100.0%	1 / 1 100.0%	5 / 5 100.0%	4 / 4 100.0%	2 / 2 100.0%	2 / 2 100.0%	1 / 1 100.0%	3 / 3 100.0%	2 / 2 100.0%	4 / 4 100.0%	6 / 6 100.0%	4 / 4 100.0%	1 / 1 100.0%	6 / 6 100.0%	1 / 1 100.0%	0 / 0 0.0%	0 / 0 0.0%	0 / 0 0.0%	44 / 73 60.3%AB	29 / 73 39.7%ABE			
1	"Desulfitobacterium dichloroeliminans" De Wildeman et al. 2003	73 / 73 100.0%	3 / 3 100.0%	3 / 3 100.0%	3 / 3 100.0%	2 / 2 100.0%	7 / 7 100.0%	4 / 4 100.0%	1 / 1 100.0%	3 / 3 100.0%	5 / 5 100.0%	1 / 1 100.0%	5 / 5 100.0%	4 / 4 100.0%	2 / 2 100.0%	2 / 2 100.0%	1 / 1 100.0%	3 / 3 100.0%	2 / 2 100.0%	4 / 4 100.0%	6 / 6 100.0%	4 / 4 100.0%	1 / 1 100.0%	6 / 6 100.0%	1 / 1 100.0%	0 / 0 0.0%	0 / 0 0.0%	0 / 0 0.0%	45 / 73 61.6%AB	28 / 73 38.4%ABE			
1	Desulfitobacterium dichloroeliminans DCA1	73 / 73 100.0%	3 / 3 100.0%	3 / 3 100.0%	3 / 3 100.0%	2 / 2 100.0%	7 / 7 100.0%	4 / 4 100.0%	1 / 1 100.0%	3 / 3 100.0%	5 / 5 100.0%	1 / 1 100.0%	5 / 5 100.0%	4 / 4 100.0%	2 / 2 100.0%	2 / 2 100.0%	1 / 1 100.0%	3 / 3 100.0%	2 / 2 100.0%	4 / 4 100.0%	6 / 6 100.0%	4 / 4 100.0%	1 / 1 100.0%	6 / 6 100.0%	1 / 1 100.0%	0 / 0 0.0%	0 / 0 0.0%	0 / 0 0.0%	45 / 73 61.6%AB	28 / 73 38.4%ABE			
1	Syntrophobotulus	54 / 54 100.0%	1 / 1 100.0%	2 / 2 100.0%	2 / 2 100.0%	2 / 2 100.0%	4 / 4 100.0%	3 / 3 100.0%	1 / 1 100.0%	1 / 1 100.0%	5 / 5 100.0%	1 / 1 100.0%	5 / 5 100.0%	3 / 3 100.0%	2 / 2 100.0%	2 / 2 100.0%	1 / 1 100.0%	3 / 3 100.0%	1 / 1 100.0%	3 / 3 100.0%	4 / 4 100.0%	2 / 2 100.0%	1 / 1 100.0%	5 / 5 100.0%	0 / 0 0.0%	0 / 0 0.0%	0 / 0 0.0%	0 / 0 0.0%	37 / 54 68.5%AB	17 / 54 31.5%ABE			
1	DSM 8271	54 / 54 100.0%	1 / 1 100.0%	2 / 2 100.0%	2 / 2 100.0%	2 / 2 100.0%	4 / 4 100.0%	3 / 3 100.0%	1 / 1 100.0%	1 / 1 100.0%	5 / 5 100.0%	1 / 1 100.0%	5 / 5 100.0%	3 / 3 100.0%	2 / 2 100.0%	2 / 2 100.0%	1 / 1 100.0%	3 / 3 100.0%	1 / 1 100.0%	3 / 3 100.0%	4 / 4 100.0%	2 / 2 100.0%	1 / 1 100.0%	5 / 5 100.0%	0 / 0 0.0%	0 / 0 0.0%	0 / 0 0.0%	0 / 0 0.0%	37 / 54 68.5%AB	17 / 54 31.5%ABE			
1	Syntrophobotulus glycolicus DSM 8271	54 / 54 100.0%	1 / 1 100.0%	2 / 2 100.0%	2 / 2 100.0%	2 / 2 100.0%	4 / 4 100.0%	3 / 3 100.0%	1 / 1 100.0%	1 / 1 100.0%	5 / 5 100.0%	1 / 1 100.0%	5 / 5 100.0%	3 / 3 100.0%	2 / 2 100.0%	2 / 2 100.0%	1 / 1 100.0%	3 / 3 100.0%	1 / 1 100.0%	3 / 3 100.0%	4 / 4 100.0%	2 / 2 100.0%	1 / 1 100.0%	5 / 5 100.0%	0 / 0 0.0%	0 / 0 0.0%	0 / 0 0.0%	0 / 0 0.0%	37 / 54 68.5%AB	17 / 54 31.5%ABE			
2	Dehalobacter	104 / 104 100.0%	2 / 2 100.0%	4 / 4 100.0%	4 / 4 100.0%	2 / 2 100.0%	8 / 8 100.0%	8 / 8 100.0%	2 / 2 100.0%	2 / 2 100.0%	6 / 6 100.0%	2 / 2 100.0%	8 / 8 100.0%	6 / 6 100.0%	4 / 4 100.0%	4 / 4 100.0%	2 / 2 100.0%	6 / 6 100.0%	2 / 2 100.0%	6 / 6 100.0%	8 / 8 100.0%	4 / 4 100.0%	2 / 2 100.0%	10 / 10 100.0%	2 / 2 100.0%	0 / 0 0.0%	0 / 0 0.0%	0 / 0 0.0%	70 / 104 67.3%AB	34 / 104 32.7%ABE			
1	Dehalobacter sp. CF	52 / 52 100.0%	1 / 1 100.0%	2 / 2 100.0%	2 / 2 100.0%	1 / 1 100.0%	4 / 4 100.0%	4 / 4 100.0%	1 / 1 100.0%	1 / 1 100.0%	3 / 3 100.0%	1 / 1 100.0%	4 / 4 100.0%	3 / 3 100.0%	2 / 2 100.0%	2 / 2 100.0%	1 / 1 100.0%	3 / 3 100.0%	1 / 1 100.0%	3 / 3 100.0%	4 / 4 100.0%	2 / 2 100.0%	1 / 1 100.0%	5 / 5 100.0%	1 / 1 100.0%	0 / 0 0.0%	0 / 0 0.0%	0 / 0 0.0%	35 / 52 67.3%AB	17 / 52 32.7%ABE			
1	Dehalobacter sp. 11DCA	52 / 52 100.0%	1 / 1 100.0%	2 / 2 100.0%	2 / 2 100.0%	1 / 1 100.0%	4 / 4 100.0%	4 / 4 100.0%	1 / 1 100.0%	1 / 1 100.0%	3 / 3 100.0%	1 / 1 100.0%	4 / 4 100.0%	3 / 3 100.0%	2 / 2 100.0%	2 / 2 100.0%	1 / 1 100.0%	3 / 3 100.0%	1 / 1 100.0%	3 / 3 100.0%	4 / 4 100.0%	2 / 2 100.0%	1 / 1 100.0%	5 / 5 100.0%	1 / 1 100.0%	0 / 0 0.0%	0 / 0 0.0%	0 / 0 0.0%	35 / 52 67.3%AB	17 / 52 32.7%ABE			
3	Desulfosporosinus	217 / 217 100.0%	5 / 5 100.0%	10 / 10 100.0%	8 / 8 100.0%	5 / 5 100.0%	19 / 19 100.0%	13 / 13 100.0%	4 / 4 100.0%	6 / 6 100.0%	15 / 15 100.0%	5 / 5 100.0%	16 / 16 100.0%	15 / 15 100.0%	6 / 6 100.0%	7 / 7 100.0%	6 / 6 100.0%	9 / 9 100.0%	4 / 4 100.0%	13 / 13 100.0%	17 / 17 100.0%	10 / 10 100.0%	3 / 3 100.0%	20 / 20 100.0%	1 / 1 100.0%	0 / 0 0.0%	0 / 0 0.0%	0 / 0 0.0%	138 / 217 63.6%AB	79 / 217 36.4%ABE			
1	"Desulfovibrio orientis" Adams and Postgate 1959	73 / 73 100.0%	2 / 2 100.0%	3 / 3 100.0%	2 / 2 100.0%	1 / 1 100.0%	7 / 7 100.0%	4 / 4 100.0%	2 / 2 100.0%	2 / 2 100.0%	7 / 7 100.0%	2 / 2 100.0%	4 / 4 100.0%	5 / 5 100.0%	2 / 2 100.0%	3 / 3 100.0%	2 / 2 100.0%	3 / 3 100.0%	1 / 1 100.0%	3 / 3 100.0%	6 / 6 100.0%	4 / 4 100.0%	1 / 1 100.0%	6 / 6 100.0%	1 / 1 100.0%	0 / 0 0.0%	0 / 0 0.0%	0 / 0 0.0%	46 / 73 63.0%AB	27 / 73 37.0%ABE			
1	Desulfosporosinus orientis DSM 765	73 / 73 100.0%	2 / 2 100.0%	3 / 3 100.0%	2 / 2 100.0%	1 / 1 100.0%	7 / 7 100.0%	4 / 4 100.0%	2 / 2 100.0%	2 / 2 100.0%	7 / 7 100.0%	2 / 2 100.0%	4 / 4 100.0%	5 / 5 100.0%	2 / 2 100.0%	3 / 3 100.0%	2 / 2 100.0%	3 / 3 100.0%	1 / 1 100.0%	3 / 3 100.0%	6 / 6 100.0%	4 / 4 100.0%	1 / 1 100.0%	6 / 6 100.0%	1 / 1 100.0%	0 / 0 0.0%	0 / 0 0.0%	0 / 0 0.0%	46 / 73 63.0%AB	27 / 73 37.0%ABE			
1	ATCC BAA-275	79 / 79 100.0%	2 / 2 100.0%	4 / 4 100.0%	3 / 3 100.0%	2 / 2 100.0%	7 / 7 100.0%	5 / 5 100.0%	1 / 1 100.0%	2 / 2 100.0%	4 / 4 100.0%	2 / 2 100.0%	6 / 6 100.0%	6 / 6 100.0%	2 / 2 100.0%	2 / 2 100.0%	2 / 2 100.0%	3 / 3 100.0%	2 / 2 100.0%	5 / 5 100.0%	6 / 6 100.0%	4 / 4 100.0%	1 / 1 100.0%	8 / 8 100.0%	0 / 0 0.0%	0 / 0 0.0%	0 / 0 0.0%	0 / 0 0.0%	50 / 79 63.3%AB	29 / 79 36.7%ABE			
1	Desulfosporosinus meridiei DSM 132567	79 / 79 100.0%	2 / 2 100.0%	4 / 4 100.0%	3 / 3 100.0%	2 / 2 100.0%	7 / 7 100.0%	5 / 5 100.0%	1 / 1 100.0%	2 / 2 100.0%	4 / 4 100.0%	2 / 2 100.0%	6 / 6 100.0%	6 / 6 100.0%	2 / 2 100.0%	2 / 2 100.0%	2 / 2 100.0%	3 / 3 100.0%	2 / 2 100.0%	5 / 5 100.0%	6 / 6 100.0%	4 / 4 100.0%	1 / 1 100.0%	8 / 8 100.0%	0 / 0 0.0%	0 / 0 0.0%	0 / 0 0.0%	0 / 0 0.0%	50 / 79 63.3%AB	29 / 79 36.7%ABE			
1	"Desulfosporosinus acidiphilus" Alazard et al. 2010	65 / 65 100.0%	1 / 1 100.0%	3 / 3 100.0%	3 / 3 100.0%	2 / 2 100.0%	5 / 5 100.0%	4 / 4 100.0%	1 / 1 100.0%	2 / 2 100.0%	4 / 4 100.0%	1 / 1 100.0%	6 / 6 100.0%	4 / 4 100.0%	2 / 2 100.0%	2 / 2 100.0%	2 / 2 100.0%	3 / 3 100.0%	1 / 1 100.0%	5 / 5 100.0%	5 / 5 100.0%	2 / 2 100.0%	1 / 1 100.0%	6 / 6 100.0%	0 / 0 0.0%	0 / 0 0.0%	0 / 0 0.0%	0 / 0 0.0%	42 / 65 64.6%AB	23 / 65 35.4%ABE			
1	Desulfosporosinus acidiphilus DSM 22704	65 / 65 100.0%	1 / 1 100.0%	3 / 3 100.0%	3 / 3 100.0%	2 / 2 100.0%	5 / 5 100.0%	4 / 4 100.0%	1 / 1 100.0%	2 / 2 100.0%	4 / 4 100.0%	1 / 1 100.0%	6 / 6 100.0%	4 / 4 100.0%	2 / 2 100.0%	2 / 2 100.0%	2 / 2 100.0%	3 / 3 100.0%	1 / 1 100.0%	5 / 5 100.0%	5 / 5 100.0%	2 / 2 100.0%	1 / 1 100.0%	6 / 6 100.0%	0 / 0 0.0%	0 / 0 0.0%	0 / 0 0.0%	0 / 0 0.0%	42 / 65 64.6%AB	23 / 65 35.4%ABE			
1	Pelotomaculum	50 / 50 100.0%	1 / 1 100.0%	1 / 1 100.0%	2 / 2 100.0%	1 / 1 100.0%	4 / 4 100.0%	3 / 3 100.0%	1 / 1 100.0%	1 / 1 100.0%	4 / 4 100.0%	1 / 1 100.0%	4 / 4 100.0%	3 / 3 100.0%	1 / 1 100.0%	2 / 2 100.0%	1 / 1 100.0%	3 / 3 100.0%	1 / 1 100.0%	3 / 3 100.0%	4 / 4 100.0%	2 / 2 100.0%	1 / 1 100.0%	5 / 5 100.0%	1 / 1 100.0%	0 / 0 0.0%	0 / 0 0.0%	0 / 0 0.0%	31 / 50 62.0%AB	18 / 50 36.0%ABE	1 / 50 2.0%BE		
1	DSM 13744	50 / 50 100.0%	1 / 1 100.0%	1 / 1 100.0%	2 / 2 100.0%	1 / 1 100.0%	4 / 4 100.0%	3 / 3 100.0%	1 / 1 100.0%	1 / 1 100.0%	4 / 4 100.0%	1 / 1 100.0%	4 / 4 100.0%	3 / 3 100.0%	1 / 1 100.0%	2 / 2 100.0%	1 / 1 100.0%	3 / 3 100.0%	1 / 1 100.0%	3 / 3 100.0%	4 / 4 100.0%	2 / 2 100.0%	1 / 1 100.0%	5 / 5 100.0%	1 / 1 100.0%	0 / 0 0.0%	0 / 0 0.0%	0 / 0 0.0%	31 / 50 62.0%AB	18 / 50 36.0%ABE	1 / 50 2.0%BE		
1	Pelotomaculum thermopropionicum SI	50 / 50 100.0%	1 / 1 100.0%	1 / 1 100.0%	2 / 2 100.0%	1 / 1 100.0%	4 / 4 100.0%	3 / 3 100.0%	1 / 1 100.0%	1 / 1 100.0%	4 / 4 100.0%	1 / 1 100.0%	4 / 4 100.0%	3 / 3 100.0%	1 / 1 100.0%	2 / 2 100.0%	1 / 1 100.0%	3 / 3 100.0%	1 / 1 100.0%	3 / 3 100.0%	4 / 4 100.0%	2 / 2 100.0%	1 / 1 100.0%	5 / 5 100.0%	1 / 1 100.0%	0 / 0 0.0%	0 / 0 0.0%	0 / 0 0.0%	31 / 50 62.0%AB	18 / 50 36.0%ABE	1 / 50 2.0%BE		
1	Thermincola	52 / 52 100.0%	1 / 1 100.0%	2 / 2 100.0%	2 / 2 100.0%	2 / 2 100.0%	4 / 4 100.0%	3 / 3 100.0%	1 / 1 100.0%	2 / 2 100.0%	3 / 3 100.0%	1 / 1 100.0%	4 / 4 100.0%	3 / 3 100.0%	1 / 1 100.0%	2 / 2 100.0%	1 / 1 100.0%	3 / 3 100.0%	1 / 1 100.0%	3 / 3 100.0%	4 / 4 100.0%	2 / 2 100.0%	1 / 1 100.0%	5 / 5 100.0%	1 / 1 100.0%	0 / 0 0.0%	0 / 0 0.0%	0 / 0 0.0%	31 / 52 59.6%AB	21 / 52 40.4%ABE			
1	"Thermincola potens" Byrne-Bailey et al. 2010	52 / 52 100.0%	1 / 1 100.0%	2 / 2 100.0%	2 / 2 100.0%	2 / 2 100.0%	4 / 4 100.0%	3 / 3 100.0%	1 / 1 100.0%	2 / 2 100.0%	3 / 3 100.0%	1 / 1 100.0%	4 / 4 100.0%	3 / 3 100.0%	1 / 1 100.0%	2 / 2 100.0%	1 / 1 100.0%	3 / 3 100.0%	1 / 1 100.0%	3 / 3 100.0%	4 / 4 100.0%	2 / 2 100.0%	1 / 1 100.0%	5 / 5 100.0%	1 / 1 100.0%	0 / 0 0.0%	0 / 0 0.0%	0 / 0 0.0%	31 / 52 59.6%AB	21 / 52 40.4%ABE			
1	Thermincola potens JR	52 / 52 100.0%	1 / 1 100.0%	2 / 2 100.0%	2 / 2 100.0%	2 / 2 100.0%	4 / 4 100.0%	3 / 3 100.0%	1 / 1 100.0%	2 / 2 100.0%	3 / 3 100.0%	1 / 1 100.0%	4 / 4 100.0%	3 / 3 100.0%	1 / 1 100.0%	2 / 2 100.0%	1 / 1 100.0%	3 / 3 100.0%	1 / 1 100.0%	3 / 3 100.0%	4 / 4 100.0%	2 / 2 100.0%	1 / 1 100.0%	5 / 5 100.0%	1 / 1 100.0%	0 / 0 0.0%	0 / 0 0.0%	0 / 0 0.0%	31 / 52 59.6%AB	21 / 52 40.4%ABE			
1	"Candidatus Desulforudis" Chivian et al. 2008	45 / 45 100.0%	1 / 1 100.0%	1 / 1 100.0%	1 / 1 100.0%	1 / 1 100.0%	3 / 3 100.0%	3 / 3 100.0%	1 / 1 100.0%	1 / 1 100.0%	3 / 3 100.0%	1 / 1 100.0%	4 / 4 100.0%	2 / 2 100.0%	1 / 1 100.0%	2 / 2 100.0%	1 / 1 100.0%	3 / 3 100.0%	1 / 1 100.0%	3 / 3 100.0%	4 / 4 100.0%	2 / 2 100.0%	0 / 0 0.0%	5 / 5 100.0%	1 / 1 100.0%	0 / 0 0.0%	0 / 0 0.0%	0 / 0 0.0%	14 / 45 31.1%B	13 / 45 28.9%AB	10 / 45 22.2%ABE	8 / 45 17.8%BE	
1	"Candidatus Desulforudis audaxviator" Chivian et al. 2008	45 / 45 100.0%	1 / 1 100.0%	1 / 1 100.0%	1 / 1 100.0%	1 / 1 100.0%	3 / 3 100.0%	3 / 3 100.0%	1 / 1 100.0%	1 / 1 100.0%	3 / 3 100.0%	1 / 1 100.0%	4 / 4 100.0%	2 / 2 100.0%	1 / 1 100.0%	2 / 2 100.0%	1 / 1 100.0%	3 / 3 100.0%	1 / 1 100.0%	3 / 3 100.0%	4 / 4 100.0%	2 / 2 100.0%	0 / 0 0.0%	5 / 5 100.0%	1 / 1 100.0%	0 / 0 0.0%	0 / 0 0.0%	0 / 0 0.0%	14 / 45 31.1%B	13 / 45 28.9%AB	10 / 45 22.2%ABE	8 / 45 17.8%BE	
1	Candidatus Desulforudis audaxviator MP104C	45 / 45 100.0%	1 / 1 100.0%	1 / 1 100.0%	1 / 1 100.0%	1 / 1 100.0%	3 / 3 100.0%	3 / 3 100.0%	1 / 1 100.0%	1 / 1 100.0%	3 / 3 100.0%	1 / 1 100.0%	4 / 4 100.0%	2 / 2 100.0%	1 / 1 100.0%	2 / 2 100.0%	1 / 1 100.0%	3 / 3 100.0%	1 / 1 100.0%	3 / 3 100.0%	4 / 4 100.0%	2 / 2 100.0%	0 / 0 0.0%	5 / 5 100.0%	1 / 1 100.0%	0 / 0 0.0%	0 / 0 0.0%	0 / 0 0.0%	14 / 45 31.1%B	13 / 45 28.9%AB	10 / 45 22.2%ABE	8 / 45 17.8%BE	
2	unclassified Clostridiales	126 / 126 100.0%	4 / 4 100.0%	5 / 5 100.0%	4 / 4 100.0%	3 / 3 100.0%	9 / 9 100.0%	8 / 8 100.0%	3 / 3 100.0%	4 / 4 100.0%	5 / 5 100.0%	2 / 2 100.0%	10 / 10 100.0%	6 / 6 100.0%	4 / 4 100.0%	5 / 5 100.0%	3 / 3 100.0%	6 / 6 100.0%	3 / 3 100.0%	8 / 8 100.0%	9 / 9 100.0%	7 / 7 100.0%	5 / 5 100.0%	12 / 12 100.0%	1 / 1 100.0%	0 / 0 0.0%	0 / 0 0.0%	0 / 0 0.0%	79 / 126 62.7%AB	47 / 126 37.3%ABE			
1	unclassified Clostridiales (miscellaneous)	45 / 45 100.0%	1 / 1 100.0%	1 / 1 100.0%	1 / 1 100.0%	1 / 1 100.0%	3 / 3 100.0%	3 / 3 100.0%	1 / 1 100.0%	1 / 1 100.0%	3 / 3 100.0%	1 / 1 100.0%	4 / 4 100.0%	2 / 2 100.0%	1 / 1 100.0%	2 / 2 100.0%	1 / 1 100.0%	3 / 3 100.0%	1 / 1 100.0%	3 / 3 100.0%	4 / 4 100.0%	2 / 2 100.0%	1 / 1 100.0%	5 / 5 100.0%	0 / 0 0.0%	0 / 0 0.0%	0 / 0 0.0%	0 / 0 0.0%	29 / 45 64.4%AB	16 / 45 35.6%ABE			
1	Clostridiales genomosp. BVAB3	45 / 45 100.0%	1 / 1 100.0%	1 / 1 100.0%	1 / 1 100.0%	1 / 1 100.0%	3 / 3 100.0%	3 / 3 100.0%	1 / 1 100.0%	1 / 1 100.0%	3 / 3 100.0%	1 / 1 100.0%	4 / 4 100.0%	2 / 2 100.0%	1 / 1 100.0%	2 / 2 100.0%	1 / 1 100.0%	3 / 3 100.0%	1 / 1 100.0%	3 / 3 100.0%	4 / 4 100.0%	2 / 2 100.0%	1 / 1 100.0%	5 / 5 100.0%	0 / 0 0.0%	0 / 0 0.0%	0 / 0 0.0%	0 / 0 0.0%	29 / 45 64.4%AB	16 / 45 35.6%ABE			
1	Clostridiales genomosp. BVAB3 str. UPII9-5	45 / 45 100.0%	1 / 1 100.0%	1 / 1 100.0%	1 / 1 100.0%	1 / 1 100.0%	3 / 3 100.0%	3 / 3 100.0%	1 / 1 100.0%	1 / 1 100.0%	3 / 3 100.0%	1 / 1 100.0%	4 / 4 100.0%	2 / 2 100.0%	1 / 1 100.0%	2 / 2 100.0%	1 / 1 100.0%	3 / 3 100.0%	1 / 1 100.0%	3 / 3 100.0%	4 / 4 100.0%	2 / 2 100.0%	1 / 1 100.0%	5 / 5 100.0%	0 / 0 0.0%	0 / 0 0.0%	0 / 0 0.0%	0 / 0 0.0%	29 / 45 64.4%AB	16 / 45 35.6%ABE			
1	"Gottschalkia" Yutin and Galperin	81 / 81 100.0%	3 / 3 100.0%	4 / 4 100.0%	3 / 3 100.0%	2 / 2 100.0%	6 / 6 100.0%	5 / 5 100.0%	2 / 2 100.0%	3 / 3 100.0%	2 / 2 100.0%	1 / 1 100.0%	6 / 6 100.0%	4 / 4 100.0%	3 / 3 100.0%	3 / 3 100.0%	2 / 2 100.0%	3 / 3 100.0%	2 / 2 100.0%	5 / 5 100.0%	5 / 5 100.0%	5 / 5 100.0%	4 / 4 100.0%	7 / 7 100.0%	1 / 1 100.0%	0 / 0 0.0%	0 / 0 0.0%	0 / 0 0.0%	50 / 81 61.7%AB	31 / 81 38.3%ABE			
1	"Bacillus acidi-urici" Liebert 1909	81 / 81 100.0%	3 / 3 100.0%	4 / 4 100.0%	3 / 3 100.0%	2 / 2 100.0%	6 / 6 100.0%	5 / 5 100.0%	2 / 2 100.0%	3 / 3 100.0%	2 / 2 100.0%	1 / 1 100.0%	6 / 6 100.0%	4 / 4 100.0%	3 / 3 100.0%	3 / 3 100.0%	2 / 2 100.0%	3 / 3 100.0%	2 / 2 100.0%	5 / 5 100.0%	5 / 5 100.0%	5 / 5 100.0%	4 / 4 100.0%	7 / 7 100.0%	1 / 1 100.0%	0 / 0 0.0%	0 / 0 0.0%	0 / 0 0.0%	50 / 81 61.7%AB	31 / 81 38.3%ABE			
1	Clostridium acidurici 9a	81 / 81 100.0%	3 / 3 100.0%	4 / 4 100.0%	3 / 3 100.0%	2 / 2 100.0%	6 / 6 100.0%	5 / 5 100.0%	2 / 2 100.0%	3 / 3 100.0%	2 / 2 100.0%	1 / 1 100.0%	6 / 6 100.0%	4 / 4 100.0%	3 / 3 100.0%	3 / 3 100.0%	2 / 2 100.0%	3 / 3 100.0%	2 / 2 100.0%	5 / 5 100.0%	5 / 5 100.0%	5 / 5 100.0%	4 / 4 100.0%	7 / 7 100.0%	1 / 1 100.0%	0 / 0 0.0%	0 / 0 0.0%	0 / 0 0.0%	50 / 81 61.7%AB	31 / 81 38.3%ABE			
1	Oscillospiraceae	57 / 58 98.3%	1 / 1 100.0%	2 / 2 100.0%	1 / 1 100.0%	3 / 3 100.0%	4 / 4 100.0%	3 / 3 100.0%	1 / 1 100.0%	1 / 1 100.0%	6 / 6 100.0%	1 / 1 100.0%	4 / 4 100.0%	4 / 4 100.0%	1 / 1 100.0%	2 / 2 100.0%	1 / 1 100.0%	3 / 3 100.0%	1 / 2 50.0%	3 / 3 100.0%	4 / 4 100.0%	4 / 4 100.0%	1 / 1 100.0%	6 / 6 100.0%	0 / 0 0.0%	0 / 0 0.0%	0 / 0 0.0%	0 / 0 0.0%	43 / 58 74.1%AB	14 / 58 24.1%ABE	1 / 58 1.7%		

1	Oscillibacter	[illegible] 98.3%	[illegible] 100.0%	[illegible] 100.0%	[illegible] 100.0%	[illegible] 100.0%	[illegible] 100.0%	[illegible] 100.0%	[illegible] 100.0%	[illegible] 100.0%	[illegible] 100.0%	[illegible] 100.0%	[illegible] 100.0%	[illegible] 100.0%	[illegible] 100.0%	[illegible] 100.0%	[illegible] 100.0%	[illegible] 100.0%	[illegible] 50.0%	[illegible] 100.0%	[illegible] 100.0%	[illegible] 100.0%	[illegible] 100.0%	[illegible] 100.0%	[illegible] 0.0%	[illegible] 0.0%	[illegible] 0.0%	[illegible] 0.0%	[illegible] 74.1%AB	[illegible] 24.1%ABE	[illegible] 1.7%			
1	Clostridiales bacterium Sjm18-20	57 / 58 98.3%	1 / 1 100.0%	2 / 2 100.0%	1 / 1 100.0%	3 / 3 100.0%	4 / 4 100.0%	3 / 3 100.0%	1 / 1 100.0%	1 / 1 100.0%	6 / 6 100.0%	1 / 1 100.0%	4 / 4 100.0%	4 / 4 100.0%	1 / 1 100.0%	2 / 2 100.0%	1 / 1 100.0%	3 / 3 100.0%	1 / 2 50.0%	3 / 3 100.0%	4 / 4 100.0%	4 / 4 100.0%	1 / 1 100.0%	6 / 6 100.0%	0 / 0 0.0%	0 / 0 0.0%	0 / 0 0.0%	0 / 0 0.0%	43 / 58 74.1%AB	14 / 58 24.1%ABE	1 / 58 1.7%			
1	Oscillibacter valericigenes NBRC 101213	57 / 58 98.3%	1 / 1 100.0%	2 / 2 100.0%	1 / 1 100.0%	3 / 3 100.0%	4 / 4 100.0%	3 / 3 100.0%	1 / 1 100.0%	1 / 1 100.0%	6 / 6 100.0%	1 / 1 100.0%	4 / 4 100.0%	4 / 4 100.0%	1 / 1 100.0%	2 / 2 100.0%	1 / 1 100.0%	3 / 3 100.0%	1 / 2 50.0%	3 / 3 100.0%	4 / 4 100.0%	4 / 4 100.0%	1 / 1 100.0%	6 / 6 100.0%	0 / 0 0.0%	0 / 0 0.0%	0 / 0 0.0%	0 / 0 0.0%	43 / 58 74.1%AB	14 / 58 24.1%ABE	1 / 58 1.7%			
3	Clostridiales incertae sedis	114 / 154 74.0%	5 / 5 100.0%	5 / 5 100.0%	3 / 3 100.0%	4 / 6 66.7%	8 / 12 66.7%	7 / 9 77.8%	2 / 3 66.7%	2 / 3 66.7%	8 / 11 72.7%	3 / 3 100.0%	10 / 14 71.4%	5 / 6 83.3%	3 / 3 100.0%	4 / 6 66.7%	2 / 3 66.7%	6 / 9 66.7%	2 / 3 66.7%	6 / 9 66.7%	11 / 14 78.6%	4 / 6 66.7%	2 / 3 66.7%	10 / 15 66.7%	2 / 3 66.7%	0 / 0 0.0%	0 / 0 0.0%	0 / 0 0.0%	72 / 154 46.8%AB	42 / 154 27.3%ABE	18 / 154 11.7%	10 / 154 6.5%E	7 / 154 4.5%C	5 / 154 3.2%CE
3	Clostridiales Family XVII. Incertae Sedis	114 / 154 74.0%	5 / 5 100.0%	5 / 5 100.0%	3 / 3 100.0%	4 / 6 66.7%	8 / 12 66.7%	7 / 9 77.8%	2 / 3 66.7%	2 / 3 66.7%	8 / 11 72.7%	3 / 3 100.0%	10 / 14 71.4%	5 / 6 83.3%	3 / 3 100.0%	4 / 6 66.7%	2 / 3 66.7%	6 / 9 66.7%	2 / 3 66.7%	6 / 9 66.7%	11 / 14 78.6%	4 / 6 66.7%	2 / 3 66.7%	10 / 15 66.7%	2 / 3 66.7%	0 / 0 0.0%	0 / 0 0.0%	0 / 0 0.0%	72 / 154 46.8%AB	42 / 154 27.3%ABE	18 / 154 11.7%	10 / 154 6.5%E	7 / 154 4.5%C	5 / 154 3.2%CE
2	Sulfobacillus	104 / 104 100.0%	4 / 4 100.0%	4 / 4 100.0%	2 / 2 100.0%	4 / 4 100.0%	8 / 8 100.0%	6 / 6 100.0%	2 / 2 100.0%	2 / 2 100.0%	6 / 6 100.0%	2 / 2 100.0%	10 / 10 100.0%	4 / 4 100.0%	2 / 2 100.0%	4 / 4 100.0%	2 / 2 100.0%	6 / 6 100.0%	2 / 2 100.0%	6 / 6 100.0%	10 / 10 100.0%	4 / 4 100.0%	2 / 2 100.0%	10 / 10 100.0%	2 / 2 100.0%	0 / 0 0.0%	0 / 0 0.0%	0 / 0 0.0%	64 / 104 61.5%AB	40 / 104 38.5%ABE				
2	ATCC 700253	104 / 104 100.0%	4 / 4 100.0%	4 / 4 100.0%	2 / 2 100.0%	4 / 4 100.0%	8 / 8 100.0%	6 / 6 100.0%	2 / 2 100.0%	2 / 2 100.0%	6 / 6 100.0%	2 / 2 100.0%	10 / 10 100.0%	4 / 4 100.0%	2 / 2 100.0%	4 / 4 100.0%	2 / 2 100.0%	6 / 6 100.0%	2 / 2 100.0%	6 / 6 100.0%	10 / 10 100.0%	4 / 4 100.0%	2 / 2 100.0%	10 / 10 100.0%	2 / 2 100.0%	0 / 0 0.0%	0 / 0 0.0%	0 / 0 0.0%	64 / 104 61.5%AB	40 / 104 38.5%ABE				
1	Sulfobacillus acidophilus DSM 10332	52 / 52 100.0%	2 / 2 100.0%	2 / 2 100.0%	1 / 1 100.0%	2 / 2 100.0%	4 / 4 100.0%	3 / 3 100.0%	1 / 1 100.0%	1 / 1 100.0%	3 / 3 100.0%	1 / 1 100.0%	5 / 5 100.0%	2 / 2 100.0%	1 / 1 100.0%	2 / 2 100.0%	1 / 1 100.0%	3 / 3 100.0%	1 / 1 100.0%	3 / 3 100.0%	5 / 5 100.0%	2 / 2 100.0%	1 / 1 100.0%	5 / 5 100.0%	1 / 1 100.0%	0 / 0 0.0%	0 / 0 0.0%	0 / 0 0.0%	32 / 52 61.5%AB	20 / 52 38.5%ABE				
1	Sulfobacillus acidophilus TPY	52 / 52 100.0%	2 / 2 100.0%	2 / 2 100.0%	1 / 1 100.0%	2 / 2 100.0%	4 / 4 100.0%	3 / 3 100.0%	1 / 1 100.0%	1 / 1 100.0%	3 / 3 100.0%	1 / 1 100.0%	5 / 5 100.0%	2 / 2 100.0%	1 / 1 100.0%	2 / 2 100.0%	1 / 1 100.0%	3 / 3 100.0%	1 / 1 100.0%	3 / 3 100.0%	5 / 5 100.0%	2 / 2 100.0%	1 / 1 100.0%	5 / 5 100.0%	1 / 1 100.0%	0 / 0 0.0%	0 / 0 0.0%	0 / 0 0.0%	32 / 52 61.5%AB	20 / 52 38.5%ABE				
1	Aerothermobacter	10 / 50 20.0%	1 / 1 100.0%	1 / 1 100.0%	1 / 1 100.0%	0 / 2 0.0%	0 / 4 0.0%	1 / 3 33.3%	0 / 1 0.0%	0 / 1 0.0%	2 / 5 40.0%	1 / 1 100.0%	0 / 4 0.0%	1 / 2 50.0%	1 / 1 100.0%	0 / 2 0.0%	0 / 1 0.0%	0 / 3 0.0%	0 / 1 0.0%	0 / 3 0.0%	1 / 4 25.0%	0 / 2 0.0%	0 / 1 0.0%	0 / 5 0.0%	0 / 1 0.0%	0 / 0 0.0%	0 / 0 0.0%	0 / 0 0.0%	18 / 50 36.0%	10 / 50 20.0%E	8 / 50 16.0%AB	7 / 50 14.0%C	5 / 50 10.0%CE	2 / 50 4.0%ABE
1	ATCC 700841	10 / 50 20.0%	1 / 1 100.0%	1 / 1 100.0%	1 / 1 100.0%	0 / 2 0.0%	0 / 4 0.0%	1 / 3 33.3%	0 / 1 0.0%	0 / 1 0.0%	2 / 5 40.0%	1 / 1 100.0%	0 / 4 0.0%	1 / 2 50.0%	1 / 1 100.0%	0 / 2 0.0%	0 / 1 0.0%	0 / 3 0.0%	0 / 1 0.0%	0 / 3 0.0%	1 / 4 25.0%	0 / 2 0.0%	0 / 1 0.0%	0 / 5 0.0%	0 / 1 0.0%	0 / 0 0.0%	0 / 0 0.0%	0 / 0 0.0%	18 / 50 36.0%	10 / 50 20.0%E	8 / 50 16.0%AB	7 / 50 14.0%C	5 / 50 10.0%CE	2 / 50 4.0%ABE
1	Thermaerobacter marianensis 7p75a	10 / 50 20.0%	1 / 1 100.0%	1 / 1 100.0%	1 / 1 100.0%	0 / 2 0.0%	0 / 4 0.0%	1 / 3 33.3%	0 / 1 0.0%	0 / 1 0.0%	2 / 5 40.0%	1 / 1 100.0%	0 / 4 0.0%	1 / 2 50.0%	1 / 1 100.0%	0 / 2 0.0%	0 / 1 0.0%	0 / 3 0.0%	0 / 1 0.0%	0 / 3 0.0%	1 / 4 25.0%	0 / 2 0.0%	0 / 1 0.0%	0 / 5 0.0%	0 / 1 0.0%	0 / 0 0.0%	0 / 0 0.0%	0 / 0 0.0%	18 / 50 36.0%	10 / 50 20.0%E	8 / 50 16.0%AB	7 / 50 14.0%C	5 / 50 10.0%CE	2 / 50 4.0%ABE
6	Ruminococcaceae	334 / 384 87.0%	7 / 7 100.0%	12 / 14 85.7%	13 / 15 86.7%	8 / 12 66.7%	25 / 29 86.2%	20 / 25 80.0%	6 / 7 85.7%	10 / 10 100.0%	22 / 23 95.7%	6 / 8 75.0%	26 / 28 92.9%	18 / 21 85.7%	11 / 11 100.0%	14 / 15 93.3%	7 / 8 87.5%	20 / 22 90.9%	7 / 7 100.0%	20 / 24 83.3%	26 / 29 89.7%	18 / 22 81.8%	9 / 11 81.8%	29 / 36 80.6%	0 / 0 0.0%	0 / 0 0.0%	0 / 0 0.0%	0 / 0 0.0%	236 / 384 61.5%AB	98 / 384 25.5%ABE	30 / 384 7.8%	20 / 384 5.2%E		
1	Ruminococcus	18 / 57 31.6%	1 / 1 100.0%	0 / 2 0.0%	0 / 2 0.0%	0 / 4 0.0%	0 / 4 0.0%	1 / 2 50.0%	0 / 1 0.0%	1 / 1 100.0%	4 / 4 100.0%	0 / 2 0.0%	2 / 4 50.0%	0 / 3 0.0%	1 / 1 100.0%	1 / 2 50.0%	0 / 1 0.0%	2 / 4 50.0%	1 / 1 100.0%	1 / 3 33.3%	1 / 4 25.0%	1 / 3 33.3%	0 / 2 0.0%	1 / 6 16.7%	0 / 0 0.0%	0 / 0 0.0%	0 / 0 0.0%	0 / 0 0.0%	24 / 57 42.1%	15 / 57 26.3%E	10 / 57 17.5%AB	8 / 57 14.0%ABE		
1	ATCC 27210	18 / 57 31.6%	1 / 1 100.0%	0 / 2 0.0%	0 / 2 0.0%	0 / 4 0.0%	0 / 4 0.0%	1 / 2 50.0%	0 / 1 0.0%	1 / 1 100.0%	4 / 4 100.0%	0 / 2 0.0%	2 / 4 50.0%	0 / 3 0.0%	1 / 1 100.0%	1 / 2 50.0%	0 / 1 0.0%	2 / 4 50.0%	1 / 1 100.0%	1 / 3 33.3%	1 / 4 25.0%	1 / 3 33.3%	0 / 2 0.0%	1 / 6 16.7%	0 / 0 0.0%	0 / 0 0.0%	0 / 0 0.0%	0 / 0 0.0%	24 / 57 42.1%	15 / 57 26.3%E	10 / 57 17.5%AB	8 / 57 14.0%ABE		
1	Ruminococcus albus 7	18 / 57 31.6%	1 / 1 100.0%	0 / 2 0.0%	0 / 2 0.0%	0 / 4 0.0%	0 / 4 0.0%	1 / 2 50.0%	0 / 1 0.0%	1 / 1 100.0%	4 / 4 100.0%	0 / 2 0.0%	2 / 4 50.0%	0 / 3 0.0%	1 / 1 100.0%	1 / 2 50.0%	0 / 1 0.0%	2 / 4 50.0%	1 / 1 100.0%	1 / 3 33.3%	1 / 4 25.0%	1 / 3 33.3%	0 / 2 0.0%	1 / 6 16.7%	0 / 0 0.0%	0 / 0 0.0%	0 / 0 0.0%	0 / 0 0.0%	24 / 57 42.1%	15 / 57 26.3%E	10 / 57 17.5%AB	8 / 57 14.0%ABE		
1	Ethanoligenens	59 / 59 100.0%	1 / 1 100.0%	2 / 2 100.0%	2 / 2 100.0%	3 / 3 100.0%	4 / 4 100.0%	3 / 3 100.0%	1 / 1 100.0%	1 / 1 100.0%	6 / 6 100.0%	1 / 1 100.0%	4 / 4 100.0%	4 / 4 100.0%	2 / 2 100.0%	2 / 2 100.0%	2 / 2 100.0%	3 / 3 100.0%	1 / 1 100.0%	3 / 3 100.0%	4 / 4 100.0%	4 / 4 100.0%	1 / 1 100.0%	5 / 5 100.0%	0 / 0 0.0%	0 / 0 0.0%	0 / 0 0.0%	0 / 0 0.0%	41 / 59 69.5%AB	18 / 59 30.5%ABE				
1	CGMCC 1.5033	59 / 59 100.0%	1 / 1 100.0%	2 / 2 100.0%	2 / 2 100.0%	3 / 3 100.0%	4 / 4 100.0%	3 / 3 100.0%	1 / 1 100.0%	1 / 1 100.0%	6 / 6 100.0%	1 / 1 100.0%	4 / 4 100.0%	4 / 4 100.0%	2 / 2 100.0%	2 / 2 100.0%	2 / 2 100.0%	3 / 3 100.0%	1 / 1 100.0%	3 / 3 100.0%	4 / 4 100.0%	4 / 4 100.0%	1 / 1 100.0%	5 / 5 100.0%	0 / 0 0.0%	0 / 0 0.0%	0 / 0 0.0%	0 / 0 0.0%	41 / 59 69.5%AB	18 / 59 30.5%ABE				
1	Ethanoligenens harbinense YUAN-3	59 / 59 100.0%	1 / 1 100.0%	2 / 2 100.0%	2 / 2 100.0%	3 / 3 100.0%	4 / 4 100.0%	3 / 3 100.0%	1 / 1 100.0%	1 / 1 100.0%	6 / 6 100.0%	1 / 1 100.0%	4 / 4 100.0%	4 / 4 100.0%	2 / 2 100.0%	2 / 2 100.0%	2 / 2 100.0%	3 / 3 100.0%	1 / 1 100.0%	3 / 3 100.0%	4 / 4 100.0%	4 / 4 100.0%	1 / 1 100.0%	5 / 5 100.0%	0 / 0 0.0%	0 / 0 0.0%	0 / 0 0.0%	0 / 0 0.0%	41 / 59 69.5%AB	18 / 59 30.5%ABE				
4	"Ruminiclostridium" Yutin and Galperin 2013	257 / 268 95.9%	5 / 5 100.0%	10 / 10 100.0%	11 / 11 100.0%	5 / 5 100.0%	21 / 21 100.0%	16 / 20 80.0%	5 / 5 100.0%	8 / 8 100.0%	12 / 13 92.3%	5 / 5 100.0%	20 / 20 100.0%	14 / 14 100.0%	8 / 8 100.0%	11 / 11 100.0%	5 / 5 100.0%	15 / 15 100.0%	5 / 5 100.0%	16 / 18 88.9%	21 / 21 100.0%	13 / 15 86.7%	8 / 8 100.0%	23 / 25 92.0%	0 / 0 0.0%	0 / 0 0.0%	0 / 0 0.0%	0 / 0 0.0%	185 / 268 69.0%AB	72 / 268 26.9%ABE	6 / 268 2.2%	5 / 268 1.9%E		
1	ATCC 35414	86 / 96 89.6%	2 / 2 100.0%	4 / 4 100.0%	4 / 4 100.0%	2 / 2 100.0%	6 / 6 100.0%	4 / 8 50.0%	2 / 2 100.0%	2 / 2 100.0%	4 / 4 100.0%	2 / 2 100.0%	8 / 8 100.0%	4 / 4 100.0%	2 / 2 100.0%	4 / 4 100.0%	2 / 2 100.0%	6 / 6 100.0%	2 / 2 100.0%	4 / 6 66.7%	8 / 8 100.0%	4 / 6 66.7%	2 / 2 100.0%	8 / 10 80.0%	0 / 0 0.0%	0 / 0 0.0%	0 / 0 0.0%	0 / 0 0.0%	58 / 96 60.4%AB	28 / 96 29.2%ABE	6 / 96 6.2%	4 / 96 4.2%E		
1	Clostridium stercorarium subsp. stercorarium	86 / 96 89.6%	2 / 2 100.0%	4 / 4 100.0%	4 / 4 100.0%	2 / 2 100.0%	6 / 6 100.0%	4 / 8 50.0%	2 / 2 100.0%	2 / 2 100.0%	4 / 4 100.0%	2 / 2 100.0%	8 / 8 100.0%	4 / 4 100.0%	2 / 2 100.0%	4 / 4 100.0%	2 / 2 100.0%	6 / 6 100.0%	2 / 2 100.0%	4 / 6 66.7%	8 / 8 100.0%	4 / 6 66.7%	2 / 2 100.0%	8 / 10 80.0%	0 / 0 0.0%	0 / 0 0.0%	0 / 0 0.0%	0 / 0 0.0%	58 / 96 60.4%AB	28 / 96 29.2%ABE	6 / 96 6.2%	4 / 96 4.2%E		
1	Clostridium stercorarium subsp. stercorarium DSM 8532	86 / 96 89.6%	2 / 2 100.0%	4 / 4 100.0%	4 / 4 100.0%	2 / 2 100.0%	6 / 6 100.0%	4 / 8 50.0%	2 / 2 100.0%	2 / 2 100.0%	4 / 4 100.0%	2 / 2 100.0%	8 / 8 100.0%	4 / 4 100.0%	2 / 2 100.0%	4 / 4 100.0%	2 / 2 100.0%	6 / 6 100.0%	2 / 2 100.0%	4 / 6 66.7%	8 / 8 100.0%	4 / 6 66.7%	2 / 2 100.0%	8 / 10 80.0%	0 / 0 0.0%	0 / 0 0.0%	0 / 0 0.0%	0 / 0 0.0%	58 / 96 60.4%AB	28 / 96 29.2%ABE	6 / 96 6.2%	4 / 96 4.2%E		
2	"Ruminiclostridium thermocellum" Yutin and Galperin 2013	112 / 112 100.0%	2 / 2 100.0%	4 / 4 100.0%	4 / 4 100.0%	2 / 2 100.0%	10 / 10 100.0%	8 / 8 100.0%	2 / 2 100.0%	4 / 4 100.0%	6 / 6 100.0%	2 / 2 100.0%	8 / 8 100.0%	6 / 6 100.0%	4 / 4 100.0%	4 / 4 100.0%	2 / 2 100.0%	6 / 6 100.0%	2 / 2 100.0%	8 / 8 100.0%	8 / 8 100.0%	6 / 6 100.0%	4 / 4 100.0%	10 / 10 100.0%	0 / 0 0.0%	0 / 0 0.0%	0 / 0 0.0%	0 / 0 0.0%	84 / 112 75.0%AB	28 / 112 25.0%ABE				
1	Clostridium thermocellum ATCC 27405	56 / 56 100.0%	1 / 1 100.0%	2 / 2 100.0%	2 / 2 100.0%	1 / 1 100.0%	5 / 5 100.0%	4 / 4 100.0%	1 / 1 100.0%	2 / 2 100.0%	3 / 3 100.0%	1 / 1 100.0%	4 / 4 100.0%	3 / 3 100.0%	2 / 2 100.0%	2 / 2 100.0%	1 / 1 100.0%	3 / 3 100.0%	1 / 1 100.0%	4 / 4 100.0%	4 / 4 100.0%	3 / 3 100.0%	2 / 2 100.0%	5 / 5 100.0%	0 / 0 0.0%	0 / 0 0.0%	0 / 0 0.0%	0 / 0 0.0%	42 / 56 75.0%AB	14 / 56 25.0%ABE				
1	Clostridium thermocellum DSM 1313	56 / 56 100.0%	1 / 1 100.0%	2 / 2 100.0%	2 / 2 100.0%	1 / 1 100.0%	5 / 5 100.0%	4 / 4 100.0%	1 / 1 100.0%	2 / 2 100.0%	3 / 3 100.0%	1 / 1 100.0%	4 / 4 100.0%	3 / 3 100.0%	2 / 2 100.0%	2 / 2 100.0%	1 / 1 100.0%	3 / 3 100.0%	1 / 1 100.0%	4 / 4 100.0%	4 / 4 100.0%	3 / 3 100.0%	2 / 2 100.0%	5 / 5 100.0%	0 / 0 0.0%	0 / 0 0.0%	0 / 0 0.0%	0 / 0 0.0%	42 / 56 75.0%AB	14 / 56 25.0%ABE				
1	Clostridium clariflavum	59 / 60 98.3%	1 / 1 100.0%	2 / 2 100.0%	3 / 3 100.0%	1 / 1 100.0%	5 / 5 100.0%	4 / 4 100.0%	1 / 1 100.0%	2 / 2 100.0%	2 / 3 66.7%	1 / 1 100.0%	4 / 4 100.0%	4 / 4 100.0%	2 / 2 100.0%	3 / 3 100.0%	1 / 1 100.0%	3 / 3 100.0%	1 / 1 100.0%	4 / 4 100.0%	5 / 5 100.0%	3 / 3 100.0%	2 / 2 100.0%	5 / 5 100.0%	0 / 0 0.0%	0 / 0 0.0%	0 / 0 0.0%	0 / 0 0.0%	43 / 60 71.7%AB	16 / 60 26.7%ABE	1 / 60 1.7%E			
1	Clostridium clariflavum DSM 19732	59 / 60 98.3%	1 / 1 100.0%	2 / 2 100.0%	3 / 3 100.0%	1 / 1 100.0%	5 / 5 100.0%	4 / 4 100.0%	1 / 1 100.0%	2 / 2 100.0%	2 / 3 66.7%	1 / 1 100.0%	4 / 4 100.0%	4 / 4 100.0%	2 / 2 100.0%	3 / 3 100.0%	1 / 1 100.0%	3 / 3 100.0%	1 / 1 100.0%	4 / 4 100.0%	5 / 5 100.0%	3 / 3 100.0%	2 / 2 100.0%	5 / 5 100.0%	0 / 0 0.0%	0 / 0 0.0%	0 / 0 0.0%	0 / 0 0.0%	43 / 60 71.7%AB	16 / 60 26.7%ABE	1 / 60 1.7%E			
1	Clostridiales Family XVIII. Incertae Sedis	97 / 98 99.0%	3 / 3 100.0%	4 / 4 100.0%	3 / 3 100.0%	4 / 4 100.0%	7 / 7 100.0%	7 / 7 100.0%	2 / 2 100.0%	3 / 3 100.0%	8 / 8 100.0%	2 / 2 100.0%	7 / 7 100.0%	5 / 5 100.0%	3 / 3 100.0%	3 / 3 100.0%	2 / 2 100.0%	5 / 5 100.0%	2 / 2 100.0%	5 / 6 83.3%	6 / 6 100.0%	5 / 5 100.0%	1 / 1 100.0%	9 / 9 100.0%	1 / 1 100.0%	0 / 0 0.0%	0 / 0 0.0%	0 / 0 0.0%	62 / 98 63.3%AB	34 / 98 34.7%ABE	1 / 98 1.0%BE	1 / 98 1.0%C		
1	Symbiobacterium	97 / 98 99.0%	3 / 3 100.0%	4 / 4 100.0%	3 / 3 100.0%	4 / 4 100.0%	7 / 7 100.0%	7 / 7 100.0%	2 / 2 100.0%	3 / 3 100.0%	8 / 8 100.0%	2 / 2 100.0%	7 / 7 100.0%	5 / 5 100.0%	3 / 3 100.0%	3 / 3 100.0%	2 / 2 100.0%	5 / 5 100.0%	2 / 2 100.0%	5 / 6 83.3%	6 / 6 100.0%	5 / 5 100.0%	1 / 1 100.0%	9 / 9 100.0%	1 / 1 100.0%	0 / 0 0.0%	0 / 0 0.0%	0 / 0 0.0%	62 / 98 63.3%AB	34 / 98 34.7%ABE	1 / 98 1.0%C	1 / 98 1.0%BE		
1	DSM 24528	97 / 98 99.0%	3 / 3 100.0%	4 / 4 100.0%	3 / 3 100.0%	4 / 4 100.0%	7 / 7 100.0%	7 / 7 100.0%	2 / 2 100.0%	3 / 3 100.0%	8 / 8 100.0%	2 / 2 100.0%	7 / 7 100.0%	5 / 5 100.0%	3 / 3 100.0%	3 / 3 100.0%	2 / 2 100.0%	5 / 5 100.0%	2 / 2 100.0%	5 / 6 83.3%	6 / 6 100.0%	5 / 5 100.0%	1 / 1 100.0%	9 / 9 100.0%	1 / 1 100.0%	0 / 0 0.0%	0 / 0 0.0%	0 / 0 0.0%	62 / 98 63.3%AB	34 / 98 34.7%ABE	1 / 98 1.0%BE	1 / 98 1.0%C		
1	Symbiobacterium thermophilum IAM 14863	97 / 98 99.0%	3 / 3 100.0%	4 / 4 100.0%	3 / 3 100.0%	4 / 4 100.0%	7 / 7 100.0%	7 / 7 100.0%	2 / 2 100.0%	3 / 3 100.0%	8 / 8 100.0%	2 / 2 100.0%	7 / 7 100.0%	5 / 5 100.0%	3 / 3 100.0%	3 / 3 100.0%	2 / 2 100.0%	5 / 5 100.0%	2 / 2 100.0%	5 / 6 83.3%	6 / 6 100.0%	5 / 5 100.0%	1 / 1 100.0%	9 / 9 100.0%	1 / 1 100.0%	0 / 0 0.0%	0 / 0 0.0%	0 / 0 0.0%	62 / 98 63.3%AB	34 / 98 34.7%ABE	1 / 98 1.0%C	1 / 98 1.0%BE		
2	Peptoniphilaceae	94 / 95 98.9%	4 / 4 100.0%	4 / 4 100.0%	4 / 4 100.0%	3 / 3 100.0%	7 / 7 100.0%	4 / 4 100.0%	2 / 2 100.0%	3 / 3 100.0%	5 / 5 100.0%	2 / 2 100.0%	7 / 7 100.0%	6 / 6 100.0%	4 / 4 100.0%	3 / 3 100.0%	2 / 2 100.0%	2 / 2 100.0%	2 / 2 100.0%	7 / 7 100.0%	7 / 7 100.0%	5 / 5 100.0%	2 / 2 100.0%	9 / 9 100.0%	0 / 1 0.0%	0 / 0 0.0%	0 / 0 0.0%	0 / 0 0.0%	64 / 95 67.4%AB	26 / 95 27.4%ABE	3 / 95 3.2%BE	1 / 95 1.1%E	1 / 95 1.1%B	
1	Finegoldia	47 / 48 97.9%	2 / 2 100.0%	2 / 2 100.0%	2 / 2 100.0%	1 / 1 100.0%	3 / 3 100.0%	2 / 2 100.0%	1 / 1 100.0%	2 / 2 100.0%	2 / 2 100.0%	1 / 1 100.0%	4 / 4 100.0%	4 / 4 100.0%	2 / 2 100.0%	1 / 1 100.0%	1 / 1 100.0%	1 / 1 100.0%	1 / 1 100.0%	3 / 3 100.0%	4 / 4 100.0%	2 / 2 100.0%	1 / 1 100.0%	5 / 5 100.0%	0 / 1 0.0%	0 / 0 0.0%	0 / 0 0.0%	0 / 0 0.0%	32 / 48 66.7%AB	11 / 48 22.9%ABE	3 / 48 6.2%BE	1 / 48 2.1%B	1 / 48 2.1%E	
1	"Diplococcus magnus" Prevot 1933	47 / 48 97.9%	2 / 2 100.0%	2 / 2 100.0%	2 / 2 100.0%	1 / 1 100.0%	3 / 3 100.0%	2 / 2 100.0%	1 / 1 100.0%	2 / 2 100.0%	2 / 2 100.0%	1 / 1 100.0%	4 / 4 100.0%	4 / 4 100.0%	2 / 2 100.0%	1 / 1 100.0%	1 / 1 100.0%	1 / 1 100.0%	1 / 1 100.0%	3 / 3 100.0%	4 / 4 100.0%	2 / 2 100.0%	1 / 1 100.0%	5 / 5 100.0%	0 / 1 0.0%	0 / 0 0.0%	0 / 0 0.0%	0 / 0 0.0%	32 / 48 66.7%AB	11 / 48 22.9%ABE	3 / 48 6.2%BE	1 / 48 2.1%B	1 / 48 2.1%E	
1	Finegoldia magna ATCC 29328	47 / 48 97.9%	2 / 2 100.0%	2 / 2 100.0%	2 / 2 100.0%	1 / 1 100.0%	3 / 3 100.0%	2 / 2 100.0%	1 / 1 100.0%	2 / 2 100.0%	2 / 2 100.0%	1 / 1 100.0%	4 / 4 100.0%	4 / 4 100.0%	2 / 2 100.0%	1 / 1 100.0%	1 / 1 100.0%	1 / 1 100.0%	1 / 1 100.0%	3 / 3 100.0%	4 / 4 100.0%	2 / 2 100.0%	1 / 1 100.0%	5 / 5 100.0%	0 / 1 0.0%	0 / 0 0.0%	0 / 0 0.0%	0 / 0 0.0%	32 / 48 66.7%AB	11 / 48 22.9%ABE	3 / 48 6.2%BE	1 / 48 2.1%E	1 / 48 2.1%B	
1	Anaerococcus	47 / 47 100.0%	2 / 2 100.0%	2 / 2 100.0%	2 / 2 100.0%	2 / 2 100.0%	4 / 4 100.0%	2 / 2 100.0%	1 / 1 100.0%	1 / 1 100.0%	3 / 3 100.0%	1 / 1 100.0%	3 / 3 100.0%	2 / 2 100.0%	2 / 2 100.0%	2 / 2 100.0%	1 / 1 100.0%	1 / 1 100.0%	1 / 1 100.0%	4 / 4 100.0%	3 / 3 100.0%	3 / 3 100.0%	1 / 1 100.0%	4 / 4 100.0%	0 / 0 0.0%	0 / 0 0.0%	0 / 0 0.0%	0 / 0 0.0%	32 / 47 68.1%AB	15 / 47 31.9%ABE				
1	"Micrococcus prevotii" Foubert and Douglas 1948	47 / 47 100.0%	2 / 2 100.0%	2 / 2 100.0%	2 / 2 100.0%	2 / 2 100.0%	4 / 4 100.0%	2 / 2 100.0%	1 / 1 100.0%	1 / 1 100.0%	3 / 3 100.0%	1 / 1 100.0%	3 / 3 100.0%	2 / 2 100.0%	2 / 2 100.0%	2 / 2 100.0%	1 / 1 100.0%	1 / 1 100.0%	1 / 1 100.0%	4 / 4 100.0%	3 / 3 100.0%	3 / 3 100.0%	1 / 1 100.0%	4 / 4 100.0%	0 / 0 0.0%	0 / 0 0.0%	0 / 0 0.0%	0 / 0 0.0%	32 / 47 68.1%AB	15 / 47 31.9%ABE				
1	Anaerococcus prevotii ATCC 9321	47 / 47 100.0%	2 / 2 100.0%	2 / 2 100.0%	2 / 2 100.0%	2 / 2 100.0%	4 / 4 100.0%	2 / 2 100.0%	1 / 1 100.0%	1 / 1 100.0%	3 / 3 100.0%	1 / 1 100.0%	3 / 3 100.0%	2 / 2 100.0%	2 / 2 100.0%	2 / 2 100.0%	1 / 1 100.0%	1 / 1 100.0%	1 / 1 100.0%	4 / 4 100.0%	3 / 3 100.0%	3 / 3 100.0%	1 / 1 100.0%	4 / 4 100.0%	0 / 0 0.0%	0 / 0 0.0%	0 / 0 0.0%	0 / 0 0.0%	32 / 47 68.1%AB	15 / 47 31.9%ABE				
1	Natranaerobiales	51 / 51 100.0%	2 / 2 100.0%	2 / 2 100.0%	2 / 2 100.0%	2 / 2 100.0%	4 / 4 100.0%	4 / 4 100.0%	1 / 1 100.0%	2 / 2 100.0%	3 / 3 100.0%	1 / 1 100.0%	4 / 4 100.0%	2 / 2 100.0%	1 / 1 100.0%	2 / 2 100.0%	1 / 1 100.0%	3 / 3 100.0%	1 / 1 100.0%	2 / 2 100.0%	4 / 4 100.0%	2 / 2 100.0%	1 / 1 100.0%	4 / 4 100.0%	1 / 1 100.0%	0 / 0 0.0%	0 / 0 0.0%	0 / 0 0.0%	20 / 51 39.2%AB	15 / 51 29.4%B	11 / 51 21.6%ABE	5 / 51 9.8%BE		
1	Natranaerobiaceae	51 / 51 100.0%	2 / 2 100.0%	2 / 2 100.0%	2 / 2 100.0%	2 / 2 100.0%	4 / 4 100.0%	4 / 4 100.0%	1 / 1 100.0%	2 / 2 100.0%	3 / 3 100.0%	1 / 1 100.0%	4 / 4 100.0%	2 / 2 100.0%	1 / 1 100.0%	2 / 2 100.0%	1 / 1 100.0%	3 / 3 100.0%	1 / 1 100.0%	2 / 2 100.0%	4 / 4 100.0%	2 / 2 100.0%	1 / 1 100.0%	4 / 4 100.0%	1 / 1 100.0%	0 / 0 0.0%	0 / 0 0.0%	0 / 0 0.0%	20 / 51 39.2%AB	15 / 51 29.4%B	11 / 51 21.6%ABE	5 / 51 9.8%BE		
1	Natranaerobius	51 / 51 100.0%	2 / 2 100.0%	2 / 2 100.0%	2 / 2 100.0%	2 / 2 100.0%	4 / 4 100.0%	4 / 4 100.0%	1 / 1 100.0%	2 / 2 100.0%	3 / 3 100.0%	1 / 1 100.0%	4 / 4 100.0%	2 / 2 100.0%	1 / 1 100.0%	2 / 2 100.0%	1 / 1 100.0%	3 / 3 100.0%	1 / 1 100.0%	2 / 2 100.0%	4 / 4 100.0%	2 / 2 100.0%	1 / 1 100.0%	4 / 4 100.0%	1 / 1 100.0%	0 / 0 0.0%	0 / 0 0.0%	0 / 0 0.0%	20 / 51 39.2%AB	15 / 51 29.4%B	11 / 51 21.6%ABE	5 / 51 9.8%BE		
1	ATCC BAA-1301	51 / 51 100.0%	2 / 2 100.0%	2 / 2 100.0%	2 / 2 100.0%	2 / 2 100.0%	4 / 4 100.0%	4 / 4 100.0%	1 / 1 100.0%	2 / 2 100.0%	3 / 3 100.0%	1 / 1 100.0%	4 / 4 100.0%	2 / 2 100.0%	1 / 1 100.0%	2 / 2 100.0%	1 / 1 100.0%	3 / 3 100.0%	1 / 1 100.0%	2 / 2 100.0%	4 / 4 100.0%	2 / 2 100.0%	1 / 1 100.0%	4 / 4 100.0%	1 / 1 100.0%	0 / 0 0.0%	0 / 0 0.0%	0 / 0 0.0%	20 / 51 39.2%AB	15 / 51 29.4%B	11 / 51 21.6%ABE	5 / 51 9.8%BE		
1	Natranaerobius thermophilus JW/NM-WN-LF	51 / 51 100.0%	2 / 2 100.0%	2 / 2 100.0%	2 / 2 100.0%	2 / 2 100.0%	4 / 4 100.0%	4 / 4 100.0%	1 / 1 100.0%	2 / 2 100.0%	3 / 3 100.0%	1 / 1 100.0%	4 / 4 100.0%	2 / 2 100.0%	1 / 1 100.0%	2 / 2 100.0%	1 / 1 100.0%	3 / 3 100.0%	1 / 1 100.0%	2 / 2 100.0%	4 / 4 100.0%	2 / 2 100.0%	1 / 1 100.0%	4 / 4 100.0%	1 / 1 100.0%	0 / 0 0.0%	0 / 0 0.0%	0 / 0 0.0%	20 / 51 39.2%AB	15 / 51 29.4%B	11 / 51 21.6%ABE	5 / 51 9.8%BE		
1	Erysipelotrichi	55 / 55 100.0%	1 / 1 100.0%	3 / 3 100.0%	3 / 3 100.0%	3 / 3 100.0%	5 / 5 100.0%	3 / 3 100.0%	2 / 2 100.0%	1 / 1 100.0%	4 / 4 100.0%	1 / 1 100.0%	4 / 4 100.0%	3 / 3 100.0%	1 / 1 100.0%	1 / 1 100.0%	1 / 1 100.0%	1 / 1 100.0%	1 / 1 100.0%	4 / 4 100.0%	5 / 5 100.0%	3 / 3 100.0%	1 / 1 100.0%	4 / 4 100.0%	0 / 0 0.0%	0 / 0 0.0%	0 / 0 0.0%	0 / 0 0.0%	41 / 55 74.5%AB	14 / 55 25.5%ABE				
1	Erysipelotrichales	55 / 55 100.0%	1 / 1 100.0%	3 / 3 100.0%	3 / 3 100.0%	3 / 3 100.0%	5 / 5 100.0%	3 / 3 100.0%	2 / 2 100.0%	1 / 1 100.0%	4 / 4 100.0%	1 / 1 100.0%	4 / 4 100.0%	3 / 3 100.0%	1 / 1 100.0%	1 / 1 100.0%	1 / 1 100.0%	1 / 1 100.0%	1 / 1 100.0%	4 / 4 100.0%	5 / 5 100.0%	3 / 3 100.0%	1 / 1 100.0%	4 / 4 100.0%	0 / 0 0.0%	0 / 0 0.0%	0 / 0 0.0%	0 / 0 0.0%	41 / 55 74.5%AB	14 / 55 25.5%ABE				
1	Erysipelothrix group	55 / 55 100.0%	1 / 1 100.0%	3 / 3 100.0%	3 / 3 100.0%	3 / 3 100.0%	5 / 5 100.0%	3 / 3 100.0%	2 / 2 100.0%	1 / 1 100.0%	4 / 4 100.0%	1 / 1 100.0%	4 / 4 100.0%	3 / 3 100.0%	1 / 1 100.0%	1 / 1 100.0%	1 / 1 100.0%	1 / 1 100.0%	1 / 1 100.0%	4 / 4 100.0%	5 / 5 100.0%	3 / 3 100.0%	1 / 1 100.0%	4 / 4 100.0%	0 / 0 0.0%	0 / 0 0.0%	0 / 0 0.0%	0 / 0 0.0%	41 / 55 74.5%AB	14 / 55 25.5%ABE				
1	Erysipelothrix	55 / 55 100.0%	1 / 1 100.0%	3 / 3 100.0%	3 / 3 100.0%	3 / 3 100.0%	5 / 5 100.0%	3 / 3 100.0%	2 / 2 100.0%	1 / 1 100.0%	4 / 4 100.0%	1 / 1 100.0%	4 / 4 100.0%	3 / 3 100.0%	1 / 1 100.0%	1 / 1 100.0%	1 / 1 100.0%	1 / 1 100.0%	1 / 1 100.0%	4 / 4 100.0%	5 / 5 100.0%	3 / 3 100.0%	1 / 1 100.0%	4 / 4 100.0%	0 / 0 0.0%	0 / 0 0.0%	0 / 0 0.0%	0 / 0 0.0%	41 / 55 74.5%AB	14 / 55 25.5%ABE				
1	"Bacillus insidiosus" Trevisan 1885	55 / 55 100.0%	1 / 1 100.0%	3 / 3 100.0%	3 / 3 100.0%	3 / 3 100.0%	5 / 5 100.0%	3 / 3 100.0%	2 / 2 100.0%	1 / 1 100.0%	4 / 4 100.0%	1 / 1 100.0%	4 / 4 100.0%	3 / 3 100.0%	1 / 1 100.0%	1 / 1 100.0%	1 / 1 100.0%	1 / 1 100.0%	1 / 1 100.0%	4 / 4 100.0%	5 / 5 100.0%	3 / 3 100.0%	1 / 1 100.0%	4 / 4 100.0%	0 / 0 0.0%	0 / 0 0.0%	0 / 0 0.0%	0 / 0 0.0%	41 / 55 74.5%AB	14 / 55 25.5%ABE				
1	Erysipelothrix rhusiopathiae Fujisawa	55 / 55 100.0%	1 / 1 100.0%	3 / 3 100.0%	3 / 3 100.0%	3 / 3 100.0%	5 / 5 100.0%	3 / 3 100.0%	2 / 2 100.0%	1 / 1 100.0%	4 / 4 100.0%	1 / 1 100.0%	4 / 4 100.0%	3 / 3 100.0%	1 / 1 100.0%	1 / 1 100.0%	1 / 1 100.0%	1 / 1 100.0%	1 / 1 100.0%	4 / 4 100.0%	5 / 5 100.0%	3 / 3 100.0%	1 / 1 100.0%	4 / 4 100.0%	0 / 0 0.0%	0 / 0 0.0%	0 / 0 0.0%	0 / 0 0.0%	41 / 55 74.5%AB	14 / 55 25.5%ABE				
5	Negativicutes	282 / 282 100.0%	7 / 7 100.0%	12 / 12 100.0%	11 / 11 100.0%	7 / 7 100.0%	27 / 27 100.0%	18 / 18 100.0%	5 / 5 100.0%	7 / 7 100.0%	15 / 15 100.0%	6 / 6 100.0%	22 / 22 100.0%	14 / 14 100.0%	8 / 8 100.0%	11 / 11 100.0%	7 / 7 100.0%	14 / 14 100.0%	7 / 7 100.0%	15 / 15 100.0%	22 / 22 100.0%	15 / 15 100.0%	5 / 5 100.0%	27 / 27 100.0%	0 / 0 0.0%	0 / 0 0.0%	0 / 0 0.0%	0 / 0 0.0%	191 / 282 67.7%AB	91 / 282 32.3%ABE				
5	Selenomonadales	282 / 282 100.0%	7 / 7 100.0%	12 / 12 100.0%	11 / 11 100.0%	7 / 7 100.0%	27 / 27 100.0%	18 / 18 100.0%	5 / 5 100.0%	7 / 7 100.0%	15 / 15 100.0%	6 / 6 100.0%	22 / 22 100.0%	14 / 14 100.0%	8 / 8 100.0%	11 / 11 100.0%	7 / 7 100.0%	14 / 14 100.0%	7 / 7 100.0%	15 / 15 100.0%	22 / 22 100.0%	15 / 15 100.0%	5 / 5 100.0%	27 / 27 100.0%	0 / 0 0.0%	0 / 0 0.0%	0 / 0 0.0%	0 / 0 0.0%	191 / 282 67.7%AB	91 / 282 32.3%ABE				
3	'Selenomonadaceae'	170 / 170 100.0%	5 / 5 100.0%	7 / 7 100.0%	7 / 7 100.0%	5 / 5 100.0%	16 / 16 100.0%	10 / 10 100.0%	3 / 3 100.0%	5 / 5 100.0%	9 / 9 100.0%	3 / 3 100.0%	13 / 13 100.0%	8 / 8 100.0%	5 / 5 100.0%	6 / 6 100.0%	4 / 4 100.0%	8 / 8 100.0%	5 / 5 100.0%	9 / 9 100.0%	14 / 14 100.0%	9 / 9 100.0%	3 / 3 100.0%	16 / 16 100.0%	0 / 0 0.0%	0 / 0 0.0%	0 / 0 0.0%	0 / 0 0.0%	113 / 170 66.5%AB	57 / 170 33.5%ABE				
2	Selenomonas	122 / 122 100.0%	4 / 4 100.0%	5 / 5 100.0%	4 / 4 100.0%	3 / 3 100.0%	11 / 11 100.0%	7 / 7 100.0%	2 / 2 100.0%	4 / 4 100.0%	7 / 7 100.0%	2 / 2 100.0%	9 / 9 100.0%	5 / 5 100.0%	3 / 3 100.0%	5 / 5 100.0%	3 / 3 100.0%	7 / 7 100.0%	4 / 4 100.0%	7 / 7 100.0%	10 / 10 100.0%	6 / 6 100.0%	2 / 2 100.0%	12 / 12 100.0%	0 / 0 0.0%	0 / 0 0.0%	0 / 0 0.0%	0 / 0 0.0%	80 / 122 65.6%AB	42 / 122 34.4%ABE				
1	"Ancyromonas ruminantium" Certes 1889	69 / 69 100.0%	2 / 2 100.0%	3 / 3 100.0%	2 / 2 100.0%	2 / 2 100.0%	6 / 6 100.0%	4 / 4 100.0%	1 / 1 100.0%	2 / 2 100.0%	4 / 4 100.0%	1 / 1 100.0%	5 / 5 100.0%	3 / 3 100.0%	2 / 2 100.0%	3 / 3 100.0%	2 / 2 100.0%	4 / 4 100.0%	2 / 2 100.0%	4 / 4 100.0%	5 / 5 100.0%	4 / 4 100.0%	1 / 1 100.0%	7 / 7 100.0%	0 / 0 0.0%	0 / 0 0.0%	0 / 0 0.0%	0 / 0 0.0%	46 / 69 66.7%AB	23 / 69 33.3%ABE				
1	"Selenomonas lactilytica" Hungate 1966	69 / 69 100.0%	2 / 2 100.0%	3 / 3 100.0%	2 / 2 100.0%	2 / 2 100.0%	6 / 6 100.0%	4 / 4 100.0%	1 / 1 100.0%	2 / 2 100.0%	4 / 4 100.0%	1 / 1 100.0%	5 / 5 100.0%	3 / 3 100.0%	2 / 2 100.0%	3 / 3 100.0%	2 / 2 100.0%	4 / 4 100.0%	2 / 2 100.0%	4 / 4 100.0%	5 / 5 100.0%	4 / 4 100.0%	1 / 1 100.0%	7 / 7 100.0%	0 / 0 0.0%	0 / 0 0.0%	0 / 0 0.0%	0 / 0 0.0%	46 / 69 66.7%AB	23 / 69 33.3%ABE				
1	Selenomonas ruminantium subsp. lactilytica TAM6421	69 / 69 100.0%	2 / 2 100.0%	3 / 3 100.0%	2 / 2 100.0%	2 / 2 100.0%	6 / 6 100.0%	4 / 4 100.0%	1 / 1 100.0%	2 / 2 100.0%	4 / 4 100.0%	1 / 1 100.0%	5 / 5 100.0%	3 / 3 100.0%	2 / 2 100.0%	3 / 3 100.0%	2 / 2 100.0%	4 / 4 100.0%	2 / 2 100.0%	4 / 4 100.0%	5 / 5 100.0%	4 / 4 100.0%	1 / 1 100.0%	7 / 7 100.0%	0 / 0 0.0%	0 / 0 0.0%	0 / 0 0.0%	0 / 0 0.0%	46 / 69 66.7%AB	23 / 69 33.3%ABE				
1	"Spirillum sputigenum" Flugge 1886	53 / 53 100.0%	2 / 2 100.0%	2 / 2 100.0%	2 / 2 100.0%	1 / 1 100.0%	5 / 5 100.0%	3 / 3 100.0%	1 / 1 100.0%	2 / 2 100.0%	3 / 3 100.0%	1 / 1 100.0%	4 / 4 100.0%	2 / 2 100.0%	1 / 1 100.0%	2 / 2 100.0%	1 / 1 100.0%	3 / 3 100.0%	2 / 2 100.0%	3 / 3 100.0%	5 / 5 100.0%	2 / 2 100.0%	1 / 1 100.0%	5 / 5 100.0%	0 / 0 0.0%	0 / 0 0.0%	0 / 0 0.0%	0 / 0 0.0%	34 / 53 64.2%AB	19 / 53 35.8%ABE				
1	Selenomonas sputigena ATCC 35185	53 / 53 100.0%	2 / 2 100.0%	2 / 2 100.0%	2 / 2 100.0%	1 / 1 100.0%	5 / 5 100.0%	3 / 3 100.0%	1 / 1 100.0%	2 / 2 100.0%	3 / 3 100.0%	1 / 1 100.0%	4 / 4 100.0%	2 / 2 100.0%	1 / 1 100.0%	2 / 2 100.0%	1 / 1 100.0%	3 / 3 100.0%	2 / 2 100.0%	3 / 3 100.0%	5 / 5 100.0%	2 / 2 100.0%	1 / 1 100.0%	5 / 5 100.0%	0 / 0 0.0%	0 / 0 0.0%	0 / 0 0.0%	0 / 0 0.0%	34 / 53 64.2%AB	19 / 53 35.8%ABE				
1	Veillonella	48 / 48 100.0%	1 / 1 100.0%	2 / 2 100.0%	3 / 3 100.0%	2 / 2 100.0%	5 / 5 100.0%	3 / 3 100.0%	1 / 1 100.0%	1 / 1 100.0%	2 / 2 100.0%	1 / 1 100.0%	4 / 4 100.0%	3 / 3 100.0%	2 / 2 100.0%	1 / 1 100.0%	1 / 1 100.0%	1 / 1 100.0%	1 / 1 100.0%	2 / 2 100.0%	4 / 4 100.0%	3 / 3 100.0%	1 / 1 100.0%	4 / 4 100.0%	0 / 0 0.0%	0 / 0 0.0%	0 / 0 0.0%	0 / 0 0.0%	33 / 48 68.8%AB	15 / 48 31.2%ABE				
1	"Micrococcus gazogenes alcalescens anaerobius" Lewkowicz 1901	48 / 48 100.0%	1 / 1 100.0%	2 / 2 100.0%	3 / 3 100.0%	2 / 2 100.0%	5 / 5 100.0%	3 / 3 100.0%	1 / 1 100.0%	1 / 1 100.0%	2 / 2 100.0%	1 / 1 100.0%	4 / 4 100.0%	3 / 3 100.0%	2 / 2 100.0%	1 / 1 100.0%	1 / 1 100.0%	1 / 1 100.0%	1 / 1 100.0%	2 / 2 100.0%	4 / 4 100.0%	3 / 3 100.0%	1 / 1 100.0%	4 / 4 100.0%	0 / 0 0.0%	0 / 0 0.0%	0 / 0 0.0%	0 / 0 0.0%	33 / 48 68.8%AB	15 / 48 31.2%ABE				
1	Veillonella parvula DSM 2008	48 / 48 100.0%	1 / 1 100.0%	2 / 2 100.0%	3 / 3 100.0%	2 / 2 100.0%	5 / 5 100.0%	3 / 3 100.0%	1 / 1 100.0%	1 / 1 100.0%	2 / 2 100.0%	1 / 1 100.0%	4 / 4 100.0%	3 / 3 100.0%	2 / 2 100.0%	1 / 1 100.0%	1 / 1 100.0%	1 / 1 100.0%	1 / 1 100.0%	2 / 2 100.0%	4 / 4 100.0%	3 / 3 100.0%	1 / 1 100.0%	4 / 4 100.0%	0 / 0 0.0%	0 / 0 0.0%	0 / 0 0.0%	0 / 0 0.0%	33 / 48 68.8%AB	15 / 48 31.2%ABE				
2	Acidaminococcaceae	112 / 112 100.0%	2 / 2 100.0%	5 / 5 100.0%	4 / 4 100.0%	2 / 2 100.0%	11 / 11 100.0%	8 / 8 100.0%	2 / 2 100.0%	2 / 2 100.0%	6 / 6 100.0%	3 / 3 100.0%	9 / 9 100.0%	6 / 6 100.0%	3 / 3 100.0%	5 / 5 100.0%	3 / 3 100.0%	6 / 6 100.0%	2 / 2 100.0%	6 / 6 100.0%	8 / 8 100.0%	6 / 6 100.0%	2 / 2 100.0%	11 / 11 100.0%	0 / 0 0.0%	0 / 0 0.0%	0 / 0 0.0%	0 / 0 0.0%	78 / 112 69.6%AB	34 / 112 30.4%ABE				
2	Acidaminococcus	112 / 112 100.0%	2 / 2 100.0%	5 / 5 100.0%	4 / 4 100.0%	2 / 2 100.0%	11 / 11 100.0%	8 / 8 100.0%	2 / 2 100.0%	2 / 2 100.0%	6 / 6 100.0%	3 / 3 100.0%	9 / 9 100.0%	6 / 6 100.0%	3 / 3 100.0%	5 / 5 100.0%	3 / 3 100.0%	6 / 6 100.0%	2 / 2 100.0%	6 / 6 100.0%	8 / 8 100.0%	6 / 6 100.0%	2 / 2 100.0%	11 / 11 100.0%	0 / 0 0.0%	0 / 0 0.0%	0 / 0 0.0%	0 / 0 0.0%	78 / 112 69.6%AB	34 / 112 30.4%ABE				
1	ATCC 25085	60 / 60 100.0%	1 / 1 100.0%	3 / 3 100.0%	2 / 2 100.0%	2 / 2 100.0%	6 / 6 100.0%	4 / 4 100.0%	1 / 1 100.0%	1 / 1 100.0%	4 / 4 100.0%	1 / 1 100.0%	4 / 4 100.0%	3 / 3 100.0%	2 / 2 100.0%	3 / 3 100.0%	2 / 2 100.0%	3 / 3 100.0%	1 / 1 100.0%	3 / 3 100.0%	4 / 4 100.0%	3 / 3 100.0%	1 / 1 100.0%	6 / 6 100.0%	0 / 0 0.0%	0 / 0 0.0%	0 / 0 0.0%	0 / 0 0.0%	43 / 60 71.7%AB	17 / 60 28.3%ABE				
1	Acidaminococcus fermentans DSM 20731	60 / 60 100.0%	1 / 1 100.0%	3 / 3 100.0%	2 / 2 100.0%	2 / 2 100.0%	6 / 6 100.0%	4 / 4 100.0%	1 / 1 100.0%	1 / 1 100.0%	4 / 4 100.0%	1 / 1 100.0%	4 / 4 100.0%	3 / 3 100.0%	2 / 2 100.0%	3 / 3 100.0%	2 / 2 100.0%	3 / 3 100.0%	1 / 1 100.0%	3 / 3 100.0%	4 / 4 100.0%	3 / 3 100.0%	1 / 1 100.0%	6 / 6 100.0%	0 / 0 0.0%	0 / 0 0.0%	0 / 0 0.0%	0 / 0 0.0%	43 / 60 71.7%AB	17 / 60 28.3%ABE				
1	AIP 283.01	52 / 52 100.0%	1 / 1 100.0%	2 / 2 100.0%	2 / 2 100.0%	0 / 0 0.0%	5 / 5 100.0%	4 / 4 100.0%	1 / 1 100.0%	1 / 1 100.0%	2 / 2 100.0%	2 / 2 100.0%	5 / 5 100.0%	3 / 3 100.0%	1 / 1 100.0%	2 / 2 100.0%	1 / 1 100.0%	3 / 3 100.0%	1 / 1 100.0%	3 / 3 100.0%	4 / 4 100.0%	3 / 3 100.0%	1 / 1 100.0%	5 / 5 100.0%	0 / 0 0.0%	0 / 0 0.0%	0 / 0 0.0%	0 / 0 0.0%	35 / 52 67.3%AB	17 / 52 32.7%ABE				
1	'Acidaminococcus intestini str. RyC-MR95	52 / 52 100.0%	1 / 1 100.0%	2 / 2 100.0%	2 / 2 100.0%	0 / 0 0.0%	5 / 5 100.0%	4 / 4 100.0%	1 / 1 100.0%	1 / 1 100.0%	2 / 2 100.0%	2 / 2 100.0%	5 / 5 100.0%	3 / 3 100.0%	1 / 1 100.0%	2 / 2 100.0%	1 / 1 100.0%	3 / 3 100.0%	1 / 1 100.0%	3 / 3 100.0%	4 / 4 100.0%	3 / 3 100.0%	1 / 1 100.0%	5 / 5 100.0%	0 / 0 0.0%	0 / 0 0.0%	0 / 0 0.0%	0 / 0 0.0%	35 / 52 67.3%AB	17 / 52 32.7%ABE				
19	'Deinococcus-Thermus'	986 / 998 98.8%	24 / 24 100.0%	36 / 36 100.0%	21 / 21 100.0%	24 / 24 100.0%	83 / 90 92.2%	64 / 64 100.0%	21 / 21 100.0%	24 / 25 96.0%	63 / 63 100.0%	21 / 21 100.0%	80 / 84 95.2%	42 / 42 100.0%	21 / 21 100.0%	42 / 42 100.0%	21 / 21 100.0%	63 / 63 100.0%	20 / 20 100.0%	63 / 63 100.0%	85 / 85 100.0%	42 / 42 100.0%	20 / 20 100.0%	106 / 106 100.0%	0 / 0 0.0%	0 / 0 0.0%	0 / 0 0.0%	0 / 0 0.0%	623 / 998 62.4%AB	363 / 998 36.4%ABE	7 / 998 0.7%	4 / 998 0.4%E	1 / 998 0.1%CE	
19	"Hadobacteria" Cavalier-Smith 1992 emend. Cavalier-Smith 1998	986 / 998 98.8%	24 / 24 100.0%	36 / 36 100.0%	21 / 21 100.0%	24 / 24 100.0%	83 / 90 92.2%	64 / 64 100.0%	21 / 21 100.0%	24 / 25 96.0%	63 / 63 100.0%	21 / 21 100.0%	80 / 84 95.2%	42 / 42 100.0%	21 / 21 100.0%	42 / 42 100.0%	21 / 21 100.0%	63 / 63 100.0%	20 / 20 100.0%	63 / 63 100.0%	85 / 85 100.0%	42 / 42 100.0%	20 / 20 100.0%	106 / 106 100.0%	0 / 0 0.0%	0 / 0 0.0%	0 / 0 0.0%	0 / 0 0.0%	623 / 998 62.4%AB	363 / 998 36.4%ABE	7 / 998 0.7%	4 / 998 0.4%E	1 / 998 0.1%CE	
11	Thermales	518 / 522 99.2%	14 / 14 100.0%	13 / 13 100.0%	11 / 11 100.0%	14 / 14 100.0%	43 / 43 100.0%	34 / 34 100.0%	11 / 11 100.0%	11 / 11 100.0%	33 / 33 100.0%	11 / 11 100.0%	40 / 44 90.9%	22 / 22 100.0%	11 / 11 100.0%	22 / 22 100.0%	11 / 11 100.0%	33 / 33 100.0%	11 / 11 100.0%	33 / 33 100.0%	44 / 44 100.0%	29 / 29 100.0%	11 / 11 100.0%	56 / 56 100.0%	0 / 0 0.0%	0 / 0 0.0%	0 / 0 0.0%	0 / 0 0.0%	314 / 522 60.2%AB	204 / 522 39.1%ABE	4 / 522 0.8%E			
11	Thermaceae	518 / 522 99.2%	14 / 14 100.0%	13 / 13 100.0%	11 / 11 100.0%	14 / 14 100.0%	43 / 43 100.0%	34 / 34 100.0%	11 / 11 100.0%	11 / 11 100.0%	33 / 33 100.0%	11 / 11 100.0%	40 / 44 90.9%	22 / 22 100.0%	11 / 11 100.0%	22 / 22 100.0%	11 / 11 100.0%	33 / 33 100.0%	11 / 11 100.0%	33 / 33 100.0%	44 / 44 100.0%	29 / 29 100.0%	11 / 11 100.0%	56 / 56 100.0%	0 / 0 0.0%	0 / 0 0.0%	0 / 0 0.0%	0 / 0 0.0%	314 / 522 60.2%AB	204 / 522 39.1%ABE	4 / 522 0.8%E			
7	Thermus	328 / 332 98.8%	7 / 7 100.0%	7 / 7 100.0%	7 / 7 100.0%	10 / 10 100.0%	28 / 28 100.0%	21 / 21 100.0%	7 / 7 100.0%	7 / 7 100.0%	21 / 21 100.0%	7 / 7 100.0%	24 / 28 85.7%	14 / 14 100.0%	7 / 7 100.0%	14 / 14 100.0%	7 / 7 100.0%	21 / 21 100.0%	7 / 7 100.0%	21 / 21 100.0%	28 / 28 100.0%	21 / 21 100.0%	7 / 7 100.0%	35 / 35 100.0%	0 / 0 0.0%	0 / 0 0.0%	0 / 0 0.0%	0 / 0 0.0%	199 / 332 59.9%AB	129 / 332 38.9%ABE	4 / 332 1.2%E			
4	"Flavobacterium thermophilum" Yoshida and Oshima 1971	186 / 188 98.9%	4 / 4 100.0%	4 / 4 100.0%	4 / 4 100.0%	4 / 4 100.0%	16 / 16 100.0%	12 / 12 100.0%	4 / 4 100.0%	4 / 4 100.0%	12 / 12 100.0%	4 / 4 100.0%	14 / 16 87.5%	8 / 8 100.0%	4 / 4 100.0%	8 / 8 100.0%	4 / 4 100.0%	12 / 12 100.0%	4 / 4 100.0%	12 / 12 100.0%	16 / 16 100.0%	12 / 12 100.0%	4 / 4 100.0%	20 / 20 100.0%	0 / 0 0.0%	0 / 0 0.0%	0 / 0 0.0%	0 / 0 0.0%	112 / 188 59.6%AB	74 / 188 39.4%ABE	2 / 188 1.1%E			
1	Thermus thermophilus HB27	47 / 47 100.0%	1 / 1 100.0%	1 / 1 100.0%	1 / 1 100.0%	1 / 1 100.0%	4 / 4 100.0%	3 / 3 100.0%	1 / 1 100.0%	1 / 1 100.0%	3 / 3 100.0%	1 / 1 100.0%	4 / 4 100.0%	2 / 2 100.0%	1 / 1 100.0%	2 / 2 100.0%	1 / 1 100.0%	3 / 3 100.0%	1 / 1 100.0%	3 / 3 100.0%	4 / 4 100.0%	3 / 3 100.0%	1 / 1 100.0%	5 / 5 100.0%	0 / 0 0.0%	0 / 0 0.0%	0 / 0 0.0%	0 / 0 0.0%	28 / 47 59.6%AB	19 / 47 40.4%ABE				
1	Thermus thermophilus ATCC 27634	47 / 47 100.0%	1 / 1 100.0%	1 / 1 100.0%	1 / 1 100.0%	1 / 1 100.0%	4 / 4 100.0%	3 / 3 100.0%	1 / 1 100.0%	1 / 1 100.0%	3 / 3 100.0%	1 / 1 100.0%	4 / 4 100.0%	2 / 2 100.0%	1 / 1 100.0%	2 / 2 100.0%	1 / 1 100.0%	3 / 3 100.0%	1 / 1 100.0%	3 / 3 100.0%	4 / 4 100.0%	3 / 3 100.0%	1 / 1 100.0%	5 / 5 100.0%	0 / 0 0.0%	0 / 0 0.0%	0 / 0 0.0%	0 / 0 0.0%	28 / 47 59.6%AB	19 / 47 40.4%ABE				
1	Thermus thermophilus SG0.5JP17-16	46 / 47 97.9%	1 / 1 100.0%	1 / 1 100.0%	1 / 1 100.0%	1 / 1 100.0%	4 / 4 100.0%	3 / 3 100.0%	1 / 1 100.0%	1 / 1 100.0%	3 / 3 100.0%	1 / 1 100.0%	3 / 4 75.0%	2 / 2 100.0%	1 / 1 100.0%	2 / 2 100.0%	1 / 1 100.0%	3 / 3 100.0%	1 / 1 100.0%	3 / 3 100.0%	4 / 4 100.0%	3 / 3 100.0%	1 / 1 100.0%	5 / 5 100.0%	0 / 0 0.0%	0 / 0 0.0%	0 / 0 0.0%	0 / 0 0.0%	28 / 47 59.6%AB	18 / 47 38.3%ABE	1 / 47 2.1%E			
1	Thermus thermophilus JL-18	46 / 47 97.9%	1 / 1 100.0%	1 / 1 100.0%	1 / 1 100.0%	1 / 1 100.0%	4 / 4 100.0%	3 / 3 100.0%	1 / 1 100.0%	1 / 1 100.0%	3 / 3 100.0%	1 / 1 100.0%	3 / 4 75.0%	2 / 2 100.0%	1 / 1 100.0%	2 / 2 100.0%	1 / 1 100.0%	3 / 3 100.0%	1 / 1 100.0%	3 / 3 100.0%	4 / 4 100.0%	3 / 3 100.0%	1 / 1 100.0%	5 / 5 100.0%	0 / 0 0.0%	0 / 0 0.0%	0 / 0 0.0%	0 / 0 0.0%	28 / 47 59.6%AB	18 / 47 38.3%ABE	1 / 47 2.1%E			
1	ATCC 51532	46 / 47 97.9%	1 / 1 100.0%	1 / 1 100.0%	1 / 1 100.0%	1 / 1 100.0%	4 / 4 100.0%	3 / 3 100.0%	1 / 1 100.0%	1 / 1 100.0%	3 / 3 100.0%	1 / 1 100.0%	3 / 4 75.0%	2 / 2 100.0%	1 / 1 100.0%	2 / 2 100.0%	1 / 1 100.0%	3 / 3 100.0%	1 / 1 100.0%	3 / 3 100.0%	4 / 4 100.0%	3 / 3 100.0%	1 / 1 100.0%	5 / 5 100.0%	0 / 0 0.0%	0 / 0 0.0%	0 / 0 0.0%	0 / 0 0.0%	28 / 47 59.6%AB	18 / 47 38.3%ABE	1 / 47 2.1%E			
1	Thermus scotoductus SA-01	46 / 47 97.9%	1 / 1 100.0%	1 / 1 100.0%	1 / 1 100.0%	1 / 1 100.0%	4 / 4 100.0%	3 / 3 100.0%	1 / 1 100.0%	1 / 1 100.0%	3 / 3 100.0%	1 / 1 100.0%	3 / 4 75.0%	2 / 2 100.0%	1 / 1 100.0%	2 / 2 100.0%	1 / 1 100.0%	3 / 3 100.0%	1 / 1 100.0%	3 / 3 100.0%	4 / 4 100.0%	3 / 3 100.0%	1 / 1 100.0%	5 / 5 100.0%	0 / 0 0.0%	0 / 0 0.0%	0 / 0 0.0%	0 / 0 0.0%	28 / 47 59.6%AB	18 / 47 38.3%ABE	1 / 47 2.1%E			
1	ATCC 700435	50 / 50 100.0%	1 / 1 100.0%	1 / 1 100.0%	1 / 1 100.0%	4 / 4 100.0%	4 / 4 100.0%	3 / 3 100.0%	1 / 1 100.0%	1 / 1 100.0%	3 / 3 100.0%	1 / 1 100.0%	4 / 4 100.0%	2 / 2 100.0%	1 / 1 100.0%	2 / 2 100.0%	1 / 1 100.0%	3 / 3 100.0%	1 / 1 100.0%	3 / 3 100.0%	4 / 4 100.0%	3 / 3 100.0%	1 / 1 100.0%	5 / 5 100.0%	0 / 0 0.0%	0 / 0 0.0%	0 / 0 0.0%	0 / 0 0.0%	31 / 50 62.0%AB	19 / 50 38.0%ABE				
1	Thermus oshimai JL-2	50 / 50 100.0%	1 / 1 100.0%	1 / 1 100.0%	1 / 1 100.0%	4 / 4 100.0%	4 / 4 100.0%	3 / 3 100.0%	1 / 1 100.0%	1 / 1 100.0%	3 / 3 100.0%	1 / 1 100.0%	4 / 4 100.0%	2 / 2 100.0%	1 / 1 100.0%	2 / 2 100.0%	1 / 1 100.0%	3 / 3 100.0%	1 / 1 100.0%	3 / 3 100.0%	4 / 4 100.0%	3 / 3 100.0%	1 / 1 100.0%	5 / 5 100.0%	0 / 0 0.0%	0 / 0 0.0%	0 / 0 0.0%	0 / 0 0.0%	31 / 50 62.0%AB	19 / 50 38.0%ABE				

1	Thermus sp. CCB_US3_UF1	46 / 47 97.9%	1 / 1 100.0%	1 / 1 100.0%	1 / 1 100.0%	1 / 1 100.0%	4 / 4 100.0%	3 / 3 100.0%	1 / 1 100.0%	1 / 1 100.0%	3 / 3 100.0%	1 / 1 100.0%	3 / 4 75.0%	2 / 2 100.0%	1 / 1 100.0%	2 / 2 100.0%	1 / 1 100.0%	3 / 3 100.0%	1 / 1 100.0%	3 / 3 100.0%	4 / 4 100.0%	3 / 3 100.0%	1 / 1 100.0%	5 / 5 100.0%	0 / 0 0.0%	0 / 0 0.0%	0 / 0 0.0%	0 / 0 0.0%	28 / 47 59.6%AB	18 / 47 38.3%ABE	1 / 47 2.1%E			
2	Meiothermus	94 / 94 100.0%	4 / 4 100.0%	2 / 2 100.0%	2 / 2 100.0%	2 / 2 100.0%	8 / 8 100.0%	6 / 6 100.0%	2 / 2 100.0%	2 / 2 100.0%	6 / 6 100.0%	2 / 2 100.0%	8 / 8 100.0%	4 / 4 100.0%	2 / 2 100.0%	4 / 4 100.0%	2 / 2 100.0%	6 / 6 100.0%	2 / 2 100.0%	6 / 6 100.0%	8 / 8 100.0%	4 / 4 100.0%	2 / 2 100.0%	10 / 10 100.0%	0 / 0 0.0%	0 / 0 0.0%	0 / 0 0.0%	0 / 0 0.0%	57 / 94 60.6%AB	37 / 94 39.4%ABE				
1	"Thermus ruber" Loginova et al. 1975	47 / 47 100.0%	2 / 2 100.0%	1 / 1 100.0%	1 / 1 100.0%	1 / 1 100.0%	4 / 4 100.0%	3 / 3 100.0%	1 / 1 100.0%	1 / 1 100.0%	3 / 3 100.0%	1 / 1 100.0%	4 / 4 100.0%	2 / 2 100.0%	1 / 1 100.0%	2 / 2 100.0%	1 / 1 100.0%	3 / 3 100.0%	1 / 1 100.0%	3 / 3 100.0%	4 / 4 100.0%	2 / 2 100.0%	1 / 1 100.0%	5 / 5 100.0%	0 / 0 0.0%	0 / 0 0.0%	0 / 0 0.0%	0 / 0 0.0%	28 / 47 59.6%AB	19 / 47 40.4%ABE				
1	Meiothermus ruber ATCC 35948	47 / 47 100.0%	2 / 2 100.0%	1 / 1 100.0%	1 / 1 100.0%	1 / 1 100.0%	4 / 4 100.0%	3 / 3 100.0%	1 / 1 100.0%	1 / 1 100.0%	3 / 3 100.0%	1 / 1 100.0%	4 / 4 100.0%	2 / 2 100.0%	1 / 1 100.0%	2 / 2 100.0%	1 / 1 100.0%	3 / 3 100.0%	1 / 1 100.0%	3 / 3 100.0%	4 / 4 100.0%	2 / 2 100.0%	1 / 1 100.0%	5 / 5 100.0%	0 / 0 0.0%	0 / 0 0.0%	0 / 0 0.0%	0 / 0 0.0%	28 / 47 59.6%AB	19 / 47 40.4%ABE				
1	ATCC 700542	47 / 47 100.0%	2 / 2 100.0%	1 / 1 100.0%	1 / 1 100.0%	1 / 1 100.0%	4 / 4 100.0%	3 / 3 100.0%	1 / 1 100.0%	1 / 1 100.0%	3 / 3 100.0%	1 / 1 100.0%	4 / 4 100.0%	2 / 2 100.0%	1 / 1 100.0%	2 / 2 100.0%	1 / 1 100.0%	3 / 3 100.0%	1 / 1 100.0%	3 / 3 100.0%	4 / 4 100.0%	2 / 2 100.0%	1 / 1 100.0%	5 / 5 100.0%	0 / 0 0.0%	0 / 0 0.0%	0 / 0 0.0%	0 / 0 0.0%	29 / 47 61.7%AB	18 / 47 38.3%ABE				
1	Meiothermus silvanus ATCC 700542	47 / 47 100.0%	2 / 2 100.0%	1 / 1 100.0%	1 / 1 100.0%	1 / 1 100.0%	4 / 4 100.0%	3 / 3 100.0%	1 / 1 100.0%	1 / 1 100.0%	3 / 3 100.0%	1 / 1 100.0%	4 / 4 100.0%	2 / 2 100.0%	1 / 1 100.0%	2 / 2 100.0%	1 / 1 100.0%	3 / 3 100.0%	1 / 1 100.0%	3 / 3 100.0%	4 / 4 100.0%	2 / 2 100.0%	1 / 1 100.0%	5 / 5 100.0%	0 / 0 0.0%	0 / 0 0.0%	0 / 0 0.0%	0 / 0 0.0%	29 / 47 61.7%AB	18 / 47 38.3%ABE				
1	Marinithermus	50 / 50 100.0%	2 / 2 100.0%	2 / 2 100.0%	1 / 1 100.0%	1 / 1 100.0%	4 / 4 100.0%	4 / 4 100.0%	1 / 1 100.0%	1 / 1 100.0%	3 / 3 100.0%	1 / 1 100.0%	4 / 4 100.0%	2 / 2 100.0%	1 / 1 100.0%	2 / 2 100.0%	1 / 1 100.0%	3 / 3 100.0%	1 / 1 100.0%	3 / 3 100.0%	4 / 4 100.0%	2 / 2 100.0%	1 / 1 100.0%	6 / 6 100.0%	0 / 0 0.0%	0 / 0 0.0%	0 / 0 0.0%	0 / 0 0.0%	30 / 50 60.0%AB	20 / 50 40.0%ABE				
1	DSM 14884	50 / 50 100.0%	2 / 2 100.0%	2 / 2 100.0%	1 / 1 100.0%	1 / 1 100.0%	4 / 4 100.0%	4 / 4 100.0%	1 / 1 100.0%	1 / 1 100.0%	3 / 3 100.0%	1 / 1 100.0%	4 / 4 100.0%	2 / 2 100.0%	1 / 1 100.0%	2 / 2 100.0%	1 / 1 100.0%	3 / 3 100.0%	1 / 1 100.0%	3 / 3 100.0%	4 / 4 100.0%	2 / 2 100.0%	1 / 1 100.0%	6 / 6 100.0%	0 / 0 0.0%	0 / 0 0.0%	0 / 0 0.0%	0 / 0 0.0%	30 / 50 60.0%AB	20 / 50 40.0%ABE				
1	Marinithermus hydrothermalis DSM 14884	50 / 50 100.0%	2 / 2 100.0%	2 / 2 100.0%	1 / 1 100.0%	1 / 1 100.0%	4 / 4 100.0%	4 / 4 100.0%	1 / 1 100.0%	1 / 1 100.0%	3 / 3 100.0%	1 / 1 100.0%	4 / 4 100.0%	2 / 2 100.0%	1 / 1 100.0%	2 / 2 100.0%	1 / 1 100.0%	3 / 3 100.0%	1 / 1 100.0%	3 / 3 100.0%	4 / 4 100.0%	2 / 2 100.0%	1 / 1 100.0%	6 / 6 100.0%	0 / 0 0.0%	0 / 0 0.0%	0 / 0 0.0%	0 / 0 0.0%	30 / 50 60.0%AB	20 / 50 40.0%ABE				
1	Oceanithermus	46 / 46 100.0%	1 / 1 100.0%	2 / 2 100.0%	1 / 1 100.0%	1 / 1 100.0%	3 / 3 100.0%	3 / 3 100.0%	1 / 1 100.0%	1 / 1 100.0%	3 / 3 100.0%	1 / 1 100.0%	4 / 4 100.0%	2 / 2 100.0%	1 / 1 100.0%	2 / 2 100.0%	1 / 1 100.0%	3 / 3 100.0%	1 / 1 100.0%	3 / 3 100.0%	4 / 4 100.0%	2 / 2 100.0%	1 / 1 100.0%	5 / 5 100.0%	0 / 0 0.0%	0 / 0 0.0%	0 / 0 0.0%	0 / 0 0.0%	28 / 46 60.9%AB	18 / 46 39.1%ABE				
1	DSM 14977	46 / 46 100.0%	1 / 1 100.0%	2 / 2 100.0%	1 / 1 100.0%	1 / 1 100.0%	3 / 3 100.0%	3 / 3 100.0%	1 / 1 100.0%	1 / 1 100.0%	3 / 3 100.0%	1 / 1 100.0%	4 / 4 100.0%	2 / 2 100.0%	1 / 1 100.0%	2 / 2 100.0%	1 / 1 100.0%	3 / 3 100.0%	1 / 1 100.0%	3 / 3 100.0%	4 / 4 100.0%	2 / 2 100.0%	1 / 1 100.0%	5 / 5 100.0%	0 / 0 0.0%	0 / 0 0.0%	0 / 0 0.0%	0 / 0 0.0%	28 / 46 60.9%AB	18 / 46 39.1%ABE				
1	Oceanithermus profundus DSM 14977	46 / 46 100.0%	1 / 1 100.0%	2 / 2 100.0%	1 / 1 100.0%	1 / 1 100.0%	3 / 3 100.0%	3 / 3 100.0%	1 / 1 100.0%	1 / 1 100.0%	3 / 3 100.0%	1 / 1 100.0%	4 / 4 100.0%	2 / 2 100.0%	1 / 1 100.0%	2 / 2 100.0%	1 / 1 100.0%	3 / 3 100.0%	1 / 1 100.0%	3 / 3 100.0%	4 / 4 100.0%	2 / 2 100.0%	1 / 1 100.0%	5 / 5 100.0%	0 / 0 0.0%	0 / 0 0.0%	0 / 0 0.0%	0 / 0 0.0%	28 / 46 60.9%AB	18 / 46 39.1%ABE				
8	Deinococcales	468 / 476 98.3%	10 / 10 100.0%	23 / 23 100.0%	10 / 10 100.0%	10 / 10 100.0%	40 / 47 85.1%	30 / 30 100.0%	10 / 10 100.0%	13 / 14 92.9%	30 / 30 100.0%	10 / 10 100.0%	40 / 40 100.0%	20 / 20 100.0%	10 / 10 100.0%	20 / 20 100.0%	10 / 10 100.0%	30 / 30 100.0%	9 / 9 100.0%	30 / 30 100.0%	41 / 41 100.0%	13 / 13 100.0%	9 / 9 100.0%	50 / 50 100.0%	0 / 0 0.0%	0 / 0 0.0%	0 / 0 0.0%	0 / 0 0.0%	309 / 476 64.9%AB	159 / 476 33.4%ABE	7 / 476 1.5%	1 / 476 0.2%CE		
7	Deinococcaceae	423 / 431 98.1%	9 / 9 100.0%	21 / 21 100.0%	9 / 9 100.0%	9 / 9 100.0%	37 / 44 84.1%	27 / 27 100.0%	9 / 9 100.0%	12 / 13 92.3%	27 / 27 100.0%	9 / 9 100.0%	36 / 36 100.0%	18 / 18 100.0%	9 / 9 100.0%	18 / 18 100.0%	9 / 9 100.0%	27 / 27 100.0%	8 / 8 100.0%	27 / 27 100.0%	37 / 37 100.0%	12 / 12 100.0%	8 / 8 100.0%	45 / 45 100.0%	0 / 0 0.0%	0 / 0 0.0%	0 / 0 0.0%	0 / 0 0.0%	280 / 431 65.0%AB	143 / 431 33.2%ABE	7 / 431 1.6%	1 / 431 0.2%CE		
7	Deinobacter	423 / 431 98.1%	9 / 9 100.0%	21 / 21 100.0%	9 / 9 100.0%	9 / 9 100.0%	37 / 44 84.1%	27 / 27 100.0%	9 / 9 100.0%	12 / 13 92.3%	27 / 27 100.0%	9 / 9 100.0%	36 / 36 100.0%	18 / 18 100.0%	9 / 9 100.0%	18 / 18 100.0%	9 / 9 100.0%	27 / 27 100.0%	8 / 8 100.0%	27 / 27 100.0%	37 / 37 100.0%	12 / 12 100.0%	8 / 8 100.0%	45 / 45 100.0%	0 / 0 0.0%	0 / 0 0.0%	0 / 0 0.0%	0 / 0 0.0%	280 / 431 65.0%AB	143 / 431 33.2%ABE	7 / 431 1.6%	1 / 431 0.2%CE		
1	"Micrococcus radiodurans" Raj et al. 1960	144 / 147 98.0%	3 / 3 100.0%	9 / 9 100.0%	3 / 3 100.0%	3 / 3 100.0%	12 / 15 80.0%	9 / 9 100.0%	3 / 3 100.0%	6 / 6 100.0%	9 / 9 100.0%	3 / 3 100.0%	12 / 12 100.0%	6 / 6 100.0%	3 / 3 100.0%	6 / 6 100.0%	3 / 3 100.0%	9 / 9 100.0%	3 / 3 100.0%	9 / 9 100.0%	12 / 12 100.0%	3 / 3 100.0%	3 / 3 100.0%	15 / 15 100.0%	0 / 0 0.0%	0 / 0 0.0%	0 / 0 0.0%	0 / 0 0.0%	93 / 147 63.3%AB	51 / 147 34.7%ABE	3 / 147 2.0%			
1	Deinococcus radiodurans R1	144 / 147 98.0%	3 / 3 100.0%	9 / 9 100.0%	3 / 3 100.0%	3 / 3 100.0%	12 / 15 80.0%	9 / 9 100.0%	3 / 3 100.0%	6 / 6 100.0%	9 / 9 100.0%	3 / 3 100.0%	12 / 12 100.0%	6 / 6 100.0%	3 / 3 100.0%	6 / 6 100.0%	3 / 3 100.0%	9 / 9 100.0%	3 / 3 100.0%	9 / 9 100.0%	12 / 12 100.0%	3 / 3 100.0%	3 / 3 100.0%	15 / 15 100.0%	0 / 0 0.0%	0 / 0 0.0%	0 / 0 0.0%	0 / 0 0.0%	93 / 147 63.3%AB	51 / 147 34.7%ABE	3 / 147 2.0%			
1	"Micrococcus radioproteolyticus" Kobatake et al. 1973	46 / 46 100.0%	1 / 1 100.0%	2 / 2 100.0%	1 / 1 100.0%	1 / 1 100.0%	4 / 4 100.0%	3 / 3 100.0%	1 / 1 100.0%	1 / 1 100.0%	3 / 3 100.0%	1 / 1 100.0%	4 / 4 100.0%	2 / 2 100.0%	1 / 1 100.0%	2 / 2 100.0%	1 / 1 100.0%	3 / 3 100.0%	1 / 1 100.0%	3 / 3 100.0%	5 / 5 100.0%	1 / 1 100.0%	0 / 0 0.0%	5 / 5 100.0%	0 / 0 0.0%	0 / 0 0.0%	0 / 0 0.0%	0 / 0 0.0%	31 / 46 67.4%AB	15 / 46 32.6%ABE				
1	Deinococcus proteolyticus DSM 20540	46 / 46 100.0%	1 / 1 100.0%	2 / 2 100.0%	1 / 1 100.0%	1 / 1 100.0%	4 / 4 100.0%	3 / 3 100.0%	1 / 1 100.0%	1 / 1 100.0%	3 / 3 100.0%	1 / 1 100.0%	4 / 4 100.0%	2 / 2 100.0%	1 / 1 100.0%	2 / 2 100.0%	1 / 1 100.0%	3 / 3 100.0%	1 / 1 100.0%	3 / 3 100.0%	5 / 5 100.0%	1 / 1 100.0%	0 / 0 0.0%	5 / 5 100.0%	0 / 0 0.0%	0 / 0 0.0%	0 / 0 0.0%	0 / 0 0.0%	31 / 46 67.4%AB	15 / 46 32.6%ABE				
1	CIP 105573	47 / 48 97.9%	1 / 1 100.0%	2 / 2 100.0%	1 / 1 100.0%	1 / 1 100.0%	4 / 5 80.0%	3 / 3 100.0%	1 / 1 100.0%	1 / 1 100.0%	3 / 3 100.0%	1 / 1 100.0%	4 / 4 100.0%	2 / 2 100.0%	1 / 1 100.0%	2 / 2 100.0%	1 / 1 100.0%	3 / 3 100.0%	1 / 1 100.0%	3 / 3 100.0%	4 / 4 100.0%	2 / 2 100.0%	1 / 1 100.0%	5 / 5 100.0%	0 / 0 0.0%	0 / 0 0.0%	0 / 0 0.0%	0 / 0 0.0%	31 / 48 64.6%AB	16 / 48 33.3%ABE	1 / 48 2.1%			
1	Deinococcus geothermalis AG-3a	47 / 48 97.9%	1 / 1 100.0%	2 / 2 100.0%	1 / 1 100.0%	1 / 1 100.0%	4 / 5 80.0%	3 / 3 100.0%	1 / 1 100.0%	1 / 1 100.0%	3 / 3 100.0%	1 / 1 100.0%	4 / 4 100.0%	2 / 2 100.0%	1 / 1 100.0%	2 / 2 100.0%	1 / 1 100.0%	3 / 3 100.0%	1 / 1 100.0%	3 / 3 100.0%	4 / 4 100.0%	2 / 2 100.0%	1 / 1 100.0%	5 / 5 100.0%	0 / 0 0.0%	0 / 0 0.0%	0 / 0 0.0%	0 / 0 0.0%	31 / 48 64.6%AB	16 / 48 33.3%ABE	1 / 48 2.1%			
1	DSM 21211	48 / 50 96.0%	1 / 1 100.0%	2 / 2 100.0%	1 / 1 100.0%	1 / 1 100.0%	4 / 5 80.0%	3 / 3 100.0%	1 / 1 100.0%	1 / 2 50.0%	3 / 3 100.0%	1 / 1 100.0%	4 / 4 100.0%	2 / 2 100.0%	1 / 1 100.0%	2 / 2 100.0%	1 / 1 100.0%	3 / 3 100.0%	1 / 1 100.0%	3 / 3 100.0%	4 / 4 100.0%	3 / 3 100.0%	1 / 1 100.0%	5 / 5 100.0%	0 / 0 0.0%	0 / 0 0.0%	0 / 0 0.0%	0 / 0 0.0%	31 / 50 62.0%AB	17 / 50 34.0%ABE	1 / 50 2.0%CE	1 / 50 2.0%		
1	Deinococcus maricopensis DSM 21211	48 / 50 96.0%	1 / 1 100.0%	2 / 2 100.0%	1 / 1 100.0%	1 / 1 100.0%	4 / 5 80.0%	3 / 3 100.0%	1 / 1 100.0%	1 / 2 50.0%	3 / 3 100.0%	1 / 1 100.0%	4 / 4 100.0%	2 / 2 100.0%	1 / 1 100.0%	2 / 2 100.0%	1 / 1 100.0%	3 / 3 100.0%	1 / 1 100.0%	3 / 3 100.0%	4 / 4 100.0%	3 / 3 100.0%	1 / 1 100.0%	5 / 5 100.0%	0 / 0 0.0%	0 / 0 0.0%	0 / 0 0.0%	0 / 0 0.0%	31 / 50 62.0%AB	17 / 50 34.0%ABE	1 / 50 2.0%	1 / 50 2.0%CE		
1	DSM 17065	46 / 47 97.9%	1 / 1 100.0%	2 / 2 100.0%	1 / 1 100.0%	1 / 1 100.0%	4 / 5 80.0%	3 / 3 100.0%	1 / 1 100.0%	1 / 1 100.0%	3 / 3 100.0%	1 / 1 100.0%	4 / 4 100.0%	2 / 2 100.0%	1 / 1 100.0%	2 / 2 100.0%	1 / 1 100.0%	3 / 3 100.0%	1 / 1 100.0%	3 / 3 100.0%	4 / 4 100.0%	1 / 1 100.0%	1 / 1 100.0%	5 / 5 100.0%	0 / 0 0.0%	0 / 0 0.0%	0 / 0 0.0%	0 / 0 0.0%	32 / 47 68.1%AB	14 / 47 29.8%ABE	1 / 47 2.1%			
1	Deinococcus deserti VCD115	46 / 47 97.9%	1 / 1 100.0%	2 / 2 100.0%	1 / 1 100.0%	1 / 1 100.0%	4 / 5 80.0%	3 / 3 100.0%	1 / 1 100.0%	1 / 1 100.0%	3 / 3 100.0%	1 / 1 100.0%	4 / 4 100.0%	2 / 2 100.0%	1 / 1 100.0%	2 / 2 100.0%	1 / 1 100.0%	3 / 3 100.0%	1 / 1 100.0%	3 / 3 100.0%	4 / 4 100.0%	1 / 1 100.0%	1 / 1 100.0%	5 / 5 100.0%	0 / 0 0.0%	0 / 0 0.0%	0 / 0 0.0%	0 / 0 0.0%	32 / 47 68.1%AB	14 / 47 29.8%ABE	1 / 47 2.1%			
1	CIP 109416	47 / 47 100.0%	1 / 1 100.0%	2 / 2 100.0%	1 / 1 100.0%	1 / 1 100.0%	5 / 5 100.0%	3 / 3 100.0%	1 / 1 100.0%	1 / 1 100.0%	3 / 3 100.0%	1 / 1 100.0%	4 / 4 100.0%	2 / 2 100.0%	1 / 1 100.0%	2 / 2 100.0%	1 / 1 100.0%	3 / 3 100.0%	1 / 1 100.0%	3 / 3 100.0%	4 / 4 100.0%	1 / 1 100.0%	1 / 1 100.0%	5 / 5 100.0%	0 / 0 0.0%	0 / 0 0.0%	0 / 0 0.0%	0 / 0 0.0%	32 / 47 68.1%AB	15 / 47 31.9%ABE				
1	Deinococcus peraridilitoris DSM 19664	47 / 47 100.0%	1 / 1 100.0%	2 / 2 100.0%	1 / 1 100.0%	1 / 1 100.0%	5 / 5 100.0%	3 / 3 100.0%	1 / 1 100.0%	1 / 1 100.0%	3 / 3 100.0%	1 / 1 100.0%	4 / 4 100.0%	2 / 2 100.0%	1 / 1 100.0%	2 / 2 100.0%	1 / 1 100.0%	3 / 3 100.0%	1 / 1 100.0%	3 / 3 100.0%	4 / 4 100.0%	1 / 1 100.0%	1 / 1 100.0%	5 / 5 100.0%	0 / 0 0.0%	0 / 0 0.0%	0 / 0 0.0%	0 / 0 0.0%	32 / 47 68.1%AB	15 / 47 31.9%ABE				
1	CGMCC 1.7299	45 / 46 97.8%	1 / 1 100.0%	2 / 2 100.0%	1 / 1 100.0%	1 / 1 100.0%	4 / 5 80.0%	3 / 3 100.0%	1 / 1 100.0%	1 / 1 100.0%	3 / 3 100.0%	1 / 1 100.0%	4 / 4 100.0%	2 / 2 100.0%	1 / 1 100.0%	2 / 2 100.0%	1 / 1 100.0%	3 / 3 100.0%	0 / 0 0.0%	3 / 3 100.0%	4 / 4 100.0%	1 / 1 100.0%	1 / 1 100.0%	5 / 5 100.0%	0 / 0 0.0%	0 / 0 0.0%	0 / 0 0.0%	0 / 0 0.0%	30 / 46 65.2%AB	15 / 46 32.6%ABE	1 / 46 2.2%			
1	Deinococcus gobiensis I-0	45 / 46 97.8%	1 / 1 100.0%	2 / 2 100.0%	1 / 1 100.0%	1 / 1 100.0%	4 / 5 80.0%	3 / 3 100.0%	1 / 1 100.0%	1 / 1 100.0%	3 / 3 100.0%	1 / 1 100.0%	4 / 4 100.0%	2 / 2 100.0%	1 / 1 100.0%	2 / 2 100.0%	1 / 1 100.0%	3 / 3 100.0%	0 / 0 0.0%	3 / 3 100.0%	4 / 4 100.0%	1 / 1 100.0%	1 / 1 100.0%	5 / 5 100.0%	0 / 0 0.0%	0 / 0 0.0%	0 / 0 0.0%	0 / 0 0.0%	30 / 46 65.2%AB	15 / 46 32.6%ABE	1 / 46 2.2%			
1	Trueperaceae	45 / 45 100.0%	1 / 1 100.0%	2 / 2 100.0%	1 / 1 100.0%	1 / 1 100.0%	3 / 3 100.0%	3 / 3 100.0%	1 / 1 100.0%	1 / 1 100.0%	3 / 3 100.0%	1 / 1 100.0%	4 / 4 100.0%	2 / 2 100.0%	1 / 1 100.0%	2 / 2 100.0%	1 / 1 100.0%	3 / 3 100.0%	1 / 1 100.0%	3 / 3 100.0%	4 / 4 100.0%	1 / 1 100.0%	1 / 1 100.0%	5 / 5 100.0%	0 / 0 0.0%	0 / 0 0.0%	0 / 0 0.0%	0 / 0 0.0%	29 / 45 64.4%AB	16 / 45 35.6%ABE				
1	Truepera	45 / 45 100.0%	1 / 1 100.0%	2 / 2 100.0%	1 / 1 100.0%	1 / 1 100.0%	3 / 3 100.0%	3 / 3 100.0%	1 / 1 100.0%	1 / 1 100.0%	3 / 3 100.0%	1 / 1 100.0%	4 / 4 100.0%	2 / 2 100.0%	1 / 1 100.0%	2 / 2 100.0%	1 / 1 100.0%	3 / 3 100.0%	1 / 1 100.0%	3 / 3 100.0%	4 / 4 100.0%	1 / 1 100.0%	1 / 1 100.0%	5 / 5 100.0%	0 / 0 0.0%	0 / 0 0.0%	0 / 0 0.0%	0 / 0 0.0%	29 / 45 64.4%AB	16 / 45 35.6%ABE				
1	CIP 108686	45 / 45 100.0%	1 / 1 100.0%	2 / 2 100.0%	1 / 1 100.0%	1 / 1 100.0%	3 / 3 100.0%	3 / 3 100.0%	1 / 1 100.0%	1 / 1 100.0%	3 / 3 100.0%	1 / 1 100.0%	4 / 4 100.0%	2 / 2 100.0%	1 / 1 100.0%	2 / 2 100.0%	1 / 1 100.0%	3 / 3 100.0%	1 / 1 100.0%	3 / 3 100.0%	4 / 4 100.0%	1 / 1 100.0%	1 / 1 100.0%	5 / 5 100.0%	0 / 0 0.0%	0 / 0 0.0%	0 / 0 0.0%	0 / 0 0.0%	29 / 45 64.4%AB	16 / 45 35.6%ABE				
1	Truepera radiovictrix DSM 17093	45 / 45 100.0%	1 / 1 100.0%	2 / 2 100.0%	1 / 1 100.0%	1 / 1 100.0%	3 / 3 100.0%	3 / 3 100.0%	1 / 1 100.0%	1 / 1 100.0%	3 / 3 100.0%	1 / 1 100.0%	4 / 4 100.0%	2 / 2 100.0%	1 / 1 100.0%	2 / 2 100.0%	1 / 1 100.0%	3 / 3 100.0%	1 / 1 100.0%	3 / 3 100.0%	4 / 4 100.0%	1 / 1 100.0%	1 / 1 100.0%	5 / 5 100.0%	0 / 0 0.0%	0 / 0 0.0%	0 / 0 0.0%	0 / 0 0.0%	29 / 45 64.4%AB	16 / 45 35.6%ABE				
3	Not yet classified (Prokaryotae)	117 / 143 81.8%	2 / 3 66.7%	2 / 3 66.7%	2 / 3 66.7%	2 / 4 50.0%	9 / 10 90.0%	6 / 9 66.7%	3 / 3 100.0%	2 / 3 66.7%	7 / 9 77.8%	3 / 3 100.0%	9 / 12 75.0%	6 / 6 100.0%	3 / 3 100.0%	6 / 6 100.0%	3 / 3 100.0%	8 / 10 80.0%	3 / 3 100.0%	8 / 9 88.9%	13 / 15 86.7%	5 / 6 83.3%	2 / 3 66.7%	11 / 15 73.3%	2 / 2 100.0%	0 / 0 0.0%	0 / 0 0.0%	0 / 0 0.0%	66 / 143 46.2%AB	51 / 143 35.7%ABE	10 / 143 7.0%C	9 / 143 6.3%CE	4 / 143 2.8%E	3 / 143 2.1%
1	"Candidatus Saccharibacteria" Albertsen et al. 2013	45 / 45 100.0%	1 / 1 100.0%	1 / 1 100.0%	1 / 1 100.0%	1 / 1 100.0%	4 / 4 100.0%	2 / 2 100.0%	1 / 1 100.0%	1 / 1 100.0%	2 / 2 100.0%	1 / 1 100.0%	4 / 4 100.0%	2 / 2 100.0%	1 / 1 100.0%	2 / 2 100.0%	1 / 1 100.0%	3 / 3 100.0%	1 / 1 100.0%	3 / 3 100.0%	5 / 5 100.0%	2 / 2 100.0%	1 / 1 100.0%	5 / 5 100.0%	0 / 0 0.0%	0 / 0 0.0%	0 / 0 0.0%	0 / 0 0.0%	23 / 45 51.1%AB	22 / 45 48.9%ABE				
1	"Candidatus Saccharimonas" Albertsen et al. 2013	45 / 45 100.0%	1 / 1 100.0%	1 / 1 100.0%	1 / 1 100.0%	1 / 1 100.0%	4 / 4 100.0%	2 / 2 100.0%	1 / 1 100.0%	1 / 1 100.0%	2 / 2 100.0%	1 / 1 100.0%	4 / 4 100.0%	2 / 2 100.0%	1 / 1 100.0%	2 / 2 100.0%	1 / 1 100.0%	3 / 3 100.0%	1 / 1 100.0%	3 / 3 100.0%	5 / 5 100.0%	2 / 2 100.0%	1 / 1 100.0%	5 / 5 100.0%	0 / 0 0.0%	0 / 0 0.0%	0 / 0 0.0%	0 / 0 0.0%	23 / 45 51.1%AB	22 / 45 48.9%ABE				
1	"Candidatus Saccharimonas aalborgensis" Albertsen et al. 2013	45 / 45 100.0%	1 / 1 100.0%	1 / 1 100.0%	1 / 1 100.0%	1 / 1 100.0%	4 / 4 100.0%	2 / 2 100.0%	1 / 1 100.0%	1 / 1 100.0%	2 / 2 100.0%	1 / 1 100.0%	4 / 4 100.0%	2 / 2 100.0%	1 / 1 100.0%	2 / 2 100.0%	1 / 1 100.0%	3 / 3 100.0%	1 / 1 100.0%	3 / 3 100.0%	5 / 5 100.0%	2 / 2 100.0%	1 / 1 100.0%	5 / 5 100.0%	0 / 0 0.0%	0 / 0 0.0%	0 / 0 0.0%	0 / 0 0.0%	23 / 45 51.1%AB	22 / 45 48.9%ABE				
1	"Thermobaculum" Boterl et al. 2004	26 / 51 51.0%	1 / 1 100.0%	0 / 1 0.0%	0 / 1 0.0%	0 / 2 0.0%	2 / 3 66.7%	1 / 4 25.0%	1 / 1 100.0%	0 / 1 0.0%	2 / 4 50.0%	1 / 1 100.0%	1 / 4 25.0%	2 / 2 100.0%	1 / 1 100.0%	2 / 2 100.0%	1 / 1 100.0%	2 / 4 50.0%	1 / 1 100.0%	2 / 3 66.7%	3 / 5 60.0%	1 / 2 50.0%	0 / 1 0.0%	1 / 5 20.0%	1 / 1 100.0%	0 / 0 0.0%	0 / 0 0.0%	0 / 0 0.0%	14 / 51 27.5%AB	12 / 51 23.5%ABE	10 / 51 19.6%C	9 / 51 17.6%CE	3 / 51 5.9%	3 / 51 5.9%E
1	"Thermobaculum terrenum" Botero et al. 2004	26 / 51 51.0%	1 / 1 100.0%	0 / 1 0.0%	0 / 1 0.0%	0 / 2 0.0%	2 / 3 66.7%	1 / 4 25.0%	1 / 1 100.0%	0 / 1 0.0%	2 / 4 50.0%	1 / 1 100.0%	1 / 4 25.0%	2 / 2 100.0%	1 / 1 100.0%	2 / 2 100.0%	1 / 1 100.0%	2 / 4 50.0%	1 / 1 100.0%	2 / 3 66.7%	3 / 5 60.0%	1 / 2 50.0%	0 / 1 0.0%	1 / 5 20.0%	1 / 1 100.0%	0 / 0 0.0%	0 / 0 0.0%	0 / 0 0.0%	14 / 51 27.5%AB	12 / 51 23.5%ABE	10 / 51 19.6%C	9 / 51 17.6%CE	3 / 51 5.9%	3 / 51 5.9%E
1	Thermobaculum terrenum ATCC BAA-798	26 / 51 51.0%	1 / 1 100.0%	0 / 1 0.0%	0 / 1 0.0%	0 / 2 0.0%	2 / 3 66.7%	1 / 4 25.0%	1 / 1 100.0%	0 / 1 0.0%	2 / 4 50.0%	1 / 1 100.0%	1 / 4 25.0%	2 / 2 100.0%	1 / 1 100.0%	2 / 2 100.0%	1 / 1 100.0%	2 / 4 50.0%	1 / 1 100.0%	2 / 3 66.7%	3 / 5 60.0%	1 / 2 50.0%	0 / 1 0.0%	1 / 5 20.0%	1 / 1 100.0%	0 / 0 0.0%	0 / 0 0.0%	0 / 0 0.0%	14 / 51 27.5%AB	12 / 51 23.5%ABE	10 / 51 19.6%C	9 / 51 17.6%CE	3 / 51 5.9%	3 / 51 5.9%E
1	NC10 phylum bacteria	46 / 47 97.9%	0 / 1 0.0%	1 / 1 100.0%	1 / 1 100.0%	1 / 1 100.0%	3 / 3 100.0%	3 / 3 100.0%	1 / 1 100.0%	1 / 1 100.0%	3 / 3 100.0%	1 / 1 100.0%	4 / 4 100.0%	2 / 2 100.0%	1 / 1 100.0%	2 / 2 100.0%	1 / 1 100.0%	3 / 3 100.0%	1 / 1 100.0%	3 / 3 100.0%	5 / 5 100.0%	2 / 2 100.0%	1 / 1 100.0%	5 / 5 100.0%	1 / 1 100.0%	0 / 0 0.0%	0 / 0 0.0%	0 / 0 0.0%	29 / 47 61.7%AB	17 / 47 36.2%ABE	1 / 47 2.1%E			
1	"Candidatus Methylomirabilis" Ettwig et al. 2010	46 / 47 97.9%	0 / 1 0.0%	1 / 1 100.0%	1 / 1 100.0%	1 / 1 100.0%	3 / 3 100.0%	3 / 3 100.0%	1 / 1 100.0%	1 / 1 100.0%	3 / 3 100.0%	1 / 1 100.0%	4 / 4 100.0%	2 / 2 100.0%	1 / 1 100.0%	2 / 2 100.0%	1 / 1 100.0%	3 / 3 100.0%	1 / 1 100.0%	3 / 3 100.0%	5 / 5 100.0%	2 / 2 100.0%	1 / 1 100.0%	5 / 5 100.0%	1 / 1 100.0%	0 / 0 0.0%	0 / 0 0.0%	0 / 0 0.0%	29 / 47 61.7%AB	17 / 47 36.2%ABE	1 / 47 2.1%E			
1	"Candidatus Methylomirabilis oxyfera" Ettwig et al. 2010	46 / 47 97.9%	0 / 1 0.0%	1 / 1 100.0%	1 / 1 100.0%	1 / 1 100.0%	3 / 3 100.0%	3 / 3 100.0%	1 / 1 100.0%	1 / 1 100.0%	3 / 3 100.0%	1 / 1 100.0%	4 / 4 100.0%	2 / 2 100.0%	1 / 1 100.0%	2 / 2 100.0%	1 / 1 100.0%	3 / 3 100.0%	1 / 1 100.0%	3 / 3 100.0%	5 / 5 100.0%	2 / 2 100.0%	1 / 1 100.0%	5 / 5 100.0%	1 / 1 100.0%	0 / 0 0.0%	0 / 0 0.0%	0 / 0 0.0%	29 / 47 61.7%AB	17 / 47 36.2%ABE	1 / 47 2.1%E			
6	Fusobacteria	263 / 263 100.0%	8 / 8 100.0%	14 / 14 100.0%	17 / 17 100.0%	11 / 11 100.0%	19 / 19 100.0%	14 / 14 100.0%	6 / 6 100.0%	6 / 6 100.0%	13 / 13 100.0%	6 / 6 100.0%	17 / 17 100.0%	16 / 16 100.0%	11 / 11 100.0%	8 / 8 100.0%	6 / 6 100.0%	9 / 9 100.0%	6 / 6 100.0%	10 / 10 100.0%	20 / 20 100.0%	14 / 14 100.0%	6 / 6 100.0%	26 / 26 100.0%	0 / 0 0.0%	0 / 0 0.0%	0 / 0 0.0%	0 / 0 0.0%	187 / 263 71.1%AB	76 / 263 28.9%ABE				
6	Fusobacteria	263 / 263 100.0%	8 / 8 100.0%	14 / 14 100.0%	17 / 17 100.0%	11 / 11 100.0%	19 / 19 100.0%	14 / 14 100.0%	6 / 6 100.0%	6 / 6 100.0%	13 / 13 100.0%	6 / 6 100.0%	17 / 17 100.0%	16 / 16 100.0%	11 / 11 100.0%	8 / 8 100.0%	6 / 6 100.0%	9 / 9 100.0%	6 / 6 100.0%	10 / 10 100.0%	20 / 20 100.0%	14 / 14 100.0%	6 / 6 100.0%	26 / 26 100.0%	0 / 0 0.0%	0 / 0 0.0%	0 / 0 0.0%	0 / 0 0.0%	187 / 263 71.1%AB	76 / 263 28.9%ABE				
6	"Fusobacteriales" Staley and Whitman 2011	263 / 263 100.0%	8 / 8 100.0%	14 / 14 100.0%	17 / 17 100.0%	11 / 11 100.0%	19 / 19 100.0%	14 / 14 100.0%	6 / 6 100.0%	6 / 6 100.0%	13 / 13 100.0%	6 / 6 100.0%	17 / 17 100.0%	16 / 16 100.0%	11 / 11 100.0%	8 / 8 100.0%	6 / 6 100.0%	9 / 9 100.0%	6 / 6 100.0%	10 / 10 100.0%	20 / 20 100.0%	14 / 14 100.0%	6 / 6 100.0%	26 / 26 100.0%	0 / 0 0.0%	0 / 0 0.0%	0 / 0 0.0%	0 / 0 0.0%	187 / 263 71.1%AB	76 / 263 28.9%ABE				
3	"Fusobacteriaceae" Staley and Whitman 2011	138 / 138 100.0%	4 / 4 100.0%	8 / 8 100.0%	7 / 7 100.0%	5 / 5 100.0%	11 / 11 100.0%	8 / 8 100.0%	3 / 3 100.0%	3 / 3 100.0%	7 / 7 100.0%	3 / 3 100.0%	9 / 9 100.0%	8 / 8 100.0%	6 / 6 100.0%	5 / 5 100.0%	3 / 3 100.0%	6 / 6 100.0%	3 / 3 100.0%	5 / 5 100.0%	12 / 12 100.0%	8 / 8 100.0%	3 / 3 100.0%	11 / 11 100.0%	0 / 0 0.0%	0 / 0 0.0%	0 / 0 0.0%	0 / 0 0.0%	99 / 138 71.7%AB	39 / 138 28.3%ABE				
2	Fusibacterium	93 / 93 100.0%	2 / 2 100.0%	6 / 6 100.0%	4 / 4 100.0%	2 / 2 100.0%	8 / 8 100.0%	6 / 6 100.0%	2 / 2 100.0%	2 / 2 100.0%	4 / 4 100.0%	2 / 2 100.0%	6 / 6 100.0%	6 / 6 100.0%	4 / 4 100.0%	4 / 4 100.0%	2 / 2 100.0%	4 / 4 100.0%	2 / 2 100.0%	4 / 4 100.0%	8 / 8 100.0%	6 / 6 100.0%	2 / 2 100.0%	7 / 7 100.0%	0 / 0 0.0%	0 / 0 0.0%	0 / 0 0.0%	0 / 0 0.0%	66 / 93 71.0%AB	27 / 93 29.0%ABE				
2	"Bacillus fusiformis" Veillon and Zuber 1898	93 / 93 100.0%	2 / 2 100.0%	6 / 6 100.0%	4 / 4 100.0%	2 / 2 100.0%	8 / 8 100.0%	6 / 6 100.0%	2 / 2 100.0%	2 / 2 100.0%	4 / 4 100.0%	2 / 2 100.0%	6 / 6 100.0%	6 / 6 100.0%	4 / 4 100.0%	4 / 4 100.0%	2 / 2 100.0%	4 / 4 100.0%	2 / 2 100.0%	4 / 4 100.0%	8 / 8 100.0%	6 / 6 100.0%	2 / 2 100.0%	7 / 7 100.0%	0 / 0 0.0%	0 / 0 0.0%	0 / 0 0.0%	0 / 0 0.0%	66 / 93 71.0%AB	27 / 93 29.0%ABE				
1	Fusobacterium nucleatum subsp. nucleatum	47 / 47 100.0%	1 / 1 100.0%	3 / 3 100.0%	2 / 2 100.0%	1 / 1 100.0%	4 / 4 100.0%	3 / 3 100.0%	1 / 1 100.0%	1 / 1 100.0%	2 / 2 100.0%	1 / 1 100.0%	3 / 3 100.0%	3 / 3 100.0%	2 / 2 100.0%	2 / 2 100.0%	1 / 1 100.0%	2 / 2 100.0%	1 / 1 100.0%	2 / 2 100.0%	4 / 4 100.0%	3 / 3 100.0%	1 / 1 100.0%	4 / 4 100.0%	0 / 0 0.0%	0 / 0 0.0%	0 / 0 0.0%	0 / 0 0.0%	33 / 47 70.2%AB	14 / 47 29.8%ABE				
1	Fusobacterium nucleatum subsp. nucleatum ATCC 25586	47 / 47 100.0%	1 / 1 100.0%	3 / 3 100.0%	2 / 2 100.0%	1 / 1 100.0%	4 / 4 100.0%	3 / 3 100.0%	1 / 1 100.0%	1 / 1 100.0%	2 / 2 100.0%	1 / 1 100.0%	3 / 3 100.0%	3 / 3 100.0%	2 / 2 100.0%	2 / 2 100.0%	1 / 1 100.0%	2 / 2 100.0%	1 / 1 100.0%	2 / 2 100.0%	4 / 4 100.0%	3 / 3 100.0%	1 / 1 100.0%	4 / 4 100.0%	0 / 0 0.0%	0 / 0 0.0%	0 / 0 0.0%	0 / 0 0.0%	33 / 47 70.2%AB	14 / 47 29.8%ABE				
1	ATCC 51191	46 / 46 100.0%	1 / 1 100.0%	3 / 3 100.0%	2 / 2 100.0%	1 / 1 100.0%	4 / 4 100.0%	3 / 3 100.0%	1 / 1 100.0%	1 / 1 100.0%	2 / 2 100.0%	1 / 1 100.0%	3 / 3 100.0%	3 / 3 100.0%	2 / 2 100.0%	2 / 2 100.0%	1 / 1 100.0%	2 / 2 100.0%	1 / 1 100.0%	2 / 2 100.0%	4 / 4 100.0%	3 / 3 100.0%	1 / 1 100.0%	3 / 3 100.0%	0 / 0 0.0%	0 / 0 0.0%	0 / 0 0.0%	0 / 0 0.0%	33 / 46 71.7%AB	13 / 46 28.3%ABE				
1	Fusobacterium nucleatum subsp. animalis 4_8	46 / 46 100.0%	1 / 1 100.0%	3 / 3 100.0%	2 / 2 100.0%	1 / 1 100.0%	4 / 4 100.0%	3 / 3 100.0%	1 / 1 100.0%	1 / 1 100.0%	2 / 2 100.0%	1 / 1 100.0%	3 / 3 100.0%	3 / 3 100.0%	2 / 2 100.0%	2 / 2 100.0%	1 / 1 100.0%	2 / 2 100.0%	1 / 1 100.0%	2 / 2 100.0%	4 / 4 100.0%	3 / 3 100.0%	1 / 1 100.0%	3 / 3 100.0%	0 / 0 0.0%	0 / 0 0.0%	0 / 0 0.0%	0 / 0 0.0%	33 / 46 71.7%AB	13 / 46 28.3%ABE				
1	Ilyobacter	45 / 45 100.0%	2 / 2 100.0%	2 / 2 100.0%	3 / 3 100.0%	3 / 3 100.0%	3 / 3 100.0%	2 / 2 100.0%	1 / 1 100.0%	1 / 1 100.0%	3 / 3 100.0%	1 / 1 100.0%	3 / 3 100.0%	2 / 2 100.0%	2 / 2 100.0%	1 / 1 100.0%	1 / 1 100.0%	2 / 2 100.0%	1 / 1 100.0%	1 / 1 100.0%	4 / 4 100.0%	2 / 2 100.0%	1 / 1 100.0%	4 / 4 100.0%	0 / 0 0.0%	0 / 0 0.0%	0 / 0 0.0%	0 / 0 0.0%	33 / 45 73.3%AB	12 / 45 26.7%ABE				
1	ATCC 51220	45 / 45 100.0%	2 / 2 100.0%	2 / 2 100.0%	3 / 3 100.0%	3 / 3 100.0%	3 / 3 100.0%	2 / 2 100.0%	1 / 1 100.0%	1 / 1 100.0%	3 / 3 100.0%	1 / 1 100.0%	3 / 3 100.0%	2 / 2 100.0%	2 / 2 100.0%	1 / 1 100.0%	1 / 1 100.0%	2 / 2 100.0%	1 / 1 100.0%	1 / 1 100.0%	4 / 4 100.0%	2 / 2 100.0%	1 / 1 100.0%	4 / 4 100.0%	0 / 0 0.0%	0 / 0 0.0%	0 / 0 0.0%	0 / 0 0.0%	33 / 45 73.3%AB	12 / 45 26.7%ABE				
1	Ilyobacter polytropus DSM 2926	45 / 45 100.0%	2 / 2 100.0%	2 / 2 100.0%	3 / 3 100.0%	3 / 3 100.0%	3 / 3 100.0%	2 / 2 100.0%	1 / 1 100.0%	1 / 1 100.0%	3 / 3 100.0%	1 / 1 100.0%	3 / 3 100.0%	2 / 2 100.0%	2 / 2 100.0%	1 / 1 100.0%	1 / 1 100.0%	2 / 2 100.0%	1 / 1 100.0%	1 / 1 100.0%	4 / 4 100.0%	2 / 2 100.0%	1 / 1 100.0%	4 / 4 100.0%	0 / 0 0.0%	0 / 0 0.0%	0 / 0 0.0%	0 / 0 0.0%	33 / 45 73.3%AB	12 / 45 26.7%ABE				
3	"Leptotrichiaceae" Staley and Whitman 2011	125 / 125 100.0%	4 / 4 100.0%	6 / 6 100.0%	10 / 10 100.0%	6 / 6 100.0%	8 / 8 100.0%	6 / 6 100.0%	3 / 3 100.0%	3 / 3 100.0%	6 / 6 100.0%	3 / 3 100.0%	8 / 8 100.0%	8 / 8 100.0%	5 / 5 100.0%	3 / 3 100.0%	3 / 3 100.0%	3 / 3 100.0%	3 / 3 100.0%	5 / 5 100.0%	8 / 8 100.0%	6 / 6 100.0%	3 / 3 100.0%	15 / 15 100.0%	0 / 0 0.0%	0 / 0 0.0%	0 / 0 0.0%	0 / 0 0.0%	88 / 125 70.4%AB	37 / 125 29.6%ABE				
1	Leptotrichia	46 / 46 100.0%	1 / 1 100.0%	2 / 2 100.0%	5 / 5 100.0%	2 / 2 100.0%	2 / 2 100.0%	2 / 2 100.0%	1 / 1 100.0%	1 / 1 100.0%	2 / 2 100.0%	1 / 1 100.0%	4 / 4 100.0%	3 / 3 100.0%	2 / 2 100.0%	1 / 1 100.0%	1 / 1 100.0%	1 / 1 100.0%	1 / 1 100.0%	2 / 2 100.0%	3 / 3 100.0%	2 / 2 100.0%	1 / 1 100.0%	6 / 6 100.0%	0 / 0 0.0%	0 / 0 0.0%	0 / 0 0.0%	0 / 0 0.0%	31 / 46 67.4%AB	15 / 46 32.6%ABE				
1	"Leptothrix buccalis" Robin 1853	46 / 46 100.0%	1 / 1 100.0%	2 / 2 100.0%	5 / 5 100.0%	2 / 2 100.0%	2 / 2 100.0%	2 / 2 100.0%	1 / 1 100.0%	1 / 1 100.0%	2 / 2 100.0%	1 / 1 100.0%	4 / 4 100.0%	3 / 3 100.0%	2 / 2 100.0%	1 / 1 100.0%	1 / 1 100.0%	1 / 1 100.0%	1 / 1 100.0%	2 / 2 100.0%	3 / 3 100.0%	2 / 2 100.0%	1 / 1 100.0%	6 / 6 100.0%	0 / 0 0.0%	0 / 0 0.0%	0 / 0 0.0%	0 / 0 0.0%	31 / 46 67.4%AB	15 / 46 32.6%ABE				
1	Leptotrichia buccalis C-1013-b	46 / 46 100.0%	1 / 1 100.0%	2 / 2 100.0%	5 / 5 100.0%	2 / 2 100.0%	2 / 2 100.0%	2 / 2 100.0%	1 / 1 100.0%	1 / 1 100.0%	2 / 2 100.0%	1 / 1 100.0%	4 / 4 100.0%	3 / 3 100.0%	2 / 2 100.0%	1 / 1 100.0%	1 / 1 100.0%	1 / 1 100.0%	1 / 1 100.0%	2 / 2 100.0%	3 / 3 100.0%	2 / 2 100.0%	1 / 1 100.0%	6 / 6 100.0%	0 / 0 0.0%	0 / 0 0.0%	0 / 0 0.0%	0 / 0 0.0%	31 / 46 67.4%AB	15 / 46 32.6%ABE				
1	Sebaldella	40 / 40 100.0%	2 / 2 100.0%	2 / 2 100.0%	2 / 2 100.0%	2 / 2 100.0%	3 / 3 100.0%	2 / 2 100.0%	1 / 1 100.0%	1 / 1 100.0%	2 / 2 100.0%	1 / 1 100.0%	2 / 2 100.0%	2 / 2 100.0%	2 / 2 100.0%	1 / 1 100.0%	1 / 1 100.0%	1 / 1 100.0%	1 / 1 100.0%	1 / 1 100.0%	3 / 3 100.0%	2 / 2 100.0%	1 / 1 100.0%	5 / 5 100.0%	0 / 0 0.0%	0 / 0 0.0%	0 / 0 0.0%	0 / 0 0.0%	27 / 40 67.5%AB	13 / 40 32.5%ABE				
1	"Sphaerophorus siccus var. termitidis" Sebald 1962	40 / 40 100.0%	2 / 2 100.0%	2 / 2 100.0%	2 / 2 100.0%	2 / 2 100.0%	3 / 3 100.0%	2 / 2 100.0%	1 / 1 100.0%	1 / 1 100.0%	2 / 2 100.0%	1 / 1 100.0%	2 / 2 100.0%	2 / 2 100.0%	2 / 2 100.0%	1 / 1 100.0%	1 / 1 100.0%	1 / 1 100.0%	1 / 1 100.0%	1 / 1 100.0%	3 / 3 100.0%	2 / 2 100.0%	1 / 1 100.0%	5 / 5 100.0%	0 / 0 0.0%	0 / 0 0.0%	0 / 0 0.0%	0 / 0 0.0%	27 / 40 67.5%AB	13 / 40 32.5%ABE				
1	Sebaldella termitidis ATCC 33386	40 / 40 100.0%	2 / 2 100.0%	2 / 2 100.0%	2 / 2 100.0%	2 / 2 100.0%	3 / 3 100.0%	2 / 2 100.0%	1 / 1 100.0%	1 / 1 100.0%	2 / 2 100.0%	1 / 1 100.0%	2 / 2 100.0%	2 / 2 100.0%	2 / 2 100.0%	1 / 1 100.0%	1 / 1 100.0%	1 / 1 100.0%	1 / 1 100.0%	1 / 1 100.0%	3 / 3 100.0%	2 / 2 100.0%	1 / 1 100.0%	5 / 5 100.0%	0 / 0 0.0%	0 / 0 0.0%	0 / 0 0.0%	0 / 0 0.0%	27 / 40 67.5%AB	13 / 40 32.5%ABE				
1	"Haverhillia" Parker and Hudson 1926	39 / 39 100.0%	1 / 1 100.0%	2 / 2 100.0%	3 / 3 100.0%	2 / 2 100.0%	3 / 3 100.0%	2 / 2 100.0%	1 / 1 100.0%	1 / 1 100.0%	2 / 2 100.0%	1 / 1 100.0%	2 / 2 100.0%	3 / 3 100.0%	1 / 1 100.0%	1 / 1 100.0%	1 / 1 100.0%	1 / 1 100.0%	1 / 1 100.0%	2 / 2 100.0%	2 / 2 100.0%	2 / 2 100.0%	1 / 1 100.0%	4 / 4 100.0%	0 / 0 0.0%	0 / 0 0.0%	0 / 0 0.0%	0 / 0 0.0%	30 / 39 76.9%AB	9 / 39 23.1%ABE				
1	"Actinobacillus muris" (de Mello and Pais 1918) Wilson and Miles 1955	39 / 39 100.0%	1 / 1 100.0%	2 / 2 100.0%	3 / 3 100.0%	2 / 2 100.0%	3 / 3 100.0%	2 / 2 100.0%	1 / 1 100.0%	1 / 1 100.0%	2 / 2 100.0%	1 / 1 100.0%	2 / 2 100.0%	3 / 3 100.0%	1 / 1 100.0%	1 / 1 100.0%	1 / 1 100.0%	1 / 1 100.0%	1 / 1 100.0%	2 / 2 100.0%	2 / 2 100.0%	2 / 2 100.0%	1 / 1 100.0%	4 / 4 100.0%	0 / 0 0.0%	0 / 0 0.0%	0 / 0 0.0%	0 / 0 0.0%	30 / 39 76.9%AB	9 / 39 23.1%ABE				
1	Streptobacillus moniliformis ATCC 14647	39 / 39 100.0%	1 / 1 100.0%	2 / 2 100.0%	3 / 3 100.0%	2 / 2 100.0%	3 / 3 100.0%	2 / 2 100.0%	1 / 1 100.0%	1 / 1 100.0%	2 / 2 100.0%	1 / 1 100.0%	2 / 2 100.0%	3 / 3 100.0%	1 / 1 100.0%	1 / 1 100.0%	1 / 1 100.0%	1 / 1 100.0%	1 / 1 100.0%	2 / 2 100.0%	2 / 2 100.0%	2 / 2 100.0%	1 / 1 100.0%	4 / 4 100.0%	0 / 0 0.0%	0 / 0 0.0%	0 / 0 0.0%	0 / 0 0.0%	30 / 39 76.9%AB	9 / 39 23.1%ABE				
4	'Nitrospirae'	148 / 193 76.7%	3 / 4 75.0%	3 / 4 75.0%	3 / 4 75.0%	6 / 7 85.7%	9 / 12 75.0%	9 / 12 75.0%	4 / 4 100.0%	3 / 4 75.0%	13 / 15 86.7%	3 / 4 75.0%	13 / 18 72.2%	6 / 8 75.0%	3 / 4 75.0%	6 / 8 75.0%	3 / 4 75.0%	9 / 13 69.2%	3 / 4 75.0%	9 / 12 75.0%	15 / 19 78.9%	6 / 8 75.0%	3 / 4 75.0%	15 / 20 75.0%	1 / 1 100.0%	0 / 0 0.0%	0 / 0 0.0%	0 / 0 0.0%	95 / 193 49.2%AB	53 / 193 27.5%ABE	26 / 193 13.5%E	19 / 193 9.8%		
4	Nitrospira	148 / 193 76.7%	3 / 4 75.0%	3 / 4 75.0%	3 / 4 75.0%	6 / 7 85.7%	9 / 12 75.0%	9 / 12 75.0%	4 / 4 100.0%	3 / 4 75.0%	13 / 15 86.7%	3 / 4 75.0%	13 / 18 72.2%	6 / 8 75.0%	3 / 4 75.0%	6 / 8 75.0%	3 / 4 75.0%	9 / 13 69.2%	3 / 4 75.0%	9 / 12 75.0%	15 / 19 78.9%	6 / 8 75.0%	3 / 4 75.0%	15 / 20 75.0%	1 / 1 100.0%	0 / 0 0.0%	0 / 0 0.0%	0 / 0 0.0%	95 / 193 49.2%AB	53 / 193 27.5%ABE	26 / 193 13.5%E	19 / 193 9.8%		
4	'Nitrospirales'	148 / 193 76.7%	3 / 4 75.0%	3 / 4 75.0%	3 / 4 75.0%	6 / 7 85.7%	9 / 12 75.0%	9 / 12 75.0%	4 / 4 100.0%	3 / 4 75.0%	13 / 15 86.7%	3 / 4 75.0%	13 / 18 72.2%	6 / 8 75.0%	3 / 4 75.0%	6 / 8 75.0%	3 / 4 75.0%	9 / 13 69.2%	3 / 4 75.0%	9 / 12 75.0%	15 / 19 78.9%	6 / 8 75.0%	3 / 4 75.0%	15 / 20 75.0%	1 / 1 100.0%	0 / 0 0.0%	0 / 0 0.0%	0 / 0 0.0%	95 / 193 49.2%AB	53 / 193 27.5%ABE	26 / 193 13.5%E	19 / 193 9.8%		
4	'Nitrospiraceae'	148 / 193 76.7%	3 / 4 75.0%	3 / 4 75.0%	3 / 4 75.0%	6 / 7 85.7%	9 / 12 75.0%	9 / 12 75.0%	4 / 4 100.0%	3 / 4 75.0%	13 / 15 86.7%	3 / 4 75.0%	13 / 18 72.2%	6 / 8 75.0%	3 / 4 75.0%	6 / 8 75.0%	3 / 4 75.0%	9 / 13 69.2%	3 / 4 75.0%	9 / 12 75.0%	15 / 19 78.9%	6 / 8 75.0%	3 / 4 75.0%	15 / 20 75.0%	1 / 1 100.0%	0 / 0 0.0%	0 / 0 0.0%	0 / 0 0.0%	95 / 193 49.2%AB	53 / 193 27.5%ABE	26 / 193 13.5%E	19 / 193 9.8%		
2	"Leptospirillum" Markosyan 1972	99 / 99 100.0%	2 / 2 100.0%	2 / 2 100.0%	2 / 2 100.0%	5 / 5 100.0%	6 / 6 100.0%	6 / 6 100.0%	2 / 2 100.0%	2 / 2 100.0%	9 / 9 100.0%	2 / 2 100.0%	9 / 9 100.0%	4 / 4 100.0%	2 / 2 100.0%	4 / 4 100.0%	2 / 2 100.0%	6 / 6 100.0%	2 / 2 100.0%	6 / 6 100.0%	10 / 10 100.0%	4 / 4 100.0%	2 / 2 100.0%	10 / 10 100.0%	0 / 0 0.0%	0 / 0 0.0%	0 / 0 0.0%	0 / 0 0.0%	63 / 99 63.6%AB	36 / 99 36.4%ABE				
1	Leptospirillum sp. Group III	48 / 48 100.0%	1 / 1 100.0%	1 / 1 100.0%	1 / 1 100.0%	2 / 2 100.0%	3 / 3 100.0%	3 / 3 100.0%	1 / 1 100.0%	1 / 1 100.0%	4 / 4 100.0%	1 / 1 100.0%	4 / 4 100.0%	2 / 2 100.0%	1 / 1 100.0%	2 / 2 100.0%	1 / 1 100.0%	3 / 3 100.0%	1 / 1 100.0%	3 / 3 100.0%	5 / 5 100.0%	2 / 2 100.0%	1 / 1 100.0%	5 / 5 100.0%	0 / 0 0.0%	0 / 0 0.0%	0 / 0 0.0%	0 / 0 0.0%	30 / 48 62.5%AB	18 / 48 37.5%ABE				
1	ATCC 49881	48 / 48 100.0%	1 / 1 100.0%	1 / 1 100.0%	1 / 1 100.0%	2 / 2 100.0%	3 / 3 100.0%	3 / 3 100.0%	1 / 1 100.0%	1 / 1 100.0%	4 / 4 100.0%	1 / 1 100.0%	4 / 4 100.0%	2 / 2 100.0%	1 / 1 100.0%	2 / 2 100.0%	1 / 1 100.0%	3 / 3 100.0%	1 / 1 100.0%	3 / 3 100.0%	5 / 5 100.0%	2 / 2 100.0%	1 / 1 100.0%	5 / 5 100.0%	0 / 0 0.0%	0 / 0 0.0%	0 / 0 0.0%	0 / 0 0.0%	30 / 48 62.5%AB	18 / 48 37.5%ABE				
1	Leptospirillum ferriphilum ML-04	48 / 48 100.0%	1 / 1 100.0%	1 / 1 100.0%	1 / 1 100.0%	2 / 2 100.0%	3 / 3 100.0%	3 / 3 100.0%	1 / 1 100.0%	1 / 1 100.0%	4 / 4 100.0%	1 / 1 100.0%	4 / 4 100.0%	2 / 2 100.0%	1 / 1 100.0%	2 / 2 100.0%	1 / 1 100.0%	3 / 3 100.0%	1 / 1 100.0%	3 / 3 100.0%	5 / 5 100.0%	2 / 2 100.0%	1 / 1 100.0%	5 / 5 100.0%	0 / 0 0.0%	0 / 0 0.0%	0 / 0 0.0%	0 / 0 0.0%	30 / 48 62.5%AB	18 / 48 37.5%ABE				
1	Leptospirillum sp. Group I	51 / 51 100.0%	1 / 1 100.0%	1 / 1 100.0%	1 / 1 100.0%	3 / 3 100.0%	3 / 3 100.0%	3 / 3 100.0%	1 / 1 100.0%	1 / 1 100.0%	5 / 5 100.0%	1 / 1 100.0%	5 / 5 100.0%	2 / 2 100.0%	1 / 1 100.0%	2 / 2 100.0%	1 / 1 100.0%	3 / 3 100.0%	1 / 1 100.0%	3 / 3 100.0%	5 / 5 100.0%	2 / 2 100.0%	1 / 1 100.0%	5 / 5 100.0%	0 / 0 0.0%	0 / 0 0.0%	0 / 0 0.0%	0 / 0 0.0%	33 / 51 64.7%AB	18 / 51 35.3%ABE				
1	"Leptospirillum ferrooxidans" Markosyan 1972	51 / 51 100.0%	1 / 1 100.0%	1 / 1 100.0%	1 / 1 100.0%	3 / 3 100.0%	3 / 3 100.0%	3 / 3 100.0%	1 / 1 100.0%	1 / 1 100.0%	5 / 5 100.0%	1 / 1 100.0%	5 / 5 100.0%	2 / 2 100.0%	1 / 1 100.0%	2 / 2 100.0%	1 / 1 100.0%	3 / 3 100.0%	1 / 1 100.0%	3 / 3 100.0%	5 / 5 100.0%	2 / 2 100.0%	1 / 1 100.0%	5 / 5 100.0%	0 / 0 0.0%	0 / 0 0.0%	0 / 0 0.0%	0 / 0 0.0%	33 / 51 64.7%AB	18 / 51 35.3%ABE				
1	Leptospirillum ferrooxidans C2-3	51 / 51 100.0%	1 / 1 100.0%	1 / 1 100.0%	1 / 1 100.0%	3 / 3 100.0%	3 / 3 100.0%	3 / 3 100.0%	1 / 1 100.0%	1 / 1 100.0%	5 / 5 100.0%	1 / 1 100.0%	5 / 5 100.0%	2 / 2 100.0%	1 / 1 100.0%	2 / 2 100.0%	1 / 1 100.0%	3 / 3 100.0%	1 / 1 100.0%	3 / 3 100.0%	5 / 5 100.0%	2 / 2 100.0%	1 / 1 100.0%	5 / 5 100.0%	0 / 0 0.0%	0 / 0 0.0%	0 / 0 0.0%	0 / 0 0.0%	33 / 51 64.7%AB	18 / 51 35.3%ABE				
1	Nitrospira	46 / 46 100.0%	1 / 1 100.0%	1 / 1 100.0%	1 / 1 100.0%	1 / 1 100.0%	3 / 3 100.0%	3 / 3 100.0%	1 / 1 100.0%	1 / 1 100.0%	3 / 3 100.0%	1 / 1 100.0%	4 / 4 100.0%	2 / 2 100.0%	1 / 1 100.0%	2 / 2 100.0%	1 / 1 100.0%	3 / 3 100.0%	1 / 1 100.0%	3 / 3 100.0%	5 / 5 100.0%	2 / 2 100.0%	1 / 1 100.0%	5 / 5 100.0%	0 / 0 0.0%	0 / 0 0.0%	0 / 0 0.0%	0 / 0 0.0%	29 / 46 63.0%AB	17 / 46 37.0%ABE				
1	"Candidatus Nitrospira defluvii" Spieck et al. 2006	46 / 46 100.0%	1 / 1 100.0%	1 / 1 100.0%	1 / 1 100.0%	1 / 1 100.0%	3 / 3 100.0%	3 / 3 100.0%	1 / 1 100.0%	1 / 1 100.0%	3 / 3 100.0%	1 / 1 100.0%	4 / 4 100.0%	2 / 2 100.0%	1 / 1 100.0%	2 / 2 100.0%	1 / 1 100.0%	3 / 3 100.0%	1 / 1 100.0%	3 / 3 100.0%	5 / 5 100.0%	2 / 2 100.0%	1 / 1 100.0%	5 / 5 100.0%	0 / 0 0.0%	0 / 0 0.0%	0 / 0 0.0%	0 / 0 0.0%	29 / 46 63.0%AB	17 / 46 37.0%ABE				
1	Thermodesulfovibrio	3 / 48 6.2%	0 / 1 0.0%	0 / 1 0.0%	0 / 1 0.0%	0 / 1 0.0%	0 / 3 0.0%	0 / 3 0.0%	1 / 1 100.0%	0 / 1 0.0%	1 / 3 33.3%	0 / 1 0.0%	0 / 5 0.0%	0 / 2 0.0%	0 / 1 0.0%	0 / 2 0.0%	0 / 1 0.0%	0 / 4 0.0%	0 / 1 0.0%	0 / 3 0.0%	0 / 4 0.0%	0 / 2 0.0%	0 / 1 0.0%	0 / 5 0.0%	1 / 1 100.0%	0 / 0 0.0%	0 / 0 0.0%	0 / 0 0.0%	26 / 48 54.2%E	19 / 48 39.6%	3 / 48 6.2%AB			
1	ATCC 51303	3 / 48 6.2%	0 / 1 0.0%	0 / 1 0.0%	0 / 1 0.0%	0 / 1 0.0%	0 / 3 0.0%	0 / 3 0.0%	1 / 1 100.0%	0 / 1 0.0%	1 / 3 33.3%	0 / 1 0.0%	0 / 5 0.0%	0 / 2 0.0%	0 / 1 0.0%	0 / 2 0.0%	0 / 1 0.0%	0 / 4 0.0%	0 / 1 0.0%	0 / 3 0.0%	0 / 4 0.0%	0 / 2 0.0%	0 / 1 0.0%	0 / 5 0.0%	1 / 1 100.0%	0 / 0 0.0%	0 / 0 0.0%	0 / 0 0.0%	26 / 48 54.2%E	19 / 48 39.6%	3 / 48 6.2%AB			

1	Thermodesulfovibrio yellowstonii DSM 11347	3 / 48 6.2%	0 / 1 0.0%	0 / 1 0.0%	0 / 1 0.0%	0 / 1 0.0%	0 / 3 0.0%	0 / 3 0.0%	1 / 1 100.0%	0 / 1 0.0%	1 / 3 33.3%	0 / 1 0.0%	0 / 5 0.0%	0 / 2 0.0%	0 / 1 0.0%	0 / 2 0.0%	0 / 1 0.0%	0 / 4 0.0%	0 / 1 0.0%	0 / 3 0.0%	0 / 4 0.0%	0 / 2 0.0%	0 / 1 0.0%	0 / 5 0.0%	1 / 1 100.0%	0 / 0 0.0%	0 / 0 0.0%	0 / 0 0.0%	26 / 48 54.2%E	19 / 48 39.6%	3 / 48 6.2%AB				
75	Chlamydiae/Verrucomicrobia group	186 / 2937 6.3%	3 / 78 3.8%	4 / 78 5.1%	5 / 78 6.4%	7 / 80 8.8%	13 / 165 7.9%	9 / 158 5.7%	4 / 77 5.2%	4 / 78 5.1%	11 / 159 6.9%	3 / 77 3.9%	32 / 305 10.5%	7 / 83 8.4%	4 / 78 5.1%	8 / 82 9.8%	4 / 78 5.1%	10 / 159 6.3%	5 / 78 6.4%	9 / 228 3.9%	16 / 270 5.9%	10 / 84 11.9%	2 / 77 2.6%	16 / 387 4.1%	0 / 0 0.0%	0 / 0 0.0%	0 / 0 0.0%	0 / 0 0.0%	1659 / 2937 56.5%E	1046 / 2937 35.6%	71 / 2937 2.4%AB	64 / 2937 2.2%ABE	46 / 2937 1.6%CE	36 / 2937 1.2%B	15 / 2937 0.5%BE
4	Verrucomicrobia	164 / 211 77.7%	3 / 4 75.0%	4 / 5 80.0%	5 / 5 100.0%	7 / 8 87.5%	13 / 16 81.2%	9 / 12 75.0%	3 / 4 75.0%	4 / 5 80.0%	11 / 14 78.6%	3 / 4 75.0%	13 / 16 81.2%	7 / 10 70.0%	4 / 5 80.0%	8 / 9 88.9%	4 / 5 80.0%	10 / 13 76.9%	4 / 5 80.0%	9 / 12 75.0%	15 / 21 71.4%	10 / 11 90.9%	2 / 4 50.0%	16 / 23 69.6%	0 / 0 0.0%	0 / 0 0.0%	0 / 0 0.0%	0 / 0 0.0%	69 / 211 32.7%AB	44 / 211 20.9%ABE	36 / 211 17.1%B	24 / 211 11.4%	21 / 211 10.0%E	15 / 211 7.1%BE	2 / 211 0.9%CE
1	Verrucomicrobiae	52 / 53 98.1%	1 / 1 100.0%	2 / 2 100.0%	1 / 1 100.0%	3 / 3 100.0%	4 / 4 100.0%	3 / 3 100.0%	1 / 1 100.0%	1 / 1 100.0%	5 / 5 100.0%	1 / 1 100.0%	4 / 4 100.0%	3 / 3 100.0%	1 / 1 100.0%	2 / 2 100.0%	1 / 1 100.0%	3 / 3 100.0%	1 / 1 100.0%	3 / 3 100.0%	4 / 4 100.0%	3 / 3 100.0%	1 / 1 100.0%	4 / 5 80.0%	0 / 0 0.0%	0 / 0 0.0%	0 / 0 0.0%	0 / 0 0.0%	20 / 53 37.7%AB	17 / 53 32.1%B	9 / 53 17.0%ABE	6 / 53 11.3%BE	1 / 53 1.9%E		
1	Verrucomicrobiales	52 / 53 98.1%	1 / 1 100.0%	2 / 2 100.0%	1 / 1 100.0%	3 / 3 100.0%	4 / 4 100.0%	3 / 3 100.0%	1 / 1 100.0%	1 / 1 100.0%	5 / 5 100.0%	1 / 1 100.0%	4 / 4 100.0%	3 / 3 100.0%	1 / 1 100.0%	2 / 2 100.0%	1 / 1 100.0%	3 / 3 100.0%	1 / 1 100.0%	3 / 3 100.0%	4 / 4 100.0%	3 / 3 100.0%	1 / 1 100.0%	4 / 5 80.0%	0 / 0 0.0%	0 / 0 0.0%	0 / 0 0.0%	0 / 0 0.0%	20 / 53 37.7%AB	17 / 53 32.1%B	9 / 53 17.0%ABE	6 / 53 11.3%BE	1 / 53 1.9%E		
1	Verrucomicrobia subdivision 1	52 / 53 98.1%	1 / 1 100.0%	2 / 2 100.0%	1 / 1 100.0%	3 / 3 100.0%	4 / 4 100.0%	3 / 3 100.0%	1 / 1 100.0%	1 / 1 100.0%	5 / 5 100.0%	1 / 1 100.0%	4 / 4 100.0%	3 / 3 100.0%	1 / 1 100.0%	2 / 2 100.0%	1 / 1 100.0%	3 / 3 100.0%	1 / 1 100.0%	3 / 3 100.0%	4 / 4 100.0%	3 / 3 100.0%	1 / 1 100.0%	4 / 5 80.0%	0 / 0 0.0%	0 / 0 0.0%	0 / 0 0.0%	0 / 0 0.0%	20 / 53 37.7%AB	17 / 53 32.1%B	9 / 53 17.0%ABE	6 / 53 11.3%BE	1 / 53 1.9%E		
1	Akkermansia	52 / 53 98.1%	1 / 1 100.0%	2 / 2 100.0%	1 / 1 100.0%	3 / 3 100.0%	4 / 4 100.0%	3 / 3 100.0%	1 / 1 100.0%	1 / 1 100.0%	5 / 5 100.0%	1 / 1 100.0%	4 / 4 100.0%	3 / 3 100.0%	1 / 1 100.0%	2 / 2 100.0%	1 / 1 100.0%	3 / 3 100.0%	1 / 1 100.0%	3 / 3 100.0%	4 / 4 100.0%	3 / 3 100.0%	1 / 1 100.0%	4 / 5 80.0%	0 / 0 0.0%	0 / 0 0.0%	0 / 0 0.0%	0 / 0 0.0%	20 / 53 37.7%AB	17 / 53 32.1%B	9 / 53 17.0%ABE	6 / 53 11.3%BE	1 / 53 1.9%E		
1	ATCC BAA-835	52 / 53 98.1%	1 / 1 100.0%	2 / 2 100.0%	1 / 1 100.0%	3 / 3 100.0%	4 / 4 100.0%	3 / 3 100.0%	1 / 1 100.0%	1 / 1 100.0%	5 / 5 100.0%	1 / 1 100.0%	4 / 4 100.0%	3 / 3 100.0%	1 / 1 100.0%	2 / 2 100.0%	1 / 1 100.0%	3 / 3 100.0%	1 / 1 100.0%	3 / 3 100.0%	4 / 4 100.0%	3 / 3 100.0%	1 / 1 100.0%	4 / 5 80.0%	0 / 0 0.0%	0 / 0 0.0%	0 / 0 0.0%	0 / 0 0.0%	20 / 53 37.7%AB	17 / 53 32.1%B	9 / 53 17.0%ABE	6 / 53 11.3%BE	1 / 53 1.9%E		
1	Akkermansia muciniphila ATCC BAA-835	52 / 53 98.1%	1 / 1 100.0%	2 / 2 100.0%	1 / 1 100.0%	3 / 3 100.0%	4 / 4 100.0%	3 / 3 100.0%	1 / 1 100.0%	1 / 1 100.0%	5 / 5 100.0%	1 / 1 100.0%	4 / 4 100.0%	3 / 3 100.0%	1 / 1 100.0%	2 / 2 100.0%	1 / 1 100.0%	3 / 3 100.0%	1 / 1 100.0%	3 / 3 100.0%	4 / 4 100.0%	3 / 3 100.0%	1 / 1 100.0%	4 / 5 80.0%	0 / 0 0.0%	0 / 0 0.0%	0 / 0 0.0%	0 / 0 0.0%	20 / 53 37.7%AB	17 / 53 32.1%B	9 / 53 17.0%ABE	6 / 53 11.3%BE	1 / 53 1.9%E		
1	unclassified Verrucomicrobia	5 / 46 10.9%	0 / 1 0.0%	0 / 1 0.0%	1 / 1 100.0%	0 / 1 0.0%	0 / 3 0.0%	0 / 3 0.0%	0 / 1 0.0%	0 / 1 0.0%	0 / 3 0.0%	0 / 1 0.0%	1 / 4 25.0%	0 / 2 0.0%	0 / 1 0.0%	1 / 2 50.0%	0 / 1 0.0%	0 / 3 0.0%	0 / 1 0.0%	0 / 3 0.0%	1 / 5 20.0%	1 / 2 50.0%	0 / 1 0.0%	0 / 5 0.0%	0 / 0 0.0%	0 / 0 0.0%	0 / 0 0.0%	0 / 0 0.0%	23 / 46 50.0%	16 / 46 34.8%E	2 / 46 4.3%ABE	2 / 46 4.3%CE	1 / 46 2.2%BE	1 / 46 2.2%AB	1 / 46 2.2%B
1	"Methylacidiphilales" Op den Camp 2009	5 / 46 10.9%	0 / 1 0.0%	0 / 1 0.0%	1 / 1 100.0%	0 / 1 0.0%	0 / 3 0.0%	0 / 3 0.0%	0 / 1 0.0%	0 / 1 0.0%	0 / 3 0.0%	0 / 1 0.0%	1 / 4 25.0%	0 / 2 0.0%	0 / 1 0.0%	1 / 2 50.0%	0 / 1 0.0%	0 / 3 0.0%	0 / 1 0.0%	0 / 3 0.0%	1 / 5 20.0%	1 / 2 50.0%	0 / 1 0.0%	0 / 5 0.0%	0 / 0 0.0%	0 / 0 0.0%	0 / 0 0.0%	0 / 0 0.0%	23 / 46 50.0%	16 / 46 34.8%E	2 / 46 4.3%CE	2 / 46 4.3%ABE	1 / 46 2.2%B	1 / 46 2.2%AB	1 / 46 2.2%BE
1	"Methylacidiphilaceae" Op den Camp 2009	5 / 46 10.9%	0 / 1 0.0%	0 / 1 0.0%	1 / 1 100.0%	0 / 1 0.0%	0 / 3 0.0%	0 / 3 0.0%	0 / 1 0.0%	0 / 1 0.0%	0 / 3 0.0%	0 / 1 0.0%	1 / 4 25.0%	0 / 2 0.0%	0 / 1 0.0%	1 / 2 50.0%	0 / 1 0.0%	0 / 3 0.0%	0 / 1 0.0%	0 / 3 0.0%	1 / 5 20.0%	1 / 2 50.0%	0 / 1 0.0%	0 / 5 0.0%	0 / 0 0.0%	0 / 0 0.0%	0 / 0 0.0%	0 / 0 0.0%	23 / 46 50.0%	16 / 46 34.8%E	2 / 46 4.3%CE	2 / 46 4.3%ABE	1 / 46 2.2%B	1 / 46 2.2%AB	1 / 46 2.2%BE
1	"Acidimethylosilex" Pol et al. 2007	5 / 46 10.9%	0 / 1 0.0%	0 / 1 0.0%	1 / 1 100.0%	0 / 1 0.0%	0 / 3 0.0%	0 / 3 0.0%	0 / 1 0.0%	0 / 1 0.0%	0 / 3 0.0%	0 / 1 0.0%	1 / 4 25.0%	0 / 2 0.0%	0 / 1 0.0%	1 / 2 50.0%	0 / 1 0.0%	0 / 3 0.0%	0 / 1 0.0%	0 / 3 0.0%	1 / 5 20.0%	1 / 2 50.0%	0 / 1 0.0%	0 / 5 0.0%	0 / 0 0.0%	0 / 0 0.0%	0 / 0 0.0%	0 / 0 0.0%	23 / 46 50.0%	16 / 46 34.8%E	2 / 46 4.3%CE	2 / 46 4.3%ABE	1 / 46 2.2%B	1 / 46 2.2%AB	1 / 46 2.2%BE
1	"Methylacidiphilum infernorum" Hou et al. 2008	5 / 46 10.9%	0 / 1 0.0%	0 / 1 0.0%	1 / 1 100.0%	0 / 1 0.0%	0 / 3 0.0%	0 / 3 0.0%	0 / 1 0.0%	0 / 1 0.0%	0 / 3 0.0%	0 / 1 0.0%	1 / 4 25.0%	0 / 2 0.0%	0 / 1 0.0%	1 / 2 50.0%	0 / 1 0.0%	0 / 3 0.0%	0 / 1 0.0%	0 / 3 0.0%	1 / 5 20.0%	1 / 2 50.0%	0 / 1 0.0%	0 / 5 0.0%	0 / 0 0.0%	0 / 0 0.0%	0 / 0 0.0%	0 / 0 0.0%	23 / 46 50.0%	16 / 46 34.8%E	2 / 46 4.3%ABE	2 / 46 4.3%CE	1 / 46 2.2%B	1 / 46 2.2%BE	1 / 46 2.2%AB
1	Methylacidiphilum infernorum V4	5 / 46 10.9%	0 / 1 0.0%	0 / 1 0.0%	1 / 1 100.0%	0 / 1 0.0%	0 / 3 0.0%	0 / 3 0.0%	0 / 1 0.0%	0 / 1 0.0%	0 / 3 0.0%	0 / 1 0.0%	1 / 4 25.0%	0 / 2 0.0%	0 / 1 0.0%	1 / 2 50.0%	0 / 1 0.0%	0 / 3 0.0%	0 / 1 0.0%	0 / 3 0.0%	1 / 5 20.0%	1 / 2 50.0%	0 / 1 0.0%	0 / 5 0.0%	0 / 0 0.0%	0 / 0 0.0%	0 / 0 0.0%	0 / 0 0.0%	23 / 46 50.0%	16 / 46 34.8%E	2 / 46 4.3%ABE	2 / 46 4.3%CE	1 / 46 2.2%BE	1 / 46 2.2%AB	1 / 46 2.2%B
2	Opitutae	107 / 112 95.5%	2 / 2 100.0%	2 / 2 100.0%	3 / 3 100.0%	4 / 4 100.0%	9 / 9 100.0%	6 / 6 100.0%	2 / 2 100.0%	3 / 3 100.0%	6 / 6 100.0%	2 / 2 100.0%	8 / 8 100.0%	4 / 5 80.0%	3 / 3 100.0%	5 / 5 100.0%	3 / 3 100.0%	7 / 7 100.0%	3 / 3 100.0%	6 / 6 100.0%	10 / 12 83.3%	6 / 6 100.0%	1 / 2 50.0%	12 / 13 92.3%	0 / 0 0.0%	0 / 0 0.0%	0 / 0 0.0%	0 / 0 0.0%	48 / 112 42.9%AB	33 / 112 29.5%ABE	18 / 112 16.1%B	8 / 112 7.1%BE	4 / 112 3.6%E	1 / 112 0.9%	
1	Opitutales	64 / 66 97.0%	1 / 1 100.0%	1 / 1 100.0%	2 / 2 100.0%	2 / 2 100.0%	4 / 4 100.0%	4 / 4 100.0%	1 / 1 100.0%	2 / 2 100.0%	3 / 3 100.0%	1 / 1 100.0%	4 / 4 100.0%	3 / 3 100.0%	2 / 2 100.0%	4 / 4 100.0%	2 / 2 100.0%	4 / 4 100.0%	2 / 2 100.0%	3 / 3 100.0%	7 / 8 87.5%	4 / 4 100.0%	0 / 1 0.0%	8 / 8 100.0%	0 / 0 0.0%	0 / 0 0.0%	0 / 0 0.0%	0 / 0 0.0%	20 / 66 30.3%ABE	18 / 66 27.3%AB	18 / 66 27.3%B	8 / 66 12.1%BE	2 / 66 3.0%E		
1	Opitutaceae	64 / 66 97.0%	1 / 1 100.0%	1 / 1 100.0%	2 / 2 100.0%	2 / 2 100.0%	4 / 4 100.0%	4 / 4 100.0%	1 / 1 100.0%	2 / 2 100.0%	3 / 3 100.0%	1 / 1 100.0%	4 / 4 100.0%	3 / 3 100.0%	2 / 2 100.0%	4 / 4 100.0%	2 / 2 100.0%	4 / 4 100.0%	2 / 2 100.0%	3 / 3 100.0%	7 / 8 87.5%	4 / 4 100.0%	0 / 1 0.0%	8 / 8 100.0%	0 / 0 0.0%	0 / 0 0.0%	0 / 0 0.0%	0 / 0 0.0%	20 / 66 30.3%ABE	18 / 66 27.3%B	18 / 66 27.3%AB	8 / 66 12.1%BE	2 / 66 3.0%E		
1	Opitutus	64 / 66 97.0%	1 / 1 100.0%	1 / 1 100.0%	2 / 2 100.0%	2 / 2 100.0%	4 / 4 100.0%	4 / 4 100.0%	1 / 1 100.0%	2 / 2 100.0%	3 / 3 100.0%	1 / 1 100.0%	4 / 4 100.0%	3 / 3 100.0%	2 / 2 100.0%	4 / 4 100.0%	2 / 2 100.0%	4 / 4 100.0%	2 / 2 100.0%	3 / 3 100.0%	7 / 8 87.5%	4 / 4 100.0%	0 / 1 0.0%	8 / 8 100.0%	0 / 0 0.0%	0 / 0 0.0%	0 / 0 0.0%	0 / 0 0.0%	20 / 66 30.3%ABE	18 / 66 27.3%B	18 / 66 27.3%AB	8 / 66 12.1%BE	2 / 66 3.0%E		
1	DSM 11246	64 / 66 97.0%	1 / 1 100.0%	1 / 1 100.0%	2 / 2 100.0%	2 / 2 100.0%	4 / 4 100.0%	4 / 4 100.0%	1 / 1 100.0%	2 / 2 100.0%	3 / 3 100.0%	1 / 1 100.0%	4 / 4 100.0%	3 / 3 100.0%	2 / 2 100.0%	4 / 4 100.0%	2 / 2 100.0%	4 / 4 100.0%	2 / 2 100.0%	3 / 3 100.0%	7 / 8 87.5%	4 / 4 100.0%	0 / 1 0.0%	8 / 8 100.0%	0 / 0 0.0%	0 / 0 0.0%	0 / 0 0.0%	0 / 0 0.0%	20 / 66 30.3%ABE	18 / 66 27.3%AB	18 / 66 27.3%B	8 / 66 12.1%BE	2 / 66 3.0%E		
1	Opitutus terrae PB-1	64 / 66 97.0%	1 / 1 100.0%	1 / 1 100.0%	2 / 2 100.0%	2 / 2 100.0%	4 / 4 100.0%	4 / 4 100.0%	1 / 1 100.0%	2 / 2 100.0%	3 / 3 100.0%	1 / 1 100.0%	4 / 4 100.0%	3 / 3 100.0%	2 / 2 100.0%	4 / 4 100.0%	2 / 2 100.0%	4 / 4 100.0%	2 / 2 100.0%	3 / 3 100.0%	7 / 8 87.5%	4 / 4 100.0%	0 / 1 0.0%	8 / 8 100.0%	0 / 0 0.0%	0 / 0 0.0%	0 / 0 0.0%	0 / 0 0.0%	20 / 66 30.3%ABE	18 / 66 27.3%B	18 / 66 27.3%AB	8 / 66 12.1%BE	2 / 66 3.0%E		
1	Puniceicoccales	43 / 46 93.5%	1 / 1 100.0%	1 / 1 100.0%	1 / 1 100.0%	2 / 2 100.0%	5 / 5 100.0%	2 / 2 100.0%	1 / 1 100.0%	1 / 1 100.0%	3 / 3 100.0%	1 / 1 100.0%	4 / 4 100.0%	1 / 2 50.0%	1 / 1 100.0%	1 / 1 100.0%	1 / 1 100.0%	3 / 3 100.0%	1 / 1 100.0%	3 / 3 100.0%	3 / 4 75.0%	2 / 2 100.0%	1 / 1 100.0%	4 / 5 80.0%	0 / 0 0.0%	0 / 0 0.0%	0 / 0 0.0%	0 / 0 0.0%	30 / 46 65.2%AB	13 / 46 28.3%ABE	2 / 46 4.3%E	1 / 46 2.2%			
1	Puniceicoccaceae	43 / 46 93.5%	1 / 1 100.0%	1 / 1 100.0%	1 / 1 100.0%	2 / 2 100.0%	5 / 5 100.0%	2 / 2 100.0%	1 / 1 100.0%	1 / 1 100.0%	3 / 3 100.0%	1 / 1 100.0%	4 / 4 100.0%	1 / 2 50.0%	1 / 1 100.0%	1 / 1 100.0%	1 / 1 100.0%	3 / 3 100.0%	1 / 1 100.0%	3 / 3 100.0%	3 / 4 75.0%	2 / 2 100.0%	1 / 1 100.0%	4 / 5 80.0%	0 / 0 0.0%	0 / 0 0.0%	0 / 0 0.0%	0 / 0 0.0%	30 / 46 65.2%AB	13 / 46 28.3%ABE	2 / 46 4.3%E	1 / 46 2.2%			
1	Coraliomargarita	43 / 46 93.5%	1 / 1 100.0%	1 / 1 100.0%	1 / 1 100.0%	2 / 2 100.0%	5 / 5 100.0%	2 / 2 100.0%	1 / 1 100.0%	1 / 1 100.0%	3 / 3 100.0%	1 / 1 100.0%	4 / 4 100.0%	1 / 2 50.0%	1 / 1 100.0%	1 / 1 100.0%	1 / 1 100.0%	3 / 3 100.0%	1 / 1 100.0%	3 / 3 100.0%	3 / 4 75.0%	2 / 2 100.0%	1 / 1 100.0%	4 / 5 80.0%	0 / 0 0.0%	0 / 0 0.0%	0 / 0 0.0%	0 / 0 0.0%	30 / 46 65.2%AB	13 / 46 28.3%ABE	2 / 46 4.3%E	1 / 46 2.2%			
1	Coraliomargarita akajimensis	43 / 46 93.5%	1 / 1 100.0%	1 / 1 100.0%	1 / 1 100.0%	2 / 2 100.0%	5 / 5 100.0%	2 / 2 100.0%	1 / 1 100.0%	1 / 1 100.0%	3 / 3 100.0%	1 / 1 100.0%	4 / 4 100.0%	1 / 2 50.0%	1 / 1 100.0%	1 / 1 100.0%	1 / 1 100.0%	3 / 3 100.0%	1 / 1 100.0%	3 / 3 100.0%	3 / 4 75.0%	2 / 2 100.0%	1 / 1 100.0%	4 / 5 80.0%	0 / 0 0.0%	0 / 0 0.0%	0 / 0 0.0%	0 / 0 0.0%	30 / 46 65.2%AB	13 / 46 28.3%ABE	2 / 46 4.3%E	1 / 46 2.2%			
1	Coraliomargarita akajimensis DSM 45221	43 / 46 93.5%	1 / 1 100.0%	1 / 1 100.0%	1 / 1 100.0%	2 / 2 100.0%	5 / 5 100.0%	2 / 2 100.0%	1 / 1 100.0%	1 / 1 100.0%	3 / 3 100.0%	1 / 1 100.0%	4 / 4 100.0%	1 / 2 50.0%	1 / 1 100.0%	1 / 1 100.0%	1 / 1 100.0%	3 / 3 100.0%	1 / 1 100.0%	3 / 3 100.0%	3 / 4 75.0%	2 / 2 100.0%	1 / 1 100.0%	4 / 5 80.0%	0 / 0 0.0%	0 / 0 0.0%	0 / 0 0.0%	0 / 0 0.0%	30 / 46 65.2%AB	13 / 46 28.3%ABE	2 / 46 4.3%E	1 / 46 2.2%			
71	'Chlamydiae'	22 / 2726 0.8%	0 / 74 0.0%	0 / 73 0.0%	0 / 73 0.0%	0 / 72 0.0%	0 / 149 0.0%	0 / 146 0.0%	1 / 73 1.4%	0 / 73 0.0%	0 / 145 0.0%	0 / 73 0.0%	19 / 289 6.6%	0 / 73 0.0%	0 / 73 0.0%	0 / 73 0.0%	0 / 73 0.0%	0 / 146 0.0%	1 / 73 1.4%	0 / 216 0.0%	1 / 249 0.4%	0 / 73 0.0%	0 / 73 0.0%	0 / 364 0.0%	0 / 0 0.0%	0 / 0 0.0%	0 / 0 0.0%	0 / 0 0.0%	1638 / 2726 60.1%E	1022 / 2726 37.5%	44 / 2726 1.6%CE	20 / 2726 0.7%ABE	2 / 2726 0.1%AB		
71	"Chlamydiia" Horn 2011	22 / 2726 0.8%	0 / 74 0.0%	0 / 73 0.0%	0 / 73 0.0%	0 / 72 0.0%	0 / 149 0.0%	0 / 146 0.0%	1 / 73 1.4%	0 / 73 0.0%	0 / 145 0.0%	0 / 73 0.0%	19 / 289 6.6%	0 / 73 0.0%	0 / 73 0.0%	0 / 73 0.0%	0 / 73 0.0%	0 / 146 0.0%	1 / 73 1.4%	0 / 216 0.0%	1 / 249 0.4%	0 / 73 0.0%	0 / 73 0.0%	0 / 364 0.0%	0 / 0 0.0%	0 / 0 0.0%	0 / 0 0.0%	0 / 0 0.0%	1638 / 2726 60.1%E	1022 / 2726 37.5%	44 / 2726 1.6%CE	20 / 2726 0.7%ABE	2 / 2726 0.1%AB		
71	Chlamydiales	22 / 2726 0.8%	0 / 74 0.0%	0 / 73 0.0%	0 / 73 0.0%	0 / 72 0.0%	0 / 149 0.0%	0 / 146 0.0%	1 / 73 1.4%	0 / 73 0.0%	0 / 145 0.0%	0 / 73 0.0%	19 / 289 6.6%	0 / 73 0.0%	0 / 73 0.0%	0 / 73 0.0%	0 / 73 0.0%	0 / 146 0.0%	1 / 73 1.4%	0 / 216 0.0%	1 / 249 0.4%	0 / 73 0.0%	0 / 73 0.0%	0 / 364 0.0%	0 / 0 0.0%	0 / 0 0.0%	0 / 0 0.0%	0 / 0 0.0%	1638 / 2726 60.1%E	1022 / 2726 37.5%	44 / 2726 1.6%CE	20 / 2726 0.7%ABE	2 / 2726 0.1%AB		
67	Chlamydiaceae	20 / 2579 0.8%	0 / 69 0.0%	0 / 69 0.0%	0 / 69 0.0%	0 / 69 0.0%	0 / 138 0.0%	0 / 138 0.0%	0 / 69 0.0%	0 / 69 0.0%	0 / 138 0.0%	0 / 69 0.0%	19 / 276 6.9%	0 / 69 0.0%	0 / 69 0.0%	0 / 69 0.0%	0 / 69 0.0%	0 / 138 0.0%	1 / 69 1.4%	0 / 207 0.0%	0 / 233 0.0%	0 / 69 0.0%	0 / 69 0.0%	0 / 345 0.0%	0 / 0 0.0%	0 / 0 0.0%	0 / 0 0.0%	0 / 0 0.0%	1557 / 2579 60.4%E	962 / 2579 37.3%	40 / 2579 1.6%CE	20 / 2579 0.8%ABE			
67	Chlamydia/Chlamydophila group	20 / 2579 0.8%	0 / 69 0.0%	0 / 69 0.0%	0 / 69 0.0%	0 / 69 0.0%	0 / 138 0.0%	0 / 138 0.0%	0 / 69 0.0%	0 / 69 0.0%	0 / 138 0.0%	0 / 69 0.0%	19 / 276 6.9%	0 / 69 0.0%	0 / 69 0.0%	0 / 69 0.0%	0 / 69 0.0%	0 / 138 0.0%	1 / 69 1.4%	0 / 207 0.0%	0 / 233 0.0%	0 / 69 0.0%	0 / 69 0.0%	0 / 345 0.0%	0 / 0 0.0%	0 / 0 0.0%	0 / 0 0.0%	0 / 0 0.0%	1557 / 2579 60.4%E	962 / 2579 37.3%	40 / 2579 1.6%CE	20 / 2579 0.8%ABE			
64	"Bedsonia" Meyer 1953	19 / 2465 0.8%	0 / 66 0.0%	0 / 66 0.0%	0 / 66 0.0%	0 / 66 0.0%	0 / 132 0.0%	0 / 132 0.0%	0 / 66 0.0%	0 / 66 0.0%	0 / 132 0.0%	0 / 66 0.0%	18 / 264 6.8%	0 / 66 0.0%	0 / 66 0.0%	0 / 66 0.0%	0 / 66 0.0%	0 / 132 0.0%	1 / 66 1.5%	0 / 198 0.0%	0 / 221 0.0%	0 / 66 0.0%	0 / 66 0.0%	0 / 330 0.0%	0 / 0 0.0%	0 / 0 0.0%	0 / 0 0.0%	0 / 0 0.0%	1525 / 2465 61.9%E	881 / 2465 35.7%	40 / 2465 1.6%CE	19 / 2465 0.8%ABE			
40	"Chlamydozoon trachomatis" (Busacca 1935) Moshkovski 1945	0 / 1517 0.0%	0 / 41 0.0%	0 / 41 0.0%	0 / 41 0.0%	0 / 41 0.0%	0 / 82 0.0%	0 / 82 0.0%	0 / 41 0.0%	0 / 41 0.0%	0 / 82 0.0%	0 / 41 0.0%	0 / 164 0.0%	0 / 41 0.0%	0 / 41 0.0%	0 / 41 0.0%	0 / 41 0.0%	0 / 82 0.0%	0 / 41 0.0%	0 / 123 0.0%	0 / 123 0.0%	0 / 41 0.0%	0 / 41 0.0%	0 / 205 0.0%	0 / 0 0.0%	0 / 0 0.0%	0 / 0 0.0%	0 / 0 0.0%	1001 / 1517 66.0%E	477 / 1517 31.4%	39 / 1517 2.6%CE				
1	Chlamydia trachomatis D/UW-3/CX	0 / 37 0.0%	0 / 1 0.0%	0 / 1 0.0%	0 / 1 0.0%	0 / 1 0.0%	0 / 2 0.0%	0 / 2 0.0%	0 / 1 0.0%	0 / 1 0.0%	0 / 2 0.0%	0 / 1 0.0%	0 / 4 0.0%	0 / 1 0.0%	0 / 1 0.0%	0 / 1 0.0%	0 / 1 0.0%	0 / 2 0.0%	0 / 1 0.0%	0 / 3 0.0%	0 / 3 0.0%	0 / 1 0.0%	0 / 1 0.0%	0 / 5 0.0%	0 / 0 0.0%	0 / 0 0.0%	0 / 0 0.0%	0 / 0 0.0%	24 / 37 64.9%	13 / 37 35.1%E					
1	Chlamydia trachomatis A/HAR-13	0 / 37 0.0%	0 / 1 0.0%	0 / 1 0.0%	0 / 1 0.0%	0 / 1 0.0%	0 / 2 0.0%	0 / 2 0.0%	0 / 1 0.0%	0 / 1 0.0%	0 / 2 0.0%	0 / 1 0.0%	0 / 4 0.0%	0 / 1 0.0%	0 / 1 0.0%	0 / 1 0.0%	0 / 1 0.0%	0 / 2 0.0%	0 / 1 0.0%	0 / 3 0.0%	0 / 3 0.0%	0 / 1 0.0%	0 / 1 0.0%	0 / 5 0.0%	0 / 0 0.0%	0 / 0 0.0%	0 / 0 0.0%	0 / 0 0.0%	24 / 37 64.9%	13 / 37 35.1%E					
1	Chlamydia trachomatis 434/Bu	0 / 37 0.0%	0 / 1 0.0%	0 / 1 0.0%	0 / 1 0.0%	0 / 1 0.0%	0 / 2 0.0%	0 / 2 0.0%	0 / 1 0.0%	0 / 1 0.0%	0 / 2 0.0%	0 / 1 0.0%	0 / 4 0.0%	0 / 1 0.0%	0 / 1 0.0%	0 / 1 0.0%	0 / 1 0.0%	0 / 2 0.0%	0 / 1 0.0%	0 / 3 0.0%	0 / 3 0.0%	0 / 1 0.0%	0 / 1 0.0%	0 / 5 0.0%	0 / 0 0.0%	0 / 0 0.0%	0 / 0 0.0%	0 / 0 0.0%	25 / 37 67.6%E	11 / 37 29.7%	1 / 37 2.7%CE				
1	Chlamydia trachomatis L2b/UCH-1/proctitis	0 / 37 0.0%	0 / 1 0.0%	0 / 1 0.0%	0 / 1 0.0%	0 / 1 0.0%	0 / 2 0.0%	0 / 2 0.0%	0 / 1 0.0%	0 / 1 0.0%	0 / 2 0.0%	0 / 1 0.0%	0 / 4 0.0%	0 / 1 0.0%	0 / 1 0.0%	0 / 1 0.0%	0 / 1 0.0%	0 / 2 0.0%	0 / 1 0.0%	0 / 3 0.0%	0 / 3 0.0%	0 / 1 0.0%	0 / 1 0.0%	0 / 5 0.0%	0 / 0 0.0%	0 / 0 0.0%	0 / 0 0.0%	0 / 0 0.0%	25 / 37 67.6%E	11 / 37 29.7%	1 / 37 2.7%CE				
1	Chlamydia trachomatis A2497	0 / 74 0.0%	0 / 2 0.0%	0 / 2 0.0%	0 / 2 0.0%	0 / 2 0.0%	0 / 4 0.0%	0 / 4 0.0%	0 / 2 0.0%	0 / 2 0.0%	0 / 4 0.0%	0 / 2 0.0%	0 / 8 0.0%	0 / 2 0.0%	0 / 2 0.0%	0 / 2 0.0%	0 / 2 0.0%	0 / 4 0.0%	0 / 2 0.0%	0 / 6 0.0%	0 / 6 0.0%	0 / 2 0.0%	0 / 2 0.0%	0 / 10 0.0%	0 / 0 0.0%	0 / 0 0.0%	0 / 0 0.0%	0 / 0 0.0%	50 / 74 67.6%E	22 / 74 29.7%	2 / 74 2.7%CE				
1	Chlamydia trachomatis E/Bour	0 / 37 0.0%	0 / 1 0.0%	0 / 1 0.0%	0 / 1 0.0%	0 / 1 0.0%	0 / 2 0.0%	0 / 2 0.0%	0 / 1 0.0%	0 / 1 0.0%	0 / 2 0.0%	0 / 1 0.0%	0 / 4 0.0%	0 / 1 0.0%	0 / 1 0.0%	0 / 1 0.0%	0 / 1 0.0%	0 / 2 0.0%	0 / 1 0.0%	0 / 3 0.0%	0 / 3 0.0%	0 / 1 0.0%	0 / 1 0.0%	0 / 5 0.0%	0 / 0 0.0%	0 / 0 0.0%	0 / 0 0.0%	0 / 0 0.0%	25 / 37 67.6%E	11 / 37 29.7%	1 / 37 2.7%CE				
1	Chlamydia trachomatis Sweden2	0 / 37 0.0%	0 / 1 0.0%	0 / 1 0.0%	0 / 1 0.0%	0 / 1 0.0%	0 / 2 0.0%	0 / 2 0.0%	0 / 1 0.0%	0 / 1 0.0%	0 / 2 0.0%	0 / 1 0.0%	0 / 4 0.0%	0 / 1 0.0%	0 / 1 0.0%	0 / 1 0.0%	0 / 1 0.0%	0 / 2 0.0%	0 / 1 0.0%	0 / 3 0.0%	0 / 3 0.0%	0 / 1 0.0%	0 / 1 0.0%	0 / 5 0.0%	0 / 0 0.0%	0 / 0 0.0%	0 / 0 0.0%	0 / 0 0.0%	25 / 37 67.6%E	11 / 37 29.7%	1 / 37 2.7%CE				
1	Chlamydia trachomatis B/TZ1A828/OT	0 / 37 0.0%	0 / 1 0.0%	0 / 1 0.0%	0 / 1 0.0%	0 / 1 0.0%	0 / 2 0.0%	0 / 2 0.0%	0 / 1 0.0%	0 / 1 0.0%	0 / 2 0.0%	0 / 1 0.0%	0 / 4 0.0%	0 / 1 0.0%	0 / 1 0.0%	0 / 1 0.0%	0 / 1 0.0%	0 / 2 0.0%	0 / 1 0.0%	0 / 3 0.0%	0 / 3 0.0%	0 / 1 0.0%	0 / 1 0.0%	0 / 5 0.0%	0 / 0 0.0%	0 / 0 0.0%	0 / 0 0.0%	0 / 0 0.0%	25 / 37 67.6%E	11 / 37 29.7%	1 / 37 2.7%CE				
1	Chlamydia trachomatis E/11023	0 / 37 0.0%	0 / 1 0.0%	0 / 1 0.0%	0 / 1 0.0%	0 / 1 0.0%	0 / 2 0.0%	0 / 2 0.0%	0 / 1 0.0%	0 / 1 0.0%	0 / 2 0.0%	0 / 1 0.0%	0 / 4 0.0%	0 / 1 0.0%	0 / 1 0.0%	0 / 1 0.0%	0 / 1 0.0%	0 / 2 0.0%	0 / 1 0.0%	0 / 3 0.0%	0 / 3 0.0%	0 / 1 0.0%	0 / 1 0.0%	0 / 5 0.0%	0 / 0 0.0%	0 / 0 0.0%	0 / 0 0.0%	0 / 0 0.0%	25 / 37 67.6%E	11 / 37 29.7%	1 / 37 2.7%CE				
1	Chlamydia trachomatis E/150	0 / 37 0.0%	0 / 1 0.0%	0 / 1 0.0%	0 / 1 0.0%	0 / 1 0.0%	0 / 2 0.0%	0 / 2 0.0%	0 / 1 0.0%	0 / 1 0.0%	0 / 2 0.0%	0 / 1 0.0%	0 / 4 0.0%	0 / 1 0.0%	0 / 1 0.0%	0 / 1 0.0%	0 / 1 0.0%	0 / 2 0.0%	0 / 1 0.0%	0 / 3 0.0%	0 / 3 0.0%	0 / 1 0.0%	0 / 1 0.0%	0 / 5 0.0%	0 / 0 0.0%	0 / 0 0.0%	0 / 0 0.0%	0 / 0 0.0%	25 / 37 67.6%E	11 / 37 29.7%	1 / 37 2.7%CE				
1	Chlamydia trachomatis G/9768	0 / 37 0.0%	0 / 1 0.0%	0 / 1 0.0%	0 / 1 0.0%	0 / 1 0.0%	0 / 2 0.0%	0 / 2 0.0%	0 / 1 0.0%	0 / 1 0.0%	0 / 2 0.0%	0 / 1 0.0%	0 / 4 0.0%	0 / 1 0.0%	0 / 1 0.0%	0 / 1 0.0%	0 / 1 0.0%	0 / 2 0.0%	0 / 1 0.0%	0 / 3 0.0%	0 / 3 0.0%	0 / 1 0.0%	0 / 1 0.0%	0 / 5 0.0%	0 / 0 0.0%	0 / 0 0.0%	0 / 0 0.0%	0 / 0 0.0%	25 / 37 67.6%E	11 / 37 29.7%	1 / 37 2.7%CE				
1	Chlamydia trachomatis G/11222	0 / 37 0.0%	0 / 1 0.0%	0 / 1 0.0%	0 / 1 0.0%	0 / 1 0.0%	0 / 2 0.0%	0 / 2 0.0%	0 / 1 0.0%	0 / 1 0.0%	0 / 2 0.0%	0 / 1 0.0%	0 / 4 0.0%	0 / 1 0.0%	0 / 1 0.0%	0 / 1 0.0%	0 / 1 0.0%	0 / 2 0.0%	0 / 1 0.0%	0 / 3 0.0%	0 / 3 0.0%	0 / 1 0.0%	0 / 1 0.0%	0 / 5 0.0%	0 / 0 0.0%	0 / 0 0.0%	0 / 0 0.0%	0 / 0 0.0%	25 / 37 67.6%E	11 / 37 29.7%	1 / 37 2.7%CE				
1	Chlamydia trachomatis G/11074	0 / 37 0.0%	0 / 1 0.0%	0 / 1 0.0%	0 / 1 0.0%	0 / 1 0.0%	0 / 2 0.0%	0 / 2 0.0%	0 / 1 0.0%	0 / 1 0.0%	0 / 2 0.0%	0 / 1 0.0%	0 / 4 0.0%	0 / 1 0.0%	0 / 1 0.0%	0 / 1 0.0%	0 / 1 0.0%	0 / 2 0.0%	0 / 1 0.0%	0 / 3 0.0%	0 / 3 0.0%	0 / 1 0.0%	0 / 1 0.0%	0 / 5 0.0%	0 / 0 0.0%	0 / 0 0.0%	0 / 0 0.0%	0 / 0 0.0%	25 / 37 67.6%E	11 / 37 29.7%	1 / 37 2.7%CE				
1	Chlamydia trachomatis G/9301	0 / 37 0.0%	0 / 1 0.0%	0 / 1 0.0%	0 / 1 0.0%	0 / 1 0.0%	0 / 2 0.0%	0 / 2 0.0%	0 / 1 0.0%	0 / 1 0.0%	0 / 2 0.0%	0 / 1 0.0%	0 / 4 0.0%	0 / 1 0.0%	0 / 1 0.0%	0 / 1 0.0%	0 / 1 0.0%	0 / 2 0.0%	0 / 1 0.0%	0 / 3 0.0%	0 / 3 0.0%	0 / 1 0.0%	0 / 1 0.0%	0 / 5 0.0%	0 / 0 0.0%	0 / 0 0.0%	0 / 0 0.0%	0 / 0 0.0%	25 / 37 67.6%E	11 / 37 29.7%	1 / 37 2.7%CE				
1	Chlamydia trachomatis D-EC	0 / 37 0.0%	0 / 1 0.0%	0 / 1 0.0%	0 / 1 0.0%	0 / 1 0.0%	0 / 2 0.0%	0 / 2 0.0%	0 / 1 0.0%	0 / 1 0.0%	0 / 2 0.0%	0 / 1 0.0%	0 / 4 0.0%	0 / 1 0.0%	0 / 1 0.0%	0 / 1 0.0%	0 / 1 0.0%	0 / 2 0.0%	0 / 1 0.0%	0 / 3 0.0%	0 / 3 0.0%	0 / 1 0.0%	0 / 1 0.0%	0 / 5 0.0%	0 / 0 0.0%	0 / 0 0.0%	0 / 0 0.0%	0 / 0 0.0%	25 / 37 67.6%E	11 / 37 29.7%	1 / 37 2.7%CE				
1	Chlamydia trachomatis D-LC	0 / 37 0.0%	0 / 1 0.0%	0 / 1 0.0%	0 / 1 0.0%	0 / 1 0.0%	0 / 2 0.0%	0 / 2 0.0%	0 / 1 0.0%	0 / 1 0.0%	0 / 2 0.0%	0 / 1 0.0%	0 / 4 0.0%	0 / 1 0.0%	0 / 1 0.0%	0 / 1 0.0%	0 / 1 0.0%	0 / 2 0.0%	0 / 1 0.0%	0 / 3 0.0%	0 / 3 0.0%	0 / 1 0.0%	0 / 1 0.0%	0 / 5 0.0%	0 / 0 0.0%	0 / 0 0.0%	0 / 0 0.0%	0 / 0 0.0%	25 / 37 67.6%E	11 / 37 29.7%	1 / 37 2.7%CE				
1	Chlamydia trachomatis L2c	0 / 37 0.0%	0 / 1 0.0%	0 / 1 0.0%	0 / 1 0.0%	0 / 1 0.0%	0 / 2 0.0%	0 / 2 0.0%	0 / 1 0.0%	0 / 1 0.0%	0 / 2 0.0%	0 / 1 0.0%	0 / 4 0.0%	0 / 1 0.0%	0 / 1 0.0%	0 / 1 0.0%	0 / 1 0.0%	0 / 2 0.0%	0 / 1 0.0%	0 / 3 0.0%	0 / 3 0.0%	0 / 1 0.0%	0 / 1 0.0%	0 / 5 0.0%	0 / 0 0.0%	0 / 0 0.0%	0 / 0 0.0%	0 / 0 0.0%	25 / 37 67.6%E	11 / 37 29.7%	1 / 37 2.7%CE				
1	Chlamydia trachomatis A/363	0 / 37 0.0%	0 / 1 0.0%	0 / 1 0.0%	0 / 1 0.0%	0 / 1 0.0%	0 / 2 0.0%	0 / 2 0.0%	0 / 1 0.0%	0 / 1 0.0%	0 / 2 0.0%	0 / 1 0.0%	0 / 4 0.0%	0 / 1 0.0%	0 / 1 0.0%	0 / 1 0.0%	0 / 1 0.0%	0 / 2 0.0%	0 / 1 0.0%	0 / 3 0.0%	0 / 3 0.0%	0 / 1 0.0%	0 / 1 0.0%	0 / 5 0.0%	0 / 0 0.0%	0 / 0 0.0%	0 / 0 0.0%	0 / 0 0.0%	25 / 37 67.6%E	11 / 37 29.7%	1 / 37 2.7%CE				
1	Chlamydia trachomatis A/5291	0 / 37 0.0%	0 / 1 0.0%	0 / 1 0.0%	0 / 1 0.0%	0 / 1 0.0%	0 / 2 0.0%	0 / 2 0.0%	0 / 1 0.0%	0 / 1 0.0%	0 / 2 0.0%	0 / 1 0.0%	0 / 4 0.0%	0 / 1 0.0%	0 / 1 0.0%	0 / 1 0.0%	0 / 1 0.0%	0 / 2 0.0%	0 / 1 0.0%	0 / 3 0.0%	0 / 3 0.0%	0 / 1 0.0%	0 / 1 0.0%	0 / 5 0.0%	0 / 0 0.0%	0 / 0 0.0%	0 / 0 0.0%	0 / 0 0.0%	25 / 37 67.6%E	11 / 37 29.7%	1 / 37 2.7%CE				
1	Chlamydia trachomatis A/7249	0 / 37 0.0%	0 / 1 0.0%	0 / 1 0.0%	0 / 1 0.0%	0 / 1 0.0%	0 / 2 0.0%	0 / 2 0.0%	0 / 1 0.0%	0 / 1 0.0%	0 / 2 0.0%	0 / 1 0.0%	0 / 4 0.0%	0 / 1 0.0%	0 / 1 0.0%	0 / 1 0.0%	0 / 1 0.0%	0 / 2 0.0%	0 / 1 0.0%	0 / 3 0.0%	0 / 3 0.0%	0 / 1 0.0%	0 / 1 0.0%	0 / 5 0.0%	0 / 0 0.0%	0 / 0 0.0%	0 / 0 0.0%	0 / 0 0.0%	25 / 37 67.6%E	11 / 37 29.7%	1 / 37 2.7%CE				
1	Chlamydia trachomatis E/SW3	0 / 37 0.0%	0 / 1 0.0%	0 / 1 0.0%	0 / 1 0.0%	0 / 1 0.0%	0 / 2 0.0%	0 / 2 0.0%	0 / 1 0.0%	0 / 1 0.0%	0 / 2 0.0%	0 / 1 0.0%	0 / 4 0.0%	0 / 1 0.0%	0 / 1 0.0%	0 / 1 0.0%	0 / 1 0.0%	0 / 2 0.0%	0 / 1 0.0%	0 / 3 0.0%	0 / 3 0.0%	0 / 1 0.0%	0 / 1 0.0%	0 / 5 0.0%	0 / 0 0.0%	0 / 0 0.0%	0 / 0 0.0%	0 / 0 0.0%	25 / 37 67.6%E	11 / 37 29.7%	1 / 37 2.7%CE				
1	Chlamydia trachomatis F/SW4	0 / 37 0.0%	0 / 1 0.0%	0 / 1 0.0%	0 / 1 0.0%	0 / 1 0.0%	0 / 2 0.0%	0 / 2 0.0%	0 / 1 0.0%	0 / 1 0.0%	0 / 2 0.0%	0 / 1 0.0%	0 / 4 0.0%	0 / 1 0.0%	0 / 1 0.0%	0 / 1 0.0%	0 / 1 0.0%	0 / 2 0.0%	0 / 1 0.0%	0 / 3 0.0%	0 / 3 0.0%	0 / 1 0.0%	0 / 1 0.0%	0 / 5 0.0%	0 / 0 0.0%	0 / 0 0.0%	0 / 0 0.0%	0 / 0 0.0%	25 / 37 67.6%E	11 / 37 29.7%	1 / 37 2.7%CE				
1	Chlamydia trachomatis F/SW5	0 / 37 0.0%	0 / 1 0.0%	0 / 1 0.0%	0 / 1 0.0%	0 / 1 0.0%	0 / 2 0.0%	0 / 2 0.0%	0 / 1 0.0%	0 / 1 0.0%	0 / 2 0.0%	0 / 1 0.0%	0 / 4 0.0%	0 / 1 0.0%	0 / 1 0.0%	0 / 1 0.0%	0 / 1 0.0%	0 / 2 0.0%	0 / 1 0.0%	0 / 3 0.0%	0 / 3 0.0%	0 / 1 0.0%	0 / 1 0.0%	0 / 5 0.0%	0 / 0 0.0%	0 / 0 0.0%	0 / 0 0.0%	0 / 0 0.0%	25 / 37 67.6%E	11 / 37 29.7%	1 / 37 2.7%CE				
1	Chlamydia trachomatis G/SotonG1	0 / 37 0.0%	0 / 1 0.0%	0 / 1 0.0%	0 / 1 0.0%	0 / 1 0.0%	0 / 2 0.0%	0 / 2 0.0%	0 / 1 0.0%	0 / 1 0.0%	0 / 2 0.0%	0 / 1 0.0%	0 / 4 0.0%	0 / 1 0.0%	0 / 1 0.0%	0 / 1 0.0%	0 / 1 0.0%	0 / 2 0.0%	0 / 1 0.0%	0 / 3 0.0%	0 / 3 0.0%	0 / 1 0.0%	0 / 1 0.0%	0 / 5 0.0%	0 / 0 0.0%	0 / 0 0.0%	0 / 0 0.0%	0 / 0 0.0%	25 / 37 67.6%E	11 / 37 29.7%	1 / 37 2.7%CE				
1	Chlamydia trachomatis Ia/SotonIa1	0 / 37 0.0%	0 / 1 0.0%	0 / 1 0.0%	0 / 1 0.0%	0 / 1 0.0%	0 / 2 0.0%	0 / 2 0.0%	0 / 1 0.0%	0 / 1 0.0%	0 / 2 0.0%	0 / 1 0.0%	0 / 4 0.0%	0 / 1 0.0%	0 / 1 0.0%	0 / 1 0.0%	0 / 1 0.0%	0 / 2 0.0%	0 / 1 0.0%	0 / 3 0.0%	0 / 3 0.0%	0 / 1 0.0%	0 / 1 0.0%	0 / 5 0.0%	0 / 0 0.0%	0 / 0 0.0%	0 / 0 0.0%	0 / 0 0.0%	25 / 37 67.6%E	11 / 37 29.7%	1 / 37 2.7%CE				
1	Chlamydia trachomatis Ia/SotonIa3	0 / 37 0.0%	0 / 1 0.0%	0 / 1 0.0%	0 / 1 0.0%	0 / 1 0.0%	0 / 2 0.0%	0 / 2 0.0%	0 / 1 0.0%	0 / 1 0.0%	0 / 2 0.0%	0 / 1 0.0%	0 / 4 0.0%	0 / 1 0.0%	0 / 1 0.0%	0 / 1 0.0%	0 / 1 0.0%	0 / 2 0.0%	0 / 1 0.0%	0 / 3 0.0%	0 / 3 0.0%	0 / 1 0.0%	0 / 1 0.0%	0 / 5 0.0%	0 / 0 0.0%	0 / 0 0.0%	0 / 0 0.0%	0 / 0 0.0%	25 / 37 67.6%E	11 / 37 29.7%	1 / 37 2.7%CE				
1	Chlamydia trachomatis K/SotonK1	0 / 37 0.0%	0 / 1 0.0%	0 / 1 0.0%	0 / 1 0.0%	0 / 1 0.0%	0 / 2 0.0%	0 / 2 0.0%	0 / 1 0.0%	0 / 1 0.0%	0 / 2 0.0%	0 / 1 0.0%	0 / 4 0.0%	0 / 1 0.0%	0 / 1 0.0%	0 / 1 0.0%	0 / 1 0.0%	0 / 2 0.0%	0 / 1 0.0%	0 / 3 0.0%	0 / 3 0.0%	0 / 1 0.0%	0 / 1 0.0%	0 / 5 0.0%	0 / 0 0.0%	0 / 0 0.0%	0 / 0 0.0%	0 / 0 0.0%	25 / 37 67.6%E	11 / 37 29.7%	1 / 37 2.7%CE				
1	Chlamydia trachomatis L1/440/LN	0 / 37 0.0%	0 / 1 0.0%	0 / 1 0.0%	0 / 1 0.0%	0 / 1 0.0%	0 / 2 0.0%	0 / 2 0.0%	0 / 1 0.0%	0 / 1 0.0%	0 / 2 0.0%	0 / 1 0.0%	0 / 4 0.0%	0 / 1 0.0%	0 / 1 0.0%	0 / 1 0.0%	0 / 1 0.0%	0 / 2 0.0%	0 / 1 0.0%	0 / 3 0.0%	0 / 3 0.0%	0 / 1 0.0%	0 / 1 0.0%	0 / 5 0.0%	0 / 0 0.0%	0 / 0 0.0%	0 / 0 0.0%	0 / 0 0.0%	25 / 37 67.6%E	11 / 37 29.7%	1 / 37 2.7%CE				
1	Chlamydia trachomatis L1/115	0 / 37 0.0%	0 / 1 0.0%	0 / 1 0.0%	0 / 1 0.0%	0 / 1 0.0%	0 / 2 0.0%	0 / 2 0.0%	0 / 1 0.0%	0 / 1 0.0%	0 / 2 0.0%	0 / 1 0.0%	0 / 4 0.0%	0 / 1 0.0%	0 / 1 0.0%	0 / 1 0.0%	0 / 1 0.0%	0 / 2 0.0%	0 / 1 0.0%	0 / 3 0.0%	0 / 3 0.0%	0 / 1 0.0%	0 / 1 0.0%	0 / 5 0.0%	0 / 0 0.0%	0 / 0 0.0%	0 / 0 0.0%	0 / 0 0.0%	25 / 37 67.6%E	11 / 37 29.7%	1 / 37 2.7%CE				
1	Chlamydia trachomatis L1/224	0 / 37 0.0%	0 / 1 0.0%	0 / 1 0.0%	0 / 1 0.0%	0 / 1 0.0%	0 / 2 0.0%	0 / 2 0.0%	0 / 1 0.0%	0 / 1 0.0%	0 / 2 0.0%	0 / 1 0.0%	0 / 4 0.0%	0 / 1 0.0%	0 / 1 0.0%	0 / 1 0.0%	0 / 1 0.0%	0 / 2 0.0%	0 / 1 0.0%	0 / 3 0.0%	0 / 3 0.0%	0 / 1 0.0%	0 / 1 0.0%	0 / 5 0.0%	0 / 0 0.0%	0 / 0 0.0%	0 / 0 0.0%	0 / 0 0.0%	25 / 37 67.6%E	11 / 37 29.7%	1 / 37 2.7%CE				
1	Chlamydia trachomatis L2/25667R	0 / 37 0.0%	0 / 1 0.0%	0 / 1 0.0%	0 / 1 0.0%	0 / 1 0.0%	0 / 2 0.0%	0 / 2 0.0%	0 / 1 0.0%	0 / 1 0.0%	0 / 2 0.0%	0 / 1 0.0%	0 / 4 0.0%	0 / 1 0.0%	0 / 1 0.0%	0 / 1 0.0%	0 / 1 0.0%	0 / 2 0.0%	0 / 1 0.0%	0 / 3 0.0%	0 / 3 0.0%	0 / 1 0.0%	0 / 1 0.0%	0 / 5 0.0%	0 / 0 0.0%	0 / 0 0.0%	0 / 0 0.0%	0 / 0 0.0%	25 / 37 67.6%E	11 / 37 29.7%	1 / 37 2.7%CE				
1	Chlamydia trachomatis L2b/8200/07	0 / 37 0.0%	0 / 1 0.0%	0 / 1 0.0%	0 / 1 0.0%	0 / 1 0.0%	0 / 2 0.0%	0 / 2 0.0%	0 / 1 0.0%	0 / 1 0.0%	0 / 2 0.0%	0 / 1 0.0%	0 / 4 0.0%	0 / 1 0.0%	0 / 1 0.0%	0 / 1 0.0%	0 / 1 0.0%	0 / 2 0.0%	0 / 1 0.0%	0 / 3 0.0%	0 / 3 0.0%	0 / 1 0.0%	0 / 1 0.0%	0 / 5 0.0%	0 / 0 0.0%	0 / 0 0.0%	0 / 0 0.0%	0 / 0 0.0%	25 / 37 67.6%E	11 / 37 29.7%	1 / 37 2.7%CE				
1	Chlamydia trachomatis L2b/UCH-2	0 / 37 0.0%	0 / 1 0.0%	0 / 1 0.0%	0 / 1 0.0%	0 / 1 0.0%	0 / 2 0.0%	0 / 2 0.0%	0 / 1 0.0%	0 / 1 0.0%	0 / 2 0.0%	0 / 1 0.0%	0 / 4 0.0%	0 / 1 0.0%	0 / 1 0.0%	0 / 1 0.0%	0 / 1 0.0%	0 / 2 0.0%	0 / 1 0.0%	0 / 3 0.0%	0 / 3 0.0%	0 / 1 0.0%	0 / 1 0.0%	0 / 5 0.0%	0 / 0 0.0%	0 / 0 0.0%	0 / 0 0.0%	0 / 0 0.0%	25 / 37 67.6%E	11 / 37 29.7%	1 / 37 2.7%CE				
1	Chlamydia trachomatis L2b/795	0 / 37 0.0%	0 / 1 0.0%	0 / 1 0.0%	0 / 1 0.0%	0 / 1 0.0%	0 / 2 0.0%	0 / 2 0.0%	0 / 1 0.0%	0 / 1 0.0%	0 / 2 0.0%	0 / 1 0.0%	0 / 4 0.0%	0 / 1 0.0%	0 / 1 0.0%	0 / 1 0.0%	0 / 1 0.0%	0 / 2 0.0%	0 / 1 0.0%	0 / 3 0.0%	0 / 3 0.0%	0 / 1 0.0%	0 / 1 0.0%	0 / 5 0.0%	0 / 0 0.0%	0 / 0 0.0%	0 / 0 0.0%	0 / 0 0.0%	25 / 37 67.6%E	11 / 37 29.7%	1 / 37 2.7%CE				
1	Chlamydia trachomatis L2b/Ams1	0 / 37 0.0%	0 / 1 0.0%	0 / 1 0.0%	0 / 1 0.0%	0 / 1 0.0%	0 / 2 0.0%	0 / 2 0.0%	0 / 1 0.0%	0 / 1 0.0%	0 / 2 0.0%	0 / 1 0.0%	0 / 4 0.0%	0 / 1 0.0%	0 / 1 0.0%	0 / 1 0.0%	0 / 1 0.0%	0 / 2 0.0%	0 / 1 0.0%	0 / 3 0.0%	0 / 3 0.0%	0 / 1 0.0%	0 / 1 0.0%	0 / 5 0.0%	0 / 0 0.0%	0 / 0 0.0%	0 / 0 0.0%	0 / 0 0.0%	25 / 37 67.6%E	11 / 37 29.7%	1 / 37 2.7%CE				
1	Chlamydia trachomatis L3/404/LN	0 / 37 0.0%	0 / 1 0.0%	0 / 1 0.0%	0 / 1 0.0%	0 / 1 0.0%	0 / 2 0.0%	0 / 2 0.0%	0 / 1 0.0%	0 / 1 0.0%	0 / 2 0.0%	0 / 1 0.0%	0 / 4 0.0%	0 / 1 0.0%	0 / 1 0.0%	0 / 1 0.0%	0 / 1 0.0%	0 / 2 0.0%	0 / 1 0.0%	0 / 3 0.0%	0 / 3 0.0%	0 / 1 0.0%	0 / 1 0.0%	0 / 5 0.0%	0 / 0 0.0%	0 / 0 0.0%	0 / 0 0.0%	0 / 0 0.0%	25 / 37 67.6%E	11 / 37 29.7%	1 / 37 2.7%CE				
1	Chlamydia trachomatis IU824	0 / 37 0.0%	0 / 1 0.0%	0 / 1 0.0%	0 / 1 0.0%	0 / 1 0.0%	0 / 2 0.0%	0 / 2 0.0%	0 / 1 0.0%	0 / 1 0.0%	0 / 2 0.0%	0 / 1 0.0%	0 / 4 0.0%	0 / 1 0.0%	0 / 1 0.0%	0 / 1 0.0%	0 / 1 0.0%	0 / 2 0.0%	0 / 1 0.0%	0 / 3 0.0%	0 / 3 0.0%	0 / 1 0.0%	0 / 1 0.0%	0 / 5 0.0%	0 / 0 0.0%	0 / 0 0.0%	0 / 0 0.0%	0 / 0 0.0%	25 / 37 67.6%E	11 / 37 29.7%	1 / 37 2.7%CE				
1	Chlamydia trachomatis IU888	0 / 37 0.0%	0 / 1 0.0%	0 / 1 0.0%	0 / 1 0.0%	0 / 1 0.0%	0 / 2 0.0%	0 / 2 0.0%	0 / 1 0.0%	0 / 1 0.0%	0 / 2 0.0%	0 / 1 0.0%	0 / 4 0.0%	0 / 1 0.0%	0 / 1 0.0%	0 / 1 0.0%	0 / 1 0.0%	0 / 2 0.0%	0 / 1 0.0%	0 / 3 0.0%	0 / 3 0.0%	0 / 1 0.0%	0 / 1 0.0%	0 / 5 0.0%	0 / 0 0.0%	0 / 0 0.0%	0 / 0 0.0%	0 / 0 0.0%	25 / 37 67.6%E	11 / 37 29.7%	1 / 37 2.7%CE				
2	Chlamydia trachomatis L2/434/Bu(i)	0 / 74 0.0%	0 / 2 0.0%	0 / 2 0.0%	0 / 2 0.0%	0 / 2 0.0%	0 / 4 0.0%	0 / 4 0.0%	0 / 2 0.0%	0 / 2 0.0%	0 / 4 0.0%	0 / 2 0.0%	0 / 8 0.0%	0 / 2 0.0%	0 / 2 0.0%	0 / 2 0.0%	0 / 2 0.0%	0 / 4 0.0%	0 / 2 0.0%	0 / 6 0.0%	0 / 6 0.0%	0 / 2 0.0%	0 / 2 0.0%	0 / 10 0.0%	0 / 0 0.0%	0 / 0 0.0%	0 / 0 0.0%	0 / 0 0.0%	50 / 74 67.6%E	22 / 74 29.7%	2 / 74 2.7%CE				
1	Chlamydia trachomatis L2/434/Bu(f)	0 / 37 0.0%	0 / 1 0.0%	0 / 1 0.0%	0 / 1 0.0%	0 / 1 0.0%	0 / 2 0.0%	0 / 2 0.0%	0 / 1 0.0%	0 / 1 0.0%	0 / 2 0.0%	0 / 1 0.0%	0 / 4 0.0%	0 / 1 0.0%	0 / 1 0.0%	0 / 1 0.0%	0 / 1 0.0%	0 / 2 0.0%	0 / 1 0.0%	0 / 3 0.0%	0 / 3 0.0%	0 / 1 0.0%	0 / 1 0.0%	0 / 5 0.0%	0 / 0 0.0%	0 / 0 0.0%	0 / 0 0.0%	0 / 0 0.0%	25 / 37 67.6%E	11 / 37 29.7%	1 / 37 2.7%CE				
17	"Chlamydozoon psittaci" (Lillie 1930) Ryzhkov 1950	18 / 683 2.6%	0 / 18 0.0%	0 / 18 0.0%	0 / 18 0.0%	0 / 18 0.0%	0 / 36 0.0%	0 / 36 0.0%	0 / 18 0.0%	0 / 18 0.0%	0 / 36 0.0%	0 / 18 0.0%	18 / 72 25.0%	0 / 18 0.0%	0 / 18 0.0%	0 / 18 0.0%	0 / 18 0.0%	0 / 36 0.0%	0 / 18 0.0%	0 / 54 0.0%	0 / 71 0.0%	0 / 18 0.0%	0 / 18 0.0%	0 / 90 0.0%	0 / 0 0.0%	0 / 0 0.0%	0 / 0 0.0%	0 / 0 0.0%	414 / 683 60.6%E	251 / 683 36.7%	18 / 683 2.6%ABE				
1	Chlamydia psittaci 6BC	2 / 76 2.6%	0 / 2 0.0%	0 / 2 0.0%	0 / 2 0.0%	0 / 2 0.0%	0 / 4 0.0%	0 / 4 0.0%	0 / 2 0.0%	0 / 2 0.0%	0 / 4 0.0%	0 / 2 0.0%	2 / 8 25.0%	0 / 2 0.0%	0 / 2 0.0%	0 / 2 0.0%	0 / 2 0.0%	0 / 4 0.0%	0 / 2 0.0%	0 / 6 0.0%	0 / 8 0.0%	0 / 2 0.0%	0 / 2 0.0%	0 / 10 0.0%	0 / 0 0.0%	0 / 0 0.0%	0 / 0 0.0%	0 / 0 0.0%	46 / 76 60.5%E	28 / 76 36.8%	2 / 76 2.6%ABE				
1	Chlamydia psittaci Mat116	1 / 38 2.6%	0 / 1 0.0%	0 / 1 0.0%	0 / 1 0.0%	0 / 1 0.0%	0 / 2 0.0%	0 / 2 0.0%	0 / 1 0.0%	0 / 1 0.0%	0 / 2 0.0%	0 / 1 0.0%	1 / 4 25.0%	0 / 1 0.0%	0 / 1 0.0%	0 / 1 0.0%	0 / 1 0.0%	0 / 2 0.0%	0 / 1 0.0%	0 / 3 0.0%	0 / 4 0.0%	0 / 1 0.0%	0 / 1 0.0%	0 / 5 0.0%	0 / 0 0.0%	0 / 0 0.0%	0 / 0 0.0%	0 / 0 0.0%	23 / 38 60.5%E	14 / 38 36.8%	1 / 38 2.6%ABE				
1	Chlamydia psittaci RD1	1 / 37 2.7%	0 / 1 0.0%	0 / 1 0.0%	0 / 1 0.0%	0 / 1 0.0%	0 / 2 0.0%	0 / 2 0.0%	0 / 1 0.0%	0 / 1 0.0%	0 / 2 0.0%	0 / 1 0.0%	1 / 4 25.0%	0 / 1 0.0%	0 / 1 0.0%	0 / 1 0.0%	0 / 1 0.0%	0 / 2 0.0%	0 / 1 0.0%	0 / 3 0.0%	0 / 3 0.0%	0 / 1 0.0%	0 / 1 0.0%	0 / 5 0.0%	0 / 0 0.0%	0 / 0 0.0%	0 / 0 0.0%	0 / 0 0.0%	22 / 37 59.5%E	14 / 37 37.8%	1 / 37 2.7%ABE				
1	Chlamydia psittaci CP3	1 / 38 2.6%	0 / 1 0.0%	0 / 1 0.0%	0 / 1 0.0%	0 / 1 0.0%	0 / 2 0.0%	0 / 2 0.0%	0 / 1 0.0%	0 / 1 0.0%	0 / 2 0.0%	0 / 1 0.0%	1 / 4 25.0%	0 / 1 0.0%	0 / 1 0.0%	0 / 1 0.0%	0 / 1 0.0%	0 / 2 0.0%	0 / 1 0.0%	0 / 3 0.0%	0 / 4 0.0%	0 / 1 0.0%	0 / 1 0.0%	0 / 5 0.0%	0 / 0 0.0%	0 / 0 0.0%	0 / 0 0.0%	0 / 0 0.0%	23 / 38 60.5%E	14 / 38 36.8%	1 / 38 2.6%ABE				
1	Chlamydia psittaci NJ1	1 / 38 2.6%	0 / 1 0.0%	0 / 1 0.0%	0 / 1 0.0%	0 / 1 0.0%	0 / 2 0.0%	0 / 2 0.0%	0 / 1 0.0%	0 / 1 0.0%	0 / 2 0.0%	0 / 1 0.0%	1 / 4 25.0%	0 / 1 0.0%	0 / 1 0.0%	0 / 1 0.0%	0 / 1 0.0%	0 / 2 0.0%	0 / 1 0.0%	0 / 3 0.0%	0 / 4 0.0%	0 / 1 0.0%	0 / 1 0.0%	0 / 5 0.0%	0 / 0 0.0%	0 / 0 0.0%	0 / 0 0.0%	0 / 0 0.0%	23 / 38 60.5%E	14 / 38 36.8%	1 / 38 2.6%ABE				
1	Chlamydia psittaci C19/98	1 / 38 2.6%	0 / 1 0.0%	0 / 1 0.0%	0 / 1 0.0%	0 / 1 0.0%	0 / 2 0.0%	0 / 2 0.0%	0 / 1 0.0%	0 / 1 0.0%	0 / 2 0.0%	0 / 1 0.0%	1 / 4 25.0%	0 / 1 0.0%	0 / 1 0.0%	0 / 1 0.0%	0 / 1 0.0%	0 / 2 0.0%	0 / 1 0.0%	0 / 3 0.0%	0 / 4 0.0%	0 / 1 0.0%	0 / 1 0.0%	0 / 5 0.0%	0 / 0 0.0%	0 / 0 0.0%	0 / 0 0.0%	0 / 0 0.0%	23 / 38 60.5%E	14 / 38 36.8%	1 / 38 2.6%ABE				
1	Chlamydia psittaci 01DC11	1 / 38 2.6%	0 / 1 0.0%	0 / 1 0.0%	0 / 1 0.0%	0 / 1 0.0%	0 / 2 0.0%	0 / 2 0.0%	0 / 1 0.0%	0 / 1 0.0%	0 / 2 0.0%	0 / 1 0.0%	1 / 4 25.0%	0 / 1 0.0%	0 / 1 0.0%	0 / 1 0.0%	0 / 1 0.0%	0 / 2 0.0%	0 / 1 0.0%	0 / 3 0.0%	0 / 4 0.0%	0 / 1 0.0%	0 / 1 0.0%	0 / 5 0.0%	0 / 0 0.0%	0 / 0 0.0%	0 / 0 0.0%	0 / 0 0.0%	23 / 38 60.5%E	14 / 38 36.8%	1 / 38 2.6%ABE				
1	Chlamydia psittaci 02DC15	1 / 38 2.6%	0 / 1 0.0%	0 / 1 0.0%	0 / 1 0.0%	0 / 1 0.0%	0 / 2 0.0%	0 / 2 0.0%	0 / 1 0.0%	0 / 1 0.0%	0 / 2 0.0%	0 / 1 0.0%	1 / 4 25.0%	0 / 1 0.0%	0 / 1 0.0%	0 / 1 0.0%	0 / 1 0.0%	0 / 2 0.0%	0 / 1 0.0%	0 / 3 0.0%	0 / 4 0.0%	0 / 1 0.0%	0 / 1 0.0%	0 / 5 0.0%	0 / 0 0.0%	0 / 0 0.0%	0 / 0 0.0%	0 / 0 0.0%	23 / 38 60.5%E	14 / 38 36.8%	1 / 38 2.6%ABE				
1	Chlamydia psittaci 08DC60	1 / 38 2.6%	0 / 1 0.0%	0 / 1 0.0%	0 / 1 0.0%	0 / 1 0.0%	0 / 2 0.0%	0 / 2 0.0%	0 / 1 0.0%	0 / 1 0.0%	0 / 2 0.0%	0 / 1 0.0%	1 / 4 25.0%	0 / 1 0.0%	0 / 1 0.0%	0 / 1 0.0%	0 / 1 0.0%	0 / 2 0.0%	0 / 1 0.0%	0 / 3 0.0%	0 / 4 0.0%	0 / 1 0.0%	0 / 1 0.0%	0 / 5 0.0%	0 / 0 0.0%	0 / 0 0.0%	0 / 0 0.0%	0 / 0 0.0%	23 / 38 60.5%E	14 / 38 36.8%	1 / 38 2.6%ABE				
1	Chlamydia psittaci 84/55	1 / 38 2.6%	0 / 1 0.0%	0 / 1 0.0%	0 / 1 0.0%	0 / 1 0.0%	0 / 2 0.0%	0 / 2 0.0%	0 / 1 0.0%	0 / 1 0.0%	0 / 2 0.0%	0 / 1 0.0%	1 / 4 25.0%	0 / 1 0.0%	0 / 1 0.0%	0 / 1 0.0%	0 / 1 0.0%	0 / 2 0.0%	0 / 1 0.0%	0 / 3 0.0%	0 / 4 0.0%	0 / 1 0.0%	0 / 1 0.0%	0 / 5 0.0%	0 / 0 0.0%	0 / 0 0.0%	0 / 0 0.0%	0 / 0 0.0%	23 / 38 60.5%E	14 / 38 36.8%	1 / 38 2.6%ABE				
1	Chlamydia psittaci GR9	1 / 38 2.6%	0 / 1 0.0%	0 / 1 0.0%	0 / 1 0.0%	0 / 1 0.0%	0 / 2 0.0%	0 / 2 0.0%	0 / 1 0.0%	0 / 1 0.0%	0 / 2 0.0%	0 / 1 0.0%	1 / 4 25.0%	0 / 1 0.0%	0 / 1 0.0%	0 / 1 0.0%	0 / 1 0.0%	0 / 2 0.0%	0 / 1 0.0%	0 / 3 0.0%	0 / 4 0.0%	0 / 1 0.0%	0 / 1 0.0%	0 / 5 0.0%	0 / 0 0.0%	0 / 0 0.0%	0 / 0 0.0%	0 / 0 0.0%	23 / 38 60.5%E	14 / 38 36.8%	1 / 38 2.6%ABE				
1	Chlamydia psittaci MN	1 / 38 2.6%	0 / 1 0.0%	0 / 1 0.0%	0 / 1 0.0%	0 / 1 0.0%	0 / 2 0.0%	0 / 2 0.0%	0 / 1 0.0%	0 / 1 0.0%	0 / 2 0.0%	0 / 1 0.0%	1 / 4 25.0%	0 / 1 0.0%	0 / 1 0.0%	0 / 1 0.0%	0 / 1 0.0%	0 / 2 0.0%	0 / 1 0.0%	0 / 3 0.0%	0 / 4 0.0%	0 / 1 0.0%	0 / 1 0.0%	0 / 5 0.0%	0 / 0 0.0%	0 / 0 0.0%	0 / 0 0.0%	0 / 0 0.0%	23 / 38 60.5%E	14 / 38 36.8%	1 / 38 2.6%ABE				
1	Chlamydia psittaci VS225	1 / 38	0 / 1	0 / 1	0 / 1	0 / 1	0 / 2	0 / 2	0 / 1	0 / 1	0 / 2	0 / 1	1 / 4	0 / 1	0 / 1	0 / 1	0 / 1	0 / 2	0 / 1	0 / 3	0 / 4	0 / 1	0 / 1	0 / 5	0 / 0	0 / 0	0 / 0	0 / 0	23 / 38	14 / 38	1 / 38				

1	Chlamydia psittaci WS/RT/E30	1 / 38 2.6%	0 / 1 0.0%	0 / 1 0.0%	0 / 1 0.0%	0 / 1 0.0%	0 / 2 0.0%	0 / 2 0.0%	0 / 1 0.0%	0 / 1 0.0%	0 / 2 0.0%	0 / 1 0.0%	1 / 4 25.0%	0 / 1 0.0%	0 / 1 0.0%	0 / 1 0.0%	0 / 1 0.0%	0 / 2 0.0%	0 / 1 0.0%	0 / 3 0.0%	0 / 4 0.0%	0 / 1 0.0%	0 / 1 0.0%	0 / 5 0.0%	0 / 0 0.0%	0 / 0 0.0%	0 / 0 0.0%	0 / 0 0.0%	23 / 38 60.5%E	14 / 38 36.8%	1 / 38 2.6%ABE							
1	Chlamydia psittaci M56	1 / 38 2.6%	0 / 1 0.0%	0 / 1 0.0%	0 / 1 0.0%	0 / 1 0.0%	0 / 2 0.0%	0 / 2 0.0%	0 / 1 0.0%	0 / 1 0.0%	0 / 2 0.0%	0 / 1 0.0%	1 / 4 25.0%	0 / 1 0.0%	0 / 1 0.0%	0 / 1 0.0%	0 / 1 0.0%	0 / 2 0.0%	0 / 1 0.0%	0 / 3 0.0%	0 / 4 0.0%	0 / 1 0.0%	0 / 1 0.0%	0 / 5 0.0%	0 / 0 0.0%	0 / 0 0.0%	0 / 0 0.0%	0 / 0 0.0%	23 / 38 60.5%E	14 / 38 36.8%	1 / 38 2.6%ABE							
1	Chlamydia psittaci WC	1 / 38 2.6%	0 / 1 0.0%	0 / 1 0.0%	0 / 1 0.0%	0 / 1 0.0%	0 / 2 0.0%	0 / 2 0.0%	0 / 1 0.0%	0 / 1 0.0%	0 / 2 0.0%	0 / 1 0.0%	1 / 4 25.0%	0 / 1 0.0%	0 / 1 0.0%	0 / 1 0.0%	0 / 1 0.0%	0 / 2 0.0%	0 / 1 0.0%	0 / 3 0.0%	0 / 4 0.0%	0 / 1 0.0%	0 / 1 0.0%	0 / 5 0.0%	0 / 0 0.0%	0 / 0 0.0%	0 / 0 0.0%	0 / 0 0.0%	24 / 38 63.2%E	13 / 38 34.2%	1 / 38 2.6%ABE							
1	Chlamydia psittaci 01DC12	1 / 38 2.6%	0 / 1 0.0%	0 / 1 0.0%	0 / 1 0.0%	0 / 1 0.0%	0 / 2 0.0%	0 / 2 0.0%	0 / 1 0.0%	0 / 1 0.0%	0 / 2 0.0%	0 / 1 0.0%	1 / 4 25.0%	0 / 1 0.0%	0 / 1 0.0%	0 / 1 0.0%	0 / 1 0.0%	0 / 2 0.0%	0 / 1 0.0%	0 / 3 0.0%	0 / 4 0.0%	0 / 1 0.0%	0 / 1 0.0%	0 / 5 0.0%	0 / 0 0.0%	0 / 0 0.0%	0 / 0 0.0%	0 / 0 0.0%	23 / 38 60.5%E	14 / 38 36.8%	1 / 38 2.6%ABE							
5	ATCC VR-2282	1 / 190 0.5%	0 / 5 0.0%	0 / 5 0.0%	0 / 5 0.0%	0 / 5 0.0%	0 / 10 0.0%	0 / 10 0.0%	0 / 5 0.0%	0 / 5 0.0%	0 / 10 0.0%	0 / 5 0.0%	0 / 20 0.0%	0 / 5 0.0%	0 / 5 0.0%	0 / 5 0.0%	0 / 5 0.0%	0 / 10 0.0%	1 / 5 20.0%	0 / 15 0.0%	0 / 20 0.0%	0 / 5 0.0%	0 / 5 0.0%	0 / 25 0.0%	0 / 0 0.0%	0 / 0 0.0%	0 / 0 0.0%	0 / 0 0.0%	119 / 190 62.6%	70 / 190 36.8%E	1 / 190 0.5%ABE							
1	Chlamydia pneumoniae AR39	0 / 38 0.0%	0 / 1 0.0%	0 / 1 0.0%	0 / 1 0.0%	0 / 1 0.0%	0 / 2 0.0%	0 / 2 0.0%	0 / 1 0.0%	0 / 1 0.0%	0 / 2 0.0%	0 / 1 0.0%	0 / 4 0.0%	0 / 1 0.0%	0 / 1 0.0%	0 / 1 0.0%	0 / 1 0.0%	0 / 2 0.0%	0 / 1 0.0%	0 / 3 0.0%	0 / 4 0.0%	0 / 1 0.0%	0 / 1 0.0%	0 / 5 0.0%	0 / 0 0.0%	0 / 0 0.0%	0 / 0 0.0%	0 / 0 0.0%	26 / 38 68.4%	12 / 38 31.6%E								
1	Chlamydia pneumoniae CWL029	0 / 38 0.0%	0 / 1 0.0%	0 / 1 0.0%	0 / 1 0.0%	0 / 1 0.0%	0 / 2 0.0%	0 / 2 0.0%	0 / 1 0.0%	0 / 1 0.0%	0 / 2 0.0%	0 / 1 0.0%	0 / 4 0.0%	0 / 1 0.0%	0 / 1 0.0%	0 / 1 0.0%	0 / 1 0.0%	0 / 2 0.0%	0 / 1 0.0%	0 / 3 0.0%	0 / 4 0.0%	0 / 1 0.0%	0 / 1 0.0%	0 / 5 0.0%	0 / 0 0.0%	0 / 0 0.0%	0 / 0 0.0%	0 / 0 0.0%	26 / 38 68.4%	12 / 38 31.6%E								
1	Chlamydia pneumoniae (strain J138)	0 / 38 0.0%	0 / 1 0.0%	0 / 1 0.0%	0 / 1 0.0%	0 / 1 0.0%	0 / 2 0.0%	0 / 2 0.0%	0 / 1 0.0%	0 / 1 0.0%	0 / 2 0.0%	0 / 1 0.0%	0 / 4 0.0%	0 / 1 0.0%	0 / 1 0.0%	0 / 1 0.0%	0 / 1 0.0%	0 / 2 0.0%	0 / 1 0.0%	0 / 3 0.0%	0 / 4 0.0%	0 / 1 0.0%	0 / 1 0.0%	0 / 5 0.0%	0 / 0 0.0%	0 / 0 0.0%	0 / 0 0.0%	0 / 0 0.0%	26 / 38 68.4%	12 / 38 31.6%E								
1	Chlamydia pneumoniae TW-183	0 / 38 0.0%	0 / 1 0.0%	0 / 1 0.0%	0 / 1 0.0%	0 / 1 0.0%	0 / 2 0.0%	0 / 2 0.0%	0 / 1 0.0%	0 / 1 0.0%	0 / 2 0.0%	0 / 1 0.0%	0 / 4 0.0%	0 / 1 0.0%	0 / 1 0.0%	0 / 1 0.0%	0 / 1 0.0%	0 / 2 0.0%	0 / 1 0.0%	0 / 3 0.0%	0 / 4 0.0%	0 / 1 0.0%	0 / 1 0.0%	0 / 5 0.0%	0 / 0 0.0%	0 / 0 0.0%	0 / 0 0.0%	0 / 0 0.0%	26 / 38 68.4%	12 / 38 31.6%E								
1	Chlamydophila pneumoniae LPCoLN	1 / 38 2.6%	0 / 1 0.0%	0 / 1 0.0%	0 / 1 0.0%	0 / 1 0.0%	0 / 2 0.0%	0 / 2 0.0%	0 / 1 0.0%	0 / 1 0.0%	0 / 2 0.0%	0 / 1 0.0%	0 / 4 0.0%	0 / 1 0.0%	0 / 1 0.0%	0 / 1 0.0%	0 / 1 0.0%	0 / 2 0.0%	1 / 1 100.0%	0 / 3 0.0%	0 / 4 0.0%	0 / 1 0.0%	0 / 1 0.0%	0 / 5 0.0%	0 / 0 0.0%	0 / 0 0.0%	0 / 0 0.0%	0 / 0 0.0%	22 / 38 57.9%E	15 / 38 39.5%	1 / 38 2.6%ABE							
1	ATCC VR-123	0 / 37 0.0%	0 / 1 0.0%	0 / 1 0.0%	0 / 1 0.0%	0 / 1 0.0%	0 / 2 0.0%	0 / 2 0.0%	0 / 1 0.0%	0 / 1 0.0%	0 / 2 0.0%	0 / 1 0.0%	0 / 4 0.0%	0 / 1 0.0%	0 / 1 0.0%	0 / 1 0.0%	0 / 1 0.0%	0 / 2 0.0%	0 / 1 0.0%	0 / 3 0.0%	0 / 3 0.0%	0 / 1 0.0%	0 / 1 0.0%	0 / 5 0.0%	0 / 0 0.0%	0 / 0 0.0%	0 / 0 0.0%	0 / 0 0.0%	24 / 37 64.9%	13 / 37 35.1%E								
1	Chlamydia muridarum Nigg	0 / 37 0.0%	0 / 1 0.0%	0 / 1 0.0%	0 / 1 0.0%	0 / 1 0.0%	0 / 2 0.0%	0 / 2 0.0%	0 / 1 0.0%	0 / 1 0.0%	0 / 2 0.0%	0 / 1 0.0%	0 / 4 0.0%	0 / 1 0.0%	0 / 1 0.0%	0 / 1 0.0%	0 / 1 0.0%	0 / 2 0.0%	0 / 1 0.0%	0 / 3 0.0%	0 / 3 0.0%	0 / 1 0.0%	0 / 1 0.0%	0 / 5 0.0%	0 / 0 0.0%	0 / 0 0.0%	0 / 0 0.0%	0 / 0 0.0%	24 / 37 64.9%	13 / 37 35.1%E								
1	ATCC VR-628	0 / 38 0.0%	0 / 1 0.0%	0 / 1 0.0%	0 / 1 0.0%	0 / 1 0.0%	0 / 2 0.0%	0 / 2 0.0%	0 / 1 0.0%	0 / 1 0.0%	0 / 2 0.0%	0 / 1 0.0%	0 / 4 0.0%	0 / 1 0.0%	0 / 1 0.0%	0 / 1 0.0%	0 / 1 0.0%	0 / 2 0.0%	0 / 1 0.0%	0 / 3 0.0%	0 / 4 0.0%	0 / 1 0.0%	0 / 1 0.0%	0 / 5 0.0%	0 / 0 0.0%	0 / 0 0.0%	0 / 0 0.0%	0 / 0 0.0%	27 / 38 71.1%E	10 / 38 26.3%	1 / 38 2.6%CE							
1	Chlamydia pecorum E58	0 / 38 0.0%	0 / 1 0.0%	0 / 1 0.0%	0 / 1 0.0%	0 / 1 0.0%	0 / 2 0.0%	0 / 2 0.0%	0 / 1 0.0%	0 / 1 0.0%	0 / 2 0.0%	0 / 1 0.0%	0 / 4 0.0%	0 / 1 0.0%	0 / 1 0.0%	0 / 1 0.0%	0 / 1 0.0%	0 / 2 0.0%	0 / 1 0.0%	0 / 3 0.0%	0 / 4 0.0%	0 / 1 0.0%	0 / 1 0.0%	0 / 5 0.0%	0 / 0 0.0%	0 / 0 0.0%	0 / 0 0.0%	0 / 0 0.0%	27 / 38 71.1%E	10 / 38 26.3%	1 / 38 2.6%CE							
3	Chlamydophila	1 / 114 0.9%	0 / 3 0.0%	0 / 3 0.0%	0 / 3 0.0%	0 / 3 0.0%	0 / 6 0.0%	0 / 6 0.0%	0 / 3 0.0%	0 / 3 0.0%	0 / 6 0.0%	0 / 3 0.0%	1 / 12 8.3%	0 / 3 0.0%	0 / 3 0.0%	0 / 3 0.0%	0 / 3 0.0%	0 / 6 0.0%	0 / 3 0.0%	0 / 9 0.0%	0 / 12 0.0%	0 / 3 0.0%	0 / 3 0.0%	0 / 15 0.0%	0 / 0 0.0%	0 / 0 0.0%	0 / 0 0.0%	0 / 0 0.0%	81 / 114 71.1%	32 / 114 28.1%E	1 / 114 0.9%ABE							
1	"Chlamydia abortus" (Everett et al. 1999) Kuo et al. 2011	1 / 38 2.6%	0 / 1 0.0%	0 / 1 0.0%	0 / 1 0.0%	0 / 1 0.0%	0 / 2 0.0%	0 / 2 0.0%	0 / 1 0.0%	0 / 1 0.0%	0 / 2 0.0%	0 / 1 0.0%	1 / 4 25.0%	0 / 1 0.0%	0 / 1 0.0%	0 / 1 0.0%	0 / 1 0.0%	0 / 2 0.0%	0 / 1 0.0%	0 / 3 0.0%	0 / 4 0.0%	0 / 1 0.0%	0 / 1 0.0%	0 / 5 0.0%	0 / 0 0.0%	0 / 0 0.0%	0 / 0 0.0%	0 / 0 0.0%	27 / 38 71.1%	10 / 38 26.3%E	1 / 38 2.6%ABE							
1	Chlamydophila abortus S26/3	1 / 38 2.6%	0 / 1 0.0%	0 / 1 0.0%	0 / 1 0.0%	0 / 1 0.0%	0 / 2 0.0%	0 / 2 0.0%	0 / 1 0.0%	0 / 1 0.0%	0 / 2 0.0%	0 / 1 0.0%	1 / 4 25.0%	0 / 1 0.0%	0 / 1 0.0%	0 / 1 0.0%	0 / 1 0.0%	0 / 2 0.0%	0 / 1 0.0%	0 / 3 0.0%	0 / 4 0.0%	0 / 1 0.0%	0 / 1 0.0%	0 / 5 0.0%	0 / 0 0.0%	0 / 0 0.0%	0 / 0 0.0%	0 / 0 0.0%	27 / 38 71.1%	10 / 38 26.3%E	1 / 38 2.6%ABE							
1	"Chlamydia felis" (Everett et al. 1999) Kuo et al. 2011	0 / 38 0.0%	0 / 1 0.0%	0 / 1 0.0%	0 / 1 0.0%	0 / 1 0.0%	0 / 2 0.0%	0 / 2 0.0%	0 / 1 0.0%	0 / 1 0.0%	0 / 2 0.0%	0 / 1 0.0%	0 / 4 0.0%	0 / 1 0.0%	0 / 1 0.0%	0 / 1 0.0%	0 / 1 0.0%	0 / 2 0.0%	0 / 1 0.0%	0 / 3 0.0%	0 / 4 0.0%	0 / 1 0.0%	0 / 1 0.0%	0 / 5 0.0%	0 / 0 0.0%	0 / 0 0.0%	0 / 0 0.0%	0 / 0 0.0%	27 / 38 71.1%	11 / 38 28.9%E								
1	Chlamydophila felis Fe/C-56	0 / 38 0.0%	0 / 1 0.0%	0 / 1 0.0%	0 / 1 0.0%	0 / 1 0.0%	0 / 2 0.0%	0 / 2 0.0%	0 / 1 0.0%	0 / 1 0.0%	0 / 2 0.0%	0 / 1 0.0%	0 / 4 0.0%	0 / 1 0.0%	0 / 1 0.0%	0 / 1 0.0%	0 / 1 0.0%	0 / 2 0.0%	0 / 1 0.0%	0 / 3 0.0%	0 / 4 0.0%	0 / 1 0.0%	0 / 1 0.0%	0 / 5 0.0%	0 / 0 0.0%	0 / 0 0.0%	0 / 0 0.0%	0 / 0 0.0%	27 / 38 71.1%	11 / 38 28.9%E								
1	"Chlamydia caviae" (Everett et al. 1999) Kuo et al. 2011	0 / 38 0.0%	0 / 1 0.0%	0 / 1 0.0%	0 / 1 0.0%	0 / 1 0.0%	0 / 2 0.0%	0 / 2 0.0%	0 / 1 0.0%	0 / 1 0.0%	0 / 2 0.0%	0 / 1 0.0%	0 / 4 0.0%	0 / 1 0.0%	0 / 1 0.0%	0 / 1 0.0%	0 / 1 0.0%	0 / 2 0.0%	0 / 1 0.0%	0 / 3 0.0%	0 / 4 0.0%	0 / 1 0.0%	0 / 1 0.0%	0 / 5 0.0%	0 / 0 0.0%	0 / 0 0.0%	0 / 0 0.0%	0 / 0 0.0%	27 / 38 71.1%	11 / 38 28.9%E								
1	Chlamydophila caviae GPIC	0 / 38 0.0%	0 / 1 0.0%	0 / 1 0.0%	0 / 1 0.0%	0 / 1 0.0%	0 / 2 0.0%	0 / 2 0.0%	0 / 1 0.0%	0 / 1 0.0%	0 / 2 0.0%	0 / 1 0.0%	0 / 4 0.0%	0 / 1 0.0%	0 / 1 0.0%	0 / 1 0.0%	0 / 1 0.0%	0 / 2 0.0%	0 / 1 0.0%	0 / 3 0.0%	0 / 4 0.0%	0 / 1 0.0%	0 / 1 0.0%	0 / 5 0.0%	0 / 0 0.0%	0 / 0 0.0%	0 / 0 0.0%	0 / 0 0.0%	27 / 38 71.1%	11 / 38 28.9%E								
1	Simkaniaceae	2 / 35 5.7%	0 / 1 0.0%	0 / 1 0.0%	0 / 1 0.0%	0 / 1 0.0%	0 / 2 0.0%	0 / 2 0.0%	1 / 1 100.0%	0 / 1 0.0%	0 / 2 0.0%	0 / 1 0.0%	0 / 3 0.0%	0 / 1 0.0%	0 / 1 0.0%	0 / 1 0.0%	0 / 1 0.0%	0 / 2 0.0%	0 / 1 0.0%	0 / 2 0.0%	1 / 4 25.0%	0 / 1 0.0%	0 / 1 0.0%	0 / 4 0.0%	0 / 0 0.0%	0 / 0 0.0%	0 / 0 0.0%	0 / 0 0.0%	22 / 35 62.9%E	9 / 35 25.7%	2 / 35 5.7%AB	2 / 35 5.7%CE						
1	Simkania	2 / 35 5.7%	0 / 1 0.0%	0 / 1 0.0%	0 / 1 0.0%	0 / 1 0.0%	0 / 2 0.0%	0 / 2 0.0%	1 / 1 100.0%	0 / 1 0.0%	0 / 2 0.0%	0 / 1 0.0%	0 / 3 0.0%	0 / 1 0.0%	0 / 1 0.0%	0 / 1 0.0%	0 / 1 0.0%	0 / 2 0.0%	0 / 1 0.0%	0 / 2 0.0%	1 / 4 25.0%	0 / 1 0.0%	0 / 1 0.0%	0 / 4 0.0%	0 / 0 0.0%	0 / 0 0.0%	0 / 0 0.0%	0 / 0 0.0%	22 / 35 62.9%E	9 / 35 25.7%	2 / 35 5.7%AB	2 / 35 5.7%CE						
1	ATCC VR-1471	2 / 35 5.7%	0 / 1 0.0%	0 / 1 0.0%	0 / 1 0.0%	0 / 1 0.0%	0 / 2 0.0%	0 / 2 0.0%	1 / 1 100.0%	0 / 1 0.0%	0 / 2 0.0%	0 / 1 0.0%	0 / 3 0.0%	0 / 1 0.0%	0 / 1 0.0%	0 / 1 0.0%	0 / 1 0.0%	0 / 2 0.0%	0 / 1 0.0%	0 / 2 0.0%	1 / 4 25.0%	0 / 1 0.0%	0 / 1 0.0%	0 / 4 0.0%	0 / 0 0.0%	0 / 0 0.0%	0 / 0 0.0%	0 / 0 0.0%	22 / 35 62.9%E	9 / 35 25.7%	2 / 35 5.7%AB	2 / 35 5.7%CE						
1	Simkania negevensis Z	2 / 35 5.7%	0 / 1 0.0%	0 / 1 0.0%	0 / 1 0.0%	0 / 1 0.0%	0 / 2 0.0%	0 / 2 0.0%	1 / 1 100.0%	0 / 1 0.0%	0 / 2 0.0%	0 / 1 0.0%	0 / 3 0.0%	0 / 1 0.0%	0 / 1 0.0%	0 / 1 0.0%	0 / 1 0.0%	0 / 2 0.0%	0 / 1 0.0%	0 / 2 0.0%	1 / 4 25.0%	0 / 1 0.0%	0 / 1 0.0%	0 / 4 0.0%	0 / 0 0.0%	0 / 0 0.0%	0 / 0 0.0%	0 / 0 0.0%	22 / 35 62.9%E	9 / 35 25.7%	2 / 35 5.7%AB	2 / 35 5.7%CE						
2	Parachlamydiaceae	0 / 75 0.0%	0 / 3 0.0%	0 / 2 0.0%	0 / 2 0.0%	0 / 1 0.0%	0 / 7 0.0%	0 / 4 0.0%	0 / 2 0.0%	0 / 2 0.0%	0 / 3 0.0%	0 / 2 0.0%	0 / 7 0.0%	0 / 2 0.0%	0 / 2 0.0%	0 / 2 0.0%	0 / 2 0.0%	0 / 4 0.0%	0 / 2 0.0%	0 / 4 0.0%	0 / 8 0.0%	0 / 2 0.0%	0 / 2 0.0%	0 / 10 0.0%	0 / 0 0.0%	0 / 0 0.0%	0 / 0 0.0%	0 / 0 0.0%	37 / 75 49.3%E	37 / 75 49.3%	1 / 75 1.3%CE							
1	"Candidatus Parachlamydia" Amann et al. 1997	0 / 40 0.0%	0 / 2 0.0%	0 / 1 0.0%	0 / 1 0.0%	0 / 1 0.0%	0 / 4 0.0%	0 / 2 0.0%	0 / 1 0.0%	0 / 1 0.0%	0 / 2 0.0%	0 / 1 0.0%	0 / 4 0.0%	0 / 1 0.0%	0 / 1 0.0%	0 / 1 0.0%	0 / 1 0.0%	0 / 2 0.0%	0 / 1 0.0%	0 / 2 0.0%	0 / 4 0.0%	0 / 1 0.0%	0 / 1 0.0%	0 / 5 0.0%	0 / 0 0.0%	0 / 0 0.0%	0 / 0 0.0%	0 / 0 0.0%	26 / 40 65.0%E	13 / 40 32.5%	1 / 40 2.5%CE							
1	"Candidatus Parachlamydia Acanthamoebae" Amann et al. 1997	0 / 40 0.0%	0 / 2 0.0%	0 / 1 0.0%	0 / 1 0.0%	0 / 1 0.0%	0 / 4 0.0%	0 / 2 0.0%	0 / 1 0.0%	0 / 1 0.0%	0 / 2 0.0%	0 / 1 0.0%	0 / 4 0.0%	0 / 1 0.0%	0 / 1 0.0%	0 / 1 0.0%	0 / 1 0.0%	0 / 2 0.0%	0 / 1 0.0%	0 / 2 0.0%	0 / 4 0.0%	0 / 1 0.0%	0 / 1 0.0%	0 / 5 0.0%	0 / 0 0.0%	0 / 0 0.0%	0 / 0 0.0%	0 / 0 0.0%	26 / 40 65.0%E	13 / 40 32.5%	1 / 40 2.5%CE							
1	Parachlamydia acanthamoebae UV-7	0 / 40 0.0%	0 / 2 0.0%	0 / 1 0.0%	0 / 1 0.0%	0 / 1 0.0%	0 / 4 0.0%	0 / 2 0.0%	0 / 1 0.0%	0 / 1 0.0%	0 / 2 0.0%	0 / 1 0.0%	0 / 4 0.0%	0 / 1 0.0%	0 / 1 0.0%	0 / 1 0.0%	0 / 1 0.0%	0 / 2 0.0%	0 / 1 0.0%	0 / 2 0.0%	0 / 4 0.0%	0 / 1 0.0%	0 / 1 0.0%	0 / 5 0.0%	0 / 0 0.0%	0 / 0 0.0%	0 / 0 0.0%	0 / 0 0.0%	26 / 40 65.0%E	13 / 40 32.5%	1 / 40 2.5%CE							
1	"Candidatus Protochlamydia" Collingro et al. 2005	0 / 35 0.0%	0 / 1 0.0%	0 / 1 0.0%	0 / 1 0.0%	0 / 0 0.0%	0 / 3 0.0%	0 / 2 0.0%	0 / 1 0.0%	0 / 1 0.0%	0 / 1 0.0%	0 / 1 0.0%	0 / 3 0.0%	0 / 1 0.0%	0 / 1 0.0%	0 / 1 0.0%	0 / 1 0.0%	0 / 2 0.0%	0 / 1 0.0%	0 / 2 0.0%	0 / 4 0.0%	0 / 1 0.0%	0 / 1 0.0%	0 / 5 0.0%	0 / 0 0.0%	0 / 0 0.0%	0 / 0 0.0%	0 / 0 0.0%	24 / 35 68.6%	11 / 35 31.4%E								
1	"Candidatus Protochlamydia amoebophila" Collingro et al. 2005	0 / 35 0.0%	0 / 1 0.0%	0 / 1 0.0%	0 / 1 0.0%	0 / 0 0.0%	0 / 3 0.0%	0 / 2 0.0%	0 / 1 0.0%	0 / 1 0.0%	0 / 1 0.0%	0 / 1 0.0%	0 / 3 0.0%	0 / 1 0.0%	0 / 1 0.0%	0 / 1 0.0%	0 / 1 0.0%	0 / 2 0.0%	0 / 1 0.0%	0 / 2 0.0%	0 / 4 0.0%	0 / 1 0.0%	0 / 1 0.0%	0 / 5 0.0%	0 / 0 0.0%	0 / 0 0.0%	0 / 0 0.0%	0 / 0 0.0%	24 / 35 68.6%	11 / 35 31.4%E								
1	Candidatus Protochlamydia amoebophila UWE25	0 / 35 0.0%	0 / 1 0.0%	0 / 1 0.0%	0 / 1 0.0%	0 / 0 0.0%	0 / 3 0.0%	0 / 2 0.0%	0 / 1 0.0%	0 / 1 0.0%	0 / 1 0.0%	0 / 1 0.0%	0 / 3 0.0%	0 / 1 0.0%	0 / 1 0.0%	0 / 1 0.0%	0 / 1 0.0%	0 / 2 0.0%	0 / 1 0.0%	0 / 2 0.0%	0 / 4 0.0%	0 / 1 0.0%	0 / 1 0.0%	0 / 5 0.0%	0 / 0 0.0%	0 / 0 0.0%	0 / 0 0.0%	0 / 0 0.0%	24 / 35 68.6%	11 / 35 31.4%E								
1	Waddliaceae	0 / 37 0.0%	0 / 1 0.0%	0 / 1 0.0%	0 / 1 0.0%	0 / 1 0.0%	0 / 2 0.0%	0 / 2 0.0%	0 / 1 0.0%	0 / 1 0.0%	0 / 2 0.0%	0 / 1 0.0%	0 / 3 0.0%	0 / 1 0.0%	0 / 1 0.0%	0 / 1 0.0%	0 / 1 0.0%	0 / 2 0.0%	0 / 1 0.0%	0 / 3 0.0%	0 / 4 0.0%	0 / 1 0.0%	0 / 1 0.0%	0 / 5 0.0%	0 / 0 0.0%	0 / 0 0.0%	0 / 0 0.0%	0 / 0 0.0%	22 / 37 59.5%E	14 / 37 37.8%	1 / 37 2.7%CE							
1	Waddlia	0 / 37 0.0%	0 / 1 0.0%	0 / 1 0.0%	0 / 1 0.0%	0 / 1 0.0%	0 / 2 0.0%	0 / 2 0.0%	0 / 1 0.0%	0 / 1 0.0%	0 / 2 0.0%	0 / 1 0.0%	0 / 3 0.0%	0 / 1 0.0%	0 / 1 0.0%	0 / 1 0.0%	0 / 1 0.0%	0 / 2 0.0%	0 / 1 0.0%	0 / 3 0.0%	0 / 4 0.0%	0 / 1 0.0%	0 / 1 0.0%	0 / 5 0.0%	0 / 0 0.0%	0 / 0 0.0%	0 / 0 0.0%	0 / 0 0.0%	22 / 37 59.5%E	14 / 37 37.8%	1 / 37 2.7%CE							
1	ATCC VR-1470	0 / 37 0.0%	0 / 1 0.0%	0 / 1 0.0%	0 / 1 0.0%	0 / 1 0.0%	0 / 2 0.0%	0 / 2 0.0%	0 / 1 0.0%	0 / 1 0.0%	0 / 2 0.0%	0 / 1 0.0%	0 / 3 0.0%	0 / 1 0.0%	0 / 1 0.0%	0 / 1 0.0%	0 / 1 0.0%	0 / 2 0.0%	0 / 1 0.0%	0 / 3 0.0%	0 / 4 0.0%	0 / 1 0.0%	0 / 1 0.0%	0 / 5 0.0%	0 / 0 0.0%	0 / 0 0.0%	0 / 0 0.0%	0 / 0 0.0%	22 / 37 59.5%E	14 / 37 37.8%	1 / 37 2.7%CE							
1	Waddlia chondrophila ATCC VR-1470	0 / 37 0.0%	0 / 1 0.0%	0 / 1 0.0%	0 / 1 0.0%	0 / 1 0.0%	0 / 2 0.0%	0 / 2 0.0%	0 / 1 0.0%	0 / 1 0.0%	0 / 2 0.0%	0 / 1 0.0%	0 / 3 0.0%	0 / 1 0.0%	0 / 1 0.0%	0 / 1 0.0%	0 / 1 0.0%	0 / 2 0.0%	0 / 1 0.0%	0 / 3 0.0%	0 / 4 0.0%	0 / 1 0.0%	0 / 1 0.0%	0 / 5 0.0%	0 / 0 0.0%	0 / 0 0.0%	0 / 0 0.0%	0 / 0 0.0%	22 / 37 59.5%E	14 / 37 37.8%	1 / 37 2.7%CE							
1	Caldiserica	46 / 46 100.0%	1 / 1 100.0%	1 / 1 100.0%	1 / 1 100.0%	1 / 1 100.0%	3 / 3 100.0%	3 / 3 100.0%	1 / 1 100.0%	1 / 1 100.0%	3 / 3 100.0%	1 / 1 100.0%	4 / 4 100.0%	2 / 2 100.0%	1 / 1 100.0%	2 / 2 100.0%	1 / 1 100.0%	3 / 3 100.0%	1 / 1 100.0%	3 / 3 100.0%	5 / 5 100.0%	2 / 2 100.0%	1 / 1 100.0%	5 / 5 100.0%	0 / 0 0.0%	0 / 0 0.0%	0 / 0 0.0%	0 / 0 0.0%	29 / 46 63.0%AB	17 / 46 37.0%ABE								
1	Caldisericia	46 / 46 100.0%	1 / 1 100.0%	1 / 1 100.0%	1 / 1 100.0%	1 / 1 100.0%	3 / 3 100.0%	3 / 3 100.0%	1 / 1 100.0%	1 / 1 100.0%	3 / 3 100.0%	1 / 1 100.0%	4 / 4 100.0%	2 / 2 100.0%	1 / 1 100.0%	2 / 2 100.0%	1 / 1 100.0%	3 / 3 100.0%	1 / 1 100.0%	3 / 3 100.0%	5 / 5 100.0%	2 / 2 100.0%	1 / 1 100.0%	5 / 5 100.0%	0 / 0 0.0%	0 / 0 0.0%	0 / 0 0.0%	0 / 0 0.0%	29 / 46 63.0%AB	17 / 46 37.0%ABE								
1	Caldisericales	46 / 46 100.0%	1 / 1 100.0%	1 / 1 100.0%	1 / 1 100.0%	1 / 1 100.0%	3 / 3 100.0%	3 / 3 100.0%	1 / 1 100.0%	1 / 1 100.0%	3 / 3 100.0%	1 / 1 100.0%	4 / 4 100.0%	2 / 2 100.0%	1 / 1 100.0%	2 / 2 100.0%	1 / 1 100.0%	3 / 3 100.0%	1 / 1 100.0%	3 / 3 100.0%	5 / 5 100.0%	2 / 2 100.0%	1 / 1 100.0%	5 / 5 100.0%	0 / 0 0.0%	0 / 0 0.0%	0 / 0 0.0%	0 / 0 0.0%	29 / 46 63.0%AB	17 / 46 37.0%ABE								
1	Caldisericaceae	46 / 46 100.0%	1 / 1 100.0%	1 / 1 100.0%	1 / 1 100.0%	1 / 1 100.0%	3 / 3 100.0%	3 / 3 100.0%	1 / 1 100.0%	1 / 1 100.0%	3 / 3 100.0%	1 / 1 100.0%	4 / 4 100.0%	2 / 2 100.0%	1 / 1 100.0%	2 / 2 100.0%	1 / 1 100.0%	3 / 3 100.0%	1 / 1 100.0%	3 / 3 100.0%	5 / 5 100.0%	2 / 2 100.0%	1 / 1 100.0%	5 / 5 100.0%	0 / 0 0.0%	0 / 0 0.0%	0 / 0 0.0%	0 / 0 0.0%	29 / 46 63.0%AB	17 / 46 37.0%ABE								
1	Caldisericum	46 / 46 100.0%	1 / 1 100.0%	1 / 1 100.0%	1 / 1 100.0%	1 / 1 100.0%	3 / 3 100.0%	3 / 3 100.0%	1 / 1 100.0%	1 / 1 100.0%	3 / 3 100.0%	1 / 1 100.0%	4 / 4 100.0%	2 / 2 100.0%	1 / 1 100.0%	2 / 2 100.0%	1 / 1 100.0%	3 / 3 100.0%	1 / 1 100.0%	3 / 3 100.0%	5 / 5 100.0%	2 / 2 100.0%	1 / 1 100.0%	5 / 5 100.0%	0 / 0 0.0%	0 / 0 0.0%	0 / 0 0.0%	0 / 0 0.0%	29 / 46 63.0%AB	17 / 46 37.0%ABE								
1	Caldisericum exile	46 / 46 100.0%	1 / 1 100.0%	1 / 1 100.0%	1 / 1 100.0%	1 / 1 100.0%	3 / 3 100.0%	3 / 3 100.0%	1 / 1 100.0%	1 / 1 100.0%	3 / 3 100.0%	1 / 1 100.0%	4 / 4 100.0%	2 / 2 100.0%	1 / 1 100.0%	2 / 2 100.0%	1 / 1 100.0%	3 / 3 100.0%	1 / 1 100.0%	3 / 3 100.0%	5 / 5 100.0%	2 / 2 100.0%	1 / 1 100.0%	5 / 5 100.0%	0 / 0 0.0%	0 / 0 0.0%	0 / 0 0.0%	0 / 0 0.0%	29 / 46 63.0%AB	17 / 46 37.0%ABE								
1	Caldisericum exile AZM16c01	46 / 46 100.0%	1 / 1 100.0%	1 / 1 100.0%	1 / 1 100.0%	1 / 1 100.0%	3 / 3 100.0%	3 / 3 100.0%	1 / 1 100.0%	1 / 1 100.0%	3 / 3 100.0%	1 / 1 100.0%	4 / 4 100.0%	2 / 2 100.0%	1 / 1 100.0%	2 / 2 100.0%	1 / 1 100.0%	3 / 3 100.0%	1 / 1 100.0%	3 / 3 100.0%	5 / 5 100.0%	2 / 2 100.0%	1 / 1 100.0%	5 / 5 100.0%	0 / 0 0.0%	0 / 0 0.0%	0 / 0 0.0%	0 / 0 0.0%	29 / 46 63.0%AB	17 / 46 37.0%ABE								
1	Armatimonadetes	1 / 47 2.1%	0 / 1 0.0%	0 / 1 0.0%	0 / 1 0.0%	0 / 1 0.0%	0 / 3 0.0%	0 / 3 0.0%	0 / 1 0.0%	0 / 1 0.0%	0 / 3 0.0%	0 / 1 0.0%	0 / 4 0.0%	0 / 2 0.0%	0 / 1 0.0%	0 / 2 0.0%	0 / 1 0.0%	0 / 3 0.0%	0 / 1 0.0%	0 / 3 0.0%	0 / 5 0.0%	0 / 2 0.0%	0 / 1 0.0%	0 / 5 0.0%	1 / 1 100.0%	0 / 0 0.0%	0 / 0 0.0%	0 / 0 0.0%	26 / 47 55.3%E	16 / 47 34.0%	2 / 47 4.3%C	2 / 47 4.3%CE	1 / 47 2.1%ABE					
1	Chthonomonadetes	1 / 47 2.1%	0 / 1 0.0%	0 / 1 0.0%	0 / 1 0.0%	0 / 1 0.0%	0 / 3 0.0%	0 / 3 0.0%	0 / 1 0.0%	0 / 1 0.0%	0 / 3 0.0%	0 / 1 0.0%	0 / 4 0.0%	0 / 2 0.0%	0 / 1 0.0%	0 / 2 0.0%	0 / 1 0.0%	0 / 3 0.0%	0 / 1 0.0%	0 / 3 0.0%	0 / 5 0.0%	0 / 2 0.0%	0 / 1 0.0%	0 / 5 0.0%	1 / 1 100.0%	0 / 0 0.0%	0 / 0 0.0%	0 / 0 0.0%	26 / 47 55.3%E	16 / 47 34.0%	2 / 47 4.3%CE	2 / 47 4.3%C	1 / 47 2.1%ABE					
1	Chthonomonadales	1 / 47 2.1%	0 / 1 0.0%	0 / 1 0.0%	0 / 1 0.0%	0 / 1 0.0%	0 / 3 0.0%	0 / 3 0.0%	0 / 1 0.0%	0 / 1 0.0%	0 / 3 0.0%	0 / 1 0.0%	0 / 4 0.0%	0 / 2 0.0%	0 / 1 0.0%	0 / 2 0.0%	0 / 1 0.0%	0 / 3 0.0%	0 / 1 0.0%	0 / 3 0.0%	0 / 5 0.0%	0 / 2 0.0%	0 / 1 0.0%	0 / 5 0.0%	1 / 1 100.0%	0 / 0 0.0%	0 / 0 0.0%	0 / 0 0.0%	26 / 47 55.3%E	16 / 47 34.0%	2 / 47 4.3%C	2 / 47 4.3%CE	1 / 47 2.1%ABE					
1	Chthonomonadaceae	1 / 47 2.1%	0 / 1 0.0%	0 / 1 0.0%	0 / 1 0.0%	0 / 1 0.0%	0 / 3 0.0%	0 / 3 0.0%	0 / 1 0.0%	0 / 1 0.0%	0 / 3 0.0%	0 / 1 0.0%	0 / 4 0.0%	0 / 2 0.0%	0 / 1 0.0%	0 / 2 0.0%	0 / 1 0.0%	0 / 3 0.0%	0 / 1 0.0%	0 / 3 0.0%	0 / 5 0.0%	0 / 2 0.0%	0 / 1 0.0%	0 / 5 0.0%	1 / 1 100.0%	0 / 0 0.0%	0 / 0 0.0%	0 / 0 0.0%	26 / 47 55.3%E	16 / 47 34.0%	2 / 47 4.3%C	2 / 47 4.3%CE	1 / 47 2.1%ABE					
1	Chthonomonas	1 / 47 2.1%	0 / 1 0.0%	0 / 1 0.0%	0 / 1 0.0%	0 / 1 0.0%	0 / 3 0.0%	0 / 3 0.0%	0 / 1 0.0%	0 / 1 0.0%	0 / 3 0.0%	0 / 1 0.0%	0 / 4 0.0%	0 / 2 0.0%	0 / 1 0.0%	0 / 2 0.0%	0 / 1 0.0%	0 / 3 0.0%	0 / 1 0.0%	0 / 3 0.0%	0 / 5 0.0%	0 / 2 0.0%	0 / 1 0.0%	0 / 5 0.0%	1 / 1 100.0%	0 / 0 0.0%	0 / 0 0.0%	0 / 0 0.0%	26 / 47 55.3%E	16 / 47 34.0%	2 / 47 4.3%CE	2 / 47 4.3%C	1 / 47 2.1%ABE					
1	Chthonomonas calidirosea	1 / 47 2.1%	0 / 1 0.0%	0 / 1 0.0%	0 / 1 0.0%	0 / 1 0.0%	0 / 3 0.0%	0 / 3 0.0%	0 / 1 0.0%	0 / 1 0.0%	0 / 3 0.0%	0 / 1 0.0%	0 / 4 0.0%	0 / 2 0.0%	0 / 1 0.0%	0 / 2 0.0%	0 / 1 0.0%	0 / 3 0.0%	0 / 1 0.0%	0 / 3 0.0%	0 / 5 0.0%	0 / 2 0.0%	0 / 1 0.0%	0 / 5 0.0%	1 / 1 100.0%	0 / 0 0.0%	0 / 0 0.0%	0 / 0 0.0%	26 / 47 55.3%E	16 / 47 34.0%	2 / 47 4.3%CE	2 / 47 4.3%C	1 / 47 2.1%ABE					
1	Armatimonadetes bacterium T49	1 / 47 2.1%	0 / 1 0.0%	0 / 1 0.0%	0 / 1 0.0%	0 / 1 0.0%	0 / 3 0.0%	0 / 3 0.0%	0 / 1 0.0%	0 / 1 0.0%	0 / 3 0.0%	0 / 1 0.0%	0 / 4 0.0%	0 / 2 0.0%	0 / 1 0.0%	0 / 2 0.0%	0 / 1 0.0%	0 / 3 0.0%	0 / 1 0.0%	0 / 3 0.0%	0 / 5 0.0%	0 / 2 0.0%	0 / 1 0.0%	0 / 5 0.0%	1 / 1 100.0%	0 / 0 0.0%	0 / 0 0.0%	0 / 0 0.0%	26 / 47 55.3%E	16 / 47 34.0%	2 / 47 4.3%CE	2 / 47 4.3%C	1 / 47 2.1%ABE					
2	'Dictyoglomi'	92 / 92 100.0%	2 / 2 100.0%	2 / 2 100.0%	2 / 2 100.0%	2 / 2 100.0%	6 / 6 100.0%	6 / 6 100.0%	2 / 2 100.0%	2 / 2 100.0%	6 / 6 100.0%	2 / 2 100.0%	8 / 8 100.0%	4 / 4 100.0%	2 / 2 100.0%	4 / 4 100.0%	2 / 2 100.0%	6 / 6 100.0%	2 / 2 100.0%	6 / 6 100.0%	10 / 10 100.0%	4 / 4 100.0%	2 / 2 100.0%	10 / 10 100.0%	0 / 0 0.0%	0 / 0 0.0%	0 / 0 0.0%	0 / 0 0.0%	58 / 92 63.0%AB	34 / 92 37.0%ABE								
2	'Dictyoglomia'	92 / 92 100.0%	2 / 2 100.0%	2 / 2 100.0%	2 / 2 100.0%	2 / 2 100.0%	6 / 6 100.0%	6 / 6 100.0%	2 / 2 100.0%	2 / 2 100.0%	6 / 6 100.0%	2 / 2 100.0%	8 / 8 100.0%	4 / 4 100.0%	2 / 2 100.0%	4 / 4 100.0%	2 / 2 100.0%	6 / 6 100.0%	2 / 2 100.0%	6 / 6 100.0%	10 / 10 100.0%	4 / 4 100.0%	2 / 2 100.0%	10 / 10 100.0%	0 / 0 0.0%	0 / 0 0.0%	0 / 0 0.0%	0 / 0 0.0%	58 / 92 63.0%AB	34 / 92 37.0%ABE								
2	'Dictyoglomales'	92 / 92 100.0%	2 / 2 100.0%	2 / 2 100.0%	2 / 2 100.0%	2 / 2 100.0%	6 / 6 100.0%	6 / 6 100.0%	2 / 2 100.0%	2 / 2 100.0%	6 / 6 100.0%	2 / 2 100.0%	8 / 8 100.0%	4 / 4 100.0%	2 / 2 100.0%	4 / 4 100.0%	2 / 2 100.0%	6 / 6 100.0%	2 / 2 100.0%	6 / 6 100.0%	10 / 10 100.0%	4 / 4 100.0%	2 / 2 100.0%	10 / 10 100.0%	0 / 0 0.0%	0 / 0 0.0%	0 / 0 0.0%	0 / 0 0.0%	58 / 92 63.0%AB	34 / 92 37.0%ABE								
2	'Dictyoglomaceae'	92 / 92 100.0%	2 / 2 100.0%	2 / 2 100.0%	2 / 2 100.0%	2 / 2 100.0%	6 / 6 100.0%	6 / 6 100.0%	2 / 2 100.0%	2 / 2 100.0%	6 / 6 100.0%	2 / 2 100.0%	8 / 8 100.0%	4 / 4 100.0%	2 / 2 100.0%	4 / 4 100.0%	2 / 2 100.0%	6 / 6 100.0%	2 / 2 100.0%	6 / 6 100.0%	10 / 10 100.0%	4 / 4 100.0%	2 / 2 100.0%	10 / 10 100.0%	0 / 0 0.0%	0 / 0 0.0%	0 / 0 0.0%	0 / 0 0.0%	58 / 92 63.0%AB	34 / 92 37.0%ABE								
2	Dictyoglomus	92 / 92 100.0%	2 / 2 100.0%	2 / 2 100.0%	2 / 2 100.0%	2 / 2 100.0%	6 / 6 100.0%	6 / 6 100.0%	2 / 2 100.0%	2 / 2 100.0%	6 / 6 100.0%	2 / 2 100.0%	8 / 8 100.0%	4 / 4 100.0%	2 / 2 100.0%	4 / 4 100.0%	2 / 2 100.0%	6 / 6 100.0%	2 / 2 100.0%	6 / 6 100.0%	10 / 10 100.0%	4 / 4 100.0%	2 / 2 100.0%	10 / 10 100.0%	0 / 0 0.0%	0 / 0 0.0%	0 / 0 0.0%	0 / 0 0.0%	58 / 92 63.0%AB	34 / 92 37.0%ABE								
1	ATCC 35947	46 / 46 100.0%	1 / 1 100.0%	1 / 1 100.0%	1 / 1 100.0%	1 / 1 100.0%	3 / 3 100.0%	3 / 3 100.0%	1 / 1 100.0%	1 / 1 100.0%	3 / 3 100.0%	1 / 1 100.0%	4 / 4 100.0%	2 / 2 100.0%	1 / 1 100.0%	2 / 2 100.0%	1 / 1 100.0%	3 / 3 100.0%	1 / 1 100.0%	3 / 3 100.0%	5 / 5 100.0%	2 / 2 100.0%	1 / 1 100.0%	5 / 5 100.0%	0 / 0 0.0%	0 / 0 0.0%	0 / 0 0.0%	0 / 0 0.0%	29 / 46 63.0%AB	17 / 46 37.0%ABE								
1	Dictyoglomus thermophilum H-6-12	46 / 46 100.0%	1 / 1 100.0%	1 / 1 100.0%	1 / 1 100.0%	1 / 1 100.0%	3 / 3 100.0%	3 / 3 100.0%	1 / 1 100.0%	1 / 1 100.0%	3 / 3 100.0%	1 / 1 100.0%	4 / 4 100.0%	2 / 2 100.0%	1 / 1 100.0%	2 / 2 100.0%	1 / 1 100.0%	3 / 3 100.0%	1 / 1 100.0%	3 / 3 100.0%	5 / 5 100.0%	2 / 2 100.0%	1 / 1 100.0%	5 / 5 100.0%	0 / 0 0.0%	0 / 0 0.0%	0 / 0 0.0%	0 / 0 0.0%	29 / 46 63.0%AB	17 / 46 37.0%ABE								
1	DSM 6724	46 / 46 100.0%	1 / 1 100.0%	1 / 1 100.0%	1 / 1 100.0%	1 / 1 100.0%	3 / 3 100.0%	3 / 3 100.0%	1 / 1 100.0%	1 / 1 100.0%	3 / 3 100.0%	1 / 1 100.0%	4 / 4 100.0%	2 / 2 100.0%	1 / 1 100.0%	2 / 2 100.0%	1 / 1 100.0%	3 / 3 100.0%	1 / 1 100.0%	3 / 3 100.0%	5 / 5 100.0%	2 / 2 100.0%	1 / 1 100.0%	5 / 5 100.0%	0 / 0 0.0%	0 / 0 0.0%	0 / 0 0.0%	0 / 0 0.0%	29 / 46 63.0%AB	17 / 46 37.0%ABE								
1	Dictyoglomus turgidum DSM 6724	46 / 46 100.0%	1 / 1 100.0%	1 / 1 100.0%	1 / 1 100.0%	1 / 1 100.0%	3 / 3 100.0%	3 / 3 100.0%	1 / 1 100.0%	1 / 1 100.0%	3 / 3 100.0%	1 / 1 100.0%	4 / 4 100.0%	2 / 2 100.0%	1 / 1 100.0%	2 / 2 100.0%	1 / 1 100.0%	3 / 3 100.0%	1 / 1 100.0%	3 / 3 100.0%	5 / 5 100.0%	2 / 2 100.0%	1 / 1 100.0%	5 / 5 100.0%	0 / 0 0.0%	0 / 0 0.0%	0 / 0 0.0%	0 / 0 0.0%	29 / 46 63.0%AB	17 / 46 37.0%ABE								
103	Bacteroidetes/Chlorobi group	473 / 4763 9.9%	9 / 116 7.8%	8 / 156 5.1%	5 / 128 3.9%	26 / 252 10.3%	34 / 330 10.3%	33 / 231 14.3%	10 / 107 9.3%	10 / 136 7.4%	47 / 345 13.6%	7 / 115 6.1%	39 / 363 10.7%	24 / 225 10.7%	20 / 120 16.7%	8 / 161 5.0%	16 / 114 14.0%	22 / 247 8.9%	16 / 106 15.1%	16 / 294 5.4%	34 / 417 8.2%	22 / 198 11.1%	7 / 105 6.7%	60 / 483 12.4%	0 / 1 0.0%	0 / 0 0.0%	0 / 13 0.0%	0 / 0 0.0%	2754 / 4763 57.8%	1318 / 4763 27.7%E	281 / 4763 5.9%AB	167 / 4763 3.5%ABE	148 / 4763 3.1%C	66 / 4763 1.4%CE	16 / 4763 0.3%BE	9 / 4763 0.2%B	3 / 4763 0.1%GG	1 / 4763 0.0%D
90	BCF group	354 / 4156 8.5%	4 / 100 4.0%	8 / 143 5.6%	2 / 115 1.7%	22 / 231 9.5%	26 / 289 9.0%	25 / 196 12.8%	9 / 94 9.6%	9 / 123 7.3%	47 / 299 15.7%	7 / 102 6.9%	28 / 311 9.0%	12 / 197 6.1%	15 / 107 14.0%	5 / 135 3.7%	9 / 101 8.9%	17 / 208 8.2%	11 / 93 11.8%	16 / 255 6.3%	23 / 363 6.3%	17 / 171 9.9%	5 / 92 5.4%	37 / 417 8.9%	0 / 1 0.0%	0 / 0 0.0%	0 / 13 0.0%	0 / 0 0.0%	2459 / 4156 59.2%	1172 / 4156 28.2%E	235 / 4156 5.7%AB	117 / 4156 2.8%ABE	114 / 4156 2.7%C	53 / 4156 1.3%CE	3 / 4156 0.1%GG	2 / 4156 0.0%B	1 / 4156 0.0%D	
42	Flavobacteria	89 / 1677 5.3%	0 / 46 0.0%	1 / 51 2.0%	1 / 46 2.2%	1 / 103 1.0%	6 / 99 6.1%	10 / 79 12.7%	0 / 44 0.0%	1 / 52 1.9%	21 / 131 16.0%	0 / 43 0.0%	8 / 126 6.3%	2 / 70 2.9%	8 / 46 17.4%	1 / 48 2.1%	3 / 46 6.5%	4 / 75 5.3%	3 / 42 7.1%	0 / 95 0.0%	7 / 143 4.9%	2 / 71 2.8%	1 / 42 2.4%	9 / 175 5.1%	0 / 1 0.0%	0 / 0 0.0%	0 / 3 0.0%	0 / 0 0.0%	1013 / 1677 60.4%	548 / 1677 32.7%E	65 / 1677 3.9%AB	24 / 1677 1.4%ABE	19 / 1677 1.1%C	7 / 1677 0.4%CE	1 / 1677 0.1%GG			
42	"Flavobacteriales" Bernardet 2011	89 / 1677 5.3%	0 / 46 0.0%	1 / 51 2.0%	1 / 46 2.2%	1 / 103 1.0%	6 / 99 6.1%	10 / 79 12.7%	0 / 44 0.0%	1 / 52 1.9%	21 / 131 16.0%	0 / 43 0.0%	8 / 126 6.3%	2 / 70 2.9%	8 / 46 17.4%	1 / 48 2.1%	3 / 46 6.5%	4 / 75 5.3%	3 / 42 7.1%	0 / 95 0.0%	7 / 143 4.9%	2 / 71 2.8%	1 / 42 2.4%	9 / 175 5.1%	0 / 1 0.0%	0 / 0 0.0%	0 / 3 0.0%	0 / 0 0.0%	1013 / 1677 60.4%	548 / 1677 32.7%E	65 / 1677 3.9%AB	24 / 1677 1.4%ABE	19 / 1677 1.1%C	7 / 1677 0.4%CE	1 / 1677 0.1%GG			
7	"Blattabacteriaceae" Kambhampati 2011	2 / 234 0.9%	0 / 7 0.0%	0 / 7 0.0%	0 / 7 0.0%	0 / 7 0.0%	2 / 14 14.3%	0 / 14 0.0%	0 / 7 0.0%	0 / 7 0.0%	0 / 7 0.0%	0 / 7 0.0%	0 / 21 0.0%	0 / 7 0.0%	0 / 7 0.0%	0 / 7 0.0%	0 / 7 0.0%	0 / 11 0.0%	0 / 7 0.0%	0 / 14 0.0%	0 / 27 0.0%	0 / 7 0.0%	0 / 7 0.0%	0 / 28 0.0%	0 / 0 0.0%	0 / 0 0.0%	0 / 0 0.0%	0 / 0 0.0%	116 / 234 49.6%E	114 / 234 48.7%	2 / 234 0.9%AB	2 / 234 0.9%C						
7	Blattabacterium	2 / 234 0.9%	0 / 7 0.0%	0 / 7 0.0%	0 / 7 0.0%	0 / 7 0.0%	2 / 14 14.3%	0 / 14 0.0%	0 / 7 0.0%	0 / 7 0.0%	0 / 7 0.0%	0 / 7 0.0%	0 / 21 0.0%	0 / 7 0.0%	0 / 7 0.0%	0 / 7 0.0%	0 / 7 0.0%	0 / 11 0.0%	0 / 7 0.0%	0 / 14 0.0%	0 / 27 0.0%	0 / 7 0.0%	0 / 7 0.0%	0 / 28 0.0%	0 / 0 0.0%	0 / 0 0.0%	0 / 0 0.0%	0 / 0 0.0%	116 / 234 49.6%E	114 / 234 48.7%	2 / 234 0.9%C	2 / 234 0.9%AB						
1	Blattabacterium sp. (Mastotermes darwiniensis)	1 / 34 2.9%	0 / 1 0.0%	0 / 1 0.0%	0 / 1 0.0%	0 / 1 0.0%	1 / 2 50.0%	0 / 2 0.0%	0 / 1 0.0%	0 / 1 0.0%	0 / 1 0.0%	0 / 1 0.0%	0 / 3 0.0%	0 / 1 0.0%	0 / 1 0.0%	0 / 1 0.0%	0 / 1 0.0%	0 / 2 0.0%	0 / 1 0.0%	0 / 2 0.0%	0 / 4 0.0%	0 / 1 0.0%	0 / 1 0.0%	0 / 4 0.0%	0 / 0 0.0%	0 / 0 0.0%	0 / 0 0.0%	0 / 0 0.0%	17 / 34 50.0%E	16 / 34 47.1%	1 / 34 2.9%AB							
1	Blattabacterium sp. (Mastotermes darwiniensis) str. MADAR	1 / 34 2.9%	0 / 1 0.0%	0 / 1 0.0%	0 / 1 0.0%	0 / 1 0.0%	1 / 2 50.0%	0 / 2 0.0%	0 / 1 0.0%	0 / 1 0.0%	0 / 1 0.0%	0 / 1 0.0%	0 / 3 0.0%	0 / 1 0.0%	0 / 1 0.0%	0 / 1 0.0%	0 / 1 0.0%	0 / 2 0.0%	0 / 1 0.0%	0 / 2 0.0%	0 / 4 0.0%	0 / 1 0.0%	0 / 1 0.0%	0 / 4 0.0%	0 / 0 0.0%	0 / 0 0.0%	0 / 0 0.0%	0 / 0 0.0%	17 / 34 50.0%E	16 / 34 47.1%	1 / 34 2.9%AB							
1	"Blattabacterium punctulatus" Clark and Kambhanpati 2003	1 / 32 3.1%	0 / 1 0.0%	0 / 1 0.0%	0 / 1 0.0%	0 / 1 0.0%	1 / 2 50.0%	0 / 2 0.0%	0 / 1 0.0%	0 / 1 0.0%	0 / 1 0.0%	0 / 1 0.0%	0 / 3 0.0%	0 / 1 0.0%	0 / 1 0.0%	0 / 1 0.0%	0 / 1 0.0%	0 / 1 0.0%	0 / 1 0.0%	0 / 2 0.0%	0 / 3 0.0%	0 / 1 0.0%	0 / 1 0.0%	0 / 4 0.0%	0 / 0 0.0%	0 / 0 0.0%	0 / 0 0.0%	0 / 0 0.0%	16 / 32 50.0%	15 / 32 46.9%E	1 / 32 3.1%AB							
1	Blattabacterium sp. (Cryptocercus punctulatus) str. Cpu	1 / 32 3.1%	0 / 1 0.0%	0 / 1 0.0%	0 / 1 0.0%	0 / 1 0.0%	1 / 2 50.0%	0 / 2 0.0%	0 / 1 0.0%	0 / 1 0.0%	0 / 1 0.0%	0 / 1 0.0%	0 / 3 0.0%	0 / 1 0.0%	0 / 1 0.0%	0 / 1 0.0%	0 / 1 0.0%	0 / 1 0.0%	0 / 1 0.0%	0 / 2 0.0%	0 / 3 0.0%	0 / 1 0.0%	0 / 1 0.0%	0 / 4 0.0%	0 / 0 0.0%	0 / 0 0.0%	0 / 0 0.0%	0 / 0 0.0%	16 / 32 50.0%	15 / 32 46.9%E	1 / 32 3.1%AB							
1	Blattabacterium sp. (Panesthia angustipennis spadica)	0 / 34 0.0%	0 / 1 0.0%	0 / 1 0.0%	0 / 1 0.0%	0 / 1 0.0%	0 / 2 0.0%	0 / 2 0.0%	0 / 1 0.0%	0 / 1 0.0%	0 / 1 0.0%	0 / 1 0.0%	0 / 3 0.0%	0 / 1 0.0%	0 / 1 0.0%	0 / 1 0.0%	0 / 1 0.0%	0 / 2 0.0%	0 / 1 0.0%	0 / 2 0.0%	0 / 4 0.0%	0 / 1 0.0%	0 / 1 0.0%	0 / 4 0.0%	0 / 0 0.0%	0 / 0 0.0%	0 / 0 0.0%	0 / 0 0.0%	19 / 34 55.9%	15 / 34 44.1%E								
1	Blattabacterium sp. (Panesthia angustipennis spadica) BPAA	0 / 34 0.0%	0 / 1 0.0%	0 / 1 0.0%	0 / 1 0.0%	0 / 1 0.0%	0 / 2 0.0%	0 / 2 0.0%	0 / 1 0.0%	0 / 1 0.0%	0 / 1 0.0%	0 / 1 0.0%	0 / 3 0.0%	0 / 1 0.0%	0 / 1 0.0%	0 / 1 0.0%	0 / 1 0.0%	0 / 2 0.0%	0 / 1 0.0%	0 / 2 0.0%	0 / 4 0.0%	0 / 1 0.0%	0 / 1 0.0%	0 / 4 0.0%	0 / 0 0.0%	0 / 0 0.0%	0 / 0 0.0%	0 / 0 0.0%	19 / 34 55.9%	15 / 34 44.1%E								
1	Blattabacterium sp. (Periplaneta americana)	0 / 33 0.0%	0 / 1 0.0%	0 / 1 0.0%	0 / 1 0.0%	0 / 1 0.0%	0 / 2 0.0%	0 / 2 0.0%	0 / 1 0.0%	0 / 1 0.0%	0 / 1 0.0%	0 / 1 0.0%	0 / 3 0.0%	0 / 1 0.0%	0 / 1 0.0%	0 / 1 0.0%	0 / 1 0.0%	0 / 1 0.0%	0 / 1 0.0%	0 / 2 0.0%	0 / 4 0.0%	0 / 1 0.0%	0 / 1 0.0%	0 / 4 0.0%	0 / 0 0.0%	0 / 0 0.0%	0 / 0 0.0%	0 / 0 0.0%	16 / 33 48.5%E	16 / 33 48.5%	1 / 33 3.0%C							
1	Blattabacterium sp. (Periplaneta americana) str. BPLAN	0 / 33 0.0%	0 / 1 0.0%	0 / 1 0.0%	0 / 1 0.0%	0 / 1 0.0%	0 / 2 0.0%	0 / 2 0.0%	0 / 1 0.0%	0 / 1 0.0%	0 / 1 0.0%	0 / 1 0.0%	0 / 3 0.0%	0 / 1 0.0%	0 / 1 0.0%	0 / 1 0.0%	0 / 1 0.0%	0 / 1 0.0%	0 / 1 0.0%	0 / 2 0.0%	0 / 4 0.0%	0 / 1 0.0%	0 / 1 0.0%	0 / 4 0.0%	0 / 0 0.0%	0 / 0 0.0%	0 / 0 0.0%	0 / 0 0.0%	16 / 33 48.5%E	16 / 33 48.5%	1 / 33 3.0%C							
1	Blattabacterium sp. (Blatta orientalis)	0 / 34 0.0%	0 / 1 0.0%	0 / 1 0.0%	0 / 1 0.0%	0 / 1 0.0%	0 / 2 0.0%	0 / 2 0.0%	0 / 1 0.0%	0 / 1 0.0%	0 / 1 0.0%	0 / 1 0.0%	0 / 3 0.0%	0 / 1 0.0%	0 / 1 0.0%	0 / 1 0.0%	0 / 1 0.0%	0 / 2 0.0%	0 / 1 0.0%	0 / 2 0.0%	0 / 4 0.0%	0 / 1 0.0%	0 / 1 0.0%	0 / 4 0.0%	0 / 0 0.0%	0 / 0 0.0%	0 / 0 0.0%	0 / 0 0.0%	18 / 34 52.9%E	15 / 34 44.1%	1 / 34 2.9%C							
1	Blattabacterium sp. (Blatta orientalis) str. Tarazona	0 / 34 0.0%	0 / 1 0.0%	0 / 1 0.0%	0 / 1 0.0%	0 / 1 0.0%	0 / 2 0.0%	0 / 2 0.0%	0 / 1 0.0%	0 / 1 0.0%	0 / 1 0.0%	0 / 1 0.0%	0 / 3 0.0%	0 / 1 0.0%	0 / 1 0.0%	0 / 1 0.0%	0 / 1 0.0%	0 / 2 0.0%	0 / 1 0.0%	0 / 2 0.0%	0 / 4 0.0%	0 / 1 0.0%	0 / 1 0.0%	0 / 4 0.0%	0 / 0 0.0%	0 / 0 0.0%	0 / 0 0.0%	0 / 0 0.0%	18 / 34 52.9%E	15 / 34 44.1%	1 / 34 2.9%C							
1	Blattabacterium sp. (Blattella germanica)	0 / 34 0.0%	0 / 1 0.0%	0 / 1 0.0%	0 / 1 0.0%	0 / 1 0.0%	0 / 2 0.0%	0 / 2 0.0%	0 / 1 0.0%	0 / 1 0.0%	0 / 1 0.0%	0 / 1 0.0%	0 / 3 0.0%	0 / 1 0.0%	0 / 1 0.0%	0 / 1 0.0%	0 / 1 0.0%	0 / 2 0.0%	0 / 1 0.0%	0 / 2 0.0%	0 / 4 0.0%	0 / 1 0.0%	0 / 1 0.0%	0 / 4 0.0%	0 / 0 0.0%	0 / 0 0.0%	0 / 0 0.0%	0 / 0 0.0%	18 / 34 52.9%E	16 / 34 47.1%								
1	Blattabacterium sp. (Blattella germanica) str. Alboraia	0 / 34 0.0%	0 / 1 0.0%	0 / 1 0.0%	0 / 1 0.0%	0 / 1 0.0%	0 / 2 0.0%	0 / 2 0.0%	0 / 1 0.0%	0 / 1 0.0%	0 / 1 0.0%	0 / 1 0.0%	0 / 3 0.0%	0 / 1 0.0%	0 / 1 0.0%	0 / 1 0.0%	0 / 1 0.0%	0 / 2 0.0%	0 / 1 0.0%	0 / 2 0.0%	0 / 4 0.0%	0 / 1 0.0%	0 / 1 0.0%	0 / 4 0.0%	0 / 0 0.0%	0 / 0 0.0%	0 / 0 0.0%	0 / 0 0.0%	18 / 34 52.9%E	16 / 34 47.1%								
1	Blattabacterium sp. (Blaberus giganteus)	0 / 33 0.0%	0 / 1 0.0%	0 / 1 0.0%	0 / 1 0.0%	0 / 1 0.0%	0 / 2 0.0%	0 / 2 0.0%	0 / 1 0.0%	0 / 1 0.0%	0 / 1 0.0%	0 / 1 0.0%	0 / 3 0.0%	0 / 1 0.0%	0 / 1 0.0%	0 / 1 0.0%	0 / 1 0.0%	0 / 1 0.0%	0 / 1 0.0%	0 / 2 0.0%	0 / 4 0.0%	0 / 1 0.0%	0 / 1 0.0%	0 / 4 0.0%	0 / 0 0.0%	0 / 0 0.0%	0 / 0 0.0%	0 / 0 0.0%	17 / 33 51.5%E	16 / 33 48.5%								
28	Flavobacteriaceae	78 / 1216 6.4%	0 / 32 0.0%	1 / 37 2.7%	1 / 32 3.1%	0 / 87 0.0%	3 / 71 4.2%	10 / 56 17.9%	0 / 30 0.0%	1 / 37 2.7%	21 / 115 18.3%	0 / 29 0.0%	6 / 86 7.0%	2 / 55 3.6%	8 / 32 25.0%	1 / 33 3.0%	2 / 30 6.7%	3 / 55 5.5%	3 / 28 10.7%	0 / 68 0.0%	7 / 97 7.2%	2 / 56 3.6%	1 / 28 3.6%	6 / 119 5.0%	0 / 1 0.0%	0 / 0 0.0%	0 / 2 0.0%	0 / 0 0.0%	765 / 1216 62.9%	356 / 1216 29.3%E	60 / 1216 4.9%AB	18 / 1216 1.5%ABE	11 / 1216 0.9%C	6 / 1216 0.5%CE				
4	Flavibacterium	17 / 227 7.5%	0 / 6 0.0%	0 / 6 0.0%	0 / 6 0.0%	0 / 20 0.0%	1 / 12 8.3%	2 / 14 14.3%	0 / 5 0.0%	0 / 9 0.0%	6 / 25 24.0%	0 / 5 0.0%	3 / 16 18.8%	2 / 12 16.7%	1 / 6 16.7%	0 / 6 0.0%	0 / 4 0.0%	0 / 10 0.0%	1 / 4 25.0%	0 / 14 0.0%	0 / 14 0.0%	0 / 10 0.0%	0 / 4 0.0%	1 / 19 5.3%	0 / 0 0.0%	0 / 0 0.0%	0 / 0 0.0%	0 / 0 0.0%	152 / 227 67.0%	56 / 227 24.7%E	13 / 227 5.7%AB	4 / 227 1.8%ABE	2 / 227 0.9%C					
1	"Cytophaga johnsonii" (sic) Stanier 1957	5 / 61 8.2%	0 / 2 0.0%	0 / 2 0.0%	0 / 2 0.0%	0 / 6 0.0%	0 / 3 0.0%	1 / 3 33.3%	0 / 1 0.0%	0 / 2 0.0%	0 / 7 0.0%	0 / 2 0.0%	2 / 4 50.0%	0 / 3 0.0%	1 / 2 50.0%	0 / 2 0.0%	0 / 1 0.0%	0 / 2 0.0%	1 / 1 100.0%	0 / 4 0.0%	0 / 3 0.0%	0 / 3 0.0%	0 / 1 0.0%	0 / 5 0.0%	0 / 0 0.0%	0 / 0 0.0%	0 / 0 0.0%	0 / 0 0.0%	41 / 61 67.2%	14 / 61 23.0%E	3 / 61 4.9%AB	2 / 61 3.3%ABE	1 / 61 1.6%C					
1	Flavobacterium johnsoniae ATCC 17061	5 / 61	0 / 2	0 / 2	0 / 2	0 / 6	0 / 3	1 / 3	0 / 1	0 / 2	0 / 7	0 / 2	2 / 4	0 / 3	1 / 2	0 / 2	0 / 1	0 / 2	1 / 1	0 / 4	0 / 3	0 / 3	0 / 1	0 / 5	0 / 0	0 / 0	0 / 0	0 / 0	41 / 61	14 / 61	3 / 61	2 / 61	1 / 61					

1	"Bacillus columnaris" Davis 1922	8 / 74 10.8%	0 / 1 0.0%	0 / 2 0.0%	0 / 2 0.0%	0 / 5 0.0%	0 / 4 0.0%	0 / 7 0.0%	0 / 2 0.0%	0 / 3 0.0%	6 / 7 85.7%	0 / 1 0.0%	0 / 6 0.0%	1 / 5 20.0%	0 / 2 0.0%	0 / 2 0.0%	0 / 1 0.0%	0 / 4 0.0%	0 / 1 0.0%	0 / 4 0.0%	0 / 5 0.0%	0 / 3 0.0%	0 / 1 0.0%	1 / 6 16.7%	0 / 0 0.0%	0 / 0 0.0%	0 / 0 0.0%	0 / 0 0.0%	46 / 74 62.2%	19 / 74 25.7%E	7 / 74 9.5%AB	1 / 74 1.4%ABE	1 / 74 1.4%C	
1	Flavobacterium columnare ATCC 49512	8 / 74 10.8%	0 / 1 0.0%	0 / 2 0.0%	0 / 2 0.0%	0 / 5 0.0%	0 / 4 0.0%	0 / 7 0.0%	0 / 2 0.0%	0 / 3 0.0%	6 / 7 85.7%	0 / 1 0.0%	0 / 6 0.0%	1 / 5 20.0%	0 / 2 0.0%	0 / 2 0.0%	0 / 1 0.0%	0 / 4 0.0%	0 / 1 0.0%	0 / 4 0.0%	0 / 5 0.0%	0 / 3 0.0%	0 / 1 0.0%	1 / 6 16.7%	0 / 0 0.0%	0 / 0 0.0%	0 / 0 0.0%	0 / 0 0.0%	46 / 74 62.2%	19 / 74 25.7%E	7 / 74 9.5%AB	1 / 74 1.4%ABE	1 / 74 1.4%C	
1	ATCC 35035	3 / 43 7.0%	0 / 1 0.0%	0 / 1 0.0%	0 / 1 0.0%	0 / 3 0.0%	1 / 3 33.3%	1 / 2 50.0%	0 / 1 0.0%	0 / 2 0.0%	0 / 4 0.0%	0 / 1 0.0%	0 / 3 0.0%	1 / 2 50.0%	0 / 1 0.0%	0 / 1 0.0%	0 / 1 0.0%	0 / 2 0.0%	0 / 1 0.0%	0 / 3 0.0%	0 / 3 0.0%	0 / 2 0.0%	0 / 1 0.0%	0 / 4 0.0%	0 / 0 0.0%	0 / 0 0.0%	0 / 0 0.0%	0 / 0 0.0%	28 / 43 65.1%	12 / 43 27.9%E	3 / 43 7.0%AB			
1	Flavobacterium branchiophilum FL-15	3 / 43 7.0%	0 / 1 0.0%	0 / 1 0.0%	0 / 1 0.0%	0 / 3 0.0%	1 / 3 33.3%	1 / 2 50.0%	0 / 1 0.0%	0 / 2 0.0%	0 / 4 0.0%	0 / 1 0.0%	0 / 3 0.0%	1 / 2 50.0%	0 / 1 0.0%	0 / 1 0.0%	0 / 1 0.0%	0 / 2 0.0%	0 / 1 0.0%	0 / 3 0.0%	0 / 3 0.0%	0 / 2 0.0%	0 / 1 0.0%	0 / 4 0.0%	0 / 0 0.0%	0 / 0 0.0%	0 / 0 0.0%	0 / 0 0.0%	28 / 43 65.1%	12 / 43 27.9%E	3 / 43 7.0%AB			
1	"Cytophaga psychrophila" Borg 1960	1 / 49 2.0%	0 / 2 0.0%	0 / 1 0.0%	0 / 1 0.0%	0 / 6 0.0%	0 / 2 0.0%	0 / 2 0.0%	0 / 1 0.0%	0 / 2 0.0%	0 / 7 0.0%	0 / 1 0.0%	1 / 3 33.3%	0 / 2 0.0%	0 / 1 0.0%	0 / 1 0.0%	0 / 1 0.0%	0 / 2 0.0%	0 / 1 0.0%	0 / 3 0.0%	0 / 3 0.0%	0 / 2 0.0%	0 / 1 0.0%	0 / 4 0.0%	0 / 0 0.0%	0 / 0 0.0%	0 / 0 0.0%	0 / 0 0.0%	37 / 49 75.5%	11 / 49 22.4%E	1 / 49 2.0%ABE			
1	Flavobacterium psychrophilum JIP02/86	1 / 49 2.0%	0 / 2 0.0%	0 / 1 0.0%	0 / 1 0.0%	0 / 6 0.0%	0 / 2 0.0%	0 / 2 0.0%	0 / 1 0.0%	0 / 2 0.0%	0 / 7 0.0%	0 / 1 0.0%	1 / 3 33.3%	0 / 2 0.0%	0 / 1 0.0%	0 / 1 0.0%	0 / 1 0.0%	0 / 2 0.0%	0 / 1 0.0%	0 / 3 0.0%	0 / 3 0.0%	0 / 2 0.0%	0 / 1 0.0%	0 / 4 0.0%	0 / 0 0.0%	0 / 0 0.0%	0 / 0 0.0%	0 / 0 0.0%	37 / 49 75.5%	11 / 49 22.4%E	1 / 49 2.0%ABE			
1	Weeksella	2 / 58 3.4%	0 / 2 0.0%	0 / 2 0.0%	0 / 2 0.0%	0 / 5 0.0%	1 / 4 25.0%	0 / 3 0.0%	0 / 2 0.0%	0 / 2 0.0%	0 / 6 0.0%	0 / 1 0.0%	0 / 3 0.0%	0 / 3 0.0%	0 / 2 0.0%	0 / 2 0.0%	1 / 1 100.0%	0 / 2 0.0%	0 / 1 0.0%	0 / 3 0.0%	0 / 3 0.0%	0 / 3 0.0%	0 / 1 0.0%	0 / 5 0.0%	0 / 0 0.0%	0 / 0 0.0%	0 / 0 0.0%	0 / 0 0.0%	39 / 58 67.2%	17 / 58 29.3%E	1 / 58 1.7%ABE	1 / 58 1.7%AB		
1	ATCC 43766	2 / 58 3.4%	0 / 2 0.0%	0 / 2 0.0%	0 / 2 0.0%	0 / 5 0.0%	1 / 4 25.0%	0 / 3 0.0%	0 / 2 0.0%	0 / 2 0.0%	0 / 6 0.0%	0 / 1 0.0%	0 / 3 0.0%	0 / 3 0.0%	0 / 2 0.0%	0 / 2 0.0%	1 / 1 100.0%	0 / 2 0.0%	0 / 1 0.0%	0 / 3 0.0%	0 / 3 0.0%	0 / 3 0.0%	0 / 1 0.0%	0 / 5 0.0%	0 / 0 0.0%	0 / 0 0.0%	0 / 0 0.0%	0 / 0 0.0%	39 / 58 67.2%	17 / 58 29.3%E	1 / 58 1.7%AB	1 / 58 1.7%ABE		
1	Weeksella virosa ATCC 43766	2 / 58 3.4%	0 / 2 0.0%	0 / 2 0.0%	0 / 2 0.0%	0 / 5 0.0%	1 / 4 25.0%	0 / 3 0.0%	0 / 2 0.0%	0 / 2 0.0%	0 / 6 0.0%	0 / 1 0.0%	0 / 3 0.0%	0 / 3 0.0%	0 / 2 0.0%	0 / 2 0.0%	1 / 1 100.0%	0 / 2 0.0%	0 / 1 0.0%	0 / 3 0.0%	0 / 3 0.0%	0 / 3 0.0%	0 / 1 0.0%	0 / 5 0.0%	0 / 0 0.0%	0 / 0 0.0%	0 / 0 0.0%	0 / 0 0.0%	39 / 58 67.2%	17 / 58 29.3%E	1 / 58 1.7%ABE	1 / 58 1.7%AB		
2	Capnocytophaga	3 / 93 3.2%	0 / 2 0.0%	0 / 2 0.0%	0 / 2 0.0%	0 / 7 0.0%	0 / 7 0.0%	1 / 5 20.0%	0 / 2 0.0%	0 / 3 0.0%	0 / 9 0.0%	0 / 2 0.0%	1 / 6 16.7%	0 / 5 0.0%	0 / 2 0.0%	0 / 2 0.0%	0 / 2 0.0%	0 / 4 0.0%	0 / 2 0.0%	0 / 5 0.0%	1 / 9 11.1%	0 / 4 0.0%	0 / 2 0.0%	0 / 8 0.0%	0 / 1 0.0%	0 / 0 0.0%	0 / 0 0.0%	0 / 0 0.0%	63 / 93 67.7%	25 / 93 26.9%E	2 / 93 2.2%AB	2 / 93 2.2%C	1 / 93 1.1%ABE	
1	"Fusiformis nucleatus var. ochraceus" Prevot et al. 1956	2 / 47 4.3%	0 / 1 0.0%	0 / 1 0.0%	0 / 1 0.0%	0 / 4 0.0%	0 / 4 0.0%	1 / 3 33.3%	0 / 1 0.0%	0 / 1 0.0%	0 / 5 0.0%	0 / 1 0.0%	1 / 3 33.3%	0 / 3 0.0%	0 / 1 0.0%	0 / 1 0.0%	0 / 1 0.0%	0 / 2 0.0%	0 / 1 0.0%	0 / 2 0.0%	0 / 4 0.0%	0 / 2 0.0%	0 / 1 0.0%	0 / 4 0.0%	0 / 0 0.0%	0 / 0 0.0%	0 / 0 0.0%	0 / 0 0.0%	32 / 47 68.1%	12 / 47 25.5%E	1 / 47 2.1%AB	1 / 47 2.1%C	1 / 47 2.1%ABE	
1	Capnocytophaga ochracea ATCC 27872	2 / 47 4.3%	0 / 1 0.0%	0 / 1 0.0%	0 / 1 0.0%	0 / 4 0.0%	0 / 4 0.0%	1 / 3 33.3%	0 / 1 0.0%	0 / 1 0.0%	0 / 5 0.0%	0 / 1 0.0%	1 / 3 33.3%	0 / 3 0.0%	0 / 1 0.0%	0 / 1 0.0%	0 / 1 0.0%	0 / 2 0.0%	0 / 1 0.0%	0 / 2 0.0%	0 / 4 0.0%	0 / 2 0.0%	0 / 1 0.0%	0 / 4 0.0%	0 / 0 0.0%	0 / 0 0.0%	0 / 0 0.0%	0 / 0 0.0%	32 / 47 68.1%	12 / 47 25.5%E	1 / 47 2.1%ABE	1 / 47 2.1%C	1 / 47 2.1%AB	
1	ATCC 35979	1 / 46 2.2%	0 / 1 0.0%	0 / 1 0.0%	0 / 1 0.0%	0 / 3 0.0%	0 / 3 0.0%	0 / 2 0.0%	0 / 1 0.0%	0 / 2 0.0%	0 / 4 0.0%	0 / 1 0.0%	0 / 3 0.0%	0 / 2 0.0%	0 / 1 0.0%	0 / 1 0.0%	0 / 1 0.0%	0 / 2 0.0%	0 / 1 0.0%	0 / 3 0.0%	1 / 5 20.0%	0 / 2 0.0%	0 / 1 0.0%	0 / 4 0.0%	0 / 1 0.0%	0 / 0 0.0%	0 / 0 0.0%	0 / 0 0.0%	31 / 46 67.4%	13 / 46 28.3%E	1 / 46 2.2%C	1 / 46 2.2%AB		
1	Capnocytophaga canimorsus Cc5	1 / 46 2.2%	0 / 1 0.0%	0 / 1 0.0%	0 / 1 0.0%	0 / 3 0.0%	0 / 3 0.0%	0 / 2 0.0%	0 / 1 0.0%	0 / 2 0.0%	0 / 4 0.0%	0 / 1 0.0%	0 / 3 0.0%	0 / 2 0.0%	0 / 1 0.0%	0 / 1 0.0%	0 / 1 0.0%	0 / 2 0.0%	0 / 1 0.0%	0 / 3 0.0%	1 / 5 20.0%	0 / 2 0.0%	0 / 1 0.0%	0 / 4 0.0%	0 / 1 0.0%	0 / 0 0.0%	0 / 0 0.0%	0 / 0 0.0%	31 / 46 67.4%	13 / 46 28.3%E	1 / 46 2.2%AB	1 / 46 2.2%C		
1	Ornithobacterium	5 / 43 11.6%	0 / 1 0.0%	0 / 2 0.0%	1 / 1 100.0%	0 / 3 0.0%	0 / 3 0.0%	2 / 2 100.0%	0 / 1 0.0%	0 / 1 0.0%	0 / 4 0.0%	0 / 1 0.0%	0 / 3 0.0%	0 / 2 0.0%	1 / 1 100.0%	0 / 2 0.0%	1 / 1 100.0%	0 / 2 0.0%	0 / 1 0.0%	0 / 2 0.0%	0 / 3 0.0%	0 / 2 0.0%	0 / 1 0.0%	0 / 4 0.0%	0 / 0 0.0%	0 / 0 0.0%	0 / 0 0.0%	0 / 0 0.0%	25 / 43 58.1%	11 / 43 25.6%E	4 / 43 9.3%AB	1 / 43 2.3%C	1 / 43 2.3%CE	1 / 43 2.3%ABE
1	ATCC 51463	5 / 43 11.6%	0 / 1 0.0%	0 / 2 0.0%	1 / 1 100.0%	0 / 3 0.0%	0 / 3 0.0%	2 / 2 100.0%	0 / 1 0.0%	0 / 1 0.0%	0 / 4 0.0%	0 / 1 0.0%	0 / 3 0.0%	0 / 2 0.0%	1 / 1 100.0%	0 / 2 0.0%	1 / 1 100.0%	0 / 2 0.0%	0 / 1 0.0%	0 / 2 0.0%	0 / 3 0.0%	0 / 2 0.0%	0 / 1 0.0%	0 / 4 0.0%	0 / 0 0.0%	0 / 0 0.0%	0 / 0 0.0%	0 / 0 0.0%	25 / 43 58.1%	11 / 43 25.6%E	4 / 43 9.3%AB	1 / 43 2.3%C	1 / 43 2.3%ABE	1 / 43 2.3%CE
1	Ornithobacterium rhinotracheale DSM 15997	5 / 43 11.6%	0 / 1 0.0%	0 / 2 0.0%	1 / 1 100.0%	0 / 3 0.0%	0 / 3 0.0%	2 / 2 100.0%	0 / 1 0.0%	0 / 1 0.0%	0 / 4 0.0%	0 / 1 0.0%	0 / 3 0.0%	0 / 2 0.0%	1 / 1 100.0%	0 / 2 0.0%	1 / 1 100.0%	0 / 2 0.0%	0 / 1 0.0%	0 / 2 0.0%	0 / 3 0.0%	0 / 2 0.0%	0 / 1 0.0%	0 / 4 0.0%	0 / 0 0.0%	0 / 0 0.0%	0 / 0 0.0%	0 / 0 0.0%	25 / 43 58.1%	11 / 43 25.6%E	4 / 43 9.3%AB	1 / 43 2.3%ABE	1 / 43 2.3%CE	1 / 43 2.3%C
5	Riemerella	13 / 197 6.6%	0 / 5 0.0%	0 / 9 0.0%	0 / 5 0.0%	0 / 15 0.0%	0 / 10 0.0%	4 / 10 40.0%	0 / 5 0.0%	0 / 5 0.0%	0 / 20 0.0%	0 / 5 0.0%	0 / 15 0.0%	0 / 9 0.0%	5 / 5 100.0%	0 / 5 0.0%	0 / 5 0.0%	0 / 5 0.0%	0 / 5 0.0%	0 / 10 0.0%	4 / 15 26.7%	0 / 10 0.0%	0 / 5 0.0%	0 / 19 0.0%	0 / 0 0.0%	0 / 0 0.0%	0 / 0 0.0%	0 / 0 0.0%	115 / 197 58.4%	65 / 197 33.0%E	13 / 197 6.6%AB	4 / 197 2.0%CE		
5	"Pasteurella anapestifer" (sic) Hauduroy et al. 1953	13 / 197 6.6%	0 / 5 0.0%	0 / 9 0.0%	0 / 5 0.0%	0 / 15 0.0%	0 / 10 0.0%	4 / 10 40.0%	0 / 5 0.0%	0 / 5 0.0%	0 / 20 0.0%	0 / 5 0.0%	0 / 15 0.0%	0 / 9 0.0%	5 / 5 100.0%	0 / 5 0.0%	0 / 5 0.0%	0 / 5 0.0%	0 / 5 0.0%	0 / 10 0.0%	4 / 15 26.7%	0 / 10 0.0%	0 / 5 0.0%	0 / 19 0.0%	0 / 0 0.0%	0 / 0 0.0%	0 / 0 0.0%	0 / 0 0.0%	115 / 197 58.4%	65 / 197 33.0%E	13 / 197 6.6%AB	4 / 197 2.0%CE		
2	Riemerella anatipestifer ATCC 11845	6 / 77 7.8%	0 / 2 0.0%	0 / 3 0.0%	0 / 2 0.0%	0 / 6 0.0%	0 / 4 0.0%	2 / 4 50.0%	0 / 2 0.0%	0 / 2 0.0%	0 / 8 0.0%	0 / 2 0.0%	0 / 6 0.0%	0 / 3 0.0%	2 / 2 100.0%	0 / 2 0.0%	0 / 2 0.0%	0 / 2 0.0%	0 / 2 0.0%	0 / 4 0.0%	2 / 6 33.3%	0 / 4 0.0%	0 / 2 0.0%	0 / 7 0.0%	0 / 0 0.0%	0 / 0 0.0%	0 / 0 0.0%	0 / 0 0.0%	44 / 77 57.1%	25 / 77 32.5%E	6 / 77 7.8%AB	2 / 77 2.6%CE		
1	Riemerella anatipestifer RA-GD	3 / 40 7.5%	0 / 1 0.0%	0 / 2 0.0%	0 / 1 0.0%	0 / 3 0.0%	0 / 2 0.0%	1 / 2 50.0%	0 / 1 0.0%	0 / 1 0.0%	0 / 4 0.0%	0 / 1 0.0%	0 / 3 0.0%	0 / 2 0.0%	1 / 1 100.0%	0 / 1 0.0%	0 / 1 0.0%	0 / 1 0.0%	0 / 1 0.0%	0 / 2 0.0%	1 / 3 33.3%	0 / 2 0.0%	0 / 1 0.0%	0 / 4 0.0%	0 / 0 0.0%	0 / 0 0.0%	0 / 0 0.0%	0 / 0 0.0%	23 / 40 57.5%	13 / 40 32.5%E	3 / 40 7.5%AB	1 / 40 2.5%CE		
1	Riemerella anatipestifer RA-CH-1	1 / 40 2.5%	0 / 1 0.0%	0 / 2 0.0%	0 / 1 0.0%	0 / 3 0.0%	0 / 2 0.0%	0 / 2 0.0%	0 / 1 0.0%	0 / 1 0.0%	0 / 4 0.0%	0 / 1 0.0%	0 / 3 0.0%	0 / 2 0.0%	1 / 1 100.0%	0 / 1 0.0%	0 / 1 0.0%	0 / 1 0.0%	0 / 1 0.0%	0 / 2 0.0%	0 / 3 0.0%	0 / 2 0.0%	0 / 1 0.0%	0 / 4 0.0%	0 / 0 0.0%	0 / 0 0.0%	0 / 0 0.0%	0 / 0 0.0%	25 / 40 62.5%	14 / 40 35.0%E	1 / 40 2.5%AB			
1	Riemerella anatipestifer RA-CH-2	3 / 40 7.5%	0 / 1 0.0%	0 / 2 0.0%	0 / 1 0.0%	0 / 3 0.0%	0 / 2 0.0%	1 / 2 50.0%	0 / 1 0.0%	0 / 1 0.0%	0 / 4 0.0%	0 / 1 0.0%	0 / 3 0.0%	0 / 2 0.0%	1 / 1 100.0%	0 / 1 0.0%	0 / 1 0.0%	0 / 1 0.0%	0 / 1 0.0%	0 / 2 0.0%	1 / 3 33.3%	0 / 2 0.0%	0 / 1 0.0%	0 / 4 0.0%	0 / 0 0.0%	0 / 0 0.0%	0 / 0 0.0%	0 / 0 0.0%	23 / 40 57.5%	13 / 40 32.5%E	3 / 40 7.5%AB	1 / 40 2.5%CE		
1	Antarcticum	0 / 35 0.0%	0 / 1 0.0%	0 / 1 0.0%	0 / 1 0.0%	0 / 2 0.0%	0 / 3 0.0%	0 / 1 0.0%	0 / 1 0.0%	0 / 1 0.0%	0 / 2 0.0%	0 / 1 0.0%	0 / 2 0.0%	0 / 2 0.0%	0 / 1 0.0%	0 / 1 0.0%	0 / 1 0.0%	0 / 2 0.0%	0 / 1 0.0%	0 / 2 0.0%	0 / 3 0.0%	0 / 1 0.0%	0 / 1 0.0%	0 / 4 0.0%	0 / 0 0.0%	0 / 0 0.0%	0 / 0 0.0%	0 / 0 0.0%	25 / 35 71.4%	10 / 35 28.6%E				
1	Polaribacter dokdonensis MED152	0 / 35 0.0%	0 / 1 0.0%	0 / 1 0.0%	0 / 1 0.0%	0 / 2 0.0%	0 / 3 0.0%	0 / 1 0.0%	0 / 1 0.0%	0 / 1 0.0%	0 / 2 0.0%	0 / 1 0.0%	0 / 2 0.0%	0 / 2 0.0%	0 / 1 0.0%	0 / 1 0.0%	0 / 1 0.0%	0 / 2 0.0%	0 / 1 0.0%	0 / 2 0.0%	0 / 3 0.0%	0 / 1 0.0%	0 / 1 0.0%	0 / 4 0.0%	0 / 0 0.0%	0 / 0 0.0%	0 / 0 0.0%	0 / 0 0.0%	25 / 35 71.4%	10 / 35 28.6%E				
1	Flavobacteriaceae bacterium	1 / 42 2.4%	0 / 2 0.0%	0 / 2 0.0%	0 / 1 0.0%	0 / 2 0.0%	0 / 2 0.0%	0 / 2 0.0%	0 / 1 0.0%	0 / 1 0.0%	0 / 3 0.0%	0 / 1 0.0%	0 / 3 0.0%	0 / 2 0.0%	0 / 1 0.0%	0 / 1 0.0%	0 / 1 0.0%	0 / 2 0.0%	0 / 1 0.0%	0 / 2 0.0%	0 / 4 0.0%	0 / 2 0.0%	0 / 1 0.0%	1 / 5 20.0%	0 / 0 0.0%	0 / 0 0.0%	0 / 0 0.0%	0 / 0 0.0%	27 / 42 64.3%	14 / 42 33.3%E	1 / 42 2.4%ABE			
1	Flavobacteriaceae bacterium 3519-10	1 / 42 2.4%	0 / 2 0.0%	0 / 2 0.0%	0 / 1 0.0%	0 / 2 0.0%	0 / 2 0.0%	0 / 2 0.0%	0 / 1 0.0%	0 / 1 0.0%	0 / 3 0.0%	0 / 1 0.0%	0 / 3 0.0%	0 / 2 0.0%	0 / 1 0.0%	0 / 1 0.0%	0 / 1 0.0%	0 / 2 0.0%	0 / 1 0.0%	0 / 2 0.0%	0 / 4 0.0%	0 / 2 0.0%	0 / 1 0.0%	1 / 5 20.0%	0 / 0 0.0%	0 / 0 0.0%	0 / 0 0.0%	0 / 0 0.0%	27 / 42 64.3%	14 / 42 33.3%E	1 / 42 2.4%ABE			
1	Psychroflexus	2 / 37 5.4%	0 / 1 0.0%	0 / 1 0.0%	0 / 1 0.0%	0 / 2 0.0%	0 / 2 0.0%	0 / 2 0.0%	0 / 1 0.0%	0 / 1 0.0%	0 / 2 0.0%	0 / 1 0.0%	1 / 3 33.3%	0 / 2 0.0%	0 / 1 0.0%	0 / 1 0.0%	0 / 1 0.0%	0 / 1 0.0%	0 / 1 0.0%	0 / 2 0.0%	0 / 4 0.0%	0 / 2 0.0%	0 / 1 0.0%	1 / 4 25.0%	0 / 0 0.0%	0 / 0 0.0%	0 / 0 0.0%	0 / 0 0.0%	25 / 37 67.6%	9 / 37 24.3%E	2 / 37 5.4%ABE	1 / 37 2.7%C		
1	ACAM 623	2 / 37 5.4%	0 / 1 0.0%	0 / 1 0.0%	0 / 1 0.0%	0 / 2 0.0%	0 / 2 0.0%	0 / 2 0.0%	0 / 1 0.0%	0 / 1 0.0%	0 / 2 0.0%	0 / 1 0.0%	1 / 3 33.3%	0 / 2 0.0%	0 / 1 0.0%	0 / 1 0.0%	0 / 1 0.0%	0 / 1 0.0%	0 / 1 0.0%	0 / 2 0.0%	0 / 4 0.0%	0 / 2 0.0%	0 / 1 0.0%	1 / 4 25.0%	0 / 0 0.0%	0 / 0 0.0%	0 / 0 0.0%	0 / 0 0.0%	25 / 37 67.6%	9 / 37 24.3%E	2 / 37 5.4%ABE	1 / 37 2.7%C		
1	Psychroflexus torquis ACAM 623	2 / 37 5.4%	0 / 1 0.0%	0 / 1 0.0%	0 / 1 0.0%	0 / 2 0.0%	0 / 2 0.0%	0 / 2 0.0%	0 / 1 0.0%	0 / 1 0.0%	0 / 2 0.0%	0 / 1 0.0%	1 / 3 33.3%	0 / 2 0.0%	0 / 1 0.0%	0 / 1 0.0%	0 / 1 0.0%	0 / 1 0.0%	0 / 1 0.0%	0 / 2 0.0%	0 / 4 0.0%	0 / 2 0.0%	0 / 1 0.0%	1 / 4 25.0%	0 / 0 0.0%	0 / 0 0.0%	0 / 0 0.0%	0 / 0 0.0%	25 / 37 67.6%	9 / 37 24.3%E	2 / 37 5.4%ABE	1 / 37 2.7%C		
2	Cellulophaga	11 / 86 12.8%	0 / 2 0.0%	1 / 2 50.0%	0 / 2 0.0%	0 / 9 0.0%	0 / 4 0.0%	0 / 2 0.0%	0 / 2 0.0%	0 / 2 0.0%	6 / 11 54.5%	0 / 2 0.0%	0 / 6 0.0%	0 / 4 0.0%	0 / 2 0.0%	0 / 2 0.0%	0 / 4 0.0%	2 / 4 50.0%	1 / 2 50.0%	0 / 4 0.0%	1 / 6 16.7%	0 / 4 0.0%	0 / 2 0.0%	0 / 8 0.0%	0 / 0 0.0%	0 / 0 0.0%	0 / 0 0.0%	0 / 0 0.0%	48 / 86 55.8%	26 / 86 30.2%E	10 / 86 11.6%AB	1 / 86 1.2%C	1 / 86 1.2%ABE	
1	ATCC 23178	5 / 41 12.2%	0 / 1 0.0%	0 / 1 0.0%	0 / 1 0.0%	0 / 4 0.0%	0 / 2 0.0%	0 / 1 0.0%	0 / 1 0.0%	0 / 1 0.0%	5 / 5 100.0%	0 / 1 0.0%	0 / 3 0.0%	0 / 2 0.0%	0 / 1 0.0%	0 / 1 0.0%	0 / 2 0.0%	0 / 1 0.0%	0 / 1 0.0%	0 / 2 0.0%	0 / 3 0.0%	0 / 2 0.0%	0 / 1 0.0%	0 / 4 0.0%	0 / 0 0.0%	0 / 0 0.0%	0 / 0 0.0%	0 / 0 0.0%	22 / 41 53.7%	14 / 41 34.1%E	5 / 41 12.2%AB			
1	Cellulophaga lytica ATCC 23178	5 / 41 12.2%	0 / 1 0.0%	0 / 1 0.0%	0 / 1 0.0%	0 / 4 0.0%	0 / 2 0.0%	0 / 1 0.0%	0 / 1 0.0%	0 / 1 0.0%	5 / 5 100.0%	0 / 1 0.0%	0 / 3 0.0%	0 / 2 0.0%	0 / 1 0.0%	0 / 1 0.0%	0 / 2 0.0%	0 / 1 0.0%	0 / 1 0.0%	0 / 2 0.0%	0 / 3 0.0%	0 / 2 0.0%	0 / 1 0.0%	0 / 4 0.0%	0 / 0 0.0%	0 / 0 0.0%	0 / 0 0.0%	0 / 0 0.0%	22 / 41 53.7%	14 / 41 34.1%E	5 / 41 12.2%AB			
1	ACAM 630	6 / 45 13.3%	0 / 1 0.0%	1 / 1 100.0%	0 / 1 0.0%	0 / 5 0.0%	0 / 2 0.0%	0 / 1 0.0%	0 / 1 0.0%	0 / 1 0.0%	1 / 6 16.7%	0 / 1 0.0%	0 / 3 0.0%	0 / 2 0.0%	0 / 1 0.0%	0 / 1 0.0%	0 / 2 0.0%	2 / 3 66.7%	1 / 1 100.0%	0 / 2 0.0%	1 / 3 33.3%	0 / 2 0.0%	0 / 1 0.0%	0 / 4 0.0%	0 / 0 0.0%	0 / 0 0.0%	0 / 0 0.0%	0 / 0 0.0%	26 / 45 57.8%	12 / 45 26.7%E	5 / 45 11.1%AB	1 / 45 2.2%C	1 / 45 2.2%ABE	
1	Cellulophaga algicola DSM 14237	6 / 45 13.3%	0 / 1 0.0%	1 / 1 100.0%	0 / 1 0.0%	0 / 5 0.0%	0 / 2 0.0%	0 / 1 0.0%	0 / 1 0.0%	0 / 1 0.0%	1 / 6 16.7%	0 / 1 0.0%	0 / 3 0.0%	0 / 2 0.0%	0 / 1 0.0%	0 / 1 0.0%	0 / 2 0.0%	2 / 3 66.7%	1 / 1 100.0%	0 / 2 0.0%	1 / 3 33.3%	0 / 2 0.0%	0 / 1 0.0%	0 / 4 0.0%	0 / 0 0.0%	0 / 0 0.0%	0 / 0 0.0%	0 / 0 0.0%	26 / 45 57.8%	12 / 45 26.7%E	5 / 45 11.1%AB	1 / 45 2.2%ABE	1 / 45 2.2%C	
1	Caudamuris	1 / 38 2.6%	0 / 1 0.0%	0 / 1 0.0%	0 / 1 0.0%	0 / 2 0.0%	0 / 2 0.0%	0 / 2 0.0%	0 / 1 0.0%	0 / 1 0.0%	0 / 3 0.0%	0 / 1 0.0%	0 / 3 0.0%	0 / 1 0.0%	0 / 1 0.0%	0 / 1 0.0%	0 / 1 0.0%	0 / 2 0.0%	0 / 1 0.0%	0 / 2 0.0%	1 / 4 25.0%	0 / 2 0.0%	0 / 1 0.0%	0 / 4 0.0%	0 / 0 0.0%	0 / 0 0.0%	0 / 0 0.0%	0 / 0 0.0%	23 / 38 60.5%	13 / 38 34.2%E	1 / 38 2.6%C	1 / 38 2.6%AB		
1	Caudamuris ruestringensis	1 / 38 2.6%	0 / 1 0.0%	0 / 1 0.0%	0 / 1 0.0%	0 / 2 0.0%	0 / 2 0.0%	0 / 2 0.0%	0 / 1 0.0%	0 / 1 0.0%	0 / 3 0.0%	0 / 1 0.0%	0 / 3 0.0%	0 / 1 0.0%	0 / 1 0.0%	0 / 1 0.0%	0 / 1 0.0%	0 / 2 0.0%	0 / 1 0.0%	0 / 2 0.0%	1 / 4 25.0%	0 / 2 0.0%	0 / 1 0.0%	0 / 4 0.0%	0 / 0 0.0%	0 / 0 0.0%	0 / 0 0.0%	0 / 0 0.0%	23 / 38 60.5%	13 / 38 34.2%E	1 / 38 2.6%C	1 / 38 2.6%AB		
1	Muricauda ruestringensis B1	1 / 38 2.6%	0 / 1 0.0%	0 / 1 0.0%	0 / 1 0.0%	0 / 2 0.0%	0 / 2 0.0%	0 / 2 0.0%	0 / 1 0.0%	0 / 1 0.0%	0 / 3 0.0%	0 / 1 0.0%	0 / 3 0.0%	0 / 1 0.0%	0 / 1 0.0%	0 / 1 0.0%	0 / 1 0.0%	0 / 2 0.0%	0 / 1 0.0%	0 / 2 0.0%	1 / 4 25.0%	0 / 2 0.0%	0 / 1 0.0%	0 / 4 0.0%	0 / 0 0.0%	0 / 0 0.0%	0 / 0 0.0%	0 / 0 0.0%	23 / 38 60.5%	13 / 38 34.2%E	1 / 38 2.6%AB	1 / 38 2.6%C		
1	Aequorivita	3 / 36 8.3%	0 / 1 0.0%	0 / 1 0.0%	0 / 1 0.0%	0 / 2 0.0%	0 / 2 0.0%	1 / 1 100.0%	0 / 1 0.0%	0 / 1 0.0%	2 / 3 66.7%	0 / 1 0.0%	0 / 3 0.0%	0 / 1 0.0%	0 / 1 0.0%	0 / 1 0.0%	0 / 1 0.0%	0 / 2 0.0%	0 / 1 0.0%	0 / 2 0.0%	0 / 4 0.0%	0 / 1 0.0%	0 / 1 0.0%	0 / 4 0.0%	0 / 0 0.0%	0 / 0 0.0%	0 / 0 0.0%	0 / 0 0.0%	21 / 36 58.3%	12 / 36 33.3%E	3 / 36 8.3%AB			
1	ACAM 643	3 / 36 8.3%	0 / 1 0.0%	0 / 1 0.0%	0 / 1 0.0%	0 / 2 0.0%	0 / 2 0.0%	1 / 1 100.0%	0 / 1 0.0%	0 / 1 0.0%	2 / 3 66.7%	0 / 1 0.0%	0 / 3 0.0%	0 / 1 0.0%	0 / 1 0.0%	0 / 1 0.0%	0 / 1 0.0%	0 / 2 0.0%	0 / 1 0.0%	0 / 2 0.0%	0 / 4 0.0%	0 / 1 0.0%	0 / 1 0.0%	0 / 4 0.0%	0 / 0 0.0%	0 / 0 0.0%	0 / 0 0.0%	0 / 0 0.0%	21 / 36 58.3%	12 / 36 33.3%E	3 / 36 8.3%AB			
1	Aequorivita sublithincola 9-3	3 / 36 8.3%	0 / 1 0.0%	0 / 1 0.0%	0 / 1 0.0%	0 / 2 0.0%	0 / 2 0.0%	1 / 1 100.0%	0 / 1 0.0%	0 / 1 0.0%	2 / 3 66.7%	0 / 1 0.0%	0 / 3 0.0%	0 / 1 0.0%	0 / 1 0.0%	0 / 1 0.0%	0 / 1 0.0%	0 / 2 0.0%	0 / 1 0.0%	0 / 2 0.0%	0 / 4 0.0%	0 / 1 0.0%	0 / 1 0.0%	0 / 4 0.0%	0 / 0 0.0%	0 / 0 0.0%	0 / 0 0.0%	0 / 0 0.0%	21 / 36 58.3%	12 / 36 33.3%E	3 / 36 8.3%AB			
1	Croceibacter	0 / 36 0.0%	0 / 1 0.0%	0 / 1 0.0%	0 / 1 0.0%	0 / 2 0.0%	0 / 2 0.0%	0 / 1 0.0%	0 / 1 0.0%	0 / 1 0.0%	0 / 3 0.0%	0 / 1 0.0%	0 / 3 0.0%	0 / 2 0.0%	0 / 1 0.0%	0 / 1 0.0%	0 / 1 0.0%	0 / 1 0.0%	0 / 1 0.0%	0 / 2 0.0%	0 / 3 0.0%	0 / 2 0.0%	0 / 1 0.0%	0 / 4 0.0%	0 / 0 0.0%	0 / 0 0.0%	0 / 0 0.0%	0 / 0 0.0%	23 / 36 63.9%	13 / 36 36.1%E				
1	ATCC BAA-628	0 / 36 0.0%	0 / 1 0.0%	0 / 1 0.0%	0 / 1 0.0%	0 / 2 0.0%	0 / 2 0.0%	0 / 1 0.0%	0 / 1 0.0%	0 / 1 0.0%	0 / 3 0.0%	0 / 1 0.0%	0 / 3 0.0%	0 / 2 0.0%	0 / 1 0.0%	0 / 1 0.0%	0 / 1 0.0%	0 / 1 0.0%	0 / 1 0.0%	0 / 2 0.0%	0 / 3 0.0%	0 / 2 0.0%	0 / 1 0.0%	0 / 4 0.0%	0 / 0 0.0%	0 / 0 0.0%	0 / 0 0.0%	0 / 0 0.0%	23 / 36 63.9%	13 / 36 36.1%E				
1	Croceibacter atlanticus HTCC2559	0 / 36 0.0%	0 / 1 0.0%	0 / 1 0.0%	0 / 1 0.0%	0 / 2 0.0%	0 / 2 0.0%	0 / 1 0.0%	0 / 1 0.0%	0 / 1 0.0%	0 / 3 0.0%	0 / 1 0.0%	0 / 3 0.0%	0 / 2 0.0%	0 / 1 0.0%	0 / 1 0.0%	0 / 1 0.0%	0 / 1 0.0%	0 / 1 0.0%	0 / 2 0.0%	0 / 3 0.0%	0 / 2 0.0%	0 / 1 0.0%	0 / 4 0.0%	0 / 0 0.0%	0 / 0 0.0%	0 / 0 0.0%	0 / 0 0.0%	23 / 36 63.9%	13 / 36 36.1%E				
1	Robiginitalea	3 / 41 7.3%	0 / 1 0.0%	0 / 1 0.0%	0 / 1 0.0%	0 / 2 0.0%	1 / 2 50.0%	0 / 2 0.0%	0 / 1 0.0%	0 / 1 0.0%	0 / 4 0.0%	0 / 1 0.0%	0 / 3 0.0%	0 / 1 0.0%	0 / 1 0.0%	0 / 1 0.0%	0 / 1 0.0%	1 / 3 33.3%	0 / 1 0.0%	0 / 2 0.0%	0 / 4 0.0%	1 / 2 50.0%	0 / 1 0.0%	0 / 5 0.0%	0 / 0 0.0%	0 / 0 0.0%	0 / 0 0.0%	0 / 0 0.0%	25 / 41 61.0%	13 / 41 31.7%E	2 / 41 4.9%AB	1 / 41 2.4%ABE		
1	ATCC BAA-864	3 / 41 7.3%	0 / 1 0.0%	0 / 1 0.0%	0 / 1 0.0%	0 / 2 0.0%	1 / 2 50.0%	0 / 2 0.0%	0 / 1 0.0%	0 / 1 0.0%	0 / 4 0.0%	0 / 1 0.0%	0 / 3 0.0%	0 / 1 0.0%	0 / 1 0.0%	0 / 1 0.0%	0 / 1 0.0%	1 / 3 33.3%	0 / 1 0.0%	0 / 2 0.0%	0 / 4 0.0%	1 / 2 50.0%	0 / 1 0.0%	0 / 5 0.0%	0 / 0 0.0%	0 / 0 0.0%	0 / 0 0.0%	0 / 0 0.0%	25 / 41 61.0%	13 / 41 31.7%E	2 / 41 4.9%AB	1 / 41 2.4%ABE		
1	Robiginitalea biformata HTCC2501	3 / 41 7.3%	0 / 1 0.0%	0 / 1 0.0%	0 / 1 0.0%	0 / 2 0.0%	1 / 2 50.0%	0 / 2 0.0%	0 / 1 0.0%	0 / 1 0.0%	0 / 4 0.0%	0 / 1 0.0%	0 / 3 0.0%	0 / 1 0.0%	0 / 1 0.0%	0 / 1 0.0%	0 / 1 0.0%	1 / 3 33.3%	0 / 1 0.0%	0 / 2 0.0%	0 / 4 0.0%	1 / 2 50.0%	0 / 1 0.0%	0 / 5 0.0%	0 / 0 0.0%	0 / 0 0.0%	0 / 0 0.0%	0 / 0 0.0%	25 / 41 61.0%	13 / 41 31.7%E	2 / 41 4.9%AB	1 / 41 2.4%ABE		
1	Maribacter	2 / 38 5.3%	0 / 1 0.0%	0 / 1 0.0%	0 / 1 0.0%	0 / 1 0.0%	0 / 2 0.0%	0 / 2 0.0%	0 / 1 0.0%	0 / 1 0.0%	0 / 3 0.0%	0 / 1 0.0%	0 / 3 0.0%	0 / 1 0.0%	0 / 1 0.0%	0 / 1 0.0%	0 / 1 0.0%	0 / 2 0.0%	1 / 1 100.0%	0 / 2 0.0%	0 / 4 0.0%	1 / 2 50.0%	0 / 1 0.0%	0 / 4 0.0%	0 / 0 0.0%	0 / 0 0.0%	0 / 1 0.0%	0 / 0 0.0%	23 / 38 60.5%	13 / 38 34.2%E	1 / 38 2.6%AB	1 / 38 2.6%ABE		
1	Flavobacteriales bacterium HTCC2170	2 / 38 5.3%	0 / 1 0.0%	0 / 1 0.0%	0 / 1 0.0%	0 / 1 0.0%	0 / 2 0.0%	0 / 2 0.0%	0 / 1 0.0%	0 / 1 0.0%	0 / 3 0.0%	0 / 1 0.0%	0 / 3 0.0%	0 / 1 0.0%	0 / 1 0.0%	0 / 1 0.0%	0 / 1 0.0%	0 / 2 0.0%	1 / 1 100.0%	0 / 2 0.0%	0 / 4 0.0%	1 / 2 50.0%	0 / 1 0.0%	0 / 4 0.0%	0 / 0 0.0%	0 / 0 0.0%	0 / 1 0.0%	0 / 0 0.0%	23 / 38 60.5%	13 / 38 34.2%E	1 / 38 2.6%AB	1 / 38 2.6%ABE		
1	Lacinutrix	1 / 36 2.8%	0 / 1 0.0%	0 / 1 0.0%	0 / 1 0.0%	0 / 2 0.0%	0 / 3 0.0%	0 / 1 0.0%	0 / 1 0.0%	0 / 2 0.0%	0 / 2 0.0%	0 / 1 0.0%	0 / 2 0.0%	0 / 1 0.0%	1 / 1 100.0%	0 / 1 0.0%	0 / 1 0.0%	0 / 2 0.0%	0 / 1 0.0%	0 / 3 0.0%	0 / 3 0.0%	0 / 1 0.0%	0 / 1 0.0%	0 / 4 0.0%	0 / 0 0.0%	0 / 0 0.0%	0 / 0 0.0%	0 / 0 0.0%	25 / 36 69.4%	10 / 36 27.8%E	1 / 36 2.8%AB			
1	Lacinutrix algicola 5H-3-7-4	1 / 36 2.8%	0 / 1 0.0%	0 / 1 0.0%	0 / 1 0.0%	0 / 2 0.0%	0 / 3 0.0%	0 / 1 0.0%	0 / 1 0.0%	0 / 2 0.0%	0 / 2 0.0%	0 / 1 0.0%	0 / 2 0.0%	0 / 1 0.0%	1 / 1 100.0%	0 / 1 0.0%	0 / 1 0.0%	0 / 2 0.0%	0 / 1 0.0%	0 / 3 0.0%	0 / 3 0.0%	0 / 1 0.0%	0 / 1 0.0%	0 / 4 0.0%	0 / 0 0.0%	0 / 0 0.0%	0 / 0 0.0%	0 / 0 0.0%	25 / 36 69.4%	10 / 36 27.8%E	1 / 36 2.8%AB			
1	Gramella	4 / 44 9.1%	0 / 1 0.0%	0 / 1 0.0%	0 / 1 0.0%	0 / 3 0.0%	0 / 3 0.0%	0 / 2 0.0%	0 / 1 0.0%	1 / 1 100.0%	0 / 4 0.0%	0 / 1 0.0%	1 / 3 33.3%	0 / 2 0.0%	0 / 1 0.0%	0 / 1 0.0%	0 / 1 0.0%	0 / 3 0.0%	0 / 1 0.0%	0 / 3 0.0%	0 / 4 0.0%	0 / 2 0.0%	0 / 1 0.0%	2 / 4 50.0%	0 / 0 0.0%	0 / 0 0.0%	0 / 0 0.0%	0 / 0 0.0%	28 / 44 63.6%	11 / 44 25.0%E	3 / 44 6.8%ABE	1 / 44 2.3%C	1 / 44 2.3%AB	
1	"Gramella forsetii" Bauer et al. 2006	4 / 44 9.1%	0 / 1 0.0%	0 / 1 0.0%	0 / 1 0.0%	0 / 3 0.0%	0 / 3 0.0%	0 / 2 0.0%	0 / 1 0.0%	1 / 1 100.0%	0 / 4 0.0%	0 / 1 0.0%	1 / 3 33.3%	0 / 2 0.0%	0 / 1 0.0%	0 / 1 0.0%	0 / 1 0.0%	0 / 3 0.0%	0 / 1 0.0%	0 / 3 0.0%	0 / 4 0.0%	0 / 2 0.0%	0 / 1 0.0%	2 / 4 50.0%	0 / 0 0.0%	0 / 0 0.0%	0 / 0 0.0%	0 / 0 0.0%	28 / 44 63.6%	11 / 44 25.0%E	3 / 44 6.8%ABE	1 / 44 2.3%C	1 / 44 2.3%AB	
1	Gramella forsetii KT0803	4 / 44 9.1%	0 / 1 0.0%	0 / 1 0.0%	0 / 1 0.0%	0 / 3 0.0%	0 / 3 0.0%	0 / 2 0.0%	0 / 1 0.0%	1 / 1 100.0%	0 / 4 0.0%	0 / 1 0.0%	1 / 3 33.3%	0 / 2 0.0%	0 / 1 0.0%	0 / 1 0.0%	0 / 1 0.0%	0 / 3 0.0%	0 / 1 0.0%	0 / 3 0.0%	0 / 4 0.0%	0 / 2 0.0%	0 / 1 0.0%	2 / 4 50.0%	0 / 0 0.0%	0 / 0 0.0%	0 / 0 0.0%	0 / 0 0.0%	28 / 44 63.6%	11 / 44 25.0%E	3 / 44 6.8%ABE	1 / 44 2.3%C	1 / 44 2.3%AB	
1	Dokdoa	4 / 42 9.5%	0 / 1 0.0%	0 / 1 0.0%	0 / 1 0.0%	0 / 3 0.0%	0 / 3 0.0%	0 / 1 0.0%	0 / 1 0.0%	0 / 1 0.0%	4 / 4 100.0%	0 / 1 0.0%	0 / 3 0.0%	0 / 2 0.0%	0 / 1 0.0%	0 / 1 0.0%	0 / 1 0.0%	0 / 3 0.0%	0 / 1 0.0%	0 / 3 0.0%	0 / 3 0.0%	0 / 2 0.0%	0 / 1 0.0%	0 / 4 0.0%	0 / 0 0.0%	0 / 0 0.0%	0 / 0 0.0%	0 / 0 0.0%	26 / 42 61.9%	12 / 42 28.6%E	4 / 42 9.5%AB			
1	Dokdonia sp. 4H-3-7-5	4 / 42 9.5%	0 / 1 0.0%	0 / 1 0.0%	0 / 1 0.0%	0 / 3 0.0%	0 / 3 0.0%	0 / 1 0.0%	0 / 1 0.0%	0 / 1 0.0%	4 / 4 100.0%	0 / 1 0.0%	0 / 3 0.0%	0 / 2 0.0%	0 / 1 0.0%	0 / 1 0.0%	0 / 1 0.0%	0 / 3 0.0%	0 / 1 0.0%	0 / 3 0.0%	0 / 3 0.0%	0 / 2 0.0%	0 / 1 0.0%	0 / 4 0.0%	0 / 0 0.0%	0 / 0 0.0%	0 / 0 0.0%	0 / 0 0.0%	26 / 42 61.9%	12 / 42 28.6%E	4 / 42 9.5%AB			
1	Donghaea	2 / 41 4.9%	0 / 1 0.0%	0 / 1 0.0%	0 / 1 0.0%	0 / 2 0.0%	0 / 2 0.0%	0 / 1 0.0%	0 / 1 0.0%	0 / 2 0.0%	0 / 3 0.0%	0 / 1 0.0%	0 / 3 0.0%	0 / 1 0.0%	0 / 1 0.0%	1 / 2 50.0%	0 / 1 0.0%	0 / 2 0.0%	0 / 1 0.0%	0 / 2 0.0%	0 / 3 0.0%	0 / 2 0.0%	0 / 1 0.0%	1 / 6 16.7%	0 / 0 0.0%	0 / 0 0.0%	0 / 1 0.0%	0 / 0 0.0%	25 / 41 61.0%	13 / 41 31.7%E	2 / 41 4.9%ABE	1 / 41 2.4%CE		
1	DSM 17205	2 / 41 4.9%	0 / 1 0.0%	0 / 1 0.0%	0 / 1 0.0%	0 / 2 0.0%	0 / 2 0.0%	0 / 1 0.0%	0 / 1 0.0%	0 / 2 0.0%	0 / 3 0.0%	0 / 1 0.0%	0 / 3 0.0%	0 / 1 0.0%	0 / 1 0.0%	1 / 2 50.0%	0 / 1 0.0%	0 / 2 0.0%	0 / 1 0.0%	0 / 2 0.0%	0 / 3 0.0%	0 / 2 0.0%	0 / 1 0.0%	1 / 6 16.7%	0 / 0 0.0%	0 / 0 0.0%	0 / 1 0.0%	0 / 0 0.0%	25 / 41 61.0%	13 / 41 31.7%E	2 / 41 4.9%ABE	1 / 41 2.4%CE		
1	Donghaeana dokdonensis DSW-6	2 / 41 4.9%	0 / 1 0.0%	0 / 1 0.0%	0 / 1 0.0%	0 / 2 0.0%	0 / 2 0.0%	0 / 1 0.0%	0 / 1 0.0%	0 / 2 0.0%	0 / 3 0.0%	0 / 1 0.0%	0 / 3 0.0%	0 / 1 0.0%	0 / 1 0.0%	1 / 2 50.0%	0 / 1 0.0%	0 / 2 0.0%	0 / 1 0.0%	0 / 2 0.0%	0 / 3 0.0%	0 / 2 0.0%	0 / 1 0.0%	1 / 6 16.7%	0 / 0 0.0%	0 / 0 0.0%	0 / 1 0.0%	0 / 0 0.0%	25 / 41 61.0%	13 / 41 31.7%E	2 / 41 4.9%ABE	1 / 41 2.4%CE		
1	"Wangia" Qin et al. 2007	4 / 46 8.7%	0 / 1 0.0%	0 / 1 0.0%	0 / 2 0.0%	0 / 3 0.0%	0 / 3 0.0%	0 / 2 0.0%	0 / 1 0.0%	0 / 1 0.0%	3 / 4 75.0%	0 / 1 0.0%	0 / 3 0.0%	0 / 2 0.0%	0 / 2 0.0%	0 / 1 0.0%	0 / 1 0.0%	0 / 3 0.0%	0 / 1 0.0%	0 / 3 0.0%	0 / 4 0.0%	0 / 2 0.0%	1 / 1 100.0%	0 / 4 0.0%	0 / 0 0.0%	0 / 0 0.0%	0 / 0 0.0%	0 / 0 0.0%	27 / 46 58.7%	13 / 46 28.3%E	4 / 46 8.7%AB	2 / 46 4.3%C		
1	"Wangia profunda" Qin et al. 2007	4 / 46 8.7%	0 / 1 0.0%	0 / 1 0.0%	0 / 2 0.0%	0 / 3 0.0%	0 / 3 0.0%	0 / 2 0.0%	0 / 1 0.0%	0 / 1 0.0%	3 / 4 75.0%	0 / 1 0.0%	0 / 3 0.0%	0 / 2 0.0%	0 / 2 0.0%	0 / 1 0.0%	0 / 1 0.0%	0 / 3 0.0%	0 / 1 0.0%	0 / 3 0.0%	0 / 4 0.0%	0 / 2 0.0%	1 / 1 100.0%	0 / 4 0.0%	0 / 0 0.0%	0 / 0 0.0%	0 / 0 0.0%	0 / 0 0.0%	27 / 46 58.7%	13 / 46 28.3%E	4 / 46 8.7%AB	2 / 46 4.3%C		
1	Zunongwangia profunda SM-A87	4 / 46 8.7%	0 / 1 0.0%	0 / 1 0.0%	0 / 2 0.0%	0 / 3 0.0%	0 / 3 0.0%	0 / 2 0.0%	0 / 1 0.0%	0 / 1 0.0%	3 / 4 75.0%	0 / 1 0.0%	0 / 3 0.0%	0 / 2 0.0%	0 / 2 0.0%	0 / 1 0.0%	0 / 1 0.0%	0 / 3 0.0%	0 / 1 0.0%	0 / 3 0.0%	0 / 4 0.0%	0 / 2 0.0%	1 / 1 100.0%	0 / 4 0.0%	0 / 0 0.0%	0 / 0 0.0%	0 / 0 0.0%	0 / 0 0.0%	27 / 46 58.7%	13 / 46 28.3%E	4 / 46 8.7%AB	2 / 46 4.3%C		
2	Cryomorphaceae	8 / 77 10.4%	0 / 2 0.0%	0 / 2 0.0%	0 / 2 0.0%	1 / 4 25.0%	1 / 4 25.0%	0 / 2 0.0%	0 / 2 0.0%	0 / 3 0.0%	0 / 4 0.0%	0 / 2 0.0%	2 / 7 28.6%	0 / 3 0.0%	0 / 2 0.0%	0 / 3 0.0%	0 / 4 0.0%	1 / 4 25.0%	0 / 2 0.0%	0 / 4 0.0%	0 / 7 0.0%	0 / 3 0.0%	0 / 2 0.0%	3 / 9 33.3%	0 / 0 0.0%	0 / 0 0.0%	0 / 0 0.0%	0 / 0 0.0%	41 / 77 53.2%	26 / 77 33.8%E	5 / 77 6.5%ABE	3 / 77 3.9%AB	1 / 77 1.3%GG	1 / 77 1.3%C
1	Owenweeksia	3 / 37 8.1%	0 / 1 0.0%	0 / 1 0.0%	0 / 1 0.0%	0 / 2 0.0%	1 / 2 50.0%	0 / 1 0.0%	0 / 1 0.0%	0 / 1 0.0%	0 / 3 0.0%	0 / 1 0.0%	0 / 3 0.0%	0 / 2 0.0%	0 / 1 0.0%	0 / 1 0.0%	0 / 1 0.0%	1 / 2 50.0%	0 / 1 0.0%	0 / 2 0.0%	0 / 4 0.0%	0 / 1 0.0%	0 / 1 0.0%	1 / 4 25.0%	0 / 0 0.0%	0 / 0 0.0%	0 / 0 0.0%	0 / 0 0.0%	21 / 37 56.8%	11 / 37 29.7%E	2 / 37 5.4%AB	1 / 37 2.7%ABE	1 / 37 2.7%GG	1 / 37 2.7%C
1	CIP 108786	3 / 37 8.1%	0 / 1 0.0%	0 / 1 0.0%	0 / 1 0.0%	0 / 2 0.0%	1 / 2 50.0%	0 / 1 0.0%	0 / 1 0.0%	0 / 1 0.0%	0 / 3 0.0%	0 / 1 0.0%	0 / 3 0.0%	0 / 2 0.0%	0 / 1 0.0%	0 / 1 0.0%	0 / 1 0.0%	1 / 2 50.0%	0 / 1 0.0%	0 / 2 0.0%	0 / 4 0.0%	0 / 1 0.0%	0 / 1 0.0%	1 / 4 25.0%	0 / 0 0.0%	0 / 0 0.0%	0 / 0 0.0%	0 / 0 0.0%	21 / 37 56.8%	11 / 37 29.7%E	2 / 37 5.4%AB	1 / 37 2.7%C	1 / 37 2.7%ABE	1 / 37 2.7%GG
1	Owenweeksia hongkongensis DSM 17368	3 / 37 8.1%	0 / 1 0.0%	0 / 1 0.0%	0 / 1 0.0%	0 / 2 0.0%	1 / 2 50.0%	0 / 1 0.0%	0 / 1 0.0%	0 / 1 0.0%	0 / 3 0.0%	0 / 1 0.0%	0 / 3 0.0%	0 / 2 0.0%	0 / 1 0.0%	0 / 1 0.0%	0 / 1 0.0%	1 / 2 50.0%	0 / 1 0.0%	0 / 2 0.0%	0 / 4 0.0%	0 / 1 0.0%	0 / 1 0.0%	1 / 4 25.0%	0 / 0 0.0%	0 / 0 0.0%	0 / 0 0.0%	0 / 0 0.0%	21 / 37 56.8%	11 / 37 29.7%E	2 / 37 5.4%AB	1 / 37 2.7%ABE	1 / 37 2.7%GG	1 / 37 2.7%C
1	Fluviicola	5 / 40 12.5%	0 / 1 0.0%	0 / 1 0.0%	0 / 1 0.0%	1 / 2 50.0%	0 / 2 0.0%	0 / 1 0.0%	0 / 1 0.0%	0 / 2 0.0%	0 / 1 0.0%	0 / 1 0.0%	2 / 4 50.0%	0 / 1 0.0%	0 / 1 0.0%	0 / 2 0.0%	0 / 3 0.0%	0 / 2 0.0%	0 / 1 0.0%	0 / 2 0.0%	0 / 3 0.0%	0 / 2 0.0%	0 / 1 0.0%	2 / 5 40.0%	0 / 0 0.0%	0 / 0 0.0%	0 / 0 0.0%	0 / 0 0.0%	20 / 40 50.0%	15 / 40 37.5%E	4 / 40 10.0%ABE	1 / 40 2.5%AB		
1	DSM 16823	5 / 40 12.5%	0 / 1 0.0%	0 / 1 0.0%	0 / 1 0.0%	1 / 2 50.0%	0 / 2 0.0%	0 / 1 0.0%	0 / 1 0.0%	0 / 2 0.0%	0 / 1 0.0%	0 / 1 0.0%	2 / 4 50.0%	0 / 1 0.0%	0 / 1 0.0%	0 / 2 0.0%	0 / 3 0.0%	0 / 2 0.0%	0 / 1 0.0%	0 / 2 0.0%	0 / 3 0.0%	0 / 2 0.0%	0 / 1 0.0%	2 / 5 40.0%	0 / 0 0.0%	0 / 0 0.0%	0 / 0 0.0%	0 / 0 0.0%	20 / 40 50.0%	15 / 40 37.5%E	4 / 40 10.0%ABE	1 / 40 2.5%AB		
1	CFB group bacterium RW262	5 / 40 12.5%	0 / 1 0.0%	0 / 1 0.0%	0 / 1 0.0%	1 / 2 50.0%	0 / 2 0.0%	0 / 1 0.0%	0 / 1 0.0%	0 / 2 0.0%	0 / 1 0.0%	0 / 1 0.0%	2 / 4 50.0%	0 / 1 0.0%	0 / 1 0.0%	0 / 2 0.0%	0 / 3 0.0%	0 / 2 0.0%	0 / 1 0.0%	0 / 2 0.0%	0 / 3 0.0%	0 / 2 0.0%	0 / 1 0.0%	2 / 5 40.0%	0 / 0 0.0%	0 / 0 0.0%	0 / 0 0.0%	0 / 0 0.0%	20 / 40 50.0%	15 / 40 37.5%E	4 / 40 10.0%ABE	1 / 40 2.5%AB		
5	unclassified Flavobacteriales	1 / 150 0.7%	0 / 5 0.0%	0 / 5 0.0%	0 / 5 0.0%	0 / 5 0.0%	0 / 10 0.0%	0 / 7 0.0%	0 / 5 0.0%	0 / 5 0.0%	0 / 5 0.0%	0 / 5 0.0%	0 / 12 0.0%	0 / 5 0.0%	0 / 5 0.0%	0 / 5 0.0%	1 / 5 20.0%	0 / 5 0.0%	0 / 5 0.0%	0 / 9 0.0%	0 / 12 0.0%	0 / 5 0.0%	0 / 5 0.0%	0 / 19 0.0%	0 / 0 0.0%	0 / 0 0.0%	0 / 1 0.0%	0 / 0 0.0%	93 / 150 62.0%	50 / 150 33.3%E	5 / 150 3.3%C	1 / 150 0.7%CE	1 / 150 0.7%ABE	
4	"Candidatus Sulcia" Moran et al. 2005	0 / 120 0.0%	0 / 4 0.0%	0 / 4 0.0%	0 / 4 0.0%	0 / 4 0.0%	0 / 8 0.0%	0 / 5 0.0%	0 / 4 0.0%	0 / 4 0.0%	0 / 4 0.0%	0 / 4 0.0%	0 / 11 0.0%	0 / 4 0.0%	0 / 4 0.0%	0 / 4 0.0%	0 / 4 0.0%	0 / 4 0.0%	0 / 4 0.0%	0 / 7 0.0%	0 / 10 0.0%	0 / 4 0.0%	0 / 4 0.0%	0 / 15 0.0%	0 / 0 0.0%	0 / 0 0.0%	0 / 0 0.0%	0 / 0 0.0%	76 / 120 63.3%	42 / 120 35.0%E	1 / 120 0.8%CE	1 / 120 0.8%C		
4	"Candidatus Sulcia muelleri" Moran et al. 2005	0 / 120 0.0%	0 / 4 0.0%	0 / 4 0.0%	0 / 4 0.0%	0 / 4 0.0%	0 / 8 0.0%	0 / 5 0.0%	0 / 4 0.0%	0 / 4 0.0%	0 / 4 0.0%	0 / 4 0.0%	0 / 11 0.0%	0 / 4 0.0%	0 / 4 0.0%	0 / 4 0.0%	0 / 4 0.0%	0 / 4 0.0%	0 / 4 0.0%	0 / 7 0.0%	0 / 10 0.0%	0 / 4 0.0%	0 / 4 0.0%	0 / 15 0.0%	0 / 0 0.0%	0 / 0 0.0%	0 / 0 0.0%	0 / 0 0.0%	76 / 120 63.3%	42 / 120 35.0%E	1 / 120 0.8%CE	1 / 120 0.8%C		
1	Candidatus Sulcia muelleri GWSS	0 / 31 0.0%	0 / 1 0.0%	0 / 1 0.0%	0 / 1 0.0%	0 / 1 0.0%	0 / 2 0.0%	0 / 1 0.0%	0 / 1 0.0%	0 / 1 0.0%	0 / 1 0.0%	0 / 1 0.0%	0 / 3 0.0%	0 / 1 0.0%	0 / 1 0.0%	0 / 1 0.0%	0 / 1 0.0%	0 / 1 0.0%	0 / 1 0.0%	0 / 2 0.0%	0 / 3 0.0%	0 / 1 0.0%	0 / 1 0.0%	0 / 4 0.0%	0 / 0 0.0%	0 / 0 0.0%	0 / 0 0.0%	0 / 0 0.0%	20 / 31 64.5%	11 / 31 35.5%E				
1	Candidatus Sulcia muelleri SMDSEM	0 / 28 0.0%	0 / 1 0.0%	0 / 1 0.0%	0 / 1 0.0%	0 / 1 0.0%	0 / 2 0.0%	0 / 1 0.0%	0 / 1 0.0%	0 / 1 0.0%	0 / 1 0.0%	0 / 1 0.0%	0 / 2 0.0%	0 / 1 0.0%	0 / 1 0.0%	0 / 1 0.0%	0 / 1 0.0%	0 / 1 0.0%	0 / 1 0.0%	0 / 1 0.0%	0 / 2 0.0%	0 / 1 0.0%	0 / 1 0.0%	0 / 4 0.0%	0 / 0 0.0%	0 / 0 0.0%	0 / 0 0.0%	0 / 0 0.0%	16 / 28 57.1%	10 / 28 35.7%E	1 / 28 3.6%C	1 / 28 3.6%CE		
1	Candidatus Sulcia muelleri DMIN	0 / 31 0.0%	0 / 1 0.0%	0 / 1 0.0%	0 / 1 0.0%	0 / 1 0.0%	0 / 2 0.0%	0 / 1 0.0%	0 / 1 0.0%	0 / 1 0.0%	0 / 1 0.0%	0 / 1 0.0%	0 / 3 0.0%	0 / 1 0.0%	0 / 1 0.0%	0 / 1 0.0%	0 / 1 0.0%	0 / 1 0.0%	0 / 1 0.0%	0 / 2 0.0%	0 / 3 0.0%	0 / 1 0.0%	0 / 1 0.0%	0 / 4 0.0%	0 / 0 0.0%	0 / 0 0.0%	0 / 0 0.0%	0 / 0 0.0%	20 / 31 64.5%	11 / 31 35.5%E				
1	Candidatus Sulcia muelleri CARI	0 / 30 0.0%	0 / 1 0.0%	0 / 1 0.0%	0 / 1 0.0%	0 / 1 0.0%	0 / 2 0.0%	0 / 2 0.0%	0 / 1 0.0%	0 / 1 0.0%	0 / 1 0.0%	0 / 1 0.0%	0 / 3 0.0%	0 / 1 0.0%	0 / 1 0.0%	0 / 1 0.0%	0 / 1 0.0%	0 / 1 0.0%	0 / 1 0.0%	0 / 2 0.0%	0 / 2 0.0%	0 / 1 0.0%	0 / 1 0.0%	0 / 3 0.0%	0 / 0 0.0%	0 / 0 0.0%	0 / 0 0.0%	0 / 0 0.0%	20 / 30 66.7%	10 / 30 33.3%E				
1	"Candidatus Uzinura" Gruwell et al. 2007	1 / 30 3.3%	0 / 1 0.0%	0 / 1 0.0%	0 / 1 0.0%	0 / 1 0.0%	0 / 2 0.0%	0 / 2 0.0%	0 / 1 0.0%	0 / 1 0.0%	0 / 1 0.0%	0 / 1 0.0%	0 / 1 0.0%	0 / 1 0.0%	0 / 1 0.0%	0 / 1 0.0%	1 / 1 100.0%	0 / 1 0.0%	0 / 1 0.0%	0 / 2 0.0%	0 / 2 0.0%	0 / 1 0.0%	0 / 1 0.0%	0 / 4 0.0%	0 / 0 0.0%	0 / 0 0.0%	0 / 1 0.0%	0 / 0 0.0%	17 / 30 56.7%	8 / 30 26.7%E	4 / 30 13.3%C	1 / 30 3.3%ABE		
1	"Candidatus Uzinura diaspidicola" Gruwell et al. 2007	1 / 30 3.3%	0 / 1 0.0%	0 / 1 0.0%	0 / 1 0.0%	0 / 1 0.0%	0 / 2 0.0%	0 / 2 0.0%	0 / 1 0.0%	0 / 1 0.0%	0 / 1 0.0%	0 / 1 0.0%	0 / 1 0.0%	0 / 1 0.0%	0 / 1 0.0%	0 / 1 0.0%	1 / 1 100.0%	0 / 1 0.0%	0 / 1 0.0%	0 / 2 0.0%	0 / 2 0.0%	0 / 1 0.0%	0 / 1 0.0%	0 / 4 0.0%	0 / 0 0.0%	0 / 0 0.0%	0 / 1 0.0%	0 / 0 0.0%	17 / 30 56.7%	8 / 30 26.7%E	4 / 30 13.3%C	1 / 30 3.3%ABE		
1	Candidatus Uzinura diaspidicola ASNER	1 / 30 3.3%	0 / 1 0.0%	0 / 1 0.0%	0 / 1 0.0%	0 / 1 0.0%	0 / 2 0.0%	0 / 2 0.0%	0 / 1 0.0%	0 / 1 0.0%	0 / 1 0.0%	0 / 1 0.0%	0 / 1 0.0%	0 / 1 0.0%	0 / 1 0.0%	0 / 1 0.0%	1 / 1 100.0%	0 / 1 0.0%	0 / 1 0.0%	0 / 2 0.0%	0 / 2 0.0%	0 / 1 0.0%	0 / 1 0.0%	0 / 4 0.0%	0 / 0 0.0%	0 / 0 0.0%	0 / 1 0.0%	0 / 0 0.0%	17 / 30 56.7%	8 / 30 26.7%E	4 / 30 13.3%C	1 / 30 3.3%ABE		
8	"Sphingobacteriia" Kampfer 2011	65 / 430 15.1%	2 / 11 18.2%	2 / 19 10.5%	1 / 12 8.3%	1 / 22 4.5%	5 / 28 17.9%	5 / 21 23.8%	1 / 10 10.0%	2 / 8 25.0%	14 / 28 50.0%	0 / 9 0.0%	6 / 32 18.8%	2 / 24 8.3%	1 / 11 9.1%	0 / 14 0.0%	2 / 14 14.3%	4 / 22 18.2%	1 / 8 12.5%	6 / 23 26.1%	2 / 35 5.7%	3 / 20 15.0%	0 / 10 0.0%	5 / 43 11.6%	0 / 0 0.0%	0 / 0 0.0%	0 / 6 0.0%	0 / 0 0.0%	226 / 430 52.6%	117 / 430 27.2%E	43 / 430 10.0%AB	22 / 430 5.1%ABE	19 / 430 4.4%C	3 / 430 0.7%CE
8	"Sphingobacteriales" Kampfer 2011	65 / 430 15.1%	2 / 11 18.2%	2 / 19 10.5%	1 / 12 8.3%	1 / 22 4.5%	5 / 28 17.9%	5 / 21 23.8%	1 / 10 10.0%	2 / 8 25.0%	14 / 28 50.0%	0 / 9 0.0%	6 / 32 18.8%	2 / 24 8.3%	1 / 11 9.1%	0 / 14 0.0%	2 / 14 14.3%	4 / 22 18.2%	1 / 8 12.5%	6 / 23 26.1%	2 / 35 5.7%	3 / 20 15.0%	0 / 10 0.0%	5 / 43 11.6%	0 / 0 0.0%	0 / 0 0.0%	0 / 6 0.0%	0 / 0 0.0%	226 / 430 52.6%	117 / 430 27.2%E	43 / 430 10.0%AB	22 / 430 5.1%ABE	19 / 430 4.4%C	3 / 430 0.7%CE
4	Sphingobacteriaceae	32 / 203 15.8%	2 / 5 40.0%	0 / 7 0.0%	0 / 5 0.0%	0 / 15 0.0%	2 / 13 15.4%	4 / 10 40.0%	1 / 4 25.0%	2 / 4 50.0%	8 / 17 47.1%	0 / 4 0.0%	3 / 14 21.4%	2 / 11 18.2%	0 / 5 0.0%	0 / 4 0.0%	1 / 7 14.3%	1 / 11 9.1%	1 / 4 25.0%	2 / 11 18.2%	0 / 16 0.0%	1 / 10 10.0%	0 / 5 0.0%	2 / 21 9.5%	0 / 0 0.0%	0 / 0 0.0%	0 / 0 0.0%	0 / 0 0.0%	111 / 203 54.7%	47 / 203 23.2%E	21 / 203 10.3%AB	11 / 203 5.4%C	11 / 203 5.4%ABE	2 / 203 1.0%CE
1	Sphingobacter	7 / 51 13.7%	0 / 1 0.0%	0 / 2 0.0%	0 / 1 0.0%	0 / 4 0.0%	1 / 3 33.3%	1 / 3 33.3%	0 / 1 0.0%	1 / 1 100.0%	0 / 3 0.0%	0 / 1 0.0%	2 / 4 50.0%	2 / 3 66.7%	0 / 1 0.0%	0 / 1 0.0%	0 / 2 0.0%	0 / 4 0.0%	0 / 1 0.0%	0 / 3 0.0%	0 / 4 0.0%	0 / 2 0.0%	0 / 1 0.0%	0 / 5 0.0%	0 / 0 0.0%	0 / 0 0.0%	0 / 0 0.0%	0 / 0 0.0%	26 / 51 51.0%	12 / 51 23.5%E	6 / 51 11.8%C	4 / 51 7.8%AB	3 / 51 5.9%ABE	
1	Sphingobacterium sp. 21	7 / 51 13.7%	0 / 1 0.0%	0 / 2 0.0%	0 / 1 0.0%	0 / 4 0.0%	1 / 3 33.3%	1 / 3 33.3%	0 / 1 0.0%	1 / 1 100.0%	0 / 3 0.0%	0 / 1 0.0%	2 / 4 50.0%	2 / 3 66.7%	0 / 1 0.0%	0 / 1 0.0%	0 / 2 0.0%	0 / 4 0.0%	0 / 1 0.0%	0 / 3 0.0%	0 / 4 0.0%	0 / 2 0.0%	0 / 1 0.0%	0 / 5 0.0%	0 / 0 0.0%	0 / 0 0.0%	0 / 0 0.0%	0 / 0 0.0%	26 / 51 51.0%	12 / 51 23.5%E	6 / 51 11.8%C	4 / 51 7.8%AB	3 / 51 5.9%ABE	
2	Pedobacter	10 / 93 10.8%	0 / 2 0.0%	0 / 3 0.0%	0 / 2 0.0%	0 / 7 0.0%	0 / 6 0.0%	1 / 4 25.0%	1 / 2 50.0%	0 / 2 0.0%	4 / 9 44.4%	0 / 2 0.0%	1 / 7 14.3%	0 / 4 0.0%	0 / 2 0.0%	0 / 2 0.0%	1 / 3 33.3%	0 / 4 0.0%	0 / 2 0.0%	0 / 5 0.0%	0 / 8 0.0%	0 / 4 0.0%	0 / 3 0.0%	2 / 10 20.0%	0 / 0 0.0%	0 / 0 0.0%	0 / 0 0.0%	0 / 0 0.0%	56 / 93 60.2%	21 / 93 22.6%E	6 / 93 6.5%AB	4 / 93 4.3%ABE	4 / 93 4.3%C	2 / 93 2.2%CE
1	ATCC 13125	5 / 44 11.4%	0 / 1 0.0%	0 / 1 0.0%	0 / 1 0.0%	0 / 3 0.0%	0 / 3 0.0%	0 / 2 0.0%	0 / 1 0.0%	0 / 1 0.0%	3 / 4 75.0%	0 / 1 0.0%	1 / 3 33.3%	0 / 2 0.0%	0 / 1 0.0%	0 / 1 0.0%	1 / 1 100.0%	0 / 2 0.0%	0 / 1 0.0%	0 / 2 0.0%	0 / 4 0.0%	0 / 2 0.0%	0 / 2 0.0%	0 / 5 0.0%	0 / 0 0.0%	0 / 0 0.0%	0 / 0 0.0%	0 / 0 0.0%	26 / 44 59.1%	9 / 44 20.5%E	3 / 44 6.8%C	3 / 44 6.8%AB	2 / 44 4.5%ABE	1 / 44 2.3%CE
1	Pedobacter heparinus DSM 2366	5 / 44 11.4%	0 / 1 0.0%	0 / 1 0.0%	0 / 1 0.0%	0 / 3 0.0%	0 / 3 0.0%	0 / 2 0.0%	0 / 1 0.0%	0 / 1 0.0%	3 / 4 75.0%	0 / 1 0.0%	1 / 3 33.3%	0 / 2 0.0%	0 / 1 0.0%	0 / 1 0.0%	1 / 1 100.0%	0 / 2 0.0%	0 / 1 0.0%	0 / 2 0.0%	0 / 4 0.0%	0 / 2 0.0%	0 / 2 0.0%	0 / 5 0.0%	0 / 0 0.0%	0 / 0 0.0%	0 / 0 0.0%	0 / 0 0.0%	26 / 44 59.1%	9 / 44 20.5%E	3 / 44 6.8%AB	3 / 44 6.8%C	2 / 44 4.5%ABE	1 / 44 2.3%CE
1	CCUG 39354	5 / 49 10.2%	0 / 1 0.0%	0 / 2 0.0%	0 / 1 0.0%	0 / 4 0.0%	0 / 3 0.0%	1 / 2 50.0%	1 / 1 100.0%	0 / 1 0.0%	1 / 5 20.0%	0 / 1 0.0%	0 / 4 0.0%	0 / 2 0.0%	0 / 1 0.0%	0 / 1 0.0%	0 / 2 0.0%	0 / 2 0.0%	0 / 1 0.0%	0 / 3 0.0%	0 / 4 0.0%	0 / 2 0.0%	0 / 1 0.0%	2 / 5 40.0%	0 / 0 0.0%	0 / 0 0.0%	0 / 0 0.0%	0 / 0 0.0%	30 / 49 61.2%	12 / 49 24.5%E	3 / 49 6.1%AB	2 / 49 4.1%ABE	1 / 49 2.0%C	1 / 49 2.0%CE

1	Pedobacter saltans DSM 12145	10.2%	0.0%	0.0%	0.0%	0.0%	0.0%	50.0%	100.0%	0.0%	20.0%	0.0%	0.0%	0.0%	0.0%	0.0%	0.0%	0.0%	0.0%	0.0%	0.0%	0.0%	0.0%	40.0%	0.0%	0.0%	0.0%	0.0%	61.2%	24.5%E	6.1%AB	4.1%ABE	2.0%CE	2.0%C			
1	Solitalea	15 / 59 25.4%	2 / 2 100.0%	0 / 2 0.0%	0 / 2 0.0%	0 / 4 0.0%	1 / 4 25.0%	2 / 3 66.7%	0 / 1 0.0%	1 / 1 100.0%	4 / 5 80.0%	0 / 1 0.0%	0 / 3 0.0%	0 / 4 0.0%	0 / 2 0.0%	0 / 1 0.0%	0 / 2 0.0%	1 / 3 33.3%	1 / 1 100.0%	2 / 3 66.7%	0 / 4 0.0%	1 / 4 25.0%	0 / 1 0.0%	0 / 6 0.0%	0 / 0 0.0%	0 / 0 0.0%	0 / 0 0.0%	0 / 0 0.0%	29 / 59 49.2%	14 / 59 23.7%E	11 / 59 18.6%AB	4 / 59 6.8%ABE	1 / 59 1.7%C				
1	ATCC 29591	15 / 59 25.4%	2 / 2 100.0%	0 / 2 0.0%	0 / 2 0.0%	0 / 4 0.0%	1 / 4 25.0%	2 / 3 66.7%	0 / 1 0.0%	1 / 1 100.0%	4 / 5 80.0%	0 / 1 0.0%	0 / 3 0.0%	0 / 4 0.0%	0 / 2 0.0%	0 / 1 0.0%	0 / 2 0.0%	1 / 3 33.3%	1 / 1 100.0%	2 / 3 66.7%	0 / 4 0.0%	1 / 4 25.0%	0 / 1 0.0%	0 / 6 0.0%	0 / 0 0.0%	0 / 0 0.0%	0 / 0 0.0%	0 / 0 0.0%	29 / 59 49.2%	14 / 59 23.7%E	11 / 59 18.6%AB	4 / 59 6.8%ABE	1 / 59 1.7%C				
1	Solitalea canadensis DSM 3403	15 / 59 25.4%	2 / 2 100.0%	0 / 2 0.0%	0 / 2 0.0%	0 / 4 0.0%	1 / 4 25.0%	2 / 3 66.7%	0 / 1 0.0%	1 / 1 100.0%	4 / 5 80.0%	0 / 1 0.0%	0 / 3 0.0%	0 / 4 0.0%	0 / 2 0.0%	0 / 1 0.0%	0 / 2 0.0%	1 / 3 33.3%	1 / 1 100.0%	2 / 3 66.7%	0 / 4 0.0%	1 / 4 25.0%	0 / 1 0.0%	0 / 6 0.0%	0 / 0 0.0%	0 / 0 0.0%	0 / 0 0.0%	0 / 0 0.0%	29 / 59 49.2%	14 / 59 23.7%E	11 / 59 18.6%AB	4 / 59 6.8%ABE	1 / 59 1.7%C				
2	"Saprospiraceae" The Editorial Board BMSB 2nd Ed. V. 4 2011	22 / 98 22.4%	0 / 3 0.0%	2 / 7 28.6%	0 / 4 0.0%	0 / 3 0.0%	3 / 7 42.9%	0 / 5 0.0%	0 / 3 0.0%	0 / 2 0.0%	3 / 5 60.0%	0 / 2 0.0%	3 / 8 37.5%	0 / 4 0.0%	1 / 3 33.3%	0 / 4 0.0%	1 / 2 50.0%	1 / 4 25.0%	0 / 2 0.0%	4 / 6 66.7%	1 / 8 12.5%	1 / 5 20.0%	0 / 3 0.0%	2 / 8 25.0%	0 / 0 0.0%	0 / 0 0.0%	0 / 0 0.0%	0 / 0 0.0%	44 / 98 44.9%	29 / 98 29.6%E	14 / 98 14.3%AB	8 / 98 8.2%ABE	3 / 98 3.1%C				
1	Saprospira	11 / 48 22.9%	0 / 2 0.0%	1 / 4 25.0%	0 / 2 0.0%	0 / 1 0.0%	1 / 3 33.3%	0 / 3 0.0%	0 / 2 0.0%	0 / 1 0.0%	1 / 2 50.0%	0 / 1 0.0%	2 / 4 50.0%	0 / 3 0.0%	0 / 2 0.0%	0 / 2 0.0%	1 / 1 100.0%	1 / 2 50.0%	0 / 1 0.0%	2 / 2 100.0%	0 / 3 0.0%	0 / 3 0.0%	0 / 1 0.0%	2 / 3 66.7%	0 / 0 0.0%	0 / 0 0.0%	0 / 0 0.0%	0 / 0 0.0%	23 / 48 47.9%	13 / 48 27.1%E	6 / 48 12.5%ABE	5 / 48 10.4%AB	1 / 48 2.1%C				
1	ATCC 23119	11 / 48 22.9%	0 / 2 0.0%	1 / 4 25.0%	0 / 2 0.0%	0 / 1 0.0%	1 / 3 33.3%	0 / 3 0.0%	0 / 2 0.0%	0 / 1 0.0%	1 / 2 50.0%	0 / 1 0.0%	2 / 4 50.0%	0 / 3 0.0%	0 / 2 0.0%	0 / 2 0.0%	1 / 1 100.0%	1 / 2 50.0%	0 / 1 0.0%	2 / 2 100.0%	0 / 3 0.0%	0 / 3 0.0%	0 / 1 0.0%	2 / 3 66.7%	0 / 0 0.0%	0 / 0 0.0%	0 / 0 0.0%	0 / 0 0.0%	23 / 48 47.9%	13 / 48 27.1%E	6 / 48 12.5%ABE	5 / 48 10.4%AB	1 / 48 2.1%C				
1	Saprospira grandis str. Lewin	11 / 48 22.9%	0 / 2 0.0%	1 / 4 25.0%	0 / 2 0.0%	0 / 1 0.0%	1 / 3 33.3%	0 / 3 0.0%	0 / 2 0.0%	0 / 1 0.0%	1 / 2 50.0%	0 / 1 0.0%	2 / 4 50.0%	0 / 3 0.0%	0 / 2 0.0%	0 / 2 0.0%	1 / 1 100.0%	1 / 2 50.0%	0 / 1 0.0%	2 / 2 100.0%	0 / 3 0.0%	0 / 3 0.0%	0 / 1 0.0%	2 / 3 66.7%	0 / 0 0.0%	0 / 0 0.0%	0 / 0 0.0%	0 / 0 0.0%	23 / 48 47.9%	13 / 48 27.1%E	6 / 48 12.5%ABE	5 / 48 10.4%AB	1 / 48 2.1%C				
1	Haliscomenobacter	11 / 50 22.0%	0 / 1 0.0%	1 / 3 33.3%	0 / 2 0.0%	0 / 2 0.0%	2 / 4 50.0%	0 / 2 0.0%	0 / 1 0.0%	0 / 1 0.0%	2 / 3 66.7%	0 / 1 0.0%	1 / 4 25.0%	0 / 1 0.0%	1 / 1 100.0%	0 / 2 0.0%	0 / 1 0.0%	0 / 2 0.0%	0 / 1 0.0%	2 / 4 50.0%	1 / 5 20.0%	1 / 2 50.0%	0 / 2 0.0%	0 / 5 0.0%	0 / 0 0.0%	0 / 0 0.0%	0 / 0 0.0%	0 / 0 0.0%	21 / 50 42.0%	16 / 50 32.0%E	9 / 50 18.0%AB	2 / 50 4.0%C	2 / 50 4.0%ABE				
1	ATCC 27775	11 / 50 22.0%	0 / 1 0.0%	1 / 3 33.3%	0 / 2 0.0%	0 / 2 0.0%	2 / 4 50.0%	0 / 2 0.0%	0 / 1 0.0%	0 / 1 0.0%	2 / 3 66.7%	0 / 1 0.0%	1 / 4 25.0%	0 / 1 0.0%	1 / 1 100.0%	0 / 2 0.0%	0 / 1 0.0%	0 / 2 0.0%	0 / 1 0.0%	2 / 4 50.0%	1 / 5 20.0%	1 / 2 50.0%	0 / 2 0.0%	0 / 5 0.0%	0 / 0 0.0%	0 / 0 0.0%	0 / 0 0.0%	0 / 0 0.0%	21 / 50 42.0%	16 / 50 32.0%E	9 / 50 18.0%AB	2 / 50 4.0%C	2 / 50 4.0%ABE				
1	Haliscomenobacter hydrossis DSM 1100	11 / 50 22.0%	0 / 1 0.0%	1 / 3 33.3%	0 / 2 0.0%	0 / 2 0.0%	2 / 4 50.0%	0 / 2 0.0%	0 / 1 0.0%	0 / 1 0.0%	2 / 3 66.7%	0 / 1 0.0%	1 / 4 25.0%	0 / 1 0.0%	1 / 1 100.0%	0 / 2 0.0%	0 / 1 0.0%	0 / 2 0.0%	0 / 1 0.0%	2 / 4 50.0%	1 / 5 20.0%	1 / 2 50.0%	0 / 2 0.0%	0 / 5 0.0%	0 / 0 0.0%	0 / 0 0.0%	0 / 0 0.0%	0 / 0 0.0%	21 / 50 42.0%	16 / 50 32.0%E	9 / 50 18.0%AB	2 / 50 4.0%ABE	2 / 50 4.0%C				
2	Chitinophagaceae	11 / 129 8.5%	0 / 3 0.0%	0 / 5 0.0%	1 / 3 33.3%	1 / 4 25.0%	0 / 8 0.0%	1 / 6 16.7%	0 / 3 0.0%	0 / 2 0.0%	3 / 6 50.0%	0 / 3 0.0%	0 / 10 0.0%	0 / 9 0.0%	0 / 3 0.0%	0 / 6 0.0%	0 / 5 0.0%	2 / 7 28.6%	0 / 2 0.0%	0 / 6 0.0%	1 / 11 9.1%	1 / 5 20.0%	0 / 2 0.0%	1 / 14 7.1%	0 / 0 0.0%	0 / 0 0.0%	0 / 6 0.0%	0 / 0 0.0%	71 / 129 55.0%	41 / 129 31.8%E	8 / 129 6.2%AB	5 / 129 3.9%C	3 / 129 2.3%ABE	1 / 129 0.8%CE			
1	Chitiniphaga	3 / 72 4.2%	0 / 2 0.0%	0 / 3 0.0%	0 / 1 0.0%	0 / 2 0.0%	0 / 4 0.0%	0 / 3 0.0%	0 / 2 0.0%	0 / 1 0.0%	2 / 3 66.7%	0 / 1 0.0%	0 / 4 0.0%	0 / 6 0.0%	0 / 1 0.0%	0 / 3 0.0%	0 / 3 0.0%	0 / 3 0.0%	0 / 1 0.0%	0 / 4 0.0%	0 / 7 0.0%	1 / 2 50.0%	0 / 1 0.0%	0 / 9 0.0%	0 / 0 0.0%	0 / 0 0.0%	0 / 6 0.0%	0 / 0 0.0%	40 / 72 55.6%	25 / 72 34.7%E	4 / 72 5.6%C	2 / 72 2.8%AB	1 / 72 1.4%ABE				
1	ACM 2034	3 / 72 4.2%	0 / 2 0.0%	0 / 3 0.0%	0 / 1 0.0%	0 / 2 0.0%	0 / 4 0.0%	0 / 3 0.0%	0 / 2 0.0%	0 / 1 0.0%	2 / 3 66.7%	0 / 1 0.0%	0 / 4 0.0%	0 / 6 0.0%	0 / 1 0.0%	0 / 3 0.0%	0 / 3 0.0%	0 / 3 0.0%	0 / 1 0.0%	0 / 4 0.0%	0 / 7 0.0%	1 / 2 50.0%	0 / 1 0.0%	0 / 9 0.0%	0 / 0 0.0%	0 / 0 0.0%	0 / 6 0.0%	0 / 0 0.0%	40 / 72 55.6%	25 / 72 34.7%E	4 / 72 5.6%C	2 / 72 2.8%AB	1 / 72 1.4%ABE				
1	Chitinophaga pinensis ACM 2034	3 / 72 4.2%	0 / 2 0.0%	0 / 3 0.0%	0 / 1 0.0%	0 / 2 0.0%	0 / 4 0.0%	0 / 3 0.0%	0 / 2 0.0%	0 / 1 0.0%	2 / 3 66.7%	0 / 1 0.0%	0 / 4 0.0%	0 / 6 0.0%	0 / 1 0.0%	0 / 3 0.0%	0 / 3 0.0%	0 / 3 0.0%	0 / 1 0.0%	0 / 4 0.0%	0 / 7 0.0%	1 / 2 50.0%	0 / 1 0.0%	0 / 9 0.0%	0 / 0 0.0%	0 / 0 0.0%	0 / 6 0.0%	0 / 0 0.0%	40 / 72 55.6%	25 / 72 34.7%E	4 / 72 5.6%C	2 / 72 2.8%AB	1 / 72 1.4%ABE				
1	Niastella	8 / 57 14.0%	0 / 1 0.0%	0 / 2 0.0%	1 / 2 50.0%	1 / 2 50.0%	0 / 4 0.0%	1 / 3 33.3%	0 / 1 0.0%	0 / 1 0.0%	1 / 3 33.3%	0 / 2 0.0%	0 / 6 0.0%	0 / 3 0.0%	0 / 2 0.0%	0 / 3 0.0%	0 / 2 0.0%	2 / 4 50.0%	0 / 1 0.0%	0 / 2 0.0%	1 / 4 25.0%	0 / 3 0.0%	0 / 1 0.0%	1 / 5 20.0%	0 / 0 0.0%	0 / 0 0.0%	0 / 0 0.0%	0 / 0 0.0%	31 / 57 54.4%	16 / 57 28.1%E	6 / 57 10.5%AB	2 / 57 3.5%ABE	1 / 57 1.8%C	1 / 57 1.8%CE			
1	DSM 17620	8 / 57 14.0%	0 / 1 0.0%	0 / 2 0.0%	1 / 2 50.0%	1 / 2 50.0%	0 / 4 0.0%	1 / 3 33.3%	0 / 1 0.0%	0 / 1 0.0%	1 / 3 33.3%	0 / 2 0.0%	0 / 6 0.0%	0 / 3 0.0%	0 / 2 0.0%	0 / 3 0.0%	0 / 2 0.0%	2 / 4 50.0%	0 / 1 0.0%	0 / 2 0.0%	1 / 4 25.0%	0 / 3 0.0%	0 / 1 0.0%	1 / 5 20.0%	0 / 0 0.0%	0 / 0 0.0%	0 / 0 0.0%	0 / 0 0.0%	31 / 57 54.4%	16 / 57 28.1%E	6 / 57 10.5%AB	2 / 57 3.5%ABE	1 / 57 1.8%CE	1 / 57 1.8%C			
1	Niastella koreensis DSM 17620	8 / 57 14.0%	0 / 1 0.0%	0 / 2 0.0%	1 / 2 50.0%	1 / 2 50.0%	0 / 4 0.0%	1 / 3 33.3%	0 / 1 0.0%	0 / 1 0.0%	1 / 3 33.3%	0 / 2 0.0%	0 / 6 0.0%	0 / 3 0.0%	0 / 2 0.0%	0 / 3 0.0%	0 / 2 0.0%	2 / 4 50.0%	0 / 1 0.0%	0 / 2 0.0%	1 / 4 25.0%	0 / 3 0.0%	0 / 1 0.0%	1 / 5 20.0%	0 / 0 0.0%	0 / 0 0.0%	0 / 0 0.0%	0 / 0 0.0%	31 / 57 54.4%	16 / 57 28.1%E	6 / 57 10.5%AB	2 / 57 3.5%ABE	1 / 57 1.8%CE	1 / 57 1.8%C			
23	"Bacteroidia" Krieg 2011	108 / 1350 8.0%	1 / 27 3.7%	1 / 50 2.0%	0 / 40 0.0%	18 / 83 21.7%	8 / 121 6.6%	5 / 55 9.1%	6 / 25 24.0%	0 / 44 0.0%	4 / 99 4.0%	2 / 33 6.1%	10 / 96 10.4%	5 / 71 7.0%	3 / 32 9.4%	2 / 49 4.1%	1 / 23 4.3%	4 / 70 5.7%	5 / 26 19.2%	6 / 91 6.6%	8 / 113 7.1%	6 / 51 11.8%	1 / 23 4.3%	12 / 124 9.7%	0 / 0 0.0%	0 / 0 0.0%	0 / 4 0.0%	0 / 0 0.0%	858 / 1350 63.6%	335 / 1350 24.8%E	69 / 1350 5.1%AB	37 / 1350 2.7%ABE	30 / 1350 2.2%C	17 / 1350 1.3%CE	2 / 1350 0.1%B	1 / 1350 0.1%GG	1 / 1350 0.1%D
23	"Bacteroidales" Krieg 2011	108 / 1350 8.0%	1 / 27 3.7%	1 / 50 2.0%	0 / 40 0.0%	18 / 83 21.7%	8 / 121 6.6%	5 / 55 9.1%	6 / 25 24.0%	0 / 44 0.0%	4 / 99 4.0%	2 / 33 6.1%	10 / 96 10.4%	5 / 71 7.0%	3 / 32 9.4%	2 / 49 4.1%	1 / 23 4.3%	4 / 70 5.7%	5 / 26 19.2%	6 / 91 6.6%	8 / 113 7.1%	6 / 51 11.8%	1 / 23 4.3%	12 / 124 9.7%	0 / 0 0.0%	0 / 0 0.0%	0 / 4 0.0%	0 / 0 0.0%	858 / 1350 63.6%	335 / 1350 24.8%E	69 / 1350 5.1%AB	37 / 1350 2.7%ABE	30 / 1350 2.2%C	17 / 1350 1.3%CE	2 / 1350 0.1%B	1 / 1350 0.1%D	1 / 1350 0.1%GG
8	Bacteroidaceae	40 / 570 7.0%	0 / 9 0.0%	0 / 23 0.0%	0 / 17 0.0%	18 / 41 43.9%	3 / 58 5.2%	0 / 21 0.0%	2 / 9 22.2%	0 / 17 0.0%	0 / 48 0.0%	0 / 16 0.0%	1 / 41 2.4%	2 / 33 6.1%	2 / 14 14.3%	1 / 17 5.9%	1 / 8 12.5%	1 / 26 3.8%	2 / 10 20.0%	1 / 38 2.6%	1 / 48 2.1%	2 / 19 10.5%	0 / 8 0.0%	3 / 49 6.1%	0 / 0 0.0%	0 / 0 0.0%	0 / 0 0.0%	0 / 0 0.0%	388 / 570 68.1%	133 / 570 23.3%E	30 / 570 5.3%AB	10 / 570 1.8%ABE	6 / 570 1.1%C	3 / 570 0.5%CE			
8	"Ristella" Prevot 1938	40 / 570 7.0%	0 / 9 0.0%	0 / 23 0.0%	0 / 17 0.0%	18 / 41 43.9%	3 / 58 5.2%	0 / 21 0.0%	2 / 9 22.2%	0 / 17 0.0%	0 / 48 0.0%	0 / 16 0.0%	1 / 41 2.4%	2 / 33 6.1%	2 / 14 14.3%	1 / 17 5.9%	1 / 8 12.5%	1 / 26 3.8%	2 / 10 20.0%	1 / 38 2.6%	1 / 48 2.1%	2 / 19 10.5%	0 / 8 0.0%	3 / 49 6.1%	0 / 0 0.0%	0 / 0 0.0%	0 / 0 0.0%	0 / 0 0.0%	388 / 570 68.1%	133 / 570 23.3%E	30 / 570 5.3%AB	10 / 570 1.8%ABE	6 / 570 1.1%C	3 / 570 0.5%CE			
3	"Bacteroides inaequalis" Eggerth and Gagnon 1933	20 / 219 9.1%	0 / 3 0.0%	0 / 9 0.0%	0 / 6 0.0%	18 / 18 100.0%	0 / 21 0.0%	0 / 9 0.0%	0 / 3 0.0%	0 / 6 0.0%	0 / 21 0.0%	0 / 6 0.0%	0 / 15 0.0%	0 / 12 0.0%	0 / 6 0.0%	0 / 6 0.0%	0 / 3 0.0%	0 / 12 0.0%	2 / 3 66.7%	0 / 15 0.0%	0 / 18 0.0%	0 / 6 0.0%	0 / 3 0.0%	0 / 18 0.0%	0 / 0 0.0%	0 / 0 0.0%	0 / 0 0.0%	0 / 0 0.0%	148 / 219 67.6%	51 / 219 23.3%E	20 / 219 9.1%AB						
1	Bacteroides fragilis ATCC 25285	7 / 73 9.6%	0 / 1 0.0%	0 / 3 0.0%	0 / 2 0.0%	6 / 6 100.0%	0 / 7 0.0%	0 / 3 0.0%	0 / 1 0.0%	0 / 2 0.0%	0 / 7 0.0%	0 / 2 0.0%	0 / 5 0.0%	0 / 4 0.0%	0 / 2 0.0%	0 / 2 0.0%	0 / 1 0.0%	0 / 4 0.0%	1 / 1 100.0%	0 / 5 0.0%	0 / 6 0.0%	0 / 2 0.0%	0 / 1 0.0%	0 / 6 0.0%	0 / 0 0.0%	0 / 0 0.0%	0 / 0 0.0%	0 / 0 0.0%	49 / 73 67.1%	17 / 73 23.3%E	7 / 73 9.6%AB						
1	Bacteroides fragilis YCH46	7 / 73 9.6%	0 / 1 0.0%	0 / 3 0.0%	0 / 2 0.0%	6 / 6 100.0%	0 / 7 0.0%	0 / 3 0.0%	0 / 1 0.0%	0 / 2 0.0%	0 / 7 0.0%	0 / 2 0.0%	0 / 5 0.0%	0 / 4 0.0%	0 / 2 0.0%	0 / 2 0.0%	0 / 1 0.0%	0 / 4 0.0%	1 / 1 100.0%	0 / 5 0.0%	0 / 6 0.0%	0 / 2 0.0%	0 / 1 0.0%	0 / 6 0.0%	0 / 0 0.0%	0 / 0 0.0%	0 / 0 0.0%	0 / 0 0.0%	49 / 73 67.1%	17 / 73 23.3%E	7 / 73 9.6%AB						
1	Bacteroides fragilis 638R	6 / 73 8.2%	0 / 1 0.0%	0 / 3 0.0%	0 / 2 0.0%	6 / 6 100.0%	0 / 7 0.0%	0 / 3 0.0%	0 / 1 0.0%	0 / 2 0.0%	0 / 7 0.0%	0 / 2 0.0%	0 / 5 0.0%	0 / 4 0.0%	0 / 2 0.0%	0 / 2 0.0%	0 / 1 0.0%	0 / 4 0.0%	0 / 1 0.0%	0 / 5 0.0%	0 / 6 0.0%	0 / 2 0.0%	0 / 1 0.0%	0 / 6 0.0%	0 / 0 0.0%	0 / 0 0.0%	0 / 0 0.0%	0 / 0 0.0%	50 / 73 68.5%	17 / 73 23.3%E	6 / 73 8.2%AB						
1	"Bacillus thetaiotaomicron" Distaso 1912	5 / 70 7.1%	0 / 1 0.0%	0 / 3 0.0%	0 / 2 0.0%	0 / 5 0.0%	0 / 7 0.0%	0 / 2 0.0%	1 / 1 100.0%	0 / 2 0.0%	0 / 6 0.0%	0 / 2 0.0%	0 / 5 0.0%	0 / 4 0.0%	0 / 2 0.0%	1 / 2 50.0%	0 / 1 0.0%	0 / 4 0.0%	0 / 1 0.0%	1 / 5 20.0%	1 / 6 16.7%	0 / 2 0.0%	0 / 1 0.0%	1 / 6 16.7%	0 / 0 0.0%	0 / 0 0.0%	0 / 0 0.0%	0 / 0 0.0%	50 / 70 71.4%	15 / 70 21.4%E	3 / 70 4.3%ABE	2 / 70 2.9%AB					
1	Bacteroides thetaiotaomicron VPI-5482	5 / 70 7.1%	0 / 1 0.0%	0 / 3 0.0%	0 / 2 0.0%	0 / 5 0.0%	0 / 7 0.0%	0 / 2 0.0%	1 / 1 100.0%	0 / 2 0.0%	0 / 6 0.0%	0 / 2 0.0%	0 / 5 0.0%	0 / 4 0.0%	0 / 2 0.0%	1 / 2 50.0%	0 / 1 0.0%	0 / 4 0.0%	0 / 1 0.0%	1 / 5 20.0%	1 / 6 16.7%	0 / 2 0.0%	0 / 1 0.0%	1 / 6 16.7%	0 / 0 0.0%	0 / 0 0.0%	0 / 0 0.0%	0 / 0 0.0%	50 / 70 71.4%	15 / 70 21.4%E	3 / 70 4.3%ABE	2 / 70 2.9%AB					
1	ATCC 8482	6 / 80 7.5%	0 / 1 0.0%	0 / 4 0.0%	0 / 2 0.0%	0 / 7 0.0%	0 / 8 0.0%	0 / 3 0.0%	0 / 1 0.0%	0 / 2 0.0%	0 / 8 0.0%	0 / 2 0.0%	0 / 5 0.0%	1 / 5 20.0%	2 / 2 100.0%	0 / 2 0.0%	1 / 1 100.0%	0 / 3 0.0%	0 / 2 0.0%	0 / 5 0.0%	0 / 7 0.0%	1 / 3 33.3%	0 / 1 0.0%	1 / 6 16.7%	0 / 0 0.0%	0 / 0 0.0%	0 / 0 0.0%	0 / 0 0.0%	55 / 80 68.8%	15 / 80 18.8%E	3 / 80 3.8%ABE	3 / 80 3.8%C	3 / 80 3.8%AB	1 / 80 1.2%CE			
1	Bacteroides vulgatus ATCC 8482	6 / 80 7.5%	0 / 1 0.0%	0 / 4 0.0%	0 / 2 0.0%	0 / 7 0.0%	0 / 8 0.0%	0 / 3 0.0%	0 / 1 0.0%	0 / 2 0.0%	0 / 8 0.0%	0 / 2 0.0%	0 / 5 0.0%	1 / 5 20.0%	2 / 2 100.0%	0 / 2 0.0%	1 / 1 100.0%	0 / 3 0.0%	0 / 2 0.0%	0 / 5 0.0%	0 / 7 0.0%	1 / 3 33.3%	0 / 1 0.0%	1 / 6 16.7%	0 / 0 0.0%	0 / 0 0.0%	0 / 0 0.0%	0 / 0 0.0%	55 / 80 68.8%	15 / 80 18.8%E	3 / 80 3.8%ABE	3 / 80 3.8%C	3 / 80 3.8%AB	1 / 80 1.2%CE			
1	ATCC 35417	1 / 67 1.5%	0 / 1 0.0%	0 / 2 0.0%	0 / 2 0.0%	0 / 5 0.0%	0 / 6 0.0%	0 / 2 0.0%	0 / 1 0.0%	0 / 2 0.0%	0 / 6 0.0%	0 / 2 0.0%	0 / 6 0.0%	0 / 4 0.0%	0 / 2 0.0%	0 / 2 0.0%	0 / 1 0.0%	0 / 3 0.0%	0 / 1 0.0%	0 / 4 0.0%	0 / 6 0.0%	1 / 2 50.0%	0 / 1 0.0%	0 / 6 0.0%	0 / 0 0.0%	0 / 0 0.0%	0 / 0 0.0%	0 / 0 0.0%	49 / 67 73.1%	17 / 67 25.4%E	1 / 67 1.5%ABE						
1	Bacteroides helcogenes DSM 20613	1 / 67 1.5%	0 / 1 0.0%	0 / 2 0.0%	0 / 2 0.0%	0 / 5 0.0%	0 / 6 0.0%	0 / 2 0.0%	0 / 1 0.0%	0 / 2 0.0%	0 / 6 0.0%	0 / 2 0.0%	0 / 6 0.0%	0 / 4 0.0%	0 / 2 0.0%	0 / 2 0.0%	0 / 1 0.0%	0 / 3 0.0%	0 / 1 0.0%	0 / 4 0.0%	0 / 6 0.0%	1 / 2 50.0%	0 / 1 0.0%	0 / 6 0.0%	0 / 0 0.0%	0 / 0 0.0%	0 / 0 0.0%	0 / 0 0.0%	49 / 67 73.1%	17 / 67 25.4%E	1 / 67 1.5%ABE						
1	Bacteroides xylanisolvens	3 / 53 5.7%	0 / 1 0.0%	0 / 3 0.0%	0 / 2 0.0%	0 / 0 0.0%	1 / 7 14.3%	0 / 2 0.0%	1 / 1 100.0%	0 / 2 0.0%	0 / 1 0.0%	0 / 2 0.0%	0 / 4 0.0%	0 / 4 0.0%	0 / 0 0.0%	0 / 2 0.0%	0 / 1 0.0%	0 / 1 0.0%	0 / 1 0.0%	0 / 5 0.0%	0 / 5 0.0%	0 / 2 0.0%	0 / 1 0.0%	1 / 6 16.7%	0 / 0 0.0%	0 / 0 0.0%	0 / 0 0.0%	0 / 0 0.0%	35 / 53 66.0%	15 / 53 28.3%E	2 / 53 3.8%ABE	1 / 53 1.9%AB					
1	Bacteroides sp. XB1A	3 / 53 5.7%	0 / 1 0.0%	0 / 3 0.0%	0 / 2 0.0%	0 / 0 0.0%	1 / 7 14.3%	0 / 2 0.0%	1 / 1 100.0%	0 / 2 0.0%	0 / 1 0.0%	0 / 2 0.0%	0 / 4 0.0%	0 / 4 0.0%	0 / 0 0.0%	0 / 2 0.0%	0 / 1 0.0%	0 / 1 0.0%	0 / 1 0.0%	0 / 5 0.0%	0 / 5 0.0%	0 / 2 0.0%	0 / 1 0.0%	1 / 6 16.7%	0 / 0 0.0%	0 / 0 0.0%	0 / 0 0.0%	0 / 0 0.0%	35 / 53 66.0%	15 / 53 28.3%E	2 / 53 3.8%ABE	1 / 53 1.9%AB					
1	Bacteroides salanitronis	5 / 81 6.2%	0 / 2 0.0%	0 / 2 0.0%	0 / 3 0.0%	0 / 6 0.0%	2 / 9 22.2%	0 / 3 0.0%	0 / 2 0.0%	0 / 3 0.0%	0 / 6 0.0%	0 / 2 0.0%	1 / 6 16.7%	1 / 4 25.0%	0 / 2 0.0%	0 / 3 0.0%	0 / 1 0.0%	1 / 3 33.3%	0 / 2 0.0%	0 / 4 0.0%	0 / 6 0.0%	0 / 4 0.0%	0 / 1 0.0%	0 / 7 0.0%	0 / 0 0.0%	0 / 0 0.0%	0 / 0 0.0%	0 / 0 0.0%	51 / 81 63.0%	20 / 81 24.7%E	4 / 81 4.9%AB	3 / 81 3.7%C	2 / 81 2.5%CE	1 / 81 1.2%ABE			
1	Bacteroides salanitronis BL78	5 / 81 6.2%	0 / 2 0.0%	0 / 2 0.0%	0 / 3 0.0%	0 / 6 0.0%	2 / 9 22.2%	0 / 3 0.0%	0 / 2 0.0%	0 / 3 0.0%	0 / 6 0.0%	0 / 2 0.0%	1 / 6 16.7%	1 / 4 25.0%	0 / 2 0.0%	0 / 3 0.0%	0 / 1 0.0%	1 / 3 33.3%	0 / 2 0.0%	0 / 4 0.0%	0 / 6 0.0%	0 / 4 0.0%	0 / 1 0.0%	0 / 7 0.0%	0 / 0 0.0%	0 / 0 0.0%	0 / 0 0.0%	0 / 0 0.0%	51 / 81 63.0%	20 / 81 24.7%E	4 / 81 4.9%AB	3 / 81 3.7%C	2 / 81 2.5%CE	1 / 81 1.2%ABE			
2	"Rikenellaceae" The Editorial Board BMSB 2nd Ed. V.4 2011	14 / 91 15.4%	1 / 3 33.3%	1 / 4 25.0%	0 / 2 0.0%	0 / 1 0.0%	1 / 8 12.5%	1 / 5 20.0%	0 / 2 0.0%	0 / 2 0.0%	1 / 3 33.3%	2 / 2 100.0%	3 / 8 37.5%	1 / 4 25.0%	0 / 3 0.0%	0 / 4 0.0%	0 / 2 0.0%	0 / 6 0.0%	0 / 2 0.0%	1 / 6 16.7%	1 / 9 11.1%	1 / 4 25.0%	0 / 2 0.0%	0 / 9 0.0%	0 / 0 0.0%	0 / 0 0.0%	0 / 0 0.0%	0 / 0 0.0%	52 / 91 57.1%	25 / 91 27.5%E	9 / 91 9.9%AB	5 / 91 5.5%ABE					
2	Alistipes	14 / 91 15.4%	1 / 3 33.3%	1 / 4 25.0%	0 / 2 0.0%	0 / 1 0.0%	1 / 8 12.5%	1 / 5 20.0%	0 / 2 0.0%	0 / 2 0.0%	1 / 3 33.3%	2 / 2 100.0%	3 / 8 37.5%	1 / 4 25.0%	0 / 3 0.0%	0 / 4 0.0%	0 / 2 0.0%	0 / 6 0.0%	0 / 2 0.0%	1 / 6 16.7%	1 / 9 11.1%	1 / 4 25.0%	0 / 2 0.0%	0 / 9 0.0%	0 / 0 0.0%	0 / 0 0.0%	0 / 0 0.0%	0 / 0 0.0%	52 / 91 57.1%	25 / 91 27.5%E	9 / 91 9.9%AB	5 / 91 5.5%ABE					
1	AHN 2437	5 / 46 10.9%	1 / 2 50.0%	1 / 2 50.0%	0 / 1 0.0%	0 / 1 0.0%	0 / 4 0.0%	0 / 2 0.0%	0 / 1 0.0%	0 / 1 0.0%	0 / 2 0.0%	1 / 1 100.0%	2 / 4 50.0%	0 / 2 0.0%	0 / 1 0.0%	0 / 2 0.0%	0 / 1 0.0%	0 / 3 0.0%	0 / 1 0.0%	0 / 3 0.0%	0 / 4 0.0%	0 / 2 0.0%	0 / 1 0.0%	0 / 5 0.0%	0 / 0 0.0%	0 / 0 0.0%	0 / 0 0.0%	0 / 0 0.0%	28 / 46 60.9%	13 / 46 28.3%E	3 / 46 6.5%ABE	2 / 46 4.3%AB					
1	Alistipes finegoldii AHN 2437	5 / 46 10.9%	1 / 2 50.0%	1 / 2 50.0%	0 / 1 0.0%	0 / 1 0.0%	0 / 4 0.0%	0 / 2 0.0%	0 / 1 0.0%	0 / 1 0.0%	0 / 2 0.0%	1 / 1 100.0%	2 / 4 50.0%	0 / 2 0.0%	0 / 1 0.0%	0 / 2 0.0%	0 / 1 0.0%	0 / 3 0.0%	0 / 1 0.0%	0 / 3 0.0%	0 / 4 0.0%	0 / 2 0.0%	0 / 1 0.0%	0 / 5 0.0%	0 / 0 0.0%	0 / 0 0.0%	0 / 0 0.0%	0 / 0 0.0%	28 / 46 60.9%	13 / 46 28.3%E	3 / 46 6.5%ABE	2 / 46 4.3%AB					
1	ATCC BAA-1179	9 / 45 20.0%	0 / 1 0.0%	0 / 2 0.0%	0 / 1 0.0%	0 / 0 0.0%	1 / 4 25.0%	1 / 3 33.3%	0 / 1 0.0%	0 / 1 0.0%	1 / 1 100.0%	1 / 1 100.0%	1 / 4 25.0%	1 / 2 50.0%	0 / 2 0.0%	0 / 2 0.0%	0 / 1 0.0%	0 / 3 0.0%	0 / 1 0.0%	1 / 3 33.3%	1 / 5 20.0%	1 / 2 50.0%	0 / 1 0.0%	0 / 4 0.0%	0 / 0 0.0%	0 / 0 0.0%	0 / 0 0.0%	0 / 0 0.0%	24 / 45 53.3%	12 / 45 26.7%E	7 / 45 15.6%AB	2 / 45 4.4%ABE					
1	Alistipes shahii WAL 8301	9 / 45 20.0%	0 / 1 0.0%	0 / 2 0.0%	0 / 1 0.0%	0 / 0 0.0%	1 / 4 25.0%	1 / 3 33.3%	0 / 1 0.0%	0 / 1 0.0%	1 / 1 100.0%	1 / 1 100.0%	1 / 4 25.0%	1 / 2 50.0%	0 / 2 0.0%	0 / 2 0.0%	0 / 1 0.0%	0 / 3 0.0%	0 / 1 0.0%	1 / 3 33.3%	1 / 5 20.0%	1 / 2 50.0%	0 / 1 0.0%	0 / 4 0.0%	0 / 0 0.0%	0 / 0 0.0%	0 / 0 0.0%	0 / 0 0.0%	24 / 45 53.3%	12 / 45 26.7%E	7 / 45 15.6%AB	2 / 45 4.4%ABE					
8	"Porphyromonadaceae" Krieg 2011	38 / 446 8.5%	0 / 10 0.0%	0 / 14 0.0%	0 / 16 0.0%	0 / 28 0.0%	4 / 36 11.1%	0 / 17 0.0%	4 / 9 44.4%	0 / 16 0.0%	3 / 36 8.3%	0 / 10 0.0%	4 / 31 12.9%	2 / 20 10.0%	1 / 10 10.0%	1 / 18 5.6%	0 / 9 0.0%	2 / 24 8.3%	3 / 9 33.3%	1 / 28 3.6%	5 / 37 13.5%	1 / 18 5.6%	1 / 8 12.5%	6 / 42 14.3%	0 / 0 0.0%	0 / 0 0.0%	0 / 0 0.0%	0 / 0 0.0%	271 / 446 60.8%	104 / 446 23.3%E	23 / 446 5.2%AB	19 / 446 4.3%C	13 / 446 2.9%CE	13 / 446 2.9%ABE	2 / 446 0.4%B	1 / 446 0.2%GG	
4	Oribaculum	19 / 206 9.2%	0 / 4 0.0%	0 / 7 0.0%	0 / 8 0.0%	0 / 14 0.0%	3 / 15 20.0%	0 / 8 0.0%	4 / 4 100.0%	0 / 8 0.0%	2 / 18 11.1%	0 / 4 0.0%	0 / 13 0.0%	0 / 8 0.0%	0 / 4 0.0%	0 / 8 0.0%	0 / 4 0.0%	1 / 12 8.3%	1 / 4 25.0%	0 / 12 0.0%	3 / 19 15.8%	0 / 8 0.0%	1 / 4 25.0%	4 / 20 20.0%	0 / 0 0.0%	0 / 0 0.0%	0 / 0 0.0%	0 / 0 0.0%	120 / 206 58.3%	48 / 206 23.3%E	13 / 206 6.3%C	12 / 206 5.8%AB	6 / 206 2.9%CE	5 / 206 2.4%ABE	2 / 206 1.0%B		
3	ATCC 33277	12 / 159 7.5%	0 / 3 0.0%	0 / 6 0.0%	0 / 6 0.0%	0 / 12 0.0%	2 / 12 16.7%	0 / 6 0.0%	3 / 3 100.0%	0 / 6 0.0%	1 / 15 6.7%	0 / 3 0.0%	0 / 9 0.0%	0 / 6 0.0%	0 / 3 0.0%	0 / 6 0.0%	0 / 3 0.0%	0 / 9 0.0%	1 / 3 33.3%	0 / 9 0.0%	3 / 15 20.0%	0 / 6 0.0%	0 / 3 0.0%	2 / 15 13.3%	0 / 0 0.0%	0 / 0 0.0%	0 / 0 0.0%	0 / 0 0.0%	93 / 159 58.5%	36 / 159 22.6%E	13 / 159 8.2%C	7 / 159 4.4%AB	5 / 159 3.1%CE	3 / 159 1.9%ABE	2 / 159 1.3%B		
1	Porphyromonas gingivalis W83	3 / 53 5.7%	0 / 1 0.0%	0 / 2 0.0%	0 / 2 0.0%	0 / 4 0.0%	1 / 4 25.0%	0 / 2 0.0%	1 / 1 100.0%	0 / 2 0.0%	0 / 5 0.0%	0 / 1 0.0%	0 / 3 0.0%	0 / 2 0.0%	0 / 1 0.0%	0 / 2 0.0%	0 / 1 0.0%	0 / 3 0.0%	0 / 1 0.0%	0 / 3 0.0%	1 / 5 20.0%	0 / 2 0.0%	0 / 1 0.0%	0 / 5 0.0%	0 / 0 0.0%	0 / 0 0.0%	0 / 0 0.0%	0 / 0 0.0%	31 / 53 58.5%	12 / 53 22.6%E	5 / 53 9.4%C	3 / 53 5.7%AB	2 / 53 3.8%CE				
1	Porphyromonas gingivalis ATCC 33277	4 / 53 7.5%	0 / 1 0.0%	0 / 2 0.0%	0 / 2 0.0%	0 / 4 0.0%	1 / 4 25.0%	0 / 2 0.0%	1 / 1 100.0%	0 / 2 0.0%	0 / 5 0.0%	0 / 1 0.0%	0 / 3 0.0%	0 / 2 0.0%	0 / 1 0.0%	0 / 2 0.0%	0 / 1 0.0%	0 / 3 0.0%	0 / 1 0.0%	0 / 3 0.0%	1 / 5 20.0%	0 / 2 0.0%	0 / 1 0.0%	1 / 5 20.0%	0 / 0 0.0%	0 / 0 0.0%	0 / 0 0.0%	0 / 0 0.0%	31 / 53 58.5%	12 / 53 22.6%E	4 / 53 7.5%C	2 / 53 3.8%CE	2 / 53 3.8%B	1 / 53 1.9%ABE	1 / 53 1.9%AB		
1	Porphyromonas gingivalis TDC60	5 / 53 9.4%	0 / 1 0.0%	0 / 2 0.0%	0 / 2 0.0%	0 / 4 0.0%	0 / 4 0.0%	0 / 2 0.0%	1 / 1 100.0%	0 / 2 0.0%	1 / 5 20.0%	0 / 1 0.0%	0 / 3 0.0%	0 / 2 0.0%	0 / 1 0.0%	0 / 2 0.0%	0 / 1 0.0%	0 / 3 0.0%	1 / 1 100.0%	0 / 3 0.0%	1 / 5 20.0%	0 / 2 0.0%	0 / 1 0.0%	1 / 5 20.0%	0 / 0 0.0%	0 / 0 0.0%	0 / 0 0.0%	0 / 0 0.0%	31 / 53 58.5%	12 / 53 22.6%E	4 / 53 7.5%C	3 / 53 5.7%AB	2 / 53 3.8%ABE	1 / 53 1.9%CE			
1	"Bacteroides melaninogenicus subsp. asaccharolyticus" Holdeman and Moore 1970	7 / 47 14.9%	0 / 1 0.0%	0 / 1 0.0%	0 / 2 0.0%	0 / 2 0.0%	1 / 3 33.3%	0 / 2 0.0%	1 / 1 100.0%	0 / 2 0.0%	1 / 3 33.3%	0 / 1 0.0%	0 / 4 0.0%	0 / 2 0.0%	0 / 1 0.0%	0 / 2 0.0%	0 / 1 0.0%	1 / 3 33.3%	0 / 1 0.0%	0 / 3 0.0%	0 / 4 0.0%	0 / 2 0.0%	1 / 1 100.0%	2 / 5 40.0%	0 / 0 0.0%	0 / 0 0.0%	0 / 0 0.0%	0 / 0 0.0%	27 / 47 57.4%	12 / 47 25.5%E	5 / 47 10.6%AB	2 / 47 4.3%ABE	1 / 47 2.1%CE				
1	Porphyromonas asaccharolytica ATCC 25260	7 / 47 14.9%	0 / 1 0.0%	0 / 1 0.0%	0 / 2 0.0%	0 / 2 0.0%	1 / 3 33.3%	0 / 2 0.0%	1 / 1 100.0%	0 / 2 0.0%	1 / 3 33.3%	0 / 1 0.0%	0 / 4 0.0%	0 / 2 0.0%	0 / 1 0.0%	0 / 2 0.0%	0 / 1 0.0%	1 / 3 33.3%	0 / 1 0.0%	0 / 3 0.0%	0 / 4 0.0%	0 / 2 0.0%	1 / 1 100.0%	2 / 5 40.0%	0 / 0 0.0%	0 / 0 0.0%	0 / 0 0.0%	0 / 0 0.0%	27 / 47 57.4%	12 / 47 25.5%E	5 / 47 10.6%AB	2 / 47 4.3%ABE	1 / 47 2.1%CE				
1	Tannerella	1 / 46 2.2%	0 / 1 0.0%	0 / 1 0.0%	0 / 1 0.0%	0 / 2 0.0%	0 / 4 0.0%	0 / 1 0.0%	0 / 1 0.0%	0 / 1 0.0%	0 / 3 0.0%	0 / 1 0.0%	0 / 4 0.0%	0 / 2 0.0%	0 / 1 0.0%	0 / 2 0.0%	0 / 1 0.0%	1 / 3 33.3%	0 / 1 0.0%	0 / 4 0.0%	0 / 4 0.0%	0 / 2 0.0%	0 / 1 0.0%	0 / 5 0.0%	0 / 0 0.0%	0 / 0 0.0%	0 / 0 0.0%	0 / 0 0.0%	29 / 46 63.0%	10 / 46 21.7%E	5 / 46 10.9%CE	1 / 46 2.2%C	1 / 46 2.2%AB				
1	ATCC 43037	1 / 46 2.2%	0 / 1 0.0%	0 / 1 0.0%	0 / 1 0.0%	0 / 2 0.0%	0 / 4 0.0%	0 / 1 0.0%	0 / 1 0.0%	0 / 1 0.0%	0 / 3 0.0%	0 / 1 0.0%	0 / 4 0.0%	0 / 2 0.0%	0 / 1 0.0%	0 / 2 0.0%	0 / 1 0.0%	1 / 3 33.3%	0 / 1 0.0%	0 / 4 0.0%	0 / 4 0.0%	0 / 2 0.0%	0 / 1 0.0%	0 / 5 0.0%	0 / 0 0.0%	0 / 0 0.0%	0 / 0 0.0%	0 / 0 0.0%	29 / 46 63.0%	10 / 46 21.7%E	5 / 46 10.9%CE	1 / 46 2.2%C	1 / 46 2.2%AB				
1	Tannerella forsythensis ATCC 43037	1 / 46 2.2%	0 / 1 0.0%	0 / 1 0.0%	0 / 1 0.0%	0 / 2 0.0%	0 / 4 0.0%	0 / 1 0.0%	0 / 1 0.0%	0 / 1 0.0%	0 / 3 0.0%	0 / 1 0.0%	0 / 4 0.0%	0 / 2 0.0%	0 / 1 0.0%	0 / 2 0.0%	0 / 1 0.0%	1 / 3 33.3%	0 / 1 0.0%	0 / 4 0.0%	0 / 4 0.0%	0 / 2 0.0%	0 / 1 0.0%	0 / 5 0.0%	0 / 0 0.0%	0 / 0 0.0%	0 / 0 0.0%	0 / 0 0.0%	29 / 46 63.0%	10 / 46 21.7%E	5 / 46 10.9%CE	1 / 46 2.2%AB	1 / 46 2.2%C				
1	Odoribacter	7 / 60 11.7%	0 / 2 0.0%	0 / 2 0.0%	0 / 2 0.0%	0 / 2 0.0%	0 / 6 0.0%	0 / 2 0.0%	0 / 1 0.0%	0 / 2 0.0%	0 / 3 0.0%	0 / 2 0.0%	1 / 4 25.0%	1 / 3 33.3%	0 / 2 0.0%	0 / 3 0.0%	0 / 1 0.0%	0 / 3 0.0%	2 / 2 100.0%	1 / 3 33.3%	1 / 4 25.0%	0 / 4 0.0%	0 / 1 0.0%	1 / 6 16.7%	0 / 0 0.0%	0 / 0 0.0%	0 / 0 0.0%	0 / 0 0.0%	34 / 60 56.7%	17 / 60 28.3%E	5 / 60 8.3%AB	2 / 60 3.3%ABE	1 / 60 1.7%CE	1 / 60 1.7%C			
1	ATCC 29572	7 / 60 11.7%	0 / 2 0.0%	0 / 2 0.0%	0 / 2 0.0%	0 / 2 0.0%	0 / 6 0.0%	0 / 2 0.0%	0 / 1 0.0%	0 / 2 0.0%	0 / 3 0.0%	0 / 2 0.0%	1 / 4 25.0%	1 / 3 33.3%	0 / 2 0.0%	0 / 3 0.0%	0 / 1 0.0%	0 / 3 0.0%	2 / 2 100.0%	1 / 3 33.3%	1 / 4 25.0%	0 / 4 0.0%	0 / 1 0.0%	1 / 6 16.7%	0 / 0 0.0%	0 / 0 0.0%	0 / 0 0.0%	0 / 0 0.0%	34 / 60 56.7%	17 / 60 28.3%E	5 / 60 8.3%AB	2 / 60 3.3%ABE	1 / 60 1.7%CE	1 / 60 1.7%C			
1	Odoribacter splanchnicus DSM 20712	7 / 60 11.7%	0 / 2 0.0%	0 / 2 0.0%	0 / 2 0.0%	0 / 2 0.0%	0 / 6 0.0%	0 / 2 0.0%	0 / 1 0.0%	0 / 2 0.0%	0 / 3 0.0%	0 / 2 0.0%	1 / 4 25.0%	1 / 3 33.3%	0 / 2 0.0%	0 / 3 0.0%	0 / 1 0.0%	0 / 3 0.0%	2 / 2 100.0%	1 / 3 33.3%	1 / 4 25.0%	0 / 4 0.0%	0 / 1 0.0%	1 / 6 16.7%	0 / 0 0.0%	0 / 0 0.0%	0 / 0 0.0%	0 / 0 0.0%	34 / 60 56.7%	17 / 60 28.3%E	5 / 60 8.3%AB	2 / 60 3.3%ABE	1 / 60 1.7%CE	1 / 60 1.7%C			
1	Paludibacter	6 / 52 11.5%	0 / 1 0.0%	0 / 1 0.0%	0 / 2 0.0%	0 / 3 0.0%	1 / 5 20.0%	0 / 2 0.0%	0 / 1 0.0%	0 / 2 0.0%	0 / 4 0.0%	0 / 1 0.0%	3 / 4 75.0%	0 / 3 0.0%	0 / 1 0.0%	1 / 2 50.0%	0 / 1 0.0%	0 / 3 0.0%	0 / 1 0.0%	0 / 4 0.0%	0 / 4 0.0%	0 / 1 0.0%	0 / 1 0.0%	1 / 5 20.0%	0 / 0 0.0%	0 / 0 0.0%	0 / 0 0.0%	0 / 0 0.0%	36 / 52 69.2%	9 / 52 17.3%E	5 / 52 9.6%ABE	1 / 52 1.9%AB	1 / 52 1.9%GG				
1	Bacteroidales str. WB4	6 / 52 11.5%	0 / 1 0.0%	0 / 1 0.0%	0 / 2 0.0%	0 / 3 0.0%	1 / 5 20.0%	0 / 2 0.0%	0 / 1 0.0%	0 / 2 0.0%	0 / 4 0.0%	0 / 1 0.0%	3 / 4 75.0%	0 / 3 0.0%	0 / 1 0.0%	1 / 2 50.0%	0 / 1 0.0%	0 / 3 0.0%	0 / 1 0.0%	0 / 4 0.0%	0 / 4 0.0%	0 / 1 0.0%	0 / 1 0.0%	1 / 5 20.0%	0 / 0 0.0%	0 / 0 0.0%	0 / 0 0.0%	0 / 0 0.0%	36 / 52 69.2%	9 / 52 17.3%E	5 / 52 9.6%ABE	1 / 52 1.9%AB	1 / 52 1.9%GG				
1	Paludibacter propionicigenes CCUG 53888	6 / 52 11.5%	0 / 1 0.0%	0 / 1 0.0%	0 / 2 0.0%	0 / 3 0.0%	1 / 5 20.0%	0 / 2 0.0%	0 / 1 0.0%	0 / 2 0.0%	0 / 4 0.0%	0 / 1 0.0%	3 / 4 75.0%	0 / 3 0.0%	0 / 1 0.0%	1 / 2 50.0%	0 / 1 0.0%	0 / 3 0.0%	0 / 1 0.0%	0 / 4 0.0%	0 / 4 0.0%	0 / 1 0.0%	0 / 1 0.0%	1 / 5 20.0%	0 / 0 0.0%	0 / 0 0.0%	0 / 0 0.0%	0 / 0 0.0%	36 / 52 69.2%	9 / 52 17.3%E	5 / 52 9.6%ABE	1 / 52 1.9%AB	1 / 52 1.9%GG				
1	Parabacteroides	5 / 82 6.1%	0 / 2 0.0%	0 / 3 0.0%	0 / 3 0.0%	0 / 7 0.0%	0 / 6 0.0%	0 / 4 0.0%	0 / 2 0.0%	0 / 3 0.0%	1 / 8 12.5%	0 / 2 0.0%	0 / 6 0.0%	1 / 4 25.0%	1 / 2 50.0%	0 / 3 0.0%	0 / 2 0.0%	0 / 3 0.0%	0 / 1 0.0%	0 / 5 0.0%	1 / 6 16.7%	1 / 3 33.3%	0 / 1 0.0%	0 / 6 0.0%	0 / 0 0.0%	0 / 0 0.0%	0 / 0 0.0%	0 / 0 0.0%	52 / 82 63.4%	20 / 82 24.4%E	4 / 82 4.9%C	4 / 82 4.9%AB	1 / 82 1.2%CE	1 / 82 1.2%ABE			
1	"Bacteroides fragilis subsp. distasonis" (Eggerth and Gagnon 1933) Holdeman and Moore 1970	5 / 82 6.1%	0 / 2 0.0%	0 / 3 0.0%	0 / 3 0.0%	0 / 7 0.0%	0 / 6 0.0%	0 / 4 0.0%	0 / 2 0.0%	0 / 3 0.0%	1 / 8 12.5%	0 / 2 0.0%	0 / 6 0.0%	1 / 4 25.0%	1 / 2 50.0%	0 / 3 0.0%	0 / 2 0.0%	0 / 3 0.0%	0 / 1 0.0%	0 / 5 0.0%	1 / 6 16.7%	1 / 3 33.3%	0 / 1 0.0%	0 / 6 0.0%	0 / 0 0.0%	0 / 0 0.0%	0 / 0 0.0%	0 / 0 0.0%	52 / 82 63.4%	20 / 82 24.4%E	4 / 82 4.9%AB	4 / 82 4.9%C	1 / 82 1.2%ABE	1 / 82 1.2%CE			
1	Parabacteroides distasonis ATCC 8503	5 / 82 6.1%	0 / 2 0.0%	0 / 3 0.0%	0 / 3 0.0%	0 / 7 0.0%	0 / 6 0.0%	0 / 4 0.0%	0 / 2 0.0%	0 / 3 0.0%	1 / 8 12.5%	0 / 2 0.0%	0 / 6 0.0%	1 / 4 25.0%	1 / 2 50.0%	0 / 3 0.0%	0 / 2 0.0%	0 / 3 0.0%	0 / 1 0.0%	0 / 5 0.0%	1 / 6 16.7%	1 / 3 33.3%	0 / 1 0.0%	0 / 6 0.0%	0 / 0 0.0%	0 / 0 0.0%	0 / 0 0.0%	0 / 0 0.0%	52 / 82 63.4%	20 / 82 24.4%E	4 / 82 4.9%AB	4 / 82 4.9%C	1 / 82 1.2%ABE	1 / 82 1.2%CE			
4	"Prevotellaceae" Krieg 2011	14 / 205 6.8%	0 / 4 0.0%	0 / 8 0.0%	0 / 4 0.0%	0 / 11 0.0%	0 / 16 0.0%	4 / 11 36.4%	0 / 4 0.0%	0 / 8 0.0%	0 / 11 0.0%	0 / 4 0.0%	1 / 13 7.7%	0 / 12 0.0%	0 / 4 0.0%	0 / 8 0.0%	0 / 3 0.0%	1 / 12 8.3%	0 / 4 0.0%	2 / 16 12.5%	1 / 15 6.7%	2 / 9 22.2%	0 / 4 0.0%	3 / 20 15.0%	0 / 0 0.0%	0 / 0 0.0%	0 / 4 0.0%	0 / 0 0.0%	122 / 205 59.5%	63 / 205 30.7%E	8 / 205 3.9%ABE	6 / 205 2.9%AB	4 / 205 2.0%C	1 / 205 0.5%CE	1 / 205 0.5%D		
4	Prevotella	14 / 205 6.8%	0 / 4 0.0%	0 / 8 0.0%	0 / 4 0.0%	0 / 11 0.0%	0 / 16 0.0%	4 / 11 36.4%	0 / 4 0.0%	0 / 8 0.0%	0 / 11 0.0%	0 / 4 0.0%	1 / 13 7.7%	0 / 12 0.0%	0 / 4 0.0%	0 / 8 0.0%	0 / 3 0.0%	1 / 12 8.3%	0 / 4 0.0%	2 / 16 12.5%	1 / 15 6.7%	2 / 9 22.2%	0 / 4 0.0%	3 / 20 15.0%	0 / 0 0.0%	0 / 0 0.0%	0 / 4 0.0%	0 / 0 0.0%	122 / 205 59.5%	63 / 205 30.7%E	8 / 205 3.9%ABE	6 / 205 2.9%AB	4 / 205 2.0%C	1 / 205 0.5%D	1 / 205 0.5%CE		
1	"Ruminobacter ruminicola" (Bryant et al. 1958) Prevot 1966	4 / 55 7.3%	0 / 1 0.0%	0 / 2 0.0%	0 / 1 0.0%	0 / 4 0.0%	0 / 4 0.0%	0 / 2 0.0%	0 / 1 0.0%	0 / 2 0.0%	0 / 4 0.0%	0 / 1 0.0%	0 / 4 0.0%	0 / 3 0.0%	0 / 1 0.0%	0 / 2 0.0%	0 / 1 0.0%	1 / 3 33.3%	0 / 1 0.0%	0 / 4 0.0%	1 / 4 25.0%	0 / 2 0.0%	0 / 1 0.0%	2 / 5 40.0%	0 / 0 0.0%	0 / 0 0.0%	0 / 2 0.0%	0 / 0 0.0%	32 / 55 58.2%	16 / 55 29.1%E	2 / 55 3.6%ABE	2 / 55 3.6%AB	1 / 55 1.8%CE	1 / 55 1.8%D	1 / 55 1.8%C		
1	Prevotella ruminicola 23	4 / 55 7.3%	0 / 1 0.0%	0 / 2 0.0%	0 / 1 0.0%	0 / 4 0.0%	0 / 4 0.0%	0 / 2 0.0%	0 / 1 0.0%	0 / 2 0.0%	0 / 4 0.0%	0 / 1 0.0%	0 / 4 0.0%	0 / 3 0.0%	0 / 1 0.0%	0 / 2 0.0%	0 / 1 0.0%	1 / 3 33.3%	0 / 1 0.0%	0 / 4 0.0%	1 / 4 25.0%	0 / 2 0.0%	0 / 1 0.0%	2 / 5 40.0%	0 / 0 0.0%	0 / 0 0.0%	0 / 2 0.0%	0 / 0 0.0%	32 / 55 58.2%	16 / 55 29.1%E	2 / 55 3.6%AB	2 / 55 3.6%ABE	1 / 55 1.8%C	1 / 55 1.8%D	1 / 55 1.8%CE		
1	ATCC 35308	4 / 51 7.8%	0 / 1 0.0%	0 / 2 0.0%	0 / 1 0.0%	0 / 2 0.0%	0 / 4 0.0%	1 / 3 33.3%	0 / 1 0.0%	0 / 2 0.0%	0 / 2 0.0%	0 / 1 0.0%	1 / 4 25.0%	0 / 3 0.0%	0 / 1 0.0%	0 / 2 0.0%	0 / 1 0.0%	0 / 3 0.0%	0 / 1 0.0%	1 / 4 25.0%	0 / 4 0.0%	0 / 3 0.0%	0 / 1 0.0%	1 / 5 20.0%	0 / 0 0.0%	0 / 0 0.0%	0 / 0 0.0%	0 / 0 0.0%	29 / 51 56.9%	16 / 51 31.4%E	3 / 51 5.9%ABE	2 / 51 3.9%C	1 / 51 2.0%AB				
1	Prevotella denticola F0289	4 / 51 7.8%	0 / 1 0.0%	0 / 2 0.0%	0 / 1 0.0%	0 / 2 0.0%	0 / 4 0.0%	1 / 3 33.3%	0 / 1 0.0%	0 / 2 0.0%	0 / 2 0.0%	0 / 1 0.0%	1 / 4 25.0%	0 / 3 0.0%	0 / 1 0.0%	0 / 2 0.0%	0 / 1 0.0%	0 / 3 0.0%	0 / 1 0.0%	1 / 4 25.0%	0 / 4 0.0%	0 / 3 0.0%	0 / 1 0.0%	1 / 5 20.0%	0 / 0 0.0%	0 / 0 0.0%	0 / 0 0.0%	0 / 0 0.0%	29 / 51 56.9%	16 / 51 31.4%E	3 / 51 5.9%ABE	2 / 51 3.9%C	1 / 51 2.0%AB				
1	ATCC 25611	1 / 49 2.0%	0 / 1 0.0%	0 / 2 0.0%	0 / 1 0.0%	0 / 3 0.0%	0 / 4 0.0%	1 / 3 33.3%	0 / 1 0.0%	0 / 2 0.0%	0 / 3 0.0%	0 / 1 0.0%	0 / 2 0.0%	0 / 3 0.0%	0 / 1 0.0%	0 / 2 0.0%	0 / 1 0.0%	0 / 3 0.0%	0 / 1 0.0%	0 / 4 0.0%	0 / 3 0.0%	0 / 2 0.0%	0 / 1 0.0%	0 / 5 0.0%	0 / 0 0.0%	0 / 0 0.0%	0 / 0 0.0%	0 / 0 0.0%	32 / 49 65.3%	16 / 49 32.7%E	1 / 49 2.0%AB						
1	Prevotella intermedia 17	1 / 49 2.0%	0 / 1 0.0%	0 / 2 0.0%	0 / 1 0.0%	0 / 3 0.0%	0 / 4 0.0%	1 / 3 33.3%	0 / 1 0.0%	0 / 2 0.0%	0 / 3 0.0%	0 / 1 0.0%	0 / 2 0.0%	0 / 3 0.0%	0 / 1 0.0%	0 / 2 0.0%	0 / 1 0.0%	0 / 3 0.0%	0 / 1 0.0%	0 / 4 0.0%	0 / 3 0.0%	0 / 2 0.0%	0 / 1 0.0%	0 / 5 0.0%	0 / 0 0.0%	0 / 0 0.0%	0 / 0 0.0%	0 / 0 0.0%	32 / 49 65.3%	16 / 49 32.7%E	1 / 49 2.0%AB						
1	"Bacterium melaninogenicum" Oliver and Wherry 1921	5 / 50 10.0%	0 / 1 0.0%	0 / 2 0.0%	0 / 1 0.0%	0 / 2 0.0%	0 / 4 0.0%	2 / 3 66.7%	0 / 1 0.0%	0 / 2 0.0%	0 / 3 0.0%	0 / 1 0.0%	0 / 3 0.0%	0 / 3 0.0%	0 / 1 0.0%	0 / 2 0.0%	0 / 1 0.0%	0 / 3 0.0%	0 / 1 0.0%	1 / 4 25.0%	0 / 3 0.0%	2 / 2 100.0%	0 / 1 0.0%	0 / 5 0.0%	0 / 0 0.0%	0 / 0 0.0%	0 / 2 0.0%	0 / 0 0.0%	29 / 50 58.0%	15 / 50 30.0%E	3 / 50 6.0%ABE	2 / 50 4.0%AB	1 / 50 2.0%C				
1	Prevotella melaninogenica ATCC 25845	5 / 50 10.0%	0 / 1 0.0%	0 / 2 0.0%	0 / 1 0.0%	0 / 2 0.0%	0 / 4 0.0%	2 / 3 66.7%	0 / 1 0.0%	0 / 2 0.0%	0 / 3 0.0%	0 / 1 0.0%	0 / 3 0.0%	0 / 3 0.0%	0 / 1 0.0%	0 / 2 0.0%	0 / 1 0.0%	0 / 3 0.0%	0 / 1 0.0%	1 / 4 25.0%	0 / 3 0.0%	2 / 2 100.0%	0 / 1 0.0%	0 / 5 0.0%	0 / 0 0.0%	0 / 0 0.0%	0 / 2 0.0%	0 / 0 0.0%	29 / 50 58.0%	15 / 50 30.0%E	3 / 50 6.0%ABE	2 / 50 4.0%AB	1 / 50 2.0%C				
1	unclassified Bacteroidales	2 / 38 5.3%	0 / 1 0.0%	0 / 1 0.0%	0 / 1 0.0%	0 / 2 0.0%	0 / 3 0.0%	0 / 1 0.0%	0 / 1 0.0%	0 / 1 0.0%	0 / 1 0.0%	0 / 1 0.0%	1 / 3 33.3%	0 / 2 0.0%	0 / 1 0.0%	0 / 2 0.0%	0 / 1 0.0%	0 / 2 0.0%	0 / 1 0.0%	1 / 3 33.3%	0 / 4 0.0%	0 / 1 0.0%	0 / 1 0.0%	0 / 4 0.0%	0 / 0 0.0%	0 / 0 0.0%	0 / 0 0.0%	0 / 0 0.0%	25 / 38 65.8%	10 / 38 26.3%E	1 / 38 2.6%AB	1 / 38 2.6%C	1 / 38 2.6%ABE				
1	'Candidatus Azobacteroides'	2 / 38 5.3%	0 / 1 0.0%	0 / 1 0.0%	0 / 1 0.0%	0 / 2 0.0%	0 / 3 0.0%	0 / 1 0.0%	0 / 1 0.0%	0 / 1 0.0%	0 / 1 0.0%	0 / 1 0.0%	1 / 3 33.3%	0 / 2 0.0%	0 / 1 0.0%	0 / 2 0.0%	0 / 1 0.0%	0 / 2 0.0%	0 / 1 0.0%	1 / 3 33.3%	0 / 4 0.0%	0 / 1 0.0%	0 / 1 0.0%	0 / 4 0.0%	0 / 0 0.0%	0 / 0 0.0%	0 / 0 0.0%	0 / 0 0.0%	25 / 38 65.8%	10 / 38 26.3%E	1 / 38 2.6%ABE	1 / 38 2.6%AB	1 / 38 2.6%C				
1	'Candidatus Azobacteroides pseudotrichonymphae'	2 / 38 5.3%	0 / 1 0.0%	0 / 1 0.0%	0 / 1 0.0%	0 / 2 0.0%	0 / 3 0.0%	0 / 1 0.0%	0 / 1 0.0%	0 / 1 0.0%	0 / 1 0.0%	0 / 1 0.0%	1 / 3 33.3%	0 / 2 0.0%	0 / 1 0.0%	0 / 2 0.0%	0 / 1 0.0%	0 / 2 0.0%	0 / 1 0.0%	1 / 3 33.3%	0 / 4 0.0%	0 / 1 0.0%	0 / 1 0.0%	0 / 4 0.0%	0 / 0 0.0%	0 / 0 0.0%	0 / 0 0.0%	0 / 0 0.0%	25 / 38 65.8%	10 / 38 26.3%E	1 / 38 2.6%ABE	1 / 38 2.6%C	1 / 38 2.6%AB				
1	Candidatus Azobacteroides pseudotrichonymphae genomovar. CFP2	2 / 38 5.3%	0 / 1 0.0%	0 / 1 0.0%	0 / 1 0.0%	0 / 2 0.0%	0 / 3 0.0%	0 / 1 0.0%	0 / 1 0.0%	0 / 1 0.0%	0 / 1 0.0%	0 / 1 0.0%	1 / 3 33.3%	0 / 2 0.0%	0 / 1 0.0%	0 / 2 0.0%	0 / 1 0.0%	0 / 2 0.0%	0 / 1 0.0%	1 / 3 33.3%	0 / 4 0.0%	0 / 1 0.0%	0 / 1 0.0%	0 / 4 0.0%	0 / 0 0.0%	0 / 0 0.0%	0 / 0 0.0%	0 / 0 0.0%	25 / 38 65.8%	10 / 38 26.3%E	1 / 38 2.6%AB	1 / 38 2.6%C	1 / 38 2.6%ABE				
13	"Cytophagia" Nakagawa 2011	49 / 520 9.4%	1 / 12 8.3%	3 / 19 15.8%	0 / 13 0.0%	2 / 19 10.5%	3 / 29 10.3%	5 / 29 17.2%	2 / 11 18.2%	4 / 15 26.7%	5 / 29 17.2%	1 / 13 7.7%	2 / 41 4.9%	1 / 24 4.2%	2 / 14 14.3%	0 / 16 0.0%	3 / 14 21.4%	0 / 29 0.0%	1 / 13 7.7%	2 / 34 5.9%	2 / 56 3.6%	3 / 21 14.3%	1 / 13 7.7%	6 / 56 10.7%	0 / 0 0.0%	0 / 0 0.0%	0 / 0 0.0%	0 / 0 0.0%	313 / 520 60.2%	139 / 520 26.7%E	31 / 520 6.0%AB	18 / 520 3.5%ABE	11 / 520 2.1%C	7 / 520 1.3%CE	1 / 520 0.2%GG		
13	Cytophagales	49 / 520 9.4%	1 / 12 8.3%	3 / 19 15.8%	0 / 13 0.0%	2 / 19 10.5%	3 / 29 10.3%	5 / 29 17.2%	2 / 11 18.2%	4 / 15 26.7%	5 / 29 17.2%	1 / 13 7.7%	2 / 41 4.9%	1 / 24 4.2%	2 / 14 14.3%	0 / 16 0.0%	3 / 14 21.4%	0 / 29 0.0%	1 / 13 7.7%	2 / 34 5.9%	2 / 56 3.6%	3 / 21 14.3%	1 / 13 7.7%	6 / 56 10.7%	0 / 0 0.0%	0 / 0 0.0%	0 / 0 0.0%	0 / 0 0.0%	313 / 520 60.2%	139 / 520 26.7%E	31 / 520 6.0%AB	18 / 520 3.5%ABE	11 / 520 2.1%C	7 / 520 1.3%CE	1 / 520 0.2%GG		
7	Cytophaga-Flexibacter group	30 / 293 10.2%	1 / 6 16.7%	3 / 10 30.0%	0 / 7 0.0%	2 / 12 16.7%	3 / 17 17.6%	2 / 16 12.5%	1 / 7 14.3%	3 / 9 33.3%	2 / 16 12.5%	1 / 7 14.3%	2 / 22 9.1%	0 / 14 0.0%	2 / 8 25.0%	0 / 10 0.0%	0 / 8 0.0%	0 / 16 0.0%	1 / 7 14.3%	1 / 21 4.8%	1 / 31 3.2%	2 / 11 18.2%	0 / 7 0.0%	3 / 31 9.7%	0 / 0 0.0%	0 / 0 0.0%	0 / 0 0.0%	0 / 0 0.0%	174 / 293 59.4%	75 / 293 25.6%E	20 / 293 6.8%AB	10 / 293 3.4%ABE	8 / 293 2.7%C	6 / 293 2.0%CE			
1	Runella	4 / 41 9.8%	0 / 1 0.0%	0 / 1 0.0%	0 / 1 0.0%	0 / 2 0.0%	1 / 3 33.3%	0 / 3 0.0%	0 / 1 0.0%	0 / 1 0.0%	0 / 2 0.0%	0 / 1 0.0%	1 / 3 33.3%	0 / 1 0.0%	0 / 1 0.0%	0 / 1 0.0%	0 / 1 0.0%	0 / 3 0.0%	0 / 1 0.0%	1 / 4 25.0%	1 / 4 25.0%	0 / 1 0.0%	0 / 1 0.0%	0 / 4 0.0%	0 / 0 0.0%	0 / 0 0.0%	0 / 0 0.0%	0 / 0 0.0%	25 / 41 61.0%	8 / 41 19.5%E	3 / 41 7.3%AB	2 / 41 4.9%C	2 / 41 4.9%CE	1 / 41 2.4%ABE			
1	ATCC 29530	4 / 41 9.8%	0 / 1 0.0%	0 / 1 0.0%	0 / 1 0.0%	0 / 2 0.0%	1 / 3 33.3%	0 / 3 0.0%	0 / 1 0.0%	0 / 1 0.0%	0 / 2 0.0%	0 / 1 0.0%	1 / 3 33.3%	0 / 1 0.0%	0 / 1 0.0%	0 / 1 0.0%	0 / 1 0.0%	0 / 3 0.0%	0 / 1 0.0%	1 / 4 25.0%	1 / 4 25.0%	0 / 1 0.0%	0 / 1 0.0%	0 / 4 0.0%	0 / 0 0.0%	0 / 0 0.0%	0 / 0 0.0%	0 / 0 0.0%	25 / 41 61.0%	8 / 41 19.5%E	3 / 41 7.3%AB	2 / 41 4.9%CE	2 / 41 4.9%C	1 / 41 2.4%ABE			

1	Runella slithyformis DSM 19594	9.8%	0.0%	0.0%	0.0%	0.0%	33.3%	0.0%	0.0%	0.0%	0.0%	0.0%	33.3%	0.0%	0.0%	0.0%	0.0%	0.0%	0.0%	25.0%	25.0%	0.0%	0.0%	0.0%	0.0%	0.0%	0.0%	0.0%	61.0%	19.5%E	7.3%AB	4.9%C	4.9%CE	2.4%ABE		
1	Spirosoma	8 / 46 17.4%	0 / 0 0.0%	1 / 1 100.0%	0 / 1 0.0%	0 / 1 0.0%	1 / 3 33.3%	1 / 2 50.0%	0 / 1 0.0%	1 / 2 50.0%	0 / 2 0.0%	1 / 1 100.0%	1 / 4 25.0%	0 / 2 0.0%	1 / 1 100.0%	0 / 3 0.0%	0 / 2 0.0%	0 / 4 0.0%	1 / 1 100.0%	0 / 3 0.0%	0 / 5 0.0%	0 / 1 0.0%	0 / 1 0.0%	0 / 5 0.0%	0 / 0 0.0%	0 / 0 0.0%	0 / 0 0.0%	0 / 0 0.0%	21 / 46 45.7%	14 / 46 30.4%E	6 / 46 13.0%AB	2 / 46 4.3%ABE	2 / 46 4.3%C	1 / 46 2.2%CE		
1	ATCC 33905	8 / 46 17.4%	0 / 0 0.0%	1 / 1 100.0%	0 / 1 0.0%	0 / 1 0.0%	1 / 3 33.3%	1 / 2 50.0%	0 / 1 0.0%	1 / 2 50.0%	0 / 2 0.0%	1 / 1 100.0%	1 / 4 25.0%	0 / 2 0.0%	1 / 1 100.0%	0 / 3 0.0%	0 / 2 0.0%	0 / 4 0.0%	1 / 1 100.0%	0 / 3 0.0%	0 / 5 0.0%	0 / 1 0.0%	0 / 1 0.0%	0 / 5 0.0%	0 / 0 0.0%	0 / 0 0.0%	0 / 0 0.0%	0 / 0 0.0%	21 / 46 45.7%	14 / 46 30.4%E	6 / 46 13.0%AB	2 / 46 4.3%C	2 / 46 4.3%ABE	1 / 46 2.2%CE		
1	Spirosoma linguale DSM 74	8 / 46 17.4%	0 / 0 0.0%	1 / 1 100.0%	0 / 1 0.0%	0 / 1 0.0%	1 / 3 33.3%	1 / 2 50.0%	0 / 1 0.0%	1 / 2 50.0%	0 / 2 0.0%	1 / 1 100.0%	1 / 4 25.0%	0 / 2 0.0%	1 / 1 100.0%	0 / 3 0.0%	0 / 2 0.0%	0 / 4 0.0%	1 / 1 100.0%	0 / 3 0.0%	0 / 5 0.0%	0 / 1 0.0%	0 / 1 0.0%	0 / 5 0.0%	0 / 0 0.0%	0 / 0 0.0%	0 / 0 0.0%	0 / 0 0.0%	21 / 46 45.7%	14 / 46 30.4%E	6 / 46 13.0%AB	2 / 46 4.3%ABE	2 / 46 4.3%C	1 / 46 2.2%CE		
1	"Promyxobacterium" Imshenetski and Solntseva 1945	7 / 39 17.9%	1 / 1 100.0%	0 / 1 0.0%	0 / 1 0.0%	0 / 1 0.0%	0 / 2 0.0%	1 / 1 100.0%	1 / 1 100.0%	2 / 2 100.0%	0 / 2 0.0%	0 / 1 0.0%	0 / 3 0.0%	0 / 2 0.0%	0 / 2 0.0%	0 / 1 0.0%	0 / 1 0.0%	0 / 2 0.0%	0 / 1 0.0%	0 / 3 0.0%	0 / 4 0.0%	2 / 2 100.0%	0 / 1 0.0%	0 / 4 0.0%	0 / 0 0.0%	0 / 0 0.0%	0 / 0 0.0%	0 / 0 0.0%	22 / 39 56.4%	9 / 39 23.1%E	4 / 39 10.3%ABE	3 / 39 7.7%AB	1 / 39 2.6%C			
1	ATCC 33406	7 / 39 17.9%	1 / 1 100.0%	0 / 1 0.0%	0 / 1 0.0%	0 / 1 0.0%	0 / 2 0.0%	1 / 1 100.0%	1 / 1 100.0%	2 / 2 100.0%	0 / 2 0.0%	0 / 1 0.0%	0 / 3 0.0%	0 / 2 0.0%	0 / 2 0.0%	0 / 1 0.0%	0 / 1 0.0%	0 / 2 0.0%	0 / 1 0.0%	0 / 3 0.0%	0 / 4 0.0%	2 / 2 100.0%	0 / 1 0.0%	0 / 4 0.0%	0 / 0 0.0%	0 / 0 0.0%	0 / 0 0.0%	0 / 0 0.0%	22 / 39 56.4%	9 / 39 23.1%E	4 / 39 10.3%ABE	3 / 39 7.7%AB	1 / 39 2.6%C			
1	Cytophaga hutchinsonii ATCC 33406	7 / 39 17.9%	1 / 1 100.0%	0 / 1 0.0%	0 / 1 0.0%	0 / 1 0.0%	0 / 2 0.0%	1 / 1 100.0%	1 / 1 100.0%	2 / 2 100.0%	0 / 2 0.0%	0 / 1 0.0%	0 / 3 0.0%	0 / 2 0.0%	0 / 2 0.0%	0 / 1 0.0%	0 / 1 0.0%	0 / 2 0.0%	0 / 1 0.0%	0 / 3 0.0%	0 / 4 0.0%	2 / 2 100.0%	0 / 1 0.0%	0 / 4 0.0%	0 / 0 0.0%	0 / 0 0.0%	0 / 0 0.0%	0 / 0 0.0%	22 / 39 56.4%	9 / 39 23.1%E	4 / 39 10.3%ABE	3 / 39 7.7%AB	1 / 39 2.6%C			
1	Flexibacter	1 / 47 2.1%	0 / 1 0.0%	1 / 2 50.0%	0 / 1 0.0%	0 / 3 0.0%	0 / 3 0.0%	0 / 3 0.0%	0 / 1 0.0%	0 / 1 0.0%	0 / 3 0.0%	0 / 1 0.0%	0 / 4 0.0%	0 / 2 0.0%	0 / 1 0.0%	0 / 2 0.0%	0 / 1 0.0%	0 / 1 0.0%	0 / 1 0.0%	0 / 3 0.0%	0 / 5 0.0%	0 / 2 0.0%	0 / 1 0.0%	0 / 5 0.0%	0 / 0 0.0%	0 / 0 0.0%	0 / 0 0.0%	0 / 0 0.0%	33 / 47 70.2%	12 / 47 25.5%E	1 / 47 2.1%AB	1 / 47 2.1%C				
1	ATCC 23117	1 / 47 2.1%	0 / 1 0.0%	1 / 2 50.0%	0 / 1 0.0%	0 / 3 0.0%	0 / 3 0.0%	0 / 3 0.0%	0 / 1 0.0%	0 / 1 0.0%	0 / 3 0.0%	0 / 1 0.0%	0 / 4 0.0%	0 / 2 0.0%	0 / 1 0.0%	0 / 2 0.0%	0 / 1 0.0%	0 / 1 0.0%	0 / 1 0.0%	0 / 3 0.0%	0 / 5 0.0%	0 / 2 0.0%	0 / 1 0.0%	0 / 5 0.0%	0 / 0 0.0%	0 / 0 0.0%	0 / 0 0.0%	0 / 0 0.0%	33 / 47 70.2%	12 / 47 25.5%E	1 / 47 2.1%C	1 / 47 2.1%AB				
1	Flexibacter litoralis ATCC 23117	1 / 47 2.1%	0 / 1 0.0%	1 / 2 50.0%	0 / 1 0.0%	0 / 3 0.0%	0 / 3 0.0%	0 / 3 0.0%	0 / 1 0.0%	0 / 1 0.0%	0 / 3 0.0%	0 / 1 0.0%	0 / 4 0.0%	0 / 2 0.0%	0 / 1 0.0%	0 / 2 0.0%	0 / 1 0.0%	0 / 1 0.0%	0 / 1 0.0%	0 / 3 0.0%	0 / 5 0.0%	0 / 2 0.0%	0 / 1 0.0%	0 / 5 0.0%	0 / 0 0.0%	0 / 0 0.0%	0 / 0 0.0%	0 / 0 0.0%	33 / 47 70.2%	12 / 47 25.5%E	1 / 47 2.1%AB	1 / 47 2.1%C				
1	Dyadobacter	2 / 38 5.3%	0 / 1 0.0%	0 / 2 0.0%	0 / 1 0.0%	0 / 1 0.0%	1 / 2 50.0%	0 / 2 0.0%	0 / 1 0.0%	0 / 1 0.0%	0 / 2 0.0%	0 / 1 0.0%	0 / 3 0.0%	0 / 2 0.0%	0 / 1 0.0%	0 / 1 0.0%	0 / 1 0.0%	0 / 2 0.0%	0 / 1 0.0%	0 / 2 0.0%	0 / 4 0.0%	0 / 1 0.0%	0 / 1 0.0%	1 / 5 20.0%	0 / 0 0.0%	0 / 0 0.0%	0 / 0 0.0%	0 / 0 0.0%	22 / 38 57.9%	10 / 38 26.3%E	3 / 38 7.9%CE	1 / 38 2.6%ABE	1 / 38 2.6%AB	1 / 38 2.6%C		
1	ATCC 700827	2 / 38 5.3%	0 / 1 0.0%	0 / 2 0.0%	0 / 1 0.0%	0 / 1 0.0%	1 / 2 50.0%	0 / 2 0.0%	0 / 1 0.0%	0 / 1 0.0%	0 / 2 0.0%	0 / 1 0.0%	0 / 3 0.0%	0 / 2 0.0%	0 / 1 0.0%	0 / 1 0.0%	0 / 1 0.0%	0 / 2 0.0%	0 / 1 0.0%	0 / 2 0.0%	0 / 4 0.0%	0 / 1 0.0%	0 / 1 0.0%	1 / 5 20.0%	0 / 0 0.0%	0 / 0 0.0%	0 / 0 0.0%	0 / 0 0.0%	22 / 38 57.9%	10 / 38 26.3%E	3 / 38 7.9%CE	1 / 38 2.6%AB	1 / 38 2.6%C	1 / 38 2.6%ABE		
1	Dyadobacter fermentans DSM 18053	2 / 38 5.3%	0 / 1 0.0%	0 / 2 0.0%	0 / 1 0.0%	0 / 1 0.0%	1 / 2 50.0%	0 / 2 0.0%	0 / 1 0.0%	0 / 1 0.0%	0 / 2 0.0%	0 / 1 0.0%	0 / 3 0.0%	0 / 2 0.0%	0 / 1 0.0%	0 / 1 0.0%	0 / 1 0.0%	0 / 2 0.0%	0 / 1 0.0%	0 / 2 0.0%	0 / 4 0.0%	0 / 1 0.0%	0 / 1 0.0%	1 / 5 20.0%	0 / 0 0.0%	0 / 0 0.0%	0 / 0 0.0%	0 / 0 0.0%	22 / 38 57.9%	10 / 38 26.3%E	3 / 38 7.9%CE	1 / 38 2.6%ABE	1 / 38 2.6%C	1 / 38 2.6%AB		
1	Emticicia	4 / 41 9.8%	0 / 1 0.0%	1 / 2 50.0%	0 / 1 0.0%	2 / 2 100.0%	0 / 2 0.0%	0 / 3 0.0%	0 / 1 0.0%	0 / 1 0.0%	0 / 3 0.0%	0 / 1 0.0%	0 / 2 0.0%	0 / 2 0.0%	0 / 1 0.0%	0 / 1 0.0%	0 / 1 0.0%	0 / 1 0.0%	0 / 1 0.0%	0 / 3 0.0%	0 / 5 0.0%	0 / 2 0.0%	0 / 1 0.0%	1 / 4 25.0%	0 / 0 0.0%	0 / 0 0.0%	0 / 0 0.0%	0 / 0 0.0%	25 / 41 61.0%	11 / 41 26.8%E	3 / 41 7.3%AB	1 / 41 2.4%ABE	1 / 41 2.4%C			
1	DSM 17448	4 / 41 9.8%	0 / 1 0.0%	1 / 2 50.0%	0 / 1 0.0%	2 / 2 100.0%	0 / 2 0.0%	0 / 3 0.0%	0 / 1 0.0%	0 / 1 0.0%	0 / 3 0.0%	0 / 1 0.0%	0 / 2 0.0%	0 / 2 0.0%	0 / 1 0.0%	0 / 1 0.0%	0 / 1 0.0%	0 / 1 0.0%	0 / 1 0.0%	0 / 3 0.0%	0 / 5 0.0%	0 / 2 0.0%	0 / 1 0.0%	1 / 4 25.0%	0 / 0 0.0%	0 / 0 0.0%	0 / 0 0.0%	0 / 0 0.0%	25 / 41 61.0%	11 / 41 26.8%E	3 / 41 7.3%AB	1 / 41 2.4%ABE	1 / 41 2.4%C			
1	Emticicia oligotrophica DSM 17448	4 / 41 9.8%	0 / 1 0.0%	1 / 2 50.0%	0 / 1 0.0%	2 / 2 100.0%	0 / 2 0.0%	0 / 3 0.0%	0 / 1 0.0%	0 / 1 0.0%	0 / 3 0.0%	0 / 1 0.0%	0 / 2 0.0%	0 / 2 0.0%	0 / 1 0.0%	0 / 1 0.0%	0 / 1 0.0%	0 / 1 0.0%	0 / 1 0.0%	0 / 3 0.0%	0 / 5 0.0%	0 / 2 0.0%	0 / 1 0.0%	1 / 4 25.0%	0 / 0 0.0%	0 / 0 0.0%	0 / 0 0.0%	0 / 0 0.0%	25 / 41 61.0%	11 / 41 26.8%E	3 / 41 7.3%AB	1 / 41 2.4%C	1 / 41 2.4%ABE			
1	Leadbetterella	4 / 41 9.8%	0 / 1 0.0%	0 / 1 0.0%	0 / 1 0.0%	0 / 2 0.0%	0 / 2 0.0%	0 / 2 0.0%	0 / 1 0.0%	0 / 1 0.0%	2 / 2 100.0%	0 / 1 0.0%	0 / 3 0.0%	0 / 3 0.0%	1 / 1 100.0%	0 / 1 0.0%	0 / 1 0.0%	0 / 3 0.0%	0 / 1 0.0%	0 / 3 0.0%	0 / 4 0.0%	0 / 2 0.0%	0 / 1 0.0%	1 / 4 25.0%	0 / 0 0.0%	0 / 0 0.0%	0 / 0 0.0%	0 / 0 0.0%	26 / 41 63.4%	11 / 41 26.8%E	3 / 41 7.3%AB	1 / 41 2.4%ABE				
1	DSM 17132	4 / 41 9.8%	0 / 1 0.0%	0 / 1 0.0%	0 / 1 0.0%	0 / 2 0.0%	0 / 2 0.0%	0 / 2 0.0%	0 / 1 0.0%	0 / 1 0.0%	2 / 2 100.0%	0 / 1 0.0%	0 / 3 0.0%	0 / 3 0.0%	1 / 1 100.0%	0 / 1 0.0%	0 / 1 0.0%	0 / 3 0.0%	0 / 1 0.0%	0 / 3 0.0%	0 / 4 0.0%	0 / 2 0.0%	0 / 1 0.0%	1 / 4 25.0%	0 / 0 0.0%	0 / 0 0.0%	0 / 0 0.0%	0 / 0 0.0%	26 / 41 63.4%	11 / 41 26.8%E	3 / 41 7.3%AB	1 / 41 2.4%ABE				
1	Leadbetterella byssophila 4M15	4 / 41 9.8%	0 / 1 0.0%	0 / 1 0.0%	0 / 1 0.0%	0 / 2 0.0%	0 / 2 0.0%	0 / 2 0.0%	0 / 1 0.0%	0 / 1 0.0%	2 / 2 100.0%	0 / 1 0.0%	0 / 3 0.0%	0 / 3 0.0%	1 / 1 100.0%	0 / 1 0.0%	0 / 1 0.0%	0 / 3 0.0%	0 / 1 0.0%	0 / 3 0.0%	0 / 4 0.0%	0 / 2 0.0%	0 / 1 0.0%	1 / 4 25.0%	0 / 0 0.0%	0 / 0 0.0%	0 / 0 0.0%	0 / 0 0.0%	26 / 41 63.4%	11 / 41 26.8%E	3 / 41 7.3%AB	1 / 41 2.4%ABE				
1	"Flammeovirgaceae" Nedashkovskaya and Ludwig 2011	1 / 37 2.7%	0 / 1 0.0%	0 / 1 0.0%	0 / 1 0.0%	0 / 2 0.0%	0 / 2 0.0%	1 / 1 100.0%	0 / 1 0.0%	0 / 1 0.0%	0 / 3 0.0%	0 / 1 0.0%	0 / 3 0.0%	0 / 2 0.0%	0 / 1 0.0%	0 / 1 0.0%	0 / 1 0.0%	0 / 1 0.0%	0 / 1 0.0%	0 / 2 0.0%	0 / 4 0.0%	0 / 2 0.0%	0 / 1 0.0%	0 / 4 0.0%	0 / 0 0.0%	0 / 0 0.0%	0 / 0 0.0%	0 / 0 0.0%	23 / 37 62.2%	12 / 37 32.4%E	1 / 37 2.7%GG	1 / 37 2.7%AB				
1	Marivirga	1 / 37 2.7%	0 / 1 0.0%	0 / 1 0.0%	0 / 1 0.0%	0 / 2 0.0%	0 / 2 0.0%	1 / 1 100.0%	0 / 1 0.0%	0 / 1 0.0%	0 / 3 0.0%	0 / 1 0.0%	0 / 3 0.0%	0 / 2 0.0%	0 / 1 0.0%	0 / 1 0.0%	0 / 1 0.0%	0 / 1 0.0%	0 / 1 0.0%	0 / 2 0.0%	0 / 4 0.0%	0 / 2 0.0%	0 / 1 0.0%	0 / 4 0.0%	0 / 0 0.0%	0 / 0 0.0%	0 / 0 0.0%	0 / 0 0.0%	23 / 37 62.2%	12 / 37 32.4%E	1 / 37 2.7%AB	1 / 37 2.7%GG				
1	"Microscilla tractuosa" Lewin 1969	1 / 37 2.7%	0 / 1 0.0%	0 / 1 0.0%	0 / 1 0.0%	0 / 2 0.0%	0 / 2 0.0%	1 / 1 100.0%	0 / 1 0.0%	0 / 1 0.0%	0 / 3 0.0%	0 / 1 0.0%	0 / 3 0.0%	0 / 2 0.0%	0 / 1 0.0%	0 / 1 0.0%	0 / 1 0.0%	0 / 1 0.0%	0 / 1 0.0%	0 / 2 0.0%	0 / 4 0.0%	0 / 2 0.0%	0 / 1 0.0%	0 / 4 0.0%	0 / 0 0.0%	0 / 0 0.0%	0 / 0 0.0%	0 / 0 0.0%	23 / 37 62.2%	12 / 37 32.4%E	1 / 37 2.7%AB	1 / 37 2.7%GG				
1	Flexibacter tractuosus DSM 4126	1 / 37 2.7%	0 / 1 0.0%	0 / 1 0.0%	0 / 1 0.0%	0 / 2 0.0%	0 / 2 0.0%	1 / 1 100.0%	0 / 1 0.0%	0 / 1 0.0%	0 / 3 0.0%	0 / 1 0.0%	0 / 3 0.0%	0 / 2 0.0%	0 / 1 0.0%	0 / 1 0.0%	0 / 1 0.0%	0 / 1 0.0%	0 / 1 0.0%	0 / 2 0.0%	0 / 4 0.0%	0 / 2 0.0%	0 / 1 0.0%	0 / 4 0.0%	0 / 0 0.0%	0 / 0 0.0%	0 / 0 0.0%	0 / 0 0.0%	23 / 37 62.2%	12 / 37 32.4%E	1 / 37 2.7%GG	1 / 37 2.7%AB				
3	Cyclobacteriaceae	12 / 118 10.2%	0 / 3 0.0%	0 / 6 0.0%	0 / 3 0.0%	0 / 3 0.0%	0 / 6 0.0%	2 / 8 25.0%	1 / 1 100.0%	0 / 3 0.0%	2 / 6 33.3%	0 / 3 0.0%	0 / 9 0.0%	1 / 6 16.7%	0 / 3 0.0%	0 / 3 0.0%	3 / 3 100.0%	0 / 8 0.0%	0 / 3 0.0%	0 / 7 0.0%	1 / 12 8.3%	1 / 6 16.7%	1 / 3 33.3%	0 / 13 0.0%	0 / 0 0.0%	0 / 0 0.0%	0 / 0 0.0%	0 / 0 0.0%	72 / 118 61.0%	33 / 118 28.0%E	8 / 118 6.8%AB	4 / 118 3.4%ABE	1 / 118 0.8%C			
1	Cyclobacterium	5 / 38 13.2%	0 / 1 0.0%	0 / 2 0.0%	0 / 1 0.0%	0 / 1 0.0%	0 / 2 0.0%	2 / 3 66.7%	0 / 0 0.0%	0 / 1 0.0%	0 / 2 0.0%	0 / 1 0.0%	0 / 3 0.0%	1 / 2 50.0%	0 / 1 0.0%	0 / 1 0.0%	1 / 1 100.0%	0 / 2 0.0%	0 / 1 0.0%	0 / 2 0.0%	0 / 4 0.0%	0 / 2 0.0%	1 / 1 100.0%	0 / 4 0.0%	0 / 0 0.0%	0 / 0 0.0%	0 / 0 0.0%	0 / 0 0.0%	22 / 38 57.9%	11 / 38 28.9%E	4 / 38 10.5%AB	1 / 38 2.6%ABE				
1	"Micrococyclus marinus" Raj 1976	5 / 38 13.2%	0 / 1 0.0%	0 / 2 0.0%	0 / 1 0.0%	0 / 1 0.0%	0 / 2 0.0%	2 / 3 66.7%	0 / 0 0.0%	0 / 1 0.0%	0 / 2 0.0%	0 / 1 0.0%	0 / 3 0.0%	1 / 2 50.0%	0 / 1 0.0%	0 / 1 0.0%	1 / 1 100.0%	0 / 2 0.0%	0 / 1 0.0%	0 / 2 0.0%	0 / 4 0.0%	0 / 2 0.0%	1 / 1 100.0%	0 / 4 0.0%	0 / 0 0.0%	0 / 0 0.0%	0 / 0 0.0%	0 / 0 0.0%	22 / 38 57.9%	11 / 38 28.9%E	4 / 38 10.5%AB	1 / 38 2.6%ABE				
1	Cyclobacterium marinum DSM 745	5 / 38 13.2%	0 / 1 0.0%	0 / 2 0.0%	0 / 1 0.0%	0 / 1 0.0%	0 / 2 0.0%	2 / 3 66.7%	0 / 0 0.0%	0 / 1 0.0%	0 / 2 0.0%	0 / 1 0.0%	0 / 3 0.0%	1 / 2 50.0%	0 / 1 0.0%	0 / 1 0.0%	1 / 1 100.0%	0 / 2 0.0%	0 / 1 0.0%	0 / 2 0.0%	0 / 4 0.0%	0 / 2 0.0%	1 / 1 100.0%	0 / 4 0.0%	0 / 0 0.0%	0 / 0 0.0%	0 / 0 0.0%	0 / 0 0.0%	22 / 38 57.9%	11 / 38 28.9%E	4 / 38 10.5%AB	1 / 38 2.6%ABE				
1	Bella	2 / 38 5.3%	0 / 1 0.0%	0 / 2 0.0%	0 / 1 0.0%	0 / 1 0.0%	0 / 2 0.0%	0 / 2 0.0%	0 / 0 0.0%	0 / 1 0.0%	1 / 2 50.0%	0 / 1 0.0%	0 / 3 0.0%	0 / 2 0.0%	0 / 1 0.0%	0 / 1 0.0%	1 / 1 100.0%	0 / 3 0.0%	0 / 1 0.0%	0 / 2 0.0%	0 / 4 0.0%	0 / 2 0.0%	0 / 1 0.0%	0 / 4 0.0%	0 / 0 0.0%	0 / 0 0.0%	0 / 0 0.0%	0 / 0 0.0%	24 / 38 63.2%	11 / 38 28.9%E	1 / 38 2.6%ABE	1 / 38 2.6%C	1 / 38 2.6%AB			
1	Bella baltica	2 / 38 5.3%	0 / 1 0.0%	0 / 2 0.0%	0 / 1 0.0%	0 / 1 0.0%	0 / 2 0.0%	0 / 2 0.0%	0 / 0 0.0%	0 / 1 0.0%	1 / 2 50.0%	0 / 1 0.0%	0 / 3 0.0%	0 / 2 0.0%	0 / 1 0.0%	0 / 1 0.0%	1 / 1 100.0%	0 / 3 0.0%	0 / 1 0.0%	0 / 2 0.0%	0 / 4 0.0%	0 / 2 0.0%	0 / 1 0.0%	0 / 4 0.0%	0 / 0 0.0%	0 / 0 0.0%	0 / 0 0.0%	0 / 0 0.0%	24 / 38 63.2%	11 / 38 28.9%E	1 / 38 2.6%AB	1 / 38 2.6%C	1 / 38 2.6%ABE			
1	Belliella baltica BA134	2 / 38 5.3%	0 / 1 0.0%	0 / 2 0.0%	0 / 1 0.0%	0 / 1 0.0%	0 / 2 0.0%	0 / 2 0.0%	0 / 0 0.0%	0 / 1 0.0%	1 / 2 50.0%	0 / 1 0.0%	0 / 3 0.0%	0 / 2 0.0%	0 / 1 0.0%	0 / 1 0.0%	1 / 1 100.0%	0 / 3 0.0%	0 / 1 0.0%	0 / 2 0.0%	0 / 4 0.0%	0 / 2 0.0%	0 / 1 0.0%	0 / 4 0.0%	0 / 0 0.0%	0 / 0 0.0%	0 / 0 0.0%	0 / 0 0.0%	24 / 38 63.2%	11 / 38 28.9%E	1 / 38 2.6%ABE	1 / 38 2.6%C	1 / 38 2.6%AB			
1	Echinicola	5 / 42 11.9%	0 / 1 0.0%	0 / 2 0.0%	0 / 1 0.0%	0 / 1 0.0%	0 / 2 0.0%	0 / 3 0.0%	1 / 1 100.0%	0 / 1 0.0%	1 / 2 50.0%	0 / 1 0.0%	0 / 3 0.0%	0 / 2 0.0%	0 / 1 0.0%	0 / 1 0.0%	1 / 1 100.0%	0 / 3 0.0%	0 / 1 0.0%	0 / 3 0.0%	1 / 4 25.0%	1 / 2 50.0%	0 / 1 0.0%	0 / 5 0.0%	0 / 0 0.0%	0 / 0 0.0%	0 / 0 0.0%	0 / 0 0.0%	26 / 42 61.9%	11 / 42 26.2%E	3 / 42 7.1%AB	2 / 42 4.8%ABE				
1	DSM 17526	5 / 42 11.9%	0 / 1 0.0%	0 / 2 0.0%	0 / 1 0.0%	0 / 1 0.0%	0 / 2 0.0%	0 / 3 0.0%	1 / 1 100.0%	0 / 1 0.0%	1 / 2 50.0%	0 / 1 0.0%	0 / 3 0.0%	0 / 2 0.0%	0 / 1 0.0%	0 / 1 0.0%	1 / 1 100.0%	0 / 3 0.0%	0 / 1 0.0%	0 / 3 0.0%	1 / 4 25.0%	1 / 2 50.0%	0 / 1 0.0%	0 / 5 0.0%	0 / 0 0.0%	0 / 0 0.0%	0 / 0 0.0%	0 / 0 0.0%	26 / 42 61.9%	11 / 42 26.2%E	3 / 42 7.1%AB	2 / 42 4.8%ABE				
1	Echinicola vietnamensis DSM 17526	5 / 42 11.9%	0 / 1 0.0%	0 / 2 0.0%	0 / 1 0.0%	0 / 1 0.0%	0 / 2 0.0%	0 / 3 0.0%	1 / 1 100.0%	0 / 1 0.0%	1 / 2 50.0%	0 / 1 0.0%	0 / 3 0.0%	0 / 2 0.0%	0 / 1 0.0%	0 / 1 0.0%	1 / 1 100.0%	0 / 3 0.0%	0 / 1 0.0%	0 / 3 0.0%	1 / 4 25.0%	1 / 2 50.0%	0 / 1 0.0%	0 / 5 0.0%	0 / 0 0.0%	0 / 0 0.0%	0 / 0 0.0%	0 / 0 0.0%	26 / 42 61.9%	11 / 42 26.2%E	3 / 42 7.1%AB	2 / 42 4.8%ABE				
2	"Amoebophilaceae" Santos-Garcia et al. 2014	6 / 72 8.3%	0 / 2 0.0%	0 / 2 0.0%	0 / 2 0.0%	0 / 2 0.0%	0 / 4 0.0%	0 / 4 0.0%	0 / 2 0.0%	1 / 2 50.0%	1 / 4 25.0%	0 / 2 0.0%	0 / 7 0.0%	0 / 2 0.0%	0 / 2 0.0%	0 / 2 0.0%	0 / 2 0.0%	0 / 4 0.0%	0 / 2 0.0%	1 / 4 25.0%	0 / 9 0.0%	0 / 2 0.0%	0 / 2 0.0%	3 / 8 37.5%	0 / 0 0.0%	0 / 0 0.0%	0 / 0 0.0%	0 / 0 0.0%	44 / 72 61.1%	19 / 72 26.4%E	4 / 72 5.6%ABE	2 / 72 2.8%C	2 / 72 2.8%AB	1 / 72 1.4%CE		
1	'Candidatus Cardinium'	5 / 37 13.5%	0 / 1 0.0%	0 / 1 0.0%	0 / 1 0.0%	0 / 1 0.0%	0 / 2 0.0%	0 / 2 0.0%	0 / 1 0.0%	0 / 1 0.0%	1 / 2 50.0%	0 / 1 0.0%	0 / 4 0.0%	0 / 1 0.0%	0 / 1 0.0%	0 / 1 0.0%	0 / 1 0.0%	0 / 2 0.0%	0 / 1 0.0%	1 / 2 50.0%	0 / 5 0.0%	0 / 1 0.0%	0 / 1 0.0%	3 / 4 75.0%	0 / 0 0.0%	0 / 0 0.0%	0 / 0 0.0%	0 / 0 0.0%	22 / 37 59.5%	8 / 37 21.6%E	3 / 37 8.1%ABE	2 / 37 5.4%AB	1 / 37 2.7%C	1 / 37 2.7%CE		
1	Cardinium endosymbiont of Encarsia pergandiella	5 / 37 13.5%	0 / 1 0.0%	0 / 1 0.0%	0 / 1 0.0%	0 / 1 0.0%	0 / 2 0.0%	0 / 2 0.0%	0 / 1 0.0%	0 / 1 0.0%	1 / 2 50.0%	0 / 1 0.0%	0 / 4 0.0%	0 / 1 0.0%	0 / 1 0.0%	0 / 1 0.0%	0 / 1 0.0%	0 / 2 0.0%	0 / 1 0.0%	1 / 2 50.0%	0 / 5 0.0%	0 / 1 0.0%	0 / 1 0.0%	3 / 4 75.0%	0 / 0 0.0%	0 / 0 0.0%	0 / 0 0.0%	0 / 0 0.0%	22 / 37 59.5%	8 / 37 21.6%E	3 / 37 8.1%ABE	2 / 37 5.4%AB	1 / 37 2.7%CE	1 / 37 2.7%C		
1	Cardinium endosymbiont cEper1 of Encarsia pergandiella	5 / 37 13.5%	0 / 1 0.0%	0 / 1 0.0%	0 / 1 0.0%	0 / 1 0.0%	0 / 2 0.0%	0 / 2 0.0%	0 / 1 0.0%	0 / 1 0.0%	1 / 2 50.0%	0 / 1 0.0%	0 / 4 0.0%	0 / 1 0.0%	0 / 1 0.0%	0 / 1 0.0%	0 / 1 0.0%	0 / 2 0.0%	0 / 1 0.0%	1 / 2 50.0%	0 / 5 0.0%	0 / 1 0.0%	0 / 1 0.0%	3 / 4 75.0%	0 / 0 0.0%	0 / 0 0.0%	0 / 0 0.0%	0 / 0 0.0%	22 / 37 59.5%	8 / 37 21.6%E	3 / 37 8.1%ABE	2 / 37 5.4%AB	1 / 37 2.7%CE	1 / 37 2.7%C		
1	'Candidatus Amoebophilus'	1 / 35 2.9%	0 / 1 0.0%	0 / 1 0.0%	0 / 1 0.0%	0 / 1 0.0%	0 / 2 0.0%	0 / 2 0.0%	0 / 1 0.0%	1 / 1 100.0%	0 / 2 0.0%	0 / 1 0.0%	0 / 3 0.0%	0 / 1 0.0%	0 / 1 0.0%	0 / 1 0.0%	0 / 1 0.0%	0 / 2 0.0%	0 / 1 0.0%	0 / 2 0.0%	0 / 4 0.0%	0 / 1 0.0%	0 / 1 0.0%	0 / 4 0.0%	0 / 0 0.0%	0 / 0 0.0%	0 / 0 0.0%	0 / 0 0.0%	22 / 35 62.9%	11 / 35 31.4%E	1 / 35 2.9%C	1 / 35 2.9%ABE				
1	"Candidatus Amoebophilus asiaticus" Horn et al. 2001	1 / 35 2.9%	0 / 1 0.0%	0 / 1 0.0%	0 / 1 0.0%	0 / 1 0.0%	0 / 2 0.0%	0 / 2 0.0%	0 / 1 0.0%	1 / 1 100.0%	0 / 2 0.0%	0 / 1 0.0%	0 / 3 0.0%	0 / 1 0.0%	0 / 1 0.0%	0 / 1 0.0%	0 / 1 0.0%	0 / 2 0.0%	0 / 1 0.0%	0 / 2 0.0%	0 / 4 0.0%	0 / 1 0.0%	0 / 1 0.0%	0 / 4 0.0%	0 / 0 0.0%	0 / 0 0.0%	0 / 0 0.0%	0 / 0 0.0%	22 / 35 62.9%	11 / 35 31.4%E	1 / 35 2.9%ABE	1 / 35 2.9%C				
1	Candidatus Amoebophilus asiaticus 5a2	1 / 35 2.9%	0 / 1 0.0%	0 / 1 0.0%	0 / 1 0.0%	0 / 1 0.0%	0 / 2 0.0%	0 / 2 0.0%	0 / 1 0.0%	1 / 1 100.0%	0 / 2 0.0%	0 / 1 0.0%	0 / 3 0.0%	0 / 1 0.0%	0 / 1 0.0%	0 / 1 0.0%	0 / 1 0.0%	0 / 2 0.0%	0 / 1 0.0%	0 / 2 0.0%	0 / 4 0.0%	0 / 1 0.0%	0 / 1 0.0%	0 / 4 0.0%	0 / 0 0.0%	0 / 0 0.0%	0 / 0 0.0%	0 / 0 0.0%	22 / 35 62.9%	11 / 35 31.4%E	1 / 35 2.9%C	1 / 35 2.9%ABE				
4	Bacteroidetes Order II. Incertae sedis	43 / 179 24.0%	0 / 4 0.0%	1 / 4 25.0%	0 / 4 0.0%	0 / 4 0.0%	4 / 12 33.3%	0 / 12 0.0%	0 / 4 0.0%	2 / 4 50.0%	3 / 12 25.0%	4 / 4 100.0%	2 / 16 12.5%	2 / 8 25.0%	1 / 4 25.0%	2 / 8 25.0%	0 / 4 0.0%	5 / 12 41.7%	1 / 4 25.0%	2 / 12 16.7%	4 / 16 25.0%	3 / 8 37.5%	2 / 4 50.0%	5 / 19 26.3%	0 / 0 0.0%	0 / 0 0.0%	0 / 0 0.0%	0 / 0 0.0%	49 / 179 27.4%	35 / 179 19.6%C	33 / 179 18.4%E	27 / 179 15.1%AB	19 / 179 10.6%CE	16 / 179 8.9%ABE		
4	"Rhodothermaceae" Ludwig et al. 2011	43 / 179 24.0%	0 / 4 0.0%	1 / 4 25.0%	0 / 4 0.0%	0 / 4 0.0%	4 / 12 33.3%	0 / 12 0.0%	0 / 4 0.0%	2 / 4 50.0%	3 / 12 25.0%	4 / 4 100.0%	2 / 16 12.5%	2 / 8 25.0%	1 / 4 25.0%	2 / 8 25.0%	0 / 4 0.0%	5 / 12 41.7%	1 / 4 25.0%	2 / 12 16.7%	4 / 16 25.0%	3 / 8 37.5%	2 / 4 50.0%	5 / 19 26.3%	0 / 0 0.0%	0 / 0 0.0%	0 / 0 0.0%	0 / 0 0.0%	49 / 179 27.4%	35 / 179 19.6%C	33 / 179 18.4%E	27 / 179 15.1%AB	19 / 179 10.6%CE	16 / 179 8.9%ABE		
2	Rhodothermus	36 / 90 40.0%	0 / 2 0.0%	1 / 2 50.0%	0 / 2 0.0%	0 / 2 0.0%	4 / 6 66.7%	0 / 6 0.0%	0 / 2 0.0%	2 / 2 100.0%	3 / 6 50.0%	2 / 2 100.0%	2 / 8 25.0%	0 / 4 0.0%	1 / 2 50.0%	0 / 4 0.0%	0 / 2 0.0%	5 / 6 83.3%	1 / 2 50.0%	2 / 6 33.3%	4 / 8 50.0%	2 / 4 50.0%	2 / 2 100.0%	5 / 10 50.0%	0 / 0 0.0%	0 / 0 0.0%	0 / 0 0.0%	0 / 0 0.0%	23 / 90 25.6%AB	21 / 90 23.3%	17 / 90 18.9%E	13 / 90 14.4%ABE	11 / 90 12.2%C	5 / 90 5.6%CE		
2	ATCC 43812	36 / 90 40.0%	0 / 2 0.0%	1 / 2 50.0%	0 / 2 0.0%	0 / 2 0.0%	4 / 6 66.7%	0 / 6 0.0%	0 / 2 0.0%	2 / 2 100.0%	3 / 6 50.0%	2 / 2 100.0%	2 / 8 25.0%	0 / 4 0.0%	1 / 2 50.0%	0 / 4 0.0%	0 / 2 0.0%	5 / 6 83.3%	1 / 2 50.0%	2 / 6 33.3%	4 / 8 50.0%	2 / 4 50.0%	2 / 2 100.0%	5 / 10 50.0%	0 / 0 0.0%	0 / 0 0.0%	0 / 0 0.0%	0 / 0 0.0%	23 / 90 25.6%AB	21 / 90 23.3%	17 / 90 18.9%E	13 / 90 14.4%ABE	11 / 90 12.2%C	5 / 90 5.6%CE		
1	Rhodothermus marinus DSM 4252	16 / 45 35.6%	0 / 1 0.0%	1 / 1 100.0%	0 / 1 0.0%	0 / 1 0.0%	2 / 3 66.7%	0 / 3 0.0%	0 / 1 0.0%	1 / 1 100.0%	1 / 3 33.3%	1 / 1 100.0%	1 / 4 25.0%	0 / 2 0.0%	1 / 1 100.0%	0 / 2 0.0%	0 / 1 0.0%	2 / 3 66.7%	0 / 1 0.0%	0 / 3 0.0%	2 / 4 50.0%	1 / 2 50.0%	1 / 1 100.0%	2 / 5 40.0%	0 / 0 0.0%	0 / 0 0.0%	0 / 0 0.0%	0 / 0 0.0%	11 / 45 24.4%	11 / 45 24.4%AB	9 / 45 20.0%E	6 / 45 13.3%C	5 / 45 11.1%ABE	3 / 45 6.7%CE		
1	Rhodothermus marinus SG0.5JP17-172	20 / 45 44.4%	0 / 1 0.0%	0 / 1 0.0%	0 / 1 0.0%	0 / 1 0.0%	2 / 3 66.7%	0 / 3 0.0%	0 / 1 0.0%	1 / 1 100.0%	2 / 3 66.7%	1 / 1 100.0%	1 / 4 25.0%	0 / 2 0.0%	0 / 1 0.0%	0 / 2 0.0%	0 / 1 0.0%	3 / 3 100.0%	1 / 1 100.0%	2 / 3 66.7%	2 / 4 50.0%	1 / 2 50.0%	1 / 1 100.0%	3 / 5 60.0%	0 / 0 0.0%	0 / 0 0.0%	0 / 0 0.0%	0 / 0 0.0%	12 / 45 26.7%AB	10 / 45 22.2%	8 / 45 17.8%ABE	8 / 45 17.8%E	5 / 45 11.1%C	2 / 45 4.4%CE		
2	"Candidatus Salinibacter" Anton et al. 2000	7 / 89 7.9%	0 / 2 0.0%	0 / 2 0.0%	0 / 2 0.0%	0 / 2 0.0%	0 / 6 0.0%	0 / 6 0.0%	0 / 2 0.0%	0 / 2 0.0%	0 / 6 0.0%	2 / 2 100.0%	0 / 8 0.0%	2 / 4 50.0%	0 / 2 0.0%	2 / 4 50.0%	0 / 2 0.0%	0 / 6 0.0%	0 / 2 0.0%	0 / 6 0.0%	0 / 8 0.0%	1 / 4 25.0%	0 / 2 0.0%	0 / 9 0.0%	0 / 0 0.0%	0 / 0 0.0%	0 / 0 0.0%	0 / 0 0.0%	28 / 89 31.5%	24 / 89 27.0%C	16 / 89 18.0%E	14 / 89 15.7%CE	4 / 89 4.5%AB	3 / 89 3.4%ABE		
2	CECT 5946	7 / 89 7.9%	0 / 2 0.0%	0 / 2 0.0%	0 / 2 0.0%	0 / 2 0.0%	0 / 6 0.0%	0 / 6 0.0%	0 / 2 0.0%	0 / 2 0.0%	0 / 6 0.0%	2 / 2 100.0%	0 / 8 0.0%	2 / 4 50.0%	0 / 2 0.0%	2 / 4 50.0%	0 / 2 0.0%	0 / 6 0.0%	0 / 2 0.0%	0 / 6 0.0%	0 / 8 0.0%	1 / 4 25.0%	0 / 2 0.0%	0 / 9 0.0%	0 / 0 0.0%	0 / 0 0.0%	0 / 0 0.0%	0 / 0 0.0%	28 / 89 31.5%	24 / 89 27.0%C	16 / 89 18.0%E	14 / 89 15.7%CE	4 / 89 4.5%AB	3 / 89 3.4%ABE		
1	Salinibacter ruber DSM 13855	4 / 45 8.9%	0 / 1 0.0%	0 / 1 0.0%	0 / 1 0.0%	0 / 1 0.0%	0 / 3 0.0%	0 / 3 0.0%	0 / 1 0.0%	0 / 1 0.0%	0 / 3 0.0%	1 / 1 100.0%	0 / 4 0.0%	1 / 2 50.0%	0 / 1 0.0%	1 / 2 50.0%	0 / 1 0.0%	0 / 3 0.0%	0 / 1 0.0%	0 / 3 0.0%	0 / 4 0.0%	1 / 2 50.0%	0 / 1 0.0%	0 / 5 0.0%	0 / 0 0.0%	0 / 0 0.0%	0 / 0 0.0%	0 / 0 0.0%	14 / 45 31.1%	12 / 45 26.7%C	8 / 45 17.8%E	7 / 45 15.6%CE	2 / 45 4.4%AB	2 / 45 4.4%ABE		
1	Salinibacter ruber M8	3 / 44 6.8%	0 / 1 0.0%	0 / 1 0.0%	0 / 1 0.0%	0 / 1 0.0%	0 / 3 0.0%	0 / 3 0.0%	0 / 1 0.0%	0 / 1 0.0%	0 / 3 0.0%	1 / 1 100.0%	0 / 4 0.0%	1 / 2 50.0%	0 / 1 0.0%	1 / 2 50.0%	0 / 1 0.0%	0 / 3 0.0%	0 / 1 0.0%	0 / 3 0.0%	0 / 4 0.0%	0 / 2 0.0%	0 / 1 0.0%	0 / 4 0.0%	0 / 0 0.0%	0 / 0 0.0%	0 / 0 0.0%	0 / 0 0.0%	14 / 44 31.8%	12 / 44 27.3%C	8 / 44 18.2%E	7 / 44 15.9%CE	2 / 44 4.5%AB	1 / 44 2.3%ABE		
11	"Chlorobi" Garrity and Holt 2001	108 / 517 20.9%	5 / 14 35.7%	0 / 11 0.0%	1 / 11 9.1%	2 / 19 10.5%	7 / 35 20.0%	8 / 31 25.8%	1 / 11 9.1%	1 / 11 9.1%	0 / 41 0.0%	0 / 11 0.0%	11 / 44 25.0%	11 / 24 45.8%	5 / 11 45.5%	2 / 22 9.1%	6 / 11 54.5%	4 / 33 12.1%	5 / 11 45.5%	0 / 33 0.0%	11 / 44 25.0%	5 / 22 22.7%	2 / 11 18.2%	21 / 56 37.5%	0 / 0 0.0%	0 / 0 0.0%	0 / 0 0.0%	0 / 0 0.0%	246 / 517 47.6%	117 / 517 22.6%E	47 / 517 9.1%ABE	38 / 517 7.4%AB	34 / 517 6.6%C	16 / 517 3.1%BE	12 / 517 2.3%CE	7 / 517 1.4%B
11	"Chlorobia" Garrity and Holt 2001	108 / 517 20.9%	5 / 14 35.7%	0 / 11 0.0%	1 / 11 9.1%	2 / 19 10.5%	7 / 35 20.0%	8 / 31 25.8%	1 / 11 9.1%	1 / 11 9.1%	0 / 41 0.0%	0 / 11 0.0%	11 / 44 25.0%	11 / 24 45.8%	5 / 11 45.5%	2 / 22 9.1%	6 / 11 54.5%	4 / 33 12.1%	5 / 11 45.5%	0 / 33 0.0%	11 / 44 25.0%	5 / 22 22.7%	2 / 11 18.2%	21 / 56 37.5%	0 / 0 0.0%	0 / 0 0.0%	0 / 0 0.0%	0 / 0 0.0%	246 / 517 47.6%	117 / 517 22.6%E	47 / 517 9.1%ABE	38 / 517 7.4%AB	34 / 517 6.6%C	16 / 517 3.1%BE	12 / 517 2.3%CE	7 / 517 1.4%B
11	Chlorobiales	108 / 517 20.9%	5 / 14 35.7%	0 / 11 0.0%	1 / 11 9.1%	2 / 19 10.5%	7 / 35 20.0%	8 / 31 25.8%	1 / 11 9.1%	1 / 11 9.1%	0 / 41 0.0%	0 / 11 0.0%	11 / 44 25.0%	11 / 24 45.8%	5 / 11 45.5%	2 / 22 9.1%	6 / 11 54.5%	4 / 33 12.1%	5 / 11 45.5%	0 / 33 0.0%	11 / 44 25.0%	5 / 22 22.7%	2 / 11 18.2%	21 / 56 37.5%	0 / 0 0.0%	0 / 0 0.0%	0 / 0 0.0%	0 / 0 0.0%	246 / 517 47.6%	117 / 517 22.6%E	47 / 517 9.1%ABE	38 / 517 7.4%AB	34 / 517 6.6%C	16 / 517 3.1%BE	12 / 517 2.3%CE	7 / 517 1.4%B
11	"Chlorobiacea" (sic) Copeland 1956	108 / 517 20.9%	5 / 14 35.7%	0 / 11 0.0%	1 / 11 9.1%	2 / 19 10.5%	7 / 35 20.0%	8 / 31 25.8%	1 / 11 9.1%	1 / 11 9.1%	0 / 41 0.0%	0 / 11 0.0%	11 / 44 25.0%	11 / 24 45.8%	5 / 11 45.5%	2 / 22 9.1%	6 / 11 54.5%	4 / 33 12.1%	5 / 11 45.5%	0 / 33 0.0%	11 / 44 25.0%	5 / 22 22.7%	2 / 11 18.2%	21 / 56 37.5%	0 / 0 0.0%	0 / 0 0.0%	0 / 0 0.0%	0 / 0 0.0%	246 / 517 47.6%	117 / 517 22.6%E	47 / 517 9.1%ABE	38 / 517 7.4%AB	34 / 517 6.6%C	16 / 517 3.1%BE	12 / 517 2.3%CE	7 / 517 1.4%B
1	Prosthecochloris	6 / 46 13.0%	0 / 2 0.0%	0 / 1 0.0%	0 / 1 0.0%	0 / 1 0.0%	0 / 3 0.0%	1 / 3 33.3%	0 / 1 0.0%	0 / 1 0.0%	0 / 3 0.0%	0 / 1 0.0%	0 / 4 0.0%	1 / 2 50.0%	1 / 1 100.0%	0 / 2 0.0%	0 / 1 0.0%	0 / 3 0.0%	0 / 1 0.0%	0 / 3 0.0%	0 / 4 0.0%	0 / 2 0.0%	0 / 1 0.0%	3 / 5 60.0%	0 / 0 0.0%	0 / 0 0.0%	0 / 0 0.0%	0 / 0 0.0%	24 / 46 52.2%	14 / 46 30.4%E	3 / 46 6.5%BE	2 / 46 4.3%C	1 / 46 2.2%AB	1 / 46 2.2%ABE	1 / 46 2.2%B	
1	DSM 271	6 / 46 13.0%	0 / 2 0.0%	0 / 1 0.0%	0 / 1 0.0%	0 / 1 0.0%	0 / 3 0.0%	1 / 3 33.3%	0 / 1 0.0%	0 / 1 0.0%	0 / 3 0.0%	0 / 1 0.0%	0 / 4 0.0%	1 / 2 50.0%	1 / 1 100.0%	0 / 2 0.0%	0 / 1 0.0%	0 / 3 0.0%	0 / 1 0.0%	0 / 3 0.0%	0 / 4 0.0%	0 / 2 0.0%	0 / 1 0.0%	3 / 5 60.0%	0 / 0 0.0%	0 / 0 0.0%	0 / 0 0.0%	0 / 0 0.0%	24 / 46 52.2%	14 / 46 30.4%E	3 / 46 6.5%BE	2 / 46 4.3%C	1 / 46 2.2%ABE	1 / 46 2.2%B	1 / 46 2.2%AB	
1	Prosthecochloris aestuarii DSM 271	6 / 46 13.0%	0 / 2 0.0%	0 / 1 0.0%	0 / 1 0.0%	0 / 1 0.0%	0 / 3 0.0%	1 / 3 33.3%	0 / 1 0.0%	0 / 1 0.0%	0 / 3 0.0%	0 / 1 0.0%	0 / 4 0.0%	1 / 2 50.0%	1 / 1 100.0%	0 / 2 0.0%	0 / 1 0.0%	0 / 3 0.0%	0 / 1 0.0%	0 / 3 0.0%	0 / 4 0.0%	0 / 2 0.0%	0 / 1 0.0%	3 / 5 60.0%	0 / 0 0.0%	0 / 0 0.0%	0 / 0 0.0%	0 / 0 0.0%	24 / 46 52.2%	14 / 46 30.4%E	3 / 46 6.5%BE	2 / 46 4.3%C	1 / 46 2.2%AB	1 / 46 2.2%ABE	1 / 46 2.2%B	
1	Chloroherpeton	7 / 44 15.9%	0 / 1 0.0%	0 / 1 0.0%	0 / 1 0.0%	0 / 1 0.0%	0 / 3 0.0%	0 / 2 0.0%	0 / 1 0.0%	0 / 1 0.0%	0 / 3 0.0%	0 / 1 0.0%	1 / 4 25.0%	1 / 2 50.0%	1 / 1 100.0%	0 / 2 0.0%	1 / 1 100.0%	1 / 3 33.3%	0 / 1 0.0%	0 / 3 0.0%	1 / 4 25.0%	1 / 2 50.0%	0 / 1 0.0%	0 / 5 0.0%	0 / 0 0.0%	0 / 0 0.0%	0 / 0 0.0%	0 / 0 0.0%	26 / 44 59.1%	8 / 44 18.2%E	3 / 44 6.8%ABE	3 / 44 6.8%CE	2 / 44 4.5%BE	2 / 44 4.5%AB		
1	ATCC 35110	7 / 44 15.9%	0 / 1 0.0%	0 / 1 0.0%	0 / 1 0.0%	0 / 1 0.0%	0 / 3 0.0%	0 / 2 0.0%	0 / 1 0.0%	0 / 1 0.0%	0 / 3 0.0%	0 / 1 0.0%	1 / 4 25.0%	1 / 2 50.0%	1 / 1 100.0%	0 / 2 0.0%	1 / 1 100.0%	1 / 3 33.3%	0 / 1 0.0%	0 / 3 0.0%	1 / 4 25.0%	1 / 2 50.0%	0 / 1 0.0%	0 / 5 0.0%	0 / 0 0.0%	0 / 0 0.0%	0 / 0 0.0%	0 / 0 0.0%	26 / 44 59.1%	8 / 44 18.2%E	3 / 44 6.8%ABE	3 / 44 6.8%CE	2 / 44 4.5%BE	2 / 44 4.5%AB		
1	Chloroherpeton thalassium ATCC 35110	7 / 44 15.9%	0 / 1 0.0%	0 / 1 0.0%	0 / 1 0.0%	0 / 1 0.0%	0 / 3 0.0%	0 / 2 0.0%	0 / 1 0.0%	0 / 1 0.0%	0 / 3 0.0%	0 / 1 0.0%	1 / 4 25.0%	1 / 2 50.0%	1 / 1 100.0%	0 / 2 0.0%	1 / 1 100.0%	1 / 3 33.3%	0 / 1 0.0%	0 / 3 0.0%	1 / 4 25.0%	1 / 2 50.0%	0 / 1 0.0%	0 / 5 0.0%	0 / 0 0.0%	0 / 0 0.0%	0 / 0 0.0%	0 / 0 0.0%	26 / 44 59.1%	8 / 44 18.2%E	3 / 44 6.8%CE	3 / 44 6.8%ABE	2 / 44 4.5%AB	2 / 44 4.5%BE		
2	Chlorobaculum	30 / 99 30.3%	3 / 4 75.0%	0 / 2 0.0%	0 / 2 0.0%	2 / 4 50.0%	2 / 8 25.0%	2 / 6 33.3%	0 / 2 0.0%	0 / 2 0.0%	0 / 8 0.0%	0 / 2 0.0%	2 / 8 25.0%	2 / 4 50.0%	2 / 2 100.0%	0 / 4 0.0%	2 / 2 100.0%	2 / 6 33.3%	1 / 2 50.0%	0 / 6 0.0%	2 / 8 25.0%	2 / 4 50.0%	2 / 2 100.0%	4 / 11 36.4%	0 / 0 0.0%	0 / 0 0.0%	0 / 0 0.0%	0 / 0 0.0%	38 / 99 38.4%	20 / 99 20.2%E	12 / 99 12.1%AB	12 / 99 12.1%ABE	11 / 99 11.1%C	3 / 99 3.0%BE	3 / 99 3.0%B	
1	ATCC 49652	13 / 50 26.0%	1 / 2 50.0%	0 / 1 0.0%	0 / 1 0.0%	2 / 2 100.0%	0 / 4 0.0%	1 / 3 33.3%	0 / 1 0.0%	0 / 1 0.0%	0 / 4 0.0%	0 / 1 0.0%	1 / 4 25.0%	1 / 2 50.0%	1 / 1 100.0%	0 / 2 0.0%	1 / 1 100.0%	1 / 3 33.3%	0 / 1 0.0%	0 / 3 0.0%	1 / 4 25.0%	1 / 2 50.0%	1 / 1 100.0%	1 / 6 16.7%	0 / 0 0.0%	0 / 0 0.0%	0 / 0 0.0%	0 / 0 0.0%	20 / 50 40.0%	11 / 50 22.0%E	7 / 50 14.0%AB	6 / 50 12.0%C	6 / 50 12.0%ABE			
1	Chlorobaculum tepidum TLS	13 / 50 26.0%	1 / 2 50.0%	0 / 1 0.0%	0 / 1 0.0%	2 / 2 100.0%	0 / 4 0.0%	1 / 3 33.3%	0 / 1 0.0%	0 / 1 0.0%	0 / 4 0.0%	0 / 1 0.0%	1 / 4 25.0%	1 / 2 50.0%	1 / 1 100.0%	0 / 2 0.0%	1 / 1 100.0%	1 / 3 33.3%	0 / 1 0.0%	0 / 3 0.0%	1 / 4 25.0%	1 / 2 50.0%	1 / 1 100.0%	1 / 6 16.7%	0 / 0 0.0%	0 / 0 0.0%	0 / 0 0.0%	0 / 0 0.0%	20 / 50 40.0%	11 / 50 22.0%E	7 / 50 14.0%AB	6 / 50 12.0%C	6 / 50 12.0%ABE			
1	Chlorobaculum parvum	17 / 49 34.7%	2 / 2 100.0%	0 / 1 0.0%	0 / 1 0.0%	0 / 2 0.0%	2 / 4 50.0%	1 / 3 33.3%	0 / 1 0.0%	0 / 1 0.0%	0 / 4 0.0%	0 / 1 0.0%	1 / 4 25.0%	1 / 2 50.0%	1 / 1 100.0%	0 / 2 0.0%	1 / 1 100.0%	1 / 3 33.3%	1 / 1 100.0%	0 / 3 0.0%	1 / 4 25.0%	1 / 2 50.0%	1 / 1 100.0%	3 / 5 60.0%	0 / 0 0.0%	0 / 0 0.0%	0 / 0 0.0%	0 / 0 0.0%	18 / 49 36.7%	9 / 49 18.4%E	6 / 49 12.2%ABE	5 / 49 10.2%AB	5 / 49 10.2%C	3 / 49 6.1%B	3 / 49 6.1%BE	
1	Chlorobaculum parvum NCIB 8327	17 / 49 34.7%	2 / 2 100.0%	0 / 1 0.0%	0 / 1 0.0%	0 / 2 0.0%	2 / 4 50.0%	1 / 3 33.3%	0 / 1 0.0%	0 / 1 0.0%	0 / 4 0.0%	0 / 1 0.0%	1 / 4 25.0%	1 / 2 50.0%	1 / 1 100.0%	0 / 2 0.0%	1 / 1 100.0%	1 / 3 33.3%	1 / 1 100.0%	0 / 3 0.0%	1 / 4 25.0%	1 / 2 50.0%	1 / 1 100.0%	3 / 5 60.0%	0 / 0 0.0%	0 / 0 0.0%	0 / 0 0.0%	0 / 0 0.0%	18 / 49 36.7%	9 / 49 18.4%E	6 / 49 12.2%ABE	5 / 49 10.2%C	5 / 49 10.2%AB	3 / 49 6.1%BE	3 / 49 6.1%B	
7	Chlorobium/Pelodictyon group	65 / 328 19.8%	2 / 7 28.6%	0 / 7 0.0%	1 / 7 14.3%	0 / 13 0.0%	5 / 21 23.8%	5 / 20 25.0%	1 / 7 14.3%	1 / 7 14.3%	0 / 27 0.0%	0 / 7 0.0%	8 / 28 28.6%	7 / 16 43.8%	1 / 7 14.3%	2 / 14 14.3%	3 / 7 42.9%	1 / 21 4.8%	4 / 7 57.1%	0 / 21 0.0%	8 / 28 28.6%	2 / 14 14.3%	0 / 7 0.0%	14 / 35 40.0%	0 / 0 0.0%	0 / 0 0.0%	0 / 0 0.0%	0 / 0 0.0%	158 / 328 48.2%	75 / 328 22.9%E	31 / 328 9.5%ABE	23 / 328 7.0%AB	21 / 328 6.4%C	9 / 328 2.7%CE	8 / 328 2.4%BE	3 / 328 0.9%B
5	Chlorobium	48 / 231 20.8%	1 / 5 20.0%	0 / 5 0.0%	1 / 5 20.0%	0 / 8 0.0%	4 / 15 26.7%	4 / 14 28.6%	1 / 5 20.0%	1 / 5 20.0%	0 / 18 0.0%	0 / 5 0.0%	5 / 20 25.0%	5 / 11 45.5%	1 / 5 20.0%	2 / 10 20.0%	2 / 5 40.0%	1 / 15 6.7%	2 / 5 40.0%	0 / 15 0.0%	7 / 20 35.0%	1 / 10 10.0%	0 / 5 0.0%	10 / 25 40.0%	0 / 0 0.0%	0 / 0 0.0%	0 / 0 0.0%	0 / 0 0.0%	109 / 231 47.2%	55 / 231 23.8%E	22 / 231 9.5%ABE	18 / 231 7.8%AB	14 / 231 6.1%C	5 / 231 2.2%CE	5 / 231 2.2%BE	3 / 231 1.3%B
1	Chlorobium limicola	10 / 48 20.8%	0 / 1 0.0%	0 / 1 0.0%	1 / 1 100.0%	0 / 2 0.0%	1 / 3 33.3%	1 / 3 33.3%	0 / 1 0.0%	0 / 1 0.0%	0 / 4 0.0%	0 / 1 0.0%	1 / 4 25.0%	1 / 3 33.3%	1 / 1 100.0%	0 / 2 0.0%	0 / 1 0.0%	0 / 3 0.0%	0 / 1 0.0%	0 / 3 0.0%	1 / 4 25.0%	0 / 2 0.0%	0 / 1 0.0%	3 / 5 60.0%	0 / 0 0.0%	0 / 0 0.0%	0 / 0 0.0%	0 / 0 0.0%	24 / 48 50.0%	12 / 48 25.0%E	4 / 48 8.3%BE	3 / 48 6.2%B	2 / 48 4.2%AB	2 / 48 4.2%C	1 / 48 2.1%ABE	
1	Chlorobium limicola 6330	10 / 48 20.8%	0 / 1 0.0%	0 / 1 0.0%	1 / 1 100.0%	0 / 2 0.0%	1 / 3 33.3%	1 / 3 33.3%	0 / 1 0.0%	0 / 1 0.0%	0 / 4 0.0%	0 / 1 0.0%	1 / 4 25.0%	1 / 3 33.3%	1 / 1 100.0%	0 / 2 0.0%	0 / 1 0.0%	0 / 3 0.0%	0 / 1 0.0%	0 / 3 0.0%	1 / 4 25.0%	0 / 2 0.0%	0 / 1 0.0%	3 / 5 60.0%	0 / 0 0.0%	0 / 0 0.0%	0 / 0 0.0%	0 / 0 0.0%	24 / 48 50.0%	12 / 48 25.0%E	4 / 48 8.3%BE	3 / 48 6.2%B	2 / 48 4.2%AB	2 / 48 4.2%C	1 / 48 2.1%ABE	
1	Chlorobium phaeovibrioides	10 / 45 22.2%	0 / 1 0.0%	0 / 1 0.0%	0 / 1 0.0%	0 / 1 0.0%	1 / 3 33.3%	1 / 3 33.3%	0 / 1 0.0%	0 / 1 0.0%	0 / 3 0.0%	0 / 1 0.0%	1 / 4 25.0%	1 / 2 50.0%	0 / 1 0.0%	1 / 2 50.0%	1 / 1 100.0%	0 / 3 0.0%	1 / 1 100.0%	0 / 3 0.0%	2 / 4 50.0%	0 / 2 0.0%	0 / 1 0.0%	1 / 5 20.0%	0 / 0 0.0%	0 / 0 0.0%	0 / 0 0.0%	0 / 0 0.0%	19 / 45 42.2%	12 / 45 26.7%E	5 / 45 11.1%AB	5 / 45 11.1%ABE	4 / 45 8.9%C			
1	Chlorobium phaeovibrioides DSM 265	10 / 45 22.2%	0 / 1 0.0%	0 / 1 0.0%	0 / 1 0.0%	0 / 1 0.0%	1 / 3 33.3%	1 / 3 33.3%	0 / 1 0.0%	0 / 1 0.0%	0 / 3 0.0%	0 / 1 0.0%	1 / 4 25.0%	1 / 2 50.0%	0 / 1 0.0%	1 / 2 50.0%	1 / 1 100.0%	0 / 3 0.0%	1 / 1 100.0%	0 / 3 0.0%	2 / 4 50.0%	0 / 2 0.0%	0 / 1 0.0%	1 / 5 20.0%	0 / 0 0.0%	0 / 0 0.0%	0 / 0 0.0%	0 / 0 0.0%	19 / 45 42.2%	12 / 45 26.7%E	5 / 45 11.1%AB	5 / 45 11.1%ABE	4 / 45 8.9%C			
2	Chlorobium phaeobacteroides	7 / 93 7.5%	0 / 2 0.0%	0 / 2 0.0%	0 / 2 0.0%	0 / 4 0.0%	0 / 6 0.0%	0 / 5 0.0%	0 / 2 0.0%	0 / 2 0.0%	0 / 8 0.0%	0 / 2 0.0%	2 / 8 25.0%	2 / 4 50.0%	0 / 2 0.0%	0 / 4 0.0%	0 / 2 0.0%	0 / 6 0.0%	0 / 2 0.0%	0 / 6 0.0%	0 / 8 0.0%	0 / 4 0.0%	0 / 2 0.0%	3 / 10 30.0%	0 / 0 0.0%	0 / 0 0.0%	0 / 0 0.0%	0 / 0 0.0%	51 / 93 54.8%	25 / 93 26.9%E	6 / 93 6.5%ABE	6 / 93 6.5%C	4 / 93 4.3%CE	1 / 93 1.1%BE		
1	Chlorobium phaeobacteroides DSM 266	5 / 47 10.6%	0 / 1 0.0%	0 / 1 0.0%	0 / 1 0.0%	0 / 2 0.0%	0 / 3 0.0%	0 / 3 0.0%	0 / 1 0.0%	0 / 1 0.0%	0 / 4 0.0%	0 / 1 0.0%	2 / 4 50.0%	1 / 2 50.0%	0 / 1 0.0%	0 / 2 0.0%	0 / 1 0.0%	0 / 3 0.0%	0 / 1 0.0%	0 / 3 0.0%	0 / 4 0.0%	0 / 2 0.0%	0 / 1 0.0%	2 / 5 40.0%	0 / 0 0.0%	0 / 0 0.0%	0 / 0 0.0%	0 / 0 0.0%	24 / 47 51.1%	12 / 47 25.5%E	6 / 47 12.8%C	5 / 47 10.6%ABE				
1	Chlorobium phaeobacteroides BS1	2 / 46 4.3%	0 / 1 0.0%	0 / 1 0.0%	0 / 1 0.0%	0 / 2 0.0%	0 / 3 0.0%	0 / 2 0.0%	0 / 1 0.0%	0 / 1 0.0%	0 / 4 0.0%	0 / 1 0.0%	0 / 4 0.0%	1 / 2 50.0%	0 / 1 0.0%	0 / 2 0.0%	0 / 1 0.0%	0 / 3 0.0%	0 / 1 0.0%	0 / 3 0.0%	0 / 4 0.0%	0 / 2 0.0%	0 / 1 0.0%	1 / 5 20.0%	0 / 0 0.0%	0 / 0 0.0%	0 / 0 0.0%	0 / 0 0.0%	27 / 46 58.7%	13 / 46 28.3%E	4 / 46 8.7%CE	1 / 46 2.2%ABE	1 / 46 2.2%BE			
1	"Chlorobium chlorochromatii" Meschner 1957	21 / 45 46.7%	1 / 1 100.0%	0 / 1 0.0%	0 / 1 0.0%	0 / 1 0.0%	2 / 3 66.7%	2 / 3 66.7%	1 / 1 100.0%	1 / 1 100.0%	0 / 3 0.0%	0 / 1 0.0%	1 / 4 25.0%	1 / 2 50.0%	0 / 1 0.0%	1 / 2 50.0%	1 / 1 100.0%	1 / 3 33.3%	1 / 1 100.0%	0 / 3 0.0%	4 / 4 100.0%	1 / 2 50.0%	0 / 1 0.0%	3 / 5 60.0%	0 / 0 0.0%	0 / 0 0.0%	0 / 0 0.0%	0 / 0 0.0%	15 / 45 33.3%	11 / 45 24.4%AB	10 / 45 22.2%ABE	6 / 45 13.3%E	2 / 45 4.4%C	1 / 45 2.2%CE		
1	Chlorobium chlorochromatii CaD3	21 / 45 46.7%	1 / 1 100.0%	0 / 1 0.0%	0 / 1 0.0%	0 / 1 0.0%	2 / 3 66.7%	2 / 3 66.7%	1 / 1 100.0%	1 / 1 100.0%	0 / 3 0.0%	0 / 1 0.0%	1 / 4 25.0%	1 / 2 50.0%	0 / 1 0.0%	1 / 2 50.0%	1 / 1 100.0%	1 / 3 33.3%	1 / 1 100.0%	0 / 3 0.0%	4 / 4 100.0%	1 / 2 50.0%	0 / 1 0.0%	3 / 5 60.0%	0 / 0 0.0%	0 / 0 0.0%	0 / 0 0.0%	0 / 0 0.0%	15 / 45 33.3%	11 / 45 24.4%AB	10 / 45 22.2%ABE	6 / 45 13.3%E	2 / 45 4.4%C	1 / 45 2.2%CE		
2	Pelodictyon	17 / 97 17.5%	1 / 2 50.0%	0 / 2 0.0%	0 / 2 0.0%	0 / 5 0.0%	1 / 6 16.7%	1 / 6 16.7%	0 / 2 0.0%	0 / 2 0.0%	0 / 9 0.0%	0 / 2 0.0%	3 / 8 37.5%	2 / 5 40.0%	0 / 2 0.0%	0 / 4 0.0%	1 / 2 50.0%	0 / 6 0.0%	2 / 2 100.0%	0 / 6 0.0%	1 / 8 12.5%	1 / 4 25.0%	0 / 2 0.0%	4 / 10 40.0%	0 / 0 0.0%	0 / 0 0.0%	0 / 0 0.0%	0 / 0 0.0%	49 / 97 50.5%	20 / 97 20.6%E	9 / 97 9.3%ABE	7 / 97 7.2%C	5 / 97 5.2%AB	4 / 97 4.1%CE	3 / 97 3.1%BE	
1	"Aphanothece luteola" Schmidle 1901	8 / 48 16.7%	0 / 1 0.0%	0 / 1 0.0%	0 / 1 0.0%	0 / 2 0.0%	0 / 3 0.0%	1 / 3 33.3%	0 / 1 0.0%	0 / 1 0.0%	0 / 4 0.0%	0 / 1 0.0%	1 / 4 25.0%	1 / 3 33.3%	0 / 1 0.0%	0 / 2 0.0%	1 / 1 100.0%	0 / 3 0.0%	1 / 1 100.0%	0 / 3 0.0%	1 / 4 25.0%	0 / 2 0.0%	0 / 1 0.0%	2 / 5 40.0%	0 / 0 0.0%	0 / 0 0.0%	0 / 0 0.0%	0 / 0 0.0%	23 / 48 47.9%	10 / 48 20.8%E	5 / 48 10.4%ABE	5 / 48 10.4%C	3 / 48 6.2%AB	2 / 48 4.2%CE		
1	Chlorobium luteolum DSM 273	8 / 48 16.7%	0 / 1 0.0%	0 / 1 0.0%	0 / 1 0.0%	0 / 2 0.0%	0 / 3 0.0%	1 / 3 33.3%	0 / 1 0.0%	0 / 1 0.0%	0 / 4 0.0%	0 / 1 0.0%	1 / 4 25.0%	1 / 3 33.3%	0 / 1 0.0%	0 / 2 0.0%	1 / 1 100.0%	0 / 3 0.0%	1 / 1 100.0%	0 / 3 0.0%	1 / 4 25.0%	0 / 2 0.0%	0 / 1 0.0%	2 / 5 40.0%	0 / 0 0.0%	0 / 0 0.0%	0 / 0 0.0%	0 / 0 0.0%	23 / 48 47.9%	10 / 48 20.8%E	5 / 48 10.4%C	5 / 48 10.4%ABE	3 / 48 6.2%AB	2 / 48 4.2%CE		
1	DSM 5477	9 / 49 18.4%	1 / 1 100.0%	0 / 1 0.0%	0 / 1 0.0%	0 / 3 0.0%	1 / 3 33.3%	0 / 3 0.0%	0 / 1 0.0%	0 / 1 0.0%	0 / 5 0.0%	0 / 1 0.0%	2 / 4 50.0%	1 / 2 50.0%	0 / 1 0.0%	0 / 2 0.0%	0 / 1 0.0%	0 / 3 0.0%	1 / 1 100.0%	0 / 3 0.0%	0 / 4 0.0%	1 / 2 50.0%	0 / 1 0.0%	2 / 5 40.0%	0 / 0 0.0%	0 / 0 0.0%	0 / 0 0.0%	0 / 0 0.0%	26 / 49 53.1%	10 / 49 20.4%E	4 / 49 8.2%ABE	3 / 49 6.1%BE	2 / 49 4.1%AB	2 / 49 4.1%C	2 / 49 4.1%CE	
1	Pelodictyon phaeoclathratiforme BU-1	9 / 49 18.4%	1 / 1 100.0%	0 / 1 0.0%	0 / 1 0.0%	0 / 3 0.0%	1 / 3 33.3%	0 / 3 0.0%	0 / 1 0.0%	0 / 1 0.0%	0 / 5 0.0%	0 / 1 0.0%	2 / 4 50.0%	1 / 2 50.0%	0 / 1 0.0%	0 / 2 0.0%	0 / 1 0.0%	0 / 3 0.0%	1 / 1 100.0%	0 / 3 0.0%	0 / 4 0.0%	1 / 2 50.0%	0 / 1 0.0%	2 / 5 40.0%	0 / 0 0.0%	0 / 0 0.0%	0 / 0 0.0%	0 / 0 0.0%	26 / 49 53.1%	10 / 49 20.4%E	4 / 49 8.2%ABE	3 / 49 6.1%BE	2 / 49 4.1%C	2 / 49 4.1%AB	2 / 49 4.1%CE	
2	Ignavibacteria (sic) Podosokorskaya et al. 2010	11 / 90 12.2%	0 / 2 0.0%	0 / 2 0.0%	2 / 2 100.0%	2 / 2 100.0%	1 / 6 16.7%	0 / 4 0.0%	0 / 2 0.0%	0 / 2 0.0%	0 / 5 0.0%	0 / 2 0.0%	0 / 8 0.0%	1 / 4 25.0%	0 / 2 0.0%	1 / 4 25.0%	1 / 2 50.0%	1 / 6 16.7%	0 / 2 0.0%	0 / 6 0.0%	0 / 10 0.0%	0 / 5 0.0%	0 / 2 0.0%	2 / 10 20.0%	0 / 0 0.0%	0 / 0 0.0%	0 / 0 0.0%	0 / 0 0.0%	49 / 90 54.4%	29 / 90 32.2%E	8 / 90 8.9%AB	3 / 90 3.3%ABE	1 / 90 1.1%CE			
2	Ignavibacteria	11 / 90 12.2%	0 / 2 0.0%	0 / 2 0.0%	2 / 2 100.0%	2 / 2 100.0%	1 / 6 16.7%	0 / 4 0.0%	0 / 2 0.0%	0 / 2 0.0%	0 / 5 0.0%	0 / 2 0.0%	0 / 8 0.0%	1 / 4 25.0%	0 / 2 0.0%	1 / 4 25.0%	1 / 2 50.0%	1 / 6 16.7%	0 / 2 0.0%	0 / 6 0.0%	0 / 10 0.0%	0 / 5 0.0%	0 / 2 0.0%	2 / 10 20.0%	0 / 0 0.0%	0 / 0 0.0%	0 / 0 0.0%	0 / 0 0.0%	49 / 90 54.4%	29 / 90 32.2%E	8 / 90 8.9%AB	3 / 90 3.3%ABE	1 / 90 1.1%CE			
2	Ignavibacteriales	11 / 90 12.2%	0 / 2 0.0%	0 / 2 0.0%	2 / 2 100.0%	2 / 2 100.0%	1 / 6 16.7%	0 / 4 0.0%	0 / 2 0.0%	0 / 2 0.0%	0 / 5 0.0%	0 / 2 0.0%	0 / 8 0.0%	1 / 4 25.0%	0 / 2 0.0%	1 / 4 25.0%	1 / 2 50.0%	1 / 6 16.7%	0 / 2 0.0%	0 / 6 0.0%	0 / 10 0.0%	0 / 5 0.0%	0 / 2 0.0%	2 / 10 20.0%	0 / 0 0.0%	0 / 0 0.0%	0 / 0 0.0%	0 / 0 0.0%	49 / 90 54.4%	29 / 90 32.2%E	8 / 90 8.9%AB	3 / 90 3.3%ABE	1 / 90 1.1%CE			
1	Ignavibacteriaceae	7 / 45 15.6%	0 / 1 0.0%	0 / 1 0.0%	1 / 1 100.0%	1 / 1 100.0%	1 / 3 33.3%	0 / 2 0.0%	0 / 1 0.0%	0 / 1 0.0%	0 / 3 0.0%	0 / 1 0.0%	0 / 4 0.0%	1 / 2 50.0%	0 / 1 0.0%	1 / 2 50.0%	1 / 1 100.0%	0 / 3 0.0%	0 / 1 0.0%	0 / 3 0.0%	0 / 5 0.0%	0 / 2 0.0%	0 / 1 0.0%	1 / 5 20.0%	0 / 0 0.0%	0 / 0 0.0%	0 / 0 0.0%	0 / 0 0.0%	23 / 45 51.1%	14 / 45 31.1%E	5 / 45 11.1%AB	2 / 45 4.4%ABE	1 / 45 2.2%CE			
1	Ignavibacterium	7 / 45	0 / 1	0 / 1	1 / 1	1 / 1	1 / 3	0 / 2	0 / 1	0 / 1	0 / 3	0 / 1	0 / 4	1 / 2	0 / 1	1 / 2	1 / 1	0 / 3	0 / 1	0 / 3	0 / 5	0 / 2	0 / 1	1 / 5	0 / 0	0 / 0	0 / 0	0 / 0	23 / 45	14 / 45	5 / 45	2 / 45	1 / 45			

1	DSM 19864	7 / 45 15.6%	0 / 1 0.0%	0 / 1 0.0%	1 / 1 100.0%	1 / 1 100.0%	1 / 3 33.3%	0 / 2 0.0%	0 / 1 0.0%	0 / 1 0.0%	0 / 3 0.0%	0 / 1 0.0%	0 / 4 0.0%	1 / 2 50.0%	0 / 1 0.0%	1 / 2 50.0%	1 / 1 100.0%	0 / 3 0.0%	0 / 1 0.0%	0 / 3 0.0%	0 / 5 0.0%	0 / 2 0.0%	0 / 1 0.0%	1 / 5 20.0%	0 / 0 0.0%	0 / 0 0.0%	0 / 0 0.0%	0 / 0 0.0%	23 / 45 51.1%	14 / 45 31.1%E	5 / 45 11.1%AB	2 / 45 4.4%ABE	1 / 45 2.2%CE			
1	Chlorobi bacterium Mat9-16	7 / 45 15.6%	0 / 1 0.0%	0 / 1 0.0%	1 / 1 100.0%	1 / 1 100.0%	1 / 3 33.3%	0 / 2 0.0%	0 / 1 0.0%	0 / 1 0.0%	0 / 3 0.0%	0 / 1 0.0%	0 / 4 0.0%	1 / 2 50.0%	0 / 1 0.0%	1 / 2 50.0%	1 / 1 100.0%	0 / 3 0.0%	0 / 1 0.0%	0 / 3 0.0%	0 / 5 0.0%	0 / 2 0.0%	0 / 1 0.0%	1 / 5 20.0%	0 / 0 0.0%	0 / 0 0.0%	0 / 0 0.0%	0 / 0 0.0%	23 / 45 51.1%	14 / 45 31.1%E	5 / 45 11.1%AB	2 / 45 4.4%ABE	1 / 45 2.2%CE			
1	"Melioribacteraceae" Podosokorskaya et al. 2013	4 / 45 8.9%	0 / 1 0.0%	0 / 1 0.0%	1 / 1 100.0%	1 / 1 100.0%	0 / 3 0.0%	0 / 2 0.0%	0 / 1 0.0%	0 / 1 0.0%	0 / 2 0.0%	0 / 1 0.0%	0 / 4 0.0%	0 / 2 0.0%	0 / 1 0.0%	0 / 2 0.0%	0 / 1 0.0%	1 / 3 33.3%	0 / 1 0.0%	0 / 3 0.0%	0 / 5 0.0%	0 / 3 0.0%	0 / 1 0.0%	1 / 5 20.0%	0 / 0 0.0%	0 / 0 0.0%	0 / 0 0.0%	0 / 0 0.0%	26 / 45 57.8%	15 / 45 33.3%E	3 / 45 6.7%AB	1 / 45 2.2%ABE				
1	"Melioribacter" Podosokorskaya et al. 2013	4 / 45 8.9%	0 / 1 0.0%	0 / 1 0.0%	1 / 1 100.0%	1 / 1 100.0%	0 / 3 0.0%	0 / 2 0.0%	0 / 1 0.0%	0 / 1 0.0%	0 / 2 0.0%	0 / 1 0.0%	0 / 4 0.0%	0 / 2 0.0%	0 / 1 0.0%	0 / 2 0.0%	0 / 1 0.0%	1 / 3 33.3%	0 / 1 0.0%	0 / 3 0.0%	0 / 5 0.0%	0 / 3 0.0%	0 / 1 0.0%	1 / 5 20.0%	0 / 0 0.0%	0 / 0 0.0%	0 / 0 0.0%	0 / 0 0.0%	26 / 45 57.8%	15 / 45 33.3%E	3 / 45 6.7%AB	1 / 45 2.2%ABE				
1	"Melioribacter roseus" Podosokorskaya et al. 2013	4 / 45 8.9%	0 / 1 0.0%	0 / 1 0.0%	1 / 1 100.0%	1 / 1 100.0%	0 / 3 0.0%	0 / 2 0.0%	0 / 1 0.0%	0 / 1 0.0%	0 / 2 0.0%	0 / 1 0.0%	0 / 4 0.0%	0 / 2 0.0%	0 / 1 0.0%	0 / 2 0.0%	0 / 1 0.0%	1 / 3 33.3%	0 / 1 0.0%	0 / 3 0.0%	0 / 5 0.0%	0 / 3 0.0%	0 / 1 0.0%	1 / 5 20.0%	0 / 0 0.0%	0 / 0 0.0%	0 / 0 0.0%	0 / 0 0.0%	26 / 45 57.8%	15 / 45 33.3%E	3 / 45 6.7%AB	1 / 45 2.2%ABE				
1	Melioribacter roseus P3M	4 / 45 8.9%	0 / 1 0.0%	0 / 1 0.0%	1 / 1 100.0%	1 / 1 100.0%	0 / 3 0.0%	0 / 2 0.0%	0 / 1 0.0%	0 / 1 0.0%	0 / 2 0.0%	0 / 1 0.0%	0 / 4 0.0%	0 / 2 0.0%	0 / 1 0.0%	0 / 2 0.0%	0 / 1 0.0%	1 / 3 33.3%	0 / 1 0.0%	0 / 3 0.0%	0 / 5 0.0%	0 / 3 0.0%	0 / 1 0.0%	1 / 5 20.0%	0 / 0 0.0%	0 / 0 0.0%	0 / 0 0.0%	0 / 0 0.0%	26 / 45 57.8%	15 / 45 33.3%E	3 / 45 6.7%AB	1 / 45 2.2%ABE				
2	Elusimicrobia	14 / 90 15.6%	0 / 2 0.0%	1 / 2 50.0%	0 / 2 0.0%	0 / 2 0.0%	0 / 6 0.0%	2 / 6 33.3%	1 / 2 50.0%	0 / 2 0.0%	0 / 6 0.0%	1 / 2 50.0%	1 / 9 11.1%	0 / 4 0.0%	0 / 2 0.0%	2 / 4 50.0%	0 / 2 0.0%	1 / 5 20.0%	1 / 2 50.0%	0 / 6 0.0%	3 / 8 37.5%	1 / 4 25.0%	0 / 2 0.0%	0 / 10 0.0%	0 / 0 0.0%	0 / 0 0.0%	0 / 0 0.0%	0 / 0 0.0%	38 / 90 42.2%E	38 / 90 42.2%	6 / 90 6.7%ABE	4 / 90 4.4%B	2 / 90 2.2%BE	2 / 90 2.2%AB		
1	environmental samples	1 / 45 2.2%	0 / 1 0.0%	0 / 1 0.0%	0 / 1 0.0%	0 / 1 0.0%	0 / 3 0.0%	0 / 3 0.0%	0 / 1 0.0%	0 / 1 0.0%	0 / 3 0.0%	0 / 1 0.0%	1 / 5 20.0%	0 / 2 0.0%	0 / 1 0.0%	0 / 2 0.0%	0 / 1 0.0%	0 / 2 0.0%	0 / 1 0.0%	0 / 3 0.0%	0 / 4 0.0%	0 / 2 0.0%	0 / 1 0.0%	0 / 5 0.0%	0 / 0 0.0%	0 / 0 0.0%	0 / 0 0.0%	0 / 0 0.0%	23 / 45 51.1%	21 / 45 46.7%E	1 / 45 2.2%ABE					
1	uncultured TG1 bacterium	1 / 45 2.2%	0 / 1 0.0%	0 / 1 0.0%	0 / 1 0.0%	0 / 1 0.0%	0 / 3 0.0%	0 / 3 0.0%	0 / 1 0.0%	0 / 1 0.0%	0 / 3 0.0%	0 / 1 0.0%	1 / 5 20.0%	0 / 2 0.0%	0 / 1 0.0%	0 / 2 0.0%	0 / 1 0.0%	0 / 2 0.0%	0 / 1 0.0%	0 / 3 0.0%	0 / 4 0.0%	0 / 2 0.0%	0 / 1 0.0%	0 / 5 0.0%	0 / 0 0.0%	0 / 0 0.0%	0 / 0 0.0%	0 / 0 0.0%	23 / 45 51.1%	21 / 45 46.7%E	1 / 45 2.2%ABE					
1	uncultured Termite group 1 bacterium phylotype Rs-D17	1 / 45 2.2%	0 / 1 0.0%	0 / 1 0.0%	0 / 1 0.0%	0 / 1 0.0%	0 / 3 0.0%	0 / 3 0.0%	0 / 1 0.0%	0 / 1 0.0%	0 / 3 0.0%	0 / 1 0.0%	1 / 5 20.0%	0 / 2 0.0%	0 / 1 0.0%	0 / 2 0.0%	0 / 1 0.0%	0 / 2 0.0%	0 / 1 0.0%	0 / 3 0.0%	0 / 4 0.0%	0 / 2 0.0%	0 / 1 0.0%	0 / 5 0.0%	0 / 0 0.0%	0 / 0 0.0%	0 / 0 0.0%	0 / 0 0.0%	23 / 45 51.1%	21 / 45 46.7%E	1 / 45 2.2%ABE					
1	Elusimicrobia	13 / 45 28.9%	0 / 1 0.0%	1 / 1 100.0%	0 / 1 0.0%	0 / 1 0.0%	0 / 3 0.0%	2 / 3 66.7%	1 / 1 100.0%	0 / 1 0.0%	0 / 3 0.0%	1 / 1 100.0%	0 / 4 0.0%	0 / 2 0.0%	0 / 1 0.0%	2 / 2 100.0%	0 / 1 0.0%	1 / 3 33.3%	1 / 1 100.0%	0 / 3 0.0%	3 / 4 75.0%	1 / 2 50.0%	0 / 1 0.0%	0 / 5 0.0%	0 / 0 0.0%	0 / 0 0.0%	0 / 0 0.0%	0 / 0 0.0%	17 / 45 37.8%E	15 / 45 33.3%	5 / 45 11.1%ABE	4 / 45 8.9%B	2 / 45 4.4%AB	2 / 45 4.4%BE		
1	Elusimicrobiales	13 / 45 28.9%	0 / 1 0.0%	1 / 1 100.0%	0 / 1 0.0%	0 / 1 0.0%	0 / 3 0.0%	2 / 3 66.7%	1 / 1 100.0%	0 / 1 0.0%	0 / 3 0.0%	1 / 1 100.0%	0 / 4 0.0%	0 / 2 0.0%	0 / 1 0.0%	2 / 2 100.0%	0 / 1 0.0%	1 / 3 33.3%	1 / 1 100.0%	0 / 3 0.0%	3 / 4 75.0%	1 / 2 50.0%	0 / 1 0.0%	0 / 5 0.0%	0 / 0 0.0%	0 / 0 0.0%	0 / 0 0.0%	0 / 0 0.0%	17 / 45 37.8%E	15 / 45 33.3%	5 / 45 11.1%ABE	4 / 45 8.9%B	2 / 45 4.4%BE	2 / 45 4.4%AB		
1	Elusimicrobiaceae	13 / 45 28.9%	0 / 1 0.0%	1 / 1 100.0%	0 / 1 0.0%	0 / 1 0.0%	0 / 3 0.0%	2 / 3 66.7%	1 / 1 100.0%	0 / 1 0.0%	0 / 3 0.0%	1 / 1 100.0%	0 / 4 0.0%	0 / 2 0.0%	0 / 1 0.0%	2 / 2 100.0%	0 / 1 0.0%	1 / 3 33.3%	1 / 1 100.0%	0 / 3 0.0%	3 / 4 75.0%	1 / 2 50.0%	0 / 1 0.0%	0 / 5 0.0%	0 / 0 0.0%	0 / 0 0.0%	0 / 0 0.0%	0 / 0 0.0%	17 / 45 37.8%E	15 / 45 33.3%	5 / 45 11.1%ABE	4 / 45 8.9%B	2 / 45 4.4%BE	2 / 45 4.4%AB		
1	"Elusimicrobium" Helreman et al. 2007	13 / 45 28.9%	0 / 1 0.0%	1 / 1 100.0%	0 / 1 0.0%	0 / 1 0.0%	0 / 3 0.0%	2 / 3 66.7%	1 / 1 100.0%	0 / 1 0.0%	0 / 3 0.0%	1 / 1 100.0%	0 / 4 0.0%	0 / 2 0.0%	0 / 1 0.0%	2 / 2 100.0%	0 / 1 0.0%	1 / 3 33.3%	1 / 1 100.0%	0 / 3 0.0%	3 / 4 75.0%	1 / 2 50.0%	0 / 1 0.0%	0 / 5 0.0%	0 / 0 0.0%	0 / 0 0.0%	0 / 0 0.0%	0 / 0 0.0%	17 / 45 37.8%E	15 / 45 33.3%	5 / 45 11.1%ABE	4 / 45 8.9%B	2 / 45 4.4%AB	2 / 45 4.4%BE		
1	"Elusimicrobium minutum" Helreman et al. 2007	13 / 45 28.9%	0 / 1 0.0%	1 / 1 100.0%	0 / 1 0.0%	0 / 1 0.0%	0 / 3 0.0%	2 / 3 66.7%	1 / 1 100.0%	0 / 1 0.0%	0 / 3 0.0%	1 / 1 100.0%	0 / 4 0.0%	0 / 2 0.0%	0 / 1 0.0%	2 / 2 100.0%	0 / 1 0.0%	1 / 3 33.3%	1 / 1 100.0%	0 / 3 0.0%	3 / 4 75.0%	1 / 2 50.0%	0 / 1 0.0%	0 / 5 0.0%	0 / 0 0.0%	0 / 0 0.0%	0 / 0 0.0%	0 / 0 0.0%	17 / 45 37.8%E	15 / 45 33.3%	5 / 45 11.1%ABE	4 / 45 8.9%B	2 / 45 4.4%BE	2 / 45 4.4%AB		
1	Elusimicrobium minutum Pei191	13 / 45 28.9%	0 / 1 0.0%	1 / 1 100.0%	0 / 1 0.0%	0 / 1 0.0%	0 / 3 0.0%	2 / 3 66.7%	1 / 1 100.0%	0 / 1 0.0%	0 / 3 0.0%	1 / 1 100.0%	0 / 4 0.0%	0 / 2 0.0%	0 / 1 0.0%	2 / 2 100.0%	0 / 1 0.0%	1 / 3 33.3%	1 / 1 100.0%	0 / 3 0.0%	3 / 4 75.0%	1 / 2 50.0%	0 / 1 0.0%	0 / 5 0.0%	0 / 0 0.0%	0 / 0 0.0%	0 / 0 0.0%	0 / 0 0.0%	17 / 45 37.8%E	15 / 45 33.3%	5 / 45 11.1%ABE	4 / 45 8.9%B	2 / 45 4.4%BE	2 / 45 4.4%AB		
9	Fibrobacter/Acidobacteria group	359 / 489 73.4%	8 / 8 100.0%	9 / 15 60.0%	8 / 12 66.7%	9 / 16 56.2%	24 / 34 70.6%	23 / 30 76.7%	8 / 10 80.0%	8 / 10 80.0%	24 / 36 66.7%	8 / 10 80.0%	33 / 42 78.6%	15 / 24 62.5%	8 / 10 80.0%	17 / 21 81.0%	13 / 15 86.7%	24 / 30 80.0%	8 / 11 72.7%	22 / 30 73.3%	30 / 40 75.0%	17 / 23 73.9%	7 / 10 70.0%	35 / 50 70.0%	1 / 2 50.0%	0 / 0 0.0%	0 / 0 0.0%	0 / 0 0.0%	210 / 489 42.9%AB	148 / 489 30.3%ABE	89 / 489 18.2%	41 / 489 8.4%E	1 / 489 0.2%B			
8	Acidobacteria	359 / 375 95.7%	8 / 8 100.0%	9 / 9 100.0%	8 / 8 100.0%	9 / 10 90.0%	24 / 24 100.0%	23 / 24 95.8%	8 / 8 100.0%	8 / 8 100.0%	24 / 26 92.3%	8 / 8 100.0%	33 / 34 97.1%	15 / 16 93.8%	8 / 8 100.0%	17 / 17 100.0%	13 / 13 100.0%	24 / 24 100.0%	8 / 9 88.9%	22 / 24 91.7%	30 / 32 93.8%	17 / 17 100.0%	7 / 8 87.5%	35 / 38 92.1%	1 / 2 50.0%	0 / 0 0.0%	0 / 0 0.0%	0 / 0 0.0%	210 / 375 56.0%AB	148 / 375 39.5%ABE	11 / 375 2.9%	5 / 375 1.3%E	1 / 375 0.3%B			
6	Acidobacteria	277 / 279 99.3%	6 / 6 100.0%	7 / 7 100.0%	6 / 6 100.0%	7 / 7 100.0%	18 / 18 100.0%	18 / 18 100.0%	6 / 6 100.0%	6 / 6 100.0%	19 / 19 100.0%	6 / 6 100.0%	25 / 26 96.2%	12 / 12 100.0%	6 / 6 100.0%	13 / 13 100.0%	11 / 11 100.0%	18 / 18 100.0%	6 / 6 100.0%	18 / 18 100.0%	22 / 22 100.0%	13 / 13 100.0%	6 / 6 100.0%	28 / 28 100.0%	0 / 1 0.0%	0 / 0 0.0%	0 / 0 0.0%	0 / 0 0.0%	165 / 279 59.1%AB	112 / 279 40.1%ABE	1 / 279 0.4%	1 / 279 0.4%E				
6	Acidobacteria subdivision 1	277 / 279 99.3%	6 / 6 100.0%	7 / 7 100.0%	6 / 6 100.0%	7 / 7 100.0%	18 / 18 100.0%	18 / 18 100.0%	6 / 6 100.0%	6 / 6 100.0%	19 / 19 100.0%	6 / 6 100.0%	25 / 26 96.2%	12 / 12 100.0%	6 / 6 100.0%	13 / 13 100.0%	11 / 11 100.0%	18 / 18 100.0%	6 / 6 100.0%	18 / 18 100.0%	22 / 22 100.0%	13 / 13 100.0%	6 / 6 100.0%	28 / 28 100.0%	0 / 1 0.0%	0 / 0 0.0%	0 / 0 0.0%	0 / 0 0.0%	165 / 279 59.1%AB	112 / 279 40.1%ABE	1 / 279 0.4%	1 / 279 0.4%E				
6	"Acidobacteriaceae" Thash and Coates 2011	277 / 279 99.3%	6 / 6 100.0%	7 / 7 100.0%	6 / 6 100.0%	7 / 7 100.0%	18 / 18 100.0%	18 / 18 100.0%	6 / 6 100.0%	6 / 6 100.0%	19 / 19 100.0%	6 / 6 100.0%	25 / 26 96.2%	12 / 12 100.0%	6 / 6 100.0%	13 / 13 100.0%	11 / 11 100.0%	18 / 18 100.0%	6 / 6 100.0%	18 / 18 100.0%	22 / 22 100.0%	13 / 13 100.0%	6 / 6 100.0%	28 / 28 100.0%	0 / 1 0.0%	0 / 0 0.0%	0 / 0 0.0%	0 / 0 0.0%	165 / 279 59.1%AB	112 / 279 40.1%ABE	1 / 279 0.4%E	1 / 279 0.4%				
1	Acidobacterium	45 / 45 100.0%	1 / 1 100.0%	1 / 1 100.0%	1 / 1 100.0%	1 / 1 100.0%	3 / 3 100.0%	3 / 3 100.0%	1 / 1 100.0%	1 / 1 100.0%	3 / 3 100.0%	1 / 1 100.0%	4 / 4 100.0%	2 / 2 100.0%	1 / 1 100.0%	2 / 2 100.0%	2 / 2 100.0%	3 / 3 100.0%	1 / 1 100.0%	3 / 3 100.0%	4 / 4 100.0%	2 / 2 100.0%	1 / 1 100.0%	4 / 4 100.0%	0 / 0 0.0%	0 / 0 0.0%	0 / 0 0.0%	0 / 0 0.0%	27 / 45 60.0%AB	18 / 45 40.0%ABE						
1	ATCC 51196	45 / 45 100.0%	1 / 1 100.0%	1 / 1 100.0%	1 / 1 100.0%	1 / 1 100.0%	3 / 3 100.0%	3 / 3 100.0%	1 / 1 100.0%	1 / 1 100.0%	3 / 3 100.0%	1 / 1 100.0%	4 / 4 100.0%	2 / 2 100.0%	1 / 1 100.0%	2 / 2 100.0%	2 / 2 100.0%	3 / 3 100.0%	1 / 1 100.0%	3 / 3 100.0%	4 / 4 100.0%	2 / 2 100.0%	1 / 1 100.0%	4 / 4 100.0%	0 / 0 0.0%	0 / 0 0.0%	0 / 0 0.0%	0 / 0 0.0%	27 / 45 60.0%AB	18 / 45 40.0%ABE						
1	Acidobacterium capsulatum ATCC 51196	45 / 45 100.0%	1 / 1 100.0%	1 / 1 100.0%	1 / 1 100.0%	1 / 1 100.0%	3 / 3 100.0%	3 / 3 100.0%	1 / 1 100.0%	1 / 1 100.0%	3 / 3 100.0%	1 / 1 100.0%	4 / 4 100.0%	2 / 2 100.0%	1 / 1 100.0%	2 / 2 100.0%	2 / 2 100.0%	3 / 3 100.0%	1 / 1 100.0%	3 / 3 100.0%	4 / 4 100.0%	2 / 2 100.0%	1 / 1 100.0%	4 / 4 100.0%	0 / 0 0.0%	0 / 0 0.0%	0 / 0 0.0%	0 / 0 0.0%	27 / 45 60.0%AB	18 / 45 40.0%ABE						
2	Terraglobus	96 / 96 100.0%	2 / 2 100.0%	3 / 3 100.0%	2 / 2 100.0%	3 / 3 100.0%	6 / 6 100.0%	6 / 6 100.0%	2 / 2 100.0%	2 / 2 100.0%	7 / 7 100.0%	2 / 2 100.0%	9 / 9 100.0%	4 / 4 100.0%	2 / 2 100.0%	5 / 5 100.0%	4 / 4 100.0%	6 / 6 100.0%	2 / 2 100.0%	6 / 6 100.0%	7 / 7 100.0%	4 / 4 100.0%	2 / 2 100.0%	10 / 10 100.0%	0 / 0 0.0%	0 / 0 0.0%	0 / 0 0.0%	0 / 0 0.0%	56 / 96 58.3%AB	40 / 96 41.7%ABE						
1	DSM 18391	51 / 51 100.0%	1 / 1 100.0%	2 / 2 100.0%	1 / 1 100.0%	2 / 2 100.0%	3 / 3 100.0%	3 / 3 100.0%	1 / 1 100.0%	1 / 1 100.0%	4 / 4 100.0%	1 / 1 100.0%	5 / 5 100.0%	2 / 2 100.0%	1 / 1 100.0%	3 / 3 100.0%	2 / 2 100.0%	3 / 3 100.0%	1 / 1 100.0%	3 / 3 100.0%	3 / 3 100.0%	2 / 2 100.0%	1 / 1 100.0%	6 / 6 100.0%	0 / 0 0.0%	0 / 0 0.0%	0 / 0 0.0%	0 / 0 0.0%	29 / 51 56.9%AB	22 / 51 43.1%ABE						
1	Terriglobus roseus DSM 18391	51 / 51 100.0%	1 / 1 100.0%	2 / 2 100.0%	1 / 1 100.0%	2 / 2 100.0%	3 / 3 100.0%	3 / 3 100.0%	1 / 1 100.0%	1 / 1 100.0%	4 / 4 100.0%	1 / 1 100.0%	5 / 5 100.0%	2 / 2 100.0%	1 / 1 100.0%	3 / 3 100.0%	2 / 2 100.0%	3 / 3 100.0%	1 / 1 100.0%	3 / 3 100.0%	3 / 3 100.0%	2 / 2 100.0%	1 / 1 100.0%	6 / 6 100.0%	0 / 0 0.0%	0 / 0 0.0%	0 / 0 0.0%	0 / 0 0.0%	29 / 51 56.9%AB	22 / 51 43.1%ABE						
1	'Terriglobus saanensis'	45 / 45 100.0%	1 / 1 100.0%	1 / 1 100.0%	1 / 1 100.0%	1 / 1 100.0%	3 / 3 100.0%	3 / 3 100.0%	1 / 1 100.0%	1 / 1 100.0%	3 / 3 100.0%	1 / 1 100.0%	4 / 4 100.0%	2 / 2 100.0%	1 / 1 100.0%	2 / 2 100.0%	2 / 2 100.0%	3 / 3 100.0%	1 / 1 100.0%	3 / 3 100.0%	4 / 4 100.0%	2 / 2 100.0%	1 / 1 100.0%	4 / 4 100.0%	0 / 0 0.0%	0 / 0 0.0%	0 / 0 0.0%	0 / 0 0.0%	27 / 45 60.0%AB	18 / 45 40.0%ABE						
1	Acidobacterium sp. SP1PR4	45 / 45 100.0%	1 / 1 100.0%	1 / 1 100.0%	1 / 1 100.0%	1 / 1 100.0%	3 / 3 100.0%	3 / 3 100.0%	1 / 1 100.0%	1 / 1 100.0%	3 / 3 100.0%	1 / 1 100.0%	4 / 4 100.0%	2 / 2 100.0%	1 / 1 100.0%	2 / 2 100.0%	2 / 2 100.0%	3 / 3 100.0%	1 / 1 100.0%	3 / 3 100.0%	4 / 4 100.0%	2 / 2 100.0%	1 / 1 100.0%	4 / 4 100.0%	0 / 0 0.0%	0 / 0 0.0%	0 / 0 0.0%	0 / 0 0.0%	27 / 45 60.0%AB	18 / 45 40.0%ABE						
1	"Candidatus Koribacter" Ward et al. 2009	46 / 47 97.9%	1 / 1 100.0%	1 / 1 100.0%	1 / 1 100.0%	1 / 1 100.0%	3 / 3 100.0%	3 / 3 100.0%	1 / 1 100.0%	1 / 1 100.0%	3 / 3 100.0%	1 / 1 100.0%	4 / 4 100.0%	2 / 2 100.0%	1 / 1 100.0%	2 / 2 100.0%	1 / 1 100.0%	3 / 3 100.0%	1 / 1 100.0%	3 / 3 100.0%	5 / 5 100.0%	2 / 2 100.0%	1 / 1 100.0%	5 / 5 100.0%	0 / 1 0.0%	0 / 0 0.0%	0 / 0 0.0%	0 / 0 0.0%	28 / 47 59.6%AB	18 / 47 38.3%ABE	1 / 47 2.1%E					
1	"Candidatus Koribacter versatilis" Ward et al. 2009	46 / 47 97.9%	1 / 1 100.0%	1 / 1 100.0%	1 / 1 100.0%	1 / 1 100.0%	3 / 3 100.0%	3 / 3 100.0%	1 / 1 100.0%	1 / 1 100.0%	3 / 3 100.0%	1 / 1 100.0%	4 / 4 100.0%	2 / 2 100.0%	1 / 1 100.0%	2 / 2 100.0%	1 / 1 100.0%	3 / 3 100.0%	1 / 1 100.0%	3 / 3 100.0%	5 / 5 100.0%	2 / 2 100.0%	1 / 1 100.0%	5 / 5 100.0%	0 / 1 0.0%	0 / 0 0.0%	0 / 0 0.0%	0 / 0 0.0%	28 / 47 59.6%AB	18 / 47 38.3%ABE	1 / 47 2.1%E					
1	Acidobacteria bacterium Ellin345	46 / 47 97.9%	1 / 1 100.0%	1 / 1 100.0%	1 / 1 100.0%	1 / 1 100.0%	3 / 3 100.0%	3 / 3 100.0%	1 / 1 100.0%	1 / 1 100.0%	3 / 3 100.0%	1 / 1 100.0%	4 / 4 100.0%	2 / 2 100.0%	1 / 1 100.0%	2 / 2 100.0%	1 / 1 100.0%	3 / 3 100.0%	1 / 1 100.0%	3 / 3 100.0%	5 / 5 100.0%	2 / 2 100.0%	1 / 1 100.0%	5 / 5 100.0%	0 / 1 0.0%	0 / 0 0.0%	0 / 0 0.0%	0 / 0 0.0%	28 / 47 59.6%AB	18 / 47 38.3%ABE	1 / 47 2.1%E					
2	Granulicella	90 / 91 98.9%	2 / 2 100.0%	2 / 2 100.0%	2 / 2 100.0%	2 / 2 100.0%	6 / 6 100.0%	6 / 6 100.0%	2 / 2 100.0%	2 / 2 100.0%	6 / 6 100.0%	2 / 2 100.0%	8 / 9 88.9%	4 / 4 100.0%	2 / 2 100.0%	4 / 4 100.0%	4 / 4 100.0%	6 / 6 100.0%	2 / 2 100.0%	6 / 6 100.0%	6 / 6 100.0%	5 / 5 100.0%	2 / 2 100.0%	9 / 9 100.0%	0 / 0 0.0%	0 / 0 0.0%	0 / 0 0.0%	0 / 0 0.0%	54 / 91 59.3%AB	36 / 91 39.6%ABE	1 / 91 1.1%					
1	ATCC BAA-1857	45 / 46 97.8%	1 / 1 100.0%	1 / 1 100.0%	1 / 1 100.0%	1 / 1 100.0%	3 / 3 100.0%	3 / 3 100.0%	1 / 1 100.0%	1 / 1 100.0%	3 / 3 100.0%	1 / 1 100.0%	4 / 5 80.0%	2 / 2 100.0%	1 / 1 100.0%	2 / 2 100.0%	2 / 2 100.0%	3 / 3 100.0%	1 / 1 100.0%	3 / 3 100.0%	3 / 3 100.0%	3 / 3 100.0%	1 / 1 100.0%	4 / 4 100.0%	0 / 0 0.0%	0 / 0 0.0%	0 / 0 0.0%	0 / 0 0.0%	27 / 46 58.7%AB	18 / 46 39.1%ABE	1 / 46 2.2%					
1	Acidobacterium sp. MP5ACTX8	45 / 46 97.8%	1 / 1 100.0%	1 / 1 100.0%	1 / 1 100.0%	1 / 1 100.0%	3 / 3 100.0%	3 / 3 100.0%	1 / 1 100.0%	1 / 1 100.0%	3 / 3 100.0%	1 / 1 100.0%	4 / 5 80.0%	2 / 2 100.0%	1 / 1 100.0%	2 / 2 100.0%	2 / 2 100.0%	3 / 3 100.0%	1 / 1 100.0%	3 / 3 100.0%	3 / 3 100.0%	3 / 3 100.0%	1 / 1 100.0%	4 / 4 100.0%	0 / 0 0.0%	0 / 0 0.0%	0 / 0 0.0%	0 / 0 0.0%	27 / 46 58.7%AB	18 / 46 39.1%ABE	1 / 46 2.2%					
1	ATCC BAA-1859	45 / 45 100.0%	1 / 1 100.0%	1 / 1 100.0%	1 / 1 100.0%	1 / 1 100.0%	3 / 3 100.0%	3 / 3 100.0%	1 / 1 100.0%	1 / 1 100.0%	3 / 3 100.0%	1 / 1 100.0%	4 / 4 100.0%	2 / 2 100.0%	1 / 1 100.0%	2 / 2 100.0%	2 / 2 100.0%	3 / 3 100.0%	1 / 1 100.0%	3 / 3 100.0%	3 / 3 100.0%	2 / 2 100.0%	1 / 1 100.0%	5 / 5 100.0%	0 / 0 0.0%	0 / 0 0.0%	0 / 0 0.0%	0 / 0 0.0%	27 / 45 60.0%AB	18 / 45 40.0%ABE						
1	Acidobacterium sp. MP5ACTX9	45 / 45 100.0%	1 / 1 100.0%	1 / 1 100.0%	1 / 1 100.0%	1 / 1 100.0%	3 / 3 100.0%	3 / 3 100.0%	1 / 1 100.0%	1 / 1 100.0%	3 / 3 100.0%	1 / 1 100.0%	4 / 4 100.0%	2 / 2 100.0%	1 / 1 100.0%	2 / 2 100.0%	2 / 2 100.0%	3 / 3 100.0%	1 / 1 100.0%	3 / 3 100.0%	3 / 3 100.0%	2 / 2 100.0%	1 / 1 100.0%	5 / 5 100.0%	0 / 0 0.0%	0 / 0 0.0%	0 / 0 0.0%	0 / 0 0.0%	27 / 45 60.0%AB	18 / 45 40.0%ABE						
1	'Solibacteres'	49 / 50 98.0%	1 / 1 100.0%	1 / 1 100.0%	1 / 1 100.0%	2 / 2 100.0%	3 / 3 100.0%	3 / 3 100.0%	1 / 1 100.0%	1 / 1 100.0%	4 / 4 100.0%	1 / 1 100.0%	4 / 4 100.0%	2 / 2 100.0%	1 / 1 100.0%	2 / 2 100.0%	1 / 1 100.0%	3 / 3 100.0%	1 / 2 50.0%	3 / 3 100.0%	5 / 5 100.0%	2 / 2 100.0%	1 / 1 100.0%	5 / 5 100.0%	1 / 1 100.0%	0 / 0 0.0%	0 / 0 0.0%	0 / 0 0.0%	29 / 50 58.0%AB	19 / 50 38.0%ABE	1 / 50 2.0%	1 / 50 2.0%B				
1	'Solibacterales'	49 / 50 98.0%	1 / 1 100.0%	1 / 1 100.0%	1 / 1 100.0%	2 / 2 100.0%	3 / 3 100.0%	3 / 3 100.0%	1 / 1 100.0%	1 / 1 100.0%	4 / 4 100.0%	1 / 1 100.0%	4 / 4 100.0%	2 / 2 100.0%	1 / 1 100.0%	2 / 2 100.0%	1 / 1 100.0%	3 / 3 100.0%	1 / 2 50.0%	3 / 3 100.0%	5 / 5 100.0%	2 / 2 100.0%	1 / 1 100.0%	5 / 5 100.0%	1 / 1 100.0%	0 / 0 0.0%	0 / 0 0.0%	0 / 0 0.0%	29 / 50 58.0%AB	19 / 50 38.0%ABE	1 / 50 2.0%B	1 / 50 2.0%				
1	'Solibacteraceae'	49 / 50 98.0%	1 / 1 100.0%	1 / 1 100.0%	1 / 1 100.0%	2 / 2 100.0%	3 / 3 100.0%	3 / 3 100.0%	1 / 1 100.0%	1 / 1 100.0%	4 / 4 100.0%	1 / 1 100.0%	4 / 4 100.0%	2 / 2 100.0%	1 / 1 100.0%	2 / 2 100.0%	1 / 1 100.0%	3 / 3 100.0%	1 / 2 50.0%	3 / 3 100.0%	5 / 5 100.0%	2 / 2 100.0%	1 / 1 100.0%	5 / 5 100.0%	1 / 1 100.0%	0 / 0 0.0%	0 / 0 0.0%	0 / 0 0.0%	29 / 50 58.0%AB	19 / 50 38.0%ABE	1 / 50 2.0%B	1 / 50 2.0%				
1	"Candidatus Solibacter" Ward et al. 2009	49 / 50 98.0%	1 / 1 100.0%	1 / 1 100.0%	1 / 1 100.0%	2 / 2 100.0%	3 / 3 100.0%	3 / 3 100.0%	1 / 1 100.0%	1 / 1 100.0%	4 / 4 100.0%	1 / 1 100.0%	4 / 4 100.0%	2 / 2 100.0%	1 / 1 100.0%	2 / 2 100.0%	1 / 1 100.0%	3 / 3 100.0%	1 / 2 50.0%	3 / 3 100.0%	5 / 5 100.0%	2 / 2 100.0%	1 / 1 100.0%	5 / 5 100.0%	1 / 1 100.0%	0 / 0 0.0%	0 / 0 0.0%	0 / 0 0.0%	29 / 50 58.0%AB	19 / 50 38.0%ABE	1 / 50 2.0%	1 / 50 2.0%B				
1	"Candidatus Solibacter usitatus" Ward et al. 2009	49 / 50 98.0%	1 / 1 100.0%	1 / 1 100.0%	1 / 1 100.0%	2 / 2 100.0%	3 / 3 100.0%	3 / 3 100.0%	1 / 1 100.0%	1 / 1 100.0%	4 / 4 100.0%	1 / 1 100.0%	4 / 4 100.0%	2 / 2 100.0%	1 / 1 100.0%	2 / 2 100.0%	1 / 1 100.0%	3 / 3 100.0%	1 / 2 50.0%	3 / 3 100.0%	5 / 5 100.0%	2 / 2 100.0%	1 / 1 100.0%	5 / 5 100.0%	1 / 1 100.0%	0 / 0 0.0%	0 / 0 0.0%	0 / 0 0.0%	29 / 50 58.0%AB	19 / 50 38.0%ABE	1 / 50 2.0%B	1 / 50 2.0%				
1	Acidobacteriaceae bacterium Ellin6076	49 / 50 98.0%	1 / 1 100.0%	1 / 1 100.0%	1 / 1 100.0%	2 / 2 100.0%	3 / 3 100.0%	3 / 3 100.0%	1 / 1 100.0%	1 / 1 100.0%	4 / 4 100.0%	1 / 1 100.0%	4 / 4 100.0%	2 / 2 100.0%	1 / 1 100.0%	2 / 2 100.0%	1 / 1 100.0%	3 / 3 100.0%	1 / 2 50.0%	3 / 3 100.0%	5 / 5 100.0%	2 / 2 100.0%	1 / 1 100.0%	5 / 5 100.0%	1 / 1 100.0%	0 / 0 0.0%	0 / 0 0.0%	0 / 0 0.0%	29 / 50 58.0%AB	19 / 50 38.0%ABE	1 / 50 2.0%B	1 / 50 2.0%				
1	unclassifed Acidobacteria	33 / 46 71.7%	1 / 1 100.0%	1 / 1 100.0%	1 / 1 100.0%	0 / 1 0.0%	3 / 3 100.0%	2 / 3 66.7%	1 / 1 100.0%	1 / 1 100.0%	1 / 3 33.3%	1 / 1 100.0%	4 / 4 100.0%	1 / 2 50.0%	1 / 1 100.0%	2 / 2 100.0%	1 / 1 100.0%	3 / 3 100.0%	1 / 1 100.0%	1 / 3 33.3%	3 / 5 60.0%	2 / 2 100.0%	0 / 1 0.0%	2 / 5 40.0%	0 / 0 0.0%	0 / 0 0.0%	0 / 0 0.0%	0 / 0 0.0%	17 / 46 37.0%ABE	16 / 46 34.8%AB	9 / 46 19.6%	4 / 46 8.7%E				
1	Acidobacteria subdivision 4	33 / 46 71.7%	1 / 1 100.0%	1 / 1 100.0%	1 / 1 100.0%	0 / 1 0.0%	3 / 3 100.0%	2 / 3 66.7%	1 / 1 100.0%	1 / 1 100.0%	1 / 3 33.3%	1 / 1 100.0%	4 / 4 100.0%	1 / 2 50.0%	1 / 1 100.0%	2 / 2 100.0%	1 / 1 100.0%	3 / 3 100.0%	1 / 1 100.0%	1 / 3 33.3%	3 / 5 60.0%	2 / 2 100.0%	0 / 1 0.0%	2 / 5 40.0%	0 / 0 0.0%	0 / 0 0.0%	0 / 0 0.0%	0 / 0 0.0%	17 / 46 37.0%ABE	16 / 46 34.8%AB	9 / 46 19.6%	4 / 46 8.7%E				
1	"Candidatus Chloracidobacterium" Bryant et al. 2007	33 / 46 71.7%	1 / 1 100.0%	1 / 1 100.0%	1 / 1 100.0%	0 / 1 0.0%	3 / 3 100.0%	2 / 3 66.7%	1 / 1 100.0%	1 / 1 100.0%	1 / 3 33.3%	1 / 1 100.0%	4 / 4 100.0%	1 / 2 50.0%	1 / 1 100.0%	2 / 2 100.0%	1 / 1 100.0%	3 / 3 100.0%	1 / 1 100.0%	1 / 3 33.3%	3 / 5 60.0%	2 / 2 100.0%	0 / 1 0.0%	2 / 5 40.0%	0 / 0 0.0%	0 / 0 0.0%	0 / 0 0.0%	0 / 0 0.0%	17 / 46 37.0%ABE	16 / 46 34.8%AB	9 / 46 19.6%	4 / 46 8.7%E				
1	"Candidatus Chloracidobacterium thermophilum" Bryant et al. 2007	33 / 46 71.7%	1 / 1 100.0%	1 / 1 100.0%	1 / 1 100.0%	0 / 1 0.0%	3 / 3 100.0%	2 / 3 66.7%	1 / 1 100.0%	1 / 1 100.0%	1 / 3 33.3%	1 / 1 100.0%	4 / 4 100.0%	1 / 2 50.0%	1 / 1 100.0%	2 / 2 100.0%	1 / 1 100.0%	3 / 3 100.0%	1 / 1 100.0%	1 / 3 33.3%	3 / 5 60.0%	2 / 2 100.0%	0 / 1 0.0%	2 / 5 40.0%	0 / 0 0.0%	0 / 0 0.0%	0 / 0 0.0%	0 / 0 0.0%	17 / 46 37.0%ABE	16 / 46 34.8%AB	9 / 46 19.6%	4 / 46 8.7%E				
1	Candidatus Chloracidobacterium thermophilum B	33 / 46 71.7%	1 / 1 100.0%	1 / 1 100.0%	1 / 1 100.0%	0 / 1 0.0%	3 / 3 100.0%	2 / 3 66.7%	1 / 1 100.0%	1 / 1 100.0%	1 / 3 33.3%	1 / 1 100.0%	4 / 4 100.0%	1 / 2 50.0%	1 / 1 100.0%	2 / 2 100.0%	1 / 1 100.0%	3 / 3 100.0%	1 / 1 100.0%	1 / 3 33.3%	3 / 5 60.0%	2 / 2 100.0%	0 / 1 0.0%	2 / 5 40.0%	0 / 0 0.0%	0 / 0 0.0%	0 / 0 0.0%	0 / 0 0.0%	17 / 46 37.0%ABE	16 / 46 34.8%AB	9 / 46 19.6%	4 / 46 8.7%E				
1	Fibrobacter group	0 / 114 0.0%	0 / 0 0.0%	0 / 6 0.0%	0 / 4 0.0%	0 / 6 0.0%	0 / 10 0.0%	0 / 6 0.0%	0 / 2 0.0%	0 / 2 0.0%	0 / 10 0.0%	0 / 2 0.0%	0 / 8 0.0%	0 / 8 0.0%	0 / 2 0.0%	0 / 4 0.0%	0 / 2 0.0%	0 / 6 0.0%	0 / 2 0.0%	0 / 6 0.0%	0 / 8 0.0%	0 / 6 0.0%	0 / 2 0.0%	0 / 12 0.0%	0 / 0 0.0%	0 / 0 0.0%	0 / 0 0.0%	0 / 0 0.0%	78 / 114 68.4%	36 / 114 31.6%E						
1	"Fibrobacteria" Spain et al. 2011	0 / 114 0.0%	0 / 0 0.0%	0 / 6 0.0%	0 / 4 0.0%	0 / 6 0.0%	0 / 10 0.0%	0 / 6 0.0%	0 / 2 0.0%	0 / 2 0.0%	0 / 10 0.0%	0 / 2 0.0%	0 / 8 0.0%	0 / 8 0.0%	0 / 2 0.0%	0 / 4 0.0%	0 / 2 0.0%	0 / 6 0.0%	0 / 2 0.0%	0 / 6 0.0%	0 / 8 0.0%	0 / 6 0.0%	0 / 2 0.0%	0 / 12 0.0%	0 / 0 0.0%	0 / 0 0.0%	0 / 0 0.0%	0 / 0 0.0%	78 / 114 68.4%	36 / 114 31.6%E						
1	"Fibrobacterales" Spain et al. 2011	0 / 114 0.0%	0 / 0 0.0%	0 / 6 0.0%	0 / 4 0.0%	0 / 6 0.0%	0 / 10 0.0%	0 / 6 0.0%	0 / 2 0.0%	0 / 2 0.0%	0 / 10 0.0%	0 / 2 0.0%	0 / 8 0.0%	0 / 8 0.0%	0 / 2 0.0%	0 / 4 0.0%	0 / 2 0.0%	0 / 6 0.0%	0 / 2 0.0%	0 / 6 0.0%	0 / 8 0.0%	0 / 6 0.0%	0 / 2 0.0%	0 / 12 0.0%	0 / 0 0.0%	0 / 0 0.0%	0 / 0 0.0%	0 / 0 0.0%	78 / 114 68.4%	36 / 114 31.6%E						
1	"Fibrobacteraceae" Spain et al. 2011	0 / 114 0.0%	0 / 0 0.0%	0 / 6 0.0%	0 / 4 0.0%	0 / 6 0.0%	0 / 10 0.0%	0 / 6 0.0%	0 / 2 0.0%	0 / 2 0.0%	0 / 10 0.0%	0 / 2 0.0%	0 / 8 0.0%	0 / 8 0.0%	0 / 2 0.0%	0 / 4 0.0%	0 / 2 0.0%	0 / 6 0.0%	0 / 2 0.0%	0 / 6 0.0%	0 / 8 0.0%	0 / 6 0.0%	0 / 2 0.0%	0 / 12 0.0%	0 / 0 0.0%	0 / 0 0.0%	0 / 0 0.0%	0 / 0 0.0%	78 / 114 68.4%	36 / 114 31.6%E						
1	Fibribacter	0 / 114 0.0%	0 / 0 0.0%	0 / 6 0.0%	0 / 4 0.0%	0 / 6 0.0%	0 / 10 0.0%	0 / 6 0.0%	0 / 2 0.0%	0 / 2 0.0%	0 / 10 0.0%	0 / 2 0.0%	0 / 8 0.0%	0 / 8 0.0%	0 / 2 0.0%	0 / 4 0.0%	0 / 2 0.0%	0 / 6 0.0%	0 / 2 0.0%	0 / 6 0.0%	0 / 8 0.0%	0 / 6 0.0%	0 / 2 0.0%	0 / 12 0.0%	0 / 0 0.0%	0 / 0 0.0%	0 / 0 0.0%	0 / 0 0.0%	78 / 114 68.4%	36 / 114 31.6%E						
1	"Ruminobacter succinogenes" (Hungate 1950) Prevot 1966	0 / 114 0.0%	0 / 0 0.0%	0 / 6 0.0%	0 / 4 0.0%	0 / 6 0.0%	0 / 10 0.0%	0 / 6 0.0%	0 / 2 0.0%	0 / 2 0.0%	0 / 10 0.0%	0 / 2 0.0%	0 / 8 0.0%	0 / 8 0.0%	0 / 2 0.0%	0 / 4 0.0%	0 / 2 0.0%	0 / 6 0.0%	0 / 2 0.0%	0 / 6 0.0%	0 / 8 0.0%	0 / 6 0.0%	0 / 2 0.0%	0 / 12 0.0%	0 / 0 0.0%	0 / 0 0.0%	0 / 0 0.0%	0 / 0 0.0%	78 / 114 68.4%	36 / 114 31.6%E						
1	Fibribacter succinicigenes subsp. succinicigenes	0 / 114 0.0%	0 / 0 0.0%	0 / 6 0.0%	0 / 4 0.0%	0 / 6 0.0%	0 / 10 0.0%	0 / 6 0.0%	0 / 2 0.0%	0 / 2 0.0%	0 / 10 0.0%	0 / 2 0.0%	0 / 8 0.0%	0 / 8 0.0%	0 / 2 0.0%	0 / 4 0.0%	0 / 2 0.0%	0 / 6 0.0%	0 / 2 0.0%	0 / 6 0.0%	0 / 8 0.0%	0 / 6 0.0%	0 / 2 0.0%	0 / 12 0.0%	0 / 0 0.0%	0 / 0 0.0%	0 / 0 0.0%	0 / 0 0.0%	78 / 114 68.4%	36 / 114 31.6%E						
1	Fibrobacter succinogenes S85	0 / 114 0.0%	0 / 0 0.0%	0 / 6 0.0%	0 / 4 0.0%	0 / 6 0.0%	0 / 10 0.0%	0 / 6 0.0%	0 / 2 0.0%	0 / 2 0.0%	0 / 10 0.0%	0 / 2 0.0%	0 / 8 0.0%	0 / 8 0.0%	0 / 2 0.0%	0 / 4 0.0%	0 / 2 0.0%	0 / 6 0.0%	0 / 2 0.0%	0 / 6 0.0%	0 / 8 0.0%	0 / 6 0.0%	0 / 2 0.0%	0 / 12 0.0%	0 / 0 0.0%	0 / 0 0.0%	0 / 0 0.0%	0 / 0 0.0%	78 / 114 68.4%	36 / 114 31.6%E						
1	Gemmatimonadetes	0 / 47 0.0%	0 / 1 0.0%	0 / 1 0.0%	0 / 2 0.0%	0 / 1 0.0%	0 / 4 0.0%	0 / 3 0.0%	0 / 1 0.0%	0 / 1 0.0%	0 / 3 0.0%	0 / 1 0.0%	0 / 4 0.0%	0 / 2 0.0%	0 / 1 0.0%	0 / 2 0.0%	0 / 1 0.0%	0 / 3 0.0%	0 / 1 0.0%	0 / 3 0.0%	0 / 4 0.0%	0 / 2 0.0%	0 / 1 0.0%	0 / 5 0.0%	0 / 0 0.0%	0 / 0 0.0%	0 / 0 0.0%	0 / 0 0.0%	31 / 47 66.0%	15 / 47 31.9%E	1 / 47 2.1%CE					
1	Gemmatimonadetes	0 / 47 0.0%	0 / 1 0.0%	0 / 1 0.0%	0 / 2 0.0%	0 / 1 0.0%	0 / 4 0.0%	0 / 3 0.0%	0 / 1 0.0%	0 / 1 0.0%	0 / 3 0.0%	0 / 1 0.0%	0 / 4 0.0%	0 / 2 0.0%	0 / 1 0.0%	0 / 2 0.0%	0 / 1 0.0%	0 / 3 0.0%	0 / 1 0.0%	0 / 3 0.0%	0 / 4 0.0%	0 / 2 0.0%	0 / 1 0.0%	0 / 5 0.0%	0 / 0 0.0%	0 / 0 0.0%	0 / 0 0.0%	0 / 0 0.0%	31 / 47 66.0%	15 / 47 31.9%E	1 / 47 2.1%CE					
1	Gemmantimonadales	0 / 47 0.0%	0 / 1 0.0%	0 / 1 0.0%	0 / 2 0.0%	0 / 1 0.0%	0 / 4 0.0%	0 / 3 0.0%	0 / 1 0.0%	0 / 1 0.0%	0 / 3 0.0%	0 / 1 0.0%	0 / 4 0.0%	0 / 2 0.0%	0 / 1 0.0%	0 / 2 0.0%	0 / 1 0.0%	0 / 3 0.0%	0 / 1 0.0%	0 / 3 0.0%	0 / 4 0.0%	0 / 2 0.0%	0 / 1 0.0%	0 / 5 0.0%	0 / 0 0.0%	0 / 0 0.0%	0 / 0 0.0%	0 / 0 0.0%	31 / 47 66.0%	15 / 47 31.9%E	1 / 47 2.1%CE					
1	Gemmantimonadaceae	0 / 47 0.0%	0 / 1 0.0%	0 / 1 0.0%	0 / 2 0.0%	0 / 1 0.0%	0 / 4 0.0%	0 / 3 0.0%	0 / 1 0.0%	0 / 1 0.0%	0 / 3 0.0%	0 / 1 0.0%	0 / 4 0.0%	0 / 2 0.0%	0 / 1 0.0%	0 / 2 0.0%	0 / 1 0.0%	0 / 3 0.0%	0 / 1 0.0%	0 / 3 0.0%	0 / 4 0.0%	0 / 2 0.0%	0 / 1 0.0%	0 / 5 0.0%	0 / 0 0.0%	0 / 0 0.0%	0 / 0 0.0%	0 / 0 0.0%	31 / 47 66.0%	15 / 47 31.9%E	1 / 47 2.1%CE					
1	Gemmatimonas	0 / 47 0.0%	0 / 1 0.0%	0 / 1 0.0%	0 / 2 0.0%	0 / 1 0.0%	0 / 4 0.0%	0 / 3 0.0%	0 / 1 0.0%	0 / 1 0.0%	0 / 3 0.0%	0 / 1 0.0%	0 / 4 0.0%	0 / 2 0.0%	0 / 1 0.0%	0 / 2 0.0%	0 / 1 0.0%	0 / 3 0.0%	0 / 1 0.0%	0 / 3 0.0%	0 / 4 0.0%	0 / 2 0.0%	0 / 1 0.0%	0 / 5 0.0%	0 / 0 0.0%	0 / 0 0.0%	0 / 0 0.0%	0 / 0 0.0%	31 / 47 66.0%	15 / 47 31.9%E	1 / 47 2.1%CE					
1	DSM 14586	0 / 47 0.0%	0 / 1 0.0%	0 / 1 0.0%	0 / 2 0.0%	0 / 1 0.0%	0 / 4 0.0%	0 / 3 0.0%	0 / 1 0.0%	0 / 1 0.0%	0 / 3 0.0%	0 / 1 0.0%	0 / 4 0.0%	0 / 2 0.0%	0 / 1 0.0%	0 / 2 0.0%	0 / 1 0.0%	0 / 3 0.0%	0 / 1 0.0%	0 / 3 0.0%	0 / 4 0.0%	0 / 2 0.0%	0 / 1 0.0%	0 / 5 0.0%	0 / 0 0.0%	0 / 0 0.0%	0 / 0 0.0%	0 / 0 0.0%	31 / 47 66.0%	15 / 47 31.9%E	1 / 47 2.1%CE					
1	Gemmatimonas aurantiaca DSM 14586	0 / 47 0.0%	0 / 1 0.0%	0 / 1 0.0%	0 / 2 0.0%	0 / 1 0.0%	0 / 4 0.0%	0 / 3 0.0%	0 / 1 0.0%	0 / 1 0.0%	0 / 3 0.0%	0 / 1 0.0%	0 / 4 0.0%	0 / 2 0.0%	0 / 1 0.0%	0 / 2 0.0%	0 / 1 0.0%	0 / 3 0.0%	0 / 1 0.0%	0 / 3 0.0%	0 / 4 0.0%	0 / 2 0.0%	0 / 1 0.0%	0 / 5 0.0%	0 / 0 0.0%	0 / 0 0.0%	0 / 0 0.0%	0 / 0 0.0%	31 / 47 66.0%	15 / 47 31.9%E	1 / 47 2.1%CE					
11	Aquificae	189 / 519 36.4%	6 / 12 50.0%	10 / 12 83.3%	6 / 12 50.0%	3 / 21 14.3%	18 / 35 51.4%	10 / 30 33.3%	4 / 12 33.3%	8 / 12 66.7%	9 / 38 23.7%	3 / 13 23.1%	23 / 45 51.1%	8 / 24 33.3%	4 / 12 33.3%	4 / 12 33.3%	4 / 12 33.3%	6 / 32 18.8%	3 / 12 25.0%	7 / 33 21.2%	13 / 47 27.7%	8 / 17 47.1%	4 / 12 33.3%	23 / 58 39.7%	5 / 6 83.3%	0 / 0 0.0%	0 / 0 0.0%	0 / 0 0.0%	187 / 519 36.0%	138 / 519 26.6%E	110 / 519 21.2%AB	73 / 519 14.1%ABE	5 / 519 1.0%BE	3 / 519 0.6%C	2 / 519 0.4%CE	1 / 519 0.2%B
11	Aquificae	189 / 519 36.4%	6 / 12 50.0%	10 / 12 83.3%	6 / 12 50.0%	3 / 21 14.3%	18 / 35 51.4%	10 / 30 33.3%	4 / 12 33.3%	8 / 12 66.7%	9 / 38 23.7%	3 / 13 23.1%	23 / 45 51.1%	8 / 24 33.3%	4 / 12 33.3%	4 / 12 33.3%	4 / 12 33.3%	6 / 32 18.8%	3 / 12 25.0%	7 / 33 21.2%	13 / 47 27.7%	8 / 17 47.1%	4 / 12 33.3%	23 / 58 39.7%	5 / 6 83.3%	0 / 0 0.0%	0 / 0 0.0%	0 / 0 0.0%	187 / 519 36.0%	138 / 519 26.6%E	110 / 519 21.2%AB	73 / 519 14.1%ABE	5 / 519 1.0%BE	3 / 519 0.6%C	2 / 519 0.4%CE	1 / 519 0.2%B
9	Aquifecales	100 / 430 23.3%	4 / 10 40.0%	8 / 10 80.0%	4 / 10 40.0%	0 / 18 0.0%	10 / 27 37.0%	6 / 26 23.1%	2 / 10 20.0%	6 / 10 60.0%	4 / 33 12.1%	1 / 11 9.1%	15 / 37 40.5%	4 / 20 20.0%	2 / 10 20.0%	2 / 10 20.0%	2 / 10 20.0%	1 / 27 3.7%	1 / 10 10.0%	1 / 27 3.7%	5 / 39 12.8%	4 / 13 30.8%	2 / 10 20.0%	13 / 48 27.1%	3 / 4 75.0%	0 / 0 0.0%	0 / 0 0.0%	0 / 0 0.0%	187 / 430 43.5%	138 / 430 32.1%E	55 / 430 12.8%AB	39 / 430 9.1%ABE	5 / 430 1.2%BE	3 / 430 0.7%C	2 / 430 0.5%CE	1 / 430 0.2%B
6	Aquificaceae	81 / 311 26.0%	1 / 7 14.3%	7 / 7 100.0%	3 / 7 42.9%	0 / 11 0.0%	8 / 21 38.1%	6 / 20 30.0%	2 / 7 28.6%	5 / 7 71.4%	4 / 24 16.7%	1 / 7 14.3%	13 / 28 46.4%	3 / 14 21.4%	1 / 7 14.3%	1 / 7 14.3%	2 / 7 28.6%	1 / 21 4.8%	1 / 7 14.3%	1 / 21 4.8%	4 / 28 14.3%	4 / 7 57.1%	1 / 7 14.3%	9 / 35 25.7%	3 / 4 75.0%	0 / 0 0.0%	0 / 0 0.0%	0 / 0 0.0%	132 / 311 42.4%	96 / 311 30.9%E	46 / 311 14.8%AB	31 / 311 10.0%ABE	3 / 311 1.0%BE	2 / 311 0.6%C	1 / 311 0.3%B	
1	Caldariibacterium	18 / 88 20.5%	0 / 2 0.0%	2 / 2 100.0%	2 / 2 100.0%	0 / 2 0.0%	2 / 6 33.3%	4 / 6 66.7%	0 / 2 0.0%	2 / 2 100.0%	2 / 6 33.3%	0 / 2 0.0%	0 / 8 0.0%	0 / 4 0.0%	0 / 2 0.0%	0 / 2 0.0%	0 / 2 0.0%	0 / 6 0.0%	0 / 2 0.0%	0 / 6 0.0%	0 / 8 0.0%	0 / 2 0.0%	0 / 2 0.0%	2 / 10 20.0%	2 / 2 100.0%	0 / 0 0.0%	0 / 0 0.0%	0 / 0 0.0%	34 / 88 38.6%E	34 / 88 38.6%	12 / 88 13.6%AB	6 / 88 6.8%ABE	2 / 88 2.3%C			
1	DSM 6534	18 / 88 20.5%	0 / 2 0.0%	2 / 2 100.0%	2 / 2 100.0%	0 / 2 0.0%	2 / 6 33.3%	4 / 6 66.7%	0 / 2 0.0%	2 / 2 100.0%	2 / 6 33.3%	0 / 2 0.0%	0 / 8 0.0%	0 / 4 0.0%	0 / 2 0.0%	0 / 2 0.0%	0 / 2 0.0%	0 / 6 0.0%	0 / 2 0.0%	0 / 6 0.0%	0 / 8 0.0%	0 / 2 0.0%	0 / 2 0.0%	2 / 10 20.0%	2 / 2 100.0%	0 / 0 0.0%	0 / 0 0.0%	0 / 0 0.0%	34 / 88 38.6%	34 / 88 38.6%E	12 / 88 13.6%AB	6 / 88 6.8%ABE	2 / 88 2.3%C			
1	Hydrogenobacter thermophilus DSM 6534	18 / 88 20.5%	0 / 2 0.0%	2 / 2 100.0%	2 / 2 100.0%	0 / 2 0.0%	2 / 6 33.3%	4 / 6 66.7%	0 / 2 0.0%	2 / 2 100.0%	2 / 6 33.3%	0 / 2 0.0%	0 / 8 0.0%	0 / 4 0.0%	0 / 2 0.0%	0 / 2 0.0%	0 / 2 0.0%	0 / 6 0.0%	0 / 2 0.0%	0 / 6 0.0%	0 / 8 0.0%	0 / 2 0.0%	0 / 2 0.0%	2 / 10 20.0%	2 / 2 100.0%	0 / 0 0.0%	0 / 0 0.0%	0 / 0 0.0%	34 / 88 38.6%	34 / 88 38.6%E	12 / 88 13.6%AB	6 / 88 6.8%ABE	2 / 88 2.3%C			
1	Aquifex	33 / 44 75.0%	1 / 1 100.0%	1 / 1 100.0%	1 / 1 100.0%	0 / 2 0.0%	3 / 3 100.0%	2 / 2 100.0%	1 / 1 100.0%	0 / 1 0.0%	1 / 3 33.3%	1 / 1 100.0%	3 / 4 75.0%	2 / 2 100.0%	1 / 1 100.0%	1 / 1 100.0%	1 / 1 100.0%	1 / 3 33.3%	1 / 1 100.0%	1 / 3 33.3%	3 / 4 75.0%	1 / 1 100.0%	1 / 1 100.0%	5 / 5 100.0%	1 / 1 100.0%	0 / 0 0.0%	0 / 0 0.0%	0 / 0 0.0%	21 / 44 47.7%AB	11 / 44 25.0%ABE	9 / 44 20.5%	2 / 44 4.5%E	1 / 44 2.3%BE			
1	"Aquifex aeolicus" Huber and Stetter 2001	33 / 44 75.0%	1 / 1 100.0%	1 / 1 100.0%	1 / 1 100.0%	0 / 2 0.0%	3 / 3 100.0%	2 / 2 100.0%	1 / 1 100.0%	0 / 1 0.0%	1 / 3 33.3%	1 / 1 100.0%	3 / 4 75.0%	2 / 2 100.0%	1 / 1 100.0%	1 / 1 100.0%	1 / 1 100.0%	1 / 3 33.3%	1 / 1 100.0%	1 / 3 33.3%	3 / 4 75.0%	1 / 1 100.0%	1 / 1 100.0%	5 / 5 100.0%	1 / 1 100.0%	0 / 0 0.0%	0 / 0 0.0%	0 / 0 0.0%	21 / 44 47.7%AB	11 / 44 25.0%ABE	9 / 44 20.5%	2 / 44 4.5%E	1 / 44 2.3%BE			
1	Aquifex aeolicus VF5	33 / 44 75.0%	1 / 1 100.0%	1 / 1 100.0%	1 / 1 100.0%	0 / 2 0.0%	3 / 3 100.0%	2 / 2 100.0%	1 / 1 100.0%	0 / 1 0.0%	1 / 3 33.3%	1 / 1 100.0%	3 / 4 75.0%	2 / 2 100.0%	1 / 1 100.0%	1 / 1 100.0%	1 / 1 100.0%	1 / 3 33.3%	1 / 1 100.0%	1 / 3 33.3%	3 / 4 75.0%	1 / 1 100.0%	1 / 1 100.0%	5 / 5 100.0%	1 / 1 100.0%	0 / 0 0.0%	0 / 0 0.0%	0 / 0 0.0%	21 / 44 47.7%AB	11 / 44 25.0%ABE	9 / 44 20.5%	2 / 44 4.5%E	1 / 44 2.3%BE			
1	Thermocrinis	9 / 44 20.5%	0 / 1 0.0%	1 / 1 100.0%	0 / 1 0.0%	0 / 1 0.0%	0 / 3 0.0%	0 / 3 0.0%	1 / 1 100.0%	0 / 1 0.0%	1 / 3 33.3%	0 / 1 0.0%	1 / 4 25.0%	1 / 2 50.0%	0 / 1 0.0%	0 / 1 0.0%	1 / 1 100.0%	0 / 3 0.0%	0 / 1 0.0%	0 / 3 0.0%	1 / 4 25.0%	0 / 1 0.0%	0 / 1 0.0%	2 / 5 40.0%	0 / 1 0.0%	0 / 0 0.0%	0 / 0 0.0%	0 / 0 0.0%	20 / 44 45.5%	15 / 44 34.1%E	5 / 44 11.4%AB	4 / 44 9.1%ABE				
1	DSM 14484	9 / 44 20.5%	0 / 1 0.0%	1 / 1 100.0%	0 / 1 0.0%	0 / 1 0.0%	0 / 3 0.0%	0 / 3 0.0%	1 / 1 100.0%	0 / 1 0.0%	1 / 3 33.3%	0 / 1 0.0%	1 / 4 25.0%	1 / 2 50.0%	0 / 1 0.0%	0 / 1 0.0%	1 / 1 100.0%	0 / 3 0.0%	0 / 1 0.0%	0 / 3 0.0%	1 / 4 25.0%	0 / 1 0.0%	0 / 1 0.0%	2 / 5 40.0%	0 / 1 0.0%	0 / 0 0.0%	0 / 0 0.0%	0 / 0 0.0%	20 / 44 45.5%	15 / 44 34.1%E	5 / 44 11.4%AB	4 / 44 9.1%ABE				
1	Thermocrinis albus DSM 14484	9 / 44 20.5%	0 / 1 0.0%	1 / 1 100.0%	0 / 1 0.0%	0 / 1 0.0%	0 / 3 0.0%	0 / 3 0.0%	1 / 1 100.0%	0 / 1 0.0%	1 / 3 33.3%	0 / 1 0.0%	1 / 4 25.0%	1 / 2 50.0%	0 / 1 0.0%	0 / 1 0.0%	1 / 1 100.0%	0 / 3 0.0%	0 / 1 0.0%	0 / 3 0.0%	1 / 4 25.0%	0 / 1 0.0%	0 / 1 0.0%	2 / 5 40.0%	0 / 1 0.0%	0 / 0 0.0%	0 / 0 0.0%	0 / 0 0.0%	20 / 44 45.5%	15 / 44 34.1%E	5 / 44 11.4%AB	4 / 44 9.1%ABE				
3	Hydrogenobaculum	21 / 135 15.6%	0 / 3 0.0%	3 / 3 100.0%	0 / 3 0.0%	0 / 6 0.0%	3 / 9 33.3%	0 / 9 0.0%	0 / 3 0.0%	3 / 3 100.0%	0 / 12 0.0%	0 / 3 0.0%	9 / 12 75.0%	0 / 6 0.0%	0 / 3 0.0%	0 / 3 0.0%	0 / 3 0.0%	0 / 9 0.0%	0 / 3 0.0%	0 / 9 0.0%	0 / 12 0.0%	3 / 3 100.0%	0 / 3 0.0%	0 / 15 0.0%	0 / 0 0.0%	0 / 0 0.0%	0 / 0 0.0%	0 / 0 0.0%	69 / 135 51.1%	45 / 135 33.3%E	10 / 135 7.4%ABE	8 / 135 5.9%AB	2 / 135 1.5%BE	1 / 135 0.7%B		
1	Hydrogenobaculum sp. Y04AAS1	7 / 45 15.6%	0 / 1 0.0%	1 / 1 100.0%	0 / 1 0.0%	0 / 2 0.0%	1 / 3 33.3%	0 / 3 0.0%	0 / 1 0.0%	1 / 1 100.0%	0 / 4 0.0%	0 / 1 0.0%	3 / 4 75.0%	0 / 2 0.0%	0 / 1 0.0%	0 / 1 0.0%	0 / 1 0.0%	0 / 3 0.0%	0 / 1 0.0%	0 / 3 0.0%	0 / 4 0.0%	1 / 1 100.0%	0 / 1 0.0%	0 / 5 0.0%	0 / 0 0.0%	0 / 0 0.0%	0 / 0 0.0%	0 / 0 0.0%	23 / 45 51.1%	15 / 45 33.3%E	2 / 45 4.4%AB	2 / 45 4.4%BE	2 / 45 4.4%ABE	1 / 45 2.2%B		
1	Hydrogenobaculum sp. HO	7 / 45 15.6%	0 / 1 0.0%	1 / 1 100.0%	0 / 1 0.0%	0 / 2 0.0%	1 / 3 33.3%	0 / 3 0.0%	0 / 1 0.0%	1 / 1 100.0%	0 / 4 0.0%	0 / 1 0.0%	3 / 4 75.0%	0 / 2 0.0%	0 / 1 0.0%	0 / 1 0.0%	0 / 1 0.0%	0 / 3 0.0%	0 / 1 0.0%	0 / 3 0.0%	0 / 4 0.0%	1 / 1 100.0%	0 / 1 0.0%	0 / 5 0.0%	0 / 0 0.0%	0 / 0 0.0%	0 / 0 0.0%	0 / 0 0.0%	23 / 45 51.1%	15 / 45 33.3%E	4 / 45 8.9%ABE	3 / 45 6.7%AB				
1	Hydrogenobaculum sp. SN	7 / 45 15.6%	0 / 1 0.0%	1 / 1 100.0%	0 / 1 0.0%	0 / 2 0.0%	1 / 3 33.3%	0 / 3 0.0%	0 / 1 0.0%	1 / 1 100.0%	0 / 4 0.0%	0 / 1 0.0%	3 / 4 75.0%	0 / 2 0.0%	0 / 1 0.0%	0 / 1 0.0%	0 / 1 0.0%	0 / 3 0.0%	0 / 1 0.0%	0 / 3 0.0%	0 / 4 0.0%	1 / 1 100.0%	0 / 1 0.0%	0 / 5 0.0%	0 / 0 0.0%	0 / 0 0.0%	0 / 0 0.0%	0 / 0 0.0%	23 / 45 51.1%	15 / 45 33.3%E	4 / 45 8.9%ABE	3 / 45 6.7%AB				
3	Hydrogenothermaceae	19 / 119 16.0%	3 / 3 100.0%	1 / 3 33.3%	1 / 3 33.3%	0 / 7 0.0%	2 / 6 33.3%	0 / 6 0.0%	0 / 3 0.0%	1 / 3 33.3%	0 / 9 0.0%	0 / 4 0.0%	2 / 9 22.2%	1 / 6 16.7%	1 / 3 33.3%	1 / 3 33.3%	0 / 3 0.0%	0 / 6 0.0%	0 / 3 0.0%	0 / 6 0.0%	1 / 11 9.1%	0 / 6 0.0%	1 / 3 33.3%	4 / 13 30.8%	0 / 0 0.0%	0 / 0 0.0%	0 / 0 0.0%	0 / 0 0.0%	55 / 119 46.2%	42 / 119 35.3%E	9 / 119 7.6%AB	8 / 119 6.7%ABE	2 / 119 1.7%BE	2 / 119 1.7%CE	1 / 119 0.8%C	
1	Persephonella	15 / 40 37.5%	1 / 1 100.0%	1 / 1 100.0%	1 / 1 100.0%	0 / 2 0.0%	2 / 2 100.0%	0 / 2 0.0%	0 / 1 0.0%	1 / 1 100.0%	0 / 3 0.0%	0 / 2 0.0%	2 / 3 66.7%	1 / 2 50.0%	1 / 1 100.0%	1 / 1 100.0%	0 / 1 0.0%	0 / 2 0.0%	0 / 1 0.0%	0 / 2 0.0%	1 / 3 33.3%	0 / 2 0.0%	1 / 1 100.0%	2 / 5 40.0%	0 / 0 0.0%	0 / 0 0.0%	0 / 0 0.0%	0 / 0 0.0%	14 / 40 35.0%	11 / 40 27.5%E	9 / 40 22.5%AB	6 / 40 15.0%ABE				
1	DSM 14350	15 / 40 37.5%	1 / 1 100.0%	1 / 1 100.0%	1 / 1 100.0%	0 / 2 0.0%	2 / 2 100.0%	0 / 2 0.0%	0 / 1 0.0%	1 / 1 100.0%	0 / 3 0.0%	0 / 2 0.0%	2 / 3 66.7%	1 / 2 50.0%	1 / 1 100.0%	1 / 1 100.0%	0 / 1 0.0%	0 / 2 0.0%	0 / 1 0.0%	0 / 2 0.0%	1 / 3 33.3%	0 / 2 0.0%	1 / 1 100.0%	2 / 5 40.0%	0 / 0 0.0%	0 / 0 0.0%	0 / 0 0.0%	0 / 0 0.0%	14 / 40 35.0%	11 / 40 27.5%E	9 / 40 22.5%AB	6 / 40 15.0%ABE				

1	Persephonella marina EX-H1	15 / 40 37.5%	1 / 1 100.0%	1 / 1 100.0%	1 / 1 100.0%	0 / 2 0.0%	2 / 2 100.0%	0 / 2 0.0%	0 / 1 0.0%	1 / 1 100.0%	0 / 3 0.0%	0 / 2 0.0%	2 / 3 66.7%	1 / 2 50.0%	1 / 1 100.0%	1 / 1 100.0%	0 / 1 0.0%	0 / 2 0.0%	0 / 1 0.0%	0 / 2 0.0%	1 / 3 33.3%	0 / 2 0.0%	1 / 1 100.0%	2 / 5 40.0%	0 / 0 0.0%	0 / 0 0.0%	0 / 0 0.0%	0 / 0 0.0%	14 / 40 35.0%	11 / 40 27.5%E	9 / 40 22.5%AB	6 / 40 15.0%ABE				
2	Sulfurihydrogenibium	4 / 79 5.1%	2 / 2 100.0%	0 / 2 0.0%	0 / 2 0.0%	0 / 5 0.0%	0 / 4 0.0%	0 / 4 0.0%	0 / 2 0.0%	0 / 2 0.0%	0 / 6 0.0%	0 / 2 0.0%	0 / 6 0.0%	0 / 4 0.0%	0 / 2 0.0%	0 / 2 0.0%	0 / 2 0.0%	0 / 4 0.0%	0 / 2 0.0%	0 / 2 0.0%	0 / 8 0.0%	0 / 4 0.0%	0 / 2 0.0%	2 / 8 25.0%	0 / 0 0.0%	0 / 0 0.0%	0 / 0 0.0%	0 / 0 0.0%	41 / 79 51.9%	31 / 79 39.2%E	2 / 79 2.5%ABE	2 / 79 2.5%CE	2 / 79 2.5%BE	1 / 79 1.3%C		
1	DSM 15241	2 / 39 5.1%	1 / 1 100.0%	0 / 1 0.0%	0 / 1 0.0%	0 / 2 0.0%	0 / 2 0.0%	0 / 2 0.0%	0 / 1 0.0%	0 / 1 0.0%	0 / 3 0.0%	0 / 1 0.0%	0 / 3 0.0%	0 / 2 0.0%	0 / 1 0.0%	0 / 1 0.0%	0 / 1 0.0%	0 / 2 0.0%	0 / 1 0.0%	0 / 2 0.0%	0 / 4 0.0%	0 / 2 0.0%	0 / 1 0.0%	1 / 4 25.0%	0 / 0 0.0%	0 / 0 0.0%	0 / 0 0.0%	0 / 0 0.0%	22 / 39 56.4%	14 / 39 35.9%E	2 / 39 5.1%ABE	1 / 39 2.6%CE				
1	Aquificaceae bacterium Az-Fu1	2 / 39 5.1%	1 / 1 100.0%	0 / 1 0.0%	0 / 1 0.0%	0 / 2 0.0%	0 / 2 0.0%	0 / 2 0.0%	0 / 1 0.0%	0 / 1 0.0%	0 / 3 0.0%	0 / 1 0.0%	0 / 3 0.0%	0 / 2 0.0%	0 / 1 0.0%	0 / 1 0.0%	0 / 1 0.0%	0 / 2 0.0%	0 / 1 0.0%	0 / 2 0.0%	0 / 4 0.0%	0 / 2 0.0%	0 / 1 0.0%	1 / 4 25.0%	0 / 0 0.0%	0 / 0 0.0%	0 / 0 0.0%	0 / 0 0.0%	22 / 39 56.4%	14 / 39 35.9%E	2 / 39 5.1%ABE	1 / 39 2.6%CE				
1	Sulfurihydrogenibium sp. YO3AOP1	2 / 40 5.0%	1 / 1 100.0%	0 / 1 0.0%	0 / 1 0.0%	0 / 3 0.0%	0 / 2 0.0%	0 / 2 0.0%	0 / 1 0.0%	0 / 1 0.0%	0 / 3 0.0%	0 / 1 0.0%	0 / 3 0.0%	0 / 2 0.0%	0 / 1 0.0%	0 / 1 0.0%	0 / 1 0.0%	0 / 2 0.0%	0 / 1 0.0%	0 / 2 0.0%	0 / 4 0.0%	0 / 2 0.0%	0 / 1 0.0%	1 / 4 25.0%	0 / 0 0.0%	0 / 0 0.0%	0 / 0 0.0%	0 / 0 0.0%	19 / 40 47.5%	17 / 40 42.5%E	2 / 40 5.0%BE	1 / 40 2.5%C	1 / 40 2.5%CE			
2	Desulfurobacteriales	89 / 89 100.0%	2 / 2 100.0%	2 / 2 100.0%	2 / 2 100.0%	3 / 3 100.0%	8 / 8 100.0%	4 / 4 100.0%	2 / 2 100.0%	2 / 2 100.0%	5 / 5 100.0%	2 / 2 100.0%	8 / 8 100.0%	4 / 4 100.0%	2 / 2 100.0%	2 / 2 100.0%	2 / 2 100.0%	5 / 5 100.0%	2 / 2 100.0%	6 / 6 100.0%	8 / 8 100.0%	4 / 4 100.0%	2 / 2 100.0%	10 / 10 100.0%	2 / 2 100.0%	0 / 0 0.0%	0 / 0 0.0%	0 / 0 0.0%	55 / 89 61.8%AB	34 / 89 38.2%ABE						
2	Desulfurobacteriaceae	89 / 89 100.0%	2 / 2 100.0%	2 / 2 100.0%	2 / 2 100.0%	3 / 3 100.0%	8 / 8 100.0%	4 / 4 100.0%	2 / 2 100.0%	2 / 2 100.0%	5 / 5 100.0%	2 / 2 100.0%	8 / 8 100.0%	4 / 4 100.0%	2 / 2 100.0%	2 / 2 100.0%	2 / 2 100.0%	5 / 5 100.0%	2 / 2 100.0%	6 / 6 100.0%	8 / 8 100.0%	4 / 4 100.0%	2 / 2 100.0%	10 / 10 100.0%	2 / 2 100.0%	0 / 0 0.0%	0 / 0 0.0%	0 / 0 0.0%	55 / 89 61.8%AB	34 / 89 38.2%ABE						
1	Desulfurobacterium	43 / 43 100.0%	1 / 1 100.0%	1 / 1 100.0%	1 / 1 100.0%	1 / 1 100.0%	4 / 4 100.0%	2 / 2 100.0%	1 / 1 100.0%	1 / 1 100.0%	2 / 2 100.0%	1 / 1 100.0%	4 / 4 100.0%	2 / 2 100.0%	1 / 1 100.0%	1 / 1 100.0%	1 / 1 100.0%	2 / 2 100.0%	1 / 1 100.0%	3 / 3 100.0%	4 / 4 100.0%	2 / 2 100.0%	1 / 1 100.0%	5 / 5 100.0%	1 / 1 100.0%	0 / 0 0.0%	0 / 0 0.0%	0 / 0 0.0%	26 / 43 60.5%AB	17 / 43 39.5%ABE						
1	DSM 11699	43 / 43 100.0%	1 / 1 100.0%	1 / 1 100.0%	1 / 1 100.0%	1 / 1 100.0%	4 / 4 100.0%	2 / 2 100.0%	1 / 1 100.0%	1 / 1 100.0%	2 / 2 100.0%	1 / 1 100.0%	4 / 4 100.0%	2 / 2 100.0%	1 / 1 100.0%	1 / 1 100.0%	1 / 1 100.0%	2 / 2 100.0%	1 / 1 100.0%	3 / 3 100.0%	4 / 4 100.0%	2 / 2 100.0%	1 / 1 100.0%	5 / 5 100.0%	1 / 1 100.0%	0 / 0 0.0%	0 / 0 0.0%	0 / 0 0.0%	26 / 43 60.5%AB	17 / 43 39.5%ABE						
1	Desulfurobacterium thermolithotrophum BSA	43 / 43 100.0%	1 / 1 100.0%	1 / 1 100.0%	1 / 1 100.0%	1 / 1 100.0%	4 / 4 100.0%	2 / 2 100.0%	1 / 1 100.0%	1 / 1 100.0%	2 / 2 100.0%	1 / 1 100.0%	4 / 4 100.0%	2 / 2 100.0%	1 / 1 100.0%	1 / 1 100.0%	1 / 1 100.0%	2 / 2 100.0%	1 / 1 100.0%	3 / 3 100.0%	4 / 4 100.0%	2 / 2 100.0%	1 / 1 100.0%	5 / 5 100.0%	1 / 1 100.0%	0 / 0 0.0%	0 / 0 0.0%	0 / 0 0.0%	26 / 43 60.5%AB	17 / 43 39.5%ABE						
1	Thermovibrio	46 / 46 100.0%	1 / 1 100.0%	1 / 1 100.0%	1 / 1 100.0%	2 / 2 100.0%	4 / 4 100.0%	2 / 2 100.0%	1 / 1 100.0%	1 / 1 100.0%	3 / 3 100.0%	1 / 1 100.0%	4 / 4 100.0%	2 / 2 100.0%	1 / 1 100.0%	1 / 1 100.0%	1 / 1 100.0%	3 / 3 100.0%	1 / 1 100.0%	3 / 3 100.0%	4 / 4 100.0%	2 / 2 100.0%	1 / 1 100.0%	5 / 5 100.0%	1 / 1 100.0%	0 / 0 0.0%	0 / 0 0.0%	0 / 0 0.0%	29 / 46 63.0%AB	17 / 46 37.0%ABE						
1	DSM 15698	46 / 46 100.0%	1 / 1 100.0%	1 / 1 100.0%	1 / 1 100.0%	2 / 2 100.0%	4 / 4 100.0%	2 / 2 100.0%	1 / 1 100.0%	1 / 1 100.0%	3 / 3 100.0%	1 / 1 100.0%	4 / 4 100.0%	2 / 2 100.0%	1 / 1 100.0%	1 / 1 100.0%	1 / 1 100.0%	3 / 3 100.0%	1 / 1 100.0%	3 / 3 100.0%	4 / 4 100.0%	2 / 2 100.0%	1 / 1 100.0%	5 / 5 100.0%	1 / 1 100.0%	0 / 0 0.0%	0 / 0 0.0%	0 / 0 0.0%	29 / 46 63.0%AB	17 / 46 37.0%ABE						
1	Thermovibrio ammonificans DSM 15698	46 / 46 100.0%	1 / 1 100.0%	1 / 1 100.0%	1 / 1 100.0%	2 / 2 100.0%	4 / 4 100.0%	2 / 2 100.0%	1 / 1 100.0%	1 / 1 100.0%	3 / 3 100.0%	1 / 1 100.0%	4 / 4 100.0%	2 / 2 100.0%	1 / 1 100.0%	1 / 1 100.0%	1 / 1 100.0%	3 / 3 100.0%	1 / 1 100.0%	3 / 3 100.0%	4 / 4 100.0%	2 / 2 100.0%	1 / 1 100.0%	5 / 5 100.0%	1 / 1 100.0%	0 / 0 0.0%	0 / 0 0.0%	0 / 0 0.0%	29 / 46 63.0%AB	17 / 46 37.0%ABE						
18	Chloroflexi	663 / 853 77.7%	20 / 23 87.0%	19 / 21 90.5%	17 / 20 85.0%	14 / 19 73.7%	48 / 58 82.8%	45 / 54 83.3%	18 / 18 100.0%	6 / 19 31.6%	39 / 54 72.2%	14 / 18 77.8%	55 / 71 77.5%	20 / 34 58.8%	14 / 18 77.8%	30 / 35 85.7%	16 / 18 88.9%	46 / 54 85.2%	15 / 17 88.2%	38 / 54 70.4%	66 / 91 72.5%	31 / 39 79.5%	14 / 20 70.0%	75 / 90 83.3%	3 / 8 37.5%	0 / 0 0.0%	0 / 0 0.0%	0 / 0 0.0%	349 / 853 40.9%AB	255 / 853 29.9%ABE	64 / 853 7.5%	62 / 853 7.3%E	42 / 853 4.9%B	37 / 853 4.3%C	27 / 853 3.2%CE	17 / 853 2.0%BE
6	"Chloroflexi" Castenholz 2001	287 / 292 98.3%	9 / 10 90.0%	7 / 7 100.0%	8 / 8 100.0%	6 / 6 100.0%	22 / 22 100.0%	18 / 18 100.0%	6 / 6 100.0%	6 / 6 100.0%	18 / 18 100.0%	6 / 6 100.0%	22 / 23 95.7%	12 / 12 100.0%	6 / 6 100.0%	11 / 11 100.0%	6 / 6 100.0%	18 / 18 100.0%	6 / 6 100.0%	18 / 18 100.0%	31 / 31 100.0%	14 / 14 100.0%	6 / 6 100.0%	30 / 30 100.0%	1 / 4 25.0%	0 / 0 0.0%	0 / 0 0.0%	0 / 0 0.0%	130 / 292 44.5%AB	98 / 292 33.6%ABE	42 / 292 14.4%B	17 / 292 5.8%BE	4 / 292 1.4%E	1 / 292 0.3%CE		
5	Chloroflexaceae group	236 / 241 97.9%	8 / 9 88.9%	5 / 5 100.0%	5 / 5 100.0%	5 / 5 100.0%	18 / 18 100.0%	15 / 15 100.0%	5 / 5 100.0%	5 / 5 100.0%	15 / 15 100.0%	5 / 5 100.0%	18 / 19 94.7%	10 / 10 100.0%	5 / 5 100.0%	10 / 10 100.0%	5 / 5 100.0%	15 / 15 100.0%	5 / 5 100.0%	15 / 15 100.0%	25 / 25 100.0%	12 / 12 100.0%	5 / 5 100.0%	25 / 25 100.0%	0 / 3 0.0%	0 / 0 0.0%	0 / 0 0.0%	0 / 0 0.0%	113 / 241 46.9%AB	82 / 241 34.0%ABE	28 / 241 11.6%B	13 / 241 5.4%BE	4 / 241 1.7%E	1 / 241 0.4%CE		
3	Chloroflexineae	141 / 145 97.2%	4 / 5 80.0%	3 / 3 100.0%	3 / 3 100.0%	3 / 3 100.0%	12 / 12 100.0%	9 / 9 100.0%	3 / 3 100.0%	3 / 3 100.0%	9 / 9 100.0%	3 / 3 100.0%	11 / 11 100.0%	6 / 6 100.0%	3 / 3 100.0%	6 / 6 100.0%	3 / 3 100.0%	9 / 9 100.0%	3 / 3 100.0%	9 / 9 100.0%	15 / 15 100.0%	6 / 6 100.0%	3 / 3 100.0%	15 / 15 100.0%	0 / 3 0.0%	0 / 0 0.0%	0 / 0 0.0%	0 / 0 0.0%	72 / 145 49.7%AB	50 / 145 34.5%ABE	15 / 145 10.3%B	4 / 145 2.8%E	4 / 145 2.8%BE			
3	"Chloroflexaceae" Pierson 2001	141 / 145 97.2%	4 / 5 80.0%	3 / 3 100.0%	3 / 3 100.0%	3 / 3 100.0%	12 / 12 100.0%	9 / 9 100.0%	3 / 3 100.0%	3 / 3 100.0%	9 / 9 100.0%	3 / 3 100.0%	11 / 11 100.0%	6 / 6 100.0%	3 / 3 100.0%	6 / 6 100.0%	3 / 3 100.0%	9 / 9 100.0%	3 / 3 100.0%	9 / 9 100.0%	15 / 15 100.0%	6 / 6 100.0%	3 / 3 100.0%	15 / 15 100.0%	0 / 3 0.0%	0 / 0 0.0%	0 / 0 0.0%	0 / 0 0.0%	72 / 145 49.7%AB	50 / 145 34.5%ABE	15 / 145 10.3%B	4 / 145 2.8%E	4 / 145 2.8%BE			
3	Chloroflexus	141 / 145 97.2%	4 / 5 80.0%	3 / 3 100.0%	3 / 3 100.0%	3 / 3 100.0%	12 / 12 100.0%	9 / 9 100.0%	3 / 3 100.0%	3 / 3 100.0%	9 / 9 100.0%	3 / 3 100.0%	11 / 11 100.0%	6 / 6 100.0%	3 / 3 100.0%	6 / 6 100.0%	3 / 3 100.0%	9 / 9 100.0%	3 / 3 100.0%	9 / 9 100.0%	15 / 15 100.0%	6 / 6 100.0%	3 / 3 100.0%	15 / 15 100.0%	0 / 3 0.0%	0 / 0 0.0%	0 / 0 0.0%	0 / 0 0.0%	72 / 145 49.7%AB	50 / 145 34.5%ABE	15 / 145 10.3%B	4 / 145 2.8%E	4 / 145 2.8%BE			
1	ATCC 29366	46 / 48 95.8%	1 / 2 50.0%	1 / 1 100.0%	1 / 1 100.0%	1 / 1 100.0%	4 / 4 100.0%	3 / 3 100.0%	1 / 1 100.0%	1 / 1 100.0%	3 / 3 100.0%	1 / 1 100.0%	3 / 3 100.0%	2 / 2 100.0%	1 / 1 100.0%	2 / 2 100.0%	1 / 1 100.0%	3 / 3 100.0%	1 / 1 100.0%	3 / 3 100.0%	5 / 5 100.0%	2 / 2 100.0%	1 / 1 100.0%	5 / 5 100.0%	0 / 1 0.0%	0 / 0 0.0%	0 / 0 0.0%	0 / 0 0.0%	15 / 48 31.2%B	14 / 48 29.2%AB	13 / 48 27.1%ABE	4 / 48 8.3%BE	2 / 48 4.2%E			
1	Chloroflexus aurantiacus ATCC 29366	46 / 48 95.8%	1 / 2 50.0%	1 / 1 100.0%	1 / 1 100.0%	1 / 1 100.0%	4 / 4 100.0%	3 / 3 100.0%	1 / 1 100.0%	1 / 1 100.0%	3 / 3 100.0%	1 / 1 100.0%	3 / 3 100.0%	2 / 2 100.0%	1 / 1 100.0%	2 / 2 100.0%	1 / 1 100.0%	3 / 3 100.0%	1 / 1 100.0%	3 / 3 100.0%	5 / 5 100.0%	2 / 2 100.0%	1 / 1 100.0%	5 / 5 100.0%	0 / 1 0.0%	0 / 0 0.0%	0 / 0 0.0%	0 / 0 0.0%	15 / 48 31.2%B	14 / 48 29.2%AB	13 / 48 27.1%ABE	4 / 48 8.3%BE	2 / 48 4.2%E			
1	Chlorocrinis aggregans	47 / 48 97.9%	1 / 1 100.0%	1 / 1 100.0%	1 / 1 100.0%	1 / 1 100.0%	4 / 4 100.0%	3 / 3 100.0%	1 / 1 100.0%	1 / 1 100.0%	3 / 3 100.0%	1 / 1 100.0%	4 / 4 100.0%	2 / 2 100.0%	1 / 1 100.0%	2 / 2 100.0%	1 / 1 100.0%	3 / 3 100.0%	1 / 1 100.0%	3 / 3 100.0%	5 / 5 100.0%	2 / 2 100.0%	1 / 1 100.0%	5 / 5 100.0%	0 / 1 0.0%	0 / 0 0.0%	0 / 0 0.0%	0 / 0 0.0%	29 / 48 60.4%AB	18 / 48 37.5%ABE	1 / 48 2.1%E					
1	Chloroflexus aggregans DSM 9485	47 / 48 97.9%	1 / 1 100.0%	1 / 1 100.0%	1 / 1 100.0%	1 / 1 100.0%	4 / 4 100.0%	3 / 3 100.0%	1 / 1 100.0%	1 / 1 100.0%	3 / 3 100.0%	1 / 1 100.0%	4 / 4 100.0%	2 / 2 100.0%	1 / 1 100.0%	2 / 2 100.0%	1 / 1 100.0%	3 / 3 100.0%	1 / 1 100.0%	3 / 3 100.0%	5 / 5 100.0%	2 / 2 100.0%	1 / 1 100.0%	5 / 5 100.0%	0 / 1 0.0%	0 / 0 0.0%	0 / 0 0.0%	0 / 0 0.0%	29 / 48 60.4%AB	18 / 48 37.5%ABE	1 / 48 2.1%E					
1	Chloroflexus sp. Y-400-fl	48 / 49 98.0%	2 / 2 100.0%	1 / 1 100.0%	1 / 1 100.0%	1 / 1 100.0%	4 / 4 100.0%	3 / 3 100.0%	1 / 1 100.0%	1 / 1 100.0%	3 / 3 100.0%	1 / 1 100.0%	4 / 4 100.0%	2 / 2 100.0%	1 / 1 100.0%	2 / 2 100.0%	1 / 1 100.0%	3 / 3 100.0%	1 / 1 100.0%	3 / 3 100.0%	5 / 5 100.0%	2 / 2 100.0%	1 / 1 100.0%	5 / 5 100.0%	0 / 1 0.0%	0 / 0 0.0%	0 / 0 0.0%	0 / 0 0.0%	29 / 49 59.2%AB	19 / 49 38.8%ABE	1 / 49 2.0%E					
2	Roseiflexineae	95 / 96 99.0%	4 / 4 100.0%	2 / 2 100.0%	2 / 2 100.0%	2 / 2 100.0%	6 / 6 100.0%	6 / 6 100.0%	2 / 2 100.0%	2 / 2 100.0%	6 / 6 100.0%	2 / 2 100.0%	7 / 8 87.5%	4 / 4 100.0%	2 / 2 100.0%	4 / 4 100.0%	2 / 2 100.0%	6 / 6 100.0%	2 / 2 100.0%	6 / 6 100.0%	10 / 10 100.0%	6 / 6 100.0%	2 / 2 100.0%	10 / 10 100.0%	0 / 0 0.0%	0 / 0 0.0%	0 / 0 0.0%	0 / 0 0.0%	41 / 96 42.7%AB	32 / 96 33.3%ABE	13 / 96 13.5%B	9 / 96 9.4%BE	1 / 96 1.0%CE			
2	Roseiflexaceae	95 / 96 99.0%	4 / 4 100.0%	2 / 2 100.0%	2 / 2 100.0%	2 / 2 100.0%	6 / 6 100.0%	6 / 6 100.0%	2 / 2 100.0%	2 / 2 100.0%	6 / 6 100.0%	2 / 2 100.0%	7 / 8 87.5%	4 / 4 100.0%	2 / 2 100.0%	4 / 4 100.0%	2 / 2 100.0%	6 / 6 100.0%	2 / 2 100.0%	6 / 6 100.0%	10 / 10 100.0%	6 / 6 100.0%	2 / 2 100.0%	10 / 10 100.0%	0 / 0 0.0%	0 / 0 0.0%	0 / 0 0.0%	0 / 0 0.0%	41 / 96 42.7%AB	32 / 96 33.3%ABE	13 / 96 13.5%B	9 / 96 9.4%BE	1 / 96 1.0%CE			
2	Roseiflexus	95 / 96 99.0%	4 / 4 100.0%	2 / 2 100.0%	2 / 2 100.0%	2 / 2 100.0%	6 / 6 100.0%	6 / 6 100.0%	2 / 2 100.0%	2 / 2 100.0%	6 / 6 100.0%	2 / 2 100.0%	7 / 8 87.5%	4 / 4 100.0%	2 / 2 100.0%	4 / 4 100.0%	2 / 2 100.0%	6 / 6 100.0%	2 / 2 100.0%	6 / 6 100.0%	10 / 10 100.0%	6 / 6 100.0%	2 / 2 100.0%	10 / 10 100.0%	0 / 0 0.0%	0 / 0 0.0%	0 / 0 0.0%	0 / 0 0.0%	41 / 96 42.7%AB	32 / 96 33.3%ABE	13 / 96 13.5%B	9 / 96 9.4%BE	1 / 96 1.0%CE			
1	DSM 13941	47 / 48 97.9%	2 / 2 100.0%	1 / 1 100.0%	1 / 1 100.0%	1 / 1 100.0%	3 / 3 100.0%	3 / 3 100.0%	1 / 1 100.0%	1 / 1 100.0%	3 / 3 100.0%	1 / 1 100.0%	3 / 4 75.0%	2 / 2 100.0%	1 / 1 100.0%	2 / 2 100.0%	1 / 1 100.0%	3 / 3 100.0%	1 / 1 100.0%	3 / 3 100.0%	5 / 5 100.0%	3 / 3 100.0%	1 / 1 100.0%	5 / 5 100.0%	0 / 0 0.0%	0 / 0 0.0%	0 / 0 0.0%	0 / 0 0.0%	14 / 48 29.2%AB	13 / 48 27.1%B	11 / 48 22.9%ABE	9 / 48 18.8%BE	1 / 48 2.1%CE			
1	Roseiflexus castenholzii DSM 13941	47 / 48 97.9%	2 / 2 100.0%	1 / 1 100.0%	1 / 1 100.0%	1 / 1 100.0%	3 / 3 100.0%	3 / 3 100.0%	1 / 1 100.0%	1 / 1 100.0%	3 / 3 100.0%	1 / 1 100.0%	3 / 4 75.0%	2 / 2 100.0%	1 / 1 100.0%	2 / 2 100.0%	1 / 1 100.0%	3 / 3 100.0%	1 / 1 100.0%	3 / 3 100.0%	5 / 5 100.0%	3 / 3 100.0%	1 / 1 100.0%	5 / 5 100.0%	0 / 0 0.0%	0 / 0 0.0%	0 / 0 0.0%	0 / 0 0.0%	14 / 48 29.2%AB	13 / 48 27.1%B	11 / 48 22.9%ABE	9 / 48 18.8%BE	1 / 48 2.1%CE			
1	Roseiflexus sp. RS-1	48 / 48 100.0%	2 / 2 100.0%	1 / 1 100.0%	1 / 1 100.0%	1 / 1 100.0%	3 / 3 100.0%	3 / 3 100.0%	1 / 1 100.0%	1 / 1 100.0%	3 / 3 100.0%	1 / 1 100.0%	4 / 4 100.0%	2 / 2 100.0%	1 / 1 100.0%	2 / 2 100.0%	1 / 1 100.0%	3 / 3 100.0%	1 / 1 100.0%	3 / 3 100.0%	5 / 5 100.0%	3 / 3 100.0%	1 / 1 100.0%	5 / 5 100.0%	0 / 0 0.0%	0 / 0 0.0%	0 / 0 0.0%	0 / 0 0.0%	27 / 48 56.2%AB	21 / 48 43.8%ABE						
1	"Herpetosiphonales" Castenholz 2001	51 / 51 100.0%	1 / 1 100.0%	2 / 2 100.0%	3 / 3 100.0%	1 / 1 100.0%	4 / 4 100.0%	3 / 3 100.0%	1 / 1 100.0%	1 / 1 100.0%	3 / 3 100.0%	1 / 1 100.0%	4 / 4 100.0%	2 / 2 100.0%	1 / 1 100.0%	1 / 1 100.0%	1 / 1 100.0%	3 / 3 100.0%	1 / 1 100.0%	3 / 3 100.0%	6 / 6 100.0%	2 / 2 100.0%	1 / 1 100.0%	5 / 5 100.0%	1 / 1 100.0%	0 / 0 0.0%	0 / 0 0.0%	0 / 0 0.0%	17 / 51 33.3%AB	16 / 51 31.4%ABE	14 / 51 27.5%B	4 / 51 7.8%BE				
1	'Herpetosiphonaceae'	51 / 51 100.0%	1 / 1 100.0%	2 / 2 100.0%	3 / 3 100.0%	1 / 1 100.0%	4 / 4 100.0%	3 / 3 100.0%	1 / 1 100.0%	1 / 1 100.0%	3 / 3 100.0%	1 / 1 100.0%	4 / 4 100.0%	2 / 2 100.0%	1 / 1 100.0%	1 / 1 100.0%	1 / 1 100.0%	3 / 3 100.0%	1 / 1 100.0%	3 / 3 100.0%	6 / 6 100.0%	2 / 2 100.0%	1 / 1 100.0%	5 / 5 100.0%	1 / 1 100.0%	0 / 0 0.0%	0 / 0 0.0%	0 / 0 0.0%	17 / 51 33.3%AB	16 / 51 31.4%ABE	14 / 51 27.5%B	4 / 51 7.8%BE				
1	Herpetosiphon	51 / 51 100.0%	1 / 1 100.0%	2 / 2 100.0%	3 / 3 100.0%	1 / 1 100.0%	4 / 4 100.0%	3 / 3 100.0%	1 / 1 100.0%	1 / 1 100.0%	3 / 3 100.0%	1 / 1 100.0%	4 / 4 100.0%	2 / 2 100.0%	1 / 1 100.0%	1 / 1 100.0%	1 / 1 100.0%	3 / 3 100.0%	1 / 1 100.0%	3 / 3 100.0%	6 / 6 100.0%	2 / 2 100.0%	1 / 1 100.0%	5 / 5 100.0%	1 / 1 100.0%	0 / 0 0.0%	0 / 0 0.0%	0 / 0 0.0%	17 / 51 33.3%AB	16 / 51 31.4%ABE	14 / 51 27.5%B	4 / 51 7.8%BE				
1	ATCC 23779	51 / 51 100.0%	1 / 1 100.0%	2 / 2 100.0%	3 / 3 100.0%	1 / 1 100.0%	4 / 4 100.0%	3 / 3 100.0%	1 / 1 100.0%	1 / 1 100.0%	3 / 3 100.0%	1 / 1 100.0%	4 / 4 100.0%	2 / 2 100.0%	1 / 1 100.0%	1 / 1 100.0%	1 / 1 100.0%	3 / 3 100.0%	1 / 1 100.0%	3 / 3 100.0%	6 / 6 100.0%	2 / 2 100.0%	1 / 1 100.0%	5 / 5 100.0%	1 / 1 100.0%	0 / 0 0.0%	0 / 0 0.0%	0 / 0 0.0%	17 / 51 33.3%AB	16 / 51 31.4%ABE	14 / 51 27.5%B	4 / 51 7.8%BE				
1	Herpetosiphon aurantiacus ATCC 23779	51 / 51 100.0%	1 / 1 100.0%	2 / 2 100.0%	3 / 3 100.0%	1 / 1 100.0%	4 / 4 100.0%	3 / 3 100.0%	1 / 1 100.0%	1 / 1 100.0%	3 / 3 100.0%	1 / 1 100.0%	4 / 4 100.0%	2 / 2 100.0%	1 / 1 100.0%	1 / 1 100.0%	1 / 1 100.0%	3 / 3 100.0%	1 / 1 100.0%	3 / 3 100.0%	6 / 6 100.0%	2 / 2 100.0%	1 / 1 100.0%	5 / 5 100.0%	1 / 1 100.0%	0 / 0 0.0%	0 / 0 0.0%	0 / 0 0.0%	17 / 51 33.3%AB	16 / 51 31.4%ABE	14 / 51 27.5%B	4 / 51 7.8%BE				
2	Thermomicrobia	37 / 96 38.5%	3 / 3 100.0%	1 / 3 33.3%	1 / 2 50.0%	0 / 2 0.0%	2 / 6 33.3%	2 / 6 33.3%	2 / 2 100.0%	0 / 3 0.0%	3 / 6 50.0%	0 / 2 0.0%	5 / 8 62.5%	1 / 2 50.0%	0 / 2 0.0%	1 / 4 25.0%	2 / 2 100.0%	1 / 6 16.7%	0 / 1 0.0%	3 / 6 50.0%	3 / 10 30.0%	1 / 5 20.0%	0 / 3 0.0%	4 / 10 40.0%	2 / 2 100.0%	0 / 0 0.0%	0 / 0 0.0%	0 / 0 0.0%	22 / 96 22.9%C	20 / 96 20.8%ABE	20 / 96 20.8%CE	17 / 96 17.7%AB	11 / 96 11.5%	6 / 96 6.2%E		
1	Sphaerobacteridae	24 / 50 48.0%	2 / 2 100.0%	1 / 2 50.0%	1 / 1 100.0%	0 / 1 0.0%	1 / 3 33.3%	2 / 3 66.7%	1 / 1 100.0%	0 / 1 0.0%	2 / 3 66.7%	0 / 1 0.0%	2 / 4 50.0%	1 / 2 50.0%	0 / 1 0.0%	1 / 2 50.0%	1 / 1 100.0%	1 / 3 33.3%	0 / 1 0.0%	2 / 3 66.7%	2 / 5 40.0%	1 / 3 33.3%	0 / 1 0.0%	2 / 5 40.0%	1 / 1 100.0%	0 / 0 0.0%	0 / 0 0.0%	0 / 0 0.0%	13 / 50 26.0%C	12 / 50 24.0%ABE	12 / 50 24.0%AB	9 / 50 18.0%CE	2 / 50 4.0%	2 / 50 4.0%E		
1	Sphaerobacterales	24 / 50 48.0%	2 / 2 100.0%	1 / 2 50.0%	1 / 1 100.0%	0 / 1 0.0%	1 / 3 33.3%	2 / 3 66.7%	1 / 1 100.0%	0 / 1 0.0%	2 / 3 66.7%	0 / 1 0.0%	2 / 4 50.0%	1 / 2 50.0%	0 / 1 0.0%	1 / 2 50.0%	1 / 1 100.0%	1 / 3 33.3%	0 / 1 0.0%	2 / 3 66.7%	2 / 5 40.0%	1 / 3 33.3%	0 / 1 0.0%	2 / 5 40.0%	1 / 1 100.0%	0 / 0 0.0%	0 / 0 0.0%	0 / 0 0.0%	13 / 50 26.0%C	12 / 50 24.0%ABE	12 / 50 24.0%AB	9 / 50 18.0%CE	2 / 50 4.0%	2 / 50 4.0%E		
1	'Sphaerobacterineae'	24 / 50 48.0%	2 / 2 100.0%	1 / 2 50.0%	1 / 1 100.0%	0 / 1 0.0%	1 / 3 33.3%	2 / 3 66.7%	1 / 1 100.0%	0 / 1 0.0%	2 / 3 66.7%	0 / 1 0.0%	2 / 4 50.0%	1 / 2 50.0%	0 / 1 0.0%	1 / 2 50.0%	1 / 1 100.0%	1 / 3 33.3%	0 / 1 0.0%	2 / 3 66.7%	2 / 5 40.0%	1 / 3 33.3%	0 / 1 0.0%	2 / 5 40.0%	1 / 1 100.0%	0 / 0 0.0%	0 / 0 0.0%	0 / 0 0.0%	13 / 50 26.0%C	12 / 50 24.0%ABE	12 / 50 24.0%AB	9 / 50 18.0%CE	2 / 50 4.0%	2 / 50 4.0%E		
1	Sphaerobacteraceae	24 / 50 48.0%	2 / 2 100.0%	1 / 2 50.0%	1 / 1 100.0%	0 / 1 0.0%	1 / 3 33.3%	2 / 3 66.7%	1 / 1 100.0%	0 / 1 0.0%	2 / 3 66.7%	0 / 1 0.0%	2 / 4 50.0%	1 / 2 50.0%	0 / 1 0.0%	1 / 2 50.0%	1 / 1 100.0%	1 / 3 33.3%	0 / 1 0.0%	2 / 3 66.7%	2 / 5 40.0%	1 / 3 33.3%	0 / 1 0.0%	2 / 5 40.0%	1 / 1 100.0%	0 / 0 0.0%	0 / 0 0.0%	0 / 0 0.0%	13 / 50 26.0%C	12 / 50 24.0%ABE	12 / 50 24.0%AB	9 / 50 18.0%CE	2 / 50 4.0%	2 / 50 4.0%E		
1	Sphaerobacter	24 / 50 48.0%	2 / 2 100.0%	1 / 2 50.0%	1 / 1 100.0%	0 / 1 0.0%	1 / 3 33.3%	2 / 3 66.7%	1 / 1 100.0%	0 / 1 0.0%	2 / 3 66.7%	0 / 1 0.0%	2 / 4 50.0%	1 / 2 50.0%	0 / 1 0.0%	1 / 2 50.0%	1 / 1 100.0%	1 / 3 33.3%	0 / 1 0.0%	2 / 3 66.7%	2 / 5 40.0%	1 / 3 33.3%	0 / 1 0.0%	2 / 5 40.0%	1 / 1 100.0%	0 / 0 0.0%	0 / 0 0.0%	0 / 0 0.0%	13 / 50 26.0%C	12 / 50 24.0%AB	12 / 50 24.0%ABE	9 / 50 18.0%CE	2 / 50 4.0%E	2 / 50 4.0%		
1	DSM 20745	24 / 50 48.0%	2 / 2 100.0%	1 / 2 50.0%	1 / 1 100.0%	0 / 1 0.0%	1 / 3 33.3%	2 / 3 66.7%	1 / 1 100.0%	0 / 1 0.0%	2 / 3 66.7%	0 / 1 0.0%	2 / 4 50.0%	1 / 2 50.0%	0 / 1 0.0%	1 / 2 50.0%	1 / 1 100.0%	1 / 3 33.3%	0 / 1 0.0%	2 / 3 66.7%	2 / 5 40.0%	1 / 3 33.3%	0 / 1 0.0%	2 / 5 40.0%	1 / 1 100.0%	0 / 0 0.0%	0 / 0 0.0%	0 / 0 0.0%	13 / 50 26.0%C	12 / 50 24.0%AB	12 / 50 24.0%ABE	9 / 50 18.0%CE	2 / 50 4.0%E	2 / 50 4.0%		
1	Sphaerobacter thermophilus DSM 20745	24 / 50 48.0%	2 / 2 100.0%	1 / 2 50.0%	1 / 1 100.0%	0 / 1 0.0%	1 / 3 33.3%	2 / 3 66.7%	1 / 1 100.0%	0 / 1 0.0%	2 / 3 66.7%	0 / 1 0.0%	2 / 4 50.0%	1 / 2 50.0%	0 / 1 0.0%	1 / 2 50.0%	1 / 1 100.0%	1 / 3 33.3%	0 / 1 0.0%	2 / 3 66.7%	2 / 5 40.0%	1 / 3 33.3%	0 / 1 0.0%	2 / 5 40.0%	1 / 1 100.0%	0 / 0 0.0%	0 / 0 0.0%	0 / 0 0.0%	13 / 50 26.0%C	12 / 50 24.0%ABE	12 / 50 24.0%AB	9 / 50 18.0%CE	2 / 50 4.0%	2 / 50 4.0%E		
1	Thermomicrobiales	13 / 46 28.3%	1 / 1 100.0%	0 / 1 0.0%	0 / 1 0.0%	0 / 1 0.0%	1 / 3 33.3%	0 / 3 0.0%	1 / 1 100.0%	0 / 2 0.0%	1 / 3 33.3%	0 / 1 0.0%	3 / 4 75.0%	0 / 0 0.0%	0 / 1 0.0%	0 / 2 0.0%	1 / 1 100.0%	0 / 3 0.0%	0 / 0 0.0%	1 / 3 33.3%	1 / 5 20.0%	0 / 2 0.0%	0 / 2 0.0%	2 / 5 40.0%	1 / 1 100.0%	0 / 0 0.0%	0 / 0 0.0%	0 / 0 0.0%	11 / 46 23.9%CE	9 / 46 19.6%	9 / 46 19.6%C	8 / 46 17.4%ABE	5 / 46 10.9%AB	4 / 46 8.7%E		
1	Thermomicrobiaceae	13 / 46 28.3%	1 / 1 100.0%	0 / 1 0.0%	0 / 1 0.0%	0 / 1 0.0%	1 / 3 33.3%	0 / 3 0.0%	1 / 1 100.0%	0 / 2 0.0%	1 / 3 33.3%	0 / 1 0.0%	3 / 4 75.0%	0 / 0 0.0%	0 / 1 0.0%	0 / 2 0.0%	1 / 1 100.0%	0 / 3 0.0%	0 / 0 0.0%	1 / 3 33.3%	1 / 5 20.0%	0 / 2 0.0%	0 / 2 0.0%	2 / 5 40.0%	1 / 1 100.0%	0 / 0 0.0%	0 / 0 0.0%	0 / 0 0.0%	11 / 46 23.9%CE	9 / 46 19.6%	9 / 46 19.6%C	8 / 46 17.4%ABE	5 / 46 10.9%AB	4 / 46 8.7%E		
1	Thermomicrobium	13 / 46 28.3%	1 / 1 100.0%	0 / 1 0.0%	0 / 1 0.0%	0 / 1 0.0%	1 / 3 33.3%	0 / 3 0.0%	1 / 1 100.0%	0 / 2 0.0%	1 / 3 33.3%	0 / 1 0.0%	3 / 4 75.0%	0 / 0 0.0%	0 / 1 0.0%	0 / 2 0.0%	1 / 1 100.0%	0 / 3 0.0%	0 / 0 0.0%	1 / 3 33.3%	1 / 5 20.0%	0 / 2 0.0%	0 / 2 0.0%	2 / 5 40.0%	1 / 1 100.0%	0 / 0 0.0%	0 / 0 0.0%	0 / 0 0.0%	11 / 46 23.9%CE	9 / 46 19.6%C	9 / 46 19.6%	8 / 46 17.4%ABE	5 / 46 10.9%AB	4 / 46 8.7%E		
1	ATCC 27502	13 / 46 28.3%	1 / 1 100.0%	0 / 1 0.0%	0 / 1 0.0%	0 / 1 0.0%	1 / 3 33.3%	0 / 3 0.0%	1 / 1 100.0%	0 / 2 0.0%	1 / 3 33.3%	0 / 1 0.0%	3 / 4 75.0%	0 / 0 0.0%	0 / 1 0.0%	0 / 2 0.0%	1 / 1 100.0%	0 / 3 0.0%	0 / 0 0.0%	1 / 3 33.3%	1 / 5 20.0%	0 / 2 0.0%	0 / 2 0.0%	2 / 5 40.0%	1 / 1 100.0%	0 / 0 0.0%	0 / 0 0.0%	0 / 0 0.0%	11 / 46 23.9%CE	9 / 46 19.6%	9 / 46 19.6%C	8 / 46 17.4%ABE	5 / 46 10.9%AB	4 / 46 8.7%E		
1	Thermomicrobium roseum DSM 5159	13 / 46 28.3%	1 / 1 100.0%	0 / 1 0.0%	0 / 1 0.0%	0 / 1 0.0%	1 / 3 33.3%	0 / 3 0.0%	1 / 1 100.0%	0 / 2 0.0%	1 / 3 33.3%	0 / 1 0.0%	3 / 4 75.0%	0 / 0 0.0%	0 / 1 0.0%	0 / 2 0.0%	1 / 1 100.0%	0 / 3 0.0%	0 / 0 0.0%	1 / 3 33.3%	1 / 5 20.0%	0 / 2 0.0%	0 / 2 0.0%	2 / 5 40.0%	1 / 1 100.0%	0 / 0 0.0%	0 / 0 0.0%	0 / 0 0.0%	11 / 46 23.9%CE	9 / 46 19.6%	9 / 46 19.6%C	8 / 46 17.4%ABE	5 / 46 10.9%AB	4 / 46 8.7%E		
1	Anaerolineae	6 / 49 12.2%	0 / 1 0.0%	2 / 2 100.0%	0 / 1 0.0%	0 / 1 0.0%	0 / 3 0.0%	1 / 3 33.3%	1 / 1 100.0%	0 / 1 0.0%	0 / 3 0.0%	0 / 1 0.0%	0 / 4 0.0%	0 / 2 0.0%	0 / 1 0.0%	0 / 2 0.0%	0 / 1 0.0%	1 / 3 33.3%	0 / 1 0.0%	1 / 3 33.3%	0 / 5 0.0%	0 / 2 0.0%	0 / 2 0.0%	0 / 5 0.0%	0 / 1 0.0%	0 / 0 0.0%	0 / 0 0.0%	0 / 0 0.0%	16 / 49 32.7%E	14 / 49 28.6%	9 / 49 18.4%C	4 / 49 8.2%CE	3 / 49 6.1%AB	3 / 49 6.1%ABE		
1	Anaerolinaeles	6 / 49 12.2%	0 / 1 0.0%	2 / 2 100.0%	0 / 1 0.0%	0 / 1 0.0%	0 / 3 0.0%	1 / 3 33.3%	1 / 1 100.0%	0 / 1 0.0%	0 / 3 0.0%	0 / 1 0.0%	0 / 4 0.0%	0 / 2 0.0%	0 / 1 0.0%	0 / 2 0.0%	0 / 1 0.0%	1 / 3 33.3%	0 / 1 0.0%	1 / 3 33.3%	0 / 5 0.0%	0 / 2 0.0%	0 / 2 0.0%	0 / 5 0.0%	0 / 1 0.0%	0 / 0 0.0%	0 / 0 0.0%	0 / 0 0.0%	16 / 49 32.7%E	14 / 49 28.6%	9 / 49 18.4%C	4 / 49 8.2%CE	3 / 49 6.1%ABE	3 / 49 6.1%AB		
1	Anaerolinaceae	6 / 49 12.2%	0 / 1 0.0%	2 / 2 100.0%	0 / 1 0.0%	0 / 1 0.0%	0 / 3 0.0%	1 / 3 33.3%	1 / 1 100.0%	0 / 1 0.0%	0 / 3 0.0%	0 / 1 0.0%	0 / 4 0.0%	0 / 2 0.0%	0 / 1 0.0%	0 / 2 0.0%	0 / 1 0.0%	1 / 3 33.3%	0 / 1 0.0%	1 / 3 33.3%	0 / 5 0.0%	0 / 2 0.0%	0 / 2 0.0%	0 / 5 0.0%	0 / 1 0.0%	0 / 0 0.0%	0 / 0 0.0%	0 / 0 0.0%	16 / 49 32.7%E	14 / 49 28.6%	9 / 49 18.4%C	4 / 49 8.2%CE	3 / 49 6.1%ABE	3 / 49 6.1%AB		
1	Anaerolinea	6 / 49 12.2%	0 / 1 0.0%	2 / 2 100.0%	0 / 1 0.0%	0 / 1 0.0%	0 / 3 0.0%	1 / 3 33.3%	1 / 1 100.0%	0 / 1 0.0%	0 / 3 0.0%	0 / 1 0.0%	0 / 4 0.0%	0 / 2 0.0%	0 / 1 0.0%	0 / 2 0.0%	0 / 1 0.0%	1 / 3 33.3%	0 / 1 0.0%	1 / 3 33.3%	0 / 5 0.0%	0 / 2 0.0%	0 / 2 0.0%	0 / 5 0.0%	0 / 1 0.0%	0 / 0 0.0%	0 / 0 0.0%	0 / 0 0.0%	16 / 49 32.7%E	14 / 49 28.6%	9 / 49 18.4%C	4 / 49 8.2%CE	3 / 49 6.1%ABE	3 / 49 6.1%AB		
1	Anaerolinea thermophila	6 / 49 12.2%	0 / 1 0.0%	2 / 2 100.0%	0 / 1 0.0%	0 / 1 0.0%	0 / 3 0.0%	1 / 3 33.3%	1 / 1 100.0%	0 / 1 0.0%	0 / 3 0.0%	0 / 1 0.0%	0 / 4 0.0%	0 / 2 0.0%	0 / 1 0.0%	0 / 2 0.0%	0 / 1 0.0%	1 / 3 33.3%	0 / 1 0.0%	1 / 3 33.3%	0 / 5 0.0%	0 / 2 0.0%	0 / 2 0.0%	0 / 5 0.0%	0 / 1 0.0%	0 / 0 0.0%	0 / 0 0.0%	0 / 0 0.0%	16 / 49 32.7%E	14 / 49 28.6%	9 / 49 18.4%C	4 / 49 8.2%CE	3 / 49 6.1%AB	3 / 49 6.1%ABE		
1	Anaerolinea thermophila UNI-1	6 / 49 12.2%	0 / 1 0.0%	2 / 2 100.0%	0 / 1 0.0%	0 / 1 0.0%	0 / 3 0.0%	1 / 3 33.3%	1 / 1 100.0%	0 / 1 0.0%	0 / 3 0.0%	0 / 1 0.0%	0 / 4 0.0%	0 / 2 0.0%	0 / 1 0.0%	0 / 2 0.0%	0 / 1 0.0%	1 / 3 33.3%	0 / 1 0.0%	1 / 3 33.3%	0 / 5 0.0%	0 / 2 0.0%	0 / 2 0.0%	0 / 5 0.0%	0 / 1 0.0%	0 / 0 0.0%	0 / 0 0.0%	0 / 0 0.0%	16 / 49 32.7%E	14 / 49 28.6%	9 / 49 18.4%C	4 / 49 8.2%CE	3 / 49 6.1%ABE	3 / 49 6.1%AB		
8	"Dehalococcoidetes" Hugenholtz and Stackebrandt 2004	319 / 368 86.7%	8 / 8 100.0%	8 / 8 100.0%	8 / 8 100.0%	8 / 8 100.0%	24 / 24 100.0%	24 / 24 100.0%	8 / 8 100.0%	0 / 8 0.0%	18 / 24 75.0%	8 / 8 100.0%	25 / 32 78.1%	7 / 16 43.8%	8 / 8 100.0%	16 / 16 100.0%	8 / 8 100.0%	24 / 24 100.0%	8 / 8 100.0%	16 / 24 66.7%	31 / 40 77.5%	15 / 16 93.8%	8 / 8 100.0%	39 / 40 97.5%	0 / 0 0.0%	0 / 0 0.0%	0 / 0 0.0%	0 / 0 0.0%	193 / 368 52.4%AB	126 / 368 34.2%ABE	29 / 368 7.9%E	20 / 368 5.4%				
1	Dehalogenimonas	39 / 46 84.8%	1 / 1 100.0%	1 / 1 100.0%	1 / 1 100.0%	1 / 1 100.0%	3 / 3 100.0%	3 / 3 100.0%	1 / 1 100.0%	0 / 1 0.0%	3 / 3 100.0%	1 / 1 100.0%	4 / 4 100.0%	1 / 2 50.0%	1 / 1 100.0%	2 / 2 100.0%	1 / 1 100.0%	3 / 3 100.0%	1 / 1 100.0%	2 / 3 66.7%	3 / 5 60.0%	1 / 2 50.0%	1 / 1 100.0%	4 / 5 80.0%	0 / 0 0.0%	0 / 0 0.0%	0 / 0 0.0%	0 / 0 0.0%	25 / 46 54.3%AB	14 / 46 30.4%ABE	4 / 46 8.7%E	3 / 46 6.5%				
1	ATCC BAA-1523	39 / 46 84.8%	1 / 1 100.0%	1 / 1 100.0%	1 / 1 100.0%	1 / 1 100.0%	3 / 3 100.0%	3 / 3 100.0%	1 / 1 100.0%	0 / 1 0.0%	3 / 3 100.0%	1 / 1 100.0%	4 / 4 100.0%	1 / 2 50.0%	1 / 1 100.0%	2 / 2 100.0%	1 / 1 100.0%	3 / 3 100.0%	1 / 1 100.0%	2 / 3 66.7%	3 / 5 60.0%	1 / 2 50.0%	1 / 1 100.0%	4 / 5 80.0%	0 / 0 0.0%	0 / 0 0.0%	0 / 0 0.0%	0 / 0 0.0%	25 / 46 54.3%AB	14 / 46 30.4%ABE	4 / 46 8.7%E	3 / 46 6.5%				
1	Chlorobi bacterium BL-DC-8	39 / 46 84.8%	1 / 1 100.0%	1 / 1 100.0%	1 / 1 100.0%	1 / 1 100.0%	3 / 3 100.0%	3 / 3 100.0%	1 / 1 100.0%	0 / 1 0.0%	3 / 3 100.0%	1 / 1 100.0%	4 / 4 100.0%	1 / 2 50.0%	1 / 1 100.0%	2 / 2 100.0%	1 / 1 100.0%	3 / 3 100.0%	1 / 1 100.0%	2 / 3 66.7%	3 / 5 60.0%	1 / 2 50.0%	1 / 1 100.0%	4 / 5 80.0%	0 / 0 0.0%	0 / 0 0.0%	0 / 0 0.0%	0 / 0 0.0%	25 / 46 54.3%AB	14 / 46 30.4%ABE	4 / 46 8.7%E	3 / 46 6.5%				
7	Dehalococcoidales	280 / 322 87.0%	7 / 7 100.0%	7 / 7 100.0%	7 / 7 100.0%	7 / 7 100.0%	21 / 21 100.0%	21 / 21 100.0%	7 / 7 100.0%	0 / 7 0.0%	15 / 21 71.4%	7 / 7 100.0%	21 / 28 75.0%	6 / 14 42.9%	7 / 7 100.0%	14 / 14 100.0%	7 / 7 100.0%	21 / 21 100.0%	7 / 7 100.0%	14 / 21 66.7%	28 / 35 80.0%	14 / 14 100.0%	7 / 7 100.0%	35 / 35 100.0%	0 / 0 0.0%	0 / 0 0.0%	0 / 0 0.0%	0 / 0 0.0%	168 / 322 52.2%AB	112 / 322 34.8%ABE	25 / 322 7.8%E	17 / 322 5.3%				
7	Dehalococcoidaceae	280 / 322 87.0%	7 / 7 100.0%	7 / 7 100.0%	7 / 7 100.0%	7 / 7 100.0%	21 / 21 100.0%	21 / 21 100.0%	7 / 7 100.0%	0 / 7 0.0%	15 / 21 71.4%	7 / 7 100.0%	21 / 28 75.0%	6 / 14 42.9%	7 / 7 100.0%	14 / 14 100.0%	7 / 7 100.0%	21 / 21 100.0%	7 / 7 100.0%	14 / 21 66.7%	28 / 35 80.0%	14 / 14 100.0%	7 / 7 100.0%	35 / 35 100.0%	0 / 0 0.0%	0 / 0 0.0%	0 / 0 0.0%	0 / 0 0.0%	168 / 322 52.2%AB	112 / 322 34.8%ABE	25 / 322 7.8%E	17 / 322 5.3%				
7	"Dehalococcoides" Maymo-Gatell et al. 1997	280 / 322 87.0%	7 / 7 100.0%	7 / 7 100.0%	7 / 7 100.0%	7 / 7 100.0%	21 / 21 100.0%	21 / 21 100.0%	7 / 7 100.0%	0 / 7 0.0%	15 / 21 71.4%	7 / 7 100.0%	21 / 28 75.0%	6 / 14 42.9%	7 / 7 100.0%	14 / 14 100.0%	7 / 7 100.0%	21 / 21 100.0%	7 / 7 100.0%	14 / 21 66.7%	28 / 35 80.0%	14 / 14 100.0%	7 / 7 100.0%	35 / 35 100.0%	0 / 0 0.0%	0 / 0 0.0%	0 / 0 0.0%	0 / 0 0.0%	168 / 322 52.2%AB	112 / 322 34.8%ABE	25 / 322 7.8%E	17 / 322 5.3%				
7	"Dehalococcoides ethenogenes" Maymo-Gatell et al. 1997	280 / 322 87.0%	7 / 7 100.0%	7 / 7 100.0%	7 / 7 100.0%	7 / 7 100.0%	21 / 21 100.0%	21 / 21 100.0%	7 / 7 100.0%	0 / 7 0.0%	15 / 21 71.4%	7 / 7 100.0%	21 / 28 75.0%	6 / 14 42.9%	7 / 7 100.0%	14 / 14 100.0%	7 / 7 100.0%	21 / 21 100.0%	7 / 7 100.0%	14 / 21 66.7%	28 / 35 80.0%	14 / 14 100.0%	7 / 7 100.0%	35 / 35 100.0%	0 / 0 0.0%	0 / 0 0.0%	0 / 0 0.0%	0 / 0 0.0%	168 / 322 52.2%AB	112 / 322 34.8%ABE	25 / 322 7.8%E	17 / 322 5.3%				
1	Dehalococcoides mccartyi BAV1	40 / 46 87.0%	1 / 1 100.0%	1 / 1 100.0%	1 / 1 100.0%	1 / 1 100.0%	3 / 3 100.0%	3 / 3 100.0%	1 / 1 100.0%	0 / 1 0.0%	2 / 3 66.7%	1 / 1 100.0%	3 / 4 75.0%	1 / 2 50.0%	1 / 1 100.0%	2 / 2 100.0%	1 / 1 100.0%	3 / 3 100.0%	1 / 1 100.0%	2 / 3 66.7%	4 / 5 80.0%	2 / 2 100.0%	1 / 1 100.0%	5 / 5 100.0%	0 / 0 0.0%	0 / 0 0.0%	0 / 0 0.0%	0 / 0 0.0%	24 / 46 52.2%AB	16 / 46 34.8%ABE	3 / 46 6.5%E	3 / 46 6.5%				
1	Dehalococcoides ethenogenes 195	40 / 46 87.0%	1 / 1 100.0%	1 / 1 100.0%	1 / 1 100.0%	1 / 1 100.0%	3 / 3 100.0%	3 / 3 100.0%	1 / 1 100.0%	0 / 1 0.0%	3 / 3 100.0%	1 / 1 100.0%	3 / 4 75.0%	0 / 2 0.0%	1 / 1 100.0%	2 / 2 100.0%	1 / 1 100.0%	3 / 3 100.0%	1 / 1 100.0%	2 / 3 66.7%	4 / 5 80.0%	2 / 2 100.0%	1 / 1 100.0%	5 / 5 100.0%	0 / 0 0.0%	0 / 0 0.0%	0 / 0 0.0%	0 / 0 0.0%	24 / 46 52.2%AB	16 / 46 34.8%ABE	3 / 46 6.5%E	3 / 46 6.5%				
1	Dehalococcoides mccartyi CBDB1	40 / 46 87.0%	1 / 1 100.0%	1 / 1 100.0%	1 / 1 100.0%	1 / 1 100.0%	3 / 3 100.0%	3 / 3 100.0%	1 / 1 100.0%	0 / 1 0.0%	2 / 3 66.7%	1 / 1 100.0%	3 / 4 75.0%	1 / 2 50.0%	1 / 1 100.0%	2 / 2 100.0%	1 / 1 100.0%	3 / 3 100.0%	1 / 1 100.0%	2 / 3 66.7%	4 / 5 80.0%	2 / 2 100.0%	1 / 1 100.0%	5 / 5 100.0%	0 / 0 0.0%	0 / 0 0.0%	0 / 0 0.0%	0 / 0 0.0%	24 / 46 52.2%AB	16 / 46 34.8%ABE	3 / 46 6.5%E	3 / 46 6.5%				
1	Dehalococcoides mccartyi VS	40 / 46 87.0%	1 / 1 100.0%	1 / 1 100.0%	1 / 1 100.0%	1 / 1 100.0%	3 / 3 100.0%	3 / 3 100.0%	1 / 1 100.0%	0 / 1 0.0%	2 / 3 66.7%	1 / 1 100.0%	3 / 4 75.0%	1 / 2 50.0%	1 / 1 100.0%	2 / 2 100.0%	1 / 1 100.0%	3 / 3 100.0%	1 / 1 100.0%	2 / 3 66.7%	4 / 5 80.0%	2 / 2 100.0%	1 / 1 100.0%	5 / 5 100.0%	0 / 0 0.0%	0 / 0 0.0%	0 / 0 0.0%	0 / 0 0.0%	24 / 46 52.2%AB	16 / 46 34.8%ABE	4 / 46 8.7%E	2 / 46 4.3%				
1	Dehalococcoides mccartyi GT	40 / 46 87.0%	1 / 1 100.0%	1 / 1 100.0%	1 / 1 100.0%	1 / 1 100.0%	3 / 3 100.0%	3 / 3 100.0%	1 / 1 100.0%	0 / 1 0.0%	2 / 3 66.7%	1 / 1 100.0%	3 / 4 75.0%	1 / 2 50.0%	1 / 1 100.0%	2 / 2 100.0%	1 / 1 100.0%	3 / 3 100.0%	1 / 1 100.0%	2 / 3 66.7%	4 / 5 80.0%	2 / 2 100.0%	1 / 1 100.0%	5 / 5 100.0%	0 / 0 0.0%	0 / 0 0.0%	0 / 0 0.0%	0 / 0 0.0%	24 / 46 52.2%AB	16 / 46 34.8%ABE	4 / 46 8.7%E	2 / 46 4.3%				
1	Dehalococcoides mccartyi BTF08	40 / 46 87.0%	1 / 1 100.0%	1 / 1 100.0%	1 / 1 100.0%	1 / 1 100.0%	3 / 3 100.0%	3 / 3 100.0%	1 / 1 100.0%	0 / 1 0.0%	2 / 3 66.7%	1 / 1 100.0%	3 / 4 75.0%	1 / 2 50.0%	1 / 1 100.0%	2 / 2 100.0%	1 / 1 100.0%	3 / 3 100.0%	1 / 1 100.0%	2 / 3 66.7%	4 / 5 80.0%	2 / 2 100.0%	1 / 1 100.0%	5 / 5 100.0%	0 / 0 0.0%	0 / 0 0.0%	0 / 0 0.0%	0 / 0 0.0%	24 / 46 52.2%AB	16 / 46 34.8%ABE	4 / 46 8.7%E	2 / 46 4.3%				
1	Dehalococcoides mccartyi DCMB5	40 / 46 87.0%	1 / 1 100.0%	1 / 1 100.0%	1 / 1 100.0%	1 / 1 100.0%	3 / 3 100.0%	3 / 3 100.0%	1 / 1 100.0%	0 / 1 0.0%	2 / 3 66.7%	1 / 1 100.0%	3 / 4 75.0%	1 / 2 50.0%	1 / 1 100.0%	2 / 2 100.0%	1 / 1 100.0%	3 / 3 100.0%	1 / 1 100.0%	2 / 3 66.7%	4 / 5 80.0%	2 / 2 100.0%	1 / 1 100.0%	5 / 5 100.0%	0 / 0 0.0%	0 / 0 0.0%	0 / 0 0.0%	0 / 0 0.0%	24 / 46 52.2%AB	16 / 46 34.8%ABE	4 / 46 8.7%E	2 / 46 4.3%				
1	Caldilineae	14 / 48 29.2%	0 / 1 0.0%	1 / 1 100.0%	0 / 1 0.0%	0 / 2 0.0%	0 / 3 0.0%	0 / 3 0.0%	1 / 1 100.0%	0 / 1 0.0%	0 / 3 0.0%	0 / 1 0.0%	3 / 4 75.0%	0 / 2 0.0%	0 / 1 0.0%	2 / 2 100.0%	0 / 1 0.0%	2 / 3 66.7%	1 / 1 100.0%	0 / 3 0.0%	1 / 5 20.0%	1 / 2 50.0%	0 / 1 0.0%	2 / 5 40.0%	0 / 1 0.0%	0 / 0 0.0%	0 / 0 0.0%	0 / 0 0.0%	19 / 48 39.6%	8 / 48 16.7%ABE	7 / 48 14.6%E	6 / 48 12.5%C	6 / 48 12.5%AB	2 / 48 4.2%CE		
1	Caldilineales	14 / 48 29.2%	0 / 1 0.0%	1 / 1 100.0%	0 / 1 0.0%	0 / 2 0.0%	0 / 3 0.0%	0 / 3 0.0%	1 / 1 100.0%	0 / 1 0.0%	0 / 3 0.0%	0 / 1 0.0%	3 / 4 75.0%	0 / 2 0.0%	0 / 1 0.0%	2 / 2 100.0%	0 / 1 0.0%	2 / 3 66.7%	1 / 1 100.0%	0 / 3 0.0%	1 / 5 20.0%	1 / 2 50.0%	0 / 1 0.0%	2 / 5 40.0%	0 / 1 0.0%	0 / 0 0.0%	0 / 0 0.0%	0 / 0 0.0%	19 / 48 39.6%	8 / 48 16.7%ABE	7 / 48 14.6%E	6 / 48 12.5%C	6 / 48 12.5%AB	2 / 48 4.2%CE		
1	Caldilineaceae	14 / 48 29.2%	0 / 1 0.0%	1 / 1 100.0%	0 / 1 0.0%	0 / 2 0.0%	0 / 3 0.0%	0 / 3 0.0%	1 / 1 100.0%	0 / 1 0.0%	0 / 3 0.0%	0 / 1 0.0%	3 / 4 75.0%	0 / 2 0.0%	0 / 1 0.0%	2 / 2 100.0%	0 / 1 0.0%	2 / 3 66.7%	1 / 1 100.0%	0 / 3 0.0%	1 / 5 20.0%	1 / 2 50.0%	0 / 1 0.0%	2 / 5 40.0%	0 / 1 0.0%	0 / 0 0.0%	0 / 0 0.0%	0 / 0 0.0%	19 / 48 39.6%	8 / 48 16.7%ABE	7 / 48 14.6%E	6 / 48 12.5%C	6 / 48 12.5%AB	2 / 48 4.2%CE		
1	Caldilinea	14 / 48 29.2%	0 / 1 0.0%	1 / 1 100.0%	0 / 1 0.0%	0 / 2 0.0%	0 / 3 0.0%	0 / 3 0.0%	1 / 1 100.0%	0 / 1 0.0%	0 / 3 0.0%	0 / 1 0.0%	3 / 4 75.0%	0 / 2 0.0%	0 / 1 0.0%	2 / 2 100.0%	0 / 1 0.0%	2 / 3 66.7%	1 / 1 100.0%	0 / 3 0.0%	1 / 5 20.0%	1 / 2 50.0%	0 / 1 0.0%	2 / 5 40.0%	0 / 1 0.0%	0 / 0 0.0%	0 / 0 0.0%	0 / 0 0.0%	19 / 48 39.6%	8 / 48 16.7%ABE	7 / 48 14.6%E	6 / 48 12.5%C	6 / 48 12.5%AB	2 / 48 4.2%CE		
1	Caldilinea aerophila	14 / 48 29.2%	0 / 1 0.0%	1 / 1 100.0%	0 / 1 0.0%	0 / 2 0.0%	0 / 3 0.0%	0 / 3 0.0%	1 / 1 100.0%	0 / 1 0.0%	0 / 3 0.0%	0 / 1 0.0%	3 / 4 75.0%	0 / 2 0.0%	0 / 1 0.0%	2 / 2 100.0%	0 / 1 0.0%	2 / 3 66.7%	1 / 1 100.0%	0 / 3 0.0%	1 / 5 20.0%	1 / 2 50.0%	0 / 1 0.0%	2 / 5 40.0%	0 / 1 0.0%	0 / 0 0.0%	0 / 0 0.0%	0 / 0 0.0%	19 / 48 39.6%	8 / 48 16.7%ABE	7 / 48 14.6%E	6 / 48 12.5%C	6 / 48 12.5%AB	2 / 48 4.2%CE		
1	Caldilinea aerophila DSM 14535	14 / 48 29.2%	0 / 1 0.0%	1 / 1 100.0%	0 / 1 0.0%	0 / 2 0.0%	0 / 3 0.0%	0 / 3 0.0%	1 / 1 100.0%	0 / 1 0.0%	0 / 3 0.0%	0 / 1 0.0%	3 / 4 75.0%	0 / 2 0.0%	0 / 1 0.0%	2 / 2 100.0%	0 / 1 0.0%	2 / 3 66.7%	1 / 1 100.0%	0 / 3 0.0%	1 / 5 20.0%	1 / 2 50.0%	0 / 1 0.0%	2 / 5 40.0%	0 / 1 0.0%	0 / 0 0.0%	0 / 0 0.0%	0 / 0 0.0%	19 / 48 39.6%	8 / 48 16.7%ABE	7 / 48 14.6%E	6 / 48 12.5%AB	6 / 48 12.5%C	2 / 48 4.2%CE		
15	Thermotogae	746 / 757 98.5%	17 / 17 100.0%	19 / 19 100.0%	19 / 19 100.0%	19 / 19 100.0%	49 / 49 100.0%	48 / 48 100.0%	17 / 17 100.0%	16 / 16 100.0%	51 / 51 100.0%	16 / 16 100.0%	64 / 64 100.0%	32 / 32 100.0%	17 / 17 100.0%	32 / 32 100.0%	16 / 16 100.0%	49 / 49 100.0%	10 / 17 58.8%	49 / 49 100.0%	77 / 80 96.2%	33 / 33 100.0%	16 / 16 100.0%	80 / 81 98.8%	0 / 0 0.0%	0 / 0 0.0%	0 / 0 0.0%	0 / 0 0.0%	433 / 757 57.2%AB	223 / 757 29.5%ABE	61 / 757 8.1%B	29 / 757 3.8%BE	7 / 757 0.9%	2 / 757 0.3%E	1 / 757 0.1%CE	1 / 757 0.1%C
15	"Togobacteria" Cavalier-Smith 1992	746 / 757 98.5%	17 / 17 100.0%	19 / 19 100.0%	19 / 19 100.0%	19 / 19 100.0%	49 / 49 100.0%	48 / 48 100.0%	17 / 17 100.0%	16 / 16 100.0%	51 / 51 100.0%	16 / 16 100.0%	64 / 64 100.0%	32 / 32 100.0%	17 / 17 100.0%	32 / 32 100.0%	16 / 16 100.0%	49 / 49 100.0%	10 / 17 58.8%	49 / 49 100.0%	77 / 80 96.2%	33 / 33 100.0%	16 / 16 100.0%	80 / 81 98.8%	0 / 0 0.0%	0 / 0 0.0%	0 / 0 0.0%	0 / 0 0.0%	433 / 757 57.2%AB	223 / 757 29.5%ABE	61 / 757 8.1%B	29 / 757 3.8%BE	7 / 757 0.9%	2 / 757 0.3%E	1 / 757 0.1%C	1 / 757 0.1%CE
15	"Thermotogales" Huber and Stetter 1992	746 / 757 98.5%	17 / 17 100.0%	19 / 19 100.0%	19 / 19 100.0%	19 / 19 100.0%	49 / 49 100.0%	48 / 48 100.0%	17 / 17 100.0%	16 / 16 100.0%	51 / 51 100.0%	16 / 16 100.0%	64 / 64 100.0%	32 / 32 100.0%	17 / 17 100.0%	32 / 32 100.0%	16 / 16 100.0%	49 / 49 100.0%	10 / 17 58.8%	49 / 49 100.0%	77 / 80 96.2%	33 / 33 100.0%	16 / 16 100.0%	80 / 81 98.8%	0 / 0 0.0%	0 / 0 0.0%	0 / 0 0.0%	0 / 0 0.0%	433 / 757 57.2%AB	223 / 757 29.5%ABE	61 / 757 8.1%B	29 / 757 3.8%BE	7 / 757 0.9%	2 / 757 0.3%E	1 / 757 0.1%CE	1 / 757 0.1%C
15	Thermotogaceae	746 / 757 98.5%	17 / 17 100.0%	19 / 19 100.0%	19 / 19 100.0%	19 / 19 100.0%	49 / 49 100.0%	48 / 48 100.0%	17 / 17 100.0%	16 / 16 100.0%	51 / 51 100.0%	16 / 16 100.0%	64 / 64 100.0%	32 / 32 100.0%	17 / 17 100.0%	32 / 32 100.0%	16 / 16 100.0%	49 / 49 100.0%	10 / 17 58.8%	49 / 49 100.0%	77 / 80 96.2%	33 / 33 100.0%	16 / 16 100.0%	80 / 81 98.8%	0 / 0 0.0%	0 / 0 0.0%	0 / 0 0.0%	0 / 0 0.0%	433 / 757 57.2%AB	223 / 757 29.5%ABE	61 / 757 8.1%B	29 / 757 3.8%BE	7 / 757 0.9%	2 / 757 0.3%E	1 / 757 0.1%C	1 / 757 0.1%CE
7	Thermotoga	361 / 370 97.6%	8 / 8 100.0%	8 / 8 100.0%	8 / 8 100.0%	8 / 8 100.0%	24 / 24 100.0%	24 / 24 100.0%	9 / 9 100.0%	8 / 8 100.0%	24 / 24 100.0%	8 / 8 100.0%	32 / 32 100.0%	16 / 16 100.0%	8 / 8 100.0%	16 / 16 100.0%	8 / 8 100.0%	24 / 24 100.0%	1 / 8 12.5%	24 / 24 100.0%	40 / 41 97.6%	16 / 16 100.0%	8 / 8 100.0%	39 / 40 97.5%	0 / 0 0.0%	0 / 0 0.0%	0 / 0 0.0%	0 / 0 0.0%	213 / 370 57.6%AB	109 / 370 29.5%ABE	25 / 370 6.8%B	14 / 370 3.8%BE	6 / 370 1.6%	1 / 370 0.3%CE	1 / 370 0.3%E	1 / 370 0.3%C
1	ATCC 43589	90 / 92 97.8%	2 / 2 100.0%	2 / 2 100.0%	2 / 2 100.0%	2 / 2 100.0%	6 / 6 100.0%	6 / 6 100.0%	2 / 2 100.0%	2 / 2 100.0%	6 / 6 100.0%	2 / 2 100.0%	8 / 8 100.0%	4 / 4 100.0%	2 / 2 100.0%	4 / 4 100.0%	2 / 2 100.0%	6 / 6 100.0%	0 / 2 0.0%	6 / 6 100.0%	10 / 10 100.0%	4 / 4 100.0%	2 / 2 100.0%	10 / 10 100.0%	0 / 0 0.0%	0 / 0 0.0%	0 / 0 0.0%	0 / 0 0.0%	60 / 92 65.2%AB	30 / 92 32.6%ABE	2 / 92 2.2%					
1	Thermotoga maritima DSM 3109	90 / 92 97.8%	2 / 2 100.0%	2 / 2 100.0%	2 / 2 100.0%	2 / 2 100.0%	6 / 6 100.0%	6 / 6 100.0%	2 / 2 100.0%	2 / 2 100.0%	6 / 6 100.0%	2 / 2 100.0%	8 / 8 100.0%	4 / 4 100.0%	2 / 2 100.0%	4 / 4 100.0%	2 / 2 100.0%	6 / 6 100.0%	0 / 2 0.0%	6 / 6 100.0%	10 / 10 100.0%	4 / 4 100.0%	2 / 2 100.0%	10 / 10 100.0%	0 / 0 0.0%	0 / 0 0.0%	0 / 0 0.0%	0 / 0 0.0%	60 / 92 65.2%AB	30 / 92 32.6%ABE	2 / 92 2.2%					
1	ATCC 49049	46 / 48 95.8%	1 / 1 100.0%	1 / 1 100.0%	1 / 1 100.0%	1 / 1 100.0%	3 / 3 100.0%	3 / 3 100.0%	2 / 2 100.0%	1 / 1 100.0%	3 / 3 100.0%	1 / 1 100.0%	4 / 4 100.0%	2 / 2 100.0%	1 / 1 100.0%	2 / 2 100.0%	1 / 1 100.0%	3 / 3 100.0%	0 / 1 0.0%	3 / 3 100.0%	5 / 6 83.3%	2 / 2 100.0%	1 / 1 100.0%	5 / 5 100.0%	0 / 0 0.0%	0 / 0 0.0%	0 / 0 0.0%	0 / 0 0.0%	31 / 48 64.6%AB	15 / 48 31.2%ABE	1 / 48 2.1%C	1 / 48 2.1%				
1	Thermotoga neapolitana ATCC 49049	46 / 48 95.8%	1 / 1 100.0%	1 / 1 100.0%	1 / 1 100.0%	1 / 1 100.0%	3 / 3 100.0%	3 / 3 100.0%	2 / 2 100.0%	1 / 1 100.0%	3 / 3 100.0%	1 / 1 100.0%	4 / 4 100.0%	2 / 2 100.0%	1 / 1 100.0%	2 / 2 100.0%	1 / 1 100.0%	3 / 3 100.0%	0 / 1 0.0%	3 / 3 100.0%	5 / 6 83.3%	2 / 2 100.0%	1 / 1 100.0%	5 / 5 100.0%	0 / 0 0.0%	0 / 0 0.0%	0 / 0 0.0%	0 / 0 0.0%	31 / 48 64.6%AB	15 / 48 31.2%ABE	1 / 48 2.1%C	1 / 48 2.1%				
1	ATCC BAA-488	45 / 46 97.8%	1 / 1 100.0%	1 / 1 100.0%	1 / 1 100.0%	1 / 1 100.0%	3 / 3 100.0%	3 / 3 100.0%	1 / 1 100.0%	1 / 1 100.0%	3 / 3 100.0%	1 / 1 100.0%	4 / 4 100.0%	2 / 2 100.0%	1 / 1 100.0%	2 / 2 100.0%	1 / 1 100.0%	3 / 3 100.0%	0 / 1 0.0%	3 / 3 100.0%	5 / 5 100.0%	2 / 2 100.0%	1 / 1 100.0%	5 / 5 100.0%	0 / 0 0.0%	0 / 0 0.0%	0 / 0 0.0%	0 / 0 0.0%	29 / 46 63.0%AB	16 / 46 34.8%ABE	1 / 46 2.2%					
1	Thermotoga petrophila RKU-1	45 / 46 97.8%	1 / 1 100.0%	1 / 1 100.0%	1 / 1 100.0%	1 / 1 100.0%	3 / 3 100.0%	3 / 3 100.0%	1 / 1 100.0%	1 / 1 100.0%	3 / 3 100.0%	1 / 1 100.0%	4 / 4 100.0%	2 / 2 100.0%	1 / 1 100.0%	2 / 2 100.0%	1 / 1 100.0%	3 / 3 100.0%	0 / 1 0.0%	3 / 3 100.0%	5 / 5 100.0%	2 / 2 100.0%	1 / 1 100.0%	5 / 5 100.0%	0 / 0 0.0%	0 / 0 0.0%	0 / 0 0.0%	0 / 0 0.0%	29 / 46 63.0%AB	16 / 46 34.8%ABE	1 / 46 2.2%					
[illegible]	DSM [illegible]	45 / 46 [illegible]	1 / 1 [illegible]	1 / 1 [illegible]	1 / 1 [illegible]	1 / 1 [illegible]	3 / 3 [illegible]	3 / 3 [illegible]	1 / 1 [illegible]	1 / 1 [illegible]	3 / 3 [illegible]	1 / 1 [illegible]	4 / 4 [illegible]	2 / 2 [illegible]	1 / 1 [illegible]	2 / 2 [illegible]	1 / 1 [illegible]	3 / 3 [illegible]	0 / 1 [illegible]	3 / 3 [illegible]	5 / 5 [illegible]	2 / 2 [illegible]	1 / 1 [illegible]	5 / 5 [illegible]	0 / 0 [illegible]	0 / 0 [illegible]	0 / 0 [illegible]	0 / 0 [illegible]	29 / 46 [illegible]	16 / 46 [illegible]	1 / 46 [illegible]					

[illegible]	DSM [illegible]	[illegible] 97.8%	[illegible] 100.0%	[illegible] 100.0%	[illegible] 100.0%	[illegible] 100.0%	[illegible] 100.0%	[illegible] 100.0%	[illegible] 100.0%	[illegible] 100.0%	[illegible] 100.0%	[illegible] 100.0%	[illegible] 100.0%	[illegible] 100.0%	[illegible] 100.0%	[illegible] 100.0%	[illegible] 100.0%	[illegible] 100.0%	[illegible] 0.0%	[illegible] 100.0%	[illegible] 100.0%	[illegible] 100.0%	[illegible] 100.0%	[illegible] 100.0%	[illegible] 0.0%	[illegible] 0.0%	[illegible] 0.0%	[illegible] 0.0%	[illegible] 63.0%AB	[illegible] 34.8%ABE	[illegible] 2.2%						
1	Thermotoga naphthophila RKU-10	45 / 46 97.8%	1 / 1 100.0%	1 / 1 100.0%	1 / 1 100.0%	1 / 1 100.0%	3 / 3 100.0%	3 / 3 100.0%	1 / 1 100.0%	1 / 1 100.0%	3 / 3 100.0%	1 / 1 100.0%	4 / 4 100.0%	2 / 2 100.0%	1 / 1 100.0%	2 / 2 100.0%	1 / 1 100.0%	3 / 3 100.0%	0 / 1 0.0%	3 / 3 100.0%	5 / 5 100.0%	2 / 2 100.0%	1 / 1 100.0%	5 / 5 100.0%	0 / 0 0.0%	0 / 0 0.0%	0 / 0 0.0%	0 / 0 0.0%	29 / 46 63.0%AB	16 / 46 34.8%ABE	1 / 46 2.2%						
1	DSM 5069	46 / 46 100.0%	1 / 1 100.0%	1 / 1 100.0%	1 / 1 100.0%	1 / 1 100.0%	3 / 3 100.0%	3 / 3 100.0%	1 / 1 100.0%	1 / 1 100.0%	3 / 3 100.0%	1 / 1 100.0%	4 / 4 100.0%	2 / 2 100.0%	1 / 1 100.0%	2 / 2 100.0%	1 / 1 100.0%	3 / 3 100.0%	1 / 1 100.0%	3 / 3 100.0%	5 / 5 100.0%	2 / 2 100.0%	1 / 1 100.0%	5 / 5 100.0%	0 / 0 0.0%	0 / 0 0.0%	0 / 0 0.0%	0 / 0 0.0%	30 / 46 65.2%AB	16 / 46 34.8%ABE							
1	Thermotoga thermarum DSM 5069	46 / 46 100.0%	1 / 1 100.0%	1 / 1 100.0%	1 / 1 100.0%	1 / 1 100.0%	3 / 3 100.0%	3 / 3 100.0%	1 / 1 100.0%	1 / 1 100.0%	3 / 3 100.0%	1 / 1 100.0%	4 / 4 100.0%	2 / 2 100.0%	1 / 1 100.0%	2 / 2 100.0%	1 / 1 100.0%	3 / 3 100.0%	1 / 1 100.0%	3 / 3 100.0%	5 / 5 100.0%	2 / 2 100.0%	1 / 1 100.0%	5 / 5 100.0%	0 / 0 0.0%	0 / 0 0.0%	0 / 0 0.0%	0 / 0 0.0%	30 / 46 65.2%AB	16 / 46 34.8%ABE							
1	Thermotoga sp. RQ2	45 / 46 97.8%	1 / 1 100.0%	1 / 1 100.0%	1 / 1 100.0%	1 / 1 100.0%	3 / 3 100.0%	3 / 3 100.0%	1 / 1 100.0%	1 / 1 100.0%	3 / 3 100.0%	1 / 1 100.0%	4 / 4 100.0%	2 / 2 100.0%	1 / 1 100.0%	2 / 2 100.0%	1 / 1 100.0%	3 / 3 100.0%	0 / 1 0.0%	3 / 3 100.0%	5 / 5 100.0%	2 / 2 100.0%	1 / 1 100.0%	5 / 5 100.0%	0 / 0 0.0%	0 / 0 0.0%	0 / 0 0.0%	0 / 0 0.0%	16 / 46 34.8%B	13 / 46 28.3%AB	10 / 46 21.7%BE	6 / 46 13.0%ABE	1 / 46 2.2%				
1	ATCC BAA-301	44 / 46 95.7%	1 / 1 100.0%	1 / 1 100.0%	1 / 1 100.0%	1 / 1 100.0%	3 / 3 100.0%	3 / 3 100.0%	1 / 1 100.0%	1 / 1 100.0%	3 / 3 100.0%	1 / 1 100.0%	4 / 4 100.0%	2 / 2 100.0%	1 / 1 100.0%	2 / 2 100.0%	1 / 1 100.0%	3 / 3 100.0%	0 / 1 0.0%	3 / 3 100.0%	5 / 5 100.0%	2 / 2 100.0%	1 / 1 100.0%	4 / 5 80.0%	0 / 0 0.0%	0 / 0 0.0%	0 / 0 0.0%	0 / 0 0.0%	21 / 46 45.7%AB	10 / 46 21.7%ABE	9 / 46 19.6%B	4 / 46 8.7%BE	1 / 46 2.2%E	1 / 46 2.2%CE			
1	Thermotoga lettingae TMO	44 / 46 95.7%	1 / 1 100.0%	1 / 1 100.0%	1 / 1 100.0%	1 / 1 100.0%	3 / 3 100.0%	3 / 3 100.0%	1 / 1 100.0%	1 / 1 100.0%	3 / 3 100.0%	1 / 1 100.0%	4 / 4 100.0%	2 / 2 100.0%	1 / 1 100.0%	2 / 2 100.0%	1 / 1 100.0%	3 / 3 100.0%	0 / 1 0.0%	3 / 3 100.0%	5 / 5 100.0%	2 / 2 100.0%	1 / 1 100.0%	4 / 5 80.0%	0 / 0 0.0%	0 / 0 0.0%	0 / 0 0.0%	0 / 0 0.0%	21 / 46 45.7%AB	10 / 46 21.7%ABE	9 / 46 19.6%B	4 / 46 8.7%BE	1 / 46 2.2%CE	1 / 46 2.2%E			
2	Thermosipho	98 / 98 100.0%	2 / 2 100.0%	4 / 4 100.0%	4 / 4 100.0%	3 / 3 100.0%	6 / 6 100.0%	6 / 6 100.0%	2 / 2 100.0%	2 / 2 100.0%	7 / 7 100.0%	2 / 2 100.0%	8 / 8 100.0%	4 / 4 100.0%	2 / 2 100.0%	4 / 4 100.0%	2 / 2 100.0%	6 / 6 100.0%	2 / 2 100.0%	6 / 6 100.0%	10 / 10 100.0%	4 / 4 100.0%	2 / 2 100.0%	10 / 10 100.0%	0 / 0 0.0%	0 / 0 0.0%	0 / 0 0.0%	0 / 0 0.0%	64 / 98 65.3%AB	34 / 98 34.7%ABE							
1	DSM 5309	48 / 48 100.0%	1 / 1 100.0%	2 / 2 100.0%	2 / 2 100.0%	1 / 1 100.0%	3 / 3 100.0%	3 / 3 100.0%	1 / 1 100.0%	1 / 1 100.0%	3 / 3 100.0%	1 / 1 100.0%	4 / 4 100.0%	2 / 2 100.0%	1 / 1 100.0%	2 / 2 100.0%	1 / 1 100.0%	3 / 3 100.0%	1 / 1 100.0%	3 / 3 100.0%	5 / 5 100.0%	2 / 2 100.0%	1 / 1 100.0%	5 / 5 100.0%	0 / 0 0.0%	0 / 0 0.0%	0 / 0 0.0%	0 / 0 0.0%	31 / 48 64.6%AB	17 / 48 35.4%ABE							
1	Thermosipho africanus TCF52B	48 / 48 100.0%	1 / 1 100.0%	2 / 2 100.0%	2 / 2 100.0%	1 / 1 100.0%	3 / 3 100.0%	3 / 3 100.0%	1 / 1 100.0%	1 / 1 100.0%	3 / 3 100.0%	1 / 1 100.0%	4 / 4 100.0%	2 / 2 100.0%	1 / 1 100.0%	2 / 2 100.0%	1 / 1 100.0%	3 / 3 100.0%	1 / 1 100.0%	3 / 3 100.0%	5 / 5 100.0%	2 / 2 100.0%	1 / 1 100.0%	5 / 5 100.0%	0 / 0 0.0%	0 / 0 0.0%	0 / 0 0.0%	0 / 0 0.0%	31 / 48 64.6%AB	17 / 48 35.4%ABE							
1	CIP 104789	50 / 50 100.0%	1 / 1 100.0%	2 / 2 100.0%	2 / 2 100.0%	2 / 2 100.0%	3 / 3 100.0%	3 / 3 100.0%	1 / 1 100.0%	1 / 1 100.0%	4 / 4 100.0%	1 / 1 100.0%	4 / 4 100.0%	2 / 2 100.0%	1 / 1 100.0%	2 / 2 100.0%	1 / 1 100.0%	3 / 3 100.0%	1 / 1 100.0%	3 / 3 100.0%	5 / 5 100.0%	2 / 2 100.0%	1 / 1 100.0%	5 / 5 100.0%	0 / 0 0.0%	0 / 0 0.0%	0 / 0 0.0%	0 / 0 0.0%	33 / 50 66.0%AB	17 / 50 34.0%ABE							
1	Thermosipho melanesiensis BI429	50 / 50 100.0%	1 / 1 100.0%	2 / 2 100.0%	2 / 2 100.0%	2 / 2 100.0%	3 / 3 100.0%	3 / 3 100.0%	1 / 1 100.0%	1 / 1 100.0%	4 / 4 100.0%	1 / 1 100.0%	4 / 4 100.0%	2 / 2 100.0%	1 / 1 100.0%	2 / 2 100.0%	1 / 1 100.0%	3 / 3 100.0%	1 / 1 100.0%	3 / 3 100.0%	5 / 5 100.0%	2 / 2 100.0%	1 / 1 100.0%	5 / 5 100.0%	0 / 0 0.0%	0 / 0 0.0%	0 / 0 0.0%	0 / 0 0.0%	33 / 50 66.0%AB	17 / 50 34.0%ABE							
2	Fervidibacterium	98 / 98 100.0%	2 / 2 100.0%	2 / 2 100.0%	3 / 3 100.0%	4 / 4 100.0%	6 / 6 100.0%	6 / 6 100.0%	2 / 2 100.0%	2 / 2 100.0%	8 / 8 100.0%	2 / 2 100.0%	8 / 8 100.0%	4 / 4 100.0%	2 / 2 100.0%	4 / 4 100.0%	2 / 2 100.0%	6 / 6 100.0%	2 / 2 100.0%	7 / 7 100.0%	10 / 10 100.0%	4 / 4 100.0%	2 / 2 100.0%	10 / 10 100.0%	0 / 0 0.0%	0 / 0 0.0%	0 / 0 0.0%	0 / 0 0.0%	50 / 98 51.0%AB	22 / 98 22.4%ABE	20 / 98 20.4%B	6 / 98 6.1%BE					
1	ATCC 35602	50 / 50 100.0%	1 / 1 100.0%	1 / 1 100.0%	2 / 2 100.0%	2 / 2 100.0%	3 / 3 100.0%	3 / 3 100.0%	1 / 1 100.0%	1 / 1 100.0%	4 / 4 100.0%	1 / 1 100.0%	4 / 4 100.0%	2 / 2 100.0%	1 / 1 100.0%	2 / 2 100.0%	1 / 1 100.0%	3 / 3 100.0%	1 / 1 100.0%	4 / 4 100.0%	5 / 5 100.0%	2 / 2 100.0%	1 / 1 100.0%	5 / 5 100.0%	0 / 0 0.0%	0 / 0 0.0%	0 / 0 0.0%	0 / 0 0.0%	20 / 50 40.0%B	16 / 50 32.0%AB	8 / 50 16.0%ABE	6 / 50 12.0%BE					
1	Fervidobacterium nodosum Rt17-B1	50 / 50 100.0%	1 / 1 100.0%	1 / 1 100.0%	2 / 2 100.0%	2 / 2 100.0%	3 / 3 100.0%	3 / 3 100.0%	1 / 1 100.0%	1 / 1 100.0%	4 / 4 100.0%	1 / 1 100.0%	4 / 4 100.0%	2 / 2 100.0%	1 / 1 100.0%	2 / 2 100.0%	1 / 1 100.0%	3 / 3 100.0%	1 / 1 100.0%	4 / 4 100.0%	5 / 5 100.0%	2 / 2 100.0%	1 / 1 100.0%	5 / 5 100.0%	0 / 0 0.0%	0 / 0 0.0%	0 / 0 0.0%	0 / 0 0.0%	20 / 50 40.0%B	16 / 50 32.0%AB	8 / 50 16.0%ABE	6 / 50 12.0%BE					
1	DSM 9078	48 / 48 100.0%	1 / 1 100.0%	1 / 1 100.0%	1 / 1 100.0%	2 / 2 100.0%	3 / 3 100.0%	3 / 3 100.0%	1 / 1 100.0%	1 / 1 100.0%	4 / 4 100.0%	1 / 1 100.0%	4 / 4 100.0%	2 / 2 100.0%	1 / 1 100.0%	2 / 2 100.0%	1 / 1 100.0%	3 / 3 100.0%	1 / 1 100.0%	3 / 3 100.0%	5 / 5 100.0%	2 / 2 100.0%	1 / 1 100.0%	5 / 5 100.0%	0 / 0 0.0%	0 / 0 0.0%	0 / 0 0.0%	0 / 0 0.0%	34 / 48 70.8%AB	14 / 48 29.2%ABE							
1	Fervidobacterium pennevorans DSM 9078	48 / 48 100.0%	1 / 1 100.0%	1 / 1 100.0%	1 / 1 100.0%	2 / 2 100.0%	3 / 3 100.0%	3 / 3 100.0%	1 / 1 100.0%	1 / 1 100.0%	4 / 4 100.0%	1 / 1 100.0%	4 / 4 100.0%	2 / 2 100.0%	1 / 1 100.0%	2 / 2 100.0%	1 / 1 100.0%	3 / 3 100.0%	1 / 1 100.0%	3 / 3 100.0%	5 / 5 100.0%	2 / 2 100.0%	1 / 1 100.0%	5 / 5 100.0%	0 / 0 0.0%	0 / 0 0.0%	0 / 0 0.0%	0 / 0 0.0%	34 / 48 70.8%AB	14 / 48 29.2%ABE							
1	Petrotoga	46 / 48 95.8%	1 / 1 100.0%	2 / 2 100.0%	1 / 1 100.0%	1 / 1 100.0%	4 / 4 100.0%	3 / 3 100.0%	1 / 1 100.0%	1 / 1 100.0%	3 / 3 100.0%	1 / 1 100.0%	4 / 4 100.0%	2 / 2 100.0%	1 / 1 100.0%	2 / 2 100.0%	1 / 1 100.0%	3 / 3 100.0%	1 / 1 100.0%	3 / 3 100.0%	3 / 5 60.0%	2 / 2 100.0%	1 / 1 100.0%	5 / 5 100.0%	0 / 0 0.0%	0 / 0 0.0%	0 / 0 0.0%	0 / 0 0.0%	16 / 48 33.3%B	14 / 48 29.2%AB	9 / 48 18.8%BE	7 / 48 14.6%ABE	1 / 48 2.1%E	1 / 48 2.1%			
1	DSM 10674	46 / 48 95.8%	1 / 1 100.0%	2 / 2 100.0%	1 / 1 100.0%	1 / 1 100.0%	4 / 4 100.0%	3 / 3 100.0%	1 / 1 100.0%	1 / 1 100.0%	3 / 3 100.0%	1 / 1 100.0%	4 / 4 100.0%	2 / 2 100.0%	1 / 1 100.0%	2 / 2 100.0%	1 / 1 100.0%	3 / 3 100.0%	1 / 1 100.0%	3 / 3 100.0%	3 / 5 60.0%	2 / 2 100.0%	1 / 1 100.0%	5 / 5 100.0%	0 / 0 0.0%	0 / 0 0.0%	0 / 0 0.0%	0 / 0 0.0%	16 / 48 33.3%B	14 / 48 29.2%AB	9 / 48 18.8%BE	7 / 48 14.6%ABE	1 / 48 2.1%	1 / 48 2.1%E			
1	Petrotoga mobilis DSM 10674	46 / 48 95.8%	1 / 1 100.0%	2 / 2 100.0%	1 / 1 100.0%	1 / 1 100.0%	4 / 4 100.0%	3 / 3 100.0%	1 / 1 100.0%	1 / 1 100.0%	3 / 3 100.0%	1 / 1 100.0%	4 / 4 100.0%	2 / 2 100.0%	1 / 1 100.0%	2 / 2 100.0%	1 / 1 100.0%	3 / 3 100.0%	1 / 1 100.0%	3 / 3 100.0%	3 / 5 60.0%	2 / 2 100.0%	1 / 1 100.0%	5 / 5 100.0%	0 / 0 0.0%	0 / 0 0.0%	0 / 0 0.0%	0 / 0 0.0%	16 / 48 33.3%B	14 / 48 29.2%AB	9 / 48 18.8%BE	7 / 48 14.6%ABE	1 / 48 2.1%	1 / 48 2.1%E			
1	Marinitoga	51 / 51 100.0%	2 / 2 100.0%	1 / 1 100.0%	1 / 1 100.0%	1 / 1 100.0%	3 / 3 100.0%	3 / 3 100.0%	1 / 1 100.0%	1 / 1 100.0%	3 / 3 100.0%	1 / 1 100.0%	4 / 4 100.0%	2 / 2 100.0%	2 / 2 100.0%	2 / 2 100.0%	1 / 1 100.0%	4 / 4 100.0%	2 / 2 100.0%	3 / 3 100.0%	4 / 4 100.0%	3 / 3 100.0%	1 / 1 100.0%	6 / 6 100.0%	0 / 0 0.0%	0 / 0 0.0%	0 / 0 0.0%	0 / 0 0.0%	31 / 51 60.8%AB	20 / 51 39.2%ABE							
1	DSM 14283	51 / 51 100.0%	2 / 2 100.0%	1 / 1 100.0%	1 / 1 100.0%	1 / 1 100.0%	3 / 3 100.0%	3 / 3 100.0%	1 / 1 100.0%	1 / 1 100.0%	3 / 3 100.0%	1 / 1 100.0%	4 / 4 100.0%	2 / 2 100.0%	2 / 2 100.0%	2 / 2 100.0%	1 / 1 100.0%	4 / 4 100.0%	2 / 2 100.0%	3 / 3 100.0%	4 / 4 100.0%	3 / 3 100.0%	1 / 1 100.0%	6 / 6 100.0%	0 / 0 0.0%	0 / 0 0.0%	0 / 0 0.0%	0 / 0 0.0%	31 / 51 60.8%AB	20 / 51 39.2%ABE							
1	Marinitoga piezophila KA3	51 / 51 100.0%	2 / 2 100.0%	1 / 1 100.0%	1 / 1 100.0%	1 / 1 100.0%	3 / 3 100.0%	3 / 3 100.0%	1 / 1 100.0%	1 / 1 100.0%	3 / 3 100.0%	1 / 1 100.0%	4 / 4 100.0%	2 / 2 100.0%	2 / 2 100.0%	2 / 2 100.0%	1 / 1 100.0%	4 / 4 100.0%	2 / 2 100.0%	3 / 3 100.0%	4 / 4 100.0%	3 / 3 100.0%	1 / 1 100.0%	6 / 6 100.0%	0 / 0 0.0%	0 / 0 0.0%	0 / 0 0.0%	0 / 0 0.0%	31 / 51 60.8%AB	20 / 51 39.2%ABE							
1	Kosmotoga	46 / 46 100.0%	1 / 1 100.0%	1 / 1 100.0%	1 / 1 100.0%	1 / 1 100.0%	3 / 3 100.0%	3 / 3 100.0%	1 / 1 100.0%	1 / 1 100.0%	3 / 3 100.0%	1 / 1 100.0%	4 / 4 100.0%	2 / 2 100.0%	1 / 1 100.0%	2 / 2 100.0%	1 / 1 100.0%	3 / 3 100.0%	1 / 1 100.0%	3 / 3 100.0%	5 / 5 100.0%	2 / 2 100.0%	1 / 1 100.0%	5 / 5 100.0%	0 / 0 0.0%	0 / 0 0.0%	0 / 0 0.0%	0 / 0 0.0%	31 / 46 67.4%AB	15 / 46 32.6%ABE							
1	ATCC BAA-1733	46 / 46 100.0%	1 / 1 100.0%	1 / 1 100.0%	1 / 1 100.0%	1 / 1 100.0%	3 / 3 100.0%	3 / 3 100.0%	1 / 1 100.0%	1 / 1 100.0%	3 / 3 100.0%	1 / 1 100.0%	4 / 4 100.0%	2 / 2 100.0%	1 / 1 100.0%	2 / 2 100.0%	1 / 1 100.0%	3 / 3 100.0%	1 / 1 100.0%	3 / 3 100.0%	5 / 5 100.0%	2 / 2 100.0%	1 / 1 100.0%	5 / 5 100.0%	0 / 0 0.0%	0 / 0 0.0%	0 / 0 0.0%	0 / 0 0.0%	31 / 46 67.4%AB	15 / 46 32.6%ABE							
1	Kosmotoga olearia TB 19.5.1	46 / 46 100.0%	1 / 1 100.0%	1 / 1 100.0%	1 / 1 100.0%	1 / 1 100.0%	3 / 3 100.0%	3 / 3 100.0%	1 / 1 100.0%	1 / 1 100.0%	3 / 3 100.0%	1 / 1 100.0%	4 / 4 100.0%	2 / 2 100.0%	1 / 1 100.0%	2 / 2 100.0%	1 / 1 100.0%	3 / 3 100.0%	1 / 1 100.0%	3 / 3 100.0%	5 / 5 100.0%	2 / 2 100.0%	1 / 1 100.0%	5 / 5 100.0%	0 / 0 0.0%	0 / 0 0.0%	0 / 0 0.0%	0 / 0 0.0%	31 / 46 67.4%AB	15 / 46 32.6%ABE							
1	'Mesotoga'	46 / 46 100.0%	1 / 1 100.0%	1 / 1 100.0%	1 / 1 100.0%	1 / 1 100.0%	3 / 3 100.0%	3 / 3 100.0%	1 / 1 100.0%	1 / 1 100.0%	3 / 3 100.0%	1 / 1 100.0%	4 / 4 100.0%	2 / 2 100.0%	1 / 1 100.0%	2 / 2 100.0%	1 / 1 100.0%	3 / 3 100.0%	1 / 1 100.0%	3 / 3 100.0%	5 / 5 100.0%	2 / 2 100.0%	1 / 1 100.0%	5 / 5 100.0%	0 / 0 0.0%	0 / 0 0.0%	0 / 0 0.0%	0 / 0 0.0%	30 / 46 65.2%AB	16 / 46 34.8%ABE							
1	'Mesotoga prima'	46 / 46 100.0%	1 / 1 100.0%	1 / 1 100.0%	1 / 1 100.0%	1 / 1 100.0%	3 / 3 100.0%	3 / 3 100.0%	1 / 1 100.0%	1 / 1 100.0%	3 / 3 100.0%	1 / 1 100.0%	4 / 4 100.0%	2 / 2 100.0%	1 / 1 100.0%	2 / 2 100.0%	1 / 1 100.0%	3 / 3 100.0%	1 / 1 100.0%	3 / 3 100.0%	5 / 5 100.0%	2 / 2 100.0%	1 / 1 100.0%	5 / 5 100.0%	0 / 0 0.0%	0 / 0 0.0%	0 / 0 0.0%	0 / 0 0.0%	30 / 46 65.2%AB	16 / 46 34.8%ABE							
1	Mesotoga prima MesG1.Ag.4.2	46 / 46 100.0%	1 / 1 100.0%	1 / 1 100.0%	1 / 1 100.0%	1 / 1 100.0%	3 / 3 100.0%	3 / 3 100.0%	1 / 1 100.0%	1 / 1 100.0%	3 / 3 100.0%	1 / 1 100.0%	4 / 4 100.0%	2 / 2 100.0%	1 / 1 100.0%	2 / 2 100.0%	1 / 1 100.0%	3 / 3 100.0%	1 / 1 100.0%	3 / 3 100.0%	5 / 5 100.0%	2 / 2 100.0%	1 / 1 100.0%	5 / 5 100.0%	0 / 0 0.0%	0 / 0 0.0%	0 / 0 0.0%	0 / 0 0.0%	30 / 46 65.2%AB	16 / 46 34.8%ABE							
4	Deferribacteres	31 / 169 18.3%	4 / 4 100.0%	0 / 4 0.0%	1 / 4 25.0%	2 / 8 25.0%	0 / 15 0.0%	0 / 8 0.0%	0 / 4 0.0%	0 / 4 0.0%	2 / 12 16.7%	4 / 4 100.0%	2 / 14 14.3%	1 / 4 25.0%	3 / 4 75.0%	3 / 4 75.0%	0 / 4 0.0%	2 / 11 18.2%	0 / 4 0.0%	0 / 10 0.0%	0 / 16 0.0%	0 / 4 0.0%	1 / 4 25.0%	3 / 20 15.0%	3 / 3 100.0%	0 / 0 0.0%	0 / 0 0.0%	0 / 0 0.0%	83 / 169 49.1%E	54 / 169 32.0%	21 / 169 12.4%AB	10 / 169 5.9%ABE	1 / 169 0.6%CE				
4	Deferribacteres	31 / 169 18.3%	4 / 4 100.0%	0 / 4 0.0%	1 / 4 25.0%	2 / 8 25.0%	0 / 15 0.0%	0 / 8 0.0%	0 / 4 0.0%	0 / 4 0.0%	2 / 12 16.7%	4 / 4 100.0%	2 / 14 14.3%	1 / 4 25.0%	3 / 4 75.0%	3 / 4 75.0%	0 / 4 0.0%	2 / 11 18.2%	0 / 4 0.0%	0 / 10 0.0%	0 / 16 0.0%	0 / 4 0.0%	1 / 4 25.0%	3 / 20 15.0%	3 / 3 100.0%	0 / 0 0.0%	0 / 0 0.0%	0 / 0 0.0%	83 / 169 49.1%E	54 / 169 32.0%	21 / 169 12.4%AB	10 / 169 5.9%ABE	1 / 169 0.6%CE				
4	Deferribacterales	31 / 169 18.3%	4 / 4 100.0%	0 / 4 0.0%	1 / 4 25.0%	2 / 8 25.0%	0 / 15 0.0%	0 / 8 0.0%	0 / 4 0.0%	0 / 4 0.0%	2 / 12 16.7%	4 / 4 100.0%	2 / 14 14.3%	1 / 4 25.0%	3 / 4 75.0%	3 / 4 75.0%	0 / 4 0.0%	2 / 11 18.2%	0 / 4 0.0%	0 / 10 0.0%	0 / 16 0.0%	0 / 4 0.0%	1 / 4 25.0%	3 / 20 15.0%	3 / 3 100.0%	0 / 0 0.0%	0 / 0 0.0%	0 / 0 0.0%	83 / 169 49.1%E	54 / 169 32.0%	21 / 169 12.4%AB	10 / 169 5.9%ABE	1 / 169 0.6%CE				
4	Deferribacteraceae	31 / 169 18.3%	4 / 4 100.0%	0 / 4 0.0%	1 / 4 25.0%	2 / 8 25.0%	0 / 15 0.0%	0 / 8 0.0%	0 / 4 0.0%	0 / 4 0.0%	2 / 12 16.7%	4 / 4 100.0%	2 / 14 14.3%	1 / 4 25.0%	3 / 4 75.0%	3 / 4 75.0%	0 / 4 0.0%	2 / 11 18.2%	0 / 4 0.0%	0 / 10 0.0%	0 / 16 0.0%	0 / 4 0.0%	1 / 4 25.0%	3 / 20 15.0%	3 / 3 100.0%	0 / 0 0.0%	0 / 0 0.0%	0 / 0 0.0%	83 / 169 49.1%E	54 / 169 32.0%	21 / 169 12.4%AB	10 / 169 5.9%ABE	1 / 169 0.6%CE				
1	Flexistipes	7 / 44 15.9%	1 / 1 100.0%	0 / 1 0.0%	0 / 1 0.0%	0 / 2 0.0%	0 / 4 0.0%	0 / 2 0.0%	0 / 1 0.0%	0 / 1 0.0%	0 / 3 0.0%	1 / 1 100.0%	1 / 4 25.0%	0 / 1 0.0%	0 / 1 0.0%	1 / 1 100.0%	0 / 1 0.0%	0 / 3 0.0%	0 / 1 0.0%	0 / 3 0.0%	0 / 4 0.0%	0 / 1 0.0%	0 / 1 0.0%	2 / 5 40.0%	1 / 1 100.0%	0 / 0 0.0%	0 / 0 0.0%	0 / 0 0.0%	19 / 44 43.2%E	17 / 44 38.6%	5 / 44 11.4%ABE	2 / 44 4.5%AB	1 / 44 2.3%CE				
1	ATCC 49648	7 / 44 15.9%	1 / 1 100.0%	0 / 1 0.0%	0 / 1 0.0%	0 / 2 0.0%	0 / 4 0.0%	0 / 2 0.0%	0 / 1 0.0%	0 / 1 0.0%	0 / 3 0.0%	1 / 1 100.0%	1 / 4 25.0%	0 / 1 0.0%	0 / 1 0.0%	1 / 1 100.0%	0 / 1 0.0%	0 / 3 0.0%	0 / 1 0.0%	0 / 3 0.0%	0 / 4 0.0%	0 / 1 0.0%	0 / 1 0.0%	2 / 5 40.0%	1 / 1 100.0%	0 / 0 0.0%	0 / 0 0.0%	0 / 0 0.0%	19 / 44 43.2%E	17 / 44 38.6%	5 / 44 11.4%ABE	2 / 44 4.5%AB	1 / 44 2.3%CE				
1	Flexistipes sinusarabici DSM 4947	7 / 44 15.9%	1 / 1 100.0%	0 / 1 0.0%	0 / 1 0.0%	0 / 2 0.0%	0 / 4 0.0%	0 / 2 0.0%	0 / 1 0.0%	0 / 1 0.0%	0 / 3 0.0%	1 / 1 100.0%	1 / 4 25.0%	0 / 1 0.0%	0 / 1 0.0%	1 / 1 100.0%	0 / 1 0.0%	0 / 3 0.0%	0 / 1 0.0%	0 / 3 0.0%	0 / 4 0.0%	0 / 1 0.0%	0 / 1 0.0%	2 / 5 40.0%	1 / 1 100.0%	0 / 0 0.0%	0 / 0 0.0%	0 / 0 0.0%	19 / 44 43.2%E	17 / 44 38.6%	5 / 44 11.4%ABE	2 / 44 4.5%AB	1 / 44 2.3%CE				
1	Deferribacter	9 / 43 20.9%	1 / 1 100.0%	0 / 1 0.0%	1 / 1 100.0%	0 / 2 0.0%	0 / 4 0.0%	0 / 2 0.0%	0 / 1 0.0%	0 / 1 0.0%	2 / 3 66.7%	1 / 1 100.0%	0 / 4 0.0%	1 / 1 100.0%	1 / 1 100.0%	0 / 1 0.0%	0 / 1 0.0%	1 / 2 50.0%	0 / 1 0.0%	0 / 3 0.0%	0 / 4 0.0%	0 / 1 0.0%	0 / 1 0.0%	0 / 5 0.0%	1 / 1 100.0%	0 / 0 0.0%	0 / 0 0.0%	0 / 0 0.0%	24 / 43 55.8%E	10 / 43 23.3%	8 / 43 18.6%AB	1 / 43 2.3%ABE					
1	DSM 14783	9 / 43 20.9%	1 / 1 100.0%	0 / 1 0.0%	1 / 1 100.0%	0 / 2 0.0%	0 / 4 0.0%	0 / 2 0.0%	0 / 1 0.0%	0 / 1 0.0%	2 / 3 66.7%	1 / 1 100.0%	0 / 4 0.0%	1 / 1 100.0%	1 / 1 100.0%	0 / 1 0.0%	0 / 1 0.0%	1 / 2 50.0%	0 / 1 0.0%	0 / 3 0.0%	0 / 4 0.0%	0 / 1 0.0%	0 / 1 0.0%	0 / 5 0.0%	1 / 1 100.0%	0 / 0 0.0%	0 / 0 0.0%	0 / 0 0.0%	24 / 43 55.8%E	10 / 43 23.3%	8 / 43 18.6%AB	1 / 43 2.3%ABE					
1	Deferribacter desulfuricans SMM1	9 / 43 20.9%	1 / 1 100.0%	0 / 1 0.0%	1 / 1 100.0%	0 / 2 0.0%	0 / 4 0.0%	0 / 2 0.0%	0 / 1 0.0%	0 / 1 0.0%	2 / 3 66.7%	1 / 1 100.0%	0 / 4 0.0%	1 / 1 100.0%	1 / 1 100.0%	0 / 1 0.0%	0 / 1 0.0%	1 / 2 50.0%	0 / 1 0.0%	0 / 3 0.0%	0 / 4 0.0%	0 / 1 0.0%	0 / 1 0.0%	0 / 5 0.0%	1 / 1 100.0%	0 / 0 0.0%	0 / 0 0.0%	0 / 0 0.0%	24 / 43 55.8%E	10 / 43 23.3%	8 / 43 18.6%AB	1 / 43 2.3%ABE					
1	Denitrovibrio	6 / 42 14.3%	1 / 1 100.0%	0 / 1 0.0%	0 / 1 0.0%	0 / 2 0.0%	0 / 4 0.0%	0 / 2 0.0%	0 / 1 0.0%	0 / 1 0.0%	0 / 3 0.0%	1 / 1 100.0%	0 / 3 0.0%	0 / 1 0.0%	1 / 1 100.0%	1 / 1 100.0%	0 / 1 0.0%	0 / 3 0.0%	0 / 1 0.0%	0 / 2 0.0%	0 / 4 0.0%	0 / 1 0.0%	0 / 1 0.0%	1 / 5 20.0%	1 / 1 100.0%	0 / 0 0.0%	0 / 0 0.0%	0 / 0 0.0%	21 / 42 50.0%E	15 / 42 35.7%	4 / 42 9.5%AB	2 / 42 4.8%ABE					
1	DSM 12809	6 / 42 14.3%	1 / 1 100.0%	0 / 1 0.0%	0 / 1 0.0%	0 / 2 0.0%	0 / 4 0.0%	0 / 2 0.0%	0 / 1 0.0%	0 / 1 0.0%	0 / 3 0.0%	1 / 1 100.0%	0 / 3 0.0%	0 / 1 0.0%	1 / 1 100.0%	1 / 1 100.0%	0 / 1 0.0%	0 / 3 0.0%	0 / 1 0.0%	0 / 2 0.0%	0 / 4 0.0%	0 / 1 0.0%	0 / 1 0.0%	1 / 5 20.0%	1 / 1 100.0%	0 / 0 0.0%	0 / 0 0.0%	0 / 0 0.0%	21 / 42 50.0%E	15 / 42 35.7%	4 / 42 9.5%AB	2 / 42 4.8%ABE					
1	Denitrovibrio acetiphilus DSM 12809	6 / 42 14.3%	1 / 1 100.0%	0 / 1 0.0%	0 / 1 0.0%	0 / 2 0.0%	0 / 4 0.0%	0 / 2 0.0%	0 / 1 0.0%	0 / 1 0.0%	0 / 3 0.0%	1 / 1 100.0%	0 / 3 0.0%	0 / 1 0.0%	1 / 1 100.0%	1 / 1 100.0%	0 / 1 0.0%	0 / 3 0.0%	0 / 1 0.0%	0 / 2 0.0%	0 / 4 0.0%	0 / 1 0.0%	0 / 1 0.0%	1 / 5 20.0%	1 / 1 100.0%	0 / 0 0.0%	0 / 0 0.0%	0 / 0 0.0%	21 / 42 50.0%E	15 / 42 35.7%	4 / 42 9.5%AB	2 / 42 4.8%ABE					
1	Calditerrivibrio	9 / 40 22.5%	1 / 1 100.0%	0 / 1 0.0%	0 / 1 0.0%	2 / 2 100.0%	0 / 3 0.0%	0 / 2 0.0%	0 / 1 0.0%	0 / 1 0.0%	0 / 3 0.0%	1 / 1 100.0%	1 / 3 33.3%	0 / 1 0.0%	1 / 1 100.0%	1 / 1 100.0%	0 / 1 0.0%	1 / 3 33.3%	0 / 1 0.0%	0 / 2 0.0%	0 / 4 0.0%	0 / 1 0.0%	1 / 1 100.0%	0 / 5 0.0%	0 / 0 0.0%	0 / 0 0.0%	0 / 0 0.0%	0 / 0 0.0%	19 / 40 47.5%E	12 / 40 30.0%	7 / 40 17.5%AB	2 / 40 5.0%ABE					
1	Calditerrivibrio nitroreducens	9 / 40 22.5%	1 / 1 100.0%	0 / 1 0.0%	0 / 1 0.0%	2 / 2 100.0%	0 / 3 0.0%	0 / 2 0.0%	0 / 1 0.0%	0 / 1 0.0%	0 / 3 0.0%	1 / 1 100.0%	1 / 3 33.3%	0 / 1 0.0%	1 / 1 100.0%	1 / 1 100.0%	0 / 1 0.0%	1 / 3 33.3%	0 / 1 0.0%	0 / 2 0.0%	0 / 4 0.0%	0 / 1 0.0%	1 / 1 100.0%	0 / 5 0.0%	0 / 0 0.0%	0 / 0 0.0%	0 / 0 0.0%	0 / 0 0.0%	19 / 40 47.5%E	12 / 40 30.0%	7 / 40 17.5%AB	2 / 40 5.0%ABE					
1	Calditerrivibrio nitroreducens DSM 19672	9 / 40 22.5%	1 / 1 100.0%	0 / 1 0.0%	0 / 1 0.0%	2 / 2 100.0%	0 / 3 0.0%	0 / 2 0.0%	0 / 1 0.0%	0 / 1 0.0%	0 / 3 0.0%	1 / 1 100.0%	1 / 3 33.3%	0 / 1 0.0%	1 / 1 100.0%	1 / 1 100.0%	0 / 1 0.0%	1 / 3 33.3%	0 / 1 0.0%	0 / 2 0.0%	0 / 4 0.0%	0 / 1 0.0%	1 / 1 100.0%	0 / 5 0.0%	0 / 0 0.0%	0 / 0 0.0%	0 / 0 0.0%	0 / 0 0.0%	19 / 40 47.5%E	12 / 40 30.0%	7 / 40 17.5%AB	2 / 40 5.0%ABE					
1	Chrysiogenetes	37 / 37 100.0%	1 / 1 100.0%	1 / 1 100.0%	1 / 1 100.0%	1 / 1 100.0%	2 / 2 100.0%	2 / 2 100.0%	1 / 1 100.0%	1 / 1 100.0%	2 / 2 100.0%	1 / 1 100.0%	3 / 3 100.0%	1 / 1 100.0%	1 / 1 100.0%	1 / 1 100.0%	1 / 1 100.0%	3 / 3 100.0%	1 / 1 100.0%	2 / 2 100.0%	4 / 4 100.0%	1 / 1 100.0%	1 / 1 100.0%	5 / 5 100.0%	0 / 0 0.0%	0 / 0 0.0%	0 / 0 0.0%	0 / 0 0.0%	24 / 37 64.9%AB	13 / 37 35.1%ABE							
1	Chrysiogenes group	37 / 37 100.0%	1 / 1 100.0%	1 / 1 100.0%	1 / 1 100.0%	1 / 1 100.0%	2 / 2 100.0%	2 / 2 100.0%	1 / 1 100.0%	1 / 1 100.0%	2 / 2 100.0%	1 / 1 100.0%	3 / 3 100.0%	1 / 1 100.0%	1 / 1 100.0%	1 / 1 100.0%	1 / 1 100.0%	3 / 3 100.0%	1 / 1 100.0%	2 / 2 100.0%	4 / 4 100.0%	1 / 1 100.0%	1 / 1 100.0%	5 / 5 100.0%	0 / 0 0.0%	0 / 0 0.0%	0 / 0 0.0%	0 / 0 0.0%	24 / 37 64.9%AB	13 / 37 35.1%ABE							
1	Chrysiogenales	37 / 37 100.0%	1 / 1 100.0%	1 / 1 100.0%	1 / 1 100.0%	1 / 1 100.0%	2 / 2 100.0%	2 / 2 100.0%	1 / 1 100.0%	1 / 1 100.0%	2 / 2 100.0%	1 / 1 100.0%	3 / 3 100.0%	1 / 1 100.0%	1 / 1 100.0%	1 / 1 100.0%	1 / 1 100.0%	3 / 3 100.0%	1 / 1 100.0%	2 / 2 100.0%	4 / 4 100.0%	1 / 1 100.0%	1 / 1 100.0%	5 / 5 100.0%	0 / 0 0.0%	0 / 0 0.0%	0 / 0 0.0%	0 / 0 0.0%	24 / 37 64.9%AB	13 / 37 35.1%ABE							
1	Chrysiogenaceae	37 / 37 100.0%	1 / 1 100.0%	1 / 1 100.0%	1 / 1 100.0%	1 / 1 100.0%	2 / 2 100.0%	2 / 2 100.0%	1 / 1 100.0%	1 / 1 100.0%	2 / 2 100.0%	1 / 1 100.0%	3 / 3 100.0%	1 / 1 100.0%	1 / 1 100.0%	1 / 1 100.0%	1 / 1 100.0%	3 / 3 100.0%	1 / 1 100.0%	2 / 2 100.0%	4 / 4 100.0%	1 / 1 100.0%	1 / 1 100.0%	5 / 5 100.0%	0 / 0 0.0%	0 / 0 0.0%	0 / 0 0.0%	0 / 0 0.0%	24 / 37 64.9%AB	13 / 37 35.1%ABE							
1	Desulfurispirillum	37 / 37 100.0%	1 / 1 100.0%	1 / 1 100.0%	1 / 1 100.0%	1 / 1 100.0%	2 / 2 100.0%	2 / 2 100.0%	1 / 1 100.0%	1 / 1 100.0%	2 / 2 100.0%	1 / 1 100.0%	3 / 3 100.0%	1 / 1 100.0%	1 / 1 100.0%	1 / 1 100.0%	1 / 1 100.0%	3 / 3 100.0%	1 / 1 100.0%	2 / 2 100.0%	4 / 4 100.0%	1 / 1 100.0%	1 / 1 100.0%	5 / 5 100.0%	0 / 0 0.0%	0 / 0 0.0%	0 / 0 0.0%	0 / 0 0.0%	24 / 37 64.9%AB	13 / 37 35.1%ABE							
1	"Selenospirillum indicus" Narasingarao and Haggblom 2006	37 / 37 100.0%	1 / 1 100.0%	1 / 1 100.0%	1 / 1 100.0%	1 / 1 100.0%	2 / 2 100.0%	2 / 2 100.0%	1 / 1 100.0%	1 / 1 100.0%	2 / 2 100.0%	1 / 1 100.0%	3 / 3 100.0%	1 / 1 100.0%	1 / 1 100.0%	1 / 1 100.0%	1 / 1 100.0%	3 / 3 100.0%	1 / 1 100.0%	2 / 2 100.0%	4 / 4 100.0%	1 / 1 100.0%	1 / 1 100.0%	5 / 5 100.0%	0 / 0 0.0%	0 / 0 0.0%	0 / 0 0.0%	0 / 0 0.0%	24 / 37 64.9%AB	13 / 37 35.1%ABE							
1	Desulfurispirillum indicum S5	37 / 37 100.0%	1 / 1 100.0%	1 / 1 100.0%	1 / 1 100.0%	1 / 1 100.0%	2 / 2 100.0%	2 / 2 100.0%	1 / 1 100.0%	1 / 1 100.0%	2 / 2 100.0%	1 / 1 100.0%	3 / 3 100.0%	1 / 1 100.0%	1 / 1 100.0%	1 / 1 100.0%	1 / 1 100.0%	3 / 3 100.0%	1 / 1 100.0%	2 / 2 100.0%	4 / 4 100.0%	1 / 1 100.0%	1 / 1 100.0%	5 / 5 100.0%	0 / 0 0.0%	0 / 0 0.0%	0 / 0 0.0%	0 / 0 0.0%	24 / 37 64.9%AB	13 / 37 35.1%ABE							
2	Thermodesulfobacteria	59 / 95 62.1%	2 / 2 100.0%	1 / 2 50.0%	1 / 2 50.0%	2 / 3 66.7%	5 / 6 83.3%	4 / 6 66.7%	2 / 2 100.0%	1 / 2 50.0%	5 / 7 71.4%	2 / 2 100.0%	5 / 8 62.5%	2 / 4 50.0%	0 / 2 0.0%	4 / 4 100.0%	1 / 2 50.0%	3 / 6 50.0%	1 / 2 50.0%	2 / 6 33.3%	5 / 9 55.6%	2 / 4 50.0%	1 / 2 50.0%	6 / 10 60.0%	2 / 2 100.0%	0 / 0 0.0%	0 / 0 0.0%	0 / 0 0.0%	36 / 95 37.9%AB	23 / 95 24.2%ABE	17 / 95 17.9%E	16 / 95 16.8%	2 / 95 2.1%C	1 / 95 1.1%CE			
2	Thermodesulfobacteria	59 / 95 62.1%	2 / 2 100.0%	1 / 2 50.0%	1 / 2 50.0%	2 / 3 66.7%	5 / 6 83.3%	4 / 6 66.7%	2 / 2 100.0%	1 / 2 50.0%	5 / 7 71.4%	2 / 2 100.0%	5 / 8 62.5%	2 / 4 50.0%	0 / 2 0.0%	4 / 4 100.0%	1 / 2 50.0%	3 / 6 50.0%	1 / 2 50.0%	2 / 6 33.3%	5 / 9 55.6%	2 / 4 50.0%	1 / 2 50.0%	6 / 10 60.0%	2 / 2 100.0%	0 / 0 0.0%	0 / 0 0.0%	0 / 0 0.0%	36 / 95 37.9%AB	23 / 95 24.2%ABE	17 / 95 17.9%E	16 / 95 16.8%	2 / 95 2.1%C	1 / 95 1.1%CE			
2	Thermodesulfobacteriales	59 / 95 62.1%	2 / 2 100.0%	1 / 2 50.0%	1 / 2 50.0%	2 / 3 66.7%	5 / 6 83.3%	4 / 6 66.7%	2 / 2 100.0%	1 / 2 50.0%	5 / 7 71.4%	2 / 2 100.0%	5 / 8 62.5%	2 / 4 50.0%	0 / 2 0.0%	4 / 4 100.0%	1 / 2 50.0%	3 / 6 50.0%	1 / 2 50.0%	2 / 6 33.3%	5 / 9 55.6%	2 / 4 50.0%	1 / 2 50.0%	6 / 10 60.0%	2 / 2 100.0%	0 / 0 0.0%	0 / 0 0.0%	0 / 0 0.0%	36 / 95 37.9%AB	23 / 95 24.2%ABE	17 / 95 17.9%E	16 / 95 16.8%	2 / 95 2.1%C	1 / 95 1.1%CE			
2	Thermodesulfobacteriaceae	59 / 95 62.1%	2 / 2 100.0%	1 / 2 50.0%	1 / 2 50.0%	2 / 3 66.7%	5 / 6 83.3%	4 / 6 66.7%	2 / 2 100.0%	1 / 2 50.0%	5 / 7 71.4%	2 / 2 100.0%	5 / 8 62.5%	2 / 4 50.0%	0 / 2 0.0%	4 / 4 100.0%	1 / 2 50.0%	3 / 6 50.0%	1 / 2 50.0%	2 / 6 33.3%	5 / 9 55.6%	2 / 4 50.0%	1 / 2 50.0%	6 / 10 60.0%	2 / 2 100.0%	0 / 0 0.0%	0 / 0 0.0%	0 / 0 0.0%	36 / 95 37.9%AB	23 / 95 24.2%ABE	17 / 95 17.9%E	16 / 95 16.8%	2 / 95 2.1%C	1 / 95 1.1%CE			
1	Thermodesulfobacterium	12 / 46 26.1%	1 / 1 100.0%	0 / 1 0.0%	0 / 1 0.0%	0 / 1 0.0%	2 / 3 66.7%	1 / 3 33.3%	1 / 1 100.0%	0 / 1 0.0%	1 / 3 33.3%	1 / 1 100.0%	1 / 4 25.0%	0 / 2 0.0%	0 / 1 0.0%	2 / 2 100.0%	0 / 1 0.0%	0 / 3 0.0%	0 / 1 0.0%	0 / 3 0.0%	0 / 4 0.0%	0 / 2 0.0%	0 / 1 0.0%	1 / 5 20.0%	1 / 1 100.0%	0 / 0 0.0%	0 / 0 0.0%	0 / 0 0.0%	17 / 46 37.0%E	15 / 46 32.6%	8 / 46 17.4%AB	4 / 46 8.7%ABE	1 / 46 2.2%CE	1 / 46 2.2%C			
1	'Thermodesulfobacterium geofontis'	12 / 46 26.1%	1 / 1 100.0%	0 / 1 0.0%	0 / 1 0.0%	0 / 1 0.0%	2 / 3 66.7%	1 / 3 33.3%	1 / 1 100.0%	0 / 1 0.0%	1 / 3 33.3%	1 / 1 100.0%	1 / 4 25.0%	0 / 2 0.0%	0 / 1 0.0%	2 / 2 100.0%	0 / 1 0.0%	0 / 3 0.0%	0 / 1 0.0%	0 / 3 0.0%	0 / 4 0.0%	0 / 2 0.0%	0 / 1 0.0%	1 / 5 20.0%	1 / 1 100.0%	0 / 0 0.0%	0 / 0 0.0%	0 / 0 0.0%	17 / 46 37.0%E	15 / 46 32.6%	8 / 46 17.4%AB	4 / 46 8.7%ABE	1 / 46 2.2%CE	1 / 46 2.2%C			
1	Thermodesulfobacterium geofontis OPF15	12 / 46 26.1%	1 / 1 100.0%	0 / 1 0.0%	0 / 1 0.0%	0 / 1 0.0%	2 / 3 66.7%	1 / 3 33.3%	1 / 1 100.0%	0 / 1 0.0%	1 / 3 33.3%	1 / 1 100.0%	1 / 4 25.0%	0 / 2 0.0%	0 / 1 0.0%	2 / 2 100.0%	0 / 1 0.0%	0 / 3 0.0%	0 / 1 0.0%	0 / 3 0.0%	0 / 4 0.0%	0 / 2 0.0%	0 / 1 0.0%	1 / 5 20.0%	1 / 1 100.0%	0 / 0 0.0%	0 / 0 0.0%	0 / 0 0.0%	17 / 46 37.0%E	15 / 46 32.6%	8 / 46 17.4%AB	4 / 46 8.7%ABE	1 / 46 2.2%CE	1 / 46 2.2%C			
1	Thermodesulfatator	47 / 49 95.9%	1 / 1 100.0%	1 / 1 100.0%	1 / 1 100.0%	2 / 2 100.0%	3 / 3 100.0%	3 / 3 100.0%	1 / 1 100.0%	1 / 1 100.0%	4 / 4 100.0%	1 / 1 100.0%	4 / 4 100.0%	2 / 2 100.0%	0 / 1 0.0%	2 / 2 100.0%	1 / 1 100.0%	3 / 3 100.0%	1 / 1 100.0%	2 / 3 66.7%	5 / 5 100.0%	2 / 2 100.0%	1 / 1 100.0%	5 / 5 100.0%	1 / 1 100.0%	0 / 0 0.0%	0 / 0 0.0%	0 / 0 0.0%	28 / 49 57.1%AB	19 / 49 38.8%ABE	1 / 49 2.0%C	1 / 49 2.0%					
1	DSM 15286	47 / 49 95.9%	1 / 1 100.0%	1 / 1 100.0%	1 / 1 100.0%	2 / 2 100.0%	3 / 3 100.0%	3 / 3 100.0%	1 / 1 100.0%	1 / 1 100.0%	4 / 4 100.0%	1 / 1 100.0%	4 / 4 100.0%	2 / 2 100.0%	0 / 1 0.0%	2 / 2 100.0%	1 / 1 100.0%	3 / 3 100.0%	1 / 1 100.0%	2 / 3 66.7%	5 / 5 100.0%	2 / 2 100.0%	1 / 1 100.0%	5 / 5 100.0%	1 / 1 100.0%	0 / 0 0.0%	0 / 0 0.0%	0 / 0 0.0%	28 / 49 57.1%AB	19 / 49 38.8%ABE	1 / 49 2.0%C	1 / 49 2.0%					
1	Thermodesulfatator indicus CIR29812	47 / 49 95.9%	1 / 1 100.0%	1 / 1 100.0%	1 / 1 100.0%	2 / 2 100.0%	3 / 3 100.0%	3 / 3 100.0%	1 / 1 100.0%	1 / 1 100.0%	4 / 4 100.0%	1 / 1 100.0%	4 / 4 100.0%	2 / 2 100.0%	0 / 1 0.0%	2 / 2 100.0%	1 / 1 100.0%	3 / 3 100.0%	1 / 1 100.0%	2 / 3 66.7%	5 / 5 100.0%	2 / 2 100.0%	1 / 1 100.0%	5 / 5 100.0%	1 / 1 100.0%	0 / 0 0.0%	0 / 0 0.0%	0 / 0 0.0%	28 / 49 57.1%AB	19 / 49 38.8%ABE	1 / 49 2.0%	1 / 49 2.0%C					
260	'Actinobacteria'	3874 / 13430 28.8%	146 / 359 40.7%	97 / 375 25.9%	77 / 337 22.8%	102 / 289 35.3%	435 / 1040 41.8%	312 / 963 32.4%	177 / 270 65.6%	95 / 277 34.3%	146 / 894 16.3%	49 / 292 16.8%	413 / 1087 38.0%	158 / 616 25.6%	148 / 275 53.8%	55 / 562 9.8%	95 / 314 30.3%	272 / 817 33.3%	48 / 270 17.8%	269 / 848 31.7%	217 / 1175 18.5%	150 / 657 22.8%	45 / 268 16.8%	328 / 1388 23.6%	38 / 45 84.4%	0 / 0 0.0%	1 / 9 11.1%	1 / 3 33.3%	4350 / 13430 32.4%	3723 / 13430 27.7%E	2136 / 13430 15.9%AB	1667 / 13430 12.4%ABE	749 / 13430 5.6%C	733 / 13430 5.5%CE	41 / 13430 0.3%B	30 / 13430 0.2%BE	1 / 13430 0.0%D
260	Actinobacteria	3874 / 13430 28.8%	146 / 359 40.7%	97 / 375 25.9%	77 / 337 22.8%	102 / 289 35.3%	435 / 1040 41.8%	312 / 963 32.4%	177 / 270 65.6%	95 / 277 34.3%	146 / 894 16.3%	49 / 292 16.8%	413 / 1087 38.0%	158 / 616 25.6%	148 / 275 53.8%	55 / 562 9.8%	95 / 314 30.3%	272 / 817 33.3%	48 / 270 17.8%	269 / 848 31.7%	217 / 1175 18.5%	150 / 657 22.8%	45 / 268 16.8%	328 / 1388 23.6%	38 / 45 84.4%	0 / 0 0.0%	1 / 9 11.1%	1 / 3 33.3%	4350 / 13430 32.4%	3723 / 13430 27.7%E	2136 / 13430 15.9%AB	1667 / 13430 12.4%ABE	749 / 13430 5.6%C	733 / 13430 5.5%CE	41 / 13430 0.3%B	30 / 13430 0.2%BE	1 / 13430 0.0%D
2	Acidimicrobidae	38 / 90 42.2%	1 / 2 50.0%	1 / 2 50.0%	1 / 2 50.0%	0 / 2 0.0%	3 / 5 60.0%	3 / 6 50.0%	2 / 2 100.0%	0 / 2 0.0%	1 / 6 16.7%	1 / 2 50.0%	4 / 7 57.1%	1 / 4 25.0%	1 / 2 50.0%	2 / 4 50.0%	1 / 2 50.0%	4 / 6 66.7%	1 / 2 50.0%	1 / 6 16.7%	4 / 8 50.0%	2 / 4 50.0%	0 / 2 0.0%	2 / 10 20.0%	1 / 1 100.0%	0 / 0 0.0%	1 / 1 100.0%	0 / 0 0.0%	21 / 90 23.3%AB	18 / 90 20.0%E	17 / 90 18.9%ABE	17 / 90 18.9%	12 / 90 13.3%C	5 / 90 5.6%CE			
2	Acidimicrobiales	38 / 90 42.2%	1 / 2 50.0%	1 / 2 50.0%	1 / 2 50.0%	0 / 2 0.0%	3 / 5 60.0%	3 / 6 50.0%	2 / 2 100.0%	0 / 2 0.0%	1 / 6 16.7%	1 / 2 50.0%	4 / 7 57.1%	1 / 4 25.0%	1 / 2 50.0%	2 / 4 50.0%	1 / 2 50.0%	4 / 6 66.7%	1 / 2 50.0%	1 / 6 16.7%	4 / 8 50.0%	2 / 4 50.0%	0 / 2 0.0%	2 / 10 20.0%	1 / 1 100.0%	0 / 0 0.0%	1 / 1 100.0%	0 / 0 0.0%	21 / 90 23.3%AB	18 / 90 20.0%E	17 / 90 18.9%ABE	17 / 90 18.9%	12 / 90 13.3%C	5 / 90 5.6%CE			
2	'Acidimicrobineae'	38 / 90 42.2%	1 / 2 50.0%	1 / 2 50.0%	1 / 2 50.0%	0 / 2 0.0%	3 / 5 60.0%	3 / 6 50.0%	2 / 2 100.0%	0 / 2 0.0%	1 / 6 16.7%	1 / 2 50.0%	4 / 7 57.1%	1 / 4 25.0%	1 / 2 50.0%	2 / 4 50.0%	1 / 2 50.0%	4 / 6 66.7%	1 / 2 50.0%	1 / 6 16.7%	4 / 8 50.0%	2 / 4 50.0%	0 / 2 0.0%	2 / 10 20.0%	1 / 1 100.0%	0 / 0 0.0%	1 / 1 100.0%	0 / 0 0.0%	21 / 90 23.3%AB	18 / 90 20.0%E	17 / 90 18.9%	17 / 90 18.9%ABE	12 / 90 13.3%C	5 / 90 5.6%CE			
2	Acidimicrobiaceae	38 / 90 42.2%	1 / 2 50.0%	1 / 2 50.0%	1 / 2 50.0%	0 / 2 0.0%	3 / 5 60.0%	3 / 6 50.0%	2 / 2 100.0%	0 / 2 0.0%	1 / 6 16.7%	1 / 2 50.0%	4 / 7 57.1%	1 / 4 25.0%	1 / 2 50.0%	2 / 4 50.0%	1 / 2 50.0%	4 / 6 66.7%	1 / 2 50.0%	1 / 6 16.7%	4 / 8 50.0%	2 / 4 50.0%	0 / 2 0.0%	2 / 10 20.0%	1 / 1 100.0%	0 / 0 0.0%	1 / 1 100.0%	0 / 0 0.0%	21 / 90 23.3%AB	18 / 90 20.0%E	17 / 90 18.9%ABE	17 / 90 18.9%	12 / 90 13.3%C	5 / 90 5.6%CE			
1	Acidimicrobium	14 / 45 31.1%	0 / 1 0.0%	0 / 1 0.0%	0 / 1 0.0%	0 / 1 0.0%	1 / 3 33.3%	2 / 3 66.7%	1 / 1 100.0%	0 / 1 0.0%	0 / 3 0.0%	0 / 1 0.0%	1 / 4 25.0%	0 / 2 0.0%	1 / 1 100.0%	1 / 2 50.0%	1 / 1 100.0%	2 / 3 66.7%	0 / 1 0.0%	0 / 3 0.0%	2 / 4 50.0%	1 / 2 50.0%	0 / 1 0.0%	1 / 5 20.0%	0 / 0 0.0%	0 / 0 0.0%	0 / 0 0.0%	0 / 0 0.0%	12 / 45 26.7%E	8 / 45 17.8%C	8 / 45 17.8%AB	7 / 45 15.6%	6 / 45 13.3%ABE	4 / 45 8.9%CE			
1	Acidimicrobium ferrooxidans	14 / 45 31.1%	0 / 1 0.0%	0 / 1 0.0%	0 / 1 0.0%	0 / 1 0.0%	1 / 3 33.3%	2 / 3 66.7%	1 / 1 100.0%	0 / 1 0.0%	0 / 3 0.0%	0 / 1 0.0%	1 / 4 25.0%	0 / 2 0.0%	1 / 1 100.0%	1 / 2 50.0%	1 / 1 100.0%	2 / 3 66.7%	0 / 1 0.0%	0 / 3 0.0%	2 / 4 50.0%	1 / 2 50.0%	0 / 1 0.0%	1 / 5 20.0%	0 / 0 0.0%	0 / 0 0.0%	0 / 0 0.0%	0 / 0 0.0%	12 / 45 26.7%E	8 / 45 17.8%C	8 / 45 17.8%AB	7 / 45 15.6%	6 / 45 13.3%ABE	4 / 45 8.9%CE			
1	Acidimicrobium ferrooxidans DSM 10331	14 / 45 31.1%	0 / 1 0.0%	0 / 1 0.0%	0 / 1 0.0%	0 / 1 0.0%	1 / 3 33.3%	2 / 3 66.7%	1 / 1 100.0%	0 / 1 0.0%	0 / 3 0.0%	0 / 1 0.0%	1 / 4 25.0%	0 / 2 0.0%	1 / 1 100.0%	1 / 2 50.0%	1 / 1 100.0%	2 / 3 66.7%	0 / 1 0.0%	0 / 3 0.0%	2 / 4 50.0%	1 / 2 50.0%	0 / 1 0.0%	1 / 5 20.0%	0 / 0 0.0%	0 / 0 0.0%	0 / 0 0.0%	0 / 0 0.0%	12 / 45 26.7%E	8 / 45 17.8%C	8 / 45 17.8%AB	7 / 45 15.6%	6 / 45 13.3%ABE	4 / 45 8.9%CE			
1	Ilumatobacter	24 / 45 53.3%	1 / 1 100.0%	1 / 1 100.0%	1 / 1 100.0%	0 / 1 0.0%	2 / 2 100.0%	1 / 3 33.3%	1 / 1 100.0%	0 / 1 0.0%	1 / 3 33.3%	1 / 1 100.0%	3 / 3 100.0%	1 / 2 50.0%	0 / 1 0.0%	1 / 2 50.0%	0 / 1 0.0%	2 / 3 66.7%	1 / 1 100.0%	1 / 3 33.3%	2 / 4 50.0%	1 / 2 50.0%	0 / 1 0.0%	1 / 5 20.0%	1 / 1 100.0%	0 / 0 0.0%	1 / 1 100.0%	0 / 0 0.0%	13 / 45 28.9%AB	11 / 45 24.4%ABE	10 / 45 22.2%	6 / 45 13.3%E	4 / 45 8.9%C	1 / 45 2.2%CE			
1	'Ilumatobacter coccineum'	24 / 45 53.3%	1 / 1 100.0%	1 / 1 100.0%	1 / 1 100.0%	0 / 1 0.0%	2 / 2 100.0%	1 / 3 33.3%	1 / 1 100.0%	0 / 1 0.0%	1 / 3 33.3%	1 / 1 100.0%	3 / 3 100.0%	1 / 2 50.0%	0 / 1 0.0%	1 / 2 50.0%	0 / 1 0.0%	2 / 3 66.7%	1 / 1 100.0%	1 / 3 33.3%	2 / 4 50.0%	1 / 2 50.0%	0 / 1 0.0%	1 / 5 20.0%	1 / 1 100.0%	0 / 0 0.0%	1 / 1 100.0%	0 / 0 0.0%	13 / 45 28.9%AB	11 / 45 24.4%ABE	10 / 45 22.2%	6 / 45 13.3%E	4 / 45 8.9%C	1 / 45 2.2%CE			
1	Acidimicrobidae bacterium YM16-304	24 / 45 53.3%	1 / 1 100.0%	1 / 1 100.0%	1 / 1 100.0%	0 / 1 0.0%	2 / 2 100.0%	1 / 3 33.3%	1 / 1 100.0%	0 / 1 0.0%	1 / 3 33.3%	1 / 1 100.0%	3 / 3 100.0%	1 / 2 50.0%	0 / 1 0.0%	1 / 2 50.0%	0 / 1 0.0%	2 / 3 66.7%	1 / 1 100.0%	1 / 3 33.3%	2 / 4 50.0%	1 / 2 50.0%	0 / 1 0.0%	1 / 5 20.0%	1 / 1 100.0%	0 / 0 0.0%	1 / 1 100.0%	0 / 0 0.0%	13 / 45 28.9%AB	11 / 45 24.4%ABE	10 / 45 22.2%	6 / 45 13.3%E	4 / 45 8.9%C	1 / 45 2.2%CE			
2	Rubrobacteridae	1 / 90 1.1%	0 / 2 0.0%	0 / 2 0.0%	0 / 2 0.0%	0 / 2 0.0%	0 / 6 0.0%	0 / 6 0.0%	0 / 2 0.0%	0 / 2 0.0%	0 / 6 0.0%	0 / 2 0.0%	0 / 8 0.0%	0 / 4 0.0%	0 / 2 0.0%	1 / 4 25.0%	0 / 2 0.0%	0 / 6 0.0%	0 / 2 0.0%	0 / 6 0.0%	0 / 8 0.0%	0 / 4 0.0%	0 / 2 0.0%	0 / 10 0.0%	0 / 0 0.0%	0 / 0 0.0%	0 / 0 0.0%	0 / 0 0.0%	48 / 90 53.3%	34 / 90 37.8%E	5 / 90 5.6%CE	2 / 90 2.2%C	1 / 90 1.1%AB				
1	Rubrobacterales	1 / 45 2.2%	0 / 1 0.0%	0 / 1 0.0%	0 / 1 0.0%	0 / 1 0.0%	0 / 3 0.0%	0 / 3 0.0%	0 / 1 0.0%	0 / 1 0.0%	0 / 3 0.0%	0 / 1 0.0%	0 / 4 0.0%	0 / 2 0.0%	0 / 1 0.0%	1 / 2 50.0%	0 / 1 0.0%	0 / 3 0.0%	0 / 1 0.0%	0 / 3 0.0%	0 / 4 0.0%	0 / 2 0.0%	0 / 1 0.0%	0 / 5 0.0%	0 / 0 0.0%	0 / 0 0.0%	0 / 0 0.0%	0 / 0 0.0%	27 / 45 60.0%	13 / 45 28.9%E	3 / 45 6.7%CE	1 / 45 2.2%AB	1 / 45 2.2%C				
1	'Rubrobacterineae'	1 / 45 2.2%	0 / 1 0.0%	0 / 1 0.0%	0 / 1 0.0%	0 / 1 0.0%	0 / 3 0.0%	0 / 3 0.0%	0 / 1 0.0%	0 / 1 0.0%	0 / 3 0.0%	0 / 1 0.0%	0 / 4 0.0%	0 / 2 0.0%	0 / 1 0.0%	1 / 2 50.0%	0 / 1 0.0%	0 / 3 0.0%	0 / 1 0.0%	0 / 3 0.0%	0 / 4 0.0%	0 / 2 0.0%	0 / 1 0.0%	0 / 5 0.0%	0 / 0 0.0%	0 / 0 0.0%	0 / 0 0.0%	0 / 0 0.0%	27 / 45 60.0%	13 / 45 28.9%E	3 / 45 6.7%CE	1 / 45 2.2%C	1 / 45 2.2%AB				
1	Rubrobacteraceae	1 / 45 2.2%	0 / 1 0.0%	0 / 1 0.0%	0 / 1 0.0%	0 / 1 0.0%	0 / 3 0.0%	0 / 3 0.0%	0 / 1 0.0%	0 / 1 0.0%	0 / 3 0.0%	0 / 1 0.0%	0 / 4 0.0%	0 / 2 0.0%	0 / 1 0.0%	1 / 2 50.0%	0 / 1 0.0%	0 / 3 0.0%	0 / 1 0.0%	0 / 3 0.0%	0 / 4 0.0%	0 / 2 0.0%	0 / 1 0.0%	0 / 5 0.0%	0 / 0 0.0%	0 / 0 0.0%	0 / 0 0.0%	0 / 0 0.0%	27 / 45 60.0%	13 / 45 28.9%E	3 / 45 6.7%CE	1 / 45 2.2%AB	1 / 45 2.2%C				
1	Rubrobacter	1 / 45 2.2%	0 / 1 0.0%	0 / 1 0.0%	0 / 1 0.0%	0 / 1 0.0%	0 / 3 0.0%	0 / 3 0.0%	0 / 1 0.0%	0 / 1 0.0%	0 / 3 0.0%	0 / 1 0.0%	0 / 4 0.0%	0 / 2 0.0%	0 / 1 0.0%	1 / 2 50.0%	0 / 1 0.0%	0 / 3 0.0%	0 / 1 0.0%	0 / 3 0.0%	0 / 4 0.0%	0 / 2 0.0%	0 / 1 0.0%	0 / 5 0.0%	0 / 0 0.0%	0 / 0 0.0%	0 / 0 0.0%	0 / 0 0.0%	27 / 45 60.0%	13 / 45 28.9%E	3 / 45 6.7%CE	1 / 45 2.2%C	1 / 45 2.2%AB				
1	CIP 105412	1 / 45 2.2%	0 / 1 0.0%	0 / 1 0.0%	0 / 1 0.0%	0 / 1 0.0%	0 / 3 0.0%	0 / 3 0.0%	0 / 1 0.0%	0 / 1 0.0%	0 / 3 0.0%	0 / 1 0.0%	0 / 4 0.0%	0 / 2 0.0%	0 / 1 0.0%	1 / 2 50.0%	0 / 1 0.0%	0 / 3 0.0%	0 / 1 0.0%	0 / 3 0.0%	0 / 4 0.0%	0 / 2 0.0%	0 / 1 0.0%	0 / 5 0.0%	0 / 0 0.0%	0 / 0 0.0%	0 / 0 0.0%	0 / 0 0.0%	27 / 45 60.0%	13 / 45 28.9%E	3 / 45 6.7%CE	1 / 45 2.2%C	1 / 45 2.2%AB				
1	Rubrobacter xylanophilus DSM 9941	1 / 45 2.2%	0 / 1 0.0%	0 / 1 0.0%	0 / 1 0.0%	0 / 1 0.0%	0 / 3 0.0%	0 / 3 0.0%	0 / 1 0.0%	0 / 1 0.0%	0 / 3 0.0%	0 / 1 0.0%	0 / 4 0.0%	0 / 2 0.0%	0 / 1 0.0%	1 / 2 50.0%	0 / 1 0.0%	0 / 3 0.0%	0 / 1 0.0%	0 / 3 0.0%	0 / 4 0.0%	0 / 2 0.0%	0 / 1 0.0%	0 / 5 0.0%	0 / 0 0.0%	0 / 0 0.0%	0 / 0 0.0%	0 / 0 0.0%	27 / 45 60.0%	13 / 45 28.9%E	3 / 45 6.7%CE	1 / 45 2.2%AB	1 / 45 2.2%C				
1	Solirubrobacterales	0 / 45 0.0%	0 / 1 0.0%	0 / 1 0.0%	0 / 1 0.0%	0 / 1 0.0%	0 / 3 0.0%	0 / 3 0.0%	0 / 1 0.0%	0 / 1 0.0%	0 / 3 0.0%	0 / 1 0.0%	0 / 4 0.0%	0 / 2 0.0%	0 / 1 0.0%	0 / 2 0.0%	0 / 1 0.0%	0 / 3 0.0%	0 / 1 0.0%	0 / 3 0.0%	0 / 4 0.0%	0 / 2 0.0%	0 / 1 0.0%	0 / 5 0.0%	0 / 0 0.0%	0 / 0 0.0%	0 / 0 0.0%	0 / 0 0.0%	21 / 45 46.7%E	21 / 45 46.7%	2 / 45 4.4%CE	1 / 45 2.2%C					
1	Conexibacteraceae	0 / 45 0.0%	0 / 1 0.0%	0 / 1 0.0%	0 / 1 0.0%	0 / 1 0.0%	0 / 3 0.0%	0 / 3 0.0%	0 / 1 0.0%	0 / 1 0.0%	0 / 3 0.0%	0 / 1 0.0%	0 / 4 0.0%	0 / 2 0.0%	0 / 1 0.0%	0 / 2 0.0%	0 / 1 0.0%	0 / 3 0.0%	0 / 1 0.0%	0 / 3 0.0%	0 / 4 0.0%	0 / 2 0.0%	0 / 1 0.0%	0 / 5 0.0%	0 / 0 0.0%	0 / 0 0.0%	0 / 0 0.0%	0 / 0 0.0%	21 / 45 46.7%E	21 / 45 46.7%	2 / 45 4.4%CE	1 / 45 2.2%C					
1	Conexibacter	0 / 45 0.0%	0 / 1 0.0%	0 / 1 0.0%	0 / 1 0.0%	0 / 1 0.0%	0 / 3 0.0%	0 / 3 0.0%	0 / 1 0.0%	0 / 1 0.0%	0 / 3 0.0%	0 / 1 0.0%	0 / 4 0.0%	0 / 2 0.0%	0 / 1 0.0%	0 / 2 0.0%	0 / 1 0.0%	0 / 3 0.0%	0 / 1 0.0%	0 / 3 0.0%	0 / 4 0.0%	0 / 2 0.0%	0 / 1 0.0%	0 / 5 0.0%	0 / 0 0.0%	0 / 0 0.0%	0 / 0 0.0%	0 / 0 0.0%	21 / 45 46.7%E	21 / 45 46.7%	2 / 45 4.4%CE	1 / 45 2.2%C					
1	Conexibacter woesei	0 / 45 0.0%	0 / 1 0.0%	0 / 1 0.0%	0 / 1 0.0%	0 / 1 0.0%	0 / 3 0.0%	0 / 3 0.0%	0 / 1 0.0%	0 / 1 0.0%	0 / 3 0.0%	0 / 1 0.0%	0 / 4 0.0%	0 / 2 0.0%	0 / 1 0.0%	0 / 2 0.0%	0 / 1 0.0%	0 / 3 0.0%	0 / 1 0.0%	0 / 3 0.0%	0 / 4 0.0%	0 / 2 0.0%	0 / 1 0.0%	0 / 5 0.0%	0 / 0 0.0%	0 / 0 0.0%	0 / 0 0.0%	0 / 0 0.0%	21 / 45 46.7%	21 / 45 46.7%E	2 / 45 4.4%CE	1 / 45 2.2%C					
1	Conexibacter woesei DSM 14684	0 / 45 0.0%	0 / 1 0.0%	0 / 1 0.0%	0 / 1 0.0%	0 / 1 0.0%	0 / 3 0.0%	0 / 3 0.0%	0 / 1 0.0%	0 / 1 0.0%	0 / 3 0.0%	0 / 1 0.0%	0 / 4 0.0%	0 / 2 0.0%	0 / 1 0.0%	0 / 2 0.0%	0 / 1 0.0%	0 / 3 0.0%	0 / 1 0.0%	0 / 3 0.0%	0 / 4 0.0%	0 / 2 0.0%	0 / 1 0.0%	0 / 5 0.0%	0 / 0 0.0%	0 / 0 0.0%	0 / 0 0.0%	0 / 0 0.0%	21 / 45 46.7%E	21 / 45 46.7%	2 / 45 4.4%CE	1 / 45 2.2%C					
7	Coriobacteridae	325 / 332 97.9%	7 / 7 100.0%	10 / 10 100.0%	7 / 7 100.0%	7 / 7 100.0%	24 / 24 100.0%	20 / 21 95.2%	7 / 7 100.0%	6 / 7 85.7%	21 / 22 95.5%	7 / 7 100.0%	27 / 28 96.4%	15 / 16 93.8%	6 / 7 85.7%	14 / 14 100.0%	7 / 7 100.0%	21 / 21 100.0%	7 / 7 100.0%	21 / 21 100.0%	29 / 29 100.0%	17 / 17 100.0%	7 / 7 100.0%	34 / 35 97.1%	4 / 4 100.0%	0 / 0 0.0%	0 / 0 0.0%	0 / 0 0.0%	182 / 332 54.8%ABE	143 / 332 43.1%AB	5 / 332 1.5%CE	2 / 332 0.6%C					
7	Coriobacteriales	325 / 332 97.9%	7 / 7 100.0%	10 / 10 100.0%	7 / 7 100.0%	7 / 7 100.0%	24 / 24 100.0%	20 / 21 95.2%	7 / 7 100.0%	6 / 7 85.7%	21 / 22 95.5%	7 / 7 100.0%	27 / 28 96.4%	15 / 16 93.8%	6 / 7 85.7%	14 / 14 100.0%	7 / 7 100.0%	21 / 21 100.0%	7 / 7 100.0%	21 / 21 100.0%	29 / 29 100.0%	17 / 17 100.0%	7 / 7 100.0%	34 / 35 97.1%	4 / 4 100.0%	0 / 0 0.0%	0 / 0 0.0%	0 / 0 0.0%	182 / 332 54.8%ABE	143 / 332 43.1%AB	5 / 332 1.5%CE	2 / 332 0.6%C					
7	'Coriobacterineae'	325 / 332 97.9%	7 / 7 100.0%	10 / 10 100.0%	7 / 7 100.0%	7 / 7 100.0%	24 / 24 100.0%	20 / 21 95.2%	7 / 7 100.0%	6 / 7 85.7%	21 / 22 95.5%	7 / 7 100.0%	27 / 28 96.4%	15 / 16 93.8%	6 / 7 85.7%	14 / 14 100.0%	7 / 7 100.0%	21 / 21 100.0%	7 / 7 100.0%	21 / 21 100.0%	29 / 29 100.0%	17 / 17 100.0%	7 / 7 100.0%	34 / 35 97.1%	4 / 4 100.0%	0 / 0 0.0%	0 / 0 0.0%	0 / 0 0.0%	182 / 332 54.8%ABE	143 / 332 43.1%AB	5 / 332 1.5%CE	2 / 332 0.6%C					
		325 /																																			

7	Coriobacteriaceae	[illegible] 97.9%	[illegible] 100.0%	[illegible] 100.0%	[illegible] 100.0%	[illegible] 100.0%	[illegible] 100.0%	[illegible] 95.2%	[illegible] 100.0%	[illegible] 85.7%	[illegible] 95.5%	[illegible] 100.0%	[illegible] 96.4%	[illegible] 93.8%	[illegible] 85.7%	[illegible] 100.0%	[illegible] 100.0%	[illegible] 100.0%	[illegible] 100.0%	[illegible] 100.0%	[illegible] 100.0%	[illegible] 100.0%	[illegible] 100.0%	[illegible] 97.1%	[illegible] 100.0%	[illegible] 0.0%	[illegible] 0.0%	[illegible] 0.0%	[illegible] 54.8%ABE	[illegible] 43.1%AB	[illegible] 1.5%CE	[illegible] 0.6%C					
1	Atopobium	45 / 45 100.0%	1 / 1 100.0%	1 / 1 100.0%	1 / 1 100.0%	1 / 1 100.0%	3 / 3 100.0%	3 / 3 100.0%	1 / 1 100.0%	1 / 1 100.0%	3 / 3 100.0%	1 / 1 100.0%	4 / 4 100.0%	2 / 2 100.0%	1 / 1 100.0%	2 / 2 100.0%	1 / 1 100.0%	3 / 3 100.0%	1 / 1 100.0%	3 / 3 100.0%	4 / 4 100.0%	2 / 2 100.0%	1 / 1 100.0%	5 / 5 100.0%	0 / 0 0.0%	0 / 0 0.0%	0 / 0 0.0%	0 / 0 0.0%	23 / 45 51.1%ABE	22 / 45 48.9%AB							
1	"Streptococcus parvulus" Weinberg et al. 1937	45 / 45 100.0%	1 / 1 100.0%	1 / 1 100.0%	1 / 1 100.0%	1 / 1 100.0%	3 / 3 100.0%	3 / 3 100.0%	1 / 1 100.0%	1 / 1 100.0%	3 / 3 100.0%	1 / 1 100.0%	4 / 4 100.0%	2 / 2 100.0%	1 / 1 100.0%	2 / 2 100.0%	1 / 1 100.0%	3 / 3 100.0%	1 / 1 100.0%	3 / 3 100.0%	4 / 4 100.0%	2 / 2 100.0%	1 / 1 100.0%	5 / 5 100.0%	0 / 0 0.0%	0 / 0 0.0%	0 / 0 0.0%	0 / 0 0.0%	23 / 45 51.1%ABE	22 / 45 48.9%AB							
1	Atopobium parvulum ATCC 33793	45 / 45 100.0%	1 / 1 100.0%	1 / 1 100.0%	1 / 1 100.0%	1 / 1 100.0%	3 / 3 100.0%	3 / 3 100.0%	1 / 1 100.0%	1 / 1 100.0%	3 / 3 100.0%	1 / 1 100.0%	4 / 4 100.0%	2 / 2 100.0%	1 / 1 100.0%	2 / 2 100.0%	1 / 1 100.0%	3 / 3 100.0%	1 / 1 100.0%	3 / 3 100.0%	4 / 4 100.0%	2 / 2 100.0%	1 / 1 100.0%	5 / 5 100.0%	0 / 0 0.0%	0 / 0 0.0%	0 / 0 0.0%	0 / 0 0.0%	23 / 45 51.1%ABE	22 / 45 48.9%AB							
1	Coriobacterium	40 / 46 87.0%	1 / 1 100.0%	1 / 1 100.0%	1 / 1 100.0%	1 / 1 100.0%	3 / 3 100.0%	2 / 3 66.7%	1 / 1 100.0%	0 / 1 0.0%	2 / 3 66.7%	1 / 1 100.0%	4 / 4 100.0%	1 / 2 50.0%	0 / 1 0.0%	2 / 2 100.0%	1 / 1 100.0%	3 / 3 100.0%	1 / 1 100.0%	3 / 3 100.0%	4 / 4 100.0%	3 / 3 100.0%	1 / 1 100.0%	4 / 5 80.0%	0 / 0 0.0%	0 / 0 0.0%	0 / 0 0.0%	0 / 0 0.0%	23 / 46 50.0%ABE	17 / 46 37.0%AB	4 / 46 8.7%CE	2 / 46 4.3%C					
1	ATCC 49209	40 / 46 87.0%	1 / 1 100.0%	1 / 1 100.0%	1 / 1 100.0%	1 / 1 100.0%	3 / 3 100.0%	2 / 3 66.7%	1 / 1 100.0%	0 / 1 0.0%	2 / 3 66.7%	1 / 1 100.0%	4 / 4 100.0%	1 / 2 50.0%	0 / 1 0.0%	2 / 2 100.0%	1 / 1 100.0%	3 / 3 100.0%	1 / 1 100.0%	3 / 3 100.0%	4 / 4 100.0%	3 / 3 100.0%	1 / 1 100.0%	4 / 5 80.0%	0 / 0 0.0%	0 / 0 0.0%	0 / 0 0.0%	0 / 0 0.0%	23 / 46 50.0%ABE	17 / 46 37.0%AB	4 / 46 8.7%CE	2 / 46 4.3%C					
1	Coriobacterium glomerans DSM 20642	40 / 46 87.0%	1 / 1 100.0%	1 / 1 100.0%	1 / 1 100.0%	1 / 1 100.0%	3 / 3 100.0%	2 / 3 66.7%	1 / 1 100.0%	0 / 1 0.0%	2 / 3 66.7%	1 / 1 100.0%	4 / 4 100.0%	1 / 2 50.0%	0 / 1 0.0%	2 / 2 100.0%	1 / 1 100.0%	3 / 3 100.0%	1 / 1 100.0%	3 / 3 100.0%	4 / 4 100.0%	3 / 3 100.0%	1 / 1 100.0%	4 / 5 80.0%	0 / 0 0.0%	0 / 0 0.0%	0 / 0 0.0%	0 / 0 0.0%	23 / 46 50.0%ABE	17 / 46 37.0%AB	4 / 46 8.7%CE	2 / 46 4.3%C					
1	Slackia	49 / 49 100.0%	1 / 1 100.0%	2 / 2 100.0%	1 / 1 100.0%	1 / 1 100.0%	4 / 4 100.0%	3 / 3 100.0%	1 / 1 100.0%	1 / 1 100.0%	3 / 3 100.0%	1 / 1 100.0%	4 / 4 100.0%	2 / 2 100.0%	1 / 1 100.0%	2 / 2 100.0%	1 / 1 100.0%	3 / 3 100.0%	1 / 1 100.0%	3 / 3 100.0%	4 / 4 100.0%	3 / 3 100.0%	1 / 1 100.0%	5 / 5 100.0%	1 / 1 100.0%	0 / 0 0.0%	0 / 0 0.0%	0 / 0 0.0%	28 / 49 57.1%ABE	21 / 49 42.9%AB							
1	ATCC 29202	49 / 49 100.0%	1 / 1 100.0%	2 / 2 100.0%	1 / 1 100.0%	1 / 1 100.0%	4 / 4 100.0%	3 / 3 100.0%	1 / 1 100.0%	1 / 1 100.0%	3 / 3 100.0%	1 / 1 100.0%	4 / 4 100.0%	2 / 2 100.0%	1 / 1 100.0%	2 / 2 100.0%	1 / 1 100.0%	3 / 3 100.0%	1 / 1 100.0%	3 / 3 100.0%	4 / 4 100.0%	3 / 3 100.0%	1 / 1 100.0%	5 / 5 100.0%	1 / 1 100.0%	0 / 0 0.0%	0 / 0 0.0%	0 / 0 0.0%	28 / 49 57.1%ABE	21 / 49 42.9%AB							
1	Slackia heliotrinireducens DSM 20476	49 / 49 100.0%	1 / 1 100.0%	2 / 2 100.0%	1 / 1 100.0%	1 / 1 100.0%	4 / 4 100.0%	3 / 3 100.0%	1 / 1 100.0%	1 / 1 100.0%	3 / 3 100.0%	1 / 1 100.0%	4 / 4 100.0%	2 / 2 100.0%	1 / 1 100.0%	2 / 2 100.0%	1 / 1 100.0%	3 / 3 100.0%	1 / 1 100.0%	3 / 3 100.0%	4 / 4 100.0%	3 / 3 100.0%	1 / 1 100.0%	5 / 5 100.0%	1 / 1 100.0%	0 / 0 0.0%	0 / 0 0.0%	0 / 0 0.0%	28 / 49 57.1%ABE	21 / 49 42.9%AB							
2	Eggerthella	96 / 96 100.0%	2 / 2 100.0%	3 / 3 100.0%	2 / 2 100.0%	2 / 2 100.0%	8 / 8 100.0%	6 / 6 100.0%	2 / 2 100.0%	2 / 2 100.0%	7 / 7 100.0%	2 / 2 100.0%	8 / 8 100.0%	4 / 4 100.0%	2 / 2 100.0%	4 / 4 100.0%	2 / 2 100.0%	6 / 6 100.0%	2 / 2 100.0%	6 / 6 100.0%	8 / 8 100.0%	4 / 4 100.0%	2 / 2 100.0%	10 / 10 100.0%	2 / 2 100.0%	0 / 0 0.0%	0 / 0 0.0%	0 / 0 0.0%	56 / 96 58.3%ABE	40 / 96 41.7%AB							
1	"Bacteroides lentus" Eggerth 1935	48 / 48 100.0%	1 / 1 100.0%	2 / 2 100.0%	1 / 1 100.0%	1 / 1 100.0%	4 / 4 100.0%	3 / 3 100.0%	1 / 1 100.0%	1 / 1 100.0%	3 / 3 100.0%	1 / 1 100.0%	4 / 4 100.0%	2 / 2 100.0%	1 / 1 100.0%	2 / 2 100.0%	1 / 1 100.0%	3 / 3 100.0%	1 / 1 100.0%	3 / 3 100.0%	4 / 4 100.0%	2 / 2 100.0%	1 / 1 100.0%	5 / 5 100.0%	1 / 1 100.0%	0 / 0 0.0%	0 / 0 0.0%	0 / 0 0.0%	28 / 48 58.3%ABE	20 / 48 41.7%AB							
1	Eggerthella lenta DSM 2243	48 / 48 100.0%	1 / 1 100.0%	2 / 2 100.0%	1 / 1 100.0%	1 / 1 100.0%	4 / 4 100.0%	3 / 3 100.0%	1 / 1 100.0%	1 / 1 100.0%	3 / 3 100.0%	1 / 1 100.0%	4 / 4 100.0%	2 / 2 100.0%	1 / 1 100.0%	2 / 2 100.0%	1 / 1 100.0%	3 / 3 100.0%	1 / 1 100.0%	3 / 3 100.0%	4 / 4 100.0%	2 / 2 100.0%	1 / 1 100.0%	5 / 5 100.0%	1 / 1 100.0%	0 / 0 0.0%	0 / 0 0.0%	0 / 0 0.0%	28 / 48 58.3%ABE	20 / 48 41.7%AB							
1	Eggerthella sp. YY7918	48 / 48 100.0%	1 / 1 100.0%	1 / 1 100.0%	1 / 1 100.0%	1 / 1 100.0%	4 / 4 100.0%	3 / 3 100.0%	1 / 1 100.0%	1 / 1 100.0%	4 / 4 100.0%	1 / 1 100.0%	4 / 4 100.0%	2 / 2 100.0%	1 / 1 100.0%	2 / 2 100.0%	1 / 1 100.0%	3 / 3 100.0%	1 / 1 100.0%	3 / 3 100.0%	4 / 4 100.0%	2 / 2 100.0%	1 / 1 100.0%	5 / 5 100.0%	1 / 1 100.0%	0 / 0 0.0%	0 / 0 0.0%	0 / 0 0.0%	28 / 48 58.3%ABE	20 / 48 41.7%AB							
1	Cryptobacterium	47 / 47 100.0%	1 / 1 100.0%	1 / 1 100.0%	1 / 1 100.0%	1 / 1 100.0%	3 / 3 100.0%	3 / 3 100.0%	1 / 1 100.0%	1 / 1 100.0%	3 / 3 100.0%	1 / 1 100.0%	4 / 4 100.0%	3 / 3 100.0%	1 / 1 100.0%	2 / 2 100.0%	1 / 1 100.0%	3 / 3 100.0%	1 / 1 100.0%	3 / 3 100.0%	4 / 4 100.0%	2 / 2 100.0%	1 / 1 100.0%	5 / 5 100.0%	1 / 1 100.0%	0 / 0 0.0%	0 / 0 0.0%	0 / 0 0.0%	27 / 47 57.4%ABE	20 / 47 42.6%AB							
1	ATCC 700683	47 / 47 100.0%	1 / 1 100.0%	1 / 1 100.0%	1 / 1 100.0%	1 / 1 100.0%	3 / 3 100.0%	3 / 3 100.0%	1 / 1 100.0%	1 / 1 100.0%	3 / 3 100.0%	1 / 1 100.0%	4 / 4 100.0%	3 / 3 100.0%	1 / 1 100.0%	2 / 2 100.0%	1 / 1 100.0%	3 / 3 100.0%	1 / 1 100.0%	3 / 3 100.0%	4 / 4 100.0%	2 / 2 100.0%	1 / 1 100.0%	5 / 5 100.0%	1 / 1 100.0%	0 / 0 0.0%	0 / 0 0.0%	0 / 0 0.0%	27 / 47 57.4%ABE	20 / 47 42.6%AB							
1	Cryptobacterium curtum 12-3	47 / 47 100.0%	1 / 1 100.0%	1 / 1 100.0%	1 / 1 100.0%	1 / 1 100.0%	3 / 3 100.0%	3 / 3 100.0%	1 / 1 100.0%	1 / 1 100.0%	3 / 3 100.0%	1 / 1 100.0%	4 / 4 100.0%	3 / 3 100.0%	1 / 1 100.0%	2 / 2 100.0%	1 / 1 100.0%	3 / 3 100.0%	1 / 1 100.0%	3 / 3 100.0%	4 / 4 100.0%	2 / 2 100.0%	1 / 1 100.0%	5 / 5 100.0%	1 / 1 100.0%	0 / 0 0.0%	0 / 0 0.0%	0 / 0 0.0%	27 / 47 57.4%ABE	20 / 47 42.6%AB							
1	Olsenella	48 / 49 98.0%	1 / 1 100.0%	2 / 2 100.0%	1 / 1 100.0%	1 / 1 100.0%	3 / 3 100.0%	3 / 3 100.0%	1 / 1 100.0%	1 / 1 100.0%	3 / 3 100.0%	1 / 1 100.0%	3 / 4 75.0%	3 / 3 100.0%	1 / 1 100.0%	2 / 2 100.0%	1 / 1 100.0%	3 / 3 100.0%	1 / 1 100.0%	3 / 3 100.0%	5 / 5 100.0%	3 / 3 100.0%	1 / 1 100.0%	5 / 5 100.0%	0 / 0 0.0%	0 / 0 0.0%	0 / 0 0.0%	0 / 0 0.0%	25 / 49 51.0%ABE	23 / 49 46.9%AB	1 / 49 2.0%CE						
1	ATCC 49627	48 / 49 98.0%	1 / 1 100.0%	2 / 2 100.0%	1 / 1 100.0%	1 / 1 100.0%	3 / 3 100.0%	3 / 3 100.0%	1 / 1 100.0%	1 / 1 100.0%	3 / 3 100.0%	1 / 1 100.0%	3 / 4 75.0%	3 / 3 100.0%	1 / 1 100.0%	2 / 2 100.0%	1 / 1 100.0%	3 / 3 100.0%	1 / 1 100.0%	3 / 3 100.0%	5 / 5 100.0%	3 / 3 100.0%	1 / 1 100.0%	5 / 5 100.0%	0 / 0 0.0%	0 / 0 0.0%	0 / 0 0.0%	0 / 0 0.0%	25 / 49 51.0%ABE	23 / 49 46.9%AB	1 / 49 2.0%CE						
1	Olsenella uli ATCC 49627	48 / 49 98.0%	1 / 1 100.0%	2 / 2 100.0%	1 / 1 100.0%	1 / 1 100.0%	3 / 3 100.0%	3 / 3 100.0%	1 / 1 100.0%	1 / 1 100.0%	3 / 3 100.0%	1 / 1 100.0%	3 / 4 75.0%	3 / 3 100.0%	1 / 1 100.0%	2 / 2 100.0%	1 / 1 100.0%	3 / 3 100.0%	1 / 1 100.0%	3 / 3 100.0%	5 / 5 100.0%	3 / 3 100.0%	1 / 1 100.0%	5 / 5 100.0%	0 / 0 0.0%	0 / 0 0.0%	0 / 0 0.0%	0 / 0 0.0%	25 / 49 51.0%ABE	23 / 49 46.9%AB	1 / 49 2.0%CE						
249	Actinobacteridae	3510 / 12918 27.2%	138 / 348 39.7%	86 / 361 23.8%	69 / 326 21.2%	95 / 278 34.2%	408 / 1005 40.6%	289 / 930 31.1%	168 / 259 64.9%	89 / 266 33.5%	124 / 860 14.4%	41 / 281 14.6%	382 / 1044 36.6%	142 / 592 24.0%	141 / 264 53.4%	38 / 540 7.0%	87 / 303 28.7%	247 / 784 31.5%	40 / 259 15.4%	247 / 815 30.3%	184 / 1130 16.3%	131 / 632 20.7%	38 / 257 14.8%	292 / 1333 21.9%	33 / 40 82.5%	0 / 0 0.0%	0 / 8 0.0%	1 / 3 33.3%	4285 / 12918 33.2%	3671 / 12918 28.4%E	1971 / 12918 15.3%AB	1468 / 12918 11.4%ABE	733 / 12918 5.7%C	718 / 12918 5.6%CE	41 / 12918 0.3%B	30 / 12918 0.2%BE	1 / 12918 0.0%D
217	Actinomycetales	3094 / 11053 28.0%	124 / 314 39.5%	72 / 298 24.2%	28 / 255 11.0%	95 / 243 39.1%	318 / 834 38.1%	274 / 781 35.1%	147 / 225 65.3%	88 / 226 38.9%	112 / 727 15.4%	11 / 233 4.7%	364 / 895 40.7%	141 / 520 27.1%	110 / 225 48.9%	22 / 468 4.7%	87 / 269 32.3%	237 / 685 34.6%	40 / 225 17.8%	219 / 690 31.7%	145 / 946 15.3%	131 / 564 23.2%	38 / 223 17.0%	257 / 1156 22.2%	33 / 40 82.5%	0 / 0 0.0%	0 / 8 0.0%	1 / 3 33.3%	3506 / 11053 31.7%	3221 / 11053 29.1%E	1692 / 11053 15.3%AB	1335 / 11053 12.1%ABE	670 / 11053 6.1%C	561 / 11053 5.1%CE	38 / 11053 0.3%B	29 / 11053 0.3%BE	1 / 11053 0.0%D
2	Actinomycetineae	27 / 96 28.1%	0 / 2 0.0%	1 / 3 33.3%	0 / 2 0.0%	1 / 2 50.0%	3 / 7 42.9%	2 / 7 28.6%	1 / 2 50.0%	2 / 2 100.0%	3 / 6 50.0%	1 / 2 50.0%	3 / 8 37.5%	1 / 5 20.0%	2 / 2 100.0%	0 / 4 0.0%	0 / 2 0.0%	1 / 6 16.7%	0 / 2 0.0%	2 / 6 33.3%	1 / 9 11.1%	1 / 5 20.0%	0 / 2 0.0%	2 / 10 20.0%	0 / 0 0.0%	0 / 0 0.0%	0 / 0 0.0%	0 / 0 0.0%	33 / 96 34.4%	31 / 96 32.3%E	17 / 96 17.7%AB	10 / 96 10.4%ABE	3 / 96 3.1%CE	2 / 96 2.1%C			
2	Actinomycetaceae	27 / 96 28.1%	0 / 2 0.0%	1 / 3 33.3%	0 / 2 0.0%	1 / 2 50.0%	3 / 7 42.9%	2 / 7 28.6%	1 / 2 50.0%	2 / 2 100.0%	3 / 6 50.0%	1 / 2 50.0%	3 / 8 37.5%	1 / 5 20.0%	2 / 2 100.0%	0 / 4 0.0%	0 / 2 0.0%	1 / 6 16.7%	0 / 2 0.0%	2 / 6 33.3%	1 / 9 11.1%	1 / 5 20.0%	0 / 2 0.0%	2 / 10 20.0%	0 / 0 0.0%	0 / 0 0.0%	0 / 0 0.0%	0 / 0 0.0%	33 / 96 34.4%	31 / 96 32.3%E	17 / 96 17.7%AB	10 / 96 10.4%ABE	3 / 96 3.1%CE	2 / 96 2.1%C			
1	Arcanibacterium	12 / 50 24.0%	0 / 1 0.0%	1 / 2 50.0%	0 / 1 0.0%	0 / 1 0.0%	1 / 4 25.0%	0 / 4 0.0%	1 / 1 100.0%	1 / 1 100.0%	2 / 3 66.7%	0 / 1 0.0%	1 / 4 25.0%	0 / 3 0.0%	1 / 1 100.0%	0 / 2 0.0%	0 / 1 0.0%	1 / 3 33.3%	0 / 1 0.0%	1 / 3 33.3%	0 / 4 0.0%	1 / 3 33.3%	0 / 1 0.0%	1 / 5 20.0%	0 / 0 0.0%	0 / 0 0.0%	0 / 0 0.0%	0 / 0 0.0%	19 / 50 38.0%	18 / 50 36.0%E	7 / 50 14.0%AB	5 / 50 10.0%ABE	1 / 50 2.0%CE				
1	"Corynebacterium haemolyticum" Mac Lean et al. 1946	12 / 50 24.0%	0 / 1 0.0%	1 / 2 50.0%	0 / 1 0.0%	0 / 1 0.0%	1 / 4 25.0%	0 / 4 0.0%	1 / 1 100.0%	1 / 1 100.0%	2 / 3 66.7%	0 / 1 0.0%	1 / 4 25.0%	0 / 3 0.0%	1 / 1 100.0%	0 / 2 0.0%	0 / 1 0.0%	1 / 3 33.3%	0 / 1 0.0%	1 / 3 33.3%	0 / 4 0.0%	1 / 3 33.3%	0 / 1 0.0%	1 / 5 20.0%	0 / 0 0.0%	0 / 0 0.0%	0 / 0 0.0%	0 / 0 0.0%	19 / 50 38.0%	18 / 50 36.0%E	7 / 50 14.0%AB	5 / 50 10.0%ABE	1 / 50 2.0%CE				
1	Arcanobacterium haemolyticum ATCC 9345	12 / 50 24.0%	0 / 1 0.0%	1 / 2 50.0%	0 / 1 0.0%	0 / 1 0.0%	1 / 4 25.0%	0 / 4 0.0%	1 / 1 100.0%	1 / 1 100.0%	2 / 3 66.7%	0 / 1 0.0%	1 / 4 25.0%	0 / 3 0.0%	1 / 1 100.0%	0 / 2 0.0%	0 / 1 0.0%	1 / 3 33.3%	0 / 1 0.0%	1 / 3 33.3%	0 / 4 0.0%	1 / 3 33.3%	0 / 1 0.0%	1 / 5 20.0%	0 / 0 0.0%	0 / 0 0.0%	0 / 0 0.0%	0 / 0 0.0%	19 / 50 38.0%	18 / 50 36.0%E	7 / 50 14.0%AB	5 / 50 10.0%ABE	1 / 50 2.0%CE				
1	Mobiluncus/Falcivibrio group	15 / 46 32.6%	0 / 1 0.0%	0 / 1 0.0%	0 / 1 0.0%	1 / 1 100.0%	2 / 3 66.7%	2 / 3 66.7%	0 / 1 0.0%	1 / 1 100.0%	1 / 3 33.3%	1 / 1 100.0%	2 / 4 50.0%	1 / 2 50.0%	1 / 1 100.0%	0 / 2 0.0%	0 / 1 0.0%	0 / 3 0.0%	0 / 1 0.0%	1 / 3 33.3%	1 / 5 20.0%	0 / 2 0.0%	0 / 1 0.0%	1 / 5 20.0%	0 / 0 0.0%	0 / 0 0.0%	0 / 0 0.0%	0 / 0 0.0%	14 / 46 30.4%	13 / 46 28.3%E	10 / 46 21.7%AB	5 / 46 10.9%ABE	2 / 46 4.3%CE	2 / 46 4.3%C			
1	Falcivibrio	15 / 46 32.6%	0 / 1 0.0%	0 / 1 0.0%	0 / 1 0.0%	1 / 1 100.0%	2 / 3 66.7%	2 / 3 66.7%	0 / 1 0.0%	1 / 1 100.0%	1 / 3 33.3%	1 / 1 100.0%	2 / 4 50.0%	1 / 2 50.0%	1 / 1 100.0%	0 / 2 0.0%	0 / 1 0.0%	0 / 3 0.0%	0 / 1 0.0%	1 / 3 33.3%	1 / 5 20.0%	0 / 2 0.0%	0 / 1 0.0%	1 / 5 20.0%	0 / 0 0.0%	0 / 0 0.0%	0 / 0 0.0%	0 / 0 0.0%	14 / 46 30.4%	13 / 46 28.3%E	10 / 46 21.7%AB	5 / 46 10.9%ABE	2 / 46 4.3%C	2 / 46 4.3%CE			
1	ATCC 35241	15 / 46 32.6%	0 / 1 0.0%	0 / 1 0.0%	0 / 1 0.0%	1 / 1 100.0%	2 / 3 66.7%	2 / 3 66.7%	0 / 1 0.0%	1 / 1 100.0%	1 / 3 33.3%	1 / 1 100.0%	2 / 4 50.0%	1 / 2 50.0%	1 / 1 100.0%	0 / 2 0.0%	0 / 1 0.0%	0 / 3 0.0%	0 / 1 0.0%	1 / 3 33.3%	1 / 5 20.0%	0 / 2 0.0%	0 / 1 0.0%	1 / 5 20.0%	0 / 0 0.0%	0 / 0 0.0%	0 / 0 0.0%	0 / 0 0.0%	14 / 46 30.4%	13 / 46 28.3%E	10 / 46 21.7%AB	5 / 46 10.9%ABE	2 / 46 4.3%CE	2 / 46 4.3%C			
1	Falcivibrio vaginalis ATCC 43063	15 / 46 32.6%	0 / 1 0.0%	0 / 1 0.0%	0 / 1 0.0%	1 / 1 100.0%	2 / 3 66.7%	2 / 3 66.7%	0 / 1 0.0%	1 / 1 100.0%	1 / 3 33.3%	1 / 1 100.0%	2 / 4 50.0%	1 / 2 50.0%	1 / 1 100.0%	0 / 2 0.0%	0 / 1 0.0%	0 / 3 0.0%	0 / 1 0.0%	1 / 3 33.3%	1 / 5 20.0%	0 / 2 0.0%	0 / 1 0.0%	1 / 5 20.0%	0 / 0 0.0%	0 / 0 0.0%	0 / 0 0.0%	0 / 0 0.0%	14 / 46 30.4%	13 / 46 28.3%E	10 / 46 21.7%AB	5 / 46 10.9%ABE	2 / 46 4.3%C	2 / 46 4.3%CE			
29	Micrococcineae	402 / 1410 28.5%	9 / 38 23.7%	4 / 34 11.8%	10 / 35 28.6%	6 / 29 20.7%	42 / 107 39.3%	42 / 97 43.3%	22 / 30 73.3%	2 / 29 6.9%	25 / 95 26.3%	3 / 30 10.0%	46 / 116 39.7%	28 / 69 40.6%	15 / 34 44.1%	6 / 60 10.0%	2 / 32 6.2%	33 / 89 37.1%	10 / 30 33.3%	26 / 90 28.9%	22 / 118 18.6%	17 / 68 25.0%	5 / 29 17.2%	25 / 148 16.9%	2 / 3 66.7%	0 / 0 0.0%	0 / 0 0.0%	0 / 0 0.0%	408 / 1410 28.9%E	357 / 1410 25.3%	210 / 1410 14.9%AB	174 / 1410 12.3%ABE	134 / 1410 9.5%C	109 / 1410 7.7%CE	11 / 1410 0.8%B	7 / 1410 0.5%BE	
11	Micrococcaceae	150 / 557 26.9%	3 / 18 16.7%	0 / 12 0.0%	3 / 16 18.8%	0 / 11 0.0%	21 / 45 46.7%	18 / 39 46.2%	7 / 12 58.3%	0 / 11 0.0%	12 / 40 30.0%	0 / 11 0.0%	15 / 44 34.1%	11 / 29 37.9%	7 / 12 58.3%	1 / 23 4.3%	0 / 13 0.0%	17 / 35 48.6%	2 / 11 18.2%	11 / 36 30.6%	8 / 45 17.8%	6 / 26 23.1%	1 / 11 9.1%	6 / 56 10.7%	1 / 1 100.0%	0 / 0 0.0%	0 / 0 0.0%	0 / 0 0.0%	183 / 557 32.9%	169 / 557 30.3%E	82 / 557 14.7%AB	56 / 557 10.1%ABE	30 / 557 5.4%C	25 / 557 4.5%CE	8 / 557 1.4%B	4 / 557 0.7%BE	
1	Micrococcus	11 / 48 22.9%	0 / 1 0.0%	0 / 1 0.0%	0 / 1 0.0%	0 / 1 0.0%	2 / 4 50.0%	1 / 3 33.3%	1 / 1 100.0%	0 / 1 0.0%	1 / 4 25.0%	0 / 1 0.0%	1 / 4 25.0%	1 / 2 50.0%	1 / 1 100.0%	0 / 2 0.0%	0 / 1 0.0%	2 / 3 66.7%	0 / 1 0.0%	1 / 3 33.3%	0 / 4 0.0%	0 / 3 0.0%	0 / 1 0.0%	0 / 5 0.0%	0 / 0 0.0%	0 / 0 0.0%	0 / 0 0.0%	0 / 0 0.0%	12 / 48 25.0%E	11 / 48 22.9%	8 / 48 16.7%CE	7 / 48 14.6%AB	6 / 48 12.5%C	4 / 48 8.3%ABE			
1	"Bacteridium luteum" Schroeter 1872	11 / 48 22.9%	0 / 1 0.0%	0 / 1 0.0%	0 / 1 0.0%	0 / 1 0.0%	2 / 4 50.0%	1 / 3 33.3%	1 / 1 100.0%	0 / 1 0.0%	1 / 4 25.0%	0 / 1 0.0%	1 / 4 25.0%	1 / 2 50.0%	1 / 1 100.0%	0 / 2 0.0%	0 / 1 0.0%	2 / 3 66.7%	0 / 1 0.0%	1 / 3 33.3%	0 / 4 0.0%	0 / 3 0.0%	0 / 1 0.0%	0 / 5 0.0%	0 / 0 0.0%	0 / 0 0.0%	0 / 0 0.0%	0 / 0 0.0%	12 / 48 25.0%E	11 / 48 22.9%	8 / 48 16.7%CE	7 / 48 14.6%AB	6 / 48 12.5%C	4 / 48 8.3%ABE			
1	Micrococcus luteus ATCC 4698	11 / 48 22.9%	0 / 1 0.0%	0 / 1 0.0%	0 / 1 0.0%	0 / 1 0.0%	2 / 4 50.0%	1 / 3 33.3%	1 / 1 100.0%	0 / 1 0.0%	1 / 4 25.0%	0 / 1 0.0%	1 / 4 25.0%	1 / 2 50.0%	1 / 1 100.0%	0 / 2 0.0%	0 / 1 0.0%	2 / 3 66.7%	0 / 1 0.0%	1 / 3 33.3%	0 / 4 0.0%	0 / 3 0.0%	0 / 1 0.0%	0 / 5 0.0%	0 / 0 0.0%	0 / 0 0.0%	0 / 0 0.0%	0 / 0 0.0%	12 / 48 25.0%E	11 / 48 22.9%	8 / 48 16.7%CE	7 / 48 14.6%AB	6 / 48 12.5%C	4 / 48 8.3%ABE			
1	Renibacterium	12 / 45 26.7%	0 / 1 0.0%	0 / 1 0.0%	0 / 1 0.0%	0 / 1 0.0%	1 / 3 33.3%	0 / 3 0.0%	1 / 1 100.0%	0 / 1 0.0%	1 / 3 33.3%	0 / 1 0.0%	2 / 4 50.0%	1 / 2 50.0%	1 / 1 100.0%	0 / 2 0.0%	0 / 1 0.0%	2 / 3 66.7%	0 / 1 0.0%	1 / 3 33.3%	0 / 4 0.0%	0 / 2 0.0%	1 / 1 100.0%	1 / 5 20.0%	0 / 0 0.0%	0 / 0 0.0%	0 / 0 0.0%	0 / 0 0.0%	15 / 45 33.3%E	13 / 45 28.9%	5 / 45 11.1%AB	3 / 45 6.7%CE	3 / 45 6.7%B	2 / 45 4.4%ABE	2 / 45 4.4%BE	2 / 45 4.4%C	
1	ATCC 33209	12 / 45 26.7%	0 / 1 0.0%	0 / 1 0.0%	0 / 1 0.0%	0 / 1 0.0%	1 / 3 33.3%	0 / 3 0.0%	1 / 1 100.0%	0 / 1 0.0%	1 / 3 33.3%	0 / 1 0.0%	2 / 4 50.0%	1 / 2 50.0%	1 / 1 100.0%	0 / 2 0.0%	0 / 1 0.0%	2 / 3 66.7%	0 / 1 0.0%	1 / 3 33.3%	0 / 4 0.0%	0 / 2 0.0%	1 / 1 100.0%	1 / 5 20.0%	0 / 0 0.0%	0 / 0 0.0%	0 / 0 0.0%	0 / 0 0.0%	15 / 45 33.3%E	13 / 45 28.9%	5 / 45 11.1%AB	3 / 45 6.7%B	3 / 45 6.7%CE	2 / 45 4.4%C	2 / 45 4.4%BE	2 / 45 4.4%ABE	
1	Renibacterium salmoninarum ATCC 33209	12 / 45 26.7%	0 / 1 0.0%	0 / 1 0.0%	0 / 1 0.0%	0 / 1 0.0%	1 / 3 33.3%	0 / 3 0.0%	1 / 1 100.0%	0 / 1 0.0%	1 / 3 33.3%	0 / 1 0.0%	2 / 4 50.0%	1 / 2 50.0%	1 / 1 100.0%	0 / 2 0.0%	0 / 1 0.0%	2 / 3 66.7%	0 / 1 0.0%	1 / 3 33.3%	0 / 4 0.0%	0 / 2 0.0%	1 / 1 100.0%	1 / 5 20.0%	0 / 0 0.0%	0 / 0 0.0%	0 / 0 0.0%	0 / 0 0.0%	15 / 45 33.3%E	13 / 45 28.9%	5 / 45 11.1%AB	3 / 45 6.7%B	3 / 45 6.7%CE	2 / 45 4.4%C	2 / 45 4.4%ABE	2 / 45 4.4%BE	
6	Arthrobacter	98 / 321 30.5%	1 / 13 7.7%	0 / 7 0.0%	2 / 11 18.2%	0 / 6 0.0%	15 / 27 55.6%	15 / 24 62.5%	4 / 7 57.1%	0 / 6 0.0%	9 / 24 37.5%	0 / 6 0.0%	8 / 24 33.3%	7 / 17 41.2%	5 / 7 71.4%	1 / 13 7.7%	0 / 8 0.0%	12 / 20 60.0%	2 / 6 33.3%	6 / 19 31.6%	5 / 25 20.0%	5 / 13 38.5%	0 / 6 0.0%	1 / 32 3.1%	0 / 0 0.0%	0 / 0 0.0%	0 / 0 0.0%	0 / 0 0.0%	108 / 321 33.6%	95 / 321 29.6%E	62 / 321 19.3%AB	36 / 321 11.2%ABE	12 / 321 3.7%C	8 / 321 2.5%CE			
1	ATCC 13344	17 / 54 31.5%	0 / 2 0.0%	0 / 1 0.0%	0 / 2 0.0%	0 / 1 0.0%	3 / 5 60.0%	4 / 4 100.0%	1 / 1 100.0%	0 / 1 0.0%	2 / 4 50.0%	0 / 1 0.0%	1 / 4 25.0%	2 / 3 66.7%	1 / 1 100.0%	0 / 2 0.0%	0 / 2 0.0%	2 / 3 66.7%	0 / 1 0.0%	1 / 3 33.3%	0 / 4 0.0%	0 / 2 0.0%	0 / 1 0.0%	0 / 6 0.0%	0 / 0 0.0%	0 / 0 0.0%	0 / 0 0.0%	0 / 0 0.0%	18 / 54 33.3%	16 / 54 29.6%E	11 / 54 20.4%AB	6 / 54 11.1%ABE	2 / 54 3.7%C	1 / 54 1.9%CE			
1	Arthrobacter aurescens TC1	17 / 54 31.5%	0 / 2 0.0%	0 / 1 0.0%	0 / 2 0.0%	0 / 1 0.0%	3 / 5 60.0%	4 / 4 100.0%	1 / 1 100.0%	0 / 1 0.0%	2 / 4 50.0%	0 / 1 0.0%	1 / 4 25.0%	2 / 3 66.7%	1 / 1 100.0%	0 / 2 0.0%	0 / 2 0.0%	2 / 3 66.7%	0 / 1 0.0%	1 / 3 33.3%	0 / 4 0.0%	0 / 2 0.0%	0 / 1 0.0%	0 / 6 0.0%	0 / 0 0.0%	0 / 0 0.0%	0 / 0 0.0%	0 / 0 0.0%	18 / 54 33.3%	16 / 54 29.6%E	11 / 54 20.4%AB	6 / 54 11.1%ABE	2 / 54 3.7%C	1 / 54 1.9%CE			
1	ATCC 700700	12 / 49 24.5%	0 / 2 0.0%	0 / 1 0.0%	0 / 1 0.0%	0 / 1 0.0%	2 / 4 50.0%	1 / 4 25.0%	0 / 1 0.0%	0 / 1 0.0%	1 / 4 25.0%	0 / 1 0.0%	2 / 4 50.0%	1 / 2 50.0%	1 / 1 100.0%	0 / 2 0.0%	0 / 1 0.0%	2 / 3 66.7%	0 / 1 0.0%	1 / 3 33.3%	0 / 4 0.0%	1 / 2 50.0%	0 / 1 0.0%	0 / 5 0.0%	0 / 0 0.0%	0 / 0 0.0%	0 / 0 0.0%	0 / 0 0.0%	16 / 49 32.7%	16 / 49 32.7%E	7 / 49 14.3%AB	5 / 49 10.2%ABE	3 / 49 6.1%C	2 / 49 4.1%CE			
1	Arthrobacter chlorophenolicus A6	12 / 49 24.5%	0 / 2 0.0%	0 / 1 0.0%	0 / 1 0.0%	0 / 1 0.0%	2 / 4 50.0%	1 / 4 25.0%	0 / 1 0.0%	0 / 1 0.0%	1 / 4 25.0%	0 / 1 0.0%	2 / 4 50.0%	1 / 2 50.0%	1 / 1 100.0%	0 / 2 0.0%	0 / 1 0.0%	2 / 3 66.7%	0 / 1 0.0%	1 / 3 33.3%	0 / 4 0.0%	1 / 2 50.0%	0 / 1 0.0%	0 / 5 0.0%	0 / 0 0.0%	0 / 0 0.0%	0 / 0 0.0%	0 / 0 0.0%	16 / 49 32.7%	16 / 49 32.7%E	7 / 49 14.3%AB	5 / 49 10.2%ABE	3 / 49 6.1%C	2 / 49 4.1%CE			
1	Arthrobacter arilaitensis	23 / 64 35.9%	0 / 3 0.0%	0 / 2 0.0%	1 / 2 50.0%	0 / 1 0.0%	3 / 6 50.0%	3 / 5 60.0%	2 / 2 100.0%	0 / 1 0.0%	2 / 4 50.0%	0 / 1 0.0%	2 / 4 50.0%	0 / 4 0.0%	0 / 1 0.0%	1 / 3 33.3%	0 / 1 0.0%	2 / 5 40.0%	1 / 1 100.0%	1 / 4 25.0%	2 / 5 40.0%	2 / 3 66.7%	0 / 1 0.0%	1 / 5 20.0%	0 / 0 0.0%	0 / 0 0.0%	0 / 0 0.0%	0 / 0 0.0%	24 / 64 37.5%	16 / 64 25.0%E	13 / 64 20.3%AB	10 / 64 15.6%ABE	1 / 64 1.6%C				
1	Arthrobacter sp. FB24	14 / 51 27.5%	1 / 2 50.0%	0 / 1 0.0%	0 / 2 0.0%	0 / 1 0.0%	2 / 4 50.0%	3 / 4 75.0%	0 / 1 0.0%	0 / 1 0.0%	1 / 4 25.0%	0 / 1 0.0%	0 / 4 0.0%	1 / 3 33.3%	1 / 1 100.0%	0 / 2 0.0%	0 / 1 0.0%	2 / 3 66.7%	1 / 1 100.0%	1 / 3 33.3%	0 / 4 0.0%	1 / 2 50.0%	0 / 1 0.0%	0 / 5 0.0%	0 / 0 0.0%	0 / 0 0.0%	0 / 0 0.0%	0 / 0 0.0%	19 / 51 37.3%	15 / 51 29.4%E	8 / 51 15.7%AB	6 / 51 11.8%ABE	2 / 51 3.9%CE	1 / 51 2.0%C			
1	Arthrobacter phenanthrenivorans	17 / 50 34.0%	0 / 2 0.0%	0 / 1 0.0%	1 / 2 50.0%	0 / 1 0.0%	2 / 4 50.0%	2 / 4 50.0%	0 / 1 0.0%	0 / 1 0.0%	1 / 4 25.0%	0 / 1 0.0%	2 / 4 50.0%	1 / 2 50.0%	1 / 1 100.0%	0 / 2 0.0%	0 / 1 0.0%	2 / 3 66.7%	0 / 1 0.0%	1 / 3 33.3%	3 / 4 75.0%	1 / 2 50.0%	0 / 1 0.0%	0 / 5 0.0%	0 / 0 0.0%	0 / 0 0.0%	0 / 0 0.0%	0 / 0 0.0%	15 / 50 30.0%E	14 / 50 28.0%	12 / 50 24.0%AB	5 / 50 10.0%ABE	2 / 50 4.0%C	2 / 50 4.0%CE			
1	Arthrobacter phenanthrenivorans Sphe3	17 / 50 34.0%	0 / 2 0.0%	0 / 1 0.0%	1 / 2 50.0%	0 / 1 0.0%	2 / 4 50.0%	2 / 4 50.0%	0 / 1 0.0%	0 / 1 0.0%	1 / 4 25.0%	0 / 1 0.0%	2 / 4 50.0%	1 / 2 50.0%	1 / 1 100.0%	0 / 2 0.0%	0 / 1 0.0%	2 / 3 66.7%	0 / 1 0.0%	1 / 3 33.3%	3 / 4 75.0%	1 / 2 50.0%	0 / 1 0.0%	0 / 5 0.0%	0 / 0 0.0%	0 / 0 0.0%	0 / 0 0.0%	0 / 0 0.0%	15 / 50 30.0%E	14 / 50 28.0%	12 / 50 24.0%AB	5 / 50 10.0%ABE	2 / 50 4.0%CE	2 / 50 4.0%C			
1	Arthrobacter nitroguajacolicus Rue61a	15 / 53 28.3%	0 / 2 0.0%	0 / 1 0.0%	0 / 2 0.0%	0 / 1 0.0%	3 / 4 75.0%	2 / 3 66.7%	1 / 1 100.0%	0 / 1 0.0%	2 / 4 50.0%	0 / 1 0.0%	1 / 4 25.0%	2 / 3 66.7%	1 / 2 50.0%	0 / 2 0.0%	0 / 2 0.0%	2 / 3 66.7%	0 / 1 0.0%	1 / 3 33.3%	0 / 4 0.0%	0 / 2 0.0%	0 / 1 0.0%	0 / 6 0.0%	0 / 0 0.0%	0 / 0 0.0%	0 / 0 0.0%	0 / 0 0.0%	17 / 53 32.1%	17 / 53 32.1%E	11 / 53 20.8%AB	4 / 53 7.5%ABE	3 / 53 5.7%C	1 / 53 1.9%CE			
2	Rothia	18 / 97 18.6%	1 / 2 50.0%	0 / 2 0.0%	0 / 2 0.0%	0 / 2 0.0%	2 / 8 25.0%	1 / 6 16.7%	0 / 2 0.0%	0 / 2 0.0%	0 / 6 0.0%	0 / 2 0.0%	3 / 8 37.5%	0 / 6 0.0%	0 / 2 0.0%	0 / 4 0.0%	0 / 2 0.0%	1 / 6 16.7%	0 / 2 0.0%	3 / 8 37.5%	2 / 8 25.0%	1 / 6 16.7%	0 / 2 0.0%	4 / 9 44.4%	0 / 0 0.0%	0 / 0 0.0%	0 / 0 0.0%	0 / 0 0.0%	38 / 97 39.2%	36 / 97 37.1%E	11 / 97 11.3%ABE	7 / 97 7.2%AB	4 / 97 4.1%C	1 / 97 1.0%CE			
1	"Actinomyces dentocariosus" Onishi 1949	7 / 49 14.3%	0 / 1 0.0%	0 / 1 0.0%	0 / 1 0.0%	0 / 1 0.0%	1 / 4 25.0%	0 / 3 0.0%	0 / 1 0.0%	0 / 1 0.0%	0 / 3 0.0%	0 / 1 0.0%	1 / 4 25.0%	0 / 3 0.0%	0 / 1 0.0%	0 / 2 0.0%	0 / 1 0.0%	1 / 3 33.3%	0 / 1 0.0%	2 / 4 50.0%	0 / 4 0.0%	0 / 3 0.0%	0 / 1 0.0%	2 / 5 40.0%	0 / 0 0.0%	0 / 0 0.0%	0 / 0 0.0%	0 / 0 0.0%	20 / 49 40.8%E	19 / 49 38.8%	4 / 49 8.2%ABE	3 / 49 6.1%AB	2 / 49 4.1%C	1 / 49 2.0%CE			
1	Rothia dentocariosa ATCC 17931	7 / 49 14.3%	0 / 1 0.0%	0 / 1 0.0%	0 / 1 0.0%	0 / 1 0.0%	1 / 4 25.0%	0 / 3 0.0%	0 / 1 0.0%	0 / 1 0.0%	0 / 3 0.0%	0 / 1 0.0%	1 / 4 25.0%	0 / 3 0.0%	0 / 1 0.0%	0 / 2 0.0%	0 / 1 0.0%	1 / 3 33.3%	0 / 1 0.0%	2 / 4 50.0%	0 / 4 0.0%	0 / 3 0.0%	0 / 1 0.0%	2 / 5 40.0%	0 / 0 0.0%	0 / 0 0.0%	0 / 0 0.0%	0 / 0 0.0%	20 / 49 40.8%E	19 / 49 38.8%	4 / 49 8.2%ABE	3 / 49 6.1%AB	2 / 49 4.1%C	1 / 49 2.0%CE			
1	"Micrococcus mucilaginosus" Migula 1900	11 / 48 22.9%	1 / 1 100.0%	0 / 1 0.0%	0 / 1 0.0%	0 / 1 0.0%	1 / 4 25.0%	1 / 3 33.3%	0 / 1 0.0%	0 / 1 0.0%	0 / 3 0.0%	0 / 1 0.0%	2 / 4 50.0%	0 / 3 0.0%	0 / 1 0.0%	0 / 2 0.0%	0 / 1 0.0%	0 / 3 0.0%	0 / 1 0.0%	1 / 4 25.0%	2 / 4 50.0%	1 / 3 33.3%	0 / 1 0.0%	2 / 4 50.0%	0 / 0 0.0%	0 / 0 0.0%	0 / 0 0.0%	0 / 0 0.0%	19 / 48 39.6%	16 / 48 33.3%E	7 / 48 14.6%ABE	4 / 48 8.3%AB	2 / 48 4.2%C				
1	Rothia mucilaginosa DY-18	11 / 48 22.9%	1 / 1 100.0%	0 / 1 0.0%	0 / 1 0.0%	0 / 1 0.0%	1 / 4 25.0%	1 / 3 33.3%	0 / 1 0.0%	0 / 1 0.0%	0 / 3 0.0%	0 / 1 0.0%	2 / 4 50.0%	0 / 3 0.0%	0 / 1 0.0%	0 / 2 0.0%	0 / 1 0.0%	0 / 3 0.0%	0 / 1 0.0%	1 / 4 25.0%	2 / 4 50.0%	1 / 3 33.3%	0 / 1 0.0%	2 / 4 50.0%	0 / 0 0.0%	0 / 0 0.0%	0 / 0 0.0%	0 / 0 0.0%	19 / 48 39.6%	16 / 48 33.3%E	7 / 48 14.6%ABE	4 / 48 8.3%AB	2 / 48 4.2%C				
1	Kocuria	11 / 46 23.9%	1 / 1 100.0%	0 / 1 0.0%	1 / 1 100.0%	0 / 1 0.0%	1 / 3 33.3%	1 / 3 33.3%	1 / 1 100.0%	0 / 1 0.0%	1 / 3 33.3%	0 / 1 0.0%	1 / 4 25.0%	2 / 2 100.0%	0 / 1 0.0%	0 / 2 0.0%	0 / 1 0.0%	0 / 3 0.0%	0 / 1 0.0%	0 / 3 0.0%	1 / 4 25.0%	0 / 2 0.0%	0 / 1 0.0%	0 / 5 0.0%	1 / 1 100.0%	0 / 0 0.0%	0 / 0 0.0%	0 / 0 0.0%	13 / 46 28.3%	11 / 46 23.9%E	6 / 46 13.0%C	5 / 46 10.9%B	5 / 46 10.9%CE	3 / 46 6.5%ABE	2 / 46 4.3%BE	1 / 46 2.2%AB	
1	ATCC BAA-50	11 / 46 23.9%	1 / 1 100.0%	0 / 1 0.0%	1 / 1 100.0%	0 / 1 0.0%	1 / 3 33.3%	1 / 3 33.3%	1 / 1 100.0%	0 / 1 0.0%	1 / 3 33.3%	0 / 1 0.0%	1 / 4 25.0%	2 / 2 100.0%	0 / 1 0.0%	0 / 2 0.0%	0 / 1 0.0%	0 / 3 0.0%	0 / 1 0.0%	0 / 3 0.0%	1 / 4 25.0%	0 / 2 0.0%	0 / 1 0.0%	0 / 5 0.0%	1 / 1 100.0%	0 / 0 0.0%	0 / 0 0.0%	0 / 0 0.0%	13 / 46 28.3%	11 / 46 23.9%E	6 / 46 13.0%C	5 / 46 10.9%B	5 / 46 10.9%CE	3 / 46 6.5%ABE	2 / 46 4.3%BE	1 / 46 2.2%AB	
1	Kocuria rhizophila DC2201	11 / 46 23.9%	1 / 1 100.0%	0 / 1 0.0%	1 / 1 100.0%	0 / 1 0.0%	1 / 3 33.3%	1 / 3 33.3%	1 / 1 100.0%	0 / 1 0.0%	1 / 3 33.3%	0 / 1 0.0%	1 / 4 25.0%	2 / 2 100.0%	0 / 1 0.0%	0 / 2 0.0%	0 / 1 0.0%	0 / 3 0.0%	0 / 1 0.0%	0 / 3 0.0%	1 / 4 25.0%	0 / 2 0.0%	0 / 1 0.0%	0 / 5 0.0%	1 / 1 100.0%	0 / 0 0.0%	0 / 0 0.0%	0 / 0 0.0%	13 / 46 28.3%	11 / 46 23.9%E	6 / 46 13.0%C	5 / 46 10.9%B	5 / 46 10.9%CE	3 / 46 6.5%ABE	2 / 46 4.3%BE	1 / 46 2.2%AB	
3	Cellulomonadaceae	26 / 135 19.3%	0 / 3 0.0%	0 / 3 0.0%	1 / 3 33.3%	0 / 3 0.0%	0 / 9 0.0%	4 / 9 44.4%	3 / 3 100.0%	0 / 3 0.0%	4 / 9 44.4%	0 / 3 0.0%	3 / 12 25.0%	3 / 6 50.0%	1 / 3 33.3%	0 / 6 0.0%	0 / 3 0.0%	0 / 9 0.0%	1 / 3 33.3%	3 / 9 33.3%	1 / 12 8.3%	0 / 6 0.0%	1 / 3 33.3%	1 / 15 6.7%	0 / 0 0.0%	0 / 0 0.0%	0 / 0 0.0%	0 / 0 0.0%	38 / 135 28.1%E	27 / 135 20.0%C	24 / 135 17.8%	20 / 135 14.8%CE	15 / 135 11.1%AB	11 / 135 8.1%ABE			
3	Cellulomonas	26 / 135 19.3%	0 / 3 0.0%	0 / 3 0.0%	1 / 3 33.3%	0 / 3 0.0%	0 / 9 0.0%	4 / 9 44.4%	3 / 3 100.0%	0 / 3 0.0%	4 / 9 44.4%	0 / 3 0.0%	3 / 12 25.0%	3 / 6 50.0%	1 / 3 33.3%	0 / 6 0.0%	0 / 3 0.0%	0 / 9 0.0%	1 / 3 33.3%	3 / 9 33.3%	1 / 12 8.3%	0 / 6 0.0%	1 / 3 33.3%	1 / 15 6.7%	0 / 0 0.0%	0 / 0 0.0%	0 / 0 0.0%	0 / 0 0.0%	38 / 135 28.1%E	27 / 135 20.0%C	24 / 135 17.8%	20 / 135 14.8%CE	15 / 135 11.1%AB	11 / 135 8.1%ABE			
1	"Cellvibrio gilvus" Hulcher and King 1958	10 / 45 22.2%	0 / 1 0.0%	0 / 1 0.0%	1 / 1 100.0%	0 / 1 0.0%	0 / 3 0.0%	2 / 3 66.7%	1 / 1 100.0%	0 / 1 0.0%	2 / 3 66.7%	0 / 1 0.0%	2 / 4 50.0%	1 / 2 50.0%	0 / 1 0.0%	0 / 2 0.0%	0 / 1 0.0%	0 / 3 0.0%	0 / 1 0.0%	0 / 3 0.0%	0 / 4 0.0%	0 / 2 0.0%	1 / 1 100.0%	0 / 5 0.0%	0 / 0 0.0%	0 / 0 0.0%	0 / 0 0.0%	0 / 0 0.0%	12 / 45 26.7%E	9 / 45 20.0%C	8 / 45 17.8%	6 / 45 13.3%CE	5 / 45 11.1%ABE	5 / 45 11.1%AB			
1	Cellulomonas gilvus ATCC 13127	10 / 45 22.2%	0 / 1 0.0%	0 / 1 0.0%	1 / 1 100.0%	0 / 1 0.0%	0 / 3 0.0%	2 / 3 66.7%	1 / 1 100.0%	0 / 1 0.0%	2 / 3 66.7%	0 / 1 0.0%	2 / 4 50.0%	1 / 2 50.0%	0 / 1 0.0%	0 / 2 0.0%	0 / 1 0.0%	0 / 3 0.0%	0 / 1 0.0%	0 / 3 0.0%	0 / 4 0.0%	0 / 2 0.0%	1 / 1 100.0%	0 / 5 0.0%	0 / 0 0.0%	0 / 0 0.0%	0 / 0 0.0%	0 / 0 0.0%	12 / 45 26.7%E	9 / 45 20.0%C	8 / 45 17.8%	6 / 45 13.3%CE	5 / 45 11.1%AB	5 / 45 11.1%ABE			
1	"Bacterium fimi" McBeth and Scales 1913	9 / 45 20.0%	0 / 1 0.0%	0 / 1 0.0%	0 / 1 0.0%	0 / 1 0.0%	0 / 3 0.0%	1 / 3 33.3%	1 / 1 100.0%	0 / 1 0.0%	2 / 3 66.7%	0 / 1 0.0%	1 / 4 25.0%	1 / 2 50.0%	1 / 1 100.0%	0 / 2 0.0%	0 / 1 0.0%	0 / 3 0.0%	0 / 1 0.0%	2 / 3 66.7%	0 / 4 0.0%	0 / 2 0.0%	0 / 1 0.0%	0 / 5 0.0%	0 / 0 0.0%	0 / 0 0.0%	0 / 0 0.0%	0 / 0 0.0%	13 / 45 28.9%E	9 / 45 20.0%C	8 / 45 17.8%	6 / 45 13.3%CE	5 / 45 11.1%AB	4 / 45 8.9%ABE			
1	Cellulomonas fimi ATCC 484	9 / 45 20.0%	0 / 1 0.0%	0 / 1 0.0%	0 / 1 0.0%	0 / 1 0.0%	0 / 3 0.0%	1 / 3 33.3%	1 / 1 100.0%	0 / 1 0.0%	2 / 3 66.7%	0 / 1 0.0%	1 / 4 25.0%	1 / 2 50.0%	1 / 1 100.0%	0 / 2 0.0%	0 / 1 0.0%	0 / 3 0.0%	0 / 1 0.0%	2 / 3 66.7%	0 / 4 0.0%	0 / 2 0.0%	0 / 1 0.0%	0 / 5 0.0%	0 / 0 0.0%	0 / 0 0.0%	0 / 0 0.0%	0 / 0 0.0%	13 / 45 28.9%E	9 / 45 20.0%C	8 / 45 17.8%	6 / 45 13.3%CE	5 / 45 11.1%AB	4 / 45 8.9%ABE			
1	"Aplanobacter flavigenum" (sic) (Kellerman and McBeth 1912) Magrou and Prevot 1948	7 / 45 15.6%	0 / 1 0.0%	0 / 1 0.0%	0 / 1 0.0%	0 / 1 0.0%	0 / 3 0.0%	1 / 3 33.3%	1 / 1 100.0%	0 / 1 0.0%	0 / 3 0.0%	0 / 1 0.0%	0 / 4 0.0%	1 / 2 50.0%	0 / 1 0.0%	0 / 2 0.0%	0 / 1 0.0%	0 / 3 0.0%	1 / 1 100.0%	1 / 3 33.3%	1 / 4 25.0%	0 / 2 0.0%	0 / 1 0.0%	1 / 5 20.0%	0 / 0 0.0%	0 / 0 0.0%	0 / 0 0.0%	0 / 0 0.0%	13 / 45 28.9%E	9 / 45 20.0%C	8 / 45 17.8%	8 / 45 17.8%CE	5 / 45 11.1%AB	2 / 45 4.4%ABE			
1	Cellulomonas flavigena DSM 20109	7 / 45 15.6%	0 / 1 0.0%	0 / 1 0.0%	0 / 1 0.0%	0 / 1 0.0%	0 / 3 0.0%	1 / 3 33.3%	1 / 1 100.0%	0 / 1 0.0%	0 / 3 0.0%	0 / 1 0.0%	0 / 4 0.0%	1 / 2 50.0%	0 / 1 0.0%	0 / 2 0.0%	0 / 1 0.0%	0 / 3 0.0%	1 / 1 100.0%	1 / 3 33.3%	1 / 4 25.0%	0 / 2 0.0%	0 / 1 0.0%	1 / 5 20.0%	0 / 0 0.0%	0 / 0 0.0%	0 / 0 0.0%	0 / 0 0.0%	13 / 45 28.9%E	9 / 45 20.0%C	8 / 45 17.8%	8 / 45 17.8%CE	5 / 45 11.1%AB	2 / 45 4.4%ABE			
2	Promicromonosporaceae	34 / 100 34.0%	2 / 2 100.0%	0 / 2 0.0%	2 / 2 100.0%	1 / 2 50.0%	1 / 8 12.5%	6 / 8 75.0%	2 / 2 100.0%	1 / 2 50.0%	1 / 7 14.3%	2 / 2 100.0%	3 / 8 37.5%	2 / 6 33.3%	0 / 2 0.0%	0 / 5 0.0%	0 / 2 0.0%	3 / 6 50.0%	2 / 2 100.0%	0 / 6 0.0%	3 / 8 37.5%	1 / 6 16.7%	0 / 2 0.0%	2 / 10 20.0%	0 / 0 0.0%	0 / 0 0.0%	0 / 0 0.0%	0 / 0 0.0%	30 / 100 30.0%E	20 / 100 20.0%ABE	18 / 100 18.0%	14 / 100 14.0%AB	9 / 100 9.0%CE	9 / 100 9.0%C			
1	Xylanimonas	18 / 51 35.3%	1 / 1 100.0%	0 / 1 0.0%	1 / 1 100.0%	1 / 1 100.0%	1 / 4 25.0%	3 / 4 75.0%	1 / 1 100.0%	1 / 1 100.0%	1 / 4 25.0%	1 / 1 100.0%	1 / 4 25.0%	1 / 3 33.3%	0 / 1 0.0%	0 / 3 0.0%	0 / 1 0.0%	1 / 3 33.3%	1 / 1 100.0%	0 / 3 0.0%	1 / 4 25.0%	0 / 3 0.0%	0 / 1 0.0%	2 / 5 40.0%	0 / 0 0.0%	0 / 0 0.0%	0 / 0 0.0%	0 / 0 0.0%	16 / 51 31.4%E	11 / 51 21.6%ABE	7 / 51 13.7%AB	6 / 51 11.8%C	6 / 51 11.8%	5 / 51 9.8%CE			
1	CECT 5975	18 / 51 35.3%	1 / 1 100.0%	0 / 1 0.0%	1 / 1 100.0%	1 / 1 100.0%	1 / 4 25.0%	3 / 4 75.0%	1 / 1 100.0%	1 / 1 100.0%	1 / 4 25.0%	1 / 1 100.0%	1 / 4 25.0%	1 / 3 33.3%	0 / 1 0.0%	0 / 3 0.0%	0 / 1 0.0%	1 / 3 33.3%	1 / 1 100.0%	0 / 3 0.0%	1 / 4 25.0%	0 / 3 0.0%	0 / 1 0.0%	2 / 5 40.0%	0 / 0 0.0%	0 / 0 0.0%	0 / 0 0.0%	0 / 0 0.0%	16 / 51 31.4%E	11 / 51 21.6%ABE	7 / 51 13.7%AB	6 / 51 11.8%C	6 / 51 11.8%	5 / 51 9.8%CE			
1	Xylanimonas cellulosilytica DSM 15894	18 / 51 35.3%	1 / 1 100.0%	0 / 1 0.0%	1 / 1 100.0%	1 / 1 100.0%	1 / 4 25.0%	3 / 4 75.0%	1 / 1 100.0%	1 / 1 100.0%	1 / 4 25.0%	1 / 1 100.0%	1 / 4 25.0%	1 / 3 33.3%	0 / 1 0.0%	0 / 3 0.0%	0 / 1 0.0%	1 / 3 33.3%	1 / 1 100.0%	0 / 3 0.0%	1 / 4 25.0%	0 / 3 0.0%	0 / 1 0.0%	2 / 5 40.0%	0 / 0 0.0%	0 / 0 0.0%	0 / 0 0.0%	0 / 0 0.0%	16 / 51 31.4%E	11 / 51 21.6%ABE	7 / 51 13.7%AB	6 / 51 11.8%C	6 / 51 11.8%	5 / 51 9.8%CE			
1	Isoptericola	16 / 49 32.7%	1 / 1 100.0%	0 / 1 0.0%	1 / 1 100.0%	0 / 1 0.0%	0 / 4 0.0%	3 / 4 75.0%	1 / 1 100.0%	0 / 1 0.0%	0 / 3 0.0%	1 / 1 100.0%	2 / 4 50.0%	1 / 3 33.3%	0 / 1 0.0%	0 / 2 0.0%	0 / 1 0.0%	2 / 3 66.7%	1 / 1 100.0%	0 / 3 0.0%	2 / 4 50.0%	1 / 3 33.3%	0 / 1 0.0%	0 / 5 0.0%	0 / 0 0.0%	0 / 0 0.0%	0 / 0 0.0%	0 / 0 0.0%	14 / 49 28.6%E	12 / 49 24.5%	9 / 49 18.4%ABE	7 / 49 14.3%AB	4 / 49 8.2%CE	3 / 49 6.1%C			
1	ATCC BAA-303	16 / 49 32.7%	1 / 1 100.0%	0 / 1 0.0%	1 / 1 100.0%	0 / 1 0.0%	0 / 4 0.0%	3 / 4 75.0%	1 / 1 100.0%	0 / 1 0.0%	0 / 3 0.0%	1 / 1 100.0%	2 / 4 50.0%	1 / 3 33.3%	0 / 1 0.0%	0 / 2 0.0%	0 / 1 0.0%	2 / 3 66.7%	1 / 1 100.0%	0 / 3 0.0%	2 / 4 50.0%	1 / 3 33.3%	0 / 1 0.0%	0 / 5 0.0%	0 / 0 0.0%	0 / 0 0.0%	0 / 0 0.0%	0 / 0 0.0%	14 / 49 28.6%E	12 / 49 24.5%	9 / 49 18.4%ABE	7 / 49 14.3%AB	4 / 49 8.2%CE	3 / 49 6.1%C			
1	Isoptericola variabilis 225	16 / 49 32.7%	1 / 1 100.0%	0 / 1 0.0%	1 / 1 100.0%	0 / 1 0.0%	0 / 4 0.0%	3 / 4 75.0%	1 / 1 100.0%	0 / 1 0.0%	0 / 3 0.0%	1 / 1 100.0%	2 / 4 50.0%	1 / 3 33.3%	0 / 1 0.0%	0 / 2 0.0%	0 / 1 0.0%	2 / 3 66.7%	1 / 1 100.0%	0 / 3 0.0%	2 / 4 50.0%	1 / 3 33.3%	0 / 1 0.0%	0 / 5 0.0%	0 / 0 0.0%	0 / 0 0.0%	0 / 0 0.0%	0 / 0 0.0%	14 / 49 28.6%E	12 / 49 24.5%	9 / 49 18.4%ABE	7 / 49 14.3%AB	4 / 49 8.2%CE	3 / 49 6.1%C			
1	Dermabacteraceae	8 / 50 16.0%	0 / 1 0.0%	0 / 1 0.0%	0 / 1 0.0%	0 / 1 0.0%	2 / 4 50.0%	0 / 4 0.0%	1 / 1 100.0%	0 / 1 0.0%	0 / 3 0.0%	0 / 1 0.0%	0 / 4 0.0%	0 / 2 0.0%	1 / 1 100.0%	0 / 2 0.0%	0 / 1 0.0%	2 / 3 66.7%	1 / 2 50.0%	0 / 3 0.0%	0 / 4 0.0%	0 / 3 0.0%	0 / 1 0.0%	1 / 5 20.0%	0 / 1 0.0%	0 / 0 0.0%	0 / 0 0.0%	0 / 0 0.0%	14 / 50 28.0%C	14 / 50 28.0%E	8 / 50 16.0%CE	6 / 50 12.0%	5 / 50 10.0%AB	3 / 50 6.0%ABE			
1	Brachybacterium	8 / 50 16.0%	0 / 1 0.0%	0 / 1 0.0%	0 / 1 0.0%	0 / 1 0.0%	2 / 4 50.0%	0 / 4 0.0%	1 / 1 100.0%	0 / 1 0.0%	0 / 3 0.0%	0 / 1 0.0%	0 / 4 0.0%	0 / 2 0.0%	1 / 1 100.0%	0 / 2 0.0%	0 / 1 0.0%	2 / 3 66.7%	1 / 2 50.0%	0 / 3 0.0%	0 / 4 0.0%	0 / 3 0.0%	0 / 1 0.0%	1 / 5 20.0%	0 / 1 0.0%	0 / 0 0.0%	0 / 0 0.0%	0 / 0 0.0%	14 / 50 28.0%E	14 / 50 28.0%C	8 / 50 16.0%CE	6 / 50 12.0%	5 / 50 10.0%AB	3 / 50 6.0%ABE			
1	ATCC 43885	8 / 50 16.0%	0 / 1 0.0%	0 / 1 0.0%	0 / 1 0.0%	0 / 1 0.0%	2 / 4 50.0%	0 / 4 0.0%	1 / 1 100.0%	0 / 1 0.0%	0 / 3 0.0%	0 / 1 0.0%	0 / 4 0.0%	0 / 2 0.0%	1 / 1 100.0%	0 / 2 0.0%	0 / 1 0.0%	2 / 3 66.7%	1 / 2 50.0%	0 / 3 0.0%	0 / 4 0.0%	0 / 3 0.0%	0 / 1 0.0%	1 / 5 20.0%	0 / 1 0.0%	0 / 0 0.0%	0 / 0 0.0%	0 / 0 0.0%	14 / 50 28.0%C	14 / 50 28.0%E	8 / 50 16.0%CE	6 / 50 12.0%	5 / 50 10.0%AB	3 / 50 6.0%ABE			
1	Brachybacterium faecium ATCC 43885	8 / 50 16.0%	0 / 1 0.0%	0 / 1 0.0%	0 / 1 0.0%	0 / 1 0.0%	2 / 4 50.0%	0 / 4 0.0%	1 / 1 100.0%	0 / 1 0.0%	0 / 3 0.0%	0 / 1 0.0%	0 / 4 0.0%	0 / 2 0.0%	1 / 1 100.0%	0 / 2 0.0%	0 / 1 0.0%	2 / 3 66.7%	1 / 2 50.0%	0 / 3 0.0%	0 / 4 0.0%	0 / 3 0.0%	0 / 1 0.0%	1 / 5 20.0%	0 / 1 0.0%	0 / 0 0.0%	0 / 0 0.0%	0 / 0 0.0%	14 / 50 28.0%E	14 / 50 28.0%C	8 / 50 16.0%CE	6 / 50 12.0%	5 / 50 10.0%AB	3 / 50 6.0%ABE			
1	Intrasporangiaceae	21 / 47 44.7%	1 / 1 100.0%	0 / 1 0.0%	0 / 1 0.0%	1 / 1 100.0%	2 / 5 40.0%	2 / 3 66.7%	1 / 1 100.0%	0 / 1 0.0%	3 / 3 100.0%	0 / 1 0.0%	4 / 4 100.0%	1 / 2 50.0%	1 / 1 100.0%	0 / 2 0.0%	0 / 1 0.0%	0 / 3 0.0%	1 / 1 100.0%	1 / 3 33.3%	1 / 4 25.0%	1 / 2 50.0%	0 / 1 0.0%	1 / 5 20.0%	0 / 0 0.0%	0 / 0 0.0%	0 / 0 0.0%	0 / 0 0.0%	11 / 47 23.4%ABE	10 / 47 21.3%AB	10 / 47 21.3%E	9 / 47 19.1%	5 / 47 10.6%C	2 / 47 4.3%CE			
1	Humihabitans	21 / 47 44.7%	1 / 1 100.0%	0 / 1 0.0%	0 / 1 0.0%	1 / 1 100.0%	2 / 5 40.0%	2 / 3 66.7%	1 / 1 100.0%	0 / 1 0.0%	3 / 3 100.0%	0 / 1 0.0%	4 / 4 100.0%	1 / 2 50.0%	1 / 1 100.0%	0 / 2 0.0%	0 / 1 0.0%	0 / 3 0.0%	1 / 1 100.0%	1 / 3 33.3%	1 / 4 25.0%	1 / 2 50.0%	0 / 1 0.0%	1 / 5 20.0%	0 / 0 0.0%	0 / 0 0.0%	0 / 0 0.0%	0 / 0 0.0%	11 / 47 23.4%ABE	10 / 47 21.3%E	10 / 47 21.3%AB	9 / 47 19.1%	5 / 47 10.6%C	2 / 47 4.3%CE			
1	ATCC 23552	21 / 47 44.7%	1 / 1 100.0%	0 / 1 0.0%	0 / 1 0.0%	1 / 1 100.0%	2 / 5 40.0%	2 / 3 66.7%	1 / 1 100.0%	0 / 1 0.0%	3 / 3 100.0%	0 / 1 0.0%	4 / 4 100.0%	1 / 2 50.0%	1 / 1 100.0%	0 / 2 0.0%	0 / 1 0.0%	0 / 3 0.0%	1 / 1 100.0%	1 / 3 33.3%	1 / 4 25.0%	1 / 2 50.0%	0 / 1 0.0%	1 / 5 20.0%	0 / 0 0.0%	0 / 0 0.0%	0 / 0 0.0%	0 / 0 0.0%	11 / 47 23.4%ABE	10 / 47 21.3%E	10 / 47 21.3%AB	9 / 47 19.1%	5 / 47 10.6%C	2 / 47 4.3%CE			
1	Intrasporangium calvum DSM 43043	21 / 47 44.7%	1 / 1 100.0%	0 / 1 0.0%	0 / 1 0.0%	1 / 1 100.0%	2 / 5 40.0%	2 / 3 66.7%	1 / 1 100.0%	0 / 1 0.0%	3 / 3 100.0%	0 / 1 0.0%	4 / 4 100.0%	1 / 2 50.0%	1 / 1 100.0%	0 / 2 0.0%	0 / 1 0.0%	0 / 3 0.0%	1 / 1 100.0%	1 / 3 33.3%	1 / 4 25.0%	1 / 2 50.0%	0 / 1 0.0%	1 / 5 20.0%	0 / 0 0.0%	0 / 0 0.0%	0 / 0 0.0%	0 / 0 0.0%	11 / 47 23.4%ABE	10 / 47 21.3%E	10 / 47 21.3%AB	9 / 47 19.1%	5 / 47 10.6%C	2 / 47 4.3%CE			
1	Jonesiaceae	29 / 54 53.7%	1 / 2 50.0%	0 / 2 0.0%	2 / 2 100.0%	0 / 1 0.0%	3 / 4 75.0%	2 / 3 66.7%	1 / 1 100.0%	0 / 1 0.0%	0 / 3 0.0%	1 / 2 50.0%	3 / 4 75.0%	3 / 3 100.0%	1 / 1 100.0%	0 / 2 0.0%	0 / 1 0.0%	1 / 3 33.3%	0 / 1 0.0%	2 / 3 66.7%	4 / 5 80.0%	2 / 3 66.7%	1 / 1 100.0%	2 / 6 33.3%	0 / 0 0.0%	0 / 0 0.0%	0 / 0 0.0%	0 / 0 0.0%	17 / 54 31.5%AB	15 / 54 27.8%E	12 / 54 22.2%ABE	9 / 54 16.7%	1 / 54 1.9%C				
1	Jonesia	29 / 54 53.7%	1 / 2 50.0%	0 / 2 0.0%	2 / 2 100.0%	0 / 1 0.0%	3 / 4 75.0%	2 / 3 66.7%	1 / 1 100.0%	0 / 1 0.0%	0 / 3 0.0%	1 / 2 50.0%	3 / 4 75.0%	3 / 3 100.0%	1 / 1 100.0%	0 / 2 0.0%	0 / 1 0.0%	1 / 3 33.3%	0 / 1 0.0%	2 / 3 66.7%	4 / 5 80.0%	2 / 3 66.7%	1 / 1 100.0%	2 / 6 33.3%	0 / 0 0.0%	0 / 0 0.0%	0 / 0 0.0%	0 / 0 0.0%	17 / 54 31.5%AB	15 / 54 27.8%E	12 / 54 22.2%ABE	9 / 54 16.7%	1 / 54 1.9%C				
1	"Listeria L26" Sohier et al.1948	29 / 54 53.7%	1 / 2 50.0%	0 / 2 0.0%	2 / 2 100.0%	0 / 1 0.0%	3 / 4 75.0%	2 / 3 66.7%	1 / 1 100.0%	0 / 1 0.0%	0 / 3 0.0%	1 / 2 50.0%	3 / 4 75.0%	3 / 3 100.0%	1 / 1 100.0%	0 / 2 0.0%	0 / 1 0.0%	1 / 3 33.3%	0 / 1 0.0%	2 / 3 66.7%	4 / 5 80.0%	2 / 3 66.7%	1 / 1 100.0%	2 / 6 33.3%	0 / 0 0.0%	0 / 0 0.0%	0 / 0 0.0%	0 / 0 0.0%	17 / 54 31.5%AB	15 / 54 27.8%E	12 / 54 22.2%ABE	9 / 54 16.7%	1 / 54 1.9%C				
1	Jonesia denitrificans DSM 20603	29 / 54 53.7%	1 / 2 50.0%	0 / 2 0.0%	2 / 2 100.0%	0 / 1 0.0%	3 / 4 75.0%	2 / 3 66.7%	1 / 1 100.0%	0 / 1 0.0%	0 / 3 0.0%	1 / 2 50.0%	3 / 4 75.0%	3 / 3 100.0%	1 / 1 100.0%	0 / 2 0.0%	0 / 1 0.0%	1 / 3 33.3%	0 / 1 0.0%	2 / 3 66.7%	4 / 5 80.0%	2 / 3 66.7%	1 / 1 100.0%	2 / 6 33.3%	0 / 0 0.0%	0 / 0 0.0%	0 / 0 0.0%	0 / 0 0.0%	17 / 54 31.5%AB	15 / 54 27.8%E	12 / 54 22.2%ABE	9 / 54 16.7%	1 / 54 1.9%C				
5	Microbacteriaceae	80 / 225 35.6%	1 / 5 20.0%	4 / 5 80.0%	0 / 5 0.0%	4 / 5 80.0%	8 / 15 53.3%	6 / 15 40.0%	5 / 5 100.0%	0 / 5 0.0%	1 / 15 6.7%	0 / 5 0.0%	13 / 20 65.0%	4 / 10 40.0%	3 / 5 60.0%	4 / 10 40.0%	2 / 5 40.0%	6 / 15 40.0%	0 / 5 0.0%	5 / 15 33.3%	0 / 20 0.0%	6 / 10 60.0%	1 / 5 20.0%	7 / 25 28.0%	0 / 0 0.0%	0 / 0 0.0%	0 / 0 0.0%	0 / 0 0.0%	49 / 225 21.8%E	45 / 225 20.0%	39 / 225 17.3%ABE	35 / 225 15.6%AB	30 / 225 13.3%C	21 / 225 9.3%CE	3 / 225 1.3%BE	3 / 225 1.3%B	
3	Clavibacter	56 / 135 41.5%	0 / 3 0.0%	3 / 3 100.0%	0 / 3 0.0%	3 / 3 100.0%	6 / 9 66.7%	3 / 9 33.3%	3 / 3 100.0%	0 / 3 0.0%	0 / 9 0.0%	0 / 3 0.0%	9 / 12 75.0%	3 / 6 50.0%	3 / 3 100.0%	3 / 6 50.0%	2 / 3 66.7%	3 / 9 33.3%	0 / 3 0.0%	3 / 9 33.3%	0 / 12 0.0%	5 / 6 83.3%	0 / 3 0.0%	7 / 15 46.7%	0 / 0 0.0%	0 / 0 0.0%	0 / 0 0.0%	0 / 0 0.0%	30 / 135 22.2%E	25 / 135 18.5%ABE	25 / 135 18.5%AB	23 / 135 17.0%	15 / 135 11.1%C	11 / 135 8.1%CE	3 / 135 2.2%BE	3 / 135 2.2%B	
	"Aplanobacter michiganense" (sic) [illegible]	56 / [illegible]	[illegible]	[illegible]	[illegible]	[illegible]	[illegible]	[illegible]	[illegible]	[illegible]	[illegible]	[illegible]	[illegible]	[illegible]	[illegible]	[illegible]	[illegible]	[illegible]	[illegible]	[illegible]	[illegible]	[illegible]	[illegible]	[illegible]	[illegible]	[illegible]	[illegible]	[illegible]	[illegible]	[illegible]	[illegible]	[illegible]	[illegible]	[illegible]	[illegible]	[illegible]	[illegible]

3	[illegible] (Smith 1910) Smith 1914	[illegible] 135 41.5%	0 / 3 0.0%	3 / 3 100.0%	0 / 3 0.0%	3 / 3 100.0%	6 / 9 66.7%	3 / 9 33.3%	3 / 3 100.0%	0 / 3 0.0%	0 / 9 0.0%	0 / 3 0.0%	9 / 12 75.0%	3 / 6 50.0%	3 / 3 100.0%	3 / 6 50.0%	2 / 3 66.7%	3 / 9 33.3%	0 / 3 0.0%	3 / 9 33.3%	0 / 12 0.0%	5 / 6 83.3%	0 / 3 0.0%	7 / 15 46.7%	0 / 0 0.0%	0 / 0 0.0%	0 / 0 0.0%	0 / 0 0.0%	30 / 135 22.2%E	25 / 135 18.5%AB	25 / 135 18.5%ABE	23 / 135 17.0%	15 / 135 11.1%C	11 / 135 8.1%CE	3 / 135 2.2%B	3 / 135 2.2%BE
1	ATCC 27794	18 / 45 40.0%	0 / 1 0.0%	1 / 1 100.0%	0 / 1 0.0%	1 / 1 100.0%	2 / 3 66.7%	1 / 3 33.3%	1 / 1 100.0%	0 / 1 0.0%	0 / 3 0.0%	0 / 1 0.0%	3 / 4 75.0%	1 / 2 50.0%	1 / 1 100.0%	1 / 2 50.0%	0 / 1 0.0%	1 / 3 33.3%	0 / 1 0.0%	1 / 3 33.3%	0 / 4 0.0%	1 / 2 50.0%	0 / 1 0.0%	3 / 5 60.0%	0 / 0 0.0%	0 / 0 0.0%	0 / 0 0.0%	0 / 0 0.0%	11 / 45 24.4%E	10 / 45 22.2%AB	8 / 45 17.8%ABE	7 / 45 15.6%	5 / 45 11.1%C	4 / 45 8.9%CE		
1	Clavibacter michiganensis subsp. nebraskensis NCPPB 2581	18 / 45 40.0%	0 / 1 0.0%	1 / 1 100.0%	0 / 1 0.0%	1 / 1 100.0%	2 / 3 66.7%	1 / 3 33.3%	1 / 1 100.0%	0 / 1 0.0%	0 / 3 0.0%	0 / 1 0.0%	3 / 4 75.0%	1 / 2 50.0%	1 / 1 100.0%	1 / 2 50.0%	0 / 1 0.0%	1 / 3 33.3%	0 / 1 0.0%	1 / 3 33.3%	0 / 4 0.0%	1 / 2 50.0%	0 / 1 0.0%	3 / 5 60.0%	0 / 0 0.0%	0 / 0 0.0%	0 / 0 0.0%	0 / 0 0.0%	11 / 45 24.4%E	10 / 45 22.2%AB	8 / 45 17.8%ABE	7 / 45 15.6%	5 / 45 11.1%C	4 / 45 8.9%CE		
1	"Aplanobacter sepedonicum" (sic) (Spieckermann and Kotthoff 1914) Smith 1920	19 / 45 42.2%	0 / 1 0.0%	1 / 1 100.0%	0 / 1 0.0%	1 / 1 100.0%	2 / 3 66.7%	1 / 3 33.3%	1 / 1 100.0%	0 / 1 0.0%	0 / 3 0.0%	0 / 1 0.0%	3 / 4 75.0%	1 / 2 50.0%	1 / 1 100.0%	1 / 2 50.0%	1 / 1 100.0%	1 / 3 33.3%	0 / 1 0.0%	1 / 3 33.3%	0 / 4 0.0%	2 / 2 100.0%	0 / 1 0.0%	2 / 5 40.0%	0 / 0 0.0%	0 / 0 0.0%	0 / 0 0.0%	0 / 0 0.0%	10 / 45 22.2%E	7 / 45 15.6%ABE	7 / 45 15.6%	6 / 45 13.3%C	6 / 45 13.3%AB	3 / 45 6.7%BE	3 / 45 6.7%CE	3 / 45 6.7%B
1	Clavibacter michiganense michiganense	19 / 45 42.2%	0 / 1 0.0%	1 / 1 100.0%	0 / 1 0.0%	1 / 1 100.0%	2 / 3 66.7%	1 / 3 33.3%	1 / 1 100.0%	0 / 1 0.0%	0 / 3 0.0%	0 / 1 0.0%	3 / 4 75.0%	1 / 2 50.0%	1 / 1 100.0%	1 / 2 50.0%	1 / 1 100.0%	1 / 3 33.3%	0 / 1 0.0%	1 / 3 33.3%	0 / 4 0.0%	2 / 2 100.0%	0 / 1 0.0%	2 / 5 40.0%	0 / 0 0.0%	0 / 0 0.0%	0 / 0 0.0%	0 / 0 0.0%	10 / 45 22.2%ABE	9 / 45 20.0%	9 / 45 20.0%AB	9 / 45 20.0%E	4 / 45 8.9%CE	4 / 45 8.9%C		
1	Clavibacter michiganensis subsp. michiganensis NCPPB 382	19 / 45 42.2%	0 / 1 0.0%	1 / 1 100.0%	0 / 1 0.0%	1 / 1 100.0%	2 / 3 66.7%	1 / 3 33.3%	1 / 1 100.0%	0 / 1 0.0%	0 / 3 0.0%	0 / 1 0.0%	3 / 4 75.0%	1 / 2 50.0%	1 / 1 100.0%	1 / 2 50.0%	1 / 1 100.0%	1 / 3 33.3%	0 / 1 0.0%	1 / 3 33.3%	0 / 4 0.0%	2 / 2 100.0%	0 / 1 0.0%	2 / 5 40.0%	0 / 0 0.0%	0 / 0 0.0%	0 / 0 0.0%	0 / 0 0.0%	10 / 45 22.2%ABE	9 / 45 20.0%	9 / 45 20.0%E	9 / 45 20.0%AB	4 / 45 8.9%CE	4 / 45 8.9%C		
1	Aureibacterium	11 / 45 24.4%	0 / 1 0.0%	0 / 1 0.0%	0 / 1 0.0%	0 / 1 0.0%	1 / 3 33.3%	2 / 3 66.7%	1 / 1 100.0%	0 / 1 0.0%	0 / 3 0.0%	0 / 1 0.0%	2 / 4 50.0%	1 / 2 50.0%	0 / 1 0.0%	0 / 2 0.0%	0 / 1 0.0%	1 / 3 33.3%	0 / 1 0.0%	1 / 3 33.3%	0 / 4 0.0%	1 / 2 50.0%	1 / 1 100.0%	0 / 5 0.0%	0 / 0 0.0%	0 / 0 0.0%	0 / 0 0.0%	0 / 0 0.0%	15 / 45 33.3%	10 / 45 22.2%E	7 / 45 15.6%CE	6 / 45 13.3%ABE	5 / 45 11.1%AB	2 / 45 4.4%C		
1	ATCC 15829	11 / 45 24.4%	0 / 1 0.0%	0 / 1 0.0%	0 / 1 0.0%	0 / 1 0.0%	1 / 3 33.3%	2 / 3 66.7%	1 / 1 100.0%	0 / 1 0.0%	0 / 3 0.0%	0 / 1 0.0%	2 / 4 50.0%	1 / 2 50.0%	0 / 1 0.0%	0 / 2 0.0%	0 / 1 0.0%	1 / 3 33.3%	0 / 1 0.0%	1 / 3 33.3%	0 / 4 0.0%	1 / 2 50.0%	1 / 1 100.0%	0 / 5 0.0%	0 / 0 0.0%	0 / 0 0.0%	0 / 0 0.0%	0 / 0 0.0%	15 / 45 33.3%	10 / 45 22.2%E	7 / 45 15.6%CE	6 / 45 13.3%ABE	5 / 45 11.1%AB	2 / 45 4.4%C		
1	Microbacterium testaceum StLB037	11 / 45 24.4%	0 / 1 0.0%	0 / 1 0.0%	0 / 1 0.0%	0 / 1 0.0%	1 / 3 33.3%	2 / 3 66.7%	1 / 1 100.0%	0 / 1 0.0%	0 / 3 0.0%	0 / 1 0.0%	2 / 4 50.0%	1 / 2 50.0%	0 / 1 0.0%	0 / 2 0.0%	0 / 1 0.0%	1 / 3 33.3%	0 / 1 0.0%	1 / 3 33.3%	0 / 4 0.0%	1 / 2 50.0%	1 / 1 100.0%	0 / 5 0.0%	0 / 0 0.0%	0 / 0 0.0%	0 / 0 0.0%	0 / 0 0.0%	15 / 45 33.3%	10 / 45 22.2%E	7 / 45 15.6%CE	6 / 45 13.3%ABE	5 / 45 11.1%AB	2 / 45 4.4%C		
1	"Leifsonia" Suzuki et al. 1999	13 / 45 28.9%	1 / 1 100.0%	1 / 1 100.0%	0 / 1 0.0%	1 / 1 100.0%	1 / 3 33.3%	1 / 3 33.3%	1 / 1 100.0%	0 / 1 0.0%	1 / 3 33.3%	0 / 1 0.0%	2 / 4 50.0%	0 / 2 0.0%	0 / 1 0.0%	1 / 2 50.0%	0 / 1 0.0%	2 / 3 66.7%	0 / 1 0.0%	1 / 3 33.3%	0 / 4 0.0%	0 / 2 0.0%	0 / 1 0.0%	0 / 5 0.0%	0 / 0 0.0%	0 / 0 0.0%	0 / 0 0.0%	0 / 0 0.0%	13 / 45 28.9%C	9 / 45 20.0%E	8 / 45 17.8%ABE	7 / 45 15.6%	5 / 45 11.1%AB	3 / 45 6.7%CE		
1	ATCC 33974	13 / 45 28.9%	1 / 1 100.0%	1 / 1 100.0%	0 / 1 0.0%	1 / 1 100.0%	1 / 3 33.3%	1 / 3 33.3%	1 / 1 100.0%	0 / 1 0.0%	1 / 3 33.3%	0 / 1 0.0%	2 / 4 50.0%	0 / 2 0.0%	0 / 1 0.0%	1 / 2 50.0%	0 / 1 0.0%	2 / 3 66.7%	0 / 1 0.0%	1 / 3 33.3%	0 / 4 0.0%	0 / 2 0.0%	0 / 1 0.0%	0 / 5 0.0%	0 / 0 0.0%	0 / 0 0.0%	0 / 0 0.0%	0 / 0 0.0%	13 / 45 28.9%C	9 / 45 20.0%E	8 / 45 17.8%ABE	7 / 45 15.6%	5 / 45 11.1%AB	3 / 45 6.7%CE		
1	Clavibacter xyli subsp. xyli	13 / 45 28.9%	1 / 1 100.0%	1 / 1 100.0%	0 / 1 0.0%	1 / 1 100.0%	1 / 3 33.3%	1 / 3 33.3%	1 / 1 100.0%	0 / 1 0.0%	1 / 3 33.3%	0 / 1 0.0%	2 / 4 50.0%	0 / 2 0.0%	0 / 1 0.0%	1 / 2 50.0%	0 / 1 0.0%	2 / 3 66.7%	0 / 1 0.0%	1 / 3 33.3%	0 / 4 0.0%	0 / 2 0.0%	0 / 1 0.0%	0 / 5 0.0%	0 / 0 0.0%	0 / 0 0.0%	0 / 0 0.0%	0 / 0 0.0%	13 / 45 28.9%C	9 / 45 20.0%E	8 / 45 17.8%ABE	7 / 45 15.6%	5 / 45 11.1%AB	3 / 45 6.7%CE		
1	Leifsonia xyli subsp. xyli CTCB07	13 / 45 28.9%	1 / 1 100.0%	1 / 1 100.0%	0 / 1 0.0%	1 / 1 100.0%	1 / 3 33.3%	1 / 3 33.3%	1 / 1 100.0%	0 / 1 0.0%	1 / 3 33.3%	0 / 1 0.0%	2 / 4 50.0%	0 / 2 0.0%	0 / 1 0.0%	1 / 2 50.0%	0 / 1 0.0%	2 / 3 66.7%	0 / 1 0.0%	1 / 3 33.3%	0 / 4 0.0%	0 / 2 0.0%	0 / 1 0.0%	0 / 5 0.0%	0 / 0 0.0%	0 / 0 0.0%	0 / 0 0.0%	0 / 0 0.0%	13 / 45 28.9%C	9 / 45 20.0%E	8 / 45 17.8%ABE	7 / 45 15.6%	5 / 45 11.1%AB	3 / 45 6.7%CE		
1	"Beutenbergiaceae" Garrity and Holt 2001	15 / 45 33.3%	0 / 1 0.0%	0 / 1 0.0%	1 / 1 100.0%	0 / 1 0.0%	0 / 3 0.0%	3 / 3 100.0%	1 / 1 100.0%	0 / 1 0.0%	2 / 3 66.7%	0 / 1 0.0%	2 / 4 50.0%	1 / 2 50.0%	1 / 1 100.0%	0 / 2 0.0%	0 / 1 0.0%	1 / 3 33.3%	0 / 1 0.0%	1 / 3 33.3%	0 / 4 0.0%	0 / 2 0.0%	0 / 1 0.0%	2 / 5 40.0%	0 / 0 0.0%	0 / 0 0.0%	0 / 0 0.0%	0 / 0 0.0%	10 / 45 22.2%E	10 / 45 22.2%C	9 / 45 20.0%ABE	6 / 45 13.3%AB	5 / 45 11.1%	5 / 45 11.1%CE		
1	Beutenbergia	15 / 45 33.3%	0 / 1 0.0%	0 / 1 0.0%	1 / 1 100.0%	0 / 1 0.0%	0 / 3 0.0%	3 / 3 100.0%	1 / 1 100.0%	0 / 1 0.0%	2 / 3 66.7%	0 / 1 0.0%	2 / 4 50.0%	1 / 2 50.0%	1 / 1 100.0%	0 / 2 0.0%	0 / 1 0.0%	1 / 3 33.3%	0 / 1 0.0%	1 / 3 33.3%	0 / 4 0.0%	0 / 2 0.0%	0 / 1 0.0%	2 / 5 40.0%	0 / 0 0.0%	0 / 0 0.0%	0 / 0 0.0%	0 / 0 0.0%	10 / 45 22.2%C	10 / 45 22.2%E	9 / 45 20.0%ABE	6 / 45 13.3%AB	5 / 45 11.1%	5 / 45 11.1%CE		
1	ATCC BAA-8	15 / 45 33.3%	0 / 1 0.0%	0 / 1 0.0%	1 / 1 100.0%	0 / 1 0.0%	0 / 3 0.0%	3 / 3 100.0%	1 / 1 100.0%	0 / 1 0.0%	2 / 3 66.7%	0 / 1 0.0%	2 / 4 50.0%	1 / 2 50.0%	1 / 1 100.0%	0 / 2 0.0%	0 / 1 0.0%	1 / 3 33.3%	0 / 1 0.0%	1 / 3 33.3%	0 / 4 0.0%	0 / 2 0.0%	0 / 1 0.0%	2 / 5 40.0%	0 / 0 0.0%	0 / 0 0.0%	0 / 0 0.0%	0 / 0 0.0%	10 / 45 22.2%C	10 / 45 22.2%E	9 / 45 20.0%ABE	6 / 45 13.3%AB	5 / 45 11.1%	5 / 45 11.1%CE		
1	Beutenbergia cavernae DSM 12333	15 / 45 33.3%	0 / 1 0.0%	0 / 1 0.0%	1 / 1 100.0%	0 / 1 0.0%	0 / 3 0.0%	3 / 3 100.0%	1 / 1 100.0%	0 / 1 0.0%	2 / 3 66.7%	0 / 1 0.0%	2 / 4 50.0%	1 / 2 50.0%	1 / 1 100.0%	0 / 2 0.0%	0 / 1 0.0%	1 / 3 33.3%	0 / 1 0.0%	1 / 3 33.3%	0 / 4 0.0%	0 / 2 0.0%	0 / 1 0.0%	2 / 5 40.0%	0 / 0 0.0%	0 / 0 0.0%	0 / 0 0.0%	0 / 0 0.0%	10 / 45 22.2%E	10 / 45 22.2%C	9 / 45 20.0%ABE	6 / 45 13.3%AB	5 / 45 11.1%CE	5 / 45 11.1%		
1	Dermacoccaceae	19 / 47 40.4%	1 / 1 100.0%	0 / 1 0.0%	0 / 1 0.0%	0 / 1 0.0%	3 / 4 75.0%	1 / 3 33.3%	1 / 1 100.0%	1 / 1 100.0%	0 / 3 0.0%	0 / 1 0.0%	2 / 4 50.0%	1 / 2 50.0%	0 / 1 0.0%	1 / 2 50.0%	0 / 1 0.0%	2 / 3 66.7%	0 / 1 0.0%	2 / 3 66.7%	1 / 4 25.0%	0 / 2 0.0%	0 / 1 0.0%	2 / 5 40.0%	1 / 1 100.0%	0 / 0 0.0%	0 / 0 0.0%	0 / 0 0.0%	11 / 47 23.4%ABE	9 / 47 19.1%	8 / 47 17.0%AB	8 / 47 17.0%E	6 / 47 12.8%CE	5 / 47 10.6%C		
1	Kytococcus	19 / 47 40.4%	1 / 1 100.0%	0 / 1 0.0%	0 / 1 0.0%	0 / 1 0.0%	3 / 4 75.0%	1 / 3 33.3%	1 / 1 100.0%	1 / 1 100.0%	0 / 3 0.0%	0 / 1 0.0%	2 / 4 50.0%	1 / 2 50.0%	0 / 1 0.0%	1 / 2 50.0%	0 / 1 0.0%	2 / 3 66.7%	0 / 1 0.0%	2 / 3 66.7%	1 / 4 25.0%	0 / 2 0.0%	0 / 1 0.0%	2 / 5 40.0%	1 / 1 100.0%	0 / 0 0.0%	0 / 0 0.0%	0 / 0 0.0%	11 / 47 23.4%ABE	9 / 47 19.1%	8 / 47 17.0%E	8 / 47 17.0%AB	6 / 47 12.8%CE	5 / 47 10.6%C		
1	ATCC 14392	19 / 47 40.4%	1 / 1 100.0%	0 / 1 0.0%	0 / 1 0.0%	0 / 1 0.0%	3 / 4 75.0%	1 / 3 33.3%	1 / 1 100.0%	1 / 1 100.0%	0 / 3 0.0%	0 / 1 0.0%	2 / 4 50.0%	1 / 2 50.0%	0 / 1 0.0%	1 / 2 50.0%	0 / 1 0.0%	2 / 3 66.7%	0 / 1 0.0%	2 / 3 66.7%	1 / 4 25.0%	0 / 2 0.0%	0 / 1 0.0%	2 / 5 40.0%	1 / 1 100.0%	0 / 0 0.0%	0 / 0 0.0%	0 / 0 0.0%	11 / 47 23.4%ABE	9 / 47 19.1%	8 / 47 17.0%E	8 / 47 17.0%AB	6 / 47 12.8%CE	5 / 47 10.6%C		
1	Kytococcus sedentarius DSM 20547	19 / 47 40.4%	1 / 1 100.0%	0 / 1 0.0%	0 / 1 0.0%	0 / 1 0.0%	3 / 4 75.0%	1 / 3 33.3%	1 / 1 100.0%	1 / 1 100.0%	0 / 3 0.0%	0 / 1 0.0%	2 / 4 50.0%	1 / 2 50.0%	0 / 1 0.0%	1 / 2 50.0%	0 / 1 0.0%	2 / 3 66.7%	0 / 1 0.0%	2 / 3 66.7%	1 / 4 25.0%	0 / 2 0.0%	0 / 1 0.0%	2 / 5 40.0%	1 / 1 100.0%	0 / 0 0.0%	0 / 0 0.0%	0 / 0 0.0%	11 / 47 23.4%ABE	9 / 47 19.1%	8 / 47 17.0%E	8 / 47 17.0%AB	6 / 47 12.8%CE	5 / 47 10.6%C		
1	Sanguibacteraceae	16 / 51 31.4%	0 / 2 0.0%	0 / 1 0.0%	1 / 1 100.0%	0 / 1 0.0%	2 / 4 50.0%	0 / 4 0.0%	0 / 1 0.0%	0 / 1 0.0%	0 / 3 0.0%	0 / 1 0.0%	1 / 4 25.0%	2 / 3 66.7%	0 / 1 0.0%	0 / 2 0.0%	0 / 2 0.0%	1 / 3 33.3%	1 / 1 100.0%	1 / 3 33.3%	4 / 4 100.0%	1 / 2 50.0%	1 / 1 100.0%	1 / 6 16.7%	0 / 0 0.0%	0 / 0 0.0%	0 / 0 0.0%	0 / 0 0.0%	23 / 51 45.1%E	14 / 51 27.5%AB	6 / 51 11.8%	3 / 51 5.9%CE	3 / 51 5.9%C	2 / 51 3.9%ABE		
1	Sanguibacter	16 / 51 31.4%	0 / 2 0.0%	0 / 1 0.0%	1 / 1 100.0%	0 / 1 0.0%	2 / 4 50.0%	0 / 4 0.0%	0 / 1 0.0%	0 / 1 0.0%	0 / 3 0.0%	0 / 1 0.0%	1 / 4 25.0%	2 / 3 66.7%	0 / 1 0.0%	0 / 2 0.0%	0 / 2 0.0%	1 / 3 33.3%	1 / 1 100.0%	1 / 3 33.3%	4 / 4 100.0%	1 / 2 50.0%	1 / 1 100.0%	1 / 6 16.7%	0 / 0 0.0%	0 / 0 0.0%	0 / 0 0.0%	0 / 0 0.0%	23 / 51 45.1%E	14 / 51 27.5%AB	6 / 51 11.8%	3 / 51 5.9%C	3 / 51 5.9%CE	2 / 51 3.9%ABE		
1	ATCC 51767	16 / 51 31.4%	0 / 2 0.0%	0 / 1 0.0%	1 / 1 100.0%	0 / 1 0.0%	2 / 4 50.0%	0 / 4 0.0%	0 / 1 0.0%	0 / 1 0.0%	0 / 3 0.0%	0 / 1 0.0%	1 / 4 25.0%	2 / 3 66.7%	0 / 1 0.0%	0 / 2 0.0%	0 / 2 0.0%	1 / 3 33.3%	1 / 1 100.0%	1 / 3 33.3%	4 / 4 100.0%	1 / 2 50.0%	1 / 1 100.0%	1 / 6 16.7%	0 / 0 0.0%	0 / 0 0.0%	0 / 0 0.0%	0 / 0 0.0%	23 / 51 45.1%E	14 / 51 27.5%AB	6 / 51 11.8%	3 / 51 5.9%CE	3 / 51 5.9%C	2 / 51 3.9%ABE		
1	Sanguibacter keddieii DSM 10542	16 / 51 31.4%	0 / 2 0.0%	0 / 1 0.0%	1 / 1 100.0%	0 / 1 0.0%	2 / 4 50.0%	0 / 4 0.0%	0 / 1 0.0%	0 / 1 0.0%	0 / 3 0.0%	0 / 1 0.0%	1 / 4 25.0%	2 / 3 66.7%	0 / 1 0.0%	0 / 2 0.0%	0 / 2 0.0%	1 / 3 33.3%	1 / 1 100.0%	1 / 3 33.3%	4 / 4 100.0%	1 / 2 50.0%	1 / 1 100.0%	1 / 6 16.7%	0 / 0 0.0%	0 / 0 0.0%	0 / 0 0.0%	0 / 0 0.0%	23 / 51 45.1%E	14 / 51 27.5%AB	6 / 51 11.8%	3 / 51 5.9%C	3 / 51 5.9%CE	2 / 51 3.9%ABE		
2	unclassified Micrococcineae	4 / 99 4.0%	0 / 2 0.0%	0 / 5 0.0%	0 / 2 0.0%	0 / 2 0.0%	0 / 6 0.0%	0 / 6 0.0%	0 / 2 0.0%	0 / 2 0.0%	2 / 6 33.3%	0 / 2 0.0%	0 / 8 0.0%	0 / 4 0.0%	0 / 6 0.0%	0 / 4 0.0%	0 / 2 0.0%	0 / 6 0.0%	2 / 2 100.0%	0 / 6 0.0%	0 / 8 0.0%	0 / 6 0.0%	0 / 2 0.0%	0 / 10 0.0%	0 / 0 0.0%	0 / 0 0.0%	0 / 0 0.0%	0 / 0 0.0%	43 / 99 43.4%	42 / 99 42.4%E	10 / 99 10.1%CE	4 / 99 4.0%AB				
2	Tropheryma	4 / 99 4.0%	0 / 2 0.0%	0 / 5 0.0%	0 / 2 0.0%	0 / 2 0.0%	0 / 6 0.0%	0 / 6 0.0%	0 / 2 0.0%	0 / 2 0.0%	2 / 6 33.3%	0 / 2 0.0%	0 / 8 0.0%	0 / 4 0.0%	0 / 6 0.0%	0 / 4 0.0%	0 / 2 0.0%	0 / 6 0.0%	2 / 2 100.0%	0 / 6 0.0%	0 / 8 0.0%	0 / 6 0.0%	0 / 2 0.0%	0 / 10 0.0%	0 / 0 0.0%	0 / 0 0.0%	0 / 0 0.0%	0 / 0 0.0%	43 / 99 43.4%	42 / 99 42.4%E	10 / 99 10.1%CE	4 / 99 4.0%AB				
2	"Tropheryma whippelii" (sic) Relman et al. 1992	4 / 99 4.0%	0 / 2 0.0%	0 / 5 0.0%	0 / 2 0.0%	0 / 2 0.0%	0 / 6 0.0%	0 / 6 0.0%	0 / 2 0.0%	0 / 2 0.0%	2 / 6 33.3%	0 / 2 0.0%	0 / 8 0.0%	0 / 4 0.0%	0 / 6 0.0%	0 / 4 0.0%	0 / 2 0.0%	0 / 6 0.0%	2 / 2 100.0%	0 / 6 0.0%	0 / 8 0.0%	0 / 6 0.0%	0 / 2 0.0%	0 / 10 0.0%	0 / 0 0.0%	0 / 0 0.0%	0 / 0 0.0%	0 / 0 0.0%	43 / 99 43.4%	42 / 99 42.4%E	10 / 99 10.1%CE	4 / 99 4.0%AB				
1	Tropheryma whipplei Twist	2 / 49 4.1%	0 / 1 0.0%	0 / 2 0.0%	0 / 1 0.0%	0 / 1 0.0%	0 / 3 0.0%	0 / 3 0.0%	0 / 1 0.0%	0 / 1 0.0%	1 / 3 33.3%	0 / 1 0.0%	0 / 4 0.0%	0 / 2 0.0%	0 / 3 0.0%	0 / 2 0.0%	0 / 1 0.0%	0 / 3 0.0%	1 / 1 100.0%	0 / 3 0.0%	0 / 4 0.0%	0 / 3 0.0%	0 / 1 0.0%	0 / 5 0.0%	0 / 0 0.0%	0 / 0 0.0%	0 / 0 0.0%	0 / 0 0.0%	21 / 49 42.9%E	21 / 49 42.9%	5 / 49 10.2%CE	2 / 49 4.1%AB				
1	Tropheryma whipplei TW08/27	2 / 50 4.0%	0 / 1 0.0%	0 / 3 0.0%	0 / 1 0.0%	0 / 1 0.0%	0 / 3 0.0%	0 / 3 0.0%	0 / 1 0.0%	0 / 1 0.0%	1 / 3 33.3%	0 / 1 0.0%	0 / 4 0.0%	0 / 2 0.0%	0 / 3 0.0%	0 / 2 0.0%	0 / 1 0.0%	0 / 3 0.0%	1 / 1 100.0%	0 / 3 0.0%	0 / 4 0.0%	0 / 3 0.0%	0 / 1 0.0%	0 / 5 0.0%	0 / 0 0.0%	0 / 0 0.0%	0 / 0 0.0%	0 / 0 0.0%	22 / 50 44.0%	21 / 50 42.0%E	5 / 50 10.0%CE	2 / 50 4.0%AB				
117	Corynebacterineae	1740 / 5783 30.1%	72 / 132 54.5%	60 / 167 35.9%	12 / 127 9.4%	73 / 139 52.5%	159 / 439 36.2%	144 / 387 37.2%	79 / 120 65.8%	68 / 121 56.2%	49 / 377 13.0%	4 / 122 3.3%	236 / 480 49.2%	74 / 254 29.1%	74 / 119 62.2%	11 / 247 4.5%	60 / 135 44.4%	131 / 360 36.4%	8 / 121 6.6%	118 / 364 32.4%	57 / 523 10.9%	77 / 293 26.3%	10 / 122 8.2%	149 / 613 24.3%	15 / 16 93.8%	0 / 0 0.0%	0 / 4 0.0%	0 / 1 0.0%	1974 / 5783 34.1%	1639 / 5783 28.3%E	932 / 5783 16.1%AB	783 / 5783 13.5%ABE	220 / 5783 3.8%C	210 / 5783 3.6%CE	15 / 5783 0.3%B	10 / 5783 0.2%BE
47	Corynebacteriaceae	671 / 2434 27.6%	18 / 52 34.6%	3 / 93 3.2%	0 / 54 0.0%	65 / 65 100.0%	99 / 212 46.7%	45 / 164 27.4%	26 / 47 55.3%	20 / 47 42.6%	2 / 159 1.3%	1 / 48 2.1%	88 / 189 46.6%	31 / 105 29.5%	22 / 47 46.8%	2 / 100 2.0%	1 / 42 2.4%	49 / 140 35.0%	3 / 48 6.2%	40 / 148 27.0%	13 / 230 5.7%	73 / 146 50.0%	3 / 47 6.4%	65 / 245 26.5%	2 / 2 100.0%	0 / 0 0.0%	0 / 4 0.0%	0 / 0 0.0%	877 / 2434 36.0%	813 / 2434 33.4%E	353 / 2434 14.5%AB	313 / 2434 12.9%ABE	43 / 2434 1.8%C	30 / 2434 1.2%CE	3 / 2434 0.1%B	2 / 2434 0.1%BE
47	Caseobacter	671 / 2434 27.6%	18 / 52 34.6%	3 / 93 3.2%	0 / 54 0.0%	65 / 65 100.0%	99 / 212 46.7%	45 / 164 27.4%	26 / 47 55.3%	20 / 47 42.6%	2 / 159 1.3%	1 / 48 2.1%	88 / 189 46.6%	31 / 105 29.5%	22 / 47 46.8%	2 / 100 2.0%	1 / 42 2.4%	49 / 140 35.0%	3 / 48 6.2%	40 / 148 27.0%	13 / 230 5.7%	73 / 146 50.0%	3 / 47 6.4%	65 / 245 26.5%	2 / 2 100.0%	0 / 0 0.0%	0 / 4 0.0%	0 / 0 0.0%	877 / 2434 36.0%	813 / 2434 33.4%E	353 / 2434 14.5%AB	313 / 2434 12.9%ABE	43 / 2434 1.8%C	30 / 2434 1.2%CE	3 / 2434 0.1%B	2 / 2434 0.1%BE
14	"Bacillus diphtheriae" Kruse in Flugge 1886	125 / 724 17.3%	0 / 14 0.0%	0 / 28 0.0%	0 / 14 0.0%	27 / 27 100.0%	21 / 64 32.8%	0 / 50 0.0%	0 / 14 0.0%	0 / 14 0.0%	0 / 55 0.0%	0 / 14 0.0%	14 / 56 25.0%	4 / 29 13.8%	0 / 14 0.0%	1 / 28 3.6%	0 / 8 0.0%	7 / 42 16.7%	0 / 14 0.0%	7 / 42 16.7%	3 / 70 4.3%	14 / 42 33.3%	0 / 14 0.0%	27 / 70 38.6%	0 / 0 0.0%	0 / 0 0.0%	0 / 1 0.0%	0 / 0 0.0%	303 / 724 41.9%	267 / 724 36.9%E	76 / 724 10.5%AB	49 / 724 6.8%ABE	15 / 724 2.1%C	14 / 724 1.9%CE		
1	Corynebacterium diphtheriae NCTC 13129	9 / 53 17.0%	0 / 1 0.0%	0 / 2 0.0%	0 / 1 0.0%	2 / 2 100.0%	2 / 5 40.0%	0 / 4 0.0%	0 / 1 0.0%	0 / 1 0.0%	0 / 4 0.0%	0 / 1 0.0%	1 / 4 25.0%	0 / 2 0.0%	0 / 1 0.0%	0 / 1 0.0%	0 / 1 0.0%	1 / 3 33.3%	0 / 1 0.0%	0 / 3 0.0%	0 / 5 0.0%	1 / 3 33.3%	0 / 1 0.0%	2 / 5 40.0%	0 / 0 0.0%	0 / 0 0.0%	0 / 1 0.0%	0 / 0 0.0%	23 / 53 43.4%	19 / 53 35.8%E	5 / 53 9.4%AB	4 / 53 7.5%ABE	1 / 53 1.9%CE	1 / 53 1.9%C		
1	Corynebacterium diphtheriae 31A	9 / 50 18.0%	0 / 1 0.0%	0 / 2 0.0%	0 / 1 0.0%	2 / 2 100.0%	2 / 4 50.0%	0 / 3 0.0%	0 / 1 0.0%	0 / 1 0.0%	0 / 4 0.0%	0 / 1 0.0%	1 / 4 25.0%	0 / 2 0.0%	0 / 1 0.0%	0 / 2 0.0%	0 / 0 0.0%	0 / 3 0.0%	0 / 1 0.0%	1 / 3 33.3%	0 / 5 0.0%	1 / 3 33.3%	0 / 1 0.0%	2 / 5 40.0%	0 / 0 0.0%	0 / 0 0.0%	0 / 0 0.0%	0 / 0 0.0%	20 / 50 40.0%	19 / 50 38.0%E	6 / 50 12.0%AB	3 / 50 6.0%ABE	1 / 50 2.0%CE	1 / 50 2.0%C		
1	Corynebacterium diphtheriae C7 (beta)	9 / 55 16.4%	0 / 1 0.0%	0 / 2 0.0%	0 / 1 0.0%	2 / 2 100.0%	1 / 5 20.0%	0 / 4 0.0%	0 / 1 0.0%	0 / 1 0.0%	0 / 4 0.0%	0 / 1 0.0%	1 / 4 25.0%	0 / 3 0.0%	0 / 1 0.0%	1 / 3 33.3%	0 / 1 0.0%	1 / 3 33.3%	0 / 1 0.0%	1 / 3 33.3%	0 / 5 0.0%	1 / 3 33.3%	0 / 1 0.0%	1 / 5 20.0%	0 / 0 0.0%	0 / 0 0.0%	0 / 0 0.0%	0 / 0 0.0%	24 / 55 43.6%	19 / 55 34.5%E	5 / 55 9.1%AB	4 / 55 7.3%ABE	2 / 55 3.6%C	1 / 55 1.8%CE		
1	Corynebacterium diphtheriae PW8	9 / 51 17.6%	0 / 1 0.0%	0 / 2 0.0%	0 / 1 0.0%	1 / 1 100.0%	1 / 5 20.0%	0 / 4 0.0%	0 / 1 0.0%	0 / 1 0.0%	0 / 3 0.0%	0 / 1 0.0%	1 / 4 25.0%	0 / 2 0.0%	0 / 1 0.0%	0 / 2 0.0%	0 / 1 0.0%	0 / 3 0.0%	0 / 1 0.0%	1 / 3 33.3%	2 / 5 40.0%	1 / 3 33.3%	0 / 1 0.0%	2 / 5 40.0%	0 / 0 0.0%	0 / 0 0.0%	0 / 0 0.0%	0 / 0 0.0%	20 / 51 39.2%	20 / 51 39.2%E	6 / 51 11.8%AB	3 / 51 5.9%ABE	1 / 51 2.0%CE	1 / 51 2.0%C		
1	Corynebacterium diphtheriae CDCE 8392	10 / 53 18.9%	0 / 1 0.0%	0 / 2 0.0%	0 / 1 0.0%	2 / 2 100.0%	1 / 5 20.0%	0 / 4 0.0%	0 / 1 0.0%	0 / 1 0.0%	0 / 4 0.0%	0 / 1 0.0%	1 / 4 25.0%	0 / 2 0.0%	0 / 1 0.0%	0 / 2 0.0%	0 / 1 0.0%	1 / 3 33.3%	0 / 1 0.0%	1 / 3 33.3%	1 / 5 20.0%	1 / 3 33.3%	0 / 1 0.0%	2 / 5 40.0%	0 / 0 0.0%	0 / 0 0.0%	0 / 0 0.0%	0 / 0 0.0%	22 / 53 41.5%	19 / 53 35.8%E	6 / 53 11.3%AB	4 / 53 7.5%ABE	1 / 53 1.9%CE	1 / 53 1.9%C		
1	Corynebacterium diphtheriae 241	9 / 50 18.0%	0 / 1 0.0%	0 / 2 0.0%	0 / 1 0.0%	2 / 2 100.0%	2 / 4 50.0%	0 / 3 0.0%	0 / 1 0.0%	0 / 1 0.0%	0 / 4 0.0%	0 / 1 0.0%	1 / 4 25.0%	1 / 2 50.0%	0 / 1 0.0%	0 / 2 0.0%	0 / 0 0.0%	0 / 3 0.0%	0 / 1 0.0%	0 / 3 0.0%	0 / 5 0.0%	1 / 3 33.3%	0 / 1 0.0%	2 / 5 40.0%	0 / 0 0.0%	0 / 0 0.0%	0 / 0 0.0%	0 / 0 0.0%	20 / 50 40.0%	19 / 50 38.0%E	6 / 50 12.0%AB	3 / 50 6.0%ABE	1 / 50 2.0%CE	1 / 50 2.0%C		
1	Corynebacterium diphtheriae HC01	9 / 53 17.0%	0 / 1 0.0%	0 / 2 0.0%	0 / 1 0.0%	2 / 2 100.0%	2 / 5 40.0%	0 / 4 0.0%	0 / 1 0.0%	0 / 1 0.0%	0 / 4 0.0%	0 / 1 0.0%	1 / 4 25.0%	1 / 2 50.0%	0 / 1 0.0%	0 / 2 0.0%	0 / 1 0.0%	0 / 3 0.0%	0 / 1 0.0%	0 / 3 0.0%	0 / 5 0.0%	1 / 3 33.3%	0 / 1 0.0%	2 / 5 40.0%	0 / 0 0.0%	0 / 0 0.0%	0 / 0 0.0%	0 / 0 0.0%	22 / 53 41.5%	20 / 53 37.7%E	6 / 53 11.3%AB	3 / 53 5.7%ABE	1 / 53 1.9%C	1 / 53 1.9%CE		
1	Corynebacterium diphtheriae HC02	8 / 53 15.1%	0 / 1 0.0%	0 / 2 0.0%	0 / 1 0.0%	2 / 2 100.0%	1 / 5 20.0%	0 / 4 0.0%	0 / 1 0.0%	0 / 1 0.0%	0 / 4 0.0%	0 / 1 0.0%	1 / 4 25.0%	0 / 2 0.0%	0 / 1 0.0%	0 / 2 0.0%	0 / 1 0.0%	0 / 3 0.0%	0 / 1 0.0%	1 / 3 33.3%	0 / 5 0.0%	1 / 3 33.3%	0 / 1 0.0%	2 / 5 40.0%	0 / 0 0.0%	0 / 0 0.0%	0 / 0 0.0%	0 / 0 0.0%	23 / 53 43.4%	20 / 53 37.7%E	5 / 53 9.4%AB	3 / 53 5.7%ABE	1 / 53 1.9%CE	1 / 53 1.9%C		
1	Corynebacterium diphtheriae HC03	9 / 50 18.0%	0 / 1 0.0%	0 / 2 0.0%	0 / 1 0.0%	2 / 2 100.0%	1 / 4 25.0%	0 / 3 0.0%	0 / 1 0.0%	0 / 1 0.0%	0 / 4 0.0%	0 / 1 0.0%	1 / 4 25.0%	1 / 2 50.0%	0 / 1 0.0%	0 / 2 0.0%	0 / 0 0.0%	1 / 3 33.3%	0 / 1 0.0%	0 / 3 0.0%	0 / 5 0.0%	1 / 3 33.3%	0 / 1 0.0%	2 / 5 40.0%	0 / 0 0.0%	0 / 0 0.0%	0 / 0 0.0%	0 / 0 0.0%	21 / 50 42.0%	18 / 50 36.0%E	5 / 50 10.0%AB	4 / 50 8.0%ABE	1 / 50 2.0%C	1 / 50 2.0%CE		
1	Corynebacterium diphtheriae HC04	8 / 50 16.0%	0 / 1 0.0%	0 / 2 0.0%	0 / 1 0.0%	2 / 2 100.0%	1 / 4 25.0%	0 / 3 0.0%	0 / 1 0.0%	0 / 1 0.0%	0 / 4 0.0%	0 / 1 0.0%	1 / 4 25.0%	0 / 2 0.0%	0 / 1 0.0%	0 / 2 0.0%	0 / 0 0.0%	1 / 3 33.3%	0 / 1 0.0%	0 / 3 0.0%	0 / 5 0.0%	1 / 3 33.3%	0 / 1 0.0%	2 / 5 40.0%	0 / 0 0.0%	0 / 0 0.0%	0 / 0 0.0%	0 / 0 0.0%	22 / 50 44.0%	18 / 50 36.0%E	4 / 50 8.0%ABE	4 / 50 8.0%AB	1 / 50 2.0%CE	1 / 50 2.0%C		
1	Corynebacterium diphtheriae VA01	8 / 50 16.0%	0 / 1 0.0%	0 / 2 0.0%	0 / 1 0.0%	2 / 2 100.0%	1 / 4 25.0%	0 / 3 0.0%	0 / 1 0.0%	0 / 1 0.0%	0 / 4 0.0%	0 / 1 0.0%	1 / 4 25.0%	0 / 2 0.0%	0 / 1 0.0%	0 / 2 0.0%	0 / 0 0.0%	1 / 3 33.3%	0 / 1 0.0%	0 / 3 0.0%	0 / 5 0.0%	1 / 3 33.3%	0 / 1 0.0%	2 / 5 40.0%	0 / 0 0.0%	0 / 0 0.0%	0 / 0 0.0%	0 / 0 0.0%	22 / 50 44.0%	18 / 50 36.0%E	4 / 50 8.0%ABE	4 / 50 8.0%AB	1 / 50 2.0%CE	1 / 50 2.0%C		
1	Corynebacterium diphtheriae INCA 402	10 / 50 20.0%	0 / 1 0.0%	0 / 2 0.0%	0 / 1 0.0%	2 / 2 100.0%	2 / 4 50.0%	0 / 3 0.0%	0 / 1 0.0%	0 / 1 0.0%	0 / 4 0.0%	0 / 1 0.0%	1 / 4 25.0%	1 / 2 50.0%	0 / 1 0.0%	0 / 2 0.0%	0 / 0 0.0%	0 / 3 0.0%	0 / 1 0.0%	1 / 3 33.3%	0 / 5 0.0%	1 / 3 33.3%	0 / 1 0.0%	2 / 5 40.0%	0 / 0 0.0%	0 / 0 0.0%	0 / 0 0.0%	0 / 0 0.0%	19 / 50 38.0%	19 / 50 38.0%E	7 / 50 14.0%AB	3 / 50 6.0%ABE	1 / 50 2.0%CE	1 / 50 2.0%C		
1	Corynebacterium diphtheriae BH8	9 / 53 17.0%	0 / 1 0.0%	0 / 2 0.0%	0 / 1 0.0%	2 / 2 100.0%	2 / 5 40.0%	0 / 4 0.0%	0 / 1 0.0%	0 / 1 0.0%	0 / 4 0.0%	0 / 1 0.0%	1 / 4 25.0%	0 / 2 0.0%	0 / 1 0.0%	0 / 2 0.0%	0 / 1 0.0%	0 / 3 0.0%	0 / 1 0.0%	1 / 3 33.3%	0 / 5 0.0%	1 / 3 33.3%	0 / 1 0.0%	2 / 5 40.0%	0 / 0 0.0%	0 / 0 0.0%	0 / 0 0.0%	0 / 0 0.0%	22 / 53 41.5%	20 / 53 37.7%E	6 / 53 11.3%AB	3 / 53 5.7%ABE	1 / 53 1.9%CE	1 / 53 1.9%C		
4	"Micrococcus glutamicus" Kinoshita et al. 1958	43 / 239 18.0%	0 / 8 0.0%	0 / 8 0.0%	0 / 8 0.0%	8 / 8 100.0%	4 / 19 21.1%	7 / 15 46.7%	0 / 4 0.0%	0 / 4 0.0%	0 / 16 0.0%	0 / 4 0.0%	4 / 16 25.0%	4 / 12 33.3%	4 / 4 100.0%	0 / 10 0.0%	0 / 4 0.0%	0 / 12 0.0%	0 / 4 0.0%	4 / 16 25.0%	0 / 20 0.0%	0 / 16 0.0%	1 / 4 25.0%	7 / 25 28.0%	0 / 0 0.0%	0 / 0 0.0%	0 / 2 0.0%	0 / 0 0.0%	105 / 239 43.9%E	79 / 239 33.1%	36 / 239 15.1%AB	12 / 239 5.0%C	7 / 239 2.9%ABE			
1	Corynebacterium glutamicum ATCC 13032	13 / 60 21.7%	0 / 2 0.0%	0 / 2 0.0%	0 / 2 0.0%	2 / 2 100.0%	1 / 5 20.0%	2 / 4 50.0%	0 / 1 0.0%	0 / 1 0.0%	0 / 4 0.0%	0 / 1 0.0%	1 / 4 25.0%	1 / 3 33.3%	1 / 1 100.0%	0 / 3 0.0%	0 / 1 0.0%	0 / 3 0.0%	0 / 1 0.0%	1 / 4 25.0%	0 / 5 0.0%	0 / 4 0.0%	1 / 1 100.0%	3 / 6 50.0%	0 / 0 0.0%	0 / 0 0.0%	0 / 0 0.0%	0 / 0 0.0%	26 / 60 43.3%E	18 / 60 30.0%	11 / 60 18.3%AB	3 / 60 5.0%C	2 / 60 3.3%ABE			
1	Corynebacterium glutamicum K051	13 / 60 21.7%	0 / 2 0.0%	0 / 2 0.0%	0 / 2 0.0%	2 / 2 100.0%	1 / 5 20.0%	2 / 4 50.0%	0 / 1 0.0%	0 / 1 0.0%	0 / 4 0.0%	0 / 1 0.0%	1 / 4 25.0%	1 / 3 33.3%	1 / 1 100.0%	0 / 3 0.0%	0 / 1 0.0%	0 / 3 0.0%	0 / 1 0.0%	1 / 4 25.0%	0 / 5 0.0%	0 / 4 0.0%	1 / 1 100.0%	3 / 6 50.0%	0 / 0 0.0%	0 / 0 0.0%	0 / 0 0.0%	0 / 0 0.0%	26 / 60 43.3%E	18 / 60 30.0%	11 / 60 18.3%AB	3 / 60 5.0%C	2 / 60 3.3%ABE			
1	Corynebacterium glutamicum R	11 / 59 18.6%	0 / 2 0.0%	0 / 2 0.0%	0 / 2 0.0%	2 / 2 100.0%	1 / 4 25.0%	2 / 3 66.7%	0 / 1 0.0%	0 / 1 0.0%	0 / 4 0.0%	0 / 1 0.0%	1 / 4 25.0%	1 / 3 33.3%	1 / 1 100.0%	0 / 3 0.0%	0 / 1 0.0%	0 / 3 0.0%	0 / 1 0.0%	1 / 4 25.0%	0 / 5 0.0%	0 / 4 0.0%	0 / 1 0.0%	2 / 7 28.6%	0 / 0 0.0%	0 / 0 0.0%	0 / 0 0.0%	0 / 0 0.0%	25 / 59 42.4%E	20 / 59 33.9%	8 / 59 13.6%AB	3 / 59 5.1%ABE	3 / 59 5.1%C			
1	Corynebacterium glutamicum SCgG1	9 / 60 15.0%	0 / 2 0.0%	0 / 2 0.0%	0 / 2 0.0%	2 / 2 100.0%	1 / 5 20.0%	1 / 4 25.0%	0 / 1 0.0%	0 / 1 0.0%	0 / 4 0.0%	0 / 1 0.0%	1 / 4 25.0%	1 / 3 33.3%	1 / 1 100.0%	0 / 2 0.0%	0 / 1 0.0%	0 / 3 0.0%	0 / 1 0.0%	1 / 4 25.0%	0 / 5 0.0%	0 / 4 0.0%	0 / 1 0.0%	1 / 6 16.7%	0 / 0 0.0%	0 / 0 0.0%	0 / 1 0.0%	0 / 0 0.0%	27 / 60 45.0%E	21 / 60 35.0%	8 / 60 13.3%AB	3 / 60 5.0%C	1 / 60 1.7%ABE			
1	Corynebacterium glutamicum SCgG2	10 / 60 16.7%	0 / 2 0.0%	0 / 2 0.0%	0 / 2 0.0%	2 / 2 100.0%	1 / 5 20.0%	2 / 4 50.0%	0 / 1 0.0%	0 / 1 0.0%	0 / 4 0.0%	0 / 1 0.0%	1 / 4 25.0%	1 / 3 33.3%	1 / 1 100.0%	0 / 2 0.0%	0 / 1 0.0%	0 / 3 0.0%	0 / 1 0.0%	1 / 4 25.0%	0 / 5 0.0%	0 / 4 0.0%	0 / 1 0.0%	1 / 6 16.7%	0 / 0 0.0%	0 / 0 0.0%	0 / 1 0.0%	0 / 0 0.0%	27 / 60 45.0%E	20 / 60 33.3%	9 / 60 15.0%AB	3 / 60 5.0%C	1 / 60 1.7%ABE			
15	"Bacillus pseudotuberculosis" Buchanan 1911	270 / 727 37.1%	14 / 14 100.0%	0 / 30 0.0%	0 / 15 0.0%	15 / 15 100.0%	30 / 60 50.0%	15 / 45 33.3%	15 / 15 100.0%	15 / 15 100.0%	0 / 44 0.0%	0 / 15 0.0%	42 / 60 70.0%	12 / 30 40.0%	15 / 15 100.0%	0 / 30 0.0%	0 / 15 0.0%	24 / 44 54.5%	0 / 15 0.0%	15 / 45 33.3%	0 / 72 0.0%	43 / 43 100.0%	0 / 15 0.0%	15 / 75 20.0%	0 / 0 0.0%	0 / 0 0.0%	0 / 0 0.0%	0 / 0 0.0%	254 / 727 34.9%	200 / 727 27.5%E	153 / 727 21.0%ABE	117 / 727 16.1%AB	3 / 727 0.4%CE			
1	Corynebacterium pseudotuberculosis 1002	18 / 48 37.5%	1 / 1 100.0%	0 / 2 0.0%	0 / 1 0.0%	1 / 1 100.0%	2 / 4 50.0%	1 / 3 33.3%	1 / 1 100.0%	1 / 1 100.0%	0 / 3 0.0%	0 / 1 0.0%	3 / 4 75.0%	1 / 2 50.0%	1 / 1 100.0%	0 / 2 0.0%	0 / 1 0.0%	2 / 3 66.7%	0 / 1 0.0%	1 / 3 33.3%	0 / 5 0.0%	2 / 2 100.0%	0 / 1 0.0%	1 / 5 20.0%	0 / 0 0.0%	0 / 0 0.0%	0 / 0 0.0%	0 / 0 0.0%	17 / 48 35.4%	13 / 48 27.1%E	10 / 48 20.8%ABE	8 / 48 16.7%AB				
1	Corynebacterium pseudotuberculosis C231	19 / 48 39.6%	1 / 1 100.0%	0 / 2 0.0%	0 / 1 0.0%	1 / 1 100.0%	2 / 4 50.0%	1 / 3 33.3%	1 / 1 100.0%	1 / 1 100.0%	0 / 2 0.0%	0 / 1 0.0%	3 / 4 75.0%	1 / 2 50.0%	1 / 1 100.0%	0 / 2 0.0%	0 / 1 0.0%	2 / 3 66.7%	0 / 1 0.0%	1 / 3 33.3%	0 / 5 0.0%	3 / 3 100.0%	0 / 1 0.0%	1 / 5 20.0%	0 / 0 0.0%	0 / 0 0.0%	0 / 0 0.0%	0 / 0 0.0%	17 / 48 35.4%	12 / 48 25.0%E	11 / 48 22.9%ABE	8 / 48 16.7%AB				
1	Corynebacterium pseudotuberculosis FRC41	19 / 49 38.8%	1 / 1 100.0%	0 / 2 0.0%	0 / 1 0.0%	1 / 1 100.0%	2 / 4 50.0%	1 / 3 33.3%	1 / 1 100.0%	1 / 1 100.0%	0 / 3 0.0%	0 / 1 0.0%	3 / 4 75.0%	1 / 2 50.0%	1 / 1 100.0%	0 / 2 0.0%	0 / 1 0.0%	2 / 3 66.7%	0 / 1 0.0%	1 / 3 33.3%	0 / 5 0.0%	3 / 3 100.0%	0 / 1 0.0%	1 / 5 20.0%	0 / 0 0.0%	0 / 0 0.0%	0 / 0 0.0%	0 / 0 0.0%	17 / 49 34.7%	13 / 49 26.5%E	11 / 49 22.4%ABE	8 / 49 16.3%AB				
1	Corynebacterium pseudotuberculosis I19	19 / 49 38.8%	1 / 1 100.0%	0 / 2 0.0%	0 / 1 0.0%	1 / 1 100.0%	2 / 4 50.0%	1 / 3 33.3%	1 / 1 100.0%	1 / 1 100.0%	0 / 3 0.0%	0 / 1 0.0%	3 / 4 75.0%	1 / 2 50.0%	1 / 1 100.0%	0 / 2 0.0%	0 / 1 0.0%	2 / 3 66.7%	0 / 1 0.0%	1 / 3 33.3%	0 / 5 0.0%	3 / 3 100.0%	0 / 1 0.0%	1 / 5 20.0%	0 / 0 0.0%	0 / 0 0.0%	0 / 0 0.0%	0 / 0 0.0%	17 / 49 34.7%	13 / 49 26.5%E	11 / 49 22.4%ABE	8 / 49 16.3%AB				
1	Corynebacterium pseudotuberculosis PAT10	18 / 48 37.5%	1 / 1 100.0%	0 / 2 0.0%	0 / 1 0.0%	1 / 1 100.0%	2 / 4 50.0%	1 / 3 33.3%	1 / 1 100.0%	1 / 1 100.0%	0 / 3 0.0%	0 / 1 0.0%	3 / 4 75.0%	1 / 2 50.0%	1 / 1 100.0%	0 / 2 0.0%	0 / 1 0.0%	2 / 3 66.7%	0 / 1 0.0%	1 / 3 33.3%	0 / 5 0.0%	2 / 2 100.0%	0 / 1 0.0%	1 / 5 20.0%	0 / 0 0.0%	0 / 0 0.0%	0 / 0 0.0%	0 / 0 0.0%	16 / 48 33.3%	14 / 48 29.2%E	10 / 48 20.8%ABE	8 / 48 16.7%AB				
1	Corynebacterium pseudotuberculosis CIP 52.97	15 / 47 31.9%	0 / 0 0.0%	0 / 2 0.0%	0 / 1 0.0%	1 / 1 100.0%	2 / 4 50.0%	1 / 3 33.3%	1 / 1 100.0%	1 / 1 100.0%	0 / 3 0.0%	0 / 1 0.0%	2 / 4 50.0%	1 / 2 50.0%	1 / 1 100.0%	0 / 2 0.0%	0 / 1 0.0%	0 / 2 0.0%	0 / 1 0.0%	1 / 3 33.3%	0 / 5 0.0%	3 / 3 100.0%	0 / 1 0.0%	1 / 5 20.0%	0 / 0 0.0%	0 / 0 0.0%	0 / 0 0.0%	0 / 0 0.0%	17 / 47 36.2%	14 / 47 29.8%E	8 / 47 17.0%AB	7 / 47 14.9%ABE	1 / 47 2.1%CE			
1	Corynebacterium pseudotuberculosis 316	17 / 48 35.4%	1 / 1 100.0%	0 / 2 0.0%	0 / 1 0.0%	1 / 1 100.0%	2 / 4 50.0%	1 / 3 33.3%	1 / 1 100.0%	1 / 1 100.0%	0 / 3 0.0%	0 / 1 0.0%	3 / 4 75.0%	0 / 2 0.0%	1 / 1 100.0%	0 / 2 0.0%	0 / 1 0.0%	1 / 3 33.3%	0 / 1 0.0%	1 / 3 33.3%	0 / 4 0.0%	3 / 3 100.0%	0 / 1 0.0%	1 / 5 20.0%	0 / 0 0.0%	0 / 0 0.0%	0 / 0 0.0%	0 / 0 0.0%	17 / 48 35.4%	14 / 48 29.2%E	10 / 48 20.8%ABE	7 / 48 14.6%AB				
1	Corynebacterium pseudotuberculosis 31	17 / 47 36.2%	1 / 1 100.0%	0 / 2 0.0%	0 / 1 0.0%	1 / 1 100.0%	2 / 4 50.0%	1 / 3 33.3%	1 / 1 100.0%	1 / 1 100.0%	0 / 3 0.0%	0 / 1 0.0%	2 / 4 50.0%	1 / 2 50.0%	1 / 1 100.0%	0 / 2 0.0%	0 / 1 0.0%	1 / 3 33.3%	0 / 1 0.0%	1 / 3 33.3%	0 / 3 0.0%	3 / 3 100.0%	0 / 1 0.0%	1 / 5 20.0%	0 / 0 0.0%	0 / 0 0.0%	0 / 0 0.0%	0 / 0 0.0%	15 / 47 31.9%	14 / 47 29.8%E	9 / 47 19.1%ABE	8 / 47 17.0%AB	1 / 47 2.1%CE			
1	Corynebacterium pseudotuberculosis 3/99-5	19 / 49 38.8%	1 / 1 100.0%	0 / 2 0.0%	0 / 1 0.0%	1 / 1 100.0%	2 / 4 50.0%	1 / 3 33.3%	1 / 1 100.0%	1 / 1 100.0%	0 / 3 0.0%	0 / 1 0.0%	3 / 4 75.0%	1 / 2 50.0%	1 / 1 100.0%	0 / 2 0.0%	0 / 1 0.0%	2 / 3 66.7%	0 / 1 0.0%	1 / 3 33.3%	0 / 5 0.0%	3 / 3 100.0%	0 / 1 0.0%	1 / 5 20.0%	0 / 0 0.0%	0 / 0 0.0%	0 / 0 0.0%	0 / 0 0.0%	17 / 49 34.7%	13 / 49 26.5%E	11 / 49 22.4%ABE	8 / 49 16.3%AB				
1	Corynebacterium pseudotuberculosis 42/02-A	19 / 49 38.8%	1 / 1 100.0%	0 / 2 0.0%	0 / 1 0.0%	1 / 1 100.0%	2 / 4 50.0%	1 / 3 33.3%	1 / 1 100.0%	1 / 1 100.0%	0 / 3 0.0%	0 / 1 0.0%	3 / 4 75.0%	1 / 2 50.0%	1 / 1 100.0%	0 / 2 0.0%	0 / 1 0.0%	2 / 3 66.7%	0 / 1 0.0%	1 / 3 33.3%	0 / 5 0.0%	3 / 3 100.0%	0 / 1 0.0%	1 / 5 20.0%	0 / 0 0.0%	0 / 0 0.0%	0 / 0 0.0%	0 / 0 0.0%	17 / 49 34.7%	13 / 49 26.5%E	11 / 49 22.4%ABE	8 / 49 16.3%AB				
1	Corynebacterium pseudotuberculosis 1/06-A	18 / 49 36.7%	1 / 1 100.0%	0 / 2 0.0%	0 / 1 0.0%	1 / 1 100.0%	2 / 4 50.0%	1 / 3 33.3%	1 / 1 100.0%	1 / 1 100.0%	0 / 3 0.0%	0 / 1 0.0%	3 / 4 75.0%	1 / 2 50.0%	1 / 1 100.0%	0 / 2 0.0%	0 / 1 0.0%	1 / 3 33.3%	0 / 1 0.0%	1 / 3 33.3%	0 / 5 0.0%	3 / 3 100.0%	0 / 1 0.0%	1 / 5 20.0%	0 / 0 0.0%	0 / 0 0.0%	0 / 0 0.0%	0 / 0 0.0%	17 / 49 34.7%	14 / 49 28.6%E	10 / 49 20.4%ABE	8 / 49 16.3%AB				
1	Corynebacterium pseudotuberculosis 267	19 / 49 38.8%	1 / 1 100.0%	0 / 2 0.0%	0 / 1 0.0%	1 / 1 100.0%	2 / 4 50.0%	1 / 3 33.3%	1 / 1 100.0%	1 / 1 100.0%	0 / 3 0.0%	0 / 1 0.0%	3 / 4 75.0%	1 / 2 50.0%	1 / 1 100.0%	0 / 2 0.0%	0 / 1 0.0%	2 / 3 66.7%	0 / 1 0.0%	1 / 3 33.3%	0 / 5 0.0%	3 / 3 100.0%	0 / 1 0.0%	1 / 5 20.0%	0 / 0 0.0%	0 / 0 0.0%	0 / 0 0.0%	0 / 0 0.0%	17 / 49 34.7%	13 / 49 26.5%E	11 / 49 22.4%ABE	8 / 49 16.3%AB				
1	Corynebacterium pseudotuberculosis P54B96	19 / 49 38.8%	1 / 1 100.0%	0 / 2 0.0%	0 / 1 0.0%	1 / 1 100.0%	2 / 4 50.0%	1 / 3 33.3%	1 / 1 100.0%	1 / 1 100.0%	0 / 3 0.0%	0 / 1 0.0%	3 / 4 75.0%	1 / 2 50.0%	1 / 1 100.0%	0 / 2 0.0%	0 / 1 0.0%	2 / 3 66.7%	0 / 1 0.0%	1 / 3 33.3%	0 / 5 0.0%	3 / 3 100.0%	0 / 1 0.0%	1 / 5 20.0%	0 / 0 0.0%	0 / 0 0.0%	0 / 0 0.0%	0 / 0 0.0%	17 / 49 34.7%	13 / 49 26.5%E	11 / 49 22.4%ABE	8 / 49 16.3%AB				
1	Corynebacterium pseudotuberculosis Cp162	18 / 49 36.7%	1 / 1 100.0%	0 / 2 0.0%	0 / 1 0.0%	1 / 1 100.0%	2 / 4 50.0%	1 / 3 33.3%	1 / 1 100.0%	1 / 1 100.0%	0 / 3 0.0%	0 / 1 0.0%	3 / 4 75.0%	0 / 2 0.0%	1 / 1 100.0%	0 / 2 0.0%	0 / 1 0.0%	2 / 3 66.7%	0 / 1 0.0%	1 / 3 33.3%	0 / 5 0.0%	3 / 3 100.0%	0 / 1 0.0%	1 / 5 20.0%	0 / 0 0.0%	0 / 0 0.0%	0 / 0 0.0%	0 / 0 0.0%	18 / 49 36.7%	13 / 49 26.5%E	11 / 49 22.4%ABE	7 / 49 14.3%AB				
1	Corynebacterium pseudotuberculosis 258	16 / 49 32.7%	1 / 1 100.0%	0 / 2 0.0%	0 / 1 0.0%	1 / 1 100.0%	2 / 4 50.0%	1 / 3 33.3%	1 / 1 100.0%	1 / 1 100.0%	0 / 3 0.0%	0 / 1 0.0%	2 / 4 50.0%	0 / 2 0.0%	1 / 1 100.0%	0 / 2 0.0%	0 / 1 0.0%	1 / 3 33.3%	0 / 1 0.0%	1 / 3 33.3%	0 / 5 0.0%	3 / 3 100.0%	0 / 1 0.0%	1 / 5 20.0%	0 / 0 0.0%	0 / 0 0.0%	0 / 0 0.0%	0 / 0 0.0%	18 / 49 36.7%	14 / 49 28.6%E	9 / 49 18.4%ABE	7 / 49 14.3%AB	1 / 49 2.0%CE			
1	ATCC 15991	9 / 61 14.8%	0 / 2 0.0%	0 / 2 0.0%	0 / 2 0.0%	2 / 2 100.0%	1 / 5 20.0%	1 / 4 25.0%	0 / 1 0.0%	0 / 1 0.0%	0 / 5 0.0%	0 / 1 0.0%	1 / 4 25.0%	1 / 3 33.3%	0 / 1 0.0%	0 / 2 0.0%	0 / 1 0.0%	1 / 3 33.3%	0 / 1 0.0%	0 / 4 0.0%	0 / 5 0.0%	0 / 4 0.0%	0 / 1 0.0%	2 / 6 33.3%	0 / 0 0.0%	0 / 0 0.0%	0 / 1 0.0%	0 / 0 0.0%	26 / 61 42.6%	24 / 61 39.3%E	5 / 61 8.2%ABE	4 / 61 6.6%AB	2 / 61 3.3%C			
1	Corynebacterium callunae DSM 20147	9 / 61 14.8%	0 / 2 0.0%	0 / 2 0.0%	0 / 2 0.0%	2 / 2 100.0%	1 / 5 20.0%	1 / 4 25.0%	0 / 1 0.0%	0 / 1 0.0%	0 / 5 0.0%	0 / 1 0.0%	1 / 4 25.0%	1 / 3 33.3%	0 / 1 0.0%	0 / 2 0.0%	0 / 1 0.0%	1 / 3 33.3%	0 / 1 0.0%	0 / 4 0.0%	0 / 5 0.0%	0 / 4 0.0%	0 / 1 0.0%	2 / 6 33.3%	0 / 0 0.0%	0 / 0 0.0%	0 / 1 0.0%	0 / 0 0.0%	26 / 61 42.6%	24 / 61 39.3%E	5 / 61 8.2%ABE	4 / 61 6.6%AB	2 / 61 3.3%C			
1	ATCC 15753	21 / 58 36.2%	0 / 1 0.0%	0 / 2 0.0%	0 / 1 0.0%	1 / 1 100.0%	2 / 5 40.0%	1 / 4 25.0%	1 / 1 100.0%	0 / 1 0.0%	0 / 4 0.0%	0 / 1 0.0%	4 / 5 80.0%	2 / 3 66.7%	0 / 1 0.0%	0 / 3 0.0%	0 / 1 0.0%	0 / 3 0.0%	1 / 1 100.0%	3 / 4 75.0%	1 / 5 20.0%	3 / 4 75.0%	0 / 1 0.0%	2 / 6 33.3%	0 / 0 0.0%	0 / 0 0.0%	0 / 0 0.0%	0 / 0 0.0%	19 / 58 32.8%E	14 / 58 24.1%	12 / 58 20.7%AB	9 / 58 15.5%ABE	3 / 58 5.2%C	1 / 58 1.7%CE		
1	Corynebacterium variabile CIP 107183	21 / 58 36.2%	0 / 1 0.0%	0 / 2 0.0%	0 / 1 0.0%	1 / 1 100.0%	2 / 5 40.0%	1 / 4 25.0%	1 / 1 100.0%	0 / 1 0.0%	0 / 4 0.0%	0 / 1 0.0%	4 / 5 80.0%	2 / 3 66.7%	0 / 1 0.0%	0 / 3 0.0%	0 / 1 0.0%	0 / 3 0.0%	1 / 1 100.0%	3 / 4 75.0%	1 / 5 20.0%	3 / 4 75.0%	0 / 1 0.0%	2 / 6 33.3%	0 / 0 0.0%	0 / 0 0.0%	0 / 0 0.0%	0 / 0 0.0%	19 / 58 32.8%E	14 / 58 24.1%	12 / 58 20.7%AB	9 / 58 15.5%ABE	3 / 58 5.2%C	1 / 58 1.7%CE		
1	ATCC 43734	18 / 50 36.0%	0 / 1 0.0%	1 / 2 50.0%	0 / 1 0.0%	1 / 1 100.0%	5 / 5 100.0%	2 / 4 50.0%	1 / 1 100.0%	0 / 1 0.0%	0 / 2 0.0%	0 / 1 0.0%	2 / 4 50.0%	0 / 2 0.0%	0 / 1 0.0%	0 / 2 0.0%	1 / 1 100.0%	1 / 3 33.3%	0 / 1 0.0%	1 / 3 33.3%	2 / 5 40.0%	1 / 3 33.3%	0 / 1 0.0%	0 / 5 0.0%	0 / 0 0.0%	0 / 0 0.0%	0 / 0 0.0%	0 / 0 0.0%	17 / 50 34.0%E	15 / 50 30.0%	11 / 50 22.0%AB	7 / 50 14.0%ABE				
1	Corynebacterium jeikeium K411	18 / 50 36.0%	0 / 1 0.0%	1 / 2 50.0%	0 / 1 0.0%	1 / 1 100.0%	5 / 5 100.0%	2 / 4 50.0%	1 / 1 100.0%	0 / 1 0.0%	0 / 2 0.0%	0 / 1 0.0%	2 / 4 50.0%	0 / 2 0.0%	0 / 1 0.0%	0 / 2 0.0%	1 / 1 100.0%	1 / 3 33.3%	0 / 1 0.0%	1 / 3 33.3%	2 / 5 40.0%	1 / 3 33.3%	0 / 1 0.0%	0 / 5 0.0%	0 / 0 0.0%	0 / 0 0.0%	0 / 0 0.0%	0 / 0 0.0%	17 / 50 34.0%E	15 / 50 30.0%	11 / 50 22.0%AB	7 / 50 14.0%ABE				
2	ATCC 43042	22 / 104 21.2%	1 / 2 50.0%	0 / 4 0.0%	0 / 4 0.0%	2 / 2 100.0%	8 / 10 80.0%	0 / 7 0.0%	2 / 2 100.0%	0 / 2 0.0%	0 / 4 0.0%	0 / 3 0.0%	0 / 8 0.0%	2 / 4 50.0%	0 / 2 0.0%	0 / 4 0.0%	0 / 3 0.0%	3 / 6 50.0%	0 / 3 0.0%	0 / 6 0.0%	0 / 10 0.0%	2 / 6 33.3%	0 / 2 0.0%	2 / 10 20.0%	0 / 0 0.0%	0 / 0 0.0%	0 / 0 0.0%	0 / 0 0.0%	43 / 104 41.3%	36 / 104 34.6%E	9 / 104 8.7%AB	8 / 104 7.7%ABE	3 / 104 2.9%B	2 / 104 1.9%BE	2 / 104 1.9%C	1 / 104 1.0%CE
1	Corynebacterium urealyticum ATCC 43042	11 / 51 21.6%	1 / 1 100.0%	0 / 2 0.0%	0 / 2 0.0%	1 / 1 100.0%	4 / 5 80.0%	0 / 4 0.0%	1 / 1 100.0%	0 / 1 0.0%	0 / 2 0.0%	0 / 1 0.0%	0 / 4 0.0%	1 / 2 50.0%	0 / 1 0.0%	0 / 2 0.0%	0 / 1 0.0%	1 / 3 33.3%	0 / 1 0.0%	0 / 3 0.0%	0 / 5 0.0%	1 / 3 33.3%	0 / 1 0.0%	1 / 5 20.0%	0 / 0 0.0%	0 / 0 0.0%	0 / 0 0.0%	0 / 0 0.0%	21 / 51 41.2%	18 / 51 35.3%E	3 / 51 5.9%ABE	3 / 51 5.9%B	3 / 51 5.9%AB	2 / 51 3.9%BE	1 / 51 2.0%C	
1	Corynebacterium urealyticum DSM 7111	11 / 53 20.8%	0 / 1 0.0%	0 / 2 0.0%	0 / 2 0.0%	1 / 1 100.0%	4 / 5 80.0%	0 / 3 0.0%	1 / 1 100.0%	0 / 1 0.0%	0 / 2 0.0%	0 / 2 0.0%	0 / 4 0.0%	1 / 2 50.0%	0 / 1 0.0%	0 / 2 0.0%	0 / 2 0.0%	2 / 3 66.7%	0 / 2 0.0%	0 / 3 0.0%	0 / 5 0.0%	1 / 3 33.3%	0 / 1 0.0%	1 / 5 20.0%	0 / 0 0.0%	0 / 0 0.0%	0 / 0 0.0%	0 / 0 0.0%	22 / 53 41.5%	18 / 53 34.0%E	6 / 53 11.3%AB	5 / 53 9.4%ABE	1 / 53 1.9%C	1 / 53 1.9%CE		
3	"Corynebacterium ulcerans" Gilbert and Stewart 1927	54 / 155 34.8%	0 / 3 0.0%	0 / 6 0.0%	0 / 3 0.0%	3 / 3 100.0%	9 / 15 60.0%	9 / 12 75.0%	3 / 3 100.0%	3 / 3 100.0%	0 / 9 0.0%	0 / 3 0.0%	7 / 12 58.3%	2 / 8 25.0%	3 / 3 100.0%	0 / 6 0.0%	0 / 3 0.0%	6 / 9 66.7%	0 / 3 0.0%	0 / 9 0.0%	0 / 15 0.0%	6 / 9 66.7%	0 / 3 0.0%	3 / 15 20.0%	0 / 0 0.0%	0 / 0 0.0%	0 / 0 0.0%	0 / 0 0.0%	57 / 155 36.8%	42 / 155 27.1%E	28 / 155 18.1%ABE	26 / 155 16.8%AB	2 / 155 1.3%CE			
[illegible]	[illegible]	[illegible]	[illegible]	[illegible]	[illegible]	[illegible]	[illegible]	[illegible]	[illegible]	[illegible]	[illegible]	[illegible]	[illegible]	[illegible]	[illegible]	[illegible]	[illegible]	[illegible]	[illegible]	[illegible]	[illegible]	[illegible]	[illegible]	[illegible]	[illegible]	[illegible]	[illegible]	[illegible]	[illegible]	[illegible]	[illegible]	[illegible]	[illegible]	[illegible]		

1	Corynebacterium ulcerans 809	34.6%	0.0%	0.0%	0.0%	100.0%	60.0%	75.0%	100.0%	100.0%	0.0%	0.0%	50.0%	33.3%	100.0%	0.0%	0.0%	66.7%	0.0%	0.0%	0.0%	66.7%	0.0%	20.0%	0.0%	0.0%	0.0%	0.0%	36.5%	26.9%E	17.3%ABE	17.3%AB	1.9%CE		
1	Corynebacterium ulcerans BR-AD22	17 / 52 32.7%	0 / 1 0.0%	0 / 2 0.0%	0 / 1 0.0%	1 / 1 100.0%	3 / 5 60.0%	3 / 4 75.0%	1 / 1 100.0%	1 / 1 100.0%	0 / 3 0.0%	0 / 1 0.0%	2 / 4 50.0%	0 / 3 0.0%	1 / 1 100.0%	0 / 2 0.0%	0 / 1 0.0%	2 / 3 66.7%	0 / 1 0.0%	0 / 3 0.0%	0 / 5 0.0%	2 / 3 66.7%	0 / 1 0.0%	1 / 5 20.0%	0 / 0 0.0%	0 / 0 0.0%	0 / 0 0.0%	0 / 0 0.0%	20 / 52 38.5%	14 / 52 26.9%E	9 / 52 17.3%ABE	8 / 52 15.4%AB	1 / 52 1.9%CE		
1	Corynebacterium ulcerans 0102	19 / 51 37.3%	0 / 1 0.0%	0 / 2 0.0%	0 / 1 0.0%	1 / 1 100.0%	3 / 5 60.0%	3 / 4 75.0%	1 / 1 100.0%	1 / 1 100.0%	0 / 3 0.0%	0 / 1 0.0%	3 / 4 75.0%	1 / 2 50.0%	1 / 1 100.0%	0 / 2 0.0%	0 / 1 0.0%	2 / 3 66.7%	0 / 1 0.0%	0 / 3 0.0%	0 / 5 0.0%	2 / 3 66.7%	0 / 1 0.0%	1 / 5 20.0%	0 / 0 0.0%	0 / 0 0.0%	0 / 0 0.0%	0 / 0 0.0%	18 / 51 35.3%	14 / 51 27.5%E	10 / 51 19.6%ABE	9 / 51 17.6%AB			
1	Arthrobacter sp. Y-11	22 / 57 38.6%	0 / 1 0.0%	0 / 2 0.0%	0 / 1 0.0%	1 / 1 100.0%	4 / 5 80.0%	1 / 4 25.0%	1 / 1 100.0%	0 / 1 0.0%	1 / 4 25.0%	0 / 1 0.0%	3 / 4 75.0%	2 / 3 66.7%	0 / 1 0.0%	0 / 3 0.0%	0 / 1 0.0%	1 / 3 33.3%	1 / 1 100.0%	3 / 4 75.0%	0 / 5 0.0%	1 / 4 25.0%	1 / 1 100.0%	2 / 6 33.3%	0 / 0 0.0%	0 / 0 0.0%	0 / 0 0.0%	0 / 0 0.0%	19 / 57 33.3%E	14 / 57 24.6%AB	12 / 57 21.1%	8 / 57 14.0%ABE	2 / 57 3.5%C	2 / 57 3.5%CE	
1	Corynebacterium terpenotabidum CIP 105927	22 / 57 38.6%	0 / 1 0.0%	0 / 2 0.0%	0 / 1 0.0%	1 / 1 100.0%	4 / 5 80.0%	1 / 4 25.0%	1 / 1 100.0%	0 / 1 0.0%	1 / 4 25.0%	0 / 1 0.0%	3 / 4 75.0%	2 / 3 66.7%	0 / 1 0.0%	0 / 3 0.0%	0 / 1 0.0%	1 / 3 33.3%	1 / 1 100.0%	3 / 4 75.0%	0 / 5 0.0%	1 / 4 25.0%	1 / 1 100.0%	2 / 6 33.3%	0 / 0 0.0%	0 / 0 0.0%	0 / 0 0.0%	0 / 0 0.0%	19 / 57 33.3%E	14 / 57 24.6%AB	12 / 57 21.1%	8 / 57 14.0%ABE	2 / 57 3.5%C	2 / 57 3.5%CE	
1	AJ 12310	12 / 56 21.4%	0 / 2 0.0%	0 / 2 0.0%	0 / 1 0.0%	1 / 1 100.0%	1 / 5 20.0%	2 / 4 50.0%	0 / 1 0.0%	0 / 1 0.0%	0 / 3 0.0%	0 / 1 0.0%	1 / 4 25.0%	2 / 3 66.7%	0 / 1 0.0%	0 / 3 0.0%	0 / 1 0.0%	1 / 3 33.3%	0 / 1 0.0%	1 / 3 33.3%	2 / 5 40.0%	0 / 4 0.0%	0 / 1 0.0%	1 / 6 16.7%	0 / 0 0.0%	0 / 0 0.0%	0 / 0 0.0%	0 / 0 0.0%	27 / 56 48.2%E	16 / 56 28.6%	10 / 56 17.9%AB	2 / 56 3.6%ABE	1 / 56 1.8%C		
1	Corynebacterium efficiens DSM 44549	12 / 56 21.4%	0 / 2 0.0%	0 / 2 0.0%	0 / 1 0.0%	1 / 1 100.0%	1 / 5 20.0%	2 / 4 50.0%	0 / 1 0.0%	0 / 1 0.0%	0 / 3 0.0%	0 / 1 0.0%	1 / 4 25.0%	2 / 3 66.7%	0 / 1 0.0%	0 / 3 0.0%	0 / 1 0.0%	1 / 3 33.3%	0 / 1 0.0%	1 / 3 33.3%	2 / 5 40.0%	0 / 4 0.0%	0 / 1 0.0%	1 / 6 16.7%	0 / 0 0.0%	0 / 0 0.0%	0 / 0 0.0%	0 / 0 0.0%	27 / 56 48.2%E	16 / 56 28.6%	10 / 56 17.9%AB	2 / 56 3.6%ABE	1 / 56 1.8%C		
1	CCUG 35717	13 / 46 28.3%	1 / 1 100.0%	0 / 1 0.0%	0 / 1 0.0%	1 / 1 100.0%	3 / 4 75.0%	1 / 3 33.3%	1 / 1 100.0%	0 / 1 0.0%	0 / 3 0.0%	0 / 1 0.0%	2 / 4 50.0%	0 / 2 0.0%	0 / 1 0.0%	1 / 2 50.0%	0 / 1 0.0%	1 / 3 33.3%	0 / 1 0.0%	1 / 3 33.3%	1 / 4 25.0%	0 / 2 0.0%	0 / 1 0.0%	0 / 5 0.0%	0 / 0 0.0%	0 / 0 0.0%	0 / 0 0.0%	0 / 0 0.0%	17 / 46 37.0%E	14 / 46 30.4%	7 / 46 15.2%AB	6 / 46 13.0%ABE	1 / 46 2.2%C	1 / 46 2.2%CE	
1	Corynebacterium kroppenstedtii CCUG 35717	13 / 46 28.3%	1 / 1 100.0%	0 / 1 0.0%	0 / 1 0.0%	1 / 1 100.0%	3 / 4 75.0%	1 / 3 33.3%	1 / 1 100.0%	0 / 1 0.0%	0 / 3 0.0%	0 / 1 0.0%	2 / 4 50.0%	0 / 2 0.0%	0 / 1 0.0%	1 / 2 50.0%	0 / 1 0.0%	1 / 3 33.3%	0 / 1 0.0%	1 / 3 33.3%	1 / 4 25.0%	0 / 2 0.0%	0 / 1 0.0%	0 / 5 0.0%	0 / 0 0.0%	0 / 0 0.0%	0 / 0 0.0%	0 / 0 0.0%	17 / 46 37.0%E	14 / 46 30.4%	7 / 46 15.2%AB	6 / 46 13.0%ABE	1 / 46 2.2%CE	1 / 46 2.2%C	
1	CCUG 47449	23 / 54 42.6%	1 / 1 100.0%	1 / 2 50.0%	0 / 1 0.0%	1 / 1 100.0%	5 / 5 100.0%	2 / 4 50.0%	0 / 1 0.0%	0 / 1 0.0%	1 / 4 25.0%	1 / 1 100.0%	3 / 4 75.0%	0 / 2 0.0%	0 / 1 0.0%	0 / 3 0.0%	0 / 1 0.0%	1 / 3 33.3%	0 / 1 0.0%	2 / 3 66.7%	1 / 4 25.0%	2 / 3 66.7%	0 / 1 0.0%	1 / 6 16.7%	1 / 1 100.0%	0 / 0 0.0%	0 / 0 0.0%	0 / 0 0.0%	15 / 54 27.8%E	13 / 54 24.1%	12 / 54 22.2%AB	11 / 54 20.4%ABE	2 / 54 3.7%C	1 / 54 1.9%CE	
1	Corynebacterium aurimucosum ATCC 700975	23 / 54 42.6%	1 / 1 100.0%	1 / 2 50.0%	0 / 1 0.0%	1 / 1 100.0%	5 / 5 100.0%	2 / 4 50.0%	0 / 1 0.0%	0 / 1 0.0%	1 / 4 25.0%	1 / 1 100.0%	3 / 4 75.0%	0 / 2 0.0%	0 / 1 0.0%	0 / 3 0.0%	0 / 1 0.0%	1 / 3 33.3%	0 / 1 0.0%	2 / 3 66.7%	1 / 4 25.0%	2 / 3 66.7%	0 / 1 0.0%	1 / 6 16.7%	1 / 1 100.0%	0 / 0 0.0%	0 / 0 0.0%	0 / 0 0.0%	15 / 54 27.8%E	13 / 54 24.1%	12 / 54 22.2%AB	11 / 54 20.4%ABE	2 / 54 3.7%C	1 / 54 1.9%CE	
1	CCTCC AA 001024	15 / 53 28.3%	1 / 1 100.0%	0 / 2 0.0%	0 / 1 0.0%	1 / 1 100.0%	2 / 5 40.0%	1 / 4 25.0%	1 / 1 100.0%	1 / 1 100.0%	0 / 4 0.0%	0 / 1 0.0%	2 / 4 50.0%	0 / 2 0.0%	0 / 1 0.0%	0 / 2 0.0%	0 / 1 0.0%	1 / 3 33.3%	0 / 1 0.0%	1 / 3 33.3%	1 / 5 20.0%	0 / 3 0.0%	0 / 1 0.0%	2 / 5 40.0%	1 / 1 100.0%	0 / 0 0.0%	0 / 0 0.0%	0 / 0 0.0%	18 / 53 34.0%	12 / 53 22.6%E	9 / 53 17.0%ABE	6 / 53 11.3%AB	5 / 53 9.4%CE	3 / 53 5.7%C	
1	Corynebacterium halotolerans DSM 44683	15 / 53 28.3%	1 / 1 100.0%	0 / 2 0.0%	0 / 1 0.0%	1 / 1 100.0%	2 / 5 40.0%	1 / 4 25.0%	1 / 1 100.0%	1 / 1 100.0%	0 / 4 0.0%	0 / 1 0.0%	2 / 4 50.0%	0 / 2 0.0%	0 / 1 0.0%	0 / 2 0.0%	0 / 1 0.0%	1 / 3 33.3%	0 / 1 0.0%	1 / 3 33.3%	1 / 5 20.0%	0 / 3 0.0%	0 / 1 0.0%	2 / 5 40.0%	1 / 1 100.0%	0 / 0 0.0%	0 / 0 0.0%	0 / 0 0.0%	18 / 53 34.0%	12 / 53 22.6%E	9 / 53 17.0%ABE	6 / 53 11.3%AB	5 / 53 9.4%CE	3 / 53 5.7%C	
1	CCUG 50093	24 / 50 48.0%	0 / 1 0.0%	1 / 2 50.0%	0 / 1 0.0%	1 / 1 100.0%	4 / 5 80.0%	3 / 4 75.0%	1 / 1 100.0%	1 / 1 100.0%	0 / 2 0.0%	0 / 1 0.0%	3 / 4 75.0%	0 / 2 0.0%	0 / 1 0.0%	0 / 2 0.0%	0 / 1 0.0%	2 / 3 66.7%	1 / 1 100.0%	2 / 3 66.7%	2 / 5 40.0%	1 / 3 33.3%	1 / 1 100.0%	1 / 5 20.0%	0 / 0 0.0%	0 / 0 0.0%	0 / 0 0.0%	0 / 0 0.0%	13 / 50 26.0%AB	13 / 50 26.0%E	13 / 50 26.0%	11 / 50 22.0%ABE			
1	Corynebacterium resistens DSM 45100	24 / 50 48.0%	0 / 1 0.0%	1 / 2 50.0%	0 / 1 0.0%	1 / 1 100.0%	4 / 5 80.0%	3 / 4 75.0%	1 / 1 100.0%	1 / 1 100.0%	0 / 2 0.0%	0 / 1 0.0%	3 / 4 75.0%	0 / 2 0.0%	0 / 1 0.0%	0 / 2 0.0%	0 / 1 0.0%	2 / 3 66.7%	1 / 1 100.0%	2 / 3 66.7%	2 / 5 40.0%	1 / 3 33.3%	1 / 1 100.0%	1 / 5 20.0%	0 / 0 0.0%	0 / 0 0.0%	0 / 0 0.0%	0 / 0 0.0%	13 / 50 26.0%E	13 / 50 26.0%AB	13 / 50 26.0%	11 / 50 22.0%ABE			
58	Mycobacteriaceae	887 / 2763 32.1%	46 / 61 75.4%	54 / 61 88.5%	11 / 61 18.0%	6 / 62 9.7%	42 / 182 23.1%	83 / 182 45.6%	48 / 61 78.7%	48 / 61 78.7%	37 / 182 20.3%	3 / 61 4.9%	113 / 243 46.5%	38 / 123 30.9%	46 / 60 76.7%	8 / 122 6.6%	52 / 73 71.2%	70 / 183 38.3%	3 / 61 4.9%	68 / 180 37.8%	27 / 244 11.1%	3 / 121 2.5%	6 / 62 9.7%	63 / 305 20.7%	12 / 12 100.0%	0 / 0 0.0%	0 / 0 0.0%	0 / 0 0.0%	932 / 2763 33.7%	671 / 2763 24.3%E	482 / 2763 17.4%AB	385 / 2763 13.9%ABE	151 / 2763 5.5%CE	122 / 2763 4.4%C	12 / 2763 0.4%B	8 / 2763 0.3%BE
57	Mycobacterium	880 / 2718 32.4%	46 / 60 76.7%	54 / 60 90.0%	10 / 60 16.7%	6 / 61 9.8%	42 / 179 23.5%	82 / 179 45.8%	48 / 60 80.0%	48 / 60 80.0%	37 / 179 20.7%	3 / 60 5.0%	111 / 239 46.4%	37 / 121 30.6%	46 / 59 78.0%	8 / 120 6.7%	52 / 72 72.2%	70 / 180 38.9%	3 / 60 5.0%	68 / 177 38.4%	27 / 240 11.2%	3 / 119 2.5%	6 / 61 9.8%	61 / 300 20.3%	12 / 12 100.0%	0 / 0 0.0%	0 / 0 0.0%	0 / 0 0.0%	914 / 2718 33.6%	657 / 2718 24.2%E	478 / 2718 17.6%AB	382 / 2718 14.1%ABE	149 / 2718 5.5%CE	118 / 2718 4.3%C	12 / 2718 0.4%B	8 / 2718 0.3%BE
2	"Bacillus leprae" Hansen 1880	22 / 90 24.4%	2 / 2 100.0%	0 / 2 0.0%	0 / 2 0.0%	0 / 2 0.0%	2 / 6 33.3%	6 / 6 100.0%	2 / 2 100.0%	2 / 2 100.0%	0 / 6 0.0%	0 / 2 0.0%	2 / 8 25.0%	0 / 4 0.0%	0 / 2 0.0%	0 / 4 0.0%	0 / 2 0.0%	2 / 6 33.3%	0 / 2 0.0%	2 / 6 33.3%	2 / 8 25.0%	0 / 4 0.0%	0 / 2 0.0%	0 / 10 0.0%	0 / 0 0.0%	0 / 0 0.0%	0 / 0 0.0%	0 / 0 0.0%	30 / 90 33.3%	26 / 90 28.9%E	14 / 90 15.6%AB	8 / 90 8.9%ABE	6 / 90 6.7%CE	6 / 90 6.7%C		
1	Mycobacterium leprae Br4923	11 / 45 24.4%	1 / 1 100.0%	0 / 1 0.0%	0 / 1 0.0%	0 / 1 0.0%	1 / 3 33.3%	3 / 3 100.0%	1 / 1 100.0%	1 / 1 100.0%	0 / 3 0.0%	0 / 1 0.0%	1 / 4 25.0%	0 / 2 0.0%	0 / 1 0.0%	0 / 2 0.0%	0 / 1 0.0%	1 / 3 33.3%	0 / 1 0.0%	1 / 3 33.3%	1 / 4 25.0%	0 / 2 0.0%	0 / 1 0.0%	0 / 5 0.0%	0 / 0 0.0%	0 / 0 0.0%	0 / 0 0.0%	0 / 0 0.0%	15 / 45 33.3%	13 / 45 28.9%E	7 / 45 15.6%AB	4 / 45 8.9%ABE	3 / 45 6.7%C	3 / 45 6.7%CE		
2	"Bacillus smegmatis" Trevisan 1889	44 / 140 31.4%	1 / 3 33.3%	3 / 3 100.0%	0 / 3 0.0%	0 / 3 0.0%	4 / 9 44.4%	7 / 9 77.8%	0 / 3 0.0%	0 / 3 0.0%	1 / 9 11.1%	0 / 3 0.0%	5 / 12 41.7%	2 / 6 33.3%	1 / 3 33.3%	3 / 6 50.0%	3 / 6 50.0%	1 / 9 11.1%	0 / 3 0.0%	3 / 9 33.3%	1 / 12 8.3%	0 / 6 0.0%	0 / 3 0.0%	7 / 15 46.7%	2 / 2 100.0%	0 / 0 0.0%	0 / 0 0.0%	0 / 0 0.0%	49 / 140 35.0%	36 / 140 25.7%E	24 / 140 17.1%AB	20 / 140 14.3%ABE	7 / 140 5.0%CE	4 / 140 2.9%CE		
1	Mycobacterium smegmatis 'MC2 155'	26 / 94 27.7%	0 / 2 0.0%	2 / 2 100.0%	0 / 2 0.0%	0 / 2 0.0%	2 / 6 33.3%	4 / 6 66.7%	0 / 2 0.0%	0 / 2 0.0%	0 / 6 0.0%	0 / 2 0.0%	4 / 8 50.0%	2 / 4 50.0%	0 / 2 0.0%	2 / 4 50.0%	2 / 4 50.0%	0 / 6 0.0%	0 / 2 0.0%	2 / 6 33.3%	0 / 8 0.0%	0 / 4 0.0%	0 / 2 0.0%	4 / 10 40.0%	2 / 2 100.0%	0 / 0 0.0%	0 / 0 0.0%	0 / 0 0.0%	36 / 94 38.3%	26 / 94 27.7%E	14 / 94 14.9%AB	12 / 94 12.8%ABE	4 / 94 4.3%C	2 / 94 2.1%CE		
1	Mycobacterium smegmatis JS623	18 / 46 39.1%	1 / 1 100.0%	1 / 1 100.0%	0 / 1 0.0%	0 / 1 0.0%	2 / 3 66.7%	3 / 3 100.0%	0 / 1 0.0%	0 / 1 0.0%	1 / 3 33.3%	0 / 1 0.0%	1 / 4 25.0%	0 / 2 0.0%	1 / 1 100.0%	1 / 2 50.0%	1 / 2 50.0%	1 / 3 33.3%	0 / 1 0.0%	1 / 3 33.3%	1 / 4 25.0%	0 / 2 0.0%	0 / 1 0.0%	3 / 5 60.0%	0 / 0 0.0%	0 / 0 0.0%	0 / 0 0.0%	0 / 0 0.0%	13 / 46 28.3%	10 / 46 21.7%AB	10 / 46 21.7%E	8 / 46 17.4%ABE	3 / 46 6.5%C	2 / 46 4.3%CE		
1	"Mycobacterium balnei" Linell and Norden 1952	19 / 46 41.3%	1 / 1 100.0%	0 / 1 0.0%	1 / 1 100.0%	0 / 1 0.0%	2 / 3 66.7%	2 / 3 66.7%	1 / 1 100.0%	1 / 1 100.0%	0 / 3 0.0%	0 / 1 0.0%	2 / 4 50.0%	0 / 2 0.0%	1 / 1 100.0%	0 / 2 0.0%	1 / 1 100.0%	1 / 3 33.3%	0 / 1 0.0%	1 / 3 33.3%	3 / 4 75.0%	0 / 2 0.0%	0 / 1 0.0%	1 / 5 20.0%	1 / 1 100.0%	0 / 0 0.0%	0 / 0 0.0%	0 / 0 0.0%	13 / 46 28.3%E	11 / 46 23.9%	7 / 46 15.2%B	6 / 46 13.0%AB	3 / 46 6.5%BE	3 / 46 6.5%ABE	2 / 46 4.3%CE	1 / 46 2.2%C
1	Mycobacterium marinum M	19 / 46 41.3%	1 / 1 100.0%	0 / 1 0.0%	1 / 1 100.0%	0 / 1 0.0%	2 / 3 66.7%	2 / 3 66.7%	1 / 1 100.0%	1 / 1 100.0%	0 / 3 0.0%	0 / 1 0.0%	2 / 4 50.0%	0 / 2 0.0%	1 / 1 100.0%	0 / 2 0.0%	1 / 1 100.0%	1 / 3 33.3%	0 / 1 0.0%	1 / 3 33.3%	3 / 4 75.0%	0 / 2 0.0%	0 / 1 0.0%	1 / 5 20.0%	1 / 1 100.0%	0 / 0 0.0%	0 / 0 0.0%	0 / 0 0.0%	13 / 46 28.3%E	11 / 46 23.9%	7 / 46 15.2%B	6 / 46 13.0%AB	3 / 46 6.5%BE	3 / 46 6.5%ABE	2 / 46 4.3%CE	1 / 46 2.2%C
1	"Mycobacterium chubuense" Tsukamura 1973	15 / 46 32.6%	0 / 1 0.0%	1 / 1 100.0%	0 / 1 0.0%	0 / 1 0.0%	2 / 3 66.7%	2 / 3 66.7%	0 / 1 0.0%	0 / 1 0.0%	0 / 3 0.0%	0 / 1 0.0%	1 / 4 25.0%	2 / 2 100.0%	0 / 1 0.0%	1 / 2 50.0%	1 / 1 100.0%	1 / 3 33.3%	0 / 1 0.0%	0 / 3 0.0%	1 / 4 25.0%	1 / 2 50.0%	0 / 1 0.0%	1 / 5 20.0%	1 / 1 100.0%	0 / 0 0.0%	0 / 0 0.0%	0 / 0 0.0%	17 / 46 37.0%	12 / 46 26.1%E	8 / 46 17.4%AB	7 / 46 15.2%ABE	1 / 46 2.2%CE	1 / 46 2.2%C		
1	Mycobacterium chubuense NBB4	15 / 46 32.6%	0 / 1 0.0%	1 / 1 100.0%	0 / 1 0.0%	0 / 1 0.0%	2 / 3 66.7%	2 / 3 66.7%	0 / 1 0.0%	0 / 1 0.0%	0 / 3 0.0%	0 / 1 0.0%	1 / 4 25.0%	2 / 2 100.0%	0 / 1 0.0%	1 / 2 50.0%	1 / 1 100.0%	1 / 3 33.3%	0 / 1 0.0%	0 / 3 0.0%	1 / 4 25.0%	1 / 2 50.0%	0 / 1 0.0%	1 / 5 20.0%	1 / 1 100.0%	0 / 0 0.0%	0 / 0 0.0%	0 / 0 0.0%	17 / 46 37.0%	12 / 46 26.1%E	8 / 46 17.4%AB	7 / 46 15.2%ABE	1 / 46 2.2%CE	1 / 46 2.2%C		
2	ATCC 43909	33 / 92 35.9%	0 / 2 0.0%	2 / 2 100.0%	2 / 2 100.0%	0 / 2 0.0%	4 / 6 66.7%	3 / 6 50.0%	0 / 2 0.0%	2 / 2 100.0%	1 / 6 16.7%	0 / 2 0.0%	4 / 8 50.0%	4 / 4 100.0%	0 / 2 0.0%	2 / 4 50.0%	2 / 3 66.7%	2 / 6 33.3%	0 / 2 0.0%	4 / 6 66.7%	0 / 8 0.0%	0 / 4 0.0%	1 / 3 33.3%	0 / 10 0.0%	0 / 0 0.0%	0 / 0 0.0%	0 / 0 0.0%	0 / 0 0.0%	29 / 92 31.5%	23 / 92 25.0%E	20 / 92 21.7%AB	13 / 92 14.1%ABE	6 / 92 6.5%C	1 / 92 1.1%CE		
1	Mycobacterium gilvum Spyr1	17 / 47 36.2%	0 / 1 0.0%	1 / 1 100.0%	1 / 1 100.0%	0 / 1 0.0%	2 / 3 66.7%	1 / 3 33.3%	0 / 1 0.0%	1 / 1 100.0%	1 / 3 33.3%	0 / 1 0.0%	2 / 4 50.0%	2 / 2 100.0%	0 / 1 0.0%	1 / 2 50.0%	1 / 2 50.0%	1 / 3 33.3%	0 / 1 0.0%	2 / 3 66.7%	0 / 4 0.0%	0 / 2 0.0%	1 / 2 50.0%	0 / 5 0.0%	0 / 0 0.0%	0 / 0 0.0%	0 / 0 0.0%	0 / 0 0.0%	15 / 47 31.9%	12 / 47 25.5%E	10 / 47 21.3%AB	7 / 47 14.9%ABE	3 / 47 6.4%C			
1	Mycobacterium flavescens ATCC 700033	16 / 45 35.6%	0 / 1 0.0%	1 / 1 100.0%	1 / 1 100.0%	0 / 1 0.0%	2 / 3 66.7%	2 / 3 66.7%	0 / 1 0.0%	1 / 1 100.0%	0 / 3 0.0%	0 / 1 0.0%	2 / 4 50.0%	2 / 2 100.0%	0 / 1 0.0%	1 / 2 50.0%	1 / 1 100.0%	1 / 3 33.3%	0 / 1 0.0%	2 / 3 66.7%	0 / 4 0.0%	0 / 2 0.0%	0 / 1 0.0%	0 / 5 0.0%	0 / 0 0.0%	0 / 0 0.0%	0 / 0 0.0%	0 / 0 0.0%	14 / 45 31.1%	11 / 45 24.4%E	10 / 45 22.2%AB	6 / 45 13.3%ABE	3 / 45 6.7%C	1 / 45 2.2%CE		
1	ATCC 19423	19 / 45 42.2%	1 / 1 100.0%	0 / 1 0.0%	1 / 1 100.0%	0 / 1 0.0%	2 / 3 66.7%	2 / 3 66.7%	1 / 1 100.0%	1 / 1 100.0%	0 / 3 0.0%	0 / 1 0.0%	2 / 4 50.0%	0 / 2 0.0%	0 / 1 0.0%	0 / 2 0.0%	1 / 1 100.0%	2 / 3 66.7%	0 / 1 0.0%	2 / 3 66.7%	3 / 4 75.0%	0 / 2 0.0%	0 / 1 0.0%	1 / 5 20.0%	0 / 0 0.0%	0 / 0 0.0%	0 / 0 0.0%	0 / 0 0.0%	13 / 45 28.9%AB	12 / 45 26.7%	12 / 45 26.7%E	6 / 45 13.3%ABE	2 / 45 4.4%CE			
1	Mycobacterium ulcerans Agy99	19 / 45 42.2%	1 / 1 100.0%	0 / 1 0.0%	1 / 1 100.0%	0 / 1 0.0%	2 / 3 66.7%	2 / 3 66.7%	1 / 1 100.0%	1 / 1 100.0%	0 / 3 0.0%	0 / 1 0.0%	2 / 4 50.0%	0 / 2 0.0%	0 / 1 0.0%	0 / 2 0.0%	1 / 1 100.0%	2 / 3 66.7%	0 / 1 0.0%	2 / 3 66.7%	3 / 4 75.0%	0 / 2 0.0%	0 / 1 0.0%	1 / 5 20.0%	0 / 0 0.0%	0 / 0 0.0%	0 / 0 0.0%	0 / 0 0.0%	13 / 45 28.9%AB	12 / 45 26.7%E	12 / 45 26.7%	6 / 45 13.3%ABE	2 / 45 4.4%CE			
1	"Mycobacterium rhodesiae" Tsukamura et al. 1971	16 / 46 34.8%	0 / 1 0.0%	1 / 1 100.0%	0 / 1 0.0%	0 / 1 0.0%	1 / 3 33.3%	1 / 3 33.3%	0 / 1 0.0%	1 / 1 100.0%	0 / 3 0.0%	0 / 1 0.0%	2 / 4 50.0%	2 / 2 100.0%	1 / 1 100.0%	1 / 2 50.0%	0 / 2 0.0%	1 / 3 33.3%	0 / 1 0.0%	2 / 3 66.7%	2 / 4 50.0%	0 / 2 0.0%	1 / 1 100.0%	0 / 5 0.0%	0 / 0 0.0%	0 / 0 0.0%	0 / 0 0.0%	0 / 0 0.0%	14 / 46 30.4%	13 / 46 28.3%E	9 / 46 19.6%AB	7 / 46 15.2%ABE	2 / 46 4.3%CE	1 / 46 2.2%C		
1	Mycobacterium rhodesiae NBB3	16 / 46 34.8%	0 / 1 0.0%	1 / 1 100.0%	0 / 1 0.0%	0 / 1 0.0%	1 / 3 33.3%	1 / 3 33.3%	0 / 1 0.0%	1 / 1 100.0%	0 / 3 0.0%	0 / 1 0.0%	2 / 4 50.0%	2 / 2 100.0%	1 / 1 100.0%	1 / 2 50.0%	0 / 2 0.0%	1 / 3 33.3%	0 / 1 0.0%	2 / 3 66.7%	2 / 4 50.0%	0 / 2 0.0%	1 / 1 100.0%	0 / 5 0.0%	0 / 0 0.0%	0 / 0 0.0%	0 / 0 0.0%	0 / 0 0.0%	14 / 46 30.4%	13 / 46 28.3%E	9 / 46 19.6%AB	7 / 46 15.2%ABE	2 / 46 4.3%CE	1 / 46 2.2%C		
29	Mycobacterium complex	398 / 1388 28.7%	31 / 31 100.0%	31 / 31 100.0%	0 / 31 0.0%	0 / 32 0.0%	0 / 92 0.0%	30 / 92 32.6%	31 / 31 100.0%	31 / 31 100.0%	29 / 92 31.5%	0 / 31 0.0%	62 / 123 50.4%	1 / 62 1.6%	31 / 31 100.0%	0 / 62 0.0%	31 / 31 100.0%	31 / 93 33.3%	0 / 31 0.0%	28 / 89 31.5%	0 / 124 0.0%	0 / 62 0.0%	0 / 31 0.0%	31 / 155 20.0%	0 / 0 0.0%	0 / 0 0.0%	0 / 0 0.0%	0 / 0 0.0%	526 / 1388 37.9%	310 / 1388 22.3%E	215 / 1388 15.5%ABE	183 / 1388 13.2%AB	92 / 1388 6.6%CE	62 / 1388 4.5%C		
7	"Mycobacterium tuberculosis typus bovinus" Lehmann and Neumann 1907	91 / 317 28.7%	7 / 7 100.0%	7 / 7 100.0%	0 / 7 0.0%	0 / 8 0.0%	0 / 21 0.0%	7 / 21 33.3%	7 / 7 100.0%	7 / 7 100.0%	7 / 22 31.8%	0 / 7 0.0%	14 / 28 50.0%	0 / 14 0.0%	7 / 7 100.0%	0 / 14 0.0%	7 / 7 100.0%	7 / 21 33.3%	0 / 7 0.0%	7 / 21 33.3%	0 / 28 0.0%	0 / 14 0.0%	0 / 7 0.0%	7 / 35 20.0%	0 / 0 0.0%	0 / 0 0.0%	0 / 0 0.0%	0 / 0 0.0%	121 / 317 38.2%	70 / 317 22.1%E	49 / 317 15.5%ABE	42 / 317 13.2%AB	21 / 317 6.6%CE	14 / 317 4.4%C		
6	BCG	78 / 272 28.7%	6 / 6 100.0%	6 / 6 100.0%	0 / 6 0.0%	0 / 7 0.0%	0 / 18 0.0%	6 / 18 33.3%	6 / 6 100.0%	6 / 6 100.0%	6 / 19 31.6%	0 / 6 0.0%	12 / 24 50.0%	0 / 12 0.0%	6 / 6 100.0%	0 / 12 0.0%	6 / 6 100.0%	6 / 18 33.3%	0 / 6 0.0%	6 / 18 33.3%	0 / 24 0.0%	0 / 12 0.0%	0 / 6 0.0%	6 / 30 20.0%	0 / 0 0.0%	0 / 0 0.0%	0 / 0 0.0%	0 / 0 0.0%	104 / 272 38.2%	60 / 272 22.1%E	42 / 272 15.4%ABE	36 / 272 13.2%AB	18 / 272 6.6%CE	12 / 272 4.4%C		
1	Mycobacterium bovis BCG Pasteur 1173P2	13 / 47 27.7%	1 / 1 100.0%	1 / 1 100.0%	0 / 1 0.0%	0 / 2 0.0%	0 / 3 0.0%	1 / 3 33.3%	1 / 1 100.0%	1 / 1 100.0%	1 / 4 25.0%	0 / 1 0.0%	2 / 4 50.0%	0 / 2 0.0%	1 / 1 100.0%	0 / 2 0.0%	1 / 1 100.0%	1 / 3 33.3%	0 / 1 0.0%	1 / 3 33.3%	0 / 4 0.0%	0 / 2 0.0%	0 / 1 0.0%	1 / 5 20.0%	0 / 0 0.0%	0 / 0 0.0%	0 / 0 0.0%	0 / 0 0.0%	19 / 47 40.4%	10 / 47 21.3%E	7 / 47 14.9%ABE	6 / 47 12.8%AB	3 / 47 6.4%CE	2 / 47 4.3%C		
1	Mycobacterium bovis BCG Moreau RDJ	13 / 45 28.9%	1 / 1 100.0%	1 / 1 100.0%	0 / 1 0.0%	0 / 1 0.0%	0 / 3 0.0%	1 / 3 33.3%	1 / 1 100.0%	1 / 1 100.0%	1 / 3 33.3%	0 / 1 0.0%	2 / 4 50.0%	0 / 2 0.0%	1 / 1 100.0%	0 / 2 0.0%	1 / 1 100.0%	1 / 3 33.3%	0 / 1 0.0%	1 / 3 33.3%	0 / 4 0.0%	0 / 2 0.0%	0 / 1 0.0%	1 / 5 20.0%	0 / 0 0.0%	0 / 0 0.0%	0 / 0 0.0%	0 / 0 0.0%	17 / 45 37.8%	10 / 45 22.2%E	7 / 45 15.6%ABE	6 / 45 13.3%AB	3 / 45 6.7%CE	2 / 45 4.4%C		
1	Mycobacterium bovis BCG Tokyo 172	13 / 45 28.9%	1 / 1 100.0%	1 / 1 100.0%	0 / 1 0.0%	0 / 1 0.0%	0 / 3 0.0%	1 / 3 33.3%	1 / 1 100.0%	1 / 1 100.0%	1 / 3 33.3%	0 / 1 0.0%	2 / 4 50.0%	0 / 2 0.0%	1 / 1 100.0%	0 / 2 0.0%	1 / 1 100.0%	1 / 3 33.3%	0 / 1 0.0%	1 / 3 33.3%	0 / 4 0.0%	0 / 2 0.0%	0 / 1 0.0%	1 / 5 20.0%	0 / 0 0.0%	0 / 0 0.0%	0 / 0 0.0%	0 / 0 0.0%	17 / 45 37.8%	10 / 45 22.2%E	7 / 45 15.6%ABE	6 / 45 13.3%AB	3 / 45 6.7%CE	2 / 45 4.4%C		
1	Mycobacterium bovis BCG Mexico	13 / 45 28.9%	1 / 1 100.0%	1 / 1 100.0%	0 / 1 0.0%	0 / 1 0.0%	0 / 3 0.0%	1 / 3 33.3%	1 / 1 100.0%	1 / 1 100.0%	1 / 3 33.3%	0 / 1 0.0%	2 / 4 50.0%	0 / 2 0.0%	1 / 1 100.0%	0 / 2 0.0%	1 / 1 100.0%	1 / 3 33.3%	0 / 1 0.0%	1 / 3 33.3%	0 / 4 0.0%	0 / 2 0.0%	0 / 1 0.0%	1 / 5 20.0%	0 / 0 0.0%	0 / 0 0.0%	0 / 0 0.0%	0 / 0 0.0%	17 / 45 37.8%	10 / 45 22.2%E	7 / 45 15.6%ABE	6 / 45 13.3%AB	3 / 45 6.7%CE	2 / 45 4.4%C		
1	Mycobacterium bovis BCG str. Korea 1168P	13 / 45 28.9%	1 / 1 100.0%	1 / 1 100.0%	0 / 1 0.0%	0 / 1 0.0%	0 / 3 0.0%	1 / 3 33.3%	1 / 1 100.0%	1 / 1 100.0%	1 / 3 33.3%	0 / 1 0.0%	2 / 4 50.0%	0 / 2 0.0%	1 / 1 100.0%	0 / 2 0.0%	1 / 1 100.0%	1 / 3 33.3%	0 / 1 0.0%	1 / 3 33.3%	0 / 4 0.0%	0 / 2 0.0%	0 / 1 0.0%	1 / 5 20.0%	0 / 0 0.0%	0 / 0 0.0%	0 / 0 0.0%	0 / 0 0.0%	17 / 45 37.8%	10 / 45 22.2%E	7 / 45 15.6%ABE	6 / 45 13.3%AB	3 / 45 6.7%CE	2 / 45 4.4%C		
1	Mycobacterium bovis AF2122/97	13 / 45 28.9%	1 / 1 100.0%	1 / 1 100.0%	0 / 1 0.0%	0 / 1 0.0%	0 / 3 0.0%	1 / 3 33.3%	1 / 1 100.0%	1 / 1 100.0%	1 / 3 33.3%	0 / 1 0.0%	2 / 4 50.0%	0 / 2 0.0%	1 / 1 100.0%	0 / 2 0.0%	1 / 1 100.0%	1 / 3 33.3%	0 / 1 0.0%	1 / 3 33.3%	0 / 4 0.0%	0 / 2 0.0%	0 / 1 0.0%	1 / 5 20.0%	0 / 0 0.0%	0 / 0 0.0%	0 / 0 0.0%	0 / 0 0.0%	17 / 45 37.8%	10 / 45 22.2%E	7 / 45 15.6%ABE	6 / 45 13.3%AB	3 / 45 6.7%CE	2 / 45 4.4%C		
20	"Bacillus tuberculosis" (Zopf 1883) Klein 1884	279 / 981 28.4%	22 / 22 100.0%	22 / 22 100.0%	0 / 22 0.0%	0 / 22 0.0%	0 / 65 0.0%	21 / 65 32.3%	22 / 22 100.0%	22 / 22 100.0%	20 / 64 31.2%	0 / 22 0.0%	44 / 87 50.6%	0 / 44 0.0%	22 / 22 100.0%	0 / 44 0.0%	22 / 22 100.0%	22 / 66 33.3%	0 / 22 0.0%	18 / 62 29.0%	0 / 88 0.0%	0 / 44 0.0%	0 / 22 0.0%	22 / 110 20.0%	0 / 0 0.0%	0 / 0 0.0%	0 / 0 0.0%	0 / 0 0.0%	374 / 981 38.1%	220 / 981 22.4%E	152 / 981 15.5%ABE	127 / 981 12.9%AB	65 / 981 6.6%CE	43 / 981 4.4%C		
1	Mycobacterium tuberculosis CDC1551	13 / 45 28.9%	1 / 1 100.0%	1 / 1 100.0%	0 / 1 0.0%	0 / 1 0.0%	0 / 3 0.0%	1 / 3 33.3%	1 / 1 100.0%	1 / 1 100.0%	1 / 3 33.3%	0 / 1 0.0%	2 / 4 50.0%	0 / 2 0.0%	1 / 1 100.0%	0 / 2 0.0%	1 / 1 100.0%	1 / 3 33.3%	0 / 1 0.0%	1 / 3 33.3%	0 / 4 0.0%	0 / 2 0.0%	0 / 1 0.0%	1 / 5 20.0%	0 / 0 0.0%	0 / 0 0.0%	0 / 0 0.0%	0 / 0 0.0%	17 / 45 37.8%	10 / 45 22.2%E	7 / 45 15.6%ABE	6 / 45 13.3%AB	3 / 45 6.7%CE	2 / 45 4.4%C		
1	Mycobacterium sp. H37Rv	26 / 90 28.9%	2 / 2 100.0%	2 / 2 100.0%	0 / 2 0.0%	0 / 2 0.0%	0 / 6 0.0%	2 / 6 33.3%	2 / 2 100.0%	2 / 2 100.0%	2 / 6 33.3%	0 / 2 0.0%	4 / 8 50.0%	0 / 4 0.0%	2 / 2 100.0%	0 / 4 0.0%	2 / 2 100.0%	2 / 6 33.3%	0 / 2 0.0%	2 / 6 33.3%	0 / 8 0.0%	0 / 4 0.0%	0 / 2 0.0%	2 / 10 20.0%	0 / 0 0.0%	0 / 0 0.0%	0 / 0 0.0%	0 / 0 0.0%	34 / 90 37.8%	20 / 90 22.2%E	14 / 90 15.6%ABE	12 / 90 13.3%AB	6 / 90 6.7%CE	4 / 90 4.4%C		
1	Mycobacterium tuberculosis F11	13 / 45 28.9%	1 / 1 100.0%	1 / 1 100.0%	0 / 1 0.0%	0 / 1 0.0%	0 / 3 0.0%	1 / 3 33.3%	1 / 1 100.0%	1 / 1 100.0%	1 / 3 33.3%	0 / 1 0.0%	2 / 4 50.0%	0 / 2 0.0%	1 / 1 100.0%	0 / 2 0.0%	1 / 1 100.0%	1 / 3 33.3%	0 / 1 0.0%	1 / 3 33.3%	0 / 4 0.0%	0 / 2 0.0%	0 / 1 0.0%	1 / 5 20.0%	0 / 0 0.0%	0 / 0 0.0%	0 / 0 0.0%	0 / 0 0.0%	17 / 45 37.8%	10 / 45 22.2%E	7 / 45 15.6%ABE	6 / 45 13.3%AB	3 / 45 6.7%CE	2 / 45 4.4%C		
1	Mycobacterium tuberculosis Haarlem	10 / 40 25.0%	1 / 1 100.0%	1 / 1 100.0%	0 / 1 0.0%	0 / 1 0.0%	0 / 2 0.0%	0 / 2 0.0%	1 / 1 100.0%	1 / 1 100.0%	0 / 2 0.0%	0 / 1 0.0%	2 / 3 66.7%	0 / 2 0.0%	1 / 1 100.0%	0 / 2 0.0%	1 / 1 100.0%	1 / 3 33.3%	0 / 1 0.0%	0 / 2 0.0%	0 / 4 0.0%	0 / 2 0.0%	0 / 1 0.0%	1 / 5 20.0%	0 / 0 0.0%	0 / 0 0.0%	0 / 0 0.0%	0 / 0 0.0%	17 / 40 42.5%	10 / 40 25.0%E	6 / 40 15.0%ABE	4 / 40 10.0%AB	2 / 40 5.0%CE	1 / 40 2.5%C		
1	Mycobacterium tuberculosis str. Beijing/NITR202	10 / 40 25.0%	1 / 1 100.0%	1 / 1 100.0%	0 / 1 0.0%	0 / 1 0.0%	0 / 2 0.0%	0 / 2 0.0%	1 / 1 100.0%	1 / 1 100.0%	0 / 2 0.0%	0 / 1 0.0%	2 / 3 66.7%	0 / 2 0.0%	1 / 1 100.0%	0 / 2 0.0%	1 / 1 100.0%	1 / 3 33.3%	0 / 1 0.0%	0 / 2 0.0%	0 / 4 0.0%	0 / 2 0.0%	0 / 1 0.0%	1 / 5 20.0%	0 / 0 0.0%	0 / 0 0.0%	0 / 0 0.0%	0 / 0 0.0%	17 / 40 42.5%	10 / 40 25.0%E	6 / 40 15.0%ABE	4 / 40 10.0%AB	2 / 40 5.0%CE	1 / 40 2.5%C		
1	Mycobacterium tuberculosis ATCC 25177	13 / 45 28.9%	1 / 1 100.0%	1 / 1 100.0%	0 / 1 0.0%	0 / 1 0.0%	0 / 3 0.0%	1 / 3 33.3%	1 / 1 100.0%	1 / 1 100.0%	1 / 3 33.3%	0 / 1 0.0%	2 / 4 50.0%	0 / 2 0.0%	1 / 1 100.0%	0 / 2 0.0%	1 / 1 100.0%	1 / 3 33.3%	0 / 1 0.0%	1 / 3 33.3%	0 / 4 0.0%	0 / 2 0.0%	0 / 1 0.0%	1 / 5 20.0%	0 / 0 0.0%	0 / 0 0.0%	0 / 0 0.0%	0 / 0 0.0%	17 / 45 37.8%	10 / 45 22.2%E	7 / 45 15.6%ABE	6 / 45 13.3%AB	3 / 45 6.7%CE	2 / 45 4.4%C		
1	Mycobacterium tuberculosis CCDC5079	26 / 90 28.9%	2 / 2 100.0%	2 / 2 100.0%	0 / 2 0.0%	0 / 2 0.0%	0 / 6 0.0%	2 / 6 33.3%	2 / 2 100.0%	2 / 2 100.0%	2 / 6 33.3%	0 / 2 0.0%	4 / 8 50.0%	0 / 4 0.0%	2 / 2 100.0%	0 / 4 0.0%	2 / 2 100.0%	2 / 6 33.3%	0 / 2 0.0%	2 / 6 33.3%	0 / 8 0.0%	0 / 4 0.0%	0 / 2 0.0%	2 / 10 20.0%	0 / 0 0.0%	0 / 0 0.0%	0 / 0 0.0%	0 / 0 0.0%	34 / 90 37.8%	20 / 90 22.2%E	14 / 90 15.6%ABE	12 / 90 13.3%AB	6 / 90 6.7%CE	4 / 90 4.4%C		
1	Mycobacterium tuberculosis CCDC5180	13 / 45 28.9%	1 / 1 100.0%	1 / 1 100.0%	0 / 1 0.0%	0 / 1 0.0%	0 / 3 0.0%	1 / 3 33.3%	1 / 1 100.0%	1 / 1 100.0%	1 / 3 33.3%	0 / 1 0.0%	2 / 4 50.0%	0 / 2 0.0%	1 / 1 100.0%	0 / 2 0.0%	1 / 1 100.0%	1 / 3 33.3%	0 / 1 0.0%	1 / 3 33.3%	0 / 4 0.0%	0 / 2 0.0%	0 / 1 0.0%	1 / 5 20.0%	0 / 0 0.0%	0 / 0 0.0%	0 / 0 0.0%	0 / 0 0.0%	17 / 45 37.8%	10 / 45 22.2%E	7 / 45 15.6%ABE	6 / 45 13.3%AB	3 / 45 6.7%CE	2 / 45 4.4%C		
1	Mycobacterium tuberculosis KZN 4207	13 / 45 28.9%	1 / 1 100.0%	1 / 1 100.0%	0 / 1 0.0%	0 / 1 0.0%	0 / 3 0.0%	1 / 3 33.3%	1 / 1 100.0%	1 / 1 100.0%	1 / 3 33.3%	0 / 1 0.0%	2 / 4 50.0%	0 / 2 0.0%	1 / 1 100.0%	0 / 2 0.0%	1 / 1 100.0%	1 / 3 33.3%	0 / 1 0.0%	1 / 3 33.3%	0 / 4 0.0%	0 / 2 0.0%	0 / 1 0.0%	1 / 5 20.0%	0 / 0 0.0%	0 / 0 0.0%	0 / 0 0.0%	0 / 0 0.0%	17 / 45 37.8%	10 / 45 22.2%E	7 / 45 15.6%ABE	6 / 45 13.3%AB	3 / 45 6.7%CE	2 / 45 4.4%C		
1	Mycobacterium tuberculosis KZN 1435	13 / 45 28.9%	1 / 1 100.0%	1 / 1 100.0%	0 / 1 0.0%	0 / 1 0.0%	0 / 3 0.0%	1 / 3 33.3%	1 / 1 100.0%	1 / 1 100.0%	1 / 3 33.3%	0 / 1 0.0%	2 / 4 50.0%	0 / 2 0.0%	1 / 1 100.0%	0 / 2 0.0%	1 / 1 100.0%	1 / 3 33.3%	0 / 1 0.0%	1 / 3 33.3%	0 / 4 0.0%	0 / 2 0.0%	0 / 1 0.0%	1 / 5 20.0%	0 / 0 0.0%	0 / 0 0.0%	0 / 0 0.0%	0 / 0 0.0%	17 / 45 37.8%	10 / 45 22.2%E	7 / 45 15.6%ABE	6 / 45 13.3%AB	3 / 45 6.7%CE	2 / 45 4.4%C		
1	Mycobacterium tuberculosis KZN 605	13 / 45 28.9%	1 / 1 100.0%	1 / 1 100.0%	0 / 1 0.0%	0 / 1 0.0%	0 / 3 0.0%	1 / 3 33.3%	1 / 1 100.0%	1 / 1 100.0%	1 / 3 33.3%	0 / 1 0.0%	2 / 4 50.0%	0 / 2 0.0%	1 / 1 100.0%	0 / 2 0.0%	1 / 1 100.0%	1 / 3 33.3%	0 / 1 0.0%	1 / 3 33.3%	0 / 4 0.0%	0 / 2 0.0%	0 / 1 0.0%	1 / 5 20.0%	0 / 0 0.0%	0 / 0 0.0%	0 / 0 0.0%	0 / 0 0.0%	17 / 45 37.8%	10 / 45 22.2%E	7 / 45 15.6%ABE	6 / 45 13.3%AB	3 / 45 6.7%CE	2 / 45 4.4%C		
1	Mycobacterium tuberculosis ATCC 35801	13 / 45 28.9%	1 / 1 100.0%	1 / 1 100.0%	0 / 1 0.0%	0 / 1 0.0%	0 / 3 0.0%	1 / 3 33.3%	1 / 1 100.0%	1 / 1 100.0%	1 / 3 33.3%	0 / 1 0.0%	2 / 4 50.0%	0 / 2 0.0%	1 / 1 100.0%	0 / 2 0.0%	1 / 1 100.0%	1 / 3 33.3%	0 / 1 0.0%	1 / 3 33.3%	0 / 4 0.0%	0 / 2 0.0%	0 / 1 0.0%	1 / 5 20.0%	0 / 0 0.0%	0 / 0 0.0%	0 / 0 0.0%	0 / 0 0.0%	17 / 45 37.8%	10 / 45 22.2%E	7 / 45 15.6%ABE	6 / 45 13.3%AB	3 / 45 6.7%CE	2 / 45 4.4%C		
1	Mycobacterium tuberculosis CTRI-2	13 / 45 28.9%	1 / 1 100.0%	1 / 1 100.0%	0 / 1 0.0%	0 / 1 0.0%	0 / 3 0.0%	1 / 3 33.3%	1 / 1 100.0%	1 / 1 100.0%	1 / 3 33.3%	0 / 1 0.0%	2 / 4 50.0%	0 / 2 0.0%	1 / 1 100.0%	0 / 2 0.0%	1 / 1 100.0%	1 / 3 33.3%	0 / 1 0.0%	1 / 3 33.3%	0 / 4 0.0%	0 / 2 0.0%	0 / 1 0.0%	1 / 5 20.0%	0 / 0 0.0%	0 / 0 0.0%	0 / 0 0.0%	0 / 0 0.0%	17 / 45 37.8%	10 / 45 22.2%E	7 / 45 15.6%ABE	6 / 45 13.3%AB	3 / 45 6.7%CE	2 / 45 4.4%C		
1	Mycobacterium tuberculosis RGTB327	13 / 45 28.9%	1 / 1 100.0%	1 / 1 100.0%	0 / 1 0.0%	0 / 1 0.0%	0 / 3 0.0%	1 / 3 33.3%	1 / 1 100.0%	1 / 1 100.0%	1 / 3 33.3%	0 / 1 0.0%	2 / 4 50.0%	0 / 2 0.0%	1 / 1 100.0%	0 / 2 0.0%	1 / 1 100.0%	1 / 3 33.3%	0 / 1 0.0%	1 / 3 33.3%	0 / 4 0.0%	0 / 2 0.0%	0 / 1 0.0%	1 / 5 20.0%	0 / 0 0.0%	0 / 0 0.0%	0 / 0 0.0%	0 / 0 0.0%	17 / 45 37.8%	10 / 45 22.2%E	7 / 45 15.6%ABE	6 / 45 13.3%AB	3 / 45 6.7%CE	2 / 45 4.4%C		
1	Mycobacterium tuberculosis RGTB423	13 / 45 28.9%	1 / 1 100.0%	1 / 1 100.0%	0 / 1 0.0%	0 / 1 0.0%	0 / 3 0.0%	1 / 3 33.3%	1 / 1 100.0%	1 / 1 100.0%	1 / 3 33.3%	0 / 1 0.0%	2 / 4 50.0%	0 / 2 0.0%	1 / 1 100.0%	0 / 2 0.0%	1 / 1 100.0%	1 / 3 33.3%	0 / 1 0.0%	1 / 3 33.3%	0 / 4 0.0%	0 / 2 0.0%	0 / 1 0.0%	1 / 5 20.0%	0 / 0 0.0%	0 / 0 0.0%	0 / 0 0.0%	0 / 0 0.0%	17 / 45 37.8%	10 / 45 22.2%E	7 / 45 15.6%ABE	6 / 45 13.3%AB	3 / 45 6.7%CE	2 / 45 4.4%C		
1	Mycobacterium tuberculosis UT205	13 / 45 28.9%	1 / 1 100.0%	1 / 1 100.0%	0 / 1 0.0%	0 / 1 0.0%	0 / 3 0.0%	1 / 3 33.3%	1 / 1 100.0%	1 / 1 100.0%	1 / 3 33.3%	0 / 1 0.0%	2 / 4 50.0%	0 / 2 0.0%	1 / 1 100.0%	0 / 2 0.0%	1 / 1 100.0%	1 / 3 33.3%	0 / 1 0.0%	1 / 3 33.3%	0 / 4 0.0%	0 / 2 0.0%	0 / 1 0.0%	1 / 5 20.0%	0 / 0 0.0%	0 / 0 0.0%	0 / 0 0.0%	0 / 0 0.0%	17 / 45 37.8%	10 / 45 22.2%E	7 / 45 15.6%ABE	6 / 45 13.3%AB	3 / 45 6.7%CE	2 / 45 4.4%C		
1	Mycobacterium tuberculosis 7199-99	13 / 45 28.9%	1 / 1 100.0%	1 / 1 100.0%	0 / 1 0.0%	0 / 1 0.0%	0 / 3 0.0%	1 / 3 33.3%	1 / 1 100.0%	1 / 1 100.0%	1 / 3 33.3%	0 / 1 0.0%	2 / 4 50.0%	0 / 2 0.0%	1 / 1 100.0%	0 / 2 0.0%	1 / 1 100.0%	1 / 3 33.3%	0 / 1 0.0%	1 / 3 33.3%	0 / 4 0.0%	0 / 2 0.0%	0 / 1 0.0%	1 / 5 20.0%	0 / 0 0.0%	0 / 0 0.0%	0 / 0 0.0%	0 / 0 0.0%	17 / 45 37.8%	10 / 45 22.2%E	7 / 45 15.6%ABE	6 / 45 13.3%AB	3 / 45 6.7%CE	2 / 45 4.4%C		
1	Mycobacterium tuberculosis str. Beijing/NITR203	12 / 44 27.3%	1 / 1 100.0%	1 / 1 100.0%	0 / 1 0.0%	0 / 1 0.0%	0 / 3 0.0%	1 / 3 33.3%	1 / 1 100.0%	1 / 1 100.0%	1 / 3 33.3%	0 / 1 0.0%	2 / 4 50.0%	0 / 2 0.0%	1 / 1 100.0%	0 / 2 0.0%	1 / 1 100.0%	1 / 3 33.3%	0 / 1 0.0%	0 / 2 0.0%	0 / 4 0.0%	0 / 2 0.0%	0 / 1 0.0%	1 / 5 20.0%	0 / 0 0.0%	0 / 0 0.0%	0 / 0 0.0%	0 / 0 0.0%	17 / 44 38.6%	10 / 44 22.7%E	7 / 44 15.9%ABE	5 / 44 11.4%AB	3 / 44 6.8%CE	2 / 44 4.5%C		
1	Mycobacterium tuberculosis CAS	11 / 43 25.6%	1 / 1 100.0%	1 / 1 100.0%	0 / 1 0.0%	0 / 1 0.0%	0 / 3 0.0%	1 / 3 33.3%	1 / 1 100.0%	1 / 1 100.0%	0 / 2 0.0%	0 / 1 0.0%	2 / 4 50.0%	0 / 2 0.0%	1 / 1 100.0%	0 / 2 0.0%	1 / 1 100.0%	1 / 3 33.3%	0 / 1 0.0%	0 / 2 0.0%	0 / 4 0.0%	0 / 2 0.0%	0 / 1 0.0%	1 / 5 20.0%	0 / 0 0.0%	0 / 0 0.0%	0 / 0 0.0%	0 / 0 0.0%	17 / 43 39.5%	10 / 43 23.3%E	6 / 43 14.0%ABE	5 / 43 11.6%AB	3 / 43 7.0%CE	2 / 43 4.7%C		
1	Mycobacterium tuberculosis CAS/NITR204	11 / 43 25.6%	1 / 1 100.0%	1 / 1 100.0%	0 / 1 0.0%	0 / 1 0.0%	0 / 3 0.0%	1 / 3 33.3%	1 / 1 100.0%	1 / 1 100.0%	0 / 2 0.0%	0 / 1 0.0%	2 / 4 50.0%	0 / 2 0.0%	1 / 1 100.0%	0 / 2 0.0%	1 / 1 100.0%	1 / 3 33.3%	0 / 1 0.0%	0 / 2 0.0%	0 / 4 0.0%	0 / 2 0.0%	0 / 1 0.0%	1 / 5 20.0%	0 / 0 0.0%	0 / 0 0.0%	0 / 0 0.0%	0 / 0 0.0%	17 / 43 39.5%	10 / 43 23.3%E	6 / 43 14.0%ABE	5 / 43 11.6%AB	3 / 43 7.0%CE	2 / 43 4.7%C		
2	Mycobacterium tuberculosis EAI5	25 / 89 28.1%	2 / 2 100.0%	2 / 2 100.0%	0 / 2 0.0%	0 / 2 0.0%	0 / 6 0.0%	2 / 6 33.3%	2 / 2 100.0%	2 / 2 100.0%	2 / 6 33.3%	0 / 2 0.0%	4 / 8 50.0%	0 / 4 0.0%	2 / 2 100.0%	0 / 4 0.0%	2 / 2 100.0%	2 / 6 33.3%	0 / 2 0.0%	1 / 5 20.0%	0 / 8 0.0%	0 / 4 0.0%	0 / 2 0.0%	2 / 10 20.0%	0 / 0 0.0%	0 / 0 0.0%	0 / 0 0.0%	0 / 0 0.0%	34 / 89 38.2%	20 / 89 22.5%E	14 / 89 15.7%ABE	11 / 89 12.4%AB	6 / 89 6.7%CE	4 / 89 4.5%C		
1	Mycobacterium tuberculosis EAI5/NITR206	12 / 44 27.3%	1 / 1 100.0%	1 / 1 100.0%	0 / 1 0.0%	0 / 1 0.0%	0 / 3 0.0%	1 / 3 33.3%	1 / 1 100.0%	1 / 1 100.0%	1 / 3 33.3%	0 / 1 0.0%	2 / 4 50.0%	0 / 2 0.0%	1 / 1 100.0%	0 / 2 0.0%	1 / 1 100.0%	1 / 3 33.3%	0 / 1 0.0%	0 / 2 0.0%	0 / 4 0.0%	0 / 2 0.0%	0 / 1 0.0%	1 / 5 20.0%	0 / 0 0.0%	0 / 0 0.0%	0 / 0 0.0%	0 / 0 0.0%	17 / 44 38.6%	10 / 44 22.7%E	7 / 44 15.9%ABE	5 / 44 11.4%AB	3 / 44 6.8%CE	2 / 44 4.5%C		
1	ATCC 25420	13 / 45 28.9%	1 / 1 100.0%	1 / 1 100.0%	0 / 1 0.0%	0 / 1 0.0%	0 / 3 0.0%	1 / 3 33.3%	1 / 1 100.0%	1 / 1 100.0%	1 / 3 33.3%	0 / 1 0.0%	2 / 4 50.0%	0 / 2 0.0%	1 / 1 100.0%	0 / 2 0.0%	1 / 1 100.0%	1 / 3 33.3%	0 / 1 0.0%	1 / 3 33.3%	0 / 4 0.0%	0 / 2 0.0%	0 / 1 0.0%	1 / 5 20.0%	0 / 0 0.0%	0 / 0 0.0%	0 / 0 0.0%	0 / 0 0.0%	17 / 45 37.8%	10 / 45 22.2%E	7 / 45 15.6%ABE	6 / 45 13.3%AB	3 / 45 6.7%CE	2 / 45 4.4%C		
1	Mycobacterium africanum GM041182	13 / 45 28.9%	1 / 1 100.0%	1 / 1 100.0%	0 / 1 0.0%	0 / 1 0.0%	0 / 3 0.0%	1 / 3 33.3%	1 / 1 100.0%	1 / 1 100.0%	1 / 3 33.3%	0 / 1 0.0%	2 / 4 50.0%	0 / 2 0.0%	1 / 1 100.0%	0 / 2 0.0%	1 / 1 100.0%	1 / 3 33.3%	0 / 1 0.0%	1 / 3 33.3%	0 / 4 0.0%	0 / 2 0.0%	0 / 1 0.0%	1 / 5 20.0%	0 / 0 0.0%	0 / 0 0.0%	0 / 0 0.0%	0 / 0 0.0%	17 / 45 37.8%	10 / 45 22.2%E	7 / 45 15.6%ABE	6 / 45 13.3%AB	3 / 45 6.7%CE	2 / 45 4.4%C		
1	"Mycobacterium canetti" van Soolingen et al. 1997	15 / 45 33.3%	1 / 1 100.0%	1 / 1 100.0%	0 / 1 0.0%	0 / 1 0.0%	0 / 3 0.0%	1 / 3 33.3%	1 / 1 100.0%	1 / 1 100.0%	1 / 3 33.3%	0 / 1 0.0%	2 / 4 50.0%	1 / 2 50.0%	1 / 1 100.0%	0 / 2 0.0%	1 / 1 100.0%	1 / 3 33.3%	0 / 1 0.0%	2 / 3 66.7%	0 / 4 0.0%	0 / 2 0.0%	0 / 1 0.0%	1 / 5 20.0%	0 / 0 0.0%	0 / 0 0.0%	0 / 0 0.0%	0 / 0 0.0%	14 / 45 31.1%	10 / 45 22.2%E	8 / 45 17.8%AB	7 / 45 15.6%ABE	3 / 45 6.7%CE	3 / 45 6.7%C		
1	Mycobacterium canetti CIPT 140010059	15 / 45 33.3%	1 / 1 100.0%	1 / 1 100.0%	0 / 1 0.0%	0 / 1 0.0%	0 / 3 0.0%	1 / 3 33.3%	1 / 1 100.0%	1 / 1 100.0%	1 / 3 33.3%	0 / 1 0.0%	2 / 4 50.0%	1 / 2 50.0%	1 / 1 100.0%	0 / 2 0.0%	1 / 1 100.0%	1 / 3 33.3%	0 / 1 0.0%	2 / 3 66.7%	0 / 4 0.0%	0 / 2 0.0%	0 / 1 0.0%	1 / 5 20.0%	0 / 0 0.0%	0 / 0 0.0%	0 / 0 0.0%	0 / 0 0.0%	14 / 45 31.1%	10 / 45 22.2%E	8 / 45 17.8%AB	7 / 45 15.6%ABE	3 / 45 6.7%CE	3 / 45 6.7%C		
1	DSM 7251	14 / 46 30.4%	0 / 1 0.0%	1 / 1 100.0%	1 / 1 100.0%	0 / 1 0.0%	2 / 3 66.7%	3 / 3 100.0%	0 / 1 0.0%	0 / 1 0.0%	0 / 3 0.0%	0 / 1 0.0%	1 / 4 25.0%	1 / 2 50.0%	0 / 1 0.0%	1 / 2 50.0%	0 / 2 0.0%	1 / 3 33.3%	0 / 1 0.0%	1 / 3 33.3%	1 / 4 25.0%	0 / 2 0.0%	0 / 1 0.0%	1 / 5 20.0%	0 / 0 0.0%	0 / 0 0.0%	0 / 0 0.0%	0 / 0 0.0%	14 / 46 30.4%E	13 / 46 28.3%	11 / 46 23.9%AB	3 / 46 6.5%C	3 / 46 6.5%ABE	2 / 46 4.3%CE		
1	Mycobacterium sp. PYR-1	14 / 46 30.4%	0 / 1 0.0%	1 / 1 100.0%	1 / 1 100.0%	0 / 1 0.0%	2 / 3 66.7%	3 / 3 100.0%	0 / 1 0.0%	0 / 1 0.0%	0 / 3 0.0%	0 / 1 0.0%	1 / 4 25.0%	1 / 2 50.0%	0 / 1 0.0%	1 / 2 50.0%	0 / 2 0.0%	1 / 3 33.3%	0 / 1 0.0%	1 / 3 33.3%	1 / 4 25.0%	0 / 2 0.0%	0 / 1 0.0%	1 / 5 20.0%	0 / 0 0.0%	0 / 0 0.0%	0 / 0 0.0%	0 / 0 0.0%	14 / 46 30.4%E	13 / 46 28.3%	11 / 46 23.9%AB	3 / 46 6.5%ABE	3 / 46 6.5%C	2 / 46 4.3%CE		
8	MAC	142 / 363 39.1%	7 / 8 87.5%	8 / 8 100.0%	0 / 8 0.0%	3 / 8 37.5%	8 / 24 33.3%	9 / 24 37.5%	8 / 8 100.0%	8 / 8 100.0%	0 / 24 0.0%	0 / 8 0.0%	16 / 32 50.0%	11 / 16 68.8%	5 / 8 62.5%	0 / 16 0.0%	8 / 8 100.0%	20 / 24 83.3%	0 / 8 0.0%	10 / 24 41.7%	5 / 32 15.6%	0 / 16 0.0%	3 / 8 37.5%	10 / 40 25.0%	3 / 3 100.0%	0 / 0 0.0%	0 / 0 0.0%	0 / 0 0.0%	98 / 363 27.0%	81 / 363 22.3%E	80 / 363 22.0%AB	60 / 363 16.5%ABE	22 / 363 6.1%C	20 / 363 5.5%CE	2 / 363 0.6%BE	
3	"Bacillus tuberculosis gallinarum" Sternberg 1892	50 / 138 36.2%	2 / 3 66.7%	3 / 3 100.0%	0 / 3 0.0%	3 / 3 100.0%	3 / 9 33.3%	3 / 9 33.3%	3 / 3 100.0%	3 / 3 100.0%	0 / 9 0.0%	0 / 3 0.0%	6 / 12 50.0%	5 / 6 83.3%	0 / 3 0.0%	0 / 6 0.0%	3 / 3 100.0%	6 / 9 66.7%	0 / 3 0.0%	1 / 9 11.1%	3 / 12 25.0%	0 / 6 0.0%	0 / 3 0.0%	3 / 15 20.0%	3 / 3 100.0%	0 / 0 0.0%	0 / 0 0.0%	0 / 0 0.0%	33 / 138 23.9%	30 / 138 21.7%E	27 / 138 19.6%AB	21 / 138 15.2%ABE	15 / 138 10.9%C	10 / 138 7.2%CE	2 / 138 1.4%BE	
2	"Bacillus paratuberculosis" (Bergey et al. 1923) Krasil'nikov 1941	34 / 92 37.0%	2 / 2 100.0%	2 / 2 100.0%	0 / 2 0.0%	2 / 2 100.0%	2 / 6 33.3%	2 / 6 33.3%	2 / 2 100.0%	2 / 2 100.0%	0 / 6 0.0%	0 / 2 0.0%	4 / 8 50.0%	4 / 4 100.0%	0 / 2 0.0%	0 / 4 0.0%	2 / 2 100.0%	4 / 6 66.7%	0 / 2 0.0%	0 / 6 0.0%	2 / 8 25.0%	0 / 4 0.0%	0 / 2 0.0%	2 / 10 20.0%	2 / 2 100.0%	0 / 0 0.0%	0 / 0 0.0%	0 / 0 0.0%	22 / 92 23.9%	20 / 92 21.7%E	18 / 92 19.6%AB	15 / 92 16.3%ABE	10 / 92 10.9%C	6 / 92 6.5%CE	1 / 92 1.1%BE	
1	Mycobacterium avium subsp. paratuberculosis K-10	17 / 46 37.0%	1 / 1 100.0%	1 / 1 100.0%	0 / 1 0.0%	1 / 1 100.0%	1 / 3 33.3%	1 / 3 33.3%	1 / 1 100.0%	1 / 1 100.0%	0 / 3 0.0%	0 / 1 0.0%	2 / 4 50.0%	2 / 2 100.0%	0 / 1 0.0%	0 / 2 0.0%	1 / 1 100.0%	2 / 3 66.7%	0 / 1 0.0%	0 / 3 0.0%	1 / 4 25.0%	0 / 2 0.0%	0 / 1 0.0%	1 / 5 20.0%	1 / 1 100.0%	0 / 0 0.0%	0 / 0 0.0%	0 / 0 0.0%	11 / 46 23.9%	10 / 46 21.7%E	9 / 46 19.6%AB	7 / 46 15.2%ABE	5 / 46 10.9%C	3 / 46 6.5%CE	1 / 46 2.2%BE	
1	Mycobacterium avium subsp. paratuberculosis MAP4	17 / 46 37.0%	1 / 1 100.0%	1 / 1 100.0%	0 / 1 0.0%	1 / 1 100.0%	1 / 3 33.3%	1 / 3 33.3%	1 / 1 100.0%	1 / 1 100.0%	0 / 3 0.0%	0 / 1 0.0%	2 / 4 50.0%	2 / 2 100.0%	0 / 1 0.0%	0 / 2 0.0%	1 / 1 100.0%	2 / 3 66.7%	0 / 1 0.0%	0 / 3 0.0%	1 / 4 25.0%	0 / 2 0.0%	0 / 1 0.0%	1 / 5 20.0%	1 / 1 100.0%	0 / 0 0.0%	0 / 0 0.0%	0 / 0 0.0%	11 / 46 23.9%	10 / 46 21.7%E	9 / 46 19.6%AB	8 / 46 17.4%ABE	5 / 46 10.9%C	3 / 46 6.5%CE		
1	Mycobacterium avium 104	16 / 46 34.8%	0 / 1 0.0%	1 / 1 100.0%	0 / 1 0.0%	1 / 1 100.0%	1 / 3 33.3%	1 / 3 33.3%	1 / 1 100.0%	1 / 1 100.0%	0 / 3 0.0%	0 / 1 0.0%	2 / 4 50.0%	1 / 2 50.0%	0 / 1 0.0%	0 / 2 0.0%	1 / 1 100.0%	2 / 3 66.7%	0 / 1 0.0%	1 / 3 33.3%	1 / 4 25.0%	0 / 2 0.0%	0 / 1 0.0%	1 / 5 20.0%	1 / 1 100.0%	0 / 0 0.0%	0 / 0 0.0%	0 / 0 0.0%	11 / 46 23.9%	10 / 46 21.7%E	9 / 46 19.6%AB	6 / 46 13.0%ABE	5 / 46 10.9%C	4 / 46 8.7%CE	1 / 46 2.2%BE	
3	"Nocardia intracellularis" Cuttino and McCabe 1949	57 / 135 42.2%	3 / 3 100.0%	3 / 3 100.0%	0 / 3 0.0%	0 / 3 0.0%	3 / 9 33.3%	3 / 9 33.3%	3 / 3 100.0%	3 / 3 100.0%	0 / 9 0.0%	0 / 3 0.0%	6 / 12 50.0%	4 / 6 66.7%	3 / 3 100.0%	0 / 6 0.0%	3 / 3 100.0%	9 / 9 100.0%	0 / 3 0.0%	6 / 9 66.7%	1 / 12 8.3%	0 / 6 0.0%	2 / 3 66.7%	5 / 15 33.3%	0 / 0 0.0%	0 / 0 0.0%	0 / 0 0.0%	0 / 0 0.0%	39 / 135 28.9%	33 / 135 24.4%AB	30 / 135 22.2%E	24 / 135 17.8%ABE	6 / 135 4.4%CE	3 / 135 2.2%C		
1	Mycobacterium intracellulare ATCC 13950	19 / 45 42.2%	1 / 1 100.0%	1 / 1 100.0%	0 / 1 0.0%	0 / 1 0.0%	1 / 3 33.3%	1 / 3 33.3%	1 / 1 100.0%	1 / 1 100.0%	0 / 3 0.0%	0 / 1 0.0%	2 / 4 50.0%	1 / 2 50.0%	1 / 1 100.0%	0 / 2 0.0%	1 / 1 100.0%	3 / 3 100.0%	0 / 1 0.0%	2 / 3 66.7%	0 / 4 0.0%	0 / 2 0.0%	1 / 1 100.0%	2 / 5 40.0%	0 / 0 0.0%	0 / 0 0.0%	0 / 0 0.0%	0 / 0 0.0%	13 / 45 28.9%	11 / 45 24.4%AB	10 / 45 22.2%E	8 / 45 17.8%ABE	2 / 45 4.4%CE	1 / 45 2.2%C		
1	Mycobacterium intracellulare MOTT-02	19 / 45 42.2%	1 / 1 100.0%	1 / 1 100.0%	0 / 1 0.0%	0 / 1 0.0%	1 / 3 33.3%	1 / 3 33.3%	1 / 1 100.0%	1 / 1 100.0%	0 / 3 0.0%	0 / 1 0.0%	2 / 4 50.0%	1 / 2 50.0%	1 / 1 100.0%	0 / 2 0.0%	1 / 1 100.0%	3 / 3 100.0%	0 / 1 0.0%	2 / 3 66.7%	1 / 4 25.0%	0 / 2 0.0%	1 / 1 100.0%	1 / 5 20.0%	0 / 0 0.0%	0 / 0 0.0%	0 / 0 0.0%	0 / 0 0.0%	13 / 45 28.9%	11 / 45 24.4%AB	10 / 45 22.2%E	8 / 45 17.8%ABE	2 / 45 4.4%CE	1 / 45 2.2%C		
1	Mycobacterium intracellulare MOTT-64	19 / 45 [illegible]	1 / 1 [illegible]	1 / 1 [illegible]	0 / 1 [illegible]	0 / 1 [illegible]	1 / 3 [illegible]	1 / 3 [illegible]	1 / 1 [illegible]	1 / 1 [illegible]	0 / 3 [illegible]	0 / 1 [illegible]	2 / 4 [illegible]	2 / 2 [illegible]	1 / 1 [illegible]	0 / 2 [illegible]	1 / 1 [illegible]	3 / 3 [illegible]	0 / 1 [illegible]	2 / 3 [illegible]	0 / 4 [illegible]	0 / 2 [illegible]	0 / 1 [illegible]	2 / 5 [illegible]	0 / 0 [illegible]	0 / 0 [illegible]	0 / 0 [illegible]	0 / 0 [illegible]	13 / 45 [illegible]	11 / 45 [illegible]	10 / 45 [illegible]	8 / 45 [illegible]	2 / 45 [illegible]	1 / 45 [illegible]		

1	"Mycobacterium indicus pranii" Ahmed et al. 2007	17 / 45 37.8%	1 / 1 100.0%	1 / 1 100.0%	0 / 1 0.0%	0 / 1 0.0%	1 / 3 33.3%	2 / 3 66.7%	1 / 1 100.0%	1 / 1 100.0%	0 / 3 0.0%	0 / 1 0.0%	2 / 4 50.0%	1 / 2 50.0%	1 / 1 100.0%	0 / 2 0.0%	1 / 1 100.0%	2 / 3 66.7%	0 / 1 0.0%	2 / 3 66.7%	0 / 4 0.0%	0 / 2 0.0%	0 / 1 0.0%	1 / 5 20.0%	0 / 0 0.0%	0 / 0 0.0%	0 / 0 0.0%	0 / 0 0.0%	13 / 45 28.9%	11 / 45 24.4%E	10 / 45 22.2%AB	7 / 45 15.6%ABE	2 / 45 4.4%C	2 / 45 4.4%CE		
1	Mycobacterium indicus pranii MTCC 9506	17 / 45 37.8%	1 / 1 100.0%	1 / 1 100.0%	0 / 1 0.0%	0 / 1 0.0%	1 / 3 33.3%	2 / 3 66.7%	1 / 1 100.0%	1 / 1 100.0%	0 / 3 0.0%	0 / 1 0.0%	2 / 4 50.0%	1 / 2 50.0%	1 / 1 100.0%	0 / 2 0.0%	1 / 1 100.0%	2 / 3 66.7%	0 / 1 0.0%	2 / 3 66.7%	0 / 4 0.0%	0 / 2 0.0%	0 / 1 0.0%	1 / 5 20.0%	0 / 0 0.0%	0 / 0 0.0%	0 / 0 0.0%	0 / 0 0.0%	13 / 45 28.9%	11 / 45 24.4%E	10 / 45 22.2%AB	7 / 45 15.6%ABE	2 / 45 4.4%C	2 / 45 4.4%CE		
1	'Mycobacterium yongonense'	18 / 45 40.0%	1 / 1 100.0%	1 / 1 100.0%	0 / 1 0.0%	0 / 1 0.0%	1 / 3 33.3%	1 / 3 33.3%	1 / 1 100.0%	1 / 1 100.0%	0 / 3 0.0%	0 / 1 0.0%	2 / 4 50.0%	1 / 2 50.0%	1 / 1 100.0%	0 / 2 0.0%	1 / 1 100.0%	3 / 3 100.0%	0 / 1 0.0%	1 / 3 33.3%	1 / 4 25.0%	0 / 2 0.0%	1 / 1 100.0%	1 / 5 20.0%	0 / 0 0.0%	0 / 0 0.0%	0 / 0 0.0%	0 / 0 0.0%	13 / 45 28.9%	10 / 45 22.2%AB	10 / 45 22.2%E	8 / 45 17.8%ABE	2 / 45 4.4%C	2 / 45 4.4%CE		
1	Mycobacterium sp. 05-1390	18 / 45 40.0%	1 / 1 100.0%	1 / 1 100.0%	0 / 1 0.0%	0 / 1 0.0%	1 / 3 33.3%	1 / 3 33.3%	1 / 1 100.0%	1 / 1 100.0%	0 / 3 0.0%	0 / 1 0.0%	2 / 4 50.0%	1 / 2 50.0%	1 / 1 100.0%	0 / 2 0.0%	1 / 1 100.0%	3 / 3 100.0%	0 / 1 0.0%	1 / 3 33.3%	1 / 4 25.0%	0 / 2 0.0%	1 / 1 100.0%	1 / 5 20.0%	0 / 0 0.0%	0 / 0 0.0%	0 / 0 0.0%	0 / 0 0.0%	13 / 45 28.9%	10 / 45 22.2%AB	10 / 45 22.2%E	8 / 45 17.8%ABE	2 / 45 4.4%C	2 / 45 4.4%CE		
1	Mycobacterium sp. MCS	14 / 47 29.8%	0 / 1 0.0%	1 / 1 100.0%	0 / 1 0.0%	0 / 1 0.0%	2 / 3 66.7%	2 / 3 66.7%	0 / 1 0.0%	0 / 1 0.0%	0 / 3 0.0%	1 / 1 100.0%	1 / 4 25.0%	2 / 2 100.0%	1 / 1 100.0%	0 / 2 0.0%	0 / 2 0.0%	0 / 3 0.0%	0 / 1 0.0%	1 / 3 33.3%	1 / 4 25.0%	0 / 2 0.0%	0 / 1 0.0%	1 / 5 20.0%	1 / 1 100.0%	0 / 0 0.0%	0 / 0 0.0%	0 / 0 0.0%	15 / 47 31.9%E	14 / 47 29.8%	12 / 47 25.5%AB	3 / 47 6.4%CE	2 / 47 4.3%ABE	1 / 47 2.1%C		
1	Mycobacterium sp. JLS	15 / 47 31.9%	1 / 1 100.0%	1 / 1 100.0%	0 / 1 0.0%	0 / 1 0.0%	2 / 3 66.7%	2 / 3 66.7%	0 / 1 0.0%	0 / 1 0.0%	0 / 3 0.0%	1 / 1 100.0%	1 / 4 25.0%	2 / 2 100.0%	1 / 1 100.0%	0 / 2 0.0%	0 / 2 0.0%	0 / 3 0.0%	0 / 1 0.0%	1 / 3 33.3%	1 / 4 25.0%	0 / 2 0.0%	0 / 1 0.0%	1 / 5 20.0%	1 / 1 100.0%	0 / 0 0.0%	0 / 0 0.0%	0 / 0 0.0%	14 / 47 29.8%E	13 / 47 27.7%	12 / 47 25.5%AB	3 / 47 6.4%ABE	3 / 47 6.4%CE	2 / 47 4.3%C		
1	Mycobacterium sp. KMS	14 / 47 29.8%	0 / 1 0.0%	1 / 1 100.0%	0 / 1 0.0%	0 / 1 0.0%	2 / 3 66.7%	2 / 3 66.7%	0 / 1 0.0%	0 / 1 0.0%	0 / 3 0.0%	1 / 1 100.0%	1 / 4 25.0%	2 / 2 100.0%	1 / 1 100.0%	0 / 2 0.0%	0 / 2 0.0%	0 / 3 0.0%	0 / 1 0.0%	1 / 3 33.3%	1 / 4 25.0%	0 / 2 0.0%	0 / 1 0.0%	1 / 5 20.0%	1 / 1 100.0%	0 / 0 0.0%	0 / 0 0.0%	0 / 0 0.0%	15 / 47 31.9%E	14 / 47 29.8%	12 / 47 25.5%AB	3 / 47 6.4%CE	2 / 47 4.3%ABE	1 / 47 2.1%C		
1	"Mycobacterium liflandii" Trott et al. 2004	21 / 47 44.7%	1 / 1 100.0%	0 / 1 0.0%	1 / 1 100.0%	0 / 1 0.0%	2 / 3 66.7%	2 / 3 66.7%	1 / 1 100.0%	1 / 1 100.0%	0 / 3 0.0%	0 / 1 0.0%	2 / 4 50.0%	0 / 2 0.0%	0 / 1 0.0%	0 / 2 0.0%	1 / 1 100.0%	2 / 3 66.7%	0 / 1 0.0%	3 / 4 75.0%	3 / 4 75.0%	0 / 2 0.0%	0 / 1 0.0%	1 / 5 20.0%	1 / 1 100.0%	0 / 0 0.0%	0 / 0 0.0%	0 / 0 0.0%	14 / 47 29.8%AB	12 / 47 25.5%E	12 / 47 25.5%	7 / 47 14.9%ABE	2 / 47 4.3%CE			
1	Mycobacterium liflandii 128FXT	21 / 47 44.7%	1 / 1 100.0%	0 / 1 0.0%	1 / 1 100.0%	0 / 1 0.0%	2 / 3 66.7%	2 / 3 66.7%	1 / 1 100.0%	1 / 1 100.0%	0 / 3 0.0%	0 / 1 0.0%	2 / 4 50.0%	0 / 2 0.0%	0 / 1 0.0%	0 / 2 0.0%	1 / 1 100.0%	2 / 3 66.7%	0 / 1 0.0%	3 / 4 75.0%	3 / 4 75.0%	0 / 2 0.0%	0 / 1 0.0%	1 / 5 20.0%	1 / 1 100.0%	0 / 0 0.0%	0 / 0 0.0%	0 / 0 0.0%	14 / 47 29.8%AB	12 / 47 25.5%E	12 / 47 25.5%	7 / 47 14.9%ABE	2 / 47 4.3%CE			
3	Mycobacterium chelonae group	62 / 137 45.3%	0 / 3 0.0%	3 / 3 100.0%	3 / 3 100.0%	3 / 3 100.0%	6 / 9 66.7%	6 / 9 66.7%	3 / 3 100.0%	0 / 3 0.0%	6 / 9 66.7%	0 / 3 0.0%	5 / 12 41.7%	6 / 7 85.7%	2 / 2 100.0%	0 / 6 0.0%	3 / 6 50.0%	2 / 9 22.2%	3 / 3 100.0%	5 / 9 55.6%	1 / 12 8.3%	2 / 5 40.0%	0 / 3 0.0%	3 / 15 20.0%	0 / 0 0.0%	0 / 0 0.0%	0 / 0 0.0%	0 / 0 0.0%	40 / 137 29.2%AB	37 / 137 27.0%E	35 / 137 25.5%	14 / 137 10.2%ABE	5 / 137 3.6%B	3 / 137 2.2%BE	2 / 137 1.5%CE	1 / 137 0.7%C
3	Mycobacterium abscessus subgroup	62 / 137 45.3%	0 / 3 0.0%	3 / 3 100.0%	3 / 3 100.0%	3 / 3 100.0%	6 / 9 66.7%	6 / 9 66.7%	3 / 3 100.0%	0 / 3 0.0%	6 / 9 66.7%	0 / 3 0.0%	5 / 12 41.7%	6 / 7 85.7%	2 / 2 100.0%	0 / 6 0.0%	3 / 6 50.0%	2 / 9 22.2%	3 / 3 100.0%	5 / 9 55.6%	1 / 12 8.3%	2 / 5 40.0%	0 / 3 0.0%	3 / 15 20.0%	0 / 0 0.0%	0 / 0 0.0%	0 / 0 0.0%	0 / 0 0.0%	40 / 137 29.2%AB	37 / 137 27.0%E	35 / 137 25.5%	14 / 137 10.2%ABE	5 / 137 3.6%B	3 / 137 2.2%BE	2 / 137 1.5%CE	1 / 137 0.7%C
3	"Mycobacterium abcessus" Moore and Frerichs 1953	62 / 137 45.3%	0 / 3 0.0%	3 / 3 100.0%	3 / 3 100.0%	3 / 3 100.0%	6 / 9 66.7%	6 / 9 66.7%	3 / 3 100.0%	0 / 3 0.0%	6 / 9 66.7%	0 / 3 0.0%	5 / 12 41.7%	6 / 7 85.7%	2 / 2 100.0%	0 / 6 0.0%	3 / 6 50.0%	2 / 9 22.2%	3 / 3 100.0%	5 / 9 55.6%	1 / 12 8.3%	2 / 5 40.0%	0 / 3 0.0%	3 / 15 20.0%	0 / 0 0.0%	0 / 0 0.0%	0 / 0 0.0%	0 / 0 0.0%	40 / 137 29.2%AB	37 / 137 27.0%E	35 / 137 25.5%	14 / 137 10.2%ABE	5 / 137 3.6%B	3 / 137 2.2%BE	2 / 137 1.5%CE	1 / 137 0.7%C
2	"Mycobacterium abscessus subsp. massiliense" Leao et al. 2009	39 / 91 42.9%	0 / 2 0.0%	2 / 2 100.0%	2 / 2 100.0%	2 / 2 100.0%	4 / 6 66.7%	4 / 6 66.7%	2 / 2 100.0%	0 / 2 0.0%	4 / 6 66.7%	0 / 2 0.0%	3 / 8 37.5%	4 / 5 80.0%	1 / 1 100.0%	0 / 4 0.0%	2 / 4 50.0%	1 / 6 16.7%	2 / 2 100.0%	3 / 6 50.0%	0 / 8 0.0%	1 / 3 33.3%	0 / 2 0.0%	2 / 10 20.0%	0 / 0 0.0%	0 / 0 0.0%	0 / 0 0.0%	0 / 0 0.0%	29 / 91 31.9%AB	25 / 91 27.5%E	24 / 91 26.4%	10 / 91 11.0%ABE	2 / 91 2.2%CE	1 / 91 1.1%C		
1	Mycobacterium abscessus subsp. bolletii GO 06	19 / 45 42.2%	0 / 1 0.0%	1 / 1 100.0%	1 / 1 100.0%	1 / 1 100.0%	2 / 3 66.7%	2 / 3 66.7%	1 / 1 100.0%	0 / 1 0.0%	2 / 3 66.7%	0 / 1 0.0%	2 / 4 50.0%	2 / 3 66.7%	0 / 0 0.0%	0 / 2 0.0%	1 / 2 50.0%	0 / 3 0.0%	1 / 1 100.0%	2 / 3 66.7%	0 / 4 0.0%	0 / 1 0.0%	0 / 1 0.0%	1 / 5 20.0%	0 / 0 0.0%	0 / 0 0.0%	0 / 0 0.0%	0 / 0 0.0%	15 / 45 33.3%AB	13 / 45 28.9%E	12 / 45 26.7%	4 / 45 8.9%ABE	1 / 45 2.2%CE			
1	Mycobacterium abscessus subsp. bolletii 50594	20 / 46 43.5%	0 / 1 0.0%	1 / 1 100.0%	1 / 1 100.0%	1 / 1 100.0%	2 / 3 66.7%	2 / 3 66.7%	1 / 1 100.0%	0 / 1 0.0%	2 / 3 66.7%	0 / 1 0.0%	1 / 4 25.0%	2 / 2 100.0%	1 / 1 100.0%	0 / 2 0.0%	1 / 2 50.0%	1 / 3 33.3%	1 / 1 100.0%	1 / 3 33.3%	0 / 4 0.0%	1 / 2 50.0%	0 / 1 0.0%	1 / 5 20.0%	0 / 0 0.0%	0 / 0 0.0%	0 / 0 0.0%	0 / 0 0.0%	14 / 46 30.4%AB	12 / 46 26.1%E	12 / 46 26.1%	6 / 46 13.0%ABE	1 / 46 2.2%C	1 / 46 2.2%CE		
1	Mycobacterium sp. 506	14 / 46 30.4%	0 / 1 0.0%	0 / 1 0.0%	1 / 1 100.0%	0 / 1 0.0%	0 / 3 0.0%	2 / 3 66.7%	0 / 1 0.0%	0 / 1 0.0%	0 / 3 0.0%	0 / 1 0.0%	2 / 4 50.0%	1 / 2 50.0%	1 / 1 100.0%	0 / 2 0.0%	0 / 1 0.0%	1 / 3 33.3%	0 / 1 0.0%	2 / 3 66.7%	2 / 4 50.0%	0 / 2 0.0%	0 / 1 0.0%	1 / 5 20.0%	1 / 1 100.0%	0 / 0 0.0%	0 / 0 0.0%	0 / 0 0.0%	14 / 46 30.4%	14 / 46 30.4%E	10 / 46 21.7%AB	4 / 46 8.7%ABE	2 / 46 4.3%CE	2 / 46 4.3%C		
1	Mycobacterium sp. MOTT36Y	18 / 45 40.0%	1 / 1 100.0%	1 / 1 100.0%	0 / 1 0.0%	0 / 1 0.0%	1 / 3 33.3%	1 / 3 33.3%	1 / 1 100.0%	1 / 1 100.0%	0 / 3 0.0%	0 / 1 0.0%	2 / 4 50.0%	1 / 2 50.0%	1 / 1 100.0%	0 / 2 0.0%	1 / 1 100.0%	3 / 3 100.0%	0 / 1 0.0%	2 / 3 66.7%	0 / 4 0.0%	0 / 2 0.0%	1 / 1 100.0%	1 / 5 20.0%	0 / 0 0.0%	0 / 0 0.0%	0 / 0 0.0%	0 / 0 0.0%	13 / 45 28.9%	10 / 45 22.2%E	10 / 45 22.2%AB	8 / 45 17.8%ABE	2 / 45 4.4%C	2 / 45 4.4%CE		
1	Amycolicicoccus	7 / 45 15.6%	0 / 1 0.0%	0 / 1 0.0%	1 / 1 100.0%	0 / 1 0.0%	0 / 3 0.0%	1 / 3 33.3%	0 / 1 0.0%	0 / 1 0.0%	0 / 3 0.0%	0 / 1 0.0%	2 / 4 50.0%	1 / 2 50.0%	0 / 1 0.0%	0 / 2 0.0%	0 / 1 0.0%	0 / 3 0.0%	0 / 1 0.0%	0 / 3 0.0%	0 / 4 0.0%	0 / 2 0.0%	0 / 1 0.0%	2 / 5 40.0%	0 / 0 0.0%	0 / 0 0.0%	0 / 0 0.0%	0 / 0 0.0%	18 / 45 40.0%	14 / 45 31.1%E	4 / 45 8.9%C	4 / 45 8.9%AB	3 / 45 6.7%ABE	2 / 45 4.4%CE		
1	Amycolicicoccus subflavus	7 / 45 15.6%	0 / 1 0.0%	0 / 1 0.0%	1 / 1 100.0%	0 / 1 0.0%	0 / 3 0.0%	1 / 3 33.3%	0 / 1 0.0%	0 / 1 0.0%	0 / 3 0.0%	0 / 1 0.0%	2 / 4 50.0%	1 / 2 50.0%	0 / 1 0.0%	0 / 2 0.0%	0 / 1 0.0%	0 / 3 0.0%	0 / 1 0.0%	0 / 3 0.0%	0 / 4 0.0%	0 / 2 0.0%	0 / 1 0.0%	2 / 5 40.0%	0 / 0 0.0%	0 / 0 0.0%	0 / 0 0.0%	0 / 0 0.0%	18 / 45 40.0%	14 / 45 31.1%E	4 / 45 8.9%C	4 / 45 8.9%AB	3 / 45 6.7%ABE	2 / 45 4.4%CE		
1	Amycolicicoccus subflavus DQS3-9A1	7 / 45 15.6%	0 / 1 0.0%	0 / 1 0.0%	1 / 1 100.0%	0 / 1 0.0%	0 / 3 0.0%	1 / 3 33.3%	0 / 1 0.0%	0 / 1 0.0%	0 / 3 0.0%	0 / 1 0.0%	2 / 4 50.0%	1 / 2 50.0%	0 / 1 0.0%	0 / 2 0.0%	0 / 1 0.0%	0 / 3 0.0%	0 / 1 0.0%	0 / 3 0.0%	0 / 4 0.0%	0 / 2 0.0%	0 / 1 0.0%	2 / 5 40.0%	0 / 0 0.0%	0 / 0 0.0%	0 / 0 0.0%	0 / 0 0.0%	18 / 45 40.0%	14 / 45 31.1%E	4 / 45 8.9%C	4 / 45 8.9%AB	3 / 45 6.7%ABE	2 / 45 4.4%CE		
7	Nocardiaceae	107 / 353 30.3%	5 / 14 35.7%	0 / 8 0.0%	0 / 7 0.0%	0 / 7 0.0%	12 / 28 42.9%	9 / 25 36.0%	3 / 7 42.9%	0 / 8 0.0%	6 / 21 28.6%	0 / 8 0.0%	24 / 28 85.7%	3 / 16 18.8%	4 / 7 57.1%	0 / 15 0.0%	2 / 14 14.3%	11 / 22 50.0%	2 / 7 28.6%	4 / 21 19.0%	10 / 29 34.5%	1 / 15 6.7%	0 / 7 0.0%	11 / 38 28.9%	0 / 0 0.0%	0 / 0 0.0%	0 / 0 0.0%	0 / 1 0.0%	103 / 353 29.2%	96 / 353 27.2%E	56 / 353 15.9%AB	51 / 353 14.4%ABE	32 / 353 9.1%C	15 / 353 4.2%CE		
3	Micropolyspora	43 / 152 28.3%	0 / 6 0.0%	0 / 3 0.0%	0 / 3 0.0%	0 / 3 0.0%	4 / 12 33.3%	3 / 9 33.3%	1 / 3 33.3%	0 / 4 0.0%	4 / 9 44.4%	0 / 4 0.0%	9 / 12 75.0%	3 / 6 50.0%	0 / 3 0.0%	0 / 7 0.0%	2 / 6 33.3%	5 / 10 50.0%	2 / 3 66.7%	2 / 9 22.2%	3 / 13 23.1%	0 / 6 0.0%	0 / 3 0.0%	5 / 17 29.4%	0 / 0 0.0%	0 / 0 0.0%	0 / 0 0.0%	0 / 1 0.0%	49 / 152 32.2%	39 / 152 25.7%E	24 / 152 15.8%AB	19 / 152 12.5%ABE	13 / 152 8.6%C	8 / 152 5.3%CE		
1	"Actinomyces brasiliensis" (Lindenberg 1909) Gomes 1923	14 / 51 27.5%	0 / 2 0.0%	0 / 1 0.0%	0 / 1 0.0%	0 / 1 0.0%	1 / 4 25.0%	1 / 3 33.3%	0 / 1 0.0%	0 / 2 0.0%	0 / 3 0.0%	0 / 1 0.0%	3 / 4 75.0%	1 / 2 50.0%	0 / 1 0.0%	0 / 2 0.0%	1 / 2 50.0%	2 / 3 66.7%	1 / 1 100.0%	1 / 3 33.3%	2 / 4 50.0%	0 / 2 0.0%	0 / 1 0.0%	1 / 6 16.7%	0 / 0 0.0%	0 / 0 0.0%	0 / 0 0.0%	0 / 1 0.0%	16 / 51 31.4%	13 / 51 25.5%E	8 / 51 15.7%AB	6 / 51 11.8%ABE	4 / 51 7.8%CE	4 / 51 7.8%C		
1	Nocardia brasiliensis ATCC 700358	14 / 51 27.5%	0 / 2 0.0%	0 / 1 0.0%	0 / 1 0.0%	0 / 1 0.0%	1 / 4 25.0%	1 / 3 33.3%	0 / 1 0.0%	0 / 2 0.0%	0 / 3 0.0%	0 / 1 0.0%	3 / 4 75.0%	1 / 2 50.0%	0 / 1 0.0%	0 / 2 0.0%	1 / 2 50.0%	2 / 3 66.7%	1 / 1 100.0%	1 / 3 33.3%	2 / 4 50.0%	0 / 2 0.0%	0 / 1 0.0%	1 / 6 16.7%	0 / 0 0.0%	0 / 0 0.0%	0 / 0 0.0%	0 / 1 0.0%	16 / 51 31.4%	13 / 51 25.5%E	8 / 51 15.7%AB	6 / 51 11.8%ABE	4 / 51 7.8%CE	4 / 51 7.8%C		
1	"Actinomyces bovis farcinicus" Gasperini 1894	14 / 52 26.9%	0 / 2 0.0%	0 / 1 0.0%	0 / 1 0.0%	0 / 1 0.0%	3 / 4 75.0%	1 / 3 33.3%	0 / 1 0.0%	0 / 1 0.0%	2 / 3 66.7%	0 / 2 0.0%	3 / 4 75.0%	2 / 2 100.0%	0 / 1 0.0%	0 / 3 0.0%	0 / 2 0.0%	1 / 4 25.0%	0 / 1 0.0%	1 / 3 33.3%	0 / 4 0.0%	0 / 2 0.0%	0 / 1 0.0%	1 / 6 16.7%	0 / 0 0.0%	0 / 0 0.0%	0 / 0 0.0%	0 / 0 0.0%	16 / 52 30.8%	16 / 52 30.8%E	9 / 52 17.3%AB	5 / 52 9.6%ABE	4 / 52 7.7%C	2 / 52 3.8%CE		
1	Nocardia farcinica IFM 10152	14 / 52 26.9%	0 / 2 0.0%	0 / 1 0.0%	0 / 1 0.0%	0 / 1 0.0%	3 / 4 75.0%	1 / 3 33.3%	0 / 1 0.0%	0 / 1 0.0%	2 / 3 66.7%	0 / 2 0.0%	3 / 4 75.0%	2 / 2 100.0%	0 / 1 0.0%	0 / 3 0.0%	0 / 2 0.0%	1 / 4 25.0%	0 / 1 0.0%	1 / 3 33.3%	0 / 4 0.0%	0 / 2 0.0%	0 / 1 0.0%	1 / 6 16.7%	0 / 0 0.0%	0 / 0 0.0%	0 / 0 0.0%	0 / 0 0.0%	16 / 52 30.8%E	16 / 52 30.8%	9 / 52 17.3%AB	5 / 52 9.6%ABE	4 / 52 7.7%C	2 / 52 3.8%CE		
1	CCUG 48295	15 / 49 30.6%	0 / 2 0.0%	0 / 1 0.0%	0 / 1 0.0%	0 / 1 0.0%	0 / 4 0.0%	1 / 3 33.3%	1 / 1 100.0%	0 / 1 0.0%	2 / 3 66.7%	0 / 1 0.0%	3 / 4 75.0%	0 / 2 0.0%	0 / 1 0.0%	0 / 2 0.0%	1 / 2 50.0%	2 / 3 66.7%	1 / 1 100.0%	0 / 3 0.0%	1 / 5 20.0%	0 / 2 0.0%	0 / 1 0.0%	3 / 5 60.0%	0 / 0 0.0%	0 / 0 0.0%	0 / 0 0.0%	0 / 0 0.0%	17 / 49 34.7%	10 / 49 20.4%E	8 / 49 16.3%ABE	7 / 49 14.3%AB	5 / 49 10.2%C	2 / 49 4.1%CE		
1	Nocardia cyriacigeorgica GUH-2	15 / 49 30.6%	0 / 2 0.0%	0 / 1 0.0%	0 / 1 0.0%	0 / 1 0.0%	0 / 4 0.0%	1 / 3 33.3%	1 / 1 100.0%	0 / 1 0.0%	2 / 3 66.7%	0 / 1 0.0%	3 / 4 75.0%	0 / 2 0.0%	0 / 1 0.0%	0 / 2 0.0%	1 / 2 50.0%	2 / 3 66.7%	1 / 1 100.0%	0 / 3 0.0%	1 / 5 20.0%	0 / 2 0.0%	0 / 1 0.0%	3 / 5 60.0%	0 / 0 0.0%	0 / 0 0.0%	0 / 0 0.0%	0 / 0 0.0%	17 / 49 34.7%	10 / 49 20.4%E	8 / 49 16.3%ABE	7 / 49 14.3%AB	5 / 49 10.2%C	2 / 49 4.1%CE		
4	Rhodococcus	64 / 201 31.8%	5 / 8 62.5%	0 / 5 0.0%	0 / 4 0.0%	0 / 4 0.0%	8 / 16 50.0%	6 / 16 37.5%	2 / 4 50.0%	0 / 4 0.0%	2 / 12 16.7%	0 / 4 0.0%	15 / 16 93.8%	0 / 10 0.0%	4 / 4 100.0%	0 / 8 0.0%	0 / 8 0.0%	6 / 12 50.0%	0 / 4 0.0%	2 / 12 16.7%	7 / 16 43.8%	1 / 9 11.1%	0 / 4 0.0%	6 / 21 28.6%	0 / 0 0.0%	0 / 0 0.0%	0 / 0 0.0%	0 / 0 0.0%	57 / 201 28.4%E	54 / 201 26.9%	32 / 201 15.9%ABE	32 / 201 15.9%AB	19 / 201 9.5%C	7 / 201 3.5%CE		
1	"Mycobacterium erythropolis" Gray and Thornton 1928	15 / 52 28.8%	0 / 2 0.0%	0 / 1 0.0%	0 / 1 0.0%	0 / 1 0.0%	3 / 4 75.0%	2 / 4 50.0%	0 / 1 0.0%	0 / 1 0.0%	1 / 3 33.3%	0 / 1 0.0%	4 / 4 100.0%	0 / 3 0.0%	1 / 1 100.0%	0 / 2 0.0%	0 / 3 0.0%	0 / 3 0.0%	0 / 1 0.0%	0 / 3 0.0%	3 / 4 75.0%	0 / 2 0.0%	0 / 1 0.0%	1 / 6 16.7%	0 / 0 0.0%	0 / 0 0.0%	0 / 0 0.0%	0 / 0 0.0%	17 / 52 32.7%E	15 / 52 28.8%	8 / 52 15.4%AB	7 / 52 13.5%ABE	3 / 52 5.8%C	2 / 52 3.8%CE		
1	Rhodococcus erythropolis (strain PR4)	15 / 52 28.8%	0 / 2 0.0%	0 / 1 0.0%	0 / 1 0.0%	0 / 1 0.0%	3 / 4 75.0%	2 / 4 50.0%	0 / 1 0.0%	0 / 1 0.0%	1 / 3 33.3%	0 / 1 0.0%	4 / 4 100.0%	0 / 3 0.0%	1 / 1 100.0%	0 / 2 0.0%	0 / 3 0.0%	0 / 3 0.0%	0 / 1 0.0%	0 / 3 0.0%	3 / 4 75.0%	0 / 2 0.0%	0 / 1 0.0%	1 / 6 16.7%	0 / 0 0.0%	0 / 0 0.0%	0 / 0 0.0%	0 / 0 0.0%	17 / 52 32.7%E	15 / 52 28.8%	8 / 52 15.4%AB	7 / 52 13.5%ABE	3 / 52 5.8%C	2 / 52 3.8%CE		
1	"Nocardia opaca" Aggag and Schlegel 1973	16 / 49 32.7%	2 / 2 100.0%	0 / 1 0.0%	0 / 1 0.0%	0 / 1 0.0%	2 / 4 50.0%	1 / 4 25.0%	1 / 1 100.0%	0 / 1 0.0%	0 / 3 0.0%	0 / 1 0.0%	3 / 4 75.0%	0 / 2 0.0%	1 / 1 100.0%	0 / 2 0.0%	0 / 2 0.0%	2 / 3 66.7%	0 / 1 0.0%	1 / 3 33.3%	2 / 4 50.0%	0 / 2 0.0%	0 / 1 0.0%	1 / 5 20.0%	0 / 0 0.0%	0 / 0 0.0%	0 / 0 0.0%	0 / 0 0.0%	14 / 49 28.6%E	11 / 49 22.4%	9 / 49 18.4%AB	7 / 49 14.3%ABE	5 / 49 10.2%C	3 / 49 6.1%CE		
1	Rhodococcus opacus B4	16 / 49 32.7%	2 / 2 100.0%	0 / 1 0.0%	0 / 1 0.0%	0 / 1 0.0%	2 / 4 50.0%	1 / 4 25.0%	1 / 1 100.0%	0 / 1 0.0%	0 / 3 0.0%	0 / 1 0.0%	3 / 4 75.0%	0 / 2 0.0%	1 / 1 100.0%	0 / 2 0.0%	0 / 2 0.0%	2 / 3 66.7%	0 / 1 0.0%	1 / 3 33.3%	2 / 4 50.0%	0 / 2 0.0%	0 / 1 0.0%	1 / 5 20.0%	0 / 0 0.0%	0 / 0 0.0%	0 / 0 0.0%	0 / 0 0.0%	14 / 49 28.6%E	11 / 49 22.4%	9 / 49 18.4%AB	7 / 49 14.3%ABE	5 / 49 10.2%C	3 / 49 6.1%CE		
1	"Bacillus hoagii" Morse 1912	15 / 51 29.4%	1 / 2 50.0%	0 / 2 0.0%	0 / 1 0.0%	0 / 1 0.0%	1 / 4 25.0%	2 / 4 50.0%	0 / 1 0.0%	0 / 1 0.0%	0 / 3 0.0%	0 / 1 0.0%	4 / 4 100.0%	0 / 3 0.0%	1 / 1 100.0%	0 / 2 0.0%	0 / 1 0.0%	2 / 3 66.7%	0 / 1 0.0%	0 / 3 0.0%	1 / 4 25.0%	1 / 3 33.3%	0 / 1 0.0%	2 / 5 40.0%	0 / 0 0.0%	0 / 0 0.0%	0 / 0 0.0%	0 / 0 0.0%	15 / 51 29.4%	12 / 51 23.5%E	9 / 51 17.6%ABE	7 / 51 13.7%C	6 / 51 11.8%AB	2 / 51 3.9%CE		
1	Rhodococcus equi 103S	15 / 51 29.4%	1 / 2 50.0%	0 / 2 0.0%	0 / 1 0.0%	0 / 1 0.0%	1 / 4 25.0%	2 / 4 50.0%	0 / 1 0.0%	0 / 1 0.0%	0 / 3 0.0%	0 / 1 0.0%	4 / 4 100.0%	0 / 3 0.0%	1 / 1 100.0%	0 / 2 0.0%	0 / 1 0.0%	2 / 3 66.7%	0 / 1 0.0%	0 / 3 0.0%	1 / 4 25.0%	1 / 3 33.3%	0 / 1 0.0%	2 / 5 40.0%	0 / 0 0.0%	0 / 0 0.0%	0 / 0 0.0%	0 / 0 0.0%	15 / 51 29.4%	12 / 51 23.5%E	9 / 51 17.6%ABE	7 / 51 13.7%C	6 / 51 11.8%AB	2 / 51 3.9%CE		
1	CCM 4760	18 / 49 36.7%	2 / 2 100.0%	0 / 1 0.0%	0 / 1 0.0%	0 / 1 0.0%	2 / 4 50.0%	1 / 4 25.0%	1 / 1 100.0%	0 / 1 0.0%	1 / 3 33.3%	0 / 1 0.0%	4 / 4 100.0%	0 / 2 0.0%	1 / 1 100.0%	0 / 2 0.0%	0 / 2 0.0%	2 / 3 66.7%	0 / 1 0.0%	1 / 3 33.3%	1 / 4 25.0%	0 / 2 0.0%	0 / 1 0.0%	2 / 5 40.0%	0 / 0 0.0%	0 / 0 0.0%	0 / 0 0.0%	0 / 0 0.0%	14 / 49 28.6%E	13 / 49 26.5%	9 / 49 18.4%AB	9 / 49 18.4%ABE	4 / 49 8.2%C			
1	Rhodococcus jostii RHA1	18 / 49 36.7%	2 / 2 100.0%	0 / 1 0.0%	0 / 1 0.0%	0 / 1 0.0%	2 / 4 50.0%	1 / 4 25.0%	1 / 1 100.0%	0 / 1 0.0%	1 / 3 33.3%	0 / 1 0.0%	4 / 4 100.0%	0 / 2 0.0%	1 / 1 100.0%	0 / 2 0.0%	0 / 2 0.0%	2 / 3 66.7%	0 / 1 0.0%	1 / 3 33.3%	1 / 4 25.0%	0 / 2 0.0%	0 / 1 0.0%	2 / 5 40.0%	0 / 0 0.0%	0 / 0 0.0%	0 / 0 0.0%	0 / 0 0.0%	14 / 49 28.6%E	13 / 49 26.5%	9 / 49 18.4%ABE	9 / 49 18.4%AB	4 / 49 8.2%C			
3	Gordoniaceae	50 / 140 35.7%	3 / 3 100.0%	2 / 3 66.7%	0 / 3 0.0%	1 / 3 33.3%	3 / 10 30.0%	6 / 10 60.0%	0 / 3 0.0%	0 / 3 0.0%	3 / 9 33.3%	0 / 3 0.0%	7 / 12 58.3%	2 / 6 33.3%	1 / 3 33.3%	0 / 6 0.0%	4 / 4 100.0%	1 / 9 11.1%	0 / 3 0.0%	3 / 9 33.3%	3 / 12 25.0%	0 / 6 0.0%	1 / 3 33.3%	9 / 15 60.0%	1 / 2 50.0%	0 / 0 0.0%	0 / 0 0.0%	0 / 0 0.0%	32 / 140 22.9%	30 / 140 21.4%E	25 / 140 17.9%ABE	25 / 140 17.9%AB	18 / 140 12.9%C	10 / 140 7.1%CE		
3	"Gordona (sic)" Tsukamura et al. 1970	50 / 140 35.7%	3 / 3 100.0%	2 / 3 66.7%	0 / 3 0.0%	1 / 3 33.3%	3 / 10 30.0%	6 / 10 60.0%	0 / 3 0.0%	0 / 3 0.0%	3 / 9 33.3%	0 / 3 0.0%	7 / 12 58.3%	2 / 6 33.3%	1 / 3 33.3%	0 / 6 0.0%	4 / 4 100.0%	1 / 9 11.1%	0 / 3 0.0%	3 / 9 33.3%	3 / 12 25.0%	0 / 6 0.0%	1 / 3 33.3%	9 / 15 60.0%	1 / 2 50.0%	0 / 0 0.0%	0 / 0 0.0%	0 / 0 0.0%	32 / 140 22.9%	30 / 140 21.4%E	25 / 140 17.9%ABE	25 / 140 17.9%AB	18 / 140 12.9%C	10 / 140 7.1%CE		
1	"Gordona (sic) bronchialis" Tsukamura et al. 1971	13 / 46 28.3%	1 / 1 100.0%	0 / 1 0.0%	0 / 1 0.0%	0 / 1 0.0%	1 / 3 33.3%	1 / 3 33.3%	0 / 1 0.0%	0 / 1 0.0%	2 / 3 66.7%	0 / 1 0.0%	2 / 4 50.0%	1 / 2 50.0%	0 / 1 0.0%	0 / 2 0.0%	1 / 1 100.0%	0 / 3 0.0%	0 / 1 0.0%	1 / 3 33.3%	0 / 4 0.0%	0 / 2 0.0%	0 / 1 0.0%	2 / 5 40.0%	1 / 1 100.0%	0 / 0 0.0%	0 / 0 0.0%	0 / 0 0.0%	12 / 46 26.1%	11 / 46 23.9%E	7 / 46 15.2%ABE	6 / 46 13.0%C	6 / 46 13.0%AB	4 / 46 8.7%CE		
1	Gordonia bronchialis ATCC 25592	13 / 46 28.3%	1 / 1 100.0%	0 / 1 0.0%	0 / 1 0.0%	0 / 1 0.0%	1 / 3 33.3%	1 / 3 33.3%	0 / 1 0.0%	0 / 1 0.0%	2 / 3 66.7%	0 / 1 0.0%	2 / 4 50.0%	1 / 2 50.0%	0 / 1 0.0%	0 / 2 0.0%	1 / 1 100.0%	0 / 3 0.0%	0 / 1 0.0%	1 / 3 33.3%	0 / 4 0.0%	0 / 2 0.0%	0 / 1 0.0%	2 / 5 40.0%	1 / 1 100.0%	0 / 0 0.0%	0 / 0 0.0%	0 / 0 0.0%	12 / 46 26.1%	11 / 46 23.9%E	7 / 46 15.2%ABE	6 / 46 13.0%C	6 / 46 13.0%AB	4 / 46 8.7%CE		
1	ATCC BAA-14	18 / 49 36.7%	1 / 1 100.0%	1 / 1 100.0%	0 / 1 0.0%	1 / 1 100.0%	0 / 4 0.0%	3 / 4 75.0%	0 / 1 0.0%	0 / 1 0.0%	0 / 3 0.0%	0 / 1 0.0%	2 / 4 50.0%	0 / 2 0.0%	1 / 1 100.0%	0 / 2 0.0%	2 / 2 100.0%	1 / 3 33.3%	0 / 1 0.0%	1 / 3 33.3%	0 / 4 0.0%	0 / 2 0.0%	1 / 1 100.0%	4 / 5 80.0%	0 / 1 0.0%	0 / 0 0.0%	0 / 0 0.0%	0 / 0 0.0%	10 / 49 20.4%	10 / 49 20.4%ABE	9 / 49 18.4%C	8 / 49 16.3%AB	8 / 49 16.3%E	4 / 49 8.2%CE		
1	Gordonia polyisoprenivorans VH2	18 / 49 36.7%	1 / 1 100.0%	1 / 1 100.0%	0 / 1 0.0%	1 / 1 100.0%	0 / 4 0.0%	3 / 4 75.0%	0 / 1 0.0%	0 / 1 0.0%	0 / 3 0.0%	0 / 1 0.0%	2 / 4 50.0%	0 / 2 0.0%	1 / 1 100.0%	0 / 2 0.0%	2 / 2 100.0%	1 / 3 33.3%	0 / 1 0.0%	1 / 3 33.3%	0 / 4 0.0%	0 / 2 0.0%	1 / 1 100.0%	4 / 5 80.0%	0 / 1 0.0%	0 / 0 0.0%	0 / 0 0.0%	0 / 0 0.0%	10 / 49 20.4%ABE	10 / 49 20.4%	9 / 49 18.4%C	8 / 49 16.3%E	8 / 49 16.3%AB	4 / 49 8.2%CE		
1	Gordonia sp. KTR9	19 / 45 42.2%	1 / 1 100.0%	1 / 1 100.0%	0 / 1 0.0%	0 / 1 0.0%	2 / 3 66.7%	2 / 3 66.7%	0 / 1 0.0%	0 / 1 0.0%	1 / 3 33.3%	0 / 1 0.0%	3 / 4 75.0%	1 / 2 50.0%	0 / 1 0.0%	0 / 2 0.0%	1 / 1 100.0%	0 / 3 0.0%	0 / 1 0.0%	1 / 3 33.3%	3 / 4 75.0%	0 / 2 0.0%	0 / 1 0.0%	3 / 5 60.0%	0 / 0 0.0%	0 / 0 0.0%	0 / 0 0.0%	0 / 0 0.0%	11 / 45 24.4%AB	11 / 45 24.4%E	10 / 45 22.2%	8 / 45 17.8%ABE	3 / 45 6.7%C	2 / 45 4.4%CE		
1	Tsukamurellaceae	14 / 48 29.2%	0 / 1 0.0%	1 / 1 100.0%	0 / 1 0.0%	1 / 1 100.0%	2 / 4 50.0%	1 / 3 33.3%	1 / 1 100.0%	0 / 1 0.0%	0 / 3 0.0%	0 / 1 0.0%	2 / 4 50.0%	0 / 2 0.0%	1 / 1 100.0%	0 / 2 0.0%	1 / 1 100.0%	0 / 3 0.0%	0 / 1 0.0%	2 / 3 66.7%	1 / 4 25.0%	0 / 3 0.0%	0 / 2 0.0%	1 / 5 20.0%	0 / 0 0.0%	0 / 0 0.0%	0 / 0 0.0%	0 / 0 0.0%	16 / 48 33.3%	13 / 48 27.1%E	9 / 48 18.8%AB	5 / 48 10.4%ABE	3 / 48 6.2%CE	2 / 48 4.2%C		
1	Tsukamurella	14 / 48 29.2%	0 / 1 0.0%	1 / 1 100.0%	0 / 1 0.0%	1 / 1 100.0%	2 / 4 50.0%	1 / 3 33.3%	1 / 1 100.0%	0 / 1 0.0%	0 / 3 0.0%	0 / 1 0.0%	2 / 4 50.0%	0 / 2 0.0%	1 / 1 100.0%	0 / 2 0.0%	1 / 1 100.0%	0 / 3 0.0%	0 / 1 0.0%	2 / 3 66.7%	1 / 4 25.0%	0 / 3 0.0%	0 / 2 0.0%	1 / 5 20.0%	0 / 0 0.0%	0 / 0 0.0%	0 / 0 0.0%	0 / 0 0.0%	16 / 48 33.3%	13 / 48 27.1%E	9 / 48 18.8%AB	5 / 48 10.4%ABE	3 / 48 6.2%CE	2 / 48 4.2%C		
1	ATCC 8368	14 / 48 29.2%	0 / 1 0.0%	1 / 1 100.0%	0 / 1 0.0%	1 / 1 100.0%	2 / 4 50.0%	1 / 3 33.3%	1 / 1 100.0%	0 / 1 0.0%	0 / 3 0.0%	0 / 1 0.0%	2 / 4 50.0%	0 / 2 0.0%	1 / 1 100.0%	0 / 2 0.0%	1 / 1 100.0%	0 / 3 0.0%	0 / 1 0.0%	2 / 3 66.7%	1 / 4 25.0%	0 / 3 0.0%	0 / 2 0.0%	1 / 5 20.0%	0 / 0 0.0%	0 / 0 0.0%	0 / 0 0.0%	0 / 0 0.0%	16 / 48 33.3%	13 / 48 27.1%E	9 / 48 18.8%AB	5 / 48 10.4%ABE	3 / 48 6.2%CE	2 / 48 4.2%C		
1	Tsukamurella paurometabola ATCC 8368	14 / 48 29.2%	0 / 1 0.0%	1 / 1 100.0%	0 / 1 0.0%	1 / 1 100.0%	2 / 4 50.0%	1 / 3 33.3%	1 / 1 100.0%	0 / 1 0.0%	0 / 3 0.0%	0 / 1 0.0%	2 / 4 50.0%	0 / 2 0.0%	1 / 1 100.0%	0 / 2 0.0%	1 / 1 100.0%	0 / 3 0.0%	0 / 1 0.0%	2 / 3 66.7%	1 / 4 25.0%	0 / 3 0.0%	0 / 2 0.0%	1 / 5 20.0%	0 / 0 0.0%	0 / 0 0.0%	0 / 0 0.0%	0 / 0 0.0%	16 / 48 33.3%	13 / 48 27.1%E	9 / 48 18.8%AB	5 / 48 10.4%ABE	3 / 48 6.2%CE	2 / 48 4.2%C		
1	Segniliparaceae	11 / 45 24.4%	0 / 1 0.0%	0 / 1 0.0%	1 / 1 100.0%	0 / 1 0.0%	1 / 3 33.3%	0 / 3 0.0%	1 / 1 100.0%	0 / 1 0.0%	1 / 3 33.3%	0 / 1 0.0%	2 / 4 50.0%	0 / 2 0.0%	0 / 1 0.0%	1 / 2 50.0%	0 / 1 0.0%	0 / 3 0.0%	0 / 1 0.0%	1 / 3 33.3%	3 / 4 75.0%	0 / 2 0.0%	0 / 1 0.0%	0 / 5 0.0%	0 / 0 0.0%	0 / 0 0.0%	0 / 0 0.0%	0 / 0 0.0%	16 / 45 35.6%E	14 / 45 31.1%	7 / 45 15.6%AB	4 / 45 8.9%ABE	3 / 45 6.7%C	1 / 45 2.2%CE		
1	Segniliparus	11 / 45 24.4%	0 / 1 0.0%	0 / 1 0.0%	1 / 1 100.0%	0 / 1 0.0%	1 / 3 33.3%	0 / 3 0.0%	1 / 1 100.0%	0 / 1 0.0%	1 / 3 33.3%	0 / 1 0.0%	2 / 4 50.0%	0 / 2 0.0%	0 / 1 0.0%	1 / 2 50.0%	0 / 1 0.0%	0 / 3 0.0%	0 / 1 0.0%	1 / 3 33.3%	3 / 4 75.0%	0 / 2 0.0%	0 / 1 0.0%	0 / 5 0.0%	0 / 0 0.0%	0 / 0 0.0%	0 / 0 0.0%	0 / 0 0.0%	16 / 45 35.6%E	14 / 45 31.1%	7 / 45 15.6%AB	4 / 45 8.9%ABE	3 / 45 6.7%C	1 / 45 2.2%CE		
1	ATCC BAA-972	11 / 45 24.4%	0 / 1 0.0%	0 / 1 0.0%	1 / 1 100.0%	0 / 1 0.0%	1 / 3 33.3%	0 / 3 0.0%	1 / 1 100.0%	0 / 1 0.0%	1 / 3 33.3%	0 / 1 0.0%	2 / 4 50.0%	0 / 2 0.0%	0 / 1 0.0%	1 / 2 50.0%	0 / 1 0.0%	0 / 3 0.0%	0 / 1 0.0%	1 / 3 33.3%	3 / 4 75.0%	0 / 2 0.0%	0 / 1 0.0%	0 / 5 0.0%	0 / 0 0.0%	0 / 0 0.0%	0 / 0 0.0%	0 / 0 0.0%	16 / 45 35.6%E	14 / 45 31.1%	7 / 45 15.6%AB	4 / 45 8.9%ABE	3 / 45 6.7%C	1 / 45 2.2%CE		
1	Segniliparus rotundus ATCC BAA-972	11 / 45 24.4%	0 / 1 0.0%	0 / 1 0.0%	1 / 1 100.0%	0 / 1 0.0%	1 / 3 33.3%	0 / 3 0.0%	1 / 1 100.0%	0 / 1 0.0%	1 / 3 33.3%	0 / 1 0.0%	2 / 4 50.0%	0 / 2 0.0%	0 / 1 0.0%	1 / 2 50.0%	0 / 1 0.0%	0 / 3 0.0%	0 / 1 0.0%	1 / 3 33.3%	3 / 4 75.0%	0 / 2 0.0%	0 / 1 0.0%	0 / 5 0.0%	0 / 0 0.0%	0 / 0 0.0%	0 / 0 0.0%	0 / 0 0.0%	16 / 45 35.6%E	14 / 45 31.1%	7 / 45 15.6%AB	4 / 45 8.9%ABE	3 / 45 6.7%C	1 / 45 2.2%CE		
8	Micromonosporineae	130 / 464 28.0%	3 / 16 18.8%	1 / 12 8.3%	1 / 10 10.0%	1 / 10 10.0%	13 / 36 36.1%	15 / 33 45.5%	6 / 9 66.7%	6 / 9 66.7%	7 / 30 23.3%	0 / 10 0.0%	8 / 35 22.9%	13 / 24 54.2%	3 / 8 37.5%	0 / 20 0.0%	0 / 11 0.0%	6 / 26 23.1%	0 / 9 0.0%	9 / 26 34.6%	7 / 38 18.4%	2 / 27 7.4%	6 / 8 75.0%	16 / 50 32.0%	7 / 7 100.0%	0 / 0 0.0%	0 / 0 0.0%	0 / 0 0.0%	142 / 464 30.6%E	135 / 464 29.1%	69 / 464 14.9%AB	57 / 464 12.3%ABE	31 / 464 6.7%CE	26 / 464 5.6%C	2 / 464 0.4%B	2 / 464 0.4%BE
8	Actinoplanaceae	130 / 464 28.0%	3 / 16 18.8%	1 / 12 8.3%	1 / 10 10.0%	1 / 10 10.0%	13 / 36 36.1%	15 / 33 45.5%	6 / 9 66.7%	6 / 9 66.7%	7 / 30 23.3%	0 / 10 0.0%	8 / 35 22.9%	13 / 24 54.2%	3 / 8 37.5%	0 / 20 0.0%	0 / 11 0.0%	6 / 26 23.1%	0 / 9 0.0%	9 / 26 34.6%	7 / 38 18.4%	2 / 27 7.4%	6 / 8 75.0%	16 / 50 32.0%	7 / 7 100.0%	0 / 0 0.0%	0 / 0 0.0%	0 / 0 0.0%	142 / 464 30.6%E	135 / 464 29.1%	69 / 464 14.9%AB	57 / 464 12.3%ABE	31 / 464 6.7%CE	26 / 464 5.6%C	2 / 464 0.4%BE	2 / 464 0.4%B
3	"Ampullaria" Couch 1963	48 / 208 23.1%	1 / 6 16.7%	1 / 7 14.3%	1 / 5 20.0%	1 / 5 20.0%	3 / 16 18.8%	6 / 13 46.2%	3 / 4 75.0%	1 / 4 25.0%	2 / 15 13.3%	0 / 5 0.0%	6 / 15 40.0%	3 / 10 30.0%	3 / 3 100.0%	0 / 10 0.0%	0 / 6 0.0%	3 / 10 30.0%	0 / 4 0.0%	4 / 11 36.4%	1 / 18 5.6%	1 / 10 10.0%	1 / 3 33.3%	4 / 25 16.0%	3 / 3 100.0%	0 / 0 0.0%	0 / 0 0.0%	0 / 0 0.0%	76 / 208 36.5%	64 / 208 30.8%E	25 / 208 12.0%ABE	23 / 208 11.1%AB	11 / 208 5.3%CE	9 / 208 4.3%C		
1	ATCC 14538	13 / 58 22.4%	0 / 2 0.0%	0 / 1 0.0%	1 / 1 100.0%	0 / 1 0.0%	1 / 4 25.0%	2 / 4 50.0%	1 / 1 100.0%	0 / 1 0.0%	0 / 3 0.0%	0 / 1 0.0%	2 / 5 40.0%	1 / 3 33.3%	1 / 1 100.0%	0 / 2 0.0%	0 / 2 0.0%	1 / 3 33.3%	0 / 2 0.0%	1 / 3 33.3%	0 / 5 0.0%	0 / 3 0.0%	0 / 1 0.0%	1 / 8 12.5%	1 / 1 100.0%	0 / 0 0.0%	0 / 0 0.0%	0 / 0 0.0%	20 / 58 34.5%	19 / 58 32.8%E	7 / 58 12.1%AB	6 / 58 10.3%ABE	4 / 58 6.9%CE	2 / 58 3.4%C		
1	Actinoplanes missouriensis 431	13 / 58 22.4%	0 / 2 0.0%	0 / 1 0.0%	1 / 1 100.0%	0 / 1 0.0%	1 / 4 25.0%	2 / 4 50.0%	1 / 1 100.0%	0 / 1 0.0%	0 / 3 0.0%	0 / 1 0.0%	2 / 5 40.0%	1 / 3 33.3%	1 / 1 100.0%	0 / 2 0.0%	0 / 2 0.0%	1 / 3 33.3%	0 / 2 0.0%	1 / 3 33.3%	0 / 5 0.0%	0 / 3 0.0%	0 / 1 0.0%	1 / 8 12.5%	1 / 1 100.0%	0 / 0 0.0%	0 / 0 0.0%	0 / 0 0.0%	20 / 58 34.5%	19 / 58 32.8%E	7 / 58 12.1%AB	6 / 58 10.3%ABE	4 / 58 6.9%CE	2 / 58 3.4%C		
1	Actinoplanes sp. (strain 50/110)	17 / 92 18.5%	1 / 2 50.0%	0 / 5 0.0%	0 / 2 0.0%	0 / 2 0.0%	1 / 7 14.3%	2 / 6 33.3%	1 / 2 50.0%	1 / 2 50.0%	1 / 7 14.3%	0 / 3 0.0%	2 / 6 33.3%	2 / 5 40.0%	1 / 1 100.0%	0 / 5 0.0%	0 / 2 0.0%	1 / 4 25.0%	0 / 1 0.0%	1 / 5 20.0%	0 / 8 0.0%	1 / 5 20.0%	0 / 1 0.0%	1 / 10 10.0%	1 / 1 100.0%	0 / 0 0.0%	0 / 0 0.0%	0 / 0 0.0%	38 / 92 41.3%	27 / 92 29.3%E	10 / 92 10.9%ABE	7 / 92 7.6%AB	5 / 92 5.4%C	5 / 92 5.4%CE		
1	Actinoplanes sp. N902-109	18 / 58 31.0%	0 / 2 0.0%	1 / 1 100.0%	0 / 2 0.0%	1 / 2 50.0%	1 / 5 20.0%	2 / 3 66.7%	1 / 1 100.0%	0 / 1 0.0%	1 / 5 20.0%	0 / 1 0.0%	2 / 4 50.0%	0 / 2 0.0%	1 / 1 100.0%	0 / 3 0.0%	0 / 2 0.0%	1 / 3 33.3%	0 / 1 0.0%	2 / 3 66.7%	1 / 5 20.0%	0 / 2 0.0%	1 / 1 100.0%	2 / 7 28.6%	1 / 1 100.0%	0 / 0 0.0%	0 / 0 0.0%	0 / 0 0.0%	18 / 58 31.0%E	18 / 58 31.0%	9 / 58 15.5%AB	9 / 58 15.5%ABE	2 / 58 3.4%C	2 / 58 3.4%CE		
2	Micromonospora	39 / 104 37.5%	2 / 4 50.0%	0 / 2 0.0%	0 / 2 0.0%	0 / 2 0.0%	4 / 8 50.0%	5 / 8 62.5%	2 / 2 100.0%	2 / 2 100.0%	2 / 6 33.3%	0 / 2 0.0%	2 / 8 25.0%	5 / 6 83.3%	0 / 2 0.0%	0 / 4 0.0%	0 / 2 0.0%	2 / 6 33.3%	0 / 2 0.0%	2 / 6 33.3%	2 / 8 25.0%	0 / 8 0.0%	2 / 2 100.0%	5 / 10 50.0%	2 / 2 100.0%	0 / 0 0.0%	0 / 0 0.0%	0 / 0 0.0%	29 / 104 27.9%E	22 / 104 21.2%AB	21 / 104 20.2%	17 / 104 16.3%ABE	8 / 104 7.7%CE	7 / 104 6.7%C		
1	ATCC 27029	21 / 52 40.4%	1 / 2 50.0%	0 / 1 0.0%	0 / 1 0.0%	0 / 1 0.0%	2 / 4 50.0%	3 / 4 75.0%	1 / 1 100.0%	1 / 1 100.0%	1 / 3 33.3%	0 / 1 0.0%	1 / 4 25.0%	3 / 3 100.0%	0 / 1 0.0%	0 / 2 0.0%	0 / 1 0.0%	1 / 3 33.3%	0 / 1 0.0%	1 / 3 33.3%	1 / 4 25.0%	0 / 4 0.0%	1 / 1 100.0%	3 / 5 60.0%	1 / 1 100.0%	0 / 0 0.0%	0 / 0 0.0%	0 / 0 0.0%	14 / 52 26.9%E	12 / 52 23.1%AB	10 / 52 19.2%	9 / 52 17.3%ABE	4 / 52 7.7%CE	3 / 52 5.8%C		
1	Micromonospora aurantiaca ATCC 27029	21 / 52 40.4%	1 / 2 50.0%	0 / 1 0.0%	0 / 1 0.0%	0 / 1 0.0%	2 / 4 50.0%	3 / 4 75.0%	1 / 1 100.0%	1 / 1 100.0%	1 / 3 33.3%	0 / 1 0.0%	1 / 4 25.0%	3 / 3 100.0%	0 / 1 0.0%	0 / 2 0.0%	0 / 1 0.0%	1 / 3 33.3%	0 / 1 0.0%	1 / 3 33.3%	1 / 4 25.0%	0 / 4 0.0%	1 / 1 100.0%	3 / 5 60.0%	1 / 1 100.0%	0 / 0 0.0%	0 / 0 0.0%	0 / 0 0.0%	14 / 52 26.9%E	12 / 52 23.1%AB	10 / 52 19.2%	9 / 52 17.3%ABE	4 / 52 7.7%CE	3 / 52 5.8%C		
1	Micromonospora sp. L5	18 / 52 34.6%	1 / 2 50.0%	0 / 1 0.0%	0 / 1 0.0%	0 / 1 0.0%	2 / 4 50.0%	2 / 4 50.0%	1 / 1 100.0%	1 / 1 100.0%	1 / 3 33.3%	0 / 1 0.0%	1 / 4 25.0%	2 / 3 66.7%	0 / 1 0.0%	0 / 2 0.0%	0 / 1 0.0%	1 / 3 33.3%	0 / 1 0.0%	1 / 3 33.3%	1 / 4 25.0%	0 / 4 0.0%	1 / 1 100.0%	2 / 5 40.0%	1 / 1 100.0%	0 / 0 0.0%	0 / 0 0.0%	0 / 0 0.0%	15 / 52 28.8%E	11 / 52 21.2%	10 / 52 19.2%AB	8 / 52 15.4%ABE	4 / 52 7.7%CE	4 / 52 7.7%C		
1	Verrucosispora	15 / 51 29.4%	0 / 2 0.0%	0 / 1 0.0%	0 / 1 0.0%	0 / 1 0.0%	2 / 4 50.0%	2 / 4 50.0%	0 / 1 0.0%	1 / 1 100.0%	1 / 3 33.3%	0 / 1 0.0%	0 / 4 0.0%	2 / 3 66.7%	0 / 1 0.0%	0 / 2 0.0%	0 / 1 0.0%	0 / 3 0.0%	0 / 1 0.0%	1 / 3 33.3%	1 / 4 25.0%	1 / 3 33.3%	1 / 1 100.0%	2 / 5 40.0%	1 / 1 100.0%	0 / 0 0.0%	0 / 0 0.0%	0 / 0 0.0%	18 / 51 35.3%E	13 / 51 25.5%	9 / 51 17.6%AB	6 / 51 11.8%ABE	3 / 51 5.9%CE	2 / 51 3.9%C		
1	"Verrucosispora maris" Fiedler et al. 2008	15 / 51 29.4%	0 / 2 0.0%	0 / 1 0.0%	0 / 1 0.0%	0 / 1 0.0%	2 / 4 50.0%	2 / 4 50.0%	0 / 1 0.0%	1 / 1 100.0%	1 / 3 33.3%	0 / 1 0.0%	0 / 4 0.0%	2 / 3 66.7%	0 / 1 0.0%	0 / 2 0.0%	0 / 1 0.0%	0 / 3 0.0%	0 / 1 0.0%	1 / 3 33.3%	1 / 4 25.0%	1 / 3 33.3%	1 / 1 100.0%	2 / 5 40.0%	1 / 1 100.0%	0 / 0 0.0%	0 / 0 0.0%	0 / 0 0.0%	18 / 51 35.3%E	13 / 51 25.5%	9 / 51 17.6%AB	6 / 51 11.8%ABE	3 / 51 5.9%CE	2 / 51 3.9%C		
1	Verrucosispora maris AB-18-032	15 / 51 29.4%	0 / 2 0.0%	0 / 1 0.0%	0 / 1 0.0%	0 / 1 0.0%	2 / 4 50.0%	2 / 4 50.0%	0 / 1 0.0%	1 / 1 100.0%	1 / 3 33.3%	0 / 1 0.0%	0 / 4 0.0%	2 / 3 66.7%	0 / 1 0.0%	0 / 2 0.0%	0 / 1 0.0%	0 / 3 0.0%	0 / 1 0.0%	1 / 3 33.3%	1 / 4 25.0%	1 / 3 33.3%	1 / 1 100.0%	2 / 5 40.0%	1 / 1 100.0%	0 / 0 0.0%	0 / 0 0.0%	0 / 0 0.0%	18 / 51 35.3%E	13 / 51 25.5%	9 / 51 17.6%AB	6 / 51 11.8%ABE	3 / 51 5.9%CE	2 / 51 3.9%C		
2	"Salinospora" Mincer et al. 2002	28 / 101 27.7%	0 / 4 0.0%	0 / 2 0.0%	0 / 2 0.0%	0 / 2 0.0%	4 / 8 50.0%	2 / 8 25.0%	1 / 2 50.0%	2 / 2 100.0%	2 / 6 33.3%	0 / 2 0.0%	0 / 8 0.0%	3 / 5 60.0%	0 / 2 0.0%	0 / 4 0.0%	0 / 2 0.0%	1 / 7 14.3%	0 / 2 0.0%	2 / 6 33.3%	3 / 8 37.5%	0 / 6 0.0%	2 / 2 100.0%	5 / 10 50.0%	1 / 1 100.0%	0 / 0 0.0%	0 / 0 0.0%	0 / 0 0.0%	31 / 101 30.7%E	25 / 101 24.8%	15 / 101 14.9%AB	9 / 101 8.9%CE	9 / 101 8.9%ABE	8 / 101 7.9%C	2 / 101 2.0%B	2 / 101 2.0%BE
1	ATCC BAA-916	15 / 50 30.0%	0 / 2 0.0%	0 / 1 0.0%	0 / 1 0.0%	0 / 1 0.0%	2 / 4 50.0%	1 / 4 25.0%	1 / 1 100.0%	1 / 1 100.0%	1 / 3 33.3%	0 / 1 0.0%	0 / 4 0.0%	2 / 2 100.0%	0 / 1 0.0%	0 / 2 0.0%	0 / 1 0.0%	1 / 3 33.3%	0 / 1 0.0%	1 / 3 33.3%	1 / 4 25.0%	0 / 3 0.0%	1 / 1 100.0%	2 / 5 40.0%	1 / 1 100.0%	0 / 0 0.0%	0 / 0 0.0%	0 / 0 0.0%	16 / 50 32.0%E	11 / 50 22.0%	8 / 50 16.0%AB	7 / 50 14.0%ABE	5 / 50 10.0%C	3 / 50 6.0%CE		
1	Salinispora sp. CNB-440	15 / 50 30.0%	0 / 2 0.0%	0 / 1 0.0%	0 / 1 0.0%	0 / 1 0.0%	2 / 4 50.0%	1 / 4 25.0%	1 / 1 100.0%	1 / 1 100.0%	1 / 3 33.3%	0 / 1 0.0%	0 / 4 0.0%	2 / 2 100.0%	0 / 1 0.0%	0 / 2 0.0%	0 / 1 0.0%	1 / 3 33.3%	0 / 1 0.0%	1 / 3 33.3%	1 / 4 25.0%	0 / 3 0.0%	1 / 1 100.0%	2 / 5 40.0%	1 / 1 100.0%	0 / 0 0.0%	0 / 0 0.0%	0 / 0 0.0%	16 / 50 32.0%E	11 / 50 22.0%	8 / 50 16.0%AB	7 / 50 14.0%ABE	5 / 50 10.0%C	3 / 50 6.0%CE		
1	ATCC BAA-917	13 / 51 25.5%	0 / 2 0.0%	0 / 1 0.0%	0 / 1 0.0%	0 / 1 0.0%	2 / 4 50.0%	1 / 4 25.0%	0 / 1 0.0%	1 / 1 100.0%	1 / 3 33.3%	0 / 1 0.0%	0 / 4 0.0%	1 / 3 33.3%	0 / 1 0.0%	0 / 2 0.0%	0 / 1 0.0%	0 / 4 0.0%	0 / 1 0.0%	1 / 3 33.3%	2 / 4 50.0%	0 / 3 0.0%	1 / 1 100.0%	3 / 5 60.0%	0 / 0 0.0%	0 / 0 0.0%	0 / 0 0.0%	0 / 0 0.0%	15 / 51 29.4%E	14 / 51 27.5%	7 / 51 13.7%AB	6 / 51 11.8%CE	3 / 51 5.9%C	2 / 51 3.9%BE	2 / 51 3.9%ABE	2 / 51 3.9%B
1	Salinispora arenicola CNS-205	13 / 51 25.5%	0 / 2 0.0%	0 / 1 0.0%	0 / 1 0.0%	0 / 1 0.0%	2 / 4 50.0%	1 / 4 25.0%	0 / 1 0.0%	1 / 1 100.0%	1 / 3 33.3%	0 / 1 0.0%	0 / 4 0.0%	1 / 3 33.3%	0 / 1 0.0%	0 / 2 0.0%	0 / 1 0.0%	0 / 4 0.0%	0 / 1 0.0%	1 / 3 33.3%	2 / 4 50.0%	0 / 3 0.0%	1 / 1 100.0%	3 / 5 60.0%	0 / 0 0.0%	0 / 0 0.0%	0 / 0 0.0%	0 / 0 0.0%	15 / 51 29.4%E	14 / 51 27.5%	7 / 51 13.7%AB	6 / 51 11.8%CE	3 / 51 5.9%C	2 / 51 3.9%BE	2 / 51 3.9%ABE	2 / 51 3.9%B
17	Propionibacterineae	249 / 783 31.8%	15 / 18 83.3%	2 / 18 11.1%	2 / 18 11.1%	2 / 17 11.8%	31 / 53 58.5%	8 / 53 15.1%	16 / 17 94.1%	1 / 17 5.9%	12 / 54 22.2%	0 / 17 0.0%	38 / 68 55.9%	4 / 35 11.4%	3 / 17 17.6%	0 / 33 0.0%	2 / 19 10.5%	26 / 51 51.0%	16 / 18 88.9%	27 / 51 52.9%	19 / 69 27.5%	17 / 34 50.0%	3 / 17 17.6%	4 / 87 4.6%	1 / 1 100.0%	0 / 0 0.0%	0 / 1 0.0%	0 / 0 0.0%	220 / 783 28.1%E	182 / 783 23.2%	146 / 783 18.6%AB	103 / 783 13.2%ABE	81 / 783 10.3%CE	51 / 783 6.5%C		
15	Propionibacteriaceae	216 / 686 31.5%	14 / 16 87.5%	0 / 16 0.0%	2 / 16 12.5%	2 / 15 13.3%	28 / 46 60.9%	7 / 46 15.2%	15 / 15 100.0%	1 / 15 6.7%	9 / 48 18.8%	0 / 15 0.0%	33 / 60 55.0%	2 / 31 6.5%	3 / 15 20.0%	0 / 30 0.0%	1 / 16 6.2%	25 / 45 55.6%	14 / 15 93.3%	24 / 45 53.3%	17 / 60 28.3%	15 / 30 50.0%	1 / 15 6.7%	2 / 75 2.7%	1 / 1 100.0%	0 / 0 0.0%	0 / 0 0.0%	0 / 0 0.0%	196 / 686 28.6%E	160 / 686 23.3%	129 / 686 18.8%AB	87 / 686 12.7%ABE	71 / 686 10.3%CE	43 / 686 6.3%C		
14	Arachnia	200 / 640 31.2%	14 / 15 93.3%	0 / 15 0.0%	2 / 15 13.3%	2 / 14 14.3%	26 / 43 60.5%	5 / 43 11.6%	14 / 14 100.0%	0 / 14 0.0%	9 / 45 20.0%	0 / 14 0.0%	30 / 56 53.6%	0 / 29 0.0%	2 / 14 14.3%	0 / 28 0.0%	1 / 15 6.7%	24 / 42 57.1%	13 / 14 92.9%	24 / 42 57.1%	17 / 56 30.4%	14 / 28 50.0%	1 / 14 7.1%	2 / 70 2.9%	0 / 0 0.0%	0 / 0 0.0%	0 / 0 0.0%	0 / 0 0.0%	184 / 640 28.8%E	150 / 640 23.4%	123 / 640 19.2%AB	77 / 640 12.0%ABE	67 / 640 10.5%CE	39 / 640 6.1%C		
1	"Bacterium acidi propionici a" von Freudenreich and Orla-Jensen 1907	12 / 45 26.7%	0 / 1 0.0%	0 / 1 0.0%	1 / 1 100.0%	0 / 1 0.0%	2 / 3 66.7%	0 / 3 0.0%	1 / 1 100.0%	0 / 1 0.0%	0 / 3 0.0%	0 / 1 0.0%	4 / 4 100.0%	0 / 2 0.0%	0 / 1 0.0%	0 / 2 0.0%	0 / 1 0.0%	0 / 3 0.0%	0 / 1 0.0%	1 / 3 33.3%	2 / 4 50.0%	0 / 2 0.0%	0 / 1 0.0%	1 / 5 20.0%	0 / 0 0.0%	0 / 0 0.0%	0 / 0 0.0%	0 / 0 0.0%	13 / 45 28.9%E	9 / 45 20.0%	6 / 45 13.3%ABE	6 / 45 13.3%C	6 / 45 13.3%AB	5 / 45 11.1%CE		
1	"Propionibacterium shermanii" van Niel 1928	12 / 45 26.7%	0 / 1 0.0%	0 / 1 0.0%	1 / 1 100.0%	0 / 1 0.0%	2 / 3 66.7%	0 / 3 0.0%	1 / 1 100.0%	0 / 1 0.0%	0 / 3 0.0%	0 / 1 0.0%	4 / 4 100.0%	0 / 2 0.0%	0 / 1 0.0%	0 / 2 0.0%	0 / 1 0.0%	0 / 3 0.0%	0 / 1 0.0%	1 / 3 33.3%	2 / 4 50.0%	0 / 2 0.0%	0 / 1 0.0%	1 / 5 20.0%	0 / 0 0.0%	0 / 0 0.0%	0 / 0 0.0%	0 / 0 0.0%	13 / 45 28.9%E	9 / 45 20.0%	6 / 45 13.3%C	6 / 45 13.3%AB	6 / 45 13.3%ABE	5 / 45 11.1%CE		
1	Propionibacterium freudenreichii subsp. shermanii ATCC 9614	12 / 45 26.7%	0 / 1 0.0%	0 / 1 0.0%	1 / 1 100.0%	0 / 1 0.0%	2 / 3 66.7%	0 / 3 0.0%	1 / 1 100.0%	0 / 1 0.0%	0 / 3 0.0%	0 / 1 0.0%	4 / 4 100.0%	0 / 2 0.0%	0 / 1 0.0%	0 / 2 0.0%	0 / 1 0.0%	0 / 3 0.0%	0 / 1 0.0%	1 / 3 33.3%	2 / 4 50.0%	0 / 2 0.0%	0 / 1 0.0%	1 / 5 20.0%	0 / 0 0.0%	0 / 0 0.0%	0 / 0 0.0%	0 / 0 0.0%	13 / 45 28.9%E	9 / 45 20.0%	6 / 45 13.3%ABE	6 / 45 13.3%AB	6 / 45 13.3%C	5 / 45 11.1%CE		
10	"Bacillus acnes" Gilchrist 1900	133 / 450 29.6%	10 / 10 100.0%	0 / 10 0.0%	0 / 10 0.0%	0 / 10 0.0%	20 / 30 66.7%	0 / 30 0.0%	10 / 10 100.0%	0 / 10 0.0%	3 / 30 10.0%	0 / 10 0.0%	20 / 40 50.0%	0 / 20 0.0%	0 / 10 0.0%	0 / 20 0.0%	0 / 10 0.0%	20 / 30 66.7%	10 / 10 100.0%	20 / 30 66.7%	10 / 40 25.0%	10 / 20 50.0%	0 / 10 0.0%	0 / 50 0.0%	0 / 0 0.0%	0 / 0 0.0%	0 / 0 0.0%	0 / 0 0.0%	133 / 450 29.6%E	111 / 450 24.7%	89 / 450 19.8%AB	53 / 450 11.8%CE	44 / 450 9.8%ABE	20 / 450 4.4%C		
1	Propionibacterium acnes DSM 16379	13 / 45 28.9%	1 / 1 100.0%	0 / 1 0.0%	0 / 1 0.0%	0 / 1 0.0%	2 / 3 66.7%	0 / 3 0.0%	1 / 1 100.0%	0 / 1 0.0%	0 / 3 0.0%	0 / 1 0.0%	2 / 4 50.0%	0 / 2 0.0%	0 / 1 0.0%	0 / 2 0.0%	0 / 1 0.0%	2 / 3 66.7%	1 / 1 100.0%	2 / 3 66.7%	1 / 4 25.0%	1 / 2 50.0%	0 / 1 0.0%	0 / 5 0.0%	0 / 0 0.0%	0 / 0 0.0%	0 / 0 0.0%	0 / 0 0.0%	13 / 45 28.9%E	11 / 45 24.4%	9 / 45 20.0%AB	6 / 45 13.3%CE	4 / 45 8.9%ABE	2 / 45 4.4%C		
1	Propionibacterium acnes SK137	14 / 45 31.1%	1 / 1 100.0%	0 / 1 0.0%	0 / 1 0.0%	0 / 1 0.0%	2 / 3 66.7%	0 / 3 0.0%	1 / 1 100.0%	0 / 1 0.0%	1 / 3 33.3%	0 / 1 0.0%	2 / 4 50.0%	0 / 2 0.0%	0 / 1 0.0%	0 / 2 0.0%	0 / 1 0.0%	2 / 3 66.7%	1 / 1 100.0%	2 / 3 66.7%	1 / 4 25.0%	1 / 2 50.0%	0 / 1 0.0%	0 / 5 0.0%	0 / 0 0.0%	0 / 0 0.0%	0 / 0 0.0%	0 / 0 0.0%	13 / 45 28.9%E	11 / 45 24.4%	9 / 45 20.0%AB	5 / 45 11.1%ABE	5 / 45 11.1%CE	2 / 45 4.4%C		

1	Propionibacterium acnes 266	[illegible] 28.9%	[illegible] 100.0%	[illegible] 0.0%	[illegible] 0.0%	[illegible] 0.0%	[illegible] 66.7%	[illegible] 0.0%	[illegible] 100.0%	[illegible] 0.0%	[illegible] 0.0%	[illegible] 0.0%	[illegible] 50.0%	[illegible] 0.0%	[illegible] 0.0%	[illegible] 0.0%	[illegible] 0.0%	[illegible] 66.7%	[illegible] 100.0%	[illegible] 66.7%	[illegible] 25.0%	[illegible] 50.0%	[illegible] 0.0%	[illegible] 0.0%	[illegible] 0.0%	[illegible] 0.0%	[illegible] 0.0%	[illegible] 0.0%	[illegible] 28.9%E	[illegible] 24.4%	[illegible] 20.0%AB	[illegible] 13.3%CE	[illegible] 8.9%ABE	[illegible] 4.4%C		
1	Propionibacterium acnes 6609	13 / 45 28.9%	1 / 1 100.0%	0 / 1 0.0%	0 / 1 0.0%	0 / 1 0.0%	2 / 3 66.7%	0 / 3 0.0%	1 / 1 100.0%	0 / 1 0.0%	0 / 3 0.0%	0 / 1 0.0%	2 / 4 50.0%	0 / 2 0.0%	0 / 1 0.0%	0 / 2 0.0%	0 / 1 0.0%	2 / 3 66.7%	1 / 1 100.0%	2 / 3 66.7%	1 / 4 25.0%	1 / 2 50.0%	0 / 1 0.0%	0 / 5 0.0%	0 / 0 0.0%	0 / 0 0.0%	0 / 0 0.0%	0 / 0 0.0%	13 / 45 28.9%E	11 / 45 24.4%	9 / 45 20.0%AB	6 / 45 13.3%CE	4 / 45 8.9%ABE	2 / 45 4.4%C		
1	Propionibacterium acnes ATCC 11828	13 / 45 28.9%	1 / 1 100.0%	0 / 1 0.0%	0 / 1 0.0%	0 / 1 0.0%	2 / 3 66.7%	0 / 3 0.0%	1 / 1 100.0%	0 / 1 0.0%	0 / 3 0.0%	0 / 1 0.0%	2 / 4 50.0%	0 / 2 0.0%	0 / 1 0.0%	0 / 2 0.0%	0 / 1 0.0%	2 / 3 66.7%	1 / 1 100.0%	2 / 3 66.7%	1 / 4 25.0%	1 / 2 50.0%	0 / 1 0.0%	0 / 5 0.0%	0 / 0 0.0%	0 / 0 0.0%	0 / 0 0.0%	0 / 0 0.0%	14 / 45 31.1%E	11 / 45 24.4%	8 / 45 17.8%AB	5 / 45 11.1%ABE	5 / 45 11.1%CE	2 / 45 4.4%C		
1	Propionibacterium acnes ST9	13 / 45 28.9%	1 / 1 100.0%	0 / 1 0.0%	0 / 1 0.0%	0 / 1 0.0%	2 / 3 66.7%	0 / 3 0.0%	1 / 1 100.0%	0 / 1 0.0%	0 / 3 0.0%	0 / 1 0.0%	2 / 4 50.0%	0 / 2 0.0%	0 / 1 0.0%	0 / 2 0.0%	0 / 1 0.0%	2 / 3 66.7%	1 / 1 100.0%	2 / 3 66.7%	1 / 4 25.0%	1 / 2 50.0%	0 / 1 0.0%	0 / 5 0.0%	0 / 0 0.0%	0 / 0 0.0%	0 / 0 0.0%	0 / 0 0.0%	14 / 45 31.1%E	11 / 45 24.4%	9 / 45 20.0%AB	5 / 45 11.1%CE	4 / 45 8.9%ABE	2 / 45 4.4%C		
1	Propionibacterium acnes ST22	13 / 45 28.9%	1 / 1 100.0%	0 / 1 0.0%	0 / 1 0.0%	0 / 1 0.0%	2 / 3 66.7%	0 / 3 0.0%	1 / 1 100.0%	0 / 1 0.0%	0 / 3 0.0%	0 / 1 0.0%	2 / 4 50.0%	0 / 2 0.0%	0 / 1 0.0%	0 / 2 0.0%	0 / 1 0.0%	2 / 3 66.7%	1 / 1 100.0%	2 / 3 66.7%	1 / 4 25.0%	1 / 2 50.0%	0 / 1 0.0%	0 / 5 0.0%	0 / 0 0.0%	0 / 0 0.0%	0 / 0 0.0%	0 / 0 0.0%	13 / 45 28.9%E	12 / 45 26.7%	9 / 45 20.0%AB	5 / 45 11.1%CE	4 / 45 8.9%ABE	2 / 45 4.4%C		
1	Propionibacterium acnes ST27	13 / 45 28.9%	1 / 1 100.0%	0 / 1 0.0%	0 / 1 0.0%	0 / 1 0.0%	2 / 3 66.7%	0 / 3 0.0%	1 / 1 100.0%	0 / 1 0.0%	0 / 3 0.0%	0 / 1 0.0%	2 / 4 50.0%	0 / 2 0.0%	0 / 1 0.0%	0 / 2 0.0%	0 / 1 0.0%	2 / 3 66.7%	1 / 1 100.0%	2 / 3 66.7%	1 / 4 25.0%	1 / 2 50.0%	0 / 1 0.0%	0 / 5 0.0%	0 / 0 0.0%	0 / 0 0.0%	0 / 0 0.0%	0 / 0 0.0%	14 / 45 31.1%E	11 / 45 24.4%	9 / 45 20.0%AB	5 / 45 11.1%CE	4 / 45 8.9%ABE	2 / 45 4.4%C		
1	Propionibacterium acnes HL096PA1	14 / 45 31.1%	1 / 1 100.0%	0 / 1 0.0%	0 / 1 0.0%	0 / 1 0.0%	2 / 3 66.7%	0 / 3 0.0%	1 / 1 100.0%	0 / 1 0.0%	1 / 3 33.3%	0 / 1 0.0%	2 / 4 50.0%	0 / 2 0.0%	0 / 1 0.0%	0 / 2 0.0%	0 / 1 0.0%	2 / 3 66.7%	1 / 1 100.0%	2 / 3 66.7%	1 / 4 25.0%	1 / 2 50.0%	0 / 1 0.0%	0 / 5 0.0%	0 / 0 0.0%	0 / 0 0.0%	0 / 0 0.0%	0 / 0 0.0%	13 / 45 28.9%E	11 / 45 24.4%	9 / 45 20.0%AB	5 / 45 11.1%ABE	5 / 45 11.1%CE	2 / 45 4.4%C		
1	Propionibacterium acnes C1	14 / 45 31.1%	1 / 1 100.0%	0 / 1 0.0%	0 / 1 0.0%	0 / 1 0.0%	2 / 3 66.7%	0 / 3 0.0%	1 / 1 100.0%	0 / 1 0.0%	1 / 3 33.3%	0 / 1 0.0%	2 / 4 50.0%	0 / 2 0.0%	0 / 1 0.0%	0 / 2 0.0%	0 / 1 0.0%	2 / 3 66.7%	1 / 1 100.0%	2 / 3 66.7%	1 / 4 25.0%	1 / 2 50.0%	0 / 1 0.0%	0 / 5 0.0%	0 / 0 0.0%	0 / 0 0.0%	0 / 0 0.0%	0 / 0 0.0%	13 / 45 28.9%E	11 / 45 24.4%	9 / 45 20.0%AB	5 / 45 11.1%ABE	5 / 45 11.1%CE	2 / 45 4.4%C		
1	"Bacillus acidi propionici" von Freudenreich and Orla-Jensen 1907	25 / 52 48.1%	2 / 2 100.0%	0 / 2 0.0%	1 / 2 50.0%	1 / 1 100.0%	2 / 4 50.0%	3 / 4 75.0%	1 / 1 100.0%	0 / 1 0.0%	3 / 4 75.0%	0 / 1 0.0%	2 / 4 50.0%	0 / 2 0.0%	1 / 1 100.0%	0 / 2 0.0%	1 / 2 50.0%	1 / 3 33.3%	1 / 1 100.0%	2 / 3 66.7%	2 / 4 50.0%	1 / 2 50.0%	1 / 1 100.0%	0 / 5 0.0%	0 / 0 0.0%	0 / 0 0.0%	0 / 0 0.0%	0 / 0 0.0%	13 / 52 25.0%AB	12 / 52 23.1%E	12 / 52 23.1%ABE	8 / 52 15.4%	4 / 52 7.7%C	3 / 52 5.8%CE		
1	Propionibacterium acidipropionici ATCC 4875	25 / 52 48.1%	2 / 2 100.0%	0 / 2 0.0%	1 / 2 50.0%	1 / 1 100.0%	2 / 4 50.0%	3 / 4 75.0%	1 / 1 100.0%	0 / 1 0.0%	3 / 4 75.0%	0 / 1 0.0%	2 / 4 50.0%	0 / 2 0.0%	1 / 1 100.0%	0 / 2 0.0%	1 / 2 50.0%	1 / 3 33.3%	1 / 1 100.0%	2 / 3 66.7%	2 / 4 50.0%	1 / 2 50.0%	1 / 1 100.0%	0 / 5 0.0%	0 / 0 0.0%	0 / 0 0.0%	0 / 0 0.0%	0 / 0 0.0%	13 / 52 25.0%AB	12 / 52 23.1%E	12 / 52 23.1%ABE	8 / 52 15.4%	4 / 52 7.7%C	3 / 52 5.8%CE		
1	"Actinomyces propionicus" Buchanan and Pine 1962	18 / 47 38.3%	1 / 1 100.0%	0 / 1 0.0%	0 / 1 0.0%	0 / 1 0.0%	2 / 3 66.7%	0 / 3 0.0%	1 / 1 100.0%	0 / 1 0.0%	2 / 4 50.0%	0 / 1 0.0%	3 / 4 75.0%	0 / 3 0.0%	1 / 1 100.0%	0 / 2 0.0%	0 / 1 0.0%	2 / 3 66.7%	1 / 1 100.0%	0 / 3 0.0%	2 / 4 50.0%	2 / 2 100.0%	0 / 1 0.0%	1 / 5 20.0%	0 / 0 0.0%	0 / 0 0.0%	0 / 0 0.0%	0 / 0 0.0%	14 / 47 29.8%E	11 / 47 23.4%	9 / 47 19.1%ABE	9 / 47 19.1%AB	3 / 47 6.4%C	1 / 47 2.1%CE		
1	Propionibacterium propionicum F0230a	18 / 47 38.3%	1 / 1 100.0%	0 / 1 0.0%	0 / 1 0.0%	0 / 1 0.0%	2 / 3 66.7%	0 / 3 0.0%	1 / 1 100.0%	0 / 1 0.0%	2 / 4 50.0%	0 / 1 0.0%	3 / 4 75.0%	0 / 3 0.0%	1 / 1 100.0%	0 / 2 0.0%	0 / 1 0.0%	2 / 3 66.7%	1 / 1 100.0%	0 / 3 0.0%	2 / 4 50.0%	2 / 2 100.0%	0 / 1 0.0%	1 / 5 20.0%	0 / 0 0.0%	0 / 0 0.0%	0 / 0 0.0%	0 / 0 0.0%	14 / 47 29.8%E	11 / 47 23.4%	9 / 47 19.1%ABE	9 / 47 19.1%AB	3 / 47 6.4%C	1 / 47 2.1%CE		
1	"Bacteroides avidus" Eggerth 1935	12 / 46 26.1%	1 / 1 100.0%	0 / 1 0.0%	0 / 1 0.0%	1 / 1 100.0%	0 / 3 0.0%	2 / 3 66.7%	1 / 1 100.0%	0 / 1 0.0%	1 / 4 25.0%	0 / 1 0.0%	1 / 4 25.0%	0 / 2 0.0%	0 / 1 0.0%	0 / 2 0.0%	0 / 1 0.0%	1 / 3 33.3%	1 / 1 100.0%	1 / 3 33.3%	1 / 4 25.0%	1 / 2 50.0%	0 / 1 0.0%	0 / 5 0.0%	0 / 0 0.0%	0 / 0 0.0%	0 / 0 0.0%	0 / 0 0.0%	12 / 46 26.1%E	11 / 46 23.9%	6 / 46 13.0%ABE	6 / 46 13.0%C	6 / 46 13.0%AB	5 / 46 10.9%CE		
1	Propionibacterium avidum 44067	12 / 46 26.1%	1 / 1 100.0%	0 / 1 0.0%	0 / 1 0.0%	1 / 1 100.0%	0 / 3 0.0%	2 / 3 66.7%	1 / 1 100.0%	0 / 1 0.0%	1 / 4 25.0%	0 / 1 0.0%	1 / 4 25.0%	0 / 2 0.0%	0 / 1 0.0%	0 / 2 0.0%	0 / 1 0.0%	1 / 3 33.3%	1 / 1 100.0%	1 / 3 33.3%	1 / 4 25.0%	1 / 2 50.0%	0 / 1 0.0%	0 / 5 0.0%	0 / 0 0.0%	0 / 0 0.0%	0 / 0 0.0%	0 / 0 0.0%	12 / 46 26.1%E	11 / 46 23.9%	6 / 46 13.0%AB	6 / 46 13.0%C	6 / 46 13.0%ABE	5 / 46 10.9%CE		
1	Microlunatus	16 / 46 34.8%	0 / 1 0.0%	0 / 1 0.0%	0 / 1 0.0%	0 / 1 0.0%	2 / 3 66.7%	2 / 3 66.7%	1 / 1 100.0%	1 / 1 100.0%	0 / 3 0.0%	0 / 1 0.0%	3 / 4 75.0%	2 / 2 100.0%	1 / 1 100.0%	0 / 2 0.0%	0 / 1 0.0%	1 / 3 33.3%	1 / 1 100.0%	0 / 3 0.0%	0 / 4 0.0%	1 / 2 50.0%	0 / 1 0.0%	0 / 5 0.0%	1 / 1 100.0%	0 / 0 0.0%	0 / 0 0.0%	0 / 0 0.0%	12 / 46 26.1%E	10 / 46 21.7%	10 / 46 21.7%ABE	6 / 46 13.0%AB	4 / 46 8.7%CE	4 / 46 8.7%C		
1	ATCC 700054	16 / 46 34.8%	0 / 1 0.0%	0 / 1 0.0%	0 / 1 0.0%	0 / 1 0.0%	2 / 3 66.7%	2 / 3 66.7%	1 / 1 100.0%	1 / 1 100.0%	0 / 3 0.0%	0 / 1 0.0%	3 / 4 75.0%	2 / 2 100.0%	1 / 1 100.0%	0 / 2 0.0%	0 / 1 0.0%	1 / 3 33.3%	1 / 1 100.0%	0 / 3 0.0%	0 / 4 0.0%	1 / 2 50.0%	0 / 1 0.0%	0 / 5 0.0%	1 / 1 100.0%	0 / 0 0.0%	0 / 0 0.0%	0 / 0 0.0%	12 / 46 26.1%E	10 / 46 21.7%	10 / 46 21.7%ABE	6 / 46 13.0%AB	4 / 46 8.7%CE	4 / 46 8.7%C		
1	Microlunatus phosphovorus NM-1	16 / 46 34.8%	0 / 1 0.0%	0 / 1 0.0%	0 / 1 0.0%	0 / 1 0.0%	2 / 3 66.7%	2 / 3 66.7%	1 / 1 100.0%	1 / 1 100.0%	0 / 3 0.0%	0 / 1 0.0%	3 / 4 75.0%	2 / 2 100.0%	1 / 1 100.0%	0 / 2 0.0%	0 / 1 0.0%	1 / 3 33.3%	1 / 1 100.0%	0 / 3 0.0%	0 / 4 0.0%	1 / 2 50.0%	0 / 1 0.0%	0 / 5 0.0%	1 / 1 100.0%	0 / 0 0.0%	0 / 0 0.0%	0 / 0 0.0%	12 / 46 26.1%E	10 / 46 21.7%ABE	10 / 46 21.7%	6 / 46 13.0%AB	4 / 46 8.7%CE	4 / 46 8.7%C		
2	Nocardioidaceae	33 / 97 34.0%	1 / 2 50.0%	2 / 2 100.0%	0 / 2 0.0%	0 / 2 0.0%	3 / 7 42.9%	1 / 7 14.3%	1 / 2 50.0%	0 / 2 0.0%	3 / 6 50.0%	0 / 2 0.0%	5 / 8 62.5%	2 / 4 50.0%	0 / 2 0.0%	0 / 3 0.0%	1 / 3 33.3%	1 / 6 16.7%	2 / 3 66.7%	3 / 6 50.0%	2 / 9 22.2%	2 / 4 50.0%	2 / 2 100.0%	2 / 12 16.7%	0 / 0 0.0%	0 / 0 0.0%	0 / 1 0.0%	0 / 0 0.0%	24 / 97 24.7%E	22 / 97 22.7%	17 / 97 17.5%AB	16 / 97 16.5%ABE	10 / 97 10.3%CE	8 / 97 8.2%C		
1	Nocardioides	17 / 45 37.8%	0 / 1 0.0%	1 / 1 100.0%	0 / 1 0.0%	0 / 1 0.0%	2 / 3 66.7%	1 / 3 33.3%	1 / 1 100.0%	0 / 1 0.0%	2 / 3 66.7%	0 / 1 0.0%	3 / 4 75.0%	0 / 2 0.0%	0 / 1 0.0%	0 / 2 0.0%	1 / 1 100.0%	1 / 3 33.3%	0 / 1 0.0%	2 / 3 66.7%	0 / 4 0.0%	1 / 2 50.0%	1 / 1 100.0%	1 / 5 20.0%	0 / 0 0.0%	0 / 0 0.0%	0 / 0 0.0%	0 / 0 0.0%	12 / 45 26.7%	9 / 45 20.0%ABE	8 / 45 17.8%AB	6 / 45 13.3%E	6 / 45 13.3%CE	4 / 45 8.9%C		
1	Nocardioides sp. JS614	17 / 45 37.8%	0 / 1 0.0%	1 / 1 100.0%	0 / 1 0.0%	0 / 1 0.0%	2 / 3 66.7%	1 / 3 33.3%	1 / 1 100.0%	0 / 1 0.0%	2 / 3 66.7%	0 / 1 0.0%	3 / 4 75.0%	0 / 2 0.0%	0 / 1 0.0%	0 / 2 0.0%	1 / 1 100.0%	1 / 3 33.3%	0 / 1 0.0%	2 / 3 66.7%	0 / 4 0.0%	1 / 2 50.0%	1 / 1 100.0%	1 / 5 20.0%	0 / 0 0.0%	0 / 0 0.0%	0 / 0 0.0%	0 / 0 0.0%	12 / 45 26.7%	9 / 45 20.0%ABE	8 / 45 17.8%AB	6 / 45 13.3%CE	6 / 45 13.3%E	4 / 45 8.9%C		
1	Hongia	16 / 52 30.8%	1 / 1 100.0%	1 / 1 100.0%	0 / 1 0.0%	0 / 1 0.0%	1 / 4 25.0%	0 / 4 0.0%	0 / 1 0.0%	0 / 1 0.0%	1 / 3 33.3%	0 / 1 0.0%	2 / 4 50.0%	2 / 2 100.0%	0 / 1 0.0%	0 / 1 0.0%	0 / 2 0.0%	0 / 3 0.0%	2 / 2 100.0%	1 / 3 33.3%	2 / 5 40.0%	1 / 2 50.0%	1 / 1 100.0%	1 / 7 14.3%	0 / 0 0.0%	0 / 0 0.0%	0 / 1 0.0%	0 / 0 0.0%	18 / 52 34.6%E	10 / 52 19.2%	9 / 52 17.3%AB	7 / 52 13.5%ABE	4 / 52 7.7%C	4 / 52 7.7%CE		
1	DSM 17836	16 / 52 30.8%	1 / 1 100.0%	1 / 1 100.0%	0 / 1 0.0%	0 / 1 0.0%	1 / 4 25.0%	0 / 4 0.0%	0 / 1 0.0%	0 / 1 0.0%	1 / 3 33.3%	0 / 1 0.0%	2 / 4 50.0%	2 / 2 100.0%	0 / 1 0.0%	0 / 1 0.0%	0 / 2 0.0%	0 / 3 0.0%	2 / 2 100.0%	1 / 3 33.3%	2 / 5 40.0%	1 / 2 50.0%	1 / 1 100.0%	1 / 7 14.3%	0 / 0 0.0%	0 / 0 0.0%	0 / 1 0.0%	0 / 0 0.0%	18 / 52 34.6%E	10 / 52 19.2%	9 / 52 17.3%AB	7 / 52 13.5%ABE	4 / 52 7.7%CE	4 / 52 7.7%C		
1	Kribbella flavida DSM 17836	16 / 52 30.8%	1 / 1 100.0%	1 / 1 100.0%	0 / 1 0.0%	0 / 1 0.0%	1 / 4 25.0%	0 / 4 0.0%	0 / 1 0.0%	0 / 1 0.0%	1 / 3 33.3%	0 / 1 0.0%	2 / 4 50.0%	2 / 2 100.0%	0 / 1 0.0%	0 / 1 0.0%	0 / 2 0.0%	0 / 3 0.0%	2 / 2 100.0%	1 / 3 33.3%	2 / 5 40.0%	1 / 2 50.0%	1 / 1 100.0%	1 / 7 14.3%	0 / 0 0.0%	0 / 0 0.0%	0 / 1 0.0%	0 / 0 0.0%	18 / 52 34.6%E	10 / 52 19.2%	9 / 52 17.3%AB	7 / 52 13.5%ABE	4 / 52 7.7%C	4 / 52 7.7%CE		
9	Pseudonocardineae	112 / 507 22.1%	3 / 20 15.0%	1 / 10 10.0%	0 / 10 0.0%	1 / 10 10.0%	8 / 41 19.5%	25 / 41 61.0%	8 / 11 72.7%	1 / 13 7.7%	3 / 31 9.7%	1 / 11 9.1%	5 / 40 12.5%	6 / 21 28.6%	4 / 10 40.0%	0 / 21 0.0%	0 / 11 0.0%	6 / 30 20.0%	2 / 10 20.0%	4 / 31 12.9%	7 / 44 15.9%	5 / 24 20.8%	4 / 10 40.0%	14 / 52 26.9%	4 / 5 80.0%	0 / 0 0.0%	0 / 0 0.0%	0 / 0 0.0%	178 / 507 35.1%	156 / 507 30.8%E	87 / 507 17.2%AB	43 / 507 8.5%C	24 / 507 4.7%ABE	18 / 507 3.6%CE	1 / 507 0.2%B	
9	Actinosynnemataceae	112 / 507 22.1%	3 / 20 15.0%	1 / 10 10.0%	0 / 10 0.0%	1 / 10 10.0%	8 / 41 19.5%	25 / 41 61.0%	8 / 11 72.7%	1 / 13 7.7%	3 / 31 9.7%	1 / 11 9.1%	5 / 40 12.5%	6 / 21 28.6%	4 / 10 40.0%	0 / 21 0.0%	0 / 11 0.0%	6 / 30 20.0%	2 / 10 20.0%	4 / 31 12.9%	7 / 44 15.9%	5 / 24 20.8%	4 / 10 40.0%	14 / 52 26.9%	4 / 5 80.0%	0 / 0 0.0%	0 / 0 0.0%	0 / 0 0.0%	178 / 507 35.1%	156 / 507 30.8%E	87 / 507 17.2%AB	43 / 507 8.5%C	24 / 507 4.7%ABE	18 / 507 3.6%CE	1 / 507 0.2%B	
3	Amycolatopsis	38 / 206 18.4%	0 / 8 0.0%	0 / 4 0.0%	0 / 4 0.0%	0 / 4 0.0%	4 / 16 25.0%	12 / 20 60.0%	4 / 4 100.0%	0 / 7 0.0%	0 / 12 0.0%	0 / 4 0.0%	1 / 16 6.2%	0 / 8 0.0%	0 / 4 0.0%	0 / 8 0.0%	0 / 4 0.0%	3 / 12 25.0%	0 / 4 0.0%	0 / 12 0.0%	3 / 19 15.8%	3 / 9 33.3%	1 / 4 25.0%	4 / 20 20.0%	3 / 3 100.0%	0 / 0 0.0%	0 / 0 0.0%	0 / 0 0.0%	78 / 206 37.9%	61 / 206 29.6%E	31 / 206 15.0%AB	21 / 206 10.2%C	8 / 206 3.9%CE	7 / 206 3.4%ABE		
1	"Streptomyces orientalis" Pittenger and Brigham 1956	8 / 50 16.0%	0 / 2 0.0%	0 / 1 0.0%	0 / 1 0.0%	0 / 1 0.0%	1 / 4 25.0%	3 / 5 60.0%	1 / 1 100.0%	0 / 1 0.0%	0 / 3 0.0%	0 / 1 0.0%	1 / 4 25.0%	0 / 2 0.0%	0 / 1 0.0%	0 / 2 0.0%	0 / 1 0.0%	0 / 3 0.0%	0 / 1 0.0%	0 / 3 0.0%	0 / 4 0.0%	0 / 3 0.0%	1 / 1 100.0%	1 / 5 20.0%	0 / 0 0.0%	0 / 0 0.0%	0 / 0 0.0%	0 / 0 0.0%	18 / 50 36.0%	16 / 50 32.0%E	7 / 50 14.0%AB	6 / 50 12.0%C	2 / 50 4.0%CE	1 / 50 2.0%ABE		
1	Amycolatopsis orientalis HCCB10007	8 / 50 16.0%	0 / 2 0.0%	0 / 1 0.0%	0 / 1 0.0%	0 / 1 0.0%	1 / 4 25.0%	3 / 5 60.0%	1 / 1 100.0%	0 / 1 0.0%	0 / 3 0.0%	0 / 1 0.0%	1 / 4 25.0%	0 / 2 0.0%	0 / 1 0.0%	0 / 2 0.0%	0 / 1 0.0%	0 / 3 0.0%	0 / 1 0.0%	0 / 3 0.0%	0 / 4 0.0%	0 / 3 0.0%	1 / 1 100.0%	1 / 5 20.0%	0 / 0 0.0%	0 / 0 0.0%	0 / 0 0.0%	0 / 0 0.0%	18 / 50 36.0%	16 / 50 32.0%E	7 / 50 14.0%AB	6 / 50 12.0%C	2 / 50 4.0%CE	1 / 50 2.0%ABE		
2	"Streptomyces mediterranei" Margalith and Beretta 1960	30 / 156 19.2%	0 / 6 0.0%	0 / 3 0.0%	0 / 3 0.0%	0 / 3 0.0%	3 / 12 25.0%	9 / 15 60.0%	3 / 3 100.0%	0 / 6 0.0%	0 / 9 0.0%	0 / 3 0.0%	0 / 12 0.0%	0 / 6 0.0%	0 / 3 0.0%	0 / 6 0.0%	0 / 3 0.0%	3 / 9 33.3%	0 / 3 0.0%	0 / 9 0.0%	3 / 15 20.0%	3 / 6 50.0%	0 / 3 0.0%	3 / 15 20.0%	3 / 3 100.0%	0 / 0 0.0%	0 / 0 0.0%	0 / 0 0.0%	60 / 156 38.5%	45 / 156 28.8%E	24 / 156 15.4%AB	15 / 156 9.6%C	6 / 156 3.8%ABE	6 / 156 3.8%CE		
1	Amycolatopsis mediterranei S699	20 / 104 19.2%	0 / 4 0.0%	0 / 2 0.0%	0 / 2 0.0%	0 / 2 0.0%	2 / 8 25.0%	6 / 10 60.0%	2 / 2 100.0%	0 / 4 0.0%	0 / 6 0.0%	0 / 2 0.0%	0 / 8 0.0%	0 / 4 0.0%	0 / 2 0.0%	0 / 4 0.0%	0 / 2 0.0%	2 / 6 33.3%	0 / 2 0.0%	0 / 6 0.0%	2 / 10 20.0%	2 / 4 50.0%	0 / 2 0.0%	2 / 10 20.0%	2 / 2 100.0%	0 / 0 0.0%	0 / 0 0.0%	0 / 0 0.0%	40 / 104 38.5%	30 / 104 28.8%E	16 / 104 15.4%AB	10 / 104 9.6%C	4 / 104 3.8%ABE	4 / 104 3.8%CE		
1	Amycolatopsis mediterranei U32	10 / 52 19.2%	0 / 2 0.0%	0 / 1 0.0%	0 / 1 0.0%	0 / 1 0.0%	1 / 4 25.0%	3 / 5 60.0%	1 / 1 100.0%	0 / 2 0.0%	0 / 3 0.0%	0 / 1 0.0%	0 / 4 0.0%	0 / 2 0.0%	0 / 1 0.0%	0 / 2 0.0%	0 / 1 0.0%	1 / 3 33.3%	0 / 1 0.0%	0 / 3 0.0%	1 / 5 20.0%	1 / 2 50.0%	0 / 1 0.0%	1 / 5 20.0%	1 / 1 100.0%	0 / 0 0.0%	0 / 0 0.0%	0 / 0 0.0%	20 / 52 38.5%	15 / 52 28.8%E	8 / 52 15.4%AB	5 / 52 9.6%C	2 / 52 3.8%CE	2 / 52 3.8%ABE		
1	Faenia	12 / 50 24.0%	1 / 2 50.0%	1 / 1 100.0%	0 / 1 0.0%	1 / 1 100.0%	0 / 4 0.0%	2 / 4 50.0%	1 / 1 100.0%	0 / 1 0.0%	0 / 3 0.0%	0 / 1 0.0%	0 / 4 0.0%	1 / 2 50.0%	1 / 1 100.0%	0 / 2 0.0%	0 / 1 0.0%	0 / 3 0.0%	1 / 1 100.0%	0 / 3 0.0%	0 / 4 0.0%	0 / 3 0.0%	1 / 1 100.0%	1 / 5 20.0%	1 / 1 100.0%	0 / 0 0.0%	0 / 0 0.0%	0 / 0 0.0%	17 / 50 34.0%	15 / 50 30.0%E	9 / 50 18.0%AB	3 / 50 6.0%CE	3 / 50 6.0%C	2 / 50 4.0%ABE	1 / 50 2.0%B	
1	"Actinomyces erythreus" (sic) Waksman 1923	12 / 50 24.0%	1 / 2 50.0%	1 / 1 100.0%	0 / 1 0.0%	1 / 1 100.0%	0 / 4 0.0%	2 / 4 50.0%	1 / 1 100.0%	0 / 1 0.0%	0 / 3 0.0%	0 / 1 0.0%	0 / 4 0.0%	1 / 2 50.0%	1 / 1 100.0%	0 / 2 0.0%	0 / 1 0.0%	0 / 3 0.0%	1 / 1 100.0%	0 / 3 0.0%	0 / 4 0.0%	0 / 3 0.0%	1 / 1 100.0%	1 / 5 20.0%	1 / 1 100.0%	0 / 0 0.0%	0 / 0 0.0%	0 / 0 0.0%	17 / 50 34.0%	15 / 50 30.0%E	9 / 50 18.0%AB	3 / 50 6.0%CE	3 / 50 6.0%C	2 / 50 4.0%ABE	1 / 50 2.0%B	
1	Saccharopolyspora erythraea ATCC 11635	12 / 50 24.0%	1 / 2 50.0%	1 / 1 100.0%	0 / 1 0.0%	1 / 1 100.0%	0 / 4 0.0%	2 / 4 50.0%	1 / 1 100.0%	0 / 1 0.0%	0 / 3 0.0%	0 / 1 0.0%	0 / 4 0.0%	1 / 2 50.0%	1 / 1 100.0%	0 / 2 0.0%	0 / 1 0.0%	0 / 3 0.0%	1 / 1 100.0%	0 / 3 0.0%	0 / 4 0.0%	0 / 3 0.0%	1 / 1 100.0%	1 / 5 20.0%	1 / 1 100.0%	0 / 0 0.0%	0 / 0 0.0%	0 / 0 0.0%	17 / 50 34.0%	15 / 50 30.0%E	9 / 50 18.0%AB	3 / 50 6.0%CE	3 / 50 6.0%C	2 / 50 4.0%ABE	1 / 50 2.0%B	
1	Actinobispora	14 / 46 30.4%	0 / 1 0.0%	0 / 1 0.0%	0 / 1 0.0%	0 / 1 0.0%	1 / 4 25.0%	1 / 3 33.3%	0 / 1 0.0%	0 / 1 0.0%	1 / 3 33.3%	1 / 1 100.0%	3 / 4 75.0%	1 / 2 50.0%	0 / 1 0.0%	0 / 2 0.0%	0 / 1 0.0%	0 / 3 0.0%	1 / 1 100.0%	2 / 3 66.7%	1 / 4 25.0%	0 / 2 0.0%	0 / 1 0.0%	2 / 5 40.0%	0 / 0 0.0%	0 / 0 0.0%	0 / 0 0.0%	0 / 0 0.0%	14 / 46 30.4%E	13 / 46 28.3%	11 / 46 23.9%AB	4 / 46 8.7%C	3 / 46 6.5%ABE	1 / 46 2.2%CE		
1	ATCC 55486	14 / 46 30.4%	0 / 1 0.0%	0 / 1 0.0%	0 / 1 0.0%	0 / 1 0.0%	1 / 4 25.0%	1 / 3 33.3%	0 / 1 0.0%	0 / 1 0.0%	1 / 3 33.3%	1 / 1 100.0%	3 / 4 75.0%	1 / 2 50.0%	0 / 1 0.0%	0 / 2 0.0%	0 / 1 0.0%	0 / 3 0.0%	1 / 1 100.0%	2 / 3 66.7%	1 / 4 25.0%	0 / 2 0.0%	0 / 1 0.0%	2 / 5 40.0%	0 / 0 0.0%	0 / 0 0.0%	0 / 0 0.0%	0 / 0 0.0%	14 / 46 30.4%E	13 / 46 28.3%	11 / 46 23.9%AB	4 / 46 8.7%C	3 / 46 6.5%ABE	1 / 46 2.2%CE		
1	Pseudonocardia dioxanivorans CB1190	14 / 46 30.4%	0 / 1 0.0%	0 / 1 0.0%	0 / 1 0.0%	0 / 1 0.0%	1 / 4 25.0%	1 / 3 33.3%	0 / 1 0.0%	0 / 1 0.0%	1 / 3 33.3%	1 / 1 100.0%	3 / 4 75.0%	1 / 2 50.0%	0 / 1 0.0%	0 / 2 0.0%	0 / 1 0.0%	0 / 3 0.0%	1 / 1 100.0%	2 / 3 66.7%	1 / 4 25.0%	0 / 2 0.0%	0 / 1 0.0%	2 / 5 40.0%	0 / 0 0.0%	0 / 0 0.0%	0 / 0 0.0%	0 / 0 0.0%	14 / 46 30.4%E	13 / 46 28.3%	11 / 46 23.9%AB	4 / 46 8.7%C	3 / 46 6.5%ABE	1 / 46 2.2%CE		
1	Saccharomonospora	9 / 47 19.1%	0 / 1 0.0%	0 / 1 0.0%	0 / 1 0.0%	0 / 1 0.0%	1 / 4 25.0%	3 / 3 100.0%	0 / 1 0.0%	0 / 1 0.0%	1 / 3 33.3%	0 / 1 0.0%	1 / 4 25.0%	1 / 2 50.0%	0 / 1 0.0%	0 / 2 0.0%	0 / 1 0.0%	0 / 3 0.0%	0 / 1 0.0%	0 / 3 0.0%	0 / 4 0.0%	0 / 2 0.0%	0 / 1 0.0%	2 / 5 40.0%	0 / 1 0.0%	0 / 0 0.0%	0 / 0 0.0%	0 / 0 0.0%	17 / 47 36.2%	13 / 47 27.7%E	7 / 47 14.9%AB	6 / 47 12.8%C	2 / 47 4.3%CE	2 / 47 4.3%ABE		
1	"Saccharomonospora internatus" (Agre et al. 1974) Greiner-Mai et al. 1988	9 / 47 19.1%	0 / 1 0.0%	0 / 1 0.0%	0 / 1 0.0%	0 / 1 0.0%	1 / 4 25.0%	3 / 3 100.0%	0 / 1 0.0%	0 / 1 0.0%	1 / 3 33.3%	0 / 1 0.0%	1 / 4 25.0%	1 / 2 50.0%	0 / 1 0.0%	0 / 2 0.0%	0 / 1 0.0%	0 / 3 0.0%	0 / 1 0.0%	0 / 3 0.0%	0 / 4 0.0%	0 / 2 0.0%	0 / 1 0.0%	2 / 5 40.0%	0 / 1 0.0%	0 / 0 0.0%	0 / 0 0.0%	0 / 0 0.0%	17 / 47 36.2%	13 / 47 27.7%E	7 / 47 14.9%AB	6 / 47 12.8%C	2 / 47 4.3%ABE	2 / 47 4.3%CE		
1	Saccharomonospora viridis ATCC 15386	9 / 47 19.1%	0 / 1 0.0%	0 / 1 0.0%	0 / 1 0.0%	0 / 1 0.0%	1 / 4 25.0%	3 / 3 100.0%	0 / 1 0.0%	0 / 1 0.0%	1 / 3 33.3%	0 / 1 0.0%	1 / 4 25.0%	1 / 2 50.0%	0 / 1 0.0%	0 / 2 0.0%	0 / 1 0.0%	0 / 3 0.0%	0 / 1 0.0%	0 / 3 0.0%	0 / 4 0.0%	0 / 2 0.0%	0 / 1 0.0%	2 / 5 40.0%	0 / 1 0.0%	0 / 0 0.0%	0 / 0 0.0%	0 / 0 0.0%	17 / 47 36.2%	13 / 47 27.7%E	7 / 47 14.9%AB	6 / 47 12.8%C	2 / 47 4.3%CE	2 / 47 4.3%ABE		
1	Saccharothrix	10 / 51 19.6%	0 / 3 0.0%	0 / 1 0.0%	0 / 1 0.0%	0 / 1 0.0%	0 / 4 0.0%	2 / 3 66.7%	1 / 1 100.0%	0 / 1 0.0%	0 / 3 0.0%	0 / 1 0.0%	0 / 4 0.0%	2 / 2 100.0%	1 / 1 100.0%	0 / 3 0.0%	0 / 1 0.0%	1 / 3 33.3%	0 / 1 0.0%	1 / 3 33.3%	0 / 5 0.0%	0 / 2 0.0%	1 / 1 100.0%	1 / 6 16.7%	0 / 0 0.0%	0 / 0 0.0%	0 / 0 0.0%	0 / 0 0.0%	20 / 51 39.2%E	19 / 51 37.3%	9 / 51 17.6%AB	2 / 51 3.9%C	1 / 51 2.0%ABE			
1	ATCC 51144	10 / 51 19.6%	0 / 3 0.0%	0 / 1 0.0%	0 / 1 0.0%	0 / 1 0.0%	0 / 4 0.0%	2 / 3 66.7%	1 / 1 100.0%	0 / 1 0.0%	0 / 3 0.0%	0 / 1 0.0%	0 / 4 0.0%	2 / 2 100.0%	1 / 1 100.0%	0 / 3 0.0%	0 / 1 0.0%	1 / 3 33.3%	0 / 1 0.0%	1 / 3 33.3%	0 / 5 0.0%	0 / 2 0.0%	1 / 1 100.0%	1 / 6 16.7%	0 / 0 0.0%	0 / 0 0.0%	0 / 0 0.0%	0 / 0 0.0%	20 / 51 39.2%E	19 / 51 37.3%	9 / 51 17.6%AB	2 / 51 3.9%C	1 / 51 2.0%ABE			
1	Saccharothrix espanaensis ATCC 51144	10 / 51 19.6%	0 / 3 0.0%	0 / 1 0.0%	0 / 1 0.0%	0 / 1 0.0%	0 / 4 0.0%	2 / 3 66.7%	1 / 1 100.0%	0 / 1 0.0%	0 / 3 0.0%	0 / 1 0.0%	0 / 4 0.0%	2 / 2 100.0%	1 / 1 100.0%	0 / 3 0.0%	0 / 1 0.0%	1 / 3 33.3%	0 / 1 0.0%	1 / 3 33.3%	0 / 5 0.0%	0 / 2 0.0%	1 / 1 100.0%	1 / 6 16.7%	0 / 0 0.0%	0 / 0 0.0%	0 / 0 0.0%	0 / 0 0.0%	20 / 51 39.2%E	19 / 51 37.3%	9 / 51 17.6%AB	2 / 51 3.9%C	1 / 51 2.0%ABE			
1	Actinosynnema	12 / 54 22.2%	1 / 3 33.3%	0 / 1 0.0%	0 / 1 0.0%	0 / 1 0.0%	1 / 4 25.0%	2 / 4 50.0%	1 / 2 50.0%	0 / 1 0.0%	0 / 3 0.0%	0 / 2 0.0%	0 / 4 0.0%	1 / 2 50.0%	1 / 1 100.0%	0 / 2 0.0%	0 / 1 0.0%	1 / 3 33.3%	0 / 1 0.0%	1 / 4 25.0%	2 / 4 50.0%	0 / 3 0.0%	0 / 1 0.0%	1 / 6 16.7%	0 / 0 0.0%	0 / 0 0.0%	0 / 0 0.0%	0 / 0 0.0%	20 / 54 37.0%E	17 / 54 31.5%	9 / 54 16.7%AB	4 / 54 7.4%C	3 / 54 5.6%ABE	1 / 54 1.9%CE		
1	ATCC 29888	12 / 54 22.2%	1 / 3 33.3%	0 / 1 0.0%	0 / 1 0.0%	0 / 1 0.0%	1 / 4 25.0%	2 / 4 50.0%	1 / 2 50.0%	0 / 1 0.0%	0 / 3 0.0%	0 / 2 0.0%	0 / 4 0.0%	1 / 2 50.0%	1 / 1 100.0%	0 / 2 0.0%	0 / 1 0.0%	1 / 3 33.3%	0 / 1 0.0%	1 / 4 25.0%	2 / 4 50.0%	0 / 3 0.0%	0 / 1 0.0%	1 / 6 16.7%	0 / 0 0.0%	0 / 0 0.0%	0 / 0 0.0%	0 / 0 0.0%	20 / 54 37.0%E	17 / 54 31.5%	9 / 54 16.7%AB	4 / 54 7.4%C	3 / 54 5.6%ABE	1 / 54 1.9%CE		
1	Actinosynnema mirum DSM 43827	12 / 54 22.2%	1 / 3 33.3%	0 / 1 0.0%	0 / 1 0.0%	0 / 1 0.0%	1 / 4 25.0%	2 / 4 50.0%	1 / 2 50.0%	0 / 1 0.0%	0 / 3 0.0%	0 / 2 0.0%	0 / 4 0.0%	1 / 2 50.0%	1 / 1 100.0%	0 / 2 0.0%	0 / 1 0.0%	1 / 3 33.3%	0 / 1 0.0%	1 / 4 25.0%	2 / 4 50.0%	0 / 3 0.0%	0 / 1 0.0%	1 / 6 16.7%	0 / 0 0.0%	0 / 0 0.0%	0 / 0 0.0%	0 / 0 0.0%	20 / 54 37.0%E	17 / 54 31.5%	9 / 54 16.7%AB	4 / 54 7.4%C	3 / 54 5.6%ABE	1 / 54 1.9%CE		
1	Thermobispora	17 / 53 32.1%	1 / 2 50.0%	0 / 1 0.0%	0 / 1 0.0%	0 / 1 0.0%	1 / 5 20.0%	3 / 4 75.0%	1 / 1 100.0%	1 / 1 100.0%	1 / 4 25.0%	0 / 1 0.0%	0 / 4 0.0%	0 / 3 0.0%	1 / 1 100.0%	0 / 2 0.0%	0 / 2 0.0%	1 / 3 33.3%	0 / 1 0.0%	0 / 3 0.0%	1 / 4 25.0%	2 / 3 66.7%	1 / 1 100.0%	3 / 5 60.0%	0 / 0 0.0%	0 / 0 0.0%	0 / 0 0.0%	0 / 0 0.0%	17 / 53 32.1%	13 / 53 24.5%E	11 / 53 20.8%AB	6 / 53 11.3%ABE	3 / 53 5.7%CE	3 / 53 5.7%C		
1	"Thermopolyspora bispora" Henssen 1957	17 / 53 32.1%	1 / 2 50.0%	0 / 1 0.0%	0 / 1 0.0%	0 / 1 0.0%	1 / 5 20.0%	3 / 4 75.0%	1 / 1 100.0%	1 / 1 100.0%	1 / 4 25.0%	0 / 1 0.0%	0 / 4 0.0%	0 / 3 0.0%	1 / 1 100.0%	0 / 2 0.0%	0 / 2 0.0%	1 / 3 33.3%	0 / 1 0.0%	0 / 3 0.0%	1 / 4 25.0%	2 / 3 66.7%	1 / 1 100.0%	3 / 5 60.0%	0 / 0 0.0%	0 / 0 0.0%	0 / 0 0.0%	0 / 0 0.0%	17 / 53 32.1%	13 / 53 24.5%E	11 / 53 20.8%AB	6 / 53 11.3%ABE	3 / 53 5.7%C	3 / 53 5.7%CE		
1	Thermobispora bispora ATCC 19993	17 / 53 32.1%	1 / 2 50.0%	0 / 1 0.0%	0 / 1 0.0%	0 / 1 0.0%	1 / 5 20.0%	3 / 4 75.0%	1 / 1 100.0%	1 / 1 100.0%	1 / 4 25.0%	0 / 1 0.0%	0 / 4 0.0%	0 / 3 0.0%	1 / 1 100.0%	0 / 2 0.0%	0 / 2 0.0%	1 / 3 33.3%	0 / 1 0.0%	0 / 3 0.0%	1 / 4 25.0%	2 / 3 66.7%	1 / 1 100.0%	3 / 5 60.0%	0 / 0 0.0%	0 / 0 0.0%	0 / 0 0.0%	0 / 0 0.0%	17 / 53 32.1%	13 / 53 24.5%E	11 / 53 20.8%AB	6 / 53 11.3%ABE	3 / 53 5.7%CE	3 / 53 5.7%C		
17	Streptomycetes	215 / 1118 19.2%	6 / 59 10.2%	0 / 34 0.0%	0 / 34 0.0%	10 / 18 55.6%	41 / 85 48.2%	21 / 103 20.4%	3 / 17 17.6%	0 / 17 0.0%	1 / 70 1.4%	0 / 23 0.0%	16 / 74 21.6%	3 / 70 4.3%	5 / 17 29.4%	1 / 49 2.0%	21 / 35 60.0%	18 / 67 26.9%	0 / 17 0.0%	13 / 68 19.1%	15 / 71 21.1%	4 / 68 5.9%	6 / 17 35.3%	31 / 102 30.4%	0 / 3 0.0%	0 / 0 0.0%	0 / 0 0.0%	0 / 0 0.0%	393 / 1118 35.2%	352 / 1118 31.5%E	110 / 1118 9.8%AB	108 / 1118 9.7%C	97 / 1118 8.7%ABE	50 / 1118 4.5%CE	4 / 1118 0.4%BE	4 / 1118 0.4%B
17	Actinoplanaceae	215 / 1118 19.2%	6 / 59 10.2%	0 / 34 0.0%	0 / 34 0.0%	10 / 18 55.6%	41 / 85 48.2%	21 / 103 20.4%	3 / 17 17.6%	0 / 17 0.0%	1 / 70 1.4%	0 / 23 0.0%	16 / 74 21.6%	3 / 70 4.3%	5 / 17 29.4%	1 / 49 2.0%	21 / 35 60.0%	18 / 67 26.9%	0 / 17 0.0%	13 / 68 19.1%	15 / 71 21.1%	4 / 68 5.9%	6 / 17 35.3%	31 / 102 30.4%	0 / 3 0.0%	0 / 0 0.0%	0 / 0 0.0%	0 / 0 0.0%	393 / 1118 35.2%	352 / 1118 31.5%E	110 / 1118 9.8%AB	108 / 1118 9.7%C	97 / 1118 8.7%ABE	50 / 1118 4.5%CE	4 / 1118 0.4%BE	4 / 1118 0.4%B
16	"Microstreptospora" Yan et al.	199 / 1047 19.0%	6 / 56 10.7%	0 / 32 0.0%	0 / 32 0.0%	10 / 17 58.8%	39 / 80 48.8%	20 / 97 20.6%	2 / 16 12.5%	0 / 16 0.0%	0 / 64 0.0%	0 / 22 0.0%	15 / 70 21.4%	3 / 65 4.6%	4 / 16 25.0%	0 / 46 0.0%	19 / 31 61.3%	18 / 64 28.1%	0 / 16 0.0%	12 / 64 18.8%	13 / 64 20.3%	4 / 64 6.2%	6 / 16 37.5%	28 / 96 29.2%	0 / 3 0.0%	0 / 0 0.0%	0 / 0 0.0%	0 / 0 0.0%	375 / 1047 35.8%	333 / 1047 31.8%E	102 / 1047 9.7%AB	97 / 1047 9.3%C	89 / 1047 8.5%ABE	43 / 1047 4.1%CE	4 / 1047 0.4%B	4 / 1047 0.4%BE
1	"Streptotrix alba" (sic) Rossi Doria 1891	13 / 66 19.7%	1 / 4 25.0%	0 / 2 0.0%	0 / 2 0.0%	0 / 1 0.0%	3 / 5 60.0%	2 / 6 33.3%	0 / 1 0.0%	0 / 1 0.0%	0 / 4 0.0%	0 / 1 0.0%	1 / 4 25.0%	0 / 4 0.0%	0 / 1 0.0%	0 / 3 0.0%	1 / 2 50.0%	1 / 4 25.0%	0 / 1 0.0%	0 / 4 0.0%	1 / 4 25.0%	0 / 4 0.0%	0 / 1 0.0%	3 / 6 50.0%	0 / 1 0.0%	0 / 0 0.0%	0 / 0 0.0%	0 / 0 0.0%	24 / 66 36.4%	21 / 66 31.8%E	7 / 66 10.6%AB	6 / 66 9.1%ABE	5 / 66 7.6%C	3 / 66 4.5%CE		
1	Streptomyces albus J1074	13 / 66 19.7%	1 / 4 25.0%	0 / 2 0.0%	0 / 2 0.0%	0 / 1 0.0%	3 / 5 60.0%	2 / 6 33.3%	0 / 1 0.0%	0 / 1 0.0%	0 / 4 0.0%	0 / 1 0.0%	1 / 4 25.0%	0 / 4 0.0%	0 / 1 0.0%	0 / 3 0.0%	1 / 2 50.0%	1 / 4 25.0%	0 / 1 0.0%	0 / 4 0.0%	1 / 4 25.0%	0 / 4 0.0%	0 / 1 0.0%	3 / 6 50.0%	0 / 1 0.0%	0 / 0 0.0%	0 / 0 0.0%	0 / 0 0.0%	24 / 66 36.4%	21 / 66 31.8%E	7 / 66 10.6%AB	6 / 66 9.1%ABE	5 / 66 7.6%C	3 / 66 4.5%CE		
2	"Actinomyces hygroscopicus" Jensen 1931	20 / 136 14.7%	0 / 10 0.0%	0 / 4 0.0%	0 / 4 0.0%	2 / 2 100.0%	0 / 10 0.0%	0 / 12 0.0%	0 / 2 0.0%	0 / 2 0.0%	0 / 8 0.0%	0 / 2 0.0%	2 / 8 25.0%	0 / 8 0.0%	0 / 2 0.0%	0 / 6 0.0%	2 / 4 50.0%	2 / 10 20.0%	0 / 2 0.0%	4 / 8 50.0%	4 / 8 50.0%	0 / 8 0.0%	2 / 2 100.0%	2 / 12 16.7%	0 / 2 0.0%	0 / 0 0.0%	0 / 0 0.0%	0 / 0 0.0%	50 / 136 36.8%	48 / 136 35.3%E	12 / 136 8.8%AB	12 / 136 8.8%C	8 / 136 5.9%ABE	6 / 136 4.4%CE		
2	'Streptomyces hygroscopicus subsp. jinggangensis'	20 / 136 14.7%	0 / 10 0.0%	0 / 4 0.0%	0 / 4 0.0%	2 / 2 100.0%	0 / 10 0.0%	0 / 12 0.0%	0 / 2 0.0%	0 / 2 0.0%	0 / 8 0.0%	0 / 2 0.0%	2 / 8 25.0%	0 / 8 0.0%	0 / 2 0.0%	0 / 6 0.0%	2 / 4 50.0%	2 / 10 20.0%	0 / 2 0.0%	4 / 8 50.0%	4 / 8 50.0%	0 / 8 0.0%	2 / 2 100.0%	2 / 12 16.7%	0 / 2 0.0%	0 / 0 0.0%	0 / 0 0.0%	0 / 0 0.0%	50 / 136 36.8%	48 / 136 35.3%E	12 / 136 8.8%AB	12 / 136 8.8%C	8 / 136 5.9%ABE	6 / 136 4.4%CE		
1	Streptomyces hygroscopicus subsp. jinggangensis 5008	10 / 68 14.7%	0 / 5 0.0%	0 / 2 0.0%	0 / 2 0.0%	1 / 1 100.0%	0 / 5 0.0%	0 / 6 0.0%	0 / 1 0.0%	0 / 1 0.0%	0 / 4 0.0%	0 / 1 0.0%	1 / 4 25.0%	0 / 4 0.0%	0 / 1 0.0%	0 / 3 0.0%	1 / 2 50.0%	1 / 5 20.0%	0 / 1 0.0%	2 / 4 50.0%	2 / 4 50.0%	0 / 4 0.0%	1 / 1 100.0%	1 / 6 16.7%	0 / 1 0.0%	0 / 0 0.0%	0 / 0 0.0%	0 / 0 0.0%	25 / 68 36.8%	24 / 68 35.3%E	6 / 68 8.8%C	6 / 68 8.8%AB	4 / 68 5.9%ABE	3 / 68 4.4%CE		
1	Streptomyces hygroscopicus subsp. jinggangensis TL01	10 / 68 14.7%	0 / 5 0.0%	0 / 2 0.0%	0 / 2 0.0%	1 / 1 100.0%	0 / 5 0.0%	0 / 6 0.0%	0 / 1 0.0%	0 / 1 0.0%	0 / 4 0.0%	0 / 1 0.0%	1 / 4 25.0%	0 / 4 0.0%	0 / 1 0.0%	0 / 3 0.0%	1 / 2 50.0%	1 / 5 20.0%	0 / 1 0.0%	2 / 4 50.0%	2 / 4 50.0%	0 / 4 0.0%	1 / 1 100.0%	1 / 6 16.7%	0 / 1 0.0%	0 / 0 0.0%	0 / 0 0.0%	0 / 0 0.0%	25 / 68 36.8%	24 / 68 35.3%E	6 / 68 8.8%C	6 / 68 8.8%AB	4 / 68 5.9%ABE	3 / 68 4.4%CE		
1	"Actinomyces scabies" (Thaxter 1891) Gussow 1914	8 / 71 11.3%	0 / 4 0.0%	0 / 2 0.0%	0 / 2 0.0%	1 / 2 50.0%	1 / 5 20.0%	0 / 6 0.0%	0 / 1 0.0%	0 / 1 0.0%	0 / 4 0.0%	0 / 3 0.0%	1 / 5 20.0%	1 / 4 25.0%	0 / 1 0.0%	0 / 3 0.0%	1 / 3 33.3%	1 / 5 20.0%	0 / 1 0.0%	1 / 4 25.0%	1 / 4 25.0%	0 / 4 0.0%	0 / 1 0.0%	0 / 6 0.0%	0 / 0 0.0%	0 / 0 0.0%	0 / 0 0.0%	0 / 0 0.0%	27 / 71 38.0%E	27 / 71 38.0%	5 / 71 7.0%C	5 / 71 7.0%AB	4 / 71 5.6%CE	3 / 71 4.2%ABE		
1	Streptomyces scabiei 87.22	8 / 71 11.3%	0 / 4 0.0%	0 / 2 0.0%	0 / 2 0.0%	1 / 2 50.0%	1 / 5 20.0%	0 / 6 0.0%	0 / 1 0.0%	0 / 1 0.0%	0 / 4 0.0%	0 / 3 0.0%	1 / 5 20.0%	1 / 4 25.0%	0 / 1 0.0%	0 / 3 0.0%	1 / 3 33.3%	1 / 5 20.0%	0 / 1 0.0%	1 / 4 25.0%	1 / 4 25.0%	0 / 4 0.0%	0 / 1 0.0%	0 / 6 0.0%	0 / 0 0.0%	0 / 0 0.0%	0 / 0 0.0%	0 / 0 0.0%	27 / 71 38.0%	27 / 71 38.0%E	5 / 71 7.0%AB	5 / 71 7.0%C	4 / 71 5.6%CE	3 / 71 4.2%ABE		
2	"Streptomyces cattleya" Kahan et al. 1979	22 / 121 18.2%	2 / 6 33.3%	0 / 4 0.0%	0 / 4 0.0%	0 / 2 0.0%	6 / 10 60.0%	4 / 12 33.3%	2 / 2 100.0%	0 / 2 0.0%	0 / 8 0.0%	0 / 2 0.0%	0 / 8 0.0%	0 / 9 0.0%	0 / 2 0.0%	0 / 4 0.0%	0 / 2 0.0%	2 / 6 33.3%	0 / 2 0.0%	2 / 8 25.0%	0 / 8 0.0%	2 / 6 33.3%	0 / 2 0.0%	2 / 12 16.7%	0 / 0 0.0%	0 / 0 0.0%	0 / 0 0.0%	0 / 0 0.0%	35 / 121 28.9%	34 / 121 28.1%E	22 / 121 18.2%C	14 / 121 11.6%AB	8 / 121 6.6%ABE	8 / 121 6.6%CE		
2	Streptomyces cattleya DSM 46488	22 / 121 18.2%	2 / 6 33.3%	0 / 4 0.0%	0 / 4 0.0%	0 / 2 0.0%	6 / 10 60.0%	4 / 12 33.3%	2 / 2 100.0%	0 / 2 0.0%	0 / 8 0.0%	0 / 2 0.0%	0 / 8 0.0%	0 / 9 0.0%	0 / 2 0.0%	0 / 4 0.0%	0 / 2 0.0%	2 / 6 33.3%	0 / 2 0.0%	2 / 8 25.0%	0 / 8 0.0%	2 / 6 33.3%	0 / 2 0.0%	2 / 12 16.7%	0 / 0 0.0%	0 / 0 0.0%	0 / 0 0.0%	0 / 0 0.0%	35 / 121 28.9%	34 / 121 28.1%E	22 / 121 18.2%C	14 / 121 11.6%AB	8 / 121 6.6%ABE	8 / 121 6.6%CE		
1	"Streptomyces avermitilis" Burg et al. 1979	14 / 68 20.6%	0 / 4 0.0%	0 / 2 0.0%	0 / 2 0.0%	1 / 1 100.0%	4 / 5 80.0%	2 / 7 28.6%	0 / 1 0.0%	0 / 1 0.0%	0 / 4 0.0%	0 / 1 0.0%	1 / 5 20.0%	0 / 4 0.0%	0 / 1 0.0%	0 / 3 0.0%	1 / 2 50.0%	1 / 5 20.0%	0 / 1 0.0%	1 / 4 25.0%	1 / 4 25.0%	0 / 4 0.0%	0 / 1 0.0%	2 / 6 33.3%	0 / 0 0.0%	0 / 0 0.0%	0 / 0 0.0%	0 / 0 0.0%	23 / 68 33.8%	22 / 68 32.4%E	9 / 68 13.2%AB	5 / 68 7.4%C	5 / 68 7.4%ABE	4 / 68 5.9%CE		
1	Streptomyces avermitilis MA-4680	14 / 68 20.6%	0 / 4 0.0%	0 / 2 0.0%	0 / 2 0.0%	1 / 1 100.0%	4 / 5 80.0%	2 / 7 28.6%	0 / 1 0.0%	0 / 1 0.0%	0 / 4 0.0%	0 / 1 0.0%	1 / 5 20.0%	0 / 4 0.0%	0 / 1 0.0%	0 / 3 0.0%	1 / 2 50.0%	1 / 5 20.0%	0 / 1 0.0%	1 / 4 25.0%	1 / 4 25.0%	0 / 4 0.0%	0 / 1 0.0%	2 / 6 33.3%	0 / 0 0.0%	0 / 0 0.0%	0 / 0 0.0%	0 / 0 0.0%	23 / 68 33.8%	22 / 68 32.4%E	9 / 68 13.2%AB	5 / 68 7.4%C	5 / 68 7.4%ABE	4 / 68 5.9%CE		
1	AS 4.1526	16 / 64 25.0%	1 / 2 50.0%	0 / 2 0.0%	0 / 2 0.0%	0 / 1 0.0%	3 / 5 60.0%	2 / 6 33.3%	0 / 1 0.0%	0 / 1 0.0%	0 / 4 0.0%	0 / 1 0.0%	1 / 4 25.0%	0 / 4 0.0%	0 / 1 0.0%	0 / 3 0.0%	2 / 2 100.0%	1 / 4 25.0%	0 / 1 0.0%	1 / 4 25.0%	0 / 4 0.0%	0 / 4 0.0%	1 / 1 100.0%	4 / 7 57.1%	0 / 0 0.0%	0 / 0 0.0%	0 / 0 0.0%	0 / 0 0.0%	26 / 64 40.6%	17 / 64 26.6%E	11 / 64 17.2%ABE	5 / 64 7.8%AB	3 / 64 4.7%CE	2 / 64 3.1%C		
1	Streptomyces venezuelae ATCC 10712	16 / 64 25.0%	1 / 2 50.0%	0 / 2 0.0%	0 / 2 0.0%	0 / 1 0.0%	3 / 5 60.0%	2 / 6 33.3%	0 / 1 0.0%	0 / 1 0.0%	0 / 4 0.0%	0 / 1 0.0%	1 / 4 25.0%	0 / 4 0.0%	0 / 1 0.0%	0 / 3 0.0%	2 / 2 100.0%	1 / 4 25.0%	0 / 1 0.0%	1 / 4 25.0%	0 / 4 0.0%	0 / 4 0.0%	1 / 1 100.0%	4 / 7 57.1%	0 / 0 0.0%	0 / 0 0.0%	0 / 0 0.0%	0 / 0 0.0%	26 / 64 40.6%	17 / 64 26.6%E	11 / 64 17.2%ABE	5 / 64 7.8%AB	3 / 64 4.7%CE	2 / 64 3.1%C		
1	"Actinomyces fulvissimus" Jensen 1930	13 / 69 18.8%	0 / 4 0.0%	0 / 2 0.0%	0 / 2 0.0%	1 / 1 100.0%	4 / 5 80.0%	2 / 6 33.3%	0 / 1 0.0%	0 / 1 0.0%	0 / 4 0.0%	0 / 1 0.0%	1 / 4 25.0%	0 / 4 0.0%	0 / 1 0.0%	0 / 4 0.0%	2 / 2 100.0%	1 / 4 25.0%	0 / 1 0.0%	0 / 4 0.0%	0 / 4 0.0%	0 / 7 0.0%	0 / 1 0.0%	2 / 6 33.3%	0 / 0 0.0%	0 / 0 0.0%	0 / 0 0.0%	0 / 0 0.0%	25 / 69 36.2%	24 / 69 34.8%E	7 / 69 10.1%AB	6 / 69 8.7%ABE	5 / 69 7.2%C	2 / 69 2.9%CE		
1	Streptomyces fulvissimus DSM 40593	13 / 69 18.8%	0 / 4 0.0%	0 / 2 0.0%	0 / 2 0.0%	1 / 1 100.0%	4 / 5 80.0%	2 / 6 33.3%	0 / 1 0.0%	0 / 1 0.0%	0 / 4 0.0%	0 / 1 0.0%	1 / 4 25.0%	0 / 4 0.0%	0 / 1 0.0%	0 / 4 0.0%	2 / 2 100.0%	1 / 4 25.0%	0 / 1 0.0%	0 / 4 0.0%	0 / 4 0.0%	0 / 7 0.0%	0 / 1 0.0%	2 / 6 33.3%	0 / 0 0.0%	0 / 0 0.0%	0 / 0 0.0%	0 / 0 0.0%	25 / 69 36.2%	24 / 69 34.8%E	7 / 69 10.1%AB	6 / 69 8.7%ABE	5 / 69 7.2%C	2 / 69 2.9%CE		
1	"Actinomyces violaceus-niger" (sic) Waksman and Curtis 1916	18 / 63 28.6%	1 / 3 33.3%	0 / 2 0.0%	0 / 2 0.0%	0 / 1 0.0%	4 / 5 80.0%	1 / 6 16.7%	0 / 1 0.0%	0 / 1 0.0%	0 / 4 0.0%	0 / 1 0.0%	1 / 4 25.0%	2 / 4 50.0%	0 / 1 0.0%	0 / 3 0.0%	1 / 2 50.0%	2 / 3 66.7%	0 / 1 0.0%	0 / 4 0.0%	1 / 4 25.0%	2 / 4 50.0%	1 / 1 100.0%	2 / 6 33.3%	0 / 0 0.0%	0 / 0 0.0%	0 / 0 0.0%	0 / 0 0.0%	21 / 63 33.3%	18 / 63 28.6%E	10 / 63 15.9%ABE	8 / 63 12.7%AB	5 / 63 7.9%C	1 / 63 1.6%CE		
1	Streptomyces violaceusniger Tu 4113	18 / 63 28.6%	1 / 3 33.3%	0 / 2 0.0%	0 / 2 0.0%	0 / 1 0.0%	4 / 5 80.0%	1 / 6 16.7%	0 / 1 0.0%	0 / 1 0.0%	0 / 4 0.0%	0 / 1 0.0%	1 / 4 25.0%	2 / 4 50.0%	0 / 1 0.0%	0 / 3 0.0%	1 / 2 50.0%	2 / 3 66.7%	0 / 1 0.0%	0 / 4 0.0%	1 / 4 25.0%	2 / 4 50.0%	1 / 1 100.0%	2 / 6 33.3%	0 / 0 0.0%	0 / 0 0.0%	0 / 0 0.0%	0 / 0 0.0%	21 / 63 33.3%	18 / 63 28.6%E	10 / 63 15.9%ABE	8 / 63 12.7%AB	5 / 63 7.9%C	1 / 63 1.6%CE		
1	"Streptomyces davawensis" Shinobu 1974	17 / 67 25.4%	0 / 3 0.0%	0 / 2 0.0%	0 / 2 0.0%	1 / 1 100.0%	2 / 5 40.0%	0 / 6 0.0%	0 / 1 0.0%	0 / 1 0.0%	0 / 4 0.0%	0 / 1 0.0%	2 / 5 40.0%	0 / 4 0.0%	1 / 1 100.0%	0 / 3 0.0%	2 / 2 100.0%	2 / 5 40.0%	0 / 1 0.0%	2 / 4 50.0%	2 / 4 50.0%	0 / 5 0.0%	0 / 1 0.0%	3 / 6 50.0%	0 / 0 0.0%	0 / 0 0.0%	0 / 0 0.0%	0 / 0 0.0%	24 / 67 35.8%	18 / 67 26.9%E	9 / 67 13.4%ABE	8 / 67 11.9%AB	4 / 67 6.0%C	4 / 67 6.0%CE		
1	Streptomyces davawensis 768	17 / 67 25.4%	0 / 3 0.0%	0 / 2 0.0%	0 / 2 0.0%	1 / 1 100.0%	2 / 5 40.0%	0 / 6 0.0%	0 / 1 0.0%	0 / 1 0.0%	0 / 4 0.0%	0 / 1 0.0%	2 / 5 40.0%	0 / 4 0.0%	1 / 1 100.0%	0 / 3 0.0%	2 / 2 100.0%	2 / 5 40.0%	0 / 1 0.0%	2 / 4 50.0%	2 / 4 50.0%	0 / 5 0.0%	0 / 1 0.0%	3 / 6 50.0%	0 / 0 0.0%	0 / 0 0.0%	0 / 0 0.0%	0 / 0 0.0%	24 / 67 35.8%	18 / 67 26.9%E	9 / 67 13.4%ABE	8 / 67 11.9%AB	4 / 67 6.0%CE	4 / 67 6.0%C		
1	"Streptomyces bingchenggensis" Xiang et al. 2007	10 / 63 15.9%	1 / 3 33.3%	0 / 2 0.0%	0 / 2 0.0%	0 / 1 0.0%	3 / 5 60.0%	1 / 6 16.7%	0 / 1 0.0%	0 / 1 0.0%	0 / 4 0.0%	0 / 2 0.0%	1 / 4 25.0%	0 / 4 0.0%	0 / 1 0.0%	0 / 3 0.0%	0 / 2 0.0%	1 / 2 50.0%	0 / 1 0.0%	1 / 4 25.0%	1 / 4 25.0%	0 / 4 0.0%	0 / 1 0.0%	1 / 6 16.7%	0 / 0 0.0%	0 / 0 0.0%	0 / 0 0.0%	0 / 0 0.0%	21 / 63 33.3%E	21 / 63 33.3%	9 / 63 14.3%C	5 / 63 7.9%AB	5 / 63 7.9%ABE	2 / 63 3.2%CE		
1	Streptomyces bingchenggensis BCW-1	10 / 63 15.9%	1 / 3 33.3%	0 / 2 0.0%	0 / 2 0.0%	0 / 1 0.0%	3 / 5 60.0%	1 / 6 16.7%	0 / 1 0.0%	0 / 1 0.0%	0 / 4 0.0%	0 / 2 0.0%	1 / 4 25.0%	0 / 4 0.0%	0 / 1 0.0%	0 / 3 0.0%	0 / 2 0.0%	1 / 2 50.0%	0 / 1 0.0%	1 / 4 25.0%	1 / 4 25.0%	0 / 4 0.0%	0 / 1 0.0%	1 / 6 16.7%	0 / 0 0.0%	0 / 0 0.0%	0 / 0 0.0%	0 / 0 0.0%	21 / 63 33.3%	21 / 63 33.3%E	9 / 63 14.3%C	5 / 63 7.9%ABE	5 / 63 7.9%AB	2 / 63 3.2%CE		
1	Streptomyces griseus clade	10 / 66 15.2%	0 / 4 0.0%	0 / 2 0.0%	0 / 2 0.0%	1 / 1 100.0%	2 / 5 40.0%	1 / 6 16.7%	0 / 1 0.0%	0 / 1 0.0%	0 / 4 0.0%	0 / 2 0.0%	1 / 4 25.0%	0 / 4 0.0%	1 / 1 100.0%	0 / 3 0.0%	1 / 2 50.0%	1 / 4 25.0%	0 / 1 0.0%	0 / 4 0.0%	0 / 4 0.0%	0 / 4 0.0%	0 / 1 0.0%	2 / 6 33.3%	0 / 0 0.0%	0 / 0 0.0%	0 / 0 0.0%	0 / 0 0.0%	26 / 66 39.4%	22 / 66 33.3%E	7 / 66 10.6%C	4 / 66 6.1%B	4 / 66 6.1%BE	1 / 66 1.5%AB	1 / 66 1.5%CE	1 / 66 1.5%ABE
1	Streptomyces griseus subgroup	10 / 66 15.2%	0 / 4 0.0%	0 / 2 0.0%	0 / 2 0.0%	1 / 1 100.0%	2 / 5 40.0%	1 / 6 16.7%	0 / 1 0.0%	0 / 1 0.0%	0 / 4 0.0%	0 / 2 0.0%	1 / 4 25.0%	0 / 4 0.0%	1 / 1 100.0%	0 / 3 0.0%	1 / 2 50.0%	1 / 4 25.0%	0 / 1 0.0%	0 / 4 0.0%	0 / 4 0.0%	0 / 4 0.0%	0 / 1 0.0%	2 / 6 33.3%	0 / 0 0.0%	0 / 0 0.0%	0 / 0 0.0%	0 / 0 0.0%	26 / 66 39.4%	22 / 66 33.3%E	7 / 66 10.6%C	4 / 66 6.1%B	4 / 66 6.1%BE	1 / 66 1.5%AB	1 / 66 1.5%ABE	1 / 66 1.5%CE
1	"Actinomyces griseus" Krainsky 1914	10 / 66 15.2%	0 / 4 0.0%	0 / 2 0.0%	0 / 2 0.0%	1 / 1 100.0%	2 / 5 40.0%	1 / 6 16.7%	0 / 1 0.0%	0 / 1 0.0%	0 / 4 0.0%	0 / 2 0.0%	1 / 4 25.0%	0 / 4 0.0%	1 / 1 100.0%	0 / 3 0.0%	1 / 2 50.0%	1 / 4 25.0%	0 / 1 0.0%	0 / 4 0.0%	0 / 4 0.0%	0 / 4 0.0%	0 / 1 0.0%	2 / 6 33.3%	0 / 0 0.0%	0 / 0 0.0%	0 / 0 0.0%	0 / 0 0.0%	26 / 66 39.4%	22 / 66 33.3%E	7 / 66 10.6%C	4 / 66 6.1%BE	4 / 66 6.1%B	1 / 66 1.5%CE	1 / 66 1.5%ABE	1 / 66 1.5%AB
1	"Nocardia italica" Spalla et al.	10 / 66 15.2%	0 / 4 0.0%	0 / 2 0.0%	0 / 2 0.0%	1 / 1 100.0%	2 / 5 40.0%	1 / 6 16.7%	0 / 1 0.0%	0 / 1 0.0%	0 / 4 0.0%	0 / 2 0.0%	1 / 4 25.0%	0 / 4 0.0%	1 / 1 100.0%	0 / 3 0.0%	1 / 2 50.0%	1 / 4 25.0%	0 / 1 0.0%	0 / 4 0.0%	0 / 4 0.0%	0 / 4 0.0%	0 / 1 0.0%	2 / 6 33.3%	0 / 0 0.0%	0 / 0 0.0%	0 / 0 0.0%	0 / 0 0.0%	26 / 66 39.4%	22 / 66 33.3%E	7 / 66 10.6%C	4 / 66 6.1%BE	4 / 66 6.1%B	1 / 66 1.5%CE	1 / 66 1.5%ABE	1 / 66 1.5%AB
1	Streptomyces griseus subsp. griseus IFO 13350	10 / 66 15.2%	0 / 4 0.0%	0 / 2 0.0%	0 / 2 0.0%	1 / 1 100.0%	2 / 5 40.0%	1 / 6 16.7%	0 / 1 0.0%	0 / 1 0.0%	0 / 4 0.0%	0 / 2 0.0%	1 / 4 25.0%	0 / 4 0.0%	1 / 1 100.0%	0 / 3 0.0%	1 / 2 50.0%	1 / 4 25.0%	0 / 1 0.0%	0 / 4 0.0%	0 / 4 0.0%	0 / 4 0.0%	0 / 1 0.0%	2 / 6 33.3%	0 / 0 0.0%	0 / 0 0.0%	0 / 0 0.0%	0 / 0 0.0%	26 / 66 39.4%	22 / 66 33.3%E	7 / 66 10.6%C	4 / 66 6.1%BE	4 / 66 6.1%B	1 / 66 1.5%ABE	1 / 66 1.5%CE	1 / 66 1.5%AB
1	Streptomyces sp. ACTE	11 / 62 17.7%	0 / 3 0.0%	0 / 2 0.0%	0 / 2 0.0%	1 / 1 100.0%	1 / 5 20.0%	1 / 6 16.7%	0 / 1 0.0%	0 / 1 0.0%	0 / 4 0.0%	0 / 1 0.0%	1 / 5 20.0%	0 / 4 0.0%	0 / 1 0.0%	0 / 2 0.0%	2 / 2 100.0%	1 / 4 25.0%	0 / 1 0.0%	0 / 4 0.0%	1 / 4 25.0%	0 / 2 0.0%	1 / 1 100.0%	2 / 6 33.3%	0 / 0 0.0%	0 / 0 0.0%	0 / 0 0.0%	0 / 0 0.0%	24 / 62 38.7%	18 / 62 29.0%E	6 / 62 9.7%ABE	6 / 62 9.7%C	5 / 62 8.1%AB	3 / 62 4.8%CE		
1	CGMCC 4.6829	14 / 66 21.2%	0 / 3 0.0%	0 / 2 0.0%	0 / 2 0.0%	1 / 1 100.0%	3 / 5 60.0%	2 / 6 33.3%	0 / 1 0.0%	0 / 1 0.0%	0 / 4 0.0%	0 / 2 0.0%	1 / 5 20.0%	0 / 4 0.0%	1 / 1 100.0%	0 / 3 0.0%	2 / 2 100.0%	1 / 4 25.0%	0 / 1 0.0%	0 / 4 0.0%	1 / 4 25.0%	0 / 4 0.0%	0 / 1 0.0%	2 / 6 33.3%	0 / 0 0.0%	0 / 0 0.0%	0 / 0 0.0%	0 / 0 0.0%	24 / 66 36.4%	22 / 66 33.3%E	8 / 66 12.1%AB	6 / 66 9.1%ABE	5 / 66 7.6%C	1 / 66 1.5%CE		
[illegible]	Streptomyces flavogriseus ATCC [illegible]	14 / 66 [illegible]	0 / 3 [illegible]	0 / 2 [illegible]	0 / 2 [illegible]	1 / 1 [illegible]	3 / 5 [illegible]	2 / 6 [illegible]	0 / 1 [illegible]	0 / 1 [illegible]	0 / 4 [illegible]	0 / 2 [illegible]	1 / 5 [illegible]	0 / 4 [illegible]	1 / 1 [illegible]	0 / 3 [illegible]	2 / 2 [illegible]	1 / 4 [illegible]	0 / 1 [illegible]	0 / 4 [illegible]	1 / 4 [illegible]	0 / 4 [illegible]	0 / 1 [illegible]	2 / 6 [illegible]	0 / 0 [illegible]	0 / 0 [illegible]	0 / 0 [illegible]	0 / 0 [illegible]	24 / 66 [illegible]	22 / 66 [illegible]	8 / 66 [illegible]	6 / 66 [illegible]	5 / 66 [illegible]	1 / 66 [illegible]		

[illegible]	[illegible]	21.2%	0.0%	0.0%	0.0%	100.0%	60.0%	33.3%	0.0%	0.0%	0.0%	0.0%	20.0%	0.0%	100.0%	0.0%	100.0%	25.0%	0.0%	0.0%	25.0%	0.0%	0.0%	33.3%	0.0%	0.0%	0.0%	0.0%	36.4%	33.3%E	12.1%AB	9.1%ABE	7.6%C	1.5%CE		
1	Streptomyces sp. PAMC26508	13 / 65 20.0%	0 / 3 0.0%	0 / 2 0.0%	0 / 2 0.0%	1 / 1 100.0%	3 / 5 60.0%	2 / 6 33.3%	0 / 1 0.0%	0 / 1 0.0%	0 / 4 0.0%	0 / 2 0.0%	1 / 5 20.0%	0 / 4 0.0%	1 / 1 100.0%	0 / 3 0.0%	2 / 2 100.0%	1 / 4 25.0%	0 / 1 0.0%	0 / 4 0.0%	0 / 4 0.0%	0 / 4 0.0%	1 / 1 100.0%	1 / 5 20.0%	0 / 0 0.0%	0 / 0 0.0%	0 / 0 0.0%	0 / 0 0.0%	25 / 65 38.5%	21 / 65 32.3%E	8 / 65 12.3%AB	5 / 65 7.7%C	5 / 65 7.7%ABE	1 / 65 1.5%CE		
1	Kitasatospora	16 / 71 22.5%	0 / 3 0.0%	0 / 2 0.0%	0 / 2 0.0%	0 / 1 0.0%	2 / 5 40.0%	1 / 6 16.7%	1 / 1 100.0%	0 / 1 0.0%	1 / 6 16.7%	0 / 1 0.0%	1 / 4 25.0%	0 / 5 0.0%	1 / 1 100.0%	1 / 3 33.3%	2 / 4 50.0%	0 / 3 0.0%	0 / 1 0.0%	1 / 4 25.0%	2 / 7 28.6%	0 / 4 0.0%	0 / 1 0.0%	3 / 6 50.0%	0 / 0 0.0%	0 / 0 0.0%	0 / 0 0.0%	0 / 0 0.0%	19 / 71 26.8%E	18 / 71 25.4%	11 / 71 15.5%C	8 / 71 11.3%ABE	8 / 71 11.3%AB	7 / 71 9.9%CE		
1	ATCC 33774	16 / 71 22.5%	0 / 3 0.0%	0 / 2 0.0%	0 / 2 0.0%	0 / 1 0.0%	2 / 5 40.0%	1 / 6 16.7%	1 / 1 100.0%	0 / 1 0.0%	1 / 6 16.7%	0 / 1 0.0%	1 / 4 25.0%	0 / 5 0.0%	1 / 1 100.0%	1 / 3 33.3%	2 / 4 50.0%	0 / 3 0.0%	0 / 1 0.0%	1 / 4 25.0%	2 / 7 28.6%	0 / 4 0.0%	0 / 1 0.0%	3 / 6 50.0%	0 / 0 0.0%	0 / 0 0.0%	0 / 0 0.0%	0 / 0 0.0%	19 / 71 26.8%E	18 / 71 25.4%	11 / 71 15.5%C	8 / 71 11.3%ABE	8 / 71 11.3%AB	7 / 71 9.9%CE		
1	Kitasatospora setae KM-6054	16 / 71 22.5%	0 / 3 0.0%	0 / 2 0.0%	0 / 2 0.0%	0 / 1 0.0%	2 / 5 40.0%	1 / 6 16.7%	1 / 1 100.0%	0 / 1 0.0%	1 / 6 16.7%	0 / 1 0.0%	1 / 4 25.0%	0 / 5 0.0%	1 / 1 100.0%	1 / 3 33.3%	2 / 4 50.0%	0 / 3 0.0%	0 / 1 0.0%	1 / 4 25.0%	2 / 7 28.6%	0 / 4 0.0%	0 / 1 0.0%	3 / 6 50.0%	0 / 0 0.0%	0 / 0 0.0%	0 / 0 0.0%	0 / 0 0.0%	19 / 71 26.8%E	18 / 71 25.4%	11 / 71 15.5%C	8 / 71 11.3%AB	8 / 71 11.3%ABE	7 / 71 9.9%CE		
5	Streptosporangineae	69 / 281 24.6%	3 / 9 33.3%	1 / 7 14.3%	1 / 6 16.7%	0 / 5 0.0%	6 / 25 24.0%	8 / 21 38.1%	3 / 6 50.0%	2 / 5 40.0%	4 / 25 16.0%	0 / 5 0.0%	5 / 21 23.8%	7 / 15 46.7%	0 / 5 0.0%	1 / 8 12.5%	1 / 10 10.0%	2 / 17 11.8%	2 / 5 40.0%	5 / 15 33.3%	6 / 21 28.6%	3 / 13 23.1%	0 / 5 0.0%	7 / 27 25.9%	2 / 2 100.0%	0 / 0 0.0%	0 / 3 0.0%	0 / 0 0.0%	99 / 281 35.2%E	75 / 281 26.7%	43 / 281 15.3%AB	27 / 281 9.6%C	26 / 281 9.3%ABE	11 / 281 3.9%CE		
1	Actinoplanaceae	18 / 61 29.5%	0 / 2 0.0%	0 / 1 0.0%	0 / 1 0.0%	0 / 1 0.0%	2 / 6 33.3%	4 / 5 80.0%	1 / 1 100.0%	1 / 1 100.0%	1 / 6 16.7%	0 / 1 0.0%	2 / 4 50.0%	1 / 4 25.0%	0 / 1 0.0%	1 / 2 50.0%	0 / 3 0.0%	1 / 3 33.3%	0 / 1 0.0%	0 / 3 0.0%	1 / 4 25.0%	1 / 3 33.3%	0 / 1 0.0%	0 / 5 0.0%	2 / 2 100.0%	0 / 0 0.0%	0 / 0 0.0%	0 / 0 0.0%	20 / 61 32.8%E	17 / 61 27.9%	10 / 61 16.4%AB	8 / 61 13.1%ABE	6 / 61 9.8%C			
1	Streptosporangium	18 / 61 29.5%	0 / 2 0.0%	0 / 1 0.0%	0 / 1 0.0%	0 / 1 0.0%	2 / 6 33.3%	4 / 5 80.0%	1 / 1 100.0%	1 / 1 100.0%	1 / 6 16.7%	0 / 1 0.0%	2 / 4 50.0%	1 / 4 25.0%	0 / 1 0.0%	1 / 2 50.0%	0 / 3 0.0%	1 / 3 33.3%	0 / 1 0.0%	0 / 3 0.0%	1 / 4 25.0%	1 / 3 33.3%	0 / 1 0.0%	0 / 5 0.0%	2 / 2 100.0%	0 / 0 0.0%	0 / 0 0.0%	0 / 0 0.0%	20 / 61 32.8%E	17 / 61 27.9%	10 / 61 16.4%AB	8 / 61 13.1%ABE	6 / 61 9.8%C			
1	"Angiococcus moliroseus" Peterson 1959	18 / 61 29.5%	0 / 2 0.0%	0 / 1 0.0%	0 / 1 0.0%	0 / 1 0.0%	2 / 6 33.3%	4 / 5 80.0%	1 / 1 100.0%	1 / 1 100.0%	1 / 6 16.7%	0 / 1 0.0%	2 / 4 50.0%	1 / 4 25.0%	0 / 1 0.0%	1 / 2 50.0%	0 / 3 0.0%	1 / 3 33.3%	0 / 1 0.0%	0 / 3 0.0%	1 / 4 25.0%	1 / 3 33.3%	0 / 1 0.0%	0 / 5 0.0%	2 / 2 100.0%	0 / 0 0.0%	0 / 0 0.0%	0 / 0 0.0%	20 / 61 32.8%E	17 / 61 27.9%	10 / 61 16.4%AB	8 / 61 13.1%ABE	6 / 61 9.8%C			
1	Streptosporangium roseum DSM 43021	18 / 61 29.5%	0 / 2 0.0%	0 / 1 0.0%	0 / 1 0.0%	0 / 1 0.0%	2 / 6 33.3%	4 / 5 80.0%	1 / 1 100.0%	1 / 1 100.0%	1 / 6 16.7%	0 / 1 0.0%	2 / 4 50.0%	1 / 4 25.0%	0 / 1 0.0%	1 / 2 50.0%	0 / 3 0.0%	1 / 3 33.3%	0 / 1 0.0%	0 / 3 0.0%	1 / 4 25.0%	1 / 3 33.3%	0 / 1 0.0%	0 / 5 0.0%	2 / 2 100.0%	0 / 0 0.0%	0 / 0 0.0%	0 / 0 0.0%	20 / 61 32.8%E	17 / 61 27.9%	10 / 61 16.4%AB	8 / 61 13.1%ABE	6 / 61 9.8%C			
1	Actinoplanaceae	11 / 57 19.3%	0 / 2 0.0%	1 / 1 100.0%	1 / 1 100.0%	0 / 1 0.0%	0 / 4 0.0%	1 / 4 25.0%	0 / 2 0.0%	1 / 1 100.0%	1 / 5 20.0%	0 / 1 0.0%	1 / 5 20.0%	1 / 2 50.0%	0 / 1 0.0%	0 / 2 0.0%	0 / 2 0.0%	0 / 3 0.0%	0 / 1 0.0%	1 / 3 33.3%	0 / 5 0.0%	0 / 2 0.0%	0 / 1 0.0%	3 / 7 42.9%	0 / 0 0.0%	0 / 0 0.0%	0 / 1 0.0%	0 / 0 0.0%	20 / 57 35.1%E	17 / 57 29.8%	6 / 57 10.5%ABE	5 / 57 8.8%C	5 / 57 8.8%AB	4 / 57 7.0%CE		
1	Thermomonospora	11 / 57 19.3%	0 / 2 0.0%	1 / 1 100.0%	1 / 1 100.0%	0 / 1 0.0%	0 / 4 0.0%	1 / 4 25.0%	0 / 2 0.0%	1 / 1 100.0%	1 / 5 20.0%	0 / 1 0.0%	1 / 5 20.0%	1 / 2 50.0%	0 / 1 0.0%	0 / 2 0.0%	0 / 2 0.0%	0 / 3 0.0%	0 / 1 0.0%	1 / 3 33.3%	0 / 5 0.0%	0 / 2 0.0%	0 / 1 0.0%	3 / 7 42.9%	0 / 0 0.0%	0 / 0 0.0%	0 / 1 0.0%	0 / 0 0.0%	20 / 57 35.1%E	17 / 57 29.8%	6 / 57 10.5%ABE	5 / 57 8.8%C	5 / 57 8.8%AB	4 / 57 7.0%CE		
1	ATCC 19995	11 / 57 19.3%	0 / 2 0.0%	1 / 1 100.0%	1 / 1 100.0%	0 / 1 0.0%	0 / 4 0.0%	1 / 4 25.0%	0 / 2 0.0%	1 / 1 100.0%	1 / 5 20.0%	0 / 1 0.0%	1 / 5 20.0%	1 / 2 50.0%	0 / 1 0.0%	0 / 2 0.0%	0 / 2 0.0%	0 / 3 0.0%	0 / 1 0.0%	1 / 3 33.3%	0 / 5 0.0%	0 / 2 0.0%	0 / 1 0.0%	3 / 7 42.9%	0 / 0 0.0%	0 / 0 0.0%	0 / 1 0.0%	0 / 0 0.0%	20 / 57 35.1%E	17 / 57 29.8%	6 / 57 10.5%ABE	5 / 57 8.8%AB	5 / 57 8.8%C	4 / 57 7.0%CE		
1	Thermomonospora curvata DSM 43183	11 / 57 19.3%	0 / 2 0.0%	1 / 1 100.0%	1 / 1 100.0%	0 / 1 0.0%	0 / 4 0.0%	1 / 4 25.0%	0 / 2 0.0%	1 / 1 100.0%	1 / 5 20.0%	0 / 1 0.0%	1 / 5 20.0%	1 / 2 50.0%	0 / 1 0.0%	0 / 2 0.0%	0 / 2 0.0%	0 / 3 0.0%	0 / 1 0.0%	1 / 3 33.3%	0 / 5 0.0%	0 / 2 0.0%	0 / 1 0.0%	3 / 7 42.9%	0 / 0 0.0%	0 / 0 0.0%	0 / 1 0.0%	0 / 0 0.0%	20 / 57 35.1%E	17 / 57 29.8%	6 / 57 10.5%ABE	5 / 57 8.8%C	5 / 57 8.8%AB	4 / 57 7.0%CE		
3	Nocardiopsaceae	40 / 163 24.5%	3 / 5 60.0%	0 / 5 0.0%	0 / 4 0.0%	0 / 3 0.0%	4 / 15 26.7%	3 / 12 25.0%	2 / 3 66.7%	0 / 3 0.0%	2 / 14 14.3%	0 / 3 0.0%	2 / 12 16.7%	5 / 9 55.6%	0 / 3 0.0%	0 / 4 0.0%	1 / 5 20.0%	1 / 11 9.1%	2 / 3 66.7%	4 / 9 44.4%	5 / 12 41.7%	2 / 8 25.0%	0 / 3 0.0%	4 / 15 26.7%	0 / 0 0.0%	0 / 0 0.0%	0 / 2 0.0%	0 / 0 0.0%	59 / 163 36.2%E	41 / 163 25.2%	28 / 163 17.2%AB	16 / 163 9.8%C	12 / 163 7.4%ABE	7 / 163 4.3%CE		
2	'Brachystreptospora'	27 / 111 24.3%	3 / 3 100.0%	0 / 4 0.0%	0 / 3 0.0%	0 / 2 0.0%	3 / 10 30.0%	1 / 8 12.5%	2 / 2 100.0%	0 / 2 0.0%	2 / 10 20.0%	0 / 2 0.0%	1 / 8 12.5%	2 / 6 33.3%	0 / 2 0.0%	0 / 2 0.0%	1 / 4 25.0%	0 / 8 0.0%	2 / 2 100.0%	3 / 6 50.0%	4 / 8 50.0%	1 / 5 20.0%	0 / 2 0.0%	2 / 10 20.0%	0 / 0 0.0%	0 / 0 0.0%	0 / 2 0.0%	0 / 0 0.0%	38 / 111 34.2%E	26 / 111 23.4%	19 / 111 17.1%AB	13 / 111 11.7%C	8 / 111 7.2%ABE	7 / 111 6.3%CE		
1	"Nocardia dassonvillei" (Brocq-Rousseau 1904) Liegard and Landrieu 1911	12 / 57 21.1%	2 / 2 100.0%	0 / 2 0.0%	0 / 2 0.0%	0 / 1 0.0%	1 / 5 20.0%	1 / 4 25.0%	1 / 1 100.0%	0 / 1 0.0%	1 / 5 20.0%	0 / 1 0.0%	0 / 4 0.0%	0 / 3 0.0%	0 / 1 0.0%	0 / 1 0.0%	0 / 2 0.0%	0 / 4 0.0%	1 / 1 100.0%	2 / 3 66.7%	2 / 4 50.0%	1 / 3 33.3%	0 / 1 0.0%	0 / 5 0.0%	0 / 0 0.0%	0 / 0 0.0%	0 / 1 0.0%	0 / 0 0.0%	20 / 57 35.1%E	15 / 57 26.3%	8 / 57 14.0%AB	6 / 57 10.5%C	4 / 57 7.0%ABE	4 / 57 7.0%CE		
1	Nocardiopsis dassonvillei subsp. dassonvillei	12 / 57 21.1%	2 / 2 100.0%	0 / 2 0.0%	0 / 2 0.0%	0 / 1 0.0%	1 / 5 20.0%	1 / 4 25.0%	1 / 1 100.0%	0 / 1 0.0%	1 / 5 20.0%	0 / 1 0.0%	0 / 4 0.0%	0 / 3 0.0%	0 / 1 0.0%	0 / 1 0.0%	0 / 2 0.0%	0 / 4 0.0%	1 / 1 100.0%	2 / 3 66.7%	2 / 4 50.0%	1 / 3 33.3%	0 / 1 0.0%	0 / 5 0.0%	0 / 0 0.0%	0 / 0 0.0%	0 / 1 0.0%	0 / 0 0.0%	20 / 57 35.1%E	15 / 57 26.3%	8 / 57 14.0%AB	6 / 57 10.5%C	4 / 57 7.0%ABE	4 / 57 7.0%CE		
1	Nocardiopsis dassonvillei DSM 43111	12 / 57 21.1%	2 / 2 100.0%	0 / 2 0.0%	0 / 2 0.0%	0 / 1 0.0%	1 / 5 20.0%	1 / 4 25.0%	1 / 1 100.0%	0 / 1 0.0%	1 / 5 20.0%	0 / 1 0.0%	0 / 4 0.0%	0 / 3 0.0%	0 / 1 0.0%	0 / 1 0.0%	0 / 2 0.0%	0 / 4 0.0%	1 / 1 100.0%	2 / 3 66.7%	2 / 4 50.0%	1 / 3 33.3%	0 / 1 0.0%	0 / 5 0.0%	0 / 0 0.0%	0 / 0 0.0%	0 / 1 0.0%	0 / 0 0.0%	20 / 57 35.1%E	15 / 57 26.3%	8 / 57 14.0%AB	6 / 57 10.5%C	4 / 57 7.0%ABE	4 / 57 7.0%CE		
1	DSM 43377	15 / 54 27.8%	1 / 1 100.0%	0 / 2 0.0%	0 / 1 0.0%	0 / 1 0.0%	2 / 5 40.0%	0 / 4 0.0%	1 / 1 100.0%	0 / 1 0.0%	1 / 5 20.0%	0 / 1 0.0%	1 / 4 25.0%	2 / 3 66.7%	0 / 1 0.0%	0 / 1 0.0%	1 / 2 50.0%	0 / 4 0.0%	1 / 1 100.0%	1 / 3 33.3%	2 / 4 50.0%	0 / 2 0.0%	0 / 1 0.0%	2 / 5 40.0%	0 / 0 0.0%	0 / 0 0.0%	0 / 1 0.0%	0 / 0 0.0%	18 / 54 33.3%E	11 / 54 20.4%	11 / 54 20.4%AB	7 / 54 13.0%C	4 / 54 7.4%ABE	3 / 54 5.6%CE		
1	Nocardiopsis alba ATCC BAA-2165	15 / 54 27.8%	1 / 1 100.0%	0 / 2 0.0%	0 / 1 0.0%	0 / 1 0.0%	2 / 5 40.0%	0 / 4 0.0%	1 / 1 100.0%	0 / 1 0.0%	1 / 5 20.0%	0 / 1 0.0%	1 / 4 25.0%	2 / 3 66.7%	0 / 1 0.0%	0 / 1 0.0%	1 / 2 50.0%	0 / 4 0.0%	1 / 1 100.0%	1 / 3 33.3%	2 / 4 50.0%	0 / 2 0.0%	0 / 1 0.0%	2 / 5 40.0%	0 / 0 0.0%	0 / 0 0.0%	0 / 1 0.0%	0 / 0 0.0%	18 / 54 33.3%E	11 / 54 20.4%AB	11 / 54 20.4%	7 / 54 13.0%C	4 / 54 7.4%ABE	3 / 54 5.6%CE		
1	Thermobifida	13 / 52 25.0%	0 / 2 0.0%	0 / 1 0.0%	0 / 1 0.0%	0 / 1 0.0%	1 / 5 20.0%	2 / 4 50.0%	0 / 1 0.0%	0 / 1 0.0%	0 / 4 0.0%	0 / 1 0.0%	1 / 4 25.0%	3 / 3 100.0%	0 / 1 0.0%	0 / 2 0.0%	0 / 1 0.0%	1 / 3 33.3%	0 / 1 0.0%	1 / 3 33.3%	1 / 4 25.0%	1 / 3 33.3%	0 / 1 0.0%	2 / 5 40.0%	0 / 0 0.0%	0 / 0 0.0%	0 / 0 0.0%	0 / 0 0.0%	21 / 52 40.4%E	15 / 52 28.8%	9 / 52 17.3%AB	4 / 52 7.7%ABE	3 / 52 5.8%C			
1	"Thermonospora fusca" Henssen 1957	13 / 52 25.0%	0 / 2 0.0%	0 / 1 0.0%	0 / 1 0.0%	0 / 1 0.0%	1 / 5 20.0%	2 / 4 50.0%	0 / 1 0.0%	0 / 1 0.0%	0 / 4 0.0%	0 / 1 0.0%	1 / 4 25.0%	3 / 3 100.0%	0 / 1 0.0%	0 / 2 0.0%	0 / 1 0.0%	1 / 3 33.3%	0 / 1 0.0%	1 / 3 33.3%	1 / 4 25.0%	1 / 3 33.3%	0 / 1 0.0%	2 / 5 40.0%	0 / 0 0.0%	0 / 0 0.0%	0 / 0 0.0%	0 / 0 0.0%	21 / 52 40.4%E	15 / 52 28.8%	9 / 52 17.3%AB	4 / 52 7.7%ABE	3 / 52 5.8%C			
1	Thermobifida fusca YX	13 / 52 25.0%	0 / 2 0.0%	0 / 1 0.0%	0 / 1 0.0%	0 / 1 0.0%	1 / 5 20.0%	2 / 4 50.0%	0 / 1 0.0%	0 / 1 0.0%	0 / 4 0.0%	0 / 1 0.0%	1 / 4 25.0%	3 / 3 100.0%	0 / 1 0.0%	0 / 2 0.0%	0 / 1 0.0%	1 / 3 33.3%	0 / 1 0.0%	1 / 3 33.3%	1 / 4 25.0%	1 / 3 33.3%	0 / 1 0.0%	2 / 5 40.0%	0 / 0 0.0%	0 / 0 0.0%	0 / 0 0.0%	0 / 0 0.0%	21 / 52 40.4%E	15 / 52 28.8%	9 / 52 17.3%AB	4 / 52 7.7%ABE	3 / 52 5.8%C			
10	Frankineae	111 / 461 24.1%	10 / 14 71.4%	2 / 10 20.0%	2 / 10 20.0%	0 / 10 0.0%	10 / 30 33.3%	6 / 30 20.0%	7 / 10 70.0%	6 / 10 60.0%	7 / 30 23.3%	2 / 10 20.0%	5 / 40 12.5%	3 / 20 15.0%	3 / 10 30.0%	2 / 20 10.0%	0 / 11 0.0%	9 / 30 30.0%	2 / 10 20.0%	10 / 30 33.3%	8 / 40 20.0%	4 / 23 17.4%	4 / 10 40.0%	7 / 50 14.0%	1 / 1 100.0%	0 / 0 0.0%	0 / 0 0.0%	1 / 2 50.0%	142 / 461 30.8%	130 / 461 28.2%E	60 / 461 13.0%AB	49 / 461 10.6%ABE	45 / 461 9.8%C	33 / 461 7.2%CE	1 / 461 0.2%BE	1 / 461 0.2%B
5	Frankiaceae	47 / 226 20.8%	5 / 6 83.3%	0 / 5 0.0%	0 / 5 0.0%	0 / 5 0.0%	4 / 15 26.7%	1 / 15 6.7%	4 / 5 80.0%	5 / 5 100.0%	3 / 15 20.0%	0 / 5 0.0%	1 / 20 5.0%	0 / 10 0.0%	1 / 5 20.0%	0 / 10 0.0%	0 / 5 0.0%	3 / 15 20.0%	2 / 5 40.0%	5 / 15 33.3%	3 / 20 15.0%	3 / 10 30.0%	3 / 5 60.0%	4 / 25 16.0%	0 / 0 0.0%	0 / 0 0.0%	0 / 0 0.0%	0 / 0 0.0%	77 / 226 34.1%	63 / 226 27.9%E	23 / 226 10.2%AB	22 / 226 9.7%C	22 / 226 9.7%ABE	17 / 226 7.5%CE	1 / 226 0.4%B	1 / 226 0.4%BE
5	"Frankiella" Maire and Tison 1909	47 / 226 20.8%	5 / 6 83.3%	0 / 5 0.0%	0 / 5 0.0%	0 / 5 0.0%	4 / 15 26.7%	1 / 15 6.7%	4 / 5 80.0%	5 / 5 100.0%	3 / 15 20.0%	0 / 5 0.0%	1 / 20 5.0%	0 / 10 0.0%	1 / 5 20.0%	0 / 10 0.0%	0 / 5 0.0%	3 / 15 20.0%	2 / 5 40.0%	5 / 15 33.3%	3 / 20 15.0%	3 / 10 30.0%	3 / 5 60.0%	4 / 25 16.0%	0 / 0 0.0%	0 / 0 0.0%	0 / 0 0.0%	0 / 0 0.0%	77 / 226 34.1%	63 / 226 27.9%E	23 / 226 10.2%AB	22 / 226 9.7%ABE	22 / 226 9.7%C	17 / 226 7.5%CE	1 / 226 0.4%BE	1 / 226 0.4%B
1	"Actinomyces alni" (Woronin 1866) Roberg 1934	5 / 45 11.1%	1 / 1 100.0%	0 / 1 0.0%	0 / 1 0.0%	0 / 1 0.0%	0 / 3 0.0%	0 / 3 0.0%	1 / 1 100.0%	1 / 1 100.0%	0 / 3 0.0%	0 / 1 0.0%	0 / 4 0.0%	0 / 2 0.0%	0 / 1 0.0%	0 / 2 0.0%	0 / 1 0.0%	1 / 3 33.3%	0 / 1 0.0%	1 / 3 33.3%	0 / 4 0.0%	0 / 2 0.0%	0 / 1 0.0%	0 / 5 0.0%	0 / 0 0.0%	0 / 0 0.0%	0 / 0 0.0%	0 / 0 0.0%	17 / 45 37.8%	14 / 45 31.1%E	6 / 45 13.3%C	3 / 45 6.7%ABE	3 / 45 6.7%CE	2 / 45 4.4%AB		
1	Frankia alni ACN14a	5 / 45 11.1%	1 / 1 100.0%	0 / 1 0.0%	0 / 1 0.0%	0 / 1 0.0%	0 / 3 0.0%	0 / 3 0.0%	1 / 1 100.0%	1 / 1 100.0%	0 / 3 0.0%	0 / 1 0.0%	0 / 4 0.0%	0 / 2 0.0%	0 / 1 0.0%	0 / 2 0.0%	0 / 1 0.0%	1 / 3 33.3%	0 / 1 0.0%	1 / 3 33.3%	0 / 4 0.0%	0 / 2 0.0%	0 / 1 0.0%	0 / 5 0.0%	0 / 0 0.0%	0 / 0 0.0%	0 / 0 0.0%	0 / 0 0.0%	17 / 45 37.8%	14 / 45 31.1%E	6 / 45 13.3%C	3 / 45 6.7%ABE	3 / 45 6.7%CE	2 / 45 4.4%AB		
1	Frankia sp. CcI3	14 / 45 31.1%	1 / 1 100.0%	0 / 1 0.0%	0 / 1 0.0%	0 / 1 0.0%	2 / 3 66.7%	0 / 3 0.0%	1 / 1 100.0%	1 / 1 100.0%	1 / 3 33.3%	0 / 1 0.0%	0 / 4 0.0%	0 / 2 0.0%	1 / 1 100.0%	0 / 2 0.0%	0 / 1 0.0%	0 / 3 0.0%	1 / 1 100.0%	2 / 3 66.7%	0 / 4 0.0%	1 / 2 50.0%	1 / 1 100.0%	2 / 5 40.0%	0 / 0 0.0%	0 / 0 0.0%	0 / 0 0.0%	0 / 0 0.0%	13 / 45 28.9%	11 / 45 24.4%E	9 / 45 20.0%AB	5 / 45 11.1%ABE	4 / 45 8.9%CE	3 / 45 6.7%C		
1	Frankia sp. EAN1pec	10 / 45 22.2%	0 / 1 0.0%	0 / 1 0.0%	0 / 1 0.0%	0 / 1 0.0%	0 / 3 0.0%	1 / 3 33.3%	1 / 1 100.0%	1 / 1 100.0%	0 / 3 0.0%	0 / 1 0.0%	1 / 4 25.0%	0 / 2 0.0%	0 / 1 0.0%	0 / 2 0.0%	0 / 1 0.0%	1 / 3 33.3%	1 / 1 100.0%	1 / 3 33.3%	1 / 4 25.0%	1 / 2 50.0%	1 / 1 100.0%	0 / 5 0.0%	0 / 0 0.0%	0 / 0 0.0%	0 / 0 0.0%	0 / 0 0.0%	15 / 45 33.3%	14 / 45 31.1%E	5 / 45 11.1%C	4 / 45 8.9%AB	4 / 45 8.9%ABE	1 / 45 2.2%B	1 / 45 2.2%BE	1 / 45 2.2%CE
1	Frankia sp. EuI1c	12 / 46 26.1%	2 / 2 100.0%	0 / 1 0.0%	0 / 1 0.0%	0 / 1 0.0%	1 / 3 33.3%	0 / 3 0.0%	1 / 1 100.0%	1 / 1 100.0%	1 / 3 33.3%	0 / 1 0.0%	0 / 4 0.0%	0 / 2 0.0%	0 / 1 0.0%	0 / 2 0.0%	0 / 1 0.0%	1 / 3 33.3%	0 / 1 0.0%	1 / 3 33.3%	2 / 4 50.0%	0 / 2 0.0%	1 / 1 100.0%	1 / 5 20.0%	0 / 0 0.0%	0 / 0 0.0%	0 / 0 0.0%	0 / 0 0.0%	14 / 46 30.4%	13 / 46 28.3%E	6 / 46 13.0%AB	6 / 46 13.0%ABE	4 / 46 8.7%C	3 / 46 6.5%CE		
1	Frankia symbiont of Datisca glomerata	6 / 45 13.3%	1 / 1 100.0%	0 / 1 0.0%	0 / 1 0.0%	0 / 1 0.0%	1 / 3 33.3%	0 / 3 0.0%	0 / 1 0.0%	1 / 1 100.0%	1 / 3 33.3%	0 / 1 0.0%	0 / 4 0.0%	0 / 2 0.0%	0 / 1 0.0%	0 / 2 0.0%	0 / 1 0.0%	0 / 3 0.0%	0 / 1 0.0%	0 / 3 0.0%	0 / 4 0.0%	1 / 2 50.0%	0 / 1 0.0%	1 / 5 20.0%	0 / 0 0.0%	0 / 0 0.0%	0 / 0 0.0%	0 / 0 0.0%	18 / 45 40.0%	11 / 45 24.4%E	6 / 45 13.3%CE	4 / 45 8.9%C	4 / 45 8.9%ABE	2 / 45 4.4%AB		
3	"Geodermatophilaceae" Normand et al. 1996 emend. Stackebrandt et al. 1997	31 / 143 21.7%	4 / 6 66.7%	1 / 3 33.3%	1 / 3 33.3%	0 / 3 0.0%	4 / 9 44.4%	2 / 9 22.2%	2 / 3 66.7%	0 / 3 0.0%	1 / 9 11.1%	2 / 3 66.7%	1 / 12 8.3%	3 / 6 50.0%	0 / 3 0.0%	1 / 6 16.7%	0 / 3 0.0%	2 / 9 22.2%	0 / 3 0.0%	1 / 9 11.1%	3 / 12 25.0%	0 / 9 0.0%	1 / 3 33.3%	1 / 15 6.7%	0 / 0 0.0%	0 / 0 0.0%	0 / 0 0.0%	1 / 2 50.0%	46 / 143 32.2%E	33 / 143 23.1%	20 / 143 14.0%C	18 / 143 12.6%AB	13 / 143 9.1%CE	13 / 143 9.1%ABE		
1	Geodermatophilus	8 / 47 17.0%	1 / 2 50.0%	0 / 1 0.0%	0 / 1 0.0%	0 / 1 0.0%	1 / 3 33.3%	1 / 3 33.3%	1 / 1 100.0%	0 / 1 0.0%	0 / 3 0.0%	0 / 1 0.0%	0 / 4 0.0%	1 / 2 50.0%	0 / 1 0.0%	0 / 2 0.0%	0 / 1 0.0%	1 / 3 33.3%	0 / 1 0.0%	1 / 3 33.3%	1 / 4 25.0%	0 / 3 0.0%	0 / 1 0.0%	0 / 5 0.0%	0 / 0 0.0%	0 / 0 0.0%	0 / 0 0.0%	0 / 0 0.0%	16 / 47 34.0%E	12 / 47 25.5%	7 / 47 14.9%C	5 / 47 10.6%AB	4 / 47 8.5%CE	3 / 47 6.4%ABE		
1	ATCC 25078	8 / 47 17.0%	1 / 2 50.0%	0 / 1 0.0%	0 / 1 0.0%	0 / 1 0.0%	1 / 3 33.3%	1 / 3 33.3%	1 / 1 100.0%	0 / 1 0.0%	0 / 3 0.0%	0 / 1 0.0%	0 / 4 0.0%	1 / 2 50.0%	0 / 1 0.0%	0 / 2 0.0%	0 / 1 0.0%	1 / 3 33.3%	0 / 1 0.0%	1 / 3 33.3%	1 / 4 25.0%	0 / 3 0.0%	0 / 1 0.0%	0 / 5 0.0%	0 / 0 0.0%	0 / 0 0.0%	0 / 0 0.0%	0 / 0 0.0%	16 / 47 34.0%E	12 / 47 25.5%	7 / 47 14.9%C	5 / 47 10.6%AB	4 / 47 8.5%CE	3 / 47 6.4%ABE		
1	Geodermatophilus obscurus ATCC 25078	8 / 47 17.0%	1 / 2 50.0%	0 / 1 0.0%	0 / 1 0.0%	0 / 1 0.0%	1 / 3 33.3%	1 / 3 33.3%	1 / 1 100.0%	0 / 1 0.0%	0 / 3 0.0%	0 / 1 0.0%	0 / 4 0.0%	1 / 2 50.0%	0 / 1 0.0%	0 / 2 0.0%	0 / 1 0.0%	1 / 3 33.3%	0 / 1 0.0%	1 / 3 33.3%	1 / 4 25.0%	0 / 3 0.0%	0 / 1 0.0%	0 / 5 0.0%	0 / 0 0.0%	0 / 0 0.0%	0 / 0 0.0%	0 / 0 0.0%	16 / 47 34.0%E	12 / 47 25.5%	7 / 47 14.9%C	5 / 47 10.6%AB	4 / 47 8.5%CE	3 / 47 6.4%ABE		
1	Blastococcus	14 / 48 29.2%	2 / 2 100.0%	0 / 1 0.0%	0 / 1 0.0%	0 / 1 0.0%	1 / 3 33.3%	1 / 3 33.3%	1 / 1 100.0%	0 / 1 0.0%	1 / 3 33.3%	1 / 1 100.0%	1 / 4 25.0%	1 / 2 50.0%	0 / 1 0.0%	0 / 2 0.0%	0 / 1 0.0%	0 / 3 0.0%	0 / 1 0.0%	0 / 3 0.0%	2 / 4 50.0%	0 / 3 0.0%	1 / 1 100.0%	1 / 5 20.0%	0 / 0 0.0%	0 / 0 0.0%	0 / 0 0.0%	1 / 1 100.0%	13 / 48 27.1%E	11 / 48 22.9%	7 / 48 14.6%ABE	7 / 48 14.6%AB	5 / 48 10.4%CE	5 / 48 10.4%C		
1	Blastococcus saxobsidens	14 / 48 29.2%	2 / 2 100.0%	0 / 1 0.0%	0 / 1 0.0%	0 / 1 0.0%	1 / 3 33.3%	1 / 3 33.3%	1 / 1 100.0%	0 / 1 0.0%	1 / 3 33.3%	1 / 1 100.0%	1 / 4 25.0%	1 / 2 50.0%	0 / 1 0.0%	0 / 2 0.0%	0 / 1 0.0%	0 / 3 0.0%	0 / 1 0.0%	0 / 3 0.0%	2 / 4 50.0%	0 / 3 0.0%	1 / 1 100.0%	1 / 5 20.0%	0 / 0 0.0%	0 / 0 0.0%	0 / 0 0.0%	1 / 1 100.0%	13 / 48 27.1%E	11 / 48 22.9%	7 / 48 14.6%AB	7 / 48 14.6%ABE	5 / 48 10.4%C	5 / 48 10.4%CE		
1	Blastococcus saxobsidens DD2	14 / 48 29.2%	2 / 2 100.0%	0 / 1 0.0%	0 / 1 0.0%	0 / 1 0.0%	1 / 3 33.3%	1 / 3 33.3%	1 / 1 100.0%	0 / 1 0.0%	1 / 3 33.3%	1 / 1 100.0%	1 / 4 25.0%	1 / 2 50.0%	0 / 1 0.0%	0 / 2 0.0%	0 / 1 0.0%	0 / 3 0.0%	0 / 1 0.0%	0 / 3 0.0%	2 / 4 50.0%	0 / 3 0.0%	1 / 1 100.0%	1 / 5 20.0%	0 / 0 0.0%	0 / 0 0.0%	0 / 0 0.0%	1 / 1 100.0%	13 / 48 27.1%E	11 / 48 22.9%	7 / 48 14.6%ABE	7 / 48 14.6%AB	5 / 48 10.4%CE	5 / 48 10.4%C		
1	Modestibacter	9 / 48 18.8%	1 / 2 50.0%	1 / 1 100.0%	1 / 1 100.0%	0 / 1 0.0%	2 / 3 66.7%	0 / 3 0.0%	0 / 1 0.0%	0 / 1 0.0%	0 / 3 0.0%	1 / 1 100.0%	0 / 4 0.0%	1 / 2 50.0%	0 / 1 0.0%	1 / 2 50.0%	0 / 1 0.0%	1 / 3 33.3%	0 / 1 0.0%	0 / 3 0.0%	0 / 4 0.0%	0 / 3 0.0%	0 / 1 0.0%	0 / 5 0.0%	0 / 0 0.0%	0 / 0 0.0%	0 / 0 0.0%	0 / 1 0.0%	17 / 48 35.4%E	10 / 48 20.8%	8 / 48 16.7%C	6 / 48 12.5%AB	4 / 48 8.3%CE	3 / 48 6.2%ABE		
1	CGMCC 4.5581	9 / 48 18.8%	1 / 2 50.0%	1 / 1 100.0%	1 / 1 100.0%	0 / 1 0.0%	2 / 3 66.7%	0 / 3 0.0%	0 / 1 0.0%	0 / 1 0.0%	0 / 3 0.0%	1 / 1 100.0%	0 / 4 0.0%	1 / 2 50.0%	0 / 1 0.0%	1 / 2 50.0%	0 / 1 0.0%	1 / 3 33.3%	0 / 1 0.0%	0 / 3 0.0%	0 / 4 0.0%	0 / 3 0.0%	0 / 1 0.0%	0 / 5 0.0%	0 / 0 0.0%	0 / 0 0.0%	0 / 0 0.0%	0 / 1 0.0%	17 / 48 35.4%E	10 / 48 20.8%	8 / 48 16.7%C	6 / 48 12.5%AB	4 / 48 8.3%CE	3 / 48 6.2%ABE		
1	Microsphaeraceae	18 / 47 38.3%	1 / 1 100.0%	0 / 1 0.0%	0 / 1 0.0%	0 / 1 0.0%	2 / 3 66.7%	2 / 3 66.7%	0 / 1 0.0%	0 / 1 0.0%	2 / 3 66.7%	0 / 1 0.0%	2 / 4 50.0%	0 / 2 0.0%	1 / 1 100.0%	0 / 2 0.0%	0 / 2 0.0%	2 / 3 66.7%	0 / 1 0.0%	2 / 3 66.7%	2 / 4 50.0%	0 / 2 0.0%	0 / 1 0.0%	1 / 5 20.0%	1 / 1 100.0%	0 / 0 0.0%	0 / 0 0.0%	0 / 0 0.0%	12 / 47 25.5%	11 / 47 23.4%E	11 / 47 23.4%AB	7 / 47 14.9%ABE	3 / 47 6.4%C	3 / 47 6.4%CE		
1	Humicoccus	18 / 47 38.3%	1 / 1 100.0%	0 / 1 0.0%	0 / 1 0.0%	0 / 1 0.0%	2 / 3 66.7%	2 / 3 66.7%	0 / 1 0.0%	0 / 1 0.0%	2 / 3 66.7%	0 / 1 0.0%	2 / 4 50.0%	0 / 2 0.0%	1 / 1 100.0%	0 / 2 0.0%	0 / 2 0.0%	2 / 3 66.7%	0 / 1 0.0%	2 / 3 66.7%	2 / 4 50.0%	0 / 2 0.0%	0 / 1 0.0%	1 / 5 20.0%	1 / 1 100.0%	0 / 0 0.0%	0 / 0 0.0%	0 / 0 0.0%	12 / 47 25.5%	11 / 47 23.4%AB	11 / 47 23.4%E	7 / 47 14.9%ABE	3 / 47 6.4%CE	3 / 47 6.4%C		
1	ATCC 700099	18 / 47 38.3%	1 / 1 100.0%	0 / 1 0.0%	0 / 1 0.0%	0 / 1 0.0%	2 / 3 66.7%	2 / 3 66.7%	0 / 1 0.0%	0 / 1 0.0%	2 / 3 66.7%	0 / 1 0.0%	2 / 4 50.0%	0 / 2 0.0%	1 / 1 100.0%	0 / 2 0.0%	0 / 2 0.0%	2 / 3 66.7%	0 / 1 0.0%	2 / 3 66.7%	2 / 4 50.0%	0 / 2 0.0%	0 / 1 0.0%	1 / 5 20.0%	1 / 1 100.0%	0 / 0 0.0%	0 / 0 0.0%	0 / 0 0.0%	12 / 47 25.5%	11 / 47 23.4%E	11 / 47 23.4%AB	7 / 47 14.9%ABE	3 / 47 6.4%CE	3 / 47 6.4%C		
1	Nakamurella multipartita DSM 44233	18 / 47 38.3%	1 / 1 100.0%	0 / 1 0.0%	0 / 1 0.0%	0 / 1 0.0%	2 / 3 66.7%	2 / 3 66.7%	0 / 1 0.0%	0 / 1 0.0%	2 / 3 66.7%	0 / 1 0.0%	2 / 4 50.0%	0 / 2 0.0%	1 / 1 100.0%	0 / 2 0.0%	0 / 2 0.0%	2 / 3 66.7%	0 / 1 0.0%	2 / 3 66.7%	2 / 4 50.0%	0 / 2 0.0%	0 / 1 0.0%	1 / 5 20.0%	1 / 1 100.0%	0 / 0 0.0%	0 / 0 0.0%	0 / 0 0.0%	12 / 47 25.5%	11 / 47 23.4%AB	11 / 47 23.4%E	7 / 47 14.9%ABE	3 / 47 6.4%CE	3 / 47 6.4%C		
1	Acidithermaceae	15 / 45 33.3%	0 / 1 0.0%	1 / 1 100.0%	1 / 1 100.0%	0 / 1 0.0%	0 / 3 0.0%	1 / 3 33.3%	1 / 1 100.0%	1 / 1 100.0%	1 / 3 33.3%	0 / 1 0.0%	1 / 4 25.0%	0 / 2 0.0%	1 / 1 100.0%	1 / 2 50.0%	0 / 1 0.0%	2 / 3 66.7%	0 / 1 0.0%	2 / 3 66.7%	0 / 4 0.0%	1 / 2 50.0%	0 / 1 0.0%	1 / 5 20.0%	0 / 0 0.0%	0 / 0 0.0%	0 / 0 0.0%	0 / 0 0.0%	20 / 45 44.4%	10 / 45 22.2%E	8 / 45 17.8%AB	7 / 45 15.6%ABE				
1	Acidithermus	15 / 45 33.3%	0 / 1 0.0%	1 / 1 100.0%	1 / 1 100.0%	0 / 1 0.0%	0 / 3 0.0%	1 / 3 33.3%	1 / 1 100.0%	1 / 1 100.0%	1 / 3 33.3%	0 / 1 0.0%	1 / 4 25.0%	0 / 2 0.0%	1 / 1 100.0%	1 / 2 50.0%	0 / 1 0.0%	2 / 3 66.7%	0 / 1 0.0%	2 / 3 66.7%	0 / 4 0.0%	1 / 2 50.0%	0 / 1 0.0%	1 / 5 20.0%	0 / 0 0.0%	0 / 0 0.0%	0 / 0 0.0%	0 / 0 0.0%	20 / 45 44.4%	10 / 45 22.2%E	8 / 45 17.8%AB	7 / 45 15.6%ABE				
1	ATCC 43068	15 / 45 33.3%	0 / 1 0.0%	1 / 1 100.0%	1 / 1 100.0%	0 / 1 0.0%	0 / 3 0.0%	1 / 3 33.3%	1 / 1 100.0%	1 / 1 100.0%	1 / 3 33.3%	0 / 1 0.0%	1 / 4 25.0%	0 / 2 0.0%	1 / 1 100.0%	1 / 2 50.0%	0 / 1 0.0%	2 / 3 66.7%	0 / 1 0.0%	2 / 3 66.7%	0 / 4 0.0%	1 / 2 50.0%	0 / 1 0.0%	1 / 5 20.0%	0 / 0 0.0%	0 / 0 0.0%	0 / 0 0.0%	0 / 0 0.0%	20 / 45 44.4%	10 / 45 22.2%E	8 / 45 17.8%AB	7 / 45 15.6%ABE				
1	Acidothermus cellulolyticus 11B	15 / 45 33.3%	0 / 1 0.0%	1 / 1 100.0%	1 / 1 100.0%	0 / 1 0.0%	0 / 3 0.0%	1 / 3 33.3%	1 / 1 100.0%	1 / 1 100.0%	1 / 3 33.3%	0 / 1 0.0%	1 / 4 25.0%	0 / 2 0.0%	1 / 1 100.0%	1 / 2 50.0%	0 / 1 0.0%	2 / 3 66.7%	0 / 1 0.0%	2 / 3 66.7%	0 / 4 0.0%	1 / 2 50.0%	0 / 1 0.0%	1 / 5 20.0%	0 / 0 0.0%	0 / 0 0.0%	0 / 0 0.0%	0 / 0 0.0%	20 / 45 44.4%	10 / 45 22.2%E	8 / 45 17.8%AB	7 / 45 15.6%ABE				
1	Glycomycetineae	20 / 45 44.4%	0 / 1 0.0%	0 / 1 0.0%	0 / 1 0.0%	1 / 1 100.0%	2 / 3 66.7%	2 / 3 66.7%	1 / 1 100.0%	0 / 1 0.0%	0 / 3 0.0%	0 / 1 0.0%	2 / 4 50.0%	1 / 2 50.0%	1 / 1 100.0%	1 / 2 50.0%	0 / 1 0.0%	2 / 3 66.7%	0 / 1 0.0%	2 / 3 66.7%	3 / 4 75.0%	1 / 2 50.0%	0 / 1 0.0%	1 / 5 20.0%	0 / 0 0.0%	0 / 0 0.0%	0 / 0 0.0%	0 / 0 0.0%	12 / 45 26.7%AB	11 / 45 24.4%	11 / 45 24.4%E	8 / 45 17.8%ABE	2 / 45 4.4%CE	1 / 45 2.2%C		
1	Glycomycetaceae	20 / 45 44.4%	0 / 1 0.0%	0 / 1 0.0%	0 / 1 0.0%	1 / 1 100.0%	2 / 3 66.7%	2 / 3 66.7%	1 / 1 100.0%	0 / 1 0.0%	0 / 3 0.0%	0 / 1 0.0%	2 / 4 50.0%	1 / 2 50.0%	1 / 1 100.0%	1 / 2 50.0%	0 / 1 0.0%	2 / 3 66.7%	0 / 1 0.0%	2 / 3 66.7%	3 / 4 75.0%	1 / 2 50.0%	0 / 1 0.0%	1 / 5 20.0%	0 / 0 0.0%	0 / 0 0.0%	0 / 0 0.0%	0 / 0 0.0%	12 / 45 26.7%AB	11 / 45 24.4%E	11 / 45 24.4%	8 / 45 17.8%ABE	2 / 45 4.4%CE	1 / 45 2.2%C		
1	Stackebrandtia	20 / 45 44.4%	0 / 1 0.0%	0 / 1 0.0%	0 / 1 0.0%	1 / 1 100.0%	2 / 3 66.7%	2 / 3 66.7%	1 / 1 100.0%	0 / 1 0.0%	0 / 3 0.0%	0 / 1 0.0%	2 / 4 50.0%	1 / 2 50.0%	1 / 1 100.0%	1 / 2 50.0%	0 / 1 0.0%	2 / 3 66.7%	0 / 1 0.0%	2 / 3 66.7%	3 / 4 75.0%	1 / 2 50.0%	0 / 1 0.0%	1 / 5 20.0%	0 / 0 0.0%	0 / 0 0.0%	0 / 0 0.0%	0 / 0 0.0%	12 / 45 26.7%AB	11 / 45 24.4%E	11 / 45 24.4%	8 / 45 17.8%ABE	2 / 45 4.4%CE	1 / 45 2.2%C		
1	DSM 44728	20 / 45 44.4%	0 / 1 0.0%	0 / 1 0.0%	0 / 1 0.0%	1 / 1 100.0%	2 / 3 66.7%	2 / 3 66.7%	1 / 1 100.0%	0 / 1 0.0%	0 / 3 0.0%	0 / 1 0.0%	2 / 4 50.0%	1 / 2 50.0%	1 / 1 100.0%	1 / 2 50.0%	0 / 1 0.0%	2 / 3 66.7%	0 / 1 0.0%	2 / 3 66.7%	3 / 4 75.0%	1 / 2 50.0%	0 / 1 0.0%	1 / 5 20.0%	0 / 0 0.0%	0 / 0 0.0%	0 / 0 0.0%	0 / 0 0.0%	12 / 45 26.7%AB	11 / 45 24.4%	11 / 45 24.4%E	8 / 45 17.8%ABE	2 / 45 4.4%CE	1 / 45 2.2%C		
1	Stackebrandtia nassauensis DSM 44728	20 / 45 44.4%	0 / 1 0.0%	0 / 1 0.0%	0 / 1 0.0%	1 / 1 100.0%	2 / 3 66.7%	2 / 3 66.7%	1 / 1 100.0%	0 / 1 0.0%	0 / 3 0.0%	0 / 1 0.0%	2 / 4 50.0%	1 / 2 50.0%	1 / 1 100.0%	1 / 2 50.0%	0 / 1 0.0%	2 / 3 66.7%	0 / 1 0.0%	2 / 3 66.7%	3 / 4 75.0%	1 / 2 50.0%	0 / 1 0.0%	1 / 5 20.0%	0 / 0 0.0%	0 / 0 0.0%	0 / 0 0.0%	0 / 0 0.0%	12 / 45 26.7%AB	11 / 45 24.4%	11 / 45 24.4%E	8 / 45 17.8%ABE	2 / 45 4.4%CE	1 / 45 2.2%C		
1	Catenulisporinae	6 / 56 10.7%	1 / 3 33.3%	0 / 1 0.0%	0 / 1 0.0%	0 / 1 0.0%	1 / 4 25.0%	0 / 3 0.0%	0 / 1 0.0%	0 / 1 0.0%	1 / 3 33.3%	0 / 1 0.0%	0 / 5 0.0%	0 / 3 0.0%	0 / 1 0.0%	0 / 2 0.0%	0 / 1 0.0%	1 / 3 33.3%	0 / 1 0.0%	1 / 3 33.3%	0 / 5 0.0%	0 / 4 0.0%	0 / 1 0.0%	1 / 7 14.3%	0 / 1 0.0%	0 / 0 0.0%	0 / 0 0.0%	0 / 0 0.0%	21 / 56 37.5%E	15 / 56 26.8%	7 / 56 12.5%C	6 / 56 10.7%CE	3 / 56 5.4%AB	3 / 56 5.4%ABE	1 / 56 1.8%D	
1	Catenulisporaceae	6 / 56 10.7%	1 / 3 33.3%	0 / 1 0.0%	0 / 1 0.0%	0 / 1 0.0%	1 / 4 25.0%	0 / 3 0.0%	0 / 1 0.0%	0 / 1 0.0%	1 / 3 33.3%	0 / 1 0.0%	0 / 5 0.0%	0 / 3 0.0%	0 / 1 0.0%	0 / 2 0.0%	0 / 1 0.0%	1 / 3 33.3%	0 / 1 0.0%	1 / 3 33.3%	0 / 5 0.0%	0 / 4 0.0%	0 / 1 0.0%	1 / 7 14.3%	0 / 1 0.0%	0 / 0 0.0%	0 / 0 0.0%	0 / 0 0.0%	21 / 56 37.5%E	15 / 56 26.8%	7 / 56 12.5%C	6 / 56 10.7%CE	3 / 56 5.4%AB	3 / 56 5.4%ABE	1 / 56 1.8%D	
1	Catenulispora	6 / 56 10.7%	1 / 3 33.3%	0 / 1 0.0%	0 / 1 0.0%	0 / 1 0.0%	1 / 4 25.0%	0 / 3 0.0%	0 / 1 0.0%	0 / 1 0.0%	1 / 3 33.3%	0 / 1 0.0%	0 / 5 0.0%	0 / 3 0.0%	0 / 1 0.0%	0 / 2 0.0%	0 / 1 0.0%	1 / 3 33.3%	0 / 1 0.0%	1 / 3 33.3%	0 / 5 0.0%	0 / 4 0.0%	0 / 1 0.0%	1 / 7 14.3%	0 / 1 0.0%	0 / 0 0.0%	0 / 0 0.0%	0 / 0 0.0%	21 / 56 37.5%E	15 / 56 26.8%	7 / 56 12.5%C	6 / 56 10.7%CE	3 / 56 5.4%AB	3 / 56 5.4%ABE	1 / 56 1.8%D	
1	Actinomycetales bacterium Neo3	6 / 56 10.7%	1 / 3 33.3%	0 / 1 0.0%	0 / 1 0.0%	0 / 1 0.0%	1 / 4 25.0%	0 / 3 0.0%	0 / 1 0.0%	0 / 1 0.0%	1 / 3 33.3%	0 / 1 0.0%	0 / 5 0.0%	0 / 3 0.0%	0 / 1 0.0%	0 / 2 0.0%	0 / 1 0.0%	1 / 3 33.3%	0 / 1 0.0%	1 / 3 33.3%	0 / 5 0.0%	0 / 4 0.0%	0 / 1 0.0%	1 / 7 14.3%	0 / 1 0.0%	0 / 0 0.0%	0 / 0 0.0%	0 / 0 0.0%	21 / 56 37.5%E	15 / 56 26.8%	7 / 56 12.5%C	6 / 56 10.7%CE	3 / 56 5.4%AB	3 / 56 5.4%ABE	1 / 56 1.8%D	
1	Catenulispora acidiphila DSM 44928	6 / 56 10.7%	1 / 3 33.3%	0 / 1 0.0%	0 / 1 0.0%	0 / 1 0.0%	1 / 4 25.0%	0 / 3 0.0%	0 / 1 0.0%	0 / 1 0.0%	1 / 3 33.3%	0 / 1 0.0%	0 / 5 0.0%	0 / 3 0.0%	0 / 1 0.0%	0 / 2 0.0%	0 / 1 0.0%	1 / 3 33.3%	0 / 1 0.0%	1 / 3 33.3%	0 / 5 0.0%	0 / 4 0.0%	0 / 1 0.0%	1 / 7 14.3%	0 / 1 0.0%	0 / 0 0.0%	0 / 0 0.0%	0 / 0 0.0%	21 / 56 37.5%E	15 / 56 26.8%	7 / 56 12.5%C	6 / 56 10.7%CE	3 / 56 5.4%AB	3 / 56 5.4%ABE	1 / 56 1.8%D	
1	Kineosporiineae	13 / 49 26.5%	2 / 2 100.0%	0 / 1 0.0%	0 / 1 0.0%	0 / 1 0.0%	2 / 4 50.0%	1 / 3 33.3%	1 / 1 100.0%	0 / 1 0.0%	0 / 3 0.0%	0 / 1 0.0%	0 / 4 0.0%	1 / 2 50.0%	0 / 1 0.0%	0 / 2 0.0%	1 / 1 100.0%	2 / 3 66.7%	0 / 1 0.0%	2 / 3 66.7%	0 / 4 0.0%	0 / 3 0.0%	0 / 1 0.0%	0 / 5 0.0%	1 / 1 100.0%	0 / 0 0.0%	0 / 0 0.0%	0 / 0 0.0%	12 / 49 24.5%E	11 / 49 22.4%	7 / 49 14.3%CE	6 / 49 12.2%C	5 / 49 10.2%BE	4 / 49 8.2%B	3 / 49 6.1%AB	1 / 49 2.0%ABE
1	"Kineosporiaceae" Garrity and Holt 2001	13 / 49 26.5%	2 / 2 100.0%	0 / 1 0.0%	0 / 1 0.0%	0 / 1 0.0%	2 / 4 50.0%	1 / 3 33.3%	1 / 1 100.0%	0 / 1 0.0%	0 / 3 0.0%	0 / 1 0.0%	0 / 4 0.0%	1 / 2 50.0%	0 / 1 0.0%	0 / 2 0.0%	1 / 1 100.0%	2 / 3 66.7%	0 / 1 0.0%	2 / 3 66.7%	0 / 4 0.0%	0 / 3 0.0%	0 / 1 0.0%	0 / 5 0.0%	1 / 1 100.0%	0 / 0 0.0%	0 / 0 0.0%	0 / 0 0.0%	12 / 49 24.5%E	11 / 49 22.4%	7 / 49 14.3%CE	6 / 49 12.2%C	5 / 49 10.2%BE	4 / 49 8.2%B	3 / 49 6.1%AB	1 / 49 2.0%ABE
1	Kineococcus	13 / 49 26.5%	2 / 2 100.0%	0 / 1 0.0%	0 / 1 0.0%	0 / 1 0.0%	2 / 4 50.0%	1 / 3 33.3%	1 / 1 100.0%	0 / 1 0.0%	0 / 3 0.0%	0 / 1 0.0%	0 / 4 0.0%	1 / 2 50.0%	0 / 1 0.0%	0 / 2 0.0%	1 / 1 100.0%	2 / 3 66.7%	0 / 1 0.0%	2 / 3 66.7%	0 / 4 0.0%	0 / 3 0.0%	0 / 1 0.0%	0 / 5 0.0%	1 / 1 100.0%	0 / 0 0.0%	0 / 0 0.0%	0 / 0 0.0%	12 / 49 24.5%E	11 / 49 22.4%	7 / 49 14.3%CE	6 / 49 12.2%C	5 / 49 10.2%BE	4 / 49 8.2%B	3 / 49 6.1%AB	1 / 49 2.0%ABE
1	ATCC BAA-149	13 / 49 26.5%	2 / 2 100.0%	0 / 1 0.0%	0 / 1 0.0%	0 / 1 0.0%	2 / 4 50.0%	1 / 3 33.3%	1 / 1 100.0%	0 / 1 0.0%	0 / 3 0.0%	0 / 1 0.0%	0 / 4 0.0%	1 / 2 50.0%	0 / 1 0.0%	0 / 2 0.0%	1 / 1 100.0%	2 / 3 66.7%	0 / 1 0.0%	2 / 3 66.7%	0 / 4 0.0%	0 / 3 0.0%	0 / 1 0.0%	0 / 5 0.0%	1 / 1 100.0%	0 / 0 0.0%	0 / 0 0.0%	0 / 0 0.0%	12 / 49 24.5%E	11 / 49 22.4%	7 / 49 14.3%CE	6 / 49 12.2%C	5 / 49 10.2%BE	4 / 49 8.2%B	3 / 49 6.1%AB	1 / 49 2.0%ABE
1	Kineococcus radiotolerans ATCC BAA-149	13 / 49 26.5%	2 / 2 100.0%	0 / 1 0.0%	0 / 1 0.0%	0 / 1 0.0%	2 / 4 50.0%	1 / 3 33.3%	1 / 1 100.0%	0 / 1 0.0%	0 / 3 0.0%	0 / 1 0.0%	0 / 4 0.0%	1 / 2 50.0%	0 / 1 0.0%	0 / 2 0.0%	1 / 1 100.0%	2 / 3 66.7%	0 / 1 0.0%	2 / 3 66.7%	0 / 4 0.0%	0 / 3 0.0%	0 / 1 0.0%	0 / 5 0.0%	1 / 1 100.0%	0 / 0 0.0%	0 / 0 0.0%	0 / 0 0.0%	12 / 49 24.5%E	11 / 49 22.4%	7 / 49 14.3%CE	6 / 49 12.2%C	5 / 49 10.2%BE	4 / 49 8.2%B	3 / 49 6.1%AB	1 / 49 2.0%ABE
32	Bifidobacteriales	416 / 1865 22.3%	14 / 34 41.2%	14 / 63 22.2%	41 / 71 57.7%	0 / 35 0.0%	90 / 171 52.6%	15 / 149 10.1%	21 / 34 61.8%	1 / 40 2.5%	12 / 133 9.0%	30 / 48 62.5%	18 / 149 12.1%	1 / 72 1.4%	31 / 39 79.5%	16 / 72 22.2%	0 / 34 0.0%	10 / 99 10.1%	0 / 34 0.0%	28 / 125 22.4%	39 / 184 21.2%	0 / 68 0.0%	0 / 34 0.0%	35 / 177 19.8%	0 / 0 0.0%	0 / 0 0.0%	0 / 0 0.0%	0 / 0 0.0%	779 / 1865 41.8%	450 / 1865 24.1%E	279 / 1865 15.0%AB	157 / 1865 8.4%CE	133 / 1865 7.1%ABE	63 / 1865 3.4%C	3 / 1865 0.2%B	1 / 1865 0.1%BE
32	Bifidobacteriaceae	416 / 1865 22.3%	14 / 34 41.2%	14 / 63 22.2%	41 / 71 57.7%	0 / 35 0.0%	90 / 171 52.6%	15 / 149 10.1%	21 / 34 61.8%	1 / 40 2.5%	12 / 133 9.0%	30 / 48 62.5%	18 / 149 12.1%	1 / 72 1.4%	31 / 39 79.5%	16 / 72 22.2%	0 / 34 0.0%	10 / 99 10.1%	0 / 34 0.0%	28 / 125 22.4%	39 / 184 21.2%	0 / 68 0.0%	0 / 34 0.0%	35 / 177 19.8%	0 / 0 0.0%	0 / 0 0.0%	0 / 0 0.0%	0 / 0 0.0%	779 / 1865 41.8%	450 / 1865 24.1%E	279 / 1865 15.0%AB	157 / 1865 8.4%CE	133 / 1865 7.1%ABE	63 / 1865 3.4%C	3 / 1865 0.2%B	1 / 1865 0.1%BE
29	"Bifidibacterium" Prevot 1938	397 / 1730 22.9%	14 / 31 45.2%	14 / 60 23.3%	38 / 68 55.9%	0 / 32 0.0%	81 / 159 50.9%	15 / 140 10.7%	18 / 31 58.1%	1 / 37 2.7%	12 / 124 9.7%	27 / 45 60.0%	18 / 137 13.1%	1 / 66 1.5%	31 / 36 86.1%	16 / 66 24.2%	0 / 31 0.0%	10 / 93 10.8%	0 / 31 0.0%	27 / 116 23.3%	39 / 172 22.7%	0 / 62 0.0%	0 / 31 0.0%	35 / 162 21.6%	0 / 0 0.0%	0 / 0 0.0%	0 / 0 0.0%	0 / 0 0.0%	715 / 1730 41.3%	398 / 1730 23.0%E	263 / 1730 15.2%AB	157 / 1730 9.1%CE	130 / 1730 7.5%ABE	63 / 1730 3.6%C	3 / 1730 0.2%B	1 / 1730 0.1%BE
1	AS 1.2190	14 / 54 25.9%	1 / 1 100.0%	1 / 2 50.0%	1 / 3 33.3%	0 / 2 0.0%	4 / 5 80.0%	0 / 5 0.0%	0 / 1 0.0%	0 / 1 0.0%	0 / 4 0.0%	1 / 1 100.0%	0 / 4 0.0%	0 / 2 0.0%	1 / 1 100.0%	1 / 2 50.0%	0 / 1 0.0%	1 / 3 33.3%	0 / 1 0.0%	1 / 3 33.3%	2 / 5 40.0%	0 / 2 0.0%	0 / 0 0.0%	0 / 5 0.0%	0 / 0 0.0%	0 / 0 0.0%	0 / 0 0.0%	0 / 0 0.0%	20 / 54 37.0%	14 / 54 25.9%E	11 / 54 20.4%AB	4 / 54 7.4%CE	3 / 54 5.6%ABE	2 / 54 3.7%C		
1	Bifidobacterium adolescentis ATCC 15703	14 / 54 25.9%	1 / 1 100.0%	1 / 2 50.0%	1 / 3 33.3%	0 / 2 0.0%	4 / 5 80.0%	0 / 5 0.0%	0 / 1 0.0%	0 / 1 0.0%	0 / 4 0.0%	1 / 1 100.0%	0 / 4 0.0%	0 / 2 0.0%	1 / 1 100.0%	1 / 2 50.0%	0 / 1 0.0%	1 / 3 33.3%	0 / 1 0.0%	1 / 3 33.3%	2 / 5 40.0%	0 / 2 0.0%	0 / 0 0.0%	0 / 5 0.0%	0 / 0 0.0%	0 / 0 0.0%	0 / 0 0.0%	0 / 0 0.0%	20 / 54 37.0%	14 / 54 25.9%E	11 / 54 20.4%AB	4 / 54 7.4%CE	3 / 54 5.6%ABE	2 / 54 3.7%C		
3	"Actinobacterium bifidum" (Tissier 1900) Puntoni 1937	45 / 157 28.7%	3 / 3 100.0%	0 / 6 0.0%	3 / 3 100.0%	0 / 3 0.0%	9 / 15 60.0%	3 / 15 20.0%	3 / 3 100.0%	0 / 4 0.0%	1 / 12 8.3%	3 / 3 100.0%	4 / 12 33.3%	0 / 6 0.0%	3 / 3 100.0%	0 / 6 0.0%	0 / 3 0.0%	1 / 9 11.1%	0 / 3 0.0%	3 / 9 33.3%	6 / 15 40.0%	0 / 6 0.0%	0 / 3 0.0%	3 / 15 20.0%	0 / 0 0.0%	0 / 0 0.0%	0 / 0 0.0%	0 / 0 0.0%	62 / 157 39.5%	40 / 157 25.5%E	25 / 157 15.9%AB	20 / 157 12.7%ABE	7 / 157 4.5%CE	3 / 157 1.9%C		
1	Bifidobacterium bifidum BGN4	14 / 52 26.9%	1 / 1 100.0%	0 / 2 0.0%	1 / 1 100.0%	0 / 1 0.0%	3 / 5 60.0%	1 / 5 20.0%	1 / 1 100.0%	0 / 1 0.0%	0 / 4 0.0%	1 / 1 100.0%	1 / 4 25.0%	0 / 2 0.0%	1 / 1 100.0%	0 / 2 0.0%	0 / 1 0.0%	0 / 3 0.0%	0 / 1 0.0%	1 / 3 33.3%	2 / 5 40.0%	0 / 2 0.0%	0 / 1 0.0%	1 / 5 20.0%	0 / 0 0.0%	0 / 0 0.0%	0 / 0 0.0%	0 / 0 0.0%	21 / 52 40.4%	14 / 52 26.9%E	8 / 52 15.4%AB	6 / 52 11.5%ABE	2 / 52 3.8%CE	1 / 52 1.9%C		
1	Bifidobacterium bifidum PRL2010	17 / 52 32.7%	1 / 1 100.0%	0 / 2 0.0%	1 / 1 100.0%	0 / 1 0.0%	3 / 5 60.0%	1 / 5 20.0%	1 / 1 100.0%	0 / 1 0.0%	1 / 4 25.0%	1 / 1 100.0%	2 / 4 50.0%	0 / 2 0.0%	1 / 1 100.0%	0 / 2 0.0%	0 / 1 0.0%	1 / 3 33.3%	0 / 1 0.0%	1 / 3 33.3%	2 / 5 40.0%	0 / 2 0.0%	0 / 1 0.0%	1 / 5 20.0%	0 / 0 0.0%	0 / 0 0.0%	0 / 0 0.0%	0 / 0 0.0%	20 / 52 38.5%	12 / 52 23.1%E	9 / 52 17.3%AB	8 / 52 15.4%ABE	2 / 52 3.8%CE	1 / 52 1.9%C		
1	Bifidobacterium bifidum S17	14 / 53 26.4%	1 / 1 100.0%	0 / 2 0.0%	1 / 1 100.0%	0 / 1 0.0%	3 / 5 60.0%	1 / 5 20.0%	1 / 1 100.0%	0 / 2 0.0%	0 / 4 0.0%	1 / 1 100.0%	1 / 4 25.0%	0 / 2 0.0%	1 / 1 100.0%	0 / 2 0.0%	0 / 1 0.0%	0 / 3 0.0%	0 / 1 0.0%	1 / 3 33.3%	2 / 5 40.0%	0 / 2 0.0%	0 / 1 0.0%	1 / 5 20.0%	0 / 0 0.0%	0 / 0 0.0%	0 / 0 0.0%	0 / 0 0.0%	21 / 53 39.6%	14 / 53 26.4%E	8 / 53 15.1%AB	6 / 53 11.3%ABE	3 / 53 5.7%CE	1 / 53 1.9%C		
1	AS 1.2266	12 / 43 27.9%	1 / 1 100.0%	0 / 1 0.0%	0 / 1 0.0%	0 / 1 0.0%	1 / 2 50.0%	0 / 3 0.0%	1 / 1 100.0%	0 / 1 0.0%	0 / 2 0.0%	1 / 1 100.0%	2 / 4 50.0%	0 / 2 0.0%	1 / 1 100.0%	0 / 2 0.0%	0 / 1 0.0%	1 / 3 33.3%	0 / 1 0.0%	0 / 3 0.0%	3 / 4 75.0%	0 / 2 0.0%	0 / 1 0.0%	1 / 5 20.0%	0 / 0 0.0%	0 / 0 0.0%	0 / 0 0.0%	0 / 0 0.0%	14 / 43 32.6%	13 / 43 30.2%E	7 / 43 16.3%AB	5 / 43 11.6%ABE	4 / 43 9.3%CE			
1	Bifidobacterium asteroides PRL2011	12 / 43 27.9%	1 / 1 100.0%	0 / 1 0.0%	0 / 1 0.0%	0 / 1 0.0%	1 / 2 50.0%	0 / 3 0.0%	1 / 1 100.0%	0 / 1 0.0%	0 / 2 0.0%	1 / 1 100.0%	2 / 4 50.0%	0 / 2 0.0%	1 / 1 100.0%	0 / 2 0.0%	0 / 1 0.0%	1 / 3 33.3%	0 / 1 0.0%	0 / 3 0.0%	3 / 4 75.0%	0 / 2 0.0%	0 / 1 0.0%	1 / 5 20.0%	0 / 0 0.0%	0 / 0 0.0%	0 / 0 0.0%	0 / 0 0.0%	14 / 43 32.6%	13 / 43 30.2%E	7 / 43 16.3%AB	5 / 43 11.6%ABE	4 / 43 9.3%CE			
2	"Bifidobacterium parvulorum" Reuter 1963	24 / 107 22.4%	2 / 2 100.0%	2 / 4 50.0%	0 / 4 0.0%	0 / 2 0.0%	6 / 10 60.0%	0 / 10 0.0%	2 / 2 100.0%	0 / 3 0.0%	0 / 8 0.0%	2 / 2 100.0%	2 / 8 25.0%	0 / 4 0.0%	2 / 2 100.0%	2 / 4 50.0%	0 / 2 0.0%	0 / 6 0.0%	0 / 2 0.0%	0 / 6 0.0%	2 / 10 20.0%	0 / 4 0.0%	0 / 2 0.0%	2 / 10 20.0%	0 / 0 0.0%	0 / 0 0.0%	0 / 0 0.0%	0 / 0 0.0%	51 / 107 47.7%	21 / 107 19.6%E	14 / 107 13.1%AB	10 / 107 9.3%ABE	8 / 107 7.5%CE	3 / 107 2.8%C		
1	Bifidobacterium breve NCIMB 8807	12 / 54 22.2%	1 / 1 100.0%	1 / 2 50.0%	0 / 2 0.0%	0 / 1 0.0%	3 / 5 60.0%	0 / 5 0.0%	1 / 1 100.0%	0 / 2 0.0%	0 / 4 0.0%	1 / 1 100.0%	1 / 4 25.0%	0 / 2 0.0%	1 / 1 100.0%	1 / 2 50.0%	0 / 1 0.0%	0 / 3 0.0%	0 / 1 0.0%	0 / 3 0.0%	1 / 5 20.0%	0 / 2 0.0%	0 / 1 0.0%	1 / 5 20.0%	0 / 0 0.0%	0 / 0 0.0%	0 / 0 0.0%	0 / 0 0.0%	26 / 54 48.1%	11 / 54 20.4%E	7 / 54 13.0%AB	5 / 54 9.3%ABE	4 / 54 7.4%CE	1 / 54 1.9%C		
1	Bifidobacterium breve ACS-071-V-Sch8b	12 / 53 22.6%	1 / 1 100.0%	1 / 2 50.0%	0 / 2 0.0%	0 / 1 0.0%	3 / 5 60.0%	0 / 5 0.0%	1 / 1 100.0%	0 / 1 0.0%	0 / 4 0.0%	1 / 1 100.0%	1 / 4 25.0%	0 / 2 0.0%	1 / 1 100.0%	1 / 2 50.0%	0 / 1 0.0%	0 / 3 0.0%	0 / 1 0.0%	0 / 3 0.0%	1 / 5 20.0%	0 / 2 0.0%	0 / 1 0.0%	1 / 5 20.0%	0 / 0 0.0%	0 / 0 0.0%	0 / 0 0.0%	0 / 0 0.0%	25 / 53 47.2%	10 / 53 18.9%E	7 / 53 13.2%AB	5 / 53 9.4%ABE	4 / 53 7.5%CE	2 / 53 3.8%C		
1	AS 1.2188	17 / 55 30.9%	1 / 1 100.0%	0 / 2 0.0%	1 / 3 33.3%	0 / 1 0.0%	4 / 5 80.0%	1 / 5 20.0%	0 / 1 0.0%	0 / 1 0.0%	0 / 4 0.0%	1 / 1 100.0%	1 / 4 25.0%	0 / 2 0.0%	2 / 2 100.0%	1 / 2 50.0%	0 / 1 0.0%	2 / 3 66.7%	0 / 1 0.0%	1 / 3 33.3%	1 / 5 20.0%	0 / 2 0.0%	0 / 1 0.0%	1 / 5 20.0%	0 / 0 0.0%	0 / 0 0.0%	0 / 0 0.0%	0 / 0 0.0%	19 / 55 34.5%	13 / 55 23.6%E	11 / 55 20.0%AB	6 / 55 10.9%ABE	3 / 55 5.5%C	3 / 55 5.5%CE		
1	Bifidobacterium dentium Bd1	17 / 55 30.9%	1 / 1 100.0%	0 / 2 0.0%	1 / 3 33.3%	0 / 1 0.0%	4 / 5 80.0%	1 / 5 20.0%	0 / 1 0.0%	0 / 1 0.0%	0 / 4 0.0%	1 / 1 100.0%	1 / 4 25.0%	0 / 2 0.0%	2 / 2 100.0%	1 / 2 50.0%	0 / 1 0.0%	2 / 3 66.7%	0 / 1 0.0%	1 / 3 33.3%	1 / 5 20.0%	0 / 2 0.0%	0 / 1 0.0%	1 / 5 20.0%	0 / 0 0.0%	0 / 0 0.0%	0 / 0 0.0%	0 / 0 0.0%	19 / 55 34.5%	13 / 55 23.6%E	11 / 55 20.0%AB	6 / 55 10.9%ABE	3 / 55 5.5%C	3 / 55 5.5%CE		
11	"Bifidobacterium longum subsp. animalis biotype a" Mitsuoka 1969	125 / 571 21.9%	1 / 11 9.1%	0 / 22 0.0%	22 / 22 100.0%	0 / 11 0.0%	23 / 55 41.8%	11 / 44 25.0%	0 / 11 0.0%	0 / 11 0.0%	11 / 43 25.6%	0 / 11 0.0%	1 / 44 2.3%	0 / 22 0.0%	11 / 11 100.0%	0 / 22 0.0%	0 / 11 0.0%	0 / 33 0.0%	0 / 11 0.0%	22 / 33 66.7%	11 / 55 20.0%	0 / 22 0.0%	0 / 11 0.0%	12 / 55 21.8%	0 / 0 0.0%	0 / 0 0.0%	0 / 0 0.0%	0 / 0 0.0%	208 / 571 36.4%	141 / 571 24.7%E	89 / 571 15.6%AB	64 / 571 11.2%CE	36 / 571 6.3%ABE	33 / 571 5.8%C		
10	Bifidobacterium animalis subsp. lactis	110 / 519 21.2%	0 / 10 0.0%	0 / 20 0.0%	20 / 20 100.0%	0 / 10 0.0%	20 / 50 40.0%	10 / 40 25.0%	0 / 10 0.0%	0 / 10 0.0%	10 / 39 25.6%	0 / 10 0.0%	0 / 40 0.0%	0 / 20 0.0%	10 / 10 100.0%	0 / 20 0.0%	0 / 10 0.0%	0 / 30 0.0%	0 / 10 0.0%	20 / 30 66.7%	10 / 50 20.0%	0 / 20 0.0%	0 / 10 0.0%	10 / 50 20.0%	0 / 0 0.0%	0 / 0 0.0%	0 / 0 0.0%	0 / 0 0.0%	190 / 519 36.6%	129 / 519 24.9%E	80 / 519 15.4%AB	60 / 519 11.6%CE	30 / 519 5.8%ABE	30 / 519 5.8%C		
1	Bifidobacterium animalis subsp. lactis AD011	11 / 52 21.2%	0 / 1 0.0%	0 / 2 0.0%	2 / 2 100.0%	0 / 1 0.0%	2 / 5 40.0%	1 / 4 25.0%	0 / 1 0.0%	0 / 1 0.0%	1 / 4 25.0%	0 / 1 0.0%	0 / 4 0.0%	0 / 2 0.0%	1 / 1 100.0%	0 / 2 0.0%	0 / 1 0.0%	0 / 3 0.0%	0 / 1 0.0%	2 / 3 66.7%	1 / 5 20.0%	0 / 2 0.0%	0 / 1 0.0%	1 / 5 20.0%	0 / 0 0.0%	0 / 0 0.0%	0 / 0 0.0%	0 / 0 0.0%	19 / 52 36.5%	13 / 52 25.0%E	8 / 52 15.4%AB	6 / 52 11.5%CE	3 / 52 5.8%ABE	3 / 52 5.8%C		

1	Bifidobacterium animalis subsp. lactis BB-12	11 / 52 21.2%	0 / 1 0.0%	0 / 2 0.0%	2 / 2 100.0%	0 / 1 0.0%	2 / 5 40.0%	1 / 4 25.0%	0 / 1 0.0%	0 / 1 0.0%	1 / 4 25.0%	0 / 1 0.0%	0 / 4 0.0%	0 / 2 0.0%	1 / 1 100.0%	0 / 2 0.0%	0 / 1 0.0%	0 / 3 0.0%	0 / 1 0.0%	2 / 3 66.7%	1 / 5 20.0%	0 / 2 0.0%	0 / 1 0.0%	1 / 5 20.0%	0 / 0 0.0%	0 / 0 0.0%	0 / 0 0.0%	0 / 0 0.0%	19 / 52 36.5%	13 / 52 25.0%E	8 / 52 15.4%AB	6 / 52 11.5%CE	3 / 52 5.8%ABE	3 / 52 5.8%C			
1	Bifidobacterium animalis subsp. lactis DSM 10140	11 / 51 21.6%	0 / 1 0.0%	0 / 2 0.0%	2 / 2 100.0%	0 / 1 0.0%	2 / 5 40.0%	1 / 4 25.0%	0 / 1 0.0%	0 / 1 0.0%	1 / 3 33.3%	0 / 1 0.0%	0 / 4 0.0%	0 / 2 0.0%	1 / 1 100.0%	0 / 2 0.0%	0 / 1 0.0%	0 / 3 0.0%	0 / 1 0.0%	2 / 3 66.7%	1 / 5 20.0%	0 / 2 0.0%	0 / 1 0.0%	1 / 5 20.0%	0 / 0 0.0%	0 / 0 0.0%	0 / 0 0.0%	0 / 0 0.0%	19 / 51 37.3%	12 / 51 23.5%E	8 / 51 15.7%AB	6 / 51 11.8%CE	3 / 51 5.9%ABE	3 / 51 5.9%C			
1	Bifidobacterium animalis subsp. lactis V9	11 / 52 21.2%	0 / 1 0.0%	0 / 2 0.0%	2 / 2 100.0%	0 / 1 0.0%	2 / 5 40.0%	1 / 4 25.0%	0 / 1 0.0%	0 / 1 0.0%	1 / 4 25.0%	0 / 1 0.0%	0 / 4 0.0%	0 / 2 0.0%	1 / 1 100.0%	0 / 2 0.0%	0 / 1 0.0%	0 / 3 0.0%	0 / 1 0.0%	2 / 3 66.7%	1 / 5 20.0%	0 / 2 0.0%	0 / 1 0.0%	1 / 5 20.0%	0 / 0 0.0%	0 / 0 0.0%	0 / 0 0.0%	0 / 0 0.0%	19 / 52 36.5%	13 / 52 25.0%E	8 / 52 15.4%AB	6 / 52 11.5%CE	3 / 52 5.8%C	3 / 52 5.8%ABE			
1	Bifidobacterium animalis subsp. lactis Bl-04	11 / 52 21.2%	0 / 1 0.0%	0 / 2 0.0%	2 / 2 100.0%	0 / 1 0.0%	2 / 5 40.0%	1 / 4 25.0%	0 / 1 0.0%	0 / 1 0.0%	1 / 4 25.0%	0 / 1 0.0%	0 / 4 0.0%	0 / 2 0.0%	1 / 1 100.0%	0 / 2 0.0%	0 / 1 0.0%	0 / 3 0.0%	0 / 1 0.0%	2 / 3 66.7%	1 / 5 20.0%	0 / 2 0.0%	0 / 1 0.0%	1 / 5 20.0%	0 / 0 0.0%	0 / 0 0.0%	0 / 0 0.0%	0 / 0 0.0%	19 / 52 36.5%	13 / 52 25.0%E	8 / 52 15.4%AB	6 / 52 11.5%CE	3 / 52 5.8%ABE	3 / 52 5.8%C			
1	Bifidobacterium animalis subsp. lactis Bi-07	11 / 52 21.2%	0 / 1 0.0%	0 / 2 0.0%	2 / 2 100.0%	0 / 1 0.0%	2 / 5 40.0%	1 / 4 25.0%	0 / 1 0.0%	0 / 1 0.0%	1 / 4 25.0%	0 / 1 0.0%	0 / 4 0.0%	0 / 2 0.0%	1 / 1 100.0%	0 / 2 0.0%	0 / 1 0.0%	0 / 3 0.0%	0 / 1 0.0%	2 / 3 66.7%	1 / 5 20.0%	0 / 2 0.0%	0 / 1 0.0%	1 / 5 20.0%	0 / 0 0.0%	0 / 0 0.0%	0 / 0 0.0%	0 / 0 0.0%	19 / 52 36.5%	13 / 52 25.0%E	8 / 52 15.4%AB	6 / 52 11.5%CE	3 / 52 5.8%C	3 / 52 5.8%ABE			
1	Bifidobacterium animalis subsp. lactis CNCM I-2494	11 / 52 21.2%	0 / 1 0.0%	0 / 2 0.0%	2 / 2 100.0%	0 / 1 0.0%	2 / 5 40.0%	1 / 4 25.0%	0 / 1 0.0%	0 / 1 0.0%	1 / 4 25.0%	0 / 1 0.0%	0 / 4 0.0%	0 / 2 0.0%	1 / 1 100.0%	0 / 2 0.0%	0 / 1 0.0%	0 / 3 0.0%	0 / 1 0.0%	2 / 3 66.7%	1 / 5 20.0%	0 / 2 0.0%	0 / 1 0.0%	1 / 5 20.0%	0 / 0 0.0%	0 / 0 0.0%	0 / 0 0.0%	0 / 0 0.0%	19 / 52 36.5%	13 / 52 25.0%E	8 / 52 15.4%AB	6 / 52 11.5%CE	3 / 52 5.8%C	3 / 52 5.8%ABE			
1	Bifidobacterium animalis subsp. lactis BLC1	11 / 52 21.2%	0 / 1 0.0%	0 / 2 0.0%	2 / 2 100.0%	0 / 1 0.0%	2 / 5 40.0%	1 / 4 25.0%	0 / 1 0.0%	0 / 1 0.0%	1 / 4 25.0%	0 / 1 0.0%	0 / 4 0.0%	0 / 2 0.0%	1 / 1 100.0%	0 / 2 0.0%	0 / 1 0.0%	0 / 3 0.0%	0 / 1 0.0%	2 / 3 66.7%	1 / 5 20.0%	0 / 2 0.0%	0 / 1 0.0%	1 / 5 20.0%	0 / 0 0.0%	0 / 0 0.0%	0 / 0 0.0%	0 / 0 0.0%	19 / 52 36.5%	13 / 52 25.0%E	8 / 52 15.4%AB	6 / 52 11.5%CE	3 / 52 5.8%C	3 / 52 5.8%ABE			
1	Bifidobacterium animalis subsp. lactis B420	11 / 52 21.2%	0 / 1 0.0%	0 / 2 0.0%	2 / 2 100.0%	0 / 1 0.0%	2 / 5 40.0%	1 / 4 25.0%	0 / 1 0.0%	0 / 1 0.0%	1 / 4 25.0%	0 / 1 0.0%	0 / 4 0.0%	0 / 2 0.0%	1 / 1 100.0%	0 / 2 0.0%	0 / 1 0.0%	0 / 3 0.0%	0 / 1 0.0%	2 / 3 66.7%	1 / 5 20.0%	0 / 2 0.0%	0 / 1 0.0%	1 / 5 20.0%	0 / 0 0.0%	0 / 0 0.0%	0 / 0 0.0%	0 / 0 0.0%	19 / 52 36.5%	13 / 52 25.0%E	8 / 52 15.4%AB	6 / 52 11.5%CE	3 / 52 5.8%C	3 / 52 5.8%ABE			
1	Bifidobacterium animalis subsp. lactis BI12	11 / 52 21.2%	0 / 1 0.0%	0 / 2 0.0%	2 / 2 100.0%	0 / 1 0.0%	2 / 5 40.0%	1 / 4 25.0%	0 / 1 0.0%	0 / 1 0.0%	1 / 4 25.0%	0 / 1 0.0%	0 / 4 0.0%	0 / 2 0.0%	1 / 1 100.0%	0 / 2 0.0%	0 / 1 0.0%	0 / 3 0.0%	0 / 1 0.0%	2 / 3 66.7%	1 / 5 20.0%	0 / 2 0.0%	0 / 1 0.0%	1 / 5 20.0%	0 / 0 0.0%	0 / 0 0.0%	0 / 0 0.0%	0 / 0 0.0%	19 / 52 36.5%	13 / 52 25.0%E	8 / 52 15.4%AB	6 / 52 11.5%CE	3 / 52 5.8%ABE	3 / 52 5.8%C			
1	Bifidobacterium animalis subsp. animalis	15 / 52 28.8%	1 / 1 100.0%	0 / 2 0.0%	2 / 2 100.0%	0 / 1 0.0%	3 / 5 60.0%	1 / 4 25.0%	0 / 1 0.0%	0 / 1 0.0%	1 / 4 25.0%	0 / 1 0.0%	1 / 4 25.0%	0 / 2 0.0%	1 / 1 100.0%	0 / 2 0.0%	0 / 1 0.0%	0 / 3 0.0%	0 / 1 0.0%	2 / 3 66.7%	1 / 5 20.0%	0 / 2 0.0%	0 / 1 0.0%	2 / 5 40.0%	0 / 0 0.0%	0 / 0 0.0%	0 / 0 0.0%	0 / 0 0.0%	18 / 52 34.6%	12 / 52 23.1%E	9 / 52 17.3%AB	6 / 52 11.5%ABE	4 / 52 7.7%CE	3 / 52 5.8%C			
1	Bifidobacterium animalis subsp. animalis ATCC 25527	15 / 52 28.8%	1 / 1 100.0%	0 / 2 0.0%	2 / 2 100.0%	0 / 1 0.0%	3 / 5 60.0%	1 / 4 25.0%	0 / 1 0.0%	0 / 1 0.0%	1 / 4 25.0%	0 / 1 0.0%	1 / 4 25.0%	0 / 2 0.0%	1 / 1 100.0%	0 / 2 0.0%	0 / 1 0.0%	0 / 3 0.0%	0 / 1 0.0%	2 / 3 66.7%	1 / 5 20.0%	0 / 2 0.0%	0 / 1 0.0%	2 / 5 40.0%	0 / 0 0.0%	0 / 0 0.0%	0 / 0 0.0%	0 / 0 0.0%	18 / 52 34.6%	12 / 52 23.1%E	9 / 52 17.3%AB	6 / 52 11.5%ABE	4 / 52 7.7%CE	3 / 52 5.8%C			
1	"Bifidobacterium ruminale" Scardovi et al. 1969	12 / 47 25.5%	1 / 1 100.0%	0 / 1 0.0%	0 / 1 0.0%	0 / 1 0.0%	2 / 4 50.0%	0 / 3 0.0%	1 / 1 100.0%	0 / 1 0.0%	0 / 3 0.0%	1 / 1 100.0%	1 / 4 25.0%	1 / 2 50.0%	0 / 1 0.0%	1 / 2 50.0%	0 / 1 0.0%	2 / 3 66.7%	0 / 1 0.0%	0 / 3 0.0%	0 / 5 0.0%	0 / 2 0.0%	0 / 1 0.0%	2 / 5 40.0%	0 / 0 0.0%	0 / 0 0.0%	0 / 0 0.0%	0 / 0 0.0%	20 / 47 42.6%	11 / 47 23.4%E	6 / 47 12.8%ABE	6 / 47 12.8%AB	2 / 47 4.3%CE	2 / 47 4.3%C			
1	Bifidobacterium thermophilum RBL67	12 / 47 25.5%	1 / 1 100.0%	0 / 1 0.0%	0 / 1 0.0%	0 / 1 0.0%	2 / 4 50.0%	0 / 3 0.0%	1 / 1 100.0%	0 / 1 0.0%	0 / 3 0.0%	1 / 1 100.0%	1 / 4 25.0%	1 / 2 50.0%	0 / 1 0.0%	1 / 2 50.0%	0 / 1 0.0%	2 / 3 66.7%	0 / 1 0.0%	0 / 3 0.0%	0 / 5 0.0%	0 / 2 0.0%	0 / 1 0.0%	2 / 5 40.0%	0 / 0 0.0%	0 / 0 0.0%	0 / 0 0.0%	0 / 0 0.0%	20 / 47 42.6%	11 / 47 23.4%E	6 / 47 12.8%AB	6 / 47 12.8%ABE	2 / 47 4.3%C	2 / 47 4.3%CE			
9	AS 1.2186	148 / 696 21.3%	4 / 11 36.4%	11 / 22 50.0%	11 / 31 35.5%	0 / 11 0.0%	32 / 63 50.8%	0 / 55 0.0%	11 / 11 100.0%	1 / 15 6.7%	0 / 48 0.0%	18 / 25 72.0%	7 / 57 12.3%	0 / 26 0.0%	11 / 15 73.3%	11 / 26 42.3%	0 / 11 0.0%	3 / 33 9.1%	0 / 11 0.0%	0 / 56 0.0%	14 / 73 19.2%	0 / 22 0.0%	0 / 12 0.0%	14 / 62 22.6%	0 / 0 0.0%	0 / 0 0.0%	0 / 0 0.0%	0 / 0 0.0%	321 / 696 46.1%	145 / 696 20.8%E	100 / 696 14.4%AB	65 / 696 9.3%CE	44 / 696 6.3%ABE	17 / 696 2.4%C	3 / 696 0.4%B	1 / 696 0.1%BE	
5	Bifidobacterium longum biovar Longum	63 / 290 21.7%	0 / 5 0.0%	5 / 10 50.0%	5 / 13 38.5%	0 / 5 0.0%	14 / 27 51.9%	0 / 25 0.0%	5 / 5 100.0%	1 / 6 16.7%	0 / 21 0.0%	6 / 6 100.0%	4 / 22 18.2%	0 / 11 0.0%	5 / 6 83.3%	5 / 11 45.5%	0 / 5 0.0%	3 / 15 20.0%	0 / 5 0.0%	0 / 23 0.0%	5 / 28 17.9%	0 / 10 0.0%	0 / 6 0.0%	5 / 25 20.0%	0 / 0 0.0%	0 / 0 0.0%	0 / 0 0.0%	0 / 0 0.0%	140 / 290 48.3%	52 / 290 17.9%E	43 / 290 14.8%AB	30 / 290 10.3%CE	20 / 290 6.9%ABE	5 / 290 1.7%C			
1	Bifidobacterium longum subsp. longum JCM 1217	14 / 69 20.3%	0 / 1 0.0%	1 / 2 50.0%	1 / 3 33.3%	0 / 1 0.0%	3 / 7 42.9%	0 / 5 0.0%	1 / 1 100.0%	1 / 2 50.0%	0 / 5 0.0%	2 / 2 100.0%	0 / 5 0.0%	0 / 3 0.0%	1 / 2 50.0%	1 / 3 33.3%	0 / 1 0.0%	1 / 3 33.3%	0 / 1 0.0%	0 / 4 0.0%	1 / 9 11.1%	0 / 2 0.0%	0 / 2 0.0%	1 / 5 20.0%	0 / 0 0.0%	0 / 0 0.0%	0 / 0 0.0%	0 / 0 0.0%	33 / 69 47.8%	15 / 69 21.7%E	10 / 69 14.5%AB	6 / 69 8.7%CE	4 / 69 5.8%ABE	1 / 69 1.4%C			
1	Bifidobacterium longum subsp. longum F8	13 / 57 22.8%	0 / 1 0.0%	1 / 2 50.0%	1 / 3 33.3%	0 / 1 0.0%	3 / 5 60.0%	0 / 5 0.0%	1 / 1 100.0%	0 / 1 0.0%	0 / 4 0.0%	1 / 1 100.0%	2 / 5 40.0%	0 / 2 0.0%	1 / 1 100.0%	1 / 2 50.0%	0 / 1 0.0%	0 / 3 0.0%	0 / 1 0.0%	0 / 5 0.0%	1 / 5 20.0%	0 / 2 0.0%	0 / 1 0.0%	1 / 5 20.0%	0 / 0 0.0%	0 / 0 0.0%	0 / 0 0.0%	0 / 0 0.0%	28 / 57 49.1%	9 / 57 15.8%E	8 / 57 14.0%AB	6 / 57 10.5%CE	5 / 57 8.8%ABE	1 / 57 1.8%C			
1	Bifidobacterium longum subsp. longum JDM301	11 / 54 20.4%	0 / 1 0.0%	1 / 2 50.0%	1 / 3 33.3%	0 / 1 0.0%	2 / 5 40.0%	0 / 5 0.0%	1 / 1 100.0%	0 / 1 0.0%	0 / 4 0.0%	1 / 1 100.0%	0 / 4 0.0%	0 / 2 0.0%	1 / 1 100.0%	1 / 2 50.0%	0 / 1 0.0%	1 / 3 33.3%	0 / 1 0.0%	0 / 4 0.0%	1 / 4 25.0%	0 / 2 0.0%	0 / 1 0.0%	1 / 5 20.0%	0 / 0 0.0%	0 / 0 0.0%	0 / 0 0.0%	0 / 0 0.0%	26 / 54 48.1%	9 / 54 16.7%E	8 / 54 14.8%AB	7 / 54 13.0%CE	3 / 54 5.6%ABE	1 / 54 1.9%C			
1	Bifidobacterium longum subsp. longum BBMN68	13 / 54 24.1%	0 / 1 0.0%	1 / 2 50.0%	1 / 1 100.0%	0 / 1 0.0%	3 / 5 60.0%	0 / 5 0.0%	1 / 1 100.0%	0 / 1 0.0%	0 / 4 0.0%	1 / 1 100.0%	1 / 4 25.0%	0 / 2 0.0%	1 / 1 100.0%	1 / 2 50.0%	0 / 1 0.0%	1 / 3 33.3%	0 / 1 0.0%	0 / 5 0.0%	1 / 5 20.0%	0 / 2 0.0%	0 / 1 0.0%	1 / 5 20.0%	0 / 0 0.0%	0 / 0 0.0%	0 / 0 0.0%	0 / 0 0.0%	25 / 54 46.3%	10 / 54 18.5%E	9 / 54 16.7%AB	5 / 54 9.3%CE	4 / 54 7.4%ABE	1 / 54 1.9%C			
1	Bifidobacterium longum subsp. longum KACC 91563	12 / 56 21.4%	0 / 1 0.0%	1 / 2 50.0%	1 / 3 33.3%	0 / 1 0.0%	3 / 5 60.0%	0 / 5 0.0%	1 / 1 100.0%	0 / 1 0.0%	0 / 4 0.0%	1 / 1 100.0%	1 / 4 25.0%	0 / 2 0.0%	1 / 1 100.0%	1 / 2 50.0%	0 / 1 0.0%	0 / 3 0.0%	0 / 1 0.0%	0 / 5 0.0%	1 / 5 20.0%	0 / 2 0.0%	0 / 1 0.0%	1 / 5 20.0%	0 / 0 0.0%	0 / 0 0.0%	0 / 0 0.0%	0 / 0 0.0%	28 / 56 50.0%	9 / 56 16.1%E	8 / 56 14.3%AB	6 / 56 10.7%CE	4 / 56 7.1%ABE	1 / 56 1.8%C			
2	"Bifidobacterium lactentis" Reuter 1963	60 / 294 20.4%	3 / 4 75.0%	4 / 8 50.0%	4 / 12 33.3%	0 / 4 0.0%	12 / 26 46.2%	0 / 20 0.0%	4 / 4 100.0%	0 / 7 0.0%	0 / 19 0.0%	10 / 17 58.8%	1 / 26 3.8%	0 / 11 0.0%	4 / 7 57.1%	4 / 11 36.4%	0 / 4 0.0%	0 / 12 0.0%	0 / 4 0.0%	0 / 24 0.0%	7 / 35 20.0%	0 / 8 0.0%	0 / 4 0.0%	7 / 27 25.9%	0 / 0 0.0%	0 / 0 0.0%	0 / 0 0.0%	0 / 0 0.0%	126 / 294 42.9%	75 / 294 25.5%E	44 / 294 15.0%AB	23 / 294 7.8%CE	16 / 294 5.4%ABE	10 / 294 3.4%C			
1	Bifidobacterium longum bv. Infantis ATCC 15697	48 / 234 20.5%	3 / 3 100.0%	3 / 6 50.0%	3 / 9 33.3%	0 / 3 0.0%	9 / 21 42.9%	0 / 15 0.0%	3 / 3 100.0%	0 / 6 0.0%	0 / 15 0.0%	9 / 15 60.0%	0 / 21 0.0%	0 / 9 0.0%	3 / 6 50.0%	3 / 9 33.3%	0 / 3 0.0%	0 / 9 0.0%	0 / 3 0.0%	0 / 18 0.0%	6 / 30 20.0%	0 / 6 0.0%	0 / 3 0.0%	6 / 21 28.6%	0 / 0 0.0%	0 / 0 0.0%	0 / 0 0.0%	0 / 0 0.0%	96 / 234 41.0%	63 / 234 26.9%E	36 / 234 15.4%AB	18 / 234 7.7%CE	12 / 234 5.1%ABE	9 / 234 3.8%C			
1	Bifidobacterium longum subsp. infantis 157F	12 / 60 20.0%	0 / 1 0.0%	1 / 2 50.0%	1 / 3 33.3%	0 / 1 0.0%	3 / 5 60.0%	0 / 5 0.0%	1 / 1 100.0%	0 / 1 0.0%	0 / 4 0.0%	1 / 2 50.0%	1 / 5 20.0%	0 / 2 0.0%	1 / 1 100.0%	1 / 2 50.0%	0 / 1 0.0%	0 / 3 0.0%	0 / 1 0.0%	0 / 6 0.0%	1 / 5 20.0%	0 / 2 0.0%	0 / 1 0.0%	1 / 6 16.7%	0 / 0 0.0%	0 / 0 0.0%	0 / 0 0.0%	0 / 0 0.0%	30 / 60 50.0%	12 / 60 20.0%E	8 / 60 13.3%AB	5 / 60 8.3%CE	4 / 60 6.7%ABE	1 / 60 1.7%C			
1	Bifidobacterium longum DJO10A	13 / 57 22.8%	0 / 1 0.0%	1 / 2 50.0%	1 / 3 33.3%	0 / 1 0.0%	3 / 5 60.0%	0 / 5 0.0%	1 / 1 100.0%	0 / 1 0.0%	0 / 4 0.0%	1 / 1 100.0%	2 / 5 40.0%	0 / 2 0.0%	1 / 1 100.0%	1 / 2 50.0%	0 / 1 0.0%	0 / 3 0.0%	0 / 1 0.0%	0 / 5 0.0%	1 / 5 20.0%	0 / 2 0.0%	0 / 1 0.0%	1 / 5 20.0%	0 / 0 0.0%	0 / 0 0.0%	0 / 0 0.0%	0 / 0 0.0%	28 / 57 49.1%	9 / 57 15.8%	6 / 57 10.5%CE	5 / 57 8.8%AB	4 / 57 7.0%ABE	3 / 57 5.3%B	1 / 57 1.8%C	1 / 57 1.8%BE	
1	Bifidobacterium longum NCC2705	12 / 55 21.8%	1 / 1 100.0%	1 / 2 50.0%	1 / 3 33.3%	0 / 1 0.0%	3 / 5 60.0%	0 / 5 0.0%	1 / 1 100.0%	0 / 1 0.0%	0 / 4 0.0%	1 / 1 100.0%	0 / 4 0.0%	0 / 2 0.0%	1 / 1 100.0%	1 / 2 50.0%	0 / 1 0.0%	0 / 3 0.0%	0 / 1 0.0%	0 / 4 0.0%	1 / 5 20.0%	0 / 2 0.0%	0 / 1 0.0%	1 / 5 20.0%	0 / 0 0.0%	0 / 0 0.0%	0 / 0 0.0%	0 / 0 0.0%	27 / 55 49.1%	9 / 55 16.4%E	8 / 55 14.5%AB	6 / 55 10.9%CE	4 / 55 7.3%ABE	1 / 55 1.8%C			
3	Gardnerella	19 / 135 14.1%	0 / 3 0.0%	0 / 3 0.0%	3 / 3 100.0%	0 / 3 0.0%	9 / 12 75.0%	0 / 9 0.0%	3 / 3 100.0%	0 / 3 0.0%	0 / 9 0.0%	3 / 3 100.0%	0 / 12 0.0%	0 / 6 0.0%	0 / 3 0.0%	0 / 6 0.0%	0 / 3 0.0%	0 / 6 0.0%	0 / 3 0.0%	1 / 9 11.1%	0 / 12 0.0%	0 / 6 0.0%	0 / 3 0.0%	0 / 15 0.0%	0 / 0 0.0%	0 / 0 0.0%	0 / 0 0.0%	0 / 0 0.0%	64 / 135 47.4%	52 / 135 38.5%E	16 / 135 11.9%AB	3 / 135 2.2%ABE					
3	"Corynebacterium vaginale" Zinnemann and Turner 1963	19 / 135 14.1%	0 / 3 0.0%	0 / 3 0.0%	3 / 3 100.0%	0 / 3 0.0%	9 / 12 75.0%	0 / 9 0.0%	3 / 3 100.0%	0 / 3 0.0%	0 / 9 0.0%	3 / 3 100.0%	0 / 12 0.0%	0 / 6 0.0%	0 / 3 0.0%	0 / 6 0.0%	0 / 3 0.0%	0 / 6 0.0%	0 / 3 0.0%	1 / 9 11.1%	0 / 12 0.0%	0 / 6 0.0%	0 / 3 0.0%	0 / 15 0.0%	0 / 0 0.0%	0 / 0 0.0%	0 / 0 0.0%	0 / 0 0.0%	64 / 135 47.4%	52 / 135 38.5%E	16 / 135 11.9%AB	3 / 135 2.2%ABE					
1	Gardnerella vaginalis ATCC 14019	6 / 45 13.3%	0 / 1 0.0%	0 / 1 0.0%	1 / 1 100.0%	0 / 1 0.0%	3 / 4 75.0%	0 / 3 0.0%	1 / 1 100.0%	0 / 1 0.0%	0 / 3 0.0%	1 / 1 100.0%	0 / 4 0.0%	0 / 2 0.0%	0 / 1 0.0%	0 / 2 0.0%	0 / 1 0.0%	0 / 2 0.0%	0 / 1 0.0%	0 / 3 0.0%	0 / 4 0.0%	0 / 2 0.0%	0 / 1 0.0%	0 / 5 0.0%	0 / 0 0.0%	0 / 0 0.0%	0 / 0 0.0%	0 / 0 0.0%	22 / 45 48.9%	17 / 45 37.8%E	5 / 45 11.1%AB	1 / 45 2.2%ABE					
1	Gardnerella vaginalis 409-05	7 / 45 15.6%	0 / 1 0.0%	0 / 1 0.0%	1 / 1 100.0%	0 / 1 0.0%	3 / 4 75.0%	0 / 3 0.0%	1 / 1 100.0%	0 / 1 0.0%	0 / 3 0.0%	1 / 1 100.0%	0 / 4 0.0%	0 / 2 0.0%	0 / 1 0.0%	0 / 2 0.0%	0 / 1 0.0%	0 / 2 0.0%	0 / 1 0.0%	1 / 3 33.3%	0 / 4 0.0%	0 / 2 0.0%	0 / 1 0.0%	0 / 5 0.0%	0 / 0 0.0%	0 / 0 0.0%	0 / 0 0.0%	0 / 0 0.0%	20 / 45 44.4%	18 / 45 40.0%E	6 / 45 13.3%AB	1 / 45 2.2%ABE					
1	Gardnerella vaginalis HMP9231	6 / 45 13.3%	0 / 1 0.0%	0 / 1 0.0%	1 / 1 100.0%	0 / 1 0.0%	3 / 4 75.0%	0 / 3 0.0%	1 / 1 100.0%	0 / 1 0.0%	0 / 3 0.0%	1 / 1 100.0%	0 / 4 0.0%	0 / 2 0.0%	0 / 1 0.0%	0 / 2 0.0%	0 / 1 0.0%	0 / 2 0.0%	0 / 1 0.0%	0 / 3 0.0%	0 / 4 0.0%	0 / 2 0.0%	0 / 1 0.0%	0 / 5 0.0%	0 / 0 0.0%	0 / 0 0.0%	0 / 0 0.0%	0 / 0 0.0%	22 / 45 48.9%	17 / 45 37.8%E	5 / 45 11.1%AB	1 / 45 2.2%ABE					
7	Planctomycetes	1 / 383 0.3%	0 / 11 0.0%	0 / 11 0.0%	0 / 10 0.0%	0 / 10 0.0%	0 / 24 0.0%	0 / 26 0.0%	0 / 9 0.0%	0 / 7 0.0%	0 / 23 0.0%	0 / 8 0.0%	0 / 25 0.0%	1 / 17 5.9%	0 / 7 0.0%	0 / 17 0.0%	0 / 11 0.0%	0 / 26 0.0%	0 / 7 0.0%	0 / 22 0.0%	0 / 41 0.0%	0 / 16 0.0%	0 / 5 0.0%	0 / 46 0.0%	0 / 1 0.0%	0 / 0 0.0%	0 / 3 0.0%	0 / 0 0.0%	212 / 383 55.4%	156 / 383 40.7%E	8 / 383 2.1%C	6 / 383 1.6%CE	1 / 383 0.3%ABE				
6	"Planctomycetacia" Garrity and Holt 2001	1 / 337 0.3%	0 / 10 0.0%	0 / 10 0.0%	0 / 9 0.0%	0 / 9 0.0%	0 / 21 0.0%	0 / 23 0.0%	0 / 8 0.0%	0 / 6 0.0%	0 / 20 0.0%	0 / 7 0.0%	0 / 21 0.0%	1 / 15 6.7%	0 / 6 0.0%	0 / 15 0.0%	0 / 10 0.0%	0 / 23 0.0%	0 / 6 0.0%	0 / 19 0.0%	0 / 36 0.0%	0 / 14 0.0%	0 / 4 0.0%	0 / 41 0.0%	0 / 1 0.0%	0 / 0 0.0%	0 / 3 0.0%	0 / 0 0.0%	182 / 337 54.0%	140 / 337 41.5%E	8 / 337 2.4%C	6 / 337 1.8%CE	1 / 337 0.3%ABE				
6	"Planctomycetales" Schlesner and Stackebrandt 1987 emend. Ward 2011	1 / 337 0.3%	0 / 10 0.0%	0 / 10 0.0%	0 / 9 0.0%	0 / 9 0.0%	0 / 21 0.0%	0 / 23 0.0%	0 / 8 0.0%	0 / 6 0.0%	0 / 20 0.0%	0 / 7 0.0%	0 / 21 0.0%	1 / 15 6.7%	0 / 6 0.0%	0 / 15 0.0%	0 / 10 0.0%	0 / 23 0.0%	0 / 6 0.0%	0 / 19 0.0%	0 / 36 0.0%	0 / 14 0.0%	0 / 4 0.0%	0 / 41 0.0%	0 / 1 0.0%	0 / 0 0.0%	0 / 3 0.0%	0 / 0 0.0%	182 / 337 54.0%	140 / 337 41.5%E	8 / 337 2.4%C	6 / 337 1.8%CE	1 / 337 0.3%ABE				
6	"Planctomycetaceae" Schlesner and Stackebrandt 1987 emend. Ward 2011	1 / 337 0.3%	0 / 10 0.0%	0 / 10 0.0%	0 / 9 0.0%	0 / 9 0.0%	0 / 21 0.0%	0 / 23 0.0%	0 / 8 0.0%	0 / 6 0.0%	0 / 20 0.0%	0 / 7 0.0%	0 / 21 0.0%	1 / 15 6.7%	0 / 6 0.0%	0 / 15 0.0%	0 / 10 0.0%	0 / 23 0.0%	0 / 6 0.0%	0 / 19 0.0%	0 / 36 0.0%	0 / 14 0.0%	0 / 4 0.0%	0 / 41 0.0%	0 / 1 0.0%	0 / 0 0.0%	0 / 3 0.0%	0 / 0 0.0%	182 / 337 54.0%	140 / 337 41.5%E	8 / 337 2.4%C	6 / 337 1.8%CE	1 / 337 0.3%ABE				
2	"Acinothrix" Novacek 1938	1 / 104 1.0%	0 / 2 0.0%	0 / 3 0.0%	0 / 2 0.0%	0 / 3 0.0%	0 / 7 0.0%	0 / 7 0.0%	0 / 2 0.0%	0 / 2 0.0%	0 / 6 0.0%	0 / 2 0.0%	0 / 8 0.0%	1 / 6 16.7%	0 / 2 0.0%	0 / 5 0.0%	0 / 2 0.0%	0 / 7 0.0%	0 / 2 0.0%	0 / 6 0.0%	0 / 12 0.0%	0 / 2 0.0%	0 / 2 0.0%	0 / 13 0.0%	0 / 0 0.0%	0 / 0 0.0%	0 / 1 0.0%	0 / 0 0.0%	52 / 104 50.0%	43 / 104 41.3%E	4 / 104 3.8%CE	4 / 104 3.8%C	1 / 104 1.0%ABE				
1	ATCC 49424	0 / 44 0.0%	0 / 1 0.0%	0 / 1 0.0%	0 / 1 0.0%	0 / 1 0.0%	0 / 3 0.0%	0 / 3 0.0%	0 / 1 0.0%	0 / 1 0.0%	0 / 3 0.0%	0 / 1 0.0%	0 / 3 0.0%	0 / 2 0.0%	0 / 1 0.0%	0 / 2 0.0%	0 / 1 0.0%	0 / 3 0.0%	0 / 1 0.0%	0 / 3 0.0%	0 / 5 0.0%	0 / 1 0.0%	0 / 1 0.0%	0 / 5 0.0%	0 / 0 0.0%	0 / 0 0.0%	0 / 0 0.0%	0 / 0 0.0%	28 / 44 63.6%	11 / 44 25.0%E	3 / 44 6.8%CE	2 / 44 4.5%C					
1	Planctomyces brasiliensis ATCC 49424	0 / 44 0.0%	0 / 1 0.0%	0 / 1 0.0%	0 / 1 0.0%	0 / 1 0.0%	0 / 3 0.0%	0 / 3 0.0%	0 / 1 0.0%	0 / 1 0.0%	0 / 3 0.0%	0 / 1 0.0%	0 / 3 0.0%	0 / 2 0.0%	0 / 1 0.0%	0 / 2 0.0%	0 / 1 0.0%	0 / 3 0.0%	0 / 1 0.0%	0 / 3 0.0%	0 / 5 0.0%	0 / 1 0.0%	0 / 1 0.0%	0 / 5 0.0%	0 / 0 0.0%	0 / 0 0.0%	0 / 0 0.0%	0 / 0 0.0%	28 / 44 63.6%	11 / 44 25.0%E	3 / 44 6.8%CE	2 / 44 4.5%C					
1	ATCC 43296	1 / 60 1.7%	0 / 1 0.0%	0 / 2 0.0%	0 / 1 0.0%	0 / 2 0.0%	0 / 4 0.0%	0 / 4 0.0%	0 / 1 0.0%	0 / 1 0.0%	0 / 3 0.0%	0 / 1 0.0%	0 / 5 0.0%	1 / 4 25.0%	0 / 1 0.0%	0 / 3 0.0%	0 / 1 0.0%	0 / 4 0.0%	0 / 1 0.0%	0 / 3 0.0%	0 / 7 0.0%	0 / 1 0.0%	0 / 1 0.0%	0 / 8 0.0%	0 / 0 0.0%	0 / 0 0.0%	0 / 1 0.0%	0 / 0 0.0%	32 / 60 53.3%E	24 / 60 40.0%	2 / 60 3.3%C	1 / 60 1.7%ABE	1 / 60 1.7%CE				
1	Planctomyces limnophilus ATCC 43296	1 / 60 1.7%	0 / 1 0.0%	0 / 2 0.0%	0 / 1 0.0%	0 / 2 0.0%	0 / 4 0.0%	0 / 4 0.0%	0 / 1 0.0%	0 / 1 0.0%	0 / 3 0.0%	0 / 1 0.0%	0 / 5 0.0%	1 / 4 25.0%	0 / 1 0.0%	0 / 3 0.0%	0 / 1 0.0%	0 / 4 0.0%	0 / 1 0.0%	0 / 3 0.0%	0 / 7 0.0%	0 / 1 0.0%	0 / 1 0.0%	0 / 8 0.0%	0 / 0 0.0%	0 / 0 0.0%	0 / 1 0.0%	0 / 0 0.0%	32 / 60 53.3%E	24 / 60 40.0%	2 / 60 3.3%C	1 / 60 1.7%CE	1 / 60 1.7%ABE				
1	Pirella	0 / 44 0.0%	0 / 1 0.0%	0 / 1 0.0%	0 / 1 0.0%	0 / 1 0.0%	0 / 3 0.0%	0 / 3 0.0%	0 / 1 0.0%	0 / 1 0.0%	0 / 3 0.0%	0 / 1 0.0%	0 / 2 0.0%	0 / 2 0.0%	0 / 1 0.0%	0 / 2 0.0%	0 / 1 0.0%	0 / 3 0.0%	0 / 1 0.0%	0 / 3 0.0%	0 / 5 0.0%	0 / 2 0.0%	0 / 1 0.0%	0 / 5 0.0%	0 / 0 0.0%	0 / 0 0.0%	0 / 0 0.0%	0 / 0 0.0%	30 / 44 68.2%	12 / 44 27.3%E	2 / 44 4.5%C						
1	ATCC 27377	0 / 44 0.0%	0 / 1 0.0%	0 / 1 0.0%	0 / 1 0.0%	0 / 1 0.0%	0 / 3 0.0%	0 / 3 0.0%	0 / 1 0.0%	0 / 1 0.0%	0 / 3 0.0%	0 / 1 0.0%	0 / 2 0.0%	0 / 2 0.0%	0 / 1 0.0%	0 / 2 0.0%	0 / 1 0.0%	0 / 3 0.0%	0 / 1 0.0%	0 / 3 0.0%	0 / 5 0.0%	0 / 2 0.0%	0 / 1 0.0%	0 / 5 0.0%	0 / 0 0.0%	0 / 0 0.0%	0 / 0 0.0%	0 / 0 0.0%	30 / 44 68.2%	12 / 44 27.3%E	2 / 44 4.5%C						
1	Pirellula staleyi ATCC 27377	0 / 44 0.0%	0 / 1 0.0%	0 / 1 0.0%	0 / 1 0.0%	0 / 1 0.0%	0 / 3 0.0%	0 / 3 0.0%	0 / 1 0.0%	0 / 1 0.0%	0 / 3 0.0%	0 / 1 0.0%	0 / 2 0.0%	0 / 2 0.0%	0 / 1 0.0%	0 / 2 0.0%	0 / 1 0.0%	0 / 3 0.0%	0 / 1 0.0%	0 / 3 0.0%	0 / 5 0.0%	0 / 2 0.0%	0 / 1 0.0%	0 / 5 0.0%	0 / 0 0.0%	0 / 0 0.0%	0 / 0 0.0%	0 / 0 0.0%	30 / 44 68.2%	12 / 44 27.3%E	2 / 44 4.5%C						
1	Isosphaera	0 / 46 0.0%	0 / 1 0.0%	0 / 1 0.0%	0 / 1 0.0%	0 / 1 0.0%	0 / 3 0.0%	0 / 3 0.0%	0 / 1 0.0%	0 / 1 0.0%	0 / 3 0.0%	0 / 1 0.0%	0 / 4 0.0%	0 / 1 0.0%	0 / 1 0.0%	0 / 2 0.0%	0 / 2 0.0%	0 / 3 0.0%	0 / 1 0.0%	0 / 3 0.0%	0 / 5 0.0%	0 / 2 0.0%	0 / 0 0.0%	0 / 5 0.0%	0 / 1 0.0%	0 / 0 0.0%	0 / 0 0.0%	0 / 0 0.0%	27 / 46 58.7%	18 / 46 39.1%E	1 / 46 2.2%CE						
1	"Isocystis pallida" Woronichin 1927 (as cyanobacterium)	0 / 46 0.0%	0 / 1 0.0%	0 / 1 0.0%	0 / 1 0.0%	0 / 1 0.0%	0 / 3 0.0%	0 / 3 0.0%	0 / 1 0.0%	0 / 1 0.0%	0 / 3 0.0%	0 / 1 0.0%	0 / 4 0.0%	0 / 1 0.0%	0 / 1 0.0%	0 / 2 0.0%	0 / 2 0.0%	0 / 3 0.0%	0 / 1 0.0%	0 / 3 0.0%	0 / 5 0.0%	0 / 2 0.0%	0 / 0 0.0%	0 / 5 0.0%	0 / 1 0.0%	0 / 0 0.0%	0 / 0 0.0%	0 / 0 0.0%	27 / 46 58.7%	18 / 46 39.1%E	1 / 46 2.2%CE						
1	Isosphaera pallida ATCC 43644	0 / 46 0.0%	0 / 1 0.0%	0 / 1 0.0%	0 / 1 0.0%	0 / 1 0.0%	0 / 3 0.0%	0 / 3 0.0%	0 / 1 0.0%	0 / 1 0.0%	0 / 3 0.0%	0 / 1 0.0%	0 / 4 0.0%	0 / 1 0.0%	0 / 1 0.0%	0 / 2 0.0%	0 / 2 0.0%	0 / 3 0.0%	0 / 1 0.0%	0 / 3 0.0%	0 / 5 0.0%	0 / 2 0.0%	0 / 0 0.0%	0 / 5 0.0%	0 / 1 0.0%	0 / 0 0.0%	0 / 0 0.0%	0 / 0 0.0%	27 / 46 58.7%	18 / 46 39.1%E	1 / 46 2.2%CE						
1	Rhodopirellula	0 / 77 0.0%	0 / 1 0.0%	0 / 3 0.0%	0 / 3 0.0%	0 / 2 0.0%	0 / 5 0.0%	0 / 5 0.0%	0 / 3 0.0%	0 / 1 0.0%	0 / 4 0.0%	0 / 2 0.0%	0 / 2 0.0%	0 / 5 0.0%	0 / 1 0.0%	0 / 3 0.0%	0 / 2 0.0%	0 / 5 0.0%	0 / 1 0.0%	0 / 4 0.0%	0 / 8 0.0%	0 / 5 0.0%	0 / 1 0.0%	0 / 11 0.0%	0 / 0 0.0%	0 / 0 0.0%	0 / 0 0.0%	0 / 0 0.0%	41 / 77 53.2%E	33 / 77 42.9%	2 / 77 2.6%C	1 / 77 1.3%CE					
1	DSM 10527	0 / 77 0.0%	0 / 1 0.0%	0 / 3 0.0%	0 / 3 0.0%	0 / 2 0.0%	0 / 5 0.0%	0 / 5 0.0%	0 / 3 0.0%	0 / 1 0.0%	0 / 4 0.0%	0 / 2 0.0%	0 / 2 0.0%	0 / 5 0.0%	0 / 1 0.0%	0 / 3 0.0%	0 / 2 0.0%	0 / 5 0.0%	0 / 1 0.0%	0 / 4 0.0%	0 / 8 0.0%	0 / 5 0.0%	0 / 1 0.0%	0 / 11 0.0%	0 / 0 0.0%	0 / 0 0.0%	0 / 0 0.0%	0 / 0 0.0%	41 / 77 53.2%E	33 / 77 42.9%	2 / 77 2.6%C	1 / 77 1.3%CE					
1	Pirellula sp. 1	0 / 77 0.0%	0 / 1 0.0%	0 / 3 0.0%	0 / 3 0.0%	0 / 2 0.0%	0 / 5 0.0%	0 / 5 0.0%	0 / 3 0.0%	0 / 1 0.0%	0 / 4 0.0%	0 / 2 0.0%	0 / 2 0.0%	0 / 5 0.0%	0 / 1 0.0%	0 / 3 0.0%	0 / 2 0.0%	0 / 5 0.0%	0 / 1 0.0%	0 / 4 0.0%	0 / 8 0.0%	0 / 5 0.0%	0 / 1 0.0%	0 / 11 0.0%	0 / 0 0.0%	0 / 0 0.0%	0 / 0 0.0%	0 / 0 0.0%	41 / 77 53.2%E	33 / 77 42.9%	2 / 77 2.6%C	1 / 77 1.3%CE					
1	Singulisphaera	0 / 66 0.0%	0 / 5 0.0%	0 / 2 0.0%	0 / 2 0.0%	0 / 2 0.0%	0 / 3 0.0%	0 / 5 0.0%	0 / 1 0.0%	0 / 1 0.0%	0 / 4 0.0%	0 / 1 0.0%	0 / 5 0.0%	0 / 1 0.0%	0 / 1 0.0%	0 / 3 0.0%	0 / 3 0.0%	0 / 5 0.0%	0 / 1 0.0%	0 / 3 0.0%	0 / 6 0.0%	0 / 3 0.0%	0 / 0 0.0%	0 / 7 0.0%	0 / 0 0.0%	0 / 0 0.0%	0 / 2 0.0%	0 / 0 0.0%	40 / 66 60.6%	26 / 66 39.4%E							
1	ATCC BAA-1392	0 / 66 0.0%	0 / 5 0.0%	0 / 2 0.0%	0 / 2 0.0%	0 / 2 0.0%	0 / 3 0.0%	0 / 5 0.0%	0 / 1 0.0%	0 / 1 0.0%	0 / 4 0.0%	0 / 1 0.0%	0 / 5 0.0%	0 / 1 0.0%	0 / 1 0.0%	0 / 3 0.0%	0 / 3 0.0%	0 / 5 0.0%	0 / 1 0.0%	0 / 3 0.0%	0 / 6 0.0%	0 / 3 0.0%	0 / 0 0.0%	0 / 7 0.0%	0 / 0 0.0%	0 / 0 0.0%	0 / 2 0.0%	0 / 0 0.0%	40 / 66 60.6%	26 / 66 39.4%E							
1	Singulisphaera acidiphila ATCC BAA-1392	0 / 66 0.0%	0 / 5 0.0%	0 / 2 0.0%	0 / 2 0.0%	0 / 2 0.0%	0 / 3 0.0%	0 / 5 0.0%	0 / 1 0.0%	0 / 1 0.0%	0 / 4 0.0%	0 / 1 0.0%	0 / 5 0.0%	0 / 1 0.0%	0 / 1 0.0%	0 / 3 0.0%	0 / 3 0.0%	0 / 5 0.0%	0 / 1 0.0%	0 / 3 0.0%	0 / 6 0.0%	0 / 3 0.0%	0 / 0 0.0%	0 / 7 0.0%	0 / 0 0.0%	0 / 0 0.0%	0 / 2 0.0%	0 / 0 0.0%	40 / 66 60.6%	26 / 66 39.4%E							
1	Phycisphaerae	0 / 46 0.0%	0 / 1 0.0%	0 / 1 0.0%	0 / 1 0.0%	0 / 1 0.0%	0 / 3 0.0%	0 / 3 0.0%	0 / 1 0.0%	0 / 1 0.0%	0 / 3 0.0%	0 / 1 0.0%	0 / 4 0.0%	0 / 2 0.0%	0 / 1 0.0%	0 / 2 0.0%	0 / 1 0.0%	0 / 3 0.0%	0 / 1 0.0%	0 / 3 0.0%	0 / 5 0.0%	0 / 2 0.0%	0 / 1 0.0%	0 / 5 0.0%	0 / 0 0.0%	0 / 0 0.0%	0 / 0 0.0%	0 / 0 0.0%	30 / 46 65.2%	16 / 46 34.8%E							
1	Phycisphaerales	0 / 46 0.0%	0 / 1 0.0%	0 / 1 0.0%	0 / 1 0.0%	0 / 1 0.0%	0 / 3 0.0%	0 / 3 0.0%	0 / 1 0.0%	0 / 1 0.0%	0 / 3 0.0%	0 / 1 0.0%	0 / 4 0.0%	0 / 2 0.0%	0 / 1 0.0%	0 / 2 0.0%	0 / 1 0.0%	0 / 3 0.0%	0 / 1 0.0%	0 / 3 0.0%	0 / 5 0.0%	0 / 2 0.0%	0 / 1 0.0%	0 / 5 0.0%	0 / 0 0.0%	0 / 0 0.0%	0 / 0 0.0%	0 / 0 0.0%	30 / 46 65.2%	16 / 46 34.8%E							
1	Phycisphaeraceae	0 / 46 0.0%	0 / 1 0.0%	0 / 1 0.0%	0 / 1 0.0%	0 / 1 0.0%	0 / 3 0.0%	0 / 3 0.0%	0 / 1 0.0%	0 / 1 0.0%	0 / 3 0.0%	0 / 1 0.0%	0 / 4 0.0%	0 / 2 0.0%	0 / 1 0.0%	0 / 2 0.0%	0 / 1 0.0%	0 / 3 0.0%	0 / 1 0.0%	0 / 3 0.0%	0 / 5 0.0%	0 / 2 0.0%	0 / 1 0.0%	0 / 5 0.0%	0 / 0 0.0%	0 / 0 0.0%	0 / 0 0.0%	0 / 0 0.0%	30 / 46 65.2%	16 / 46 34.8%E							
1	Phycisphaera	0 / 46 0.0%	0 / 1 0.0%	0 / 1 0.0%	0 / 1 0.0%	0 / 1 0.0%	0 / 3 0.0%	0 / 3 0.0%	0 / 1 0.0%	0 / 1 0.0%	0 / 3 0.0%	0 / 1 0.0%	0 / 4 0.0%	0 / 2 0.0%	0 / 1 0.0%	0 / 2 0.0%	0 / 1 0.0%	0 / 3 0.0%	0 / 1 0.0%	0 / 3 0.0%	0 / 5 0.0%	0 / 2 0.0%	0 / 1 0.0%	0 / 5 0.0%	0 / 0 0.0%	0 / 0 0.0%	0 / 0 0.0%	0 / 0 0.0%	30 / 46 65.2%	16 / 46 34.8%E							
1	KCTC 22515	0 / 46 0.0%	0 / 1 0.0%	0 / 1 0.0%	0 / 1 0.0%	0 / 1 0.0%	0 / 3 0.0%	0 / 3 0.0%	0 / 1 0.0%	0 / 1 0.0%	0 / 3 0.0%	0 / 1 0.0%	0 / 4 0.0%	0 / 2 0.0%	0 / 1 0.0%	0 / 2 0.0%	0 / 1 0.0%	0 / 3 0.0%	0 / 1 0.0%	0 / 3 0.0%	0 / 5 0.0%	0 / 2 0.0%	0 / 1 0.0%	0 / 5 0.0%	0 / 0 0.0%	0 / 0 0.0%	0 / 0 0.0%	0 / 0 0.0%	30 / 46 65.2%	16 / 46 34.8%E							
1	Phycisphaera mikrensis NBRC 102666	0 / 46 0.0%	0 / 1 0.0%	0 / 1 0.0%	0 / 1 0.0%	0 / 1 0.0%	0 / 3 0.0%	0 / 3 0.0%	0 / 1 0.0%	0 / 1 0.0%	0 / 3 0.0%	0 / 1 0.0%	0 / 4 0.0%	0 / 2 0.0%	0 / 1 0.0%	0 / 2 0.0%	0 / 1 0.0%	0 / 3 0.0%	0 / 1 0.0%	0 / 3 0.0%	0 / 5 0.0%	0 / 2 0.0%	0 / 1 0.0%	0 / 5 0.0%	0 / 0 0.0%	0 / 0 0.0%	0 / 0 0.0%	0 / 0 0.0%	30 / 46 65.2%	16 / 46 34.8%E							
53	Spirochaetae	89 / 2198 4.0%	0 / 57 0.0%	2 / 60 3.3%	5 / 56 8.9%	0 / 60 0.0%	9 / 126 7.1%	5 / 112 4.5%	5 / 56 8.9%	3 / 56 5.4%	1 / 126 0.8%	2 / 56 3.6%	2 / 192 1.0%	0 / 96 0.0%	0 / 67 0.0%	7 / 81 8.6%	0 / 56 0.0%	12 / 114 10.5%	2 / 56 3.6%	2 / 147 1.4%	15 / 231 6.5%	9 / 78 11.5%	0 / 56 0.0%	7 / 256 2.7%	1 / 1 100.0%	0 / 0 0.0%	0 / 2 0.0%	0 / 0 0.0%	1095 / 2198 49.8%E	983 / 2198 44.7%	45 / 2198 2.0%AB	30 / 2198 1.4%ABE	17 / 2198 0.8%CE	13 / 2198 0.6%C	10 / 2198 0.5%B	4 / 2198 0.2%BE	1 / 2198 0.0%D
53	"Spirochaetia" Paster 2011	89 / 2198 4.0%	0 / 57 0.0%	2 / 60 3.3%	5 / 56 8.9%	0 / 60 0.0%	9 / 126 7.1%	5 / 112 4.5%	5 / 56 8.9%	3 / 56 5.4%	1 / 126 0.8%	2 / 56 3.6%	2 / 192 1.0%	0 / 96 0.0%	0 / 67 0.0%	7 / 81 8.6%	0 / 56 0.0%	12 / 114 10.5%	2 / 56 3.6%	2 / 147 1.4%	15 / 231 6.5%	9 / 78 11.5%	0 / 56 0.0%	7 / 256 2.7%	1 / 1 100.0%	0 / 0 0.0%	0 / 2 0.0%	0 / 0 0.0%	1095 / 2198 49.8%E	983 / 2198 44.7%	45 / 2198 2.0%AB	30 / 2198 1.4%ABE	17 / 2198 0.8%CE	13 / 2198 0.6%C	10 / 2198 0.5%B	4 / 2198 0.2%BE	1 / 2198 0.0%D
53	Spirochaetales	89 / 2198 4.0%	0 / 57 0.0%	2 / 60 3.3%	5 / 56 8.9%	0 / 60 0.0%	9 / 126 7.1%	5 / 112 4.5%	5 / 56 8.9%	3 / 56 5.4%	1 / 126 0.8%	2 / 56 3.6%	2 / 192 1.0%	0 / 96 0.0%	0 / 67 0.0%	7 / 81 8.6%	0 / 56 0.0%	12 / 114 10.5%	2 / 56 3.6%	2 / 147 1.4%	15 / 231 6.5%	9 / 78 11.5%	0 / 56 0.0%	7 / 256 2.7%	1 / 1 100.0%	0 / 0 0.0%	0 / 2 0.0%	0 / 0 0.0%	1095 / 2198 49.8%E	983 / 2198 44.7%	45 / 2198 2.0%AB	30 / 2198 1.4%ABE	17 / 2198 0.8%CE	13 / 2198 0.6%C	10 / 2198 0.5%B	4 / 2198 0.2%BE	1 / 2198 0.0%D
38	"Spirochaetaceae" Swellengrebel 1907 emend. Abt et al. 2012	20 / 1673 1.2%	0 / 42 0.0%	0 / 45 0.0%	0 / 41 0.0%	0 / 44 0.0%	3 / 96 3.1%	0 / 89 0.0%	0 / 41 0.0%	0 / 41 0.0%	1 / 99 1.0%	0 / 41 0.0%	0 / 147 0.0%	0 / 81 0.0%	0 / 52 0.0%	0 / 66 0.0%	0 / 41 0.0%	12 / 91 13.2%	0 / 41 0.0%	0 / 107 0.0%	1 / 171 0.6%	2 / 63 3.2%	0 / 41 0.0%	0 / 190 0.0%	1 / 1 100.0%	0 / 0 0.0%	0 / 2 0.0%	0 / 0 0.0%	895 / 1673 53.5%E	729 / 1673 43.6%	15 / 1673 0.9%CE	13 / 1673 0.8%AB	13 / 1673 0.8%C	7 / 1673 0.4%ABE	1 / 1673 0.1%D		
15	Borrelia	0 / 522 0.0%	0 / 16 0.0%	0 / 16 0.0%	0 / 16 0.0%	0 / 16 0.0%	0 / 32 0.0%	0 / 16 0.0%	0 / 16 0.0%	0 / 16 0.0%	0 / 16 0.0%	0 / 16 0.0%	0 / 48 0.0%	0 / 31 0.0%	0 / 27 0.0%	0 / 16 0.0%	0 / 16 0.0%	0 / 16 0.0%	0 / 16 0.0%	0 / 32 0.0%	0 / 48 0.0%	0 / 14 0.0%	0 / 16 0.0%	0 / 64 0.0%	0 / 0 0.0%	0 / 0 0.0%	0 / 2 0.0%	0 / 0 0.0%	283 / 522 54.2%E	229 / 522 43.9%	10 / 522 1.9%C						
1	"Spirochaeta hermsi" (sic) Davis 1942	0 / 32 0.0%	0 / 1 0.0%	0 / 1 0.0%	0 / 1 0.0%	0 / 1 0.0%	0 / 2 0.0%	0 / 1 0.0%	0 / 1 0.0%	0 / 1 0.0%	0 / 1 0.0%	0 / 1 0.0%	0 / 3 0.0%	0 / 2 0.0%	0 / 1 0.0%	0 / 1 0.0%	0 / 1 0.0%	0 / 1 0.0%	0 / 1 0.0%	0 / 2 0.0%	0 / 3 0.0%	0 / 1 0.0%	0 / 1 0.0%	0 / 4 0.0%	0 / 0 0.0%	0 / 0 0.0%	0 / 0 0.0%	0 / 0 0.0%	18 / 32 56.2%E	13 / 32 40.6%	1 / 32 3.1%C						
1	Borrelia hermsii DAH	0 / 32 0.0%	0 / 1 0.0%	0 / 1 0.0%	0 / 1 0.0%	0 / 1 0.0%	0 / 2 0.0%	0 / 1 0.0%	0 / 1 0.0%	0 / 1 0.0%	0 / 1 0.0%	0 / 1 0.0%	0 / 3 0.0%	0 / 2 0.0%	0 / 1 0.0%	0 / 1 0.0%	0 / 1 0.0%	0 / 1 0.0%	0 / 1 0.0%	0 / 2 0.0%	0 / 3 0.0%	0 / 1 0.0%	0 / 1 0.0%	0 / 4 0.0%	0 / 0 0.0%	0 / 0 0.0%	0 / 0 0.0%	0 / 0 0.0%	18 / 32 56.2%E	13 / 32 40.6%	1 / 32 3.1%C						
1	"Spirochaeta turicatae" Brumpt 1933	0 / 31 0.0%	0 / 1 0.0%	0 / 1 0.0%	0 / 1 0.0%	0 / 1 0.0%	0 / 2 0.0%	0 / 1 0.0%	0 / 1 0.0%	0 / 1 0.0%	0 / 1 0.0%	0 / 1 0.0%	0 / 3 0.0%	0 / 1 0.0%	0 / 1 0.0%	0 / 1 0.0%	0 / 1 0.0%	0 / 1 0.0%	0 / 1 0.0%	0 / 2 0.0%	0 / 3 0.0%	0 / 1 0.0%	0 / 1 0.0%	0 / 4 0.0%	0 / 0 0.0%	0 / 0 0.0%	0 / 0 0.0%	0 / 0 0.0%	19 / 31 61.3%	11 / 31 35.5%E	1 / 31 3.2%C						
1	Borrelia turicatae 91E135	0 / 31 0.0%	0 / 1 0.0%	0 / 1 0.0%	0 / 1 0.0%	0 / 1 0.0%	0 / 2 0.0%	0 / 1 0.0%	0 / 1 0.0%	0 / 1 0.0%	0 / 1 0.0%	0 / 1 0.0%	0 / 3 0.0%	0 / 1 0.0%	0 / 1 0.0%	0 / 1 0.0%	0 / 1 0.0%	0 / 1 0.0%	0 / 1 0.0%	0 / 2 0.0%	0 / 3 0.0%	0 / 1 0.0%	0 / 1 0.0%	0 / 4 0.0%	0 / 0 0.0%	0 / 0 0.0%	0 / 0 0.0%	0 / 0 0.0%	19 / 31 61.3%	11 / 31 35.5%E	1 / 31 3.2%C						
1	"Spirochaeta crocidurae" Leger 1917	0 / 32 0.0%	0 / 1 0.0%	0 / 1 0.0%	0 / 1 0.0%	0 / 1 0.0%	0 / 2 0.0%	0 / 1 0.0%	0 / 1 0.0%	0 / 1 0.0%	0 / 1 0.0%	0 / 1 0.0%	0 / 3 0.0%	0 / 2 0.0%	0 / 1 0.0%	0 / 1 0.0%	0 / 1 0.0%	0 / 1 0.0%	0 / 1 0.0%	0 / 2 0.0%	0 / 3 0.0%	0 / 1 0.0%	0 / 1 0.0%	0 / 4 0.0%	0 / 0 0.0%	0 / 0 0.0%	0 / 0 0.0%	0 / 0 0.0%	19 / 32 59.4%E	13 / 32 40.6%							
1	Borrelia crocidurae str. Achema	0 / 32 0.0%	0 / 1 0.0%	0 / 1 0.0%	0 / 1 0.0%	0 / 1 0.0%	0 / 2 0.0%	0 / 1 0.0%	0 / 1 0.0%	0 / 1 0.0%	0 / 1 0.0%	0 / 1 0.0%	0 / 3 0.0%	0 / 2 0.0%	0 / 1 0.0%	0 / 1 0.0%	0 / 1 0.0%	0 / 1 0.0%	0 / 1 0.0%	0 / 2 0.0%	0 / 3 0.0%	0 / 1 0.0%	0 / 1 0.0%	0 / 4 0.0%	0 / 0 0.0%	0 / 0 0.0%	0 / 0 0.0%	0 / 0 0.0%	19 / 32 59.4%E	13 / 32 40.6%							
1	"Spirillum duttoni" (sic) Novy and Knapp 1906	0 / 32 0.0%	0 / 1 0.0%	0 / 1 0.0%	0 / 1 0.0%	0 / 1 0.0%	0 / 2 0.0%	0 / 1 0.0%	0 / 1 0.0%	0 / 1 0.0%	0 / 1 0.0%	0 / 1 0.0%	0 / 3 0.0%	0 / 2 0.0%	0 / 1 0.0%	0 / 1 0.0%	0 / 1 0.0%	0 / 1 0.0%	0 / 1 0.0%	0 / 2 0.0%	0 / 3 0.0%	0 / 1 0.0%	0 / 1 0.0%	0 / 4 0.0%	0 / 0 0.0%	0 / 0 0.0%	0 / 0 0.0%	0 / 0 0.0%	18 / 32 56.2%E	14 / 32 43.8%							
1	Borrelia duttonii Ly	0 / 32 0.0%	0 / 1 0.0%	0 / 1 0.0%	0 / 1 0.0%	0 / 1 0.0%	0 / 2 0.0%	0 / 1 0.0%	0 / 1 0.0%	0 / 1 0.0%	0 / 1 0.0%	0 / 1 0.0%	0 / 3 0.0%	0 / 2 0.0%	0 / 1 0.0%	0 / 1 0.0%	0 / 1 0.0%	0 / 1 0.0%	0 / 1 0.0%	0 / 2 0.0%	0 / 3 0.0%	0 / 1 0.0%	0 / 1 0.0%	0 / 4 0.0%	0 / 0 0.0%	0 / 0 0.0%	0 / 0 0.0%	0 / 0 0.0%	18 / 32 56.2%E	14 / 32 43.8%							
1	"Spirochaete obermeieri" Cohn 1875	0 / 32 0.0%	0 / 1 0.0%	0 / 1 0.0%	0 / 1 0.0%	0 / 1 0.0%	0 / 2 0.0%	0 / 1 0.0%	0 / 1 0.0%	0 / 1 0.0%	0 / 1 0.0%	0 / 1 0.0%	0 / 3 0.0%	0 / 2 0.0%	0 / 1 0.0%	0 / 1 0.0%	0 / 1 0.0%	0 / 1 0.0%	0 / 1 0.0%	0 / 2 0.0%	0 / 3 0.0%	0 / 1 0.0%	0 / 1 0.0%	0 / 4 0.0%	0 / 0 0.0%	0 / 0 0.0%	0 / 0 0.0%	0 / 0 0.0%	19 / 32 59.4%E	13 / 32 40.6%							
1	Borrelia recurrentis A1	0 / 32 0.0%	0 / 1 0.0%	0 / 1 0.0%	0 / 1 0.0%	0 / 1 0.0%	0 / 2 0.0%	0 / 1 0.0%	0 / 1 0.0%	0 / 1 0.0%	0 / 1 0.0%	0 / 1 0.0%	0 / 3 0.0%	0 / 2 0.0%	0 / 1 0.0%	0 / 1 0.0%	0 / 1 0.0%	0 / 1 0.0%	0 / 1 0.0%	0 / 2 0.0%	0 / 3 0.0%	0 / 1 0.0%	0 / 1 0.0%	0 / 4 0.0%	0 / 0 0.0%	0 / 0 0.0%	0 / 0 0.0%	0 / 0 0.0%	19 / 32 59.4%E	13 / 32 40.6%							
10	Borrelia burgdorferi group	0 / 363 0.0%	0 / 11 0.0%	0 / 11 0.0%	0 / 11 0.0%	0 / 11 0.0%	0 / 22 0.0%	0 / 11 0.0%	0 / 11 0.0%	0 / 11 0.0%	0 / 11 0.0%	0 / 11 0.0%	0 / 33 0.0%	0 / 22 0.0%	0 / 22 0.0%	0 / 11 0.0%	0 / 11 0.0%	0 / 11 0.0%	0 / 11 0.0%	0 / 22 0.0%	0 / 33 0.0%	0 / 9 0.0%	0 / 11 0.0%	0 / 44 0.0%	0 / 0 0.0%	0 / 0 0.0%	0 / 2 0.0%	0 / 0 0.0%	198 / 363 54.5%E	157 / 363 43.3%	8 / 363 2.2%C						
4	ATCC 35210	0 / 132 0.0%	0 / 4 0.0%	0 / 4 0.0%	0 / 4 0.0%	0 / 4 0.0%	0 / 8 0.0%	0 / 4 0.0%	0 / 4 0.0%	0 / 4 0.0%	0 / 4 0.0%	0 / 4 0.0%	0 / 12 0.0%	0 / 8 0.0%	0 / 8 0.0%	0 / 4 0.0%	0 / 4 0.0%	0 / 4 0.0%	0 / 4 0.0%	0 / 8 0.0%	0 / 12 0.0%	0 / 4 0.0%	0 / 4 0.0%	0 / 16 0.0%	0 / 0 0.0%	0 / 0 0.0%	0 / 0 0.0%	0 / 0 0.0%	75 / 132 56.8%E	56 / 132 42.4%	1 / 132 0.8%C						
1	Borrelia burgdorferi B31	0 / 33 0.0%	0 / 1 0.0%	0 / 1 0.0%	0 / 1 0.0%	0 / 1 0.0%	0 / 2 0.0%	0 / 1 0.0%	0 / 1 0.0%	0 / 1 0.0%	0 / 1 0.0%	0 / 1 0.0%	0 / 3 0.0%	0 / 2 0.0%	0 / 2 0.0%	0 / 1 0.0%	0 / 1 0.0%	0 / 1 0.0%	0 / 1 0.0%	0 / 2 0.0%	0 / 3 0.0%	0 / 1 0.0%	0 / 1 0.0%	0 / 4 0.0%	0 / 0 0.0%	0 / 0 0.0%	0 / 0 0.0%	0 / 0 0.0%	21 / 33 63.6%	11 / 33 33.3%E	1 / 33 3.0%C						
1	Borrelia burgdorferi ZS7	0 / 33 0.0%	0 / 1 0.0%	0 / 1 0.0%	0 / 1 0.0%	0 / 1 0.0%	0 / 2 0.0%	0 / 1 0.0%	0 / 1 0.0%	0 / 1 0.0%	0 / 1 0.0%	0 / 1 0.0%	0 / 3 0.0%	0 / 2 0.0%	0 / 2 0.0%	0 / 1 0.0%	0 / 1 0.0%	0 / 1 0.0%	0 / 1 0.0%	0 / 2 0.0%	0 / 3 0.0%	0 / 1 0.0%	0 / 1 0.0%	0 / 4 0.0%	0 / 0 0.0%	0 / 0 0.0%	0 / 0 0.0%	0 / 0 0.0%	21 / 33 63.6%E	12 / 33 36.4%							
1	Borrelia burgdorferi N40	0 / 33 0.0%	0 / 1 0.0%	0 / 1 0.0%	0 / 1 0.0%	0 / 1 0.0%	0 / 2 0.0%	0 / 1 0.0%	0 / 1 0.0%	0 / 1 0.0%	0 / 1 0.0%	0 / 1 0.0%	0 / 3 0.0%	0 / 2 0.0%	0 / 2 0.0%	0 / 1 0.0%	0 / 1 0.0%	0 / 1 0.0%	0 / 1 0.0%	0 / 2 0.0%	0 / 3 0.0%	0 / 1 0.0%	0 / 1 0.0%	0 / 4 0.0%	0 / 0 0.0%	0 / 0 0.0%	0 / 0 0.0%	0 / 0 0.0%	21 / 33 63.6%E	12 / 33 36.4%							
1	Borrelia burgdorferi JD1	0 / 33 0.0%	0 / 1 0.0%	0 / 1 0.0%	0 / 1 0.0%	0 / 1 0.0%	0 / 2 0.0%	0 / 1 0.0%	0 / 1 0.0%	0 / 1 0.0%	0 / 1 0.0%	0 / 1 0.0%	0 / 3 0.0%	0 / 2 0.0%	0 / 2 0.0%	0 / 1 0.0%	0 / 1 0.0%	0 / 1 0.0%	0 / 1 0.0%	0 / 2 0.0%	0 / 3 0.0%	0 / 1 0.0%	0 / 1 0.0%	0 / 4 0.0%	0 / 0 0.0%	0 / 0 0.0%	0 / 0 0.0%	0 / 0 0.0%	22 / 33 66.7%E	11 / 33 33.3%							
2	ATCC 51567	0 / 99 0.0%	0 / 3 0.0%	0 / 3 0.0%	0 / 3 0.0%	0 / 3 0.0%	0 / 6 0.0%	0 / 3 0.0%	0 / 3 0.0%	0 / 3 0.0%	0 / 3 0.0%	0 / 3 0.0%	0 / 9 0.0%	0 / 6 0.0%	0 / 6 0.0%	0 / 3 0.0%	0 / 3 0.0%	0 / 3 0.0%	0 / 3 0.0%	0 / 6 0.0%	0 / 9 0.0%	0 / 2 0.0%	0 / 3 0.0%	0 / 12 0.0%	0 / 0 0.0%	0 / 0 0.0%	0 / 1 0.0%	0 / 0 0.0%	51 / 99 51.5%E	45 / 99 45.5%	3 / 99 3.0%C						
1	Borrelia afzelii PKo	0 / 66 0.0%	0 / 2 0.0%	0 / 2 0.0%	0 / 2 0.0%	0 / 2 0.0%	0 / 4 0.0%	0 / 2 0.0%	0 / 2 0.0%	0 / 2 0.0%	0 / 2 0.0%	0 / 2 0.0%	0 / 6 0.0%	0 / 4 0.0%	0 / 4 0.0%	0 / 2 0.0%	0 / 2 0.0%	0 / 2 0.0%	0 / 2 0.0%	0 / 4 0.0%	0 / 6 0.0%	0 / 2 0.0%	0 / 2 0.0%	0 / 8 0.0%	0 / 0 0.0%	0 / 0 0.0%	0 / 0 0.0%	0 / 0 0.0%	33 / 66 50.0%	31 / 66 47.0%E	2 / 66 3.0%C						
1	Borrelia afzelii HLJ01	0 / 33 0.0%	0 / 1 0.0%	0 / 1 0.0%	0 / 1 0.0%	0 / 1 0.0%	0 / 2 0.0%	0 / 1 0.0%	0 / 1 0.0%	0 / 1 0.0%	0 / 1 0.0%	0 / 1 0.0%	0 / 3 0.0%	0 / 2 0.0%	0 / 2 0.0%	0 / 1 0.0%	0 / 1 0.0%	0 / 1 0.0%	0 / 1 0.0%	0 / 2 0.0%	0 / 3 0.0%	0 / 0 0.0%	0 / 1 0.0%	0 / 4 0.0%	0 / 0 0.0%	0 / 0 0.0%	0 / 1 0.0%	0 / 0 0.0%	20 / 33 60.6%E	12 / 33 36.4%	1 / 33 3.0%C						

2	ATCC 51383	0 / 66 0.0%	0 / 2 0.0%	0 / 2 0.0%	0 / 2 0.0%	0 / 2 0.0%	0 / 4 0.0%	0 / 2 0.0%	0 / 2 0.0%	0 / 2 0.0%	0 / 2 0.0%	0 / 2 0.0%	0 / 6 0.0%	0 / 4 0.0%	0 / 4 0.0%	0 / 2 0.0%	0 / 2 0.0%	0 / 2 0.0%	0 / 2 0.0%	0 / 4 0.0%	0 / 6 0.0%	0 / 1 0.0%	0 / 2 0.0%	0 / 8 0.0%	0 / 0 0.0%	0 / 0 0.0%	0 / 1 0.0%	0 / 0 0.0%	40 / 66 60.6%E	24 / 66 36.4%	2 / 66 3.0%C				
1	Borrelia garinii BgVir	0 / 33 0.0%	0 / 1 0.0%	0 / 1 0.0%	0 / 1 0.0%	0 / 1 0.0%	0 / 2 0.0%	0 / 1 0.0%	0 / 1 0.0%	0 / 1 0.0%	0 / 1 0.0%	0 / 1 0.0%	0 / 3 0.0%	0 / 2 0.0%	0 / 2 0.0%	0 / 1 0.0%	0 / 1 0.0%	0 / 1 0.0%	0 / 1 0.0%	0 / 2 0.0%	0 / 3 0.0%	0 / 1 0.0%	0 / 1 0.0%	0 / 4 0.0%	0 / 0 0.0%	0 / 0 0.0%	0 / 0 0.0%	0 / 0 0.0%	20 / 33 60.6%E	12 / 33 36.4%	1 / 33 3.0%C				
1	Borrelia garinii NMJW1	0 / 33 0.0%	0 / 1 0.0%	0 / 1 0.0%	0 / 1 0.0%	0 / 1 0.0%	0 / 2 0.0%	0 / 1 0.0%	0 / 1 0.0%	0 / 1 0.0%	0 / 1 0.0%	0 / 1 0.0%	0 / 3 0.0%	0 / 2 0.0%	0 / 2 0.0%	0 / 1 0.0%	0 / 1 0.0%	0 / 1 0.0%	0 / 1 0.0%	0 / 2 0.0%	0 / 3 0.0%	0 / 0 0.0%	0 / 1 0.0%	0 / 4 0.0%	0 / 0 0.0%	0 / 0 0.0%	0 / 1 0.0%	0 / 0 0.0%	20 / 33 60.6%E	12 / 33 36.4%	1 / 33 3.0%C				
1	"Borrelia bissettii" Postic et al. 1998	0 / 33 0.0%	0 / 1 0.0%	0 / 1 0.0%	0 / 1 0.0%	0 / 1 0.0%	0 / 2 0.0%	0 / 1 0.0%	0 / 1 0.0%	0 / 1 0.0%	0 / 1 0.0%	0 / 1 0.0%	0 / 3 0.0%	0 / 2 0.0%	0 / 2 0.0%	0 / 1 0.0%	0 / 1 0.0%	0 / 1 0.0%	0 / 1 0.0%	0 / 2 0.0%	0 / 3 0.0%	0 / 1 0.0%	0 / 1 0.0%	0 / 4 0.0%	0 / 0 0.0%	0 / 0 0.0%	0 / 0 0.0%	0 / 0 0.0%	21 / 33 63.6%E	11 / 33 33.3%	1 / 33 3.0%C				
1	Borrelia bissettii DN127	0 / 33 0.0%	0 / 1 0.0%	0 / 1 0.0%	0 / 1 0.0%	0 / 1 0.0%	0 / 2 0.0%	0 / 1 0.0%	0 / 1 0.0%	0 / 1 0.0%	0 / 1 0.0%	0 / 1 0.0%	0 / 3 0.0%	0 / 2 0.0%	0 / 2 0.0%	0 / 1 0.0%	0 / 1 0.0%	0 / 1 0.0%	0 / 1 0.0%	0 / 2 0.0%	0 / 3 0.0%	0 / 1 0.0%	0 / 1 0.0%	0 / 4 0.0%	0 / 0 0.0%	0 / 0 0.0%	0 / 0 0.0%	0 / 0 0.0%	21 / 33 63.6%E	11 / 33 33.3%	1 / 33 3.0%C				
1	"Borrelia bavariensis" Margos et al. 2009	0 / 33 0.0%	0 / 1 0.0%	0 / 1 0.0%	0 / 1 0.0%	0 / 1 0.0%	0 / 2 0.0%	0 / 1 0.0%	0 / 1 0.0%	0 / 1 0.0%	0 / 1 0.0%	0 / 1 0.0%	0 / 3 0.0%	0 / 2 0.0%	0 / 2 0.0%	0 / 1 0.0%	0 / 1 0.0%	0 / 1 0.0%	0 / 1 0.0%	0 / 2 0.0%	0 / 3 0.0%	0 / 1 0.0%	0 / 1 0.0%	0 / 4 0.0%	0 / 0 0.0%	0 / 0 0.0%	0 / 0 0.0%	0 / 0 0.0%	21 / 33 63.6%	11 / 33 33.3%E	1 / 33 3.0%C				
1	Borrelia bavariensis PBi	0 / 33 0.0%	0 / 1 0.0%	0 / 1 0.0%	0 / 1 0.0%	0 / 1 0.0%	0 / 2 0.0%	0 / 1 0.0%	0 / 1 0.0%	0 / 1 0.0%	0 / 1 0.0%	0 / 1 0.0%	0 / 3 0.0%	0 / 2 0.0%	0 / 2 0.0%	0 / 1 0.0%	0 / 1 0.0%	0 / 1 0.0%	0 / 1 0.0%	0 / 2 0.0%	0 / 3 0.0%	0 / 1 0.0%	0 / 1 0.0%	0 / 4 0.0%	0 / 0 0.0%	0 / 0 0.0%	0 / 0 0.0%	0 / 0 0.0%	21 / 33 63.6%	11 / 33 33.3%E	1 / 33 3.0%C				
4	"Ehrenbergia" Gieszczykiewiez 1939	4 / 185 2.2%	0 / 5 0.0%	0 / 5 0.0%	0 / 4 0.0%	0 / 5 0.0%	2 / 12 16.7%	0 / 11 0.0%	0 / 4 0.0%	0 / 4 0.0%	1 / 12 8.3%	0 / 4 0.0%	0 / 16 0.0%	0 / 8 0.0%	0 / 4 0.0%	0 / 8 0.0%	0 / 4 0.0%	0 / 12 0.0%	0 / 4 0.0%	0 / 12 0.0%	0 / 20 0.0%	1 / 7 14.3%	0 / 4 0.0%	0 / 20 0.0%	0 / 0 0.0%	0 / 0 0.0%	0 / 0 0.0%	0 / 0 0.0%	93 / 185 50.3%E	73 / 185 39.5%	13 / 185 7.0%CE	3 / 185 1.6%ABE	2 / 185 1.1%C	1 / 185 0.5%AB	
2	ATCC 700085	3 / 92 3.3%	0 / 2 0.0%	0 / 2 0.0%	0 / 2 0.0%	0 / 2 0.0%	2 / 6 33.3%	0 / 6 0.0%	0 / 2 0.0%	0 / 2 0.0%	1 / 6 16.7%	0 / 2 0.0%	0 / 8 0.0%	0 / 4 0.0%	0 / 2 0.0%	0 / 4 0.0%	0 / 2 0.0%	0 / 6 0.0%	0 / 2 0.0%	0 / 6 0.0%	0 / 10 0.0%	0 / 4 0.0%	0 / 2 0.0%	0 / 10 0.0%	0 / 0 0.0%	0 / 0 0.0%	0 / 0 0.0%	0 / 0 0.0%	44 / 92 47.8%E	31 / 92 33.7%	12 / 92 13.0%CE	2 / 92 2.2%ABE	2 / 92 2.2%C	1 / 92 1.1%AB	
1	Spirochaeta thermophila DSM 6192	1 / 46 2.2%	0 / 1 0.0%	0 / 1 0.0%	0 / 1 0.0%	0 / 1 0.0%	1 / 3 33.3%	0 / 3 0.0%	0 / 1 0.0%	0 / 1 0.0%	0 / 3 0.0%	0 / 1 0.0%	0 / 4 0.0%	0 / 2 0.0%	0 / 1 0.0%	0 / 2 0.0%	0 / 1 0.0%	0 / 3 0.0%	0 / 1 0.0%	0 / 3 0.0%	0 / 5 0.0%	0 / 2 0.0%	0 / 1 0.0%	0 / 5 0.0%	0 / 0 0.0%	0 / 0 0.0%	0 / 0 0.0%	0 / 0 0.0%	22 / 46 47.8%E	16 / 46 34.8%	6 / 46 13.0%CE	1 / 46 2.2%C	1 / 46 2.2%ABE		
1	Spirochaeta thermophila DSM 6578	2 / 46 4.3%	0 / 1 0.0%	0 / 1 0.0%	0 / 1 0.0%	0 / 1 0.0%	1 / 3 33.3%	0 / 3 0.0%	0 / 1 0.0%	0 / 1 0.0%	1 / 3 33.3%	0 / 1 0.0%	0 / 4 0.0%	0 / 2 0.0%	0 / 1 0.0%	0 / 2 0.0%	0 / 1 0.0%	0 / 3 0.0%	0 / 1 0.0%	0 / 3 0.0%	0 / 5 0.0%	0 / 2 0.0%	0 / 1 0.0%	0 / 5 0.0%	0 / 0 0.0%	0 / 0 0.0%	0 / 0 0.0%	0 / 0 0.0%	22 / 46 47.8%E	15 / 46 32.6%	6 / 46 13.0%CE	1 / 46 2.2%ABE	1 / 46 2.2%AB	1 / 46 2.2%C	
1	ATCC 700263	1 / 45 2.2%	0 / 1 0.0%	0 / 1 0.0%	0 / 1 0.0%	0 / 2 0.0%	0 / 3 0.0%	0 / 2 0.0%	0 / 1 0.0%	0 / 1 0.0%	0 / 3 0.0%	0 / 1 0.0%	0 / 4 0.0%	0 / 2 0.0%	0 / 1 0.0%	0 / 2 0.0%	0 / 1 0.0%	0 / 3 0.0%	0 / 1 0.0%	0 / 3 0.0%	0 / 5 0.0%	1 / 1 100.0%	0 / 1 0.0%	0 / 5 0.0%	0 / 0 0.0%	0 / 0 0.0%	0 / 0 0.0%	0 / 0 0.0%	23 / 45 51.1%	21 / 45 46.7%E	1 / 45 2.2%ABE				
1	Spirochaeta africana DSM 8902	1 / 45 2.2%	0 / 1 0.0%	0 / 1 0.0%	0 / 1 0.0%	0 / 2 0.0%	0 / 3 0.0%	0 / 2 0.0%	0 / 1 0.0%	0 / 1 0.0%	0 / 3 0.0%	0 / 1 0.0%	0 / 4 0.0%	0 / 2 0.0%	0 / 1 0.0%	0 / 2 0.0%	0 / 1 0.0%	0 / 3 0.0%	0 / 1 0.0%	0 / 3 0.0%	0 / 5 0.0%	1 / 1 100.0%	0 / 1 0.0%	0 / 5 0.0%	0 / 0 0.0%	0 / 0 0.0%	0 / 0 0.0%	0 / 0 0.0%	23 / 45 51.1%	21 / 45 46.7%E	1 / 45 2.2%ABE				
1	DSM 11293	0 / 48 0.0%	0 / 2 0.0%	0 / 2 0.0%	0 / 1 0.0%	0 / 1 0.0%	0 / 3 0.0%	0 / 3 0.0%	0 / 1 0.0%	0 / 1 0.0%	0 / 3 0.0%	0 / 1 0.0%	0 / 4 0.0%	0 / 2 0.0%	0 / 1 0.0%	0 / 2 0.0%	0 / 1 0.0%	0 / 3 0.0%	0 / 1 0.0%	0 / 3 0.0%	0 / 5 0.0%	0 / 2 0.0%	0 / 1 0.0%	0 / 5 0.0%	0 / 0 0.0%	0 / 0 0.0%	0 / 0 0.0%	0 / 0 0.0%	28 / 48 58.3%E	19 / 48 39.6%	1 / 48 2.1%CE				
1	Spirochaeta smaragdinae DSM 11293	0 / 48 0.0%	0 / 2 0.0%	0 / 2 0.0%	0 / 1 0.0%	0 / 1 0.0%	0 / 3 0.0%	0 / 3 0.0%	0 / 1 0.0%	0 / 1 0.0%	0 / 3 0.0%	0 / 1 0.0%	0 / 4 0.0%	0 / 2 0.0%	0 / 1 0.0%	0 / 2 0.0%	0 / 1 0.0%	0 / 3 0.0%	0 / 1 0.0%	0 / 3 0.0%	0 / 5 0.0%	0 / 2 0.0%	0 / 1 0.0%	0 / 5 0.0%	0 / 0 0.0%	0 / 0 0.0%	0 / 0 0.0%	0 / 0 0.0%	28 / 48 58.3%E	19 / 48 39.6%	1 / 48 2.1%CE				
16	"Microspironema" Stiles and Pfender 1905	15 / 822 1.8%	0 / 18 0.0%	0 / 19 0.0%	0 / 18 0.0%	0 / 20 0.0%	1 / 43 2.3%	0 / 53 0.0%	0 / 18 0.0%	0 / 18 0.0%	0 / 58 0.0%	0 / 18 0.0%	0 / 71 0.0%	0 / 36 0.0%	0 / 18 0.0%	0 / 36 0.0%	0 / 18 0.0%	12 / 54 22.2%	0 / 18 0.0%	0 / 54 0.0%	0 / 88 0.0%	1 / 36 2.8%	0 / 18 0.0%	0 / 91 0.0%	1 / 1 100.0%	0 / 0 0.0%	0 / 0 0.0%	0 / 0 0.0%	453 / 822 55.1%E	351 / 822 42.7%	12 / 822 1.5%AB	3 / 822 0.4%ABE	2 / 822 0.2%CE	1 / 822 0.1%C	
1	"Spirillum dentium" (Miller 1889) Sternberg 1892	0 / 43 0.0%	0 / 1 0.0%	0 / 1 0.0%	0 / 1 0.0%	0 / 1 0.0%	0 / 2 0.0%	0 / 2 0.0%	0 / 1 0.0%	0 / 1 0.0%	0 / 3 0.0%	0 / 1 0.0%	0 / 4 0.0%	0 / 2 0.0%	0 / 1 0.0%	0 / 2 0.0%	0 / 1 0.0%	0 / 3 0.0%	0 / 1 0.0%	0 / 3 0.0%	0 / 4 0.0%	0 / 2 0.0%	0 / 1 0.0%	0 / 5 0.0%	0 / 0 0.0%	0 / 0 0.0%	0 / 0 0.0%	0 / 0 0.0%	28 / 43 65.1%	15 / 43 34.9%E					
1	Treponema denticola ATCC 35405	0 / 43 0.0%	0 / 1 0.0%	0 / 1 0.0%	0 / 1 0.0%	0 / 1 0.0%	0 / 2 0.0%	0 / 2 0.0%	0 / 1 0.0%	0 / 1 0.0%	0 / 3 0.0%	0 / 1 0.0%	0 / 4 0.0%	0 / 2 0.0%	0 / 1 0.0%	0 / 2 0.0%	0 / 1 0.0%	0 / 3 0.0%	0 / 1 0.0%	0 / 3 0.0%	0 / 4 0.0%	0 / 2 0.0%	0 / 1 0.0%	0 / 5 0.0%	0 / 0 0.0%	0 / 0 0.0%	0 / 0 0.0%	0 / 0 0.0%	28 / 43 65.1%	15 / 43 34.9%E					
9	"Microspironema pallidum" (Schaudinn and Hoffmann 1905) Stiles and Pfender 1905	11 / 495 2.2%	0 / 11 0.0%	0 / 11 0.0%	0 / 11 0.0%	0 / 11 0.0%	0 / 22 0.0%	0 / 33 0.0%	0 / 11 0.0%	0 / 11 0.0%	0 / 33 0.0%	0 / 11 0.0%	0 / 44 0.0%	0 / 22 0.0%	0 / 11 0.0%	0 / 22 0.0%	0 / 11 0.0%	11 / 33 33.3%	0 / 11 0.0%	0 / 33 0.0%	0 / 55 0.0%	0 / 22 0.0%	0 / 11 0.0%	0 / 55 0.0%	0 / 0 0.0%	0 / 0 0.0%	0 / 0 0.0%	0 / 0 0.0%	279 / 495 56.4%E	205 / 495 41.4%	11 / 495 2.2%AB				
5	"Treponema pallidum subsp. pallidum" (Schaudinn and Hoffmann 1905) Schaudinn 1905	7 / 315 2.2%	0 / 7 0.0%	0 / 7 0.0%	0 / 7 0.0%	0 / 7 0.0%	0 / 14 0.0%	0 / 21 0.0%	0 / 7 0.0%	0 / 7 0.0%	0 / 21 0.0%	0 / 7 0.0%	0 / 28 0.0%	0 / 14 0.0%	0 / 7 0.0%	0 / 14 0.0%	0 / 7 0.0%	7 / 21 33.3%	0 / 7 0.0%	0 / 21 0.0%	0 / 35 0.0%	0 / 14 0.0%	0 / 7 0.0%	0 / 35 0.0%	0 / 0 0.0%	0 / 0 0.0%	0 / 0 0.0%	0 / 0 0.0%	175 / 315 55.6%E	133 / 315 42.2%	7 / 315 2.2%AB				
1	Treponema pallidum Nichols	2 / 90 2.2%	0 / 2 0.0%	0 / 2 0.0%	0 / 2 0.0%	0 / 2 0.0%	0 / 4 0.0%	0 / 6 0.0%	0 / 2 0.0%	0 / 2 0.0%	0 / 6 0.0%	0 / 2 0.0%	0 / 8 0.0%	0 / 4 0.0%	0 / 2 0.0%	0 / 4 0.0%	0 / 2 0.0%	2 / 6 33.3%	0 / 2 0.0%	0 / 6 0.0%	0 / 10 0.0%	0 / 4 0.0%	0 / 2 0.0%	0 / 10 0.0%	0 / 0 0.0%	0 / 0 0.0%	0 / 0 0.0%	0 / 0 0.0%	45 / 90 50.0%E	43 / 90 47.8%	2 / 90 2.2%AB				
1	Treponema pallidum subsp. pallidum SS14	2 / 90 2.2%	0 / 2 0.0%	0 / 2 0.0%	0 / 2 0.0%	0 / 2 0.0%	0 / 4 0.0%	0 / 6 0.0%	0 / 2 0.0%	0 / 2 0.0%	0 / 6 0.0%	0 / 2 0.0%	0 / 8 0.0%	0 / 4 0.0%	0 / 2 0.0%	0 / 4 0.0%	0 / 2 0.0%	2 / 6 33.3%	0 / 2 0.0%	0 / 6 0.0%	0 / 10 0.0%	0 / 4 0.0%	0 / 2 0.0%	0 / 10 0.0%	0 / 0 0.0%	0 / 0 0.0%	0 / 0 0.0%	0 / 0 0.0%	52 / 90 57.8%E	36 / 90 40.0%	2 / 90 2.2%AB				
1	Treponema pallidum subsp. pallidum DAL-1	1 / 45 2.2%	0 / 1 0.0%	0 / 1 0.0%	0 / 1 0.0%	0 / 1 0.0%	0 / 2 0.0%	0 / 3 0.0%	0 / 1 0.0%	0 / 1 0.0%	0 / 3 0.0%	0 / 1 0.0%	0 / 4 0.0%	0 / 2 0.0%	0 / 1 0.0%	0 / 2 0.0%	0 / 1 0.0%	1 / 3 33.3%	0 / 1 0.0%	0 / 3 0.0%	0 / 5 0.0%	0 / 2 0.0%	0 / 1 0.0%	0 / 5 0.0%	0 / 0 0.0%	0 / 0 0.0%	0 / 0 0.0%	0 / 0 0.0%	26 / 45 57.8%E	18 / 45 40.0%	1 / 45 2.2%AB				
1	Treponema pallidum subsp. pallidum str. Chicago	1 / 45 2.2%	0 / 1 0.0%	0 / 1 0.0%	0 / 1 0.0%	0 / 1 0.0%	0 / 2 0.0%	0 / 3 0.0%	0 / 1 0.0%	0 / 1 0.0%	0 / 3 0.0%	0 / 1 0.0%	0 / 4 0.0%	0 / 2 0.0%	0 / 1 0.0%	0 / 2 0.0%	0 / 1 0.0%	1 / 3 33.3%	0 / 1 0.0%	0 / 3 0.0%	0 / 5 0.0%	0 / 2 0.0%	0 / 1 0.0%	0 / 5 0.0%	0 / 0 0.0%	0 / 0 0.0%	0 / 0 0.0%	0 / 0 0.0%	26 / 45 57.8%E	18 / 45 40.0%	1 / 45 2.2%AB				
1	Treponema pallidum subsp. pallidum Mexico A	1 / 45 2.2%	0 / 1 0.0%	0 / 1 0.0%	0 / 1 0.0%	0 / 1 0.0%	0 / 2 0.0%	0 / 3 0.0%	0 / 1 0.0%	0 / 1 0.0%	0 / 3 0.0%	0 / 1 0.0%	0 / 4 0.0%	0 / 2 0.0%	0 / 1 0.0%	0 / 2 0.0%	0 / 1 0.0%	1 / 3 33.3%	0 / 1 0.0%	0 / 3 0.0%	0 / 5 0.0%	0 / 2 0.0%	0 / 1 0.0%	0 / 5 0.0%	0 / 0 0.0%	0 / 0 0.0%	0 / 0 0.0%	0 / 0 0.0%	26 / 45 57.8%E	18 / 45 40.0%	1 / 45 2.2%AB				
3	"Treponema pallidum subsp. pertenue" Smibert 1984	3 / 135 2.2%	0 / 3 0.0%	0 / 3 0.0%	0 / 3 0.0%	0 / 3 0.0%	0 / 6 0.0%	0 / 9 0.0%	0 / 3 0.0%	0 / 3 0.0%	0 / 9 0.0%	0 / 3 0.0%	0 / 12 0.0%	0 / 6 0.0%	0 / 3 0.0%	0 / 6 0.0%	0 / 3 0.0%	3 / 9 33.3%	0 / 3 0.0%	0 / 9 0.0%	0 / 15 0.0%	0 / 6 0.0%	0 / 3 0.0%	0 / 15 0.0%	0 / 0 0.0%	0 / 0 0.0%	0 / 0 0.0%	0 / 0 0.0%	78 / 135 57.8%E	54 / 135 40.0%	3 / 135 2.2%AB				
1	Treponema pallidum subsp. pertenue SamoaD	1 / 45 2.2%	0 / 1 0.0%	0 / 1 0.0%	0 / 1 0.0%	0 / 1 0.0%	0 / 2 0.0%	0 / 3 0.0%	0 / 1 0.0%	0 / 1 0.0%	0 / 3 0.0%	0 / 1 0.0%	0 / 4 0.0%	0 / 2 0.0%	0 / 1 0.0%	0 / 2 0.0%	0 / 1 0.0%	1 / 3 33.3%	0 / 1 0.0%	0 / 3 0.0%	0 / 5 0.0%	0 / 2 0.0%	0 / 1 0.0%	0 / 5 0.0%	0 / 0 0.0%	0 / 0 0.0%	0 / 0 0.0%	0 / 0 0.0%	26 / 45 57.8%E	18 / 45 40.0%	1 / 45 2.2%AB				
1	Treponema pallidum subsp. pertenue CDC2	1 / 45 2.2%	0 / 1 0.0%	0 / 1 0.0%	0 / 1 0.0%	0 / 1 0.0%	0 / 2 0.0%	0 / 3 0.0%	0 / 1 0.0%	0 / 1 0.0%	0 / 3 0.0%	0 / 1 0.0%	0 / 4 0.0%	0 / 2 0.0%	0 / 1 0.0%	0 / 2 0.0%	0 / 1 0.0%	1 / 3 33.3%	0 / 1 0.0%	0 / 3 0.0%	0 / 5 0.0%	0 / 2 0.0%	0 / 1 0.0%	0 / 5 0.0%	0 / 0 0.0%	0 / 0 0.0%	0 / 0 0.0%	0 / 0 0.0%	26 / 45 57.8%E	18 / 45 40.0%	1 / 45 2.2%AB				
1	Treponema pallidum subsp. pertenue Gauthier	1 / 45 2.2%	0 / 1 0.0%	0 / 1 0.0%	0 / 1 0.0%	0 / 1 0.0%	0 / 2 0.0%	0 / 3 0.0%	0 / 1 0.0%	0 / 1 0.0%	0 / 3 0.0%	0 / 1 0.0%	0 / 4 0.0%	0 / 2 0.0%	0 / 1 0.0%	0 / 2 0.0%	0 / 1 0.0%	1 / 3 33.3%	0 / 1 0.0%	0 / 3 0.0%	0 / 5 0.0%	0 / 2 0.0%	0 / 1 0.0%	0 / 5 0.0%	0 / 0 0.0%	0 / 0 0.0%	0 / 0 0.0%	0 / 0 0.0%	26 / 45 57.8%E	18 / 45 40.0%	1 / 45 2.2%AB				
1	Treponema pallidum str. Fribourg-Blanc	1 / 45 2.2%	0 / 1 0.0%	0 / 1 0.0%	0 / 1 0.0%	0 / 1 0.0%	0 / 2 0.0%	0 / 3 0.0%	0 / 1 0.0%	0 / 1 0.0%	0 / 3 0.0%	0 / 1 0.0%	0 / 4 0.0%	0 / 2 0.0%	0 / 1 0.0%	0 / 2 0.0%	0 / 1 0.0%	1 / 3 33.3%	0 / 1 0.0%	0 / 3 0.0%	0 / 5 0.0%	0 / 2 0.0%	0 / 1 0.0%	0 / 5 0.0%	0 / 0 0.0%	0 / 0 0.0%	0 / 0 0.0%	0 / 0 0.0%	26 / 45 57.8%E	18 / 45 40.0%	1 / 45 2.2%AB				
1	ATCC 33096	0 / 48 0.0%	0 / 1 0.0%	0 / 2 0.0%	0 / 1 0.0%	0 / 2 0.0%	0 / 4 0.0%	0 / 3 0.0%	0 / 1 0.0%	0 / 1 0.0%	0 / 4 0.0%	0 / 1 0.0%	0 / 3 0.0%	0 / 2 0.0%	0 / 1 0.0%	0 / 2 0.0%	0 / 1 0.0%	0 / 3 0.0%	0 / 1 0.0%	0 / 3 0.0%	0 / 4 0.0%	0 / 2 0.0%	0 / 1 0.0%	0 / 5 0.0%	0 / 0 0.0%	0 / 0 0.0%	0 / 0 0.0%	0 / 0 0.0%	28 / 48 58.3%E	19 / 48 39.6%	1 / 48 2.1%CE				
1	Treponema succinifaciens 6091	0 / 48 0.0%	0 / 1 0.0%	0 / 2 0.0%	0 / 1 0.0%	0 / 2 0.0%	0 / 4 0.0%	0 / 3 0.0%	0 / 1 0.0%	0 / 1 0.0%	0 / 4 0.0%	0 / 1 0.0%	0 / 3 0.0%	0 / 2 0.0%	0 / 1 0.0%	0 / 2 0.0%	0 / 1 0.0%	0 / 3 0.0%	0 / 1 0.0%	0 / 3 0.0%	0 / 4 0.0%	0 / 2 0.0%	0 / 1 0.0%	0 / 5 0.0%	0 / 0 0.0%	0 / 0 0.0%	0 / 0 0.0%	0 / 0 0.0%	28 / 48 58.3%E	19 / 48 39.6%	1 / 48 2.1%CE				
1	"Spirochaeta cuniculi" Levaditi, Marie and Isaicu 1921	1 / 45 2.2%	0 / 1 0.0%	0 / 1 0.0%	0 / 1 0.0%	0 / 1 0.0%	0 / 2 0.0%	0 / 3 0.0%	0 / 1 0.0%	0 / 1 0.0%	0 / 3 0.0%	0 / 1 0.0%	0 / 4 0.0%	0 / 2 0.0%	0 / 1 0.0%	0 / 2 0.0%	0 / 1 0.0%	1 / 3 33.3%	0 / 1 0.0%	0 / 3 0.0%	0 / 5 0.0%	0 / 2 0.0%	0 / 1 0.0%	0 / 5 0.0%	0 / 0 0.0%	0 / 0 0.0%	0 / 0 0.0%	0 / 0 0.0%	26 / 45 57.8%E	18 / 45 40.0%	1 / 45 2.2%AB				
1	Treponema paraluiscuniculi Cuniculi A	1 / 45 2.2%	0 / 1 0.0%	0 / 1 0.0%	0 / 1 0.0%	0 / 1 0.0%	0 / 2 0.0%	0 / 3 0.0%	0 / 1 0.0%	0 / 1 0.0%	0 / 3 0.0%	0 / 1 0.0%	0 / 4 0.0%	0 / 2 0.0%	0 / 1 0.0%	0 / 2 0.0%	0 / 1 0.0%	1 / 3 33.3%	0 / 1 0.0%	0 / 3 0.0%	0 / 5 0.0%	0 / 2 0.0%	0 / 1 0.0%	0 / 5 0.0%	0 / 0 0.0%	0 / 0 0.0%	0 / 0 0.0%	0 / 0 0.0%	26 / 45 57.8%E	18 / 45 40.0%	1 / 45 2.2%AB				
1	CIP 105900	0 / 47 0.0%	0 / 1 0.0%	0 / 1 0.0%	0 / 1 0.0%	0 / 2 0.0%	0 / 2 0.0%	0 / 3 0.0%	0 / 1 0.0%	0 / 1 0.0%	0 / 4 0.0%	0 / 1 0.0%	0 / 4 0.0%	0 / 2 0.0%	0 / 1 0.0%	0 / 2 0.0%	0 / 1 0.0%	0 / 3 0.0%	0 / 1 0.0%	0 / 3 0.0%	0 / 5 0.0%	0 / 2 0.0%	0 / 1 0.0%	0 / 5 0.0%	0 / 0 0.0%	0 / 0 0.0%	0 / 0 0.0%	0 / 0 0.0%	28 / 47 59.6%E	17 / 47 36.2%	1 / 47 2.1%C	1 / 47 2.1%CE			
1	Treponema brennaborense DSM 12168	0 / 47 0.0%	0 / 1 0.0%	0 / 1 0.0%	0 / 1 0.0%	0 / 2 0.0%	0 / 2 0.0%	0 / 3 0.0%	0 / 1 0.0%	0 / 1 0.0%	0 / 4 0.0%	0 / 1 0.0%	0 / 4 0.0%	0 / 2 0.0%	0 / 1 0.0%	0 / 2 0.0%	0 / 1 0.0%	0 / 3 0.0%	0 / 1 0.0%	0 / 3 0.0%	0 / 5 0.0%	0 / 2 0.0%	0 / 1 0.0%	0 / 5 0.0%	0 / 0 0.0%	0 / 0 0.0%	0 / 0 0.0%	0 / 0 0.0%	28 / 47 59.6%E	17 / 47 36.2%	1 / 47 2.1%CE	1 / 47 2.1%C			
1	ATCC BAA-887	1 / 50 2.0%	0 / 1 0.0%	0 / 1 0.0%	0 / 1 0.0%	0 / 2 0.0%	0 / 4 0.0%	0 / 3 0.0%	0 / 1 0.0%	0 / 1 0.0%	0 / 3 0.0%	0 / 1 0.0%	0 / 4 0.0%	0 / 2 0.0%	0 / 1 0.0%	0 / 2 0.0%	0 / 1 0.0%	0 / 3 0.0%	0 / 1 0.0%	0 / 3 0.0%	0 / 5 0.0%	0 / 2 0.0%	0 / 1 0.0%	0 / 6 0.0%	1 / 1 100.0%	0 / 0 0.0%	0 / 0 0.0%	0 / 0 0.0%	30 / 50 60.0%E	19 / 50 38.0%	1 / 50 2.0%ABE				
1	Treponema primitia ZAS-2	1 / 50 2.0%	0 / 1 0.0%	0 / 1 0.0%	0 / 1 0.0%	0 / 2 0.0%	0 / 4 0.0%	0 / 3 0.0%	0 / 1 0.0%	0 / 1 0.0%	0 / 3 0.0%	0 / 1 0.0%	0 / 4 0.0%	0 / 2 0.0%	0 / 1 0.0%	0 / 2 0.0%	0 / 1 0.0%	0 / 3 0.0%	0 / 1 0.0%	0 / 3 0.0%	0 / 5 0.0%	0 / 2 0.0%	0 / 1 0.0%	0 / 6 0.0%	1 / 1 100.0%	0 / 0 0.0%	0 / 0 0.0%	0 / 0 0.0%	30 / 50 60.0%E	19 / 50 38.0%	1 / 50 2.0%ABE				
1	ATCC BAA-888	0 / 46 0.0%	0 / 1 0.0%	0 / 1 0.0%	0 / 1 0.0%	0 / 0 0.0%	0 / 3 0.0%	0 / 3 0.0%	0 / 1 0.0%	0 / 1 0.0%	0 / 4 0.0%	0 / 1 0.0%	0 / 4 0.0%	0 / 2 0.0%	0 / 1 0.0%	0 / 2 0.0%	0 / 1 0.0%	0 / 3 0.0%	0 / 1 0.0%	0 / 3 0.0%	0 / 5 0.0%	0 / 2 0.0%	0 / 1 0.0%	0 / 5 0.0%	0 / 0 0.0%	0 / 0 0.0%	0 / 0 0.0%	0 / 0 0.0%	26 / 46 56.5%	20 / 46 43.5%E					
1	Treponema azotonutricium ZAS-9	0 / 46 0.0%	0 / 1 0.0%	0 / 1 0.0%	0 / 1 0.0%	0 / 0 0.0%	0 / 3 0.0%	0 / 3 0.0%	0 / 1 0.0%	0 / 1 0.0%	0 / 4 0.0%	0 / 1 0.0%	0 / 4 0.0%	0 / 2 0.0%	0 / 1 0.0%	0 / 2 0.0%	0 / 1 0.0%	0 / 3 0.0%	0 / 1 0.0%	0 / 3 0.0%	0 / 5 0.0%	0 / 2 0.0%	0 / 1 0.0%	0 / 5 0.0%	0 / 0 0.0%	0 / 0 0.0%	0 / 0 0.0%	0 / 0 0.0%	26 / 46 56.5%	20 / 46 43.5%E					
1	"Treponema caldaria" (Pohlschroeder et al. 19950) Abt et al. 2013	2 / 48 4.2%	0 / 1 0.0%	0 / 1 0.0%	0 / 1 0.0%	0 / 1 0.0%	1 / 4 25.0%	0 / 3 0.0%	0 / 1 0.0%	0 / 1 0.0%	0 / 4 0.0%	0 / 1 0.0%	0 / 4 0.0%	0 / 2 0.0%	0 / 1 0.0%	0 / 2 0.0%	0 / 1 0.0%	0 / 3 0.0%	0 / 1 0.0%	0 / 3 0.0%	0 / 5 0.0%	1 / 2 50.0%	0 / 1 0.0%	0 / 5 0.0%	0 / 0 0.0%	0 / 0 0.0%	0 / 0 0.0%	0 / 0 0.0%	27 / 48 56.2%E	19 / 48 39.6%	2 / 48 4.2%ABE				
1	Spirochaeta caldaria DSM 7334	2 / 48 4.2%	0 / 1 0.0%	0 / 1 0.0%	0 / 1 0.0%	0 / 1 0.0%	1 / 4 25.0%	0 / 3 0.0%	0 / 1 0.0%	0 / 1 0.0%	0 / 4 0.0%	0 / 1 0.0%	0 / 4 0.0%	0 / 2 0.0%	0 / 1 0.0%	0 / 2 0.0%	0 / 1 0.0%	0 / 3 0.0%	0 / 1 0.0%	0 / 3 0.0%	0 / 5 0.0%	1 / 2 50.0%	0 / 1 0.0%	0 / 5 0.0%	0 / 0 0.0%	0 / 0 0.0%	0 / 0 0.0%	0 / 0 0.0%	27 / 48 56.2%E	19 / 48 39.6%	2 / 48 4.2%ABE				
3	"Sphaerochaeta" Ritalahti et al. 2012 emend. Abt et al. 2012	1 / 144 0.7%	0 / 3 0.0%	0 / 5 0.0%	0 / 3 0.0%	0 / 3 0.0%	0 / 9 0.0%	0 / 9 0.0%	0 / 3 0.0%	0 / 3 0.0%	0 / 13 0.0%	0 / 3 0.0%	0 / 12 0.0%	0 / 6 0.0%	0 / 3 0.0%	0 / 6 0.0%	0 / 3 0.0%	0 / 9 0.0%	0 / 3 0.0%	0 / 9 0.0%	1 / 15 6.7%	0 / 6 0.0%	0 / 3 0.0%	0 / 15 0.0%	0 / 0 0.0%	0 / 0 0.0%	0 / 0 0.0%	0 / 0 0.0%	76 / 144 52.8%	66 / 144 45.8%E	1 / 144 0.7%D	1 / 144 0.7%ABE			
1	"Spirochaeta coccoides" (Droge et al. 2006) Abt et al. 2012	0 / 47 0.0%	0 / 1 0.0%	0 / 1 0.0%	0 / 1 0.0%	0 / 1 0.0%	0 / 3 0.0%	0 / 3 0.0%	0 / 1 0.0%	0 / 1 0.0%	0 / 4 0.0%	0 / 1 0.0%	0 / 4 0.0%	0 / 2 0.0%	0 / 1 0.0%	0 / 2 0.0%	0 / 1 0.0%	0 / 3 0.0%	0 / 1 0.0%	0 / 3 0.0%	0 / 5 0.0%	0 / 2 0.0%	0 / 1 0.0%	0 / 5 0.0%	0 / 0 0.0%	0 / 0 0.0%	0 / 0 0.0%	0 / 0 0.0%	24 / 47 51.1%	23 / 47 48.9%E					
1	Sphaerochaeta coccoides DSM 17374	0 / 47 0.0%	0 / 1 0.0%	0 / 1 0.0%	0 / 1 0.0%	0 / 1 0.0%	0 / 3 0.0%	0 / 3 0.0%	0 / 1 0.0%	0 / 1 0.0%	0 / 4 0.0%	0 / 1 0.0%	0 / 4 0.0%	0 / 2 0.0%	0 / 1 0.0%	0 / 2 0.0%	0 / 1 0.0%	0 / 3 0.0%	0 / 1 0.0%	0 / 3 0.0%	0 / 5 0.0%	0 / 2 0.0%	0 / 1 0.0%	0 / 5 0.0%	0 / 0 0.0%	0 / 0 0.0%	0 / 0 0.0%	0 / 0 0.0%	24 / 47 51.1%	23 / 47 48.9%E					
1	'Sphaerochaeta globosa'	0 / 48 0.0%	0 / 1 0.0%	0 / 2 0.0%	0 / 1 0.0%	0 / 1 0.0%	0 / 3 0.0%	0 / 3 0.0%	0 / 1 0.0%	0 / 1 0.0%	0 / 4 0.0%	0 / 1 0.0%	0 / 4 0.0%	0 / 2 0.0%	0 / 1 0.0%	0 / 2 0.0%	0 / 1 0.0%	0 / 3 0.0%	0 / 1 0.0%	0 / 3 0.0%	0 / 5 0.0%	0 / 2 0.0%	0 / 1 0.0%	0 / 5 0.0%	0 / 0 0.0%	0 / 0 0.0%	0 / 0 0.0%	0 / 0 0.0%	24 / 48 50.0%E	24 / 48 50.0%					
1	Sphaerochaeta globosa str. Buddy	0 / 48 0.0%	0 / 1 0.0%	0 / 2 0.0%	0 / 1 0.0%	0 / 1 0.0%	0 / 3 0.0%	0 / 3 0.0%	0 / 1 0.0%	0 / 1 0.0%	0 / 4 0.0%	0 / 1 0.0%	0 / 4 0.0%	0 / 2 0.0%	0 / 1 0.0%	0 / 2 0.0%	0 / 1 0.0%	0 / 3 0.0%	0 / 1 0.0%	0 / 3 0.0%	0 / 5 0.0%	0 / 2 0.0%	0 / 1 0.0%	0 / 5 0.0%	0 / 0 0.0%	0 / 0 0.0%	0 / 0 0.0%	0 / 0 0.0%	24 / 48 50.0%	24 / 48 50.0%E					
1	ATCC BAA-1885	1 / 49 2.0%	0 / 1 0.0%	0 / 2 0.0%	0 / 1 0.0%	0 / 1 0.0%	0 / 3 0.0%	0 / 3 0.0%	0 / 1 0.0%	0 / 1 0.0%	0 / 5 0.0%	0 / 1 0.0%	0 / 4 0.0%	0 / 2 0.0%	0 / 1 0.0%	0 / 2 0.0%	0 / 1 0.0%	0 / 3 0.0%	0 / 1 0.0%	0 / 3 0.0%	1 / 5 20.0%	0 / 2 0.0%	0 / 1 0.0%	0 / 5 0.0%	0 / 0 0.0%	0 / 0 0.0%	0 / 0 0.0%	0 / 0 0.0%	28 / 49 57.1%	19 / 49 38.8%E	1 / 49 2.0%D	1 / 49 2.0%ABE			
1	Sphaerochaeta pleomorpha str. Grapes	1 / 49 2.0%	0 / 1 0.0%	0 / 2 0.0%	0 / 1 0.0%	0 / 1 0.0%	0 / 3 0.0%	0 / 3 0.0%	0 / 1 0.0%	0 / 1 0.0%	0 / 5 0.0%	0 / 1 0.0%	0 / 4 0.0%	0 / 2 0.0%	0 / 1 0.0%	0 / 2 0.0%	0 / 1 0.0%	0 / 3 0.0%	0 / 1 0.0%	0 / 3 0.0%	1 / 5 20.0%	0 / 2 0.0%	0 / 1 0.0%	0 / 5 0.0%	0 / 0 0.0%	0 / 0 0.0%	0 / 0 0.0%	0 / 0 0.0%	28 / 49 57.1%	19 / 49 38.8%E	1 / 49 2.0%D	1 / 49 2.0%ABE			
8	Leptospiraceae	63 / 293 21.5%	0 / 8 0.0%	2 / 8 25.0%	5 / 8 62.5%	0 / 9 0.0%	0 / 16 0.0%	5 / 16 31.2%	5 / 8 62.5%	3 / 8 37.5%	0 / 17 0.0%	2 / 8 25.0%	2 / 24 8.3%	0 / 8 0.0%	0 / 8 0.0%	7 / 8 87.5%	0 / 8 0.0%	0 / 16 0.0%	2 / 8 25.0%	2 / 21 9.5%	14 / 32 43.8%	7 / 8 87.5%	0 / 8 0.0%	7 / 38 18.4%	0 / 0 0.0%	0 / 0 0.0%	0 / 0 0.0%	0 / 0 0.0%	141 / 293 48.1%	87 / 293 29.7%E	32 / 293 10.9%AB	17 / 293 5.8%ABE	10 / 293 3.4%B	4 / 293 1.4%BE	2 / 293 0.7%CE
7	Ancona	63 / 255 24.7%	0 / 7 0.0%	2 / 7 28.6%	5 / 7 71.4%	0 / 7 0.0%	0 / 14 0.0%	5 / 14 35.7%	5 / 7 71.4%	3 / 7 42.9%	0 / 14 0.0%	2 / 7 28.6%	2 / 21 9.5%	0 / 7 0.0%	0 / 7 0.0%	7 / 7 100.0%	0 / 7 0.0%	0 / 14 0.0%	2 / 7 28.6%	2 / 19 10.5%	14 / 28 50.0%	7 / 7 100.0%	0 / 7 0.0%	7 / 33 21.2%	0 / 0 0.0%	0 / 0 0.0%	0 / 0 0.0%	0 / 0 0.0%	117 / 255 45.9%	73 / 255 28.6%E	32 / 255 12.5%AB	17 / 255 6.7%ABE	10 / 255 3.9%B	4 / 255 1.6%BE	2 / 255 0.8%CE
2	"Spirochaeta biflexa" Wolbach and Binger 1914	18 / 70 25.7%	0 / 2 0.0%	2 / 2 100.0%	0 / 2 0.0%	0 / 2 0.0%	0 / 4 0.0%	0 / 4 0.0%	0 / 2 0.0%	0 / 2 0.0%	0 / 4 0.0%	2 / 2 100.0%	2 / 6 33.3%	0 / 2 0.0%	0 / 2 0.0%	2 / 2 100.0%	0 / 2 0.0%	0 / 4 0.0%	2 / 2 100.0%	2 / 4 50.0%	2 / 8 25.0%	2 / 2 100.0%	0 / 2 0.0%	2 / 8 25.0%	0 / 0 0.0%	0 / 0 0.0%	0 / 0 0.0%	0 / 0 0.0%	30 / 70 42.9%	20 / 70 28.6%E	10 / 70 14.3%B	4 / 70 5.7%BE	2 / 70 2.9%CE	2 / 70 2.9%ABE	2 / 70 2.9%AB
2	Leptospira biflexa serovar Patoc	18 / 70 25.7%	0 / 2 0.0%	2 / 2 100.0%	0 / 2 0.0%	0 / 2 0.0%	0 / 4 0.0%	0 / 4 0.0%	0 / 2 0.0%	0 / 2 0.0%	0 / 4 0.0%	2 / 2 100.0%	2 / 6 33.3%	0 / 2 0.0%	0 / 2 0.0%	2 / 2 100.0%	0 / 2 0.0%	0 / 4 0.0%	2 / 2 100.0%	2 / 4 50.0%	2 / 8 25.0%	2 / 2 100.0%	0 / 2 0.0%	2 / 8 25.0%	0 / 0 0.0%	0 / 0 0.0%	0 / 0 0.0%	0 / 0 0.0%	30 / 70 42.9%	20 / 70 28.6%E	10 / 70 14.3%B	4 / 70 5.7%BE	2 / 70 2.9%ABE	2 / 70 2.9%CE	2 / 70 2.9%AB
1	Leptospira biflexa serovar Patoc str. 'Patoc1 (Ames)'	9 / 35 25.7%	0 / 1 0.0%	1 / 1 100.0%	0 / 1 0.0%	0 / 1 0.0%	0 / 2 0.0%	0 / 2 0.0%	0 / 1 0.0%	0 / 1 0.0%	0 / 2 0.0%	1 / 1 100.0%	1 / 3 33.3%	0 / 1 0.0%	0 / 1 0.0%	1 / 1 100.0%	0 / 1 0.0%	0 / 2 0.0%	1 / 1 100.0%	1 / 2 50.0%	1 / 4 25.0%	1 / 1 100.0%	0 / 1 0.0%	1 / 4 25.0%	0 / 0 0.0%	0 / 0 0.0%	0 / 0 0.0%	0 / 0 0.0%	15 / 35 42.9%	10 / 35 28.6%E	5 / 35 14.3%B	2 / 35 5.7%BE	1 / 35 2.9%ABE	1 / 35 2.9%CE	1 / 35 2.9%AB
1	Leptospira biflexa serovar Patoc str. 'Patoc 1 (Paris)'	9 / 35 25.7%	0 / 1 0.0%	1 / 1 100.0%	0 / 1 0.0%	0 / 1 0.0%	0 / 2 0.0%	0 / 2 0.0%	0 / 1 0.0%	0 / 1 0.0%	0 / 2 0.0%	1 / 1 100.0%	1 / 3 33.3%	0 / 1 0.0%	0 / 1 0.0%	1 / 1 100.0%	0 / 1 0.0%	0 / 2 0.0%	1 / 1 100.0%	1 / 2 50.0%	1 / 4 25.0%	1 / 1 100.0%	0 / 1 0.0%	1 / 4 25.0%	0 / 0 0.0%	0 / 0 0.0%	0 / 0 0.0%	0 / 0 0.0%	15 / 35 42.9%	10 / 35 28.6%E	5 / 35 14.3%B	2 / 35 5.7%BE	1 / 35 2.9%AB	1 / 35 2.9%CE	1 / 35 2.9%ABE
3	"Leptospira icteroides" Noguchi 1919	27 / 111 24.3%	0 / 3 0.0%	0 / 3 0.0%	3 / 3 100.0%	0 / 3 0.0%	0 / 6 0.0%	3 / 6 50.0%	3 / 3 100.0%	3 / 3 100.0%	0 / 6 0.0%	0 / 3 0.0%	0 / 9 0.0%	0 / 3 0.0%	0 / 3 0.0%	3 / 3 100.0%	0 / 3 0.0%	0 / 6 0.0%	0 / 3 0.0%	0 / 9 0.0%	6 / 12 50.0%	3 / 3 100.0%	0 / 3 0.0%	3 / 15 20.0%	0 / 0 0.0%	0 / 0 0.0%	0 / 0 0.0%	0 / 0 0.0%	51 / 111 45.9%	33 / 111 29.7%E	18 / 111 16.2%AB	9 / 111 8.1%ABE			
1	Leptospira interrogans copenhageni	9 / 37 24.3%	0 / 1 0.0%	0 / 1 0.0%	1 / 1 100.0%	0 / 1 0.0%	0 / 2 0.0%	1 / 2 50.0%	1 / 1 100.0%	1 / 1 100.0%	0 / 2 0.0%	0 / 1 0.0%	0 / 3 0.0%	0 / 1 0.0%	0 / 1 0.0%	1 / 1 100.0%	0 / 1 0.0%	0 / 2 0.0%	0 / 1 0.0%	0 / 3 0.0%	2 / 4 50.0%	1 / 1 100.0%	0 / 1 0.0%	1 / 5 20.0%	0 / 0 0.0%	0 / 0 0.0%	0 / 0 0.0%	0 / 0 0.0%	18 / 37 48.6%	10 / 37 27.0%E	6 / 37 16.2%AB	3 / 37 8.1%ABE			
1	Leptospira interrogans serovar Copenhageni 'Fiocruz L1-130'	9 / 37 24.3%	0 / 1 0.0%	0 / 1 0.0%	1 / 1 100.0%	0 / 1 0.0%	0 / 2 0.0%	1 / 2 50.0%	1 / 1 100.0%	1 / 1 100.0%	0 / 2 0.0%	0 / 1 0.0%	0 / 3 0.0%	0 / 1 0.0%	0 / 1 0.0%	1 / 1 100.0%	0 / 1 0.0%	0 / 2 0.0%	0 / 1 0.0%	0 / 3 0.0%	2 / 4 50.0%	1 / 1 100.0%	0 / 1 0.0%	1 / 5 20.0%	0 / 0 0.0%	0 / 0 0.0%	0 / 0 0.0%	0 / 0 0.0%	18 / 37 48.6%	10 / 37 27.0%E	6 / 37 16.2%AB	3 / 37 8.1%ABE			
2	Leptospira interrogans serovar Lai	18 / 74 24.3%	0 / 2 0.0%	0 / 2 0.0%	2 / 2 100.0%	0 / 2 0.0%	0 / 4 0.0%	2 / 4 50.0%	2 / 2 100.0%	2 / 2 100.0%	0 / 4 0.0%	0 / 2 0.0%	0 / 6 0.0%	0 / 2 0.0%	0 / 2 0.0%	2 / 2 100.0%	0 / 2 0.0%	0 / 4 0.0%	0 / 2 0.0%	0 / 6 0.0%	4 / 8 50.0%	2 / 2 100.0%	0 / 2 0.0%	2 / 10 20.0%	0 / 0 0.0%	0 / 0 0.0%	0 / 0 0.0%	0 / 0 0.0%	33 / 74 44.6%	23 / 74 31.1%E	12 / 74 16.2%AB	6 / 74 8.1%ABE			
1	Leptospira interrogans serovar Lai str. 56601	9 / 37 24.3%	0 / 1 0.0%	0 / 1 0.0%	1 / 1 100.0%	0 / 1 0.0%	0 / 2 0.0%	1 / 2 50.0%	1 / 1 100.0%	1 / 1 100.0%	0 / 2 0.0%	0 / 1 0.0%	0 / 3 0.0%	0 / 1 0.0%	0 / 1 0.0%	1 / 1 100.0%	0 / 1 0.0%	0 / 2 0.0%	0 / 1 0.0%	0 / 3 0.0%	2 / 4 50.0%	1 / 1 100.0%	0 / 1 0.0%	1 / 5 20.0%	0 / 0 0.0%	0 / 0 0.0%	0 / 0 0.0%	0 / 0 0.0%	18 / 37 48.6%	10 / 37 27.0%E	6 / 37 16.2%AB	3 / 37 8.1%ABE			
1	Leptospira interrogans serovar Lai str. IPAV	9 / 37 24.3%	0 / 1 0.0%	0 / 1 0.0%	1 / 1 100.0%	0 / 1 0.0%	0 / 2 0.0%	1 / 2 50.0%	1 / 1 100.0%	1 / 1 100.0%	0 / 2 0.0%	0 / 1 0.0%	0 / 3 0.0%	0 / 1 0.0%	0 / 1 0.0%	1 / 1 100.0%	0 / 1 0.0%	0 / 2 0.0%	0 / 1 0.0%	0 / 3 0.0%	2 / 4 50.0%	1 / 1 100.0%	0 / 1 0.0%	1 / 5 20.0%	0 / 0 0.0%	0 / 0 0.0%	0 / 0 0.0%	0 / 0 0.0%	15 / 37 40.5%	13 / 37 35.1%E	6 / 37 16.2%AB	3 / 37 8.1%ABE			
2	ATCC 43292	18 / 74 24.3%	0 / 2 0.0%	0 / 2 0.0%	2 / 2 100.0%	0 / 2 0.0%	0 / 4 0.0%	2 / 4 50.0%	2 / 2 100.0%	0 / 2 0.0%	0 / 4 0.0%	0 / 2 0.0%	0 / 6 0.0%	0 / 2 0.0%	0 / 2 0.0%	2 / 2 100.0%	0 / 2 0.0%	0 / 4 0.0%	0 / 2 0.0%	0 / 6 0.0%	6 / 8 75.0%	2 / 2 100.0%	0 / 2 0.0%	2 / 10 20.0%	0 / 0 0.0%	0 / 0 0.0%	0 / 0 0.0%	0 / 0 0.0%	36 / 74 48.6%	20 / 74 27.0%E	12 / 74 16.2%AB	6 / 74 8.1%ABE			
2	Leptospira borgpetersenii serovar Hardjo-bovis	18 / 74 24.3%	0 / 2 0.0%	0 / 2 0.0%	2 / 2 100.0%	0 / 2 0.0%	0 / 4 0.0%	2 / 4 50.0%	2 / 2 100.0%	0 / 2 0.0%	0 / 4 0.0%	0 / 2 0.0%	0 / 6 0.0%	0 / 2 0.0%	0 / 2 0.0%	2 / 2 100.0%	0 / 2 0.0%	0 / 4 0.0%	0 / 2 0.0%	0 / 6 0.0%	6 / 8 75.0%	2 / 2 100.0%	0 / 2 0.0%	2 / 10 20.0%	0 / 0 0.0%	0 / 0 0.0%	0 / 0 0.0%	0 / 0 0.0%	36 / 74 48.6%	20 / 74 27.0%E	12 / 74 16.2%AB	6 / 74 8.1%ABE			
1	Leptospira borgpetersenii serovar Hardjo-bovis L550	9 / 37 24.3%	0 / 1 0.0%	0 / 1 0.0%	1 / 1 100.0%	0 / 1 0.0%	0 / 2 0.0%	1 / 2 50.0%	1 / 1 100.0%	0 / 1 0.0%	0 / 2 0.0%	0 / 1 0.0%	0 / 3 0.0%	0 / 1 0.0%	0 / 1 0.0%	1 / 1 100.0%	0 / 1 0.0%	0 / 2 0.0%	0 / 1 0.0%	0 / 3 0.0%	3 / 4 75.0%	1 / 1 100.0%	0 / 1 0.0%	1 / 5 20.0%	0 / 0 0.0%	0 / 0 0.0%	0 / 0 0.0%	0 / 0 0.0%	18 / 37 48.6%	10 / 37 27.0%E	6 / 37 16.2%AB	3 / 37 8.1%ABE			
1	Leptospira borgpetersenii serovar Hardjo-bovis JB197	9 / 37 24.3%	0 / 1 0.0%	0 / 1 0.0%	1 / 1 100.0%	0 / 1 0.0%	0 / 2 0.0%	1 / 2 50.0%	1 / 1 100.0%	0 / 1 0.0%	0 / 2 0.0%	0 / 1 0.0%	0 / 3 0.0%	0 / 1 0.0%	0 / 1 0.0%	1 / 1 100.0%	0 / 1 0.0%	0 / 2 0.0%	0 / 1 0.0%	0 / 3 0.0%	3 / 4 75.0%	1 / 1 100.0%	0 / 1 0.0%	1 / 5 20.0%	0 / 0 0.0%	0 / 0 0.0%	0 / 0 0.0%	0 / 0 0.0%	18 / 37 48.6%	10 / 37 27.0%E	6 / 37 16.2%AB	3 / 37 8.1%ABE			
1	"Turneria" Hookey et al. 1993	0 / 38 0.0%	0 / 1 0.0%	0 / 1 0.0%	0 / 1 0.0%	0 / 2 0.0%	0 / 2 0.0%	0 / 2 0.0%	0 / 1 0.0%	0 / 1 0.0%	0 / 3 0.0%	0 / 1 0.0%	0 / 3 0.0%	0 / 1 0.0%	0 / 1 0.0%	0 / 1 0.0%	0 / 1 0.0%	0 / 2 0.0%	0 / 1 0.0%	0 / 2 0.0%	0 / 4 0.0%	0 / 1 0.0%	0 / 1 0.0%	0 / 5 0.0%	0 / 0 0.0%	0 / 0 0.0%	0 / 0 0.0%	0 / 0 0.0%	24 / 38 63.2%	14 / 38 36.8%E					
1	"Turneria parva" (Hovind-Hougen et al. 1982) Hookey et al. 1993	0 / 38 0.0%	0 / 1 0.0%	0 / 1 0.0%	0 / 1 0.0%	0 / 2 0.0%	0 / 2 0.0%	0 / 2 0.0%	0 / 1 0.0%	0 / 1 0.0%	0 / 3 0.0%	0 / 1 0.0%	0 / 3 0.0%	0 / 1 0.0%	0 / 1 0.0%	0 / 1 0.0%	0 / 1 0.0%	0 / 2 0.0%	0 / 1 0.0%	0 / 2 0.0%	0 / 4 0.0%	0 / 1 0.0%	0 / 1 0.0%	0 / 5 0.0%	0 / 0 0.0%	0 / 0 0.0%	0 / 0 0.0%	0 / 0 0.0%	24 / 38 63.2%	14 / 38 36.8%E					
1	Turneriella parva DSM 21527	0 / 38 0.0%	0 / 1 0.0%	0 / 1 0.0%	0 / 1 0.0%	0 / 2 0.0%	0 / 2 0.0%	0 / 2 0.0%	0 / 1 0.0%	0 / 1 0.0%	0 / 3 0.0%	0 / 1 0.0%	0 / 3 0.0%	0 / 1 0.0%	0 / 1 0.0%	0 / 1 0.0%	0 / 1 0.0%	0 / 2 0.0%	0 / 1 0.0%	0 / 2 0.0%	0 / 4 0.0%	0 / 1 0.0%	0 / 1 0.0%	0 / 5 0.0%	0 / 0 0.0%	0 / 0 0.0%	0 / 0 0.0%	0 / 0 0.0%	24 / 38 63.2%	14 / 38 36.8%E					
7	"Brachyspiraceae" Paster and Dewhirst 2000	6 / 232 2.6%	0 / 7 0.0%	0 / 7 0.0%	0 / 7 0.0%	0 / 7 0.0%	6 / 14 42.9%	0 / 7 0.0%	0 / 7 0.0%	0 / 7 0.0%	0 / 10 0.0%	0 / 7 0.0%	0 / 21 0.0%	0 / 7 0.0%	0 / 7 0.0%	0 / 7 0.0%	0 / 7 0.0%	0 / 7 0.0%	0 / 7 0.0%	0 / 19 0.0%	0 / 28 0.0%	0 / 7 0.0%	0 / 7 0.0%	0 / 28 0.0%	0 / 0 0.0%	0 / 0 0.0%	0 / 0 0.0%	0 / 0 0.0%	113 / 232 48.7%	113 / 232 48.7%E	6 / 232 2.6%ABE				
7	Brachyspira	6 / 232 2.6%	0 / 7 0.0%	0 / 7 0.0%	0 / 7 0.0%	0 / 7 0.0%	6 / 14 42.9%	0 / 7 0.0%	0 / 7 0.0%	0 / 7 0.0%	0 / 10 0.0%	0 / 7 0.0%	0 / 21 0.0%	0 / 7 0.0%	0 / 7 0.0%	0 / 7 0.0%	0 / 7 0.0%	0 / 7 0.0%	0 / 7 0.0%	0 / 19 0.0%	0 / 28 0.0%	0 / 7 0.0%	0 / 7 0.0%	0 / 28 0.0%	0 / 0 0.0%	0 / 0 0.0%	0 / 0 0.0%	0 / 0 0.0%	113 / 232 48.7%	113 / 232 48.7%E	6 / 232 2.6%ABE				
1	ATCC 27164	1 / 33 3.0%	0 / 1 0.0%	0 / 1 0.0%	0 / 1 0.0%	0 / 1 0.0%	1 / 2 50.0%	0 / 1 0.0%	0 / 1 0.0%	0 / 1 0.0%	0 / 2 0.0%	0 / 1 0.0%	0 / 3 0.0%	0 / 1 0.0%	0 / 1 0.0%	0 / 1 0.0%	0 / 1 0.0%	0 / 1 0.0%	0 / 1 0.0%	0 / 2 0.0%	0 / 4 0.0%	0 / 1 0.0%	0 / 1 0.0%	0 / 4 0.0%	0 / 0 0.0%	0 / 0 0.0%	0 / 0 0.0%	0 / 0 0.0%	16 / 33 48.5%	16 / 33 48.5%E	1 / 33 3.0%ABE				
1	Brachyspira hyodysenteriae WA1	1 / 33 3.0%	0 / 1 0.0%	0 / 1 0.0%	0 / 1 0.0%	0 / 1 0.0%	1 / 2 50.0%	0 / 1 0.0%	0 / 1 0.0%	0 / 1 0.0%	0 / 2 0.0%	0 / 1 0.0%	0 / 3 0.0%	0 / 1 0.0%	0 / 1 0.0%	0 / 1 0.0%	0 / 1 0.0%	0 / 1 0.0%	0 / 1 0.0%	0 / 2 0.0%	0 / 4 0.0%	0 / 1 0.0%	0 / 1 0.0%	0 / 4 0.0%	0 / 0 0.0%	0 / 0 0.0%	0 / 0 0.0%	0 / 0 0.0%	16 / 33 48.5%	16 / 33 48.5%E	1 / 33 3.0%ABE				
4	"Anguillina coli" Lee et al. 1993	4 / 132 3.0%	0 / 4 0.0%	0 / 4 0.0%	0 / 4 0.0%	0 / 4 0.0%	4 / 8 50.0%	0 / 4 0.0%	0 / 4 0.0%	0 / 4 0.0%	0 / 4 0.0%	0 / 4 0.0%	0 / 12 0.0%	0 / 4 0.0%	0 / 4 0.0%	0 / 4 0.0%	0 / 4 0.0%	0 / 4 0.0%	0 / 4 0.0%	0 / 12 0.0%	0 / 16 0.0%	0 / 4 0.0%	0 / 4 0.0%	0 / 16 0.0%	0 / 0 0.0%	0 / 0 0.0%	0 / 0 0.0%	0 / 0 0.0%	64 / 132 48.5%	64 / 132 48.5%E	4 / 132 3.0%ABE				
1	Brachyspira pilosicoli 95/1000	1 / 33 3.0%	0 / 1 0.0%	0 / 1 0.0%	0 / 1 0.0%	0 / 1 0.0%	1 / 2 50.0%	0 / 1 0.0%	0 / 1 0.0%	0 / 1 0.0%	0 / 1 0.0%	0 / 1 0.0%	0 / 3 0.0%	0 / 1 0.0%	0 / 1 0.0%	0 / 1 0.0%	0 / 1 0.0%	0 / 1 0.0%	0 / 1 0.0%	0 / 3 0.0%	0 / 4 0.0%	0 / 1 0.0%	0 / 1 0.0%	0 / 4 0.0%	0 / 0 0.0%	0 / 0 0.0%	0 / 0 0.0%	0 / 0 0.0%	16 / 33 48.5%	16 / 33 48.5%E	1 / 33 3.0%ABE				
1	Brachyspira pilosicoli ATCC 51139	1 / 33 3.0%	0 / 1 0.0%	0 / 1 0.0%	0 / 1 0.0%	0 / 1 0.0%	1 / 2 50.0%	0 / 1 0.0%	0 / 1 0.0%	0 / 1 0.0%	0 / 1 0.0%	0 / 1 0.0%	0 / 3 0.0%	0 / 1 0.0%	0 / 1 0.0%	0 / 1 0.0%	0 / 1 0.0%	0 / 1 0.0%	0 / 1 0.0%	0 / 3 0.0%	0 / 4 0.0%	0 / 1 0.0%	0 / 1 0.0%	0 / 4 0.0%	0 / 0 0.0%	0 / 0 0.0%	0 / 0 0.0%	0 / 0 0.0%	16 / 33 48.5%	16 / 33 48.5%E	1 / 33 3.0%ABE				
1	Brachyspira pilosicoli B2904	1 / 33 3.0%	0 / 1 0.0%	0 / 1 0.0%	0 / 1 0.0%	0 / 1 0.0%	1 / 2 50.0%	0 / 1 0.0%	0 / 1 0.0%	0 / 1 0.0%	0 / 1 0.0%	0 / 1 0.0%	0 / 3 0.0%	0 / 1 0.0%	0 / 1 0.0%	0 / 1 0.0%	0 / 1 0.0%	0 / 1 0.0%	0 / 1 0.0%	0 / 3 0.0%	0 / 4 0.0%	0 / 1 0.0%	0 / 1 0.0%	0 / 4 0.0%	0 / 0 0.0%	0 / 0 0.0%	0 / 0 0.0%	0 / 0 0.0%	16 / 33 48.5%	16 / 33 48.5%E	1 / 33 3.0%ABE				
1	Brachyspira pilosicoli WesB	1 / 33 3.0%	0 / 1 0.0%	0 / 1 0.0%	0 / 1 0.0%	0 / 1 0.0%	1 / 2 50.0%	0 / 1 0.0%	0 / 1 0.0%	0 / 1 0.0%	0 / 1 0.0%	0 / 1 0.0%	0 / 3 0.0%	0 / 1 0.0%	0 / 1 0.0%	0 / 1 0.0%	0 / 1 0.0%	0 / 1 0.0%	0 / 1 0.0%	0 / 3 0.0%	0 / 4 0.0%	0 / 1 0.0%	0 / 1 0.0%	0 / 4 0.0%	0 / 0 0.0%	0 / 0 0.0%	0 / 0 0.0%	0 / 0 0.0%	16 / 33 48.5%E	16 / 33 48.5%	1 / 33 3.0%ABE				
1	ATCC 51140	1 / 33 3.0%	0 / 1 0.0%	0 / 1 0.0%	0 / 1 0.0%	0 / 1 0.0%	1 / 2 50.0%	0 / 1 0.0%	0 / 1 0.0%	0 / 1 0.0%	0 / 2 0.0%	0 / 1 0.0%	0 / 3 0.0%	0 / 1 0.0%	0 / 1 0.0%	0 / 1 0.0%	0 / 1 0.0%	0 / 1 0.0%	0 / 1 0.0%	0 / 2 0.0%	0 / 4 0.0%	0 / 1 0.0%	0 / 1 0.0%	0 / 4 0.0%	0 / 0 0.0%	0 / 0 0.0%	0 / 0 0.0%	0 / 0 0.0%	16 / 33 48.5%	16 / 33 48.5%E	1 / 33 3.0%ABE				
1	Brachyspira intermedia ATCC 51140	1 / 33 3.0%	0 / 1 0.0%	0 / 1 0.0%	0 / 1 0.0%	0 / 1 0.0%	1 / 2 50.0%	0 / 1 0.0%	0 / 1 0.0%	0 / 1 0.0%	0 / 2 0.0%	0 / 1 0.0%	0 / 3 0.0%	0 / 1 0.0%	0 / 1 0.0%	0 / 1 0.0%	0 / 1 0.0%	0 / 1 0.0%	0 / 1 0.0%	0 / 2 0.0%	0 / 4 0.0%	0 / 1 0.0%	0 / 1 0.0%	0 / 4 0.0%	0 / 0 0.0%	0 / 0 0.0%	0 / 0 0.0%	0 / 0 0.0%	16 / 33 48.5%E	16 / 33 48.5%	1 / 33 3.0%ABE				
1	ATCC 51284	0 / 34 0.0%	0 / 1 0.0%	0 / 1 0.0%	0 / 1 0.0%	0 / 1 0.0%	0 / 2 0.0%	0 / 1 0.0%	0 / 1 0.0%	0 / 1 0.0%	0 / 2 0.0%	0 / 1 0.0%	0 / 3 0.0%	0 / 1 0.0%	0 / 1 0.0%	0 / 1 0.0%	0 / 1 0.0%	0 / 1 0.0%	0 / 1 0.0%	0 / 3 0.0%	0 / 4 0.0%	0 / 1 0.0%	0 / 1 0.0%	0 / 4 0.0%	0 / 0 0.0%	0 / 0 0.0%	0 / 0 0.0%	0 / 0 0.0%	17 / 34 50.0%	17 / 34 50.0%E					

1	Brachyspira murdochii 56-150	0.0%	0.0%	0.0%	0.0%	0.0%	0.0%	0.0%	0.0%	0.0%	0.0%	0.0%	0.0%	0.0%	0.0%	0.0%	0.0%	0.0%	0.0%	0.0%	0.0%	0.0%	0.0%	0.0%	0.0%	0.0%	0.0%	0.0%	50.0%E	50.0%				
4	"Aminanaerobia" Hugenholtz	191 / 191 100.0%	5 / 5 100.0%	4 / 4 100.0%	4 / 4 100.0%	4 / 4 100.0%	12 / 12 100.0%	12 / 12 100.0%	4 / 4 100.0%	4 / 4 100.0%	12 / 12 100.0%	4 / 4 100.0%	16 / 16 100.0%	10 / 10 100.0%	4 / 4 100.0%	8 / 8 100.0%	4 / 4 100.0%	12 / 12 100.0%	4 / 4 100.0%	12 / 12 100.0%	20 / 20 100.0%	9 / 9 100.0%	4 / 4 100.0%	20 / 20 100.0%	3 / 3 100.0%	0 / 0 0.0%	0 / 0 0.0%	0 / 0 0.0%	127 / 191 66.5%AB	64 / 191 33.5%ABE				
4	Synergistia	191 / 191 100.0%	5 / 5 100.0%	4 / 4 100.0%	4 / 4 100.0%	4 / 4 100.0%	12 / 12 100.0%	12 / 12 100.0%	4 / 4 100.0%	4 / 4 100.0%	12 / 12 100.0%	4 / 4 100.0%	16 / 16 100.0%	10 / 10 100.0%	4 / 4 100.0%	8 / 8 100.0%	4 / 4 100.0%	12 / 12 100.0%	4 / 4 100.0%	12 / 12 100.0%	20 / 20 100.0%	9 / 9 100.0%	4 / 4 100.0%	20 / 20 100.0%	3 / 3 100.0%	0 / 0 0.0%	0 / 0 0.0%	0 / 0 0.0%	127 / 191 66.5%AB	64 / 191 33.5%ABE				
4	Synergistales	191 / 191 100.0%	5 / 5 100.0%	4 / 4 100.0%	4 / 4 100.0%	4 / 4 100.0%	12 / 12 100.0%	12 / 12 100.0%	4 / 4 100.0%	4 / 4 100.0%	12 / 12 100.0%	4 / 4 100.0%	16 / 16 100.0%	10 / 10 100.0%	4 / 4 100.0%	8 / 8 100.0%	4 / 4 100.0%	12 / 12 100.0%	4 / 4 100.0%	12 / 12 100.0%	20 / 20 100.0%	9 / 9 100.0%	4 / 4 100.0%	20 / 20 100.0%	3 / 3 100.0%	0 / 0 0.0%	0 / 0 0.0%	0 / 0 0.0%	127 / 191 66.5%AB	64 / 191 33.5%ABE				
4	Clostridiales Family XV. Incertae Sedis	191 / 191 100.0%	5 / 5 100.0%	4 / 4 100.0%	4 / 4 100.0%	4 / 4 100.0%	12 / 12 100.0%	12 / 12 100.0%	4 / 4 100.0%	4 / 4 100.0%	12 / 12 100.0%	4 / 4 100.0%	16 / 16 100.0%	10 / 10 100.0%	4 / 4 100.0%	8 / 8 100.0%	4 / 4 100.0%	12 / 12 100.0%	4 / 4 100.0%	12 / 12 100.0%	20 / 20 100.0%	9 / 9 100.0%	4 / 4 100.0%	20 / 20 100.0%	3 / 3 100.0%	0 / 0 0.0%	0 / 0 0.0%	0 / 0 0.0%	127 / 191 66.5%AB	64 / 191 33.5%ABE				
1	Anaerobaculum	47 / 47 100.0%	1 / 1 100.0%	1 / 1 100.0%	1 / 1 100.0%	1 / 1 100.0%	3 / 3 100.0%	3 / 3 100.0%	1 / 1 100.0%	1 / 1 100.0%	3 / 3 100.0%	1 / 1 100.0%	4 / 4 100.0%	2 / 2 100.0%	1 / 1 100.0%	2 / 2 100.0%	1 / 1 100.0%	3 / 3 100.0%	1 / 1 100.0%	3 / 3 100.0%	5 / 5 100.0%	2 / 2 100.0%	1 / 1 100.0%	5 / 5 100.0%	1 / 1 100.0%	0 / 0 0.0%	0 / 0 0.0%	0 / 0 0.0%	32 / 47 68.1%AB	15 / 47 31.9%ABE				
1	ATCC BAA-54	47 / 47 100.0%	1 / 1 100.0%	1 / 1 100.0%	1 / 1 100.0%	1 / 1 100.0%	3 / 3 100.0%	3 / 3 100.0%	1 / 1 100.0%	1 / 1 100.0%	3 / 3 100.0%	1 / 1 100.0%	4 / 4 100.0%	2 / 2 100.0%	1 / 1 100.0%	2 / 2 100.0%	1 / 1 100.0%	3 / 3 100.0%	1 / 1 100.0%	3 / 3 100.0%	5 / 5 100.0%	2 / 2 100.0%	1 / 1 100.0%	5 / 5 100.0%	1 / 1 100.0%	0 / 0 0.0%	0 / 0 0.0%	0 / 0 0.0%	32 / 47 68.1%AB	15 / 47 31.9%ABE				
1	Anaerobaculum mobile ATCC BAA-54	47 / 47 100.0%	1 / 1 100.0%	1 / 1 100.0%	1 / 1 100.0%	1 / 1 100.0%	3 / 3 100.0%	3 / 3 100.0%	1 / 1 100.0%	1 / 1 100.0%	3 / 3 100.0%	1 / 1 100.0%	4 / 4 100.0%	2 / 2 100.0%	1 / 1 100.0%	2 / 2 100.0%	1 / 1 100.0%	3 / 3 100.0%	1 / 1 100.0%	3 / 3 100.0%	5 / 5 100.0%	2 / 2 100.0%	1 / 1 100.0%	5 / 5 100.0%	1 / 1 100.0%	0 / 0 0.0%	0 / 0 0.0%	0 / 0 0.0%	32 / 47 68.1%AB	15 / 47 31.9%ABE				
1	Thermanaerovibrio	50 / 50 100.0%	2 / 2 100.0%	1 / 1 100.0%	1 / 1 100.0%	1 / 1 100.0%	3 / 3 100.0%	3 / 3 100.0%	1 / 1 100.0%	1 / 1 100.0%	3 / 3 100.0%	1 / 1 100.0%	4 / 4 100.0%	3 / 3 100.0%	1 / 1 100.0%	2 / 2 100.0%	1 / 1 100.0%	3 / 3 100.0%	1 / 1 100.0%	3 / 3 100.0%	5 / 5 100.0%	3 / 3 100.0%	1 / 1 100.0%	5 / 5 100.0%	1 / 1 100.0%	0 / 0 0.0%	0 / 0 0.0%	0 / 0 0.0%	31 / 50 62.0%AB	19 / 50 38.0%ABE				
1	ATCC 49978	50 / 50 100.0%	2 / 2 100.0%	1 / 1 100.0%	1 / 1 100.0%	1 / 1 100.0%	3 / 3 100.0%	3 / 3 100.0%	1 / 1 100.0%	1 / 1 100.0%	3 / 3 100.0%	1 / 1 100.0%	4 / 4 100.0%	3 / 3 100.0%	1 / 1 100.0%	2 / 2 100.0%	1 / 1 100.0%	3 / 3 100.0%	1 / 1 100.0%	3 / 3 100.0%	5 / 5 100.0%	3 / 3 100.0%	1 / 1 100.0%	5 / 5 100.0%	1 / 1 100.0%	0 / 0 0.0%	0 / 0 0.0%	0 / 0 0.0%	31 / 50 62.0%AB	19 / 50 38.0%ABE				
1	Thermanaerovibrio acidaminovorans DSM 6589	50 / 50 100.0%	2 / 2 100.0%	1 / 1 100.0%	1 / 1 100.0%	1 / 1 100.0%	3 / 3 100.0%	3 / 3 100.0%	1 / 1 100.0%	1 / 1 100.0%	3 / 3 100.0%	1 / 1 100.0%	4 / 4 100.0%	3 / 3 100.0%	1 / 1 100.0%	2 / 2 100.0%	1 / 1 100.0%	3 / 3 100.0%	1 / 1 100.0%	3 / 3 100.0%	5 / 5 100.0%	3 / 3 100.0%	1 / 1 100.0%	5 / 5 100.0%	1 / 1 100.0%	0 / 0 0.0%	0 / 0 0.0%	0 / 0 0.0%	31 / 50 62.0%AB	19 / 50 38.0%ABE				
1	Aminobacterium	46 / 46 100.0%	1 / 1 100.0%	1 / 1 100.0%	1 / 1 100.0%	1 / 1 100.0%	3 / 3 100.0%	3 / 3 100.0%	1 / 1 100.0%	1 / 1 100.0%	3 / 3 100.0%	1 / 1 100.0%	4 / 4 100.0%	2 / 2 100.0%	1 / 1 100.0%	2 / 2 100.0%	1 / 1 100.0%	3 / 3 100.0%	1 / 1 100.0%	3 / 3 100.0%	5 / 5 100.0%	2 / 2 100.0%	1 / 1 100.0%	5 / 5 100.0%	0 / 0 0.0%	0 / 0 0.0%	0 / 0 0.0%	0 / 0 0.0%	32 / 46 69.6%AB	14 / 46 30.4%ABE				
1	Aminobacterium colombiense	46 / 46 100.0%	1 / 1 100.0%	1 / 1 100.0%	1 / 1 100.0%	1 / 1 100.0%	3 / 3 100.0%	3 / 3 100.0%	1 / 1 100.0%	1 / 1 100.0%	3 / 3 100.0%	1 / 1 100.0%	4 / 4 100.0%	2 / 2 100.0%	1 / 1 100.0%	2 / 2 100.0%	1 / 1 100.0%	3 / 3 100.0%	1 / 1 100.0%	3 / 3 100.0%	5 / 5 100.0%	2 / 2 100.0%	1 / 1 100.0%	5 / 5 100.0%	0 / 0 0.0%	0 / 0 0.0%	0 / 0 0.0%	0 / 0 0.0%	32 / 46 69.6%AB	14 / 46 30.4%ABE				
1	Aminobacterium colombiense DSM 12261	46 / 46 100.0%	1 / 1 100.0%	1 / 1 100.0%	1 / 1 100.0%	1 / 1 100.0%	3 / 3 100.0%	3 / 3 100.0%	1 / 1 100.0%	1 / 1 100.0%	3 / 3 100.0%	1 / 1 100.0%	4 / 4 100.0%	2 / 2 100.0%	1 / 1 100.0%	2 / 2 100.0%	1 / 1 100.0%	3 / 3 100.0%	1 / 1 100.0%	3 / 3 100.0%	5 / 5 100.0%	2 / 2 100.0%	1 / 1 100.0%	5 / 5 100.0%	0 / 0 0.0%	0 / 0 0.0%	0 / 0 0.0%	0 / 0 0.0%	32 / 46 69.6%AB	14 / 46 30.4%ABE				
1	Thermovirga	48 / 48 100.0%	1 / 1 100.0%	1 / 1 100.0%	1 / 1 100.0%	1 / 1 100.0%	3 / 3 100.0%	3 / 3 100.0%	1 / 1 100.0%	1 / 1 100.0%	3 / 3 100.0%	1 / 1 100.0%	4 / 4 100.0%	3 / 3 100.0%	1 / 1 100.0%	2 / 2 100.0%	1 / 1 100.0%	3 / 3 100.0%	1 / 1 100.0%	3 / 3 100.0%	5 / 5 100.0%	2 / 2 100.0%	1 / 1 100.0%	5 / 5 100.0%	1 / 1 100.0%	0 / 0 0.0%	0 / 0 0.0%	0 / 0 0.0%	32 / 48 66.7%AB	16 / 48 33.3%ABE				
1	ATCC BAA-1197	48 / 48 100.0%	1 / 1 100.0%	1 / 1 100.0%	1 / 1 100.0%	1 / 1 100.0%	3 / 3 100.0%	3 / 3 100.0%	1 / 1 100.0%	1 / 1 100.0%	3 / 3 100.0%	1 / 1 100.0%	4 / 4 100.0%	3 / 3 100.0%	1 / 1 100.0%	2 / 2 100.0%	1 / 1 100.0%	3 / 3 100.0%	1 / 1 100.0%	3 / 3 100.0%	5 / 5 100.0%	2 / 2 100.0%	1 / 1 100.0%	5 / 5 100.0%	1 / 1 100.0%	0 / 0 0.0%	0 / 0 0.0%	0 / 0 0.0%	32 / 48 66.7%AB	16 / 48 33.3%ABE				
1	Thermovirga lienii DSM 17291	48 / 48 100.0%	1 / 1 100.0%	1 / 1 100.0%	1 / 1 100.0%	1 / 1 100.0%	3 / 3 100.0%	3 / 3 100.0%	1 / 1 100.0%	1 / 1 100.0%	3 / 3 100.0%	1 / 1 100.0%	4 / 4 100.0%	3 / 3 100.0%	1 / 1 100.0%	2 / 2 100.0%	1 / 1 100.0%	3 / 3 100.0%	1 / 1 100.0%	3 / 3 100.0%	5 / 5 100.0%	2 / 2 100.0%	1 / 1 100.0%	5 / 5 100.0%	1 / 1 100.0%	0 / 0 0.0%	0 / 0 0.0%	0 / 0 0.0%	32 / 48 66.7%AB	16 / 48 33.3%ABE				
76	Tenericutes	2469 / 2495 99.0%	78 / 79 98.7%	79 / 79 100.0%	78 / 78 100.0%	92 / 92 100.0%	127 / 128 99.2%	84 / 84 100.0%	80 / 80 100.0%	75 / 78 96.2%	62 / 81 76.5%	109 / 109 100.0%	213 / 213 100.0%	144 / 144 100.0%	78 / 79 98.7%	80 / 80 100.0%	78 / 78 100.0%	78 / 78 100.0%	78 / 78 100.0%	172 / 172 100.0%	225 / 225 100.0%	79 / 79 100.0%	70 / 70 100.0%	270 / 271 99.6%	40 / 40 100.0%	0 / 0 0.0%	0 / 0 0.0%	0 / 0 0.0%	1816 / 2495 72.8%AB	576 / 2495 23.1%ABE	58 / 2495 2.3%B	24 / 2495 1.0%	19 / 2495 0.8%BE	2 / 2495 0.1%E
76	"Paramycetes" Sabin 1941	2469 / 2495 99.0%	78 / 79 98.7%	79 / 79 100.0%	78 / 78 100.0%	92 / 92 100.0%	127 / 128 99.2%	84 / 84 100.0%	80 / 80 100.0%	75 / 78 96.2%	62 / 81 76.5%	109 / 109 100.0%	213 / 213 100.0%	144 / 144 100.0%	78 / 79 98.7%	80 / 80 100.0%	78 / 78 100.0%	78 / 78 100.0%	78 / 78 100.0%	172 / 172 100.0%	225 / 225 100.0%	79 / 79 100.0%	70 / 70 100.0%	270 / 271 99.6%	40 / 40 100.0%	0 / 0 0.0%	0 / 0 0.0%	0 / 0 0.0%	1816 / 2495 72.8%AB	576 / 2495 23.1%ABE	58 / 2495 2.3%B	24 / 2495 1.0%	19 / 2495 0.8%BE	2 / 2495 0.1%E
65	"Borrelomycetales" Turner 1935	2119 / 2144 98.8%	67 / 68 98.5%	68 / 68 100.0%	67 / 67 100.0%	76 / 76 100.0%	110 / 110 100.0%	67 / 67 100.0%	67 / 67 100.0%	64 / 67 95.5%	48 / 67 71.6%	97 / 97 100.0%	183 / 183 100.0%	127 / 127 100.0%	67 / 68 98.5%	67 / 67 100.0%	67 / 67 100.0%	67 / 67 100.0%	67 / 67 100.0%	150 / 150 100.0%	202 / 202 100.0%	67 / 67 100.0%	59 / 59 100.0%	229 / 230 99.6%	36 / 36 100.0%	0 / 0 0.0%	0 / 0 0.0%	0 / 0 0.0%	1598 / 2144 74.5%AB	487 / 2144 22.7%ABE	29 / 2144 1.4%B	24 / 2144 1.1%	5 / 2144 0.2%BE	1 / 2144 0.0%E
65	"Borrelomycetaceae" Turner 1935	2119 / 2144 98.8%	67 / 68 98.5%	68 / 68 100.0%	67 / 67 100.0%	76 / 76 100.0%	110 / 110 100.0%	67 / 67 100.0%	67 / 67 100.0%	64 / 67 95.5%	48 / 67 71.6%	97 / 97 100.0%	183 / 183 100.0%	127 / 127 100.0%	67 / 68 98.5%	67 / 67 100.0%	67 / 67 100.0%	67 / 67 100.0%	67 / 67 100.0%	150 / 150 100.0%	202 / 202 100.0%	67 / 67 100.0%	59 / 59 100.0%	229 / 230 99.6%	36 / 36 100.0%	0 / 0 0.0%	0 / 0 0.0%	0 / 0 0.0%	1598 / 2144 74.5%AB	487 / 2144 22.7%ABE	29 / 2144 1.4%B	24 / 2144 1.1%	5 / 2144 0.2%BE	1 / 2144 0.0%E
62	"Asterococcus" Borrel et al. 1910	2030 / 2054 98.8%	65 / 65 100.0%	65 / 65 100.0%	64 / 64 100.0%	73 / 73 100.0%	104 / 104 100.0%	64 / 64 100.0%	64 / 64 100.0%	61 / 64 95.3%	45 / 64 70.3%	91 / 91 100.0%	177 / 177 100.0%	121 / 121 100.0%	64 / 65 98.5%	64 / 64 100.0%	64 / 64 100.0%	64 / 64 100.0%	64 / 64 100.0%	147 / 147 100.0%	193 / 193 100.0%	64 / 64 100.0%	56 / 56 100.0%	220 / 221 99.5%	36 / 36 100.0%	0 / 0 0.0%	0 / 0 0.0%	0 / 0 0.0%	1542 / 2054 75.1%AB	471 / 2054 22.9%ABE	23 / 2054 1.1%	14 / 2054 0.7%B	3 / 2054 0.1%BE	1 / 2054 0.0%E
11	ATCC 19610	352 / 352 100.0%	11 / 11 100.0%	11 / 11 100.0%	11 / 11 100.0%	11 / 11 100.0%	22 / 22 100.0%	11 / 11 100.0%	11 / 11 100.0%	11 / 11 100.0%	11 / 11 100.0%	12 / 12 100.0%	22 / 22 100.0%	22 / 22 100.0%	11 / 11 100.0%	11 / 11 100.0%	11 / 11 100.0%	11 / 11 100.0%	11 / 11 100.0%	22 / 22 100.0%	44 / 44 100.0%	11 / 11 100.0%	11 / 11 100.0%	33 / 33 100.0%	10 / 10 100.0%	0 / 0 0.0%	0 / 0 0.0%	0 / 0 0.0%	286 / 352 81.2%AB	66 / 352 18.8%ABE				
2	Mycoplasma gallisepticum R	64 / 64 100.0%	2 / 2 100.0%	2 / 2 100.0%	2 / 2 100.0%	2 / 2 100.0%	4 / 4 100.0%	2 / 2 100.0%	2 / 2 100.0%	2 / 2 100.0%	2 / 2 100.0%	3 / 3 100.0%	4 / 4 100.0%	4 / 4 100.0%	2 / 2 100.0%	2 / 2 100.0%	2 / 2 100.0%	2 / 2 100.0%	2 / 2 100.0%	4 / 4 100.0%	8 / 8 100.0%	2 / 2 100.0%	2 / 2 100.0%	6 / 6 100.0%	1 / 1 100.0%	0 / 0 0.0%	0 / 0 0.0%	0 / 0 0.0%	52 / 64 81.2%AB	12 / 64 18.8%ABE				
1	Mycoplasma gallisepticum R(high)	32 / 32 100.0%	1 / 1 100.0%	1 / 1 100.0%	1 / 1 100.0%	1 / 1 100.0%	2 / 2 100.0%	1 / 1 100.0%	1 / 1 100.0%	1 / 1 100.0%	1 / 1 100.0%	1 / 1 100.0%	2 / 2 100.0%	2 / 2 100.0%	1 / 1 100.0%	1 / 1 100.0%	1 / 1 100.0%	1 / 1 100.0%	1 / 1 100.0%	2 / 2 100.0%	4 / 4 100.0%	1 / 1 100.0%	1 / 1 100.0%	3 / 3 100.0%	1 / 1 100.0%	0 / 0 0.0%	0 / 0 0.0%	0 / 0 0.0%	26 / 32 81.2%AB	6 / 32 18.8%ABE				
1	Mycoplasma gallisepticum F	32 / 32 100.0%	1 / 1 100.0%	1 / 1 100.0%	1 / 1 100.0%	1 / 1 100.0%	2 / 2 100.0%	1 / 1 100.0%	1 / 1 100.0%	1 / 1 100.0%	1 / 1 100.0%	1 / 1 100.0%	2 / 2 100.0%	2 / 2 100.0%	1 / 1 100.0%	1 / 1 100.0%	1 / 1 100.0%	1 / 1 100.0%	1 / 1 100.0%	2 / 2 100.0%	4 / 4 100.0%	1 / 1 100.0%	1 / 1 100.0%	3 / 3 100.0%	1 / 1 100.0%	0 / 0 0.0%	0 / 0 0.0%	0 / 0 0.0%	26 / 32 81.2%AB	6 / 32 18.8%ABE				
1	Mycoplasma gallisepticum VA94_7994-1-7P	32 / 32 100.0%	1 / 1 100.0%	1 / 1 100.0%	1 / 1 100.0%	1 / 1 100.0%	2 / 2 100.0%	1 / 1 100.0%	1 / 1 100.0%	1 / 1 100.0%	1 / 1 100.0%	1 / 1 100.0%	2 / 2 100.0%	2 / 2 100.0%	1 / 1 100.0%	1 / 1 100.0%	1 / 1 100.0%	1 / 1 100.0%	1 / 1 100.0%	2 / 2 100.0%	4 / 4 100.0%	1 / 1 100.0%	1 / 1 100.0%	3 / 3 100.0%	1 / 1 100.0%	0 / 0 0.0%	0 / 0 0.0%	0 / 0 0.0%	26 / 32 81.2%AB	6 / 32 18.8%ABE				
1	Mycoplasma gallisepticum NC95_13295-2-2P	32 / 32 100.0%	1 / 1 100.0%	1 / 1 100.0%	1 / 1 100.0%	1 / 1 100.0%	2 / 2 100.0%	1 / 1 100.0%	1 / 1 100.0%	1 / 1 100.0%	1 / 1 100.0%	1 / 1 100.0%	2 / 2 100.0%	2 / 2 100.0%	1 / 1 100.0%	1 / 1 100.0%	1 / 1 100.0%	1 / 1 100.0%	1 / 1 100.0%	2 / 2 100.0%	4 / 4 100.0%	1 / 1 100.0%	1 / 1 100.0%	3 / 3 100.0%	1 / 1 100.0%	0 / 0 0.0%	0 / 0 0.0%	0 / 0 0.0%	26 / 32 81.2%AB	6 / 32 18.8%ABE				
1	Mycoplasma gallisepticum NC96_1596-4-2P	32 / 32 100.0%	1 / 1 100.0%	1 / 1 100.0%	1 / 1 100.0%	1 / 1 100.0%	2 / 2 100.0%	1 / 1 100.0%	1 / 1 100.0%	1 / 1 100.0%	1 / 1 100.0%	1 / 1 100.0%	2 / 2 100.0%	2 / 2 100.0%	1 / 1 100.0%	1 / 1 100.0%	1 / 1 100.0%	1 / 1 100.0%	1 / 1 100.0%	2 / 2 100.0%	4 / 4 100.0%	1 / 1 100.0%	1 / 1 100.0%	3 / 3 100.0%	1 / 1 100.0%	0 / 0 0.0%	0 / 0 0.0%	0 / 0 0.0%	26 / 32 81.2%AB	6 / 32 18.8%ABE				
1	Mycoplasma gallisepticum NY01_2001.047-5-1P	32 / 32 100.0%	1 / 1 100.0%	1 / 1 100.0%	1 / 1 100.0%	1 / 1 100.0%	2 / 2 100.0%	1 / 1 100.0%	1 / 1 100.0%	1 / 1 100.0%	1 / 1 100.0%	1 / 1 100.0%	2 / 2 100.0%	2 / 2 100.0%	1 / 1 100.0%	1 / 1 100.0%	1 / 1 100.0%	1 / 1 100.0%	1 / 1 100.0%	2 / 2 100.0%	4 / 4 100.0%	1 / 1 100.0%	1 / 1 100.0%	3 / 3 100.0%	1 / 1 100.0%	0 / 0 0.0%	0 / 0 0.0%	0 / 0 0.0%	26 / 32 81.2%AB	6 / 32 18.8%ABE				
1	Mycoplasma gallisepticum WI01_2001.043-13-2P	32 / 32 100.0%	1 / 1 100.0%	1 / 1 100.0%	1 / 1 100.0%	1 / 1 100.0%	2 / 2 100.0%	1 / 1 100.0%	1 / 1 100.0%	1 / 1 100.0%	1 / 1 100.0%	1 / 1 100.0%	2 / 2 100.0%	2 / 2 100.0%	1 / 1 100.0%	1 / 1 100.0%	1 / 1 100.0%	1 / 1 100.0%	1 / 1 100.0%	2 / 2 100.0%	4 / 4 100.0%	1 / 1 100.0%	1 / 1 100.0%	3 / 3 100.0%	1 / 1 100.0%	0 / 0 0.0%	0 / 0 0.0%	0 / 0 0.0%	26 / 32 81.2%AB	6 / 32 18.8%ABE				
1	Mycoplasma gallisepticum NC06_2006.080-5-2P	32 / 32 100.0%	1 / 1 100.0%	1 / 1 100.0%	1 / 1 100.0%	1 / 1 100.0%	2 / 2 100.0%	1 / 1 100.0%	1 / 1 100.0%	1 / 1 100.0%	1 / 1 100.0%	1 / 1 100.0%	2 / 2 100.0%	2 / 2 100.0%	1 / 1 100.0%	1 / 1 100.0%	1 / 1 100.0%	1 / 1 100.0%	1 / 1 100.0%	2 / 2 100.0%	4 / 4 100.0%	1 / 1 100.0%	1 / 1 100.0%	3 / 3 100.0%	1 / 1 100.0%	0 / 0 0.0%	0 / 0 0.0%	0 / 0 0.0%	26 / 32 81.2%AB	6 / 32 18.8%ABE				
1	Mycoplasma gallisepticum CA06_2006.052-5-2P	32 / 32 100.0%	1 / 1 100.0%	1 / 1 100.0%	1 / 1 100.0%	1 / 1 100.0%	2 / 2 100.0%	1 / 1 100.0%	1 / 1 100.0%	1 / 1 100.0%	1 / 1 100.0%	1 / 1 100.0%	2 / 2 100.0%	2 / 2 100.0%	1 / 1 100.0%	1 / 1 100.0%	1 / 1 100.0%	1 / 1 100.0%	1 / 1 100.0%	2 / 2 100.0%	4 / 4 100.0%	1 / 1 100.0%	1 / 1 100.0%	3 / 3 100.0%	1 / 1 100.0%	0 / 0 0.0%	0 / 0 0.0%	0 / 0 0.0%	26 / 32 81.2%AB	6 / 32 18.8%ABE				
1	Mycoplasma gallisepticum NC08_2008.031-4-3P	32 / 32 100.0%	1 / 1 100.0%	1 / 1 100.0%	1 / 1 100.0%	1 / 1 100.0%	2 / 2 100.0%	1 / 1 100.0%	1 / 1 100.0%	1 / 1 100.0%	1 / 1 100.0%	1 / 1 100.0%	2 / 2 100.0%	2 / 2 100.0%	1 / 1 100.0%	1 / 1 100.0%	1 / 1 100.0%	1 / 1 100.0%	1 / 1 100.0%	2 / 2 100.0%	4 / 4 100.0%	1 / 1 100.0%	1 / 1 100.0%	3 / 3 100.0%	1 / 1 100.0%	0 / 0 0.0%	0 / 0 0.0%	0 / 0 0.0%	26 / 32 81.2%AB	6 / 32 18.8%ABE				
5	ATCC 33530	180 / 180 100.0%	5 / 5 100.0%	5 / 5 100.0%	5 / 5 100.0%	5 / 5 100.0%	10 / 10 100.0%	5 / 5 100.0%	5 / 5 100.0%	5 / 5 100.0%	5 / 5 100.0%	6 / 6 100.0%	20 / 20 100.0%	10 / 10 100.0%	5 / 5 100.0%	5 / 5 100.0%	5 / 5 100.0%	5 / 5 100.0%	5 / 5 100.0%	15 / 15 100.0%	20 / 20 100.0%	5 / 5 100.0%	5 / 5 100.0%	20 / 20 100.0%	4 / 4 100.0%	0 / 0 0.0%	0 / 0 0.0%	0 / 0 0.0%	135 / 180 75.0%AB	45 / 180 25.0%ABE				
1	Mycoplasma genitalium ATCC 33530	36 / 36 100.0%	1 / 1 100.0%	1 / 1 100.0%	1 / 1 100.0%	1 / 1 100.0%	2 / 2 100.0%	1 / 1 100.0%	1 / 1 100.0%	1 / 1 100.0%	1 / 1 100.0%	2 / 2 100.0%	4 / 4 100.0%	2 / 2 100.0%	1 / 1 100.0%	1 / 1 100.0%	1 / 1 100.0%	1 / 1 100.0%	1 / 1 100.0%	3 / 3 100.0%	4 / 4 100.0%	1 / 1 100.0%	1 / 1 100.0%	4 / 4 100.0%	0 / 0 0.0%	0 / 0 0.0%	0 / 0 0.0%	0 / 0 0.0%	27 / 36 75.0%AB	9 / 36 25.0%ABE				
1	Mycoplasma genitalium M6320	36 / 36 100.0%	1 / 1 100.0%	1 / 1 100.0%	1 / 1 100.0%	1 / 1 100.0%	2 / 2 100.0%	1 / 1 100.0%	1 / 1 100.0%	1 / 1 100.0%	1 / 1 100.0%	1 / 1 100.0%	4 / 4 100.0%	2 / 2 100.0%	1 / 1 100.0%	1 / 1 100.0%	1 / 1 100.0%	1 / 1 100.0%	1 / 1 100.0%	3 / 3 100.0%	4 / 4 100.0%	1 / 1 100.0%	1 / 1 100.0%	4 / 4 100.0%	1 / 1 100.0%	0 / 0 0.0%	0 / 0 0.0%	0 / 0 0.0%	27 / 36 75.0%AB	9 / 36 25.0%ABE				
1	Mycoplasma genitalium M6282	36 / 36 100.0%	1 / 1 100.0%	1 / 1 100.0%	1 / 1 100.0%	1 / 1 100.0%	2 / 2 100.0%	1 / 1 100.0%	1 / 1 100.0%	1 / 1 100.0%	1 / 1 100.0%	1 / 1 100.0%	4 / 4 100.0%	2 / 2 100.0%	1 / 1 100.0%	1 / 1 100.0%	1 / 1 100.0%	1 / 1 100.0%	1 / 1 100.0%	3 / 3 100.0%	4 / 4 100.0%	1 / 1 100.0%	1 / 1 100.0%	4 / 4 100.0%	1 / 1 100.0%	0 / 0 0.0%	0 / 0 0.0%	0 / 0 0.0%	27 / 36 75.0%AB	9 / 36 25.0%ABE				
1	Mycoplasma genitalium M2288	36 / 36 100.0%	1 / 1 100.0%	1 / 1 100.0%	1 / 1 100.0%	1 / 1 100.0%	2 / 2 100.0%	1 / 1 100.0%	1 / 1 100.0%	1 / 1 100.0%	1 / 1 100.0%	1 / 1 100.0%	4 / 4 100.0%	2 / 2 100.0%	1 / 1 100.0%	1 / 1 100.0%	1 / 1 100.0%	1 / 1 100.0%	1 / 1 100.0%	3 / 3 100.0%	4 / 4 100.0%	1 / 1 100.0%	1 / 1 100.0%	4 / 4 100.0%	1 / 1 100.0%	0 / 0 0.0%	0 / 0 0.0%	0 / 0 0.0%	27 / 36 75.0%AB	9 / 36 25.0%ABE				
1	Mycoplasma genitalium M2321	36 / 36 100.0%	1 / 1 100.0%	1 / 1 100.0%	1 / 1 100.0%	1 / 1 100.0%	2 / 2 100.0%	1 / 1 100.0%	1 / 1 100.0%	1 / 1 100.0%	1 / 1 100.0%	1 / 1 100.0%	4 / 4 100.0%	2 / 2 100.0%	1 / 1 100.0%	1 / 1 100.0%	1 / 1 100.0%	1 / 1 100.0%	1 / 1 100.0%	3 / 3 100.0%	4 / 4 100.0%	1 / 1 100.0%	1 / 1 100.0%	4 / 4 100.0%	1 / 1 100.0%	0 / 0 0.0%	0 / 0 0.0%	0 / 0 0.0%	27 / 36 75.0%AB	9 / 36 25.0%ABE				
1	"Asterococcus hominis" (Freundt 1953) Prevot 1961	32 / 33 97.0%	1 / 1 100.0%	1 / 1 100.0%	1 / 1 100.0%	1 / 1 100.0%	2 / 2 100.0%	1 / 1 100.0%	1 / 1 100.0%	1 / 1 100.0%	0 / 1 0.0%	1 / 1 100.0%	3 / 3 100.0%	2 / 2 100.0%	1 / 1 100.0%	1 / 1 100.0%	1 / 1 100.0%	1 / 1 100.0%	1 / 1 100.0%	3 / 3 100.0%	3 / 3 100.0%	1 / 1 100.0%	1 / 1 100.0%	3 / 3 100.0%	1 / 1 100.0%	0 / 0 0.0%	0 / 0 0.0%	0 / 0 0.0%	25 / 33 75.8%AB	7 / 33 21.2%ABE	1 / 33 3.0%			
1	Mycoplasma hominis ATCC 23114	32 / 33 97.0%	1 / 1 100.0%	1 / 1 100.0%	1 / 1 100.0%	1 / 1 100.0%	2 / 2 100.0%	1 / 1 100.0%	1 / 1 100.0%	1 / 1 100.0%	0 / 1 0.0%	1 / 1 100.0%	3 / 3 100.0%	2 / 2 100.0%	1 / 1 100.0%	1 / 1 100.0%	1 / 1 100.0%	1 / 1 100.0%	1 / 1 100.0%	3 / 3 100.0%	3 / 3 100.0%	1 / 1 100.0%	1 / 1 100.0%	3 / 3 100.0%	1 / 1 100.0%	0 / 0 0.0%	0 / 0 0.0%	0 / 0 0.0%	25 / 33 75.8%AB	7 / 33 21.2%ABE	1 / 33 3.0%			
6	"Mycoplasma suipneumoniae" Goodwin et al. 1965	174 / 180 96.7%	6 / 6 100.0%	6 / 6 100.0%	6 / 6 100.0%	6 / 6 100.0%	6 / 6 100.0%	6 / 6 100.0%	6 / 6 100.0%	6 / 6 100.0%	0 / 6 0.0%	10 / 10 100.0%	18 / 18 100.0%	6 / 6 100.0%	6 / 6 100.0%	6 / 6 100.0%	6 / 6 100.0%	6 / 6 100.0%	6 / 6 100.0%	12 / 12 100.0%	12 / 12 100.0%	6 / 6 100.0%	6 / 6 100.0%	24 / 24 100.0%	2 / 2 100.0%	0 / 0 0.0%	0 / 0 0.0%	0 / 0 0.0%	126 / 180 70.0%AB	48 / 180 26.7%ABE	6 / 180 3.3%			
1	Mycoplasma hyopneumoniae ATCC 25934	29 / 30 96.7%	1 / 1 100.0%	1 / 1 100.0%	1 / 1 100.0%	1 / 1 100.0%	1 / 1 100.0%	1 / 1 100.0%	1 / 1 100.0%	1 / 1 100.0%	0 / 1 0.0%	2 / 2 100.0%	3 / 3 100.0%	1 / 1 100.0%	1 / 1 100.0%	1 / 1 100.0%	1 / 1 100.0%	1 / 1 100.0%	1 / 1 100.0%	2 / 2 100.0%	2 / 2 100.0%	1 / 1 100.0%	1 / 1 100.0%	4 / 4 100.0%	0 / 0 0.0%	0 / 0 0.0%	0 / 0 0.0%	0 / 0 0.0%	21 / 30 70.0%AB	8 / 30 26.7%ABE	1 / 30 3.3%			
1	Mycoplasma hyopneumoniae 7448	29 / 30 96.7%	1 / 1 100.0%	1 / 1 100.0%	1 / 1 100.0%	1 / 1 100.0%	1 / 1 100.0%	1 / 1 100.0%	1 / 1 100.0%	1 / 1 100.0%	0 / 1 0.0%	2 / 2 100.0%	3 / 3 100.0%	1 / 1 100.0%	1 / 1 100.0%	1 / 1 100.0%	1 / 1 100.0%	1 / 1 100.0%	1 / 1 100.0%	2 / 2 100.0%	2 / 2 100.0%	1 / 1 100.0%	1 / 1 100.0%	4 / 4 100.0%	0 / 0 0.0%	0 / 0 0.0%	0 / 0 0.0%	0 / 0 0.0%	21 / 30 70.0%AB	8 / 30 26.7%ABE	1 / 30 3.3%			
1	Mycoplasma hyopneumoniae 232	29 / 30 96.7%	1 / 1 100.0%	1 / 1 100.0%	1 / 1 100.0%	1 / 1 100.0%	1 / 1 100.0%	1 / 1 100.0%	1 / 1 100.0%	1 / 1 100.0%	0 / 1 0.0%	2 / 2 100.0%	3 / 3 100.0%	1 / 1 100.0%	1 / 1 100.0%	1 / 1 100.0%	1 / 1 100.0%	1 / 1 100.0%	1 / 1 100.0%	2 / 2 100.0%	2 / 2 100.0%	1 / 1 100.0%	1 / 1 100.0%	4 / 4 100.0%	0 / 0 0.0%	0 / 0 0.0%	0 / 0 0.0%	0 / 0 0.0%	21 / 30 70.0%AB	8 / 30 26.7%ABE	1 / 30 3.3%			
1	Mycoplasma hyopneumoniae 7422	29 / 30 96.7%	1 / 1 100.0%	1 / 1 100.0%	1 / 1 100.0%	1 / 1 100.0%	1 / 1 100.0%	1 / 1 100.0%	1 / 1 100.0%	1 / 1 100.0%	0 / 1 0.0%	1 / 1 100.0%	3 / 3 100.0%	1 / 1 100.0%	1 / 1 100.0%	1 / 1 100.0%	1 / 1 100.0%	1 / 1 100.0%	1 / 1 100.0%	2 / 2 100.0%	2 / 2 100.0%	1 / 1 100.0%	1 / 1 100.0%	4 / 4 100.0%	1 / 1 100.0%	0 / 0 0.0%	0 / 0 0.0%	0 / 0 0.0%	21 / 30 70.0%AB	8 / 30 26.7%ABE	1 / 30 3.3%			
2	Mycoplasma hyopneumoniae 168	58 / 60 96.7%	2 / 2 100.0%	2 / 2 100.0%	2 / 2 100.0%	2 / 2 100.0%	2 / 2 100.0%	2 / 2 100.0%	2 / 2 100.0%	2 / 2 100.0%	0 / 2 0.0%	3 / 3 100.0%	6 / 6 100.0%	2 / 2 100.0%	2 / 2 100.0%	2 / 2 100.0%	2 / 2 100.0%	2 / 2 100.0%	2 / 2 100.0%	4 / 4 100.0%	4 / 4 100.0%	2 / 2 100.0%	2 / 2 100.0%	8 / 8 100.0%	1 / 1 100.0%	0 / 0 0.0%	0 / 0 0.0%	0 / 0 0.0%	42 / 60 70.0%AB	16 / 60 26.7%ABE	2 / 60 3.3%			
1	Mycoplasma hyopneumoniae 168-L	29 / 30 96.7%	1 / 1 100.0%	1 / 1 100.0%	1 / 1 100.0%	1 / 1 100.0%	1 / 1 100.0%	1 / 1 100.0%	1 / 1 100.0%	1 / 1 100.0%	0 / 1 0.0%	1 / 1 100.0%	3 / 3 100.0%	1 / 1 100.0%	1 / 1 100.0%	1 / 1 100.0%	1 / 1 100.0%	1 / 1 100.0%	1 / 1 100.0%	2 / 2 100.0%	2 / 2 100.0%	1 / 1 100.0%	1 / 1 100.0%	4 / 4 100.0%	1 / 1 100.0%	0 / 0 0.0%	0 / 0 0.0%	0 / 0 0.0%	21 / 30 70.0%AB	8 / 30 26.7%ABE	1 / 30 3.3%			
4	"Asterococcus hyorhinis" (Switzer 1955) Prevot 1961	116 / 120 96.7%	4 / 4 100.0%	4 / 4 100.0%	4 / 4 100.0%	4 / 4 100.0%	4 / 4 100.0%	4 / 4 100.0%	4 / 4 100.0%	4 / 4 100.0%	0 / 4 0.0%	5 / 5 100.0%	12 / 12 100.0%	4 / 4 100.0%	4 / 4 100.0%	4 / 4 100.0%	4 / 4 100.0%	4 / 4 100.0%	4 / 4 100.0%	8 / 8 100.0%	8 / 8 100.0%	4 / 4 100.0%	4 / 4 100.0%	16 / 16 100.0%	3 / 3 100.0%	0 / 0 0.0%	0 / 0 0.0%	0 / 0 0.0%	84 / 120 70.0%AB	32 / 120 26.7%ABE	4 / 120 3.3%			
1	Mycoplasma hyorhinis HUB-1	29 / 30 96.7%	1 / 1 100.0%	1 / 1 100.0%	1 / 1 100.0%	1 / 1 100.0%	1 / 1 100.0%	1 / 1 100.0%	1 / 1 100.0%	1 / 1 100.0%	0 / 1 0.0%	1 / 1 100.0%	3 / 3 100.0%	1 / 1 100.0%	1 / 1 100.0%	1 / 1 100.0%	1 / 1 100.0%	1 / 1 100.0%	1 / 1 100.0%	2 / 2 100.0%	2 / 2 100.0%	1 / 1 100.0%	1 / 1 100.0%	4 / 4 100.0%	1 / 1 100.0%	0 / 0 0.0%	0 / 0 0.0%	0 / 0 0.0%	21 / 30 70.0%AB	8 / 30 26.7%ABE	1 / 30 3.3%			
1	Mycoplasma hyorhinis MCLD	29 / 30 96.7%	1 / 1 100.0%	1 / 1 100.0%	1 / 1 100.0%	1 / 1 100.0%	1 / 1 100.0%	1 / 1 100.0%	1 / 1 100.0%	1 / 1 100.0%	0 / 1 0.0%	2 / 2 100.0%	3 / 3 100.0%	1 / 1 100.0%	1 / 1 100.0%	1 / 1 100.0%	1 / 1 100.0%	1 / 1 100.0%	1 / 1 100.0%	2 / 2 100.0%	2 / 2 100.0%	1 / 1 100.0%	1 / 1 100.0%	4 / 4 100.0%	0 / 0 0.0%	0 / 0 0.0%	0 / 0 0.0%	0 / 0 0.0%	21 / 30 70.0%AB	8 / 30 26.7%ABE	1 / 30 3.3%			
1	Mycoplasma hyorhinis SK76	29 / 30 96.7%	1 / 1 100.0%	1 / 1 100.0%	1 / 1 100.0%	1 / 1 100.0%	1 / 1 100.0%	1 / 1 100.0%	1 / 1 100.0%	1 / 1 100.0%	0 / 1 0.0%	1 / 1 100.0%	3 / 3 100.0%	1 / 1 100.0%	1 / 1 100.0%	1 / 1 100.0%	1 / 1 100.0%	1 / 1 100.0%	1 / 1 100.0%	2 / 2 100.0%	2 / 2 100.0%	1 / 1 100.0%	1 / 1 100.0%	4 / 4 100.0%	1 / 1 100.0%	0 / 0 0.0%	0 / 0 0.0%	0 / 0 0.0%	21 / 30 70.0%AB	8 / 30 26.7%ABE	1 / 30 3.3%			
1	Mycoplasma hyorhinis GDL-1	29 / 30 96.7%	1 / 1 100.0%	1 / 1 100.0%	1 / 1 100.0%	1 / 1 100.0%	1 / 1 100.0%	1 / 1 100.0%	1 / 1 100.0%	1 / 1 100.0%	0 / 1 0.0%	1 / 1 100.0%	3 / 3 100.0%	1 / 1 100.0%	1 / 1 100.0%	1 / 1 100.0%	1 / 1 100.0%	1 / 1 100.0%	1 / 1 100.0%	2 / 2 100.0%	2 / 2 100.0%	1 / 1 100.0%	1 / 1 100.0%	4 / 4 100.0%	1 / 1 100.0%	0 / 0 0.0%	0 / 0 0.0%	0 / 0 0.0%	21 / 30 70.0%AB	8 / 30 26.7%ABE	1 / 30 3.3%			
4	"Filterable agent of primary atypical pneumonia" Eaton et al. 1944	144 / 144 100.0%	4 / 4 100.0%	4 / 4 100.0%	4 / 4 100.0%	4 / 4 100.0%	8 / 8 100.0%	4 / 4 100.0%	4 / 4 100.0%	4 / 4 100.0%	4 / 4 100.0%	5 / 5 100.0%	16 / 16 100.0%	8 / 8 100.0%	4 / 4 100.0%	4 / 4 100.0%	4 / 4 100.0%	4 / 4 100.0%	4 / 4 100.0%	12 / 12 100.0%	16 / 16 100.0%	4 / 4 100.0%	4 / 4 100.0%	16 / 16 100.0%	3 / 3 100.0%	0 / 0 0.0%	0 / 0 0.0%	0 / 0 0.0%	112 / 144 77.8%AB	32 / 144 22.2%ABE				
1	Mycoplasma pneumoniae ATCC 29342	36 / 36 100.0%	1 / 1 100.0%	1 / 1 100.0%	1 / 1 100.0%	1 / 1 100.0%	2 / 2 100.0%	1 / 1 100.0%	1 / 1 100.0%	1 / 1 100.0%	1 / 1 100.0%	2 / 2 100.0%	4 / 4 100.0%	2 / 2 100.0%	1 / 1 100.0%	1 / 1 100.0%	1 / 1 100.0%	1 / 1 100.0%	1 / 1 100.0%	3 / 3 100.0%	4 / 4 100.0%	1 / 1 100.0%	1 / 1 100.0%	4 / 4 100.0%	0 / 0 0.0%	0 / 0 0.0%	0 / 0 0.0%	0 / 0 0.0%	28 / 36 77.8%AB	8 / 36 22.2%ABE				
1	Mycoplasma pneumoniae ATCC 15531	36 / 36 100.0%	1 / 1 100.0%	1 / 1 100.0%	1 / 1 100.0%	1 / 1 100.0%	2 / 2 100.0%	1 / 1 100.0%	1 / 1 100.0%	1 / 1 100.0%	1 / 1 100.0%	1 / 1 100.0%	4 / 4 100.0%	2 / 2 100.0%	1 / 1 100.0%	1 / 1 100.0%	1 / 1 100.0%	1 / 1 100.0%	1 / 1 100.0%	3 / 3 100.0%	4 / 4 100.0%	1 / 1 100.0%	1 / 1 100.0%	4 / 4 100.0%	1 / 1 100.0%	0 / 0 0.0%	0 / 0 0.0%	0 / 0 0.0%	28 / 36 77.8%AB	8 / 36 22.2%ABE				
1	Mycoplasma pneumoniae 309	36 / 36 100.0%	1 / 1 100.0%	1 / 1 100.0%	1 / 1 100.0%	1 / 1 100.0%	2 / 2 100.0%	1 / 1 100.0%	1 / 1 100.0%	1 / 1 100.0%	1 / 1 100.0%	1 / 1 100.0%	4 / 4 100.0%	2 / 2 100.0%	1 / 1 100.0%	1 / 1 100.0%	1 / 1 100.0%	1 / 1 100.0%	1 / 1 100.0%	3 / 3 100.0%	4 / 4 100.0%	1 / 1 100.0%	1 / 1 100.0%	4 / 4 100.0%	1 / 1 100.0%	0 / 0 0.0%	0 / 0 0.0%	0 / 0 0.0%	28 / 36 77.8%AB	8 / 36 22.2%ABE				
1	Mycoplasma pneumoniae M129-B7	36 / 36 100.0%	1 / 1 100.0%	1 / 1 100.0%	1 / 1 100.0%	1 / 1 100.0%	2 / 2 100.0%	1 / 1 100.0%	1 / 1 100.0%	1 / 1 100.0%	1 / 1 100.0%	1 / 1 100.0%	4 / 4 100.0%	2 / 2 100.0%	1 / 1 100.0%	1 / 1 100.0%	1 / 1 100.0%	1 / 1 100.0%	1 / 1 100.0%	3 / 3 100.0%	4 / 4 100.0%	1 / 1 100.0%	1 / 1 100.0%	4 / 4 100.0%	1 / 1 100.0%	0 / 0 0.0%	0 / 0 0.0%	0 / 0 0.0%	28 / 36 77.8%AB	8 / 36 22.2%ABE				
1	"Asterococcus pulmonis" (Sabin 1941) Prevot 1961	27 / 29 93.1%	1 / 1 100.0%	1 / 1 100.0%	1 / 1 100.0%	1 / 1 100.0%	1 / 1 100.0%	1 / 1 100.0%	1 / 1 100.0%	0 / 1 0.0%	0 / 1 0.0%	2 / 2 100.0%	2 / 2 100.0%	1 / 1 100.0%	1 / 1 100.0%	1 / 1 100.0%	1 / 1 100.0%	1 / 1 100.0%	1 / 1 100.0%	2 / 2 100.0%	3 / 3 100.0%	1 / 1 100.0%	1 / 1 100.0%	3 / 3 100.0%	0 / 0 0.0%	0 / 0 0.0%	0 / 0 0.0%	0 / 0 0.0%	20 / 29 69.0%AB	7 / 29 24.1%ABE	2 / 29 6.9%			
1	ATCC 25204	32 / 34 94.1%	2 / 2 100.0%	2 / 2 100.0%	1 / 1 100.0%	1 / 1 100.0%	1 / 1 100.0%	1 / 1 100.0%	1 / 1 100.0%	0 / 1 0.0%	1 / 1 100.0%	2 / 2 100.0%	3 / 3 100.0%	1 / 1 100.0%	1 / 2 50.0%	1 / 1 100.0%	1 / 1 100.0%	1 / 1 100.0%	1 / 1 100.0%	2 / 2 100.0%	2 / 2 100.0%	1 / 1 100.0%	2 / 2 100.0%	4 / 4 100.0%	0 / 0 0.0%	0 / 0 0.0%	0 / 0 0.0%	0 / 0 0.0%	23 / 34 67.6%AB	9 / 34 26.5%ABE	2 / 34 5.9%			
1	Mycoplasma synoviae 53	32 / 34 94.1%	2 / 2 100.0%	2 / 2 100.0%	1 / 1 100.0%	1 / 1 100.0%	1 / 1 100.0%	1 / 1 100.0%	1 / 1 100.0%	0 / 1 0.0%	1 / 1 100.0%	2 / 2 100.0%	3 / 3 100.0%	1 / 1 100.0%	1 / 2 50.0%	1 / 1 100.0%	1 / 1 100.0%	1 / 1 100.0%	1 / 1 100.0%	2 / 2 100.0%	2 / 2 100.0%	1 / 1 100.0%	2 / 2 100.0%	4 / 4 100.0%	0 / 0 0.0%	0 / 0 0.0%	0 / 0 0.0%	0 / 0 0.0%	23 / 34 67.6%AB	9 / 34 26.5%ABE	2 / 34 5.9%			
1	"Anulomyces agalaxiae" (sic) Wroblewski 1931	68 / 68 100.0%	2 / 2 100.0%	2 / 2 100.0%	2 / 2 100.0%	2 / 2 100.0%	4 / 4 100.0%	2 / 2 100.0%	2 / 2 100.0%	2 / 2 100.0%	2 / 2 100.0%	3 / 3 100.0%	6 / 6 100.0%	4 / 4 100.0%	2 / 2 100.0%	2 / 2 100.0%	2 / 2 100.0%	2 / 2 100.0%	2 / 2 100.0%	6 / 6 100.0%	6 / 6 100.0%	2 / 2 100.0%	2 / 2 100.0%	8 / 8 100.0%	1 / 1 100.0%	0 / 0 0.0%	0 / 0 0.0%	0 / 0 0.0%	48 / 68 70.6%AB	20 / 68 29.4%ABE				
1	"Asterococcus arthritidis" (Sabin 1941) Prevot 1961	32 / 33 97.0%	1 / 1 100.0%	1 / 1 100.0%	1 / 1 100.0%	1 / 1 100.0%	2 / 2 100.0%	1 / 1 100.0%	1 / 1 100.0%	1 / 1 100.0%	0 / 1 0.0%	1 / 1 100.0%	3 / 3 100.0%	2 / 2 100.0%	1 / 1 100.0%	1 / 1 100.0%	1 / 1 100.0%	1 / 1 100.0%	1 / 1 100.0%	3 / 3 100.0%	3 / 3 100.0%	1 / 1 100.0%	1 / 1 100.0%	3 / 3 100.0%	1 / 1 100.0%	0 / 0 0.0%	0 / 0 0.0%	0 / 0 0.0%	14 / 33 42.4%B	11 / 33 33.3%AB	4 / 33 12.1%ABE	3 / 33 9.1%BE	1 / 33 3.0%	
1	Mycoplasma arthritidis 158L3-1	32 / 33 97.0%	1 / 1 100.0%	1 / 1 100.0%	1 / 1 100.0%	1 / 1 100.0%	2 / 2 100.0%	1 / 1 100.0%	1 / 1 100.0%	1 / 1 100.0%	0 / 1 0.0%	1 / 1 100.0%	3 / 3 100.0%	2 / 2 100.0%	1 / 1 100.0%	1 / 1 100.0%	1 / 1 100.0%	1 / 1 100.0%	1 / 1 100.0%	3 / 3 100.0%	3 / 3 100.0%	1 / 1 100.0%	1 / 1 100.0%	3 / 3 100.0%	1 / 1 100.0%	0 / 0 0.0%	0 / 0 0.0%	0 / 0 0.0%	14 / 33 42.4%B	11 / 33 33.3%AB	4 / 33 12.1%ABE	3 / 33 9.1%BE	1 / 33 3.0%	
3	"Asterococcus fermentans" (Edward 1955) Prevot 1961	105 / 105 100.0%	3 / 3 100.0%	3 / 3 100.0%	3 / 3 100.0%	3 / 3 100.0%	6 / 6 100.0%	3 / 3 100.0%	3 / 3 100.0%	3 / 3 100.0%	3 / 3 100.0%	5 / 5 100.0%	9 / 9 100.0%	6 / 6 100.0%	3 / 3 100.0%	3 / 3 100.0%	3 / 3 100.0%	3 / 3 100.0%	3 / 3 100.0%	9 / 9 100.0%	12 / 12 100.0%	3 / 3 100.0%	3 / 3 100.0%	12 / 12 100.0%	1 / 1 100.0%	0 / 0 0.0%	0 / 0 0.0%	0 / 0 0.0%	75 / 105 71.4%AB	30 / 105 28.6%ABE				
1	Mycoplasma fermentans PG18	35 / 35 100.0%	1 / 1 100.0%	1 / 1 100.0%	1 / 1 100.0%	1 / 1 100.0%	2 / 2 100.0%	1 / 1 100.0%	1 / 1 100.0%	1 / 1 100.0%	1 / 1 100.0%	1 / 1 100.0%	3 / 3 100.0%	2 / 2 100.0%	1 / 1 100.0%	1 / 1 100.0%	1 / 1 100.0%	1 / 1 100.0%	1 / 1 100.0%	3 / 3 100.0%	4 / 4 100.0%	1 / 1 100.0%	1 / 1 100.0%	4 / 4 100.0%	1 / 1 100.0%	0 / 0 0.0%	0 / 0 0.0%	0 / 0 0.0%	25 / 35 71.4%AB	10 / 35 28.6%ABE				
1	Mycoplasma fermentans JER	35 / 35 100.0%	1 / 1 100.0%	1 / 1 100.0%	1 / 1 100.0%	1 / 1 100.0%	2 / 2 100.0%	1 / 1 100.0%	1 / 1 100.0%	1 / 1 100.0%	1 / 1 100.0%	2 / 2 100.0%	3 / 3 100.0%	2 / 2 100.0%	1 / 1 100.0%	1 / 1 100.0%	1 / 1 100.0%	1 / 1 100.0%	1 / 1 100.0%	3 / 3 100.0%	4 / 4 100.0%	1 / 1 100.0%	1 / 1 100.0%	4 / 4 100.0%	0 / 0 0.0%	0 / 0 0.0%	0 / 0 0.0%	0 / 0 0.0%	25 / 35 71.4%AB	10 / 35 28.6%ABE				
1	Mycoplasma fermentans M64	35 / 35 100.0%	1 / 1 100.0%	1 / 1 100.0%	1 / 1 100.0%	1 / 1 100.0%	2 / 2 100.0%	1 / 1 100.0%	1 / 1 100.0%	1 / 1 100.0%	1 / 1 100.0%	2 / 2 100.0%	3 / 3 100.0%	2 / 2 100.0%	1 / 1 100.0%	1 / 1 100.0%	1 / 1 100.0%	1 / 1 100.0%	1 / 1 100.0%	3 / 3 100.0%	4 / 4 100.0%	1 / 1 100.0%	1 / 1 100.0%	4 / 4 100.0%	0 / 0 0.0%	0 / 0 0.0%	0 / 0 0.0%	0 / 0 0.0%	25 / 35 71.4%AB	10 / 35 28.6%ABE				
1	ATCC 43663	26 / 28 92.9%	1 / 1 100.0%	1 / 1 100.0%	1 / 1 100.0%	2 / 2 100.0%	1 / 1 100.0%	1 / 1 100.0%	1 / 1 100.0%	0 / 1 0.0%	0 / 1 0.0%	2 / 2 100.0%	2 / 2 100.0%	1 / 1 100.0%	1 / 1 100.0%	1 / 1 100.0%	1 / 1 100.0%	1 / 1 100.0%	1 / 1 100.0%	2 / 2 100.0%	2 / 2 100.0%	1 / 1 100.0%	0 / 0 0.0%	3 / 3 100.0%	0 / 0 0.0%	0 / 0 0.0%	0 / 0 0.0%	0 / 0 0.0%	20 / 28 71.4%AB	6 / 28 21.4%ABE	2 / 28 7.1%			
1	Mycoplasma mobile 163K	26 / 28 92.9%	1 / 1 100.0%	1 / 1 100.0%	1 / 1 100.0%	2 / 2 100.0%	1 / 1 100.0%	1 / 1 100.0%	1 / 1 100.0%	0 / 1 0.0%	0 / 1 0.0%	2 / 2 100.0%	2 / 2 100.0%	1 / 1 100.0%	1 / 1 100.0%	1 / 1 100.0%	1 / 1 100.0%	1 / 1 100.0%	1 / 1 100.0%	2 / 2 100.0%	2 / 2 100.0%	1 / 1 100.0%	0 / 0 0.0%	3 / 3 100.0%	0 / 0 0.0%	0 / 0 0.0%	0 / 0 0.0%	0 / 0 0.0%	20 / 28 71.4%AB	6 / 28 21.4%ABE	2 / 28 7.1%			
2	ATCC 15718	60 / 60 100.0%	2 / 2 100.0%	2 / 2 100.0%	2 / 2 100.0%	2 / 2 100.0%	2 / 2 100.0%	2 / 2 100.0%	2 / 2 100.0%	2 / 2 100.0%	2 / 2 100.0%	3 / 3 100.0%	4 / 4 100.0%	6 / 6 100.0%	2 / 2 100.0%	2 / 2 100.0%	2 / 2 100.0%	2 / 2 100.0%	2 / 2 100.0%	4 / 4 100.0%	4 / 4 100.0%	2 / 2 100.0%	2 / 2 100.0%	6 / 6 100.0%	1 / 1 100.0%	0 / 0 0.0%	0 / 0 0.0%	0 / 0 0.0%	48 / 60 80.0%AB	12 / 60 20.0%ABE				
1	Mycoplasma putrefaciens KS1	30 / 30 100.0%	1 / 1 100.0%	1 / 1 100.0%	1 / 1 100.0%	1 / 1 100.0%	1 / 1 100.0%	1 / 1 100.0%	1 / 1 100.0%	1 / 1 100.0%	1 / 1 100.0%	2 / 2 100.0%	2 / 2 100.0%	3 / 3 100.0%	1 / 1 100.0%	1 / 1 100.0%	1 / 1 100.0%	1 / 1 100.0%	1 / 1 100.0%	2 / 2 100.0%	2 / 2 100.0%	1 / 1 100.0%	1 / 1 100.0%	3 / 3 100.0%	0 / 0 0.0%	0 / 0 0.0%	0 / 0 0.0%	0 / 0 0.0%	24 / 30 80.0%AB	6 / 30 20.0%ABE				
1	Mycoplasma putrefaciens Mput9231	30 / 30 100.0%	1 / 1 100.0%	1 / 1 100.0%	1 / 1 100.0%	1 / 1 100.0%	1 / 1 100.0%	1 / 1 100.0%	1 / 1 100.0%	1 / 1 100.0%	1 / 1 100.0%	1 / 1 100.0%	2 / 2 100.0%	3 / 3 100.0%	1 / 1 100.0%	1 / 1 100.0%	1 / 1 100.0%	1 / 1 100.0%	1 / 1 100.0%	2 / 2 100.0%	2 / 2 100.0%	1 / 1 100.0%	1 / 1 100.0%	3 / 3 100.0%	1 / 1 100.0%	0 / 0 0.0%	0 / 0 0.0%	0 / 0 0.0%	24 / 30 80.0%AB	6 / 30 20.0%ABE				
1	ATCC 55252	30 / 30 100.0%	1 / 1 100.0%	1 / 1 100.0%	1 / 1 100.0%	1 / 1 100.0%	2 / 2 100.0%	1 / 1 100.0%	1 / 1 100.0%	1 / 1 100.0%	1 / 1 100.0%	2 / 2 100.0%	2 / 2 100.0%	2 / 2 100.0%	1 / 1 100.0%	1 / 1 100.0%	1 / 1 100.0%	1 / 1 100.0%	1 / 1 100.0%	1 / 1 100.0%	3 / 3 100.0%	1 / 1 100.0%	1 / 1 100.0%	3 / 3 100.0%	0 / 0 0.0%	0 / 0 0.0%	0 / 0 0.0%	0 / 0 0.0%	22 / 30 73.3%AB	8 / 30 26.7%ABE				
1	Mycoplasma penetrans HF-2	30 / 30 100.0%	1 / 1 100.0%	1 / 1 100.0%	1 / 1 100.0%	1 / 1 100.0%	2 / 2 100.0%	1 / 1 100.0%	1 / 1 100.0%	1 / 1 100.0%	1 / 1 100.0%	2 / 2 100.0%	2 / 2 100.0%	2 / 2 100.0%	1 / 1 100.0%	1 / 1 100.0%	1 / 1 100.0%	1 / 1 100.0%	1 / 1 100.0%	1 / 1 100.0%	3 / 3 100.0%	1 / 1 100.0%	1 / 1 100.0%	3 / 3 100.0%	0 / 0 0.0%	0 / 0 0.0%	0 / 0 0.0%	0 / 0 0.0%	22 / 30 73.3%AB	8 / 30 26.7%ABE				
3	"Bovine serotype 5" Leach 1967	102 / 102 100.0%	3 / 3 100.0%	3 / 3 100.0%	3 / 3 100.0%	3 / 3 100.0%	6 / 6 100.0%	3 / 3 100.0%	3 / 3 100.0%	3 / 3 100.0%	3 / 3 100.0%	5 / 5 100.0%	9 / 9 100.0%	6 / 6 100.0%	3 / 3 100.0%	3 / 3 100.0%	3 / 3 100.0%	3 / 3 100.0%	3 / 3 100.0%	9 / 9 100.0%	9 / 9 100.0%	3 / 3 100.0%	3 / 3 100.0%	12 / 12 100.0%	1 / 1 100.0%	0 / 0 0.0%	0 / 0 0.0%	0 / 0 0.0%	72 / 102 70.6%AB	30 / 102 29.4%ABE				
1	Mycoplasma bovis PG45	34 / 34 100.0%	1 / 1 100.0%	1 / 1 100.0%	1 / 1 100.0%	1 / 1 100.0%	2 / 2 100.0%	1 / 1 100.0%	1 / 1 100.0%	1 / 1 100.0%	1 / 1 100.0%	2 / 2 100.0%	3 / 3 100.0%	2 / 2 100.0%	1 / 1 100.0%	1 / 1 100.0%	1 / 1 100.0%	1 / 1 100.0%	1 / 1 100.0%	3 / 3 100.0%	3 / 3 100.0%	1 / 1 100.0%	1 / 1 100.0%	4 / 4 100.0%	0 / 0 0.0%	0 / 0 0.0%	0 / 0 0.0%	0 / 0 0.0%	24 / 34 70.6%AB	10 / 34 29.4%ABE				
1	Mycoplasma bovis HB0801	34 / 34 100.0%	1 / 1 100.0%	1 / 1 100.0%	1 / 1 100.0%	1 / 1 100.0%	2 / 2 100.0%	1 / 1 100.0%	1 / 1 100.0%	1 / 1 100.0%	1 / 1 100.0%	1 / 1 100.0%	3 / 3 100.0%	2 / 2 100.0%	1 / 1 100.0%	1 / 1 100.0%	1 / 1 100.0%	1 / 1 100.0%	1 / 1 100.0%	3 / 3 100.0%	3 / 3 100.0%	1 / 1 100.0%	1 / 1 100.0%	4 / 4 100.0%	1 / 1 100.0%	0 / 0 0.0%	0 / 0 0.0%	0 / 0 0.0%	24 / 34 70.6%AB	10 / 34 29.4%ABE				
1	Mycoplasma bovis Hubei-1	34 / 34 100.0%	1 / 1 100.0%	1 / 1 100.0%	1 / 1 100.0%	1 / 1 100.0%	2 / 2 100.0%	1 / 1 100.0%	1 / 1 100.0%	1 / 1 100.0%	1 / 1 100.0%	2 / 2 100.0%	3 / 3 100.0%	2 / 2 100.0%	1 / 1 100.0%	1 / 1 100.0%	1 / 1 100.0%	1 / 1 100.0%	1 / 1 100.0%	3 / 3 100.0%	3 / 3 100.0%	1 / 1 100.0%	1 / 1 100.0%	4 / 4 100.0%	0 / 0 0.0%	0 / 0 0.0%	0 / 0 0.0%	0 / 0 0.0%	24 / 34 70.6%AB	10 / 34 29.4%ABE				
2	"Candidatus Mycoplasma haemofelis" Neimark et al. 2001	60 / 62 96.8%	2 / 2 100.0%	2 / 2 100.0%	2 / 2 100.0%	4 / 4 100.0%	4 / 4 100.0%	2 / 2 100.0%	2 / 2 100.0%	2 / 2 100.0%	0 / 2 0.0%	4 / 4 100.0%	6 / 6 100.0%	2 / 2 100.0%	2 / 2 100.0%	2 / 2 100.0%	2 / 2 100.0%	2 / 2 100.0%	2 / 2 100.0%	4 / 4 100.0%	6 / 6 100.0%	2 / 2 100.0%	0 / 0 0.0%	6 / 6 100.0%	0 / 0 0.0%	0 / 0 0.0%	0 / 0 0.0%	0 / 0 0.0%	48 / 62 77.4%AB	12 / 62 19.4%ABE	2 / 62 3.2%			
[illegible]	[illegible]	30 / 31	1 / 1	1 / 1	1 / 1	2 / 2	2 / 2	1 / 1	1 / 1	1 / 1	0 / 1	2 / 2	3 / 3	1 / 1	1 / 1	1 / 1	1 / 1	1 / 1	1 / 1	2 / 2	3 / 3	1 / 1	0 / 0	3 / 3	0 / 0	0 / 0	0 / 0	0 / 0	24 / 31	6 / 31	1 / 31			

1	Mycoplasma haemofelis Ohio2	96.8%	100.0%	100.0%	100.0%	100.0%	100.0%	100.0%	100.0%	100.0%	0.0%	100.0%	100.0%	100.0%	100.0%	100.0%	100.0%	100.0%	100.0%	100.0%	100.0%	100.0%	0.0%	100.0%	0.0%	0.0%	0.0%	0.0%	77.4%AB	19.4%ABE	3.2%			
1	Mycoplasma haemofelis str. Langford 1	30 / 31 96.8%	1 / 1 100.0%	1 / 1 100.0%	1 / 1 100.0%	2 / 2 100.0%	2 / 2 100.0%	1 / 1 100.0%	1 / 1 100.0%	1 / 1 100.0%	0 / 1 0.0%	2 / 2 100.0%	3 / 3 100.0%	1 / 1 100.0%	1 / 1 100.0%	1 / 1 100.0%	1 / 1 100.0%	1 / 1 100.0%	1 / 1 100.0%	2 / 2 100.0%	3 / 3 100.0%	1 / 1 100.0%	0 / 0 0.0%	3 / 3 100.0%	0 / 0 0.0%	0 / 0 0.0%	0 / 0 0.0%	0 / 0 0.0%	24 / 31 77.4%AB	6 / 31 19.4%ABE	1 / 31 3.2%			
1	ATCC 25834	27 / 28 96.4%	1 / 1 100.0%	1 / 1 100.0%	1 / 1 100.0%	1 / 1 100.0%	1 / 1 100.0%	1 / 1 100.0%	1 / 1 100.0%	1 / 1 100.0%	0 / 1 0.0%	1 / 1 100.0%	3 / 3 100.0%	1 / 1 100.0%	1 / 1 100.0%	1 / 1 100.0%	1 / 1 100.0%	1 / 1 100.0%	1 / 1 100.0%	2 / 2 100.0%	2 / 2 100.0%	1 / 1 100.0%	1 / 1 100.0%	3 / 3 100.0%	0 / 0 0.0%	0 / 0 0.0%	0 / 0 0.0%	0 / 0 0.0%	20 / 28 71.4%AB	7 / 28 25.0%ABE	1 / 28 3.6%			
1	ATCC 51981	33 / 34 97.1%	1 / 1 100.0%	1 / 1 100.0%	1 / 1 100.0%	1 / 1 100.0%	1 / 1 100.0%	1 / 1 100.0%	1 / 1 100.0%	1 / 1 100.0%	0 / 1 0.0%	1 / 1 100.0%	3 / 3 100.0%	2 / 2 100.0%	1 / 1 100.0%	1 / 1 100.0%	1 / 1 100.0%	1 / 1 100.0%	1 / 1 100.0%	3 / 3 100.0%	4 / 4 100.0%	1 / 1 100.0%	1 / 1 100.0%	4 / 4 100.0%	1 / 1 100.0%	0 / 0 0.0%	0 / 0 0.0%	0 / 0 0.0%	25 / 34 73.5%AB	8 / 34 23.5%ABE	1 / 34 2.9%			
1	Mycoplasma crocodyli ATCC 51981	33 / 34 97.1%	1 / 1 100.0%	1 / 1 100.0%	1 / 1 100.0%	1 / 1 100.0%	1 / 1 100.0%	1 / 1 100.0%	1 / 1 100.0%	1 / 1 100.0%	0 / 1 0.0%	1 / 1 100.0%	3 / 3 100.0%	2 / 2 100.0%	1 / 1 100.0%	1 / 1 100.0%	1 / 1 100.0%	1 / 1 100.0%	1 / 1 100.0%	3 / 3 100.0%	4 / 4 100.0%	1 / 1 100.0%	1 / 1 100.0%	4 / 4 100.0%	1 / 1 100.0%	0 / 0 0.0%	0 / 0 0.0%	0 / 0 0.0%	25 / 34 73.5%AB	8 / 34 23.5%ABE	1 / 34 2.9%			
2	"Candidatus Mycoplasma haemosuis" Neimark et al. 2001	64 / 64 100.0%	2 / 2 100.0%	2 / 2 100.0%	2 / 2 100.0%	4 / 4 100.0%	4 / 4 100.0%	2 / 2 100.0%	2 / 2 100.0%	2 / 2 100.0%	2 / 2 100.0%	4 / 4 100.0%	6 / 6 100.0%	4 / 4 100.0%	2 / 2 100.0%	2 / 2 100.0%	2 / 2 100.0%	2 / 2 100.0%	2 / 2 100.0%	4 / 4 100.0%	6 / 6 100.0%	2 / 2 100.0%	0 / 0 0.0%	6 / 6 100.0%	0 / 0 0.0%	0 / 0 0.0%	0 / 0 0.0%	0 / 0 0.0%	52 / 64 81.2%AB	12 / 64 18.8%ABE				
1	Mycoplasma suis Illinois	32 / 32 100.0%	1 / 1 100.0%	1 / 1 100.0%	1 / 1 100.0%	2 / 2 100.0%	2 / 2 100.0%	1 / 1 100.0%	1 / 1 100.0%	1 / 1 100.0%	1 / 1 100.0%	2 / 2 100.0%	3 / 3 100.0%	2 / 2 100.0%	1 / 1 100.0%	1 / 1 100.0%	1 / 1 100.0%	1 / 1 100.0%	1 / 1 100.0%	2 / 2 100.0%	3 / 3 100.0%	1 / 1 100.0%	0 / 0 0.0%	3 / 3 100.0%	0 / 0 0.0%	0 / 0 0.0%	0 / 0 0.0%	0 / 0 0.0%	26 / 32 81.2%AB	6 / 32 18.8%ABE				
1	"Candidatus Mycoplasma wenyonii" Neimark et al. 2001	32 / 32 100.0%	1 / 1 100.0%	1 / 1 100.0%	1 / 1 100.0%	2 / 2 100.0%	2 / 2 100.0%	1 / 1 100.0%	1 / 1 100.0%	1 / 1 100.0%	1 / 1 100.0%	1 / 1 100.0%	3 / 3 100.0%	2 / 2 100.0%	1 / 1 100.0%	1 / 1 100.0%	1 / 1 100.0%	1 / 1 100.0%	1 / 1 100.0%	2 / 2 100.0%	3 / 3 100.0%	1 / 1 100.0%	0 / 0 0.0%	3 / 3 100.0%	1 / 1 100.0%	0 / 0 0.0%	0 / 0 0.0%	0 / 0 0.0%	27 / 32 84.4%AB	5 / 32 15.6%ABE				
1	Mycoplasma wenyonii str. Massachusetts	32 / 32 100.0%	1 / 1 100.0%	1 / 1 100.0%	1 / 1 100.0%	2 / 2 100.0%	2 / 2 100.0%	1 / 1 100.0%	1 / 1 100.0%	1 / 1 100.0%	1 / 1 100.0%	1 / 1 100.0%	3 / 3 100.0%	2 / 2 100.0%	1 / 1 100.0%	1 / 1 100.0%	1 / 1 100.0%	1 / 1 100.0%	1 / 1 100.0%	2 / 2 100.0%	3 / 3 100.0%	1 / 1 100.0%	0 / 0 0.0%	3 / 3 100.0%	1 / 1 100.0%	0 / 0 0.0%	0 / 0 0.0%	0 / 0 0.0%	27 / 32 84.4%AB	5 / 32 15.6%ABE				
1	"Bartonella canis" Kikuth 1928	30 / 31 96.8%	1 / 1 100.0%	1 / 1 100.0%	1 / 1 100.0%	2 / 2 100.0%	2 / 2 100.0%	1 / 1 100.0%	1 / 1 100.0%	1 / 1 100.0%	0 / 1 0.0%	1 / 1 100.0%	3 / 3 100.0%	1 / 1 100.0%	1 / 1 100.0%	1 / 1 100.0%	1 / 1 100.0%	1 / 1 100.0%	1 / 1 100.0%	2 / 2 100.0%	3 / 3 100.0%	1 / 1 100.0%	0 / 0 0.0%	3 / 3 100.0%	1 / 1 100.0%	0 / 0 0.0%	0 / 0 0.0%	0 / 0 0.0%	24 / 31 77.4%AB	6 / 31 19.4%ABE	1 / 31 3.2%			
1	Mycoplasma haemocanis str. Illinois	30 / 31 96.8%	1 / 1 100.0%	1 / 1 100.0%	1 / 1 100.0%	2 / 2 100.0%	2 / 2 100.0%	1 / 1 100.0%	1 / 1 100.0%	1 / 1 100.0%	0 / 1 0.0%	1 / 1 100.0%	3 / 3 100.0%	1 / 1 100.0%	1 / 1 100.0%	1 / 1 100.0%	1 / 1 100.0%	1 / 1 100.0%	1 / 1 100.0%	2 / 2 100.0%	3 / 3 100.0%	1 / 1 100.0%	0 / 0 0.0%	3 / 3 100.0%	1 / 1 100.0%	0 / 0 0.0%	0 / 0 0.0%	0 / 0 0.0%	24 / 31 77.4%AB	6 / 31 19.4%ABE	1 / 31 3.2%			
1	"Candidatus Mycoplasma haemolamae" Messick et al. 2002	33 / 33 100.0%	1 / 1 100.0%	1 / 1 100.0%	1 / 1 100.0%	2 / 2 100.0%	3 / 3 100.0%	1 / 1 100.0%	1 / 1 100.0%	1 / 1 100.0%	1 / 1 100.0%	1 / 1 100.0%	3 / 3 100.0%	2 / 2 100.0%	1 / 1 100.0%	1 / 1 100.0%	1 / 1 100.0%	1 / 1 100.0%	1 / 1 100.0%	2 / 2 100.0%	3 / 3 100.0%	1 / 1 100.0%	0 / 0 0.0%	3 / 3 100.0%	1 / 1 100.0%	0 / 0 0.0%	0 / 0 0.0%	0 / 0 0.0%	27 / 33 81.8%AB	6 / 33 18.2%ABE				
1	Candidatus Mycoplasma haemolamae str. Purdue	33 / 33 100.0%	1 / 1 100.0%	1 / 1 100.0%	1 / 1 100.0%	2 / 2 100.0%	3 / 3 100.0%	1 / 1 100.0%	1 / 1 100.0%	1 / 1 100.0%	1 / 1 100.0%	1 / 1 100.0%	3 / 3 100.0%	2 / 2 100.0%	1 / 1 100.0%	1 / 1 100.0%	1 / 1 100.0%	1 / 1 100.0%	1 / 1 100.0%	2 / 2 100.0%	3 / 3 100.0%	1 / 1 100.0%	0 / 0 0.0%	3 / 3 100.0%	1 / 1 100.0%	0 / 0 0.0%	0 / 0 0.0%	0 / 0 0.0%	27 / 33 81.8%AB	6 / 33 18.2%ABE				
1	"Candidatus Mycoplasma haemominutum" Foley and Pedersen 2001	32 / 32 100.0%	1 / 1 100.0%	1 / 1 100.0%	1 / 1 100.0%	2 / 2 100.0%	2 / 2 100.0%	1 / 1 100.0%	1 / 1 100.0%	1 / 1 100.0%	1 / 1 100.0%	1 / 1 100.0%	3 / 3 100.0%	2 / 2 100.0%	1 / 1 100.0%	1 / 1 100.0%	1 / 1 100.0%	1 / 1 100.0%	1 / 1 100.0%	2 / 2 100.0%	3 / 3 100.0%	1 / 1 100.0%	0 / 0 0.0%	3 / 3 100.0%	1 / 1 100.0%	0 / 0 0.0%	0 / 0 0.0%	0 / 0 0.0%	25 / 32 78.1%AB	7 / 32 21.9%ABE				
1	Candidatus Mycoplasma haemominutum 'Birmingham 1'	32 / 32 100.0%	1 / 1 100.0%	1 / 1 100.0%	1 / 1 100.0%	2 / 2 100.0%	2 / 2 100.0%	1 / 1 100.0%	1 / 1 100.0%	1 / 1 100.0%	1 / 1 100.0%	1 / 1 100.0%	3 / 3 100.0%	2 / 2 100.0%	1 / 1 100.0%	1 / 1 100.0%	1 / 1 100.0%	1 / 1 100.0%	1 / 1 100.0%	2 / 2 100.0%	3 / 3 100.0%	1 / 1 100.0%	0 / 0 0.0%	3 / 3 100.0%	1 / 1 100.0%	0 / 0 0.0%	0 / 0 0.0%	0 / 0 0.0%	25 / 32 78.1%AB	7 / 32 21.9%ABE				
7	Mycoplasma mycoides cluster	239 / 240 99.6%	8 / 8 100.0%	8 / 8 100.0%	8 / 8 100.0%	8 / 8 100.0%	8 / 8 100.0%	8 / 8 100.0%	8 / 8 100.0%	8 / 8 100.0%	8 / 8 100.0%	13 / 13 100.0%	16 / 16 100.0%	24 / 24 100.0%	8 / 8 100.0%	8 / 8 100.0%	8 / 8 100.0%	8 / 8 100.0%	8 / 8 100.0%	16 / 16 100.0%	16 / 16 100.0%	8 / 8 100.0%	8 / 8 100.0%	23 / 24 95.8%	3 / 3 100.0%	0 / 0 0.0%	0 / 0 0.0%	0 / 0 0.0%	187 / 240 77.9%AB	52 / 240 21.7%ABE	1 / 240 0.4%E			
1	ATCC 27343	30 / 30 100.0%	1 / 1 100.0%	1 / 1 100.0%	1 / 1 100.0%	1 / 1 100.0%	1 / 1 100.0%	1 / 1 100.0%	1 / 1 100.0%	1 / 1 100.0%	1 / 1 100.0%	2 / 2 100.0%	2 / 2 100.0%	3 / 3 100.0%	1 / 1 100.0%	1 / 1 100.0%	1 / 1 100.0%	1 / 1 100.0%	1 / 1 100.0%	2 / 2 100.0%	2 / 2 100.0%	1 / 1 100.0%	1 / 1 100.0%	3 / 3 100.0%	0 / 0 0.0%	0 / 0 0.0%	0 / 0 0.0%	0 / 0 0.0%	23 / 30 76.7%AB	7 / 30 23.3%ABE				
1	Mycoplasma capricolum capricolum	30 / 30 100.0%	1 / 1 100.0%	1 / 1 100.0%	1 / 1 100.0%	1 / 1 100.0%	1 / 1 100.0%	1 / 1 100.0%	1 / 1 100.0%	1 / 1 100.0%	1 / 1 100.0%	2 / 2 100.0%	2 / 2 100.0%	3 / 3 100.0%	1 / 1 100.0%	1 / 1 100.0%	1 / 1 100.0%	1 / 1 100.0%	1 / 1 100.0%	2 / 2 100.0%	2 / 2 100.0%	1 / 1 100.0%	1 / 1 100.0%	3 / 3 100.0%	0 / 0 0.0%	0 / 0 0.0%	0 / 0 0.0%	0 / 0 0.0%	23 / 30 76.7%AB	7 / 30 23.3%ABE				
1	Mycoplasma capricolum subsp. capricolum 'California kid'	30 / 30 100.0%	1 / 1 100.0%	1 / 1 100.0%	1 / 1 100.0%	1 / 1 100.0%	1 / 1 100.0%	1 / 1 100.0%	1 / 1 100.0%	1 / 1 100.0%	1 / 1 100.0%	2 / 2 100.0%	2 / 2 100.0%	3 / 3 100.0%	1 / 1 100.0%	1 / 1 100.0%	1 / 1 100.0%	1 / 1 100.0%	1 / 1 100.0%	2 / 2 100.0%	2 / 2 100.0%	1 / 1 100.0%	1 / 1 100.0%	3 / 3 100.0%	0 / 0 0.0%	0 / 0 0.0%	0 / 0 0.0%	0 / 0 0.0%	23 / 30 76.7%AB	7 / 30 23.3%ABE				
4	"Asterococcus mycoides" Borrel et al. 1910	150 / 150 100.0%	5 / 5 100.0%	5 / 5 100.0%	5 / 5 100.0%	5 / 5 100.0%	5 / 5 100.0%	5 / 5 100.0%	5 / 5 100.0%	5 / 5 100.0%	5 / 5 100.0%	7 / 7 100.0%	10 / 10 100.0%	15 / 15 100.0%	5 / 5 100.0%	5 / 5 100.0%	5 / 5 100.0%	5 / 5 100.0%	5 / 5 100.0%	10 / 10 100.0%	10 / 10 100.0%	5 / 5 100.0%	5 / 5 100.0%	15 / 15 100.0%	3 / 3 100.0%	0 / 0 0.0%	0 / 0 0.0%	0 / 0 0.0%	118 / 150 78.7%AB	32 / 150 21.3%ABE				
2	Mycoplasma mycoides mycoides	60 / 60 100.0%	2 / 2 100.0%	2 / 2 100.0%	2 / 2 100.0%	2 / 2 100.0%	2 / 2 100.0%	2 / 2 100.0%	2 / 2 100.0%	2 / 2 100.0%	2 / 2 100.0%	3 / 3 100.0%	4 / 4 100.0%	6 / 6 100.0%	2 / 2 100.0%	2 / 2 100.0%	2 / 2 100.0%	2 / 2 100.0%	2 / 2 100.0%	4 / 4 100.0%	4 / 4 100.0%	2 / 2 100.0%	2 / 2 100.0%	6 / 6 100.0%	1 / 1 100.0%	0 / 0 0.0%	0 / 0 0.0%	0 / 0 0.0%	46 / 60 76.7%AB	14 / 60 23.3%ABE				
2	Mycoplasma mycoides mycoides SC	60 / 60 100.0%	2 / 2 100.0%	2 / 2 100.0%	2 / 2 100.0%	2 / 2 100.0%	2 / 2 100.0%	2 / 2 100.0%	2 / 2 100.0%	2 / 2 100.0%	2 / 2 100.0%	3 / 3 100.0%	4 / 4 100.0%	6 / 6 100.0%	2 / 2 100.0%	2 / 2 100.0%	2 / 2 100.0%	2 / 2 100.0%	2 / 2 100.0%	4 / 4 100.0%	4 / 4 100.0%	2 / 2 100.0%	2 / 2 100.0%	6 / 6 100.0%	1 / 1 100.0%	0 / 0 0.0%	0 / 0 0.0%	0 / 0 0.0%	46 / 60 76.7%AB	14 / 60 23.3%ABE				
1	Mycoplasma mycoides subsp. mycoides SC str. PG1	30 / 30 100.0%	1 / 1 100.0%	1 / 1 100.0%	1 / 1 100.0%	1 / 1 100.0%	1 / 1 100.0%	1 / 1 100.0%	1 / 1 100.0%	1 / 1 100.0%	1 / 1 100.0%	2 / 2 100.0%	2 / 2 100.0%	3 / 3 100.0%	1 / 1 100.0%	1 / 1 100.0%	1 / 1 100.0%	1 / 1 100.0%	1 / 1 100.0%	2 / 2 100.0%	2 / 2 100.0%	1 / 1 100.0%	1 / 1 100.0%	3 / 3 100.0%	0 / 0 0.0%	0 / 0 0.0%	0 / 0 0.0%	0 / 0 0.0%	23 / 30 76.7%AB	7 / 30 23.3%ABE				
1	Mycoplasma mycoides subsp. mycoides SC str. Gladysdale	30 / 30 100.0%	1 / 1 100.0%	1 / 1 100.0%	1 / 1 100.0%	1 / 1 100.0%	1 / 1 100.0%	1 / 1 100.0%	1 / 1 100.0%	1 / 1 100.0%	1 / 1 100.0%	1 / 1 100.0%	2 / 2 100.0%	3 / 3 100.0%	1 / 1 100.0%	1 / 1 100.0%	1 / 1 100.0%	1 / 1 100.0%	1 / 1 100.0%	2 / 2 100.0%	2 / 2 100.0%	1 / 1 100.0%	1 / 1 100.0%	3 / 3 100.0%	1 / 1 100.0%	0 / 0 0.0%	0 / 0 0.0%	0 / 0 0.0%	23 / 30 76.7%AB	7 / 30 23.3%ABE				
2	"Asterococcus mycoides subsp. capri" Edward 1953	90 / 90 100.0%	3 / 3 100.0%	3 / 3 100.0%	3 / 3 100.0%	3 / 3 100.0%	3 / 3 100.0%	3 / 3 100.0%	3 / 3 100.0%	3 / 3 100.0%	3 / 3 100.0%	4 / 4 100.0%	6 / 6 100.0%	9 / 9 100.0%	3 / 3 100.0%	3 / 3 100.0%	3 / 3 100.0%	3 / 3 100.0%	3 / 3 100.0%	6 / 6 100.0%	6 / 6 100.0%	3 / 3 100.0%	3 / 3 100.0%	9 / 9 100.0%	2 / 2 100.0%	0 / 0 0.0%	0 / 0 0.0%	0 / 0 0.0%	72 / 90 80.0%AB	18 / 90 20.0%ABE				
2	Mycoplasma mycoides mycoides LC	90 / 90 100.0%	3 / 3 100.0%	3 / 3 100.0%	3 / 3 100.0%	3 / 3 100.0%	3 / 3 100.0%	3 / 3 100.0%	3 / 3 100.0%	3 / 3 100.0%	3 / 3 100.0%	4 / 4 100.0%	6 / 6 100.0%	9 / 9 100.0%	3 / 3 100.0%	3 / 3 100.0%	3 / 3 100.0%	3 / 3 100.0%	3 / 3 100.0%	6 / 6 100.0%	6 / 6 100.0%	3 / 3 100.0%	3 / 3 100.0%	9 / 9 100.0%	2 / 2 100.0%	0 / 0 0.0%	0 / 0 0.0%	0 / 0 0.0%	72 / 90 80.0%AB	18 / 90 20.0%ABE				
1	Mycoplasma mycoides subsp. capri GM12	60 / 60 100.0%	2 / 2 100.0%	2 / 2 100.0%	2 / 2 100.0%	2 / 2 100.0%	2 / 2 100.0%	2 / 2 100.0%	2 / 2 100.0%	2 / 2 100.0%	2 / 2 100.0%	3 / 3 100.0%	4 / 4 100.0%	6 / 6 100.0%	2 / 2 100.0%	2 / 2 100.0%	2 / 2 100.0%	2 / 2 100.0%	2 / 2 100.0%	4 / 4 100.0%	4 / 4 100.0%	2 / 2 100.0%	2 / 2 100.0%	6 / 6 100.0%	1 / 1 100.0%	0 / 0 0.0%	0 / 0 0.0%	0 / 0 0.0%	48 / 60 80.0%AB	12 / 60 20.0%ABE				
1	Mycoplasma mycoides subsp. capri LC str. 95010	30 / 30 100.0%	1 / 1 100.0%	1 / 1 100.0%	1 / 1 100.0%	1 / 1 100.0%	1 / 1 100.0%	1 / 1 100.0%	1 / 1 100.0%	1 / 1 100.0%	1 / 1 100.0%	1 / 1 100.0%	2 / 2 100.0%	3 / 3 100.0%	1 / 1 100.0%	1 / 1 100.0%	1 / 1 100.0%	1 / 1 100.0%	1 / 1 100.0%	2 / 2 100.0%	2 / 2 100.0%	1 / 1 100.0%	1 / 1 100.0%	3 / 3 100.0%	1 / 1 100.0%	0 / 0 0.0%	0 / 0 0.0%	0 / 0 0.0%	24 / 30 80.0%AB	6 / 30 20.0%ABE				
2	DSM 21131	59 / 60 98.3%	2 / 2 100.0%	2 / 2 100.0%	2 / 2 100.0%	2 / 2 100.0%	2 / 2 100.0%	2 / 2 100.0%	2 / 2 100.0%	2 / 2 100.0%	2 / 2 100.0%	4 / 4 100.0%	4 / 4 100.0%	6 / 6 100.0%	2 / 2 100.0%	2 / 2 100.0%	2 / 2 100.0%	2 / 2 100.0%	2 / 2 100.0%	4 / 4 100.0%	4 / 4 100.0%	2 / 2 100.0%	2 / 2 100.0%	5 / 6 83.3%	0 / 0 0.0%	0 / 0 0.0%	0 / 0 0.0%	0 / 0 0.0%	46 / 60 76.7%AB	13 / 60 21.7%ABE	1 / 60 1.7%E			
1	Mycoplasma leachii 99/014/6	29 / 30 96.7%	1 / 1 100.0%	1 / 1 100.0%	1 / 1 100.0%	1 / 1 100.0%	1 / 1 100.0%	1 / 1 100.0%	1 / 1 100.0%	1 / 1 100.0%	1 / 1 100.0%	2 / 2 100.0%	2 / 2 100.0%	3 / 3 100.0%	1 / 1 100.0%	1 / 1 100.0%	1 / 1 100.0%	1 / 1 100.0%	1 / 1 100.0%	2 / 2 100.0%	2 / 2 100.0%	1 / 1 100.0%	1 / 1 100.0%	2 / 3 66.7%	0 / 0 0.0%	0 / 0 0.0%	0 / 0 0.0%	0 / 0 0.0%	23 / 30 76.7%AB	6 / 30 20.0%ABE	1 / 30 3.3%E			
1	Mycoplasma PG50	30 / 30 100.0%	1 / 1 100.0%	1 / 1 100.0%	1 / 1 100.0%	1 / 1 100.0%	1 / 1 100.0%	1 / 1 100.0%	1 / 1 100.0%	1 / 1 100.0%	1 / 1 100.0%	2 / 2 100.0%	2 / 2 100.0%	3 / 3 100.0%	1 / 1 100.0%	1 / 1 100.0%	1 / 1 100.0%	1 / 1 100.0%	1 / 1 100.0%	2 / 2 100.0%	2 / 2 100.0%	1 / 1 100.0%	1 / 1 100.0%	3 / 3 100.0%	0 / 0 0.0%	0 / 0 0.0%	0 / 0 0.0%	0 / 0 0.0%	23 / 30 76.7%AB	7 / 30 23.3%ABE				
3	Ureaplasma	89 / 90 98.9%	2 / 3 66.7%	3 / 3 100.0%	3 / 3 100.0%	3 / 3 100.0%	6 / 6 100.0%	3 / 3 100.0%	3 / 3 100.0%	3 / 3 100.0%	3 / 3 100.0%	6 / 6 100.0%	6 / 6 100.0%	6 / 6 100.0%	3 / 3 100.0%	3 / 3 100.0%	3 / 3 100.0%	3 / 3 100.0%	3 / 3 100.0%	3 / 3 100.0%	9 / 9 100.0%	3 / 3 100.0%	3 / 3 100.0%	9 / 9 100.0%	0 / 0 0.0%	0 / 0 0.0%	0 / 0 0.0%	0 / 0 0.0%	56 / 90 62.2%AB	16 / 90 17.8%ABE	15 / 90 16.7%B	2 / 90 2.2%BE	1 / 90 1.1%	
1	ATCC 27618	30 / 30 100.0%	1 / 1 100.0%	1 / 1 100.0%	1 / 1 100.0%	1 / 1 100.0%	2 / 2 100.0%	1 / 1 100.0%	1 / 1 100.0%	1 / 1 100.0%	1 / 1 100.0%	2 / 2 100.0%	2 / 2 100.0%	2 / 2 100.0%	1 / 1 100.0%	1 / 1 100.0%	1 / 1 100.0%	1 / 1 100.0%	1 / 1 100.0%	1 / 1 100.0%	3 / 3 100.0%	1 / 1 100.0%	1 / 1 100.0%	3 / 3 100.0%	0 / 0 0.0%	0 / 0 0.0%	0 / 0 0.0%	0 / 0 0.0%	24 / 30 80.0%AB	6 / 30 20.0%ABE				
1	Ureaplasma urealyticum serovar 10	30 / 30 100.0%	1 / 1 100.0%	1 / 1 100.0%	1 / 1 100.0%	1 / 1 100.0%	2 / 2 100.0%	1 / 1 100.0%	1 / 1 100.0%	1 / 1 100.0%	1 / 1 100.0%	2 / 2 100.0%	2 / 2 100.0%	2 / 2 100.0%	1 / 1 100.0%	1 / 1 100.0%	1 / 1 100.0%	1 / 1 100.0%	1 / 1 100.0%	1 / 1 100.0%	3 / 3 100.0%	1 / 1 100.0%	1 / 1 100.0%	3 / 3 100.0%	0 / 0 0.0%	0 / 0 0.0%	0 / 0 0.0%	0 / 0 0.0%	24 / 30 80.0%AB	6 / 30 20.0%ABE				
1	Ureaplasma urealyticum serovar 10 str. ATCC 33699	30 / 30 100.0%	1 / 1 100.0%	1 / 1 100.0%	1 / 1 100.0%	1 / 1 100.0%	2 / 2 100.0%	1 / 1 100.0%	1 / 1 100.0%	1 / 1 100.0%	1 / 1 100.0%	2 / 2 100.0%	2 / 2 100.0%	2 / 2 100.0%	1 / 1 100.0%	1 / 1 100.0%	1 / 1 100.0%	1 / 1 100.0%	1 / 1 100.0%	1 / 1 100.0%	3 / 3 100.0%	1 / 1 100.0%	1 / 1 100.0%	3 / 3 100.0%	0 / 0 0.0%	0 / 0 0.0%	0 / 0 0.0%	0 / 0 0.0%	24 / 30 80.0%AB	6 / 30 20.0%ABE				
2	ATCC 27815	59 / 60 98.3%	1 / 2 50.0%	2 / 2 100.0%	2 / 2 100.0%	2 / 2 100.0%	4 / 4 100.0%	2 / 2 100.0%	2 / 2 100.0%	2 / 2 100.0%	2 / 2 100.0%	4 / 4 100.0%	4 / 4 100.0%	4 / 4 100.0%	2 / 2 100.0%	2 / 2 100.0%	2 / 2 100.0%	2 / 2 100.0%	2 / 2 100.0%	2 / 2 100.0%	6 / 6 100.0%	2 / 2 100.0%	2 / 2 100.0%	6 / 6 100.0%	0 / 0 0.0%	0 / 0 0.0%	0 / 0 0.0%	0 / 0 0.0%	32 / 60 53.3%AB	15 / 60 25.0%B	10 / 60 16.7%ABE	2 / 60 3.3%BE	1 / 60 1.7%	
2	Ureaplasma parvum serovar 3	59 / 60 98.3%	1 / 2 50.0%	2 / 2 100.0%	2 / 2 100.0%	2 / 2 100.0%	4 / 4 100.0%	2 / 2 100.0%	2 / 2 100.0%	2 / 2 100.0%	2 / 2 100.0%	4 / 4 100.0%	4 / 4 100.0%	4 / 4 100.0%	2 / 2 100.0%	2 / 2 100.0%	2 / 2 100.0%	2 / 2 100.0%	2 / 2 100.0%	2 / 2 100.0%	6 / 6 100.0%	2 / 2 100.0%	2 / 2 100.0%	6 / 6 100.0%	0 / 0 0.0%	0 / 0 0.0%	0 / 0 0.0%	0 / 0 0.0%	32 / 60 53.3%AB	15 / 60 25.0%B	10 / 60 16.7%ABE	2 / 60 3.3%BE	1 / 60 1.7%	
1	Ureaplasma parvum serovar 3 'ATCC 700970'	30 / 30 100.0%	1 / 1 100.0%	1 / 1 100.0%	1 / 1 100.0%	1 / 1 100.0%	2 / 2 100.0%	1 / 1 100.0%	1 / 1 100.0%	1 / 1 100.0%	1 / 1 100.0%	2 / 2 100.0%	2 / 2 100.0%	2 / 2 100.0%	1 / 1 100.0%	1 / 1 100.0%	1 / 1 100.0%	1 / 1 100.0%	1 / 1 100.0%	1 / 1 100.0%	3 / 3 100.0%	1 / 1 100.0%	1 / 1 100.0%	3 / 3 100.0%	0 / 0 0.0%	0 / 0 0.0%	0 / 0 0.0%	0 / 0 0.0%	24 / 30 80.0%AB	6 / 30 20.0%ABE				
1	Ureaplasma parvum serovar 3 str. ATCC 27815	29 / 30 96.7%	0 / 1 0.0%	1 / 1 100.0%	1 / 1 100.0%	1 / 1 100.0%	2 / 2 100.0%	1 / 1 100.0%	1 / 1 100.0%	1 / 1 100.0%	1 / 1 100.0%	2 / 2 100.0%	2 / 2 100.0%	2 / 2 100.0%	1 / 1 100.0%	1 / 1 100.0%	1 / 1 100.0%	1 / 1 100.0%	1 / 1 100.0%	1 / 1 100.0%	3 / 3 100.0%	1 / 1 100.0%	1 / 1 100.0%	3 / 3 100.0%	0 / 0 0.0%	0 / 0 0.0%	0 / 0 0.0%	0 / 0 0.0%	15 / 30 50.0%B	8 / 30 26.7%AB	4 / 30 13.3%ABE	2 / 30 6.7%BE	1 / 30 3.3%	
5	Entomoplasmatales	151 / 152 99.3%	5 / 5 100.0%	5 / 5 100.0%	5 / 5 100.0%	5 / 5 100.0%	5 / 6 83.3%	7 / 7 100.0%	5 / 5 100.0%	5 / 5 100.0%	7 / 7 100.0%	6 / 6 100.0%	12 / 12 100.0%	10 / 10 100.0%	5 / 5 100.0%	5 / 5 100.0%	5 / 5 100.0%	5 / 5 100.0%	5 / 5 100.0%	10 / 10 100.0%	10 / 10 100.0%	5 / 5 100.0%	5 / 5 100.0%	15 / 15 100.0%	4 / 4 100.0%	0 / 0 0.0%	0 / 0 0.0%	0 / 0 0.0%	115 / 152 75.7%AB	36 / 152 23.7%ABE	1 / 152 0.7%E			
4	Spiroplasmataceae	122 / 123 99.2%	4 / 4 100.0%	4 / 4 100.0%	4 / 4 100.0%	4 / 4 100.0%	4 / 5 80.0%	6 / 6 100.0%	4 / 4 100.0%	4 / 4 100.0%	6 / 6 100.0%	4 / 4 100.0%	10 / 10 100.0%	8 / 8 100.0%	4 / 4 100.0%	4 / 4 100.0%	4 / 4 100.0%	4 / 4 100.0%	4 / 4 100.0%	8 / 8 100.0%	8 / 8 100.0%	4 / 4 100.0%	4 / 4 100.0%	12 / 12 100.0%	4 / 4 100.0%	0 / 0 0.0%	0 / 0 0.0%	0 / 0 0.0%	92 / 123 74.8%AB	30 / 123 24.4%ABE	1 / 123 0.8%E			
4	Spiroplasma	122 / 123 99.2%	4 / 4 100.0%	4 / 4 100.0%	4 / 4 100.0%	4 / 4 100.0%	4 / 5 80.0%	6 / 6 100.0%	4 / 4 100.0%	4 / 4 100.0%	6 / 6 100.0%	4 / 4 100.0%	10 / 10 100.0%	8 / 8 100.0%	4 / 4 100.0%	4 / 4 100.0%	4 / 4 100.0%	4 / 4 100.0%	4 / 4 100.0%	8 / 8 100.0%	8 / 8 100.0%	4 / 4 100.0%	4 / 4 100.0%	12 / 12 100.0%	4 / 4 100.0%	0 / 0 0.0%	0 / 0 0.0%	0 / 0 0.0%	92 / 123 74.8%AB	30 / 123 24.4%ABE	1 / 123 0.8%E			
1	ATCC 43302	29 / 29 100.0%	1 / 1 100.0%	1 / 1 100.0%	1 / 1 100.0%	1 / 1 100.0%	1 / 1 100.0%	1 / 1 100.0%	1 / 1 100.0%	1 / 1 100.0%	1 / 1 100.0%	1 / 1 100.0%	2 / 2 100.0%	2 / 2 100.0%	1 / 1 100.0%	1 / 1 100.0%	1 / 1 100.0%	1 / 1 100.0%	1 / 1 100.0%	2 / 2 100.0%	2 / 2 100.0%	1 / 1 100.0%	1 / 1 100.0%	3 / 3 100.0%	1 / 1 100.0%	0 / 0 0.0%	0 / 0 0.0%	0 / 0 0.0%	21 / 29 72.4%AB	8 / 29 27.6%ABE				
1	Spiroplasma taiwanense ATCC 43302	29 / 29 100.0%	1 / 1 100.0%	1 / 1 100.0%	1 / 1 100.0%	1 / 1 100.0%	1 / 1 100.0%	1 / 1 100.0%	1 / 1 100.0%	1 / 1 100.0%	1 / 1 100.0%	1 / 1 100.0%	2 / 2 100.0%	2 / 2 100.0%	1 / 1 100.0%	1 / 1 100.0%	1 / 1 100.0%	1 / 1 100.0%	1 / 1 100.0%	2 / 2 100.0%	2 / 2 100.0%	1 / 1 100.0%	1 / 1 100.0%	3 / 3 100.0%	1 / 1 100.0%	0 / 0 0.0%	0 / 0 0.0%	0 / 0 0.0%	21 / 29 72.4%AB	8 / 29 27.6%ABE				
1	ATCC 43209	32 / 33 97.0%	1 / 1 100.0%	1 / 1 100.0%	1 / 1 100.0%	1 / 1 100.0%	1 / 2 50.0%	2 / 2 100.0%	1 / 1 100.0%	1 / 1 100.0%	2 / 2 100.0%	1 / 1 100.0%	3 / 3 100.0%	2 / 2 100.0%	1 / 1 100.0%	1 / 1 100.0%	1 / 1 100.0%	1 / 1 100.0%	1 / 1 100.0%	2 / 2 100.0%	2 / 2 100.0%	1 / 1 100.0%	1 / 1 100.0%	3 / 3 100.0%	1 / 1 100.0%	0 / 0 0.0%	0 / 0 0.0%	0 / 0 0.0%	25 / 33 75.8%AB	7 / 33 21.2%ABE	1 / 33 3.0%E			
1	Spiroplasma chrysopicola ATCC 43209	32 / 33 97.0%	1 / 1 100.0%	1 / 1 100.0%	1 / 1 100.0%	1 / 1 100.0%	1 / 2 50.0%	2 / 2 100.0%	1 / 1 100.0%	1 / 1 100.0%	2 / 2 100.0%	1 / 1 100.0%	3 / 3 100.0%	2 / 2 100.0%	1 / 1 100.0%	1 / 1 100.0%	1 / 1 100.0%	1 / 1 100.0%	1 / 1 100.0%	2 / 2 100.0%	2 / 2 100.0%	1 / 1 100.0%	1 / 1 100.0%	3 / 3 100.0%	1 / 1 100.0%	0 / 0 0.0%	0 / 0 0.0%	0 / 0 0.0%	25 / 33 75.8%AB	7 / 33 21.2%ABE	1 / 33 3.0%E			
1	ATCC 49235	29 / 29 100.0%	1 / 1 100.0%	1 / 1 100.0%	1 / 1 100.0%	1 / 1 100.0%	1 / 1 100.0%	1 / 1 100.0%	1 / 1 100.0%	1 / 1 100.0%	1 / 1 100.0%	1 / 1 100.0%	2 / 2 100.0%	2 / 2 100.0%	1 / 1 100.0%	1 / 1 100.0%	1 / 1 100.0%	1 / 1 100.0%	1 / 1 100.0%	2 / 2 100.0%	2 / 2 100.0%	1 / 1 100.0%	1 / 1 100.0%	3 / 3 100.0%	1 / 1 100.0%	0 / 0 0.0%	0 / 0 0.0%	0 / 0 0.0%	21 / 29 72.4%AB	8 / 29 27.6%ABE				
1	Spiroplasma diminutum ATCC 49235	29 / 29 100.0%	1 / 1 100.0%	1 / 1 100.0%	1 / 1 100.0%	1 / 1 100.0%	1 / 1 100.0%	1 / 1 100.0%	1 / 1 100.0%	1 / 1 100.0%	1 / 1 100.0%	1 / 1 100.0%	2 / 2 100.0%	2 / 2 100.0%	1 / 1 100.0%	1 / 1 100.0%	1 / 1 100.0%	1 / 1 100.0%	1 / 1 100.0%	2 / 2 100.0%	2 / 2 100.0%	1 / 1 100.0%	1 / 1 100.0%	3 / 3 100.0%	1 / 1 100.0%	0 / 0 0.0%	0 / 0 0.0%	0 / 0 0.0%	21 / 29 72.4%AB	8 / 29 27.6%ABE				
1	ATCC 33826	32 / 32 100.0%	1 / 1 100.0%	1 / 1 100.0%	1 / 1 100.0%	1 / 1 100.0%	1 / 1 100.0%	2 / 2 100.0%	1 / 1 100.0%	1 / 1 100.0%	2 / 2 100.0%	1 / 1 100.0%	3 / 3 100.0%	2 / 2 100.0%	1 / 1 100.0%	1 / 1 100.0%	1 / 1 100.0%	1 / 1 100.0%	1 / 1 100.0%	2 / 2 100.0%	2 / 2 100.0%	1 / 1 100.0%	1 / 1 100.0%	3 / 3 100.0%	1 / 1 100.0%	0 / 0 0.0%	0 / 0 0.0%	0 / 0 0.0%	25 / 32 78.1%AB	7 / 32 21.9%ABE				
1	Spiroplasma syrphidicola ATCC 33826	32 / 32 100.0%	1 / 1 100.0%	1 / 1 100.0%	1 / 1 100.0%	1 / 1 100.0%	1 / 1 100.0%	2 / 2 100.0%	1 / 1 100.0%	1 / 1 100.0%	2 / 2 100.0%	1 / 1 100.0%	3 / 3 100.0%	2 / 2 100.0%	1 / 1 100.0%	1 / 1 100.0%	1 / 1 100.0%	1 / 1 100.0%	1 / 1 100.0%	2 / 2 100.0%	2 / 2 100.0%	1 / 1 100.0%	1 / 1 100.0%	3 / 3 100.0%	1 / 1 100.0%	0 / 0 0.0%	0 / 0 0.0%	0 / 0 0.0%	25 / 32 78.1%AB	7 / 32 21.9%ABE				
1	Entomoplasma group	29 / 29 100.0%	1 / 1 100.0%	1 / 1 100.0%	1 / 1 100.0%	1 / 1 100.0%	1 / 1 100.0%	1 / 1 100.0%	1 / 1 100.0%	1 / 1 100.0%	1 / 1 100.0%	2 / 2 100.0%	2 / 2 100.0%	2 / 2 100.0%	1 / 1 100.0%	1 / 1 100.0%	1 / 1 100.0%	1 / 1 100.0%	1 / 1 100.0%	2 / 2 100.0%	2 / 2 100.0%	1 / 1 100.0%	1 / 1 100.0%	3 / 3 100.0%	0 / 0 0.0%	0 / 0 0.0%	0 / 0 0.0%	0 / 0 0.0%	23 / 29 79.3%AB	6 / 29 20.7%ABE				
1	Mesoplasma	29 / 29 100.0%	1 / 1 100.0%	1 / 1 100.0%	1 / 1 100.0%	1 / 1 100.0%	1 / 1 100.0%	1 / 1 100.0%	1 / 1 100.0%	1 / 1 100.0%	1 / 1 100.0%	2 / 2 100.0%	2 / 2 100.0%	2 / 2 100.0%	1 / 1 100.0%	1 / 1 100.0%	1 / 1 100.0%	1 / 1 100.0%	1 / 1 100.0%	2 / 2 100.0%	2 / 2 100.0%	1 / 1 100.0%	1 / 1 100.0%	3 / 3 100.0%	0 / 0 0.0%	0 / 0 0.0%	0 / 0 0.0%	0 / 0 0.0%	23 / 29 79.3%AB	6 / 29 20.7%ABE				
1	ATCC 33453	29 / 29 100.0%	1 / 1 100.0%	1 / 1 100.0%	1 / 1 100.0%	1 / 1 100.0%	1 / 1 100.0%	1 / 1 100.0%	1 / 1 100.0%	1 / 1 100.0%	1 / 1 100.0%	2 / 2 100.0%	2 / 2 100.0%	2 / 2 100.0%	1 / 1 100.0%	1 / 1 100.0%	1 / 1 100.0%	1 / 1 100.0%	1 / 1 100.0%	2 / 2 100.0%	2 / 2 100.0%	1 / 1 100.0%	1 / 1 100.0%	3 / 3 100.0%	0 / 0 0.0%	0 / 0 0.0%	0 / 0 0.0%	0 / 0 0.0%	23 / 29 79.3%AB	6 / 29 20.7%ABE				
1	Mesoplasma florum L1	29 / 29 100.0%	1 / 1 100.0%	1 / 1 100.0%	1 / 1 100.0%	1 / 1 100.0%	1 / 1 100.0%	1 / 1 100.0%	1 / 1 100.0%	1 / 1 100.0%	1 / 1 100.0%	2 / 2 100.0%	2 / 2 100.0%	2 / 2 100.0%	1 / 1 100.0%	1 / 1 100.0%	1 / 1 100.0%	1 / 1 100.0%	1 / 1 100.0%	2 / 2 100.0%	2 / 2 100.0%	1 / 1 100.0%	1 / 1 100.0%	3 / 3 100.0%	0 / 0 0.0%	0 / 0 0.0%	0 / 0 0.0%	0 / 0 0.0%	23 / 29 79.3%AB	6 / 29 20.7%ABE				
6	Acholeplasmatales	199 / 199 100.0%	6 / 6 100.0%	6 / 6 100.0%	6 / 6 100.0%	11 / 11 100.0%	12 / 12 100.0%	10 / 10 100.0%	8 / 8 100.0%	6 / 6 100.0%	7 / 7 100.0%	6 / 6 100.0%	18 / 18 100.0%	7 / 7 100.0%	6 / 6 100.0%	8 / 8 100.0%	6 / 6 100.0%	6 / 6 100.0%	6 / 6 100.0%	12 / 12 100.0%	13 / 13 100.0%	7 / 7 100.0%	6 / 6 100.0%	26 / 26 100.0%	0 / 0 0.0%	0 / 0 0.0%	0 / 0 0.0%	0 / 0 0.0%	103 / 199 51.8%AB	53 / 199 26.6%ABE	29 / 199 14.6%B	14 / 199 7.0%BE		
6	"Sapromycetaceae" Sabin 1941	199 / 199 100.0%	6 / 6 100.0%	6 / 6 100.0%	6 / 6 100.0%	11 / 11 100.0%	12 / 12 100.0%	10 / 10 100.0%	8 / 8 100.0%	6 / 6 100.0%	7 / 7 100.0%	6 / 6 100.0%	18 / 18 100.0%	7 / 7 100.0%	6 / 6 100.0%	8 / 8 100.0%	6 / 6 100.0%	6 / 6 100.0%	6 / 6 100.0%	12 / 12 100.0%	13 / 13 100.0%	7 / 7 100.0%	6 / 6 100.0%	26 / 26 100.0%	0 / 0 0.0%	0 / 0 0.0%	0 / 0 0.0%	0 / 0 0.0%	103 / 199 51.8%AB	53 / 199 26.6%ABE	29 / 199 14.6%B	14 / 199 7.0%BE		
1	"Sapromyces" Sabin 1941	35 / 35 100.0%	1 / 1 100.0%	1 / 1 100.0%	1 / 1 100.0%	1 / 1 100.0%	2 / 2 100.0%	2 / 2 100.0%	1 / 1 100.0%	1 / 1 100.0%	2 / 2 100.0%	1 / 1 100.0%	3 / 3 100.0%	2 / 2 100.0%	1 / 1 100.0%	1 / 1 100.0%	1 / 1 100.0%	1 / 1 100.0%	1 / 1 100.0%	2 / 2 100.0%	3 / 3 100.0%	2 / 2 100.0%	1 / 1 100.0%	4 / 4 100.0%	0 / 0 0.0%	0 / 0 0.0%	0 / 0 0.0%	0 / 0 0.0%	14 / 35 40.0%AB	9 / 35 25.7%B	6 / 35 17.1%ABE	6 / 35 17.1%BE		
1	"Asterococcus laidlawii" (Sabin 1941) Prevot 1961	35 / 35 100.0%	1 / 1 100.0%	1 / 1 100.0%	1 / 1 100.0%	1 / 1 100.0%	2 / 2 100.0%	2 / 2 100.0%	1 / 1 100.0%	1 / 1 100.0%	2 / 2 100.0%	1 / 1 100.0%	3 / 3 100.0%	2 / 2 100.0%	1 / 1 100.0%	1 / 1 100.0%	1 / 1 100.0%	1 / 1 100.0%	1 / 1 100.0%	2 / 2 100.0%	3 / 3 100.0%	2 / 2 100.0%	1 / 1 100.0%	4 / 4 100.0%	0 / 0 0.0%	0 / 0 0.0%	0 / 0 0.0%	0 / 0 0.0%	14 / 35 40.0%AB	9 / 35 25.7%B	6 / 35 17.1%BE	6 / 35 17.1%ABE		
1	Acholeplasma laidlawii PG-8A	35 / 35 100.0%	1 / 1 100.0%	1 / 1 100.0%	1 / 1 100.0%	1 / 1 100.0%	2 / 2 100.0%	2 / 2 100.0%	1 / 1 100.0%	1 / 1 100.0%	2 / 2 100.0%	1 / 1 100.0%	3 / 3 100.0%	2 / 2 100.0%	1 / 1 100.0%	1 / 1 100.0%	1 / 1 100.0%	1 / 1 100.0%	1 / 1 100.0%	2 / 2 100.0%	3 / 3 100.0%	2 / 2 100.0%	1 / 1 100.0%	4 / 4 100.0%	0 / 0 0.0%	0 / 0 0.0%	0 / 0 0.0%	0 / 0 0.0%	14 / 35 40.0%AB	9 / 35 25.7%B	6 / 35 17.1%BE	6 / 35 17.1%ABE		
5	"Candidatus Phytoplasma" Firrao et al. 2004	164 / 164 100.0%	5 / 5 100.0%	5 / 5 100.0%	5 / 5 100.0%	10 / 10 100.0%	10 / 10 100.0%	8 / 8 100.0%	7 / 7 100.0%	5 / 5 100.0%	5 / 5 100.0%	5 / 5 100.0%	15 / 15 100.0%	5 / 5 100.0%	5 / 5 100.0%	7 / 7 100.0%	5 / 5 100.0%	5 / 5 100.0%	5 / 5 100.0%	10 / 10 100.0%	10 / 10 100.0%	5 / 5 100.0%	5 / 5 100.0%	22 / 22 100.0%	0 / 0 0.0%	0 / 0 0.0%	0 / 0 0.0%	0 / 0 0.0%	89 / 164 54.3%AB	47 / 164 28.7%ABE	20 / 164 12.2%B	8 / 164 4.9%BE		
2	"Candidatus Phytoplasma asteris" Lee et al. 2004	64 / 64 100.0%	2 / 2 100.0%	2 / 2 100.0%	2 / 2 100.0%	4 / 4 100.0%	4 / 4 100.0%	4 / 4 100.0%	2 / 2 100.0%	2 / 2 100.0%	2 / 2 100.0%	2 / 2 100.0%	6 / 6 100.0%	2 / 2 100.0%	2 / 2 100.0%	2 / 2 100.0%	2 / 2 100.0%	2 / 2 100.0%	2 / 2 100.0%	4 / 4 100.0%	4 / 4 100.0%	2 / 2 100.0%	2 / 2 100.0%	8 / 8 100.0%	0 / 0 0.0%	0 / 0 0.0%	0 / 0 0.0%	0 / 0 0.0%	43 / 64 67.2%AB	21 / 64 32.8%ABE				
1	Onion yellows phytoplasma	32 / 32 100.0%	1 / 1 100.0%	1 / 1 100.0%	1 / 1 100.0%	2 / 2 100.0%	2 / 2 100.0%	2 / 2 100.0%	1 / 1 100.0%	1 / 1 100.0%	1 / 1 100.0%	1 / 1 100.0%	3 / 3 100.0%	1 / 1 100.0%	1 / 1 100.0%	1 / 1 100.0%	1 / 1 100.0%	1 / 1 100.0%	1 / 1 100.0%	2 / 2 100.0%	2 / 2 100.0%	1 / 1 100.0%	1 / 1 100.0%	4 / 4 100.0%	0 / 0 0.0%	0 / 0 0.0%	0 / 0 0.0%	0 / 0 0.0%	22 / 32 68.8%AB	10 / 32 31.2%ABE				
1	Onion yellows phytoplasma OY-M	32 / 32 100.0%	1 / 1 100.0%	1 / 1 100.0%	1 / 1 100.0%	2 / 2 100.0%	2 / 2 100.0%	2 / 2 100.0%	1 / 1 100.0%	1 / 1 100.0%	1 / 1 100.0%	1 / 1 100.0%	3 / 3 100.0%	1 / 1 100.0%	1 / 1 100.0%	1 / 1 100.0%	1 / 1 100.0%	1 / 1 100.0%	1 / 1 100.0%	2 / 2 100.0%	2 / 2 100.0%	1 / 1 100.0%	1 / 1 100.0%	4 / 4 100.0%	0 / 0 0.0%	0 / 0 0.0%	0 / 0 0.0%	0 / 0 0.0%	22 / 32 68.8%AB	10 / 32 31.2%ABE				
1	Aster yellows witches'-broom phytoplasma	32 / 32 100.0%	1 / 1 100.0%	1 / 1 100.0%	1 / 1 100.0%	2 / 2 100.0%	2 / 2 100.0%	2 / 2 100.0%	1 / 1 100.0%	1 / 1 100.0%	1 / 1 100.0%	1 / 1 100.0%	3 / 3 100.0%	1 / 1 100.0%	1 / 1 100.0%	1 / 1 100.0%	1 / 1 100.0%	1 / 1 100.0%	1 / 1 100.0%	2 / 2 100.0%	2 / 2 100.0%	1 / 1 100.0%	1 / 1 100.0%	4 / 4 100.0%	0 / 0 0.0%	0 / 0 0.0%	0 / 0 0.0%	0 / 0 0.0%	21 / 32 65.6%AB	11 / 32 34.4%ABE				
1	Aster yellows witches'-broom phytoplasma AYWB	32 / 32 100.0%	1 / 1 100.0%	1 / 1 100.0%	1 / 1 100.0%	2 / 2 100.0%	2 / 2 100.0%	2 / 2 100.0%	1 / 1 100.0%	1 / 1 100.0%	1 / 1 100.0%	1 / 1 100.0%	3 / 3 100.0%	1 / 1 100.0%	1 / 1 100.0%	1 / 1 100.0%	1 / 1 100.0%	1 / 1 100.0%	1 / 1 100.0%	2 / 2 100.0%	2 / 2 100.0%	1 / 1 100.0%	1 / 1 100.0%	4 / 4 100.0%	0 / 0 0.0%	0 / 0 0.0%	0 / 0 0.0%	0 / 0 0.0%	21 / 32 65.6%AB	11 / 32 34.4%ABE				
1	16SrX (Apple proliferation group)	32 / 32 100.0%	1 / 1 100.0%	1 / 1 100.0%	1 / 1 100.0%	2 / 2 100.0%	2 / 2 100.0%	2 / 2 100.0%	1 / 1 100.0%	1 / 1 100.0%	1 / 1 100.0%	1 / 1 100.0%	3 / 3 100.0%	1 / 1 100.0%	1 / 1 100.0%	1 / 1 100.0%	1 / 1 100.0%	1 / 1 100.0%	1 / 1 100.0%	2 / 2 100.0%	2 / 2 100.0%	1 / 1 100.0%	1 / 1 100.0%	4 / 4 100.0%	0 / 0 0.0%	0 / 0 0.0%	0 / 0 0.0%	0 / 0 0.0%	19 / 32 59.4%AB	5 / 32 15.6%BE	5 / 32 15.6%ABE	3 / 32 9.4%B		
1	"Candidatus Phytoplasma mali" Seemuller and Schneider 2004	32 / 32 100.0%	1 / 1 100.0%	1 / 1 100.0%	1 / 1 100.0%	2 / 2 100.0%	2 / 2 100.0%	2 / 2 100.0%	1 / 1 100.0%	1 / 1 100.0%	1 / 1 100.0%	1 / 1 100.0%	3 / 3 100.0%	1 / 1 100.0%	1 / 1 100.0%	1 / 1 100.0%	1 / 1 100.0%	1 / 1 100.0%	1 / 1 100.0%	2 / 2 100.0%	2 / 2 100.0%	1 / 1 100.0%	1 / 1 100.0%	4 / 4 100.0%	0 / 0 0.0%	0 / 0 0.0%	0 / 0 0.0%	0 / 0 0.0%	19 / 32 59.4%AB	5 / 32 15.6%ABE	5 / 32 15.6%BE	3 / 32 9.4%B		
2	16SrXII (Stolbur group)	68 / 68 100.0%	2 / 2 100.0%	2 / 2 100.0%	2 / 2 100.0%	4 / 4 100.0%	4 / 4 100.0%	2 / 2 100.0%	4 / 4 100.0%	2 / 2 100.0%	2 / 2 100.0%	2 / 2 100.0%	6 / 6 100.0%	2 / 2 100.0%	2 / 2 100.0%	4 / 4 100.0%	2 / 2 100.0%	2 / 2 100.0%	2 / 2 100.0%	4 / 4 100.0%	4 / 4 100.0%	2 / 2 100.0%	2 / 2 100.0%	10 / 10 100.0%	0 / 0 0.0%	0 / 0 0.0%	0 / 0 0.0%	0 / 0 0.0%	27 / 68 39.7%AB	21 / 68 30.9%ABE	17 / 68 25.0%B	3 / 68 4.4%BE		
2	"Candidatus Phytoplasma australiense" Davis et al. 1997	68 / 68 100.0%	2 / 2 100.0%	2 / 2 100.0%	2 / 2 100.0%	4 / 4 100.0%	4 / 4 100.0%	2 / 2 100.0%	4 / 4 100.0%	2 / 2 100.0%	2 / 2 100.0%	2 / 2 100.0%	6 / 6 100.0%	2 / 2 100.0%	2 / 2 100.0%	4 / 4 100.0%	2 / 2 100.0%	2 / 2 100.0%	2 / 2 100.0%	4 / 4 100.0%	4 / 4 100.0%	2 / 2 100.0%	2 / 2 100.0%	10 / 10 100.0%	0 / 0 0.0%	0 / 0 0.0%	0 / 0 0.0%	0 / 0 0.0%	27 / 68 39.7%AB	21 / 68 30.9%ABE	17 / 68 25.0%B	3 / 68 4.4%BE		
1	Strawberry lethal yellows phytoplasma (CPA)	34 / 34 100.0%	1 / 1 100.0%	1 / 1 100.0%	1 / 1 100.0%	2 / 2 100.0%	2 / 2 100.0%	1 / 1 100.0%	2 / 2 100.0%	1 / 1 100.0%	1 / 1 100.0%	1 / 1 100.0%	3 / 3 100.0%	1 / 1 100.0%	1 / 1 100.0%	2 / 2 100.0%	1 / 1 100.0%	1 / 1 100.0%	1 / 1 100.0%	2 / 2 100.0%	2 / 2 100.0%	1 / 1 100.0%	1 / 1 100.0%	5 / 5 100.0%	0 / 0 0.0%	0 / 0 0.0%	0 / 0 0.0%	0 / 0 0.0%	22 / 34 64.7%AB	12 / 34 35.3%ABE				
1	Strawberry lethal yellows phytoplasma (CPA) str. NZSb11	34 / 34 100.0%	1 / 1 100.0%	1 / 1 100.0%	1 / 1 100.0%	2 / 2 100.0%	2 / 2 100.0%	1 / 1 100.0%	2 / 2 100.0%	1 / 1 100.0%	1 / 1 100.0%	1 / 1 100.0%	3 / 3 100.0%	1 / 1 100.0%	1 / 1 100.0%	2 / 2 100.0%	1 / 1 100.0%	1 / 1 100.0%	1 / 1 100.0%	2 / 2 100.0%	2 / 2 100.0%	1 / 1 100.0%	1 / 1 100.0%	5 / 5 100.0%	0 / 0 0.0%	0 / 0 0.0%	0 / 0 0.0%	0 / 0 0.0%	22 / 34 64.7%AB	12 / 34 35.3%ABE				
47	"Archaea" Woese et al. 1990	650 / 2140 30.4%	16 / 56 28.6%	14 / 53 26.4%	17 / 51 33.3%	16 / 51 31.4%	35 / 141 24.8%	43 / 136 31.6%	19 / 47 40.4%	14 / 47 29.8%	41 / 146 28.1%	15 / 48 31.2%	58 / 180 32.2%	30 / 90 33.3%	20 / 51 39.2%	29 / 85 34.1%	13 / 59 22.0%	45 / 127 35.4%	11 / 47 23.4%	35 / 137 25.5%	63 / 213 29.6%	32 / 95 33.7%	10 / 47 21.3%	70 / 222 31.5%	0 / 7 0.0%	4 / 4 100.0%	0 / 0 0.0%	0 / 0 0.0%	761 / 2140 35.6%	665 / 2140 31.1%E	350 / 2140 16.4%AB	300 / 2140 14.0%ABE	35 / 2140 1.6%C	29 / 2140 1.4%CE
13	"Crenarchaeota" Woese et al. 1990	302 / 598 50.5%	6 / 13 46.2%	7 / 13 53.8%	6 / 13 46.2%	5 / 13 38.5%	18 / 39 46.2%	17 / 39 43.6%	5 / 13 38.5%	5 / 13 38.5%	22 / 39 56.4%	9 / 13 69.2%	28 / 52 53.8%	16 / 26 61.5%	6 / 13 46.2%	15 / 26 57.7%	7 / 13 53.8%	16 / 39 41.0%	5 / 13 38.5%	17 / 39 43.6%	38 / 65 58.5%	13 / 26 50.0%	5 / 13 38.5%	36 / 65 55.4%	0 / 0 0.0%	0 / 0 0.0%	0 / 0 0.0%	0 / 0 0.0%	185 / 598 30.9%	170 / 598 28.4%AB	132 / 598 22.1%ABE	84 / 598 14.0%E	15 / 598 2.5%C	12 / 598 2.0%CE
13	Crenarchaeota	302 / 598 50.5%	6 / 13 46.2%	7 / 13 53.8%	6 / 13 46.2%	5 / 13 38.5%	18 / 39 46.2%	17 / 39 43.6%	5 / 13 38.5%	5 / 13 38.5%	22 / 39 56.4%	9 / 13 69.2%	28 / 52 53.8%	16 / 26 61.5%	6 / 13 46.2%	15 / 26 57.7%	7 / 13 53.8%	16 / 39 41.0%	5 / 13 38.5%	17 / 39 43.6%	38 / 65 58.5%	13 / 26 50.0%	5 / 13 38.5%	36 / 65 55.4%	0 / 0 0.0%	0 / 0 0.0%	0 / 0 0.0%	0 / 0 0.0%	185 / 598 30.9%	170 / 598 28.4%AB	132 / 598 22.1%ABE	84 / 598 14.0%E	15 / 598 2.5%C	12 / 598 2.0%CE
5	Thermoproteales	139 / 230 60.4%	1 / 5 20.0%	4 / 5 80.0%	2 / 5 40.0%	3 / 5 60.0%	6 / 15 40.0%	7 / 15 46.7%	2 / 5 40.0%	1 / 5 20.0%	10 / 15 66.7%	5 / 5 100.0%	11 / 20 55.0%	8 / 10 80.0%	4 / 5 80.0%	7 / 10 70.0%	3 / 5 60.0%	8 / 15 53.3%	1 / 5 20.0%	8 / 15 53.3%	23 / 25 92.0%	7 / 10 70.0%	2 / 5 40.0%	16 / 25 64.0%	0 / 0 0.0%	0 / 0 0.0%	0 / 0 0.0%	0 / 0 0.0%	88 / 230 38.3%AB	62 / 230 27.0%	51 / 230 22.2%ABE	20 / 230 8.7%E	5 / 230 2.2%C	4 / 230 1.7%CE
4	Thermoproteaceae	95 / 184 51.6%	1 / 4 25.0%	3 / 4 75.0%	1 / 4 25.0%	2 / 4 50.0%	3 / 12 25.0%	4 / 12 33.3%	1 / 4 25.0%	1 / 4 25.0%	7 / 12 58.3%	4 / 4 100.0%	7 / 16 43.8%	6 / 8 75.0%	3 / 4 75.0%	5 / 8 62.5%	2 / 4 50.0%	5 / 12 41.7%	0 / 4 0.0%	5 / 12 41.7%	18 / 20 90.0%	5 / 8 62.5%	1 / 4 25.0%	11 / 20 55.0%	0 / 0 0.0%	0 / 0 0.0%	0 / 0 0.0%	0 / 0 0.0%	68 / 184 37.0%AB	61 / 184 33.2%	27 / 184 14.7%ABE	19 / 184 10.3%E	5 / 184 2.7%C	4 / 184 2.2%CE
4	Pyrobaculum	95 / 184 51.6%	1 / 4 25.0%	3 / 4 75.0%	1 / 4 25.0%	2 / 4 50.0%	3 / 12 25.0%	4 / 12 33.3%	1 / 4 25.0%	1 / 4 25.0%	7 / 12 58.3%	4 / 4 100.0%	7 / 16 43.8%	6 / 8 75.0%	3 / 4 75.0%	5 / 8 62.5%	2 / 4 50.0%	5 / 12 41.7%	0 / 4 0.0%	5 / 12 41.7%	18 / 20 90.0%	5 / 8 62.5%	1 / 4 25.0%	11 / 20 55.0%	0 / 0 0.0%	0 / 0 0.0%	0 / 0 0.0%	0 / 0 0.0%	68 / 184 37.0%AB	61 / 184 33.2%	27 / 184 14.7%ABE	19 / 184 10.3%E	5 / 184 2.7%C	4 / 184 2.2%CE
1	DSM 4184	26 / 46 56.5%	1 / 1 100.0%	1 / 1 100.0%	0 / 1 0.0%	1 / 1 100.0%	1 / 3 33.3%	1 / 3 33.3%	0 / 1 0.0%	0 / 1 0.0%	1 / 3 33.3%	1 / 1 100.0%	4 / 4 100.0%	1 / 2 50.0%	1 / 1 100.0%	2 / 2 100.0%	1 / 1 100.0%	2 / 3 66.7%	0 / 1 0.0%	0 / 3 0.0%	3 / 5 60.0%	2 / 2 100.0%	0 / 1 0.0%	3 / 5 60.0%	0 / 0 0.0%	0 / 0 0.0%	0 / 0 0.0%	0 / 0 0.0%	17 / 46 37.0%	16 / 46 34.8%AB	10 / 46 21.7%ABE	2 / 46 4.3%E	1 / 46 2.2%C	
1	Pyrobaculum islandicum DSM 4184	26 / 46 56.5%	1 / 1 100.0%	1 / 1 100.0%	0 / 1 0.0%	1 / 1 100.0%	1 / 3 33.3%	1 / 3 33.3%	0 / 1 0.0%	0 / 1 0.0%	1 / 3 33.3%	1 / 1 100.0%	4 / 4 100.0%	1 / 2 50.0%	1 / 1 100.0%	2 / 2 100.0%	1 / 1 100.0%	2 / 3 66.7%	0 / 1 0.0%	0 / 3 0.0%	3 / 5 60.0%	2 / 2 100.0%	0 / 1 0.0%	3 / 5 60.0%	0 / 0 0.0%	0 / 0 0.0%	0 / 0 0.0%	0 / 0 0.0%	17 / 46 37.0%	16 / 46 34.8%AB	10 / 46 21.7%ABE	2 / 46 4.3%E	1 / 46 2.2%C	

1	ATCC 51768	52.2%	0.0%	100.0%	0.0%	0.0%	33.3%	0.0%	100.0%	0.0%	66.7%	100.0%	25.0%	50.0%	100.0%	50.0%	100.0%	0.0%	0.0%	66.7%	100.0%	50.0%	0.0%	100.0%	0.0%	0.0%	0.0%	0.0%	34.8%AB	30.4%	17.4%ABE	6.5%C	6.5%E	4.3%CE
1	Pyrobaculum aerophilum IM2	24 / 46 52.2%	0 / 1 0.0%	1 / 1 100.0%	0 / 1 0.0%	0 / 1 0.0%	1 / 3 33.3%	0 / 3 0.0%	1 / 1 100.0%	0 / 1 0.0%	2 / 3 66.7%	1 / 1 100.0%	1 / 4 25.0%	1 / 2 50.0%	1 / 1 100.0%	1 / 2 50.0%	1 / 1 100.0%	0 / 3 0.0%	0 / 1 0.0%	2 / 3 66.7%	5 / 5 100.0%	1 / 2 50.0%	0 / 1 0.0%	5 / 5 100.0%	0 / 0 0.0%	0 / 0 0.0%	0 / 0 0.0%	0 / 0 0.0%	16 / 46 34.8%AB	14 / 46 30.4%	8 / 46 17.4%ABE	3 / 46 6.5%C	3 / 46 6.5%E	2 / 46 4.3%CE
1	ATCC 700994	17 / 46 37.0%	0 / 1 0.0%	1 / 1 100.0%	0 / 1 0.0%	0 / 1 0.0%	1 / 3 33.3%	0 / 3 0.0%	0 / 1 0.0%	0 / 1 0.0%	1 / 3 33.3%	1 / 1 100.0%	2 / 4 50.0%	2 / 2 100.0%	1 / 1 100.0%	0 / 2 0.0%	0 / 1 0.0%	0 / 3 0.0%	0 / 1 0.0%	0 / 3 0.0%	5 / 5 100.0%	0 / 2 0.0%	0 / 1 0.0%	3 / 5 60.0%	0 / 0 0.0%	0 / 0 0.0%	0 / 0 0.0%	0 / 0 0.0%	21 / 46 45.7%	11 / 46 23.9%AB	6 / 46 13.0%ABE	5 / 46 10.9%E	2 / 46 4.3%CE	1 / 46 2.2%C
1	Pyrobaculum arsenaticum DSM 13514	17 / 46 37.0%	0 / 1 0.0%	1 / 1 100.0%	0 / 1 0.0%	0 / 1 0.0%	1 / 3 33.3%	0 / 3 0.0%	0 / 1 0.0%	0 / 1 0.0%	1 / 3 33.3%	1 / 1 100.0%	2 / 4 50.0%	2 / 2 100.0%	1 / 1 100.0%	0 / 2 0.0%	0 / 1 0.0%	0 / 3 0.0%	0 / 1 0.0%	0 / 3 0.0%	5 / 5 100.0%	0 / 2 0.0%	0 / 1 0.0%	3 / 5 60.0%	0 / 0 0.0%	0 / 0 0.0%	0 / 0 0.0%	0 / 0 0.0%	21 / 46 45.7%	11 / 46 23.9%AB	6 / 46 13.0%ABE	5 / 46 10.9%E	2 / 46 4.3%CE	1 / 46 2.2%C
1	DSM 21063	28 / 46 60.9%	0 / 1 0.0%	0 / 1 0.0%	1 / 1 100.0%	1 / 1 100.0%	0 / 3 0.0%	3 / 3 100.0%	0 / 1 0.0%	1 / 1 100.0%	3 / 3 100.0%	1 / 1 100.0%	0 / 4 0.0%	2 / 2 100.0%	0 / 1 0.0%	2 / 2 100.0%	0 / 1 0.0%	3 / 3 100.0%	0 / 1 0.0%	3 / 3 100.0%	5 / 5 100.0%	2 / 2 100.0%	1 / 1 100.0%	0 / 5 0.0%	0 / 0 0.0%	0 / 0 0.0%	0 / 0 0.0%	0 / 0 0.0%	25 / 46 54.3%AB	9 / 46 19.6%E	9 / 46 19.6%	3 / 46 6.5%ABE		
1	Pyrobaculum calidifontis JCM 11548	28 / 46 60.9%	0 / 1 0.0%	0 / 1 0.0%	1 / 1 100.0%	1 / 1 100.0%	0 / 3 0.0%	3 / 3 100.0%	0 / 1 0.0%	1 / 1 100.0%	3 / 3 100.0%	1 / 1 100.0%	0 / 4 0.0%	2 / 2 100.0%	0 / 1 0.0%	2 / 2 100.0%	0 / 1 0.0%	3 / 3 100.0%	0 / 1 0.0%	3 / 3 100.0%	5 / 5 100.0%	2 / 2 100.0%	1 / 1 100.0%	0 / 5 0.0%	0 / 0 0.0%	0 / 0 0.0%	0 / 0 0.0%	0 / 0 0.0%	25 / 46 54.3%AB	9 / 46 19.6%E	9 / 46 19.6%	3 / 46 6.5%ABE		
1	Thermofilaceae	44 / 46 95.7%	0 / 1 0.0%	1 / 1 100.0%	1 / 1 100.0%	1 / 1 100.0%	3 / 3 100.0%	3 / 3 100.0%	1 / 1 100.0%	0 / 1 0.0%	3 / 3 100.0%	1 / 1 100.0%	4 / 4 100.0%	2 / 2 100.0%	1 / 1 100.0%	2 / 2 100.0%	1 / 1 100.0%	3 / 3 100.0%	1 / 1 100.0%	3 / 3 100.0%	5 / 5 100.0%	2 / 2 100.0%	1 / 1 100.0%	5 / 5 100.0%	0 / 0 0.0%	0 / 0 0.0%	0 / 0 0.0%	0 / 0 0.0%	24 / 46 52.2%ABE	20 / 46 43.5%AB	1 / 46 2.2%	1 / 46 2.2%E		
1	Thermofilum	44 / 46 95.7%	0 / 1 0.0%	1 / 1 100.0%	1 / 1 100.0%	1 / 1 100.0%	3 / 3 100.0%	3 / 3 100.0%	1 / 1 100.0%	0 / 1 0.0%	3 / 3 100.0%	1 / 1 100.0%	4 / 4 100.0%	2 / 2 100.0%	1 / 1 100.0%	2 / 2 100.0%	1 / 1 100.0%	3 / 3 100.0%	1 / 1 100.0%	3 / 3 100.0%	5 / 5 100.0%	2 / 2 100.0%	1 / 1 100.0%	5 / 5 100.0%	0 / 0 0.0%	0 / 0 0.0%	0 / 0 0.0%	0 / 0 0.0%	24 / 46 52.2%ABE	20 / 46 43.5%AB	1 / 46 2.2%	1 / 46 2.2%E		
1	DSM 2475	44 / 46 95.7%	0 / 1 0.0%	1 / 1 100.0%	1 / 1 100.0%	1 / 1 100.0%	3 / 3 100.0%	3 / 3 100.0%	1 / 1 100.0%	0 / 1 0.0%	3 / 3 100.0%	1 / 1 100.0%	4 / 4 100.0%	2 / 2 100.0%	1 / 1 100.0%	2 / 2 100.0%	1 / 1 100.0%	3 / 3 100.0%	1 / 1 100.0%	3 / 3 100.0%	5 / 5 100.0%	2 / 2 100.0%	1 / 1 100.0%	5 / 5 100.0%	0 / 0 0.0%	0 / 0 0.0%	0 / 0 0.0%	0 / 0 0.0%	24 / 46 52.2%ABE	20 / 46 43.5%AB	1 / 46 2.2%E	1 / 46 2.2%		
1	Thermofilum pendens Hrk 5	44 / 46 95.7%	0 / 1 0.0%	1 / 1 100.0%	1 / 1 100.0%	1 / 1 100.0%	3 / 3 100.0%	3 / 3 100.0%	1 / 1 100.0%	0 / 1 0.0%	3 / 3 100.0%	1 / 1 100.0%	4 / 4 100.0%	2 / 2 100.0%	1 / 1 100.0%	2 / 2 100.0%	1 / 1 100.0%	3 / 3 100.0%	1 / 1 100.0%	3 / 3 100.0%	5 / 5 100.0%	2 / 2 100.0%	1 / 1 100.0%	5 / 5 100.0%	0 / 0 0.0%	0 / 0 0.0%	0 / 0 0.0%	0 / 0 0.0%	24 / 46 52.2%ABE	20 / 46 43.5%AB	1 / 46 2.2%	1 / 46 2.2%E		
4	Sulfolobales	7 / 184 3.8%	1 / 4 25.0%	0 / 4 0.0%	1 / 4 25.0%	0 / 4 0.0%	1 / 12 8.3%	0 / 12 0.0%	0 / 4 0.0%	0 / 4 0.0%	0 / 12 0.0%	0 / 4 0.0%	2 / 16 12.5%	1 / 8 12.5%	0 / 4 0.0%	0 / 8 0.0%	0 / 4 0.0%	0 / 12 0.0%	1 / 4 25.0%	0 / 12 0.0%	0 / 20 0.0%	0 / 8 0.0%	0 / 4 0.0%	0 / 20 0.0%	0 / 0 0.0%	0 / 0 0.0%	0 / 0 0.0%	0 / 0 0.0%	102 / 184 55.4%	59 / 184 32.1%E	10 / 184 5.4%C	6 / 184 3.3%CE	4 / 184 2.2%AB	3 / 184 1.6%ABE
4	Sulfolobaceae	7 / 184 3.8%	1 / 4 25.0%	0 / 4 0.0%	1 / 4 25.0%	0 / 4 0.0%	1 / 12 8.3%	0 / 12 0.0%	0 / 4 0.0%	0 / 4 0.0%	0 / 12 0.0%	0 / 4 0.0%	2 / 16 12.5%	1 / 8 12.5%	0 / 4 0.0%	0 / 8 0.0%	0 / 4 0.0%	0 / 12 0.0%	1 / 4 25.0%	0 / 12 0.0%	0 / 20 0.0%	0 / 8 0.0%	0 / 4 0.0%	0 / 20 0.0%	0 / 0 0.0%	0 / 0 0.0%	0 / 0 0.0%	0 / 0 0.0%	102 / 184 55.4%	59 / 184 32.1%E	10 / 184 5.4%C	6 / 184 3.3%CE	4 / 184 2.2%AB	3 / 184 1.6%ABE
3	Sulfolobus	4 / 138 2.9%	1 / 3 33.3%	0 / 3 0.0%	1 / 3 33.3%	0 / 3 0.0%	0 / 9 0.0%	0 / 9 0.0%	0 / 3 0.0%	0 / 3 0.0%	0 / 9 0.0%	0 / 3 0.0%	2 / 12 16.7%	0 / 6 0.0%	0 / 3 0.0%	0 / 6 0.0%	0 / 3 0.0%	0 / 9 0.0%	0 / 3 0.0%	0 / 9 0.0%	0 / 15 0.0%	0 / 6 0.0%	0 / 3 0.0%	0 / 15 0.0%	0 / 0 0.0%	0 / 0 0.0%	0 / 0 0.0%	0 / 0 0.0%	81 / 138 58.7%	46 / 138 33.3%E	4 / 138 2.9%CE	3 / 138 2.2%C	3 / 138 2.2%ABE	1 / 138 0.7%AB
1	ATCC 33909	1 / 46 2.2%	0 / 1 0.0%	0 / 1 0.0%	0 / 1 0.0%	0 / 1 0.0%	0 / 3 0.0%	0 / 3 0.0%	0 / 1 0.0%	0 / 1 0.0%	0 / 3 0.0%	0 / 1 0.0%	1 / 4 25.0%	0 / 2 0.0%	0 / 1 0.0%	0 / 2 0.0%	0 / 1 0.0%	0 / 3 0.0%	0 / 1 0.0%	0 / 3 0.0%	0 / 5 0.0%	0 / 2 0.0%	0 / 1 0.0%	0 / 5 0.0%	0 / 0 0.0%	0 / 0 0.0%	0 / 0 0.0%	0 / 0 0.0%	29 / 46 63.0%	14 / 46 30.4%E	2 / 46 4.3%CE	1 / 46 2.2%ABE		
1	Sulfolobus acidocaldarius ATCC 33909	1 / 46 2.2%	0 / 1 0.0%	0 / 1 0.0%	0 / 1 0.0%	0 / 1 0.0%	0 / 3 0.0%	0 / 3 0.0%	0 / 1 0.0%	0 / 1 0.0%	0 / 3 0.0%	0 / 1 0.0%	1 / 4 25.0%	0 / 2 0.0%	0 / 1 0.0%	0 / 2 0.0%	0 / 1 0.0%	0 / 3 0.0%	0 / 1 0.0%	0 / 3 0.0%	0 / 5 0.0%	0 / 2 0.0%	0 / 1 0.0%	0 / 5 0.0%	0 / 0 0.0%	0 / 0 0.0%	0 / 0 0.0%	0 / 0 0.0%	29 / 46 63.0%	14 / 46 30.4%E	2 / 46 4.3%CE	1 / 46 2.2%ABE		
1	ATCC 35091	1 / 46 2.2%	1 / 1 100.0%	0 / 1 0.0%	0 / 1 0.0%	0 / 1 0.0%	0 / 3 0.0%	0 / 3 0.0%	0 / 1 0.0%	0 / 1 0.0%	0 / 3 0.0%	0 / 1 0.0%	0 / 4 0.0%	0 / 2 0.0%	0 / 1 0.0%	0 / 2 0.0%	0 / 1 0.0%	0 / 3 0.0%	0 / 1 0.0%	0 / 3 0.0%	0 / 5 0.0%	0 / 2 0.0%	0 / 1 0.0%	0 / 5 0.0%	0 / 0 0.0%	0 / 0 0.0%	0 / 0 0.0%	0 / 0 0.0%	26 / 46 56.5%	16 / 46 34.8%E	3 / 46 6.5%C	1 / 46 2.2%ABE		
1	Sulfolobus solfataricus P2	1 / 46 2.2%	1 / 1 100.0%	0 / 1 0.0%	0 / 1 0.0%	0 / 1 0.0%	0 / 3 0.0%	0 / 3 0.0%	0 / 1 0.0%	0 / 1 0.0%	0 / 3 0.0%	0 / 1 0.0%	0 / 4 0.0%	0 / 2 0.0%	0 / 1 0.0%	0 / 2 0.0%	0 / 1 0.0%	0 / 3 0.0%	0 / 1 0.0%	0 / 3 0.0%	0 / 5 0.0%	0 / 2 0.0%	0 / 1 0.0%	0 / 5 0.0%	0 / 0 0.0%	0 / 0 0.0%	0 / 0 0.0%	0 / 0 0.0%	26 / 46 56.5%	16 / 46 34.8%E	3 / 46 6.5%C	1 / 46 2.2%ABE		
1	DSM 16993	2 / 46 4.3%	0 / 1 0.0%	0 / 1 0.0%	1 / 1 100.0%	0 / 1 0.0%	0 / 3 0.0%	0 / 3 0.0%	0 / 1 0.0%	0 / 1 0.0%	0 / 3 0.0%	0 / 1 0.0%	1 / 4 25.0%	0 / 2 0.0%	0 / 1 0.0%	0 / 2 0.0%	0 / 1 0.0%	0 / 3 0.0%	0 / 1 0.0%	0 / 3 0.0%	0 / 5 0.0%	0 / 2 0.0%	0 / 1 0.0%	0 / 5 0.0%	0 / 0 0.0%	0 / 0 0.0%	0 / 0 0.0%	0 / 0 0.0%	26 / 46 56.5%	16 / 46 34.8%E	2 / 46 4.3%CE	1 / 46 2.2%ABE	1 / 46 2.2%AB	
1	Sulfolobus sp. (strain 7)	2 / 46 4.3%	0 / 1 0.0%	0 / 1 0.0%	1 / 1 100.0%	0 / 1 0.0%	0 / 3 0.0%	0 / 3 0.0%	0 / 1 0.0%	0 / 1 0.0%	0 / 3 0.0%	0 / 1 0.0%	1 / 4 25.0%	0 / 2 0.0%	0 / 1 0.0%	0 / 2 0.0%	0 / 1 0.0%	0 / 3 0.0%	0 / 1 0.0%	0 / 3 0.0%	0 / 5 0.0%	0 / 2 0.0%	0 / 1 0.0%	0 / 5 0.0%	0 / 0 0.0%	0 / 0 0.0%	0 / 0 0.0%	0 / 0 0.0%	26 / 46 56.5%	16 / 46 34.8%E	2 / 46 4.3%CE	1 / 46 2.2%ABE	1 / 46 2.2%AB	
1	Metallosphaera	3 / 46 6.5%	0 / 1 0.0%	0 / 1 0.0%	0 / 1 0.0%	0 / 1 0.0%	1 / 3 33.3%	0 / 3 0.0%	0 / 1 0.0%	0 / 1 0.0%	0 / 3 0.0%	0 / 1 0.0%	0 / 4 0.0%	1 / 2 50.0%	0 / 1 0.0%	0 / 2 0.0%	0 / 1 0.0%	0 / 3 0.0%	1 / 1 100.0%	0 / 3 0.0%	0 / 5 0.0%	0 / 2 0.0%	0 / 1 0.0%	0 / 5 0.0%	0 / 0 0.0%	0 / 0 0.0%	0 / 0 0.0%	0 / 0 0.0%	21 / 46 45.7%	13 / 46 28.3%E	7 / 46 15.2%C	3 / 46 6.5%AB	2 / 46 4.3%CE	
1	ATCC 51363	3 / 46 6.5%	0 / 1 0.0%	0 / 1 0.0%	0 / 1 0.0%	0 / 1 0.0%	1 / 3 33.3%	0 / 3 0.0%	0 / 1 0.0%	0 / 1 0.0%	0 / 3 0.0%	0 / 1 0.0%	0 / 4 0.0%	1 / 2 50.0%	0 / 1 0.0%	0 / 2 0.0%	0 / 1 0.0%	0 / 3 0.0%	1 / 1 100.0%	0 / 3 0.0%	0 / 5 0.0%	0 / 2 0.0%	0 / 1 0.0%	0 / 5 0.0%	0 / 0 0.0%	0 / 0 0.0%	0 / 0 0.0%	0 / 0 0.0%	21 / 46 45.7%	13 / 46 28.3%E	7 / 46 15.2%C	3 / 46 6.5%AB	2 / 46 4.3%CE	
1	Metallosphaera sedula DSM 5348	3 / 46 6.5%	0 / 1 0.0%	0 / 1 0.0%	0 / 1 0.0%	0 / 1 0.0%	1 / 3 33.3%	0 / 3 0.0%	0 / 1 0.0%	0 / 1 0.0%	0 / 3 0.0%	0 / 1 0.0%	0 / 4 0.0%	1 / 2 50.0%	0 / 1 0.0%	0 / 2 0.0%	0 / 1 0.0%	0 / 3 0.0%	1 / 1 100.0%	0 / 3 0.0%	0 / 5 0.0%	0 / 2 0.0%	0 / 1 0.0%	0 / 5 0.0%	0 / 0 0.0%	0 / 0 0.0%	0 / 0 0.0%	0 / 0 0.0%	21 / 46 45.7%	13 / 46 28.3%E	7 / 46 15.2%C	3 / 46 6.5%AB	2 / 46 4.3%CE	
4	Desulfurococcales	156 / 184 84.8%	4 / 4 100.0%	3 / 4 75.0%	3 / 4 75.0%	2 / 4 50.0%	11 / 12 91.7%	10 / 12 83.3%	3 / 4 75.0%	4 / 4 100.0%	12 / 12 100.0%	4 / 4 100.0%	15 / 16 93.8%	7 / 8 87.5%	2 / 4 50.0%	8 / 8 100.0%	4 / 4 100.0%	8 / 12 66.7%	3 / 4 75.0%	9 / 12 75.0%	15 / 20 75.0%	6 / 8 75.0%	3 / 4 75.0%	20 / 20 100.0%	0 / 0 0.0%	0 / 0 0.0%	0 / 0 0.0%	0 / 0 0.0%	78 / 184 42.4%ABE	78 / 184 42.4%AB	21 / 184 11.4%	5 / 184 2.7%E	2 / 184 1.1%CE	
3	Desulfurococcaceae	110 / 138 79.7%	3 / 3 100.0%	2 / 3 66.7%	2 / 3 66.7%	1 / 3 33.3%	8 / 9 88.9%	7 / 9 77.8%	2 / 3 66.7%	3 / 3 100.0%	9 / 9 100.0%	3 / 3 100.0%	11 / 12 91.7%	5 / 6 83.3%	1 / 3 33.3%	6 / 6 100.0%	3 / 3 100.0%	5 / 9 55.6%	2 / 3 66.7%	6 / 9 66.7%	10 / 15 66.7%	4 / 6 66.7%	2 / 3 66.7%	15 / 15 100.0%	0 / 0 0.0%	0 / 0 0.0%	0 / 0 0.0%	0 / 0 0.0%	57 / 138 41.3%ABE	53 / 138 38.4%AB	21 / 138 15.2%	5 / 138 3.6%E	2 / 138 1.4%CE	
1	Staphylothermus	20 / 45 44.4%	1 / 1 100.0%	0 / 1 0.0%	0 / 1 0.0%	0 / 1 0.0%	2 / 3 66.7%	1 / 3 33.3%	0 / 1 0.0%	1 / 1 100.0%	3 / 3 100.0%	1 / 1 100.0%	3 / 4 75.0%	0 / 1 0.0%	0 / 1 0.0%	2 / 2 100.0%	1 / 1 100.0%	0 / 3 0.0%	0 / 1 0.0%	0 / 3 0.0%	0 / 5 0.0%	0 / 2 0.0%	0 / 1 0.0%	5 / 5 100.0%	0 / 0 0.0%	0 / 0 0.0%	0 / 0 0.0%	0 / 0 0.0%	19 / 45 42.2%	14 / 45 31.1%ABE	6 / 45 13.3%AB	5 / 45 11.1%E	1 / 45 2.2%CE	
1	ATCC 43588	20 / 45 44.4%	1 / 1 100.0%	0 / 1 0.0%	0 / 1 0.0%	0 / 1 0.0%	2 / 3 66.7%	1 / 3 33.3%	0 / 1 0.0%	1 / 1 100.0%	3 / 3 100.0%	1 / 1 100.0%	3 / 4 75.0%	0 / 1 0.0%	0 / 1 0.0%	2 / 2 100.0%	1 / 1 100.0%	0 / 3 0.0%	0 / 1 0.0%	0 / 3 0.0%	0 / 5 0.0%	0 / 2 0.0%	0 / 1 0.0%	5 / 5 100.0%	0 / 0 0.0%	0 / 0 0.0%	0 / 0 0.0%	0 / 0 0.0%	19 / 45 42.2%	14 / 45 31.1%ABE	6 / 45 13.3%AB	5 / 45 11.1%E	1 / 45 2.2%CE	
1	Staphylothermus marinus ATCC 43588	20 / 45 44.4%	1 / 1 100.0%	0 / 1 0.0%	0 / 1 0.0%	0 / 1 0.0%	2 / 3 66.7%	1 / 3 33.3%	0 / 1 0.0%	1 / 1 100.0%	3 / 3 100.0%	1 / 1 100.0%	3 / 4 75.0%	0 / 1 0.0%	0 / 1 0.0%	2 / 2 100.0%	1 / 1 100.0%	0 / 3 0.0%	0 / 1 0.0%	0 / 3 0.0%	0 / 5 0.0%	0 / 2 0.0%	0 / 1 0.0%	5 / 5 100.0%	0 / 0 0.0%	0 / 0 0.0%	0 / 0 0.0%	0 / 0 0.0%	19 / 45 42.2%	14 / 45 31.1%ABE	6 / 45 13.3%AB	5 / 45 11.1%E	1 / 45 2.2%CE	
1	Igneococcus	44 / 47 93.6%	1 / 1 100.0%	1 / 1 100.0%	1 / 1 100.0%	0 / 1 0.0%	3 / 3 100.0%	3 / 3 100.0%	1 / 1 100.0%	1 / 1 100.0%	3 / 3 100.0%	1 / 1 100.0%	4 / 4 100.0%	3 / 3 100.0%	0 / 1 0.0%	2 / 2 100.0%	1 / 1 100.0%	2 / 3 66.7%	1 / 1 100.0%	3 / 3 100.0%	5 / 5 100.0%	2 / 2 100.0%	1 / 1 100.0%	5 / 5 100.0%	0 / 0 0.0%	0 / 0 0.0%	0 / 0 0.0%	0 / 0 0.0%	22 / 47 46.8%AB	22 / 47 46.8%ABE	2 / 47 4.3%	1 / 47 2.1%CE		
1	DSM 18386	44 / 47 93.6%	1 / 1 100.0%	1 / 1 100.0%	1 / 1 100.0%	0 / 1 0.0%	3 / 3 100.0%	3 / 3 100.0%	1 / 1 100.0%	1 / 1 100.0%	3 / 3 100.0%	1 / 1 100.0%	4 / 4 100.0%	3 / 3 100.0%	0 / 1 0.0%	2 / 2 100.0%	1 / 1 100.0%	2 / 3 66.7%	1 / 1 100.0%	3 / 3 100.0%	5 / 5 100.0%	2 / 2 100.0%	1 / 1 100.0%	5 / 5 100.0%	0 / 0 0.0%	0 / 0 0.0%	0 / 0 0.0%	0 / 0 0.0%	22 / 47 46.8%AB	22 / 47 46.8%ABE	2 / 47 4.3%	1 / 47 2.1%CE		
1	Ignicoccus hospitalis DSM 18386	44 / 47 93.6%	1 / 1 100.0%	1 / 1 100.0%	1 / 1 100.0%	0 / 1 0.0%	3 / 3 100.0%	3 / 3 100.0%	1 / 1 100.0%	1 / 1 100.0%	3 / 3 100.0%	1 / 1 100.0%	4 / 4 100.0%	3 / 3 100.0%	0 / 1 0.0%	2 / 2 100.0%	1 / 1 100.0%	2 / 3 66.7%	1 / 1 100.0%	3 / 3 100.0%	5 / 5 100.0%	2 / 2 100.0%	1 / 1 100.0%	5 / 5 100.0%	0 / 0 0.0%	0 / 0 0.0%	0 / 0 0.0%	0 / 0 0.0%	22 / 47 46.8%AB	22 / 47 46.8%ABE	2 / 47 4.3%	1 / 47 2.1%CE		
1	Aeropyrum	46 / 46 100.0%	1 / 1 100.0%	1 / 1 100.0%	1 / 1 100.0%	1 / 1 100.0%	3 / 3 100.0%	3 / 3 100.0%	1 / 1 100.0%	1 / 1 100.0%	3 / 3 100.0%	1 / 1 100.0%	4 / 4 100.0%	2 / 2 100.0%	1 / 1 100.0%	2 / 2 100.0%	1 / 1 100.0%	3 / 3 100.0%	1 / 1 100.0%	3 / 3 100.0%	5 / 5 100.0%	2 / 2 100.0%	1 / 1 100.0%	5 / 5 100.0%	0 / 0 0.0%	0 / 0 0.0%	0 / 0 0.0%	0 / 0 0.0%	25 / 46 54.3%AB	21 / 46 45.7%ABE				
1	ATCC 700893	46 / 46 100.0%	1 / 1 100.0%	1 / 1 100.0%	1 / 1 100.0%	1 / 1 100.0%	3 / 3 100.0%	3 / 3 100.0%	1 / 1 100.0%	1 / 1 100.0%	3 / 3 100.0%	1 / 1 100.0%	4 / 4 100.0%	2 / 2 100.0%	1 / 1 100.0%	2 / 2 100.0%	1 / 1 100.0%	3 / 3 100.0%	1 / 1 100.0%	3 / 3 100.0%	5 / 5 100.0%	2 / 2 100.0%	1 / 1 100.0%	5 / 5 100.0%	0 / 0 0.0%	0 / 0 0.0%	0 / 0 0.0%	0 / 0 0.0%	25 / 46 54.3%AB	21 / 46 45.7%ABE				
1	Aeropyrum pernix K1	46 / 46 100.0%	1 / 1 100.0%	1 / 1 100.0%	1 / 1 100.0%	1 / 1 100.0%	3 / 3 100.0%	3 / 3 100.0%	1 / 1 100.0%	1 / 1 100.0%	3 / 3 100.0%	1 / 1 100.0%	4 / 4 100.0%	2 / 2 100.0%	1 / 1 100.0%	2 / 2 100.0%	1 / 1 100.0%	3 / 3 100.0%	1 / 1 100.0%	3 / 3 100.0%	5 / 5 100.0%	2 / 2 100.0%	1 / 1 100.0%	5 / 5 100.0%	0 / 0 0.0%	0 / 0 0.0%	0 / 0 0.0%	0 / 0 0.0%	25 / 46 54.3%AB	21 / 46 45.7%ABE				
1	Pyrodictiaceae	46 / 46 100.0%	1 / 1 100.0%	1 / 1 100.0%	1 / 1 100.0%	1 / 1 100.0%	3 / 3 100.0%	3 / 3 100.0%	1 / 1 100.0%	1 / 1 100.0%	3 / 3 100.0%	1 / 1 100.0%	4 / 4 100.0%	2 / 2 100.0%	1 / 1 100.0%	2 / 2 100.0%	1 / 1 100.0%	3 / 3 100.0%	1 / 1 100.0%	3 / 3 100.0%	5 / 5 100.0%	2 / 2 100.0%	1 / 1 100.0%	5 / 5 100.0%	0 / 0 0.0%	0 / 0 0.0%	0 / 0 0.0%	0 / 0 0.0%	25 / 46 54.3%AB	21 / 46 45.7%ABE				
1	Hyperthermus	46 / 46 100.0%	1 / 1 100.0%	1 / 1 100.0%	1 / 1 100.0%	1 / 1 100.0%	3 / 3 100.0%	3 / 3 100.0%	1 / 1 100.0%	1 / 1 100.0%	3 / 3 100.0%	1 / 1 100.0%	4 / 4 100.0%	2 / 2 100.0%	1 / 1 100.0%	2 / 2 100.0%	1 / 1 100.0%	3 / 3 100.0%	1 / 1 100.0%	3 / 3 100.0%	5 / 5 100.0%	2 / 2 100.0%	1 / 1 100.0%	5 / 5 100.0%	0 / 0 0.0%	0 / 0 0.0%	0 / 0 0.0%	0 / 0 0.0%	25 / 46 54.3%AB	21 / 46 45.7%ABE				
1	ATCC 700455	46 / 46 100.0%	1 / 1 100.0%	1 / 1 100.0%	1 / 1 100.0%	1 / 1 100.0%	3 / 3 100.0%	3 / 3 100.0%	1 / 1 100.0%	1 / 1 100.0%	3 / 3 100.0%	1 / 1 100.0%	4 / 4 100.0%	2 / 2 100.0%	1 / 1 100.0%	2 / 2 100.0%	1 / 1 100.0%	3 / 3 100.0%	1 / 1 100.0%	3 / 3 100.0%	5 / 5 100.0%	2 / 2 100.0%	1 / 1 100.0%	5 / 5 100.0%	0 / 0 0.0%	0 / 0 0.0%	0 / 0 0.0%	0 / 0 0.0%	25 / 46 54.3%AB	21 / 46 45.7%ABE				
1	Hyperthermus butylicus DSM 5456	46 / 46 100.0%	1 / 1 100.0%	1 / 1 100.0%	1 / 1 100.0%	1 / 1 100.0%	3 / 3 100.0%	3 / 3 100.0%	1 / 1 100.0%	1 / 1 100.0%	3 / 3 100.0%	1 / 1 100.0%	4 / 4 100.0%	2 / 2 100.0%	1 / 1 100.0%	2 / 2 100.0%	1 / 1 100.0%	3 / 3 100.0%	1 / 1 100.0%	3 / 3 100.0%	5 / 5 100.0%	2 / 2 100.0%	1 / 1 100.0%	5 / 5 100.0%	0 / 0 0.0%	0 / 0 0.0%	0 / 0 0.0%	0 / 0 0.0%	25 / 46 54.3%AB	21 / 46 45.7%ABE				
32	"Euryarchaeota" Woese et al. 1990	331 / 1454 22.8%	10 / 41 24.4%	6 / 38 15.8%	10 / 36 27.8%	11 / 36 30.6%	17 / 96 17.7%	25 / 91 27.5%	14 / 32 43.8%	8 / 32 25.0%	16 / 101 15.8%	6 / 32 18.8%	27 / 120 22.5%	14 / 61 23.0%	13 / 36 36.1%	14 / 56 25.0%	6 / 44 13.6%	27 / 82 32.9%	6 / 32 18.8%	17 / 92 18.5%	25 / 140 17.9%	18 / 65 27.7%	5 / 32 15.6%	32 / 148 21.6%	0 / 7 0.0%	4 / 4 100.0%	0 / 0 0.0%	0 / 0 0.0%	554 / 1454 38.1%E	541 / 1454 37.2%	172 / 1454 11.8%AB	159 / 1454 10.9%ABE	15 / 1454 1.0%C	13 / 1454 0.9%CE
3	Archaeobacteria	3 / 116 2.6%	0 / 3 0.0%	0 / 3 0.0%	0 / 4 0.0%	0 / 3 0.0%	0 / 7 0.0%	0 / 7 0.0%	3 / 3 100.0%	0 / 3 0.0%	0 / 9 0.0%	0 / 3 0.0%	0 / 12 0.0%	0 / 3 0.0%	0 / 4 0.0%	0 / 4 0.0%	0 / 3 0.0%	0 / 4 0.0%	0 / 3 0.0%	0 / 9 0.0%	0 / 12 0.0%	0 / 4 0.0%	0 / 3 0.0%	0 / 10 0.0%	0 / 0 0.0%	0 / 0 0.0%	0 / 0 0.0%	0 / 0 0.0%	68 / 116 58.6%	45 / 116 38.8%E	2 / 116 1.7%AB	1 / 116 0.9%ABE		
3	Methanobacteriales	3 / 116 2.6%	0 / 3 0.0%	0 / 3 0.0%	0 / 4 0.0%	0 / 3 0.0%	0 / 7 0.0%	0 / 7 0.0%	3 / 3 100.0%	0 / 3 0.0%	0 / 9 0.0%	0 / 3 0.0%	0 / 12 0.0%	0 / 3 0.0%	0 / 4 0.0%	0 / 4 0.0%	0 / 3 0.0%	0 / 4 0.0%	0 / 3 0.0%	0 / 9 0.0%	0 / 12 0.0%	0 / 4 0.0%	0 / 3 0.0%	0 / 10 0.0%	0 / 0 0.0%	0 / 0 0.0%	0 / 0 0.0%	0 / 0 0.0%	68 / 116 58.6%	45 / 116 38.8%E	2 / 116 1.7%AB	1 / 116 0.9%ABE		
3	Methanobacteriaceae	3 / 116 2.6%	0 / 3 0.0%	0 / 3 0.0%	0 / 4 0.0%	0 / 3 0.0%	0 / 7 0.0%	0 / 7 0.0%	3 / 3 100.0%	0 / 3 0.0%	0 / 9 0.0%	0 / 3 0.0%	0 / 12 0.0%	0 / 3 0.0%	0 / 4 0.0%	0 / 4 0.0%	0 / 3 0.0%	0 / 4 0.0%	0 / 3 0.0%	0 / 9 0.0%	0 / 12 0.0%	0 / 4 0.0%	0 / 3 0.0%	0 / 10 0.0%	0 / 0 0.0%	0 / 0 0.0%	0 / 0 0.0%	0 / 0 0.0%	68 / 116 58.6%	45 / 116 38.8%E	2 / 116 1.7%AB	1 / 116 0.9%ABE		
1	Methanobrevibacter	1 / 36 2.8%	0 / 1 0.0%	0 / 1 0.0%	0 / 1 0.0%	0 / 1 0.0%	0 / 2 0.0%	0 / 2 0.0%	1 / 1 100.0%	0 / 1 0.0%	0 / 2 0.0%	0 / 1 0.0%	0 / 5 0.0%	0 / 1 0.0%	0 / 1 0.0%	0 / 1 0.0%	0 / 1 0.0%	0 / 1 0.0%	0 / 1 0.0%	0 / 3 0.0%	0 / 4 0.0%	0 / 1 0.0%	0 / 1 0.0%	0 / 3 0.0%	0 / 0 0.0%	0 / 0 0.0%	0 / 0 0.0%	0 / 0 0.0%	20 / 36 55.6%	15 / 36 41.7%E	1 / 36 2.8%AB			
1	ATCC 35061	1 / 36 2.8%	0 / 1 0.0%	0 / 1 0.0%	0 / 1 0.0%	0 / 1 0.0%	0 / 2 0.0%	0 / 2 0.0%	1 / 1 100.0%	0 / 1 0.0%	0 / 2 0.0%	0 / 1 0.0%	0 / 5 0.0%	0 / 1 0.0%	0 / 1 0.0%	0 / 1 0.0%	0 / 1 0.0%	0 / 1 0.0%	0 / 1 0.0%	0 / 3 0.0%	0 / 4 0.0%	0 / 1 0.0%	0 / 1 0.0%	0 / 3 0.0%	0 / 0 0.0%	0 / 0 0.0%	0 / 0 0.0%	0 / 0 0.0%	20 / 36 55.6%	15 / 36 41.7%E	1 / 36 2.8%AB			
1	Methanobrevibacter smithii ATCC 35061	1 / 36 2.8%	0 / 1 0.0%	0 / 1 0.0%	0 / 1 0.0%	0 / 1 0.0%	0 / 2 0.0%	0 / 2 0.0%	1 / 1 100.0%	0 / 1 0.0%	0 / 2 0.0%	0 / 1 0.0%	0 / 5 0.0%	0 / 1 0.0%	0 / 1 0.0%	0 / 1 0.0%	0 / 1 0.0%	0 / 1 0.0%	0 / 1 0.0%	0 / 3 0.0%	0 / 4 0.0%	0 / 1 0.0%	0 / 1 0.0%	0 / 3 0.0%	0 / 0 0.0%	0 / 0 0.0%	0 / 0 0.0%	0 / 0 0.0%	20 / 36 55.6%	15 / 36 41.7%E	1 / 36 2.8%AB			
1	Methanosphaera	1 / 41 2.4%	0 / 1 0.0%	0 / 1 0.0%	0 / 2 0.0%	0 / 1 0.0%	0 / 3 0.0%	0 / 2 0.0%	1 / 1 100.0%	0 / 1 0.0%	0 / 4 0.0%	0 / 1 0.0%	0 / 3 0.0%	0 / 1 0.0%	0 / 2 0.0%	0 / 1 0.0%	0 / 1 0.0%	0 / 1 0.0%	0 / 1 0.0%	0 / 3 0.0%	0 / 4 0.0%	0 / 2 0.0%	0 / 1 0.0%	0 / 4 0.0%	0 / 0 0.0%	0 / 0 0.0%	0 / 0 0.0%	0 / 0 0.0%	25 / 41 61.0%	15 / 41 36.6%E	1 / 41 2.4%AB			
1	ATCC 43021	1 / 41 2.4%	0 / 1 0.0%	0 / 1 0.0%	0 / 2 0.0%	0 / 1 0.0%	0 / 3 0.0%	0 / 2 0.0%	1 / 1 100.0%	0 / 1 0.0%	0 / 4 0.0%	0 / 1 0.0%	0 / 3 0.0%	0 / 1 0.0%	0 / 2 0.0%	0 / 1 0.0%	0 / 1 0.0%	0 / 1 0.0%	0 / 1 0.0%	0 / 3 0.0%	0 / 4 0.0%	0 / 2 0.0%	0 / 1 0.0%	0 / 4 0.0%	0 / 0 0.0%	0 / 0 0.0%	0 / 0 0.0%	0 / 0 0.0%	25 / 41 61.0%	15 / 41 36.6%E	1 / 41 2.4%AB			
1	Methanosphaera stadtmanae ATCC 43021	1 / 41 2.4%	0 / 1 0.0%	0 / 1 0.0%	0 / 2 0.0%	0 / 1 0.0%	0 / 3 0.0%	0 / 2 0.0%	1 / 1 100.0%	0 / 1 0.0%	0 / 4 0.0%	0 / 1 0.0%	0 / 3 0.0%	0 / 1 0.0%	0 / 2 0.0%	0 / 1 0.0%	0 / 1 0.0%	0 / 1 0.0%	0 / 1 0.0%	0 / 3 0.0%	0 / 4 0.0%	0 / 2 0.0%	0 / 1 0.0%	0 / 4 0.0%	0 / 0 0.0%	0 / 0 0.0%	0 / 0 0.0%	0 / 0 0.0%	25 / 41 61.0%	15 / 41 36.6%E	1 / 41 2.4%AB			
1	"Methanobacter" Boone et al. 1993	1 / 39 2.6%	0 / 1 0.0%	0 / 1 0.0%	0 / 1 0.0%	0 / 1 0.0%	0 / 2 0.0%	0 / 3 0.0%	1 / 1 100.0%	0 / 1 0.0%	0 / 3 0.0%	0 / 1 0.0%	0 / 4 0.0%	0 / 1 0.0%	0 / 1 0.0%	0 / 2 0.0%	0 / 1 0.0%	0 / 2 0.0%	0 / 1 0.0%	0 / 3 0.0%	0 / 4 0.0%	0 / 1 0.0%	0 / 1 0.0%	0 / 3 0.0%	0 / 0 0.0%	0 / 0 0.0%	0 / 0 0.0%	0 / 0 0.0%	23 / 39 59.0%	15 / 39 38.5%E	1 / 39 2.6%ABE			
1	"Methanobacterium thermoautotrophicus" (sic) Zeikus and Wolfe 1972	1 / 39 2.6%	0 / 1 0.0%	0 / 1 0.0%	0 / 1 0.0%	0 / 1 0.0%	0 / 2 0.0%	0 / 3 0.0%	1 / 1 100.0%	0 / 1 0.0%	0 / 3 0.0%	0 / 1 0.0%	0 / 4 0.0%	0 / 1 0.0%	0 / 1 0.0%	0 / 2 0.0%	0 / 1 0.0%	0 / 2 0.0%	0 / 1 0.0%	0 / 3 0.0%	0 / 4 0.0%	0 / 1 0.0%	0 / 1 0.0%	0 / 3 0.0%	0 / 0 0.0%	0 / 0 0.0%	0 / 0 0.0%	0 / 0 0.0%	23 / 39 59.0%	15 / 39 38.5%E	1 / 39 2.6%ABE			
1	Methanobacterium thermoautotrophicum str. Delta H	1 / 39 2.6%	0 / 1 0.0%	0 / 1 0.0%	0 / 1 0.0%	0 / 1 0.0%	0 / 2 0.0%	0 / 3 0.0%	1 / 1 100.0%	0 / 1 0.0%	0 / 3 0.0%	0 / 1 0.0%	0 / 4 0.0%	0 / 1 0.0%	0 / 1 0.0%	0 / 2 0.0%	0 / 1 0.0%	0 / 2 0.0%	0 / 1 0.0%	0 / 3 0.0%	0 / 4 0.0%	0 / 1 0.0%	0 / 1 0.0%	0 / 3 0.0%	0 / 0 0.0%	0 / 0 0.0%	0 / 0 0.0%	0 / 0 0.0%	23 / 39 59.0%	15 / 39 38.5%E	1 / 39 2.6%ABE			
6	Methanococci	64 / 228 28.1%	5 / 11 45.5%	1 / 11 9.1%	5 / 6 83.3%	1 / 6 16.7%	2 / 13 15.4%	10 / 13 76.9%	5 / 6 83.3%	2 / 6 33.3%	1 / 13 7.7%	1 / 6 16.7%	6 / 18 33.3%	0 / 11 0.0%	1 / 6 16.7%	4 / 6 66.7%	1 / 6 16.7%	8 / 12 66.7%	1 / 6 16.7%	2 / 12 16.7%	4 / 18 22.2%	0 / 12 0.0%	1 / 6 16.7%	3 / 18 16.7%	0 / 6 0.0%	0 / 0 0.0%	0 / 0 0.0%	0 / 0 0.0%	108 / 228 47.4%E	49 / 228 21.5%	33 / 228 14.5%AB	31 / 228 13.6%ABE	4 / 228 1.8%CE	3 / 228 1.3%C
6	Methanococcales	64 / 228 28.1%	5 / 11 45.5%	1 / 11 9.1%	5 / 6 83.3%	1 / 6 16.7%	2 / 13 15.4%	10 / 13 76.9%	5 / 6 83.3%	2 / 6 33.3%	1 / 13 7.7%	1 / 6 16.7%	6 / 18 33.3%	0 / 11 0.0%	1 / 6 16.7%	4 / 6 66.7%	1 / 6 16.7%	8 / 12 66.7%	1 / 6 16.7%	2 / 12 16.7%	4 / 18 22.2%	0 / 12 0.0%	1 / 6 16.7%	3 / 18 16.7%	0 / 6 0.0%	0 / 0 0.0%	0 / 0 0.0%	0 / 0 0.0%	108 / 228 47.4%E	49 / 228 21.5%	33 / 228 14.5%AB	31 / 228 13.6%ABE	4 / 228 1.8%CE	3 / 228 1.3%C
5	Methanococcaceae	39 / 191 20.4%	5 / 10 50.0%	0 / 10 0.0%	4 / 5 80.0%	0 / 5 0.0%	0 / 11 0.0%	8 / 10 80.0%	5 / 5 100.0%	1 / 5 20.0%	0 / 10 0.0%	0 / 5 0.0%	4 / 15 26.7%	0 / 10 0.0%	0 / 5 0.0%	3 / 5 60.0%	0 / 5 0.0%	6 / 10 60.0%	0 / 5 0.0%	0 / 10 0.0%	3 / 15 20.0%	0 / 10 0.0%	0 / 5 0.0%	0 / 15 0.0%	0 / 5 0.0%	0 / 0 0.0%	0 / 0 0.0%	0 / 0 0.0%	100 / 191 52.4%E	45 / 191 23.6%	23 / 191 12.0%AB	16 / 191 8.4%ABE	4 / 191 2.1%CE	3 / 191 1.6%C
5	Methanococcus	39 / 191 20.4%	5 / 10 50.0%	0 / 10 0.0%	4 / 5 80.0%	0 / 5 0.0%	0 / 11 0.0%	8 / 10 80.0%	5 / 5 100.0%	1 / 5 20.0%	0 / 10 0.0%	0 / 5 0.0%	4 / 15 26.7%	0 / 10 0.0%	0 / 5 0.0%	3 / 5 60.0%	0 / 5 0.0%	6 / 10 60.0%	0 / 5 0.0%	0 / 10 0.0%	3 / 15 20.0%	0 / 10 0.0%	0 / 5 0.0%	0 / 15 0.0%	0 / 5 0.0%	0 / 0 0.0%	0 / 0 0.0%	0 / 0 0.0%	100 / 191 52.4%E	45 / 191 23.6%	23 / 191 12.0%AB	16 / 191 8.4%ABE	4 / 191 2.1%CE	3 / 191 1.6%C
1	ATCC 35089	5 / 38 13.2%	0 / 2 0.0%	0 / 2 0.0%	1 / 1 100.0%	0 / 1 0.0%	0 / 2 0.0%	1 / 2 50.0%	1 / 1 100.0%	0 / 1 0.0%	0 / 2 0.0%	0 / 1 0.0%	1 / 3 33.3%	0 / 2 0.0%	0 / 1 0.0%	0 / 1 0.0%	0 / 1 0.0%	1 / 2 50.0%	0 / 1 0.0%	0 / 2 0.0%	0 / 3 0.0%	0 / 2 0.0%	0 / 1 0.0%	0 / 3 0.0%	0 / 1 0.0%	0 / 0 0.0%	0 / 0 0.0%	0 / 0 0.0%	21 / 38 55.3%E	12 / 38 31.6%	3 / 38 7.9%AB	2 / 38 5.3%ABE		
1	Methanococcus vannielii SB	5 / 38 13.2%	0 / 2 0.0%	0 / 2 0.0%	1 / 1 100.0%	0 / 1 0.0%	0 / 2 0.0%	1 / 2 50.0%	1 / 1 100.0%	0 / 1 0.0%	0 / 2 0.0%	0 / 1 0.0%	1 / 3 33.3%	0 / 2 0.0%	0 / 1 0.0%	0 / 1 0.0%	0 / 1 0.0%	1 / 2 50.0%	0 / 1 0.0%	0 / 2 0.0%	0 / 3 0.0%	0 / 2 0.0%	0 / 1 0.0%	0 / 3 0.0%	0 / 1 0.0%	0 / 0 0.0%	0 / 0 0.0%	0 / 0 0.0%	21 / 38 55.3%E	12 / 38 31.6%	3 / 38 7.9%AB	2 / 38 5.3%ABE		
3	ATCC 43000	29 / 114 25.4%	5 / 6 83.3%	0 / 6 0.0%	3 / 3 100.0%	0 / 3 0.0%	0 / 6 0.0%	6 / 6 100.0%	3 / 3 100.0%	0 / 3 0.0%	0 / 6 0.0%	0 / 3 0.0%	3 / 9 33.3%	0 / 6 0.0%	0 / 3 0.0%	3 / 3 100.0%	0 / 3 0.0%	3 / 6 50.0%	0 / 3 0.0%	0 / 6 0.0%	3 / 9 33.3%	0 / 6 0.0%	0 / 3 0.0%	0 / 9 0.0%	0 / 3 0.0%	0 / 0 0.0%	0 / 0 0.0%	0 / 0 0.0%	56 / 114 49.1%E	22 / 114 19.3%	17 / 114 14.9%AB	12 / 114 10.5%ABE	4 / 114 3.5%CE	3 / 114 2.6%C
1	Methanococcus maripaludis LL	10 / 38 26.3%	2 / 2 100.0%	0 / 2 0.0%	1 / 1 100.0%	0 / 1 0.0%	0 / 2 0.0%	2 / 2 100.0%	1 / 1 100.0%	0 / 1 0.0%	0 / 2 0.0%	0 / 1 0.0%	1 / 3 33.3%	0 / 2 0.0%	0 / 1 0.0%	1 / 1 100.0%	0 / 1 0.0%	1 / 2 50.0%	0 / 1 0.0%	0 / 2 0.0%	1 / 3 33.3%	0 / 2 0.0%	0 / 1 0.0%	0 / 3 0.0%	0 / 1 0.0%	0 / 0 0.0%	0 / 0 0.0%	0 / 0 0.0%	18 / 38 47.4%E	7 / 38 18.4%	6 / 38 15.8%AB	4 / 38 10.5%ABE	2 / 38 5.3%CE	1 / 38 2.6%C
1	Methanococcus maripaludis C5	10 / 38 26.3%	2 / 2 100.0%	0 / 2 0.0%	1 / 1 100.0%	0 / 1 0.0%	0 / 2 0.0%	2 / 2 100.0%	1 / 1 100.0%	0 / 1 0.0%	0 / 2 0.0%	0 / 1 0.0%	1 / 3 33.3%	0 / 2 0.0%	0 / 1 0.0%	1 / 1 100.0%	0 / 1 0.0%	1 / 2 50.0%	0 / 1 0.0%	0 / 2 0.0%	1 / 3 33.3%	0 / 2 0.0%	0 / 1 0.0%	0 / 3 0.0%	0 / 1 0.0%	0 / 0 0.0%	0 / 0 0.0%	0 / 0 0.0%	19 / 38 50.0%E	7 / 38 18.4%	6 / 38 15.8%AB	4 / 38 10.5%ABE	1 / 38 2.6%CE	1 / 38 2.6%C
1	Methanococcus maripaludis C7	9 / 38 23.7%	1 / 2 50.0%	0 / 2 0.0%	1 / 1 100.0%	0 / 1 0.0%	0 / 2 0.0%	2 / 2 100.0%	1 / 1 100.0%	0 / 1 0.0%	0 / 2 0.0%	0 / 1 0.0%	1 / 3 33.3%	0 / 2 0.0%	0 / 1 0.0%	1 / 1 100.0%	0 / 1 0.0%	1 / 2 50.0%	0 / 1 0.0%	0 / 2 0.0%	1 / 3 33.3%	0 / 2 0.0%	0 / 1 0.0%	0 / 3 0.0%	0 / 1 0.0%	0 / 0 0.0%	0 / 0 0.0%	0 / 0 0.0%	19 / 38 50.0%E	8 / 38 21.1%	5 / 38 13.2%AB	4 / 38 10.5%ABE	1 / 38 2.6%C	1 / 38 2.6%CE
1	"Methanococcus aeolicus" Schmid et al. 1984	5 / 39 12.8%	0 / 2 0.0%	0 / 2 0.0%	0 / 1 0.0%	0 / 1 0.0%	0 / 3 0.0%	1 / 2 50.0%	1 / 1 100.0%	1 / 1 100.0%	0 / 2 0.0%	0 / 1 0.0%	0 / 3 0.0%	0 / 2 0.0%	0 / 1 0.0%	0 / 1 0.0%	0 / 1 0.0%	2 / 2 100.0%	0 / 1 0.0%	0 / 2 0.0%	0 / 3 0.0%	0 / 2 0.0%	0 / 1 0.0%	0 / 3 0.0%	0 / 1 0.0%	0 / 0 0.0%	0 / 0 0.0%	0 / 0 0.0%	23 / 39 59.0%E	11 / 39 28.2%	3 / 39 7.7%AB	2 / 39 5.1%ABE		
1	Methanococcus aeolicus Nankai-3	5 / 39 12.8%	0 / 2 0.0%	0 / 2 0.0%	0 / 1 0.0%	0 / 1 0.0%	0 / 3 0.0%	1 / 2 50.0%	1 / 1 100.0%	1 / 1 100.0%	0 / 2 0.0%	0 / 1 0.0%	0 / 3 0.0%	0 / 2 0.0%	0 / 1 0.0%	0 / 1 0.0%	0 / 1 0.0%	2 / 2 100.0%	0 / 1 0.0%	0 / 2 0.0%	0 / 3 0.0%	0 / 2 0.0%	0 / 1 0.0%	0 / 3 0.0%	0 / 1 0.0%	0 / 0 0.0%	0 / 0 0.0%	0 / 0 0.0%	23 / 39 59.0%E	11 / 39 28.2%	3 / 39 7.7%AB	2 / 39 5.1%ABE		
1	Methanocaldococcaceae	25 / 37 67.6%	0 / 1 0.0%	1 / 1 100.0%	1 / 1 100.0%	1 / 1 100.0%	2 / 2 100.0%	2 / 3 66.7%	0 / 1 0.0%	1 / 1 100.0%	1 / 3 33.3%	1 / 1 100.0%	2 / 3 66.7%	0 / 1 0.0%	1 / 1 100.0%	1 / 1 100.0%	1 / 1 100.0%	2 / 2 100.0%	1 / 1 100.0%	2 / 2 100.0%	1 / 3 33.3%	0 / 2 0.0%	1 / 1 100.0%	3 / 3 100.0%	0 / 1 0.0%	0 / 0 0.0%	0 / 0 0.0%	0 / 0 0.0%	15 / 37 40.5%ABE	10 / 37 27.0%AB	8 / 37 21.6%E	4 / 37 10.8%		
1	Methanocaldococcus	25 / 37 67.6%	0 / 1 0.0%	1 / 1 100.0%	1 / 1 100.0%	1 / 1 100.0%	2 / 2 100.0%	2 / 3 66.7%	0 / 1 0.0%	1 / 1 100.0%	1 / 3 33.3%	1 / 1 100.0%	2 / 3 66.7%	0 / 1 0.0%	1 / 1 100.0%	1 / 1 100.0%	1 / 1 100.0%	2 / 2 100.0%	1 / 1 100.0%	2 / 2 100.0%	1 / 3 33.3%	0 / 2 0.0%	1 / 1 100.0%	3 / 3 100.0%	0 / 1 0.0%	0 / 0 0.0%	0 / 0 0.0%	0 / 0 0.0%	15 / 37 40.5%ABE	10 / 37 27.0%AB	8 / 37 21.6%E	4 / 37 10.8%		
1	ATCC 43067	25 / 37 67.6%	0 / 1 0.0%	1 / 1 100.0%	1 / 1 100.0%	1 / 1 100.0%	2 / 2 100.0%	2 / 3 66.7%	0 / 1 0.0%	1 / 1 100.0%	1 / 3 33.3%	1 / 1 100.0%	2 / 3 66.7%	0 / 1 0.0%	1 / 1 100.0%	1 / 1 100.0%	1 / 1 100.0%	2 / 2 100.0%	1 / 1 100.0%	2 / 2 100.0%	1 / 3 33.3%	0 / 2 0.0%	1 / 1 100.0%	3 / 3 100.0%	0 / 1 0.0%	0 / 0 0.0%	0 / 0 0.0%	0 / 0 0.0%	15 / 37 40.5%ABE	10 / 37 27.0%AB	8 / 37 21.6%E	4 / 37 10.8%		
1	Methanocaldococcus jannaschii DSM 2661	25 / 37 67.6%	0 / 1 0.0%	1 / 1 100.0%	1 / 1 100.0%	1 / 1 100.0%	2 / 2 100.0%	2 / 3 66.7%	0 / 1 0.0%	1 / 1 100.0%	1 / 3 33.3%	1 / 1 100.0%	2 / 3 66.7%	0 / 1 0.0%	1 / 1 100.0%	1 / 1 100.0%	1 / 1 100.0%	2 / 2 100.0%	1 / 1 100.0%	2 / 2 100.0%	1 / 3 33.3%	0 / 2 0.0%	1 / 1 100.0%	3 / 3 100.0%	0 / 1 0.0%	0 / 0 0.0%	0 / 0 0.0%	0 / 0 0.0%	15 / 37 40.5%ABE	10 / 37 27.0%AB	8 / 37 21.6%E	4 / 37 10.8%		
4	"Halomebacteria" Cavalier-Smith 1986	0 / 184 0.0%	0 / 4 0.0%	0 / 4 0.0%	0 / 4 0.0%	0 / 4 0.0%	0 / 12 0.0%	0 / 12 0.0%	0 / 4 0.0%	0 / 4 0.0%	0 / 14 0.0%	0 / 4 0.0%	0 / 16 0.0%	0 / 9 0.0%	0 / 4 0.0%	0 / 8 0.0%	0 / 4 0.0%	0 / 11 0.0%	0 / 4 0.0%	0 / 12 0.0%	0 / 18 0.0%	0 / 8 0.0%	0 / 4 0.0%	0 / 20 0.0%	0 / 0 0.0%	0 / 0 0.0%	0 / 0 0.0%	0 / 0 0.0%	93 / 184 50.5%	88 / 184 47.8%E	2 / 184 1.1%C	1 / 184 0.5%CE		
4	"Haloarchaeales" DasSarma and DasSarma 2008	0 / 184 0.0%	0 / 4 0.0%	0 / 4 0.0%	0 / 4 0.0%	0 / 4 0.0%	0 / 12 0.0%	0 / 12 0.0%	0 / 4 0.0%	0 / 4 0.0%	0 / 14 0.0%	0 / 4 0.0%	0 / 16 0.0%	0 / 9 0.0%	0 / 4 0.0%	0 / 8 0.0%	0 / 4 0.0%	0 / 11 0.0%	0 / 4 0.0%	0 / 12 0.0%	0 / 18 0.0%	0 / 8 0.0%	0 / 4 0.0%	0 / 20 0.0%	0 / 0 0.0%	0 / 0 0.0%	0 / 0 0.0%	0 / 0 0.0%	93 / 184 50.5%	88 / 184 47.8%E	2 / 184 1.1%C	1 / 184 0.5%CE		
4	"Haloarchaeaceae" DasSarma and DasSarma 2008	0 / 184 0.0%	0 / 4 0.0%	0 / 4 0.0%	0 / 4 0.0%	0 / 4 0.0%	0 / 12 0.0%	0 / 12 0.0%	0 / 4 0.0%	0 / 4 0.0%	0 / 14 0.0%	0 / 4 0.0%	0 / 16 0.0%	0 / 9 0.0%	0 / 4 0.0%	0 / 8 0.0%	0 / 4 0.0%	0 / 11 0.0%	0 / 4 0.0%	0 / 12 0.0%	0 / 18 0.0%	0 / 8 0.0%	0 / 4 0.0%	0 / 20 0.0%	0 / 0 0.0%	0 / 0 0.0%	0 / 0 0.0%	0 / 0 0.0%	93 / 184 50.5%	88 / 184 47.8%E	2 / 184 1.1%C	1 / 184 0.5%CE		
1	Haloarcula	0 / 47 0.0%	0 / 1 0.0%	0 / 1 0.0%	0 / 1 0.0%	0 / 1 0.0%	0 / 3 0.0%	0 / 3 0.0%	0 / 1 0.0%	0 / 1 0.0%	0 / 3 0.0%	0 / 1 0.0%	0 / 4 0.0%	0 / 3 0.0%	0 / 1 0.0%	0 / 2 0.0%	0 / 1 0.0%	0 / 3 0.0%	0 / 1 0.0%	0 / 3 0.0%	0 / 5 0.0%	0 / 2 0.0%	0 / 1 0.0%	0 / 5 0.0%	0 / 0 0.0%	0 / 0 0.0%	0 / 0 0.0%	0 / 0 0.0%	24 / 47 51.1%E	23 / 47 48.9%				
1	"Flavobacterium (Halobacterium) maris-mortui (sic)" Elazari-Volcani 1940	0 / 47 0.0%	0 / 1 0.0%	0 / 1 0.0%	0 / 1 0.0%	0 / 1 0.0%	0 / 3 0.0%	0 / 3 0.0%	0 / 1 0.0%	0 / 1 0.0%	0 / 3 0.0%	0 / 1 0.0%	0 / 4 0.0%	0 / 3 0.0%	0 / 1 0.0%	0 / 2 0.0%	0 / 1 0.0%	0 / 3 0.0%	0 / 1 0.0%	0 / 3 0.0%	0 / 5 0.0%	0 / 2 0.0%	0 / 1 0.0%	0 / 5 0.0%	0 / 0 0.0%	0 / 0 0.0%	0 / 0 0.0%	0 / 0 0.0%	24 / 47 51.1%E	23 / 47 48.9%				
1	Haloarcula marismortui ATCC 43049	0 / 47 0.0%	0 / 1 0.0%	0 / 1 0.0%	0 / 1 0.0%	0 / 1 0.0%	0 / 3 0.0%	0 / 3 0.0%	0 / 1 0.0%	0 / 1 0.0%	0 / 3 0.0%	0 / 1 0.0%	0 / 4 0.0%	0 / 3 0.0%	0 / 1 0.0%	0 / 2 0.0%	0 / 1 0.0%	0 / 3 0.0%	0 / 1 0.0%	0 / 3 0.0%	0 / 5 0.0%	0 / 2 0.0%	0 / 1 0.0%	0 / 5 0.0%	0 / 0 0.0%	0 / 0 0.0%	0 / 0 0.0%	0 / 0 0.0%	24 / 47 51.1%E	23 / 47 48.9%				
1	"Flavobacterium (subgen. Halobacterium)" Elazari-Volcani 1940	0 / 47 0.0%	0 / 1 0.0%	0 / 1 0.0%	0 / 1 0.0%	0 / 1 0.0%	0 / 3 0.0%	0 / 3 0.0%	0 / 1 0.0%	0 / 1 0.0%	0 / 4 0.0%	0 / 1 0.0%	0 / 4 0.0%	0 / 2 0.0%	0 / 1 0.0%	0 / 2 0.0%	0 / 1 0.0%	0 / 3 0.0%	0 / 1 0.0%	0 / 3 0.0%	0 / 5 0.0%	0 / 2 0.0%	0 / 1 0.0%	0 / 5 0.0%	0 / 0 0.0%	0 / 0 0.0%	0 / 0 0.0%	0 / 0 0.0%	27 / 47 57.4%	19 / 47 40.4%E	1 / 47 2.1%C			
1	"Bacillus halobius ruber" Klebahn 1919	0 / 47 0.0%	0 / 1 0.0%	0 / 1 0.0%	0 / 1 0.0%	0 / 1 0.0%	0 / 3 0.0%	0 / 3 0.0%	0 / 1 0.0%	0 / 1 0.0%	0 / 4 0.0%	0 / 1 0.0%	0 / 4 0.0%	0 / 2 0.0%	0 / 1 0.0%	0 / 2 0.0%	0 / 1 0.0%	0 / 3 0.0%	0 / 1 0.0%	0 / 3 0.0%	0 / 5 0.0%	0 / 2 0.0%	0 / 1 0.0%	0 / 5 0.0%	0 / 0 0.0%	0 / 0 0.0%	0 / 0 0.0%	0 / 0 0.0%	27 / 47 57.4%	19 / 47 40.4%E	1 / 47 2.1%C			
1	Halobacterium salinarum NRC-1	0 / 47 0.0%	0 / 1 0.0%	0 / 1 0.0%	0 / 1 0.0%	0 / 1 0.0%	0 / 3 0.0%	0 / 3 0.0%	0 / 1 0.0%	0 / 1 0.0%	0 / 4 0.0%	0 / 1 0.0%	0 / 4 0.0%	0 / 2 0.0%	0 / 1 0.0%	0 / 2 0.0%	0 / 1 0.0%	0 / 3 0.0%	0 / 1 0.0%	0 / 3 0.0%	0 / 5 0.0%	0 / 2 0.0%	0 / 1 0.0%	0 / 5 0.0%	0 / 0 0.0%	0 / 0 0.0%	0 / 0 0.0%	0 / 0 0.0%	27 / 47 57.4%	19 / 47 40.4%E	1 / 47 2.1%C			
1	Natronomonas	0 / 45 0.0%	0 / 1 0.0%	0 / 1 0.0%	0 / 1 0.0%	0 / 1 0.0%	0 / 3 0.0%	0 / 3 0.0%	0 / 1 0.0%	0 / 1 0.0%	0 / 3 0.0%	0 / 1 0.0%	0 / 4 0.0%	0 / 2 0.0%	0 / 1 0.0%	0 / 2 0.0%	0 / 1 0.0%	0 / 3 0.0%	0 / 1 0.0%	0 / 3 0.0%	0 / 4 0.0%	0 / 2 0.0%	0 / 1 0.0%	0 / 5 0.0%	0 / 0 0.0%	0 / 0 0.0%	0 / 0 0.0%	0 / 0 0.0%	23 / 45 51.1%E	21 / 45 46.7%	1 / 45 2.2%CE			
1	ATCC 35678	0 / 45 0.0%	0 / 1 0.0%	0 / 1 0.0%	0 / 1 0.0%	0 / 1 0.0%	0 / 3 0.0%	0 / 3 0.0%	0 / 1 0.0%	0 / 1 0.0%	0 / 3 0.0%	0 / 1 0.0%	0 / 4 0.0%	0 / 2 0.0%	0 / 1 0.0%	0 / 2 0.0%	0 / 1 0.0%	0 / 3 0.0%	0 / 1 0.0%	0 / 3 0.0%	0 / 4 0.0%	0 / 2 0.0%	0 / 1 0.0%	0 / 5 0.0%	0 / 0 0.0%	0 / 0 0.0%	0 / 0 0.0%	0 / 0 0.0%	23 / 45 51.1%E	21 / 45 46.7%	1 / 45 2.2%CE			
1	Natronomonas pharaonis ATCC 35678	0 / 45 0.0%	0 / 1 0.0%	0 / 1 0.0%	0 / 1 0.0%	0 / 1 0.0%	0 / 3 0.0%	0 / 3 0.0%	0 / 1 0.0%	0 / 1 0.0%	0 / 3 0.0%	0 / 1 0.0%	0 / 4 0.0%	0 / 2 0.0%	0 / 1 0.0%	0 / 2 0.0%	0 / 1 0.0%	0 / 3 0.0%	0 / 1 0.0%	0 / 3 0.0%	0 / 4 0.0%	0 / 2 0.0%	0 / 1 0.0%	0 / 5 0.0%	0 / 0 0.0%	0 / 0 0.0%	0 / 0 0.0%	0 / 0 0.0%	23 / 45 51.1%E	21 / 45 46.7%	1 / 45 2.2%CE			
1	Haloquadra	0 / 45 0.0%	0 / 1 0.0%	0 / 1 0.0%	0 / 1 0.0%	0 / 1 0.0%	0 / 3 0.0%	0 / 3 0.0%	0 / 1 0.0%	0 / 1 0.0%	0 / 4 0.0%	0 / 1 0.0%	0 / 4 0.0%	0 / 2 0.0%	0 / 1 0.0%	0 / 2 0.0%	0 / 1 0.0%	0 / 2 0.0%	0 / 1 0.0%	0 / 3 0.0%	0 / 4 0.0%	0 / 2 0.0%	0 / 1 0.0%	0 / 5 0.0%	0 / 0 0.0%	0 / 0 0.0%	0 / 0 0.0%	0 / 0 0.0%	22 / 45 48.9%	22 / 45 48.9%E	1 / 45 2.2%C			
1	"Haloquadratum walsbyi" Bolhuis et al. 2004	0 / 45 0.0%	0 / 1 0.0%	0 / 1 0.0%	0 / 1 0.0%	0 / 1 0.0%	0 / 3 0.0%	0 / 3 0.0%	0 / 1 0.0%	0 / 1 0.0%	0 / 4 0.0%	0 / 1 0.0%	0 / 4 0.0%	0 / 2 0.0%	0 / 1 0.0%	0 / 2 0.0%	0 / 1 0.0%	0 / 2 0.0%	0 / 1 0.0%	0 / 3 0.0%	0 / 4 0.0%	0 / 2 0.0%	0 / 1 0.0%	0 / 5 0.0%	0 / 0 0.0%	0 / 0 0.0%	0 / 0 0.0%	0 / 0 0.0%	22 / 45 48.9%	22 / 45 48.9%E	1 / 45 2.2%C			
3	Thermoplasmata	2 / 138 1.4%	0 / 3 0.0%	0 / 3 0.0%	0 / 3 0.0%	0 / 3 0.0%	0 / 9 0.0%	0 / 9 0.0%	0 / 3 0.0%	0 / 3 0.0%	0 / 9 0.0%	0 / 3 0.0%	1 / 12 8.3%	0 / 6 0.0%	0 / 3 0.0%	0 / 6 0.0%	0 / 3 0.0%	0 / 9 0.0%	0 / 3 0.0%	0 / 9 0.0%	1 / 15 6.7%	0 / 6 0.0%	0 / 3 0.0%	0 / 15 0.0%	0 / 0 0.0%	0 / 0 0.0%	0 / 0 0.0%	0 / 0 0.0%	76 / 138 55.1%E	56 / 138 40.6%	2 / 138 1.4%AB	2 / 138 1.4%C	2 / 138 1.4%CE	
3	Thermoplasmales	2 / 138 1.4%	0 / 3 0.0%	0 / 3 0.0%	0 / 3 0.0%	0 / 3 0.0%	0 / 9 0.0%	0 / 9 0.0%	0 / 3 0.0%	0 / 3 0.0%	0 / 9 0.0%	0 / 3 0.0%	1 / 12 8.3%	0 / 6 0.0%	0 / 3 0.0%	0 / 6 0.0%	0 / 3 0.0%	0 / 9 0.0%	0 / 3 0.0%	0 / 9 0.0%	1 / 15 6.7%	0 / 6 0.0%	0 / 3 0.0%	0 / 15 0.0%	0 / 0 0.0%	0 / 0 0.0%	0 / 0 0.0%	0 / 0 0.0%	76 / 138 55.1%E	56 / 138 40.6%	2 / 138 1.4%C	2 / 138 1.4%AB	2 / 138 1.4%CE	
1	Picrophilaceae	1 / 46 2.2%	0 / 1 0.0%	0 / 1 0.0%	0 / 1 0.0%	0 / 1 0.0%	0 / 3 0.0%	0 / 3 0.0%	0 / 1 0.0%	0 / 1 0.0%	0 / 3 0.0%	0 / 1 0.0%	1 / 4 25.0%	0 / 2 0.0%	0 / 1 0.0%	0 / 2 0.0%	0 / 1 0.0%	0 / 3 0.0%	0 / 1 0.0%	0 / 3 0.0%	0 / 5 0.0%	0 / 2 0.0%	0 / 1 0.0%	0 / 5 0.0%	0 / 0 0.0%	0 / 0 0.0%	0 / 0 0.0%	0 / 0 0.0%	26 / 46 56.5%E	18 / 46 39.1%	1 / 46 2.2%AB	1 / 46 2.2%CE		
1	Picrophilus	1 / 46 2.2%	0 / 1 0.0%	0 / 1 0.0%	0 / 1 0.0%	0 / 1 0.0%	0 / 3 0.0%	0 / 3 0.0%	0 / 1 0.0%	0 / 1 0.0%	0 / 3 0.0%	0 / 1 0.0%	1 / 4 25.0%	0 / 2 0.0%	0 / 1 0.0%	0 / 2 0.0%	0 / 1 0.0%	0 / 3 0.0%	0 / 1 0.0%	0 / 3 0.0%	0 / 5 0.0%	0 / 2 0.0%	0 / 1 0.0%	0 / 5 0.0%	0 / 0 0.0%	0 / 0 0.0%	0 / 0 0.0%	0 / 0 0.0%	26 / 46 56.5%E	18 / 46 39.1%	1 / 46 2.2%CE	1 / 46 2.2%AB		
1	ATCC 700027	1 / 46 2.2%	0 / 1 0.0%	0 / 1 0.0%	0 / 1 0.0%	0 / 1 0.0%	0 / 3 0.0%	0 / 3 0.0%	0 / 1 0.0%	0 / 1 0.0%	0 / 3 0.0%	0 / 1 0.0%	1 / 4 25.0%	0 / 2 0.0%	0 / 1 0.0%	0 / 2 0.0%	0 / 1 0.0%	0 / 3 0.0%	0 / 1 0.0%	0 / 3 0.0%	0 / 5 0.0%	0 / 2 0.0%	0 / 1 0.0%	0 / 5 0.0%	0 / 0 0.0%	0 / 0 0.0%	0 / 0 0.0%	0 / 0 0.0%	26 / 46 56.5%E	18 / 46 39.1%	1 / 46 2.2%CE	1 / 46 2.2%AB		
1	Picrophilus torridus DSM 9790	1 / 46 2.2%	0 / 1 0.0%	0 / 1 0.0%	0 / 1 0.0%	0 / 1 0.0%	0 / 3 0.0%	0 / 3 0.0%	0 / 1 0.0%	0 / 1 0.0%	0 / 3 0.0%	0 / 1 0.0%	1 / 4 25.0%	0 / 2 0.0%	0 / 1 0.0%	0 / 2 0.0%	0 / 1 0.0%	0 / 3 0.0%	0 / 1 0.0%	0 / 3 0.0%	0 / 5 0.0%	0 / 2 0.0%	0 / 1 0.0%	0 / 5 0.0%	0 / 0 0.0%	0 / 0 0.0%	0 / 0 0.0%	0 / 0 0.0%	26 / 46 56.5%E	18 / 46 39.1%	1 / 46 2.2%CE	1 / 46 2.2%AB		

2	Thermoplasmaceae	1.1%	0.0%	0.0%	0.0%	0.0%	0.0%	0.0%	0.0%	0.0%	0.0%	0.0%	0.0%	0.0%	0.0%	0.0%	0.0%	0.0%	0.0%	0.0%	10.0%	0.0%	0.0%	0.0%	0.0%	0.0%	0.0%	0.0%	54.3%E	41.3%	2.2%C	1.1%CE	1.1%AB	
2	Thermoplasma	1 / 92 1.1%	0 / 2 0.0%	0 / 2 0.0%	0 / 2 0.0%	0 / 2 0.0%	0 / 6 0.0%	0 / 6 0.0%	0 / 2 0.0%	0 / 2 0.0%	0 / 6 0.0%	0 / 2 0.0%	0 / 8 0.0%	0 / 4 0.0%	0 / 2 0.0%	0 / 4 0.0%	0 / 2 0.0%	0 / 6 0.0%	0 / 2 0.0%	0 / 6 0.0%	1 / 10 10.0%	0 / 4 0.0%	0 / 2 0.0%	0 / 10 0.0%	0 / 0 0.0%	0 / 0 0.0%	0 / 0 0.0%	0 / 0 0.0%	50 / 92 54.3%E	38 / 92 41.3%	2 / 92 2.2%C	1 / 92 1.1%AB	1 / 92 1.1%CE	
1	"Thermoplasma acidophila" (sic) Darland et al. 1970	1 / 46 2.2%	0 / 1 0.0%	0 / 1 0.0%	0 / 1 0.0%	0 / 1 0.0%	0 / 3 0.0%	0 / 3 0.0%	0 / 1 0.0%	0 / 1 0.0%	0 / 3 0.0%	0 / 1 0.0%	0 / 4 0.0%	0 / 2 0.0%	0 / 1 0.0%	0 / 2 0.0%	0 / 1 0.0%	0 / 3 0.0%	0 / 1 0.0%	0 / 3 0.0%	1 / 5 20.0%	0 / 2 0.0%	0 / 1 0.0%	0 / 5 0.0%	0 / 0 0.0%	0 / 0 0.0%	0 / 0 0.0%	0 / 0 0.0%	25 / 46 54.3%E	19 / 46 41.3%	1 / 46 2.2%CE	1 / 46 2.2%AB		
1	Thermoplasma acidophilum DSM 1728	1 / 46 2.2%	0 / 1 0.0%	0 / 1 0.0%	0 / 1 0.0%	0 / 1 0.0%	0 / 3 0.0%	0 / 3 0.0%	0 / 1 0.0%	0 / 1 0.0%	0 / 3 0.0%	0 / 1 0.0%	0 / 4 0.0%	0 / 2 0.0%	0 / 1 0.0%	0 / 2 0.0%	0 / 1 0.0%	0 / 3 0.0%	0 / 1 0.0%	0 / 3 0.0%	1 / 5 20.0%	0 / 2 0.0%	0 / 1 0.0%	0 / 5 0.0%	0 / 0 0.0%	0 / 0 0.0%	0 / 0 0.0%	0 / 0 0.0%	25 / 46 54.3%E	19 / 46 41.3%	1 / 46 2.2%CE	1 / 46 2.2%AB		
1	ATCC 51530	0 / 46 0.0%	0 / 1 0.0%	0 / 1 0.0%	0 / 1 0.0%	0 / 1 0.0%	0 / 3 0.0%	0 / 3 0.0%	0 / 1 0.0%	0 / 1 0.0%	0 / 3 0.0%	0 / 1 0.0%	0 / 4 0.0%	0 / 2 0.0%	0 / 1 0.0%	0 / 2 0.0%	0 / 1 0.0%	0 / 3 0.0%	0 / 1 0.0%	0 / 3 0.0%	0 / 5 0.0%	0 / 2 0.0%	0 / 1 0.0%	0 / 5 0.0%	0 / 0 0.0%	0 / 0 0.0%	0 / 0 0.0%	0 / 0 0.0%	25 / 46 54.3%E	19 / 46 41.3%	2 / 46 4.3%C			
1	Thermoplasma volcanium GSS1	0 / 46 0.0%	0 / 1 0.0%	0 / 1 0.0%	0 / 1 0.0%	0 / 1 0.0%	0 / 3 0.0%	0 / 3 0.0%	0 / 1 0.0%	0 / 1 0.0%	0 / 3 0.0%	0 / 1 0.0%	0 / 4 0.0%	0 / 2 0.0%	0 / 1 0.0%	0 / 2 0.0%	0 / 1 0.0%	0 / 3 0.0%	0 / 1 0.0%	0 / 3 0.0%	0 / 5 0.0%	0 / 2 0.0%	0 / 1 0.0%	0 / 5 0.0%	0 / 0 0.0%	0 / 0 0.0%	0 / 0 0.0%	0 / 0 0.0%	25 / 46 54.3%E	19 / 46 41.3%	2 / 46 4.3%C			
4	Protoarchaea	181 / 184 98.4%	4 / 4 100.0%	4 / 4 100.0%	4 / 4 100.0%	4 / 4 100.0%	12 / 12 100.0%	12 / 12 100.0%	4 / 4 100.0%	4 / 4 100.0%	12 / 12 100.0%	4 / 4 100.0%	16 / 16 100.0%	8 / 8 100.0%	4 / 4 100.0%	8 / 8 100.0%	4 / 4 100.0%	12 / 12 100.0%	4 / 4 100.0%	12 / 12 100.0%	17 / 20 85.0%	8 / 8 100.0%	4 / 4 100.0%	20 / 20 100.0%	0 / 0 0.0%	0 / 0 0.0%	0 / 0 0.0%	0 / 0 0.0%	97 / 184 52.7%AB	84 / 184 45.7%ABE	2 / 184 1.1%E	1 / 184 0.5%		
4	Thermococcales	181 / 184 98.4%	4 / 4 100.0%	4 / 4 100.0%	4 / 4 100.0%	4 / 4 100.0%	12 / 12 100.0%	12 / 12 100.0%	4 / 4 100.0%	4 / 4 100.0%	12 / 12 100.0%	4 / 4 100.0%	16 / 16 100.0%	8 / 8 100.0%	4 / 4 100.0%	8 / 8 100.0%	4 / 4 100.0%	12 / 12 100.0%	4 / 4 100.0%	12 / 12 100.0%	17 / 20 85.0%	8 / 8 100.0%	4 / 4 100.0%	20 / 20 100.0%	0 / 0 0.0%	0 / 0 0.0%	0 / 0 0.0%	0 / 0 0.0%	97 / 184 52.7%AB	84 / 184 45.7%ABE	2 / 184 1.1%E	1 / 184 0.5%		
4	Thermococcaceae	181 / 184 98.4%	4 / 4 100.0%	4 / 4 100.0%	4 / 4 100.0%	4 / 4 100.0%	12 / 12 100.0%	12 / 12 100.0%	4 / 4 100.0%	4 / 4 100.0%	12 / 12 100.0%	4 / 4 100.0%	16 / 16 100.0%	8 / 8 100.0%	4 / 4 100.0%	8 / 8 100.0%	4 / 4 100.0%	12 / 12 100.0%	4 / 4 100.0%	12 / 12 100.0%	17 / 20 85.0%	8 / 8 100.0%	4 / 4 100.0%	20 / 20 100.0%	0 / 0 0.0%	0 / 0 0.0%	0 / 0 0.0%	0 / 0 0.0%	97 / 184 52.7%AB	84 / 184 45.7%ABE	2 / 184 1.1%E	1 / 184 0.5%		
3	Pyrococcus	135 / 138 97.8%	3 / 3 100.0%	3 / 3 100.0%	3 / 3 100.0%	3 / 3 100.0%	9 / 9 100.0%	9 / 9 100.0%	3 / 3 100.0%	3 / 3 100.0%	9 / 9 100.0%	3 / 3 100.0%	12 / 12 100.0%	6 / 6 100.0%	3 / 3 100.0%	6 / 6 100.0%	3 / 3 100.0%	9 / 9 100.0%	3 / 3 100.0%	9 / 9 100.0%	12 / 15 80.0%	6 / 6 100.0%	3 / 3 100.0%	15 / 15 100.0%	0 / 0 0.0%	0 / 0 0.0%	0 / 0 0.0%	0 / 0 0.0%	74 / 138 53.6%AB	61 / 138 44.2%ABE	2 / 138 1.4%E	1 / 138 0.7%		
1	ATCC 43587	45 / 46 97.8%	1 / 1 100.0%	1 / 1 100.0%	1 / 1 100.0%	1 / 1 100.0%	3 / 3 100.0%	3 / 3 100.0%	1 / 1 100.0%	1 / 1 100.0%	3 / 3 100.0%	1 / 1 100.0%	4 / 4 100.0%	2 / 2 100.0%	1 / 1 100.0%	2 / 2 100.0%	1 / 1 100.0%	3 / 3 100.0%	1 / 1 100.0%	3 / 3 100.0%	4 / 5 80.0%	2 / 2 100.0%	1 / 1 100.0%	5 / 5 100.0%	0 / 0 0.0%	0 / 0 0.0%	0 / 0 0.0%	0 / 0 0.0%	25 / 46 54.3%AB	20 / 46 43.5%ABE	1 / 46 2.2%E			
1	Pyrococcus furiosus DSM 3638	45 / 46 97.8%	1 / 1 100.0%	1 / 1 100.0%	1 / 1 100.0%	1 / 1 100.0%	3 / 3 100.0%	3 / 3 100.0%	1 / 1 100.0%	1 / 1 100.0%	3 / 3 100.0%	1 / 1 100.0%	4 / 4 100.0%	2 / 2 100.0%	1 / 1 100.0%	2 / 2 100.0%	1 / 1 100.0%	3 / 3 100.0%	1 / 1 100.0%	3 / 3 100.0%	4 / 5 80.0%	2 / 2 100.0%	1 / 1 100.0%	5 / 5 100.0%	0 / 0 0.0%	0 / 0 0.0%	0 / 0 0.0%	0 / 0 0.0%	25 / 46 54.3%AB	20 / 46 43.5%ABE	1 / 46 2.2%E			
1	"Pyrococcus abyssi" Erauso et al. 1993	44 / 46 95.7%	1 / 1 100.0%	1 / 1 100.0%	1 / 1 100.0%	1 / 1 100.0%	3 / 3 100.0%	3 / 3 100.0%	1 / 1 100.0%	1 / 1 100.0%	3 / 3 100.0%	1 / 1 100.0%	4 / 4 100.0%	2 / 2 100.0%	1 / 1 100.0%	2 / 2 100.0%	1 / 1 100.0%	3 / 3 100.0%	1 / 1 100.0%	3 / 3 100.0%	3 / 5 60.0%	2 / 2 100.0%	1 / 1 100.0%	5 / 5 100.0%	0 / 0 0.0%	0 / 0 0.0%	0 / 0 0.0%	0 / 0 0.0%	24 / 46 52.2%AB	20 / 46 43.5%ABE	1 / 46 2.2%E	1 / 46 2.2%		
1	'Pyrococcus shinkaii'	46 / 46 100.0%	1 / 1 100.0%	1 / 1 100.0%	1 / 1 100.0%	1 / 1 100.0%	3 / 3 100.0%	3 / 3 100.0%	1 / 1 100.0%	1 / 1 100.0%	3 / 3 100.0%	1 / 1 100.0%	4 / 4 100.0%	2 / 2 100.0%	1 / 1 100.0%	2 / 2 100.0%	1 / 1 100.0%	3 / 3 100.0%	1 / 1 100.0%	3 / 3 100.0%	5 / 5 100.0%	2 / 2 100.0%	1 / 1 100.0%	5 / 5 100.0%	0 / 0 0.0%	0 / 0 0.0%	0 / 0 0.0%	0 / 0 0.0%	25 / 46 54.3%AB	21 / 46 45.7%ABE				
1	Pyrococcus OT3	46 / 46 100.0%	1 / 1 100.0%	1 / 1 100.0%	1 / 1 100.0%	1 / 1 100.0%	3 / 3 100.0%	3 / 3 100.0%	1 / 1 100.0%	1 / 1 100.0%	3 / 3 100.0%	1 / 1 100.0%	4 / 4 100.0%	2 / 2 100.0%	1 / 1 100.0%	2 / 2 100.0%	1 / 1 100.0%	3 / 3 100.0%	1 / 1 100.0%	3 / 3 100.0%	5 / 5 100.0%	2 / 2 100.0%	1 / 1 100.0%	5 / 5 100.0%	0 / 0 0.0%	0 / 0 0.0%	0 / 0 0.0%	0 / 0 0.0%	25 / 46 54.3%AB	21 / 46 45.7%ABE				
1	Thermococcus	46 / 46 100.0%	1 / 1 100.0%	1 / 1 100.0%	1 / 1 100.0%	1 / 1 100.0%	3 / 3 100.0%	3 / 3 100.0%	1 / 1 100.0%	1 / 1 100.0%	3 / 3 100.0%	1 / 1 100.0%	4 / 4 100.0%	2 / 2 100.0%	1 / 1 100.0%	2 / 2 100.0%	1 / 1 100.0%	3 / 3 100.0%	1 / 1 100.0%	3 / 3 100.0%	5 / 5 100.0%	2 / 2 100.0%	1 / 1 100.0%	5 / 5 100.0%	0 / 0 0.0%	0 / 0 0.0%	0 / 0 0.0%	0 / 0 0.0%	23 / 46 50.0%AB	23 / 46 50.0%ABE				
1	"Thermococcus kodakaraensis" Atomi et al. 2004	46 / 46 100.0%	1 / 1 100.0%	1 / 1 100.0%	1 / 1 100.0%	1 / 1 100.0%	3 / 3 100.0%	3 / 3 100.0%	1 / 1 100.0%	1 / 1 100.0%	3 / 3 100.0%	1 / 1 100.0%	4 / 4 100.0%	2 / 2 100.0%	1 / 1 100.0%	2 / 2 100.0%	1 / 1 100.0%	3 / 3 100.0%	1 / 1 100.0%	3 / 3 100.0%	5 / 5 100.0%	2 / 2 100.0%	1 / 1 100.0%	5 / 5 100.0%	0 / 0 0.0%	0 / 0 0.0%	0 / 0 0.0%	0 / 0 0.0%	23 / 46 50.0%ABE	23 / 46 50.0%AB				
1	Pyrococcus sp. (strain KOD1)	46 / 46 100.0%	1 / 1 100.0%	1 / 1 100.0%	1 / 1 100.0%	1 / 1 100.0%	3 / 3 100.0%	3 / 3 100.0%	1 / 1 100.0%	1 / 1 100.0%	3 / 3 100.0%	1 / 1 100.0%	4 / 4 100.0%	2 / 2 100.0%	1 / 1 100.0%	2 / 2 100.0%	1 / 1 100.0%	3 / 3 100.0%	1 / 1 100.0%	3 / 3 100.0%	5 / 5 100.0%	2 / 2 100.0%	1 / 1 100.0%	5 / 5 100.0%	0 / 0 0.0%	0 / 0 0.0%	0 / 0 0.0%	0 / 0 0.0%	23 / 46 50.0%ABE	23 / 46 50.0%AB				
1	Archaeoglobea	0 / 46 0.0%	0 / 1 0.0%	0 / 1 0.0%	0 / 1 0.0%	0 / 1 0.0%	0 / 3 0.0%	0 / 3 0.0%	0 / 1 0.0%	0 / 1 0.0%	0 / 3 0.0%	0 / 1 0.0%	0 / 4 0.0%	0 / 2 0.0%	0 / 1 0.0%	0 / 2 0.0%	0 / 1 0.0%	0 / 3 0.0%	0 / 1 0.0%	0 / 3 0.0%	0 / 5 0.0%	0 / 2 0.0%	0 / 1 0.0%	0 / 5 0.0%	0 / 0 0.0%	0 / 0 0.0%	0 / 0 0.0%	0 / 0 0.0%	31 / 46 67.4%E	15 / 46 32.6%				
1	"Archaeoglobales" Stetter 1989	0 / 46 0.0%	0 / 1 0.0%	0 / 1 0.0%	0 / 1 0.0%	0 / 1 0.0%	0 / 3 0.0%	0 / 3 0.0%	0 / 1 0.0%	0 / 1 0.0%	0 / 3 0.0%	0 / 1 0.0%	0 / 4 0.0%	0 / 2 0.0%	0 / 1 0.0%	0 / 2 0.0%	0 / 1 0.0%	0 / 3 0.0%	0 / 1 0.0%	0 / 3 0.0%	0 / 5 0.0%	0 / 2 0.0%	0 / 1 0.0%	0 / 5 0.0%	0 / 0 0.0%	0 / 0 0.0%	0 / 0 0.0%	0 / 0 0.0%	31 / 46 67.4%E	15 / 46 32.6%				
1	"Archaeoglobaceae" Stetter 1989	0 / 46 0.0%	0 / 1 0.0%	0 / 1 0.0%	0 / 1 0.0%	0 / 1 0.0%	0 / 3 0.0%	0 / 3 0.0%	0 / 1 0.0%	0 / 1 0.0%	0 / 3 0.0%	0 / 1 0.0%	0 / 4 0.0%	0 / 2 0.0%	0 / 1 0.0%	0 / 2 0.0%	0 / 1 0.0%	0 / 3 0.0%	0 / 1 0.0%	0 / 3 0.0%	0 / 5 0.0%	0 / 2 0.0%	0 / 1 0.0%	0 / 5 0.0%	0 / 0 0.0%	0 / 0 0.0%	0 / 0 0.0%	0 / 0 0.0%	31 / 46 67.4%E	15 / 46 32.6%				
1	Archaeoglobus	0 / 46 0.0%	0 / 1 0.0%	0 / 1 0.0%	0 / 1 0.0%	0 / 1 0.0%	0 / 3 0.0%	0 / 3 0.0%	0 / 1 0.0%	0 / 1 0.0%	0 / 3 0.0%	0 / 1 0.0%	0 / 4 0.0%	0 / 2 0.0%	0 / 1 0.0%	0 / 2 0.0%	0 / 1 0.0%	0 / 3 0.0%	0 / 1 0.0%	0 / 3 0.0%	0 / 5 0.0%	0 / 2 0.0%	0 / 1 0.0%	0 / 5 0.0%	0 / 0 0.0%	0 / 0 0.0%	0 / 0 0.0%	0 / 0 0.0%	31 / 46 67.4%E	15 / 46 32.6%				
1	ATCC 49558	0 / 46 0.0%	0 / 1 0.0%	0 / 1 0.0%	0 / 1 0.0%	0 / 1 0.0%	0 / 3 0.0%	0 / 3 0.0%	0 / 1 0.0%	0 / 1 0.0%	0 / 3 0.0%	0 / 1 0.0%	0 / 4 0.0%	0 / 2 0.0%	0 / 1 0.0%	0 / 2 0.0%	0 / 1 0.0%	0 / 3 0.0%	0 / 1 0.0%	0 / 3 0.0%	0 / 5 0.0%	0 / 2 0.0%	0 / 1 0.0%	0 / 5 0.0%	0 / 0 0.0%	0 / 0 0.0%	0 / 0 0.0%	0 / 0 0.0%	31 / 46 67.4%E	15 / 46 32.6%				
1	Archaeoglobus fulgidus DSM 4304	0 / 46 0.0%	0 / 1 0.0%	0 / 1 0.0%	0 / 1 0.0%	0 / 1 0.0%	0 / 3 0.0%	0 / 3 0.0%	0 / 1 0.0%	0 / 1 0.0%	0 / 3 0.0%	0 / 1 0.0%	0 / 4 0.0%	0 / 2 0.0%	0 / 1 0.0%	0 / 2 0.0%	0 / 1 0.0%	0 / 3 0.0%	0 / 1 0.0%	0 / 3 0.0%	0 / 5 0.0%	0 / 2 0.0%	0 / 1 0.0%	0 / 5 0.0%	0 / 0 0.0%	0 / 0 0.0%	0 / 0 0.0%	0 / 0 0.0%	31 / 46 67.4%E	15 / 46 32.6%				
1	Methanopyri	29 / 35 82.9%	1 / 1 100.0%	1 / 1 100.0%	1 / 1 100.0%	1 / 1 100.0%	2 / 2 100.0%	1 / 2 50.0%	1 / 1 100.0%	1 / 1 100.0%	2 / 2 100.0%	1 / 1 100.0%	3 / 3 100.0%	1 / 1 100.0%	1 / 1 100.0%	1 / 1 100.0%	1 / 1 100.0%	1 / 2 50.0%	1 / 1 100.0%	2 / 2 100.0%	1 / 3 33.3%	2 / 2 100.0%	0 / 1 0.0%	3 / 3 100.0%	0 / 1 0.0%	0 / 0 0.0%	0 / 0 0.0%	0 / 0 0.0%	28 / 35 80.0%ABE	4 / 35 11.4%E	2 / 35 5.7%CE	1 / 35 2.9%AB		
1	Methanopyrales	29 / 35 82.9%	1 / 1 100.0%	1 / 1 100.0%	1 / 1 100.0%	1 / 1 100.0%	2 / 2 100.0%	1 / 2 50.0%	1 / 1 100.0%	1 / 1 100.0%	2 / 2 100.0%	1 / 1 100.0%	3 / 3 100.0%	1 / 1 100.0%	1 / 1 100.0%	1 / 1 100.0%	1 / 1 100.0%	1 / 2 50.0%	1 / 1 100.0%	2 / 2 100.0%	1 / 3 33.3%	2 / 2 100.0%	0 / 1 0.0%	3 / 3 100.0%	0 / 1 0.0%	0 / 0 0.0%	0 / 0 0.0%	0 / 0 0.0%	28 / 35 80.0%ABE	4 / 35 11.4%E	2 / 35 5.7%CE	1 / 35 2.9%AB		
1	Methanopyraceae	29 / 35 82.9%	1 / 1 100.0%	1 / 1 100.0%	1 / 1 100.0%	1 / 1 100.0%	2 / 2 100.0%	1 / 2 50.0%	1 / 1 100.0%	1 / 1 100.0%	2 / 2 100.0%	1 / 1 100.0%	3 / 3 100.0%	1 / 1 100.0%	1 / 1 100.0%	1 / 1 100.0%	1 / 1 100.0%	1 / 2 50.0%	1 / 1 100.0%	2 / 2 100.0%	1 / 3 33.3%	2 / 2 100.0%	0 / 1 0.0%	3 / 3 100.0%	0 / 1 0.0%	0 / 0 0.0%	0 / 0 0.0%	0 / 0 0.0%	28 / 35 80.0%ABE	4 / 35 11.4%E	2 / 35 5.7%CE	1 / 35 2.9%AB		
1	Methanopyrus	29 / 35 82.9%	1 / 1 100.0%	1 / 1 100.0%	1 / 1 100.0%	1 / 1 100.0%	2 / 2 100.0%	1 / 2 50.0%	1 / 1 100.0%	1 / 1 100.0%	2 / 2 100.0%	1 / 1 100.0%	3 / 3 100.0%	1 / 1 100.0%	1 / 1 100.0%	1 / 1 100.0%	1 / 1 100.0%	1 / 2 50.0%	1 / 1 100.0%	2 / 2 100.0%	1 / 3 33.3%	2 / 2 100.0%	0 / 1 0.0%	3 / 3 100.0%	0 / 1 0.0%	0 / 0 0.0%	0 / 0 0.0%	0 / 0 0.0%	28 / 35 80.0%ABE	4 / 35 11.4%E	2 / 35 5.7%CE	1 / 35 2.9%AB		
1	DSM 6324	29 / 35 82.9%	1 / 1 100.0%	1 / 1 100.0%	1 / 1 100.0%	1 / 1 100.0%	2 / 2 100.0%	1 / 2 50.0%	1 / 1 100.0%	1 / 1 100.0%	2 / 2 100.0%	1 / 1 100.0%	3 / 3 100.0%	1 / 1 100.0%	1 / 1 100.0%	1 / 1 100.0%	1 / 1 100.0%	1 / 2 50.0%	1 / 1 100.0%	2 / 2 100.0%	1 / 3 33.3%	2 / 2 100.0%	0 / 1 0.0%	3 / 3 100.0%	0 / 1 0.0%	0 / 0 0.0%	0 / 0 0.0%	0 / 0 0.0%	28 / 35 80.0%ABE	4 / 35 11.4%E	2 / 35 5.7%CE	1 / 35 2.9%AB		
1	Methanopyrus kandleri AV19	29 / 35 82.9%	1 / 1 100.0%	1 / 1 100.0%	1 / 1 100.0%	1 / 1 100.0%	2 / 2 100.0%	1 / 2 50.0%	1 / 1 100.0%	1 / 1 100.0%	2 / 2 100.0%	1 / 1 100.0%	3 / 3 100.0%	1 / 1 100.0%	1 / 1 100.0%	1 / 1 100.0%	1 / 1 100.0%	1 / 2 50.0%	1 / 1 100.0%	2 / 2 100.0%	1 / 3 33.3%	2 / 2 100.0%	0 / 1 0.0%	3 / 3 100.0%	0 / 1 0.0%	0 / 0 0.0%	0 / 0 0.0%	0 / 0 0.0%	28 / 35 80.0%ABE	4 / 35 11.4%E	2 / 35 5.7%CE	1 / 35 2.9%AB		
10	'Methanomicrobia'	52 / 523 9.9%	0 / 14 0.0%	0 / 11 0.0%	0 / 13 0.0%	5 / 14 35.7%	1 / 38 2.6%	2 / 33 6.1%	1 / 10 10.0%	1 / 10 10.0%	1 / 39 2.6%	0 / 10 0.0%	1 / 39 2.6%	5 / 21 23.8%	7 / 13 53.8%	1 / 21 4.8%	0 / 22 0.0%	6 / 29 20.7%	0 / 10 0.0%	1 / 33 3.0%	2 / 49 4.1%	8 / 23 34.8%	0 / 10 0.0%	6 / 57 10.5%	0 / 0 0.0%	4 / 4 100.0%	0 / 0 0.0%	0 / 0 0.0%	259 / 523 49.5%	200 / 523 38.2%E	37 / 523 7.1%AB	15 / 523 2.9%ABE	8 / 523 1.5%C	4 / 523 0.8%CE
4	Methanomicrobiales	2 / 198 1.0%	0 / 4 0.0%	0 / 5 0.0%	0 / 4 0.0%	0 / 4 0.0%	0 / 13 0.0%	0 / 12 0.0%	0 / 4 0.0%	1 / 4 25.0%	0 / 17 0.0%	0 / 4 0.0%	1 / 16 6.2%	0 / 9 0.0%	0 / 4 0.0%	0 / 8 0.0%	0 / 8 0.0%	0 / 12 0.0%	0 / 4 0.0%	0 / 13 0.0%	0 / 20 0.0%	0 / 8 0.0%	0 / 4 0.0%	0 / 21 0.0%	0 / 0 0.0%	0 / 0 0.0%	0 / 0 0.0%	0 / 0 0.0%	122 / 198 61.6%	73 / 198 36.9%E	2 / 198 1.0%AB	1 / 198 0.5%CE		
1	Methanomicrobiaceae	0 / 49 0.0%	0 / 1 0.0%	0 / 1 0.0%	0 / 1 0.0%	0 / 1 0.0%	0 / 3 0.0%	0 / 3 0.0%	0 / 1 0.0%	0 / 1 0.0%	0 / 3 0.0%	0 / 1 0.0%	0 / 4 0.0%	0 / 3 0.0%	0 / 1 0.0%	0 / 2 0.0%	0 / 1 0.0%	0 / 3 0.0%	0 / 1 0.0%	0 / 4 0.0%	0 / 5 0.0%	0 / 2 0.0%	0 / 1 0.0%	0 / 6 0.0%	0 / 0 0.0%	0 / 0 0.0%	0 / 0 0.0%	0 / 0 0.0%	27 / 49 55.1%	22 / 49 44.9%E				
1	Methaniculleus	0 / 49 0.0%	0 / 1 0.0%	0 / 1 0.0%	0 / 1 0.0%	0 / 1 0.0%	0 / 3 0.0%	0 / 3 0.0%	0 / 1 0.0%	0 / 1 0.0%	0 / 3 0.0%	0 / 1 0.0%	0 / 4 0.0%	0 / 3 0.0%	0 / 1 0.0%	0 / 2 0.0%	0 / 1 0.0%	0 / 3 0.0%	0 / 1 0.0%	0 / 4 0.0%	0 / 5 0.0%	0 / 2 0.0%	0 / 1 0.0%	0 / 6 0.0%	0 / 0 0.0%	0 / 0 0.0%	0 / 0 0.0%	0 / 0 0.0%	27 / 49 55.1%	22 / 49 44.9%E				
1	ATCC 35101	0 / 49 0.0%	0 / 1 0.0%	0 / 1 0.0%	0 / 1 0.0%	0 / 1 0.0%	0 / 3 0.0%	0 / 3 0.0%	0 / 1 0.0%	0 / 1 0.0%	0 / 3 0.0%	0 / 1 0.0%	0 / 4 0.0%	0 / 3 0.0%	0 / 1 0.0%	0 / 2 0.0%	0 / 1 0.0%	0 / 3 0.0%	0 / 1 0.0%	0 / 4 0.0%	0 / 5 0.0%	0 / 2 0.0%	0 / 1 0.0%	0 / 6 0.0%	0 / 0 0.0%	0 / 0 0.0%	0 / 0 0.0%	0 / 0 0.0%	27 / 49 55.1%	22 / 49 44.9%E				
1	Methanoculleus marisnigri JR1	0 / 49 0.0%	0 / 1 0.0%	0 / 1 0.0%	0 / 1 0.0%	0 / 1 0.0%	0 / 3 0.0%	0 / 3 0.0%	0 / 1 0.0%	0 / 1 0.0%	0 / 3 0.0%	0 / 1 0.0%	0 / 4 0.0%	0 / 3 0.0%	0 / 1 0.0%	0 / 2 0.0%	0 / 1 0.0%	0 / 3 0.0%	0 / 1 0.0%	0 / 4 0.0%	0 / 5 0.0%	0 / 2 0.0%	0 / 1 0.0%	0 / 6 0.0%	0 / 0 0.0%	0 / 0 0.0%	0 / 0 0.0%	0 / 0 0.0%	27 / 49 55.1%	22 / 49 44.9%E				
1	Methanicorpusculaceae	2 / 49 4.1%	0 / 1 0.0%	0 / 1 0.0%	0 / 1 0.0%	0 / 1 0.0%	0 / 4 0.0%	0 / 3 0.0%	0 / 1 0.0%	1 / 1 100.0%	0 / 5 0.0%	0 / 1 0.0%	1 / 4 25.0%	0 / 2 0.0%	0 / 1 0.0%	0 / 2 0.0%	0 / 1 0.0%	0 / 3 0.0%	0 / 1 0.0%	0 / 3 0.0%	0 / 5 0.0%	0 / 2 0.0%	0 / 1 0.0%	0 / 5 0.0%	0 / 0 0.0%	0 / 0 0.0%	0 / 0 0.0%	0 / 0 0.0%	32 / 49 65.3%	15 / 49 30.6%E	2 / 49 4.1%AB			
1	Methanocorpusculum	2 / 49 4.1%	0 / 1 0.0%	0 / 1 0.0%	0 / 1 0.0%	0 / 1 0.0%	0 / 4 0.0%	0 / 3 0.0%	0 / 1 0.0%	1 / 1 100.0%	0 / 5 0.0%	0 / 1 0.0%	1 / 4 25.0%	0 / 2 0.0%	0 / 1 0.0%	0 / 2 0.0%	0 / 1 0.0%	0 / 3 0.0%	0 / 1 0.0%	0 / 3 0.0%	0 / 5 0.0%	0 / 2 0.0%	0 / 1 0.0%	0 / 5 0.0%	0 / 0 0.0%	0 / 0 0.0%	0 / 0 0.0%	0 / 0 0.0%	32 / 49 65.3%	15 / 49 30.6%E	2 / 49 4.1%AB			
1	ATCC 43576	2 / 49 4.1%	0 / 1 0.0%	0 / 1 0.0%	0 / 1 0.0%	0 / 1 0.0%	0 / 4 0.0%	0 / 3 0.0%	0 / 1 0.0%	1 / 1 100.0%	0 / 5 0.0%	0 / 1 0.0%	1 / 4 25.0%	0 / 2 0.0%	0 / 1 0.0%	0 / 2 0.0%	0 / 1 0.0%	0 / 3 0.0%	0 / 1 0.0%	0 / 3 0.0%	0 / 5 0.0%	0 / 2 0.0%	0 / 1 0.0%	0 / 5 0.0%	0 / 0 0.0%	0 / 0 0.0%	0 / 0 0.0%	0 / 0 0.0%	32 / 49 65.3%	15 / 49 30.6%E	2 / 49 4.1%AB			
1	Methanocorpusculum labreanum Z	2 / 49 4.1%	0 / 1 0.0%	0 / 1 0.0%	0 / 1 0.0%	0 / 1 0.0%	0 / 4 0.0%	0 / 3 0.0%	0 / 1 0.0%	1 / 1 100.0%	0 / 5 0.0%	0 / 1 0.0%	1 / 4 25.0%	0 / 2 0.0%	0 / 1 0.0%	0 / 2 0.0%	0 / 1 0.0%	0 / 3 0.0%	0 / 1 0.0%	0 / 3 0.0%	0 / 5 0.0%	0 / 2 0.0%	0 / 1 0.0%	0 / 5 0.0%	0 / 0 0.0%	0 / 0 0.0%	0 / 0 0.0%	0 / 0 0.0%	32 / 49 65.3%	15 / 49 30.6%E	2 / 49 4.1%AB			
1	Methanospirillaceae	0 / 51 0.0%	0 / 1 0.0%	0 / 2 0.0%	0 / 1 0.0%	0 / 1 0.0%	0 / 3 0.0%	0 / 3 0.0%	0 / 1 0.0%	0 / 1 0.0%	0 / 6 0.0%	0 / 1 0.0%	0 / 4 0.0%	0 / 2 0.0%	0 / 1 0.0%	0 / 2 0.0%	0 / 2 0.0%	0 / 3 0.0%	0 / 1 0.0%	0 / 3 0.0%	0 / 5 0.0%	0 / 2 0.0%	0 / 1 0.0%	0 / 5 0.0%	0 / 0 0.0%	0 / 0 0.0%	0 / 0 0.0%	0 / 0 0.0%	34 / 51 66.7%	16 / 51 31.4%E	1 / 51 2.0%CE			
1	Methanospirillum	0 / 51 0.0%	0 / 1 0.0%	0 / 2 0.0%	0 / 1 0.0%	0 / 1 0.0%	0 / 3 0.0%	0 / 3 0.0%	0 / 1 0.0%	0 / 1 0.0%	0 / 6 0.0%	0 / 1 0.0%	0 / 4 0.0%	0 / 2 0.0%	0 / 1 0.0%	0 / 2 0.0%	0 / 2 0.0%	0 / 3 0.0%	0 / 1 0.0%	0 / 3 0.0%	0 / 5 0.0%	0 / 2 0.0%	0 / 1 0.0%	0 / 5 0.0%	0 / 0 0.0%	0 / 0 0.0%	0 / 0 0.0%	0 / 0 0.0%	34 / 51 66.7%	16 / 51 31.4%E	1 / 51 2.0%CE			
1	ATCC 27890	0 / 51 0.0%	0 / 1 0.0%	0 / 2 0.0%	0 / 1 0.0%	0 / 1 0.0%	0 / 3 0.0%	0 / 3 0.0%	0 / 1 0.0%	0 / 1 0.0%	0 / 6 0.0%	0 / 1 0.0%	0 / 4 0.0%	0 / 2 0.0%	0 / 1 0.0%	0 / 2 0.0%	0 / 2 0.0%	0 / 3 0.0%	0 / 1 0.0%	0 / 3 0.0%	0 / 5 0.0%	0 / 2 0.0%	0 / 1 0.0%	0 / 5 0.0%	0 / 0 0.0%	0 / 0 0.0%	0 / 0 0.0%	0 / 0 0.0%	34 / 51 66.7%	16 / 51 31.4%E	1 / 51 2.0%CE			
1	Methanospirillum hungatei DSM 864	0 / 51 0.0%	0 / 1 0.0%	0 / 2 0.0%	0 / 1 0.0%	0 / 1 0.0%	0 / 3 0.0%	0 / 3 0.0%	0 / 1 0.0%	0 / 1 0.0%	0 / 6 0.0%	0 / 1 0.0%	0 / 4 0.0%	0 / 2 0.0%	0 / 1 0.0%	0 / 2 0.0%	0 / 2 0.0%	0 / 3 0.0%	0 / 1 0.0%	0 / 3 0.0%	0 / 5 0.0%	0 / 2 0.0%	0 / 1 0.0%	0 / 5 0.0%	0 / 0 0.0%	0 / 0 0.0%	0 / 0 0.0%	0 / 0 0.0%	34 / 51 66.7%	16 / 51 31.4%E	1 / 51 2.0%CE			
1	Methanoregulaceae	0 / 49 0.0%	0 / 1 0.0%	0 / 1 0.0%	0 / 1 0.0%	0 / 1 0.0%	0 / 3 0.0%	0 / 3 0.0%	0 / 1 0.0%	0 / 1 0.0%	0 / 3 0.0%	0 / 1 0.0%	0 / 4 0.0%	0 / 2 0.0%	0 / 1 0.0%	0 / 2 0.0%	0 / 4 0.0%	0 / 3 0.0%	0 / 1 0.0%	0 / 3 0.0%	0 / 5 0.0%	0 / 2 0.0%	0 / 1 0.0%	0 / 5 0.0%	0 / 0 0.0%	0 / 0 0.0%	0 / 0 0.0%	0 / 0 0.0%	29 / 49 59.2%	20 / 49 40.8%E				
1	"Candidatus Methanoregula" Brauer et al. 2006	0 / 49 0.0%	0 / 1 0.0%	0 / 1 0.0%	0 / 1 0.0%	0 / 1 0.0%	0 / 3 0.0%	0 / 3 0.0%	0 / 1 0.0%	0 / 1 0.0%	0 / 3 0.0%	0 / 1 0.0%	0 / 4 0.0%	0 / 2 0.0%	0 / 1 0.0%	0 / 2 0.0%	0 / 4 0.0%	0 / 3 0.0%	0 / 1 0.0%	0 / 3 0.0%	0 / 5 0.0%	0 / 2 0.0%	0 / 1 0.0%	0 / 5 0.0%	0 / 0 0.0%	0 / 0 0.0%	0 / 0 0.0%	0 / 0 0.0%	29 / 49 59.2%	20 / 49 40.8%E				
1	"Candidatus Methanoregula boonei" Brauer et al. 2006	0 / 49 0.0%	0 / 1 0.0%	0 / 1 0.0%	0 / 1 0.0%	0 / 1 0.0%	0 / 3 0.0%	0 / 3 0.0%	0 / 1 0.0%	0 / 1 0.0%	0 / 3 0.0%	0 / 1 0.0%	0 / 4 0.0%	0 / 2 0.0%	0 / 1 0.0%	0 / 2 0.0%	0 / 4 0.0%	0 / 3 0.0%	0 / 1 0.0%	0 / 3 0.0%	0 / 5 0.0%	0 / 2 0.0%	0 / 1 0.0%	0 / 5 0.0%	0 / 0 0.0%	0 / 0 0.0%	0 / 0 0.0%	0 / 0 0.0%	29 / 49 59.2%	20 / 49 40.8%E				
1	Candidatus Methanoregula boonei 6A8	0 / 49 0.0%	0 / 1 0.0%	0 / 1 0.0%	0 / 1 0.0%	0 / 1 0.0%	0 / 3 0.0%	0 / 3 0.0%	0 / 1 0.0%	0 / 1 0.0%	0 / 3 0.0%	0 / 1 0.0%	0 / 4 0.0%	0 / 2 0.0%	0 / 1 0.0%	0 / 2 0.0%	0 / 4 0.0%	0 / 3 0.0%	0 / 1 0.0%	0 / 3 0.0%	0 / 5 0.0%	0 / 2 0.0%	0 / 1 0.0%	0 / 5 0.0%	0 / 0 0.0%	0 / 0 0.0%	0 / 0 0.0%	0 / 0 0.0%	29 / 49 59.2%	20 / 49 40.8%E				
5	"Halomebacteria" Cavalier-Smith 1986	45 / 274 16.4%	0 / 9 0.0%	0 / 5 0.0%	0 / 8 0.0%	5 / 8 62.5%	0 / 21 0.0%	2 / 18 11.1%	1 / 5 20.0%	0 / 5 0.0%	1 / 18 5.6%	0 / 5 0.0%	0 / 19 0.0%	4 / 10 40.0%	7 / 8 87.5%	1 / 11 9.1%	0 / 12 0.0%	4 / 14 28.6%	0 / 5 0.0%	0 / 16 0.0%	2 / 24 8.3%	8 / 13 61.5%	0 / 5 0.0%	6 / 31 19.4%	0 / 0 0.0%	4 / 4 100.0%	0 / 0 0.0%	0 / 0 0.0%	112 / 274 40.9%	108 / 274 39.4%E	33 / 274 12.0%AB	12 / 274 4.4%ABE	7 / 274 2.6%C	2 / 274 0.7%CE
4	Methanosarcinaceae	45 / 228 19.7%	0 / 8 0.0%	0 / 4 0.0%	0 / 7 0.0%	5 / 7 71.4%	0 / 18 0.0%	2 / 15 13.3%	1 / 4 25.0%	0 / 4 0.0%	1 / 14 7.1%	0 / 4 0.0%	0 / 16 0.0%	4 / 8 50.0%	7 / 7 100.0%	1 / 9 11.1%	0 / 10 0.0%	4 / 11 36.4%	0 / 4 0.0%	0 / 13 0.0%	2 / 20 10.0%	8 / 11 72.7%	0 / 4 0.0%	6 / 26 23.1%	0 / 0 0.0%	4 / 4 100.0%	0 / 0 0.0%	0 / 0 0.0%	88 / 228 38.6%	87 / 228 38.2%E	33 / 228 14.5%AB	12 / 228 5.3%ABE	7 / 228 3.1%C	1 / 228 0.4%CE
3	Kluyver and van Niel 1936 emend. Barker 1956 (Approved Lists 1980), nom. cons., emend. Mah and Kuhn 1984	28 / 177 15.8%	0 / 7 0.0%	0 / 3 0.0%	0 / 6 0.0%	5 / 6 83.3%	0 / 15 0.0%	0 / 12 0.0%	0 / 3 0.0%	0 / 3 0.0%	0 / 9 0.0%	0 / 3 0.0%	0 / 12 0.0%	3 / 6 50.0%	6 / 6 100.0%	0 / 7 0.0%	0 / 7 0.0%	3 / 9 33.3%	0 / 3 0.0%	0 / 10 0.0%	0 / 15 0.0%	6 / 9 66.7%	0 / 3 0.0%	2 / 20 10.0%	0 / 0 0.0%	3 / 3 100.0%	0 / 0 0.0%	0 / 0 0.0%	71 / 177 40.1%E	70 / 177 39.5%	23 / 177 13.0%AB	7 / 177 4.0%C	5 / 177 2.8%ABE	1 / 177 0.6%CE
1	"Methanosarcina barkerii" (sic) Schnellen 1947	9 / 62 14.5%	0 / 3 0.0%	0 / 1 0.0%	0 / 2 0.0%	2 / 2 100.0%	0 / 5 0.0%	0 / 4 0.0%	0 / 1 0.0%	0 / 1 0.0%	0 / 3 0.0%	0 / 1 0.0%	0 / 4 0.0%	1 / 2 50.0%	2 / 2 100.0%	0 / 2 0.0%	0 / 3 0.0%	1 / 3 33.3%	0 / 1 0.0%	0 / 4 0.0%	0 / 5 0.0%	2 / 3 66.7%	0 / 1 0.0%	0 / 8 0.0%	0 / 0 0.0%	1 / 1 100.0%	0 / 0 0.0%	0 / 0 0.0%	26 / 62 41.9%E	25 / 62 40.3%	8 / 62 12.9%AB	2 / 62 3.2%C	1 / 62 1.6%ABE	
1	Methanosarcina barkeri 'Fusaro'	9 / 62 14.5%	0 / 3 0.0%	0 / 1 0.0%	0 / 2 0.0%	2 / 2 100.0%	0 / 5 0.0%	0 / 4 0.0%	0 / 1 0.0%	0 / 1 0.0%	0 / 3 0.0%	0 / 1 0.0%	0 / 4 0.0%	1 / 2 50.0%	2 / 2 100.0%	0 / 2 0.0%	0 / 3 0.0%	1 / 3 33.3%	0 / 1 0.0%	0 / 4 0.0%	0 / 5 0.0%	2 / 3 66.7%	0 / 1 0.0%	0 / 8 0.0%	0 / 0 0.0%	1 / 1 100.0%	0 / 0 0.0%	0 / 0 0.0%	26 / 62 41.9%E	25 / 62 40.3%	8 / 62 12.9%AB	2 / 62 3.2%C	1 / 62 1.6%ABE	
1	DSM 2053	9 / 58 15.5%	0 / 2 0.0%	0 / 1 0.0%	0 / 2 0.0%	1 / 2 50.0%	0 / 5 0.0%	0 / 4 0.0%	0 / 1 0.0%	0 / 1 0.0%	0 / 3 0.0%	0 / 1 0.0%	0 / 4 0.0%	1 / 2 50.0%	2 / 2 100.0%	0 / 2 0.0%	0 / 3 0.0%	1 / 3 33.3%	0 / 1 0.0%	0 / 3 0.0%	0 / 5 0.0%	2 / 3 66.7%	0 / 1 0.0%	1 / 6 16.7%	0 / 0 0.0%	1 / 1 100.0%	0 / 0 0.0%	0 / 0 0.0%	24 / 58 41.4%E	22 / 58 37.9%	7 / 58 12.1%AB	3 / 58 5.2%C	2 / 58 3.4%ABE	
1	Methanosarcina mazei Go1	9 / 58 15.5%	0 / 2 0.0%	0 / 1 0.0%	0 / 2 0.0%	1 / 2 50.0%	0 / 5 0.0%	0 / 4 0.0%	0 / 1 0.0%	0 / 1 0.0%	0 / 3 0.0%	0 / 1 0.0%	0 / 4 0.0%	1 / 2 50.0%	2 / 2 100.0%	0 / 2 0.0%	0 / 3 0.0%	1 / 3 33.3%	0 / 1 0.0%	0 / 3 0.0%	0 / 5 0.0%	2 / 3 66.7%	0 / 1 0.0%	1 / 6 16.7%	0 / 0 0.0%	1 / 1 100.0%	0 / 0 0.0%	0 / 0 0.0%	24 / 58 41.4%E	22 / 58 37.9%	7 / 58 12.1%AB	3 / 58 5.2%C	2 / 58 3.4%ABE	
1	ATCC 35395	10 / 57 17.5%	0 / 2 0.0%	0 / 1 0.0%	0 / 2 0.0%	2 / 2 100.0%	0 / 5 0.0%	0 / 4 0.0%	0 / 1 0.0%	0 / 1 0.0%	0 / 3 0.0%	0 / 1 0.0%	0 / 4 0.0%	1 / 2 50.0%	2 / 2 100.0%	0 / 3 0.0%	0 / 1 0.0%	1 / 3 33.3%	0 / 1 0.0%	0 / 3 0.0%	0 / 5 0.0%	2 / 3 66.7%	0 / 1 0.0%	1 / 6 16.7%	0 / 0 0.0%	1 / 1 100.0%	0 / 0 0.0%	0 / 0 0.0%	23 / 57 40.4%	21 / 57 36.8%E	8 / 57 14.0%AB	2 / 57 3.5%C	2 / 57 3.5%ABE	1 / 57 1.8%CE
1	Methanosarcina acetivorans C2A	10 / 57 17.5%	0 / 2 0.0%	0 / 1 0.0%	0 / 2 0.0%	2 / 2 100.0%	0 / 5 0.0%	0 / 4 0.0%	0 / 1 0.0%	0 / 1 0.0%	0 / 3 0.0%	0 / 1 0.0%	0 / 4 0.0%	1 / 2 50.0%	2 / 2 100.0%	0 / 3 0.0%	0 / 1 0.0%	1 / 3 33.3%	0 / 1 0.0%	0 / 3 0.0%	0 / 5 0.0%	2 / 3 66.7%	0 / 1 0.0%	1 / 6 16.7%	0 / 0 0.0%	1 / 1 100.0%	0 / 0 0.0%	0 / 0 0.0%	23 / 57 40.4%	21 / 57 36.8%E	8 / 57 14.0%AB	2 / 57 3.5%C	2 / 57 3.5%ABE	1 / 57 1.8%CE
1	Methanococcoides	17 / 51 33.3%	0 / 1 0.0%	0 / 1 0.0%	0 / 1 0.0%	0 / 1 0.0%	0 / 3 0.0%	2 / 3 66.7%	1 / 1 100.0%	0 / 1 0.0%	1 / 5 20.0%	0 / 1 0.0%	0 / 4 0.0%	1 / 2 50.0%	1 / 1 100.0%	1 / 2 50.0%	0 / 3 0.0%	1 / 2 50.0%	0 / 1 0.0%	0 / 3 0.0%	2 / 5 40.0%	2 / 2 100.0%	0 / 1 0.0%	4 / 6 66.7%	0 / 0 0.0%	1 / 1 100.0%	0 / 0 0.0%	0 / 0 0.0%	18 / 51 35.3%	16 / 51 31.4%E	10 / 51 19.6%AB	7 / 51 13.7%ABE		
1	DSM 6242	17 / 51 33.3%	0 / 1 0.0%	0 / 1 0.0%	0 / 1 0.0%	0 / 1 0.0%	0 / 3 0.0%	2 / 3 66.7%	1 / 1 100.0%	0 / 1 0.0%	1 / 5 20.0%	0 / 1 0.0%	0 / 4 0.0%	1 / 2 50.0%	1 / 1 100.0%	1 / 2 50.0%	0 / 3 0.0%	1 / 2 50.0%	0 / 1 0.0%	0 / 3 0.0%	2 / 5 40.0%	2 / 2 100.0%	0 / 1 0.0%	4 / 6 66.7%	0 / 0 0.0%	1 / 1 100.0%	0 / 0 0.0%	0 / 0 0.0%	18 / 51 35.3%	16 / 51 31.4%E	10 / 51 19.6%AB	7 / 51 13.7%ABE		
1	Methanococcoides burtonii DSM 6242	17 / 51 33.3%	0 / 1 0.0%	0 / 1 0.0%	0 / 1 0.0%	0 / 1 0.0%	0 / 3 0.0%	2 / 3 66.7%	1 / 1 100.0%	0 / 1 0.0%	1 / 5 20.0%	0 / 1 0.0%	0 / 4 0.0%	1 / 2 50.0%	1 / 1 100.0%	1 / 2 50.0%	0 / 3 0.0%	1 / 2 50.0%	0 / 1 0.0%	0 / 3 0.0%	2 / 5 40.0%	2 / 2 100.0%	0 / 1 0.0%	4 / 6 66.7%	0 / 0 0.0%	1 / 1 100.0%	0 / 0 0.0%	0 / 0 0.0%	18 / 51 35.3%	16 / 51 31.4%E	10 / 51 19.6%AB	7 / 51 13.7%ABE		
1	Methanosaetaceae	0 / 46 0.0%	0 / 1 0.0%	0 / 1 0.0%	0 / 1 0.0%	0 / 1 0.0%	0 / 3 0.0%	0 / 3 0.0%	0 / 1 0.0%	0 / 1 0.0%	0 / 4 0.0%	0 / 1 0.0%	0 / 3 0.0%	0 / 2 0.0%	0 / 1 0.0%	0 / 2 0.0%	0 / 2 0.0%	0 / 3 0.0%	0 / 1 0.0%	0 / 3 0.0%	0 / 4 0.0%	0 / 2 0.0%	0 / 1 0.0%	0 / 5 0.0%	0 / 0 0.0%	0 / 0 0.0%	0 / 0 0.0%	0 / 0 0.0%	24 / 46 52.2%	21 / 46 45.7%E	1 / 46 2.2%CE			
1	"Methanothrix" Huser et al. 1982	0 / 46 0.0%	0 / 1 0.0%	0 / 1 0.0%	0 / 1 0.0%	0 / 1 0.0%	0 / 3 0.0%	0 / 3 0.0%	0 / 1 0.0%	0 / 1 0.0%	0 / 4 0.0%	0 / 1 0.0%	0 / 3 0.0%	0 / 2 0.0%	0 / 1 0.0%	0 / 2 0.0%	0 / 2 0.0%	0 / 3 0.0%	0 / 1 0.0%	0 / 3 0.0%	0 / 4 0.0%	0 / 2 0.0%	0 / 1 0.0%	0 / 5 0.0%	0 / 0 0.0%	0 / 0 0.0%	0 / 0 0.0%	0 / 0 0.0%	24 / 46 52.2%	21 / 46 45.7%E	1 / 46 2.2%CE			
1	DSM 6194	0 / 46 0.0%	0 / 1 0.0%	0 / 1 0.0%	0 / 1 0.0%	0 / 1 0.0%	0 / 3 0.0%	0 / 3 0.0%	0 / 1 0.0%	0 / 1 0.0%	0 / 4 0.0%	0 / 1 0.0%	0 / 3 0.0%	0 / 2 0.0%	0 / 1 0.0%	0 / 2 0.0%	0 / 2 0.0%	0 / 3 0.0%	0 / 1 0.0%	0 / 3 0.0%	0 / 4 0.0%	0 / 2 0.0%	0 / 1 0.0%	0 / 5 0.0%	0 / 0 0.0%	0 / 0 0.0%	0 / 0 0.0%	0 / 0 0.0%	24 / 46 52.2%	21 / 46 45.7%E	1 / 46 2.2%CE			
1	Methanosaeta thermophila PT	0 / 46 0.0%	0 / 1 0.0%	0 / 1 0.0%	0 / 1 0.0%	0 / 1 0.0%	0 / 3 0.0%	0 / 3 0.0%	0 / 1 0.0%	0 / 1 0.0%	0 / 4 0.0%	0 / 1 0.0%	0 / 3 0.0%	0 / 2 0.0%	0 / 1 0.0%	0 / 2 0.0%	0 / 2 0.0%	0 / 3 0.0%	0 / 1 0.0%	0 / 3 0.0%	0 / 4 0.0%	0 / 2 0.0%	0 / 1 0.0%	0 / 5 0.0%	0 / 0 0.0%	0 / 0 0.0%	0 / 0 0.0%	0 / 0 0.0%	24 / 46 52.2%	21 / 46 45.7%E	1 / 46 2.2%CE			
1	Methanocellales	5 / 51 9.8%	0 / 1 0.0%	0 / 1 0.0%	0 / 1 0.0%	0 / 2 0.0%	1 / 4 25.0%	0 / 3 0.0%	0 / 1 0.0%	0 / 1 0.0%	0 / 4 0.0%	0 / 1 0.0%	0 / 4 0.0%	1 / 2 50.0%	0 / 1 0.0%	0 / 2 0.0%	0 / 2 0.0%	2 / 3 66.7%	0 / 1 0.0%	1 / 4 25.0%	0 / 5 0.0%	0 / 2 0.0%	0 / 1 0.0%	0 / 5 0.0%	0 / 0 0.0%	0 / 0 0.0%	0 / 0 0.0%	0 / 0 0.0%	25 / 51 49.0%	19 / 51 37.3%E	3 / 51 5.9%ABE	2 / 51 3.9%AB	1 / 51 2.0%C	1 / 51 2.0%CE
1	Methanocellaceae	5 / 51 9.8%	0 / 1 0.0%	0 / 1 0.0%	0 / 1 0.0%	0 / 2 0.0%	1 / 4 25.0%	0 / 3 0.0%	0 / 1 0.0%	0 / 1 0.0%	0 / 4 0.0%	0 / 1 0.0%	0 / 4 0.0%	1 / 2 50.0%	0 / 1 0.0%	0 / 2 0.0%	0 / 2 0.0%	2 / 3 66.7%	0 / 1 0.0%	1 / 4 25.0%	0 / 5 0.0%	0 / 2 0.0%	0 / 1 0.0%	0 / 5 0.0%	0 / 0 0.0%	0 / 0 0.0%	0 / 0 0.0%	0 / 0 0.0%	25 / 51 49.0%	19 / 51 37.3%E	3 / 51 5.9%ABE	2 / 51 3.9%AB	1 / 51 2.0%C	1 / 51 2.0%CE
1	Methanocella	5 / 51 9.8%	0 / 1 0.0%	0 / 1 0.0%	0 / 1 0.0%	0 / 2 0.0%	1 / 4 25.0%	0 / 3 0.0%	0 / 1 0.0%	0 / 1 0.0%	0 / 4 0.0%	0 / 1 0.0%	0 / 4 0.0%	1 / 2 50.0%	0 / 1 0.0%	0 / 2 0.0%	0 / 2 0.0%	2 / 3 66.7%	0 / 1 0.0%	1 / 4 25.0%	0 / 5 0.0%	0 / 2 0.0%	0 / 1 0.0%	0 / 5 0.0%	0 / 0 0.0%	0 / 0 0.0%	0 / 0 0.0%	0 / 0 0.0%	25 / 51 49.0%	19 / 51 37.3%E	3 / 51 5.9%ABE	2 / 51 3.9%AB	1 / 51 2.0%C	1 / 51 2.0%CE
1	DSM 22066	5 / 51 9.8%	0 / 1 0.0%	0 / 1 0.0%	0 / 1 0.0%	0 / 2 0.0%	1 / 4 25.0%	0 / 3 0.0%	0 / 1 0.0%	0 / 1 0.0%	0 / 4 0.0%	0 / 1 0.0%	0 / 4 0.0%	1 / 2 50.0%	0 / 1 0.0%	0 / 2 0.0%	0 / 2 0.0%	2 / 3 66.7%	0 / 1 0.0%	1 / 4 25.0%	0 / 5 0.0%	0 / 2 0.0%	0 / 1 0.0%	0 / 5 0.0%	0 / 0 0.0%	0 / 0 0.0%	0 / 0 0.0%	0 / 0 0.0%	25 / 51 49.0%	19 / 51 37.3%E	3 / 51 5.9%ABE	2 / 51 3.9%AB	1 / 51 2.0%CE	1 / 51 2.0%C
1	Methanocella arvoryzae MRE50	5 / 51 9.8%	0 / 1 0.0%	0 / 1 0.0%	0 / 1 0.0%	0 / 2 0.0%	1 / 4 25.0%	0 / 3 0.0%	0 / 1 0.0%	0 / 1 0.0%	0 / 4 0.0%	0 / 1 0.0%	0 / 4 0.0%	1 / 2 50.0%	0 / 1 0.0%	0 / 2 0.0%	0 / 2 0.0%	2 / 3 66.7%	0 / 1 0.0%	1 / 4 25.0%	0 / 5 0.0%	0 / 2 0.0%	0 / 1 0.0%	0 / 5 0.0%	0 / 0 0.0%	0 / 0 0.0%	0 / 0 0.0%	0 / 0 0.0%	25 / 51 49.0%	19 / 51 37.3%E	3 / 51 5.9%ABE	2 / 51 3.9%AB	1 / 51 2.0%C	1 / 51 2.0%CE
1	Nanoarchaeota	0 / 43 0.0%	0 / 1 0.0%	0 / 1 0.0%	0 / 1 0.0%	0 / 1 0.0%	0 / 3 0.0%	0 / 3 0.0%	0 / 1 0.0%	0 / 1 0.0%	0 / 3 0.0%	0 / 2 0.0%	0 / 4 0.0%	0 / 1 0.0%	0 / 1 0.0%	0 / 1 0.0%	0 / 1 0.0%	0 / 3 0.0%	0 / 1 0.0%	0 / 3 0.0%	0 / 4 0.0%	0 / 2 0.0%	0 / 1 0.0%	0 / 4 0.0%	0 / 0 0.0%	0 / 0 0.0%	0 / 0 0.0%	0 / 0 0.0%	25 / 43 58.1%	18 / 43 41.9%E				
1	"Nanoarchaeum" Huber et al. 2002	0 / 43 0.0%	0 / 1 0.0%	0 / 1 0.0%	0 / 1 0.0%	0 / 1 0.0%	0 / 3 0.0%	0 / 3 0.0%	0 / 1 0.0%	0 / 1 0.0%	0 / 3 0.0%	0 / 2 0.0%	0 / 4 0.0%	0 / 1 0.0%	0 / 1 0.0%	0 / 1 0.0%	0 / 1 0.0%	0 / 3 0.0%	0 / 1 0.0%	0 / 3 0.0%	0 / 4 0.0%	0 / 2 0.0%	0 / 1 0.0%	0 / 4 0.0%	0 / 0 0.0%	0 / 0 0.0%	0 / 0 0.0%	0 / 0 0.0%	25 / 43 58.1%	18 / 43 41.9%E				
1	"Nanoarchaeum equitans" Huber et al. 2002	0 / 43 0.0%	0 / 1 0.0%	0 / 1 0.0%	0 / 1 0.0%	0 / 1 0.0%	0 / 3 0.0%	0 / 3 0.0%	0 / 1 0.0%	0 / 1 0.0%	0 / 3 0.0%	0 / 2 0.0%	0 / 4 0.0%	0 / 1 0.0%	0 / 1 0.0%	0 / 1 0.0%	0 / 1 0.0%	0 / 3 0.0%	0 / 1 0.0%	0 / 3 0.0%	0 / 4 0.0%	0 / 2 0.0%	0 / 1 0.0%	0 / 4 0.0%	0 / 0 0.0%	0 / 0 0.0%	0 / 0 0.0%	0 / 0 0.0%	25 / 43 58.1%	18 / 43 41.9%E				
1	Microarchaeum sp. Kin4-M	0 / 43 0.0%	0 / 1 0.0%	0 / 1 0.0%	0 / 1 0.0%	0 / 1 0.0%	0 / 3 0.0%	0 / 3 0.0%	0 / 1 0.0%	0 / 1 0.0%	0 / 3 0.0%	0 / 2 0.0%	0 / 4 0.0%	0 / 1 0.0%	0 / 1 0.0%	0 / 1 0.0%	0 / 1 0.0%	0 / 3 0.0%	0 / 1 0.0%	0 / 3 0.0%	0 / 4 0.0%	0 / 2 0.0%	0 / 1 0.0%	0 / 4 0.0%	0 / 0 0.0%	0 / 0 0.0%	0 / 0 0.0%	0 / 0 0.0%	25 / 43 58.1%	18 / 43 41.9%E				
1	"Aigarchaeota" Nunoura et al. 2011	17 / 45 37.8%	0 / 1 0.0%	1 / 1 100.0%	1 / 1 100.0%	0 / 1 0.0%	0 / 3 0.0%	1 / 3 33.3%	0 / 1 0.0%	1 / 1 100.0%	3 / 3 100.0%	0 / 1 0.0%	3 / 4 75.0%	0 / 2 0.0%	1 / 1 100.0%	0 / 2 0.0%	0 / 1 0.0%	2 / 3 66.7%	0 / 1 0.0%	1 / 3 33.3%	0 / 4 0.0%	1 / 2 50.0%	0 / 1 0.0%	2 / 5 40.0%	0 / 0 0.0%	0 / 0 0.0%	0 / 0 0.0%	0 / 0 0.0%	10 / 45 22.2%	9 / 45 20.0%E	9 / 45 20.0%ABE	8 / 45 17.8%AB	5 / 45 11.1%C	4 / 45 8.9%CE
1	"Cenarchaeales" DeLong and Preston 1996	17 / 45 37.8%	0 / 1 0.0%	1 / 1 100.0%	1 / 1 100.0%	0 / 1 0.0%	0 / 3 0.0%	1 / 3 33.3%	0 / 1 0.0%	1 / 1 100.0%	3 / 3 100.0%	0 / 1 0.0%	3 / 4 75.0%	0 / 2 0.0%	1 / 1 100.0%	0 / 2 0.0%	0 / 1 0.0%	2 / 3 66.7%	0 / 1 0.0%	1 / 3 33.3%	0 / 4 0.0%	1 / 2 50.0%	0 / 1 0.0%	2 / 5 40.0%	0 / 0 0.0%	0 / 0 0.0%	0 / 0 0.0%	0 / 0 0.0%	10 / 45 22.2%	9 / 45 20.0%E	9 / 45 20.0%ABE	8 / 45 17.8%AB	5 / 45 11.1%C	4 / 45 8.9%CE
1	"Cenarchaeaceae" DeLong and Preston 1996	17 / 45 37.8%	0 / 1 0.0%	1 / 1 100.0%	1 / 1 100.0%	0 / 1 0.0%	0 / 3 0.0%	1 / 3 33.3%	0 / 1 0.0%	1 / 1 100.0%	3 / 3 100.0%	0 / 1 0.0%	3 / 4 75.0%	0 / 2 0.0%	1 / 1 100.0%	0 / 2 0.0%	0 / 1 0.0%	2 / 3 66.7%	0 / 1 0.0%	1 / 3 33.3%	0 / 4 0.0%	1 / 2 50.0%	0 / 1 0.0%	2 / 5 40.0%	0 / 0 0.0%	0 / 0 0.0%	0 / 0 0.0%	0 / 0 0.0%	10 / 45 22.2%	9 / 45 20.0%E	9 / 45 20.0%ABE	8 / 45 17.8%AB	5 / 45 11.1%C	4 / 45 8.9%CE
1	"Cenarchaeum" DeLong and Preston 1996	17 / 45 37.8%	0 / 1 0.0%	1 / 1 100.0%	1 / 1 100.0%	0 / 1 0.0%	0 / 3 0.0%	1 / 3 33.3%	0 / 1 0.0%	1 / 1 100.0%	3 / 3 100.0%	0 / 1 0.0%	3 / 4 75.0%	0 / 2 0.0%	1 / 1 100.0%	0 / 2 0.0%	0 / 1 0.0%	2 / 3 66.7%	0 / 1 0.0%	1 / 3 33.3%	0 / 4 0.0%	1 / 2 50.0%	0 / 1 0.0%	2 / 5 40.0%	0 / 0 0.0%	0 / 0 0.0%	0 / 0 0.0%	0 / 0 0.0%	10 / 45 22.2%	9 / 45 20.0%E	9 / 45 20.0%ABE	8 / 45 17.8%AB	5 / 45 11.1%C	4 / 45 8.9%CE
1	"Cenarchaeum symbiosum" DeLong and Preston 1996	17 / 45 37.8%	0 / 1 0.0%	1 / 1 100.0%	1 / 1 100.0%	0 / 1 0.0%	0 / 3 0.0%	1 / 3 33.3%	0 / 1 0.0%	1 / 1 100.0%	3 / 3 100.0%	0 / 1 0.0%	3 / 4 75.0%	0 / 2 0.0%	1 / 1 100.0%	0 / 2 0.0%	0 / 1 0.0%	2 / 3 66.7%	0 / 1 0.0%	1 / 3 33.3%	0 / 4 0.0%	1 / 2 50.0%	0 / 1 0.0%	2 / 5 40.0%	0 / 0 0.0%	0 / 0 0.0%	0 / 0 0.0%	0 / 0 0.0%	10 / 45 22.2%	9 / 45 20.0%ABE	9 / 45 20.0%E	8 / 45 17.8%AB	5 / 45 11.1%C	4 / 45 8.9%CE
